# Lost in plasmids: next generation sequencing and the complex genome of the tick-borne pathogen *Borrelia burgdorferi*

**DOI:** 10.1186/s12864-017-3804-5

**Published:** 2017-05-30

**Authors:** G. Margos, S. Hepner, C. Mang, D. Marosevic, S. E. Reynolds, S. Krebs, A. Sing, M. Derdakova, M. A. Reiter, V. Fingerle

**Affiliations:** 1 German National Reference Centre for Borrelia (NRZ), Bavarian Health and Food Safety Authority (LGL), Veterinärstrasse 2, 85764 Oberschleissheim, Germany; 2 Bavarian Health and Food Safety Authority (LGL), Veterinärstrasse 2, 85764 Oberschleissheim, Germany; 3 European Programme for Public Health Microbiology Training, European Centre of Disease Prevention and Control (ECDC), Stockholm, Sweden; 4 Department of Biology and Biochemistry, University of Bath, Claverton Down, BA2 7AY Bath, UK; 5 Gene Centre, Laboratory for Functional Genome Analysis, LMU Munich, Feodor-Lynen-Strasse 25, 81377 Munich, Germany; 6 Institute of Zoology, Slovak Academy of Sciences, Bratislava, Slovakia; 7 Institut für Hygiene und Angewandte Immunologie, Medizinische Universität Wien, Kinderspitalgasse 15, A-1090 Wien, Austria

**Keywords:** *Borrelia burgdorferi*, Genomics, Plasmids, Next generation sequencing, *De novo* assembly

## Abstract

**Background:**

*Borrelia* (*B.*) *burgdorferi* sensu lato, including the tick-transmitted agents of human Lyme borreliosis, have particularly complex genomes, consisting of a linear main chromosome and numerous linear and circular plasmids. The number and structure of plasmids is variable even in strains within a single genospecies. Genes on these plasmids are known to play essential roles in virulence and pathogenicity as well as host and vector associations. For this reason, it is essential to explore methods for rapid and reliable characterisation of molecular level changes on plasmids.

In this study we used three strains: a low passage isolate of *B. burgdorferi* sensu stricto strain B31(−NRZ) and two closely related strains (PAli and PAbe) that were isolated from human patients. Sequences of these strains were compared to the previously sequenced reference strain B31 (available in GenBank) to obtain proof-of-principle information on the suitability of next generation sequencing (NGS) library construction and sequencing methods on the assembly of bacterial plasmids. We tested the effectiveness of different short read assemblers on Illumina sequences, and of long read generation methods on sequence data from Pacific Bioscience single-molecule real-time (SMRT) and nanopore (Oxford Nanopore Technologies) sequencing technology.

**Results:**

Inclusion of mate pair library reads improved the assembly in some plasmids as did prior enrichment of plasmids. While cp32 plasmids remained refractory to assembly using only short reads they were effectively assembled by long read sequencing methods. The long read SMRT and nanopore sequences came, however, at the cost of indels (insertions or deletions) appearing in an unpredictable manner. Using long and short read technologies together allowed us to show that the three *B. burgdorferi* s.s. strains investigated here, whilst having similar plasmid structures to each other (apart from fusion of cp32 plasmids), differed significantly from the reference strain B31-GB, especially in the case of cp32 plasmids.

**Conclusion:**

Short read methods are sufficient to assemble the main chromosome and many of the plasmids in *B. burgdorferi*. However, a combination of short and long read sequencing methods is essential for proper assembly of all plasmids including cp32 and thus, for gaining an understanding of host- or vector adaptations. An important conclusion from our work is that the evolution of *Borrelia* plasmids appears to be dynamic. This has important implications for the development of useful research strategies to monitor the risk of Lyme disease occurrence and how to medically manage it.

**Electronic supplementary material:**

The online version of this article (doi:10.1186/s12864-017-3804-5) contains supplementary material, which is available to authorized users.

## Background

Comparative analyses of whole genome sequences have been shown to be vital for understanding the correlation between infecting pathogen and disease manifestation (e.g. [1–3]). A pre-requisite for comparative genomics is the identification of strains of differing pathogenicity and also the ability to assemble the whole of the genome, including accessory regions in genomes of pathogenic bacteria. Bacterial genomes can be divided into conserved core and less conserved accessory (or non-core) regions [4]. Importantly, accessory regions often undergo horizontal gene transfer, probably promoted by mobile genome elements (phages, transposons, plasmids) [5]. This may change pathogenicity as well as host, or vector specificity (e.g. [6]).

Next generation sequencing (NGS) technologies have allowed a big step to be taken towards the goal of whole genome analysis. Different sequencing methods are currently on the market with Illumina being notable for short sequencing read technology [7–9], but assembly of accessory regions of the genome may be particularly challenging from short read NGS data [10, 11]. On the other hand Pacific Bioscience Systems’ Single Molecule Real-Time (SMRT) DNA sequencing and Oxford Nanopore Technologies’ nanopore sequencing methods generate long sequence reads (maximum length 20 kb and >100 kb, respectively) with enormous advantages for genome assembly and in particular for plasmids (e.g. [10]). Although long read methods such as nanopore or SMRT sequencing may have less precision with respect to single nucleotide polymorphism accuracy [9], high sequence coverage with the latter method (>100×) has been reported to provide more accuracy at the single nucleotide [12]. Costs of the sequencing methods vary considerably and thus, precision and economics are important factors for deciding which sequencing technology is best suited to investigate the pathogen in question.

**Fig. 1:**
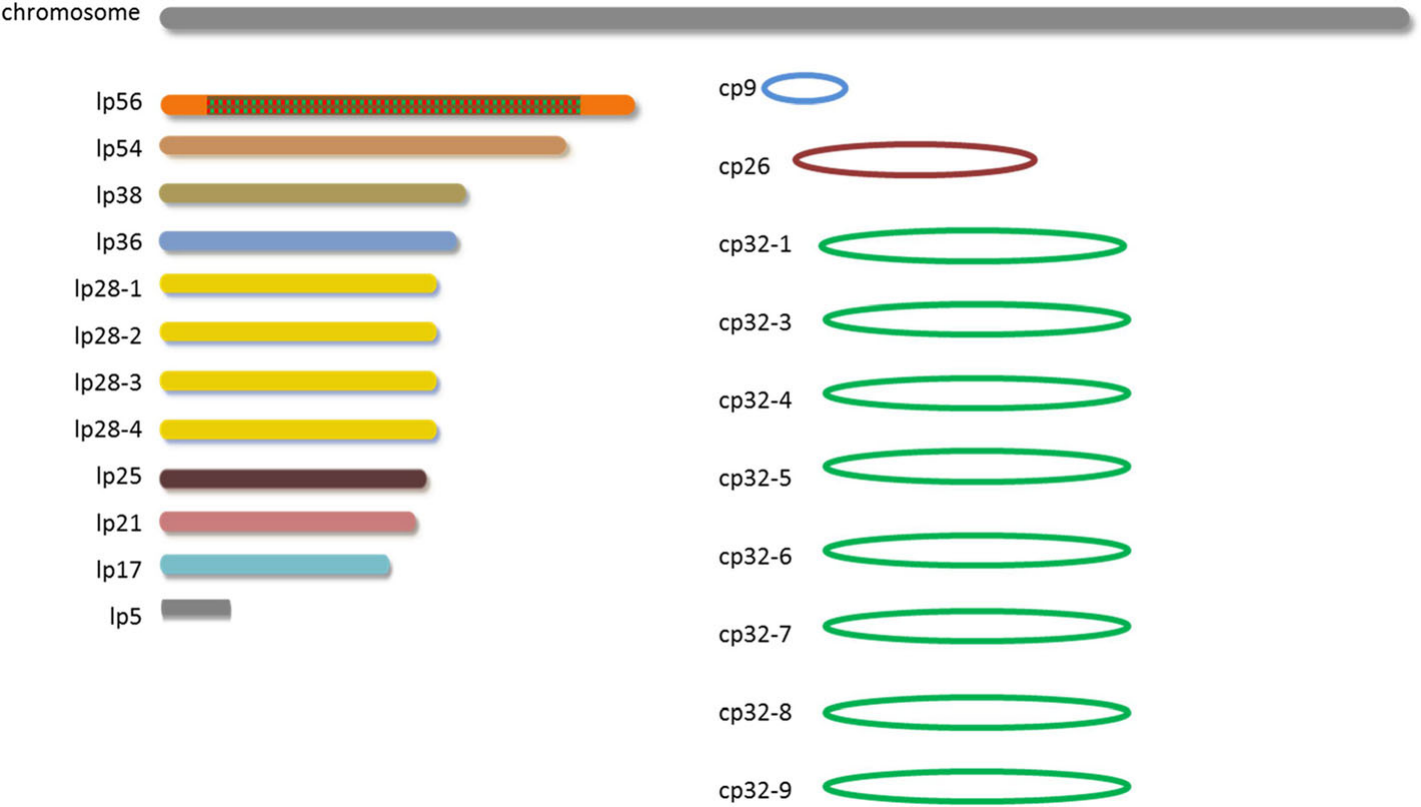
Schematic drawing of the genome organization of B. burgdorferi s.s. strain B31-GB. Strain B31-GB has been described to possess >20 linear and circular plasmids (Casjens et al. 2012). Similar color indicates plasmids with high sequence similarity. Note that lp56 has an insertion of a cp32 plasmid

In the present work we compared different NGS methodologies for their utility in analyzing the very complex genome of *Borrelia burgdorferi* sensu stricto (s.s.). *B. burgdorferi* s.s. belongs to a bacterial species complex that consists of more than 20 genospecies with differing propensity to cause Lyme borreliosis in humans. The bacteria are maintained in natural transmission cycles between vertebrate reservoir hosts and tick vectors. The *Borrelia* genome is highly fragmented consisting of a linear main chromosome and numerous linear and circular plasmids [13, 14]. Figure 1 shows a schematic drawing of the published genome of strain B31, the first strain of *B. burgdorferi* s.s. ever sequenced. The size of the whole genome amounts to 1.5 Mb with plasmids constituting appr. 40% of it. The GC content is about 28%. It is known that plasmids encode the majority of outer surface proteins [14] that are located at the vector-host-pathogen interface (reviewed by [15]) playing a critical role in the ecology of *B. burgdorferi* sensu lato (s.l.) and in their ability to cause disease. Therefore, information on plasmid content and structure is an absolute requirement for understanding host or vector adaptation of vector-borne disease agents such as *Borrelia*. The number and structure of plasmids harbored by *Borrelia* strains can vary considerably [13, 16] and for many European *Borrelia* species only limited information on plasmid content and structure is available (see http://borreliabase.org/). Furthermore, difficulties in assembling plasmid sequences currently hamper efforts to understand the interaction of this organism with human and animal reservoir hosts as well as its arthropod (tick) vector [17].

Molecular level changes in the genomes of bacteria with significant consequences for virulence and pathogenicity, together with host and vector associations can be accelerated through horizontal gene transfer and recombination instigated by mobile genetic elements (e.g. transposons, phages). To explore molecular level changes in the genome of vector-borne bacterial pathogens and to understand how best to identify them, we investigated the genomes of closely related strains of the tick-transmitted bacterium *B. burgdorferi* s. s. using a variety of library construction methods, enrichment of plasmids and different sequencing technologies (Illumina, Pacific Bioscience Systems SMRT technology, Oxford Nanopore technology). Previous work has shown that linear and circular plasmids can have a mosaic structure and differ even between strains of a given species [13], thus we especially focused on the plasmid fraction, which is known to be important in influencing host specificity and virulence in *Borrelia* [18, 19]. It is well recognized in other bacteria that gain or loss of genetic elements (plasmid acquisition/loss, phage transduction) can lead to adaptation to a new environment including host specialization and may change pathogenicity of bacterial pathogens with consequences for human or animal health (e.g. [6, 10]).

We sequenced a low passage strain B31 of *B. burgdorferi* s. s., available at the German National Reference Centre for Borrelia (here termed B31-NRZ), and two additional closely related strains PAli and PAbe [20, 21], comparing these with the previously sequenced reference strain B31, available in GenBank (accession number AE000783.1) (here termed B31-GB). These strains were chosen because their close relatedness to the B31 reference strain allowed addressing questions of performance of library construction methods, of assembly software and sequencing technology. One of the main aims was to address the question of what are appropriate requirements for future reference genome assembly in other *Borrelia* species.

Our data show that short read methods are sufficient to assemble the main chromosome and many of the plasmids. However, both long and short read methods are required to obtain reliable data on cp32 plasmids. The *B. burgdorferi* s.s. strains investigated here showed in general a high degree of similarity to the reference strain B31-GB but, intriguingly, both long read technologies revealed remarkable differences between strains in some cp32 plasmids. These data together with previous work indicate that *B. burgdorferi* s.s. strains that show high similarity at the core genome may show structural differences in some of their plasmids suggesting a dynamic system for rapid evolution.

## Methods

### Strains

We used a B31 low passage strain of *B. burgdorferi* s.s. (4th passage after receiving the strain in 1983), provided as a kind gift of W. Burgdorfer to the German National Reference Centre (NRZ). The strain was originally isolated in 1981 from an *Ixodes scapularis* tick on Shelter Island [22]. Throughout the paper, this B31 strain is termed B31-NRZ and will be compared to B31-GB, the strain that had been sequenced by Fraser and co-authors [14] and deposited to GenBank under accession number AE000783.1. The strains sequenced by Fraser and co-authors originated from the same tick isolate as B31-NRZ but was cloned and passed through a mouse before sequencing using a whole-genome random approach utilizing small insert plasmid libraries [14, 23].

PAli and PAbe are strains isolated from patients in Germany in the 1990s (5th and 6th in vitro passage, respectively). Based on Multilocus Sequence Typing (MLST) and chromosomal single nucleotide polymorphisms (SNPs), they have been shown to belong to the same MLST sequence type (ST) as B31 (ST1) and both these strains differ in approximately 60 SNPs from B31-GB on the main chromosome, thus they are very closely related to B31 [20, 21].

### PCR analyses

To probe which plasmids are present in the strains B31-NRZ, PAbe and PAli, primers were designed to sequence regions that were unique for individual plasmids in B31-GB (Additional file 1: Tables S5 and S6).

PCR primer and conditions used to verify the presence/absence of gaps in Illumina alignments are given in Additional file 1: Tables S7.

### DNA purification, plasmid enrichment and library construction

To generate genomic DNA for Illumina sequencing, *Borrelia* strains were cultured in MKP medium using conditions as described previously [24]. Genomic DNA was extracted via a Maxwell® 16 using a Maxwell LEV Blood DNA kit (Promega, Germany) and libraries of whole genomic DNA (total DNA) were prepared according to the manufacturer’s recommendation.

Following DNA quantification, libraries were prepared according to Illumina DNA sample preparation guide (Illumina, San Diego CA, USA). Three different library preparations were used for total DNA for Illumina MiSeq sequencing: Nextera (NX) (cat. no. FC-102-2003, FC-121-1011), TruSeq (TS) V2 (cat.no. RS-102-2003) and Nextera mate pair library prep kit (MP) (cat. no. FC-132-1001). Library prep kits, Library index kits and MiSeq Reagent kits were purchased from Illumina (Munich, Germany). To generate libraries, Nextera required 50 ng of DNA, TS and mate pair libraries required 1–2 μg of DNA. For TS libraries genomic DNA was fragmented using ultrasound in a Covaris M220 (Covaris Ltd, Brighton, UK). Settings were as follows: DNA_550 bp protocol, peak power 50.0, duty factor 20.1, cycles/burst 200, duration 45 s. To shear the circularized fragments during mate-pair library construction, the following Covaris settings were used: DNA_750 bp, peak power 50.0, duty factor 20.1, cycles/burst 200, duration 36 s. MP library construction yielded the following sizes after circularization: B31 8400 bp; PAbe 3600 bp; PAli 7500 bp. Purified biotinylated fragments of 500 bp, 644 bp and 687 bp were obtained for B31, PAbe and PAli, respectively, after shearing of circularized DNA.

To investigate whether sequence assembly would improve for plasmids if the main linear chromosome was removed prior to library construction, we used plasmid purification kits. For an initial assessment of suitability of plasmid purification kits for separation of the *Borrelia* main chromosome from plasmids, three different plasmid purification kits were run in duplicate, two from Qiagen and one from Promega (QIAGEN® Plasmid Mini kit (QIAGEN®, cat.nr.: 12123); QIAprep® Spin Miniprep kit (QIAGEN®, cat.nr.: 27104); Wizard® Plus SV Minipreps DNA Purification System (Promega, cat.nr.: A1270)). Based on the results of this assessment, a larger scale plasmid purification of B31-NRZ, PAli und PAbe DNA was performed using the QIAGEN® Plasmid Midi kit (QIAGEN®, cat.nr.: 12143) according to a protocol provided by the manufacturer.

Libraries on enriched plasmids were prepared using the Nextera® XT Library Prep Kit (Illumina, cat.no. 15032350, 15032352), the Nextera® XT Index Kit (Illumina, cat.no. 15055294) and libraries run with a MiSeq® Reagent Nano Kit v2 (Illumina, cat.no. 15036714). Extracted DNA was diluted to a concentration of 0.28 ng/μL and four μL were used as starting material for library construction according to the manufacturer’s recommendations.

All libraries were validated using genomic DNA analysis screen tapes (cat no. 5067–5365) and genomics DNA reagents (cat. no. 5067–5366) (Agilent, Santa Clara, CA, USA).

A quality check on Illumina sequencing reads was performed either in Galaxy [25] or in the CLC Genomics Workbench and reads that did not pass the filter were not included in the analyses. Sequence reads were mapped on the B31 reference genome (GenBank accession no. NC_001318.1) in the CLC Genomics Workbench using default settings: Mismatch cost = 2; Cost of insertions and deletions = Linear gap cost; Length fraction = 0,5; Similarity fraction = 0,8; Auto-detect paired distances = yes; Non-specific match handling = Map randomly. Variant calls were generated using the fixed ploidy variant detection option with ploidy = 1; required variant probability (%) = 90; minimum coverage = 10; minimum count = 2; minimum frequency (%) = 80; base quality filter = yes; neighborhood radium = 5; minimum central quality = 20; minimum neighborhood quality = 15. Read tracks and reports were generated and saved as well as un-mapped reads.

*De novo* assembly was also done in the CLC Genomics Workbench using the following parameters: mapping mode = map reads back to contigs; mismatch cost = 2; insertion cost = 3; delection cost = 3; colorspace error cost = 3; length fraction = 0.5; similarity fraction = 0.8; colorspace alignment; alignment mode = local; match mode = random.

For *de novo* assembly of reads in Galaxy [26], SGA [27] and Velvet [28] were used with the following settings in SGA: minimum overlap for the final assembly = 85; correction k-mer = 31; min read length = 40; coverage filter = 2; overlap parameter used for FM-merge = 55; number of pairs required to link two contigs = 5; minimum length of contigs to include into scaffold = 200. Settings for Velvetoptimiser were: start hash length = 35, end hash length = 129, Kmer optimisation metric = N50 size; coverage optimisation metric = length of single longest contig.

For B31-NRZ *de novo* assembly was also conducted using SPAdes [29] on a virtual BioLinux v.1.8. machine [30] using the following command line: −k 21, 33, 55, 77, 99 --careful --pe1-1 B31_R1.fastq --pe1-2 B31_R2.fastq -o B31_spades_output.

For SMRT sequencing 10 μg of DNA were purified via Maxwell® 16 using a Maxwell LEV Blood DNA kit (Promega, Germany). The quality and quantity of DNA was validated using a Qubit 3.0 fluorimeter (Life Technologies, Germany) and a genomic DNA screen tape (Agilent). DNA was sent to the Norwegian Sequencing Centre, University of Oslo, Norway for library construction, sequencing and assembly. Libraries were constructed using Pacific Biosciences 20 kb library preparation protocol. Size selection of the final library was performed using BluePippin with a 7 kb cut-off. Libraries were sequenced on Pacific Biosciences RS II instrument using P6-C4 chemistry with 360 min movie time.

Assembled sequences and consensus sequences of the read mapping conducted in CLC were aligned using MEGA5 [31]. Genetic distance analyses between sequences and multiple alignments of sequences were created in MEGA5.

For nanopore sequencing 1 μg of DNA was end-repaired and dA-tailed without previous fragmentation (NEBNext End repair / dA-tailing Module; New England Biolabs, USA) and ligated to 1D sequencing adapters (LSK108 kit, Oxford Nanopore Technologies, Great Britain). Prepared library was loaded to a MinION flowcell (R9.4 version, Oxford Nanopore Technologies, Great Britain) and sequenced for 6 h with offline basecalling. For nanopore reads error correction, trimming and assembly was performed with the CANU assembler [32] using the command options genomeSize = 0.94 M, CorOutCoverage = 1000, corMinCoverage = 0, errorRate = 0.035, minReadLength = 750.

### Sequencing and sequence analysis

Short read sequencing was performed on an Illumina MiSeq platform (Illumina, San Diego CA, USA). Reads were demultiplexed and tags removed as part of the MiSeq run. SMRT sequences were assembled and polished using the Hierarchical Genome Assembly Process (HGAP). Overlapping regions due to circularization of plasmids were removed. Nanopore sequences were processed employing the long read assembler Canu [32] available through a Galaxy platform [25, 33].

The software package Mauve [34] was used to order pre-assembled contigs and to align them to a reference genome.

BLAST ring image generator (BRIG) was used to display similarities between genomes [35]. BLAST [36] was performed with default settings to search for similarities between contigs and sequences available in GenBank.

## Results

### Impact of Library construction and assembler on Illumina MiSeq sequence reads

**Fig. 2:**
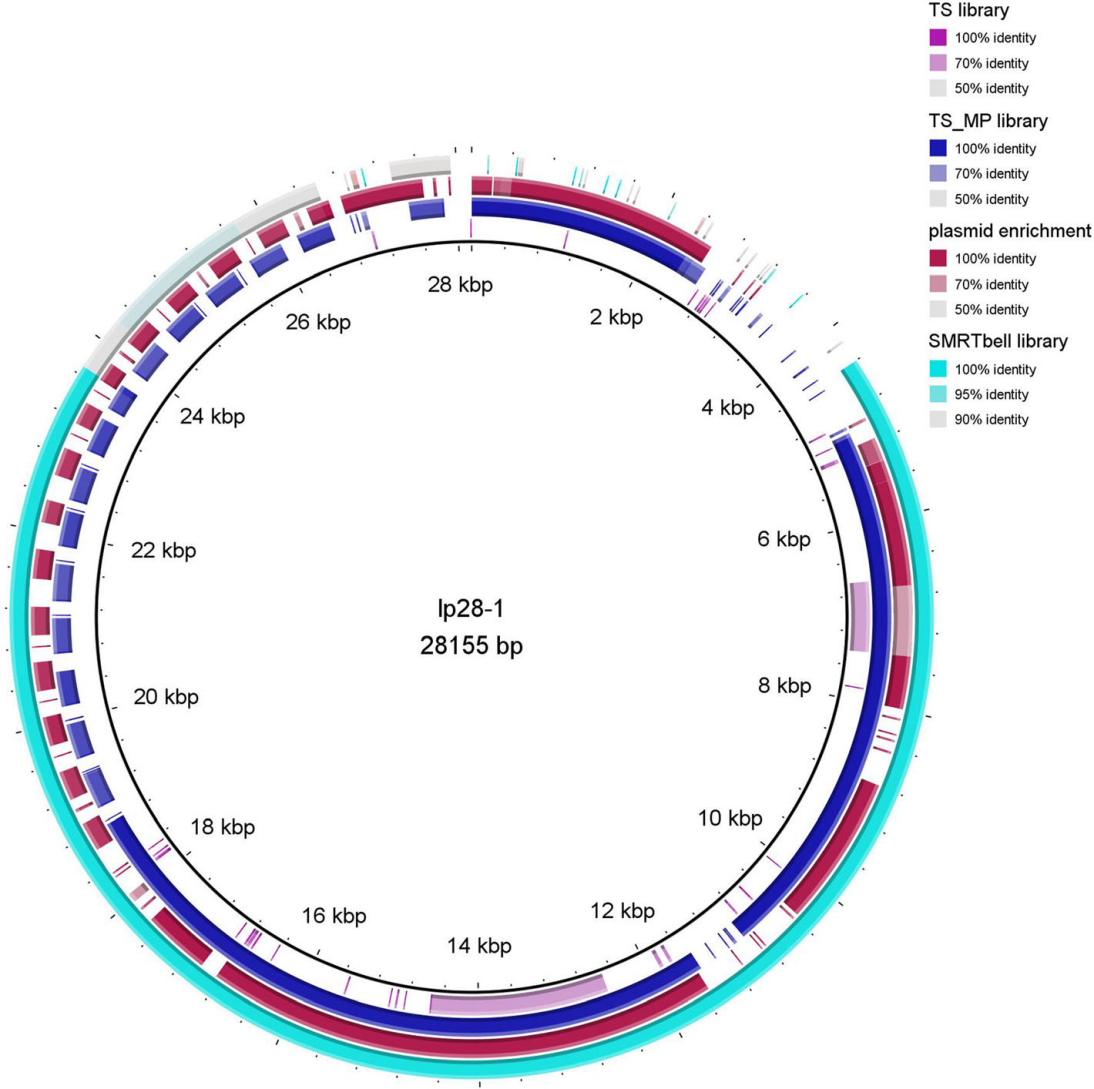
Visualization of *de novo* assembly of lp28-1 in B31-NRZ. Poor assembly was observed using TS libraries only (*inner most ring*, *purple color*, labelled TS library in legend). A combination of TS and MP libraries considerably improved the assembly of lp28-1 (*blue color*, labelled TS-MP library in legend). Also the use of enriched plasmid DNA for NX library construction improved the assembly (*red color*, plasmid enrichment). Pacific Bioscience SMRT sequences also provided a good assembly (*turquois color*, SMRT Bell library). It is noteworthy that in all cases (TS-MP, plasmid enrichment, SMRT Bell) the plasmid of B31-NRZ was shorter than that of B31-GB. Different shades of coloration in one panel indicate different identities between aligned sequences. Regions with lighter shades correspond to less sequence similarity (see region app. 7 kbp to 7.8 kbp in plasmid enrichment)

A summary of the sequencing statistics, i.e. total bp, average read length, GC content, revealed that for all strains sequencing results were of comparative, sufficiently high quantity and quality to obtain a representative view of the genome (Table 1). Comparison of the different methods using *de novo* assembly revealed little differences in assembly success when either NX or TS were used on their own. MP libraries have been developed to provide scaffolding for short reads especially for regions carrying repetitive sequences [37]. In our study, when combining NX and TS reads with MP reads the number of contigs (counts) was only slightly reduced from 65 to 62 with NX or NX_MP, respectively, and 63 to 62 for TS and TS_MP for strain PAbe (not PAli or B31-NRZ). Similarly, N50 values changed only slightly when libraries were constructed using TS only or including MP libraries in the assembly (B31-NRZ-TS, N50 value 433.943 and B31-NRZ-TS_MP, N50 value 434.762; PAbe-TS, N50 405.884 and PAbe-TS_MP, N50 value 434.591, Additional file 1: Table S1). For B31-NRZ *de novo* assembly of lp28-1 was improved using a combination of TS and MP library reads compared to TS reads alone (Fig. 2).

**Table 1:** Sequencing statistics for all samples and sequencers

Sequencer/Strain		B31-NRZ		PAli		PAbe		
Illumina	Library prep	TS	MP	NX	MP	NX	TS	MP
	Total bp (Mb)	564.6	174.4	779	2,140	514	527	106.5
	Average read length after trimming	249	239	221	243	218	228	242
	GC content %	28.1	28.1	28.2	28.2	28.2	28.2	28.2
	Total sequences in pairs	2,264,058	725,744	3,092,326	8,984,852	2,040,798	2,178,498	437,050
PacBio	Library prep	BluePippin		BluePippin		BluePippin		
	Total bp	794.6		429.3		2,188.6		
	Average read length	13,987		13,632		21,727		
	GC content	28.4		28.3		28.3		
	Average insert size (bp)	11,332		7,997		4,177		
Nanopore	Library prep	Ligation 1D						
	Total bp (Mb)	77.5						
	Average read length	4000						
	GC content	28.8

**Fig. 3:**
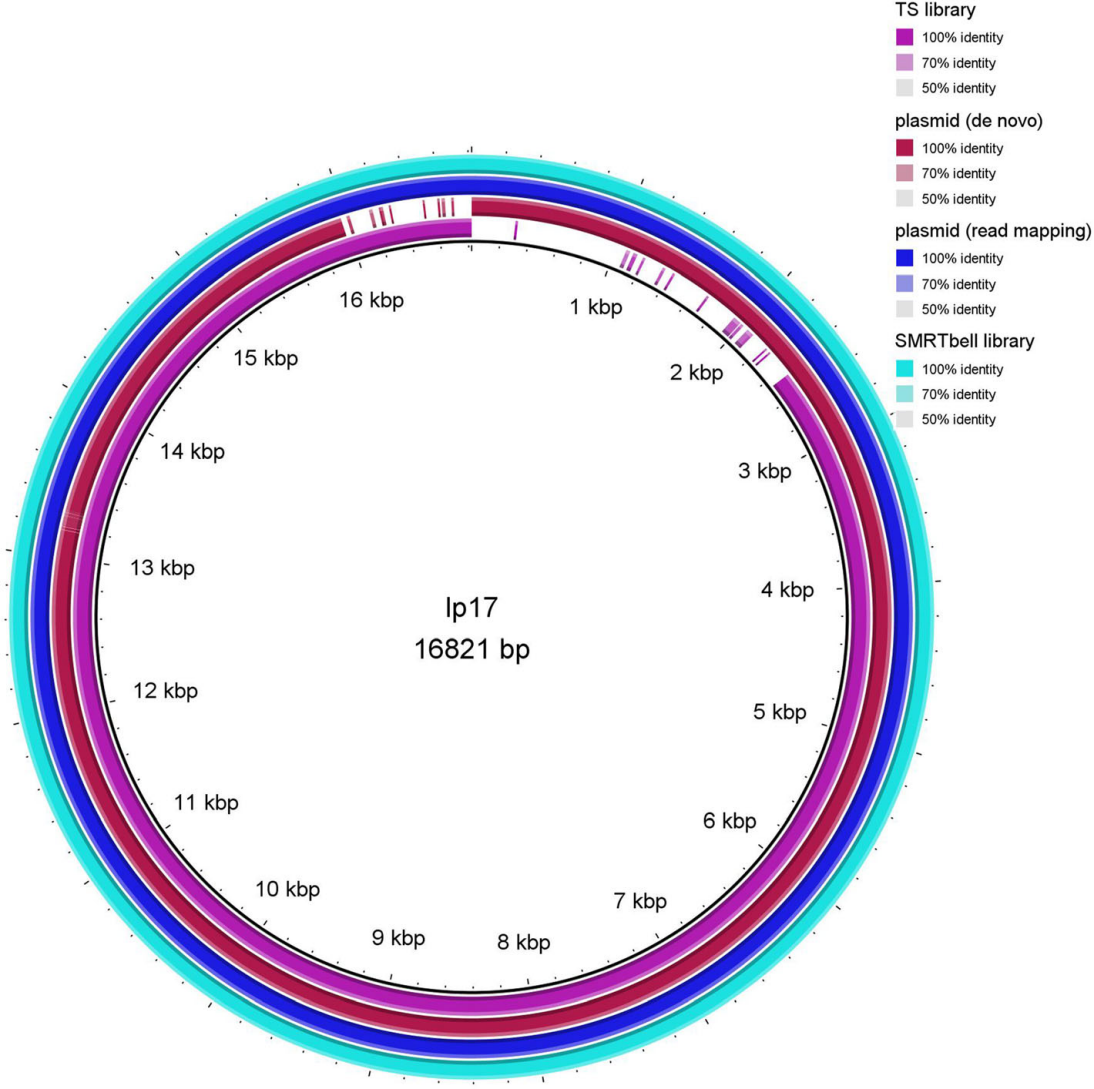
Visualization of assemblies of lp17 in PAbe aligned to the reference B31-GB using BRIG. Total genomic DNA was used for TS library preparation (*purple color*, labelled TS library). A spurious gap in the region from 0 to 2.5 kb is visible. Using enriched plasmid DNA for NX library construction improved the assembly and a smaller gap at 16–17 kb is visible (*red color*, labelled plasmid (*de novo*)). Not surprinsingly, read mapping of Illumina reads on B31-GB using enriched plasmid DNA for library construction showed complete coverage suggesting that reads for the complete plasmid existed but were not assembled de novo (*blue color*, labelled plasmid readmapping). *De novo* assemblies of Pacific Bioscience SMRT sequences showed complete coverage of lp17 confirming that the complete plasmid was present in PAbe and that the gaps presenting in *de novo* assemblies of short reads were artefacts either of library construction or short read assembly (*torquois color*, labelled SMRT Bell library)

In some cases, when *de novo* assemblies of plasmids of B31-NRZ, PAli and PAbe were aligned to the B31-GB reference, gaps in the alignment were observed e.g. lp17 (B31-NRZ) (Fig. 3), lp28-1 (Fig. 2), lp36 (PAbe) and lp38 (PAli). To examine whether these gaps represented true differences between strains or whether these gaps may have appeared as a result of problems in library construction or assembly, PCR was employed. PCR with primers (Additional file 1: Table S8) designed to bridge the border between neighboring contigs and the gapped regions resulted in PCR products, suggesting that these gaps are artefacts likely to be results of library construction methods or assembly of short reads (data not shown). Sequences of PCR products were identical to B31-GB sequences further confirming the artefactual nature of these gaps (e.g. lp38, Additional file 1: Figure S8). The artefactual nature of these gaps was further confirmed by long read assemblies (see below).

Comparison of different assemblers revealed that using SPAdes for assembly gave the longest contigs. A single contig covered the whole of the main chromosome (910,137 bp), while several others covered whole plasmids such as lp36, lp38, lp54 or cp26 (Table 2, Additional file 1: Table S2). However, as shown by BLAST [36] searches, cp32 plasmids were difficult to assemble *de novo* using any assembler including SPAdes and only short contigs that matched several B31-GB cp32 sequences were generated (Additional file 1: Table S2).

**Table 2:** Summary statistics of B31-NRZ TS paired-end Illumina reads using different *de novo* assemblers

Value/Assembler	CLC Genomic Workbench	SGA	Velvetoptimiser	SPAdes
Number of contigs	71	3930	304	145
Length of longest contig	442,339	174,427	434,181	910,137
Total length	1,255,479	2,307,446	1,173,546	1,251,637
N50	359,624	663	353,278	910,137

As expected, read mapping to the B31-GB reference genome (including plasmids) provided evidence that the different library preparation methods gave equally good results with numbers of SNPs being very similar regardless of the library construction method (Additional file 1: Table S3).

PCR with plasmid specific primers (Additional file 1: Tables S5–S7) on strain B31-NRZ confirmed the presence of eight plasmids, six linear plasmids, lp17, lp28-1, lp36, lp38, lp54, lp56, and two circular plasmids cp26 and cp32-3. Primers for plasmid lp28-3 turned out to cross-react with lp56 (data not shown). For cp32’s (other than cp32-3) it was not possible to design specific primers due to sequence similarities between various cp32 plasmids. SMRT sequences (see below) revealed more subtle differences in plasmid content between B31-NRZ and B31-GB.

### Plasmids of NRZ strains B31-NRZ, PAli and PAbe

**Fig. 4:**
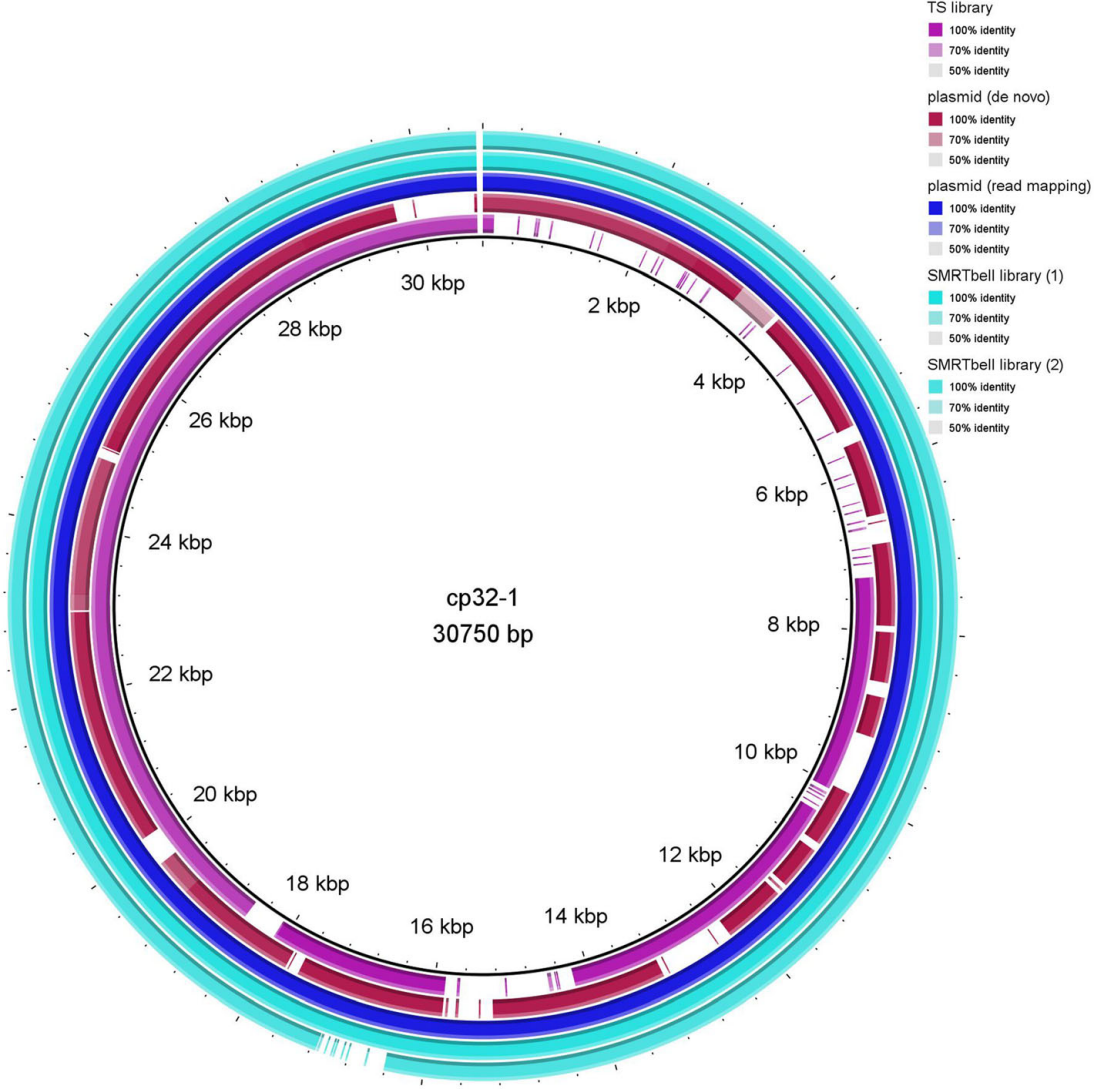
Visualization of assemblies of cp32-1 of B31-NRZ aligned to the reference B31-GB. *De novo* assembly of total genomic DNA sequenced from a TS library preparation (*purple color*, labelled TS library). The alignment shows several gaps covering only three quarter of the plasmid. Using enriched plasmid DNA for NX library construction followed by *de novo* assembly in the CLC Genomics workbench (*red color*, plasmid (*de novo*)), the size of gaps was reduced. Read mapping of Illumina reads on B31-GB using enriched plasmid DNA for NX library construction (*blue color*, plasmid (read mapping)) produced a gapless alignment of reads to the reference. *De novo* assemblies of Pacific Bioscience SMRT sequences (*torquois color*, SMRT Bell library) showed complete coverage of the cp32-1 plasmid. The image shows the larger size of the SMRT assembly with a gap appearing around 17 kb which likely reflect the differences observed between B31-NRZ and B31-GB. Different shades of coloration in one panel indicate different identities between aligned sequences. Regions with lighter shades correspond to less sequence similarity (see region app. 3.5 kbp to 4.0 kbp in *de novo* assembly using enriched plasmids)

Using commercially available plasmid mini kits, it was possible to enrich *Borrelia* linear and circular plasmids of all sizes, and even the large linear plasmids lp54 and lp56 were found by PCR in the preparation. The yield of DNA from the main linear chromosome was drastically reduced by this procedure (Additional file 1: Table S1, Figures S1 and S2,) permitting a high read coverage of plasmids and a slightly better *de novo* assembly of some plasmids of *B. burgdorferi* strains B31, PAli und PAbe. Graphical representation generated using BRIG [35] and comparison of the different methods underlined the suitability of plasmid enrichment for plasmid assembly in *Borrelia* (Figs. 2, 3 and 4). These images show that gaps that were present with *de novo* assemblies of total genomic DNA were reduced when enriched plasmid DNA was used for MiSeq sequencing (Figs. 3 and 4). Pacific Bioscience sequences performed even better resulting in fewer gaps in plasmids assemblies (Figs. 2, 3 and 4).

### Pacific Biosciences SMRT sequences

*De novo* assemblies obtained from Pacific Biosciences SMRT sequences resulted in contigs which indicated the presence of one main chromosome and12 plasmids in B31-NRZ, 12 plasmids in PAli and ten plasmids in PAbe. SMRT sequence assemblies correctly displayed the duplicated 5S-23S locus on the main chromosome and repetitive sequences, for example on plasmid lp17 (Additional file 1: Figure S3). In all three strains, differences in plasmid content compared to B31-GB were found; especially some of the cp32 plasmids appeared to have merged (e.g. cp32-1 and cp32-5 in B31-NRZ, cp32-1 and cp32-5 as well as cp-32-4 and cp32-9 in PAbe).

The 12 plasmids found in strain B31-NRZ were six linear plasmids lp17, lp28-1, lp36, lp38, lp54, lp56, and six circular plasmids, cp26, cp32-5 + 1, cp32-3, cp32-4, cp32-9 and cp32-2 (Table 3, Additional file 1: Table S4). Differences in B31-NRZ compared to B31-GB included (i) that contig4 matching plasmid cp32-1 in this strain seemed to be fusion of cp32-5 and cp32-1 as its PFam32 sequences were 100% identical to that of cp32-5 and cp32-1. Furthermore, it differed to B31-GB’s cp32-1 in sequence stretches of one or several kb that showed higher similarity to cp32-5 and cp32-6 of strains 64b and 156a, respectively (Table 3 and below, see also Additional file 1: Figure S4); (ii) that plasmid lp28-1 was shorter (21,885 bp) than in B31-GB (28,115 bp) and (iii) that plasmid cp32-2 was present and had >3000 SNPs compared to cp32-7. The PFam32 sequence of B31-NRZ contig6 showed 100% identity to PFam32 of plasmid cp32-2 and 99% similarity to PFam32 of plasmids cp32-7 of several *B. burgdorferi* s. s. strains. Plasmid cp32-2 was described originally in B31 [23] and in some B31 cultures it appears to have been replaced by cp32-7 [13].

**Table 3:** Plasmids present in B31-NRZ, PAli and PAbe

GenBank	PCR verification	PacBioscience SMRT sequences		
B31 or other Bbss^a^ strains	B31 NRZ	B31 NRZ	PAli	PAbe
cp26	cp26	cp26 (con9)	cp26 (con6)	cp26 (con8)
cp32-1		cp32-1_i5_i6 (con4)^b^ (61,436 bp)	cp32-1^c^ (con14) cp32-1_i6 (con15)	cp32-1_i5_i6^d^ (con2) (56,633 bp)
cp32-3	cp32-3	cp32-3 (con8)	cp32-3 (con4)	cp32-3 (con5)
cp32-4		cp32-4 (con10)	cp32-4 (con9)	(fusion cp32-9)
cp32-2		cp32-2 (con6)^e^	-	cp32-2 (con6)^e^
cp32-5		(fusion cp32-1)	cp32-5 (con13)	(fusion cp32-1)
cp32-9		cp32-9 (con7)	cp32-9 (con7)	cp32-9 (con3)^f^ (62,240 bp)
lp17	lp17	lp17 (con5)	lp17 (con8)	lp17 (con7)
lp28-1	lp28-1^g^	lp28-1 (con12, 21,885 bp)	-	lp28-1 (4800 bp)
lp36	lp36	lp36 (con11)	lp36 (con5)	lp36 (part; con11: 2313 bp; con12: 10,472 bp; con20: 2825 bp; con22: 3.932 bp)
lp38	lp38	lp38 (con2)	lp38 (con10)	-
lp54	lp54	lp54 (con1)	lp54 (con2)	lp54 (con1)
lp56	lp56	lp56 (con3)	lp56 (con3)	lp56 (con42: 10,951 bp, con43: 48,674 bp; overlap 200 bp)

^a^Bbss = *B. burgdorferi* s.s.; the full complement of B31-GB plasmids can be found in Table S3

^b^In B31-NRZ contig4 (con4) of the SMRT sequences was likely a hybrid plasmid as the first app. 33,000 bp showed 1138 SNPs to cp32-1 while the sequence starting from 33,000 had 773 SNPs to cp32-1. The regions showing variation to cp32-1 of B31 showed similarity to cp32-5 of strain 64b (21–26 kb) and to cp32-6 of strain 156a (47–48.5 kb) (see text for details). Contig4 had 6631 SNPs compared to cp32-1 + 5 of JD1. Its PFam32 sequences showed 100% similarity to cp32-5 and cp32-1

^c^In PAli, three contigs were found that matched cp32-1 in BLAST searches. Contig14 showed 20 SNPs to cp32-1 but was short (20,004 bp) and did not have a PFam32 locus. Contig13 matched cp32-1 from 1 to 18,740 bp, 18.7–23 kb the similarity was higher to cp32-5 of *B. burgdorferi *s.s. strain 64b , while the remaining sequence to 32,285 matched cp32-1 again. Its PFam32 sequences showed 100% similarity to cp32-5 of several *B. burgdorferi* s. s. strains. Contig15 between 16.5 and 18 kb showed a closer match to cp32-6 of strain 156a while the remaining sequence matched cp32-1. PFam32 sequence of contig15 was identical to cp32-1 of B31-GB

^d^In PAbe one contig was present (contig2) that partially matched cp32-1 but part of the sequence also matched cp32-5 (appr. 14.5–19 kb) and cp32-6 (appr. 40–42 kb). In contig2, the PFam32 of cp32-1 showed 100% identity

^e^A plasmid was found in B31-NRZ that was not present in the B31-GB reference. Its PFam32 sequence was identical to cp32-2 and showed high similarity (99%) to cp32-7 of several *B. burgdorferi* s.s. strains. An almost identical plasmid was found in PAbe with a PFam32 matching that of cp32-2

^f^In BLAST searches contig3 of PAbe showed similarity to cp32-9 . However, the first part of the sequence was highly similar to cp32-9 while the second part was more similar to cp32-4 of B31 suggesting that this plasmid represents a hybrid plasmid. The PFam32 sequences were identical to PFam32 of cp32-9 and cp32-4

^g^In B31 plasmid lp28-1 is short (21,885 bp). In PAbe the presence of lp28-1 is questionable as there were only two contigs of approximately 4800 bp that showed high similarity to lp28-1

PAli contained seven circular plasmids and five out of the six linear plasmids as identified in B31-NRZ, lp28-1 was missing completely. Three contigs showed similarities to plasmid cp32-1, and to varying degrees similarities to other cp32 plasmids (Table 3, and discussed below in further detail).

In strain PAbe contigs matching seven complete plasmids were identified including lp17, lp54, cp26, cp32-5/cp32-1, cp32-3, cp32-4/cp32-9, cp32-2, of which two appeared to be fusion plasmids, i.e. contig2 fusion cp32-5 and cp32-1 and contig3 fusion of plasmids cp32-9 and cp32-4. Plasmid lp38 was missing from PAbe.

Contig4 of B31-NRZ seemed to be a mosaic plasmid of cp32-1 and other plasmids. It contained in the region from 21 kb – 26 kb sequences with high similarity to Bbu64b_V0030, and Bbu64b_V0032 to _V0037 that correspond to region 20,971-21,744 and 23,338-26,211 of cp32-5 of *B. burgdorferi* s.s. strain 64b. Bbu64b_V0030 to Bbu64b_V0037 encode hypothetical proteins, putative plasmid partition genes or KID repeat protein. The region between 47 and 49 kb in contig4 of B31-NRZ showed the highest similarity to BbU156a_M27 and _M28 (corresponding to regions 16,558 – 17,133 and 17,239-17,676 of cp32-6 of *B. burgdorferi* s.s. strain 156a). M27 of strain 156a codes for a repeat motif-containing protein whose nucleotide sequences was 91% similar to the *Borrelia* direct repeat (*BdrN*) gene of cp32-7 in B31.

In PAli, there were three different contigs that had similarity to cp32-1 of B31-GB. Contig14 had highest similarity to cp32-1 but was truncated (20,004 bp) compared to cp32-1 of B31-GB (30,681 bp) and no PFam32 sequence was found. Contig13 and 15 were partially highly similar to cp32-1 but had sequence stretches of higher similarity to cp32-5 or cp32-6, respectively (Table 3 and Additional file 1: Tables S3 and S4), similar to what was found in contig4 of B31-NRZ. Similarity searches with PFam32 sequences revealed that contig13 was likely a member of cp32-5, its PFam32 was identical to PFam32 of cp32-5 plasmids of other *B. burgdorferi* s. s. strains. The PFam32 sequence of contig15 had 100% similarity to cp32-1 of B31-GB, in spite of this plasmid containing a sequence stretch that was identical to cp32-6 of *B. burgdorferi* s.s. strain 156a. The cp32-6 sequences mapped to the same region as those in B31-NRZ (BbU156a_M27 and _M28), indicating that amongst the strains investigated here, the plasmids and plasmid parts showed a high degree of similarity to each other (Additional file 1: Figure S4).

In PAbe, plasmid cp32-2 was found being 99% identical to cp32-2 of B31-NRZ. The size of contig3 in PAbe was 62 kb. It showed sequence similarities to cp32-4 and cp32-9 suggesting that this plasmid represents a hybrid plasmid in this strain. To this point, its two PFam32 sequences showed 100% identity to cp32-9 and cp32-4.

In PAbe, several contigs were obtained that partially matched three plasmids (lp28-1, lp36 and lp56). Lp36 was represented by 4 short contigs (2.3–10 kb, Table 3) that aligned in a gapped fashion to lp36 of B31-GB which may suggest that the plasmid was in the process of degradation. For lp56 two overlapping contigs were obtained. It is conceivable that different cells of the population contained the different fragments of the plasmid which was perhaps also in the process of degradation. Discrepancies concerning plasmid lp28-1 existed between different assemblies. *De novo* assemblies of Illumina reads using total genomic DNA suggested the presence of an lp28-1 in PAbe. A protein BLAST search in the Illumina *de novo* assembly of PAbe with the lp28-1 partitioning protein of B31-GB revealed a 100% identical hit supporting the presence of lp28-1. However, read mapping of plasmid enriched Illumina reads showed a surprisingly low coverage of lp28-1 of only 14× compared to >100× coverage for other plasmids. In SMRT assemblies two short contigs (total appr. 4800 bp) were recovered one of which showed high similarity (99%) to the VlsE-locus that is located on lp28-1 in B31-GB. Taken together, the data suggest the presence of lp28-1 in PAbe albeit perhaps in low abundance and perhaps in the process of decay.

Analysis of coverage of SMRT contigs suggested it was likely that not all the plasmids were present in each individual cell of the population, but that individual plasmids were present in only a subset of cells. For example in PAbe, coverage of the main chromosome was nearly 1,000fold while plasmids cp32-2 and lp17 showed a coverage of 500fold and 250fold, respectively suggesting a presence of the respective plasmids in either one half or one quarter of the cells.

A drawback of Pacific Biosciences SMRT and nanopore sequences was the presence of (presumably artefactual) insertions and deletions (indels) (Additional file 1: Figure S5) that were neither present in the B31-GB reference sequence nor in consensus sequences of read mapping of the respective Illumina sequences (Additional file 2, Alignments_B31_PAli_PAbe).

### Nanopore sequencing

Sequences obtained using the MinION device of Oxford Nanopore Technology were trimmed and assembled using the single read assembler Canu [32]. Both, assembled and unassembled trimmed reads had an average coverage of 40fold. BLAST [36] searches of nanopore contigs and trimmed reads against sequences available in GenBank returned matches to the main chromosome and plasmids as detailed in Table 4. The main chromosome as well as five plasmids (lp54, lp56, lp38, cp26, and cp32-1) returned from Canu assemblies while plasmids lp17, lp28-1, lp36, cp32-3, cp32-4, cp32-9 and cp32-2 were recovered from trimmed read sequences indicating that the (almost) complete length of these plasmids was sequenced as a single length of DNA. Contig 241245 was longer in size than the B31-GB cp26. However, the first 13,431 bases matched to position 12,626-26,496 of cp26 and bases 39,932-46,144 matched to the first 7358 bp of cp26 suggesting an overlap of sequences. While most plasmids were comparable in size to the GenBank reference, some showed only partial matches, e.g. plasmid lp28-1 (15,787 bp) and cp32-4 (22,112 bp) were shorter than plasmids found in the GenBank reference of B31. Interestingly, shorter sequences for plasmid lp28-1 were also found in Illumina and SMRT sequences (Table 3). Intriguingly, nanopore sequences contained a single plasmid of approximately 60 kb in size that matched (in large parts) to contig4 of SMRT sequences (Additional file 1: Figure S6). In addition, unassembled trimmed reads of 18,058 bp showed highest similarity to contig6 of B31-NRZ, a plasmid resembling cp32-2 that was originally described in B31 [23]. The *parA* gene present in contig6 and the nanopore reads showed highest similarity in BLAST searches to the *parA* gene of cp32-2.

**Table 4:** Comparison of nanopore contigs and trimmed reads with GenBank and determination of single nucleotide differences (SNPs) in MEGA

B31-GB genome (length)	Nanopore contig name	Nanopore contig length	Identity with B31-GB	Id %	Gaps	Gaps %	Start query (subject)	Stop query (subject)	SNPs NP MEGA	%	SNPs PB MEGA	%
main (910,724)	241244	878,078	659,561/683,765	96	24,016/683,765	3	205,813 (213,067)	865,577 (897,715	nd	nd		
cp26 (26,498)	241245	46,144	25,597/26,496	97	8,951/26,496	3	13,432 (1)	39,032 (26,496)	15/26,498	0.05	4/26,498	0.01
lp54 (53,657)	241246	51,693	51,381/53,655	96	2,222/53,655	4	199 (53,651)	51,635 (1)	199/53,651	0.2	7/53,915	0.01
lp56 (52,971)	241248	51,105	50,156/52,909	95	2,152/52,909	4	1 (65)	51,105 (52,578)	837/53,318	1.6	7/53,318	0.01
lp38 (38,829)	241249	37,824	37,356/38,823	96	1,429/38,823	3	54 (38,821)	37,454 (6)	176/38,821	0.45	1/38,821	0.002
cp32-1 (30,750)	241247	67,311	20,520/21,493	95	663/21,493	3	36,733 (1)	57,597 (21,458)	2694/60,973^b^	4.4		
lp17 (16,821)	ch_216^a^	16,238	16,067/16,821	96	751/16,821	4	42 (1)	16,111 (16,821)	80/16,989	0.47	0/16,989	0.0
lp28-1 (28,155)	ch_17^a^	15,787	15,638/16,873	93	1,117/16,873	6	1 (1866)	15,785 (18,709)	1911/19,082	10.0	171/21,885	0.78
lp36 (36,849)	ch_474^a^	34,829	34,690/36,856	94	2,094/36,856	5	1 (8)	34,777 (36,848)	281/36,856	0.7	0/36,856	0.0
cp32-3 (30,223)	ch_176^a^	30,596	23,314/24,600	95	1,195/24,600	4	7187 (5629)	30,596 (30,223)	286/31,974	0.9	2/30,223	0.006
cp32-4 (30,299)	ch_152^a^	22,112	21,907/23,209	94	1,105/23,209	4	1 (4046)	22,111 (27,247)	339/27,247	1.2	3/27,247	0.01
cp32-9 (30,651)	ch_212^a^	28,699	17,790/19,416	92	1,325/19,416	6	1 (11,245)	18,100 (30,651)	491/30,651	2.7	2/30,651	0.006
cp32-2	ch_293^a^	18,058	nd	nd	nd	nd	nd	nd	181/18,058^b^	1.0		

^a^unassembled trimmed reads

^b^aligned to Pacific Bioscience SMRT sequence unitig4 (cp32-1 fused plasmid) and unitig6 (cp32-2), respectively

Determination of single nucleotide differences (SNPs) in MEGA between nanopore sequences and B31-GB sequences resulted in a number of SNPs some of which were due to alignment errors resulting from the fact that gap extension is less costly in alignments than opening of new gaps (see Additional file 1: Figure S5). In comparison, fewer SNPs were observed in SMRT sequences (Table 4) which can be attributed to higher accuracy of SMRT sequences and fewer sequencing gaps in homopolymer regions.

## Discussion

We chose *B. burgdorferi* s.s. strain B31 that was available at the German NRZ for *Borrelia* and two closely related strains (PAli and PAbe) that were isolated from patients with different clinical manifestations, i.e. erythema migrans and Lyme neuroborreliosis, respectively, to compare various next generation sequencing methods. These strains were chosen to obtain proof-of-principle data on the suitability of methods and to understand how similar or different the plasmid content and structure of very closely related strains in *B. burgdorferi* s.s. are. Previous studies of genomic differences between *Borrelia* strains investigated markedly divergent strains of *B. burgdorferi* s.s., i.e. representing different MLST STs. These studies revealed a substantial rearrangement of plasmid located sequences likely due to homologous and non-homologous recombination [13].

### Short read methods provide a good representation of the core genome but not of all plasmids

TS libraries have been described to give a more even coverage of genomes due to the mechanical fragmentation of DNA by ultrasound [9]. The NX library construction method, based on enzymatic fragmentation and tagmentation of DNA in a single step, represents a fast and very convenient way of constructing sequencing libraries and requires very little DNA input (1-50 ng). MP libraries give approximate distance and orientation of reads and can serve as a scaffold for reads of other library preparation methods [37, 38]. A combination of NX or TS with MP libraries may improve *de novo* assembly of genomes, especially in regions with sequence repeats [37].

NX libraries have been reported to be especially suited for genomes with a balanced GC content. However, this method performed very well for the AT-rich (70%) [14] *Borrelia* genome in our study. We did not observe major differences in *de novo* assemblies whether TS or NX libraries were used, many plasmids being well represented in alignments. For B31-NRZ the assembly of plasmid lp28-1 was considerably improved using a combination of TS and MP library reads compared to TS reads alone (Fig. 2), while in PAbe no difference was observed in lp28-1 assembly. Enrichment of plasmids using commercially available plasmid purification kits also resulted in improvement of *de novo* assemblies of some plasmids (e.g. reduction of gapped regions in lp17or cp32-1, Figs. 3 and 4) suggesting that higher depth of coverage may have improved assembly of these regions.

Of the different *de novo* assemblers used in this study, SPAdes produced the longest contigs, at least for the main chromosome and several of the plasmids but not for cp32 plasmids. Plasmid assembly of cp32 plasmids proved very difficult with short sequence reads, regardless of library construction, plasmid enrichment or assembly software used. *Borrelia* cp32 plasmids are of particular interest, as they harbor *erp* loci which encode gene products that confer protection of the bacteria against the mammalian innate immunity [39]. Unfortunately, however, many *Borrelia* strains contain multiple versions of highly similar cp32s, thereby impeding sequence assembly. Obtaining accurate sequences for such genetic elements is of crucial importance and might provide new insights in to ecology and pathogenicity of these spirochetes in the future. To assemble cp32 plasmids properly, long read sequencing was required and revealed subtle differences that were not detected using combinations of library preparations and Illumina sequencing only.

### Plasmids in *B. burgdorferi* appear dynamic

The strains included in our study showed differences in plasmid numbers compared to B31-GB which may have reflected the loss of *B. burgdorferi* s.s. plasmids during cultivation or freeze/thaw cycles of strains [18, 40, 41]. The plasmids reported to be lost most frequently in cultures of B31 include cp9, lp5, lp21, lp25, lp28-1, lp28-4 and lp56 [18, 41]. Of these cp9, lp5, lp21, lp25, and lp28-4 were not found in any of our strains, either with plasmid specific PCR, or in Illumina sequences of whole genomic DNA, enriched plasmid preparations or in either of the long read sequence assemblies. In addition, plasmids cp32-6, −7, −8, lp28-2, lp28-3, lp28-5, lp28-6 and lp28-7 were also absent. For the remaining plasmids, our data show that not all cells in a given population may harbor every plasmid that was present in the population. There were obvious differences in the frequency of plasmids and some plasmids (e.g. lp36 and lp28-1 in PAbe) may have been present in the population in such low frequencies that they were not captured by all methods. For example, whilst Illumina MiSeq sequences suggested the presence of lp28-1 in PAbe, neither plasmid-enriched preparations nor Pacific Bioscience sequencing captured the full lp28-1 plasmid. These differences in sequencing success point to methodological weaknesses or drawbacks. For example, Pacific Bioscience sequencing requires large amounts of DNA and plasmids that are present in a subset of the population may not be represented well in sequence assemblies. One has to consider that the strains investigated here were very closely related and we assumed that their plasmid content was identical or highly similar. The advantages and disadvantages of sequencing methods described here combined with economics and sample handling (Additional file 1: Table S9) need to be considered when sequencing unknown strains or species for which no reference sequence is available.

Apart from quantitative information, we aimed to explore methods to qualitatively investigate the plasmid structure of closely related *Borrelia* strains to obtain information on requirements for future reference genomes. It had been shown in *Borrelia* that plasmids may be inserted into the main linear chromosome or other plasmids, and merging of plasmids has also been observed [13, 42]. Linear plasmids have been shown to contain a large number of pseudogenes that may have emerged through gene duplications, accumulation of deleterious mutations and decay [42]. As the insertion of a gene into a different genomic environment may change its expression pattern, and such changes may influence host- or vector-interaction of bacterial pathogens, it is important to investigate the structural changes that may occur in the plasmids of closely related bacterial strains.

The close relationship of the strains investigated here is already shown by identical MLST types [21] and is further demonstrated by the very low number of SNPs that are found in the main chromosome [20] and linear extrachromosomal elements. Of the circular plasmids, cp26 and three cp32s, i.e. cp32-3, cp32-4 and cp32-9, showed also very few SNPs or rearrangements among the strains studied here (although cp32-4 and cp32-9 presented as fusion plasmid in PAbe), further emphasizing the close relationship of the strains. The remaining cp32s showed similarity amongst the three NRZ strains, but revealed differences to B31-GB. Variant segments in the cp32-1 or cp32-5 plasmids had higher BLAST similarity to regions in plasmids cp32-6 of *B. burgdorferi* s.s. strain156a or cp32-5 of *B. burgdorferi* s.s. strain 64b. Interestingly, the variable region in contig4 (cp32-1) of B31-NRZ, in contigs15 (cp32-1) and 13 (cp32-5) of PAli and contig2 of PAbe corresponded to regions 1 and 2 described by Casjens and co-workers in 2012. Although homologous recombination may contribute to re-shuffling of sequences in cp32 plasmids within strains [13] and although cp32 plasmids have been shown to contain prophage sequences [43, 44] and may therefore be particularly prone to horizontal gene transfer, the mechanisms behind the rearrangements observed here are difficult to understand because the inserted variable sequences had high similarity to *B. burgdorferi* s.s. strains 156a and 64b, but not B31. It can only be speculated that: (i) the original strain B31 may not have been a clonal isolate and actually consisted of several different *Borrelia* clones; each clone maintaining different genetic elements during sub-passage of cultures; (ii) the plasmid content of a single *Borrelia* strain differs between different individual cells; (iii) some plasmids may have been present in our strain population in such a low frequency that no data were obtained. Support for the latter hypothesis comes from our Pacific Bioscience data which showed that some plasmids occur in the population with low frequency, i.e. the data suggest that only every tenth cell may contain these plasmids as the average coverage of plasmids tended to be variable ranging from >300fold for the chromosome and some plasmids to 30fold for other plasmids (data not shown).

Contig6 in B31-NRZ and PAbe corresponded most likely to the elusive cp32-2 plasmid in B31-GB [13]. Casjens et al. [13] suggested the use of PFam32 sequences for plasmid classification as they are homologous to plasmid partitioning proteins (ParA) in other systems and were shown to allow categorization of plasmids in *Borrelia*. We used this system to classify plasmids in the closely related strains investigated here. Similarity searches with the PFam32 gene of cp32-2 that had been initially described by [23] revealed that it was identical to sequences in both contig6 and in reads obtained by nanopore sequencing. Cp32-2 also possessed a gene region containing an *erpCD* locus [23, 45] and sequences in the contigs6 of B31-NRZ and PAbe had 99% similarity with it.

Despite the fact that Pacific Biosciences SMRT assemblies showed generally >100fold and nanopore sequences a 40fold coverage, a drawback of these assemblies was the occurrence of indels that may provoke frameshifts in coding sequences. This tendency was more pronounced in nanopore than in SMRT sequences. This problem has been noticed for Pacific Bioscience sequence assemblies before and different strategies have been proposed to address the issue including hybrid approaches [46, 47] or long read correction tools [48]. It demonstrates that although long read sequencing has hugely improved the assembly of bacterial genomes and in particular plasmids, there are still hurdles to overcome. In view of the fact that in *B. burgdorferi* s.s. linear plasmids may contain a large number of non-functional pseudogenes [42], in the case of *Borrelia* the use of a hybrid strategy employing short and long reads may be prudent to generate accurate genome sequences.

The demonstrated merging of cp32 plasmids in the *B. burgdorferi* s. s. strains investigated here shows that some elements in the genome are dynamic even between strains that are almost identical on the main chromosome [20]. It suggests that even for such closely related strains, the use of reference genomes for read mapping may give only a distorted view of reality. *B. burgdorferi* s. s. belongs to a bacterial species complex (*B. burgdorferi* s. l.) which currently comprises >20 named and proposed species. Individual genospecies within the complex differ in their ecological niches in terms of reservoir host and vector associations [49–52] and in their ability to cause human disease [53, 54]. The dynamic nature of the *B. burgdorferi* s.s. genome (that has been demonstrated previously and here) may be important in terms of understanding the ecology and pathogenicity of *B. burgdorferi* s.l. in general and for individual strains in particular. PAli and PAbe were isolated from patients with different clinical manifestations and further investigations including a larger number of strains may reveal whether or not a common pattern in plasmid content or structure can be found in strains isolated from patients with different clinical manifestations.

## Conclusion

The data accumulated in the present study are in agreement with previous studies that showed that the plasmid content and structure may vary between even closely related *Borrelia* strains. Importantly, it was noted that different next generation sequencing methods provide different details on the genome structure of the bacterial pathogens, even those belonging to the same MLST sequence type. A combination of different sequencing methods will therefore be the method of choice for investigating genetic elements that confer host- and vector-adaptations as well as differing levels of pathogenicity to tick-borne pathogens such as *Borrelia*.

The genomic complexity shown in this and previous studies [13, 14] may reflect the ecological [52, 55] and/or humanpathogenic complexity of *Borrelia* [56]. It raises the question of whether this is also the case in other human pathogenic or vector-borne bacteria, and may perhaps indicate that *Borrelia* is an interesting paradigm for comparative genomics studies.

An important conclusion from our work is that some of *Borrelia’s* plasmids, as opposed to its chromosome, appear dynamic. While it remains important to investigate the great variety of human pathogenic and non-pathogenic *B. burgdorferi* s.l. genospecies, it is already evident that the many plasmids of this spirochete are subject to a much greater degree of within-strain genetic variation than is the chromosomal component of its genome, even in strains that appear to be closely related in terms of chromosomal genes. Since proteins conferring both virulence against mammalian hosts and competence to be transmitted by tick vectors are known to be encoded on plasmids [57, 58], methods for typing *Borrelia* strains on the basis of their plasmids will be increasingly important in determining the risk of, and developing strategies to manage, Lyme borreliosis.

## Additional files

Additional file 1: **Tables S1–S8** and **Figures S1–S8.** (PDF 1274 kb)

Additional file 2: Multiple Plasmid Alignments_B31_PAbe_PAli. (PDF 2284 kb)

## References

[1] Holmes A, Allison L, Ward M, Dallman TJ, Clark R, Fawkes A, Murphy L, Hanson M (2015). Utility of whole-genome Sequencing of *Escherichia coli* O157 for outbreak detection and epidemiological surveillance. J Clin Microbiol.

[2] Mather AE, Reid SW, Maskell DJ, Parkhill J, Fookes MC, Harris SR, Brown DJ, Coia JE, Mulvey MR, Gilmour MW (2013). Distinguishable epidemics of multidrug-resistant Salmonella Typhimurium DT104 in different hosts. Science.

[3] Suez J, Porwollik S, Dagan A, Marzel A, Schorr YI, Desai PT, Agmon V, McClelland M, Rahav G, Gal-Mor O (2013). Virulence gene profiling and pathogenicity characterization of non-typhoidal Salmonella accounted for invasive disease in humans. PLoS One.

[4] Guttman DS, Stavrinides J. Population genomics of bacteria. In: Bacterial population genetics in infectious disease. Edited by Robinson DA, Falush D, Feil EJ. Hoboken: John Wiley & Sons, Inc.; 2010.

[5] Mikalsen T, Pedersen T, Willems R, Coque TM, Werner G, Sadowy E, van Schaik W, Jensen LB, Sundsfjord A, Hegstad K (2015). Investigating the mobilome in clinically important lineages of *Enterococcus faecium* and *Enterococcus faecalis*. BMC Genomics.

[6] Drancourt M (2012). Plague in the genomic area. Clin Microbiol Infect.

[7] Junemann S, Sedlazeck FJ, Prior K, Albersmeier A, John U, Kalinowski J, Mellmann A, Goesmann A, von Haeseler A, Stoye J (2013). Updating benchtop sequencing performance comparison. Nat Biotechnol.

[8] Loman NJ, Misra RV, Dallman TJ, Constantinidou C, Gharbia SE, Wain J, Pallen MJ (2012). Performance comparison of benchtop high-throughput sequencing platforms. Nat Biotechnol.

[9] Quail MA, Smith M, Coupland P, Otto TD, Harris SR, Connor TR, Bertoni A, Swerdlow HP, Gu Y (2012). A tale of three next generation sequencing platforms: comparison of Ion Torrent, Pacific Biosciences and Illumina MiSeq sequencers. BMC Genomics.

[10] de Been M, Lanza VF, de Toro M, Scharringa J, Dohmen W, Du Y, Hu J, Lei Y, Li N, Tooming-Klunderud A (2014). Dissemination of cephalosporin resistance genes between *Escherichia coli* strains from farm animals and humans by specific plasmid lineages. PLoS Genet.

[11] Gatzmann F, Metzler D, Krebs S, Blum H, Sing A, Takano A, Kawabata H, Fingerle V, Margos G, Becker NS (2015). NGS population genetics analyses reveal divergent evolution of a Lyme Borreliosis agent in Europe and Asia. Ticks Tick Borne Dis.

[12] Chin CS, Alexander DH, Marks P, Klammer AA, Drake J, Heiner C, Clum A, Copeland A, Huddleston J, Eichler EE (2013). Nonhybrid, finished microbial genome assemblies from long-read SMRT sequencing data. Nat Methods.

[13] Casjens SR, Mongodin EF, Qiu WG, Luft BJ, Schutzer SE, Gilcrease EB, Huang WM, Vujadinovic M, Aron JK, Vargas LC (2012). Genome stability of Lyme disease spirochetes: comparative genomics of Borrelia burgdorferi plasmids. PLoS One.

[14] Fraser CM, Casjens S, Huang WM, Sutton GG, Clayton R, Lathigra R, White O, Ketchum KA, Dodson R, Hickey EK (1997). Genomic sequence of a Lyme disease spirochaete, *Borrelia burgdorferi*. Nature.

[15] Kenedy MR, Lenhart TR, Akins DR (2012). The role of *Borrelia burgdorferi* outer surface proteins. FEMS Immunol Med Microbiol.

[16] Di L, Pagan PE, Packer D, Martin CL, Akther S, Ramrattan G, Mongodin EF, Fraser CM, Schutzer SE, Luft BJ (2014). BorreliaBase: a phylogeny-centered browser of Borrelia genomes. BMC Bioinformatics.

[17] Becker NS, Margos G, Blum H, Krebs S, Graf A, Lane RS, Castillo-Ramirez S, Sing A, Fingerle V (2016). Recurrent evolution of host and vector association in bacteria of the *Borrelia burgdorferi* sensu lato species complex. BMC Genomics.

[18] Grimm D, Elias AF, Tilly K, Rosa PA (2003). Plasmid stability during in vitro propagation of Borrelia burgdorferi assessed at a clonal level. Infect Immun.

[19] Purser JE, Norris SJ (2000). Correlation between plasmid content and infectivity in Borrelia burgdorferi. Proc Natl Acad Sci U S A.

[20] Castillo-Ramirez S, Fingerle V, Jungnick S, Straubinger RK, Krebs S, Blum H, Meinel DM, Hofmann H, Guertler P, Sing A (2016). Trans-Atlantic exchanges have shaped the population structure of the Lyme disease agent Borrelia burgdorferi sensu stricto. Sci Rep.

[21] Jungnick S, Margos G, Rieger M, Dzaferovic E, Bent SJ, Overzier E, Silaghi C, Walder G, Wex F, Koloczek J (2015). *Borrelia burgdorferi* sensu stricto and *Borrelia afzelii*: Population structure and differential pathogenicity. Int J Med Microbiol.

[22] Burgdorfer W, Barbour AG, Hayes SF, Benach JL, Grunwaldt E, Davis JP (1982). Lyme disease-a tick-borne spirochetosis?. Science.

[23] Casjens S, van Vugt R, Tilly K, Rosa PA, Stevenson B. Homology throughout the multiple 32-kilobase circular plasmids present in Lyme disease spirochetes. J Bacteriol. 1997b;179(1):217–27. 10.1128/jb.179.1.217-227.1997 PMC178682 8982001

[24] Preac-Mursic V, Wilske B, Schierz G (1986). European *Borrelia burgdorferi* isolated from humans and ticks culture conditions and antibiotic susceptibility. Zentralbl Bakteriol Mikrobiol Hyg A.

[25] Goecks J, Nekrutenko A, Taylor J (2010). Galaxy: a comprehensive approach for supporting accessible, reproducible, and transparent computational research in the life sciences. Genome Biol.

[26] Blankenberg D, Von Kuster G, Coraor N, Ananda G, Lazarus R, Mangan M, Nekrutenko A, Taylor J (2010). Galaxy: a web-based genome analysis tool for experimentalists. Curr Protoc Mol Biol.

[27] Simpson JT, Durbin R (2012). Efficient *de novo* assembly of large genomes using compressed data structures. Genome Res.

[28] Zerbino DR, Birney E (2008). Velvet: algorithms for *de novo* short read assembly using de Bruijn graphs. Genome Res.

[29] Nurk S, Bankevich A, Antipov D, Gurevich AA, Korobeynikov A, Lapidus A, Prjibelski AD, Pyshkin A, Sirotkin A, Sirotkin Y (2013). Assembling single-cell genomes and mini-metagenomes from chimeric MDA products. J Comput Biol.

[30] Field D, Feil EJ, Wilson GA (2005). Databases and software for the comparison of prokaryotic genomes. Microbiology.

[31] Tamura K, Peterson D, Peterson N, Stecher G, Nei M, Kumar S (2011). MEGA5: molecular evolutionary genetics analysis using maximum likelihood, evolutionary distance, and maximum parsimony methods. Mol Biol Evol.

[32] Koren S, Walenz BP, Berlin K, Miller JR, Bergman NH, Phillippy AM. Canu: scalable and accurate long-read assembly via adaptive k-mer weighting and repeat separation. Genome Res. 2017;27(5):722-36. 10.1101/gr.215087.116 PMC5411767 28298431

[33] Giardine B, Riemer C, Hardison RC, Burhans R, Elnitski L, Shah P, Zhang Y, Blankenberg D, Albert I, Taylor J (2005). Galaxy: a platform for interactive large-scale genome analysis. Genome Res.

[34] Darling AC, Mau B, Blattner FR, Perna NT (2004). Mauve: multiple alignment of conserved genomic sequence with rearrangements. Genome Res.

[35] Alikhan NF, Petty NK, Ben Zakour NL, Beatson SA (2011). BLAST Ring Image Generator (BRIG): simple prokaryote genome comparisons. BMC Genomics.

[36] Altschul SF, Gish W, Miller W, Myers EW, Lipman DJ (1990). Basic local alignment search tool. J Mol Biol.

[37] Wetzel J, Kingsford C, Pop M (2011). Assessing the benefits of using mate-pairs to resolve repeats in *de novo* short-read prokaryotic assemblies. BMC Bioinformatics.

[38] Park N, Shirley L, Gu Y, Keane TM, Swerdlow H, Quail MA (2013). An improved approach to mate-paired library preparation for Illumina sequencing. Methods Next Gener Seq.

[39] Brissette CA, Cooley AE, Burns LH, Riley SP, Verma A, Woodman ME, Bykowski T, Stevenson B (2008). Lyme borreliosis spirochete Erp proteins, their known host ligands, and potential roles in mammalian infection. Int J Med Microbiol.

[40] Iyer R, Kalu O, Purser J, Norris S, Stevenson B, Schwartz I (2003). Linear and circular plasmid content in Borrelia burgdorferi clinical isolates. Infect Immun.

[41] Norris SJ, Howell JK, Odeh EA, Lin T, Gao L, Edmondson DG (2011). High-throughput plasmid content analysis of *Borrelia burgdorferi* B31 by using Luminex multiplex technology. Appl Environ Microbiol.

[42] Casjens S, Palmer N, van Vugt R, Huang WM, Stevenson B, Rosa P, Lathigra R, Sutton G, Peterson J, Dodson RJ (2000). A bacterial genome in flux: the twelve linear and nine circular extrachromosomal DNAs in an infectious isolate of the Lyme disease spirochete *Borrelia burgdorferi*. Mol Microbiol.

[43] Chenail AM, Jutras BL, Adams CA, Burns LH, Bowman A, Verma A, Stevenson B (2012). Borrelia burgdorferi cp32 BpaB modulates expression of the prophage NucP nuclease and SsbP single-stranded DNA-binding protein. J Bacteriol.

[44] Eggers CH, Samuels DS (1999). Molecular evidence for a new bacteriophage of Borrelia burgdorferi. J Bacteriol.

[45] Stevenson B, Bono JL, Schwan TG, Rosa P (1998). *Borrelia burgdorferi* erp proteins are immunogenic in mammals infected by tick bite, and their synthesis is inducible in cultured bacteria. Infect Immun.

[46] Koren S, Harhay GP, Smith TPL, Bono J, Harhay DM, Mcvey SD, Radune D, Bergman NH, Phillippy AM (2013). Reducing assembly complexity of microbial genomes with single-molecule sequencing. Genome Biol.

[47] Lin HH, Liao YC (2015). Evaluation and validation of assembling corrected pacbio long reads for microbial genome completion via hybrid approaches. PLoS One.

[48] Du N, Sun Y (2016). Improve homology search sensitivity of PacBio data by correcting frameshifts. Bioinformatics.

[49] Brisson D, Dykhuizen DE, Ostfeld RS (2008). Conspicuous impacts of inconspicuous hosts on the Lyme disease epidemic. Proc Biol Sci.

[50] Brown RN, Lane RS (1992). Lyme disease in California: a novel enzootic transmission cycle of *Borrelia burgdorferi*. Science.

[51] Gern L, Humair P, Gray JS, Kahl O, Lane RS, Stanek G (2002). Ecology of *Borrelia burgdorferi* sensu lato in Europe. Lyme Borreliosis: biology of the infectious agents and epidemiology of disease.

[52] Kurtenbach K, Hanincova K, Tsao JI, Margos G, Fish D, Ogden NH (2006). Fundamental processes in the evolutionary ecology of Lyme borreliosis. Nat Rev Microbiol.

[53] Stanek G, Fingerle V, Hunfeld KP, Jaulhac B, Kaiser R, Krause A, Kristoferitsch W, O’Connell S, Ornstein K, Strle F (2010). Lyme borreliosis: clinical case definitions for diagnosis and management in Europe. Clin Microbiol Infect.

[54] Stanek G, Strle F (2009). Lyme borreliosis: a European perspective on diagnosis and clinical management. Curr Opin Infect Dis.

[55] Hanincova K, Kurtenbach K, Diuk-Wasser M, Brei B, Fish D (2006). Epidemic spread of Lyme borreliosis, northeastern United States. Emerg Infect Dis.

[56] Hanincova K, Mukherjee P, Ogden NH, Margos G, Wormser GP, Reed KD, Meece JK, Vandermause MF, Schwartz I (2013). Multilocus sequence typing of Borrelia burgdorferi suggests existence of lineages with differential pathogenic properties in humans. PLoS One.

[57] Norris SJ (2012). How do lyme borrelia organisms cause disease? The quest for virulence determinants(). Open Neurol J.

[58] Pal U, Fikrig E. Tick Interactions. In: Borrelia - Molecular Biology, Host Interaction and Pathogenesis. Edited by Samuels DS, Radolf J: Caister Academic Press, Poole, UK; 2010: 279–98.

