# Supplementary material for: Lost in plasmids: next generation sequencing and the complex genome of the tick-borne pathogen Borrelia burgdorferi
Source: BMC Genomics. 2017 May 30;18:422. doi: 10.1186/s12864-017-3804-5 (PMC5450258; doi:10.1186/s12864-017-3804-5)
Supplement: Supplementary file 1 — Tables S1–S8 and Figures S1–S8. (PDF 1274 kb) [file 12864_2017_3804_MOESM1_ESM.pdf]

Margos et al. **Additional file 1** - **Tables S1-S9 and Figures S1-S8**

Table S1. Statistics of *de novo* assemblies and read mapping using different library methods conducted in the CLC Genomic Workbench

De novo	B31-NRZ TS	B31-NRZ TS_MP	PAli NX	PAli NX_MP	PAbe NX	PAbe NX_MP	PAbe TS	PAbe TS_MP
N50	433,943	434,752	434,744	434,814	434,703	434,752	405,884	434,591
Minimum	654	872	702	855	727	872	679	760
Maximum	472,020	469,072	468,323	469,038	469,051	469,072	434,372	471,864
Average	22,586	19,577	22,807	21,394	18,644	19,577	19,192	19,383
Count	54	62	53	57	65	62	63	62

**Read mapping**	B31-NRZ TS	B31-NRZ TS_MP	PAli NX	PAli NX_MP	PAbe NX	PAbe NX_MP	PAbe TS	PAbe TS_MP
Total read number	2,264,058	2,989,802	3,092,326	12,077,178	2,040,798	2,477,848	2,178,088	2,926,680
No mapped reads	2,215,365	2,924,437	2,944,475	11,422,798	1,923,048	2,348,522	2,144,311	2,871,490
Average coverage main chromosome	379	459	452	1,518	225	277	273	
Average coverage confirmed plasmids	> 250 (except lp28-1: 1.5x)	>300 (lp28-1: 120x)	>250	>1,000	>350	>350	>300	

**Enriched plasmids**	B31-NRZ TS	B31-NRZ TS_MP	PAli NX	PAli NX_MP	PAbe NX	PAbe NX_MP	PAbe TS	PAbe TS_MP
Total number reads	1,125,153		713,198		678,522			
Mapped reads	1,119,268		685,144		656,910			
Average coverage main chromosome	3		17		7			
Average coverage plasmids	>200 (except lp28-1: 56x)		Appr. 100		>100			

Table S2. SPAdes *de novo* assemblies of B31-NRZ TS_MP. Contigs shorter than 500 bp were not considered

Length in bp	Name	Plasmid	Strain	Ident in %
910137	SPAdes_1	complete genome	B31, Bbss	99
53797	SPAdes_2	lp54	B31, Bbss	99
38882	SPAdes_3	lp38	B31, Bbss	99
36997	SPAdes_4	lp36	B31, Bbss	99
26625	SPAdes_5	cp26	B31, Bbss	99
14541	SPAdes_6	lp17	B31, Bbss	100
13664	SPAdes_7	lp56	B31, Bbss	99
10543	SPAdes_8	cp32-4	B31, Bbss	99
6927	SPAdes_9	lp56	B31, Bbss	99
6388	SPAdes_10	cp32-1	B31, Bbss	99
5513	SPAdes_11		Echinostoma caproni genome assembly E_caproni_Egypt, scaffold ECPE_contig0001929	100
4893	SPAdes_12	cp32-9	B31, Bbss	99
4818	SPAdes_13	cp32-1	B31, Bbss	99
4606	SPAdes_14	lp56	B31, Bbss	100
3901	SPAdes_15	cp32-3	B31, Bbss	99
3804	SPAdes_16	lp56	Bol26, Bbss	99 (91% c)#
3675	SPAdes_17	lp56	B31, Bbss	99
3725	SPAdes_18	cp32-1	B31, Bbss	100
3451	SPAdes_19	cp32-3 ????	B31, Bbss	100
3187	SPAdes_20	cp32-9	B31, Bbss	99
3147	SPAdes_21	cp32-9	B31, Bbss	100
3088	SPAdes_22	cp32-4	B31, Bbss	100
2964	SPAdes_23	cp32-1	B31, Bbss	99
2960	SPAdes_24	lp56	B31, Bbss	99
2895	SPAdes_25	cp32-3	B31, Bbss	100
2684	SPAdes_26	lp17	B31, Bbss	100 (97%c)
2659	SPAdes_27	cp32-4	B31, Bbss	99
2611	SPAdes_28	cp32-5	64b, Bbss	99
2582	SPAdes_29	cp32-5	64b, Bbss	99
2582	SPAdes_30	cp32-7	64b, Bbss	99
2260	SPAdes_31	cp32-2	Bbss	99
2043	SPAdes_32	cp32-9	B31, Bbss	99
2025	SPAdes_33	lp56	B31, Bbss	100
1770	SPAdes_34	cp32-4	B31, Bbss	100
1667	SPAdes_35	cp32-4	B31, Bbss	98

1658	SPAdes_36	cp32-8	B31, Bbss	99
1648	SPAdes_37	cp32-9	B31, Bbss	99
1577	SPAdes_38	cp32-3-8	64b, Bbss	99
1468	SPAdes_39	cp32-9	B31, Bbss	99
1320	SPAdes_40	cp32-3	B31, Bbss	100
1182	SPAdes_41	cp32-1	B31, Bbss	100
1003	SPAdes_42	cp32-1	B31, Bbss	100
984	SPAdes_43	lp56	B31, Bbss	99
968	SPAdes_44	cp32-5	64b, Bbss	99
951	SPAdes_45	cp32-1	B31, Bbss	100
949	SPAdes_46	cp32-3	B31, Bbss	99
860	SPAdes_47	cp32-6	156a, Bbss	98
846	SPAdes_48	cp32-3	B31, Bbss	100
811	SPAdes_49	cp32-1	B31, Bbss	100
757	SPAdes_50	cp32-1	B31, Bbss	100
716	SPAdes_51	cp32-1	B31, Bbss	100
680	SPAdes_52	cp32-4	B31, Bbss	100
679	SPAdes_53		Corynebacterium diphtheriae genome assembly NCTC11397, chromosome : 1	97
621	SPAdes_54	cp32-4	B31, Bbss	100
621	SPAdes_55	cp32-4	B31, Bbss	100
600	SPAdes_56	cp32-4	B31, Bbss	99
594	SPAdes_57	cp32-6	JD1, Bbss	100
587	SPAdes_58	cp32-10	VS116, *B. valaisiana*	88
581	SPAdes_59	cp32-5	64b, Bbss	99
580	SPAdes_60	cp32-1	B31, Bbss	100
571	SPAdes_61	cp32-1	B31, Bbss	100
571	SPAdes_62	cp32-5	64b, Bbss	99
567	SPAdes_63	cp32-5	64b, Bbss	100
566	SPAdes_64	cp32-1	B31, Bbss	100
543	SPAdes_65	lp17	VS116, *B. valaisiana*	95
541	SPAdes_66	cp32-9	B31, Bbss	99
535	SPAdes_67	lp56	B31, Bbss	99
533	SPAdes_68	cp32-4	B31, Bbss	100
510	SPAdes_69	lp25	JD1, Bbss	98

c=coverage

Table S3. Number of SNPs in *B. burgdorferi* s.s. Illumina and SMRT sequences compared to B31-GB.

B31-GB	B31 NRZ read mapping[a)]				B31 NRZ de novo[b)]		PAli read mapping			PAli de novo	PAbe read mapping					PAbe de novo
	TS	TS_MP	NX_Pl	NX_Pl_MP	SMRT*	SPADes	NX	NX_MP	NX_Pl	SMRT*	NX	NX_MP	TS	TS_MP	NX_Pl	SMRT*
main chromosom	72	73	nd	nd	45	Nd	90	90	nd	58	92	92	90	91	nd	2
cp9	nd	nd	nd	nd	nd	Nd	nd	nd	nd	nd	nd	nd	nd	nd	nd	nd
cp26	3	3	1	1	6	3	1	1	0	3	1	1	1	1	2	4
cp32-1	20	21	3	6	1911 (un4)	Nd	17	18	20	20 (un14)	12	12	18	16	4	465
cp32-1+5/JD1	nd	nd	nd	nd	6631 (un4)	Nd	nd	nd	nd	nd	nd	nd	nd	nd	nd	nd
cp32-3	0	0	0	0	2	Nd	1	1	1	1 (195 bp no match)	0	0	0	0	0	6
cp32-4	0	0	0	0	3	Nd	0	0	0	0	1	0	0	0	0	144
cp32-6	nd	nd	nd	nd	nd	Nd	nd	nd	nd	nd	nd	nd	nd	nd	nd	nd
cp32-7	nd	nd	nd	nd	3322 (un6, cp32-2)	Nd	nd	nd	nd	nd	nd	nd	nd	nd	nd	3320 (un6, cp32-2)
cp32-8	nd	nd	nd	nd	nd	Nd	nd	nd	nd	nd	nd	nd	nd	nd	nd	nd
cp32-9	2	2	2	2	2	Nd	6	5	5	10410/Bol26	3	2	2	2	2	35
cp32-12/ZS7	nd	nd	nd	nd	2353 (un6, cp32-2)	Nd	nd	nd	nd	nd	nd	nd	nd	nd	nd	2348 (un6, cp32-2)
cp32-1	nd	nd	nd	nd	nd	Nd	nd	nd	nd	1073 (un13)	nd	nd	nd	nd	nd	nd
cp32-1	nd	nd	nd	nd	nd	Nd	nd	nd	nd	337(un15)	nd	nd	nd	nd	nd	nd
lp5	nd	nd	nd	nd	nd	Nd	nd	nd	nd	nd	nd	nd	nd	nd	nd	nd
lp17	0	0	0	0	0	0	0	0	0	0	3	3	3	3	1	1

lp21	nd	nd	nd	nd	nd	Nd	nd	nd	nd	nd	nd	nd	nd	nd	nd	nd
lp25	nd	nd	nd	nd	nd	Nd	nd	nd	nd	nd	nd	nd	nd	nd	nd	nd
lp28-1	nd	10	30	32	179	Nd	nd	nd	nd	nd	41	41	20	20	3	see text
lp28-2	nd	nd	nd	nd	nd	Nd	nd	nd	nd	nd	nd	nd	nd	nd	nd	nd
lp28-3	nd	nd	nd	nd	nd	Nd	nd	nd	nd	nd	nd	nd	nd	nd	nd	nd
lp28-4	nd	nd	nd	nd	nd	Nd	nd	nd	nd	nd	nd	nd	nd	nd	nd	nd
lp36	0	0	0	0	0	0	0	0	0	nd	0	0	0	0	0	0
lp38	1	2	2	2	1	5	2	2	2	1	nd	nd	nd	nd	nd	nd
lp54	10	10	10	10	7	7	11	11	11	8	10	10	10	10	10	7
lp56	1	1	12	1	2	37§	1	1	1	2	1	1	1	1	1	2 (un43)<br>0 (un42)

nd = no data

*indels are not considered

a) data from CLC Genomic Workbench

b) data determined in MEGA5

§plasmid incomplete assembled, 8 nodes

Table S4. Correlation of genome elements of B31-GB to SMRT conitigs

genome element	B31-NRZ	PAli	PAbe
Chromosom	contig0	contig0	contig0
lp5	absent	Absent	absent
lp17	contig5	contig8	contig7
lp21	absent	Absent	absent
lp25	absent	Absent	absent
lp28-1	contig12/short	Absent	contig13/contig32 - 4800 bp
lp28-2	absent	Absent	absent
lp28-3	absent	Absent	absent
lp28-4	absent	Absent	absent
lp28-7	absent	Absent	absent
lp28-9	absent	Absent	absent
lp36	contig11	contig5	contigs11/12/20/22
lp38	contig2	contig10	absent
lp54	contig1	contig2	contig1
lp56	contig3	contig3	contig42/43
cp9	absent	absent	absent
cp26	contig9	contig6	contig8
cp32-1_i5_i6*	contig4**	contig14 (truncated)	contig2**
cp32-3	contig8	contig4	contig5
cp32-4	contig10	contig9	contig3#
cp32-6	absent	absent	absent
cp32-7/cp32-2	contig6	absent	contig6
cp32-8	absent	absent	absent
cp32-9	contig7	contig7	contig3#
cp32-1_i5*		contig13 (cp32-5)	
cp32-1_i6*		contig15 (cp32-1)	

*insertion of sequence stretches from cp32-5 Bbss strain 64b and cp32-6 Bbss strain 156a

**fusion of cp32-5 and cp32-1

#fusion cp32-9 and cp32-4

Table S5. Primer and PCR conditions for plasmid determination in *B. burgdorferi* s. s.

Plasmid	Primer	DNA-Sequence 5‘ to 3‘	Tm in °C	Annealingtemp. in °C
lp17	for	TGATTTAGTAGCCTGTTGTTGTGA	60	55
	rev	AATGGGAAAAAGGCTGGATAGAT	59	
lp21	for	AAGATCGATTAAAGCTCCAACATA	58	55
	rev	GCGGGGTCAGAAATAAACAA	56	
lp25	for	AAGCTGAAGGCCCGTTTGTTT	59	55
	rev	GGGGTATTTGGCCTGAGCACT	63	
lp28-1	for	GGCGGCTGGTGCTGTTAGTGC	67	55
	rev	AGCCCCCTCAGCCTTCTCTTTCTC	69	
lp28-2	for	GAATTTGAGCGCTTGTTGACT	57	55
	rev	TTTGACTGGCAGGGTAATGT	56	
lp28-3	for	CTGGCATTAGACACATCATT	54	52
	rev	AAAAGGCCAAGTATTATTCTA	52	
lp28-4	for	TGGTAGAAAGCAAACATCAAA	54	52
	rev	GTCAATCGTGCCAGTGCTAATA	60	
lp36	for	TTAGCGGTGAAAGTGGTGAA	56	55
	rev	CATTTGGGTTAGCTTTGTTGAA	57	
lp38	for	TACGGGGAGCGGCAAGACAA	63	55
	rev	CCCCCACTAAAGACAGCACTCCAT	67	
lp56	for	ATGCCTCTGAATGTGATGTT	54	52
	rev	TTAAAGATATTGCAAAAGAGACG	56	
cp9-1	for	TATTGAGCGTATTTTGAGTTTGTT	57	55
	rev	TTAATGATGAGGCCGATGAAG	57	
cp32-3*	for	CAAGATTGATGCGAGTAGTG	56	52
	rev	TCAATATTTTTAAGAGCATCATC	54	

*For cp32 plasmids it was only possible to design plasmid-specific primers for cp32-3 but not for all cp32 present in B31-GB.

Table S6. Published primer for plasmids cp26 and lp54 [12, 13] Bunikis / Michel

Plasmid	Primer	DNA-Sequenz von 5‘ nach 3‘	Tm in °C	Annealingtemp. in °C
cp26	for	ATGAAAAAGAATACATTAAGTGC	54	52
	rev	ATTAATCTTATAATATTGATTTTAATTAAGG	58	
lp54	for 1	GGGAATAGGTCTAATATTAGC	55	55
	for 2	GGGGATAGGTCTAATATTAGC	57	
	rev 1	CATAAATTCTCTTTATTTTAAAGC	53	
	rev 2	CCTTATTTTAAAGCGGC	48	

Table S7. PCR conditions for plasmid-specific primer

Cycles	Temperature in °C	Time in min	Step
1	94	15	Activation of Taq-Polymerase
30	94	0,5	Denaturation
	52 / 55	0,5	Primer-Annealing
	72	1	Elongation
1	72	5	Final elongation
1	10	∞	Hold

A conventional PCR was performed with 30 cycles using conditions as detailed above. PCR products were visualized on an agarose gel stained with GelRed Nucleic Acid Gel stain (Biotium, CA, USA , cat. no. 41003).

Table S8 PCR Primer and PCR conditions to investigate gaps in lp36 and lp38 of PAbe and PAli, respectively

Strain name, plamid	forward primer 5' – 3'	Tm °C	reverse primer 5' – 3'	Tm °C	expected size of PCR product
PAbe lp36_1.1	TTATCACCATTGCTAC TTAC	51,1	AACTCCGGAAGAG CTAG	55,2	204
PAbe lp36_1.2	CGGAAGAGCTAGAA AATTTAG	51,7	GGGTACTTTTATCA CCATTG	52,6	204
PAbe lp36_2	GAATAAGAATTCATC AAAAG	41,6	TTATATTCTAAATTA ATCAT	34,9	1033
PAbe lp36_3	AGTCAGAAGTAGTTT GCATG	53,1	TAAATAAGGAAGCT TTTGGA	49,2	959
PAli lp38_1	GGCGCAGATAAAATT ATAAG	54,0	TCTAACAAGCATAT TCTACC	54,0	635
PAli lp38_2	GGGCAAAAAGATAG ATATTG	54,0	TCACAGCTTCTAAT ATCTTG	54,0	637

PCR conditions (Qiagen HotStarTaq Mastermix, 0.05 pmol final primer concentration)

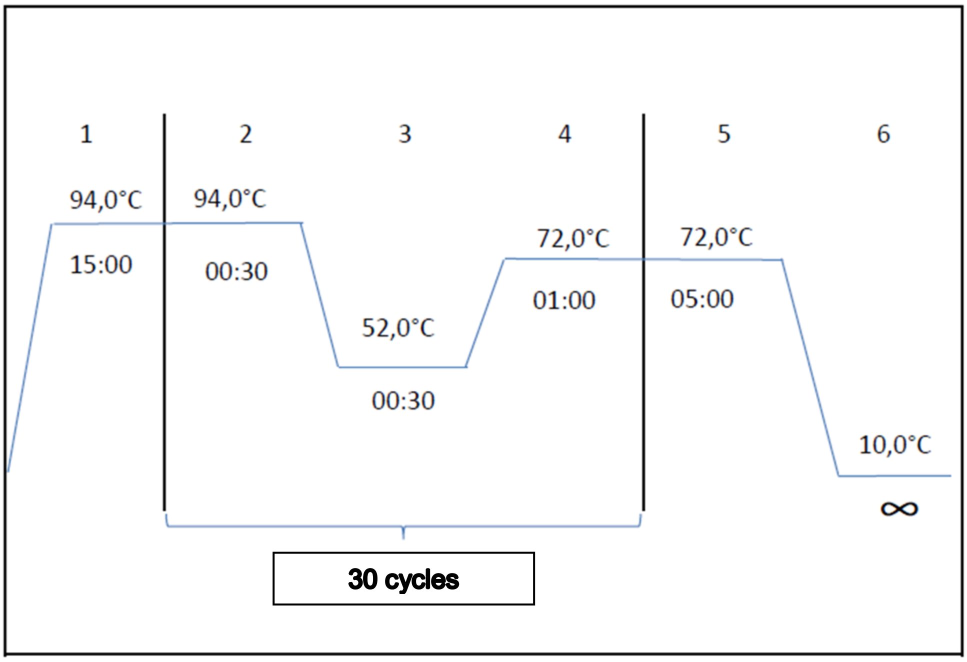

Table S9: Sequencing cost, sample preparation and data assembly

Technology	library	approximate cost per sample (library prep and run, w/o assembly)	time required for library preparation	ease of library preparation	DNA requirements	de novo plasmid assembly
Illumina	Nextera XT	100 Euro	0.5 day	simple	1 ng	difficult
Illumina	TruSeq*	120 Euro	1-2 days	easy	1 µg	difficult
Illumina	Mate-pair*	160 Euro	2 days	laborious	2 µg	difficult
PacBio	BluePippin	1,300 Euro	n/d	n/d	10 µg	easy
Nanopore		280 Euro	1 h	simple	0.5-1 µg	easy

*these two library preparation methods require the use of ultrasound for DNA fragmentation which adds additional hands-on time and costs to sample preparation.

Additional file 1 – Figures S1-S6

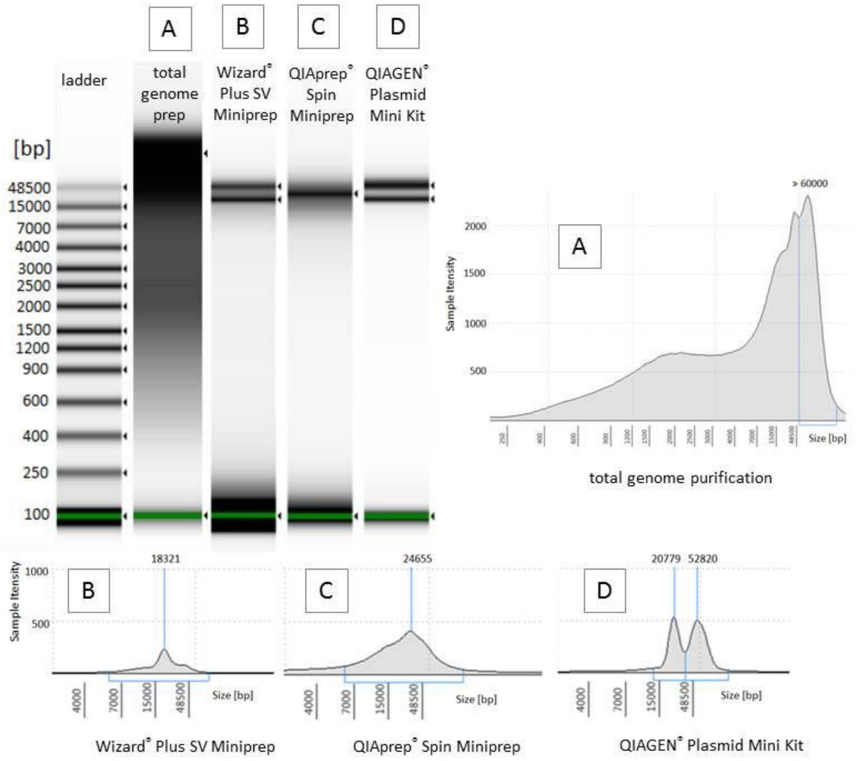

Figure S1. Plasmid enrichment in *B. burgdorferi* s.s.. Commercial kits were used to enrich plasmids of *B. burgdorferi* s.s. strain B31-NRZ. Genomic DNA gel electrophoresis was conducted in the Agilent TapeStation. Gel images and electropherogramms are shown below. Compared to total genomic DNA (A), all kits used, i.e. Wizard® Plus SV Miniprep kit from Promega (B), QIAprep® Spin Miniprep (C) and QIAGEN® Plasmid Mini kit (D) enriched the plasmid DNA of strain B31. However, the QIAGEN® Plasmid Mini kit gave best results in term of DNA quantity and sharpness of plasmid peaks. The QIAGEN® Plasmid Midi kit was therefore used to prepare plasmid-enriched preparations for NGS library construction.

A) B)

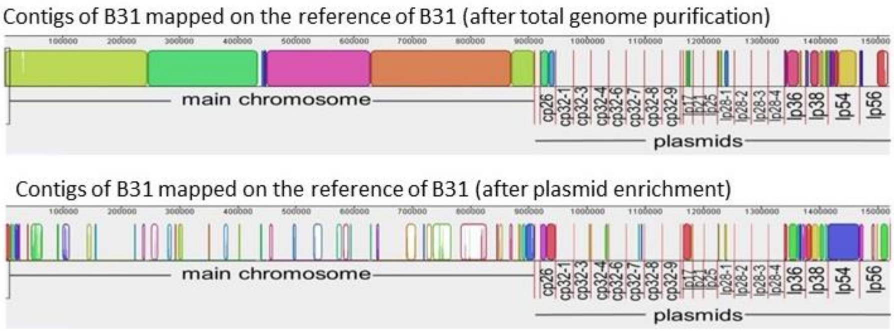

Figure S2. Alignment image of *de novo* assembled Illumina contigs using plasmid enriched DNA preparations for Nextera XT library construction. Panel A shows the alignment when total DNA was used for library construction and sequencing. Panel B when plasmid-enriched preparations were used for library construction and sequencing. It is obvious that much of the main chromosome was separated from the plasmid fraction during the Mini prep procedure.

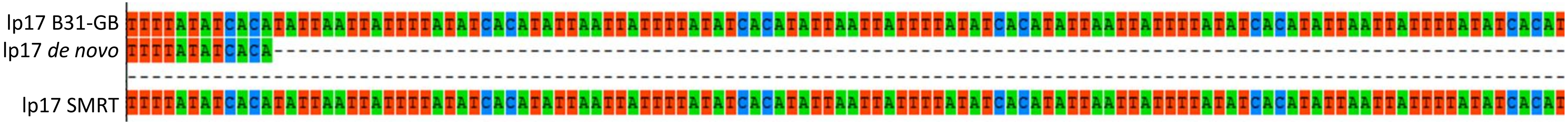

Figure S3: Assembly of repetitive sequences in lp17. B31-GB = reference sequence; *de novo* = Illumina *de novo* assembly; SMRT = Pacific Bioscience SMRT sequences *de novo* assembly. The figure illustrates that with short reads repetitive sequences may not be represented in alignments

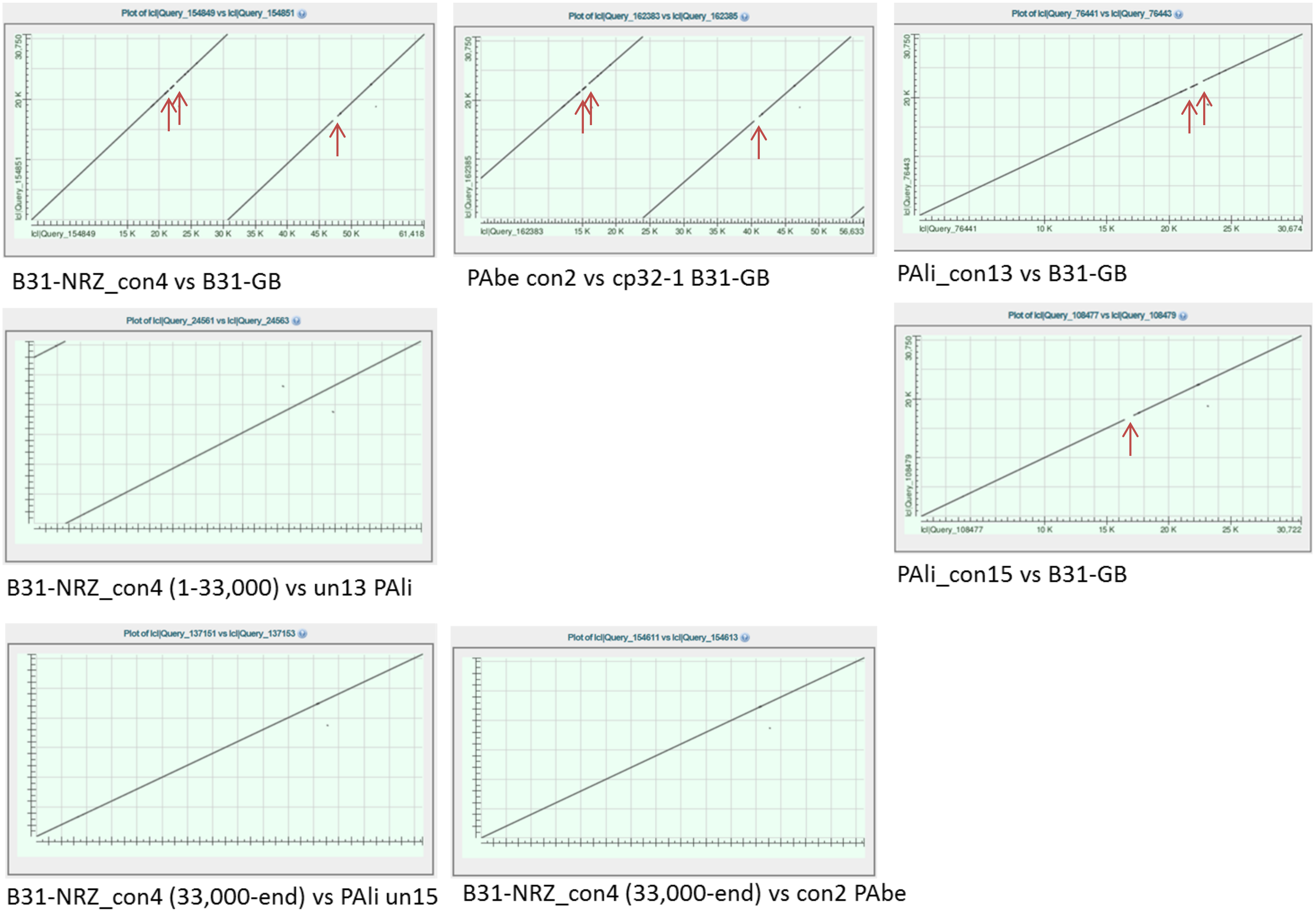

Figure S4: Dotplots of B31-NRZ SMRT contig4, contigs13 and 15 of PAli and contig2 of PAbe vs B31-GB. Gaps in the alignments to B31-GB indicate variant sequences with identity to cp32-5 and cp32-6 of strains 64b and 156a, respectively, while there are not gaps visible when comparing B31-NRZ contigs with contigs of PAli and PAbe.

Similar mismatches were observed when contig13 of PAli and contig2 of PAbe were blasted against cp32-1 of B31-GB.

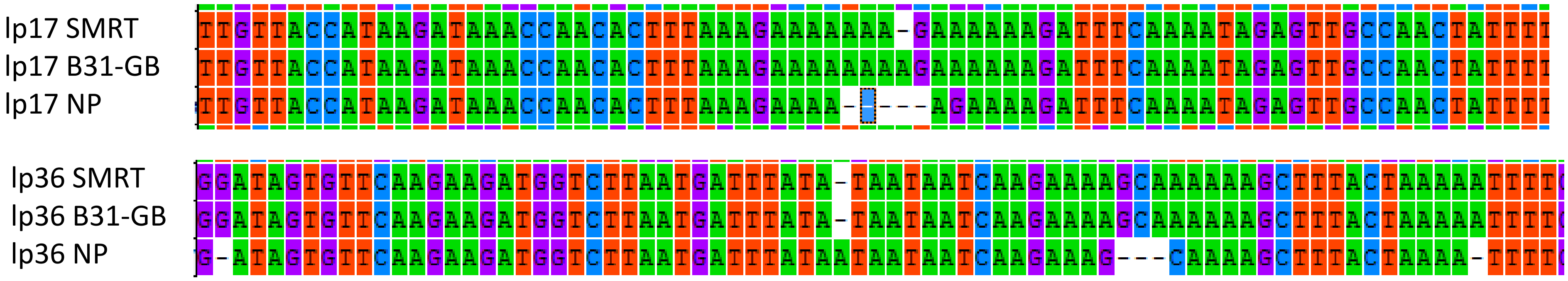

Figure S5. Illustrates mismatches due to alignment errors in nanopore sequences. B31-GB = reference sequence; *de novo* = Illumina *de novo* assembly; SMRT = Pacific Bioscience SMRT sequences *de novo* assembly; NP = nanopore sequences

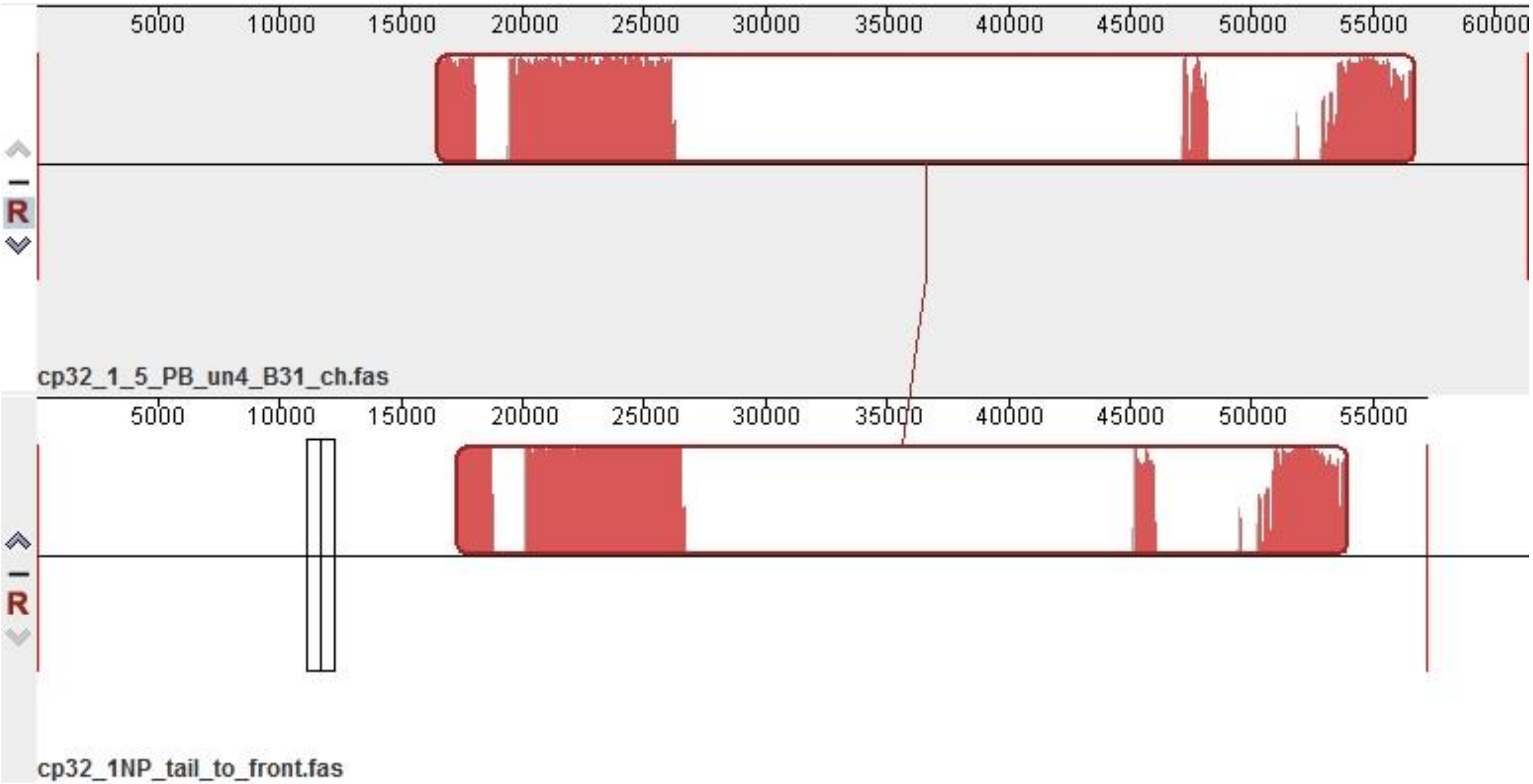

Figure S6: Mauve alignment of B31-NRZ SMRT contig4 (corresponding to cp32-1 of B31-GB) and contig241247 (rearranged tail-to-head; bp 26343–end / bp 1-26342) of nanopore sequences. Both show similarity to plasmid cp32-1 but are >60 kb in size suggestion a fusion of cp32 plasmids. The Mauve alignment demonstrates the similarity in at least in a large part of the two sequence contigs. This data strongly support the notion that in B31-NRZ cp32-1 is of larger size than in B31-GB.

Figure S7: PCR analysis confirmed the fusion of plasmids cp32-5 and cp32-1 in B31-NRZ

cp32-1: contig 4 B31-NRZ (PacBio), PCR product for cp32-1, cp32-1 B31-GB, contig 4 (1-33000 bp), PCR product cp32-1, contig4 (33000 bp to end)

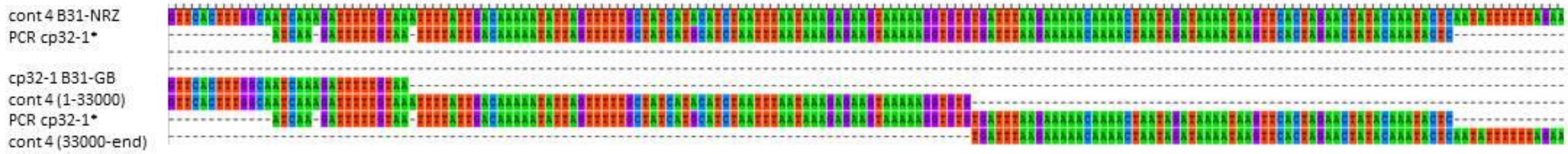

*there are two indels and one mismatch in the Sanger sequence. The PCR product spans the region where the B31-GB cp32-1 ends confirming that in B31-NRZ the corresponding plasmid is a fusion of two plasmids

Primer used for PCR

forwarts_primer_32-1-5	5'-TGTCTTTAGGATATAGAGAGC-3'
reverse_primer_32-1-5	5'-TCACTAGAACTATACAAATACTC-3'

Figure S8: Partial alignment of PCR product of the lp38 gap in PAli to B31. The dots below the PCR sequence indicate identical bases; there are no differences discernible as indicated by the dots in lp38_gi.

```
lp38_PCR               ------------------------------------TTTAAGCTTTAA [22272]
lp38_gi|11496831|B31 TGATCAATATTTTAGTTTTAATAAATTATTCTTCAA............ [22272]

lp38_PCR             TGATTTTATTTTTAAATTAGGTAGGCAAAATTATTATTTAGGAAATGG [22320]
lp38_gi|11496831|B31 ................................................ [22320]

lp38_PCR             ATTAATTGAAAATATTGTTTTAAAAAGGACCACAATAGAACCAGAATG [22368]
lp38_gi|11496831|B31 ................................................ [22368]

lp38_PCR             GTTTTTTGAGTTTTATTATTTTATTTCTAATTATTCTGTCTCTTTGGG [22416]
lp38_gi|11496831|B31 ................................................ [22416]

lp38_PCR             TTCTATGTTAGACAAAGAAAGCTTAGATAAATTTTCATCTCCCAAATA [22464]
lp38_gi|11496831|B31 ................................................ [22464]

lp38_PCR             TTTATCCCCTTGGCTTTATTTTCAAGCATCTTTTAATGAAGTTGATTT [22512]
lp38_gi|11496831|B31 ................................................ [22512]

lp38_PCR             ACTTGCAATGGTTGAATTCCCATTTAGCATTGAAAATAAAACTTTTGA [22560]
lp38_gi|11496831|B31 ................................................ [22560]

lp38_PCR             TATTACTGTTATTTTTGATTTTTTATTTGAAATTTATAGTGGAATATT [22608]
lp38_gi|11496831|B31 ................................................ [22608]

lp38_PCR             TTTTTACTCTACGTTAAGACAAGACCTACTTTGGTCTAAAAATTATAA [22656]
lp38_gi|11496831|B31 ................................................ [22656]

lp38_PCR             ATTTGATGATGATGACAATAGATTTTTGCTTGGTCTTAGATATTATAT [22704]
lp38_gi|11496831|B31 ................................................ [22704]

lp38_PCR             TAGCTTTGAAAATGACGTTTTTAATGATTTGTCAATAGTATTTGAGAG [22752]
lp38_gi|11496831|B31 ................................................ [22752]

lp38_PCR             TTATTCGAAGAATAATAATTATTTTTTGGGAGCTGGGATCAAACTAGC [22800]
lp38_gi|11496831|B31 ................................................ [22800]

lp38_PCR             ATGGATTTATGAGCTACTCCAAACGACGGTTTCTTTAAGAACCAATCT [22848]
lp38_gi|11496831|B31 ................................................ [22848]

lp38_PCR             AAATACTCATTCCTTGCAATTCTATTTTGAAAATAATTTTTTAATTCT [22896]
lp38_gi|11496831|B31 ................................................ [22896]

lp38_PCR             TAAAGAATGTTCTTTAAAGGTGTCAAATATATTGGAATTTAATAAAAA [22944]
lp38_gi|11496831|B31 ................................................ [22944]

lp38_PCR             GATAAATCTTAAGGATACTCCTTTTTACAATATGTTTAGTATTGAACT [22992]
lp38_gi|11496831|B31 ................................................ [22992]

lp38_PCR             AAAAATTGAATTATAAGATTTCTAGTTGGTATATTATATTTTTTCATG [23040]
lp38_gi|11496831|B31 ................................................ [23040]

lp38_PCR             TTATATTCAAGATGAGTGTATTATGAAGCTGGGCGCAGATAAAATTAT [23088]
lp38_gi|11496831|B31 ................................................ [23088]

lp38_PCR             AAGGCAAAAAGATAGATATTGTTTAAAAACTTAAAAACATAAAGCTAA [23136]
lp38_gi|11496831|B31 ................................................ [23136]
```
