# Supplementary material for: Lost in plasmids: next generation sequencing and the complex genome of the tick-borne pathogen Borrelia burgdorferi
Source: BMC Genomics. 2017 May 30;18:422. doi: 10.1186/s12864-017-3804-5 (PMC5450258; doi:10.1186/s12864-017-3804-5)
Supplement: Supplementary file 2 — Multiple Plasmid Alignments_B31_PAbe_PAli. (PDF 2284 kb) [file 12864_2017_3804_MOESM2_ESM.pdf]

```
#NEXUS
[ Title ]
begin data;
   dimensions ntax=8 nchar=48911;
   format missing=? gap=- matchchar=. datatype=nucleotide interleave=yes;
   matrix

[!Domain=Data property=Coding CodonStart=1;]
cp26_gi|11497007|B31_GB      TTTAAAACTTTTCTATTGGATAGATTTTATACAAAGAAGGTAATAATGTATAAACAACAATATTTTATTTCTGGCAAG [   78]
cp26_un9_B31_PacBio          TTTAAAACTTTTCTATTGGATAGATTTTATACAAAGAAGGTAATAATGTATAAACAACAATATTTTATTTCTGGCAAG [   78]
cp26_B31_TS_MP_FG_consensus  TTTAAAACTTTTCTATTGGATAGATTTTATACAAAGAAGGTAATAATGTATAAACAACAATATTTTATTTCTGGCAAG [   78]
cp26_B31_NX_Pl_consensus     TTTAAAACTTTTCTATTGGATAGATTTTATACAAAGAAGGTAATAATGTATAAACAACAATATTTTATTTCTGGCAAG [   78]
cp26_PAli_NX_MP_FG_consensus TTTAAAACTTTTCTATTGGATAGATTTTATACAAAGAAGGTAATAATGTATAAACAACAATATTTTATTTCTGGCAAG [   78]
cp26_PAli_NX_Pl_consensus    TTTAAAACTTTTCTATTGGATAGATTTTATACAAAGAAGGTAATAATGTATAAACAACAATATTTTATTTCTGGCAAG [   78]
cp26_PAbe_NX_MP_FG_consensus TTTAAAACTTTTCTATTGGATAGATTTTATACAAAGAAGGTAATAATGTATAAACAACAATATTTTATTTCTGGCAAG [   78]
cp26_PAbe_NX_Pl_consensus    TTTAAAACTTTTCTATTGGATAGATTTTATACAAAGAAGGTAATAATGTATAAACAACAATATTTTATTTCTGGCAAG [   78]

cp26_gi|11497007|B31_GB      GTGCAAGGTGTTGGTTTTAGATTTTTCACAGAGCAAATAGCAAATAATATGAAACTAAAAGGATTTGTAAAAAATCTC [  156]
cp26_un9_B31_PacBio          GTGCAAGGTGTTGGTTTTAGATTTTTCACAGAGCAAATAGCAAATAATATGAAACTAAAAGGATTTGTAAAAAATCTC [  156]
cp26_B31_TS_MP_FG_consensus  GTGCAAGGTGTTGGTTTTAGATTTTTCACAGAGCAAATAGCAAATAATATGAAACTAAAAGGATTTGTAAAAAATCTC [  156]
cp26_B31_NX_Pl_consensus     GTGCAAGGTGTTGGTTTTAGATTTTTCACAGAGCAAATAGCAAATAATATGAAACTAAAAGGATTTGTAAAAAATCTC [  156]
cp26_PAli_NX_MP_FG_consensus GTGCAAGGTGTTGGTTTTAGATTTTTCACAGAGCAAATAGCAAATAATATGAAACTAAAAGGATTTGTAAAAAATCTC [  156]
cp26_PAli_NX_Pl_consensus    GTGCAAGGTGTTGGTTTTAGATTTTTCACAGAGCAAATAGCAAATAATATGAAACTAAAAGGATTTGTAAAAAATCTC [  156]
cp26_PAbe_NX_MP_FG_consensus GTGCAAGGTGTTGGTTTTAGATTTTTCACAGAGCAAATAGCAAATAATATGAAACTAAAAGGATTTGTAAAAAATCTC [  156]
cp26_PAbe_NX_Pl_consensus    GTGCAAGGTGTTGGTTTTAGATTTTTCACAGAGCAAATAGCAAATAATATGAAACTAAAAGGATTTGTAAAAAATCTC [  156]

cp26_gi|11497007|B31_GB      AACGATGGAAGGGTAGAAATTGTAGCTTTCTTTAATACTAAAGAACAAATGAAAAAAATTTGAAAAATTATTAAATGGG [  234]
cp26_un9_B31_PacBio          AACGATGGAAGGGTAGAAATTGTAGCTTTCTTTAATACTAAAGAACAAATGAAAAA--TTGAAAAATTATTAAATGGG [  234]
cp26_B31_TS_MP_FG_consensus  AACGATGGAAGGGTAGAAATTGTAGCTTTCTTTAATACTAAAGAACAAATGAAAAAAATTTGAAAAATTATTAAATGGG [  234]
cp26_B31_NX_Pl_consensus     AACGATGGAAGGGTAGAAATTGTAGCTTTCTTTAATACTAAAGAACAAATGAAAAAAATTTGAAAAATTATTAAATGGG [  234]
cp26_PAli_NX_MP_FG_consensus AACGATGGAAGGGTAGAAATTGTAGCTTTCTTTAATACTAAAGAACAAATGAAAAAAATTTGAAAAATTATTAAATGGG [  234]
cp26_PAli_NX_Pl_consensus    AACGATGGAAGGGTAGAAATTGTAGCTTTCTTTAATACTAAAGAACAAATGAAAAAAATTTGAAAAATTATTAAATGGG [  234]
cp26_PAbe_NX_MP_FG_consensus AACGATGGAAGGGTAGAAATTGTAGCTTTCTTTAATACTAAAGAACAAATGAAAAAAATTTGAAAAATTATTAAATGGG [  234]
cp26_PAbe_NX_Pl_consensus    AACGATGGAAGGGTAGAAATTGTAGCTTTCTTTAATACTAAAGAACAAATGAAAAAAATTTGAAAAATTATTAAATGGG [  234]

cp26_gi|11497007|B31_GB      AATAAGTATTCAAACATTAAAAACATTGAAAAAATAGTTTTAGATGAAAATTATCCTTTTCAATTTAATGATTTTAAA [  312]
cp26_un9_B31_PacBio          AATAAGTATTCAAACATTAAAAACATTGAAAAAATAGTTTTAGATGAAAATTATCCTTT-CAATTTAATGATTTTAAA [  312]
cp26_B31_TS_MP_FG_consensus  AATAAGTATTCAAACATTAAAAACATTGAAAAAATAGTTTTAGATGAAAATTATCCTTTTCAATTTAATGATTTTAAA [  312]
cp26_B31_NX_Pl_consensus     AATAAGTATTCAAACATTAAAAACATTGAAAAAATAGTTTTAGATGAAAATTATCCTTTTCAATTTAATGATTTTAAA [  312]
cp26_PAli_NX_MP_FG_consensus AATAAGTATTCAAACATTAAAAACATTGAAAAAATAGTTTTAGATGAAAATTATCCTTTTCAATTTAATGATTTTAAA [  312]
cp26_PAli_NX_Pl_consensus    AATAAGTATTCAAACATTAAAAACATTGAAAAAATAGTTTTAGATGAAAATTATCCTTTTCAATTTAATGATTTTAAA [  312]
cp26_PAbe_NX_MP_FG_consensus AATAAGTATTCAAACATTAAAAACATTGAAAAAATAGTTTTAGATGAAAATTATCCTTTTCAATTTAATGATTTTAAA [  312]
cp26_PAbe_NX_Pl_consensus    AATAAGTATTCAAACATTAAAAACATTGAAAAAATAGTTTTAGATGAAAATTATCCTTTTCAATTTAATGATTTTAAA [  312]

cp26_gi|11497007|B31_GB      ATTTATTATTAGGGCTTGCCTCTCGTTTAACAAGTACCTTAACCTTATTTTTTGGTTTAATATTGTGCACATAAGAAT [  390]
cp26_un9_B31_PacBio          ATTTATTATTAGGGCTTGCCTCTCGTTTAACAAGTAC-TTAACCTTATTTTTTGGTTTAATATTGTGCACATAAGAAT [  390]
cp26_B31_TS_MP_FG_consensus  ATTTATTATTAGGGCTTGCCTCTCGTTTAACAAGTACCTTAACCTTATTTTTTGGTTTAATATTGTGCACATAAGAAT [  390]
cp26_B31_NX_Pl_consensus     ATTTATTATTAGGGCTTGCCTCTCGTTTAACAAGTACCTTAACCTTATTTTTTGGTTTAATATTGTGCACATAAGAAT [  390]
cp26_PAli_NX_MP_FG_consensus ATTTATTATTAGGGCTTGCCTCTCGTTTAACAAGTACCTTAACCTTATTTTTTGGTTTAATATTGTGCACATAAGAAT [  390]
cp26_PAli_NX_Pl_consensus    ATTTATTATTAGGGCTTGCCTCTCGTTTAACAAGTACCTTAACCTTATTTTTTGGTTTAATATTGTGCACATAAGAAT [  390]
cp26_PAbe_NX_MP_FG_consensus ATTTATTATTAGGGCTTGCCTCTCGTTTAACAAGTACCTTAACCTTATTTTTTGGTTTAATATTGTGCACATAAGAAT [  390]
cp26_PAbe_NX_Pl_consensus    ATTTATTATTAGGGCTTGCCTCTCGTTTAACAAGTACCTTAACCTTATTTTTTGGTTTAATATTGTGCACATAAGAAT [  390]

cp26_gi|11497007|B31_GB      TGTTATTCTTATAGCAAGACACACTAGTTCT--AAAAAAATGTTCGACTTTAAATTTCAAAAACTCTAAAGACTTTCT [  468]
cp26_un9_B31_PacBio          TGTTATTCTTATAGCAAGACACACTAGTTTCTAAAAAAAATGTTCGACTTTAAATTTCAAAAACTCTAAAGACTTTCT [  468]
cp26_B31_TS_MP_FG_consensus  TGTTATTCTTATAGCAAGACACACTAGTTCT--AAAAAAATGTTCGACTTTAAATTTCAAAAACTCTAAAGACTTTCT [  468]
cp26_B31_NX_Pl_consensus     TGTTATTCTTATAGCAAGACACACTAGTTCT--AAAAAAATGTTCGACTTTAAATTTCAAAAACTCTAAAGACTTTCT [  468]
cp26_PAli_NX_MP_FG_consensus TGTTATTCTTATAGCAAGACACACTAGTTCT--AAAAAAATGTTCGACTTTAAATTTCAAAAACTCTAAAGACTTTCT [  468]
cp26_PAli_NX_Pl_consensus    TGTTATTCTTATAGCAAGACACACTAGTTCT--AAAAAAATGTTCGACTTTAAATTTCAAAAACTCTAAAGACTTTCT [  468]
cp26_PAbe_NX_MP_FG_consensus TGTTATTCTTATAGCAAGACACACTAGTTCT--AAAAAAATGTTCGACTTTAAATTTCAAAAACTCTAAAGACTTTCT [  468]
cp26_PAbe_NX_Pl_consensus    TGTTATTCTTATAGCAAGACACACTAGTTCT--AAAAAAATGTTCGACTTTAAATTTCAAAAACTCTAAAGACTTTCT [  468]

cp26_gi|11497007|B31_GB      GTTTCTACAAAAAATATTCAAATTGCCATCAGAGAATTTAATATCAAGCCCACAATTACAACTTGTTTTTAAAAAACT [  546]
cp26_un9_B31_PacBio          GTTTCTACAAAAA-TATTCAAATTGCCATCAGAGAATTTAATATCAAGCCCACAATTACAACTTGTTTTTAAAAAACT [  546]
cp26_B31_TS_MP_FG_consensus  GTTTCTACAAAAAATATTCAAATTGCCATCAGAGAATTTAATATCAAGCCCACAATTACAACTTGTTTTTAAAAAACT [  546]
cp26_B31_NX_Pl_consensus     GTTTCTACAAAAAATATTCAAATTGCCATCAGAGAATTTAATATCAAGCCCACAATTACAACTTGTTTTTAAAAAACT [  546]
cp26_PAli_NX_MP_FG_consensus GTTTCTACAAAAAATATTCAAATTGCCATCAGAGAATTTAATATCAAGCCCACAATTACAACTTGTTTTTAAAAAACT [  546]
cp26_PAli_NX_Pl_consensus    GTTTCTACAAAAAATATTCAAATTGCCATCAGAGAATTTAATATCAAGCCCACAATTACAACTTGTTTTTAAAAAACT [  546]
cp26_PAbe_NX_MP_FG_consensus GTTTCTACAAAAAATATTCAAATTGCCATCAGAGAATTTAATATCAAGCCCACAATTACAACTTGTTTTTAAAAAACT [  546]
cp26_PAbe_NX_Pl_consensus    GTTTCTACAAAAAATATTCAAATTGCCATCAGAGAATTTAATATCAAGCCCACAATTACAACTTGTTTTTAAAAAACT [  546]

cp26_gi|11497007|B31_GB      GCACGAAATAAAACTACCATTTGAACTATTCCTATTAAAAAGAATAAAATATTTTTTACCTCTTTTGGAGTCTTTAAA [  624]
cp26_un9_B31_PacBio          GCACGAAATAAA-CTACCATTTGAACTATTCCTATTAAAAAGAATAAAATATTTTTTACCTCTTTTGGAGTCTTTAAA [  624]
cp26_B31_TS_MP_FG_consensus  GCACGAAATAAAACTACCATTTGAACTATTCCTATTAAAAAGAATAAAATATTTTTTACCTCTTTTGGAGTCTTTAAA [  624]
cp26_B31_NX_Pl_consensus     GCACGAAATAAAACTACCATTTGAACTATTCCTATTAAAAAGAATAAAATATTTTTTACCTCTTTTGGAGTCTTTAAA [  624]
cp26_PAli_NX_MP_FG_consensus GCACGAAATAAAACTACCATTTGAACTATTCCTATTAAAAAGAATAAAATATTTTTTACCTCTTTTGGAGTCTTTAAA [  624]
cp26_PAli_NX_Pl_consensus    GCACGAAATAAAACTACCATTTGAACTATTCCTATTAAAAAGAATAAAATATTTTTTACCTCTTTTGGAGTCTTTAAA [  624]
cp26_PAbe_NX_MP_FG_consensus GCACGAAATAAAACTACCATTTGAACTATTCCTATTAAAAAGAATAAAATATTTTTTACCTCTTTTGGAGTCTTTAAA [  624]
cp26_PAbe_NX_Pl_consensus    GCACGAAATAAAACTACCATTTGAACTATTCCTATTAAAAAGAATAAAATATTTTTTACCTCTTTTGGAGTCTTTAAA [  624]

cp26_gi|11497007|B31_GB      ATAAATTCTAAGCCATTCTCCTGCTGGCTTAACTGAAGCCAATCTATCATAAAGATAAGAAATGTAAATCGTTCTATT [  702]
cp26_un9_B31_PacBio          ATAAATTCTAAGCCATTCTCCTGCTGGCTTAACTGAAGCCAATCTATCATAAAGATAAGAAATGTAAATCGTTCTATT [  702]
cp26_B31_TS_MP_FG_consensus  ATAAATTCTAAGCCATTCTCCTGCTGGCTTAACTGAAGCCAATCTATCATAAAGATAAGAAATGTAAATCGTTCTATT [  702]
cp26_B31_NX_Pl_consensus     ATAAATTCTAAGCCATTCTCCTGCTGGCTTAACTGAAGCCAATCTATCATAAAGATAAGAAATGTAAATCGTTCTATT [  702]
cp26_PAli_NX_MP_FG_consensus ATAAATTCTAAGCCATTCTCCTGCTGGCTTAACTGAAGCCAATCTATCATAAAGATAAGAAATGTAAATCGTTCTATT [  702]
cp26_PAli_NX_Pl_consensus    ATAAATTCTAAGCCATTCTCCTGCTGGCTTAACTGAAGCCAATCTATCATAAAGATAAGAAATGTAAATCGTTCTATT [  702]
cp26_PAbe_NX_MP_FG_consensus ATAAATTCTAAGCCATTCTCCTGCTGGCTTAACTGAAGCCAATCTATCATAAAGATAAGAAATGTAAATCGTTCTATT [  702]
cp26_PAbe_NX_Pl_consensus    ATAAATTCTAAGCCATTCTCCTGCTGGCTTAACTGAAGCCAATCTATCATAAAGATAAGAAATGTAAATCGTTCTATT [  702]

cp26_gi|11497007|B31_GB      GTCATTAGATTTTAAAATTTTTTTAAAAAAATAATGAGGACCTATTTTCATACAAATTATATAATATCTTATTAATAA [  780]
cp26_un9_B31_PacBio          GTCATTAGATTTTAAAATTTTTTTAAAAAAATAATGAGGACCTATTTTCATACAA-TTATATAATATCTTATTAATAA [  780]
cp26_B31_TS_MP_FG_consensus  GTCATTAGATTTTAAAATTTTTTTAAAAAAATAATGAGGACCTATTTTCATACAAATTATATAATATCTTATTAATAA [  780]
cp26_B31_NX_Pl_consensus     GTCATTAGATTTTAAAATTTTTTTAAAAAAATAATGAGGACCTATTTTCATACAAATTATATAATATCTTATTAATAA [  780]
cp26_PAli_NX_MP_FG_consensus GTCATTAGATTTTAAAATTTTTTTAAAAAAATAATGAGGACCTATTTTCATACAAATTATATAATATCTTATTAATAA [  780]
cp26_PAli_NX_Pl_consensus    GTCATTAGATTTTAAAATTTTTTTAAAAAAATAATGAGGACCTATTTTCATACAAATTATATAATATCTTATTAATAA [  780]
cp26_PAbe_NX_MP_FG_consensus GTCATTAGATTTTAAAATTTTTTTAAAAAAATAATGAGGACCTATTTTCATACAAATTATATAATATCTTATTAATAA [  780]
cp26_PAbe_NX_Pl_consensus    GTCATTAGATTTTAAAATTTTTTTAAAAAAATAATGAGGACCTATTTTCATACAAATTATATAATATCTTATTAATAA [  780]

cp26_gi|11497007|B31_GB      AATATTTCCTAATATTTCCCAAATATATTGATAATGCCTGAATTTAAAAAAACAAAAACTATAGCTTATAATTAAAAA [  858]
cp26_un9_B31_PacBio          AATATTTCCTAATATTTCCCCAATATATTGATAATGCCTGAATTTAAAAAAACAAAAACTATAGCTTATAATTAAAAA [  858]
cp26_B31_TS_MP_FG_consensus  AATATTTCCTAATATTTCCCAAATATATTGATAATGCCTGAATTTAAAAAAACAAAAACTATAGCTTATAATTAAAAA [  858]
cp26_B31_NX_Pl_consensus     AATATTTCCTAATATTTCCCAAATATATTGATAATGCCTGAATTTAAAAAAACAAAAACTATAGCTTATAATTAAAAA [  858]
cp26_PAli_NX_MP_FG_consensus AATATTTCCTAATATTTCCCAAATATATTGATAATGCCTGAATTTAAAAAAACAAAAACTATAGCTTATAATTAAAAA [  858]
cp26_PAli_NX_Pl_consensus    AATATTTCCTAATATTTCCCAAATATATTGATAATGCCTGAATTTAAAAAAACAAAAACTATAGCTTATAATTAAAAA [  858]
cp26_PAbe_NX_MP_FG_consensus AATATTTCCTAATATTTCCCAAATATATTGATAATGCCTGAATTTAAAAAAACAAAAACTATAGCTTATAATTAAAAA [  858]
cp26_PAbe_NX_Pl_consensus    AATATTTCCTAATATTTCCCAAATATATTGATAATGCCTGAATTTAAAAAAACAAAAACTATAGCTTATAATTAAAAA [  858]
```

```
cp26_gi|11497007|B31_GB      TTATTGATAAGTATTCTGTAAGTTTTATATAGTTATAATTGTATCCATATGTGGCTTTAAAAGCTTTGCGAATTTTAA [  936]
cp26_un9_B31_PacBio          TTATTGATAAGTATTCTGTAAGTTTTATATAGTTATAATTGTATCCATATGTGGCTTTAAAAGCTTTGCGAATTTTAA [  936]
cp26_B31_TS_MP_FG_consensus  TTATTGATAAGTATTCTGTAAGTTTTATATAGTTATAATTGTATCCATATGTGGCTTTAAAAGCTTTGCGAATTTTAA [  936]
cp26_B31_NX_Pl_consensus     TTATTGATAAGTATTCTGTAAGTTTTATATAGTTATAATTGTATCCATATGTGGCTTTAAAAGCTTTGCGAATTTTAA [  936]
cp26_PAli_NX_MP_FG_consensus TTATTGATAAGTATTCTGTAAGTTTTATATAGTTATAATTGTATCCATATGTGGCTTTAAAAGCTTTGCGAATTTTAA [  936]
cp26_PAli_NX_Pl_consensus    TTATTGATAAGTATTCTGTAAGTTTTATATAGTTATAATTGTATCCATATGTGGCTTTAAAAGCTTTGCGAATTTTAA [  936]
cp26_PAbe_NX_MP_FG_consensus TTATTGATAAGTATTCTGTAAGTTTTATATAGTTATAATTGTATCCATATGTGGCTTTAAAAGCTTTGCGAATTTTAA [  936]
cp26_PAbe_NX_Pl_consensus    TTATTGATAAGTATTCTGTAAGTTTTATATAGTTATAATTGTATCCATATGTGGCTTTAAAAGCTTTGCGAATTTTAA [  936]

cp26_gi|11497007|B31_GB      CATTCAATCCTCTAACCATTGCCAAAGTTTCTAAATTATCCTTTAACATCAAATTTTTAATTACAATCAACGTTTTAA [ 1014]
cp26_un9_B31_PacBio          CATTCAATCCTCTAACCATTGCCAAAGTTTCTAAATTATCCTTTAACATCAAATTTTTAATTACAATCAACGTTTTAA [ 1014]
cp26_B31_TS_MP_FG_consensus  CATTCAATCCTCTAACCATTGCCAAAGTTTCTAAATTATCCTTTAACATCAAATTTTTAATTACAATCAACGTTTTAA [ 1014]
cp26_B31_NX_Pl_consensus     CATTCAATCCTCTAACCATTGCCAAAGTTTCTAAATTATCCTTTAACATCAAATTTTTAATTACAATCAACGTTTTAA [ 1014]
cp26_PAli_NX_MP_FG_consensus CATTCAATCCTCTAACCATTGCCAAAGTTTCTAAATTATCCTTTAACATCAAATTTTTAATTACAATCAACGTTTTAA [ 1014]
cp26_PAli_NX_Pl_consensus    CATTCAATCCTCTAACCATTGCCAAAGTTTCTAAATTATCCTTTAACATCAAATTTTTAATTACAATCAACGTTTTAA [ 1014]
cp26_PAbe_NX_MP_FG_consensus CATTCAATCCTCTAACCATTGCCAAAGTTTCTAAATTATCCTTTAACATCAAATTTTTAATTACAATCAACGTTTTAA [ 1014]
cp26_PAbe_NX_Pl_consensus    CATTCAATCCTCTAACCATTGCCAAAGTTTCTAAATTATCCTTTAACATCAAATTTTTAATTACAATCAACGTTTTAA [ 1014]

cp26_gi|11497007|B31_GB      TATAATTATCATCAAATATATGATGTTCTTTTATCAAACTTTCTAAACGATCCATTGTAAGAGTAGAATAAGTAGCTT [ 1092]
cp26_un9_B31_PacBio          TATAATTATCATCAAATATATGATGTTCTTTTATCAAACTTTCTAAACGATCCATTGTAAGAGTAGAATAAGTAGCTT [ 1092]
cp26_B31_TS_MP_FG_consensus  TATAATTATCATCAAATATATGATGTTCTTTTATCAAACTTTCTAAACGATCCATTGTAAGAGTAGAATAAGTAGCTT [ 1092]
cp26_B31_NX_Pl_consensus     TATAATTATCATCAAATATATGATGTTCTTTTATCAAACTTTCTAAACGATCCATTGTAAGAGTAGAATAAGTAGCTT [ 1092]
cp26_PAli_NX_MP_FG_consensus TATAATTATCATCAAATATATGATGTTCTTTTATCAAACTTTCTAAACGATCCATTGTAAGAGTAGAATAAGTAGCTT [ 1092]
cp26_PAli_NX_Pl_consensus    TATAATTATCATCAAATATATGATGTTCTTTTATCAAACTTTCTAAACGATCCATTGTAAGAGTAGAATAAGTAGCTT [ 1092]
cp26_PAbe_NX_MP_FG_consensus TATAATTATCATCAAATATATGATGTTCTTTTATCAAACTTTCTAAACGATCCATTGTAAGAGTAGAATAAGTAGCTT [ 1092]
cp26_PAbe_NX_Pl_consensus    TATAATTATCATCAAATATATGATGTTCTTTTATCAAACTTTCTAAACGATCCATTGTAAGAGTAGAATAAGTAGCTT [ 1092]

cp26_gi|11497007|B31_GB      TACGTCTAACATATGTATAAATTCTTTGATTAAGCAATGTTAATAAGCTATTATCTGCCTTATCGTCTAAATTATCTA [ 1170]
cp26_un9_B31_PacBio          TACGTCTAACATATGTATAAATTCTTTGATTAAGCAATGTTAATAAGCTATTATCTGCCTTATCGTCTAAATTATCTA [ 1170]
cp26_B31_TS_MP_FG_consensus  TACGTCTAACATATGTATAAATTCTTTGATTAAGCAATGTTAATAAGCTATTATCTGCCTTATCGTCTAAATTATCTA [ 1170]
cp26_B31_NX_Pl_consensus     TACGTCTAACATATGTATAAATTCTTTGATTAAGCAATGTTAATAAGCTATTATCTGCCTTATCGTCTAAATTATCTA [ 1170]
cp26_PAli_NX_MP_FG_consensus TACGTCTAACATATGTATAAATTCTTTGATTAAGCAATGTTAATAAGCTATTATCTGCCTTATCGTCTAAATTATCTA [ 1170]
cp26_PAli_NX_Pl_consensus    TACGTCTAACATATGTATAAATTCTTTGATTAAGCAATGTTAATAAGCTATTATCTGCCTTATCGTCTAAATTATCTA [ 1170]
cp26_PAbe_NX_MP_FG_consensus TACGTCTAACATATGTATAAATTCTTTGATTAAGCAATGTTAATAAGCTATTATCTGCCTTATCGTCTAAATTATCTA [ 1170]
cp26_PAbe_NX_Pl_consensus    TACGTCTAACATATGTATAAATTCTTTGATTAAGCAATGTTAATAAGCTATTATCTGCCTTATCGTCTAAATTATCTA [ 1170]

cp26_gi|11497007|B31_GB      AAACATACCGATTATAATGAAAGGCCGTTGTTATGTCATTTGGCTCGTGCCCCAAAACTTTTGTTATCCAATAATTCA [ 1248]
cp26_un9_B31_PacBio          AAACATACCGATTATAATGAAAGGCCGTTGTTATGTCATTTGGCTCGTGCCCCAAAACTTTTGTTATCCAATAATTCA [ 1248]
cp26_B31_TS_MP_FG_consensus  AAACATACCGATTATAATGAAAGGCCGTTGTTATGTCATTTGGCTCGTGCCCCAAAACTTTTGTTATCCAATAATTCA [ 1248]
cp26_B31_NX_Pl_consensus     AAACATACCGATTATAATGAAAGGCCGTTGTTATGTCATTTGGCTCGTGCCCCAAAACTTTTGTTATCCAATAATTCA [ 1248]
cp26_PAli_NX_MP_FG_consensus AAACATACCGATTATAATGAAAGGCCGTTGTTATGTCATTTGGCTCGTGCCCCAAAACTTTTGTTATCCAATAATTCA [ 1248]
cp26_PAli_NX_Pl_consensus    AAACATACCGATTATAATGAAAGGCCGTTGTTATGTCATTTGGCTCGTGCCCCAAAACTTTTGTTATCCAATAATTCA [ 1248]
cp26_PAbe_NX_MP_FG_consensus AAACATACCGATTATAATGAAAGGCCGTTGTTATGTCATTTGGCTCGTGCCCCAAAACTTTTGTTATCCAATAATTCA [ 1248]
cp26_PAbe_NX_Pl_consensus    AAACATACCGATTATAATGAAAGGCCGTTGTTATGTCATTTGGCTCGTGCCCCAAAACTTTTGTTATCCAATAATTCA [ 1248]

cp26_gi|11497007|B31_GB      TTTCCATGTTTTTAGGCGCAAATGCAAGATAAGAAAATTTACAATAAATTGCTCTGCAAAAATAGACAGACTCTTCAG [ 1326]
cp26_un9_B31_PacBio          TTTCCATGTTTTTAGGCGCAAATGCAAGATAAGAAAATTTACAATAAATTGCTCTGCAAAAATAGACAGACTCTTCAG [ 1326]
cp26_B31_TS_MP_FG_consensus  TTTCCATGTTTTTAGGCGCAAATGCAAGATAAGAAAATTTACAATAAATTGCTCTGCAAAAATAGACAGACTCTTCAG [ 1326]
cp26_B31_NX_Pl_consensus     TTTCCATGTTTTTAGGCGCAAATGCAAGATAAGAAAATTTACAATAAATTGCTCTGCAAAAATAGACAGACTCTTCAG [ 1326]
cp26_PAli_NX_MP_FG_consensus TTTCCATGTTTTTAGGCGCAAATGCAAGATAAGAAAATTTACAATAAATTGCTCTGCAAAAATAGACAGACTCTTCAG [ 1326]
cp26_PAli_NX_Pl_consensus    TTTCCATGTTTTTAGGCGCAAATGCAAGATAAGAAAATTTACAATAAATTGCTCTGCAAAAATAGACAGACTCTTCAG [ 1326]
cp26_PAbe_NX_MP_FG_consensus TTTCCATGTTTTTAGGCGCAAATGCAAGATAAGAAAATTTACAATAAATTGCTCTGCAAAAATAGACAGACTCTTCAG [ 1326]
cp26_PAbe_NX_Pl_consensus    TTTCCATGTTTTTAGGCGCAAATGCAAGATAAGAAAATTTACAATAAATTGCTCTGCAAAAATAGACAGACTCTTCAG [ 1326]

cp26_gi|11497007|B31_GB      GAGCAAAAATATTATTAAAAATTTGACGAAACAATCTATTATAACTGTATGCAAGATTAGAAGAGATTATCTCTTTAG [ 1404]
cp26_un9_B31_PacBio          GAGCAAAAATATTATTAAAAATTTGACGAAACAATCTATTATAACTGTATGCAAGATTAGAAGAGATTATCTCTTTAG [ 1404]
cp26_B31_TS_MP_FG_consensus  GAGCAAAAATATTATTAAAAATTTGACGAAACAATCTATTATAACTGTATGCAAGATTAGAAGAGATTATCTCTTTAG [ 1404]
cp26_B31_NX_Pl_consensus     GAGCAAAAATATTATTAAAAATTTGACGAAACAATCTATTATAACTGTATGCAAGATTAGAAGAGATTATCTCTTTAG [ 1404]
cp26_PAli_NX_MP_FG_consensus GAGCAAAAATATTATTAAAAATTTGACGAAACAATCTATTATAACTGTATGCAAGATTAGAAGAGATTATCTCTTTAG [ 1404]
cp26_PAli_NX_Pl_consensus    GAGCAAAAATATTATTAAAAATTTGACGAAACAATCTATTATAACTGTATGCAAGATTAGAAGAGATTATCTCTTTAG [ 1404]
cp26_PAbe_NX_MP_FG_consensus GAGCAAAAATATTATTAAAAATTTGACGAAACAATCTATTATAACTGTATGCAAGATTAGAAGAGATTATCTCTTTAG [ 1404]
cp26_PAbe_NX_Pl_consensus    GAGCAAAAATATTATTAAAAATTTGACGAAACAATCTATTATAACTGTATGCAAGATTAGAAGAGATTATCTCTTTAG [ 1404]

cp26_gi|11497007|B31_GB      TAAGATTCTCAGTCTGCTCCATATAGCGTATTTCTTTTATGGAATTAATTATTAATTCAGGATCTGCAAAAACTGGAA [ 1482]
cp26_un9_B31_PacBio          TAAGATTCTCAGTCTGCTCCATATAGCGTATTTCTTTTATGGAATTAATTATTAATTCAGGATCTGCAAAAACTGGAA [ 1482]
cp26_B31_TS_MP_FG_consensus  TAAGATTCTCAGTCTGCTCCATATAGCGTATTTCTTTTATGGAATTAATTATTAATTCAGGATCTGCAAAAACTGGAA [ 1482]
cp26_B31_NX_Pl_consensus     TAAGATTCTCAGTCTGCTCCATATAGCGTATTTCTTTTATGGAATTAATTATTAATTCAGGATCTGCAAAAACTGGAA [ 1482]
cp26_PAli_NX_MP_FG_consensus TAAGATTCTCAGTCTGCTCCATATAGCGTATTTCTTTTATGGAATTAATTATTAATTCAGGATCTGCAAAAACTGGAA [ 1482]
cp26_PAli_NX_Pl_consensus    TAAGATTCTCAGTCTGCTCCATATAGCGTATTTCTTTTATGGAATTAATTATTAATTCAGGATCTGCAAAAACTGGAA [ 1482]
cp26_PAbe_NX_MP_FG_consensus TAAGATTCTCAGTCTGCTCCATATAGCGTATTTCTTTTATGGAATTAATTATTAATTCAGGATCTGCAAAAACTGGAA [ 1482]
cp26_PAbe_NX_Pl_consensus    TAAGATTCTCAGTCTGCTCCATATAGCGTATTTCTTTTATGGAATTAATTATTAATTCAGGATCTGCAAAAACTGGAA [ 1482]

cp26_gi|11497007|B31_GB      AAACAACTTCATTAACAATATTATTTTCTCGCTTTTTTGCAATAAACTCCATACGAATATGATTTTTATCTGCGATAT [ 1560]
cp26_un9_B31_PacBio          AAACAACTTCATTAACAATATTATTTTCTCGCTTTTTTGCAATAAACTCCATACGAATATGATTTTTATCTGCGATAT [ 1560]
cp26_B31_TS_MP_FG_consensus  AAACAACTTCATTAACAATATTATTTTCTCGCTTTTTTGCAATAAACTCCATACGAATATGATTTTTATCTGCGATAT [ 1560]
cp26_B31_NX_Pl_consensus     AAACAACTTCATTAACAATATTATTTTCTCGCTTTTTTGCAATAAACTCCATACGAATATGATTTTTATCTGCGATAT [ 1560]
cp26_PAli_NX_MP_FG_consensus AAACAACTTCATTAACAATATTATTTTCTCGCTTTTTTGCAATAAACTCCATACGAATATGATTTTTATCTGCGATAT [ 1560]
cp26_PAli_NX_Pl_consensus    AAACAACTTCATTAACAATATTATTTTCTCGCTTTTTTGCAATAAACTCCATACGAATATGATTTTTATCTGCGATAT [ 1560]
cp26_PAbe_NX_MP_FG_consensus AAACAACTTCATTAACAATATTATTTTCTCGCTTTTTTGCAATAAACTCCATACGAATATGATTTTTATCTGCGATAT [ 1560]
cp26_PAbe_NX_Pl_consensus    AAACAACTTCATTAACAATATTATTTTCTCGCTTTTTTGCAATAAACTCCATACGAATATGATTTTTATCTGCGATAT [ 1560]

cp26_gi|11497007|B31_GB      AAAATTGTGAAAGCTTCATTACTTCAACAGGACGACGTCCTATTGCCATTAAAACTCCATAAAATTTTAATCTAATAT [ 1638]
cp26_un9_B31_PacBio          AAAATTGTGAAAGCTTCATTACTTCAACAGGACGACGTCCTATTGCCATTAAAACTCCATAAAATTTTAATCTAATAT [ 1638]
cp26_B31_TS_MP_FG_consensus  AAAATTGTGAAAGCTTCATTACTTCAACAGGACGACGTCCTATTGCCATTAAAACTCCATAAAATTTTAATCTAATAT [ 1638]
cp26_B31_NX_Pl_consensus     AAAATTGTGAAAGCTTCATTACTTCAACAGGACGACGTCCTATTGCCATTAAAACTCCATAAAATTTTAATCTAATAT [ 1638]
cp26_PAli_NX_MP_FG_consensus AAAATTGTGAAAGCTTCATTACTTCAACAGGACGACGTCCTATTGCCATTAAAACTCCATAAAATTTTAATCTAATAT [ 1638]
cp26_PAli_NX_Pl_consensus    AAAATTGTGAAAGCTTCATTACTTCAACAGGACGACGTCCTATTGCCATTAAAACTCCATAAAATTTTAATCTAATAT [ 1638]
cp26_PAbe_NX_MP_FG_consensus AAAATTGTGAAAGCTTCATTACTTCAACAGGACGACGTCCTATTGCCATTAAAACTCCATAAAATTTTAATCTAATAT [ 1638]
cp26_PAbe_NX_Pl_consensus    AAAATTGTGAAAGCTTCATTACTTCAACAGGACGACGTCCTATTGCCATTAAAACTCCATAAAATTTTAATCTAATAT [ 1638]

cp26_gi|11497007|B31_GB      CTCGACTTTGATTCAGTAGAAGCTTAATTATTTCAATATAGGTATTTAAATTAATTTTAACAAATATCTGCTCTTTCC [ 1716]
cp26_un9_B31_PacBio          CTCGACTTTGATTCAGTAGAAGCTTAATTATTTCAATATAGGTATTTAAATTAATTTTAACAAATATCTGCTCTTTCC [ 1716]
cp26_B31_TS_MP_FG_consensus  CTCGACTTTGATTCAGTAGAAGCTTAATTATTTCAATATAGGTATTTAAATTAATTTTAACAAATATCTGCTCTTTCC [ 1716]
cp26_B31_NX_Pl_consensus     CTCGACTTTGATTCAGTAGAAGCTTAATTATTTCAATATAGGTATTTAAATTAATTTTAACAAATATCTGCTCTTTCC [ 1716]
cp26_PAli_NX_MP_FG_consensus CTCGACTTTGATTCAGTAGAAGCTTAATTATTTCAATATAGGTATTTAAATTAATTTTAACAAATATCTGCTCTTTCC [ 1716]
cp26_PAli_NX_Pl_consensus    CTCGACTTTGATTCAGTAGAAGCTTAATTATTTCAATATAGGTATTTAAATTAATTTTAACAAATATCTGCTCTTTCC [ 1716]
cp26_PAbe_NX_MP_FG_consensus CTCGACTTTGATTCAGTAGAAGCTTAATTATTTCAATATAGGTATTTAAATTAATTTTAACAAATATCTGCTCTTTCC [ 1716]
cp26_PAbe_NX_Pl_consensus    CTCGACTTTGATTCAGTAGAAGCTTAATTATTTCAATATAGGTATTTAAATTAATTTTAACAAATATCTGCTCTTTCC [ 1716]

cp26_gi|11497007|B31_GB      TATAACTATTAATTTTTTCAATTTTATATTTATGAGCATAATCATTTAACCATTTTGGAGTTACAAACAAATCTATAA [ 1794]
cp26_un9_B31_PacBio          TATAACTATTAATTTTTTCAATTTTATATTTATGAGCATAATCATTTAACCATTTTGGAGTTACAAACAAATCTATAA [ 1794]
cp26_B31_TS_MP_FG_consensus  TATAACTATTAATTTTTTCAATTTTATATTTATGAGCATAATCATTTAACCATTTTGGAGTTACAAACAAATCTATAA [ 1794]
cp26_B31_NX_Pl_consensus     TATAACTATTAATTTTTTCAATTTTATATTTATGAGCATAATCATTTAACCATTTTGGAGTTACAAACAAATCTATAA [ 1794]
cp26_PAli_NX_MP_FG_consensus TATAACTATTAATTTTTTCAATTTTATATTTATGAGCATAATCATTTAACCATTTTGGAGTTACAAACAAATCTATAA [ 1794]
cp26_PAli_NX_Pl_consensus    TATAACTATTAATTTTTTCAATTTTATATTTATGAGCATAATCATTTAACCATTTTGGAGTTACAAACAAATCTATAA [ 1794]
cp26_PAbe_NX_MP_FG_consensus TATAACTATTAATTTTTTCAATTTTATATTTATGAGCATAATCATTTAACCATTTTGGAGTTACAAACAAATCTATAA [ 1794]
cp26_PAbe_NX_Pl_consensus    TATAACTATTAATTTTTTCAATTTTATATTTATGAGCATAATCATTTAACCATTTTGGAGTTACAAACAAATCTATAA [ 1794]
```

```
cp26_gi|11497007|B31_GB       AATATTGAAAATAGGACTTCTCATCATTCATTTGATTTGAAAATATCAACTCTTCTATTTTTTTTCGATCTTTAATTC [ 1872]
cp26_un9_B31_PacBio           AATATTGAAAATAGGACTTCTCATCATTCATTTGATTTGAAAATATCAACTCTTCTATTTTTTTTCGATCTTTAATTC [ 1872]
cp26_B31_TS_MP_FG_consensus   AATATTGAAAATAGGACTTCTCATCATTCATTTGATTTGAAAATATCAACTCTTCTATTTTTTTTCGATCTTTAATTC [ 1872]
cp26_B31_NX_Pl_consensus      AATATTGAAAATAGGACTTCTCATCATTCATTTGATTTGAAAATATCAACTCTTCTATTTTTTTTCGATCTTTAATTC [ 1872]
cp26_PAli_NX_MP_FG_consensus AATATTGAAAATAGGACTTCTCATCATTCATTTGATTTGAAAATATCAACTCTTCTATTTTTTTTCGATCTTTAATTC [ 1872]
cp26_PAli_NX_Pl_consensus     AATATTGAAAATAGGACTTCTCATCATTCATTTGATTTGAAAATATCAACTCTTCTATTTTTTTTCGATCTTTAATTC [ 1872]
cp26_PAbe_NX_MP_FG_consensus AATATTGAAAATAGGACTTCTCATCATTCATTTGATTTGAAAATATCAACTCTTCTATTTTTTTTCGATCTTTAATTC [ 1872]
cp26_PAbe_NX_Pl_consensus     AATATTGAAAATAGGACTTCTCATCATTCATTTGATTTGAAAATATCAACTCTTCTATTTTTTTTCGATCTTTAATTC [ 1872]

cp26_gi|11497007|B31_GB       CTATATTTTTTAATTCTTTTATTCTTCTTGATTTCCAAAAAAATAAAAAAGTATTATCTCTTCTAATCCTTTCAATTA [ 1950]
cp26_un9_B31_PacBio           CTATATTTTTTAATTCTTTTATTCTTCTTGATTTCCAAAAAAATAAAAAAGTATTATCTCTTCTAATCCTTTCAATTA [ 1950]
cp26_B31_TS_MP_FG_consensus   CTATATTTTTTAATTCTTTTATTCTTCTTGATTTCCAAAAAAATAAAAAAGTATTATCTCTTCTAATCCTTTCAATTA [ 1950]
cp26_B31_NX_Pl_consensus      CTATATTTTTTAATTCTTTTATTCTTCTTGATTTCCAAAAAAATAAAAAAGTATTATCTCTTCTAATCCTTTCAATTA [ 1950]
cp26_PAli_NX_MP_FG_consensus CTATATTTTTTAATTCTTTTATTCTTCTTGATTTCCAAAAAAATAAAAAAGTATTATCTCTTCTAATCCTTTCAATTA [ 1950]
cp26_PAli_NX_Pl_consensus     CTATATTTTTTAATTCTTTTATTCTTCTTGATTTCCAAAAAAATAAAAAAGTATTATCTCTTCTAATCCTTTCAATTA [ 1950]
cp26_PAbe_NX_MP_FG_consensus CTATATTTTTTAATTCTTTTATTCTTCTTGATTTCCAAAAAAATAAAAAAGTATTATCTCTTCTAATCCTTTCAATTA [ 1950]
cp26_PAbe_NX_Pl_consensus     CTATATTTTTTAATTCTTTTATTCTTCTTGATTTCCAAAAAAATAAAAAAGTATTATCTCTTCTAATCCTTTCAATTA [ 1950]

cp26_gi|11497007|B31_GB       CTGAAAGATTAATATATTCTTTAATTATTTTACGAGTCTTTGAAAGATTTAAAATTATTGAACGATTTGTAAATTTAT [ 2028]
cp26_un9_B31_PacBio           CTGAAAGATTAATATATTCTTTAATTATTTTACGAGTCTTTGAAAGATTTAAAATTATTGAACGATTTGTAAATTTAT [ 2028]
cp26_B31_TS_MP_FG_consensus   CTGAAAGATTAATATATTCTTTAATTATTTTACGAGTCTTTGAAAGATTTAAAATTATTGAACGATTTGTAAATTTAT [ 2028]
cp26_B31_NX_Pl_consensus      CTGAAAGATTAATATATTCTTTAATTATTTTACGAGTCTTTGAAAGATTTAAAATTATTGAACGATTTGTAAATTTAT [ 2028]
cp26_PAli_NX_MP_FG_consensus CTGAAAGATTAATATATTCTTTAATTATTTTACGAGTCTTTGAAAGATTTAAAATTATTGAACGATTTGTAAATTTAT [ 2028]
cp26_PAli_NX_Pl_consensus     CTGAAAGATTAATATATTCTTTAATTATTTTACGAGTCTTTGAAAGATTTAAAATTATTGAACGATTTGTAAATTTAT [ 2028]
cp26_PAbe_NX_MP_FG_consensus CTGAAAGATTAATATATTCTTTAATTATTTTACGAGTCTTTGAAAGATTTAAAATTATTGAACGATTTGTAAATTTAT [ 2028]
cp26_PAbe_NX_Pl_consensus     CTGAAAGATTAATATATTCTTTAATTATTTTACGAGTCTTTGAAAGATTTAAAATTATTGAACGATTTGTAAATTTAT [ 2028]

cp26_gi|11497007|B31_GB       CTTTTCTAAAAAGAATAGCTTTGTGATTCTCTGCAAGAATTTTTAGTTTTTCTTTCAATTTTAAATATGAAAGCTCGT [ 2106]
cp26_un9_B31_PacBio           CTTTTCTAAAAAGAATAGCTTTGTGATTCTCTGCAAGAATTTTTAGTTTTTCTTTCAATTTTAAATATGAAAGCTCGT [ 2106]
cp26_B31_TS_MP_FG_consensus   CTTTTCTAAAAAGAATAGCTTTGTGATTCTCTGCAAGAATTTTTAGTTTTTCTTTCAATTTTAAATATGAAAGCTCGT [ 2106]
cp26_B31_NX_Pl_consensus      CTTTTCTAAAAAGAATAGCTTTGTGATTCTCTGCAAGAATTTTTAGTTTTTCTTTCAATTTTAAATATGAAAGCTCGT [ 2106]
cp26_PAli_NX_MP_FG_consensus CTTTTCTAAAAAGAATAGCTTTGTGATTCTCTGCAAGAATTTTTAGTTTTTCTTTCAATTTTAAATATGAAAGCTCGT [ 2106]
cp26_PAli_NX_Pl_consensus     CTTTTCTAAAAAGAATAGCTTTGTGATTCTCTGCAAGAATTTTTAGTTTTTCTTTCAATTTTAAATATGAAAGCTCGT [ 2106]
cp26_PAbe_NX_MP_FG_consensus CTTTTCTAAAAAGAATAGCTTTGTGATTCTCTGCAAGAATTTTTAGTTTTTCTTTCAATTTTAAATATGAAAGCTCGT [ 2106]
cp26_PAbe_NX_Pl_consensus     CTTTTCTAAAAAGAATAGCTTTGTGATTCTCTGCAAGAATTTTTAGTTTTTCTTTCAATTTTAAATATGAAAGCTCGT [ 2106]

cp26_gi|11497007|B31_GB       TATTGAGATATTTTTTATATAGAATTTCTAATTCTTTTCTAAATATTTCAAAATCATTTTTTATCTTCACTTTTGGAG [ 2184]
cp26_un9_B31_PacBio           TATTGAGATATTTTTTATATAGAATTTCTAATTCTTTTCTAAATATTTCAAAATCATTTTTTATCTTCACTTTTGGAG [ 2184]
cp26_B31_TS_MP_FG_consensus   TATTGAGATATTTTTTATATAGAATTTCTAATTCTTTTCTAAATATTTCAAAATCATTTTTTATCTTCACTTTTGGAG [ 2184]
cp26_B31_NX_Pl_consensus      TATTGAGATATTTTTTATATAGAATTTCTAATTCTTTTCTAAATATTTCAAAATCATTTTTTATCTTCACTTTTGGAG [ 2184]
cp26_PAli_NX_MP_FG_consensus TATTGAGATATTTTTTATATAGAATTTCTAATTCTTTTCTAAATATTTCAAAATCATTTTTTATCTTCACTTTTGGAG [ 2184]
cp26_PAli_NX_Pl_consensus     TATTGAGATATTTTTTATATAGAATTTCTAATTCTTTTCTAAATATTTCAAAATCATTTTTTATCTTCACTTTTGGAG [ 2184]
cp26_PAbe_NX_MP_FG_consensus TATTGAGATATTTTTTATATAGAATTTCTAATTCTTTTCTAAATATTTCAAAATCATTTTTTATCTTCACTTTTGGAG [ 2184]
cp26_PAbe_NX_Pl_consensus     TATTGAGATATTTTTTATATAGAATTTCTAATTCTTTTCTAAATATTTCAAAATCATTTTTTATCTTCACTTTTGGAG [ 2184]

cp26_gi|11497007|B31_GB       GCATATTAATTCAATTATACCAAGTTTAAAATCTTTAGCTAAGTAAAAAATTTCTGATTTTAAAAAAATATGCTTATA [ 2262]
cp26_un9_B31_PacBio           GCATATTAATTCAATTATACCAAGTTTAAAATCTTTAGCTAAGTAAAAAATTTCTGATTTTAAAAAAATATGCTTATA [ 2262]
cp26_B31_TS_MP_FG_consensus   GCATATTAATTCAATTATACCAAGTTTAAAATCTTTAGCTAAGTAAAAAATTTCTGATTTTAAAAAAATATGCTTATA [ 2262]
cp26_B31_NX_Pl_consensus      GCATATTAATTCAATTATACCAAGTTTAAAATCTTTAGCTAAGTAAAAAATTTCTGATTTTAAAAAAATATGCTTATA [ 2262]
cp26_PAli_NX_MP_FG_consensus GCATATTAATTCAATTATACCAAGTTTAAAATCTTTAGCTAAGTAAAAAATTTCTGATTTTAAAAAAATATGCTTATA [ 2262]
cp26_PAli_NX_Pl_consensus     GCATATTAATTCAATTATACCAAGTTTAAAATCTTTAGCTAAGTAAAAAATTTCTGATTTTAAAAAAATATGCTTATA [ 2262]
cp26_PAbe_NX_MP_FG_consensus GCATATTAATTCAATTATACCAAGTTTAAAATCTTTAGCTAAGTAAAAAATTTCTGATTTTAAAAAAATATGCTTATA [ 2262]
cp26_PAbe_NX_Pl_consensus     GCATATTAATTCAATTATACCAAGTTTAAAATCTTTAGCTAAGTAAAAAATTTCTGATTTTAAAAAAATATGCTTATA [ 2262]

cp26_gi|11497007|B31_GB       TATTAAATTAAATTATATAATATGTACTAGGATGCATAATAAACCAAAGAAATATTCTATTCACACCGATAAATCTTT [ 2340]
cp26_un9_B31_PacBio           TATTAAATTAAATTATATAATATGTACTAGGATGCATAATAAACCAAAGAAATATTCTATTCACACCGATAAATCTTT [ 2340]
cp26_B31_TS_MP_FG_consensus   TATTAAATTAAATTATATAATATGTACTAGGATGCATAATAAACCAAAGAAATATTCTATTCACACCGATAAATCTTT [ 2340]
cp26_B31_NX_Pl_consensus      TATTAAATTAAATTATATAATATGTACTAGGATGCATAATAAACCAAAGAAATATTCTATTCACACCGATAAATCTTT [ 2340]
cp26_PAli_NX_MP_FG_consensus TATTAAATTAAATTATATAATATGTACTAGGATGCATAATAAACCAAAGAAATATTCTATTCACACCGATAAATCTTT [ 2340]
cp26_PAli_NX_Pl_consensus     TATTAAATTAAATTATATAATATGTACTAGGATGCATAATAAACCAAAGAAATATTCTATTCACACCGATAAATCTTT [ 2340]
cp26_PAbe_NX_MP_FG_consensus TATTAAATTAAATTATATAATATGTACTAGGATGCATAATAAACCAAAGAAATATTCTATTCACACCGATAAATCTTT [ 2340]
cp26_PAbe_NX_Pl_consensus     TATTAAATTAAATTATATAATATGTACTAGGATGCATAATAAACCAAAGAAATATTCTATTCACACCGATAAATCTTT [ 2340]

cp26_gi|11497007|B31_GB       TTATTAATTTAAATCATAAAATAATAAACTTCGAATTTTAAATAAATTCTTTTATCTCCCAAAAAAAGAGCTATTTAT [ 2418]
cp26_un9_B31_PacBio           TTATTAATTTAAATCATAAAATAATAAACTTCGAATTTTAAATAAATTCTTTTATCTCCCAAAAAAAGAGCTATTTAT [ 2418]
cp26_B31_TS_MP_FG_consensus   TTATTAATTTAAATCATAAAATAATAAACTTCGAATTTTAAATAAATTCTTTTATCTCCCAAAAAAAGAGCTATTTAT [ 2418]
cp26_B31_NX_Pl_consensus      TTATTAATTTAAATCATAAAATAATAAACTTCGAATTTTAAATAAATTCTTTTATCTCCCAAAAAAAGAGCTATTTAT [ 2418]
cp26_PAli_NX_MP_FG_consensus TTATTAATTTAAATCATAAAATAATAAACTTCGAATTTTAAATAAATTCTTTTATCTCCCAAAAAAAGAGCTATTTAT [ 2418]
cp26_PAli_NX_Pl_consensus     TTATTAATTTAAATCATAAAATAATAAACTTCGAATTTTAAATAAATTCTTTTATCTCCCAAAAAAAGAGCTATTTAT [ 2418]
cp26_PAbe_NX_MP_FG_consensus TTATTAATTTAAATCATAAAATAATAAACTTCGAATTTTAAATAAATTCTTTTATCTCCCAAAAAAAGAGCTATTTAT [ 2418]
cp26_PAbe_NX_Pl_consensus     TTATTAATTTAAATCATAAAATAATAAACTTCGAATTTTAAATAAATTCTTTTATCTCCCAAAAAAAGAGCTATTTAT [ 2418]

cp26_gi|11497007|B31_GB       AAATAGCTCTCTAATATTGTGTATCACAAGTTTTGCAATAGCAATTCTAAAAAAATTTCCTATTCTTTTTCCTGCAAA [ 2496]
cp26_un9_B31_PacBio           AAATAGCTCTCTAATATTGTGTATCACAAGTTTTGCAATAGCAATTCTAAAAAAATTTCCTATTCTTTTTCCTGCAAA [ 2496]
cp26_B31_TS_MP_FG_consensus   AAATAGCTCTCTAATATTGTGTATCACAAGTTTTGCAATAGCAATTCTAAAAAAATTTCCTATTCTTTTTCCTGCAAA [ 2496]
cp26_B31_NX_Pl_consensus      AAATAGCTCTCTAATATTGTGTATCACAAGTTTTGCAATAGCAATTCTAAAAAAATTTCCTATTCTTTTTCCTGCAAA [ 2496]
cp26_PAli_NX_MP_FG_consensus AAATAGCTCTCTAATATTGTGTATCACAAGTTTTGCAATAGCAATTCTAAAAAAATTTCCTATTCTTTTTCCTGCAAA [ 2496]
cp26_PAli_NX_Pl_consensus     AAATAGCTCTCTAATATTGTGTATCACAAGTTTTGCAATAGCAATTCTAAAAAAATTTCCTATTCTTTTTCCTGCAAA [ 2496]
cp26_PAbe_NX_MP_FG_consensus AAATAGCTCTCTAATATTGTGTATCACAAGTTTTGCAATAGCAATTCTAAAAAAATTTCCTATTCTTTTTCCTGCAAA [ 2496]
cp26_PAbe_NX_Pl_consensus     AAATAGCTCTCTAATATTGTGTATCACAAGTTTTGCAATAGCAATTCTAAAAAAATTTCCTATTCTTTTTCCTGCAAA [ 2496]

cp26_gi|11497007|B31_GB       AGTAGAGCCTTATCATACGCTTTAATAAAGGGTAAATACATAAATACTGAAATAATCAATAATAATAAAACTAGAACA [ 2574]
cp26_un9_B31_PacBio           AGTAGAGCCTTATCATACGCTTTAATAAAGGGTAAATACATAAATACTGAAATAATCAATAATAATAAAACTAGAACA [ 2574]
cp26_B31_TS_MP_FG_consensus   AGTAGAGCCTTATCATACGCTTTAATAAAGGGTAAATACATAAATACTGAAATAATCAATAATAATAAAACTAGAACA [ 2574]
cp26_B31_NX_Pl_consensus      AGTAGAGCCTTATCATACGCTTTAATAAAGGGTAAATACATAAATACTGAAATAATCAATAATAATAAAACTAGAACA [ 2574]
cp26_PAli_NX_MP_FG_consensus AGTAGAGCCTTATCATACGCTTTAATAAAGGGTAAATACATAAATACTGAAATAATCAATAATAATAAAACTAGAACA [ 2574]
cp26_PAli_NX_Pl_consensus     AGTAGAGCCTTATCATACGCTTTAATAAAGGGTAAATACATAAATACTGAAATAATCAATAATAATAAAACTAGAACA [ 2574]
cp26_PAbe_NX_MP_FG_consensus AGTAGAGCCTTATCATACGCTTTAATAAAGGGTAAATACATAAATACTGAAATAATCAATAATAATAAAACTAGAACA [ 2574]
cp26_PAbe_NX_Pl_consensus     AGTAGAGCCTTATCATACGCTTTAATAAAGGGTAAATACATAAATACTGAAATAATCAATAATAATAAAACTAGAACA [ 2574]

cp26_gi|11497007|B31_GB       AACGATTTAATATCAAGCCCTGTAGAAAAAAAAGCTGCAATAGGAGCAGGGGTTGTCCATGGAACCAGAGTTCTAACC [ 2652]
cp26_un9_B31_PacBio           AACGATTTAATATCAAGCCCTGTAGAAAAAAAAGCTGCAATAGGAGCAGGGGTTGTCCATGGAACCAGAGTTCTAACC [ 2652]
cp26_B31_TS_MP_FG_consensus   AACGATTTAATATCAAGCCCTGTAGAAAAAAAAGCTGCAATAGGAGCAGGGGTTGTCCATGGAACCAGAGTTCTAACC [ 2652]
cp26_B31_NX_Pl_consensus      AACGATTTAATATCAAGCCCTGTAGAAAAAAAAGCTGCAATAGGAGCAGGGGTTGTCCATGGAACCAGAGTTCTAACC [ 2652]
cp26_PAli_NX_MP_FG_consensus AACGATTTAATATCAAGCCCTGTAGAAAAAAAAGCTGCAATAGGAGCAGGGGTTGTCCATGGAACCAGAGTTCTAACC [ 2652]
cp26_PAli_NX_Pl_consensus     AACGATTTAATATCAAGCCCTGTAGAAAAAAAAGCTGCAATAGGAGCAGGGGTTGTCCATGGAACCAGAGTTCTAACC [ 2652]
cp26_PAbe_NX_MP_FG_consensus AACGATTTAATATCAAGCCCTGTAGAAAAAAAAGCTGCAATAGGAGCAGGGGTTGTCCATGGAACCAGAGTTCTAACC [ 2652]
cp26_PAbe_NX_Pl_consensus     AACGATTTAATATCAAGCCCTGTAGAAAAAAAAGCTGCAATAGGAGCAGGGGTTGTCCATGGAACCAGAGTTCTAACC [ 2652]

cp26_gi|11497007|B31_GB       CTTTCAATAATTCCAAAATTAGTAAGAGTATATGCAACAATTATATTAAATATAGGGATAAGTAAAAAAGGAATGCCT [ 2730]
cp26_un9_B31_PacBio           CTTTCAATAATTCCAAAATTAGTAAGAGTATATGCAACAATTATATTAAATATAGGGATAAGTAAAAAAGGAATGCCT [ 2730]
cp26_B31_TS_MP_FG_consensus   CTTTCAATAATTCCAAAATTAGTAAGAGTATATGCAACAATTATATTAAATATAGGGATAAGTAAAAAAGGAATGCCT [ 2730]
cp26_B31_NX_Pl_consensus      CTTTCAATAATTCCAAAATTAGTAAGAGTATATGCAACAATTATATTAAATATAGGGATAAGTAAAAAAGGAATGCCT [ 2730]
cp26_PAli_NX_MP_FG_consensus CTTTCAATAATTCCAAAATTAGTAAGAGTATATGCAACAATTATATTAAATATAGGGATAAGTAAAAAAGGAATGCCT [ 2730]
cp26_PAli_NX_Pl_consensus     CTTTCAATAATTCCAAAATTAGTAAGAGTATATGCAACAATTATATTAAATATAGGGATAAGTAAAAAAGGAATGCCT [ 2730]
cp26_PAbe_NX_MP_FG_consensus CTTTCAATAATTCCAAAATTAGTAAGAGTATATGCAACAATTATATTAAATATAGGGATAAGTAAAAAAGGAATGCCT [ 2730]
cp26_PAbe_NX_Pl_consensus     CTTTCAATAATTCCAAAATTAGTAAGAGTATATGCAACAATTATATTAAATATAGGGATAAGTAAAAAAGGAATGCCT [ 2730]
```

```
cp26_gi|11497007|B31_GB      AATATAGGATTTAAAACTATTGGTGCACCGAACATAATAGGTTCATTAATATTAAAAAGACCAGGCGCAAATGAAAGT [ 2808]
cp26_un9_B31_PacBio          AATATAGGATTTAAAACTATTGGTGCACCGAACATAATAGGTTCATTAATATTAAAAAGACCAGGCGCAAATGAAAGT [ 2808]
cp26_B31_TS_MP_FG_consensus  AATATAGGATTTAAAACTATTGGTGCACCGAACATAATAGGTTCATTAATATTAAAAAGACCAGGCGCAAATGAAAGT [ 2808]
cp26_B31_NX_Pl_consensus     AATATAGGATTTAAAACTATTGGTGCACCGAACATAATAGGTTCATTAATATTAAAAAGACCAGGCGCAAATGAAAGT [ 2808]
cp26_PAli_NX_MP_FG_consensus AATATAGGATTTAAAACTATTGGTGCACCGAACATAATAGGTTCATTAATATTAAAAAGACCAGGCGCAAATGAAAGT [ 2808]
cp26_PAli_NX_Pl_consensus    AATATAGGATTTAAAACTATTGGTGCACCGAACATAATAGGTTCATTAATATTAAAAAGACCAGGCGCAAATGAAAGT [ 2808]
cp26_PAbe_NX_MP_FG_consensus AATATAGGATTTAAAACTATTGGTGCACCGAACATAATAGGTTCATTAATATTAAAAAGACCAGGCGCAAATGAAAGT [ 2808]
cp26_PAbe_NX_Pl_consensus    AATATAGGATTTAAAACTATTGGTGCACCGAACATAATAGGTTCATTAATATTAAAAAGACCAGGCGCAAATGAAAGT [ 2808]

cp26_gi|11497007|B31_GB      CTACCTATAGCCTTTAGATGTTGGGATTTACTAAGCATCATAGCAATAGCAAGCCCTAGGGTTGCGCCAGCACCACCA [ 2886]
cp26_un9_B31_PacBio          CTACCTATAGCCTTTAGATGTTGGGATTTACTAAGCATCATAGCAATAGCAAGCCCTAGGGTTGCGCCAGCACCACCA [ 2886]
cp26_B31_TS_MP_FG_consensus  CTACCTATAGCCTTTAGATGTTGGGATTTACTAAGCATCATAGCAATAGCAAGCCCTAGGGTTGCGCCAGCACCACCA [ 2886]
cp26_B31_NX_Pl_consensus     CTACCTATAGCCTTTAGATGTTGGGATTTACTAAGCATCATAGCAATAGCAAGCCCTAGGGTTGCGCCAGCACCACCA [ 2886]
cp26_PAli_NX_MP_FG_consensus CTACCTATAGCCTTTAGATGTTGGGATTTACTAAGCATCATAGCAATAGCAAGCCCTAGGGTTGCGCCAGCACCACCA [ 2886]
cp26_PAli_NX_Pl_consensus    CTACCTATAGCCTTTAGATGTTGGGATTTACTAAGCATCATAGCAATAGCAAGCCCTAGGGTTGCGCCAGCACCACCA [ 2886]
cp26_PAbe_NX_MP_FG_consensus CTACCTATAGCCTTTAGATGTTGGGATTTACTAAGCATCATAGCAATAGCAAGCCCTAGGGTTGCGCCAGCACCACCA [ 2886]
cp26_PAbe_NX_Pl_consensus    CTACCTATAGCCTTTAGATGTTGGGATTTACTAAGCATCATAGCAATAGCAAGCCCTAGGGTTGCGCCAGCACCACCA [ 2886]

cp26_gi|11497007|B31_GB      ATATACACAAATGAATCAAGAAATCCCCCCGCTAAAATATGAGGAAGTGGAAGATTGTCAGAAAGAGCCCTAATATTA [ 2964]
cp26_un9_B31_PacBio          ATATACACAAATGAATCAAGAAATCCCCCCGCTAAAATATGAGGAAGTGGAAGATTGTCAGAAAGAGCCCTAATATTA [ 2964]
cp26_B31_TS_MP_FG_consensus  ATATACACAAATGAATCAAGAAATCCCCCCGCTAAAATATGAGGAAGTGGAAGATTGTCAGAAAGAGCCCTAATATTA [ 2964]
cp26_B31_NX_Pl_consensus     ATATACACAAATGAATCAAGAAATCCCCCCGCTAAAATATGAGGAAGTGGAAGATTGTCAGAAAGAGCCCTAATATTA [ 2964]
cp26_PAli_NX_MP_FG_consensus ATATACACAAATGAATCAAGAAATCCCCCCGCTAAAATATGAGGAAGTGGAAGATTGTCAGAAAGAGCCCTAATATTA [ 2964]
cp26_PAli_NX_Pl_consensus    ATATACACAAATGAATCAAGAAATCCCCCCGCTAAAATATGAGGAAGTGGAAGATTGTCAGAAAGAGCCCTAATATTA [ 2964]
cp26_PAbe_NX_MP_FG_consensus ATATACACAAATGAATCAAGAAATCCCCCCGCTAAAATATGAGGAAGTGGAAGATTGTCAGAAAGAGCCCTAATATTA [ 2964]
cp26_PAbe_NX_Pl_consensus    ATATACACAAATGAATCAAGAAATCCCCCCGCTAAAATATGAGGAAGTGGAAGATTGTCAGAAAGAGCCCTAATATTA [ 2964]

cp26_gi|11497007|B31_GB      GAATCAAGATTTGACAAAATTATAGGATTAAGAAGAGCAATAATAACATTGGTACCATGAAGACCACAAAACCATAAT [ 3042]
cp26_un9_B31_PacBio          GAATCAAGATTTGACAAAATTATAGGATTAAGAAGAGCAATAATAACATTGGTACCATGAAGACCACAAAACCATAAT [ 3042]
cp26_B31_TS_MP_FG_consensus  GAATCAAGATTTGACAAAATTATAGGATTAAGAAGAGCAATAATAACATTGGTACCATGAAGACCACAAAACCATAAT [ 3042]
cp26_B31_NX_Pl_consensus     GAATCAAGATTTGACAAAATTATAGGATTAAGAAGAGCAATAATAACATTGGTACCATGAAGACCACAAAACCATAAT [ 3042]
cp26_PAli_NX_MP_FG_consensus GAATCAAGATTTGACAAAATTATAGGATTAAGAAGAGCAATAATAACATTGGTACCATGAAGACCACAAAACCATAAT [ 3042]
cp26_PAli_NX_Pl_consensus    GAATCAAGATTTGACAAAATTATAGGATTAAGAAGAGCAATAATAACATTGGTACCATGAAGACCACAAAACCATAAT [ 3042]
cp26_PAbe_NX_MP_FG_consensus GAATCAAGATTTGACAAAATTATAGGATTAAGAAGAGCAATAATAACATTGGTACCATGAAGACCACAAAACCATAAT [ 3042]
cp26_PAbe_NX_Pl_consensus    GAATCAAGATTTGACAAAATTATAGGATTAAGAAGAGCAATAATAACATTGGTACCATGAAGACCACAAAACCATAAT [ 3042]

cp26_gi|11497007|B31_GB      ATATGAACAATAAAAGAAATTGTTAAAGTCCCAACTAAAGTATCGCTAATTTGCAAAACAGGCCTAAACATGCTCATA [ 3120]
cp26_un9_B31_PacBio          ATATGAACAATAAAAGAAATTGTTAAAGTCCCAACTAAAGTATCGCTAATTTGCAAAACAGGCCTAAACATGCTCATA [ 3120]
cp26_B31_TS_MP_FG_consensus  ATATGAACAATAAAAGAAATTGTTAAAGTCCCAACTAAAGTATCGCTAATTTGCAAAACAGGCCTAAACATGCTCATA [ 3120]
cp26_B31_NX_Pl_consensus     ATATGAACAATAAAAGAAATTGTTAAAGTCCCAACTAAAGTATCGCTAATTTGCAAAACAGGCCTAAACATGCTCATA [ 3120]
cp26_PAli_NX_MP_FG_consensus ATATGAACAATAAAAGAAATTGTTAAAGTCCCAACTAAAGTATCGCTAATTTGCAAAACAGGCCTAAACATGCTCATA [ 3120]
cp26_PAli_NX_Pl_consensus    ATATGAACAATAAAAGAAATTGTTAAAGTCCCAACTAAAGTATCGCTAATTTGCAAAACAGGCCTAAACATGCTCATA [ 3120]
cp26_PAbe_NX_MP_FG_consensus ATATGAACAATAAAAGAAATTGTTAAAGTCCCAACTAAAGTATCGCTAATTTGCAAAACAGGCCTAAACATGCTCATA [ 3120]
cp26_PAbe_NX_Pl_consensus    ATATGAACAATAAAAGAAATTGTTAAAGTCCCAACTAAAGTATCGCTAATTTGCAAAACAGGCCTAAACATGCTCATA [ 3120]

cp26_gi|11497007|B31_GB      ATTATTTCGGGAAAAAGGCTTCCTAAAGAACTTTGAATAGCAATATTAACACTTTGAGCTACAATTGAAAGCGCAACA [ 3198]
cp26_un9_B31_PacBio          ATTATTTCGGGAAAAAGGCTTCCTAAAGAACTTTGAATAGCAATATTAACACTTTGAGCTACAATTGAAAGCGCAACA [ 3198]
cp26_B31_TS_MP_FG_consensus  ATTATTTCGGGAAAAAGGCTTCCTAAAGAACTTTGAATAGCAATATTAACACTTTGAGCTACAATTGAAAGCGCAACA [ 3198]
cp26_B31_NX_Pl_consensus     ATTATTTCGGGAAAAAGGCTTCCTAAAGAACTTTGAATAGCAATATTAACACTTTGAGCTACAATTGAAAGCGCAACA [ 3198]
cp26_PAli_NX_MP_FG_consensus ATTATTTCGGGAAAAAGGCTTCCTAAAGAACTTTGAATAGCAATATTAACACTTTGAGCTACAATTGAAAGCGCAACA [ 3198]
cp26_PAli_NX_Pl_consensus    ATTATTTCGGGAAAAAGGCTTCCTAAAGAACTTTGAATAGCAATATTAACACTTTGAGCTACAATTGAAAGCGCAACA [ 3198]
cp26_PAbe_NX_MP_FG_consensus ATTATTTCGGGAAAAAGGCTTCCTAAAGAACTTTGAATAGCAATATTAACACTTTGAGCTACAATTGAAAGCGCAACA [ 3198]
cp26_PAbe_NX_Pl_consensus    ATTATTTCGGGAAAAAGGCTTCCTAAAGAACTTTGAATAGCAATATTAACACTTTGAGCTACAATTGAAAGCGCAACA [ 3198]

cp26_gi|11497007|B31_GB      ACAGGAATTAAAGCTTCAAAAGATTTTAAAACAGCAGGCGGAACAGACTCTGGAAGCTTAATTGCCATATTTTTTTGA [ 3276]
cp26_un9_B31_PacBio          ACAGGAATTAAAGCTTCAAAAGATTTTAAAACAGCAGGCGGAACAGACTCTGGAAGCTTAATTGCCATATTTTTTTGA [ 3276]
cp26_B31_TS_MP_FG_consensus  ACAGGAATTAAAGCTTCAAAAGATTTTAAAACAGCAGGCGGAACAGACTCTGGAAGCTTAATTGCCATATTTTTTTGA [ 3276]
cp26_B31_NX_Pl_consensus     ACAGGAATTAAAGCTTCAAAAGATTTTAAAACAGCAGGCGGAACAGACTCTGGAAGCTTAATTGCCATATTTTTTTGA [ 3276]
cp26_PAli_NX_MP_FG_consensus ACAGGAATTAAAGCTTCAAAAGATTTTAAAACAGCAGGCGGAACAGACTCTGGAAGCTTAATTGCCATATTTTTTTGA [ 3276]
cp26_PAli_NX_Pl_consensus    ACAGGAATTAAAGCTTCAAAAGATTTTAAAACAGCAGGCGGAACAGACTCTGGAAGCTTAATTGCCATATTTTTTTGA [ 3276]
cp26_PAbe_NX_MP_FG_consensus ACAGGAATTAAAGCTTCAAAAGATTTTAAAACAGCAGGCGGAACAGACTCTGGAAGCTTAATTGCCATATTTTTTTGA [ 3276]
cp26_PAbe_NX_Pl_consensus    ACAGGAATTAAAGCTTCAAAAGATTTTAAAACAGCAGGCGGAACAGACTCTGGAAGCTTAATTGCCATATTTTTTTGA [ 3276]

cp26_gi|11497007|B31_GB      ACTAAAAATTTATAAACCTCAACAGAAAAAATAGCAGCAATAATAGCCGTAAATACTCCTTGAGCACTAAAATATCTT [ 3354]
cp26_un9_B31_PacBio          ACTAAAAATTTATAAACCTCAACAGAAAAAATAGCAGCAATAATAGCCGTAAATACTCCTTGAGCACTAAAATATCTT [ 3354]
cp26_B31_TS_MP_FG_consensus  ACTAAAAATTTATAAACCTCAACAGAAAAAATAGCAGCAATAATAGCCGTAAATACTCCTTGAGCACTAAAATATCTT [ 3354]
cp26_B31_NX_Pl_consensus     ACTAAAAATTTATAAACCTCAACAGAAAAAATAGCAGCAATAATAGCCGTAAATACTCCTTGAGCACTAAAATATCTT [ 3354]
cp26_PAli_NX_MP_FG_consensus ACTAAAAATTTATAAACCTCAACAGAAAAAATAGCAGCAATAATAGCCGTAAATACTCCTTGAGCACTAAAATATCTT [ 3354]
cp26_PAli_NX_Pl_consensus    ACTAAAAATTTATAAACCTCAACAGAAAAAATAGCAGCAATAATAGCCGTAAATACTCCTTGAGCACTAAAATATCTT [ 3354]
cp26_PAbe_NX_MP_FG_consensus ACTAAAAATTTATAAACCTCAACAGAAAAAATAGCAGCAATAATAGCCGTAAATACTCCTTGAGCACTAAAATATCTT [ 3354]
cp26_PAbe_NX_Pl_consensus    ACTAAAAATTTATAAACCTCAACAGAAAAAATAGCAGCAATAATAGCCGTAAATACTCCTTGAGCACTAAAATATCTT [ 3354]

cp26_gi|11497007|B31_GB      GCATCAATTACAGGAAACCATGAATTGGGCTGAATTCCCCATTTAGCAGCATCTCCACCGTAAGGTATCCAATCTGAT [ 3432]
cp26_un9_B31_PacBio          GCATCAATTACAGGAAACCATGAATTGGGCTGAATTCCCCATTTAGCAGCATCTCCACCGTAAGGTATCCAATCTGAT [ 3432]
cp26_B31_TS_MP_FG_consensus  GCATCAATTACAGGAAACCATGAATTGGGCTGAATTCCCCATTTAGCAGCATCTCCACCGTAAGGTATCCAATCTGAT [ 3432]
cp26_B31_NX_Pl_consensus     GCATCAATTACAGGAAACCATGAATTGGGCTGAATTCCCCATTTAGCAGCATCTCCACCGTAAGGTATCCAATCTGAT [ 3432]
cp26_PAli_NX_MP_FG_consensus GCATCAATTACAGGAAACCATGAATTGGGCTGAATTCCCCATTTAGCAGCATCTCCACCGTAAGGTATCCAATCTGAT [ 3432]
cp26_PAli_NX_Pl_consensus    GCATCAATTACAGGAAACCATGAATTGGGCTGAATTCCCCATTTAGCAGCATCTCCACCGTAAGGTATCCAATCTGAT [ 3432]
cp26_PAbe_NX_MP_FG_consensus GCATCAATTACAGGAAACCATGAATTGGGCTGAATTCCCCATTTAGCAGCATCTCCACCGTAAGGTATCCAATCTGAT [ 3432]
cp26_PAbe_NX_Pl_consensus    GCATCAATTACAGGAAACCATGAATTGGGCTGAATTCCCCATTTAGCAGCATCTCCACCGTAAGGTATCCAATCTGAT [ 3432]

cp26_gi|11497007|B31_GB      TGTCCAGCTAAAATTAAAAATGTATAAAGAGATAAAAATCCTCCTGTAATCCCACTAAGTTTATAATGATTAGATAAG [ 3510]
cp26_un9_B31_PacBio          TGTCCAGCTAAAATTAAAAATGTATAAAGAGATAAAAATCCTCCTGTAATCCCACTAAGTTTATAATGATTAGATAAG [ 3510]
cp26_B31_TS_MP_FG_consensus  TGTCCAGCTAAAATTAAAAATGTATAAAGAGATAAAAATCCTCCTGTAATCCCACTAAGTTTATAATGATTAGATAAG [ 3510]
cp26_B31_NX_Pl_consensus     TGTCCAGCTAAAATTAAAAATGTATAAAGAGATAAAAATCCTCCTGTAATCCCACTAAGTTTATAATGATTAGATAAG [ 3510]
cp26_PAli_NX_MP_FG_consensus TGTCCAGCTAAAATTAAAAATGTATAAAGAGATAAAAATCCTCCTGTAATCCCACTAAGTTTATAATGATTAGATAAG [ 3510]
cp26_PAli_NX_Pl_consensus    TGTCCAGCTAAAATTAAAAATGTATAAAGAGATAAAAATCCTCCTGTAATCCCACTAAGTTTATAATGATTAGATAAG [ 3510]
cp26_PAbe_NX_MP_FG_consensus TGTCCAGCTAAAATTAAAAATGTATAAAGAGATAAAAATCCTCCTGTAATCCCACTAAGTTTATAATGATTAGATAAG [ 3510]
cp26_PAbe_NX_Pl_consensus    TGTCCAGCTAAAATTAAAAATGTATAAAGAGATAAAAATCCTCCTGTAATCCCACTAAGTTTATAATGATTAGATAAG [ 3510]

cp26_gi|11497007|B31_GB      TTATAACCAATACCAAAAACAACAAATATAGACATAATACCCATACTTACATAAAATGGCTGAACAAGATTTCCTTTA [ 3588]
cp26_un9_B31_PacBio          TTATAACCAATACCAAAAACAACAAATATAGACATAATACCCATACTTACATAAAATGGCTGAACAAGATTTCCTTTA [ 3588]
cp26_B31_TS_MP_FG_consensus  TTATAACCAATACCAAAAACAACAAATATAGACATAATACCCATACTTACATAAAATGGCTGAACAAGATTTCCTTTA [ 3588]
cp26_B31_NX_Pl_consensus     TTATAACCAATACCAAAAACAACAAATATAGACATAATACCCATACTTACATAAAATGGCTGAACAAGATTTCCTTTA [ 3588]
cp26_PAli_NX_MP_FG_consensus TTATAACCAATACCAAAAACAACAAATATAGACATAATACCCATACTTACATAAAATGGCTGAACAAGATTTCCTTTA [ 3588]
cp26_PAli_NX_Pl_consensus    TTATAACCAATACCAAAAACAACAAATATAGACATAATACCCATACTTACATAAAATGGCTGAACAAGATTTCCTTTA [ 3588]
cp26_PAbe_NX_MP_FG_consensus TTATAACCAATACCAAAAACAACAAATATAGACATAATACCCATACTTACATAAAATGGCTGAACAAGATTTCCTTTA [ 3588]
cp26_PAbe_NX_Pl_consensus    TTATAACCAATACCAAAAACAACAAATATAGACATAATACCCATACTTACATAAAATGGCTGAACAAGATTTCCTTTA [ 3588]

cp26_gi|11497007|B31_GB      TATTTAGCCATTAAATCAACATACCACTGTTGGTATAACAATGTTTGAGAATCTGTAAAGGGCAAATTAACTAAAAGT [ 3666]
cp26_un9_B31_PacBio          TATTTAGCCATTAAATCAACATACCACTGTTGGTATAACAATGTTTGAGAATCTGTAAAGGGCAAATTAACTAAAAGT [ 3666]
cp26_B31_TS_MP_FG_consensus  TATTTAGCCATTAAATCAACATACCACTGTTGGTATAACAATGTTTGAGAATCTGTAAAGGGCAAATTAACTAAAAGT [ 3666]
cp26_B31_NX_Pl_consensus     TATTTAGCCATTAAATCAACATACCACTGTTGGTATAACAATGTTTGAGAATCTGTAAAGGGCAAATTAACTAAAAGT [ 3666]
cp26_PAli_NX_MP_FG_consensus TATTTAGCCATTAAATCAACATACCACTGTTGGTATAACAATGTTTGAGAATCTGTAAAGGGCAAATTAACTAAAAGT [ 3666]
cp26_PAli_NX_Pl_consensus    TATTTAGCCATTAAATCAACATACCACTGTTGGTATAACAATGTTTGAGAATCTGTAAAGGGCAAATTAACTAAAAGT [ 3666]
cp26_PAbe_NX_MP_FG_consensus TATTTAGCCATTAAATCAACATACCACTGTTGGTATAACAATGTTTGAGAATCTGTAAAGGGCAAATTAACTAAAAGT [ 3666]
cp26_PAbe_NX_Pl_consensus    TATTTAGCCATTAAATCAACATACCACTGTTGGTATAACAATGTTTGAGAATCTGTAAAGGGCAAATTAACTAAAAGT [ 3666]
```

```
cp26_gi|11497007|B31_GB      AAAATAAAAGAACCAACTATTAAAAAGGGCATAGAAAAAGTAAAGCCATCTCTTAAAGCAATTAAATATCTATTTGAA [ 3744]
cp26_un9_B31_PacBio          AAAATAAAAGAACCAACTATTAAAAAGGGCATAGAAAAAGTAAAGCCATCTCTTAAAGCAATTAAATATCTATTTGAA [ 3744]
cp26_B31_TS_MP_FG_consensus  AAAATAAAAGAACCAACTATTAAAAAGGGCATAGAAAAAGTAAAGCCATCTCTTAAAGCAATTAAATATCTATTTGAA [ 3744]
cp26_B31_NX_Pl_consensus     AAAATAAAAGAACCAACTATTAAAAAGGGCATAGAAAAAGTAAAGCCATCTCTTAAAGCAATTAAATATCTATTTGAA [ 3744]
cp26_PAli_NX_MP_FG_consensus AAAATAAAAGAACCAACTATTAAAAAGGGCATAGAAAAAGTAAAGCCATCTCTTAAAGCAATTAAATATCTATTTGAA [ 3744]
cp26_PAli_NX_Pl_consensus    AAAATAAAAGAACCAACTATTAAAAAGGGCATAGAAAAAGTAAAGCCATCTCTTAAAGCAATTAAATATCTATTTGAA [ 3744]
cp26_PAbe_NX_MP_FG_consensus AAAATAAAAGAACCAACTATTAAAAAGGGCATAGAAAAAGTAAAGCCATCTCTTAAAGCAATTAAATATCTATTTGAA [ 3744]
cp26_PAbe_NX_Pl_consensus    AAAATAAAAGAACCAACTATTAAAAAGGGCATAGAAAAAGTAAAGCCATCTCTTAAAGCAATTAAATATCTATTTGAA [ 3744]

cp26_gi|11497007|B31_GB      CCAATTTTGCTAGCAATAGGAACTAAAGTAGTTTCAATAAAATCTTGAAAATTCATGAAAACCATCCTCCAATAAAAA [ 3822]
cp26_un9_B31_PacBio          CCAATTTTGCTAGCAATAGGAACTAAAGTAGTTTCAATAAAATCTTGAAAATTCATGAAAACCATCCTCCAATAAAAA [ 3822]
cp26_B31_TS_MP_FG_consensus  CCAATTTTGCTAGCAATAGGAACTAAAGTAGTTTCAATAAAATCTTGAAAATTCATGAAAACCATCCTCCAATAAAAA [ 3822]
cp26_B31_NX_Pl_consensus     CCAATTTTGCTAGCAATAGGAACTAAAGTAGTTTCAATAAAATCTTGAAAATTCATGAAAACCATCCTCCAATAAAAA [ 3822]
cp26_PAli_NX_MP_FG_consensus CCAATTTTGCTAGCAATAGGAACTAAAGTAGTTTCAATAAAATCTTGAAAATTCATGAAAACCATCCTCCAATAAAAA [ 3822]
cp26_PAli_NX_Pl_consensus    CCAATTTTGCTAGCAATAGGAACTAAAGTAGTTTCAATAAAATCTTGAAAATTCATGAAAACCATCCTCCAATAAAAA [ 3822]
cp26_PAbe_NX_MP_FG_consensus CCAATTTTGCTAGCAATAGGAACTAAAGTAGTTTCAATAAAATCTTGAAAATTCATGAAAACCATCCTCCAATAAAAA [ 3822]
cp26_PAbe_NX_Pl_consensus    CCAATTTTGCTAGCAATAGGAACTAAAGTAGTTTCAATAAAATCTTGAAAATTCATGAAAACCATCCTCCAATAAAAA [ 3822]

cp26_gi|11497007|B31_GB      CTAATTAAAAGTTATAATTAATAACTTACAATATTTATTATAATATAATTTATCTCTCAATAAAAACTAATTAAAAGT [ 3900]
cp26_un9_B31_PacBio          CTAATTAAAAGTTATAATTAATAACTTACAATATTTATTATAATATAATTTATCTCTCAATAAAAACTAATTAAAAGT [ 3900]
cp26_B31_TS_MP_FG_consensus  CTAATTAAAAGTTATAATTAATAACTTACAATATTTATTATAATATAATTTATCTCTCAATAAAAACTAATTAAAAGT [ 3900]
cp26_B31_NX_Pl_consensus     CTAATTAAAAGTTATAATTAATAACTTACAATATTTATTATAATATAATTTATCTCTCAATAAAAACTAATTAAAAGT [ 3900]
cp26_PAli_NX_MP_FG_consensus CTAATTAAAAGTTATAATTAATAACTTACAATATTTATTATAATATAATTTATCTCTCAATAAAAACTAATTAAAAGT [ 3900]
cp26_PAli_NX_Pl_consensus    CTAATTAAAAGTTATAATTAATAACTTACAATATTTATTATAATATAATTTATCTCTCAATAAAAACTAATTAAAAGT [ 3900]
cp26_PAbe_NX_MP_FG_consensus CTAATTAAAAGTTATAATTAATAACTTACAATATTTATTATAATATAATTTATCTCTCAATAAAAACTAATTAAAAGT [ 3900]
cp26_PAbe_NX_Pl_consensus    CTAATTAAAAGTTATAATTAATAACTTACAATATTTATTATAATATAATTTATCTCTCAATAAAAACTAATTAAAAGT [ 3900]

cp26_gi|11497007|B31_GB      TATAATTAATGACTTACAATATTTATAATAACATGGTATAATTAATAGCAATGATAATAAAATCCTTATAAGATATAT [ 3978]
cp26_un9_B31_PacBio          TATAATTAATGACTTACAATATTTATAATAACATGGTATAATTAATAGCAATGATAATAAAATCCTTATAAGATATAT [ 3978]
cp26_B31_TS_MP_FG_consensus  TATAATTAATGACTTACAATATTTATAATAACATGGTATAATTAATAGCAATGATAATAAAATCCTTATAAGATATAT [ 3978]
cp26_B31_NX_Pl_consensus     TATAATTAATGACTTACAATATTTATAATAACATGGTATAATTAATAGCAATGATAATAAAATCCTTATAAGATATAT [ 3978]
cp26_PAli_NX_MP_FG_consensus TATAATTAATGACTTACAATATTTATAATAACATGGTATAATTAATAGCAATGATAATAAAATCCTTATAAGATATAT [ 3978]
cp26_PAli_NX_Pl_consensus    TATAATTAATGACTTACAATATTTATAATAACATGGTATAATTAATAGCAATGATAATAAAATCCTTATAAGATATAT [ 3978]
cp26_PAbe_NX_MP_FG_consensus TATAATTAATGACTTACAATATTTATAATAACATGGTATAATTAATAGCAATGATAATAAAATCCTTATAAGATATAT [ 3978]
cp26_PAbe_NX_Pl_consensus    TATAATTAATGACTTACAATATTTATAATAACATGGTATAATTAATAGCAATGATAATAAAATCCTTATAAGATATAT [ 3978]

cp26_gi|11497007|B31_GB      TTATAATAAAATTATTTATTTTAAATATATCTTATAATATAATATTTGTAAGATATATTAAATAATATTAATTATTGC [ 4056]
cp26_un9_B31_PacBio          TTATAATAAAATTATTTATTTTAAATATATCTTATAATATAATATTTGTAAGATATATTAAATAATATTAATTATTGC [ 4056]
cp26_B31_TS_MP_FG_consensus  TTATAATAAAATTATTTATTTTAAATATATCTTATAATATAATATTTGTAAGATATATTAAATAATATTAATTATTGC [ 4056]
cp26_B31_NX_Pl_consensus     TTATAATAAAATTATTTATTTTAAATATATCTTATAATATAATATTTGTAAGATATATTAAATAATATTAATTATTGC [ 4056]
cp26_PAli_NX_MP_FG_consensus TTATAATAAAATTATTTATTTTAAATATATCTTATAATATAATATTTGTAAGATATATTAAATAATATTAATTATTGC [ 4056]
cp26_PAli_NX_Pl_consensus    TTATAATAAAATTATTTATTTTAAATATATCTTATAATATAATATTTGTAAGATATATTAAATAATATTAATTATTGC [ 4056]
cp26_PAbe_NX_MP_FG_consensus TTATAATAAAATTATTTATTTTAAATATATCTTATAATATAATATTTGTAAGATATATTAAATAATATTAATTATTGC [ 4056]
cp26_PAbe_NX_Pl_consensus    TTATAATAAAATTATTTATTTTAAATATATCTTATAATATAATATTTGTAAGATATATTAAATAATATTAATTATTGC [ 4056]

cp26_gi|11497007|B31_GB      TTTATAAATAAAAGGAGTATATTTTATACATGAATAAAAAAATATATAGCATAGAAGAATTAATAGACAAAATAAGCA [ 4134]
cp26_un9_B31_PacBio          TTTATAAATAAAAGGAGTATATTTTATACATGAATAAAAAAATATATAGCATAGAAGAATTAATAGACAAAATAAGCA [ 4134]
cp26_B31_TS_MP_FG_consensus  TTTATAAATAAAAGGAGTATATTTTATACATGAATAAAAAAATATATAGCATAGAAGAATTAATAGACAAAATAAGCA [ 4134]
cp26_B31_NX_Pl_consensus     TTTATAAATAAAAGGAGTATATTTTATACATGAATAAAAAAATATATAGCATAGAAGAATTAATAGACAAAATAAGCA [ 4134]
cp26_PAli_NX_MP_FG_consensus TTTATAAATAAAAGGAGTATATTTTATACATGAATAAAAAAATATATAGCATAGAAGAATTAATAGACAAAATAAGCA [ 4134]
cp26_PAli_NX_Pl_consensus    TTTATAAATAAAAGGAGTATATTTTATACATGAATAAAAAAATATATAGCATAGAAGAATTAATAGACAAAATAAGCA [ 4134]
cp26_PAbe_NX_MP_FG_consensus TTTATAAATAAAAGGAGTATATTTTATACATGAATAAAAAAATATATAGCATAGAAGAATTAATAGACAAAATAAGCA [ 4134]
cp26_PAbe_NX_Pl_consensus    TTTATAAATAAAAGGAGTATATTTTATACATGAATAAAAAAATATATAGCATAGAAGAATTAATAGACAAAATAAGCA [ 4134]

cp26_gi|11497007|B31_GB      TGCCTGTTGTAGCTTACTCTGGTGAGGCTAAAAGCTTTCTAAGAGAAGCTTTAGAATATGCCAAAAACAAAGAATATG [ 4212]
cp26_un9_B31_PacBio          TGCCTGTTGTAGCTTACTCTGGTGAGGCTAAAAGCTTTCTAAGAGAAGCTTTAGAATATGCCAAAAACAAAGAATATG [ 4212]
cp26_B31_TS_MP_FG_consensus  TGCCTGTTGTAGCTTACTCTGGTGAGGCTAAAAGCTTTCTAAGAGAAGCTTTAGAATATGCCAAAAACAAAGAATATG [ 4212]
cp26_B31_NX_Pl_consensus     TGCCTGTTGTAGCTTACTCTGGTGAGGCTAAAAGCTTTCTAAGAGAAGCTTTAGAATATGCCAAAAACAAAGAATATG [ 4212]
cp26_PAli_NX_MP_FG_consensus TGCCTGTTGTAGCTTACTCTGGTGAGGCTAAAAGCTTTCTAAGAGAAGCTTTAGAATATGCCAAAAACAAAGAATATG [ 4212]
cp26_PAli_NX_Pl_consensus    TGCCTGTTGTAGCTTACTCTGGTGAGGCTAAAAGCTTTCTAAGAGAAGCTTTAGAATATGCCAAAAACAAAGAATATG [ 4212]
cp26_PAbe_NX_MP_FG_consensus TGCCTGTTGTAGCTTACTCTGGTGAGGCTAAAAGCTTTCTAAGAGAAGCTTTAGAATATGCCAAAAACAAAGAATATG [ 4212]
cp26_PAbe_NX_Pl_consensus    TGCCTGTTGTAGCTTACTCTGGTGAGGCTAAAAGCTTTCTAAGAGAAGCTTTAGAATATGCCAAAAACAAAGAATATG [ 4212]

cp26_gi|11497007|B31_GB      ACAAGGCAGAGCTTACTATACAAGAAAGTAAAAAATCCATTGCAAAGGCCCATGAAGCACACAGAGAAATAATACATC [ 4290]
cp26_un9_B31_PacBio          ACAAGGCAGAGCTTACTATACAAGAAAGTAAAAAATCCATTGCAAAGGCCCATGAAGCACACAGAGAAATAATACATC [ 4290]
cp26_B31_TS_MP_FG_consensus  ACAAGGCAGAGCTTACTATACAAGAAAGTAAAAAATCCATTGCAAAGGCCCATGAAGCACACAGAGAAATAATACATC [ 4290]
cp26_B31_NX_Pl_consensus     ACAAGGCAGAGCTTACTATACAAGAAAGTAAAAAATCCATTGCAAAGGCCCATGAAGCACACAGAGAAATAATACATC [ 4290]
cp26_PAli_NX_MP_FG_consensus ACAAGGCAGAGCTTACTATACAAGAAAGTAAAAAATCCATTGCAAAGGCCCATGAAGCACACAGAGAAATAATACATC [ 4290]
cp26_PAli_NX_Pl_consensus    ACAAGGCAGAGCTTACTATACAAGAAAGTAAAAAATCCATTGCAAAGGCCCATGAAGCACACAGAGAAATAATACATC [ 4290]
cp26_PAbe_NX_MP_FG_consensus ACAAGGCAGAGCTTACTATACAAGAAAGTAAAAAATCCATTGCAAAGGCCCATGAAGCACACAGAGAAATAATACATC [ 4290]
cp26_PAbe_NX_Pl_consensus    ACAAGGCAGAGCTTACTATACAAGAAAGTAAAAAATCCATTGCAAAGGCCCATGAAGCACACAGAGAAATAATACATC [ 4290]

cp26_gi|11497007|B31_GB      AATCAGCCACTAATCCAAACTCTGTCAAAACGCCTTTTATTTTAATTCATGCTGAAGATCATTTAATGTCTGCAATTT [ 4368]
cp26_un9_B31_PacBio          AATCAGCCACTAATCCAAACTCTGTCAAAACGCCTTTTATTTTAATTCATGCTGAAGATCATTTAATGTCTGCAATTT [ 4368]
cp26_B31_TS_MP_FG_consensus  AATCAGCCACTAATCCAAACTCTGTCAAAACGCCTTTTATTTTAATTCATGCTGAAGATCATTTAATGTCTGCAATTT [ 4368]
cp26_B31_NX_Pl_consensus     AATCAGCCACTAATCCAAACTCTGTCAAAACGCCTTTTATTTTAATTCATGCTGAAGATCATTTAATGTCTGCAATTT [ 4368]
cp26_PAli_NX_MP_FG_consensus AATCAGCCACTAATCCAAACTCTGTCAAAACGCCTTTTATTTTAATTCATGCTGAAGATCATTTAATGTCTGCAATTT [ 4368]
cp26_PAli_NX_Pl_consensus    AATCAGCCACTAATCCAAACTCTGTCAAAACGCCTTTTATTTTAATTCATGCTGAAGATCATTTAATGTCTGCAATTT [ 4368]
cp26_PAbe_NX_MP_FG_consensus AATCAGCCACTAATCCAAACTCTGTCAAAACGCCTTTTATTTTAATTCATGCTGAAGATCATTTAATGTCTGCAATTT [ 4368]
cp26_PAbe_NX_Pl_consensus    AATCAGCCACTAATCCAAACTCTGTCAAAACGCCTTTTATTTTAATTCATGCTGAAGATCATTTAATGTCTGCAATTT [ 4368]

cp26_gi|11497007|B31_GB      CAGAATTAAGCATTTTTGAAGAATTAATCAATGTTTACAAAATAATAAATGAAATAAAAAAATAGGAGAAAATATGAA [ 4446]
cp26_un9_B31_PacBio          CAGAATTAAGCATTTTTGAAGAATTAATCAATGTTTACAAAATAATAAATGAAATAAAAAAATAGGAGAAAATATGAA [ 4446]
cp26_B31_TS_MP_FG_consensus  CAGAATTAAGCATTTTTGAAGAATTAATCAATGTTTACAAAATAATAAATGAAATAAAAAAATAGGAGAAAATATGAA [ 4446]
cp26_B31_NX_Pl_consensus     CAGAATTAAGCATTTTTGAAGAATTAATCAATGTTTACAAAATAATAAATGAAATAAAAAAATAGGAGAAAATATGAA [ 4446]
cp26_PAli_NX_MP_FG_consensus CAGAATTAAGCATTTTTGAAGAATTAATCAATGTTTACAAAATAATAAATGAAATAAAAAAATAGGAGAAAATATGAA [ 4446]
cp26_PAli_NX_Pl_consensus    CAGAATTAAGCATTTTTGAAGAATTAATCAATGTTTACAAAATAATAAATGAAATAAAAAAATAGGAGAAAATATGAA [ 4446]
cp26_PAbe_NX_MP_FG_consensus CAGAATTAAGCATTTTTGAAGAATTAATCAATGTTTACAAAATAATAAATGAAATAAAAAAATAGGAGAAAATATGAA [ 4446]
cp26_PAbe_NX_Pl_consensus    CAGAATTAAGCATTTTTGAAGAATTAATCAATGTTTACAAAATAATAAATGAAATAAAAAAATAGGAGAAAATATGAA [ 4446]

cp26_gi|11497007|B31_GB      CATACTACTTGTATGTGGAGCTGGGATGTCCACAAGTATGCTGGTACAAAGAATTGAAAAATATGCCAAATCAAAAAA [ 4524]
cp26_un9_B31_PacBio          CATACTACTTGTATGTGGAGCTGGGATGTCCACAAGTATGCTGGTACAAAGAATTGAAAAATATGCCAAATCAAAAAA [ 4524]
cp26_B31_TS_MP_FG_consensus  CATACTACTTGTATGTGGAGCTGGGATGTCCACAAGTATGCTGGTACAAAGAATTGAAAAATATGCCAAATCAAAAAA [ 4524]
cp26_B31_NX_Pl_consensus     CATACTACTTGTATGTGGAGCTGGGATGTCCACAAGTATGCTGGTACAAAGAATTGAAAAATATGCCAAATCAAAAAA [ 4524]
cp26_PAli_NX_MP_FG_consensus CATACTACTTGTATGTGGAGCTGGGATGTCCACAAGTATGCTGGTACAAAGAATTGAAAAATATGCCAAATCAAAAAA [ 4524]
cp26_PAli_NX_Pl_consensus    CATACTACTTGTATGTGGAGCTGGGATGTCCACAAGTATGCTGGTACAAAGAATTGAAAAATATGCCAAATCAAAAAA [ 4524]
cp26_PAbe_NX_MP_FG_consensus CATACTACTTGTATGTGGAGCTGGGATGTCCACAAGTATGCTGGTACAAAGAATTGAAAAATATGCCAAATCAAAAAA [ 4524]
cp26_PAbe_NX_Pl_consensus    CATACTACTTGTATGTGGAGCTGGGATGTCCACAAGTATGCTGGTACAAAGAATTGAAAAATATGCCAAATCAAAAAA [ 4524]

cp26_gi|11497007|B31_GB      TATAAATGCAACAATTGAAGCTATTGCTGAGACACGCCTTAGCGAAGTTGTTGACCGATTTGACGTTGTTTTACTTGC [ 4602]
cp26_un9_B31_PacBio          TATAAATGCAACAATTGAAGCTATTGCTGAGACACGCCTTAGCGAAGTTGTTGACCGATTTGACGTTGTTTTACTTGC [ 4602]
cp26_B31_TS_MP_FG_consensus  TATAAATGCAACAATTGAAGCTATTGCTGAGACACGCCTTAGCGAAGTTGTTGACCGATTTGACGTTGTTTTACTTGC [ 4602]
cp26_B31_NX_Pl_consensus     TATAAATGCAACAATTGAAGCTATTGCTGAGACACGCCTTAGCGAAGTTGTTGACCGATTTGACGTTGTTTTACTTGC [ 4602]
cp26_PAli_NX_MP_FG_consensus TATAAATGCAACAATTGAAGCTATTGCTGAGACACGCCTTAGCGAAGTTGTTGACCGATTTGACGTTGTTTTACTTGC [ 4602]
cp26_PAli_NX_Pl_consensus    TATAAATGCAACAATTGAAGCTATTGCTGAGACACGCCTTAGCGAAGTTGTTGACCGATTTGACGTTGTTTTACTTGC [ 4602]
cp26_PAbe_NX_MP_FG_consensus TATAAATGCAACAATTGAAGCTATTGCTGAGACACGCCTTAGCGAAGTTGTTGACCGATTTGACGTTGTTTTACTTGC [ 4602]
cp26_PAbe_NX_Pl_consensus    TATAAATGCAACAATTGAAGCTATTGCTGAGACACGCCTTAGCGAAGTTGTTGACCGATTTGACGTTGTTTTACTTGC [ 4602]
```

```
cp26_gi|11497007|B31_GB      ACCACAGTCAAGATTTAATAAAAAAAGACTTGAAGAAATCACAAAACCCAAAGGAATTCCAATCGAAATAATTAACAC [ 4680]
cp26_un9_B31_PacBio          ACCACAGTCAAGATTTAATAAAAAAAGACTTGAAGAAATCACAAAACCCAAAGGAATTCCAATCGAAATAATTAACAC [ 4680]
cp26_B31_TS_MP_FG_consensus  ACCACAGTCAAGATTTAATAAAAAAAGACTTGAAGAAATCACAAAACCCAAAGGAATTCCAATCGAAATAATTAACAC [ 4680]
cp26_B31_NX_Pl_consensus     ACCACAGTCAAGATTTAATAAAAAAAGACTTGAAGAAATCACAAAACCCAAAGGAATTCCAATCGAAATAATTAACAC [ 4680]
cp26_PAli_NX_MP_FG_consensus ACCACAGTCAAGATTTAATAAAAAAAGACTTGAAGAAATCACAAAACCCAAAGGAATTCCAATCGAAATAATTAACAC [ 4680]
cp26_PAli_NX_Pl_consensus    ACCACAGTCAAGATTTAATAAAAAAAGACTTGAAGAAATCACAAAACCCAAAGGAATTCCAATCGAAATAATTAACAC [ 4680]
cp26_PAbe_NX_MP_FG_consensus ACCACAGTCAAGATTTAATAAAAAAAGACTTGAAGAAATCACAAAACCCAAAGGAATTCCAATCGAAATAATTAACAC [ 4680]
cp26_PAbe_NX_Pl_consensus    ACCACAGTCAAGATTTAATAAAAAAAGACTTGAAGAAATCACAAAACCCAAAGGAATTCCAATCGAAATAATTAACAC [ 4680]

cp26_gi|11497007|B31_GB      AATCGATTATGGAACAATGAATGGCGAAAAAGTATTACAACTTGCAATCAATGCATTTAACAATAAAAGTTCAGTTTA [ 4758]
cp26_un9_B31_PacBio          AATCGATTATGGAACAATGAATGGCGAAAAAGTATTACAACTTGCAATCAATGCATTTAACAATAAAAGTTCAGTTTA [ 4758]
cp26_B31_TS_MP_FG_consensus  AATCGATTATGGAACAATGAATGGCGAAAAAGTATTACAACTTGCAATCAATGCATTTAACAATAAAAGTTCAGTTTA [ 4758]
cp26_B31_NX_Pl_consensus     AATCGATTATGGAACAATGAATGGCGAAAAAGTATTACAACTTGCAATCAATGCATTTAACAATAAAAGTTCAGTTTA [ 4758]
cp26_PAli_NX_MP_FG_consensus AATCGATTATGGAACAATGAATGGCGAAAAAGTATTACAACTTGCAATCAATGCATTTAACAATAAAAGTTCAGTTTA [ 4758]
cp26_PAli_NX_Pl_consensus    AATCGATTATGGAACAATGAATGGCGAAAAAGTATTACAACTTGCAATCAATGCATTTAACAATAAAAGTTCAGTTTA [ 4758]
cp26_PAbe_NX_MP_FG_consensus AATCGATTATGGAACAATGAATGGCGAAAAAGTATTACAACTTGCAATCAATGCATTTAACAATAAAAGTTCAGTTTA [ 4758]
cp26_PAbe_NX_Pl_consensus    AATCGATTATGGAACAATGAATGGCGAAAAAGTATTACAACTTGCAATCAATGCATTTAACAATAAAAGTTCAGTTTA [ 4758]

cp26_gi|11497007|B31_GB      AGGAAGGCTAAAATGAAAGAAATTGGAATATCCATATACCCCAATGTAAGTCCTAAAAATAAAATTATTAAATATCTT [ 4836]
cp26_un9_B31_PacBio          AGGAAGGCTAAAATGAAAGAAATTGGAATATCCATATACCCCAATGTAAGTCCTAAAAATAAAATTATTAAATATCTT [ 4836]
cp26_B31_TS_MP_FG_consensus  AGGAAGGCTAAAATGAAAGAAATTGGAATATCCATATACCCCAATGTAAGTCCTAAAAATAAAATTATTAAATATCTT [ 4836]
cp26_B31_NX_Pl_consensus     AGGAAGGCTAAAATGAAAGAAATTGGAATATCCATATACCCCAATGTAAGTCCTAAAAATAAAATTATTAAATATCTT [ 4836]
cp26_PAli_NX_MP_FG_consensus AGGAAGGCTAAAATGAAAGAAATTGGAATATCCATATACCCCAATGTAAGTCCTAAAAATAAAATTATTAAATATCTT [ 4836]
cp26_PAli_NX_Pl_consensus    AGGAAGGCTAAAATGAAAGAAATTGGAATATCCATATACCCCAATGTAAGTCCTAAAAATAAAATTATTAAATATCTT [ 4836]
cp26_PAbe_NX_MP_FG_consensus AGGAAGGCTAAAATGAAAGAAATTGGAATATCCATATACCCCAATGTAAGTCCTAAAAATAAAATTATTAAATATCTT [ 4836]
cp26_PAbe_NX_Pl_consensus    AGGAAGGCTAAAATGAAAGAAATTGGAATATCCATATACCCCAATGTAAGTCCTAAAAATAAAATTATTAAATATCTT [ 4836]

cp26_gi|11497007|B31_GB      GAAAAAAGTGCTCATTTTGGATTTACTCAAGTATTTACCTCTTTGCTCTATATTAACGGAAATGAATTTGACATATTC [ 4914]
cp26_un9_B31_PacBio          GAAAAAAGTGCTCATTTTGGATTTACTCAAGTATTTACCTCTTTGCTCTATATTAACGGAAATGAATTTGACATATTC [ 4914]
cp26_B31_TS_MP_FG_consensus  GAAAAAAGTGCTCATTTTGGATTTACTCAAGTATTTACCTCTTTGCTCTATATTAACGGAAATGAATTTGACATATTC [ 4914]
cp26_B31_NX_Pl_consensus     GAAAAAAGTGCTCATTTTGGATTTACTCAAGTATTTACCTCTTTGCTCTATATTAACGGAAATGAATTTGACATATTC [ 4914]
cp26_PAli_NX_MP_FG_consensus GAAAAAAGTGCTCATTTTGGATTTACTCAAGTATTTACCTCTTTGCTCTATATTAACGGAAATGAATTTGACATATTC [ 4914]
cp26_PAli_NX_Pl_consensus    GAAAAAAGTGCTCATTTTGGATTTACTCAAGTATTTACCTCTTTGCTCTATATTAACGGAAATGAATTTGACATATTC [ 4914]
cp26_PAbe_NX_MP_FG_consensus GAAAAAAGTGCTCATTTTGGATTTACTCAAGTATTTACCTCTTTGCTCTATATTAACGGAAATGAATTTGACATATTC [ 4914]
cp26_PAbe_NX_Pl_consensus    GAAAAAAGTGCTCATTTTGGATTTACTCAAGTATTTACCTCTTTGCTCTATATTAACGGAAATGAATTTGACATATTC [ 4914]

cp26_gi|11497007|B31_GB      AAAGAATTACTTAGCATTGCAAATAAAAACGGAATGAAACCTATTATTGACGTAAGTCCTGAAATTTTCAAAGAATTA [ 4992]
cp26_un9_B31_PacBio          AAAGAATTACTTAGCATTGCAAATAAAAACGGAATGAAACCTATTATTGACGTAAGTCCTGAAATTTTCAAAGAATTA [ 4992]
cp26_B31_TS_MP_FG_consensus  AAAGAATTACTTAGCATTGCAAATAAAAACGGAATGAAACCTATTATTGACGTAAGTCCTGAAATTTTCAAAGAATTA [ 4992]
cp26_B31_NX_Pl_consensus     AAAGAATTACTTAGCATTGCAAATAAAAACGGAATGAAACCTATTATTGACGTAAGTCCTGAAATTTTCAAAGAATTA [ 4992]
cp26_PAli_NX_MP_FG_consensus AAAGAATTACTTAGCATTGCAAATAAAAACGGAATGAAACCTATTATTGACGTAAGTCCTGAAATTTTCAAAGAATTA [ 4992]
cp26_PAli_NX_Pl_consensus    AAAGAATTACTTAGCATTGCAAATAAAAACGGAATGAAACCTATTATTGACGTAAGTCCTGAAATTTTCAAAGAATTA [ 4992]
cp26_PAbe_NX_MP_FG_consensus AAAGAATTACTTAGCATTGCAAATAAAAACGGAATGAAACCTATTATTGACGTAAGTCCTGAAATTTTCAAAGAATTA [ 4992]
cp26_PAbe_NX_Pl_consensus    AAAGAATTACTTAGCATTGCAAATAAAAACGGAATGAAACCTATTATTGACGTAAGTCCTGAAATTTTCAAAGAATTA [ 4992]

cp26_gi|11497007|B31_GB      GGCATTGATCTTTCAAATCTTAGAAATTGTCCAAAACTAGATTATTTTAAAAAGCTTGGCGCCTGGGCAATTAGATTA [ 5070]
cp26_un9_B31_PacBio          GGCATTGATCTTTCAAATCTTAGAAATTGTCCAAAACTAGATTATTTTAAAAAGCTTGGCGCCTGGGCAATTAGATTA [ 5070]
cp26_B31_TS_MP_FG_consensus  GGCATTGATCTTTCAAATCTTAGAAATTGTCCAAAACTAGATTATTTTAAAAAGCTTGGCGCCTGGGCAATTAGATTA [ 5070]
cp26_B31_NX_Pl_consensus     GGCATTGATCTTTCAAATCTTAGAAATTGTCCAAAACTAGATTATTTTAAAAAGCTTGGCGCCTGGGCAATTAGATTA [ 5070]
cp26_PAli_NX_MP_FG_consensus GGCATTGATCTTTCAAATCTTAGAAATTGTCCAAAACTAGATTATTTTAAAAAGCTTGGCGCCTGGGCAATTAGATTA [ 5070]
cp26_PAli_NX_Pl_consensus    GGCATTGATCTTTCAAATCTTAGAAATTGTCCAAAACTAGATTATTTTAAAAAGCTTGGCGCCTGGGCAATTAGATTA [ 5070]
cp26_PAbe_NX_MP_FG_consensus GGCATTGATCTTTCAAATCTTAGAAATTGTCCAAAACTAGATTATTTTAAAAAGCTTGGCGCCTGGGCAATTAGATTA [ 5070]
cp26_PAbe_NX_Pl_consensus    GGCATTGATCTTTCAAATCTTAGAAATTGTCCAAAACTAGATTATTTTAAAAAGCTTGGCGCCTGGGCAATTAGATTA [ 5070]

cp26_gi|11497007|B31_GB      GACAATACATTCACAGGCATTGAAGAATCATTAATGACTTTTAACGACTCTGACCTTAAGATTCAACTTAATATAAGC [ 5148]
cp26_un9_B31_PacBio          GACAATACATTCACAGGCATTGAAGAATCATTAATGACTTTTAACGACTCTGACCTTAAGATTCAACTTAATATAAGC [ 5148]
cp26_B31_TS_MP_FG_consensus  GACAATACATTCACAGGCATTGAAGAATCATTAATGACTTTTAACGACTCTGACCTTAAGATTCAACTTAATATAAGC [ 5148]
cp26_B31_NX_Pl_consensus     GACAATACATTCACAGGCATTGAAGAATCATTAATGACTTTTAACGACTCTGACCTTAAGATTCAACTTAATATAAGC [ 5148]
cp26_PAli_NX_MP_FG_consensus GACAATACATTCACAGGCATTGAAGAATCATTAATGACTTTTAACGACTCTGACCTTAAGATTCAACTTAATATAAGC [ 5148]
cp26_PAli_NX_Pl_consensus    GACAATACATTCACAGGCATTGAAGAATCATTAATGACTTTTAACGACTCTGACCTTAAGATTCAACTTAATATAAGC [ 5148]
cp26_PAbe_NX_MP_FG_consensus GACAATACATTCACAGGCATTGAAGAATCATTAATGACTTTTAACGACTCTGACCTTAAGATTCAACTTAATATAAGC [ 5148]
cp26_PAbe_NX_Pl_consensus    GACAATACATTCACAGGCATTGAAGAATCATTAATGACTTTTAACGACTCTGACCTTAAGATTCAACTTAATATAAGC [ 5148]

cp26_gi|11497007|B31_GB      AATATAAATAAACATATTGATACAATAATGTATTTTAAGCCTAATATAAAAAATTTACTTGGGTGTCATAATTTTTAT [ 5226]
cp26_un9_B31_PacBio          AATATAAATAAACATATTGATACAATAATGTATTTTAAGCCTAATATAAAAAATTTACTTGGGTGTCATAATTTTTAT [ 5226]
cp26_B31_TS_MP_FG_consensus  AATATAAATAAACATATTGATACAATAATGTATTTTAAGCCTAATATAAAAAATTTACTTGGGTGTCATAATTTTTAT [ 5226]
cp26_B31_NX_Pl_consensus     AATATAAATAAACATATTGATACAATAATGTATTTTAAGCCTAATATAAAAAATTTACTTGGGTGTCATAATTTTTAT [ 5226]
cp26_PAli_NX_MP_FG_consensus AATATAAATAAACATATTGATACAATAATGTATTTTAAGCCTAATATAAAAAATTTACTTGGGTGTCATAATTTTTAT [ 5226]
cp26_PAli_NX_Pl_consensus    AATATAAATAAACATATTGATACAATAATGTATTTTAAGCCTAATATAAAAAATTTACTTGGGTGTCATAATTTTTAT [ 5226]
cp26_PAbe_NX_MP_FG_consensus AATATAAATAAACATATTGATACAATAATGTATTTTAAGCCTAATATAAAAAATTTACTTGGGTGTCATAATTTTTAT [ 5226]
cp26_PAbe_NX_Pl_consensus    AATATAAATAAACATATTGATACAATAATGTATTTTAAGCCTAATATAAAAAATTTACTTGGGTGTCATAATTTTTAT [ 5226]

cp26_gi|11497007|B31_GB      CCTCACAAATATACAGGACTTTCAAGAAATTTCTTTAAAGAAACAACAAAAATATTTAAACATTATTCAATCCCAACA [ 5304]
cp26_un9_B31_PacBio          CCTCACAAATATACAGGACTTTCAAGAAATTTCTTTAAAGAAACAACAAAAATATTTAAACATTATTCAATCCCAACA [ 5304]
cp26_B31_TS_MP_FG_consensus  CCTCACAAATATACAGGACTTTCAAGAAATTTCTTTAAAGAAACAACAAAAATATTTAAACATTATTCAATCCCAACA [ 5304]
cp26_B31_NX_Pl_consensus     CCTCACAAATATACAGGACTTTCAAGAAATTTCTTTAAAGAAACAACAAAAATATTTAAACATTATTCAATCCCAACA [ 5304]
cp26_PAli_NX_MP_FG_consensus CCTCACAAATATACAGGACTTTCAAGAAATTTCTTTAAAGAAACAACAAAAATATTTAAACATTATTCAATCCCAACA [ 5304]
cp26_PAli_NX_Pl_consensus    CCTCACAAATATACAGGACTTTCAAGAAATTTCTTTAAAGAAACAACAAAAATATTTAAACATTATTCAATCCCAACA [ 5304]
cp26_PAbe_NX_MP_FG_consensus CCTCACAAATATACAGGACTTTCAAGAAATTTCTTTAAAGAAACAACAAAAATATTTAAACATTATTCAATCCCAACA [ 5304]
cp26_PAbe_NX_Pl_consensus    CCTCACAAATATACAGGACTTTCAAGAAATTTCTTTAAAGAAACAACAAAAATATTTAAACATTATTCAATCCCAACA [ 5304]

cp26_gi|11497007|B31_GB      GCCGCATTTATTAGCTCTAATAATGCAGAAGAATGCGCACGAGGGAAAGAAAAAGAAGGTGTTCCTACGCTAGAATCA [ 5382]
cp26_un9_B31_PacBio          GCCGCATTTATTAGCTCTAATAATGCAGAAGAATGCGCACGAGGGAAAGAAAAAGAAGGTGTTCCTACGCTAGAATCA [ 5382]
cp26_B31_TS_MP_FG_consensus  GCCGCATTTATTAGCTCTAATAATGCAGAAGAATGCGCACGAGGGAAAGAAAAAGAAGGTGTTCCTACGCTAGAATCA [ 5382]
cp26_B31_NX_Pl_consensus     GCCGCATTTATTAGCTCTAATAATGCAGAAGAATGCGCACGAGGGAAAGAAAAAGAAGGTGTTCCTACGCTAGAATCA [ 5382]
cp26_PAli_NX_MP_FG_consensus GCCGCATTTATTAGCTCTAATAATGCAGAAGAATGCGCACGAGGGAAAGAAAAAGAAGGTGTTCCTACGCTAGAATCA [ 5382]
cp26_PAli_NX_Pl_consensus    GCCGCATTTATTAGCTCTAATAATGCAGAAGAATGCGCACGAGGGAAAGAAAAAGAAGGTGTTCCTACGCTAGAATCA [ 5382]
cp26_PAbe_NX_MP_FG_consensus GCCGCATTTATTAGCTCTAATAATGCAGAAGAATGCGCACGAGGGAAAGAAAAAGAAGGTGTTCCTACGCTAGAATCA [ 5382]
cp26_PAbe_NX_Pl_consensus    GCCGCATTTATTAGCTCTAATAATGCAGAAGAATGCGCACGAGGGAAAGAAAAAGAAGGTGTTCCTACGCTAGAATCA [ 5382]

cp26_gi|11497007|B31_GB      CATAGATCTAAAGATATAGAAACCCAAGCAAAAGATCTTTTCAAAGAAGGAATAGATACAGTCCTAATTTCTAATTGT [ 5460]
cp26_un9_B31_PacBio          CATAGATCTAAAGATATAGAAACCCAAGCAAAAGATCTTTTCAAAGAAGGAATAGATACAGTCCTAATTTCTAATTGT [ 5460]
cp26_B31_TS_MP_FG_consensus  CATAGATCTAAAGATATAGAAACCCAAGCAAAAGATCTTTTCAAAGAAGGAATAGATACAGTCCTAATTTCTAATTGT [ 5460]
cp26_B31_NX_Pl_consensus     CATAGATCTAAAGATATAGAAACCCAAGCAAAAGATCTTTTCAAAGAAGGAATAGATACAGTCCTAATTTCTAATTGT [ 5460]
cp26_PAli_NX_MP_FG_consensus CATAGATCTAAAGATATAGAAACCCAAGCAAAAGATCTTTTCAAAGAAGGAATAGATACAGTCCTAATTTCTAATTGT [ 5460]
cp26_PAli_NX_Pl_consensus    CATAGATCTAAAGATATAGAAACCCAAGCAAAAGATCTTTTCAAAGAAGGAATAGATACAGTCCTAATTTCTAATTGT [ 5460]
cp26_PAbe_NX_MP_FG_consensus CATAGATCTAAAGATATAGAAACCCAAGCAAAAGATCTTTTCAAAGAAGGAATAGATACAGTCCTAATTTCTAATTGT [ 5460]
cp26_PAbe_NX_Pl_consensus    CATAGATCTAAAGATATAGAAACCCAAGCAAAAGATCTTTTCAAAGAAGGAATAGATACAGTCCTAATTTCTAATTGT [ 5460]

cp26_gi|11497007|B31_GB      TTTCCAAGTGAAACAGAACTAAAAAAAGTATCAAAGGTAAACAGAAATATTTTAGAGCTCAAAGCAGACCTAAATCCA [ 5538]
cp26_un9_B31_PacBio          TTTCCAAGTGAAACAGAACTAAAAAAAGTATCAAAGGTAAACAGAAATATTTTAGAGCTCAAAGCAGACCTAAATCCA [ 5538]
cp26_B31_TS_MP_FG_consensus  TTTCCAAGTGAAACAGAACTAAAAAAAGTATCAAAGGTAAACAGAAATATTTTAGAGCTCAAAGCAGACCTAAATCCA [ 5538]
cp26_B31_NX_Pl_consensus     TTTCCAAGTGAAACAGAACTAAAAAAAGTATCAAAGGTAAACAGAAATATTTTAGAGCTCAAAGCAGACCTAAATCCA [ 5538]
cp26_PAli_NX_MP_FG_consensus TTTCCAAGTGAAACAGAACTAAAAAAAGTATCAAAGGTAAACAGAAATATTTTAGAGCTCAAAGCAGACCTAAATCCA [ 5538]
cp26_PAli_NX_Pl_consensus    TTTCCAAGTGAAACAGAACTAAAAAAAGTATCAAAGGTAAACAGAAATATTTTAGAGCTCAAAGCAGACCTAAATCCA [ 5538]
cp26_PAbe_NX_MP_FG_consensus TTTCCAAGTGAAACAGAACTAAAAAAAGTATCAAAGGTAAACAGAAATATTTTAGAGCTCAAAGCAGACCTAAATCCA [ 5538]
cp26_PAbe_NX_Pl_consensus    TTTCCAAGTGAAACAGAACTAAAAAAAGTATCAAAGGTAAACAGAAATATTTTAGAGCTCAAAGCAGACCTAAATCCA [ 5538]
```

```
cp26_gi|11497007|B31_GB      GATGCAAATTCAGTAGAAAAAGAAATAATCCTTGAAAATTTACATTTTAATAGAGGTGACATTAATTCCTATAGAATT [ 5616]
cp26_un9_B31_PacBio          GATGCAAATTCAGTAGAAAAAGAAATAATCCTTGAAAATTTACATTTTAATAGAGGTGACATTAATTCCTATAGAATT [ 5616]
cp26_B31_TS_MP_FG_consensus  GATGCAAATTCAGTAGAAAAAGAAATAATCCTTGAAAATTTACATTTTAATAGAGGTGACATTAATTCCTATAGAATT [ 5616]
cp26_B31_NX_Pl_consensus     GATGCAAATTCAGTAGAAAAAGAAATAATCCTTGAAAATTTACATTTTAATAGAGGTGACATTAATTCCTATAGAATT [ 5616]
cp26_PAli_NX_MP_FG_consensus GATGCAAATTCAGTAGAAAAAGAAATAATCCTTGAAAATTTACATTTTAATAGAGGTGACATTAATTCCTATAGAATT [ 5616]
cp26_PAli_NX_Pl_consensus    GATGCAAATTCAGTAGAAAAAGAAATAATCCTTGAAAATTTACATTTTAATAGAGGTGACATTAATTCCTATAGAATT [ 5616]
cp26_PAbe_NX_MP_FG_consensus GATGCAAATTCAGTAGAAAAAGAAATAATCCTTGAAAATTTACATTTTAATAGAGGTGACATTAATTCCTATAGAATT [ 5616]
cp26_PAbe_NX_Pl_consensus    GATGCAAATTCAGTAGAAAAAGAAATAATCCTTGAAAATTTACATTTTAATAGAGGTGACATTAATTCCTATAGAATT [ 5616]

cp26_gi|11497007|B31_GB      CGATCAACTATGCCAAGAGTGTACTACAATAATAAAAAATTTCCTGTACATTCTCCAAATGAAATTAAAAAAGGAGAC [ 5694]
cp26_un9_B31_PacBio          CGATCAACTATGCCAAGAGTGTACTACAATAATAAAAAATTTCCTGTACATTCTCCAAATGAAATTAAAAAAGGAGAC [ 5694]
cp26_B31_TS_MP_FG_consensus  CGATCAACTATGCCAAGAGTGTACTACAATAATAAAAAATTTCCTGTACATTCTCCAAATGAAATTAAAAAAGGAGAC [ 5694]
cp26_B31_NX_Pl_consensus     CGATCAACTATGCCAAGAGTGTACTACAATAATAAAAAATTTCCTGTACATTCTCCAAATGAAATTAAAAAAGGAGAC [ 5694]
cp26_PAli_NX_MP_FG_consensus CGATCAACTATGCCAAGAGTGTACTACAATAATAAAAAATTTCCTGTACATTCTCCAAATGAAATTAAAAAAGGAGAC [ 5694]
cp26_PAli_NX_Pl_consensus    CGATCAACTATGCCAAGAGTGTACTACAATAATAAAAAATTTCCTGTACATTCTCCAAATGAAATTAAAAAAGGAGAC [ 5694]
cp26_PAbe_NX_MP_FG_consensus CGATCAACTATGCCAAGAGTGTACTACAATAATAAAAAATTTCCTGTACATTCTCCAAATGAAATTAAAAAAGGAGAC [ 5694]
cp26_PAbe_NX_Pl_consensus    CGATCAACTATGCCAAGAGTGTACTACAATAATAAAAAATTTCCTGTACATTCTCCAAATGAAATTAAAAAAGGAGAC [ 5694]

cp26_gi|11497007|B31_GB      ATCCTAATAGATTCTTCAGAATACTTGGGTTATACAGGAGAACTTCAAATAGCACTAAAAGATACCCCAAATAATGGT [ 5772]
cp26_un9_B31_PacBio          ATCCTAATAGATTCTTCAGAATACTTGGGTTATACAGGAGAACTTCAAATAGCACTAAAAGATACCCCAAATAATGGT [ 5772]
cp26_B31_TS_MP_FG_consensus  ATCCTAATAGATTCTTCAGAATACTTGGGTTATACAGGAGAACTTCAAATAGCACTAAAAGATACCCCAAATAATGGT [ 5772]
cp26_B31_NX_Pl_consensus     ATCCTAATAGATTCTTCAGAATACTTGGGTTATACAGGAGAACTTCAAATAGCACTAAAAGATACCCCAAATAATGGT [ 5772]
cp26_PAli_NX_MP_FG_consensus ATCCTAATAGATTCTTCAGAATACTTGGGTTATACAGGAGAACTTCAAATAGCACTAAAAGATACCCCAAATAATGGT [ 5772]
cp26_PAli_NX_Pl_consensus    ATCCTAATAGATTCTTCAGAATACTTGGGTTATACAGGAGAACTTCAAATAGCACTAAAAGATACCCCAAATAATGGT [ 5772]
cp26_PAbe_NX_MP_FG_consensus ATCCTAATAGATTCTTCAGAATACTTGGGTTATACAGGAGAACTTCAAATAGCACTAAAAGATACCCCAAATAATGGT [ 5772]
cp26_PAbe_NX_Pl_consensus    ATCCTAATAGATTCTTCAGAATACTTGGGTTATACAGGAGAACTTCAAATAGCACTAAAAGATACCCCAAATAATGGT [ 5772]

cp26_gi|11497007|B31_GB      CTTGTAAACGTAGTTGGGAAAATAATCAATGATGAAATATATCTACTTGAAAAAATAGAACCTTGGGAAAAATTCAAA [ 5850]
cp26_un9_B31_PacBio          CTTGTAAACGTAGTTGGGAAAATAATCAATGATGAAATATATCTACTTGAAAAAATAGAACCTTGGGAAAAATTCAAA [ 5850]
cp26_B31_TS_MP_FG_consensus  CTTGTAAACGTAGTTGGGAAAATAATCAATGATGAAATATATCTACTTGAAAAAATAGAACCTTGGGAAAAATTCAAA [ 5850]
cp26_B31_NX_Pl_consensus     CTTGTAAACGTAGTTGGGAAAATAATCAATGATGAAATATATCTACTTGAAAAAATAGAACCTTGGGAAAAATTCAAA [ 5850]
cp26_PAli_NX_MP_FG_consensus CTTGTAAACGTAGTTGGGAAAATAATCAATGATGAAATATATCTACTTGAAAAAATAGAACCTTGGGAAAAATTCAAA [ 5850]
cp26_PAli_NX_Pl_consensus    CTTGTAAACGTAGTTGGGAAAATAATCAATGATGAAATATATCTACTTGAAAAAATAGAACCTTGGGAAAAATTCAAA [ 5850]
cp26_PAbe_NX_MP_FG_consensus CTTGTAAACGTAGTTGGGAAAATAATCAATGATGAAATATATCTACTTGAAAAAATAGAACCTTGGGAAAAATTCAAA [ 5850]
cp26_PAbe_NX_Pl_consensus    CTTGTAAACGTAGTTGGGAAAATAATCAATGATGAAATATATCTACTTGAAAAAATAGAACCTTGGGAAAAATTCAAA [ 5850]

cp26_gi|11497007|B31_GB      ATAATAGAAAATAAATAAATGCACTAATAAAAAGTTTCTTTATTGTAAAAATTTTTCAATTGCTTTAAGGACATAATA [ 5928]
cp26_un9_B31_PacBio          ATAATAGAAAATAAATAAATGCACTAATAAAAAGTTTCTTTATTGTAAAAATTTTTCAATTGCTTTAAGGACATAATA [ 5928]
cp26_B31_TS_MP_FG_consensus  ATAATAGAAAATAAATAAATGCACTAATAAAAAGTTTCTTTATTGTAAAAATTTTTCAATTGCTTTAAGGACATAATA [ 5928]
cp26_B31_NX_Pl_consensus     ATAATAGAAAATAAATAAATGCACTAATAAAAAGTTTCTTTATTGTAAAAATTTTTCAATTGCTTTAAGGACATAATA [ 5928]
cp26_PAli_NX_MP_FG_consensus ATAATAGAAAATAAATAAATGCACTAATAAAAAGTTTCTTTATTGTAAAAATTTTTCAATTGCTTTAAGGACATAATA [ 5928]
cp26_PAli_NX_Pl_consensus    ATAATAGAAAATAAATAAATGCACTAATAAAAAGTTTCTTTATTGTAAAAATTTTTCAATTGCTTTAAGGACATAATA [ 5928]
cp26_PAbe_NX_MP_FG_consensus ATAATAGAAAATAAATAAATGCACTAATAAAAAGTTTCTTTATTGTAAAAATTTTTCAATTGCTTTAAGGACATAATA [ 5928]
cp26_PAbe_NX_Pl_consensus    ATAATAGAAAATAAATAAATGCACTAATAAAAAGTTTCTTTATTGTAAAAATTTTTCAATTGCTTTAAGGACATAATA [ 5928]

cp26_gi|11497007|B31_GB      ATAAACAGTAATGTAAGACTCTCTTAAATCAAGATTTTTAGAACTATCAAGAAAATATATAAATTCATTGGTAGAAGA [ 6006]
cp26_un9_B31_PacBio          ATAAACAGTAATGTAAGACTCTCTTAAATCAAGATTTTTAGAACTATCAAGAAAATATATAAATTCATTGGTAGAAGA [ 6006]
cp26_B31_TS_MP_FG_consensus  ATAAACAGTAATGTAAGACTCTCTTAAATCAAGATTTTTAGAACTATCAAGAAAATATATAAATTCATTGGTAGAAGA [ 6006]
cp26_B31_NX_Pl_consensus     ATAAACAGTAATGTAAGACTCTCTTAAATCAAGATTTTTAGAACTATCAAGAAAATATATAAATTCATTGGTAGAAGA [ 6006]
cp26_PAli_NX_MP_FG_consensus ATAAACAGTAATGTAAGACTCTCTTAAATCAAGATTTTTAGAACTATCAAGAAAATATATAAATTCATTGGTAGAAGA [ 6006]
cp26_PAli_NX_Pl_consensus    ATAAACAGTAATGTAAGACTCTCTTAAATCAAGATTTTTAGAACTATCAAGAAAATATATAAATTCATTGGTAGAAGA [ 6006]
cp26_PAbe_NX_MP_FG_consensus ATAAACAGTAATGTAAGACTCTCTTAAATCAAGATTTTTAGAACTATCAAGAAAATATATAAATTCATTGGTAGAAGA [ 6006]
cp26_PAbe_NX_Pl_consensus    ATAAACAGTAATGTAAGACTCTCTTAAATCAAGATTTTTAGAACTATCAAGAAAATATATAAATTCATTGGTAGAAGA [ 6006]

cp26_gi|11497007|B31_GB      ATTAAGATCCTCAATATTATTTTTAAGTTCAAACTCCCGATTAATAGCCCTTATGTCGTCCAAATTAAAAATAATAGT [ 6084]
cp26_un9_B31_PacBio          ATTAAGATCCTCAATATTATTTTTAAGTTCAAACTCCCGATTAATAGCCCTTATGTCGTCCAAATTAAAAATAATAGT [ 6084]
cp26_B31_TS_MP_FG_consensus  ATTAAGATCCTCAATATTATTTTTAAGTTCAAACTCCCGATTAATAGCCCTTATGTCGTCCAAATTAAAAATAATAGT [ 6084]
cp26_B31_NX_Pl_consensus     ATTAAGATCCTCAATATTATTTTTAAGTTCAAACTCCCGATTAATAGCCCTTATGTCGTCCAAATTAAAAATAATAGT [ 6084]
cp26_PAli_NX_MP_FG_consensus ATTAAGATCCTCAATATTATTTTTAAGTTCAAACTCCCGATTAATAGCCCTTATGTCGTCCAAATTAAAAATAATAGT [ 6084]
cp26_PAli_NX_Pl_consensus    ATTAAGATCCTCAATATTATTTTTAAGTTCAAACTCCCGATTAATAGCCCTTATGTCGTCCAAATTAAAAATAATAGT [ 6084]
cp26_PAbe_NX_MP_FG_consensus ATTAAGATCCTCAATATTATTTTTAAGTTCAAACTCCCGATTAATAGCCCTTATGTCGTCCAAATTAAAAATAATAGT [ 6084]
cp26_PAbe_NX_Pl_consensus    ATTAAGATCCTCAATATTATTTTTAAGTTCAAACTCCCGATTAATAGCCCTTATGTCGTCCAAATTAAAAATAATAGT [ 6084]

cp26_gi|11497007|B31_GB      GGCAAAATTCTGAATATTACCATTGATTCTATTTTTAAAAGAAATTTTTTTGTCAAAACTATCAATACCGCTAAATTC [ 6162]
cp26_un9_B31_PacBio          GGCAAAATTCTGAATATTACCATTGATTCTATTTTTAAAAGAAATTTTTTTGTCAAAACTATCAATACCGCTAAATTC [ 6162]
cp26_B31_TS_MP_FG_consensus  GGCAAAATTCTGAATATTACCATTGATTCTATTTTTAAAAGAAATTTTTTTGTCAAAACTATCAATACCGCTAAATTC [ 6162]
cp26_B31_NX_Pl_consensus     GGCAAAATTCTGAATATTACCATTGATTCTATTTTTAAAAGAAATTTTTTTGTCAAAACTATCAATACCGCTAAATTC [ 6162]
cp26_PAli_NX_MP_FG_consensus GGCAAAATTCTGAATATTACCATTGATTCTATTTTTAAAAGAAATTTTTTTGTCAAAACTATCAATACCGCTAAATTC [ 6162]
cp26_PAli_NX_Pl_consensus    GGCAAAATTCTGAATATTACCATTGATTCTATTTTTAAAAGAAATTTTTTTGTCAAAACTATCAATACCGCTAAATTC [ 6162]
cp26_PAbe_NX_MP_FG_consensus GGCAAAATTCTGAATATTACCATTGATTCTATTTTTAAAAGAAATTTTTTTGTCAAAACTATCAATACCGCTAAATTC [ 6162]
cp26_PAbe_NX_Pl_consensus    GGCAAAATTCTGAATATTACCATTGATTCTATTTTTAAAAGAAATTTTTTTGTCAAAACTATCAATACCGCTAAATTC [ 6162]

cp26_gi|11497007|B31_GB      AGGTTCATTTGAATTTAAATTAACAATTTCTAATTTAAATTTACGATCAAAAAAAATACCAAAATCATAGGTTAATAA [ 6240]
cp26_un9_B31_PacBio          AGGTTCATTTGAATTTAAATTAACAATTTCTAATTTAAATTTACGATCAAAAAAAATACCAAAATCATAGGTTAATAA [ 6240]
cp26_B31_TS_MP_FG_consensus  AGGTTCATTTGAATTTAAATTAACAATTTCTAATTTAAATTTACGATCAAAAAAAATACCAAAATCATAGGTTAATAA [ 6240]
cp26_B31_NX_Pl_consensus     AGGTTCATTTGAATTTAAATTAACAATTTCTAATTTAAATTTACGATCAAAAAAAATACCAAAATCATAGGTTAATAA [ 6240]
cp26_PAli_NX_MP_FG_consensus AGGTTCATTTGAATTTAAATTAACAATTTCTAATTTAAATTTACGATCAAAAAAAATACCAAAATCATAGGTTAATAA [ 6240]
cp26_PAli_NX_Pl_consensus    AGGTTCATTTGAATTTAAATTAACAATTTCTAATTTAAATTTACGATCAAAAAAAATACCAAAATCATAGGTTAATAA [ 6240]
cp26_PAbe_NX_MP_FG_consensus AGGTTCATTTGAATTTAAATTAACAATTTCTAATTTAAATTTACGATCAAAAAAAATACCAAAATCATAGGTTAATAA [ 6240]
cp26_PAbe_NX_Pl_consensus    AGGTTCATTTGAATTTAAATTAACAATTTCTAATTTAAATTTACGATCAAAAAAAATACCAAAATCATAGGTTAATAA [ 6240]

cp26_gi|11497007|B31_GB      GTTTGCTTTTTTAGAACTTAAAGTCAATTTCATTGTAATATTATTGCTAAATTTATCTAAAATAAAAAAATAAGAATA [ 6318]
cp26_un9_B31_PacBio          GTTTGCTTTTTTAGAACTTAAAGTCAATTTCATTGTAATATTATTGCTAAATTTATCTAAAATAAAAAAATAAGAATA [ 6318]
cp26_B31_TS_MP_FG_consensus  GTTTGCTTTTTTAGAACTTAAAGTCAATTTCATTGTAATATTATTGCTAAATTTATCTAAAATAAAAAAATAAGAATA [ 6318]
cp26_B31_NX_Pl_consensus     GTTTGCTTTTTTAGAACTTAAAGTCAATTTCATTGTAATATTATTGCTAAATTTATCTAAAATAAAAAAATAAGAATA [ 6318]
cp26_PAli_NX_MP_FG_consensus GTTTGCTTTTTTAGAACTTAAAGTCAATTTCATTGTAATATTATTGCTAAATTTATCTAAAATAAAAAAATAAGAATA [ 6318]
cp26_PAli_NX_Pl_consensus    GTTTGCTTTTTTAGAACTTAAAGTCAATTTCATTGTAATATTATTGCTAAATTTATCTAAAATAAAAAAATAAGAATA [ 6318]
cp26_PAbe_NX_MP_FG_consensus GTTTGCTTTTTTAGAACTTAAAGTCAATTTCATTGTAATATTATTGCTAAATTTATCTAAAATAAAAAAATAAGAATA [ 6318]
cp26_PAbe_NX_Pl_consensus    GTTTGCTTTTTTAGAACTTAAAGTCAATTTCATTGTAATATTATTGCTAAATTTATCTAAAATAAAAAAATAAGAATA [ 6318]

cp26_gi|11497007|B31_GB      ATAACTAATATCTTTAATAGAATCAAAGTAATTCTTAGCATGAATGCCGGTTTTAAATTTACCATCTCCTAACGATTC [ 6396]
cp26_un9_B31_PacBio          ATAACTAATATCTTTAATAGAATCAAAGTAATTCTTAGCATGAATGCCGGTTTTAAATTTACCATCTCCTAACGATTC [ 6396]
cp26_B31_TS_MP_FG_consensus  ATAACTAATATCTTTAATAGAATCAAAGTAATTCTTAGCATGAATGCCGGTTTTAAATTTACCATCTCCTAACGATTC [ 6396]
cp26_B31_NX_Pl_consensus     ATAACTAATATCTTTAATAGAATCAAAGTAATTCTTAGCATGAATGCCGGTTTTAAATTTACCATCTCCTAACGATTC [ 6396]
cp26_PAli_NX_MP_FG_consensus ATAACTAATATCTTTAATAGAATCAAAGTAATTCTTAGCATGAATGCCGGTTTTAAATTTACCATCTCCTAACGATTC [ 6396]
cp26_PAli_NX_Pl_consensus    ATAACTAATATCTTTAATAGAATCAAAGTAATTCTTAGCATGAATGCCGGTTTTAAATTTACCATCTCCTAACGATTC [ 6396]
cp26_PAbe_NX_MP_FG_consensus ATAACTAATATCTTTAATAGAATCAAAGTAATTCTTAGCATGAATGCCGGTTTTAAATTTACCATCTCCTAACGATTC [ 6396]
cp26_PAbe_NX_Pl_consensus    ATAACTAATATCTTTAATAGAATCAAAGTAATTCTTAGCATGAATGCCGGTTTTAAATTTACCATCTCCTAACGATTC [ 6396]

cp26_gi|11497007|B31_GB      ATACAAAGCAATCTCACTTTTTATTACCTCATCCAAAGGTCTAATAAAAATTGAAGAAACACTAATATTGCATGAAAA [ 6474]
cp26_un9_B31_PacBio          ATACAAAGCAATCTCACTTTTTATTACCTCATCCAAAGGTCTAATAAAAATTGAAGAAACACTAATATTGCATGAAAA [ 6474]
cp26_B31_TS_MP_FG_consensus  ATACAAAGCAATCTCACTTTTTATTACCTCATCCAAAGGTCTAATAAAAATTGAAGAAACACTAATATTGCATGAAAA [ 6474]
cp26_B31_NX_Pl_consensus     ATACAAAGCAATCTCACTTTTTATTACCTCATCCAAAGGTCTAATAAAAATTGAAGAAACACTAATATTGCATGAAAA [ 6474]
cp26_PAli_NX_MP_FG_consensus ATACAAAGCAATCTCACTTTTTATTACCTCATCCAAAGGTCTAATAAAAATTGAAGAAACACTAATATTGCATGAAAA [ 6474]
cp26_PAli_NX_Pl_consensus    ATACAAAGCAATCTCACTTTTTATTACCTCATCCAAAGGTCTAATAAAAATTGAAGAAACACTAATATTGCATGAAAA [ 6474]
cp26_PAbe_NX_MP_FG_consensus ATACAAAGCAATCTCACTTTTTATTACCTCATCCAAAGGTCTAATAAAAATTGAAGAAACACTAATATTGCATGAAAA [ 6474]
cp26_PAbe_NX_Pl_consensus    ATACAAAGCAATCTCACTTTTTATTACCTCATCCAAAGGTCTAATAAAAATTGAAGAAACACTAATATTGCATGAAAA [ 6474]
```

```
cp26_gi|11497007|B31_GB      CAAAAAAATAATAAAGGGAAAAATAAAATTAAACTTTTTTTTCATCTAAAATTTCTCATTAAATATACTAAAATATAT [ 6552]
cp26_un9_B31_PacBio          CAAAAAAATAATAAAGGGAAAAATAAAATTAAACTTTTTTTTCATCTAAAATTTCTCATTAAATATACTAAAATATAT [ 6552]
cp26_B31_TS_MP_FG_consensus  CAAAAAAATAATAAAGGGAAAAATAAAATTAAACTTTTTTTTCATCTAAAATTTCTCATTAAATATACTAAAATATAT [ 6552]
cp26_B31_NX_Pl_consensus     CAAAAAAATAATAAAGGGAAAAATAAAATTAAACTTTTTTTTCATCTAAAATTTCTCATTAAATATACTAAAATATAT [ 6552]
cp26_PAli_NX_MP_FG_consensus CAAAAAAATAATAAAGGGAAAAATAAAATTAAACTTTTTTTTCATCTAAAATTTCTCATTAAATATACTAAAATATAT [ 6552]
cp26_PAli_NX_Pl_consensus    CAAAAAAATAATAAAGGGAAAAATAAAATTAAACTTTTTTTTCATCTAAAATTTCTCATTAAATATACTAAAATATAT [ 6552]
cp26_PAbe_NX_MP_FG_consensus CAAAAAAATAATAAAGGGAAAAATAAAATTAAACTTTTTTTTCATCTAAAATTTCTCATTAAATATACTAAAATATAT [ 6552]
cp26_PAbe_NX_Pl_consensus    CAAAAAAATAATAAAGGGAAAAATAAAATTAAACTTTTTTTTCATCTAAAATTTCTCATTAAATATACTAAAATATAT [ 6552]

cp26_gi|11497007|B31_GB      TAATATTAAAGAAAAGCTTGCATAAAAAGTATAACAAATTCTTTAATAATTAAAATCAAAAAGAATATAATTATTGCA [ 6630]
cp26_un9_B31_PacBio          TAATATTAAAGAAAAGCTTGCATAAAAAGTATAACAAATTCTTTAATAATTAAAATCAAAAAGAATATAATTATTGCA [ 6630]
cp26_B31_TS_MP_FG_consensus  TAATATTAAAGAAAAGCTTGCATAAAAAGTATAACAAATTCTTTAATAATTAAAATCAAAAAGAATATAATTATTGCA [ 6630]
cp26_B31_NX_Pl_consensus     TAATATTAAAGAAAAGCTTGCATAAAAAGTATAACAAATTCTTTAATAATTAAAATCAAAAAGAATATAATTATTGCA [ 6630]
cp26_PAli_NX_MP_FG_consensus TAATATTAAAGAAAAGCTTGCATAAAAAGTATAACAAATTCTTTAATAATTAAAATCAAAAAGAATATAATTATTGCA [ 6630]
cp26_PAli_NX_Pl_consensus    TAATATTAAAGAAAAGCTTGCATAAAAAGTATAACAAATTCTTTAATAATTAAAATCAAAAAGAATATAATTATTGCA [ 6630]
cp26_PAbe_NX_MP_FG_consensus TAATATTAAAGAAAAGCTTGCATAAAAAGTATAACAAATTCTTTAATAATTAAAATCAAAAAGAATATAATTATTGCA [ 6630]
cp26_PAbe_NX_Pl_consensus    TAATATTAAAGAAAAGCTTGCATAAAAAGTATAACAAATTCTTTAATAATTAAAATCAAAAAGAATATAATTATTGCA [ 6630]

cp26_gi|11497007|B31_GB      CTAAAATTAAATTTATACAGTTATATAGAATCACTTAAGGAACAAAAAATGAAATACCTTAAAAACATTTCCTTATTT [ 6708]
cp26_un9_B31_PacBio          CTAAAATTAAATTTATACAGTTATATAGAATCACTTAAGGAACAAAAAATGAAATACCTTAAAAACATTTCCTTATTT [ 6708]
cp26_B31_TS_MP_FG_consensus  CTAAAATTAAATTTATACAGTTATATAGAATCACTTAAGGAACAAAAAATGAAATACCTTAAAAACATTTCCTTATTT [ 6708]
cp26_B31_NX_Pl_consensus     CTAAAATTAAATTTATACAGTTATATAGAATCACTTAAGGAACAAAAAATGAAATACCTTAAAAACATTTCCTTATTT [ 6708]
cp26_PAli_NX_MP_FG_consensus CTAAAATTAAATTTATACAGTTATATAGAATCACTTAAGGAACAAAAAATGAAATACCTTAAAAACATTTCCTTATTT [ 6708]
cp26_PAli_NX_Pl_consensus    CTAAAATTAAATTTATACAGTTATATAGAATCACTTAAGGAACAAAAAATGAAATACCTTAAAAACATTTCCTTATTT [ 6708]
cp26_PAbe_NX_MP_FG_consensus CTAAAATTAAATTTATACAGTTATATAGAATCACTTAAGGAACAAAAAATGAAATACCTTAAAAACATTTCCTTATTT [ 6708]
cp26_PAbe_NX_Pl_consensus    CTAAAATTAAATTTATACAGTTATATAGAATCACTTAAGGAACAAAAAATGAAATACCTTAAAAACATTTCCTTATTT [ 6708]

cp26_gi|11497007|B31_GB      TTGTTAATTTTAGGTTGCAAATCCATCCCAAATGGTAATTTCAATCTACACGATACAAACCATAAATTAGGAAAACTA [ 6786]
cp26_un9_B31_PacBio          TTGTTAATTTTAGGTTGCAAATCCATCCCAAATGGTAATTTCAATCTACACGATACAAACCATAAATTAGGAAAACTA [ 6786]
cp26_B31_TS_MP_FG_consensus  TTGTTAATTTTAGGTTGCAAATCCATCCCAAATGGTAATTTCAATCTACACGATACAAACCATAAATTAGGAAAACTA [ 6786]
cp26_B31_NX_Pl_consensus     TTGTTAATTTTAGGTTGCAAATCCATCCCAAATGGTAATTTCAATCTACACGATACAAACCATAAATTAGGAAAACTA [ 6786]
cp26_PAli_NX_MP_FG_consensus TTGTTAATTTTAGGTTGCAAATCCATCCCAAATGGTAATTTCAATCTACACGATACAAACCATAAATTAGGAAAACTA [ 6786]
cp26_PAli_NX_Pl_consensus    TTGTTAATTTTAGGTTGCAAATCCATCCCAAATGGTAATTTCAATCTACACGATACAAACCATAAATTAGGAAAACTA [ 6786]
cp26_PAbe_NX_MP_FG_consensus TTGTTAATTTTAGGTTGCAAATCCATCCCAAATGGTAATTTCAATCTACACGATACAAACCATAAATTAGGAAAACTA [ 6786]
cp26_PAbe_NX_Pl_consensus    TTGTTAATTTTAGGTTGCAAATCCATCCCAAATGGTAATTTCAATCTACACGATACAAACCATAAATTAGGAAAACTA [ 6786]

cp26_gi|11497007|B31_GB      AAATTTCAAGAAGACTCGATAATAAGCAGAAATTATGATAATAAAATATCCATTGTGGGAGTATACAACCCTTTAACA [ 6864]
cp26_un9_B31_PacBio          AAATTTCAAGAAGACTCGATAATAAGCAGAAATTATGATAATAAAATATCCATTGTGGGAGTATACAACCCTTTAACA [ 6864]
cp26_B31_TS_MP_FG_consensus  AAATTTCAAGAAGACTCGATAATAAGCAGAAATTATGATAATAAAATATCCATTGTGGGAGTATACAACCCTTTAACA [ 6864]
cp26_B31_NX_Pl_consensus     AAATTTCAAGAAGACTCGATAATAAGCAGAAATTATGATAATAAAATATCCATTGTGGGAGTATACAACCCTTTAACA [ 6864]
cp26_PAli_NX_MP_FG_consensus AAATTTCAAGAAGACTCGATAATAAGCAGAAATTATGATAATAAAATATCCATTGTGGGAGTATACAACCCTTTAACA [ 6864]
cp26_PAli_NX_Pl_consensus    AAATTTCAAGAAGACTCGATAATAAGCAGAAATTATGATAATAAAATATCCATTGTGGGAGTATACAACCCTTTAACA [ 6864]
cp26_PAbe_NX_MP_FG_consensus AAATTTCAAGAAGACTCGATAATAAGCAGAAATTATGATAATAAAATATCCATTGTGGGAGTATACAACCCTTTAACA [ 6864]
cp26_PAbe_NX_Pl_consensus    AAATTTCAAGAAGACTCGATAATAAGCAGAAATTATGATAATAAAATATCCATTGTGGGAGTATACAACCCTTTAACA [ 6864]

cp26_gi|11497007|B31_GB      GAAAAAGAAAATTTTAAAGTCAATATTTTCATCAAAAAAAAAGGATTACAAATAGATCCTGAAAATATTTTGATAAAT [ 6942]
cp26_un9_B31_PacBio          GAAAAAGAAAATTTTAAAGTCAATATTTTCATCAAAAAAAAAGGATTACAAATAGATCCTGAAAATATTTTGATAAAT [ 6942]
cp26_B31_TS_MP_FG_consensus  GAAAAAGAAAATTTTAAAGTCAATATTTTCATCAAAAAAAAAGGATTACAAATAGATCCTGAAAATATTTTGATAAAT [ 6942]
cp26_B31_NX_Pl_consensus     GAAAAAGAAAATTTTAAAGTCAATATTTTCATCAAAAAAAAAGGATTACAAATAGATCCTGAAAATATTTTGATAAAT [ 6942]
cp26_PAli_NX_MP_FG_consensus GAAAAAGAAAATTTTAAAGTCAATATTTTCATCAAAAAAAAAGGATTACAAATAGATCCTGAAAATATTTTGATAAAT [ 6942]
cp26_PAli_NX_Pl_consensus    GAAAAAGAAAATTTTAAAGTCAATATTTTCATCAAAAAAAAAGGATTACAAATAGATCCTGAAAATATTTTGATAAAT [ 6942]
cp26_PAbe_NX_MP_FG_consensus GAAAAAGAAAATTTTAAAGTCAATATTTTCATCAAAAAAAAAGGATTACAAATAGATCCTGAAAATATTTTGATAAAT [ 6942]
cp26_PAbe_NX_Pl_consensus    GAAAAAGAAAATTTTAAAGTCAATATTTTCATCAAAAAAAAAGGATTACAAATAGATCCTGAAAATATTTTGATAAAT [ 6942]

cp26_gi|11497007|B31_GB      GAAGAAAAAATTAATTATTCAAAATATAAAGCAGAACTCAAAGTAAAATCTAGCTTTAATAAAAGCATTATCAGTATT [ 7020]
cp26_un9_B31_PacBio          GAAGAAAAAATTAATTATTCAAAATATAAAGCAGAACTCAAAGTAAAATCTAGCTTTAATAAAAGCATTATCAGTATT [ 7020]
cp26_B31_TS_MP_FG_consensus  GAAGAAAAAATTAATTATTCAAAATATAAAGCAGAACTCAAAGTAAAATCTAGCTTTAATAAAAGCATTATCAGTATT [ 7020]
cp26_B31_NX_Pl_consensus     GAAGAAAAAATTAATTATTCAAAATATAAAGCAGAACTCAAAGTAAAATCTAGCTTTAATAAAAGCATTATCAGTATT [ 7020]
cp26_PAli_NX_MP_FG_consensus GAAGAAAAAATTAATTATTCAAAATATAAAGCAGAACTCAAAGTAAAATCTAGCTTTAATAAAAGCATTATCAGTATT [ 7020]
cp26_PAli_NX_Pl_consensus    GAAGAAAAAATTAATTATTCAAAATATAAAGCAGAACTCAAAGTAAAATCTAGCTTTAATAAAAGCATTATCAGTATT [ 7020]
cp26_PAbe_NX_MP_FG_consensus GAAGAAAAAATTAATTATTCAAAATATAAAGCAGAACTCAAAGTAAAATCTAGCTTTAATAAAAGCATTATCAGTATT [ 7020]
cp26_PAbe_NX_Pl_consensus    GAAGAAAAAATTAATTATTCAAAATATAAAGCAGAACTCAAAGTAAAATCTAGCTTTAATAAAAGCATTATCAGTATT [ 7020]

cp26_gi|11497007|B31_GB      TCACTAACTAATTCAAGAGATCTATTAACCTACATTTACGATAAAAGCACAGGGAAATACATTAACATTGACTTTAAG [ 7098]
cp26_un9_B31_PacBio          TCACTAACTAATTCAAGAGATCTATTAACCTACATTTACGATAAAAGCACAGGGAAATACATTAACATTGACTTTAAG [ 7098]
cp26_B31_TS_MP_FG_consensus  TCACTAACTAATTCAAGAGATCTATTAACCTACATTTACGATAAAAGCACAGGGAAATACATTAACATTGACTTTAAG [ 7098]
cp26_B31_NX_Pl_consensus     TCACTAACTAATTCAAGAGATCTATTAACCTACATTTACGATAAAAGCACAGGGAAATACATTAACATTGACTTTAAG [ 7098]
cp26_PAli_NX_MP_FG_consensus TCACTAACTAATTCAAGAGATCTATTAACCTACATTTACGATAAAAGCACAGGGAAATACATTAACATTGACTTTAAG [ 7098]
cp26_PAli_NX_Pl_consensus    TCACTAACTAATTCAAGAGATCTATTAACCTACATTTACGATAAAAGCACAGGGAAATACATTAACATTGACTTTAAG [ 7098]
cp26_PAbe_NX_MP_FG_consensus TCACTAACTAATTCAAGAGATCTATTAACCTACATTTACGATAAAAGCACAGGGAAATACATTAACATTGACTTTAAG [ 7098]
cp26_PAbe_NX_Pl_consensus    TCACTAACTAATTCAAGAGATCTATTAACCTACATTTACGATAAAAGCACAGGGAAATACATTAACATTGACTTTAAG [ 7098]

cp26_gi|11497007|B31_GB      GACAATTGGAACGTATCGCACAGTATAAAATTTAATAAGGAGTATATTTTAGCATATATAACAGATTTTGATAAAGAA [ 7176]
cp26_un9_B31_PacBio          GACAATTGGAACGTATCGCACAGTATAAAATTTAATAAGGAGTATATTTTAGCATATATAACAGATTTTGATAAAGAA [ 7176]
cp26_B31_TS_MP_FG_consensus  GACAATTGGAACGTATCGCACAGTATAAAATTTAATAAGGAGTATATTTTAGCATATATAACAGATTTTGATAAAGAA [ 7176]
cp26_B31_NX_Pl_consensus     GACAATTGGAACGTATCGCACAGTATAAAATTTAATAAGGAGTATATTTTAGCATATATAACAGATTTTGATAAAGAA [ 7176]
cp26_PAli_NX_MP_FG_consensus GACAATTGGAACGTATCGCACAGTATAAAATTTAATAAGGAGTATATTTTAGCATATATAACAGATTTTGATAAAGAA [ 7176]
cp26_PAli_NX_Pl_consensus    GACAATTGGAACGTATCGCACAGTATAAAATTTAATAAGGAGTATATTTTAGCATATATAACAGATTTTGATAAAGAA [ 7176]
cp26_PAbe_NX_MP_FG_consensus GACAATTGGAACGTATCGCACAGTATAAAATTTAATAAGGAGTATATTTTAGCATATATAACAGATTTTGATAAAGAA [ 7176]
cp26_PAbe_NX_Pl_consensus    GACAATTGGAACGTATCGCACAGTATAAAATTTAATAAGGAGTATATTTTAGCATATATAACAGATTTTGATAAAGAA [ 7176]

cp26_gi|11497007|B31_GB      ATTAAAATATCTAAAAATATTTTGCAAAAACGTATTGATAATAGAAAAATTGAAATTGAAAAAACAGAGCTTAAAACA [ 7254]
cp26_un9_B31_PacBio          ATTAAAATATCTAAAAATATTTTGCAAAAACGTATTGATAATAGAAAAATTGAAATTGAAAAAACAGAGCTTAAAACA [ 7254]
cp26_B31_TS_MP_FG_consensus  ATTAAAATATCTAAAAATATTTTGCAAAAACGTATTGATAATAGAAAAATTGAAATTGAAAAAACAGAGCTTAAAACA [ 7254]
cp26_B31_NX_Pl_consensus     ATTAAAATATCTAAAAATATTTTGCAAAAACGTATTGATAATAGAAAAATTGAAATTGAAAAAACAGAGCTTAAAACA [ 7254]
cp26_PAli_NX_MP_FG_consensus ATTAAAATATCTAAAAATATTTTGCAAAAACGTATTGATAATAGAAAAATTGAAATTGAAAAAACAGAGCTTAAAACA [ 7254]
cp26_PAli_NX_Pl_consensus    ATTAAAATATCTAAAAATATTTTGCAAAAACGTATTGATAATAGAAAAATTGAAATTGAAAAAACAGAGCTTAAAACA [ 7254]
cp26_PAbe_NX_MP_FG_consensus ATTAAAATATCTAAAAATATTTTGCAAAAACGTATTGATAATAGAAAAATTGAAATTGAAAAAACAGAGCTTAAAACA [ 7254]
cp26_PAbe_NX_Pl_consensus    ATTAAAATATCTAAAAATATTTTGCAAAAACGTATTGATAATAGAAAAATTGAAATTGAAAAAACAGAGCTTAAAACA [ 7254]

cp26_gi|11497007|B31_GB      GAATATAATGAAATAGAGGATTATTACATCTACAGTATGAAAATTCCAAAATTATTTGAAAAATCAGACGCTCCCTCT [ 7332]
cp26_un9_B31_PacBio          GAATATAATGAAATAGAGGATTATTACATCTACAGTATGAAAATTCCAAAATTATTTGAAAAATCAGACGCTCCCTCT [ 7332]
cp26_B31_TS_MP_FG_consensus  GAATATAATGAAATAGAGGATTATTACATCTACAGTATGAAAATTCCAAAATTATTTGAAAAATCAGACGCTCCCTCT [ 7332]
cp26_B31_NX_Pl_consensus     GAATATAATGAAATAGAGGATTATTACATCTACAGTATGAAAATTCCAAAATTATTTGAAAAATCAGACGCTCCCTCT [ 7332]
cp26_PAli_NX_MP_FG_consensus GAATATAATGAAATAGAGGATTATTACATCTACAGTATGAAAATTCCAAAATTATTTGAAAAATCAGACGCTCCCTCT [ 7332]
cp26_PAli_NX_Pl_consensus    GAATATAATGAAATAGAGGATTATTACATCTACAGTATGAAAATTCCAAAATTATTTGAAAAATCAGACGCTCCCTCT [ 7332]
cp26_PAbe_NX_MP_FG_consensus GAATATAATGAAATAGAGGATTATTACATCTACAGTATGAAAATTCCAAAATTATTTGAAAAATCAGACGCTCCCTCT [ 7332]
cp26_PAbe_NX_Pl_consensus    GAATATAATGAAATAGAGGATTATTACATCTACAGTATGAAAATTCCAAAATTATTTGAAAAATCAGACGCTCCCTCT [ 7332]

cp26_gi|11497007|B31_GB      GAAACTTACGAAACATTTGTTATAGCAAATTATTACCCCTGTGAAAATTTAAATATACTGTTTTTGAATTTAAGCTTA [ 7410]
cp26_un9_B31_PacBio          GAAACTTACGAAACATTTGTTATAGCAAATTATTACCCCTGTGAAAATTTAAATATACTGTTTTTGAATTTAAGCTTA [ 7410]
cp26_B31_TS_MP_FG_consensus  GAAACTTACGAAACATTTGTTATAGCAAATTATTACCCCTGTGAAAATTTAAATATACTGTTTTTGAATTTAAGCTTA [ 7410]
cp26_B31_NX_Pl_consensus     GAAACTTACGAAACATTTGTTATAGCAAATTATTACCCCTGTGAAAATTTAAATATACTGTTTTTGAATTTAAGCTTA [ 7410]
cp26_PAli_NX_MP_FG_consensus GAAACTTACGAAACATTTGTTATAGCAAATTATTACCCCTGTGAAAATTTAAATATACTGTTTTTGAATTTAAGCTTA [ 7410]
cp26_PAli_NX_Pl_consensus    GAAACTTACGAAACATTTGTTATAGCAAATTATTACCCCTGTGAAAATTTAAATATACTGTTTTTGAATTTAAGCTTA [ 7410]
cp26_PAbe_NX_MP_FG_consensus GAAACTTACGAAACATTTGTTATAGCAAATTATTACCCCTGTGAAAATTTAAATATACTGTTTTTGAATTTAAGCTTA [ 7410]
cp26_PAbe_NX_Pl_consensus    GAAACTTACGAAACATTTGTTATAGCAAATTATTACCCCTGTGAAAATTTAAATATACTGTTTTTGAATTTAAGCTTA [ 7410]
```

```
cp26_gi|11497007|B31_GB      TACTCTGATAAATTACGCTTTCTAAACTCTATTTATGATGAGAATGATAGAAAATTAAAAATGGAGCCTCCTGTGAGA [ 7488]
cp26_un9_B31_PacBio          TACTCTGATAAATTACGCTTTCTAAACTCTATTTATGATGAGAATGATAGAAAATTAAAAATGGAGCCTCCTGTGAGA [ 7488]
cp26_B31_TS_MP_FG_consensus  TACTCTGATAAATTACGCTTTCTAAACTCTATTTATGATGAGAATGATAGAAAATTAAAAATGGAGCCTCCTGTGAGA [ 7488]
cp26_B31_NX_Pl_consensus     TACTCTGATAAATTACGCTTTCTAAACTCTATTTATGATGAGAATGATAGAAAATTAAAAATGGAGCCTCCTGTGAGA [ 7488]
cp26_PAli_NX_MP_FG_consensus TACTCTGATAAATTACGCTTTCTAAACTCTATTTATGATGAGAATGATAGAAAATTAAAAATGGAGCCTCCTGTGAGA [ 7488]
cp26_PAli_NX_Pl_consensus    TACTCTGATAAATTACGCTTTCTAAACTCTATTTATGATGAGAATGATAGAAAATTAAAAATGGAGCCTCCTGTGAGA [ 7488]
cp26_PAbe_NX_MP_FG_consensus TACTCTGATAAATTACGCTTTCTAAACTCTATTTATGATGAGAATGATAGAAAATTAAAAATGGAGCCTCCTGTGAGA [ 7488]
cp26_PAbe_NX_Pl_consensus    TACTCTGATAAATTACGCTTTCTAAACTCTATTTATGATGAGAATGATAGAAAATTAAAAATGGAGCCTCCTGTGAGA [ 7488]

cp26_gi|11497007|B31_GB      GCCTTAAAGAATTCAAAAACAATAAAAGAAACATTAAATATAGTATTAAGTCCTCAAAAAATAATAGAGCTAGCAAAA [ 7566]
cp26_un9_B31_PacBio          GCCTTAAAGAATTCAAAAACAATAAAAGAAACATTAAATATAGTATTAAGTCCTCAAAAAATAATAGAGCTAGCAAAA [ 7566]
cp26_B31_TS_MP_FG_consensus  GCCTTAAAGAATTCAAAAACAATAAAAGAAACATTAAATATAGTATTAAGTCCTCAAAAAATAATAGAGCTAGCAAAA [ 7566]
cp26_B31_NX_Pl_consensus     GCCTTAAAGAATTCAAAAACAATAAAAGAAACATTAAATATAGTATTAAGTCCTCAAAAAATAATAGAGCTAGCAAAA [ 7566]
cp26_PAli_NX_MP_FG_consensus GCCTTAAAGAATTCAAAAACAATAAAAGAAACATTAAATATAGTATTAAGTCCTCAAAAAATAATAGAGCTAGCAAAA [ 7566]
cp26_PAli_NX_Pl_consensus    GCCTTAAAGAATTCAAAAACAATAAAAGAAACATTAAATATAGTATTAAGTCCTCAAAAAATAATAGAGCTAGCAAAA [ 7566]
cp26_PAbe_NX_MP_FG_consensus GCCTTAAAGAATTCAAAAACAATAAAAGAAACATTAAATATAGTATTAAGTCCTCAAAAAATAATAGAGCTAGCAAAA [ 7566]
cp26_PAbe_NX_Pl_consensus    GCCTTAAAGAATTCAAAAACAATAAAAGAAACATTAAATATAGTATTAAGTCCTCAAAAAATAATAGAGCTAGCAAAA [ 7566]

cp26_gi|11497007|B31_GB      AACATTGAAAAAGATATTACTCTAAAATTAAAATCTTACGGAGAAAAGGGAGAATTCACATTTGAAATATATAAACCA [ 7644]
cp26_un9_B31_PacBio          AACATTGAAAAAGATATTACTCTAAAATTAAAATCTTACGGAGAAAAGGGAGAATTCACATTTGAAATATATAAACCA [ 7644]
cp26_B31_TS_MP_FG_consensus  AACATTGAAAAAGATATTACTCTAAAATTAAAATCTTACGGAGAAAAGGGAGAATTCACATTTGAAATATATAAACCA [ 7644]
cp26_B31_NX_Pl_consensus     AACATTGAAAAAGATATTACTCTAAAATTAAAATCTTACGGAGAAAAGGGAGAATTCACATTTGAAATATATAAACCA [ 7644]
cp26_PAli_NX_MP_FG_consensus AACATTGAAAAAGATATTACTCTAAAATTAAAATCTTACGGAGAAAAGGGAGAATTCACATTTGAAATATATAAACCA [ 7644]
cp26_PAli_NX_Pl_consensus    AACATTGAAAAAGATATTACTCTAAAATTAAAATCTTACGGAGAAAAGGGAGAATTCACATTTGAAATATATAAACCA [ 7644]
cp26_PAbe_NX_MP_FG_consensus AACATTGAAAAAGATATTACTCTAAAATTAAAATCTTACGGAGAAAAGGGAGAATTCACATTTGAAATATATAAACCA [ 7644]
cp26_PAbe_NX_Pl_consensus    AACATTGAAAAAGATATTACTCTAAAATTAAAATCTTACGGAGAAAAGGGAGAATTCACATTTGAAATATATAAACCA [ 7644]

cp26_gi|11497007|B31_GB      CTTCTTTTAAAATTCTTAAAAGAAGTAGATCATTGCATAAAAAATTTGCAATCAAGTAGGCATAAATTTTAACTTATT [ 7722]
cp26_un9_B31_PacBio          CTTCTTTTAAAATTCTTAAAAGAAGTAGATCATTGCATAAAAAATTTGCAATCAAGTAGGCATAAATTTTAACTTATT [ 7722]
cp26_B31_TS_MP_FG_consensus  CTTCTTTTAAAATTCTTAAAAGAAGTAGATCATTGCATAAAAAATTTGCAATCAAGTAGGCATAAATTTTAACTTATT [ 7722]
cp26_B31_NX_Pl_consensus     CTTCTTTTAAAATTCTTAAAAGAAGTAGATCATTGCATAAAAAATTTGCAATCAAGTAGGCATAAATTTTAACTTATT [ 7722]
cp26_PAli_NX_MP_FG_consensus CTTCTTTTAAAATTCTTAAAAGAAGTAGATCATTGCATAAAAAATTTGCAATCAAGTAGGCATAAATTTTAACTTATT [ 7722]
cp26_PAli_NX_Pl_consensus    CTTCTTTTAAAATTCTTAAAAGAAGTAGATCATTGCATAAAAAATTTGCAATCAAGTAGGCATAAATTTTAACTTATT [ 7722]
cp26_PAbe_NX_MP_FG_consensus CTTCTTTTAAAATTCTTAAAAGAAGTAGATCATTGCATAAAAAATTTGCAATCAAGTAGGCATAAATTTTAACTTATT [ 7722]
cp26_PAbe_NX_Pl_consensus    CTTCTTTTAAAATTCTTAAAAGAAGTAGATCATTGCATAAAAAATTTGCAATCAAGTAGGCATAAATTTTAACTTATT [ 7722]

cp26_gi|11497007|B31_GB      GACTCTTACACATTTGAATGCAAATTCACCAACAAAACTTTACTTTTTTAAATAAGTGTATTACTATTAATTTTAGTG [ 7800]
cp26_un9_B31_PacBio          GACTCTTACACATTTGAATGCAAATTCACCAACAAAACTTTACTTTTTTAAATAAGTGTATTACTATTAATTTTAGTG [ 7800]
cp26_B31_TS_MP_FG_consensus  GACTCTTACACATTTGAATGCAAATTCACCAACAAAACTTTACTTTTTTAAATAAGTGTATTACTATTAATTTTAGTG [ 7800]
cp26_B31_NX_Pl_consensus     GACTCTTACACATTTGAATGCAAATTCACCAACAAAACTTTACTTTTTTAAATAAGTGTATTACTATTAATTTTAGTG [ 7800]
cp26_PAli_NX_MP_FG_consensus GACTCTTACACATTTGAATGCAAATTCACCAACAAAACTTTACTTTTTTAAATAAGTGTATTACTATTAATTTTAGTG [ 7800]
cp26_PAli_NX_Pl_consensus    GACTCTTACACATTTGAATGCAAATTCACCAACAAAACTTTACTTTTTTAAATAAGTGTATTACTATTAATTTTAGTG [ 7800]
cp26_PAbe_NX_MP_FG_consensus GACTCTTACACATTTGAATGCAAATTCACCAACAAAACTTTACTTTTTTAAATAAGTGTATTACTATTAATTTTAGTG [ 7800]
cp26_PAbe_NX_Pl_consensus    GACTCTTACACATTTGAATGCAAATTCACCAACAAAACTTTACTTTTTTAAATAAGTGTATTACTATTAATTTTAGTG [ 7800]

cp26_gi|11497007|B31_GB      CTTAATGCAATAAAAGATCAAACGATAGGAGCTTAAAATGTATCAAATAAAAACCAACAAGATGCCTTTTAATAAAGT [ 7878]
cp26_un9_B31_PacBio          CTTAATGCAATAAAAGATCAAACGATAGGAGCTTAAAATGTATCAAATAAAAACCAACAAGATGCCTTTTAATAAAGT [ 7878]
cp26_B31_TS_MP_FG_consensus  CTTAATGCAATAAAAGATCAAACGATAGGAGCTTAAAATGTATCAAATAAAAACCAACAAGATGCCTTTTAATAAAGT [ 7878]
cp26_B31_NX_Pl_consensus     CTTAATGCAATAAAAGATCAAACGATAGGAGCTTAAAATGTATCAAATAAAAACCAACAAGATGCCTTTTAATAAAGT [ 7878]
cp26_PAli_NX_MP_FG_consensus CTTAATGCAATAAAAGATCAAACGATAGGAGCTTAAAATGTATCAAATAAAAACCAACAAGATGCCTTTTAATAAAGT [ 7878]
cp26_PAli_NX_Pl_consensus    CTTAATGCAATAAAAGATCAAACGATAGGAGCTTAAAATGTATCAAATAAAAACCAACAAGATGCCTTTTAATAAAGT [ 7878]
cp26_PAbe_NX_MP_FG_consensus CTTAATGCAATAAAAGATCAAACGATAGGAGCTTAAAATGTATCAAATAAAAACCAACAAGATGCCTTTTAATAAAGT [ 7878]
cp26_PAbe_NX_Pl_consensus    CTTAATGCAATAAAAGATCAAACGATAGGAGCTTAAAATGTATCAAATAAAAACCAACAAGATGCCTTTTAATAAAGT [ 7878]

cp26_gi|11497007|B31_GB      AGTAGACCGAAGATTGAAAATATTTTGGGTCATCCAAAAACTAAGCGCCAACTACTTCATATCTAAAAAAAAATACTC [ 7956]
cp26_un9_B31_PacBio          AGTAGACCGAAGATTGAAAATATTTTGGGTCATCCAAAAACTAAGCGCCAACTACTTCATATCTAAAAAAAAATACTC [ 7956]
cp26_B31_TS_MP_FG_consensus  AGTAGACCGAAGATTGAAAATATTTTGGGTCATCCAAAAACTAAGCGCCAACTACTTCATATCTAAAAAAAAATACTC [ 7956]
cp26_B31_NX_Pl_consensus     AGTAGACCGAAGATTGAAAATATTTTGGGTCATCCAAAAACTAAGCGCCAACTACTTCATATCTAAAAAAAAATACTC [ 7956]
cp26_PAli_NX_MP_FG_consensus AGTAGACCGAAGATTGAAAATATTTTGGGTCATCCAAAAACTAAGCGCCAACTACTTCATATCTAAAAAAAAATACTC [ 7956]
cp26_PAli_NX_Pl_consensus    AGTAGACCGAAGATTGAAAATATTTTGGGTCATCCAAAAACTAAGCGCCAACTACTTCATATCTAAAAAAAAATACTC [ 7956]
cp26_PAbe_NX_MP_FG_consensus AGTAGACCGAAGATTGAAAATATTTTGGGTCATCCAAAAACTAAGCGCCAACTACTTCATATCTAAAAAAAAATACTC [ 7956]
cp26_PAbe_NX_Pl_consensus    AGTAGACCGAAGATTGAAAATATTTTGGGTCATCCAAAAACTAAGCGCCAACTACTTCATATCTAAAAAAAAATACTC [ 7956]

cp26_gi|11497007|B31_GB      TCTAAGCAATGTTGTAGCAATGACAAATTCTATATTGGAAAAAAAAGGATTCAAAAGGGTCACTAAAAGGACGATACA [ 8034]
cp26_un9_B31_PacBio          TCTAAGCAATGTTGTAGCAATGACAAATTCTATATTGGAAAAAAAAGGATTCAAAAGGGTCACTAAAAGGACGATACA [ 8034]
cp26_B31_TS_MP_FG_consensus  TCTAAGCAATGTTGTAGCAATGACAAATTCTATATTGGAAAAAAAAGGATTCAAAAGGGTCACTAAAAGGACGATACA [ 8034]
cp26_B31_NX_Pl_consensus     TCTAAGCAATGTTGTAGCAATGACAAATTCTATATTGGAAAAAAAAGGATTCAAAAGGGTCACTAAAAGGACGATACA [ 8034]
cp26_PAli_NX_MP_FG_consensus TCTAAGCAATGTTGTAGCAATGACAAATTCTATATTGGAAAAAAAAGGATTCAAAAGGGTCACTAAAAGGACGATACA [ 8034]
cp26_PAli_NX_Pl_consensus    TCTAAGCAATGTTGTAGCAATGACAAATTCTATATTGGAAAAAAAAGGATTCAAAAGGGTCACTAAAAGGACGATACA [ 8034]
cp26_PAbe_NX_MP_FG_consensus TCTAAGCAATGTTGTAGCAATGACAAATTCTATATTGGAAAAAAAAGGATTCAAAAGGGTCACTAAAAGGACGATACA [ 8034]
cp26_PAbe_NX_Pl_consensus    TCTAAGCAATGTTGTAGCAATGACAAATTCTATATTGGAAAAAAAAGGATTCAAAAGGGTCACTAAAAGGACGATACA [ 8034]

cp26_gi|11497007|B31_GB      AAATGATATAAAAATTTTTGAAACTTTAGGATTAATTAAAAGTCATTTCAATCCACTTGGAAAAAACAATGGAAGTTT [ 8112]
cp26_un9_B31_PacBio          AAATGATATAAAAATTTTTGAAACTTTAGGATTAATTAAAAGTCATTTCAATCCACTTGGAAAAAACAATGGAAGTTT [ 8112]
cp26_B31_TS_MP_FG_consensus  AAATGATATAAAAATTTTTGAAACTTTAGGATTAATTAAAAGTCATTTCAATCCACTTGGAAAAAACAATGGAAGTTT [ 8112]
cp26_B31_NX_Pl_consensus     AAATGATATAAAAATTTTTGAAACTTTAGGATTAATTAAAAGTCATTTCAATCCACTTGGAAAAAACAATGGAAGTTT [ 8112]
cp26_PAli_NX_MP_FG_consensus AAATGATATAAAAATTTTTGAAACTTTAGGATTAATTAAAAGTCATTTCAATCCACTTGGAAAAAACAATGGAAGTTT [ 8112]
cp26_PAli_NX_Pl_consensus    AAATGATATAAAAATTTTTGAAACTTTAGGATTAATTAAAAGTCATTTCAATCCACTTGGAAAAAACAATGGAAGTTT [ 8112]
cp26_PAbe_NX_MP_FG_consensus AAATGATATAAAAATTTTTGAAACTTTAGGATTAATTAAAAGTCATTTCAATCCACTTGGAAAAAACAATGGAAGTTT [ 8112]
cp26_PAbe_NX_Pl_consensus    AAATGATATAAAAATTTTTGAAACTTTAGGATTAATTAAAAGTCATTTCAATCCACTTGGAAAAAACAATGGAAGTTT [ 8112]

cp26_gi|11497007|B31_GB      TACTTACTATACAATAAATAAAGCCCTTGAAAAATTAGCTAAAAAAATTATCAGTACAGCATATTTTATTGACAAAAA [ 8190]
cp26_un9_B31_PacBio          TACTTACTATACAATAAATAAAGCCCTTGAAAAATTAGCTAAAAAAATTATCAGTACAGCATATTTTATTGACAAAAA [ 8190]
cp26_B31_TS_MP_FG_consensus  TACTTACTATACAATAAATAAAGCCCTTGAAAAATTAGCTAAAAAAATTATCAGTACAGCATATTTTATTGACAAAAA [ 8190]
cp26_B31_NX_Pl_consensus     TACTTACTATACAATAAATAAAGCCCTTGAAAAATTAGCTAAAAAAATTATCAGTACAGCATATTTTATTGACAAAAA [ 8190]
cp26_PAli_NX_MP_FG_consensus TACTTACTATACAATAAATAAAGCCCTTGAAAAATTAGCTAAAAAAATTATCAGTACAGCATATTTTATTGACAAAAA [ 8190]
cp26_PAli_NX_Pl_consensus    TACTTACTATACAATAAATAAAGCCCTTGAAAAATTAGCTAAAAAAATTATCAGTACAGCATATTTTATTGACAAAAA [ 8190]
cp26_PAbe_NX_MP_FG_consensus TACTTACTATACAATAAATAAAGCCCTTGAAAAATTAGCTAAAAAAATTATCAGTACAGCATATTTTATTGACAAAAA [ 8190]
cp26_PAbe_NX_Pl_consensus    TACTTACTATACAATAAATAAAGCCCTTGAAAAATTAGCTAAAAAAATTATCAGTACAGCATATTTTATTGACAAAAA [ 8190]

cp26_gi|11497007|B31_GB      AACAAAACATGAAAAATCAAAAAATAAGCAACTAAAAAAAATTAAAATAATAGAAGAATCTCAAAAATACAAAATTTC [ 8268]
cp26_un9_B31_PacBio          AACAAAACATGAAAAATCAAAAAATAAGCAACTAAAAAAAATTAAAATAATAGAAGAATCTCAAAAATACAAAATTTC [ 8268]
cp26_B31_TS_MP_FG_consensus  AACAAAACATGAAAAATCAAAAAATAAGCAACTAAAAAAAATTAAAATAATAGAAGAATCTCAAAAATACAAAATTTC [ 8268]
cp26_B31_NX_Pl_consensus     AACAAAACATGAAAAATCAAAAAATAAGCAACTAAAAAAAATTAAAATAATAGAAGAATCTCAAAAATACAAAATTTC [ 8268]
cp26_PAli_NX_MP_FG_consensus AACAAAACATGAAAAATCAAAAAATAAGCAACTAAAAAAAATTAAAATAATAGAAGAATCTCAAAAATACAAAATTTC [ 8268]
cp26_PAli_NX_Pl_consensus    AACAAAACATGAAAAATCAAAAAATAAGCAACTAAAAAAAATTAAAATAATAGAAGAATCTCAAAAATACAAAATTTC [ 8268]
cp26_PAbe_NX_MP_FG_consensus AACAAAACATGAAAAATCAAAAAATAAGCAACTAAAAAAAATTAAAATAATAGAAGAATCTCAAAAATACAAAATTTC [ 8268]
cp26_PAbe_NX_Pl_consensus    AACAAAACATGAAAAATCAAAAAATAAGCAACTAAAAAAAATTAAAATAATAGAAGAATCTCAAAAATACAAAATTTC [ 8268]

cp26_gi|11497007|B31_GB      ACATCAAATAACTTCACATGTATTAAGTAATAATATAAGTAAAAAATATAAGAATTCTAAATATTCTTTTAGAAGAAA [ 8346]
cp26_un9_B31_PacBio          ACATCAAATAACTTCACATGTATTAAGTAATAATATAAGTAAAAAATATAAGAATTCTAAATATTCTTTTAGAAGAAA [ 8346]
cp26_B31_TS_MP_FG_consensus  ACATCAAATAACTTCACATGTATTAAGTAATAATATAAGTAAAAAATATAAGAATTCTAAATATTCTTTTAGAAGAAA [ 8346]
cp26_B31_NX_Pl_consensus     ACATCAAATAACTTCACATGTATTAAGTAATAATATAAGTAAAAAATATAAGAATTCTAAATATTCTTTTAGAAGAAA [ 8346]
cp26_PAli_NX_MP_FG_consensus ACATCAAATAACTTCACATGTATTAAGTAATAATATAAGTAAAAAATATAAGAATTCTAAATATTCTTTTAGAAGAAA [ 8346]
cp26_PAli_NX_Pl_consensus    ACATCAAATAACTTCACATGTATTAAGTAATAATATAAGTAAAAAATATAAGAATTCTAAATATTCTTTTAGAAGAAA [ 8346]
cp26_PAbe_NX_MP_FG_consensus ACATCAAATAACTTCACATGTATTAAGTAATAATATAAGTAAAAAATATAAGAATTCTAAATATTCTTTTAGAAGAAA [ 8346]
cp26_PAbe_NX_Pl_consensus    ACATCAAATAACTTCACATGTATTAAGTAATAATATAAGTAAAAAATATAAGAATTCTAAATATTCTTTTAGAAGAAA [ 8346]
```

```
cp26_gi|11497007|B31_GB      AAATCAAAACATAAAAAAAACTATAAATTTCTTAGAAAAAGAAATAAAAAAAAAATCAAAATCAATAAATCTAGAAGA [ 8424]
cp26_un9_B31_PacBio          AAATCAAAACATAAAAAAAACTATAAATTTCTTAGAAAAAGAAATAAAAAAAAAATCAAAATCAATAAATCTAGAAGA [ 8424]
cp26_B31_TS_MP_FG_consensus  AAATCAAAACATAAAAAAAACTATAAATTTCTTAGAAAAAGAAATAAAAAAAAAATCAAAATCAATAAATCTAGAAGA [ 8424]
cp26_B31_NX_Pl_consensus     AAATCAAAACATAAAAAAAACTATAAATTTCTTAGAAAAAGAAATAAAAAAAAAATCAAAATCAATAAATCTAGAAGA [ 8424]
cp26_PAli_NX_MP_FG_consensus AAATCAAAACATAAAAAAAACTATAAATTTCTTAGAAAAAGAAATAAAAAAAAAATCAAAATCAATAAATCTAGAAGA [ 8424]
cp26_PAli_NX_Pl_consensus    AAATCAAAACATAAAAAAAACTATAAATTTCTTAGAAAAAGAAATAAAAAAAAAATCAAAATCAATAAATCTAGAAGA [ 8424]
cp26_PAbe_NX_MP_FG_consensus AAATCAAAACATAAAAAAAACTATAAATTTCTTAGAAAAAGAAATAAAAAAAAAATCAAAATCAATAAATCTAGAAGA [ 8424]
cp26_PAbe_NX_Pl_consensus    AAATCAAAACATAAAAAAAACTATAAATTTCTTAGAAAAAGAAATAAAAAAAAAATCAAAATCAATAAATCTAGAAGA [ 8424]

cp26_gi|11497007|B31_GB      AATAAAAAAAATAACAGAAAACGACATAACTTATAAGAATTCATTATGGAACTTAAAAGATTTTATGGAAGAATTAAA [ 8502]
cp26_un9_B31_PacBio          AATAAAAAAAATAACAGAAAACGACATAACTTATAAGAATTCATTATGGAACTTAAAAGATTTTATGGAAGAATTAAA [ 8502]
cp26_B31_TS_MP_FG_consensus  AATAAAAAAAATAACAGAAAACGACATAACTTATAAGAATTCATTATGGAACTTAAAAGATTTTATGGAAGAATTAAA [ 8502]
cp26_B31_NX_Pl_consensus     AATAAAAAAAATAACAGAAAACGACATAACTTATAAGAATTCATTATGGAACTTAAAAGATTTTATGGAAGAATTAAA [ 8502]
cp26_PAli_NX_MP_FG_consensus AATAAAAAAAATAACAGAAAACGACATAACTTATAAGAATTCATTATGGAACTTAAAAGATTTTATGGAAGAATTAAA [ 8502]
cp26_PAli_NX_Pl_consensus    AATAAAAAAAATAACAGAAAACGACATAACTTATAAGAATTCATTATGGAACTTAAAAGATTTTATGGAAGAATTAAA [ 8502]
cp26_PAbe_NX_MP_FG_consensus AATAAAAAAAATAACAGAAAACGACATAACTTATAAGAATTCATTATGGAACTTAAAAGATTTTATGGAAGAATTAAA [ 8502]
cp26_PAbe_NX_Pl_consensus    AATAAAAAAAATAACAGAAAACGACATAACTTATAAGAATTCATTATGGAACTTAAAAGATTTTATGGAAGAATTAAA [ 8502]

cp26_gi|11497007|B31_GB      AGAATATGAAGAAAAAAAATTATAAAATTTTATAAAAAAAATCTAGAAAAAAAGAAACAAAAAAATATGGTTTATGGC [ 8580]
cp26_un9_B31_PacBio          AGAATATGAAGAAAAAAAATTATAAAATTTTATAAAAAAAATCTAGAAAAAAAGAAACAAAAAAATATGGTTTATGGC [ 8580]
cp26_B31_TS_MP_FG_consensus  AGAATATGAAGAAAAAAAATTATAAAATTTTATAAAAAAAATCTAGAAAAAAAGAAACAAAAAAATATGGTTTATGGC [ 8580]
cp26_B31_NX_Pl_consensus     AGAATATGAAGAAAAAAAATTATAAAATTTTATAAAAAAAATCTAGAAAAAAAGAAACAAAAAAATATGGTTTATGGC [ 8580]
cp26_PAli_NX_MP_FG_consensus AGAATATGAAGAAAAAAAATTATAAAATTTTATAAAAAAAATCTAGAAAAAAAGAAACAAAAAAATATGGTTTATGGC [ 8580]
cp26_PAli_NX_Pl_consensus    AGAATATGAAGAAAAAAAATTATAAAATTTTATAAAAAAAATCTAGAAAAAAAGAAACAAAAAAATATGGTTTATGGC [ 8580]
cp26_PAbe_NX_MP_FG_consensus AGAATATGAAGAAAAAAAATTATAAAATTTTATAAAAAAAATCTAGAAAAAAAGAAACAAAAAAATATGGTTTATGGC [ 8580]
cp26_PAbe_NX_Pl_consensus    AGAATATGAAGAAAAAAAATTATAAAATTTTATAAAAAAAATCTAGAAAAAAAGAAACAAAAAAATATGGTTTATGGC [ 8580]

cp26_gi|11497007|B31_GB      AAAAAAATTCAAAAATACAGACTTTGATAAACTAATAAAAAAATTTAAAATTAAAAACAAAATGGAAAGAGAAAAAAA [ 8658]
cp26_un9_B31_PacBio          AAAAAAATTCAAAAATACAGACTTTGATAAACTAATAAAAAAATTTAAAATTAAAAACAAAATGGAAAGAGAAAAAAA [ 8658]
cp26_B31_TS_MP_FG_consensus  AAAAAAATTCAAAAATACAGACTTTGATAAACTAATAAAAAAATTTAAAATTAAAAACAAAATGGAAAGAGAAAAAAA [ 8658]
cp26_B31_NX_Pl_consensus     AAAAAAATTCAAAAATACAGACTTTGATAAACTAATAAAAAAATTTAAAATTAAAAACAAAATGGAAAGAGAAAAAAA [ 8658]
cp26_PAli_NX_MP_FG_consensus AAAAAAATTCAAAAATACAGACTTTGATAAACTAATAAAAAAATTTAAAATTAAAAACAAAATGGAAAGAGAAAAAAA [ 8658]
cp26_PAli_NX_Pl_consensus    AAAAAAATTCAAAAATACAGACTTTGATAAACTAATAAAAAAATTTAAAATTAAAAACAAAATGGAAAGAGAAAAAAA [ 8658]
cp26_PAbe_NX_MP_FG_consensus AAAAAAATTCAAAAATACAGACTTTGATAAACTAATAAAAAAATTTAAAATTAAAAACAAAATGGAAAGAGAAAAAAA [ 8658]
cp26_PAbe_NX_Pl_consensus    AAAAAAATTCAAAAATACAGACTTTGATAAACTAATAAAAAAATTTAAAATTAAAAACAAAATGGAAAGAGAAAAAAA [ 8658]

cp26_gi|11497007|B31_GB      TTATGAAAACGAGAACCAAATACATACATCAAATAACATAAAAAAATGCTATTGTATTAATGAAGACTCTAATAAAAA [ 8736]
cp26_un9_B31_PacBio          TTATGAAAACGAGAACCAAATACATACATCAAATAACATAAAAAAATGCTATTGTATTAATGAAGACTCTAATAAAAA [ 8736]
cp26_B31_TS_MP_FG_consensus  TTATGAAAACGAGAACCAAATACATACATCAAATAACATAAAAAAATGCTATTGTATTAATGAAGACTCTAATAAAAA [ 8736]
cp26_B31_NX_Pl_consensus     TTATGAAAACGAGAACCAAATACATACATCAAATAACATAAAAAAATGCTATTGTATTAATGAAGACTCTAATAAAAA [ 8736]
cp26_PAli_NX_MP_FG_consensus TTATGAAAACGAGAACCAAATACATACATCAAATAACATAAAAAAATGCTATTGTATTAATGAAGACTCTAATAAAAA [ 8736]
cp26_PAli_NX_Pl_consensus    TTATGAAAACGAGAACCAAATACATACATCAAATAACATAAAAAAATGCTATTGTATTAATGAAGACTCTAATAAAAA [ 8736]
cp26_PAbe_NX_MP_FG_consensus TTATGAAAACGAGAACCAAATACATACATCAAATAACATAAAAAAATGCTATTGTATTAATGAAGACTCTAATAAAAA [ 8736]
cp26_PAbe_NX_Pl_consensus    TTATGAAAACGAGAACCAAATACATACATCAAATAACATAAAAAAATGCTATTGTATTAATGAAGACTCTAATAAAAA [ 8736]

cp26_gi|11497007|B31_GB      ACAAAAATATGACAAAAAAATTAAAAAGTAAAGCAAAAATAATATAATGAAAAATAAAGAAACAAAAAAAAATTTTTT [ 8814]
cp26_un9_B31_PacBio          ACAAAAATATGACAAAAAAATTAAAAAGTAAAGCAAAAATAATATAATGAAAAATAAAGAAACAAAAAAAAATTTTTT [ 8814]
cp26_B31_TS_MP_FG_consensus  ACAAAAATATGACAAAAAAATTAAAAAGTAAAGCAAAAATAATATAATGAAAAATAAAGAAACAAAAAAAAATTTTTT [ 8814]
cp26_B31_NX_Pl_consensus     ACAAAAATATGACAAAAAAATTAAAAAGTAAAGCAAAAATAATATAATGAAAAATAAAGAAACAAAAAAAAATTTTTT [ 8814]
cp26_PAli_NX_MP_FG_consensus ACAAAAATATGACAAAAAAATTAAAAAGTAAAGCAAAAATAATATAATGAAAAATAAAGAAACAAAAAAAAATTTTTT [ 8814]
cp26_PAli_NX_Pl_consensus    ACAAAAATATGACAAAAAAATTAAAAAGTAAAGCAAAAATAATATAATGAAAAATAAAGAAACAAAAAAAAATTTTTT [ 8814]
cp26_PAbe_NX_MP_FG_consensus ACAAAAATATGACAAAAAAATTAAAAAGTAAAGCAAAAATAATATAATGAAAAATAAAGAAACAAAAAAAAATTTTTT [ 8814]
cp26_PAbe_NX_Pl_consensus    ACAAAAATATGACAAAAAAATTAAAAAGTAAAGCAAAAATAATATAATGAAAAATAAAGAAACAAAAAAAAATTTTTT [ 8814]

cp26_gi|11497007|B31_GB      CAACAAAATTGAAAAATTAGAAAACAAAATTATTTATCACACAAAAATCTTTAGCATGATAAATAATTTTGAAGCAAA [ 8892]
cp26_un9_B31_PacBio          CAACAAAATTGAAAAATTAGAAAACAAAATTATTTATCACACAAAAATCTTTAGCATGATAAATAATTTTGAAGCAAA [ 8892]
cp26_B31_TS_MP_FG_consensus  CAACAAAATTGAAAAATTAGAAAACAAAATTATTTATCACACAAAAATCTTTAGCATGATAAATAATTTTGAAGCAAA [ 8892]
cp26_B31_NX_Pl_consensus     CAACAAAATTGAAAAATTAGAAAACAAAATTATTTATCACACAAAAATCTTTAGCATGATAAATAATTTTGAAGCAAA [ 8892]
cp26_PAli_NX_MP_FG_consensus CAACAAAATTGAAAAATTAGAAAACAAAATTATTTATCACACAAAAATCTTTAGCATGATAAATAATTTTGAAGCAAA [ 8892]
cp26_PAli_NX_Pl_consensus    CAACAAAATTGAAAAATTAGAAAACAAAATTATTTATCACACAAAAATCTTTAGCATGATAAATAATTTTGAAGCAAA [ 8892]
cp26_PAbe_NX_MP_FG_consensus CAACAAAATTGAAAAATTAGAAAACAAAATTATTTATCACACAAAAATCTTTAGCATGATAAATAATTTTGAAGCAAA [ 8892]
cp26_PAbe_NX_Pl_consensus    CAACAAAATTGAAAAATTAGAAAACAAAATTATTTATCACACAAAAATCTTTAGCATGATAAATAATTTTGAAGCAAA [ 8892]

cp26_gi|11497007|B31_GB      ACCCAAAAAAGGAAAATTTTGGCTATGTCTGAGAAATATATTTAATAATAAAAAATACGAAAGTTTTCATTTATTTTC [ 8970]
cp26_un9_B31_PacBio          ACCCAAAAAAGGAAAATTTTGGCTATGTCTGAGAAATATATTTAATAATAAAAAATACGAAAGTTTTCATTTATTTTC [ 8970]
cp26_B31_TS_MP_FG_consensus  ACCCAAAAAAGGAAAATTTTGGCTATGTCTGAGAAATATATTTAATAATAAAAAATACGAAAGTTTTCATTTATTTTC [ 8970]
cp26_B31_NX_Pl_consensus     ACCCAAAAAAGGAAAATTTTGGCTATGTCTGAGAAATATATTTAATAATAAAAAATACGAAAGTTTTCATTTATTTTC [ 8970]
cp26_PAli_NX_MP_FG_consensus ACCCAAAAAAGGAAAATTTTGGCTATGTCTGAGAAATATATTTAATAATAAAAAATACGAAAGTTTTCATTTATTTTC [ 8970]
cp26_PAli_NX_Pl_consensus    ACCCAAAAAAGGAAAATTTTGGCTATGTCTGAGAAATATATTTAATAATAAAAAATACGAAAGTTTTCATTTATTTTC [ 8970]
cp26_PAbe_NX_MP_FG_consensus ACCCAAAAAAGGAAAATTTTGGCTATGTCTGAGAAATATATTTAATAATAAAAAATACGAAAGTTTTCATTTATTTTC [ 8970]
cp26_PAbe_NX_Pl_consensus    ACCCAAAAAAGGAAAATTTTGGCTATGTCTGAGAAATATATTTAATAATAAAAAATACGAAAGTTTTCATTTATTTTC [ 8970]

cp26_gi|11497007|B31_GB      AGTAAAAGAAAACGATAAATTTTTAGGAATTTTTTATGGTTTTAAAAACCTACCAAGACCATTTGTTATAAATTATTC [ 9048]
cp26_un9_B31_PacBio          AGTAAAAGAAAACGATAAATTTTTAGGAATTTTTTATGGTTTTAAAAACCTACCAAGACCATTTGTTATAAATTATTC [ 9048]
cp26_B31_TS_MP_FG_consensus  AGTAAAAGAAAACGATAAATTTTTAGGAATTTTTTATGGTTTTAAAAACCTACCAAGACCATTTGTTATAAATTATTC [ 9048]
cp26_B31_NX_Pl_consensus     AGTAAAAGAAAACGATAAATTTTTAGGAATTTTTTATGGTTTTAAAAACCTACCAAGACCATTTGTTATAAATTATTC [ 9048]
cp26_PAli_NX_MP_FG_consensus AGTAAAAGAAAACGATAAATTTTTAGGAATTTTTTATGGTTTTAAAAACCTACCAAGACCATTTGTTATAAATTATTC [ 9048]
cp26_PAli_NX_Pl_consensus    AGTAAAAGAAAACGATAAATTTTTAGGAATTTTTTATGGTTTTAAAAACCTACCAAGACCATTTGTTATAAATTATTC [ 9048]
cp26_PAbe_NX_MP_FG_consensus AGTAAAAGAAAACGATAAATTTTTAGGAATTTTTTATGGTTTTAAAAACCTACCAAGACCATTTGTTATAAATTATTC [ 9048]
cp26_PAbe_NX_Pl_consensus    AGTAAAAGAAAACGATAAATTTTTAGGAATTTTTTATGGTTTTAAAAACCTACCAAGACCATTTGTTATAAATTATTC [ 9048]

cp26_gi|11497007|B31_GB      AGAAAAAGGAACAAAAAAAACAATAAGATTAAAAAAAATATTTTATATGGAATTCAAATTTAAAAAAGGAAGCGTTTT [ 9126]
cp26_un9_B31_PacBio          AGAAAAAGGAACAAAAAAAACAATAAGATTAAAAAAAATATTTTATATGGAATTCAAATTTAAAAAAGGAAGCGTTTT [ 9126]
cp26_B31_TS_MP_FG_consensus  AGAAAAAGGAACAAAAAAAACAATAAGATTAAAAAAAATATTTTATATGGAATTCAAATTTAAAAAAGGAAGCGTTTT [ 9126]
cp26_B31_NX_Pl_consensus     AGAAAAAGGAACAAAAAAAACAATAAGATTAAAAAAAATATTTTATATGGAATTCAAATTTAAAAAAGGAAGCGTTTT [ 9126]
cp26_PAli_NX_MP_FG_consensus AGAAAAAGGAACAAAAAAAACAATAAGATTAAAAAAAATATTTTATATGGAATTCAAATTTAAAAAAGGAAGCGTTTT [ 9126]
cp26_PAli_NX_Pl_consensus    AGAAAAAGGAACAAAAAAAACAATAAGATTAAAAAAAATATTTTATATGGAATTCAAATTTAAAAAAGGAAGCGTTTT [ 9126]
cp26_PAbe_NX_MP_FG_consensus AGAAAAAGGAACAAAAAAAACAATAAGATTAAAAAAAATATTTTATATGGAATTCAAATTTAAAAAAGGAAGCGTTTT [ 9126]
cp26_PAbe_NX_Pl_consensus    AGAAAAAGGAACAAAAAAAACAATAAGATTAAAAAAAATATTTTATATGGAATTCAAATTTAAAAAAGGAAGCGTTTT [ 9126]

cp26_gi|11497007|B31_GB      TTGTTATTTAAGAAGCTTATACATATTCACCAAGATAAAAAATAAAAATAAACTTTTTTATAAATCTCTTTTAGAAAG [ 9204]
cp26_un9_B31_PacBio          TTGTTATTTAAGAAGCTTATACATATTCACCAAGATAAAAAATAAAAATAAACTTTTTTATAAATCTCTTTTAGAAAG [ 9204]
cp26_B31_TS_MP_FG_consensus  TTGTTATTTAAGAAGCTTATACATATTCACCAAGATAAAAAATAAAAATAAACTTTTTTATAAATCTCTTTTAGAAAG [ 9204]
cp26_B31_NX_Pl_consensus     TTGTTATTTAAGAAGCTTATACATATTCACCAAGATAAAAAATAAAAATAAACTTTTTTATAAATCTCTTTTAGAAAG [ 9204]
cp26_PAli_NX_MP_FG_consensus TTGTTATTTAAGAAGCTTATACATATTCACCAAGATAAAAAATAAAAATAAACTTTTTTATAAATCTCTTTTAGAAAG [ 9204]
cp26_PAli_NX_Pl_consensus    TTGTTATTTAAGAAGCTTATACATATTCACCAAGATAAAAAATAAAAATAAACTTTTTTATAAATCTCTTTTAGAAAG [ 9204]
cp26_PAbe_NX_MP_FG_consensus TTGTTATTTAAGAAGCTTATACATATTCACCAAGATAAAAAATAAAAATAAACTTTTTTATAAATCTCTTTTAGAAAG [ 9204]
cp26_PAbe_NX_Pl_consensus    TTGTTATTTAAGAAGCTTATACATATTCACCAAGATAAAAAATAAAAATAAACTTTTTTATAAATCTCTTTTAGAAAG [ 9204]

cp26_gi|11497007|B31_GB      AACTCTAAAAATTGAAGAAGAAATACATAAATTTTATGGTAAAAAATATGAAAGTAATAAAGGGATAGTAAATTGGAT [ 9282]
cp26_un9_B31_PacBio          AACTCTAAAAATTGAAGAAGAAATACATAAATTTTATGGTAAAAAATATGAAAGTAATAAAGGGATAGTAAATTGGAT [ 9282]
cp26_B31_TS_MP_FG_consensus  AACTCTAAAAATTGAAGAAGAAATACATAAATTTTATGGTAAAAAATATGAAAGTAATAAAGGGATAGTAAATTGGAT [ 9282]
cp26_B31_NX_Pl_consensus     AACTCTAAAAATTGAAGAAGAAATACATAAATTTTATGGTAAAAAATATGAAAGTAATAAAGGGATAGTAAATTGGAT [ 9282]
cp26_PAli_NX_MP_FG_consensus AACTCTAAAAATTGAAGAAGAAATACATAAATTTTATGGTAAAAAATATGAAAGTAATAAAGGGATAGTAAATTGGAT [ 9282]
cp26_PAli_NX_Pl_consensus    AACTCTAAAAATTGAAGAAGAAATACATAAATTTTATGGTAAAAAATATGAAAGTAATAAAGGGATAGTAAATTGGAT [ 9282]
cp26_PAbe_NX_MP_FG_consensus AACTCTAAAAATTGAAGAAGAAATACATAAATTTTATGGTAAAAAATATGAAAGTAATAAAGGGATAGTAAATTGGAT [ 9282]
cp26_PAbe_NX_Pl_consensus    AACTCTAAAAATTGAAGAAGAAATACATAAATTTTATGGTAAAAAATATGAAAGTAATAAAGGGATAGTAAATTGGAT [ 9282]
```

```
cp26_gi|11497007|B31_GB      AAAAAAAACACAAAAATAATAACAATTGCATCAATAAAAGGGGGGGTGGGAAAAAGTACAACATCATTAATGTTTACA [ 9360]
cp26_un9_B31_PacBio          AAAAAAAACACAAAAATAATAACAATTGCATCAATAAAAGGGGGGGTGGGAAAAAGTACAACATCATTAATGTTTACA [ 9360]
cp26_B31_TS_MP_FG_consensus  AAAAAAAACACAAAAATAATAACAATTGCATCAATAAAAGGGGGGGTGGGAAAAAGTACAACATCATTAATGTTTACA [ 9360]
cp26_B31_NX_Pl_consensus     AAAAAAAACACAAAAATAATAACAATTGCATCAATAAAAGGGGGGGTGGGAAAAAGTACAACATCATTAATGTTTACA [ 9360]
cp26_PAli_NX_MP_FG_consensus AAAAAAAACACAAAAATAATAACAATTGCATCAATAAAAGGGGGGGTGGGAAAAAGTACAACATCATTAATGTTTACA [ 9360]
cp26_PAli_NX_Pl_consensus    AAAAAAAACACAAAAATAATAACAATTGCATCAATAAAAGGGGGGGTGGGAAAAAGTACAACATCATTAATGTTTACA [ 9360]
cp26_PAbe_NX_MP_FG_consensus AAAAAAAACACAAAAATAATAACAATTGCATCAATAAAAGGGGGGGTGGGAAAAAGTACAACATCATTAATGTTTACA [ 9360]
cp26_PAbe_NX_Pl_consensus    AAAAAAAACACAAAAATAATAACAATTGCATCAATAAAAGGGGGGGTGGGAAAAAGTACAACATCATTAATGTTTACA [ 9360]

cp26_gi|11497007|B31_GB      AACATACTCTCAAAAAAAAATAATAAAATATTATTAATAGATTTAGATCCACAAGCAAGTAGTACAAGTTTTTATATT [ 9438]
cp26_un9_B31_PacBio          AACATACTCTCAAAAAAAAATAATAAAATATTATTAATAGATTTAGATCCACAAGCAAGTAGTACAAGTTTTTATATT [ 9438]
cp26_B31_TS_MP_FG_consensus  AACATACTCTCAAAAAAAAATAATAAAATATTATTAATAGATTTAGATCCACAAGCAAGTAGTACAAGTTTTTATATT [ 9438]
cp26_B31_NX_Pl_consensus     AACATACTCTCAAAAAAAAATAATAAAATATTATTAATAGATTTAGATCCACAAGCAAGTAGTACAAGTTTTTATATT [ 9438]
cp26_PAli_NX_MP_FG_consensus AACATACTCTCAAAAAAAAATAATAAAATATTATTAATAGATTTAGATCCACAAGCAAGTAGTACAAGTTTTTATATT [ 9438]
cp26_PAli_NX_Pl_consensus    AACATACTCTCAAAAAAAAATAATAAAATATTATTAATAGATTTAGATCCACAAGCAAGTAGTACAAGTTTTTATATT [ 9438]
cp26_PAbe_NX_MP_FG_consensus AACATACTCTCAAAAAAAAATAATAAAATATTATTAATAGATTTAGATCCACAAGCAAGTAGTACAAGTTTTTATATT [ 9438]
cp26_PAbe_NX_Pl_consensus    AACATACTCTCAAAAAAAAATAATAAAATATTATTAATAGATTTAGATCCACAAGCAAGTAGTACAAGTTTTTATATT [ 9438]

cp26_gi|11497007|B31_GB      AATATATTAAGAAAAAAAAACCTTAGTCCAAAAGATATCAATATATATAAAGTTCTTAAAAAAGAAATAGACATAGAA [ 9516]
cp26_un9_B31_PacBio          AATATATTAAGAAAAAAAAACCTTAGTCCAAAAGATATCAATATATATAAAGTTCTTAAAAAAGAAATAGACATAGAA [ 9516]
cp26_B31_TS_MP_FG_consensus  AATATATTAAGAAAAAAAAACCTTAGTCCAAAAGATATCAATATATATAAAGTTCTTAAAAAAGAAATAGACATAGAA [ 9516]
cp26_B31_NX_Pl_consensus     AATATATTAAGAAAAAAAAACCTTAGTCCAAAAGATATCAATATATATAAAGTTCTTAAAAAAGAAATAGACATAGAA [ 9516]
cp26_PAli_NX_MP_FG_consensus AATATATTAAGAAAAAAAAACCTTAGTCCAAAAGATATCAATATATATAAAGTTCTTAAAAAAGAAATAGACATAGAA [ 9516]
cp26_PAli_NX_Pl_consensus    AATATATTAAGAAAAAAAAACCTTAGTCCAAAAGATATCAATATATATAAAGTTCTTAAAAAAGAAATAGACATAGAA [ 9516]
cp26_PAbe_NX_MP_FG_consensus AATATATTAAGAAAAAAAAACCTTAGTCCAAAAGATATCAATATATATAAAGTTCTTAAAAAAGAAATAGACATAGAA [ 9516]
cp26_PAbe_NX_Pl_consensus    AATATATTAAGAAAAAAAAACCTTAGTCCAAAAGATATCAATATATATAAAGTTCTTAAAAAAGAAATAGACATAGAA [ 9516]

cp26_gi|11497007|B31_GB      AATTCAACAATCAAAATAGATAATAATACAGATTTCATAGCAAGCCACATAAACCTGAGTAGATTTAATGAAGAAAGT [ 9594]
cp26_un9_B31_PacBio          AATTCAACAATCAAAATAGATAATAATACAGATTTCATAGCAAGCCACATAAACCTGAGTAGATTTAATGAAGAAAGT [ 9594]
cp26_B31_TS_MP_FG_consensus  AATTCAACAATCAAAATAGATAATAATACAGATTTCATAGCAAGCCACATAAACCTGAGTAGATTTAATGAAGAAAGT [ 9594]
cp26_B31_NX_Pl_consensus     AATTCAACAATCAAAATAGATAATAATACAGATTTCATAGCAAGCCACATAAACCTGAGTAGATTTAATGAAGAAAGT [ 9594]
cp26_PAli_NX_MP_FG_consensus AATTCAACAATCAAAATAGATAATAATACAGATTTCATAGCAAGCCACATAAACCTGAGTAGATTTAATGAAGAAAGT [ 9594]
cp26_PAli_NX_Pl_consensus    AATTCAACAATCAAAATAGATAATAATACAGATTTCATAGCAAGCCACATAAACCTGAGTAGATTTAATGAAGAAAGT [ 9594]
cp26_PAbe_NX_MP_FG_consensus AATTCAACAATCAAAATAGATAATAATACAGATTTCATAGCAAGCCACATAAACCTGAGTAGATTTAATGAAGAAAGT [ 9594]
cp26_PAbe_NX_Pl_consensus    AATTCAACAATCAAAATAGATAATAATACAGATTTCATAGCAAGCCACATAAACCTGAGTAGATTTAATGAAGAAAGT [ 9594]

cp26_gi|11497007|B31_GB      ATTTCATTGAAAGAAAATTTACTCAAAATTTTCTTAAGTTTTATTCAAAACAGATACGACTTTATTATAATGGATACT [ 9672]
cp26_un9_B31_PacBio          ATTTCATTGAAAGAAAATTTACTCAAAATTTTCTTAAGTTTTATTCAAAACAGATACGACTTTATTATAATGGATACT [ 9672]
cp26_B31_TS_MP_FG_consensus  ATTTCATTGAAAGAAAATTTACTCAAAATTTTCTTAAGTTTTATTCAAAACAGATACGACTTTATTATAATGGATACT [ 9672]
cp26_B31_NX_Pl_consensus     ATTTCATTGAAAGAAAATTTACTCAAAATTTTCTTAAGTTTTATTCAAAACAGATACGACTTTATTATAATGGATACT [ 9672]
cp26_PAli_NX_MP_FG_consensus ATTTCATTGAAAGAAAATTTACTCAAAATTTTCTTAAGTTTTATTCAAAACAGATACGACTTTATTATAATGGATACT [ 9672]
cp26_PAli_NX_Pl_consensus    ATTTCATTGAAAGAAAATTTACTCAAAATTTTCTTAAGTTTTATTCAAAACAGATACGACTTTATTATAATGGATACT [ 9672]
cp26_PAbe_NX_MP_FG_consensus ATTTCATTGAAAGAAAATTTACTCAAAATTTTCTTAAGTTTTATTCAAAACAGATACGACTTTATTATAATGGATACT [ 9672]
cp26_PAbe_NX_Pl_consensus    ATTTCATTGAAAGAAAATTTACTCAAAATTTTCTTAAGTTTTATTCAAAACAGATACGACTTTATTATAATGGATACT [ 9672]

cp26_gi|11497007|B31_GB      GCACCCACATTAGGAAGCTTGCTCAATAATAGTTTAATCATCTCAGATTACTTAATCATACCTTTGCCAACAGACCAA [ 9750]
cp26_un9_B31_PacBio          GCACCCACATTAGGAAGCTTGCTCAATAATAGTTTAATCATCTCAGATTACTTAATCATACCTTTGCCAACAGACCAA [ 9750]
cp26_B31_TS_MP_FG_consensus  GCACCCACATTAGGAAGCTTGCTCAATAATAGTTTAATCATCTCAGATTACTTAATCATACCTTTGCCAACAGACCAA [ 9750]
cp26_B31_NX_Pl_consensus     GCACCCACATTAGGAAGCTTGCTCAATAATAGTTTAATCATCTCAGATTACTTAATCATACCTTTGCCAACAGACCAA [ 9750]
cp26_PAli_NX_MP_FG_consensus GCACCCACATTAGGAAGCTTGCTCAATAATAGTTTAATCATCTCAGATTACTTAATCATACCTTTGCCAACAGACCAA [ 9750]
cp26_PAli_NX_Pl_consensus    GCACCCACATTAGGAAGCTTGCTCAATAATAGTTTAATCATCTCAGATTACTTAATCATACCTTTGCCAACAGACCAA [ 9750]
cp26_PAbe_NX_MP_FG_consensus GCACCCACATTAGGAAGCTTGCTCAATAATAGTTTAATCATCTCAGATTACTTAATCATACCTTTGCCAACAGACCAA [ 9750]
cp26_PAbe_NX_Pl_consensus    GCACCCACATTAGGAAGCTTGCTCAATAATAGTTTAATCATCTCAGATTACTTAATCATACCTTTGCCAACAGACCAA [ 9750]

cp26_gi|11497007|B31_GB      TGGGCAATTGAAAGTTTAGACCTAATAATTAACAGGCTTGATGATCTTTTTAGAAAGGATTTGCCAATATTTTATTTA [ 9828]
cp26_un9_B31_PacBio          TGGGCAATTGAAAGTTTAGACCTAATAATTAACAGGCTTGATGATCTTTTTAGAAAGGATTTGCCAATATTTTATTTA [ 9828]
cp26_B31_TS_MP_FG_consensus  TGGGCAATTGAAAGTTTAGACCTAATAATTAACAGGCTTGATGATCTTTTTAGAAAGGATTTGCCAATATTTTATTTA [ 9828]
cp26_B31_NX_Pl_consensus     TGGGCAATTGAAAGTTTAGACCTAATAATTAACAGGCTTGATGATCTTTTTAGAAAGGATTTGCCAATATTTTATTTA [ 9828]
cp26_PAli_NX_MP_FG_consensus TGGGCAATTGAAAGTTTAGACCTAATAATTAACAGGCTTGATGATCTTTTTAGAAAGGATTTGCCAATATTTTATTTA [ 9828]
cp26_PAli_NX_Pl_consensus    TGGGCAATTGAAAGTTTAGACCTAATAATTAACAGGCTTGATGATCTTTTTAGAAAGGATTTGCCAATATTTTATTTA [ 9828]
cp26_PAbe_NX_MP_FG_consensus TGGGCAATTGAAAGTTTAGACCTAATAATTAACAGGCTTGATGATCTTTTTAGAAAGGATTTGCCAATATTTTATTTA [ 9828]
cp26_PAbe_NX_Pl_consensus    TGGGCAATTGAAAGTTTAGACCTAATAATTAACAGGCTTGATGATCTTTTTAGAAAGGATTTGCCAATATTTTATTTA [ 9828]

cp26_gi|11497007|B31_GB      ATAACCAAATTTATTGAAAGACAAAATATTGATAAAGAACTTAAAAAGTTTATTGAATGCGAATATAAAGGCAAATTT [ 9906]
cp26_un9_B31_PacBio          ATAACCAAATTTATTGAAAGACAAAATATTGATAAAGAACTTAAAAAGTTTATTGAATGCGAATATAAAGGCAAATTT [ 9906]
cp26_B31_TS_MP_FG_consensus  ATAACCAAATTTATTGAAAGACAAAATATTGATAAAGAACTTAAAAAGTTTATTGAATGCGAATATAAAGGCAAATTT [ 9906]
cp26_B31_NX_Pl_consensus     ATAACCAAATTTATTGAAAGACAAAATATTGATAAAGAACTTAAAAAGTTTATTGAATGCGAATATAAAGGCAAATTT [ 9906]
cp26_PAli_NX_MP_FG_consensus ATAACCAAATTTATTGAAAGACAAAATATTGATAAAGAACTTAAAAAGTTTATTGAATGCGAATATAAAGGCAAATTT [ 9906]
cp26_PAli_NX_Pl_consensus    ATAACCAAATTTATTGAAAGACAAAATATTGATAAAGAACTTAAAAAGTTTATTGAATGCGAATATAAAGGCAAATTT [ 9906]
cp26_PAbe_NX_MP_FG_consensus ATAACCAAATTTATTGAAAGACAAAATATTGATAAAGAACTTAAAAAGTTTATTGAATGCGAATATAAAGGCAAATTT [ 9906]
cp26_PAbe_NX_Pl_consensus    ATAACCAAATTTATTGAAAGACAAAATATTGATAAAGAACTTAAAAAGTTTATTGAATGCGAATATAAAGGCAAATTT [ 9906]

cp26_gi|11497007|B31_GB      TTAGGAAGTGTTCCAAAAAGAGATAATTTACGTAAAACCATTTTTTACAGAGAAGAATTCAATTCTAATGAAGATTAT [ 9984]
cp26_un9_B31_PacBio          TTAGGAAGTGTTCCAAAAAGAGATAATTTACGTAAAACCATTTTTTACAGAGAAGAATTCAATTCTAATGAAGATTAT [ 9984]
cp26_B31_TS_MP_FG_consensus  TTAGGAAGTGTTCCAAAAAGAGATAATTTACGTAAAACCATTTTTTACAGAGAAGAATTCAATTCTAATGAAGATTAT [ 9984]
cp26_B31_NX_Pl_consensus     TTAGGAAGTGTTCCAAAAAGAGATAATTTACGTAAAACCATTTTTTACAGAGAAGAATTCAATTCTAATGAAGATTAT [ 9984]
cp26_PAli_NX_MP_FG_consensus TTAGGAAGTGTTCCAAAAAGAGATAATTTACGTAAAACCATTTTTTACAGAGAAGAATTCAATTCTAATGAAGATTAT [ 9984]
cp26_PAli_NX_Pl_consensus    TTAGGAAGTGTTCCAAAAAGAGATAATTTACGTAAAACCATTTTTTACAGAGAAGAATTCAATTCTAATGAAGATTAT [ 9984]
cp26_PAbe_NX_MP_FG_consensus TTAGGAAGTGTTCCAAAAAGAGATAATTTACGTAAAACCATTTTTTACAGAGAAGAATTCAATTCTAATGAAGATTAT [ 9984]
cp26_PAbe_NX_Pl_consensus    TTAGGAAGTGTTCCAAAAAGAGATAATTTACGTAAAACCATTTTTTACAGAGAAGAATTCAATTCTAATGAAGATTAT [ 9984]

cp26_gi|11497007|B31_GB      TATAAAGCTTATGAAAATATACTTGAAAATTTTTTAAGCATAATTTCAAATTAGTTCCATATGGAACTAAAAGTTCCA [10062]
cp26_un9_B31_PacBio          TATAAAGCTTATGAAAATATACTTGAAAATTTTTTAAGCATAATTTCAAATTAGTTCCATATGGAACTTTTAGTTCCA [10062]
cp26_B31_TS_MP_FG_consensus  TATAAAGCTTATGAAAATATACTTGAAAATTTTTTAAGCATAATTTCAAATTAGTTCCATATGGAACTTTTAGTTCCA [10062]
cp26_B31_NX_Pl_consensus     TATAAAGCTTATGAAAATATACTTGAAAATTTTTTAAGCATAATTTCAAATTAGTTCCATATGGAACTTTTAGTTCCA [10062]
cp26_PAli_NX_MP_FG_consensus TATAAAGCTTATGAAAATATACTTGAAAATTTTTTAAGCATAATTTCAAATTAGTTCCATATGGAACTTTTAGTTCCA [10062]
cp26_PAli_NX_Pl_consensus    TATAAAGCTTATGAAAATATACTTGAAAATTTTTTAAGCATAATTTCAAATTAGTTCCATATGGAACTTTTAGTTCCA [10062]
cp26_PAbe_NX_MP_FG_consensus TATAAAGCTTATGAAAATATACTTGAAAATTTTTTAAGCATAATTTCAAATTAGTTCCATATGGAACTTTTAGTTCCA [10062]
cp26_PAbe_NX_Pl_consensus    TATAAAGCTTATGAAAATATACTTGAAAATTTTTTAAGCATAATTTCAAATTAGTTCCATATGGAACTTTTAGTTCCA [10062]

cp26_gi|11497007|B31_GB      TATGGAACTAATTTGAAATTATGCAATGGAGTAAAAATAAAAGATGAAATTAAATAAAAAAAATAGAAATTGTCAAAAG [10140]
cp26_un9_B31_PacBio          TATGGAACTAATTTGAAATTATGCAATGGAGTAAAAATAAAAGATGAAATTAAATAAAAAAAATAGAAATTGTCAAAAG [10140]
cp26_B31_TS_MP_FG_consensus  TATGGAACTAATTTGAAATTATGCAATGGAGTAAAAATAAAAGATGAAATTAAATAAAAAAAATAGAAATTGTCAAAAG [10140]
cp26_B31_NX_Pl_consensus     TATGGAACTAATTTGAAATTATGCAATGGAGTAAAAATAAAAGATGAAATTAAATAAAAAAAATAGAAATTGTCAAAAG [10140]
cp26_PAli_NX_MP_FG_consensus TATGGAACTAATTTGAAATTATGCAATGGAGTAAAAATAAAAGATGAAATTAAATAAAAAAAATAGAAATTGTCAAAAG [10140]
cp26_PAli_NX_Pl_consensus    TATGGAACTAATTTGAAATTATGCAATGGAGTAAAAATAAAAGATGAAATTAAATAAAAAAAATAGAAATTGTCAAAAG [10140]
cp26_PAbe_NX_MP_FG_consensus TATGGAACTAATTTGAAATTATGCAATGGAGTAAAAATAAAAGATGAAATTAAATAAAAAAAATAGAAATTGTCAAAAG [10140]
cp26_PAbe_NX_Pl_consensus    TATGGAACTAATTTGAAATTATGCAATGGAGTAAAAATAAAAGATGAAATTAAATAAAAAAAATAGAAATTGTCAAAAG [10140]

cp26_gi|11497007|B31_GB      AATTGAGCTTAGTGGAAATGAAATAAAAAAAAGCAGAGAATCTAGATATTTAAAATTGAAAGAAAAACTAAAAATTTT [10218]
cp26_un9_B31_PacBio          AATTGAGCTTAGTGGAAATGAAATAAAAAAAAGCAGAGAATCTAGATATTTAAAATTGAAAGAAAAACTAAAAATTTT [10218]
cp26_B31_TS_MP_FG_consensus  AATTGAGCTTAGTGGAAATGAAATAAAAAAAAGCAGAGAATCTAGATATTTAAAATTGAAAGAAAAACTAAAAATTTT [10218]
cp26_B31_NX_Pl_consensus     AATTGAGCTTAGTGGAAATGAAATAAAAAAAAGCAGAGAATCTAGATATTTAAAATTGAAAGAAAAACTAAAAATTTT [10218]
cp26_PAli_NX_MP_FG_consensus AATTGAGCTTAGTGGAAATGAAATAAAAAAAAGCAGAGAATCTAGATATTTAAAATTGAAAGAAAAACTAAAAATTTT [10218]
cp26_PAli_NX_Pl_consensus    AATTGAGCTTAGTGGAAATGAAATAAAAAAAAGCAGAGAATCTAGATATTTAAAATTGAAAGAAAAACTAAAAATTTT [10218]
cp26_PAbe_NX_MP_FG_consensus AATTGAGCTTAGTGGAAATGAAATAAAAAAAAGCAGAGAATCTAGATATTTAAAATTGAAAGAAAAACTAAAAATTTT [10218]
cp26_PAbe_NX_Pl_consensus    AATTGAGCTTAGTGGAAATGAAATAAAAAAAAGCAGAGAATCTAGATATTTAAAATTGAAAGAAAAACTAAAAATTTT [10218]

cp26_gi|11497007|B31_GB      GATAAAAGAGGAATCTTACAATAAAATTGAAACAGCTAGAATTCTAAAAGAAATTAATGATAATAAGTACTATGTTAT [10296]
```

```
cp26_un9_B31_PacBio          GATAAAAGAGGAATCTTACAATAAAATTGAAACAGCTAGAATTCTAAAAGAAATTAATGATAATAAGTACTATGTTAT [10296]
cp26_B31_TS_MP_FG_consensus  GATAAAAGAGGAATCTTACAATAAAATTGAAACAGCTAGAATTCTAAAAGAAATTAATGATAATAAGTACTATGTTAT [10296]
cp26_B31_NX_Pl_consensus     GATAAAAGAGGAATCTTACAATAAAATTGAAACAGCTAGAATTCTAAAAGAAATTAATGATAATAAGTACTATGTTAT [10296]
cp26_PAli_NX_MP_FG_consensus GATAAAAGAGGAATCTTACAATAAAATTGAAACAGCTAGAATTCTAAAAGAAATTAATGATAATAAGTACTATGTTAT [10296]
cp26_PAli_NX_Pl_consensus    GATAAAAGAGGAATCTTACAATAAAATTGAAACAGCTAGAATTCTAAAAGAAATTAATGATAATAAGTACTATGTTAT [10296]
cp26_PAbe_NX_MP_FG_consensus GATAAAAGAGGAATCTTACAATAAAATTGAAACAGCTAGAATTCTAAAAGAAATTAATGATAATAAGTACTATGTTAT [10296]
cp26_PAbe_NX_Pl_consensus    GATAAAAGAGGAATCTTACAATAAAATTGAAACAGCTAGAATTCTAAAAGAAATTAATGATAATAAGTACTATGTTAT [10296]

cp26_gi|11497007|B31_GB      AGACAAATATAAAAGCTTTGGTCATTTTATAAAAGATTATAATATGGCAAAAACTACTGTTTATAGATATATTAAACT [10374]
cp26_un9_B31_PacBio          AGACAAATATAAAAGCTTTGGTCATTTTATAAAAGATTATAATATGGCAAAAACTACTGTTTATAGATATATTAAACT [10374]
cp26_B31_TS_MP_FG_consensus  AGACAAATATAAAAGCTTTGGTCATTTTATAAAAGATTATAATATGGCAAAAACTACTGTTTATAGATATATTAAACT [10374]
cp26_B31_NX_Pl_consensus     AGACAAATATAAAAGCTTTGGTCATTTTATAAAAGATTATAATATGGCAAAAACTACTGTTTATAGATATATTAAACT [10374]
cp26_PAli_NX_MP_FG_consensus AGACAAATATAAAAGCTTTGGTCATTTTATAAAAGATTATAATATGGCAAAAACTACTGTTTATAGATATATTAAACT [10374]
cp26_PAli_NX_Pl_consensus    AGACAAATATAAAAGCTTTGGTCATTTTATAAAAGATTATAATATGGCAAAAACTACTGTTTATAGATATATTAAACT [10374]
cp26_PAbe_NX_MP_FG_consensus AGACAAATATAAAAGCTTTGGTCATTTTATAAAAGATTATAATATGGCAAAAACTACTGTTTATAGATATATTAAACT [10374]
cp26_PAbe_NX_Pl_consensus    AGACAAATATAAAAGCTTTGGTCATTTTATAAAAGATTATAATATGGCAAAAACTACTGTTTATAGATATATTAAACT [10374]

cp26_gi|11497007|B31_GB      AGCAATAGGAATTGACAGTGGTAAAATCAATTATGAATTAATTTTAAAAAAAGGAATATATTATGCTATGCAATTTTT [10452]
cp26_un9_B31_PacBio          AGCAATAGGAATTGACAGTGGTAAAATCAATTATGAATTAATTTTAAAAAAAGGAATATATTATGCTATGCAATTTTT [10452]
cp26_B31_TS_MP_FG_consensus  AGCAATAGGAATTGACAGTGGTAAAATCAATTATGAATTAATTTTAAAAAAAGGAATATATTATGCTATGCAATTTTT [10452]
cp26_B31_NX_Pl_consensus     AGCAATAGGAATTGACAGTGGTAAAATCAATTATGAATTAATTTTAAAAAAAGGAATATATTATGCTATGCAATTTTT [10452]
cp26_PAli_NX_MP_FG_consensus AGCAATAGGAATTGACAGTGGTAAAATCAATTATGAATTAATTTTAAAAAAAGGAATATATTATGCTATGCAATTTTT [10452]
cp26_PAli_NX_Pl_consensus    AGCAATAGGAATTGACAGTGGTAAAATCAATTATGAATTAATTTTAAAAAAAGGAATATATTATGCTATGCAATTTTT [10452]
cp26_PAbe_NX_MP_FG_consensus AGCAATAGGAATTGACAGTGGTAAAATCAATTATGAATTAATTTTAAAAAAAGGAATATATTATGCTATGCAATTTTT [10452]
cp26_PAbe_NX_Pl_consensus    AGCAATAGGAATTGACAGTGGTAAAATCAATTATGAATTAATTTTAAAAAAAGGAATATATTATGCTATGCAATTTTT [10452]

cp26_gi|11497007|B31_GB      AGAAAATAATGAGACTATAGTTAATTCAAAAAGGATATTAAATAGATCACTTAGGCTAAAAATTGAAGACGAAAAAAG [10530]
cp26_un9_B31_PacBio          AGAAAATAATGAGACTATAGTTAATTCAAAAAGGATATTAAATAGATCACTTAGGCTAAAAATTGAAGACGAAAAAAG [10530]
cp26_B31_TS_MP_FG_consensus  AGAAAATAATGAGACTATAGTTAATTCAAAAAGGATATTAAATAGATCACTTAGGCTAAAAATTGAAGACGAAAAAAG [10530]
cp26_B31_NX_Pl_consensus     AGAAAATAATGAGACTATAGTTAATTCAAAAAGGATATTAAATAGATCACTTAGGCTAAAAATTGAAGACGAAAAAAG [10530]
cp26_PAli_NX_MP_FG_consensus AGAAAATAATGAGACTATAGTTAATTCAAAAAGGATATTAAATAGATCACTTAGGCTAAAAATTGAAGACGAAAAAAG [10530]
cp26_PAli_NX_Pl_consensus    AGAAAATAATGAGACTATAGTTAATTCAAAAAGGATATTAAATAGATCACTTAGGCTAAAAATTGAAGACGAAAAAAG [10530]
cp26_PAbe_NX_MP_FG_consensus AGAAAATAATGAGACTATAGTTAATTCAAAAAGGATATTAAATAGATCACTTAGGCTAAAAATTGAAGACGAAAAAAG [10530]
cp26_PAbe_NX_Pl_consensus    AGAAAATAATGAGACTATAGTTAATTCAAAAAGGATATTAAATAGATCACTTAGGCTAAAAATTGAAGACGAAAAAAG [10530]

cp26_gi|11497007|B31_GB      CCTTAATTTTTATAAATCAAATACAAACTTTGTAAGCTTCTTATTAAAAGAGCTATATTATGATAATCAAAAACTACT [10608]
cp26_un9_B31_PacBio          CCTTAATTTTTATAAATCAAATACAAACTTTGTAAGCTTCTTATTAAAAGAGCTATATTATGATAATCAAAAACTACT [10608]
cp26_B31_TS_MP_FG_consensus  CCTTAATTTTTATAAATCAAATACAAACTTTGTAAGCTTCTTATTAAAAGAGCTATATTATGATAATCAAAAACTACT [10608]
cp26_B31_NX_Pl_consensus     CCTTAATTTTTATAAATCAAATACAAACTTTGTAAGCTTCTTATTAAAAGAGCTATATTATGATAATCAAAAACTACT [10608]
cp26_PAli_NX_MP_FG_consensus CCTTAATTTTTATAAATCAAATACAAACTTTGTAAGCTTCTTATTAAAAGAGCTATATTATGATAATCAAAAACTACT [10608]
cp26_PAli_NX_Pl_consensus    CCTTAATTTTTATAAATCAAATACAAACTTTGTAAGCTTCTTATTAAAAGAGCTATATTATGATAATCAAAAACTACT [10608]
cp26_PAbe_NX_MP_FG_consensus CCTTAATTTTTATAAATCAAATACAAACTTTGTAAGCTTCTTATTAAAAGAGCTATATTATGATAATCAAAAACTACT [10608]
cp26_PAbe_NX_Pl_consensus    CCTTAATTTTTATAAATCAAATACAAACTTTGTAAGCTTCTTATTAAAAGAGCTATATTATGATAATCAAAAACTACT [10608]

cp26_gi|11497007|B31_GB      TATAAATATTAAAAATAAATATGACAATTTGAAAAATAAAAAATAATATTCTATATTTATAAAATAGTACTATATTTA [10686]
cp26_un9_B31_PacBio          TATAAATATTAAAAATAAATATGACAATTTGAAAAATAAAAAATAATATTCTATATTTATAAAATAGTACTATATTTA [10686]
cp26_B31_TS_MP_FG_consensus  TATAAATATTAAAAATAAATATGACAATTTGAAAAATAAAAAATAATATTCTATATTTATAAAATAGTACTATATTTA [10686]
cp26_B31_NX_Pl_consensus     TATAAATATTAAAAATAAATATGACAATTTGAAAAATAAAAAATAATATTCTATATTTATAAAATAGTACTATATTTA [10686]
cp26_PAli_NX_MP_FG_consensus TATAAATATTAAAAATAAATATGACAATTTGAAAAATAAAAAATAATATTCTATATTTATAAAATAGTACTATATTTA [10686]
cp26_PAli_NX_Pl_consensus    TATAAATATTAAAAATAAATATGACAATTTGAAAAATAAAAAATAATATTCTATATTTATAAAATAGTACTATATTTA [10686]
cp26_PAbe_NX_MP_FG_consensus TATAAATATTAAAAATAAATATGACAATTTGAAAAATAAAAAATAATATTCTATATTTATAAAATAGTACTATATTTA [10686]
cp26_PAbe_NX_Pl_consensus    TATAAATATTAAAAATAAATATGACAATTTGAAAAATAAAAAATAATATTCTATATTTATAAAATAGTACTATATTTA [10686]

cp26_gi|11497007|B31_GB      AAAAATGTTTCAAAAACACTTTACAAAAAAATAAAATACTATATAATAATCTATATAGATTGAGATGCAATGTCTCGC [10764]
cp26_un9_B31_PacBio          AAAAATGTTTCAAAAACACTTTACAAAAAAATAAAATACTATATAATAATCTATATAGATTGAGATGCAATGTCTCGC [10764]
cp26_B31_TS_MP_FG_consensus  AAAAATGTTTCAAAAACACTTTACAAAAAAATAAAATACTATATAATAATCTATATAGATTGAGATGCAATGTCTCGC [10764]
cp26_B31_NX_Pl_consensus     AAAAATGTTTCAAAAACACTTTACAAAAAAATAAAATACTATATAATAATCTATATAGATTGAGATGCAATGTCTCGC [10764]
cp26_PAli_NX_MP_FG_consensus AAAAATGTTTCAAAAACACTTTACAAAAAAATAAAATACTATATAATAATCTATATAGATTGAGATGCAATGTCTCGC [10764]
cp26_PAli_NX_Pl_consensus    AAAAATGTTTCAAAAACACTTTACAAAAAAATAAAATACTATATAATAATCTATATAGATTGAGATGCAATGTCTCGC [10764]
cp26_PAbe_NX_MP_FG_consensus AAAAATGTTTCAAAAACACTTTACAAAAAAATAAAATACTATATAATAATCTATATAGATTGAGATGCAATGTCTCGC [10764]
cp26_PAbe_NX_Pl_consensus    AAAAATGTTTCAAAAACACTTTACAAAAAAATAAAATACTATATAATAATCTATATAGATTGAGATGCAATGTCTCGC [10764]

cp26_gi|11497007|B31_GB      TCTTGTTGTTTGATTAGAATAATAAGTTGGCTACCACAAGGTAGCCTTTGTCATTTTTGCAAAGAATTAAAATAGGAA [10842]
cp26_un9_B31_PacBio          TCTTGTTGTTTGATTAGAATAATAAGTTGGCTACCACAAGGTAGCCTTTGTCATTTTTGCAAAGAATTAAAATAGGAA [10842]
cp26_B31_TS_MP_FG_consensus  TCTTGTTGTTTGATTAGAATAATAAGTTGGCTACCACAAGGTAGCCTTTGTCATTTTTGCAAAGAATTAAAATAGGAA [10842]
cp26_B31_NX_Pl_consensus     TCTTGTTGTTTGATTAGAATAATAAGTTGGCTACCACAAGGTAGCCTTTGTCATTTTTGCAAAGAATTAAAATAGGAA [10842]
cp26_PAli_NX_MP_FG_consensus TCTTGTTGTTTGATTAGAATAATAAGTTGGCTACCACAAGGTAGCCTTTGTCATTTTTGCAAAGAATTAAAATAGGAA [10842]
cp26_PAli_NX_Pl_consensus    TCTTGTTGTTTGATTAGAATAATAAGTTGGCTACCACAAGGTAGCCTTTGTCATTTTTGCAAAGAATTAAAATAGGAA [10842]
cp26_PAbe_NX_MP_FG_consensus TCTTGTTGTTTGATTAGAATAATAAGTTGGCTACCACAAGGTAGCCTTTGTCATTTTTGCAAAGAATTAAAATAGGAA [10842]
cp26_PAbe_NX_Pl_consensus    TCTTGTTGTTTGATTAGAATAATAAGTTGGCTACCACAAGGTAGCCTTTGTCATTTTTGCAAAGAATTAAAATAGGAA [10842]

cp26_gi|11497007|B31_GB      TAATATAGCCTCTATATCAATTCTATTTTGCAATAAAAATTTATTTTAAACTGAAAATAAATACCAATAAATTAAAAA [10920]
cp26_un9_B31_PacBio          TAATATAGCCTCTATATCAATTCTATTTTGCAATAAAAATTTATTTTAAACTGAAAATAAATACCAATAAATTAAAAA [10920]
cp26_B31_TS_MP_FG_consensus  TAATATAGCCTCTATATCAATTCTATTTTGCAATAAAAATTTATTTTAAACTGAAAATAAATACCAATAAATTAAAAA [10920]
cp26_B31_NX_Pl_consensus     TAATATAGCCTCTATATCAATTCTATTTTGCAATAAAAATTTATTTTAAACTGAAAATAAATACCAATAAATTAAAAA [10920]
cp26_PAli_NX_MP_FG_consensus TAATATAGCCTCTATATCAATTCTATTTTGCAATAAAAATTTATTTTAAACTGAAAATAAATACCAATAAATTAAAAA [10920]
cp26_PAli_NX_Pl_consensus    TAATATAGCCTCTATATCAATTCTATTTTGCAATAAAAATTTATTTTAAACTGAAAATAAATACCAATAAATTAAAAA [10920]
cp26_PAbe_NX_MP_FG_consensus TAATATAGCCTCTATATCAATTCTATTTTGCAATAAAAATTTATTTTAAACTGAAAATAAATACCAATAAATTAAAAA [10920]
cp26_PAbe_NX_Pl_consensus    TAATATAGCCTCTATATCAATTCTATTTTGCAATAAAAATTTATTTTAAACTGAAAATAAATACCAATAAATTAAAAA [10920]

cp26_gi|11497007|B31_GB      ATTAACTTTTATAATCTTTATTTTCAATCGATTCAACCATTACTAGAAAGTCCAAAATTTTATATTTTTTCAAGCTTA [10998]
cp26_un9_B31_PacBio          ATTAACTTTTATAATCTTTATTTTCAATCGATTCAACCATTACTAGAAAGTCCAAAATTTTATATTTTTTCAAGCTTA [10998]
cp26_B31_TS_MP_FG_consensus  ATTAACTTTTATAATCTTTATTTTCAATCGATTCAACCATTACTAGAAAGTCCAAAATTTTATATTTTTTCAAGCTTA [10998]
cp26_B31_NX_Pl_consensus     ATTAACTTTTATAATCTTTATTTTCAATCGATTCAACCATTACTAGAAAGTCCAAAATTTTATATTTTTTCAAGCTTA [10998]
cp26_PAli_NX_MP_FG_consensus ATTAACTTTTATAATCTTTATTTTCAATCGATTCAACCATTACTAGAAAGTCCAAAATTTTATATTTTTTCAAGCTTA [10998]
cp26_PAli_NX_Pl_consensus    ATTAACTTTTATAATCTTTATTTTCAATCGATTCAACCATTACTAGAAAGTCCAAAATTTTATATTTTTTCAAGCTTA [10998]
cp26_PAbe_NX_MP_FG_consensus ATTAACTTTTATAATCTTTATTTTCAATCGATTCAACCATTACTAGAAAGTCCAAAATTTTATATTTTTTCAAGCTTA [10998]
cp26_PAbe_NX_Pl_consensus    ATTAACTTTTATAATCTTTATTTTCAATCGATTCAACCATTACTAGAAAGTCCAAAATTTTATATTTTTTCAAGCTTA [10998]

cp26_gi|11497007|B31_GB      AATCTTCTAATCCAATTCTAATTTTAACCTCCTTATTCTTATAAGAAGCATCTTCTTCTTTAATCATCCTTTCCAATA [11076]
cp26_un9_B31_PacBio          AATCTTCTAATCCAATTCTAATTTTAACCTCCTTATTCTTATAAGAAGCATCTTCTTCTTTAATCATCCTTTCCAATA [11076]
cp26_B31_TS_MP_FG_consensus  AATCTTCTAATCCAATTCTAATTTTAACCTCCTTATTCTTATAAGAAGCATCTTCTTCTTTAATCATCCTTTCCAATA [11076]
cp26_B31_NX_Pl_consensus     AATCTTCTAATCCAATTCTAATTTTAACCTCCTTATTCTTATAAGAAGCATCTTCTTCTTTAATCATCCTTTCCAATA [11076]
cp26_PAli_NX_MP_FG_consensus AATCTTCTAATCCAATTCTAATTTTAACCTCCTTATTCTTATAAGAAGCATCTTCTTCTTTAATCATCCTTTCCAATA [11076]
cp26_PAli_NX_Pl_consensus    AATCTTCTAATCCAATTCTAATTTTAACCTCCTTATTCTTATAAGAAGCATCTTCTTCTTTAATCATCCTTTCCAATA [11076]
cp26_PAbe_NX_MP_FG_consensus AATCTTCTAATCCAATTCTAATTTTAACCTCCTTATTCTTATAAGAAGCATCTTCTTCTTTAATCATCCTTTCCAATA [11076]
cp26_PAbe_NX_Pl_consensus    AATCTTCTAATCCAATTCTAATTTTAACCTCCTTATTCTTATAAGAAGCATCTTCTTCTTTAATCATCCTTTCCAATA [11076]

cp26_gi|11497007|B31_GB      AAGATTTGGTCAAAACAAATGATGAATAATTAATCCAGTTGCCAAAAAAAATGGTTATTATCTCTTTTGTAGCTTACTT [11154]
cp26_un9_B31_PacBio          AAGATTTGGTCAAAACAAATGATGAATAATTAATCCAGTTGCCAAAAAAAATGGTTATTATCTCTTTTGTAGCTTACTT [11154]
cp26_B31_TS_MP_FG_consensus  AAGATTTGGTCAAAACAAATGATGAATAATTAATCCAGTTGCCAAAAAAAATGGTTATTATCTCTTTTGTAGCTTACTT [11154]
cp26_B31_NX_Pl_consensus     AAGATTTGGTCAAAACAAATGATGAATAATTAATCCAGTTGCCAAAAAAAATGGTTATTATCTCTTTTGTAGCTTACTT [11154]
cp26_PAli_NX_MP_FG_consensus AAGATTTGGTCAAAACAAATGATGAATAATTAATCCAGTTGCCAAAAAAAATGGTTATTATCTCTTTTGTAGCTTACTT [11154]
cp26_PAli_NX_Pl_consensus    AAGATTTGGTCAAAACAAATGATGAATAATTAATCCAGTTGCCAAAAAAAATGGTTATTATCTCTTTTGTAGCTTACTT [11154]
cp26_PAbe_NX_MP_FG_consensus AAGATTTGGTCAAAACAAATGATGAATAATTAATCCAGTTGCCAAAAAAAATGGTTATTATCTCTTTTGTAGCTTACTT [11154]
cp26_PAbe_NX_Pl_consensus    AAGATTTGGTCAAAACAAATGATGAATAATTAATCCAGTTGCCAAAAAAAATGGTTATTATCTCTTTTGTAGCTTACTT [11154]

cp26_gi|11497007|B31_GB      TTCCCTCAATTTTAGTTCCATCCAGCAGCAATAAAGGATTCTGTTCTGTTATACTATGGTTTCTAAACCTTGTGTATA [11232]
cp26_un9_B31_PacBio          TTCCCTCAATTTTAGTTCCATCCAGCAGCAATAAAGGATTCTGTTCTGTTATACTATGGTTTCTAAACCTTGTGTATA [11232]
```

```
cp26_B31_TS_MP_FG_consensus  TTCCCTCAATTTTAGTTCCATCCAGCAGCAATAAAGGATTCTGTTCTGTTATACTATGGTTTCTAAACCTTGTGTATA [11232]
cp26_B31_NX_Pl_consensus     TTCCCTCAATTTTAGTTCCATCCAGCAGCAATAAAGGATTCTGTTCTGTTATACTATGGTTTCTAAACCTTGTGTATA [11232]
cp26_PAli_NX_MP_FG_consensus TTCCCTCAATTTTAGTTCCATCCAGCAGCAATAAAGGATTCTGTTCTGTTATACTATGGTTTCTAAACCTTGTGTATA [11232]
cp26_PAli_NX_Pl_consensus    TTCCCTCAATTTTAGTTCCATCCAGCAGCAATAAAGGATTCTGTTCTGTTATACTATGGTTTCTAAACCTTGTGTATA [11232]
cp26_PAbe_NX_MP_FG_consensus TTCCCTCAATTTTAGTTCCATCCAGCAGCAATAAAGGATTCTGTTCTGTTATACTATGGTTTCTAAACCTTGTGTATA [11232]
cp26_PAbe_NX_Pl_consensus    TTCCCTCAATTTTAGTTCCATCCAGCAGCAATAAAGGATTCTGTTCTGTTATACTATGGTTTCTAAACCTTGTGTATA [11232]

cp26_gi|11497007|B31_GB      ATACTATTTTAGTTATTCCTGTTTTATAATTAAAAATACCCTTAAGTTCTGCATTTTTATTTAAGCTTTGGTATATTC [11310]
cp26_un9_B31_PacBio          ATACTATTTTAGTTATTCCTGTTTTATAATTAAAAATACCCTTAAGTTCTGCATTTTTATTTAAGCTTTGGTATATTC [11310]
cp26_B31_TS_MP_FG_consensus  ATACTATTTTAGTTATTCCTGTTTTATAATTAAAAATACCCTTAAGTTCTGCATTTTTATTTAAGCTTTGGTATATTC [11310]
cp26_B31_NX_Pl_consensus     ATACTATTTTAGTTATTCCTGTTTTATAATTAAAAATACCCTTAAGTTCTGCATTTTTATTTAAGCTTTGGTATATTC [11310]
cp26_PAli_NX_MP_FG_consensus ATACTATTTTAGTTATTCCTGTTTTATAATTAAAAATACCCTTAAGTTCTGCATTTTTATTTAAGCTTTGGTATATTC [11310]
cp26_PAli_NX_Pl_consensus    ATACTATTTTAGTTATTCCTGTTTTATAATTAAAAATACCCTTAAGTTCTGCATTTTTATTTAAGCTTTGGTATATTC [11310]
cp26_PAbe_NX_MP_FG_consensus ATACTATTTTAGTTATTCCTGTTTTATAATTAAAAATACCCTTAAGTTCTGCATTTTTATTTAAGCTTTGGTATATTC [11310]
cp26_PAbe_NX_Pl_consensus    ATACTATTTTAGTTATTCCTGTTTTATAATTAAAAATACCCTTAAGTTCTGCATTTTTATTTAAGCTTTGGTATATTC [11310]

cp26_gi|11497007|B31_GB      TAAATGTTTTTCCAAATTGATCGTGCTCAACGTTTAAGGAAGTGCAAGATATTAATAAAAAAAAATACCAACAATTTTA [11388]
cp26_un9_B31_PacBio          TAAATGTTTTTCCAAATTGATCGTGCTCAACGTTTAAGGAAGTGCAAGATATTAATAAAAAAAAATACCAACAATTTTA [11388]
cp26_B31_TS_MP_FG_consensus  TAAATGTTTTTCCAAATTGATCGTGCTCAACGTTTAAGGAAGTGCAAGATATTAATAAAAAAAAATACCAACAATTTTA [11388]
cp26_B31_NX_Pl_consensus     TAAATGTTTTTCCAAATTGATCGTGCTCAACGTTTAAGGAAGTGCAAGATATTAATAAAAAAAAATACCAACAATTTTA [11388]
cp26_PAli_NX_MP_FG_consensus TAAATGTTTTTCCAAATTGATCGTGCTCAACGTTTAAGGAAGTGCAAGATATTAATAAAAAAAAATACCAACAATTTTA [11388]
cp26_PAli_NX_Pl_consensus    TAAATGTTTTTCCAAATTGATCGTGCTCAACGTTTAAGGAAGTGCAAGATATTAATAAAAAAAAATACCAACAATTTTA [11388]
cp26_PAbe_NX_MP_FG_consensus TAAATGTTTTTCCAAATTGATCGTGCTCAACGTTTAAGGAAGTGCAAGATATTAATAAAAAAAAATACCAACAATTTTA [11388]
cp26_PAbe_NX_Pl_consensus    TAAATGTTTTTCCAAATTGATCGTGCTCAACGTTTAAGGAAGTGCAAGATATTAATAAAAAAAAATACCAACAATTTTA [11388]

cp26_gi|11497007|B31_GB      AAAATTTTAATTGATTTTGATATAATATCATATTCCTCTCCTTTGATAAAAAAAATTATAAACTAGTGAACATAATAAT [11466]
cp26_un9_B31_PacBio          AAAATTTTAATTGATTTTGATATAATATCATATTCCTCTCCTTTGATAAAAAAAATTATAAACTAGTGAACATAATAAT [11466]
cp26_B31_TS_MP_FG_consensus  AAAATTTTAATTGATTTTGATATAATATCATATTCCTCTCCTTTGATAAAAAAAATTATAAACTAGTGAACATAATAAT [11466]
cp26_B31_NX_Pl_consensus     AAAATTTTAATTGATTTTGATATAATATCATATTCCTCTCCTTTGATAAAAAAAATTATAAACTAGTGAACATAATAAT [11466]
cp26_PAli_NX_MP_FG_consensus AAAATTTTAATTGATTTTGATATAATATCATATTCCTCTCCTTTGATAAAAAAAATTATAAACTAGTGAACATAATAAT [11466]
cp26_PAli_NX_Pl_consensus    AAAATTTTAATTGATTTTGATATAATATCATATTCCTCTCCTTTGATAAAAAAAATTATAAACTAGTGAACATAATAAT [11466]
cp26_PAbe_NX_MP_FG_consensus AAAATTTTAATTGATTTTGATATAATATCATATTCCTCTCCTTTGATAAAAAAAATTATAAACTAGTGAACATAATAAT [11466]
cp26_PAbe_NX_Pl_consensus    AAAATTTTAATTGATTTTGATATAATATCATATTCCTCTCCTTTGATAAAAAAAATTATAAACTAGTGAACATAATAAT [11466]

cp26_gi|11497007|B31_GB      ATATTTTTTATTAGATCATTGCAAACAATTTTTGATAAATTAATTTTTTATTTCTTTAAATCATTATTTTAGGGATTT [11544]
cp26_un9_B31_PacBio          ATATTTTTTATTAGATCATTGCAAACAATTTTTGATAAATTAATTTTTTATTTCTTTAAATCATTATTTTAGGGATTT [11544]
cp26_B31_TS_MP_FG_consensus  ATATTTTTTATTAGATCATTGCAAACAATTTTTGATAAATTAATTTTTTATTTCTTTAAATCATTATTTTAGGGATTT [11544]
cp26_B31_NX_Pl_consensus     ATATTTTTTATTAGATCATTGCAAACAATTTTTGATAAATTAATTTTTTATTTCTTTAAATCATTATTTTAGGGATTT [11544]
cp26_PAli_NX_MP_FG_consensus ATATTTTTTATTAGATCATTGCAAACAATTTTTGATAAATTAATTTTTTATTTCTTTAAATCATTATTTTAGGGATTT [11544]
cp26_PAli_NX_Pl_consensus    ATATTTTTTATTAGATCATTGCAAACAATTTTTGATAAATTAATTTTTTATTTCTTTAAATCATTATTTTAGGGATTT [11544]
cp26_PAbe_NX_MP_FG_consensus ATATTTTTTATTAGATCATTGCAAACAATTTTTGATAAATTAATTTTTTATTTCTTTAAATCATTATTTTAGGGATTT [11544]
cp26_PAbe_NX_Pl_consensus    ATATTTTTTATTAGATCATTGCAAACAATTTTTGATAAATTAATTTTTTATTTCTTTAAATCATTATTTTAGGGATTT [11544]

cp26_gi|11497007|B31_GB      TTGGTTGCTACTTTTGTTTAGCATTGCTTTTGTTAGAAATATATAAGAAAGTTTTTAAACTCTTTAAAAGGTGTTTTA [11622]
cp26_un9_B31_PacBio          TTGGTTGCTACTTTTGTTTAGCATTGCTTTTGTTAGAAATATATAAGAAAGTTTTTAAACTCTTTAAAAGGTGTTTTA [11622]
cp26_B31_TS_MP_FG_consensus  TTGGTTGCTACTTTTGTTTAGCATTGCTTTTGTTAGAAATATATAAGAAAGTTTTTAAACTCTTTAAAAGGTGTTTTA [11622]
cp26_B31_NX_Pl_consensus     TTGGTTGCTACTTTTGTTTAGCATTGCTTTTGTTAGAAATATATAAGAAAGTTTTTAAACTCTTTAAAAGGTGTTTTA [11622]
cp26_PAli_NX_MP_FG_consensus TTGGTTGCTACTTTTGTTTAGCATTGCTTTTGTTAGAAATATATAAGAAAGTTTTTAAACTCTTTAAAAGGTGTTTTA [11622]
cp26_PAli_NX_Pl_consensus    TTGGTTGCTACTTTTGTTTAGCATTGCTTTTGTTAGAAATATATAAGAAAGTTTTTAAACTCTTTAAAAGGTGTTTTA [11622]
cp26_PAbe_NX_MP_FG_consensus TTGGTTGCTACTTTTGTTTAGCATTGCTTTTGTTAGAAATATATAAGAAAGTTTTTAAACTCTTTAAAAGGTGTTTTA [11622]
cp26_PAbe_NX_Pl_consensus    TTGGTTGCTACTTTTGTTTAGCATTGCTTTTGTTAGAAATATATAAGAAAGTTTTTAAACTCTTTAAAAGGTGTTTTA [11622]

cp26_gi|11497007|B31_GB      AAAGCAAGAAAGTTCTTGTATAGAACTTTTATTGCCACAAAGGAAGTGTTAAATATGAAATTTAGTTTAGAATTTAAT [11700]
cp26_un9_B31_PacBio          AAAGCAAGAAAGTTCTTGTATAGAACTTTTATTGCCACAAAGGAAGTGTTAAATATGAAATTTAGTTTAGAATTTAAT [11700]
cp26_B31_TS_MP_FG_consensus  AAAGCAAGAAAGTTCTTGTATAGAACTTTTATTGCCACAAAGGAAGTGTTAAATATGAAATTTAGTTTAGAATTTAAT [11700]
cp26_B31_NX_Pl_consensus     AAAGCAAGAAAGTTCTTGTATAGAACTTTTATTGCCACAAAGGAAGTGTTAAATATGAAATTTAGTTTAGAATTTAAT [11700]
cp26_PAli_NX_MP_FG_consensus AAAGCAAGAAAGTTCTTGTATAGAACTTTTATTGCCACAAAGGAAGTGTTAAATATGAAATTTAGTTTAGAATTTAAT [11700]
cp26_PAli_NX_Pl_consensus    AAAGCAAGAAAGTTCTTGTATAGAACTTTTATTGCCACAAAGGAAGTGTTAAATATGAAATTTAGTTTAGAATTTAAT [11700]
cp26_PAbe_NX_MP_FG_consensus AAAGCAAGAAAGTTCTTGTATAGAACTTTTATTGCCACAAAGGAAGTGTTAAATATGAAATTTAGTTTAGAATTTAAT [11700]
cp26_PAbe_NX_Pl_consensus    AAAGCAAGAAAGTTCTTGTATAGAACTTTTATTGCCACAAAGGAAGTGTTAAATATGAAATTTAGTTTAGAATTTAAT [11700]

cp26_gi|11497007|B31_GB      TCTAATTTTTTAAAATTCAATATTAATTTATTTTTTTATTAAAAAAATAATAAATCTAAATAAATCTAATTTACAAATA [11778]
cp26_un9_B31_PacBio          TCTAATTTTTTAAAATTCAATATTAATTTATTTTTTTATTAAAAAAATAATAAATCTAAATAAATCTAATTTACAAATA [11778]
cp26_B31_TS_MP_FG_consensus  TCTAATTTTTTAAAATTCAATATTAATTTATTTTTTTATTAAAAAAATAATAAATCTAAATAAATCTAATTTACAAATA [11778]
cp26_B31_NX_Pl_consensus     TCTAATTTTTTAAAATTCAATATTAATTTATTTTTTTATTAAAAAAATAATAAATCTAAATAAATCTAATTTACAAATA [11778]
cp26_PAli_NX_MP_FG_consensus TCTAATTTTTTAAAATTCAATATTAATTTATTTTTTTATTAAAAAAATAATAAATCTAAATAAATCTAATTTACAAATA [11778]
cp26_PAli_NX_Pl_consensus    TCTAATTTTTTAAAATTCAATATTAATTTATTTTTTTATTAAAAAAATAATAAATCTAAATAAATCTAATTTACAAATA [11778]
cp26_PAbe_NX_MP_FG_consensus TCTAATTTTTTAAAATTCAATATTAATTTATTTTTTTATTAAAAAAATAATAAATCTAAATAAATCTAATTTACAAATA [11778]
cp26_PAbe_NX_Pl_consensus    TCTAATTTTTTAAAATTCAATATTAATTTATTTTTTTATTAAAAAAATAATAAATCTAAATAAATCTAATTTACAAATA [11778]

cp26_gi|11497007|B31_GB      GATAAATAATTAAATTTAAAATTATTTTTTATATGTATAATAACCATTGGAGAGCAAGCAATAAAAGATTATAATCTT [11856]
cp26_un9_B31_PacBio          GATAAATAATTAAATTTAAAATTATTTTTTATATGTATAATAACCATTGGAGAGCAAGCAATAAAAGATTATAATCTT [11856]
cp26_B31_TS_MP_FG_consensus  GATAAATAATTAAATTTAAAATTATTTTTTATATGTATAATAACCATTGGAGAGCAAGCAATAAAAGATTATAATCTT [11856]
cp26_B31_NX_Pl_consensus     GATAAATAATTAAATTTAAAATTATTTTTTATATGTATAATAACCATTGGAGAGCAAGCAATAAAAGATTATAATCTT [11856]
cp26_PAli_NX_MP_FG_consensus GATAAATAATTAAATTTAAAATTATTTTTTATATGTATAATAACCATTGGAGAGCAAGCAATAAAAGATTATAATCTT [11856]
cp26_PAli_NX_Pl_consensus    GATAAATAATTAAATTTAAAATTATTTTTTATATGTATAATAACCATTGGAGAGCAAGCAATAAAAGATTATAATCTT [11856]
cp26_PAbe_NX_MP_FG_consensus GATAAATAATTAAATTTAAAATTATTTTTTATATGTATAATAACCATTGGAGAGCAAGCAATAAAAGATTATAATCTT [11856]
cp26_PAbe_NX_Pl_consensus    GATAAATAATTAAATTTAAAATTATTTTTTATATGTATAATAACCATTGGAGAGCAAGCAATAAAAGATTATAATCTT [11856]

cp26_gi|11497007|B31_GB      TTCAATATAAGATCAATTATTTTGCTTATTTAATGTAAAATGCTAATTTTATTTTAGAATAATTTAAGTGCTTTTAAT [11934]
cp26_un9_B31_PacBio          TTCAATATAAGATCAATTATTTTGCTTATTTAATGTAAAATGCTAATTTTATTTTAGAATAATTTAAGTGCTTTTAAT [11934]
cp26_B31_TS_MP_FG_consensus  TTCAATATAAGATCAATTATTTTGCTTATTTAATGTAAAATGCTAATTTTATTTTAGAATAATTTAAGTGCTTTTAAT [11934]
cp26_B31_NX_Pl_consensus     TTCAATATAAGATCAATTATTTTGCTTATTTAATGTAAAATGCTAATTTTATTTTAGAATAATTTAAGTGCTTTTAAT [11934]
cp26_PAli_NX_MP_FG_consensus TTCAATATAAGATCAATTATTTTGCTTATTTAATGTAAAATGCTAATTTTATTTTAGAATAATTTAAGTGCTTTTAAT [11934]
cp26_PAli_NX_Pl_consensus    TTCAATATAAGATCAATTATTTTGCTTATTTAATGTAAAATGCTAATTTTATTTTAGAATAATTTAAGTGCTTTTAAT [11934]
cp26_PAbe_NX_MP_FG_consensus TTCAATATAAGATCAATTATTTTGCTTATTTAATGTAAAATGCTAATTTTATTTTAGAATAATTTAAGTGCTTTTAAT [11934]
cp26_PAbe_NX_Pl_consensus    TTCAATATAAGATCAATTATTTTGCTTATTTAATGTAAAATGCTAATTTTATTTTAGAATAATTTAAGTGCTTTTAAT [11934]

cp26_gi|11497007|B31_GB      AACGTTAATGTGAAATTATATTTCATATATAATAAAATATAGGCAAAATTTAAATTTATAAAAACTTGTAAGGATGCT [12012]
cp26_un9_B31_PacBio          AACGTTAATGTGAAATTATATTTCATATATAATAAAATATAGGCAAAATTTAAATTTATAAAAACTTGTAAGGATGCT [12012]
cp26_B31_TS_MP_FG_consensus  AACGTTAATGTGAAATTATATTTCATATATAATAAAATATAGGCAAAATTTAAATTTATAAAAACTTGTAAGGATGCT [12012]
cp26_B31_NX_Pl_consensus     AACGTTAATGTGAAATTATATTTCATATATAATAAAATATAGGCAAAATTTAAATTTATAAAAACTTGTAAGGATGCT [12012]
cp26_PAli_NX_MP_FG_consensus AACGTTAATGTGAAATTATATTTCATATATAATAAAATATAGGCAAAATTTAAATTTATAAAAACTTGTAAGGATGCT [12012]
cp26_PAli_NX_Pl_consensus    AACGTTAATGTGAAATTATATTTCATATATAATAAAATATAGGCAAAATTTAAATTTATAAAAACTTGTAAGGATGCT [12012]
cp26_PAbe_NX_MP_FG_consensus AACGTTAATGTGAAATTATATTTCATATATAATAAAATATAGGCAAAATTTAAATTTATAAAAACTTGTAAGGATGCT [12012]
cp26_PAbe_NX_Pl_consensus    AACGTTAATGTGAAATTATATTTCATATATAATAAAATATAGGCAAAATTTAAATTTATAAAAACTTGTAAGGATGCT [12012]

cp26_gi|11497007|B31_GB      TGTATGAAAATATTGATAAAAAAGTTAAAAGTTGTATTATTTCTCAATTTAATTTTACTTATTTCTTGTGTTAATGAA [12090]
cp26_un9_B31_PacBio          TGTATGAAAATATTGATAAAAAAGTTAAAAGTTGTATTATTTCTCAATTTAATTTTACTTATTTCTTGTGTTAATGAA [12090]
cp26_B31_TS_MP_FG_consensus  TGTATGAAAATATTGATAAAAAAGTTAAAAGTTGTATTATTTCTCAATTTAATTTTACTTATTTCTTGTGTTAATGAA [12090]
cp26_B31_NX_Pl_consensus     TGTATGAAAATATTGATAAAAAAGTTAAAAGTTGTATTATTTCTCAATTTAATTTTACTTATTTCTTGTGTTAATGAA [12090]
cp26_PAli_NX_MP_FG_consensus TGTATGAAAATATTGATAAAAAAGTTAAAAGTTGTATTATTTCTCAATTTAATTTTACTTATTTCTTGTGTTAATGAA [12090]
cp26_PAli_NX_Pl_consensus    TGTATGAAAATATTGATAAAAAAGTTAAAAGTTGTATTATTTCTCAATTTAATTTTACTTATTTCTTGTGTTAATGAA [12090]
cp26_PAbe_NX_MP_FG_consensus TGTATGAAAATATTGATAAAAAAGTTAAAAGTTGTATTATTTCTCAATTTAATTTTACTTATTTCTTGTGTTAATGAA [12090]
cp26_PAbe_NX_Pl_consensus    TGTATGAAAATATTGATAAAAAAGTTAAAAGTTGTATTATTTCTCAATTTAATTTTACTTATTTCTTGTGTTAATGAA [12090]

cp26_gi|11497007|B31_GB      AGTAATAGAAACAAATTGGTTTTTAAGCTAAATATTGGAAGTGAGCCTGCTACTTTAGATGCTCAATTAATAAACGAT [12168]
cp26_un9_B31_PacBio          AGTAATAGAAACAAATTGGTTTTTAAGCTAAATATTGGAAGTGAGCCTGCTACTTTAGATGCTCAATTAATAAACGAT [12168]
cp26_B31_TS_MP_FG_consensus  AGTAATAGAAACAAATTGGTTTTTAAGCTAAATATTGGAAGTGAGCCTGCTACTTTAGATGCTCAATTAATAAACGAT [12168]
```

```
cp26_B31_NX_Pl_consensus     AGTAATAGAAACAAATTGGTTTTTAAGCTAAATATTGGAAGTGAGCCTGCTACTTTAGATGCTCAATTAATAAACGAT [12168]
cp26_PAli_NX_MP_FG_consensus AGTAATAGAAACAAATTGGTTTTTAAGCTAAATATTGGAAGTGAGCCTGCTACTTTAGATGCTCAATTAATAAACGAT [12168]
cp26_PAli_NX_Pl_consensus    AGTAATAGAAACAAATTGGTTTTTAAGCTAAATATTGGAAGTGAGCCTGCTACTTTAGATGCTCAATTAATAAACGAT [12168]
cp26_PAbe_NX_MP_FG_consensus AGTAATAGAAACAAATTGGTTTTTAAGCTAAATATTGGAAGTGAGCCTGCTACTTTAGATGCTCAATTAATAAACGAT [12168]
cp26_PAbe_NX_Pl_consensus    AGTAATAGAAACAAATTGGTTTTTAAGCTAAATATTGGAAGTGAGCCTGCTACTTTAGATGCTCAATTAATAAACGAT [12168]

cp26_gi|11497007|B31_GB      ACGGTTGGATCAGGGATTGTAAGCCAAATGTTTCTTGGCATTTTAGATGGAGATCCCAGGACTGGAGGATACAGACCG [12246]
cp26_un9_B31_PacBio          ACGGTTGGATCAGGGATTGTAAGCCAAATGTTTCTTGGCATTTTAGATGGAGATCCCAGGACTGGAGGATACAGACCG [12246]
cp26_B31_TS_MP_FG_consensus  ACGGTTGGATCAGGGATTGTAAGCCAAATGTTTCTTGGCATTTTAGATGGAGATCCCAGGACTGGAGGATACAGACCG [12246]
cp26_B31_NX_Pl_consensus     ACGGTTGGATCAGGGATTGTAAGCCAAATGTTTCTTGGCATTTTAGATGGAGATCCCAGGACTGGAGGATACAGACCG [12246]
cp26_PAli_NX_MP_FG_consensus ACGGTTGGATCAGGGATTGTAAGCCAAATGTTTCTTGGCATTTTAGATGGAGATCCCAGGACTGGAGGATACAGACCG [12246]
cp26_PAli_NX_Pl_consensus    ACGGTTGGATCAGGGATTGTAAGCCAAATGTTTCTTGGCATTTTAGATGGAGATCCCAGGACTGGAGGATACAGACCG [12246]
cp26_PAbe_NX_MP_FG_consensus ACGGTTGGATCAGGGATTGTAAGCCAAATGTTTCTTGGCATTTTAGATGGAGATCCCAGGACTGGAGGATACAGACCG [12246]
cp26_PAbe_NX_Pl_consensus    ACGGTTGGATCAGGGATTGTAAGCCAAATGTTTCTTGGCATTTTAGATGGAGATCCCAGGACTGGAGGATACAGACCG [12246]

cp26_gi|11497007|B31_GB      GGACTTGCTAAAAGTTGGGATATTTCTGATGACGGAGTAGTTTATACGTTTCATTTAAGAGATAATCTTGTTTGGAGT [12324]
cp26_un9_B31_PacBio          GGACTTGCTAAAAGTTGGGATATTTCTGATGACGGAGTAGTTTATACGTTTCATTTAAGAGATAATCTTGTTTGGAGT [12324]
cp26_B31_TS_MP_FG_consensus  GGACTTGCTAAAAGTTGGGATATTTCTGATGACGGAGTAGTTTATACGTTTCATTTAAGAGATAATCTTGTTTGGAGT [12324]
cp26_B31_NX_Pl_consensus     GGACTTGCTAAAAGTTGGGATATTTCTGATGACGGAGTAGTTTATACGTTTCATTTAAGAGATAATCTTGTTTGGAGT [12324]
cp26_PAli_NX_MP_FG_consensus GGACTTGCTAAAAGTTGGGATATTTCTGATGACGGAGTAGTTTATACGTTTCATTTAAGAGATAATCTTGTTTGGAGT [12324]
cp26_PAli_NX_Pl_consensus    GGACTTGCTAAAAGTTGGGATATTTCTGATGACGGAGTAGTTTATACGTTTCATTTAAGAGATAATCTTGTTTGGAGT [12324]
cp26_PAbe_NX_MP_FG_consensus GGACTTGCTAAAAGTTGGGATATTTCTGATGACGGAGTAGTTTATACGTTTCATTTAAGAGATAATCTTGTTTGGAGT [12324]
cp26_PAbe_NX_Pl_consensus    GGACTTGCTAAAAGTTGGGATATTTCTGATGACGGAGTAGTTTATACGTTTCATTTAAGAGATAATCTTGTTTGGAGT [12324]

cp26_gi|11497007|B31_GB      GATGGAGTTTCCATTACTGCCGAAGGAATAAGAAAATCTTATCTTAGAATTTTAGATAAAGAAACCGGCTCATCTTTT [12402]
cp26_un9_B31_PacBio          GATGGAGTTTCCATTACTGCCGAAGGAATAAGAAAATCTTATCTTAGAATTTTAGATAAAGAAACCGGCTCATCTTTT [12402]
cp26_B31_TS_MP_FG_consensus  GATGGAGTTTCCATTACTGCCGAAGGAATAAGAAAATCTTATCTTAGAATTTTAGATAAAGAAACCGGCTCATCTTTT [12402]
cp26_B31_NX_Pl_consensus     GATGGAGTTTCCATTACTGCCGAAGGAATAAGAAAATCTTATCTTAGAATTTTAGATAAAGAAACCGGCTCATCTTTT [12402]
cp26_PAli_NX_MP_FG_consensus GATGGAGTTTCCATTACTGCCGAAGGAATAAGAAAATCTTATCTTAGAATTTTAGATAAAGAAACCGGCTCATCTTTT [12402]
cp26_PAli_NX_Pl_consensus    GATGGAGTTTCCATTACTGCCGAAGGAATAAGAAAATCTTATCTTAGAATTTTAGATAAAGAAACCGGCTCATCTTTT [12402]
cp26_PAbe_NX_MP_FG_consensus GATGGAGTTTCCATTACTGCCGAAGGAATAAGAAAATCTTATCTTAGAATTTTAGATAAAGAAACCGGCTCATCTTTT [12402]
cp26_PAbe_NX_Pl_consensus    GATGGAGTTTCCATTACTGCCGAAGGAATAAGAAAATCTTATCTTAGAATTTTAGATAAAGAAACCGGCTCATCTTTT [12402]

cp26_gi|11497007|B31_GB      GTTAACATGATTAAGTCCGTTATTAAAAATGCAGAAGAGTATTTTGACGGCAAAGCAAATGAGTCTGAGCTTGGAATT [12480]
cp26_un9_B31_PacBio          GTTAACATGATTAAGTCCGTTATTAAAAATGCAGAAGAGTATTTTGACGGCAAAGCAAATGAGTCTGAGCTTGGAATT [12480]
cp26_B31_TS_MP_FG_consensus  GTTAACATGATTAAGTCCGTTATTAAAAATGCAGAAGAGTATTTTGACGGCAAAGCAAATGAGTCTGAGCTTGGAATT [12480]
cp26_B31_NX_Pl_consensus     GTTAACATGATTAAGTCCGTTATTAAAAATGCAGAAGAGTATTTTGACGGCAAAGCAAATGAGTCTGAGCTTGGAATT [12480]
cp26_PAli_NX_MP_FG_consensus GTTAACATGATTAAGTCCGTTATTAAAAATGCAGAAGAGTATTTTGACGGCAAAGCAAATGAGTCTGAGCTTGGAATT [12480]
cp26_PAli_NX_Pl_consensus    GTTAACATGATTAAGTCCGTTATTAAAAATGCAGAAGAGTATTTTGACGGCAAAGCAAATGAGTCTGAGCTTGGAATT [12480]
cp26_PAbe_NX_MP_FG_consensus GTTAACATGATTAAGTCCGTTATTAAAAATGCAGAAGAGTATTTTGACGGCAAAGCAAATGAGTCTGAGCTTGGAATT [12480]
cp26_PAbe_NX_Pl_consensus    GTTAACATGATTAAGTCCGTTATTAAAAATGCAGAAGAGTATTTTGACGGCAAAGCAAATGAGTCTGAGCTTGGAATT [12480]

cp26_gi|11497007|B31_GB      AAAGCTCTTGATGAAAAAACTTTAGAAATAACGCTAAAATCTCCAAAGCCATATTTTCTTGATATGTTAGTACATCAA [12558]
cp26_un9_B31_PacBio          AAAGCTCTTGATGAAAAAACTTTAGAAATAACGCTAAAATCTCCAAAGCCATATTTTCTTGATATGTTAGTACATCAA [12558]
cp26_B31_TS_MP_FG_consensus  AAAGCTCTTGATGAAAAAACTTTAGAAATAACGCTAAAATCTCCAAAGCCATATTTTCTTGATATGTTAGTACATCAA [12558]
cp26_B31_NX_Pl_consensus     AAAGCTCTTGATGAAAAAACTTTAGAAATAACGCTAAAATCTCCAAAGCCATATTTTCTTGATATGTTAGTACATCAA [12558]
cp26_PAli_NX_MP_FG_consensus AAAGCTCTTGATGAAAAAACTTTAGAAATAACGCTAAAATCTCCAAAGCCATATTTTCTTGATATGTTAGTACATCAA [12558]
cp26_PAli_NX_Pl_consensus    AAAGCTCTTGATGAAAAAACTTTAGAAATAACGCTAAAATCTCCAAAGCCATATTTTCTTGATATGTTAGTACATCAA [12558]
cp26_PAbe_NX_MP_FG_consensus AAAGCTCTTGATGAAAAAACTTTAGAAATAACGCTAAAATCTCCAAAGCCATATTTTCTTGATATGTTAGTACATCAA [12558]
cp26_PAbe_NX_Pl_consensus    AAAGCTCTTGATGAAAAAACTTTAGAAATAACGCTAAAATCTCCAAAGCCATATTTTCTTGATATGTTAGTACATCAA [12558]

cp26_gi|11497007|B31_GB      ACATTTATTCCTGTACCAATGCACGTTATTGAAAAGTATGGGCAAAGGTGGACAGACCCCGAGAATATGGTTGTTAGT [12636]
cp26_un9_B31_PacBio          ACATTTATTCCTGTACCAATGCACGTTATTGAAAAGTATGGGCAAAGGTGGACAGACCCCGAGAATATGGTTGTTAGT [12636]
cp26_B31_TS_MP_FG_consensus  ACATTTATTCCTGTACCAATGCACGTTATTGAAAAGTATGGGCAAAGGTGGACAGACCCCGAGAATATGGTTGTTAGT [12636]
cp26_B31_NX_Pl_consensus     ACATTTATTCCTGTACCAATGCACGTTATTGAAAAGTATGGGCAAAGGTGGACAGACCCCGAGAATATGGTTGTTAGT [12636]
cp26_PAli_NX_MP_FG_consensus ACATTTATTCCTGTACCAATGCACGTTATTGAAAAGTATGGGCAAAGGTGGACAGACCCCGAGAATATGGTTGTTAGT [12636]
cp26_PAli_NX_Pl_consensus    ACATTTATTCCTGTACCAATGCACGTTATTGAAAAGTATGGGCAAAGGTGGACAGACCCCGAGAATATGGTTGTTAGT [12636]
cp26_PAbe_NX_MP_FG_consensus ACATTTATTCCTGTACCAATGCACGTTATTGAAAAGTATGGGCAAAGGTGGACAGACCCCGAGAATATGGTTGTTAGT [12636]
cp26_PAbe_NX_Pl_consensus    ACATTTATTCCTGTACCAATGCACGTTATTGAAAAGTATGGGCAAAGGTGGACAGACCCCGAGAATATGGTTGTTAGT [12636]

cp26_gi|11497007|B31_GB      GGTCCTTTCAAATTAAAATCTAGAGTTTTAAATGAAAAGGTTGTTCTTGAAAAGAATAATAAATATTATAATTCCAAA [12714]
cp26_un9_B31_PacBio          GGTCCTTTCAAATTAAAATCTAGAGTTTTAAATGAAAAGGTTGTTCTTGAAAAGAATAATAAATATTATAATTCCAAA [12714]
cp26_B31_TS_MP_FG_consensus  GGTCCTTTCAAATTAAAATCTAGAGTTTTAAATGAAAAGGTTGTTCTTGAAAAGAATAATAAATATTATAATTCCAAA [12714]
cp26_B31_NX_Pl_consensus     GGTCCTTTCAAATTAAAATCTAGAGTTTTAAATGAAAAGGTTGTTCTTGAAAAGAATAATAAATATTATAATTCCAAA [12714]
cp26_PAli_NX_MP_FG_consensus GGTCCTTTCAAATTAAAATCTAGAGTTTTAAATGAAAAGGTTGTTCTTGAAAAGAATAATAAATATTATAATTCCAAA [12714]
cp26_PAli_NX_Pl_consensus    GGTCCTTTCAAATTAAAATCTAGAGTTTTAAATGAAAAGGTTGTTCTTGAAAAGAATAATAAATATTATAATTCCAAA [12714]
cp26_PAbe_NX_MP_FG_consensus GGTCCTTTCAAATTAAAATCTAGAGTTTTAAATGAAAAGGTTGTTCTTGAAAAGAATAATAAATATTATAATTCCAAA [12714]
cp26_PAbe_NX_Pl_consensus    GGTCCTTTCAAATTAAAATCTAGAGTTTTAAATGAAAAGGTTGTTCTTGAAAAGAATAATAAATATTATAATTCCAAA [12714]

cp26_gi|11497007|B31_GB      GATGTTGTTCTTGACAGTATTATATTTTTTGTCACAGATAATAGCATTACAGCTTATAATATGTATTTAAATGATGAG [12792]
cp26_un9_B31_PacBio          GATGTTGTTCTTGACAGTATTATATTTTTTGTCACAGATAATAGCATTACAGCTTATAATATGTATTTAAATGATGAG [12792]
cp26_B31_TS_MP_FG_consensus  GATGTTGTTCTTGACAGTATTATATTTTTTGTCACAGATAATAGCATTACAGCTTATAATATGTATTTAAATGATGAG [12792]
cp26_B31_NX_Pl_consensus     GATGTTGTTCTTGACAGTATTATATTTTTTGTCACAGATAATAGCATTACAGCTTATAATATGTATTTAAATGATGAG [12792]
cp26_PAli_NX_MP_FG_consensus GATGTTGTTCTTGACAGTATTATATTTTTTGTCACAGATAATAGCATTACAGCTTATAATATGTATTTAAATGATGAG [12792]
cp26_PAli_NX_Pl_consensus    GATGTTGTTCTTGACAGTATTATATTTTTTGTCACAGATAATAGCATTACAGCTTATAATATGTATTTAAATGATGAG [12792]
cp26_PAbe_NX_MP_FG_consensus GATGTTGTTCTTGACAGTATTATATTTTTTGTCACAGATAATAGCATTACAGCTTATAATATGTATTTAAATGATGAG [12792]
cp26_PAbe_NX_Pl_consensus    GATGTTGTTCTTGACAGTATTATATTTTTTGTCACAGATAATAGCATTACAGCTTATAATATGTATTTAAATGATGAG [12792]

cp26_gi|11497007|B31_GB      CTGGATGCAATTTTTAAGAATGTTCCACCAGATTTGCTTAAGGATCTTAAGCTTAGGGACGATTATTATTCAATGGGT [12870]
cp26_un9_B31_PacBio          CTGGATGCAATTTTTAAGAATGTTCCACCAGATTTGCTTAAGGATCTTAAGCTTAGGGACGATTATTATTCAATGGGT [12870]
cp26_B31_TS_MP_FG_consensus  CTGGATGCAATTTTTAAGAATGTTCCACCAGATTTGCTTAAGGATCTTAAGCTTAGGGACGATTATTATTCAATGGGT [12870]
cp26_B31_NX_Pl_consensus     CTGGATGCAATTTTTAAGAATGTTCCACCAGATTTGCTTAAGGATCTTAAGCTTAGGGACGATTATTATTCAATGGGT [12870]
cp26_PAli_NX_MP_FG_consensus CTGGATGCAATTTTTAAGAATGTTCCACCAGATTTGCTTAAGGATCTTAAGCTTAGGGACGATTATTATTCAATGGGT [12870]
cp26_PAli_NX_Pl_consensus    CTGGATGCAATTTTTAAGAATGTTCCACCAGATTTGCTTAAGGATCTTAAGCTTAGGGACGATTATTATTCAATGGGT [12870]
cp26_PAbe_NX_MP_FG_consensus CTGGATGCAATTTTTAAGAATGTTCCACCAGATTTGCTTAAGGATCTTAAGCTTAGGGACGATTATTATTCAATGGGT [12870]
cp26_PAbe_NX_Pl_consensus    CTGGATGCAATTTTTAAGAATGTTCCACCAGATTTGCTTAAGGATCTTAAGCTTAGGGACGATTATTATTCAATGGGT [12870]

cp26_gi|11497007|B31_GB      ATTAATTCAACCTCTTTTTATTCTTTGAACATGAAAGTAAAACCGCTTGACAATGTTAAAGTTAGAAAGGCGCTGTCT [12948]
cp26_un9_B31_PacBio          ATTAATTCAACCTCTTTTTATTCTTTGAACATGAAAGTAAAACCGCTTGACAATGTTAAAGTTAGAAAGGCGCTGTCT [12948]
cp26_B31_TS_MP_FG_consensus  ATTAATTCAACCTCTTTTTATTCTTTGAACATGAAAGTAAAACCGCTTGACAATGTTAAAGTTAGAAAGGCGCTGTCT [12948]
cp26_B31_NX_Pl_consensus     ATTAATTCAACCTCTTTTTATTCTTTGAACATGAAAGTAAAACCGCTTGACAATGTTAAAGTTAGAAAGGCGCTGTCT [12948]
cp26_PAli_NX_MP_FG_consensus ATTAATTCAACCTCTTTTTATTCTTTGAACATGAAAGTAAAACCGCTTGACAATGTTAAAGTTAGAAAGGCGCTGTCT [12948]
cp26_PAli_NX_Pl_consensus    ATTAATTCAACCTCTTTTTATTCTTTGAACATGAAAGTAAAACCGCTTGACAATGTTAAAGTTAGAAAGGCGCTGTCT [12948]
cp26_PAbe_NX_MP_FG_consensus ATTAATTCAACCTCTTTTTATTCTTTGAACATGAAAGTAAAACCGCTTGACAATGTTAAAGTTAGAAAGGCGCTGTCT [12948]
cp26_PAbe_NX_Pl_consensus    ATTAATTCAACCTCTTTTTATTCTTTGAACATGAAAGTAAAACCGCTTGACAATGTTAAAGTTAGAAAGGCGCTGTCT [12948]

cp26_gi|11497007|B31_GB      TTTGCTATTGATAGAAAAACCTTAACAGAGAGCGTTCTTAATGATAGTTCTATTCCTACAAGAAGAGCAACTCCAGAT [13026]
cp26_un9_B31_PacBio          TTTGCTATTGATAGAAAAACCTTAACAGAGAGCGTTCTTAATGATAGTTCTATTCCTACAAGAAGAGCAACTCCAGAT [13026]
cp26_B31_TS_MP_FG_consensus  TTTGCTATTGATAGAAAAACCTTAACAGAGAGCGTTCTTAATGATAGTTCTATTCCTACAAGAAGAGCAACTCCAGAT [13026]
cp26_B31_NX_Pl_consensus     TTTGCTATTGATAGAAAAACCTTAACAGAGAGCGTTCTTAATGATAGTTCTATTCCTACAAGAAGAGCAACTCCAGAT [13026]
cp26_PAli_NX_MP_FG_consensus TTTGCTATTGATAGAAAAACCTTAACAGAGAGCGTTCTTAATGATAGTTCTATTCCTACAAGAAGAGCAACTCCAGAT [13026]
cp26_PAli_NX_Pl_consensus    TTTGCTATTGATAGAAAAACCTTAACAGAGAGCGTTCTTAATGATAGTTCTATTCCTACAAGAAGAGCAACTCCAGAT [13026]
cp26_PAbe_NX_MP_FG_consensus TTTGCTATTGATAGAAAAACCTTAACAGAGAGCGTTCTTAATGATAGTTCTATTCCTACAAGAAGAGCAACTCCAGAT [13026]
cp26_PAbe_NX_Pl_consensus    TTTGCTATTGATAGAAAAACCTTAACAGAGAGCGTTCTTAATGATAGTTCTATTCCTACAAGAAGAGCAACTCCAGAT [13026]

cp26_gi|11497007|B31_GB      TATATTGATTACTCTTATAAAAGCAATTTGAGCTTATTTGATGCTGAAATGGCAAAGAAGCTTTTGGCAGATGCAGGA [13104]
cp26_un9_B31_PacBio          TATATTGATTACTCTTATAAAAGCAATTTGAGCTTATTTGATGCTGAAATGGCAAAGAAGCTTTTGGCAGATGCAGGA [13104]
cp26_B31_TS_MP_FG_consensus  TATATTGATTACTCTTATAAAAGCAATTTGAGCTTATTTGATGCTGAAATGGCAAAGAAGCTTTTGGCAGATGCAGGA [13104]
cp26_B31_NX_Pl_consensus     TATATTGATTACTCTTATAAAAGCAATTTGAGCTTATTTGATGCTGAAATGGCAAAGAAGCTTTTGGCAGATGCAGGA [13104]
```

```
cp26_PAli_NX_MP_FG_consensus TATATTGATTACTCTTATAAAAGCAATTTGAGCTTATTTGATGCTGAAATGGCAAAGAAGCTTTTGGCAGATGCAGGA [13104]
cp26_PAli_NX_Pl_consensus    TATATTGATTACTCTTATAAAAGCAATTTGAGCTTATTTGATGCTGAAATGGCAAAGAAGCTTTTGGCAGATGCAGGA [13104]
cp26_PAbe_NX_MP_FG_consensus TATATTGATTACTCTTATAAAAGCAATTTGAGCTTATTTGATGCTGAAATGGCAAAGAAGCTTTTGGCAGATGCAGGA [13104]
cp26_PAbe_NX_Pl_consensus    TATATTGATTACTCTTATAAAAGCAATTTGAGCTTATTTGATGCTGAAATGGCAAAGAAGCTTTTGGCAGATGCAGGA [13104]

cp26_gi|11497007|B31_GB      TATCCCAATGGTAATAATTTTCCTTTATTAAAAGTAAAGTACAATACAAGTGATAGCCAGAGAAAAATTGCTGAATTT [13182]
cp26_un9_B31_PacBio          TATCCCAATGGTAATAATTTTCCTTTATTAAAAGTAAAGTACAATACAAGTGATAGCCAGAGAAAAATTGCTGAATTT [13182]
cp26_B31_TS_MP_FG_consensus  TATCCCAATGGTAATAATTTTCCTTTATTAAAAGTAAAGTACAATACAAGTGATAGCCAGAGAAAAATTGCTGAATTT [13182]
cp26_B31_NX_Pl_consensus     TATCCCAATGGTAATAATTTTCCTTTATTAAAAGTAAAGTACAATACAAGTGATAGCCAGAGAAAAATTGCTGAATTT [13182]
cp26_PAli_NX_MP_FG_consensus TATCCCAATGGTAATAATTTTCCTTTATTAAAAGTAAAGTACAATACAAGTGATAGCCAGAGAAAAATTGCTGAATTT [13182]
cp26_PAli_NX_Pl_consensus    TATCCCAATGGTAATAATTTTCCTTTATTAAAAGTAAAGTACAATACAAGTGATAGCCAGAGAAAAATTGCTGAATTT [13182]
cp26_PAbe_NX_MP_FG_consensus TATCCCAATGGTAATAATTTTCCTTTATTAAAAGTAAAGTACAATACAAGTGATAGCCAGAGAAAAATTGCTGAATTT [13182]
cp26_PAbe_NX_Pl_consensus    TATCCCAATGGTAATAATTTTCCTTTATTAAAAGTAAAGTACAATACAAGTGATAGCCAGAGAAAAATTGCTGAATTT [13182]

cp26_gi|11497007|B31_GB      ATTCAAAATCAGTGGAAGAAAAACTTAAATATTAATGTACAGCTTGAGAATGAAGAATGGTCAACATATATAAATAGT [13260]
cp26_un9_B31_PacBio          ATTCAAAATCAGTGGAAGAAAAACTTAAATATTAATGTACAGCTTGAGAATGAAGAATGGTCAACATATATAAATAGT [13260]
cp26_B31_TS_MP_FG_consensus  ATTCAAAATCAGTGGAAGAAAAACTTAAATATTAATGTACAGCTTGAGAATGAAGAATGGTCAACATATATAAATAGT [13260]
cp26_B31_NX_Pl_consensus     ATTCAAAATCAGTGGAAGAAAAACTTAAATATTAATGTACAGCTTGAGAATGAAGAATGGTCAACATATATAAATAGT [13260]
cp26_PAli_NX_MP_FG_consensus ATTCAAAATCAGTGGAAGAAAAACTTAAATATTAATGTACAGCTTGAGAATGAAGAATGGTCAACATATATAAATAGT [13260]
cp26_PAli_NX_Pl_consensus    ATTCAAAATCAGTGGAAGAAAAACTTAAATATTAATGTACAGCTTGAGAATGAAGAATGGTCAACATATATAAATAGT [13260]
cp26_PAbe_NX_MP_FG_consensus ATTCAAAATCAGTGGAAGAAAAACTTAAATATTAATGTACAGCTTGAGAATGAAGAATGGTCAACATATATAAATAGT [13260]
cp26_PAbe_NX_Pl_consensus    ATTCAAAATCAGTGGAAGAAAAACTTAAATATTAATGTACAGCTTGAGAATGAAGAATGGTCAACATATATAAATAGT [13260]

cp26_gi|11497007|B31_GB      AGAGTAAATGGTAATTATGAAATAATAAGATCAGGATGGTCAGGAGATTATGCTGATCCTATGACGTTCTTAAGCATC [13338]
cp26_un9_B31_PacBio          AGAGTAAATGGTAATTATGAAATAATAAGATCAGGATGGTCAGGAGATTATGCTGATCCTATGACGTTCTTAAGCATC [13338]
cp26_B31_TS_MP_FG_consensus  AGAGTAAATGGTAATTATGAAATAATAAGATCAGGATGGTCAGGAGATTATGCTGATCCTATGACGTTCTTAAGCATC [13338]
cp26_B31_NX_Pl_consensus     AGAGTAAATGGTAATTATGAAATAATAAGATCAGGATGGTCAGGAGATTATGCTGATCCTATGACGTTCTTAAGCATC [13338]
cp26_PAli_NX_MP_FG_consensus AGAGTAAATGGTAATTATGAAATAATAAGATCAGGATGGTCAGGAGATTATGCTGATCCTATGACGTTCTTAAGCATC [13338]
cp26_PAli_NX_Pl_consensus    AGAGTAAATGGTAATTATGAAATAATAAGATCAGGATGGTCAGGAGATTATGCTGATCCTATGACGTTCTTAAGCATC [13338]
cp26_PAbe_NX_MP_FG_consensus AGAGTAAATGGTAATTATGAAATAATAAGATCAGGATGGTCAGGAGATTATGCTGATCCTATGACGTTCTTAAGCATC [13338]
cp26_PAbe_NX_Pl_consensus    AGAGTAAATGGTAATTATGAAATAATAAGATCAGGATGGTCAGGAGATTATGCTGATCCTATGACGTTCTTAAGCATC [13338]

cp26_gi|11497007|B31_GB      TTTCAAACTGAAAATACATCTTTTTCATCTTATGGATATTCAAATTCTGAATATGATGAACTTTTAATAAAATCAGAT [13416]
cp26_un9_B31_PacBio          TTTCAAACTGAAAATACATCTTTTTCATCTTATGGATATTCAAATTCTGAATATGATGAACTTTTAATAAAATCAGAT [13416]
cp26_B31_TS_MP_FG_consensus  TTTCAAACTGAAAATACATCTTTTTCATCTTATGGATATTCAAATTCTGAATATGATGAACTTTTAATAAAATCAGAT [13416]
cp26_B31_NX_Pl_consensus     TTTCAAACTGAAAATACATCTTTTTCATCTTATGGATATTCAAATTCTGAATATGATGAACTTTTAATAAAATCAGAT [13416]
cp26_PAli_NX_MP_FG_consensus TTTCAAACTGAAAATACATCTTTTTCATCTTATGGATATTCAAATTCTGAATATGATGAACTTTTAATAAAATCAGAT [13416]
cp26_PAli_NX_Pl_consensus    TTTCAAACTGAAAATACATCTTTTTCATCTTATGGATATTCAAATTCTGAATATGATGAACTTTTAATAAAATCAGAT [13416]
cp26_PAbe_NX_MP_FG_consensus TTTCAAACTGAAAATACATCTTTTTCATCTTATGGATATTCAAATTCTGAATATGATGAACTTTTAATAAAATCAGAT [13416]
cp26_PAbe_NX_Pl_consensus    TTTCAAACTGAAAATACATCTTTTTCATCTTATGGATATTCAAATTCTGAATATGATGAACTTTTAATAAAATCAGAT [13416]

cp26_gi|11497007|B31_GB      AATGAGAGAGATATTTTTAAAAGACAGGAAATTCTAAAAAAAGCAGAAGCAATAATAATTGAAAGAGATTTTCCGGCT [13494]
cp26_un9_B31_PacBio          AATGAGAGAGATATTTTTAAAAGACAGGAAATTCTAAAAAAAGCAGAAGCAATAATAATTGAAAGAGATTTTCCGGCT [13494]
cp26_B31_TS_MP_FG_consensus  AATGAGAGAGATATTTTTAAAAGACAGGAAATTCTAAAAAAAGCAGAAGCAATAATAATTGAAAGAGATTTTCCGGCT [13494]
cp26_B31_NX_Pl_consensus     AATGAGAGAGATATTTTTAAAAGACAGGAAATTCTAAAAAAAGCAGAAGCAATAATAATTGAAAGAGATTTTCCGGCT [13494]
cp26_PAli_NX_MP_FG_consensus AATGAGAGAGATATTTTTAAAAGACAGGAAATTCTAAAAAAAGCAGAAGCAATAATAATTGAAAGAGATTTTCCGGCT [13494]
cp26_PAli_NX_Pl_consensus    AATGAGAGAGATATTTTTAAAAGACAGGAAATTCTAAAAAAAGCAGAAGCAATAATAATTGAAAGAGATTTTCCGGCT [13494]
cp26_PAbe_NX_MP_FG_consensus AATGAGAGAGATATTTTTAAAAGACAGGAAATTCTAAAAAAAGCAGAAGCAATAATAATTGAAAGAGATTTTCCGGCT [13494]
cp26_PAbe_NX_Pl_consensus    AATGAGAGAGATATTTTTAAAAGACAGGAAATTCTAAAAAAAGCAGAAGCAATAATAATTGAAAGAGATTTTCCGGCT [13494]

cp26_gi|11497007|B31_GB      GTATTTCTTAATATAACTTCTTCTAGTTATCTTTTTAGAAACGACAAGTGGAAAGGTTGGGAGCCAAATATTTCGGAA [13572]
cp26_un9_B31_PacBio          GTATTTCTTAATATAACTTCTTCTAGTTATCTTTTTAGAAACGACAAGTGGAAAGGTTGGGAGCCAAATATTTCGGAA [13572]
cp26_B31_TS_MP_FG_consensus  GTATTTCTTAATATAACTTCTTCTAGTTATCTTTTTAGAAACGACAAGTGGAAAGGTTGGGAGCCAAATATTTCGGAA [13572]
cp26_B31_NX_Pl_consensus     GTATTTCTTAATATAACTTCTTCTAGTTATCTTTTTAGAAACGACAAGTGGAAAGGTTGGGAGCCAAATATTTCGGAA [13572]
cp26_PAli_NX_MP_FG_consensus GTATTTCTTAATATAACTTCTTCTAGTTATCTTTTTAGAAACGACAAGTGGAAAGGTTGGGAGCCAAATATTTCGGAA [13572]
cp26_PAli_NX_Pl_consensus    GTATTTCTTAATATAACTTCTTCTAGTTATCTTTTTAGAAACGACAAGTGGAAAGGTTGGGAGCCAAATATTTCGGAA [13572]
cp26_PAbe_NX_MP_FG_consensus GTATTTCTTAATATAACTTCTTCTAGTTATCTTTTTAGAAACGACAAGTGGAAAGGTTGGGAGCCAAATATTTCGGAA [13572]
cp26_PAbe_NX_Pl_consensus    GTATTTCTTAATATAACTTCTTCTAGTTATCTTTTTAGAAACGACAAGTGGAAAGGTTGGGAGCCAAATATTTCGGAA [13572]

cp26_gi|11497007|B31_GB      AGATTTAATTTATCTGAAATAAAACCAATTAAATGATTTAAGTAAAAATGGTTTATAGCTAGATCTTTTGATTTGGCT [13650]
cp26_un9_B31_PacBio          AGATTTAATTTATCTGAAATAAAACCAATTAAATGATTTAAGTAAAAATGGTTTATAGCTAGATCTTTTGATTTGGCT [13650]
cp26_B31_TS_MP_FG_consensus  AGATTTAATTTATCTGAAATAAAACCAATTAAATGATTTAAGTAAAAATGGTTTATAGCTAGATCTTTTGATTTGGCT [13650]
cp26_B31_NX_Pl_consensus     AGATTTAATTTATCTGAAATAAAACCAATTAAATGATTTAAGTAAAAATGGTTTATAGCTAGATCTTTTGATTTGGCT [13650]
cp26_PAli_NX_MP_FG_consensus AGATTTAATTTATCTGAAATAAAACCAATTAAATGATTTAAGTAAAAATGGTTTATAGCTAGATCTTTTGATTTGGCT [13650]
cp26_PAli_NX_Pl_consensus    AGATTTAATTTATCTGAAATAAAACCAATTAAATGATTTAAGTAAAAATGGTTTATAGCTAGATCTTTTGATTTGGCT [13650]
cp26_PAbe_NX_MP_FG_consensus AGATTTAATTTATCTGAAATAAAACCAATTAAATGATTTAAGTAAAAATGGTTTATAGCTAGATCTTTTGATTTGGCT [13650]
cp26_PAbe_NX_Pl_consensus    AGATTTAATTTATCTGAAATAAAACCAATTAAATGATTTAAGTAAAAATGGTTTATAGCTAGATCTTTTGATTTGGCT [13650]

cp26_gi|11497007|B31_GB      ATAAACAAAAATAAGAAATGAATTCTTAAATTAAGAATTCATTTCTTTAACTTAATTATTTTAGAATTATTATTTTTA [13728]
cp26_un9_B31_PacBio          ATAAACAAAAATAAGAAATGAATTCTTAAATTAAGAATTCATTTCTTTAACTTAATTATTTTAGAATTATTATTTTTA [13728]
cp26_B31_TS_MP_FG_consensus  ATAAACAAAAATAAGAAATGAATTCTTAAATTAAGAATTCATTTCTTTAACTTAATTATTTTAGAATTATTATTTTTA [13728]
cp26_B31_NX_Pl_consensus     ATAAACAAAAATAAGAAATGAATTCTTAAATTAAGAATTCATTTCTTTAACTTAATTATTTTAGAATTATTATTTTTA [13728]
cp26_PAli_NX_MP_FG_consensus ATAAACAAAAATAAGAAATGAATTCTTAAATTAAGAATTCATTTCTTTAACTTAATTATTTTAGAATTATTATTTTTA [13728]
cp26_PAli_NX_Pl_consensus    ATAAACAAAAATAAGAAATGAATTCTTAAATTAAGAATTCATTTCTTTAACTTAATTATTTTAGAATTATTATTTTTA [13728]
cp26_PAbe_NX_MP_FG_consensus ATAAACAAAAATAAGAAATGAATTCTTAAATTAAGAATTCATTTCTTTAACTTAATTATTTTAGAATTATTATTTTTA [13728]
cp26_PAbe_NX_Pl_consensus    ATAAACAAAAATAAGAAATGAATTCTTAAATTAAGAATTCATTTCTTTAACTTAATTATTTTAGAATTATTATTTTTA [13728]

cp26_gi|11497007|B31_GB      AATAGAGTAAGCATTAATATATTAATACTAGAAAAAGCATTACAAAAACAGAAAGCTATATTTATTATTCTTTTCCTA [13806]
cp26_un9_B31_PacBio          AATAGAGTAAGCATTAATATATTAATACTAGAAAAAGCATTACAAAAACAGAAAGCTATATTTATTATTCTTTTCCTA [13806]
cp26_B31_TS_MP_FG_consensus  AATAGAGTAAGCATTAATATATTAATACTAGAAAAAGCATTACAAAAACAGAAAGCTATATTTATTATTCTTTTCCTA [13806]
cp26_B31_NX_Pl_consensus     AATAGAGTAAGCATTAATATATTAATACTAGAAAAAGCATTACAAAAACAGAAAGCTATATTTATTATTCTTTTCCTA [13806]
cp26_PAli_NX_MP_FG_consensus AATAGAGTAAGCATTAATATATTAATACTAGAAAAAGCATTACAAAAACAGAAAGCTATATTTATTATTCTTTTCCTA [13806]
cp26_PAli_NX_Pl_consensus    AATAGAGTAAGCATTAATATATTAATACTAGAAAAAGCATTACAAAAACAGAAAGCTATATTTATTATTCTTTTCCTA [13806]
cp26_PAbe_NX_MP_FG_consensus AATAGAGTAAGCATTAATATATTAATACTAGAAAAAGCATTACAAAAACAGAAAGCTATATTTATTATTCTTTTCCTA [13806]
cp26_PAbe_NX_Pl_consensus    AATAGAGTAAGCATTAATATATTAATACTAGAAAAAGCATTACAAAAACAGAAAGCTATATTTATTATTCTTTTCCTA [13806]

cp26_gi|11497007|B31_GB      CCAAGGTTGTTATTTCTTTAAAAGAAGGATGCAATATTTTGATCTTTTAAAGATGTTAGTTATTTAATTTGATAAATG [13884]
cp26_un9_B31_PacBio          CCAAGGTTGTTATTTCTTTAAAAGAAGGATGCAATATTTTGATCTTTTAAAGATGTTAGTTATTTAATTTGATAAATG [13884]
cp26_B31_TS_MP_FG_consensus  CCAAGGTTGTTATTTCTTTAAAAGAAGGATGCAATATTTTGATCTTTTAAAGATGTTAGTTATTTAATTTGATAAATG [13884]
cp26_B31_NX_Pl_consensus     CCAAGGTTGTTATTTCTTTAAAAGAAGGATGCAATATTTTGATCTTTTAAAGATGTTAGTTATTTAATTTGATAAATG [13884]
cp26_PAli_NX_MP_FG_consensus CCAAGGTTGTTATTTCTTTAAAAGAAGGATGCAATATTTTGATCTTTTAAAGATGTTAGTTATTTAATTTGATAAATG [13884]
cp26_PAli_NX_Pl_consensus    CCAAGGTTGTTATTTCTTTAAAAGAAGGATGCAATATTTTGATCTTTTAAAGATGTTAGTTATTTAATTTGATAAATG [13884]
cp26_PAbe_NX_MP_FG_consensus CCAAGGTTGTTATTTCTTTAAAAGAAGGATGCAATATTTTGATCTTTTAAAGATGTTAGTTATTTAATTTGATAAATG [13884]
cp26_PAbe_NX_Pl_consensus    CCAAGGTTGTTATTTCTTTAAAAGAAGGATGCAATATTTTGATCTTTTAAAGATGTTAGTTATTTAATTTGATAAATG [13884]

cp26_gi|11497007|B31_GB      TTTTTTATTTTTATGTTATGCTAAAAACATCGTGAGGATGGGATTCTTTTAATGAAGAATGGCTTATTTTTACAAACT [13962]
cp26_un9_B31_PacBio          TTTTTTATTTTTATGTTATGCTAAAAACATCGTGAGGATGGGATTCTTTTAATGAAGAATGGCTTATTTTTACAAACT [13962]
cp26_B31_TS_MP_FG_consensus  TTTTTTATTTTTATGTTATGCTAAAAACATCGTGAGGATGGGATTCTTTTAATGAAGAATGGCTTATTTTTACAAACT [13962]
cp26_B31_NX_Pl_consensus     TTTTTTATTTTTATGTTATGCTAAAAACATCGTGAGGATGGGATTCTTTTAATGAAGAATGGCTTATTTTTACAAACT [13962]
cp26_PAli_NX_MP_FG_consensus TTTTTTATTTTTATGTTATGCTAAAAACATCGTGAGGATGGGATTCTTTTAATGAAGAATGGCTTATTTTTACAAACT [13962]
cp26_PAli_NX_Pl_consensus    TTTTTTATTTTTATGTTATGCTAAAAACATCGTGAGGATGGGATTCTTTTAATGAAGAATGGCTTATTTTTACAAACT [13962]
cp26_PAbe_NX_MP_FG_consensus TTTTTTATTTTTATGTTATGCTAAAAACATCGTGAGGATGGGATTCTTTTAATGAAGAATGGCTTATTTTTACAAACT [13962]
cp26_PAbe_NX_Pl_consensus    TTTTTTATTTTTATGTTATGCTAAAAACATCGTGAGGATGGGATTCTTTTAATGAAGAATGGCTTATTTTTACAAACT [13962]

cp26_gi|11497007|B31_GB      TAGAATTTATTTTTAAATCAGATATTGTTGCTGCTCCTAAATAGCCCATTCCAGACATTAAACCGCCTTTTAATTGAG [14040]
cp26_un9_B31_PacBio          TAGAATTTATTTTTAAATCAGATATTGTTGCTGCTCCTAAATAGCCCATTCCAGACATTAAACCGCCTTTTAATTGAG [14040]
cp26_B31_TS_MP_FG_consensus  TAGAATTTATTTTTAAATCAGATATTGTTGCTGCTCCTAAATAGCCCATTCCAGACATTAAACCGCCTTTTAATTGAG [14040]
cp26_B31_NX_Pl_consensus     TAGAATTTATTTTTAAATCAGATATTGTTGCTGCTCCTAAATAGCCCATTCCAGACATTAAACCGCCTTTTAATTGAG [14040]
cp26_PAli_NX_MP_FG_consensus TAGAATTTATTTTTAAATCAGATATTGTTGCTGCTCCTAAATAGCCCATTCCAGACATTAAACCGCCTTTTAATTGAG [14040]
```

```
cp26_PAli_NX_Pl_consensus    TAGAATTTATTTTTAAATCAGATATTGTTGCTGCTCCTAAATAGCCCATTCCAGACATTAAACCGCCTTTTAATTGAG [14040]
cp26_PAbe_NX_MP_FG_consensus TAGAATTTATTTTTAAATCAGATATTGTTGCTGCTCCTAAATAGCCCATTCCAGACATTAAACCGCCTTTTAATTGAG [14040]
cp26_PAbe_NX_Pl_consensus    TAGAATTTATTTTTAAATCAGATATTGTTGCTGCTCCTAAATAGCCCATTCCAGACATTAAACCGCCTTTTAATTGAG [14040]

cp26_gi|11497007|B31_GB      TCAAAATATCTTTTAATTTTCCAGAATACGGTACCATGCCTTCAATTCCTTCGGGGACTAATTTTTTAGGTTCGTTGT [14118]
cp26_un9_B31_PacBio          TCAAAATATCTTTTAATTTTCCAGAATACGGTACCATGCCTTCAATTCCTTCGGGGACTAATTTTTTAGGTTCGTTGT [14118]
cp26_B31_TS_MP_FG_consensus  TCAAAATATCTTTTAATTTTCCAGAATACGGTACCATGCCTTCAATTCCTTCGGGGACTAATTTTTTAGGTTCGTTGT [14118]
cp26_B31_NX_Pl_consensus     TCAAAATATCTTTTAATTTTCCAGAATACGGTACCATGCCTTCAATTCCTTCGGGGACTAATTTTTTAGGTTCGTTGT [14118]
cp26_PAli_NX_MP_FG_consensus TCAAAATATCTTTTAATTTTCCAGAATACGGTACCATGCCTTCAATTCCTTCGGGGACTAATTTTTTAGGTTCGTTGT [14118]
cp26_PAli_NX_Pl_consensus    TCAAAATATCTTTTAATTTTCCAGAATACGGTACCATGCCTTCAATTCCTTCGGGGACTAATTTTTTAGGTTCGTTGT [14118]
cp26_PAbe_NX_MP_FG_consensus TCAAAATATCTTTTAATTTTCCAGAATACGGTACCATGCCTTCAATTCCTTCGGGGACTAATTTTTTAGGTTCGTTGT [14118]
cp26_PAbe_NX_Pl_consensus    TCAAAATATCTTTTAATTTTCCAGAATACGGTACCATGCCTTCAATTCCTTCGGGGACTAATTTTTTAGGTTCGTTGT [14118]

cp26_gi|11497007|B31_GB      TTTCTAGTTGAAAATATCTTGATTTGGAGCCTCTTTTCATAGCAGAAATAGAGCCCATTCCAACGTAAGATTTGAATT [14196]
cp26_un9_B31_PacBio          TTTCTAGTTGAAAATATCTTGATTTGGAGCCTCTTTTCATAGCAGAAATAGAGCCCATTCCAACGTAAGATTTGAATT [14196]
cp26_B31_TS_MP_FG_consensus  TTTCTAGTTGAAAATATCTTGATTTGGAGCCTCTTTTCATAGCAGAAATAGAGCCCATTCCAACGTAAGATTTGAATT [14196]
cp26_B31_NX_Pl_consensus     TTTCTAGTTGAAAATATCTTGATTTGGAGCCTCTTTTCATAGCAGAAATAGAGCCCATTCCAACGTAAGATTTGAATT [14196]
cp26_PAli_NX_MP_FG_consensus TTTCTAGTTGAAAATATCTTGATTTGGAGCCTCTTTTCATAGCAGAAATAGAGCCCATTCCAACGTAAGATTTGAATT [14196]
cp26_PAli_NX_Pl_consensus    TTTCTAGTTGAAAATATCTTGATTTGGAGCCTCTTTTCATAGCAGAAATAGAGCCCATTCCAACGTAAGATTTGAATT [14196]
cp26_PAbe_NX_MP_FG_consensus TTTCTAGTTGAAAATATCTTGATTTGGAGCCTCTTTTCATAGCAGAAATAGAGCCCATTCCAACGTAAGATTTGAATT [14196]
cp26_PAbe_NX_Pl_consensus    TTTCTAGTTGAAAATATCTTGATTTGGAGCCTCTTTTCATAGCAGAAATAGAGCCCATTCCAACGTAAGATTTGAATT [14196]

cp26_gi|11497007|B31_GB      TTTTTCCATTGTAAATTATTTCTTCAGAAGGAGATTCTTTTGTGCCTGCAAAGAGATTGCCTATCATTACGCTATCAG [14274]
cp26_un9_B31_PacBio          TTTTTCCATTGTAAATTATTTCTTCAGAAGGAGATTCTTTTGTGCCTGCAAAGAGATTGCCTATCATTACGCTATCAG [14274]
cp26_B31_TS_MP_FG_consensus  TTTTTCCATTGTAAATTATTTCTTCAGAAGGAGATTCTTTTGTGCCTGCAAAGAGATTGCCTATCATTACGCTATCAG [14274]
cp26_B31_NX_Pl_consensus     TTTTTCCATTGTAAATTATTTCTTCAGAAGGAGATTCTTTTGTGCCTGCAAAGAGATTGCCTATCATTACGCTATCAG [14274]
cp26_PAli_NX_MP_FG_consensus TTTTTCCATTGTAAATTATTTCTTCAGAAGGAGATTCTTTTGTGCCTGCAAAGAGATTGCCTATCATTACGCTATCAG [14274]
cp26_PAli_NX_Pl_consensus    TTTTTCCATTGTAAATTATTTCTTCAGAAGGAGATTCTTTTGTGCCTGCAAAGAGATTGCCTATCATTACGCTATCAG [14274]
cp26_PAbe_NX_MP_FG_consensus TTTTTCCATTGTAAATTATTTCTTCAGAAGGAGATTCTTTTGTGCCTGCAAAGAGATTGCCTATCATTACGCTATCAG [14274]
cp26_PAbe_NX_Pl_consensus    TTTTTCCATTGTAAATTATTTCTTCAGAAGGAGATTCTTTTGTGCCTGCAAAGAGATTGCCTATCATTACGCTATCAG [14274]

cp26_gi|11497007|B31_GB      CTCCTGCTGCGATGGCTTTAACCACATCTCCTGAAAACCTAATTCCGCCATCTGCTATAATACAAATATTTGTATTAT [14352]
cp26_un9_B31_PacBio          CTCCTGCTGCGATGGCTTTAACCACATCTCCTGAAAACCTAATTCCGCCATCTGCTATAATACAAATATTTGTATTAT [14352]
cp26_B31_TS_MP_FG_consensus  CTCCTGCTGCGATGGCTTTAACCACATCTCCTGAAAACCTAATTCCGCCATCTGCTATAATACAAATATTTGTATTAT [14352]
cp26_B31_NX_Pl_consensus     CTCCTGCTGCGATGGCTTTAACCACATCTCCTGAAAACCTAATTCCGCCATCTGCTATAATACAAATATTTGTATTAT [14352]
cp26_PAli_NX_MP_FG_consensus CTCCTGCTGCGATGGCTTTAACCACATCTCCTGAAAACCTAATTCCGCCATCTGCTATAATACAAATATTTGTATTAT [14352]
cp26_PAli_NX_Pl_consensus    CTCCTGCTGCGATGGCTTTAACCACATCTCCTGAAAACCTAATTCCGCCATCTGCTATAATACAAATATTTGTATTAT [14352]
cp26_PAbe_NX_MP_FG_consensus CTCCTGCTGCGATGGCTTTAACCACATCTCCTGAAAACCTAATTCCGCCATCTGCTATAATACAAATATTTGTATTAT [14352]
cp26_PAbe_NX_Pl_consensus    CTCCTGCTGCGATGGCTTTAACCACATCTCCTGAAAACCTAATTCCGCCATCTGCTATAATACAAATATTTGTATTAT [14352]

cp26_gi|11497007|B31_GB      TACAAGCCTCATAGACATCGCAGATTGCTGTTATTTGGGGAACTCCAACTCCCGCAACGATTCTTGTTGTGCATATAC [14430]
cp26_un9_B31_PacBio          TACAAGCCTCATAGACATCGCAGATTGCTGTTATTTGGGGAACTCCAACTCCCGCAACGATTCTTGTTGTGCATATAC [14430]
cp26_B31_TS_MP_FG_consensus  TACAAGCCTCATAGACATCGCAGATTGCTGTTATTTGGGGAACTCCAACTCCCGCAACGATTCTTGTTGTGCATATAC [14430]
cp26_B31_NX_Pl_consensus     TACAAGCCTCATAGACATCGCAGATTGCTGTTATTTGGGGAACTCCAACTCCCGCAACGATTCTTGTTGTGCATATAC [14430]
cp26_PAli_NX_MP_FG_consensus TACAAGCCTCATAGACATCGCAGATTGCTGTTATTTGGGGAACTCCAACTCCCGCAACGATTCTTGTTGTGCATATAC [14430]
cp26_PAli_NX_Pl_consensus    TACAAGCCTCATAGACATCGCAGATTGCTGTTATTTGGGGAACTCCAACTCCCGCAACGATTCTTGTTGTGCATATAC [14430]
cp26_PAbe_NX_MP_FG_consensus TACAAGCCTCATAGACATCGCAGATTGCTGTTATTTGGGGAACTCCAACTCCCGCAACGATTCTTGTTGTGCATATAC [14430]
cp26_PAbe_NX_Pl_consensus    TACAAGCCTCATAGACATCGCAGATTGCTGTTATTTGGGGAACTCCAACTCCCGCAACGATTCTTGTTGTGCATATAC [14430]

cp26_gi|11497007|B31_GB      TACCCGGACCTATTCCTACTTTTAAACAATCTGCTCCTACACTTATTAAATCTAATGCAGCTTCTTTAGTTACTATGT [14508]
cp26_un9_B31_PacBio          TACCCGGACCTATTCCTACTTTTAAACAATCTGCTCCTACACTTATTAAATCTAATGCAGCTTCTTTAGTTACTATGT [14508]
cp26_B31_TS_MP_FG_consensus  TACCCGGACCTATTCCTACTTTTAAACAATCTGCTCCTACACTTATTAAATCTAATGCAGCTTCTTTAGTTACTATGT [14508]
cp26_B31_NX_Pl_consensus     TACCCGGACCTATTCCTACTTTTAAACAATCTGCTCCTACACTTATTAAATCTAATGCAGCTTCTTTAGTTACTATGT [14508]
cp26_PAli_NX_MP_FG_consensus TACCCGGACCTATTCCTACTTTTAAACAATCTGCTCCTACACTTATTAAATCTAATGCAGCTTCTTTAGTTACTATGT [14508]
cp26_PAli_NX_Pl_consensus    TACCCGGACCTATTCCTACTTTTAAACAATCTGCTCCTACACTTATTAAATCTAATGCAGCTTCTTTAGTTACTATGT [14508]
cp26_PAbe_NX_MP_FG_consensus TACCCGGACCTATTCCTACTTTTAAACAATCTGCTCCTACACTTATTAAATCTAATGCAGCTTCTTTAGTTACTATGT [14508]
cp26_PAbe_NX_Pl_consensus    TACCCGGACCTATTCCTACTTTTAAACAATCTGCTCCTACACTTATTAAATCTAATGCAGCTTCTTTAGTTACTATGT [14508]

cp26_gi|11497007|B31_GB      TGCCAGCAATAAGGTCTAAGTTTGGGTACTTGGTTTTAATTTTTTTGATAAGCTCTATTATTCTTGTAGAATGTCCAT [14586]
cp26_un9_B31_PacBio          TGCCAGCAATAAGGTCTAAGTTTGGGTACTTGGTTTTAATTTTTTTGATAAGCTCTATTATTCTTGTAGAATGTCCAT [14586]
cp26_B31_TS_MP_FG_consensus  TGCCAGCAATAAGGTCTAAGTTTGGGTACTTGGTTTTAATTTTTTTGATAAGCTCTATTATTCTTGTAGAATGTCCAT [14586]
cp26_B31_NX_Pl_consensus     TGCCAGCAATAAGGTCTAAGTTTGGGTACTTGGTTTTAATTTTTTTGATAAGCTCTATTATTCTTGTAGAATGTCCAT [14586]
cp26_PAli_NX_MP_FG_consensus TGCCAGCAATAAGGTCTAAGTTTGGGTACTTGGTTTTAATTTTTTTGATAAGCTCTATTATTCTTGTAGAATGTCCAT [14586]
cp26_PAli_NX_Pl_consensus    TGCCAGCAATAAGGTCTAAGTTTGGGTACTTGGTTTTAATTTTTTTGATAAGCTCTATTATTCTTGTAGAATGTCCAT [14586]
cp26_PAbe_NX_MP_FG_consensus TGCCAGCAATAAGGTCTAAGTTTGGGTACTTGGTTTTAATTTTTTTGATAAGCTCTATTATTCTTGTAGAATGTCCAT [14586]
cp26_PAbe_NX_Pl_consensus    TGCCAGCAATAAGGTCTAAGTTTGGGTACTTGGTTTTAATTTTTTTGATAAGCTCTATTATTCTTGTAGAATGTCCAT [14586]

cp26_gi|11497007|B31_GB      GGGCAGAGTCTATGACAAGTATATCTACATGTGCTTTTACAAGCTCTTCAACTCGTTCTATGGTATCAATATCAATAG [14664]
cp26_un9_B31_PacBio          GGGCAGAGTCTATGACAAGTATATCTACATGTGCTTTTACAAGCTCTTCAACTCGTTCTATGGTATCAATATCAATAG [14664]
cp26_B31_TS_MP_FG_consensus  GGGCAGAGTCTATGACAAGTATATCTACATGTGCTTTTACAAGCTCTTCAACTCGTTCTATGGTATCAATATCAATAG [14664]
cp26_B31_NX_Pl_consensus     GGGCAGAGTCTATGACAAGTATATCTACATGTGCTTTTACAAGCTCTTCAACTCGTTCTATGGTATCAATATCAATAG [14664]
cp26_PAli_NX_MP_FG_consensus GGGCAGAGTCTATGACAAGTATATCTACATGTGCTTTTACAAGCTCTTCAACTCGTTCTATGGTATCAATATCAATAG [14664]
cp26_PAli_NX_Pl_consensus    GGGCAGAGTCTATGACAAGTATATCTACATGTGCTTTTACAAGCTCTTCAACTCGTTCTATGGTATCAATATCAATAG [14664]
cp26_PAbe_NX_MP_FG_consensus GGGCAGAGTCTATGACAAGTATATCTACATGTGCTTTTACAAGCTCTTCAACTCGTTCTATGGTATCAATATCAATAG [14664]
cp26_PAbe_NX_Pl_consensus    GGGCAGAGTCTATGACAAGTATATCTACATGTGCTTTTACAAGCTCTTCAACTCGTTCTATGGTATCAATATCAATAG [14664]

cp26_gi|11497007|B31_GB      AAACAGCAGCACCTACTCTTAGCTTGTTATTTAAATCTTTGCATGCATTAGGAAAATCTTCTTTGTGTTCTGCATTTT [14742]
cp26_un9_B31_PacBio          AAACAGCAGCACCTACTCTTAGCTTGTTATTTAAATCTTTGCATGCATTAGGAAAATCTTCTTTGTGTTCTGCATTTT [14742]
cp26_B31_TS_MP_FG_consensus  AAACAGCAGCACCTACTCTTAGCTTGTTATTTAAATCTTTGCATGCATTAGGAAAATCTTCTTTGTGTTCTGCATTTT [14742]
cp26_B31_NX_Pl_consensus     AAACAGCAGCACCTACTCTTAGCTTGTTATTTAAATCTTTGCATGCATTAGGAAAATCTTCTTTGTGTTCTGCATTTT [14742]
cp26_PAli_NX_MP_FG_consensus AAACAGCAGCACCTACTCTTAGCTTGTTATTTAAATCTTTGCATGCATTAGGAAAATCTTCTTTGTGTTCTGCATTTT [14742]
cp26_PAli_NX_Pl_consensus    AAACAGCAGCACCTACTCTTAGCTTGTTATTTAAATCTTTGCATGCATTAGGAAAATCTTCTTTGTGTTCTGCATTTT [14742]
cp26_PAbe_NX_MP_FG_consensus AAACAGCAGCACCTACTCTTAGCTTGTTATTTAAATCTTTGCATGCATTAGGAAAATCTTCTTTGTGTTCTGCATTTT [14742]
cp26_PAbe_NX_Pl_consensus    AAACAGCAGCACCTACTCTTAGCTTGTTATTTAAATCTTTGCATGCATTAGGAAAATCTTCTTTGTGTTCTGCATTTT [14742]

cp26_gi|11497007|B31_GB      TGTATGCATCGGATTTTTCTAGATGTTGTTTTGCTGTAAATATTTCGGGTTTTTGCTCATTTGTATCTCCATTAGTGT [14820]
cp26_un9_B31_PacBio          TGTATGCATCGGATTTTTCTAGATGTTGTTTTGCTGTAAATATTTCGGGTTTTTGCTCATTTGTATCTCCATTAGTGT [14820]
cp26_B31_TS_MP_FG_consensus  TGTATGCATCGGATTTTTCTAGATGTTGTTTTGCTGTAAATATTTCGGGTTTTTGCTCATTTGTATCTCCATTAGTGT [14820]
cp26_B31_NX_Pl_consensus     TGTATGCATCGGATTTTTCTAGATGTTGTTTTGCTGTAAATATTTCGGGTTTTTGCTCATTTGTATCTCCATTAGTGT [14820]
cp26_PAli_NX_MP_FG_consensus TGTATGCATCGGATTTTTCTAGATGTTGTTTTGCTGTAAATATTTCGGGTTTTTGCTCATTTGTATCTCCATTAGTGT [14820]
cp26_PAli_NX_Pl_consensus    TGTATGCATCGGATTTTTCTAGATGTTGTTTTGCTGTAAATATTTCGGGTTTTTGCTCATTTGTATCTCCATTAGTGT [14820]
cp26_PAbe_NX_MP_FG_consensus TGTATGCATCGGATTTTTCTAGATGTTGTTTTGCTGTAAATATTTCGGGTTTTTGCTCATTTGTATCTCCATTAGTGT [14820]
cp26_PAbe_NX_Pl_consensus    TGTATGCATCGGATTTTTCTAGATGTTGTTTTGCTGTAAATATTTCGGGTTTTTGCTCATTTGTATCTCCATTAGTGT [14820]

cp26_gi|11497007|B31_GB      TAATAGTCTTTTGGAATTTATATGTTTTTACTTTTTCTATCTCTTTTCTTTGAGCTTCTATTGACATATTTTTATGTA [14898]
cp26_un9_B31_PacBio          TAATAGTCTTTTGGAATTTATATGTTTTTACTTTTTCTATCTCTTTTCTTTGAGCTTCTATTGACATATTTTTATGTA [14898]
cp26_B31_TS_MP_FG_consensus  TAATAGTCTTTTGGAATTTATATGTTTTTACTTTTTCTATCTCTTTTCTTTGAGCTTCTATTGACATATTTTTATGTA [14898]
cp26_B31_NX_Pl_consensus     TAATAGTCTTTTGGAATTTATATGTTTTTACTTTTTCTATCTCTTTTCTTTGAGCTTCTATTGACATATTTTTATGTA [14898]
cp26_PAli_NX_MP_FG_consensus TAATAGTCTTTTGGAATTTATATGTTTTTACTTTTTCTATCTCTTTTCTTTGAGCTTCTATTGACATATTTTTATGTA [14898]
cp26_PAli_NX_Pl_consensus    TAATAGTCTTTTGGAATTTATATGTTTTTACTTTTTCTATCTCTTTTCTTTGAGCTTCTATTGACATATTTTTATGTA [14898]
cp26_PAbe_NX_MP_FG_consensus TAATAGTCTTTTGGAATTTATATGTTTTTACTTTTTCTATCTCTTTTCTTTGAGCTTCTATTGACATATTTTTATGTA [14898]
cp26_PAbe_NX_Pl_consensus    TAATAGTCTTTTGGAATTTATATGTTTTTACTTTTTCTATCTCTTTTCTTTGAGCTTCTATTGACATATTTTTATGTA [14898]

cp26_gi|11497007|B31_GB      TAATTCCTATTCCACCCTCTTTAGCAATGGCTATTGCCATTTGGCTTTCTGTAACAGTATCCATTGCTGAGCTTAAAA [14976]
cp26_un9_B31_PacBio          TAATTCCTATTCCACCCTCTTTAGCAATGGCTATTGCCATTTGGCTTTCTGTAACAGTATCCATTGCTGAGCTTAAAA [14976]
cp26_B31_TS_MP_FG_consensus  TAATTCCTATTCCACCCTCTTTAGCAATGGCTATTGCCATTTGGCTTTCTGTAACAGTATCCATTGCTGAGCTTAAAA [14976]
cp26_B31_NX_Pl_consensus     TAATTCCTATTCCACCCTCTTTAGCAATGGCTATTGCCATTTGGCTTTCTGTAACAGTATCCATTGCTGAGCTTAAAA [14976]
cp26_PAli_NX_MP_FG_consensus TAATTCCTATTCCACCCTCTTTAGCAATGGCTATTGCCATTTGGCTTTCTGTAACAGTATCCATTGCTGAGCTTAAAA [14976]
cp26_PAli_NX_Pl_consensus    TAATTCCTATTCCACCCTCTTTAGCAATGGCTATTGCCATTTGGCTTTCTGTAACAGTATCCATTGCTGAGCTTAAAA [14976]
```

```
cp26_PAbe_NX_MP_FG_consensus TAATTCCTATTCCACCCTCTTTAGCAATGGCTATTGCCATTTGGCTTTCTGTAACAGTATCCATTGCTGAGCTTAAAA [14976]
cp26_PAbe_NX_Pl_consensus    TAATTCCTATTCCACCCTCTTTAGCAATGGCTATTGCCATTTGGCTTTCTGTAACAGTATCCATTGCTGAGCTTAAAA [14976]

cp26_gi|11497007|B31_GB      ATGGTATGTTTAGGGATATGTTTTTTGTTAATTGTGTTTTTAAACTAACCTCACTAGGTAATACAGATGATTTTCTTG [15054]
cp26_un9_B31_PacBio          ATGGTATGTTTAGGGATATGTTTTTTGTTAATTGTGTTTTTAAACTAACCTCACTAGGTAATACAGATGATTTTCTTG [15054]
cp26_B31_TS_MP_FG_consensus  ATGGTATGTTTAGGGATATGTTTTTTGTTAATTGTGTTTTTAAACTAACCTCACTAGGTAATACAGATGATTTTCTTG [15054]
cp26_B31_NX_Pl_consensus     ATGGTATGTTTAGGGATATGTTTTTTGTTAATTGTGTTTTTAAACTAACCTCACTAGGTAATACAGATGATTTTCTTG [15054]
cp26_PAli_NX_MP_FG_consensus ATGGTATGTTTAGGGATATGTTTTTTGTTAATTGTGTTTTTAAACTAACCTCACTAGGTAATACAGATGATTTTCTTG [15054]
cp26_PAli_NX_Pl_consensus    ATGGTATGTTTAGGGATATGTTTTTTGTTAATTGTGTTTTTAAACTAACCTCACTAGGTAATACAGATGATTTTCTTG [15054]
cp26_PAbe_NX_MP_FG_consensus ATGGTATGTTTAGGGATATGTTTTTTGTTAATTGTGTTTTTAAACTAACCTCACTAGGTAATACAGATGATTTTCTTG [15054]
cp26_PAbe_NX_Pl_consensus    ATGGTATGTTTAGGGATATGTTTTTTGTTAATTGTGTTTTTAAACTAACCTCACTAGGTAATACAGATGATTTTCTTG [15054]

cp26_gi|11497007|B31_GB      GAATTAAAGACACATCATCAAAAGTTAAAGCTTCTTTTGTTATCTTATTTGGCATAAATTTTCCTTTTTATTGTTCTT [15132]
cp26_un9_B31_PacBio          GAATTAAAGACACATCATCAAAAGTTAAAGCTTCTTTTGTTATCTTATTTGGCATAAATTTTCCTTTTTATTGTTCTT [15132]
cp26_B31_TS_MP_FG_consensus  GAATTAAAGACACATCATCAAAAGTTAAAGCTTCTTTTGTTATCTTATTTGGCATAAATTTTCCTTTTTATTGTTCTT [15132]
cp26_B31_NX_Pl_consensus     GAATTAAAGACACATCATCAAAAGTTAAAGCTTCTTTTGTTATCTTATTTGGCATAAATTTTCCTTTTTATTGTTCTT [15132]
cp26_PAli_NX_MP_FG_consensus GAATTAAAGACACATCATCAAAAGTTAAAGCTTCTTTTGTTATCTTATTTGGCATAAATTTTCCTTTTTATTGTTCTT [15132]
cp26_PAli_NX_Pl_consensus    GAATTAAAGACACATCATCAAAAGTTAAAGCTTCTTTTGTTATCTTATTTGGCATAAATTTTCCTTTTTATTGTTCTT [15132]
cp26_PAbe_NX_MP_FG_consensus GAATTAAAGACACATCATCAAAAGTTAAAGCTTCTTTTGTTATCTTATTTGGCATAAATTTTCCTTTTTATTGTTCTT [15132]
cp26_PAbe_NX_Pl_consensus    GAATTAAAGACACATCATCAAAAGTTAAAGCTTCTTTTGTTATCTTATTTGGCATAAATTTTCCTTTTTATTGTTCTT [15132]

cp26_gi|11497007|B31_GB      ATTATTCCCATTCTATGGTTGATGGAGGCTTAGAAGATATATCATAACAAACTCTATTTATACCCCGAACTTCATTAA [15210]
cp26_un9_B31_PacBio          ATTATTCCCATTCTATGGTTGATGGAGGCTTAGAAGATATATCATAACAAACTCTATTTATACCCCGAACTTCATTAA [15210]
cp26_B31_TS_MP_FG_consensus  ATTATTCCCATTCTATGGTTGATGGAGGCTTAGAAGATATATCATAACAAACTCTATTTATACCCCGAACTTCATTAA [15210]
cp26_B31_NX_Pl_consensus     ATTATTCCCATTCTATGGTTGATGGAGGCTTAGAAGATATATCATAACAAACTCTATTTATACCCCGAACTTCATTAA [15210]
cp26_PAli_NX_MP_FG_consensus ATTATTCCCATTCTATGGTTGATGGAGGCTTAGAAGATATATCATAACAAACTCTATTTATACCCCGAACTTCATTAA [15210]
cp26_PAli_NX_Pl_consensus    ATTATTCCCATTCTATGGTTGATGGAGGCTTAGAAGATATATCATAACAAACTCTATTTATACCCCGAACTTCATTAA [15210]
cp26_PAbe_NX_MP_FG_consensus ATTATTCCCATTCTATGGTTGATGGAGGCTTAGAAGATATATCATAACAAACTCTATTTATACCCCGAACTTCATTAA [15210]
cp26_PAbe_NX_Pl_consensus    ATTATTCCCATTCTATGGTTGATGGAGGCTTAGAAGATATATCATAACAAACTCTATTTATACCCCGAACTTCATTAA [15210]

cp26_gi|11497007|B31_GB      TTATTCTTGAAGAAACTTTTTTTAAAAAACTGTAAGGAAGTTCAGTCCATTCTGCAGTCATGAAGTCTTGGGTATTTA [15288]
cp26_un9_B31_PacBio          TTATTCTTGAAGAAACTTTTTTTAAAAAACTGTAAGGAAGTTCAGTCCATTCTGCAGTCATGAAGTCTTGGGTATTTA [15288]
cp26_B31_TS_MP_FG_consensus  TTATTCTTGAAGAAACTTTTTTTAAAAAACTGTAAGGAAGTTCAGTCCATTCTGCAGTCATGAAGTCTTGGGTATTTA [15288]
cp26_B31_NX_Pl_consensus     TTATTCTTGAAGAAACTTTTTTTAAAAAACTGTAAGGAAGTTCAGTCCATTCTGCAGTCATGAAGTCTTGGGTATTTA [15288]
cp26_PAli_NX_MP_FG_consensus TTATTCTTGAAGAAACTTTTTTTAAAAAACTGTAAGGAAGTTCAGTCCATTCTGCAGTCATGAAGTCTTGGGTATTTA [15288]
cp26_PAli_NX_Pl_consensus    TTATTCTTGAAGAAACTTTTTTTAAAAAACTGTAAGGAAGTTCAGTCCATTCTGCAGTCATGAAGTCTTGGGTATTTA [15288]
cp26_PAbe_NX_MP_FG_consensus TTATTCTTGAAGAAACTTTTTTTAAAAAACTGTAAGGAAGTTCAGTCCATTCTGCAGTCATGAAGTCTTGGGTATTTA [15288]
cp26_PAbe_NX_Pl_consensus    TTATTCTTGAAGAAACTTTTTTTAAAAAACTGTAAGGAAGTTCAGTCCATTCTGCAGTCATGAAGTCTTGGGTATTTA [15288]

cp26_gi|11497007|B31_GB      CACATCTAATTACAGCTGTATATTCGTATGTCCTTTGATCTCCCATTACGCCTACAGATTTGACAGGAAGCAATACAA [15366]
cp26_un9_B31_PacBio          CACATCTAATTACAGCTGTATATTCGTATGTCCTTTGATCTCCCATTACGCCTACAGATTTGACAGGAAGCAATACAA [15366]
cp26_B31_TS_MP_FG_consensus  CACATCTAATTACAGCTGTATATTCGTATGTCCTTTGATCTCCCATTACGCCTACAGATTTGACAGGAAGCAATACAA [15366]
cp26_B31_NX_Pl_consensus     CACATCTAATTACAGCTGTATATTCGTATGTCCTTTGATCTCCCATTACGCCTACAGATTTGACAGGAAGCAATACAA [15366]
cp26_PAli_NX_MP_FG_consensus CACATCTAATTACAGCTGTATATTCGTATGTCCTTTGATCTCCCATTACGCCTACAGATTTGACAGGAAGCAATACAA [15366]
cp26_PAli_NX_Pl_consensus    CACATCTAATTACAGCTGTATATTCGTATGTCCTTTGATCTCCCATTACGCCTACAGATTTGACAGGAAGCAATACAA [15366]
cp26_PAbe_NX_MP_FG_consensus CACATCTAATTACAGCTGTATATTCGTATGTCCTTTGATCTCCCATTACGCCTACAGATTTGACAGGAAGCAATACAA [15366]
cp26_PAbe_NX_Pl_consensus    CACATCTAATTACAGCTGTATATTCGTATGTCCTTTGATCTCCCATTACGCCTACAGATTTGACAGGAAGCAATACAA [15366]

cp26_gi|11497007|B31_GB      CAAATGCTTGTCTTATTTGATAATATAAATCATTTATAAAAAGCTCCTCTGTTAGAATATTGTCTGCTTCTTGTAAGA [15444]
cp26_un9_B31_PacBio          CAAATGCTTGTCTTATTTGATAATATAAATCATTTATAAAAAGCTCCTCTGTTAGAATATTGTCTGCTTCTTGTAAGA [15444]
cp26_B31_TS_MP_FG_consensus  CAAATGCTTGTCTTATTTGATAATATAAATCATTTATAAAAAGCTCCTCTGTTAGAATATTGTCTGCTTCTTGTAAGA [15444]
cp26_B31_NX_Pl_consensus     CAAATGCTTGTCTTATTTGATAATATAAATCATTTATAAAAAGCTCCTCTGTTAGAATATTGTCTGCTTCTTGTAAGA [15444]
cp26_PAli_NX_MP_FG_consensus CAAATGCTTGTCTTATTTGATAATATAAATCATTTATAAAAAGCTCCTCTGTTAGAATATTGTCTGCTTCTTGTAAGA [15444]
cp26_PAli_NX_Pl_consensus    CAAATGCTTGTCTTATTTGATAATATAAATCATTTATAAAAAGCTCCTCTGTTAGAATATTGTCTGCTTCTTGTAAGA [15444]
cp26_PAbe_NX_MP_FG_consensus CAAATGCTTGTCTTATTTGATAATATAAATCATTTATAAAAAGCTCCTCTGTTAGAATATTGTCTGCTTCTTGTAAGA [15444]
cp26_PAbe_NX_Pl_consensus    CAAATGCTTGTCTTATTTGATAATATAAATCATTTATAAAAAGCTCCTCTGTTAGAATATTGTCTGCTTCTTGTAAGA [15444]

cp26_gi|11497007|B31_GB      TATTTATCTTTTCTTGTGTTACTTCTCCAATTATTCTTATAGCTAGTCCTGGGCCGGGAAATGGATGTCTGTAAAGAG [15522]
cp26_un9_B31_PacBio          TATTTATCTTTTCTTGTGTTACTTCTCCAATTATTCTTATAGCTAGTCCTGGGCCGGGAAATGGATGTCTGTAAAGAG [15522]
cp26_B31_TS_MP_FG_consensus  TATTTATCTTTTCTTGTGTTACTTCTCCAATTATTCTTATAGCTAGTCCTGGGCCGGGAAATGGATGTCTGTAAAGAG [15522]
cp26_B31_NX_Pl_consensus     TATTTATCTTTTCTTGTGTTACTTCTCCAATTATTCTTATAGCTAGTCCTGGGCCGGGAAATGGATGTCTGTAAAGAG [15522]
cp26_PAli_NX_MP_FG_consensus TATTTATCTTTTCTTGTGTTACTTCTCCAATTATTCTTATAGCTAGTCCTGGGCCGGGAAATGGATGTCTGTAAAGAG [15522]
cp26_PAli_NX_Pl_consensus    TATTTATCTTTTCTTGTGTTACTTCTCCAATTATTCTTATAGCTAGTCCTGGGCCGGGAAATGGATGTCTGTAAAGAG [15522]
cp26_PAbe_NX_MP_FG_consensus TATTTATCTTTTCTTGTGTTACTTCTCCAATTATTCTTATAGCTAGTCCTGGGCCGGGAAATGGATGTCTGTAAAGAG [15522]
cp26_PAbe_NX_Pl_consensus    TATTTATCTTTTCTTGTGTTACTTCTCCAATTATTCTTATAGCTAGTCCTGGGCCGGGAAATGGATGTCTGTAAAGAG [15522]

cp26_gi|11497007|B31_GB      ATTCTTTTTTAATACCTAGATTTATTCCGATTTGAATTATTTCATCTTTAAAAAATTCATTCAAAGGTTCTAAGAGTT [15600]
cp26_un9_B31_PacBio          ATTCTTTTTTAATACCTAGATTTATTCCGATTTGAATTATTTCATCTTTAAAAAATTCATTCAAAGGTTCTAAGAGTT [15600]
cp26_B31_TS_MP_FG_consensus  ATTCTTTTTTAATACCTAGATTTATTCCGATTTGAATTATTTCATCTTTAAAAAATTCATTCAAAGGTTCTAAGAGTT [15600]
cp26_B31_NX_Pl_consensus     ATTCTTTTTTAATACCTAGATTTATTCCGATTTGAATTATTTCATCTTTAAAAAATTCATTCAAAGGTTCTAAGAGTT [15600]
cp26_PAli_NX_MP_FG_consensus ATTCTTTTTTAATACCTAGATTTATTCCGATTTGAATTATTTCATCTTTAAAAAATTCATTCAAAGGTTCTAAGAGTT [15600]
cp26_PAli_NX_Pl_consensus    ATTCTTTTTTAATACCTAGATTTATTCCGATTTGAATTATTTCATCTTTAAAAAATTCATTCAAAGGTTCTAAGAGTT [15600]
cp26_PAbe_NX_MP_FG_consensus ATTCTTTTTTAATACCTAGATTTATTCCGATTTGAATTATTTCATCTTTAAAAAATTCATTCAAAGGTTCTAAGAGTT [15600]
cp26_PAbe_NX_Pl_consensus    ATTCTTTTTTAATACCTAGATTTATTCCGATTTGAATTATTTCATCTTTAAAAAATTCATTCAAAGGTTCTAAGAGTT [15600]

cp26_gi|11497007|B31_GB      TTAAACTCATCTTATCTGGGAGTCCCCCTACGTTGTGATGAGACTTGATTTTTGAAGAAGAGCTGTCTTTTGATTTAG [15678]
cp26_un9_B31_PacBio          TTAAACTCATCTTATCTGGGAGTCCCCCTACGTTGTGATGAGACTTGATTTTTGAAGAAGAGCTGTCTTTTGATTTAG [15678]
cp26_B31_TS_MP_FG_consensus  TTAAACTCATCTTATCTGGGAGTCCCCCTACGTTGTGATGAGACTTGATTTTTGAAGAAGAGCTGTCTTTTGATTTAG [15678]
cp26_B31_NX_Pl_consensus     TTAAACTCATCTTATCTGGGAGTCCCCCTACGTTGTGATGAGACTTGATTTTTGAAGAAGAGCTGTCTTTTGATTTAG [15678]
cp26_PAli_NX_MP_FG_consensus TTAAACTCATCTTATCTGGGAGTCCCCCTACGTTGTGATGAGACTTGATTTTTGAAGAAGAGCTGTCTTTTGATTTAG [15678]
cp26_PAli_NX_Pl_consensus    TTAAACTCATCTTATCTGGGAGTCCCCCTACGTTGTGATGAGACTTGATTTTTGAAGAAGAGCTGTCTTTTGATTTAG [15678]
cp26_PAbe_NX_MP_FG_consensus TTAAACTCATCTTATCTGGGAGTCCCCCTACGTTGTGATGAGACTTGATTTTTGAAGAAGAGCTGTCTTTTGATTTAG [15678]
cp26_PAbe_NX_Pl_consensus    TTAAACTCATCTTATCTGGGAGTCCCCCTACGTTGTGATGAGACTTGATTTTTGAAGAAGAGCTGTCTTTTGATTTAG [15678]

cp26_gi|11497007|B31_GB      ATTCAATTACGTCAGAATAAATTGTTCCTTGAGCTAAATATTCTATATTTTGATCTTCTAGAGTAATTTTTTCAAAAA [15756]
cp26_un9_B31_PacBio          ATTCAATTACGTCAGAATAAATTGTTCCTTGAGCTAAATATTCTATATTTTGATCTTCTAGAGTAATTTTTTCAAAAA [15756]
cp26_B31_TS_MP_FG_consensus  ATTCAATTACGTCAGAATAAATTGTTCCTTGAGCTAAATATTCTATATTTTGATCTTCTAGAGTAATTTTTTCAAAAA [15756]
cp26_B31_NX_Pl_consensus     ATTCAATTACGTCAGAATAAATTGTTCCTTGAGCTAAATATTCTATATTTTGATCTTCTAGAGTAATTTTTTCAAAAA [15756]
cp26_PAli_NX_MP_FG_consensus ATTCAATTACGTCAGAATAAATTGTTCCTTGAGCTAAATATTCTATATTTTGATCTTCTAGAGTAATTTTTTCAAAAA [15756]
cp26_PAli_NX_Pl_consensus    ATTCAATTACGTCAGAATAAATTGTTCCTTGAGCTAAATATTCTATATTTTGATCTTCTAGAGTAATTTTTTCAAAAA [15756]
cp26_PAbe_NX_MP_FG_consensus ATTCAATTACGTCAGAATAAATTGTTCCTTGAGCTAAATATTCTATATTTTGATCTTCTAGAGTAATTTTTTCAAAAA [15756]
cp26_PAbe_NX_Pl_consensus    ATTCAATTACGTCAGAATAAATTGTTCCTTGAGCTAAATATTCTATATTTTGATCTTCTAGAGTAATTTTTTCAAAAA [15756]

cp26_gi|11497007|B31_GB      CATCTACAAATTCTTTTCCTATTATTTTTCTCTTTTCCTCAGGATCACTTATATTTTTTAAACGGTTCAAGAATTTTG [15834]
cp26_un9_B31_PacBio          CATCTACAAATTCTTTTCCTATTATTTTTCTCTTTTCCTCAGGATCACTTATATTTTTTAAACGGTTCAAGAATTTTG [15834]
cp26_B31_TS_MP_FG_consensus  CATCTACAAATTCTTTTCCTATTATTTTTCTCTTTTCCTCAGGATCACTTATATTTTTTAAACGGTTCAAGAATTTTG [15834]
cp26_B31_NX_Pl_consensus     CATCTACAAATTCTTTTCCTATTATTTTTCTCTTTTCCTCAGGATCACTTATATTTTTTAAACGGTTCAAGAATTTTG [15834]
cp26_PAli_NX_MP_FG_consensus CATCTACAAATTCTTTTCCTATTATTTTTCTCTTTTCCTCAGGATCACTTATATTTTTTAAACGGTTCAAGAATTTTG [15834]
cp26_PAli_NX_Pl_consensus    CATCTACAAATTCTTTTCCTATTATTTTTCTCTTTTCCTCAGGATCACTTATATTTTTTAAACGGTTCAAGAATTTTG [15834]
cp26_PAbe_NX_MP_FG_consensus CATCTACAAATTCTTTTCCTATTATTTTTCTCTTTTCCTCAGGATCACTTATATTTTTTAAACGGTTCAAGAATTTTG [15834]
cp26_PAbe_NX_Pl_consensus    CATCTACAAATTCTTTTCCTATTATTTTTCTCTTTTCCTCAGGATCACTTATATTTTTTAAACGGTTCAAGAATTTTG [15834]

cp26_gi|11497007|B31_GB      TAGAAGCATCAATATATTTTATATTTAAATCATATTGATGCTTTAATTCTAGTATTTTTTTATCTTCATTTTTGCGCA [15912]
cp26_un9_B31_PacBio          TAGAAGCATCAATATATTTTATATTTAAATCATATTGATGCTTTAATTCTAGTATTTTTTTATCTTCATTTTTGCGCA [15912]
cp26_B31_TS_MP_FG_consensus  TAGAAGCATCAATATATTTTATATTTAAATCATATTGATGCTTTAATTCTAGTATTTTTTTATCTTCATTTTTGCGCA [15912]
cp26_B31_NX_Pl_consensus     TAGAAGCATCAATATATTTTATATTTAAATCATATTGATGCTTTAATTCTAGTATTTTTTTATCTTCATTTTTGCGCA [15912]
cp26_PAli_NX_MP_FG_consensus TAGAAGCATCAATATATTTTATATTTAAATCATATTGATGCTTTAATTCTAGTATTTTTTTATCTTCATTTTTGCGCA [15912]
cp26_PAli_NX_Pl_consensus    TAGAAGCATCAATATATTTTATATTTAAATCATATTGATGCTTTAATTCTAGTATTTTTTTATCTTCATTTTTGCGCA [15912]
cp26_PAbe_NX_MP_FG_consensus TAGAAGCATCAATATATTTTATATTTAAATCATATTGATGCTTTAATTCTAGTATTTTTTTATCTTCATTTTTGCGCA [15912]
```

```
cp26_PAbe_NX_Pl_consensus    TAGAAGCATCAATATATTTTATATTTAAATCATATTGATGCTTTAATTCTAGTATTTTTTTATCTTCATTTTTGCGCA [15912]

cp26_gi|11497007|B31_GB      ACAATCCAGTGTTTACAAAAACGCAGATCAAATTTTCGTTTATAGCTTTTTTTATAAGCAATGCGCAAACTAAAGAGT [15990]
cp26_un9_B31_PacBio          ACAATCCAGTGTTTACAAAAACGCAGATCAAATTTTCGTTTATAGCTTTTTTTATAAGCAATGCGCAAACTAAAGAGT [15990]
cp26_B31_TS_MP_FG_consensus  ACAATCCAGTGTTTACAAAAACGCAGATCAAATTTTCGTTTATAGCTTTTTTTATAAGCAATGCGCAAACTAAAGAGT [15990]
cp26_B31_NX_Pl_consensus     ACAATCCAGTGTTTACAAAAACGCAGATCAAATTTTCGTTTATAGCTTTTTTTATAAGCAATGCGCAAACTAAAGAGT [15990]
cp26_PAli_NX_MP_FG_consensus ACAATCCAGTGTTTACAAAAACGCAGATCAAATTTTCGTTTATAGCTTTTTTTATAAGCAATGCGCAAACTAAAGAGT [15990]
cp26_PAli_NX_Pl_consensus    ACAATCCAGTGTTTACAAAAACGCAGATCAAATTTTCGTTTATAGCTTTTTTTATAAGCAATGCGCAAACTAAAGAGT [15990]
cp26_PAbe_NX_MP_FG_consensus ACAATCCAGTGTTTACAAAAACGCAGATCAAATTTTCGTTTATAGCTTTTTTTATAAGCAATGCGCAAACTAAAGAGT [15990]
cp26_PAbe_NX_Pl_consensus    ACAATCCAGTGTTTACAAAAACGCAGATCAAATTTTCGTTTATAGCTTTTTTTATAAGCAATGCGCAAACTAAAGAGT [15990]

cp26_gi|11497007|B31_GB      CTGTGCCACCAGAAAGTCCTAAAATAACCTTTTTGCTTCCCACTTTAAGCTTAATTTTTTTCACAATGGTTTCTAGAT [16068]
cp26_un9_B31_PacBio          CTGTGCCACCAGAAAGTCCTAAAATAACCTTTTTGCTTCCCACTTTAAGCTTAATTTTTTTCACAATGGTTTCTAGAT [16068]
cp26_B31_TS_MP_FG_consensus  CTGTGCCACCAGAAAGTCCTAAAATAACCTTTTTGCTTCCCACTTTAAGCTTAATTTTTTTCACAATGGTTTCTAGAT [16068]
cp26_B31_NX_Pl_consensus     CTGTGCCACCAGAAAGTCCTAAAATAACCTTTTTGCTTCCCACTTTAAGCTTAATTTTTTTCACAATGGTTTCTAGAT [16068]
cp26_PAli_NX_MP_FG_consensus CTGTGCCACCAGAAAGTCCTAAAATAACCTTTTTGCTTCCCACTTTAAGCTTAATTTTTTTCACAATGGTTTCTAGAT [16068]
cp26_PAli_NX_Pl_consensus    CTGTGCCACCAGAAAGTCCTAAAATAACCTTTTTGCTTCCCACTTTAAGCTTAATTTTTTTCACAATGGTTTCTAGAT [16068]
cp26_PAbe_NX_MP_FG_consensus CTGTGCCACCAGAAAGTCCTAAAATAACCTTTTTGCTTCCCACTTTAAGCTTAATTTTTTTCACAATGGTTTCTAGAT [16068]
cp26_PAbe_NX_Pl_consensus    CTGTGCCACCAGAAAGTCCTAAAATAACCTTTTTGCTTCCCACTTTAAGCTTAATTTTTTTCACAATGGTTTCTAGAT [16068]

cp26_gi|11497007|B31_GB      TGCCTTCTAATGACCAATTAATTTGGGCTTGGCAAATTTTAAAAACAAAATTTTTAATTATTTGATCACCAAATTCAG [16146]
cp26_un9_B31_PacBio          TGCCTTCTAATGACCAATTAATTTGGGCTTGGCAAATTTTAAAAACAAAATTTTTAATTATTTGATCACCAAATTCAG [16146]
cp26_B31_TS_MP_FG_consensus  TGCCTTCTAATGACCAATTAATTTGGGCTTGGCAAATTTTAAAAACAAAATTTTTAATTATTTGATCACCAAATTCAG [16146]
cp26_B31_NX_Pl_consensus     TGCCTTCTAATGACCAATTAATTTGGGCTTGGCAAATTTTAAAAACAAAATTTTTAATTATTTGATCACCAAATTCAG [16146]
cp26_PAli_NX_MP_FG_consensus TGCCTTCTAATGACCAATTAATTTGGGCTTGGCAAATTTTAAAAACAAAATTTTTAATTATTTGATCACCAAATTCAG [16146]
cp26_PAli_NX_Pl_consensus    TGCCTTCTAATGACCAATTAATTTGGGCTTGGCAAATTTTAAAAACAAAATTTTTAATTATTTGATCACCAAATTCAG [16146]
cp26_PAbe_NX_MP_FG_consensus TGCCTTCTAATGACCAATTAATTTGGGCTTGGCAAATTTTAAAAACAAAATTTTTAATTATTTGATCACCAAATTCAG [16146]
cp26_PAbe_NX_Pl_consensus    TGCCTTCTAATGACCAATTAATTTGGGCTTGGCAAATTTTAAAAACAAAATTTTTAATTATTTGATCACCAAATTCAG [16146]

cp26_gi|11497007|B31_GB      AATGAGTTACTTCTGGATGGAATTGTAGGCCGTAAATTTTTTGAGTTTCATTTGATATAGAAGCAATACAATTTTTTG [16224]
cp26_un9_B31_PacBio          AATGAGTTACTTCTGGATGGAATTGTAGGCCGTAAATTTTTTGAGTTTCATTTGATATAGAAGCAATACAATTTTTTG [16224]
cp26_B31_TS_MP_FG_consensus  AATGAGTTACTTCTGGATGGAATTGTAGGCCGTAAATTTTTTGAGTTTCATTTGATATAGAAGCAATACAATTTTTTG [16224]
cp26_B31_NX_Pl_consensus     AATGAGTTACTTCTGGATGGAATTGTAGGCCGTAAATTTTTTGAGTTTCATTTGATATAGAAGCAATACAATTTTTTG [16224]
cp26_PAli_NX_MP_FG_consensus AATGAGTTACTTCTGGATGGAATTGTAGGCCGTAAATTTTTTGAGTTTCATTTGATATAGAAGCAATACAATTTTTTG [16224]
cp26_PAli_NX_Pl_consensus    AATGAGTTACTTCTGGATGGAATTGTAGGCCGTAAATTTTTTGAGTTTCATTTGATATAGAAGCAATACAATTTTTTG [16224]
cp26_PAbe_NX_MP_FG_consensus AATGAGTTACTTCTGGATGGAATTGTAGGCCGTAAATTTTTTGAGTTTCATTTGATATAGAAGCAATACAATTTTTTG [16224]
cp26_PAbe_NX_Pl_consensus    AATGAGTTACTTCTGGATGGAATTGTAGGCCGTAAATTTTTTGAGTTTCATTTGATATAGAAGCAATACAATTTTTTG [16224]

cp26_gi|11497007|B31_GB      TAAAAGCTAATTGTTTGAAATTATCAGGAATTTTTTCAATACTGTCTCCATGACTCATGATAATTTGAAATTTGTTTG [16302]
cp26_un9_B31_PacBio          TAAAAGCTAATTGTTTGAAATTATCAGGAATTTTTTCAATACTGTCTCCATGACTCATGATAATTTGAAATTTGTTTG [16302]
cp26_B31_TS_MP_FG_consensus  TAAAAGCTAATTGTTTGAAATTATCAGGAATTTTTTCAATACTGTCTCCATGACTCATGATAATTTGAAATTTGTTTG [16302]
cp26_B31_NX_Pl_consensus     TAAAAGCTAATTGTTTGAAATTATCAGGAATTTTTTCAATACTGTCTCCATGACTCATGATAATTTGAAATTTGTTTG [16302]
cp26_PAli_NX_MP_FG_consensus TAAAAGCTAATTGTTTGAAATTATCAGGAATTTTTTCAATACTGTCTCCATGACTCATGATAATTTGAAATTTGTTTG [16302]
cp26_PAli_NX_Pl_consensus    TAAAAGCTAATTGTTTGAAATTATCAGGAATTTTTTCAATACTGTCTCCATGACTCATGATAATTTGAAATTTGTTTG [16302]
cp26_PAbe_NX_MP_FG_consensus TAAAAGCTAATTGTTTGAAATTATCAGGAATTTTTTCAATACTGTCTCCATGACTCATGATAATTTGAAATTTGTTTG [16302]
cp26_PAbe_NX_Pl_consensus    TAAAAGCTAATTGTTTGAAATTATCAGGAATTTTTTCAATACTGTCTCCATGACTCATGATAATTTGAAATTTGTTTG [16302]

cp26_gi|11497007|B31_GB      GAAGTTCTGAAAATAAAAGAGATTTTTCATCTCTTAGAAAGATTTCAGAGCTCCCATATTCTTGCTTAGAGTCTTTAG [16380]
cp26_un9_B31_PacBio          GAAGTTCTGAAAATAAAAGAGATTTTTCATCTCTTAGAAAGATTTCAGAGCTCCCATATTCTTGCTTAGAGTCTTTAG [16380]
cp26_B31_TS_MP_FG_consensus  GAAGTTCTGAAAATAAAAGAGATTTTTCATCTCTTAGAAAGATTTCAGAGCTCCCATATTCTTGCTTAGAGTCTTTAG [16380]
cp26_B31_NX_Pl_consensus     GAAGTTCTGAAAATAAAAGAGATTTTTCATCTCTTAGAAAGATTTCAGAGCTCCCATATTCTTGCTTAGAGTCTTTAG [16380]
cp26_PAli_NX_MP_FG_consensus GAAGTTCTGAAAATAAAAGAGATTTTTCATCTCTTAGAAAGATTTCAGAGCTCCCATATTCTTGCTTAGAGTCTTTAG [16380]
cp26_PAli_NX_Pl_consensus    GAAGTTCTGAAAATAAAAGAGATTTTTCATCTCTTAGAAAGATTTCAGAGCTCCCATATTCTTGCTTAGAGTCTTTAG [16380]
cp26_PAbe_NX_MP_FG_consensus GAAGTTCTGAAAATAAAAGAGATTTTTCATCTCTTAGAAAGATTTCAGAGCTCCCATATTCTTGCTTAGAGTCTTTAG [16380]
cp26_PAbe_NX_Pl_consensus    GAAGTTCTGAAAATAAAAGAGATTTTTCATCTCTTAGAAAGATTTCAGAGCTCCCATATTCTTGCTTAGAGTCTTTAG [16380]

cp26_gi|11497007|B31_GB      ATACTAGGCCCCCAAATAATTTAACAATTATTTGCATTCCATAACATATACCCAAAATAGGTATTTTCAAATTAAAAA [16458]
cp26_un9_B31_PacBio          ATACTAGGCCCCCAAATAATTTAACAATTATTTGCATTCCATAACATATACCCAAAATAGGTATTTTCAAATTAAAAA [16458]
cp26_B31_TS_MP_FG_consensus  ATACTAGGCCCCCAAATAATTTAACAATTATTTGCATTCCATAACATATACCCAAAATAGGTATTTTCAAATTAAAAA [16458]
cp26_B31_NX_Pl_consensus     ATACTAGGCCCCCAAATAATTTAACAATTATTTGCATTCCATAACATATACCCAAAATAGGTATTTTCAAATTAAAAA [16458]
cp26_PAli_NX_MP_FG_consensus ATACTAGGCCCCCAAATAATTTAACAATTATTTGCATTCCATAACATATACCCAAAATAGGTATTTTCAAATTAAAAA [16458]
cp26_PAli_NX_Pl_consensus    ATACTAGGCCCCCAAATAATTTAACAATTATTTGCATTCCATAACATATACCCAAAATAGGTATTTTCAAATTAAAAA [16458]
cp26_PAbe_NX_MP_FG_consensus ATACTAGGCCCCCAAATAATTTAACAATTATTTGCATTCCATAACATATACCCAAAATAGGTATTTTCAAATTAAAAA [16458]
cp26_PAbe_NX_Pl_consensus    ATACTAGGCCCCCAAATAATTTAACAATTATTTGCATTCCATAACATATACCCAAAATAGGTATTTTCAAATTAAAAA [16458]

cp26_gi|11497007|B31_GB      TTTCCATATTCAAGGTAGGAGCTTCTTTTGAATAAACAGAAGCAGGACTTCCACTTAGTATTATTCCTGAGATATTCA [16536]
cp26_un9_B31_PacBio          TTTCCATATTCAAGGTAGGAGCTTCTTTTGAATAAACAGAAGCAGGACTTCCACTTAGTATTATTCCTGAGATATTCA [16536]
cp26_B31_TS_MP_FG_consensus  TTTCCATATTCAAGGTAGGAGCTTCTTTTGAATAAACAGAAGCAGGACTTCCACTTAGTATTATTCCTGAGATATTCA [16536]
cp26_B31_NX_Pl_consensus     TTTCCATATTCAAGGTAGGAGCTTCTTTTGAATAAACAGAAGCAGGACTTCCACTTAGTATTATTCCTGAGATATTCA [16536]
cp26_PAli_NX_MP_FG_consensus TTTCCATATTCAAGGTAGGAGCTTCTTTTGAATAAACAGAAGCAGGACTTCCACTTAGTATTATTCCTGAGATATTCA [16536]
cp26_PAli_NX_Pl_consensus    TTTCCATATTCAAGGTAGGAGCTTCTTTTGAATAAACAGAAGCAGGACTTCCACTTAGTATTATTCCTGAGATATTCA [16536]
cp26_PAbe_NX_MP_FG_consensus TTTCCATATTCAAGGTAGGAGCTTCTTTTGAATAAACAGAAGCAGGACTTCCACTTAGTATTATTCCTGAGATATTCA [16536]
cp26_PAbe_NX_Pl_consensus    TTTCCATATTCAAGGTAGGAGCTTCTTTTGAATAAACAGAAGCAGGACTTCCACTTAGTATTATTCCTGAGATATTCA [16536]

cp26_gi|11497007|B31_GB      TATTTTTAATTTCTTTTAAAGGAGTATAGTAAGGTATTACTTTTGTATAAACGCCAATTTCTCTAATTCTTCTTGCAA [16614]
cp26_un9_B31_PacBio          TATTTTTAATTTCTTTTAAAGGAGTATAGTAAGGTATTACTTTTGTATAAACGCCAATTTCTCTAATTCTTCTTGCAA [16614]
cp26_B31_TS_MP_FG_consensus  TATTTTTAATTTCTTTTAAAGGAGTATAGTAAGGTATTACTTTTGTATAAACGCCAATTTCTCTAATTCTTCTTGCAA [16614]
cp26_B31_NX_Pl_consensus     TATTTTTAATTTCTTTTAAAGGAGTATAGTAAGGTATTACTTTTGTATAAACGCCAATTTCTCTAATTCTTCTTGCAA [16614]
cp26_PAli_NX_MP_FG_consensus TATTTTTAATTTCTTTTAAAGGAGTATAGTAAGGTATTACTTTTGTATAAACGCCAATTTCTCTAATTCTTCTTGCAA [16614]
cp26_PAli_NX_Pl_consensus    TATTTTTAATTTCTTTTAAAGGAGTATAGTAAGGTATTACTTTTGTATAAACGCCAATTTCTCTAATTCTTCTTGCAA [16614]
cp26_PAbe_NX_MP_FG_consensus TATTTTTAATTTCTTTTAAAGGAGTATAGTAAGGTATTACTTTTGTATAAACGCCAATTTCTCTAATTCTTCTTGCAA [16614]
cp26_PAbe_NX_Pl_consensus    TATTTTTAATTTCTTTTAAAGGAGTATAGTAAGGTATTACTTTTGTATAAACGCCAATTTCTCTAATTCTTCTTGCAA [16614]

cp26_gi|11497007|B31_GB      TTAGTTGGCTATATTGGGATCCAAAATCTAATACAAGTATTGCCTGAGTATTCATTATATAAGTCCTTAAATAAATTA [16692]
cp26_un9_B31_PacBio          TTAGTTGGCTATATTGGGATCCAAAATCTAATACAAGTATTGCCTGAGTATTCATTATATAAGTCCTTAAATAAATTA [16692]
cp26_B31_TS_MP_FG_consensus  TTAGTTGGCTATATTGGGATCCAAAATCTAATACAAGTATTGCCTGAGTATTCATTATATAAGTCCTTAAATAAATTA [16692]
cp26_B31_NX_Pl_consensus     TTAGTTGGCTATATTGGGATCCAAAATCTAATACAAGTATTGCCTGAGTATTCATTATATAAGTCCTTAAATAAATTA [16692]
cp26_PAli_NX_MP_FG_consensus TTAGTTGGCTATATTGGGATCCAAAATCTAATACAAGTATTGCCTGAGTATTCATTATATAAGTCCTTAAATAAATTA [16692]
cp26_PAli_NX_Pl_consensus    TTAGTTGGCTATATTGGGATCCAAAATCTAATACAAGTATTGCCTGAGTATTCATTATATAAGTCCTTAAATAAATTA [16692]
cp26_PAbe_NX_MP_FG_consensus TTAGTTGGCTATATTGGGATCCAAAATCTAATACAAGTATTGCCTGAGTATTCATTATATAAGTCCTTAAATAAATTA [16692]
cp26_PAbe_NX_Pl_consensus    TTAGTTGGCTATATTGGGATCCAAAATCTAATACAAGTATTGCCTGAGTATTCATTATATAAGTCCTTAAATAAATTA [16692]

cp26_gi|11497007|B31_GB      AAACTTTTTTTATTAAAGTATACTTCATTTAATTTTAGCATATTTGGCTTTGCTTATGTCGATTTTAAAATCAAATTA [16770]
cp26_un9_B31_PacBio          AAACTTTTTTTATTAAAGTATACTTCATTTAATTTTAGCATATTTGGCTTTGCTTATGTCGATTTTAAAATCAAATTA [16770]
cp26_B31_TS_MP_FG_consensus  AAACTTTTTTTATTAAAGTATACTTCATTTAATTTTAGCATATTTGGCTTTGCTTATGTCGATTTTAAAATCAAATTA [16770]
cp26_B31_NX_Pl_consensus     AAACTTTTTTTATTAAAGTATACTTCATTTAATTTTAGCATATTTGGCTTTGCTTATGTCGATTTTAAAATCAAATTA [16770]
cp26_PAli_NX_MP_FG_consensus AAACTTTTTTTATTAAAGTATACTTCATTTAATTTTAGCATATTTGGCTTTGCTTATGTCGATTTTAAAATCAAATTA [16770]
cp26_PAli_NX_Pl_consensus    AAACTTTTTTTATTAAAGTATACTTCATTTAATTTTAGCATATTTGGCTTTGCTTATGTCGATTTTAAAATCAAATTA [16770]
cp26_PAbe_NX_MP_FG_consensus AAACTTTTTTTATTAAAGTATACTTCATTTAATTTTAGCATATTTGGCTTTGCTTATGTCGATTTTAAAATCAAATTA [16770]
cp26_PAbe_NX_Pl_consensus    AAACTTTTTTTATTAAAGTATACTTCATTTAATTTTAGCATATTTGGCTTTGCTTATGTCGATTTTAAAATCAAATTA [16770]

cp26_gi|11497007|B31_GB      AGACAATATTTTTCAAATTCTTCAATATTTATTCAAGATATTGAAGAATTTGAAAAAATTATTTTTTCAAATAAAAAA [16848]
cp26_un9_B31_PacBio          AGACAATATTTTTCAAATTCTTCAATATTTATTCAAGATATTGAAGAATTTGAAAAAATTATTTTTTCAAATAAAAAA [16848]
cp26_B31_TS_MP_FG_consensus  AGACAATATTTTTCAAATTCTTCAATATTTATTCAAGATATTGAAGAATTTGAAAAAATTATTTTTTCAAATAAAAAA [16848]
cp26_B31_NX_Pl_consensus     AGACAATATTTTTCAAATTCTTCAATATTTATTCAAGATATTGAAGAATTTGAAAAAATTATTTTTTCAAATAAAAAA [16848]
cp26_PAli_NX_MP_FG_consensus AGACAATATTTTTCAAATTCTTCAATATTTATTCAAGATATTGAAGAATTTGAAAAAATTATTTTTTCAAATAAAAAA [16848]
cp26_PAli_NX_Pl_consensus    AGACAATATTTTTCAAATTCTTCAATATTTATTCAAGATATTGAAGAATTTGAAAAAATTATTTTTTCAAATAAAAAA [16848]
cp26_PAbe_NX_MP_FG_consensus AGACAATATTTTTCAAATTCTTCAATATTTATTCAAGATATTGAAGAATTTGAAAAAATTATTTTTTCAAATAAAAAA [16848]
cp26_PAbe_NX_Pl_consensus    AGACAATATTTTTCAAATTCTTCAATATTTATTCAAGATATTGAAGAATTTGAAAAAATTATTTTTTCAAATAAAAAA [16848]
```

```
cp26_gi|11497007|B31_GB       TTGAAAAACAAAATTGTTGGACTAATAATTCATAAATAAAAAGGAGGCACAAATTAATGAAAAAGAATACATTAAGTG [16926]
cp26_un9_B31_PacBio           TTGAAAAACAAAATTGTTGGACTAATAATTCATAAATAAAAAGGAGGCACAAATTAATGAAAAAGAATACATTAAGTG [16926]
cp26_B31_TS_MP_FG_consensus   TTGAAAAACAAAATTGTTGGACTAATAATTCATAAATAAAAAGGAGGCACAAATTAATGAAAAAGAATACATTAAGTG [16926]
cp26_B31_NX_Pl_consensus      TTGAAAAACAAAATTGTTGGACTAATAATTCATAAATAAAAAGGAGGCACAAATTAATGAAAAAGAATACATTAAGTG [16926]
cp26_PAli_NX_MP_FG_consensus TTGAAAAACAAAATTGTTGGACTAATAATTCATAAATAAAAAGGAGGCACAAATTAATGAAAAAGAATACATTAAGTG [16926]
cp26_PAli_NX_Pl_consensus     TTGAAAAACAAAATTGTTGGACTAATAATTCATAAATAAAAAGGAGGCACAAATTAATGAAAAAGAATACATTAAGTG [16926]
cp26_PAbe_NX_MP_FG_consensus TTGAAAAACAAAATTGTTGGACTAATAATTCATAAATAAAAAGGAGGCACAAATTAATGAAAAAGAATACATTAAGTG [16926]
cp26_PAbe_NX_Pl_consensus     TTGAAAAACAAAATTGTTGGACTAATAATTCATAAATAAAAAGGAGGCACAAATTAATGAAAAAGAATACATTAAGTG [16926]

cp26_gi|11497007|B31_GB       CAATATTAATGACTTTATTTTTATTTATATCTTGTAATAATTCAGGGAAAGATGGGAATACATCTGCAAATTCTGCTG [17004]
cp26_un9_B31_PacBio           CAATATTAATGACTTTATTTTTATTTATATCTTGTAATAATTCAGGGAAAGATGGGAATACATCTGCAAATTCTGCTG [17004]
cp26_B31_TS_MP_FG_consensus   CAATATTAATGACTTTATTTTTATTTATATCTTGTAATAATTCAGGGAAAGATGGGAATACATCTGCAAATTCTGCTG [17004]
cp26_B31_NX_Pl_consensus      CAATATTAATGACTTTATTTTTATTTATATCTTGTAATAATTCAGGGAAAGATGGGAATACATCTGCAAATTCTGCTG [17004]
cp26_PAli_NX_MP_FG_consensus CAATATTAATGACTTTATTTTTATTTATATCTTGTAATAATTCAGGGAAAGATGGGAATACATCTGCAAATTCTGCTG [17004]
cp26_PAli_NX_Pl_consensus     CAATATTAATGACTTTATTTTTATTTATATCTTGTAATAATTCAGGGAAAGATGGGAATACATCTGCAAATTCTGCTG [17004]
cp26_PAbe_NX_MP_FG_consensus CAATATTAATGACTTTATTTTTATTTATATCTTGTAATAATTCAGGGAAAGATGGGAATACATCTGCAAATTCTGCTG [17004]
cp26_PAbe_NX_Pl_consensus     CAATATTAATGACTTTATTTTTATTTATATCTTGTAATAATTCAGGGAAAGATGGGAATACATCTGCAAATTCTGCTG [17004]

cp26_gi|11497007|B31_GB       ATGAGTCTGTTAAAGGGCCTAATCTTACAGAAATAAGTAAAAAAATTACGGATTCTAATGCGGTTTTACTTGCTGTGA [17082]
cp26_un9_B31_PacBio           ATGAGTCTGTTAAAGGGCCTAATCTTACAGAAATAAGTAAAAAAATTACGGATTCTAATGCGGTTTTACTTGCTGTGA [17082]
cp26_B31_TS_MP_FG_consensus   ATGAGTCTGTTAAAGGGCCTAATCTTACAGAAATAAGTAAAAAAATTACGGATTCTAATGCGGTTTTACTTGCTGTGA [17082]
cp26_B31_NX_Pl_consensus      ATGAGTCTGTTAAAGGGCCTAATCTTACAGAAATAAGTAAAAAAATTACGGATTCTAATGCGGTTTTACTTGCTGTGA [17082]
cp26_PAli_NX_MP_FG_consensus ATGAGTCTGTTAAAGGGCCTAATCTTACAGAAATAAGTAAAAAAATTACGGATTCTAATGCGGTTTTACTTGCTGTGA [17082]
cp26_PAli_NX_Pl_consensus     ATGAGTCTGTTAAAGGGCCTAATCTTACAGAAATAAGTAAAAAAATTACGGATTCTAATGCGGTTTTACTTGCTGTGA [17082]
cp26_PAbe_NX_MP_FG_consensus ATGAGTCTGTTAAAGGGCCTAATCTTACAGAAATAAGTAAAAAAATTACGGATTCTAATGCGGTTTTACTTGCTGTGA [17082]
cp26_PAbe_NX_Pl_consensus     ATGAGTCTGTTAAAGGGCCTAATCTTACAGAAATAAGTAAAAAAATTACGGATTCTAATGCGGTTTTACTTGCTGTGA [17082]

cp26_gi|11497007|B31_GB       AAGAGGTTGAAGCGTTGCTGTCATCTATAGATGAAATTGCTGCTAAAGCTATTGGTAAAAAAATACACCAAAATAATG [17160]
cp26_un9_B31_PacBio           AAGAGGTTGAAGCGTTGCTGTCATCTATAGATGAAATTGCTGCTAAAGCTATTGGTAAAAAAATACACCAAAATAATG [17160]
cp26_B31_TS_MP_FG_consensus   AAGAGGTTGAAGCGTTGCTGTCATCTATAGATGAAATTGCTGCTAAAGCTATTGGTAAAAAAATACACCAAAATAATG [17160]
cp26_B31_NX_Pl_consensus      AAGAGGTTGAAGCGTTGCTGTCATCTATAGATGAAATTGCTGCTAAAGCTATTGGTAAAAAAATACACCAAAATAATG [17160]
cp26_PAli_NX_MP_FG_consensus AAGAGGTTGAAGCGTTGCTGTCATCTATAGATGAAATTGCTGCTAAAGCTATTGGTAAAAAAATACACCAAAATAATG [17160]
cp26_PAli_NX_Pl_consensus     AAGAGGTTGAAGCGTTGCTGTCATCTATAGATGAAATTGCTGCTAAAGCTATTGGTAAAAAAATACACCAAAATAATG [17160]
cp26_PAbe_NX_MP_FG_consensus AAGAGGTTGAAGCGTTGCTGTCATCTATAGATGAAATTGCTGCTAAAGCTATTGGTAAAAAAATACACCAAAATAATG [17160]
cp26_PAbe_NX_Pl_consensus     AAGAGGTTGAAGCGTTGCTGTCATCTATAGATGAAATTGCTGCTAAAGCTATTGGTAAAAAAATACACCAAAATAATG [17160]

cp26_gi|11497007|B31_GB       GTTTGGATACCGAAAATAATCACAATGGATCATTGTTAGCGGGAGCTTATGCAATATCAACCCTAATAAAACAAAAAT [17238]
cp26_un9_B31_PacBio           GTTTGGATACCGAAAATAATCACAATGGATCATTGTTAGCGGGAGCTTATGCAATATCAACCCTAATAAAACAAAAAT [17238]
cp26_B31_TS_MP_FG_consensus   GTTTGGATACCGAAAATAATCACAATGGATCATTGTTAGCGGGAGCTTATGCAATATCAACCCTAATAAAACAAAAAT [17238]
cp26_B31_NX_Pl_consensus      GTTTGGATACCGAAAATAATCACAATGGATCATTGTTAGCGGGAGCTTATGCAATATCAACCCTAATAAAACAAAAAT [17238]
cp26_PAli_NX_MP_FG_consensus GTTTGGATACCGAAAATAATCACAATGGATCATTGTTAGCGGGAGCTTATGCAATATCAACCCTAATAAAACAAAAAT [17238]
cp26_PAli_NX_Pl_consensus     GTTTGGATACCGAAAATAATCACAATGGATCATTGTTAGCGGGAGCTTATGCAATATCAACCCTAATAAAACAAAAAT [17238]
cp26_PAbe_NX_MP_FG_consensus GTTTGGATACCGAAAATAATCACAATGGATCATTGTTAGCGGGAGCTTATGCAATATCAACCCTAATAAAACAAAAAT [17238]
cp26_PAbe_NX_Pl_consensus     GTTTGGATACCGAAAATAATCACAATGGATCATTGTTAGCGGGAGCTTATGCAATATCAACCCTAATAAAACAAAAAT [17238]

cp26_gi|11497007|B31_GB       TAGATGGATTGAAAAATGAAGGATTAAAGGAAAAAATTGATGCGGCTAAGAAATGTTCTGAAACATTTACTAATAAAT [17316]
cp26_un9_B31_PacBio           TAGATGGATTGAAAAATGAAGGATTAAAGGAAAAAATTGATGCGGCTAAGAAATGTTCTGAAACATTTACTAATAAAT [17316]
cp26_B31_TS_MP_FG_consensus   TAGATGGATTGAAAAATGAAGGATTAAAGGAAAAAATTGATGCGGCTAAGAAATGTTCTGAAACATTTACTAATAAAT [17316]
cp26_B31_NX_Pl_consensus      TAGATGGATTGAAAAATGAAGGATTAAAGGAAAAAATTGATGCGGCTAAGAAATGTTCTGAAACATTTACTAATAAAT [17316]
cp26_PAli_NX_MP_FG_consensus TAGATGGATTGAAAAATGAAGGATTAAAGGAAAAAATTGATGCGGCTAAGAAATGTTCTGAAACATTTACTAATAAAT [17316]
cp26_PAli_NX_Pl_consensus     TAGATGGATTGAAAAATGAAGGATTAAAGGAAAAAATTGATGCGGCTAAGAAATGTTCTGAAACATTTACTAATAAAT [17316]
cp26_PAbe_NX_MP_FG_consensus TAGATGGATTGAAAAATGAAGGATTAAAGGAAAAAATTGATGCGGCTAAGAAATGTTCTGAAACATTTACTAATAAAT [17316]
cp26_PAbe_NX_Pl_consensus     TAGATGGATTGAAAAATGAAGGATTAAAGGAAAAAATTGATGCGGCTAAGAAATGTTCTGAAACATTTACTAATAAAT [17316]

cp26_gi|11497007|B31_GB       TAAAAGAAAAACACACAGATCTTGGTAAAGAAGGTGTTACTGATGCTGATGCAAAAGAAGCCATTTTAAAAACAAATG [17394]
cp26_un9_B31_PacBio           TAAAAGAAAAACACACAGATCTTGGTAAAGAAGGTGTTACTGATGCTGATGCAAAAGAAGCCATTTTAAAAACAAATG [17394]
cp26_B31_TS_MP_FG_consensus   TAAAAGAAAAACACACAGATCTTGGTAAAGAAGGTGTTACTGATGCTGATGCAAAAGAAGCCATTTTAAAAACAAATG [17394]
cp26_B31_NX_Pl_consensus      TAAAAGAAAAACACACAGATCTTGGTAAAGAAGGTGTTACTGATGCTGATGCAAAAGAAGCCATTTTAAAAACAAATG [17394]
cp26_PAli_NX_MP_FG_consensus TAAAAGAAAAACACACAGATCTTGGTAAAGAAGGTGTTACTGATGCTGATGCAAAAGAAGCCATTTTAAAAACAAATG [17394]
cp26_PAli_NX_Pl_consensus     TAAAAGAAAAACACACAGATCTTGGTAAAGAAGGTGTTACTGATGCTGATGCAAAAGAAGCCATTTTAAAAACAAATG [17394]
cp26_PAbe_NX_MP_FG_consensus TAAAAGAAAAACACACAGATCTTGGTAAAGAAGGTGTTACTGATGCTGATGCAAAAGAAGCCATTTTAAAAACAAATG [17394]
cp26_PAbe_NX_Pl_consensus     TAAAAGAAAAACACACAGATCTTGGTAAAGAAGGTGTTACTGATGCTGATGCAAAAGAAGCCATTTTAAAAACAAATG [17394]

cp26_gi|11497007|B31_GB       GTACTAAAACTAAAGGTGCTGAAGAACTTGGAAAATTATTTGAATCAGTAGAGGTCTTGTCAAAAGCAGCTAAAGAGA [17472]
cp26_un9_B31_PacBio           GTACTAAAACTAAAGGTGCTGAAGAACTTGGAAAATTATTTGAATCAGTAGAGGTCTTGTCAAAAGCAGCTAAAGAGA [17472]
cp26_B31_TS_MP_FG_consensus   GTACTAAAACTAAAGGTGCTGAAGAACTTGGAAAATTATTTGAATCAGTAGAGGTCTTGTCAAAAGCAGCTAAAGAGA [17472]
cp26_B31_NX_Pl_consensus      GTACTAAAACTAAAGGTGCTGAAGAACTTGGAAAATTATTTGAATCAGTAGAGGTCTTGTCAAAAGCAGCTAAAGAGA [17472]
cp26_PAli_NX_MP_FG_consensus GTACTAAAACTAAAGGTGCTGAAGAACTTGGAAAATTATTTGAATCAGTAGAGGTCTTGTCAAAAGCAGCTAAAGAGA [17472]
cp26_PAli_NX_Pl_consensus     GTACTAAAACTAAAGGTGCTGAAGAACTTGGAAAATTATTTGAATCAGTAGAGGTCTTGTCAAAAGCAGCTAAAGAGA [17472]
cp26_PAbe_NX_MP_FG_consensus GTACTAAAACTAAAGGTGCTGAAGAACTTGGAAAATTATTTGAATCAGTAGAGGTCTTGTCAAAAGCAGCTAAAGAGA [17472]
cp26_PAbe_NX_Pl_consensus     GTACTAAAACTAAAGGTGCTGAAGAACTTGGAAAATTATTTGAATCAGTAGAGGTCTTGTCAAAAGCAGCTAAAGAGA [17472]

cp26_gi|11497007|B31_GB       TGCTTGCTAATTCAGTTAAAGAGCTTACAAGCCCTGTTGTGGCAGAAAGTCCAAAAAAACCTTAATTAAGATCAATAT [17550]
cp26_un9_B31_PacBio           TGCTTGCTAATTCAGTTAAAGAGCTTACAAGCCCTGTTGTGGCAGAAAGTCCAAAAAAACCTTAATTAAGATCAATAT [17550]
cp26_B31_TS_MP_FG_consensus   TGCTTGCTAATTCAGTTAAAGAGCTTACAAGCCCTGTTGTGGCAGAAAGTCCAAAAAAACCTTAATTAAGATCAATAT [17550]
cp26_B31_NX_Pl_consensus      TGCTTGCTAATTCAGTTAAAGAGCTTACAAGCCCTGTTGTGGCAGAAAGTCCAAAAAAACCTTAATTAAGATCAATAT [17550]
cp26_PAli_NX_MP_FG_consensus TGCTTGCTAATTCAGTTAAAGAGCTTACAAGCCCTGTTGTGGCAGAAAGTCCAAAAAAACCTTAATTAAGATCAATAT [17550]
cp26_PAli_NX_Pl_consensus     TGCTTGCTAATTCAGTTAAAGAGCTTACAAGCCCTGTTGTGGCAGAAAGTCCAAAAAAACCTTAATTAAGATCAATAT [17550]
cp26_PAbe_NX_MP_FG_consensus TGCTTGCTAATTCAGTTAAAGAGCTTACAAGCCCTGTTGTGGCAGAAAGTCCAAAAAAACCTTAATTAAGATCAATAT [17550]
cp26_PAbe_NX_Pl_consensus     TGCTTGCTAATTCAGTTAAAGAGCTTACAAGCCCTGTTGTGGCAGAAAGTCCAAAAAAACCTTAATTAAGATCAATAT [17550]

cp26_gi|11497007|B31_GB       TATAAGATTAATTTGTTTTAAAAAAGTAACTGGAAAAATAAAGTCAATATAAGTCAAGAAAGATTTCTTGGCTTTTAT [17628]
cp26_un9_B31_PacBio           TATAAGATTAATTTGTTTTAAAAAAGTAACTGGAAAAATAAAGTCAATATAAGTCAAGAAAGATTTCTTGGCTTTTAT [17628]
cp26_B31_TS_MP_FG_consensus   TATAAGATTAATTTGTTTTAAAAAAGTAACTGGAAAAATAAAGTCAATATAAGTCAAGAAAGATTTCTTGGCTTTTAT [17628]
cp26_B31_NX_Pl_consensus      TATAAGATTAATTTGTTTTAAAAAAGTAACTGGAAAAATAAAGTCAATATAAGTCAAGAAAGATTTCTTGGCTTTTAT [17628]
cp26_PAli_NX_MP_FG_consensus TATAAGATTAATTTGTTTTAAAAAAGTAACTGGAAAAATAAAGTCAATATAAGTCAAGAAAGATTTCTTGGCTTTTAT [17628]
cp26_PAli_NX_Pl_consensus     TATAAGATTAATTTGTTTTAAAAAAGTAACTGGAAAAATAAAGTCAATATAAGTCAAGAAAGATTTCTTGGCTTTTAT [17628]
cp26_PAbe_NX_MP_FG_consensus TATAAGATTAATTTGTTTTAAAAAAGTAACTGGAAAAATAAAGTCAATATAAGTCAAGAAAGATTTCTTGGCTTTTAT [17628]
cp26_PAbe_NX_Pl_consensus     TATAAGATTAATTTGTTTTAAAAAAGTAACTGGAAAAATAAAGTCAATATAAGTCAAGAAAGATTTCTTGGCTTTTAT [17628]

cp26_gi|11497007|B31_GB       TTTTTTATTTTCTCAAAAAATTTCTTAAAATTCATTTTAACTTTAAGCTCTTCTCTTAAAGAATAAAAACTTATTACA [17706]
cp26_un9_B31_PacBio           TTTTTTATTTTCTCAAAAAATTTCTTAAAATTCATTTTAACTTTAAGCTCTTCTCTTAAAGAATAAAAACTTATTACA [17706]
cp26_B31_TS_MP_FG_consensus   TTTTTTATTTTCTCAAAAAATTTCTTAAAATTCATTTTAACTTTAAGCTCTTCTCTTAAAGAATAAAAACTTATTACA [17706]
cp26_B31_NX_Pl_consensus      TTTTTTATTTTCTCAAAAAATTTCTTAAAATTCATTTTAACTTTAAGCTCTTCTCTTAAAGAATAAAAACTTATTACA [17706]
cp26_PAli_NX_MP_FG_consensus TTTTTTATTTTCTCAAAAAATTTCTTAAAATTCATTTTAACTTTAAGCTCTTCTCTTAAAGAATAAAAACTTATTACA [17706]
cp26_PAli_NX_Pl_consensus     TTTTTTATTTTCTCAAAAAATTTCTTAAAATTCATTTTAACTTTAAGCTCTTCTCTTAAAGAATAAAAACTTATTACA [17706]
cp26_PAbe_NX_MP_FG_consensus TTTTTTATTTTCTCAAAAAATTTCTTAAAATTCATTTTAACTTTAAGCTCTTCTCTTAAAGAATAAAAACTTATTACA [17706]
cp26_PAbe_NX_Pl_consensus     TTTTTTATTTTCTCAAAAAATTTCTTAAAATTCATTTTAACTTTAAGCTCTTCTCTTAAAGAATAAAAACTTATTACA [17706]

cp26_gi|11497007|B31_GB       AAAGCCGTTAATAAATTGAATTTAAGCATTTAAATATAAACAAAATTGGGTGCTAGAAATTTGATTTTAAAGACTTTA [17784]
cp26_un9_B31_PacBio           AAAGCCGTTAATAAATTGAATTTAAGCATTTAAATATAAACAAAATTGGGTGCTAGAAATTTGATTTTAAAGACTTTA [17784]
cp26_B31_TS_MP_FG_consensus   AAAGCCGTTAATAAATTGAATTTAAGCATTTAAATATAAACAAAATTGGGTGCTAGAAATTTGATTTTAAAGACTTTA [17784]
cp26_B31_NX_Pl_consensus      AAAGCCGTTAATAAATTGAATTTAAGCATTTAAATATAAACAAAATTGGGTGCTAGAAATTTGATTTTAAAGACTTTA [17784]
cp26_PAli_NX_MP_FG_consensus AAAGCCGTTAATAAATTGAATTTAAGCATTTAAATATAAACAAAATTGGGTGCTAGAAATTTGATTTTAAAGACTTTA [17784]
cp26_PAli_NX_Pl_consensus     AAAGCCGTTAATAAATTGAATTTAAGCATTTAAATATAAACAAAATTGGGTGCTAGAAATTTGATTTTAAAGACTTTA [17784]
cp26_PAbe_NX_MP_FG_consensus AAAGCCGTTAATAAATTGAATTTAAGCATTTAAATATAAACAAAATTGGGTGCTAGAAATTTGATTTTAAAGACTTTA [17784]
cp26_PAbe_NX_Pl_consensus     AAAGCCGTTAATAAATTGAATTTAAGCATTTAAATATAAACAAAATTGGGTGTTAGAAATTTGATTTTAAAGACTTTA [17784]
```

```
cp26_gi|11497007|B31_GB       AGTAGGCTTAAACGGTTTTGGCAAATTTTTAAAGATAATAGTAGTTTCTTTGGTTTTATGTGAGATTTTAAAAAGTTG [17862]
cp26_un9_B31_PacBio           AGTAGGCTTAAACGGTTTTGGCAAATTTTTAAAGATAATAGTAGTTTCTTTGGTTTTATGTGAGATTTTAAAAAGTTG [17862]
cp26_B31_TS_MP_FG_consensus   AGTAGGCTTAAACGGTTTTGGCAAATTTTTAAAGATAATAGTAGTTTCTTTGGTTTTATGTGAGATTTTAAAAAGTTG [17862]
cp26_B31_NX_Pl_consensus      AGTAGGCTTAAACGGTTTTGGCAAATTTTTAAAGATAATAGTAGTTTCTTTGGTTTTATGTGAGATTTTAAAAAGTTG [17862]
cp26_PAli_NX_MP_FG_consensus AGTAGGCTTAAACGGTTTTGGCAAATTTTTAAAGATAATAGTAGTTTCTTTGGTTTTATGTGAGATTTTAAAAAGTTG [17862]
cp26_PAli_NX_Pl_consensus     AGTAGGCTTAAACGGTTTTGGCAAATTTTTAAAGATAATAGTAGTTTCTTTGGTTTTATGTGAGATTTTAAAAAGTTG [17862]
cp26_PAbe_NX_MP_FG_consensus AGTAGGCTTAAACGGTTTTGGCAAATTTTTAAAGATAATAGTAGTTTCTTTGGTTTTATGTGAGATTTTAAAAAGTTG [17862]
cp26_PAbe_NX_Pl_consensus     AGTAGGCTTAAACGGTTTTGGCAAATTTTTAAAGATAATAGTAGTTTCTTTGGTTTTATGTGAGATTTTAAAAAGTTG [17862]

cp26_gi|11497007|B31_GB       TTAAATAGACTTAACTATTTAAAAAATAAAGACTCTTTTTTATGATTTATAATTTAGGTTTTTGAAATTGCAAATTTT [17940]
cp26_un9_B31_PacBio           TTAAATAGACTTAACTATTTAAAAAATAAAGACTCTTTTTTATGATTTATAATTTAGGTTTTTGAAATTGCAAATTTT [17940]
cp26_B31_TS_MP_FG_consensus   TTAAATAGACTTAACTATTTAAAAAATAAAGACTCTTTTTTATGATTTATAATTTAGGTTTTTGAAATTGCAAATTTT [17940]
cp26_B31_NX_Pl_consensus      TTAAATAGACTTAACTATTTAAAAAATAAAGACTCTTTTTTATGATTTATAATTTAGGTTTTTGAAATTGCAAATTTT [17940]
cp26_PAli_NX_MP_FG_consensus TTAAATAGACTTAACTATTTAAAAAATAAAGACTCTTTTTTATGATTTATAATTTAGGTTTTTGAAATTGCAAATTTT [17940]
cp26_PAli_NX_Pl_consensus     TTAAATAGACTTAACTATTTAAAAAATAAAGACTCTTTTTTATGATTTATAATTTAGGTTTTTGAAATTGCAAATTTT [17940]
cp26_PAbe_NX_MP_FG_consensus TTAAATAGACTTAACTATTTAAAAAATAAAGACTCTTTTTTATGATTTATAATTTAGGTTTTTGAAATTGCAAATTTT [17940]
cp26_PAbe_NX_Pl_consensus     TTAAATAGACTTAACTATTTAAAAAATAAAGACTCTTTTTTATGATTTATAATTTAGGTTTTTGAAATTGCAAATTTT [17940]

cp26_gi|11497007|B31_GB       AAATTTTTTTTATAAATAACAAATCGGTTAATAAATATAAATAAATTTAATAATAAAAACTAGGCACAGTATTATCAT [18018]
cp26_un9_B31_PacBio           AAATTTTTTTTATAAATAACAAATCGGTTAATAAATATAAATAAATTTAATAATAAAAACTAGGCACAGTATTATCAT [18018]
cp26_B31_TS_MP_FG_consensus   AAATTTTTTTTATAAATAACAAATCGGTTAATAAATATAAATAAATTTAATAATAAAAACTAGGCACAGTATTATCAT [18018]
cp26_B31_NX_Pl_consensus      AAATTTTTTTTATAAATAACAAATCGGTTAATAAATATAAATAAATTTAATAATAAAAACTAGGCACAGTATTATCAT [18018]
cp26_PAli_NX_MP_FG_consensus AAATTTTTTTTATAAATAACAAATCGGTTAATAAATATAAATAAATTTAATAATAAAAACTAGGCACAGTATTATCAT [18018]
cp26_PAli_NX_Pl_consensus     AAATTTTTTTTATAAATAACAAATCGGTTAATAAATATAAATAAATTTAATAATAAAAACTAGGCACAGTATTATCAT [18018]
cp26_PAbe_NX_MP_FG_consensus AAATTTTTTTTATAAATAACAAATCGGTTAATAAATATAAATAAATTTAATAATAAAAACTAGGCACAGTATTATCAT [18018]
cp26_PAbe_NX_Pl_consensus     AAATTTTTTTTATAAATAACAAATCGGTTAATAAATATAAATAAATTTAATAATAAAAACTAGGCACAGTATTATCAT [18018]

cp26_gi|11497007|B31_GB       TACTGGAGAGATTTTATTTTTTTTATTTTCCAATAAATTAAGTATTATGTTTATTAAAACATAAAATATTATTCCAAT [18096]
cp26_un9_B31_PacBio           TACTGGAGAGATTTTATTTTTTTTATTTTCCAATAAATTAAGTATTATGTTTATTAAAACATAAAATATTATTCCAAT [18096]
cp26_B31_TS_MP_FG_consensus   TACTGGAGAGATTTTATTTTTTTTATTTTCCAATAAATTAAGTATTATGTTTATTAAAACATAAAATATTATTCCAAT [18096]
cp26_B31_NX_Pl_consensus      TACTGGAGAGATTTTATTTTTTTTATTTTCCAATAAATTAAGTATTATGTTTATTAAAACATAAAATATTATTCCAAT [18096]
cp26_PAli_NX_MP_FG_consensus TACTGGAGAGATTTTATTTTTTTTATTTTCCAATAAATTAAGTATTATGTTTATTAAAACATAAAATATTATTCCAAT [18096]
cp26_PAli_NX_Pl_consensus     TACTGGAGAGATTTTATTTTTTTTATTTTCCAATAAATTAAGTATTATGTTTATTAAAACATAAAATATTATTCCAAT [18096]
cp26_PAbe_NX_MP_FG_consensus TACTGGAGAGATTTTATTTTTTTTATTTTCCAATAAATTAAGTATTATGTTTATTAAAACATAAAATATTATTCCAAT [18096]
cp26_PAbe_NX_Pl_consensus     TACTGGAGAGATTTTATTTTTTTTATTTTCCAATAAATTAAGTATTATGTTTATTAAAACATAAAATATTATTCCAAT [18096]

cp26_gi|11497007|B31_GB       ACTTATCCCCGAAGAGATATTGTATGTTAAGGGGATTAGAAAAAGTATTAAAAAACTGGGAATATTTTCTCTTATATT [18174]
cp26_un9_B31_PacBio           ACTTATCCCCGAAGAGATATTGTATGTTAAGGGGATTAGAAAAAGTATTAAAAAACTGGGAATATTTTCTCTTATATT [18174]
cp26_B31_TS_MP_FG_consensus   ACTTATCCCCGAAGAGATATTGTATGTTAAGGGGATTAGAAAAAGTATTAAAAAACTGGGAATATTTTCTCTTATATT [18174]
cp26_B31_NX_Pl_consensus      ACTTATCCCCGAAGAGATATTGTATGTTAAGGGGATTAGAAAAAGTATTAAAAAACTGGGAATATTTTCTCTTATATT [18174]
cp26_PAli_NX_MP_FG_consensus ACTTATCCCCGAAGAGATATTGTATGTTAAGGGGATTAGAAAAAGTATTAAAAAACTGGGAATATTTTCTCTTATATT [18174]
cp26_PAli_NX_Pl_consensus     ACTTATCCCCGAAGAGATATTGTATGTTAAGGGGATTAGAAAAAGTATTAAAAAACTGGGAATATTTTCTCTTATATT [18174]
cp26_PAbe_NX_MP_FG_consensus ACTTATCCCCGAAGAGATATTGTATGTTAAGGGGATTAGAAAAAGTATTAAAAAACTGGGAATATTTTCTCTTATATT [18174]
cp26_PAbe_NX_Pl_consensus     ACTTATCCCCGAAGAGATATTGTATGTTAAGGGGATTAGAAAAAGTATTAAAAAACTGGGAATATTTTCTCTTATATT [18174]

cp26_gi|11497007|B31_GB       AGAGAAATTAATTTTCAATATTTCTCTGCACATTGAAAATCCTACATATATTAGTGCTGCAGCAGTTGCACTAGCAGG [18252]
cp26_un9_B31_PacBio           AGAGAAATTAATTTTCAATATTTCTCTGCACATTGAAAATCCTACATATATTAGTGCTGCAGCAGTTGCACTAGCAGG [18252]
cp26_B31_TS_MP_FG_consensus   AGAGAAATTAATTTTCAATATTTCTCTGCACATTGAAAATCCTACATATATTAGTGCTGCAGCAGTTGCACTAGCAGG [18252]
cp26_B31_NX_Pl_consensus      AGAGAAATTAATTTTCAATATTTCTCTGCACATTGAAAATCCTACATATATTAGTGCTGCAGCAGTTGCACTAGCAGG [18252]
cp26_PAli_NX_MP_FG_consensus AGAGAAATTAATTTTCAATATTTCTCTGCACATTGAAAATCCTACATATATTAGTGCTGCAGCAGTTGCACTAGCAGG [18252]
cp26_PAli_NX_Pl_consensus     AGAGAAATTAATTTTCAATATTTCTCTGCACATTGAAAATCCTACATATATTAGTGCTGCAGCAGTTGCACTAGCAGG [18252]
cp26_PAbe_NX_MP_FG_consensus AGAGAAATTAATTTTCAATATTTCTCTGCACATTGAAAATCCTACATATATTAGTGCTGCAGCAGTTGCACTAGCAGG [18252]
cp26_PAbe_NX_Pl_consensus     AGAGAAATTAATTTTCAATATTTCTCTGCACATTGAAAATCCTACATATATTAGTGCTGCAGCAGTTGCACTAGCAGG [18252]

cp26_gi|11497007|B31_GB       AACTGCAATAAATAATGGTGAAAGGAATATTGCAATAAAGAACATTATTCCCGTTACTATTGTTGTAAGTCCGGTTTT [18330]
cp26_un9_B31_PacBio           AACTGCAATAAATAATGGTGAAAGGAATATTGCAATAAAGAACATTATTCCCGTTACTATTGTTGTAAGTCCGGTTTT [18330]
cp26_B31_TS_MP_FG_consensus   AACTGCAATAAATAATGGTGAAAGGAATATTGCAATAAAGAACATTATTCCCGTTACTATTGTTGTAAGTCCGGTTTT [18330]
cp26_B31_NX_Pl_consensus      AACTGCAATAAATAATGGTGAAAGGAATATTGCAATAAAGAACATTATTCCCGTTACTATTGTTGTAAGTCCGGTTTT [18330]
cp26_PAli_NX_MP_FG_consensus AACTGCAATAAATAATGGTGAAAGGAATATTGCAATAAAGAACATTATTCCCGTTACTATTGTTGTAAGTCCGGTTTT [18330]
cp26_PAli_NX_Pl_consensus     AACTGCAATAAATAATGGTGAAAGGAATATTGCAATAAAGAACATTATTCCCGTTACTATTGTTGTAAGTCCGGTTTT [18330]
cp26_PAbe_NX_MP_FG_consensus AACTGCAATAAATAATGGTGAAAGGAATATTGCAATAAAGAACATTATTCCCGTTACTATTGTTGTAAGTCCGGTTTT [18330]
cp26_PAbe_NX_Pl_consensus     AACTGCAATAAATAATGGTGAAAGGAATATTGCAATAAAGAACATTATTCCCGTTACTATTGTTGTAAGTCCGGTTTT [18330]

cp26_gi|11497007|B31_GB       ACCACCTTCTTCTATTCCTGTGCAACTTTCAATGTATGCGGTTACGGTTGAAACTCCCATTATTGCTCCAACAGTAGT [18408]
cp26_un9_B31_PacBio           ACCACCTTCTTCTATTCCTGTGCAACTTTCAATGTATGCGGTTACGGTTGAAACTCCCATTATTGCTCCAACAGTAGT [18408]
cp26_B31_TS_MP_FG_consensus   ACCACCTTCTTCTATTCCTGTGCAACTTTCAATGTATGCGGTTACGGTTGAAACTCCCATTATTGCTCCAACAGTAGT [18408]
cp26_B31_NX_Pl_consensus      ACCACCTTCTTCTATTCCTGTGCAACTTTCAATGTATGCGGTTACGGTTGAAACTCCCATTATTGCTCCAACAGTAGT [18408]
cp26_PAli_NX_MP_FG_consensus ACCACCTTCTTCTATTCCTGTGCAACTTTCAATGTATGCGGTTACGGTTGAAACTCCCATTATTGCTCCAACAGTAGT [18408]
cp26_PAli_NX_Pl_consensus     ACCACCTTCTTCTATTCCTGTGCAACTTTCAATGTATGCGGTTACGGTTGAAACTCCCATTATTGCTCCAACAGTAGT [18408]
cp26_PAbe_NX_MP_FG_consensus ACCACCTTCTTCTATTCCTGTGCAACTTTCAATGTATGCGGTTACGGTTGAAACTCCCATTATTGCTCCAACAGTAGT [18408]
cp26_PAbe_NX_Pl_consensus     ACCACCTTCTTCTATTCCTGTGCAACTTTCAATGTATGCGGTTACGGTTGAAACTCCCATTATTGCTCCAACAGTAGT [18408]

cp26_gi|11497007|B31_GB       AGAAATGGCATCAATTAAAAATATTTTGCCGACATTAGGAATTTTTCCATTTTTGTCTAACATATTTCCTTTTGCTGC [18486]
cp26_un9_B31_PacBio           AGAAATGGCATCAATTAAAAATATTTTGCCGACATTAGGAATTTTTCCATTTTTGTCTAACATATTTCCTTTTGCTGC [18486]
cp26_B31_TS_MP_FG_consensus   AGAAATGGCATCAATTAAAAATATTTTGCCGACATTAGGAATTTTTCCATTTTTGTCTAACATATTTCCTTTTGCTGC [18486]
cp26_B31_NX_Pl_consensus      AGAAATGGCATCAATTAAAAATATTTTGCCGACATTAGGAATTTTTCCATTTTTGTCTAACATATTTCCTTTTGCTGC [18486]
cp26_PAli_NX_MP_FG_consensus AGAAATGGCATCAATTAAAAATATTTTGCCGACATTAGGAATTTTTCCATTTTTGTCTAACATATTTCCTTTTGCTGC [18486]
cp26_PAli_NX_Pl_consensus     AGAAATGGCATCAATTAAAAATATTTTGCCGACATTAGGAATTTTTCCATTTTTGTCTAACATATTTCCTTTTGCTGC [18486]
cp26_PAbe_NX_MP_FG_consensus AGAAATGGCATCAATTAAAAATATTTTGCCGACATTAGGAATTTTTCCATTTTTGTCTAACATATTTCCTTTTGCTGC [18486]
cp26_PAbe_NX_Pl_consensus     AGAAATGGCATCAATTAAAAATATTTTGCCGACATTAGGAATTTTTCCATTTTTGTCTAACATATTTCCTTTTGCTGC [18486]

cp26_gi|11497007|B31_GB       CACTGCTATTAAAGTGCCCAAAGTATCAAATAAATCGTTAAACAGCAATACCAATACAATTGTAATAAAGCTCCAAAA [18564]
cp26_un9_B31_PacBio           CACTGCTATTAAAGTGCCCAAAGTATCAAATAAATCGTTAAACAGCAATACCAATACAATTGTAATAAAGCTCCAAAA [18564]
cp26_B31_TS_MP_FG_consensus   CACTGCTATTAAAGTGCCCAAAGTATCAAATAAATCGTTAAACAGCAATACCAATACAATTGTAATAAAGCTCCAAAA [18564]
cp26_B31_NX_Pl_consensus      CACTGCTATTAAAGTGCCCAAAGTATCAAATAAATCGTTAAACAGCAATACCAATACAATTGTAATAAAGCTCCAAAA [18564]
cp26_PAli_NX_MP_FG_consensus CACTGCTATTAAAGTGCCCAAAGTATCAAATAAATCGTTAAACAGCAATACCAATACAATTGTAATAAAGCTCCAAAA [18564]
cp26_PAli_NX_Pl_consensus     CACTGCTATTAAAGTGCCCAAAGTATCAAATAAATCGTTAAACAGCAATACCAATACAATTGTAATAAAGCTCCAAAA [18564]
cp26_PAbe_NX_MP_FG_consensus CACTGCTATTAAAGTGCCCAAAGTATCAAATAAATCGTTAAACAGCAATACCAATACAATTGTAATAAAGCTCCAAAA [18564]
cp26_PAbe_NX_Pl_consensus     CACTGCTATTAAAGTGCCCAAAGTATCAAATAAATCGTTAAACAGCAATACCAATACAATTGTAATAAAGCTCCAAAA [18564]

cp26_gi|11497007|B31_GB       ATGTTTGCTTAATATGTAGGAAAAATCTAACTGGTTAAATATTGGCCCAATAGATTCGAATCTTAAAATTCCATCTGG [18642]
cp26_un9_B31_PacBio           ATGTTTGCTTAATATGTAGGAAAAATCTAACTGGTTAAATATTGGCCCAATAGATTCGAATCTTAAAATTCCATCTGG [18642]
cp26_B31_TS_MP_FG_consensus   ATGTTTGCTTAATATGTAGGAAAAATCTAACTGGTTAAATATTGGCCCAATAGATTCGAATCTTAAAATTCCATCTGG [18642]
cp26_B31_NX_Pl_consensus      ATGTTTGCTTAATATGTAGGAAAAATCTAACTGGTTAAATATTGGCCCAATAGATTCGAATCTTAAAATTCCATCTGG [18642]
cp26_PAli_NX_MP_FG_consensus ATGTTTGCTTAATATGTAGGAAAAATCTAACTGGTTAAATATTGGCCCAATAGATTCGAATCTTAAAATTCCATCTGG [18642]
cp26_PAli_NX_Pl_consensus     ATGTTTGCTTAATATGTAGGAAAAATCTAACTGGTTAAATATTGGCCCAATAGATTCGAATCTTAAAATTCCATCTGG [18642]
cp26_PAbe_NX_MP_FG_consensus ATGTTTGCTTAATATGTAGGAAAAATCTAACTGGTTAAATATTGGCCCAATAGATTCGAATCTTAAAATTCCATCTGG [18642]
cp26_PAbe_NX_Pl_consensus     ATGTTTGCTTAATATGTAGGAAAAATCTAACTGGTTAAATATTGGCCCAATAGATTCGAATCTTAAAATTCCATCTGG [18642]

cp26_gi|11497007|B31_GB       AAAACGTATTCCAGCAGCTACTGCGCTTTCTGGACTAAAGATTGCATATATCCAAGCTATGGCAGTGACTGAGCAAAT [18720]
cp26_un9_B31_PacBio           AAAACGTATTCCAGCAGCTACTGCGCTTTCTGGACTAAAGATTGCATATATCCAAGCTATGGCAGTGACTGAGCAAAT [18720]
cp26_B31_TS_MP_FG_consensus   AAAACGTATTCCAGCAGCTACTGCGCTTTCTGGACTAAAGATTGCATATATCCAAGCTATGGCAGTGACTGAGCAAAT [18720]
cp26_B31_NX_Pl_consensus      AAAACGTATTCCAGCAGCTACTGCGCTTTCTGGACTAAAGATTGCATATATCCAAGCTATGGCAGTGACTGAGCAAAT [18720]
cp26_PAli_NX_MP_FG_consensus AAAACGTATTCCAGCAGCTACTGCGCTTTCTGGACTAAAGATTGCATATATCCAAGCTATGGCAGTGACTGAGCAAAT [18720]
cp26_PAli_NX_Pl_consensus     AAAACGTATTCCAGCAGCTACTGCGCTTTCTGGACTAAAGATTGCATATATCCAAGCTATGGCAGTGACTGAGCAAAT [18720]
cp26_PAbe_NX_MP_FG_consensus AAAACGTATTCCAGCAGCTACTGCGCTTTCTGGACTAAAGATTGCATATATCCAAGCTATGGCAGTGACTGAGCAAAT [18720]
cp26_PAbe_NX_Pl_consensus     AAAACGTATTCCAGCAGCTACTGCGCTTTCTGGACTAAAGATTGCATATATCCAAGCTATGGCAGTGACTGAGCAAAT [18720]

cp26_gi|11497007|B31_GB       TGCCCAAAGTATACTTCCCCTTATATTTAATTGTTCAAAAATTACAATAAAAAATAATCCTAAAAATGTAAATAAAAC [18798]
```

```
cp26_un9_B31_PacBio          TGCCCAAAGTATACTTCCCCTTATATTTAATTGTTCAAAAATTACAATAAAAAATAATCCTAAAAATGTAAATAAAAC [18798]
cp26_B31_TS_MP_FG_consensus  TGCCCAAAGTATACTTCCCCTTATATTTAATTGTTCAAAAATTACAATAAAAAATAATCCTAAAAATGTAAATAAAAC [18798]
cp26_B31_NX_Pl_consensus     TGCCCAAAGTATACTTCCCCTTATATTTAATTGTTCAAAAATTACAATAAAAAATAATCCTAAAAATGTAAATAAAAC [18798]
cp26_PAli_NX_MP_FG_consensus TGCCCAAAGTATACTTCCCCTTATATTTAATTGTTCAAAAATTACAATAAAAAATAATCCTAAAAATGTAAATAAAAC [18798]
cp26_PAli_NX_Pl_consensus    TGCCCAAAGTATACTTCCCCTTATATTTAATTGTTCAAAAATTACAATAAAAAATAATCCTAAAAATGTAAATAAAAC [18798]
cp26_PAbe_NX_MP_FG_consensus TGCCCAAAGTATACTTCCCCTTATATTTAATTGTTCAAAAATTACAATAAAAAATAATCCTAAAAATGTAAATAAAAC [18798]
cp26_PAbe_NX_Pl_consensus    TGCCCAAAGTATACTTCCCCTTATATTTAATTGTTCAAAAATTACAATAAAAAATAATCCTAAAAATGTAAATAAAAC [18798]

cp26_gi|11497007|B31_GB      TTTCAAGTCAATGAATGATCCAATTCCAACCAATGTAGCATCATTTTTAACAATAATTCCCCCATTGACAAAGCCAAT [18876]
cp26_un9_B31_PacBio          TTTCAAGTCAATGAATGATCCAATTCCAACCAATGTAGCATCATTTTTAACAATAATTCCCCCATTGACAAAGCCAAT [18876]
cp26_B31_TS_MP_FG_consensus  TTTCAAGTCAATGAATGATCCAATTCCAACCAATGTAGCATCATTTTTAACAATAATTCCCCCATTGACAAAGCCAAT [18876]
cp26_B31_NX_Pl_consensus     TTTCAAGTCAATGAATGATCCAATTCCAACCAATGTAGCATCATTTTTAACAATAATTCCCCCATTGACAAAGCCAAT [18876]
cp26_PAli_NX_MP_FG_consensus TTTCAAGTCAATGAATGATCCAATTCCAACCAATGTAGCATCATTTTTAACAATAATTCCCCCATTGACAAAGCCAAT [18876]
cp26_PAli_NX_Pl_consensus    TTTCAAGTCAATGAATGATCCAATTCCAACCAATGTAGCATCATTTTTAACAATAATTCCCCCATTGACAAAGCCAAT [18876]
cp26_PAbe_NX_MP_FG_consensus TTTCAAGTCAATGAATGATCCAATTCCAACCAATGTAGCATCATTTTTAACAATAATTCCCCCATTGACAAAGCCAAT [18876]
cp26_PAbe_NX_Pl_consensus    TTTCAAGTCAATGAATGATCCAATTCCAACCAATGTAGCATCATTTTTAACAATAATTCCCCCATTGACAAAGCCAAT [18876]

cp26_gi|11497007|B31_GB      AAAGGCAATAAAAAGCCCTATTCCAACCGTAATAGAGTATTTTAAATTTACCGGTATGGAATTTGCAATACTTTCCCT [18954]
cp26_un9_B31_PacBio          AAAGGCAATAAAAAGCCCTATTCCAACCGTAATAGAGTATTTTAAATTTACCGGTATGGAATTTGCAATACTTTCCCT [18954]
cp26_B31_TS_MP_FG_consensus  AAAGGCAATAAAAAGCCCTATTCCAACCGTAATAGAGTATTTTAAATTTACCGGTATGGAATTTGCAATACTTTCCCT [18954]
cp26_B31_NX_Pl_consensus     AAAGGCAATAAAAAGCCCTATTCCAACCGTAATAGAGTATTTTAAATTTACCGGTATGGAATTTGCAATACTTTCCCT [18954]
cp26_PAli_NX_MP_FG_consensus AAAGGCAATAAAAAGCCCTATTCCAACCGTAATAGAGTATTTTAAATTTACCGGTATGGAATTTGCAATACTTTCCCT [18954]
cp26_PAli_NX_Pl_consensus    AAAGGCAATAAAAAGCCCTATTCCAACCGTAATAGAGTATTTTAAATTTACCGGTATGGAATTTGCAATACTTTCCCT [18954]
cp26_PAbe_NX_MP_FG_consensus AAAGGCAATAAAAAGCCCTATTCCAACCGTAATAGAGTATTTTAAATTTACCGGTATGGAATTTGCAATACTTTCCCT [18954]
cp26_PAbe_NX_Pl_consensus    AAAGGCAATAAAAAGCCCTATTCCAACCGTAATAGAGTATTTTAAATTTACCGGTATGGAATTTGCAATACTTTCCCT [18954]

cp26_gi|11497007|B31_GB      AGCTCTTGATAAAGATAGCACAATAAAGATTAGCCCCTCGATAAAAACAGCAGCTAGTGCAACTTGCCAAGGAATATT [19032]
cp26_un9_B31_PacBio          AGCTCTTGATAAAGATAGCACAATAAAGATTAGCCCCTCGATAAAAACAGCAGCTAGTGCAACTTGCCAAGGAATATT [19032]
cp26_B31_TS_MP_FG_consensus  AGCTCTTGATAAAGATAGCACAATAAAGATTAGCCCCTCGATAAAAACAGCAGCTAGTGCAACTTGCCAAGGAATATT [19032]
cp26_B31_NX_Pl_consensus     AGCTCTTGATAAAGATAGCACAATAAAGATTAGCCCCTCGATAAAAACAGCAGCTAGTGCAACTTGCCAAGGAATATT [19032]
cp26_PAli_NX_MP_FG_consensus AGCTCTTGATAAAGATAGCACAATAAAGATTAGCCCCTCGATAAAAACAGCAGCTAGTGCAACTTGCCAAGGAATATT [19032]
cp26_PAli_NX_Pl_consensus    AGCTCTTGATAAAGATAGCACAATAAAGATTAGCCCCTCGATAAAAACAGCAGCTAGTGCAACTTGCCAAGGAATATT [19032]
cp26_PAbe_NX_MP_FG_consensus AGCTCTTGATAAAGATAGCACAATAAAGATTAGCCCCTCGATAAAAACAGCAGCTAGTGCAACTTGCCAAGGAATATT [19032]
cp26_PAbe_NX_Pl_consensus    AGCTCTTGATAAAGATAGCACAATAAAGATTAGCCCCTCGATAAAAACAGCAGCTAGTGCAACTTGCCAAGGAATATT [19032]

cp26_gi|11497007|B31_GB      CATTCCAATTACTACAGAAAATGCAAAAAATGCATTAAGACCCATACCTGGTGCTAGTGATATAGGGGTATTAGTATA [19110]
cp26_un9_B31_PacBio          CATTCCAATTACTACAGAAAATGCAAAAAATGCATTAAGACCCATACCTGGTGCTAGTGATATAGGGGTATTAGTATA [19110]
cp26_B31_TS_MP_FG_consensus  CATTCCAATTACTACAGAAAATGCAAAAAATGCATTAAGACCCATACCTGGTGCTAGTGATATAGGGGTATTAGTATA [19110]
cp26_B31_NX_Pl_consensus     CATTCCAATTACTACAGAAAATGCAAAAAATGCATTAAGACCCATACCTGGTGCTAGTGATATAGGGGTATTAGTATA [19110]
cp26_PAli_NX_MP_FG_consensus CATTCCAATTACTACAGAAAATGCAAAAAATGCATTAAGACCCATACCTGGTGCTAGTGATATAGGGGTATTAGTATA [19110]
cp26_PAli_NX_Pl_consensus    CATTCCAATTACTACAGAAAATGCAAAAAATGCATTAAGACCCATACCTGGTGCTAGTGATATAGGGGTATTAGTATA [19110]
cp26_PAbe_NX_MP_FG_consensus CATTCCAATTACTACAGAAAATGCAAAAAATGCATTAAGACCCATACCTGGTGCTAGTGATATAGGGGTATTAGTATA [19110]
cp26_PAbe_NX_Pl_consensus    CATTCCAATTACTACAGAAAATGCAAAAAATGCATTAAGACCCATACCTGGTGCTAGTGATATAGGGGTATTAGTATA [19110]

cp26_gi|11497007|B31_GB      AAGCCCCATTAATATACTAGAAAATGCTGATGTTAAGCAAGTTGCAGTAACTAATGCGCCAATTGGCATACCTGTGCT [19188]
cp26_un9_B31_PacBio          AAGCCCCATTAATATACTAGAAAATGCTGATGTTAAGCAAGTTGCAGTAACTAATGCGCCAATTGGCATACCTGTGCT [19188]
cp26_B31_TS_MP_FG_consensus  AAGCCCCATTAATATACTAGAAAATGCTGATGTTAAGCAAGTTGCAGTAACTAATGCGCCAATTGGCATACCTGTGCT [19188]
cp26_B31_NX_Pl_consensus     AAGCCCCATTAATATACTAGAAAATGCTGATGTTAAGCAAGTTGCAGTAACTAATGCGCCAATTGGCATACCTGTGCT [19188]
cp26_PAli_NX_MP_FG_consensus AAGCCCCATTAATATACTAGAAAATGCTGATGTTAAGCAAGTTGCAGTAACTAATGCGCCAATTGGCATACCTGTGCT [19188]
cp26_PAli_NX_Pl_consensus    AAGCCCCATTAATATACTAGAAAATGCTGATGTTAAGCAAGTTGCAGTAACTAATGCGCCAATTGGCATACCTGTGCT [19188]
cp26_PAbe_NX_MP_FG_consensus AAGCCCCATTAATATACTAGAAAATGCTGATGTTAAGCAAGTTGCAGTAACTAATGCGCCAATTGGCATACCTGTGCT [19188]
cp26_PAbe_NX_Pl_consensus    AAGCCCCATTAATATACTAGAAAATGCTGATGTTAAGCAAGTTGCAGTAACTAATGCGCCAATTGGCATACCTGTGCT [19188]

cp26_gi|11497007|B31_GB      AGATAATATTGCCGGATTAACAGCTATTATGTATGCCATGCTTAAAAAAGTGGTAATACCACCGATAATTTCTTTTTT [19266]
cp26_un9_B31_PacBio          AGATAATATTGCCGGATTAACAGCTATTATGTATGCCATGCTTAAAAAAGTGGTAATACCACCGATAATTTCTTTTTT [19266]
cp26_B31_TS_MP_FG_consensus  AGATAATATTGCCGGATTAACAGCTATTATGTATGCCATGCTTAAAAAAGTGGTAATACCACCGATAATTTCTTTTTT [19266]
cp26_B31_NX_Pl_consensus     AGATAATATTGCCGGATTAACAGCTATTATGTATGCCATGCTTAAAAAAGTGGTAATACCACCGATAATTTCTTTTTT [19266]
cp26_PAli_NX_MP_FG_consensus AGATAATATTGCCGGATTAACAGCTATTATGTATGCCATGCTTAAAAAAGTGGTAATACCACCGATAATTTCTTTTTT [19266]
cp26_PAli_NX_Pl_consensus    AGATAATATTGCCGGATTAACAGCTATTATGTATGCCATGCTTAAAAAAGTGGTAATACCACCGATAATTTCTTTTTT [19266]
cp26_PAbe_NX_MP_FG_consensus AGATAATATTGCCGGATTAACAGCTATTATGTATGCCATGCTTAAAAAAGTGGTAATACCACCGATAATTTCTTTTTT [19266]
cp26_PAbe_NX_Pl_consensus    AGATAATATTGCCGGATTAACAGCTATTATGTATGCCATGCTTAAAAAAGTGGTAATACCACCGATAATTTCTTTTTT [19266]

cp26_gi|11497007|B31_GB      ATAATCAATGGTTTTATTTTTAAATTGAAATAACAATGTTTCTTTGGATTGATTCATTACAAATTACTCCTCAAATTT [19344]
cp26_un9_B31_PacBio          ATAATCAATGGTTTTATTTTTAAATTGAAATAACAATGTTTCTTTGGATTGATTCATTACAAATTACTCCTCAAATTT [19344]
cp26_B31_TS_MP_FG_consensus  ATAATCAATGGTTTTATTTTTAAATTGAAATAACAATGTTTCTTTGGATTGATTCATTACAAATTACTCCTCAAATTT [19344]
cp26_B31_NX_Pl_consensus     ATAATCAATGGTTTTATTTTTAAATTGAAATAACAATGTTTCTTTGGATTGATTCATTACAAATTACTCCTCAAATTT [19344]
cp26_PAli_NX_MP_FG_consensus ATAATCAATGGTTTTATTTTTAAATTGAAATAACAATGTTTCTTTGGATTGATTCATTACAAATTACTCCTCAAATTT [19344]
cp26_PAli_NX_Pl_consensus    ATAATCAATGGTTTTATTTTTAAATTGAAATAACAATGTTTCTTTGGATTGATTCATTACAAATTACTCCTCAAATTT [19344]
cp26_PAbe_NX_MP_FG_consensus ATAATCAATGGTTTTATTTTTAAATTGAAATAACAATGTTTCTTTGGATTGATTCATTACAAATTACTCCTCAAATTT [19344]
cp26_PAbe_NX_Pl_consensus    ATAATCAATGGTTTTATTTTTAAATTGAAATAACAATGTTTCTTTGGATTGATTCATTACAAATTACTCCTCAAATTT [19344]

cp26_gi|11497007|B31_GB      TATTTTATATCAAAAATAAAACCAACGCTATTTATTGGATTTTAATCTAATAATAAAATATATTGATAAATCAAATTT [19422]
cp26_un9_B31_PacBio          TATTTTATATCAAAAATAAAACCAACGCTATTTATTGGATTTTAATCTAATAATAAAATATATTGATAAATCAAATTT [19422]
cp26_B31_TS_MP_FG_consensus  TATTTTATATCAAAAATAAAACCAACGCTATTTATTGGATTTTAATCTAATAATAAAATATATTGATAAATCAAATTT [19422]
cp26_B31_NX_Pl_consensus     TATTTTATATCAAAAATAAAACCAACGCTATTTATTGGATTTTAATCTAATAATAAAATATATTGATAAATCAAATTT [19422]
cp26_PAli_NX_MP_FG_consensus TATTTTATATCAAAAATAAAACCAACGCTATTTATTGGATTTTAATCTAATAATAAAATATATTGATAAATCAAATTT [19422]
cp26_PAli_NX_Pl_consensus    TATTTTATATCAAAAATAAAACCAACGCTATTTATTGGATTTTAATCTAATAATAAAATATATTGATAAATCAAATTT [19422]
cp26_PAbe_NX_MP_FG_consensus TATTTTATATCAAAAATAAAACCAACGCTATTTATTGGATTTTAATCTAATAATAAAATATATTGATAAATCAAATTT [19422]
cp26_PAbe_NX_Pl_consensus    TATTTTATATCAAAAATAAAACCAACGCTATTTATTGGATTTTAATCTAATAATAAAATATATTGATAAATCAAATTT [19422]

cp26_gi|11497007|B31_GB      TATAGAAATATTAATAGCCATAAATAAATTTAATAATAAAAATTAAGCATAATATTAGCAGTACAGGAGAAATTTTAA [19500]
cp26_un9_B31_PacBio          TATAGAAATATTAATAGCCATAAATAAATTTAATAATAAAAATTAAGCATAATATTAGCAGTACAGGAGAAATTTTAA [19500]
cp26_B31_TS_MP_FG_consensus  TATAGAAATATTAATAGCCATAAATAAATTTAATAATAAAAATTAAGCATAATATTAGCAGTACAGGAGAAATTTTAA [19500]
cp26_B31_NX_Pl_consensus     TATAGAAATATTAATAGCCATAAATAAATTTAATAATAAAAATTAAGCATAATATTAGCAGTACAGGAGAAATTTTAA [19500]
cp26_PAli_NX_MP_FG_consensus TATAGAAATATTAATAGCCATAAATAAATTTAATAATAAAAATTAAGCATAATATTAGCAGTACAGGAGAAATTTTAA [19500]
cp26_PAli_NX_Pl_consensus    TATAGAAATATTAATAGCCATAAATAAATTTAATAATAAAAATTAAGCATAATATTAGCAGTACAGGAGAAATTTTAA [19500]
cp26_PAbe_NX_MP_FG_consensus TATAGAAATATTAATAGCCATAAATAAATTTAATAATAAAAATTAAGCATAATATTAGCAGTACAGGAGAAATTTTAA [19500]
cp26_PAbe_NX_Pl_consensus    TATAGAAATATTAATAGCCATAAATAAATTTAATAATAAAAATTAAGCATAATATTAGCAGTACAGGAGAAATTTTAA [19500]

cp26_gi|11497007|B31_GB      TCTTTTCTTTGCTAAAAAAATTAAATGATACATTTACTAAAATATAAAATGCTGCGCCAACAAAAAATCCTGAAGAAA [19578]
cp26_un9_B31_PacBio          TCTTTTCTTTGCTAAAAAAATTAAATGATACATTTACTAAAATATAAAATGCTGCGCCAACAAAAAATCCTGAAGAAA [19578]
cp26_B31_TS_MP_FG_consensus  TCTTTTCTTTGCTAAAAAAATTAAATGATACATTTACTAAAATATAAAATGCTGCGCCAACAAAAAATCCTGAAGAAA [19578]
cp26_B31_NX_Pl_consensus     TCTTTTCTTTGCTAAAAAAATTAAATGATACATTTACTAAAATATAAAATGCTGCGCCAACAAAAAATCCTGAAGAAA [19578]
cp26_PAli_NX_MP_FG_consensus TCTTTTCTTTGCTAAAAAAATTAAATGATACATTTACTAAAATATAAAATGCTGCGCCAACAAAAAATCCTGAAGAAA [19578]
cp26_PAli_NX_Pl_consensus    TCTTTTCTTTGCTAAAAAAATTAAATGATACATTTACTAAAATATAAAATGCTGCGCCAACAAAAAATCCTGAAGAAA [19578]
cp26_PAbe_NX_MP_FG_consensus TCTTTTCTTTGCTAAAAAAATTAAATGATACATTTACTAAAATATAAAATGCTGCGCCAACAAAAAATCCTGAAGAAA [19578]
cp26_PAbe_NX_Pl_consensus    TCTTTTCTTTGCTAAAAAAATTAAATGATACATTTACTAAAATATAAAATGCTGCGCCAACAAAAAATCCTGAAGAAA [19578]

cp26_gi|11497007|B31_GB      TGCTATAAGCTAAAGGAATCAAAAAAAATATTAAAAAGCTGGAAATATTTTCTCTAATATTAAAGAATCAATTTTAA [19656]
cp26_un9_B31_PacBio          TGCTATAAGCTAAAGGAATCAAAAAAAATATTAAAAAGCTGGAAATATTTTCTCTAATATTAAAGAATCAATTTTAA [19656]
cp26_B31_TS_MP_FG_consensus  TGCTATAAGCTAAAGGAATCAAAAAAAATATTAAAAAGCTGGAAATATTTTCTCTAATATTAAAGAATCAATTTTAA [19656]
cp26_B31_NX_Pl_consensus     TGCTATAAGCTAAAGGAATCAAAAAAAATATTAAAAAGCTGGAAATATTTTCTCTAATATTAAAGAATCAATTTTAA [19656]
cp26_PAli_NX_MP_FG_consensus TGCTATAAGCTAAAGGAATCAAAAAAAATATTAAAAAGCTGGAAATATTTTCTCTAATATTAAAGAATCAATTTTAA [19656]
cp26_PAli_NX_Pl_consensus    TGCTATAAGCTAAAGGAATCAAAAAAAATATTAAAAAGCTGGAAATATTTTCTCTAATATTAAAGAATCAATTTTAA [19656]
cp26_PAbe_NX_MP_FG_consensus TGCTATAAGCTAAAGGAATCAAAAAAAATATTAAAAAGCTGGAAATATTTTCTCTAATATTAAAGAATCAATTTTAA [19656]
cp26_PAbe_NX_Pl_consensus    TGCTATAAGCTAAAGGAATCAAAAAAAATATTAAAAAGCTGGAAATATTTTCTCTAATATTAAAGAATCAATTTTAA [19656]

cp26_gi|11497007|B31_GB      TTATTTCTCTACACATTGAAAATCCTACATATATTAATGCTGCAGCAGTTGCGCTAGCAGGAACGGCAATAAACAATG [19734]
cp26_un9_B31_PacBio          TTATTTCTCTACACATTGAAAATCCTACATATATTAATGCTGCAGCAGTTGCGCTAGCAGGAACGGCAATAAACAATG [19734]
```

```
cp26_B31_TS_MP_FG_consensus  TTATTTCTCTACACATTGAAAATCCTACATATATTAATGCTGCAGCAGTTGCGCTAGCAGGAACGGCAATAAACAATG [19734]
cp26_B31_NX_Pl_consensus     TTATTTCTCTACACATTGAAAATCCTACATATATTAATGCTGCAGCAGTTGCGCTAGCAGGAACGGCAATAAACAATG [19734]
cp26_PAli_NX_MP_FG_consensus TTATTTCTCTACACATTGAAAATCCTACATATATTAATGCTGCAGCAGTTGCGCTAGCAGGAACGGCAATAAACAATG [19734]
cp26_PAli_NX_Pl_consensus    TTATTTCTCTACACATTGAAAATCCTACATATATTAATGCTGCAGCAGTTGCGCTAGCAGGAACGGCAATAAACAATG [19734]
cp26_PAbe_NX_MP_FG_consensus TTATTTCTCTACACATTGAAAATCCTACATATATTAATGCTGCAGCAGTTGCGCTAGCAGGAACGGCAATAAACAATG [19734]
cp26_PAbe_NX_Pl_consensus    TTATTTCTCTACACATTGAAAATCCTACATATATTAATGCTGCAGCAGTTGCGCTAGCAGGAACGGCAATAAACAATG [19734]

cp26_gi|11497007|B31_GB      GGGCAAAAAAAACTGCAAATAGAAATAATGTTCCAGTTACAATTGAAGTAAGGCCCGTTTTTCCACCCTCAGCAATTC [19812]
cp26_un9_B31_PacBio          GGGCAAAAAAAACTGCAAATAGAAATAATGTTCCAGTTACAATTGAAGTAAGGCCCGTTTTTCCACCCTCAGCAATTC [19812]
cp26_B31_TS_MP_FG_consensus  GGGCAAAAAAAACTGCAAATAGAAATAATGTTCCAGTTACAATTGAAGTAAGGCCCGTTTTTCCACCCTCAGCAATTC [19812]
cp26_B31_NX_Pl_consensus     GGGCAAAAAAAACTGCAAATAGAAATAATGTTCCAGTTACAATTGAAGTAAGGCCCGTTTTTCCACCCTCAGCAATTC [19812]
cp26_PAli_NX_MP_FG_consensus GGGCAAAAAAAACTGCAAATAGAAATAATGTTCCAGTTACAATTGAAGTAAGGCCCGTTTTTCCACCCTCAGCAATTC [19812]
cp26_PAli_NX_Pl_consensus    GGGCAAAAAAAACTGCAAATAGAAATAATGTTCCAGTTACAATTGAAGTAAGGCCCGTTTTTCCACCCTCAGCAATTC [19812]
cp26_PAbe_NX_MP_FG_consensus GGGCAAAAAAAACTGCAAATAGAAATAATGTTCCAGTTACAATTGAAGTAAGGCCCGTTTTTCCACCCTCAGCAATTC [19812]
cp26_PAbe_NX_Pl_consensus    GGGCAAAAAAAACTGCAAATAGAAATAATGTTCCAGTTACAATTGAAGTAAGGCCCGTTTTTCCACCCTCAGCAATTC [19812]

cp26_gi|11497007|B31_GB      CTGTAAAACTTTCAATATAAGTAGTAACAGTGGAAACACCCATTATTGCTCCAAAAGTAGTAGCAATGCCGTCTACCA [19890]
cp26_un9_B31_PacBio          CTGTAAAACTTTCAATATAAGTAGTAACAGTGGAAACACCCATTATTGCTCCAAAAGTAGTAGCAATGCCGTCTACCA [19890]
cp26_B31_TS_MP_FG_consensus  CTGTAAAACTTTCAATATAAGTAGTAACAGTGGAAACACCCATTATTGCTCCAAAAGTAGTAGCAATGCCGTCTACCA [19890]
cp26_B31_NX_Pl_consensus     CTGTAAAACTTTCAATATAAGTAGTAACAGTGGAAACACCCATTATTGCTCCAAAAGTAGTAGCAATGCCGTCTACCA [19890]
cp26_PAli_NX_MP_FG_consensus CTGTAAAACTTTCAATATAAGTAGTAACAGTGGAAACACCCATTATTGCTCCAAAAGTAGTAGCAATGCCGTCTACCA [19890]
cp26_PAli_NX_Pl_consensus    CTGTAAAACTTTCAATATAAGTAGTAACAGTGGAAACACCCATTATTGCTCCAAAAGTAGTAGCAATGCCGTCTACCA [19890]
cp26_PAbe_NX_MP_FG_consensus CTGTAAAACTTTCAATATAAGTAGTAACAGTGGAAACACCCATTATTGCTCCAAAAGTAGTAGCAATGCCGTCTACCA [19890]
cp26_PAbe_NX_Pl_consensus    CTGTAAAACTTTCAATATAAGTAGTAACAGTGGAAACACCCATTATTGCTCCAAAAGTAGTAGCAATGCCGTCTACCA [19890]

cp26_gi|11497007|B31_GB      GTAATATTTTTTTTGCATTAGGAATTTTTCCATTTTTATCCAACATGCCACCTTTTGTTGTAACGCTTATTAAAATAC [19968]
cp26_un9_B31_PacBio          GTAATATTTTTTTTGCATTAGGAATTTTTCCATTTTTATCCAACATGCCACCTTTTGTTGTAACGCTTATTAAAATAC [19968]
cp26_B31_TS_MP_FG_consensus  GTAATATTTTTTTTGCATTAGGAATTTTTCCATTTTTATCCAACATGCCACCTTTTGTTGTAACGCTTATTAAAATAC [19968]
cp26_B31_NX_Pl_consensus     GTAATATTTTTTTTGCATTAGGAATTTTTCCATTTTTATCCAACATGCCACCTTTTGTTGTAACGCTTATTAAAATAC [19968]
cp26_PAli_NX_MP_FG_consensus GTAATATTTTTTTTGCATTAGGAATTTTTCCATTTTTATCCAACATGCCACCTTTTGTTGTAACGCTTATTAAAATAC [19968]
cp26_PAli_NX_Pl_consensus    GTAATATTTTTTTTGCATTAGGAATTTTTCCATTTTTATCCAACATGCCACCTTTTGTTGTAACGCTTATTAAAATAC [19968]
cp26_PAbe_NX_MP_FG_consensus GTAATATTTTTTTTGCATTAGGAATTTTTCCATTTTTATCCAACATGCCACCTTTTGTTGTAACGCTTATTAAAATAC [19968]
cp26_PAbe_NX_Pl_consensus    GTAATATTTTTTTTGCATTAGGAATTTTTCCATTTTTATCCAACATGCCACCTTTTGTTGTAACGCTTATTAAAATAC [19968]

cp26_gi|11497007|B31_GB      CCACAGTATCAAATAAATCATTGAACAAGAGAATAAAAACTATTGATATAAAAGTCCAAAAATGCTCATTTAAAACAT [20046]
cp26_un9_B31_PacBio          CCACAGTATCAAATAAATCATTGAACAAGAGAATAAAAACTATTGATATAAAAGTCCAAAAATGCTCATTTAAAACAT [20046]
cp26_B31_TS_MP_FG_consensus  CCACAGTATCAAATAAATCATTGAACAAGAGAATAAAAACTATTGATATAAAAGTCCAAAAATGCTCATTTAAAACAT [20046]
cp26_B31_NX_Pl_consensus     CCACAGTATCAAATAAATCATTGAACAAGAGAATAAAAACTATTGATATAAAAGTCCAAAAATGCTCATTTAAAACAT [20046]
cp26_PAli_NX_MP_FG_consensus CCACAGTATCAAATAAATCATTGAACAAGAGAATAAAAACTATTGATATAAAAGTCCAAAAATGCTCATTTAAAACAT [20046]
cp26_PAli_NX_Pl_consensus    CCACAGTATCAAATAAATCATTGAACAAGAGAATAAAAACTATTGATATAAAAGTCCAAAAATGCTCATTTAAAACAT [20046]
cp26_PAbe_NX_MP_FG_consensus CCACAGTATCAAATAAATCATTGAACAAGAGAATAAAAACTATTGATATAAAAGTCCAAAAATGCTCATTTAAAACAT [20046]
cp26_PAbe_NX_Pl_consensus    CCACAGTATCAAATAAATCATTGAACAAGAGAATAAAAACTATTGATATAAAAGTCCAAAAATGCTCATTTAAAACAT [20046]

cp26_gi|11497007|B31_GB      AAGAAAAATCTAATTGATTAAATATTGGTCCAATGGATTCAAATTTTAAAATCCTGCTGGGAAGTTGTATTCCAATAG [20124]
cp26_un9_B31_PacBio          AAGAAAAATCTAATTGATTAAATATTGGTCCAATGGATTCAAATTTTAAAATCCTGCTGGGAAGTTGTATTCCAATAG [20124]
cp26_B31_TS_MP_FG_consensus  AAGAAAAATCTAATTGATTAAATATTGGTCCAATGGATTCAAATTTTAAAATCCTGCTGGGAAGTTGTATTCCAATAG [20124]
cp26_B31_NX_Pl_consensus     AAGAAAAATCTAATTGATTAAATATTGGTCCAATGGATTCAAATTTTAAAATCCTGCTGGGAAGTTGTATTCCAATAG [20124]
cp26_PAli_NX_MP_FG_consensus AAGAAAAATCTAATTGATTAAATATTGGTCCAATGGATTCAAATTTTAAAATCCTGCTGGGAAGTTGTATTCCAATAG [20124]
cp26_PAli_NX_Pl_consensus    AAGAAAAATCTAATTGATTAAATATTGGTCCAATGGATTCAAATTTTAAAATCCTGCTGGGAAGTTGTATTCCAATAG [20124]
cp26_PAbe_NX_MP_FG_consensus AAGAAAAATCTAATTGATTAAATATTGGTCCAATGGATTCAAATTTTAAAATCCTGCTGGGAAGTTGTATTCCAATAG [20124]
cp26_PAbe_NX_Pl_consensus    AAGAAAAATCTAATTGATTAAATATTGGTCCAATGGATTCAAATTTTAAAATCCTGCTGGGAAGTTGTATTCCAATAG [20124]

cp26_gi|11497007|B31_GB      CCTGGGCACCTTCTAAATTAAAGATTGCATATATCCAAGCTATGGCAGTGACTGAGCAAATTGCCCAAAGTATACTTC [20202]
cp26_un9_B31_PacBio          CCTGGGCACCTTCTAAATTAAAGATTGCATATATCCAAGCTATGGCAGTGACTGAGCAAATTGCCCAAAGTATACTTC [20202]
cp26_B31_TS_MP_FG_consensus  CCTGGGCACCTTCTAAATTAAAGATTGCATATATCCAAGCTATGGCAGTGACTGAGCAAATTGCCCAAAGTATACTTC [20202]
cp26_B31_NX_Pl_consensus     CCTGGGCACCTTCTAAATTAAAGATTGCATATATCCAAGCTATGGCAGTGACTGAGCAAATTGCCCAAAGTATACTTC [20202]
cp26_PAli_NX_MP_FG_consensus CCTGGGCACCTTCTAAATTAAAGATTGCATATATCCAAGCTATGGCAGTGACTGAGCAAATTGCCCAAAGTATACTTC [20202]
cp26_PAli_NX_Pl_consensus    CCTGGGCACCTTCTAAATTAAAGATTGCATATATCCAAGCTATGGCAGTGACTGAGCAAATTGCCCAAAGTATACTTC [20202]
cp26_PAbe_NX_MP_FG_consensus CCTGGGCACCTTCTAAATTAAAGATTGCATATATCCAAGCTATGGCAGTGACTGAGCAAATTGCCCAAAGTATACTTC [20202]
cp26_PAbe_NX_Pl_consensus    CCTGGGCACCTTCTAAATTAAAGATTGCATATATCCAAGCTATGGCAGTGACTGAGCAAATTGCCCAAAGTATACTTC [20202]

cp26_gi|11497007|B31_GB      CCCTTACATTTAATTGTTCAAAAATTACAATAAAAAATAATCCTAAAAATGTAAATAAAACTTTCAAGTCAACAAATG [20280]
cp26_un9_B31_PacBio          CCCTTACATTTAATTGTTCAAAAATTACAATAAAAAATAATCCTAAAAATGTAAATAAAACTTTCAAGTCAACAAATG [20280]
cp26_B31_TS_MP_FG_consensus  CCCTTACATTTAATTGTTCAAAAATTACAATAAAAAATAATCCTAAAAATGTAAATAAAACTTTCAAGTCAACAAATG [20280]
cp26_B31_NX_Pl_consensus     CCCTTACATTTAATTGTTCAAAAATTACAATAAAAAATAATCCTAAAAATGTAAATAAAACTTTCAAGTCAACAAATG [20280]
cp26_PAli_NX_MP_FG_consensus CCCTTACATTTAATTGTTCAAAAATTACAATAAAAAATAATCCTAAAAATGTAAATAAAACTTTCAAGTCAACAAATG [20280]
cp26_PAli_NX_Pl_consensus    CCCTTACATTTAATTGTTCAAAAATTACAATAAAAAATAATCCTAAAAATGTAAATAAAACTTTCAAGTCAACAAATG [20280]
cp26_PAbe_NX_MP_FG_consensus CCCTTACATTTAATTGTTCAAAAATTACAATAAAAAATAATCCTAAAAATGTAAATAAAACTTTCAAGTCAACAAATG [20280]
cp26_PAbe_NX_Pl_consensus    CCCTTACATTTAATTGTTCAAAAATTACAATAAAAAATAATCCTAAAAATGTAAATAAAACTTTCAAGTCAACAAATG [20280]

cp26_gi|11497007|B31_GB      ATCCGATTCCAACCAATGTAGCATCATTTTTAATGATAATTCCCCCGCTGACAAAGCCAATAAAAGCAATAAAAAGCC [20358]
cp26_un9_B31_PacBio          ATCCGATTCCAACCAATGTAGCATCATTTTTAATGATAATTCCCCCGCTGACAAAGCCAATAAAAGCAATAAAAAGCC [20358]
cp26_B31_TS_MP_FG_consensus  ATCCGATTCCAACCAATGTAGCATCATTTTTAATGATAATTCCCCCGCTGACAAAGCCAATAAAAGCAATAAAAAGCC [20358]
cp26_B31_NX_Pl_consensus     ATCCGATTCCAACCAATGTAGCATCATTTTTAATGATAATTCCCCCGCTGACAAAGCCAATAAAAGCAATAAAAAGCC [20358]
cp26_PAli_NX_MP_FG_consensus ATCCGATTCCAACCAATGTAGCATCATTTTTAATGATAATTCCCCCGCTGACAAAGCCAATAAAAGCAATAAAAAGCC [20358]
cp26_PAli_NX_Pl_consensus    ATCCGATTCCAACCAATGTAGCATCATTTTTAATGATAATTCCCCCGCTGACAAAGCCAATAAAAGCAATAAAAAGCC [20358]
cp26_PAbe_NX_MP_FG_consensus ATCCGATTCCAACCAATGTAGCATCATTTTTAATGATAATTCCCCCGCTGACAAAGCCAATAAAAGCAATAAAAAGCC [20358]
cp26_PAbe_NX_Pl_consensus    ATCCGATTCCAACCAATGTAGCATCATTTTTAATGATAATTCCCCCGCTGACAAAGCCAATAAAAGCAATAAAAAGCC [20358]

cp26_gi|11497007|B31_GB      CTATTCCAACCGAAATAGAATATTTTAAATTTATCGGAATAGAGTTTATAATTTGCTCCCTTACTCTTAAAAAAGATA [20436]
cp26_un9_B31_PacBio          CTATTCCAACCGAAATAGAATATTTTAAATTTATCGGAATAGAGTTTATAATTTGCTCCCTTACTCTTAAAAAAGATA [20436]
cp26_B31_TS_MP_FG_consensus  CTATTCCAACCGAAATAGAATATTTTAAATTTATCGGAATAGAGTTTATAATTTGCTCCCTTACTCTTAAAAAAGATA [20436]
cp26_B31_NX_Pl_consensus     CTATTCCAACCGAAATAGAATATTTTAAATTTATCGGAATAGAGTTTATAATTTGCTCCCTTACTCTTAAAAAAGATA [20436]
cp26_PAli_NX_MP_FG_consensus CTATTCCAACCGAAATAGAATATTTTAAATTTATCGGAATAGAGTTTATAATTTGCTCCCTTACTCTTAAAAAAGATA [20436]
cp26_PAli_NX_Pl_consensus    CTATTCCAACCGAAATAGAATATTTTAAATTTATCGGAATAGAGTTTATAATTTGCTCCCTTACTCTTAAAAAAGATA [20436]
cp26_PAbe_NX_MP_FG_consensus CTATTCCAACCGAAATAGAATATTTTAAATTTATCGGAATAGAGTTTATAATTTGCTCCCTTACTCTTAAAAAAGATA [20436]
cp26_PAbe_NX_Pl_consensus    CTATTCCAACCGAAATAGAATATTTTAAATTTATCGGAATAGAGTTTATAATTTGCTCCCTTACTCTTAAAAAAGATA [20436]

cp26_gi|11497007|B31_GB      AGAGAATGAAAATTAGTCCTTCAATAAAAACAGCAGCTAATGCAACTTGCCAAGGTATATTCATCCCAATTACTACAG [20514]
cp26_un9_B31_PacBio          AGAGAATGAAAATTAGTCCTTCAATAAAAACAGCAGCTAATGCAACTTGCCAAGGTATATTCATCCCAATTACTACAG [20514]
cp26_B31_TS_MP_FG_consensus  AGAGAATGAAAATTAGTCCTTCAATAAAAACAGCAGCTAATGCAACTTGCCAAGGTATATTCATCCCAATTACTACAG [20514]
cp26_B31_NX_Pl_consensus     AGAGAATGAAAATTAGTCCTTCAATAAAAACAGCAGCTAATGCAACTTGCCAAGGTATATTCATCCCAATTACTACAG [20514]
cp26_PAli_NX_MP_FG_consensus AGAGAATGAAAATTAGTCCTTCAATAAAAACAGCAGCTAATGCAACTTGCCAAGGTATATTCATCCCAATTACTACAG [20514]
cp26_PAli_NX_Pl_consensus    AGAGAATGAAAATTAGTCCTTCAATAAAAACAGCAGCTAATGCAACTTGCCAAGGTATATTCATCCCAATTACTACAG [20514]
cp26_PAbe_NX_MP_FG_consensus AGAGAATGAAAATTAGTCCTTCAATAAAAACAGCAGCTAATGCAACTTGCCAAGGTATATTCATCCCAATTACTACAG [20514]
cp26_PAbe_NX_Pl_consensus    AGAGAATGAAAATTAGTCCTTCAATAAAAACAGCAGCTAATGCAACTTGCCAAGGTATATTCATCCCAATTACTACAG [20514]

cp26_gi|11497007|B31_GB      AAAATGCAAAAAACGCATTCAAGCTCATTCCAGAAGCCAATGCTAAAGGCGTATTGGTATAAAGTCCCATTAGTATAG [20592]
cp26_un9_B31_PacBio          AAAATGCAAAAAACGCATTCAAGCTCATTCCAGAAGCCAATGCTAAAGGCGTATTGGTATAAAGTCCCATTAGTATAG [20592]
cp26_B31_TS_MP_FG_consensus  AAAATGCAAAAAACGCATTCAAGCTCATTCCAGAAGCCAATGCTAAAGGCGTATTGGTATAAAGTCCCATTAGTATAG [20592]
cp26_B31_NX_Pl_consensus     AAAATGCAAAAAACGCATTCAAGCTCATTCCAGAAGCCAATGCTAAAGGCGTATTGGTATAAAGTCCCATTAGTATAG [20592]
cp26_PAli_NX_MP_FG_consensus AAAATGCAAAAAACGCATTCAAGCTCATTCCAGAAGCCAATGCTAAAGGCGTATTGGTATAAAGTCCCATTAGTATAG [20592]
cp26_PAli_NX_Pl_consensus    AAAATGCAAAAAACGCATTCAAGCTCATTCCAGAAGCCAATGCTAAAGGCGTATTGGTATAAAGTCCCATTAGTATAG [20592]
cp26_PAbe_NX_MP_FG_consensus AAAATGCAAAAAACGCATTCAAGCTCATTCCAGAAGCCAATGCTAAAGGCGTATTGGTATAAAGTCCCATTAGTATAG [20592]
cp26_PAbe_NX_Pl_consensus    AAAATGCAAAAAACGCATTCAAGCTCATTCCAGAAGCCAATGCTAAAGGCGTATTGGTATAAAGTCCCATTAGTATAG [20592]

cp26_gi|11497007|B31_GB      TAGAAAATGCTGCTGTTAGACAGGTTGCGGTAACTAGTGCACCAATTGGCATGCCTGTGTTAGACAGTATTGCTGGGT [20670]
cp26_un9_B31_PacBio          TAGAAAATGCTGCTGTTAGACAGGTTGCGGTAACTAGTGCACCAATTGGCATGCCTGTGTTAGACAGTATTGCTGGGT [20670]
cp26_B31_TS_MP_FG_consensus  TAGAAAATGCTGCTGTTAGACAGGTTGCGGTAACTAGTGCACCAATTGGCATGCCTGTGTTAGACAGTATTGCTGGGT [20670]
```

```
cp26_B31_NX_Pl_consensus     TAGAAAATGCTGCTGTTAGACAGGTTGCGGTAACTAGTGCACCAATTGGCATGCCTGTGTTAGACAGTATTGCTGGGT [20670]
cp26_PAli_NX_MP_FG_consensus TAGAAAATGCTGCTGTTAGACAGGTTGCGGTAACTAGTGCACCAATTGGCATGCCTGTGTTAGACAGTATTGCTGGGT [20670]
cp26_PAli_NX_Pl_consensus    TAGAAAATGCTGCTGTTAGACAGGTTGCGGTAACTAGTGCACCAATTGGCATGCCTGTGTTAGACAGTATTGCTGGGT [20670]
cp26_PAbe_NX_MP_FG_consensus TAGAAAATGCTGCTGTTAGACAGGTTGCGGTAACTAGTGCACCAATTGGCATGCCTGTGTTAGACAGTATTGCTGGGT [20670]
cp26_PAbe_NX_Pl_consensus    TAGAAAATGCTGCTGTTAGACAGGTTGCGGTAACTAGTGCACCAATTGGCATGCCTGTGTTAGACAGTATTGCTGGGT [20670]

cp26_gi|11497007|B31_GB      TAACAGCTATAATATATGACATACTCAAAAAAGTAGTAATGCCCGCAAGAATTTCCTTTTTATAGTTAATATCACTGT [20748]
cp26_un9_B31_PacBio          TAACAGCTATAATATATGACATACTCAAAAAAGTAGTAATGCCCGCAAGAATTTCCTTTTTATAGTTAATATCACTGT [20748]
cp26_B31_TS_MP_FG_consensus  TAACAGCTATAATATATGACATACTCAAAAAAGTAGTAATGCCCGCAAGAATTTCCTTTTTATAGTTAATATCACTGT [20748]
cp26_B31_NX_Pl_consensus     TAACAGCTATAATATATGACATACTCAAAAAAGTAGTAATGCCCGCAAGAATTTCCTTTTTATAGTTAATATCACTGT [20748]
cp26_PAli_NX_MP_FG_consensus TAACAGCTATAATATATGACATACTCAAAAAAGTAGTAATGCCCGCAAGAATTTCCTTTTTATAGTTAATATCACTGT [20748]
cp26_PAli_NX_Pl_consensus    TAACAGCTATAATATATGACATACTCAAAAAAGTAGTAATGCCCGCAAGAATTTCCTTTTTATAGTTAATATCACTGT [20748]
cp26_PAbe_NX_MP_FG_consensus TAACAGCTATAATATATGACATACTCAAAAAAGTAGTAATGCCCGCAAGAATTTCCTTTTTATAGTTAATATCACTGT [20748]
cp26_PAbe_NX_Pl_consensus    TAACAGCTATAATATATGACATACTCAAAAAAGTAGTAATGCCCGCAAGAATTTCCTTTTTATAGTTAATATCACTGT [20748]

cp26_gi|11497007|B31_GB      TTTTAAATTGAAAAAATAAACCTTTTACATACTTACCCATAAAACTTTCCTTTTTATTGTTTAAAAATATTTTCAATA [20826]
cp26_un9_B31_PacBio          TTTTAAATTGAAAAAATAAACCTTTTACATACTTACCCATAAAACTTTCCTTTTTATTGTTTAAAAATATTTTCAATA [20826]
cp26_B31_TS_MP_FG_consensus  TTTTAAATTGAAAAAATAAACCTTTTACATACTTACCCATAAAACTTTCCTTTTTATTGTTTAAAAATATTTTCAATA [20826]
cp26_B31_NX_Pl_consensus     TTTTAAATTGAAAAAATAAACCTTTTACATACTTACCCATAAAACTTTCCTTTTTATTGTTTAAAAATATTTTCAATA [20826]
cp26_PAli_NX_MP_FG_consensus TTTTAAATTGAAAAAATAAACCTTTTACATACTTACCCATAAAACTTTCCTTTTTATTGTTTAAAAATATTTTCAATA [20826]
cp26_PAli_NX_Pl_consensus    TTTTAAATTGAAAAAATAAACCTTTTACATACTTACCCATAAAACTTTCCTTTTTATTGTTTAAAAATATTTTCAATA [20826]
cp26_PAbe_NX_MP_FG_consensus TTTTAAATTGAAAAAATAAACCTTTTACATACTTACCCATAAAACTTTCCTTTTTATTGTTTAAAAATATTTTCAATA [20826]
cp26_PAbe_NX_Pl_consensus    TTTTAAATTGAAAAAATAAACCTTTTACATACTTACCCATAAAACTTTCCTTTTTATTGTTTAAAAATATTTTCAATA [20826]

cp26_gi|11497007|B31_GB      ATAATTATTAGGCTTTTAATTCTTTTTAAAGGCCTATTGTGCCTTATTAAGCATATTGTTCTGGAAAAGAATATCTTC [20904]
cp26_un9_B31_PacBio          ATAATTATTAGGCTTTTAATTCTTTTTAAAGGCCTATTGTGCCTTATTAAGCATATTGTTCTGGAAAAGAATATCTTC [20904]
cp26_B31_TS_MP_FG_consensus  ATAATTATTAGGCTTTTAATTCTTTTTAAAGGCCTATTGTGCCTTATTAAGCATATTGTTCTGGAAAAGAATATCTTC [20904]
cp26_B31_NX_Pl_consensus     ATAATTATTAGGCTTTTAATTCTTTTTAAAGGCCTATTGTGCCTTATTAAGCATATTGTTCTGGAAAAGAATATCTTC [20904]
cp26_PAli_NX_MP_FG_consensus ATAATTATTAGGCTTTTAATTCTTTTTAAAGGCCTATTGTGCCTTATTAAGCATATTGTTCTGGAAAAGAATATCTTC [20904]
cp26_PAli_NX_Pl_consensus    ATAATTATTAGGCTTTTAATTCTTTTTAAAGGCCTATTGTGCCTTATTAAGCATATTGTTCTGGAAAAGAATATCTTC [20904]
cp26_PAbe_NX_MP_FG_consensus ATAATTATTAGGCTTTTAATTCTTTTTAAAGGCCTATTGTGCCTTATTAAGCATATTGTTCTGGAAAAGAATATCTTC [20904]
cp26_PAbe_NX_Pl_consensus    ATAATTATTAGGCTTTTAATTCTTTTTAAAGGCCTATTGTGCCTTATTAAGCATATTGTTCTGGAAAAGAATATCTTC [20904]

cp26_gi|11497007|B31_GB      GCTGTCAGGTAAGTTTGAAAATACAATGGTTTTAATAATCTTTTCAGAACTATTTGTAATAACAAATTTTCTCGCTTT [20982]
cp26_un9_B31_PacBio          GCTGTCAGGTAAGTTTGAAAATACAATGGTTTTAATAATCTTTTCAGAACTATTTGTAATAACAAATTTTCTCGCTTT [20982]
cp26_B31_TS_MP_FG_consensus  GCTGTCAGGTAAGTTTGAAAATACAATGGTTTTAATAATCTTTTCAGAACTATTTGTAATAACAAATTTTCTCGCTTT [20982]
cp26_B31_NX_Pl_consensus     GCTGTCAGGTAAGTTTGAAAATACAATGGTTTTAATAATCTTTTCAGAACTATTTGTAATAACAAATTTTCTCGCTTT [20982]
cp26_PAli_NX_MP_FG_consensus GCTGTCAGGTAAGTTTGAAAATACAATGGTTTTAATAATCTTTTCAGAACTATTTGTAATAACAAATTTTCTCGCTTT [20982]
cp26_PAli_NX_Pl_consensus    GCTGTCAGGTAAGTTTGAAAATACAATGGTTTTAATAATCTTTTCAGAACTATTTGTAATAACAAATTTTCTCGCTTT [20982]
cp26_PAbe_NX_MP_FG_consensus GCTGTCAGGTAAGTTTGAAAATACAATGGTTTTAATAATCTTTTCAGAACTATTTGTAATAACAAATTTTCTCGCTTT [20982]
cp26_PAbe_NX_Pl_consensus    GCTGTCAGGTAAGTTTGAAAATACAATGGTTTTAATAATCTTTTCAGAACTATTTGTAATAACAAATTTTCTCGCTTT [20982]

cp26_gi|11497007|B31_GB      AATAAGCTTTAAATAAGCTTTTAAAGAAATGTCTCTTCCAACGCTAAAAAAGGTTTGAAGATATTTATTATCAATTAG [21060]
cp26_un9_B31_PacBio          AATAAGCTTTAAATAAGCTTTTAAAGAAATGTCTCTTCCAACGCTAAAAAAGGTTTGAAGATATTTATTATCAATTAG [21060]
cp26_B31_TS_MP_FG_consensus  AATAAGCTTTAAATAAGCTTTTAAAGAAATGTCTCTTCCAACGCTAAAAAAGGTTTGAAGATATTTATTATCAATTAG [21060]
cp26_B31_NX_Pl_consensus     AATAAGCTTTAAATAAGCTTTTAAAGAAATGTCTCTTCCAACGCTAAAAAAGGTTTGAAGATATTTATTATCAATTAG [21060]
cp26_PAli_NX_MP_FG_consensus AATAAGCTTTAAATAAGCTTTTAAAGAAATGTCTCTTCCAACGCTAAAAAAGGTTTGAAGATATTTATTATCAATTAG [21060]
cp26_PAli_NX_Pl_consensus    AATAAGCTTTAAATAAGCTTTTAAAGAAATGTCTCTTCCAACGCTAAAAAAGGTTTGAAGATATTTATTATCAATTAG [21060]
cp26_PAbe_NX_MP_FG_consensus AATAAGCTTTAAATAAGCTTTTAAAGAAATGTCTCTTCCAACGCTAAAAAAGGTTTGAAGATATTTATTATCAATTAG [21060]
cp26_PAbe_NX_Pl_consensus    AATAAGCTTTAAATAAGCTTTTAAAGAAATGTCTCTTCCAACGCTAAAAAAGGTTTGAAGATATTTATTATCAATTAG [21060]

cp26_gi|11497007|B31_GB      CTTATGCCGATTAAATAGAAATAAGGCAATTATATCATTTCCAACTTTTAAAAATAAAGGTTCTTTGTACCTTAAGTT [21138]
cp26_un9_B31_PacBio          CTTATGCCGATTAAATAGAAATAAGGCAATTATATCATTTCCAACTTTTAAAAATAAAGGTTCTTTGTACCTTAAGTT [21138]
cp26_B31_TS_MP_FG_consensus  CTTATGCCGATTAAATAGAAATAAGGCAATTATATCATTTCCAACTTTTAAAAATAAAGGTTCTTTGTACCTTAAGTT [21138]
cp26_B31_NX_Pl_consensus     CTTATGCCGATTAAATAGAAATAAGGCAATTATATCATTTCCAACTTTTAAAAATAAAGGTTCTTTGTACCTTAAGTT [21138]
cp26_PAli_NX_MP_FG_consensus CTTATGCCGATTAAATAGAAATAAGGCAATTATATCATTTCCAACTTTTAAAAATAAAGGTTCTTTGTACCTTAAGTT [21138]
cp26_PAli_NX_Pl_consensus    CTTATGCCGATTAAATAGAAATAAGGCAATTATATCATTTCCAACTTTTAAAAATAAAGGTTCTTTGTACCTTAAGTT [21138]
cp26_PAbe_NX_MP_FG_consensus CTTATGCCGATTAAATAGAAATAAGGCAATTATATCATTTCCAACTTTTAAAAATAAAGGTTCTTTGTACCTTAAGTT [21138]
cp26_PAbe_NX_Pl_consensus    CTTATGCCGATTAAATAGAAATAAGGCAATTATATCATTTCCAACTTTTAAAAATAAAGGTTCTTTGTACCTTAAGTT [21138]

cp26_gi|11497007|B31_GB      CCAAGCATTAGAAACAGTAAAATAATGTCTTAAAGAAATTTTATTATTCTCTTGAATTAATTTAATATATTTGGAATC [21216]
cp26_un9_B31_PacBio          CCAAGCATTAGAAACAGTAAAATAATGTCTTAAAGAAATTTTATTATTCTCTTGAATTAATTTAATATATTTGGAATC [21216]
cp26_B31_TS_MP_FG_consensus  CCAAGCATTAGAAACAGTAAAATAATGTCTTAAAGAAATTTTATTATTCTCTTGAATTAATTTAATATATTTGGAATC [21216]
cp26_B31_NX_Pl_consensus     CCAAGCATTAGAAACAGTAAAATAATGTCTTAAAGAAATTTTATTATTCTCTTGAATTAATTTAATATATTTGGAATC [21216]
cp26_PAli_NX_MP_FG_consensus CCAAGCATTAGAAACAGTAAAATAATGTCTTAAAGAAATTTTATTATTCTCTTGAATTAATTTAATATATTTGGAATC [21216]
cp26_PAli_NX_Pl_consensus    CCAAGCATTAGAAACAGTAAAATAATGTCTTAAAGAAATTTTATTATTCTCTTGAATTAATTTAATATATTTGGAATC [21216]
cp26_PAbe_NX_MP_FG_consensus CCAAGCATTAGAAACAGTAAAATAATGTCTTAAAGAAATTTTATTATTCTCTTGAATTAATTTAATATATTTGGAATC [21216]
cp26_PAbe_NX_Pl_consensus    CCAAGCATTAGAAACAGTAAAATAATGTCTTAAAGAAATTTTATTATTCTCTTGAATTAATTTAATATATTTGGAATC [21216]

cp26_gi|11497007|B31_GB      TTTTTTTAATTCGGGAATGCCATCAAAATAGATTGTAGAACAAGACAATGTTAAAAATAGCAAATAAAAAAGTAATAA [21294]
cp26_un9_B31_PacBio          TTTTTTTAATTCGGGAATGCCATCAAAATAGATTGTAGAACAAGACAATGTTAAAAATAGCAAATAAAAAAGTAATAA [21294]
cp26_B31_TS_MP_FG_consensus  TTTTTTTAATTCGGGAATGCCATCAAAATAGATTGTAGAACAAGACAATGTTAAAAATAGCAAATAAAAAAGTAATAA [21294]
cp26_B31_NX_Pl_consensus     TTTTTTTAATTCGGGAATGCCATCAAAATAGATTGTAGAACAAGACAATGTTAAAAATAGCAAATAAAAAAGTAATAA [21294]
cp26_PAli_NX_MP_FG_consensus TTTTTTTAATTCGGGAATGCCATCAAAATAGATTGTAGAACAAGACAATGTTAAAAATAGCAAATAAAAAAGTAATAA [21294]
cp26_PAli_NX_Pl_consensus    TTTTTTTAATTCGGGAATGCCATCAAAATAGATTGTAGAACAAGACAATGTTAAAAATAGCAAATAAAAAAGTAATAA [21294]
cp26_PAbe_NX_MP_FG_consensus TTTTTTTAATTCGGGAATGCCATCAAAATAGATTGTAGAACAAGACAATGTTAAAAATAGCAAATAAAAAAGTAATAA [21294]
cp26_PAbe_NX_Pl_consensus    TTTTTTTAATTCGGGAATGCCATCAAAATAGATTGTAGAACAAGACAATGTTAAAAATAGCAAATAAAAAAGTAATAA [21294]

cp26_gi|11497007|B31_GB      ATATTTTTTAAAAATTTTCAAGAAAGTCCATAATATGTAAGTTTTTTTATAAAATTTTGTTTTTAAATGCATATTCTA [21372]
cp26_un9_B31_PacBio          ATATTTTTTAAAAATTTTCAAGAAAGTCCATAATATGTAAGTTTTTTTATAAAATTTTGTTTTTAAATGCATATTCTA [21372]
cp26_B31_TS_MP_FG_consensus  ATATTTTTTAAAAATTTTCAAGAAAGTCCATAATATGTAAGTTTTTTTATAAAATTTTGTTTTTAAATGCATATTCTA [21372]
cp26_B31_NX_Pl_consensus     ATATTTTTTAAAAATTTTCAAGAAAGTCCATAATATGTAAGTTTTTTTATAAAATTTTGTTTTTAAATGCATATTCTA [21372]
cp26_PAli_NX_MP_FG_consensus ATATTTTTTAAAAATTTTCAAGAAAGTCCATAATATGTAAGTTTTTTTATAAAATTTTGTTTTTAAATGCATATTCTA [21372]
cp26_PAli_NX_Pl_consensus    ATATTTTTTAAAAATTTTCAAGAAAGTCCATAATATGTAAGTTTTTTTATAAAATTTTGTTTTTAAATGCATATTCTA [21372]
cp26_PAbe_NX_MP_FG_consensus ATATTTTTTAAAAATTTTCAAGAAAGTCCATAATATGTAAGTTTTTTTATAAAATTTTGTTTTTAAATGCATATTCTA [21372]
cp26_PAbe_NX_Pl_consensus    ATATTTTTTAAAAATTTTCAAGAAAGTCCATAATATGTAAGTTTTTTTATAAAATTTTGTTTTTAAATGCATATTCTA [21372]

cp26_gi|11497007|B31_GB      AAGTATTTTTTGTATTCTTGAAATTAACATCAATAAATCTTGGATCATATTGCAAGGTATCCTTAGTCCAAACCTGAA [21450]
cp26_un9_B31_PacBio          AAGTATTTTTTGTATTCTTGAAATTAACATCAATAAATCTTGGATCATATTGCAAGGTATCCTTAGTCCAAACCTGAA [21450]
cp26_B31_TS_MP_FG_consensus  AAGTATTTTTTGTATTCTTGAAATTAACATCAATAAATCTTGGATCATATTGCAAGGTATCCTTAGTCCAAACCTGAA [21450]
cp26_B31_NX_Pl_consensus     AAGTATTTTTTGTATTCTTGAAATTAACATCAATAAATCTTGGATCATATTGCAAGGTATCCTTAGTCCAAACCTGAA [21450]
cp26_PAli_NX_MP_FG_consensus AAGTATTTTTTGTATTCTTGAAATTAACATCAATAAATCTTGGATCATATTGCAAGGTATCCTTAGTCCAAACCTGAA [21450]
cp26_PAli_NX_Pl_consensus    AAGTATTTTTTGTATTCTTGAAATTAACATCAATAAATCTTGGATCATATTGCAAGGTATCCTTAGTCCAAACCTGAA [21450]
cp26_PAbe_NX_MP_FG_consensus AAGTATTTTTTGTATTCTTGAAATTAACATCAATAAATCTTGGATCATATTGCAAGGTATCCTTAGTCCAAACCTGAA [21450]
cp26_PAbe_NX_Pl_consensus    AAGTATTTTTTGTATTCTTGAAATTAACATCAATAAATCTTGGATCATATTGCAAGGTATCCTTAGTCCAAACCTGAA [21450]

cp26_gi|11497007|B31_GB      AAGAATCCTCATTAAATAAAAAGACTAGCTTTTTGTAAGGGGTTAAAGCTTTTGACATAAGAGTAAAATTATCTTTAT [21528]
cp26_un9_B31_PacBio          AAGAATCCTCATTAAATAAAAAGACTAGCTTTTTGTAAGGGGTTAAAGCTTTTGACATAAGAGTAAAATTATCTTTAT [21528]
cp26_B31_TS_MP_FG_consensus  AAGAATCCTCATTAAATAAAAAGACTAGCTTTTTGTAAGGGGTTAAAGCTTTTGACATAAGAGTAAAATTATCTTTAT [21528]
cp26_B31_NX_Pl_consensus     AAGAATCCTCATTAAATAAAAAGACTAGCTTTTTGTAAGGGGTTAAAGCTTTTGACATAAGAGTAAAATTATCTTTAT [21528]
cp26_PAli_NX_MP_FG_consensus AAGAATCCTCATTAAATAAAAAGACTAGCTTTTTGTAAGGGGTTAAAGCTTTTGACATAAGAGTAAAATTATCTTTAT [21528]
cp26_PAli_NX_Pl_consensus    AAGAATCCTCATTAAATAAAAAGACTAGCTTTTTGTAAGGGGTTAAAGCTTTTGACATAAGAGTAAAATTATCTTTAT [21528]
cp26_PAbe_NX_MP_FG_consensus AAGAATCCTCATTAAATAAAAAGACTAGCTTTTTGTAAGGGGTTAAAGCTTTTGACATAAGAGTAAAATTATCTTTAT [21528]
cp26_PAbe_NX_Pl_consensus    AAGAATCCTCATTAAATAAAAAGACTAGCTTTTTGTAAGGGGTTAAAGCTTTTGACATAAGAGTAAAATTATCTTTAT [21528]

cp26_gi|11497007|B31_GB      CAAGATTAATGTACAAAAACTTAAAATTTCCAATACTAACTACCTTAGAAATAGCAATGCTTCCAATATATTTATTGT [21606]
cp26_un9_B31_PacBio          CAAGATTAATGTACAAAAACTTAAAATTTCCAATACTAACTACCTTAGAAATAGCAATGCTTCCAATATATTTATTGT [21606]
cp26_B31_TS_MP_FG_consensus  CAAGATTAATGTACAAAAACTTAAAATTTCCAATACTAACTACCTTAGAAATAGCAATGCTTCCAATATATTTATTGT [21606]
cp26_B31_NX_Pl_consensus     CAAGATTAATGTACAAAAACTTAAAATTTCCAATACTAACTACCTTAGAAATAGCAATGCTTCCAATATATTTATTGT [21606]
```

```
cp26_PAli_NX_MP_FG_consensus CAAGATTAATGTACAAAAACTTAAAATTTCCAATACTAACTACCTTAGAAATAGCAATGCTTCCAATATATTTATTGT [21606]
cp26_PAli_NX_Pl_consensus    CAAGATTAATGTACAAAAACTTAAAATTTCCAATACTAACTACCTTAGAAATAGCAATGCTTCCAATATATTTATTGT [21606]
cp26_PAbe_NX_MP_FG_consensus CAAGATTAATGTACAAAAACTTAAAATTTCCAATACTAACTACCTTAGAAATAGCAATGCTTCCAATATATTTATTGT [21606]
cp26_PAbe_NX_Pl_consensus    CAAGATTAATGTACAAAAACTTAAAATTTCCAATACTAACTACCTTAGAAATAGCAATGCTTCCAATATATTTATTGT [21606]
```

```
cp26_gi|11497007|B31_GB      CTTGAATTAATTTTAAATGACCTTTAGTAAAATTTAAATTTGAATCTAACATAACATTTAAAAAAACACTAAATTTAT [21684]
cp26_un9_B31_PacBio          CTTGAATTAATTTTAAATGACCTTTAGTAAAATTTAAATTTGAATCTAACATAACATTTAAAAAAACACTAAATTTAT [21684]
cp26_B31_TS_MP_FG_consensus  CTTGAATTAATTTTAAATGACCTTTAGTAAAATTTAAATTTGAATCTAACATAACATTTAAAAAAACACTAAATTTAT [21684]
cp26_B31_NX_Pl_consensus     CTTGAATTAATTTTAAATGACCTTTAGTAAAATTTAAATTTGAATCTAACATAACATTTAAAAAAACACTAAATTTAT [21684]
cp26_PAli_NX_MP_FG_consensus CTTGAATTAATTTTAAATGACCTTTAGTAAAATTTAAATTTGAATCTAACATAACATTTAAAAAAACACTAAATTTAT [21684]
cp26_PAli_NX_Pl_consensus    CTTGAATTAATTTTAAATGACCTTTAGTAAAATTTAAATTTGAATCTAACATAACATTTAAAAAAACACTAAATTTAT [21684]
cp26_PAbe_NX_MP_FG_consensus CTTGAATTAATTTTAAATGACCTTTAGTAAAATTTAAATTTGAATCTAACATAACATTTAAAAAAACACTAAATTTAT [21684]
cp26_PAbe_NX_Pl_consensus    CTTGAATTAATTTTAAATGACCTTTAGTAAAATTTAAATTTGAATCTAACATAACATTTAAAAAAACACTAAATTTAT [21684]
```

```
cp26_gi|11497007|B31_GB      TGCTCGATTTATCTTTTTTAATGTATATTTGACTGTCTGGATAAAAATAAAGAAAATAATCTGCTCTTTCAATTTTTT [21762]
cp26_un9_B31_PacBio          TGCTCGATTTATCTTTTTTAATGTATATTTGACTGTCTGGATAAAAATAAAGAAAATAATCTGCTCTTTCAATTTTTT [21762]
cp26_B31_TS_MP_FG_consensus  TGCTCGATTTATCTTTTTTAATGTATATTTGACTGTCTGGATAAAAATAAAGAAAATAATCTGCTCTTTCAATTTTTT [21762]
cp26_B31_NX_Pl_consensus     TGCTCGATTTATCTTTTTTAATGTATATTTGACTGTCTGGATAAAAATAAAGAAAATAATCTGCTCTTTCAATTTTTT [21762]
cp26_PAli_NX_MP_FG_consensus TGCTCGATTTATCTTTTTTAATGTATATTTGACTGTCTGGATAAAAATAAAGAAAATAATCTGCTCTTTCAATTTTTT [21762]
cp26_PAli_NX_Pl_consensus    TGCTCGATTTATCTTTTTTAATGTATATTTGACTGTCTGGATAAAAATAAAGAAAATAATCTGCTCTTTCAATTTTTT [21762]
cp26_PAbe_NX_MP_FG_consensus TGCTCGATTTATCTTTTTTAATGTATATTTGACTGTCTGGATAAAAATAAAGAAAATAATCTGCTCTTTCAATTTTTT [21762]
cp26_PAbe_NX_Pl_consensus    TGCTCGATTTATCTTTTTTAATGTATATTTGACTGTCTGGATAAAAATAAAGAAAATAATCTGCTCTTTCAATTTTTT [21762]
```

```
cp26_gi|11497007|B31_GB      CAAAATTCTTTTCTAAATCTTCAGCAATATTTAAAATCTTGCAAGAAGAGCAAATAAAAACCATTAAAATCAAAGAAA [21840]
cp26_un9_B31_PacBio          CAAAATTCTTTTCTAAATCTTCAGCAATATTTAAAATCTTGCAAGAAGAGCAAATAAAAACCATTAAAATCAAAGAAA [21840]
cp26_B31_TS_MP_FG_consensus  CAAAATTCTTTTCTAAATCTTCAGCAATATTTAAAATCTTGCAAGAAGAGCAAATAAAAACCATTAAAATCAAAGAAA [21840]
cp26_B31_NX_Pl_consensus     CAAAATTCTTTTCTAAATCTTCAGCAATATTTAAAATCTTGCAAGAAGAGCAAATAAAAACCATTAAAATCAAAGAAA [21840]
cp26_PAli_NX_MP_FG_consensus CAAAATTCTTTTCTAAATCTTCAGCAATATTTAAAATCTTGCAAGAAGAGCAAATAAAAACCATTAAAATCAAAGAAA [21840]
cp26_PAli_NX_Pl_consensus    CAAAATTCTTTTCTAAATCTTCAGCAATATTTAAAATCTTGCAAGAAGAGCAAATAAAAACCATTAAAATCAAAGAAA [21840]
cp26_PAbe_NX_MP_FG_consensus CAAAATTCTTTTCTAAATCTTCAGCAATATTTAAAATCTTGCAAGAAGAGCAAATAAAAACCATTAAAATCAAAGAAA [21840]
cp26_PAbe_NX_Pl_consensus    CAAAATTCTTTTCTAAATCTTCAGCAATATTTAAAATCTTGCAAGAAGAGCAAATAAAAACCATTAAAATCAAAGAAA [21840]
```

```
cp26_gi|11497007|B31_GB      AACAGTATTTCATAATTAACTTATATTATACAATACAGAAAGGAGAGTATTTATATTAAATGAATGAAAACAAATTCA [21918]
cp26_un9_B31_PacBio          AACAGTATTTCATAATTAACTTATATTATACAATACAGAAAGGAGAGTATTTATATTAAATGAATGAAAACAAATTCA [21918]
cp26_B31_TS_MP_FG_consensus  AACAGTATTTCATAATTAACTTATATTATACAATACAGAAAGGAGAGTATTTATATTAAATGAATGAAAACAAATTCA [21918]
cp26_B31_NX_Pl_consensus     AACAGTATTTCATAATTAACTTATATTATACAATACAGAAAGGAGAGTATTTATATTAAATGAATGAAAACAAATTCA [21918]
cp26_PAli_NX_MP_FG_consensus AACAGTATTTCATAATTAACTTATATTATACAATACAGAAAGGAGAGTATTTATATTAAATGAATGAAAACAAATTCA [21918]
cp26_PAli_NX_Pl_consensus    AACAGTATTTCATAATTAACTTATATTATACAATACAGAAAGGAGAGTATTTATATTAAATGAATGAAAACAAATTCA [21918]
cp26_PAbe_NX_MP_FG_consensus AACAGTATTTCATAATTAACTTATATTATACAATACAGAAAGGAGAGTATTTATATTAAATGAATGAAAACAAATTCA [21918]
cp26_PAbe_NX_Pl_consensus    AACAGTATTTCATAATTAACTTATATTATACAATACAGAAAGGAGAGTATTTATATTAAATGAATGAAAACAAATTCA [21918]
```

```
cp26_gi|11497007|B31_GB      GCTCTTGTATTGAAAATTCTAAAGCCTATTCGGATTATATAACATCAAAACATAAAATATTACTTATTCCTGTTCCTA [21996]
cp26_un9_B31_PacBio          GCTCTTGTATTGAAAATTCTAAAGCCTATTCGGATTATATAACATCAAAACATAAAATATTACTTATTCCTGTTCCTA [21996]
cp26_B31_TS_MP_FG_consensus  GCTCTTGTATTGAAAATTCTAAAGCCTATTCGGATTATATAACATCAAAACATAAAATATTACTTATTCCTGTTCCTA [21996]
cp26_B31_NX_Pl_consensus     GCTCTTGTATTGAAAATTCTAAAGCCTATTCGGATTATATAACATCAAAACATAAAATATTACTTATTCCTGTTCCTA [21996]
cp26_PAli_NX_MP_FG_consensus GCTCTTGTATTGAAAATTCTAAAGCCTATTCGGATTATATAACATCAAAACATAAAATATTACTTATTCCTGTTCCTA [21996]
cp26_PAli_NX_Pl_consensus    GCTCTTGTATTGAAAATTCTAAAGCCTATTCGGATTATATAACATCAAAACATAAAATATTACTTATTCCTGTTCCTA [21996]
cp26_PAbe_NX_MP_FG_consensus GCTCTTGTATTGAAAATTCTAAAGCCTATTCGGATTATATAACATCAAAACATAAAATATTACTTATTCCTGTTCCTA [21996]
cp26_PAbe_NX_Pl_consensus    GCTCTTGTATTGAAAATTCTAAAGCCTATTCGGATTATATAACATCAAAACATAAAATATTACTTATTCCTGTTCCTA [21996]
```

```
cp26_gi|11497007|B31_GB      TAGTAAAAATTGCCATGGGAGAAGATCTTAAAATTTTCGAAATAAGTTTTCAAGATAAGAATAAAGATTTTCCAGGAT [22074]
cp26_un9_B31_PacBio          TAGTAAAAATTGCCATGGGAGAAGATCTTAAAATTTTCGAAATAAGTTTTCAAGATAAGAATAAAGATTTTCCAGGAT [22074]
cp26_B31_TS_MP_FG_consensus  TAGTAAAAATTGCCATGGGAGAAGATCTTAAAATTTTCGAAATAAGTTTTCAAGATAAGAATAAAGATTTTCCAGGAT [22074]
cp26_B31_NX_Pl_consensus     TAGTAAAAATTGCCATGGGAGAAGATCTTAAAATTTTCGAAATAAGTTTTCAAGATAAGAATAAAGATTTTCCAGGAT [22074]
cp26_PAli_NX_MP_FG_consensus TAGTAAAAATTGCCATGGGAGAAGATCTTAAAATTTTCGAAATAAGTTTTCAAGATAAGAATAAAGATTTTCCAGGAT [22074]
cp26_PAli_NX_Pl_consensus    TAGTAAAAATTGCCATGGGAGAAGATCTTAAAATTTTCGAAATAAGTTTTCAAGATAAGAATAAAGATTTTCCAGGAT [22074]
cp26_PAbe_NX_MP_FG_consensus TAGTAAAAATTGCCATGGGAGAAGATCTTAAAATTTTCGAAATAAGTTTTCAAGATAAGAATAAAGATTTTCCAGGAT [22074]
cp26_PAbe_NX_Pl_consensus    TAGTAAAAATTGCCATGGGAGAAGATCTTAAAATTTTCGAAATAAGTTTTCAAGATAAGAATAAAGATTTTCCAGGAT [22074]
```

```
cp26_gi|11497007|B31_GB      ATATTCAATTAAATAAAAAATCTTTATATATAAATGAAAACATGAGCCTTGAAAATAAAAGATTTACAATAGCAAAAT [22152]
cp26_un9_B31_PacBio          ATATTCAATTAAATAAAAAATCTTTATATATAAATGAAAACATGAGCCTTGAAAATAAAAGATTTACAATAGCAAAAT [22152]
cp26_B31_TS_MP_FG_consensus  ATATTCAATTAAATAAAAAATCTTTATATATAAATGAAAACATGAGCCTTGAAAATAAAAGATTTACAATAGCAAAAT [22152]
cp26_B31_NX_Pl_consensus     ATATTCAATTAAATAAAAAATCTTTATATATAAATGAAAACATGAGCCTTGAAAATAAAAGATTTACAATAGCAAAAT [22152]
cp26_PAli_NX_MP_FG_consensus ATATTCAATTAAATAAAAAATCTTTATATATAAATGAAAACATGAGCCTTGAAAATAAAAGATTTACAATAGCAAAAT [22152]
cp26_PAli_NX_Pl_consensus    ATATTCAATTAAATAAAAAATCTTTATATATAAATGAAAACATGAGCCTTGAAAATAAAAGATTTACAATAGCAAAAT [22152]
cp26_PAbe_NX_MP_FG_consensus ATATTCAATTAAATAAAAAATCTTTATATATAAATGAAAACATGAGCCTTGAAAATAAAAGATTTACAATAGCAAAAT [22152]
cp26_PAbe_NX_Pl_consensus    ATATTCAATTAAATAAAAAATCTTTATATATAAATGAAAACATGAGCCTTGAAAATAAAAGATTTACAATAGCAAAAT [22152]
```

```
cp26_gi|11497007|B31_GB      ACTTGGGGCATTATTTGATGCATCAAGATCAAATAAAAAATCTATCTAAAAATGAAAACTATTATAACAACATTCAAG [22230]
cp26_un9_B31_PacBio          ACTTGGGGCATTATTTGATGCATCAAGATCAAATAAAAAATCTATCTAAAAATGAAAACTATTATAACAACATTCAAG [22230]
cp26_B31_TS_MP_FG_consensus  ACTTGGGGCATTATTTGATGCATCAAGATCAAATAAAAAATCTATCTAAAAATGAAAACTATTATAACAACATTCAAG [22230]
cp26_B31_NX_Pl_consensus     ACTTGGGGCATTATTTGATGCATCAAGATCAAATAAAAAATCTATCTAAAAATGAAAACTATTATAACAACATTCAAG [22230]
cp26_PAli_NX_MP_FG_consensus ACTTGGGGCATTATTTGATGCATCAAGATCAAATAAAAAATCTATCTAAAAATGAAAACTATTATAACAACATTCAAG [22230]
cp26_PAli_NX_Pl_consensus    ACTTGGGGCATTATTTGATGCATCAAGATCAAATAAAAAATCTATCTAAAAATGAAAACTATTATAACAACATTCAAG [22230]
cp26_PAbe_NX_MP_FG_consensus ACTTGGGGCATTATTTGATGCATCAAGATCAAATAAAAAATCTATCTAAAAATGAAAACTATTATAACAACATTCAAG [22230]
cp26_PAbe_NX_Pl_consensus    ACTTGGGGCATTATTTGATGCATCAAGATCAAATAAAAAATCTATCTAAAAATGAAAACTATTATAACAACATTCAAG [22230]
```

```
cp26_gi|11497007|B31_GB      ATAGTCAAATGGTAACAGAAGCTAACATATTTGCAGCAAACATATTGATCCCAACAACCACATTAAAATTAAAATTAT [22308]
cp26_un9_B31_PacBio          ATAGTCAAATGGTAACAGAAGCTAACATATTTGCAGCAAACATATTGATCCCAACAACCACATTAAAATTAAAATTAT [22308]
cp26_B31_TS_MP_FG_consensus  ATAGTCAAATGGTAACAGAAGCTAACATATTTGCAGCAAACATATTGATCCCAACAACCACATTAAAATTAAAATTAT [22308]
cp26_B31_NX_Pl_consensus     ATAGTCAAATGGTAACAGAAGCTAACATATTTGCAGCAAACATATTGATCCCAACAACCACATTAAAATTAAAATTAT [22308]
cp26_PAli_NX_MP_FG_consensus ATAGTCAAATGGTAACAGAAGCTAACATATTTGCAGCAAACATATTGATCCCAACAACCACATTAAAATTAAAATTAT [22308]
cp26_PAli_NX_Pl_consensus    ATAGTCAAATGGTAACAGAAGCTAACATATTTGCAGCAAACATATTGATCCCAACAACCACATTAAAATTAAAATTAT [22308]
cp26_PAbe_NX_MP_FG_consensus ATAGTCAAATGGTAACAGAAGCTAACATATTTGCAGCAAACATATTGATCCCAACAACCACATTAAAATTAAAATTAT [22308]
cp26_PAbe_NX_Pl_consensus    ATAGTCAAATGGTAACAGAAGCTAACATATTTGCAGCAAACATATTGATCCCAACAACCACATTAAAATTAAAATTAT [22308]
```

```
cp26_gi|11497007|B31_GB      CTCAATATAAATCTAAAGAGTATCCCCAAAAAACAATAGCAAAAGAATTCCAAGTAACTGAAAATATAATTTATTTAA [22386]
cp26_un9_B31_PacBio          CTCAATATAAATCTAAAGAGTATCCCCAAAAAACAATAGCAAAAGAATTCCAAGTAACTGAAAATATAATTTATTTAA [22386]
cp26_B31_TS_MP_FG_consensus  CTCAATATAAATCTAAAGAGTATCCCCAAAAAACAATAGCAAAAGAATTCCAAGTAACTGAAAATATAATTTATTTAA [22386]
cp26_B31_NX_Pl_consensus     CTCAATATAAATCTAAAGAGTATCCCCAAAAAACAATAGCAAAAGAATTCCAAGTAACTGAAAATATAATTTATTTAA [22386]
cp26_PAli_NX_MP_FG_consensus CTCAATATAAATCTAAAGAGTATCCCCAAAAAACAATAGCAAAAGAATTCCAAGTAACTGAAAATATAATTTATTTAA [22386]
cp26_PAli_NX_Pl_consensus    CTCAATATAAATCTAAAGAGTATCCCCAAAAAACAATAGCAAAAGAATTCCAAGTAACTGAAAATATAATTTATTTAA [22386]
cp26_PAbe_NX_MP_FG_consensus CTCAATATAAATCTAAAGAGTATCCCCAAAAAACAATAGCAAAAGAATTCCAAGTAACTGAAAATATAATTTATTTAA [22386]
cp26_PAbe_NX_Pl_consensus    CTCAATATAAATCTAAAGAGTATCCCCAAAAAACAATAGCAAAAGAATTCCAAGTAACTGAAAATATAATTTATTTAA [22386]
```

```
cp26_gi|11497007|B31_GB      AATTAAGCATAATTAATGATCTTGTCAAAATAGATAAAATAAAAAAGAGTAAAAAATTTTTAAAAATTAAAAACACAA [22464]
cp26_un9_B31_PacBio          AATTAAGCATAATTAATGATCTTGTCAAAATAGATAAAATAAAAAAGAGTAAAAAATTTTTAAAAATTAAAAACACAA [22464]
cp26_B31_TS_MP_FG_consensus  AATTAAGCATAATTAATGATCTTGTCAAAATAGATAAAATAAAAAAGAGTAAAAAATTTTTAAAAATTAAAAACACAA [22464]
cp26_B31_NX_Pl_consensus     AATTAAGCATAATTAATGATCTTGTCAAAATAGATAAAATAAAAAAGAGTAAAAAATTTTTAAAAATTAAAAACACAA [22464]
cp26_PAli_NX_MP_FG_consensus AATTAAGCATAATTAATGATCTTGTCAAAATAGATAAAATAAAAAAGAGTAAAAAATTTTTAAAAATTAAAAACACAA [22464]
cp26_PAli_NX_Pl_consensus    AATTAAGCATAATTAATGATCTTGTCAAAATAGATAAAATAAAAAAGAGTAAAAAATTTTTAAAAATTAAAAACACAA [22464]
cp26_PAbe_NX_MP_FG_consensus AATTAAGCATAATTAATGATCTTGTCAAAATAGATAAAATAAAAAAGAGTAAAAAATTTTTAAAAATTAAAAACACAA [22464]
cp26_PAbe_NX_Pl_consensus    AATTAAGCATAATTAATGATCTTGTCAAAATAGATAAAATAAAAAAGAGTAAAAAATTTTTAAAAATTAAAAACACAA [22464]
```

```
cp26_gi|11497007|B31_GB      AAGAAAAAAATAGAAGCCAACATCTACCTCCACAATACAGATAAAATTAAAGAATCAATAGCTCTTGATTTGGAAAAAT [22542]
cp26_un9_B31_PacBio          AAGAAAAAAATAGAAGCCAACATCTACCTCCACAATACAGATAAAATTAAAGAATCAATAGCTCTTGATTTGGAAAAAT [22542]
cp26_B31_TS_MP_FG_consensus  AAGAAAAAAATAGAAGCCAACATCTACCTCCACAATACAGATAAAATTAAAGAATCAATAGCTCTTGATTTGGAAAAAT [22542]
cp26_B31_NX_Pl_consensus     AAGAAAAAAATAGAAGCCAACATCTACCTCCACAATACAGATAAAATTAAAGAATCAATAGCTCTTGATTTGGAAAAAT [22542]
cp26_PAli_NX_MP_FG_consensus AAGAAAAAAATAGAAGCCAACATCTACCTCCACAATACAGATAAAATTAAAGAATCAATAGCTCTTGATTTGGAAAAAT [22542]
```

```
cp26_PAli_NX_Pl_consensus    AAGAAAAAATAGAAGCCAACATCTACCTCCACAATACAGATAAAATTAAAGAATCAATAGCTCTTGATTTGGAAAAAT [22542]
cp26_PAbe_NX_MP_FG_consensus AAGAAAAAATAGAAGCCAACATCTACCTCCACAATACAGATAAAATTAAAGAATCAATAGCTCTTGATTTGGAAAAAT [22542]
cp26_PAbe_NX_Pl_consensus    AAGAAAAAATAGAAGCCAACATCTACCTCCACAATACAGATAAAATTAAAGAATCAATAGCTCTTGATTTGGAAAAAT [22542]

cp26_gi|11497007|B31_GB      ATGAACTTGAAAAAAAAGAACGAATTAAAAAAATATTTGAAGATCTAGAGTAAAAAGCTTTCTCAAGTTAAAAACTTT [22620]
cp26_un9_B31_PacBio          ATGAACTTGAAAAAAAAGAACGAATTAAAAAAATATTTGAAGATCTAGAGTAAAAAGCTTTCTCAAGTTAAAAACTTT [22620]
cp26_B31_TS_MP_FG_consensus  ATGAACTTGAAAAAAAAGAACGAATTAAAAAAATATTTGAAGATCTAGAGTAAAAAGCTTTCTCAAGTTAAAAACTTT [22620]
cp26_B31_NX_Pl_consensus     ATGAACTTGAAAAAAAAGAACGAATTAAAAAAATATTTGAAGATCTAGAGTAAAAAGCTTTCTCAAGTTAAAAACTTT [22620]
cp26_PAli_NX_MP_FG_consensus ATGAACTTGAAAAAAAAGAACGAATTAAAAAAATATTTGAAGATCTAGAGTAAAAAGCTTTCTCAAGTTAAAAACTTT [22620]
cp26_PAli_NX_Pl_consensus    ATGAACTTGAAAAAAAAGAACGAATTAAAAAAATATTTGAAGATCTAGAGTAAAAAGCTTTCTCAAGTTAAAAACTTT [22620]
cp26_PAbe_NX_MP_FG_consensus ATGAACTTGAAAAAAAAGAACGAATTAAAAAAATATTTGAAGATCTAGAGTAAAAAGCTTTCTCAAGTTAAAAACTTT [22620]
cp26_PAbe_NX_Pl_consensus    ATGAACTTGAAAAAAAAGAACGAATTAAAAAAATATTTGAAGATCTAGAGTAAAAAGCTTTCTCAAGTTAAAAACTTT [22620]

cp26_gi|11497007|B31_GB      TTGAGTATATATTCCATCTTTACATTCGCCAAGACTAAATAATTTTTACTCTTAACAAGAGAATCTAAATGGTTTTTT [22698]
cp26_un9_B31_PacBio          TTGAGTATATATTCCATCTTTACATTCGCCAAGACTAAATAATTTTTACTCTTAACAAGAGAATCTAAATGGTTTTTT [22698]
cp26_B31_TS_MP_FG_consensus  TTGAGTATATATTCCATCTTTACATTCGCCAAGACTAAATAATTTTTACTCTTAACAAGAGAATCTAAATGGTTTTTT [22698]
cp26_B31_NX_Pl_consensus     TTGAGTATATATTCCATCTTTACATTCGCCAAGACTAAATAATTTTTACTCTTAACAAGAGAATCTAAATGGTTTTTT [22698]
cp26_PAli_NX_MP_FG_consensus TTGAGTATATATTCCATCTTTACATTCGCCAAGACTAAATAATTTTTACTCTTAACAAGAGAATCTAAATGGTTTTTT [22698]
cp26_PAli_NX_Pl_consensus    TTGAGTATATATTCCATCTTTACATTCGCCAAGACTAAATAATTTTTACTCTTAACAAGAGAATCTAAATGGTTTTTT [22698]
cp26_PAbe_NX_MP_FG_consensus TTGAGTATATATTCCATCTTTACATTCGCCAAGACTAAATAATTTTTACTCTTAACAAGAGAATCTAAATGGTTTTTT [22698]
cp26_PAbe_NX_Pl_consensus    TTGAGTATATATTCCATCTTTACATTCGCCAAGACTAAATAATTTTTACTCTTAACAAGAGAATCTAAATGGTTTTTT [22698]

cp26_gi|11497007|B31_GB      GCAATTACAATCCAATACTTATTATCCATAAATTTTTTAGCAGTTGGAAAATTTATTTTCAATGAAAAATTTTTCATA [22776]
cp26_un9_B31_PacBio          GCAATTACAATCCAATACTTATTATCCATAAATTTTTTAGCAGTTGGAAAATTTATTTTCAATGAAAAATTTTTCATA [22776]
cp26_B31_TS_MP_FG_consensus  GCAATTACAATCCAATACTTATTATCCATAAATTTTTTAGCAGTTGGAAAATTTATTTTCAATGAAAAATTTTTCATA [22776]
cp26_B31_NX_Pl_consensus     GCAATTACAATCCAATACTTATTATCCATAAATTTTTTAGCAGTTGGAAAATTTATTTTCAATGAAAAATTTTTCATA [22776]
cp26_PAli_NX_MP_FG_consensus GCAATTACAATCCAATACTTATTATCCATAAATTTTTTAGCAGTTGGAAAATTTATTTTCAATGAAAAATTTTTCATA [22776]
cp26_PAli_NX_Pl_consensus    GCAATTACAATCCAATACTTATTATCCATAAATTTTTTAGCAGTTGGAAAATTTATTTTCAATGAAAAATTTTTCATA [22776]
cp26_PAbe_NX_MP_FG_consensus GCAATTACAATCCAATACTTATTATCCATAAATTTTTTAGCAGTTGGAAAATTTATTTTCAATGAAAAATTTTTCATA [22776]
cp26_PAbe_NX_Pl_consensus    GCAATTACAATCCAATACTTATTATCCATAAATTTTTTAGCAGTTGGAAAATTTATTTTCAATGAAAAATTTTTCATA [22776]

cp26_gi|11497007|B31_GB      ATTTTTTGTTTTTCAAGATAAGATAAAACTTCTGCCTTAAAAGATGCACCTTGGGTCAAGCTATAAGCAATAAGTATT [22854]
cp26_un9_B31_PacBio          ATTTTTTGTTTTTCAAGATAAGATAAAACTTCTGCCTTAAAAGATGCACCTTGGGTCAAGCTATAAGCAATAAGTATT [22854]
cp26_B31_TS_MP_FG_consensus  ATTTTTTGTTTTTCAAGATAAGATAAAACTTCTGCCTTAAAAGATGCACCTTGGGTCAAGCTATAAGCAATAAGTATT [22854]
cp26_B31_NX_Pl_consensus     ATTTTTTGTTTTTCAAGATAAGATAAAACTTCTGCCTTAAAAGATGCACCTTGGGTCAAGCTATAAGCAATAAGTATT [22854]
cp26_PAli_NX_MP_FG_consensus ATTTTTTGTTTTTCAAGATAAGATAAAACTTCTGCCTTAAAAGATGCACCTTGGGTCAAGCTATAAGCAATAAGTATT [22854]
cp26_PAli_NX_Pl_consensus    ATTTTTTGTTTTTCAAGATAAGATAAAACTTCTGCCTTAAAAGATGCACCTTGGGTCAAGCTATAAGCAATAAGTATT [22854]
cp26_PAbe_NX_MP_FG_consensus ATTTTTTGTTTTTCAAGATAAGATAAAACTTCTGCCTTAAAAGATGCACCTTGGGTCAAGCTATAAGCAATAAGTATT [22854]
cp26_PAbe_NX_Pl_consensus    ATTTTTTGTTTTTCAAGATAAGATAAAACTTCTGCCTTAAAAGATGCACCTTGGGTCAAGCTATAAGCAATAAGTATT [22854]

cp26_gi|11497007|B31_GB      CTATATAATTTCTTATTATAAATAAAAAGATCTTTAATAAAATTAATATTATTATTGAAACTAGATTCATCAACGTAA [22932]
cp26_un9_B31_PacBio          CTATATAATTTCTTATTATAAATAAAAAGATCTTTAATAAAATTAATATTATTATTGAAACTAGATTCATCAACGTAA [22932]
cp26_B31_TS_MP_FG_consensus  CTATATAATTTCTTATTATAAATAAAAAGATCTTTAATAAAATTAATATTATTATTGAAACTAGATTCATCAACGTAA [22932]
cp26_B31_NX_Pl_consensus     CTATATAATTTCTTATTATAAATAAAAAGATCTTTAATAAAATTAATATTATTATTGAAACTAGATTCATCAACGTAA [22932]
cp26_PAli_NX_MP_FG_consensus CTATATAATTTCTTATTATAAATAAAAAGATCTTTAATAAAATTAATATTATTATTGAAACTAGATTCATCAACGTAA [22932]
cp26_PAli_NX_Pl_consensus    CTATATAATTTCTTATTATAAATAAAAAGATCTTTAATAAAATTAATATTATTATTGAAACTAGATTCATCAACGTAA [22932]
cp26_PAbe_NX_MP_FG_consensus CTATATAATTTCTTATTATAAATAAAAAGATCTTTAATAAAATTAATATTATTATTGAAACTAGATTCATCAACGTAA [22932]
cp26_PAbe_NX_Pl_consensus    CTATATAATTTCTTATTATAAATAAAAAGATCTTTAATAAAATTAATATTATTATTGAAACTAGATTCATCAACGTAA [22932]

cp26_gi|11497007|B31_GB      ATATAAGCACTATTTTTAGTATCACTCAAGCTTTCAAAATCAGATGAATAATGCAAATGTTCGTAGTCTTTTATTTTA [23010]
cp26_un9_B31_PacBio          ATATAAGCACTATTTTTAGTATCACTCAAGCTTTCAAAATCAGATGAATAATGCAAATGTTCGTAGTCTTTTATTTTA [23010]
cp26_B31_TS_MP_FG_consensus  ATATAAGCACTATTTTTAGTATCACTCAAGCTTTCAAAATCAGATGAATAATGCAAATGTTCGTAGTCTTTTATTTTA [23010]
cp26_B31_NX_Pl_consensus     ATATAAGCACTATTTTTAGTATCACTCAAGCTTTCAAAATCAGATGAATAATGCAAATGTTCGTAGTCTTTTATTTTA [23010]
cp26_PAli_NX_MP_FG_consensus ATATAAGCACTATTTTTAGTATCACTCAAGCTTTCAAAATCAGATGAATAATGCAAATGTTCGTAGTCTTTTATTTTA [23010]
cp26_PAli_NX_Pl_consensus    ATATAAGCACTATTTTTAGTATCACTCAAGCTTTCAAAATCAGATGAATAATGCAAATGTTCGTAGTCTTTTATTTTA [23010]
cp26_PAbe_NX_MP_FG_consensus ATATAAGCACTATTTTTAGTATCACTCAAGCTTTCAAAATCAGATGAATAATGCAAATGTTCGTAGTCTTTTATTTTA [23010]
cp26_PAbe_NX_Pl_consensus    ATATAAGCACTATTTTTAGTATCACTCAAGCTTTCAAAATCAGATGAATAATGCAAATGTTCGTAGTCTTTTATTTTA [23010]

cp26_gi|11497007|B31_GB      AAATTTGAAATCTTCAAATCAGGATAATTCATTAAAGATGATAAAACAGTTAAATTTATTTTATCTTTTTCTGGTATT [23088]
cp26_un9_B31_PacBio          AAATTTGAAATCTTCAAATCAGGATAATTCATTAAAGATGATAAAACAGTTAAATTTATTTTATCTTTTTCTGGTATT [23088]
cp26_B31_TS_MP_FG_consensus  AAATTTGAAATCTTCAAATCAGGATAATTCATTAAAGATGATAAAACAGTTAAATTTATTTTATCTTTTTCTGGTATT [23088]
cp26_B31_NX_Pl_consensus     AAATTTGAAATCTTCAAATCAGGATAATTCATTAAAGATGATAAAACAGTTAAATTTATTTTATCTTTTTCTGGTATT [23088]
cp26_PAli_NX_MP_FG_consensus AAATTTGAAATCTTCAAATCAGGATAATTCATTAAAGATGATAAAACAGTTAAATTTATTTTATCTTTTTCTGGTATT [23088]
cp26_PAli_NX_Pl_consensus    AAATTTGAAATCTTCAAATCAGGATAATTCATTAAAGATGATAAAACAGTTAAATTTATTTTATCTTTTTCTGGTATT [23088]
cp26_PAbe_NX_MP_FG_consensus AAATTTGAAATCTTCAAATCAGGATAATTCATTAAAGATGATAAAACAGTTAAATTTATTTTATCTTTTTCTGGTATT [23088]
cp26_PAbe_NX_Pl_consensus    AAATTTGAAATCTTCAAATCAGGATAATTCATTAAAGATGATAAAACAGTTAAATTTATTTTATCTTTTTCTGGTATT [23088]

cp26_gi|11497007|B31_GB      TTTACCAAAGATATAGTTGAACAACCATAAAACAATAAAAAAATAAACGGATATTAAAAACTTCTTCATTTAATGCCTA [23166]
cp26_un9_B31_PacBio          TTTACCAAAGATATAGTTGAACAACCATAAAACAATAAAAAAATAAACGGATATTAAAAACTTCTTCATTTAATGCCTA [23166]
cp26_B31_TS_MP_FG_consensus  TTTACCAAAGATATAGTTGAACAACCATAAAACAATAAAAAAATAAACGGATATTAAAAACTTCTTCATTTAATGCCTA [23166]
cp26_B31_NX_Pl_consensus     TTTACCAAAGATATAGTTGAACAACCATAAAACAATAAAAAAATAAACGGATATTAAAAACTTCTTCATTTAATGCCTA [23166]
cp26_PAli_NX_MP_FG_consensus TTTACCAAAGATATAGTTGAACAACCATAAAACAATAAAAAAATAAACGGATATTAAAAACTTCTTCATTTAATGCCTA [23166]
cp26_PAli_NX_Pl_consensus    TTTACCAAAGATATAGTTGAACAACCATAAAACAATAAAAAAATAAACGGATATTAAAAACTTCTTCATTTAATGCCTA [23166]
cp26_PAbe_NX_MP_FG_consensus TTTACCAAAGATATAGTTGAACAACCATAAAACAATAAAAAAATAAACGGATATTAAAAACTTCTTCATTTAATGCCTA [23166]
cp26_PAbe_NX_Pl_consensus    TTTACCAAAGATATAGTTGAACAACCATAAAACAATAAAAAAATAAACGGATATTAAAAACTTCTTCATTTAATGCCTA [23166]

cp26_gi|11497007|B31_GB      ATTGTATGCTTTTAGTAAAATAAAGTAAAGTAAATAAAAATATAGAAATATTTTGATATAATTTAAAAATAAACTTTA [23244]
cp26_un9_B31_PacBio          ATTGTATGCTTTTAGTAAAATAAAGTAAAGTAAATAAAAATATAGAAATATTTTGATATAATTTAAAAATAAACTTTA [23244]
cp26_B31_TS_MP_FG_consensus  ATTGTATGCTTTTAGTAAAATAAAGTAAAGTAAATAAAAATATAGAAATATTTTGATATAATTTAAAAATAAACTTTA [23244]
cp26_B31_NX_Pl_consensus     ATTGTATGCTTTTAGTAAAATAAAGTAAAGTAAATAAAAATATAGAAATATTTTGATATAATTTAAAAATAAACTTTA [23244]
cp26_PAli_NX_MP_FG_consensus ATTGTATGCTTTTAGTAAAATAAAGTAAAGTAAATAAAAATATAGAAATATTTTGATATAATTTAAAAATAAACTTTA [23244]
cp26_PAli_NX_Pl_consensus    ATTGTATGCTTTTAGTAAAATAAAGTAAAGTAAATAAAAATATAGAAATATTTTGATATAATTTAAAAATAAACTTTA [23244]
cp26_PAbe_NX_MP_FG_consensus ATTGTATGCTTTTAGTAAAATAAAGTAAAGTAAATAAAAATATAGAAATATTTTGATATAATTTAAAAATAAACTTTA [23244]
cp26_PAbe_NX_Pl_consensus    ATTGTATGCTTTTAGTAAAATAAAGTAAAGTAAATAAAAATATAGAAATATTTTGATATAATTTAAAAATAAACTTTA [23244]

cp26_gi|11497007|B31_GB      AAAGGATGTAAAATGAGTTATTATGTGCTAAGCAAAATATTTCTATATTCTGGGTACCTTGTTATTGGATTTTTATCT [23322]
cp26_un9_B31_PacBio          AAAGGATGTAAAATGAGTTATTATGTGCTAAGCAAAATATTTCTATATTCTGGGTACCTTGTTATTGGATTTTTATCT [23322]
cp26_B31_TS_MP_FG_consensus  AAAGGATGTAAAATGAGTTATTATGTGCTAAGCAAAATATTTCTATATTCTGGGTACCTTGTTATTGGATTTTTATCT [23322]
cp26_B31_NX_Pl_consensus     AAAGGATGTAAAATGAGTTATTATGTGCTAAGCAAAATATTTCTATATTCTGGGTACCTTGTTATTGGATTTTTATCT [23322]
cp26_PAli_NX_MP_FG_consensus AAAGGATGTAAAATGAGTTATTATGTGCTAAGCAAAATATTTCTATATTCTGGGTACCTTGTTATTGGATTTTTATCT [23322]
cp26_PAli_NX_Pl_consensus    AAAGGATGTAAAATGAGTTATTATGTGCTAAGCAAAATATTTCTATATTCTGGGTACCTTGTTATTGGATTTTTATCT [23322]
cp26_PAbe_NX_MP_FG_consensus AAAGGATGTAAAATGAGTTATTATGTGCTAAGCAAAATATTTCTATATTCTGGGTACCTTGTTATTGGATTTTTATCT [23322]
cp26_PAbe_NX_Pl_consensus    AAAGGATGTAAAATGAGTTATTATGTGCTAAGCAAAATATTTCTATATTCTGGGTACCTTGTTATTGGATTTTTATCT [23322]

cp26_gi|11497007|B31_GB      TTTACAATTTTCAATAAAAATTTAAGAAATAAAATCAGAGATAAATTAAAAAATTTATACTTTTTATATTATTTAACT [23400]
cp26_un9_B31_PacBio          TTTACAATTTTCAATAAAAATTTAAGAAATAAAATCAGAGATAAATTAAAAAATTTATACTTTTTATATTATTTAACT [23400]
cp26_B31_TS_MP_FG_consensus  TTTACAATTTTCAATAAAAATTTAAGAAATAAAATCAGAGATAAATTAAAAAATTTATACTTTTTATATTATTTAACT [23400]
cp26_B31_NX_Pl_consensus     TTTACAATTTTCAATAAAAATTTAAGAAATAAAATCAGAGATAAATTAAAAAATTTATACTTTTTATATTATTTAACT [23400]
cp26_PAli_NX_MP_FG_consensus TTTACAATTTTCAATAAAAATTTAAGAAATAAAATCAGAGATAAATTAAAAAATTTATACTTTTTATATTATTTAACT [23400]
cp26_PAli_NX_Pl_consensus    TTTACAATTTTCAATAAAAATTTAAGAAATAAAATCAGAGATAAATTAAAAAATTTATACTTTTTATATTATTTAACT [23400]
cp26_PAbe_NX_MP_FG_consensus TTTACAATTTTCAATAAAAATTTAAGAAATAAAATCAGAGATAAATTAAAAAATTTATACTTTTTATATTATTTAACT [23400]
cp26_PAbe_NX_Pl_consensus    TTTACAATTTTCAATAAAAATTTAAGAAATAAAATCAGAGATAAATTAAAAAATTTATACTTTTTATATTATTTAACT [23400]

cp26_gi|11497007|B31_GB      TTTTTTACTCTTTTTATTATTAGTTCAAATTTATCTTATTACTTCACCGAAAAGCAATTATTAGAAGATTTTAATATT [23478]
cp26_un9_B31_PacBio          TTTTTTACTCTTTTTATTATTAGTTCAAATTTATCTTATTACTTCACCGAAAAGCAATTATTAGAAGATTTTAATATT [23478]
cp26_B31_TS_MP_FG_consensus  TTTTTTACTCTTTTTATTATTAGTTCAAATTTATCTTATTACTTCACCGAAAAGCAATTATTAGAAGATTTTAATATT [23478]
cp26_B31_NX_Pl_consensus     TTTTTTACTCTTTTTATTATTAGTTCAAATTTATCTTATTACTTCACCGAAAAGCAATTATTAGAAGATTTTAATATT [23478]
cp26_PAli_NX_MP_FG_consensus TTTTTTACTCTTTTTATTATTAGTTCAAATTTATCTTATTACTTCACCGAAAAGCAATTATTAGAAGATTTTAATATT [23478]
cp26_PAli_NX_Pl_consensus    TTTTTTACTCTTTTTATTATTAGTTCAAATTTATCTTATTACTTCACCGAAAAGCAATTATTAGAAGATTTTAATATT [23478]
```

```
cp26_PAbe_NX_MP_FG_consensus TTTTTTACTCTTTTTATTATTAGTTCAAATTTATCTTATTACTTCACCGAAAAGCAATTATTAGAAGATTTTAATATT [23478]
cp26_PAbe_NX_Pl_consensus    TTTTTTACTCTTTTTATTATTAGTTCAAATTTATCTTATTACTTCACCGAAAAGCAATTATTAGAAGATTTTAATATT [23478]

cp26_gi|11497007|B31_GB      TTCGAAAAAGAATTTTTCGAAATACATAAAATAAACGAACAATTTTTTCAAAAATACCTACTAAATTTTCCAGTACCA [23556]
cp26_un9_B31_PacBio          TTCGAAAAAGAATTTTTCGAAATACATAAAATAAACGAACAATTTTTTCAAAAATACCTACTAAATTTTCCAGTACCA [23556]
cp26_B31_TS_MP_FG_consensus  TTCGAAAAAGAATTTTTCGAAATACATAAAATAAACGAACAATTTTTTCAAAAATACCTACTAAATTTTCCAGTACCA [23556]
cp26_B31_NX_Pl_consensus     TTCGAAAAAGAATTTTTCGAAATACATAAAATAAACGAACAATTTTTTCAAAAATACCTACTAAATTTTCCAGTACCA [23556]
cp26_PAli_NX_MP_FG_consensus TTCGAAAAAGAATTTTTCGAAATACATAAAATAAACGAACAATTTTTTCAAAAATACCTACTAAATTTTCCAGTACCA [23556]
cp26_PAli_NX_Pl_consensus    TTCGAAAAAGAATTTTTCGAAATACATAAAATAAACGAACAATTTTTTCAAAAATACCTACTAAATTTTCCAGTACCA [23556]
cp26_PAbe_NX_MP_FG_consensus TTCGAAAAAGAATTTTTCGAAATACATAAAATAAACGAACAATTTTTTCAAAAATACCTACTAAATTTTCCAGTACCA [23556]
cp26_PAbe_NX_Pl_consensus    TTCGAAAAAGAATTTTTCGAAATACATAAAATAAACGAACAATTTTTTCAAAAATACCTACTAAATTTTCCAGTACCA [23556]

cp26_gi|11497007|B31_GB      ATAAGAATGGAATTGATGTCAAAATTTGATCCACTATATACAGTGTTTAATGCTAGTTTTGAGAAATATGCTAAAAAC [23634]
cp26_un9_B31_PacBio          ATAAGAATGGAATTGATGTCAAAATTTGATCCACTATATACAGTGTTTAATGCTAGTTTTGAGAAATATGCTAAAAAC [23634]
cp26_B31_TS_MP_FG_consensus  ATAAGAATGGAATTGATGTCAAAATTTGATCCACTATATACAGTGTTTAATGCTAGTTTTGAGAAATATGCTAAAAAC [23634]
cp26_B31_NX_Pl_consensus     ATAAGAATGGAATTGATGTCAAAATTTGATCCACTATATACAGTGTTTAATGCTAGTTTTGAGAAATATGCTAAAAAC [23634]
cp26_PAli_NX_MP_FG_consensus ATAAGAATGGAATTGATGTCAAAATTTGATCCACTATATACAGTGTTTAATGCTAGTTTTGAGAAATATGCTAAAAAC [23634]
cp26_PAli_NX_Pl_consensus    ATAAGAATGGAATTGATGTCAAAATTTGATCCACTATATACAGTGTTTAATGCTAGTTTTGAGAAATATGCTAAAAAC [23634]
cp26_PAbe_NX_MP_FG_consensus ATAAGAATGGAATTGATGTCAAAATTTGATCCACTATATACAGTGTTTAATGCTAGTTTTGAGAAATATGCTAAAAAC [23634]
cp26_PAbe_NX_Pl_consensus    ATAAGAATGGAATTGATGTCAAAATTTGATCCACTATATACAGTGTTTAATGCTAGTTTTGAGAAATATGCTAAAAAC [23634]

cp26_gi|11497007|B31_GB      ATTGGAAAAAGTTCTTATGAAATTCAAACAAATTATAAAAATTATATTAGGACAACAAATTCAGAAATTAAAGAAAAA [23712]
cp26_un9_B31_PacBio          ATTGGAAAAAGTTCTTATGAAATTCAAACAAATTATAAAAATTATATTAGGACAACAAATTCAGAAATTAAAGAAAAA [23712]
cp26_B31_TS_MP_FG_consensus  ATTGGAAAAAGTTCTTATGAAATTCAAACAAATTATAAAAATTATATTAGGACAACAAATTCAGAAATTAAAGAAAAA [23712]
cp26_B31_NX_Pl_consensus     ATTGGAAAAAGTTCTTATGAAATTCAAACAAATTATAAAAATTATATTAGGACAACAAATTCAGAAATTAAAGAAAAA [23712]
cp26_PAli_NX_MP_FG_consensus ATTGGAAAAAGTTCTTATGAAATTCAAACAAATTATAAAAATTATATTAGGACAACAAATTCAGAAATTAAAGAAAAA [23712]
cp26_PAli_NX_Pl_consensus    ATTGGAAAAAGTTCTTATGAAATTCAAACAAATTATAAAAATTATATTAGGACAACAAATTCAGAAATTAAAGAAAAA [23712]
cp26_PAbe_NX_MP_FG_consensus ATTGGAAAAAGTTCTTATGAAATTCAAACAAATTATAAAAATTATATTAGGACAACAAATTCAGAAATTAAAGAAAAA [23712]
cp26_PAbe_NX_Pl_consensus    ATTGGAAAAAGTTCTTATGAAATTCAAACAAATTATAAAAATTATATTAGGACAACAAATTCAGAAATTAAAGAAAAA [23712]

cp26_gi|11497007|B31_GB      CTAGAACAAATAAAAGAAAATATTACCCCTATTTATAATAAATATAAATTACCTATTCTAAATGGAGAAAATACAGAA [23790]
cp26_un9_B31_PacBio          CTAGAACAAATAAAAGAAAATATTACCCCTATTTATAATAAATATAAATTACCTATTCTAAATGGAGAAAATACAGAA [23790]
cp26_B31_TS_MP_FG_consensus  CTAGAACAAATAAAAGAAAATATTACCCCTATTTATAATAAATATAAATTACCTATTCTAAATGGAGAAAATACAGAA [23790]
cp26_B31_NX_Pl_consensus     CTAGAACAAATAAAAGAAAATATTACCCCTATTTATAATAAATATAAATTACCTATTCTAAATGGAGAAAATACAGAA [23790]
cp26_PAli_NX_MP_FG_consensus CTAGAACAAATAAAAGAAAATATTACCCCTATTTATAATAAATATAAATTACCTATTCTAAATGGAGAAAATACAGAA [23790]
cp26_PAli_NX_Pl_consensus    CTAGAACAAATAAAAGAAAATATTACCCCTATTTATAATAAATATAAATTACCTATTCTAAATGGAGAAAATACAGAA [23790]
cp26_PAbe_NX_MP_FG_consensus CTAGAACAAATAAAAGAAAATATTACCCCTATTTATAATAAATATAAATTACCTATTCTAAATGGAGAAAATACAGAA [23790]
cp26_PAbe_NX_Pl_consensus    CTAGAACAAATAAAAGAAAATATTACCCCTATTTATAATAAATATAAATTACCTATTCTAAATGGAGAAAATACAGAA [23790]

cp26_gi|11497007|B31_GB      ATAAGTATTGATGAGAATGGAAATATTATTCCTGTTATAAAAAATACAAACGGACAAATAACAGAATTACTGTTTTAC [23868]
cp26_un9_B31_PacBio          ATAAGTATTGATGAGAATGGAAATATTATTCCTGTTATAAAAAATACAAACGGACAAATAACAGAATTACTGTTTTAC [23868]
cp26_B31_TS_MP_FG_consensus  ATAAGTATTGATGAGAATGGAAATATTATTCCTGTTATAAAAAATACAAACGGACAAATAACAGAATTACTGTTTTAC [23868]
cp26_B31_NX_Pl_consensus     ATAAGTATTGATGAGAATGGAAATATTATTCCTGTTATAAAAAATACAAACGGACAAATAACAGAATTACTGTTTTAC [23868]
cp26_PAli_NX_MP_FG_consensus ATAAGTATTGATGAGAATGGAAATATTATTCCTGTTATAAAAAATACAAACGGACAAATAACAGAATTACTGTTTTAC [23868]
cp26_PAli_NX_Pl_consensus    ATAAGTATTGATGAGAATGGAAATATTATTCCTGTTATAAAAAATACAAACGGACAAATAACAGAATTACTGTTTTAC [23868]
cp26_PAbe_NX_MP_FG_consensus ATAAGTATTGATGAGAATGGAAATATTATTCCTGTTATAAAAAATACAAACGGACAAATAACAGAATTACTGTTTTAC [23868]
cp26_PAbe_NX_Pl_consensus    ATAAGTATTGATGAGAATGGAAATATTATTCCTGTTATAAAAAATACAAACGGACAAATAACAGAATTACTGTTTTAC [23868]

cp26_gi|11497007|B31_GB      GATCAAAATTATAATTTAATCCCTTTTAAAAAATTTGAAAGCTATAAAGTTAGATTTGATCTAATCCCAGAAAATAAA [23946]
cp26_un9_B31_PacBio          GATCAAAATTATAATTTAATCCCTTTTAAAAAATTTGAAAGCTATAAAGTTAGATTTGATCTAATCCCAGAAAATAAA [23946]
cp26_B31_TS_MP_FG_consensus  GATCAAAATTATAATTTAATCCCTTTTAAAAAATTTGAAAGCTATAAAGTTAGATTTGATCTAATCCCAGAAAATAAA [23946]
cp26_B31_NX_Pl_consensus     GATCAAAATTATAATTTAATCCCTTTTAAAAAATTTGAAAGCTATAAAGTTAGATTTGATCTAATCCCAGAAAATAAA [23946]
cp26_PAli_NX_MP_FG_consensus GATCAAAATTATAATTTAATCCCTTTTAAAAAATTTGAAAGCTATAAAGTTAGATTTGATCTAATCCCAGAAAATAAA [23946]
cp26_PAli_NX_Pl_consensus    GATCAAAATTATAATTTAATCCCTTTTAAAAAATTTGAAAGCTATAAAGTTAGATTTGATCTAATCCCAGAAAATAAA [23946]
cp26_PAbe_NX_MP_FG_consensus GATCAAAATTATAATTTAATCCCTTTTAAAAAATTTGAAAGCTATAAAGTTAGATTTGATCTAATCCCAGAAAATAAA [23946]
cp26_PAbe_NX_Pl_consensus    GATCAAAATTATAATTTAATCCCTTTTAAAAAATTTGAAAGCTATAAAGTTAGATTTGATCTAATCCCAGAAAATAAA [23946]

cp26_gi|11497007|B31_GB      AACATAAACTTCAAAGAACTAATAAACGTTTACTATCTTGATGAAAAAAATATTATCACTCCCATAGAATATTATAAA [24024]
cp26_un9_B31_PacBio          AACATAAACTTCAAAGAACTAATAAACGTTTACTATCTTGATGAAAAAAATATTATCACTCCCATAGAATATTATAAA [24024]
cp26_B31_TS_MP_FG_consensus  AACATAAACTTCAAAGAACTAATAAACGTTTACTATCTTGATGAAAAAAATATTATCACTCCCATAGAATATTATAAA [24024]
cp26_B31_NX_Pl_consensus     AACATAAACTTCAAAGAACTAATAAACGTTTACTATCTTGATGAAAAAAATATTATCACTCCCATAGAATATTATAAA [24024]
cp26_PAli_NX_MP_FG_consensus AACATAAACTTCAAAGAACTAATAAACGTTTACTATCTTGATGAAAAAAATATTATCACTCCCATAGAATATTATAAA [24024]
cp26_PAli_NX_Pl_consensus    AACATAAACTTCAAAGAACTAATAAACGTTTACTATCTTGATGAAAAAAATATTATCACTCCCATAGAATATTATAAA [24024]
cp26_PAbe_NX_MP_FG_consensus AACATAAACTTCAAAGAACTAATAAACGTTTACTATCTTGATGAAAAAAATATTATCACTCCCATAGAATATTATAAA [24024]
cp26_PAbe_NX_Pl_consensus    AACATAAACTTCAAAGAACTAATAAACGTTTACTATCTTGATGAAAAAAATATTATCACTCCCATAGAATATTATAAA [24024]

cp26_gi|11497007|B31_GB      AATAATATAGACATGAGCCCTTATTACATAGACTTGCAAGAAAATAAAGATGATTTTCTTAAGGCAATAAAAATCAAA [24102]
cp26_un9_B31_PacBio          AATAATATAGACATGAGCCCTTATTACATAGACTTGCAAGAAAATAAAGATGATTTTCTTAAGGCAATAAAAATCAAA [24102]
cp26_B31_TS_MP_FG_consensus  AATAATATAGACATGAGCCCTTATTACATAGACTTGCAAGAAAATAAAGATGATTTTCTTAAGGCAATAAAAATCAAA [24102]
cp26_B31_NX_Pl_consensus     AATAATATAGACATGAGCCCTTATTACATAGACTTGCAAGAAAATAAAGATGATTTTCTTAAGGCAATAAAAATCAAA [24102]
cp26_PAli_NX_MP_FG_consensus AATAATATAGACATGAGCCCTTATTACATAGACTTGCAAGAAAATAAAGATGATTTTCTTAAGGCAATAAAAATCAAA [24102]
cp26_PAli_NX_Pl_consensus    AATAATATAGACATGAGCCCTTATTACATAGACTTGCAAGAAAATAAAGATGATTTTCTTAAGGCAATAAAAATCAAA [24102]
cp26_PAbe_NX_MP_FG_consensus AATAATATAGACATGAGCCCTTATTACATAGACTTGCAAGAAAATAAAGATGATTTTCTTAAGGCAATAAAAATCAAA [24102]
cp26_PAbe_NX_Pl_consensus    AATAATATAGACATGAGCCCTTATTACATAGACTTGCAAGAAAATAAAGATGATTTTCTTAAGGCAATAAAAATCAAA [24102]

cp26_gi|11497007|B31_GB      AAAGAATATGGTTTATACATTGAGAAAAAAAAAGCAACTACAAAATTTAACTGAAAATGATAAACTTGATGATTTTAAA [24180]
cp26_un9_B31_PacBio          AAAGAATATGGTTTATACATTGAGAAAAAAAAAGCAACTACAAAATTTAACTGAAAATGATAAACTTGATGATTTTAAA [24180]
cp26_B31_TS_MP_FG_consensus  AAAGAATATGGTTTATACATTGAGAAAAAAAAAGCAACTACAAAATTTAACTGAAAATGATAAACTTGATGATTTTAAA [24180]
cp26_B31_NX_Pl_consensus     AAAGAATATGGTTTATACATTGAGAAAAAAAAAGCAACTACAAAATTTAACTGAAAATGATAAACTTGATGATTTTAAA [24180]
cp26_PAli_NX_MP_FG_consensus AAAGAATATGGTTTATACATTGAGAAAAAAAAAGCAACTACAAAATTTAACTGAAAATGATAAACTTGATGATTTTAAA [24180]
cp26_PAli_NX_Pl_consensus    AAAGAATATGGTTTATACATTGAGAAAAAAAAAGCAACTACAAAATTTAACTGAAAATGATAAACTTGATGATTTTAAA [24180]
cp26_PAbe_NX_MP_FG_consensus AAAGAATATGGTTTATACATTGAGAAAAAAAAAGCAACTACAAAATTTAACTGAAAATGATAAACTTGATGATTTTAAA [24180]
cp26_PAbe_NX_Pl_consensus    AAAGAATATGGTTTATACATTGAGAAAAAAAAAGCAACTACAAAATTTAACTGAAAATGATAAACTTGATGATTTTAAA [24180]

cp26_gi|11497007|B31_GB      GAATTTTTATTAAAAAATAATAATATTTTTTCACTAAATACAATATTTTCCAACGGCAATCCAATATTTACTTATGCC [24258]
cp26_un9_B31_PacBio          GAATTTTTATTAAAAAATAATAATATTTTTTCACTAAATACAATATTTTCCAACGGCAATCCAATATTTACTTATGCC [24258]
cp26_B31_TS_MP_FG_consensus  GAATTTTTATTAAAAAATAATAATATTTTTTCACTAAATACAATATTTTCCAACGGCAATCCAATATTTACTTATGCC [24258]
cp26_B31_NX_Pl_consensus     GAATTTTTATTAAAAAATAATAATATTTTTTCACTAAATACAATATTTTCCAACGGCAATCCAATATTTACTTATGCC [24258]
cp26_PAli_NX_MP_FG_consensus GAATTTTTATTAAAAAATAATAATATTTTTTCACTAAATACAATATTTTCCAACGGCAATCCAATATTTACTTATGCC [24258]
cp26_PAli_NX_Pl_consensus    GAATTTTTATTAAAAAATAATAATATTTTTTCACTAAATACAATATTTTCCAACGGCAATCCAATATTTACTTATGCC [24258]
cp26_PAbe_NX_MP_FG_consensus GAATTTTTATTAAAAAATAATAATATTTTTTCACTAAATACAATATTTTCCAACGGCAATCCAATATTTACTTATGCC [24258]
cp26_PAbe_NX_Pl_consensus    GAATTTTTATTAAAAAATAATAATATTTTTTCACTAAATACAATATTTTCCAACGGCAATCCAATATTTACTTATGCC [24258]

cp26_gi|11497007|B31_GB      ATAAATGTAAAAGCAAAAAGTATTATAAATTATTTAATAACAAAAGAATTTAATATTAATTTAACAAATCAAAATTCT [24336]
cp26_un9_B31_PacBio          ATAAATGTAAAAGCAAAAAGTATTATAAATTATTTAATAACAAAAGAATTTAATATTAATTTAACAAATCAAAATTCT [24336]
cp26_B31_TS_MP_FG_consensus  ATAAATGTAAAAGCAAAAAGTATTATAAATTATTTAATAACAAAAGAATTTAATATTAATTTAACAAATCAAAATTCT [24336]
cp26_B31_NX_Pl_consensus     ATAAATGTAAAAGCAAAAAGTATTATAAATTATTTAATAACAAAAGAATTTAATATTAATTTAACAAATCAAAATTCT [24336]
cp26_PAli_NX_MP_FG_consensus ATAAATGTAAAAGCAAAAAGTATTATAAATTATTTAATAACAAAAGAATTTAATATTAATTTAACAAATCAAAATTCT [24336]
cp26_PAli_NX_Pl_consensus    ATAAATGTAAAAGCAAAAAGTATTATAAATTATTTAATAACAAAAGAATTTAATATTAATTTAACAAATCAAAATTCT [24336]
cp26_PAbe_NX_MP_FG_consensus ATAAATGTAAAAGCAAAAAGTATTATAAATTATTTAATAACAAAAGAATTTAATATTAATTTAACAAATCAAAATTCT [24336]
cp26_PAbe_NX_Pl_consensus    ATAAATGTAAAAGCAAAAAGTATTATAAATTATTTAATAACAAAAGAATTTAATATTAATTTAACAAATCAAAATTCT [24336]

cp26_gi|11497007|B31_GB      CAAACAGCTCTTCATAGTGCCATAATTCAAAAATATGATTTAAATTTTATTAAATCACTTATCGAAAAAGGCGCTAAT [24414]
cp26_un9_B31_PacBio          CAAACAGCTCTTCATAGTGCCATAATTCAAAAATATGATTTAAATTTTATTAAATCACTTATCGAAAAAGGCGCTAAT [24414]
cp26_B31_TS_MP_FG_consensus  CAAACAGCTCTTCATAGTGCCATAATTCAAAAATATGATTTAAATTTTATTAAATCACTTATCGAAAAAGGCGCTAAT [24414]
cp26_B31_NX_Pl_consensus     CAAACAGCTCTTCATAGTGCCATAATTCAAAAATATGATTTAAATTTTATTAAATCACTTATCGAAAAAGGCGCTAAT [24414]
cp26_PAli_NX_MP_FG_consensus CAAACAGCTCTTCATAGTGCCATAATTCAAAAATATGATTTAAATTTTATTAAATCACTTATCGAAAAAGGCGCTAAT [24414]
cp26_PAli_NX_Pl_consensus    CAAACAGCTCTTCATAGTGCCATAATTCAAAAATATGATTTAAATTTTATTAAATCACTTATCGAAAAAGGCGCTAAT [24414]
cp26_PAbe_NX_MP_FG_consensus CAAACAGCTCTTCATAGTGCCATAATTCAAAAATATGATTTAAATTTTATTAAATCACTTATCGAAAAAGGCGCTAAT [24414]
```

```
cp26_PAbe_NX_Pl_consensus    CAAACAGCTCTTCATAGTGCCATAATTCAAAAATATGATTTAAATTTTATTAAATCACTTATCGAAAAAGGCGCTAAT [24414]

cp26_gi|11497007|B31_GB      CCAAATATCAGAGATGGCGACAATAAACTGCCCATAGATTACTCTGACAAAACTAGTGAAATCTATAAATATTTAATC [24492]
cp26_un9_B31_PacBio          CCAAATATCAGAGATGGCGACAATAAACTGCCCATAGATTACTCTGACAAAACTAGTGAAATCTATAAATATTTAATC [24492]
cp26_B31_TS_MP_FG_consensus  CCAAATATCAGAGATGGCGACAATAAACTGCCCATAGATTACTCTGACAAAACTAGTGAAATCTATAAATATTTAATC [24492]
cp26_B31_NX_Pl_consensus     CCAAATATCAGAGATGGCGACAATAAACTGCCCATAGATTACTCTGACAAAACTAGTGAAATCTATAAATATTTAATC [24492]
cp26_PAli_NX_MP_FG_consensus CCAAATATCAGAGATGGCGACAATAAACTGCCCATAGATTACTCTGACAAAACTAGTGAAATCTATAAATATTTAATC [24492]
cp26_PAli_NX_Pl_consensus    CCAAATATCAGAGATGGCGACAATAAACTGCCCATAGATTACTCTGACAAAACTAGTGAAATCTATAAATATTTAATC [24492]
cp26_PAbe_NX_MP_FG_consensus CCAAATATCAGAGATGGCGACAATAAACTGCCCATAGATTACTCTGACAAAACTAGTGAAATCTATAAATATTTAATC [24492]
cp26_PAbe_NX_Pl_consensus    CCAAATATCAGAGATGGCGACAATAAACTGCCCATAGATTACTCTGACAAAACTAGTGAAATCTATAAATATTTAATC [24492]

cp26_gi|11497007|B31_GB      GGTATTTAACTTTACAATGCTAATTGATGATTAATTCCATTGCAAACACCCTAATCCAAATTGATCCGATTTATTTCT [24570]
cp26_un9_B31_PacBio          GGTATTTAACTTTACAATGCTAATTGATGATTAATTCCATTGCAAACACCCTAATCCAAATTGATCCGATTTATTTCT [24570]
cp26_B31_TS_MP_FG_consensus  GGTATTTAACTTTACAATGCTAATTGATGATTAATTCCATTGCAAACACCCTAATCCAAATTGATCCGATTTATTTCT [24570]
cp26_B31_NX_Pl_consensus     GGTATTTAACTTTACAATGCTAATTGATGATTAATTCCATTGCAAACACCCTAATCCAAATTGATCCGATTTATTTCT [24570]
cp26_PAli_NX_MP_FG_consensus GGTATTTAACTTTACAATGCTAATTGATGATTAATTCCATTGCAAACACCCTAATCCAAATTGATCCGATTTATTTCT [24570]
cp26_PAli_NX_Pl_consensus    GGTATTTAACTTTACAATGCTAATTGATGATTAATTCCATTGCAAACACCCTAATCCAAATTGATCCGATTTATTTCT [24570]
cp26_PAbe_NX_MP_FG_consensus GGTATTTAACTTTACAATGCTAATTGATGATTAATTCCATTGCAAACACCCTAATCCAAATTGATCCGATTTATTTCT [24570]
cp26_PAbe_NX_Pl_consensus    GGTATTTAACTTTACAATGCTAATTGATGATTAATTCCATTGCAAACACCCTAATCCAAATTGATCCGATTTATTTCT [24570]

cp26_gi|11497007|B31_GB      AAATTTAATTTAAAATAATTTCAATGAAAACGAAGATTATAAGCAATGCTTTATAACAAATGCCATGAAATTAAAGGG [24648]
cp26_un9_B31_PacBio          AAATTTAATTTAAAATAATTTCAATGAAAACGAAGATTATAAGCAATGCTTTATAACAAATGCCATGAAATTAAAGGG [24648]
cp26_B31_TS_MP_FG_consensus  AAATTTAATTTAAAATAATTTCAATGAAAACGAAGATTATAAGCAATGCTTTATAACAAATGCCATGAAATTAAAGGG [24648]
cp26_B31_NX_Pl_consensus     AAATTTAATTTAAAATAATTTCAATGAAAACGAAGATTATAAGCAATGCTTTATAACAAATGCCATGAAATTAAAGGG [24648]
cp26_PAli_NX_MP_FG_consensus AAATTTAATTTAAAATAATTTCAATGAAAACGAAGATTATAAGCAATGCTTTATAACAAATGCCATGAAATTAAAGGG [24648]
cp26_PAli_NX_Pl_consensus    AAATTTAATTTAAAATAATTTCAATGAAAACGAAGATTATAAGCAATGCTTTATAACAAATGCCATGAAATTAAAGGG [24648]
cp26_PAbe_NX_MP_FG_consensus AAATTTAATTTAAAATAATTTCAATGAAAACGAAGATTATAAGCAATGCTTTATAACAAATGCCATGAAATTAAAGGG [24648]
cp26_PAbe_NX_Pl_consensus    AAATTTAATTTAAAATAATTTCAATGAAAACGAAGATTATAAGCAATGCTTTATAACAAATGCCATGAAATTAAAGGG [24648]

cp26_gi|11497007|B31_GB      AAAAACAATCTTTAATGTTTTTTATAAATACAAAATTTTATAAACAGCAATTCAATATACTATCATTTATTCTATACT [24726]
cp26_un9_B31_PacBio          AAAAACAATCTTTAATGTTTTTTATAAATACAAAATTTTATAAACAGCAATTCAATATACTATCATTTATTCTATACT [24726]
cp26_B31_TS_MP_FG_consensus  AAAAACAATCTTTAATGTTTTTTATAAATACAAAATTTTATAAACAGCAATTCAATATACTATCATTTATTCTATACT [24726]
cp26_B31_NX_Pl_consensus     AAAAACAATCTTTAATGTTTTTTATAAATACAAAATTTTATAAACAGCAATTCAATATACTATCATTTATTCTATACT [24726]
cp26_PAli_NX_MP_FG_consensus AAAAACAATCTTTAATGTTTTTTATAAATACAAAATTTTATAAACAGCAATTCAATATACTATCATTTATTCTATACT [24726]
cp26_PAli_NX_Pl_consensus    AAAAACAATCTTTAATGTTTTTTATAAATACAAAATTTTATAAACAGCAATTCAATATACTATCATTTATTCTATACT [24726]
cp26_PAbe_NX_MP_FG_consensus AAAAACAATCTTTAATGTTTTTTATAAATACAAAATTTTATAAACAGCAATTCAATATACTATCATTTATTCTATACT [24726]
cp26_PAbe_NX_Pl_consensus    AAAAACAATCTTTAATGTTTTTTATAAATACAAAATTTTATAAACAGCAATTCAATATACTATCATTTATTCTATACT [24726]

cp26_gi|11497007|B31_GB      GATATTTACAAGATTAAAAAATCTTGTTAAGATATTAAGCGTATTATTCAAATTAATTATTTTAATTAATTAATTTGC [24804]
cp26_un9_B31_PacBio          GATATTTACAAGATTAAAAAATCTTGTTAAGATATTAAGCGTATTATTCAAATTAATTATTTTAATTAATTAATTTGC [24804]
cp26_B31_TS_MP_FG_consensus  GATATTTACAAGATTAAAAAATCTTGTTAAGATATTAAGCGTATTATTCAAATTAATTATTTTAATTAATTAATTTGC [24804]
cp26_B31_NX_Pl_consensus     GATATTTACAAGATTAAAAAATCTTGTTAAGATATTAAGCGTATTATTCAAATTAATTATTTTAATTAATTAATTTGC [24804]
cp26_PAli_NX_MP_FG_consensus GATATTTACAAGATTAAAAAATCTTGTTAAGATATTAAGCGTATTATTCAAATTAATTATTTTAATTAATTAATTTGC [24804]
cp26_PAli_NX_Pl_consensus    GATATTTACAAGATTAAAAAATCTTGTTAAGATATTAAGCGTATTATTCAAATTAATTATTTTAATTAATTAATTTGC [24804]
cp26_PAbe_NX_MP_FG_consensus GATATTTACAAGATTAAAAAATCTTGTTAAGATATTAAGCGTATTATTCAAATTAATTATTTTAATTAATTAATTTGC [24804]
cp26_PAbe_NX_Pl_consensus    GATATTTACAAGATTAAAAAATCTTGTTAAGATATTAAGCGTATTATTCAAATTAATTATTTTAATTAATTAATTTGC [24804]

cp26_gi|11497007|B31_GB      ACTTAATAAAAAGGGGAAAATTATGTCAACATCATCAGCATCTATATCTATATTTACAATATTACAAAAAATAGGAAA [24882]
cp26_un9_B31_PacBio          ACTTAATAAAAAGGGGAAAATTATGTCAACATCATCAGCATCTATATCTATATTTACAATATTACAAAAAATAGGAAA [24882]
cp26_B31_TS_MP_FG_consensus  ACTTAATAAAAAGGGGAAAATTATGTCAACATCATCAGCATCTATATCTATATTTACAATATTACAAAAAATAGGAAA [24882]
cp26_B31_NX_Pl_consensus     ACTTAATAAAAAGGGGAAAATTATGTCAACATCATCAGCATCTATATCTATATTTACAATATTACAAAAAATAGGAAA [24882]
cp26_PAli_NX_MP_FG_consensus ACTTAATAAAAAGGGGAAAATTATGTCAACATCATCAGCATCTATATCTATATTTACAATATTACAAAAAATAGGAAA [24882]
cp26_PAli_NX_Pl_consensus    ACTTAATAAAAAGGGGAAAATTATGTCAACATCATCAGCATCTATATCTATATTTACAATATTACAAAAAATAGGAAA [24882]
cp26_PAbe_NX_MP_FG_consensus ACTTAATAAAAAGGGGAAAATTATGTCAACATCATCAGCATCTATATCTATATTTACAATATTACAAAAAATAGGAAA [24882]
cp26_PAbe_NX_Pl_consensus    ACTTAATAAAAAGGGGAAAATTATGTCAACATCATCAGCATCTATATCTATATTTACAATATTACAAAAAATAGGAAA [24882]

cp26_gi|11497007|B31_GB      AGCCTTCATGCTTCCAATAGCACTTTTACCAGCAGCTGGAATTTTATTGGGAATTGGGGGAGCATTTACTAATGAAAC [24960]
cp26_un9_B31_PacBio          AGCCTTCATGCTTCCAATAGCACTTTTACCAGCAGCTGGAATTTTATTGGGAATTGGGGGAGCATTTACTAATGAAAC [24960]
cp26_B31_TS_MP_FG_consensus  AGCCTTCATGCTTCCAATAGCACTTTTACCAGCAGCTGGAATTTTATTGGGAATTGGGGGAGCATTTACTAATGAAAC [24960]
cp26_B31_NX_Pl_consensus     AGCCTTCATGCTTCCAATAGCACTTTTACCAGCAGCTGGAATTTTATTGGGAATTGGGGGAGCATTTACTAATGAAAC [24960]
cp26_PAli_NX_MP_FG_consensus AGCCTTCATGCTTCCAATAGCACTTTTACCAGCAGCTGGAATTTTATTGGGAATTGGGGGAGCATTTACTAATGAAAC [24960]
cp26_PAli_NX_Pl_consensus    AGCCTTCATGCTTCCAATAGCACTTTTACCAGCAGCTGGAATTTTATTGGGAATTGGGGGAGCATTTACTAATGAAAC [24960]
cp26_PAbe_NX_MP_FG_consensus AGCCTTCATGCTTCCAATAGCACTTTTACCAGCAGCTGGAATTTTATTGGGAATTGGGGGAGCATTTACTAATGAAAC [24960]
cp26_PAbe_NX_Pl_consensus    AGCCTTCATGCTTCCAATAGCACTTTTACCAGCAGCTGGAATTTTATTGGGAATTGGGGGAGCATTTACTAATGAAAC [24960]

cp26_gi|11497007|B31_GB      AATGATTCAGGCTTATGGATTAGAAGAAGTCCTTGGAAAAGGAACTGTTGCTAACTCAATACTTTATCTTATGAAATA [25038]
cp26_un9_B31_PacBio          AATGATTCAGGCTTATGGATTAGAAGAAGTCCTTGGAAAAGGAACTGTTGCTAACTCAATACTTTATCTTATGAAATA [25038]
cp26_B31_TS_MP_FG_consensus  AATGATTCAGGCTTATGGATTAGAAGAAGTCCTTGGAAAAGGAACTGTTGCTAACTCAATACTTTATCTTATGAAATA [25038]
cp26_B31_NX_Pl_consensus     AATGATTCAGGCTTATGGATTAGAAGAAGTCCTTGGAAAAGGAACTGTTGCTAACTCAATACTTTATCTTATGAAATA [25038]
cp26_PAli_NX_MP_FG_consensus AATGATTCAGGCTTATGGATTAGAAGAAGTCCTTGGAAAAGGAACTGTTGCTAACTCAATACTTTATCTTATGAAATA [25038]
cp26_PAli_NX_Pl_consensus    AATGATTCAGGCTTATGGATTAGAAGAAGTCCTTGGAAAAGGAACTGTTGCTAACTCAATACTTTATCTTATGAAATA [25038]
cp26_PAbe_NX_MP_FG_consensus AATGATTCAGGCTTATGGATTAGAAGAAGTCCTTGGAAAAGGAACTGTTGCTAACTCAATACTTTATCTTATGAAATA [25038]
cp26_PAbe_NX_Pl_consensus    AATGATTCAGGCTTATGGATTAGAAGAAGTCCTTGGAAAAGGAACTGTTGCTAACTCAATACTTTATCTTATGAAATA [25038]

cp26_gi|11497007|B31_GB      TACAGGGGAAGTAATTTTTGCTAATCTGCCCTTAATGTTTGCAGCGGCAATTCCAATCGGACTTGCTAAAGTAGAAAA [25116]
cp26_un9_B31_PacBio          TACAGGGGAAGTAATTTTTGCTAATCTGCCCTTAATGTTTGCAGCGGCAATTCCAATCGGACTTGCTAAAGTAGAAAA [25116]
cp26_B31_TS_MP_FG_consensus  TACAGGGGAAGTAATTTTTGCTAATCTGCCCTTAATGTTTGCAGCGGCAATTCCAATCGGACTTGCTAAAGTAGAAAA [25116]
cp26_B31_NX_Pl_consensus     TACAGGGGAAGTAATTTTTGCTAATCTGCCCTTAATGTTTGCAGCGGCAATTCCAATCGGACTTGCTAAAGTAGAAAA [25116]
cp26_PAli_NX_MP_FG_consensus TACAGGGGAAGTAATTTTTGCTAATCTGCCCTTAATGTTTGCAGCGGCAATTCCAATCGGACTTGCTAAAGTAGAAAA [25116]
cp26_PAli_NX_Pl_consensus    TACAGGGGAAGTAATTTTTGCTAATCTGCCCTTAATGTTTGCAGCGGCAATTCCAATCGGACTTGCTAAAGTAGAAAA [25116]
cp26_PAbe_NX_MP_FG_consensus TACAGGGGAAGTAATTTTTGCTAATCTGCCCTTAATGTTTGCAGCGGCAATTCCAATCGGACTTGCTAAAGTAGAAAA [25116]
cp26_PAbe_NX_Pl_consensus    TACAGGGGAAGTAATTTTTGCTAATCTGCCCTTAATGTTTGCAGCGGCAATTCCAATCGGACTTGCTAAAGTAGAAAA [25116]

cp26_gi|11497007|B31_GB      AGGAACAGCTGCTTTAGCAGGAGTAGTGGGATTTTTGGTTATGCATCAAACTATAAATGGGATTTTGTACATACAAGG [25194]
cp26_un9_B31_PacBio          AGGAACAGCTGCTTTAGCAGGAGTAGTGGGATTTTTGGTTATGCATCAAACTATAAATGGGATTTTGTACATACAAGG [25194]
cp26_B31_TS_MP_FG_consensus  AGGAACAGCTGCTTTAGCAGGAGTAGTGGGATTTTTGGTTATGCATCAAACTATAAATGGGATTTTGTACATACAAGG [25194]
cp26_B31_NX_Pl_consensus     AGGAACAGCTGCTTTAGCAGGAGTAGTGGGATTTTTGGTTATGCATCAAACTATAAATGGGATTTTGTACATACAAGG [25194]
cp26_PAli_NX_MP_FG_consensus AGGAACAGCTGCTTTAGCAGGAGTAGTGGGATTTTTGGTTATGCATCAAACTATAAATGGGATTTTGTACATACAAGG [25194]
cp26_PAli_NX_Pl_consensus    AGGAACAGCTGCTTTAGCAGGAGTAGTGGGATTTTTGGTTATGCATCAAACTATAAATGGGATTTTGTACATACAAGG [25194]
cp26_PAbe_NX_MP_FG_consensus AGGAACAGCTGCTTTAGCAGGAGTAGTGGGATTTTTGGTTATGCATCAAACTATAAATGGGATTTTGTACATACAAGG [25194]
cp26_PAbe_NX_Pl_consensus    AGGAACAGCTGCTTTAGCAGGAGTAGTGGGATTTTTGGTTATGCATCAAACTATAAATGGGATTTTGTACATACAAGG [25194]

cp26_gi|11497007|B31_GB      AATCACACCAGAAAGCGCAAGCTTAAAAGCACTATTGGAATTAGGAATGCCTGAAACAGCTGCAACTGCAAAAAGCCA [25272]
cp26_un9_B31_PacBio          AATCACACCAGAAAGCGCAAGCTTAAAAGCACTATTGGAATTAGGAATGCCTGAAACAGCTGCAACTGCAAAAAGCCA [25272]
cp26_B31_TS_MP_FG_consensus  AATCACACCAGAAAGCGCAAGCTTAAAAGCACTATTGGAATTAGGAATGCCTGAAACAGCTGCAACTGCAAAAAGCCA [25272]
cp26_B31_NX_Pl_consensus     AATCACACCAGAAAGCGCAAGCTTAAAAGCACTATTGGAATTAGGAATGCCTGAAACAGCTGCAACTGCAAAAAGCCA [25272]
cp26_PAli_NX_MP_FG_consensus AATCACACCAGAAAGCGCAAGCTTAAAAGCACTATTGGAATTAGGAATGCCTGAAACAGCTGCAACTGCAAAAAGCCA [25272]
cp26_PAli_NX_Pl_consensus    AATCACACCAGAAAGCGCAAGCTTAAAAGCACTATTGGAATTAGGAATGCCTGAAACAGCTGCAACTGCAAAAAGCCA [25272]
cp26_PAbe_NX_MP_FG_consensus AATCACACCAGAAAGCGCAAGCTTAAAAGCACTATTGGAATTAGGAATGCCTGAAACAGCTGCAACTGCAAAAAGCCA [25272]
cp26_PAbe_NX_Pl_consensus    AATCACACCAGAAAGCGCAAGCTTAAAAGCACTATTGGAATTAGGAATGCCTGAAACAGCTGCAACTGCAAAAAGCCA [25272]

cp26_gi|11497007|B31_GB      GGAATATACAAGTGTACTTGGAATATTCTCTCTTCAAATGAGCGTAATGGGAGGATTGATAGCAGGATTTGTTGCTGT [25350]
cp26_un9_B31_PacBio          GGAATATACAAGTGTACTTGGAATATTCTCTCTTCAAATGAGCGTAATGGGAGGATTGATAGCAGGATTTGTTGCTGT [25350]
cp26_B31_TS_MP_FG_consensus  GGAATATACAAGTGTACTTGGAATATTCTCTCTTCAAATGAGCGTAATGGGAGGATTGATAGCAGGATTTGTTGCTGT [25350]
cp26_B31_NX_Pl_consensus     GGAATATACAAGTGTACTTGGAATATTCTCTCTTCAAATGAGCGTAATGGGAGGATTGATAGCAGGATTTGTTGCTGT [25350]
cp26_PAli_NX_MP_FG_consensus GGAATATACAAGTGTACTTGGAATATTCTCTCTTCAAATGAGCGTAATGGGAGGATTGATAGCAGGATTTGTTGCTGT [25350]
cp26_PAli_NX_Pl_consensus    GGAATATACAAGTGTACTTGGAATATTCTCTCTTCAAATGAGCGTAATGGGAGGATTGATAGCAGGATTTGTTGCTGT [25350]
cp26_PAbe_NX_MP_FG_consensus GGAATATACAAGTGTACTTGGAATATTCTCTCTTCAAATGAGCGTAATGGGAGGATTGATAGCAGGATTTGTTGCTGT [25350]
cp26_PAbe_NX_Pl_consensus    GGAATATACAAGTGTACTTGGAATATTCTCTCTTCAAATGAGCGTAATGGGAGGATTGATAGCAGGATTTGTTGCTGT [25350]
```

```
cp26_gi|11497007|B31_GB      CTTTCTTCACAACAGATTCCATAATATTCAATTACCCACATTCTTAGCATTTTTTGGGGGCACAAGATTTGTGCCAAT [25428]
cp26_un9_B31_PacBio          CTTTCTTCACAACAGATTCCATAATATTCAATTACCCACATTCTTAGCATTTTTTGGGGGCACAAGATTTGTGCCAAT [25428]
cp26_B31_TS_MP_FG_consensus  CTTTCTTCACAACAGATTCCATAATATTCAATTACCCACATTCTTAGCATTTTTTGGGGGCACAAGATTTGTGCCAAT [25428]
cp26_B31_NX_Pl_consensus     CTTTCTTCACAACAGATTCCATAATATTCAATTACCCACATTCTTAGCATTTTTTGGGGGCACAAGATTTGTGCCAAT [25428]
cp26_PAli_NX_MP_FG_consensus CTTTCTTCACAACAGATTCCATAATATTCAATTACCCACATTCTTAGCATTTTTTGGGGGCACAAGATTTGTGCCAAT [25428]
cp26_PAli_NX_Pl_consensus    CTTTCTTCACAACAGATTCCATAATATTCAATTACCCACATTCTTAGCATTTTTTGGGGGCACAAGATTTGTGCCAAT [25428]
cp26_PAbe_NX_MP_FG_consensus CTTTCTTCACAACAGATTCCATAATATTCAATTACCCACATTCTTAGCATTTTTTGGGGGCACAAGATTTGTGCCAAT [25428]
cp26_PAbe_NX_Pl_consensus    CTTTCTTCACAACAGATTCCATAATATTCAATTACCCACATTCTTAGCATTTTTTGGGGGCACAAGATTTGTGCCAAT [25428]

cp26_gi|11497007|B31_GB      TGTAACTACAATGGCAATGTTTGTGGTAGGAATATTTTTAACATTTATTTGGCCTTTTATCCAAGGTGCAATGACTTC [25506]
cp26_un9_B31_PacBio          TGTAACTACAATGGCAATGTTTGTGGTAGGAATATTTTTAACATTTATTTGGCCTTTTATCCAAGGTGCAATGACTTC [25506]
cp26_B31_TS_MP_FG_consensus  TGTAACTACAATGGCAATGTTTGTGGTAGGAATATTTTTAACATTTATTTGGCCTTTTATCCAAGGTGCAATGACTTC [25506]
cp26_B31_NX_Pl_consensus     TGTAACTACAATGGCAATGTTTGTGGTAGGAATATTTTTAACATTTATTTGGCCTTTTATCCAAGGTGCAATGACTTC [25506]
cp26_PAli_NX_MP_FG_consensus TGTAACTACAATGGCAATGTTTGTGGTAGGAATATTTTTAACATTTATTTGGCCTTTTATCCAAGGTGCAATGACTTC [25506]
cp26_PAli_NX_Pl_consensus    TGTAACTACAATGGCAATGTTTGTGGTAGGAATATTTTTAACATTTATTTGGCCTTTTATCCAAGGTGCAATGACTTC [25506]
cp26_PAbe_NX_MP_FG_consensus TGTAACTACAATGGCAATGTTTGTGGTAGGAATATTTTTAACATTTATTTGGCCTTTTATCCAAGGTGCAATGACTTC [25506]
cp26_PAbe_NX_Pl_consensus    TGTAACTACAATGGCAATGTTTGTGGTAGGAATATTTTTAACATTTATTTGGCCTTTTATCCAAGGTGCAATGACTTC [25506]

cp26_gi|11497007|B31_GB      GTTTGGAAGAATTGTAGAACAATCAGGACTTTTTGGAACATTTGCATATGGAGCAATAAAAAGATCTTTAATTCCATT [25584]
cp26_un9_B31_PacBio          GTTTGGAAGAATTGTAGAACAATCAGGACTTTTTGGAACATTTGCATATGGAGCAATAAAAAGATCTTTAATTCCATT [25584]
cp26_B31_TS_MP_FG_consensus  GTTTGGAAGAATTGTAGAACAATCAGGACTTTTTGGAACATTTGCATATGGAGCAATAAAAAGATCTTTAATTCCATT [25584]
cp26_B31_NX_Pl_consensus     GTTTGGAAGAATTGTAGAACAATCAGGACTTTTTGGAACATTTGCATATGGAGCAATAAAAAGATCTTTAATTCCATT [25584]
cp26_PAli_NX_MP_FG_consensus GTTTGGAAGAATTGTAGAACAATCAGGACTTTTTGGAACATTTGCATATGGAGCAATAAAAAGATCTTTAATTCCATT [25584]
cp26_PAli_NX_Pl_consensus    GTTTGGAAGAATTGTAGAACAATCAGGACTTTTTGGAACATTTGCATATGGAGCAATAAAAAGATCTTTAATTCCATT [25584]
cp26_PAbe_NX_MP_FG_consensus GTTTGGAAGAATTGTAGAACAATCAGGACTTTTTGGAACATTTGCATATGGAGCAATAAAAAGATCTTTAATTCCATT [25584]
cp26_PAbe_NX_Pl_consensus    GTTTGGAAGAATTGTAGAACAATCAGGACTTTTTGGAACATTTGCATATGGAGCAATAAAAAGATCTTTAATTCCATT [25584]

cp26_gi|11497007|B31_GB      TGGACTCCACCACATATTTTATTTGCCATTTTGGCAAACAGCTGTTGGGGGAACATTGGAAATAAATAACGAATTAAT [25662]
cp26_un9_B31_PacBio          TGGACTCCACCACATATTTTATTTGCCATTTTGGCAAACAGCTGTTGGGGGAACATTGGAAATAAATAACGAATTAAT [25662]
cp26_B31_TS_MP_FG_consensus  TGGACTCCACCACATATTTTATTTGCCATTTTGGCAAACAGCTGTTGGGGGAACATTGGAAATAAATAACGAATTAAT [25662]
cp26_B31_NX_Pl_consensus     TGGACTCCACCACATATTTTATTTGCCATTTTGGCAAACAGCTGTTGGGGGAACATTGGAAATAAATAACGAATTAAT [25662]
cp26_PAli_NX_MP_FG_consensus TGGACTCCACCACATATTTTATTTGCCATTTTGGCAAACAGCTGTTGGGGGAACATTGGAAATAAATAACGAATTAAT [25662]
cp26_PAli_NX_Pl_consensus    TGGACTCCACCACATATTTTATTTGCCATTTTGGCAAACAGCTGTTGGGGGAACATTGGAAATAAATAACGAATTAAT [25662]
cp26_PAbe_NX_MP_FG_consensus TGGACTCCACCACATATTTTATTTGCCATTTTGGCAAACAGCTGTTGGGGGAACATTGGAAATAAATAACGAATTAAT [25662]
cp26_PAbe_NX_Pl_consensus    TGGACTCCACCACATATTTTATTTGCCATTTTGGCAAACAGCTGTTGGGGGAACATTGGAAATAAATAACGAATTAAT [25662]

cp26_gi|11497007|B31_GB      CTCAGGAGCACAAAATATATTTTTCAAACAACTTGCAGATCCCAATACTGTACACTTTGAAGTTGCAAAAGGAACAAG [25740]
cp26_un9_B31_PacBio          CTCAGGAGCACAAAATATATTTTTCAAACAACTTGCAGATCCCAATACTGTACACTTTGAAGTTGCAAAAGGAACAAG [25740]
cp26_B31_TS_MP_FG_consensus  CTCAGGAGCACAAAATATATTTTTCAAACAACTTGCAGATCCCAATACTGTACACTTTGAAGTTGCAAAAGGAACAAG [25740]
cp26_B31_NX_Pl_consensus     CTCAGGAGCACAAAATATATTTTTCAAACAACTTGCAGATCCCAATACTGTACACTTTGAAGTTGCAAAAGGAACAAG [25740]
cp26_PAli_NX_MP_FG_consensus CTCAGGAGCACAAAATATATTTTTCAAACAACTTGCAGATCCCAATACTGTACACTTTGAAGTTGCAAAAGGAACAAG [25740]
cp26_PAli_NX_Pl_consensus    CTCAGGAGCACAAAATATATTTTTCAAACAACTTGCAGATCCCAATACTGTACACTTTGAAGTTGCAAAAGGAACAAG [25740]
cp26_PAbe_NX_MP_FG_consensus CTCAGGAGCACAAAATATATTTTTCAAACAACTTGCAGATCCCAATACTGTACACTTTGAAGTTGCAAAAGGAACAAG [25740]
cp26_PAbe_NX_Pl_consensus    CTCAGGAGCACAAAATATATTTTTCAAACAACTTGCAGATCCCAATACTGTACACTTTGAAGTTGCAAAAGGAACAAG [25740]

cp26_gi|11497007|B31_GB      ATTTTTTAGCGGGGAATTTGTTGTTATGATTTTTGGATTACCTGGGGCGGCGCTTGCTATGTACCATACATCAAAACC [25818]
cp26_un9_B31_PacBio          ATTTTTTAGCGGGGAATTTGTTGTTATGATTTTTGGATTACCTGGGGCGGCGCTTGCTATGTACCATACATCAAAACC [25818]
cp26_B31_TS_MP_FG_consensus  ATTTTTTAGCGGGGAATTTGTTGTTATGATTTTTGGATTACCTGGGGCGGCGCTTGCTATGTACCATACATCAAAACC [25818]
cp26_B31_NX_Pl_consensus     ATTTTTTAGCGGGGAATTTGTTGTTATGATTTTTGGATTACCTGGGGCGGCGCTTGCTATGTACCATACATCAAAACC [25818]
cp26_PAli_NX_MP_FG_consensus ATTTTTTAGCGGGGAATTTGTTGTTATGATTTTTGGATTACCTGGGGCGGCGCTTGCTATGTACCATACATCAAAACC [25818]
cp26_PAli_NX_Pl_consensus    ATTTTTTAGCGGGGAATTTGTTGTTATGATTTTTGGATTACCTGGGGCGGCGCTTGCTATGTACCATACATCAAAACC [25818]
cp26_PAbe_NX_MP_FG_consensus ATTTTTTAGCGGGGAATTTGTTGTTATGATTTTTGGATTACCTGGGGCGGCGCTTGCTATGTACCATACATCAAAACC [25818]
cp26_PAbe_NX_Pl_consensus    ATTTTTTAGCGGGGAATTTGTTGTTATGATTTTTGGATTACCTGGGGCGGCGCTTGCTATGTACCATACATCAAAACC [25818]

cp26_gi|11497007|B31_GB      TGAAAATAAAAAAAACGTAGCTTCATTGCTACTATCTGCTAGCTTTACATCAATGTTAACAGGAATTACAGAGCCTCT [25896]
cp26_un9_B31_PacBio          TGAAAATAAAAAAAACGTAGCTTCATTGCTACTATCTGCTAGCTTTACATCAATGTTAACAGGAATTACAGAGCCTCT [25896]
cp26_B31_TS_MP_FG_consensus  TGAAAATAAAAAAAACGTAGCTTCATTGCTACTATCTGCTAGCTTTACATCAATGTTAACAGGAATTACAGAGCCTCT [25896]
cp26_B31_NX_Pl_consensus     TGAAAATAAAAAAAACGTAGCTTCATTGCTACTATCTGCTAGCTTTACATCAATGTTAACAGGAATTACAGAGCCTCT [25896]
cp26_PAli_NX_MP_FG_consensus TGAAAATAAAAAAAACGTAGCTTCATTGCTACTATCTGCTAGCTTTACATCAATGTTAACAGGAATTACAGAGCCTCT [25896]
cp26_PAli_NX_Pl_consensus    TGAAAATAAAAAAAACGTAGCTTCATTGCTACTATCTGCTAGCTTTACATCAATGTTAACAGGAATTACAGAGCCTCT [25896]
cp26_PAbe_NX_MP_FG_consensus TGAAAATAAAAAAAACGTAGCTTCATTGCTACTATCTGCTAGCTTTACATCAATGTTAACAGGAATTACAGAGCCTCT [25896]
cp26_PAbe_NX_Pl_consensus    TGAAAATAAAAAAAACGTAGCTTCATTGCTACTATCTGCTAGCTTTACATCAATGTTAACAGGAATTACAGAGCCTCT [25896]

cp26_gi|11497007|B31_GB      TGAATTTGCATTCCTTTTTGCAGCACCAGCACTTTATTACTTTATATATGTTCCTCTTTTTGGACTAGCATATCTTTT [25974]
cp26_un9_B31_PacBio          TGAATTTGCATTCCTTTTTGCAGCACCAGCACTTTATTACTTTATATATGTTCCTCTTTTTGGACTAGCATATCTTTT [25974]
cp26_B31_TS_MP_FG_consensus  TGAATTTGCATTCCTTTTTGCAGCACCAGCACTTTATTACTTTATATATGTTCCTCTTTTTGGACTAGCATATCTTTT [25974]
cp26_B31_NX_Pl_consensus     TGAATTTGCATTCCTTTTTGCAGCACCAGCACTTTATTACTTTATATATGTTCCTCTTTTTGGACTAGCATATCTTTT [25974]
cp26_PAli_NX_MP_FG_consensus TGAATTTGCATTCCTTTTTGCAGCACCAGCACTTTATTACTTTATATATGTTCCTCTTTTTGGACTAGCATATCTTTT [25974]
cp26_PAli_NX_Pl_consensus    TGAATTTGCATTCCTTTTTGCAGCACCAGCACTTTATTACTTTATATATGTTCCTCTTTTTGGACTAGCATATCTTTT [25974]
cp26_PAbe_NX_MP_FG_consensus TGAATTTGCATTCCTTTTTGCAGCACCAGCACTTTATTACTTTATATATGTTCCTCTTTTTGGACTAGCATATCTTTT [25974]
cp26_PAbe_NX_Pl_consensus    TGAATTTGCATTCCTTTTTGCAGCACCAGCACTTTATTACTTTATATATGTTCCTCTTTTTGGACTAGCATATCTTTT [25974]

cp26_gi|11497007|B31_GB      AACACACCTTTTAAACGTAGGAGTTGGACTAACATTTTCTGGCGGATTTATAGATATGTTCCTATTTGGAATACTTCA [26052]
cp26_un9_B31_PacBio          AACACACCTTTTAAACGTAGGAGTTGGACTAACATTTTCTGGCGGATTTATAGATATGTTCCTATTTGGAATACTTCA [26052]
cp26_B31_TS_MP_FG_consensus  AACACACCTTTTAAACGTAGGAGTTGGACTAACATTTTCTGGCGGATTTATAGATATGTTCCTATTTGGAATACTTCA [26052]
cp26_B31_NX_Pl_consensus     AACACACCTTTTAAACGTAGGAGTTGGACTAACATTTTCTGGCGGATTTATAGATATGTTCCTATTTGGAATACTTCA [26052]
cp26_PAli_NX_MP_FG_consensus AACACACCTTTTAAACGTAGGAGTTGGACTAACATTTTCTGGCGGATTTATAGATATGTTCCTATTTGGAATACTTCA [26052]
cp26_PAli_NX_Pl_consensus    AACACACCTTTTAAACGTAGGAGTTGGACTAACATTTTCTGGCGGATTTATAGATATGTTCCTATTTGGAATACTTCA [26052]
cp26_PAbe_NX_MP_FG_consensus AACACACCTTTTAAACGTAGGAGTTGGACTAACATTTTCTGGCGGATTTATAGATATGTTCCTATTTGGAATACTTCA [26052]
cp26_PAbe_NX_Pl_consensus    AACACACCTTTTAAACGTAGGAGTTGGACTAACATTTTCTGGCGGATTTATAGATATGTTCCTATTTGGAATACTTCA [26052]

cp26_gi|11497007|B31_GB      GGGAAATAGCAAAACAAATTGGATAGCAATTCCTATCTTGGGAATCTTCTACTTTATTGGATTTTACTTTATATTTAA [26130]
cp26_un9_B31_PacBio          GGGAAATAGCAAAACAAATTGGATAGCAATTCCTATCTTGGGAATCTTCTACTTTATTGGATTTTACTTTATATTTAA [26130]
cp26_B31_TS_MP_FG_consensus  GGGAAATAGCAAAACAAATTGGATAGCAATTCCTATCTTGGGAATCTTCTACTTTATTGGATTTTACTTTATATTTAA [26130]
cp26_B31_NX_Pl_consensus     GGGAAATAGCAAAACAAATTGGATAGCAATTCCTATCTTGGGAATCTTCTACTTTATTGGATTTTACTTTATATTTAA [26130]
cp26_PAli_NX_MP_FG_consensus GGGAAATAGCAAAACAAATTGGATAGCAATTCCTATCTTGGGAATCTTCTACTTTATTGGATTTTACTTTATATTTAA [26130]
cp26_PAli_NX_Pl_consensus    GGGAAATAGCAAAACAAATTGGATAGCAATTCCTATCTTGGGAATCTTCTACTTTATTGGATTTTACTTTATATTTAA [26130]
cp26_PAbe_NX_MP_FG_consensus GGGAAATAGCAAAACAAATTGGATAGCAATTCCTATCTTGGGAATCTTCTACTTTATTGGATTTTACTTTATATTTAA [26130]
cp26_PAbe_NX_Pl_consensus    GGGAAATAGCAAAACAAATTGGATAGCAATTCCTATCTTGGGAATCTTCTACTTTATTGGATTTTACTTTATATTTAA [26130]

cp26_gi|11497007|B31_GB      ATTTGCAATCATGAAATTCAATCTTAAAACAATAGGAAGAGAAGACGAAGAGATGGAAAAAACCATGATGAGTTCAGA [26208]
cp26_un9_B31_PacBio          ATTTGCAATCATGAAATTCAATCTTAAAACAATAGGAAGAGAAGACGAAGAGATGGAAAAAACCATGATGAGTTCAGA [26208]
cp26_B31_TS_MP_FG_consensus  ATTTGCAATCATGAAATTCAATCTTAAAACAATAGGAAGAGAAGACGAAGAGATGGAAAAAACCATGATGAGTTCAGA [26208]
cp26_B31_NX_Pl_consensus     ATTTGCAATCATGAAATTCAATCTTAAAACAATAGGAAGAGAAGACGAAGAGATGGAAAAAACCATGATGAGTTCAGA [26208]
cp26_PAli_NX_MP_FG_consensus ATTTGCAATCATGAAATTCAATCTTAAAACAATAGGAAGAGAAGACGAAGAGATGGAAAAAACCATGATGAGTTCAGA [26208]
cp26_PAli_NX_Pl_consensus    ATTTGCAATCATGAAATTCAATCTTAAAACAATAGGAAGAGAAGACGAAGAGATGGAAAAAACCATGATGAGTTCAGA [26208]
cp26_PAbe_NX_MP_FG_consensus ATTTGCAATCATGAAATTCAATCTTAAAACAATAGGAAGAGAAGACGAAGAGATGGAAAAAACCATGATGAGTTCAGA [26208]
cp26_PAbe_NX_Pl_consensus    ATTTGCAATCATGAAATTCAATCTTAAAACAATAGGAAGAGAAGACGAAGAGATGGAAAAAACCATGATGAGTTCAGA [26208]

cp26_gi|11497007|B31_GB      AAAAACAAGCTTATCAGAAACCGCTTCAAAAGTATTAGAGGGTCTTGGAGGAAAAGATAATATTACATACATTGATGC [26286]
cp26_un9_B31_PacBio          AAAAACAAGCTTATCAGAAACCGCTTCAAAAGTATTAGAGGGTCTTGGAGGAAAAGATAATATTACATACATTGATGC [26286]
cp26_B31_TS_MP_FG_consensus  AAAAACAAGCTTATCAGAAACCGCTTCAAAAGTATTAGAGGGTCTTGGAGGAAAAGATAATATTACATACATTGATGC [26286]
cp26_B31_NX_Pl_consensus     AAAAACAAGCTTATCAGAAACCGCTTCAAAAGTATTAGAGGGTCTTGGAGGAAAAGATAATATTACATACATTGATGC [26286]
cp26_PAli_NX_MP_FG_consensus AAAAACAAGCTTATCAGAAACCGCTTCAAAAGTATTAGAGGGTCTTGGAGGAAAAGATAATATTACATACATTGATGC [26286]
cp26_PAli_NX_Pl_consensus    AAAAACAAGCTTATCAGAAACCGCTTCAAAAGTATTAGAGGGTCTTGGAGGAAAAGATAATATTACATACATTGATGC [26286]
cp26_PAbe_NX_MP_FG_consensus AAAAACAAGCTTATCAGAAACCGCTTCAAAAGTATTAGAGGGTCTTGGAGGAAAAGATAATATTACATACATTGATGC [26286]
cp26_PAbe_NX_Pl_consensus    AAAAACAAGCTTATCAGAAACCGCTTCAAAAGTATTAGAGGGTCTTGGAGGAAAAGATAATATTACATACATTGATGC [26286]
```

```
cp26_gi|11497007|B31_GB      ATGTGCATCAAGATTAAGAGTCAATCTAAAACAAATAGAAGTCATTAAATCAGACGCCTATTTCAAAAATCTAGGTGC [26364]
cp26_un9_B31_PacBio          ATGTGCATCAAGATTAAGAGTCAATCTAAAACAAATAGAAGTCATTAAATCAGACGCCTATTTCAAAAATCTAGGTGC [26364]
cp26_B31_TS_MP_FG_consensus  ATGTGCATCAAGATTAAGAGTCAATCTAAAACAAATAGAAGTCATTAAATCAGACGCCTATTTCAAAAATCTAGGTGC [26364]
cp26_B31_NX_Pl_consensus     ATGTGCATCAAGATTAAGAGTCAATCTAAAACAAATAGAAGTCATTAAATCAGACGCCTATTTCAAAAATCTAGGTGC [26364]
cp26_PAli_NX_MP_FG_consensus ATGTGCATCAAGATTAAGAGTCAATCTAAAACAAATAGAAGTCATTAAATCAGACGCCTATTTCAAAAATCTAGGTGC [26364]
cp26_PAli_NX_Pl_consensus    ATGTGCATCAAGATTAAGAGTCAATCTAAAACAAATAGAAGTCATTAAATCAGACGCCTATTTCAAAAATCTAGGTGC [26364]
cp26_PAbe_NX_MP_FG_consensus ATGTGCATCAAGATTAAGAGTCAATCTAAAACAAATAGAAGTCATTAAATCAGACGCCTATTTCAAAAATCTAGGTGC [26364]
cp26_PAbe_NX_Pl_consensus    ATGTGCATCAAGATTAAGAGTCAATCTAAAACAAATAGAAGTCATTAAATCAGACGCCTATTTCAAAAATCTAGGTGC [26364]

cp26_gi|11497007|B31_GB      CAGTGGAATATTAAAAAAAGGAAATAGTGTTCAAATTGTATTTGGAGGATTGTCCGATAATATAAAAATGGAAATCGA [26442]
cp26_un9_B31_PacBio          CAGTGGAATATTAAAAAAAGGAAATAGTGTTCAAATTGTATTTGGAGGATTGTCCGATAATATAAAAATGGAAATCGA [26442]
cp26_B31_TS_MP_FG_consensus  CAGTGGAATATTAAAAAAAGGAAATAGTGTTCAAATTGTATTTGGAGGATTGTCCGATAATATAAAAATGGAAATCGA [26442]
cp26_B31_NX_Pl_consensus     CAGTGGAATATTAAAAAAAGGAAATAGTGTTCAAATTGTATTTGGAGGATTGTCCGATAATATAAAAATGGAAATCGA [26442]
cp26_PAli_NX_MP_FG_consensus CAGTGGAATATTAAAAAAAGGAAATAGTGTTCAAATTGTATTTGGAGGATTGTCCGATAATATAAAAATGGAAATCGA [26442]
cp26_PAli_NX_Pl_consensus    CAGTGGAATATTAAAAAAAGGAAATAGTGTTCAAATTGTATTTGGAGGATTGTCCGATAATATAAAAATGGAAATCGA [26442]
cp26_PAbe_NX_MP_FG_consensus CAGTGGAATATTAAAAAAAGGAAATAGTGTTCAAATTGTATTTGGAGGATTGTCCGATAATATAAAAATGGAAATCGA [26442]
cp26_PAbe_NX_Pl_consensus    CAGTGGAATATTAAAAAAAGGAAATAGTGTTCAAATTGTATTTGGAGGATTGTCCGATAATATAAAAATGGAAATCGA [26442]

cp26_gi|11497007|B31_GB      CAAGCTTATGTAAAATTTTTAAAATATAATATAAAAACAGCTAATCCAATAGAAAAAT-------------------- [26520]
cp26_un9_B31_PacBio          CAAGCTTATGTAAAATTTTTAAAATATAATATAAAAACAGCTAATCCAATAGAAAAAT-------------------- [26520]
cp26_B31_TS_MP_FG_consensus  CAAGCTTATGTAAAATTTTTAAAATATAATATAAAAACAGCTAATCCAATAGAAAAAT-------------------- [26520]
cp26_B31_NX_Pl_consensus     CAAGCTTATGTAAAATTTTTAAAATATAATATAAAAACAGCTAATCCAATAGAAAAAT-------------------- [26520]
cp26_PAli_NX_MP_FG_consensus CAAGCTTATGTAAAATTTTTAAAATATAATATAAAAACAGCTAATCCAATAGAAAAAT-------------------- [26520]
cp26_PAli_NX_Pl_consensus    CAAGCTTATGTAAAATTTTTAAAATATAATATAAAAACAGCTAATCCAATAGAAAAAT-------------------- [26520]
cp26_PAbe_NX_MP_FG_consensus CAAGCTTATGTAAAATTTTTAAAATATAATATAAAAACAGCTAATCCAATAGAAAAAT-------------------- [26520]
cp26_PAbe_NX_Pl_consensus    CAAGCTTATGTAAAATTTTTAAAATATAATATAAAAACAGCTAATCCAATAGAAAAAT-------------------- [26520]
```

```
#NEXUS
[ Title ]
begin data;
   dimensions ntax=8 nchar=30441;
   format missing=? gap=- matchchar=. datatype=nucleotide interleave=yes;
   matrix

[!Domain=Data property=Coding CodonStart=1;]
cp32-3_gi|11497103|B31_GB      ATTTTATTGACAAAAATATTAGTTTTTGCTATCATGCATCTAATTTAATAAAGAGAAGTAAAAAGGTGTGTGATTTAA [   78]
cp32-3_un8_B31_PacBio          ATTTTATTGACAAAAATATTAGTTTTTGCTATCATGCATCTAATTTAATAAAGAGAAGTAAAAAGGTGTGTGATTTAA [   78]
cp32-3_B31_TS_MP_FG_consensus  ATTTTATTGACAAAAATATTAGTTTTTGCTATCATGCATCTAATTTAATAAAGAGAAGTAAAAAGGTGTGTGATTTAA [   78]
cp32-3_B31_NX_Pl_consensus     ATTTTATTGACAAAAATATTAGTTTTTGCTATCATGCATCTAATTTAATAAAGAGAAGTAAAAAGGTGTGTGATTTAA [   78]
cp32-3_PAli_NX_MP_FG_consensus ATTTTATTGACAAAAATATTAGTTTTTGCTATCATGCATCTAATTTAATAAAGAGAAGTAAAAAGGTGTGTGATTTAA [   78]
cp32-3_PAli_NX_Pl_consensus    ATTTTATTGACAAAAATATTAGTTTTTGCTATCATGCATCTAATTTAATAAAGAGAAGTAAAAAGGTGTGTGATTTAA [   78]
cp32-3_PAbe_NX_MP_FG_consensus ATTTTATTGACAAAAATATTAGTTTTTGCTATCATGCATCTAATTTAATAAAGAGAAGTAAAAAGGTGTGTGATTTAA [   78]
cp32-3_PAbe_NX_Pl_consensus    ATTTTATTGACAAAAATATTAGTTTTTGCTATCATGCATCTAATTTAATAAAGAGAAGTAAAAAGGTGTGTGATTTAA [   78]

cp32-3_gi|11497103|B31_GB      GAAAAACAAAACTAATAGATAAAATAAGTTCACTAGAACTATACAAATACTCAATATTTTTTAGAAATTACATTGAAA [  156]
cp32-3_un8_B31_PacBio          GAAAAACAAAACTAATAGATAAAATAAGTTCACTAGAACTATACAAATACTCAATATTTTTTAGAAATTACATTGAAA [  156]
cp32-3_B31_TS_MP_FG_consensus  GAAAAACAAAACTAATAGATAAAATAAGTTCACTAGAACTATACAAATACTCAATATTTTTTAGAAATTACATTGAAA [  156]
cp32-3_B31_NX_Pl_consensus     GAAAAACAAAACTAATAGATAAAATAAGTTCACTAGAACTATACAAATACTCAATATTTTTTAGAAATTACATTGAAA [  156]
cp32-3_PAli_NX_MP_FG_consensus GAAAAACAAAACTAATAGATAAAATAAGTTCACTAGAACTATACAAATACTCAATATTTTTTAGAAATTACATTGAAA [  156]
cp32-3_PAli_NX_Pl_consensus    GAAAAACAAAACTAATAGATAAAATAAGTTCACTAGAACTATACAAATACTCAATATTTTTTAGAAATTACATTGAAA [  156]
cp32-3_PAbe_NX_MP_FG_consensus GAAAAACAAAACTAATAGATAAAATAAGTTCACTAGAACTATACAAATACTCAATATTTTTTAGAAATTACATTGAAA [  156]
cp32-3_PAbe_NX_Pl_consensus    GAAAAACAAAACTAATAGATAAAATAAGTTCACTAGAACTATACAAATACTCAATATTTTTTAGAAATTACATTGAAA [  156]

cp32-3_gi|11497103|B31_GB      ATGTAGCAGAAGATTGTCTCAAGAACGGACTTATTCTTGAGAGTGCTGCCCACAATGTTAGTGAGGTTGAACTTGCTA [  234]
cp32-3_un8_B31_PacBio          ATGTAGCAGAAGATTGTCTCAAGAACGGACTTATTCTTGAGAGTGCTGCCCACAATGTTAGTGAGGTTGAACTTGCTA [  234]
cp32-3_B31_TS_MP_FG_consensus  ATGTAGCAGAAGATTGTCTCAAGAACGGACTTATTCTTGAGAGTGCTGCCCACAATGTTAGTGAGGTTGAACTTGCTA [  234]
cp32-3_B31_NX_Pl_consensus     ATGTAGCAGAAGATTGTCTCAAGAACGGACTTATTCTTGAGAGTGCTGCCCACAATGTTAGTGAGGTTGAACTTGCTA [  234]
cp32-3_PAli_NX_MP_FG_consensus ATGTAGCAGAAGATTGTCTCAAGAACGGACTTATTCTTGAGAGTGCTGCCCACAATGTTAGTGAGGTTGAACTTGCTA [  234]
cp32-3_PAli_NX_Pl_consensus    ATGTAGCAGAAGATTGTCTCAAGAACGGACTTATTCTTGAGAGTGCTGCCCACAATGTTAGTGAGGTTGAACTTGCTA [  234]
cp32-3_PAbe_NX_MP_FG_consensus ATGTAGCAGAAGATTGTCTCAAGAACGGACTTATTCTTGAGAGTGCTGCCCACAATGTTAGTGAGGTTGAACTTGCTA [  234]
cp32-3_PAbe_NX_Pl_consensus    ATGTAGCAGAAGATTGTCTCAAGAACGGACTTATTCTTGAGAGTGCTGCCCACAATGTTAGTGAGGTTGAACTTGCTA [  234]

cp32-3_gi|11497103|B31_GB      GGTTAAAGGTACAGCTTAAGAATGCTCTGCTTAATTGTATTATAAGCTACCGTTTTCATGGGATTGGCTATGTTTTAG [  312]
cp32-3_un8_B31_PacBio          GGTTAAAGGTACAGCTTAAGAATGCTCTGCTTAATTGTATTATAAGCTACCGTTTTCATGGGATTGGCTATGTTTTAG [  312]
cp32-3_B31_TS_MP_FG_consensus  GGTTAAAGGTACAGCTTAAGAATGCTCTGCTTAATTGTATTATAAGCTACCGTTTTCATGGGATTGGCTATGTTTTAG [  312]
cp32-3_B31_NX_Pl_consensus     GGTTAAAGGTACAGCTTAAGAATGCTCTGCTTAATTGTATTATAAGCTACCGTTTTCATGGGATTGGCTATGTTTTAG [  312]
cp32-3_PAli_NX_MP_FG_consensus GGTTAAAGGTACAGCTTAAGAATGCTCTGCTTAATTGTATTATAAGCTACCGTTTTCATGGGATTGGCTATGTTTTAG [  312]
cp32-3_PAli_NX_Pl_consensus    GGTTAAAGGTACAGCTTAAGAATGCTCTGCTTAATTGTATTATAAGCTACCGTTTTCATGGGATTGGCTATGTTTTAG [  312]
cp32-3_PAbe_NX_MP_FG_consensus GGTTAAAGGTACAGCTTAAGAATGCTCTGCTTAATTGTATTATAAGCTACCGTTTTCATGGGATTGGCTATGTTTTAG [  312]
cp32-3_PAbe_NX_Pl_consensus    GGTTAAAGGTACAGCTTAAGAATGCTCTGCTTAATTGTATTATAAGCTACCGTTTTCATGGGATTGGCTATGTTTTAG [  312]

cp32-3_gi|11497103|B31_GB      TAAAAACCAAAGATACCCTAATAGATCTCGAACAACCCGTTAATATAGAATTACCTATTGGTTTTGAATACCTTGATT [  390]
cp32-3_un8_B31_PacBio          TAAAAACCAAAGATACCCTAATAGATCTCGAACAACCCGTTAATATAGAATTACCTATTGGTTTTGAATACCTTGATT [  390]
cp32-3_B31_TS_MP_FG_consensus  TAAAAACCAAAGATACCCTAATAGATCTCGAACAACCCGTTAATATAGAATTACCTATTGGTTTTGAATACCTTGATT [  390]
cp32-3_B31_NX_Pl_consensus     TAAAAACCAAAGATACCCTAATAGATCTCGAACAACCCGTTAATATAGAATTACCTATTGGTTTTGAATACCTTGATT [  390]
cp32-3_PAli_NX_MP_FG_consensus TAAAAACCAAAGATACCCTAATAGATCTCGAACAACCCGTTAATATAGAATTACCTATTGGTTTTGAATACCTTGATT [  390]
cp32-3_PAli_NX_Pl_consensus    TAAAAACCAAAGATACCCTAATAGATCTCGAACAACCCGTTAATATAGAATTACCTATTGGTTTTGAATACCTTGATT [  390]
cp32-3_PAbe_NX_MP_FG_consensus TAAAAACCAAAGATACCCTAATAGATCTCGAACAACCCGTTAATATAGAATTACCTATTGGTTTTGAATACCTTGATT [  390]
cp32-3_PAbe_NX_Pl_consensus    TAAAAACCAAAGATACCCTAATAGATCTCGAACAACCCGTTAATATAGAATTACCTATTGGTTTTGAATACCTTGATT [  390]

cp32-3_gi|11497103|B31_GB      ATGAATATGTAAGAGATTTGGGAGTTGATTTTGATCATATAACCTATAAAGTAAAATCCAACAATAAGAACAATTCTT [  468]
cp32-3_un8_B31_PacBio          ATGAATATGTAAGAGATTTGGGAGTTGATTTTGATCATATAACCTATAAAGTAAAATCCAACAATAAGAACAATTCTT [  468]
cp32-3_B31_TS_MP_FG_consensus  ATGAATATGTAAGAGATTTGGGAGTTGATTTTGATCATATAACCTATAAAGTAAAATCCAACAATAAGAACAATTCTT [  468]
cp32-3_B31_NX_Pl_consensus     ATGAATATGTAAGAGATTTGGGAGTTGATTTTGATCATATAACCTATAAAGTAAAATCCAACAATAAGAACAATTCTT [  468]
cp32-3_PAli_NX_MP_FG_consensus ATGAATATGTAAGAGATTTGGGAGTTGATTTTGATCATATAACCTATAAAGTAAAATCCAACAATAAGAACAATTCTT [  468]
cp32-3_PAli_NX_Pl_consensus    ATGAATATGTAAGAGATTTGGGAGTTGATTTTGATCATATAACCTATAAAGTAAAATCCAACAATAAGAACAATTCTT [  468]
cp32-3_PAbe_NX_MP_FG_consensus ATGAATATGTAAGAGATTTGGGAGTTGATTTTGATCATATAACCTATAAAGTAAAATCCAACAATAAGAACAATTCTT [  468]
cp32-3_PAbe_NX_Pl_consensus    ATGAATATGTAAGAGATTTGGGAGTTGATTTTGATCATATAACCTATAAAGTAAAATCCAACAATAAGAACAATTCTT [  468]

cp32-3_gi|11497103|B31_GB      TAGATGCAGTTAAAATACATAAAAGTCGACTTATCATATATGAAAACTTTGATTATATCTTAAAAAGATATGTTCCGT [  546]
cp32-3_un8_B31_PacBio          TAGATGCAGTTAAAATACATAAAAGTCGACTTATCATATATGAAAACTTTGATTATATCTTAAAAAGATATGTTCCGT [  546]
cp32-3_B31_TS_MP_FG_consensus  TAGATGCAGTTAAAATACATAAAAGTCGACTTATCATATATGAAAACTTTGATTATATCTTAAAAAGATATGTTCCGT [  546]
cp32-3_B31_NX_Pl_consensus     TAGATGCAGTTAAAATACATAAAAGTCGACTTATCATATATGAAAACTTTGATTATATCTTAAAAAGATATGTTCCGT [  546]
cp32-3_PAli_NX_MP_FG_consensus TAGATGCAGTTAAAATACATAAAAGTCGACTTATCATATATGAAAACTTTGATTATATCTTAAAAAGATATGTTCCGT [  546]
cp32-3_PAli_NX_Pl_consensus    TAGATGCAGTTAAAATACATAAAAGTCGACTTATCATATATGAAAACTTTGATTATATCTTAAAAAGATATGTTCCGT [  546]
cp32-3_PAbe_NX_MP_FG_consensus TAGATGCAGTTAAAATACATAAAAGTCGACTTATCATATATGAAAACTTTGATTATATCTTAAAAAGATATGTTCCGT [  546]
cp32-3_PAbe_NX_Pl_consensus    TAGATGCAGTTAAAATACATAAAAGTCGACTTATCATATATGAAAACTTTGATTATATCTTAAAAAGATATGTTCCGT [  546]

cp32-3_gi|11497103|B31_GB      GCTATACCGAAAGCTTTTTACTAGATATTTATTTATTTGAAAAGATATACGTAGAAATAGAAAGACGTATTGAAAACC [  624]
cp32-3_un8_B31_PacBio          GCTATACCGAAAGCTTTTTACTAGATATTTATTTATTTGAAAAGATATACGTAGAAATAGAAAGACGTATTGAAAACC [  624]
cp32-3_B31_TS_MP_FG_consensus  GCTATACCGAAAGCTTTTTACTAGATATTTATTTATTTGAAAAGATATACGTAGAAATAGAAAGACGTATTGAAAACC [  624]
cp32-3_B31_NX_Pl_consensus     GCTATACCGAAAGCTTTTTACTAGATATTTATTTATTTGAAAAGATATACGTAGAAATAGAAAGACGTATTGAAAACC [  624]
cp32-3_PAli_NX_MP_FG_consensus GCTATACCGAAAGCTTTTTACTAGATATTTATTTATTTGAAAAGATATACGTAGAAATAGAAAGACGTATTGAAAACC [  624]
cp32-3_PAli_NX_Pl_consensus    GCTATACCGAAAGCTTTTTACTAGATATTTATTTATTTGAAAAGATATACGTAGAAATAGAAAGACGTATTGAAAACC [  624]
cp32-3_PAbe_NX_MP_FG_consensus GCTATACCGAAAGCTTTTTACTAGATATTTATTTATTTGAAAAGATATACGTAGAAATAGAAAGACGTATTGAAAACC [  624]
cp32-3_PAbe_NX_Pl_consensus    GCTATACCGAAAGCTTTTTACTAGATATTTATTTATTTGAAAAGATATACGTAGAAATAGAAAGACGTATTGAAAACC [  624]

cp32-3_gi|11497103|B31_GB      ACAATTTTTTGTTTTACAAAGATGAATCTTTAGTACAACTACAAGACGCACTTTCTAGTGCAACAACTTCTTTAAGTG [  702]
cp32-3_un8_B31_PacBio          ACAATTTTTTGTTTTACAAAGATGAATCTTTAGTACAACTACAAGACGCACTTTCTAGTGCAACAACTTCTTTAAGTG [  702]
cp32-3_B31_TS_MP_FG_consensus  ACAATTTTTTGTTTTACAAAGATGAATCTTTAGTACAACTACAAGACGCACTTTCTAGTGCAACAACTTCTTTAAGTG [  702]
cp32-3_B31_NX_Pl_consensus     ACAATTTTTTGTTTTACAAAGATGAATCTTTAGTACAACTACAAGACGCACTTTCTAGTGCAACAACTTCTTTAAGTG [  702]
cp32-3_PAli_NX_MP_FG_consensus ACAATTTTTTGTTTTACAAAGATGAATCTTTAGTACAACTACAAGACGCACTTTCTAGTGCAACAACTTCTTTAAGTG [  702]
cp32-3_PAli_NX_Pl_consensus    ACAATTTTTTGTTTTACAAAGATGAATCTTTAGTACAACTACAAGACGCACTTTCTAGTGCAACAACTTCTTTAAGTG [  702]
cp32-3_PAbe_NX_MP_FG_consensus ACAATTTTTTGTTTTACAAAGATGAATCTTTAGTACAACTACAAGACGCACTTTCTAGTGCAACAACTTCTTTAAGTG [  702]
cp32-3_PAbe_NX_Pl_consensus    ACAATTTTTTGTTTTACAAAGATGAATCTTTAGTACAACTACAAGACGCACTTTCTAGTGCAACAACTTCTTTAAGTG [  702]

cp32-3_gi|11497103|B31_GB      CACTTACTCAGAGCAATAATGATAGGGGAAGTGGCATTTTATCTTCTTTTTTGAGAAAACAAAATTCAAACAATCATA [  780]
cp32-3_un8_B31_PacBio          CACTTACTCAGAGCAATAATGATAGGGGAAGTGGCATTTTATCTTCTTTTTTGAGAAAACAAAATTCAAACAATCATA [  780]
cp32-3_B31_TS_MP_FG_consensus  CACTTACTCAGAGCAATAATGATAGGGGAAGTGGCATTTTATCTTCTTTTTTGAGAAAACAAAATTCAAACAATCATA [  780]
cp32-3_B31_NX_Pl_consensus     CACTTACTCAGAGCAATAATGATAGGGGAAGTGGCATTTTATCTTCTTTTTTGAGAAAACAAAATTCAAACAATCATA [  780]
cp32-3_PAli_NX_MP_FG_consensus CACTTACTCAGAGCAATAATGATAGGGGAAGTGGCATTTTATCTTCTTTTTTGAGAAAACAAAATTCAAACAATCATA [  780]
cp32-3_PAli_NX_Pl_consensus    CACTTACTCAGAGCAATAATGATAGGGGAAGTGGCATTTTATCTTCTTTTTTGAGAAAACAAAATTCAAACAATCATA [  780]
cp32-3_PAbe_NX_MP_FG_consensus CACTTACTCAGAGCAATAATGATAGGGGAAGTGGCATTTTATCTTCTTTTTTGAGAAAACAAAATTCAAACAATCATA [  780]
cp32-3_PAbe_NX_Pl_consensus    CACTTACTCAGAGCAATAATGATAGGGGAAGTGGCATTTTATCTTCTTTTTTGAGAAAACAAAATTCAAACAATCATA [  780]

cp32-3_gi|11497103|B31_GB      GTAAAGATATTTCTAATTTAAGAAACCTTAATGACTCATTGTCACAGGAGCTTGCCAGGCTAAAAAGCAATCTAAATA [  858]
cp32-3_un8_B31_PacBio          GTAAAGATATTTCTAATTTAAGAAACCTTAATGACTCATTGTCACAGGAGCTTGCCAGGCTAAAAAGCAATCTAAATA [  858]
cp32-3_B31_TS_MP_FG_consensus  GTAAAGATATTTCTAATTTAAGAAACCTTAATGACTCATTGTCACAGGAGCTTGCCAGGCTAAAAAGCAATCTAAATA [  858]
cp32-3_B31_NX_Pl_consensus     GTAAAGATATTTCTAATTTAAGAAACCTTAATGACTCATTGTCACAGGAGCTTGCCAGGCTAAAAAGCAATCTAAATA [  858]
cp32-3_PAli_NX_MP_FG_consensus GTAAAGATATTTCTAATTTAAGAAACCTTAATGACTCATTGTCACAGGAGCTTGCCAGGCTAAAAAGCAATCTAAATA [  858]
cp32-3_PAli_NX_Pl_consensus    GTAAAGATATTTCTAATTTAAGAAACCTTAATGACTCATTGTCACAGGAGCTTGCCAGGCTAAAAAGCAATCTAAATA [  858]
cp32-3_PAbe_NX_MP_FG_consensus GTAAAGATATTTCTAATTTAAGAAACCTTAATGACTCATTGTCACAGGAGCTTGCCAGGCTAAAAAGCAATCTAAATA [  858]
cp32-3_PAbe_NX_Pl_consensus    GTAAAGATATTTCTAATTTAAGAAACCTTAATGACTCATTGTCACAGGAGCTTGCCAGGCTAAAAAGCAATCTAAATA [  858]

cp32-3_gi|11497103|B31_GB      ATGAGGGAATGTTTTATACAGCTACTCCTAGTGCTAGTTTAGAGGTTATTAAATACGATCTTAGTTACTTAAAGGAGG [  936]
cp32-3_un8_B31_PacBio          ATGAGGGAATGTTTTATACAGCTACTCCTAGTGCTAGTTTAGAGGTTATTAAATACGATCTTAGTTACTTAAAGGAGG [  936]
```

```
cp32-3_B31_TS_MP_FG_consensus  ATGAGGGAATGTTTTATACAGCTACTCCTAGTGCTAGTTTAGAGGTTATTAAATACGATCTTAGTTACTTAAAGGAGG [  936]
cp32-3_B31_NX_Pl_consensus     ATGAGGGAATGTTTTATACAGCTACTCCTAGTGCTAGTTTAGAGGTTATTAAATACGATCTTAGTTACTTAAAGGAGG [  936]
cp32-3_PAli_NX_MP_FG_consensus ATGAGGGAATGTTTTATACAGCTACTCCTAGTGCTAGTTTAGAGGTTATTAAATACGATCTTAGTTACTTAAAGGAGG [  936]
cp32-3_PAli_NX_Pl_consensus    ATGAGGGAATGTTTTATACAGCTACTCCTAGTGCTAGTTTAGAGGTTATTAAATACGATCTTAGTTACTTAAAGGAGG [  936]
cp32-3_PAbe_NX_MP_FG_consensus ATGAGGGAATGTTTTATACAGCTACTCCTAGTGCTAGTTTAGAGGTTATTAAATACGATCTTAGCTACTTAAAGGAGG [  936]
cp32-3_PAbe_NX_Pl_consensus    ATGAGGGAATGTTTTATACAGCTACTCCTAGTGCTAGTTTAGAGGTTATTAAATACGATCTTAGTTACTTAAAGGAGG [  936]

cp32-3_gi|11497103|B31_GB      CTTTAGCATTAATTAAGGCAAAAATTGGTGCAGATACTAAAGAGCCCTTAACCAGAAGTTTTAATGAACAGGCTAAAG [ 1014]
cp32-3_un8_B31_PacBio          CTTTAGCATTAATTAAGGCAAAAATTGGTGCAGATACTAAAGAGCCCTTAACCAGAAGTTTTAATGAACAGGCTAAAG [ 1014]
cp32-3_B31_TS_MP_FG_consensus  CTTTAGCATTAATTAAGGCAAAAATTGGTGCAGATACTAAAGAGCCCTTAACCAGAAGTTTTAATGAACAGGCTAAAG [ 1014]
cp32-3_B31_NX_Pl_consensus     CTTTAGCATTAATTAAGGCAAAAATTGGTGCAGATACTAAAGAGCCCTTAACCAGAAGTTTTAATGAACAGGCTAAAG [ 1014]
cp32-3_PAli_NX_MP_FG_consensus CTTTAGCATTAATTAAGGCAAAAATTGGTGCAGATACTAAAGAGCCCTTAACCAGAAGTTTTAATGAACAGGCTAAAG [ 1014]
cp32-3_PAli_NX_Pl_consensus    CTTTAGCATTAATTAAGGCAAAAATTGGTGCAGATACTAAAGAGCCCTTAACCAGAAGTTTTAATGAACAGGCTAAAG [ 1014]
cp32-3_PAbe_NX_MP_FG_consensus CTTTAGCATTAATTAAGGCAAAAATTGGTGCAGATACTAAAGAGCCCTTAACCAGAAGTTTTAATGAACAGGCTAAAG [ 1014]
cp32-3_PAbe_NX_Pl_consensus    CTTTAGCATTAATTAAGGCAAAAATTGGTGCAGATACTAAAGAGCCCTTAACCAGAAGTTTTAATGAACAGGCTAAAG [ 1014]

cp32-3_gi|11497103|B31_GB      GACTGGGAAATGATGGTAAAGGTGATAGGAGTAATTATTACGATTTTCTCAAAGGTGTACAAGAACAAGTTGAGAACT [ 1092]
cp32-3_un8_B31_PacBio          GACTGGGAAATGATGGTAAAGGTGATAGGAGTAATTATTACGATTTTCTCAAAGGTGTACAAGAACAAGTTGAGAACT [ 1092]
cp32-3_B31_TS_MP_FG_consensus  GACTGGGAAATGATGGTAAAGGTGATAGGAGTAATTATTACGATTTTCTCAAAGGTGTACAAGAACAAGTTGAGAACT [ 1092]
cp32-3_B31_NX_Pl_consensus     GACTGGGAAATGATGGTAAAGGTGATAGGAGTAATTATTACGATTTTCTCAAAGGTGTACAAGAACAAGTTGAGAACT [ 1092]
cp32-3_PAli_NX_MP_FG_consensus GACTGGGAAATGATGGTAAAGGTGATAGGAGTAATTATTACGATTTTCTCAAAGGTGTACAAGAACAAGTTGAGAACT [ 1092]
cp32-3_PAli_NX_Pl_consensus    GACTGGGAAATGATGGTAAAGGTGATAGGAGTAATTATTACGATTTTCTCAAAGGTGTACAAGAACAAGTTGAGAACT [ 1092]
cp32-3_PAbe_NX_MP_FG_consensus GACTGGGAAATGATGGTAAAGGTGATAGGAGTAATTATTACGATTTTCTCAAAGGTGTACAAGAACAAGTTGAGAACT [ 1092]
cp32-3_PAbe_NX_Pl_consensus    GACTGGGAAATGATGGTAAAGGTGATAGGAGTAATTATTACGATTTTCTCAAAGGTGTACAAGAACAAGTTGAGAACT [ 1092]

cp32-3_gi|11497103|B31_GB      CTTGTAATTTAAAACTTACAAAGTATTTTGGACTTGATATGAAGTTTAATTCGCTGATTATGTTAAGTGAAGAACAAA [ 1170]
cp32-3_un8_B31_PacBio          CTTGTAATTTAAAACTTACAAAGTATTTTGGACTTGATATGAAGTTTAATTCGCTGATTATGTTAAGTGAAGAACAAA [ 1170]
cp32-3_B31_TS_MP_FG_consensus  CTTGTAATTTAAAACTTACAAAGTATTTTGGACTTGATATGAAGTTTAATTCGCTGATTATGTTAAGTGAAGAACAAA [ 1170]
cp32-3_B31_NX_Pl_consensus     CTTGTAATTTAAAACTTACAAAGTATTTTGGACTTGATATGAAGTTTAATTCGCTGATTATGTTAAGTGAAGAACAAA [ 1170]
cp32-3_PAli_NX_MP_FG_consensus CTTGTAATTTAAAACTTACAAAGTATTTTGGACTTGATATGAAGTTTAATTCGCTGATTATGTTAAGTGAAGAACAAA [ 1170]
cp32-3_PAli_NX_Pl_consensus    CTTGTAATTTAAAACTTACAAAGTATTTTGGACTTGATATGAAGTTTAATTCGCTGATTATGTTAAGTGAAGAACAAA [ 1170]
cp32-3_PAbe_NX_MP_FG_consensus CTTGTAATTTAAAACTTACAAAGTATTTTGGACTTGATATGAAGTTTAATTCGCTGATTATGTTAAGTGAAGAACAAA [ 1170]
cp32-3_PAbe_NX_Pl_consensus    CTTGTAATTTAAAACTTACAAAGTATTTTGGACTTGATATGAAGTTTAATTCGCTGATTATGTTAAGTGAAGAACAAA [ 1170]

cp32-3_gi|11497103|B31_GB      AAGTAGAAAGAGATATAAAGCTAATTGAGCTTTACAGTAAATATAACCAGCTTATACAAAGTAGCTCATTTAATAATG [ 1248]
cp32-3_un8_B31_PacBio          AAGTAGAAAGAGATATAAAGCTAATTGAGCTTTACAGTAAATATAACCAGCTTATACAAAGTAGCTCATTTAATAATG [ 1248]
cp32-3_B31_TS_MP_FG_consensus  AAGTAGAAAGAGATATAAAGCTAATTGAGCTTTACAGTAAATATAACCAGCTTATACAAAGTAGCTCATTTAATAATG [ 1248]
cp32-3_B31_NX_Pl_consensus     AAGTAGAAAGAGATATAAAGCTAATTGAGCTTTACAGTAAATATAACCAGCTTATACAAAGTAGCTCATTTAATAATG [ 1248]
cp32-3_PAli_NX_MP_FG_consensus AAGTAGAAAGAGATATAAAGCTAATTGAGCTTTACAGTAAATATAACCAGCTTATACAAAGTAGCTCATTTAATAATG [ 1248]
cp32-3_PAli_NX_Pl_consensus    AAGTAGAAAGAGATATAAAGCTAATTGAGCTTTACAGTAAATATAACCAGCTTATACAAAGTAGCTCATTTAATAATG [ 1248]
cp32-3_PAbe_NX_MP_FG_consensus AAGTAGAAAGAGATATAAAGCTAATTGAGCTTTACAGTAAATATAACCAGCTTATACAAAGTAGCTCATTTAATAATG [ 1248]
cp32-3_PAbe_NX_Pl_consensus    AAGTAGAAAGAGATATAAAGCTAATTGAGCTTTACAGTAAATATAACCAGCTTATACAAAGTAGCTCATTTAATAATG [ 1248]

cp32-3_gi|11497103|B31_GB      AGGAGCTAGCGATGTTAAAAGAGAAATTATTCTCATTTTGAGAAAAGGAGTTAAAAGATGACTGAGAAAGAAGAAAAA [ 1326]
cp32-3_un8_B31_PacBio          AGGAGCTAGCGATGTTAAAAGAGAAATTATTCTCATTTTGAGAAAAGGAGTTAAAAGATGACTGAGAAAGAAGAAAAA [ 1326]
cp32-3_B31_TS_MP_FG_consensus  AGGAGCTAGCGATGTTAAAAGAGAAATTATTCTCATTTTGAGAAAAGGAGTTAAAAGATGACTGAGAAAGAAGAAAAA [ 1326]
cp32-3_B31_NX_Pl_consensus     AGGAGCTAGCGATGTTAAAAGAGAAATTATTCTCATTTTGAGAAAAGGAGTTAAAAGATGACTGAGAAAGAAGAAAAA [ 1326]
cp32-3_PAli_NX_MP_FG_consensus AGGAGCTAGCGATGTTAAAAGAGAAATTATTCTCATTTTGAGAAAAGGAGTTAAAAGATGACTGAGAAAGAAGAAAAA [ 1326]
cp32-3_PAli_NX_Pl_consensus    AGGAGCTAGCGATGTTAAAAGAGAAATTATTCTCATTTTGAGAAAAGGAGTTAAAAGATGACTGAGAAAGAAGAAAAA [ 1326]
cp32-3_PAbe_NX_MP_FG_consensus AGGAGCTAGCGATGTTAAAAGAGAAATTATTCTCATTTTGAGAAAAGGAGTTAAAAGATGACTGAGAAAGAAGAAAAA [ 1326]
cp32-3_PAbe_NX_Pl_consensus    AGGAGCTAGCGATGTTAAAAGAGAAATTATTCTCATTTTGAGAAAAGGAGTTAAAAGATGACTGAGAAAGAAGAAAAA [ 1326]

cp32-3_gi|11497103|B31_GB      GAAGACCTGCAGGCACAAGATAAAGAAGAGCAGCAAATTAAGGCTGATACTAAAGTTATCAGTGTGCAGGAATTTGAA [ 1404]
cp32-3_un8_B31_PacBio          GAAGACCTGCAGGCACAAGATAAAGAAGAGCAGCAAATTAAGGCTGATACTAAAGTTATCAGTGTGCAGGAATTTGAA [ 1404]
cp32-3_B31_TS_MP_FG_consensus  GAAGACCTGCAGGCACAAGATAAAGAAGAGCAGCAAATTAAGGCTGATACTAAAGTTATCAGTGTGCAGGAATTTGAA [ 1404]
cp32-3_B31_NX_Pl_consensus     GAAGACCTGCAGGCACAAGATAAAGAAGAGCAGCAAATTAAGGCTGATACTAAAGTTATCAGTGTGCAGGAATTTGAA [ 1404]
cp32-3_PAli_NX_MP_FG_consensus GAAGACCTGCAGGCACAAGATAAAGAAGAGCAGCAAATTAAGGCTGATACTAAAGTTATCAGTGTGCAGGAATTTGAA [ 1404]
cp32-3_PAli_NX_Pl_consensus    GAAGACCTGCAGGCACAAGATAAAGAAGAGCAGCAAATTAAGGCTGATACTAAAGTTATCAGTGTGCAGGAATTTGAA [ 1404]
cp32-3_PAbe_NX_MP_FG_consensus GAAGACCTGCAGGCACAAGATAAAGAAGAGCAGCAAATTAAGGCTGATACTAAAGTTATCAGTGTGCAGGAATTTGAA [ 1404]
cp32-3_PAbe_NX_Pl_consensus    GAAGACCTGCAGGCACAAGATAAAGAAGAGCAGCAAATTAAGGCTGATACTAAAGTTATCAGTGTGCAGGAATTTGAA [ 1404]

cp32-3_gi|11497103|B31_GB      GAGTACATGCGTTTTAAAGAGCAGGCAAATAGTAAATCTAAAGAGACAAGTCGAGATTTAAGTATAAATGAACGAATA [ 1482]
cp32-3_un8_B31_PacBio          GAGTACATGCGTTTTAAAGAGCAGGCAAATAGTAAATCTAAAGAGACAAGTCGAGATTTAAGTATAAATGAACGAATA [ 1482]
cp32-3_B31_TS_MP_FG_consensus  GAGTACATGCGTTTTAAAGAGCAGGCAAATAGTAAATCTAAAGAGACAAGTCGAGATTTAAGTATAAATGAACGAATA [ 1482]
cp32-3_B31_NX_Pl_consensus     GAGTACATGCGTTTTAAAGAGCAGGCAAATAGTAAATCTAAAGAGACAAGTCGAGATTTAAGTATAAATGAACGAATA [ 1482]
cp32-3_PAli_NX_MP_FG_consensus GAGTACATGCGTTTTAAAGAGCAGGCAAATAGTAAATCTAAAGAGACAAGTCGAGATTTAAGTATAAATGAACGAATA [ 1482]
cp32-3_PAli_NX_Pl_consensus    GAGTACATGCGTTTTAAAGAGCAGGCAAATAGTAAATCTAAAGAGACAAGTCGAGATTTAAGTATAAATGAACGAATA [ 1482]
cp32-3_PAbe_NX_MP_FG_consensus GAGTACATGCGTTTTAAAGAGCAGGCAAATAGTAAATCTAAAGAGACAAGTCGAGATTTAAGTATAAATGAACGAATA [ 1482]
cp32-3_PAbe_NX_Pl_consensus    GAGTACATGCGTTTTAAAGAGCAGGCAAATAGTAAATCTAAAGAGACAAGTCGAGATTTAAGTATAAATGAACGAATA [ 1482]

cp32-3_gi|11497103|B31_GB      ACAAAAGAACTTGCAGAAGTTGAAGAGCGGGAGCGTATTGAAAAGCAATTGTTACTAGAGGCTGAGCGAATTAATGAA [ 1560]
cp32-3_un8_B31_PacBio          ACAAAAGAACTTGCAGAAGTTGAAGAGCGGGAGCGTATTGAAAAGCAATTGTTACTAGAGGCTGAGCGAATTAATGAA [ 1560]
cp32-3_B31_TS_MP_FG_consensus  ACAAAAGAACTTGCAGAAGTTGAAGAGCGGGAGCGTATTGAAAAGCAATTGTTACTAGAGGCTGAGCGAATTAATGAA [ 1560]
cp32-3_B31_NX_Pl_consensus     ACAAAAGAACTTGCAGAAGTTGAAGAGCGGGAGCGTATTGAAAAGCAATTGTTACTAGAGGCTGAGCGAATTAATGAA [ 1560]
cp32-3_PAli_NX_MP_FG_consensus ACAAAAGAACTTGCAGAAGTTGAAGAGCGGGAGCGTATTGAAAAGCAATTGTTACTAGAGGCTGAGCGAATTAATGAA [ 1560]
cp32-3_PAli_NX_Pl_consensus    ACAAAAGAACTTGCAGAAGTTGAAGAGCGGGAGCGTATTGAAAAGCAATTGTTACTAGAGGCTGAGCGAATTAATGAA [ 1560]
cp32-3_PAbe_NX_MP_FG_consensus ACAAAAGAACTTGCAGAAGTTGAAGAGCGGGAGCGTATTGAAAAGCAATTGTTACTAGAGGCTGAGCGAATTAATGAA [ 1560]
cp32-3_PAbe_NX_Pl_consensus    ACAAAAGAACTTGCAGAAGTTGAAGAGCGGGAGCGTATTGAAAAGCAATTGTTACTAGAGGCTGAGCGAATTAATGAA [ 1560]

cp32-3_gi|11497103|B31_GB      ATTGATACACTTGCAAAAGCACATCTTAGCAATCATTTTAACAAAGAGGTGCTACTTGCAAAAGGATATACATTAAAA [ 1638]
cp32-3_un8_B31_PacBio          ATTGATACACTTGCAAAAGCACATCTTAGCAATCATTTTAACAAAGAGGTGCTACTTGCAAAAGGATATACATTAAAA [ 1638]
cp32-3_B31_TS_MP_FG_consensus  ATTGATACACTTGCAAAAGCACATCTTAGCAATCATTTTAACAAAGAGGTGCTACTTGCAAAAGGATATACATTAAAA [ 1638]
cp32-3_B31_NX_Pl_consensus     ATTGATACACTTGCAAAAGCACATCTTAGCAATCATTTTAACAAAGAGGTGCTACTTGCAAAAGGATATACATTAAAA [ 1638]
cp32-3_PAli_NX_MP_FG_consensus ATTGATACACTTGCAAAAGCACATCTTAGCAATCATTTTAACAAAGAGGTGCTACTTGCAAAAGGATATACATTAAAA [ 1638]
cp32-3_PAli_NX_Pl_consensus    ATTGATACACTTGCAAAAGCACATCTTAGCAATCATTTTAACAAAGAGGTGCTACTTGCAAAAGGATATACATTAAAA [ 1638]
cp32-3_PAbe_NX_MP_FG_consensus ATTGATACACTTGCAAAAGCACATCTTAGCAATCATTTTAACAAAGAGGTGCTACTTGCAAAAGGATATACATTAAAA [ 1638]
cp32-3_PAbe_NX_Pl_consensus    ATTGATACACTTGCAAAAGCACATCTTAGCAATCATTTTAACAAAGAGGTGCTACTTGCAAAAGGATATACATTAAAA [ 1638]

cp32-3_gi|11497103|B31_GB      GACATTATGCAAGCACAACGTAGAGAACTTGTACGCAAGTTCGTTCCAATTGAGCAAATTAAAGCTATTGCCAAAGTA [ 1716]
cp32-3_un8_B31_PacBio          GACATTATGCAAGCACAACGTAGAGAACTTGTACGCAAGTTCGTTCCAATTGAGCAAATTAAAGCTATTGCCAAAGTA [ 1716]
cp32-3_B31_TS_MP_FG_consensus  GACATTATGCAAGCACAACGTAGAGAACTTGTACGCAAGTTCGTTCCAATTGAGCAAATTAAAGCTATTGCCAAAGTA [ 1716]
cp32-3_B31_NX_Pl_consensus     GACATTATGCAAGCACAACGTAGAGAACTTGTACGCAAGTTCGTTCCAATTGAGCAAATTAAAGCTATTGCCAAAGTA [ 1716]
cp32-3_PAli_NX_MP_FG_consensus GACATTATGCAAGCACAACGTAGAGAACTTGTACGCAAGTTCGTTCCAATTGAGCAAATTAAAGCTATTGCCAAAGTA [ 1716]
cp32-3_PAli_NX_Pl_consensus    GACATTATGCAAGCACAACGTAGAGAACTTGTACGCAAGTTCGTTCCAATTGAGCAAATTAAAGCTATTGCCAAAGTA [ 1716]
cp32-3_PAbe_NX_MP_FG_consensus GACATTATGCAAGCACAACGTAGAGAACTTGTACGCAAGTTCGTTCCAATTGAGCAAATTAAAGCTATTGCCAAAGTA [ 1716]
cp32-3_PAbe_NX_Pl_consensus    GACATTATGCAAGCACAACGTAGAGAACTTGTACGCAAGTTCGTTCCAATTGAGCAAATTAAAGCTATTGCCAAAGTA [ 1716]

cp32-3_gi|11497103|B31_GB      TCAGACATAAGTCATATCGATGGAGAGATATTAGAGCAACTTGTTTCTTTAGCAAAAGTGAATATTAAATTAAGAAAA [ 1794]
cp32-3_un8_B31_PacBio          TCAGACATAAGTCATATCGATGGAGAGATATTAGAGCAACTTGTTTCTTTAGCAAAAGTGAATATTAAATTAAGAAAA [ 1794]
cp32-3_B31_TS_MP_FG_consensus  TCAGACATAAGTCATATCGATGGAGAGATATTAGAGCAACTTGTTTCTTTAGCAAAAGTGAATATTAAATTAAGAAAA [ 1794]
cp32-3_B31_NX_Pl_consensus     TCAGACATAAGTCATATCGATGGAGAGATATTAGAGCAACTTGTTTCTTTAGCAAAAGTGAATATTAAATTAAGAAAA [ 1794]
cp32-3_PAli_NX_MP_FG_consensus TCAGACATAAGTCATATCGATGGAGAGATATTAGAGCAACTTGTTTCTTTAGCAAAAGTGAATATTAAATTAAGAAAA [ 1794]
cp32-3_PAli_NX_Pl_consensus    TCAGACATAAGTCATATCGATGGAGAGATATTAGAGCAACTTGTTTCTTTAGCAAAAGTGAATATTAAATTAAGAAAA [ 1794]
cp32-3_PAbe_NX_MP_FG_consensus TCAGACATAAGTCATATCGATGGAGAGATATTAGAGCAACTTGTTTCTTTAGCAAAAGTGAATATTAAATTAAGAAAA [ 1794]
cp32-3_PAbe_NX_Pl_consensus    TCAGACATAAGTCATATCGATGGAGAGATATTAGAGCAACTTGTTTCTTTAGCAAAAGTGAATATTAAATTAAGAAAA [ 1794]

cp32-3_gi|11497103|B31_GB      AATGCGAGTAGCAGCTCTTCTTCTGTTGACTCTATTAAGGGGAATATTGCTATTAAATCAGAAGAAAGAGCAAGTTTG [ 1872]
cp32-3_un8_B31_PacBio          AATGCGAGTAGCAGCTCTTCTTCTGTTGACTCTATTAAGGGGAATATTGCTATTAAATCAGAAGAAAGAGCAAGTTTG [ 1872]
cp32-3_B31_TS_MP_FG_consensus  AATGCGAGTAGCAGCTCTTCTTCTGTTGACTCTATTAAGGGGAATATTGCTATTAAATCAGAAGAAAGAGCAAGTTTG [ 1872]
```

```
cp32-3_B31_NX_Pl_consensus     AATGCGAGTAGCAGCTCTTCTTCTGTTGACTCTATTAAGGGGAATATTGCTATTAAATCAGAAGAAAGAGCAAGTTTG [ 1872]
cp32-3_PAli_NX_MP_FG_consensus AATGCGAGTAGCAGCTCTTCTTCTGTTGACTCTATTAAGGGGAATATTGCTATTAAATCAGAAGAAAGAGCAAGTTTG [ 1872]
cp32-3_PAli_NX_Pl_consensus    AATGCGAGTAGCAGCTCTTCTTCTGTTGACTCTATTAAGGGGAATATTGCTATTAAATCAGAAGAAAGAGCAAGTTTG [ 1872]
cp32-3_PAbe_NX_MP_FG_consensus AATGCGAGTAGCAGCTCTTCTTCTGTTGACTCTATTAAGGGGAATATTGCTATTAAATCAGAAGAAAGAGCAAGTTTG [ 1872]
cp32-3_PAbe_NX_Pl_consensus    AATGCGAGTAGCAGCTCTTCTTCTGTTGACTCTATTAAGGGGAATATTGCTATTAAATCAGAAGAAAGAGCAAGTTTG [ 1872]

cp32-3_gi|11497103|B31_GB      CTTGATTCTAATTTTGTACCTATTAATTTCACAGAATTTGTACAAGCGATAAGTAATACATACAAGCAAAGACGAATT [ 1950]
cp32-3_un8_B31_PacBio          CTTGATTCTAATTTTGTACCTATTAATTTCACAGAATTTGTACAAGCGATAAGTAATACATACAAGCAAAGACGAATT [ 1950]
cp32-3_B31_TS_MP_FG_consensus  CTTGATTCTAATTTTGTACCTATTAATTTCACAGAATTTGTACAAGCGATAAGTAATACATACAAGCAAAGACGAATT [ 1950]
cp32-3_B31_NX_Pl_consensus     CTTGATTCTAATTTTGTACCTATTAATTTCACAGAATTTGTACAAGCGATAAGTAATACATACAAGCAAAGACGAATT [ 1950]
cp32-3_PAli_NX_MP_FG_consensus CTTGATTCTAATTTTGTACCTATTAATTTCACAGAATTTGTACAAGCGATAAGTAATACATACAAGCAAAGACGAATT [ 1950]
cp32-3_PAli_NX_Pl_consensus    CTTGATTCTAATTTTGTACCTATTAATTTCACAGAATTTGTACAAGCGATAAGTAATACATACAAGCAAAGACGAATT [ 1950]
cp32-3_PAbe_NX_MP_FG_consensus CTTGATTCTAATTTTGTACCTATTAATTTCACAGAATTTGTACAAGCGATAAGTAATACATACAAGCAAAGACGAATT [ 1950]
cp32-3_PAbe_NX_Pl_consensus    CTTGATTCTAATTTTGTACCTATTAATTTCACAGAATTTGTACAAGCGATAAGTAATACATACAAGCAAAGACGAATT [ 1950]

cp32-3_gi|11497103|B31_GB      CAATTTTATGAAAATCTAAAAAGACATAAAAGAACAAGTATTGCCTAAAGGAGTTTTTAAATGAGTGATATAACAAAA [ 2028]
cp32-3_un8_B31_PacBio          CAATTTTATGAAAATCTAAAAAGACATAAAAGAACAAGTATTGCCTAAAGGAGTTTTTAAATGAGTGATATAACAAAA [ 2028]
cp32-3_B31_TS_MP_FG_consensus  CAATTTTATGAAAATCTAAAAAGACATAAAAGAACAAGTATTGCCTAAAGGAGTTTTTAAATGAGTGATATAACAAAA [ 2028]
cp32-3_B31_NX_Pl_consensus     CAATTTTATGAAAATCTAAAAAGACATAAAAGAACAAGTATTGCCTAAAGGAGTTTTTAAATGAGTGATATAACAAAA [ 2028]
cp32-3_PAli_NX_MP_FG_consensus CAATTTTATGAAAATCTAAAAAGACATAAAAGAACAAGTATTGCCTAAAGGAGTTTTTAAATGAGTGATATAACAAAA [ 2028]
cp32-3_PAli_NX_Pl_consensus    CAATTTTATGAAAATCTAAAAAGACATAAAAGAACAAGTATTGCCTAAAGGAGTTTTTAAATGAGTGATATAACAAAA [ 2028]
cp32-3_PAbe_NX_MP_FG_consensus CAATTTTATGAAAATCTAAAAAGACATAAAAGAACAAGTATTGCCTAAAGGAGTTTTTAAATGAGTGATATAACAAAA [ 2028]
cp32-3_PAbe_NX_Pl_consensus    CAATTTTATGAAAATCTAAAAAGACATAAAAGAACAAGTATTGCCTAAAGGAGTTTTTAAATGAGTGATATAACAAAA [ 2028]

cp32-3_gi|11497103|B31_GB      ATTAAACAAGAATTTGATAAGAAAGTTGCAGAAATTCAAGCATTAATGAAAAATCCCCAACAAGACTCAGGATTGCTT [ 2106]
cp32-3_un8_B31_PacBio          ATTAAACAAGAATTTGATAAGAAAGTTGCAGAAATTCAAGCATTAATGAAAAATCCCCAACAAGACTCAGGATTGCTT [ 2106]
cp32-3_B31_TS_MP_FG_consensus  ATTAAACAAGAATTTGATAAGAAAGTTGCAGAAATTCAAGCATTAATGAAAAATCCCCAACAAGACTCAGGATTGCTT [ 2106]
cp32-3_B31_NX_Pl_consensus     ATTAAACAAGAATTTGATAAGAAAGTTGCAGAAATTCAAGCATTAATGAAAAATCCCCAACAAGACTCAGGATTGCTT [ 2106]
cp32-3_PAli_NX_MP_FG_consensus ATTAAACAAGAATTTGATAAGAAAGTTGCAGAAATTCAAGCATTAATGAAAAATCCCCAACAAGACTCAGGATTGCTT [ 2106]
cp32-3_PAli_NX_Pl_consensus    ATTAAACAAGAATTTGATAAGAAAGTTGCAGAAATTCAAGCATTAATGAAAAATCCCCAACAAGACTCAGGATTGCTT [ 2106]
cp32-3_PAbe_NX_MP_FG_consensus ATTAAACAAGAATTTGATAAGAAAGTTGCAGAAATTCAAGCATTAATGAAAAATCCCCAACAAGACTCAGGATTGCTT [ 2106]
cp32-3_PAbe_NX_Pl_consensus    ATTAAACAAGAATTTGATAAGAAAGTTGCAGAAATTCAAGCATTAATGAAAAATCCCCAACAAGACTCAGGATTGCTT [ 2106]

cp32-3_gi|11497103|B31_GB      AGCAATTCTATTGATTTTAGAGACCAAAATCTAATTTTTTCCAATTCTGGTGGGGTTTGCACTAGCAGTAAAGACAAA [ 2184]
cp32-3_un8_B31_PacBio          AGCAATTCTATTGATTTTAGAGACCAAAATCTAATTTTTTCCAATTCTGGTGGGGTTTGCACTAGCAGTAAAGACAAA [ 2184]
cp32-3_B31_TS_MP_FG_consensus  AGCAATTCTATTGATTTTAGAGACCAAAATCTAATTTTTTCCAATTCTGGTGGGGTTTGCACTAGCAGTAAAGACAAA [ 2184]
cp32-3_B31_NX_Pl_consensus     AGCAATTCTATTGATTTTAGAGACCAAAATCTAATTTTTTCCAATTCTGGTGGGGTTTGCACTAGCAGTAAAGACAAA [ 2184]
cp32-3_PAli_NX_MP_FG_consensus AGCAATTCTATTGATTTTAGAGACCAAAATCTAATTTTTTCCAATTCTGGTGGGGTTTGCACTAGCAGTAAAGACAAA [ 2184]
cp32-3_PAli_NX_Pl_consensus    AGCAATTCTATTGATTTTAGAGACCAAAATCTAATTTTTTCCAATTCTGGTGGGGTTTGCACTAGCAGTAAAGACAAA [ 2184]
cp32-3_PAbe_NX_MP_FG_consensus AGCAATTCTATTGATTTTAGAGACCAAAATCTAATTTTTTCCAATTCTGGTGGGGTTTGCACTAGCAGTAAAGACAAA [ 2184]
cp32-3_PAbe_NX_Pl_consensus    AGCAATTCTATTGATTTTAGAGACCAAAATCTAATTTTTTCCAATTCTGGTGGGGTTTGCACTAGCAGTAAAGACAAA [ 2184]

cp32-3_gi|11497103|B31_GB      ATAGAGAATTACCCTGCTAAAGGGTATCCGTATAAACGGGGTGTTAAGCTTAGTTTTGGAGATGGAACAACCGAACTA [ 2262]
cp32-3_un8_B31_PacBio          ATAGAGAATTACCCTGCTAAAGGGTATCCGTATAAACGGGGTGTTAAGCTTAGTTTTGGAGATGGAACAACCGAACTA [ 2262]
cp32-3_B31_TS_MP_FG_consensus  ATAGAGAATTACCCTGCTAAAGGGTATCCGTATAAACGGGGTGTTAAGCTTAGTTTTGGAGATGGAACAACCGAACTA [ 2262]
cp32-3_B31_NX_Pl_consensus     ATAGAGAATTACCCTGCTAAAGGGTATCCGTATAAACGGGGTGTTAAGCTTAGTTTTGGAGATGGAACAACCGAACTA [ 2262]
cp32-3_PAli_NX_MP_FG_consensus ATAGAGAATTACCCTGCTAAAGGGTATCCGTATAAACGGGGTGTTAAGCTTAGTTTTGGAGATGGAACAACCGAACTA [ 2262]
cp32-3_PAli_NX_Pl_consensus    ATAGAGAATTACCCTGCTAAAGGGTATCCGTATAAACGGGGTGTTAAGCTTAGTTTTGGAGATGGAACAACCGAACTA [ 2262]
cp32-3_PAbe_NX_MP_FG_consensus ATAGAGAATTACCCTGCTAAAGGGTATCCGTATAAACGGGGTGTTAAGCTTAGTTTTGGAGATGGAACAACCGAACTA [ 2262]
cp32-3_PAbe_NX_Pl_consensus    ATAGAGAATTACCCTGCTAAAGGGTATCCGTATAAACGGGGTGTTAAGCTTAGTTTTGGAGATGGAACAACCGAACTA [ 2262]

cp32-3_gi|11497103|B31_GB      GAAGTTGAGGCTGGTGGTGGAGACGATTTATATGGAGTGTGTTCCGATATAGATGAGTTTAGCGGTATGGCAACTGTT [ 2340]
cp32-3_un8_B31_PacBio          GAAGTTGAGGCTGGTGGTGGAGACGATTTATATGGAGTGTGTTCCGATATAGATGAGTTTAGCGGTATGGCAACTGTT [ 2340]
cp32-3_B31_TS_MP_FG_consensus  GAAGTTGAGGCTGGTGGTGGAGACGATTTATATGGAGTGTGTTCCGATATAGATGAGTTTAGCGGTATGGCAACTGTT [ 2340]
cp32-3_B31_NX_Pl_consensus     GAAGTTGAGGCTGGTGGTGGAGACGATTTATATGGAGTGTGTTCCGATATAGATGAGTTTAGCGGTATGGCAACTGTT [ 2340]
cp32-3_PAli_NX_MP_FG_consensus GAAGTTGAGGCTGGTGGTGGAGACGATTTATATGGAGTGTGTTCCGATATAGATGAGTTTAGCGGTATGGCAACTGTT [ 2340]
cp32-3_PAli_NX_Pl_consensus    GAAGTTGAGGCTGGTGGTGGAGACGATTTATATGGAGTGTGTTCCGATATAGATGAGTTTAGCGGTATGGCAACTGTT [ 2340]
cp32-3_PAbe_NX_MP_FG_consensus GAAGTTGAGGCTGGTGGTGGAGACGATTTATATGGAGTGTGTTCCGATATAGATGAGTTTAGCGGTATGGCAACTGTT [ 2340]
cp32-3_PAbe_NX_Pl_consensus    GAAGTTGAGGCTGGTGGTGGAGACGATTTATATGGAGTGTGTTCCGATATAGATGAGTTTAGCGGTATGGCAACTGTT [ 2340]

cp32-3_gi|11497103|B31_GB      ATACCAATTACAAATAACTTTACTGGGTATTTAACGCTTAAGAAAGAAGGACAAAATGGTGTAAATCCAGGAGATAAA [ 2418]
cp32-3_un8_B31_PacBio          ATACCAATTACAAATAACTTTACTGGGTATTTAACGCTTAAGAAAGAAGGACAAAATGGTGTAAATCCAGGAGATAAA [ 2418]
cp32-3_B31_TS_MP_FG_consensus  ATACCAATTACAAATAACTTTACTGGGTATTTAACGCTTAAGAAAGAAGGACAAAATGGTGTAAATCCAGGAGATAAA [ 2418]
cp32-3_B31_NX_Pl_consensus     ATACCAATTACAAATAACTTTACTGGGTATTTAACGCTTAAGAAAGAAGGACAAAATGGTGTAAATCCAGGAGATAAA [ 2418]
cp32-3_PAli_NX_MP_FG_consensus ATACCAATTACAAATAACTTTACTGGGTATTTAACGCTTAAGAAAGAAGGACAAAATGGTGTAAATCCAGGAGATAAA [ 2418]
cp32-3_PAli_NX_Pl_consensus    ATACCAATTACAAATAACTTTACTGGGTATTTAACGCTTAAGAAAGAAGGACAAAATGGTGTAAATCCAGGAGATAAA [ 2418]
cp32-3_PAbe_NX_MP_FG_consensus ATACCAATTACAAATAACTTTACTGGGTATTTAACGCTTAAGAAAGAAGGACAAAATGGTGTAAATCCAGGAGATAAA [ 2418]
cp32-3_PAbe_NX_Pl_consensus    ATACCAATTACAAATAACTTTACTGGGTATTTAACGCTTAAGAAAGAAGGACAAAATGGTGTAAATCCAGGAGATAAA [ 2418]

cp32-3_gi|11497103|B31_GB      TTAAATTTTAACCAACATGGGGAACTTGAAAAGGTCACTGGGGCTCAAAAATCTGTTAATGCAATAGCACTTTCAAAG [ 2496]
cp32-3_un8_B31_PacBio          TTAAATTTTAACCAACATGGGGAACTTGAAAAGGTCACTGGGGCTCAAAAATCTGTTAATGCAATAGCACTTTCAAAG [ 2496]
cp32-3_B31_TS_MP_FG_consensus  TTAAATTTTAACCAACATGGGGAACTTGAAAAGGTCACTGGGGCTCAAAAATCTGTTAATGCAATAGCACTTTCAAAG [ 2496]
cp32-3_B31_NX_Pl_consensus     TTAAATTTTAACCAACATGGGGAACTTGAAAAGGTCACTGGGGCTCAAAAATCTGTTAATGCAATAGCACTTTCAAAG [ 2496]
cp32-3_PAli_NX_MP_FG_consensus TTAAATTTTAACCAACATGGGGAACTTGAAAAGGTCACTGGGGCTCAAAAATCTGTTAATGCAATAGCACTTTCAAAG [ 2496]
cp32-3_PAli_NX_Pl_consensus    TTAAATTTTAACCAACATGGGGAACTTGAAAAGGTCACTGGGGCTCAAAAATCTGTTAATGCAATAGCACTTTCAAAG [ 2496]
cp32-3_PAbe_NX_MP_FG_consensus TTAAATTTTAACCAACATGGGGAACTTGAAAAGGTCACTGGGGCTCAAAAATCTGTTAATGCAATAGCACTTTCAAAG [ 2496]
cp32-3_PAbe_NX_Pl_consensus    TTAAATTTTAACCAACATGGGGAACTTGAAAAGGTCACTGGGGCTCAAAAATCTGTTAATGCAATAGCACTTTCAAAG [ 2496]

cp32-3_gi|11497103|B31_GB      GCACACAAATTAACTGAAGATTTATTTATAGTGCTTGCTAGTGTATTTGGGAATAGAGCAATAAAAGGGTAATAAATT [ 2574]
cp32-3_un8_B31_PacBio          GCACACAAATTAACTGAAGATTTATTTATAGTGCTTGCTAGTGTATTTGGGAATAGAGCAATAAAAGGGTAATAAATT [ 2574]
cp32-3_B31_TS_MP_FG_consensus  GCACACAAATTAACTGAAGATTTATTTATAGTGCTTGCTAGTGTATTTGGGAATAGAGCAATAAAAGGGTAATAAATT [ 2574]
cp32-3_B31_NX_Pl_consensus     GCACACAAATTAACTGAAGATTTATTTATAGTGCTTGCTAGTGTATTTGGGAATAGAGCAATAAAAGGGTAATAAATT [ 2574]
cp32-3_PAli_NX_MP_FG_consensus GCACACAAATTAACTGAAGATTTATTTATAGTGCTTGCTAGTGTATTTGGGAATAGAGCAATAAAAGGGTAATAAATT [ 2574]
cp32-3_PAli_NX_Pl_consensus    GCACACAAATTAACTGAAGATTTATTTATAGTGCTTGCTAGTGTATTTGGGAATAGAGCAATAAAAGGGTAATAAATT [ 2574]
cp32-3_PAbe_NX_MP_FG_consensus GCACACAAATTAACTGAAGATTTATTTATAGTGCTTGCTAGTGTATTTGGGAATAGAGCAATAAAAGGGTAATAAATT [ 2574]
cp32-3_PAbe_NX_Pl_consensus    GCACACAAATTAACTGAAGATTTATTTATAGTGCTTGCTAGTGTATTTGGGAATAGAGCAATAAAAGGGTAATAAATT [ 2574]

cp32-3_gi|11497103|B31_GB      ATGGCTTTAAAAGGCAACATGCAAGTAGAAAATCTTGAGGCTGTTGAGGACCCACAGGTAGATTTAGGGGCACAAGTT [ 2652]
cp32-3_un8_B31_PacBio          ATGGCTTTAAAAGGCAACATGCAAGTAGAAAATCTTGAGGCTGTTGAGGACCCACAGGTAGATTTAGGGGCACAAGTT [ 2652]
cp32-3_B31_TS_MP_FG_consensus  ATGGCTTTAAAAGGCAACATGCAAGTAGAAAATCTTGAGGCTGTTGAGGACCCACAGGTAGATTTAGGGGCACAAGTT [ 2652]
cp32-3_B31_NX_Pl_consensus     ATGGCTTTAAAAGGCAACATGCAAGTAGAAAATCTTGAGGCTGTTGAGGACCCACAGGTAGATTTAGGGGCACAAGTT [ 2652]
cp32-3_PAli_NX_MP_FG_consensus ATGGCTTTAAAAGGCAACATGCAAGTAGAAAATCTTGAGGCTGTTGAGGACCCACAGGTAGATTTAGGGGCACAAGTT [ 2652]
cp32-3_PAli_NX_Pl_consensus    ATGGCTTTAAAAGGCAACATGCAAGTAGAAAATCTTGAGGCTGTTGAGGACCCACAGGTAGATTTAGGGGCACAAGTT [ 2652]
cp32-3_PAbe_NX_MP_FG_consensus ATGGCTTTAAAAGGCAACATGCAAGTAGAAAATCTTGAGGCTGTTGAGGACCCACAGGTAGATTTAGGGGCACAAGTT [ 2652]
cp32-3_PAbe_NX_Pl_consensus    ATGGCTTTAAAAGGCAACATGCAAGTAGAAAATCTTGAGGCTGTTGAGGACCCACAGGTAGATTTAGGGGCACAAGTT [ 2652]

cp32-3_gi|11497103|B31_GB      TCCGCTGCTCCTAGAGCTAAACGGCAAGCAAGACAAGCTGAGGATGCACAAGGGGAAGATCCCTATTTGGAGGCAATT [ 2730]
cp32-3_un8_B31_PacBio          TCCGCTGCTCCTAGAGCTAAACGGCAAGCAAGACAAGCTGAGGATGCACAAGGGGAAGATCCCTATTTGGAGGCAATT [ 2730]
cp32-3_B31_TS_MP_FG_consensus  TCCGCTGCTCCTAGAGCTAAACGGCAAGCAAGACAAGCTGAGGATGCACAAGGGGAAGATCCCTATTTGGAGGCAATT [ 2730]
cp32-3_B31_NX_Pl_consensus     TCCGCTGCTCCTAGAGCTAAACGGCAAGCAAGACAAGCTGAGGATGCACAAGGGGAAGATCCCTATTTGGAGGCAATT [ 2730]
cp32-3_PAli_NX_MP_FG_consensus TCCGCTGCTCCTAGAGCTAAACGGCAAGCAAGACAAGCTGAGGATGCACAAGGGGAAGATCCCTATTTGGAGGCAATT [ 2730]
cp32-3_PAli_NX_Pl_consensus    TCCGCTGCTCCTAGAGCTAAACGGCAAGCAAGACAAGCTGAGGATGCACAAGGGGAAGATCCCTATTTGGAGGCAATT [ 2730]
cp32-3_PAbe_NX_MP_FG_consensus TCCGCTGCTCCTAGAGCTAAACGGCAAGCAAGACAAGCTGAGGATGCACAAGGGGAAGATCCCTATTTGGAGGCAATT [ 2730]
cp32-3_PAbe_NX_Pl_consensus    TCCGCTGCTCCTAGAGCTAAACGGCAAGCAAGACAAGCTGAGGATGCACAAGGGGAAGATCCCTATTTGGAGGCAATT [ 2730]

cp32-3_gi|11497103|B31_GB      AACGAGCTTGATGATGTCCTTTTGAAATTCAAGAAATATGTAAAATCGATGAGTTCAATTGAAAATAAGGTTTTTGGC [ 2808]
cp32-3_un8_B31_PacBio          AACGAGCTTGATGATGTCCTTTTGAAATTCAAGAAATATGTAAAATCGATGAGTTCAATTGAAAATAAGGTTTTTGGC [ 2808]
cp32-3_B31_TS_MP_FG_consensus  AACGAGCTTGATGATGTCCTTTTGAAATTCAAGAAATATGTAAAATCGATGAGTTCAATTGAAAATAAGGTTTTTGGC [ 2808]
cp32-3_B31_NX_Pl_consensus     AACGAGCTTGATGATGTCCTTTTGAAATTCAAGAAATATGTAAAATCGATGAGTTCAATTGAAAATAAGGTTTTTGGC [ 2808]
```

```
cp32-3_PAli_NX_MP_FG_consensus AACGAGCTTGATGATGTCCTTTTGAAATTCAAGAAATATGTAAAATCGATGAGTTCAATTGAAAATAAGGTTTTTGGC [ 2808]
cp32-3_PAli_NX_Pl_consensus    AACGAGCTTGATGATGTCCTTTTGAAATTCAAGAAATATGTAAAATCGATGAGTTCAATTGAAAATAAGGTTTTTGGC [ 2808]
cp32-3_PAbe_NX_MP_FG_consensus AACGAGCTTGATGATGTCCTTTTGAAATTCAAGAAATATGTAAAATCGATGAGTTCAATTGAAAATAAGGTTTTTGGC [ 2808]
cp32-3_PAbe_NX_Pl_consensus    AACGAGCTTGATGATGTCCTTTTGAAATTCAAGAAATATGTAAAATCGATGAGTTCAATTGAAAATAAGGTTTTTGGC [ 2808]

cp32-3_gi|11497103|B31_GB      GGTTTAAGTAGTTGTTTTAAATCTAAGAATGAGCGAGTTGATGCATATTCATTTGCATGTTCAAGTTATACAGACAAA [ 2886]
cp32-3_un8_B31_PacBio          GGTTTAAGTAGTTGTTTTAAATCTAAGAATGAGCGAGTTGATGCATATTCATTTGCATGTTCAAGTTATACAGACAAA [ 2886]
cp32-3_B31_TS_MP_FG_consensus  GGTTTAAGTAGTTGTTTTAAATCTAAGAATGAGCGAGTTGATGCATATTCATTTGCATGTTCAAGTTATACAGACAAA [ 2886]
cp32-3_B31_NX_Pl_consensus     GGTTTAAGTAGTTGTTTTAAATCTAAGAATGAGCGAGTTGATGCATATTCATTTGCATGTTCAAGTTATACAGACAAA [ 2886]
cp32-3_PAli_NX_MP_FG_consensus GGTTTAAGTAGTTGTTTTAAATCTAAGAATGAGCGAGTTGATGCATATTCATTTGCATGTTCAAGTTATACAGACAAA [ 2886]
cp32-3_PAli_NX_Pl_consensus    GGTTTAAGTAGTTGTTTTAAATCTAAGAATGAGCGAGTTGATGCATATTCATTTGCATGTTCAAGTTATACAGACAAA [ 2886]
cp32-3_PAbe_NX_MP_FG_consensus GGTTTAAGTAGTTGTTTTAAATCTAAGAATGAGCGAGTTGATGCATATTCATTTGCATGTTCAAGTTATACAGACAAA [ 2886]
cp32-3_PAbe_NX_Pl_consensus    GGTTTAAGTAGTTGTTTTAAATCTAAGAATGAGCGAGTTGATGCATATTCATTTGCATGTTCAAGTTATACAGACAAA [ 2886]

cp32-3_gi|11497103|B31_GB      ATAGAGGAATACCTTTACGACCCAGCAAATAGTTTTCCATACAAGCGTGGGGTTAAACTTGTTCCAAAAGAGAACTCT [ 2964]
cp32-3_un8_B31_PacBio          ATAGAGGAATACCTTTACGACCCAGCAAATAGTTTTCCATACAAGCGTGGGGTTAAACTTGTTCCAAAAGAGAACTCT [ 2964]
cp32-3_B31_TS_MP_FG_consensus  ATAGAGGAATACCTTTACGACCCAGCAAATAGTTTTCCATACAAGCGTGGGGTTAAACTTGTTCCAAAAGAGAACTCT [ 2964]
cp32-3_B31_NX_Pl_consensus     ATAGAGGAATACCTTTACGACCCAGCAAATAGTTTTCCATACAAGCGTGGGGTTAAACTTGTTCCAAAAGAGAACTCT [ 2964]
cp32-3_PAli_NX_MP_FG_consensus ATAGAGGAATACCTTTACGACCCAGCAAATAGTTTTCCATACAAGCGTGGGGTTAAACTTGTTCCAAAAGAGAACTCT [ 2964]
cp32-3_PAli_NX_Pl_consensus    ATAGAGGAATACCTTTACGACCCAGCAAATAGTTTTCCATACAAGCGTGGGGTTAAACTTGTTCCAAAAGAGAACTCT [ 2964]
cp32-3_PAbe_NX_MP_FG_consensus ATAGAGGAATACCTTTACGACCCAGCAAATAGTTTTCCATACAAGCGTGGGGTTAAACTTGTTCCAAAAGAGAACTCT [ 2964]
cp32-3_PAbe_NX_Pl_consensus    ATAGAGGAATACCTTTACGACCCAGCAAATAGTTTTCCATACAAGCGTGGGGTTAAACTTGTTCCAAAAGAGAACTCT [ 2964]

cp32-3_gi|11497103|B31_GB      ATATATGTAGAAGTTGGAGCTGATACTGATATGTATGGGATATGTGTAGATGTATGTGAGTTTAGTTGTACCGCGTAT [ 3042]
cp32-3_un8_B31_PacBio          ATATATGTAGAAGTTGGAGCTGATACTGATATGTATGGGATATGTGTAGATGTATGTGAGTTTAGTTGTACCGCGTAT [ 3042]
cp32-3_B31_TS_MP_FG_consensus  ATATATGTAGAAGTTGGAGCTGATACTGATATGTATGGGATATGTGTAGATGTATGTGAGTTTAGTTGTACCGCGTAT [ 3042]
cp32-3_B31_NX_Pl_consensus     ATATATGTAGAAGTTGGAGCTGATACTGATATGTATGGGATATGTGTAGATGTATGTGAGTTTAGTTGTACCGCGTAT [ 3042]
cp32-3_PAli_NX_MP_FG_consensus ATATATGTAGAAGTTGGAGCTGATACTGATATGTATGGGATATGTGTAGATGTATGTGAGTTTAGTTGTACCGCGTAT [ 3042]
cp32-3_PAli_NX_Pl_consensus    ATATATGTAGAAGTTGGAGCTGATACTGATATGTATGGGATATGTGTAGATGTATGTGAGTTTAGTTGTACCGCGTAT [ 3042]
cp32-3_PAbe_NX_MP_FG_consensus ATATATGTAGAAGTTGGAGCTGATACTGATATGTATGGGATATGTGTAGATGTATGTGAGTTTAGTTGTACCGCGTAT [ 3042]
cp32-3_PAbe_NX_Pl_consensus    ATATATGTAGAAGTTGGAGCTGATACTGATATGTATGGGATATGTGTAGATGTATGTGAGTTTAGTTGTACCGCGTAT [ 3042]

cp32-3_gi|11497103|B31_GB      GTATTGCCAATTACTAACAATTTTGAAGGGTACCTTGTCACAAGGAATCCAAGTATAAAAATAGGAGAAATCCTAGAC [ 3120]
cp32-3_un8_B31_PacBio          GTATTGCCAATTACTAACAATTTTGAAGGGTACCTTGTCACAAGGAATCCAAGTATAAAAATAGGAGAAATCCTAGAC [ 3120]
cp32-3_B31_TS_MP_FG_consensus  GTATTGCCAATTACTAACAATTTTGAAGGGTACCTTGTCACAAGGAATCCAAGTATAAAAATAGGAGAAATCCTAGAC [ 3120]
cp32-3_B31_NX_Pl_consensus     GTATTGCCAATTACTAACAATTTTGAAGGGTACCTTGTCACAAGGAATCCAAGTATAAAAATAGGAGAAATCCTAGAC [ 3120]
cp32-3_PAli_NX_MP_FG_consensus GTATTGCCAATTACTAACAATTTTGAAGGGTACCTTGTCACAAGGAATCCAAGTATAAAAATAGGAGAAATCCTAGAC [ 3120]
cp32-3_PAli_NX_Pl_consensus    GTATTGCCAATTACTAACAATTTTGAAGGGTACCTTGTCACAAGGAATCCAAGTATAAAAATAGGAGAAATCCTAGAC [ 3120]
cp32-3_PAbe_NX_MP_FG_consensus GTATTGCCAATTACTAACAATTTTGAAGGGTACCTTGTCACAAGGAATCCAAGTATAAAAATAGGAGAAATCCTAGAC [ 3120]
cp32-3_PAbe_NX_Pl_consensus    GTATTGCCAATTACTAACAATTTTGAAGGGTACCTTGTCACAAGGAATCCAAGTATAAAAATAGGAGAAATCCTAGAC [ 3120]

cp32-3_gi|11497103|B31_GB      ATAAATAATAACGGTGTTATTATCAAGGCTGGAGGTGGGCCACCAACCGTAATTAACATATATGCTCTATCTGATTCA [ 3198]
cp32-3_un8_B31_PacBio          ATAAATAATAACGGTGTTATTATCAAGGCTGGAGGTGGGCCACCAACCGTAATTAACATATATGCTCTATCTGATTCA [ 3198]
cp32-3_B31_TS_MP_FG_consensus  ATAAATAATAACGGTGTTATTATCAAGGCTGGAGGTGGGCCACCAACCGTAATTAACATATATGCTCTATCTGATTCA [ 3198]
cp32-3_B31_NX_Pl_consensus     ATAAATAATAACGGTGTTATTATCAAGGCTGGAGGTGGGCCACCAACCGTAATTAACATATATGCTCTATCTGATTCA [ 3198]
cp32-3_PAli_NX_MP_FG_consensus ATAAATAATAACGGTGTTATTATCAAGGCTGGAGGTGGGCCACCAACCGTAATTAACATATATGCTCTATCTGATTCA [ 3198]
cp32-3_PAli_NX_Pl_consensus    ATAAATAATAACGGTGTTATTATCAAGGCTGGAGGTGGGCCACCAACCGTAATTAACATATATGCTCTATCTGATTCA [ 3198]
cp32-3_PAbe_NX_MP_FG_consensus ATAAATAATAACGGTGTTATTATCAAGGCTGGAGGTGGGCCACCAACCGTAATTAACATATATGCTCTATCTGATTCA [ 3198]
cp32-3_PAbe_NX_Pl_consensus    ATAAATAATAACGGTGTTATTATCAAGGCTGGAGGTGGGCCACCAACCGTAATTAACATATATGCTCTATCTGATTCA [ 3198]

cp32-3_gi|11497103|B31_GB      TTTACAATCAATTTTGCACCCGAAGATGGAAATCAAGATCAAAATAGATATCCTAGGCAAGAGTATTCTATTAATTTG [ 3276]
cp32-3_un8_B31_PacBio          TTTACAATCAATTTTGCACCCGAAGATGGAAATCAAGATCAAAATAGATATCCTAGGCAAGAGTATTCTATTAATTTG [ 3276]
cp32-3_B31_TS_MP_FG_consensus  TTTACAATCAATTTTGCACCCGAAGATGGAAATCAAGATCAAAATAGATATCCTAGGCAAGAGTATTCTATTAATTTG [ 3276]
cp32-3_B31_NX_Pl_consensus     TTTACAATCAATTTTGCACCCGAAGATGGAAATCAAGATCAAAATAGATATCCTAGGCAAGAGTATTCTATTAATTTG [ 3276]
cp32-3_PAli_NX_MP_FG_consensus TTTACAATCAATTTTGCACCCGAAGATGGAAATCAAGATCAAAATAGATATCCTAGGCAAGAGTATTCTATTAATTTG [ 3276]
cp32-3_PAli_NX_Pl_consensus    TTTACAATCAATTTTGCACCCGAAGATGGAAATCAAGATCAAAATAGATATCCTAGGCAAGAGTATTCTATTAATTTG [ 3276]
cp32-3_PAbe_NX_MP_FG_consensus TTTACAATCAATTTTGCACCCGAAGATGGAAATCAAGATCAAAATAGATATCCTAGGCAAGAGTATTCTATTAATTTG [ 3276]
cp32-3_PAbe_NX_Pl_consensus    TTTACAATCAATTTTGCACCCGAAGATGGAAATCAAGATCAAAATAGATATCCTAGGCAAGAGTATTCTATTAATTTG [ 3276]

cp32-3_gi|11497103|B31_GB      ATAAAAGTTGCAATTTTTGGAAATAGAGGCCTTGAGAAGATAGTAATACCTGATGGTGGTTAAACTTTGGGCGAATAA [ 3354]
cp32-3_un8_B31_PacBio          ATAAAAGTTGCAATTTTTGGAAATAGAGGCCTTGAGAAGATAGTAATACCTGATGGTGGTTAAACTTTGGGCGAATAA [ 3354]
cp32-3_B31_TS_MP_FG_consensus  ATAAAAGTTGCAATTTTTGGAAATAGAGGCCTTGAGAAGATAGTAATACCTGATGGTGGTTAAACTTTGGGCGAATAA [ 3354]
cp32-3_B31_NX_Pl_consensus     ATAAAAGTTGCAATTTTTGGAAATAGAGGCCTTGAGAAGATAGTAATACCTGATGGTGGTTAAACTTTGGGCGAATAA [ 3354]
cp32-3_PAli_NX_MP_FG_consensus ATAAAAGTTGCAATTTTTGGAAATAGAGGCCTTGAGAAGATAGTAATACCTGATGGTGGTTAAACTTTGGGCGAATAA [ 3354]
cp32-3_PAli_NX_Pl_consensus    ATAAAAGTTGCAATTTTTGGAAATAGAGGCCTTGAGAAGATAGTAATACCTGATGGTGGTTAAACTTTGGGCGAATAA [ 3354]
cp32-3_PAbe_NX_MP_FG_consensus ATAAAAGTTGCAATTTTTGGAAATAGAGGCCTTGAGAAGATAGTAATACCTGATGGTGGTTAAACTTTGGGCGAATAA [ 3354]
cp32-3_PAbe_NX_Pl_consensus    ATAAAAGTTGCAATTTTTGGAAATAGAGGCCTTGAGAAGATAGTAATACCTGATGGTGGTTAAACTTTGGGCGAATAA [ 3354]

cp32-3_gi|11497103|B31_GB      AAGGAGGTAAATAAATGGGAGATACAACGCAATTAGTAAAAGAGTATCAAGAGAAAAGAAGTAAACTGGAAAAGTTTA [ 3432]
cp32-3_un8_B31_PacBio          AAGGAGGTAAATAAATGGGAGATACAACGCAATTAGTAAAAGAGTATCAAGAGAAAAGAAGTAAACTGGAAAAGTTTA [ 3432]
cp32-3_B31_TS_MP_FG_consensus  AAGGAGGTAAATAAATGGGAGATACAACGCAATTAGTAAAAGAGTATCAAGAGAAAAGAAGTAAACTGGAAAAGTTTA [ 3432]
cp32-3_B31_NX_Pl_consensus     AAGGAGGTAAATAAATGGGAGATACAACGCAATTAGTAAAAGAGTATCAAGAGAAAAGAAGTAAACTGGAAAAGTTTA [ 3432]
cp32-3_PAli_NX_MP_FG_consensus AAGGAGGTAAATAAATGGGAGATACAACGCAATTAGTAAAAGAGTATCAAGAGAAAAGAAGTAAACTGGAAAAGTTTA [ 3432]
cp32-3_PAli_NX_Pl_consensus    AAGGAGGTAAATAAATGGGAGATACAACGCAATTAGTAAAAGAGTATCAAGAGAAAAGAAGTAAACTGGAAAAGTTTA [ 3432]
cp32-3_PAbe_NX_MP_FG_consensus AAGGAGGTAAATAAATGGGAGATACAACGCAATTAGTAAAAGAGTATCAAGAGAAAAGAAGTAAACTGGAAAAGTTTA [ 3432]
cp32-3_PAbe_NX_Pl_consensus    AAGGAGGTAAATAAATGGGAGATACAACGCAATTAGTAAAAGAGTATCAAGAGAAAAGAAGTAAACTGGAAAAGTTTA [ 3432]

cp32-3_gi|11497103|B31_GB      TGAAAAATCCCCAACATGACGCTAGTTTGCTTAGCAATTCTAATGAATTTAGAGACAAAAATGTGAAATTTTTTGCTT [ 3510]
cp32-3_un8_B31_PacBio          TGAAAAATCCCCAACATGACGCTAGTTTGCTTAGCAATTCTAATGAATTTAGAGACAAAAATGTGAAATTTTTTGCTT [ 3510]
cp32-3_B31_TS_MP_FG_consensus  TGAAAAATCCCCAACATGACGCTAGTTTGCTTAGCAATTCTAATGAATTTAGAGACAAAAATGTGAAATTTTTTGCTT [ 3510]
cp32-3_B31_NX_Pl_consensus     TGAAAAATCCCCAACATGACGCTAGTTTGCTTAGCAATTCTAATGAATTTAGAGACAAAAATGTGAAATTTTTTGCTT [ 3510]
cp32-3_PAli_NX_MP_FG_consensus TGAAAAATCCCCAACATGACGCTAGTTTGCTTAGCAATTCTAATGAATTTAGAGACAAAAATGTGAAATTTTTTGCTT [ 3510]
cp32-3_PAli_NX_Pl_consensus    TGAAAAATCCCCAACATGACGCTAGTTTGCTTAGCAATTCTAATGAATTTAGAGACAAAAATGTGAAATTTTTTGCTT [ 3510]
cp32-3_PAbe_NX_MP_FG_consensus TGAAAAATCCCCAACATGACGCTAGTTTGCTTAGCAATTCTAATGAATTTAGAGACAAAAATGTGAAATTTTTTGCTT [ 3510]
cp32-3_PAbe_NX_Pl_consensus    TGAAAAATCCCCAACATGACGCTAGTTTGCTTAGCAATTCTAATGAATTTAGAGACAAAAATGTGAAATTTTTTGCTT [ 3510]

cp32-3_gi|11497103|B31_GB      CTGGAGGCACTAGAACTAGTAAGTTTGATAAATTGGAAAATCATCCATTTTTGGGGTATCCGTACAAGCGTGGAGTAA [ 3588]
cp32-3_un8_B31_PacBio          CTGGAGGCACTAGAACTAGTAAGTTTGATAAATTGGAAAATCATCCATTTTTGGGGTATCCGTACAAGCGTGGAGTAA [ 3588]
cp32-3_B31_TS_MP_FG_consensus  CTGGAGGCACTAGAACTAGTAAGTTTGATAAATTGGAAAATCATCCATTTTTGGGGTATCCGTACAAGCGTGGAGTAA [ 3588]
cp32-3_B31_NX_Pl_consensus     CTGGAGGCACTAGAACTAGTAAGTTTGATAAATTGGAAAATCATCCATTTTTGGGGTATCCGTACAAGCGTGGAGTAA [ 3588]
cp32-3_PAli_NX_MP_FG_consensus CTGGAGGCACTAGAACTAGTAAGTTTGATAAATTGGAAAATCATCCATTTTTGGGGTATCCGTACAAGCGTGGAGTAA [ 3588]
cp32-3_PAli_NX_Pl_consensus    CTGGAGGCACTAGAACTAGTAAGTTTGATAAATTGGAAAATCATCCATTTTTGGGGTATCCGTACAAGCGTGGAGTAA [ 3588]
cp32-3_PAbe_NX_MP_FG_consensus CTGGAGGCACTAGAACTAGTAAGTTTGATAAATTGGAAAATCATCCATTTTTGGGGTATCCGTACAAGCGTGGAGTAA [ 3588]
cp32-3_PAbe_NX_Pl_consensus    CTGGAGGCACTAGAACTAGTAAGTTTGATAAATTGGAAAATCATCCATTTTTGGGGTATCCGTACAAGCGTGGAGTAA [ 3588]

cp32-3_gi|11497103|B31_GB      AAAGAGTTATTCAAGAGGCTCAAGATAATCAAAGTCACTATGAGCCACATGTTGAGGCTGGTGGAGGTGAAGACTTAT [ 3666]
cp32-3_un8_B31_PacBio          AAAGAGTTATTCAAGAGGCTCAAGATAATCAAAGTCACTATGAGCCACATGTTGAGGCTGGTGGAGGTGAAGACTTAT [ 3666]
cp32-3_B31_TS_MP_FG_consensus  AAAGAGTTATTCAAGAGGCTCAAGATAATCAAAGTCACTATGAGCCACATGTTGAGGCTGGTGGAGGTGAAGACTTAT [ 3666]
cp32-3_B31_NX_Pl_consensus     AAAGAGTTATTCAAGAGGCTCAAGATAATCAAAGTCACTATGAGCCACATGTTGAGGCTGGTGGAGGTGAAGACTTAT [ 3666]
cp32-3_PAli_NX_MP_FG_consensus AAAGAGTTATTCAAGAGGCTCAAGATAATCAAAGTCACTATGAGCCACATGTTGAGGCTGGTGGAGGTGAAGACTTAT [ 3666]
cp32-3_PAli_NX_Pl_consensus    AAAGAGTTATTCAAGAGGCTCAAGATAATCAAAGTCACTATGAGCCACATGTTGAGGCTGGTGGAGGTGAAGACTTAT [ 3666]
cp32-3_PAbe_NX_MP_FG_consensus AAAGAGTTATTCAAGAGGCTCAAGATAATCAAAGTCACTATGAGCCACATGTTGAGGCTGGTGGAGGTGAAGACTTAT [ 3666]
cp32-3_PAbe_NX_Pl_consensus    AAAGAGTTATTCAAGAGGCTCAAGATAATCAAAGTCACTATGAGCCACATGTTGAGGCTGGTGGAGGTGAAGACTTAT [ 3666]

cp32-3_gi|11497103|B31_GB      ATGGAATATGCATTGACATAGATGAGTTTAGTAAAACAGCTACTATTGTGCCAATTACCAATAATTTTGAGGGATATT [ 3744]
cp32-3_un8_B31_PacBio          ATGGAATATGCATTGACATAGATGAGTTTAGTAAAACAGCTACTATTGTGCCAATTACCAATAATTTTGAGGGATATT [ 3744]
cp32-3_B31_TS_MP_FG_consensus  ATGGAATATGCATTGACATAGATGAGTTTAGTAAAACAGCTACTATTGTGCCAATTACCAATAATTTTGAGGGATATT [ 3744]
cp32-3_B31_NX_Pl_consensus     ATGGAATATGCATTGACATAGATGAGTTTAGTAAAACAGCTACTATTGTGCCAATTACCAATAATTTTGAGGGATATT [ 3744]
cp32-3_PAli_NX_MP_FG_consensus ATGGAATATGCATTGACATAGATGAGTTTAGTAAAACAGCTACTATTGTGCCAATTACCAATAATTTTGAGGGATATT [ 3744]
```

```
cp32-3_PAli_NX_Pl_consensus    ATGGAATATGCATTGACATAGATGAGTTTAGTAAAACAGCTACTATTGTGCCAATTACCAATAATTTTGAGGGATATT [ 3744]
cp32-3_PAbe_NX_MP_FG_consensus ATGGAATATGCATTGACATAGATGAGTTTAGTAAAACAGCTACTATTGTGCCAATTACCAATAATTTTGAGGGATATT [ 3744]
cp32-3_PAbe_NX_Pl_consensus    ATGGAATATGCATTGACATAGATGAGTTTAGTAAAACAGCTACTATTGTGCCAATTACCAATAATTTTGAGGGATATT [ 3744]

cp32-3_gi|11497103|B31_GB      TAGTGGCAAAAGATTCTACGGTTAAAGTAAAAGACAAACTTGTTTTTAATAAAGACGGTGCTCTTGAAAAGGTGACTG [ 3822]
cp32-3_un8_B31_PacBio          TAGTGGCAAAAGATTCTACGGTTAAAGTAAAAGACAAACTTGTTTTTAATAAAGACGGTGCTCTTGAAAAGGTGACTG [ 3822]
cp32-3_B31_TS_MP_FG_consensus  TAGTGGCAAAAGATTCTACGGTTAAAGTAAAAGACAAACTTGTTTTTAATAAAGACGGTGCTCTTGAAAAGGTGACTG [ 3822]
cp32-3_B31_NX_Pl_consensus     TAGTGGCAAAAGATTCTACGGTTAAAGTAAAAGACAAACTTGTTTTTAATAAAGACGGTGCTCTTGAAAAGGTGACTG [ 3822]
cp32-3_PAli_NX_MP_FG_consensus TAGTGGCAAAAGATTCTACGGTTAAAGTAAAAGACAAACTTGTTTTTAATAAAGACGGTGCTCTTGAAAAGGTGACTG [ 3822]
cp32-3_PAli_NX_Pl_consensus    TAGTGGCAAAAGATTCTACGGTTAAAGTAAAAGACAAACTTGTTTTTAATAAAGACGGTGCTCTTGAAAAGGTGACTG [ 3822]
cp32-3_PAbe_NX_MP_FG_consensus TAGTGGCAAAAGATTCTACGGTTAAAGTAAAAGACAAACTTGTTTTTAATAAAGACGGTGCTCTTGAAAAGGTGACTG [ 3822]
cp32-3_PAbe_NX_Pl_consensus    TAGTGGCAAAAGATTCTACGGTTAAAGTAAAAGACAAACTTGTTTTTAATAAAGACGGTGCTCTTGAAAAGGTGACTG [ 3822]

cp32-3_gi|11497103|B31_GB      GAGCACCAAATAAAGCAACTATTAATGCAACAGCATTGACTGATGCGAAACAAATTAGCAATGAGGTTTATTTAGTAA [ 3900]
cp32-3_un8_B31_PacBio          GAGCACCAAATAAAGCAACTATTAATGCAACAGCATTGACTGATGCGAAACAAATTAGCAATGAGGTTTATTTAGTAA [ 3900]
cp32-3_B31_TS_MP_FG_consensus  GAGCACCAAATAAAGCAACTATTAATGCAACAGCATTGACTGATGCGAAACAAATTAGCAATGAGGTTTATTTAGTAA [ 3900]
cp32-3_B31_NX_Pl_consensus     GAGCACCAAATAAAGCAACTATTAATGCAACAGCATTGACTGATGCGAAACAAATTAGCAATGAGGTTTATTTAGTAA [ 3900]
cp32-3_PAli_NX_MP_FG_consensus GAGCACCAAATAAAGCAACTATTAATGCAACAGCATTGACTGATGCGAAACAAATTAGCAATGAGGTTTATTTAGTAA [ 3900]
cp32-3_PAli_NX_Pl_consensus    GAGCACCAAATAAAGCAACTATTAATGCAACAGCATTGACTGATGCGAAACAAATTAGCAATGAGGTTTATTTAGTAA [ 3900]
cp32-3_PAbe_NX_MP_FG_consensus GAGCACCAAATAAAGCAACTATTAATGCAACAGCATTGACTGATGCGAAACAAATTAGCAATGAGGTTTATTTAGTAA [ 3900]
cp32-3_PAbe_NX_Pl_consensus    GAGCACCAAATAAAGCAACTATTAATGCAACAGCATTGACTGATGCGAAACAAATTAGCAATGAGGTTTATTTAGTAA [ 3900]

cp32-3_gi|11497103|B31_GB      AAGTAGCTGTATTTGGGAATAAAGCTATGAGTAGAAATTAATAATATTTAGGAGGATTTAATATGGAATTATTTGATG [ 3978]
cp32-3_un8_B31_PacBio          AAGTAGCTGTATTTGGGAATAAAGCTATGAGTAGAAATTAATAATATTTAGGAGGATTTAATATGGAATTATTTGATG [ 3978]
cp32-3_B31_TS_MP_FG_consensus  AAGTAGCTGTATTTGGGAATAAAGCTATGAGTAGAAATTAATAATATTTAGGAGGATTTAATATGGAATTATTTGATG [ 3978]
cp32-3_B31_NX_Pl_consensus     AAGTAGCTGTATTTGGGAATAAAGCTATGAGTAGAAATTAATAATATTTAGGAGGATTTAATATGGAATTATTTGATG [ 3978]
cp32-3_PAli_NX_MP_FG_consensus AAGTAGCTGTATTTGGGAATAAAGCTATGAGTAGAAATTAATAATATTTAGGAGGATTTAATATGGAATTATTTGATG [ 3978]
cp32-3_PAli_NX_Pl_consensus    AAGTAGCTGTATTTGGGAATAAAGCTATGAGTAGAAATTAATAATATTTAGGAGGATTTAATATGGAATTATTTGATG [ 3978]
cp32-3_PAbe_NX_MP_FG_consensus AAGTAGCTGTATTTGGGAATAAAGCTATGAGTAGAAATTAATAATATTTAGGAGGATTTAATATGGAATTATTTGATG [ 3978]
cp32-3_PAbe_NX_Pl_consensus    AAGTAGCTGTATTTGGGAATAAAGCTATGAGTAGAAATTAATAATATTTAGGAGGATTTAATATGGAATTATTTGATG [ 3978]

cp32-3_gi|11497103|B31_GB      AAAATTATTATGCAAAAGCTGTGGCAAATATCATAGGAGAAGTTAAAGATCCTATTATGTATAAATGGTTTTCGCCCG [ 4056]
cp32-3_un8_B31_PacBio          AAAATTATTATGCAAAAGCTGTGGCAAATATCATAGGAGAAGTTAAAGATCCTATTATGTATAAATGGTTTTCGCCCG [ 4056]
cp32-3_B31_TS_MP_FG_consensus  AAAATTATTATGCAAAAGCTGTGGCAAATATCATAGGAGAAGTTAAAGATCCTATTATGTATAAATGGTTTTCGCCCG [ 4056]
cp32-3_B31_NX_Pl_consensus     AAAATTATTATGCAAAAGCTGTGGCAAATATCATAGGAGAAGTTAAAGATCCTATTATGTATAAATGGTTTTCGCCCG [ 4056]
cp32-3_PAli_NX_MP_FG_consensus AAAATTATTATGCAAAAGCTGTGGCAAATATCATAGGAGAAGTTAAAGATCCTATTATGTATAAATGGTTTTCGCCCG [ 4056]
cp32-3_PAli_NX_Pl_consensus    AAAATTATTATGCAAAAGCTGTGGCAAATATCATAGGAGAAGTTAAAGATCCTATTATGTATAAATGGTTTTCGCCCG [ 4056]
cp32-3_PAbe_NX_MP_FG_consensus AAAATTATTATGCAAAAGCTGTGGCAAATATCATAGGAGAAGTTAAAGATCCTATTATGTATAAATGGTTTTCGCCCG [ 4056]
cp32-3_PAbe_NX_Pl_consensus    AAAATTATTATGCAAAAGCTGTGGCAAATATCATAGGAGAAGTTAAAGATCCTATTATGTATAAATGGTTTTCGCCCG [ 4056]

cp32-3_gi|11497103|B31_GB      ATCAAATTGAAGATGTTGATCTACAAATGGGATATCAAAAAACCGTAAAATGGGACGCGTTTTTAAATGCTAATCCTA [ 4134]
cp32-3_un8_B31_PacBio          ATCAAATTGAAGATGTTGATCTACAAATGGGATATCAAAAAACCGTAAAATGGGACGCGTTTTTAAATGCTAATCCTA [ 4134]
cp32-3_B31_TS_MP_FG_consensus  ATCAAATTGAAGATGTTGATCTACAAATGGGATATCAAAAAACCGTAAAATGGGACGCGTTTTTAAATGCTAATCCTA [ 4134]
cp32-3_B31_NX_Pl_consensus     ATCAAATTGAAGATGTTGATCTACAAATGGGATATCAAAAAACCGTAAAATGGGACGCGTTTTTAAATGCTAATCCTA [ 4134]
cp32-3_PAli_NX_MP_FG_consensus ATCAAATTGAAGATGTTGATCTACAAATGGGATATCAAAAAACCGTAAAATGGGACGCGTTTTTAAATGCTAATCCTA [ 4134]
cp32-3_PAli_NX_Pl_consensus    ATCAAATTGAAGATGTTGATCTACAAATGGGATATCAAAAAACCGTAAAATGGGACGCGTTTTTAAATGCTAATCCTA [ 4134]
cp32-3_PAbe_NX_MP_FG_consensus ATCAAATTGAAGATGTTGATCTACAAATGGGATATCAAAAAACCGTAAAATGGGACGCGTTTTTAAATGCTAATCCTA [ 4134]
cp32-3_PAbe_NX_Pl_consensus    ATCAAATTGAAGATGTTGATCTACAAATGGGATATCAAAAAACCGTAAAATGGGACGCGTTTTTAAATGCTAATCCTA [ 4134]

cp32-3_gi|11497103|B31_GB      CAACAATTGCCAATGAGGTTAATACTATCTCAACTATTGGATTTAGTTCTGAAGTGGTAAGACTTAATTATTTGAAAT [ 4212]
cp32-3_un8_B31_PacBio          CAACAATTGCCAATGAGGTTAATACTATCTCAACTATTGGATTTAGTTCTGAAGTGGTAAGACTTAATTATTTGAAAT [ 4212]
cp32-3_B31_TS_MP_FG_consensus  CAACAATTGCCAATGAGGTTAATACTATCTCAACTATTGGATTTAGTTCTGAAGTGGTAAGACTTAATTATTTGAAAT [ 4212]
cp32-3_B31_NX_Pl_consensus     CAACAATTGCCAATGAGGTTAATACTATCTCAACTATTGGATTTAGTTCTGAAGTGGTAAGACTTAATTATTTGAAAT [ 4212]
cp32-3_PAli_NX_MP_FG_consensus CAACAATTGCCAATGAGGTTAATACTATCTCAACTATTGGATTTAGTTCTGAAGTGGTAAGACTTAATTATTTGAAAT [ 4212]
cp32-3_PAli_NX_Pl_consensus    CAACAATTGCCAATGAGGTTAATACTATCTCAACTATTGGATTTAGTTCTGAAGTGGTAAGACTTAATTATTTGAAAT [ 4212]
cp32-3_PAbe_NX_MP_FG_consensus CAACAATTGCCAATGAGGTTAATACTATCTCAACTATTGGATTTAGTTCTGAAGTGGTAAGACTTAATTATTTGAAAT [ 4212]
cp32-3_PAbe_NX_Pl_consensus    CAACAATTGCCAATGAGGTTAATACTATCTCAACTATTGGATTTAGTTCTGAAGTGGTAAGACTTAATTATTTGAAAT [ 4212]

cp32-3_gi|11497103|B31_GB      TACAGTATAAATTCAGACATTTAAAGCAGACTTCTGAGAAATTTTATACTTCAGATTCATATATTGGGGACATTAATA [ 4290]
cp32-3_un8_B31_PacBio          TACAGTATAAATTCAGACATTTAAAGCAGACTTCTGAGAAATTTTATACTTCAGATTCATATATTGGGGACATTAATA [ 4290]
cp32-3_B31_TS_MP_FG_consensus  TACAGTATAAATTCAGACATTTAAAGCAGACTTCTGAGAAATTTTATACTTCAGATTCATATATTGGGGACATTAATA [ 4290]
cp32-3_B31_NX_Pl_consensus     TACAGTATAAATTCAGACATTTAAAGCAGACTTCTGAGAAATTTTATACTTCAGATTCATATATTGGGGACATTAATA [ 4290]
cp32-3_PAli_NX_MP_FG_consensus TACAGTATAAATTCAGACATTTAAAGCAGACTTCTGAGAAATTTTATACTTCAGATTCATATATTGGGGACATTAATA [ 4290]
cp32-3_PAli_NX_Pl_consensus    TACAGTATAAATTCAGACATTTAAAGCAGACTTCTGAGAAATTTTATACTTCAGATTCATATATTGGGGACATTAATA [ 4290]
cp32-3_PAbe_NX_MP_FG_consensus TACAGTATAAATTCAGACATTTAAAGCAGACTTCTGAGAAATTTTATACTTCAGATTCATATATTGGGGACATTAATA [ 4290]
cp32-3_PAbe_NX_Pl_consensus    TACAGTATAAATTCAGACATTTAAAGCAGACTTCTGAGAAATTTTATACTTCAGATTCATATATTGGGGACATTAATA [ 4290]

cp32-3_gi|11497103|B31_GB      ATAATTTACTTCCTTTTGCTCAAGCGTATAAGCTTGCAAGTAGTGAAATTATTAAACTTATTAATCACTTTGTATTAA [ 4368]
cp32-3_un8_B31_PacBio          ATAATTTACTTCCTTTTGCTCAAGCGTATAAGCTTGCAAGTAGTGAAATTATTAAACTTATTAATCACTTTGTATTAA [ 4368]
cp32-3_B31_TS_MP_FG_consensus  ATAATTTACTTCCTTTTGCTCAAGCGTATAAGCTTGCAAGTAGTGAAATTATTAAACTTATTAATCACTTTGTATTAA [ 4368]
cp32-3_B31_NX_Pl_consensus     ATAATTTACTTCCTTTTGCTCAAGCGTATAAGCTTGCAAGTAGTGAAATTATTAAACTTATTAATCACTTTGTATTAA [ 4368]
cp32-3_PAli_NX_MP_FG_consensus ATAATTTACTTCCTTTTGCTCAAGCGTATAAGCTTGCAAGTAGTGAAATTATTAAACTTATTAATCACTTTGTATTAA [ 4368]
cp32-3_PAli_NX_Pl_consensus    ATAATTTACTTCCTTTTGCTCAAGCGTATAAGCTTGCAAGTAGTGAAATTATTAAACTTATTAATCACTTTGTATTAA [ 4368]
cp32-3_PAbe_NX_MP_FG_consensus ATAATTTACTTCCTTTTGCTCAAGCGTATAAGCTTGCAAGTAGTGAAATTATTAAACTTATTAATCACTTTGTATTAA [ 4368]
cp32-3_PAbe_NX_Pl_consensus    ATAATTTACTTCCTTTTGCTCAAGCGTATAAGCTTGCAAGTAGTGAAATTATTAAACTTATTAATCACTTTGTATTAA [ 4368]

cp32-3_gi|11497103|B31_GB      CCGGGACTGTTTCGATTCAAAAAGATGGGAAAAATCAAAAACGCCTGCTTCCAAATATGTATGGGCTGCTTAATATGC [ 4446]
cp32-3_un8_B31_PacBio          CCGGGACTGTTTCGATTCAAAAAGATGGGAAAAATCAAAAACGCCTGCTTCCAAATATGTATGGGCTGCTTAATATGC [ 4446]
cp32-3_B31_TS_MP_FG_consensus  CCGGGACTGTTTCGATTCAAAAAGATGGGAAAAATCAAAAACGCCTGCTTCCAAATATGTATGGGCTGCTTAATATGC [ 4446]
cp32-3_B31_NX_Pl_consensus     CCGGGACTGTTTCGATTCAAAAAGATGGGAAAAATCAAAAACGCCTGCTTCCAAATATGTATGGGCTGCTTAATATGC [ 4446]
cp32-3_PAli_NX_MP_FG_consensus CCGGGACTGTTTCGATTCAAAAAGATGGGAAAAATCAAAAACGCCTGCTTCCAAATATGTATGGGCTGCTTAATATGC [ 4446]
cp32-3_PAli_NX_Pl_consensus    CCGGGACTGTTTCGATTCAAAAAGATGGGAAAAATCAAAAACGCCTGCTTCCAAATATGTATGGGCTGCTTAATATGC [ 4446]
cp32-3_PAbe_NX_MP_FG_consensus CCGGGACTGTTTCGATTCAAAAAGATGGGAAAAATCAAAAACGCCTGCTTCCAAATATGTATGGGCTGCTTAATATGC [ 4446]
cp32-3_PAbe_NX_Pl_consensus    CCGGGACTGTTTCGATTCAAAAAGATGGGAAAAATCAAAAACGCCTGCTTCCAAATATGTATGGGCTGCTTAATATGC [ 4446]

cp32-3_gi|11497103|B31_GB      CCGAGCAGATAAAAGAAGAGGTTGCTAGTGGTGATAAAGATAAAATGGATAAAATCTTTGAAAAGATTGAGGCTGGAC [ 4524]
cp32-3_un8_B31_PacBio          CCGAGCAGATAAAAGAAGAGGTTGCTAGTGGTGATAAAGATAAAATGGATAAAATCTTTGAAAAGATTGAGGCTGGAC [ 4524]
cp32-3_B31_TS_MP_FG_consensus  CCGAGCAGATAAAAGAAGAGGTTGCTAGTGGTGATAAAGATAAAATGGATAAAATCTTTGAAAAGATTGAGGCTGGAC [ 4524]
cp32-3_B31_NX_Pl_consensus     CCGAGCAGATAAAAGAAGAGGTTGCTAGTGGTGATAAAGATAAAATGGATAAAATCTTTGAAAAGATTGAGGCTGGAC [ 4524]
cp32-3_PAli_NX_MP_FG_consensus CCGAGCAGATAAAAGAAGAGGTTGCTAGTGGTGATAAAGATAAAATGGATAAAATCTTTGAAAAGATTGAGGCTGGAC [ 4524]
cp32-3_PAli_NX_Pl_consensus    CCGAGCAGATAAAAGAAGAGGTTGCTAGTGGTGATAAAGATAAAATGGATAAAATCTTTGAAAAGATTGAGGCTGGAC [ 4524]
cp32-3_PAbe_NX_MP_FG_consensus CCGAGCAGATAAAAGAAGAGGTTGCTAGTGGTGATAAAGATAAAATGGATAAAATCTTTGAAAAGATTGAGGCTGGAC [ 4524]
cp32-3_PAbe_NX_Pl_consensus    CCGAGCAGATAAAAGAAGAGGTTGCTAGTGGTGATAAAGATAAAATGGATAAAATCTTTGAAAAGATTGAGGCTGGAC [ 4524]

cp32-3_gi|11497103|B31_GB      TTTCAAAGTTAGAACTGGGCGACGAATTTTCCACCCCGATGATGGTAATAGTTGACCCAGCAACGTCACTTAAACTAG [ 4602]
cp32-3_un8_B31_PacBio          TTTCAAAGTTAGAACTGGGCGACGAATTTTCCACCCCGATGATGGTAATAGTTGACCCAGCAACGTCACTTAAACTAG [ 4602]
cp32-3_B31_TS_MP_FG_consensus  TTTCAAAGTTAGAACTGGGCGACGAATTTTCCACCCCGATGATGGTAATAGTTGACCCAGCAACGTCACTTAAACTAG [ 4602]
cp32-3_B31_NX_Pl_consensus     TTTCAAAGTTAGAACTGGGCGACGAATTTTCCACCCCGATGATGGTAATAGTTGACCCAGCAACGTCACTTAAACTAG [ 4602]
cp32-3_PAli_NX_MP_FG_consensus TTTCAAAGTTAGAACTGGGCGACGAATTTTCCACCCCGATGATGGTAATAGTTGACCCAGCAACGTCACTTAAACTAG [ 4602]
cp32-3_PAli_NX_Pl_consensus    TTTCAAAGTTAGAACTGGGCGACGAATTTTCCACCCCGATGATGGTAATAGTTGACCCAGCAACGTCACTTAAACTAG [ 4602]
cp32-3_PAbe_NX_MP_FG_consensus TTTCAAAGTTAGAACTGGGCGACGAATTTTCCACCCCGATGATGGTAATAGTTGACCCAGCAACGTCACTTAAACTAG [ 4602]
cp32-3_PAbe_NX_Pl_consensus    TTTCAAAGTTAGAACTGGGCGACGAATTTTCCACCCCGATGATGGTAATAGTTGACCCAGCAACGTCACTTAAACTAG [ 4602]

cp32-3_gi|11497103|B31_GB      TAAAACCATACGCAGCAGCACAGGGTGCAGCAAGTAGTTGTGAAAAATGGGAAGATGTTTTAATTCAAACTATTAAGG [ 4680]
cp32-3_un8_B31_PacBio          TAAAACCATACGCAGCAGCACAGGGTGCAGCAAGTAGTTGTGAAAAATGGGAAGATGTTTTAATTCAAACTATTAAGG [ 4680]
cp32-3_B31_TS_MP_FG_consensus  TAAAACCATACGCAGCAGCACAGGGTGCAGCAAGTAGTTGTGAAAAATGGGAAGATGTTTTAATTCAAACTATTAAGG [ 4680]
cp32-3_B31_NX_Pl_consensus     TAAAACCATACGCAGCAGCACAGGGTGCAGCAAGTAGTTGTGAAAAATGGGAAGATGTTTTAATTCAAACTATTAAGG [ 4680]
cp32-3_PAli_NX_MP_FG_consensus TAAAACCATACGCAGCAGCACAGGGTGCAGCAAGTAGTTGTGAAAAATGGGAAGATGTTTTAATTCAAACTATTAAGG [ 4680]
cp32-3_PAli_NX_Pl_consensus    TAAAACCATACGCAGCAGCACAGGGTGCAGCAAGTAGTTGTGAAAAATGGGAAGATGTTTTAATTCAAACTATTAAGG [ 4680]
```

```
cp32-3_PAbe_NX_MP_FG_consensus TAAAACCATACGCAGCAGCACAGGGTGCAGCAAGTAGTTGTGAAAAATGGGAAGATGTTTTAATTCAAACTATTAAGG [ 4680]
cp32-3_PAbe_NX_Pl_consensus    TAAAACCATACGCAGCAGCACAGGGTGCAGCAAGTAGTTGTGAAAAATGGGAAGATGTTTTAATTCAAACTATTAAGG [ 4680]

cp32-3_gi|11497103|B31_GB      CTATTAATAATAGAGAAGATGTTTACATTGAAACTTCAAACTTGCTGAAACATAAAATACTCATTTATCCACTAAATT [ 4758]
cp32-3_un8_B31_PacBio          CTATTAATAATAGAGAAGATGTTTACATTGAAACTTCAAACTTGCTGAAACATAAAATACTCATTTATCCACTAAATT [ 4758]
cp32-3_B31_TS_MP_FG_consensus  CTATTAATAATAGAGAAGATGTTTACATTGAAACTTCAAACTTGCTGAAACATAAAATACTCATTTATCCACTAAATT [ 4758]
cp32-3_B31_NX_Pl_consensus     CTATTAATAATAGAGAAGATGTTTACATTGAAACTTCAAACTTGCTGAAACATAAAATACTCATTTATCCACTAAATT [ 4758]
cp32-3_PAli_NX_MP_FG_consensus CTATTAATAATAGAGAAGATGTTTACATTGAAACTTCAAACTTGCTGAAACATAAAATACTCATTTATCCACTAAATT [ 4758]
cp32-3_PAli_NX_Pl_consensus    CTATTAATAATAGAGAAGATGTTTACATTGAAACTTCAAACTTGCTGAAACATAAAATACTCATTTATCCACTAAATT [ 4758]
cp32-3_PAbe_NX_MP_FG_consensus CTATTAATAATAGAGAAGATGTTTACATTGAAACTTCAAACTTGCTGAAACATAAAATACTCATTTATCCACTAAATT [ 4758]
cp32-3_PAbe_NX_Pl_consensus    CTATTAATAATAGAGAAGATGTTTACATTGAAACTTCAAACTTGCTGAAACATAAAATACTCATTTATCCACTAAATT [ 4758]

cp32-3_gi|11497103|B31_GB      CTGAACTTATTAAATTTAAACCTAGCAAGTATATGCTACCTACACCGAATGAACAAGTTGATAAAGACTCAACCGATG [ 4836]
cp32-3_un8_B31_PacBio          CTGAACTTATTAAATTTAAACCTAGCAAGTATATGCTACCTACACCGAATGAACAAGTTGATAAAGACTCAACCGATG [ 4836]
cp32-3_B31_TS_MP_FG_consensus  CTGAACTTATTAAATTTAAACCTAGCAAGTATATGCTACCTACACCGAATGAACAAGTTGATAAAGACTCAACCGATG [ 4836]
cp32-3_B31_NX_Pl_consensus     CTGAACTTATTAAATTTAAACCTAGCAAGTATATGCTACCTACACCGAATGAACAAGTTGATAAAGACTCAACCGATG [ 4836]
cp32-3_PAli_NX_MP_FG_consensus CTGAACTTATTAAATTTAAACCTAGCAAGTATATGCTACCTACACCGAATGAACAAGTTGATAAAGACTCAACCGATG [ 4836]
cp32-3_PAli_NX_Pl_consensus    CTGAACTTATTAAATTTAAACCTAGCAAGTATATGCTACCTACACCGAATGAACAAGTTGATAAAGACTCAACCGATG [ 4836]
cp32-3_PAbe_NX_MP_FG_consensus CTGAACTTATTAAATTTAAACCTAGCAAGTATATGCTACCTACACCGAATGAACAAGTTGATAAAGACTCAACCGATG [ 4836]
cp32-3_PAbe_NX_Pl_consensus    CTGAACTTATTAAATTTAAACCTAGCAAGTATATGCTACCTACACCGAATGAACAAGTTGATAAAGACTCAACCGATG [ 4836]

cp32-3_gi|11497103|B31_GB      TAGCTCATTCATACATTGATTTTGTTTTAGGCGGTCTACTTGCTACTAGAAAAACTATTTTGCAAGTTAACATAAAGC [ 4914]
cp32-3_un8_B31_PacBio          TAGCTCATTCATACATTGATTTTGTTTTAGGCGGTCTACTTGCTACTAGAAAAACTATTTTGCAAGTTAACATAAAGC [ 4914]
cp32-3_B31_TS_MP_FG_consensus  TAGCTCATTCATACATTGATTTTGTTTTAGGCGGTCTACTTGCTACTAGAAAAACTATTTTGCAAGTTAACATAAAGC [ 4914]
cp32-3_B31_NX_Pl_consensus     TAGCTCATTCATACATTGATTTTGTTTTAGGCGGTCTACTTGCTACTAGAAAAACTATTTTGCAAGTTAACATAAAGC [ 4914]
cp32-3_PAli_NX_MP_FG_consensus TAGCTCATTCATACATTGATTTTGTTTTAGGCGGTCTACTTGCTACTAGAAAAACTATTTTGCAAGTTAACATAAAGC [ 4914]
cp32-3_PAli_NX_Pl_consensus    TAGCTCATTCATACATTGATTTTGTTTTAGGCGGTCTACTTGCTACTAGAAAAACTATTTTGCAAGTTAACATAAAGC [ 4914]
cp32-3_PAbe_NX_MP_FG_consensus TAGCTCATTCATACATTGATTTTGTTTTAGGCGGTCTACTTGCTACTAGAAAAACTATTTTGCAAGTTAACATAAAGC [ 4914]
cp32-3_PAbe_NX_Pl_consensus    TAGCTCATTCATACATTGATTTTGTTTTAGGCGGTCTACTTGCTACTAGAAAAACTATTTTGCAAGTTAACATAAAGC [ 4914]

cp32-3_gi|11497103|B31_GB      AAAGTTAAAAGTATAAGGTAAGTGAAAATGAGTGAACAAGAAAGCTTACAAGCACAAGTTGCGGGAGAAGAAGAACTT [ 4992]
cp32-3_un8_B31_PacBio          AAAGTTAAAAGTATAAGGTAAGTGAAAATGAGTGAACAAGAAAGCTTACAAGCACAAGTTGCGGGAGAAGAAGAACTT [ 4992]
cp32-3_B31_TS_MP_FG_consensus  AAAGTTAAAAGTATAAGGTAAGTGAAAATGAGTGAACAAGAAAGCTTACAAGCACAAGTTGCGGGAGAAGAAGAACTT [ 4992]
cp32-3_B31_NX_Pl_consensus     AAAGTTAAAAGTATAAGGTAAGTGAAAATGAGTGAACAAGAAAGCTTACAAGCACAAGTTGCGGGAGAAGAAGAACTT [ 4992]
cp32-3_PAli_NX_MP_FG_consensus AAAGTTAAAAGTATAAGGTAAGTGAAAATGAGTGAACAAGAAAGCTTACAAGCACAAGTTGCGGGAGAAGAAGAACTT [ 4992]
cp32-3_PAli_NX_Pl_consensus    AAAGTTAAAAGTATAAGGTAAGTGAAAATGAGTGAACAAGAAAGCTTACAAGCACAAGTTGCGGGAGAAGAAGAACTT [ 4992]
cp32-3_PAbe_NX_MP_FG_consensus AAAGTTAAAAGTATAAGGTAAGTGAAAATGAGTGAACAAGAAAGCTTACAAGCACAAGTTGCGGGAGAAGAAGAACTT [ 4992]
cp32-3_PAbe_NX_Pl_consensus    AAAGTTAAAAGTATAAGGTAAGTGAAAATGAGTGAACAAGAAAGCTTACAAGCACAAGTTGCGGGAGAAGAAGAACTT [ 4992]

cp32-3_gi|11497103|B31_GB      TTAGTAACAAAACTCCATTCAGAAGTGTTATTGCTATTAGGAATAGACAAATTTGCACTAAGCAGGCAAAATTTTCTA [ 5070]
cp32-3_un8_B31_PacBio          TTAGTAACAAAACTCCATTCAGAAGTGTTATTGCTATTAGGAATAGACAAATTTGCACTAAGCAGGCAAAATTTTCTA [ 5070]
cp32-3_B31_TS_MP_FG_consensus  TTAGTAACAAAACTCCATTCAGAAGTGTTATTGCTATTAGGAATAGACAAATTTGCACTAAGCAGGCAAAATTTTCTA [ 5070]
cp32-3_B31_NX_Pl_consensus     TTAGTAACAAAACTCCATTCAGAAGTGTTATTGCTATTAGGAATAGACAAATTTGCACTAAGCAGGCAAAATTTTCTA [ 5070]
cp32-3_PAli_NX_MP_FG_consensus TTAGTAACAAAACTCCATTCAGAAGTGTTATTGCTATTAGGAATAGACAAATTTGCACTAAGCAGGCAAAATTTTCTA [ 5070]
cp32-3_PAli_NX_Pl_consensus    TTAGTAACAAAACTCCATTCAGAAGTGTTATTGCTATTAGGAATAGACAAATTTGCACTAAGCAGGCAAAATTTTCTA [ 5070]
cp32-3_PAbe_NX_MP_FG_consensus TTAGTAACAAAACTCCATTCAGAAGTGTTATTGCTATTAGGAATAGACAAATTTGCACTAAGCAGGCAAAATTTTCTA [ 5070]
cp32-3_PAbe_NX_Pl_consensus    TTAGTAACAAAACTCCATTCAGAAGTGTTATTGCTATTAGGAATAGACAAATTTGCACTAAGCAGGCAAAATTTTCTA [ 5070]

cp32-3_gi|11497103|B31_GB      CTTCATTTATCCTTACTTCAAGCTATTCTAGTAACACGCGGTATTGATGCCAGTTCACTGACGTATGAACAAATATTT [ 5148]
cp32-3_un8_B31_PacBio          CTTCATTTATCCTTACTTCAAGCTATTCTAGTAACACGCGGTATTGATGCCAGTTCACTGACGTATGAACAAATATTT [ 5148]
cp32-3_B31_TS_MP_FG_consensus  CTTCATTTATCCTTACTTCAAGCTATTCTAGTAACACGCGGTATTGATGCCAGTTCACTGACGTATGAACAAATATTT [ 5148]
cp32-3_B31_NX_Pl_consensus     CTTCATTTATCCTTACTTCAAGCTATTCTAGTAACACGCGGTATTGATGCCAGTTCACTGACGTATGAACAAATATTT [ 5148]
cp32-3_PAli_NX_MP_FG_consensus CTTCATTTATCCTTACTTCAAGCTATTCTAGTAACACGCGGTATTGATGCCAGTTCACTGACGTATGAACAAATATTT [ 5148]
cp32-3_PAli_NX_Pl_consensus    CTTCATTTATCCTTACTTCAAGCTATTCTAGTAACACGCGGTATTGATGCCAGTTCACTGACGTATGAACAAATATTT [ 5148]
cp32-3_PAbe_NX_MP_FG_consensus CTTCATTTATCCTTACTTCAAGCTATTCTAGTAACACGCGGTATTGATGCCAGTTCACTGACGTATGAACAAATATTT [ 5148]
cp32-3_PAbe_NX_Pl_consensus    CTTCATTTATCCTTACTTCAAGCTATTCTAGTAACACGCGGTATTGATGCCAGTTCACTGACGTATGAACAAATATTT [ 5148]

cp32-3_gi|11497103|B31_GB      TTACTTACTTTCTACCATATGGGCTGCCAATTAAGAAAACAGGGAGTTGTTCGAGAATTTGAATTTGATAGGATCAAA [ 5226]
cp32-3_un8_B31_PacBio          TTACTTACTTTCTACCATATGGGCTGCCAATTAAGAAAACAGGGAGTTGTTCGAGAATTTGAATTTGATAGGATCAAA [ 5226]
cp32-3_B31_TS_MP_FG_consensus  TTACTTACTTTCTACCATATGGGCTGCCAATTAAGAAAACAGGGAGTTGTTCGAGAATTTGAATTTGATAGGATCAAA [ 5226]
cp32-3_B31_NX_Pl_consensus     TTACTTACTTTCTACCATATGGGCTGCCAATTAAGAAAACAGGGAGTTGTTCGAGAATTTGAATTTGATAGGATCAAA [ 5226]
cp32-3_PAli_NX_MP_FG_consensus TTACTTACTTTCTACCATATGGGCTGCCAATTAAGAAAACAGGGAGTTGTTCGAGAATTTGAATTTGATAGGATCAAA [ 5226]
cp32-3_PAli_NX_Pl_consensus    TTACTTACTTTCTACCATATGGGCTGCCAATTAAGAAAACAGGGAGTTGTTCGAGAATTTGAATTTGATAGGATCAAA [ 5226]
cp32-3_PAbe_NX_MP_FG_consensus TTACTTACTTTCTACCATATGGGCTGCCAATTAAGAAAACAGGGAGTTGTTCGAGAATTTGAATTTGATAGGATCAAA [ 5226]
cp32-3_PAbe_NX_Pl_consensus    TTACTTACTTTCTACCATATGGGCTGCCAATTAAGAAAACAGGGAGTTGTTCGAGAATTTGAATTTGATAGGATCAAA [ 5226]

cp32-3_gi|11497103|B31_GB      AAAGAGAAATTCAATGAACTTGAACTTGATTATTATCCTAGTAGCAGTGGAGGCGAAGAAGGCGGCGAGGGGAGTTGT [ 5304]
cp32-3_un8_B31_PacBio          AAAGAGAAATTCAATGAACTTGAACTTGATTATTATCCTAGTAGCAGTGGAGGCGAAGAAGGCGGCGAGGGGAGTTGT [ 5304]
cp32-3_B31_TS_MP_FG_consensus  AAAGAGAAATTCAATGAACTTGAACTTGATTATTATCCTAGTAGCAGTGGAGGCGAAGAAGGCGGCGAGGGGAGTTGT [ 5304]
cp32-3_B31_NX_Pl_consensus     AAAGAGAAATTCAATGAACTTGAACTTGATTATTATCCTAGTAGCAGTGGAGGCGAAGAAGGCGGCGAGGGGAGTTGT [ 5304]
cp32-3_PAli_NX_MP_FG_consensus AAAGAGAAATTCAATGAACTTGAACTTGATTATTATCCTAGTAGCAGTGGAGGCGAAGAAGGCGGCGAGGGGAGTTGT [ 5304]
cp32-3_PAli_NX_Pl_consensus    AAAGAGAAATTCAATGAACTTGAACTTGATTATTATCCTAGTAGCAGTGGAGGCGAAGAAGGCGGCGAGGGGAGTTGT [ 5304]
cp32-3_PAbe_NX_MP_FG_consensus AAAGAGAAATTCAATGAACTTGAACTTGATTATTATCCTAGTAGCAGTGGAGGCGAAGAAGGCGGCGAGGGGAGTTGT [ 5304]
cp32-3_PAbe_NX_Pl_consensus    AAAGAGAAATTCAATGAACTTGAACTTGATTATTATCCTAGTAGCAGTGGAGGCGAAGAAGGCGGCGAGGGGAGTTGT [ 5304]

cp32-3_gi|11497103|B31_GB      GGCTCAAACAAGAATTTTTGTTCACAACTTGATGCATTTTTAGAAAAACTAAAAAGAGAAACTTCAACGCCATCTTGT [ 5382]
cp32-3_un8_B31_PacBio          GGCTCAAACAAGAATTTTTGTTCACAACTTGATGCATTTTTAGAAAAACTAAAAAGAGAAACTTCAACGCCATCTTGT [ 5382]
cp32-3_B31_TS_MP_FG_consensus  GGCTCAAACAAGAATTTTTGTTCACAACTTGATGCATTTTTAGAAAAACTAAAAAGAGAAACTTCAACGCCATCTTGT [ 5382]
cp32-3_B31_NX_Pl_consensus     GGCTCAAACAAGAATTTTTGTTCACAACTTGATGCATTTTTAGAAAAACTAAAAAGAGAAACTTCAACGCCATCTTGT [ 5382]
cp32-3_PAli_NX_MP_FG_consensus GGCTCAAACAAGAATTTTTGTTCACAACTTGATGCATTTTTAGAAAAACTAAAAAGAGAAACTTCAACGCCATCTTGT [ 5382]
cp32-3_PAli_NX_Pl_consensus    GGCTCAAACAAGAATTTTTGTTCACAACTTGATGCATTTTTAGAAAAACTAAAAAGAGAAACTTCAACGCCATCTTGT [ 5382]
cp32-3_PAbe_NX_MP_FG_consensus GGCTCAAACAAGAATTTTTGTTCACAACTTGATGCATTTTTAGAAAAACTAAAAAGAGAAACTTCAACGCCATCTTGT [ 5382]
cp32-3_PAbe_NX_Pl_consensus    GGCTCAAACAAGAATTTTTGTTCACAACTTGATGCATTTTTAGAAAAACTAAAAAGAGAAACTTCAACGCCATCTTGT [ 5382]

cp32-3_gi|11497103|B31_GB      GTGGGGGTTGTCTAATGAATGGTGTTAGGAAAAGACTTTCTGATATGTCATTTCGCATGATCAACGTATTTAAGGATC [ 5460]
cp32-3_un8_B31_PacBio          GTGGGGGTTGTCTAATGAATGGTGTTAGGAAAAGACTTTCTGATATGTCATTTCGCATGATCAACGTATTTAAGGATC [ 5460]
cp32-3_B31_TS_MP_FG_consensus  GTGGGGGTTGTCTAATGAATGGTGTTAGGAAAAGACTTTCTGATATGTCATTTCGCATGATCAACGTATTTAAGGATC [ 5460]
cp32-3_B31_NX_Pl_consensus     GTGGGGGTTGTCTAATGAATGGTGTTAGGAAAAGACTTTCTGATATGTCATTTCGCATGATCAACGTATTTAAGGATC [ 5460]
cp32-3_PAli_NX_MP_FG_consensus GTGGGGGTTGTCTAATGAATGGTGTTAGGAAAAGACTTTCTGATATGTCATTTCGCATGATCAACGTATTTAAGGATC [ 5460]
cp32-3_PAli_NX_Pl_consensus    GTGGGGGTTGTCTAATGAATGGTGTTAGGAAAAGACTTTCTGATATGTCATTTCGCATGATCAACGTATTTAAGGATC [ 5460]
cp32-3_PAbe_NX_MP_FG_consensus GTGGGGGTTGTCTAATGAATGGTGTTAGGAAAAGACTTTCTGATATGTCATTTCGCATGATCAACGTATTTAAGGATC [ 5460]
cp32-3_PAbe_NX_Pl_consensus    GTGGGGGTTGTCTAATGAATGGTGTTAGGAAAAGACTTTCTGATATGTCATTTCGCATGATCAACGTATTTAAGGATC [ 5460]

cp32-3_gi|11497103|B31_GB      CTAAACCCTTAAAGTTTTATAAAGGTACTGTTGTAAAGCTTGAAAATGATTCTTCTTATCAGAGAGTATTTGATAAAA [ 5538]
cp32-3_un8_B31_PacBio          CTAAACCCTTAAAGTTTTATAAAGGTACTGTTGTAAAGCTTGAAAATGATTCTTCTTATCAGAGAGTATTTGATAAAA [ 5538]
cp32-3_B31_TS_MP_FG_consensus  CTAAACCCTTAAAGTTTTATAAAGGTACTGTTGTAAAGCTTGAAAATGATTCTTCTTATCAGAGAGTATTTGATAAAA [ 5538]
cp32-3_B31_NX_Pl_consensus     CTAAACCCTTAAAGTTTTATAAAGGTACTGTTGTAAAGCTTGAAAATGATTCTTCTTATCAGAGAGTATTTGATAAAA [ 5538]
cp32-3_PAli_NX_MP_FG_consensus CTAAACCCTTAAAGTTTTATAAAGGTACTGTTGTAAAGCTTGAAAATGATTCTTCTTATCAGAGAGTATTTGATAAAA [ 5538]
cp32-3_PAli_NX_Pl_consensus    CTAAACCCTTAAAGTTTTATAAAGGTACTGTTGTAAAGCTTGAAAATGATTCTTCTTATCAGAGAGTATTTGATAAAA [ 5538]
cp32-3_PAbe_NX_MP_FG_consensus CTAAACCCTTAAAGTTTTATAAAGGTACTGTTGTAAAGCTTGAAAATGATTCTTCTTATCAGAGAGTATTTGATAAAA [ 5538]
cp32-3_PAbe_NX_Pl_consensus    CTAAACCCTTAAAGTTTTATAAAGGTACTGTTGTAAAGCTTGAAAATGATTCTTCTTATCAGAGAGTATTTGATAAAA [ 5538]

cp32-3_gi|11497103|B31_GB      CTAAGTACATTGAATTTGCAGGAGTTATTATTGACATAAAGCCACAAGAACTTGCAATTCTTTATGATTCTGATATGT [ 5616]
cp32-3_un8_B31_PacBio          CTAAGTACATTGAATTTGCAGGAGTTATTATTGACATAAAGCCACAAGAACTTGCAATTCTTTATGATTCTGATATGT [ 5616]
cp32-3_B31_TS_MP_FG_consensus  CTAAGTACATTGAATTTGCAGGAGTTATTATTGACATAAAGCCACAAGAACTTGCAATTCTTTATGATTCTGATATGT [ 5616]
cp32-3_B31_NX_Pl_consensus     CTAAGTACATTGAATTTGCAGGAGTTATTATTGACATAAAGCCACAAGAACTTGCAATTCTTTATGATTCTGATATGT [ 5616]
cp32-3_PAli_NX_MP_FG_consensus CTAAGTACATTGAATTTGCAGGAGTTATTATTGACATAAAGCCACAAGAACTTGCAATTCTTTATGATTCTGATATGT [ 5616]
cp32-3_PAli_NX_Pl_consensus    CTAAGTACATTGAATTTGCAGGAGTTATTATTGACATAAAGCCACAAGAACTTGCAATTCTTTATGATTCTGATATGT [ 5616]
cp32-3_PAbe_NX_MP_FG_consensus CTAAGTACATTGAATTTGCAGGAGTTATTATTGACATAAAGCCACAAGAACTTGCAATTCTTTATGATTCTGATATGT [ 5616]
```

```
cp32-3_PAbe_NX_Pl_consensus    CTAAGTACATTGAATTTGCAGGAGTTATTATTGACATAAAGCCACAAGAACTTGCAATTCTTTATGATTCTGATATGT [ 5616]

cp32-3_gi|11497103|B31_GB      CTGATATTCAAGGATATTCCAAACTTTACACATATCAAGACCTTAACTATGAACCAAAAGACCGAATATCAATTGCAG [ 5694]
cp32-3_un8_B31_PacBio          CTGATATTCAAGGATATTCCAAACTTTACACATATCAAGACCTTAACTATGAACCAAAAGACCGAATATCAATTGCAG [ 5694]
cp32-3_B31_TS_MP_FG_consensus  CTGATATTCAAGGATATTCCAAACTTTACACATATCAAGACCTTAACTATGAACCAAAAGACCGAATATCAATTGCAG [ 5694]
cp32-3_B31_NX_Pl_consensus     CTGATATTCAAGGATATTCCAAACTTTACACATATCAAGACCTTAACTATGAACCAAAAGACCGAATATCAATTGCAG [ 5694]
cp32-3_PAli_NX_MP_FG_consensus CTGATATTCAAGGATATTCCAAACTTTACACATATCAAGACCTTAACTATGAACCAAAAGACCGAATATCAATTGCAG [ 5694]
cp32-3_PAli_NX_Pl_consensus    CTGATATTCAAGGATATTCCAAACTTTACACATATCAAGACCTTAACTATGAACCAAAAGACCGAATATCAATTGCAG [ 5694]
cp32-3_PAbe_NX_MP_FG_consensus CTGATATTCAAGGATATTCCAAACTTTACACATATCAAGACCTTAACTATGAACCAAAAGACCGAATATCAATTGCAG [ 5694]
cp32-3_PAbe_NX_Pl_consensus    CTGATATTCAAGGATATTCCAAACTTTACACATATCAAGACCTTAACTATGAACCAAAAGACCGAATATCAATTGCAG [ 5694]

cp32-3_gi|11497103|B31_GB      ATTTAGTTTACTTTGAAATATTTAGTATTGACTCTTCAATAGGATATTTTACTTTGGTTTTAAAGGAATTTATATGGA [ 5772]
cp32-3_un8_B31_PacBio          ATTTAGTTTACTTTGAAATATTTAGTATTGACTCTTCAATAGGATATTTTACTTTGGTTTTAAAGGAATTTATATGGA [ 5772]
cp32-3_B31_TS_MP_FG_consensus  ATTTAGTTTACTTTGAAATATTTAGTATTGACTCTTCAATAGGATATTTTACTTTGGTTTTAAAGGAATTTATATGGA [ 5772]
cp32-3_B31_NX_Pl_consensus     ATTTAGTTTACTTTGAAATATTTAGTATTGACTCTTCAATAGGATATTTTACTTTGGTTTTAAAGGAATTTATATGGA [ 5772]
cp32-3_PAli_NX_MP_FG_consensus ATTTAGTTTACTTTGAAATATTTAGTATTGACTCTTCAATAGGATATTTTACTTTGGTTTTAAAGGAATTTATATGGA [ 5772]
cp32-3_PAli_NX_Pl_consensus    ATTTAGTTTACTTTGAAATATTTAGTATTGACTCTTCAATAGGATATTTTACTTTGGTTTTAAAGGAATTTATATGGA [ 5772]
cp32-3_PAbe_NX_MP_FG_consensus ATTTAGTTTACTTTGAAATATTTAGTATTGACTCTTCAATAGGATATTTTACTTTGGTTTTAAAGGAATTTATATGGA [ 5772]
cp32-3_PAbe_NX_Pl_consensus    ATTTAGTTTACTTTGAAATATTTAGTATTGACTCTTCAATAGGATATTTTACTTTGGTTTTAAAGGAATTTATATGGA [ 5772]

cp32-3_gi|11497103|B31_GB      CAAACTAGAATTTAAAATGGAATTGGAAATTGGGTGGTTTGGTGGTCGTGCAGGTATTGCTAAAATGCATGAAAAAGG [ 5850]
cp32-3_un8_B31_PacBio          CAAACTAGAATTTAAAATGGAATTGGAAATTGGGTGGTTTGGTGGTCGTGCAGGTATTGCTAAAATGCATGAAAAAGG [ 5850]
cp32-3_B31_TS_MP_FG_consensus  CAAACTAGAATTTAAAATGGAATTGGAAATTGGGTGGTTTGGTGGTCGTGCAGGTATTGCTAAAATGCATGAAAAAGG [ 5850]
cp32-3_B31_NX_Pl_consensus     CAAACTAGAATTTAAAATGGAATTGGAAATTGGGTGGTTTGGTGGTCGTGCAGGTATTGCTAAAATGCATGAAAAAGG [ 5850]
cp32-3_PAli_NX_MP_FG_consensus CAAACTAGAATTTAAAATGGAATTGGAAATTGGGTGGTTTGGTGGTCGTGCAGGTATTGCTAAAATGCATGAAAAAGG [ 5850]
cp32-3_PAli_NX_Pl_consensus    CAAACTAGAATTTAAAATGGAATTGGAAATTGGGTGGTTTGGTGGTCGTGCAGGTATTGCTAAAATGCATGAAAAAGG [ 5850]
cp32-3_PAbe_NX_MP_FG_consensus CAAACTAGAATTTAAAATGGAATTGGAAATTGGGTGGTTTGGTGGTCGTGCAGGTATTGCTAAAATGCATGAAAAAGG [ 5850]
cp32-3_PAbe_NX_Pl_consensus    CAAACTAGAATTTAAAATGGAATTGGAAATTGGGTGGTTTGGTGGTCGTGCAGGTATTGCTAAAATGCATGAAAAAGG [ 5850]

cp32-3_gi|11497103|B31_GB      GAGTAGCAATTTACCAGCAAGAAAACATTTAACCAAAATTGCTAGTAGTTCTGAGTTTAGAGAATATATCAATAATAG [ 5928]
cp32-3_un8_B31_PacBio          GAGTAGCAATTTACCAGCAAGAAAACATTTAACCAAAATTGCTAGTAGTTCTGAGTTTAGAGAATATATCAATAATAG [ 5928]
cp32-3_B31_TS_MP_FG_consensus  GAGTAGCAATTTACCAGCAAGAAAACATTTAACCAAAATTGCTAGTAGTTCTGAGTTTAGAGAATATATCAATAATAG [ 5928]
cp32-3_B31_NX_Pl_consensus     GAGTAGCAATTTACCAGCAAGAAAACATTTAACCAAAATTGCTAGTAGTTCTGAGTTTAGAGAATATATCAATAATAG [ 5928]
cp32-3_PAli_NX_MP_FG_consensus GAGTAGCAATTTACCAGCAAGAAAACATTTAACCAAAATTGCTAGTAGTTCTGAGTTTAGAGAATATATCAATAATAG [ 5928]
cp32-3_PAli_NX_Pl_consensus    GAGTAGCAATTTACCAGCAAGAAAACATTTAACCAAAATTGCTAGTAGTTCTGAGTTTAGAGAATATATCAATAATAG [ 5928]
cp32-3_PAbe_NX_MP_FG_consensus GAGTAGCAATTTACCAGCAAGAAAACATTTAACCAAAATTGCTAGTAGTTCTGAGTTTAGAGAATATATCAATAATAG [ 5928]
cp32-3_PAbe_NX_Pl_consensus    GAGTAGCAATTTACCAGCAAGAAAACATTTAACCAAAATTGCTAGTAGTTCTGAGTTTAGAGAATATATCAATAATAG [ 5928]

cp32-3_gi|11497103|B31_GB      CTATATAAATTCTAAGTTTAATCTTGACCCTAAATCGGGAATGGAGGCTATTGGACAAGCTTTTATAAGGTACTATGA [ 6006]
cp32-3_un8_B31_PacBio          CTATATAAATTCTAAGTTTAATCTTGACCCTAAATCGGGAATGGAGGCTATTGGACAAGCTTTTATAAGGTACTATGA [ 6006]
cp32-3_B31_TS_MP_FG_consensus  CTATATAAATTCTAAGTTTAATCTTGACCCTAAATCGGGAATGGAGGCTATTGGACAAGCTTTTATAAGGTACTATGA [ 6006]
cp32-3_B31_NX_Pl_consensus     CTATATAAATTCTAAGTTTAATCTTGACCCTAAATCGGGAATGGAGGCTATTGGACAAGCTTTTATAAGGTACTATGA [ 6006]
cp32-3_PAli_NX_MP_FG_consensus CTATATAAATTCTAAGTTTAATCTTGACCCTAAATCGGGAATGGAGGCTATTGGACAAGCTTTTATAAGGTACTATGA [ 6006]
cp32-3_PAli_NX_Pl_consensus    CTATATAAATTCTAAGTTTAATCTTGACCCTAAATCGGGAATGGAGGCTATTGGACAAGCTTTTATAAGGTACTATGA [ 6006]
cp32-3_PAbe_NX_MP_FG_consensus CTATATAAATTCTAAGTTTAATCTTGACCCTAAATCGGGAATGGAGGCTATTGGACAAGCTTTTATAAGGTACTATGA [ 6006]
cp32-3_PAbe_NX_Pl_consensus    CTATATAAATTCTAAGTTTAATCTTGACCCTAAATCGGGAATGGAGGCTATTGGACAAGCTTTTATAAGGTACTATGA [ 6006]

cp32-3_gi|11497103|B31_GB      AAATTATCTACTATCAGCACAAGTCACTCCAGCCTTAAAGGCTAATACAATCAAAAGTAAGTTTAAAAGGGGTAGTAA [ 6084]
cp32-3_un8_B31_PacBio          AAATTATCTACTATCAGCACAAGTCACTCCAGCCTTAAAGGCTAATACAATCAAAAGTAAGTTTAAAAGGGGTAGTAA [ 6084]
cp32-3_B31_TS_MP_FG_consensus  AAATTATCTACTATCAGCACAAGTCACTCCAGCCTTAAAGGCTAATACAATCAAAAGTAAGTTTAAAAGGGGTAGTAA [ 6084]
cp32-3_B31_NX_Pl_consensus     AAATTATCTACTATCAGCACAAGTCACTCCAGCCTTAAAGGCTAATACAATCAAAAGTAAGTTTAAAAGGGGTAGTAA [ 6084]
cp32-3_PAli_NX_MP_FG_consensus AAATTATCTACTATCAGCACAAGTCACTCCAGCCTTAAAGGCTAATACAATCAAAAGTAAGTTTAAAAGGGGTAGTAA [ 6084]
cp32-3_PAli_NX_Pl_consensus    AAATTATCTACTATCAGCACAAGTCACTCCAGCCTTAAAGGCTAATACAATCAAAAGTAAGTTTAAAAGGGGTAGTAA [ 6084]
cp32-3_PAbe_NX_MP_FG_consensus AAATTATCTACTATCAGCACAAGTCACTCCAGCCTTAAAGGCTAATACAATCAAAAGTAAGTTTAAAAGGGGTAGTAA [ 6084]
cp32-3_PAbe_NX_Pl_consensus    AAATTATCTACTATCAGCACAAGTCACTCCAGCCTTAAAGGCTAATACAATCAAAAGTAAGTTTAAAAGGGGTAGTAA [ 6084]

cp32-3_gi|11497103|B31_GB      CACTGCAGCAATTCCACTTGTTGATACAGCCAAAATGTTATCAGAGATTACTTATAAGGTAACACTTGAATGATTTTC [ 6162]
cp32-3_un8_B31_PacBio          CACTGCAGCAATTCCACTTGTTGATACAGCCAAAATGTTATCAGAGATTACTTATAAGGTAACACTTGAATGATTTTC [ 6162]
cp32-3_B31_TS_MP_FG_consensus  CACTGCAGCAATTCCACTTGTTGATACAGCCAAAATGTTATCAGAGATTACTTATAAGGTAACACTTGAATGATTTTC [ 6162]
cp32-3_B31_NX_Pl_consensus     CACTGCAGCAATTCCACTTGTTGATACAGCCAAAATGTTATCAGAGATTACTTATAAGGTAACACTTGAATGATTTTC [ 6162]
cp32-3_PAli_NX_MP_FG_consensus CACTGCAGCAATTCCACTTGTTGATACAGCCAAAATGTTATCAGAGATTACTTATAAGGTAACACTTGAATGATTTTC [ 6162]
cp32-3_PAli_NX_Pl_consensus    CACTGCAGCAATTCCACTTGTTGATACAGCCAAAATGTTATCAGAGATTACTTATAAGGTAACACTTGAATGATTTTC [ 6162]
cp32-3_PAbe_NX_MP_FG_consensus CACTGCAGCAATTCCACTTGTTGATACAGCCAAAATGTTATCAGAGATTACTTATAAGGTAACACTTGAATGATTTTC [ 6162]
cp32-3_PAbe_NX_Pl_consensus    CACTGCAGCAATTCCACTTGTTGATACAGCCAAAATGTTATCAGAGATTACTTATAAGGTAACACTTGAATGATTTTC [ 6162]

cp32-3_gi|11497103|B31_GB      ACTTTAGATATGGTATTAAATCATTTAACCCAAATATTTAAAGGGTTTAAGGCATATGCAACTGAAAATAATTTTGAG [ 6240]
cp32-3_un8_B31_PacBio          ACTTTAGATATGGTATTAAATCATTTAACCCAAATATTTAAAGGGTTTAAGGCATATGCAACTGAAAATAATTTTGAG [ 6240]
cp32-3_B31_TS_MP_FG_consensus  ACTTTAGATATGGTATTAAATCATTTAACCCAAATATTTAAAGGGTTTAAGGCATATGCAACTGAAAATAATTTTGAG [ 6240]
cp32-3_B31_NX_Pl_consensus     ACTTTAGATATGGTATTAAATCATTTAACCCAAATATTTAAAGGGTTTAAGGCATATGCAACTGAAAATAATTTTGAG [ 6240]
cp32-3_PAli_NX_MP_FG_consensus ACTTTAGATATGGTATTAAATCATTTAACCCAAATATTTAAAGGGTTTAAGGCATATGCAACTGAAAATAATTTTGAG [ 6240]
cp32-3_PAli_NX_Pl_consensus    ACTTTAGATATGGTATTAAATCATTTAACCCAAATATTTAAAGGGTTTAAGGCATATGCAACTGAAAATAATTTTGAG [ 6240]
cp32-3_PAbe_NX_MP_FG_consensus ACTTTAGATATGGTATTAAATCATTTAACCCAAATATTTAAAGGGTTTAAGGCATATGCAACTGAAAATAATTTTGAG [ 6240]
cp32-3_PAbe_NX_Pl_consensus    ACTTTAGATATGGTATTAAATCATTTAACCCAAATATTTAAAGGGTTTAAGGCATATGCAACTGAAAATAATTTTGAG [ 6240]

cp32-3_gi|11497103|B31_GB      TGCGATATCATAAATACCTACAATCATCCATATCTTTCAAAAATCACAGCTGCTAGCTCAAATATAATAGCATTGAAA [ 6318]
cp32-3_un8_B31_PacBio          TGCGATATCATAAATACCTACAATCATCCATATCTTTCAAAAATCACAGCTGCTAGCTCAAATATAATAGCATTGAAA [ 6318]
cp32-3_B31_TS_MP_FG_consensus  TGCGATATCATAAATACCTACAATCATCCATATCTTTCAAAAATCACAGCTGCTAGCTCAAATATAATAGCATTGAAA [ 6318]
cp32-3_B31_NX_Pl_consensus     TGCGATATCATAAATACCTACAATCATCCATATCTTTCAAAAATCACAGCTGCTAGCTCAAATATAATAGCATTGAAA [ 6318]
cp32-3_PAli_NX_MP_FG_consensus TGCGATATCATAAATACCTACAATCATCCATATCTTTCAAAAATCACAGCTGCTAGCTCAAATATAATAGCATTGAAA [ 6318]
cp32-3_PAli_NX_Pl_consensus    TGCGATATCATAAATACCTACAATCATCCATATCTTTCAAAAATCACAGCTGCTAGCTCAAATATAATAGCATTGAAA [ 6318]
cp32-3_PAbe_NX_MP_FG_consensus TGCGATATCATAAATACCTACAATCATCCATATCTTTCAAAAATCACAGCTGCTAGCTCAAATATAATAGCATTGAAA [ 6318]
cp32-3_PAbe_NX_Pl_consensus    TGCGATATCATAAATACCTACAATCATCCATATCTTTCAAAAATCACAGCTGCTAGCTCAAATATAATAGCATTGAAA [ 6318]

cp32-3_gi|11497103|B31_GB      TTTGATGGTACAGAAAATCTATTTGATCATAATTATAGAGCCGGTGTATTTTATGAAAATGCTTTGGAATTTAGTATA [ 6396]
cp32-3_un8_B31_PacBio          TTTGATGGTACAGAAAATCTATTTGATCATAATTATAGAGCCGGTGTATTTTATGAAAATGCTTTGGAATTTAGTATA [ 6396]
cp32-3_B31_TS_MP_FG_consensus  TTTGATGGTACAGAAAATCTATTTGATCATAATTATAGAGCCGGTGTATTTTATGAAAATGCTTTGGAATTTAGTATA [ 6396]
cp32-3_B31_NX_Pl_consensus     TTTGATGGTACAGAAAATCTATTTGATCATAATTATAGAGCCGGTGTATTTTATGAAAATGCTTTGGAATTTAGTATA [ 6396]
cp32-3_PAli_NX_MP_FG_consensus TTTGATGGTACAGAAAATCTATTTGATCATAATTATAGAGCCGGTGTATTTTATGAAAATGCTTTGGAATTTAGTATA [ 6396]
cp32-3_PAli_NX_Pl_consensus    TTTGATGGTACAGAAAATCTATTTGATCATAATTATAGAGCCGGTGTATTTTATGAAAATGCTTTGGAATTTAGTATA [ 6396]
cp32-3_PAbe_NX_MP_FG_consensus TTTGATGGTACAGAAAATCTATTTGATCATAATTATAGAGCCGGTGTATTTTATGAAAATGCTTTGGAATTTAGTATA [ 6396]
cp32-3_PAbe_NX_Pl_consensus    TTTGATGGTACAGAAAATCTATTTGATCATAATTATAGAGCCGGTGTATTTTATGAAAATGCTTTGGAATTTAGTATA [ 6396]

cp32-3_gi|11497103|B31_GB      AATTTTCAAATATATATTATTGCAATAGTGTTAAACGCCAAAGACTTTGACGCTAATTCACGCATGTTAATGCTTTAT [ 6474]
cp32-3_un8_B31_PacBio          AATTTTCAAATATATATTATTGCAATAGTGTTAAACGCCAAAGACTTTGACGCTAATTCACGCATGTTAATGCTTTAT [ 6474]
cp32-3_B31_TS_MP_FG_consensus  AATTTTCAAATATATATTATTGCAATAGTGTTAAACGCCAAAGACTTTGACGCTAATTCACGCATGTTAATGCTTTAT [ 6474]
cp32-3_B31_NX_Pl_consensus     AATTTTCAAATATATATTATTGCAATAGTGTTAAACGCCAAAGACTTTGACGCTAATTCACGCATGTTAATGCTTTAT [ 6474]
cp32-3_PAli_NX_MP_FG_consensus AATTTTCAAATATATATTATTGCAATAGTGTTAAACGCCAAAGACTTTGACGCTAATTCACGCATGTTAATGCTTTAT [ 6474]
cp32-3_PAli_NX_Pl_consensus    AATTTTCAAATATATATTATTGCAATAGTGTTAAACGCCAAAGACTTTGACGCTAATTCACGCATGTTAATGCTTTAT [ 6474]
cp32-3_PAbe_NX_MP_FG_consensus AATTTTCAAATATATATTATTGCAATAGTGTTAAACGCCAAAGACTTTGACGCTAATTCACGCATGTTAATGCTTTAT [ 6474]
cp32-3_PAbe_NX_Pl_consensus    AATTTTCAAATATATATTATTGCAATAGTGTTAAACGCCAAAGACTTTGACGCTAATTCACGCATGTTAATGCTTTAT [ 6474]

cp32-3_gi|11497103|B31_GB      AGTATGCTTAGTGACTTTCTACACAATAAAGCTCATAAGTATACTTTGCCCAGTCTACAACCCGACTATATTAATAAA [ 6552]
cp32-3_un8_B31_PacBio          AGTATGCTTAGTGACTTTCTACACAATAAAGCTCATAAGTATACTTTGCCCAGTCTACAACCCGACTATATTAATAAA [ 6552]
cp32-3_B31_TS_MP_FG_consensus  AGTATGCTTAGTGACTTTCTACACAATAAAGCTCATAAGTATACTTTGCCCAGTCTACAACCCGACTATATTAATAAA [ 6552]
cp32-3_B31_NX_Pl_consensus     AGTATGCTTAGTGACTTTCTACACAATAAAGCTCATAAGTATACTTTGCCCAGTCTACAACCCGACTATATTAATAAA [ 6552]
cp32-3_PAli_NX_MP_FG_consensus AGTATGCTTAGTGACTTTCTACACAATAAAGCTCATAAGTATACTTTGCCCAGTCTACAACCCGACTATATTAATAAA [ 6552]
cp32-3_PAli_NX_Pl_consensus    AGTATGCTTAGTGACTTTCTACACAATAAAGCTCATAAGTATACTTTGCCCAGTCTACAACCCGACTATATTAATAAA [ 6552]
cp32-3_PAbe_NX_MP_FG_consensus AGTATGCTTAGTGACTTTCTACACAATAAAGCTCATAAGTATACTTTGCCCAGTCTACAACCCGACTATATTAATAAA [ 6552]
cp32-3_PAbe_NX_Pl_consensus    AGTATGCTTAGTGACTTTCTACACAATAAAGCTCATAAGTATACTTTGCCCAGTCTACAACCCGACTATATTAATAAA [ 6552]
```

```
cp32-3_gi|11497103|B31_GB       ATTAACTTCTACATTTACCCAACATCTAATATGCAAACAGTTGGACTGATTAATTTAGGCACAAAATATAGCAACCAT [ 6630]
cp32-3_un8_B31_PacBio           ATTAACTTCTACATTTACCCAACATCTAATATGCAAACAGTTGGACTGATTAATTTAGGCACAAAATATAGCAACCAT [ 6630]
cp32-3_B31_TS_MP_FG_consensus   ATTAACTTCTACATTTACCCAACATCTAATATGCAAACAGTTGGACTGATTAATTTAGGCACAAAATATAGCAACCAT [ 6630]
cp32-3_B31_NX_Pl_consensus      ATTAACTTCTACATTTACCCAACATCTAATATGCAAACAGTTGGACTGATTAATTTAGGCACAAAATATAGCAACCAT [ 6630]
cp32-3_PAli_NX_MP_FG_consensus  ATTAACTTCTACATTTACCCAACATCTAATATGCAAACAGTTGGACTGATTAATTTAGGCACAAAATATAGCAACCAT [ 6630]
cp32-3_PAli_NX_Pl_consensus     ATTAACTTCTACATTTACCCAACATCTAATATGCAAACAGTTGGACTGATTAATTTAGGCACAAAATATAGCAACCAT [ 6630]
cp32-3_PAbe_NX_MP_FG_consensus  ATTAACTTCTACATTTACCCAACATCTAATATGCAAACAGTTGGACTGATTAATTTAGGCACAAAATATAGCAACCAT [ 6630]
cp32-3_PAbe_NX_Pl_consensus     ATTAACTTCTACATTTACCCAACATCTAATATGCAAACAGTTGGACTGATTAATTTAGGCACAAAATATAGCAACCAT [ 6630]

cp32-3_gi|11497103|B31_GB       GCATACAGTGCATCTATAGCATTTAATGCTAGTGTAAAAGCAATTGAAATTTTAAAGGAGGAATACGAAATTGCCGCA [ 6708]
cp32-3_un8_B31_PacBio           GCATACAGTGCATCTATAGCATTTAATGCTAGTGTAAAAGCAATTGAAATTTTAAAGGAGGAATACGAAATTGCCGCA [ 6708]
cp32-3_B31_TS_MP_FG_consensus   GCATACAGTGCATCTATAGCATTTAATGCTAGTGTAAAAGCAATTGAAATTTTAAAGGAGGAATACGAAATTGCCGCA [ 6708]
cp32-3_B31_NX_Pl_consensus      GCATACAGTGCATCTATAGCATTTAATGCTAGTGTAAAAGCAATTGAAATTTTAAAGGAGGAATACGAAATTGCCGCA [ 6708]
cp32-3_PAli_NX_MP_FG_consensus  GCATACAGTGCATCTATAGCATTTAATGCTAGTGTAAAAGCAATTGAAATTTTAAAGGAGGAATACGAAATTGCCGCA [ 6708]
cp32-3_PAli_NX_Pl_consensus     GCATACAGTGCATCTATAGCATTTAATGCTAGTGTAAAAGCAATTGAAATTTTAAAGGAGGAATACGAAATTGCCGCA [ 6708]
cp32-3_PAbe_NX_MP_FG_consensus  GCATACAGTGCATCTATAGCATTTAATGCTAGTGTAAAAGCAATTGAAATTTTAAAGGAGGAATACGAAATTGCCGCA [ 6708]
cp32-3_PAbe_NX_Pl_consensus     GCATACAGTGCATCTATAGCATTTAATGCTAGTGTAAAAGCAATTGAAATTTTAAAGGAGGAATACGAAATTGCCGCA [ 6708]

cp32-3_gi|11497103|B31_GB       AGATACAATTAGTGTAAGTTTGCTTGACTCTAGAATTCAAGCTAGTAGGCCTAATTATTATAATCCACTTTTGGTTTA [ 6786]
cp32-3_un8_B31_PacBio           AGATACAATTAGTGTAAGTTTGCTTGACTCTAGAATTCAAGCTAGTAGGCCTAATTATTATAATCCACTTTTGGTTTA [ 6786]
cp32-3_B31_TS_MP_FG_consensus   AGATACAATTAGTGTAAGTTTGCTTGACTCTAGAATTCAAGCTAGTAGGCCTAATTATTATAATCCACTTTTGGTTTA [ 6786]
cp32-3_B31_NX_Pl_consensus      AGATACAATTAGTGTAAGTTTGCTTGACTCTAGAATTCAAGCTAGTAGGCCTAATTATTATAATCCACTTTTGGTTTA [ 6786]
cp32-3_PAli_NX_MP_FG_consensus  AGATACAATTAGTGTAAGTTTGCTTGACTCTAGAATTCAAGCTAGTAGGCCTAATTATTATAATCCACTTTTGGTTTA [ 6786]
cp32-3_PAli_NX_Pl_consensus     AGATACAATTAGTGTAAGTTTGCTTGACTCTAGAATTCAAGCTAGTAGGCCTAATTATTATAATCCACTTTTGGTTTA [ 6786]
cp32-3_PAbe_NX_MP_FG_consensus  AGATACAATTAGTGTAAGTTTGCTTGACTCTAGAATTCAAGCTAGTAGGCCTAATTATTATAATCCACTTTTGGTTTA [ 6786]
cp32-3_PAbe_NX_Pl_consensus     AGATACAATTAGTGTAAGTTTGCTTGACTCTAGAATTCAAGCTAGTAGGCCTAATTATTATAATCCACTTTTGGTTTA [ 6786]

cp32-3_gi|11497103|B31_GB       CAAAACAGCTAAAATCAAAGTTAATAAAGATGCTGCTAGCTATAAAATATTGAATTTAACCGTTAATAATTATGAAAA [ 6864]
cp32-3_un8_B31_PacBio           CAAAACAGCTAAAATCAAAGTTAATAAAGATGCTGCTAGCTATAAAATATTGAATTTAACCGTTAATAATTATGAAAA [ 6864]
cp32-3_B31_TS_MP_FG_consensus   CAAAACAGCTAAAATCAAAGTTAATAAAGATGCTGCTAGCTATAAAATATTGAATTTAACCGTTAATAATTATGAAAA [ 6864]
cp32-3_B31_NX_Pl_consensus      CAAAACAGCTAAAATCAAAGTTAATAAAGATGCTGCTAGCTATAAAATATTGAATTTAACCGTTAATAATTATGAAAA [ 6864]
cp32-3_PAli_NX_MP_FG_consensus  CAAAACAGCTAAAATCAAAGTTAATAAAGATGCTGCTAGCTATAAAATATTGAATTTAACCGTTAATAATTATGAAAA [ 6864]
cp32-3_PAli_NX_Pl_consensus     CAAAACAGCTAAAATCAAAGTTAATAAAGATGCTGCTAGCTATAAAATATTGAATTTAACCGTTAATAATTATGAAAA [ 6864]
cp32-3_PAbe_NX_MP_FG_consensus  CAAAACAGCTAAAATCAAAGTTAATAAAGATGCTGCTAGCTATAAAATATTGAATTTAACCGTTAATAATTATGAAAA [ 6864]
cp32-3_PAbe_NX_Pl_consensus     CAAAACAGCTAAAATCAAAGTTAATAAAGATGCTGCTAGCTATAAAATATTGAATTTAACCGTTAATAATTATGAAAA [ 6864]

cp32-3_gi|11497103|B31_GB       ACAAATTGAAACTTTAGAAAAAGAGAATGGAAATGGAGAAGATCAGTTTGGAAAAGAAAAAACACTGCTTAAAACTGC [ 6942]
cp32-3_un8_B31_PacBio           ACAAATTGAAACTTTAGAAAAAGAGAATGGAAATGGAGAAGATCAGTTTGGAAAAGAAAAAACACTGCTTAAAACTGC [ 6942]
cp32-3_B31_TS_MP_FG_consensus   ACAAATTGAAACTTTAGAAAAAGAGAATGGAAATGGAGAAGATCAGTTTGGAAAAGAAAAAACACTGCTTAAAACTGC [ 6942]
cp32-3_B31_NX_Pl_consensus      ACAAATTGAAACTTTAGAAAAAGAGAATGGAAATGGAGAAGATCAGTTTGGAAAAGAAAAAACACTGCTTAAAACTGC [ 6942]
cp32-3_PAli_NX_MP_FG_consensus  ACAAATTGAAACTTTAGAAAAAGAGAATGGAAATGGAGAAGATCAGTTTGGAAAAGAAAAAACACTGCTTAAAACTGC [ 6942]
cp32-3_PAli_NX_Pl_consensus     ACAAATTGAAACTTTAGAAAAAGAGAATGGAAATGGAGAAGATCAGTTTGGAAAAGAAAAAACACTGCTTAAAACTGC [ 6942]
cp32-3_PAbe_NX_MP_FG_consensus  ACAAATTGAAACTTTAGAAAAAGAGAATGGAAATGGAGAAGATCAGTTTGGAAAAGAAAAAACACTGCTTAAAACTGC [ 6942]
cp32-3_PAbe_NX_Pl_consensus     ACAAATTGAAACTTTAGAAAAAGAGAATGGAAATGGAGAAGATCAGTTTGGAAAAGAAAAAACACTGCTTAAAACTGC [ 6942]

cp32-3_gi|11497103|B31_GB       AATGTCAAATTTTTTCAATTCAAGCGAAGAATCGTTAAAATCAGCCGATCTTTTCATTTATAAGGATAAGCCTGAAGA [ 7020]
cp32-3_un8_B31_PacBio           AATGTCAAATTTTTTCAATTCAAGCGAAGAATCGTTAAAATCAGCCGATCTTTTCATTTATAAGGATAAGCCTGAAGA [ 7020]
cp32-3_B31_TS_MP_FG_consensus   AATGTCAAATTTTTTCAATTCAAGCGAAGAATCGTTAAAATCAGCCGATCTTTTCATTTATAAGGATAAGCCTGAAGA [ 7020]
cp32-3_B31_NX_Pl_consensus      AATGTCAAATTTTTTCAATTCAAGCGAAGAATCGTTAAAATCAGCCGATCTTTTCATTTATAAGGATAAGCCTGAAGA [ 7020]
cp32-3_PAli_NX_MP_FG_consensus  AATGTCAAATTTTTTCAATTCAAGCGAAGAATCGTTAAAATCAGCCGATCTTTTCATTTATAAGGATAAGCCTGAAGA [ 7020]
cp32-3_PAli_NX_Pl_consensus     AATGTCAAATTTTTTCAATTCAAGCGAAGAATCGTTAAAATCAGCCGATCTTTTCATTTATAAGGATAAGCCTGAAGA [ 7020]
cp32-3_PAbe_NX_MP_FG_consensus  AATGTCAAATTTTTTCAATTCAAGCGAAGAATCGTTAAAATCAGCCGATCTTTTCATTTATAAGGATAAGCCTGAAGA [ 7020]
cp32-3_PAbe_NX_Pl_consensus     AATGTCAAATTTTTTCAATTCAAGCGAAGAATCGTTAAAATCAGCCGATCTTTTCATTTATAAGGATAAGCCTGAAGA [ 7020]

cp32-3_gi|11497103|B31_GB       GCTAAAAAATTATCTTAAAGTACATAGACACACTTTTGTTGTACTTATTAACACTGAGGGAGATGCGTCAGATGATGG [ 7098]
cp32-3_un8_B31_PacBio           GCTAAAAAATTATCTTAAAGTACATAGACACACTTTTGTTGTACTTATTAACACTGAGGGAGATGCGTCAGATGATGG [ 7098]
cp32-3_B31_TS_MP_FG_consensus   GCTAAAAAATTATCTTAAAGTACATAGACACACTTTTGTTGTACTTATTAACACTGAGGGAGATGCGTCAGATGATGG [ 7098]
cp32-3_B31_NX_Pl_consensus      GCTAAAAAATTATCTTAAAGTACATAGACACACTTTTGTTGTACTTATTAACACTGAGGGAGATGCGTCAGATGATGG [ 7098]
cp32-3_PAli_NX_MP_FG_consensus  GCTAAAAAATTATCTTAAAGTACATAGACACACTTTTGTTGTACTTATTAACACTGAGGGAGATGCGTCAGATGATGG [ 7098]
cp32-3_PAli_NX_Pl_consensus     GCTAAAAAATTATCTTAAAGTACATAGACACACTTTTGTTGTACTTATTAACACTGAGGGAGATGCGTCAGATGATGG [ 7098]
cp32-3_PAbe_NX_MP_FG_consensus  GCTAAAAAATTATCTTAAAGTACATAGACACACTTTTGTTGTACTTATTAACACTGAGGGAGATGCGTCAGATGATGG [ 7098]
cp32-3_PAbe_NX_Pl_consensus     GCTAAAAAATTATCTTAAAGTACATAGACACACTTTTGTTGTACTTATTAACACTGAGGGAGATGCGTCAGATGATGG [ 7098]

cp32-3_gi|11497103|B31_GB       ACTTAAAATTTACAAAGATGACTATAATAAATTCAAAAAGCCTTCAACTTTTTTTGTATTCTCGACTAAAGAACAAGA [ 7176]
cp32-3_un8_B31_PacBio           ACTTAAAATTTACAAAGATGACTATAATAAATTCAAAAAGCCTTCAACTTTTTTTGTATTCTCGACTAAAGAACAAGA [ 7176]
cp32-3_B31_TS_MP_FG_consensus   ACTTAAAATTTACAAAGATGACTATAATAAATTCAAAAAGCCTTCAACTTTTTTTGTATTCTCGACTAAAGAACAAGA [ 7176]
cp32-3_B31_NX_Pl_consensus      ACTTAAAATTTACAAAGATGACTATAATAAATTCAAAAAGCCTTCAACTTTTTTTGTATTCTCGACTAAAGAACAAGA [ 7176]
cp32-3_PAli_NX_MP_FG_consensus  ACTTAAAATTTACAAAGATGACTATAATAAATTCAAAAAGCCTTCAACTTTTTTTGTATTCTCGACTAAAGAACAAGA [ 7176]
cp32-3_PAli_NX_Pl_consensus     ACTTAAAATTTACAAAGATGACTATAATAAATTCAAAAAGCCTTCAACTTTTTTTGTATTCTCGACTAAAGAACAAGA [ 7176]
cp32-3_PAbe_NX_MP_FG_consensus  ACTTAAAATTTACAAAGATGACTATAATAAATTCAAAAAGCCTTCAACTTTTTTTGTATTCTCGACTAAAGAACAAGA [ 7176]
cp32-3_PAbe_NX_Pl_consensus     ACTTAAAATTTACAAAGATGACTATAATAAATTCAAAAAGCCTTCAACTTTTTTTGTATTCTCGACTAAAGAACAAGA [ 7176]

cp32-3_gi|11497103|B31_GB       AATAAAAGAACTATTTAAAGATAAAGGCAATACTGAAAAAGAAAGAAATATTGCTGTTTACAGCAACAATAAAGACAA [ 7254]
cp32-3_un8_B31_PacBio           AATAAAAGAACTATTTAAAGATAAAGGCAATACTGAAAAAGAAAGAAATATTGCTGTTTACAGCAACAATAAAGACAA [ 7254]
cp32-3_B31_TS_MP_FG_consensus   AATAAAAGAACTATTTAAAGATAAAGGCAATACTGAAAAAGAAAGAAATATTGCTGTTTACAGCAACAATAAAGACAA [ 7254]
cp32-3_B31_NX_Pl_consensus      AATAAAAGAACTATTTAAAGATAAAGGCAATACTGAAAAAGAAAGAAATATTGCTGTTTACAGCAACAATAAAGACAA [ 7254]
cp32-3_PAli_NX_MP_FG_consensus  AATAAAAGAACTATTTAAAGATAAAGGCAATACTGAAAAAGAAAGAAATATTGCTGTTTACAGCAACAATAAAGACAA [ 7254]
cp32-3_PAli_NX_Pl_consensus     AATAAAAGAACTATTTAAAGATAAAGGCAATACTGAAAAAGAAAGAAATATTGCTGTTTACAGCAACAATAAAGACAA [ 7254]
cp32-3_PAbe_NX_MP_FG_consensus  AATAAAAGAACTATTTAAAGATAAAGGCAATACTGAAAAAGAAAGAAATATTGCTGTTTACAGCAACAATAAAGACAA [ 7254]
cp32-3_PAbe_NX_Pl_consensus     AATAAAAGAACTATTTAAAGATAAAGGCAATACTGAAAAAGAAAGAAATATTGCTGTTTACAGCAACAATAAAGACAA [ 7254]

cp32-3_gi|11497103|B31_GB       TTTACACCTTAAATTTATAAGTCAATATCTCCATCAAGCTAGTATTTTCCATGCTGTAAATCCTTATGGCATGCCGCT [ 7332]
cp32-3_un8_B31_PacBio           TTTACACCTTAAATTTATAAGTCAATATCTCCATCAAGCTAGTATTTTCCATGCTGTAAATCCTTATGGCATGCCGCT [ 7332]
cp32-3_B31_TS_MP_FG_consensus   TTTACACCTTAAATTTATAAGTCAATATCTCCATCAAGCTAGTATTTTCCATGCTGTAAATCCTTATGGCATGCCGCT [ 7332]
cp32-3_B31_NX_Pl_consensus      TTTACACCTTAAATTTATAAGTCAATATCTCCATCAAGCTAGTATTTTCCATGCTGTAAATCCTTATGGCATGCCGCT [ 7332]
cp32-3_PAli_NX_MP_FG_consensus  TTTACACCTTAAATTTATAAGTCAATATCTCCATCAAGCTAGTATTTTCCATGCTGTAAATCCTTATGGCATGCCGCT [ 7332]
cp32-3_PAli_NX_Pl_consensus     TTTACACCTTAAATTTATAAGTCAATATCTCCATCAAGCTAGTATTTTCCATGCTGTAAATCCTTATGGCATGCCGCT [ 7332]
cp32-3_PAbe_NX_MP_FG_consensus  TTTACACCTTAAATTTATAAGTCAATATCTCCATCAAGCTAGTATTTTCCATGCTGTAAATCCTTATGGCATGCCGCT [ 7332]
cp32-3_PAbe_NX_Pl_consensus     TTTACACCTTAAATTTATAAGTCAATATCTCCATCAAGCTAGTATTTTCCATGCTGTAAATCCTTATGGCATGCCGCT [ 7332]

cp32-3_gi|11497103|B31_GB       GGCTGCTACACCACTTGTTGATGATACTGTAATTGGAAAGTTGCGAACTGCAAAAATCAACTTTTATTCACTTCTTAA [ 7410]
cp32-3_un8_B31_PacBio           GGCTGCTACACCACTTGTTGATGATACTGTAATTGGAAAGTTGCGAACTGCAAAAATCAACTTTTATTCACTTCTTAA [ 7410]
cp32-3_B31_TS_MP_FG_consensus   GGCTGCTACACCACTTGTTGATGATACTGTAATTGGAAAGTTGCGAACTGCAAAAATCAACTTTTATTCACTTCTTAA [ 7410]
cp32-3_B31_NX_Pl_consensus      GGCTGCTACACCACTTGTTGATGATACTGTAATTGGAAAGTTGCGAACTGCAAAAATCAACTTTTATTCACTTCTTAA [ 7410]
cp32-3_PAli_NX_MP_FG_consensus  GGCTGCTACACCACTTGTTGATGATACTGTAATTGGAAAGTTGCGAACTGCAAAAATCAACTTTTATTCACTTCTTAA [ 7410]
cp32-3_PAli_NX_Pl_consensus     GGCTGCTACACCACTTGTTGATGATACTGTAATTGGAAAGTTGCGAACTGCAAAAATCAACTTTTATTCACTTCTTAA [ 7410]
cp32-3_PAbe_NX_MP_FG_consensus  GGCTGCTACACCACTTGTTGATGATACTGTAATTGGAAAGTTGCGAACTGCAAAAATCAACTTTTATTCACTTCTTAA [ 7410]
cp32-3_PAbe_NX_Pl_consensus     GGCTGCTACACCACTTGTTGATGATACTGTAATTGGAAAGTTGCGAACTGCAAAAATCAACTTTTATTCACTTCTTAA [ 7410]

cp32-3_gi|11497103|B31_GB       TGAAACTGGGCTTGATGGTGTACCTGCCTTTAAAGAAGGTGTTGACCTAGCTGGAGGTGCAATAGACGAACAATTTAC [ 7488]
cp32-3_un8_B31_PacBio           TGAAACTGGGCTTGATGGTGTACCTGCCTTTAAAGAAGGTGTTGACCTAGCTGGAGGTGCAATAGACGAACAATTTAC [ 7488]
cp32-3_B31_TS_MP_FG_consensus   TGAAACTGGGCTTGATGGTGTACCTGCCTTTAAAGAAGGTGTTGACCTAGCTGGAGGTGCAATAGACGAACAATTTAC [ 7488]
cp32-3_B31_NX_Pl_consensus      TGAAACTGGGCTTGATGGTGTACCTGCCTTTAAAGAAGGTGTTGACCTAGCTGGAGGTGCAATAGACGAACAATTTAC [ 7488]
cp32-3_PAli_NX_MP_FG_consensus  TGAAACTGGGCTTGATGGTGTACCTGCCTTTAAAGAAGGTGTTGACCTAGCTGGAGGTGCAATAGACGAACAATTTAC [ 7488]
cp32-3_PAli_NX_Pl_consensus     TGAAACTGGGCTTGATGGTGTACCTGCCTTTAAAGAAGGTGTTGACCTAGCTGGAGGTGCAATAGACGAACAATTTAC [ 7488]
cp32-3_PAbe_NX_MP_FG_consensus  TGAAACTGGGCTTGATGGTGTACCTGCCTTTAAAGAAGGTGTTGACCTAGCTGGAGGTGCAATAGACGAACAATTTAC [ 7488]
cp32-3_PAbe_NX_Pl_consensus     TGAAACTGGGCTTGATGGTGTACCTGCCTTTAAAGAAGGTGTTGACCTAGCTGGAGGTGCAATAGACGAACAATTTAC [ 7488]
```

```
cp32-3_gi|11497103|B31_GB      ATACCACTATATAAAAAACGAAGCGATTATTGAGCTTATTAGAATTTGGAACAAAAACAATAGGCAAAATAGCAAATT [ 7566]
cp32-3_un8_B31_PacBio          ATACCACTATATAAAAAACGAAGCGATTATTGAGCTTATTAGAATTTGGAACAAAAACAATAGGCAAAATAGCAAATT [ 7566]
cp32-3_B31_TS_MP_FG_consensus  ATACCACTATATAAAAAACGAAGCGATTATTGAGCTTATTAGAATTTGGAACAAAAACAATAGGCAAAATAGCAAATT [ 7566]
cp32-3_B31_NX_Pl_consensus     ATACCACTATATAAAAAACGAAGCGATTATTGAGCTTATTAGAATTTGGAACAAAAACAATAGGCAAAATAGCAAATT [ 7566]
cp32-3_PAli_NX_MP_FG_consensus ATACCACTATATAAAAAACGAAGCGATTATTGAGCTTATTAGAATTTGGAACAAAAACAATAGGCAAAATAGCAAATT [ 7566]
cp32-3_PAli_NX_Pl_consensus    ATACCACTATATAAAAAACGAAGCGATTATTGAGCTTATTAGAATTTGGAACAAAAACAATAGGCAAAATAGCAAATT [ 7566]
cp32-3_PAbe_NX_MP_FG_consensus ATACCACTATATAAAAAACGAAGCGATTATTGAGCTTATTAGAATTTGGAACAAAAACAATAGGCAAAATAGCAAATT [ 7566]
cp32-3_PAbe_NX_Pl_consensus    ATACCACTATATAAAAAACGAAGCGATTATTGAGCTTATTAGAATTTGGAACAAAAACAATAGGCAAAATAGCAAATT [ 7566]

cp32-3_gi|11497103|B31_GB      ATCTGCACTACAACTTAGTGGAGCTAGAGACAATGCATATACTTCAGCAATTGAATGTTTACTGAAAAGGTTTGTGGA [ 7644]
cp32-3_un8_B31_PacBio          ATCTGCACTACAACTTAGTGGAGCTAGAGACAATGCATATACTTCAGCAATTGAATGTTTACTGAAAAGGTTTGTGGA [ 7644]
cp32-3_B31_TS_MP_FG_consensus  ATCTGCACTACAACTTAGTGGAGCTAGAGACAATGCATATACTTCAGCAATTGAATGTTTACTGAAAAGGTTTGTGGA [ 7644]
cp32-3_B31_NX_Pl_consensus     ATCTGCACTACAACTTAGTGGAGCTAGAGACAATGCATATACTTCAGCAATTGAATGTTTACTGAAAAGGTTTGTGGA [ 7644]
cp32-3_PAli_NX_MP_FG_consensus ATCTGCACTACAACTTAGTGGAGCTAGAGACAATGCATATACTTCAGCAATTGAATGTTTACTGAAAAGGTTTGTGGA [ 7644]
cp32-3_PAli_NX_Pl_consensus    ATCTGCACTACAACTTAGTGGAGCTAGAGACAATGCATATACTTCAGCAATTGAATGTTTACTGAAAAGGTTTGTGGA [ 7644]
cp32-3_PAbe_NX_MP_FG_consensus ATCTGCACTACAACTTAGTGGAGCTAGAGACAATGCATATACTTCAGCAATTGAATGTTTACTGAAAAGGTTTGTGGA [ 7644]
cp32-3_PAbe_NX_Pl_consensus    ATCTGCACTACAACTTAGTGGAGCTAGAGACAATGCATATACTTCAGCAATTGAATGTTTACTGAAAAGGTTTGTGGA [ 7644]

cp32-3_gi|11497103|B31_GB      TAGAGGACTGATAATAGAGTATAAAAATTTAAGGCTTACTCTTTCTCCTACGCCACAACTTAAATTAGAACTTAGCGT [ 7722]
cp32-3_un8_B31_PacBio          TAGAGGACTGATAATAGAGTATAAAAATTTAAGGCTTACTCTTTCTCCTACGCCACAACTTAAATTAGAACTTAGCGT [ 7722]
cp32-3_B31_TS_MP_FG_consensus  TAGAGGACTGATAATAGAGTATAAAAATTTAAGGCTTACTCTTTCTCCTACGCCACAACTTAAATTAGAACTTAGCGT [ 7722]
cp32-3_B31_NX_Pl_consensus     TAGAGGACTGATAATAGAGTATAAAAATTTAAGGCTTACTCTTTCTCCTACGCCACAACTTAAATTAGAACTTAGCGT [ 7722]
cp32-3_PAli_NX_MP_FG_consensus TAGAGGACTGATAATAGAGTATAAAAATTTAAGGCTTACTCTTTCTCCTACGCCACAACTTAAATTAGAACTTAGCGT [ 7722]
cp32-3_PAli_NX_Pl_consensus    TAGAGGACTGATAATAGAGTATAAAAATTTAAGGCTTACTCTTTCTCCTACGCCACAACTTAAATTAGAACTTAGCGT [ 7722]
cp32-3_PAbe_NX_MP_FG_consensus TAGAGGACTGATAATAGAGTATAAAAATTTAAGGCTTACTCTTTCTCCTACGCCACAACTTAAATTAGAACTTAGCGT [ 7722]
cp32-3_PAbe_NX_Pl_consensus    TAGAGGACTGATAATAGAGTATAAAAATTTAAGGCTTACTCTTTCTCCTACGCCACAACTTAAATTAGAACTTAGCGT [ 7722]

cp32-3_gi|11497103|B31_GB      GAATATTACTTATAACTTTAGCATTAATGCTGTTGCTTTAGTAATTACTACTCAAGATATAGTTGATTATCAAAACAG [ 7800]
cp32-3_un8_B31_PacBio          GAATATTACTTATAACTTTAGCATTAATGCTGTTGCTTTAGTAATTACTACTCAAGATATAGTTGATTATCAAAACAG [ 7800]
cp32-3_B31_TS_MP_FG_consensus  GAATATTACTTATAACTTTAGCATTAATGCTGTTGCTTTAGTAATTACTACTCAAGATATAGTTGATTATCAAAACAG [ 7800]
cp32-3_B31_NX_Pl_consensus     GAATATTACTTATAACTTTAGCATTAATGCTGTTGCTTTAGTAATTACTACTCAAGATATAGTTGATTATCAAAACAG [ 7800]
cp32-3_PAli_NX_MP_FG_consensus GAATATTACTTATAACTTTAGCATTAATGCTGTTGCTTTAGTAATTACTACTCAAGATATAGTTGATTATCAAAACAG [ 7800]
cp32-3_PAli_NX_Pl_consensus    GAATATTACTTATAACTTTAGCATTAATGCTGTTGCTTTAGTAATTACTACTCAAGATATAGTTGATTATCAAAACAG [ 7800]
cp32-3_PAbe_NX_MP_FG_consensus GAATATTACTTATAACTTTAGCATTAATGCTGTTGCTTTAGTAATTACTACTCAAGATATAGTTGATTATCAAAACAG [ 7800]
cp32-3_PAbe_NX_Pl_consensus    GAATATTACTTATAACTTTAGCATTAATGCTGTTGCTTTAGTAATTACTACTCAAGATATAGTTGATTATCAAAACAG [ 7800]

cp32-3_gi|11497103|B31_GB      CTTAAGTGCTTAAAAGGGGGGCTAAAAATGCAATTTTATGATTTAAGAGAAGTTTATTTTTCAATTGGTGGTACGCAG [ 7878]
cp32-3_un8_B31_PacBio          CTTAAGTGCTTAAAAGGGGGGCTAAAAATGCAATTTTATGATTTAAGAGAAGTTTATTTTTCAATTGGTGGTACGCAG [ 7878]
cp32-3_B31_TS_MP_FG_consensus  CTTAAGTGCTTAAAAGGGGGGCTAAAAATGCAATTTTATGATTTAAGAGAAGTTTATTTTTCAATTGGTGGTACGCAG [ 7878]
cp32-3_B31_NX_Pl_consensus     CTTAAGTGCTTAAAAGGGGGGCTAAAAATGCAATTTTATGATTTAAGAGAAGTTTATTTTTCAATTGGTGGTACGCAG [ 7878]
cp32-3_PAli_NX_MP_FG_consensus CTTAAGTGCTTAAAAGGGGGGCTAAAAATGCAATTTTATGATTTAAGAGAAGTTTATTTTTCAATTGGTGGTACGCAG [ 7878]
cp32-3_PAli_NX_Pl_consensus    CTTAAGTGCTTAAAAGGGGGGCTAAAAATGCAATTTTATGATTTAAGAGAAGTTTATTTTTCAATTGGTGGTACGCAG [ 7878]
cp32-3_PAbe_NX_MP_FG_consensus CTTAAGTGCTTAAAAGGGGGGCTAAAAATGCAATTTTATGATTTAAGAGAAGTTTATTTTTCAATTGGTGGTACGCAG [ 7878]
cp32-3_PAbe_NX_Pl_consensus    CTTAAGTGCTTAAAAGGGGGGCTAAAAATGCAATTTTATGATTTAAGAGAAGTTTATTTTTCAATTGGTGGTACGCAG [ 7878]

cp32-3_gi|11497103|B31_GB      TTACATAGTGGCAAGCTAGAGCTTACAAGCGAACCTACAACAAGAGCAGTGATTAGTAGTGAAGATAAAGGTATGCCT [ 7956]
cp32-3_un8_B31_PacBio          TTACATAGTGGCAAGCTAGAGCTTACAAGCGAACCTACAACAAGAGCAGTGATTAGTAGTGAAGATAAAGGTATGCCT [ 7956]
cp32-3_B31_TS_MP_FG_consensus  TTACATAGTGGCAAGCTAGAGCTTACAAGCGAACCTACAACAAGAGCAGTGATTAGTAGTGAAGATAAAGGTATGCCT [ 7956]
cp32-3_B31_NX_Pl_consensus     TTACATAGTGGCAAGCTAGAGCTTACAAGCGAACCTACAACAAGAGCAGTGATTAGTAGTGAAGATAAAGGTATGCCT [ 7956]
cp32-3_PAli_NX_MP_FG_consensus TTACATAGTGGCAAGCTAGAGCTTACAAGCGAACCTACAACAAGAGCAGTGATTAGTAGTGAAGATAAAGGTATGCCT [ 7956]
cp32-3_PAli_NX_Pl_consensus    TTACATAGTGGCAAGCTAGAGCTTACAAGCGAACCTACAACAAGAGCAGTGATTAGTAGTGAAGATAAAGGTATGCCT [ 7956]
cp32-3_PAbe_NX_MP_FG_consensus TTACATAGTGGCAAGCTAGAGCTTACAAGCGAACCTACAACAAGAGCAGTGATTAGTAGTGAAGATAAAGGTATGCCT [ 7956]
cp32-3_PAbe_NX_Pl_consensus    TTACATAGTGGCAAGCTAGAGCTTACAAGCGAACCTACAACAAGAGCAGTGATTAGTAGTGAAGATAAAGGTATGCCT [ 7956]

cp32-3_gi|11497103|B31_GB      GTAATAAGCTTAAGAGATCCCAAAACAATAACTTATGTTTTCAACATTGAAGTGACACTAGGTAGTCATGACTACATT [ 8034]
cp32-3_un8_B31_PacBio          GTAATAAGCTTAAGAGATCCCAAAACAATAACTTATGTTTTCAACATTGAAGTGACACTAGGTAGTCATGACTACATT [ 8034]
cp32-3_B31_TS_MP_FG_consensus  GTAATAAGCTTAAGAGATCCCAAAACAATAACTTATGTTTTCAACATTGAAGTGACACTAGGTAGTCATGACTACATT [ 8034]
cp32-3_B31_NX_Pl_consensus     GTAATAAGCTTAAGAGATCCCAAAACAATAACTTATGTTTTCAACATTGAAGTGACACTAGGTAGTCATGACTACATT [ 8034]
cp32-3_PAli_NX_MP_FG_consensus GTAATAAGCTTAAGAGATCCCAAAACAATAACTTATGTTTTCAACATTGAAGTGACACTAGGTAGTCATGACTACATT [ 8034]
cp32-3_PAli_NX_Pl_consensus    GTAATAAGCTTAAGAGATCCCAAAACAATAACTTATGTTTTCAACATTGAAGTGACACTAGGTAGTCATGACTACATT [ 8034]
cp32-3_PAbe_NX_MP_FG_consensus GTAATAAGCTTAAGAGATCCCAAAACAATAACTTATGTTTTCAACATTGAAGTGACACTAGGTAGTCATGACTACATT [ 8034]
cp32-3_PAbe_NX_Pl_consensus    GTAATAAGCTTAAGAGATCCCAAAACAATAACTTATGTTTTCAACATTGAAGTGACACTAGGTAGTCATGACTACATT [ 8034]

cp32-3_gi|11497103|B31_GB      TTGTTAACTGAACTTTCTGATGAACAGTTTTACAACATGGATGTGAGAAAAGAGGATAAAATGCTTGATTTAGCATTC [ 8112]
cp32-3_un8_B31_PacBio          TTGTTAACTGAACTTTCTGATGAACAGTTTTACAACATGGATGTGAGAAAAGAGGATAAAATGCTTGATTTAGCATTC [ 8112]
cp32-3_B31_TS_MP_FG_consensus  TTGTTAACTGAACTTTCTGATGAACAGTTTTACAACATGGATGTGAGAAAAGAGGATAAAATGCTTGATTTAGCATTC [ 8112]
cp32-3_B31_NX_Pl_consensus     TTGTTAACTGAACTTTCTGATGAACAGTTTTACAACATGGATGTGAGAAAAGAGGATAAAATGCTTGATTTAGCATTC [ 8112]
cp32-3_PAli_NX_MP_FG_consensus TTGTTAACTGAACTTTCTGATGAACAGTTTTACAACATGGATGTGAGAAAAGAGGATAAAATGCTTGATTTAGCATTC [ 8112]
cp32-3_PAli_NX_Pl_consensus    TTGTTAACTGAACTTTCTGATGAACAGTTTTACAACATGGATGTGAGAAAAGAGGATAAAATGCTTGATTTAGCATTC [ 8112]
cp32-3_PAbe_NX_MP_FG_consensus TTGTTAACTGAACTTTCTGATGAACAGTTTTACAACATGGATGTGAGAAAAGAGGATAAAATGCTTGATTTAGCATTC [ 8112]
cp32-3_PAbe_NX_Pl_consensus    TTGTTAACTGAACTTTCTGATGAACAGTTTTACAACATGGATGTGAGAAAAGAGGATAAAATGCTTGATTTAGCATTC [ 8112]

cp32-3_gi|11497103|B31_GB      AATGATAGAATTGCTACCAAAATTATTTCTAACTATGCAATTTTTACTGAAGAGCCTTCAAGAAGTTATTCTGCTGAG [ 8190]
cp32-3_un8_B31_PacBio          AATGATAGAATTGCTACCAAAATTATTTCTAACTATGCAATTTTTACTGAAGAGCCTTCAAGAAGTTATTCTGCTGAG [ 8190]
cp32-3_B31_TS_MP_FG_consensus  AATGATAGAATTGCTACCAAAATTATTTCTAACTATGCAATTTTTACTGAAGAGCCTTCAAGAAGTTATTCTGCTGAG [ 8190]
cp32-3_B31_NX_Pl_consensus     AATGATAGAATTGCTACCAAAATTATTTCTAACTATGCAATTTTTACTGAAGAGCCTTCAAGAAGTTATTCTGCTGAG [ 8190]
cp32-3_PAli_NX_MP_FG_consensus AATGATAGAATTGCTACCAAAATTATTTCTAACTATGCAATTTTTACTGAAGAGCCTTCAAGAAGTTATTCTGCTGAG [ 8190]
cp32-3_PAli_NX_Pl_consensus    AATGATAGAATTGCTACCAAAATTATTTCTAACTATGCAATTTTTACTGAAGAGCCTTCAAGAAGTTATTCTGCTGAG [ 8190]
cp32-3_PAbe_NX_MP_FG_consensus AATGATAGAATTGCTACCAAAATTATTTCTAACTATGCAATTTTTACTGAAGAGCCTTCAAGAAGTTATTCTGCTGAG [ 8190]
cp32-3_PAbe_NX_Pl_consensus    AATGATAGAATTGCTACCAAAATTATTTCTAACTATGCAATTTTTACTGAAGAGCCTTCAAGAAGTTATTCTGCTGAG [ 8190]

cp32-3_gi|11497103|B31_GB      GCCGAAAAAGTATCTTTTGAAATTAGGGCTATTAATTGCCAAAAATCTAAACCAAACAACTCTTAAAAGGAGATTCTT [ 8268]
cp32-3_un8_B31_PacBio          GCCGAAAAAGTATCTTTTGAAATTAGGGCTATTAATTGCCAAAAATCTAAACCAAACAACTCTTAAAAGGAGATTCTT [ 8268]
cp32-3_B31_TS_MP_FG_consensus  GCCGAAAAAGTATCTTTTGAAATTAGGGCTATTAATTGCCAAAAATCTAAACCAAACAACTCTTAAAAGGAGATTCTT [ 8268]
cp32-3_B31_NX_Pl_consensus     GCCGAAAAAGTATCTTTTGAAATTAGGGCTATTAATTGCCAAAAATCTAAACCAAACAACTCTTAAAAGGAGATTCTT [ 8268]
cp32-3_PAli_NX_MP_FG_consensus GCCGAAAAAGTATCTTTTGAAATTAGGGCTATTAATTGCCAAAAATCTAAACCAAACAACTCTTAAAAGGAGATTCTT [ 8268]
cp32-3_PAli_NX_Pl_consensus    GCCGAAAAAGTATCTTTTGAAATTAGGGCTATTAATTGCCAAAAATCTAAACCAAACAACTCTTAAAAGGAGATTCTT [ 8268]
cp32-3_PAbe_NX_MP_FG_consensus GCCGAAAAAGTATCTTTTGAAATTAGGGCTATTAATTGCCAAAAATCTAAACCAAACAACTCTTAAAAGGAGATTCTT [ 8268]
cp32-3_PAbe_NX_Pl_consensus    GCCGAAAAAGTATCTTTTGAAATTAGGGCTATTAATTGCCAAAAATCTAAACCAAACAACTCTTAAAAGGAGATTCTT [ 8268]

cp32-3_gi|11497103|B31_GB      ATTATGATAATGAGATATAAAATGAAAATTTTAACTAAAAATAAAACTTATGAATATCCGCTGAGAGTACTTCCCGTC [ 8346]
cp32-3_un8_B31_PacBio          ATTATGATAATGAGATATAAAATGAAAATTTTAACTAAAAATAAAACTTATGAATATCCGCTGAGAGTACTTCCCGTC [ 8346]
cp32-3_B31_TS_MP_FG_consensus  ATTATGATAATGAGATATAAAATGAAAATTTTAACTAAAAATAAAACTTATGAATATCCGCTGAGAGTACTTCCCGTC [ 8346]
cp32-3_B31_NX_Pl_consensus     ATTATGATAATGAGATATAAAATGAAAATTTTAACTAAAAATAAAACTTATGAATATCCGCTGAGAGTACTTCCCGTC [ 8346]
cp32-3_PAli_NX_MP_FG_consensus ATTATGATAATGAGATATAAAATGAAAATTTTAACTAAAAATAAAACTTATGAATATCCGCTGAGAGTACTTCCCGTC [ 8346]
cp32-3_PAli_NX_Pl_consensus    ATTATGATAATGAGATATAAAATGAAAATTTTAACTAAAAATAAAACTTATGAATATCCGCTGAGAGTACTTCCCGTC [ 8346]
cp32-3_PAbe_NX_MP_FG_consensus ATTATGATAATGAGATATAAAATGAAAATTTTAACTAAAAATAAAACTTATGAATATCCGCTGAGAGTACTTCCCGTC [ 8346]
cp32-3_PAbe_NX_Pl_consensus    ATTATGATAATGAGATATAAAATGAAAATTTTAACTAAAAATAAAACTTATGAATATCCGCTGAGAGTACTTCCCGTC [ 8346]

cp32-3_gi|11497103|B31_GB      TATGAATGGGATAAAGTGCTAGGATTTAATCAAAGTGACGCTGTTTTAAAGCTTAATGAGGTTAAATACTTAAGAGAA [ 8424]
cp32-3_un8_B31_PacBio          TATGAATGGGATAAAGTGCTAGGATTTAATCAAAGTGACGCTGTTTTAAAGCTTAATGAGGTTAAATACTTAAGAGAA [ 8424]
cp32-3_B31_TS_MP_FG_consensus  TATGAATGGGATAAAGTGCTAGGATTTAATCAAAGTGACGCTGTTTTAAAGCTTAATGAGGTTAAATACTTAAGAGAA [ 8424]
cp32-3_B31_NX_Pl_consensus     TATGAATGGGATAAAGTGCTAGGATTTAATCAAAGTGACGCTGTTTTAAAGCTTAATGAGGTTAAATACTTAAGAGAA [ 8424]
cp32-3_PAli_NX_MP_FG_consensus TATGAATGGGATAAAGTGCTAGGATTTAATCAAAGTGACGCTGTTTTAAAGCTTAATGAGGTTAAATACTTAAGAGAA [ 8424]
cp32-3_PAli_NX_Pl_consensus    TATGAATGGGATAAAGTGCTAGGATTTAATCAAAGTGACGCTGTTTTAAAGCTTAATGAGGTTAAATACTTAAGAGAA [ 8424]
cp32-3_PAbe_NX_MP_FG_consensus TATGAATGGGATAAAGTGCTAGGATTTAATCAAAGTGACGCTGTTTTAAAGCTTAATGAGGTTAAATACTTAAGAGAA [ 8424]
cp32-3_PAbe_NX_Pl_consensus    TATGAATGGGATAAAGTGCTAGGATTTAATCAAAGTGACGCTGTTTTAAAGCTTAATGAGGTTAAATACTTAAGAGAA [ 8424]

cp32-3_gi|11497103|B31_GB      ATCACAAGCTTAATGATAAGTCCAAAATTTTTAGACGAATTCTATGTGATTTTGGATCAAAATAGAGAATTTATTTCT [ 8502]
```

```
cp32-3_un8_B31_PacBio          ATCACAAGCTTAATGATAAGTCCAAAATTTTTAGACGAATTCTATGTGATTTTGGATCAAAATAGAGAATTTATTTCT [ 8502]
cp32-3_B31_TS_MP_FG_consensus  ATCACAAGCTTAATGATAAGTCCAAAATTTTTAGACGAATTCTATGTGATTTTGGATCAAAATAGAGAATTTATTTCT [ 8502]
cp32-3_B31_NX_Pl_consensus     ATCACAAGCTTAATGATAAGTCCAAAATTTTTAGACGAATTCTATGTGATTTTGGATCAAAATAGAGAATTTATTTCT [ 8502]
cp32-3_PAli_NX_MP_FG_consensus ATCACAAGCTTAATGATAAGTCCAAAATTTTTAGACGAATTCTATGTGATTTTGGATCAAAATAGAGAATTTATTTCT [ 8502]
cp32-3_PAli_NX_Pl_consensus    ATCACAAGCTTAATGATAAGTCCAAAATTTTTAGACGAATTCTATGTGATTTTGGATCAAAATAGAGAATTTATTTCT [ 8502]
cp32-3_PAbe_NX_MP_FG_consensus ATCACAAGCTTAATGATAAGTCCAAAATTTTTAGACGAATTCTATGTGATTTTGGATCAAAATAGAGAATTTATTTCT [ 8502]
cp32-3_PAbe_NX_Pl_consensus    ATCACAAGCTTAATGATAAGTCCAAAATTTTTAGACGAATTCTATGTGATTTTGGATCAAAATAGAGAATTTATTTCT [ 8502]

cp32-3_gi|11497103|B31_GB      TATTATAAGGACTATCTTGTTGCAATAATTTACACTGCACAATTTAATACTTTTCATTTAGACAATGATCTAAAAAAG [ 8580]
cp32-3_un8_B31_PacBio          TATTATAAGGACTATCTTGTTGCAATAATTTACACTGCACAATTTAATACTTTTCATTTAGACAATGATCTAAAAAAG [ 8580]
cp32-3_B31_TS_MP_FG_consensus  TATTATAAGGACTATCTTGTTGCAATAATTTACACTGCACAATTTAATACTTTTCATTTAGACAATGATCTAAAAAAG [ 8580]
cp32-3_B31_NX_Pl_consensus     TATTATAAGGACTATCTTGTTGCAATAATTTACACTGCACAATTTAATACTTTTCATTTAGACAATGATCTAAAAAAG [ 8580]
cp32-3_PAli_NX_MP_FG_consensus TATTATAAGGACTATCTTGTTGCAATAATTTACACTGCACAATTTAATACTTTTCATTTAGACAATGATCTAAAAAAG [ 8580]
cp32-3_PAli_NX_Pl_consensus    TATTATAAGGACTATCTTGTTGCAATAATTTACACTGCACAATTTAATACTTTTCATTTAGACAATGATCTAAAAAAG [ 8580]
cp32-3_PAbe_NX_MP_FG_consensus TATTATAAGGACTATCTTGTTGCAATAATTTACACTGCACAATTTAATACTTTTCATTTAGACAATGATCTAAAAAAG [ 8580]
cp32-3_PAbe_NX_Pl_consensus    TATTATAAGGACTATCTTGTTGCAATAATTTACACTGCACAATTTAATACTTTTCATTTAGACAATGATCTAAAAAAG [ 8580]

cp32-3_gi|11497103|B31_GB      CCCGCTTTAGTATATTTGAGTGAGTATGAAAATAATGTTGGTGATTTTGTTGCTTTTGACTATATTAATGAAAATTTT [ 8658]
cp32-3_un8_B31_PacBio          CCCGCTTTAGTATATTTGAGTGAGTATGAAAATAATGTTGGTGATTTTGTTGCTTTTGACTATATTAATGAAAATTTT [ 8658]
cp32-3_B31_TS_MP_FG_consensus  CCCGCTTTAGTATATTTGAGTGAGTATGAAAATAATGTTGGTGATTTTGTTGCTTTTGACTATATTAATGAAAATTTT [ 8658]
cp32-3_B31_NX_Pl_consensus     CCCGCTTTAGTATATTTGAGTGAGTATGAAAATAATGTTGGTGATTTTGTTGCTTTTGACTATATTAATGAAAATTTT [ 8658]
cp32-3_PAli_NX_MP_FG_consensus CCCGCTTTAGTATATTTGAGTGAGTATGAAAATAATGTTGGTGATTTTGTTGCTTTTGACTATATTAATGAAAATTTT [ 8658]
cp32-3_PAli_NX_Pl_consensus    CCCGCTTTAGTATATTTGAGTGAGTATGAAAATAATGTTGGTGATTTTGTTGCTTTTGACTATATTAATGAAAATTTT [ 8658]
cp32-3_PAbe_NX_MP_FG_consensus CCCGCTTTAGTATATTTGAGTGAGTATGAAAATAATGTTGGTGATTTTGTTGCTTTTGACTATATTAATGAAAATTTT [ 8658]
cp32-3_PAbe_NX_Pl_consensus    CCCGCTTTAGTATATTTGAGTGAGTATGAAAATAATGTTGGTGATTTTGTTGCTTTTGACTATATTAATGAAAATTTT [ 8658]

cp32-3_gi|11497103|B31_GB      GATTATGAAAAAGTAGCCACTTCACTTTCATCAAGTACATCAAATTCCAATGAGCTGGTTGCTAAATGAACAAAAGAA [ 8736]
cp32-3_un8_B31_PacBio          GATTATGAAAAAGTAGCCACTTCACTTTCATCAAGTACATCAAATTCCAATGAGCTGGTTGCTAAATGAACAAAAGAA [ 8736]
cp32-3_B31_TS_MP_FG_consensus  GATTATGAAAAAGTAGCCACTTCACTTTCATCAAGTACATCAAATTCCAATGAGCTGGTTGCTAAATGAACAAAAGAA [ 8736]
cp32-3_B31_NX_Pl_consensus     GATTATGAAAAAGTAGCCACTTCACTTTCATCAAGTACATCAAATTCCAATGAGCTGGTTGCTAAATGAACAAAAGAA [ 8736]
cp32-3_PAli_NX_MP_FG_consensus GATTATGAAAAAGTAGCCACTTCACTTTCATCAAGTACATCAAATTCCAATGAGCTGGTTGCTAAATGAACAAAAGAA [ 8736]
cp32-3_PAli_NX_Pl_consensus    GATTATGAAAAAGTAGCCACTTCACTTTCATCAAGTACATCAAATTCCAATGAGCTGGTTGCTAAATGAACAAAAGAA [ 8736]
cp32-3_PAbe_NX_MP_FG_consensus GATTATGAAAAAGTAGCCACTTCACTTTCATCAAGTACATCAAATTCCAATGAGCTGGTTGCTAAATGAACAAAAGAA [ 8736]
cp32-3_PAbe_NX_Pl_consensus    GATTATGAAAAAGTAGCCACTTCACTTTCATCAAGTACATCAAATTCCAATGAGCTGGTTGCTAAATGAACAAAAGAA [ 8736]

cp32-3_gi|11497103|B31_GB      ATAGAGATATTGATAAAGCTATTGCAAGTCTTGATGAGACTAGAAAAAAATATTTTAACTTGCTTGACGAGATTAAGA [ 8814]
cp32-3_un8_B31_PacBio          ATAGAGATATTGATAAAGCTATTGCAAGTCTTGATGAGACTAGAAAAAAATATTTTAACTTGCTTGACGAGATTAAGA [ 8814]
cp32-3_B31_TS_MP_FG_consensus  ATAGAGATATTGATAAAGCTATTGCAAGTCTTGATGAGACTAGAAAAAAATATTTTAACTTGCTTGACGAGATTAAGA [ 8814]
cp32-3_B31_NX_Pl_consensus     ATAGAGATATTGATAAAGCTATTGCAAGTCTTGATGAGACTAGAAAAAAATATTTTAACTTGCTTGACGAGATTAAGA [ 8814]
cp32-3_PAli_NX_MP_FG_consensus ATAGAGATATTGATAAAGCTATTGCAAGTCTTGATGAGACTAGAAAAAAATATTTTAACTTGCTTGACGAGATTAAGA [ 8814]
cp32-3_PAli_NX_Pl_consensus    ATAGAGATATTGATAAAGCTATTGCAAGTCTTGATGAGACTAGAAAAAAATATTTTAACTTGCTTGACGAGATTAAGA [ 8814]
cp32-3_PAbe_NX_MP_FG_consensus ATAGAGATATTGATAAAGCTATTGCAAGTCTTGATGAGACTAGAAAAAAATATTTTAACTTGCTTGACGAGATTAAGA [ 8814]
cp32-3_PAbe_NX_Pl_consensus    ATAGAGATATTGATAAAGCTATTGCAAGTCTTGATGAGACTAGAAAAAAATATTTTAACTTGCTTGACGAGATTAAGA [ 8814]

cp32-3_gi|11497103|B31_GB      ACGATAAATACTATTTTCCAGTAATTATGAATATTTGCTCATACGACTCGGTTAAAAAATTGCCTTATGACGAGCTTT [ 8892]
cp32-3_un8_B31_PacBio          ACGATAAATACTATTTTCCAGTAATTATGAATATTTGCTCATACGACTCGGTTAAAAAATTGCCTTATGACGAGCTTT [ 8892]
cp32-3_B31_TS_MP_FG_consensus  ACGATAAATACTATTTTCCAGTAATTATGAATATTTGCTCATACGACTCGGTTAAAAAATTGCCTTATGACGAGCTTT [ 8892]
cp32-3_B31_NX_Pl_consensus     ACGATAAATACTATTTTCCAGTAATTATGAATATTTGCTCATACGACTCGGTTAAAAAATTGCCTTATGACGAGCTTT [ 8892]
cp32-3_PAli_NX_MP_FG_consensus ACGATAAATACTATTTTCCAGTAATTATGAATATTTGCTCATACGACTCGGTTAAAAAATTGCCTTATGACGAGCTTT [ 8892]
cp32-3_PAli_NX_Pl_consensus    ACGATAAATACTATTTTCCAGTAATTATGAATATTTGCTCATACGACTCGGTTAAAAAATTGCCTTATGACGAGCTTT [ 8892]
cp32-3_PAbe_NX_MP_FG_consensus ACGATAAATACTATTTTCCAGTAATTATGAATATTTGCTCATACGACTCGGTTAAAAAATTGCCTTATGACGAGCTTT [ 8892]
cp32-3_PAbe_NX_Pl_consensus    ACGATAAATACTATTTTCCAGTAATTATGAATATTTGCTCATACGACTCGGTTAAAAAATTGCCTTATGACGAGCTTT [ 8892]

cp32-3_gi|11497103|B31_GB      TAGAAGTCAATAGACTTGCTGAGATTAAATTAGAAAAAGAATTGTATGAATTAATTTTAAGCAAGTGAGGACTTAGTG [ 8970]
cp32-3_un8_B31_PacBio          TAGAAGTCAATAGACTTGCTGAGATTAAATTAGAAAAAGAATTGTATGAATTAATTTTAAGCAAGTGAGGACTTAGTG [ 8970]
cp32-3_B31_TS_MP_FG_consensus  TAGAAGTCAATAGACTTGCTGAGATTAAATTAGAAAAAGAATTGTATGAATTAATTTTAAGCAAGTGAGGACTTAGTG [ 8970]
cp32-3_B31_NX_Pl_consensus     TAGAAGTCAATAGACTTGCTGAGATTAAATTAGAAAAAGAATTGTATGAATTAATTTTAAGCAAGTGAGGACTTAGTG [ 8970]
cp32-3_PAli_NX_MP_FG_consensus TAGAAGTCAATAGACTTGCTGAGATTAAATTAGAAAAAGAATTGTATGAATTAATTTTAAGCAAGTGAGGACTTAGTG [ 8970]
cp32-3_PAli_NX_Pl_consensus    TAGAAGTCAATAGACTTGCTGAGATTAAATTAGAAAAAGAATTGTATGAATTAATTTTAAGCAAGTGAGGACTTAGTG [ 8970]
cp32-3_PAbe_NX_MP_FG_consensus TAGAAGTCAATAGACTTGCTGAGATTAAATTAGAAAAAGAATTGTATGAATTAATTTTAAGCAAGTGAGGACTTAGTG [ 8970]
cp32-3_PAbe_NX_Pl_consensus    TAGAAGTCAATAGACTTGCTGAGATTAAATTAGAAAAAGAATTGTATGAATTAATTTTAAGCAAGTGAGGACTTAGTG [ 8970]

cp32-3_gi|11497103|B31_GB      AGCGACAAATTCACCATTAAATTTAAAGGTATTCTTGATCATGCTGCAACAAAAAAGGCCATTGAACAAGATATTTCT [ 9048]
cp32-3_un8_B31_PacBio          AGCGACAAATTCACCATTAAATTTAAAGGTATTCTTGATCATGCTGCAACAAAAAAGGCCATTGAACAAGATATTTCT [ 9048]
cp32-3_B31_TS_MP_FG_consensus  AGCGACAAATTCACCATTAAATTTAAAGGTATTCTTGATCATGCTGCAACAAAAAAGGCCATTGAACAAGATATTTCT [ 9048]
cp32-3_B31_NX_Pl_consensus     AGCGACAAATTCACCATTAAATTTAAAGGTATTCTTGATCATGCTGCAACAAAAAAGGCCATTGAACAAGATATTTCT [ 9048]
cp32-3_PAli_NX_MP_FG_consensus AGCGACAAATTCACCATTAAATTTAAAGGTATTCTTGATCATGCTGCAACAAAAAAGGCCATTGAACAAGATATTTCT [ 9048]
cp32-3_PAli_NX_Pl_consensus    AGCGACAAATTCACCATTAAATTTAAAGGTATTCTTGATCATGCTGCAACAAAAAAGGCCATTGAACAAGATATTTCT [ 9048]
cp32-3_PAbe_NX_MP_FG_consensus AGCGACAAATTCACCATTAAATTTAAAGGTATTCTTGATCATGCTGCAACAAAAAAGGCCATTGAACAAGATATTTCT [ 9048]
cp32-3_PAbe_NX_Pl_consensus    AGCGACAAATTCACCATTAAATTTAAAGGTATTCTTGATCATGCTGCAACAAAAAAGGCCATTGAACAAGATATTTCT [ 9048]

cp32-3_gi|11497103|B31_GB      AAAATGGAAAAATATCTTAAACCTAAAAAATCTAGTTTGGGTAGCACTAAAGATATTGTAAAAAATAATTTGTCGGAC [ 9126]
cp32-3_un8_B31_PacBio          AAAATGGAAAAATATCTTAAACCTAAAAAATCTAGTTTGGGTAGCACTAAAGATATTGTAAAAAATAATTTGTCGGAC [ 9126]
cp32-3_B31_TS_MP_FG_consensus  AAAATGGAAAAATATCTTAAACCTAAAAAATCTAGTTTGGGTAGCACTAAAGATATTGTAAAAAATAATTTGTCGGAC [ 9126]
cp32-3_B31_NX_Pl_consensus     AAAATGGAAAAATATCTTAAACCTAAAAAATCTAGTTTGGGTAGCACTAAAGATATTGTAAAAAATAATTTGTCGGAC [ 9126]
cp32-3_PAli_NX_MP_FG_consensus AAAATGGAAAAATATCTTAAACCTAAAAAATCTAGTTTGGGTAGCACTAAAGATATTGTAAAAAATAATTTGTCGGAC [ 9126]
cp32-3_PAli_NX_Pl_consensus    AAAATGGAAAAATATCTTAAACCTAAAAAATCTAGTTTGGGTAGCACTAAAGATATTGTAAAAAATAATTTGTCGGAC [ 9126]
cp32-3_PAbe_NX_MP_FG_consensus AAAATGGAAAAATATCTTAAACCTAAAAAATCTAGTTTGGGTAGCACTAAAGATATTGTAAAAAATAATTTGTCGGAC [ 9126]
cp32-3_PAbe_NX_Pl_consensus    AAAATGGAAAAATATCTTAAACCTAAAAAATCTAGTTTGGGTAGCACTAAAGATATTGTAAAAAATAATTTGTCGGAC [ 9126]

cp32-3_gi|11497103|B31_GB      AAGAAAAAAGAACTTAGTAGACAATCTAAATTTGAAAGCTTAAGAGAGCGTGTTGAGAAATATAGACTTACACAAACT [ 9204]
cp32-3_un8_B31_PacBio          AAGAAAAAAGAACTTAGTAGACAATCTAAATTTGAAAGCTTAAGAGAGCGTGTTGAGAAATATAGACTTACACAAACT [ 9204]
cp32-3_B31_TS_MP_FG_consensus  AAGAAAAAAGAACTTAGTAGACAATCTAAATTTGAAAGCTTAAGAGAGCGTGTTGAGAAATATAGACTTACACAAACT [ 9204]
cp32-3_B31_NX_Pl_consensus     AAGAAAAAAGAACTTAGTAGACAATCTAAATTTGAAAGCTTAAGAGAGCGTGTTGAGAAATATAGACTTACACAAACT [ 9204]
cp32-3_PAli_NX_MP_FG_consensus AAGAAAAAAGAACTTAGTAGACAATCTAAATTTGAAAGCTTAAGAGAGCGTGTTGAGAAATATAGACTTACACAAACT [ 9204]
cp32-3_PAli_NX_Pl_consensus    AAGAAAAAAGAACTTAGTAGACAATCTAAATTTGAAAGCTTAAGAGAGCGTGTTGAGAAATATAGACTTACACAAACT [ 9204]
cp32-3_PAbe_NX_MP_FG_consensus AAGAAAAAAGAACTTAGTAGACAATCTAAATTTGAAAGCTTAAGAGAGCGTGTTGAGAAATATAGACTTACACAAACT [ 9204]
cp32-3_PAbe_NX_Pl_consensus    AAGAAAAAAGAACTTAGTAGACAATCTAAATTTGAAAGCTTAAGAGAGCGTGTTGAGAAATATAGACTTACACAAACT [ 9204]

cp32-3_gi|11497103|B31_GB      AAAAAACTTATAAAACAGGGCATGGGGTTTGAGAAAGCTAGAAAAGAGGCTTTCAGAAGATCTTTAATGTCTGATAGA [ 9282]
cp32-3_un8_B31_PacBio          AAAAAACTTATAAAACAGGGCATGGGGTTTGAGAAAGCTAGAAAAGAGGCTTTCAGAAGATCTTTAATGTCTGATAGA [ 9282]
cp32-3_B31_TS_MP_FG_consensus  AAAAAACTTATAAAACAGGGCATGGGGTTTGAGAAAGCTAGAAAAGAGGCTTTCAGAAGATCTTTAATGTCTGATAGA [ 9282]
cp32-3_B31_NX_Pl_consensus     AAAAAACTTATAAAACAGGGCATGGGGTTTGAGAAAGCTAGAAAAGAGGCTTTCAGAAGATCTTTAATGTCTGATAGA [ 9282]
cp32-3_PAli_NX_MP_FG_consensus AAAAAACTTATAAAACAGGGCATGGGGTTTGAGAAAGCTAGAAAAGAGGCTTTCAGAAGATCTTTAATGTCTGATAGA [ 9282]
cp32-3_PAli_NX_Pl_consensus    AAAAAACTTATAAAACAGGGCATGGGGTTTGAGAAAGCTAGAAAAGAGGCTTTCAGAAGATCTTTAATGTCTGATAGA [ 9282]
cp32-3_PAbe_NX_MP_FG_consensus AAAAAACTTATAAAACAGGGCATGGGGTTTGAGAAAGCTAGAAAAGAGGCTTTCAGAAGATCTTTAATGTCTGATAGA [ 9282]
cp32-3_PAbe_NX_Pl_consensus    AAAAAACTTATAAAACAGGGCATGGGGTTTGAGAAAGCTAGAAAAGAGGCTTTCAGAAGATCTTTAATGTCTGATAGA [ 9282]

cp32-3_gi|11497103|B31_GB      GACAAAAGGCGTCTTGAGTATAAAGAACTTGCAAAAGAATCAAAAGCAAAAAGTAAAATGTTAGCGGCCTCTCAAGGA [ 9360]
cp32-3_un8_B31_PacBio          GACAAAAGGCGTCTTGAGTATAAAGAACTTGCAAAAGAATCAAAAGCAAAAAGTAAAATGTTAGCGGCCTCTCAAGGA [ 9360]
cp32-3_B31_TS_MP_FG_consensus  GACAAAAGGCGTCTTGAGTATAAAGAACTTGCAAAAGAATCAAAAGCAAAAAGTAAAATGTTAGCGGCCTCTCAAGGA [ 9360]
cp32-3_B31_NX_Pl_consensus     GACAAAAGGCGTCTTGAGTATAAAGAACTTGCAAAAGAATCAAAAGCAAAAAGTAAAATGTTAGCGGCCTCTCAAGGA [ 9360]
cp32-3_PAli_NX_MP_FG_consensus GACAAAAGGCGTCTTGAGTATAAAGAACTTGCAAAAGAATCAAAAGCAAAAAGTAAAATGTTAGCGGCCTCTCAAGGA [ 9360]
cp32-3_PAli_NX_Pl_consensus    GACAAAAGGCGTCTTGAGTATAAAGAACTTGCAAAAGAATCAAAAGCAAAAAGTAAAATGTTAGCGGCCTCTCAAGGA [ 9360]
cp32-3_PAbe_NX_MP_FG_consensus GACAAAAGGCGTCTTGAGTATAAAGAACTTGCAAAAGAATCAAAAGCAAAAAGTAAAATGTTAGCGGCCTCTCAAGGA [ 9360]
cp32-3_PAbe_NX_Pl_consensus    GACAAAAGGCGTCTTGAGTATAAAGAACTTGCAAAAGAATCAAAAGCAAAAAGTAAAATGTTAGCGGCCTCTCAAGGA [ 9360]

cp32-3_gi|11497103|B31_GB      AAAGGACTTGTTGCCAAAATTGCTATAGGTAGTGCCCTAGGGAATATCATTAGCAACGCTATGAGTAAAGTTGGAGGA [ 9438]
cp32-3_un8_B31_PacBio          AAAGGACTTGTTGCCAAAATTGCTATAGGTAGTGCCCTAGGGAATATCATTAGCAACGCTATGAGTAAAGTTGGAGGA [ 9438]
```

```
cp32-3_B31_TS_MP_FG_consensus  AAAGGACTTGTTGCCAAAATTGCTATAGGTAGTGCCCTAGGGAATATCATTAGCAACGCTATGAGTAAAGTTGGAGGA [ 9438]
cp32-3_B31_NX_Pl_consensus     AAAGGACTTGTTGCCAAAATTGCTATAGGTAGTGCCCTAGGGAATATCATTAGCAACGCTATGAGTAAAGTTGGAGGA [ 9438]
cp32-3_PAli_NX_MP_FG_consensus AAAGGACTTGTTGCCAAAATTGCTATAGGTAGTGCCCTAGGGAATATCATTAGCAACGCTATGAGTAAAGTTGGAGGA [ 9438]
cp32-3_PAli_NX_Pl_consensus    AAAGGACTTGTTGCCAAAATTGCTATAGGTAGTGCCCTAGGGAATATCATTAGCAACGCTATGAGTAAAGTTGGAGGA [ 9438]
cp32-3_PAbe_NX_MP_FG_consensus AAAGGACTTGTTGCCAAAATTGCTATAGGTAGTGCCCTAGGGAATATCATTAGCAACGCTATGAGTAAAGTTGGAGGA [ 9438]
cp32-3_PAbe_NX_Pl_consensus    AAAGGACTTGTTGCCAAAATTGCTATAGGTAGTGCCCTAGGGAATATCATTAGCAACGCTATGAGTAAAGTTGGAGGA [ 9438]

cp32-3_gi|11497103|B31_GB      GGCCTTTTAGGTTTTGCTAAAAAAGCGGTTGAAGAAGACACCAAAACAAAAAGAACACAACTTCTCAATAAAGCGTTT [ 9516]
cp32-3_un8_B31_PacBio          GGCCTTTTAGGTTTTGCTAAAAAAGCGGTTGAAGAAGACACCAAAACAAAAAGAACACAACTTCTCAATAAAGCGTTT [ 9516]
cp32-3_B31_TS_MP_FG_consensus  GGCCTTTTAGGTTTTGCTAAAAAAGCGGTTGAAGAAGACACCAAAACAAAAAGAACACAACTTCTCAATAAAGCGTTT [ 9516]
cp32-3_B31_NX_Pl_consensus     GGCCTTTTAGGTTTTGCTAAAAAAGCGGTTGAAGAAGACACCAAAACAAAAAGAACACAACTTCTCAATAAAGCGTTT [ 9516]
cp32-3_PAli_NX_MP_FG_consensus GGCCTTTTAGGTTTTGCTAAAAAAGCGGTTGAAGAAGACACCAAAACAAAAAGAACACAACTTCTCAATAAAGCGTTT [ 9516]
cp32-3_PAli_NX_Pl_consensus    GGCCTTTTAGGTTTTGCTAAAAAAGCGGTTGAAGAAGACACCAAAACAAAAAGAACACAACTTCTCAATAAAGCGTTT [ 9516]
cp32-3_PAbe_NX_MP_FG_consensus GGCCTTTTAGGTTTTGCTAAAAAAGCGGTTGAAGAAGACACCAAAACAAAAAGAACACAACTTCTCAATAAAGCGTTT [ 9516]
cp32-3_PAbe_NX_Pl_consensus    GGCCTTTTAGGTTTTGCTAAAAAAGCGGTTGAAGAAGACACCAAAACAAAAAGAACACAACTTCTCAATAAAGCGTTT [ 9516]

cp32-3_gi|11497103|B31_GB      TATGGTGATCCAAAAGAGAAAGAGGGTCTTTTAAAGATTATTGGCGGAATGAAGGGATTTGAGCGCGACCTAGAAAAA [ 9594]
cp32-3_un8_B31_PacBio          TATGGTGATCCAAAAGAGAAAGAGGGTCTTTTAAAGATTATTGGCGGAATGAAGGGATTTGAGCGCGACCTAGAAAAA [ 9594]
cp32-3_B31_TS_MP_FG_consensus  TATGGTGATCCAAAAGAGAAAGAGGGTCTTTTAAAGATTATTGGCGGAATGAAGGGATTTGAGCGCGACCTAGAAAAA [ 9594]
cp32-3_B31_NX_Pl_consensus     TATGGTGATCCAAAAGAGAAAGAGGGTCTTTTAAAGATTATTGGCGGAATGAAGGGATTTGAGCGCGACCTAGAAAAA [ 9594]
cp32-3_PAli_NX_MP_FG_consensus TATGGTGATCCAAAAGAGAAAGAGGGTCTTTTAAAGATTATTGGCGGAATGAAGGGATTTGAGCGCGACCTAGAAAAA [ 9594]
cp32-3_PAli_NX_Pl_consensus    TATGGTGATCCAAAAGAGAAAGAGGGTCTTTTAAAGATTATTGGCGGAATGAAGGGATTTGAGCGCGACCTAGAAAAA [ 9594]
cp32-3_PAbe_NX_MP_FG_consensus TATGGTGATCCAAAAGAGAAAGAGGGTCTTTTAAAGATTATTGGCGGAATGAAGGGATTTGAGCGCGACCTAGAAAAA [ 9594]
cp32-3_PAbe_NX_Pl_consensus    TATGGTGATCCAAAAGAGAAAGAGGGTCTTTTAAAGATTATTGGCGGAATGAAGGGATTTGAGCGCGACCTAGAAAAA [ 9594]

cp32-3_gi|11497103|B31_GB      GAAGAATTCTTAAATCAAGCAAGTGTCTTTAAGGGTACTTTAAGGGATTTAGATATGTTAAATGAAACTAATTTGAAA [ 9672]
cp32-3_un8_B31_PacBio          GAAGAATTCTTAAATCAAGCAAGTGTCTTTAAGGGTACTTTAAGGGATTTAGATATGTTAAATGAAACTAATTTGAAA [ 9672]
cp32-3_B31_TS_MP_FG_consensus  GAAGAATTCTTAAATCAAGCAAGTGTCTTTAAGGGTACTTTAAGGGATTTAGATATGTTAAATGAAACTAATTTGAAA [ 9672]
cp32-3_B31_NX_Pl_consensus     GAAGAATTCTTAAATCAAGCAAGTGTCTTTAAGGGTACTTTAAGGGATTTAGATATGTTAAATGAAACTAATTTGAAA [ 9672]
cp32-3_PAli_NX_MP_FG_consensus GAAGAATTCTTAAATCAAGCAAGTGTCTTTAAGGGTACTTTAAGGGATTTAGATATGTTAAATGAAACTAATTTGAAA [ 9672]
cp32-3_PAli_NX_Pl_consensus    GAAGAATTCTTAAATCAAGCAAGTGTCTTTAAGGGTACTTTAAGGGATTTAGATATGTTAAATGAAACTAATTTGAAA [ 9672]
cp32-3_PAbe_NX_MP_FG_consensus GAAGAATTCTTAAATCAAGCAAGTGTCTTTAAGGGTACTTTAAGGGATTTAGATATGTTAAATGAAACTAATTTGAAA [ 9672]
cp32-3_PAbe_NX_Pl_consensus    GAAGAATTCTTAAATCAAGCAAGTGTCTTTAAGGGTACTTTAAGGGATTTAGATATGTTAAATGAAACTAATTTGAAA [ 9672]

cp32-3_gi|11497103|B31_GB      AACGCAGTAGAATTTGCAGCTATGCTTAAATCCAGTGGTGCTATGAGCAGCGAAGATGCAGTAAAGGCTGTTAATAGT [ 9750]
cp32-3_un8_B31_PacBio          AACGCAGTAGAATTTGCAGCTATGCTTAAATCCAGTGGTGCTATGAGCAGCGAAGATGCAGTAAAGGCTGTTAATAGT [ 9750]
cp32-3_B31_TS_MP_FG_consensus  AACGCAGTAGAATTTGCAGCTATGCTTAAATCCAGTGGTGCTATGAGCAGCGAAGATGCAGTAAAGGCTGTTAATAGT [ 9750]
cp32-3_B31_NX_Pl_consensus     AACGCAGTAGAATTTGCAGCTATGCTTAAATCCAGTGGTGCTATGAGCAGCGAAGATGCAGTAAAGGCTGTTAATAGT [ 9750]
cp32-3_PAli_NX_MP_FG_consensus AACGCAGTAGAATTTGCAGCTATGCTTAAATCCAGTGGTGCTATGAGCAGCGAAGATGCAGTAAAGGCTGTTAATAGT [ 9750]
cp32-3_PAli_NX_Pl_consensus    AACGCAGTAGAATTTGCAGCTATGCTTAAATCCAGTGGTGCTATGAGCAGCGAAGATGCAGTAAAGGCTGTTAATAGT [ 9750]
cp32-3_PAbe_NX_MP_FG_consensus AACGCAGTAGAATTTGCAGCTATGCTTAAATCCAGTGGTGCTATGAGCAGCGAAGATGCAGTAAAGGCTGTTAATAGT [ 9750]
cp32-3_PAbe_NX_Pl_consensus    AACGCAGTAGAATTTGCAGCTATGCTTAAATCCAGTGGTGCTATGAGCAGCGAAGATGCAGTAAAGGCTGTTAATAGT [ 9750]

cp32-3_gi|11497103|B31_GB      GTTCTTGGGGGTGATGGAAGTGAGCTTTTTGATCTATTAAAGAAGTCAGGTGTTGGAGACAAATATATAGAAGATGCC [ 9828]
cp32-3_un8_B31_PacBio          GTTCTTGGGGGTGATGGAAGTGAGCTTTTTGATCTATTAAAGAAGTCAGGTGTTGGAGACAAATATATAGAAGATGCC [ 9828]
cp32-3_B31_TS_MP_FG_consensus  GTTCTTGGGGGTGATGGAAGTGAGCTTTTTGATCTATTAAAGAAGTCAGGTGTTGGAGACAAATATATAGAAGATGCC [ 9828]
cp32-3_B31_NX_Pl_consensus     GTTCTTGGGGGTGATGGAAGTGAGCTTTTTGATCTATTAAAGAAGTCAGGTGTTGGAGACAAATATATAGAAGATGCC [ 9828]
cp32-3_PAli_NX_MP_FG_consensus GTTCTTGGGGGTGATGGAAGTGAGCTTTTTGATCTATTAAAGAAGTCAGGTGTTGGAGACAAATATATAGAAGATGCC [ 9828]
cp32-3_PAli_NX_Pl_consensus    GTTCTTGGGGGTGATGGAAGTGAGCTTTTTGATCTATTAAAGAAGTCAGGTGTTGGAGACAAATATATAGAAGATGCC [ 9828]
cp32-3_PAbe_NX_MP_FG_consensus GTTCTTGGGGGTGATGGAAGTGAGCTTTTTGATCTATTAAAGAAGTCAGGTGTTGGAGACAAATATATAGAAGATGCC [ 9828]
cp32-3_PAbe_NX_Pl_consensus    GTTCTTGGGGGTGATGGAAGTGAGCTTTTTGATCTATTAAAGAAGTCAGGTGTTGGAGACAAATATATAGAAGATGCC [ 9828]

cp32-3_gi|11497103|B31_GB      AAAATGGCCTGGCAAAGCGGGGCTCAAGTAGATCTAGATTCCAGAATTACCAAGATGATGGAAATGTTCAAGGATTTT [ 9906]
cp32-3_un8_B31_PacBio          AAAATGGCCTGGCAAAGCGGGGCTCAAGTAGATCTAGATTCCAGAATTACCAAGATGATGGAAATGTTCAAGGATTTT [ 9906]
cp32-3_B31_TS_MP_FG_consensus  AAAATGGCCTGGCAAAGCGGGGCTCAAGTAGATCTAGATTCCAGAATTACCAAGATGATGGAAATGTTCAAGGATTTT [ 9906]
cp32-3_B31_NX_Pl_consensus     AAAATGGCCTGGCAAAGCGGGGCTCAAGTAGATCTAGATTCCAGAATTACCAAGATGATGGAAATGTTCAAGGATTTT [ 9906]
cp32-3_PAli_NX_MP_FG_consensus AAAATGGCCTGGCAAAGCGGGGCTCAAGTAGATCTAGATTCCAGAATTACCAAGATGATGGAAATGTTCAAGGATTTT [ 9906]
cp32-3_PAli_NX_Pl_consensus    AAAATGGCCTGGCAAAGCGGGGCTCAAGTAGATCTAGATTCCAGAATTACCAAGATGATGGAAATGTTCAAGGATTTT [ 9906]
cp32-3_PAbe_NX_MP_FG_consensus AAAATGGCCTGGCAAAGCGGGGCTCAAGTAGATCTAGATTCCAGAATTACCAAGATGATGGAAATGTTCAAGGATTTT [ 9906]
cp32-3_PAbe_NX_Pl_consensus    AAAATGGCCTGGCAAAGCGGGGCTCAAGTAGATCTAGATTCCAGAATTACCAAGATGATGGAAATGTTCAAGGATTTT [ 9906]

cp32-3_gi|11497103|B31_GB      AAATCTTTCGGCCTTACAAAAAAAGTCAATAATGCTGAGAGTATTCAAAGTAATTTGGCCTCAGCTGAGCAAACTCTT [ 9984]
cp32-3_un8_B31_PacBio          AAATCTTTCGGCCTTACAAAAAAAGTCAATAATGCTGAGAGTATTCAAAGTAATTTGGCCTCAGCTGAGCAAACTCTT [ 9984]
cp32-3_B31_TS_MP_FG_consensus  AAATCTTTCGGCCTTACAAAAAAAGTCAATAATGCTGAGAGTATTCAAAGTAATTTGGCCTCAGCTGAGCAAACTCTT [ 9984]
cp32-3_B31_NX_Pl_consensus     AAATCTTTCGGCCTTACAAAAAAAGTCAATAATGCTGAGAGTATTCAAAGTAATTTGGCCTCAGCTGAGCAAACTCTT [ 9984]
cp32-3_PAli_NX_MP_FG_consensus AAATCTTTCGGCCTTACAAAAAAAGTCAATAATGCTGAGAGTATTCAAAGTAATTTGGCCTCAGCTGAGCAAACTCTT [ 9984]
cp32-3_PAli_NX_Pl_consensus    AAATCTTTCGGCCTTACAAAAAAAGTCAATAATGCTGAGAGTATTCAAAGTAATTTGGCCTCAGCTGAGCAAACTCTT [ 9984]
cp32-3_PAbe_NX_MP_FG_consensus AAATCTTTCGGCCTTACAAAAAAAGCCAATACTGCTGAGAGTATTCAAAGTAATTTGGCCTCAGCTGAGCAAACTCTT [ 9984]
cp32-3_PAbe_NX_Pl_consensus    AAATCTTTCGGCCTTACAAAAAAAGTCAATAATGCTGAGAGTATTCAAAGTAATTTGGCCTCAGCTGAGCAAACTCTT [ 9984]

cp32-3_gi|11497103|B31_GB      TCAAATTTGACCACTACTGTTTTGGACCCATTACTTAATATCATTAACTGGATCACCGCTAAAATTAAAAATTTTGAT [10062]
cp32-3_un8_B31_PacBio          TCAAATTTGACCACTACTGTTTTGGACCCATTACTTAATATCATTAACTGGATCACCGCTAAAATTAAAAATTTTGAT [10062]
cp32-3_B31_TS_MP_FG_consensus  TCAAATTTGACCACTACTGTTTTGGACCCATTACTTAATATCATTAACTGGATCACCGCTAAAATTAAAAATTTTGAT [10062]
cp32-3_B31_NX_Pl_consensus     TCAAATTTGACCACTACTGTTTTGGACCCATTACTTAATATCATTAACTGGATCACCGCTAAAATTAAAAATTTTGAT [10062]
cp32-3_PAli_NX_MP_FG_consensus TCAAATTTGACCACTACTGTTTTGGACCCATTACTTAATATCATTAACTGGATCACCGCTAAAATTAAAAATTTTGAT [10062]
cp32-3_PAli_NX_Pl_consensus    TCAAATTTGACCACTACTGTTTTGGACCCATTACTTAATATCATTAACTGGATCACCGCTAAAATTAAAAATTTTGAT [10062]
cp32-3_PAbe_NX_MP_FG_consensus TCAAATTTGACCACTACTGTTTTGGACCCATTACTTAATATCATTAACTGGATCACCGCTAAAATTAAAAATTTTGAT [10062]
cp32-3_PAbe_NX_Pl_consensus    TCAAATTTGACCACTACTGTTTTGGACCCATTACTTAATATCATTAACTGGATCACCGCTAAAATTAAAAATTTTGAT [10062]

cp32-3_gi|11497103|B31_GB      TTTACCAAAGATATTATCAATCCCATAATTAATGGCATTAAAAGTATTTTTAATCTTAATTATTTCTTTGCCAAATTA [10140]
cp32-3_un8_B31_PacBio          TTTACCAAAGATATTATCAATCCCATAATTAATGGCATTAAAAGTATTTTTAATCTTAATTATTTCTTTGCCAAATTA [10140]
cp32-3_B31_TS_MP_FG_consensus  TTTACCAAAGATATTATCAATCCCATAATTAATGGCATTAAAAGTATTTTTAATCTTAATTATTTCTTTGCCAAATTA [10140]
cp32-3_B31_NX_Pl_consensus     TTTACCAAAGATATTATCAATCCCATAATTAATGGCATTAAAAGTATTTTTAATCTTAATTATTTCTTTGCCAAATTA [10140]
cp32-3_PAli_NX_MP_FG_consensus TTTACCAAAGATATTATCAATCCCATAATTAATGGCATTAAAAGTATTTTTAATCTTAATTATTTCTTTGCCAAATTA [10140]
cp32-3_PAli_NX_Pl_consensus    TTTACCAAAGATATTATCAATCCCATAATTAATGGCATTAAAAGTATTTTTAATCTTAATTATTTCTTTGCCAAATTA [10140]
cp32-3_PAbe_NX_MP_FG_consensus TTTACCAAAGATATTATCAATCCCATAATTAATGGCATTAAAAGTATTTTTAATCTTAATTATTTCTTTGCCAAATTA [10140]
cp32-3_PAbe_NX_Pl_consensus    TTTACCAAAGATATTATCAATCCCATAATTAATGGCATTAAAAGTATTTTTAATCTTAATTATTTCTTTGCCAAATTA [10140]

cp32-3_gi|11497103|B31_GB      AAATCGGTTCTACCTACAATGTTCGGCGGAGATGGCGGCGAAGCTTTAAAAAAACTGCAAGAACAGGCTGAGAATCAA [10218]
cp32-3_un8_B31_PacBio          AAATCGGTTCTACCTACAATGTTCGGCGGAGATGGCGGCGAAGCTTTAAAAAAACTGCAAGAACAGGCTGAGAATCAA [10218]
cp32-3_B31_TS_MP_FG_consensus  AAATCGGTTCTACCTACAATGTTCGGCGGAGATGGCGGCGAAGCTTTAAAAAAACTGCAAGAACAGGCTGAGAATCAA [10218]
cp32-3_B31_NX_Pl_consensus     AAATCGGTTCTACCTACAATGTTCGGCGGAGATGGCGGCGAAGCTTTAAAAAAACTGCAAGAACAGGCTGAGAATCAA [10218]
cp32-3_PAli_NX_MP_FG_consensus AAATCGGTTCTACCTACAATGTTCGGCGGAGATGGCGGCGAAGCTTTAAAAAAACTGCAAGAACAGGCTGAGAATCAA [10218]
cp32-3_PAli_NX_Pl_consensus    AAATCGGTTCTACCTACAATGTTCGGCGGAGATGGCGGCGAAGCTTTAAAAAAACTGCAAGAACAGGCTGAGAATCAA [10218]
cp32-3_PAbe_NX_MP_FG_consensus AAATCGGTTCTACCTACAATGTTCGGCGGAGATGGCGGCGAAGCTTTAAAAAAACTGCAAGAACAGGCTGAGAATCAA [10218]
cp32-3_PAbe_NX_Pl_consensus    AAATCGGTTCTACCTACAATGTTCGGCGGAGATGGCGGCGAAGCTTTAAAAAAACTGCAAGAACAGGCTGAGAATCAA [10218]

cp32-3_gi|11497103|B31_GB      GACAATGCTAACAACACTCCATAATTTTGGTAAAAGGTAATTACTTATGACAAGTAACAAAAAAATTGCTAACAATGC [10296]
cp32-3_un8_B31_PacBio          GACAATGCTAACAACACTCCATAATTTTGGTAAAAGGTAATTACTTATGACAAGTAACAAAAAAATTGCTAACAATGC [10296]
cp32-3_B31_TS_MP_FG_consensus  GACAATGCTAACAACACTCCATAATTTTGGTAAAAGGTAATTACTTATGACAAGTAACAAAAAAATTGCTAACAATGC [10296]
cp32-3_B31_NX_Pl_consensus     GACAATGCTAACAACACTCCATAATTTTGGTAAAAGGTAATTACTTATGACAAGTAACAAAAAAATTGCTAACAATGC [10296]
cp32-3_PAli_NX_MP_FG_consensus GACAATGCTAACAACACTCCATAATTTTGGTAAAAGGTAATTACTTATGACAAGTAACAAAAAAATTGCTAACAATGC [10296]
cp32-3_PAli_NX_Pl_consensus    GACAATGCTAACAACACTCCATAATTTTGGTAAAAGGTAATTACTTATGACAAGTAACAAAAAAATTGCTAACAATGC [10296]
cp32-3_PAbe_NX_MP_FG_consensus GACAATGCTAACAACACTCCATAATTTTGGTAAAAGGTAATTACTTATGACAAGTAACAAAAAAATTGCTAACAATGC [10296]
cp32-3_PAbe_NX_Pl_consensus    GACAATGCTAACAACACTCCATAATTTTGGTAAAAGGTAATTACTTATGACAAGTAACAAAAAAATTGCTAACAATGC [10296]

cp32-3_gi|11497103|B31_GB      AGCTAACAAAATAGATATTAATAATAAAATTACTAACAATCATGATATTGAAAAGAAAAAAATCAAGGAAAAAATTAA [10374]
cp32-3_un8_B31_PacBio          AGCTAACAAAATAGATATTAATAATAAAATTACTAACAATCATGATATTGAAAAGAAAAAAATCAAGGAAAAAATTAA [10374]
cp32-3_B31_TS_MP_FG_consensus  AGCTAACAAAATAGATATTAATAATAAAATTACTAACAATCATGATATTGAAAAGAAAAAAATCAAGGAAAAAATTAA [10374]
```

```
cp32-3_B31_NX_Pl_consensus     AGCTAACAAAATAGATATTAATAATAAAATTACTAACAATCATGATATTGAAAAGAAAAAAATCAAGGAAAAAATTAA [10374]
cp32-3_PAli_NX_MP_FG_consensus AGCTAACAAAATAGATATTAATAATAAAATTACTAACAATCATGATATTGAAAAGAAAAAAATCAAGGAAAAAATTAA [10374]
cp32-3_PAli_NX_Pl_consensus    AGCTAACAAAATAGATATTAATAATAAAATTACTAACAATCATGATATTGAAAAGAAAAAAATCAAGGAAAAAATTAA [10374]
cp32-3_PAbe_NX_MP_FG_consensus AGCTAACAAAATAGATATTAATAATAAAATTACTAACAATCATGATATTGAAAAGAAAAAAATCAAGGAAAAAATTAA [10374]
cp32-3_PAbe_NX_Pl_consensus    AGCTAACAAAATAGATATTAATAATAAAATTACTAACAATCATGATATTGAAAAGAAAAAAATCAAGGAAAAAATTAA [10374]

cp32-3_gi|11497103|B31_GB      TGATATTGAAAAGAAAGAAATCAGGGAAATTACTCGAATAATAAGAGATGTAATAACCCAAATATTTGCTCTTTTTGG [10452]
cp32-3_un8_B31_PacBio          TGATATTGAAAAGAAAGAAATCAGGGAAATTACTCGAATAATAAGAGATGTAATAACCCAAATATTTGCTCTTTTTGG [10452]
cp32-3_B31_TS_MP_FG_consensus  TGATATTGAAAAGAAAGAAATCAGGGAAATTACTCGAATAATAAGAGATGTAATAACCCAAATATTTGCTCTTTTTGG [10452]
cp32-3_B31_NX_Pl_consensus     TGATATTGAAAAGAAAGAAATCAGGGAAATTACTCGAATAATAAGAGATGTAATAACCCAAATATTTGCTCTTTTTGG [10452]
cp32-3_PAli_NX_MP_FG_consensus TGATATTGAAAAGAAAGAAATCAGGGAAATTACTCGAATAATAAGAGATGTAATAACCCAAATATTTGCTCTTTTTGG [10452]
cp32-3_PAli_NX_Pl_consensus    TGATATTGAAAAGAAAGAAATCAGGGAAATTACTCGAATAATAAGAGATGTAATAACCCAAATATTTGCTCTTTTTGG [10452]
cp32-3_PAbe_NX_MP_FG_consensus TGATATTGAAAAGAAAGAAATCAGGGAAATTACTCGAATAATAAGAGATGTAATAACCCAAATATTTGCTCTTTTTGG [10452]
cp32-3_PAbe_NX_Pl_consensus    TGATATTGAAAAGAAAGAAATCAGGGAAATTACTCGAATAATAAGAGATGTAATAACCCAAATATTTGCTCTTTTTGG [10452]

cp32-3_gi|11497103|B31_GB      AGCAGATAATTTTTTAGTGTTATTTCCTAGAATGGATCTAAAAGGTTTTGGATATATTCCTCAATTGTTTTTTATAAA [10530]
cp32-3_un8_B31_PacBio          AGCAGATAATTTTTTAGTGTTATTTCCTAGAATGGATCTAAAAGGTTTTGGATATATTCCTCAATTGTTTTTTATAAA [10530]
cp32-3_B31_TS_MP_FG_consensus  AGCAGATAATTTTTTAGTGTTATTTCCTAGAATGGATCTAAAAGGTTTTGGATATATTCCTCAATTGTTTTTTATAAA [10530]
cp32-3_B31_NX_Pl_consensus     AGCAGATAATTTTTTAGTGTTATTTCCTAGAATGGATCTAAAAGGTTTTGGATATATTCCTCAATTGTTTTTTATAAA [10530]
cp32-3_PAli_NX_MP_FG_consensus AGCAGATAATTTTTTAGTGTTATTTCCTAGAATGGATCTAAAAGGTTTTGGATATATTCCTCAATTGTTTTTTATAAA [10530]
cp32-3_PAli_NX_Pl_consensus    AGCAGATAATTTTTTAGTGTTATTTCCTAGAATGGATCTAAAAGGTTTTGGATATATTCCTCAATTGTTTTTTATAAA [10530]
cp32-3_PAbe_NX_MP_FG_consensus AGCAGATAATTTTTTAGTGTTATTTCCTAGAATGGATCTAAAAGGTTTTGGATATATTCCTCAATTGTTTTTTATAAA [10530]
cp32-3_PAbe_NX_Pl_consensus    AGCAGATAATTTTTTAGTGTTATTTCCTAGAATGGATCTAAAAGGTTTTGGATATATTCCTCAATTGTTTTTTATAAA [10530]

cp32-3_gi|11497103|B31_GB      ACCAAAAAATGAACTCATAACACGCACTTATAATACTAATTGTTCTAAAAGACCAGTTATCAATTATTATGATAGAAA [10608]
cp32-3_un8_B31_PacBio          ACCAAAAAATGAACTCATAACACGCACTTATAATACTAATTGTTCTAAAAGACCAGTTATCAATTATTATGATAGAAA [10608]
cp32-3_B31_TS_MP_FG_consensus  ACCAAAAAATGAACTCATAACACGCACTTATAATACTAATTGTTCTAAAAGACCAGTTATCAATTATTATGATAGAAA [10608]
cp32-3_B31_NX_Pl_consensus     ACCAAAAAATGAACTCATAACACGCACTTATAATACTAATTGTTCTAAAAGACCAGTTATCAATTATTATGATAGAAA [10608]
cp32-3_PAli_NX_MP_FG_consensus ACCAAAAAATGAACTCATAACACGCACTTATAATACTAATTGTTCTAAAAGACCAGTTATCAATTATTATGATAGAAA [10608]
cp32-3_PAli_NX_Pl_consensus    ACCAAAAAATGAACTCATAACACGCACTTATAATACTAATTGTTCTAAAAGACCAGTTATCAATTATTATGATAGAAA [10608]
cp32-3_PAbe_NX_MP_FG_consensus ACCAAAAAATGAACTCATAACACGCACTTATAATACTAATTGTTCTAAAAGACCAGTTATCAATTATTATGATAGAAA [10608]
cp32-3_PAbe_NX_Pl_consensus    ACCAAAAAATGAACTCATAACACGCACTTATAATACTAATTGTTCTAAAAGACCAGTTATCAATTATTATGATAGAAA [10608]

cp32-3_gi|11497103|B31_GB      AGCGGAATATGTAAGCTACAATCCGGTAATGACTGGTGAACATATCTCATTAAACGGTGGAATACTAACATCCTTATA [10686]
cp32-3_un8_B31_PacBio          AGCGGAATATGTAAGCTACAATCCGGTAATGACTGGTGAACATATCTCATTAAACGGTGGAATACTAACATCCTTATA [10686]
cp32-3_B31_TS_MP_FG_consensus  AGCGGAATATGTAAGCTACAATCCGGTAATGACTGGTGAACATATCTCATTAAACGGTGGAATACTAACATCCTTATA [10686]
cp32-3_B31_NX_Pl_consensus     AGCGGAATATGTAAGCTACAATCCGGTAATGACTGGTGAACATATCTCATTAAACGGTGGAATACTAACATCCTTATA [10686]
cp32-3_PAli_NX_MP_FG_consensus AGCGGAATATGTAAGCTACAATCCGGTAATGACTGGTGAACATATCTCATTAAACGGTGGAATACTAACATCCTTATA [10686]
cp32-3_PAli_NX_Pl_consensus    AGCGGAATATGTAAGCTACAATCCGGTAATGACTGGTGAACATATCTCATTAAACGGTGGAATACTAACATCCTTATA [10686]
cp32-3_PAbe_NX_MP_FG_consensus AGCGGAATATGTAAGCTACAATCCGGTAATGACTGGTGAACATATCTCATTAAACGGTGGAATACTAACATCCTTATA [10686]
cp32-3_PAbe_NX_Pl_consensus    AGCGGAATATGTAAGCTACAATCCGGTAATGACTGGTGAACATATCTCATTAAACGGTGGAATACTAACATCCTTATA [10686]

cp32-3_gi|11497103|B31_GB      TAAGGATATGCTTTCTTTACTTAAAATGACTGTTTTTGGCAATACTATGCTACATTTTGACGCGCATCTTGTAAAAGA [10764]
cp32-3_un8_B31_PacBio          TAAGGATATGCTTTCTTTACTTAAAATGACTGTTTTTGGCAATACTATGCTACATTTTGACGCGCATCTTGTAAAAGA [10764]
cp32-3_B31_TS_MP_FG_consensus  TAAGGATATGCTTTCTTTACTTAAAATGACTGTTTTTGGCAATACTATGCTACATTTTGACGCGCATCTTGTAAAAGA [10764]
cp32-3_B31_NX_Pl_consensus     TAAGGATATGCTTTCTTTACTTAAAATGACTGTTTTTGGCAATACTATGCTACATTTTGACGCGCATCTTGTAAAAGA [10764]
cp32-3_PAli_NX_MP_FG_consensus TAAGGATATGCTTTCTTTACTTAAAATGACTGTTTTTGGCAATACTATGCTACATTTTGACGCGCATCTTGTAAAAGA [10764]
cp32-3_PAli_NX_Pl_consensus    TAAGGATATGCTTTCTTTACTTAAAATGACTGTTTTTGGCAATACTATGCTACATTTTGACGCGCATCTTGTAAAAGA [10764]
cp32-3_PAbe_NX_MP_FG_consensus TAAGGATATGCTTTCTTTACTTAAAATGACTGTTTTTGGCAATACTATGCTACATTTTGACGCGCATCTTGTAAAAGA [10764]
cp32-3_PAbe_NX_Pl_consensus    TAAGGATATGCTTTCTTTACTTAAAATGACTGTTTTTGGCAATACTATGCTACATTTTGACGCGCATCTTGTAAAAGA [10764]

cp32-3_gi|11497103|B31_GB      ACAACTAGCCAATAGAATACAAGCACAAGTCCCTTTTAGTATATATAGTCCAACTTTTGGCCTTAAAGAATTAGCTGT [10842]
cp32-3_un8_B31_PacBio          ACAACTAGCCAATAGAATACAAGCACAAGTCCCTTTTAGTATATATAGTCCAACTTTTGGCCTTAAAGAATTAGCTGT [10842]
cp32-3_B31_TS_MP_FG_consensus  ACAACTAGCCAATAGAATACAAGCACAAGTCCCTTTTAGTATATATAGTCCAACTTTTGGCCTTAAAGAATTAGCTGT [10842]
cp32-3_B31_NX_Pl_consensus     ACAACTAGCCAATAGAATACAAGCACAAGTCCCTTTTAGTATATATAGTCCAACTTTTGGCCTTAAAGAATTAGCTGT [10842]
cp32-3_PAli_NX_MP_FG_consensus ACAACTAGCCAATAGAATACAAGCACAAGTCCCTTTTAGTATATATAGTCCAACTTTTGGCCTTAAAGAATTAGCTGT [10842]
cp32-3_PAli_NX_Pl_consensus    ACAACTAGCCAATAGAATACAAGCACAAGTCCCTTTTAGTATATATAGTCCAACTTTTGGCCTTAAAGAATTAGCTGT [10842]
cp32-3_PAbe_NX_MP_FG_consensus ACAACTAGCCAATAGAATACAAGCACAAGTCCCTTTTAGTATATATAGTCCAACTTTTGGCCTTAAAGAATTAGCTGT [10842]
cp32-3_PAbe_NX_Pl_consensus    ACAACTAGCCAATAGAATACAAGCACAAGTCCCTTTTAGTATATATAGTCCAACTTTTGGCCTTAAAGAATTAGCTGT [10842]

cp32-3_gi|11497103|B31_GB      AATTACAAGTCTTTCGTTTAAAGATACTCCTTTCATTGACGAAGTTGAGGTTAGTCTATCAATAGAAATAGTAAAAAC [10920]
cp32-3_un8_B31_PacBio          AATTACAAGTCTTTCGTTTAAAGATACTCCTTTCATTGACGAAGTTGAGGTTAGTCTATCAATAGAAATAGTAAAAAC [10920]
cp32-3_B31_TS_MP_FG_consensus  AATTACAAGTCTTTCGTTTAAAGATACTCCTTTCATTGACGAAGTTGAGGTTAGTCTATCAATAGAAATAGTAAAAAC [10920]
cp32-3_B31_NX_Pl_consensus     AATTACAAGTCTTTCGTTTAAAGATACTCCTTTCATTGACGAAGTTGAGGTTAGTCTATCAATAGAAATAGTAAAAAC [10920]
cp32-3_PAli_NX_MP_FG_consensus AATTACAAGTCTTTCGTTTAAAGATACTCCTTTCATTGACGAAGTTGAGGTTAGTCTATCAATAGAAATAGTAAAAAC [10920]
cp32-3_PAli_NX_Pl_consensus    AATTACAAGTCTTTCGTTTAAAGATACTCCTTTCATTGACGAAGTTGAGGTTAGTCTATCAATAGAAATAGTAAAAAC [10920]
cp32-3_PAbe_NX_MP_FG_consensus AATTACAAGTCTTTCGTTTAAAGATACTCCTTTCATTGACGAAGTTGAGGTTAGTCTATCAATAGAAATAGTAAAAAC [10920]
cp32-3_PAbe_NX_Pl_consensus    AATTACAAGTCTTTCGTTTAAAGATACTCCTTTCATTGACGAAGTTGAGGTTAGTCTATCAATAGAAATAGTAAAAAC [10920]

cp32-3_gi|11497103|B31_GB      ATTCGCATTAGAAAAATATAAAGGATAAAAAAATGCTGTTACTACAATATGATTTTAAAATTGAGTTCTACAATGTAGA [10998]
cp32-3_un8_B31_PacBio          ATTCGCATTAGAAAAATATAAAGGATAAAAAAATGCTGTTACTACAATATGATTTTAAAATTGAGTTCTACAATGTAGA [10998]
cp32-3_B31_TS_MP_FG_consensus  ATTCGCATTAGAAAAATATAAAGGATAAAAAAATGCTGTTACTACAATATGATTTTAAAATTGAGTTCTACAATGTAGA [10998]
cp32-3_B31_NX_Pl_consensus     ATTCGCATTAGAAAAATATAAAGGATAAAAAAATGCTGTTACTACAATATGATTTTAAAATTGAGTTCTACAATGTAGA [10998]
cp32-3_PAli_NX_MP_FG_consensus ATTCGCATTAGAAAAATATAAAGGATAAAAAAATGCTGTTACTACAATATGATTTTAAAATTGAGTTCTACAATGTAGA [10998]
cp32-3_PAli_NX_Pl_consensus    ATTCGCATTAGAAAAATATAAAGGATAAAAAAATGCTGTTACTACAATATGATTTTAAAATTGAGTTCTACAATGTAGA [10998]
cp32-3_PAbe_NX_MP_FG_consensus ATTCGCATTAGAAAAATATAAAGGATAAAAAAATGCTGTTACTACAATATGATTTTAAAATTGAGTTCTACAATGTAGA [10998]
cp32-3_PAbe_NX_Pl_consensus    ATTCGCATTAGAAAAATATAAAGGATAAAAAAATGCTGTTACTACAATATGATTTTAAAATTGAGTTCTACAATGTAGA [10998]

cp32-3_gi|11497103|B31_GB      TACATCAAAAAAATCCCCTGATGGAATTCCTTTCGCCGAAGAAATTCCTAAAATTATCATCAATACACAAGATGGAAT [11076]
cp32-3_un8_B31_PacBio          TACATCAAAAAAATCCCCTGATGGAATTCCTTTCGCCGAAGAAATTCCTAAAATTATCATCAATACACAAGATGGAAT [11076]
cp32-3_B31_TS_MP_FG_consensus  TACATCAAAAAAATCCCCTGATGGAATTCCTTTCGCCGAAGAAATTCCTAAAATTATCATCAATACACAAGATGGAAT [11076]
cp32-3_B31_NX_Pl_consensus     TACATCAAAAAAATCCCCTGATGGAATTCCTTTCGCCGAAGAAATTCCTAAAATTATCATCAATACACAAGATGGAAT [11076]
cp32-3_PAli_NX_MP_FG_consensus TACATCAAAAAAATCCCCTGATGGAATTCCTTTCGCCGAAGAAATTCCTAAAATTATCATCAATACACAAGATGGAAT [11076]
cp32-3_PAli_NX_Pl_consensus    TACATCAAAAAAATCCCCTGATGGAATTCCTTTCGCCGAAGAAATTCCTAAAATTATCATCAATACACAAGATGGAAT [11076]
cp32-3_PAbe_NX_MP_FG_consensus TACATCAAAAAAATCCCCTGATGGAATTCCTTTCGCCGAAGAAATTCCTAAAATTATCATCAATACACAAGATGGAAT [11076]
cp32-3_PAbe_NX_Pl_consensus    TACATCAAAAAAATCCCCTGATGGAATTCCTTTCGCCGAAGAAATTCCTAAAATTATCATCAATACACAAGATGGAAT [11076]

cp32-3_gi|11497103|B31_GB      TCATATTGATATTTCCATATCCAACATGTATTCAAATATTCATACTATAAGTTCCAAACAAGCAAAAGTCGTACTTTG [11154]
cp32-3_un8_B31_PacBio          TCATATTGATATTTCCATATCCAACATGTATTCAAATATTCATACTATAAGTTCCAAACAAGCAAAAGTCGTACTTTG [11154]
cp32-3_B31_TS_MP_FG_consensus  TCATATTGATATTTCCATATCCAACATGTATTCAAATATTCATACTATAAGTTCCAAACAAGCAAAAGTCGTACTTTG [11154]
cp32-3_B31_NX_Pl_consensus     TCATATTGATATTTCCATATCCAACATGTATTCAAATATTCATACTATAAGTTCCAAACAAGCAAAAGTCGTACTTTG [11154]
cp32-3_PAli_NX_MP_FG_consensus TCATATTGATATTTCCATATCCAACATGTATTCAAATATTCATACTATAAGTTCCAAACAAGCAAAAGTCGTACTTTG [11154]
cp32-3_PAli_NX_Pl_consensus    TCATATTGATATTTCCATATCCAACATGTATTCAAATATTCATACTATAAGTTCCAAACAAGCAAAAGTCGTACTTTG [11154]
cp32-3_PAbe_NX_MP_FG_consensus TCATATTGATATTTCCATATCCAACATGTATTCAAATATTCATACTATAAGTTCCAAACAAGCAAAAGTCGTACTTTG [11154]
cp32-3_PAbe_NX_Pl_consensus    TCATATTGATATTTCCATATCCAACATGTATTCAAATATTCATACTATAAGTTCCAAACAAGCAAAAGTCGTACTTTG [11154]

cp32-3_gi|11497103|B31_GB      GAATCTTCCCTTAGACTTCACCGACGACATTAAATTTGGAGATATAGTAAAAATATATTATAAGAAATTTGCTCATGA [11232]
cp32-3_un8_B31_PacBio          GAATCTTCCCTTAGACTTCACCGACGACATTAAATTTGGAGATATAGTAAAAATATATTATAAGAAATTTGCTCATGA [11232]
cp32-3_B31_TS_MP_FG_consensus  GAATCTTCCCTTAGACTTCACCGACGACATTAAATTTGGAGATATAGTAAAAATATATTATAAGAAATTTGCTCATGA [11232]
cp32-3_B31_NX_Pl_consensus     GAATCTTCCCTTAGACTTCACCGACGACATTAAATTTGGAGATATAGTAAAAATATATTATAAGAAATTTGCTCATGA [11232]
cp32-3_PAli_NX_MP_FG_consensus GAATCTTCCCTTAGACTTCACCGACGACATTAAATTTGGAGATATAGTAAAAATATATTATAAGAAATTTGCTCATGA [11232]
cp32-3_PAli_NX_Pl_consensus    GAATCTTCCCTTAGACTTCACCGACGACATTAAATTTGGAGATATAGTAAAAATATATTATAAGAAATTTGCTCATGA [11232]
cp32-3_PAbe_NX_MP_FG_consensus GAATCTTCCCTTAGACTTCACCGACGACATTAAATTTGGAGATATAGTAAAAATATATTATAAGAAATTTGCTCATGA [11232]
cp32-3_PAbe_NX_Pl_consensus    GAATCTTCCCTTAGACTTCACCGACGACATTAAATTTGGAGATATAGTAAAAATATATTATAAGAAATTTGCTCATGA [11232]

cp32-3_gi|11497103|B31_GB      AAAAAATTTTGATTTCATAATGGCAGGAACTTTAGGACCTCCTATGAGCACTGATTATCCGGGTGGGGATTTTAGTGT [11310]
cp32-3_un8_B31_PacBio          AAAAAATTTTGATTTCATAATGGCAGGAACTTTAGGACCTCCTATGAGCACTGATTATCCGGGTGGGGATTTTAGTGT [11310]
cp32-3_B31_TS_MP_FG_consensus  AAAAAATTTTGATTTCATAATGGCAGGAACTTTAGGACCTCCTATGAGCACTGATTATCCGGGTGGGGATTTTAGTGT [11310]
cp32-3_B31_NX_Pl_consensus     AAAAAATTTTGATTTCATAATGGCAGGAACTTTAGGACCTCCTATGAGCACTGATTATCCGGGTGGGGATTTTAGTGT [11310]
```

```
cp32-3_PAli_NX_MP_FG_consensus AAAAAATTTTGATTTCATAATGGCAGGAACTTTAGGACCTCCTATGAGCACTGATTATCCGGGTGGGGATTTTAGTGT [11310]
cp32-3_PAli_NX_Pl_consensus    AAAAAATTTTGATTTCATAATGGCAGGAACTTTAGGACCTCCTATGAGCACTGATTATCCGGGTGGGGATTTTAGTGT [11310]
cp32-3_PAbe_NX_MP_FG_consensus AAAAAATTTTGATTTCATAATGGCAGGAACTTTAGGACCTCCTATGAGCACTGATTATCCGGGTGGGGATTTTAGTGT [11310]
cp32-3_PAbe_NX_Pl_consensus    AAAAAATTTTGATTTCATAATGGCAGGAACTTTAGGACCTCCTATGAGCACTGATTATCCGGGTGGGGATTTTAGTGT [11310]

cp32-3_gi|11497103|B31_GB      AGATCTTGATGTTCGTTTATTAACTAAAAGCAATTTCTTCAATCGCGAATTGGCAGGCAAAGAAGGCAAAAACTTTAA [11388]
cp32-3_un8_B31_PacBio          AGATCTTGATGTTCGTTTATTAACTAAAAGCAATTTCTTCAATCGCGAATTGGCAGGCAAAGAAGGCAAAAACTTTAA [11388]
cp32-3_B31_TS_MP_FG_consensus  AGATCTTGATGTTCGTTTATTAACTAAAAGCAATTTCTTCAATCGCGAATTGGCAGGCAAAGAAGGCAAAAACTTTAA [11388]
cp32-3_B31_NX_Pl_consensus     AGATCTTGATGTTCGTTTATTAACTAAAAGCAATTTCTTCAATCGCGAATTGGCAGGCAAAGAAGGCAAAAACTTTAA [11388]
cp32-3_PAli_NX_MP_FG_consensus AGATCTTGATGTTCGTTTATTAACTAAAAGCAATTTCTTCAATCGCGAATTGGCAGGCAAAGAAGGCAAAAACTTTAA [11388]
cp32-3_PAli_NX_Pl_consensus    AGATCTTGATGTTCGTTTATTAACTAAAAGCAATTTCTTCAATCGCGAATTGGCAGGCAAAGAAGGCAAAAACTTTAA [11388]
cp32-3_PAbe_NX_MP_FG_consensus AGATCTTGATGTTCGTTTATTAACTAAAAGCAATTTCTTCAATCGCGAATTGGCAGGCAAAGAAGGCAAAAACTTTAA [11388]
cp32-3_PAbe_NX_Pl_consensus    AGATCTTGATGTTCGTTTATTAACTAAAAGCAATTTCTTCAATCGCGAATTGGCAGGCAAAGAAGGCAAAAACTTTAA [11388]

cp32-3_gi|11497103|B31_GB      AGGCAAAACGGTGAAGGAGGCAATAGAATCTGTATTTCCCAATCGCAATATCCTTAATATGGATGAAAAAGATTGTCT [11466]
cp32-3_un8_B31_PacBio          AGGCAAAACGGTGAAGGAGGCAATAGAATCTGTATTTCCCAATCGCAATATCCTTAATATGGATGAAAAAGATTGTCT [11466]
cp32-3_B31_TS_MP_FG_consensus  AGGCAAAACGGTGAAGGAGGCAATAGAATCTGTATTTCCCAATCGCAATATCCTTAATATGGATGAAAAAGATTGTCT [11466]
cp32-3_B31_NX_Pl_consensus     AGGCAAAACGGTGAAGGAGGCAATAGAATCTGTATTTCCCAATCGCAATATCCTTAATATGGATGAAAAAGATTGTCT [11466]
cp32-3_PAli_NX_MP_FG_consensus AGGCAAAACGGTGAAGGAGGCAATAGAATCTGTATTTCCCAATCGCAATATCCTTAATATGGATGAAAAAGATTGTCT [11466]
cp32-3_PAli_NX_Pl_consensus    AGGCAAAACGGTGAAGGAGGCAATAGAATCTGTATTTCCCAATCGCAATATCCTTAATATGGATGAAAAAGATTGTCT [11466]
cp32-3_PAbe_NX_MP_FG_consensus AGGCAAAACGGTGAAGGAGGCAATAGAATCTGTATTTCCCAATCGCAATATCCTTAATATGGATGAAAAAGATTGTCT [11466]
cp32-3_PAbe_NX_Pl_consensus    AGGCAAAACGGTGAAGGAGGCAATAGAATCTGTATTTCCCAATCGCAATATCCTTAATATGGATGAAAAAGATTGTCT [11466]

cp32-3_gi|11497103|B31_GB      TAAAATTATTGACAAAGATATTTATGCCACAACACCAAAAGAGTTTATTGACAAAATTAAAGGAACATATGTTCATAA [11544]
cp32-3_un8_B31_PacBio          TAAAATTATTGACAAAGATATTTATGCCACAACACCAAAAGAGTTTATTGACAAAATTAAAGGAACATATGTTCATAA [11544]
cp32-3_B31_TS_MP_FG_consensus  TAAAATTATTGACAAAGATATTTATGCCACAACACCAAAAGAGTTTATTGACAAAATTAAAGGAACATATGTTCATAA [11544]
cp32-3_B31_NX_Pl_consensus     TAAAATTATTGACAAAGATATTTATGCCACAACACCAAAAGAGTTTATTGACAAAATTAAAGGAACATATGTTCATAA [11544]
cp32-3_PAli_NX_MP_FG_consensus TAAAATTATTGACAAAGATATTTATGCCACAACACCAAAAGAGTTTATTGACAAAATTAAAGGAACATATGTTCATAA [11544]
cp32-3_PAli_NX_Pl_consensus    TAAAATTATTGACAAAGATATTTATGCCACAACACCAAAAGAGTTTATTGACAAAATTAAAGGAACATATGTTCATAA [11544]
cp32-3_PAbe_NX_MP_FG_consensus TAAAATTATTGACAAAGATATTTATGCCACAACACCAAAAGAGTTTATTGACAAAATTAAAGGAACATATGTTCATAA [11544]
cp32-3_PAbe_NX_Pl_consensus    TAAAATTATTGACAAAGATATTTATGCCACAACACCAAAAGAGTTTATTGACAAAATTAAAGGAACATATGTTCATAA [11544]

cp32-3_gi|11497103|B31_GB      CGTAATAGCCGATATTGGTACTGGTTTACGGGGATATGAATGCTATCTGATATTTACTAATTATATAAAAAAGGGGGA [11622]
cp32-3_un8_B31_PacBio          CGTAATAGCCGATATTGGTACTGGTTTACGGGGATATGAATGCTATCTGATATTTACTAATTATATAAAAAAGGGGGA [11622]
cp32-3_B31_TS_MP_FG_consensus  CGTAATAGCCGATATTGGTACTGGTTTACGGGGATATGAATGCTATCTGATATTTACTAATTATATAAAAAAGGGGGA [11622]
cp32-3_B31_NX_Pl_consensus     CGTAATAGCCGATATTGGTACTGGTTTACGGGGATATGAATGCTATCTGATATTTACTAATTATATAAAAAAGGGGGA [11622]
cp32-3_PAli_NX_MP_FG_consensus CGTAATAGCCGATATTGGTACTGGTTTACGGGGATATGAATGCTATCTGATATTTACTAATTATATAAAAAAGGGGGA [11622]
cp32-3_PAli_NX_Pl_consensus    CGTAATAGCCGATATTGGTACTGGTTTACGGGGATATGAATGCTATCTGATATTTACTAATTATATAAAAAAGGGGGA [11622]
cp32-3_PAbe_NX_MP_FG_consensus CGTAATAGCCGATATTGGTACTGGTTTACGGGGATATGAATGCTATCTGATATTTACTAATTATATAAAAAAGGGGGA [11622]
cp32-3_PAbe_NX_Pl_consensus    CGTAATAGCCGATATTGGTACTGGTTTACGGGGATATGAATGCTATCTGATATTTACTAATTATATAAAAAAGGGGGA [11622]

cp32-3_gi|11497103|B31_GB      AAATGTCCACTACGAGGCATTAGAAGACTATGGGCTTGAATTTATACCACAACAAGAAATTACTTTGGGCACAACACT [11700]
cp32-3_un8_B31_PacBio          AAATGTCCACTACGAGGCATTAGAAGACTATGGGCTTGAATTTATACCACAACAAGAAATTACTTTGGGCACAACACT [11700]
cp32-3_B31_TS_MP_FG_consensus  AAATGTCCACTACGAGGCATTAGAAGACTATGGGCTTGAATTTATACCACAACAAGAAATTACTTTGGGCACAACACT [11700]
cp32-3_B31_NX_Pl_consensus     AAATGTCCACTACGAGGCATTAGAAGACTATGGGCTTGAATTTATACCACAACAAGAAATTACTTTGGGCACAACACT [11700]
cp32-3_PAli_NX_MP_FG_consensus AAATGTCCACTACGAGGCATTAGAAGACTATGGGCTTGAATTTATACCACAACAAGAAATTACTTTGGGCACAACACT [11700]
cp32-3_PAli_NX_Pl_consensus    AAATGTCCACTACGAGGCATTAGAAGACTATGGGCTTGAATTTATACCACAACAAGAAATTACTTTGGGCACAACACT [11700]
cp32-3_PAbe_NX_MP_FG_consensus AAATGTCCACTACGAGGCATTAGAAGACTATGGGCTTGAATTTATACCACAACAAGAAATTACTTTGGGCACAACACT [11700]
cp32-3_PAbe_NX_Pl_consensus    AAATGTCCACTACGAGGCATTAGAAGACTATGGGCTTGAATTTATACCACAACAAGAAATTACTTTGGGCACAACACT [11700]

cp32-3_gi|11497103|B31_GB      CAAAAAAAATCTTATATTTTGGAACGCAAAAACATTTTTCACACATAAGTTAAATGTTGGAGATAAAGTCTCATTTAT [11778]
cp32-3_un8_B31_PacBio          CAAAAAAAATCTTATATTTTGGAACGCAAAAACATTTTTCACACATAAGTTAAATGTTGGAGATAAAGTCTCATTTAT [11778]
cp32-3_B31_TS_MP_FG_consensus  CAAAAAAAATCTTATATTTTGGAACGCAAAAACATTTTTCACACATAAGTTAAATGTTGGAGATAAAGTCTCATTTAT [11778]
cp32-3_B31_NX_Pl_consensus     CAAAAAAAATCTTATATTTTGGAACGCAAAAACATTTTTCACACATAAGTTAAATGTTGGAGATAAAGTCTCATTTAT [11778]
cp32-3_PAli_NX_MP_FG_consensus CAAAAAAAATCTTATATTTTGGAACGCAAAAACATTTTTCACACATAAGTTAAATGTTGGAGATAAAGTCTCATTTAT [11778]
cp32-3_PAli_NX_Pl_consensus    CAAAAAAAATCTTATATTTTGGAACGCAAAAACATTTTTCACACATAAGTTAAATGTTGGAGATAAAGTCTCATTTAT [11778]
cp32-3_PAbe_NX_MP_FG_consensus CAAAAAAAATCTTATATTTTGGAACGCAAAAACATTTTTCACACATAAGTTAAATGTTGGAGATAAAGTCTCATTTAT [11778]
cp32-3_PAbe_NX_Pl_consensus    CAAAAAAAATCTTATATTTTGGAACGCAAAAACATTTTTCACACATAAGTTAAATGTTGGAGATAAAGTCTCATTTAT [11778]

cp32-3_gi|11497103|B31_GB      TGATGGACTAGGGAAAATGATAAAAACCACTATAAAAGAAACAAGTGCAAGGCTTAGCAATACAGGAGAGTGTTCATT [11856]
cp32-3_un8_B31_PacBio          TGATGGACTAGGGAAAATGATAAAAACCACTATAAAAGAAACAAGTGCAAGGCTTAGCAATACAGGAGAGTGTTCATT [11856]
cp32-3_B31_TS_MP_FG_consensus  TGATGGACTAGGGAAAATGATAAAAACCACTATAAAAGAAACAAGTGCAAGGCTTAGCAATACAGGAGAGTGTTCATT [11856]
cp32-3_B31_NX_Pl_consensus     TGATGGACTAGGGAAAATGATAAAAACCACTATAAAAGAAACAAGTGCAAGGCTTAGCAATACAGGAGAGTGTTCATT [11856]
cp32-3_PAli_NX_MP_FG_consensus TGATGGACTAGGGAAAATGATAAAAACCACTATAAAAGAAACAAGTGCAAGGCTTAGCAATACAGGAGAGTGTTCATT [11856]
cp32-3_PAli_NX_Pl_consensus    TGATGGACTAGGGAAAATGATAAAAACCACTATAAAAGAAACAAGTGCAAGGCTTAGCAATACAGGAGAGTGTTCATT [11856]
cp32-3_PAbe_NX_MP_FG_consensus TGATGGACTAGGGAAAATGATAAAAACCACTATAAAAGAAACAAGTGCAAGGCTTAGCAATACAGGAGAGTGTTCATT [11856]
cp32-3_PAbe_NX_Pl_consensus    TGATGGACTAGGGAAAATGATAAAAACCACTATAAAAGAAACAAGTGCAAGGCTTAGCAATACAGGAGAGTGTTCATT [11856]

cp32-3_gi|11497103|B31_GB      AATATTAAAGTTAGAAGATGATTCTAATACAAAACGTAAAAAATAAAGGGGACTAGAATTAGAATGAATGAAGACTAT [11934]
cp32-3_un8_B31_PacBio          AATATTAAAGTTAGAAGATGATTCTAATACAAAACGTAAAAAATAAAGGGGACTAGAATTAGAATGAATGAAGACTAT [11934]
cp32-3_B31_TS_MP_FG_consensus  AATATTAAAGTTAGAAGATGATTCTAATACAAAACGTAAAAAATAAAGGGGACTAGAATTAGAATGAATGAAGACTAT [11934]
cp32-3_B31_NX_Pl_consensus     AATATTAAAGTTAGAAGATGATTCTAATACAAAACGTAAAAAATAAAGGGGACTAGAATTAGAATGAATGAAGACTAT [11934]
cp32-3_PAli_NX_MP_FG_consensus AATATTAAAGTTAGAAGATGATTCTAATACAAAACGTAAAAAATAAAGGGGACTAGAATTAGAATGAATGAAGACTAT [11934]
cp32-3_PAli_NX_Pl_consensus    AATATTAAAGTTAGAAGATGATTCTAATACAAAACGTAAAAAATAAAGGGGACTAGAATTAGAATGAATGAAGACTAT [11934]
cp32-3_PAbe_NX_MP_FG_consensus AATATTAAAGTTAGAAGATGATTCTAATACAAAACGTAAAAAATAAAGGGGACTAGAATTAGAATGAATGAAGACTAT [11934]
cp32-3_PAbe_NX_Pl_consensus    AATATTAAAGTTAGAAGATGATTCTAATACAAAACGTAAAAAATAAAGGGGACTAGAATTAGAATGAATGAAGACTAT [11934]

cp32-3_gi|11497103|B31_GB      GAAATTTACAGAATGAATCAACGCCTTTATGGCCAGGCATTGGCTCAAGAAGACCTTAAAAATTGGATTTATTCAAAC [12012]
cp32-3_un8_B31_PacBio          GAAATTTACAGAATGAATCAACGCCTTTATGGCCAGGCATTGGCTCAAGAAGACCTTAAAAATTGGATTTATTCAAAC [12012]
cp32-3_B31_TS_MP_FG_consensus  GAAATTTACAGAATGAATCAACGCCTTTATGGCCAGGCATTGGCTCAAGAAGACCTTAAAAATTGGATTTATTCAAAC [12012]
cp32-3_B31_NX_Pl_consensus     GAAATTTACAGAATGAATCAACGCCTTTATGGCCAGGCATTGGCTCAAGAAGACCTTAAAAATTGGATTTATTCAAAC [12012]
cp32-3_PAli_NX_MP_FG_consensus GAAATTTACAGAATGAATCAACGCCTTTATGGCCAGGCATTGGCTCAAGAAGACCTTAAAAATTGGATTTATTCAAAC [12012]
cp32-3_PAli_NX_Pl_consensus    GAAATTTACAGAATGAATCAACGCCTTTATGGCCAGGCATTGGCTCAAGAAGACCTTAAAAATTGGATTTATTCAAAC [12012]
cp32-3_PAbe_NX_MP_FG_consensus GAAATTTACAGAATGAATCAACGCCTTTATGGCCAGGCATTGGCTCAAGAAGACCTTAAAAATTGGATTTATTCAAAC [12012]
cp32-3_PAbe_NX_Pl_consensus    GAAATTTACAGAATGAATCAACGCCTTTATGGCCAGGCATTGGCTCAAGAAGACCTTAAAAATTGGATTTATTCAAAC [12012]

cp32-3_gi|11497103|B31_GB      ATTTTTATAATTAAAATTGGCACTGTAAAGGAGTTTAAACATCAAACTCAAGAAGCTATTGTTACAATACCCGAATTT [12090]
cp32-3_un8_B31_PacBio          ATTTTTATAATTAAAATTGGCACTGTAAAGGAGTTTAAACATCAAACTCAAGAAGCTATTGTTACAATACCCGAATTT [12090]
cp32-3_B31_TS_MP_FG_consensus  ATTTTTATAATTAAAATTGGCACTGTAAAGGAGTTTAAACATCAAACTCAAGAAGCTATTGTTACAATACCCGAATTT [12090]
cp32-3_B31_NX_Pl_consensus     ATTTTTATAATTAAAATTGGCACTGTAAAGGAGTTTAAACATCAAACTCAAGAAGCTATTGTTACAATACCCGAATTT [12090]
cp32-3_PAli_NX_MP_FG_consensus ATTTTTATAATTAAAATTGGCACTGTAAAGGAGTTTAAACATCAAACTCAAGAAGCTATTGTTACAATACCCGAATTT [12090]
cp32-3_PAli_NX_Pl_consensus    ATTTTTATAATTAAAATTGGCACTGTAAAGGAGTTTAAACATCAAACTCAAGAAGCTATTGTTACAATACCCGAATTT [12090]
cp32-3_PAbe_NX_MP_FG_consensus ATTTTTATAATTAAAATTGGCACTGTAAAGGAGTTTAAACATCAAACTCAAGAAGCTATTGTTACAATACCCGAATTT [12090]
cp32-3_PAbe_NX_Pl_consensus    ATTTTTATAATTAAAATTGGCACTGTAAAGGAGTTTAAACATCAAACTCAAGAAGCTATTGTTACAATACCCGAATTT [12090]

cp32-3_gi|11497103|B31_GB      GAAGATTTAGAAATTCACACAAAAAATATCTCTAATATCAGTTTAGAACTATCAAAAGGTGATTGCGTTTTACTACTT [12168]
cp32-3_un8_B31_PacBio          GAAGATTTAGAAATTCACACAAAAAATATCTCTAATATCAGTTTAGAACTATCAAAAGGTGATTGCGTTTTACTACTT [12168]
cp32-3_B31_TS_MP_FG_consensus  GAAGATTTAGAAATTCACACAAAAAATATCTCTAATATCAGTTTAGAACTATCAAAAGGTGATTGCGTTTTACTACTT [12168]
cp32-3_B31_NX_Pl_consensus     GAAGATTTAGAAATTCACACAAAAAATATCTCTAATATCAGTTTAGAACTATCAAAAGGTGATTGCGTTTTACTACTT [12168]
cp32-3_PAli_NX_MP_FG_consensus GAAGATTTAGAAATTCACACAAAAAATATCTCTAATATCAGTTTAGAACTATCAAAAGGTGATTGCGTTTTACTACTT [12168]
cp32-3_PAli_NX_Pl_consensus    GAAGATTTAGAAATTCACACAAAAAATATCTCTAATATCAGTTTAGAACTATCAAAAGGTGATTGCGTTTTACTACTT [12168]
cp32-3_PAbe_NX_MP_FG_consensus GAAGATTTAGAAATTCACACAAAAAATATCTCTAATATCAGTTTAGAACTATCAAAAGGTGATTGCGTTTTACTACTT [12168]
cp32-3_PAbe_NX_Pl_consensus    GAAGATTTAGAAATTCACACAAAAAATATCTCTAATATCAGTTTAGAACTATCAAAAGGTGATTGCGTTTTACTACTT [12168]

cp32-3_gi|11497103|B31_GB      CAATCAAGCATTAATATTTTTGATAAAAATAACGATATTCACTTTGACAAACATCATTTTTATATACTTAGTGCAATT [12246]
cp32-3_un8_B31_PacBio          CAATCAAGCATTAATATTTTTGATAAAAATAACGATATTCACTTTGACAAACATCATTTTTATATACTTAGTGCAATT [12246]
cp32-3_B31_TS_MP_FG_consensus  CAATCAAGCATTAATATTTTTGATAAAAATAACGATATTCACTTTGACAAACATCATTTTTATATACTTAGTGCAATT [12246]
cp32-3_B31_NX_Pl_consensus     CAATCAAGCATTAATATTTTTGATAAAAATAACGATATTCACTTTGACAAACATCATTTTTATATACTTAGTGCAATT [12246]
cp32-3_PAli_NX_MP_FG_consensus CAATCAAGCATTAATATTTTTGATAAAAATAACGATATTCACTTTGACAAACATCATTTTTATATACTTAGTGCAATT [12246]
```

```
cp32-3_PAli_NX_Pl_consensus    CAATCAAGCATTAATATTTTTGATAAAAATAACGATATTCACTTTGACAAACATCATTTTTATATACTTAGTGCAATT [12246]
cp32-3_PAbe_NX_MP_FG_consensus CAATCAAGCATTAATATTTTTGATAAAAATAACGATATTCACTTTGACAAACATCATTTTTATATACTTAGTGCAATT [12246]
cp32-3_PAbe_NX_Pl_consensus    CAATCAAGCATTAATATTTTTGATAAAAATAACGATATTCACTTTGACAAACATCATTTTTATATACTTAGTGCAATT [12246]

cp32-3_gi|11497103|B31_GB      AGCCCAAAGACTTTAAATCTAATCTCTGATACTGTTAAAATTAGAGCGAATAATAAAATTGAAATAGCAAACCAAACA [12324]
cp32-3_un8_B31_PacBio          AGCCCAAAGACTTTAAATCTAATCTCTGATACTGTTAAAATTAGAGCGAATAATAAAATTGAAATAGCAAACCAAACA [12324]
cp32-3_B31_TS_MP_FG_consensus  AGCCCAAAGACTTTAAATCTAATCTCTGATACTGTTAAAATTAGAGCGAATAATAAAATTGAAATAGCAAACCAAACA [12324]
cp32-3_B31_NX_Pl_consensus     AGCCCAAAGACTTTAAATCTAATCTCTGATACTGTTAAAATTAGAGCGAATAATAAAATTGAAATAGCAAACCAAACA [12324]
cp32-3_PAli_NX_MP_FG_consensus AGCCCAAAGACTTTAAATCTAATCTCTGATACTGTTAAAATTAGAGCGAATAATAAAATTGAAATAGCAAACCAAACA [12324]
cp32-3_PAli_NX_Pl_consensus    AGCCCAAAGACTTTAAATCTAATCTCTGATACTGTTAAAATTAGAGCGAATAATAAAATTGAAATAGCAAACCAAACA [12324]
cp32-3_PAbe_NX_MP_FG_consensus AGCCCAAAGACTTTAAATCTAATCTCTGATACTGTTAAAATTAGAGCGAATAATAAAATTGAAATAGCAAACCAAACA [12324]
cp32-3_PAbe_NX_Pl_consensus    AGCCCAAAGACTTTAAATCTAATCTCTGATACTGTTAAAATTAGAGCGAATAATAAAATTGAAATAGCAAACCAAACA [12324]

cp32-3_gi|11497103|B31_GB      ACTAGCTTAAAAAAAATTCTAGATAATATTGTTAGTGCTATCAATGGTATAAAAATTATAGGGGACTCAGTAATTGAC [12402]
cp32-3_un8_B31_PacBio          ACTAGCTTAAAAAAAATTCTAGATAATATTGTTAGTGCTATCAATGGTATAAAAATTATAGGGGACTCAGTAATTGAC [12402]
cp32-3_B31_TS_MP_FG_consensus  ACTAGCTTAAAAAAAATTCTAGATAATATTGTTAGTGCTATCAATGGTATAAAAATTATAGGGGACTCAGTAATTGAC [12402]
cp32-3_B31_NX_Pl_consensus     ACTAGCTTAAAAAAAATTCTAGATAATATTGTTAGTGCTATCAATGGTATAAAAATTATAGGGGACTCAGTAATTGAC [12402]
cp32-3_PAli_NX_MP_FG_consensus ACTAGCTTAAAAAAAATTCTAGATAATATTGTTAGTGCTATCAATGGTATAAAAATTATAGGGGACTCAGTAATTGAC [12402]
cp32-3_PAli_NX_Pl_consensus    ACTAGCTTAAAAAAAATTCTAGATAATATTGTTAGTGCTATCAATGGTATAAAAATTATAGGGGACTCAGTAATTGAC [12402]
cp32-3_PAbe_NX_MP_FG_consensus ACTAGCTTAAAAAAAATTCTAGATAATATTGTTAGTGCTATCAATGGTATAAAAATTATAGGGGACTCAGTAATTGAC [12402]
cp32-3_PAbe_NX_Pl_consensus    ACTAGCTTAAAAAAAATTCTAGATAATATTGTTAGTGCTATCAATGGTATAAAAATTATAGGGGACTCAGTAATTGAC [12402]

cp32-3_gi|11497103|B31_GB      GAATCAAGCTTAAAAATAGCAACCGCTCAAATTAATTCTGATATTAATAGTTTGTTTAAGTAATTTTTGCTAAATATG [12480]
cp32-3_un8_B31_PacBio          GAATCAAGCTTAAAAATAGCAACCGCTCAAATTAATTCTGATATTAATAGTTTGTTTAAGTAATTTTTGCTAAATATG [12480]
cp32-3_B31_TS_MP_FG_consensus  GAATCAAGCTTAAAAATAGCAACCGCTCAAATTAATTCTGATATTAATAGTTTGTTTAAGTAATTTTTGCTAAATATG [12480]
cp32-3_B31_NX_Pl_consensus     GAATCAAGCTTAAAAATAGCAACCGCTCAAATTAATTCTGATATTAATAGTTTGTTTAAGTAATTTTTGCTAAATATG [12480]
cp32-3_PAli_NX_MP_FG_consensus GAATCAAGCTTAAAAATAGCAACCGCTCAAATTAATTCTGATATTAATAGTTTGTTTAAGTAATTTTTGCTAAATATG [12480]
cp32-3_PAli_NX_Pl_consensus    GAATCAAGCTTAAAAATAGCAACCGCTCAAATTAATTCTGATATTAATAGTTTGTTTAAGTAATTTTTGCTAAATATG [12480]
cp32-3_PAbe_NX_MP_FG_consensus GAATCAAGCTTAAAAATAGCAACCGCTCAAATTAATTCTGATATTAATAGTTTGTTTAAGTAATTTTTGCTAAATATG [12480]
cp32-3_PAbe_NX_Pl_consensus    GAATCAAGCTTAAAAATAGCAACCGCTCAAATTAATTCTGATATTAATAGTTTGTTTAAGTAATTTTTGCTAAATATG [12480]

cp32-3_gi|11497103|B31_GB      GTATAATTACTAGTATGGATTTAAGATTAGGCAATAATTTTGAATTGGTATTTAATAACGATTTATCACTTGTTGATG [12558]
cp32-3_un8_B31_PacBio          GTATAATTACTAGTATGGATTTAAGATTAGGCAATAATTTTGAATTGGTATTTAATAACGATTTATCACTTGTTGATG [12558]
cp32-3_B31_TS_MP_FG_consensus  GTATAATTACTAGTATGGATTTAAGATTAGGCAATAATTTTGAATTGGTATTTAATAACGATTTATCACTTGTTGATG [12558]
cp32-3_B31_NX_Pl_consensus     GTATAATTACTAGTATGGATTTAAGATTAGGCAATAATTTTGAATTGGTATTTAATAACGATTTATCACTTGTTGATG [12558]
cp32-3_PAli_NX_MP_FG_consensus GTATAATTACTAGTATGGATTTAAGATTAGGCAATAATTTTGAATTGGTATTTAATAACGATTTATCACTTGTTGATG [12558]
cp32-3_PAli_NX_Pl_consensus    GTATAATTACTAGTATGGATTTAAGATTAGGCAATAATTTTGAATTGGTATTTAATAACGATTTATCACTTGTTGATG [12558]
cp32-3_PAbe_NX_MP_FG_consensus GTATAATTACTAGTATGGATTTAAGATTAGGCAATAATTTTGAATTGGTATTTAATAACGATTTATCACTTGTTGATG [12558]
cp32-3_PAbe_NX_Pl_consensus    GTATAATTACTAGTATGGATTTAAGATTAGGCAATAATTTTGAATTGGTATTTAATAACGATTTATCACTTGTTGATG [12558]

cp32-3_gi|11497103|B31_GB      GAATTGATGAACAAAAACAAAGATTTTTGATATTTTTAAAAACCTTAAGGGGTAGTTTAAGCTATGCTCCTCATTGGG [12636]
cp32-3_un8_B31_PacBio          GAATTGATGAACAAAAACAAAGATTTTTGATATTTTTAAAAACCTTAAGGGGTAGTTTAAGCTATGCTCCTCATTGGG [12636]
cp32-3_B31_TS_MP_FG_consensus  GAATTGATGAACAAAAACAAAGATTTTTGATATTTTTAAAAACCTTAAGGGGTAGTTTAAGCTATGCTCCTCATTGGG [12636]
cp32-3_B31_NX_Pl_consensus     GAATTGATGAACAAAAACAAAGATTTTTGATATTTTTAAAAACCTTAAGGGGTAGTTTAAGCTATGCTCCTCATTGGG [12636]
cp32-3_PAli_NX_MP_FG_consensus GAATTGATGAACAAAAACAAAGATTTTTGATATTTTTAAAAACCTTAAGGGGTAGTTTAAGCTATGCTCCTCATTGGG [12636]
cp32-3_PAli_NX_Pl_consensus    GAATTGATGAACAAAAACAAAGATTTTTGATATTTTTAAAAACCTTAAGGGGTAGTTTAAGCTATGCTCCTCATTGGG [12636]
cp32-3_PAbe_NX_MP_FG_consensus GAATTGATGAACAAAAACAAAGATTTTTGATATTTTTAAAAACCTTAAGGGGTAGTTTAAGCTATGCTCCTCATTGGG [12636]
cp32-3_PAbe_NX_Pl_consensus    GAATTGATGAACAAAAACAAAGATTTTTGATATTTTTAAAAACCTTAAGGGGTAGTTTAAGCTATGCTCCTCATTGGG [12636]

cp32-3_gi|11497103|B31_GB      GACTGGACTATTTCTTGCTTTTAAAGCTGTTAAAAATTAACAATCTTCACGCTGTAAAAAATTATTTTCATGAAATAT [12714]
cp32-3_un8_B31_PacBio          GACTGGACTATTTCTTGCTTTTAAAGCTGTTAAAAATTAACAATCTTCACGCTGTAAAAAATTATTTTCATGAAATAT [12714]
cp32-3_B31_TS_MP_FG_consensus  GACTGGACTATTTCTTGCTTTTAAAGCTGTTAAAAATTAACAATCTTCACGCTGTAAAAAATTATTTTCATGAAATAT [12714]
cp32-3_B31_NX_Pl_consensus     GACTGGACTATTTCTTGCTTTTAAAGCTGTTAAAAATTAACAATCTTCACGCTGTAAAAAATTATTTTCATGAAATAT [12714]
cp32-3_PAli_NX_MP_FG_consensus GACTGGACTATTTCTTGCTTTTAAAGCTGTTAAAAATTAACAATCTTCACGCTGTAAAAAATTATTTTCATGAAATAT [12714]
cp32-3_PAli_NX_Pl_consensus    GACTGGACTATTTCTTGCTTTTAAAGCTGTTAAAAATTAACAATCTTCACGCTGTAAAAAATTATTTTCATGAAATAT [12714]
cp32-3_PAbe_NX_MP_FG_consensus GACTGGACTATTTCTTGCTTTTAAAGCTGTTAAAAATTAACAATCTTCACGCTGTAAAAAATTATTTTCATGAAATAT [12714]
cp32-3_PAbe_NX_Pl_consensus    GACTGGACTATTTCTTGCTTTTAAAGCTGTTAAAAATTAACAATCTTCACGCTGTAAAAAATTATTTTCATGAAATAT [12714]

cp32-3_gi|11497103|B31_GB      CTAAAGAGCTTAACTTAGATTTAATAAATATTTCAACTACTATACAAGACAACAAAGCACATATATCCTTTTTTTTCT [12792]
cp32-3_un8_B31_PacBio          CTAAAGAGCTTAACTTAGATTTAATAAATATTTCAACTACTATACAAGACAACAAAGCACATATATCCTTTTTTTTCT [12792]
cp32-3_B31_TS_MP_FG_consensus  CTAAAGAGCTTAACTTAGATTTAATAAATATTTCAACTACTATACAAGACAACAAAGCACATATATCCTTTTTTTTCT [12792]
cp32-3_B31_NX_Pl_consensus     CTAAAGAGCTTAACTTAGATTTAATAAATATTTCAACTACTATACAAGACAACAAAGCACATATATCCTTTTTTTTCT [12792]
cp32-3_PAli_NX_MP_FG_consensus CTAAAGAGCTTAACTTAGATTTAATAAATATTTCAACTACTATACAAGACAACAAAGCACATATATCCTTTTTTTTCT [12792]
cp32-3_PAli_NX_Pl_consensus    CTAAAGAGCTTAACTTAGATTTAATAAATATTTCAACTACTATACAAGACAACAAAGCACATATATCCTTTTTTTTCT [12792]
cp32-3_PAbe_NX_MP_FG_consensus CTAAAGAGCTTAACTTAGATTTAATAAATATTTCAACTACTATACAAGACAACAAAGCACATATATCCTTTTTTTTCT [12792]
cp32-3_PAbe_NX_Pl_consensus    CTAAAGAGCTTAACTTAGATTTAATAAATATTTCAACTACTATACAAGACAACAAAGCACATATATCCTTTTTTTTCT [12792]

cp32-3_gi|11497103|B31_GB      CGGGCGATGTTTTAAATATGGAGTTTAATTTATGAGCATAGTTTTTGATTCTGATTTTGGCATTTTAAAACGTACAAT [12870]
cp32-3_un8_B31_PacBio          CGGGCGATGTTTTAAATATGGAGTTTAATTTATGAGCATAGTTTTTGATTCTGATTTTGGCATTTTAAAACGTACAAT [12870]
cp32-3_B31_TS_MP_FG_consensus  CGGGCGATGTTTTAAATATGGAGTTTAATTTATGAGCATAGTTTTTGATTCTGATTTTGGCATTTTAAAACGTACAAT [12870]
cp32-3_B31_NX_Pl_consensus     CGGGCGATGTTTTAAATATGGAGTTTAATTTATGAGCATAGTTTTTGATTCTGATTTTGGCATTTTAAAACGTACAAT [12870]
cp32-3_PAli_NX_MP_FG_consensus CGGGCGATGTTTTAAATATGGAGTTTAATTTATGAGCATAGTTTTTGATTCTGATTTTGGCATTTTAAAACGTACAAT [12870]
cp32-3_PAli_NX_Pl_consensus    CGGGCGATGTTTTAAATATGGAGTTTAATTTATGAGCATAGTTTTTGATTCTGATTTTGGCATTTTAAAACGTACAAT [12870]
cp32-3_PAbe_NX_MP_FG_consensus CGGGCGATGTTTTAAATATGGAGTTTAATTTATGAGCATAGTTTTTGATTCTGATTTTGGCATTTTAAAACGTACAAT [12870]
cp32-3_PAbe_NX_Pl_consensus    CGGGCGATGTTTTAAATATGGAGTTTAATTTATGAGCATAGTTTTTGATTCTGATTTTGGCATTTTAAAACGTACAAT [12870]

cp32-3_gi|11497103|B31_GB      TAAGGATATTGTAAGAGCTAAAAGAGAATATTTGCGTGTAAATTATGGTATTAATATTGACGACAATCAAAGCTCAAT [12948]
cp32-3_un8_B31_PacBio          TAAGGATATTGTAAGAGCTAAAAGAGAATATTTGCGTGTAAATTATGGTATTAATATTGACGACAATCAAAGCTCAAT [12948]
cp32-3_B31_TS_MP_FG_consensus  TAAGGATATTGTAAGAGCTAAAAGAGAATATTTGCGTGTAAATTATGGTATTAATATTGACGACAATCAAAGCTCAAT [12948]
cp32-3_B31_NX_Pl_consensus     TAAGGATATTGTAAGAGCTAAAAGAGAATATTTGCGTGTAAATTATGGTATTAATATTGACGACAATCAAAGCTCAAT [12948]
cp32-3_PAli_NX_MP_FG_consensus TAAGGATATTGTAAGAGCTAAAAGAGAATATTTGCGTGTAAATTATGGTATTAATATTGACGACAATCAAAGCTCAAT [12948]
cp32-3_PAli_NX_Pl_consensus    TAAGGATATTGTAAGAGCTAAAAGAGAATATTTGCGTGTAAATTATGGTATTAATATTGACGACAATCAAAGCTCAAT [12948]
cp32-3_PAbe_NX_MP_FG_consensus TAAGGATATTGTAAGAGCTAAAAGAGAATATTTGCGTGTAAATTATGGTATTAATATTGACGACAATCAAAGCTCAAT [12948]
cp32-3_PAbe_NX_Pl_consensus    TAAGGATATTGTAAGAGCTAAAAGAGAATATTTGCGTGTAAATTATGGTATTAATATTGACGACAATCAAAGCTCAAT [12948]

cp32-3_gi|11497103|B31_GB      TTATAACATTATTGCGTCTTCTTTGGCATTAATTGAAGAAGAAATAATTAATGAGCTCAATCTCTTTTTTTCTAAAAT [13026]
cp32-3_un8_B31_PacBio          TTATAACATTATTGCGTCTTCTTTGGCATTAATTGAAGAAGAAATAATTAATGAGCTCAATCTCTTTTTTTCTAAAAT [13026]
cp32-3_B31_TS_MP_FG_consensus  TTATAACATTATTGCGTCTTCTTTGGCATTAATTGAAGAAGAAATAATTAATGAGCTCAATCTCTTTTTTTCTAAAAT [13026]
cp32-3_B31_NX_Pl_consensus     TTATAACATTATTGCGTCTTCTTTGGCATTAATTGAAGAAGAAATAATTAATGAGCTCAATCTCTTTTTTTCTAAAAT [13026]
cp32-3_PAli_NX_MP_FG_consensus TTATAACATTATTGCGTCTTCTTTGGCATTAATTGAAGAAGAAATAATTAATGAGCTCAATCTCTTTTTTTCTAAAAT [13026]
cp32-3_PAli_NX_Pl_consensus    TTATAACATTATTGCGTCTTCTTTGGCATTAATTGAAGAAGAAATAATTAATGAGCTCAATCTCTTTTTTTCTAAAAT [13026]
cp32-3_PAbe_NX_MP_FG_consensus TTATAACATTATTGCGTCTTCTTTGGCATTAATTGAAGAAGAAATAATTAATGAGCTCAATCTCTTTTTTTCTAAAAT [13026]
cp32-3_PAbe_NX_Pl_consensus    TTATAACATTATTGCGTCTTCTTTGGCATTAATTGAAGAAGAAATAATTAATGAGCTCAATCTCTTTTTTTCTAAAAT [13026]

cp32-3_gi|11497103|B31_GB      GAAACCTGGTGGTACTTATTGGGCTGCTATTGAAGAACACATTTCTTCTAAAAGCACAACTTACAGTGCTGTTCGCAA [13104]
cp32-3_un8_B31_PacBio          GAAACCTGGTGGTACTTATTGGGCTGCTATTGAAGAACACATTTCTTCTAAAAGCACAACTTACAGTGCTGTTCGCAA [13104]
cp32-3_B31_TS_MP_FG_consensus  GAAACCTGGTGGTACTTATTGGGCTGCTATTGAAGAACACATTTCTTCTAAAAGCACAACTTACAGTGCTGTTCGCAA [13104]
cp32-3_B31_NX_Pl_consensus     GAAACCTGGTGGTACTTATTGGGCTGCTATTGAAGAACACATTTCTTCTAAAAGCACAACTTACAGTGCTGTTCGCAA [13104]
cp32-3_PAli_NX_MP_FG_consensus GAAACCTGGTGGTACTTATTGGGCTGCTATTGAAGAACACATTTCTTCTAAAAGCACAACTTACAGTGCTGTTCGCAA [13104]
cp32-3_PAli_NX_Pl_consensus    GAAACCTGGTGGTACTTATTGGGCTGCTATTGAAGAACACATTTCTTCTAAAAGCACAACTTACAGTGCTGTTCGCAA [13104]
cp32-3_PAbe_NX_MP_FG_consensus GAAACCTGGTGGTACTTATTGGGCTGCTATTGAAGAACACATTTCTTCTAAAAGCACAACTTACAGTGCTGTTCGCAA [13104]
cp32-3_PAbe_NX_Pl_consensus    GAAACCTGGTGGTACTTATTGGGCTGCTATTGAAGAACACATTTCTTCTAAAAGCACAACTTACAGTGCTGTTCGCAA [13104]

cp32-3_gi|11497103|B31_GB      TGCTTTACTTAATCTTGATGGGATTGAGTACACTAATATTAAAAGTGCAGCTGGTAAAGCCAACATATATCTAATTCT [13182]
cp32-3_un8_B31_PacBio          TGCTTTACTTAATCTTGATGGGATTGAGTACACTAATATTAAAAGTGCAGCTGGTAAAGCCAACATATATCTAATTCT [13182]
cp32-3_B31_TS_MP_FG_consensus  TGCTTTACTTAATCTTGATGGGATTGAGTACACTAATATTAAAAGTGCAGCTGGTAAAGCCAACATATATCTAATTCT [13182]
cp32-3_B31_NX_Pl_consensus     TGCTTTACTTAATCTTGATGGGATTGAGTACACTAATATTAAAAGTGCAGCTGGTAAAGCCAACATATATCTAATTCT [13182]
cp32-3_PAli_NX_MP_FG_consensus TGCTTTACTTAATCTTGATGGGATTGAGTACACTAATATTAAAAGTGCAGCTGGTAAAGCCAACATATATCTAATTCT [13182]
cp32-3_PAli_NX_Pl_consensus    TGCTTTACTTAATCTTGATGGGATTGAGTACACTAATATTAAAAGTGCAGCTGGTAAAGCCAACATATATCTAATTCT [13182]
```

```
cp32-3_PAbe_NX_MP_FG_consensus TGCTTTACTTAATCTTGATGGGATTGAGTACACTAATATTAAAAGTGCAGCTGGTAAAGCCAACATATATCTAATTCT [13182]
cp32-3_PAbe_NX_Pl_consensus    TGCTTTACTTAATCTTGATGGGATTGAGTACACTAATATTAAAAGTGCAGCTGGTAAAGCCAACATATATCTAATTCT [13182]

cp32-3_gi|11497103|B31_GB      AAAGGAAACTTTACTAGACACTAGTAAATCTAATATTAATAGTCCTGAATTTAAAGCAAAACTTTGGGAAACATTATA [13260]
cp32-3_un8_B31_PacBio          AAAGGAAACTTTACTAGACACTAGTAAATCTAATATTAATAGTCCTGAATTTAAAGCAAAACTTTGGGAAACATTATA [13260]
cp32-3_B31_TS_MP_FG_consensus  AAAGGAAACTTTACTAGACACTAGTAAATCTAATATTAATAGTCCTGAATTTAAAGCAAAACTTTGGGAAACATTATA [13260]
cp32-3_B31_NX_Pl_consensus     AAAGGAAACTTTACTAGACACTAGTAAATCTAATATTAATAGTCCTGAATTTAAAGCAAAACTTTGGGAAACATTATA [13260]
cp32-3_PAli_NX_MP_FG_consensus AAAGGAAACTTTACTAGACACTAGTAAATCTAATATTAATAGTCCTGAATTTAAAGCAAAACTTTGGGAAACATTATA [13260]
cp32-3_PAli_NX_Pl_consensus    AAAGGAAACTTTACTAGACACTAGTAAATCTAATATTAATAGTCCTGAATTTAAAGCAAAACTTTGGGAAACATTATA [13260]
cp32-3_PAbe_NX_MP_FG_consensus AAAGGAAACTTTACTAGACACTAGTAAATCTAATATTAATAGTCCTGAATTTAAAGCAAAACTTTGGGAAACATTATA [13260]
cp32-3_PAbe_NX_Pl_consensus    AAAGGAAACTTTACTAGACACTAGTAAATCTAATATTAATAGTCCTGAATTTAAAGCAAAACTTTGGGAAACATTATA [13260]

cp32-3_gi|11497103|B31_GB      TCTAACAACTCCTAGTGGTACTTTACTTGAGGGAGACATAGAAATTGATGGTCTCAATTCAACTGGACAACGTAAATC [13338]
cp32-3_un8_B31_PacBio          TCTAACAACTCCTAGTGGTACTTTACTTGAGGGAGACATAGAAATTGATGGTCTCAATTCAACTGGACAACGTAAATC [13338]
cp32-3_B31_TS_MP_FG_consensus  TCTAACAACTCCTAGTGGTACTTTACTTGAGGGAGACATAGAAATTGATGGTCTCAATTCAACTGGACAACGTAAATC [13338]
cp32-3_B31_NX_Pl_consensus     TCTAACAACTCCTAGTGGTACTTTACTTGAGGGAGACATAGAAATTGATGGTCTCAATTCAACTGGACAACGTAAATC [13338]
cp32-3_PAli_NX_MP_FG_consensus TCTAACAACTCCTAGTGGTACTTTACTTGAGGGAGACATAGAAATTGATGGTCTCAATTCAACTGGACAACGTAAATC [13338]
cp32-3_PAli_NX_Pl_consensus    TCTAACAACTCCTAGTGGTACTTTACTTGAGGGAGACATAGAAATTGATGGTCTCAATTCAACTGGACAACGTAAATC [13338]
cp32-3_PAbe_NX_MP_FG_consensus TCTAACAACTCCTAGTGGTACTTTACTTGAGGGAGACATAGAAATTGATGGTCTCAATTCAACTGGACAACGTAAATC [13338]
cp32-3_PAbe_NX_Pl_consensus    TCTAACAACTCCTAGTGGTACTTTACTTGAGGGAGACATAGAAATTGATGGTCTCAATTCAACTGGACAACGTAAATC [13338]

cp32-3_gi|11497103|B31_GB      CTATAAAATATCACTAGGGAAAAGAAAATATGTTTACATGAAAGTAAAGTATAAACTTGACCTTAAAAACTATCTCTA [13416]
cp32-3_un8_B31_PacBio          CTATAAAATATCACTAGGGAAAAGAAAATATGTTTACATGAAAGTAAAGTATAAACTTGACCTTAAAAACTATCTCTA [13416]
cp32-3_B31_TS_MP_FG_consensus  CTATAAAATATCACTAGGGAAAAGAAAATATGTTTACATGAAAGTAAAGTATAAACTTGACCTTAAAAACTATCTCTA [13416]
cp32-3_B31_NX_Pl_consensus     CTATAAAATATCACTAGGGAAAAGAAAATATGTTTACATGAAAGTAAAGTATAAACTTGACCTTAAAAACTATCTCTA [13416]
cp32-3_PAli_NX_MP_FG_consensus CTATAAAATATCACTAGGGAAAAGAAAATATGTTTACATGAAAGTAAAGTATAAACTTGACCTTAAAAACTATCTCTA [13416]
cp32-3_PAli_NX_Pl_consensus    CTATAAAATATCACTAGGGAAAAGAAAATATGTTTACATGAAAGTAAAGTATAAACTTGACCTTAAAAACTATCTCTA [13416]
cp32-3_PAbe_NX_MP_FG_consensus CTATAAAATATCACTAGGGAAAAGAAAATATGTTTACATGAAAGTAAAGTATAAACTTGACCTTAAAAACTATCTCTA [13416]
cp32-3_PAbe_NX_Pl_consensus    CTATAAAATATCACTAGGGAAAAGAAAATATGTTTACATGAAAGTAAAGTATAAACTTGACCTTAAAAACTATCTCTA [13416]

cp32-3_gi|11497103|B31_GB      CTTAAACATAGACTCTCAAATTAGGGACATTTATTCTAGGATTATTTCAAATAACTATTCTGATATGGGAATTAGCTT [13494]
cp32-3_un8_B31_PacBio          CTTAAACATAGACTCTCAAATTAGGGACATTTATTCTAGGATTATTTCAAATAACTATTCTGATATGGGAATTAGCTT [13494]
cp32-3_B31_TS_MP_FG_consensus  CTTAAACATAGACTCTCAAATTAGGGACATTTATTCTAGGATTATTTCAAATAACTATTCTGATATGGGAATTAGCTT [13494]
cp32-3_B31_NX_Pl_consensus     CTTAAACATAGACTCTCAAATTAGGGACATTTATTCTAGGATTATTTCAAATAACTATTCTGATATGGGAATTAGCTT [13494]
cp32-3_PAli_NX_MP_FG_consensus CTTAAACATAGACTCTCAAATTAGGGACATTTATTCTAGGATTATTTCAAATAACTATTCTGATATGGGAATTAGCTT [13494]
cp32-3_PAli_NX_Pl_consensus    CTTAAACATAGACTCTCAAATTAGGGACATTTATTCTAGGATTATTTCAAATAACTATTCTGATATGGGAATTAGCTT [13494]
cp32-3_PAbe_NX_MP_FG_consensus CTTAAACATAGACTCTCAAATTAGGGACATTTATTCTAGGATTATTTCAAATAACTATTCTGATATGGGAATTAGCTT [13494]
cp32-3_PAbe_NX_Pl_consensus    CTTAAACATAGACTCTCAAATTAGGGACATTTATTCTAGGATTATTTCAAATAACTATTCTGATATGGGAATTAGCTT [13494]

cp32-3_gi|11497103|B31_GB      TGAATATCAAGACTTTTTTGCTCCAGTTAATGAAGTTAAAGGAATTAAGTTTATGGAAATAAGTGCTTGTATTAAAGA [13572]
cp32-3_un8_B31_PacBio          TGAATATCAAGACTTTTTTGCTCCAGTTAATGAAGTTAAAGGAATTAAGTTTATGGAAATAAGTGCTTGTATTAAAGA [13572]
cp32-3_B31_TS_MP_FG_consensus  TGAATATCAAGACTTTTTTGCTCCAGTTAATGAAGTTAAAGGAATTAAGTTTATGGAAATAAGTGCTTGTATTAAAGA [13572]
cp32-3_B31_NX_Pl_consensus     TGAATATCAAGACTTTTTTGCTCCAGTTAATGAAGTTAAAGGAATTAAGTTTATGGAAATAAGTGCTTGTATTAAAGA [13572]
cp32-3_PAli_NX_MP_FG_consensus TGAATATCAAGACTTTTTTGCTCCAGTTAATGAAGTTAAAGGAATTAAGTTTATGGAAATAAGTGCTTGTATTAAAGA [13572]
cp32-3_PAli_NX_Pl_consensus    TGAATATCAAGACTTTTTTGCTCCAGTTAATGAAGTTAAAGGAATTAAGTTTATGGAAATAAGTGCTTGTATTAAAGA [13572]
cp32-3_PAbe_NX_MP_FG_consensus TGAATATCAAGACTTTTTTGCTCCAGTTAATGAAGTTAAAGGAATTAAGTTTATGGAAATAAGTGCTTGTATTAAAGA [13572]
cp32-3_PAbe_NX_Pl_consensus    TGAATATCAAGACTTTTTTGCTCCAGTTAATGAAGTTAAAGGAATTAAGTTTATGGAAATAAGTGCTTGTATTAAAGA [13572]

cp32-3_gi|11497103|B31_GB      TACAGACACTGAGAGTATTACAAAAATTGGTGATAGCGATTTTAAAAAAAATCAAGATATTGCCATTAATGATGACAC [13650]
cp32-3_un8_B31_PacBio          TACAGACACTGAGAGTATTACAAAAATTGGTGATAGCGATTTTAAAAAAAATCAAGATATTGCCATTAATGATGACAC [13650]
cp32-3_B31_TS_MP_FG_consensus  TACAGACACTGAGAGTATTACAAAAATTGGTGATAGCGATTTTAAAAAAAATCAAGATATTGCCATTAATGATGACAC [13650]
cp32-3_B31_NX_Pl_consensus     TACAGACACTGAGAGTATTACAAAAATTGGTGATAGCGATTTTAAAAAAAATCAAGATATTGCCATTAATGATGACAC [13650]
cp32-3_PAli_NX_MP_FG_consensus TACAGACACTGAGAGTATTACAAAAATTGGTGATAGCGATTTTAAAAAAAATCAAGATATTGCCATTAATGATGACAC [13650]
cp32-3_PAli_NX_Pl_consensus    TACAGACACTGAGAGTATTACAAAAATTGGTGATAGCGATTTTAAAAAAAATCAAGATATTGCCATTAATGATGACAC [13650]
cp32-3_PAbe_NX_MP_FG_consensus TACAGACACTGAGAGTATTACAAAAATTGGTGATAGCGATTTTAAAAAAAATCAAGATATTGCCATTAATGATGACAC [13650]
cp32-3_PAbe_NX_Pl_consensus    TACAGACACTGAGAGTATTACAAAAATTGGTGATAGCGATTTTAAAAAAAATCAAGATATTGCCATTAATGATGACAC [13650]

cp32-3_gi|11497103|B31_GB      AATGCTACTTTTCAATATGACAGATAGATTGCTTATTGATATTGGATAGTTAACAAATATGAAAATACCCAATCTTTT [13728]
cp32-3_un8_B31_PacBio          AATGCTACTTTTCAATATGACAGATAGATTGCTTATTGATATTGGATAGTTAACAAATATGAAAATACCCAATCTTTT [13728]
cp32-3_B31_TS_MP_FG_consensus  AATGCTACTTTTCAATATGACAGATAGATTGCTTATTGATATTGGATAGTTAACAAATATGAAAATACCCAATCTTTT [13728]
cp32-3_B31_NX_Pl_consensus     AATGCTACTTTTCAATATGACAGATAGATTGCTTATTGATATTGGATAGTTAACAAATATGAAAATACCCAATCTTTT [13728]
cp32-3_PAli_NX_MP_FG_consensus AATGCTACTTTTCAATATGACAGATAGATTGCTTATTGATATTGGATAGTTAACAAATATGAAAATACCCAATCTTTT [13728]
cp32-3_PAli_NX_Pl_consensus    AATGCTACTTTTCAATATGACAGATAGATTGCTTATTGATATTGGATAGTTAACAAATATGAAAATACCCAATCTTTT [13728]
cp32-3_PAbe_NX_MP_FG_consensus AATGCTACTTTTCAATATGACAGATAGATTGCTTATTGATATTGGATAGTTAACAAATATGAAAATACCCAATCTTTT [13728]
cp32-3_PAbe_NX_Pl_consensus    AATGCTACTTTTCAATATGACAGATAGATTGCTTATTGATATTGGATAGTTAACAAATATGAAAATACCCAATCTTTT [13728]

cp32-3_gi|11497103|B31_GB      CAAAAACACCGAAATTCATAAATTTATACGTACAGAAACAGAATATGCACAAGCATTGCTTAATGAACTTAAGTCCCT [13806]
cp32-3_un8_B31_PacBio          CAAAAACACCGAAATTCATAAATTTATACGTACAGAAACAGAATATGCACAAGCATTGCTTAATGAACTTAAGTCCCT [13806]
cp32-3_B31_TS_MP_FG_consensus  CAAAAACACCGAAATTCATAAATTTATACGTACAGAAACAGAATATGCACAAGCATTGCTTAATGAACTTAAGTCCCT [13806]
cp32-3_B31_NX_Pl_consensus     CAAAAACACCGAAATTCATAAATTTATACGTACAGAAACAGAATATGCACAAGCATTGCTTAATGAACTTAAGTCCCT [13806]
cp32-3_PAli_NX_MP_FG_consensus CAAAAACACCGAAATTCATAAATTTATACGTACAGAAACAGAATATGCACAAGCATTGCTTAATGAACTTAAGTCCCT [13806]
cp32-3_PAli_NX_Pl_consensus    CAAAAACACCGAAATTCATAAATTTATACGTACAGAAACAGAATATGCACAAGCATTGCTTAATGAACTTAAGTCCCT [13806]
cp32-3_PAbe_NX_MP_FG_consensus CAAAAACACCGAAATTCATAAATTTATACGTACAGAAACAGAATATGCACAAGCATTGCTTAATGAACTTAAGTCCCT [13806]
cp32-3_PAbe_NX_Pl_consensus    CAAAAACACCGAAATTCATAAATTTATACGTACAGAAACAGAATATGCACAAGCATTGCTTAATGAACTTAAGTCCCT [13806]

cp32-3_gi|11497103|B31_GB      TAATTCCAACTTCATATCCATTAATGTAATAGAAAATATAAAATCAAGATATATTGCAATATGGATATCTCAAGTTTT [13884]
cp32-3_un8_B31_PacBio          TAATTCCAACTTCATATCCATTAATGTAATAGAAAATATAAAATCAAGATATATTGCAATATGGATATCTCAAGTTTT [13884]
cp32-3_B31_TS_MP_FG_consensus  TAATTCCAACTTCATATCCATTAATGTAATAGAAAATATAAAATCAAGATATATTGCAATATGGATATCTCAAGTTTT [13884]
cp32-3_B31_NX_Pl_consensus     TAATTCCAACTTCATATCCATTAATGTAATAGAAAATATAAAATCAAGATATATTGCAATATGGATATCTCAAGTTTT [13884]
cp32-3_PAli_NX_MP_FG_consensus TAATTCCAACTTCATATCCATTAATGTAATAGAAAATATAAAATCAAGATATATTGCAATATGGATATCTCAAGTTTT [13884]
cp32-3_PAli_NX_Pl_consensus    TAATTCCAACTTCATATCCATTAATGTAATAGAAAATATAAAATCAAGATATATTGCAATATGGATATCTCAAGTTTT [13884]
cp32-3_PAbe_NX_MP_FG_consensus TAATTCCAACTTCATATCCATTAATGTAATAGAAAATATAAAATCAAGATATATTGCAATATGGATATCTCAAGTTTT [13884]
cp32-3_PAbe_NX_Pl_consensus    TAATTCCAACTTCATATCCATTAATGTAATAGAAAATATAAAATCAAGATATATTGCAATATGGATATCTCAAGTTTT [13884]

cp32-3_gi|11497103|B31_GB      ATCTATCTTTTATGCAAAAACTCAAACTTTACAAAGTATTACAAGCAATATTAATAGTGTTATTTTTGCTTTACGTCA [13962]
cp32-3_un8_B31_PacBio          ATCTATCTTTTATGCAAAAACTCAAACTTTACAAAGTATTACAAGCAATATTAATAGTGTTATTTTTGCTTTACGTCA [13962]
cp32-3_B31_TS_MP_FG_consensus  ATCTATCTTTTATGCAAAAACTCAAACTTTACAAAGTATTACAAGCAATATTAATAGTGTTATTTTTGCTTTACGTCA [13962]
cp32-3_B31_NX_Pl_consensus     ATCTATCTTTTATGCAAAAACTCAAACTTTACAAAGTATTACAAGCAATATTAATAGTGTTATTTTTGCTTTACGTCA [13962]
cp32-3_PAli_NX_MP_FG_consensus ATCTATCTTTTATGCAAAAACTCAAACTTTACAAAGTATTACAAGCAATATTAATAGTGTTATTTTTGCTTTACGTCA [13962]
cp32-3_PAli_NX_Pl_consensus    ATCTATCTTTTATGCAAAAACTCAAACTTTACAAAGTATTACAAGCAATATTAATAGTGTTATTTTTGCTTTACGTCA [13962]
cp32-3_PAbe_NX_MP_FG_consensus ATCTATCTTTTATGCAAAAACTCAAACTTTACAAAGTATTACAAGCAATATTAATAGTGTTATTTTTGCTTTACGTCA [13962]
cp32-3_PAbe_NX_Pl_consensus    ATCTATCTTTTATGCAAAAACTCAAACTTTACAAAGTATTACAAGCAATATTAATAGTGTTATTTTTGCTTTACGTCA [13962]

cp32-3_gi|11497103|B31_GB      TATTGGCACTGACGAGTCATTTAGACTGATTTTCAAGACCTTTTTAAATGTGGACATTGAAGTTACTACTCCTGAAGC [14040]
cp32-3_un8_B31_PacBio          TATTGGCACTGACGAGTCATTTAGACTGATTTTCAAGACCTTTTTAAATGTGGACATTGAAGTTACTACTCCTGAAGC [14040]
cp32-3_B31_TS_MP_FG_consensus  TATTGGCACTGACGAGTCATTTAGACTGATTTTCAAGACCTTTTTAAATGTGGACATTGAAGTTACTACTCCTGAAGC [14040]
cp32-3_B31_NX_Pl_consensus     TATTGGCACTGACGAGTCATTTAGACTGATTTTCAAGACCTTTTTAAATGTGGACATTGAAGTTACTACTCCTGAAGC [14040]
cp32-3_PAli_NX_MP_FG_consensus TATTGGCACTGACGAGTCATTTAGACTGATTTTCAAGACCTTTTTAAATGTGGACATTGAAGTTACTACTCCTGAAGC [14040]
cp32-3_PAli_NX_Pl_consensus    TATTGGCACTGACGAGTCATTTAGACTGATTTTCAAGACCTTTTTAAATGTGGACATTGAAGTTACTACTCCTGAAGC [14040]
cp32-3_PAbe_NX_MP_FG_consensus TATTGGCACTGACGAGTCATTTAGACTGATTTTCAAGACCTTTTTAAATGTGGACATTGAAGTTACTACTCCTGAAGC [14040]
cp32-3_PAbe_NX_Pl_consensus    TATTGGCACTGACGAGTCATTTAGACTGATTTTCAAGACCTTTTTAAATGTGGACATTGAAGTTACTACTCCTGAAGC [14040]

cp32-3_gi|11497103|B31_GB      TGGGGTTATTGATATCTCTTTAAAAGGGGTAATAAAAACAAACTTTACTACATTTATTTCGCCTAACACTAAGAAAGG [14118]
cp32-3_un8_B31_PacBio          TGGGGTTATTGATATCTCTTTAAAAGGGGTAATAAAAACAAACTTTACTACATTTATTTCGCCTAACACTAAGAAAGG [14118]
cp32-3_B31_TS_MP_FG_consensus  TGGGGTTATTGATATCTCTTTAAAAGGGGTAATAAAAACAAACTTTACTACATTTATTTCGCCTAACACTAAGAAAGG [14118]
cp32-3_B31_NX_Pl_consensus     TGGGGTTATTGATATCTCTTTAAAAGGGGTAATAAAAACAAACTTTACTACATTTATTTCGCCTAACACTAAGAAAGG [14118]
cp32-3_PAli_NX_MP_FG_consensus TGGGGTTATTGATATCTCTTTAAAAGGGGTAATAAAAACAAACTTTACTACATTTATTTCGCCTAACACTAAGAAAGG [14118]
cp32-3_PAli_NX_Pl_consensus    TGGGGTTATTGATATCTCTTTAAAAGGGGTAATAAAAACAAACTTTACTACATTTATTTCGCCTAACACTAAGAAAGG [14118]
cp32-3_PAbe_NX_MP_FG_consensus TGGGGTTATTGATATCTCTTTAAAAGGGGTAATAAAAACAAACTTTACTACATTTATTTCGCCTAACACTAAGAAAGG [14118]
```

```
cp32-3_PAbe_NX_Pl_consensus    TGGGGTTATTGATATCTCTTTAAAAGGGGTAATAAAAACAAACTTTACTACATTTATTTCGCCTAACACTAAGAAAGG [14118]

cp32-3_gi|11497103|B31_GB      AAAACGACTAAAAAAGATAATTCTTAGAGAAAAGAAGCCGGGATATGCTGCATCTAAAAAGGCTTTAGTATTCAACTC [14196]
cp32-3_un8_B31_PacBio          AAAACGACTAAAAAAGATAATTCTTAGAGAAAAGAAGCCGGGATATGCTGCATCTAAAAAGGCTTTAGTATTCAACTC [14196]
cp32-3_B31_TS_MP_FG_consensus  AAAACGACTAAAAAAGATAATTCTTAGAGAAAAGAAGCCGGGATATGCTGCATCTAAAAAGGCTTTAGTATTCAACTC [14196]
cp32-3_B31_NX_Pl_consensus     AAAACGACTAAAAAAGATAATTCTTAGAGAAAAGAAGCCGGGATATGCTGCATCTAAAAAGGCTTTAGTATTCAACTC [14196]
cp32-3_PAli_NX_MP_FG_consensus AAAACGACTAAAAAAGATAATTCTTAGAGAAAAGAAGCCGGGATATGCTGCATCTAAAAAGGCTTTAGTATTCAACTC [14196]
cp32-3_PAli_NX_Pl_consensus    AAAACGACTAAAAAAGATAATTCTTAGAGAAAAGAAGCCGGGATATGCTGCATCTAAAAAGGCTTTAGTATTCAACTC [14196]
cp32-3_PAbe_NX_MP_FG_consensus AAAACGACTAAAAAAGATAATTCTTAGAGAAAAGAAGCCGGGATATGCTGCATCTAAAAAGGCTTTAGTATTCAACTC [14196]
cp32-3_PAbe_NX_Pl_consensus    AAAACGACTAAAAAAGATAATTCTTAGAGAAAAGAAGCCGGGATATGCTGCATCTAAAAAGGCTTTAGTATTCAACTC [14196]

cp32-3_gi|11497103|B31_GB      ACTTCCTAAGGGCTATGATCATTCAATTTATGCTTTTATTAAGAGAATTATTCCTATTGGTAGAGTTCTCAAAATTAA [14274]
cp32-3_un8_B31_PacBio          ACTTCCTAAGGGCTATGATCATTCAATTTATGCTTTTATTAAGAGAATTATTCCTATTGGTAGAGTTCTCAAAATTAA [14274]
cp32-3_B31_TS_MP_FG_consensus  ACTTCCTAAGGGCTATGATCATTCAATTTATGCTTTTATTAAGAGAATTATTCCTATTGGTAGAGTTCTCAAAATTAA [14274]
cp32-3_B31_NX_Pl_consensus     ACTTCCTAAGGGCTATGATCATTCAATTTATGCTTTTATTAAGAGAATTATTCCTATTGGTAGAGTTCTCAAAATTAA [14274]
cp32-3_PAli_NX_MP_FG_consensus ACTTCCTAAGGGCTATGATCATTCAATTTATGCTTTTATTAAGAGAATTATTCCTATTGGTAGAGTTCTCAAAATTAA [14274]
cp32-3_PAli_NX_Pl_consensus    ACTTCCTAAGGGCTATGATCATTCAATTTATGCTTTTATTAAGAGAATTATTCCTATTGGTAGAGTTCTCAAAATTAA [14274]
cp32-3_PAbe_NX_MP_FG_consensus ACTTCCTAAGGGCTATGATCATTCAATTTATGCTTTTATTAAGAGAATTATTCCTATTGGTAGAGTTCTCAAAATTAA [14274]
cp32-3_PAbe_NX_Pl_consensus    ACTTCCTAAGGGCTATGATCATTCAATTTATGCTTTTATTAAGAGAATTATTCCTATTGGTAGAGTTCTCAAAATTAA [14274]

cp32-3_gi|11497103|B31_GB      TAATACAGATGGTAACAATATTATAACTTTTAACAACTAAGGAGGTTTTATGGCTGATGATCAAGAAAAATTACTAAT [14352]
cp32-3_un8_B31_PacBio          TAATACAGATGGTAACAATATTATAACTTTTAACAACTAAGGAGGTTTTATGGCTGATGATCAAGAAAAATTACTAAT [14352]
cp32-3_B31_TS_MP_FG_consensus  TAATACAGATGGTAACAATATTATAACTTTTAACAACTAAGGAGGTTTTATGGCTGATGATCAAGAAAAATTACTAAT [14352]
cp32-3_B31_NX_Pl_consensus     TAATACAGATGGTAACAATATTATAACTTTTAACAACTAAGGAGGTTTTATGGCTGATGATCAAGAAAAATTACTAAT [14352]
cp32-3_PAli_NX_MP_FG_consensus TAATACAGATGGTAACAATATTATAACTTTTAACAACTAAGGAGGTTTTATGGCTGATGATCAAGAAAAATTACTAAT [14352]
cp32-3_PAli_NX_Pl_consensus    TAATACAGATGGTAACAATATTATAACTTTTAACAACTAAGGAGGTTTTATGGCTGATGATCAAGAAAAATTACTAAT [14352]
cp32-3_PAbe_NX_MP_FG_consensus TAATACAGATGGTAACAATATTATAACTTTTAACAACTAAGGAGGTTTTATGGCTGATGATCAAGAAAAATTACTAAT [14352]
cp32-3_PAbe_NX_Pl_consensus    TAATACAGATGGTAACAATATTATAACTTTTAACAACTAAGGAGGTTTTATGGCTGATGATCAAGAAAAATTACTAAT [14352]

cp32-3_gi|11497103|B31_GB      TGATGAAGAAGAAACGGTTCAAATAAAAGATTTAAATAAGGTTACGACCGTTAACGATACTGATCTTTTACTGCTTGA [14430]
cp32-3_un8_B31_PacBio          TGATGAAGAAGAAACGGTTCAAATAAAAGATTTAAATAAGGTTACGACCGTTAACGATACTGATCTTTTACTGCTTGA [14430]
cp32-3_B31_TS_MP_FG_consensus  TGATGAAGAAGAAACGGTTCAAATAAAAGATTTAAATAAGGTTACGACCGTTAACGATACTGATCTTTTACTGCTTGA [14430]
cp32-3_B31_NX_Pl_consensus     TGATGAAGAAGAAACGGTTCAAATAAAAGATTTAAATAAGGTTACGACCGTTAACGATACTGATCTTTTACTGCTTGA [14430]
cp32-3_PAli_NX_MP_FG_consensus TGATGAAGAAGAAACGGTTCAAATAAAAGATTTAAATAAGGTTACGACCGTTAACGATACTGATCTTTTACTGCTTGA [14430]
cp32-3_PAli_NX_Pl_consensus    TGATGAAGAAGAAACGGTTCAAATAAAAGATTTAAATAAGGTTACGACCGTTAACGATACTGATCTTTTACTGCTTGA [14430]
cp32-3_PAbe_NX_MP_FG_consensus TGATGAAGAAGAAACGGTTCAAATAAAAGATTTAAATAAGGTTACGACCGTTAACGATACTGATCTTTTACTGCTTGA [14430]
cp32-3_PAbe_NX_Pl_consensus    TGATGAAGAAGAAACGGTTCAAATAAAAGATTTAAATAAGGTTACGACCGTTAACGATACTGATCTTTTACTGCTTGA [14430]

cp32-3_gi|11497103|B31_GB      TGATGGAGCTGCAAGCAGTAATGCTATCACCTTTAAAAACTTTTTAGATGCTTCTAAAGACAAAATATTTAAAGGAGA [14508]
cp32-3_un8_B31_PacBio          TGATGGAGCTGCAAGCAGTAATGCTATCACCTTTAAAAACTTTTTAGATGCTTCTAAAGACAAAATATTTAAAGGAGA [14508]
cp32-3_B31_TS_MP_FG_consensus  TGATGGAGCTGCAAGCAGTAATGCTATCACCTTTAAAAACTTTTTAGATGCTTCTAAAGACAAAATATTTAAAGGAGA [14508]
cp32-3_B31_NX_Pl_consensus     TGATGGAGCTGCAAGCAGTAATGCTATCACCTTTAAAAACTTTTTAGATGCTTCTAAAGACAAAATATTTAAAGGAGA [14508]
cp32-3_PAli_NX_MP_FG_consensus TGATGGAGCTGCAAGCAGTAATGCTATCACCTTTAAAAACTTTTTAGATGCTTCTAAAGACAAAATATTTAAAGGAGA [14508]
cp32-3_PAli_NX_Pl_consensus    TGATGGAGCTGCAAGCAGTAATGCTATCACCTTTAAAAACTTTTTAGATGCTTCTAAAGACAAAATATTTAAAGGAGA [14508]
cp32-3_PAbe_NX_MP_FG_consensus TGATGGAGCTGCAAGCAGTAATGCTATCACCTTTAAAAACTTTTTAGATGCTTCTAAAGACAAAATATTTAAAGGAGA [14508]
cp32-3_PAbe_NX_Pl_consensus    TGATGGAGCTGCAAGCAGTAATGCTATCACCTTTAAAAACTTTTTAGATGCTTCTAAAGACAAAATATTTAAAGGAGA [14508]

cp32-3_gi|11497103|B31_GB      GGGATTAGACTATTTTAAGCAGATAATTAAGTCTACAATTGCCGAAGAACTTGCAGCTGATAAAGATTTTGTAGAAAA [14586]
cp32-3_un8_B31_PacBio          GGGATTAGACTATTTTAAGCAGATAATTAAGTCTACAATTGCCGAAGAACTTGCAGCTGATAAAGATTTTGTAGAAAA [14586]
cp32-3_B31_TS_MP_FG_consensus  GGGATTAGACTATTTTAAGCAGATAATTAAGTCTACAATTGCCGAAGAACTTGCAGCTGATAAAGATTTTGTAGAAAA [14586]
cp32-3_B31_NX_Pl_consensus     GGGATTAGACTATTTTAAGCAGATAATTAAGTCTACAATTGCCGAAGAACTTGCAGCTGATAAAGATTTTGTAGAAAA [14586]
cp32-3_PAli_NX_MP_FG_consensus GGGATTAGACTATTTTAAGCAGATAATTAAGTCTACAATTGCCGAAGAACTTGCAGCTGATAAAGATTTTGTAGAAAA [14586]
cp32-3_PAli_NX_Pl_consensus    GGGATTAGACTATTTTAAGCAGATAATTAAGTCTACAATTGCCGAAGAACTTGCAGCTGATAAAGATTTTGTAGAAAA [14586]
cp32-3_PAbe_NX_MP_FG_consensus GGGATTAGACTATTTTAAGCAGATAATTAAGTCTACAATTGCCGAAGAACTTGCAGCTGATAAAGATTTTGTAGAAAA [14586]
cp32-3_PAbe_NX_Pl_consensus    GGGATTAGACTATTTTAAGCAGATAATTAAGTCTACAATTGCCGAAGAACTTGCAGCTGATAAAGATTTTGTAGAAAA [14586]

cp32-3_gi|11497103|B31_GB      AATTTATGCTAAAATAACGGACAAATTAATTAACAACGATTCTACTAATATTTCTAACCTTTTTAGTAAAATTAAATC [14664]
cp32-3_un8_B31_PacBio          AATTTATGCTAAAATAACGGACAAATTAATTAACAACGATTCTACTAATATTTCTAACCTTTTTAGTAAAATTAAATC [14664]
cp32-3_B31_TS_MP_FG_consensus  AATTTATGCTAAAATAACGGACAAATTAATTAACAACGATTCTACTAATATTTCTAACCTTTTTAGTAAAATTAAATC [14664]
cp32-3_B31_NX_Pl_consensus     AATTTATGCTAAAATAACGGACAAATTAATTAACAACGATTCTACTAATATTTCTAACCTTTTTAGTAAAATTAAATC [14664]
cp32-3_PAli_NX_MP_FG_consensus AATTTATGCTAAAATAACGGACAAATTAATTAACAACGATTCTACTAATATTTCTAACCTTTTTAGTAAAATTAAATC [14664]
cp32-3_PAli_NX_Pl_consensus    AATTTATGCTAAAATAACGGACAAATTAATTAACAACGATTCTACTAATATTTCTAACCTTTTTAGTAAAATTAAATC [14664]
cp32-3_PAbe_NX_MP_FG_consensus AATTTATGCTAAAATAACGGACAAATTAATTAACAACGATTCTACTAATATTTCTAACCTTTTTAGTAAAATTAAATC [14664]
cp32-3_PAbe_NX_Pl_consensus    AATTTATGCTAAAATAACGGACAAATTAATTAACAACGATTCTACTAATATTTCTAACCTTTTTAGTAAAATTAAATC [14664]

cp32-3_gi|11497103|B31_GB      ACGCCTTACAGATAGCATATCATCAGCCACTTTATCTAGAAGTGATGATCTTTTGATAATGCCTTCATCAGATACTAT [14742]
cp32-3_un8_B31_PacBio          ACGCCTTACAGATAGCATATCATCAGCCACTTTATCTAGAAGTGATGATCTTTTGATAATGCCTTCATCAGATACTAT [14742]
cp32-3_B31_TS_MP_FG_consensus  ACGCCTTACAGATAGCATATCATCAGCCACTTTATCTAGAAGTGATGATCTTTTGATAATGCCTTCATCAGATACTAT [14742]
cp32-3_B31_NX_Pl_consensus     ACGCCTTACAGATAGCATATCATCAGCCACTTTATCTAGAAGTGATGATCTTTTGATAATGCCTTCATCAGATACTAT [14742]
cp32-3_PAli_NX_MP_FG_consensus ACGCCTTACAGATAGCATATCATCAGCCACTTTATCTAGAAGTGATGATCTTTTGATAATGCCTTCATCAGATACTAT [14742]
cp32-3_PAli_NX_Pl_consensus    ACGCCTTACAGATAGCATATCATCAGCCACTTTATCTAGAAGTGATGATCTTTTGATAATGCCTTCATCAGATACTAT [14742]
cp32-3_PAbe_NX_MP_FG_consensus ACGCCTTACAGATAGCATATCATCAGCCACTTTATCTAGAAGTGATGATCTTTTGATAATGCCTTCATCAGATACTAT [14742]
cp32-3_PAbe_NX_Pl_consensus    ACGCCTTACAGATAGCATATCATCAGCCACTTTATCTAGAAGTGATGATCTTTTGATAATGCCTTCATCAGATACTAT [14742]

cp32-3_gi|11497103|B31_GB      TCAAAAAACACCCGTTCCTAAACATATACTTGGAGTACCATCAAATTTTGCTTATGGCAGAACTAGAAGTACTACACT [14820]
cp32-3_un8_B31_PacBio          TCAAAAAACACCCGTTCCTAAACATATACTTGGAGTACCATCAAATTTTGCTTATGGCAGAACTAGAAGTACTACACT [14820]
cp32-3_B31_TS_MP_FG_consensus  TCAAAAAACACCCGTTCCTAAACATATACTTGGAGTACCATCAAATTTTGCTTATGGCAGAACTAGAAGTACTACACT [14820]
cp32-3_B31_NX_Pl_consensus     TCAAAAAACACCCGTTCCTAAACATATACTTGGAGTACCATCAAATTTTGCTTATGGCAGAACTAGAAGTACTACACT [14820]
cp32-3_PAli_NX_MP_FG_consensus TCAAAAAACACCCGTTCCTAAACATATACTTGGAGTACCATCAAATTTTGCTTATGGCAGAACTAGAAGTACTACACT [14820]
cp32-3_PAli_NX_Pl_consensus    TCAAAAAACACCCGTTCCTAAACATATACTTGGAGTACCATCAAATTTTGCTTATGGCAGAACTAGAAGTACTACACT [14820]
cp32-3_PAbe_NX_MP_FG_consensus TCAAAAAACACCCGTTCCTAAACATATACTTGGAGTACCATCAAATTTTGCTTATGGCAGAACTAGAAGTACTACACT [14820]
cp32-3_PAbe_NX_Pl_consensus    TCAAAAAACACCCGTTCCTAAACATATACTTGGAGTACCATCAAATTTTGCTTATGGCAGAACTAGAAGTACTACACT [14820]

cp32-3_gi|11497103|B31_GB      TTATCCTTCTGACTATGAGAATAACGCGATATCTATTAATATGGAATACAATGATGATGTAACTCTTATTTTTTCTAA [14898]
cp32-3_un8_B31_PacBio          TTATCCTTCTGACTATGAGAATAACGCGATATCTATTAATATGGAATACAATGATGATGTAACTCTTATTTTTTCTAA [14898]
cp32-3_B31_TS_MP_FG_consensus  TTATCCTTCTGACTATGAGAATAACGCGATATCTATTAATATGGAATACAATGATGATGTAACTCTTATTTTTTCTAA [14898]
cp32-3_B31_NX_Pl_consensus     TTATCCTTCTGACTATGAGAATAACGCGATATCTATTAATATGGAATACAATGATGATGTAACTCTTATTTTTTCTAA [14898]
cp32-3_PAli_NX_MP_FG_consensus TTATCCTTCTGACTATGAGAATAACGCGATATCTATTAATATGGAATACAATGATGATGTAACTCTTATTTTTTCTAA [14898]
cp32-3_PAli_NX_Pl_consensus    TTATCCTTCTGACTATGAGAATAACGCGATATCTATTAATATGGAATACAATGATGATGTAACTCTTATTTTTTCTAA [14898]
cp32-3_PAbe_NX_MP_FG_consensus TTATCCTTCTGACTATGAGAATAACGCGATATCTATTAATATGGAATACAATGATGATGTAACTCTTATTTTTTCTAA [14898]
cp32-3_PAbe_NX_Pl_consensus    TTATCCTTCTGACTATGAGAATAACGCGATATCTATTAATATGGAATACAATGATGATGTAACTCTTATTTTTTCTAA [14898]

cp32-3_gi|11497103|B31_GB      AAGTTACGATAATTCTCCCGTTTATCTTGATATTGAAATTCAAGTAAAATCAATGATAATAGGATGCAGAAAAAAATC [14976]
cp32-3_un8_B31_PacBio          AAGTTACGATAATTCTCCCGTTTATCTTGATATTGAAATTCAAGTAAAATCAATGATAATAGGATGCAGAAAAAAATC [14976]
cp32-3_B31_TS_MP_FG_consensus  AAGTTACGATAATTCTCCCGTTTATCTTGATATTGAAATTCAAGTAAAATCAATGATAATAGGATGCAGAAAAAAATC [14976]
cp32-3_B31_NX_Pl_consensus     AAGTTACGATAATTCTCCCGTTTATCTTGATATTGAAATTCAAGTAAAATCAATGATAATAGGATGCAGAAAAAAATC [14976]
cp32-3_PAli_NX_MP_FG_consensus AAGTTACGATAATTCTCCCGTTTATCTTGATATTGAAATTCAAGTAAAATCAATGATAATAGGATGCAGAAAAAAATC [14976]
cp32-3_PAli_NX_Pl_consensus    AAGTTACGATAATTCTCCCGTTTATCTTGATATTGAAATTCAAGTAAAATCAATGATAATAGGATGCAGAAAAAAATC [14976]
cp32-3_PAbe_NX_MP_FG_consensus AAGTTACGATAATTCTCCCGTTTATCTTGATATTGAAATTCAAGTAAAATCAATGATAATAGGATGCAGAAAAAAATC [14976]
cp32-3_PAbe_NX_Pl_consensus    AAGTTACGATAATTCTCCCGTTTATCTTGATATTGAAATTCAAGTAAAATCAATGATAATAGGATGCAGAAAAAAATC [14976]

cp32-3_gi|11497103|B31_GB      ATTAAAACTTCAGTATTCTGATGAAACTACATACAATAGGGTTTATGAAATTGCGGGCCCCCGCGGACTATTCACCAG [15054]
cp32-3_un8_B31_PacBio          ATTAAAACTTCAGTATTCTGATGAAACTACATACAATAGGGTTTATGAAATTGCGGGCCCCCGCGGACTATTCACCAG [15054]
cp32-3_B31_TS_MP_FG_consensus  ATTAAAACTTCAGTATTCTGATGAAACTACATACAATAGGGTTTATGAAATTGCGGGCCCCCGCGGACTATTCACCAG [15054]
cp32-3_B31_NX_Pl_consensus     ATTAAAACTTCAGTATTCTGATGAAACTACATACAATAGGGTTTATGAAATTGCGGGCCCCCGCGGACTATTCACCAG [15054]
cp32-3_PAli_NX_MP_FG_consensus ATTAAAACTTCAGTATTCTGATGAAACTACATACAATAGGGTTTATGAAATTGCGGGCCCCCGCGGACTATTCACCAG [15054]
cp32-3_PAli_NX_Pl_consensus    ATTAAAACTTCAGTATTCTGATGAAACTACATACAATAGGGTTTATGAAATTGCGGGCCCCCGCGGACTATTCACCAG [15054]
cp32-3_PAbe_NX_MP_FG_consensus ATTAAAACTTCAGTATTCTGATGAAACTACATACAATAGGGTTTATGAAATTGCGGGCCCCCGCGGACTATTCACCAG [15054]
cp32-3_PAbe_NX_Pl_consensus    ATTAAAACTTCAGTATTCTGATGAAACTACATACAATAGGGTTTATGAAATTGCGGGCCCCCGCGGACTATTCACCAG [15054]
```

```
cp32-3_gi|11497103|B31_GB      AATTCCCATTTATAAAGGATGGTATGTCCAAAAAAGAGCCTCCTTGTATGGAGATCCATTCCCAGATCTTTTAAAACT [15132]
cp32-3_un8_B31_PacBio          AATTCCCATTTATAAAGGATGGTATGTCCAAAAAAGAGCCTCCTTGTATGGAGATCCATTCCCAGATCTTTTAAAACT [15132]
cp32-3_B31_TS_MP_FG_consensus  AATTCCCATTTATAAAGGATGGTATGTCCAAAAAAGAGCCTCCTTGTATGGAGATCCATTCCCAGATCTTTTAAAACT [15132]
cp32-3_B31_NX_Pl_consensus     AATTCCCATTTATAAAGGATGGTATGTCCAAAAAAGAGCCTCCTTGTATGGAGATCCATTCCCAGATCTTTTAAAACT [15132]
cp32-3_PAli_NX_MP_FG_consensus AATTCCCATTTATAAAGGATGGTATGTCCAAAAAAGAGCCTCCTTGTATGGAGATCCATTCCCAGATCTTTTAAAACT [15132]
cp32-3_PAli_NX_Pl_consensus    AATTCCCATTTATAAAGGATGGTATGTCCAAAAAAGAGCCTCCTTGTATGGAGATCCATTCCCAGATCTTTTAAAACT [15132]
cp32-3_PAbe_NX_MP_FG_consensus AATTCCCATTTATAAAGGATGGTATGTCCAAAAAAGAGCCTCCTTGTATGGAGATCCATTCCCAGATCTTTTAAAACT [15132]
cp32-3_PAbe_NX_Pl_consensus    AATTCCCATTTATAAAGGATGGTATGTCCAAAAAAGAGCCTCCTTGTATGGAGATCCATTCCCAGATCTTTTAAAACT [15132]

cp32-3_gi|11497103|B31_GB      GTAAACTTTTTAGCAAAAATTATGATTTTGGTATATAATATACGTATAAAAATAAAAAATTAAAATGAAGGATTAAAA [15210]
cp32-3_un8_B31_PacBio          GTAAACTTTTTAGCAAAAATTATGATTTTGGTATATAATATACGTATAAAAATAAAAAATTAAAATGAAGGATTAAAA [15210]
cp32-3_B31_TS_MP_FG_consensus  GTAAACTTTTTAGCAAAAATTATGATTTTGGTATATAATATACGTATAAAAATAAAAAATTAAAATGAAGGATTAAAA [15210]
cp32-3_B31_NX_Pl_consensus     GTAAACTTTTTAGCAAAAATTATGATTTTGGTATATAATATACGTATAAAAATAAAAAATTAAAATGAAGGATTAAAA [15210]
cp32-3_PAli_NX_MP_FG_consensus GTAAACTTTTTAGCAAAAATTATGATTTTGGTATATAATATACGTATAAAAATAAAAAATTAAAATGAAGGATTAAAA [15210]
cp32-3_PAli_NX_Pl_consensus    GTAAACTTTTTAGCAAAAATTATGATTTTGGTATATAATATACGTATAAAAATAAAAAATTAAAATGAAGGATTAAAA [15210]
cp32-3_PAbe_NX_MP_FG_consensus GTAAACTTTTTAGCAAAAATTATGATTTTGGTATATAATATACGTATAAAAATAAAAAATTAAAATGAAGGATTAAAA [15210]
cp32-3_PAbe_NX_Pl_consensus    GTAAACTTTTTAGCAAAAATTATGATTTTGGTATATAATATACGTATAAAAATAAAAAATTAAAATGAAGGATTAAAA [15210]

cp32-3_gi|11497103|B31_GB      AATGGATACTATTAAATTAACCGAACTTCTTATCAATTTAAACGAAATTAAACTTATAGCGGTAATGATTTTTGTAAC [15288]
cp32-3_un8_B31_PacBio          AATGGATACTATTAAATTAACCGAACTTCTTATCAATTTAAACGAAATTAAACTTATAGCGGTAATGATTTTTGTAAC [15288]
cp32-3_B31_TS_MP_FG_consensus  AATGGATACTATTAAATTAACCGAACTTCTTATCAATTTAAACGAAATTAAACTTATAGCGGTAATGATTTTTGTAAC [15288]
cp32-3_B31_NX_Pl_consensus     AATGGATACTATTAAATTAACCGAACTTCTTATCAATTTAAACGAAATTAAACTTATAGCGGTAATGATTTTTGTAAC [15288]
cp32-3_PAli_NX_MP_FG_consensus AATGGATACTATTAAATTAACCGAACTTCTTATCAATTTAAACGAAATTAAACTTATAGCGGTAATGATTTTTGTAAC [15288]
cp32-3_PAli_NX_Pl_consensus    AATGGATACTATTAAATTAACCGAACTTCTTATCAATTTAAACGAAATTAAACTTATAGCGGTAATGATTTTTGTAAC [15288]
cp32-3_PAbe_NX_MP_FG_consensus AATGGATACTATTAAATTAACCGAACTTCTTATCAATTTAAACGAAATTAAACTTATAGCGGTAATGATTTTTGTAAC [15288]
cp32-3_PAbe_NX_Pl_consensus    AATGGATACTATTAAATTAACCGAACTTCTTATCAATTTAAACGAAATTAAACTTATAGCGGTAATGATTTTTGTAAC [15288]

cp32-3_gi|11497103|B31_GB      AGTGCTGGTTTTAGGAGTATTAATTCTTCTCAAGCCTTTACTAAAAGACATATTGACTATTGTAATAGGCAAGATTTT [15366]
cp32-3_un8_B31_PacBio          AGTGCTGGTTTTAGGAGTATTAATTCTTCTCAAGCCTTTACTAAAAGACATATTGACTATTGTAATAGGCAAGATTTT [15366]
cp32-3_B31_TS_MP_FG_consensus  AGTGCTGGTTTTAGGAGTATTAATTCTTCTCAAGCCTTTACTAAAAGACATATTGACTATTGTAATAGGCAAGATTTT [15366]
cp32-3_B31_NX_Pl_consensus     AGTGCTGGTTTTAGGAGTATTAATTCTTCTCAAGCCTTTACTAAAAGACATATTGACTATTGTAATAGGCAAGATTTT [15366]
cp32-3_PAli_NX_MP_FG_consensus AGTGCTGGTTTTAGGAGTATTAATTCTTCTCAAGCCTTTACTAAAAGACATATTGACTATTGTAATAGGCAAGATTTT [15366]
cp32-3_PAli_NX_Pl_consensus    AGTGCTGGTTTTAGGAGTATTAATTCTTCTCAAGCCTTTACTAAAAGACATATTGACTATTGTAATAGGCAAGATTTT [15366]
cp32-3_PAbe_NX_MP_FG_consensus AGTGCTGGTTTTAGGAGTATTAATTCTTCTCAAGCCTTTACTAAAAGACATATTGACTATTGTAATAGGCAAGATTTT [15366]
cp32-3_PAbe_NX_Pl_consensus    AGTGCTGGTTTTAGGAGTATTAATTCTTCTCAAGCCTTTACTAAAAGACATATTGACTATTGTAATAGGCAAGATTTT [15366]

cp32-3_gi|11497103|B31_GB      TAAGAATGGTAATGGTAATGGCAAAAATCACATTAAAAAAAGAGATTAACTTATGAAATTATCCAAAGATAATGTTGA [15444]
cp32-3_un8_B31_PacBio          TAAGAATGGTAATGGTAATGGCAAAAATCACATTAAAAAAAGAGATTAACTTATGAAATTATCCAAAGATAATGTTGA [15444]
cp32-3_B31_TS_MP_FG_consensus  TAAGAATGGTAATGGTAATGGCAAAAATCACATTAAAAAAAGAGATTAACTTATGAAATTATCCAAAGATAATGTTGA [15444]
cp32-3_B31_NX_Pl_consensus     TAAGAATGGTAATGGTAATGGCAAAAATCACATTAAAAAAAGAGATTAACTTATGAAATTATCCAAAGATAATGTTGA [15444]
cp32-3_PAli_NX_MP_FG_consensus TAAGAATGGTAATGGTAATGGCAAAAATCACATTAAAAAAAGAGATTAACTTATGAAATTATCCAAAGATAATGTTGA [15444]
cp32-3_PAli_NX_Pl_consensus    TAAGAATGGTAATGGTAATGGCAAAAATCACATTAAAAAAAGAGATTAACTTATGAAATTATCCAAAGATAATGTTGA [15444]
cp32-3_PAbe_NX_MP_FG_consensus TAAGAATGGTAATGGTAATGGCAAAAATCACATTAAAAAAAGAGATTAACTTATGAAATTATCCAAAGATAATGTTGA [15444]
cp32-3_PAbe_NX_Pl_consensus    TAAGAATGGTAATGGTAATGGCAAAAATCACATTAAAAAAAGAGATTAACTTATGAAATTATCCAAAGATAATGTTGA [15444]

cp32-3_gi|11497103|B31_GB      GCTTGGACTTACGTCTTTATCAACCCTTATTGATATATTTTCTAAATTTGAAGATGAATTTGATGAAATTGCACATAA [15522]
cp32-3_un8_B31_PacBio          GCTTGGACTTACGTCTTTATCAACCCTTATTGATATATTTTCTAAATTTGAAGATGAATTTGATGAAATTGCACATAA [15522]
cp32-3_B31_TS_MP_FG_consensus  GCTTGGACTTACGTCTTTATCAACCCTTATTGATATATTTTCTAAATTTGAAGATGAATTTGATGAAATTGCACATAA [15522]
cp32-3_B31_NX_Pl_consensus     GCTTGGACTTACGTCTTTATCAACCCTTATTGATATATTTTCTAAATTTGAAGATGAATTTGATGAAATTGCACATAA [15522]
cp32-3_PAli_NX_MP_FG_consensus GCTTGGACTTACGTCTTTATCAACCCTTATTGATATATTTTCTAAATTTGAAGATGAATTTGATGAAATTGCACATAA [15522]
cp32-3_PAli_NX_Pl_consensus    GCTTGGACTTACGTCTTTATCAACCCTTATTGATATATTTTCTAAATTTGAAGATGAATTTGATGAAATTGCACATAA [15522]
cp32-3_PAbe_NX_MP_FG_consensus GCTTGGACTTACGTCTTTATCAACCCTTATTGATATATTTTCTAAATTTGAAGATGAATTTGATGAAATTGCACATAA [15522]
cp32-3_PAbe_NX_Pl_consensus    GCTTGGACTTACGTCTTTATCAACCCTTATTGATATATTTTCTAAATTTGAAGATGAATTTGATGAAATTGCACATAA [15522]

cp32-3_gi|11497103|B31_GB      AGGATTCTTTTTGGTTTATGAGCTGTATTCTCATTATAAATTAATCTATACAGCAAATATGGAAAGACTTGAGAGTGC [15600]
cp32-3_un8_B31_PacBio          AGGATTCTTTTTGGTTTATGAGCTGTATTCTCATTATAAATTAATCTATACAGCAAATATGGAAAGACTTGAGAGTGC [15600]
cp32-3_B31_TS_MP_FG_consensus  AGGATTCTTTTTGGTTTATGAGCTGTATTCTCATTATAAATTAATCTATACAGCAAATATGGAAAGACTTGAGAGTGC [15600]
cp32-3_B31_NX_Pl_consensus     AGGATTCTTTTTGGTTTATGAGCTGTATTCTCATTATAAATTAATCTATACAGCAAATATGGAAAGACTTGAGAGTGC [15600]
cp32-3_PAli_NX_MP_FG_consensus AGGATTCTTTTTGGTTTATGAGCTGTATTCTCATTATAAATTAATCTATACAGCAAATATGGAAAGACTTGAGAGTGC [15600]
cp32-3_PAli_NX_Pl_consensus    AGGATTCTTTTTGGTTTATGAGCTGTATTCTCATTATAAATTAATCTATACAGCAAATATGGAAAGACTTGAGAGTGC [15600]
cp32-3_PAbe_NX_MP_FG_consensus AGGATTCTTTTTGGTTTATGAGCTGTATTCTCATTATAAATTAATCTATACAGCAAATATGGAAAGACTTGAGAGTGC [15600]
cp32-3_PAbe_NX_Pl_consensus    AGGATTCTTTTTGGTTTATGAGCTGTATTCTCATTATAAATTAATCTATACAGCAAATATGGAAAGACTTGAGAGTGC [15600]

cp32-3_gi|11497103|B31_GB      ATTAACCCCAGCAATAAATAAAGCACTCGCTCCATTAAATGAAAAAATCAATCAATGCATTGACTTAGTTAATTCTGA [15678]
cp32-3_un8_B31_PacBio          ATTAACCCCAGCAATAAATAAAGCACTCGCTCCATTAAATGAAAAAATCAATCAATGCATTGACTTAGTTAATTCTGA [15678]
cp32-3_B31_TS_MP_FG_consensus  ATTAACCCCAGCAATAAATAAAGCACTCGCTCCATTAAATGAAAAAATCAATCAATGCATTGACTTAGTTAATTCTGA [15678]
cp32-3_B31_NX_Pl_consensus     ATTAACCCCAGCAATAAATAAAGCACTCGCTCCATTAAATGAAAAAATCAATCAATGCATTGACTTAGTTAATTCTGA [15678]
cp32-3_PAli_NX_MP_FG_consensus ATTAACCCCAGCAATAAATAAAGCACTCGCTCCATTAAATGAAAAAATCAATCAATGCATTGACTTAGTTAATTCTGA [15678]
cp32-3_PAli_NX_Pl_consensus    ATTAACCCCAGCAATAAATAAAGCACTCGCTCCATTAAATGAAAAAATCAATCAATGCATTGACTTAGTTAATTCTGA [15678]
cp32-3_PAbe_NX_MP_FG_consensus ATTAACCCCAGCAATAAATAAAGCACTCGCTCCATTAAATGAAAAAATCAATCAATGCATTGACTTAGTTAATTCTGA [15678]
cp32-3_PAbe_NX_Pl_consensus    ATTAACCCCAGCAATAAATAAAGCACTCGCTCCATTAAATGAAAAAATCAATCAATGCATTGACTTAGTTAATTCTGA [15678]

cp32-3_gi|11497103|B31_GB      TGAAAAAAATCTCAAAATATCTAATGATCTGAAATTCAATCAGGAAGGAAAACCTATCTATAAGGAAAGAACAAATAA [15756]
cp32-3_un8_B31_PacBio          TGAAAAAAATCTCAAAATATCTAATGATCTGAAATTCAATCAGGAAGGAAAACCTATCTATAAGGAAAGAACAAATAA [15756]
cp32-3_B31_TS_MP_FG_consensus  TGAAAAAAATCTCAAAATATCTAATGATCTGAAATTCAATCAGGAAGGAAAACCTATCTATAAGGAAAGAACAAATAA [15756]
cp32-3_B31_NX_Pl_consensus     TGAAAAAAATCTCAAAATATCTAATGATCTGAAATTCAATCAGGAAGGAAAACCTATCTATAAGGAAAGAACAAATAA [15756]
cp32-3_PAli_NX_MP_FG_consensus TGAAAAAAATCTCAAAATATCTAATGATCTGAAATTCAATCAGGAAGGAAAACCTATCTATAAGGAAAGAACAAATAA [15756]
cp32-3_PAli_NX_Pl_consensus    TGAAAAAAATCTCAAAATATCTAATGATCTGAAATTCAATCAGGAAGGAAAACCTATCTATAAGGAAAGAACAAATAA [15756]
cp32-3_PAbe_NX_MP_FG_consensus TGAAAAAAATCTCAAAATATCTAATGATCTGAAATTCAATCAGGAAGGAAAACCTATCTATAAGGAAAGAACAAATAA [15756]
cp32-3_PAbe_NX_Pl_consensus    TGAAAAAAATCTCAAAATATCTAATGATCTGAAATTCAATCAGGAAGGAAAACCTATCTATAAGGAAAGAACAAATAA [15756]

cp32-3_gi|11497103|B31_GB      TGCAAAATAACACTATTGGTTTAGGACTTAATTTACTATCCAGCTTAACTAACATAGCTAAAACTGATACAAACATAG [15834]
cp32-3_un8_B31_PacBio          TGCAAAATAACACTATTGGTTTAGGACTTAATTTACTATCCAGCTTAACTAACATAGCTAAAACTGATACAAACATAG [15834]
cp32-3_B31_TS_MP_FG_consensus  TGCAAAATAACACTATTGGTTTAGGACTTAATTTACTATCCAGCTTAACTAACATAGCTAAAACTGATACAAACATAG [15834]
cp32-3_B31_NX_Pl_consensus     TGCAAAATAACACTATTGGTTTAGGACTTAATTTACTATCCAGCTTAACTAACATAGCTAAAACTGATACAAACATAG [15834]
cp32-3_PAli_NX_MP_FG_consensus TGCAAAATAACACTATTGGTTTAGGACTTAATTTACTATCCAGCTTAACTAACATAGCTAAAACTGATACAAACATAG [15834]
cp32-3_PAli_NX_Pl_consensus    TGCAAAATAACACTATTGGTTTAGGACTTAATTTACTATCCAGCTTAACTAACATAGCTAAAACTGATACAAACATAG [15834]
cp32-3_PAbe_NX_MP_FG_consensus TGCAAAATAACACTATTGGTTTAGGACTTAATTTACTATCCAGCTTAACTAACATAGCTAAAACTGATACAAACATAG [15834]
cp32-3_PAbe_NX_Pl_consensus    TGCAAAATAACACTATTGGTTTAGGACTTAATTTACTATCCAGCTTAACTAACATAGCTAAAACTGATACAAACATAG [15834]

cp32-3_gi|11497103|B31_GB      ATCATAATTACATTAATACTTTTAGTAAAGTAATAGATTTTTTCTACAAAACATATATAAGCACACTAAAATCTATGG [15912]
cp32-3_un8_B31_PacBio          ATCATAATTACATTAATACTTTTAGTAAAGTAATAGATTTTTTCTACAAAACATATATAAGCACACTAAAATCTATGG [15912]
cp32-3_B31_TS_MP_FG_consensus  ATCATAATTACATTAATACTTTTAGTAAAGTAATAGATTTTTTCTACAAAACATATATAAGCACACTAAAATCTATGG [15912]
cp32-3_B31_NX_Pl_consensus     ATCATAATTACATTAATACTTTTAGTAAAGTAATAGATTTTTTCTACAAAACATATATAAGCACACTAAAATCTATGG [15912]
cp32-3_PAli_NX_MP_FG_consensus ATCATAATTACATTAATACTTTTAGTAAAGTAATAGATTTTTTCTACAAAACATATATAAGCACACTAAAATCTATGG [15912]
cp32-3_PAli_NX_Pl_consensus    ATCATAATTACATTAATACTTTTAGTAAAGTAATAGATTTTTTCTACAAAACATATATAAGCACACTAAAATCTATGG [15912]
cp32-3_PAbe_NX_MP_FG_consensus ATCATAATTACATTAATACTTTTAGTAAAGTAATAGATTTTTTCTACAAAACATATATAAGCACACTAAAATCTATGG [15912]
cp32-3_PAbe_NX_Pl_consensus    ATCATAATTACATTAATACTTTTAGTAAAGTAATAGATTTTTTCTACAAAACATATATAAGCACACTAAAATCTATGG [15912]

cp32-3_gi|11497103|B31_GB      AAACAGCTGAGTCAACTAAAATATTTGAAGAAATACAAGACATTTTAAAATACAACATTGAGATAATAGAGGCTATCT [15990]
cp32-3_un8_B31_PacBio          AAACAGCTGAGTCAACTAAAATATTTGAAGAAATACAAGACATTTTAAAATACAACATTGAGATAATAGAGGCTATCT [15990]
cp32-3_B31_TS_MP_FG_consensus  AAACAGCTGAGTCAACTAAAATATTTGAAGAAATACAAGACATTTTAAAATACAACATTGAGATAATAGAGGCTATCT [15990]
cp32-3_B31_NX_Pl_consensus     AAACAGCTGAGTCAACTAAAATATTTGAAGAAATACAAGACATTTTAAAATACAACATTGAGATAATAGAGGCTATCT [15990]
cp32-3_PAli_NX_MP_FG_consensus AAACAGCTGAGTCAACTAAAATATTTGAAGAAATACAAGACATTTTAAAATACAACATTGAGATAATAGAGGCTATCT [15990]
cp32-3_PAli_NX_Pl_consensus    AAACAGCTGAGTCAACTAAAATATTTGAAGAAATACAAGACATTTTAAAATACAACATTGAGATAATAGAGGCTATCT [15990]
cp32-3_PAbe_NX_MP_FG_consensus AAACAGCTGAGTCAACTAAAATATTTGAAGAAATACAAGACATTTTAAAATACAACATTGAGATAATAGAGGCTATCT [15990]
cp32-3_PAbe_NX_Pl_consensus    AAACAGCTGAGTCAACTAAAATATTTGAAGAAATACAAGACATTTTAAAATACAACATTGAGATAATAGAGGCTATCT [15990]
```

```
cp32-3_gi|11497103|B31_GB      CTACTGACAAAAGCAAAAGAATTATCACTTCACTTAAATCAACACGTAACAAAATCATGAAAGAATATATCAAAATAC [16068]
cp32-3_un8_B31_PacBio          CTACTGACAAAAGCAAAAGAATTATCACTTCACTTAAATCAACACGTAACAAAATCATGAAAGAATATATCAAAATAC [16068]
cp32-3_B31_TS_MP_FG_consensus  CTACTGACAAAAGCAAAAGAATTATCACTTCACTTAAATCAACACGTAACAAAATCATGAAAGAATATATCAAAATAC [16068]
cp32-3_B31_NX_Pl_consensus     CTACTGACAAAAGCAAAAGAATTATCACTTCACTTAAATCAACACGTAACAAAATCATGAAAGAATATATCAAAATAC [16068]
cp32-3_PAli_NX_MP_FG_consensus CTACTGACAAAAGCAAAAGAATTATCACTTCACTTAAATCAACACGTAACAAAATCATGAAAGAATATATCAAAATAC [16068]
cp32-3_PAli_NX_Pl_consensus    CTACTGACAAAAGCAAAAGAATTATCACTTCACTTAAATCAACACGTAACAAAATCATGAAAGAATATATCAAAATAC [16068]
cp32-3_PAbe_NX_MP_FG_consensus CTACTGACAAAAGCAAAAGAATTATCACTTCACTTAAATCAACACGTAACAAAATCATGAAAGAATATATCAAAATAC [16068]
cp32-3_PAbe_NX_Pl_consensus    CTACTGACAAAAGCAAAAGAATTATCACTTCACTTAAATCAACACGTAACAAAATCATGAAAGAATATATCAAAATAC [16068]

cp32-3_gi|11497103|B31_GB      TTAAAAGAGGTGAAAATGCTTAAAAGATTGCATTGTCTGCTAATTGTTTTGTTGTTATGTTGCACCACTATTGCTAAC [16146]
cp32-3_un8_B31_PacBio          TTAAAAGAGGTGAAAATGCTTAAAAGATTGCATTGTCTGCTAATTGTTTTGTTGTTATGTTGCACCACTATTGCTAAC [16146]
cp32-3_B31_TS_MP_FG_consensus  TTAAAAGAGGTGAAAATGCTTAAAAGATTGCATTGTCTGCTAATTGTTTTGTTGTTATGTTGCACCACTATTGCTAAC [16146]
cp32-3_B31_NX_Pl_consensus     TTAAAAGAGGTGAAAATGCTTAAAAGATTGCATTGTCTGCTAATTGTTTTGTTGTTATGTTGCACCACTATTGCTAAC [16146]
cp32-3_PAli_NX_MP_FG_consensus TTAAAAGAGGTGAAAATGCTTAAAAGATTGCATTGTCTGCTAATTGTTTTGTTGTTATGTTGCACCACTATTGCTAAC [16146]
cp32-3_PAli_NX_Pl_consensus    TTAAAAGAGGTGAAAATGCTTAAAAGATTGCATTGTCTGCTAATTGTTTTGTTGTTATGTTGCACCACTATTGCTAAC [16146]
cp32-3_PAbe_NX_MP_FG_consensus TTAAAAGAGGTGAAAATGCTTAAAAGATTGCATTGTCTGCTAATTGTTTTGTTGTTATGTTGCACCACTATTGCTAAC [16146]
cp32-3_PAbe_NX_Pl_consensus    TTAAAAGAGGTGAAAATGCTTAAAAGATTGCATTGTCTGCTAATTGTTTTGTTGTTATGTTGCACCACTATTGCTAAC [16146]

cp32-3_gi|11497103|B31_GB      CTACCAGAAGAGCCAAAACCTCCAATTATTCAAACACTAAAATCTTTAGCTAAATATGAAACACAACTTTCAGAGTAT [16224]
cp32-3_un8_B31_PacBio          CTACCAGAAGAGCCAAAACCTCCAATTATTCAAACACTAAAATCTTTAGCTAAATATGAAACACAACTTTCAGAGTAT [16224]
cp32-3_B31_TS_MP_FG_consensus  CTACCAGAAGAGCCAAAACCTCCAATTATTCAAACACTAAAATCTTTAGCTAAATATGAAACACAACTTTCAGAGTAT [16224]
cp32-3_B31_NX_Pl_consensus     CTACCAGAAGAGCCAAAACCTCCAATTATTCAAACACTAAAATCTTTAGCTAAATATGAAACACAACTTTCAGAGTAT [16224]
cp32-3_PAli_NX_MP_FG_consensus CTACCAGAAGAGCCAAAACCTCCAATTATTCAAACACTAAAATCTTTAGCTAAATATGAAACACAACTTTCAGAGTAT [16224]
cp32-3_PAli_NX_Pl_consensus    CTACCAGAAGAGCCAAAACCTCCAATTATTCAAACACTAAAATCTTTAGCTAAATATGAAACACAACTTTCAGAGTAT [16224]
cp32-3_PAbe_NX_MP_FG_consensus CTACCAGAAGAGCCAAAACCTCCAATTATTCAAACACTAAAATCTTTAGCTAAATATGAAACACAACTTTCAGAGTAT [16224]
cp32-3_PAbe_NX_Pl_consensus    CTACCAGAAGAGCCAAAACCTCCAATTATTCAAACACTAAAATCTTTAGCTAAATATGAAACACAACTTTCAGAGTAT [16224]

cp32-3_gi|11497103|B31_GB      GTTATGTACCTAGTAACATTTTTAGCTAAAACAAAAGTTAAAGTTAATGATCCAAATTATCCAGAATATCCTTATCCA [16302]
cp32-3_un8_B31_PacBio          GTTATGTACCTAGTAACATTTTTAGCTAAAACAAAAGTTAAAGTTAATGATCCAAATTATCCAGAATATCCTTATCCA [16302]
cp32-3_B31_TS_MP_FG_consensus  GTTATGTACCTAGTAACATTTTTAGCTAAAACAAAAGTTAAAGTTAATGATCCAAATTATCCAGAATATCCTTATCCA [16302]
cp32-3_B31_NX_Pl_consensus     GTTATGTACCTAGTAACATTTTTAGCTAAAACAAAAGTTAAAGTTAATGATCCAAATTATCCAGAATATCCTTATCCA [16302]
cp32-3_PAli_NX_MP_FG_consensus GTTATGTACCTAGTAACATTTTTAGCTAAAACAAAAGTTAAAGTTAATGATCCAAATTATCCAGAATATCCTTATCCA [16302]
cp32-3_PAli_NX_Pl_consensus    GTTATGTACCTAGTAACATTTTTAGCTAAAACAAAAGTTAAAGTTAATGATCCAAATTATCCAGAATATCCTTATCCA [16302]
cp32-3_PAbe_NX_MP_FG_consensus GTTATGTACCTAGTAACATTTTTAGCTAAAACAAAAGTTAAAGTTAATGATCCAAATTATCCAGAATATCCTTATCCA [16302]
cp32-3_PAbe_NX_Pl_consensus    GTTATGTACCTAGTAACATTTTTAGCTAAAACAAAAGTTAAAGTTAATGATCCAAATTATCCAGAATATCCTTATCCA [16302]

cp32-3_gi|11497103|B31_GB      GATTTATCAACACTAAAAGACGAACACTCCATAACTGCTGTAAAACATAATATCAACATATATTTAGAGTACATTAAA [16380]
cp32-3_un8_B31_PacBio          GATTTATCAACACTAAAAGACGAACACTCCATAACTGCTGTAAAACATAATATCAACATATATTTAGAGTACATTAAA [16380]
cp32-3_B31_TS_MP_FG_consensus  GATTTATCAACACTAAAAGACGAACACTCCATAACTGCTGTAAAACATAATATCAACATATATTTAGAGTACATTAAA [16380]
cp32-3_B31_NX_Pl_consensus     GATTTATCAACACTAAAAGACGAACACTCCATAACTGCTGTAAAACATAATATCAACATATATTTAGAGTACATTAAA [16380]
cp32-3_PAli_NX_MP_FG_consensus GATTTATCAACACTAAAAGACGAACACTCCATAACTGCTGTAAAACATAATATCAACATATATTTAGAGTACATTAAA [16380]
cp32-3_PAli_NX_Pl_consensus    GATTTATCAACACTAAAAGACGAACACTCCATAACTGCTGTAAAACATAATATCAACATATATTTAGAGTACATTAAA [16380]
cp32-3_PAbe_NX_MP_FG_consensus GATTTATCAACACTAAAAGACGAACACTCCATAACTGCTGTAAAACATAATATCAACATATATTTAGAGTACATTAAA [16380]
cp32-3_PAbe_NX_Pl_consensus    GATTTATCAACACTAAAAGACGAACACTCCATAACTGCTGTAAAACATAATATCAACATATATTTAGAGTACATTAAA [16380]

cp32-3_gi|11497103|B31_GB      AAAACAAAACCAATAGCGGAAAAAGTCTATAATAAATATTCTCAATTAAAAATGTAAATTACAAAAAGGTTTTTCTTG [16458]
cp32-3_un8_B31_PacBio          AAAACAAAACCAATAGCGGAAAAAGTCTATAATAAATATTCTCAATTAAAAATGTAAATTACAAAAAGGTTTTTCTTG [16458]
cp32-3_B31_TS_MP_FG_consensus  AAAACAAAACCAATAGCGGAAAAAGTCTATAATAAATATTCTCAATTAAAAATGTAAATTACAAAAAGGTTTTTCTTG [16458]
cp32-3_B31_NX_Pl_consensus     AAAACAAAACCAATAGCGGAAAAAGTCTATAATAAATATTCTCAATTAAAAATGTAAATTACAAAAAGGTTTTTCTTG [16458]
cp32-3_PAli_NX_MP_FG_consensus AAAACAAAACCAATAGCGGAAAAAGTCTATAATAAATATTCTCAATTAAAAATGTAAATTACAAAAAGGTTTTTCTTG [16458]
cp32-3_PAli_NX_Pl_consensus    AAAACAAAACCAATAGCGGAAAAAGTCTATAATAAATATTCTCAATTAAAAATGTAAATTACAAAAAGGTTTTTCTTG [16458]
cp32-3_PAbe_NX_MP_FG_consensus AAAACAAAACCAATAGCGGAAAAAGTCTATAATAAATATTCTCAATTAAAAATGTAAATTACAAAAAGGTTTTTCTTG [16458]
cp32-3_PAbe_NX_Pl_consensus    AAAACAAAACCAATAGCGGAAAAAGTCTATAATAAATATTCTCAATTAAAAATGTAAATTACAAAAAGGTTTTTCTTG [16458]

cp32-3_gi|11497103|B31_GB      CAAGAAATTCCCATTTATATTCTTAAAGTCGTTTATTACTGCTATATTTTAATAAAATTTTATATTTTAGGGCAAATC [16536]
cp32-3_un8_B31_PacBio          CAAGAAATTCCCATTTATATTCTTAAAGTCGTTTATTACTGCTATATTTTAATAAAATTTTATATTTTAGGGCAAATC [16536]
cp32-3_B31_TS_MP_FG_consensus  CAAGAAATTCCCATTTATATTCTTAAAGTCGTTTATTACTGCTATATTTTAATAAAATTTTATATTTTAGGGCAAATC [16536]
cp32-3_B31_NX_Pl_consensus     CAAGAAATTCCCATTTATATTCTTAAAGTCGTTTATTACTGCTATATTTTAATAAAATTTTATATTTTAGGGCAAATC [16536]
cp32-3_PAli_NX_MP_FG_consensus CAAGAAATTCCCATTTATATTCTTAAAGTCGTTTATTACTGCTATATTTTAATAAAATTTTATATTTTAGGGCAAATC [16536]
cp32-3_PAli_NX_Pl_consensus    CAAGAAATTCCCATTTATATTCTTAAAGTCGTTTATTACTGCTATATTTTAATAAAATTTTATATTTTAGGGCAAATC [16536]
cp32-3_PAbe_NX_MP_FG_consensus CAAGAAATTCCCATTTATATTCTTAAAGTCGTTTATTACTGCTATATTTTAATAAAATTTTATATTTTAGGGCAAATC [16536]
cp32-3_PAbe_NX_Pl_consensus    CAAGAAATTCCCATTTATATTCTTAAAGTCGTTTATTACTGCTATATTTTAATAAAATTTTATATTTTAGGGCAAATC [16536]

cp32-3_gi|11497103|B31_GB      TTAATAAAGATATAATATTGTTAATATTCTATTTATTAAGGAACACAATGTGCAAAAAATTAATTTATTAATGTTATT [16614]
cp32-3_un8_B31_PacBio          TTAATAAAGATATAATATTGTTAATATTCTATTTATTAAGGAACACAATGTGCAAAAAATTAATTTATTAATGTTATT [16614]
cp32-3_B31_TS_MP_FG_consensus  TTAATAAAGATATAATATTGTTAATATTCTATTTATTAAGGAACACAATGTGCAAAAAATTAATTTATTAATGTTATT [16614]
cp32-3_B31_NX_Pl_consensus     TTAATAAAGATATAATATTGTTAATATTCTATTTATTAAGGAACACAATGTGCAAAAAATTAATTTATTAATGTTATT [16614]
cp32-3_PAli_NX_MP_FG_consensus TTAATAAAGATATAATATTGTTAATATTCTATTTATTAAGGAACACAATGTGCAAAAAATTAATTTATTAATGTTATT [16614]
cp32-3_PAli_NX_Pl_consensus    TTAATAAAGATATAATATTGTTAATATTCTATTTATTAAGGAACACAATGTGCAAAAAATTAATTTATTAATGTTATT [16614]
cp32-3_PAbe_NX_MP_FG_consensus TTAATAAAGATATAATATTGTTAATATTCTATTTATTAAGGAACACAATGTGCAAAAAATTAATTTATTAATGTTATT [16614]
cp32-3_PAbe_NX_Pl_consensus    TTAATAAAGATATAATATTGTTAATATTCTATTTATTAAGGAACACAATGTGCAAAAAATTAATTTATTAATGTTATT [16614]

cp32-3_gi|11497103|B31_GB      TATGTTTATTATCAGTATTGGTAATTTAATTTCATATGAAATTAAAAAAGATGAAATTATAAAAGTTAAAACTTTAAT [16692]
cp32-3_un8_B31_PacBio          TATGTTTATTATCAGTATTGGTAATTTAATTTCATATGAAATTAAAAAAGATGAAATTATAAAAGTTAAAACTTTAAT [16692]
cp32-3_B31_TS_MP_FG_consensus  TATGTTTATTATCAGTATTGGTAATTTAATTTCATATGAAATTAAAAAAGATGAAATTATAAAAGTTAAAACTTTAAT [16692]
cp32-3_B31_NX_Pl_consensus     TATGTTTATTATCAGTATTGGTAATTTAATTTCATATGAAATTAAAAAAGATGAAATTATAAAAGTTAAAACTTTAAT [16692]
cp32-3_PAli_NX_MP_FG_consensus TATGTTTATTATCAGTATTGGTAATTTAATTTCATATGAAATTAAAAAAGATGAAATTATAAAAGTTAAAACTTTAAT [16692]
cp32-3_PAli_NX_Pl_consensus    TATGTTTATTATCAGTATTGGTAATTTAATTTCATATGAAATTAAAAAAGATGAAATTATAAAAGTTAAAACTTTAAT [16692]
cp32-3_PAbe_NX_MP_FG_consensus TATGTTTATTATCAGTATTGGTAATTTAATTTCATATGAAATTAAAAAAGATGAAATTATAAAAGTTAAAACTTTAAT [16692]
cp32-3_PAbe_NX_Pl_consensus    TATGTTTATTATCAGTATTGGTAATTTAATTTCATATGAAATTAAAAAAGATGAAATTATAAAAGTTAAAACTTTAAT [16692]

cp32-3_gi|11497103|B31_GB      AGCTTTAGATCAAGGCGGTGATGATCATAAGCATAACATTAACTTTCCTATAAAAAAAGAAGATAAAAACAAAACTCT [16770]
cp32-3_un8_B31_PacBio          AGCTTTAGATCAAGGCGGTGATGATCATAAGCATAACATTAACTTTCCTATAAAAAAAGAAGATAAAAACAAAACTCT [16770]
cp32-3_B31_TS_MP_FG_consensus  AGCTTTAGATCAAGGCGGTGATGATCATAAGCATAACATTAACTTTCCTATAAAAAAAGAAGATAAAAACAAAACTCT [16770]
cp32-3_B31_NX_Pl_consensus     AGCTTTAGATCAAGGCGGTGATGATCATAAGCATAACATTAACTTTCCTATAAAAAAAGAAGATAAAAACAAAACTCT [16770]
cp32-3_PAli_NX_MP_FG_consensus AGCTTTAGATCAAGGCGGTGATGATCATAAGCATAACATTAACTTTCCTATAAAAAAAGAAGATAAAAACAAAACTCT [16770]
cp32-3_PAli_NX_Pl_consensus    AGCTTTAGATCAAGGCGGTGATGATCATAAGCATAACATTAACTTTCCTATAAAAAAAGAAGATAAAAACAAAACTCT [16770]
cp32-3_PAbe_NX_MP_FG_consensus AGCTTTAGATCAAGGCGGTGATGATCATAAGCATAACATTAACTTTCCTATAAAAAAAGAAGATAAAAACAAAACTCT [16770]
cp32-3_PAbe_NX_Pl_consensus    AGCTTTAGATCAAGGCGGTGATGATCATAAGCATAACATTAACTTTCCTATAAAAAAAGAAGATAAAAACAAAACTCT [16770]

cp32-3_gi|11497103|B31_GB      ATTCGAAATTATAGGGATAGGGTTAGAAAATTTTATTAACCAAATAGGTGCCGAAAATATTCTTGAGGTTAAATCACT [16848]
cp32-3_un8_B31_PacBio          ATTCGAAATTATAGGGATAGGGTTAGAAAATTTTATTAACCAAATAGGTGCCGAAAATATTCTTGAGGTTAAATCACT [16848]
cp32-3_B31_TS_MP_FG_consensus  ATTCGAAATTATAGGGATAGGGTTAGAAAATTTTATTAACCAAATAGGTGCCGAAAATATTCTTGAGGTTAAATCACT [16848]
cp32-3_B31_NX_Pl_consensus     ATTCGAAATTATAGGGATAGGGTTAGAAAATTTTATTAACCAAATAGGTGCCGAAAATATTCTTGAGGTTAAATCACT [16848]
cp32-3_PAli_NX_MP_FG_consensus ATTCGAAATTATAGGGATAGGGTTAGAAAATTTTATTAACCAAATAGGTGCCGAAAATATTCTTGAGGTTAAATCACT [16848]
cp32-3_PAli_NX_Pl_consensus    ATTCGAAATTATAGGGATAGGGTTAGAAAATTTTATTAACCAAATAGGTGCCGAAAATATTCTTGAGGTTAAATCACT [16848]
cp32-3_PAbe_NX_MP_FG_consensus ATTCGAAATTATAGGGATAGGGTTAGAAAATTTTATTAACCAAATAGGTGCCGAAAATATTCTTGAGGTTAAATCACT [16848]
cp32-3_PAbe_NX_Pl_consensus    ATTCGAAATTATAGGGATAGGGTTAGAAAATTTTATTAACCAAATAGGTGCCGAAAATATTCTTGAGGTTAAATCACT [16848]

cp32-3_gi|11497103|B31_GB      AAACCATTTAGTGGGTTTTGGAGTTATTGTAGTATACAAAGAAAAGAAAAAGTAATCTAAGAGTTTTTGCAATACTGT [16926]
cp32-3_un8_B31_PacBio          AAACCATTTAGTGGGTTTTGGAGTTATTGTAGTATACAAAGAAAAGAAAAAGTAATCTAAGAGTTTTTGCAATACTGT [16926]
cp32-3_B31_TS_MP_FG_consensus  AAACCATTTAGTGGGTTTTGGAGTTATTGTAGTATACAAAGAAAAGAAAAAGTAATCTAAGAGTTTTTGCAATACTGT [16926]
cp32-3_B31_NX_Pl_consensus     AAACCATTTAGTGGGTTTTGGAGTTATTGTAGTATACAAAGAAAAGAAAAAGTAATCTAAGAGTTTTTGCAATACTGT [16926]
cp32-3_PAli_NX_MP_FG_consensus AAACCATTTAGTGGGTTTTGGAGTTATTGTAGTATACAAAGAAAAGAAAAAGTAATCTAAGAGTTTTTGCAATACTGT [16926]
cp32-3_PAli_NX_Pl_consensus    AAACCATTTAGTGGGTTTTGGAGTTATTGTAGTATACAAAGAAAAGAAAAAGTAATCTAAGAGTTTTTGCAATACTGT [16926]
cp32-3_PAbe_NX_MP_FG_consensus AAACCATTTAGTGGGTTTTGGAGTTATTGTAGTATACAAAGAAAAGAAAAAGTAATCTAAGAGTTTTTGCAATACTGT [16926]
cp32-3_PAbe_NX_Pl_consensus    AAACCATTTAGTGGGTTTTGGAGTTATTGTAGTATACAAAGAAAAGAAAAAGTAATCTAAGAGTTTTTGCAATACTGT [16926]

cp32-3_gi|11497103|B31_GB      TTTTGTAAAAAATCTACAGTAAAAAATTTAAAACTGATATCTGAAGTTAAAAATGTAAATTACAAAAAGGTTTTTCTT [17004]
```

```
cp32-3_un8_B31_PacBio          TTTTGTAAAAAATCTACAGTAAAAAATTTAAAACTGATATCTGAAGTTAAAAATGTAAATTACAAAAAGGTTTTTCTT [17004]
cp32-3_B31_TS_MP_FG_consensus  TTTTGTAAAAAATCTACAGTAAAAAATTTAAAACTGATATCTGAAGTTAAAAATGTAAATTACAAAAAGGTTTTTCTT [17004]
cp32-3_B31_NX_Pl_consensus     TTTTGTAAAAAATCTACAGTAAAAAATTTAAAACTGATATCTGAAGTTAAAAATGTAAATTACAAAAAGGTTTTTCTT [17004]
cp32-3_PAli_NX_MP_FG_consensus TTTTGTAAAAAATCTACAGTAAAAAATTTAAAACTGATATCTGAAGTTAAAAATGTAAATTACAAAAAGGTTTTTCTT [17004]
cp32-3_PAli_NX_Pl_consensus    TTTTGTAAAAAATCTACAGTAAAAAATTTAAAACTGATATCTGAAGTTAAAAATGTAAATTACAAAAAGGTTTTTCTT [17004]
cp32-3_PAbe_NX_MP_FG_consensus TTTTGTAAAAAATCTACAGTAAAAAATTTAAAACTGATATCTGAAGTTAAAAATGTAAATTACAAAAAGGTTTTTCTT [17004]
cp32-3_PAbe_NX_Pl_consensus    TTTTGTAAAAAATCTACAGTAAAAAATTTAAAACTGATATCTGAAGTTAAAAATGTAAATTACAAAAAGGTTTTTCTT [17004]

cp32-3_gi|11497103|B31_GB      GCAAGAAATTCTACTTTACATATAAAAATCTCTAAAGCCAATTAATCTAAAATAGTATATAATATGACCATAAGGGAA [17082]
cp32-3_un8_B31_PacBio          GCAAGAAATTCTACTTTACATATAAAAATCTCTAAAGCCAATTAATCTAAAATAGTATATAATATGACCATAAGGGAA [17082]
cp32-3_B31_TS_MP_FG_consensus  GCAAGAAATTCTACTTTACATATAAAAATCTCTAAAGCCAATTAATCTAAAATAGTATATAATATGACCATAAGGGAA [17082]
cp32-3_B31_NX_Pl_consensus     GCAAGAAATTCTACTTTACATATAAAAATCTCTAAAGCCAATTAATCTAAAATAGTATATAATATGACCATAAGGGAA [17082]
cp32-3_PAli_NX_MP_FG_consensus GCAAGAAATTCTACTTTACATATAAAAATCTCTAAAGCCAATTAATCTAAAATAGTATATAATATGACCATAAGGGAA [17082]
cp32-3_PAli_NX_Pl_consensus    GCAAGAAATTCTACTTTACATATAAAAATCTCTAAAGCCAATTAATCTAAAATAGTATATAATATGACCATAAGGGAA [17082]
cp32-3_PAbe_NX_MP_FG_consensus GCAAGAAATTCTACTTTACATATAAAAATCTCTAAAGCCAATTAATCTAAAATAGTATATAATATGACCATAAGGGAA [17082]
cp32-3_PAbe_NX_Pl_consensus    GCAAGAAATTCTACTTTACATATAAAAATCTCTAAAGCCAATTAATCTAAAATAGTATATAATATGACCATAAGGGAA [17082]

cp32-3_gi|11497103|B31_GB      AATTTTATGGAAACAGTGTCAACAAATATTGCAAGTGTAACTCAAGAACAAATATATAAAGAATTTATTAGACTGGGC [17160]
cp32-3_un8_B31_PacBio          AATTTTATGGAAACAGTGTCAACAAATATTGCAAGTGTAACTCAAGAACAAATATATAAAGAATTTATTAGACTGGGC [17160]
cp32-3_B31_TS_MP_FG_consensus  AATTTTATGGAAACAGTGTCAACAAATATTGCAAGTGTAACTCAAGAACAAATATATAAAGAATTTATTAGACTGGGC [17160]
cp32-3_B31_NX_Pl_consensus     AATTTTATGGAAACAGTGTCAACAAATATTGCAAGTGTAACTCAAGAACAAATATATAAAGAATTTATTAGACTGGGC [17160]
cp32-3_PAli_NX_MP_FG_consensus AATTTTATGGAAACAGTGTCAACAAATATTGCAAGTGTAACTCAAGAACAAATATATAAAGAATTTATTAGACTGGGC [17160]
cp32-3_PAli_NX_Pl_consensus    AATTTTATGGAAACAGTGTCAACAAATATTGCAAGTGTAACTCAAGAACAAATATATAAAGAATTTATTAGACTGGGC [17160]
cp32-3_PAbe_NX_MP_FG_consensus AATTTTATGGAAACAGTGTCAACAAATATTGCAAGTGTAACTCAAGAACAAATATATAAAGAATTTATTAGACTGGGC [17160]
cp32-3_PAbe_NX_Pl_consensus    AATTTTATGGAAACAGTGTCAACAAATATTGCAAGTGTAACTCAAGAACAAATATATAAAGAATTTATTAGACTGGGC [17160]

cp32-3_gi|11497103|B31_GB      ATGGAACAATTAATAGCACAAGATTTATCTAAAAGATATTATCACAATGAACTAACATATAGAGATTTAGAAAATTTA [17238]
cp32-3_un8_B31_PacBio          ATGGAACAATTAATAGCACAAGATTTATCTAAAAGATATTATCACAATGAACTAACATATAGAGATTTAGAAAATTTA [17238]
cp32-3_B31_TS_MP_FG_consensus  ATGGAACAATTAATAGCACAAGATTTATCTAAAAGATATTATCACAATGAACTAACATATAGAGATTTAGAAAATTTA [17238]
cp32-3_B31_NX_Pl_consensus     ATGGAACAATTAATAGCACAAGATTTATCTAAAAGATATTATCACAATGAACTAACATATAGAGATTTAGAAAATTTA [17238]
cp32-3_PAli_NX_MP_FG_consensus ATGGAACAATTAATAGCACAAGATTTATCTAAAAGATATTATCACAATGAACTAACATATAGAGATTTAGAAAATTTA [17238]
cp32-3_PAli_NX_Pl_consensus    ATGGAACAATTAATAGCACAAGATTTATCTAAAAGATATTATCACAATGAACTAACATATAGAGATTTAGAAAATTTA [17238]
cp32-3_PAbe_NX_MP_FG_consensus ATGGAACAATTAATAGCACAAGATTTATCTAAAAGATATTATCACAATGAACTAACATATAGAGATTTAGAAAATTTA [17238]
cp32-3_PAbe_NX_Pl_consensus    ATGGAACAATTAATAGCACAAGATTTATCTAAAAGATATTATCACAATGAACTAACATATAGAGATTTAGAAAATTTA [17238]

cp32-3_gi|11497103|B31_GB      GAAAAACAATTTGATATAAAATTTGATAATCTTGTTTCTAAAATAGATACTGTAGAAAAGAATTTAAATGTTAAGATT [17316]
cp32-3_un8_B31_PacBio          GAAAAACAATTTGATATAAAATTTGATAATCTTGTTTCTAAAATAGATACTGTAGAAAAGAATTTAAATGTTAAGATT [17316]
cp32-3_B31_TS_MP_FG_consensus  GAAAAACAATTTGATATAAAATTTGATAATCTTGTTTCTAAAATAGATACTGTAGAAAAGAATTTAAATGTTAAGATT [17316]
cp32-3_B31_NX_Pl_consensus     GAAAAACAATTTGATATAAAATTTGATAATCTTGTTTCTAAAATAGATACTGTAGAAAAGAATTTAAATGTTAAGATT [17316]
cp32-3_PAli_NX_MP_FG_consensus GAAAAACAATTTGATATAAAATTTGATAATCTTGTTTCTAAAATAGATACTGTAGAAAAGAATTTAAATGTTAAGATT [17316]
cp32-3_PAli_NX_Pl_consensus    GAAAAACAATTTGATATAAAATTTGATAATCTTGTTTCTAAAATAGATACTGTAGAAAAGAATTTAAATGTTAAGATT [17316]
cp32-3_PAbe_NX_MP_FG_consensus GAAAAACAATTTGATATAAAATTTGATAATCTTGTTTCTAAAATAGATACTGTAGAAAAGAATTTAAATGTTAAGATT [17316]
cp32-3_PAbe_NX_Pl_consensus    GAAAAACAATTTGATATAAAATTTGATAATCTTGTTTCTAAAATAGATACTGTAGAAAAGAATTTAAATGTTAAGATT [17316]

cp32-3_gi|11497103|B31_GB      GATGCTGTTAAAAGTGAACTTAATACCAAGATAGATAATGTAGAAAAGAATTTACAAAAAGATATATCTAATTTAGAT [17394]
cp32-3_un8_B31_PacBio          GATGCTGTTAAAAGTGAACTTAATACCAAGATAGATAATGTAGAAAAGAATTTACAAAAA-ATATATCTAATTTAGAT [17394]
cp32-3_B31_TS_MP_FG_consensus  GATGCTGTTAAAAGTGAACTTAATACCAAGATAGATAATGTAGAAAAGAATTTACAAAAAGATATATCTAATTTAGAT [17394]
cp32-3_B31_NX_Pl_consensus     GATGCTGTTAAAAGTGAACTTAATACCAAGATAGATAATGTAGAAAAGAATTTACAAAAAGATATATCTAATTTAGAT [17394]
cp32-3_PAli_NX_MP_FG_consensus GATGCTGTTAAAAGTGAACTTAATACCAAGATAGATAATGTAGAAAAGAATTTACAAAAAGATATATCTAATTTAGAT [17394]
cp32-3_PAli_NX_Pl_consensus    GATGCTGTTAAAAGTGAACTTAATACCAAGATAGATAATGTAGAAAAGAATTTACAAAAAGATATATCTAATTTAGAT [17394]
cp32-3_PAbe_NX_MP_FG_consensus GATGCTGTTAAAAGTGAACTTAATACCAAGATAGATAATGTAGAAAAGAATTTACAAAAAGATATATCTAATTTAGAT [17394]
cp32-3_PAbe_NX_Pl_consensus    GATGCTGTTAAAAGTGAACTTAATACCAAGATAGATAATGTAGAAAAGAATTTACAAAAAGATATATCTAATTTAGAT [17394]

cp32-3_gi|11497103|B31_GB      ACTAAGATTGATAATGTAGAGAAGAATTTACAAAAAGATATATCTAATTTAGATACTAAGATTGATAATGTAGAGAAG [17472]
cp32-3_un8_B31_PacBio          ACTAAGA----TAATGTAGAGAAGAATTTACAAAAAGATATATCTAATTTAGATACTAAGATTGATAATGTAGAGAAG [17472]
cp32-3_B31_TS_MP_FG_consensus  ACTAAGATTGATAATGTAGAGAAGAATTTACAAAAAGATATATCTAATTTAGATACTAAGATTGATAATGTAGAGAAG [17472]
cp32-3_B31_NX_Pl_consensus     ACTAAGATTGATAATGTAGAGAAGAATTTACAAAAAGATATATCTAATTTAGATACTAAGATTGATAATGTAGAGAAG [17472]
cp32-3_PAli_NX_MP_FG_consensus ACTAAGATTGATAATGTAGAGAAGAATTTACAAAAAGATATATCTAATTTAGATACTAAGATTGATAATGTAGAGAAG [17472]
cp32-3_PAli_NX_Pl_consensus    ACTAAGATTGATAATGTAGAGAAGAATTTACAAAAAGATATATCTAATTTAGATACTAAGATTGATAATGTAGAGAAG [17472]
cp32-3_PAbe_NX_MP_FG_consensus ACTAAGATTGATAATGTAGAGAAGAATTTACAAAAAGATATATCTAATTTAGATACTAAGATTGATAATGTAGAGAAG [17472]
cp32-3_PAbe_NX_Pl_consensus    ACTAAGATTGATAATGTAGAGAAGAATTTACAAAAAGATATATCTAATTTAGATACTAAGATTGATAATGTAGAGAAG [17472]

cp32-3_gi|11497103|B31_GB      AATTTACAAAAAGATATATCTAATTTAGATACTAAGATTGATAATGTAGAGAAGAATTTAAATGCCAAAATAGATACT [17550]
cp32-3_un8_B31_PacBio          AATTTACAAAAAGATATATCTAATTTAGATACTAAGATTGATAATGTAGAGAAGAATTTAAATGCCAAAATAGATACT [17550]
cp32-3_B31_TS_MP_FG_consensus  AATTTACAAAAAGATATATCTAATTTAGATACTAAGATTGATAATGTAGAGAAGAATTTAAATGCCAAAATAGATACT [17550]
cp32-3_B31_NX_Pl_consensus     AATTTACAAAAAGATATATCTAATTTAGATACTAAGATTGATAATGTAGAGAAGAATTTAAATGCCAAAATAGATACT [17550]
cp32-3_PAli_NX_MP_FG_consensus AATTTACAAAAAGATATATCTAATTTAGATACTAAGATTGATAATGTAGAGAAGAATTTAAATGCCAAAATAGATACT [17550]
cp32-3_PAli_NX_Pl_consensus    AATTTACAAAAAGATATATCTAATTTAGATACTAAGATTGATAATGTAGAGAAGAATTTAAATGCCAAAATAGATACT [17550]
cp32-3_PAbe_NX_MP_FG_consensus AATTTACAAAAAGATATATCTAATTTAGATACTAAGATTGATAATGTAGAGAAGAATTTAAATGCCAAAATAGATACT [17550]
cp32-3_PAbe_NX_Pl_consensus    AATTTACAAAAAGATATATCTAATTTAGATACTAAGATTGATAATGTAGAGAAGAATTTAAATGCCAAAATAGATACT [17550]

cp32-3_gi|11497103|B31_GB      GTAGAAAAGAATTTAAATGCCAAAATAGACAATGTTGAAAAGAATTTAATGTCTCTTTCAGAAATGCTTAAATGGGTA [17628]
cp32-3_un8_B31_PacBio          GTAGAAAAGAATTTAAATGCCAAAATAGACAATGTTGAAAAGAATTTAATGTCTCTTTCAGAAATGCTTAAATGGGTA [17628]
cp32-3_B31_TS_MP_FG_consensus  GTAGAAAAGAATTTAAATGCCAAAATAGACAATGTTGAAAAGAATTTAATGTCTCTTTCAGAAATGCTTAAATGGGTA [17628]
cp32-3_B31_NX_Pl_consensus     GTAGAAAAGAATTTAAATGCCAAAATAGACAATGTTGAAAAGAATTTAATGTCTCTTTCAGAAATGCTTAAATGGGTA [17628]
cp32-3_PAli_NX_MP_FG_consensus GTAGAAAAGAATTTAAATGCCAAAATAGACAATGTTGAAAAGAATTTAATGTCTCTTTCAGAAATGCTTAAATGGGTA [17628]
cp32-3_PAli_NX_Pl_consensus    GTAGAAAAGAATTTAAATGCCAAAATAGACAATGTTGAAAAGAATTTAATGTCTCTTTCAGAAATGCTTAAATGGGTA [17628]
cp32-3_PAbe_NX_MP_FG_consensus GTAGAAAAGAATTTAAATGCCAAAATAGACAATGTTGAAAAGAATTTAATGTCTCTTTCAGAAATGCTTAAATGGGTA [17628]
cp32-3_PAbe_NX_Pl_consensus    GTAGAAAAGAATTTAAATGCCAAAATAGACAATGTTGAAAAGAATTTAATGTCTCTTTCAGAAATGCTTAAATGGGTA [17628]

cp32-3_gi|11497103|B31_GB      TTGGGAATTATGGGGGCAATGTCTATCACAATGATAGCCGGGCTAATATTTGCTTTCATTTCTAAATAGCTACTACCT [17706]
cp32-3_un8_B31_PacBio          TTGGGAATTATGGGGGCAATGTCTATCACAATGATAGCCGGGCTAATATTTGCTTTCATTTCTAAATAGCTACTACCT [17706]
cp32-3_B31_TS_MP_FG_consensus  TTGGGAATTATGGGGGCAATGTCTATCACAATGATAGCCGGGCTAATATTTGCTTTCATTTCTAAATAGCTACTACCT [17706]
cp32-3_B31_NX_Pl_consensus     TTGGGAATTATGGGGGCAATGTCTATCACAATGATAGCCGGGCTAATATTTGCTTTCATTTCTAAATAGCTACTACCT [17706]
cp32-3_PAli_NX_MP_FG_consensus TTGGGAATTATGGGGGCAATGTCTATCACAATGATAGCCGGGCTAATATTTGCTTTCATTTCTAAATAGCTACTACCT [17706]
cp32-3_PAli_NX_Pl_consensus    TTGGGAATTATGGGGGCAATGTCTATCACAATGATAGCCGGGCTAATATTTGCTTTCATTTCTAAATAGCTACTACCT [17706]
cp32-3_PAbe_NX_MP_FG_consensus TTGGGAATTATGGGGGCAATGTCTATCACAATGATAGCCGGGCTAATATTTGCTTTCATTTCTAAATAGCTACTACCT [17706]
cp32-3_PAbe_NX_Pl_consensus    TTGGGAATTATGGGGGCAATGTCTATCACAATGATAGCCGGGCTAATATTTGCTTTCATTTCTAAATAGCTACTACCT [17706]

cp32-3_gi|11497103|B31_GB      CACTTAAAGAATATCAATTCAAATAATAATTGTTTATCCAATATTTAGTATTAATACTTAGTTAAGTGAATATTAATA [17784]
cp32-3_un8_B31_PacBio          CACTTAAAGAATATCAATTCAAATAATAATTGTTTATCCAATATTTAGTATTAATACTTAGTTAAGTGAATATTAATA [17784]
cp32-3_B31_TS_MP_FG_consensus  CACTTAAAGAATATCAATTCAAATAATAATTGTTTATCCAATATTTAGTATTAATACTTAGTTAAGTGAATATTAATA [17784]
cp32-3_B31_NX_Pl_consensus     CACTTAAAGAATATCAATTCAAATAATAATTGTTTATCCAATATTTAGTATTAATACTTAGTTAAGTGAATATTAATA [17784]
cp32-3_PAli_NX_MP_FG_consensus CACTTAAAGAATATCAATTCAAATAATAATTGTTTATCCAATATTTAGTATTAATACTTAGTTAAGTGAATATTAATA [17784]
cp32-3_PAli_NX_Pl_consensus    CACTTAAAGAATATCAATTCAAATAATAATTGTTTATCCAATATTTAGTATTAATACTTAGTTAAGTGAATATTAATA [17784]
cp32-3_PAbe_NX_MP_FG_consensus CACTTAAAGAATATCAATTCAAATAATAATTGTTTATCCAATATTTAGTATTAATACTTAGTTAAGTGAATATTAATA [17784]
cp32-3_PAbe_NX_Pl_consensus    CACTTAAAGAATATCAATTCAAATAATAATTGTTTATCCAATATTTAGTATTAATACTTAGTTAAGTGAATATTAATA [17784]

cp32-3_gi|11497103|B31_GB      ATAAAAAAAGGAGTAACAATGAAAATCATCAACATATTATTTTGTTTATTTTTACTAATGCTAAACGGCTGTAATTCT [17862]
cp32-3_un8_B31_PacBio          ATAAAAAAAGGAGTAACAATGAAAATCATCAACATATTATTTTGTTTATTTTTACTAATGCTAAACGGCTGTAATTCT [17862]
cp32-3_B31_TS_MP_FG_consensus  ATAAAAAAAGGAGTAACAATGAAAATCATCAACATATTATTTTGTTTATTTTTACTAATGCTAAACGGCTGTAATTCT [17862]
cp32-3_B31_NX_Pl_consensus     ATAAAAAAAGGAGTAACAATGAAAATCATCAACATATTATTTTGTTTATTTTTACTAATGCTAAACGGCTGTAATTCT [17862]
cp32-3_PAli_NX_MP_FG_consensus ATAAAAAAAGGAGTAACAATGAAAATCATCAACATATTATTTTGTTTATTTTTACTAATGCTAAACGGCTGTAATTCT [17862]
cp32-3_PAli_NX_Pl_consensus    ATAAAAAAAGGAGTAACAATGAAAATCATCAACATATTATTTTGTTTATTTTTACTAATGCTAAACGGCTGTAATTCT [17862]
cp32-3_PAbe_NX_MP_FG_consensus ATAAAAAAAGGAGTAACAATGAAAATCATCAACATATTATTTTGTTTATTTTTACTAATGCTAAACGGCTGTAATTCT [17862]
cp32-3_PAbe_NX_Pl_consensus    ATAAAAAAAGGAGTAACAATGAAAATCATCAACATATTATTTTGTTTATTTTTACTAATGCTAAACGGCTGTAATTCT [17862]

cp32-3_gi|11497103|B31_GB      AATGATAATGACACTTTAAAAAACAATGCCCAACAAACAAAAAGACGGGGAAAGCGTGATTTAACCCAAAAAGAAACA [17940]
cp32-3_un8_B31_PacBio          AATGATAATGACACTTTAAAAAACAATGCCCAACAAACAAAAAGACGGGGAAAGCGTGATTTAACCCAAAAAGAAACA [17940]
```

```
cp32-3_B31_TS_MP_FG_consensus  AATGATAATGACACTTTAAAAAACAATGCCCAACAAACAAAAAGACGGGGAAAGCGTGATTTAACCCAAAAAGAAACA [17940]
cp32-3_B31_NX_Pl_consensus     AATGATAATGACACTTTAAAAAACAATGCCCAACAAACAAAAAGACGGGGAAAGCGTGATTTAACCCAAAAAGAAACA [17940]
cp32-3_PAli_NX_MP_FG_consensus AATGATAATGACACTTTAAAAAACAATGCCCAACAAACAAAAAGACGGGGAAAGCGTGATTTAACCCAAAAAGAAACA [17940]
cp32-3_PAli_NX_Pl_consensus    AATGATAATGACACTTTAAAAAACAATGCCCAACAAACAAAAAGACGGGGAAAGCGTGATTTAACCCAAAAAGAAACA [17940]
cp32-3_PAbe_NX_MP_FG_consensus AATGATAATGACACTTTAAAAAACAATGCCCAACAAACAAAAAGACGGGGAAAGCGTGATTTAACCCAAAAAGAAACA [17940]
cp32-3_PAbe_NX_Pl_consensus    AATGATAATGACACTTTAAAAAACAATGCCCAACAAACAAAAAGACGGGGAAAGCGTGATTTAACCCAAAAAGAAACA [17940]

cp32-3_gi|11497103|B31_GB      ACACAAGAAAAACCAAAATCTAAAGAAGAACTACTTAGAGAAAAGCTATCTGACGATCAAAAAACACATCTTGACTGG [18018]
cp32-3_un8_B31_PacBio          ACACAAGAAAAACCAAAATCTAAAGAAGAACTACTTAGAGAAAAGCTATCTGACGATCAAAAAACACATCTTGACTGG [18018]
cp32-3_B31_TS_MP_FG_consensus  ACACAAGAAAAACCAAAATCTAAAGAAGAACTACTTAGAGAAAAGCTATCTGACGATCAAAAAACACATCTTGACTGG [18018]
cp32-3_B31_NX_Pl_consensus     ACACAAGAAAAACCAAAATCTAAAGAAGAACTACTTAGAGAAAAGCTATCTGACGATCAAAAAACACATCTTGACTGG [18018]
cp32-3_PAli_NX_MP_FG_consensus ACACAAGAAAAACCAAAATCTAAAGAAGAACTACTTAGAGAAAAGCTATCTGACGATCAAAAAACACATCTTAACTGG [18018]
cp32-3_PAli_NX_Pl_consensus    ACACAAGAAAAACCAAAATCTAAAGAAGAACTACTTAGAGAAAAGCTATCTGACGATCAAAAAACACATCTTAACTGG [18018]
cp32-3_PAbe_NX_MP_FG_consensus ACACAAGAAAAACCAAAATCTAAAGAAGAACTACTTAGAGAAAAGCTATCTGACGATCAAAAAACACATCTTGACTGG [18018]
cp32-3_PAbe_NX_Pl_consensus    ACACAAGAAAAACCAAAATCTAAAGAAGAACTACTTAGAGAAAAGCTATCTGACGATCAAAAAACACATCTTGACTGG [18018]

cp32-3_gi|11497103|B31_GB      TTAAAACCCGCTTTAACTGGTGCTGGAGAATTTGACAAATTCTTAGAAAATGATGATGATAAAATAAAATCAGCACTT [18096]
cp32-3_un8_B31_PacBio          TTAAAACCCGCTTTAACTGGTGCTGGAGAATTTGACAAATTCTTAGAAAATGATGATGATAAAATAAAATCAGCACTT [18096]
cp32-3_B31_TS_MP_FG_consensus  TTAAAACCCGCTTTAACTGGTGCTGGAGAATTTGACAAATTCTTAGAAAATGATGATGATAAAATAAAATCAGCACTT [18096]
cp32-3_B31_NX_Pl_consensus     TTAAAACCCGCTTTAACTGGTGCTGGAGAATTTGACAAATTCTTAGAAAATGATGATGATAAAATAAAATCAGCACTT [18096]
cp32-3_PAli_NX_MP_FG_consensus TTAAAACCCGCTTTAACTGGTGCTGGAGAATTTGACAAATTCTTAGAAAATGATGATGATAAAATAAAATCAGCACTT [18096]
cp32-3_PAli_NX_Pl_consensus    TTAAAACCCGCTTTAACTGGTGCTGGAGAATTTGACAAATTCTTAGAAAATGATGATGATAAAATAAAATCAGCACTT [18096]
cp32-3_PAbe_NX_MP_FG_consensus TTAAAACCCGCTTTAACTGGTGCTGGAGAATTTGACAAATTCTTAGAAAATGATGATGATAAAATAAAATCAGCACTT [18096]
cp32-3_PAbe_NX_Pl_consensus    TTAAAACCCGCTTTAACTGGTGCTGGAGAATTTGACAAATTCTTAGAAAATGATGATGATAAAATAAAATCAGCACTT [18096]

cp32-3_gi|11497103|B31_GB      GATCATATAAAAACTCAACTTGATAGTTGTAATGGTGATCAAGCAGAACAACAAAAAACCACTTTCAAAACTGTGGTT [18174]
cp32-3_un8_B31_PacBio          GATCATATAAAAACTCAACTTGATAGTTGTAATGGTGATCAAGCAGAACAACAAAAAACCACTTTCAAAACTGTGGTT [18174]
cp32-3_B31_TS_MP_FG_consensus  GATCATATAAAAACTCAACTTGATAGTTGTAATGGTGATCAAGCAGAACAACAAAAAACCACTTTCAAAACTGTGGTT [18174]
cp32-3_B31_NX_Pl_consensus     GATCATATAAAAACTCAACTTGATAGTTGTAATGGTGATCAAGCAGAACAACAAAAAACCACTTTCAAAACTGTGGTT [18174]
cp32-3_PAli_NX_MP_FG_consensus GATCATATAAAAACTCAACTTGATAGTTGTAATGGTGATCAAGCAGAACAACAAAAAACCACTTTCAAAACTGTGGTT [18174]
cp32-3_PAli_NX_Pl_consensus    GATCATATAAAAACTCAACTTGATAGTTGTAATGGTGATCAAGCAGAACAACAAAAAACCACTTTCAAAACTGTGGTT [18174]
cp32-3_PAbe_NX_MP_FG_consensus GATCATATAAAAACTCAACTTGATAGTTGTAATGGTGATCAAGCAGAACAACAAAAAACCACTTTCAAAACTGTGGTT [18174]
cp32-3_PAbe_NX_Pl_consensus    GATCATATAAAAACTCAACTTGATAGTTGTAATGGTGATCAAGCAGAACAACAAAAAACCACTTTCAAAACTGTGGTT [18174]

cp32-3_gi|11497103|B31_GB      ACAGAATTCTTTAAAAATGGTGATATAGATAATTTTGCAACTGGAGCGGTTAGTAACTGCAATAATGGTGGCTAATAA [18252]
cp32-3_un8_B31_PacBio          ACAGAATTCTTTAAAAATGGTGATATAGATAATTTTGCAACTGGAGCGGTTAGTAACTGCAATAATGGTGGCTAATAA [18252]
cp32-3_B31_TS_MP_FG_consensus  ACAGAATTCTTTAAAAATGGTGATATAGATAATTTTGCAACTGGAGCGGTTAGTAACTGCAATAATGGTGGCTAATAA [18252]
cp32-3_B31_NX_Pl_consensus     ACAGAATTCTTTAAAAATGGTGATATAGATAATTTTGCAACTGGAGCGGTTAGTAACTGCAATAATGGTGGCTAATAA [18252]
cp32-3_PAli_NX_MP_FG_consensus ACAGAATTCTTTAAAAATGGTGATATAGATAATTTTGCAACTGGAGCGGTTAGTAACTGCAATAATGGTGGCTAATAA [18252]
cp32-3_PAli_NX_Pl_consensus    ACAGAATTCTTTAAAAATGGTGATATAGATAATTTTGCAACTGGAGCGGTTAGTAACTGCAATAATGGTGGCTAATAA [18252]
cp32-3_PAbe_NX_MP_FG_consensus ACAGAATTCTTTAAAAATGGTGATATAGATAATTTTGCAACTGGAGCGGTTAGTAACTGCAATAATGGTGGCTAATAA [18252]
cp32-3_PAbe_NX_Pl_consensus    ACAGAATTCTTTAAAAATGGTGATATAGATAATTTTGCAACTGGAGCGGTTAGTAACTGCAATAATGGTGGCTAATAA [18252]

cp32-3_gi|11497103|B31_GB      TCTAACAGCCCCCTATTTGGGGGCTTTAATATTGCTACACTGCAAATATATAATTAAATTGTCTTTCTTTTTTTAACA [18330]
cp32-3_un8_B31_PacBio          TCTAACAGCCCCCTATTTGGGGGCTTTAATATTGCTACACTGCAAATATATAATTAAATTGTCTTTCTTTTTTTAACA [18330]
cp32-3_B31_TS_MP_FG_consensus  TCTAACAGCCCCCTATTTGGGGGCTTTAATATTGCTACACTGCAAATATATAATTAAATTGTCTTTCTTTTTTTAACA [18330]
cp32-3_B31_NX_Pl_consensus     TCTAACAGCCCCCTATTTGGGGGCTTTAATATTGCTACACTGCAAATATATAATTAAATTGTCTTTCTTTTTTTAACA [18330]
cp32-3_PAli_NX_MP_FG_consensus TCTAACAGCCCCCTATTTGGGGGCTTTAATATTGCTACACTGCAAATATATAATTAAATTGTCTTTCTTTTTTTAACA [18330]
cp32-3_PAli_NX_Pl_consensus    TCTAACAGCCCCCTATTTGGGGGCTTTAATATTGCTACACTGCAAATATATAATTAAATTGTCTTTCTTTTTTTAACA [18330]
cp32-3_PAbe_NX_MP_FG_consensus TCTAACAGCCCCCTATTTGGGGGCTTTAATATTGCTACACTGCAAATATATAATTAAATTGTCTTTCTTTTTTTAACA [18330]
cp32-3_PAbe_NX_Pl_consensus    TCTAACAGCCCCCTATTTGGGGGCTTTAATATTGCTACACTGCAAATATATAATTAAATTGTCTTTCTTTTTTTAACA [18330]

cp32-3_gi|11497103|B31_GB      TGCAAAAGAATTTTAATACTTTTGTTTTATAAACATCCCGATTATCAGACATAAATACTTTAAGCTTATTTAGATCAT [18408]
cp32-3_un8_B31_PacBio          TGCAAAAGAATTTTAATACTTTTGTTTTATAAACATCCCGATTATCAGACATAAATACTTTAAGCTTATTTAGATCAT [18408]
cp32-3_B31_TS_MP_FG_consensus  TGCAAAAGAATTTTAATACTTTTGTTTTATAAACATCCCGATTATCAGACATAAATACTTTAAGCTTATTTAGATCAT [18408]
cp32-3_B31_NX_Pl_consensus     TGCAAAAGAATTTTAATACTTTTGTTTTATAAACATCCCGATTATCAGACATAAATACTTTAAGCTTATTTAGATCAT [18408]
cp32-3_PAli_NX_MP_FG_consensus TGCAAAAGAATTTTAATACTTTTGTTTTATAAACATCCCGATTATCAGACATAAATACTTTAAGCTTATTTAGATCAT [18408]
cp32-3_PAli_NX_Pl_consensus    TGCAAAAGAATTTTAATACTTTTGTTTTATAAACATCCCGATTATCAGACATAAATACTTTAAGCTTATTTAGATCAT [18408]
cp32-3_PAbe_NX_MP_FG_consensus TGCAAAAGAATTTTAATACTTTTGTTTTATAAACATCCCGATTATCAGACATAAATACTTTAAGCTTATTTAGATCAT [18408]
cp32-3_PAbe_NX_Pl_consensus    TGCAAAAGAATTTTAATACTTTTGTTTTATAAACATCCCGATTATCAGACATAAATACTTTAAGCTTATTTAGATCAT [18408]

cp32-3_gi|11497103|B31_GB      CAAATCTATTGGTTTTATTGAAATATTTTTTTAGAATATTTACCCAATATTCTATACTCTTAAGCTCAGCATTCTTTT [18486]
cp32-3_un8_B31_PacBio          CAAATCTATTGGTTTTATTGAAATATTTTTTTAGAATATTTACCCAATATTCTATACTCTTAAGCTCAGCATTCTTTT [18486]
cp32-3_B31_TS_MP_FG_consensus  CAAATCTATTGGTTTTATTGAAATATTTTTTTAGAATATTTACCCAATATTCTATACTCTTAAGCTCAGCATTCTTTT [18486]
cp32-3_B31_NX_Pl_consensus     CAAATCTATTGGTTTTATTGAAATATTTTTTTAGAATATTTACCCAATATTCTATACTCTTAAGCTCAGCATTCTTTT [18486]
cp32-3_PAli_NX_MP_FG_consensus CAAATCTATTGGTTTTATTGAAATATTTTTTTAGAATATTTACCCAATATTCTATACTCTTAAGCTCAGCATTCTTTT [18486]
cp32-3_PAli_NX_Pl_consensus    CAAATCTATTGGTTTTATTGAAATATTTTTTTAGAATATTTACCCAATATTCTATACTCTTAAGCTCAGCATTCTTTT [18486]
cp32-3_PAbe_NX_MP_FG_consensus CAAATCTATTGGTTTTATTGAAATATTTTTTTAGAATATTTACCCAATATTCTATACTCTTAAGCTCAGCATTCTTTT [18486]
cp32-3_PAbe_NX_Pl_consensus    CAAATCTATTGGTTTTATTGAAATATTTTTTTAGAATATTTACCCAATATTCTATACTCTTAAGCTCAGCATTCTTTT [18486]

cp32-3_gi|11497103|B31_GB      CAAAAAGATCCTCTAAAGCATCATCATTATCAGTACATAATGCCCGAATAAGAGCATTTATATTATTTTTTTCTTCAC [18564]
cp32-3_un8_B31_PacBio          CAAAAAGATCCTCTAAAGCATCATCATTATCAGTACATAATGCCCGAATAAGAGCATTTATATTATTTTTTTCTTCAC [18564]
cp32-3_B31_TS_MP_FG_consensus  CAAAAAGATCCTCTAAAGCATCATCATTATCAGTACATAATGCCCGAATAAGAGCATTTATATTATTTTTTTCTTCAC [18564]
cp32-3_B31_NX_Pl_consensus     CAAAAAGATCCTCTAAAGCATCATCATTATCAGTACATAATGCCCGAATAAGAGCATTTATATTATTTTTTTCTTCAC [18564]
cp32-3_PAli_NX_MP_FG_consensus CAAAAAGATCCTCTAAAGCATCATCATTATCAGTACATAATGCCCGAATAAGAGCATTTATATTATTTTTTTCTTCAC [18564]
cp32-3_PAli_NX_Pl_consensus    CAAAAAGATCCTCTAAAGCATCATCATTATCAGTACATAATGCCCGAATAAGAGCATTTATATTATTTTTTTCTTCAC [18564]
cp32-3_PAbe_NX_MP_FG_consensus CAAAAAGATCCTCTAAAGCATCATCATTATCAGTACATAATGCCCGAATAAGAGCATTTATATTATTTTTTTCTTCAC [18564]
cp32-3_PAbe_NX_Pl_consensus    CAAAAAGATCCTCTAAAGCATCATCATTATCAGTACATAATGCCCGAATAAGAGCATTTATATTATTTTTTTCTTCAC [18564]

cp32-3_gi|11497103|B31_GB      CTTCATTTACCCAATTTTTTATATTAAACAGTGCGTCTCTAAAAATTTCATAGCAATAAGCCTTGCCAGATTTTAAGT [18642]
cp32-3_un8_B31_PacBio          CTTCATTTACCCAATTTTT-ATATTAAACAGTGCGTCTCTAAAAATTTCATAGCAATAAGCCTTGCCAGATTTTAAGT [18642]
cp32-3_B31_TS_MP_FG_consensus  CTTCATTTACCCAATTTTTTATATTAAACAGTGCGTCTCTAAAAATTTCATAGCAATAAGCCTTGCCAGATTTTAAGT [18642]
cp32-3_B31_NX_Pl_consensus     CTTCATTTACCCAATTTTTTATATTAAACAGTGCGTCTCTAAAAATTTCATAGCAATAAGCCTTGCCAGATTTTAAGT [18642]
cp32-3_PAli_NX_MP_FG_consensus CTTCATTTACCCAATTTTTTATATTAAACAGTGCGTCTCTAAAAATTTCATAGCAATAAGCCTTGCCAGATTTTAAGT [18642]
cp32-3_PAli_NX_Pl_consensus    CTTCATTTACCCAATTTTTTATATTAAACAGTGCGTCTCTAAAAATTTCATAGCAATAAGCCTTGCCAGATTTTAAGT [18642]
cp32-3_PAbe_NX_MP_FG_consensus CTTCATTTACCCAATTTTTTATATTAAACAGTGCGTCTCTAAAAATTTCATAGCAATAAGCCTTGCCAGATTTTAAGT [18642]
cp32-3_PAbe_NX_Pl_consensus    CTTCATTTACCCAATTTTTTATATTAAACAGTGCGTCTCTAAAAATTTCATAGCAATAAGCCTTGCCAGATTTTAAGT [18642]

cp32-3_gi|11497103|B31_GB      TTTTATTAAAGTTGTTTAGTTGATTTTTTTCTTGTTTTTGTTCTCTTTTTTGTTCTTGCTTTTGATTAACACTAACTT [18720]
cp32-3_un8_B31_PacBio          TTTTATTAAAGTTGTTTAGTTGATTTTTTTCTTGTTTTTGTTCTCTTTTTTGTTCTTGCTTTTGATTAACACTAACTT [18720]
cp32-3_B31_TS_MP_FG_consensus  TTTTATTAAAGTTGTTTAGTTGATTTTTTTCTTGTTTTTGTTCTCTTTTTTGTTCTTGCTTTTGATTAACACTAACTT [18720]
cp32-3_B31_NX_Pl_consensus     TTTTATTAAAGTTGTTTAGTTGATTTTTTTCTTGTTTTTGTTCTCTTTTTTGTTCTTGCTTTTGATTAACACTAACTT [18720]
cp32-3_PAli_NX_MP_FG_consensus TTTTATTAAAGTTGTTTAGTTGATTTTTTTCTTGTTTTTGTTCTCTTTTTTGTTCTTGCTTTTGATTAACACTAACTT [18720]
cp32-3_PAli_NX_Pl_consensus    TTTTATTAAAGTTGTTTAGTTGATTTTTTTCTTGTTTTTGTTCTCTTTTTTGTTCTTGCTTTTGATTAACACTAACTT [18720]
cp32-3_PAbe_NX_MP_FG_consensus TTTTATTAAAGTTGTTTAGTTGATTTTTTTCTTGTTTTTGTTCTCTTTTTTGTTCTTGCTTTTGATTAACACTAACTT [18720]
cp32-3_PAbe_NX_Pl_consensus    TTTTATTAAAGTTGTTTAGTTGATTTTTTTCTTGTTTTTGTTCTCTTTTTTGTTCTTGCTTTTGATTAACACTAACTT [18720]

cp32-3_gi|11497103|B31_GB      GCTTGCTAGGCATAGAATTTTCGTTTTCGTGGTTATTGTAAATAGGAGCTGCATCAGTATCTATTTCACTTTCTATAC [18798]
cp32-3_un8_B31_PacBio          GCTTGCTAGGCATAGAATTTTCGTTTTCGTGGTTATTGTAAATAGGAGCTGCATCAGTATCTATTTCACTTTCTATAC [18798]
cp32-3_B31_TS_MP_FG_consensus  GCTTGCTAGGCATAGAATTTTCGTTTTCGTGGTTATTGTAAATAGGAGCTGCATCAGTATCTATTTCACTTTCTATAC [18798]
cp32-3_B31_NX_Pl_consensus     GCTTGCTAGGCATAGAATTTTCGTTTTCGTGGTTATTGTAAATAGGAGCTGCATCAGTATCTATTTCACTTTCTATAC [18798]
cp32-3_PAli_NX_MP_FG_consensus GCTTGCTAGGCATAGAATTTTCGTTTTCGTGGTTATTGTAAATAGGAGCTGCATCAGTATCTATTTCACTTTCTATAC [18798]
cp32-3_PAli_NX_Pl_consensus    GCTTGCTAGGCATAGAATTTTCGTTTTCGTGGTTATTGTAAATAGGAGCTGCATCAGTATCTATTTCACTTTCTATAC [18798]
cp32-3_PAbe_NX_MP_FG_consensus GCTTGCTAGGCATAGAATTTTCGTTTTCGTGGTTATTGTAAATAGGAGCTGCATCAGTATCTATTTCACTTTCTATAC [18798]
cp32-3_PAbe_NX_Pl_consensus    GCTTGCTAGGCATAGAATTTTCGTTTTCGTGGTTATTGTAAATAGGAGCTGCATCAGTATCTATTTCACTTTCTATAC [18798]

cp32-3_gi|11497103|B31_GB      CAAGAGCTGCAACTAAAGCATACCTTTTGACATAAGTAATGCCTGAACCAAACATCTGATACACTGTATTTGTAACTT [18876]
cp32-3_un8_B31_PacBio          CAAGAGCTGCAACTAAAGCATACCTTTTGACATAAGTAATGCCTGAACCAAACATCTGATACACTGTATTTGTAACTT [18876]
cp32-3_B31_TS_MP_FG_consensus  CAAGAGCTGCAACTAAAGCATACCTTTTGACATAAGTAATGCCTGAACCAAACATCTGATACACTGTATTTGTAACTT [18876]
```

```
cp32-3_B31_NX_Pl_consensus     CAAGAGCTGCAACTAAAGCATACCTTTTGACATAAGTAATGCCTGAACCAAACATCTGATACACTGTATTTGTAACTT [18876]
cp32-3_PAli_NX_MP_FG_consensus CAAGAGCTGCAACTAAAGCATACCTTTTGACATAAGTAATGCCTGAACCAAACATCTGATACACTGTATTTGTAACTT [18876]
cp32-3_PAli_NX_Pl_consensus    CAAGAGCTGCAACTAAAGCATACCTTTTGACATAAGTAATGCCTGAACCAAACATCTGATACACTGTATTTGTAACTT [18876]
cp32-3_PAbe_NX_MP_FG_consensus CAAGAGCTGCAACTAAAGCATACCTTTTGACATAAGTAATGCCTGAACCAAACATCTGATACACTGTATTTGTAACTT [18876]
cp32-3_PAbe_NX_Pl_consensus    CAAGAGCTGCAACTAAAGCATACCTTTTGACATAAGTAATGCCTGAACCAAACATCTGATACACTGTATTTGTAACTT [18876]

cp32-3_gi|11497103|B31_GB      TAGACCCATTTTCATTGTTCCATTGTAAATTTTCTGTAGGAATTCGCGTATCAAAAGAAAATTCATATCCAGTACTTG [18954]
cp32-3_un8_B31_PacBio          TAGACCCATTTTCATTGTTCCATTGTAAATTTTCTGTAGGAATTCGCGTATCAAAAGAAA-TTCATATCCAGTACTTG [18954]
cp32-3_B31_TS_MP_FG_consensus  TAGACCCATTTTCATTGTTCCATTGTAAATTTTCTGTAGGAATTCGCGTATCAAAAGAAAATTCATATCCAGTACTTG [18954]
cp32-3_B31_NX_Pl_consensus     TAGACCCATTTTCATTGTTCCATTGTAAATTTTCTGTAGGAATTCGCGTATCAAAAGAAAATTCATATCCAGTACTTG [18954]
cp32-3_PAli_NX_MP_FG_consensus TAGACCCATTTTCATTGTTCCATTGTAAATTTTCTGTAGGAATTCGCGTATCAAAAGAAAATTCATATCCAGTACTTG [18954]
cp32-3_PAli_NX_Pl_consensus    TAGACCCATTTTCATTGTTCCATTGTAAATTTTCTGTAGGAATTCGCGTATCAAAAGAAAATTCATATCCAGTACTTG [18954]
cp32-3_PAbe_NX_MP_FG_consensus TAGACCCATTTTCATTGTTCCATTGTAAATTTTCTGTAGGAATTCGCGTATCAAAAGAAAATTCATATCCAGTACTTG [18954]
cp32-3_PAbe_NX_Pl_consensus    TAGACCCATTTTCATTGTTCCATTGTAAATTTTCTGTAGGAATTCGCGTATCAAAAGAAAATTCATATCCAGTACTTG [18954]

cp32-3_gi|11497103|B31_GB      TACTGTAGAATGTAGTCCTAATATAATCAACTATGCCATATTGACCCTCTATAGAAATTGGATATTGCTCAATATCAA [19032]
cp32-3_un8_B31_PacBio          TACTGTAGAATGTAGTCCTAATATAATCAACTATGCCATATTGACCCTCTATAGAAATTGGATATTGCTCAATATCAA [19032]
cp32-3_B31_TS_MP_FG_consensus  TACTGTAGAATGTAGTCCTAATATAATCAACTATGCCATATTGACCCTCTATAGAAATTGGATATTGCTCAATATCAA [19032]
cp32-3_B31_NX_Pl_consensus     TACTGTAGAATGTAGTCCTAATATAATCAACTATGCCATATTGACCCTCTATAGAAATTGGATATTGCTCAATATCAA [19032]
cp32-3_PAli_NX_MP_FG_consensus TACTGTAGAATGTAGTCCTAATATAATCAACTATGCCATATTGACCCTCTATAGAAATTGGATATTGCTCAATATCAA [19032]
cp32-3_PAli_NX_Pl_consensus    TACTGTAGAATGTAGTCCTAATATAATCAACTATGCCATATTGACCCTCTATAGAAATTGGATATTGCTCAATATCAA [19032]
cp32-3_PAbe_NX_MP_FG_consensus TACTGTAGAATGTAGTCCTAATATAATCAACTATGCCATATTGACCCTCTATAGAAATTGGATATTGCTCAATATCAA [19032]
cp32-3_PAbe_NX_Pl_consensus    TACTGTAGAATGTAGTCCTAATATAATCAACTATGCCATATTGACCCTCTATAGAAATTGGATATTGCTCAATATCAA [19032]

cp32-3_gi|11497103|B31_GB      GCTCCAAATTGTGCTTTTTAATAACATTTTTAATTTCTCTAACTATTTCATTGAAATTTTGATATTTATATCCATATC [19110]
cp32-3_un8_B31_PacBio          GCTCCAAATTGTGCTTTTTAATAACATTTTTAATTTCTCTAACTATTTCATTGAAATTTTGATATTTATATCCATATC [19110]
cp32-3_B31_TS_MP_FG_consensus  GCTCCAAATTGTGCTTTTTAATAACATTTTTAATTTCTCTAACTATTTCATTGAAATTTTGATATTTATATCCATATC [19110]
cp32-3_B31_NX_Pl_consensus     GCTCCAAATTGTGCTTTTTAATAACATTTTTAATTTCTCTAACTATTTCATTGAAATTTTGATATTTATATCCATATC [19110]
cp32-3_PAli_NX_MP_FG_consensus GCTCCAAATTGTGCTTTTTAATAACATTTTTAATTTCTCTAACTATTTCATTGAAATTTTGATATTTATATCCATATC [19110]
cp32-3_PAli_NX_Pl_consensus    GCTCCAAATTGTGCTTTTTAATAACATTTTTAATTTCTCTAACTATTTCATTGAAATTTTGATATTTATATCCATATC [19110]
cp32-3_PAbe_NX_MP_FG_consensus GCTCCAAATTGTGCTTTTTAATAACATTTTTAATTTCTCTAACTATTTCATTGAAATTTTGATATTTATATCCATATC [19110]
cp32-3_PAbe_NX_Pl_consensus    GCTCCAAATTGTGCTTTTTAATAACATTTTTAATTTCTCTAACTATTTCATTGAAATTTTGATATTTATATCCATATC [19110]

cp32-3_gi|11497103|B31_GB      CTTTAAGACTTTTGTCAATCCCTGGTAAATTCATTTTTAGGGTTTTCATATCTTTTCTGAAGCTTATTTTTGCTTGAA [19188]
cp32-3_un8_B31_PacBio          CTTTAAGACTTTTGTCAATCCCTGGTAAATTCATTTTTAGGGTTTTCATATCTTTTCTGAAGCTTATTTTTGCTTGAA [19188]
cp32-3_B31_TS_MP_FG_consensus  CTTTAAGACTTTTGTCAATCCCTGGTAAATTCATTTTTAGGGTTTTCATATCTTTTCTGAAGCTTATTTTTGCTTGAA [19188]
cp32-3_B31_NX_Pl_consensus     CTTTAAGACTTTTGTCAATCCCTGGTAAATTCATTTTTAGGGTTTTCATATCTTTTCTGAAGCTTATTTTTGCTTGAA [19188]
cp32-3_PAli_NX_MP_FG_consensus CTTTAAGACTTTTGTCAATCCCTGGTAAATTCATTTTTAGGGTTTTCATATCTTTTCTGAAGCTTATTTTTGCTTGAA [19188]
cp32-3_PAli_NX_Pl_consensus    CTTTAAGACTTTTGTCAATCCCTGGTAAATTCATTTTTAGGGTTTTCATATCTTTTCTGAAGCTTATTTTTGCTTGAA [19188]
cp32-3_PAbe_NX_MP_FG_consensus CTTTAAGACTTTTGTCAATCCCTGGTAAATTCATTTTTAGGGTTTTCATATCTTTTCTGAAGCTTATTTTTGCTTGAA [19188]
cp32-3_PAbe_NX_Pl_consensus    CTTTAAGACTTTTGTCAATCCCTGGTAAATTCATTTTTAGGGTTTTCATATCTTTTCTGAAGCTTATTTTTGCTTGAA [19188]

cp32-3_gi|11497103|B31_GB      TATTTTCTTGTGGATTATTATTGTTTGAAAGATTTTCCATCTTTTTTCTCCTTATATTTGTATAAAAATAAATATATA [19266]
cp32-3_un8_B31_PacBio          TATTTTCTTGTGGATTATTATTGTTTGAAAGATTTTCCATCTTTTTTCTCCTTATATTTGTATAAAAATAAATATATA [19266]
cp32-3_B31_TS_MP_FG_consensus  TATTTTCTTGTGGATTATTATTGTTTGAAAGATTTTCCATCTTTTTTCTCCTTATATTTGTATAAAAATAAATATATA [19266]
cp32-3_B31_NX_Pl_consensus     TATTTTCTTGTGGATTATTATTGTTTGAAAGATTTTCCATCTTTTTTCTCCTTATATTTGTATAAAAATAAATATATA [19266]
cp32-3_PAli_NX_MP_FG_consensus TATTTTCTTGTGGATTATTATTGTTTGAAAGATTTTCCATCTTTTTTCTCCTTATATTTGTATAAAAATAAATATATA [19266]
cp32-3_PAli_NX_Pl_consensus    TATTTTCTTGTGGATTATTATTGTTTGAAAGATTTTCCATCTTTTTTCTCCTTATATTTGTATAAAAATAAATATATA [19266]
cp32-3_PAbe_NX_MP_FG_consensus TATTTTCTTGTGGATTATTATTGTTTGAAAGATTTTCCATCTTTTTTCTCCTTATATTTGTATAAAAATAAATATATA [19266]
cp32-3_PAbe_NX_Pl_consensus    TATTTTCTTGTGGATTATTATTGTTTGAAAGATTTTCCATCTTTTTTCTCCTTATATTTGTATAAAAATAAATATATA [19266]

cp32-3_gi|11497103|B31_GB      GCAAAAACTATTTTTGCCAACTTTTTTACAAAAATCTTTACAAAAAGGTGCGGGCTTAGCTACGTTCTCTTGTTAAAG [19344]
cp32-3_un8_B31_PacBio          GCAAAAACTATTTTTGCCAACTTTTTTACAAAAATCTTTACAAAAAGGTGCGGGCTTAGCTACGTTCTCTTGTTAAAG [19344]
cp32-3_B31_TS_MP_FG_consensus  GCAAAAACTATTTTTGCCAACTTTTTTACAAAAATCTTTACAAAAAGGTGCGGGCTTAGCTACGTTCTCTTGTTAAAG [19344]
cp32-3_B31_NX_Pl_consensus     GCAAAAACTATTTTTGCCAACTTTTTTACAAAAATCTTTACAAAAAGGTGCGGGCTTAGCTACGTTCTCTTGTTAAAG [19344]
cp32-3_PAli_NX_MP_FG_consensus GCAAAAACTATTTTTGCCAACTTTTTTACAAAAATCTTTACAAAAAGGTGCGGGCTTAGCTACGTTCTCTTGTTAAAG [19344]
cp32-3_PAli_NX_Pl_consensus    GCAAAAACTATTTTTGCCAACTTTTTTACAAAAATCTTTACAAAAAGGTGCGGGCTTAGCTACGTTCTCTTGTTAAAG [19344]
cp32-3_PAbe_NX_MP_FG_consensus GCAAAAACTATTTTTGCCAACTTTTTTACAAAAATCTTTACAAAAAGGTGCGGGCTTAGCTACGTTCTCTTGTTAAAG [19344]
cp32-3_PAbe_NX_Pl_consensus    GCAAAAACTATTTTTGCCAACTTTTTTACAAAAATCTTTACAAAAAGGTGCGGGCTTAGCTACGTTCTCTTGTTAAAG [19344]

cp32-3_gi|11497103|B31_GB      AACTTAGCTAAGCCCATATCTTTTTTTCTTGTAAATTCAAATATAAGGTAGAAAAAATGAAGATATTTTTCTACACTG [19422]
cp32-3_un8_B31_PacBio          AACTTAGCTAAGCCCATATCTTTTTTTCTTGTAAATTCAAATATAAGGTAGAAAAAATGAAGATATTTTTCTACACTG [19422]
cp32-3_B31_TS_MP_FG_consensus  AACTTAGCTAAGCCCATATCTTTTTTTCTTGTAAATTCAAATATAAGGTAGAAAAAATGAAGATATTTTTCTACACTG [19422]
cp32-3_B31_NX_Pl_consensus     AACTTAGCTAAGCCCATATCTTTTTTTCTTGTAAATTCAAATATAAGGTAGAAAAAATGAAGATATTTTTCTACACTG [19422]
cp32-3_PAli_NX_MP_FG_consensus AACTTAGCTAAGCCCATATCTTTTTTTCTTGTAAATTCAAATATAAGGTAGAAAAAATGAAGATATTTTTCTACACTG [19422]
cp32-3_PAli_NX_Pl_consensus    AACTTAGCTAAGCCCATATCTTTTTTTCTTGTAAATTCAAATATAAGGTAGAAAAAATGAAGATATTTTTCTACACTG [19422]
cp32-3_PAbe_NX_MP_FG_consensus AACTTAGCTAAGCCCATATCTTTTTTTCTTGTAAATTCAAATATAAGGTAGAAAAAATGAAGATATTTTTCTACACTG [19422]
cp32-3_PAbe_NX_Pl_consensus    AACTTAGCTAAGCCCATATCTTTTTTTCTTGTAAATTCAAATATAAGGTAGAAAAAATGAAGATATTTTTCTACACTG [19422]

cp32-3_gi|11497103|B31_GB      TTTGTAGTATAGCCTAACTTTGGATTAAAATCAATTTATATTTTCACTGAATTAAAAATTTCTATATTAATTTAACAA [19500]
cp32-3_un8_B31_PacBio          TTTGTAGTATAGCCTAACTTTGGATTAAAATCAATTTATATTTTCACTGAATTAAAAATTTCTATATTAATTTAACAA [19500]
cp32-3_B31_TS_MP_FG_consensus  TTTGTAGTATAGCCTAACTTTGGATTAAAATCAATTTATATTTTCACTGAATTAAAAATTTCTATATTAATTTAACAA [19500]
cp32-3_B31_NX_Pl_consensus     TTTGTAGTATAGCCTAACTTTGGATTAAAATCAATTTATATTTTCACTGAATTAAAAATTTCTATATTAATTTAACAA [19500]
cp32-3_PAli_NX_MP_FG_consensus TTTGTAGTATAGCCTAACTTTGGATTAAAATCAATTTATATTTTCACTGAATTAAAAATTTCTATATTAATTTAACAA [19500]
cp32-3_PAli_NX_Pl_consensus    TTTGTAGTATAGCCTAACTTTGGATTAAAATCAATTTATATTTTCACTGAATTAAAAATTTCTATATTAATTTAACAA [19500]
cp32-3_PAbe_NX_MP_FG_consensus TTTGTAGTATAGCCTAACTTTGGATTAAAATCAATTTATATTTTCACTGAATTAAAAATTTCTATATTAATTTAACAA [19500]
cp32-3_PAbe_NX_Pl_consensus    TTTGTAGTATAGCCTAACTTTGGATTAAAATCAATTTATATTTTCACTGAATTAAAAATTTCTATATTAATTTAACAA [19500]

cp32-3_gi|11497103|B31_GB      AATTAATAATTAAAATTTAATATTTTTTTAGAAAAGTATTTACTTTTAAATCAAAATTTTGCATTATAATACTAAGCA [19578]
cp32-3_un8_B31_PacBio          AATTAATAATTAAAATTTAATATTTTTTTAGAAAAGTATTTACTTTTAAATCAAAATTTTGCATTATAATACTAAGCA [19578]
cp32-3_B31_TS_MP_FG_consensus  AATTAATAATTAAAATTTAATATTTTTTTAGAAAAGTATTTACTTTTAAATCAAAATTTTGCATTATAATACTAAGCA [19578]
cp32-3_B31_NX_Pl_consensus     AATTAATAATTAAAATTTAATATTTTTTTAGAAAAGTATTTACTTTTAAATCAAAATTTTGCATTATAATACTAAGCA [19578]
cp32-3_PAli_NX_MP_FG_consensus AATTAATAATTAAAATTTAATATTTTTTTAGAAAAGTATTTACTTTTAAATCAAAATTTTGCATTATAATACTAAGCA [19578]
cp32-3_PAli_NX_Pl_consensus    AATTAATAATTAAAATTTAATATTTTTTTAGAAAAGTATTTACTTTTAAATCAAAATTTTGCATTATAATACTAAGCA [19578]
cp32-3_PAbe_NX_MP_FG_consensus AATTAATAATTAAAATTTAATATTTTTTTAGAAAAGTATTTACTTTTAAATCAAAATTTTGCATTATAATACTAAGCA [19578]
cp32-3_PAbe_NX_Pl_consensus    AATTAATAATTAAAATTTAATATTTTTTTAGAAAAGTATTTACTTTTAAATCAAAATTTTGCATTATAATACTAAGCA [19578]

cp32-3_gi|11497103|B31_GB      TTAATACAAATATAAGGAGAAAAAACATGAAAGGTTTTTCAAATACCACAAAAAATCCCACTTGCTACAACAAACACC [19656]
cp32-3_un8_B31_PacBio          TTAATACAAATATAAGGAGAAAAAACATGAAAGGTTTTTCAAATACCACAAAAAATCCCACTTGCTACAACAAACACC [19656]
cp32-3_B31_TS_MP_FG_consensus  TTAATACAAATATAAGGAGAAAAAACATGAAAGGTTTTTCAAATACCACAAAAAATCCCACTTGCTACAACAAACACC [19656]
cp32-3_B31_NX_Pl_consensus     TTAATACAAATATAAGGAGAAAAAACATGAAAGGTTTTTCAAATACCACAAAAAATCCCACTTGCTACAACAAACACC [19656]
cp32-3_PAli_NX_MP_FG_consensus TTAATACAAATATAAGGAGAAAAAACATGAAAGGTTTTTCAAATACCACAAAAAATCCCACTTGCTACAACAAACACC [19656]
cp32-3_PAli_NX_Pl_consensus    TTAATACAAATATAAGGAGAAAAAACATGAAAGGTTTTTCAAATACCACAAAAAATCCCACTTGCTACAACAAACACC [19656]
cp32-3_PAbe_NX_MP_FG_consensus TTAATACAAATATAAGGAGAAAAAACATGAAAGGTTTTTCAAATACCACAAAAAATCCCACTTGCTACAACAAACACC [19656]
cp32-3_PAbe_NX_Pl_consensus    TTAATACAAATATAAGGAGAAAAAACATGAAAGGTTTTTCAAATACCACAAAAAATCCCACTTGCTACAACAAACACC [19656]

cp32-3_gi|11497103|B31_GB      AACACAAATTAATATATCTTACATCAACAGTAGATTTTCTAAACAAAAAAGATAAGAAATATACACAACAAAACATAC [19734]
cp32-3_un8_B31_PacBio          AACACAAATTAATATATCTTACATCAACAGTAGATTTTCTAAACAAAAAAGATAAGAAATATACACAACAAAACATAC [19734]
cp32-3_B31_TS_MP_FG_consensus  AACACAAATTAATATATCTTACATCAACAGTAGATTTTCTAAACAAAAAAGATAAGAAATATACACAACAAAACATAC [19734]
cp32-3_B31_NX_Pl_consensus     AACACAAATTAATATATCTTACATCAACAGTAGATTTTCTAAACAAAAAAGATAAGAAATATACACAACAAAACATAC [19734]
cp32-3_PAli_NX_MP_FG_consensus AACACAAATTAATATATCTTACATCAACAGTAGATTTTCTAAACAAAAAAGATAAGAAATATACACAACAAAACATAC [19734]
cp32-3_PAli_NX_Pl_consensus    AACACAAATTAATATATCTTACATCAACAGTAGATTTTCTAAACAAAAAAGATAAGAAATATACACAACAAAACATAC [19734]
cp32-3_PAbe_NX_MP_FG_consensus AACACAAATTAATATATCTTACATCAACAGTAGATTTTCTAAACAAAAAAGATAAGAAATATACACAACAAAACATAC [19734]
cp32-3_PAbe_NX_Pl_consensus    AACACAAATTAATATATCTTACATCAACAGTAGATTTTCTAAACAAAAAAGATAAGAAATATACACAACAAAACATAC [19734]

cp32-3_gi|11497103|B31_GB      TCTATTACTATAATAAAAATCTAAAAAGAAATGGTTTGGCTCCCACTACACTGAGAACAATGCAAAATTATCTTTATA [19812]
cp32-3_un8_B31_PacBio          TCTATTACTATAATAAAAATCTAAAAAGAAATGGTTTGGCTCCCACTACACTGAGAACAATGCAAAATTATCTTTATA [19812]
cp32-3_B31_TS_MP_FG_consensus  TCTATTACTATAATAAAAATCTAAAAAGAAATGGTTTGGCTCCCACTACACTGAGAACAATGCAAAATTATCTTTATA [19812]
cp32-3_B31_NX_Pl_consensus     TCTATTACTATAATAAAAATCTAAAAAGAAATGGTTTGGCTCCCACTACACTGAGAACAATGCAAAATTATCTTTATA [19812]
```

```
cp32-3_PAli_NX_MP_FG_consensus TCTATTACTATAATAAAAATCTAAAAAGAAATGGTTTGGCTCCCACTACACTGAGAACAATGCAAAATTATCTTTATA [19812]
cp32-3_PAli_NX_Pl_consensus    TCTATTACTATAATAAAAATCTAAAAAGAAATGGTTTGGCTCCCACTACACTGAGAACAATGCAAAATTATCTTTATA [19812]
cp32-3_PAbe_NX_MP_FG_consensus TCTATTACTATAATAAAAATCTAAAAAGAAATGGTTTGGCTCCCACTACACTGAGAACAATGCAAAATTATCTTTATA [19812]
cp32-3_PAbe_NX_Pl_consensus    TCTATTACTATAATAAAAATCTAAAAAGAAATGGTTTGGCTCCCACTACACTGAGAACAATGCAAAATTATCTTTATA [19812]

cp32-3_gi|11497103|B31_GB      AATTAGAAAAAGTATTAAAAGTCACAACGAATTACTACCAACATATGGGTGTAAATTGTGGGACTGAAATTTATTATA [19890]
cp32-3_un8_B31_PacBio          AATTAGAAAAAGTATTAAAAGTCACAACGAATTACTACCAACATATGGGTGTAAATTGTGGGACTGAAATTTATTATA [19890]
cp32-3_B31_TS_MP_FG_consensus  AATTAGAAAAAGTATTAAAAGTCACAACGAATTACTACCAACATATGGGTGTAAATTGTGGGACTGAAATTTATTATA [19890]
cp32-3_B31_NX_Pl_consensus     AATTAGAAAAAGTATTAAAAGTCACAACGAATTACTACCAACATATGGGTGTAAATTGTGGGACTGAAATTTATTATA [19890]
cp32-3_PAli_NX_MP_FG_consensus AATTAGAAAAAGTATTAAAAGTCACAACGAATTACTACCAACATATGGGTGTAAATTGTGGGACTGAAATTTATTATA [19890]
cp32-3_PAli_NX_Pl_consensus    AATTAGAAAAAGTATTAAAAGTCACAACGAATTACTACCAACATATGGGTGTAAATTGTGGGACTGAAATTTATTATA [19890]
cp32-3_PAbe_NX_MP_FG_consensus AATTAGAAAAAGTATTAAAAGTCACAACGAATTACTACCAACATATGGGTGTAAATTGTGGGACTGAAATTTATTATA [19890]
cp32-3_PAbe_NX_Pl_consensus    AATTAGAAAAAGTATTAAAAGTCACAACGAATTACTACCAACATATGGGTGTAAATTGTGGGACTGAAATTTATTATA [19890]

cp32-3_gi|11497103|B31_GB      AACTAAAGTATCCTAAAAAAGAATGTTACCAGAAAATCAACAAGTACTTTAAAGAACGAAAAAA-CTCTAGATTTAAA [19968]
cp32-3_un8_B31_PacBio          AACTAAAGTATCCTAAAAAAGAATGTTACCAGAAAATCAACAAGTACTTTAAAGAACGAAAAAAACTCTAGATTTAAA [19968]
cp32-3_B31_TS_MP_FG_consensus  AACTAAAGTATCCTAAAAAAGAATGTTACCAGAAAATCAACAAGTACTTTAAAGAACGAAAAAA-CTCTAGATTTAAA [19968]
cp32-3_B31_NX_Pl_consensus     AACTAAAGTATCCTAAAAAAGAATGTTACCAGAAAATCAACAAGTACTTTAAAGAACGAAAAAA-CTCTAGATTTAAA [19968]
cp32-3_PAli_NX_MP_FG_consensus AACTAAAGTATCCTAAAAAAGAATGTTACCAGAAAATCAACAAGTACTTTAAAGAACGAAAAAA-CTCTAGATTTAAA [19968]
cp32-3_PAli_NX_Pl_consensus    AACTAAAGTATCCTAAAAAAGAATGTTACCAGAAAATCAACAAGTACTTTAAAGAACGAAAAAA-CTCTAGATTTAAA [19968]
cp32-3_PAbe_NX_MP_FG_consensus AACTAAAGTATCCTAAAAAAGAATGTTACCAGAAAATCAACAAGTACTTTAAAGAACGAAAAAA-CTCTAGATTTAAA [19968]
cp32-3_PAbe_NX_Pl_consensus    AACTAAAGTATCCTAAAAAAGAATGTTACCAGAAAATCAACAAGTACTTTAAAGAACGAAAAAA-CTCTAGATTTAAA [19968]

cp32-3_gi|11497103|B31_GB      TCTAGAGTTAATAACCATTTTAAAGACAATGTTTCTAAAAATAGTAGTGTAAATTCAGTGGAGTGCTTAAGTAATAAA [20046]
cp32-3_un8_B31_PacBio          TCTAGAGTTAATAACCATTTTAAAGACAATGTTTCTAAAAATAGTAGTGTAAATTCAGTGGAGTGCTTAAGTAATAAA [20046]
cp32-3_B31_TS_MP_FG_consensus  TCTAGAGTTAATAACCATTTTAAAGACAATGTTTCTAAAAATAGTAGTGTAAATTCAGTGGAGTGCTTAAGTAATAAA [20046]
cp32-3_B31_NX_Pl_consensus     TCTAGAGTTAATAACCATTTTAAAGACAATGTTTCTAAAAATAGTAGTGTAAATTCAGTGGAGTGCTTAAGTAATAAA [20046]
cp32-3_PAli_NX_MP_FG_consensus TCTAGAGTTAATAACCATTTTAAAGACAATGTTTCTAAAAATAGTAGTGTAAATTCAGTGGAGTGCTTAAGTAATAAA [20046]
cp32-3_PAli_NX_Pl_consensus    TCTAGAGTTAATAACCATTTTAAAGACAATGTTTCTAAAAATAGTAGTGTAAATTCAGTGGAGTGCTTAAGTAATAAA [20046]
cp32-3_PAbe_NX_MP_FG_consensus TCTAGAGTTAATAACCATTTTAAAGACAATGTTTCTAAAAATAGTAGTGTAAATTCAGTGGAGTGCTTAAGTAATAAA [20046]
cp32-3_PAbe_NX_Pl_consensus    TCTAGAGTTAATAACCATTTTAAAGACAATGTTTCTAAAAATAGTAGTGTAAATTCAGTGGAGTGCTTAAGTAATAAA [20046]

cp32-3_gi|11497103|B31_GB      AATAATATAAAAGAAGAAAGAAAGATTAAAGAAATAGAAAAATATCAAGTAATAAAGTACTTCAACAAAAGTAACTTC [20124]
cp32-3_un8_B31_PacBio          AATAATATAAAAGAAGAAAGAAAGATTAAAGAAATAGAAAAATATCAAGTAATAAAGTACTTCAACAAAAGTAACTTC [20124]
cp32-3_B31_TS_MP_FG_consensus  AATAATATAAAAGAAGAAAGAAAGATTAAAGAAATAGAAAAATATCAAGTAATAAAGTACTTCAACAAAAGTAACTTC [20124]
cp32-3_B31_NX_Pl_consensus     AATAATATAAAAGAAGAAAGAAAGATTAAAGAAATAGAAAAATATCAAGTAATAAAGTACTTCAACAAAAGTAACTTC [20124]
cp32-3_PAli_NX_MP_FG_consensus AATAATATAAAAGAAGAAAGAAAGATTAAAGAAATAGAAAAATATCAAGTAATAAAGTACTTCAACAAAAGTAACTTC [20124]
cp32-3_PAli_NX_Pl_consensus    AATAATATAAAAGAAGAAAGAAAGATTAAAGAAATAGAAAAATATCAAGTAATAAAGTACTTCAACAAAAGTAACTTC [20124]
cp32-3_PAbe_NX_MP_FG_consensus AATAATATAAAAGAAGAAAGAAAGATTAAAGAAATAGAAAAATATCAAGTAATAAAGTACTTCAACAAAAGTAACTTC [20124]
cp32-3_PAbe_NX_Pl_consensus    AATAATATAAAAGAAGAAAGAAAGATTAAAGAAATAGAAAAATATCAAGTAATAAAGTACTTCAACAAAAGTAACTTC [20124]

cp32-3_gi|11497103|B31_GB      TTATGTAAAGAAATTCTTCCATTTTTATTAACATTAAATGTTGACAAAGATACTATGATTAAAATAATAAAAAACACA [20202]
cp32-3_un8_B31_PacBio          TTATGTAAAGAAATTCTTCCATTTTTATTAACATTAAATGTTGACAAAGATACTATGATTAAAATAATAAAAAACACA [20202]
cp32-3_B31_TS_MP_FG_consensus  TTATGTAAAGAAATTCTTCCATTTTTATTAACATTAAATGTTGACAAAGATACTATGATTAAAATAATAAAAAACACA [20202]
cp32-3_B31_NX_Pl_consensus     TTATGTAAAGAAATTCTTCCATTTTTATTAACATTAAATGTTGACAAAGATACTATGATTAAAATAATAAAAAACACA [20202]
cp32-3_PAli_NX_MP_FG_consensus TTATGTAAAGAAATTCTTCCATTTTTATTAACATTAAATGTTGACAAAGATACTATGATTAAAATAATAAAAAACACA [20202]
cp32-3_PAli_NX_Pl_consensus    TTATGTAAAGAAATTCTTCCATTTTTATTAACATTAAATGTTGACAAAGATACTATGATTAAAATAATAAAAAACACA [20202]
cp32-3_PAbe_NX_MP_FG_consensus TTATGTAAAGAAATTCTTCCATTTTTATTAACATTAAATGTTGACAAAGATACTATGATTAAAATAATAAAAAACACA [20202]
cp32-3_PAbe_NX_Pl_consensus    TTATGTAAAGAAATTCTTCCATTTTTATTAACATTAAATGTTGACAAAGATACTATGATTAAAATAATAAAAAACACA [20202]

cp32-3_gi|11497103|B31_GB      AAAAGAGTTGAAAATAAATTGCTAAAAAACACAAATTTAAATAAATCTTGCTTTAAAGAAAAGCAAGAGAAACTAAAG [20280]
cp32-3_un8_B31_PacBio          AAAAGAGTTGAAAATAAATTGCTAAAAAACACAAATTTAAATAAATCTTGCTTTAAAGAAAAGCAAGAGAAACTAAAG [20280]
cp32-3_B31_TS_MP_FG_consensus  AAAAGAGTTGAAAATAAATTGCTAAAAAACACAAATTTAAATAAATCTTGCTTTAAAGAAAAGCAAGAGAAACTAAAG [20280]
cp32-3_B31_NX_Pl_consensus     AAAAGAGTTGAAAATAAATTGCTAAAAAACACAAATTTAAATAAATCTTGCTTTAAAGAAAAGCAAGAGAAACTAAAG [20280]
cp32-3_PAli_NX_MP_FG_consensus AAAAGAGTTGAAAATAAATTGCTAAAAAACACAAATTTAAATAAATCTTGCTTTAAAGAAAAGCAAGAGAAACTAAAG [20280]
cp32-3_PAli_NX_Pl_consensus    AAAAGAGTTGAAAATAAATTGCTAAAAAACACAAATTTAAATAAATCTTGCTTTAAAGAAAAGCAAGAGAAACTAAAG [20280]
cp32-3_PAbe_NX_MP_FG_consensus AAAAGAGTTGAAAATAAATTGCTAAAAAACACAAATTTAAATAAATCTTGCTTTAAAGAAAAGCAAGAGAAACTAAAG [20280]
cp32-3_PAbe_NX_Pl_consensus    AAAAGAGTTGAAAATAAATTGCTAAAAAACACAAATTTAAATAAATCTTGCTTTAAAGAAAAGCAAGAGAAACTAAAG [20280]

cp32-3_gi|11497103|B31_GB      AAAATTCTAAATAACACTCAGAAAGAATTCGAACAAAATGGATATAATCCCGAACAATTAAAGACAAATTTACAAAAA [20358]
cp32-3_un8_B31_PacBio          AAAATTCTAAATAACACTCAGAAAGAATTCGAACAAAATGGATATAATCCCGAACAATTAAAGACAAATTTACAAAAA [20358]
cp32-3_B31_TS_MP_FG_consensus  AAAATTCTAAATAACACTCAGAAAGAATTCGAACAAAATGGATATAATCCCGAACAATTAAAGACAAATTTACAAAAA [20358]
cp32-3_B31_NX_Pl_consensus     AAAATTCTAAATAACACTCAGAAAGAATTCGAACAAAATGGATATAATCCCGAACAATTAAAGACAAATTTACAAAAA [20358]
cp32-3_PAli_NX_MP_FG_consensus AAAATTCTAAATAACACTCAGAAAGAATTCGAACAAAATGGATATAATCCCGAACAATTAAAGACAAATTTACAAAAA [20358]
cp32-3_PAli_NX_Pl_consensus    AAAATTCTAAATAACACTCAGAAAGAATTCGAACAAAATGGATATAATCCCGAACAATTAAAGACAAATTTACAAAAA [20358]
cp32-3_PAbe_NX_MP_FG_consensus AAAATTCTAAATAACACTCAGAAAGAATTCGAACAAAATGGATATAATCCCGAACAATTAAAGACAAATTTACAAAAA [20358]
cp32-3_PAbe_NX_Pl_consensus    AAAATTCTAAATAACACTCAGAAAGAATTCGAACAAAATGGATATAATCCCGAACAATTAAAGACAAATTTACAAAAA [20358]

cp32-3_gi|11497103|B31_GB      GTATACGAAAATTACAAATTTAAGCCCCATTTTATTATTGAAAGGCATAAATATAGCGATTTAAACAACATAAAACGT [20436]
cp32-3_un8_B31_PacBio          GTATACGAAAATTACAAATTTAAGCCCCATTTTATTATTGAAAGGCATAAATATAGCGATTTAAACAACATAAAACGT [20436]
cp32-3_B31_TS_MP_FG_consensus  GTATACGAAAATTACAAATTTAAGCCCCATTTTATTATTGAAAGGCATAAATATAGCGATTTAAACAACATAAAACGT [20436]
cp32-3_B31_NX_Pl_consensus     GTATACGAAAATTACAAATTTAAGCCCCATTTTATTATTGAAAGGCATAAATATAGCGATTTAAACAACATAAAACGT [20436]
cp32-3_PAli_NX_MP_FG_consensus GTATACGAAAATTACAAATTTAAGCCCCATTTTATTATTGAAAGGCATAAATATAGCGATTTAAACAACATAAAACGT [20436]
cp32-3_PAli_NX_Pl_consensus    GTATACGAAAATTACAAATTTAAGCCCCATTTTATTATTGAAAGGCATAAATATAGCGATTTAAACAACATAAAACGT [20436]
cp32-3_PAbe_NX_MP_FG_consensus GTATACGAAAATTACAAATTTAAGCCCCATTTTATTATTGAAAGGCATAAATATAGCGATTTAAACAACATAAAACGT [20436]
cp32-3_PAbe_NX_Pl_consensus    GTATACGAAAATTACAAATTTAAGCCCCATTTTATTATTGAAAGGCATAAATATAGCGATTTAAACAACATAAAACGT [20436]

cp32-3_gi|11497103|B31_GB      AAATTAGAAAAGTCGATTGAAAGGGAAAAGCAAAATTCTCAACAAAATTATCAAAATTTAAAGGAAAATATTTTCAAT [20514]
cp32-3_un8_B31_PacBio          AAATTAGAAAAGTCGATTGAAAGGGAAAAGCAAAATTCTCAACAAAATTATCAAAATTTAAAGGAAAATATTTTCAAT [20514]
cp32-3_B31_TS_MP_FG_consensus  AAATTAGAAAAGTCGATTGAAAGGGAAAAGCAAAATTCTCAACAAAATTATCAAAATTTAAAGGAAAATATTTTCAAT [20514]
cp32-3_B31_NX_Pl_consensus     AAATTAGAAAAGTCGATTGAAAGGGAAAAGCAAAATTCTCAACAAAATTATCAAAATTTAAAGGAAAATATTTTCAAT [20514]
cp32-3_PAli_NX_MP_FG_consensus AAATTAGAAAAGTCGATTGAAAGAAAAAAGGAAAATCCTCAACAAAATTATCAAAATTTAAAGGAAAATATTTTCAAT [20514]
cp32-3_PAli_NX_Pl_consensus    AAATTAGAAAAGTCGATTGAAAGGGAAAAGCAAAATTCTCAACAAAATTATCAAAATTTAAAGGAAAATATTTTCAAT [20514]
cp32-3_PAbe_NX_MP_FG_consensus AAATTAGAAAAGTCGATTGAAAGGGAAAAGCAAAATTCTCAACAAAATTATCAAAATTTAAAGGAAAATATTTTCAAT [20514]
cp32-3_PAbe_NX_Pl_consensus    AAATTAGAAAAGTCGATTGAAAGGGAAAAGCAAAATTCTCAACAAAATTATCAAAATTTAAAGGAAAATATTTTCAAT [20514]

cp32-3_gi|11497103|B31_GB      ATCCTTATTGAACAACTAAAAAAAGAAACAAATATTGAAATTCTAAAGCCAATTATCAAAAAATATTTGAATAACCAA [20592]
cp32-3_un8_B31_PacBio          ATCCTTATTGAACAACTAAAAAAAGAAACAAATATTGAAATTCTAAAGCCAATTATCAAAAAATATTTGAATAACCAA [20592]
cp32-3_B31_TS_MP_FG_consensus  ATCCTTATTGAACAACTAAAAAAAGAAACAAATATTGAAATTCTAAAGCCAATTATCAAAAAATATTTGAATAACCAA [20592]
cp32-3_B31_NX_Pl_consensus     ATCCTTATTGAACAACTAAAAAAAGAAACAAATATTGAAATTCTAAAGCCAATTATCAAAAAATATTTGAATAACCAA [20592]
cp32-3_PAli_NX_MP_FG_consensus ATCCTTATTGAACAACTAAAAAAAGAAACAAATATTGAAATTCTAAAGCCAATTATCAAAAAATATTTGAATAACCAA [20592]
cp32-3_PAli_NX_Pl_consensus    ATCCTTATTGAACAACTAAAAAAAGAAACAAATATTGAAATTCTAAAGCCAATTATCAAAAAATATTTGAATAACCAA [20592]
cp32-3_PAbe_NX_MP_FG_consensus ATCCTTATTGAACAACTAAAAAAAGAAACAAATATTGAAATTCTAAAGCCAATTATCAAAAAATATTTGAATAACCAA [20592]
cp32-3_PAbe_NX_Pl_consensus    ATCCTTATTGAACAACTAAAAAAAGAAACAAATATTGAAATTCTAAAGCCAATTATCAAAAAATATTTGAATAACCAA [20592]

cp32-3_gi|11497103|B31_GB      AAGAAAATAGAATACAATAAAGTATTTGGTATATATCATCTTGAATTATTAGAAATAATAAAAAATGAAAAAAATTCT [20670]
cp32-3_un8_B31_PacBio          AAGAAAATAGAATACAATAAAGTATTTGGTATATATCATCTTGAATTATTAGAAATAATAAAAAATGAAAAAAATTCT [20670]
cp32-3_B31_TS_MP_FG_consensus  AAGAAAATAGAATACAATAAAGTATTTGGTATATATCATCTTGAATTATTAGAAATAATAAAAAATGAAAAAAATTCT [20670]
cp32-3_B31_NX_Pl_consensus     AAGAAAATAGAATACAATAAAGTATTTGGTATATATCATCTTGAATTATTAGAAATAATAAAAAATGAAAAAAATTCT [20670]
cp32-3_PAli_NX_MP_FG_consensus AAGAAAATAGAATACAATAAAGTATTTGGTATATATCATCTTGAATTATTAGAAATAATAAAAAATGAAAAAAATTCT [20670]
cp32-3_PAli_NX_Pl_consensus    AAGAAAATAGAATACAATAAAGTATTTGGTATATATCATCTTGAATTATTAGAAATAATAAAAAATGAAAAAAATTCT [20670]
cp32-3_PAbe_NX_MP_FG_consensus AAGAAAATAGAATACAATAAAGTATTTGGTATATATCATCTTGAATTATTAGAAATAATAAAAAATGAAAAAAATTCT [20670]
cp32-3_PAbe_NX_Pl_consensus    AAGAAAATAGAATACAATAAAGTATTTGGTATATATCATCTTGAATTATTAGAAATAATAAAAAATGAAAAAAATTCT [20670]

cp32-3_gi|11497103|B31_GB      TTAACCACAGAAGAATTTAGCATAAAGGCCGTATAAGGATTTAACATGGAAAATGCACCAGAACCTATTGAAACTGTA [20748]
cp32-3_un8_B31_PacBio          TTAACCACAGAAGAATTTAGCATAAAGGCCGTATAAGGATTTAACATGGAAAATGCACCAGAACCTATTGAAACTGTA [20748]
cp32-3_B31_TS_MP_FG_consensus  TTAACCACAGAAGAATTTAGCATAAGGGCCGTATAAGGATTTAACATGGAAAATGCACCAGAACCTATTGAAACTGTA [20748]
cp32-3_B31_NX_Pl_consensus     TTAACCACAGAAGAATTTAGCATAAAGGCCGTATAAGGATTTAACATGGAAAATGCACCAGAACCTATTGAAACTGTA [20748]
cp32-3_PAli_NX_MP_FG_consensus TTAACCACAGAAGAATTTAGCATAAGGGCCGTATAAGGATTTAACATGGAAAATGCACCAGAACCTATTGAAACTGTA [20748]
```

```
cp32-3_PAli_NX_Pl_consensus    TTAACCACAGAAGAATTTAGCATAAAGGCCGTATAAGGATTTAACATGGAAAATGCACCAGAACCTATTGAAACTGTA [20748]
cp32-3_PAbe_NX_MP_FG_consensus TTAACCACAGAAGAATTTAGCATAAAGGCCGTATAAGGATTTAACATGGAAAATGCACCAGAACCTATTGAAACTGTA [20748]
cp32-3_PAbe_NX_Pl_consensus    TTAACCACAGAAGAATTTAGCATAAAGGCCGTATAAGGATTTAACATGGAAAATGCACCAGAACCTATTGAAACTGTA [20748]

cp32-3_gi|11497103|B31_GB      AAAAAAGGTAAATGTAAAGTTGAATGCCAAAATAAAGAACGCTTTATTTTAATTGAAAAAGAAAATGGTAAAGCAATG [20826]
cp32-3_un8_B31_PacBio          AAAAAAGGTAAATGTAAAGTTGAATGCCAAAATAAAGAACGCTTTATTTTAATTGAAAAAGAAAATGGTAAAGCAATG [20826]
cp32-3_B31_TS_MP_FG_consensus  AAAAAAGGTAAATGTAAAGTTGAATGCCAAAATAAAGAACGCTTTATTTTAATTGAAAAAGAAAATGGTAAAGCAATG [20826]
cp32-3_B31_NX_Pl_consensus     AAAAAAGGTAAATGTAAAGTTGAATGCCAAAATAAAGAACGCTTTATTTTAATTGAAAAAGAAAATGGTAAAGCAATG [20826]
cp32-3_PAli_NX_MP_FG_consensus AAAAAAGGTAAATGTAAAGTTGAATGCCAAAATAAAGAACGCTTTATTTTAATTGAAAAAGAAAATGGTAAAGCAATG [20826]
cp32-3_PAli_NX_Pl_consensus    AAAAAAGGTAAATGTAAAGTTGAATGCCAAAATAAAGAACGCTTTATTTTAATTGAAAAAGAAAATGGTAAAGCAATG [20826]
cp32-3_PAbe_NX_MP_FG_consensus AAAAAAGGTAAATGTAAAGTTGAATGCCAAAATAAAGAACGCTTTATTTTAATTGAAAAAGAAAATGGTAAAGCAATG [20826]
cp32-3_PAbe_NX_Pl_consensus    AAAAAAGGTAAATGTAAAGTTGAATGCCAAAATAAAGAACGCTTTATTTTAATTGAAAAAGAAAATGGTAAAGCAATG [20826]

cp32-3_gi|11497103|B31_GB      TACCATACAAAAATAATGATGGATATTTATAAATTTGGAGTTTATGAGAAAAAACACGAATTTAGATTATCATTGAGG [20904]
cp32-3_un8_B31_PacBio          TACCATACAAAAATAATGATGGATATTTATAAATTTGGAGTTTATGAGAAAAAACACGAATTTAGATTATCATTGAGG [20904]
cp32-3_B31_TS_MP_FG_consensus  TACCATACAAAAATAATGATGGATATTTATAAATTTGGAGTTTATGAGAAAAAACACGAATTTAGATTATCATTGAGG [20904]
cp32-3_B31_NX_Pl_consensus     TACCATACAAAAATAATGATGGATATTTATAAATTTGGAGTTTATGAGAAAAAACACGAATTTAGATTATCATTGAGG [20904]
cp32-3_PAli_NX_MP_FG_consensus TACCATACAAAAATAATGATGGATATTTATAAATTTGGAGTTTATGAGAAAAAACACGAATTTAGATTATCATTGAGG [20904]
cp32-3_PAli_NX_Pl_consensus    TACCATACAAAAATAATGATGGATATTTATAAATTTGGAGTTTATGAGAAAAAACACGAATTTAGATTATCATTGAGG [20904]
cp32-3_PAbe_NX_MP_FG_consensus TACCATACAAAAATAATGATGGATATTTATAAATTTGGAGTTTATGAGAAAAAACACGAATTTAGATTATCATTGAGG [20904]
cp32-3_PAbe_NX_Pl_consensus    TACCATACAAAAATAATGATGGATATTTATAAATTTGGAGTTTATGAGAAAAAACACGAATTTAGATTATCATTGAGG [20904]

cp32-3_gi|11497103|B31_GB      GCCTTATTTAATGGAGAAAGAATTGTTGAAGAAACTCATTTGTACCCAATTAAAGAAGGAGATAAATTTATTGGTATT [20982]
cp32-3_un8_B31_PacBio          GCCTTATTTAATGGAGAAAGAATTGTTGAAGAAACTCATTTGTACCCAATTAAAGAAGGAGATAAATTTATTGGTATT [20982]
cp32-3_B31_TS_MP_FG_consensus  GCCTTATTTAATGGAGAAAGAATTGTTGAAGAAACTCATTTGTACCCAATTAAAGAAGGAGATAAATTTATTGGTATT [20982]
cp32-3_B31_NX_Pl_consensus     GCCTTATTTAATGGAGAAAGAATTGTTGAAGAAACTCATTTGTACCCAATTAAAGAAGGAGATAAATTTATTGGTATT [20982]
cp32-3_PAli_NX_MP_FG_consensus GCCTTATTTAATGGAGAAAGAATTGTTGAAGAAACTCATTTGTACCCAATTAAAGAAGGAGATAAATTTATTGGTATT [20982]
cp32-3_PAli_NX_Pl_consensus    GCCTTATTTAATGGAGAAAGAATTGTTGAAGAAACTCATTTGTACCCAATTAAAGAAGGAGATAAATTTATTGGTATT [20982]
cp32-3_PAbe_NX_MP_FG_consensus GCCTTATTTAATGGAGAAAGAATTGTTGAAGAAACTCATTTGTACCCAATTAAAGAAGGAGATAAATTTATTGGTATT [20982]
cp32-3_PAbe_NX_Pl_consensus    GCCTTATTTAATGGAGAAAGAATTGTTGAAGAAACTCATTTGTACCCAATTAAAGAAGGAGATAAATTTATTGGTATT [20982]

cp32-3_gi|11497103|B31_GB      TTTTATGGCTATAGAAAGCCGATTAAAAAGCCGTTAATAAAGTATCAAATAAACGGGGCTAGAAAAGCATATGCATTA [21060]
cp32-3_un8_B31_PacBio          TTTTATGGCTATAGAAAGCCGATTAAAAAGCCGTTAATAAAGTATCAAATAAACGGGGCTAGAAAAGCATATGCATTA [21060]
cp32-3_B31_TS_MP_FG_consensus  TTTTATGGCTATAGAAAGCCGATTAAAAAGCCGTTAATAAAGTATCAAATAAACGGGGCTAGAAAAGCATATGCATTA [21060]
cp32-3_B31_NX_Pl_consensus     TTTTATGGCTATAGAAAGCCGATTAAAAAGCCGTTAATAAAGTATCAAATAAATGGGACTAGAAAAGCATATGCATTA [21060]
cp32-3_PAli_NX_MP_FG_consensus TTTTATGGCTATAGAAAGCCGATTAAAAAGCCGTTAATAAAGTATCAAATAAACGGGGCTAGAAAAGCATATGCATTA [21060]
cp32-3_PAli_NX_Pl_consensus    TTTTATGGCTATAGAAAGCCGATTAAAAAGCCGTTAATAAAGTATCAAATAAACGGGGCTAGAAAAGCATATGCATTA [21060]
cp32-3_PAbe_NX_MP_FG_consensus TTTTATGGCTATAGAAAGCCGATTAAAAAGCCGTTAATAAAGTATCAAATAAATGGGACTAGAAAAGCATATGCATTA [21060]
cp32-3_PAbe_NX_Pl_consensus    TTTTATGGCTATAGAAAGCCGATTAAAAAGCCGTTAATAAAGTATCAAATAAATGGGACTAGAAAAGCATATGCATTA [21060]

cp32-3_gi|11497103|B31_GB      GCAAGGGCATATTATATGGAATTTAGATTTAAAGCAGGAAGTGTTTTTTGCTATTTCAAGGGATTATATCGTTTATTA [21138]
cp32-3_un8_B31_PacBio          GCAAGGGCATATTATATGGAATTTAGATTTAAAGCAGGAAGTGTTTTTTGCTATTTCAAGGGATTATATCGTTTATTA [21138]
cp32-3_B31_TS_MP_FG_consensus  GCAAGGGCATATTATATGGAATTTAGATTTAAAGCAGGAAGTGTTTTTTGCTATTTCAAGGGATTATATCGTTTATTA [21138]
cp32-3_B31_NX_Pl_consensus     GCAAGGGCATATTATATGGAATTTAGATTTAAAGCAGGAAGTGTCTTTTGCTATTTTAAGGGGCTATATCGTTTATTA [21138]
cp32-3_PAli_NX_MP_FG_consensus GCAAGGGCATATTATATGGAATTTAGATTTAAAGCAGGAAGTGTTTTTTGCTATTTCAAGGGATTATATCGTTTATTA [21138]
cp32-3_PAli_NX_Pl_consensus    GCAAGGGCATATTATATGGAATTTAGATTTAAAGCAGGAAGTGTTTTTTGCTATTTCAAGGGATTATATCGTTTATTA [21138]
cp32-3_PAbe_NX_MP_FG_consensus GCAAGGGCATATTATATGGAATTTAGATTTAAAGCAGGAAGTGTTTTTTGCTATTTCAAGGGATTATATCGTTTATTA [21138]
cp32-3_PAbe_NX_Pl_consensus    GCAAGGGCATATTATATGGAATTTAGATTTAAAGCAGGAAGTGTCTTTTGCTATTTTAAGGGGCTATATCGTTTATTA [21138]

cp32-3_gi|11497103|B31_GB      GATAAAAAAAGAACAAACAATCACTACAACAAAGTGTTATTTAGTATGTTTACAGATTTAGAACAACAAGTATATAAA [21216]
cp32-3_un8_B31_PacBio          GATAAAAAAAGAACAAACAATCACTACAACAAAGTGTTATTTAGTATGTTTACAGATTTAGAACAACAAGTATATAAA [21216]
cp32-3_B31_TS_MP_FG_consensus  GATAAAAAAAGAACAAACAATCACTACAACAAAGTGTTATTTAGTATGTTTACAGATTTAGAACAACAAGTATATAAA [21216]
cp32-3_B31_NX_Pl_consensus     GATAAAAAAAGAACAAACAATCACTACAACAAAGTGTTATTTAGTATGTTTACAGATTTAGAACAACAAGTATATAAA [21216]
cp32-3_PAli_NX_MP_FG_consensus GATAAAAAAAGAACAAACAATCACTACAACAAAGTGTTATTTAGTATGTTTACAGATTTAGAACAACAAGTATATAAA [21216]
cp32-3_PAli_NX_Pl_consensus    GATAAAAAAAGAACAAACAATCACTACAACAAAGTGTTATTTAGTATGTTTACAGATTTAGAACAACAAGTATATAAA [21216]
cp32-3_PAbe_NX_MP_FG_consensus GATAAAAAAAGAACAAACAATCACTACAACAAAGTGTTATTTAGTATGTTTACAGATTTAGAACAACAAGTATATAAA [21216]
cp32-3_PAbe_NX_Pl_consensus    GATAAAAAAAGAACAAACAATCACTACAACAAAGTGTTATTTAGTATGTTTACAGATTTAGAACAACAAGTATATAAA [21216]

cp32-3_gi|11497103|B31_GB      TTTTATGGGAAGAAATACCCGGAACAAGGACCACTAATAAAATGGATAATAAAAAACCTAAAATAATAACAATAGCGT [21294]
cp32-3_un8_B31_PacBio          TTTTATGGGAAGAAATACCCGGAACAAGGACCACTAATAAAATGGATAATAAAAAACCTAAAATAATAACAATAGCGT [21294]
cp32-3_B31_TS_MP_FG_consensus  TTTTATGGGAAGAAATACCCGGAACAAGGACCACTAATAAAATGGATAATAAAAAACCTAAAATAATAACAATAGCGT [21294]
cp32-3_B31_NX_Pl_consensus     TTTTATGGGAAGAAATACCCGGAACAAGGACCACTAATAAAATGGATAATAAAAAACCTAAAATAATAACAATAGCGT [21294]
cp32-3_PAli_NX_MP_FG_consensus TTTTATGGGAAGAAATACCCGGAACAAGGACCACTAATAAAATGGATAATAAAAAACCTAAAATAATAACAATAGCGT [21294]
cp32-3_PAli_NX_Pl_consensus    TTTTATGGGAAGAAATACCCGGAACAAGGACCACTAATAAAATGGATAATAAAAAACCTAAAATAATAACAATAGCGT [21294]
cp32-3_PAbe_NX_MP_FG_consensus TTTTATGGGAAGAAATACCCGGAACAAGGACCACTAATAAAATGGATAATAAAAAACCTAAAATAATAACAATAGCGT [21294]
cp32-3_PAbe_NX_Pl_consensus    TTTTATGGGAAGAAATACCCGGAACAAGGACCACTAATAAAATGGATAATAAAAAACCTAAAATAATAACAATAGCGT [21294]

cp32-3_gi|11497103|B31_GB      CAATTAAGGGCGGTGTAGGTAAAAGCACAAGTGCGATAATTTTGGCTACTTTATTATCAAAAAATAATAAAGTTCTTT [21372]
cp32-3_un8_B31_PacBio          CAATTAAGGGCGGTGTAGGTAAAAGCACAAGTGCGATAATTTTGGCTACTTTATTATCAAAAAATAATAAAGTTCTTT [21372]
cp32-3_B31_TS_MP_FG_consensus  CAATTAAGGGCGGTGTAGGTAAAAGCACAAGTGCGATAATTTTGGCTACTTTATTATCAAAAAATAATAAAGTTCTTT [21372]
cp32-3_B31_NX_Pl_consensus     CAATTAAGGGCGGTGTAGGTAAAAGCACAACTTCAATAATTCTTGCCACTCTTTTATCAAAAAATAATAAAGTTCTTT [21372]
cp32-3_PAli_NX_MP_FG_consensus CAATTAAGGGCGGTGTAGGTAAAAGCACAAGTGCGATAATTTTGGCTACTTTATTATCAAAAAATAATAAAGTTCTTT [21372]
cp32-3_PAli_NX_Pl_consensus    CAATTAAGGGCGGTGTAGGTAAAAGCACAAGTGCGATAATTTTGGCTACTTTATTATCAAAAAATAATAAAGTTCTTT [21372]
cp32-3_PAbe_NX_MP_FG_consensus CAATTAAGGGCGGTGTAGGTAAAAGCACAAGTGCGATAATTTTGGCTACTTTATTATCAAAAAATAATAAAGTTCTTT [21372]
cp32-3_PAbe_NX_Pl_consensus    CAATTAAGGGCGGTGTAGGTAAAAGCACAAGTGCAATAATTTTTGCTACTCTTTTATCAAAAAATAATAAAGTTCTTT [21372]

cp32-3_gi|11497103|B31_GB      TAATTGATATGGATACTCAAGCATCTATTACTAGTTATTTTTACGAAAAAATAGAAAAACTAGGTATTAATTTTACCA [21450]
cp32-3_un8_B31_PacBio          TAATTGATATGGATACTCAAGCATCTATTACTAGTTATTTTTACGAAAAAATAGAAAAACTAGGTATTAATTTTACCA [21450]
cp32-3_B31_TS_MP_FG_consensus  TAATTGATATGGATACTCAAGCATCTATTACTAGTTATTTTTACGAAAAAATAGAAAAACTAGGTATTAATTTTACCA [21450]
cp32-3_B31_NX_Pl_consensus     TAATTGATATGGATACTCAAGCATCTATTACTAGTTATTTTTACGAAAAAATAGAAAAACTAGGTATTAATTTTACCA [21450]
cp32-3_PAli_NX_MP_FG_consensus TAATTGATATGGATACTCAAGCATCTATTACTAGTTATTTTTACGAAAAAATAGAAAAACTAGGTATTAATTTTACCA [21450]
cp32-3_PAli_NX_Pl_consensus    TAATTGATATGGATACTCAAGCATCTATTACTAGTTATTTTTACGAAAAAATAGAAAAACTAGGTATTAATTTTACCA [21450]
cp32-3_PAbe_NX_MP_FG_consensus TAATTGATATGGATACTCAAGCATCTATTACTAGTTATTTTTACGAAAAAATAGAAAAACTAGGTATTAATTTTACCA [21450]
cp32-3_PAbe_NX_Pl_consensus    TAATTGATATGGATACTCAAGCATCTATTACTAGTTATTTTTACGAAAAAATAGAAAAACTAGGTATTAATTTTACCA [21450]

cp32-3_gi|11497103|B31_GB      AATTTAATATTTATGAGATTTTGAAAGAAAATGTAGATATCGATAGTACCATTATAAATGTAGATAATAATCTTGATC [21528]
cp32-3_un8_B31_PacBio          AATTTAATATTTATGAGATTTTGAAAGAAA-TGTAGATATCGATAGTACCATTATAAATGTAGATAATAATCTTGATC [21528]
cp32-3_B31_TS_MP_FG_consensus  AATTTAATATTTATGAGATTTTGAAAGAAAATGTAGATATCGATAGTACCATTATAAATGTAGATAATAATCTTGATC [21528]
cp32-3_B31_NX_Pl_consensus     AATTTAATATTTATGAGATTTTGAAAGAAAATGTAGATATCGATAGTACCATTATAAATGTAGATAATAATCTTGATC [21528]
cp32-3_PAli_NX_MP_FG_consensus AATTTAATATTTATGAGATTTTGAAAGAAAATGTAGATATCGATAGTACCATTATAAATGTAGATAATAATCTTGATC [21528]
cp32-3_PAli_NX_Pl_consensus    AATTTAATATTTATGAGATTTTGAAAGAAAATGTAGATATCGATAGTACCATTATAAATGTAGATAATAATCTTGATC [21528]
cp32-3_PAbe_NX_MP_FG_consensus AATTTAATATTTATGAGATTTTGAAAGAAAATGTAGATATCGATAGTACCATTATAAATGTAGATAATAATCTTGATC [21528]
cp32-3_PAbe_NX_Pl_consensus    AATTTAATATTTATGAGATTTTGAAAGAAAATGTAGATATCGATAGTACCATTATAAATGTAGATAATAATCTTGATC [21528]

cp32-3_gi|11497103|B31_GB      TTATACCTAGCTATCTTACGCTACATAATTTTAGTGAAGATAAAATCGAACATAAAGATTTTTTGTTAAAAACCAGCC [21606]
cp32-3_un8_B31_PacBio          TTATACCTAGCTATCTTACGCTACATAATTTTAGTGAAGATAAAATCGAACATAAAGATTTTTTGTTAAAAACCAGCC [21606]
cp32-3_B31_TS_MP_FG_consensus  TTATACCTAGCTATCTTACGCTACATAATTTTAGTGAAGATAAAATCGAACATAAAGATTTTTTGTTAAAAACCAGCC [21606]
cp32-3_B31_NX_Pl_consensus     TTATACCTAGCTATCTTACGCTACATAATTTTAGTGAAGATAAAATCGAACATAAAGATTTTTTGTTAAAAACCAGCC [21606]
cp32-3_PAli_NX_MP_FG_consensus TTATACCTAGCTATCTTACGCTACATAATTTTAGTGAAGATAAAATCGAACATAAAGATTTTTTGTTAAAAACCAGCC [21606]
cp32-3_PAli_NX_Pl_consensus    TTATACCTAGCTATCTTACGCTACATAATTTTAGTGAAGATAAAATCGAACATAAAGATTTTTTGTTAAAAACCAGCC [21606]
cp32-3_PAbe_NX_MP_FG_consensus TTATACCTAGCTATCTTACGCTACATAATTTTAGTGAAGATAAAATCGAACATAAAGATTTTTTGTTAAAAACCAGCC [21606]
cp32-3_PAbe_NX_Pl_consensus    TTATACCTAGCTATCTTACGCTACATAATTTTAGTGAAGATAAAATCGAACATAAAGATTTTTTGTTAAAAACCAGCC [21606]

cp32-3_gi|11497103|B31_GB      TGGGAACTTTGTATTATAAATATGACTATATAGTAATCGATACAAATCCCAGTTTAGATGTTACGTTAAAAAATGCTC [21684]
cp32-3_un8_B31_PacBio          TGGGAACTTTGTATTATAAATATGACTATATAGTAATCGATACAAATCCCAGTTTAGATGTTACGTTAAAAAATGCTC [21684]
cp32-3_B31_TS_MP_FG_consensus  TGGGAACTTTGTATTATAAATATGACTATATAGTAATCGATACAAATCCCAGTTTAGATGTTACGTTAAAAAATGCTC [21684]
cp32-3_B31_NX_Pl_consensus     TGGGAACTTTGTATTATAAATATGACTATATAGTAATCGATACAAATCCCAGTTTAGATGTTACGTTAAAAAATGCTC [21684]
cp32-3_PAli_NX_MP_FG_consensus TGGGAACTTTGTATTATAAATATGACTATATAGTAATCGATACAAATCCCAGTTTAGATGTTACGTTAAAAAATGCTC [21684]
cp32-3_PAli_NX_Pl_consensus    TGGGAACTTTGTATTATAAATATGACTATATAGTAATCGATACAAATCCCAGTTTAGATGTTACGTTAAAAAATGCTC [21684]
```

```
cp32-3_PAbe_NX_MP_FG_consensus TGGGAACTTTGTATTATAAATATGACTATATAGTAATCGATACAAATCCCAGTTTAGATGTTACGTTAAAAAATGCTC [21684]
cp32-3_PAbe_NX_Pl_consensus    TGGGAACTTTGTATTATAAATATGACTATATAGTAATCGATACAAATCCCAGTTTAGATGTTACGTTAAAAAATGCTC [21684]

cp32-3_gi|11497103|B31_GB      TTTTATGTAGTGACTATGTAATAATTCCAATGACGGCTGAAAAGTGGGCAGTGGAAAGTTTAGATTTATTTAATTTCT [21762]
cp32-3_un8_B31_PacBio          TTTTATGTAGTGACTATGTAATAATTCCAATGACGGCTGAAAAGTGGGCAGTGGAAAGTTTAGATTTATTTAATTTCT [21762]
cp32-3_B31_TS_MP_FG_consensus  TTTTATGTAGTGACTATGTAATAATTCCAATGACGGCTGAAAAGTGGGCAGTGGAAAGTTTAGATTTATTTAATTTCT [21762]
cp32-3_B31_NX_Pl_consensus     TTTTATGTAGTGACTATGTAATAATTCCAATGACGGCTGAAAAGTGGGCAGTGGAAAGTTTAGATTTATTTAATTTCT [21762]
cp32-3_PAli_NX_MP_FG_consensus TTTTATGTAGTGACTATGTAATAATTCCAATGACGGCTGAAAAGTGGGCAGTGGAAAGTTTAGATTTATTTAATTTCT [21762]
cp32-3_PAli_NX_Pl_consensus    TTTTATGTAGTGACTATGTAATAATTCCAATGACGGCTGAAAAGTGGGCAGTGGAAAGTTTAGATTTATTTAATTTCT [21762]
cp32-3_PAbe_NX_MP_FG_consensus TTTTATGTAGTGACTATGTAATAATTCCAATGACGGCTGAAAAGTGGGCAGTGGAAAGTTTAGATTTATTTAATTTCT [21762]
cp32-3_PAbe_NX_Pl_consensus    TTTTATGTAGTGACTATGTAATAATTCCAATGACGGCTGAAAAGTGGGCAGTGGAAAGTTTAGATTTATTTAATTTCT [21762]

cp32-3_gi|11497103|B31_GB      TTGTAAGAAAATTAAATTTATTTTTACCTATATTTTTGATAATAACAAGATTTAAAAAAAATAGAACACATAAAACAT [21840]
cp32-3_un8_B31_PacBio          TTGTAAGAAAATTAAATTTATTTTTACCTATATTTTTGATAATAACAAGATTTAAAAAAAATAGAACACATAAAACAT [21840]
cp32-3_B31_TS_MP_FG_consensus  TTGTAAGAAAATTAAATTTATTTTTACCTATATTTTTGATAATAACAAGATTTAAAAAAAATAGAACACATAAAACAT [21840]
cp32-3_B31_NX_Pl_consensus     TTGTAAGAAAATTAAATTTATTTTTACCTATATTTTTGATAATAACAAGATTTAAAAAAAATAGAACACATAAAACAT [21840]
cp32-3_PAli_NX_MP_FG_consensus TTGTAAGAAAATTAAATTTATTTTTACCTATATTTTTGATAATAACAAGATTTAAAAAAAATAGAACACATAAAACAT [21840]
cp32-3_PAli_NX_Pl_consensus    TTGTAAGAAAATTAAATTTATTTTTACCTATATTTTTGATAATAACAAGATTTAAAAAAAATAGAACACATAAAACAT [21840]
cp32-3_PAbe_NX_MP_FG_consensus TTGTAAGAAAATTAAATTTATTTTTACCTATATTTTTGATAATAACAAGATTTAAAAAAAATAGAACACATAAAACAT [21840]
cp32-3_PAbe_NX_Pl_consensus    TTGTAAGAAAATTAAATTTATTTTTACCTATATTTTTGATAATAACAAGATTTAAAAAAAATAGAACACATAAAACAT [21840]

cp32-3_gi|11497103|B31_GB      TATTTGAAATATTAAAAACAAAAGATAGATTTTTAGGAACAATATCTGAAAGAGAAGACTTAAATAGACGAATAGCAG [21918]
cp32-3_un8_B31_PacBio          TATTTGAAATATTAAAA-CAAAAGATAGATTTTTAGGAACAATATCTGAAAGAGAAGACTTAAATAGACGAATAGCAG [21918]
cp32-3_B31_TS_MP_FG_consensus  TATTTGAAATATTAAAAACAAAAGATAGATTTTTAGGAACAATATCTGAAAGAGAAGACTTAAATAGACGAATAGCAG [21918]
cp32-3_B31_NX_Pl_consensus     TATTTGAAATATTAAAAACAAAAGATAGATTTTTAGGAACAATATCTGAAAGAGAAGACTTAAATAGACGAATAGCAG [21918]
cp32-3_PAli_NX_MP_FG_consensus TATTTGAAATATTAAAAACAAAAGATAGATTTTTAGGAACAATATCTGAAAGAGAAGACTTAAATAGACGAATAGCAG [21918]
cp32-3_PAli_NX_Pl_consensus    TATTTGAAATATTAAAAACAAAAGATAGATTTTTAGGAACAATATCTGAAAGAGAAGACTTAAATAGACGAATAGCAG [21918]
cp32-3_PAbe_NX_MP_FG_consensus TATTTGAAATATTAAAAACAAAAGATAGATTTTTAGGAACAATATCTGAAAGAGAAGACTTAAATAGACGAATAGCAG [21918]
cp32-3_PAbe_NX_Pl_consensus    TATTTGAAATATTAAAAACAAAAGATAGATTTTTAGGAACAATATCTGAAAGAGAAGACTTAAATAGACGAATAGCAG [21918]

cp32-3_gi|11497103|B31_GB      AAAATAATAATTTTGATTTAAATAAAGATTATATAAAAGAATATGAAAATATTTTAGAAATTTTTTTAAAAAAAAATAT [21996]
cp32-3_un8_B31_PacBio          AAAATAATAATTTTGATTTAAATAAAGATTATATAAAAGAATATGAAAATATTTTAGAAATTTTTTTAAAAAAAA-TAT [21996]
cp32-3_B31_TS_MP_FG_consensus  AAAATAATAATTTTGATTTAAATAAAGATTATATAAAAGAATATGAAAATATTTTAGAAATTTTTTTAAAAAAAAATAT [21996]
cp32-3_B31_NX_Pl_consensus     AAAATAATAATTTTGATTTAAATAAAGATTATATAAAAGAATATGAAAATATTTTAGAAATTTTTTTAAAAAAAAATAT [21996]
cp32-3_PAli_NX_MP_FG_consensus AAAATAATAATTTTGATTTAAATAAAGATTATATAAAAGAATATGAAAATATTTTAGAAATTTTTTTAAAAAAAAATAT [21996]
cp32-3_PAli_NX_Pl_consensus    AAAATAATAATTTTGATTTAAATAAAGATTATATAAAAGAATATGAAAATATTTTAGAAATTTTTTTAAAAAAAAATAT [21996]
cp32-3_PAbe_NX_MP_FG_consensus AAAATAATAATTTTGATTTAAATAAAGATTATATAAAAGAATATGAAAATATTTTAGAAATTTTTTTAAAAAAAAATAT [21996]
cp32-3_PAbe_NX_Pl_consensus    AAAATAATAATTTTGATTTAAATAAAGATTATATAAAAGAATATGAAAATATTTTAGAAATTTTTTTAAAAAAAAATAT [21996]

cp32-3_gi|11497103|B31_GB      AAAGTCTAATAATTAGACAGTTTAATGTAAAAGGAGTTTAAATTGGATATTAAGGTTAATAAAAGAGATTTAAGTGAT [22074]
cp32-3_un8_B31_PacBio          AAAGTCTAATAATTAGACAGTTTAATGTAAAAGGAGTTTAAATTGGATATTAAGGTTAATAAAAGAGATTTAAGTGAT [22074]
cp32-3_B31_TS_MP_FG_consensus  AAAGTCTAATAATTAGACAGTTTAATGTAAAAGGAGTTTAAATTGGATATTAAGGTTAATAAAAGAGATTTAAGTGAT [22074]
cp32-3_B31_NX_Pl_consensus     AAAGTCTAATAATTAGACAGTTTAATGTAAAAGGAGTTTAAATTGGATATTAAGGTTAATAAAAGAGATTTAAGTGAT [22074]
cp32-3_PAli_NX_MP_FG_consensus AAAGTCTAATAATTAGACAGTTTAATGTAAAAGGAGTTTAAATTGGATATTAAGGTTAATAAAAGAGATTTAAGTGAT [22074]
cp32-3_PAli_NX_Pl_consensus    AAAGTCTAATAATTAGACAGTTTAATGTAAAAGGAGTTTAAATTGGATATTAAGGTTAATAAAAGAGATTTAAGTGAT [22074]
cp32-3_PAbe_NX_MP_FG_consensus AAAGTCTAATAATTAGACAGTTTAATGTAAAAGGAGTTTAAATTGGATATTAAGGTTAATAAAAGAGATTTAAGTGAT [22074]
cp32-3_PAbe_NX_Pl_consensus    AAAGTCTAATAATTAGACAGTTTAATGTAAAAGGAGTTTAAATTGGATATTAAGGTTAATAAAAGAGATTTAAGTGAT [22074]

cp32-3_gi|11497103|B31_GB      AATGAAAATTATATTATTACTGATAGTGTATTAAATCATTATAATTCTTTAAAAGAAAAGTTAAAAATTAATTCTAAA [22152]
cp32-3_un8_B31_PacBio          AATGAAAATTATATTATTACTGATAGTGTATTAAATCATTATAATTCTTTAAAAGAAAAGTTAAAAATTAATTCTAAA [22152]
cp32-3_B31_TS_MP_FG_consensus  AATGAAAATTATATTATTACTGATAGTGTATTAAATCATTATAATTCTTTAAAAGAAAAGTTAAAAATTAATTCTAAA [22152]
cp32-3_B31_NX_Pl_consensus     AATGAAAATTATATTATTACTGATAGTGTATTAAATCATTATAATTCTTTAAAAGAAAAGTTAAAAATTAATTCTAAA [22152]
cp32-3_PAli_NX_MP_FG_consensus AATGAAAATTATATTATTACTGATAGTGTATTAAATCATTATAATTCTTTAAAAGAAAAGTTAAAAATTAATTCTAAA [22152]
cp32-3_PAli_NX_Pl_consensus    AATGAAAATTATATTATTACTGATAGTGTATTAAATCATTATAATTCTTTAAAAGAAAAGTTAAAAATTAATTCTAAA [22152]
cp32-3_PAbe_NX_MP_FG_consensus AATGAAAATTATATTATTACTGATAGTGTATTAAATCATTATAATTCTTTAAAAGAAAAGTTAAAAATTAATTCTAAA [22152]
cp32-3_PAbe_NX_Pl_consensus    AATGAAAATTATATTATTACTGATAGTGTATTAAATCATTATAATTCTTTAAAAGAAAAGTTAAAAATTAATTCTAAA [22152]

cp32-3_gi|11497103|B31_GB      AAGGAAATCTATTGTAAGTTAGAAACTTTAAAAATTTTAAAAGAAATTAAAGACAATCATTATTATAGGTTTGATGGT [22230]
cp32-3_un8_B31_PacBio          AAGGAAATCTATTGTAAGTTAGAAACTTTAAAA-TTTTAAAAGAAATTAAAGACAATCATTATTATAGGTTTGATGGT [22230]
cp32-3_B31_TS_MP_FG_consensus  AAGGAAATCTATTGTAAGTTAGAAACTTTAAAAATTTTAAAAGAAATTAAAGACAATCATTATTATAGGTTTGATGGT [22230]
cp32-3_B31_NX_Pl_consensus     AAGGAAATCTATTGTAAGTTAGAAACTTTAAAAATTTTAAAAGAAATTAAAGACAATCATTATTATAGGTTTGATGGT [22230]
cp32-3_PAli_NX_MP_FG_consensus AAGGAAATCTATTGTAAGTTAGAAACTTTAAAAATTTTAAAAGAAATTAAAGACAATCATTATTATAGGTTTGATGGT [22230]
cp32-3_PAli_NX_Pl_consensus    AAGGAAATCTATTGTAAGTTAGAAACTTTAAAAATTTTAAAAGAAATTAAAGACAATCATTATTATAGGTTTGATGGT [22230]
cp32-3_PAbe_NX_MP_FG_consensus AAGGAAATCTATTGTAAGTTAGAAACTTTAAAAATTTTAAAAGAAATTAAAGACAATCATTATTATAGGTTTGATGGT [22230]
cp32-3_PAbe_NX_Pl_consensus    AAGGAAATCTATTGTAAGTTAGAAACTTTAAAAATTTTAAAAGAAATTAAAGACAATCATTATTATAGGTTTGATGGT [22230]

cp32-3_gi|11497103|B31_GB      TATAAAAGTTTTGAAGCTTTTTCTAAAGATTATAGACTTGCAAGAGCACAGGTCTATAATTACTTAAAAATTGCTAAT [22308]
cp32-3_un8_B31_PacBio          TATAAAAGTTTTGAAGCTTTTTCTAAAGATTATAGACTTGCAAGAGCACAGGTCTATAATTACTTAAAAATTGCTAAT [22308]
cp32-3_B31_TS_MP_FG_consensus  TATAAAAGTTTTGAAGCTTTTTCTAAAGATTATAGACTTGCAAGAGCACAGGTCTATAATTACTTAAAAATTGCTAAT [22308]
cp32-3_B31_NX_Pl_consensus     TATAAAAGTTTTGAAGCTTTTTCTAAAGATTATAGACTTGCAAGAGCACAGGTCTATAATTACTTAAAAATTGCTAAT [22308]
cp32-3_PAli_NX_MP_FG_consensus TATAAAAGTTTTGAAGCTTTTTCTAAAGATTATAGACTTGCAAGAGCACAGGTCTATAATTACTTAAAAATTGCTAAT [22308]
cp32-3_PAli_NX_Pl_consensus    TATAAAAGTTTTGAAGCTTTTTCTAAAGATTATAGACTTGCAAGAGCACAGGTCTATAATTACTTAAAAATTGCTAAT [22308]
cp32-3_PAbe_NX_MP_FG_consensus TATAAAAGTTTTGAAGCTTTTTCTAAAGATTATAGACTTGCAAGAGCACAGGCCTATGATTATCTAAGAATTGCTAAT [22308]
cp32-3_PAbe_NX_Pl_consensus    TATAAAAGTTTTGAAGCTTTTTCTAAAGATTATAGACTTGCAAGAGCACAGGTCTATAATTACTTAAAAATTGCTAAT [22308]

cp32-3_gi|11497103|B31_GB      GCAATAGAAGATGGGATTATACAAGAAGAATTTCTAATAAAAAATGG-TATACTGGAAACGTTAATTGTATTAAGAAA [22386]
cp32-3_un8_B31_PacBio          GCAATAGAAGATGGGATTATACAAGAAGAATTTCTAATAAAAAATGG-TATACTGGAAACGTTAATTGTAT-AAGAAA [22386]
cp32-3_B31_TS_MP_FG_consensus  GCTATAGAAGAGGGGATAATAGAGGAAGAATTTTTAGTACAAAATGGATTTA-GACAAACTCTTTTTTTACTAAGAAA [22386]
cp32-3_B31_NX_Pl_consensus     GCAATAGAAGAGGGGATAATAGAGGAAGAATTTTTAGTACAAAATGGATTTA-GACAAACTCTTTTTTTACTAAGAAA [22386]
cp32-3_PAli_NX_MP_FG_consensus GCAATAGAAGATGGGATTATACAAGAAGAATTTCTAATAAAAAATGG-TATACTGGAAACGTTAATTGTATTAAGAAA [22386]
cp32-3_PAli_NX_Pl_consensus    GCAATAGAAGATGGGATTATACAAGAAGAATTTCTAATAAAAAATGG-TATACTGGAAACGTTAATTGTATTAAGAAA [22386]
cp32-3_PAbe_NX_MP_FG_consensus GCTATAGAAGAGGGGATAATAGAGGAAGAATTTTTAGTACAAAATGGATTTA-GACAAACTCTTTTTTTACTAAGAAA [22386]
cp32-3_PAbe_NX_Pl_consensus    GCAATAGAAGATGGGATAATAGAGGAAGAATTTTTAGTACAAAATGGATTTACGGCAAACGCTTTTTTTACTAAGAAA [22386]

cp32-3_gi|11497103|B31_GB      TAAAGAGAGCAAAACAATAAAAAAAATCAAAACAAAACCCGATAAAGCCCTTAAGATTTCAGCTTAAAAGACAAGAAAG [22464]
cp32-3_un8_B31_PacBio          TAAAGAGAGCAAAACAATAAAAAAAATCAAAACAAAACCCGATAAAGCCCTTAAGATTTCAGCTTAAAAGACAAGAAAG [22464]
cp32-3_B31_TS_MP_FG_consensus  CAAAGAAAGCGTAACAATAAAAAAAATCCAAACAAAACCCGATAAAGCCCTTAAGATTTCAGCTTAAAAGACAAGAAAG [22464]
cp32-3_B31_NX_Pl_consensus     CAAAGAAAGCGTAACAATAAAAAAAATCCAAACAAAATTGGATAAAACCCCTAAGATTTCAGCTTAAGAAACAAGAAAG [22464]
cp32-3_PAli_NX_MP_FG_consensus TAAAGAGAGCAAAACAATAAAAAAAATCAAAACAAAACCCGATAAAGCCCTTAAGATTTCAGCTTAAAAGACAAGAAAG [22464]
cp32-3_PAli_NX_Pl_consensus    TAAAGAGAGCAAAACAATAAAAAAAATCAAAACAAAACCCGATAAAGCCCTTAAGATTTCAGCTTAAAAGACAAGAAAG [22464]
cp32-3_PAbe_NX_MP_FG_consensus CAAAGAAAGCGTAACAATAAAAAAAATCCAAACAAAATTGGATAAAACCCCTAAGATTTCAGCTTAAAAGACAAGAAAG [22464]
cp32-3_PAbe_NX_Pl_consensus    CAAAGAAAGCGTAACAATAAAAAAAATCCAAACAAAATTGGATAAAACCCCTAAGATTTCAGCTTAAGAAACAAGAAAG [22464]

cp32-3_gi|11497103|B31_GB      CTATGATTTTTATAAAAGTAATGCTAAATTTACAGGATTTATGTTAGATAAATTATTTAGTGATGGAAAAGAAATAAT [22542]
cp32-3_un8_B31_PacBio          CTATGATTTTTATAAAAGTAATGCTAAATTTACAGGATTTATGTTAGATAAATTATTTAGTGATGGAAAAGAAATAAT [22542]
cp32-3_B31_TS_MP_FG_consensus  CTATGATTTTTATAAAAGTAATGCTAAATTTACAGGATTTATGTTAGATAAATTATTTAGTGATGGAAAAGAAATAAT [22542]
cp32-3_B31_NX_Pl_consensus     CTATGATTTTTATAAAAGTAATGCTAAATTTACAGGATTTATGTTAGATAAATTATTTAGTGATGGAAAAGAAATAAT [22542]
cp32-3_PAli_NX_MP_FG_consensus CTATGATTTTTATAAAAGTAATGCTAAATTTACAGGATTTATGTTAGATAAATTATTTAGTGATGGAAAAGAAATAAT [22542]
cp32-3_PAli_NX_Pl_consensus    CTATGATTTTTATAAAAGTAATGCTAAATTTACAGGATTTATGTTAGATAAATTATTTAGTGATGGAAAAGAAATAAT [22542]
cp32-3_PAbe_NX_MP_FG_consensus CTATGATTTTTATAAAAGTAATGCTAAATTTACAGGATTTATGTTAGATAAATTATTTAGTGATGGAAAAGAAATAAT [22542]
cp32-3_PAbe_NX_Pl_consensus    CTATGATTTTTATAAAAGTAATGCTAAATTTACAGGATTTATGTTAGATAAATTATTTAGTGATGGAAAAGAAATAAT [22542]

cp32-3_gi|11497103|B31_GB      TAAAAAACTTATGAAAGAATATAAAGAATTAAAAGGATAATATGGAAATTTTATGAATAGTTTGACTTACAGAACATA [22620]
cp32-3_un8_B31_PacBio          TAAAAAACTTATGAAAGAATATAAAGAATTAAAAGGATAATATGGAAATTTTATGAATAGTTTGACTTACAGAACATA [22620]
cp32-3_B31_TS_MP_FG_consensus  TAAAAAACTTATGAAAGAATATAAAGAATTAAAAGGATAATATGGAAATTTTATGAATAGTTTGACTTACAGAACATA [22620]
cp32-3_B31_NX_Pl_consensus     TAAAAAACTTATGAAAGAATATAAAGAATTAAAAGGATAATATGGAAATTTTATGAATAGTTTGACTTACAGAACATA [22620]
cp32-3_PAli_NX_MP_FG_consensus TAAAAAACTTATGAAAGAATATAAAGAATTAAAAGGATAATATGGAAATTTTATGAATAGTTTGACTTACAGAACATA [22620]
cp32-3_PAli_NX_Pl_consensus    TAAAAAACTTATGAAAGAATATAAAGAATTAAAAGGATAATATGGAAATTTTATGAATAGTTTGACTTACAGAACATA [22620]
cp32-3_PAbe_NX_MP_FG_consensus TAAAAAACTTATGAAAGAATATAAAGAATTAAAAGGATAATATGGAAATTTTATGAATAGTTTGACTTACAGAACATA [22620]
```

```
cp32-3_PAbe_NX_Pl_consensus    TAAAAAACTTATGAAAGAATATAAAGAATTAAAAGGATAATATGGAAATTTTATGAATAGTTTGACTTACAGAACATA [22620]

cp32-3_gi|11497103|B31_GB      CAATATAGAAAGTATAAAAAAATGAATTTTTAAAGATAGGATTTAGTGAAGAAGCAATAGATTTTGTTTTTCTGCATAA [22698]
cp32-3_un8_B31_PacBio          CAATATAGAAAGTATAAAAAAATGAATTTTTAAAGATAGGATTTAGTGAAGAAGCAATAGATTTTGTTTTTCTGCATAA [22698]
cp32-3_B31_TS_MP_FG_consensus  CAATATAGAAAGTATAAAAAAATGAATTTTTAAAGATAGGATTTAGTGAAGAAGCAATAGATTTTGTTTTTCTGCATAA [22698]
cp32-3_B31_NX_Pl_consensus     CAATATAGAAAGTATAAAAAAATGAATTTTTAAAGATAGGATTTAGTGAAGAAGCAATAGATTTTGTTTTTCTGCATAA [22698]
cp32-3_PAli_NX_MP_FG_consensus CAATATAGAAAGTATAAAAAAATGAATTTTTAAAGATAGGATTTAGTGAAGAAGCAATAGATTTTGTTTTTCTGCATAA [22698]
cp32-3_PAli_NX_Pl_consensus    CAATATAGAAAGTATAAAAAAATGAATTTTTAAAGATAGGATTTAGTGAAGAAGCAATAGATTTTGTTTTTCTGCATAA [22698]
cp32-3_PAbe_NX_MP_FG_consensus CAATATAGAAAGTATAAAAAAATGAATTTTTAAAGATAGGATTTAGTGAAGAAGCAATAGATTTTGTTTTTCTGCATAA [22698]
cp32-3_PAbe_NX_Pl_consensus    CAATATAGAAAGTATAAAAAAATGAATTTTTAAAGATAGGATTTAGTGAAGAAGCAATAGATTTTGTTTTTCTGCATAA [22698]

cp32-3_gi|11497103|B31_GB      TGATAATTACAACTTTGAATTTTTAAAAGAAAAATTGATTAATGTAGAAAAGAATTTACAAAAGGATATATCTAATTT [22776]
cp32-3_un8_B31_PacBio          TGATAATTACAACTTTGAATTTTTAAAAGAAAAATTGATTAATGTAGAAAAGAATTTACAAAAGGATATATCTAATTT [22776]
cp32-3_B31_TS_MP_FG_consensus  TGATAATTACAACTTTGAATTTTTAAAAGAAAAATTGATTAATGTAGAAAAGAATTTACAAAAGGATATATCTAATTT [22776]
cp32-3_B31_NX_Pl_consensus     TGATAATTACAACTTTGAATTTTTAAAAGAAAAATTGATTAATGTAGAAAAGAATTTACAAAAGGATATATCTAATTT [22776]
cp32-3_PAli_NX_MP_FG_consensus TGATAATTACAACTTTGAATTTTTAAAAGAAAAATTGATTAATGTAGAAAAGAATTTACAAAAGGATATATCTAATTT [22776]
cp32-3_PAli_NX_Pl_consensus    TGATAATTACAACTTTGAATTTTTAAAAGAAAAATTGATTAATGTAGAAAAGAATTTACAAAAGGATATATCTAATTT [22776]
cp32-3_PAbe_NX_MP_FG_consensus TGATAATTACAACTTTGAATTTTTAAAAGAAAAATTGATTAATGTAGAAAAGAATTTACAAAAGGATATATCTAATTT [22776]
cp32-3_PAbe_NX_Pl_consensus    TGATAATTACAACTTTGAATTTTTAAAAGAAAAATTGATTAATGTAGAAAAGAATTTACAAAAGGATATATCTAATTT [22776]

cp32-3_gi|11497103|B31_GB      AGACATTAAGATTGATACTGTAGAAAAGAATTTAAACACTAAAATAGACAATGTTGAAAAGAATTTAAATCTAAAAAT [22854]
cp32-3_un8_B31_PacBio          AGACATTAAGATTGATACTGTAGAAAAGAATTTAAACACTAAAATAGACAATGTTGAAAAGAATTTAAATCTAAAAAT [22854]
cp32-3_B31_TS_MP_FG_consensus  AGACATTAAGATTGATACTGTAGAAAAGAATTTAAACACTAAAATAGACAATGTTGAAAAGAATTTAAATCTAAAAAT [22854]
cp32-3_B31_NX_Pl_consensus     AGACATTAAGATTGATACTGTAGAAAAGAATTTAAACACTAAAATAGACAATGTTGAAAAGAATTTAAATCTAAAAAT [22854]
cp32-3_PAli_NX_MP_FG_consensus AGACATTAAGATTGATACTGTAGAAAAGAATTTAAACACTAAAATAGACAATGTTGAAAAGAATTTAAATCTAAAAAT [22854]
cp32-3_PAli_NX_Pl_consensus    AGACATTAAGATTGATACTGTAGAAAAGAATTTAAACACTAAAATAGACAATGTTGAAAAGAATTTAAATCTAAAAAT [22854]
cp32-3_PAbe_NX_MP_FG_consensus AGACATTAAGATTGATACTGTAGAAAAGAATTTAAACACTAAAATAGACAATGTTGAAAAGAATTTAAATCTAAAAAT [22854]
cp32-3_PAbe_NX_Pl_consensus    AGACATTAAGATTGATACTGTAGAAAAGAATTTAAACACTAAAATAGACAATGTTGAAAAGAATTTAAATCTAAAAAT [22854]

cp32-3_gi|11497103|B31_GB      AGATAATTTAGACTCTAAAATAGATACTGTAGAAAAGAATTTAAACACTAAAATAGACAATGTTGAAAAGAATTTAAA [22932]
cp32-3_un8_B31_PacBio          AGATAATTTAGACTCTAAAATAGATACTGTAGAAAAGAATTTAAACACTAAAATAGACAATGTTGAAAAGAATTTAAA [22932]
cp32-3_B31_TS_MP_FG_consensus  AGATAATTTAGACTCTAAAATAGATACTGTAGAAAAGAATTTAAACACTAAAATAGACAATGTTGAAAAGAATTTAAA [22932]
cp32-3_B31_NX_Pl_consensus     AGATAATTTAGACTCTAAAATAGATACTGTAGAAAAGAATTTAAACACTAAAATAGACAATGTTGAAAAGAATTTAAA [22932]
cp32-3_PAli_NX_MP_FG_consensus AGATAATTTAGACTCTAAAATAGATACTGTAGAAAAGAATTTAAACACTAAAATAGACAATGTTGAAAAGAATTTAAA [22932]
cp32-3_PAli_NX_Pl_consensus    AGATAATTTAGACTCTAAAATAGATACTGTAGAAAAGAATTTAAACACTAAAATAGACAATGTTGAAAAGAATTTAAA [22932]
cp32-3_PAbe_NX_MP_FG_consensus AGATAATTTAGACTCTAAAATAGATACTGTAGAAAAGAATTTAAACACTAAAATAGACAATGTTGAAAAGAATTTAAA [22932]
cp32-3_PAbe_NX_Pl_consensus    AGATAATTTAGACTCTAAAATAGATACTGTAGAAAAGAATTTAAACACTAAAATAGACAATGTTGAAAAGAATTTAAA [22932]

cp32-3_gi|11497103|B31_GB      CCTAAAAATGGATAATTTAGACTCTAAAATAGATACTGTAGAAAAGAATTTAAACACTAAAATAGACAATGTTGAAAA [23010]
cp32-3_un8_B31_PacBio          CCTAAAAATGGATAATTTAGACTCTAAAATAGATACTGTAGAAAAGAATTTAAACACTAAAATAGACAATGTTGAAAA [23010]
cp32-3_B31_TS_MP_FG_consensus  CCTAAAAATGGATAATTTAGACTCTAAAATAGATACTGTAGAAAAGAATTTAAACACTAAAATAGACAATGTTGAAAA [23010]
cp32-3_B31_NX_Pl_consensus     CCTAAAAATGGATAATTTAGACTCTAAAATAGATACTGTAGAAAAGAATTTAAACACTAAAATAGACAATGTTGAAAA [23010]
cp32-3_PAli_NX_MP_FG_consensus CCTAAAAATGGATAATTTAGACTCTAAAATAGATACTGTAGAAAAGAATTTAAACACTAAAATAGACAATGTTGAAAA [23010]
cp32-3_PAli_NX_Pl_consensus    CCTAAAAATGGATAATTTAGACTCTAAAATAGATACTGTAGAAAAGAATTTAAACACTAAAATAGACAATGTTGAAAA [23010]
cp32-3_PAbe_NX_MP_FG_consensus CCTAAAAATGGATAATTTAGACTCTAAAATAGATACTGTAGAAAAGAATTTAAACACTAAAATAGACAATGTTGAAAA [23010]
cp32-3_PAbe_NX_Pl_consensus    CCTAAAAATGGATAATTTAGACTCTAAAATAGATACTGTAGAAAAGAATTTAAACACTAAAATAGACAATGTTGAAAA [23010]

cp32-3_gi|11497103|B31_GB      GAATTTAAACCTAAAAATGGATGGCTTGAATATCAAAATAGACAATGTAGAAAAGAGTTTAAATCAAAAACTTAGCAT [23088]
cp32-3_un8_B31_PacBio          GAATTTAAACCTAAAAATGGATGGCTTGAATATCAAAATAGACAATGTAGAAAAGAGTTTAAATCAAAAACTTAGCAT [23088]
cp32-3_B31_TS_MP_FG_consensus  GAATTTAAACCTAAAAATGGATGGCTTGAATATCAAAATAGACAATGTAGAAAAGAGTTTAAATCAAAAACTTAGCAT [23088]
cp32-3_B31_NX_Pl_consensus     GAATTTAAACCTAAAAATGGATGGCTTGAATATCAAAATAGACAATGTAGAAAAGAGTTTAAATCAAAAACTTAGCAT [23088]
cp32-3_PAli_NX_MP_FG_consensus GAATTTAAACCTAAAAATGGATGGCTTGAATATCAAAATAGACAATGTAGAAAAGAGTTTAAATCAAAAACTTAGCAT [23088]
cp32-3_PAli_NX_Pl_consensus    GAATTTAAACCTAAAAATGGATGGCTTGAATATCAAAATAGACAATGTAGAAAAGAGTTTAAATCAAAAACTTAGCAT [23088]
cp32-3_PAbe_NX_MP_FG_consensus GAATTTAAACCTAAAAATGGATGGCTTGAATATCAAAATAGACAATGTAGAAAAGAGTTTAAATCAAAAACTTAGCAT [23088]
cp32-3_PAbe_NX_Pl_consensus    GAATTTAAACCTAAAAATGGATGGCTTGAATATCAAAATAGACAATGTAGAAAAGAGTTTAAATCAAAAACTTAGCAT [23088]

cp32-3_gi|11497103|B31_GB      GGGTAACAGACTAGTACATTTTATGATAATAACAGCAGCAATTCTAGGGCCAATTTTGAATGCCCTATTTATGAAATA [23166]
cp32-3_un8_B31_PacBio          GGGTAACAGACTAGTACATTTTATGATAATAACAGCAGCAATTCTAGGGCCAATTTTGAATGCCCTATTTATGAAATA [23166]
cp32-3_B31_TS_MP_FG_consensus  GGGTAACAGACTAGTACATTTTATGATAATAACAGCAGCAATTCTAGGGCCAATTTTGAATGCCCTATTTATGAAATA [23166]
cp32-3_B31_NX_Pl_consensus     GGGTAACAGACTAGTACATTTTATGATAATAACAGCAGCAATTCTAGGGCCAATTTTGAATGCCCTATTTATGAAATA [23166]
cp32-3_PAli_NX_MP_FG_consensus GGGTAACAGACTAGTACATTTTATGATAATAACAGCAGCAATTCTAGGGCCAATTTTGAATGCCCTATTTATGAAATA [23166]
cp32-3_PAli_NX_Pl_consensus    GGGTAACAGACTAGTACATTTTATGATAATAACAGCAGCAATTCTAGGGCCAATTTTGAATGCCCTATTTATGAAATA [23166]
cp32-3_PAbe_NX_MP_FG_consensus GGGTAACAGACTAGTACATTTTATGATAATAACAGCAGCAATTCTAGGGCCAATTTTGAATGCCCTATTTATGAAATA [23166]
cp32-3_PAbe_NX_Pl_consensus    GGGTAACAGACTAGTACATTTTATGATAATAACAGCAGCAATTCTAGGGCCAATTTTGAATGCCCTATTTATGAAATA [23166]

cp32-3_gi|11497103|B31_GB      TTTACAAGGTGGCAAATAATAATGTATTGTAAAATTTGATTTTTTAAAATGGCACATTATAATATTGATGAAGAGTAT [23244]
cp32-3_un8_B31_PacBio          TTTACAAGGTGGCAAATAATAATGTATTGTAAAATTTGATTTTTTAAAATGGCACATTATAATATTGATGAAGAGTAT [23244]
cp32-3_B31_TS_MP_FG_consensus  TTTACAAGGTGGCAAATAATAATGTATTGTAAAATTTGATTTTTTAAAATGGCACATTATAATATTGATGAAGAGTAT [23244]
cp32-3_B31_NX_Pl_consensus     TTTACAAGGTGGCAAATAATAATGTATTGTAAAATTTGATTTTTTAAAATGGCACATTATAATATTGATGAAGAGTAT [23244]
cp32-3_PAli_NX_MP_FG_consensus TTTACAAGGTGGCAAATAATAATGTATTGTAAAATTTGATTTTTTAAAATGGCACATTATAATATTGATGAAGAGTAT [23244]
cp32-3_PAli_NX_Pl_consensus    TTTACAAGGTGGCAAATAATAATGTATTGTAAAATTTGATTTTTTAAAATGGCACATTATAATATTGATGAAGAGTAT [23244]
cp32-3_PAbe_NX_MP_FG_consensus TTTACAAGGTGGCAAATAATAATGTATTGTAAAATTTGATTTTTTAAAATGGCACATTATAATATTGATGAAGAGTAT [23244]
cp32-3_PAbe_NX_Pl_consensus    TTTACAAGGTGGCAAATAATAATGTATTGTAAAATTTGATTTTTTAAAATGGCACATTATAATATTGATGAAGAGTAT [23244]

cp32-3_gi|11497103|B31_GB      TATTAATTAACACTTAATTTTTGCTTTTTCATAAATTAGAACTTATTTGAATTTTTTAACAAGAGAATTTAAATAGGT [23322]
cp32-3_un8_B31_PacBio          TATTAATTAACACTTAATTTTTGCTTTTTCATAAATTAGAACTTATTTGAATTTTTTAACAAGAGAATTTAAATAGGT [23322]
cp32-3_B31_TS_MP_FG_consensus  TATTAATTAACACTTAATTTTTGCTTTTTCATAAATTAGAACTTATTTGAATTTTTTAACAAGAGAATTTAAATAGGT [23322]
cp32-3_B31_NX_Pl_consensus     TATTAATTAACACTTAATTTTTGCTTTTTCATAAATTAGAACTTATTTGAATTTTTTAACAAGAGAATTTAAATAGGT [23322]
cp32-3_PAli_NX_MP_FG_consensus TATTAATTAACACTTAATTTTTGCTTTTTCATAAATTAGAACTTATTTGAATTTTTTAACAAGAGAATTTAAATAGGT [23322]
cp32-3_PAli_NX_Pl_consensus    TATTAATTAACACTTAATTTTTGCTTTTTCATAAATTAGAACTTATTTGAATTTTTTAACAAGAGAATTTAAATAGGT [23322]
cp32-3_PAbe_NX_MP_FG_consensus TATTAATTAACACTTAATTTTTGCTTTTTCATAAATTAGAACTTATTTGAATTTTTTAACAAGAGAATTTAAATAGGT [23322]
cp32-3_PAbe_NX_Pl_consensus    TATTAATTAACACTTAATTTTTGCTTTTTCATAAATTAGAACTTATTTGAATTTTTTAACAAGAGAATTTAAATAGGT [23322]

cp32-3_gi|11497103|B31_GB      TCTTTTATTTTAACAAATACAAATTGATTTTAATTCTAAATTATAATATATTCAATTATTGAAAAGCTTATTTAAATT [23400]
cp32-3_un8_B31_PacBio          TCTTTTATTTTAACAAATACAAATTGATTT-AATTCTAAATTATA-TATATTCAATTATTGAAAAGCTTATTTAAATT [23400]
cp32-3_B31_TS_MP_FG_consensus  TCTTTTATTTTAACAAATACAAATTGATTTTAATTCTAAATTATAATATATTCAATTATTGAAAAGCTTATTTAAATT [23400]
cp32-3_B31_NX_Pl_consensus     TCTTTTATTTTAACAAATACAAATTGATTTTAATTCTAAATTATAATATATTCAATTATTGAAAAGCTTATTTAAATT [23400]
cp32-3_PAli_NX_MP_FG_consensus TCTTTTATTTTAACAAATACAAATTGATTTTAATTCTAAATTATAATATATTCAATTATTGAAAAGCTTATTTAAATT [23400]
cp32-3_PAli_NX_Pl_consensus    TCTTTTATTTTAACAAATACAAATTGATTTTAATTCTAAATTATAATATATTCAATTATTGAAAAGCTTATTTAAATT [23400]
cp32-3_PAbe_NX_MP_FG_consensus TCTTTTATTTTAACAAATACAAATTGATTTTAATTCTAAATTATAATATATTCAATTATTGAAAAGCTTATTTAAATT [23400]
cp32-3_PAbe_NX_Pl_consensus    TCTTTTATTTTAACAAATACAAATTGATTTTAATTCTAAATTATAATATATTCAATTATTGAAAAGCTTATTTAAATT [23400]

cp32-3_gi|11497103|B31_GB      ATTTTAATAAGCAAATTTGATTAAACCCTAACTTTATTAAAATAATTTATGTAAAAAGTTGTCAAAAATAGTTTTTGT [23478]
cp32-3_un8_B31_PacBio          ATTTTAATAAGCAAATTTGATTAAACCCTAACTTTATTAAAATAATTTATGTAAAAAGTTGTCAAAA-TAGTTTTTGT [23478]
cp32-3_B31_TS_MP_FG_consensus  ATTTTAATAAGCAAATTTGATTAAACCCTAACTTTATTAAAATAATTTATGTAAAAAGTTGTCAAAAATAGTTTTTGT [23478]
cp32-3_B31_NX_Pl_consensus     ATTTTAATAAGCAAATTTGATTAAACCCTAACTTTATTAAAATAATTTATGTAAAAAGTTGTCAAAAATAGTTTTTGT [23478]
cp32-3_PAli_NX_MP_FG_consensus ATTTTAATAAGCAAATTTGATTAAACCCTAACTTTATTAAAATAATTTATGTAAAAAGTTGTCAAAAATAGTTTTTGT [23478]
cp32-3_PAli_NX_Pl_consensus    ATTTTAATAAGCAAATTTGATTAAACCCTAACTTTATTAAAATAATTTATGTAAAAAGTTGTCAAAAATAGTTTTTGT [23478]
cp32-3_PAbe_NX_MP_FG_consensus ATTTTAATAAGCAAATTTGATTAAACCCTAACTTTATTAAAATAATTTATGTAAAAAGTTGTCAAAAATAGTTTTTGT [23478]
cp32-3_PAbe_NX_Pl_consensus    ATTTTAATAAGCAAATTTGATTAAACCCTAACTTTATTAAAATAATTTATGTAAAAAGTTGTCAAAAATAGTTTTTGT [23478]

cp32-3_gi|11497103|B31_GB      TATACATATATATACATATATATATGTATGTAAATAGCTAAAAAAGTTTATTGCTATCAAAACAATCCAATCAAGTTG [23556]
cp32-3_un8_B31_PacBio          TATACATATATATACATATATATATGTATGTAAATAGCTAAAAAAGTTTATTGCTATCAAAACAATCCAATCAAGTTG [23556]
cp32-3_B31_TS_MP_FG_consensus  TATACATATATATACATATATATATGTATGTAAATAGCTAAAAAAGTTTATTGCTATCAAAACAATCCAATCAAGTTG [23556]
cp32-3_B31_NX_Pl_consensus     TATACATATATATACATATATATATGTATGTAAATAGCTAAAAAAGTTTATTGCTATCAAAACAATCCAATCAAGTTG [23556]
cp32-3_PAli_NX_MP_FG_consensus TATACATATATATACATATATATATGTATGTAAATAGCTAAAAAAGTTTATTGCTATCAAAACAATCCAATCAAGTTG [23556]
cp32-3_PAli_NX_Pl_consensus    TATACATATATATACATATATATATGTATGTAAATAGCTAAAAAAGTTTATTGCTATCAAAACAATCCAATCAAGTTG [23556]
cp32-3_PAbe_NX_MP_FG_consensus TATACATATATATACATATATATATGTATGTAAATAGCTAAAAAAGTTTATTGCTATCAAAACAATCCAATCAAGTTG [23556]
cp32-3_PAbe_NX_Pl_consensus    TATACATATATATACATATATATATGTATGTAAATAGCTAAAAAAGTTTATTGCTATCAAAACAATCCAATCAAGTTG [23556]
```

```
cp32-3_gi|11497103|B31_GB      GGTTTAGCTAAGTTCTTGGATAAGAGAATTTAAATAAACCCAACTATTTTTTTACAAAAATTTTTGTAAAAAAAAAAG [23634]
cp32-3_un8_B31_PacBio          GGTTTAGCTAAGTTCTTGGATAAGAGAATTTAAATAAACCCAACTATTTTTTTACAAAAATTTTTGTAAAAAAAAAAG [23634]
cp32-3_B31_TS_MP_FG_consensus  GGTTTAGCTAAGTTCTTGGATAAGAGAATTTAAATAAACCCAACTATTTTTTTACAAAAATTTTTGTAAAAAAAAAAG [23634]
cp32-3_B31_NX_Pl_consensus     GGTTTAGCTAAGTTCTTGGATAAGAGAATTTAAATAAACCCAACTATTTTTTTACAAAAATTTTTGTAAAAAAAAAAG [23634]
cp32-3_PAli_NX_MP_FG_consensus GGTTTAGCTAAGTTCTTGGATAAGAGAATTTAAATAAACCCAACTATTTTTTTACAAAAATTTTTGTAAAAAAAAAAG [23634]
cp32-3_PAli_NX_Pl_consensus    GGTTTAGCTAAGTTCTTGGATAAGAGAATTTAAATAAACCCAACTATTTTTTTACAAAAATTTTTGTAAAAAAAAAAG [23634]
cp32-3_PAbe_NX_MP_FG_consensus GGTTTAGCTAAGTTCTTGGATAAGAGAATTTAAATAAACCCAACTATTTTTTTACAAAAATTTTTGTAAAAAAAAAAG [23634]
cp32-3_PAbe_NX_Pl_consensus    GGTTTAGCTAAGTTCTTGGATAAGAGAATTTAAATAAACCCAACTATTTTTTTACAAAAATTTTTGTAAAAAAAAAAG [23634]

cp32-3_gi|11497103|B31_GB      TTGGCAAAAATAGTTTTTGCTATATATTTATTTTTTATACAAATATAAGGAGAAAAAAGATGGAAAATCTTTCAAACAA [23712]
cp32-3_un8_B31_PacBio          TTGGCAAAAATAGTTTTTGCTATATATTTATTTTTTATACAAATATAAGGAGAAAAAAGATGGAAAATCTTTCAAACAA [23712]
cp32-3_B31_TS_MP_FG_consensus  TTGGCAAAAATAGTTTTTGCTATATATTTATTTTTTATACAAATATAAGGAGAAAAAAGATGGAAAATCTTTCAAACAA [23712]
cp32-3_B31_NX_Pl_consensus     TTGGCAAAAATAGTTTTTGCTATATATTTATTTTTTATACAAATATAAGGAGAAAAAAGATGGAAAATCTTTCAAACAA [23712]
cp32-3_PAli_NX_MP_FG_consensus TTGGCAAAAATAGTTTTTGCTATATATTTATTTTTTATACAAATATAAGGAGAAAAAAGATGGAAAATCTTTCAAACAA [23712]
cp32-3_PAli_NX_Pl_consensus    TTGGCAAAAATAGTTTTTGCTATATATTTATTTTTTATACAAATATAAGGAGAAAAAAGATGGAAAATCTTTCAAACAA [23712]
cp32-3_PAbe_NX_MP_FG_consensus TTGGCAAAAATAGTTTTTGCTATATATTTATTTTTTATACAAATATAAGGAGGAAAAAGATGGAAAATCTTTCAAACAA [23712]
cp32-3_PAbe_NX_Pl_consensus    TTGGCAAAAATAGTTTTTGCTATATATTTATTTTTTATACAAATATAAGGAGAAAAAAGATGGAAAATCTTTCAAACAA [23712]

cp32-3_gi|11497103|B31_GB      TAATAATCCACAAGAAAATATTCAAGGAGATCTCAAAATGATAAGTGTTAATCAACAAAGTTTTACTGGTTGTGAAAT [23790]
cp32-3_un8_B31_PacBio          TAATAATCCACAAGAAAATATTCAAGGAGATCTCAAAATGATAAGTGTTAATCAACAAAGTTTTACTGGT-GTGAAAT [23790]
cp32-3_B31_TS_MP_FG_consensus  TAATAATCCACAAGAAAATATTCAAGGAGATCTCAAAATGATAAGTGTTAATCAAAAAAGTTTTACTGGATGTGAAAT [23790]
cp32-3_B31_NX_Pl_consensus     TAATAATCCACAAGAAAATATTCAAGGAGATCTCAATATGACAAGAATTAATCAAAAAAGTTTTACTGGATGTGAAAT [23790]
cp32-3_PAli_NX_MP_FG_consensus TAATAATCCACAAGAAAATATTCAAGGAGATCTCAATATGACAAGAATTAATCAAAAAAGTTTTACTGGATGTGAAAT [23790]
cp32-3_PAli_NX_Pl_consensus    TAATAATCCACAAGAAAATATTCAAGGAGATCTCAAAATGATAAGTGTTAATCAACAAAGTTTTACTGGTTGTGAAAT [23790]
cp32-3_PAbe_NX_MP_FG_consensus TAATAATCCACAAGAAAATATTCAAGGAGATCTCAATATGACAAGAATTAATCAAAAAAGTTTTACTGGATGTGAAAT [23790]
cp32-3_PAbe_NX_Pl_consensus    TAATAATCCACAAGAAAATATTCAAGGAGATCTCAAAATGATAAGTGTTAATCAACAAAGTTTTACTGGTTGTGAAAT [23790]

cp32-3_gi|11497103|B31_GB      AATTGAGGAAAAATCTTCTCCCATTAAAGAAAAAAGTAAATTAAGCAAGATAGGTAAGAAATTACCAGGAATAAGTAG [23868]
cp32-3_un8_B31_PacBio          AATTGAGGAAAAATCTTCTCCCATTAAAGAAAAAAGTAAATTAAGCAAGATAGGTAAGAAATTACCAGGAATAAGTAG [23868]
cp32-3_B31_TS_MP_FG_consensus  AATTGAGGAAAAATCTTCTCCCATTAAAGAAAAAAGTAAATTAAGCAAGATAGGTAAGAAATTACCAGGAATAAGTAG [23868]
cp32-3_B31_NX_Pl_consensus     AATTGAGGAAAAATCTTCTCCCATTAAAGAAAAAAGTAAATTAAGCAAGATAGGTAAGAAATTACCAGGAATAAGTAG [23868]
cp32-3_PAli_NX_MP_FG_consensus AATTGAGGAAAAATCTTCTCCCATTAAAGAAAAAAGTAAATTAAGCAAGATAGGTAAGAAATTACCAGGAATAAGTAG [23868]
cp32-3_PAli_NX_Pl_consensus    AATTGAGGAAAAATCTTCTCCCATTAAAGAAAAAAGTAAATTAAGCAAGATAGGTAAGAAATTACCAGGAATAAGTAG [23868]
cp32-3_PAbe_NX_MP_FG_consensus AATTGAGGAAAAATCTTCTCCCATTAAAGAAAAAAGTAAATTAAGCAAGATAGGTAAGAAATTACCAGGAATAAGTAG [23868]
cp32-3_PAbe_NX_Pl_consensus    AATTGAGGAAAAATCTTCTCCCATTAAAGAAAAAAGTAAATTAAGCAAGATAGGTAAGAAATTACCAGGAATAAGTAG [23868]

cp32-3_gi|11497103|B31_GB      TCAAGAATGTTTTAGATTTAATCGAAATATTGATTTTAGTGTGCAAAGAAACAAGTTAGATAAATACGGTGCTAGTGA [23946]
cp32-3_un8_B31_PacBio          TCAAGAATGTTTTAGATTTAATCGAAATATTGATTTTAGTGTGCAAAGAAACAAGTTAGATAAATACGGTGCTAGTGA [23946]
cp32-3_B31_TS_MP_FG_consensus  TCAAGAGTGTTTTAAATTTAATCGAAATATTGATTTTAGTACACAAAGAAACAAGTTAGATAAATACGGTGCTAGTGA [23946]
cp32-3_B31_NX_Pl_consensus     TCAAGAGTGTTTTAAATTTAATCGAAATATTGATTTTAGTACACAAAGAAACAAGTTAGATAAATACGGTGCTAGTGA [23946]
cp32-3_PAli_NX_MP_FG_consensus TCAAGAGTGTTTTAAATTTAATCGAAATATTGATTTTAGTACACAAAGAAATGAACTTGATAGATACGGAGCTAGTGA [23946]
cp32-3_PAli_NX_Pl_consensus    TCAAGAATGTTTTAGATTTAATCGAAATATTGATTTTAGTGTGCAAAGAAACAAGTTAGATAAATACGGTGCTAGTGA [23946]
cp32-3_PAbe_NX_MP_FG_consensus TCAAGAGTGTTTTAAATTTAATCGAAATATTGATTTTAGTACACAAAGAAATGAACTTGATAGATACGGAGCTAGTGA [23946]
cp32-3_PAbe_NX_Pl_consensus    TCAAGAGTGTTTTAAATTTAATCGAAATATTGATTTTAGTGTGCAAAGAAACAAGTTAGATAAATACGGTGCTAGTGA [23946]

cp32-3_gi|11497103|B31_GB      AGTAGGCAATATTCTTGTTGGAGGTGCTGGGCTGAAAGATTTAATGATAAACAGAGTGCTTAAATATTTTAATATGAG [24024]
cp32-3_un8_B31_PacBio          AGTAGGCAATATTCTTGTTGGAGGTGCTGGGCTGAAAGATTTAATGATAA-CAGAGTGCTTAAATATTTTAATATGAG [24024]
cp32-3_B31_TS_MP_FG_consensus  AGTAGGCAATATTCTTGTTGGAGGTGCTGGGCTGAAAGATTTAATGATAAACAGAGTGCTTAAATATTTTAATATGAG [24024]
cp32-3_B31_NX_Pl_consensus     AGTAGGCAATATTCTTGTTGGAGGTGCTGGGCTGAAAGATTTAATGATAAACAGAGTGCTTAAATATTTTAATATGAG [24024]
cp32-3_PAli_NX_MP_FG_consensus AGTAGGCAATATTCTTGTTGGAGGTGCTGGGCTGAAAGATTTAATGATAAACAGAGTGCTTAAATATTTTAATATGAG [24024]
cp32-3_PAli_NX_Pl_consensus    AGTAGGCAATATTCTTGTTGGAGGTGCTGGGCTGAAAGATTTAATGATAAACAGAGTGCTTAAATATTTTAATATGAG [24024]
cp32-3_PAbe_NX_MP_FG_consensus AGTAGGCAATATTCTTGTTGGAGGTGCTGGGCTGAAAGATTTAATGATAAACAGAGTGCTTAAATATTTTAATATGAG [24024]
cp32-3_PAbe_NX_Pl_consensus    AGTAGGCAATATTCTTGTTGGAGGTGCTGGGCTGAAAGATTTAATGATAAACAGAGTGCTTAAATATTTTAATATGAG [24024]

cp32-3_gi|11497103|B31_GB      ACTACCTTTTGAAGAGAATTTATATATGCTCAAGGGCAAAGAGTTAGAGAATTTAGGATTTAGAGAGTTTGTTAAAGC [24102]
cp32-3_un8_B31_PacBio          ACTACCTTTTGAAGAGAATTTATATATGCTCAAGGGCAAAGAGTTAGAGAATTTAGGATTTAGAGAGTTTGTTAAAGC [24102]
cp32-3_B31_TS_MP_FG_consensus  ACTACCTTTTGAAGAGAATTTATATATGCTCAAGGGCAAAGAGTTAGAGAATTTAGGATTTAGAGAGTTTGTTAAAGC [24102]
cp32-3_B31_NX_Pl_consensus     ACTACCTTTTGAAGAGAATTTATATATGCTCAAGGGCAAAGAGTTAGAGAATTTAGGATTTAGAGAGTTTGTTAAAGC [24102]
cp32-3_PAli_NX_MP_FG_consensus ACTACCTTTTGAAGAGAATTTATATATGCTCAAGGGCAAAGAGTTAGAGAATTTAGGATTTAGAGAGTTTGTTAAAGC [24102]
cp32-3_PAli_NX_Pl_consensus    ACTACCTTTTGAAGAGAATTTATATATGCTCAAGGGCAAAGAGTTAGAGAATTTAGGATTTAGAGAGTTTGTTAAAGC [24102]
cp32-3_PAbe_NX_MP_FG_consensus ACTACCTTTTGAAGAGAATTTATATATGCTCAAGGGCAAAGAGTTAGAGAATTTAGGATTTAGAGAGTTTGTTAAAGC [24102]
cp32-3_PAbe_NX_Pl_consensus    ACTACCTTTTGAAGAGAATTTATATATGCTCAAGGGCAAAGAGTTAGAGAATTTAGGATTTAGAGAGTTTGTTAAAGC [24102]

cp32-3_gi|11497103|B31_GB      ATACGGTGATAATATTGATATTTTATATAAAAATAAATATGCCAACGGTGTTGATAAGTATAATTATTTCAAAAAAAT [24180]
cp32-3_un8_B31_PacBio          ATACGGTGATAATATTGATATTTTATATAAAA-TAAATATGCCAACGGTGTTGATAAGTATAATTATTTCAAAAAAAT [24180]
cp32-3_B31_TS_MP_FG_consensus  ATACGGTGATAATATTGATATTTTATATAAAAATAAATATGCCAACGGTGTTGATAAGTATAATTATTTCAAAAAAAT [24180]
cp32-3_B31_NX_Pl_consensus     ATACGGTGATAATATTGATATTTTATATAAAAATAAATATGCCAACGGTGTTGATAAGTATAATTATTTCAAGAAAAC [24180]
cp32-3_PAli_NX_MP_FG_consensus ATACGGTGATAATATTGATATTTTATATAAAAATAAATATGCCAACGGTGTTGATAAGTATAATTATTTCAAAAAAAT [24180]
cp32-3_PAli_NX_Pl_consensus    ATACGGTGATAATATTGATATTTTATATAAAAATAAATATGCCAACGGTGTTGATAAGTATAATTATTTCAAAAAAAT [24180]
cp32-3_PAbe_NX_MP_FG_consensus ATACGGTGATAATATTGATATTTTATATAAAAATAAATATGCCAACGGTGTTGATAAGTATAATTATTTCAAAAAAAT [24180]
cp32-3_PAbe_NX_Pl_consensus    ATACGGTGATAATATTGATATTTTATATAAAAATAAATATGCCAACGGTGTTGATAAGTATAATTATTTCAAAAAAAT [24180]

cp32-3_gi|11497103|B31_GB      GGGCAGTTCAGAAACTTTAGTGGGCTCAACAATTGATGGCTGGTTTATTAATAATAATGGCGATTTAGAACTATTAGA [24258]
cp32-3_un8_B31_PacBio          GGGCAGTTCAGAAACTTTAGTGGGCTCAACAATTGATGGCTGGTTTATTAATAATAATGGCGATTTAGAACTATTAGA [24258]
cp32-3_B31_TS_MP_FG_consensus  GGGCAGTTCAGAAACTTTAGTGGGCTCAACAATTGATGGCTGGTTTATTAATAATAATGGCGATTTAGAACTTTTAGA [24258]
cp32-3_B31_NX_Pl_consensus     TGGTAAATCAGAAATGTTTGTTGGATCAACAATTGATGGGTGGTTTATAAATAGTAACGGCAATTAGAACTATTAGA [24258]
cp32-3_PAli_NX_MP_FG_consensus GGGCAGTTCAGAAACTTTAGTGGGCTCAACAATTGATGGCTGGTTTATTAATAATAATGGCGATTTAGAACTATTAGA [24258]
cp32-3_PAli_NX_Pl_consensus    GGGCAGTTCAGAAACTTTAGTGGGCTCAACAATTGATGGCTGGTTTATTAATAATAATGGCGATTTAGAACTATTAGA [24258]
cp32-3_PAbe_NX_MP_FG_consensus GGGCAGTTCAGAAACTTTAGTGGGCTCAACAATTGATGGCTGGTTTATAAATAGTAACGGCACCGCAGAGCTTTTAGA [24258]
cp32-3_PAbe_NX_Pl_consensus    GGGCAGTTCAGAAACTTTAGTGGGCTCAACAATTGATGGCTGGTTTATTAATAATAATGGCGATTTAGAACTATTAGA [24258]

cp32-3_gi|11497103|B31_GB      GATTAAAAGTAGCGACTCTAATTATATATGAGTAGTGCTATTGCTGAGTACAATAAAAATGGCAATTTTTTAAGTAGTAA [24336]
cp32-3_un8_B31_PacBio          GATTAAAAGTAGCGACTCTAATTATATATGAGTAGTGCTATTGCTGAGTACAATAAAAATGGCAATTTTTTAAGTAGTAA [24336]
cp32-3_B31_TS_MP_FG_consensus  AATTAAAAGCAGTGATTCTAATTATATATGAGCAGTGCTATTGCTGAGTACAATAAAAATGGCAATTTTTTAAGCAGCAA [24336]
cp32-3_B31_NX_Pl_consensus     GATTAAAAGCAGTGATTCTAATTATATATGAGCAGTGCTATTGCTGAGTACAATAAAAATGGCAATTTTTTAAGCAGCAA [24336]
cp32-3_PAli_NX_MP_FG_consensus GATTAAAAGCAGTGATTCTAATTATATATGAGCAGTGCTATTGCTGAGTACAATAAAAATGGCAATTTTTTAAGCAGCAA [24336]
cp32-3_PAli_NX_Pl_consensus    GATTAAAAGTAGCGACTCTAATTATATATGAGTAGTGCTATTGCTGAGTACAATAAAAATGGCAATTTTTTAAGTAGTAA [24336]
cp32-3_PAbe_NX_MP_FG_consensus AATTAAAAGCAGTGATTCTAATTATATATGAGCAGTGCTATTGCTGAGTACAATAAAAATGGCAATTTTTTAAGCAGCAA [24336]
cp32-3_PAbe_NX_Pl_consensus    GATTAAAAGTAGCGACTCTAATTATATATGAGTAGTGCTATTGCTGAGTACAATAAAAATGGCAATTTTTTAAGTAGTAA [24336]

cp32-3_gi|11497103|B31_GB      ATATTTTTTCAAATATTATGTACAAGCACAAATGCAGCTAGCATGTACTGGACTTGAGTATTGTAATTTGTTCTTTTT [24414]
cp32-3_un8_B31_PacBio          ATATTTTTTCAAATATTATGTACAAGCACAAATGCAGCTAGCATGTACTGGACTTGAGTATTGTAATTTGTTCTTTTT [24414]
cp32-3_B31_TS_MP_FG_consensus  ATATTTTTTCAAATATTATGTGCAGGCGCAAATGCAACTAGCATGTACTGGACTTGAGTATTGTAATTTATTCTTTTT [24414]
cp32-3_B31_NX_Pl_consensus     ATATTTTTTCAAATATTATGTGCAGGCGCAAATGCAACTAGCATGTACTGGACTTGAGTATTGTAATTTATTCTTTTT [24414]
cp32-3_PAli_NX_MP_FG_consensus ATATTTTTTCAAATATTATGTGCAGGCGCAAATGCAACTAGCATGTACTGGACTTGAGTATTGTAATTTATTCTTTTT [24414]
cp32-3_PAli_NX_Pl_consensus    ATATTTTTTCAAATATTATGTACAAGCACAAATGCAGCTAGCATGTACTGGACTTGAGTATTGTAATTTGTTCTTTTT [24414]
cp32-3_PAbe_NX_MP_FG_consensus ATATTTTTTCAAATATTATGTGCAGGCGCAAATGCAACTAGCATGTACTGGACTTGAGTATTGTAATTTATTCTTTTT [24414]
cp32-3_PAbe_NX_Pl_consensus    ATATTTTTTCAAATATTATGTGCAGGCGCAAATGCAACTAGCATGTACTGGACTTGAGTATTGTAATTTATTCTTTTT [24414]

cp32-3_gi|11497103|B31_GB      AATAGATGCTGCACCAATTAACTGTAAGATTAAAAGAGATGAGGCCTTAATATCAAAAGTGTTTGAATTTGTTAATAA [24492]
cp32-3_un8_B31_PacBio          AATAGATGCTGCACCAATTAACTGTAAGATTAAAAGAGATGAGGCCTTAATATCAAAAGTGTTTGAATTTGTTAATAA [24492]
cp32-3_B31_TS_MP_FG_consensus  AATAGATGCAGCACCAGTTAACTGTAAGATTAAGAGAAATTATGACTTAATAGCAAAAGTGCTTGCATGTGTTAATAA [24492]
cp32-3_B31_NX_Pl_consensus     AATAGATGCAGCACCAGTTAACTGTAAGATTAAGAGAAATTATGACTTAATAGCAAAAGTGCTTGCATGTGTTCACAA [24492]
cp32-3_PAli_NX_MP_FG_consensus AATAGATGCAGCACCAGTTAACTGTAAGATTAAGAGAAATTATGACTTAATAGCAAAAGTGCTTGCATGTGTTAATAA [24492]
cp32-3_PAli_NX_Pl_consensus    AATAGATGCTGCACCAATTAACTGTAAGATTAAAAGAGATGAGGCCTTAATATCAAAAGTGTTTGAATTTGTTAATAA [24492]
cp32-3_PAbe_NX_MP_FG_consensus AATAGATGCAGCACCAGTTAACTGTAAGATTAAGAGAAATTATGACTTAATAGCAAAAGTGCTTGCATGTGTTCACAA [24492]
cp32-3_PAbe_NX_Pl_consensus    AATAGATGCAGCACCAGTTAACTGTAAGATTAAGAGAAATTATGACTTAATAGCAAAAGTGCTTGCATGTGTTAATAA [24492]
```

```
cp32-3_gi|11497103|B31_GB      ATGTGAATTAGAAATTATAAATTTAAAAAAAGATATTTATAGTAACTATAGAGAGGAATACTTAATGGCACATAATTT [24570]
cp32-3_un8_B31_PacBio          ATGTGAATTAGAAATTATAAATTTAAAAAAAGATATTTATAGTAACTATAGAGAGGAATACTTAATGGCACATAATTT [24570]
cp32-3_B31_TS_MP_FG_consensus  ATGTGAATTAGAAATTATAAATTTAAAAAAAGATATTTATAGTAACTATAGAGAGGAATACTTAATGGCACATAATTT [24570]
cp32-3_B31_NX_Pl_consensus     ATGTGAATTAGAAATTATAAATTTAAAAAAAGATATTTATAGTAACTATAGAGAGGAATACTTAATGGCACATAATTT [24570]
cp32-3_PAli_NX_MP_FG_consensus ATGTGAATTAGAAATTATAAATTTAAAAAAAGATATTTATAGTAACTATAGAGAGGAATACTTAATGGCACATAATTT [24570]
cp32-3_PAli_NX_Pl_consensus    ATGTGAATTAGAAATTATAAATTTAAAAAAAGATATTTATAGTAACTATAGAGAGGAATACTTAATGGCACATAATTT [24570]
cp32-3_PAbe_NX_MP_FG_consensus ATGTGAATTAGAAATTATAAATTTAAAAAAAGATATTTATAGTAACTATAGAGAGGAATACTTAATGGCACATAATTT [24570]
cp32-3_PAbe_NX_Pl_consensus    ATGTGAATTAGAAATTATAAATTTAAAAAAAGATATTTATAGTAACTATAGAGAGGAATACTTAATGGCACATAATTT [24570]

cp32-3_gi|11497103|B31_GB      TAACGAGGATACGTTTATAAAACTTGTTGAAGATTTAGTAGAAAGGAGTGATTTTTATAGTTCTGGAGTTGAGTTTGA [24648]
cp32-3_un8_B31_PacBio          TAACGAGGATACGTTTATAAAACTTGTTGAAGATTTAGTAGAA-GGAGTGATTTTTATAGTTCTGGAGTTGAGTTTGA [24648]
cp32-3_B31_TS_MP_FG_consensus  TAACGAGGATACGTTTATAAAACTTGTTGAAGATTTAGTAGAAAGGAGTGATTTTTATAGTTCTGGAGTTGAGTTTGA [24648]
cp32-3_B31_NX_Pl_consensus     TAACGAGGATACGTTTATAAAACTTGTTGAAGATTTAGTAGAAAGGAGTGATTTTTATAGTTCTGGAGTTGAGTTTGA [24648]
cp32-3_PAli_NX_MP_FG_consensus TAACGAGGATACGTTTATAAAACTTGTTGAAGATTTAGTAGAAAGGAGTGATTTTTATAGTTCTGGAGTTGAGTTTGA [24648]
cp32-3_PAli_NX_Pl_consensus    TAACGAGGATACGTTTATAAAACTTGTTGAAGATTTAGTAGAAAGGAGTGATTTTTATAGTTCTGGAGTTGAGTTTGA [24648]
cp32-3_PAbe_NX_MP_FG_consensus TAACGAGGATACGTTTATAAAACTTGTTGAAGATTTAGTAGAAAGGAGTGATTTTTATAGTTCTGGAGTTGAGTTTGA [24648]
cp32-3_PAbe_NX_Pl_consensus    TAACGAGGATACGTTTATAAAACTTGTTGAAGATTTAGTAGAAAGGAGTGATTTTTATAGTTCTGGAGTTGAGTTTGA [24648]

cp32-3_gi|11497103|B31_GB      TTGGGCAAGAGAATTTATAGAATATGTTGATTGTGCAGATCTTGAAATTAAGGATAATCAATCTGCTGAAAATCTTGC [24726]
cp32-3_un8_B31_PacBio          TTGGGCAAGAGAATTTATAGAATATGTTGATTGTGCAGATCTTGAAATTAAGGATAATCAATCTGCTGAAAATCTTGC [24726]
cp32-3_B31_TS_MP_FG_consensus  TTGGGCAAGAGAATTTATAGAATATGTTGATTGTGCAGATCTTGAAATTAAGGATAATCAATCTGCTGAAAATCTTGC [24726]
cp32-3_B31_NX_Pl_consensus     TTGGGCAAGAGAATTTATAGAATATGTTGATTGTGCAGATCTTGAAATTAAGGATAATCAATCTGCTGAAAATCTTGC [24726]
cp32-3_PAli_NX_MP_FG_consensus TTGGGCAAGAGAATTTATAGAATATGTTGATTGTGCAGATCTTGAAATTAAGGATAATCAATCTGCTGAAAATCTTGC [24726]
cp32-3_PAli_NX_Pl_consensus    TTGGGCAAGAGAATTTATAGAATATGTTGATTGTGCAGATCTTGAAATTAAGGATAATCAATCTGCTGAAAATCTTGC [24726]
cp32-3_PAbe_NX_MP_FG_consensus TTGGGCAAGAGAATTTATAGAATATGTTGATTGTGCAGATCTTGAAATTAAGGATAATCAATCTGCTGAAAATCTTGC [24726]
cp32-3_PAbe_NX_Pl_consensus    TTGGGCAAGAGAATTTATAGAATATGTTGATTGTGCAGATCTTGAAATTAAGGATAATCAATCTGCTGAAAATCTTGC [24726]

cp32-3_gi|11497103|B31_GB      GTATGATTTAATGGAGATTGATAGTCTACAAAAAGAATTAAATAGAATCCAAAACGAAAATAAAAAAAGAGAAAAGCC [24804]
cp32-3_un8_B31_PacBio          GTATGATTTAATGGAGATTGATAGTCTACAAAAAGAATTAAATAGAATCCAAAACGAAAT--AAAAAAGAGAAAAGCC [24804]
cp32-3_B31_TS_MP_FG_consensus  GTATGATTTAATGGAGATTGATAGTCTACAAAAAGAATTAAATAGAATCCAAAACGAAAATAAAAAAAGAGAAAAGCC [24804]
cp32-3_B31_NX_Pl_consensus     GTATGATTTAATGGAGATTGATAGTCTACAAAAAGAATTAAATAGAATCCAAAACGAAAATAAAAAAAGAGAAAAGCC [24804]
cp32-3_PAli_NX_MP_FG_consensus GTATGATTTAATGGAGATTGATAGTCTACAAAAAGAATTAAATAGAATCCAAAACGAAAATAAAAAAAGAGAAAAGCC [24804]
cp32-3_PAli_NX_Pl_consensus    GTATGATTTAATGGAGATTGATAGTCTACAAAAAGAATTAAATAGAATCCAAAACGAAAATAAAAAAAGAGAAAAGCC [24804]
cp32-3_PAbe_NX_MP_FG_consensus GTATGATTTAATGGAGATTGATAGTCTACAAAAAGAATTAAATAGAATCCAAAACGAAAATAAAAAAAGAGAAAAGCC [24804]
cp32-3_PAbe_NX_Pl_consensus    GTATGATTTAATGGAGATTGATAGTCTACAAAAAGAATTAAATAGAATCCAAAACGAAAATAAAAAAAGAGAAAAGCC [24804]

cp32-3_gi|11497103|B31_GB      CATTAAAGATAGGTTGAAGATGCTAATTTACAATATTACAAATACATATCCACTAATTGAGCAGTTAAATTATAAATT [24882]
cp32-3_un8_B31_PacBio          CATTAA-GATAGGTTGAAGATGCTAATTTACAATATTACAAATACATATCCACTAATTGAGCAGTTAAATTATAAATT [24882]
cp32-3_B31_TS_MP_FG_consensus  CATTAAAGATAGGTTGAAGATGCTAATTTACAATATTACAAATACATATCCACTAATTGAGCAGTTAAATTATAAATT [24882]
cp32-3_B31_NX_Pl_consensus     CATTAAAGATAGGTTGAAGATGCTAATTTACAATATTACAAATACATATCCACTAATTGAGCAGTTAAATTATAAATT [24882]
cp32-3_PAli_NX_MP_FG_consensus CATTAAAGATAGGTTGAAGATGCTAATTTACAATATTACAAATACATATCCACTAATTGAGCAGTTAAATTATAAATT [24882]
cp32-3_PAli_NX_Pl_consensus    CATTAAAGATAGGTTGAAGATGCTAATTTACAATATTACAAATACATATCCACTAATTGAGCAGTTAAATTATAAATT [24882]
cp32-3_PAbe_NX_MP_FG_consensus CATTAAAGATAGGTTGAAGATGCTAATTTACAATATTACAAATACATATCCACTAATTGAGCAGTTAAATTATAAATT [24882]
cp32-3_PAbe_NX_Pl_consensus    CATTAAAGATAGGTTGAAGATGCTAATTTACAATATTACAAATACATATCCACTAATTGAGCAGTTAAATTATAAATT [24882]

cp32-3_gi|11497103|B31_GB      TGGAGAGTTTGTGTTTACTCTTGACCCTAAGAAAAGGGCAATATCAGATAGATTAAAGGGACTACTACCAACAAGTGG [24960]
cp32-3_un8_B31_PacBio          TGGAGAGTTTGTGTTTACTCTTGACCCTAAGAAAAGGGCAATATCAGATAGATTAAAGGGACTACTACCAACA-GTGG [24960]
cp32-3_B31_TS_MP_FG_consensus  TGGAGAGTTTGTGTTTACTCTTGACCCTAAGAAAAGGGCAATATCAGATAGATTAAAGGGACTACTACCAACAAGTGG [24960]
cp32-3_B31_NX_Pl_consensus     TGGAGAGTTTGTGTTTACTCTTGACCCTAAGAAAAGGGCAATATCAGATAGATTAAAGGGACTACTACCAACAAGTGG [24960]
cp32-3_PAli_NX_MP_FG_consensus TGGAGAGTTTGTGTTTACTCTTGACCCTAAGAAAAGGGCAATATCAGATAGATTAAAGGGACTACTACCAACAAGTGG [24960]
cp32-3_PAli_NX_Pl_consensus    TGGAGAGTTTGTGTTTACTCTTGACCCTAAGAAAAGGGCAATATCAGATAGATTAAAGGGACTACTACCAACAAGTGG [24960]
cp32-3_PAbe_NX_MP_FG_consensus TGGAGAGTTTGTGTTTACTCTTGACCCTAAGAAAAGGGCAATATCAGATAGATTAAAGGGACTACTACCAACAAGTGG [24960]
cp32-3_PAbe_NX_Pl_consensus    TGGAGAGTTTGTGTTTACTCTTGACCCTAAGAAAAGGGCAATATCAGATAGATTAAAGGGACTACTACCAACAAGTGG [24960]

cp32-3_gi|11497103|B31_GB      TACAGTATTTTTCCCTAGCAATATAGCATTTACAAATAGTGTTAGTGTCCCCATGTGAATGGGTGCACTAAAAAAATTA [25038]
cp32-3_un8_B31_PacBio          TACAGTATTTTTCCCTAGCAATATAGCATTTACAAATAGTGTTAGTGTCCCCATGTGAATGGGTGCACTAAAAA-TTA [25038]
cp32-3_B31_TS_MP_FG_consensus  TACAGTATTTTTCCCTAGCAATATAGCATTTACAAATAGTGTTAGTGTCCCCATGTGAATGGGTGCACTAAAAAAATTA [25038]
cp32-3_B31_NX_Pl_consensus     TACAGTATTTTTCCCTAGCAATATAGCATTTACAAATAGTGTTAGTGTCCCCATGTGAATGGGTGCACTAAAAAAATTA [25038]
cp32-3_PAli_NX_MP_FG_consensus TACAGTATTTTTCCCTAGCAATATAGCATTTACAAATAGTGTTAGTGTCCCCATGTGAATGGGTGCACTAAAAAAATTA [25038]
cp32-3_PAli_NX_Pl_consensus    TACAGTATTTTTCCCTAGCAATATAGCATTTACAAATAGTGTTAGTGTCCCCATGTGAATGGGTGCACTAAAAAAATTA [25038]
cp32-3_PAbe_NX_MP_FG_consensus TACAGTATTTTTCCCTAGCAATATAGCATTTACAAATAGTGTTAGTGTCCCCATGTGAATGGGTGCACTAAAAAAATTA [25038]
cp32-3_PAbe_NX_Pl_consensus    TACAGTATTTTTCCCTAGCAATATAGCATTTACAAATAGTGTTAGTGTCCCCATGTGAATGGGTGCACTAAAAAAATTA [25038]

cp32-3_gi|11497103|B31_GB      AAAAAATAAATTTAATATAGGAGGATTAATTAATGCTTATTAATAAAATAAAACAAGATAATAGAACTTTAAGACCGG [25116]
cp32-3_un8_B31_PacBio          AAAAAATAAATTTAATATAGGAGGATTAATTAATGCTTATTAATAAAATAAAACAAGATAATAGAACTTTAAGACCGG [25116]
cp32-3_B31_TS_MP_FG_consensus  AAAAAATAAATTTAATATAGGAGGATTAATTAATGCTTATTAATAAAATAAAACAAGATAATAGAACTTTAAGACCGG [25116]
cp32-3_B31_NX_Pl_consensus     AAAAAATAAATTTAATATAGGAGGATTAATTAATGCTTATTAATAAAATAAAACAAGATAATAGAACTTTAAGACCGG [25116]
cp32-3_PAli_NX_MP_FG_consensus AAAAAATAAATTTAATATAGGAGGATTAATTAATGCTTATTAATAAAATAAAACAAGATAATAGAACTTTAAGACCGG [25116]
cp32-3_PAli_NX_Pl_consensus    AAAAAATAAATTTAATATAGGAGGATTAATTAATGCTTATTAATAAAATAAAACAAGATAATAGAACTTTAAGACCGG [25116]
cp32-3_PAbe_NX_MP_FG_consensus AAAAAATAAATTTAATATAGGAGGATTAATTAATGCTTATTAATAAAATAAAACAAGATAATAGAACTTTAAGACCGG [25116]
cp32-3_PAbe_NX_Pl_consensus    AAAAAATAAATTTAATATAGGAGGATTAATTAATGCTTATTAATAAAATAAAACAAGATAATAGAACTTTAAGACCGG [25116]

cp32-3_gi|11497103|B31_GB      AGATACAAAAATGGGGTTGTTACTTTTTGTGTCTGCATTATTATACAAGTCTATTTAAGCAACGTGAATTTAATGCAT [25194]
cp32-3_un8_B31_PacBio          AGATACAAAA-TGGGGTTGTTACTTTTTGTGTCTGCATTATTATACAAGTCTATTTAAGCAACGTGAATTTAATGCAT [25194]
cp32-3_B31_TS_MP_FG_consensus  AGATACAAAAATGGGGTTGTTACTTTTTGTGTCTGCATTATTATACAAGTCTATTTAAGCAACGTGAATTTAATGCAT [25194]
cp32-3_B31_NX_Pl_consensus     AGATACAAAAATGGGGTTGTTACTTTTTGTGTCTGCATTATTATACAAGTCTATTTAAGCAACGTGAATTTAATGCAT [25194]
cp32-3_PAli_NX_MP_FG_consensus AGATACAAAAATGGGGTTGTTACTTTTTGTGTCTGCATTATTATACAAGTCTATTTAAGCAACGTGAATTTAATGCAT [25194]
cp32-3_PAli_NX_Pl_consensus    AGATACAAAAATGGGGTTGTTACTTTTTGTGTCTGCATTATTATACAAGTCTATTTAAGCAACGTGAATTTAATGCAT [25194]
cp32-3_PAbe_NX_MP_FG_consensus AGATACAAAAATGGGGTTGTTACTTTTTGTGTCTGCATTATTATACAAGTCTATTTAAGCAACGTGAATTTAATGCAT [25194]
cp32-3_PAbe_NX_Pl_consensus    AGATACAAAAATGGGGTTGTTACTTTTTGTGTCTGCATTATTATACAAGTCTATTTAAGCAACGTGAATTTAATGCAT [25194]

cp32-3_gi|11497103|B31_GB      ATGAAATAAATACAGCGTATTATAGATTTATAGGACTTGGTTATATCAAGAGCAATTGTTTTATTATAAATCCATGTA [25272]
cp32-3_un8_B31_PacBio          ATGAAATAA-TACAGCGTATTATAGATTTATAGGACTTGGTTATATCAAGAGCAATTGTTTTATTATAAATCCATGTA [25272]
cp32-3_B31_TS_MP_FG_consensus  ATGAAATAAATACAGCGTATTATAGATTTATAGGACTTGGTTATATCAAGAGCAATTGTTTTATTATAAATCCATGTA [25272]
cp32-3_B31_NX_Pl_consensus     ATGAAATAAATACAGCGTATTATAGATTTATAGGACTTGGTTATATCAAGAGCAATTGTTTTATTATAAATCCATGTA [25272]
cp32-3_PAli_NX_MP_FG_consensus ATGAAATAAATACAGCGTATTATAGATTTATAGGACTTGGTTATATCAAGAGCAATTGTTTTATTATAAATCCATGTA [25272]
cp32-3_PAli_NX_Pl_consensus    ATGAAATAAATACAGCGTATTATAGATTTATAGGACTTGGTTATATCAAGAGCAATTGTTTTATTATAAATCCATGTA [25272]
cp32-3_PAbe_NX_MP_FG_consensus ATGAAATAAATACAGCGTATTATAGATTTATAGGACTTGGTTATATCAAGAGCAATTGTTTTATTATAAATCCATGTA [25272]
cp32-3_PAbe_NX_Pl_consensus    ATGAAATAAATACAGCGTATTATAGATTTATAGGACTTGGTTATATCAAGAGCAATTGTTTTATTATAAATCCATGTA [25272]

cp32-3_gi|11497103|B31_GB      TGATACTTAATTATTACGGAATTAGAAGTAGCGTGAGATATGAAACTGCAAATTATTTGGGTGCAGCAAATGAATTTG [25350]
cp32-3_un8_B31_PacBio          TGATACTTAATTATTACGGAATTAGAAGTAGCGTGAGATATGAAACTGCAAATTATTTGGGTGCAGCAAATGAATTTG [25350]
cp32-3_B31_TS_MP_FG_consensus  TGATACTTAATTATTACGGAATTAGAAGTAGCGTGAGATATGAAACTGCAAATTATTTGGGTGCAGCAAATGAATTTG [25350]
cp32-3_B31_NX_Pl_consensus     TGATACTTAATTATTACGGAATTAGAAGTAGCGTGAGATATGAAACTGCAAATTATTTGGGTGCAGCAAATGAATTTG [25350]
cp32-3_PAli_NX_MP_FG_consensus TGATACTTAATTATTACGGAATTAGAAGTAGCGTGAGATATGAAACTGCAAATTATTTGGGTGCAGCAAATGAATTTG [25350]
cp32-3_PAli_NX_Pl_consensus    TGATACTTAATTATTACGGAATTAGAAGTAGCGTGAGATATGAAACTGCAAATTATTTGGGTGCAGCAAATGAATTTG [25350]
cp32-3_PAbe_NX_MP_FG_consensus TGATACTTAATTATTACGGAATTAGAAGTAGCGTGAGATATGAAACTGCAAATTATTTGGGTGCAGCAAATGAATTTG [25350]
cp32-3_PAbe_NX_Pl_consensus    TGATACTTAATTATTACGGAATTAGAAGTAGCGTGAGATATGAAACTGCAAATTATTTGGGTGCAGCAAATGAATTTG [25350]

cp32-3_gi|11497103|B31_GB      AAATAAGTGAAGTTAAAATCGATAAGGTTAATGGATATCACTTTATATCAACAAAAAATAAAGAAATATTATATGATT [25428]
cp32-3_un8_B31_PacBio          AAATAAGTGAAGTTAAA-TCGATAAGGTTAATGGATATCACTTTATATCAACAAAAAATAAAGAAATATTATATGATT [25428]
cp32-3_B31_TS_MP_FG_consensus  AAATAAGTGAAGTTAAAATCGATAAGGTTAATGGATATCACTTTATATCAACAAAAAATAAAGAAATATTATATGATT [25428]
cp32-3_B31_NX_Pl_consensus     AAATAAGTGAAGTTAAAATCGATAAGGTTAATGGATATCACTTTATATCAACAAAAAATAAAGAAATATTATATGATT [25428]
cp32-3_PAli_NX_MP_FG_consensus AAATAAGTGAAGTTAAAATCGATAAGGTTAATGGATATCACTTTATATCAACAAAAAATAAAGAAATATTATATGATT [25428]
cp32-3_PAli_NX_Pl_consensus    AAATAAGTGAAGTTAAAATCGATAAGGTTAATGGATATCACTTTATATCAACAAAAAATAAAGAAATATTATATGATT [25428]
cp32-3_PAbe_NX_MP_FG_consensus AAATAAGTGAAGTTAAAATCGATAAGGTTAATGGATATCACTTTATATCAACAAAAAATAAAGAAATATTATATGATT [25428]
cp32-3_PAbe_NX_Pl_consensus    AAATAAGTGAAGTTAAAATCGATAAGGTTAATGGATATCACTTTATATCAACAAAAAATAAAGAAATATTATATGATT [25428]

cp32-3_gi|11497103|B31_GB      CACTTGATTTAAAGCCACGTGGAAAAATATTTAAAGTAACTTCAAAACGTATATTTAAACTGAAATAGTTTTACTAAA [25506]
```

```
cp32-3_un8_B31_PacBio          CACTTGATTTAAAGC-ACGTGGAAAA-TATTTAAAGTAACTTCAAAACGTATATTTAAACTGAAATAGTTTTACTAAA [25506]
cp32-3_B31_TS_MP_FG_consensus  CACTTGATTTAAAGCCACGTGGAAAAATATTTAAAGTAACTTCAAAACGTATATTTAAACTGAAATAGTTTTACTAAA [25506]
cp32-3_B31_NX_Pl_consensus     CACTTGATTTAAAGCCACGTGGAAAAATATTTAAAGTAACTTCAAAACGTATATTTAAACTGAAATAGTTTTACTAAA [25506]
cp32-3_PAli_NX_MP_FG_consensus CACTTGATTTAAAGCCACGTGGAAAAATATTTAAAGTAACTTCAAAACGTATATTTAAACTGAAATAGTTTTACTAAA [25506]
cp32-3_PAli_NX_Pl_consensus    CACTTGATTTAAAGCCACGTGGAAAAATATTTAAAGTAACTTCAAAACGTATATTTAAACTGAAATAGTTTTACTAAA [25506]
cp32-3_PAbe_NX_MP_FG_consensus CACTTGATTTAAAGCCACGTGGAAAAATATTTAAAGTAACTTCAAAACGTATATTTAAACTGAAATAGTTTTACTAAA [25506]
cp32-3_PAbe_NX_Pl_consensus    CACTTGATTTAAAGCCACGTGGAAAAATATTTAAAGTAACTTCAAAACGTATATTTAAACTGAAATAGTTTTACTAAA [25506]

cp32-3_gi|11497103|B31_GB      GTTTAAGGCACTTTTTAGCACATTCATAAGCTTGAATTTTATTAGCAGAAGATAGGCCGTAGATATTATC-AATTTCA [25584]
cp32-3_un8_B31_PacBio          GTTTAAGGCACTTTT-AGCACATTCATAAGCTTGAATTTTATTAGCAGAAGATAGGCCGTAGATATTATCCAATTTCA [25584]
cp32-3_B31_TS_MP_FG_consensus  GTTTAAGGCACTTTTTAGCACATTCATAAGCTTGAATTTTATTAGCAGAAGATAGGCCGTAGATATTATC-AATTTCA [25584]
cp32-3_B31_NX_Pl_consensus     GTTTAAGGCACTTTTTAGCACATTCATAAGCTTGAATTTTATTAGCAGAAGATAGGCCGTAGATATTATC-AATTTCA [25584]
cp32-3_PAli_NX_MP_FG_consensus GTTTAAGGCACTTTTTAGCACATTCATAAGCTTGAATTTTATTAGCAGAAGATAGGCCGTAGATATTATC-AATTTCA [25584]
cp32-3_PAli_NX_Pl_consensus    GTTTAAGGCACTTTTTAGCACATTCATAAGCTTGAATTTTATTAGCAGAAGATAGGCCGTAGATATTATC-AATTTCA [25584]
cp32-3_PAbe_NX_MP_FG_consensus GTTTAAGGCACTTTTTAGCACATTCATAAGCTTGAATTTTATTAGCAGAAGATAGGCCGTAGATATTATC-AATTTCA [25584]
cp32-3_PAbe_NX_Pl_consensus    GTTTAAGGCACTTTTTAGCACATTCATAAGCTTGAATTTTATTAGCAGAAGATAGGCCGTAGATATTATC-AATTTCA [25584]

cp32-3_gi|11497103|B31_GB      TTGGTTGAAGAATATTTCATAAGTTCTTTAATTTGAAAAGAATTGTAGCCGTTAGATTTCAAATTTGAAATAAATAAA [25662]
cp32-3_un8_B31_PacBio          TTGGTTGAAGAATATTTCATAAGTTCTTTAATTTGAAAAGAATTGTAGCCGTTAGATTTCAAATTTGAAATAAATAAA [25662]
cp32-3_B31_TS_MP_FG_consensus  TTGGTTGAAGAATATTTCATAAGTTCTTTAATTTGAAAAGAATTGTAGCCGTTAGATTTCAAATTTGAAATAAATAAA [25662]
cp32-3_B31_NX_Pl_consensus     TTGGTTGAAGAATATTTCATAAGTTCTTTAATTTGAAAAGAATTGTAGCCGTTAGATTTCAAATTTGAAATAAATAAA [25662]
cp32-3_PAli_NX_MP_FG_consensus TTGGTTGAAGAATATTTCATAAGTTCTTTAATTTGAAAAGAATTGTAGCCGTTAGATTTCAAATTTGAAATAAATAAA [25662]
cp32-3_PAli_NX_Pl_consensus    TTGGTTGAAGAATATTTCATAAGTTCTTTAATTTGAAAAGAATTGTAGCCGTTAGATTTCAAATTTGAAATAAATAAA [25662]
cp32-3_PAbe_NX_MP_FG_consensus TTGGTTGAAGAATATTTCATAAGTTCTTTAATTTGAAAAGAATTGTAGCCGTTAGATTTCAAATTTGAAATAAATAAA [25662]
cp32-3_PAbe_NX_Pl_consensus    TTGGTTGAAGAATATTTCATAAGTTCTTTAATTTGAAAAGAATTGTAGCCGTTAGATTTCAAATTTGAAATAAATAAA [25662]

cp32-3_gi|11497103|B31_GB      TTTCGACATAGATGGAGAGATTTATTTACACGAAACCCCGACTTTTTAAGAAGATTTTTGAATTTTTTAGAAATATGG [25740]
cp32-3_un8_B31_PacBio          TTTCGACATAGATGGAGAGATTTATTTACACGAAACCCCGACTTTTTAAGAAGATTTTTGAATTTTT-AGAAATATGG [25740]
cp32-3_B31_TS_MP_FG_consensus  TTTCGACATAGATGGAGAGATTTATTTACACGAAACCCCGACTTTTTAAGAAGATTTTTGAATTTTTTAGAAATATGG [25740]
cp32-3_B31_NX_Pl_consensus     TTTCGACATAGATGGAGAGATTTATTTACACGAAACCCCGACTTTTTAAGAAGATTTTTGAATTTTTTAGAAATATGG [25740]
cp32-3_PAli_NX_MP_FG_consensus TTTCGACATAGATGGAGAGATTTATTTACACGAAACCCCGACTTTTTAAGAAGATTTTTGAATTTTTTAGAAATATGG [25740]
cp32-3_PAli_NX_Pl_consensus    TTTCGACATAGATGGAGAGATTTATTTACACGAAACCCCGACTTTTTAAGAAGATTTTTGAATTTTTTAGAAATATGG [25740]
cp32-3_PAbe_NX_MP_FG_consensus TTTCGACATAGATGGAGAGATTTATTTACACGAAACCCCGACTTTTTAAGAAGATTTTTGAATTTTTTAGAAATATGG [25740]
cp32-3_PAbe_NX_Pl_consensus    TTTCGACATAGATGGAGAGATTTATTTACACGAAACCCCGACTTTTTAAGAAGATTTTTGAATTTTTTAGAAATATGG [25740]

cp32-3_gi|11497103|B31_GB      ACAATATCAATTTGGTTATCTTTAAACTTATGTTTGCTCTTTTGGAAAAGATAAGTACGCCTTGAGTCAAGAGTTTTT [25818]
cp32-3_un8_B31_PacBio          ACAATATCAATTTGGTTATCTTTAA-CTTATGTTTGCTCTTTTGGAAAAGATAAGTACGCCTTGAGTCAAGAGTTTTT [25818]
cp32-3_B31_TS_MP_FG_consensus  ACAATATCAATTTGGTTATCTTTAAACTTATGTTTGCTCTTTTGGAAAAGATAAGTACGCCTTGAGTCAAGAGTTTTT [25818]
cp32-3_B31_NX_Pl_consensus     ACAATATCAATTTGGTTATCTTTAAACTTATGTTTGCTCTTTTGGAAAAGATAAGTACGCCTTGAGTCAAGAGTTTTT [25818]
cp32-3_PAli_NX_MP_FG_consensus ACAATATCAATTTGGTTATCTTTAAACTTATGTTTGCTCTTTTGGAAAAGATAAGTACGCCTTGAGTCAAGAGTTTTT [25818]
cp32-3_PAli_NX_Pl_consensus    ACAATATCAATTTGGTTATCTTTAAACTTATGTTTGCTCTTTTGGAAAAGATAAGTACGCCTTGAGTCAAGAGTTTTT [25818]
cp32-3_PAbe_NX_MP_FG_consensus ACAATATCAATTTGGTTATCTTTAAACTTATGTTTGCTCTTTTGGAAAAGATAAGTACGCCTTGAGTCAAGAGTTTTT [25818]
cp32-3_PAbe_NX_Pl_consensus    ACAATATCAATTTGGTTATCTTTAAACTTATGTTTGCTCTTTTGGAAAAGATAAGTACGCCTTGAGTCAAGAGTTTTT [25818]

cp32-3_gi|11497103|B31_GB      TCTTGGAAATGATTTTTATGTGCTGTTTGGATAGCCTCGAACTCTTCTGAGTTGATGACAATTTCTCTAATACAAGAG [25896]
cp32-3_un8_B31_PacBio          TCTTGGAAATGATTTTTATGTGCTGTTTGGATAGCCTCGAACTCTTCTGAGTTGATGACAATTTCTCTAATACAAGAG [25896]
cp32-3_B31_TS_MP_FG_consensus  TCTTGGAAATGATTTTTATGTGCTGTTTGGATAGCCTCGAACTCTTCTGAGTTGATGACAATTTCTCTAATACAAGAG [25896]
cp32-3_B31_NX_Pl_consensus     TCTTGGAAATGATTTTTATGTGCTGTTTGGATAGCCTCGAACTCTTCTGAGTTGATGACAATTTCTCTAATACAAGAG [25896]
cp32-3_PAli_NX_MP_FG_consensus TCTTGGAAATGATTTTTATGTGCTGTTTGGATAGCCTCGAACTCTTCTGAGTTGATGACAATTTCTCTAATACAAGAG [25896]
cp32-3_PAli_NX_Pl_consensus    TCTTGGAAATGATTTTTATGTGCTGTTTGGATAGCCTCGAACTCTTCTGAGTTGATGACAATTTCTCTAATACAAGAG [25896]
cp32-3_PAbe_NX_MP_FG_consensus TCTTGGAAATGATTTTTATGTGCTGTTTGGATAGCCTCGAACTCTTCTGAGTTGATGACAATTTCTCTAATACAAGAG [25896]
cp32-3_PAbe_NX_Pl_consensus    TCTTGGAAATGATTTTTATGTGCTGTTTGGATAGCCTCGAACTCTTCTGAGTTGATGACAATTTCTCTAATACAAGAG [25896]

cp32-3_gi|11497103|B31_GB      GTATTTCTTTTTTTTGCCACATTTACTTTAATAGTATATAAAGTTTTTCCGGTTTTGCTTAAAAAAGTTGAAATATCT [25974]
cp32-3_un8_B31_PacBio          GTATTTCTTTTTTT-GCCACATTTACTTTAATAGTATATAAAGTTTTTCCGGTTTTGCTTAAAAAAGTTGAAATATCT [25974]
cp32-3_B31_TS_MP_FG_consensus  GTATTTCTTTTTTTTGCCACATTTACTTTAATAGTATATAAAGTTTTTCCGGTTTTGCTTAAAAAAGTTGAAATATCT [25974]
cp32-3_B31_NX_Pl_consensus     GTATTTCTTTTTTTTGCCACATTTACTTTAATAGTATATAAAGTTTTTCCGGTTTTGCTTAAAAAAGTTGAAATATCT [25974]
cp32-3_PAli_NX_MP_FG_consensus GTATTTCTTTTTTTTGCCACATTTACTTTAATAGTATATAAAGTTTTTCCGGTTTTGCTTAAAAAAGTTGAAATATCT [25974]
cp32-3_PAli_NX_Pl_consensus    GTATTTCTTTTTTTTGCCACATTTACTTTAATAGTATATAAAGTTTTTCCGGTTTTGCTTAAAAAAGTTGAAATATCT [25974]
cp32-3_PAbe_NX_MP_FG_consensus GTATTTCTTTTTTTTGCCACATTTACTTTAATAGTATATAAAGTTTTTCCGGTTTTGCTTAAAAAAGTTGAAATATCT [25974]
cp32-3_PAbe_NX_Pl_consensus    GTATTTCTTTTTTTTGCCACATTTACTTTAATAGTATATAAAGTTTTTCCGGTTTTGCTTAAAAAAGTTGAAATATCT [25974]

cp32-3_gi|11497103|B31_GB      TGCATTTTTACTTTTTGCATTTCAGTGCCTCTACATCCACTTATTAAGAGTAGATGCACAAACCAACCAGAAATTGGG [26052]
cp32-3_un8_B31_PacBio          TGCATTTTTACTTTTTGCATTTCAGTGCCTCTACATCCACTTATTAAGAGTAGATGCACAAACCAACCAGAAATTGGG [26052]
cp32-3_B31_TS_MP_FG_consensus  TGCATTTTTACTTTTTGCATTTCAGTGCCTCTACATCCACTTATTAAGAGTAGATGCACAAACCAACCAGAAATTGGG [26052]
cp32-3_B31_NX_Pl_consensus     TGCATTTTTACTTTTTGCATTTCAGTGCCTCTACATCCACTTATTAAGAGTAGATGCACAAACCAACCAGAAATTGGG [26052]
cp32-3_PAli_NX_MP_FG_consensus TGCATTTTTACTTTTTGCATTTCAGTGCCTCTACATCCACTTATTAAGAGTAGATGCACAAACCAACCAGAAATTGGG [26052]
cp32-3_PAli_NX_Pl_consensus    TGCATTTTTACTTTTTGCATTTCAGTGCCTCTACATCCACTTATTAAGAGTAGATGCACAAACCAACCAGAAATTGGG [26052]
cp32-3_PAbe_NX_MP_FG_consensus TGCATTTTTACTTTTTGCATTTCAGTGCCTCTACATCCACTTATTAAGAGTAGATGCACAAACCAACCAGAAATTGGG [26052]
cp32-3_PAbe_NX_Pl_consensus    TGCATTTTTACTTTTTGCATTTCAGTGCCTCTACATCCACTTATTAAGAGTAGATGCACAAACCAACCAGAAATTGGG [26052]

cp32-3_gi|11497103|B31_GB      TCAATTTGTTTTAAGGTTTTGGCACATTTTAGAATTAATTTAATACTTTTAGGGGTTAAATAAAACTTAGGAGTTGGT [26130]
cp32-3_un8_B31_PacBio          TCAATTTGTTTTAAGGTTTTGGCACATTTTAGAATTAATTTAATACTTTTAGGGGTTAAATAAAACTTAGGAGTTGGT [26130]
cp32-3_B31_TS_MP_FG_consensus  TCAATTTGTTTTAAGGTTTTGGCACATTTTAGAATTAATTTAATACTTTTAGGGGTTAAATAAAACTTAGGAGTTGGT [26130]
cp32-3_B31_NX_Pl_consensus     TCAATTTGTTTTAAGGTTTTGGCACATTTTAGAATTAATTTAATACTTTTAGGGGTTAAATAAAACTTAGGAGTTGGT [26130]
cp32-3_PAli_NX_MP_FG_consensus TCAATTTGTTTTAAGGTTTTGGCACATTTTAGAATTAATTTAATACTTTTAGGGGTTAAATAAAACTTAGGAGTTGGT [26130]
cp32-3_PAli_NX_Pl_consensus    TCAATTTGTTTTAAGGTTTTGGCACATTTTAGAATTAATTTAATACTTTTAGGGGTTAAATAAAACTTAGGAGTTGGT [26130]
cp32-3_PAbe_NX_MP_FG_consensus TCAATTTGTTTTAAGGTTTTGGCACATTTTAGAATTAATTTAATACTTTTAGGGGTTAAATAAAACTTAGGAGTTGGT [26130]
cp32-3_PAbe_NX_Pl_consensus    TCAATTTGTTTTAAGGTTTTGGCACATTTTAGAATTAATTTAATACTTTTAGGGGTTAAATAAAACTTAGGAGTTGGT [26130]

cp32-3_gi|11497103|B31_GB      TTTGAATTTTCTTTTTTACTTTTAGATGAATTTTTTAGTGAATTTTTAAGAATTTTGTTTTCATTTATTAGTTTTTGG [26208]
cp32-3_un8_B31_PacBio          TTTGAATTTTCTTTTTTACTTTTAGATGAATTTTTTAGTGAATTTTTAAGAATTTTGTTTTCATTTATTAGTTTT-GG [26208]
cp32-3_B31_TS_MP_FG_consensus  TTTGAATTTTCTTTTTTACTTTTAGATGAATTTTTTAGTGAATTTTTAAGAATTTTGTTTTCATTTATTAGTTTTTGG [26208]
cp32-3_B31_NX_Pl_consensus     TTTGAATTTTCTTTTTTACTTTTAGATGAATTTTTTAGTGAATTTTTAAGAATTTTGTTTTCATTTATTAGTTTTTGG [26208]
cp32-3_PAli_NX_MP_FG_consensus TTTGAATTTTCTTTTTTACTTTTAGATGAATTTTTTAGTGAATTTTTAAGAATTTTGTTTTCATTTATTAGTTTTTGG [26208]
cp32-3_PAli_NX_Pl_consensus    TTTGAATTTTCTTTTTTACTTTTAGATGAATTTTTTAGTGAATTTTTAAGAATTTTGTTTTCATTTATTAGTTTTTGG [26208]
cp32-3_PAbe_NX_MP_FG_consensus TTTGAATTTTCTTTTTTACTTTTAGATGAATTTTTTAGTGAATTTTTAAGAATTTTGTTTTCATTTATTAGTTTTTGG [26208]
cp32-3_PAbe_NX_Pl_consensus    TTTGAATTTTCTTTTTTACTTTTAGATGAATTTTTTAGTGAATTTTTAAGAATTTTGTTTTCATTTATTAGTTTTTGG [26208]

cp32-3_gi|11497103|B31_GB      TATTCTTGAAATAGTTTGAGCGTAAAATCCATATTGAAATTATTTAAATTAAAAGAATTATTATTGTCCATAAAATCC [26286]
cp32-3_un8_B31_PacBio          TATTCTTGAAATAGTTTGAGCGTAAAATCCATATTGAAATTATTTAAATTAAAAGAATTATTATTGTCCATAAAATCC [26286]
cp32-3_B31_TS_MP_FG_consensus  TATTCTTGAAATAGTTTGAGCGTAAAATCCATATTGAAATTATTTAAATTAAAAGAATTATTATTGTCCATAAAATCC [26286]
cp32-3_B31_NX_Pl_consensus     TATTCTTGAAATAGTTTGAGCGTAAAATCCATATTGAAATTATTTAAATTAAAAGAATTATTATTGTCCATAAAATCC [26286]
cp32-3_PAli_NX_MP_FG_consensus TATTCTTGAAATAGTTTGAGCGTAAAATCCATATTGAAATTATTTAAATTAAAAGAATTATTATTGTCCATAAAATCC [26286]
cp32-3_PAli_NX_Pl_consensus    TATTCTTGAAATAGTTTGAGCGTAAAATCCATATTGAAATTATTTAAATTAAAAGAATTATTATTGTCCATAAAATCC [26286]
cp32-3_PAbe_NX_MP_FG_consensus TATTCTTGAAATAGTTTGAGCGTAAAATCCATATTGAAATTATTTAAATTAAAAGAATTATTATTGTCCATAAAATCC [26286]
cp32-3_PAbe_NX_Pl_consensus    TATTCTTGAAATAGTTTGAGCGTAAAATCCATATTGAAATTATTTAAATTAAAAGAATTATTATTGTCCATAAAATCC [26286]

cp32-3_gi|11497103|B31_GB      TCTCCTTGAAGTTGTTACTTTTAAATTAAGTAAAAGTAATAAAAATAGATAAAAAAATGTAAATTATATTTTACTAAAA [26364]
cp32-3_un8_B31_PacBio          TCTCCTTGAAGTTGTTACTTTTAAATTAAGTAAAAGTAATAAAAATAGATAAAAAAATGTAAATTATATTTTACTAAAA [26364]
cp32-3_B31_TS_MP_FG_consensus  TCTCCTTGAAGTTGTTACTTTTAAATTAAGTAAAAGTAATAAAAATAGATAAAAAAATGTAAATTATATTTTACTAAAA [26364]
cp32-3_B31_NX_Pl_consensus     TCTCCTTGAAGTTGTTACTTTTAAATTAAGTAAAAGTAATAAAAATAGATAAAAAAATGTAAATTATATTTTACTAAAA [26364]
cp32-3_PAli_NX_MP_FG_consensus TCTCCTTGAAGTTGTTACTTTTAAATTAAGTAAAAGTAATAAAAATAGATAAAAAAATGTAAATTATATTTTACTAAAA [26364]
cp32-3_PAli_NX_Pl_consensus    TCTCCTTGAAGTTGTTACTTTTAAATTAAGTAAAAGTAATAAAAATAGATAAAAAAATGTAAATTATATTTTACTAAAA [26364]
cp32-3_PAbe_NX_MP_FG_consensus TCTCCTTGAAGTTGTTACTTTTAAATTAAGTAAAAGTAATAAAAATAGATAAAAAAATGTAAATTATATTTTACTAAAA [26364]
cp32-3_PAbe_NX_Pl_consensus    TCTCCTTGAAGTTGTTACTTTTAAATTAAGTAAAAGTAATAAAAATAGATAAAAAAATGTAAATTATATTTTACTAAAA [26364]

cp32-3_gi|11497103|B31_GB      ACGAAAAAATTTTAGTCAAATTTGGAGTGTTCTCATTGCATGCAAAATCTGGGCTGTAGAGTGGGTGTAATAAACAGAA [26442]
cp32-3_un8_B31_PacBio          ACGAAAAAATTTTAGTCAAATTTGGAGTGTTCTCATTGCATGCAAAATCTGGGCTGTAGAGTGGGTGTAATAAACAGAA [26442]
```

```
cp32-3_B31_TS_MP_FG_consensus  ACGAAAAAATTTTAGTCAAATTTGGAGTGTTCTCATTGCATGCAAAATCTGGGCTGTAGAGTGGGTGTAATAAACAGAA [26442]
cp32-3_B31_NX_Pl_consensus     ACGAAAAAATTTTAGTCAAATTTGGAGTGTTCTCATTGCATGCAAAATCTGGGCTGTAGAGTGGGTGTAATAAACAGAA [26442]
cp32-3_PAli_NX_MP_FG_consensus ACGAAAAAATTTTAGTCAAATTTGGAGTGTTCTCATTGCATGCAAAATCTGGGCTGTAGAGTGGGTGTAATAAACAGAA [26442]
cp32-3_PAli_NX_Pl_consensus    ACGAAAAAATTTTAGTCAAATTTGGAGTGTTCTCATTGCATGCAAAATCTGGGCTGTAGAGTGGGTGTAATAAACAGAA [26442]
cp32-3_PAbe_NX_MP_FG_consensus ACGAAAAAATTTTAGTCAAATTTGGAGTGTTCTCATTGCATGCAAAATCTGGGCTGTAGAGTGGGTGTAATAAACAGAA [26442]
cp32-3_PAbe_NX_Pl_consensus    ACGAAAAAATTTTAGTCAAATTTGGAGTGTTCTCATTGCATGCAAAATCTGGGCTGTAGAGTGGGTGTAATAAACAGAA [26442]

cp32-3_gi|11497103|B31_GB      GTAGTGATTTTTGAGGGTGGTACACAAGAAAGATAGTATACTTTGTGTAATATATAGCAAAGACTTGAAAATTTAATT [26520]
cp32-3_un8_B31_PacBio          GTAGTGATTTTTGAGGGTGGTACACAAGAAAGATAGTATACTTTGTGTAATATATAGCAAAGACTTGAAAATTTAATT [26520]
cp32-3_B31_TS_MP_FG_consensus  GTAGTGATTTTTGAGGGTGGTACACAAGAAAGATAGTATACTTTGTGTAATATATAGCAAAGACTTGAAAATTTAATT [26520]
cp32-3_B31_NX_Pl_consensus     GTAGTGATTTTTGAGGGTGGTACACAAGAAAGATAGTATACTTTGTGTAATATATAGCAAAGACTTGAAAATTTAATT [26520]
cp32-3_PAli_NX_MP_FG_consensus GTAGTGATTTTTGAGGGTGGTACACAAGAAAGATAGTATACTTTGTGTAATATATAGCAAAGACTTGAAAATTTAATT [26520]
cp32-3_PAli_NX_Pl_consensus    GTAGTGATTTTTGAGGGTGGTACACAAGAAAGATAGTATACTTTGTGTAATATATAGCAAAGACTTGAAAATTTAATT [26520]
cp32-3_PAbe_NX_MP_FG_consensus GTAGTGATTTTTGAGGGTGGTACACAAGAAAGATAGTATACTTTGTGTAATATATAGCAAAGACTTGAAAATTTAATT [26520]
cp32-3_PAbe_NX_Pl_consensus    GTAGTGATTTTTGAGGGTGGTACACAAGAAAGATAGTATACTTTGTGTAATATATAGCAAAGACTTGAAAATTTAATT [26520]

cp32-3_gi|11497103|B31_GB      TGTATGTATTTTATAATCTTTTGTAATGAGTAGAGCATTTGCAATGGAGAGATTTTGGCGAGTTGGTTAAAATTACAT [26598]
cp32-3_un8_B31_PacBio          TGTATGTATTT-ATAATCTTT-GTAATGAGTAGAGCATTTGCAATGGAGAGATTTTGGCGAGTTGGTTAAAATTACAT [26598]
cp32-3_B31_TS_MP_FG_consensus  TGTATGTATTTTATAATCTTTTGTAATGAGTAGAGCATTTGCAATGGAGAGATTTTGGCGAGTTGGTTAAAATTACAT [26598]
cp32-3_B31_NX_Pl_consensus     TGTATGTATTTTATAATCTTTTGTAATGAGTAGAGCATTTGCAATGGAGAGATTTTGGCGAGTTGGTTAAAATTACAT [26598]
cp32-3_PAli_NX_MP_FG_consensus TGTATGTATTTTATAATCTTTTGTAATGAGTAGAGCATTTGCAATGGAGAGATTTTGGCGAGTTGGTTAAAATTACAT [26598]
cp32-3_PAli_NX_Pl_consensus    TGTATGTATTTTATAATCTTTTGTAATGAGTAGAGCATTTGCAATGGAGAGATTTTGGCGAGTTGGTTAAAATTACAT [26598]
cp32-3_PAbe_NX_MP_FG_consensus TGTATGTATTTTATAATCTTTTGTAATGAGTAGAGCATTTGCAATGGAGAGATTTTGGCGAGTTGGTTAAAATTACAT [26598]
cp32-3_PAbe_NX_Pl_consensus    TGTATGTATTTTATAATCTTTTGTAATGAGTAGAGCATTTGCAATGGAGAGATTTTGGCGAGTTGGTTAAAATTACAT [26598]

cp32-3_gi|11497103|B31_GB      TTGTGTTTTGTTAAAATGTAACAGCTGAATGTAACAAAATTATATATTTAAATCTTTGAAATATTGCAATTATTTGGT [26676]
cp32-3_un8_B31_PacBio          TTGTGTTTTGTTAAAATGTAACAGCTGAATGTAACAAAATTATATATTTAAATCTTTGAAATATTGCAATTATTTGGT [26676]
cp32-3_B31_TS_MP_FG_consensus  TTGTGTTTTGTTAAAATGTAACAGCTGAATGTAACAAAATTATATATTTAAATCTTTGAAATATTGCAATTATTTGGT [26676]
cp32-3_B31_NX_Pl_consensus     TTGTGTTTTGTTAAAATGTAACAGCTGAATGTAACAAAATTATATATTTAAATCTTTGAAATATTGCAATTATTTGGT [26676]
cp32-3_PAli_NX_MP_FG_consensus TTGTGTTTTGTTAAAATGTAACAGCTGAATGTAACAAAATTATATATTTAAATCTTTGAAATATTGCAATTATTTGGT [26676]
cp32-3_PAli_NX_Pl_consensus    TTGTGTTTTGTTAAAATGTAACAGCTGAATGTAACAAAATTATATATTTAAATCTTTGAAATATTGCAATTATTTGGT [26676]
cp32-3_PAbe_NX_MP_FG_consensus TTGTGTTTTGTTAAAATGTAACAGCTGAATGTAACAAAATTATATATTTAAATCTTTGAAATATTGCAATTATTTGGT [26676]
cp32-3_PAbe_NX_Pl_consensus    TTGTGTTTTGTTAAAATGTAACAGCTGAATGTAACAAAATTATATATTTAAATCTTTGAAATATTGCAATTATTTGGT [26676]

cp32-3_gi|11497103|B31_GB      GTTGTGGTAATATTAGTACTTTATGGAGTAACTTATGAATAAGAAAATGAAAAATTTAATTATTTGTGCAGTTTTTGT [26754]
cp32-3_un8_B31_PacBio          GTTGTGGTAATATTAGTACTT-ATGGAGTAACTTATGAATAAGAAAATGAAAAATTTAATTATTTGTGCAGTTTT--G [26754]
cp32-3_B31_TS_MP_FG_consensus  GTTGTGGTAATATTAGTACTTTATGGAGTAACTTATGAATAAGAAAATGAAAAATTTAATTATTTGTGCAGTTTTTGT [26754]
cp32-3_B31_NX_Pl_consensus     GTTGTGGTAATATTAGTACTTTATGGAGTAACTTATGAATAAGAAAATGAAAAATTTAATTATTTGTGCAGTTTTTGT [26754]
cp32-3_PAli_NX_MP_FG_consensus GTTGTGGTAATATTAGTACTTTATGGAGTAACTTATGAATAAGAAAATGAAAAATTTAATTATTTGTGCAGTTTTTGT [26754]
cp32-3_PAli_NX_Pl_consensus    GTTGTGGTAATATTAGTACTTTATGGAGTAACTTATGAATAAGAAAATGAAAAATTTAATTATTTGTGCAGTTTTTGT [26754]
cp32-3_PAbe_NX_MP_FG_consensus GTTGTGGTAATATTAGTACTTTATGGAGTAACTTATGAATAAGAAAATGAAAAATTTAATTATTTGTGCAGTTTTTGT [26754]
cp32-3_PAbe_NX_Pl_consensus    GTTGTGGTAATATTAGTACTTTATGGAGTAACTTATGAATAAGAAAATGAAAAATTTAATTATTTGTGCAGTTTTTGT [26754]

cp32-3_gi|11497103|B31_GB      TTTGATAATTTCTTGCAAGATTGATGCGAGTAGTGAAGATTTAAAACAAAATGTAAAAGAAAAAGTTGAAGGATTTTT [26832]
cp32-3_un8_B31_PacBio          TTTGATAATTTCTTGCAAGATTGATGCGAGTAGTGAAGATTTAAAACAAAATGTAAA-GAAAAAGTTGAAGGATTTTT [26832]
cp32-3_B31_TS_MP_FG_consensus  TTTGATAATTTCTTGCAAGATTGATGCGAGTAGTGAAGATTTAAAACAAAATGTAAAAGAAAAAGTTGAAGGATTTTT [26832]
cp32-3_B31_NX_Pl_consensus     TTTGATAATTTCTTGCAAGATTGATGCGAGTAGTGAAGATTTAAAACAAAATGTAAAAGAAAAAGTTGAAGGATTTTT [26832]
cp32-3_PAli_NX_MP_FG_consensus TTTGATAATTTCTTGCAAGATTGATGCGAGTAGTGAAGATTTAAAACAAAATGTAAAAGAAAAAGTTGAAGGATTTTT [26832]
cp32-3_PAli_NX_Pl_consensus    TTTGATAATTTCTTGCAAGATTGATGCGAGTAGTGAAGATTTAAAACAAAATGTAAAAGAAAAAGTTGAAGGATTTTT [26832]
cp32-3_PAbe_NX_MP_FG_consensus TTTGATAATTTCTTGCAAGATTGATGCGAGTAGTGAAGATTTAAAACAAAATGTAAAAGAAAAAGTTGAAGGATTTTT [26832]
cp32-3_PAbe_NX_Pl_consensus    TTTGATAATTTCTTGCAAGATTGATGCGAGTAGTGAAGATTTAAAACAAAATGTAAAAGAAAAAGTTGAAGGATTTTT [26832]

cp32-3_gi|11497103|B31_GB      AGATAAAGAGTTAATGCAAGGTGACGATCCTAATAACAGTCTGTTTAATCCACCACCAGTATTGCCGGCAAGTTCCCA [26910]
cp32-3_un8_B31_PacBio          AGATAAAGAGTTAATGCAAGGTGACGATCCTAATAACAGTCTGTTTAATCCACCACCAGTATTGCCGGCAAGTTCCCA [26910]
cp32-3_B31_TS_MP_FG_consensus  AGATAAAGAGTTAATGCAAGGTGACGATCCTAATAACAGTCTGTTTAATCCACCACCAGTATTGCCGGCAAGTTCCCA [26910]
cp32-3_B31_NX_Pl_consensus     AGATAAAGAGTTAATGCAAGGTGACGATCCTAATAACAGTCTGTTTAATCCACCACCAGTATTGCCGGCAAGTTCCCA [26910]
cp32-3_PAli_NX_MP_FG_consensus AGATAAAGAGTTAATGCAAGGTGACGATCCTAATAACAGTCTGTTTAATCCACCACCAGTATTGCCGGCAAGTTCCCA [26910]
cp32-3_PAli_NX_Pl_consensus    AGATAAAGAGTTAATGCAAGGTGACGATCCTAATAACAGTCTGTTTAATCCACCACCAGTATTGCCGGCAAGTTCCCA [26910]
cp32-3_PAbe_NX_MP_FG_consensus AGATAAAGAGTTAATGCAAGGTGACGATCCTAATAACAGTCTGTTTAATCCACCACCAGTATTGCCGGCAAGTTCCCA [26910]
cp32-3_PAbe_NX_Pl_consensus    AGATAAAGAGTTAATGCAAGGTGACGATCCTAATAACAGTCTGTTTAATCCACCACCAGTATTGCCGGCAAGTTCCCA [26910]

cp32-3_gi|11497103|B31_GB      CGATAACACACCCGTATTAAAAGCGGTGCAAGCAAAAGATGGTGGTCAACAAGAAGGAAAAGAAGAGAAAGAAAAAGA [26988]
cp32-3_un8_B31_PacBio          CGATAACACACCCGTATTAAAAGCGGTGCAAGCAAAAGATG-TGGTCAACAAGAAGGAAAAGAAGAGAAAGAAAAAGA [26988]
cp32-3_B31_TS_MP_FG_consensus  CGATAACACACCCGTATTAAAAGCGGTGCAAGCAAAAGATGGTGGTCAACAAGAAGGAAAAGAAGAGAAAGAAAAAGA [26988]
cp32-3_B31_NX_Pl_consensus     CGATAACACACCCGTATTAAAAGCGGTGCAAGCAAAAGATGGTGGTCAACAAGAAGGAAAAGAAGAGAAAGAAAAAGA [26988]
cp32-3_PAli_NX_MP_FG_consensus CGATAACACACCCGTATTAAAAGCGGTGCAAGCAAAAGATGGTGGTCAACAAGAAGGAAAAGAAGAGAAAGAAAAAGA [26988]
cp32-3_PAli_NX_Pl_consensus    CGATAACACACCCGTATTAAAAGCGGTGCAAGCAAAAGATGGTGGTCAACAAGAAGGAAAAGAAGAGAAAGAAAAAGA [26988]
cp32-3_PAbe_NX_MP_FG_consensus CGATAACACACCCGTATTAAAAGCGGTGCAAGCAAAAGATGGTGGTCAACAAGAAGGAAAAGAAGAGAAAGAAAAAGA [26988]
cp32-3_PAbe_NX_Pl_consensus    CGATAACACACCCGTATTAAAAGCGGTGCAAGCAAAAGATGGTGGTCAACAAGAAGGAAAAGAAGAGAAAGAAAAAGA [26988]

cp32-3_gi|11497103|B31_GB      AATTCAAGAATTAAAGGATAAAATAGATAAAAGAAAAAAAGAATTAGAAGAGGCTAGAAAGAAATTTCAAGAATTTAA [27066]
cp32-3_un8_B31_PacBio          AATTCAAGAATTAAAGGATAAAATAGATAAAAGAAAAAAAGAATTAGAAGAGGCTAGAAAGAAATTTCAAGAATTTAA [27066]
cp32-3_B31_TS_MP_FG_consensus  AATTCAAGAATTAAAGGATAAAATAGATAAAAGAAAAAAAGAATTAGAAGAGGCTAGAAAGAAATTTCAAGAATTTAA [27066]
cp32-3_B31_NX_Pl_consensus     AATTCAAGAATTAAAGGATAAAATAGATAAAAGAAAAAAAGAATTAGAAGAGGCTAGAAAGAAATTTCAAGAATTTAA [27066]
cp32-3_PAli_NX_MP_FG_consensus AATTCAAGAATTAAAGGATAAAATAGATAAAAGAAAAAAAGAATTAGAAGAGGCTAGAAAGAAATTTCAAGAATTTAA [27066]
cp32-3_PAli_NX_Pl_consensus    AATTCAAGAATTAAAGGATAAAATAGATAAAAGAAAAAAAGAATTAGAAGAGGCTAGAAAGAAATTTCAAGAATTTAA [27066]
cp32-3_PAbe_NX_MP_FG_consensus AATTCAAGAATTAAAGGATAAAATAGATAAAAGAAAAAAAGAATTAGAAGAGGCTAGAAAGAAATTTCAAGAATTTAA [27066]
cp32-3_PAbe_NX_Pl_consensus    AATTCAAGAATTAAAGGATAAAATAGATAAAAGAAAAAAAGAATTAGAAGAGGCTAGAAAGAAATTTCAAGAATTTAA [27066]

cp32-3_gi|11497103|B31_GB      AGAACAAGTTGAATCTGCAACTGGAGAAAGTACTGAGAAAGTTAAAAAACAAGGAAATATTGGACAAAAAGCTTTAAA [27144]
cp32-3_un8_B31_PacBio          AGAACAAGTTGAATCTGCAACTGGAGAAAGTACTGAGAAAGTTAAAAAACAAGGAAATATTGGACAAAAAGCTTTAAA [27144]
cp32-3_B31_TS_MP_FG_consensus  AGAACAAGTTGAATCTGCAACTGGAGAAAGTACTGAGAAAGTTAAAAAACAAGGAAATATTGGACAAAAAGCTTTAAA [27144]
cp32-3_B31_NX_Pl_consensus     AGAACAAGTTGAATCTGCAACTGGAGAAAGTACTGAGAAAGTTAAAAAACAAGGAAATATTGGACAAAAAGCTTTAAA [27144]
cp32-3_PAli_NX_MP_FG_consensus AGAACAAGTTGAATCTGCAACTGGAGAAAGTACTGAGAAAGTTAAAAAACAAGGAAATATTGGACAAAAAGCTTTAAA [27144]
cp32-3_PAli_NX_Pl_consensus    AGAACAAGTTGAATCTGCAACTGGAGAAAGTACTGAGAAAGTTAAAAAACAAGGAAATATTGGACAAAAAGCTTTAAA [27144]
cp32-3_PAbe_NX_MP_FG_consensus AGAACAAGTTGAATCTGCAACTGGAGAAAGTACTGAGAAAGTTAAAAAACAAGGAAATATTGGACAAAAAGCTTTAAA [27144]
cp32-3_PAbe_NX_Pl_consensus    AGAACAAGTTGAATCTGCAACTGGAGAAAGTACTGAGAAAGTTAAAAAACAAGGAAATATTGGACAAAAAGCTTTAAA [27144]

cp32-3_gi|11497103|B31_GB      GTATGCTAAAGAATTGGGTGTAAATGGAAGTTATTCTGTTAATGATGGTACTAATACTAATGATTTTGTAAAAAAGGT [27222]
cp32-3_un8_B31_PacBio          GTATGCTAAAGAATTGGGTGTAAATGGAAGTTATTCTGTTAATGATGGTACTAATACTAATGATTTTGTAAAAAAGGT [27222]
cp32-3_B31_TS_MP_FG_consensus  GTATGCTAAAGAATTGGGTGTAAATGGAAGTTATTCTGTTAATGATGGTACTAATACTAATGATTTTGTAAAAAAGGT [27222]
cp32-3_B31_NX_Pl_consensus     GTATGCTAAAGAATTGGGTGTAAATGGAAGTTATTCTGTTAATGATGGTACTAATACTAATGATTTTGTAAAAAAGGT [27222]
cp32-3_PAli_NX_MP_FG_consensus GTATGCTAAAGAATTGGGTGTAAATGGAAGTTATTCTGTTAATGATGGTACTAATACTAATGATTTTGTAAAAAAGGT [27222]
cp32-3_PAli_NX_Pl_consensus    GTATGCTAAAGAATTGGGTGTAAATGGAAGTTATTCTGTTAATGATGGTACTAATACTAATGATTTTGTAAAAAAGGT [27222]
cp32-3_PAbe_NX_MP_FG_consensus GTATGCTAAAGAATTGGGTGTAAATGGAAGTTATTCTGTTAATGATGGTACTAATACTAATGATTTTGTAAAAAAGGT [27222]
cp32-3_PAbe_NX_Pl_consensus    GTATGCTAAAGAATTGGGTGTAAATGGAAGTTATTCTGTTAATGATGGTACTAATACTAATGATTTTGTAAAAAAGGT [27222]

cp32-3_gi|11497103|B31_GB      TATAGATGATGCTCTTAAAAATATTGAGGAAGAACTTGAAAAGCTAGCAGAGCCTCAAAATATAGAAGATAAAAAATA [27300]
cp32-3_un8_B31_PacBio          TATAGATGATGCTCTTAAAAATATTGAGGAAGAACTTGAAAAGCTAGCAGAGCCTCAAAATATAGAAGATAAAAAATA [27300]
cp32-3_B31_TS_MP_FG_consensus  TATAGATGATGCTCTTAAAAATATTGAGGAAGAACTTGAAAAGCTAGCAGAGCCTCAAAATATAGAAGATAAAAAATA [27300]
cp32-3_B31_NX_Pl_consensus     TATAGATGATGCTCTTAAAAATATTGAGGAAGAACTTGAAAAGCTAGCAGAGCCTCAAAATATAGAAGATAAAAAATA [27300]
cp32-3_PAli_NX_MP_FG_consensus TATAGATGATGCTCTTAAAAATATTGAGGAAGAACTTGAAAAGCTAGCAGAGCCTCAAAATATAGAAGATAAAAAATA [27300]
cp32-3_PAli_NX_Pl_consensus    TATAGATGATGCTCTTAAAAATATTGAGGAAGAACTTGAAAAGCTAGCAGAGCCTCAAAATATAGAAGATAAAAAATA [27300]
cp32-3_PAbe_NX_MP_FG_consensus TATAGATGATGCTCTTAAAAATATTGAGGAAGAACTTGAAAAGCTAGCAGAGCCTCAAAATATAGAAGATAAAAAATA [27300]
cp32-3_PAbe_NX_Pl_consensus    TATAGATGATGCTCTTAAAAATATTGAGGAAGAACTTGAAAAGCTAGCAGAGCCTCAAAATATAGAAGATAAAAAATA [27300]

cp32-3_gi|11497103|B31_GB      ATAAAAAATGGGTTTTGATAGTAAAAATTATAAACAACAAGACTAACAATCAGTCTTATTGTTTATTAGCATTTACAT [27378]
cp32-3_un8_B31_PacBio          ATAAAAAATGGGTTTTGATAGTAAAAATTATAAACAACAAGACTAACAATCAGTCTTATTGTTTATTAGCATTTACAT [27378]
cp32-3_B31_TS_MP_FG_consensus  ATAAAAAATGGGTTTTGATAGTAAAAATTATAAACAACAAGACTAACAATCAGTCTTATTGTTTATTAGCATTTACAT [27378]
```

```
cp32-3_B31_NX_Pl_consensus     ATAAAAAATGGGTTTTGATAGTAAAAATTATAAACAACAAGACTAACAATCAGTCTTATTGTTTATTAGCATTTACAT [27378]
cp32-3_PAli_NX_MP_FG_consensus ATAAAAAATGGGTTTTGATAGTAAAAATTATAAACAACAAGACTAACAATCAGTCTTATTGTTTATTAGCATTTACAT [27378]
cp32-3_PAli_NX_Pl_consensus    ATAAAAAATGGGTTTTGATAGTAAAAATTATAAACAACAAGACTAACAATCAGTCTTATTGTTTATTAGCATTTACAT [27378]
cp32-3_PAbe_NX_MP_FG_consensus ATAAAAAATGGGTTTTGATAGTAAAAATTATAAACAACAAGACTAACAATCAGTCTTATTGTTTATTAGCATTTACAT [27378]
cp32-3_PAbe_NX_Pl_consensus    ATAAAAAATGGGTTTTGATAGTAAAAATTATAAACAACAAGACTAACAATCAGTCTTATTGTTTATTAGCATTTACAT [27378]

cp32-3_gi|11497103|B31_GB      TGCTGAAATTTAGGCAATAAAGCTAGGAGGGGATATGAAAAAAATTAGTCTGTTAATATTTTTGTTTTTATTTGTAGT [27456]
cp32-3_un8_B31_PacBio          TGCTGAAATTTAGGCAATAAAGCTAGGAGGGGATATGAAAAAAATTAGTCTGTTAATATTTTTGTTTTTATTTGTAGT [27456]
cp32-3_B31_TS_MP_FG_consensus  TGCTGAAATTTAGGCAATAAAGCTAGGAGGGGATATGAAAAAAATTAGTCTGTTAATATTTTTGTTTTTATTTGTAGT [27456]
cp32-3_B31_NX_Pl_consensus     TGCTGAAATTTAGGCAATAAAGCTAGGAGGGGATATGAAAAAAATTAGTCTGTTAATATTTTTGTTTTTATTTGTAGT [27456]
cp32-3_PAli_NX_MP_FG_consensus TGCTGAAATTTAGGCAATAAAGCTAGGAGGGGATATGAAAAAAATTAGTCTGTTAATATTTTTGTTTTTATTTGTAGT [27456]
cp32-3_PAli_NX_Pl_consensus    TGCTGAAATTTAGGCAATAAAGCTAGGAGGGGATATGAAAAAAATTAGTCTGTTAATATTTTTGTTTTTATTTGTAGT [27456]
cp32-3_PAbe_NX_MP_FG_consensus TGCTGAAATTTAGGCAATAAAGCTAGGAGGGGATATGAAAAAAATTAGTCTGTTAATATTTTTGTTTTTATTTGTAGT [27456]
cp32-3_PAbe_NX_Pl_consensus    TGCTGAAATTTAGGCAATAAAGCTAGGAGGGGATATGAAAAAAATTAGTCTGTTAATATTTTTGTTTTTATTTGTAGT [27456]

cp32-3_gi|11497103|B31_GB      AAGTTTAAGTGCAAATATAGAAGAAAATTATACAGAAACTAAAAGAGCTTTTTCTAAAGAAGATTTTAACCTAATAAA [27534]
cp32-3_un8_B31_PacBio          AAGTTTAAGTGCAAATATAGAAGAAAATTATACAGAAACTAAAAGAGCTTTTTCTAAAGAAGATTTTAACCTAATAAA [27534]
cp32-3_B31_TS_MP_FG_consensus  AAGTTTAAGTGCAAATATAGAAGAAAATTATACAGAAACTAAAAGAGCTTTTTCTAAAGAAGATTTTAACCTAATAAA [27534]
cp32-3_B31_NX_Pl_consensus     AAGTTTAAGTGCAAATATAGAAGAAAATTATACAGAAACTAAAAGAGCTTTTTCTAAAGAAGATTTTAACCTAATAAA [27534]
cp32-3_PAli_NX_MP_FG_consensus AAGTTTAAGTGCAAATATAGAAGAAAATTATACAGAAACTAAAAGAGCTTTTTCTAAAGAAGATTTTAACCTAATAAA [27534]
cp32-3_PAli_NX_Pl_consensus    AAGTTTAAGTGCAAATATAGAAGAAAATTATACAGAAACTAAAAGAGCTTTTTCTAAAGAAGATTTTAACCTAATAAA [27534]
cp32-3_PAbe_NX_MP_FG_consensus AAGTTTAAGTGCAAATATAGAAGAAAATTATACAGAAACTAAAAGAGCTTTTTCTAAAGAAGATTTTAACCTAATAAA [27534]
cp32-3_PAbe_NX_Pl_consensus    AAGTTTAAGTGCAAATATAGAAGAAAATTATACAGAAACTAAAAGAGCTTTTTCTAAAGAAGATTTTAACCTAATAAA [27534]

cp32-3_gi|11497103|B31_GB      TAAAAGACTAGATAATTATGATTTTAAAAA-TGAGTATGAAAAAAGTCATGTTTTTTCCGATGCTCCTAGAATTAGAG [27612]
cp32-3_un8_B31_PacBio          TAAAAGACTAGATAATTATGATTTTAAAAAATGAGTATGAAAAAAGTCATGTTTTTTCCGATGCTCCTAGAATTAGAG [27612]
cp32-3_B31_TS_MP_FG_consensus  TAAAAGACTAGATAATTATGATTTTAAAAA-TGAGTATGAAAAAAGTCATGTTTTTTCCGATGCTCCTAGAATTAGAG [27612]
cp32-3_B31_NX_Pl_consensus     TAAAAGACTAGATAATTATGATTTTAAAAA-TGAGTATGAAAAAAGTCATGTTTTTTCCGATGCTCCTAGAATTAGAG [27612]
cp32-3_PAli_NX_MP_FG_consensus TAAAAGACTAGATAATTATGATTTTAAAAA-TGAGTATGAAAAAAGTCATGTTTTTTCCGATGCTCCTAGAATTAGAG [27612]
cp32-3_PAli_NX_Pl_consensus    TAAAAGACTAGATAATTATGATTTTAAAAA-TGAGTATGAAAAAAGTCATGTTTTTTCCGATGCTCCTAGAATTAGAG [27612]
cp32-3_PAbe_NX_MP_FG_consensus TAAAAGACTAGATAATTATGATTTTAAAAA-TGAGTATGAAAAAAGTCATGTTTTTTCCGATGCTCCTAGAATTAGAG [27612]
cp32-3_PAbe_NX_Pl_consensus    TAAAAGACTAGATAATTATGATTTTAAAAA-TGAGTATGAAAAAAGTCATGTTTTTTCCGATGCTCCTAGAATTAGAG [27612]

cp32-3_gi|11497103|B31_GB      GAGATTTAAGAAAAAATTGGAATTAAAGAAAAAAGTGTTTTTTTGGACGCTCTTGAGGCTATTGAATATCTTATAAAAA [27690]
cp32-3_un8_B31_PacBio          GAGATTTAAGAAAAAATTGGAATTAAAGAAAAAAGTGTTTTTTTGGACGCTCTTGAGGCTATTGAATATCTTATAAAAA [27690]
cp32-3_B31_TS_MP_FG_consensus  GAGATTTAAGAAAAAATTGGAATTAAAGAAAAAAGTGTTTTTTTGGACGCTCTTGAGGCTATTGAATATCTTATAAAAA [27690]
cp32-3_B31_NX_Pl_consensus     GAGATTTAAGAAAAAATTGGAATTAAAGAAAAAAGTGTTTTTTTGGACGCTCTTGAGGCTATTGAATATCTTATAAAAA [27690]
cp32-3_PAli_NX_MP_FG_consensus GAGATTTAAGAAAAAATTGGAATTAAAGAAAAAAGTGTTTTTTTGGACGCTCTTGAGGCTATTGAATATCTTATAAAAA [27690]
cp32-3_PAli_NX_Pl_consensus    GAGATTTAAGAAAAAATTGGAATTAAAGAAAAAAGTGTTTTTTTGGACGCTCTTGAGGCTATTGAATATCTTATAAAAA [27690]
cp32-3_PAbe_NX_MP_FG_consensus GAGATTTAAGAAAAAATTGGAATTAAAGAAAAAAGTGTTTTTTTGGACGCTCTTGAGGCTATTGAATATCTTATAAAAA [27690]
cp32-3_PAbe_NX_Pl_consensus    GAGATTTAAGAAAAAATTGGAATTAAAGAAAAAAGTGTTTTTTTGGACGCTCTTGAGGCTATTGAATATCTTATAAAAA [27690]

cp32-3_gi|11497103|B31_GB      TTAAGATAAGTACTGATAGTATTTTCCTGTCTGAGGATATGATTAGACTTATAGGGAGTTATCCAGATTCGATTTTTA [27768]
cp32-3_un8_B31_PacBio          TTAAGATAAGTACTGATAGTATTTTCCTGTCTGAGGATATGATTAGACTTATAGGGAGTTATCCAGATTCGATTTT-A [27768]
cp32-3_B31_TS_MP_FG_consensus  TTAAGATAAGTACTGATAGTATTTTCCTGTCTGAGGATATGATTAGACTTATAGGGAGTTATCCAGATTCGATTTTTA [27768]
cp32-3_B31_NX_Pl_consensus     TTAAGATAAGTACTGATAGTATTTTCCTGTCTGAGGATATGATTAGACTTATAGGGAGTTATCCAGATTCGATTTTTA [27768]
cp32-3_PAli_NX_MP_FG_consensus TTAAGATAAGTACTGATAGTATTTTCCTGTCTGAGGATATGATTAGACTTATAGGGAGTTATCCAGATTCGATTTTTA [27768]
cp32-3_PAli_NX_Pl_consensus    TTAAGATAAGTACTGATAGTATTTTCCTGTCTGAGGATATGATTAGACTTATAGGGAGTTATCCAGATTCGATTTTTA [27768]
cp32-3_PAbe_NX_MP_FG_consensus TTAAGATAAGTACTGATAGTATTTTCCTGTCTGAGGATATGATTAGACTTATAGGGAGTTATCCAGATTCGATTTTTA [27768]
cp32-3_PAbe_NX_Pl_consensus    TTAAGATAAGTACTGATAGTATTTTCCTGTCTGAGGATATGATTAGACTTATAGGGAGTTATCCAGATTCGATTTTTA [27768]

cp32-3_gi|11497103|B31_GB      ATTATTTGATACAATTAAATTCGGATAAAATTGATTATGCTGAGAAATATGGAGATAACGCCAGAAATAATTTTAAAA [27846]
cp32-3_un8_B31_PacBio          ATTATTTGATACAATTAAATTCGGATAAAATTGATTATGCTGAGAAATATGGAGATAACGCCAGAAATAATTTTAAAA [27846]
cp32-3_B31_TS_MP_FG_consensus  ATTATTTGATACAATTAAATTCGGATAAAATTGATTATGCTGAGAAATATGGAGATAACGCCAGAAATAATTTTAAAA [27846]
cp32-3_B31_NX_Pl_consensus     ATTATTTGATACAATTAAATTCGGATAAAATTGATTATGCTGAGAAATATGGAGATAACGCCAGAAATAATTTTAAAA [27846]
cp32-3_PAli_NX_MP_FG_consensus ATTATTTGATACAATTAAATTCGGATAAAATTGATTATGCTGAGAAATATGGAGATAACGCCAGAAATAATTTTAAAA [27846]
cp32-3_PAli_NX_Pl_consensus    ATTATTTGATACAATTAAATTCGGATAAAATTGATTATGCTGAGAAATATGGAGATAACGCCAGAAATAATTTTAAAA [27846]
cp32-3_PAbe_NX_MP_FG_consensus ATTATTTGATACAATTAAATTCGGATAAAATTGATTATGCTGAGAAATATGGAGATAACGCCAGAAATAATTTTAAAA [27846]
cp32-3_PAbe_NX_Pl_consensus    ATTATTTGATACAATTAAATTCGGATAAAATTGATTATGCTGAGAAATATGGAGATAACGCCAGAAATAATTTTAAAA [27846]

cp32-3_gi|11497103|B31_GB      AAGATTATTCTGAAGATAAAGCAAA-TACAGTTAAACAAATTTTAAAACAAATTTTGGCTGATTTGCCCAAAGATTAG [27924]
cp32-3_un8_B31_PacBio          AAGATTATTCTGAAGATAAAGCAAAATACAGTTAAACAAATTTTAAAACAAATTTTGGCTGATTTGCCCAAAGATTAG [27924]
cp32-3_B31_TS_MP_FG_consensus  AAGATTATTCTGAAGATAAAGCAAA-TACAGTTAAACAAATTTTAAAACAAATTTTGGCTGATTTGCCCAAAGATTAG [27924]
cp32-3_B31_NX_Pl_consensus     AAGATTATTCTGAAGATAAAGCAAA-TACAGTTAAACAAATTTTAAAACAAATTTTGGCTGATTTGCCCAAAGATTAG [27924]
cp32-3_PAli_NX_MP_FG_consensus AAGATTATTCTGAAGATAAAGCAAA-TACAGTTAAACAAATTTTAAAACAAATTTTGGCTGATTTGCCCAAAGATTAG [27924]
cp32-3_PAli_NX_Pl_consensus    AAGATTATTCTGAAGATAAAGCAAA-TACAGTTAAACAAATTTTAAAACAAATTTTGGCTGATTTGCCCAAAGATTAG [27924]
cp32-3_PAbe_NX_MP_FG_consensus AAGATTATTCTGAAGATAAAGCAAA-TACAGTTAAACAAATTTTAAAACAAATTTTGGCTGATTTGCCCAAAGATTAG [27924]
cp32-3_PAbe_NX_Pl_consensus    AAGATTATTCTGAAGATAAAGCAAA-TACAGTTAAACAAATTTTAAAACAAATTTTGGCTGATTTGCCCAAAGATTAG [27924]

cp32-3_gi|11497103|B31_GB      ATTTTAATTTGAAAAAAGAATCTTATAAG-ATATTTATTAAGAAAAGCTAAAAAA-GATTTCGAAATAAAATAAATGG [28002]
cp32-3_un8_B31_PacBio          ATTTTAATTTGAAAAAAGAATCTTATAAGGATATTTATTAAGAAAAGCTAAAAAAAGATTTCGAAATAAAATAAATGG [28002]
cp32-3_B31_TS_MP_FG_consensus  ATTTTAATTTGAAAAAAGAATCTTATAAG-ATATTTATTAAGAAAAGCTAAAAAA-GATTTCGAAATAAAATAAATGG [28002]
cp32-3_B31_NX_Pl_consensus     ATTTTAATTTGAAAAAAGAATCTTATAAG-ATATTTATTAAGAAAAGCTAAAAAA-GATTTCGAAATAAAATAAATGG [28002]
cp32-3_PAli_NX_MP_FG_consensus ATTTTAATTTGAAAAAAGAATCTTATAAG-ATATTTATTAAGAAAAGCTAAAAAA-GATTTCGAAATAAAATAAATGG [28002]
cp32-3_PAli_NX_Pl_consensus    ATTTTAATTTGAAAAAAGAATCTTATAAG-ATATTTATTAAGAAAAGCTAAAAAA-GATTTCGAAATAAAATAAATGG [28002]
cp32-3_PAbe_NX_MP_FG_consensus ATTTTAATTTGAAAAAAGAATCTTATAAG-ATATTTATTAAGAAAAGCTAAAAAA-GATTTCGAAATAAAATAAATGG [28002]
cp32-3_PAbe_NX_Pl_consensus    ATTTTAATTTGAAAAAAGAATCTTATAAG-ATATTTATTAAGAAAAGCTAAAAAA-GATTTCGAAATAAAATAAATGG [28002]

cp32-3_gi|11497103|B31_GB      TTTTTATAAGCATAAATTATGAATAACAAGATTAGCTACTAGTCTTGTTATTTTATTAATATTGGAGAATCATGATAA [28080]
cp32-3_un8_B31_PacBio          TTTT-ATAAGCATAAATTATGAATAACAAGATTAGCTACTAGTCTTGTTATTTTATTAATATTGGAGAATCATGATAA [28080]
cp32-3_B31_TS_MP_FG_consensus  TTTTTATAAGCATAAATTATGAATAACAAGATTAGCTACTAGTCTTGTTATTTTATTAATATTGGAGAATCATGATAA [28080]
cp32-3_B31_NX_Pl_consensus     TTTTTATAAGCATAAATTATGAATAACAAGATTAGCTACTAGTCTTGTTATTTTATTAATATTGGAGAATCATGATAA [28080]
cp32-3_PAli_NX_MP_FG_consensus TTTTTATAAGCATAAATTATGAATAACAAGATTAGCTACTAGTCTTGTTATTTTATTAATATTGGAGAATCATGATAA [28080]
cp32-3_PAli_NX_Pl_consensus    TTTTTATAAGCATAAATTATGAATAACAAGATTAGCTACTAGTCTTGTTATTTTATTAATATTGGAGAATCATGATAA [28080]
cp32-3_PAbe_NX_MP_FG_consensus TTTTTATAAGCATAAATTATGAATAACAAGATTAGCTACTAGTCTTGTTATTTTATTAATATTGGAGAATCATGATAA [28080]
cp32-3_PAbe_NX_Pl_consensus    TTTTTATAAGCATAAATTATGAATAACAAGATTAGCTACTAGTCTTGTTATTTTATTAATATTGGAGAATCATGATAA [28080]

cp32-3_gi|11497103|B31_GB      ATAAAAAGAATTTATATTATTTTATATTTGCTTTATTTTCTTTAAATGCTAACGAATTTATGAGGGGTCTTCTTAATG [28158]
cp32-3_un8_B31_PacBio          ATAAAAAGAATTTATATTATTTTATATTTGCTTTATTTTCTTTAAATGCTAACGAATTTATGAGGGGTCTTCTTAATG [28158]
cp32-3_B31_TS_MP_FG_consensus  ATAAAAAGAATTTATATTATTTTATATTTGCTTTATTTTCTTTAAATGCTAACGAATTTATGAGGGGTCTTCTTAATG [28158]
cp32-3_B31_NX_Pl_consensus     ATAAAAAGAATTTATATTATTTTATATTTGCTTTATTTTCTTTAAATGCTAACGAATTTATGAGGGGTCTTCTTAATG [28158]
cp32-3_PAli_NX_MP_FG_consensus ATAAAAAGAATTTATATTATTTTATATTTGCTTTATTTTCTTTAAATGCTAACGAATTTATGAGGGGTCTTCTTAATG [28158]
cp32-3_PAli_NX_Pl_consensus    ATAAAAAGAATTTATATTATTTTATATTTGCTTTATTTTCTTTAAATGCTAACGAATTTATGAGGGGTCTTCTTAATG [28158]
cp32-3_PAbe_NX_MP_FG_consensus ATAAAAAGAATTTATATTATTTTATATTTGCTTTATTTTCTTTAAATGCTAACGAATTTATGAGGGGTCTTCTTAATG [28158]
cp32-3_PAbe_NX_Pl_consensus    ATAAAAAGAATTTATATTATTTTATATTTGCTTTATTTTCTTTAAATGCTAACGAATTTATGAGGGGTCTTCTTAATG [28158]

cp32-3_gi|11497103|B31_GB      GCGACAATAATGATACTGGTTCATGTTTAATAAGAATTGTAAAGATAGATCTGTGGTCAATAGATATCAACAAGACAA [28236]
cp32-3_un8_B31_PacBio          GCGACAATAATGATACTGGTTCATGTTTAATAAGAATTGTAA-GATAGATCTGTGGTCAATAGATATCAACAAGACAA [28236]
cp32-3_B31_TS_MP_FG_consensus  GCGACAATAATGATACTGGTTCATGTTTAATAAGAATTGTAAAGATAGATCTGTGGTCAATAGATATCAACAAGACAA [28236]
cp32-3_B31_NX_Pl_consensus     GCGACAATAATGATACTGGTTCATGTTTAATAAGAATTGTAAAGATAGATCTGTGGTCAATAGATATCAACAAGACAA [28236]
cp32-3_PAli_NX_MP_FG_consensus GCGACAATAATGATACTGGTTCATGTTTAATAAGAATTGTAAAGATAGATCTGTGGTCAATAGATATCAACAAGACAA [28236]
cp32-3_PAli_NX_Pl_consensus    GCGACAATAATGATACTGGTTCATGTTTAATAAGAATTGTAAAGATAGATCTGTGGTCAATAGATATCAACAAGACAA [28236]
cp32-3_PAbe_NX_MP_FG_consensus GCGACAATAATGATACTGGTTCATGTTTAATAAGAATTGTAAAGATAGATCTGTGGTCAATAGATATCAACAAGACAA [28236]
cp32-3_PAbe_NX_Pl_consensus    GCGACAATAATGATACTGGTTCATGTTTAATAAGAATTGTAAAGATAGATCTGTGGTCAATAGATATCAACAAGACAA [28236]

cp32-3_gi|11497103|B31_GB      TAGTTAATGTTAACAATTGATAAATTGCTTTACTATTTAGAGCAACAATTTGTTAATTTTGTTATTTTAGAGGATTTT [28314]
cp32-3_un8_B31_PacBio          TAGTTAATGTTAACAATTGATAAATTGCTTTACTATTTAGAGCAACAATTTGTTAATTTTGTTATTTTAGAGGATTTT [28314]
cp32-3_B31_TS_MP_FG_consensus  TAGTTAATGTTAACAATTGATAAATTGCTTTACTATTTAGAGCAACAATTTGTTAATTTTGTTATTTTAGAGGATTTT [28314]
cp32-3_B31_NX_Pl_consensus     TAGTTAATGTTAACAATTGATAAATTGCTTTACTATTTAGAGCAACAATTTGTTAATTTTGTTATTTTAGAGGATTTT [28314]
```

```
cp32-3_PAli_NX_MP_FG_consensus TAGTTAATGTTAACAATTGATAAATTGCTTTACTATTTAGAGCAACAATTTGTTAATTTTGTTATTTTAGAGGATTTT [28314]
cp32-3_PAli_NX_Pl_consensus    TAGTTAATGTTAACAATTGATAAATTGCTTTACTATTTAGAGCAACAATTTGTTAATTTTGTTATTTTAGAGGATTTT [28314]
cp32-3_PAbe_NX_MP_FG_consensus TAGTTAATGTTAACAATTGATAAATTGCTTTACTATTTAGAGCAACAATTTGTTAATTTTGTTATTTTAGAGGATTTT [28314]
cp32-3_PAbe_NX_Pl_consensus    TAGTTAATGTTAACAATTGATAAATTGCTTTACTATTTAGAGCAACAATTTGTTAATTTTGTTATTTTAGAGGATTTT [28314]

cp32-3_gi|11497103|B31_GB      TTGAAAAAAGTTAAAAGGTCTTTTGATGATTATGTTGCATATTTTAGAGAAGGATTGTTAGATGATAGGGAAATAGCG [28392]
cp32-3_un8_B31_PacBio          T-GAAAAAAGTTAAAAGGTCTTTTGATGATTATGTTGCATATTTTAGAGAAGGATTGTTAGATGATAGGGAAATAGCG [28392]
cp32-3_B31_TS_MP_FG_consensus  TTGAAAAAAGTTAAAAGGTCTTTTGATGATTATGTTGCATATTTTAGAGAAGGATTGTTAGATGATAGGGAAATAGCG [28392]
cp32-3_B31_NX_Pl_consensus     TTGAAAAAAGTTAAAAGGTCTTTTGATGATTATGTTGCATATTTTAGAGAAGGATTGTTAGATGATAGGGAAATAGCG [28392]
cp32-3_PAli_NX_MP_FG_consensus TTGAAAAAAGTTAAAAGGTCTTTTGATGATTATGTTGCATATTTTAGAGAAGGATTGTTAGATGATAGGGAAATAGCG [28392]
cp32-3_PAli_NX_Pl_consensus    TTGAAAAAAGTTAAAAGGTCTTTTGATGATTATGTTGCATATTTTAGAGAAGGATTGTTAGATGATAGGGAAATAGCG [28392]
cp32-3_PAbe_NX_MP_FG_consensus TTGAAAAAAGTTAAAAGGTCTTTTGATGATTATGTTGCATATTTTAGAGAAGGATTGTTAGATGATAGGGAAATAGCG [28392]
cp32-3_PAbe_NX_Pl_consensus    TTGAAAAAAGTTAAAAGGTCTTTTGATGATTATGTTGCATATTTTAGAGAAGGATTGTTAGATGATAGGGAAATAGCG [28392]

cp32-3_gi|11497103|B31_GB      GATAAATTGGGGGTTTCTAGGGTAAATGTGTGGAGGATGAGGAAAAAAATGGGAAAGTGGGGAAAATTCTGTTAGCGAT [28470]
cp32-3_un8_B31_PacBio          GATAAATTGGGGGTTTCTAGGGTAAATGTGTGGAGGATGAGGAAAAA-TGGGAAAGTGGGGAAAATTCTGTTAGCGAT [28470]
cp32-3_B31_TS_MP_FG_consensus  GATAAATTGGGGGTTTCTAGGGTAAATGTGTGGAGGATGAGGAAAAAAATGGGAAAGTGGGGAAAATTCTGTTAGCGAT [28470]
cp32-3_B31_NX_Pl_consensus     GATAAATTGGGGGTTTCTAGGGTAAATGTGTGGAGGATGAGGAAAAAAATGGGAAAGTGGGGAAAATTCTGTTAGCGAT [28470]
cp32-3_PAli_NX_MP_FG_consensus GATAAATTGGGGGTTTCTAGGGTAAATGTGTGGAGGATGAGGAAAAAAATGGGAAAGTGGGGAAAATTCTGTTAGCGAT [28470]
cp32-3_PAli_NX_Pl_consensus    GATAAATTGGGGGTTTCTAGGGTAAATGTGTGGAGGATGAGGAAAAAAATGGGAAAGTGGGGAAAATTCTGTTAGCGAT [28470]
cp32-3_PAbe_NX_MP_FG_consensus GATAAATTGGGGGTTTCTAGGGTAAATGTGTGGAGGATGAGGAAAAAAATGGGAAAGTGGGGAAAATTCTGTTAGCGAT [28470]
cp32-3_PAbe_NX_Pl_consensus    GATAAATTGGGGGTTTCTAGGGTAAATGTGTGGAGGATGAGGAAAAAAATGGGAAAGTGGGGAAAATTCTGTTAGCGAT [28470]

cp32-3_gi|11497103|B31_GB      GACTCTAGATTATCAATTAGTGAAGACACTTTTGAACACCTTTTGTCGCAAACTTTTAAATCAGAAGTTAACGCTAGG [28548]
cp32-3_un8_B31_PacBio          GACTCTAGATTATCAATTAGTGAAGACACTTTTGAACACCTTTTGTCGCAAACTTTTAAATCAGAAGTTAACGCTAGG [28548]
cp32-3_B31_TS_MP_FG_consensus  GACTCTAGATTATCAATTAGTGAAGACACTTTTGAACACCTTTTGTCGCAAACTTTTAAATCAGAAGTTAACGCTAGG [28548]
cp32-3_B31_NX_Pl_consensus     GACTCTAGATTATCAATTAGTGAAGACACTTTTGAACACCTTTTGTCGCAAACTTTTAAATCAGAAGTTAACGCTAGG [28548]
cp32-3_PAli_NX_MP_FG_consensus GACTCTAGATTATCAATTAGTGAAGACACTTTTGAACACCTTTTGTCGCAAACTTTTAAATCAGAAGTTAACGCTAGG [28548]
cp32-3_PAli_NX_Pl_consensus    GACTCTAGATTATCAATTAGTGAAGACACTTTTGAACACCTTTTGTCGCAAACTTTTAAATCAGAAGTTAACGCTAGG [28548]
cp32-3_PAbe_NX_MP_FG_consensus GACTCTAGATTATCAATTAGTGAAGACACTTTTGAACACCTTTTGTCGCAAACTTTTAAATCAGAAGTTAACGCTAGG [28548]
cp32-3_PAbe_NX_Pl_consensus    GACTCTAGATTATCAATTAGTGAAGACACTTTTGAACACCTTTTGTCGCAAACTTTTAAATCAGAAGTTAACGCTAGG [28548]

cp32-3_gi|11497103|B31_GB      AAAGTTAGAAGCGAATTGGATTTAGAGCGGGCTAATTTAGAATTAGGATTTATAAATGCATTTAAGCAATATTCTAGT [28626]
cp32-3_un8_B31_PacBio          AAAGTTAGAAGCGAATTGGATTTAGAGCGGGCTAATTTAGAATTAGGATTTATAAATGCATTTAAGCAATATTCTAGT [28626]
cp32-3_B31_TS_MP_FG_consensus  AAAGTTAGAAGCGAATTGGATTTAGAGCGGGCTAATTTAGAATTAGGATTTATAAATGCATTTAAGCAATATTCTAGT [28626]
cp32-3_B31_NX_Pl_consensus     AAAGTTAGAAGCGAATTGGATTTAGAGCGGGCTAATTTAGAATTAGGATTTATAAATGCATTTAAGCAATATTCTAGT [28626]
cp32-3_PAli_NX_MP_FG_consensus AAAGTTAGAAGCGAATTGGATTTAGAGCGGGCTAATTTAGAATTAGGATTTATAAATGCATTTAAGCAATATTCTAGT [28626]
cp32-3_PAli_NX_Pl_consensus    AAAGTTAGAAGCGAATTGGATTTAGAGCGGGCTAATTTAGAATTAGGATTTATAAATGCATTTAAGCAATATTCTAGT [28626]
cp32-3_PAbe_NX_MP_FG_consensus AAAGTTAGAAGCGAATTGGATTTAGAGCGGGCTAATTTAGAATTAGGATTTATAAATGCATTTAAGCAATATTCTAGT [28626]
cp32-3_PAbe_NX_Pl_consensus    AAAGTTAGAAGCGAATTGGATTTAGAGCGGGCTAATTTAGAATTAGGATTTATAAATGCATTTAAGCAATATTCTAGT [28626]

cp32-3_gi|11497103|B31_GB      GTTGAGCTTTTTAGTATGCATACTA-AAATAGAAAATTTAAGAGCTGAAATTGACGCTTTAAATAAAGCAAGTAGTAA [28704]
cp32-3_un8_B31_PacBio          GTTGAGCTTTTTAGTATGCATACTATAAATAGAAAATTTAAGAGCTGAAATTGACGCTTTAAATAAAGCAAGTAGTAA [28704]
cp32-3_B31_TS_MP_FG_consensus  GTTGAGCTTTTTAGTATGCATACTA-AAATAGAAAATTTAAGAGCTGAAATTGACGCTTTAAATAAAGCAAGTAGTAA [28704]
cp32-3_B31_NX_Pl_consensus     GTTGAGCTTTTTAGTATGCATACTA-AAATAGAAAATTTAAGAGCTGAAATTGACGCTTTAAATAAAGCAAGTAGTAA [28704]
cp32-3_PAli_NX_MP_FG_consensus GTTGAGCTTTTTAGTATGCATACTA-AAATAGAAAATTTAAGAGCTGAAATTGACGCTTTAAATAAAGCAAGTAGTAA [28704]
cp32-3_PAli_NX_Pl_consensus    GTTGAGCTTTTTAGTATGCATACTA-AAATAGAAAATTTAAGAGCTGAAATTGACGCTTTAAATAAAGCAAGTAGTAA [28704]
cp32-3_PAbe_NX_MP_FG_consensus GTTGAGCTTTTTAGTATGCATACTA-AAATAGAAAATTTAAGAGCTGAAATTGACGCTTTAAATAAAGCAAGTAGTAA [28704]
cp32-3_PAbe_NX_Pl_consensus    GTTGAGCTTTTTAGTATGCATACTA-AAATAGAAAATTTAAGAGCTGAAATTGACGCTTTAAATAAAGCAAGTAGTAA [28704]

cp32-3_gi|11497103|B31_GB      AAAAAATAAGCAAGTTGTTAATGGAGAAATTAATTCTTTAAAAAGCGAGCTTGATGAATATATAAAGG-AGTGTTCAA [28782]
cp32-3_un8_B31_PacBio          AAAAA-TAAGCAAGTTGTTAATGGAGAAATTAATTCTTTAAAAAGCGAGCTTGATGAATATATAAAGGGAGTGTTCAA [28782]
cp32-3_B31_TS_MP_FG_consensus  AAAAAATAAGCAAGTTGTTAATGGAGAAATTAATTCTTTAAAAAGCGAGCTTGATGAATATATAAAGG-AGTGTTCAA [28782]
cp32-3_B31_NX_Pl_consensus     AAAAAATAAGCAAGTTGTTAATGGAGAAATTAATTCTTTAAAAAGCGAGCTTGATGAATATATAAAGG-AGTGTTCAA [28782]
cp32-3_PAli_NX_MP_FG_consensus AAAAAATAAGCAAGTTGTTAATGGAGAAATTAATTCTTTAAAAAGCGAGCTTGATGAATATATAAAGG-AGTGTTCAA [28782]
cp32-3_PAli_NX_Pl_consensus    AAAAAATAAGCAAGTTGTTAATGGAGAAATTAATTCTTTAAAAAGCGAGCTTGATGAATATATAAAGG-AGTGTTCAA [28782]
cp32-3_PAbe_NX_MP_FG_consensus AAAAAATAAGCAAGTTGTTAATGGAGAAATTAATTCTTTAAAAAGCGAGCTTGATGAATATATAAAGG-AGTGTTCAA [28782]
cp32-3_PAbe_NX_Pl_consensus    AAAAAATAAGCAAGTTGTTAATGGAGAAATTAATTCTTTAAAAAGCGAGCTTGATGAATATATAAAGG-AGTGTTCAA [28782]

cp32-3_gi|11497103|B31_GB      TAAGAGAAATGGAGCTTTACTATGAATGTATGAAAAAACTT-GCAACTGCTAATGAAGCTGAAAGTAAAAGCAACTAC [28860]
cp32-3_un8_B31_PacBio          TAAGAGAAATGGAGCTTTACTATGAATGTATGAAAAAACTTTGCAACTGCTAATGAAGCTGAAAGTAAAAGCAACTAC [28860]
cp32-3_B31_TS_MP_FG_consensus  TAAGAGAAATGGAGCTTTACTATGAATGTATGAAAAAACTT-GCAACTGCTAATGAAGCTGAAAGTAAAAGCAACTAC [28860]
cp32-3_B31_NX_Pl_consensus     TAAGAGAAATGGAGCTTTACTATGAATGTATGAAAAAACTT-GCAACTGCTAATGAAGCTGAAAGTAAAAGCAACTAC [28860]
cp32-3_PAli_NX_MP_FG_consensus TAAGAGAAATGGAGCTTTACTATGAATGTATGAAAAAACTT-GCAACTGCTAATGAAGCTGAAAGTAAAAGCAACTAC [28860]
cp32-3_PAli_NX_Pl_consensus    TAAGAGAAATGGAGCTTTACTATGAATGTATGAAAAAACTT-GCAACTGCTAATGAAGCTGAAAGTAAAAGCAACTAC [28860]
cp32-3_PAbe_NX_MP_FG_consensus TAAGAGAAATGGAGCTTTACTATGAATGTATGAAAAAACTT-GCAACTGCTAATGAAGCTGAAAGTAAAAGCAACTAC [28860]
cp32-3_PAbe_NX_Pl_consensus    TAAGAGAAATGGAGCTTTACTATGAATGTATGAAAAAACTT-GCAACTGCTAATGAAGCTGAAAGTAAAAGCAACTAC [28860]

cp32-3_gi|11497103|B31_GB      AAAAATAGTAAAGGGCACAAGTGAACTTATATCAAACAAAACTTTTTACAACACTACAAAAGGAATACAAAAATAAAT [28938]
cp32-3_un8_B31_PacBio          AAAAATAGTAAAGGGCACAAGTGAACTTATATCAAACAAAACTTTTTACAACACTACAAAAGGAATACAAAAATAAAT [28938]
cp32-3_B31_TS_MP_FG_consensus  AAAAATAGTAAAGGGCACAAGTGAACTTATATCAAACAAAACTTTTTACAACACTACAAAAGGAATACAAAAATAAAT [28938]
cp32-3_B31_NX_Pl_consensus     AAAAATAGTAAAGGGCACAAGTGAACTTATATCAAACAAAACTTTTTACAACACTACAAAAGGAATACAAAAATAAAT [28938]
cp32-3_PAli_NX_MP_FG_consensus AAAAATAGTAAAGGGCACAAGTGAACTTATATCAAACAAAACTTTTTACAACACTACAAAAGGAATACAAAAATAAAT [28938]
cp32-3_PAli_NX_Pl_consensus    AAAAATAGTAAAGGGCACAAGTGAACTTATATCAAACAAAACTTTTTACAACACTACAAAAGGAATACAAAAATAAAT [28938]
cp32-3_PAbe_NX_MP_FG_consensus AAAAATAGTAAAGGGCACAAGTGAACTTATATCAAACAAAACTTTTTACAACACTACAAAAGGAATACAAAAATAAAT [28938]
cp32-3_PAbe_NX_Pl_consensus    AAAAATAGTAAAGGGCACAAGTGAACTTATATCAAACAAAACTTTTTACAACACTACAAAAGGAATACAAAAATAAAT [28938]

cp32-3_gi|11497103|B31_GB      ATGGAGTTGATATATCACAATTTGTAAAGCTAACAAATTCTTCAATTAATTTTGATAAGTTTGAAGAAAAACAGTTAA [29016]
cp32-3_un8_B31_PacBio          ATGGAGTTGATATATCACAATTTGTAAAGCTAACAAATTCTTCAATTAATTTTGATAAGTTTGAAGAAAAACAGTTAA [29016]
cp32-3_B31_TS_MP_FG_consensus  ATGGAGTTGATATATCACAATTTGTAAAGCTAACAAATTCTTCAATTAATTTTGATAAGTTTGAAGAAAAACAGTTAA [29016]
cp32-3_B31_NX_Pl_consensus     ATGGAGTTGATATATCACAATTTGTAAAGCTAACAAATTCTTCAATTAATTTTGATAAGTTTGAAGAAAAACAGTTAA [29016]
cp32-3_PAli_NX_MP_FG_consensus ATGGAGTTGATATATCACAATTTGTAAAGCTAACAAATTCTTCAATTAATTTTGATAAGTTTGAAGAAAAACAGTTAA [29016]
cp32-3_PAli_NX_Pl_consensus    ATGGAGTTGATATATCACAATTTGTAAAGCTAACAAATTCTTCAATTAATTTTGATAAGTTTGAAGAAAAACAGTTAA [29016]
cp32-3_PAbe_NX_MP_FG_consensus ATGGAGTTGATATATCACAATTTGTAAAGCTAACAAATTCTTCAATTAATTTTGATAAGTTTGAAGAAAAACAGTTAA [29016]
cp32-3_PAbe_NX_Pl_consensus    ATGGAGTTGATATATCACAATTTGTAAAGCTAACAAATTCTTCAATTAATTTTGATAAGTTTGAAGAAAAACAGTTAA [29016]

cp32-3_gi|11497103|B31_GB      CTTTAAAACAAAAAAATGTGATAAAAAGTATTAAAAAGAATAATGAAAAAAAGATTATATTCAGCGGCGGCATAGCTA [29094]
cp32-3_un8_B31_PacBio          CTTTAAAACAAAAAAATGTGATAAAAAGTATTAAAAAGAATAATGAAAAAAAGATTATATTCAGCGGCGGCATAGCTA [29094]
cp32-3_B31_TS_MP_FG_consensus  CTTTAAAACAAAAAAATGTGATAAAAAGTATTAAAAAGAATAATGAAAAAAAGATTATATTCAGCGGCGGCATAGCTA [29094]
cp32-3_B31_NX_Pl_consensus     CTTTAAAACAAAAAAATGTGATAAAAAGTATTAAAAAGAATAATGAAAAAAAGATTATATTCAGCGGCGGCATAGCTA [29094]
cp32-3_PAli_NX_MP_FG_consensus CTTTAAAACAAAAAAATGTGATAAAAAGTATTAAAAAGAATAATGAAAAAAAGATTATATTCAGCGGCGGCATAGCTA [29094]
cp32-3_PAli_NX_Pl_consensus    CTTTAAAACAAAAAAATGTGATAAAAAGTATTAAAAAGAATAATGAAAAAAAGATTATATTCAGCGGCGGCATAGCTA [29094]
cp32-3_PAbe_NX_MP_FG_consensus CTTTAAAACAAAAAAATGTGATAAAAAGTATTAAAAAGAATAATGAAAAAAAGATTATATTCAGCGGCGGCATAGCTA [29094]
cp32-3_PAbe_NX_Pl_consensus    CTTTAAAACAAAAAAATGTGATAAAAAGTATTAAAAAGAATAATGAAAAAAAGATTATATTCAGCGGCGGCATAGCTA [29094]

cp32-3_gi|11497103|B31_GB      GCGGCAAAACGTATCTTGCATGCTATCTTTTTCTCAAAAGTTTAATTGAAAATAAAAAGTTATATTCTAGCGATACGA [29172]
cp32-3_un8_B31_PacBio          GCGGCAAAACGTATCTTGCATGCTATCTTTTTCTCAAAAGTTTAATTGAAAATAAAAAGTTATATTCTAGCGATACGA [29172]
cp32-3_B31_TS_MP_FG_consensus  GCGGCAAAACGTATCTTGCATGCTATCTTTTTCTCAAAAGTTTAATTGAAAATAAAAAGTTATATTCTAGCGATACGA [29172]
cp32-3_B31_NX_Pl_consensus     GCGGCAAAACGTATCTTGCATGCTATCTTTTTCTCAAAAGTTTAATTGAAAATAAAAAGTTATATTCTAGCGATACGA [29172]
cp32-3_PAli_NX_MP_FG_consensus GCGGCAAAACGTATCTTGCATGCTATCTTTTTCTCAAAAGTTTAATTGAAAATAAAAAGTTATATTCTAGCGATACGA [29172]
cp32-3_PAli_NX_Pl_consensus    GCGGCAAAACGTATCTTGCATGCTATCTTTTTCTCAAAAGTTTAATTGAAAATAAAAAGTTATATTCTAGCGATACGA [29172]
cp32-3_PAbe_NX_MP_FG_consensus GCGGCAAAACGTATCTTGCATGCTATCTTTTTCTCAAAAGTTTAATTGAAAATAAAAAGTTATATTCTAGCGATACGA [29172]
cp32-3_PAbe_NX_Pl_consensus    GCGGCAAAACGTATCTTGCATGCTATCTTTTTCTCAAAAGTTTAATTGAAAATAAAAAGTTATATTCTAGCGATACGA [29172]

cp32-3_gi|11497103|B31_GB      ATAATTTTATTATTGGGAATTCACAACGCTCAGTTGAAGTTAATGTTTTGGGACAATTTGAAAAGCTATGTAAACTTC [29250]
cp32-3_un8_B31_PacBio          ATAATTTTATTATTGGGAATTCACAACGCTCAGTTGAAGTTAATGTTTTGGGACAATTTGAAAAGCTATGTAAACTTC [29250]
cp32-3_B31_TS_MP_FG_consensus  ATAATTTTATTATTGGGAATTCACAACGCTCAGTTGAAGTTAATGTTTTGGGACAATTTGAAAAGCTATGTAAACTTC [29250]
cp32-3_B31_NX_Pl_consensus     ATAATTTTATTATTGGGAATTCACAACGCTCAGTTGAAGTTAATGTTTTGGGACAATTTGAAAAGCTATGTAAACTTC [29250]
cp32-3_PAli_NX_MP_FG_consensus ATAATTTTATTATTGGGAATTCACAACGCTCAGTTGAAGTTAATGTTTTGGGACAATTTGAAAAGCTATGTAAACTTC [29250]
```

```
cp32-3_PAli_NX_Pl_consensus    ATAATTTTATTATTGGGAATTCACAACGCTCAGTTGAAGTTAATGTTTTGGGACAATTTGAAAAGCTATGTAAACTTC [29250]
cp32-3_PAbe_NX_MP_FG_consensus ATAATTTTATTATTGGGAATTCACAACGCTCAGTTGAAGTTAATGTTTTGGGACAATTTGAAAAGCTATGTAAACTTC [29250]
cp32-3_PAbe_NX_Pl_consensus    ATAATTTTATTATTGGGAATTCACAACGCTCAGTTGAAGTTAATGTTTTGGGACAATTTGAAAAGCTATGTAAACTTC [29250]

cp32-3_gi|11497103|B31_GB      TTAAAATTCCTTATATTCCAAGACATACAAATAATTCATATATTCTGATTGATTCACTACGTATTAATCTATATGGTG [29328]
cp32-3_un8_B31_PacBio          TTAAAATTCCTTATATTCCAAGACATACAAATAATTCATATATTCTGATTGATTCACTACGTATTAATCTATATGGTG [29328]
cp32-3_B31_TS_MP_FG_consensus  TTAAAATTCCTTATATTCCAAGACATACAAATAATTCATATATTCTGATTGATTCACTACGTATTAATCTATATGGTG [29328]
cp32-3_B31_NX_Pl_consensus     TTAAAATTCCTTATATTCCAAGACATACAAATAATTCATATATTCTGATTGATTCACTACGTATTAATCTATATGGTG [29328]
cp32-3_PAli_NX_MP_FG_consensus TTAAAATTCCTTATATTCCAAGACATACAAATAATTCATATATTCTGATTGATTCACTACGTATTAATCTATATGGTG [29328]
cp32-3_PAli_NX_Pl_consensus    TTAAAATTCCTTATATTCCAAGACATACAAATAATTCATATATTCTGATTGATTCACTACGTATTAATCTATATGGTG [29328]
cp32-3_PAbe_NX_MP_FG_consensus TTAAAATTCCTTATATTCCAAGACATACAAATAATTCATATATTCTGATTGATTCACTACGTATTAATCTATATGGTG [29328]
cp32-3_PAbe_NX_Pl_consensus    TTAAAATTCCTTATATTCCAAGACATACAAATAATTCATATATTCTGATTGATTCACTACGTATTAATCTATATGGTG [29328]

cp32-3_gi|11497103|B31_GB      GAGATAAGGCAAGTGATTTTGAAAGATTTAGGGGAAGTAATTCGGCACTTATTTTTGTTAATGAGGCTACAACTTTAC [29406]
cp32-3_un8_B31_PacBio          GAGATAAGGCAAGTGATTTTGAAAGATTTAGGGGAAGTAATTCGGCACTTATTTTTGTTAATGAGGCTACAACTTTAC [29406]
cp32-3_B31_TS_MP_FG_consensus  GAGATAAGGCAAGTGATTTTGAAAGATTTAGGGGAAGTAATTCGGCACTTATTTTTGTTAATGAGGCTACAACTTTAC [29406]
cp32-3_B31_NX_Pl_consensus     GAGATAAGGCAAGTGATTTTGAAAGATTTAGGGGAAGTAATTCGGCACTTATTTTTGTTAATGAGGCTACAACTTTAC [29406]
cp32-3_PAli_NX_MP_FG_consensus GAGATAAGGCAAGTGATTTTGAAAGATTTAGGGGAAGTAATTCGGCACTTATTTTTGTTAATGAGGCTACAACTTTAC [29406]
cp32-3_PAli_NX_Pl_consensus    GAGATAAGGCAAGTGATTTTGAAAGATTTAGGGGAAGTAATTCGGCACTTATTTTTGTTAATGAGGCTACAACTTTAC [29406]
cp32-3_PAbe_NX_MP_FG_consensus GAGATAAGGCAAGTGATTTTGAAAGATTTAGGGGAAGTAATTCGGCACTTATTTTTGTTAATGAGGCTACAACTTTAC [29406]
cp32-3_PAbe_NX_Pl_consensus    GAGATAAGGCAAGTGATTTTGAAAGATTTAGGGGAAGTAATTCGGCACTTATTTTTGTTAATGAGGCTACAACTTTAC [29406]

cp32-3_gi|11497103|B31_GB      ACAAGCAAACTTTAGAGGAGGTCCTAAAAAGACTAAGATGCGGGCAAGAAACTATTATTTTTGATACTAATCCTGATC [29484]
cp32-3_un8_B31_PacBio          ACAAGCAAACTTTAGAGGAGGTCCTAAAAAGACTAAGATGCGGGCAAGAA-CTATTATTTTTGATACTAATCCTGATC [29484]
cp32-3_B31_TS_MP_FG_consensus  ACAAGCAAACTTTAGAGGAGGTCCTAAAAAGACTAAGATGCGGGCAAGAAACTATTATTTTTGATACTAATCCTGATC [29484]
cp32-3_B31_NX_Pl_consensus     ACAAGCAAACTTTAGAGGAGGTCCTAAAAAGACTAAGATGCGGGCAAGAAACTATTATTTTTGATACTAATCCTGATC [29484]
cp32-3_PAli_NX_MP_FG_consensus ACAAGCAAACTTTAGAGGAGGTCCTAAAAAGACTAAGATGCGGGCAAGAAACTATTATTTTTGATACTAATCCTGATC [29484]
cp32-3_PAli_NX_Pl_consensus    ACAAGCAAACTTTAGAGGAGGTCCTAAAAAGACTAAGATGCGGGCAAGAAACTATTATTTTTGATACTAATCCTGATC [29484]
cp32-3_PAbe_NX_MP_FG_consensus ACAAGCAAACTTTAGAGGAGGTCCTAAAAAGACTAAGATGCGGGCAAGAAACTATTATTTTTGATACTAATCCTGATC [29484]
cp32-3_PAbe_NX_Pl_consensus    ACAAGCAAACTTTAGAGGAGGTCCTAAAAAGACTAAGATGCGGGCAAGAAACTATTATTTTTGATACTAATCCTGATC [29484]

cp32-3_gi|11497103|B31_GB      ATCCAGAACACTATTTTAAAACCGATTATATTGATAATATAGCGACATTTAAGACATATAATTTTACAACTTATGATA [29562]
cp32-3_un8_B31_PacBio          ATCCAGAACACTATTTTAAAACCGATTATATTGATAATATAGCGACATTTAAGACATATAATTTTACAACTTATGATA [29562]
cp32-3_B31_TS_MP_FG_consensus  ATCCAGAACACTATTTTAAAACCGATTATATTGATAATATAGCGACATTTAAGACATATAATTTTACAACTTATGATA [29562]
cp32-3_B31_NX_Pl_consensus     ATCCAGAACACTATTTTAAAACCGATTATATTGATAATATAGCGACATTTAAGACATATAATTTTACAACTTATGATA [29562]
cp32-3_PAli_NX_MP_FG_consensus ATCCAGAACACTATTTTAAAACCGATTATATTGATAATATAGCGACATTTAAGACATATAATTTTACAACTTATGATA [29562]
cp32-3_PAli_NX_Pl_consensus    ATCCAGAACACTATTTTAAAACCGATTATATTGATAATATAGCGACATTTAAGACATATAATTTTACAACTTATGATA [29562]
cp32-3_PAbe_NX_MP_FG_consensus ATCCAGAACACTATTTTAAAACCGATTATATTGATAATATAGCGACATTTAAGACATATAATTTTACAACTTATGATA [29562]
cp32-3_PAbe_NX_Pl_consensus    ATCCAGAACACTATTTTAAAACCGATTATATTGATAATATAGCGACATTTAAGACATATAATTTTACAACTTATGATA [29562]

cp32-3_gi|11497103|B31_GB      ATGTTCTACTTAGTAAAGGATTTATCGAAACACAAGAAAAACTCTATAAAGATATACCATCATATAAAGCAAGAGTTT [29640]
cp32-3_un8_B31_PacBio          ATGTTCTACTTAGTAAAGGATTTATCGAAACACAAGAAAAACTCTATAAAGATATACCATCATATAAAGCAAGAGTTT [29640]
cp32-3_B31_TS_MP_FG_consensus  ATGTTCTACTTAGTAAAGGATTTATCGAAACACAAGAAAAACTCTATAAAGATATACCATCATATAAAGCAAGAGTTT [29640]
cp32-3_B31_NX_Pl_consensus     ATGTTCTACTTAGTAAAGGATTTATCGAAACACAAGAAAAACTCTATAAAGATATACCATCATATAAAGCAAGAGTTT [29640]
cp32-3_PAli_NX_MP_FG_consensus ATGTTCTACTTAGTAAAGGATTTATCGAAACACAAGAAAAACTCTATAAAGATATACCATCATATAAAGCAAGAGTTT [29640]
cp32-3_PAli_NX_Pl_consensus    ATGTTCTACTTAGTAAAGGATTTATCGAAACACAAGAAAAACTCTATAAAGATATACCATCATATAAAGCAAGAGTTT [29640]
cp32-3_PAbe_NX_MP_FG_consensus ATGTTCTACTTAGTAAAGGATTTATCGAAACACAAGAAAAACTCTATAAAGATATACCATCATATAAAGCAAGAGTTT [29640]
cp32-3_PAbe_NX_Pl_consensus    ATGTTCTACTTAGTAAAGGATTTATCGAAACACAAGAAAAACTCTATAAAGATATACCATCATATAAAGCAAGAGTTT [29640]

cp32-3_gi|11497103|B31_GB      TGCTAGGTGAGTGGATAGCAAGCACTGATTCAATTTTTACACAAATAAATATTACTGATGATTATGTATTTACTAGTC [29718]
cp32-3_un8_B31_PacBio          TGCTAGGTGAGTGGATAGCAAGCACTGATTCAATTTTTACACAAATAAATATTACTGATGATTATGTATTTACTAGTC [29718]
cp32-3_B31_TS_MP_FG_consensus  TGCTAGGTGAGTGGATAGCAAGCACTGATTCAATTTTTACACAAATAAATATTACTGATGATTATGTATTTACTAGTC [29718]
cp32-3_B31_NX_Pl_consensus     TGCTAGGTGAGTGGATAGCAAGCACTGATTCAATTTTTACACAAATAAATATTACTGATGATTATGTATTTACTAGTC [29718]
cp32-3_PAli_NX_MP_FG_consensus TGCTAGGTGAGTGGATAGCAAGCACTGATTCAATTTTTACACAAATAAATATTACTGATGATTATGTATTTACTAGTC [29718]
cp32-3_PAli_NX_Pl_consensus    TGCTAGGTGAGTGGATAGCAAGCACTGATTCAATTTTTACACAAATAAATATTACTGATGATTATGTATTTACTAGTC [29718]
cp32-3_PAbe_NX_MP_FG_consensus TGCTAGGTGAGTGGATAGCAAGCACTGATTCAATTTTTACACAAATAAATATTACTGATGATTATGTATTTACTAGTC [29718]
cp32-3_PAbe_NX_Pl_consensus    TGCTAGGTGAGTGGATAGCAAGCACTGATTCAATTTTTACACAAATAAATATTACTGATGATTATGTATTTACTAGTC [29718]

cp32-3_gi|11497103|B31_GB      CAATAGCATATTTAGACCCAGCATTTAGTGTTGGAGGAGATAACACTGCATTATGTGTTATGGAGCGAGTTGATGATA [29796]
cp32-3_un8_B31_PacBio          CAATAGCATATTTAGACCCAGCATTTAGTGTTGGAGGAGA-AACACTGCATTATGTGTTATGGAGCGAGTTGATGATA [29796]
cp32-3_B31_TS_MP_FG_consensus  CAATAGCATATTTAGACCCAGCATTTAGTGTTGGAGGAGATAACACTGCATTATGTGTTATGGAGCGAGTTGATGATA [29796]
cp32-3_B31_NX_Pl_consensus     CAATAGCATATTTAGACCCAGCATTTAGTGTTGGAGGAGATAACACTGCATTATGTGTTATGGAGCGAGTTGATGATA [29796]
cp32-3_PAli_NX_MP_FG_consensus CAATAGCATATTTAGACCCAGCATTTAGTGTTGGAGGAGATAACACTGCATTATGTGTTATGGAGCGAGTTGATGATA [29796]
cp32-3_PAli_NX_Pl_consensus    CAATAGCATATTTAGACCCAGCATTTAGTGTTGGAGGAGATAACACTGCATTATGTGTTATGGAGCGAGTTGATGATA [29796]
cp32-3_PAbe_NX_MP_FG_consensus CAATAGCATATTTAGACCCAGCATTTAGTGTTGGAGGAGATAACACTGCATTATGTGTTATGGAGCGAGTTGATGATA [29796]
cp32-3_PAbe_NX_Pl_consensus    CAATAGCATATTTAGACCCAGCATTTAGTGTTGGAGGAGATAACACTGCATTATGTGTTATGGAGCGAGTTGATGATA [29796]

cp32-3_gi|11497103|B31_GB      AGTATTATGCTTTTGTATTTCAAGACCAACGACCAGCCAATGATCCTTATATTATGAATATGGTAAAGACCGTTATAG [29874]
cp32-3_un8_B31_PacBio          AGTATTATGCTTTTGTATTTCAAGACCAACGACCAGCCAATGATCCTTATATTATGAATATGGTAAAGACCGTTATAG [29874]
cp32-3_B31_TS_MP_FG_consensus  AGTATTATGCTTTTGTATTTCAAGACCAACGACCAGCCAATGATCCTTATATTATGAATATGGTAAAGACCGTTATAG [29874]
cp32-3_B31_NX_Pl_consensus     AGTATTATGCTTTTGTATTTCAAGACCAACGACCAGCCAATGATCCTTATATTATGAATATGGTAAAGACCGTTATAG [29874]
cp32-3_PAli_NX_MP_FG_consensus AGTATTATGCTTTTGTATTTCAAGACCAACGACCAGCCAATGATCCTTATATTATGAATATGGTAAAGACCGTTATAG [29874]
cp32-3_PAli_NX_Pl_consensus    AGTATTATGCTTTTGTATTTCAAGACCAACGACCAGCCAATGATCCTTATATTATGAATATGGTAAAGACCGTTATAG [29874]
cp32-3_PAbe_NX_MP_FG_consensus AGTATTATGCTTTTGTATTTCAAGACCAACGACCAGCCAATGATCCTTATATTATGAATATGGTAAAGACCGTTATAG [29874]
cp32-3_PAbe_NX_Pl_consensus    AGTATTATGCTTTTGTATTTCAAGACCAACGACCAGCCAATGATCCTTATATTATGAATATGGTAAAGACCGTTATAG [29874]

cp32-3_gi|11497103|B31_GB      AAAATTTCAATGTGCATACACTGTATTTAGAGGATAGAGATAATACAAAAGGTGCTGGTGGATTGACCCGCGAATACA [29952]
cp32-3_un8_B31_PacBio          AAAATTTCAATGTGCATACACTGTATTTAGAGGATAGAGATAATACAAAAGGTGCTGGTGGATTGACCCGCGAATACA [29952]
cp32-3_B31_TS_MP_FG_consensus  AAAATTTCAATGTGCATACACTGTATTTAGAGGATAGAGATAATACAAAAGGTGCTGGTGGATTGACCCGCGAATACA [29952]
cp32-3_B31_NX_Pl_consensus     AAAATTTCAATGTGCATACACTGTATTTAGAGGATAGAGATAATACAAAAGGTGCTGGTGGATTGACCCGCGAATACA [29952]
cp32-3_PAli_NX_MP_FG_consensus AAAATTTCAATGTGCATACACTGTATTTAGAGGATAGAGATAATACAAAAGGTGCTGGTGGATTGACCCGCGAATACA [29952]
cp32-3_PAli_NX_Pl_consensus    AAAATTTCAATGTGCATACACTGTATTTAGAGGATAGAGATAATACAAAAGGTGCTGGTGGATTGACCCGCGAATACA [29952]
cp32-3_PAbe_NX_MP_FG_consensus AAAATTTCAATGTGCATACACTGTATTTAGAGGATAGAGATAATACAAAAGGTGCTGGTGGATTGACCCGCGAATACA [29952]
cp32-3_PAbe_NX_Pl_consensus    AAAATTTCAATGTGCATACACTGTATTTAGAGGATAGAGATAATACAAAAGGTGCTGGTGGATTGACCCGCGAATACA [29952]

cp32-3_gi|11497103|B31_GB      TCTTACTAAGAAATAATATAAGCCAATATTTTAGAATTGTTCCAGTTAAGCCAAAGTCTAATAAATTTAGCAGAATAA [30030]
cp32-3_un8_B31_PacBio          TCTTACTAAGAAATAATATAAGCCAATATTTTAGAATTGTTCCAGTTAAGCCAAAGTCTAATAAATTTAGCAGAATAA [30030]
cp32-3_B31_TS_MP_FG_consensus  TCTTACTAAGAAATAATATAAGCCAATATTTTAGAATTGTTCCAGTTAAGCCAAAGTCTAATAAATTTAGCAGAATAA [30030]
cp32-3_B31_NX_Pl_consensus     TCTTACTAAGAAATAATATAAGCCAATATTTTAGAATTGTTCCAGTTAAGCCAAAGTCTAATAAATTTAGCAGAATAA [30030]
cp32-3_PAli_NX_MP_FG_consensus TCTTACTAAGAAATAATATAAGCCAATATTTTAGAATTGTTCCAGTTAAGCCAAAGTCTAATAAATTTAGCAGAATAA [30030]
cp32-3_PAli_NX_Pl_consensus    TCTTACTAAGAAATAATATAAGCCAATATTTTAGAATTGTTCCAGTTAAGCCAAAGTCTAATAAATTTAGCAGAATAA [30030]
cp32-3_PAbe_NX_MP_FG_consensus TCTTACTAAGAAATAATATAAGCCAATATTTTAGAATTGTTCCAGTTAAGCCAAAGTCTAATAAATTTAGCAGAATAA [30030]
cp32-3_PAbe_NX_Pl_consensus    TCTTACTAAGAAATAATATAAGCCAATATTTTAGAATTGTTCCAGTTAAGCCAAAGTCTAATAAATTTAGCAGAATAA [30030]

cp32-3_gi|11497103|B31_GB      CAACGTTAATTACGCCGTTTACTTATAAGAAACTTTACATTACAAAGTACAGTAGTTCTTCTGTATTTAATGATATTT [30108]
cp32-3_un8_B31_PacBio          CAACGTTAATTACGCCGTTTACTTATAAGAAACTTTACATTACAAAGTACAGTAGTTCTTCTGTATTTAATGATATTT [30108]
cp32-3_B31_TS_MP_FG_consensus  CAACGTTAATTACGCCGTTTACTTATAAGAAACTTTACATTACAAAGTACAGTAGTTCTTCTGTATTTAATGATATTT [30108]
cp32-3_B31_NX_Pl_consensus     CAACGTTAATTACGCCGTTTACTTATAAGAAACTTTACATTACAAAGTACAGTAGTTCTTCTGTATTTAATGATATTT [30108]
cp32-3_PAli_NX_MP_FG_consensus CAACGTTAATTACGCCGTTTACTTATAAGAAACTTTACATTACAAAGTACAGTAGTTCTTCTGTATTTAATGATATTT [30108]
cp32-3_PAli_NX_Pl_consensus    CAACGTTAATTACGCCGTTTACTTATAAGAAACTTTACATTACAAAGTACAGTAGTTCTTCTGTATTTAATGATATTT [30108]
cp32-3_PAbe_NX_MP_FG_consensus CAACGTTAATTACGCCGTTTACTTATAAGAAACTTTACATTACAAAGTACAGTAGTTCTTCTGTATTTAATGATATTT [30108]
cp32-3_PAbe_NX_Pl_consensus    CAACGTTAATTACGCCGTTTACTTATAAGAAACTTTACATTACAAAGTACAGTAGTTCTTCTGTATTTAATGATATTT [30108]

cp32-3_gi|11497103|B31_GB      ATTCGTATAAGGGGGATAGCAAAACCCATGATGATGCTCTTGATGCAATGTCTGCAGCATATTTGATGTTGTCTTTAG [30186]
cp32-3_un8_B31_PacBio          ATTCGTATAAGGGGGATAGCAAAACCCATGATGATGCTCTTGATGCAATGTCTGCAGCATATTTGATGTTGTCTTTAG [30186]
cp32-3_B31_TS_MP_FG_consensus  ATTCGTATAAGGGGGATAGCAAAACCCATGATGATGCTCTTGATGCAATGTCTGCAGCATATTTGATGTTGTCTTTAG [30186]
cp32-3_B31_NX_Pl_consensus     ATTCGTATAAGGGGGATAGCAAAACCCATGATGATGCTCTTGATGCAATGTCTGCAGCATATTTGATGTTGTCTTTAG [30186]
cp32-3_PAli_NX_MP_FG_consensus ATTCGTATAAGGGGGATAGCAAAACCCATGATGATGCTCTTGATGCAATGTCTGCAGCATATTTGATGTTGTCTTTAG [30186]
cp32-3_PAli_NX_Pl_consensus    ATTCGTATAAGGGGGATAGCAAAACCCATGATGATGCTCTTGATGCAATGTCTGCAGCATATTTGATGTTGTCTTTAG [30186]
```

```
cp32-3_PAbe_NX_MP_FG_consensus ATTCGTATAAGGGGGATAGCAAAACCCATGATGATGCTCTTGATGCAATGTCTGCAGCATATTTGATGTTGTCTTTAG [30186]
cp32-3_PAbe_NX_Pl_consensus    ATTCGTATAAGGGGGATAGCAAAACCCATGATGATGCTCTTGATGCAATGTCTGCAGCATATTTGATGTTGTCTTTAG [30186]

cp32-3_gi|11497103|B31_GB      GATATAGAGAGCGAAGTGTTCACTTTGGCAATCAAAGATTTTTGTAA------------------------------- [30264]
cp32-3_un8_B31_PacBio          GATATAGAGAGCGAAGTGTTCACTTTGGCAATCAAAGATTTTTGTAA------------------------------- [30264]
cp32-3_B31_TS_MP_FG_consensus  GATATAGAGAGCGAAGTGTTCACTTTGGCAATCAAAGATTTTTGTAA------------------------------- [30264]
cp32-3_B31_NX_Pl_consensus     GATATAGAGAGCGAAGTGTTCACTTTGGCAATCAAAGATTTTTGTAA------------------------------- [30264]
cp32-3_PAli_NX_MP_FG_consensus GATATAGAGAGCGAAGTGTTCACTTTGGCAATCAAAGATTTTTGTAA------------------------------- [30264]
cp32-3_PAli_NX_Pl_consensus    GATATAGAGAGCGAAGTGTTCACTTTGGCAATCAAAGATTTTTGTAA------------------------------- [30264]
cp32-3_PAbe_NX_MP_FG_consensus GATATAGAGAGCGAAGTGTTCACTTTGGCAATCAAAGATTTTTGTAA------------------------------- [30264]
cp32-3_PAbe_NX_Pl_consensus    GATATAGAGAGCGAAGTGTTCACTTTGGCAATCAAAGATTTTTGTAA------------------------------- [30264]
```

```
#NEXUS
[ Title ]
begin data;
   dimensions ntax=8 nchar=57807;
   format missing=? gap=- matchchar=. datatype=nucleotide interleave=yes;
   matrix

[!Domain=Data property=Coding CodonStart=1;]
cp32-4_gi|11497149|B31_GB       ATTTTATTGACAAAAATATTAGTTTTTGCTATCATGCATCTAATTTAATAAAGAGAAGTAAAAAGGTGTGTGATTTAA [   78]
cp32-4_un10_B31_PacBio          ATTTTATTGACAAAAATATTAGTTTTTGCTATCATGCATCTAATTTAATAAAGAGAAGTAAAAAGGTGTGTGATTTAA [   78]
cp32-4_B31_TS_MP_FG_consensus   ATTTTATTGACAAAAATATTAGTTTTTGCTATCATGCATCTAATTTAATAAAGAGAAGTAAAAAGGTGTGTGATTTAA [   78]
cp32-4_B31_NX_Pl_consensus      ATTTTATTGACAAAAATATTAGTTTTTGCTATCATGCATCTAATTTAATAAAGAGAAGTAAAAAGGTGTGTGATTTAA [   78]
cp32-4_PAli_NX_MP_FG_consensus ATTTTATTGACAAAAATATTAGTTTTTGCTATCATGCATCTAATTTAATAAAGAGAAGTAAAAAGGTGTGTGATTTAA [   78]
cp32-4_PAli_NX_Pl_consensus     ATTTTATTGACAAAAATATTAGTTTTTGCTATCATGCATCTAATTTAATAAAGAGAAGTAAAAAGGTGTGTGATTTAA [   78]
cp32-4_PAbe_NX_MP_FG_consensus ATTTTATTGACAAAAATATTAGTTTTTGCTATCATGCATCTAATTTAATAAAGAGAAGTAAAAAGGTGTGTGATTTAA [   78]
cp32-4_PAbe_NX_Pl_consensus     ATTTTATTGACAAAAATATTAGTTTTTGCTATCATGCATCTAATTTAATAAAGAGAAGTAAAAAGGTGTGTGATTTAA [   78]

cp32-4_gi|11497149|B31_GB       GAAAAACAAAACTAATAGATAAAATAAGTTCACTAGAACTATACAAATACTCAATATTTTTTAGAAATTACATTGAAA [  156]
cp32-4_un10_B31_PacBio          GAAAAACAAAACTAATAGATAAAATAAGTTCACTAGAACTATACAAATACTCAATATTTTTTAGAAATTACATTGAAA [  156]
cp32-4_B31_TS_MP_FG_consensus   GAAAAACAAAACTAATAGATAAAATAAGTTCACTAGAACTATACAAATACTCAATATTTTTTAGAAATTACATTGAAA [  156]
cp32-4_B31_NX_Pl_consensus      GAAAAACAAAACTAATAGATAAAATAAGTTCACTAGAACTATACAAATACTCAATATTTTTTAGAAATTACATTGAAA [  156]
cp32-4_PAli_NX_MP_FG_consensus GAAAAACAAAACTAATAGATAAAATAAGTTCACTAGAACTATACAAATACTCAATATTTTTTAGAAATTACATTGAAA [  156]
cp32-4_PAli_NX_Pl_consensus     GAAAAACAAAACTAATAGATAAAATAAGTTCACTAGAACTATACAAATACTCAATATTTTTTAGAAATTACATTGAAA [  156]
cp32-4_PAbe_NX_MP_FG_consensus GAAAAACAAAACTAATAGATAAAATAAGTTCACTAGAACTATACAAATACTCAATATTTTTTAGAAATTACATTGAAA [  156]
cp32-4_PAbe_NX_Pl_consensus     GAAAAACAAAACTAATAGATAAAATAAGTTCACTAGAACTATACAAATACTCAATATTTTTTAGAAATTACATTGAAA [  156]

cp32-4_gi|11497149|B31_GB       ATGTAGCAGAAGATTGTCTCAAGAACGGACTTATTCTTGAGAGTGCTGCCCACAATGTTAGTGAGGTTGAACTTGCTA [  234]
cp32-4_un10_B31_PacBio          ATGTAGCAGAAGATTGTCTCAAGAACGGACTTATTCTTGAGAGTGCTGCCCACAATGTTAGTGAGGTTGAACTTGCTA [  234]
cp32-4_B31_TS_MP_FG_consensus   ATGTAGCAGAAGATTGTCTCAAGAACGGACTTATTCTTGAGAGTGCTGCCCACAATGTTAGTGAGGTTGAACTTGCTA [  234]
cp32-4_B31_NX_Pl_consensus      ATGTAGCAGAAGATTGTCTCAAGAACGGACTTATTCTTGAGAGTGCTGCCCACAATGTTAGTGAGGTTGAACTTGCTA [  234]
cp32-4_PAli_NX_MP_FG_consensus ATGTAGCAGAAGATTGTCTCAAGAACGGACTTATTCTTGAGAGTGCTGCCCACAATGTTAGTGAGGTTGAACTTGCTA [  234]
cp32-4_PAli_NX_Pl_consensus     ATGTAGCAGAAGATTGTCTCAAGAACGGACTTATTCTTGAGAGTGCTGCCCACAATGTTAGTGAGGTTGAACTTGCTA [  234]
cp32-4_PAbe_NX_MP_FG_consensus ATGTAGCAGAAGATTGTCTCAAGAACGGACTTATTCTTGAGAGTGCTGCCCACAATGTTAGTGAGGTTGAACTTGCTA [  234]
cp32-4_PAbe_NX_Pl_consensus     ATGTAGCAGAAGATTGTCTCAAGAACGGACTTATTCTTGAGAGTGCTGCCCACAATGTTAGTGAGGTTGAACTTGCTA [  234]

cp32-4_gi|11497149|B31_GB       GGTTAAAGGTACAGCTTAAGAATGCTCTGCTTAATTGTATTATAAGCTACCGTTTTCATGGGATTGGCTATGTTTTAG [  312]
cp32-4_un10_B31_PacBio          GGTTAAAGGTACAGCTTAAGAATGCTCTGCTTAATTGTATTATAAGCTACCGTTTTCATGGGATTGGCTATGTTTTAG [  312]
cp32-4_B31_TS_MP_FG_consensus   GGTTAAAGGTACAGCTTAAGAATGCTCTGCTTAATTGTATTATAAGCTACCGTTTTCATGGGATTGGCTATGTTTTAG [  312]
cp32-4_B31_NX_Pl_consensus      GGTTAAAGGTACAGCTTAAGAATGCTCTGCTTAATTGTATTATAAGCTACCGTTTTCATGGGATTGGCTATGTTTTAG [  312]
cp32-4_PAli_NX_MP_FG_consensus GGTTAAAGGTACAGCTTAAGAATGCTCTGCTTAATTGTATTATAAGCTACCGTTTTCATGGGATTGGCTATGTTTTAG [  312]
cp32-4_PAli_NX_Pl_consensus     GGTTAAAGGTACAGCTTAAGAATGCTCTGCTTAATTGTATTATAAGCTACCGTTTTCATGGGATTGGCTATGTTTTAG [  312]
cp32-4_PAbe_NX_MP_FG_consensus GGTTAAAGGTACAGCTTAAGAATGCTCTGCTTAATTGTATTATAAGCTACCGTTTTCATGGGATTGGCTATGTTTTAG [  312]
cp32-4_PAbe_NX_Pl_consensus     GGTTAAAGGTACAGCTTAAGAATGCTCTGCTTAATTGTATTATAAGCTACCGTTTTCATGGGATTGGCTATGTTTTAG [  312]

cp32-4_gi|11497149|B31_GB       TAAAAACCAAAGATACCCTAATAGATCTCGAACAACCCGTTAATATAGAATTACCTATTGGTTTTGAATACCTTGATT [  390]
cp32-4_un10_B31_PacBio          TAAAAACCAAAGATACCCTAATAGATCTCGAACAACCCGTTAATATAGAATTACCTATTGGTTTTGAATACCTTGATT [  390]
cp32-4_B31_TS_MP_FG_consensus   TAAAAACCAAAGATACCCTAATAGATCTCGAACAACCCGTTAATATAGAATTACCTATTGGTTTTGAATACCTTGATT [  390]
cp32-4_B31_NX_Pl_consensus      TAAAAACCAAAGATACCCTAATAGATCTCGAACAACCCGTTAATATAGAATTACCTATTGGTTTTGAATACCTTGATT [  390]
cp32-4_PAli_NX_MP_FG_consensus TAAAAACCAAAGATACCCTAATAGATCTCGAACAACCCGTTAATATAGAATTACCTATTGGTTTTGAATACCTTGATT [  390]
cp32-4_PAli_NX_Pl_consensus     TAAAAACCAAAGATACCCTAATAGATCTCGAACAACCCGTTAATATAGAATTACCTATTGGTTTTGAATACCTTGATT [  390]
cp32-4_PAbe_NX_MP_FG_consensus TAAAAACCAAAGATACCCTAATAGATCTCGAACAACCCGTTAATATAGAATTACCTATTGGTTTTGAATACCTTGATT [  390]
cp32-4_PAbe_NX_Pl_consensus     TAAAAACCAAAGATACCCTAATAGATCTCGAACAACCCGTTAATATAGAATTACCTATTGGTTTTGAATACCTTGATT [  390]

cp32-4_gi|11497149|B31_GB       ATGAATATGTAAGAGATTTGGGAGTTGATTTTGATCATATAACCTATAAAGTAAAATCCAACAATAAGAACAATTCTT [  468]
cp32-4_un10_B31_PacBio          ATGAATATGTAAGAGATTTGGGAGTTGATTTTGATCATATAACCTATAAAGTAAAATCCAACAATAAGAACAATTCTT [  468]
cp32-4_B31_TS_MP_FG_consensus   ATGAATATGTAAGAGATTTGGGAGTTGATTTTGATCATATAACCTATAAAGTAAAATCCAACAATAAGAACAATTCTT [  468]
cp32-4_B31_NX_Pl_consensus      ATGAATATGTAAGAGATTTGGGAGTTGATTTTGATCATATAACCTATAAAGTAAAATCCAACAATAAGAACAATTCTT [  468]
cp32-4_PAli_NX_MP_FG_consensus ATGAATATGTAAGAGATTTGGGAGTTGATTTTGATCATATAACCTATAAAGTAAAATCCAACAATAAGAACAATTCTT [  468]
cp32-4_PAli_NX_Pl_consensus     ATGAATATGTAAGAGATTTGGGAGTTGATTTTGATCATATAACCTATAAAGTAAAATCCAACAATAAGAACAATTCTT [  468]
cp32-4_PAbe_NX_MP_FG_consensus ATGAATATGTAAGAGATTTGGGAGTTGATTTTGATCATATAACCTATAAAGTAAAATCCAACAATAAGAACAATTCTT [  468]
cp32-4_PAbe_NX_Pl_consensus     ATGAATATGTAAGAGATTTGGGAGTTGATTTTGATCATATAACCTATAAAGTAAAATCCAACAATAAGAACAATTCTT [  468]

cp32-4_gi|11497149|B31_GB       TAGACGCAGTTAAAATACATAAAAGTCGACTTATCATATATGAAAACTTTGATTATATCTTAAAAAGATATGTTCCGT [  546]
cp32-4_un10_B31_PacBio          TAGACGCAGTTAAAATACATAAAAGTCGACTTATCATATATGAAAACTTTGATTATATCTTAAAAAGATATGTTCCGT [  546]
cp32-4_B31_TS_MP_FG_consensus   TAGACGCAGTTAAAATACATAAAAGTCGACTTATCATATATGAAAACTTTGATTATATCTTAAAAAGATATGTTCCGT [  546]
cp32-4_B31_NX_Pl_consensus      TAGACGCAGTTAAAATACATAAAAGTCGACTTATCATATATGAAAACTTTGATTATATCTTAAAAAGATATGTTCCGT [  546]
cp32-4_PAli_NX_MP_FG_consensus TAGACGCAGTTAAAATACATAAAAGTCGACTTATCATATATGAAAACTTTGATTATATCTTAAAAAGATATGTTCCGT [  546]
cp32-4_PAli_NX_Pl_consensus     TAGACGCAGTTAAAATACATAAAAGTCGACTTATCATATATGAAAACTTTGATTATATCTTAAAAAGATATGTTCCGT [  546]
cp32-4_PAbe_NX_MP_FG_consensus TAGACGCAGTTAAAATACATAAAAGTCGACTTATCATATATGAAAACTTTGATTATATCTTAAAAAGATATGTTCCGT [  546]
cp32-4_PAbe_NX_Pl_consensus     TAGACGCAGTTAAAATACATAAAAGTCGACTTATCATATATGAAAACTTTGATTATATCTTAAAAAGATATGTTCCGT [  546]

cp32-4_gi|11497149|B31_GB       GTTATACCGAAAGCTTTTTACTAGATATTTATTTATTTGAAAAGATATACGTAGAAATAGAAAGACGTATTGAAAACC [  624]
cp32-4_un10_B31_PacBio          GTTATACCGAAAGCTTTTTACTAGATATTTATTTATTTGAAAAGATATACGTAGAAATAGAAAGACGTATTGAAAACC [  624]
cp32-4_B31_TS_MP_FG_consensus   GTTATACCGAAAGCTTTTTACTAGATATTTATTTATTTGAAAAGATATACGTAGAAATAGAAAGACGTATTGAAAACC [  624]
cp32-4_B31_NX_Pl_consensus      GTTATACCGAAAGCTTTTTACTAGATATTTATTTATTTGAAAAGATATACGTAGAAATAGAAAGACGTATTGAAAACC [  624]
cp32-4_PAli_NX_MP_FG_consensus GTTATACCGAAAGCTTTTTACTAGATATTTATTTATTTGAAAAGATATACGTAGAAATAGAAAGACGTATTGAAAACC [  624]
cp32-4_PAli_NX_Pl_consensus     GTTATACCGAAAGCTTTTTACTAGATATTTATTTATTTGAAAAGATATACGTAGAAATAGAAAGACGTATTGAAAACC [  624]
cp32-4_PAbe_NX_MP_FG_consensus GTTATACCGAAAGCTTTTTACTAGATATTTATTTATTTGAAAAGATATACGTAGAAATAGAAAGACGTATTGAAAACC [  624]
cp32-4_PAbe_NX_Pl_consensus     GTTATACCGAAAGCTTTTTACTAGATATTTATTTATTTGAAAAGATATACGTAGAAATAGAAAGACGTATTGAAAACC [  624]

cp32-4_gi|11497149|B31_GB       ACAATTTTTTGTTTTACAAAGATGAATCTTTAGTACAACTACAAGACGCACTTTCTAGTGCAACAACTTCTTTAAGTG [  702]
cp32-4_un10_B31_PacBio          ACAATTTTTTGTTTTACAAAGATGAATCTTTAGTACAACTACAAGACGCACTTTCTAGTGCAACAACTTCTTTAAGTG [  702]
cp32-4_B31_TS_MP_FG_consensus   ACAATTTTTTGTTTTACAAAGATGAATCTTTAGTACAACTACAAGACGCACTTTCTAGTGCAACAACTTCTTTAAGTG [  702]
cp32-4_B31_NX_Pl_consensus      ACAATTTTTTGTTTTACAAAGATGAATCTTTAGTACAACTACAAGACGCACTTTCTAGTGCAACAACTTCTTTAAGTG [  702]
cp32-4_PAli_NX_MP_FG_consensus ACAATTTTTTGTTTTACAAAGATGAATCTTTAGTACAACTACAAGACGCACTTTCTAGTGCAACAACTTCTTTAAGTG [  702]
cp32-4_PAli_NX_Pl_consensus     ACAATTTTTTGTTTTACAAAGATGAATCTTTAGTACAACTACAAGACGCACTTTCTAGTGCAACAACTTCTTTAAGTG [  702]
cp32-4_PAbe_NX_MP_FG_consensus ACAATTTTTTGTTTTACAAAGATGAATCTTTAGTACAACTACAAGACGCACTTTCTAGTGCAACAACTTCTTTAAGTG [  702]
cp32-4_PAbe_NX_Pl_consensus     ACAATTTTTTGTTTTACAAAGATGAATCTTTAGTACAACTACAAGACGCACTTTCTAGTGCAACAACTTCTTTAAGTG [  702]

cp32-4_gi|11497149|B31_GB       CACTTACTCAGAGTAATAATGATAGGGGAAGTGGCATTTTATCTTCTTTTTTGAGAAAACAAAATTCAAACAATCATA [  780]
cp32-4_un10_B31_PacBio          CACTTACTCAGAGTAATAATGATAGGGGAAGTGGCATTTTATCTTCTTTTTTGAGAAAACAAAATTCAAACAATCATA [  780]
cp32-4_B31_TS_MP_FG_consensus   CACTTACTCAGAGTAATAATGATAGGGGAAGTGGCATTTTATCTTCTTTTTTGAGAAAACAAAATTCAAACAATCATA [  780]
cp32-4_B31_NX_Pl_consensus      CACTTACTCAGAGTAATAATGATAGGGGAAGTGGCATTTTATCTTCTTTTTTGAGAAAACAAAATTCAAACAATCATA [  780]
cp32-4_PAli_NX_MP_FG_consensus CACTTACTCAGAGTAATAATGATAGGGGAAGTGGCATTTTATCTTCTTTTTTGAGAAAACAAAATTCAAACAATCATA [  780]
cp32-4_PAli_NX_Pl_consensus     CACTTACTCAGAGTAATAATGATAGGGGAAGTGGCATTTTATCTTCTTTTTTGAGAAAACAAAATTCAAACAATCATA [  780]
cp32-4_PAbe_NX_MP_FG_consensus CACTTACTCAGAGTAATAATGATAGGGGAAGTGGCATTTTATCTTCTTTTTTGAGAAAACAAAATTCAAACAATCATA [  780]
cp32-4_PAbe_NX_Pl_consensus     CACTTACTCAGAGTAATAATGATAGGGGAAGTGGCATTTTATCTTCTTTTTTGAGAAAACAAAATTCAAACAATCATA [  780]

cp32-4_gi|11497149|B31_GB       GTAAAGATATTTCTAATTTAAGAAACCTTAATGACTCATTATCACAGGAGCTTGCTAGGCTAAAAAGTAATCTAAATA [  858]
cp32-4_un10_B31_PacBio          GTAAAGATATTTCTAATTTAAGAAACCTTAATGACTCATTATCACAGGAGCTTGCTAGGCTAAAAAGTAATCTAAATA [  858]
cp32-4_B31_TS_MP_FG_consensus   GTAAAGATATTTCTAATTTAAGAAACCTTAATGACTCATTATCACAGGAGCTTGCTAGGCTAAAAAGTAATCTAAATA [  858]
cp32-4_B31_NX_Pl_consensus      GTAAAGATATTTCTAATTTAAGAAACCTTAATGACTCATTATCACAGGAGCTTGCTAGGCTAAAAAGTAATCTAAATA [  858]
cp32-4_PAli_NX_MP_FG_consensus GTAAAGATATTTCTAATTTAAGAAACCTTAATGACTCATTATCACAGGAGCTTGCTAGGCTAAAAAGTAATCTAAATA [  858]
cp32-4_PAli_NX_Pl_consensus     GTAAAGATATTTCTAATTTAAGAAACCTTAATGACTCATTATCACAGGAGCTTGCTAGGCTAAAAAGTAATCTAAATA [  858]
cp32-4_PAbe_NX_MP_FG_consensus GTAAAGATATTTCTAATTTAAGAAACCTTAATGACTCATTATCACAGGAGCTTGCTAGGCTAAAAAGTAATCTAAATA [  858]
cp32-4_PAbe_NX_Pl_consensus     GTAAAGATATTTCTAATTTAAGAAACCTTAATGACTCATTATCACAGGAGCTTGCTAGGCTAAAAAGTAATCTAAATA [  858]

cp32-4_gi|11497149|B31_GB       ATGAGGGAATGTTTTATACGGCCACCCCTAGTGCTAGTTTAGAGGTTATTAAATACGACCTTAGTTACTTAAAGGAGG [  936]
cp32-4_un10_B31_PacBio          ATGAGGGAATGTTTTATACGGCCACCCCTAGTGCTAGTTTAGAGGTTATTAAATACGACCTTAGTTACTTAAAGGAGG [  936]
```

```
cp32-4_B31_TS_MP_FG_consensus  ATGAGGGAATGTTTTATACGGCCACCCCTAGTGCTAGTTTAGAGGTTATTAAATACGACCTTAGTTACTTAAAGGAGG [  936]
cp32-4_B31_NX_Pl_consensus     ATGAGGGAATGTTTTATACGGCCACCCCTAGTGCTAGTTTAGAGGTTATTAAATACGACCTTAGTTACTTAAAGGAGG [  936]
cp32-4_PAli_NX_MP_FG_consensus ATGAGGGAATGTTTTATACGGCCACCCCTAGTGCTAGTTTAGAGGTTATTAAATACGACCTTAGTTACTTAAAGGAGG [  936]
cp32-4_PAli_NX_Pl_consensus    ATGAGGGAATGTTTTATACGGCCACCCCTAGTGCTAGTTTAGAGGTTATTAAATACGACCTTAGTTACTTAAAGGAGG [  936]
cp32-4_PAbe_NX_MP_FG_consensus ATGAGGGAATGTTTTATACGGCCACCCCTAGTGCTAGTTTAGAGGTTATTAAATACGACCTTAGTTACTTAAAGGAGG [  936]
cp32-4_PAbe_NX_Pl_consensus    ATGAGGGAATGTTTTATACGGCCACCCCTAGTGCTAGTTTAGAGGTTATTAAATACGACCTTAGTTACTTAAAGGAGG [  936]

cp32-4_gi|11497149|B31_GB      CTTTAGCATTAATTAAGGCAAAAATTGGTGCAGATACTAAAGAGCCCTTAACCAGAAGTTTTAACGAACAGGCTAAAG [ 1014]
cp32-4_un10_B31_PacBio         CTTTAGCATTAATTAAGGCAAAAATTGGTGCAGATACTAAAGAGCCCTTAACCAGAAGTTTTAACGAACAGGCTAAAG [ 1014]
cp32-4_B31_TS_MP_FG_consensus  CTTTAGCATTAATTAAGGCAAAAATTGGTGCAGATACTAAAGAGCCCTTAACCAGAAGTTTTAACGAACAGGCTAAAG [ 1014]
cp32-4_B31_NX_Pl_consensus     CTTTAGCATTAATTAAGGCAAAAATTGGTGCAGATACTAAAGAGCCCTTAACCAGAAGTTTTAACGAACAGGCTAAAG [ 1014]
cp32-4_PAli_NX_MP_FG_consensus CTTTAGCATTAATTAAGGCAAAAATTGGTGCAGATACTAAAGAGCCCTTAACCAGAAGTTTTAACGAACAGGCTAAAG [ 1014]
cp32-4_PAli_NX_Pl_consensus    CTTTAGCATTAATTAAGGCAAAAATTGGTGCAGATACTAAAGAGCCCTTAACCAGAAGTTTTAACGAACAGGCTAAAG [ 1014]
cp32-4_PAbe_NX_MP_FG_consensus CTTTAGCATTAATTAAGGCAAAAATTGGTGCAGATACTAAAGAGCCCTTAACCAGAAGTTTTAACGAACAGGCTAAAG [ 1014]
cp32-4_PAbe_NX_Pl_consensus    CTTTAGCATTAATTAAGGCAAAAATTGGTGCAGATACTAAAGAGCCCTTAACCAGAAGTTTTAACGAACAGGCTAAAG [ 1014]

cp32-4_gi|11497149|B31_GB      GGCTAGGAAATGATGGTAAAGGGGATAGGAGTAATTATTACGATTTTTTAAAGGGTGTGCAAGAACAAGTTGAGAACG [ 1092]
cp32-4_un10_B31_PacBio         GGCTAGGAAATGATGGTAAAGGGGATAGGAGTAATTATTACGATTTTTTAAAGGGTGTGCAAGAACAAGTTGAGAACG [ 1092]
cp32-4_B31_TS_MP_FG_consensus  GGCTAGGAAATGATGGTAAAGGGGATAGGAGTAATTATTACGATTTTTTAAAGGGTGTGCAAGAACAAGTTGAGAACG [ 1092]
cp32-4_B31_NX_Pl_consensus     GGCTAGGAAATGATGGTAAAGGGGATAGGAGTAATTATTACGATTTTTTAAAGGGTGTGCAAGAACAAGTTGAGAACG [ 1092]
cp32-4_PAli_NX_MP_FG_consensus GGCTAGGAAATGATGGTAAAGGGGATAGGAGTAATTATTACGATTTTTTAAAGGGTGTGCAAGAACAAGTTGAGAACG [ 1092]
cp32-4_PAli_NX_Pl_consensus    GGCTAGGAAATGATGGTAAAGGGGATAGGAGTAATTATTACGATTTTTTAAAGGGTGTGCAAGAACAAGTTGAGAACG [ 1092]
cp32-4_PAbe_NX_MP_FG_consensus GGCTAGGAAATGATGGTAAAGGGGATAGGAGTAATTATTACGATTTTTTAAAGGGTGTGCAAGAACAAGTTGAGAACG [ 1092]
cp32-4_PAbe_NX_Pl_consensus    GGCTAGGAAATGATGGTAAAGGGGATAGGAGTAATTATTACGATTTTTTAAAGGGTGTGCAAGAACAAGTTGAGAACG [ 1092]

cp32-4_gi|11497149|B31_GB      CTTGTAACTTGAAACTTACAAAGTATTTCGGGCTTGATATGAAGTTTAATTCTCTGATTATGTTAAGTGAAGAGCAAA [ 1170]
cp32-4_un10_B31_PacBio         CTTGTAACTTGAAACTTACAAAGTATTTCGGGCTTGATATGAAGTTTAATTCTCTGATTATGTTAAGTGAAGAGCAAA [ 1170]
cp32-4_B31_TS_MP_FG_consensus  CTTGTAACTTGAAACTTACAAAGTATTTCGGGCTTGATATGAAGTTTAATTCTCTGATTATGTTAAGTGAAGAGCAAA [ 1170]
cp32-4_B31_NX_Pl_consensus     CTTGTAACTTGAAACTTACAAAGTATTTCGGGCTTGATATGAAGTTTAATTCTCTGATTATGTTAAGTGAAGAGCAAA [ 1170]
cp32-4_PAli_NX_MP_FG_consensus CTTGTAACTTGAAACTTACAAAGTATTTCGGGCTTGATATGAAGTTTAATTCTCTGATTATGTTAAGTGAAGAGCAAA [ 1170]
cp32-4_PAli_NX_Pl_consensus    CTTGTAACTTGAAACTTACAAAGTATTTCGGGCTTGATATGAAGTTTAATTCTCTGATTATGTTAAGTGAAGAGCAAA [ 1170]
cp32-4_PAbe_NX_MP_FG_consensus CTTGTAACTTGAAACTTACAAAGTATTTCGGGCTTGATATGAAGTTTAATTCTCTGATTATGTTAAGTGAAGAGCAAA [ 1170]
cp32-4_PAbe_NX_Pl_consensus    CTTGTAACTTGAAACTTACAAAGTATTTCGGGCTTGATATGAAGTTTAATTCTCTGATTATGTTAAGTGAAGAGCAAA [ 1170]

cp32-4_gi|11497149|B31_GB      AAGTGGAAAGAGATATAAAGCTAATTGAGCTTTACAGTAAATATAACCAGCTTATACAAAGTAGCTCCTTTGATAATG [ 1248]
cp32-4_un10_B31_PacBio         AAGTGGAAAGAGATATAAAGCTAATTGAGCTTTACAGTAAATATAACCAGCTTATACAAAGTAGCTCCTTTGATAATG [ 1248]
cp32-4_B31_TS_MP_FG_consensus  AAGTGGAAAGAGATATAAAGCTAATTGAGCTTTACAGTAAATATAACCAGCTTATACAAAGTAGCTCCTTTGATAATG [ 1248]
cp32-4_B31_NX_Pl_consensus     AAGTGGAAAGAGATATAAAGCTAATTGAGCTTTACAGTAAATATAACCAGCTTATACAAAGTAGCTCCTTTGATAATG [ 1248]
cp32-4_PAli_NX_MP_FG_consensus AAGTGGAAAGAGATATAAAGCTAATTGAGCTTTACAGTAAATATAACCAGCTTATACAAAGTAGCTCCTTTGATAATG [ 1248]
cp32-4_PAli_NX_Pl_consensus    AAGTGGAAAGAGATATAAAGCTAATTGAGCTTTACAGTAAATATAACCAGCTTATACAAAGTAGCTCCTTTGATAATG [ 1248]
cp32-4_PAbe_NX_MP_FG_consensus AAGTGGAAAGAGATATAAAGCTAATTGAGCTTTACAGTAAATATAACCAGCTTATACAAAGTAGCTCCTTTGATAATG [ 1248]
cp32-4_PAbe_NX_Pl_consensus    AAGTGGAAAGAGATATAAAGCTAATTGAGCTTTACAGTAAATATAACCAGCTTATACAAAGTAGCTCCTTTGATAATG [ 1248]

cp32-4_gi|11497149|B31_GB      AGGAGCTAGCGATTTTAAAAGAGAAATTATTCTCATTTTGAGAAAAGGAGTTAAAAGATGACTGAGAAAAAAGAAAAA [ 1326]
cp32-4_un10_B31_PacBio         AGGAGCTAGCGATTTTAAAAGAGAAATTATTCTCATTTTGAGAAAAGGAGTTAAAAGATGACTGAGAAAAAAGAAAAA [ 1326]
cp32-4_B31_TS_MP_FG_consensus  AGGAGCTAGCGATTTTAAAAGAGAAATTATTCTCATTTTGAGAAAAGGAGTTAAAAGATGACTGAGAAAAAAGAAAAA [ 1326]
cp32-4_B31_NX_Pl_consensus     AGGAGCTAGCGATTTTAAAAGAGAAATTATTCTCATTTTGAGAAAAGGAGTTAAAAGATGACTGAGAAAAAAGAAAAA [ 1326]
cp32-4_PAli_NX_MP_FG_consensus AGGAGCTAGCGATTTTAAAAGAGAAATTATTCTCATTTTGAGAAAAGGAGTTAAAAGATGACTGAGAAAAAAGAAAAA [ 1326]
cp32-4_PAli_NX_Pl_consensus    AGGAGCTAGCGATTTTAAAAGAGAAATTATTCTCATTTTGAGAAAAGGAGTTAAAAGATGACTGAGAAAAAAGAAAAA [ 1326]
cp32-4_PAbe_NX_MP_FG_consensus AGGAGCTAGCGATTTTAAAAGAGAAATTATTCTCATTTTGAGAAAAGGAGTTAAAAGATGACTGAGAAAAAAGAAAAA [ 1326]
cp32-4_PAbe_NX_Pl_consensus    AGGAGCTAGCGATTTTAAAAGAGAAATTATTCTCATTTTGAGAAAAGGAGTTAAAAGATGACTGAGAAAAAAGAAAAA [ 1326]

cp32-4_gi|11497149|B31_GB      GAAGACCTGCAAGCACAAGATAAAGAAGAGCAGCAAAATAAGGCTGATACTAAAGTTATAAGTGCGCAGGAATTTGAA [ 1404]
cp32-4_un10_B31_PacBio         GAAGACCTGCAAGCACAAGATAAAGAAGAGCAGCAAAATAAGGCTGATACTAAAGTTATAAGTGCGCAGGAATTTGAA [ 1404]
cp32-4_B31_TS_MP_FG_consensus  GAAGACCTGCAAGCACAAGATAAAGAAGAGCAGCAAAATAAGGCTGATACTAAAGTTATAAGTGCGCAGGAATTTGAA [ 1404]
cp32-4_B31_NX_Pl_consensus     GAAGACCTGCAAGCACAAGATAAAGAAGAGCAGCAAAATAAGGCTGATACTAAAGTTATAAGTGCGCAGGAATTTGAA [ 1404]
cp32-4_PAli_NX_MP_FG_consensus GAAGACCTGCAAGCACAAGATAAAGAAGAGCAGCAAAATAAGGCTGATACTAAAGTTATAAGTGCGCAGGAATTTGAA [ 1404]
cp32-4_PAli_NX_Pl_consensus    GAAGACCTGCAAGCACAAGATAAAGAAGAGCAGCAAAATAAGGCTGATACTAAAGTTATAAGTGCGCAGGAATTTGAA [ 1404]
cp32-4_PAbe_NX_MP_FG_consensus GAAGACCTGCAAGCACAAGATAAAGAAGAGCAGCAAAATAAGGCTGATACTAAAGTTATAAGTGCGCAGGAATTTGAA [ 1404]
cp32-4_PAbe_NX_Pl_consensus    GAAGACCTGCAAGCACAAGATAAAGAAGAGCAGCAAAATAAGGCTGATACTAAAGTTATAAGTGCGCAGGAATTTGAA [ 1404]

cp32-4_gi|11497149|B31_GB      GAGTACATGCGCTTTAAAGAGCAAACAGACAATGTAAACCCCCTAAAGAGGTAAGGCGTGATGAGTATAAATGAGCGA [ 1482]
cp32-4_un10_B31_PacBio         GAGTACATGCGCTTTAAAGAGCAAACAGACAATGTAAACCCCCTAAAGAGGTAAGGCGTGATGAGTATAAATGAGCGA [ 1482]
cp32-4_B31_TS_MP_FG_consensus  GAGTACATGCGCTTTAAAGAGCAAACAGACAATGTAAACCCCCTAAAGAGGTAAGGCGTGATGAGTATAAATGAGCGA [ 1482]
cp32-4_B31_NX_Pl_consensus     GAGTACATGCGCTTTAAAGAGCAAACAGACAATGTAAACCCCCTAAAGAGGTAAGGCGTGATGAGTATAAATGAGCGA [ 1482]
cp32-4_PAli_NX_MP_FG_consensus GAGTACATGCGCTTTAAAGAGCAAACAGACAATGTAAACCCCCTAAAGAGGTAAGGCGTGATGAGTATAAATGAGCGA [ 1482]
cp32-4_PAli_NX_Pl_consensus    GAGTACATGCGCTTTAAAGAGCAAACAGACAATGTAAACCCCCTAAAGAGGTAAGGCGTGATGAGTATAAATGAGCGA [ 1482]
cp32-4_PAbe_NX_MP_FG_consensus GAGTACATGCGCTTTAAAGAGCAAACAGACAATGTAAACCCCCTAAAGAGGTAAGGCGTGATGAGTATAAATGAGCGA [ 1482]
cp32-4_PAbe_NX_Pl_consensus    GAGTACATGCGCTTTAAAGAGCAAACAGACAATGTAAACCCCCTAAAGAGGTAAGGCGTGATGAGTATAAATGAGCGA [ 1482]

cp32-4_gi|11497149|B31_GB      ATAACAAAAGAACTTGCAGAAGTTGAAGATCGAGAGCGTGTTGAAAAGCAATTGTTACTAGAGGCTGAGCGAATTAAT [ 1560]
cp32-4_un10_B31_PacBio         ATAACAAAAGAACTTGCAGAAGTTGAAGATCGAGAGCGTGTTGAAAAGCAATTGTTACTAGAGGCTGAGCGAATTAAT [ 1560]
cp32-4_B31_TS_MP_FG_consensus  ATAACAAAAGAACTTGCAGAAGTTGAAGATCGAGAGCGTGTTGAAAAGCAATTGTTACTAGAGGCTGAGCGAATTAAT [ 1560]
cp32-4_B31_NX_Pl_consensus     ATAACAAAAGAACTTGCAGAAGTTGAAGATCGAGAGCGTGTTGAAAAGCAATTGTTACTAGAGGCTGAGCGAATTAAT [ 1560]
cp32-4_PAli_NX_MP_FG_consensus ATAACAAAAGAACTTGCAGAAGTTGAAGATCGAGAGCGTGTTGAAAAGCAATTGTTACTAGAGGCTGAGCGAATTAAT [ 1560]
cp32-4_PAli_NX_Pl_consensus    ATAACAAAAGAACTTGCAGAAGTTGAAGATCGAGAGCGTGTTGAAAAGCAATTGTTACTAGAGGCTGAGCGAATTAAT [ 1560]
cp32-4_PAbe_NX_MP_FG_consensus ATAACAAAAGAACTTGCAGAAGTTGAAGATCGAGAGCGTGTTGAAAAGCAATTGTTACTAGAGGCTGAGCGAATTAAT [ 1560]
cp32-4_PAbe_NX_Pl_consensus    ATAACAAAAGAACTTGCAGAAGTTGAAGATCGAGAGCGTGTTGAAAAGCAATTGTTACTAGAGGCTGAGCGAATTAAT [ 1560]

cp32-4_gi|11497149|B31_GB      GAAATTGATACACTTGTAAAAGCGCATCTTAGCAACCATTTTAACAAGGAAGTGTTACTTGCAAAAGGATACACCCTA [ 1638]
cp32-4_un10_B31_PacBio         GAAATTGATACACTTGTAAAAGCGCATCTTAGCAACCATTTTAACAAGGAAGTGTTACTTGCAAAAGGATACACCCTA [ 1638]
cp32-4_B31_TS_MP_FG_consensus  GAAATTGATACACTTGTAAAAGCGCATCTTAGCAACCATTTTAACAAGGAAGTGTTACTTGCAAAAGGATACACCCTA [ 1638]
cp32-4_B31_NX_Pl_consensus     GAAATTGATACACTTGTAAAAGCGCATCTTAGCAACCATTTTAACAAGGAAGTGTTACTTGCAAAAGGATACACCCTA [ 1638]
cp32-4_PAli_NX_MP_FG_consensus GAAATTGATACACTTGTAAAAGCGCATCTTAGCAACCATTTTAACAAGGAAGTGTTACTTGCAAAAGGATACACCCTA [ 1638]
cp32-4_PAli_NX_Pl_consensus    GAAATTGATACACTTGTAAAAGCGCATCTTAGCAACCATTTTAACAAGGAAGTGTTACTTGCAAAAGGATACACCCTA [ 1638]
cp32-4_PAbe_NX_MP_FG_consensus GAAATTGATACACTTGTAAAAGCGCATCTTAGCAACCATTTTAACAAGGAAGTGTTACTTGCAAAAGGATACACCCTA [ 1638]
cp32-4_PAbe_NX_Pl_consensus    GAAATTGATACACTTGTAAAAGCGCATCTTAGCAACCATTTTAACAAGGAAGTGTTACTTGCAAAAGGATACACCCTA [ 1638]

cp32-4_gi|11497149|B31_GB      AAAGACATTATGCAGGCACAACGTAGAGAGCTTGTTCGTAAGTTTGTTCCAATTGAGCAAATTAAAGCTATTGCTAAA [ 1716]
cp32-4_un10_B31_PacBio         AAAGACATTATGCAGGCACAACGTAGAGAGCTTGTTCGTAAGTTTGTTCCAATTGAGCAAATTAAAGCTATTGCTAAA [ 1716]
cp32-4_B31_TS_MP_FG_consensus  AAAGACATTATGCAGGCACAACGTAGAGAGCTTGTTCGTAAGTTTGTTCCAATTGAGCAAATTAAAGCTATTGCTAAA [ 1716]
cp32-4_B31_NX_Pl_consensus     AAAGACATTATGCAGGCACAACGTAGAGAGCTTGTTCGTAAGTTTGTTCCAATTGAGCAAATTAAAGCTATTGCTAAA [ 1716]
cp32-4_PAli_NX_MP_FG_consensus AAAGACATTATGCAGGCACAACGTAGAGAGCTTGTTCGTAAGTTTGTTCCAATTGAGCAAATTAAAGCTATTGCTAAA [ 1716]
cp32-4_PAli_NX_Pl_consensus    AAAGACATTATGCAGGCACAACGTAGAGAGCTTGTTCGTAAGTTTGTTCCAATTGAGCAAATTAAAGCTATTGCTAAA [ 1716]
cp32-4_PAbe_NX_MP_FG_consensus AAAGACATTATGCAGGCACAACGTAGAGAGCTTGTTCGTAAGTTTGTTCCAATTGAGCAAATTAAAGCTATTGCTAAA [ 1716]
cp32-4_PAbe_NX_Pl_consensus    AAAGACATTATGCAGGCACAACGTAGAGAGCTTGTTCGTAAGTTTGTTCCAATTGAGCAAATTAAAGCTATTGCTAAA [ 1716]

cp32-4_gi|11497149|B31_GB      GCGTCAGATATAAGTCATATCGATGGAGAGATACTAGAACAACTTGTTTCTTTAGCAAAAGTGAATATTAAATTAAGA [ 1794]
cp32-4_un10_B31_PacBio         GCGTCAGATATAAGTCATATCGATGGAGAGATACTAGAACAACTTGTTTCTTTAGCAAAAGTGAATATTAAATTAAGA [ 1794]
cp32-4_B31_TS_MP_FG_consensus  GCGTCAGATATAAGTCATATCGATGGAGAGATACTAGAACAACTTGTTTCTTTAGCAAAAGTGAATATTAAATTAAGA [ 1794]
cp32-4_B31_NX_Pl_consensus     GCGTCAGATATAAGTCATATCGATGGAGAGATACTAGAACAACTTGTTTCTTTAGCAAAAGTGAATATTAAATTAAGA [ 1794]
cp32-4_PAli_NX_MP_FG_consensus GCGTCAGATATAAGTCATATCGATGGAGAGATACTAGAACAACTTGTTTCTTTAGCAAAAGTGAATATTAAATTAAGA [ 1794]
cp32-4_PAli_NX_Pl_consensus    GCGTCAGATATAAGTCATATCGATGGAGAGATACTAGAACAACTTGTTTCTTTAGCAAAAGTGAATATTAAATTAAGA [ 1794]
cp32-4_PAbe_NX_MP_FG_consensus GCGTCAGATATAAGTCATATCGATGGAGAGATACTAGAACAACTTGTTTCTTTAGCAAAAGTGAATATTAAATTAAGA [ 1794]
cp32-4_PAbe_NX_Pl_consensus    GCGTCAGATATAAGTCATATCGATGGAGAGATACTAGAACAACTTGTTTCTTTAGCAAAAGTGAATATTAAATTAAGA [ 1794]

cp32-4_gi|11497149|B31_GB      AAAACTGGGAGTAGCAATTCTTCTTCTGTTGACTCTGTCAGAGGCAATATTGTTGTTAAATCAGAAGAGAGAGCAAGT [ 1872]
cp32-4_un10_B31_PacBio         AAAACTGGGAGTAGCAATTCTTCTTCTGTTGACTCTGTCAGAGGCAATATTGTTGTTAAATCAGAAGAGAGAGCAAGT [ 1872]
cp32-4_B31_TS_MP_FG_consensus  AAAACTGGGAGTAGCAATTCTTCTTCTGTTGACTCTGTCAGAGGCAATATTGTTGTTAAATCAGAAGAGAGAGCAAGT [ 1872]
```

```
cp32-4_B31_NX_Pl_consensus     AAAACTGGGAGTAGCAATTCTTCTTCTGTTGACTCTGTCAGAGGCAATATTGTTGTTAAATCAGAAGAGAGAGCAAGT [ 1872]
cp32-4_PAli_NX_MP_FG_consensus AAAACTGGGAGTAGCAATTCTTCTTCTGTTGACTCTGTCAGAGGCAATATTGTTGTTAAATCAGAAGAGAGAGCAAGT [ 1872]
cp32-4_PAli_NX_Pl_consensus    AAAACTGGGAGTAGCAATTCTTCTTCTGTTGACTCTGTCAGAGGCAATATTGTTGTTAAATCAGAAGAGAGAGCAAGT [ 1872]
cp32-4_PAbe_NX_MP_FG_consensus AAAACTGGGAGTAGCAATTCTTCTTCTGTTGACTCTGTCAGAGGCAATATTGTTGTTAAATCAGAAGAGAGAGCAAGT [ 1872]
cp32-4_PAbe_NX_Pl_consensus    AAAACTGGGAGTAGCAATTCTTCTTCTGTTGACTCTGTCAGAGGCAATATTGTTGTTAAATCAGAAGAGAGAGCAAGT [ 1872]

cp32-4_gi|11497149|B31_GB      TTGCTTGATTCTAATTTTGTGCCCATTAATTTCACGGAGTTTGTACAAGCGATAAGTAATACTTACAAGCAAAGACGA [ 1950]
cp32-4_un10_B31_PacBio         TTGCTTGATTCTAATTTTGTGCCCATTAATTTCACGGAGTTTGTACAAGCGATAAGTAATACTTACAAGCAAAGACGA [ 1950]
cp32-4_B31_TS_MP_FG_consensus  TTGCTTGATTCTAATTTTGTGCCCATTAATTTCACGGAGTTTGTACAAGCGATAAGTAATACTTACAAGCAAAGACGA [ 1950]
cp32-4_B31_NX_Pl_consensus     TTGCTTGATTCTAATTTTGTGCCCATTAATTTCACGGAGTTTGTACAAGCGATAAGTAATACTTACAAGCAAAGACGA [ 1950]
cp32-4_PAli_NX_MP_FG_consensus TTGCTTGATTCTAATTTTGTGCCCATTAATTTCACGGAGTTTGTACAAGCGATAAGTAATACTTACAAGCAAAGACGA [ 1950]
cp32-4_PAli_NX_Pl_consensus    TTGCTTGATTCTAATTTTGTGCCCATTAATTTCACGGAGTTTGTACAAGCGATAAGTAATACTTACAAGCAAAGACGA [ 1950]
cp32-4_PAbe_NX_MP_FG_consensus TTGCTTGATTCTAATTTTGTGCCCATTAATTTCACGGAGTTTGTACAAGCGATAAGTAATACTTACAAGCAAAGACGA [ 1950]
cp32-4_PAbe_NX_Pl_consensus    TTGCTTGATTCTAATTTTGTGCCCATTAATTTCACGGAGTTTGTACAAGCGATAAGTAATACTTACAAGCAAAGACGA [ 1950]

cp32-4_gi|11497149|B31_GB      GCTCAATTTTATGAGAATCTAAAAAGACATAAAAGAACAAGTATTGCTTAAAGGAGTTTTTAATGAGCGATGGTATTA [ 2028]
cp32-4_un10_B31_PacBio         GCTCAATTTTATGAGAATCTAAAAAGACATAAAAGAACAAGTATTGCTTAAAGGAGTTTTTAATGAGCGATGGTATTA [ 2028]
cp32-4_B31_TS_MP_FG_consensus  GCTCAATTTTATGAGAATCTAAAAAGACATAAAAGAACAAGTATTGCTTAAAGGAGTTTTTAATGAGCGATGGTATTA [ 2028]
cp32-4_B31_NX_Pl_consensus     GCTCAATTTTATGAGAATCTAAAAAGACATAAAAGAACAAGTATTGCTTAAAGGAGTTTTTAATGAGCGATGGTATTA [ 2028]
cp32-4_PAli_NX_MP_FG_consensus GCTCAATTTTATGAGAATCTAAAAAGACATAAAAGAACAAGTATTGCTTAAAGGAGTTTTTAATGAGCGATGGTATTA [ 2028]
cp32-4_PAli_NX_Pl_consensus    GCTCAATTTTATGAGAATCTAAAAAGACATAAAAGAACAAGTATTGCTTAAAGGAGTTTTTAATGAGCGATGGTATTA [ 2028]
cp32-4_PAbe_NX_MP_FG_consensus GCTCAATTTTATGAGAATCTAAAAAGACATAAAAGAACAAGTATTGCTTAAAGGAGTTTTTAATGAGCGATGGTATTA [ 2028]
cp32-4_PAbe_NX_Pl_consensus    GCTCAATTTTATGAGAATCTAAAAAGACATAAAAGAACAAGTATTGCTTAAAGGAGTTTTTAATGAGCGATGGTATTA [ 2028]

cp32-4_gi|11497149|B31_GB      CAAAAATAAAAGAAGATTTTGATAAAAAAGTTGCAGAAATTAAAGCATTAATGAAAAATCCTCAGCAAGATGCTGGTT [ 2106]
cp32-4_un10_B31_PacBio         CAAAAATAAAAGAAGATTTTGATAAAAAAGTTGCAGAAATTAAAGCATTAATGAAAAATCCTCAGCAAGATGCTGGTT [ 2106]
cp32-4_B31_TS_MP_FG_consensus  CAAAAATAAAAGAAGATTTTGATAAAAAAGTTGCAGAAATTAAAGCATTAATGAAAAATCCTCAGCAAGATGCTGGTT [ 2106]
cp32-4_B31_NX_Pl_consensus     CAAAAATAAAAGAAGATTTTGATAAAAAAGTTGCAGAAATTAAAGCATTAATGAAAAATCCTCAGCAAGATGCTGGTT [ 2106]
cp32-4_PAli_NX_MP_FG_consensus CAAAAATAAAAGAAGATTTTGATAAAAAAGTTGCAGAAATTAAAGCATTAATGAAAAATCCTCAGCAAGATGCTGGTT [ 2106]
cp32-4_PAli_NX_Pl_consensus    CAAAAATAAAAGAAGATTTTGATAAAAAAGTTGCAGAAATTAAAGCATTAATGAAAAATCCTCAGCAAGATGCTGGTT [ 2106]
cp32-4_PAbe_NX_MP_FG_consensus CAAAAATAAAAGAAGATTTTGATAAAAAAGTTGCAGAAATTAAAGCATTAATGAAAAATCCTCAGCAAGATGCTGGTT [ 2106]
cp32-4_PAbe_NX_Pl_consensus    CAAAAATAAAAGAAGATTTTGATAAAAAAGTTGCAGAAATTAAAGCATTAATGAAAAATCCTCAGCAAGATGCTGGTT [ 2106]

cp32-4_gi|11497149|B31_GB      TGCTTAGTAATTCTGTAGATTTTAGAGATAAAAATCTAATTTACTCCAATTCGGATGGAGTTTTTACTAGTAGTAAAG [ 2184]
cp32-4_un10_B31_PacBio         TGCTTAGTAATTCTGTAGATTTTAGAGATAAAAATCTAATTTACTCCAATTCGGATGGAGTTTTTACTAGTAGTAAAG [ 2184]
cp32-4_B31_TS_MP_FG_consensus  TGCTTAGTAATTCTGTAGATTTTAGAGATAAAAATCTAATTTACTCCAATTCGGATGGAGTTTTTACTAGTAGTAAAG [ 2184]
cp32-4_B31_NX_Pl_consensus     TGCTTAGTAATTCTGTAGATTTTAGAGATAAAAATCTAATTTACTCCAATTCGGATGGAGTTTTTACTAGTAGTAAAG [ 2184]
cp32-4_PAli_NX_MP_FG_consensus TGCTTAGTAATTCTGTAGATTTTAGAGATAAAAATCTAATTTACTCCAATTCGGATGGAGTTTTTACTAGTAGTAAAG [ 2184]
cp32-4_PAli_NX_Pl_consensus    TGCTTAGTAATTCTGTAGATTTTAGAGATAAAAATCTAATTTACTCCAATTCGGATGGAGTTTTTACTAGTAGTAAAG [ 2184]
cp32-4_PAbe_NX_MP_FG_consensus TGCTTAGTAATTCTGTAGATTTTAGAGATAAAAATCTAATTTACTCCAATTCGGATGGAGTTTTTACTAGTAGTAAAG [ 2184]
cp32-4_PAbe_NX_Pl_consensus    TGCTTAGTAATTCTGTAGATTTTAGAGATAAAAATCTAATTTACTCCAATTCGGATGGAGTTTTTACTAGTAGTAAAG [ 2184]

cp32-4_gi|11497149|B31_GB      ATAAAATAGAAAATTATCCTGCTAAAGGGTATCCATACAAGCGTGGAGTCAAGCTTAGTTTTAGTGCAGATAGTACTA [ 2262]
cp32-4_un10_B31_PacBio         ATAAAATAGAAAATTATCCTGCTAAAGGGTATCCATACAAGCGTGGAGTCAAGCTTAGTTTTAGTGCAGATAGTACTA [ 2262]
cp32-4_B31_TS_MP_FG_consensus  ATAAAATAGAAAATTATCCTGCTAAAGGGTATCCATACAAGCGTGGAGTCAAGCTTAGTTTTAGTGCAGATAGTACTA [ 2262]
cp32-4_B31_NX_Pl_consensus     ATAAAATAGAAAATTATCCTGCTAAAGGGTATCCATACAAGCGTGGAGTCAAGCTTAGTTTTAGTGCAGATAGTACTA [ 2262]
cp32-4_PAli_NX_MP_FG_consensus ATAAAATAGAAAATTATCCTGCTAAAGGGTATCCATACAAGCGTGGAGTCAAGCTTAGTTTTAGTGCAGATAGTACTA [ 2262]
cp32-4_PAli_NX_Pl_consensus    ATAAAATAGAAAATTATCCTGCTAAAGGGTATCCATACAAGCGTGGAGTCAAGCTTAGTTTTAGTGCAGATAGTACTA [ 2262]
cp32-4_PAbe_NX_MP_FG_consensus ATAAAATAGAAAATTATCCTGCTAAAGGGTATCCATACAAGCGTGGAGTCAAGCTTAGTTTTAGTGCAGATAGTACTA [ 2262]
cp32-4_PAbe_NX_Pl_consensus    ATAAAATAGAAAATTATCCTGCTAAAGGGTATCCATACAAGCGTGGAGTCAAGCTTAGTTTTAGTGCAGATAGTACTA [ 2262]

cp32-4_gi|11497149|B31_GB      CAGAGCTAGAAGTTGAGGCTGGTGGTGGGGATGACTTGTACGGAATATGCACTGATATAGATGAGTTTACTGGCATGG [ 2340]
cp32-4_un10_B31_PacBio         CAGAGCTAGAAGTTGAGGCTGGTGGTGGGGATGACTTGTACGGAATATGCACTGATATAGATGAGTTTACTGGCATGG [ 2340]
cp32-4_B31_TS_MP_FG_consensus  CAGAGCTAGAAGTTGAGGCTGGTGGTGGGGATGACTTGTACGGAATATGCACTGATATAGATGAGTTTACTGGCATGG [ 2340]
cp32-4_B31_NX_Pl_consensus     CAGAGCTAGAAGTTGAGGCTGGTGGTGGGGATGACTTGTACGGAATATGCACTGATATAGATGAGTTTACTGGCATGG [ 2340]
cp32-4_PAli_NX_MP_FG_consensus CAGAGCTAGAAGTTGAGGCTGGTGGTGGGGATGACTTGTACGGAATATGCACTGATATAGATGAGTTTACTGGCATGG [ 2340]
cp32-4_PAli_NX_Pl_consensus    CAGAGCTAGAAGTTGAGGCTGGTGGTGGGGATGACTTGTACGGAATATGCACTGATATAGATGAGTTTACTGGCATGG [ 2340]
cp32-4_PAbe_NX_MP_FG_consensus CAGAGCTAGAAGTTGAGGCTGGTGGTGGGGATGACTTGTACGGAATATGCACTGATATAGATGAGTTTACTGGCATGG [ 2340]
cp32-4_PAbe_NX_Pl_consensus    CAGAGCTAGAAGTTGAGGCTGGTGGTGGGGATGACTTGTACGGAATATGCACTGATATAGATGAGTTTACTGGCATGG [ 2340]

cp32-4_gi|11497149|B31_GB      CAACTGTAGTTCCAATTACAAATAACTTCACGGGGTATTTAACATTTAAGAAAAATGGAAATGGTGTAAACCCAGGTG [ 2418]
cp32-4_un10_B31_PacBio         CAACTGTAGTTCCAATTACAAATAACTTCACGGGGTATTTAACATTTAAGAAAAATGGAAATGGTGTAAACCCAGGTG [ 2418]
cp32-4_B31_TS_MP_FG_consensus  CAACTGTAGTTCCAATTACAAATAACTTCACGGGGTATTTAACATTTAAGAAAAATGGAAATGGTGTAAACCCAGGTG [ 2418]
cp32-4_B31_NX_Pl_consensus     CAACTGTAGTTCCAATTACAAATAACTTCACGGGGTATTTAACATTTAAGAAAAATGGAAATGGTGTAAACCCAGGTG [ 2418]
cp32-4_PAli_NX_MP_FG_consensus CAACTGTAGTTCCAATTACAAATAACTTCACGGGGTATTTAACATTTAAGAAAAATGGAAATGGTGTAAACCCAGGTG [ 2418]
cp32-4_PAli_NX_Pl_consensus    CAACTGTAGTTCCAATTACAAATAACTTCACGGGGTATTTAACATTTAAGAAAAATGGAAATGGTGTAAACCCAGGTG [ 2418]
cp32-4_PAbe_NX_MP_FG_consensus CAACTGTAGTTCCAATTACAAATAACTTCACGGGGTATTTAACATTTAAGAAAAATGGAAATGGTGTAAACCCAGGTG [ 2418]
cp32-4_PAbe_NX_Pl_consensus    CAACTGTAGTTCCAATTACAAATAACTTCACGGGGTATTTAACATTTAAGAAAAATGGAAATGGTGTAAACCCAGGTG [ 2418]

cp32-4_gi|11497149|B31_GB      ATAAGCTGCATTTTAATGCACAAGGAGAGCTTGAAAAGAATGGGGGAAATGATAAATCTGTTAATGCTATAGCACTTT [ 2496]
cp32-4_un10_B31_PacBio         ATAAGCTGCATTTTAATGCACAAGGAGAGCTTGAAAAGAATGGGGGAAATGATAAATCTGTTAATGCTATAGCACTTT [ 2496]
cp32-4_B31_TS_MP_FG_consensus  ATAAGCTGCATTTTAATGCACAAGGAGAGCTTGAAAAGAATGGGGGAAATGATAAATCTGTTAATGCTATAGCACTTT [ 2496]
cp32-4_B31_NX_Pl_consensus     ATAAGCTGCATTTTAATGCACAAGGAGAGCTTGAAAAGAATGGGGGAAATGATAAATCTGTTAATGCTATAGCACTTT [ 2496]
cp32-4_PAli_NX_MP_FG_consensus ATAAGCTGCATTTTAATGCACAAGGAGAGCTTGAAAAGAATGGGGGAAATGATAAATCTGTTAATGCTATAGCACTTT [ 2496]
cp32-4_PAli_NX_Pl_consensus    ATAAGCTGCATTTTAATGCACAAGGAGAGCTTGAAAAGAATGGGGGAAATGATAAATCTGTTAATGCTATAGCACTTT [ 2496]
cp32-4_PAbe_NX_MP_FG_consensus ATAAGCTGCATTTTAATGCACAAGGAGAGCTTGAAAAGAATGGGGGAAATGATAAATCTGTTAATGCTATAGCACTTT [ 2496]
cp32-4_PAbe_NX_Pl_consensus    ATAAGCTGCATTTTAATGCACAAGGAGAGCTTGAAAAGAATGGGGGAAATGATAAATCTGTTAATGCTATAGCACTTT [ 2496]

cp32-4_gi|11497149|B31_GB      CAAAAGTACATAAATTAACCGAAGAGTTATCTATAGTGCTTGCTAGTGTTTTTGGGAATAGAGCTTTAAAAGGTAATT [ 2574]
cp32-4_un10_B31_PacBio         CAAAAGTACATAAATTAACCGAAGAGTTATCTATAGTGCTTGCTAGTGTTTTTGGGAATAGAGCTTTAAAAGGTAATT [ 2574]
cp32-4_B31_TS_MP_FG_consensus  CAAAAGTACATAAATTAACCGAAGAGTTATCTATAGTGCTTGCTAGTGTTTTTGGGAATAGAGCTTTAAAAGGTAATT [ 2574]
cp32-4_B31_NX_Pl_consensus     CAAAAGTACATAAATTAACCGAAGAGTTATCTATAGTGCTTGCTAGTGTTTTTGGGAATAGAGCTTTAAAAGGTAATT [ 2574]
cp32-4_PAli_NX_MP_FG_consensus CAAAAGTACATAAATTAACCGAAGAGTTATCTATAGTGCTTGCTAGTGTTTTTGGGAATAGAGCTTTAAAAGGTAATT [ 2574]
cp32-4_PAli_NX_Pl_consensus    CAAAAGTACATAAATTAACCGAAGAGTTATCTATAGTGCTTGCTAGTGTTTTTGGGAATAGAGCTTTAAAAGGTAATT [ 2574]
cp32-4_PAbe_NX_MP_FG_consensus CAAAAGTACATAAATTAACCGAAGAGTTATCTATAGTGCTTGCTAGTGTTTTTGGGAATAGAGCTTTAAAAGGTAATT [ 2574]
cp32-4_PAbe_NX_Pl_consensus    CAAAAGTACATAAATTAACCGAAGAGTTATCTATAGTGCTTGCTAGTGTTTTTGGGAATAGAGCTTTAAAAGGTAATT [ 2574]

cp32-4_gi|11497149|B31_GB      AAATTATGGCTTTAAAAGGCAAAGGGCAAGCTAAATCTCCTAATGTTGATGATAATCCACAATTAGGGTTAGAATCAG [ 2652]
cp32-4_un10_B31_PacBio         AAATTATGGCTTTAAAAGGCAAAGGGCAAGCTAAATCTCCTAATGTTGATGATAATCCACAATTAGGGTTAGAATCAG [ 2652]
cp32-4_B31_TS_MP_FG_consensus  AAATTATGGCTTTAAAAGGCAAAGGGCAAGCTAAATCTCCTAATGTTGATGATAATCCACAATTAGGGTTAGAATCAG [ 2652]
cp32-4_B31_NX_Pl_consensus     AAATTATGGCTTTAAAAGGCAAAGGGCAAGCTAAATCTCCTAATGTTGATGATAATCCACAATTAGGGTTAGAATCAG [ 2652]
cp32-4_PAli_NX_MP_FG_consensus AAATTATGGCTTTAAAAGGCAAAGGGCAAGCTAAATCTCCTAATGTTGATGATAATCCACAATTAGGGTTAGAATCAG [ 2652]
cp32-4_PAli_NX_Pl_consensus    AAATTATGGCTTTAAAAGGCAAAGGGCAAGCTAAATCTCCTAATGTTGATGATAATCCACAATTAGGGTTAGAATCAG [ 2652]
cp32-4_PAbe_NX_MP_FG_consensus AAATTATGGCTTTAAAAGGCAAAGGGCAAGCTAAATCTCCTAATGTTGATGATAATCCACAATTAGGGTTAGAATCAG [ 2652]
cp32-4_PAbe_NX_Pl_consensus    AAATTATGGCTTTAAAAGGCAAAGGGCAAGCTAAATCTCCTAATGTTGATGATAATCCACAATTAGGGTTAGAATCAG [ 2652]

cp32-4_gi|11497149|B31_GB      AAATTCCAGTTGCTCCTAGATCTAAACGTCAAACAAGACAGGCTGAAGAAGTACAAGCAAAAGATCCTTATTTAGATT [ 2730]
cp32-4_un10_B31_PacBio         AAATTCCAGTTGCTCCTAGATCTAAACGTCAAACAAGACAGGCTGAAGAAGTACAAGCAAAAGATCCTTATTTAGATT [ 2730]
cp32-4_B31_TS_MP_FG_consensus  AAATTCCAGTTGCTCCTAGATCTAAACGTCAAACAAGACAGGCTGAAGAAGTACAAGCAAAAGATCCTTATTTAGATT [ 2730]
cp32-4_B31_NX_Pl_consensus     AAATTCCAGTTGCTCCTAGATCTAAACGTCAAACAAGACAGGCTGAAGAAGTACAAGCAAAAGATCCTTATTTAGATT [ 2730]
cp32-4_PAli_NX_MP_FG_consensus AAATTCCAGTTGCTCCTAGATCTAAACGTCAAACAAGACAGGCTGAAGAAGTACAAGCAAAAGATCCTTATTTAGATT [ 2730]
cp32-4_PAli_NX_Pl_consensus    AAATTCCAGTTGCTCCTAGATCTAAACGTCAAACAAGACAGGCTGAAGAAGTACAAGCAAAAGATCCTTATTTAGATT [ 2730]
cp32-4_PAbe_NX_MP_FG_consensus AAATTCCAGTTGCTCCTAGATCTAAACGTCAAACAAGACAGGCTGAAGAAGTACAAGCAAAAGATCCTTATTTAGATT [ 2730]
cp32-4_PAbe_NX_Pl_consensus    AAATTCCAGTTGCTCCTAGATCTAAACGTCAAACAAGACAGGCTGAAGAAGTACAAGCAAAAGATCCTTATTTAGATT [ 2730]

cp32-4_gi|11497149|B31_GB      CGGTTAAAGAACTTGACGATGTTCTTTTAAAATTTAAAAAATATTCAAAATCAATGAGTTCGATTGAAAATAAGGTTT [ 2808]
cp32-4_un10_B31_PacBio         CGGTTAAAGAACTTGACGATGTTCTTTTAAAATTTAAAAAATATTCAAAATCAATGAGTTCGATTGAAAATAAGGTTT [ 2808]
cp32-4_B31_TS_MP_FG_consensus  CGGTTAAAGAACTTGACGATGTTCTTTTAAAATTTAAAAAATATTCAAAATCAATGAGTTCGATTGAAAATAAGGTTT [ 2808]
cp32-4_B31_NX_Pl_consensus     CGGTTAAAGAACTTGACGATGTTCTTTTAAAATTTAAAAAATATTCAAAATCAATGAGTTCGATTGAAAATAAGGTTT [ 2808]
```

```
cp32-4_PAli_NX_MP_FG_consensus CGGTTAAAGAACTTGACGATGTTCTTTTAAAATTTAAAAAATATTCAAAATCAATGAGTTCGATTGAAAATAAGGTTT [ 2808]
cp32-4_PAli_NX_Pl_consensus    CGGTTAAAGAACTTGACGATGTTCTTTTAAAATTTAAAAAATATTCAAAATCAATGAGTTCGATTGAAAATAAGGTTT [ 2808]
cp32-4_PAbe_NX_MP_FG_consensus CGGTTAAAGAACTTGACGATGTTCTTTTAAAATTTAAAAAATATTCAAAATCAATGAGTTCGATTGAAAATAAGGTTT [ 2808]
cp32-4_PAbe_NX_Pl_consensus    CGGTTAAAGAACTTGACGATGTTCTTTTAAAATTTAAAAAATATTCAAAATCAATGAGTTCGATTGAAAATAAGGTTT [ 2808]

cp32-4_gi|11497149|B31_GB      TTAGTAGTTCGGGCGGTTGTTTTAAATCAAAGAATGAGCGAGTTAATGCTTATTCTTTTACATGTTCAAGCTTTGCAG [ 2886]
cp32-4_un10_B31_PacBio         TTAGTAGTTCGGGCGGTTGTTTTAAATCAAAGAATGAGCGAGTTAATGCTTATTCTTTTACATGTTCAAGCTTTGCAG [ 2886]
cp32-4_B31_TS_MP_FG_consensus  TTAGTAGTTCGGGCGGTTGTTTTAAATCAAAGAATGAGCGAGTTAATGCTTATTCTTTTACATGTTCAAGCTTTGCAG [ 2886]
cp32-4_B31_NX_Pl_consensus     TTAGTAGTTCGGGCGGTTGTTTTAAATCAAAGAATGAGCGAGTTAATGCTTATTCTTTTACATGTTCAAGCTTTGCAG [ 2886]
cp32-4_PAli_NX_MP_FG_consensus TTAGTAGTTCGGGCGGTTGTTTTAAATCAAAGAATGAGCGAGTTAATGCTTATTCTTTTACATGTTCAAGCTTTGCAG [ 2886]
cp32-4_PAli_NX_Pl_consensus    TTAGTAGTTCGGGCGGTTGTTTTAAATCAAAGAATGAGCGAGTTAATGCTTATTCTTTTACATGTTCAAGCTTTGCAG [ 2886]
cp32-4_PAbe_NX_MP_FG_consensus TTAGTAGTTCGGGCGGTTGTTTTAAATCAAAGAATGAGCGAGTTAATGCTTATTCTTTTACATGTTCAAGCTTTGCAG [ 2886]
cp32-4_PAbe_NX_Pl_consensus    TTAGTAGTTCGGGCGGTTGTTTTAAATCAAAGAATGAGCGAGTTAATGCTTATTCTTTTACATGTTCAAGCTTTGCAG [ 2886]

cp32-4_gi|11497149|B31_GB      ACAAAATAGAAGAATATTCTTACGACCCAGCAAATAGTTTTCCATATAAGCGTGGGGTTAAACTTGTTCTAAAAGAGA [ 2964]
cp32-4_un10_B31_PacBio         ACAAAATAGAAGAATATTCTTACGACCCAGCAAATAGTTTTCCATATAAGCGTGGGGTTAAACTTGTTCTAAAAGAGA [ 2964]
cp32-4_B31_TS_MP_FG_consensus  ACAAAATAGAAGAATATTCTTACGACCCAGCAAATAGTTTTCCATATAAGCGTGGGGTTAAACTTGTTCTAAAAGAGA [ 2964]
cp32-4_B31_NX_Pl_consensus     ACAAAATAGAAGAATATTCTTACGACCCAGCAAATAGTTTTCCATATAAGCGTGGGGTTAAACTTGTTCTAAAAGAGA [ 2964]
cp32-4_PAli_NX_MP_FG_consensus ACAAAATAGAAGAATATTCTTACGACCCAGCAAATAGTTTTCCATATAAGCGTGGGGTTAAACTTGTTCTAAAAGAGA [ 2964]
cp32-4_PAli_NX_Pl_consensus    ACAAAATAGAAGAATATTCTTACGACCCAGCAAATAGTTTTCCATATAAGCGTGGGGTTAAACTTGTTCTAAAAGAGA [ 2964]
cp32-4_PAbe_NX_MP_FG_consensus ACAAAATAGAAGAATATTCTTACGACCCAGCAAATAGTTTTCCATATAAGCGTGGGGTTAAACTTGTTCTAAAAGAGA [ 2964]
cp32-4_PAbe_NX_Pl_consensus    ACAAAATAGAAGAATATTCTTACGACCCAGCAAATAGTTTTCCATATAAGCGTGGGGTTAAACTTGTTCTAAAAGAGA [ 2964]

cp32-4_gi|11497149|B31_GB      GCTCTATATATGTTGAAGTTGGTGCTGATACTGATATGTATGGGATATGTGTAGATGTATGTGAGTTTAGTAGTACTG [ 3042]
cp32-4_un10_B31_PacBio         GCTCTATATATGTTGAAGTTGGTGCTGATACTGATATGTATGGGATATGTGTAGATGTATGTGAGTTTAGTAGTACTG [ 3042]
cp32-4_B31_TS_MP_FG_consensus  GCTCTATATATGTTGAAGTTGGTGCTGATACTGATATGTATGGGATATGTGTAGATGTATGTGAGTTTAGTAGTACTG [ 3042]
cp32-4_B31_NX_Pl_consensus     GCTCTATATATGTTGAAGTTGGTGCTGATACTGATATGTATGGGATATGTGTAGATGTATGTGAGTTTAGTAGTACTG [ 3042]
cp32-4_PAli_NX_MP_FG_consensus GCTCTATATATGTTGAAGTTGGTGCTGATACTGATATGTATGGGATATGTGTAGATGTATGTGAGTTTAGTAGTACTG [ 3042]
cp32-4_PAli_NX_Pl_consensus    GCTCTATATATGTTGAAGTTGGTGCTGATACTGATATGTATGGGATATGTGTAGATGTATGTGAGTTTAGTAGTACTG [ 3042]
cp32-4_PAbe_NX_MP_FG_consensus GCTCTATATATGTTGAAGTTGGTGCTGATACTGATATGTATGGGATATGTGTAGATGTATGTGAGTTTAGTAGTACTG [ 3042]
cp32-4_PAbe_NX_Pl_consensus    GCTCTATATATGTTGAAGTTGGTGCTGATACTGATATGTATGGGATATGTGTAGATGTATGTGAGTTTAGTAGTACTG [ 3042]

cp32-4_gi|11497149|B31_GB      CGTATGTATTGCCAATTACGAATAACTTTGAAGGGTATCTTGTTACAAGAAATCCGAGTATAAAAATGGGAGAAATAT [ 3120]
cp32-4_un10_B31_PacBio         CGTATGTATTGCCAATTACGAATAACTTTGAAGGGTATCTTGTTACAAGAAATCCGAGTATAAAAATGGGAGAAATAT [ 3120]
cp32-4_B31_TS_MP_FG_consensus  CGTATGTATTGCCAATTACGAATAACTTTGAAGGGTATCTTGTTACAAGAAATCCGAGTATAAAAATGGGAGAAATAT [ 3120]
cp32-4_B31_NX_Pl_consensus     CGTATGTATTGCCAATTACGAATAACTTTGAAGGGTATCTTGTTACAAGAAATCCGAGTATAAAAATGGGAGAAATAT [ 3120]
cp32-4_PAli_NX_MP_FG_consensus CGTATGTATTGCCAATTACGAATAACTTTGAAGGGTATCTTGTTACAAGAAATCCGAGTATAAAAATGGGAGAAATAT [ 3120]
cp32-4_PAli_NX_Pl_consensus    CGTATGTATTGCCAATTACGAATAACTTTGAAGGGTATCTTGTTACAAGAAATCCGAGTATAAAAATGGGAGAAATAT [ 3120]
cp32-4_PAbe_NX_MP_FG_consensus CGTATGTATTGCCAATTACGAATAACTTTGAAGGGTATCTTGTTACAAGAAATCCGAGTATAAAAATGGGAGAAATAT [ 3120]
cp32-4_PAbe_NX_Pl_consensus    CGTATGTATTGCCAATTACGAATAACTTTGAAGGGTATCTTGTTACAAGAAATCCGAGTATAAAAATGGGAGAAATAT [ 3120]

cp32-4_gi|11497149|B31_GB      TGGATATAAATAACAATGGGGTTATTATCAAGGCTGGTGGTGGGCCACCAACCGTAATTAATGCATATGCCCTCTCTG [ 3198]
cp32-4_un10_B31_PacBio         TGGATATAAATAACAATGGGGTTATTATCAAGGCTGGTGGTGGGCCACCAACCGTAATTAATGCATATGCCCTCTCTG [ 3198]
cp32-4_B31_TS_MP_FG_consensus  TGGATATAAATAACAATGGGGTTATTATCAAGGCTGGTGGTGGGCCACCAACCGTAATTAATGCATATGCCCTCTCTG [ 3198]
cp32-4_B31_NX_Pl_consensus     TGGATATAAATAACAATGGGGTTATTATCAAGGCTGGTGGTGGGCCACCAACCGTAATTAATGCATATGCCCTCTCTG [ 3198]
cp32-4_PAli_NX_MP_FG_consensus TGGATATAAATAACAATGGGGTTATTATCAAGGCTGGTGGTGGGCCACCAACCGTAATTAATGCATATGCCCTCTCTG [ 3198]
cp32-4_PAli_NX_Pl_consensus    TGGATATAAATAACAATGGGGTTATTATCAAGGCTGGTGGTGGGCCACCAACCGTAATTAATGCATATGCCCTCTCTG [ 3198]
cp32-4_PAbe_NX_MP_FG_consensus TGGATATAAATAACAATGGGGTTATTATCAAGGCTGGTGGTGGGCCACCAACCGTAATTAATGCATATGCCCTCTCTG [ 3198]
cp32-4_PAbe_NX_Pl_consensus    TGGATATAAATAACAATGGGGTTATTATCAAGGCTGGTGGTGGGCCACCAACCGTAATTAATGCATATGCCCTCTCTG [ 3198]

cp32-4_gi|11497149|B31_GB      ATTCATTTACAATCAATTTTGCACCCGAAGATGAACATCAAGATCAAGCTAGATATCCCAAACAAGAGTATTCTATTA [ 3276]
cp32-4_un10_B31_PacBio         ATTCATTTACAATCAATTTTGCACCCGAAGATGAACATCAAGATCAAGCTAGATATCCCAAACAAGAGTATTCTATTA [ 3276]
cp32-4_B31_TS_MP_FG_consensus  ATTCATTTACAATCAATTTTGCACCCGAAGATGAACATCAAGATCAAGCTAGATATCCCAAACAAGAGTATTCTATTA [ 3276]
cp32-4_B31_NX_Pl_consensus     ATTCATTTACAATCAATTTTGCACCCGAAGATGAACATCAAGATCAAGCTAGATATCCCAAACAAGAGTATTCTATTA [ 3276]
cp32-4_PAli_NX_MP_FG_consensus ATTCATTTACAATCAATTTTGCACCCGAAGATGAACATCAAGATCAAGCTAGATATCCCAAACAAGAGTATTCTATTA [ 3276]
cp32-4_PAli_NX_Pl_consensus    ATTCATTTACAATCAATTTTGCACCCGAAGATGAACATCAAGATCAAGCTAGATATCCCAAACAAGAGTATTCTATTA [ 3276]
cp32-4_PAbe_NX_MP_FG_consensus ATTCATTTACAATCAATTTTGCACCCGAAGATGAACATCAAGATCAAGCTAGATATCCCAAACAAGAGTATTCTATTA [ 3276]
cp32-4_PAbe_NX_Pl_consensus    ATTCATTTACAATCAATTTTGCACCCGAAGATGAACATCAAGATCAAGCTAGATATCCCAAACAAGAGTATTCTATTA [ 3276]

cp32-4_gi|11497149|B31_GB      ATTTGATAAAAGTTGCAATTTTTGGCAATAGAGGCCTTGAGAAAACAGTAACACCTGAAGCTGGTGGTTAAGCCTTGG [ 3354]
cp32-4_un10_B31_PacBio         ATTTGATAAAAGTTGCAATTTTTGGCAATAGAGGCCTTGAGAAAACAGTAACACCTGAAGCTGGTGGTTAAGCCTTGG [ 3354]
cp32-4_B31_TS_MP_FG_consensus  ATTTGATAAAAGTTGCAATTTTTGGCAATAGAGGCCTTGAGAAAACAGTAACACCTGAAGCTGGTGGTTAAGCCTTGG [ 3354]
cp32-4_B31_NX_Pl_consensus     ATTTGATAAAAGTTGCAATTTTTGGCAATAGAGGCCTTGAGAAAACAGTAACACCTGAAGCTGGTGGTTAAGCCTTGG [ 3354]
cp32-4_PAli_NX_MP_FG_consensus ATTTGATAAAAGTTGCAATTTTTGGCAATAGAGGCCTTGAGAAAACAGTAACACCTGAAGCTGGTGGTTAAGCCTTGG [ 3354]
cp32-4_PAli_NX_Pl_consensus    ATTTGATAAAAGTTGCAATTTTTGGCAATAGAGGCCTTGAGAAAACAGTAACACCTGAAGCTGGTGGTTAAGCCTTGG [ 3354]
cp32-4_PAbe_NX_MP_FG_consensus ATTTGATAAAAGTTGCAATTTTTGGCAATAGAGGCCTTGAGAAAACAGTAACACCTGAAGCTGGTGGTTAAGCCTTGG [ 3354]
cp32-4_PAbe_NX_Pl_consensus    ATTTGATAAAAGTTGCAATTTTTGGCAATAGAGGCCTTGAGAAAACAGTAACACCTGAAGCTGGTGGTTAAGCCTTGG [ 3354]

cp32-4_gi|11497149|B31_GB      GCAATAAAAGGAGTTAAACAAATGGCAGACACAACGCAATTATTAAAAGATTATCAAGAGAAGAGAAGTAAACTTGAA [ 3432]
cp32-4_un10_B31_PacBio         GCAATAAAAGGAGTTAAACAAATGGCAGACACAACGCAATTATTAAAAGATTATCAAGAGAAGAGAAGTAAACTTGAA [ 3432]
cp32-4_B31_TS_MP_FG_consensus  GCAATAAAAGGAGTTAAACAAATGGCAGACACAACGCAATTATTAAAAGATTATCAAGAGAAGAGAAGTAAACTTGAA [ 3432]
cp32-4_B31_NX_Pl_consensus     GCAATAAAAGGAGTTAAACAAATGGCAGACACAACGCAATTATTAAAAGATTATCAAGAGAAGAGAAGTAAACTTGAA [ 3432]
cp32-4_PAli_NX_MP_FG_consensus GCAATAAAAGGAGTTAAACAAATGGCAGACACAACGCAATTATTAAAAGATTATCAAGAGAAGAGAAGTAAACTTGAA [ 3432]
cp32-4_PAli_NX_Pl_consensus    GCAATAAAAGGAGTTAAACAAATGGCAGACACAACGCAATTATTAAAAGATTATCAAGAGAAGAGAAGTAAACTTGAA [ 3432]
cp32-4_PAbe_NX_MP_FG_consensus GCAATAAAAGGAGTTAAACAAATGGCAGACACAACGCAATTATTAAAAGATTATCAAGAGAAGAGAAGTAAACTTGAA [ 3432]
cp32-4_PAbe_NX_Pl_consensus    GCAATAAAAGGAGTTAAACAAATGGCAGACACAACGCAATTATTAAAAGATTATCAAGAGAAGAGAAGTAAACTTGAA [ 3432]

cp32-4_gi|11497149|B31_GB      AAGTTTATGAAAAATCCCCAGCATGACGCTGGTTTGCTTAGCAATTCTGTAGAGTTTAGAGATAAAAACATACAATTT [ 3510]
cp32-4_un10_B31_PacBio         AAGTTTATGAAAAATCCCCAGCATGACGCTGGTTTGCTTAGCAATTCTGTAGAGTTTAGAGATAAAAACATACAATTT [ 3510]
cp32-4_B31_TS_MP_FG_consensus  AAGTTTATGAAAAATCCCCAGCATGACGCTGGTTTGCTTAGCAATTCTGTAGAGTTTAGAGATAAAAACATACAATTT [ 3510]
cp32-4_B31_NX_Pl_consensus     AAGTTTATGAAAAATCCCCAGCATGACGCTGGTTTGCTTAGCAATTCTGTAGAGTTTAGAGATAAAAACATACAATTT [ 3510]
cp32-4_PAli_NX_MP_FG_consensus AAGTTTATGAAAAATCCCCAGCATGACGCTGGTTTGCTTAGCAATTCTGTAGAGTTTAGAGATAAAAACATACAATTT [ 3510]
cp32-4_PAli_NX_Pl_consensus    AAGTTTATGAAAAATCCCCAGCATGACGCTGGTTTGCTTAGCAATTCTGTAGAGTTTAGAGATAAAAACATACAATTT [ 3510]
cp32-4_PAbe_NX_MP_FG_consensus AAGTTTATGAAAAATCCCCAGCATGACGCTGGTTTGCTTAGCAATTCTGTAGAGTTTAGAGATAAAAACATACAATTT [ 3510]
cp32-4_PAbe_NX_Pl_consensus    AAGTTTATGAAAAATCCCCAGCATGACGCTGGTTTGCTTAGCAATTCTGTAGAGTTTAGAGATAAAAACATACAATTT [ 3510]

cp32-4_gi|11497149|B31_GB      TTTGCCTCTGGAGGCACTAGAACCAGCAAATTTGACAAATTAGAAAATCATCCATTTTCTGGGTATCCATACAAGCGT [ 3588]
cp32-4_un10_B31_PacBio         TTTGCCTCTGGAGGCACTAGAACCAGCAAATTTGACAAATTAGAAAATCATCCATTTTCTGGGTATCCATACAAGCGT [ 3588]
cp32-4_B31_TS_MP_FG_consensus  TTTGCCTCTGGAGGCACTAGAACCAGCAAATTTGACAAATTAGAAAATCATCCATTTTCTGGGTATCCATACAAGCGT [ 3588]
cp32-4_B31_NX_Pl_consensus     TTTGCCTCTGGAGGCACTAGAACCAGCAAATTTGACAAATTAGAAAATCATCCATTTTCTGGGTATCCATACAAGCGT [ 3588]
cp32-4_PAli_NX_MP_FG_consensus TTTGCCTCTGGAGGCACTAGAACCAGCAAATTTGACAAATTAGAAAATCATCCATTTTCTGGGTATCCATACAAGCGT [ 3588]
cp32-4_PAli_NX_Pl_consensus    TTTGCCTCTGGAGGCACTAGAACCAGCAAATTTGACAAATTAGAAAATCATCCATTTTCTGGGTATCCATACAAGCGT [ 3588]
cp32-4_PAbe_NX_MP_FG_consensus TTTGCCTCTGGAGGCACTAGAACCAGCAAATTTGACAAATTAGAAAATCATCCATTTTCTGGGTATCCATACAAGCGT [ 3588]
cp32-4_PAbe_NX_Pl_consensus    TTTGCCTCTGGAGGCACTAGAACCAGCAAATTTGACAAATTAGAAAATCATCCATTTTCTGGGTATCCATACAAGCGT [ 3588]

cp32-4_gi|11497149|B31_GB      GGGGTAAAAAGAGTTATTCAAGAGGAAAAAGCTGATCAAATTCACTATGAACCCCACGTTGAGGCTGGTGGTAAAGAT [ 3666]
cp32-4_un10_B31_PacBio         GGGGTAAAAAGAGTTATTCAAGAGGAAAAAGCTGATCAAATTCACTATGAACCCCACGTTGAGGCTGGTGGTAAAGAT [ 3666]
cp32-4_B31_TS_MP_FG_consensus  GGGGTAAAAAGAGTTATTCAAGAGGAAAAAGCTGATCAAATTCACTATGAACCCCACGTTGAGGCTGGTGGTAAAGAT [ 3666]
cp32-4_B31_NX_Pl_consensus     GGGGTAAAAAGAGTTATTCAAGAGGAAAAAGCTGATCAAATTCACTATGAACCCCACGTTGAGGCTGGTGGTAAAGAT [ 3666]
cp32-4_PAli_NX_MP_FG_consensus GGGGTAAAAAGAGTTATTCAAGAGGAAAAAGCTGATCAAATTCACTATGAACCCCACGTTGAGGCTGGTGGTAAAGAT [ 3666]
cp32-4_PAli_NX_Pl_consensus    GGGGTAAAAAGAGTTATTCAAGAGGAAAAAGCTGATCAAATTCACTATGAACCCCACGTTGAGGCTGGTGGTAAAGAT [ 3666]
cp32-4_PAbe_NX_MP_FG_consensus GGGGTAAAAAGAGTTATTCAAGAGGAAAAAGCTGATCAAATTCACTATGAACCCCACGTTGAGGCTGGTGGTAAAGAT [ 3666]
cp32-4_PAbe_NX_Pl_consensus    GGGGTAAAAAGAGTTATTCAAGAGGAAAAAGCTGATCAAATTCACTATGAACCCCACGTTGAGGCTGGTGGTAAAGAT [ 3666]

cp32-4_gi|11497149|B31_GB      GATTTATACGGAATATGCATTGATATAGATGAGTTTAGCAAAACAGCCACTATTGTTCCAATTACAAATAATTTTGAG [ 3744]
cp32-4_un10_B31_PacBio         GATTTATACGGAATATGCATTGATATAGATGAGTTTAGCAAAACAGCCACTATTGTTCCAATTACAAATAATTTTGAG [ 3744]
cp32-4_B31_TS_MP_FG_consensus  GATTTATACGGAATATGCATTGATATAGATGAGTTTAGCAAAACAGCCACTATTGTTCCAATTACAAATAATTTTGAG [ 3744]
cp32-4_B31_NX_Pl_consensus     GATTTATACGGAATATGCATTGATATAGATGAGTTTAGCAAAACAGCCACTATTGTTCCAATTACAAATAATTTTGAG [ 3744]
cp32-4_PAli_NX_MP_FG_consensus GATTTATACGGAATATGCATTGATATAGATGAGTTTAGCAAAACAGCCACTATTGTTCCAATTACAAATAATTTTGAG [ 3744]
```

```
cp32-4_PAli_NX_Pl_consensus    GATTTATACGGAATATGCATTGATATAGATGAGTTTAGCAAAACAGCCACTATTGTTCCAATTACAAATAATTTTGAG [ 3744]
cp32-4_PAbe_NX_MP_FG_consensus GATTTATACGGAATATGCATTGATATAGATGAGTTTAGCAAAACAGCCACTATTGTTCCAATTACAAATAATTTTGAG [ 3744]
cp32-4_PAbe_NX_Pl_consensus    GATTTATACGGAATATGCATTGATATAGATGAGTTTAGCAAAACAGCCACTATTGTTCCAATTACAAATAATTTTGAG [ 3744]

cp32-4_gi|11497149|B31_GB      GGTTATTTAGTAGCAAAAGATTCTACGCTTAAAGTAAAAGACAAACTTGTTTTTAATAAAGACGGTGCTCTTGAAAAG [ 3822]
cp32-4_un10_B31_PacBio         GGTTATTTAGTAGCAAAAGATTCTACGCTTAAAGTAAAAGACAAACTTGTTTTTAATAAAGACGGTGCTCTTGAAAAG [ 3822]
cp32-4_B31_TS_MP_FG_consensus  GGTTATTTAGTAGCAAAAGATTCTACGCTTAAAGTAAAAGACAAACTTGTTTTTAATAAAGACGGTGCTCTTGAAAAG [ 3822]
cp32-4_B31_NX_Pl_consensus     GGTTATTTAGTAGCAAAAGATTCTACGCTTAAAGTAAAAGACAAACTTGTTTTTAATAAAGACGGTGCTCTTGAAAAG [ 3822]
cp32-4_PAli_NX_MP_FG_consensus GGTTATTTAGTAGCAAAAGATTCTACGCTTAAAGTAAAAGACAAACTTGTTTTTAATAAAGACGGTGCTCTTGAAAAG [ 3822]
cp32-4_PAli_NX_Pl_consensus    GGTTATTTAGTAGCAAAAGATTCTACGCTTAAAGTAAAAGACAAACTTGTTTTTAATAAAGACGGTGCTCTTGAAAAG [ 3822]
cp32-4_PAbe_NX_MP_FG_consensus GGTTATTTAGTAGCAAAAGATTCTACGCTTAAAGTAAAAGACAAACTTGTTTTTAATAAAGACGGTGCTCTTGAAAAG [ 3822]
cp32-4_PAbe_NX_Pl_consensus    GGTTATTTAGTAGCAAAAGATTCTACGCTTAAAGTAAAAGACAAACTTGTTTTTAATAAAGACGGTGCTCTTGAAAAG [ 3822]

cp32-4_gi|11497149|B31_GB      GTGACTGGAGCACCACCAAATAAGGCAACTATTAATGCGATAGCGTTGTCTGATGCAAAACAAATTAGTAACGATGTT [ 3900]
cp32-4_un10_B31_PacBio         GTGACTGGAGCACCACCAAATAAGGCAACTATTAATGCGATAGCGTTGTCTGATGCAAAACAAATTAGTAACGATGTT [ 3900]
cp32-4_B31_TS_MP_FG_consensus  GTGACTGGAGCACCACCAAATAAGGCAACTATTAATGCGATAGCGTTGTCTGATGCAAAACAAATTAGTAACGATGTT [ 3900]
cp32-4_B31_NX_Pl_consensus     GTGACTGGAGCACCACCAAATAAGGCAACTATTAATGCGATAGCGTTGTCTGATGCAAAACAAATTAGTAACGATGTT [ 3900]
cp32-4_PAli_NX_MP_FG_consensus GTGACTGGAGCACCACCAAATAAGGCAACTATTAATGCGATAGCGTTGTCTGATGCAAAACAAATTAGTAACGATGTT [ 3900]
cp32-4_PAli_NX_Pl_consensus    GTGACTGGAGCACCACCAAATAAGGCAACTATTAATGCGATAGCGTTGTCTGATGCAAAACAAATTAGTAACGATGTT [ 3900]
cp32-4_PAbe_NX_MP_FG_consensus GTGACTGGAGCACCACCAAATAAGGCAACTATTAATGCGATAGCGTTGTCTGATGCAAAACAAATTAGTAACGATGTT [ 3900]
cp32-4_PAbe_NX_Pl_consensus    GTGACTGGAGCACCACCAAATAAGGCAACTATTAATGCGATAGCGTTGTCTGATGCAAAACAAATTAGTAACGATGTT [ 3900]

cp32-4_gi|11497149|B31_GB      TTTTTAATAAAAGTAGCAGTATTTGGAAATAAAGCTGTAAGTAAAAATTAATGATATTTGGGAGGATTTAATATGGAA [ 3978]
cp32-4_un10_B31_PacBio         TTTTTAATAAAAGTAGCAGTATTTGGAAATAAAGCTGTAAGTAAAAATTAATGATATTTGGGAGGATTTAATATGGAA [ 3978]
cp32-4_B31_TS_MP_FG_consensus  TTTTTAATAAAAGTAGCAGTATTTGGAAATAAAGCTGTAAGTAAAAATTAATGATATTTGGGAGGATTTAATATGGAA [ 3978]
cp32-4_B31_NX_Pl_consensus     TTTTTAATAAAAGTAGCAGTATTTGGAAATAAAGCTGTAAGTAAAAATTAATGATATTTGGGAGGATTTAATATGGAA [ 3978]
cp32-4_PAli_NX_MP_FG_consensus TTTTTAATAAAAGTAGCAGTATTTGGAAATAAAGCTGTAAGTAAAAATTAATGATATTTGGGAGGATTTAATATGGAA [ 3978]
cp32-4_PAli_NX_Pl_consensus    TTTTTAATAAAAGTAGCAGTATTTGGAAATAAAGCTGTAAGTAAAAATTAATGATATTTGGGAGGATTTAATATGGAA [ 3978]
cp32-4_PAbe_NX_MP_FG_consensus TTTTTAATAAAAGTAGCAGTATTTGGAAATAAAGCTGTAAGTAAAAATTAATGATATTTGGGAGGATTTAATATGGAA [ 3978]
cp32-4_PAbe_NX_Pl_consensus    TTTTTAATAAAAGTAGCAGTATTTGGAAATAAAGCTGTAAGTAAAAATTAATGATATTTGGGAGGATTTAATATGGAA [ 3978]

cp32-4_gi|11497149|B31_GB      TTATTTGATGAAAATTATTATGCAAAAGCTGTGGCTAATATTATAGGGGAAGTTAAAGATCCTATAATGTATAAATGG [ 4056]
cp32-4_un10_B31_PacBio         TTATTTGATGAAAATTATTATGCAAAAGCTGTGGCTAATATTATAGGGGAAGTTAAAGATCCTATAATGTATAAATGG [ 4056]
cp32-4_B31_TS_MP_FG_consensus  TTATTTGATGAAAATTATTATGCAAAAGCTGTGGCTAATATTATAGGGGAAGTTAAAGATCCTATAATGTATAAATGG [ 4056]
cp32-4_B31_NX_Pl_consensus     TTATTTGATGAAAATTATTATGCAAAAGCTGTGGCTAATATTATAGGGGAAGTTAAAGATCCTATAATGTATAAATGG [ 4056]
cp32-4_PAli_NX_MP_FG_consensus TTATTTGATGAAAATTATTATGCAAAAGCTGTGGCTAATATTATAGGGGAAGTTAAAGATCCTATAATGTATAAATGG [ 4056]
cp32-4_PAli_NX_Pl_consensus    TTATTTGATGAAAATTATTATGCAAAAGCTGTGGCTAATATTATAGGGGAAGTTAAAGATCCTATAATGTATAAATGG [ 4056]
cp32-4_PAbe_NX_MP_FG_consensus TTATTTGATGAAAATTATTATGCAAAAGCTGTGGCTAATATTATAGGGGAAGTTAAAGATCCTATAATGTATAAATGG [ 4056]
cp32-4_PAbe_NX_Pl_consensus    TTATTTGATGAAAATTATTATGCAAAAGCTGTGGCTAATATTATAGGGGAAGTTAAAGATCCTATAATGTATAAATGG [ 4056]

cp32-4_gi|11497149|B31_GB      TTTTCGCCTGATCAAATTGAAGATGTTGATCTTCAGATGGGCTACCAAAGAACTGTAAAGTGGGATGCGTTTTTAAAT [ 4134]
cp32-4_un10_B31_PacBio         TTTTCGCCTGATCAAATTGAAGATGTTGATCTTCAGATGGGCTACCAAAGAACTGTAAAGTGGGATGCGTTTTTAAAT [ 4134]
cp32-4_B31_TS_MP_FG_consensus  TTTTCGCCTGATCAAATTGAAGATGTTGATCTTCAGATGGGCTACCAAAGAACTGTAAAGTGGGATGCGTTTTTAAAT [ 4134]
cp32-4_B31_NX_Pl_consensus     TTTTCGCCTGATCAAATTGAAGATGTTGATCTTCAGATGGGCTACCAAAGAACTGTAAAGTGGGATGCGTTTTTAAAT [ 4134]
cp32-4_PAli_NX_MP_FG_consensus TTTTCGCCTGATCAAATTGAAGATGTTGATCTTCAGATGGGCTACCAAAGAACTGTAAAGTGGGATGCGTTTTTAAAT [ 4134]
cp32-4_PAli_NX_Pl_consensus    TTTTCGCCTGATCAAATTGAAGATGTTGATCTTCAGATGGGCTACCAAAGAACTGTAAAGTGGGATGCGTTTTTAAAT [ 4134]
cp32-4_PAbe_NX_MP_FG_consensus TTTTCGCCTGATCAAATTGAAGATGTTGATCTTCAGATGGGCTACCAAAGAACTGTAAAGTGGGATGCGTTTTTAAAT [ 4134]
cp32-4_PAbe_NX_Pl_consensus    TTTTCGCCTGATCAAATTGAAGATGTTGATCTTCAGATGGGCTACCAAAGAACTGTAAAGTGGGATGCGTTTTTAAAT [ 4134]

cp32-4_gi|11497149|B31_GB      GCTAATCCTACAACGATTGCTAATGAAGTTAATACTATTTCAACTATTGGATTTAGTTCTGAAGTGGTAAGACTTAAT [ 4212]
cp32-4_un10_B31_PacBio         GCTAATCCTACAACGATTGCTAATGAAGTTAATACTATTTCAACTATTGGATTTAGTTCTGAAGTGGTAAGACTTAAT [ 4212]
cp32-4_B31_TS_MP_FG_consensus  GCTAATCCTACAACGATTGCTAATGAAGTTAATACTATTTCAACTATTGGATTTAGTTCTGAAGTGGTAAGACTTAAT [ 4212]
cp32-4_B31_NX_Pl_consensus     GCTAATCCTACAACGATTGCTAATGAAGTTAATACTATTTCAACTATTGGATTTAGTTCTGAAGTGGTAAGACTTAAT [ 4212]
cp32-4_PAli_NX_MP_FG_consensus GCTAATCCTACAACGATTGCTAATGAAGTTAATACTATTTCAACTATTGGATTTAGTTCTGAAGTGGTAAGACTTAAT [ 4212]
cp32-4_PAli_NX_Pl_consensus    GCTAATCCTACAACGATTGCTAATGAAGTTAATACTATTTCAACTATTGGATTTAGTTCTGAAGTGGTAAGACTTAAT [ 4212]
cp32-4_PAbe_NX_MP_FG_consensus GCTAATCCTACAACGATTGCTAATGAAGTTAATACTATTTCAACTATTGGATTTAGTTCTGAAGTGGTAAGACTTAAT [ 4212]
cp32-4_PAbe_NX_Pl_consensus    GCTAATCCTACAACGATTGCTAATGAAGTTAATACTATTTCAACTATTGGATTTAGTTCTGAAGTGGTAAGACTTAAT [ 4212]

cp32-4_gi|11497149|B31_GB      TACTTAAAATTACAGTACAAATTTAGGCACTTAAAGCAGGCGTCTGAAAAATTTTACACTTCAGACTCATATCTTGGT [ 4290]
cp32-4_un10_B31_PacBio         TACTTAAAATTACAGTACAAATTTAGGCACTTAAAGCAGGCGTCTGAAAAATTTTACACTTCAGACTCATATCTTGGT [ 4290]
cp32-4_B31_TS_MP_FG_consensus  TACTTAAAATTACAGTACAAATTTAGGCACTTAAAGCAGGCGTCTGAAAAATTTTACACTTCAGACTCATATCTTGGT [ 4290]
cp32-4_B31_NX_Pl_consensus     TACTTAAAATTACAGTACAAATTTAGGCACTTAAAGCAGGCGTCTGAAAAATTTTACACTTCAGACTCATATCTTGGT [ 4290]
cp32-4_PAli_NX_MP_FG_consensus TACTTAAAATTACAGTACAAATTTAGGCACTTAAAGCAGGCGTCTGAAAAATTTTACACTTCAGACTCATATCTTGGT [ 4290]
cp32-4_PAli_NX_Pl_consensus    TACTTAAAATTACAGTACAAATTTAGGCACTTAAAGCAGGCGTCTGAAAAATTTTACACTTCAGACTCATATCTTGGT [ 4290]
cp32-4_PAbe_NX_MP_FG_consensus TACTTAAAATTACAGTACAAATTTAGGCACTTAAAGCAGGCGTCTGAAAAATTTTACACTTCAGACTCATATCTTGGT [ 4290]
cp32-4_PAbe_NX_Pl_consensus    TACTTAAAATTACAGTACAAATTTAGGCACTTAAAGCAGGCGTCTGAAAAATTTTACACTTCAGACTCATATCTTGGT [ 4290]

cp32-4_gi|11497149|B31_GB      GACATTAATAACAACTTACTTCCTTTCTCTCAAGCCTATAAGCTTGCAAGTAGCGAGATTATTAAACTTATTAATCAC [ 4368]
cp32-4_un10_B31_PacBio         GACATTAATAACAACTTACTTCCTTTCTCTCAAGCCTATAAGCTTGCAAGTAGCGAGATTATTAAACTTATTAATCAC [ 4368]
cp32-4_B31_TS_MP_FG_consensus  GACATTAATAACAACTTACTTCCTTTCTCTCAAGCCTATAAGCTTGCAAGTAGCGAGATTATTAAACTTATTAATCAC [ 4368]
cp32-4_B31_NX_Pl_consensus     GACATTAATAACAACTTACTTCCTTTCTCTCAAGCCTATAAGCTTGCAAGTAGCGAGATTATTAAACTTATTAATCAC [ 4368]
cp32-4_PAli_NX_MP_FG_consensus GACATTAATAACAACTTACTTCCTTTCTCTCAAGCCTATAAGCTTGCAAGTAGCGAGATTATTAAACTTATTAATCAC [ 4368]
cp32-4_PAli_NX_Pl_consensus    GACATTAATAACAACTTACTTCCTTTCTCTCAAGCCTATAAGCTTGCAAGTAGCGAGATTATTAAACTTATTAATCAC [ 4368]
cp32-4_PAbe_NX_MP_FG_consensus GACATTAATAACAACTTACTTCCTTTCTCTCAAGCCTATAAGCTTGCAAGTAGCGAGATTATTAAACTTATTAATCAC [ 4368]
cp32-4_PAbe_NX_Pl_consensus    GACATTAATAACAACTTACTTCCTTTCTCTCAAGCCTATAAGCTTGCAAGTAGCGAGATTATTAAACTTATTAATCAC [ 4368]

cp32-4_gi|11497149|B31_GB      TTTGTACTAACAGGCACTGTTTCAATTCAAAAAGATGGAAAAAATCAAAAACGTCTACTTCCCAATATGTATGGACTT [ 4446]
cp32-4_un10_B31_PacBio         TTTGTACTAACAGGCACTGTTTCAATTCAAAAAGATGGAAAAAATCAAAAACGTCTACTTCCCAATATGTATGGACTT [ 4446]
cp32-4_B31_TS_MP_FG_consensus  TTTGTACTAACAGGCACTGTTTCAATTCAAAAAGATGGAAAAAATCAAAAACGTCTACTTCCCAATATGTATGGACTT [ 4446]
cp32-4_B31_NX_Pl_consensus     TTTGTACTAACAGGCACTGTTTCAATTCAAAAAGATGGAAAAAATCAAAAACGTCTACTTCCCAATATGTATGGACTT [ 4446]
cp32-4_PAli_NX_MP_FG_consensus TTTGTACTAACAGGCACTGTTTCAATTCAAAAAGATGGAAAAAATCAAAAACGTCTACTTCCCAATATGTATGGACTT [ 4446]
cp32-4_PAli_NX_Pl_consensus    TTTGTACTAACAGGCACTGTTTCAATTCAAAAAGATGGAAAAAATCAAAAACGTCTACTTCCCAATATGTATGGACTT [ 4446]
cp32-4_PAbe_NX_MP_FG_consensus TTTGTACTAACAGGCACTGTTTCAATTCAAAAAGATGGAAAAAATCAAAAACGTCTACTTCCCAATATGTATGGACTT [ 4446]
cp32-4_PAbe_NX_Pl_consensus    TTTGTACTAACAGGCACTGTTTCAATTCAAAAAGATGGAAAAAATCAAAAACGTCTACTTCCCAATATGTATGGACTT [ 4446]

cp32-4_gi|11497149|B31_GB      CTTAATATGCCACATCAAGTAAAAGAAGAGGTTGAAAGTGCTAATAAGGACAAAATGGATAAAATATTTGAAAAAATT [ 4524]
cp32-4_un10_B31_PacBio         CTTAATATGCCACATCAAGTAAAAGAAGAGGTTGAAAGTGCTAATAAGGACAAAATGGATAAAATATTTGAAAAAATT [ 4524]
cp32-4_B31_TS_MP_FG_consensus  CTTAATATGCCACATCAAGTAAAAGAAGAGGTTGAAAGTGCTAATAAGGACAAAATGGATAAAATATTTGAAAAAATT [ 4524]
cp32-4_B31_NX_Pl_consensus     CTTAATATGCCACATCAAGTAAAAGAAGAGGTTGAAAGTGCTAATAAGGACAAAATGGATAAAATATTTGAAAAAATT [ 4524]
cp32-4_PAli_NX_MP_FG_consensus CTTAATATGCCACATCAAGTAAAAGAAGAGGTTGAAAGTGCTAATAAGGACAAAATGGATAAAATATTTGAAAAAATT [ 4524]
cp32-4_PAli_NX_Pl_consensus    CTTAATATGCCACATCAAGTAAAAGAAGAGGTTGAAAGTGCTAATAAGGACAAAATGGATAAAATATTTGAAAAAATT [ 4524]
cp32-4_PAbe_NX_MP_FG_consensus CTTAATATGCCACATCAAGTAAAAGAAGAGGTTGAAAGTGCTAATAAGGACAAAATGGATAAAATATTTGAAAAAATT [ 4524]
cp32-4_PAbe_NX_Pl_consensus    CTTAATATGCCACATCAAGTAAAAGAAGAGGTTGAAAGTGCTAATAAGGACAAAATGGATAAAATATTTGAAAAAATT [ 4524]

cp32-4_gi|11497149|B31_GB      GAGGCGGGACTTTCAAAGTTAGAGCTAGGAGACGAATTTTCTACACCTATGATGGTAATAGTTGACCCTACAACTTCG [ 4602]
cp32-4_un10_B31_PacBio         GAGGCGGGACTTTCAAAGTTAGAGCTAGGAGACGAATTTTCTACACCTATGATGGTAATAGTTGACCCTACAACTTCG [ 4602]
cp32-4_B31_TS_MP_FG_consensus  GAGGCGGGACTTTCAAAGTTAGAGCTAGGAGACGAATTTTCTACACCTATGATGGTAATAGTTGACCCTACAACTTCG [ 4602]
cp32-4_B31_NX_Pl_consensus     GAGGCGGGACTTTCAAAGTTAGAGCTAGGAGACGAATTTTCTACACCTATGATGGTAATAGTTGACCCTACAACTTCG [ 4602]
cp32-4_PAli_NX_MP_FG_consensus GAGGCGGGACTTTCAAAGTTAGAGCTAGGAGACGAATTTTCTACACCTATGATGGTAATAGTTGACCCTACAACTTCG [ 4602]
cp32-4_PAli_NX_Pl_consensus    GAGGCGGGACTTTCAAAGTTAGAGCTAGGAGACGAATTTTCTACACCTATGATGGTAATAGTTGACCCTACAACTTCG [ 4602]
cp32-4_PAbe_NX_MP_FG_consensus GAGGCGGGACTTTCAAAGTTAGAGCTAGGAGACGAATTTTCTACACCTATGATGGTAATAGTTGACCCTACAACTTCG [ 4602]
cp32-4_PAbe_NX_Pl_consensus    GAGGCGGGACTTTCAAAGTTAGAGCTAGGAGACGAATTTTCTACACCTATGATGGTAATAGTTGACCCTACAACTTCG [ 4602]

cp32-4_gi|11497149|B31_GB      CTCAAACTCGTAAAGCCCTACGCAGCAGCACAGGGTGCAGCAAGTAGCTGCGAAAAATGGGAGGATGTTTTGATTCAA [ 4680]
cp32-4_un10_B31_PacBio         CTCAAACTCGTAAAGCCCTACGCAGCAGCACAGGGTGCAGCAAGTAGCTGCGAAAAATGGGAGGATGTTTTGATTCAA [ 4680]
cp32-4_B31_TS_MP_FG_consensus  CTCAAACTCGTAAAGCCCTACGCAGCAGCACAGGGTGCAGCAAGTAGCTGCGAAAAATGGGAGGATGTTTTGATTCAA [ 4680]
cp32-4_B31_NX_Pl_consensus     CTCAAACTCGTAAAGCCCTACGCAGCAGCACAGGGTGCAGCAAGTAGCTGCGAAAAATGGGAGGATGTTTTGATTCAA [ 4680]
cp32-4_PAli_NX_MP_FG_consensus CTCAAACTCGTAAAGCCCTACGCAGCAGCACAGGGTGCAGCAAGTAGCTGCGAAAAATGGGAGGATGTTTTGATTCAA [ 4680]
cp32-4_PAli_NX_Pl_consensus    CTCAAACTCGTAAAGCCCTACGCAGCAGCACAGGGTGCAGCAAGTAGCTGCGAAAAATGGGAGGATGTTTTGATTCAA [ 4680]
```

```
cp32-4_PAbe_NX_MP_FG_consensus CTCAAACTCGTAAAGCCCTACGCAGCAGCACAGGGTGCAGCAAGTAGCTGCGAAAAATGGGAGGATGTTTTGATTCAA [ 4680]
cp32-4_PAbe_NX_Pl_consensus    CTCAAACTCGTAAAGCCCTACGCAGCAGCACAGGGTGCAGCAAGTAGCTGCGAAAAATGGGAGGATGTTTTGATTCAA [ 4680]

cp32-4_gi|11497149|B31_GB      ACTATTAAGGCTATTAATAATAGAGAAGATGTCTACATTGAAACTTCAAACTTGCTAAAACATCAAATACTTATTTAC [ 4758]
cp32-4_un10_B31_PacBio         ACTATTAAGGCTATTAATAATAGAGAAGATGTCTACATTGAAACTTCAAACTTGCTAAAACATCAAATACTTATTTAC [ 4758]
cp32-4_B31_TS_MP_FG_consensus  ACTATTAAGGCTATTAATAATAGAGAAGATGTCTACATTGAAACTTCAAACTTGCTAAAACATCAAATACTTATTTAC [ 4758]
cp32-4_B31_NX_Pl_consensus     ACTATTAAGGCTATTAATAATAGAGAAGATGTCTACATTGAAACTTCAAACTTGCTAAAACATCAAATACTTATTTAC [ 4758]
cp32-4_PAli_NX_MP_FG_consensus ACTATTAAGGCTATTAATAATAGAGAAGATGTCTACATTGAAACTTCAAACTTGCTAAAACATCAAATACTTATTTAC [ 4758]
cp32-4_PAli_NX_Pl_consensus    ACTATTAAGGCTATTAATAATAGAGAAGATGTCTACATTGAAACTTCAAACTTGCTAAAACATCAAATACTTATTTAC [ 4758]
cp32-4_PAbe_NX_MP_FG_consensus ACTATTAAGGCTATTAATAATAGAGAAGATGTCTACATTGAAACTTCAAACTTGCTAAAACATCAAATACTTATTTAC [ 4758]
cp32-4_PAbe_NX_Pl_consensus    ACTATTAAGGCTATTAATAATAGAGAAGATGTCTACATTGAAACTTCAAACTTGCTAAAACATCAAATACTTATTTAC [ 4758]

cp32-4_gi|11497149|B31_GB      CCATTAAACCCAGAGCTTATTAAGTTTAAACCTAGCAAGTATATGTTGCCTACACCAAATGAACAAGTGGATAAAGAT [ 4836]
cp32-4_un10_B31_PacBio         CCATTAAACCCAGAGCTTATTAAGTTTAAACCTAGCAAGTATATGTTGCCTACACCAAATGAACAAGTGGATAAAGAT [ 4836]
cp32-4_B31_TS_MP_FG_consensus  CCATTAAACCCAGAGCTTATTAAGTTTAAACCTAGCAAGTATATGTTGCCTACACCAAATGAACAAGTGGATAAAGAT [ 4836]
cp32-4_B31_NX_Pl_consensus     CCATTAAACCCAGAGCTTATTAAGTTTAAACCTAGCAAGTATATGTTGCCTACACCAAATGAACAAGTGGATAAAGAT [ 4836]
cp32-4_PAli_NX_MP_FG_consensus CCATTAAACCCAGAGCTTATTAAGTTTAAACCTAGCAAGTATATGTTGCCTACACCAAATGAACAAGTGGATAAAGAT [ 4836]
cp32-4_PAli_NX_Pl_consensus    CCATTAAACCCAGAGCTTATTAAGTTTAAACCTAGCAAGTATATGTTGCCTACACCAAATGAACAAGTGGATAAAGAT [ 4836]
cp32-4_PAbe_NX_MP_FG_consensus CCATTAAACCCAGAGCTTATTAAGTTTAAACCTAGCAAGTATATGTTGCCTACACCAAATGAACAAGTGGATAAAGAT [ 4836]
cp32-4_PAbe_NX_Pl_consensus    CCATTAAACCCAGAGCTTATTAAGTTTAAACCTAGCAAGTATATGTTGCCTACACCAAATGAACAAGTGGATAAAGAT [ 4836]

cp32-4_gi|11497149|B31_GB      TCAACTGATATTGCTCATTCATACATTGATTTTGTTTTAGGAGGGCTACTTGCTACTAGAAAAACTATTTTGCAAGTA [ 4914]
cp32-4_un10_B31_PacBio         TCAACTGATATTGCTCATTCATACATTGATTTTGTTTTAGGAGGGCTACTTGCTACTAGAAAAACTATTTTGCAAGTA [ 4914]
cp32-4_B31_TS_MP_FG_consensus  TCAACTGATATTGCTCATTCATACATTGATTTTGTTTTAGGAGGGCTACTTGCTACTAGAAAAACTATTTTGCAAGTA [ 4914]
cp32-4_B31_NX_Pl_consensus     TCAACTGATATTGCTCATTCATACATTGATTTTGTTTTAGGAGGGCTACTTGCTACTAGAAAAACTATTTTGCAAGTA [ 4914]
cp32-4_PAli_NX_MP_FG_consensus TCAACTGATATTGCTCATTCATACATTGATTTTGTTTTAGGAGGGCTACTTGCTACTAGAAAAACTATTTTGCAAGTA [ 4914]
cp32-4_PAli_NX_Pl_consensus    TCAACTGATATTGCTCATTCATACATTGATTTTGTTTTAGGAGGGCTACTTGCTACTAGAAAAACTATTTTGCAAGTA [ 4914]
cp32-4_PAbe_NX_MP_FG_consensus TCAACTGATATTGCTCATTCATACATTGATTTTGTTTTAGGAGGGCTACTTGCTACTAGAAAAACTATTTTGCAAGTA [ 4914]
cp32-4_PAbe_NX_Pl_consensus    TCAACTGATATTGCTCATTCATACATTGATTTTGTTTTAGGAGGGCTACTTGCTACTAGAAAAACTATTTTGCAAGTA [ 4914]

cp32-4_gi|11497149|B31_GB      CATATTAAACAAAGTTAAAAACATAAGGTAAATCGAAGTGAGCGAACAAAAAAACTTACAAACACAAGTTGAGGCTGA [ 4992]
cp32-4_un10_B31_PacBio         CATATTAAACAAAGTTAAAAACATAAGGTAAATCGAAGTGAGCGAACAAAAAAACTTACAAACACAAGTTGAGGCTGA [ 4992]
cp32-4_B31_TS_MP_FG_consensus  CATATTAAACAAAGTTAAAAACATAAGGTAAATCGAAGTGAGCGAACAAAAAAACTTACAAACACAAGTTGAGGCTGA [ 4992]
cp32-4_B31_NX_Pl_consensus     CATATTAAACAAAGTTAAAAACATAAGGTAAATCGAAGTGAGCGAACAAAAAAACTTACAAACACAAGTTGAGGCTGA [ 4992]
cp32-4_PAli_NX_MP_FG_consensus CATATTAAACAAAGTTAAAAACATAAGGTAAATCGAAGTGAGCGAACAAAAAAACTTACAAACACAAGTTGAGGCTGA [ 4992]
cp32-4_PAli_NX_Pl_consensus    CATATTAAACAAAGTTAAAAACATAAGGTAAATCGAAGTGAGCGAACAAAAAAACTTACAAACACAAGTTGAGGCTGA [ 4992]
cp32-4_PAbe_NX_MP_FG_consensus CATATTAAACAAAGTTAAAAACATAAGGTAAATCGAAGTGAGCGAACAAAAAAACTTACAAACACAAGTTGAGGCTGA [ 4992]
cp32-4_PAbe_NX_Pl_consensus    CATATTAAACAAAGTTAAAAACATAAGGTAAATCGAAGTGAGCGAACAAAAAAACTTACAAACACAAGTTGAGGCTGA [ 4992]

cp32-4_gi|11497149|B31_GB      AGAAGAACTTTTGGTAACAAAACTTCATTCTGAAGTGTTATTGTTACTAGGAATAGACAAACTTGCATTAAGTAGGCA [ 5070]
cp32-4_un10_B31_PacBio         AGAAGAACTTTTGGTAACAAAACTTCATTCTGAAGTGTTATTGTTACTAGGAATAGACAAACTTGCATTAAGTAGGCA [ 5070]
cp32-4_B31_TS_MP_FG_consensus  AGAAGAACTTTTGGTAACAAAACTTCATTCTGAAGTGTTATTGTTACTAGGAATAGACAAACTTGCATTAAGTAGGCA [ 5070]
cp32-4_B31_NX_Pl_consensus     AGAAGAACTTTTGGTAACAAAACTTCATTCTGAAGTGTTATTGTTACTAGGAATAGACAAACTTGCATTAAGTAGGCA [ 5070]
cp32-4_PAli_NX_MP_FG_consensus AGAAGAACTTTTGGTAACAAAACTTCATTCTGAAGTGTTATTGTTACTAGGAATAGACAAACTTGCATTAAGTAGGCA [ 5070]
cp32-4_PAli_NX_Pl_consensus    AGAAGAACTTTTGGTAACAAAACTTCATTCTGAAGTGTTATTGTTACTAGGAATAGACAAACTTGCATTAAGTAGGCA [ 5070]
cp32-4_PAbe_NX_MP_FG_consensus AGAAGAACTTTTGGTAACAAAACTTCATTCTGAAGTGTTATTGTTACTAGGAATAGACAAACTTGCATTAAGTAGGCA [ 5070]
cp32-4_PAbe_NX_Pl_consensus    AGAAGAACTTTTGGTAACAAAACTTCATTCTGAAGTGTTATTGTTACTAGGAATAGACAAACTTGCATTAAGTAGGCA [ 5070]

cp32-4_gi|11497149|B31_GB      AAATTTTCTACTTCATTTATCTTTACTTCAAGCTATTCTAGTAACACGTGGGATTGATGCTAGCTCACTTACATATGA [ 5148]
cp32-4_un10_B31_PacBio         AAATTTTCTACTTCATTTATCTTTACTTCAAGCTATTCTAGTAACACGTGGGATTGATGCTAGCTCACTTACATATGA [ 5148]
cp32-4_B31_TS_MP_FG_consensus  AAATTTTCTACTTCATTTATCTTTACTTCAAGCTATTCTAGTAACACGTGGGATTGATGCTAGCTCACTTACATATGA [ 5148]
cp32-4_B31_NX_Pl_consensus     AAATTTTCTACTTCATTTATCTTTACTTCAAGCTATTCTAGTAACACGTGGGATTGATGCTAGCTCACTTACATATGA [ 5148]
cp32-4_PAli_NX_MP_FG_consensus AAATTTTCTACTTCATTTATCTTTACTTCAAGCTATTCTAGTAACACGTGGGATTGATGCTAGCTCACTTACATATGA [ 5148]
cp32-4_PAli_NX_Pl_consensus    AAATTTTCTACTTCATTTATCTTTACTTCAAGCTATTCTAGTAACACGTGGGATTGATGCTAGCTCACTTACATATGA [ 5148]
cp32-4_PAbe_NX_MP_FG_consensus AAATTTTCTACTTCATTTATCTTTACTTCAAGCTATTCTAGTAACACGTGGGATTGATGCTAGCTCACTTACATATGA [ 5148]
cp32-4_PAbe_NX_Pl_consensus    AAATTTTCTACTTCATTTATCTTTACTTCAAGCTATTCTAGTAACACGTGGGATTGATGCTAGCTCACTTACATATGA [ 5148]

cp32-4_gi|11497149|B31_GB      ACAAATATTTTTACTTACCCTTTACCATATGGGTTGTCAATTAAGAAAACAGGGAGTTGTTCAAGAATTTGAATTTGA [ 5226]
cp32-4_un10_B31_PacBio         ACAAATATTTTTACTTACCCTTTACCATATGGGTTGTCAATTAAGAAAACAGGGAGTTGTTCAAGAATTTGAATTTGA [ 5226]
cp32-4_B31_TS_MP_FG_consensus  ACAAATATTTTTACTTACCCTTTACCATATGGGTTGTCAATTAAGAAAACAGGGAGTTGTTCAAGAATTTGAATTTGA [ 5226]
cp32-4_B31_NX_Pl_consensus     ACAAATATTTTTACTTACCCTTTACCATATGGGTTGTCAATTAAGAAAACAGGGAGTTGTTCAAGAATTTGAATTTGA [ 5226]
cp32-4_PAli_NX_MP_FG_consensus ACAAATATTTTTACTTACCCTTTACCATATGGGTTGTCAATTAAGAAAACAGGGAGTTGTTCAAGAATTTGAATTTGA [ 5226]
cp32-4_PAli_NX_Pl_consensus    ACAAATATTTTTACTTACCCTTTACCATATGGGTTGTCAATTAAGAAAACAGGGAGTTGTTCAAGAATTTGAATTTGA [ 5226]
cp32-4_PAbe_NX_MP_FG_consensus ACAAATATTTTTACTTACCCTTTACCATATGGGTTGTCAATTAAGAAAACAGGGAGTTGTTCAAGAATTTGAATTTGA [ 5226]
cp32-4_PAbe_NX_Pl_consensus    ACAAATATTTTTACTTACCCTTTACCATATGGGTTGTCAATTAAGAAAACAGGGAGTTGTTCAAGAATTTGAATTTGA [ 5226]

cp32-4_gi|11497149|B31_GB      TAGGATCAAAAAAGAGAAATTCAATGAACTTGAACTTAATTATTATCCTAGTAGCAGTGGAGGCGAAGAAGGTGGCGA [ 5304]
cp32-4_un10_B31_PacBio         TAGGATCAAAAAAGAGAAATTCAATGAACTTGAACTTAATTATTATCCTAGTAGCAGTGGAGGCGAAGAAGGTGGCGA [ 5304]
cp32-4_B31_TS_MP_FG_consensus  TAGGATCAAAAAAGAGAAATTCAATGAACTTGAACTTAATTATTATCCTAGTAGCAGTGGAGGCGAAGAAGGTGGCGA [ 5304]
cp32-4_B31_NX_Pl_consensus     TAGGATCAAAAAAGAGAAATTCAATGAACTTGAACTTAATTATTATCCTAGTAGCAGTGGAGGCGAAGAAGGTGGCGA [ 5304]
cp32-4_PAli_NX_MP_FG_consensus TAGGATCAAAAAAGAGAAATTCAATGAACTTGAACTTAATTATTATCCTAGTAGCAGTGGAGGCGAAGAAGGTGGCGA [ 5304]
cp32-4_PAli_NX_Pl_consensus    TAGGATCAAAAAAGAGAAATTCAATGAACTTGAACTTAATTATTATCCTAGTAGCAGTGGAGGCGAAGAAGGTGGCGA [ 5304]
cp32-4_PAbe_NX_MP_FG_consensus TAGGATCAAAAAAGAGAAATTCAATGAACTTGAACTTAATTATTATCCTAGTAGCAGTGGAGGCGAAGAAGGTGGCGA [ 5304]
cp32-4_PAbe_NX_Pl_consensus    TAGGATCAAAAAAGAGAAATTCAATGAACTTGAACTTAATTATTATCCTAGTAGCAGTGGAGGCGAAGAAGGTGGCGA [ 5304]

cp32-4_gi|11497149|B31_GB      GGGGGGTTGTGGCTCAAACAAGAATTTTTGTTCACAACTTGATGCATTTTTAGAAAAATTAAAAAGAGAAACTTCAAC [ 5382]
cp32-4_un10_B31_PacBio         GGGGGGTTGTGGCTCAAACAAGAATTTTTGTTCACAACTTGATGCATTTTTAGAAAAATTAAAAAGAGAAACTTCAAC [ 5382]
cp32-4_B31_TS_MP_FG_consensus  GGGGGGTTGTGGCTCAAACAAGAATTTTTGTTCACAACTTGATGCATTTTTAGAAAAATTAAAAAGAGAAACTTCAAC [ 5382]
cp32-4_B31_NX_Pl_consensus     GGGGGGTTGTGGCTCAAACAAGAATTTTTGTTCACAACTTGATGCATTTTTAGAAAAATTAAAAAGAGAAACTTCAAC [ 5382]
cp32-4_PAli_NX_MP_FG_consensus GGGGGGTTGTGGCTCAAACAAGAATTTTTGTTCACAACTTGATGCATTTTTAGAAAAATTAAAAAGAGAAACTTCAAC [ 5382]
cp32-4_PAli_NX_Pl_consensus    GGGGGGTTGTGGCTCAAACAAGAATTTTTGTTCACAACTTGATGCATTTTTAGAAAAATTAAAAAGAGAAACTTCAAC [ 5382]
cp32-4_PAbe_NX_MP_FG_consensus GGGGGGTTGTGGCTCAAACAAGAATTTTTGTTCACAACTTGATGCATTTTTAGAAAAATTAAAAAGAGAAACTTCAAC [ 5382]
cp32-4_PAbe_NX_Pl_consensus    GGGGGGTTGTGGCTCAAACAAGAATTTTTGTTCACAACTTGATGCATTTTTAGAAAAATTAAAAAGAGAAACTTCAAC [ 5382]

cp32-4_gi|11497149|B31_GB      GCCATCTTGTGTGGGAGTTGTCTAATGAATGGTGTTAGAAAAAGACTTTCTGATATGTCATTTCGTATGATCAACGTA [ 5460]
cp32-4_un10_B31_PacBio         GCCATCTTGTGTGGGAGTTGTCTAATGAATGGTGTTAGAAAAAGACTTTCTGATATGTCATTTCGTATGATCAACGTA [ 5460]
cp32-4_B31_TS_MP_FG_consensus  GCCATCTTGTGTGGGAGTTGTCTAATGAATGGTGTTAGAAAAAGACTTTCTGATATGTCATTTCGTATGATCAACGTA [ 5460]
cp32-4_B31_NX_Pl_consensus     GCCATCTTGTGTGGGAGTTGTCTAATGAATGGTGTTAGAAAAAGACTTTCTGATATGTCATTTCGTATGATCAACGTA [ 5460]
cp32-4_PAli_NX_MP_FG_consensus GCCATCTTGTGTGGGAGTTGTCTAATGAATGGTGTTAGAAAAAGACTTTCTGATATGTCATTTCGTATGATCAACGTA [ 5460]
cp32-4_PAli_NX_Pl_consensus    GCCATCTTGTGTGGGAGTTGTCTAATGAATGGTGTTAGAAAAAGACTTTCTGATATGTCATTTCGTATGATCAACGTA [ 5460]
cp32-4_PAbe_NX_MP_FG_consensus GCCATCTTGTGTGGGAGTTGTCTAATGAATGGTGTTAGAAAAAGACTTTCTGATATGTCATTTCGTATGATCAACGTA [ 5460]
cp32-4_PAbe_NX_Pl_consensus    GCCATCTTGTGTGGGAGTTGTCTAATGAATGGTGTTAGAAAAAGACTTTCTGATATGTCATTTCGTATGATCAACGTA [ 5460]

cp32-4_gi|11497149|B31_GB      TTTAAGGATCCTAAACCCTTAAAGTTTTATAAAGGTACTGTTGTAAAGCTTGAAAATGATTCTTCTTATCAGAGAGTA [ 5538]
cp32-4_un10_B31_PacBio         TTTAAGGATCCTAAACCCTTAAAGTTTTATAAAGGTACTGTTGTAAAGCTTGAAAATGATTCTTCTTATCAGAGAGTA [ 5538]
cp32-4_B31_TS_MP_FG_consensus  TTTAAGGATCCTAAACCCTTAAAGTTTTATAAAGGTACTGTTGTAAAGCTTGAAAATGATTCTTCTTATCAGAGAGTA [ 5538]
cp32-4_B31_NX_Pl_consensus     TTTAAGGATCCTAAACCCTTAAAGTTTTATAAAGGTACTGTTGTAAAGCTTGAAAATGATTCTTCTTATCAGAGAGTA [ 5538]
cp32-4_PAli_NX_MP_FG_consensus TTTAAGGATCCTAAACCCTTAAAGTTTTATAAAGGTACTGTTGTAAAGCTTGAAAATGATTCTTCTTATCAGAGAGTA [ 5538]
cp32-4_PAli_NX_Pl_consensus    TTTAAGGATCCTAAACCCTTAAAGTTTTATAAAGGTACTGTTGTAAAGCTTGAAAATGATTCTTCTTATCAGAGAGTA [ 5538]
cp32-4_PAbe_NX_MP_FG_consensus TTTAAGGATCCTAAACCCTTAAAGTTTTATAAAGGTACTGTTGTAAAGCTTGAAAATGATTCTTCTTATCAGAGAGTA [ 5538]
cp32-4_PAbe_NX_Pl_consensus    TTTAAGGATCCTAAACCCTTAAAGTTTTATAAAGGTACTGTTGTAAAGCTTGAAAATGATTCTTCTTATCAGAGAGTA [ 5538]

cp32-4_gi|11497149|B31_GB      TTTGATAAAAAATAAGTACACTGAATTCGCAGGAGTTATTATTGACATAAAGCCACAAGAACTTGCAATTCTTTATGAT [ 5616]
cp32-4_un10_B31_PacBio         TTTGATAAAAAATAAGTACACTGAATTCGCAGGAGTTATTATTGACATAAAGCCACAAGAACTTGCAATTCTTTATGAT [ 5616]
cp32-4_B31_TS_MP_FG_consensus  TTTGATAAAAAATAAGTACACTGAATTCGCAGGAGTTATTATTGACATAAAGCCACAAGAACTTGCAATTCTTTATGAT [ 5616]
cp32-4_B31_NX_Pl_consensus     TTTGATAAAAAATAAGTACACTGAATTCGCAGGAGTTATTATTGACATAAAGCCACAAGAACTTGCAATTCTTTATGAT [ 5616]
cp32-4_PAli_NX_MP_FG_consensus TTTGATAAAAAATAAGTACACTGAATTCGCAGGAGTTATTATTGACATAAAGCCACAAGAACTTGCAATTCTTTATGAT [ 5616]
cp32-4_PAli_NX_Pl_consensus    TTTGATAAAAAATAAGTACACTGAATTCGCAGGAGTTATTATTGACATAAAGCCACAAGAACTTGCAATTCTTTATGAT [ 5616]
cp32-4_PAbe_NX_MP_FG_consensus TTTGATAAAAAATAAGTACACTGAATTCGCAGGAGTTATTATTGACATAAAGCCACAAGAACTTGCAATTCTTTATGAT [ 5616]
```

```
cp32-4_PAbe_NX_Pl_consensus    TTTGATAAAAATAAGTACACTGAATTCGCAGGAGTTATTATTGACATAAAGCCACAAGAACTTGCAATTCTTTATGAT [ 5616]

cp32-4_gi|11497149|B31_GB      TCTGATATGTCTGATATTCAAGGATATTCCAAACTTTACACATATCAAGACCTTAACTATGAACTAAAAGACCGAATA [ 5694]
cp32-4_un10_B31_PacBio         TCTGATATGTCTGATATTCAAGGATATTCCAAACTTTACACATATCAAGACCTTAACTATGAACTAAAAGACCGAATA [ 5694]
cp32-4_B31_TS_MP_FG_consensus  TCTGATATGTCTGATATTCAAGGATATTCCAAACTTTACACATATCAAGACCTTAACTATGAACTAAAAGACCGAATA [ 5694]
cp32-4_B31_NX_Pl_consensus     TCTGATATGTCTGATATTCAAGGATATTCCAAACTTTACACATATCAAGACCTTAACTATGAACTAAAAGACCGAATA [ 5694]
cp32-4_PAli_NX_MP_FG_consensus TCTGATATGTCTGATATTCAAGGATATTCCAAACTTTACACATATCAAGACCTTAACTATGAACTAAAAGACCGAATA [ 5694]
cp32-4_PAli_NX_Pl_consensus    TCTGATATGTCTGATATTCAAGGATATTCCAAACTTTACACATATCAAGACCTTAACTATGAACTAAAAGACCGAATA [ 5694]
cp32-4_PAbe_NX_MP_FG_consensus TCTGATATGTCTGATATTCAAGGATATTCCAAACTTTACACATATCAAGACCTTAACTATGAACTAAAAGACCGAATA [ 5694]
cp32-4_PAbe_NX_Pl_consensus    TCTGATATGTCTGATATTCAAGGATATTCCAAACTTTACACATATCAAGACCTTAACTATGAACTAAAAGACCGAATA [ 5694]

cp32-4_gi|11497149|B31_GB      TCAATTGCAGATTTAGTTTACTTTGAAATATTTAGTATTGACTCTTCAATCGGATATTTTACTTTGGTTTTAAAGGAA [ 5772]
cp32-4_un10_B31_PacBio         TCAATTGCAGATTTAGTTTACTTTGAAATATTTAGTATTGACTCTTCAATCGGATATTTTACTTTGGTTTTAAAGGAA [ 5772]
cp32-4_B31_TS_MP_FG_consensus  TCAATTGCAGATTTAGTTTACTTTGAAATATTTAGTATTGACTCTTCAATCGGATATTTTACTTTGGTTTTAAAGGAA [ 5772]
cp32-4_B31_NX_Pl_consensus     TCAATTGCAGATTTAGTTTACTTTGAAATATTTAGTATTGACTCTTCAATCGGATATTTTACTTTGGTTTTAAAGGAA [ 5772]
cp32-4_PAli_NX_MP_FG_consensus TCAATTGCAGATTTAGTTTACTTTGAAATATTTAGTATTGACTCTTCAATCGGATATTTTACTTTGGTTTTAAAGGAA [ 5772]
cp32-4_PAli_NX_Pl_consensus    TCAATTGCAGATTTAGTTTACTTTGAAATATTTAGTATTGACTCTTCAATCGGATATTTTACTTTGGTTTTAAAGGAA [ 5772]
cp32-4_PAbe_NX_MP_FG_consensus TCAATTGCAGATTTAGTTTACTTTGAAATATTTAGTATTGACTCTTCAATCGGATATTTTACTTTGGTTTTAAAGGAA [ 5772]
cp32-4_PAbe_NX_Pl_consensus    TCAATTGCAGATTTAGTTTACTTTGAAATATTTAGTATTGACTCTTCAATCGGATATTTTACTTTGGTTTTAAAGGAA [ 5772]

cp32-4_gi|11497149|B31_GB      TTTATATGGACAAACTAGAATTTAAAATGGAATTGGAAATTGGGTGGTTTGGTGGTCGTGCAGGTATTGCTAAAATGC [ 5850]
cp32-4_un10_B31_PacBio         TTTATATGGACAAACTAGAATTTAAAATGGAATTGGAAATTGGGTGGTTTGGTGGTCGTGCAGGTATTGCTAAAATGC [ 5850]
cp32-4_B31_TS_MP_FG_consensus  TTTATATGGACAAACTAGAATTTAAAATGGAATTGGAAATTGGGTGGTTTGGTGGTCGTGCAGGTATTGCTAAAATGC [ 5850]
cp32-4_B31_NX_Pl_consensus     TTTATATGGACAAACTAGAATTTAAAATGGAATTGGAAATTGGGTGGTTTGGTGGTCGTGCAGGTATTGCTAAAATGC [ 5850]
cp32-4_PAli_NX_MP_FG_consensus TTTATATGGACAAACTAGAATTTAAAATGGAATTGGAAATTGGGTGGTTTGGTGGTCGTGCAGGTATTGCTAAAATGC [ 5850]
cp32-4_PAli_NX_Pl_consensus    TTTATATGGACAAACTAGAATTTAAAATGGAATTGGAAATTGGGTGGTTTGGTGGTCGTGCAGGTATTGCTAAAATGC [ 5850]
cp32-4_PAbe_NX_MP_FG_consensus TTTATATGGACAAACTAGAATTTAAAATGGAATTGGAAATTGGGTGGTTTGGTGGTCGTGCAGGTATTGCTAAAATGC [ 5850]
cp32-4_PAbe_NX_Pl_consensus    TTTATATGGACAAACTAGAATTTAAAATGGAATTGGAAATTGGGTGGTTTGGTGGTCGTGCAGGTATTGCTAAAATGC [ 5850]

cp32-4_gi|11497149|B31_GB      ATGAAAAAGGGAGTAGCAATTTACCAGCAAGAAAACATTTAACCAAAATTGCTAGTAGTTCTGAGTTTAGAGAATATA [ 5928]
cp32-4_un10_B31_PacBio         ATGAAAAAGGGAGTAGCAATTTACCAGCAAGAAAACATTTAACCAAAATTGCTAGTAGTTCTGAGTTTAGAGAATATA [ 5928]
cp32-4_B31_TS_MP_FG_consensus  ATGAAAAAGGGAGTAGCAATTTACCAGCAAGAAAACATTTAACCAAAATTGCTAGTAGTTCTGAGTTTAGAGAATATA [ 5928]
cp32-4_B31_NX_Pl_consensus     ATGAAAAAGGGAGTAGCAATTTACCAGCAAGAAAACATTTAACCAAAATTGCTAGTAGTTCTGAGTTTAGAGAATATA [ 5928]
cp32-4_PAli_NX_MP_FG_consensus ATGAAAAAGGGAGTAGCAATTTACCAGCAAGAAAACATTTAACCAAAATTGCTAGTAGTTCTGAGTTTAGAGAATATA [ 5928]
cp32-4_PAli_NX_Pl_consensus    ATGAAAAAGGGAGTAGCAATTTACCAGCAAGAAAACATTTAACCAAAATTGCTAGTAGTTCTGAGTTTAGAGAATATA [ 5928]
cp32-4_PAbe_NX_MP_FG_consensus ATGAAAAAGGGAGTAGCAATTTACCAGCAAGAAAACATTTAACCAAAATTGCTAGTAGTTCTGAGTTTAGAGAATATA [ 5928]
cp32-4_PAbe_NX_Pl_consensus    ATGAAAAAGGGAGTAGCAATTTACCAGCAAGAAAACATTTAACCAAAATTGCTAGTAGTTCTGAGTTTAGAGAATATA [ 5928]

cp32-4_gi|11497149|B31_GB      TCAATAATAGCTATATAAATTCTAAGTTTAATCTTGACCCTAAATCGGGAATGGAGGCTATTGGACAAGCTTTTATAA [ 6006]
cp32-4_un10_B31_PacBio         TCAATAATAGCTATATAAATTCTAAGTTTAATCTTGACCCTAAATCGGGAATGGAGGCTATTGGACAAGCTTTTATAA [ 6006]
cp32-4_B31_TS_MP_FG_consensus  TCAATAATAGCTATATAAATTCTAAGTTTAATCTTGACCCTAAATCGGGAATGGAGGCTATTGGACAAGCTTTTATAA [ 6006]
cp32-4_B31_NX_Pl_consensus     TCAATAATAGCTATATAAATTCTAAGTTTAATCTTGACCCTAAATCGGGAATGGAGGCTATTGGACAAGCTTTTATAA [ 6006]
cp32-4_PAli_NX_MP_FG_consensus TCAATAATAGCTATATAAATTCTAAGTTTAATCTTGACCCTAAATCGGGAATGGAGGCTATTGGACAAGCTTTTATAA [ 6006]
cp32-4_PAli_NX_Pl_consensus    TCAATAATAGCTATATAAATTCTAAGTTTAATCTTGACCCTAAATCGGGAATGGAGGCTATTGGACAAGCTTTTATAA [ 6006]
cp32-4_PAbe_NX_MP_FG_consensus TCAATAATAGCTATATAAATTCTAAGTTTAATCTTGACCCTAAATCGGGAATGGAGGCTATTGGACAAGCTTTTATAA [ 6006]
cp32-4_PAbe_NX_Pl_consensus    TCAATAATAGCTATATAAATTCTAAGTTTAATCTTGACCCTAAATCGGGAATGGAGGCTATTGGACAAGCTTTTATAA [ 6006]

cp32-4_gi|11497149|B31_GB      GATACTATGAAAATTATCTACTATCAGCACAAGTCACTCCAGCCTTAAAGGCTAATACAATCAAAAGTAAGTTTAAAA [ 6084]
cp32-4_un10_B31_PacBio         GATACTATGAAAATTATCTACTATCAGCACAAGTCACTCCAGCCTTAAAGGCTAATACAATCAAAAGTAAGTTTAAAA [ 6084]
cp32-4_B31_TS_MP_FG_consensus  GATACTATGAAAATTATCTACTATCAGCACAAGTCACTCCAGCCTTAAAGGCTAATACAATCAAAAGTAAGTTTAAAA [ 6084]
cp32-4_B31_NX_Pl_consensus     GATACTATGAAAATTATCTACTATCAGCACAAGTCACTCCAGCCTTAAAGGCTAATACAATCAAAAGTAAGTTTAAAA [ 6084]
cp32-4_PAli_NX_MP_FG_consensus GATACTATGAAAATTATCTACTATCAGCACAAGTCACTCCAGCCTTAAAGGCTAATACAATCAAAAGTAAGTTTAAAA [ 6084]
cp32-4_PAli_NX_Pl_consensus    GATACTATGAAAATTATCTACTATCAGCACAAGTCACTCCAGCCTTAAAGGCTAATACAATCAAAAGTAAGTTTAAAA [ 6084]
cp32-4_PAbe_NX_MP_FG_consensus GATACTATGAAAATTATCTACTATCAGCACAAGTCACTCCAGCCTTAAAGGCTAATACAATCAAAAGTAAGTTTAAAA [ 6084]
cp32-4_PAbe_NX_Pl_consensus    GATACTATGAAAATTATCTACTATCAGCACAAGTCACTCCAGCCTTAAAGGCTAATACAATCAAAAGTAAGTTTAAAA [ 6084]

cp32-4_gi|11497149|B31_GB      GGGGTAGTAACACTGCAGCAATTCCACTTGTTGATACAGCCAAAATGTTATCAGAGATTACTTATAAGGTAACACTTG [ 6162]
cp32-4_un10_B31_PacBio         GGGGTAGTAACACTGCAGCAATTCCACTTGTTGATACAGCCAAAATGTTATCAGAGATTACTTATAAGGTAACACTTG [ 6162]
cp32-4_B31_TS_MP_FG_consensus  GGGGTAGTAACACTGCAGCAATTCCACTTGTTGATACAGCCAAAATGTTATCAGAGATTACTTATAAGGTAACACTTG [ 6162]
cp32-4_B31_NX_Pl_consensus     GGGGTAGTAACACTGCAGCAATTCCACTTGTTGATACAGCCAAAATGTTATCAGAGATTACTTATAAGGTAACACTTG [ 6162]
cp32-4_PAli_NX_MP_FG_consensus GGGGTAGTAACACTGCAGCAATTCCACTTGTTGATACAGCCAAAATGTTATCAGAGATTACTTATAAGGTAACACTTG [ 6162]
cp32-4_PAli_NX_Pl_consensus    GGGGTAGTAACACTGCAGCAATTCCACTTGTTGATACAGCCAAAATGTTATCAGAGATTACTTATAAGGTAACACTTG [ 6162]
cp32-4_PAbe_NX_MP_FG_consensus GGGGTAGTAACACTGCAGCAATTCCACTTGTTGATACAGCCAAAATGTTATCAGAGATTACTTATAAGGTAACACTTG [ 6162]
cp32-4_PAbe_NX_Pl_consensus    GGGGTAGTAACACTGCAGCAATTCCACTTGTTGATACAGCCAAAATGTTATCAGAGATTACTTATAAGGTAACACTTG [ 6162]

cp32-4_gi|11497149|B31_GB      AATGATTTTCACTTTAGATATGGTATTAAATCATTTAACTAAAATATTTAAAGGGTTTAAGGCGTATGCAACTGAAAA [ 6240]
cp32-4_un10_B31_PacBio         AATGATTTTCACTTTAGATATGGTATTAAATCATTTAACTAAAATATTTAAAGGGTTTAAGGCGTATGCAACTGAAAA [ 6240]
cp32-4_B31_TS_MP_FG_consensus  AATGATTTTCACTTTAGATATGGTATTAAATCATTTAACTAAAATATTTAAAGGGTTTAAGGCGTATGCAACTGAAAA [ 6240]
cp32-4_B31_NX_Pl_consensus     AATGATTTTCACTTTAGATATGGTATTAAATCATTTAACTAAAATATTTAAAGGGTTTAAGGCGTATGCAACTGAAAA [ 6240]
cp32-4_PAli_NX_MP_FG_consensus AATGATTTTCACTTTAGATATGGTATTAAATCATTTAACTAAAATATTTAAAGGGTTTAAGGCGTATGCAACTGAAAA [ 6240]
cp32-4_PAli_NX_Pl_consensus    AATGATTTTCACTTTAGATATGGTATTAAATCATTTAACTAAAATATTTAAAGGGTTTAAGGCGTATGCAACTGAAAA [ 6240]
cp32-4_PAbe_NX_MP_FG_consensus AATGATTTTCACTTTAGATATGGTATTAAATCATTTAACTAAAATATTTAAAGGGTTTAAGGCGTATGCAACTGAAAA [ 6240]
cp32-4_PAbe_NX_Pl_consensus    AATGATTTTCACTTTAGATATGGTATTAAATCATTTAACTAAAATATTTAAAGGGTTTAAGGCGTATGCAACTGAAAA [ 6240]

cp32-4_gi|11497149|B31_GB      TAATTTTGAGTGCGATATCATAAATACCTACAATCATCCATATCTTTCAAAAATCACAGCTGCTAGCTCAAATATAAT [ 6318]
cp32-4_un10_B31_PacBio         TAATTTTGAGTGCGATATCATAAATACCTACAATCATCCATATCTTTCAAAAATCACAGCTGCTAGCTCAAATATAAT [ 6318]
cp32-4_B31_TS_MP_FG_consensus  TAATTTTGAGTGCGATATCATAAATACCTACAATCATCCATATCTTTCAAAAATCACAGCTGCTAGCTCAAATATAAT [ 6318]
cp32-4_B31_NX_Pl_consensus     TAATTTTGAGTGCGATATCATAAATACCTACAATCATCCATATCTTTCAAAAATCACAGCTGCTAGCTCAAATATAAT [ 6318]
cp32-4_PAli_NX_MP_FG_consensus TAATTTTGAGTGCGATATCATAAATACCTACAATCATCCATATCTTTCAAAAATCACAGCTGCTAGCTCAAATATAAT [ 6318]
cp32-4_PAli_NX_Pl_consensus    TAATTTTGAGTGCGATATCATAAATACCTACAATCATCCATATCTTTCAAAAATCACAGCTGCTAGCTCAAATATAAT [ 6318]
cp32-4_PAbe_NX_MP_FG_consensus TAATTTTGAGTGCGATATCATAAATACCTACAATCATCCATATCTTTCAAAAATCACAGCTGCTAGCTCAAATATAAT [ 6318]
cp32-4_PAbe_NX_Pl_consensus    TAATTTTGAGTGCGATATCATAAATACCTACAATCATCCATATCTTTCAAAAATCACAGCTGCTAGCTCAAATATAAT [ 6318]

cp32-4_gi|11497149|B31_GB      AGCATTGAAATTTGCTGGTACAGAAAATCTATTTGATCATAATTCTAGAGCAGGTGTATTTTATGAGAATGCTTTGGA [ 6396]
cp32-4_un10_B31_PacBio         AGCATTGAAATTTGCTGGTACAGAAAATCTATTTGATCATAATTCTAGAGCAGGTGTATTTTATGAGAATGCTTTGGA [ 6396]
cp32-4_B31_TS_MP_FG_consensus  AGCATTGAAATTTGCTGGTACAGAAAATCTATTTGATCATAATTCTAGAGCAGGTGTATTTTATGAGAATGCTTTGGA [ 6396]
cp32-4_B31_NX_Pl_consensus     AGCATTGAAATTTGCTGGTACAGAAAATCTATTTGATCATAATTCTAGAGCAGGTGTATTTTATGAGAATGCTTTGGA [ 6396]
cp32-4_PAli_NX_MP_FG_consensus AGCATTGAAATTTGCTGGTACAGAAAATCTATTTGATCATAATTCTAGAGCAGGTGTATTTTATGAGAATGCTTTGGA [ 6396]
cp32-4_PAli_NX_Pl_consensus    AGCATTGAAATTTGCTGGTACAGAAAATCTATTTGATCATAATTCTAGAGCAGGTGTATTTTATGAGAATGCTTTGGA [ 6396]
cp32-4_PAbe_NX_MP_FG_consensus AGCATTGAAATTTGCTGGTACAGAAAATCTATTTGATCATAATTCTAGAGCAGGTGTATTTTATGAGAATGCTTTGGA [ 6396]
cp32-4_PAbe_NX_Pl_consensus    AGCATTGAAATTTGCTGGTACAGAAAATCTATTTGATCATAATTCTAGAGCAGGTGTATTTTATGAGAATGCTTTGGA [ 6396]

cp32-4_gi|11497149|B31_GB      ATTTAGTATAAATTTTCAAATATATATTATTGCAATAGTGTTAAACGCCAAAGACTTTGATGCTAATTCACGCATGTT [ 6474]
cp32-4_un10_B31_PacBio         ATTTAGTATAAATTTTCAAATATATATTATTGCAATAGTGTTAAACGCCAAAGACTTTGATGCTAATTCACGCATGTT [ 6474]
cp32-4_B31_TS_MP_FG_consensus  ATTTAGTATAAATTTTCAAATATATATTATTGCAATAGTGTTAAACGCCAAAGACTTTGATGCTAATTCACGCATGTT [ 6474]
cp32-4_B31_NX_Pl_consensus     ATTTAGTATAAATTTTCAAATATATATTATTGCAATAGTGTTAAACGCCAAAGACTTTGATGCTAATTCACGCATGTT [ 6474]
cp32-4_PAli_NX_MP_FG_consensus ATTTAGTATAAATTTTCAAATATATATTATTGCAATAGTGTTAAACGCCAAAGACTTTGATGCTAATTCACGCATGTT [ 6474]
cp32-4_PAli_NX_Pl_consensus    ATTTAGTATAAATTTTCAAATATATATTATTGCAATAGTGTTAAACGCCAAAGACTTTGATGCTAATTCACGCATGTT [ 6474]
cp32-4_PAbe_NX_MP_FG_consensus ATTTAGTATAAATTTTCAAATATATATTATTGCAATAGTGTTAAACGCCAAAGACTTTGATGCTAATTCACGCATGTT [ 6474]
cp32-4_PAbe_NX_Pl_consensus    ATTTAGTATAAATTTTCAAATATATATTATTGCAATAGTGTTAAACGCCAAAGACTTTGATGCTAATTCACGCATGTT [ 6474]

cp32-4_gi|11497149|B31_GB      AATGCTTTATAGTATGCTTAGTGACTTTCTACACAATAAAGCTCATAAGTATACTTTGCCCAGTCTACAACCCGAATA [ 6552]
cp32-4_un10_B31_PacBio         AATGCTTTATAGTATGCTTAGTGACTTTCTACACAATAAAGCTCATAAGTATACTTTGCCCAGTCTACAACCCGAATA [ 6552]
cp32-4_B31_TS_MP_FG_consensus  AATGCTTTATAGTATGCTTAGTGACTTTCTACACAATAAAGCTCATAAGTATACTTTGCCCAGTCTACAACCCGAATA [ 6552]
cp32-4_B31_NX_Pl_consensus     AATGCTTTATAGTATGCTTAGTGACTTTCTACACAATAAAGCTCATAAGTATACTTTGCCCAGTCTACAACCCGAATA [ 6552]
cp32-4_PAli_NX_MP_FG_consensus AATGCTTTATAGTATGCTTAGTGACTTTCTACACAATAAAGCTCATAAGTATACTTTGCCCAGTCTACAACCCGAATA [ 6552]
cp32-4_PAli_NX_Pl_consensus    AATGCTTTATAGTATGCTTAGTGACTTTCTACACAATAAAGCTCATAAGTATACTTTGCCCAGTCTACAACCCGAATA [ 6552]
cp32-4_PAbe_NX_MP_FG_consensus AATGCTTTATAGTATGCTTAGTGACTTTCTACACAATAAAGCTCATAAGTATACTTTGCCCAGTCTACAACCCGAATA [ 6552]
cp32-4_PAbe_NX_Pl_consensus    AATGCTTTATAGTATGCTTAGTGACTTTCTACACAATAAAGCTCATAAGTATACTTTGCCCAGTCTACAACCCGAATA [ 6552]
```

```
cp32-4_gi|11497149|B31_GB      TATTAATAAAATTAACTTCTACATTTACCCAACATCTAATATGCAAACAGTTGGACTGATTAATTTAGGCACAAAATA [ 6630]
cp32-4_un10_B31_PacBio         TATTAATAAAATTAACTTCTACATTTACCCAACATCTAATATGCAAACAGTTGGACTGATTAATTTAGGCACAAAATA [ 6630]
cp32-4_B31_TS_MP_FG_consensus  TATTAATAAAATTAACTTCTACATTTACCCAACATCTAATATGCAAACAGTTGGACTGATTAATTTAGGCACAAAATA [ 6630]
cp32-4_B31_NX_Pl_consensus     TATTAATAAAATTAACTTCTACATTTACCCAACATCTAATATGCAAACAGTTGGACTGATTAATTTAGGCACAAAATA [ 6630]
cp32-4_PAli_NX_MP_FG_consensus TATTAATAAAATTAACTTCTACATTTACCCAACATCTAATATGCAAACAGTTGGACTGATTAATTTAGGCACAAAATA [ 6630]
cp32-4_PAli_NX_Pl_consensus    TATTAATAAAATTAACTTCTACATTTACCCAACATCTAATATGCAAACAGTTGGACTGATTAATTTAGGCACAAAATA [ 6630]
cp32-4_PAbe_NX_MP_FG_consensus TATTAATAAAATTAACTTCTACATTTACCCAACATCTAATATGCAAACAGTTGGACTGATTAATTTAGGCACAAAATA [ 6630]
cp32-4_PAbe_NX_Pl_consensus    TATTAATAAAATTAACTTCTACATTTACCCAACATCTAATATGCAAACAGTTGGACTGATTAATTTAGGCACAAAATA [ 6630]

cp32-4_gi|11497149|B31_GB      TAGCAACCATGCATACAGTGCATCTATAGCATTCAATGCTAGTGTAAAAGCAATTGAAATTTTAAAGGAGGAATACGA [ 6708]
cp32-4_un10_B31_PacBio         TAGCAACCATGCATACAGTGCATCTATAGCATTCAATGCTAGTGTAAAAGCAATTGAAATTTTAAAGGAGGAATACGA [ 6708]
cp32-4_B31_TS_MP_FG_consensus  TAGCAACCATGCATACAGTGCATCTATAGCATTCAATGCTAGTGTAAAAGCAATTGAAATTTTAAAGGAGGAATACGA [ 6708]
cp32-4_B31_NX_Pl_consensus     TAGCAACCATGCATACAGTGCATCTATAGCATTCAATGCTAGTGTAAAAGCAATTGAAATTTTAAAGGAGGAATACGA [ 6708]
cp32-4_PAli_NX_MP_FG_consensus TAGCAACCATGCATACAGTGCATCTATAGCATTCAATGCTAGTGTAAAAGCAATTGAAATTTTAAAGGAGGAATACGA [ 6708]
cp32-4_PAli_NX_Pl_consensus    TAGCAACCATGCATACAGTGCATCTATAGCATTCAATGCTAGTGTAAAAGCAATTGAAATTTTAAAGGAGGAATACGA [ 6708]
cp32-4_PAbe_NX_MP_FG_consensus TAGCAACCATGCATACAGTGCATCTATAGCATTCAATGCTAGTGTAAAAGCAATTGAAATTTTAAAGGAGGAATACGA [ 6708]
cp32-4_PAbe_NX_Pl_consensus    TAGCAACCATGCATACAGTGCATCTATAGCATTCAATGCTAGTGTAAAAGCAATTGAAATTTTAAAGGAGGAATACGA [ 6708]

cp32-4_gi|11497149|B31_GB      AATTGCCGCAAGATACAATTAGTGTAAGTTTTATTGACTCTAGGATTCAGACTAGTAGGCCCAATTATTATAATCCAC [ 6786]
cp32-4_un10_B31_PacBio         AATTGCCGCAAGATACAATTAGTGTAAGTTTTATTGACTCTAGGATTCAGACTAGTAGGCCCAATTATTATAATCCAC [ 6786]
cp32-4_B31_TS_MP_FG_consensus  AATTGCCGCAAGATACAATTAGTGTAAGTTTTATTGACTCTAGGATTCAGACTAGTAGGCCCAATTATTATAATCCAC [ 6786]
cp32-4_B31_NX_Pl_consensus     AATTGCCGCAAGATACAATTAGTGTAAGTTTTATTGACTCTAGGATTCAGACTAGTAGGCCCAATTATTATAATCCAC [ 6786]
cp32-4_PAli_NX_MP_FG_consensus AATTGCCGCAAGATACAATTAGTGTAAGTTTTATTGACTCTAGGATTCAGACTAGTAGGCCCAATTATTATAATCCAC [ 6786]
cp32-4_PAli_NX_Pl_consensus    AATTGCCGCAAGATACAATTAGTGTAAGTTTTATTGACTCTAGGATTCAGACTAGTAGGCCCAATTATTATAATCCAC [ 6786]
cp32-4_PAbe_NX_MP_FG_consensus AATTGCCGCAAGATACAATTAGTGTAAGTTTTATTGACTCTAGGATTCAGACTAGTAGGCCCAATTATTATAATCCAC [ 6786]
cp32-4_PAbe_NX_Pl_consensus    AATTGCCGCAAGATACAATTAGTGTAAGTTTTATTGACTCTAGGATTCAGACTAGTAGGCCCAATTATTATAATCCAC [ 6786]

cp32-4_gi|11497149|B31_GB      TTTTGGTTTACAAAACAGCTAAAATCAAAGTTAACAAAGATACTGCTAGCTATAAAATATTGAATTTAACCGTTAATA [ 6864]
cp32-4_un10_B31_PacBio         TTTTGGTTTACAAAACAGCTAAAATCAAAGTTAACAAAGATACTGCTAGCTATAAAATATTGAATTTAACCGTTAATA [ 6864]
cp32-4_B31_TS_MP_FG_consensus  TTTTGGTTTACAAAACAGCTAAAATCAAAGTTAACAAAGATACTGCTAGCTATAAAATATTGAATTTAACCGTTAATA [ 6864]
cp32-4_B31_NX_Pl_consensus     TTTTGGTTTACAAAACAGCTAAAATCAAAGTTAACAAAGATACTGCTAGCTATAAAATATTGAATTTAACCGTTAATA [ 6864]
cp32-4_PAli_NX_MP_FG_consensus TTTTGGTTTACAAAACAGCTAAAATCAAAGTTAACAAAGATACTGCTAGCTATAAAATATTGAATTTAACCGTTAATA [ 6864]
cp32-4_PAli_NX_Pl_consensus    TTTTGGTTTACAAAACAGCTAAAATCAAAGTTAACAAAGATACTGCTAGCTATAAAATATTGAATTTAACCGTTAATA [ 6864]
cp32-4_PAbe_NX_MP_FG_consensus TTTTGGTTTACAAAACAGCTAAAATCAAAGTTAACAAAGATACTGCTAGCTATAAAATATTGAATTTAACCGTTAATA [ 6864]
cp32-4_PAbe_NX_Pl_consensus    TTTTGGTTTACAAAACAGCTAAAATCAAAGTTAACAAAGATACTGCTAGCTATAAAATATTGAATTTAACCGTTAATA [ 6864]

cp32-4_gi|11497149|B31_GB      ATTATGAAAAACAAATTGAAACTTTAGAAAAAGAGAATGGAAATGGAGAAGATCAGTTTGGAAAAGAAAAAACACTGC [ 6942]
cp32-4_un10_B31_PacBio         ATTATGAAAAACAAATTGAAACTTTAGAAAAAGAGAATGGAAATGGAGAAGATCAGTTTGGAAAAGAAAAAACACTGC [ 6942]
cp32-4_B31_TS_MP_FG_consensus  ATTATGAAAAACAAATTGAAACTTTAGAAAAAGAGAATGGAAATGGAGAAGATCAGTTTGGAAAAGAAAAAACACTGC [ 6942]
cp32-4_B31_NX_Pl_consensus     ATTATGAAAAACAAATTGAAACTTTAGAAAAAGAGAATGGAAATGGAGAAGATCAGTTTGGAAAAGAAAAAACACTGC [ 6942]
cp32-4_PAli_NX_MP_FG_consensus ATTATGAAAAACAAATTGAAACTTTAGAAAAAGAGAATGGAAATGGAGAAGATCAGTTTGGAAAAGAAAAAACACTGC [ 6942]
cp32-4_PAli_NX_Pl_consensus    ATTATGAAAAACAAATTGAAACTTTAGAAAAAGAGAATGGAAATGGAGAAGATCAGTTTGGAAAAGAAAAAACACTGC [ 6942]
cp32-4_PAbe_NX_MP_FG_consensus ATTATGAAAAACAAATTGAAACTTTAGAAAAAGAGAATGGAAATGGAGAAGATCAGTTTGGAAAAGAAAAAACACTGC [ 6942]
cp32-4_PAbe_NX_Pl_consensus    ATTATGAAAAACAAATTGAAACTTTAGAAAAAGAGAATGGAAATGGAGAAGATCAGTTTGGAAAAGAAAAAACACTGC [ 6942]

cp32-4_gi|11497149|B31_GB      TTAAAACTGCAATGTCAAATTTTTTCAATTCAAGCGAAGAATCGTTAAAATCAGCCGATCTTTTCATTTATAAGGATA [ 7020]
cp32-4_un10_B31_PacBio         TTAAAACTGCAATGTCAAATTTTTTCAATTCAAGCGAAGAATCGTTAAAATCAGCCGATCTTTTCATTTATAAGGATA [ 7020]
cp32-4_B31_TS_MP_FG_consensus  TTAAAACTGCAATGTCAAATTTTTTCAATTCAAGCGAAGAATCGTTAAAATCAGCCGATCTTTTCATTTATAAGGATA [ 7020]
cp32-4_B31_NX_Pl_consensus     TTAAAACTGCAATGTCAAATTTTTTCAATTCAAGCGAAGAATCGTTAAAATCAGCCGATCTTTTCATTTATAAGGATA [ 7020]
cp32-4_PAli_NX_MP_FG_consensus TTAAAACTGCAATGTCAAATTTTTTCAATTCAAGCGAAGAATCGTTAAAATCAGCCGATCTTTTCATTTATAAGGATA [ 7020]
cp32-4_PAli_NX_Pl_consensus    TTAAAACTGCAATGTCAAATTTTTTCAATTCAAGCGAAGAATCGTTAAAATCAGCCGATCTTTTCATTTATAAGGATA [ 7020]
cp32-4_PAbe_NX_MP_FG_consensus TTAAAACTGCAATGTCAAATTTTTTCAATTCAAGCGAAGAATCGTTAAAATCAGCCGATCTTTTCATTTATAAGGATA [ 7020]
cp32-4_PAbe_NX_Pl_consensus    TTAAAACTGCAATGTCAAATTTTTTCAATTCAAGCGAAGAATCGTTAAAATCAGCCGATCTTTTCATTTATAAGGATA [ 7020]

cp32-4_gi|11497149|B31_GB      AGCCTGAAGAGCTAAAAAATTATCTTAAAGTACATAGACACACTTTTGTTGTACTTATTAACACTGAGGGGGATGCGT [ 7098]
cp32-4_un10_B31_PacBio         AGCCTGAAGAGCTAAAAAATTATCTTAAAGTACATAGACACACTTTTGTTGTACTTATTAACACTGAGGGGGATGCGT [ 7098]
cp32-4_B31_TS_MP_FG_consensus  AGCCTGAAGAGCTAAAAAATTATCTTAAAGTACATAGACACACTTTTGTTGTACTTATTAACACTGAGGGGGATGCGT [ 7098]
cp32-4_B31_NX_Pl_consensus     AGCCTGAAGAGCTAAAAAATTATCTTAAAGTACATAGACACACTTTTGTTGTACTTATTAACACTGAGGGGGATGCGT [ 7098]
cp32-4_PAli_NX_MP_FG_consensus AGCCTGAAGAGCTAAAAAATTATCTTAAAGTACATAGACACACTTTTGTTGTACTTATTAACACTGAGGGGGATGCGT [ 7098]
cp32-4_PAli_NX_Pl_consensus    AGCCTGAAGAGCTAAAAAATTATCTTAAAGTACATAGACACACTTTTGTTGTACTTATTAACACTGAGGGGGATGCGT [ 7098]
cp32-4_PAbe_NX_MP_FG_consensus AGCCTGAAGAGCTAAAAAATTATCTTAAAGTACATAGACACACTTTTGTTGTACTTATTAACACTGAGGGGGATGCGT [ 7098]
cp32-4_PAbe_NX_Pl_consensus    AGCCTGAAGAGCTAAAAAATTATCTTAAAGTACATAGACACACTTTTGTTGTACTTATTAACACTGAGGGGGATGCGT [ 7098]

cp32-4_gi|11497149|B31_GB      CAGATGATGGACTTAAAATTTACAAAGATGACTATAATAAATTCAAAATGCCTTCAACTTTTTTTGTATTCTCGACTA [ 7176]
cp32-4_un10_B31_PacBio         CAGATGATGGACTTAAAATTTACAAAGATGACTATAATAAATTCAAAATGCCTTCAACTTTTTTTGTATTCTCGACTA [ 7176]
cp32-4_B31_TS_MP_FG_consensus  CAGATGATGGACTTAAAATTTACAAAGATGACTATAATAAATTCAAAATGCCTTCAACTTTTTTTGTATTCTCGACTA [ 7176]
cp32-4_B31_NX_Pl_consensus     CAGATGATGGACTTAAAATTTACAAAGATGACTATAATAAATTCAAAATGCCTTCAACTTTTTTTGTATTCTCGACTA [ 7176]
cp32-4_PAli_NX_MP_FG_consensus CAGATGATGGACTTAAAATTTACAAAGATGACTATAATAAATTCAAAATGCCTTCAACTTTTTTTGTATTCTCGACTA [ 7176]
cp32-4_PAli_NX_Pl_consensus    CAGATGATGGACTTAAAATTTACAAAGATGACTATAATAAATTCAAAATGCCTTCAACTTTTTTTGTATTCTCGACTA [ 7176]
cp32-4_PAbe_NX_MP_FG_consensus CAGATGATGGACTTAAAATTTACAAAGATGACTATAATAAATTCAAAATGCCTTCAACTTTTTTTGTATTCTCGACTA [ 7176]
cp32-4_PAbe_NX_Pl_consensus    CAGATGATGGACTTAAAATTTACAAAGATGACTATAATAAATTCAAAATGCCTTCAACTTTTTTTGTATTCTCGACTA [ 7176]

cp32-4_gi|11497149|B31_GB      AAGAACAAGAAATAAAAGAACTATTTAAAGATAAAGGCAATACTGAAAAAGAAAGAAATATTGCTG-TTTACAGTAAC [ 7254]
cp32-4_un10_B31_PacBio         AAGAACAAGAAATAAAAGAACTATTTAAAGATAAAGGCAATACTGAAAAAGAAAGAAATATTGCTGGTTTACAGTAAC [ 7254]
cp32-4_B31_TS_MP_FG_consensus  AAGAACAAGAAATAAAAGAACTATTTAAAGATAAAGGCAATACTGAAAAAGAAAGAAATATTGCTG-TTTACAGTAAC [ 7254]
cp32-4_B31_NX_Pl_consensus     AAGAACAAGAAATAAAAGAACTATTTAAAGATAAAGGCAATACTGAAAAAGAAAGAAATATTGCTG-TTTACAGTAAC [ 7254]
cp32-4_PAli_NX_MP_FG_consensus AAGAACAAGAAATAAAAGAACTATTTAAAGATAAAGGCAATACTGAAAAAGAAAGAAATATTGCTG-TTTACAGTAAC [ 7254]
cp32-4_PAli_NX_Pl_consensus    AAGAACAAGAAATAAAAGAACTATTTAAAGATAAAGGCAATACTGAAAAAGAAAGAAATATTGCTG-TTTACAGTAAC [ 7254]
cp32-4_PAbe_NX_MP_FG_consensus AAGAACAAGAAATAAAAGAACTATTTAAAGATAAAGGCAATACTGAAAAAGAAAGAAATATTGCTG-TTTACAGTAAC [ 7254]
cp32-4_PAbe_NX_Pl_consensus    AAGAACAAGAAATAAAAGAACTATTTAAAGATAAAGGCAATACTGAAAAAGAAAGAAATATTGCTG-TTTACAGTAAC [ 7254]

cp32-4_gi|11497149|B31_GB      AATAAAGACAATTTACACCTTAAATTTATAAGTCAATATCTCCATCAAGCTAGTATTTTTCATGCTGTAAATCCTTAT [ 7332]
cp32-4_un10_B31_PacBio         AATAAAGACAATTTACACCTTAAATTTATAAGTCAATATCTCCATCAAGCTAGTATTTTTCATGCTGTAAATCCTTAT [ 7332]
cp32-4_B31_TS_MP_FG_consensus  AATAAAGACAATTTACACCTTAAATTTATAAGTCAATATCTCCATCAAGCTAGTATTTTTCATGCTGTAAATCCTTAT [ 7332]
cp32-4_B31_NX_Pl_consensus     AATAAAGACAATTTACACCTTAAATTTATAAGTCAATATCTCCATCAAGCTAGTATTTTTCATGCTGTAAATCCTTAT [ 7332]
cp32-4_PAli_NX_MP_FG_consensus AATAAAGACAATTTACACCTTAAATTTATAAGTCAATATCTCCATCAAGCTAGTATTTTTCATGCTGTAAATCCTTAT [ 7332]
cp32-4_PAli_NX_Pl_consensus    AATAAAGACAATTTACACCTTAAATTTATAAGTCAATATCTCCATCAAGCTAGTATTTTTCATGCTGTAAATCCTTAT [ 7332]
cp32-4_PAbe_NX_MP_FG_consensus AATAAAGACAATTTACACCTTAAATTTATAAGTCAATATCTCCATCAAGCTAGTATTTTTCATGCTGTAAATCCTTAT [ 7332]
cp32-4_PAbe_NX_Pl_consensus    AATAAAGACAATTTACACCTTAAATTTATAAGTCAATATCTCCATCAAGCTAGTATTTTTCATGCTGTAAATCCTTAT [ 7332]

cp32-4_gi|11497149|B31_GB      GGTATGCCGCTGGCTGCTACACCACTTGTTGATGATACTGTAATTGGAAAGTTGCGAACTGCAAAAATCAACTTTTAT [ 7410]
cp32-4_un10_B31_PacBio         GGTATGCCGCTGGCTGCTACACCACTTGTTGATGATACTGTAATTGGAAAGTTGCGAACTGCAAAAATCAACTTTTAT [ 7410]
cp32-4_B31_TS_MP_FG_consensus  GGTATGCCGCTGGCTGCTACACCACTTGTTGATGATACTGTAATTGGAAAGTTGCGAACTGCAAAAATCAACTTTTAT [ 7410]
cp32-4_B31_NX_Pl_consensus     GGTATGCCGCTGGCTGCTACACCACTTGTTGATGATACTGTAATTGGAAAGTTGCGAACTGCAAAAATCAACTTTTAT [ 7410]
cp32-4_PAli_NX_MP_FG_consensus GGTATGCCGCTGGCTGCTACACCACTTGTTGATGATACTGTAATTGGAAAGTTGCGAACTGCAAAAATCAACTTTTAT [ 7410]
cp32-4_PAli_NX_Pl_consensus    GGTATGCCGCTGGCTGCTACACCACTTGTTGATGATACTGTAATTGGAAAGTTGCGAACTGCAAAAATCAACTTTTAT [ 7410]
cp32-4_PAbe_NX_MP_FG_consensus GGTATGCCGCTGGCTGCTACACCACTTGTTGATGATACTGTAATTGGAAAGTTGCGAACTGCAAAAATCAACTTTTAT [ 7410]
cp32-4_PAbe_NX_Pl_consensus    GGTATGCCGCTGGCTGCTACACCACTTGTTGATGATACTGTAATTGGAAAGTTGCGAACTGCAAAAATCAACTTTTAT [ 7410]

cp32-4_gi|11497149|B31_GB      TCACTTCTTAATGAAACTGGGCTTGATGGTGTACCTGCCTTTAAAGAAGGTGTTGACCTAGCTGGAGGTGCAATAGAC [ 7488]
cp32-4_un10_B31_PacBio         TCACTTCTTAATGAAACTGGGCTTGATG-TGTACCTGCCTTTAAAGAAGGTGTTGACCTAGCTGGAGGTGCAATAGAC [ 7488]
cp32-4_B31_TS_MP_FG_consensus  TCACTTCTTAATGAAACTGGGCTTGATGGTGTACCTGCCTTTAAAGAAGGTGTTGACCTAGCTGGAGGTGCAATAGAC [ 7488]
cp32-4_B31_NX_Pl_consensus     TCACTTCTTAATGAAACTGGGCTTGATGGTGTACCTGCCTTTAAAGAAGGTGTTGACCTAGCTGGAGGTGCAATAGAC [ 7488]
cp32-4_PAli_NX_MP_FG_consensus TCACTTCTTAATGAAACTGGGCTTGATGGTGTACCTGCCTTTAAAGAAGGTGTTGACCTAGCTGGAGGTGCAATAGAC [ 7488]
cp32-4_PAli_NX_Pl_consensus    TCACTTCTTAATGAAACTGGGCTTGATGGTGTACCTGCCTTTAAAGAAGGTGTTGACCTAGCTGGAGGTGCAATAGAC [ 7488]
cp32-4_PAbe_NX_MP_FG_consensus TCACTTCTTAATGAAACTGGGCTTGATGGTGTACCTGCCTTTAAAGAAGGTGTTGACCTAGCTGGAGGTGCAATAGAC [ 7488]
cp32-4_PAbe_NX_Pl_consensus    TCACTTCTTAATGAAACTGGGCTTGATGGTGTACCTGCCTTTAAAGAAGGTGTTGACCTAGCTGGAGGTGCAATAGAC [ 7488]
```

```
cp32-4_gi|11497149|B31_GB      GAACAATTTACATACCACTATATAAAAAACGAAGCGATTATTGAGCTTATTAGAATTTGGAACAAAAA-CAATAGGCA [ 7566]
cp32-4_un10_B31_PacBio         GAACAATTTACATACCACTATATAAAAAACGAAGCGATT-TTGAGCTTATTAGAATTTGGAACAAAAAACAATAGGCA [ 7566]
cp32-4_B31_TS_MP_FG_consensus  GAACAATTTACATACCACTATATAAAAAACGAAGCGATTATTGAGCTTATTAGAATTTGGAACAAAAA-CAATAGGCA [ 7566]
cp32-4_B31_NX_Pl_consensus     GAACAATTTACATACCACTATATAAAAAACGAAGCGATTATTGAGCTTATTAGAATTTGGAACAAAAA-CAATAGGCA [ 7566]
cp32-4_PAli_NX_MP_FG_consensus GAACAATTTACATACCACTATATAAAAAACGAAGCGATTATTGAGCTTATTAGAATTTGGAACAAAAA-CAATAGGCA [ 7566]
cp32-4_PAli_NX_Pl_consensus    GAACAATTTACATACCACTATATAAAAAACGAAGCGATTATTGAGCTTATTAGAATTTGGAACAAAAA-CAATAGGCA [ 7566]
cp32-4_PAbe_NX_MP_FG_consensus GAACAATTTACATACCACTATATAAAAAACGAAGCGATTATTGAGCTTATTAGAATTTGGAACAAAAA-CAATAGGCA [ 7566]
cp32-4_PAbe_NX_Pl_consensus    GAACAATTTACATACCACTATATAAAAAACGAAGCGATTATTGAGCTTATTAGAATTTGGAACAAAAA-CAATAGGCA [ 7566]

cp32-4_gi|11497149|B31_GB      AAATAGCAAATTATCTGCACTGCAGCTTAGTGGAGCTAGAGACAATGCATATACTTCAGCAATTGAATGTTTACTGAA [ 7644]
cp32-4_un10_B31_PacBio         AAATAGCAAATTATCTGCACTGCAGCTTAGTGGAGCTAGAGACAATGCATATACTTCAGCAATTGAATGTTTACTGAA [ 7644]
cp32-4_B31_TS_MP_FG_consensus  AAATAGCAAATTATCTGCACTGCAGCTTAGTGGAGCTAGAGACAATGCATATACTTCAGCAATTGAATGTTTACTGAA [ 7644]
cp32-4_B31_NX_Pl_consensus     AAATAGCAAATTATCTGCACTGCAGCTTAGTGGAGCTAGAGACAATGCATATACTTCAGCAATTGAATGTTTACTGAA [ 7644]
cp32-4_PAli_NX_MP_FG_consensus AAATAGCAAATTATCTGCACTGCAGCTTAGTGGAGCTAGAGACAATGCATATACTTCAGCAATTGAATGTTTACTGAA [ 7644]
cp32-4_PAli_NX_Pl_consensus    AAATAGCAAATTATCTGCACTGCAGCTTAGTGGAGCTAGAGACAATGCATATACTTCAGCAATTGAATGTTTACTGAA [ 7644]
cp32-4_PAbe_NX_MP_FG_consensus AAATAGCAAATTATCTGCACTGCAGCTTAGTGGAGCTAGAGACAATGCATATACTTCAGCAATTGAATGTTTACTGAA [ 7644]
cp32-4_PAbe_NX_Pl_consensus    AAATAGCAAATTATCTGCACTGCAGCTTAGTGGAGCTAGAGACAATGCATATACTTCAGCAATTGAATGTTTACTGAA [ 7644]

cp32-4_gi|11497149|B31_GB      AAGGTTTGTGGATAGAGGACTGATAATAGAGTATAAAAATTTAAGTCTTACTCTTTCTCCTACACCACAACTTAAATT [ 7722]
cp32-4_un10_B31_PacBio         AAGGTTTGTGGATAGAGGACTGATAATAGAGTATAAAAATTTAAGTCTTACTCTTTCTCCTACACCACAACTTAAATT [ 7722]
cp32-4_B31_TS_MP_FG_consensus  AAGGTTTGTGGATAGAGGACTGATAATAGAGTATAAAAATTTAAGTCTTACTCTTTCTCCTACACCACAACTTAAATT [ 7722]
cp32-4_B31_NX_Pl_consensus     AAGGTTTGTGGATAGAGGACTGATAATAGAGTATAAAAATTTAAGTCTTACTCTTTCTCCTACACCACAACTTAAATT [ 7722]
cp32-4_PAli_NX_MP_FG_consensus AAGGTTTGTGGATAGAGGACTGATAATAGAGTATAAAAATTTAAGTCTTACTCTTTCTCCTACACCACAACTTAAATT [ 7722]
cp32-4_PAli_NX_Pl_consensus    AAGGTTTGTGGATAGAGGACTGATAATAGAGTATAAAAATTTAAGTCTTACTCTTTCTCCTACACCACAACTTAAATT [ 7722]
cp32-4_PAbe_NX_MP_FG_consensus AAGGTTTGTGGATAGAGGACTGATAATAGAGTATAAAAATTTAAGTCTTACTCTTTCTCCTACACCACAACTTAAATT [ 7722]
cp32-4_PAbe_NX_Pl_consensus    AAGGTTTGTGGATAGAGGACTGATAATAGAGTATAAAAATTTAAGTCTTACTCTTTCTCCTACACCACAACTTAAATT [ 7722]

cp32-4_gi|11497149|B31_GB      AGAACTTAGCGTGAATATTACTTATAACTTTAGCATTAATGCTGTTGCTTTAGTAATTACTACTCAAGATATAGTTGA [ 7800]
cp32-4_un10_B31_PacBio         AGAACTTAGCGTGAATATTACTTATAACTTTAGCATTAATGCTGTTGCTTTAGTAATTACTACTCAAGATATAGTTGA [ 7800]
cp32-4_B31_TS_MP_FG_consensus  AGAACTTAGCGTGAATATTACTTATAACTTTAGCATTAATGCTGTTGCTTTAGTAATTACTACTCAAGATATAGTTGA [ 7800]
cp32-4_B31_NX_Pl_consensus     AGAACTTAGCGTGAATATTACTTATAACTTTAGCATTAATGCTGTTGCTTTAGTAATTACTACTCAAGATATAGTTGA [ 7800]
cp32-4_PAli_NX_MP_FG_consensus AGAACTTAGCGTGAATATTACTTATAACTTTAGCATTAATGCTGTTGCTTTAGTAATTACTACTCAAGATATAGTTGA [ 7800]
cp32-4_PAli_NX_Pl_consensus    AGAACTTAGCGTGAATATTACTTATAACTTTAGCATTAATGCTGTTGCTTTAGTAATTACTACTCAAGATATAGTTGA [ 7800]
cp32-4_PAbe_NX_MP_FG_consensus AGAACTTAGCGTGAATATTACTTATAACTTTAGCATTAATGCTGTTGCTTTAGTAATTACTACTCAAGATATAGTTGA [ 7800]
cp32-4_PAbe_NX_Pl_consensus    AGAACTTAGCGTGAATATTACTTATAACTTTAGCATTAATGCTGTTGCTTTAGTAATTACTACTCAAGATATAGTTGA [ 7800]

cp32-4_gi|11497149|B31_GB      TTATCAAAACAGCTTAAGTGCTTAAAAGGGGGTCTAAAAATGCAATTTTATGATTTAAGAGAAGTTTATTTTTCAATT [ 7878]
cp32-4_un10_B31_PacBio         TTATCAAAACAGCTTAAGTGCTTAAAAGGGGGTCTAAAAATGCAATTTTATGATTTAAGAGAAGTTTATTTTTCAATT [ 7878]
cp32-4_B31_TS_MP_FG_consensus  TTATCAAAACAGCTTAAGTGCTTAAAAGGGGGTCTAAAAATGCAATTTTATGATTTAAGAGAAGTTTATTTTTCAATT [ 7878]
cp32-4_B31_NX_Pl_consensus     TTATCAAAACAGCTTAAGTGCTTAAAAGGGGGTCTAAAAATGCAATTTTATGATTTAAGAGAAGTTTATTTTTCAATT [ 7878]
cp32-4_PAli_NX_MP_FG_consensus TTATCAAAACAGCTTAAGTGCTTAAAAGGGGGTCTAAAAATGCAATTTTATGATTTAAGAGAAGTTTATTTTTCAATT [ 7878]
cp32-4_PAli_NX_Pl_consensus    TTATCAAAACAGCTTAAGTGCTTAAAAGGGGGTCTAAAAATGCAATTTTATGATTTAAGAGAAGTTTATTTTTCAATT [ 7878]
cp32-4_PAbe_NX_MP_FG_consensus TTATCAAAACAGCTTAAGTGCTTAAAAGGGGGTCTAAAAATGCAATTTTATGATTTAAGAGAAGTTTATTTTTCAATT [ 7878]
cp32-4_PAbe_NX_Pl_consensus    TTATCAAAACAGCTTAAGTGCTTAAAAGGGGGTCTAAAAATGCAATTTTATGATTTAAGAGAAGTTTATTTTTCAATT [ 7878]

cp32-4_gi|11497149|B31_GB      GGTGGTACGCAGTTACATAGTGGCAAGCTAGAGCTTACAAGCGAACCTACAACAAGAGCAGTGATTAGTAGTGAAGAT [ 7956]
cp32-4_un10_B31_PacBio         GGTGGTACGCAGTTACATAGTGGCAAGCTAGAGCTTACAAGCGAACCTACAACAAGAGCAGTGATTAGTAGTGAAGAT [ 7956]
cp32-4_B31_TS_MP_FG_consensus  GGTGGTACGCAGTTACATAGTGGCAAGCTAGAGCTTACAAGCGAACCTACAACAAGAGCAGTGATTAGTAGTGAAGAT [ 7956]
cp32-4_B31_NX_Pl_consensus     GGTGGTACGCAGTTACATAGTGGCAAGCTAGAGCTTACAAGCGAACCTACAACAAGAGCAGTGATTAGTAGTGAAGAT [ 7956]
cp32-4_PAli_NX_MP_FG_consensus GGTGGTACGCAGTTACATAGTGGCAAGCTAGAGCTTACAAGCGAACCTACAACAAGAGCAGTGATTAGTAGTGAAGAT [ 7956]
cp32-4_PAli_NX_Pl_consensus    GGTGGTACGCAGTTACATAGTGGCAAGCTAGAGCTTACAAGCGAACCTACAACAAGAGCAGTGATTAGTAGTGAAGAT [ 7956]
cp32-4_PAbe_NX_MP_FG_consensus GGTGGTACGCAGTTACATAGTGGCAAGCTAGAGCTTACAAGCGAACCTACAACAAGAGCAGTGATTAGTAGTGAAGAT [ 7956]
cp32-4_PAbe_NX_Pl_consensus    GGTGGTACGCAGTTACATAGTGGCAAGCTAGAGCTTACAAGCGAACCTACAACAAGAGCAGTGATTAGTAGTGAAGAT [ 7956]

cp32-4_gi|11497149|B31_GB      AAAGGTATGCCTGTAATAAGCTTAAGAGATCCCAAAACAATAACTTATGTTTTCAATATTGAAGTGACACTAGGTAGT [ 8034]
cp32-4_un10_B31_PacBio         AAAGGTATGCCTGTAATAAGCTTAAGAGATCCCAAAACAATAACTTATGTTTTCAATATTGAAGTGACACTAGGTAGT [ 8034]
cp32-4_B31_TS_MP_FG_consensus  AAAGGTATGCCTGTAATAAGCTTAAGAGATCCCAAAACAATAACTTATGTTTTCAATATTGAAGTGACACTAGGTAGT [ 8034]
cp32-4_B31_NX_Pl_consensus     AAAGGTATGCCTGTAATAAGCTTAAGAGATCCCAAAACAATAACTTATGTTTTCAATATTGAAGTGACACTAGGTAGT [ 8034]
cp32-4_PAli_NX_MP_FG_consensus AAAGGTATGCCTGTAATAAGCTTAAGAGATCCCAAAACAATAACTTATGTTTTCAATATTGAAGTGACACTAGGTAGT [ 8034]
cp32-4_PAli_NX_Pl_consensus    AAAGGTATGCCTGTAATAAGCTTAAGAGATCCCAAAACAATAACTTATGTTTTCAATATTGAAGTGACACTAGGTAGT [ 8034]
cp32-4_PAbe_NX_MP_FG_consensus AAAGGTATGCCTGTAATAAGCTTAAGAGATCCCAAAACAATAACTTATGTTTTCAATATTGAAGTGACACTAGGTAGT [ 8034]
cp32-4_PAbe_NX_Pl_consensus    AAAGGTATGCCTGTAATAAGCTTAAGAGATCCCAAAACAATAACTTATGTTTTCAATATTGAAGTGACACTAGGTAGT [ 8034]

cp32-4_gi|11497149|B31_GB      CATGACTACATTTTGTTAACTGAACTTTCTGATGAACAGTTTTACAATATGGACGTGAGAAAAGAGGATAAAATGCTT [ 8112]
cp32-4_un10_B31_PacBio         CATGACTACATTTTGTTAACTGAACTTTCTGATGAACAGTTTTACAATATGGACGTGAGAAAAGAGGATAAAATGCTT [ 8112]
cp32-4_B31_TS_MP_FG_consensus  CATGACTACATTTTGTTAACTGAACTTTCTGATGAACAGTTTTACAATATGGACGTGAGAAAAGAGGATAAAATGCTT [ 8112]
cp32-4_B31_NX_Pl_consensus     CATGACTACATTTTGTTAACTGAACTTTCTGATGAACAGTTTTACAATATGGACGTGAGAAAAGAGGATAAAATGCTT [ 8112]
cp32-4_PAli_NX_MP_FG_consensus CATGACTACATTTTGTTAACTGAACTTTCTGATGAACAGTTTTACAATATGGACGTGAGAAAAGAGGATAAAATGCTT [ 8112]
cp32-4_PAli_NX_Pl_consensus    CATGACTACATTTTGTTAACTGAACTTTCTGATGAACAGTTTTACAATATGGACGTGAGAAAAGAGGATAAAATGCTT [ 8112]
cp32-4_PAbe_NX_MP_FG_consensus CATGACTACATTTTGTTAACTGAACTTTCTGATGAACAGTTTTACAATATGGACGTGAGAAAAGAGGATAAAATGCTT [ 8112]
cp32-4_PAbe_NX_Pl_consensus    CATGACTACATTTTGTTAACTGAACTTTCTGATGAACAGTTTTACAATATGGACGTGAGAAAAGAGGATAAAATGCTT [ 8112]

cp32-4_gi|11497149|B31_GB      GATTTAGCATTCAATGATAGAATTGCTACCAAAATTATTTCTAACTATGCAATTTTTACTGAAGAGCCTTCAAGAAGT [ 8190]
cp32-4_un10_B31_PacBio         GATTTAGCATTCAATGATAGAATTGCTACCAAAATTATTTCTAACTATGCAATTTT-ACTGAAGAGCCTTCAAGAAGT [ 8190]
cp32-4_B31_TS_MP_FG_consensus  GATTTAGCATTCAATGATAGAATTGCTACCAAAATTATTTCTAACTATGCAATTTTTACTGAAGAGCCTTCAAGAAGT [ 8190]
cp32-4_B31_NX_Pl_consensus     GATTTAGCATTCAATGATAGAATTGCTACCAAAATTATTTCTAACTATGCAATTTTTACTGAAGAGCCTTCAAGAAGT [ 8190]
cp32-4_PAli_NX_MP_FG_consensus GATTTAGCATTCAATGATAGAATTGCTACCAAAATTATTTCTAACTATGCAATTTTTACTGAAGAGCCTTCAAGAAGT [ 8190]
cp32-4_PAli_NX_Pl_consensus    GATTTAGCATTCAATGATAGAATTGCTACCAAAATTATTTCTAACTATGCAATTTTTACTGAAGAGCCTTCAAGAAGT [ 8190]
cp32-4_PAbe_NX_MP_FG_consensus GATTTAGCATTCAATGATAGAATTGCTACCAAAATTATTTCTAACTATGCAATTTTTACTGAAGAGCCTTCAAGAAGT [ 8190]
cp32-4_PAbe_NX_Pl_consensus    GATTTAGCATTCAATGATAGAATTGCTACCAAAATTATTTCTAACTATGCAATTTTTACTGAAGAGCCTTCAAGAAGT [ 8190]

cp32-4_gi|11497149|B31_GB      TATTCTGCTGAGGCCGAAAAAGTATCTTTTGAAATTAGGGCTATTAATTGCCAAAAATCTAAACCAAACAACACTTAA [ 8268]
cp32-4_un10_B31_PacBio         TATTCTGCTGAGGCCGAAAAAGTATCTTTTGAAATTAGGGCTATTAATTGCCAAAAATCTAAACCAAACAACACTTAA [ 8268]
cp32-4_B31_TS_MP_FG_consensus  TATTCTGCTGAGGCCGAAAAAGTATCTTTTGAAATTAGGGCTATTAATTGCCAAAAATCTAAACCAAACAACACTTAA [ 8268]
cp32-4_B31_NX_Pl_consensus     TATTCTGCTGAGGCCGAAAAAGTATCTTTTGAAATTAGGGCTATTAATTGCCAAAAATCTAAACCAAACAACACTTAA [ 8268]
cp32-4_PAli_NX_MP_FG_consensus TATTCTGCTGAGGCCGAAAAAGTATCTTTTGAAATTAGGGCTATTAATTGCCAAAAATCTAAACCAAACAACACTTAA [ 8268]
cp32-4_PAli_NX_Pl_consensus    TATTCTGCTGAGGCCGAAAAAGTATCTTTTGAAATTAGGGCTATTAATTGCCAAAAATCTAAACCAAACAACACTTAA [ 8268]
cp32-4_PAbe_NX_MP_FG_consensus TATTCTGCTGAGGCCGAAAAAGTATCTTTTGAAATTAGGGCTATTAATTGCCAAAAATCTAAACCAAACAACACTTAA [ 8268]
cp32-4_PAbe_NX_Pl_consensus    TATTCTGCTGAGGCCGAAAAAGTATCTTTTGAAATTAGGGCTATTAATTGCCAAAAATCTAAACCAAACAACACTTAA [ 8268]

cp32-4_gi|11497149|B31_GB      AAGGAGGGTCTTATTATGATAATGAGATA-TAAAATGAAAATTTTAACTAAAAATAAAACTTATGAATATCCGCTGAG [ 8346]
cp32-4_un10_B31_PacBio         AAGGAGGGTCTTATTATGATAATGAGATAATAAAATGAAAATTTTAACTAAAAATAAAACTTATGAATATCCGCTGAG [ 8346]
cp32-4_B31_TS_MP_FG_consensus  AAGGAGGGTCTTATTATGATAATGAGATA-TAAAATGAAAATTTTAACTAAAAATAAAACTTATGAATATCCGCTGAG [ 8346]
cp32-4_B31_NX_Pl_consensus     AAGGAGGGTCTTATTATGATAATGAGATA-TAAAATGAAAATTTTAACTAAAAATAAAACTTATGAATATCCGCTGAG [ 8346]
cp32-4_PAli_NX_MP_FG_consensus AAGGAGGGTCTTATTATGATAATGAGATA-TAAAATGAAAATTTTAACTAAAAATAAAACTTATGAATATCCGCTGAG [ 8346]
cp32-4_PAli_NX_Pl_consensus    AAGGAGGGTCTTATTATGATAATGAGATA-TAAAATGAAAATTTTAACTAAAAATAAAACTTATGAATATCCGCTGAG [ 8346]
cp32-4_PAbe_NX_MP_FG_consensus AAGGAGGGTCTTATTATGATAATGAGATA-TAAAATGAAAATTTTAACTAAAAATAAAACTTATGAATATCCGCTGAG [ 8346]
cp32-4_PAbe_NX_Pl_consensus    AAGGAGGGTCTTATTATGATAATGAGATA-TAAAATGAAAATTTTAACTAAAAATAAAACTTATGAATATCCGCTGAG [ 8346]

cp32-4_gi|11497149|B31_GB      AGTACTTCCGGTCTATGAATGGGATAAAGTGCTAGGATTTAATCAAAGTGACGCTGTTTTAAAGCTTAATGAGGTTAA [ 8424]
cp32-4_un10_B31_PacBio         AGTACTTCCGGTCTATGAATGGGATAAAGTGCTAGGATTTAATCAAAGTGACGCTGTTTTAAAGCTTAATGAGGTTAA [ 8424]
cp32-4_B31_TS_MP_FG_consensus  AGTACTTCCGGTCTATGAATGGGATAAAGTGCTAGGATTTAATCAAAGTGACGCTGTTTTAAAGCTTAATGAGGTTAA [ 8424]
cp32-4_B31_NX_Pl_consensus     AGTACTTCCGGTCTATGAATGGGATAAAGTGCTAGGATTTAATCAAAGTGACGCTGTTTTAAAGCTTAATGAGGTTAA [ 8424]
cp32-4_PAli_NX_MP_FG_consensus AGTACTTCCGGTCTATGAATGGGATAAAGTGCTAGGATTTAATCAAAGTGACGCTGTTTTAAAGCTTAATGAGGTTAA [ 8424]
cp32-4_PAli_NX_Pl_consensus    AGTACTTCCGGTCTATGAATGGGATAAAGTGCTAGGATTTAATCAAAGTGACGCTGTTTTAAAGCTTAATGAGGTTAA [ 8424]
cp32-4_PAbe_NX_MP_FG_consensus AGTACTTCCGGTCTATGAATGGGATAAAGTGCTAGGATTTAATCAAAGTGACGCTGTTTTAAAGCTTAATGAGGTTAA [ 8424]
cp32-4_PAbe_NX_Pl_consensus    AGTACTTCCGGTCTATGAATGGGATAAAGTGCTAGGATTTAATCAAAGTGACGCTGTTTTAAAGCTTAATGAGGTTAA [ 8424]

cp32-4_gi|11497149|B31_GB      ATACTTAAGAGAAATCACAAGCTTAATGATAAGTCCAAAATTTTTAGACGAATTCTATGTGATTTTGGATCAAAATAG [ 8502]
```

```
cp32-4_un10_B31_PacBio         ATACTTAAGAGAAATCACAAGCTTAATGATAAGTCCAAAATTTTTAGACGAATTCTATGTGATTTTGGATCAAAATAG [ 8502]
cp32-4_B31_TS_MP_FG_consensus  ATACTTAAGAGAAATCACAAGCTTAATGATAAGTCCAAAATTTTTAGACGAATTCTATGTGATTTTGGATCAAAATAG [ 8502]
cp32-4_B31_NX_Pl_consensus     ATACTTAAGAGAAATCACAAGCTTAATGATAAGTCCAAAATTTTTAGACGAATTCTATGTGATTTTGGATCAAAATAG [ 8502]
cp32-4_PAli_NX_MP_FG_consensus ATACTTAAGAGAAATCACAAGCTTAATGATAAGTCCAAAATTTTTAGACGAATTCTATGTGATTTTGGATCAAAATAG [ 8502]
cp32-4_PAli_NX_Pl_consensus    ATACTTAAGAGAAATCACAAGCTTAATGATAAGTCCAAAATTTTTAGACGAATTCTATGTGATTTTGGATCAAAATAG [ 8502]
cp32-4_PAbe_NX_MP_FG_consensus ATACTTAAGAGAAATCACAAGCTTAATGATAAGTCCAAAATTTTTAGACGAATTCTATGTGATTTTGGATCAAAATAG [ 8502]
cp32-4_PAbe_NX_Pl_consensus    ATACTTAAGAGAAATCACAAGCTTAATGATAAGTCCAAAATTTTTAGACGAATTCTATGTGATTTTGGATCAAAATAG [ 8502]

cp32-4_gi|11497149|B31_GB      AGAA-TTTATTTCTTATTATAAGGACTATCTTGTTGCAATAATTTACACTGCACAATTTAATACTTTTCATTTAGACA [ 8580]
cp32-4_un10_B31_PacBio         AGAAATTTATT-CTTATTATAAGGACTATCTTGTTGCAATAATTTACACTGCACAATTTAATACTTTTCATTTAGACA [ 8580]
cp32-4_B31_TS_MP_FG_consensus  AGAA-TTTATTTCTTATTATAAGGACTATCTTGTTGCAATAATTTACACTGCACAATTTAATACTTTTCATTTAGACA [ 8580]
cp32-4_B31_NX_Pl_consensus     AGAA-TTTATTTCTTATTATAAGGACTATCTTGTTGCAATAATTTACACTGCACAATTTAATACTTTTCATTTAGACA [ 8580]
cp32-4_PAli_NX_MP_FG_consensus AGAA-TTTATTTCTTATTATAAGGACTATCTTGTTGCAATAATTTACACTGCACAATTTAATACTTTTCATTTAGACA [ 8580]
cp32-4_PAli_NX_Pl_consensus    AGAA-TTTATTTCTTATTATAAGGACTATCTTGTTGCAATAATTTACACTGCACAATTTAATACTTTTCATTTAGACA [ 8580]
cp32-4_PAbe_NX_MP_FG_consensus AGAA-TTTATTTCTTATTATAAGGACTATCTTGTTGCAATAATTTACACTGCACAATTTAATACTTTTCATTTAGACA [ 8580]
cp32-4_PAbe_NX_Pl_consensus    AGAA-TTTATTTCTTATTATAAGGACTATCTTGTTGCAATAATTTACACTGCACAATTTAATACTTTTCATTTAGACA [ 8580]

cp32-4_gi|11497149|B31_GB      ATGATCTAAAAAAGCCCGCTTTAGTATATTTGAGTGAGTATGAAAATAATGTTGGTGATTTTGTTGCTTTTGACTATA [ 8658]
cp32-4_un10_B31_PacBio         ATGATCTAAAAAAGCCCGCTTTAGTATATTTGAGTGAGTATGAAAATAATGTTGGTGATTTTGTTGCTTTTGACTATA [ 8658]
cp32-4_B31_TS_MP_FG_consensus  ATGATCTAAAAAAGCCCGCTTTAGTATATTTGAGTGAGTATGAAAATAATGTTGGTGATTTTGTTGCTTTTGACTATA [ 8658]
cp32-4_B31_NX_Pl_consensus     ATGATCTAAAAAAGCCCGCTTTAGTATATTTGAGTGAGTATGAAAATAATGTTGGTGATTTTGTTGCTTTTGACTATA [ 8658]
cp32-4_PAli_NX_MP_FG_consensus ATGATCTAAAAAAGCCCGCTTTAGTATATTTGAGTGAGTATGAAAATAATGTTGGTGATTTTGTTGCTTTTGACTATA [ 8658]
cp32-4_PAli_NX_Pl_consensus    ATGATCTAAAAAAGCCCGCTTTAGTATATTTGAGTGAGTATGAAAATAATGTTGGTGATTTTGTTGCTTTTGACTATA [ 8658]
cp32-4_PAbe_NX_MP_FG_consensus ATGATCTAAAAAAGCCCGCTTTAGTATATTTGAGTGAGTATGAAAATAATGTTGGTGATTTTGTTGCTTTTGACTATA [ 8658]
cp32-4_PAbe_NX_Pl_consensus    ATGATCTAAAAAAGCCCGCTTTAGTATATTTGAGTGAGTATGAAAATAATGTTGGTGATTTTGTTGCTTTTGACTATA [ 8658]

cp32-4_gi|11497149|B31_GB      TTAATGAAAATTTTGATTATGAAAAAGTAGCCACTTCGCTTTCATCAATTACATCAAATTCCAATGAGCTGGTTGCTA [ 8736]
cp32-4_un10_B31_PacBio         TTAATGAAAATTTTGATTATGAAAAAGTAGCCACTTCGCTTTCATCAATTACATCAAATTCCAATGAGCTGGTTGCTA [ 8736]
cp32-4_B31_TS_MP_FG_consensus  TTAATGAAAATTTTGATTATGAAAAAGTAGCCACTTCGCTTTCATCAATTACATCAAATTCCAATGAGCTGGTTGCTA [ 8736]
cp32-4_B31_NX_Pl_consensus     TTAATGAAAATTTTGATTATGAAAAAGTAGCCACTTCGCTTTCATCAATTACATCAAATTCCAATGAGCTGGTTGCTA [ 8736]
cp32-4_PAli_NX_MP_FG_consensus TTAATGAAAATTTTGATTATGAAAAAGTAGCCACTTCGCTTTCATCAATTACATCAAATTCCAATGAGCTGGTTGCTA [ 8736]
cp32-4_PAli_NX_Pl_consensus    TTAATGAAAATTTTGATTATGAAAAAGTAGCCACTTCGCTTTCATCAATTACATCAAATTCCAATGAGCTGGTTGCTA [ 8736]
cp32-4_PAbe_NX_MP_FG_consensus TTAATGAAAATTTTGATTATGAAAAAGTAGCCACTTCGCTTTCATCAATTACATCAAATTCCAATGAGCTGGTTGCTA [ 8736]
cp32-4_PAbe_NX_Pl_consensus    TTAATGAAAATTTTGATTATGAAAAAGTAGCCACTTCGCTTTCATCAATTACATCAAATTCCAATGAGCTGGTTGCTA [ 8736]

cp32-4_gi|11497149|B31_GB      AATGAGCAAAAGAAATAGAGATATTGATAAAGCTATTGCAAGTCTTGATGAGACTAGAAAAAAATATTTTAACTTGCT [ 8814]
cp32-4_un10_B31_PacBio         AATGAGCAAAAGAAATAGAGATATTGATAAAGCTATTGCAAGTCTTGATGAGACTAGAAAAAAATATTTTAACTTGCT [ 8814]
cp32-4_B31_TS_MP_FG_consensus  AATGAGCAAAAGAAATAGAGATATTGATAAAGCTATTGCAAGTCTTGATGAGACTAGAAAAAAATATTTTAACTTGCT [ 8814]
cp32-4_B31_NX_Pl_consensus     AATGAGCAAAAGAAATAGAGATATTGATAAAGCTATTGCAAGTCTTGATGAGACTAGAAAAAAATATTTTAACTTGCT [ 8814]
cp32-4_PAli_NX_MP_FG_consensus AATGAGCAAAAGAAATAGAGATATTGATAAAGCTATTGCAAGTCTTGATGAGACTAGAAAAAAATATTTTAACTTGCT [ 8814]
cp32-4_PAli_NX_Pl_consensus    AATGAGCAAAAGAAATAGAGATATTGATAAAGCTATTGCAAGTCTTGATGAGACTAGAAAAAAATATTTTAACTTGCT [ 8814]
cp32-4_PAbe_NX_MP_FG_consensus AATGAGCAAAAGAAATAGAGATATTGATAAAGCTATTGCAAGTCTTGATGAGACTAGAAAAAAATATTTTAACTTGCT [ 8814]
cp32-4_PAbe_NX_Pl_consensus    AATGAGCAAAAGAAATAGAGATATTGATAAAGCTATTGCAAGTCTTGATGAGACTAGAAAAAAATATTTTAACTTGCT [ 8814]

cp32-4_gi|11497149|B31_GB      TGACGAGATTAAGAACGATAAATACTTTTTCCCAGTAATTATGAATATTTGCTCATACTACTCGGTTAAAAAATTGCC [ 8892]
cp32-4_un10_B31_PacBio         TGACGAGATTAAGAACGATAAATACTTTTTCCCAGTAATTATGAATATTTGCTCATACTACTCGGTTAAAAAATTGCC [ 8892]
cp32-4_B31_TS_MP_FG_consensus  TGACGAGATTAAGAACGATAAATACTTTTTCCCAGTAATTATGAATATTTGCTCATACTACTCGGTTAAAAAATTGCC [ 8892]
cp32-4_B31_NX_Pl_consensus     TGACGAGATTAAGAACGATAAATACTTTTTCCCAGTAATTATGAATATTTGCTCATACTACTCGGTTAAAAAATTGCC [ 8892]
cp32-4_PAli_NX_MP_FG_consensus TGACGAGATTAAGAACGATAAATACTTTTTCCCAGTAATTATGAATATTTGCTCATACTACTCGGTTAAAAAATTGCC [ 8892]
cp32-4_PAli_NX_Pl_consensus    TGACGAGATTAAGAACGATAAATACTTTTTCCCAGTAATTATGAATATTTGCTCATACTACTCGGTTAAAAAATTGCC [ 8892]
cp32-4_PAbe_NX_MP_FG_consensus TGACGAGATTAAGAACGATAAATACTTTTTCCCAGTAATTATGAATATTTGCTCATACTACTCGGTTAAAAAATTGCC [ 8892]
cp32-4_PAbe_NX_Pl_consensus    TGACGAGATTAAGAACGATAAATACTTTTTCCCAGTAATTATGAATATTTGCTCATACTACTCGGTTAAAAAATTGCC [ 8892]

cp32-4_gi|11497149|B31_GB      TTATGACGAGCTTTTAGAAGTCAATAGACTTGCTGAGATTAAATTAGAAAAAGAATTGTATGAATTAATTTTAAGCAA [ 8970]
cp32-4_un10_B31_PacBio         TTATGACGAGCTTTTAGAAGTCAATAGACTTGCTGAGATTAAATTAGAAAAAGAATTGTATGAATTAATTTTAAGCAA [ 8970]
cp32-4_B31_TS_MP_FG_consensus  TTATGACGAGCTTTTAGAAGTCAATAGACTTGCTGAGATTAAATTAGAAAAAGAATTGTATGAATTAATTTTAAGCAA [ 8970]
cp32-4_B31_NX_Pl_consensus     TTATGACGAGCTTTTAGAAGTCAATAGACTTGCTGAGATTAAATTAGAAAAAGAATTGTATGAATTAATTTTAAGCAA [ 8970]
cp32-4_PAli_NX_MP_FG_consensus TTATGACGAGCTTTTAGAAGTCAATAGACTTGCTGAGATTAAATTAGAAAAAGAATTGTATGAATTAATTTTAAGCAA [ 8970]
cp32-4_PAli_NX_Pl_consensus    TTATGACGAGCTTTTAGAAGTCAATAGACTTGCTGAGATTAAATTAGAAAAAGAATTGTATGAATTAATTTTAAGCAA [ 8970]
cp32-4_PAbe_NX_MP_FG_consensus TTATGACGAGCTTTTAGAAGTCAATAGACTTGCTGAGATTAAATTAGAAAAAGAATTGTATGAATTAATTTTAAGCAA [ 8970]
cp32-4_PAbe_NX_Pl_consensus    TTATGACGAGCTTTTAGAAGTCAATAGACTTGCTGAGATTAAATTAGAAAAAGAATTGTATGAATTAATTTTAAGCAA [ 8970]

cp32-4_gi|11497149|B31_GB      GTGAGGACTTAGTGAGCGACAAATTCACCATTAAATTTAAAGGTATTCTTGATCATGC-TGCAACAAAAAAGGCCATT [ 9048]
cp32-4_un10_B31_PacBio         GTGAGGACTTAGTGAGCGACAAATTCACCATTAAATTTAAAGGTATTCTTGATCATGCCTGCAACAAAAAAGGCCATT [ 9048]
cp32-4_B31_TS_MP_FG_consensus  GTGAGGACTTAGTGAGCGACAAATTCACCATTAAATTTAAAGGTATTCTTGATCATGC-TGCAACAAAAAAGGCCATT [ 9048]
cp32-4_B31_NX_Pl_consensus     GTGAGGACTTAGTGAGCGACAAATTCACCATTAAATTTAAAGGTATTCTTGATCATGC-TGCAACAAAAAAGGCCATT [ 9048]
cp32-4_PAli_NX_MP_FG_consensus GTGAGGACTTAGTGAGCGACAAATTCACCATTAAATTTAAAGGTATTCTTGATCATGC-TGCAACAAAAAAGGCCATT [ 9048]
cp32-4_PAli_NX_Pl_consensus    GTGAGGACTTAGTGAGCGACAAATTCACCATTAAATTTAAAGGTATTCTTGATCATGC-TGCAACAAAAAAGGCCATT [ 9048]
cp32-4_PAbe_NX_MP_FG_consensus GTGAGGACTTAGTGAGCGACAAATTCACCATTAAATTTAAAGGTATTCTTGATCATGC-TGCAACAAAAAAGGCCATT [ 9048]
cp32-4_PAbe_NX_Pl_consensus    GTGAGGACTTAGTGAGCGACAAATTCACCATTAAATTTAAAGGTATTCTTGATCATGC-TGCAACAAAAAAGGCCATT [ 9048]

cp32-4_gi|11497149|B31_GB      GAACAAGATATTTCTAAAATGGAAAAATATCTTAAACCTAAAAAATCTAGTTTGGGTAGCACTAAAGATATTGTAAAA [ 9126]
cp32-4_un10_B31_PacBio         GAACAAGATATTTCTAAAATGGAAAAATATCTTAAACCTAAAAAATCTAGTTTGGGTAGCACTAAAGATATTGTAAAA [ 9126]
cp32-4_B31_TS_MP_FG_consensus  GAACAAGATATTTCTAAAATGGAAAAATATCTTAAACCTAAAAAATCTAGTTTGGGTAGCACTAAAGATATTGTAAAA [ 9126]
cp32-4_B31_NX_Pl_consensus     GAACAAGATATTTCTAAAATGGAAAAATATCTTAAACCTAAAAAATCTAGTTTGGGTAGCACTAAAGATATTGTAAAA [ 9126]
cp32-4_PAli_NX_MP_FG_consensus GAACAAGATATTTCTAAAATGGAAAAATATCTTAAACCTAAAAAATCTAGTTTGGGTAGCACTAAAGATATTGTAAAA [ 9126]
cp32-4_PAli_NX_Pl_consensus    GAACAAGATATTTCTAAAATGGAAAAATATCTTAAACCTAAAAAATCTAGTTTGGGTAGCACTAAAGATATTGTAAAA [ 9126]
cp32-4_PAbe_NX_MP_FG_consensus GAACAAGATATTTCTAAAATGGAAAAATATCTTAAACCTAAAAAATCTAGTTTGGGTAGCACTAAAGATATTGTAAAA [ 9126]
cp32-4_PAbe_NX_Pl_consensus    GAACAAGATATTTCTAAAATGGAAAAATATCTTAAACCTAAAAAATCTAGTTTGGGTAGCACTAAAGATATTGTAAAA [ 9126]

cp32-4_gi|11497149|B31_GB      AATAATTTGTCGGACAAGAAAAAAGAACTTAGTAGACAATCTAAATTTGAAAGCTTAAGAGAGCGTGTTGAGAAATAT [ 9204]
cp32-4_un10_B31_PacBio         AATAATTTGTCGGACAAGAAAAAAGAACTTAGTAGACAATCTAAATTTGAAAGCTTAAGAGAGCGTGTTGAGAAATAT [ 9204]
cp32-4_B31_TS_MP_FG_consensus  AATAATTTGTCGGACAAGAAAAAAGAACTTAGTAGACAATCTAAATTTGAAAGCTTAAGAGAGCGTGTTGAGAAATAT [ 9204]
cp32-4_B31_NX_Pl_consensus     AATAATTTGTCGGACAAGAAAAAAGAACTTAGTAGACAATCTAAATTTGAAAGCTTAAGAGAGCGTGTTGAGAAATAT [ 9204]
cp32-4_PAli_NX_MP_FG_consensus AATAATTTGTCGGACAAGAAAAAAGAACTTAGTAGACAATCTAAATTTGAAAGCTTAAGAGAGCGTGTTGAGAAATAT [ 9204]
cp32-4_PAli_NX_Pl_consensus    AATAATTTGTCGGACAAGAAAAAAGAACTTAGTAGACAATCTAAATTTGAAAGCTTAAGAGAGCGTGTTGAGAAATAT [ 9204]
cp32-4_PAbe_NX_MP_FG_consensus AATAATTTGTCGGACAAGAAAAAAGAACTTAGTAGACAATCTAAATTTGAAAGCTTAAGAGAGCGTGTTGAGAAATAT [ 9204]
cp32-4_PAbe_NX_Pl_consensus    AATAATTTGTCGGACAAGAAAAAAGAACTTAGTAGACAATCTAAATTTGAAAGCTTAAGAGAGCGTGTTGAGAAATAT [ 9204]

cp32-4_gi|11497149|B31_GB      AGACTTACACAAACTAAAAAA-CTTATAAAACAGGGCATGGGATTTGAGAAAGCTAGAAAAGAGGCTTTCAGAAGATC [ 9282]
cp32-4_un10_B31_PacBio         AGACTTACACAAACTAAAAAAACTTATAAAACAGGGCATGGGATTTGAGAAAGCTAGAAAAGAGGCTTTCAGAAGATC [ 9282]
cp32-4_B31_TS_MP_FG_consensus  AGACTTACACAAACTAAAAAA-CTTATAAAACAGGGCATGGGATTTGAGAAAGCTAGAAAAGAGGCTTTCAGAAGATC [ 9282]
cp32-4_B31_NX_Pl_consensus     AGACTTACACAAACTAAAAAA-CTTATAAAACAGGGCATGGGATTTGAGAAAGCTAGAAAAGAGGCTTTCAGAAGATC [ 9282]
cp32-4_PAli_NX_MP_FG_consensus AGACTTACACAAACTAAAAAA-CTTATAAAACAGGGCATGGGATTTGAGAAAGCTAGAAAAGAGGCTTTCAGAAGATC [ 9282]
cp32-4_PAli_NX_Pl_consensus    AGACTTACACAAACTAAAAAA-CTTATAAAACAGGGCATGGGATTTGAGAAAGCTAGAAAAGAGGCTTTCAGAAGATC [ 9282]
cp32-4_PAbe_NX_MP_FG_consensus AGACTTACACAAACTAAAAAA-CTTATAAAACAGGGCATGGGATTTGAGAAAGCTAGAAAAGAGGCTTTCAGAAGATC [ 9282]
cp32-4_PAbe_NX_Pl_consensus    AGACTTACACAAACTAAAAAA-CTTATAAAACAGGGCATGGGATTTGAGAAAGCTAGAAAAGAGGCTTTCAGAAGATC [ 9282]

cp32-4_gi|11497149|B31_GB      TTTAATGTCTGATAGAGACAAAAGGCATCTTGAGTATAAAGAACTTGCAAAAGAATCAAAAGCAAAAAGTAAAATGTT [ 9360]
cp32-4_un10_B31_PacBio         TTTAATGTCTGATAGAGACAAAAGGCATCTTGAGTATAAAGAACTTGCAAAAGAATCAAAAGCAAAA-GTAAAATGTT [ 9360]
cp32-4_B31_TS_MP_FG_consensus  TTTAATGTCTGATAGAGACAAAAGGCATCTTGAGTATAAAGAACTTGCAAAAGAATCAAAAGCAAAAAGTAAAATGTT [ 9360]
cp32-4_B31_NX_Pl_consensus     TTTAATGTCTGATAGAGACAAAAGGCATCTTGAGTATAAAGAACTTGCAAAAGAATCAAAAGCAAAAAGTAAAATGTT [ 9360]
cp32-4_PAli_NX_MP_FG_consensus TTTAATGTCTGATAGAGACAAAAGGCATCTTGAGTATAAAGAACTTGCAAAAGAATCAAAAGCAAAAAGTAAAATGTT [ 9360]
cp32-4_PAli_NX_Pl_consensus    TTTAATGTCTGATAGAGACAAAAGGCATCTTGAGTATAAAGAACTTGCAAAAGAATCAAAAGCAAAAAGTAAAATGTT [ 9360]
cp32-4_PAbe_NX_MP_FG_consensus TTTAATGTCTGATAGAGACAAAAGGCATCTTGAGTATAAAGAACTTGCAAAAGAATCAAAAGCAAAAAGTAAAATGTT [ 9360]
cp32-4_PAbe_NX_Pl_consensus    TTTAATGTCTGATAGAGACAAAAGGCATCTTGAGTATAAAGAACTTGCAAAAGAATCAAAAGCAAAAAGTAAAATGTT [ 9360]

cp32-4_gi|11497149|B31_GB      AGCGGCCTCTCAAGGAAAAGGACTTGTTGCCAAAATTGCAATAGGTAGTGCCCTAAGGAATATCATTAGCAACGCTAT [ 9438]
cp32-4_un10_B31_PacBio         AGCGGCCTCTCAAGGAAAAGGACTTGTTGCCAAAATTGCAATAGGTAGTGCCCTAAGGAATATCATTAGCAACGCTAT [ 9438]
```

```
cp32-4_B31_TS_MP_FG_consensus  AGCGGCCTCTCAAGGAAAAGGACTTGTTGCCAAAATTGCAATAGGTAGTGCCCTAAGGAATATCATTAGCAACGCTAT [ 9438]
cp32-4_B31_NX_Pl_consensus     AGCGGCCTCTCAAGGAAAAGGACTTGTTGCCAAAATTGCAATAGGTAGTGCCCTAAGGAATATCATTAGCAACGCTAT [ 9438]
cp32-4_PAli_NX_MP_FG_consensus AGCGGCCTCTCAAGGAAAAGGACTTGTTGCCAAAATTGCAATAGGTAGTGCCCTAAGGAATATCATTAGCAACGCTAT [ 9438]
cp32-4_PAli_NX_Pl_consensus    AGCGGCCTCTCAAGGAAAAGGACTTGTTGCCAAAATTGCAATAGGTAGTGCCCTAAGGAATATCATTAGCAACGCTAT [ 9438]
cp32-4_PAbe_NX_MP_FG_consensus AGCGGCCTCTCAAGGAAAAGGACTTGTTGCCAAAATTGCAATAGGTAGTGCCCTAAGGAATATCATTAGCAACGCTAT [ 9438]
cp32-4_PAbe_NX_Pl_consensus    AGCGGCCTCTCAAGGAAAAGGACTTGTTGCCAAAATTGCAATAGGTAGTGCCCTAAGGAATATCATTAGCAACGCTAT [ 9438]

cp32-4_gi|11497149|B31_GB      GAGTAAAGTTGGAGGAGGCCTTTTAGGTTTTGCTAAAAAAGCAGTTGAAGAAGACACCAAAACAAAAAGAACAAAACT [ 9516]
cp32-4_un10_B31_PacBio         GAGTAAAGTTGGAGGAGGCCTTTTAGGTTTTGCTAAAAAAGCAGTTGAAGAAGACACCAAAACAAAAAGAACAAAACT [ 9516]
cp32-4_B31_TS_MP_FG_consensus  GAGTAAAGTTGGAGGAGGCCTTTTAGGTTTTGCTAAAAAAGCAGTTGAAGAAGACACCAAAACAAAAAGAACAAAACT [ 9516]
cp32-4_B31_NX_Pl_consensus     GAGTAAAGTTGGAGGAGGCCTTTTAGGTTTTGCTAAAAAAGCAGTTGAAGAAGACACCAAAACAAAAAGAACAAAACT [ 9516]
cp32-4_PAli_NX_MP_FG_consensus GAGTAAAGTTGGAGGAGGCCTTTTAGGTTTTGCTAAAAAAGCAGTTGAAGAAGACACCAAAACAAAAAGAACAAAACT [ 9516]
cp32-4_PAli_NX_Pl_consensus    GAGTAAAGTTGGAGGAGGCCTTTTAGGTTTTGCTAAAAAAGCAGTTGAAGAAGACACCAAAACAAAAAGAACAAAACT [ 9516]
cp32-4_PAbe_NX_MP_FG_consensus GAGTAAAGTTGGAGGAGGCCTTTTAGGTTTTGCTAAAAAAGCAGTTGAAGAAGACACCAAAACAAAAAGAACAAAACT [ 9516]
cp32-4_PAbe_NX_Pl_consensus    GAGTAAAGTTGGAGGAGGCCTTTTAGGTTTTGCTAAAAAAGCAGTTGAAGAAGACACCAAAACAAAAAGAACAAAACT [ 9516]

cp32-4_gi|11497149|B31_GB      TCTCAATAGTGCATTTTTTACAGATAACGAACGAAATATGATTATGGGAAATAAAGACAAGAATACTAAGGGAATTCT [ 9594]
cp32-4_un10_B31_PacBio         TCTCAATAGTGCATTTTTTACAGATAACGAACGAAATATGATTATGGGAAATAAAGACAAGAATACTAAGGGAATTCT [ 9594]
cp32-4_B31_TS_MP_FG_consensus  TCTCAATAGTGCATTTTTTACAGATAACGAACGAAATATGATTATGGGAAATAAAGACAAGAATACTAAGGGAATTCT [ 9594]
cp32-4_B31_NX_Pl_consensus     TCTCAATAGTGCATTTTTTACAGATAACGAACGAAATATGATTATGGGAAATAAAGACAAGAATACTAAGGGAATTCT [ 9594]
cp32-4_PAli_NX_MP_FG_consensus TCTCAATAGTGCATTTTTTACAGATAACGAACGAAATATGATTATGGGAAATAAAGACAAGAATACTAAGGGAATTCT [ 9594]
cp32-4_PAli_NX_Pl_consensus    TCTCAATAGTGCATTTTTTACAGATAACGAACGAAATATGATTATGGGAAATAAAGACAAGAATACTAAGGGAATTCT [ 9594]
cp32-4_PAbe_NX_MP_FG_consensus TCTCAATAGTGCATTTTTTACAGATAACGAACGAAATATGATTATGGGAAATAAAGACAAGAATACTAAGGGAATTCT [ 9594]
cp32-4_PAbe_NX_Pl_consensus    TCTCAATAGTGCATTTTTTACAGATAACGAACGAAATATGATTATGGGAAATAAAGACAAGAATACTAAGGGAATTCT [ 9594]

cp32-4_gi|11497149|B31_GB      TGACGGAATGAAGGGATTTGAGCGCGACTTAGAAAAAGAAGAATTCTTAAATCAAGCAAGTGTCTTTAAGGGTACTTT [ 9672]
cp32-4_un10_B31_PacBio         TGACGGAATGAAGGGATTTGAGCGCGACTTAGAAAAAGAAGAATTCTTAAATCAAGCAAGTGTCTTTAAGGGTACTTT [ 9672]
cp32-4_B31_TS_MP_FG_consensus  TGACGGAATGAAGGGATTTGAGCGCGACTTAGAAAAAGAAGAATTCTTAAATCAAGCAAGTGTCTTTAAGGGTACTTT [ 9672]
cp32-4_B31_NX_Pl_consensus     TGACGGAATGAAGGGATTTGAGCGCGACTTAGAAAAAGAAGAATTCTTAAATCAAGCAAGTGTCTTTAAGGGTACTTT [ 9672]
cp32-4_PAli_NX_MP_FG_consensus TGACGGAATGAAGGGATTTGAGCGCGACTTAGAAAAAGAAGAATTCTTAAATCAAGCAAGTGTCTTTAAGGGTACTTT [ 9672]
cp32-4_PAli_NX_Pl_consensus    TGACGGAATGAAGGGATTTGAGCGCGACTTAGAAAAAGAAGAATTCTTAAATCAAGCAAGTGTCTTTAAGGGTACTTT [ 9672]
cp32-4_PAbe_NX_MP_FG_consensus TGACGGAATGAAGGGATTTGAGCGCGACTTAGAAAAAGAAGAATTCTTAAATCAAGCAAGTGTCTTTAAGGGTACTTT [ 9672]
cp32-4_PAbe_NX_Pl_consensus    TGACGGAATGAAGGGATTTGAGCGCGACTTAGAAAAAGAAGAATTCTTAAATCAAGCAAGTGTCTTTAAGGGTACTTT [ 9672]

cp32-4_gi|11497149|B31_GB      AAGGGACTTAGATATGTTAAATGAAACTAATTTGAAAAACGCAGTAGAATTTGCAGCTATGCTTAAATCCAGTGGTGC [ 9750]
cp32-4_un10_B31_PacBio         AAGG-ACTTAGATATGTTAAATGAAACTAATTTGAAAAACGCAGTAGAATTTGCAGCTATGCTTAAATCCAGTGGTGC [ 9750]
cp32-4_B31_TS_MP_FG_consensus  AAGGGACTTAGATATGTTAAATGAAACTAATTTGAAAAACGCAGTAGAATTTGCAGCTATGCTTAAATCCAGTGGTGC [ 9750]
cp32-4_B31_NX_Pl_consensus     AAGGGACTTAGATATGTTAAATGAAACTAATTTGAAAAACGCAGTAGAATTTGCAGCTATGCTTAAATCCAGTGGTGC [ 9750]
cp32-4_PAli_NX_MP_FG_consensus AAGGGACTTAGATATGTTAAATGAAACTAATTTGAAAAACGCAGTAGAATTTGCAGCTATGCTTAAATCCAGTGGTGC [ 9750]
cp32-4_PAli_NX_Pl_consensus    AAGGGACTTAGATATGTTAAATGAAACTAATTTGAAAAACGCAGTAGAATTTGCAGCTATGCTTAAATCCAGTGGTGC [ 9750]
cp32-4_PAbe_NX_MP_FG_consensus AAGGGACTTAGATATGTTAAATGAAACTAATTTGAAAAACGCAGTAGAATTTGCAGCTATGCTTAAATCCAGTGGTGC [ 9750]
cp32-4_PAbe_NX_Pl_consensus    AAGGGACTTAGATATGTTAAATGAAACTAATTTGAAAAACGCAGTAGAATTTGCAGCTATGCTTAAATCCAGTGGTGC [ 9750]

cp32-4_gi|11497149|B31_GB      TATGAGCAGCGAAGATGCAGTAAAGGCTGTTAATAGTGTTCTTGGGGGTGATGGAAGTGAGCTTTTTGATCTATTAAA [ 9828]
cp32-4_un10_B31_PacBio         TATGAGCAGCGAAGATGCAGTAAAGGCTGTTAATAGTGTTCTTGGGGGTGATGGAAGTGAGCTTTT-GATCTATTAAA [ 9828]
cp32-4_B31_TS_MP_FG_consensus  TATGAGCAGCGAAGATGCAGTAAAGGCTGTTAATAGTGTTCTTGGGGGTGATGGAAGTGAGCTTTTTGATCTATTAAA [ 9828]
cp32-4_B31_NX_Pl_consensus     TATGAGCAGCGAAGATGCAGTAAAGGCTGTTAATAGTGTTCTTGGGGGTGATGGAAGTGAGCTTTTTGATCTATTAAA [ 9828]
cp32-4_PAli_NX_MP_FG_consensus TATGAGCAGCGAAGATGCAGTAAAGGCTGTTAATAGTGTTCTTGGGGGTGATGGAAGTGAGCTTTTTGATCTATTAAA [ 9828]
cp32-4_PAli_NX_Pl_consensus    TATGAGCAGCGAAGATGCAGTAAAGGCTGTTAATAGTGTTCTTGGGGGTGATGGAAGTGAGCTTTTTGATCTATTAAA [ 9828]
cp32-4_PAbe_NX_MP_FG_consensus TATGAGCAGCGAAGATGCAGTAAAGGCTGTTAATAGTGTTCTTGGGGGTGATGGAAGTGAGCTTTTTGATCTATTAAA [ 9828]
cp32-4_PAbe_NX_Pl_consensus    TATGAGCAGCGAAGATGCAGTAAAGGCTGTTAATAGTGTTCTTGGGGGTGATGGAAGTGAGCTTTTTGATCTATTAAA [ 9828]

cp32-4_gi|11497149|B31_GB      GAAGTCAGGTGTTGGAGACAAATATATAGAAGATGCCAAAATGGCCTGGCAAAGCGGGGCTCAAGTAGATCTAGATTC [ 9906]
cp32-4_un10_B31_PacBio         GAAGTCAGGTGTTGGAGACAAATATATAGAAGATGCCAAAATGGCCTGGCAAAGCGGGGCTCAAGTAGATCTAGATTC [ 9906]
cp32-4_B31_TS_MP_FG_consensus  GAAGTCAGGTGTTGGAGACAAATATATAGAAGATGCCAAAATGGCCTGGCAAAGCGGGGCTCAAGTAGATCTAGATTC [ 9906]
cp32-4_B31_NX_Pl_consensus     GAAGTCAGGTGTTGGAGACAAATATATAGAAGATGCCAAAATGGCCTGGCAAAGCGGGGCTCAAGTAGATCTAGATTC [ 9906]
cp32-4_PAli_NX_MP_FG_consensus GAAGTCAGGTGTTGGAGACAAATATATAGAAGATGCCAAAATGGCCTGGCAAAGCGGGGCTCAAGTAGATCTAGATTC [ 9906]
cp32-4_PAli_NX_Pl_consensus    GAAGTCAGGTGTTGGAGACAAATATATAGAAGATGCCAAAATGGCCTGGCAAAGCGGGGCTCAAGTAGATCTAGATTC [ 9906]
cp32-4_PAbe_NX_MP_FG_consensus GAAGTCAGGTGTTGGAGACAAATATATAGAAGATGCCAAAATGGCCTGGCAAAGCGGGGCTCAAGTAGATCTAGATTC [ 9906]
cp32-4_PAbe_NX_Pl_consensus    GAAGTCAGGTGTTGGAGACAAATATATAGAAGATGCCAAAATGGCCTGGCAAAGCGGGGCTCAAGTAGATCTAGATTC [ 9906]

cp32-4_gi|11497149|B31_GB      CAGAATTACCAAGATGATGGAAATGTTCAAGGATTTTAAATCTTTCGGCCTTACAAAAAAAGTCAATAATGCTGAAAG [ 9984]
cp32-4_un10_B31_PacBio         CAGAATTACCAAGATGATGGAAATGTTCAAGGATTTTAAATCTTTCGGCCTTACAAAAAAAGTCAATAATGCTGAAAG [ 9984]
cp32-4_B31_TS_MP_FG_consensus  CAGAATTACCAAGATGATGGAAATGTTCAAGGATTTTAAATCTTTCGGCCTTACAAAAAAAGTCAATAATGCTGAAAG [ 9984]
cp32-4_B31_NX_Pl_consensus     CAGAATTACCAAGATGATGGAAATGTTCAAGGATTTTAAATCTTTCGGCCTTACAAAAAAAGTCAATAATGCTGAAAG [ 9984]
cp32-4_PAli_NX_MP_FG_consensus CAGAATTACCAAGATGATGGAAATGTTCAAGGATTTTAAATCTTTCGGCCTTACAAAAAAAGTCAATAATGCTGAAAG [ 9984]
cp32-4_PAli_NX_Pl_consensus    CAGAATTACCAAGATGATGGAAATGTTCAAGGATTTTAAATCTTTCGGCCTTACAAAAAAAGTCAATAATGCTGAAAG [ 9984]
cp32-4_PAbe_NX_MP_FG_consensus CAGAATTACCAAGATGATGGAAATGTTCAAGGATTTTAAATCTTTCGGCCTTACAAAAAAAGTCAATAATGCTGAAAG [ 9984]
cp32-4_PAbe_NX_Pl_consensus    CAGAATTACCAAGATGATGGAAATGTTCAAGGATTTTAAATCTTTCGGCCTTACAAAAAAAGTCAATAATGCTGAAAG [ 9984]

cp32-4_gi|11497149|B31_GB      TATTCAAAGTAATTTGGCTTCAGCTGAGCAAACTCTTCAAAACTTAACCACCACTGTCTTAGACCCAATACTTGGCCT [10062]
cp32-4_un10_B31_PacBio         TATTCAAAGTAATTTGGCTTCAGCTGAGCAAACTCTTCAAAACTTAACCACCACTGTCTTAGACCCAATACTTGGCCT [10062]
cp32-4_B31_TS_MP_FG_consensus  TATTCAAAGTAATTTGGCTTCAGCTGAGCAAACTCTTCAAAACTTAACCACCACTGTCTTAGACCCAATACTTGGCCT [10062]
cp32-4_B31_NX_Pl_consensus     TATTCAAAGTAATTTGGCTTCAGCTGAGCAAACTCTTCAAAACTTAACCACCACTGTCTTAGACCCAATACTTGGCCT [10062]
cp32-4_PAli_NX_MP_FG_consensus TATTCAAAGTAATTTGGCTTCAGCTGAGCAAACTCTTCAAAACTTAACCACCACTGTCTTAGACCCAATACTTGGCCT [10062]
cp32-4_PAli_NX_Pl_consensus    TATTCAAAGTAATTTGGCTTCAGCTGAGCAAACTCTTCAAAACTTAACCACCACTGTCTTAGACCCAATACTTGGCCT [10062]
cp32-4_PAbe_NX_MP_FG_consensus TATTCAAAGTAATTTGGCTTCAGCTGAGCAAACTCTTCAAAACTTAACCACCACTGTCTTAGACCCAATACTTGGCCT [10062]
cp32-4_PAbe_NX_Pl_consensus    TATTCAAAGTAATTTGGCTTCAGCTGAGCAAACTCTTCAAAACTTAACCACCACTGTCTTAGACCCAATACTTGGCCT [10062]

cp32-4_gi|11497149|B31_GB      CGTTAATAAGATAACTAAATACTTTGAAGGCTTTAAGTTTGAAACACACATTATTAATCCCATAATTGATGGCATTAA [10140]
cp32-4_un10_B31_PacBio         CGTTAATAAGATAACTAAATACTTTGAAGGCTTTAAGTTTGAAACACACATTATTAATCCCATAATTGATGGCATTAA [10140]
cp32-4_B31_TS_MP_FG_consensus  CGTTAATAAGATAACTAAATACTTTGAAGGCTTTAAGTTTGAAACACACATTATTAATCCCATAATTGATGGCATTAA [10140]
cp32-4_B31_NX_Pl_consensus     CGTTAATAAGATAACTAAATACTTTGAAGGCTTTAAGTTTGAAACACACATTATTAATCCCATAATTGATGGCATTAA [10140]
cp32-4_PAli_NX_MP_FG_consensus CGTTAATAAGATAACTAAATACTTTGAAGGCTTTAAGTTTGAAACACACATTATTAATCCCATAATTGATGGCATTAA [10140]
cp32-4_PAli_NX_Pl_consensus    CGTTAATAAGATAACTAAATACTTTGAAGGCTTTAAGTTTGAAACACACATTATTAATCCCATAATTGATGGCATTAA [10140]
cp32-4_PAbe_NX_MP_FG_consensus CGTTAATAAGATAACTAAATACTTTGAAGGCTTTAAGTTTGAAACACACATTATTAATCCCATAATTGATGGCATTAA [10140]
cp32-4_PAbe_NX_Pl_consensus    CGTTAATAAGATAACTAAATACTTTGAAGGCTTTAAGTTTGAAACACACATTATTAATCCCATAATTGATGGCATTAA [10140]

cp32-4_gi|11497149|B31_GB      AAGTATTTTTAATCTTAATTATTTCTTTGCAAAATTAAAATCGATGCTACCTGGATGGATGGGCGGAGATGAGGGTGC [10218]
cp32-4_un10_B31_PacBio         AAGTATTTTTAATCTTAATTATTTCTTTGCAAAATTAAAATCGATGCTACCTGGATGGATGGGCGGAGATGAGGGTGC [10218]
cp32-4_B31_TS_MP_FG_consensus  AAGTATTTTTAATCTTAATTATTTCTTTGCAAAATTAAAATCGATGCTACCTGGATGGATGGGCGGAGATGAGGGTGC [10218]
cp32-4_B31_NX_Pl_consensus     AAGTATTTTTAATCTTAATTATTTCTTTGCAAAATTAAAATCGATGCTACCTGGATGGATGGGCGGAGATGAGGGTGC [10218]
cp32-4_PAli_NX_MP_FG_consensus AAGTATTTTTAATCTTAATTATTTCTTTGCAAAATTAAAATCGATGCTACCTGGATGGATGGGCGGAGATGAGGGTGC [10218]
cp32-4_PAli_NX_Pl_consensus    AAGTATTTTTAATCTTAATTATTTCTTTGCAAAATTAAAATCGATGCTACCTGGATGGATGGGCGGAGATGAGGGTGC [10218]
cp32-4_PAbe_NX_MP_FG_consensus AAGTATTTTTAATCTTAATTATTTCTTTGCAAAATTAAAATCGATGCTACCTGGATGGATGGGCGGAGATGAGGGTGC [10218]
cp32-4_PAbe_NX_Pl_consensus    AAGTATTTTTAATCTTAATTATTTCTTTGCAAAATTAAAATCGATGCTACCTGGATGGATGGGCGGAGATGAGGGTGC [10218]

cp32-4_gi|11497149|B31_GB      GGCTCTAAAAAAACTACAAGAAGAAGTTCAAAATCAAGACAATGCTAACAACACTCCATAATTTTGGTAAAAGGTAAT [10296]
cp32-4_un10_B31_PacBio         GGCTCTAAAAAAACTACAAGAAGAAGTTCAAAATCAAGACAATGCTAACAACACTCCATAATTTTGGTAAAAGGTAAT [10296]
cp32-4_B31_TS_MP_FG_consensus  GGCTCTAAAAAAACTACAAGAAGAAGTTCAAAATCAAGACAATGCTAACAACACTCCATAATTTTGGTAAAAGGTAAT [10296]
cp32-4_B31_NX_Pl_consensus     GGCTCTAAAAAAACTACAAGAAGAAGTTCAAAATCAAGACAATGCTAACAACACTCCATAATTTTGGTAAAAGGTAAT [10296]
cp32-4_PAli_NX_MP_FG_consensus GGCTCTAAAAAAACTACAAGAAGAAGTTCAAAATCAAGACAATGCTAACAACACTCCATAATTTTGGTAAAAGGTAAT [10296]
cp32-4_PAli_NX_Pl_consensus    GGCTCTAAAAAAACTACAAGAAGAAGTTCAAAATCAAGACAATGCTAACAACACTCCATAATTTTGGTAAAAGGTAAT [10296]
cp32-4_PAbe_NX_MP_FG_consensus GGCTCTAAAAAAACTACAAGAAGAAGTTCAAAATCAAGACAATGCTAACAACACTCCATAATTTTGGTAAAAGGTAAT [10296]
cp32-4_PAbe_NX_Pl_consensus    GGCTCTAAAAAAACTACAAGAAGAAGTTCAAAATCAAGACAATGCTAACAACACTCCATAATTTTGGTAAAAGGTAAT [10296]

cp32-4_gi|11497149|B31_GB      TACTTATGGATATTAACAATAAAAATACTAACAATAAAACAATGTTACCCCAGGAGATTTCTCAAATAATAAGAGATG [10374]
cp32-4_un10_B31_PacBio         TACTTATGGATATTAACAATAAAAATACTAACAATAAAACAATGTTACCCCAGGAGATTTCTCAAATAATAAGAGATG [10374]
cp32-4_B31_TS_MP_FG_consensus  TACTTATGGATATTAACAATAAAAATACTAACAATAAAACAATGTTACCCCAGGAGATTTCTCAAATAATAAGAGATG [10374]
```

```
cp32-4_B31_NX_Pl_consensus     TACTTATGGATATTAACAATAAAAATACTAACAATAAAACAATGTTACCCCAGGAGATTTCTCAAATAATAAGAGATG [10374]
cp32-4_PAli_NX_MP_FG_consensus TACTTATGGATATTAACAATAAAAATACTAACAATAAAACAATGTTACCCCAGGAGATTTCTCAAATAATAAGAGATG [10374]
cp32-4_PAli_NX_Pl_consensus    TACTTATGGATATTAACAATAAAAATACTAACAATAAAACAATGTTACCCCAGGAGATTTCTCAAATAATAAGAGATG [10374]
cp32-4_PAbe_NX_MP_FG_consensus TACTTATGGATATTAACAATAAAAATACTAACAATAAAACAATGTTACCCCAGGAGATTTCTCAAATAATAAGAGATG [10374]
cp32-4_PAbe_NX_Pl_consensus    TACTTATGGATATTAACAATAAAAATACTAACAATAAAACAATGTTACCCCAGGAGATTTCTCAAATAATAAGAGATG [10374]

cp32-4_gi|11497149|B31_GB      TAATAACCCAAATATTTGCTCTTTTTGGAGCAGATAATTTTTTAGTGTTATTTCCTAGAATGGATCTAAAAGGTTTTG [10452]
cp32-4_un10_B31_PacBio         TAATAACCCAAATATTTGCTCTTTTTGGAGCAGATAATTTTTTAGTGTTATTTCCTAGAATGGATCTAAAAGGTTTTG [10452]
cp32-4_B31_TS_MP_FG_consensus  TAATAACCCAAATATTTGCTCTTTTTGGAGCAGATAATTTTTTAGTGTTATTTCCTAGAATGGATCTAAAAGGTTTTG [10452]
cp32-4_B31_NX_Pl_consensus     TAATAACCCAAATATTTGCTCTTTTTGGAGCAGATAATTTTTTAGTGTTATTTCCTAGAATGGATCTAAAAGGTTTTG [10452]
cp32-4_PAli_NX_MP_FG_consensus TAATAACCCAAATATTTGCTCTTTTTGGAGCAGATAATTTTTTAGTGTTATTTCCTAGAATGGATCTAAAAGGTTTTG [10452]
cp32-4_PAli_NX_Pl_consensus    TAATAACCCAAATATTTGCTCTTTTTGGAGCAGATAATTTTTTAGTGTTATTTCCTAGAATGGATCTAAAAGGTTTTG [10452]
cp32-4_PAbe_NX_MP_FG_consensus TAATAACCCAAATATTTGCTCTTTTTGGAGCAGATAATTTTTTAGTGTTATTTCCTAGAATGGATCTAAAAGGTTTTG [10452]
cp32-4_PAbe_NX_Pl_consensus    TAATAACCCAAATATTTGCTCTTTTTGGAGCAGATAATTTTTTAGTGTTATTTCCTAGAATGGATCTAAAAGGTTTTG [10452]

cp32-4_gi|11497149|B31_GB      GATATGTTCCTCAATTGTTTTTTATAAAACCAAAAACCGAACTCATAACACGCACTTACAATACTAGTTGTTCTAAAA [10530]
cp32-4_un10_B31_PacBio         GATATGTTCCTCAATTGTTTTTTATAAAACCAAAAACCGAACTCATAACACGCACTTACAATACTAGTTGTTCTAAAA [10530]
cp32-4_B31_TS_MP_FG_consensus  GATATGTTCCTCAATTGTTTTTTATAAAACCAAAAACCGAACTCATAACACGCACTTACAATACTAGTTGTTCTAAAA [10530]
cp32-4_B31_NX_Pl_consensus     GATATGTTCCTCAATTGTTTTTTATAAAACCAAAAACCGAACTCATAACACGCACTTACAATACTAGTTGTTCTAAAA [10530]
cp32-4_PAli_NX_MP_FG_consensus GATATGTTCCTCAATTGTTTTTTATAAAACCAAAAACCGAACTCATAACACGCACTTACAATACTAGTTGTTCTAAAA [10530]
cp32-4_PAli_NX_Pl_consensus    GATATGTTCCTCAATTGTTTTTTATAAAACCAAAAACCGAACTCATAACACGCACTTACAATACTAGTTGTTCTAAAA [10530]
cp32-4_PAbe_NX_MP_FG_consensus GATATGTTCCTCAATTGTTTTTTATAAAACCAAAAACCGAACTCATAACACGCACTTACAATACTAGTTGTTCTAAAA [10530]
cp32-4_PAbe_NX_Pl_consensus    GATATGTTCCTCAATTGTTTTTTATAAAACCAAAAACCGAACTCATAACACGCACTTACAATACTAGTTGTTCTAAAA [10530]

cp32-4_gi|11497149|B31_GB      GACCGGTCATCAATTATTATGATAGAAAAGCGGAATATGTCAGCTACAATCCGGTAATGACTGGTGAACATATATCAT [10608]
cp32-4_un10_B31_PacBio         GACCGGTCATCAATTATTATGATAGAAAAGCGGAATATGTCAGCTACAATCCGGTAATGACTGGTGAACATATATCAT [10608]
cp32-4_B31_TS_MP_FG_consensus  GACCGGTCATCAATTATTATGATAGAAAAGCGGAATATGTCAGCTACAATCCGGTAATGACTGGTGAACATATATCAT [10608]
cp32-4_B31_NX_Pl_consensus     GACCGGTCATCAATTATTATGATAGAAAAGCGGAATATGTCAGCTACAATCCGGTAATGACTGGTGAACATATATCAT [10608]
cp32-4_PAli_NX_MP_FG_consensus GACCGGTCATCAATTATTATGATAGAAAAGCGGAATATGTCAGCTACAATCCGGTAATGACTGGTGAACATATATCAT [10608]
cp32-4_PAli_NX_Pl_consensus    GACCGGTCATCAATTATTATGATAGAAAAGCGGAATATGTCAGCTACAATCCGGTAATGACTGGTGAACATATATCAT [10608]
cp32-4_PAbe_NX_MP_FG_consensus GACCGGTCATCAATTATTATGATAGAAAAGCGGAATATGTCAGCTACAATCCGGTAATGACTGGTGAACATATATCAT [10608]
cp32-4_PAbe_NX_Pl_consensus    GACCGGTCATCAATTATTATGATAGAAAAGCGGAATATGTCAGCTACAATCCGGTAATGACTGGTGAACATATATCAT [10608]

cp32-4_gi|11497149|B31_GB      TAAACGGTGGAATACTAACATCCTTATATAAGGATATGCTTTCTTTACTCAAAATGACTGTTTTTGGCAATACTATGC [10686]
cp32-4_un10_B31_PacBio         TAAACGGTGGAATACTAACATCCTTATATAAGGATATGCTTTCTTTACTCAAAATGACTGTTTTTGGCAATACTATGC [10686]
cp32-4_B31_TS_MP_FG_consensus  TAAACGGTGGAATACTAACATCCTTATATAAGGATATGCTTTCTTTACTCAAAATGACTGTTTTTGGCAATACTATGC [10686]
cp32-4_B31_NX_Pl_consensus     TAAACGGTGGAATACTAACATCCTTATATAAGGATATGCTTTCTTTACTCAAAATGACTGTTTTTGGCAATACTATGC [10686]
cp32-4_PAli_NX_MP_FG_consensus TAAACGGTGGAATACTAACATCCTTATATAAGGATATGCTTTCTTTACTCAAAATGACTGTTTTTGGCAATACTATGC [10686]
cp32-4_PAli_NX_Pl_consensus    TAAACGGTGGAATACTAACATCCTTATATAAGGATATGCTTTCTTTACTCAAAATGACTGTTTTTGGCAATACTATGC [10686]
cp32-4_PAbe_NX_MP_FG_consensus TAAACGGTGGAATACTAACATCCTTATATAAGGATATGCTTTCTTTACTCAAAATGACTGTTTTTGGCAATACTATGC [10686]
cp32-4_PAbe_NX_Pl_consensus    TAAACGGTGGAATACTAACATCCTTATATAAGGATATGCTTTCTTTACTCAAAATGACTGTTTTTGGCAATACTATGC [10686]

cp32-4_gi|11497149|B31_GB      TACGCTTTGACGCTCATCTTGTAAAAGAACAACTAGCCAATAGAATACAAGCACAAGTCCCTTTTAGTATATATAGTC [10764]
cp32-4_un10_B31_PacBio         TACGCTTTGACGCTCATCTTGTAAAAGAACAACTAGCCAATAGAATACAAGCACAAGTCCCTTTTAGTATATATAGTC [10764]
cp32-4_B31_TS_MP_FG_consensus  TACGCTTTGACGCTCATCTTGTAAAAGAACAACTAGCCAATAGAATACAAGCACAAGTCCCTTTTAGTATATATAGTC [10764]
cp32-4_B31_NX_Pl_consensus     TACGCTTTGACGCTCATCTTGTAAAAGAACAACTAGCCAATAGAATACAAGCACAAGTCCCTTTTAGTATATATAGTC [10764]
cp32-4_PAli_NX_MP_FG_consensus TACGCTTTGACGCTCATCTTGTAAAAGAACAACTAGCCAATAGAATACAAGCACAAGTCCCTTTTAGTATATATAGTC [10764]
cp32-4_PAli_NX_Pl_consensus    TACGCTTTGACGCTCATCTTGTAAAAGAACAACTAGCCAATAGAATACAAGCACAAGTCCCTTTTAGTATATATAGTC [10764]
cp32-4_PAbe_NX_MP_FG_consensus TACGCTTTGACGCTCATCTTGTAAAAGAACAACTAGCCAATAGAATACAAGCACAAGTCCCTTTTAGTATATATAGTC [10764]
cp32-4_PAbe_NX_Pl_consensus    TACGCTTTGACGCTCATCTTGTAAAAGAACAACTAGCCAATAGAATACAAGCACAAGTCCCTTTTAGTATATATAGTC [10764]

cp32-4_gi|11497149|B31_GB      CAACTTTTGGCCTTAAAGAATTAGCTGTAATTACAAGTCTTTCGTTTAAGGACACTCCTTTCATTGACGAAGTTGAGG [10842]
cp32-4_un10_B31_PacBio         CAACTTTTGGCCTTAAAGAATTAGCTGTAATTACAAGTCTTTCGTTTAAGGACACTCCTTTCATTGACGAAGTTGAGG [10842]
cp32-4_B31_TS_MP_FG_consensus  CAACTTTTGGCCTTAAAGAATTAGCTGTAATTACAAGTCTTTCGTTTAAGGACACTCCTTTCATTGACGAAGTTGAGG [10842]
cp32-4_B31_NX_Pl_consensus     CAACTTTTGGCCTTAAAGAATTAGCTGTAATTACAAGTCTTTCGTTTAAGGACACTCCTTTCATTGACGAAGTTGAGG [10842]
cp32-4_PAli_NX_MP_FG_consensus CAACTTTTGGCCTTAAAGAATTAGCTGTAATTACAAGTCTTTCGTTTAAGGACACTCCTTTCATTGACGAAGTTGAGG [10842]
cp32-4_PAli_NX_Pl_consensus    CAACTTTTGGCCTTAAAGAATTAGCTGTAATTACAAGTCTTTCGTTTAAGGACACTCCTTTCATTGACGAAGTTGAGG [10842]
cp32-4_PAbe_NX_MP_FG_consensus CAACTTTTGGCCTTAAAGAATTAGCTGTAATTACAAGTCTTTCGTTTAAGGACACTCCTTTCATTGACGAAGTTGAGG [10842]
cp32-4_PAbe_NX_Pl_consensus    CAACTTTTGGCCTTAAAGAATTAGCTGTAATTACAAGTCTTTCGTTTAAGGACACTCCTTTCATTGACGAAGTTGAGG [10842]

cp32-4_gi|11497149|B31_GB      TTAGTTTATCAATAGAAATAGTAAAAACATTTGCATTGGAAAAATATAAAGGATAAAAAATGCTGTTACTACAATATG [10920]
cp32-4_un10_B31_PacBio         TTAGTTTATCAATAGAAATAGTAAAAACATTTGCATTGGAAAAATATAAAGGATAAAAAATGCTGTTACTACAATATG [10920]
cp32-4_B31_TS_MP_FG_consensus  TTAGTTTATCAATAGAAATAGTAAAAACATTTGCATTGGAAAAATATAAAGGATAAAAAATGCTGTTACTACAATATG [10920]
cp32-4_B31_NX_Pl_consensus     TTAGTTTATCAATAGAAATAGTAAAAACATTTGCATTGGAAAAATATAAAGGATAAAAAATGCTGTTACTACAATATG [10920]
cp32-4_PAli_NX_MP_FG_consensus TTAGTTTATCAATAGAAATAGTAAAAACATTTGCATTGGAAAAATATAAAGGATAAAAAATGCTGTTACTACAATATG [10920]
cp32-4_PAli_NX_Pl_consensus    TTAGTTTATCAATAGAAATAGTAAAAACATTTGCATTGGAAAAATATAAAGGATAAAAAATGCTGTTACTACAATATG [10920]
cp32-4_PAbe_NX_MP_FG_consensus TTAGTTTATCAATAGAAATAGTAAAAACATTTGCATTGGAAAAATATAAAGGATAAAAAATGCTGTTACTACAATATG [10920]
cp32-4_PAbe_NX_Pl_consensus    TTAGTTTATCAATAGAAATAGTAAAAACATTTGCATTGGAAAAATATAAAGGATAAAAAATGCTGTTACTACAATATG [10920]

cp32-4_gi|11497149|B31_GB      ATTTTAAAATTGAGTTCTACAATGTAGATACATCAAAAAAATCACCTGATGGAATTCCTTTCGCCGAAGAAATTCCTA [10998]
cp32-4_un10_B31_PacBio         ATTTTAAAATTGAGTTCTACAATGTAGATACATCAAAAAAATCACCTGATGGAATTCCTTTCGCCGAAGAAATTCCTA [10998]
cp32-4_B31_TS_MP_FG_consensus  ATTTTAAAATTGAGTTCTACAATGTAGATACATCAAAAAAATCACCTGATGGAATTCCTTTCGCCGAAGAAATTCCTA [10998]
cp32-4_B31_NX_Pl_consensus     ATTTTAAAATTGAGTTCTACAATGTAGATACATCAAAAAAATCACCTGATGGAATTCCTTTCGCCGAAGAAATTCCTA [10998]
cp32-4_PAli_NX_MP_FG_consensus ATTTTAAAATTGAGTTCTACAATGTAGATACATCAAAAAAATCACCTGATGGAATTCCTTTCGCCGAAGAAATTCCTA [10998]
cp32-4_PAli_NX_Pl_consensus    ATTTTAAAATTGAGTTCTACAATGTAGATACATCAAAAAAATCACCTGATGGAATTCCTTTCGCCGAAGAAATTCCTA [10998]
cp32-4_PAbe_NX_MP_FG_consensus ATTTTAAAATTGAGTTCTACAATGTAGATACATCAAAAAAATCACCTGATGGAATTCCTTTCGCCGAAGAAATTCCTA [10998]
cp32-4_PAbe_NX_Pl_consensus    ATTTTAAAATTGAGTTCTACAATGTAGATACATCAAAAAAATCACCTGATGGAATTCCTTTCGCCGAAGAAATTCCTA [10998]

cp32-4_gi|11497149|B31_GB      AAATTATCATCAATACACAAGATGGAATTCATATTGATATTTCAATATCCAACGTGTATTCAAATATTCATACTATAA [11076]
cp32-4_un10_B31_PacBio         AAATTATCATCAATACACAAGATGGAATTCATATTGATATTTCAATATCCAACGTGTATTCAAATATTCATACTATAA [11076]
cp32-4_B31_TS_MP_FG_consensus  AAATTATCATCAATACACAAGATGGAATTCATATTGATATTTCAATATCCAACGTGTATTCAAATATTCATACTATAA [11076]
cp32-4_B31_NX_Pl_consensus     AAATTATCATCAATACACAAGATGGAATTCATATTGATATTTCAATATCCAACGTGTATTCAAATATTCATACTATAA [11076]
cp32-4_PAli_NX_MP_FG_consensus AAATTATCATCAATACACAAGATGGAATTCATATTGATATTTCAATATCCAACGTGTATTCAAATATTCATACTATAA [11076]
cp32-4_PAli_NX_Pl_consensus    AAATTATCATCAATACACAAGATGGAATTCATATTGATATTTCAATATCCAACGTGTATTCAAATATTCATACTATAA [11076]
cp32-4_PAbe_NX_MP_FG_consensus AAATTATCATCAATACACAAGATGGAATTCATATTGATATTTCAATATCCAACGTGTATTCAAATATTCATACTATAA [11076]
cp32-4_PAbe_NX_Pl_consensus    AAATTATCATCAATACACAAGATGGAATTCATATTGATATTTCAATATCCAACGTGTATTCAAATATTCATACTATAA [11076]

cp32-4_gi|11497149|B31_GB      GTTCCAAACAAGCAAAAGTCGTACTTTGGAATCTTCCCTTAGACTTCACCGACGACATTAAATTTGGAGATATAGTAA [11154]
cp32-4_un10_B31_PacBio         GTTCCAAACAAGCAAAAGTCGTACTTTGGAATCTTCCCTTAGACTTCACCGACGACATTAAATTTGGAGATATAGTAA [11154]
cp32-4_B31_TS_MP_FG_consensus  GTTCCAAACAAGCAAAAGTCGTACTTTGGAATCTTCCCTTAGACTTCACCGACGACATTAAATTTGGAGATATAGTAA [11154]
cp32-4_B31_NX_Pl_consensus     GTTCCAAACAAGCAAAAGTCGTACTTTGGAATCTTCCCTTAGACTTCACCGACGACATTAAATTTGGAGATATAGTAA [11154]
cp32-4_PAli_NX_MP_FG_consensus GTTCCAAACAAGCAAAAGTCGTACTTTGGAATCTTCCCTTAGACTTCACCGACGACATTAAATTTGGAGATATAGTAA [11154]
cp32-4_PAli_NX_Pl_consensus    GTTCCAAACAAGCAAAAGTCGTACTTTGGAATCTTCCCTTAGACTTCACCGACGACATTAAATTTGGAGATATAGTAA [11154]
cp32-4_PAbe_NX_MP_FG_consensus GTTCCAAACAAGCAAAAGTCGTACTTTGGAATCTTCCCTTAGACTTCACCGACGACATTAAATTTGGAGATATAGTAA [11154]
cp32-4_PAbe_NX_Pl_consensus    GTTCCAAACAAGCAAAAGTCGTACTTTGGAATCTTCCCTTAGACTTCACCGACGACATTAAATTTGGAGATATAGTAA [11154]

cp32-4_gi|11497149|B31_GB      AAATATATTATAAGAAATTTGCTCATGAAAAAAATTTTGATTTCATAATGGCAGGAACTTTAGGACCTCCTATGAGTA [11232]
cp32-4_un10_B31_PacBio         AAATATATTATAAGAAATTTGCTCATGAAAAAAATTTTGATTTCATAATGGCAGGAACTTTAGGACCTCCTATGAGTA [11232]
cp32-4_B31_TS_MP_FG_consensus  AAATATATTATAAGAAATTTGCTCATGAAAAAAATTTTGATTTCATAATGGCAGGAACTTTAGGACCTCCTATGAGTA [11232]
cp32-4_B31_NX_Pl_consensus     AAATATATTATAAGAAATTTGCTCATGAAAAAAATTTTGATTTCATAATGGCAGGAACTTTAGGACCTCCTATGAGTA [11232]
cp32-4_PAli_NX_MP_FG_consensus AAATATATTATAAGAAATTTGCTCATGAAAAAAATTTTGATTTCATAATGGCAGGAACTTTAGGACCTCCTATGAGTA [11232]
cp32-4_PAli_NX_Pl_consensus    AAATATATTATAAGAAATTTGCTCATGAAAAAAATTTTGATTTCATAATGGCAGGAACTTTAGGACCTCCTATGAGTA [11232]
cp32-4_PAbe_NX_MP_FG_consensus AAATATATTATAAGAAATTTGCTCATGAAAAAAATTTTGATTTCATAATGGCAGGAACTTTAGGACCTCCTATGAGTA [11232]
cp32-4_PAbe_NX_Pl_consensus    AAATATATTATAAGAAATTTGCTCATGAAAAAAATTTTGATTTCATAATGGCAGGAACTTTAGGACCTCCTATGAGTA [11232]

cp32-4_gi|11497149|B31_GB      CTGATTATCCGGGTGGGGATTTTAGTGTAGATCTTGATGTTCGTTTATTAACTAAAAGCAACTTCTTCAATCGTAAGT [11310]
cp32-4_un10_B31_PacBio         CTGATTATCCGGGTGGGGATTTTAGTGTAGATCTTGATGTTCGTTTATTAACTAAAAGCAACTTCTTCAATCGTAAGT [11310]
cp32-4_B31_TS_MP_FG_consensus  CTGATTATCCGGGTGGGGATTTTAGTGTAGATCTTGATGTTCGTTTATTAACTAAAAGCAACTTCTTCAATCGTAAGT [11310]
cp32-4_B31_NX_Pl_consensus     CTGATTATCCGGGTGGGGATTTTAGTGTAGATCTTGATGTTCGTTTATTAACTAAAAGCAACTTCTTCAATCGTAAGT [11310]
```

```
cp32-4_PAli_NX_MP_FG_consensus CTGATTATCCGGGTGGGGATTTTAGTGTAGATCTTGATGTTCGTTTATTAACTAAAAGCAACTTCTTCAATCGTAAGT [11310]
cp32-4_PAli_NX_Pl_consensus    CTGATTATCCGGGTGGGGATTTTAGTGTAGATCTTGATGTTCGTTTATTAACTAAAAGCAACTTCTTCAATCGTAAGT [11310]
cp32-4_PAbe_NX_MP_FG_consensus CTGATTATCCGGGTGGGGATTTTAGTGTAGATCTTGATGTTCGTTTATTAACTAAAAGCAACTTCTTCAATCGTAAGT [11310]
cp32-4_PAbe_NX_Pl_consensus    CTGATTATCCGGGTGGGGATTTTAGTGTAGATCTTGATGTTCGTTTATTAACTAAAAGCAACTTCTTCAATCGTAAGT [11310]

cp32-4_gi|11497149|B31_GB      TGGCAGGCAAAGAAGGCAAAAACTTTAAAGGCAAAACGGTGCAGGAGGCAATAGAATCTGTATTTCCCAATCGCAATA [11388]
cp32-4_un10_B31_PacBio         TGGCAGGCAAAGAAGGCAAAAACTTTAAAGGCAAAACGGTGCAGGAGGCAATAGAATCTGTATTTCCCAATCGCAATA [11388]
cp32-4_B31_TS_MP_FG_consensus  TGGCAGGCAAAGAAGGCAAAAACTTTAAAGGCAAAACGGTGCAGGAGGCAATAGAATCTGTATTTCCCAATCGCAATA [11388]
cp32-4_B31_NX_Pl_consensus     TGGCAGGCAAAGAAGGCAAAAACTTTAAAGGCAAAACGGTGCAGGAGGCAATAGAATCTGTATTTCCCAATCGCAATA [11388]
cp32-4_PAli_NX_MP_FG_consensus TGGCAGGCAAAGAAGGCAAAAACTTTAAAGGCAAAACGGTGCAGGAGGCAATAGAATCTGTATTTCCCAATCGCAATA [11388]
cp32-4_PAli_NX_Pl_consensus    TGGCAGGCAAAGAAGGCAAAAACTTTAAAGGCAAAACGGTGCAGGAGGCAATAGAATCTGTATTTCCCAATCGCAATA [11388]
cp32-4_PAbe_NX_MP_FG_consensus TGGCAGGCAAAGAAGGCAAAAACTTTAAAGGCAAAACGGTGCAGGAGGCAATAGAATCTGTATTTCCCAATCGCAATA [11388]
cp32-4_PAbe_NX_Pl_consensus    TGGCAGGCAAAGAAGGCAAAAACTTTAAAGGCAAAACGGTGCAGGAGGCAATAGAATCTGTATTTCCCAATCGCAATA [11388]

cp32-4_gi|11497149|B31_GB      TCCTTAATATGGATGAAAAAGATTGTCTTAAAATTATTGACAAAGATATTTATGCCACAACACCAAAAGAGTTTATTG [11466]
cp32-4_un10_B31_PacBio         TCCTTAATATGGATGAAAAAGATTGTCTTAAAATTATTGACAAAGATATTTATGCCACAACACCAAAAGAGTTTATTG [11466]
cp32-4_B31_TS_MP_FG_consensus  TCCTTAATATGGATGAAAAAGATTGTCTTAAAATTATTGACAAAGATATTTATGCCACAACACCAAAAGAGTTTATTG [11466]
cp32-4_B31_NX_Pl_consensus     TCCTTAATATGGATGAAAAAGATTGTCTTAAAATTATTGACAAAGATATTTATGCCACAACACCAAAAGAGTTTATTG [11466]
cp32-4_PAli_NX_MP_FG_consensus TCCTTAATATGGATGAAAAAGATTGTCTTAAAATTATTGACAAAGATATTTATGCCACAACACCAAAAGAGTTTATTG [11466]
cp32-4_PAli_NX_Pl_consensus    TCCTTAATATGGATGAAAAAGATTGTCTTAAAATTATTGACAAAGATATTTATGCCACAACACCAAAAGAGTTTATTG [11466]
cp32-4_PAbe_NX_MP_FG_consensus TCCTTAATATGGATGAAAAAGATTGTCTTAAAATTATTGACAAAGATATTTATGCCACAACACCAAAAGAGTTTATTG [11466]
cp32-4_PAbe_NX_Pl_consensus    TCCTTAATATAGATGAAAAAGATTGTCTTAAAATTATTGACAAAGATATTTATGCCACAACACCAAAAGAGTTTATTG [11466]

cp32-4_gi|11497149|B31_GB      ACAAAATAAAAGGAACATATGTTCATAACGTAATAGCCGATATTGGTACTGGGTTACGGGGATATGAATGCTATCTGA [11544]
cp32-4_un10_B31_PacBio         ACAAAATAAAAGGAACATATGTTCATAACGTAATAGCCGATATTGGTACTGGGTTACGGGGATATGAATGCTATCTGA [11544]
cp32-4_B31_TS_MP_FG_consensus  ACAAAATAAAAGGAACATATGTTCATAACGTAATAGCCGATATTGGTACTGGGTTACGGGGATATGAATGCTATCTGA [11544]
cp32-4_B31_NX_Pl_consensus     ACAAAATAAAAGGAACATATGTTCATAACGTAATAGCCGATATTGGTACTGGGTTACGGGGATATGAATGCTATCTGA [11544]
cp32-4_PAli_NX_MP_FG_consensus ACAAAATAAAAGGAACATATGTTCATAACGTAATAGCCGATATTGGTACTGGGTTACGGGGATATGAATGCTATCTGA [11544]
cp32-4_PAli_NX_Pl_consensus    ACAAAATAAAAGGAACATATGTTCATAACGTAATAGCCGATATTGGTACTGGGTTACGGGGATATGAATGCTATCTGA [11544]
cp32-4_PAbe_NX_MP_FG_consensus ACAAAATAAAAGGAACATATGTTCATAACGTAATAGCCGATATTGGTACTGGGTTACGGGGATATGAATGCTATCTGA [11544]
cp32-4_PAbe_NX_Pl_consensus    ACAAAATAAAAGGAACATATGTTCATAACGTAATAGCCGATATTGGTACTGGGTTACGGGGATATGAATGCTATCTGA [11544]

cp32-4_gi|11497149|B31_GB      TATTTACTAATTATATAAAAAAGGGGGAAAATGTCCACTACGAGGCATTAGAAGACTATGGACTTGAATTTATACCAC [11622]
cp32-4_un10_B31_PacBio         TATTTACTAATTATATAAAAAAGGGGGAAAATGTCCACTACGAGGCATTAGAAGACTATGGACTTGAATTTATACCAC [11622]
cp32-4_B31_TS_MP_FG_consensus  TATTTACTAATTATATAAAAAAGGGGGAAAATGTCCACTACGAGGCATTAGAAGACTATGGACTTGAATTTATACCAC [11622]
cp32-4_B31_NX_Pl_consensus     TATTTACTAATTATATAAAAAAGGGGGAAAATGTCCACTACGAGGCATTAGAAGACTATGGACTTGAATTTATACCAC [11622]
cp32-4_PAli_NX_MP_FG_consensus TATTTACTAATTATATAAAAAAGGGGGAAAATGTCCACTACGAGGCATTAGAAGACTATGGACTTGAATTTATACCAC [11622]
cp32-4_PAli_NX_Pl_consensus    TATTTACTAATTATATAAAAAAGGGGGAAAATGTCCACTACGAGGCATTAGAAGACTATGGACTTGAATTTATACCAC [11622]
cp32-4_PAbe_NX_MP_FG_consensus TATTTACTAATTATATAAAAAAGGGGGAAAATGTCCACTACGAGGCATTAGAAGACTATGGACTTGAATTTATACCAC [11622]
cp32-4_PAbe_NX_Pl_consensus    TATTTACTAATTATATAAAAAAGGGGGAAAATGTCCACTACGAGGCATTAGAAGACTATGGACTTGAATTTATACCAC [11622]

cp32-4_gi|11497149|B31_GB      AACAAGAAATTACTTTGGGCACAACACTCAAAAAAAAATCTTATATTTTGGAACGCAAAAACATTTTTCACACATAAGT [11700]
cp32-4_un10_B31_PacBio         AACAAGAAATTACTTTGGGCACAACACTCAAAAAAAAATCTTATATTTTGGAACGCAAAAACATTTTTCACACATAAGT [11700]
cp32-4_B31_TS_MP_FG_consensus  AACAAGAAATTACTTTGGGCACAACACTCAAAAAAAAATCTTATATTTTGGAACGCAAAAACATTTTTCACACATAAGT [11700]
cp32-4_B31_NX_Pl_consensus     AACAAGAAATTACTTTGGGCACAACACTCAAAAAAAAATCTTATATTTTGGAACGCAAAAACATTTTTCACACATAAGT [11700]
cp32-4_PAli_NX_MP_FG_consensus AACAAGAAATTACTTTGGGCACAACACTCAAAAAAAAATCTTATATTTTGGAACGCAAAAACATTTTTCACACATAAGT [11700]
cp32-4_PAli_NX_Pl_consensus    AACAAGAAATTACTTTGGGCACAACACTCAAAAAAAAATCTTATATTTTGGAACGCAAAAACATTTTTCACACATAAGT [11700]
cp32-4_PAbe_NX_MP_FG_consensus AACAAGAAATTACTTTGGGCACAACACTCAAAAAAAAATCTTATATTTTGGAACGCAAAAACATTTTTCACACATAAGT [11700]
cp32-4_PAbe_NX_Pl_consensus    AACAAGAAATTACTTTGGGCACAACACTCAAAAAAAAATCTTATATTTTGGAACGCAAAAACATTTTTCACACATAAGT [11700]

cp32-4_gi|11497149|B31_GB      TAAATGTTGGAGATAAAGTCTCATTTATTGATGGACTAGGGAAAATGATAAAAACCACTATAAAAGAAACAAGTGCAA [11778]
cp32-4_un10_B31_PacBio         TAAATGTTGGAGATAAAGTCTCATTTATTGATGGACTAGGGAAAATGATAAAAACCACTATAAAAGAAACAAGTGCAA [11778]
cp32-4_B31_TS_MP_FG_consensus  TAAATGTTGGAGATAAAGTCTCATTTATTGATGGACTAGGGAAAATGATAAAAACCACTATAAAAGAAACAAGTGCAA [11778]
cp32-4_B31_NX_Pl_consensus     TAAATGTTGGAGATAAAGTCTCATTTATTGATGGACTAGGGAAAATGATAAAAACCACTATAAAAGAAACAAGTGCAA [11778]
cp32-4_PAli_NX_MP_FG_consensus TAAATGTTGGAGATAAAGTCTCATTTATTGATGGACTAGGGAAAATGATAAAAACCACTATAAAAGAAACAAGTGCAA [11778]
cp32-4_PAli_NX_Pl_consensus    TAAATGTTGGAGATAAAGTCTCATTTATTGATGGACTAGGGAAAATGATAAAAACCACTATAAAAGAAACAAGTGCAA [11778]
cp32-4_PAbe_NX_MP_FG_consensus TAAATGTTGGAGATAAAGTCTCATTTATTGATGGACTAGGGAAAATGATAAAAACCACTATAAAAGAAACAAGTGCAA [11778]
cp32-4_PAbe_NX_Pl_consensus    TAAATGTTGGAGATAAAGTCTCATTTATTGATGGACTAGGGAAAATGATAAAAACCACTATAAAAGAAACAAGTGCAA [11778]

cp32-4_gi|11497149|B31_GB      GGCTTAGCAATACAGGAGAGTGTTCATTAATATTAAAGTTAGACGATGATTCTAATACAAAACGTAAAAAATAAAGGG [11856]
cp32-4_un10_B31_PacBio         GGCTTAGCAATACAGGAGAGTGTTCATTAATATTAAAGTTAGACGATGATTCTAATACAAAACGTAAAAAATAAAGGG [11856]
cp32-4_B31_TS_MP_FG_consensus  GGCTTAGCAATACAGGAGAGTGTTCATTAATATTAAAGTTAGACGATGATTCTAATACAAAACGTAAAAAATAAAGGG [11856]
cp32-4_B31_NX_Pl_consensus     GGCTTAGCAATACAGGAGAGTGTTCATTAATATTAAAGTTAGACGATGATTCTAATACAAAACGTAAAAAATAAAGGG [11856]
cp32-4_PAli_NX_MP_FG_consensus GGCTTAGCAATACAGGAGAGTGTTCATTAATATTAAAGTTAGACGATGATTCTAATACAAAACGTAAAAAATAAAGGG [11856]
cp32-4_PAli_NX_Pl_consensus    GGCTTAGCAATACAGGAGAGTGTTCATTAATATTAAAGTTAGACGATGATTCTAATACAAAACGTAAAAAATAAAGGG [11856]
cp32-4_PAbe_NX_MP_FG_consensus GGCTTAGCAATACAGGAGAGTGTTCATTAATATTAAAGTTAGACGATGATTCTAATACAAAACGTAAAAAATAAAGGG [11856]
cp32-4_PAbe_NX_Pl_consensus    GGCTTAGCAATACAGGAGAGTGTTCATTAATATTAAAGTTAGACGATGATTCTAATACAAAACGTAAAAAATAAAGGG [11856]

cp32-4_gi|11497149|B31_GB      GACTAGAATTAGAATGAATGAAGACTATGAAATTTACAGAATGAATCAACGCCTTTATGGCCAGGCATTGGCTCAAGA [11934]
cp32-4_un10_B31_PacBio         GACTAGAATTAGAATGAATGAAGACTATGAAATTTACAGAATGAATCAACGCCTTTATGGCCAGGCATTGGCTCAAGA [11934]
cp32-4_B31_TS_MP_FG_consensus  GACTAGAATTAGAATGAATGAAGACTATGAAATTTACAGAATGAATCAACGCCTTTATGGCCAGGCATTGGCTCAAGA [11934]
cp32-4_B31_NX_Pl_consensus     GACTAGAATTAGAATGAATGAAGACTATGAAATTTACAGAATGAATCAACGCCTTTATGGCCAGGCATTGGCTCAAGA [11934]
cp32-4_PAli_NX_MP_FG_consensus GACTAGAATTAGAATGAATGAAGACTATGAAATTTACAGAATGAATCAACGCCTTTATGGCCAGGCATTGGCTCAAGA [11934]
cp32-4_PAli_NX_Pl_consensus    GACTAGAATTAGAATGAATGAAGACTATGAAATTTACAGAATGAATCAACGCCTTTATGGCCAGGCATTGGCTCAAGA [11934]
cp32-4_PAbe_NX_MP_FG_consensus GACTAGAATTAGAATGAATGAAGACTATGAAATTTACAGAATGAATCAACGCCTTTATGGCCAGGCATTGGCTCAAGA [11934]
cp32-4_PAbe_NX_Pl_consensus    GACTAGAATTAGAATGAATGAAGACTATGAAATTTACAGAATGAATCAACGCCTTTATGGCCAGGCATTGGCTCAAGA [11934]

cp32-4_gi|11497149|B31_GB      AGACCTTAAAAATTGGATTTATTCAAACATTTTTATAATTAAAATTGGCACTGTAAAGGAGTTTAAACATCAAACTCA [12012]
cp32-4_un10_B31_PacBio         AGACCTTAAAAATTGGATTTATTCAAACATTTTTATAATTAAAATTGGCACTGTAAAGGAGTTTAAACATCAAACTCA [12012]
cp32-4_B31_TS_MP_FG_consensus  AGACCTTAAAAATTGGATTTATTCAAACATTTTTATAATTAAAATTGGCACTGTAAAGGAGTTTAAACATCAAACTCA [12012]
cp32-4_B31_NX_Pl_consensus     AGACCTTAAAAATTGGATTTATTCAAACATTTTTATAATTAAAATTGGCACTGTAAAGGAGTTTAAACATCAAACTCA [12012]
cp32-4_PAli_NX_MP_FG_consensus AGACCTTAAAAATTGGATTTATTCAAACATTTTTATAATTAAAATTGGCACTGTAAAGGAGTTTAAACATCAAACTCA [12012]
cp32-4_PAli_NX_Pl_consensus    AGACCTTAAAAATTGGATTTATTCAAACATTTTTATAATTAAAATTGGCACTGTAAAGGAGTTTAAACATCAAACTCA [12012]
cp32-4_PAbe_NX_MP_FG_consensus AGACCTTAAAAATTGGATTTATTCAAACATTTTTATAATTAAAATTGGCACTGTAAAGGAGTTTAAACATCAAACTCA [12012]
cp32-4_PAbe_NX_Pl_consensus    AGACCTTAAAAATTGGATTTATTCAAACATTTTTATAATTAAAATTGGCACTGTAAAGGAGTTTAAACATCAAACTCA [12012]

cp32-4_gi|11497149|B31_GB      AGAAGCTATTGTTACAATACCCGAATTTGAAGATTTAGAAATTCACACAAAAAATATCTCTAATATCAGTTTAGAACT [12090]
cp32-4_un10_B31_PacBio         AGAAGCTATTGTTACAATACCCGAATTTGAAGATTTAGAAATTCACACAAAAAATATCTCTAATATCAGTTTAGAACT [12090]
cp32-4_B31_TS_MP_FG_consensus  AGAAGCTATTGTTACAATACCCGAATTTGAAGATTTAGAAATTCACACAAAAAATATCTCTAATATCAGTTTAGAACT [12090]
cp32-4_B31_NX_Pl_consensus     AGAAGCTATTGTTACAATACCCGAATTTGAAGATTTAGAAATTCACACAAAAAATATCTCTAATATCAGTTTAGAACT [12090]
cp32-4_PAli_NX_MP_FG_consensus AGAAGCTATTGTTACAATACCCGAATTTGAAGATTTAGAAATTCACACAAAAAATATCTCTAATATCAGTTTAGAACT [12090]
cp32-4_PAli_NX_Pl_consensus    AGAAGCTATTGTTACAATACCCGAATTTGAAGATTTAGAAATTCACACAAAAAATATCTCTAATATCAGTTTAGAACT [12090]
cp32-4_PAbe_NX_MP_FG_consensus AGAAGCTATTGTTACAATACCCGAATTTGAAGATTTAGAAATTCACACAAAAAATATCTCTAATATCAGTTTAGAACT [12090]
cp32-4_PAbe_NX_Pl_consensus    AGAAGCTATTGTTACAATACCCGAATTTGAAGATTTAGAAATTCACACAAAAAATATCTCTAATATCAGTTTAGAACT [12090]

cp32-4_gi|11497149|B31_GB      ATCAAAAGGTGATTGCGTTTTACTACTTCAATCAAGCATTAATATTTTTGATAAAAATAACGACATTCACTTTGACAA [12168]
cp32-4_un10_B31_PacBio         ATCAAAAGGTGATTGCGTTTTACTACTTCAATCAAGCATTAATATTTTTGATAAAAATAACGACATTCACTTTGACAA [12168]
cp32-4_B31_TS_MP_FG_consensus  ATCAAAAGGTGATTGCGTTTTACTACTTCAATCAAGCATTAATATTTTTGATAAAAATAACGACATTCACTTTGACAA [12168]
cp32-4_B31_NX_Pl_consensus     ATCAAAAGGTGATTGCGTTTTACTACTTCAATCAAGCATTAATATTTTTGATAAAAATAACGACATTCACTTTGACAA [12168]
cp32-4_PAli_NX_MP_FG_consensus ATCAAAAGGTGATTGCGTTTTACTACTTCAATCAAGCATTAATATTTTTGATAAAAATAACGACATTCACTTTGACAA [12168]
cp32-4_PAli_NX_Pl_consensus    ATCAAAAGGTGATTGCGTTTTACTACTTCAATCAAGCATTAATATTTTTGATAAAAATAACGACATTCACTTTGACAA [12168]
cp32-4_PAbe_NX_MP_FG_consensus ATCAAAAGGTGATTGCGTTTTACTACTTCAATCAAGCATTAATATTTTTGATAAAAATAACGACATTCACTTTGACAA [12168]
cp32-4_PAbe_NX_Pl_consensus    ATCAAAAGGTGATTGCGTTTTACTACTTCAATCAAGCATTAATATTTTTGATAAAAATAACGACATTCACTTTGACAA [12168]

cp32-4_gi|11497149|B31_GB      ACATCATTTTTATATACTTAGTGCAATTAGCCCAAAGACTTTAAATCTAATCTCTGATACTGTTAAAATTAAAGCAAA [12246]
cp32-4_un10_B31_PacBio         ACATCATTTTTATATACTTAGTGCAATTAGCCCAAAGACTTTAAATCTAATCTCTGATACTGTTAAAATTAAAGCAAA [12246]
cp32-4_B31_TS_MP_FG_consensus  ACATCATTTTTATATACTTAGTGCAATTAGCCCAAAGACTTTAAATCTAATCTCTGATACTGTTAAAATTAAAGCAAA [12246]
cp32-4_B31_NX_Pl_consensus     ACATCATTTTTATATACTTAGTGCAATTAGCCCAAAGACTTTAAATCTAATCTCTGATACTGTTAAAATTAAAGCAAA [12246]
cp32-4_PAli_NX_MP_FG_consensus ACATCATTTTTATATACTTAGTGCAATTAGCCCAAAGACTTTAAATCTAATCTCTGATACTGTTAAAATTAAAGCAAA [12246]
```

```
cp32-4_PAli_NX_Pl_consensus    ACATCATTTTTATATACTTAGTGCAATTAGCCCAAAGACTTTAAATCTAATCTCTGATACTGTTAAAATTAAAGCAAA [12246]
cp32-4_PAbe_NX_MP_FG_consensus ACATCATTTTTATATACTTAGTGCAATTAGCCCAAAGACTTTAAATCTAATCTCTGATACTGTTAAAATTAAAGCAAA [12246]
cp32-4_PAbe_NX_Pl_consensus    ACATCATTTTTATATACTTAGTGCAATTAGCCCAAAGACTTTAAATCTAATCTCTGATACTGTTAAAATTAAAGCAAA [12246]

cp32-4_gi|11497149|B31_GB      CAATAACATTGAAATAGCTAACCAAACAACTAGCTTAAAAACAATTCTCAAAAATATTGTAAGTGCTATTGAGGGTAT [12324]
cp32-4_un10_B31_PacBio         CAATAACATTGAAATAGCTAACCAAACAACTAGCTTAAAAACAATTCTCAAAAATATTGTAAGTGCTATTGAGGGTAT [12324]
cp32-4_B31_TS_MP_FG_consensus  CAATAACATTGAAATAGCTAACCAAACAACTAGCTTAAAAACAATTCTCAAAAATATTGTAAGTGCTATTGAGGGTAT [12324]
cp32-4_B31_NX_Pl_consensus     CAATAACATTGAAATAGCTAACCAAACAACTAGCTTAAAAACAATTCTCAAAAATATTGTAAGTGCTATTGAGGGTAT [12324]
cp32-4_PAli_NX_MP_FG_consensus CAATAACATTGAAATAGCTAACCAAACAACTAGCTTAAAAACAATTCTCAAAAATATTGTAAGTGCTATTGAGGGTAT [12324]
cp32-4_PAli_NX_Pl_consensus    CAATAACATTGAAATAGCTAACCAAACAACTAGCTTAAAAACAATTCTCAAAAATATTGTAAGTGCTATTGAGGGTAT [12324]
cp32-4_PAbe_NX_MP_FG_consensus CAATAACATTGAAATAGCTAACCAAACAACTAGCTTAAAAACAATTCTCAAAAATATTGTAAGTGCTATTGAGGGTAT [12324]
cp32-4_PAbe_NX_Pl_consensus    CAATAACATTGAAATAGCTAACCAAACAACTAGCTTAAAAACAATTCTCAAAAATATTGTAAGTGCTATTGAGGGTAT [12324]

cp32-4_gi|11497149|B31_GB      AAAAGTCGTACCCGCACAAGGAGGCCCAGTAATTGAATCAGTCAGTCTAAAAATAGCAACCACTAAAATTAATTCTGA [12402]
cp32-4_un10_B31_PacBio         AAAAGTCGTACCCGCACAAGGAGGCCCAGTAATTGAATCAGTCAGTCTAAAAATAGCAACCACTAAAATTAATTCTGA [12402]
cp32-4_B31_TS_MP_FG_consensus  AAAAGTCGTACCCGCACAAGGAGGCCCAGTAATTGAATCAGTCAGTCTAAAAATAGCAACCACTAAAATTAATTCTGA [12402]
cp32-4_B31_NX_Pl_consensus     AAAAGTCGTACCCGCACAAGGAGGCCCAGTAATTGAATCAGTCAGTCTAAAAATAGCAACCACTAAAATTAATTCTGA [12402]
cp32-4_PAli_NX_MP_FG_consensus AAAAGTCGTACCCGCACAAGGAGGCCCAGTAATTGAATCAGTCAGTCTAAAAATAGCAACCACTAAAATTAATTCTGA [12402]
cp32-4_PAli_NX_Pl_consensus    AAAAGTCGTACCCGCACAAGGAGGCCCAGTAATTGAATCAGTCAGTCTAAAAATAGCAACCACTAAAATTAATTCTGA [12402]
cp32-4_PAbe_NX_MP_FG_consensus AAAAGTCGTACCCGCACAAGGAGGCCCAGTAATTGAATCAGTCAGTCTAAAAATAGCAACCACTAAAATTAATTCTGA [12402]
cp32-4_PAbe_NX_Pl_consensus    AAAAGTCGTACCCGCACAAGGAGGCCCAGTAATTGAATCAGTCAGTCTAAAAATAGCAACCACTAAAATTAATTCTGA [12402]

cp32-4_gi|11497149|B31_GB      TATTAATAGCTTGTTTAAGTAATTTTTGCTAAATATGGTATAATTACTAGTATGGATTTAAGATTAGGCAATAATTTT [12480]
cp32-4_un10_B31_PacBio         TATTAATAGCTTGTTTAAGTAATTTTTGCTAAATATGGTATAATTACTAGTATGGATTTAAGATTAGGCAATAATTTT [12480]
cp32-4_B31_TS_MP_FG_consensus  TATTAATAGCTTGTTTAAGTAATTTTTGCTAAATATGGTATAATTACTAGTATGGATTTAAGATTAGGCAATAATTTT [12480]
cp32-4_B31_NX_Pl_consensus     TATTAATAGCTTGTTTAAGTAATTTTTGCTAAATATGGTATAATTACTAGTATGGATTTAAGATTAGGCAATAATTTT [12480]
cp32-4_PAli_NX_MP_FG_consensus TATTAATAGCTTGTTTAAGTAATTTTTGCTAAATATGGTATAATTACTAGTATGGATTTAAGATTAGGCAATAATTTT [12480]
cp32-4_PAli_NX_Pl_consensus    TATTAATAGCTTGTTTAAGTAATTTTTGCTAAATATGGTATAATTACTAGTATGGATTTAAGATTAGGCAATAATTTT [12480]
cp32-4_PAbe_NX_MP_FG_consensus TATTAATAGCTTGTTTAAGTAATTTTTGCTAAATATGGTATAATTACTAGTATGGATTTAAGATTAGGCAATAATTTT [12480]
cp32-4_PAbe_NX_Pl_consensus    TATTAATAGCTTGTTTAAGTAATTTTTGCTAAATATGGTATAATTACTAGTATGGATTTAAGATTAGGCAATAATTTT [12480]

cp32-4_gi|11497149|B31_GB      GAATTGGTATTTAATAACGATTTATCACTTGTTGATGGAATTGATGAACAAAAACAAAGATTTTTGATATTTTTAAAA [12558]
cp32-4_un10_B31_PacBio         GAATTGGTATTTAATAACGATTTATCACTTGTTGATGGAATTGATGAACAAAAACAAAGATTTTTGATATTTTTAAAA [12558]
cp32-4_B31_TS_MP_FG_consensus  GAATTGGTATTTAATAACGATTTATCACTTGTTGATGGAATTGATGAACAAAAACAAAGATTTTTGATATTTTTAAAA [12558]
cp32-4_B31_NX_Pl_consensus     GAATTGGTATTTAATAACGATTTATCACTTGTTGATGGAATTGATGAACAAAAACAAAGATTTTTGATATTTTTAAAA [12558]
cp32-4_PAli_NX_MP_FG_consensus GAATTGGTATTTAATAACGATTTATCACTTGTTGATGGAATTGATGAACAAAAACAAAGATTTTTGATATTTTTAAAA [12558]
cp32-4_PAli_NX_Pl_consensus    GAATTGGTATTTAATAACGATTTATCACTTGTTGATGGAATTGATGAACAAAAACAAAGATTTTTGATATTTTTAAAA [12558]
cp32-4_PAbe_NX_MP_FG_consensus GAATTGGTATTTAATAACGATTTATCACTTGTTGATGGAATTGATGAACAAAAACAAAGATTTTTGATATTTTTAAAA [12558]
cp32-4_PAbe_NX_Pl_consensus    GAATTGGTATTTAATAACGATTTATCACTTGTTGATGGAATTGATGAACAAAAACAAAGATTTTTGATATTTTTAAAA [12558]

cp32-4_gi|11497149|B31_GB      ACCTTAAGGGGTAGTTTAAGCTATGCTCCTCATTGGGGACTGGACTATTTCTTGCTTTTAAAACTGTTAAAAATTAAC [12636]
cp32-4_un10_B31_PacBio         ACCTTAAGGGGTAGTTTAAGCTATGCTCCTCATTGGGGACTGGACTATTTCTTGCTTTTAAAACTGTTAAAAATTAAC [12636]
cp32-4_B31_TS_MP_FG_consensus  ACCTTAAGGGGTAGTTTAAGCTATGCTCCTCATTGGGGACTGGACTATTTCTTGCTTTTAAAACTGTTAAAAATTAAC [12636]
cp32-4_B31_NX_Pl_consensus     ACCTTAAGGGGTAGTTTAAGCTATGCTCCTCATTGGGGACTGGACTATTTCTTGCTTTTAAAACTGTTAAAAATTAAC [12636]
cp32-4_PAli_NX_MP_FG_consensus ACCTTAAGGGGTAGTTTAAGCTATGCTCCTCATTGGGGACTGGACTATTTCTTGCTTTTAAAACTGTTAAAAATTAAC [12636]
cp32-4_PAli_NX_Pl_consensus    ACCTTAAGGGGTAGTTTAAGCTATGCTCCTCATTGGGGACTGGACTATTTCTTGCTTTTAAAACTGTTAAAAATTAAC [12636]
cp32-4_PAbe_NX_MP_FG_consensus ACCTTAAGGGGTAGTTTAAGCTATGCTCCTCATTGGGGACTGGACTATTTCTTGCTTTTAAAACTGTTAAAAATTAAC [12636]
cp32-4_PAbe_NX_Pl_consensus    ACCTTAAGGGGTAGTTTAAGCTATGCTCCTCATTGGGGACTGGACTATTTCTTGCTTTTAAAACTGTTAAAAATTAAC [12636]

cp32-4_gi|11497149|B31_GB      AATCTTCACGCTGTAAAAAATTATTTTCACGAAATATCTAAAGAACTTAACTTAGATTTAATAAATATTTCAACTACT [12714]
cp32-4_un10_B31_PacBio         AATCTTCACGCTGTAAAAAATTATTTTCACGAAATATCTAAAGAACTTAACTTAGATTTAATAAATATTTCAACTACT [12714]
cp32-4_B31_TS_MP_FG_consensus  AATCTTCACGCTGTAAAAAATTATTTTCACGAAATATCTAAAGAACTTAACTTAGATTTAATAAATATTTCAACTACT [12714]
cp32-4_B31_NX_Pl_consensus     AATCTTCACGCTGTAAAAAATTATTTTCACGAAATATCTAAAGAACTTAACTTAGATTTAATAAATATTTCAACTACT [12714]
cp32-4_PAli_NX_MP_FG_consensus AATCTTCACGCTGTAAAAAATTATTTTCACGAAATATCTAAAGAACTTAACTTAGATTTAATAAATATTTCAACTACT [12714]
cp32-4_PAli_NX_Pl_consensus    AATCTTCACGCTGTAAAAAATTATTTTCACGAAATATCTAAAGAACTTAACTTAGATTTAATAAATATTTCAACTACT [12714]
cp32-4_PAbe_NX_MP_FG_consensus AATCTTCACGCTGTAAAAAATTATTTTCACGAAATATCTAAAGAACTTAACTTAGATTTAATAAATATTTCAACTACT [12714]
cp32-4_PAbe_NX_Pl_consensus    AATCTTCACGCTGTAAAAAATTATTTTCACGAAATATCTAAAGAACTTAACTTAGATTTAATAAATATTTCAACTACT [12714]

cp32-4_gi|11497149|B31_GB      ATACAAGACAACAAAGCACACATATCCTTTTTTTTCTCAGGCGATGTTTTGAATATGGAGTTTAATTTATGAGTATAG [12792]
cp32-4_un10_B31_PacBio         ATACAAGACAACAAAGCACACATATCCTTTTTTTTCTCAGGCGATGTTTTGAATATGGAGTTTAATTTATGAGTATAG [12792]
cp32-4_B31_TS_MP_FG_consensus  ATACAAGACAACAAAGCACACATATCCTTTTTTTTCTCAGGCGATGTTTTGAATATGGAGTTTAATTTATGAGTATAG [12792]
cp32-4_B31_NX_Pl_consensus     ATACAAGACAACAAAGCACACATATCCTTTTTTTTCTCAGGCGATGTTTTGAATATGGAGTTTAATTTATGAGTATAG [12792]
cp32-4_PAli_NX_MP_FG_consensus ATACAAGACAACAAAGCACACATATCCTTTTTTTTCTCAGGCGATGTTTTGAATATGGAGTTTAATTTATGAGTATAG [12792]
cp32-4_PAli_NX_Pl_consensus    ATACAAGACAACAAAGCACACATATCCTTTTTTTTCTCAGGCGATGTTTTGAATATGGAGTTTAATTTATGAGTATAG [12792]
cp32-4_PAbe_NX_MP_FG_consensus ATACAAGACAACAAAGCACACATATCCTTTTTTTTCTCAGGCGATGTTTTGAATATGGAGTTTAATTTATGAGTATAG [12792]
cp32-4_PAbe_NX_Pl_consensus    ATACAAGACAACAAAGCACACATATCCTTTTTTTTCTCAGGCGATGTTTTGAATATGGAGTTTAATTTATGAGTATAG [12792]

cp32-4_gi|11497149|B31_GB      TTTTTGATTCTGATTTTGGCATTTTAAAACGTACAATTAAGGATATTGTAAGATCGAAAAGAGAATATTTGCGTGTAA [12870]
cp32-4_un10_B31_PacBio         TTTTTGATTCTGATTTTGGCATTTTAAAACGTACAATTAAGGATATTGTAAGATCGAAAAGAGAATATTTGCGTGTAA [12870]
cp32-4_B31_TS_MP_FG_consensus  TTTTTGATTCTGATTTTGGCATTTTAAAACGTACAATTAAGGATATTGTAAGATCGAAAAGAGAATATTTGCGTGTAA [12870]
cp32-4_B31_NX_Pl_consensus     TTTTTGATTCTGATTTTGGCATTTTAAAACGTACAATTAAGGATATTGTAAGATCGAAAAGAGAATATTTGCGTGTAA [12870]
cp32-4_PAli_NX_MP_FG_consensus TTTTTGATTCTGATTTTGGCATTTTAAAACGTACAATTAAGGATATTGTAAGATCGAAAAGAGAATATTTGCGTGTAA [12870]
cp32-4_PAli_NX_Pl_consensus    TTTTTGATTCTGATTTTGGCATTTTAAAACGTACAATTAAGGATATTGTAAGATCGAAAAGAGAATATTTGCGTGTAA [12870]
cp32-4_PAbe_NX_MP_FG_consensus TTTTTGATTCTGATTTTGGCATTTTAAAACGTACAATTAAGGATATTGTAAGATCGAAAAGAGAATATTTGCGTGTAA [12870]
cp32-4_PAbe_NX_Pl_consensus    TTTTTGATTCTGATTTTGGCATTTTAAAACGTACAATTAAGGATATTGTAAGATCGAAAAGAGAATATTTGCGTGTAA [12870]

cp32-4_gi|11497149|B31_GB      ATTATGGGATTAATATTGATGATAACCAAAGCTCAATTTATAACATTATTGCGTCTTCTTTAGCATTAATTGAAGAAG [12948]
cp32-4_un10_B31_PacBio         ATTATGGGATTAATATTGATGATAACCAAAGCTCAATTTATAACATTATTGCGTCTTCTTTAGCATTAATTGAAGAAG [12948]
cp32-4_B31_TS_MP_FG_consensus  ATTATGGGATTAATATTGATGATAACCAAAGCTCAATTTATAACATTATTGCGTCTTCTTTAGCATTAATTGAAGAAG [12948]
cp32-4_B31_NX_Pl_consensus     ATTATGGGATTAATATTGATGATAACCAAAGCTCAATTTATAACATTATTGCGTCTTCTTTAGCATTAATTGAAGAAG [12948]
cp32-4_PAli_NX_MP_FG_consensus ATTATGGGATTAATATTGATGATAACCAAAGCTCAATTTATAACATTATTGCGTCTTCTTTAGCATTAATTGAAGAAG [12948]
cp32-4_PAli_NX_Pl_consensus    ATTATGGGATTAATATTGATGATAACCAAAGCTCAATTTATAACATTATTGCGTCTTCTTTAGCATTAATTGAAGAAG [12948]
cp32-4_PAbe_NX_MP_FG_consensus ATTATGGGATTAATATTGATGATAACCAAAGCTCAATTTATAACATTATTGCGTCTTCTTTAGCATTAATTGAAGAAG [12948]
cp32-4_PAbe_NX_Pl_consensus    ATTATGGGATTAATATTGATGATAACCAAAGCTCAATTTATAACATTATTGCGTCTTCTTTAGCATTAATTGAAGAAG [12948]

cp32-4_gi|11497149|B31_GB      AAATAATTAATGAGCTTAATCTCTTTTTTTCTAAAATGAAACCGGGTGGCACTTATTGGGCTGCTATTGAAGAACACA [13026]
cp32-4_un10_B31_PacBio         AAATAATTAATGAGCTTAATCTCTTTTTTTCTAAAATGAAACCGGGTGGCACTTATTGGGCTGCTATTGAAGAACACA [13026]
cp32-4_B31_TS_MP_FG_consensus  AAATAATTAATGAGCTTAATCTCTTTTTTTCTAAAATGAAACCGGGTGGCACTTATTGGGCTGCTATTGAAGAACACA [13026]
cp32-4_B31_NX_Pl_consensus     AAATAATTAATGAGCTTAATCTCTTTTTTTCTAAAATGAAACCGGGTGGCACTTATTGGGCTGCTATTGAAGAACACA [13026]
cp32-4_PAli_NX_MP_FG_consensus AAATAATTAATGAGCTTAATCTCTTTTTTTCTAAAATGAAACCGGGTGGCACTTATTGGGCTGCTATTGAAGAACACA [13026]
cp32-4_PAli_NX_Pl_consensus    AAATAATTAATGAGCTTAATCTCTTTTTTTCTAAAATGAAACCGGGTGGCACTTATTGGGCTGCTATTGAAGAACACA [13026]
cp32-4_PAbe_NX_MP_FG_consensus AAATAATTAATGAGCTTAATCTCTTTTTTTCTAAAATGAAACCGGGTGGCACTTATTGGGCTGCTATTGAAGAACACA [13026]
cp32-4_PAbe_NX_Pl_consensus    AAATAATTAATGAGCTTAATCTCTTTTTTTCTAAAATGAAACCGGGTGGCACTTATTGGGCTGCTATTGAAGAACACA [13026]

cp32-4_gi|11497149|B31_GB      TTTCTTCTAAAAGCACAACTTACAGCGCGGTTCGCACGGCTTTACTTAATCTTGATGGGGTTGAGTACACTAATATTA [13104]
cp32-4_un10_B31_PacBio         TTTCTTCTAAAAGCACAACTTACAGCGCGGTTCGCACGGCTTTACTTAATCTTGATGGGGTTGAGTACACTAATATTA [13104]
cp32-4_B31_TS_MP_FG_consensus  TTTCTTCTAAAAGCACAACTTACAGCGCGGTTCGCACGGCTTTACTTAATCTTGATGGGGTTGAGTACACTAATATTA [13104]
cp32-4_B31_NX_Pl_consensus     TTTCTTCTAAAAGCACAACTTACAGCGCGGTTCGCACGGCTTTACTTAATCTTGATGGGGTTGAGTACACTAATATTA [13104]
cp32-4_PAli_NX_MP_FG_consensus TTTCTTCTAAAAGCACAACTTACAGCGCGGTTCGCACGGCTTTACTTAATCTTGATGGGGTTGAGTACACTAATATTA [13104]
cp32-4_PAli_NX_Pl_consensus    TTTCTTCTAAAAGCACAACTTACAGCGCGGTTCGCACGGCTTTACTTAATCTTGATGGGGTTGAGTACACTAATATTA [13104]
cp32-4_PAbe_NX_MP_FG_consensus TTTCTTCTAAAAGCACAACTTACAGCGCGGTTCGCACGGCTTTACTTAATCTTGATGGGGTTGAGTACACTAATATTA [13104]
cp32-4_PAbe_NX_Pl_consensus    TTTCTTCTAAAAGCACAACTTACAGCGCGGTTCGCACGGCTTTACTTAATCTTGATGGGGTTGAGTACACTAATATTA [13104]

cp32-4_gi|11497149|B31_GB      AAAGTGCAGCTGGTAAAGCCAACATATATCTAATTTTAAAGGAAACTTTACTAGACGATAGTAAATCTAACATTAATA [13182]
cp32-4_un10_B31_PacBio         AAAGTGCAGCTGGTAAAGCCAACATATATCTAATTTTAAAGGAAACTTTACTAGACGATAGTAAATCTAACATTAATA [13182]
cp32-4_B31_TS_MP_FG_consensus  AAAGTGCAGCTGGTAAAGCCAACATATATCTAATTTTAAAGGAAACTTTACTAGACGATAGTAAATCTAACATTAATA [13182]
cp32-4_B31_NX_Pl_consensus     AAAGTGCAGCTGGTAAAGCCAACATATATCTAATTTTAAAGGAAACTTTACTAGACGATAGTAAATCTAACATTAATA [13182]
cp32-4_PAli_NX_MP_FG_consensus AAAGTGCAGCTGGTAAAGCCAACATATATCTAATTTTAAAGGAAACTTTACTAGACGATAGTAAATCTAACATTAATA [13182]
cp32-4_PAli_NX_Pl_consensus    AAAGTGCAGCTGGTAAAGCCAACATATATCTAATTTTAAAGGAAACTTTACTAGACGATAGTAAATCTAACATTAATA [13182]
```

```
cp32-4_PAbe_NX_MP_FG_consensus AAAGTGCAGCTGGTAAAGCCAACATATATCTAATTTTAAAGGAAACTTTACTAGACGATAGTAAATCTAACATTAATA [13182]
cp32-4_PAbe_NX_Pl_consensus    AAAGTGCAGCTGGTAAAGCCAACATATATCTAATTTTAAAGGAAACTTTACTAGACGATAGTAAATCTAACATTAATA [13182]

cp32-4_gi|11497149|B31_GB      GTCCTGAATTTAAAGCAAAACTTTGGGAAACATTATATCTAACAACTCCTAGTGGGACTTTACTTGAGGGAGACATCG [13260]
cp32-4_un10_B31_PacBio         GTCCTGAATTTAAAGCAAAACTTTGGGAAACATTATATCTAACAACTCCTAGTGGGACTTTACTTGAGGGAGACATCG [13260]
cp32-4_B31_TS_MP_FG_consensus  GTCCTGAATTTAAAGCAAAACTTTGGGAAACATTATATCTAACAACTCCTAGTGGGACTTTACTTGAGGGAGACATCG [13260]
cp32-4_B31_NX_Pl_consensus     GTCCTGAATTTAAAGCAAAACTTTGGGAAACATTATATCTAACAACTCCTAGTGGGACTTTACTTGAGGGAGACATCG [13260]
cp32-4_PAli_NX_MP_FG_consensus GTCCTGAATTTAAAGCAAAACTTTGGGAAACATTATATCTAACAACTCCTAGTGGGACTTTACTTGAGGGAGACATCG [13260]
cp32-4_PAli_NX_Pl_consensus    GTCCTGAATTTAAAGCAAAACTTTGGGAAACATTATATCTAACAACTCCTAGTGGGACTTTACTTGAGGGAGACATCG [13260]
cp32-4_PAbe_NX_MP_FG_consensus GTCCTGAATTTAAAGCAAAACTTTGGGAAACATTATATCTAACAACTCCTAGTGGGACTTTACTTGAGGGAGACATCG [13260]
cp32-4_PAbe_NX_Pl_consensus    GTCCTGAATTTAAAGCAAAACTTTGGGAAACATTATATCTAACAACTCCTAGTGGGACTTTACTTGAGGGAGACATCG [13260]

cp32-4_gi|11497149|B31_GB      AAATTGATGGCCTCAATTCAACTGGACAACGGAAATCCTATAAAATATCGCTAGGGAAAAGAAAATATGTTTATATGA [13338]
cp32-4_un10_B31_PacBio         AAATTGATGGCCTCAATTCAACTGGACAACGGAAATCCTATAAAATATCGCTAGGGAAAAGAAAATATGTTTATATGA [13338]
cp32-4_B31_TS_MP_FG_consensus  AAATTGATGGCCTCAATTCAACTGGACAACGGAAATCCTATAAAATATCGCTAGGGAAAAGAAAATATGTTTATATGA [13338]
cp32-4_B31_NX_Pl_consensus     AAATTGATGGCCTCAATTCAACTGGACAACGGAAATCCTATAAAATATCGCTAGGGAAAAGAAAATATGTTTATATGA [13338]
cp32-4_PAli_NX_MP_FG_consensus AAATTGATGGCCTCAATTCAACTGGACAACGGAAATCCTATAAAATATCGCTAGGGAAAAGAAAATATGTTTATATGA [13338]
cp32-4_PAli_NX_Pl_consensus    AAATTGATGGCCTCAATTCAACTGGACAACGGAAATCCTATAAAATATCGCTAGGGAAAAGAAAATATGTTTATATGA [13338]
cp32-4_PAbe_NX_MP_FG_consensus AAATTGATGGCCTCAATTCAACTGGACAACGGAAATCCTATAAAATATCGCTAGGGAAAAGAAAATATGTTTATATGA [13338]
cp32-4_PAbe_NX_Pl_consensus    AAATTGATGGCCTCAATTCAACTGGACAACGGAAATCCTATAAAATATCGCTAGGGAAAAGAAAATATGTTTATATGA [13338]

cp32-4_gi|11497149|B31_GB      AAGTAAAGTATAAACTTGACCTTAAAAACTACCTCTACTTAAATATTGACTCTCAAATTAGGGATATATATTCTAGGA [13416]
cp32-4_un10_B31_PacBio         AAGTAAAGTATAAACTTGACCTTAAAAACTACCTCTACTTAAATATTGACTCTCAAATTAGGGATATATATTCTAGGA [13416]
cp32-4_B31_TS_MP_FG_consensus  AAGTAAAGTATAAACTTGACCTTAAAAACTACCTCTACTTAAATATTGACTCTCAAATTAGGGATATATATTCTAGGA [13416]
cp32-4_B31_NX_Pl_consensus     AAGTAAAGTATAAACTTGACCTTAAAAACTACCTCTACTTAAATATTGACTCTCAAATTAGGGATATATATTCTAGGA [13416]
cp32-4_PAli_NX_MP_FG_consensus AAGTAAAGTATAAACTTGACCTTAAAAACTACCTCTACTTAAATATTGACTCTCAAATTAGGGATATATATTCTAGGA [13416]
cp32-4_PAli_NX_Pl_consensus    AAGTAAAGTATAAACTTGACCTTAAAAACTACCTCTACTTAAATATTGACTCTCAAATTAGGGATATATATTCTAGGA [13416]
cp32-4_PAbe_NX_MP_FG_consensus AAGTAAAGTATAAACTTGACCTTAAAAACTACCTCTACTTAAATATTGACTCTCAAATTAGGGATATATATTCTAGGA [13416]
cp32-4_PAbe_NX_Pl_consensus    AAGTAAAGTATAAACTTGACCTTAAAAACTACCTCTACTTAAATATTGACTCTCAAATTAGGGATATATATTCTAGGA [13416]

cp32-4_gi|11497149|B31_GB      TTATTTCAAATAACTATTCTGATATGGGAATTAGCTTTGAATATCAAGACTTTTTTGCTCCAGTTAATGAAGTTAAAG [13494]
cp32-4_un10_B31_PacBio         TTATTTCAAATAACTATTCTGATATGGGAATTAGCTTTGAATATCAAGACTTTTTTGCTCCAGTTAATGAAGTTAAAG [13494]
cp32-4_B31_TS_MP_FG_consensus  TTATTTCAAATAACTATTCTGATATGGGAATTAGCTTTGAATATCAAGACTTTTTTGCTCCAGTTAATGAAGTTAAAG [13494]
cp32-4_B31_NX_Pl_consensus     TTATTTCAAATAACTATTCTGATATGGGAATTAGCTTTGAATATCAAGACTTTTTTGCTCCAGTTAATGAAGTTAAAG [13494]
cp32-4_PAli_NX_MP_FG_consensus TTATTTCAAATAACTATTCTGATATGGGAATTAGCTTTGAATATCAAGACTTTTTTGCTCCAGTTAATGAAGTTAAAG [13494]
cp32-4_PAli_NX_Pl_consensus    TTATTTCAAATAACTATTCTGATATGGGAATTAGCTTTGAATATCAAGACTTTTTTGCTCCAGTTAATGAAGTTAAAG [13494]
cp32-4_PAbe_NX_MP_FG_consensus TTATTTCAAATAACTATTCTGATATGGGAATTAGCTTTGAATATCAAGACTTTTTTGCTCCAGTTAATGAAGTTAAAG [13494]
cp32-4_PAbe_NX_Pl_consensus    TTATTTCAAATAACTATTCTGATATGGGAATTAGCTTTGAATATCAAGACTTTTTTGCTCCAGTTAATGAAGTTAAAG [13494]

cp32-4_gi|11497149|B31_GB      GAATTAAGTTTATGGAAATAAGTGCTTGTATTAAAGATACAGACACTGAGAGTATTACAAAAATTGGTGATAGCGATT [13572]
cp32-4_un10_B31_PacBio         GAATTAAGTTTATGGAAATAAGTGCTTGTATTAAAGATACAGACACTGAGAGTATTACAAAAATTGGTGATAGCGATT [13572]
cp32-4_B31_TS_MP_FG_consensus  GAATTAAGTTTATGGAAATAAGTGCTTGTATTAAAGATACAGACACTGAGAGTATTACAAAAATTGGTGATAGCGATT [13572]
cp32-4_B31_NX_Pl_consensus     GAATTAAGTTTATGGAAATAAGTGCTTGTATTAAAGATACAGACACTGAGAGTATTACAAAAATTGGTGATAGCGATT [13572]
cp32-4_PAli_NX_MP_FG_consensus GAATTAAGTTTATGGAAATAAGTGCTTGTATTAAAGATACAGACACTGAGAGTATTACAAAAATTGGTGATAGCGATT [13572]
cp32-4_PAli_NX_Pl_consensus    GAATTAAGTTTATGGAAATAAGTGCTTGTATTAAAGATACAGACACTGAGAGTATTACAAAAATTGGTGATAGCGATT [13572]
cp32-4_PAbe_NX_MP_FG_consensus GAATTAAGTTTATGGAAATAAGTGCTTGTATTAAAGATACAGACACTGAGAGTATTACAAAAATTGGTGATAGCGATT [13572]
cp32-4_PAbe_NX_Pl_consensus    GAATTAAGTTTATGGAAATAAGTGCTTGTATTAAAGATACAGACACTGAGAGTATTACAAAAATTGGTGATAGCGATT [13572]

cp32-4_gi|11497149|B31_GB      TTAAAAAAAATCAAGATATTGCCATTAATGATGACACAATGCTACTTTTCAATATGACAGATAGATTGCTTATTGATA [13650]
cp32-4_un10_B31_PacBio         TTAAAAAAAATCAAGATATTGCCATTAATGATGACACAATGCTACTTTTCAATATGACAGATAGATTGCTTATTGATA [13650]
cp32-4_B31_TS_MP_FG_consensus  TTAAAAAAAATCAAGATATTGCCATTAATGATGACACAATGCTACTTTTCAATATGACAGATAGATTGCTTATTGATA [13650]
cp32-4_B31_NX_Pl_consensus     TTAAAAAAAATCAAGATATTGCCATTAATGATGACACAATGCTACTTTTCAATATGACAGATAGATTGCTTATTGATA [13650]
cp32-4_PAli_NX_MP_FG_consensus TTAAAAAAAATCAAGATATTGCCATTAATGATGACACAATGCTACTTTTCAATATGACAGATAGATTGCTTATTGATA [13650]
cp32-4_PAli_NX_Pl_consensus    TTAAAAAAAATCAAGATATTGCCATTAATGATGACACAATGCTACTTTTCAATATGACAGATAGATTGCTTATTGATA [13650]
cp32-4_PAbe_NX_MP_FG_consensus TTAAAAAAAATCAAGATATTGCCATTAATGATGACACAATGCTACTTTTCAATATGACAGATAGATTGCTTATTGATA [13650]
cp32-4_PAbe_NX_Pl_consensus    TTAAAAAAAATCAAGATATTGCCATTAATGATGACACAATGCTACTTTTCAATATGACAGATAGATTGCTTATTGATA [13650]

cp32-4_gi|11497149|B31_GB      TTGGATAGTTAACAAATATGAAAATACCCAATCTTTTCAAAAACACCGAAATTCATAAATTTATACGTACAGAAACAG [13728]
cp32-4_un10_B31_PacBio         TTGGATAGTTAACAAATATGAAAATACCCAATCTTTTCAAAAACACCGAAATTCATAAATTTATACGTACAGAAACAG [13728]
cp32-4_B31_TS_MP_FG_consensus  TTGGATAGTTAACAAATATGAAAATACCCAATCTTTTCAAAAACACCGAAATTCATAAATTTATACGTACAGAAACAG [13728]
cp32-4_B31_NX_Pl_consensus     TTGGATAGTTAACAAATATGAAAATACCCAATCTTTTCAAAAACACCGAAATTCATAAATTTATACGTACAGAAACAG [13728]
cp32-4_PAli_NX_MP_FG_consensus TTGGATAGTTAACAAATATGAAAATACCCAATCTTTTCAAAAACACCGAAATTCATAAATTTATACGTACAGAAACAG [13728]
cp32-4_PAli_NX_Pl_consensus    TTGGATAGTTAACAAATATGAAAATACCCAATCTTTTCAAAAACACCGAAATTCATAAATTTATACGTACAGAAACAG [13728]
cp32-4_PAbe_NX_MP_FG_consensus TTGGATAGTTAACAAATATGAAAATACCCAATCTTTTCAAAAACACCGAAATTCATAAATTTATACGTACAGAAACAG [13728]
cp32-4_PAbe_NX_Pl_consensus    TTGGATAGTTAACAAATATGAAAATACCCAATCTTTTCAAAAACACCGAAATTCATAAATTTATACGTACAGAAACAG [13728]

cp32-4_gi|11497149|B31_GB      AATATGCACAAGCATTGCTTAATGAACTTAAGTCCCTTAATTCCAACTTCATATCCATTAATGTAATAGAAAATATAA [13806]
cp32-4_un10_B31_PacBio         AATATGCACAAGCATTGCTTAATGAACTTAAGTCCCTTAATTCCAACTTCATATCCATTAATGTAATAGAAAATATAA [13806]
cp32-4_B31_TS_MP_FG_consensus  AATATGCACAAGCATTGCTTAATGAACTTAAGTCCCTTAATTCCAACTTCATATCCATTAATGTAATAGAAAATATAA [13806]
cp32-4_B31_NX_Pl_consensus     AATATGCACAAGCATTGCTTAATGAACTTAAGTCCCTTAATTCCAACTTCATATCCATTAATGTAATAGAAAATATAA [13806]
cp32-4_PAli_NX_MP_FG_consensus AATATGCACAAGCATTGCTTAATGAACTTAAGTCCCTTAATTCCAACTTCATATCCATTAATGTAATAGAAAATATAA [13806]
cp32-4_PAli_NX_Pl_consensus    AATATGCACAAGCATTGCTTAATGAACTTAAGTCCCTTAATTCCAACTTCATATCCATTAATGTAATAGAAAATATAA [13806]
cp32-4_PAbe_NX_MP_FG_consensus AATATGCACAAGCATTGCTTAATGAACTTAAGTCCCTTAATTCCAACTTCATATCCATTAATGTAATAGAAAATATAA [13806]
cp32-4_PAbe_NX_Pl_consensus    AATATGCACAAGCATTGCTTAATGAACTTAAGTCCCTTAATTCCAACTTCATATCCATTAATGTAATAGAAAATATAA [13806]

cp32-4_gi|11497149|B31_GB      AATCAAGATATATTGCAATATGGATATCTCAAGTTTTATCTATCTTTTATGCAAAAACTCAAACTTTACAAAGTATTA [13884]
cp32-4_un10_B31_PacBio         AATCAAGATATATTGCAATATGGATATCTCAAGTTTTATCTATCTTTTATGCAAAAACTCAAACTTTACAAAGTATTA [13884]
cp32-4_B31_TS_MP_FG_consensus  AATCAAGATATATTGCAATATGGATATCTCAAGTTTTATCTATCTTTTATGCAAAAACTCAAACTTTACAAAGTATTA [13884]
cp32-4_B31_NX_Pl_consensus     AATCAAGATATATTGCAATATGGATATCTCAAGTTTTATCTATCTTTTATGCAAAAACTCAAACTTTACAAAGTATTA [13884]
cp32-4_PAli_NX_MP_FG_consensus AATCAAGATATATTGCAATATGGATATCTCAAGTTTTATCTATCTTTTATGCAAAAACTCAAACTTTACAAAGTATTA [13884]
cp32-4_PAli_NX_Pl_consensus    AATCAAGATATATTGCAATATGGATATCTCAAGTTTTATCTATCTTTTATGCAAAAACTCAAACTTTACAAAGTATTA [13884]
cp32-4_PAbe_NX_MP_FG_consensus AATCAAGATATATTGCAATATGGATATCTCAAGTTTTATCTATCTTTTATGCAAAAACTCAAACTTTACAAAGTATTA [13884]
cp32-4_PAbe_NX_Pl_consensus    AATCAAGATATATTGCAATATGGATATCTCAAGTTTTATCTATCTTTTATGCAAAAACTCAAACTTTACAAAGTATTA [13884]

cp32-4_gi|11497149|B31_GB      CAAGCAATATTAATAGTGTTATTTTTGCTTTACGTCATATTGGCACTGACGAGTCATTTAGACTGATTTTCAAGACCT [13962]
cp32-4_un10_B31_PacBio         CAAGCAATATTAATAGTGTTATTTTTGCTTTACGTCATATTGGCACTGACGAGTCATTTAGACTGATTTTCAAGACCT [13962]
cp32-4_B31_TS_MP_FG_consensus  CAAGCAATATTAATAGTGTTATTTTTGCTTTACGTCATATTGGCACTGACGAGTCATTTAGACTGATTTTCAAGACCT [13962]
cp32-4_B31_NX_Pl_consensus     CAAGCAATATTAATAGTGTTATTTTTGCTTTACGTCATATTGGCACTGACGAGTCATTTAGACTGATTTTCAAGACCT [13962]
cp32-4_PAli_NX_MP_FG_consensus CAAGCAATATTAATAGTGTTATTTTTGCTTTACGTCATATTGGCACTGACGAGTCATTTAGACTGATTTTCAAGACCT [13962]
cp32-4_PAli_NX_Pl_consensus    CAAGCAATATTAATAGTGTTATTTTTGCTTTACGTCATATTGGCACTGACGAGTCATTTAGACTGATTTTCAAGACCT [13962]
cp32-4_PAbe_NX_MP_FG_consensus CAAGCAATATTAATAGTGTTATTTTTGCTTTACGTCATATTGGCACTGACGAGTCATTTAGACTGATTTTCAAGACCT [13962]
cp32-4_PAbe_NX_Pl_consensus    CAAGCAATATTAATAGTGTTATTTTTGCTTTACGTCATATTGGCACTGACGAGTCATTTAGACTGATTTTCAAGACCT [13962]

cp32-4_gi|11497149|B31_GB      TTTTAAATGTAGACATTGAAGTTACTACTCCTGAAGCTGGGGTTATTGATATCTCTTTAAAAGGGGTAATAAAAACAA [14040]
cp32-4_un10_B31_PacBio         TTTTAAATGTAGACATTGAAGTTACTACTCCTGAAGCTGGGGTTATTGATATCTCTTTAAAAGGGGTAATAAAAACAA [14040]
cp32-4_B31_TS_MP_FG_consensus  TTTTAAATGTAGACATTGAAGTTACTACTCCTGAAGCTGGGGTTATTGATATCTCTTTAAAAGGGGTAATAAAAACAA [14040]
cp32-4_B31_NX_Pl_consensus     TTTTAAATGTAGACATTGAAGTTACTACTCCTGAAGCTGGGGTTATTGATATCTCTTTAAAAGGGGTAATAAAAACAA [14040]
cp32-4_PAli_NX_MP_FG_consensus TTTTAAATGTAGACATTGAAGTTACTACTCCTGAAGCTGGGGTTATTGATATCTCTTTAAAAGGGGTAATAAAAACAA [14040]
cp32-4_PAli_NX_Pl_consensus    TTTTAAATGTAGACATTGAAGTTACTACTCCTGAAGCTGGGGTTATTGATATCTCTTTAAAAGGGGTAATAAAAACAA [14040]
cp32-4_PAbe_NX_MP_FG_consensus TTTTAAATGTAGACATTGAAGTTACTACTCCTGAAGCTGGGGTTATTGATATCTCTTTAAAAGGGGTAATAAAAACAA [14040]
cp32-4_PAbe_NX_Pl_consensus    TTTTAAATGTAGACATTGAAGTTACTACTCCTGAAGCTGGGGTTATTGATATCTCTTTAAAAGGGGTAATAAAAACAA [14040]

cp32-4_gi|11497149|B31_GB      ACTTTACTACATTTATTTCGCCTAGCACTAAGAAAGGAAAACGACTAAAAAAGATAATTCTTAGAGAAAAGAAGCCGG [14118]
cp32-4_un10_B31_PacBio         ACTTTACTACATTTATTTCGCCTAGCACTAAGAAAGGAAAACGACTAAAAAAGATAATTCTTAGAGAAAAGAAGCCGG [14118]
cp32-4_B31_TS_MP_FG_consensus  ACTTTACTACATTTATTTCGCCTAGCACTAAGAAAGGAAAACGACTAAAAAAGATAATTCTTAGAGAAAAGAAGCCGG [14118]
cp32-4_B31_NX_Pl_consensus     ACTTTACTACATTTATTTCGCCTAGCACTAAGAAAGGAAAACGACTAAAAAAGATAATTCTTAGAGAAAAGAAGCCGG [14118]
cp32-4_PAli_NX_MP_FG_consensus ACTTTACTACATTTATTTCGCCTAGCACTAAGAAAGGAAAACGACTAAAAAAGATAATTCTTAGAGAAAAGAAGCCGG [14118]
cp32-4_PAli_NX_Pl_consensus    ACTTTACTACATTTATTTCGCCTAGCACTAAGAAAGGAAAACGACTAAAAAAGATAATTCTTAGAGAAAAGAAGCCGG [14118]
cp32-4_PAbe_NX_MP_FG_consensus ACTTTACTACATTTATTTCGCCTAGCACTAAGAAAGGAAAACGACTAAAAAAGATAATTCTTAGAGAAAAGAAGCCGG [14118]
```

```
cp32-4_PAbe_NX_Pl_consensus    ACTTTACTACATTTATTTCGCCTAGCACTAAGAAAGGAAAACGACTAAAAAAGATAATTCTTAGAGAAAAGAAGCCGG [14118]

cp32-4_gi|11497149|B31_GB      GATACGCTGCATCTAAAAAAGCTTTAGTATTTAACTCACTTCCTAAAGGCTATGATCATTCAATTTATGCTTTTATTA [14196]
cp32-4_un10_B31_PacBio         GATACGCTGCATCTAAAAAAGCTTTAGTATTTAACTCACTTCCTAAAGGCTATGATCATTCAATTTATGCTTTTATTA [14196]
cp32-4_B31_TS_MP_FG_consensus  GATACGCTGCATCTAAAAAAGCTTTAGTATTTAACTCACTTCCTAAAGGCTATGATCATTCAATTTATGCTTTTATTA [14196]
cp32-4_B31_NX_Pl_consensus     GATACGCTGCATCTAAAAAAGCTTTAGTATTTAACTCACTTCCTAAAGGCTATGATCATTCAATTTATGCTTTTATTA [14196]
cp32-4_PAli_NX_MP_FG_consensus GATACGCTGCATCTAAAAAAGCTTTAGTATTTAACTCACTTCCTAAAGGCTATGATCATTCAATTTATGCTTTTATTA [14196]
cp32-4_PAli_NX_Pl_consensus    GATACGCTGCATCTAAAAAAGCTTTAGTATTTAACTCACTTCCTAAAGGCTATGATCATTCAATTTATGCTTTTATTA [14196]
cp32-4_PAbe_NX_MP_FG_consensus GATACGCTGCATCTAAAAAAGCTTTAGTATTTAACTCACTTCCTAAAGGCTATGATCATTCAATTTATGCTTTTATTA [14196]
cp32-4_PAbe_NX_Pl_consensus    GATACGCTGCATCTAAAAAAGCTTTAGTATTTAACTCACTTCCTAAAGGCTATGATCATTCAATTTATGCTTTTATTA [14196]

cp32-4_gi|11497149|B31_GB      AGAGAATTATTCCTATTGGTAGAGTTCTCAAAATTAATAATACAGATGGTAACAATATTATTACTTTCAATAACTAAG [14274]
cp32-4_un10_B31_PacBio         AGAGAATTATTCCTATTGGTAGAGTTCTCAAAATTAATAATACAGATGGTAACAATATTATTACTTTCAATAACTAAG [14274]
cp32-4_B31_TS_MP_FG_consensus  AGAGAATTATTCCTATTGGTAGAGTTCTCAAAATTAATAATACAGATGGTAACAATATTATTACTTTCAATAACTAAG [14274]
cp32-4_B31_NX_Pl_consensus     AGAGAATTATTCCTATTGGTAGAGTTCTCAAAATTAATAATACAGATGGTAACAATATTATTACTTTCAATAACTAAG [14274]
cp32-4_PAli_NX_MP_FG_consensus AGAGAATTATTCCTATTGGTAGAGTTCTCAAAATTAATAATACAGATGGTAACAATATTATTACTTTCAATAACTAAG [14274]
cp32-4_PAli_NX_Pl_consensus    AGAGAATTATTCCTATTGGTAGAGTTCTCAAAATTAATAATACAGATGGTAACAATATTATTACTTTCAATAACTAAG [14274]
cp32-4_PAbe_NX_MP_FG_consensus AGAGAATTATTCCTATTGGTAGAGTTCTCAAAATTAATAATACAGATGGTAACAATATTATTACTTTCAATAACTAAG [14274]
cp32-4_PAbe_NX_Pl_consensus    AGAGAATTATTCCTATTGGTAGAGTTCTCAAAATTAATAATACAGATGGTAACAATATTATTACTTTCAATAACTAAG [14274]

cp32-4_gi|11497149|B31_GB      GAGGTTTTATGGCTGATGATCAAGAAAAAATTACTGATTGATGAAGAAGAAACGGTTCAAATTAAAGATTTAAATAAGG [14352]
cp32-4_un10_B31_PacBio         GAGGTTTTATGGCTGATGATCAAGAAAAAATTACTGATTGATGAAGAAGAAACGGTTCAAATTAAAGATTTAAATAAGG [14352]
cp32-4_B31_TS_MP_FG_consensus  GAGGTTTTATGGCTGATGATCAAGAAAAAATTACTGATTGATGAAGAAGAAACGGTTCAAATTAAAGATTTAAATAAGG [14352]
cp32-4_B31_NX_Pl_consensus     GAGGTTTTATGGCTGATGATCAAGAAAAAATTACTGATTGATGAAGAAGAAACGGTTCAAATTAAAGATTTAAATAAGG [14352]
cp32-4_PAli_NX_MP_FG_consensus GAGGTTTTATGGCTGATGATCAAGAAAAAATTACTGATTGATGAAGAAGAAACGGTTCAAATTAAAGATTTAAATAAGG [14352]
cp32-4_PAli_NX_Pl_consensus    GAGGTTTTATGGCTGATGATCAAGAAAAAATTACTGATTGATGAAGAAGAAACGGTTCAAATTAAAGATTTAAATAAGG [14352]
cp32-4_PAbe_NX_MP_FG_consensus GAGGTTTTATGGCTGATGATCAAGAAAAAATTACTGATTGATGAAGAAGAAACGGTTCAAATTAAAGATTTAAATAAGG [14352]
cp32-4_PAbe_NX_Pl_consensus    GAGGTTTTATGGCTGATGATCAAGAAAAAATTACTGATTGATGAAGAAGAAACGGTTCAAATTAAAGATTTAAATAAGG [14352]

cp32-4_gi|11497149|B31_GB      TTACGACCGTTAACAATACTGATCTTTTACTGCTTGATGATGGAGCTGCAAGCAGCAATGCTATCACCTTTAAAAACT [14430]
cp32-4_un10_B31_PacBio         TTACGACCGTTAACAATACTGATCTTTTACTGCTTGATGATGGAGCTGCAAGCAGCAATGCTATCACCTTTAAAAACT [14430]
cp32-4_B31_TS_MP_FG_consensus  TTACGACCGTTAACAATACTGATCTTTTACTGCTTGATGATGGAGCTGCAAGCAGCAATGCTATCACCTTTAAAAACT [14430]
cp32-4_B31_NX_Pl_consensus     TTACGACCGTTAACAATACTGATCTTTTACTGCTTGATGATGGAGCTGCAAGCAGCAATGCTATCACCTTTAAAAACT [14430]
cp32-4_PAli_NX_MP_FG_consensus TTACGACCGTTAACAATACTGATCTTTTACTGCTTGATGATGGAGCTGCAAGCAGCAATGCTATCACCTTTAAAAACT [14430]
cp32-4_PAli_NX_Pl_consensus    TTACGACCGTTAACAATACTGATCTTTTACTGCTTGATGATGGAGCTGCAAGCAGCAATGCTATCACCTTTAAAAACT [14430]
cp32-4_PAbe_NX_MP_FG_consensus TTACGACCGTTAACAATACTGATCTTTTACTGCTTGATGATGGAGCTGCAAGCAGCAATGCTATCACCTTTAAAAACT [14430]
cp32-4_PAbe_NX_Pl_consensus    TTACGACCGTTAACAATACTGATCTTTTACTGCTTGATGATGGAGCTGCAAGCAGCAATGCTATCACCTTTAAAAACT [14430]

cp32-4_gi|11497149|B31_GB      TCTTAAAAACCGTTAATCACCAAACATTTAAAGGCGAAGAGCTAGGCTATTTTAAAGAGATAATTAAATCTACAATCG [14508]
cp32-4_un10_B31_PacBio         TCTTAAAAACCGTTAATCACCAAACATTTAAAGGCGAAGAGCTAGGCTATTTTAAAGAGATAATTAAATCTACAATCG [14508]
cp32-4_B31_TS_MP_FG_consensus  TCTTAAAAACCGTTAATCACCAAACATTTAAAGGCGAAGAGCTAGGCTATTTTAAAGAGATAATTAAATCTACAATCG [14508]
cp32-4_B31_NX_Pl_consensus     TCTTAAAAACCGTTAATCACCAAACATTTAAAGGCGAAGAGCTAGGCTATTTTAAAGAGATAATTAAATCTACAATCG [14508]
cp32-4_PAli_NX_MP_FG_consensus TCTTAAAAACCGTTAATCACCAAACATTTAAAGGCGAAGAGCTAGGCTATTTTAAAGAGATAATTAAATCTACAATCG [14508]
cp32-4_PAli_NX_Pl_consensus    TCTTAAAAACCGTTAATCACCAAACATTTAAAGGCGAAGAGCTAGGCTATTTTAAAGAGATAATTAAATCTACAATCG [14508]
cp32-4_PAbe_NX_MP_FG_consensus TCTTAAAAACCGTTAATCACCAAACATTTAAAGGCGAAGAGCTAGGCTATTTTAAAGAGATAATTAAATCTACAATCG [14508]
cp32-4_PAbe_NX_Pl_consensus    TCTTAAAAACCGTTAATCACCAAACATTTAAAGGCGAAGAGCTAGGCTATTTTAAAGAGATAATTAAATCTACAATCG [14508]

cp32-4_gi|11497149|B31_GB      CTACTGAACTTGCAGCTGATAAAGATTTTATAAAAAGCATTTACGATTTAATCGTTGACAAGCTAATTGAGAATGAAT [14586]
cp32-4_un10_B31_PacBio         CTACTGAACTTGCAGCTGATAAAGATTTTATAAAAAGCATTTACGATTTAATCGTTGACAAGCTAATTGAGAATGAAT [14586]
cp32-4_B31_TS_MP_FG_consensus  CTACTGAACTTGCAGCTGATAAAGATTTTATAAAAAGCATTTACGATTTAATCGTTGACAAGCTAATTGAGAATGAAT [14586]
cp32-4_B31_NX_Pl_consensus     CTACTGAACTTGCAGCTGATAAAGATTTTATAAAAAGCATTTACGATTTAATCGTTGACAAGCTAATTGAGAATGAAT [14586]
cp32-4_PAli_NX_MP_FG_consensus CTACTGAACTTGCAGCTGATAAAGATTTTATAAAAAGCATTTACGATTTAATCGTTGACAAGCTAATTGAGAATGAAT [14586]
cp32-4_PAli_NX_Pl_consensus    CTACTGAACTTGCAGCTGATAAAGATTTTATAAAAAGCATTTACGATTTAATCGTTGACAAGCTAATTGAGAATGAAT [14586]
cp32-4_PAbe_NX_MP_FG_consensus CTACTGAACTTGCAGCTGATAAAGATTTTATAAAAAGCATTTACGATTTAATCGTTGACAAGCTAATTGAGAATGAAT [14586]
cp32-4_PAbe_NX_Pl_consensus    CTACTGAACTTGCAGCTGATAAAGATTTTATAAAAAGCATTTACGATTTAATCGTTGACAAGCTAATTGAGAATGAAT [14586]

cp32-4_gi|11497149|B31_GB      CTAGTAAACTTTCAAATCTTTTTAGTAAAATCAAATCGCGCCTTACAGATAGCATATCATCAGCCACTTTATCTAGAA [14664]
cp32-4_un10_B31_PacBio         CTAGTAAACTTTCAAATCTTTTTAGTAAAATCAAATCGCGCCTTACAGATAGCATATCATCAGCCACTTTATCTAGAA [14664]
cp32-4_B31_TS_MP_FG_consensus  CTAGTAAACTTTCAAATCTTTTTAGTAAAATCAAATCGCGCCTTACAGATAGCATATCATCAGCCACTTTATCTAGAA [14664]
cp32-4_B31_NX_Pl_consensus     CTAGTAAACTTTCAAATCTTTTTAGTAAAATCAAATCGCGCCTTACAGATAGCATATCATCAGCCACTTTATCTAGAA [14664]
cp32-4_PAli_NX_MP_FG_consensus CTAGTAAACTTTCAAATCTTTTTAGTAAAATCAAATCGCGCCTTACAGATAGCATATCATCAGCCACTTTATCTAGAA [14664]
cp32-4_PAli_NX_Pl_consensus    CTAGTAAACTTTCAAATCTTTTTAGTAAAATCAAATCGCGCCTTACAGATAGCATATCATCAGCCACTTTATCTAGAA [14664]
cp32-4_PAbe_NX_MP_FG_consensus CTAGTAAACTTTCAAATCTTTTTAGTAAAATCAAATCGCGCCTTACAGATAGCATATCATCAGCCACTTTATCTAGAA [14664]
cp32-4_PAbe_NX_Pl_consensus    CTAGTAAACTTTCAAATCTTTTTAGTAAAATCAAATCGCGCCTTACAGATAGCATATCATCAGCCACTTTATCTAGAA [14664]

cp32-4_gi|11497149|B31_GB      GTGATGATCTTTTGATAATGCCTTCATCAGATACTATTCAAAAAACACCCGTTCCTAAACATATACTTGGAGTACCAT [14742]
cp32-4_un10_B31_PacBio         GTGATGATCTTTTGATAATGCCTTCATCAGATACTATTCAAAAAACACCCGTTCCTAAACATATACTTGGAGTACCAT [14742]
cp32-4_B31_TS_MP_FG_consensus  GTGATGATCTTTTGATAATGCCTTCATCAGATACTATTCAAAAAACACCCGTTCCTAAACATATACTTGGAGTACCAT [14742]
cp32-4_B31_NX_Pl_consensus     GTGATGATCTTTTGATAATGCCTTCATCAGATACTATTCAAAAAACACCCGTTCCTAAACATATACTTGGAGTACCAT [14742]
cp32-4_PAli_NX_MP_FG_consensus GTGATGATCTTTTGATAATGCCTTCATCAGATACTATTCAAAAAACACCCGTTCCTAAACATATACTTGGAGTACCAT [14742]
cp32-4_PAli_NX_Pl_consensus    GTGATGATCTTTTGATAATGCCTTCATCAGATACTATTCAAAAAACACCCGTTCCTAAACATATACTTGGAGTACCAT [14742]
cp32-4_PAbe_NX_MP_FG_consensus GTGATGATCTTTTGATAATGCCTTCATCAGATACTATTCAAAAAACACCCGTTCCTAAACATATACTTGGAGTACCAT [14742]
cp32-4_PAbe_NX_Pl_consensus    GTGATGATCTTTTGATAATGCCTTCATCAGATACTATTCAAAAAACACCCGTTCCTAAACATATACTTGGAGTACCAT [14742]

cp32-4_gi|11497149|B31_GB      CAAATTTTACTTATGGCAGCATAACTAGAAGTACTACACTTTATCCTTCTGACTATGAGAATAAAGCGATATCTATTA [14820]
cp32-4_un10_B31_PacBio         CAAATTTTACTTATGGCAGCATAACTAGAAGTACTACACTTTATCCTTCTGACTATGAGAATAAAGCGATATCTATTA [14820]
cp32-4_B31_TS_MP_FG_consensus  CAAATTTTACTTATGGCAGCATAACTAGAAGTACTACACTTTATCCTTCTGACTATGAGAATAAAGCGATATCTATTA [14820]
cp32-4_B31_NX_Pl_consensus     CAAATTTTACTTATGGCAGCATAACTAGAAGTACTACACTTTATCCTTCTGACTATGAGAATAAAGCGATATCTATTA [14820]
cp32-4_PAli_NX_MP_FG_consensus CAAATTTTACTTATGGCAGCATAACTAGAAGTACTACACTTTATCCTTCTGACTATGAGAATAAAGCGATATCTATTA [14820]
cp32-4_PAli_NX_Pl_consensus    CAAATTTTACTTATGGCAGCATAACTAGAAGTACTACACTTTATCCTTCTGACTATGAGAATAAAGCGATATCTATTA [14820]
cp32-4_PAbe_NX_MP_FG_consensus CAAATTTTACTTATGGCAGCATAACTAGAAGTACTACACTTTATCCTTCTGACTATGAGAATAAAGCGATATCTATTA [14820]
cp32-4_PAbe_NX_Pl_consensus    CAAATTTTACTTATGGCAGCATAACTAGAAGTACTACACTTTATCCTTCTGACTATGAGAATAAAGCGATATCTATTA [14820]

cp32-4_gi|11497149|B31_GB      ATATGGAAGACAATGATGATGTAACTCTTATTTTTTACAAAAATTACGATAATGATCCCATTTATCTTGATATTGAGA [14898]
cp32-4_un10_B31_PacBio         ATATGGAAGACAATGATGATGTAACTCTTATTTTTTACAAAAATTACGATAATGATCCCATTTATCTTGATATTGAGA [14898]
cp32-4_B31_TS_MP_FG_consensus  ATATGGAAGACAATGATGATGTAACTCTTATTTTTTACAAAAATTACGATAATGATCCCATTTATCTTGATATTGAGA [14898]
cp32-4_B31_NX_Pl_consensus     ATATGGAAGACAATGATGATGTAACTCTTATTTTTTACAAAAATTACGATAATGATCCCATTTATCTTGATATTGAGA [14898]
cp32-4_PAli_NX_MP_FG_consensus ATATGGAAGACAATGATGATGTAACTCTTATTTTTTACAAAAATTACGATAATGATCCCATTTATCTTGATATTGAGA [14898]
cp32-4_PAli_NX_Pl_consensus    ATATGGAAGACAATGATGATGTAACTCTTATTTTTTACAAAAATTACGATAATGATCCCATTTATCTTGATATTGAGA [14898]
cp32-4_PAbe_NX_MP_FG_consensus ATATGGAAGACAATGATGATGTAACTCTTATTTTTTACAAAAATTACGATAATGATCCCATTTATCTTGATATTGAGA [14898]
cp32-4_PAbe_NX_Pl_consensus    ATATGGAAGACAATGATGATGTAACTCTTATTTTTTACAAAAATTACGATAATGATCCCATTTATCTTGATATTGAGA [14898]

cp32-4_gi|11497149|B31_GB      TTCAAGTAAAAATCAATGATAATAGGATGCAGAAAAAATCATTAAAACTTATGTATTCTGATGAAATTACATACAATT [14976]
cp32-4_un10_B31_PacBio         TTCAAGTAAAAATCAATGATAATAGGATGCAGAAAAAATCATTAAAACTTATGTATTCTGATGAAATTACATACAATT [14976]
cp32-4_B31_TS_MP_FG_consensus  TTCAAGTAAAAATCAATGATAATAGGATGCAGAAAAAATCATTAAAACTTATGTATTCTGATGAAATTACATACAATT [14976]
cp32-4_B31_NX_Pl_consensus     TTCAAGTAAAAATCAATGATAATAGGATGCAGAAAAAATCATTAAAACTTATGTATTCTGATGAAATTACATACAATT [14976]
cp32-4_PAli_NX_MP_FG_consensus TTCAAGTAAAAATCAATGATAATAGGATGCAGAAAAAATCATTAAAACTTATGTATTCTGATGAAATTACATACAATT [14976]
cp32-4_PAli_NX_Pl_consensus    TTCAAGTAAAAATCAATGATAATAGGATGCAGAAAAAATCATTAAAACTTATGTATTCTGATGAAATTACATACAATT [14976]
cp32-4_PAbe_NX_MP_FG_consensus TTCAAGTAAAAATCAATGATAATAGGATGCAGAAAAAATCATTAAAACTTATGTATTCTGATGAAATTACATACAATT [14976]
cp32-4_PAbe_NX_Pl_consensus    TTCAAGTAAAAATCAATGATAATAGGATGCAGAAAAAATCATTAAAACTTATGTATTCTGATGAAATTACATACAATT [14976]

cp32-4_gi|11497149|B31_GB      GGGTTTATGAAATTACGGGCCCTCGCGGACTATTCACCAGAACTCCCATTTATAACGGATGGTATATCCAAAAAAGAG [15054]
cp32-4_un10_B31_PacBio         GGGTTTATGAAATTACGGGCCCTCGCGGACTATTCACCAGAACTCCCATTTATAACGGATGGTATATCCAAAAAAGAG [15054]
cp32-4_B31_TS_MP_FG_consensus  GGGTTTATGAAATTACGGGCCCTCGCGGACTATTCACCAGAACTCCCATTTATAACGGATGGTATATCCAAAAAAGAG [15054]
cp32-4_B31_NX_Pl_consensus     GGGTTTATGAAATTACGGGCCCTCGCGGACTATTCACCAGAACTCCCATTTATAACGGATGGTATATCCAAAAAAGAG [15054]
cp32-4_PAli_NX_MP_FG_consensus GGGTTTATGAAATTACGGGCCCTCGCGGACTATTCACCAGAACTCCCATTTATAACGGATGGTATATCCAAAAAAGAG [15054]
cp32-4_PAli_NX_Pl_consensus    GGGTTTATGAAATTACGGGCCCTCGCGGACTATTCACCAGAACTCCCATTTATAACGGATGGTATATCCAAAAAAGAG [15054]
cp32-4_PAbe_NX_MP_FG_consensus GGGTTTATGAAATTACGGGCCCTCGCGGACTATTCACCAGAACTCCCATTTATAACGGATGGTATATCCAAAAAAGAG [15054]
cp32-4_PAbe_NX_Pl_consensus    GGGTTTATGAAATTACGGGCCCTCGCGGACTATTCACCAGAACTCCCATTTATAACGGATGGTATATCCAAAAAAGAG [15054]
```

```
cp32-4_gi|11497149|B31_GB      CCTCCTTGTATGGAGATTCAGTCCCAGATCTTTTAAAACTGTAAGCTTTTTTAGCAAAAATTACGATTTTGATATATA [15132]
cp32-4_un10_B31_PacBio         CCTCCTTGTATGGAGATTCAGTCCCAGATCTTTTAAAACTGTAAGCTTTTTTAGCAAAAATTACGATTTTGATATATA [15132]
cp32-4_B31_TS_MP_FG_consensus  CCTCCTTGTATGGAGATTCAGTCCCAGATCTTTTAAAACTGTAAGCTTTTTTAGCAAAAATTACGATTTTGATATATA [15132]
cp32-4_B31_NX_Pl_consensus     CCTCCTTGTATGGAGATTCAGTCCCAGATCTTTTAAAACTGTAAGCTTTTTTAGCAAAAATTACGATTTTGATATATA [15132]
cp32-4_PAli_NX_MP_FG_consensus CCTCCTTGTATGGAGATTCAGTCCCAGATCTTTTAAAACTGTAAGCTTTTTTAGCAAAAATTACGATTTTGATATATA [15132]
cp32-4_PAli_NX_Pl_consensus    CCTCCTTGTATGGAGATTCAGTCCCAGATCTTTTAAAACTGTAAGCTTTTTTAGCAAAAATTACGATTTTGATATATA [15132]
cp32-4_PAbe_NX_MP_FG_consensus CCTCCTTGTATGGAGATTCAGTCCCAGATCTTTTAAAACTGTAAGCTTTTT-AGCAAAAATTACGATTTTGATATATA [15132]
cp32-4_PAbe_NX_Pl_consensus    CCTCCTTGTATGGAGATTCAGTCCCAGATCTTTTAAAACTGTAAGCTTTTT-AGCAAAAATTACGATTTTGATATATA [15132]

cp32-4_gi|11497149|B31_GB      ATGTATATACAAATAAAAAATTAAAATAAAGGATTAAAAAATGGATACTATTAAATTAACAGAACTTCTTATCAATTT [15210]
cp32-4_un10_B31_PacBio         ATGTATATACAAATAAAAAATTAAAATAAAGGATTAAAAAATGGATACTATTAAATTAACAGAACTTCTTATCAATTT [15210]
cp32-4_B31_TS_MP_FG_consensus  ATGTATATACAAATAAAAAATTAAAATAAAGGATTAAAAAATGGATACTATTAAATTAACAGAACTTCTTATCAATTT [15210]
cp32-4_B31_NX_Pl_consensus     ATGTATATACAAATAAAAAATTAAAATAAAGGATTAAAAAATGGATACTATTAAATTAACAGAACTTCTTATCAATTT [15210]
cp32-4_PAli_NX_MP_FG_consensus ATGTATATACAAATAAAAAATTAAAATAAAGGATTAAAAAATGGATACTATTAAATTAACAGAACTTCTTATCAATTT [15210]
cp32-4_PAli_NX_Pl_consensus    ATGTATATACAAATAAAAAATTAAAATAAAGGATTAAAAAATGGATACTATTAAATTAACAGAACTTCTTATCAATTT [15210]
cp32-4_PAbe_NX_MP_FG_consensus ATGTATATACAAATAAAAAATTAAAATAAAGGATTAAAAAATGGATACTATTAAATTAACAGAACTTCTTATCAATTT [15210]
cp32-4_PAbe_NX_Pl_consensus    ATGTATATACAAATAAAAAATTAAAATAAAGGATTAAAAAATGGATACTATTAAATTAACAGAACTTCTTATCAATTT [15210]

cp32-4_gi|11497149|B31_GB      AAACGAAATTAAACTTATAGCCGTAATGATTTTTGTAACAGTGTTGGTTTTAGGAGTATTAATTCTTCTCAAGCCTTT [15288]
cp32-4_un10_B31_PacBio         AAACGAAATTAAACTTATAGCCGTAATGATTTTTGTAACAGTGTTGGTTTTAGGAGTATTAATTCTTCTCAAGCCTTT [15288]
cp32-4_B31_TS_MP_FG_consensus  AAACGAAATTAAACTTATAGCCGTAATGATTTTTGTAACAGTGTTGGTTTTAGGAGTATTAATTCTTCTCAAGCCTTT [15288]
cp32-4_B31_NX_Pl_consensus     AAACGAAATTAAACTTATAGCCGTAATGATTTTTGTAACAGTGTTGGTTTTAGGAGTATTAATTCTTCTCAAGCCTTT [15288]
cp32-4_PAli_NX_MP_FG_consensus AAACGAAATTAAACTTATAGCCGTAATGATTTTTGTAACAGTGTTGGTTTTAGGAGTATTAATTCTTCTCAAGCCTTT [15288]
cp32-4_PAli_NX_Pl_consensus    AAACGAAATTAAACTTATAGCCGTAATGATTTTTGTAACAGTGTTGGTTTTAGGAGTATTAATTCTTCTCAAGCCTTT [15288]
cp32-4_PAbe_NX_MP_FG_consensus AAACGAAATTAAACTTATAGCCGTAATGATTTTTGTAACAGTGTTGGTTTTAGGAGTATTAATTCTTCTCAAGCCTTT [15288]
cp32-4_PAbe_NX_Pl_consensus    AAACGAAATTAAACTTATAGCCGTAATGATTTTTGTAACAGTGTTGGTTTTAGGAGTATTAATTCTTCTCAAGCCTTT [15288]

cp32-4_gi|11497149|B31_GB      ACTAAAAGACATATTGACTATTGTAATAGGCAAGATTTTTAAGAATGGTAATGGTAATGGCAAAAATCACATTAAAAA [15366]
cp32-4_un10_B31_PacBio         ACTAAAAGACATATTGACTATTGTAATAGGCAAGATTTTTAAGAATGGTAATGGTAATGGCAAAAATCACATTAAAAA [15366]
cp32-4_B31_TS_MP_FG_consensus  ACTAAAAGACATATTGACTATTGTAATAGGCAAGATTTTTAAGAATGGTAATGGTAATGGCAAAAATCACATTAAAAA [15366]
cp32-4_B31_NX_Pl_consensus     ACTAAAAGACATATTGACTATTGTAATAGGCAAGATTTTTAAGAATGGTAATGGTAATGGCAAAAATCACATTAAAAA [15366]
cp32-4_PAli_NX_MP_FG_consensus ACTAAAAGACATATTGACTATTGTAATAGGCAAGATTTTTAAGAATGGTAATGGTAATGGCAAAAATCACATTAAAAA [15366]
cp32-4_PAli_NX_Pl_consensus    ACTAAAAGACATATTGACTATTGTAATAGGCAAGATTTTTAAGAATGGTAATGGTAATGGCAAAAATCACATTAAAAA [15366]
cp32-4_PAbe_NX_MP_FG_consensus ACTAAAAGACATATTGACTATTGTAATAGGCAAGATTTTTAAGAATGGTAATGGTAATGGCAAAAATCACATTAAAAA [15366]
cp32-4_PAbe_NX_Pl_consensus    ACTAAAAGACATATTGACTATTGTAATAGGCAAGATTTTTAAGAATGGTAATGGTAATGGCAAAAATCACATTAAAAA [15366]

cp32-4_gi|11497149|B31_GB      AAGAGATTAACTTATGAAATTATCCAAAGATAATGTTGAGCTTGGACTTACGTCTTTATCAACCCTTATTGATATATT [15444]
cp32-4_un10_B31_PacBio         AAGAGATTAACTTATGAAATTATCCAAAGATAATGTTGAGCTTGGACTTACGTCTTTATCAACCCTTATTGATATATT [15444]
cp32-4_B31_TS_MP_FG_consensus  AAGAGATTAACTTATGAAATTATCCAAAGATAATGTTGAGCTTGGACTTACGTCTTTATCAACCCTTATTGATATATT [15444]
cp32-4_B31_NX_Pl_consensus     AAGAGATTAACTTATGAAATTATCCAAAGATAATGTTGAGCTTGGACTTACGTCTTTATCAACCCTTATTGATATATT [15444]
cp32-4_PAli_NX_MP_FG_consensus AAGAGATTAACTTATGAAATTATCCAAAGATAATGTTGAGCTTGGACTTACGTCTTTATCAACCCTTATTGATATATT [15444]
cp32-4_PAli_NX_Pl_consensus    AAGAGATTAACTTATGAAATTATCCAAAGATAATGTTGAGCTTGGACTTACGTCTTTATCAACCCTTATTGATATATT [15444]
cp32-4_PAbe_NX_MP_FG_consensus AAGAGATTAACTTATGAAATTATCCAAAGATAATGTTGAGCTTGGACTTACGTCTTTATCAACCCTTATTGATATATT [15444]
cp32-4_PAbe_NX_Pl_consensus    AAGAGATTAACTTATGAAATTATCCAAAGATAATGTTGAGCTTGGACTTACGTCTTTATCAACCCTTATTGATATATT [15444]

cp32-4_gi|11497149|B31_GB      TTCTAAATTTGAAGATGAATTTGATGAAATTGCACATAAAGGATTCTTTTTGGTTTATGAGCTGTATTCTCATTATAA [15522]
cp32-4_un10_B31_PacBio         TTCTAAATTTGAAGATGAATTTGATGAAATTGCACATAAAGGATTCTTTTTGGTTTATGAGCTGTATTCTCATTATAA [15522]
cp32-4_B31_TS_MP_FG_consensus  TTCTAAATTTGAAGATGAATTTGATGAAATTGCACATAAAGGATTCTTTTTGGTTTATGAGCTGTATTCTCATTATAA [15522]
cp32-4_B31_NX_Pl_consensus     TTCTAAATTTGAAGATGAATTTGATGAAATTGCACATAAAGGATTCTTTTTGGTTTATGAGCTGTATTCTCATTATAA [15522]
cp32-4_PAli_NX_MP_FG_consensus TTCTAAATTTGAAGATGAATTTGATGAAATTGCACATAAAGGATTCTTTTTGGTTTATGAGCTGTATTCTCATTATAA [15522]
cp32-4_PAli_NX_Pl_consensus    TTCTAAATTTGAAGATGAATTTGATGAAATTGCACATAAAGGATTCTTTTTGGTTTATGAGCTGTATTCTCATTATAA [15522]
cp32-4_PAbe_NX_MP_FG_consensus TTCTAAATTTGAAGATGAATTTGATGAAATTGCACATAAAGGATTCTTTTTGGTTTATGAGCTGTATTCTCATTATAA [15522]
cp32-4_PAbe_NX_Pl_consensus    TTCTAAATTTGAAGATGAATTTGATGAAATTGCACATAAAGGATTCTTTTTGGTTTATGAGCTGTATTCTCATTATAA [15522]

cp32-4_gi|11497149|B31_GB      ATTAATCTATACAGCAAATATGGAAAGACTTGAGAGTGCATTAACCCCAGCAATAAATAAAGCACTCGCTCCATTAAA [15600]
cp32-4_un10_B31_PacBio         ATTAATCTATACAGCAAATATGGAAAGACTTGAGAGTGCATTAACCCCAGCAATAAATAAAGCACTCGCTCCATTAAA [15600]
cp32-4_B31_TS_MP_FG_consensus  ATTAATCTATACAGCAAATATGGAAAGACTTGAGAGTGCATTAACCCCAGCAATAAATAAAGCACTCGCTCCATTAAA [15600]
cp32-4_B31_NX_Pl_consensus     ATTAATCTATACAGCAAATATGGAAAGACTTGAGAGTGCATTAACCCCAGCAATAAATAAAGCACTCGCTCCATTAAA [15600]
cp32-4_PAli_NX_MP_FG_consensus ATTAATCTATACAGCAAATATGGAAAGACTTGAGAGTGCATTAACCCCAGCAATAAATAAAGCACTCGCTCCATTAAA [15600]
cp32-4_PAli_NX_Pl_consensus    ATTAATCTATACAGCAAATATGGAAAGACTTGAGAGTGCATTAACCCCAGCAATAAATAAAGCACTCGCTCCATTAAA [15600]
cp32-4_PAbe_NX_MP_FG_consensus ATTAATCTATACAGCAAATATGGAAAGACTTGAGAGTGCATTAACCCCAGCAATAAATAAAGCACTCGCTCCATTAAA [15600]
cp32-4_PAbe_NX_Pl_consensus    ATTAATCTATACAGCAAATATGGAAAGACTTGAGAGTGCATTAACCCCAGCAATAAATAAAGCACTCGCTCCATTAAA [15600]

cp32-4_gi|11497149|B31_GB      TGAAAAAATCAATCAATGCATTGACTTAGTTAATTCTGATGAAAAAAATCTCAAAATATCTAATGATCTGAAATTCAA [15678]
cp32-4_un10_B31_PacBio         TGAAAAAATCAATCAATGCATTGACTTAGTTAATTCTGATGAAAAAAATCTCAAAATATCTAATGATCTGAAATTCAA [15678]
cp32-4_B31_TS_MP_FG_consensus  TGAAAAAATCAATCAATGCATTGACTTAGTTAATTCTGATGAAAAAAATCTCAAAATATCTAATGATCTGAAATTCAA [15678]
cp32-4_B31_NX_Pl_consensus     TGAAAAAATCAATCAATGCATTGACTTAGTTAATTCTGATGAAAAAAATCTCAAAATATCTAATGATCTGAAATTCAA [15678]
cp32-4_PAli_NX_MP_FG_consensus TGAAAAAATCAATCAATGCATTGACTTAGTTAATTCTGATGAAAAAAATCTCAAAATATCTAATGATCTGAAATTCAA [15678]
cp32-4_PAli_NX_Pl_consensus    TGAAAAAATCAATCAATGCATTGACTTAGTTAATTCTGATGAAAAAAATCTCAAAATATCTAATGATCTGAAATTCAA [15678]
cp32-4_PAbe_NX_MP_FG_consensus TGAAAAAATCAATCAATGCATTGACTTAGTTAATTCTGATGAAAAAAATCTCAAAATATCTAATGATCTGAAATTCAA [15678]
cp32-4_PAbe_NX_Pl_consensus    TGAAAAAATCAATCAATGCATTGACTTAGTTAATTCTGATGAAAAAAATCTCAAAATATCTAATGATCTGAAATTCAA [15678]

cp32-4_gi|11497149|B31_GB      TCAGGAAGGAAAACCTATCTATAAGGAAAGAACAAATAATGCAAAATAACACTATTGGTTTAGGACTTAATTTACTAT [15756]
cp32-4_un10_B31_PacBio         TCAGGAAGGAAAACCTATCTATAAGGAAAGAACAAATAATGCAAAATAACACTATTGGTTTAGGACTTAATTTACTAT [15756]
cp32-4_B31_TS_MP_FG_consensus  TCAGGAAGGAAAACCTATCTATAAGGAAAGAACAAATAATGCAAAATAACACTATTGGTTTAGGACTTAATTTACTAT [15756]
cp32-4_B31_NX_Pl_consensus     TCAGGAAGGAAAACCTATCTATAAGGAAAGAACAAATAATGCAAAATAACACTATTGGTTTAGGACTTAATTTACTAT [15756]
cp32-4_PAli_NX_MP_FG_consensus TCAGGAAGGAAAACCTATCTATAAGGAAAGAACAAATAATGCAAAATAACACTATTGGTTTAGGACTTAATTTACTAT [15756]
cp32-4_PAli_NX_Pl_consensus    TCAGGAAGGAAAACCTATCTATAAGGAAAGAACAAATAATGCAAAATAACACTATTGGTTTAGGACTTAATTTACTAT [15756]
cp32-4_PAbe_NX_MP_FG_consensus TCAGGAAGGAAAACCTATCTATAAGGAAAGAACAAATAATGCAAAATAACACTATTGGTTTAGGACTTAATTTACTAT [15756]
cp32-4_PAbe_NX_Pl_consensus    TCAGGAAGGAAAACCTATCTATAAGGAAAGAACAAATAATGCAAAATAACACTATTGGTTTAGGACTTAATTTACTAT [15756]

cp32-4_gi|11497149|B31_GB      CCAGCTTAACTAACATAGCTAAAACTGATACAAACATAGATCATAATTACATTAATACTTTTAGTAAAGTAATAGATT [15834]
cp32-4_un10_B31_PacBio         CCAGCTTAACTAACATAGCTAAAACTGATACAAACATAGATCATAATTACATTAATACTTTTAGTAAAGTAATAGATT [15834]
cp32-4_B31_TS_MP_FG_consensus  CCAGCTTAACTAACATAGCTAAAACTGATACAAACATAGATCATAATTACATTAATACTTTTAGTAAAGTAATAGATT [15834]
cp32-4_B31_NX_Pl_consensus     CCAGCTTAACTAACATAGCTAAAACTGATACAAACATAGATCATAATTACATTAATACTTTTAGTAAAGTAATAGATT [15834]
cp32-4_PAli_NX_MP_FG_consensus CCAGCTTAACTAACATAGCTAAAACTGATACAAACATAGATCATAATTACATTAATACTTTTAGTAAAGTAATAGATT [15834]
cp32-4_PAli_NX_Pl_consensus    CCAGCTTAACTAACATAGCTAAAACTGATACAAACATAGATCATAATTACATTAATACTTTTAGTAAAGTAATAGATT [15834]
cp32-4_PAbe_NX_MP_FG_consensus CCAGCTTAACTAACATAGCTAAAACTGATACAAACATAGATCATAATTACATTAATACTTTTAGTAAAGTAATAGATT [15834]
cp32-4_PAbe_NX_Pl_consensus    CCAGCTTAACTAACATAGCTAAAACTGATACAAACATAGATCATAATTACATTAATACTTTTAGTAAAGTAATAGATT [15834]

cp32-4_gi|11497149|B31_GB      TTTTCTACAAAACATATATAAGCACACTAAAATCTATGGAAACAGCTGAGTCAACTAAAATATTTGAAGAAATACAAG [15912]
cp32-4_un10_B31_PacBio         TTTTCTACAAAACATATATAAGCACACTAAAATCTATGGAAACAGCTGAGTCAACTAAAATATTTGAAGAAATACAAG [15912]
cp32-4_B31_TS_MP_FG_consensus  TTTTCTACAAAACATATATAAGCACACTAAAATCTATGGAAACAGCTGAGTCAACTAAAATATTTGAAGAAATACAAG [15912]
cp32-4_B31_NX_Pl_consensus     TTTTCTACAAAACATATATAAGCACACTAAAATCTATGGAAACAGCTGAGTCAACTAAAATATTTGAAGAAATACAAG [15912]
cp32-4_PAli_NX_MP_FG_consensus TTTTCTACAAAACATATATAAGCACACTAAAATCTATGGAAACAGCTGAGTCAACTAAAATATTTGAAGAAATACAAG [15912]
cp32-4_PAli_NX_Pl_consensus    TTTTCTACAAAACATATATAAGCACACTAAAATCTATGGAAACAGCTGAGTCAACTAAAATATTTGAAGAAATACAAG [15912]
cp32-4_PAbe_NX_MP_FG_consensus TTTTCTACAAAACATATATAAGCACACTAAAATCTATGGAAACAGCTGAGTCAACTAAAATATTTGAAGAAATACAAG [15912]
cp32-4_PAbe_NX_Pl_consensus    TTTTCTACAAAACATATATAAGCACACTAAAATCTATGGAAACAGCTGAGTCAACTAAAATATTTGAAGAAATACAAG [15912]

cp32-4_gi|11497149|B31_GB      ACATTTTAAAATACAACATTGAGATAATAGAGGCTATCTCTACTGATAAAAGCAAAAGAATTATCACTTCACTTAAAG [15990]
cp32-4_un10_B31_PacBio         ACATTTTAAAATACAACATTGAGATAATAGAGGCTATCTCTACTGATAAAAGCAAAAGAATTATCACTTCACTTAAAG [15990]
cp32-4_B31_TS_MP_FG_consensus  ACATTTTAAAATACAACATTGAGATAATAGAGGCTATCTCTACTGATAAAAGCAAAAGAATTATCACTTCACTTAAAG [15990]
cp32-4_B31_NX_Pl_consensus     ACATTTTAAAATACAACATTGAGATAATAGAGGCTATCTCTACTGATAAAAGCAAAAGAATTATCACTTCACTTAAAG [15990]
cp32-4_PAli_NX_MP_FG_consensus ACATTTTAAAATACAACATTGAGATAATAGAGGCTATCTCTACTGATAAAAGCAAAAGAATTATCACTTCACTTAAAG [15990]
cp32-4_PAli_NX_Pl_consensus    ACATTTTAAAATACAACATTGAGATAATAGAGGCTATCTCTACTGATAAAAGCAAAAGAATTATCACTTCACTTAAAG [15990]
cp32-4_PAbe_NX_MP_FG_consensus ACATTTTAAAATACAACATTGAGATAATAGAGGCTATCTCTACTGATAAAAGCAAAAGAATTATCACTTCACTTAAAG [15990]
cp32-4_PAbe_NX_Pl_consensus    ACATTTTAAAATACAACATTGAGATAATAGAGGCTATCTCTACTGATAAAAGCAAAAGAATTATCACTTCACTTAAAG [15990]
```

```
cp32-4_gi|11497149|B31_GB       CAACACGTAACAAAATCATGAAAGAATATATCAAAATACTTAAAAGAGGTGAAAATGCTTAAAAGATTGCATTGTCTA [16068]
cp32-4_un10_B31_PacBio          CAACACGTAACAAAATCATGAAAGAATATATCAAAATACTTAAAAGAGGTGAAAATGCTTAAAAGATTGCATTGTCTA [16068]
cp32-4_B31_TS_MP_FG_consensus   CAACACGTAACAAAATCATGAAAGAATATATCAAAATACTTAAAAGAGGTGAAAATGCTTAAAAGATTGCATTGTCTA [16068]
cp32-4_B31_NX_Pl_consensus      CAACACGTAACAAAATCATGAAAGAATATATCAAAATACTTAAAAGAGGTGAAAATGCTTAAAAGATTGCATTGTCTA [16068]
cp32-4_PAli_NX_MP_FG_consensus  CAACACGTAACAAAATCATGAAAGAATATATCAAAATACTTAAAAGAGGTGAAAATGCTTAAAAGATTGCATTGTCTA [16068]
cp32-4_PAli_NX_Pl_consensus     CAACACGTAACAAAATCATGAAAGAATATATCAAAATACTTAAAAGAGGTGAAAATGCTTAAAAGATTGCATTGTCTA [16068]
cp32-4_PAbe_NX_MP_FG_consensus  CAACACGTAACAAAATCATGAAAGAATATATCAAAATACTTAAAAGAGGTGAAAATGCTTAAAAGATTGCATTGTCTA [16068]
cp32-4_PAbe_NX_Pl_consensus     CAACACGTAACAAAATCATGAAAGAATATATCAAAATACTTAAAAGAGGTGAAAATGCTTAAAAGATTGCATTGTCTA [16068]

cp32-4_gi|11497149|B31_GB       CTAATTGCTTTGCTACTATGTTGCACCACTATTGCCAACCTACCAGAAGAGCCAAAACCGCCAATTATTCCAACACTA [16146]
cp32-4_un10_B31_PacBio          CTAATTGCTTTGCTACTATGTTGCACCACTATTGCCAACCTACCAGAAGAGCCAAAACCGCCAATTATTCCAACACTA [16146]
cp32-4_B31_TS_MP_FG_consensus   CTAATTGCTTTGCTACTATGTTGCACCACTATTGCCAACCTACCAGAAGAGCCAAAACCGCCAATTATTCCAACACTA [16146]
cp32-4_B31_NX_Pl_consensus      CTAATTGCTTTGCTACTATGTTGCACCACTATTGCCAACCTACCAGAAGAGCCAAAACCGCCAATTATTCCAACACTA [16146]
cp32-4_PAli_NX_MP_FG_consensus  CTAATTGCTTTGCTACTATGTTGCACCACTATTGCCAACCTACCAGAAGAGCCAAAACCGCCAATTATTCCAACACTA [16146]
cp32-4_PAli_NX_Pl_consensus     CTAATTGCTTTGCTACTATGTTGCACCACTATTGCCAACCTACCAGAAGAGCCAAAACCGCCAATTATTCCAACACTA [16146]
cp32-4_PAbe_NX_MP_FG_consensus  CTAATTGCTTTGCTACTATGTTGCACCACTATTGCCAACCTACCAGAAGAGCCAAAACCGCCAATTATTCCAACACTA [16146]
cp32-4_PAbe_NX_Pl_consensus     CTAATTGCTTTGCTACTATGTTGCACCACTATTGCCAACCTACCAGAAGAGCCAAAACCGCCAATTATTCCAACACTA [16146]

cp32-4_gi|11497149|B31_GB       AAATCTTTAGCTAAATATGAAACACAACTTTCAGAGCATGTTATGTACCTAGTAACATTTTTAGCTAAAACAAAAGTC [16224]
cp32-4_un10_B31_PacBio          AAATCTTTAGCTAAATATGAAACACAACTTTCAGAGCATGTTATGTACCTAGTAACATTTTTAGCTAAAACAAAAGTC [16224]
cp32-4_B31_TS_MP_FG_consensus   AAATCTTTAGCTAAATATGAAACACAACTTTCAGAGCATGTTATGTACCTAGTAACATTTTTAGCTAAAACAAAAGTC [16224]
cp32-4_B31_NX_Pl_consensus      AAATCTTTAGCTAAATATGAAACACAACTTTCAGAGCATGTTATGTACCTAGTAACATTTTTAGCTAAAACAAAAGTC [16224]
cp32-4_PAli_NX_MP_FG_consensus  AAATCTTTAGCTAAATATGAAACACAACTTTCAGAGCATGTTATGTACCTAGTAACATTTTTAGCTAAAACAAAAGTC [16224]
cp32-4_PAli_NX_Pl_consensus     AAATCTTTAGCTAAATATGAAACACAACTTTCAGAGCATGTTATGTACCTAGTAACATTTTTAGCTAAAACAAAAGTC [16224]
cp32-4_PAbe_NX_MP_FG_consensus  AAATCTTTAGCTAAATATGAAACACAACTTTCAGAGCATGTTATGTACCTAGTAACATTTTTAGCTAAAACAAAAGTC [16224]
cp32-4_PAbe_NX_Pl_consensus     AAATCTTTAGCTAAATATGAAACACAACTTTCAGAGCATGTTATGTACCTAGTAACATTTTTAGCTAAAACAAAAGTC [16224]

cp32-4_gi|11497149|B31_GB       AAAGTTAATGACCCAAATTATCCAGAATATCCTTATCCAGACTTATCAACACTAAAAGACGAACACTCCATAACTGCA [16302]
cp32-4_un10_B31_PacBio          AAAGTTAATGACCCAAATTATCCAGAATATCCTTATCCAGACTTATCAACACTAAAAGACGAACACTCCATAACTGCA [16302]
cp32-4_B31_TS_MP_FG_consensus   AAAGTTAATGACCCAAATTATCCAGAATATCCTTATCCAGACTTATCAACACTAAAAGACGAACACTCCATAACTGCA [16302]
cp32-4_B31_NX_Pl_consensus      AAAGTTAATGACCCAAATTATCCAGAATATCCTTATCCAGACTTATCAACACTAAAAGACGAACACTCCATAACTGCA [16302]
cp32-4_PAli_NX_MP_FG_consensus  AAAGTTAATGACCCAAATTATCCAGAATATCCTTATCCAGACTTATCAACACTAAAAGACGAACACTCCATAACTGCA [16302]
cp32-4_PAli_NX_Pl_consensus     AAAGTTAATGACCCAAATTATCCAGAATATCCTTATCCAGACTTATCAACACTAAAAGACGAACACTCCATAACTGCA [16302]
cp32-4_PAbe_NX_MP_FG_consensus  AAAGTTAATGACCCAAATTATCCAGAATATCCTTATCCAGACTTATCAACACTAAAAGACGAACACTCCATAACTGCA [16302]
cp32-4_PAbe_NX_Pl_consensus     AAAGTTAATGACCCAAATTATCCAGAATATCCTTATCCAGACTTATCAACACTAAAAGACGAACACTCCATAACTGCA [16302]

cp32-4_gi|11497149|B31_GB       GTAAAACATAATATCAACATATATTTAGAGTACATTAAAAAAACAAAACCAATAGCGGAAAAAGTCTATAATAAATAT [16380]
cp32-4_un10_B31_PacBio          GTAAAACATAATATCAACATATATTTAGAGTACATTAAAAAAACAAAACCAATAGCGGAAAAAGTCTATAATAAATAT [16380]
cp32-4_B31_TS_MP_FG_consensus   GTAAAACATAATATCAACATATATTTAGAGTACATTAAAAAAACAAAACCAATAGCGGAAAAAGTCTATAATAAATAT [16380]
cp32-4_B31_NX_Pl_consensus      GTAAAACATAATATCAACATATATTTAGAGTACATTAAAAAAACAAAACCAATAGCGGAAAAAGTCTATAATAAATAT [16380]
cp32-4_PAli_NX_MP_FG_consensus  GTAAAACATAATATCAACATATATTTAGAGTACATTAAAAAAACAAAACCAATAGCGGAAAAAGTCTATAATAAATAT [16380]
cp32-4_PAli_NX_Pl_consensus     GTAAAACATAATATCAACATATATTTAGAGTACATTAAAAAAACAAAACCAATAGCGGAAAAAGTCTATAATAAATAT [16380]
cp32-4_PAbe_NX_MP_FG_consensus  GTAAAACATAATATCAACATATATTTAGAGTACATTAAAAAAACAAAACCAATAGCGGAAAAAGTCTATAATAAATAT [16380]
cp32-4_PAbe_NX_Pl_consensus     GTAAAACATAATATCAACATATATTTAGAGTACATTAAAAAAACAAAACCAATAGCGGAAAAAGTCTATAATAAATAT [16380]

cp32-4_gi|11497149|B31_GB       TCCCAATTAAAAATGTAAATTACAAAAAGGTTTTTCTTGCAAGAAATTCTATTTTACATATAAAAATCTCTAAAGCCA [16458]
cp32-4_un10_B31_PacBio          TCCCAATTAAAAATGTAAATTACAAAAAGGTTTTTCTTGCAAGAAATTCTATTTTACATATAAAAATCTCTAAAGCCA [16458]
cp32-4_B31_TS_MP_FG_consensus   TCCCAATTAAAAATGTAAATTACAAAAAGGTTTTTCTTGCAAGAAATTCTATTTTACATATAAAAATCTCTAAAGCCA [16458]
cp32-4_B31_NX_Pl_consensus      TCCCAATTAAAAATGTAAATTACAAAAAGGTTTTTCTTGCAAGAAATTCTATTTTACATATAAAAATCTCTAAAGCCA [16458]
cp32-4_PAli_NX_MP_FG_consensus  TCCCAATTAAAAATGTAAATTACAAAAAGGTTTTTCTTGCAAGAAATTCTATTTTACATATAAAAATCTCTAAAGCCA [16458]
cp32-4_PAli_NX_Pl_consensus     TCCCAATTAAAAATGTAAATTACAAAAAGGTTTTTCTTGCAAGAAATTCTATTTTACATATAAAAATCTCTAAAGCCA [16458]
cp32-4_PAbe_NX_MP_FG_consensus  TCCCAATTAAAAATGTAAATTACAAAAAGGTTTTTCTTGCAAGAAATTCTATTTTACATATAAAAATCTCTAAAGCCA [16458]
cp32-4_PAbe_NX_Pl_consensus     TCCCAATTAAAAATGTAAATTACAAAAAGGTTTTTCTTGCAAGAAATTCTATTTTACATATAAAAATCTCTAAAGCCA [16458]

cp32-4_gi|11497149|B31_GB       ATTAATCTAAAATAGTGTATAATATGACCATAAGGGAAAATTTTATGGAAACAGTGTCAACAAATATTGCAGGTGTAA [16536]
cp32-4_un10_B31_PacBio          ATTAATCTAAAATAGTGTATAATATGACCATAAGGGAAAATTTTATGGAAACAGTGTCAACAAATATTGCAGGTGTAA [16536]
cp32-4_B31_TS_MP_FG_consensus   ATTAATCTAAAATAGTGTATAATATGACCATAAGGGAAAATTTTATGGAAACAGTGTCAACAAATATTGCAGGTGTAA [16536]
cp32-4_B31_NX_Pl_consensus      ATTAATCTAAAATAGTGTATAATATGACCATAAGGGAAAATTTTATGGAAACAGTGTCAACAAATATTGCAGGTGTAA [16536]
cp32-4_PAli_NX_MP_FG_consensus  ATTAATCTAAAATAGTGTATAATATGACCATAAGGGAAAATTTTATGGAAACAGTGTCAACAAATATTGCAGGTGTAA [16536]
cp32-4_PAli_NX_Pl_consensus     ATTAATCTAAAATAGTGTATAATATGACCATAAGGGAAAATTTTATGGAAACAGTGTCAACAAATATTGCAGGTGTAA [16536]
cp32-4_PAbe_NX_MP_FG_consensus  ATTAATCTAAAATAGTGTATAATATGACCATAAGGGAAAATTTTATGGAAACAGTGTCAACAAATATTGCAGGTGTAA [16536]
cp32-4_PAbe_NX_Pl_consensus     ATTAATCTAAAATAGTGTATAATATGACCATAAGGGAAAATTTTATGGAAACAGTGTCAACAAATATTGCAGGTGTAA [16536]

cp32-4_gi|11497149|B31_GB       CTCAAGAACAAATATATAAAGAATTTATTAGACTGGGCATGGAACAACTAATAGCACAAGATTTATCTAAAAGATATT [16614]
cp32-4_un10_B31_PacBio          CTCAAGAACAAATATATAAAGAATTTATTAGACTGGGCATGGAACAACTAATAGCACAAGATTTATCTAAAAGATATT [16614]
cp32-4_B31_TS_MP_FG_consensus   CTCAAGAACAAATATATAAAGAATTTATTAGACTGGGCATGGAACAACTAATAGCACAAGATTTATCTAAAAGATATT [16614]
cp32-4_B31_NX_Pl_consensus      CTCAAGAACAAATATATAAAGAATTTATTAGACTGGGCATGGAACAACTAATAGCACAAGATTTATCTAAAAGATATT [16614]
cp32-4_PAli_NX_MP_FG_consensus  CTCAAGAACAAATATATAAAGAATTTATTAGACTGGGCATGGAACAACTAATAGCACAAGATTTATCTAAAAGATATT [16614]
cp32-4_PAli_NX_Pl_consensus     CTCAAGAACAAATATATAAAGAATTTATTAGACTGGGCATGGAACAACTAATAGCACAAGATTTATCTAAAAGATATT [16614]
cp32-4_PAbe_NX_MP_FG_consensus  CTCAAGAACAAATATATAAAGAATTTATTAGACTGGGCATGGAACAACTAATAGCACAAGATTTATCTAAAAGATATT [16614]
cp32-4_PAbe_NX_Pl_consensus     CTCAAGAACAAATATATAAAGAATTTATTAGACTGGGCATGGAACAACTAATAGCACAAGATTTATCTAAAAGATATT [16614]

cp32-4_gi|11497149|B31_GB       ATCACAATGAACTAACATATAGAGATTTAGAAAATTTAGAAAAACAATTTGATATAAAATTTGATAATCTTATTTCTA [16692]
cp32-4_un10_B31_PacBio          ATCACAATGAACTAACATATAGAGATTTAGAAAATTTAGAAAAACAATTTGATATAAAATTTGATAATCTTATTTCTA [16692]
cp32-4_B31_TS_MP_FG_consensus   ATCACAATGAACTAACATATAGAGATTTAGAAAATTTAGAAAAACAATTTGATATAAAATTTGATAATCTTATTTCTA [16692]
cp32-4_B31_NX_Pl_consensus      ATCACAATGAACTAACATATAGAGATTTAGAAAATTTAGAAAAACAATTTGATATAAAATTTGATAATCTTATTTCTA [16692]
cp32-4_PAli_NX_MP_FG_consensus  ATCACAATGAACTAACATATAGAGATTTAGAAAATTTAGAAAAACAATTTGATATAAAATTTGATAATCTTATTTCTA [16692]
cp32-4_PAli_NX_Pl_consensus     ATCACAATGAACTAACATATAGAGATTTAGAAAATTTAGAAAAACAATTTGATATAAAATTTGATAATCTTATTTCTA [16692]
cp32-4_PAbe_NX_MP_FG_consensus  ATCACAATGAACTAACATATAGAGATTTAGAAAATTTAGAAAAACAATTTGATATAAAATTTGATAATCTTATTTCTA [16692]
cp32-4_PAbe_NX_Pl_consensus     ATCACAATGAACTAACATATAGAGATTTAGAAAATTTAGAAAAACAATTTGATATAAAATTTGATAATCTTATTTCTA [16692]

cp32-4_gi|11497149|B31_GB       AAATAGATACTGTAGAAAAGAATTTAAATGTTAAGATTGATGCTGTTAAAAGCGAACTTAATACTAAGATTGACAATG [16770]
cp32-4_un10_B31_PacBio          AAATAGATACTGTAGAAAAGAATTTAAATGTTAAGATTGATGCTGTTAAAAGCGAACTTAATACTAAGATTGACAATG [16770]
cp32-4_B31_TS_MP_FG_consensus   AAATAGATACTGTAGAAAAGAATTTAAATGTTAAGATTGATGCTGTTAAAAGCGAACTTAATACTAAGATTGACAATG [16770]
cp32-4_B31_NX_Pl_consensus      AAATAGATACTGTAGAAAAGAATTTAAATGTTAAGATTGATGCTGTTAAAAGCGAACTTAATACTAAGATTGACAATG [16770]
cp32-4_PAli_NX_MP_FG_consensus  AAATAGATACTGTAGAAAAGAATTTAAATGTTAAGATTGATGCTGTTAAAAGCGAACTTAATACTAAGATTGACAATG [16770]
cp32-4_PAli_NX_Pl_consensus     AAATAGATACTGTAGAAAAGAATTTACATGTTAAGATTGATGCTGTTAAAAGCGAACTTAATACTAAGATTGACAATG [16770]
cp32-4_PAbe_NX_MP_FG_consensus  AAATAGATACTGTAGAAAAGAATTTAAATGTTAAGATTGATGCTGTTAAAAGCGAACTTAATACTAAGATTGACAATG [16770]
cp32-4_PAbe_NX_Pl_consensus     AAATAGATACTGTAGAAAAGAATTTAAATGTTAAGATTGATGCTGTTAAAAGCGAACTTAATACTAAGATTGACAATG [16770]

cp32-4_gi|11497149|B31_GB       TGGAAAAGAATTTAAATCTAAAAATAGATAGTTTAGATACTAAGATAGATACTGTAGAAAAGAATTTAAATTTAAAAA [16848]
cp32-4_un10_B31_PacBio          TGGAAAAGAATTTAAATCTAAAAATAGATAGTTTAGATACTAAGATAGATACTGTAGAAAAGAATTTAAATTTAAAAA [16848]
cp32-4_B31_TS_MP_FG_consensus   TGGAAAAGAATTTAAATCTAAAAATAGATAGTTTAGATACTAAGATAGATACTGTAGAAAAGAATTTAAATTTAAAAA [16848]
cp32-4_B31_NX_Pl_consensus      TGGAAAAGAATTTAAATCTAAAAATAGATAGTTTAGATACTAAGATTGATAATGTAGAAAAGAATTTAAATCTAAAAA [16848]
cp32-4_PAli_NX_MP_FG_consensus  TGGAAAAGAATTTAAATCTAAAAATAGATAGTTTAGATACTAAGATAGATACTGTAGAAAAGAATTTAAATTTAAAAA [16848]
cp32-4_PAli_NX_Pl_consensus     TGGAAAAGAATTTAAATCTAAAAATAGATAGTTTAGATACTAAGATTGATAATGTAGAAAAGAATTTAAATCTAAAAA [16848]
cp32-4_PAbe_NX_MP_FG_consensus  TGGAAAAGAATTTAAATCTAAAAATAGATAGTTTAGATACTAAGATAGATACTGTAGAAAAGAATTTAAATCTAAAAA [16848]
cp32-4_PAbe_NX_Pl_consensus     TGGAAAAGAATTTAAATCTAAAAATAGATAGTTTAGATACTAAGATTGATACTGTAGAAAAGAATTTAAATTTAAAAA [16848]

cp32-4_gi|11497149|B31_GB       TAGATAGTGTTAAAAATGAACTTACTGCTAAAATAGACAATGTGGAAAAGAATTTAATGTCTCTTTCAGAAATGCTTA [16926]
cp32-4_un10_B31_PacBio          TAGATAGTGTTAAAAATGAACTTACTGCTAAAATAGACAATGTGGAAAAGAATTTAATGTCTCTTTCAGAAATGCTTA [16926]
cp32-4_B31_TS_MP_FG_consensus   TAGATAGTGTTAAAAATGAACTTACTGCTAAAATAGACAATGTGGAAAAGAATTTAATGTCTCTTTCAGAAATGCTTA [16926]
cp32-4_B31_NX_Pl_consensus      TAGAT-GTGTTAAAAATGAACTTACTGCTAAAATAGACAATGTGGAAAAGAATTTAATGTCTCTTTCAGAAATGCTTA [16926]
cp32-4_PAli_NX_MP_FG_consensus  TAGATAGTGTTAAAAATGAACTTACTGCTAAAATAGACAATGTGGAAAAGAATTTAATGTCTCTTTCAGAAATGCTTA [16926]
cp32-4_PAli_NX_Pl_consensus     TAGATGGCTTGAA------------TATCAAAATAGACAATGTGGAAAAGAATTTAATGTCTCTTTCAGAAATGCTTA [16926]
cp32-4_PAbe_NX_MP_FG_consensus  TAGATAGTGTTAAAAATGAACTTACTGCTAAAATAGACAATGTGGAAAAGAATTTAATGTCTCTTTCAGAAATGCTTA [16926]
cp32-4_PAbe_NX_Pl_consensus     TAGATAGTGTTAAAAATGAACTTACTGCTAAAATAGACAATGTGGAAAAGAATTTAATGTCTCTTTCAGAAATGCTTA [16926]

cp32-4_gi|11497149|B31_GB       AATGGGTATTAGGAATTATGGGAGCAATGTCTATAACAATGATAGCCGGGCTAATATTTGCTTTCATTTCTAAATAGC [17004]
```

```
cp32-4_un10_B31_PacBio         AATGGGTATTAGGAATTATGGGAGCAATGTCTATAACAATGATAGCCGGGCTAATATTTGCTTTCATTTCTAAATAGC [17004]
cp32-4_B31_TS_MP_FG_consensus  AATGGGTATTAGGAATTATGGGAGCAATGTCTATAACAATGATAGCCGGGCTAATATTTGCTTTCATTTCTAAATAGC [17004]
cp32-4_B31_NX_Pl_consensus     AATGGGTATTGGGAATCATGGGAGCAATGTCTATAACAATGATAGCCGGGCTAATATTTGCTTTCATTTCTAAATAGC [17004]
cp32-4_PAli_NX_MP_FG_consensus AATGGGTATTAGGAATTATGGGAGCAATGTCTATAACAATGATAGCCGGGCTAATATTTGCTTTCATTTCTAAATAGC [17004]
cp32-4_PAli_NX_Pl_consensus    AATGGGTATTGGGAATCATGGGAGCAATGTCTATAACAATGATAGCCGGGCTAATATTTGCTTTCATTTCTAAATAGC [17004]
cp32-4_PAbe_NX_MP_FG_consensus AATGGGTATTAGGAATTATGGGAGCAATGTCTATAACAATGATAGCCGGGCTAATATTTGCTTTCATTTCTAAATAGC [17004]
cp32-4_PAbe_NX_Pl_consensus    AATGGGTATTAGGAATTATGGGAGCAATGTCTATAACAATGATAGCCGGGCTAATATTTGCTTTCATTTCTAAATAGC [17004]

cp32-4_gi|11497149|B31_GB      TACTACCTCACTTAAAGAATATCAATTCAAATAATAATTGTTTATCCAATATTTAGTATTAATAATTGATTAAGTGAA [17082]
cp32-4_un10_B31_PacBio         TACTACCTCACTTAAAGAATATCAATTCAAATAATAATTGTTTATCCAATATTTAGTATTAATAATTGATTAAGTGAA [17082]
cp32-4_B31_TS_MP_FG_consensus  TACTACCTCACTTAAAGAATATCAATTCAAATAATAATTGTTTATCCAATATTTAGTATTAATAATTGATTAAGTGAA [17082]
cp32-4_B31_NX_Pl_consensus     TACTACCTCACTTAAAGAATATCAATTCAAATAATAATTGTTTATCCAATATTTAGTATTAATAATTGATTAAGTGAA [17082]
cp32-4_PAli_NX_MP_FG_consensus TACTACCTCACTTAAAGAATATCAATTCAAATAATAATTGTTTATCCAATATTTAGTATTAATAATTGATTAAGTGAA [17082]
cp32-4_PAli_NX_Pl_consensus    TACTACCTCACTTAAAGAATATCAATTCAAATAATAATTGTTTATCCAATATTTAGTATTAATAATTGATTAAGTGAA [17082]
cp32-4_PAbe_NX_MP_FG_consensus TACTACCTCACTTAAAGAATATCAATTCAAATAATAATTGTTTATCCAATATTTAGTATTAATAATTGATTAAGTGAA [17082]
cp32-4_PAbe_NX_Pl_consensus    TACTACCTCACTTAAAGAATATCAATTCAAATAATAATTGTTTATCCAATATTTAGTATTAATAATTGATTAAGTGAA [17082]

cp32-4_gi|11497149|B31_GB      TATTAATAATAAAAAAAGGAATAATAATGAAAATTATCAACATATTATTTTGTTTATTTTTACTAATGCTAAACGGCT [17160]
cp32-4_un10_B31_PacBio         TATTAATAATAAAAAAAGGAATAATAATGAAAATTATCAACATATTATTTTGTTTATTTTTACTAATGCTAAACGGCT [17160]
cp32-4_B31_TS_MP_FG_consensus  TATTAATAATAAAAAAAGGAATAATAATGAAAATTATCAACATATTATTTTGTTTATTTTTACTAATGCTAAACGGCT [17160]
cp32-4_B31_NX_Pl_consensus     TATTAATAATAAAAAAAGGAATAATAATGAAAATTATCAACATATTATTTTGTTTATTTTTACTAATGCTAAACGGCT [17160]
cp32-4_PAli_NX_MP_FG_consensus TATTAATAATAAAAAAAGGAATAATAATGAAAATTATCAACATATTATTTTGTTTATTTTTACTAATGCTAAACGGCT [17160]
cp32-4_PAli_NX_Pl_consensus    TATTAATAATAAAAAAAGGAATAATAATGAAAATTATCAACATATTATTTTGTTTATTTTTACTAATGCTAAACGGCT [17160]
cp32-4_PAbe_NX_MP_FG_consensus TATTAATAATAAAAAAAGGAATAATAATGAAAATTATCAACATATTATTTTGTTTATTTTTACTAATGCTAAACGGCT [17160]
cp32-4_PAbe_NX_Pl_consensus    TATTAATAATAAAAAAAGGAATAATAATGAAAATTATCAACATATTATTTTGTTTATTTTTACTAATGCTAAACGGCT [17160]

cp32-4_gi|11497149|B31_GB      GTAATTCTAATGATACTAATAATAGCCAAACAAAAAGTAGACAAAAACGTGATTTAACCCAAAAAGAAGCAACACAAG [17238]
cp32-4_un10_B31_PacBio         GTAATTCTAATGATACTAATAATAGCCAAACAAAAAGTAGACAAAAACGTGATTTAACCCAAAAAGAAGCAACACAAG [17238]
cp32-4_B31_TS_MP_FG_consensus  GTAATTCTAATGATACTAATAATAGCCAAACAAAAAGTAGACAAAAACGTGATTTAACCCAAAAAGAAGCAACACAAG [17238]
cp32-4_B31_NX_Pl_consensus     GTAATTCTAATGATACTAATAATAGCCAAACAAAAAGTAGACAAAAACGTGATTTAACCCAAAAAGAAGCAACACAAG [17238]
cp32-4_PAli_NX_MP_FG_consensus GTAATTCTAATGATACTAATAATAGCCAAACAAAAAGTAGACAAAAACGTGATTTAACCCAAAAAGAAGCAACACAAG [17238]
cp32-4_PAli_NX_Pl_consensus    GTAATTCTAATGATACTAATAATAGCCAAACAAAAAGTAGACAAAAACGTGATTTAACCCAAAAAGAAGCAACACAAG [17238]
cp32-4_PAbe_NX_MP_FG_consensus GTAATTCTAATGATACTAATAATAGCCAAACAAAAAGTAGACAAAAACGTGATTTAACCCAAAAAGAAGCAACACAAG [17238]
cp32-4_PAbe_NX_Pl_consensus    GTAATTCTAATGATACTAATAATAGCCAAACAAAAAGTAGACAAAAACGTGATTTAACCCAAAAAGAAGCAACACAAG [17238]

cp32-4_gi|11497149|B31_GB      AAAAACCTAAATCTAAAGAAGAACTTCTTAGAGAAAAGCTAAATGATAATCAAAAAACACACCTTGACTGGTTAAAAG [17316]
cp32-4_un10_B31_PacBio         AAAAACCTAAATCTAAAGAAGAACTTCTTAGAGAAAAGCTAAATGATAATCAAAAAACACACCTTGACTGGTTAAAAG [17316]
cp32-4_B31_TS_MP_FG_consensus  AAAAACCTAAATCTAAAGAAGAACTTCTTAGAGAAAAGCTAAATGATAATCAAAAAACACACCTTGACTGGTTAAAAG [17316]
cp32-4_B31_NX_Pl_consensus     AAAAACCTAAATCTAAAGAAGAACTTCTTAGAGAAAAGCTAAATGATAATCAAAAAACACACCTTGACTGGTTAAAAG [17316]
cp32-4_PAli_NX_MP_FG_consensus AAAAACCTAAATCTAAAGAAGAACTTCTTAGAGAAAAGCTAAATGATAATCAAAAAACACACCTTGACTGGTTAAAAG [17316]
cp32-4_PAli_NX_Pl_consensus    AAAAACCTAAATCTAAAGAAGAACTTCTTAGAGAAAAGCTAAATGATAATCAAAAAACACACCTTGACTGGTTAAAAG [17316]
cp32-4_PAbe_NX_MP_FG_consensus AAAAACCTAAATCTAAAGAAGAACTTCTTAGAGAAAAGCTAAATGATAATCAAAAAACACACCTTGACTGGTTAAAAG [17316]
cp32-4_PAbe_NX_Pl_consensus    AAAAACCTAAATCTAAAGAAGAACTTCTTAGAGAAAAGCTAAATGATAATCAAAAAACACACCTTGACTGGTTAAAAG [17316]

cp32-4_gi|11497149|B31_GB      AAGCTCTGGGCAATGATGGAGAATTTAATAAATTTTTAGGATATGATGAAAGCAAAATAAAATCTGCACTTGATCATA [17394]
cp32-4_un10_B31_PacBio         AAGCTCTGGGCAATGATGGAGAATTTAATAAATTTTTAGGATATGATGAAAGCAAAATAAAATCTGCACTTGATCATA [17394]
cp32-4_B31_TS_MP_FG_consensus  AAGCTCTGGGCAATGATGGAGAATTTAATAAATTTTTAGGATATGATGAAAGCAAAATAAAATCTGCACTTGATCATA [17394]
cp32-4_B31_NX_Pl_consensus     AAGCTCTGGGCAATGATGGAGAATTTAATAAATTTTTAGGATATGATGAAAGCAAAATAAAATCTGCACTTGATCATA [17394]
cp32-4_PAli_NX_MP_FG_consensus AAGCTCTGGGCAATGATGGAGAATTTAATAAATTTTTAGGATATGATGAAAGCAAAATAAAATCTGCACTTGATCATA [17394]
cp32-4_PAli_NX_Pl_consensus    AAGCTCTGGGCAATGATGGAGAATTTAATAAATTTTTAGGATATGATGAAAGCAAAATAAAATCTGCACTTGATCATA [17394]
cp32-4_PAbe_NX_MP_FG_consensus AAGCTCTGGGCAATGATGGAGAATTTAATAAATTTTTAGGATATGATGAAAGCAAAATAAAATCTGCACTTGATCATA [17394]
cp32-4_PAbe_NX_Pl_consensus    AAGCTCTGGGCAATGATGGAGAATTTAATAAATTTTTAGGATATGATGAAAGCAAAATAAAATCTGCACTTGATCATA [17394]

cp32-4_gi|11497149|B31_GB      TAAAGAGTGAACTTGACAGTTGTACTGGAGATAAGGTTGAAAATAAAAATACCTTCAAGCAGGTCGTTCAGGAGGCCC [17472]
cp32-4_un10_B31_PacBio         TAAAGAGTGAACTTGACAGTTGTACTGGAGATAAGGTTGAAAATAAAAATACCTTCAAGCAGGTCGTTCAGGAGGCCC [17472]
cp32-4_B31_TS_MP_FG_consensus  TAAAGAGTGAACTTGACAGTTGTACTGGAGATAAGGTTGAAAATAAAAATACCTTCAAGCAGGTCGTTCAGGAGGCCC [17472]
cp32-4_B31_NX_Pl_consensus     TAAAGAGTGAACTTGACAGTTGTACTGGAGATAAGGTTGAAAATAAAAATACCTTCAAGCAGGTCGTTCAGGAGGCCC [17472]
cp32-4_PAli_NX_MP_FG_consensus TAAAGAGTGAACTTGACAGTTGTACTGGAGATAAGGTTGAAAATAAAAATACCTTCAAGCAGGTCGTTCAGGAGGCCC [17472]
cp32-4_PAli_NX_Pl_consensus    TAAAGAGTGAACTTGACAGTTGTACTGGAGATAAGGTTGAAAATAAAAATACCTTCAAGCAGGTCGTTCAGGAGGCCC [17472]
cp32-4_PAbe_NX_MP_FG_consensus TAAAGAGTGAACTTGACAGTTGTACTGGAGATAAGGTTGAAAATAAAAATACCTTCAAGCAGGTCGTTCAGGAGGCCC [17472]
cp32-4_PAbe_NX_Pl_consensus    TAAAGAGTGAACTTGACAGTTGTACTGGAGATAAGGTTGAAAATAAAAATACCTTCAAGCAGGTCGTTCAGGAGGCCC [17472]

cp32-4_gi|11497149|B31_GB      TTAAAGGGGGCATAGACGGCTTTGAAAATACTGCAAGTAGTACGTGCAAAAATTCATAATATATAGCAGCCCCCAAAT [17550]
cp32-4_un10_B31_PacBio         TTAAAGGGGGCATAGACGGCTTTGAAAATACTGCAAGTAGTACGTGCAAAAATTCATAATATATAGCAGCCCCCAAAT [17550]
cp32-4_B31_TS_MP_FG_consensus  TTAAAGGGGGCATAGACGGCTTTGAAAATACTGCAAGTAGTACGTGCAAAAATTCATAATATATAGCAGCCCCCAAAT [17550]
cp32-4_B31_NX_Pl_consensus     TTAAAGGGGGCATAGACGGCTTTGAAAATACTGCAAGTAGTACGTGCAAAAATTCATAATATATAGCAGCCCCCAAAT [17550]
cp32-4_PAli_NX_MP_FG_consensus TTAAAGGGGGCATAGACGGCTTTGAAAATACTGCAAGTAGTACGTGCAAAAATTCATAATATATAGCAGCCCCCAAAT [17550]
cp32-4_PAli_NX_Pl_consensus    TTAAAGGGGGCATAGACGGCTTTGAAAATACTGCAAGTAGTACGTGCAAAAATTCATAATATATAGCAGCCCCCAAAT [17550]
cp32-4_PAbe_NX_MP_FG_consensus TTAAAGGGGGCATAGACGGCTTTGAAAATACTGCAAGTAGTACGTGCAAAAATTCATAATATATAGCAGCCCCCAAAT [17550]
cp32-4_PAbe_NX_Pl_consensus    TTAAAGGGGGCATAGACGGCTTTGAAAATACTGCAAGTAGTACGTGCAAAAATTCATAATATATAGCAGCCCCCAAAT [17550]

cp32-4_gi|11497149|B31_GB      AGGGGGGCTTTACCATGTAAGAATCTTGCTATGCTGCAAATATCTCATCAAACCGTTCATATTCTTTTAAGACACTAA [17628]
cp32-4_un10_B31_PacBio         AGGGGGGCTTTACCATGTAAGAATCTTGCTATGCTGCAAATATCTCATCAAACCGTTCATATTCTTTTAAGACACTAA [17628]
cp32-4_B31_TS_MP_FG_consensus  AGGGGGGCTTTACCATGTAAGAATCTTGCTATGCTGCAAATATCTCATCAAACCGTTCATATTCTTTTAAGACACTAA [17628]
cp32-4_B31_NX_Pl_consensus     AGGGGGGCTTTACCATGTAAGAATCTTGCTATGCTGCAAATATCTCATCAAACCGTTCATATTCTTTTAAGACACTAA [17628]
cp32-4_PAli_NX_MP_FG_consensus AGGGGGGCTTTACCATGTAAGAATCTTGCTATGCTGCAAATATCTCATCAAACCGTTCATATTCTTTTAAGACACTAA [17628]
cp32-4_PAli_NX_Pl_consensus    AGGGGGGCTTTACCATGTAAGAATCTTGCTATGCTGCAAATATCTCATCAAACCGTTCATATTCTTTTAAGACACTAA [17628]
cp32-4_PAbe_NX_MP_FG_consensus AGGGGGGCTTTACCATGTAAGAATCTTGCTATGCTGCAAATATCTCATCAAACCGTTCATATTCTTTTAAGACACTAA [17628]
cp32-4_PAbe_NX_Pl_consensus    AGGGGGGCTTTACCATGTAAGAATCTTGCTATGCTGCAAATATCTCATCAAACCGTTCATATTCTTTTAAGACACTAA [17628]

cp32-4_gi|11497149|B31_GB      AGAATATTAATGGGCTAGGACCATAAGCAGTCCTCTTAAACACCACAAACTTTTCAAAATCTTGTAAATCCTTTAATT [17706]
cp32-4_un10_B31_PacBio         AGAATATTAATGGGCTAGGACCATAAGCAGTCCTCTTAAACACCACAAACTTTTCAAAATCTTGTAAATCCTTTAATT [17706]
cp32-4_B31_TS_MP_FG_consensus  AGAATATTAATGGGCTAGGACCATAAGCAGTCCTCTTAAACACCACAAACTTTTCAAAATCTTGTAAATCCTTTAATT [17706]
cp32-4_B31_NX_Pl_consensus     AGAATATTAATGGGCTAGGACCATAAGCAGTCCTCTTAAACACCACAAACTTTTCAAAATCTTGTAAATCCTTTAATT [17706]
cp32-4_PAli_NX_MP_FG_consensus AGAATATTAATGGGCTAGGACCATAAGCAGTCCTCTTAAACACCACAAACTTTTCAAAATCTTGTAAATCCTTTAATT [17706]
cp32-4_PAli_NX_Pl_consensus    AGAATATTAATGGGCTAGGACCATAAGCAGTCCTCTTAAACACCACAAACTTTTCAAAATCTTGTAAATCCTTTAATT [17706]
cp32-4_PAbe_NX_MP_FG_consensus AGAATATTAATGGGCTAGGACCATAAGCAGTCCTCTTAAACACCACAAACTTTTCAAAATCTTGTAAATCCTTTAATT [17706]
cp32-4_PAbe_NX_Pl_consensus    AGAATATTAATGGGCTAGGACCATAAGCAGTCCTCTTAAACACCACAAACTTTTCAAAATCTTGTAAATCCTTTAATT [17706]

cp32-4_gi|11497149|B31_GB      TATTATTTTTCTTGAAATAGTTTTTTATAATCTCCGCCCAATAGTTTATACTCTTAAAATCACTATTTTTCTCAAAAT [17784]
cp32-4_un10_B31_PacBio         TATTATTTTTCTTGAAATAGTTTTTTATAATCTCCGCCCAATAGTTTATACTCTTAAAATCACTATTTTTCTCAAAAT [17784]
cp32-4_B31_TS_MP_FG_consensus  TATTATTTTTCTTGAAATAGTTTTTTATAATCTCCGCCCAATAGTTTATACTCTTAAAATCACTATTTTTCTCAAAAT [17784]
cp32-4_B31_NX_Pl_consensus     TATTATTTTTCTTGAAATAGTTTTTTATAATCTCCGCCCAATAGTTTATACTCTTAAAATCACTATTTTTCTCAAAAT [17784]
cp32-4_PAli_NX_MP_FG_consensus TATTATTTTTCTTGAAATAGTTTTTTATAATCTCCGCCCAATAGTTTATACTCTTAAAATCACTATTTTTCTCAAAAT [17784]
cp32-4_PAli_NX_Pl_consensus    TATTATTTTTCTTGAAATAGTTTTTTATAATCTCCGCCCAATAGTTTATACTCTTAAAATCACTATTTTTCTCAAAAT [17784]
cp32-4_PAbe_NX_MP_FG_consensus TATTATTTTTCTTGAAATAGTTTTTTATAATCTCCGCCCAATAGTTTATACTCTTAAAATCACTATTTTTCTCAAAAT [17784]
cp32-4_PAbe_NX_Pl_consensus    TATTATTTTTCTTGAAATAGTTTTTTATAATCTCCGCCCAATAGTTTATACTCTTAAAATCACTATTTTTCTCAAAAT [17784]

cp32-4_gi|11497149|B31_GB      ACGAGATTAAATCAGATTCAATTTTCTTGATATCATCAACATTATTGGGGTCTATATTCTGAATAAAGCTTATTTTTT [17862]
cp32-4_un10_B31_PacBio         ACGAGATTAAATCAGATTCAATTTTCTTGATATCATCAACATTATTGGGGTCTATATTCTGAATAAAGCTTATTTTTT [17862]
cp32-4_B31_TS_MP_FG_consensus  ACGAGATTAAATCAGATTCAATTTTCTTGATATCATCAACATTATTGGGGTCTATATTCTGAATAAAGCTTATTTTTT [17862]
cp32-4_B31_NX_Pl_consensus     ACGAGATTAAATCAGATTCAATTTTCTTGATATCATCAACATTATTGGGGTCTATATTCTGAATAAAGCTTATTTTTT [17862]
cp32-4_PAli_NX_MP_FG_consensus ACGAGATTAAATCAGATTCAATTTTCTTGATATCATCAACATTATTGGGGTCTATATTCTGAATAAAGCTTATTTTTT [17862]
cp32-4_PAli_NX_Pl_consensus    ACGAGATTAAATCAGATTCAATTTTCTTGATATCATCAACATTATTGGGGTCTATATTCTGAATAAAGCTTATTTTTT [17862]
cp32-4_PAbe_NX_MP_FG_consensus ACGAGATTAAATCAGATTCAATTTTCTTGATATCATCAACATTATTGGGGTCTATATTCTGAATAAAGCTTATTTTTT [17862]
cp32-4_PAbe_NX_Pl_consensus    ACGAGATTAAATCAGATTCAATTTTCTTGATATCATCAACATTATTGGGGTCTATATTCTGAATAAAGCTTATTTTTT [17862]

cp32-4_gi|11497149|B31_GB      GAATAATTGAGTTTATATTATCTTTTATTGTAGGGCTATTTACCCAATCTTTTATATTAGACAACGCTTCTTTAAAAA [17940]
cp32-4_un10_B31_PacBio         GAATAATTGAGTTTATATTATCTTTTATTGTAGGGCTATTTACCCAATCTTTTATATTAGACAACGCTTCTTTAAAAA [17940]
```

```
cp32-4_B31_TS_MP_FG_consensus  GAATAATTGAGTTTATATTATCTTTTATTGTAGGGCTATTTACCCAATCTTTTATATTAGACAACGCTTCTTTAAAAA [17940]
cp32-4_B31_NX_Pl_consensus     GAATAATTGAGTTTATATTATCTTTTATTGTAGGGCTATTTACCCAATCTTTTATATTAGACAACGCTTCTTTAAAAA [17940]
cp32-4_PAli_NX_MP_FG_consensus GAATAATTGAGTTTATATTATCTTTTATTGTAGGGCTATTTACCCAATCTTTTATATTAGACAACGCTTCTTTAAAAA [17940]
cp32-4_PAli_NX_Pl_consensus    GAATAATTGAGTTTATATTATCTTTTATTGTAGGGCTATTTACCCAATCTTTTATATTAGACAACGCTTCTTTAAAAA [17940]
cp32-4_PAbe_NX_MP_FG_consensus GAATAATTGAGTTTATATTATCTTTTATTGTAGGGCTATTTACCCAATCTTTTATATTAGACAACGCTTCTTTAAAAA [17940]
cp32-4_PAbe_NX_Pl_consensus    GAATAATTGAGTTTATATTATCTTTTATTGTAGGGCTATTTACCCAATCTTTTATATTAGACAACGCTTCTTTAAAAA [17940]

cp32-4_gi|11497149|B31_GB      CACCGTAATAATAAAACCTATCTTTTTTTTGTTCTTGCTTAATGTCTCTTTTCTGGTTTTGAATAGTGTTATTTTTTT [18018]
cp32-4_un10_B31_PacBio         CACCGTAATAATAAAACCTATCTTTTTTTTGTTCTTGCTTAATGTCTCTTTTCTGGTTTTGAATAGTGTTATTTTTTT [18018]
cp32-4_B31_TS_MP_FG_consensus  CACCGTAATAATAAAACCTATCTTTTTTTTGTTCTTGCTTAATGTCTCTTTTCTGGTTTTGAATAGTGTTATTTTTTT [18018]
cp32-4_B31_NX_Pl_consensus     CACCGTAATAATAAAACCTATCTTTTTTTTGTTCTTGCTTAATGTCTCTTTTCTGGTTTTGAATAGTGTTATTTTTTT [18018]
cp32-4_PAli_NX_MP_FG_consensus CACCGTAATAATAAAACCTATCTTTTTTTTGTTCTTGCTTAATGTCTCTTTTCTGGTTTTGAATAGTGTTATTTTTTT [18018]
cp32-4_PAli_NX_Pl_consensus    CACCGTAATAATAAAACCTATCTTTTTTTTGTTCTTGCTTAATGTCTCTTTTCTGGTTTTGAATAGTGTTATTTTTTT [18018]
cp32-4_PAbe_NX_MP_FG_consensus CACCGTAATAATAAAACCTATCTTTTTTTTGTTCTTGCTTAATGTCTCTTTTCTGGTTTTGAATAGTGTTATTTTTTT [18018]
cp32-4_PAbe_NX_Pl_consensus    CACCGTAATAATAAAACCTATCTTTTTTTTGTTCTTGCTTAATGTCTCTTTTCTGGTTTTGAATAGTGTTATTTTTTT [18018]

cp32-4_gi|11497149|B31_GB      GATTTTGATTAATCTCTTGTTTTTGCTCTCTTTTTTGTTCTTGCTTTTGATTAACACTAGATTGTTTGCTAGGCATAG [18096]
cp32-4_un10_B31_PacBio         GATTTTGATTAATCTCTTGTTTTTGCTCTCTTTTTTGTTCTTGCTTTTGATTAACACTAGATTGTTTGCTAGGCATAG [18096]
cp32-4_B31_TS_MP_FG_consensus  GATTTTGATTAATCTCTTGTTTTTGCTCTCTTTTTTGTTCTTGCTTTTGATTAACACTAGATTGTTTGCTAGGCATAG [18096]
cp32-4_B31_NX_Pl_consensus     GATTTTGATTAATCTCTTGTTTTTGCTCTCTTTTTTGTTCTTGCTTTTGATTAACACTAGATTGTTTGCTAGGCATAG [18096]
cp32-4_PAli_NX_MP_FG_consensus GATTTTGATTAATCTCTTGTTTTTGCTCTCTTTTTTGTTCTTGCTTTTGATTAACACTAGATTGTTTGCTAGGCATAG [18096]
cp32-4_PAli_NX_Pl_consensus    GATTTTGATTAATCTCTTGTTTTTGCTCTCTTTTTTGTTCTTGCTTTTGATTAACACTAGATTGTTTGCTAGGCATAG [18096]
cp32-4_PAbe_NX_MP_FG_consensus GATTTTGATTAATCTCTTGTTTTTGCTCTCTTTTTTGTTCTTGCTTTTGATTAACACTAGATTGTTTGCTAGGCATAG [18096]
cp32-4_PAbe_NX_Pl_consensus    GATTTTGATTAATCTCTTGTTTTTGCTCTCTTTTTTGTTCTTGCTTTTGATTAACACTAGATTGTTTGCTAGGCATAG [18096]

cp32-4_gi|11497149|B31_GB      AATTTCTGTTTTCATAATTATTGTAAATAGGTGCTGCATCAGTATCCATTTCGCTTCTTATGCAAAGATGCCCTACTA [18174]
cp32-4_un10_B31_PacBio         AATTTCTGTTTTCATAATTATTGTAAATAGGTGCTGCATCAGTATCCATTTCGCTTCTTATGCAAAGATGCCCTACTA [18174]
cp32-4_B31_TS_MP_FG_consensus  AATTTCTGTTTTCATAATTATTGTAAATAGGTGCTGCATCAGTATCCATTTCGCTTCTTATGCAAAGATGCCCTACTA [18174]
cp32-4_B31_NX_Pl_consensus     AATTTCTGTTTTCATAATTATTGTAAATAGGTGCTGCATCAGTATCCATTTCGCTTCTTATGCAAAGATGCCCTACTA [18174]
cp32-4_PAli_NX_MP_FG_consensus AATTTCTGTTTTCATAATTATTGTAAATAGGTGCTGCATCAGTATCCATTTCGCTTCTTATGCAAAGATGCCCTACTA [18174]
cp32-4_PAli_NX_Pl_consensus    AATTTCTGTTTTCATAATTATTGTAAATAGGTGCTGCATCAGTATCCATTTCGCTTCTTATGCAAAGATGCCCTACTA [18174]
cp32-4_PAbe_NX_MP_FG_consensus AATTTCTGTTTTCATAATTATTGTAAATAGGTGCTGCATCAGTATCCATTTCGCTTCTTATGCAAAGATGCCCTACTA [18174]
cp32-4_PAbe_NX_Pl_consensus    AATTTCTGTTTTCATAATTATTGTAAATAGGTGCTGCATCAGTATCCATTTCGCTTCTTATGCAAAGATGCCCTACTA [18174]

cp32-4_gi|11497149|B31_GB      AAGCGTACCTTTTGAAATAAGTAATAGCTGATCCAACAAATTGTGGCATCGTATTCATATTTTTAGACCCATTTTCAC [18252]
cp32-4_un10_B31_PacBio         AAGCGTACCTTTTGAAATAAGTAATAGCTGATCCAACAAATTGTGGCATCGTATTCATATTTTTAGACCCATTTTCAC [18252]
cp32-4_B31_TS_MP_FG_consensus  AAGCGTACCTTTTGAAATAAGTAATAGCTGATCCAACAAATTGTGGCATCGTATTCATATTTTTAGACCCATTTTCAC [18252]
cp32-4_B31_NX_Pl_consensus     AAGCGTACCTTTTGAAATAAGTAATAGCTGATCCAACAAATTGTGGCATCGTATTCATATTTTTAGACCCATTTTCAC [18252]
cp32-4_PAli_NX_MP_FG_consensus AAGCGTACCTTTTGAAATAAGTAATAGCTGATCCAACAAATTGTGGCATCGTATTCATATTTTTAGACCCATTTTCAC [18252]
cp32-4_PAli_NX_Pl_consensus    AAGCGTACCTTTTGAAATAAGTAATAGCTGATCCAACAAATTGTGGCATCGTATTCATATTTTTAGACCCATTTTCAC [18252]
cp32-4_PAbe_NX_MP_FG_consensus AAGCGTACCTTTTGAAATAAGTAATAGCTGATCCAACAAATTGTGGCATCGTATTCATATTTTTAGACCCATTTTCAC [18252]
cp32-4_PAbe_NX_Pl_consensus    AAGCGTACCTTTTGAAATAAGTAATAGCTGATCCAACAAATTGTGGCATCGTATTCATATTTTTAGACCCATTTTCAC [18252]

cp32-4_gi|11497149|B31_GB      TGTTCCATTGTAATTTATCTGTATGAATTCGCGTATCAAATGAGTATTTGTACACAGTGCTTGTACTGTAGAATGTAG [18330]
cp32-4_un10_B31_PacBio         TGTTCCATTGTAATTTATCTGTATGAATTCGCGTATCAAATGAGTATTTGTACACAGTGCTTGTACTGTAGAATGTAG [18330]
cp32-4_B31_TS_MP_FG_consensus  TGTTCCATTGTAATTTATCTGTATGAATTCGCGTATCAAATGAGTATTTGTACACAGTGCTTGTACTGTAGAATGTAG [18330]
cp32-4_B31_NX_Pl_consensus     TGTTCCATTGTAATTTATCTGTATGAATTCGCGTATCAAATGAGTATTTGTACACAGTGCTTGTACTGTAGAATGTAG [18330]
cp32-4_PAli_NX_MP_FG_consensus TGTTCCATTGTAATTTATCTGTATGAATTCGCGTATCAAATGAGTATTTGTACACAGTGCTTGTACTGTAGAATGTAG [18330]
cp32-4_PAli_NX_Pl_consensus    TGTTCCATTGTAATTTATCTGTATGAATTCGCGTATCAAATGAGTATTTGTACACAGTGCTTGTACTGTAGAATGTAG [18330]
cp32-4_PAbe_NX_MP_FG_consensus TGTTCCATTGTAATTTATCTGTATGAATTCGCGTATCAAATGAGTATTTGTACACAGTGCTTGTACTGTAGAATGTAG [18330]
cp32-4_PAbe_NX_Pl_consensus    TGTTCCATTGTAATTTATCTGTATGAATTCGCGTATCAAATGAGTATTTGTACACAGTGCTTGTACTGTAGAATGTAG [18330]

cp32-4_gi|11497149|B31_GB      TCCTAATATAATCAACTATGCCATATTGCCCCTCTACAGAAATTGGGGCTTGCGTAAAAAAAAGATCCAAATTATGCT [18408]
cp32-4_un10_B31_PacBio         TCCTAATATAATCAACTATGCCATATTGCCCCTCTACAGAAATTGGGGCTTGCGTAAAAAAAAGATCCAAATTATGCT [18408]
cp32-4_B31_TS_MP_FG_consensus  TCCTAATATAATCAACTATGCCATATTGCCCCTCTACAGAAATTGGGGCTTGCGTAAAAAAAAGATCCAAATTATGCT [18408]
cp32-4_B31_NX_Pl_consensus     TCCTAATATAATCAACTATGCCATATTGCCCCTCTACAGAAATTGGGGCTTGCGTAAAAAAAAGATCCAAATTATGCT [18408]
cp32-4_PAli_NX_MP_FG_consensus TCCTAATATAATCAACTATGCCATATTGCCCCTCTACAGAAATTGGGGCTTGCGTAAAAAAAAGATCCAAATTATGCT [18408]
cp32-4_PAli_NX_Pl_consensus    TCCTAATATAATCAACTATGCCATATTGCCCCTCTACAGAAATTGGGGCTTGCGTAAAAAAAAGATCCAAATTATGCT [18408]
cp32-4_PAbe_NX_MP_FG_consensus TCCTAATATAATCAACTATGCCATATTGCCCCTCTACAGAAATTGGGGCTTGCGTAAAAAAAAGATCCAAATTATGCT [18408]
cp32-4_PAbe_NX_Pl_consensus    TCCTAATATAATCAACTATGCCATATTGCCCCTCTACAGAAATTGGGGCTTGCGTAAAAAAAAGATCCAAATTATGCT [18408]

cp32-4_gi|11497149|B31_GB      TATCAATAACACTATAAATTACTTCTACTATGTCATTGAAATCTTGATACTTATACCCATACCCTTTAAGACTTTTGT [18486]
cp32-4_un10_B31_PacBio         TATCAATAACACTATAAATTACTTCTACTATGTCATTGAAATCTTGATACTTATACCCATACCCTTTAAGACTTTTGT [18486]
cp32-4_B31_TS_MP_FG_consensus  TATCAATAACACTATAAATTACTTCTACTATGTCATTGAAATCTTGATACTTATACCCATACCCTTTAAGACTTTTGT [18486]
cp32-4_B31_NX_Pl_consensus     TATCAATAACACTATAAATTACTTCTACTATGTCATTGAAATCTTGATACTTATACCCATACCCTTTAAGACTTTTGT [18486]
cp32-4_PAli_NX_MP_FG_consensus TATCAATAACACTATAAATTACTTCTACTATGTCATTGAAATCTTGATACTTATACCCATACCCTTTAAGACTTTTGT [18486]
cp32-4_PAli_NX_Pl_consensus    TATCAATAACACTATAAATTACTTCTACTATGTCATTGAAATCTTGATACTTATACCCATACCCTTTAAGACTTTTGT [18486]
cp32-4_PAbe_NX_MP_FG_consensus TATCAATAACACTATAAATTACTTCTACTATGTCATTGAAATCTTGATACTTATACCCATACCCTTTAAGACTTTTGT [18486]
cp32-4_PAbe_NX_Pl_consensus    TATCAATAACACTATAAATTACTTCTACTATGTCATTGAAATCTTGATACTTATACCCATACCCTTTAAGACTTTTGT [18486]

cp32-4_gi|11497149|B31_GB      CAATACGCGGCAAGTTTCTGATTAGGGTGCTCATATCTTTTCTGAATTTAATTTCTCCTTGAATATTTTCTTGTGGAT [18564]
cp32-4_un10_B31_PacBio         CAATACGCGGCAAGTTTCTGATTAGGGTGCTCATATCTTTTCTGAATTTAATTTCTCCTTGAATATTTTCTTGTGGAT [18564]
cp32-4_B31_TS_MP_FG_consensus  CAATACGCGGCAAGTTTCTGATTAGGGTGCTCATATCTTTTCTGAATTTAATTTCTCCTTGAATATTTTCTTGTGGAT [18564]
cp32-4_B31_NX_Pl_consensus     CAATACGCGGCAAGTTTCTGATTAGGGTGCTCATATCTTTTCTGAATTTAATTTCTCCTTGAATATTTTCTTGTGGAT [18564]
cp32-4_PAli_NX_MP_FG_consensus CAATACGCGGCAAGTTTCTGATTAGGGTGCTCATATCTTTTCTGAATTTAATTTCTCCTTGAATATTTTCTTGTGGAT [18564]
cp32-4_PAli_NX_Pl_consensus    CAATACGCGGCAAGTTTCTGATTAGGGTGCTCATATCTTTTCTGAATTTAATTTCTCCTTGAATATTTTCTTGTGGAT [18564]
cp32-4_PAbe_NX_MP_FG_consensus CAATACGCGGCAAGTTTCTGATTAGGGTGCTCATATCTTTTCTGAATTTAATTTCTCCTTGAATATTTTCTTGTGGAT [18564]
cp32-4_PAbe_NX_Pl_consensus    CAATACGCGGCAAGTTTCTGATTAGGGTGCTCATATCTTTTCTGAATTTAATTTCTCCTTGAATATTTTCTTGTGGAT [18564]

cp32-4_gi|11497149|B31_GB      TATTATTGTTTGAAAGATTTTCCATCTTTTTCCTCCTTATTTTGTAATAAATAATTATATAGCAAAAACTATTTTTGC [18642]
cp32-4_un10_B31_PacBio         TATTATTGTTTGAAAGATTTTCCATCTTTTTCCTCCTTATTTTGTAATAAATAATTATATAGCAAAAACTATTTTTGC [18642]
cp32-4_B31_TS_MP_FG_consensus  TATTATTGTTTGAAAGATTTTCCATCTTTTTCCTCCTTATTTTGTAATAAATAATTATATAGCAAAAACTATTTTTGC [18642]
cp32-4_B31_NX_Pl_consensus     TATTATTGTTTGAAAGATTTTCCATCTTTTTCCTCCTTATTTTGTAATAAATAATTATATAGCAAAAACTATTTTTGC [18642]
cp32-4_PAli_NX_MP_FG_consensus TATTATTGTTTGAAAGATTTTCCATCTTTTTCCTCCTTATTTTGTAATAAATAATTATATAGCAAAAACTATTTTTGC [18642]
cp32-4_PAli_NX_Pl_consensus    TATTATTGTTTGAAAGATTTTCCATCTTTTTCCTCCTTATTTTGTAATAAATAATTATATAGCAAAAACTATTTTTGC [18642]
cp32-4_PAbe_NX_MP_FG_consensus TATTATTGTTTGAAAGATTTTCCATCTTTTTCCTCCTTATTTTGTAATAAATAATTATATAGCAAAAACTATTTTTGC [18642]
cp32-4_PAbe_NX_Pl_consensus    TATTATTGTTTGAAAGATTTTCCATCTTTTTCCTCCTTATTTTGTAATAAATAATTATATAGCAAAAACTATTTTTGC [18642]

cp32-4_gi|11497149|B31_GB      CAACTTTTTTACAAAAAATTTTACACAAAAGAAGTGGGACTTAACCAAATTCCCTTTAAAAAGAATTTCGCCAAGTTC [18720]
cp32-4_un10_B31_PacBio         CAACTTTTTTACAAAAAATTTTACACAAAAGAAGTGGGACTTAACCAAATTCCCTTTAAAAAGAATTTCGCCAAGTTC [18720]
cp32-4_B31_TS_MP_FG_consensus  CAACTTTTTTACAAAAAATTTTACACAAAAGAAGTGGGACTTAACCAAATTCCCTTTAAAAAGAATTTCGCCAAGTTC [18720]
cp32-4_B31_NX_Pl_consensus     CAACTTTTTTACAAAAAATTTTACACAAAAGAAGTGGGACTTAACCAAATTCCCTTTAAAAAGAATTTCGCCAAGTTC [18720]
cp32-4_PAli_NX_MP_FG_consensus CAACTTTTTTACAAAAAATTTTACACAAAAGAAGTGGGACTTAACCAAATTCCCTTTAAAAAGAATTTCGCCAAGTTC [18720]
cp32-4_PAli_NX_Pl_consensus    CAACTTTTTTACAAAAAATTTTACACAAAAGAAGTGGGACTTAACCAAATTCCCTTTAAAAAGAATTTCGCCAAGTTC [18720]
cp32-4_PAbe_NX_MP_FG_consensus CAACTTTTTTACAAAAAATTTTACACAAAAGAAGTGGGACTTAACCAAATTCCCTTTAAAAAGAATTTCGCCAAGTTC [18720]
cp32-4_PAbe_NX_Pl_consensus    CAACTTTTTTACAAAAAATTTTACACAAAAGAAGTGGGACTTAACCAAATTCCCTTTAAAAAGAATTTCGCCAAGTTC [18720]

cp32-4_gi|11497149|B31_GB      CCACTATGATTTTAGAAAACTACAAAATAAGGTAGGTAAAAATATGAAGATATTTTCCTACGCTATTTGCAGTATAGT [18798]
cp32-4_un10_B31_PacBio         CCACTATGATTTTAGAAAACTACAAAATAAGGTAGGTAAAAATATGAAGATATTTTCCTACGCTATTTGCAGTATAGT [18798]
cp32-4_B31_TS_MP_FG_consensus  CCACTATGATTTTAGAAAACTACAAAATAAGGTAGGTAAAAATATGAAGATATTTTCCTACGCTATTTGCAGTATAGT [18798]
cp32-4_B31_NX_Pl_consensus     CCACTATGATTTTAGAAAACTACAAAATAAGGTAGGTAAAAATATGAAGATATTTTCCTACGCTATTTGCAGTATAGT [18798]
cp32-4_PAli_NX_MP_FG_consensus CCACTATGATTTTAGAAAACTACAAAATAAGGTAGGTAAAAATATGAAGATATTTTCCTACGCTATTTGCAGTATAGT [18798]
cp32-4_PAli_NX_Pl_consensus    CCACTATGATTTTAGAAAACTACAAAATAAGGTAGGTAAAAATATGAAGATATTTTCCTACGCTATTTGCAGTATAGT [18798]
cp32-4_PAbe_NX_MP_FG_consensus CCACTATGATTTTAGAAAACTACAAAATAAGGTAGGTAAAAATATGAAGATATTTTCCTACGCTATTTGCAGTATAGT [18798]
cp32-4_PAbe_NX_Pl_consensus    CCACTATGATTTTAGAAAACTACAAAATAAGGTAGGTAAAAATATGAAGATATTTTCCTACGCTATTTGCAGTATAGT [18798]

cp32-4_gi|11497149|B31_GB      CTAATTTGTGATTAAAATCAATAAATCTTTTTACCAAATTACAAAAAGTGTATTTAATTAATAAGATTAATAATTAAA [18876]
cp32-4_un10_B31_PacBio         CTAATTTGTGATTAAAATCAATAAATCTTTTTACCAAATTACAAAAAGTGTATTTAATTAATAAGATTAATAATTAAA [18876]
cp32-4_B31_TS_MP_FG_consensus  CTAATTTGTGATTAAAATCAATAAATCTTTTTACCAAATTACAAAAAGTGTATTTAATTAATAAGATTAATAATTAAA [18876]
```

```
cp32-4_B31_NX_Pl_consensus     CTAATTTGTGATTAAAATCAATAAATCTTTTTACCAAATTACAAAAAGTGTATTTAATTAATAAGATTAATAATTAAA [18876]
cp32-4_PAli_NX_MP_FG_consensus CTAATTTGTGATTAAAATCAATAAATCTTTTTACCAAATTACAAAAAGTGTATTTAATTAATAAGATTAATAATTAAA [18876]
cp32-4_PAli_NX_Pl_consensus    CTAATTTGTGATTAAAATCAATAAATCTTTTTACCAAATTACAAAAAGTGTATTTAATTAATAAGATTAATAATTAAA [18876]
cp32-4_PAbe_NX_MP_FG_consensus CTAATTTGTGATTAAAATCAATAAATCTTTTTACCAAATTACAAAAAGTGTATTTAATTAATAAGATTAATAATTAAA [18876]
cp32-4_PAbe_NX_Pl_consensus    CTAATTTGTGATTAAAATCAATAAATCTTTTTACCAAATTACAAAAAGTGTATTTAATTAATAAGATTAATAATTAAA [18876]

cp32-4_gi|11497149|B31_GB      ATTTAATATTTTTTTAGAAAAGTATTTACTTTTAAATCAAAATTTTGCATTATAATAGTAATTGTTAATAACAAAATA [18954]
cp32-4_un10_B31_PacBio         ATTTAATATTTTTTTAGAAAAGTATTTACTTTTAAATCAAAATTTTGCATTATAATAGTAATTGTTAATAACAAAATA [18954]
cp32-4_B31_TS_MP_FG_consensus  ATTTAATATTTTTTTAGAAAAGTATTTACTTTTAAATCAAAATTTTGCATTATAATAGTAATTGTTAATAACAAAATA [18954]
cp32-4_B31_NX_Pl_consensus     ATTTAATATTTTTTTAGAAAAGTATTTACTTTTAAATCAAAATTTTGCATTATAATAGTAATTGTTAATAACAAAATA [18954]
cp32-4_PAli_NX_MP_FG_consensus ATTTAATATTTTTTTAGAAAAGTATTTACTTTTAAATCAAAATTTTGCATTATAATAGTAATTGTTAATAACAAAATA [18954]
cp32-4_PAli_NX_Pl_consensus    ATTTAATATTTTTTTAGAAAAGTATTTACTTTTAAATCAAAATTTTGCATTATAATAGTAATTGTTAATAACAAAATA [18954]
cp32-4_PAbe_NX_MP_FG_consensus ATTTAATATTTTTTTAGAAAAGTATTTACTTTTAAATCAAAATTTTGCATTATAATAGTAATTGTTAATAACAAAATA [18954]
cp32-4_PAbe_NX_Pl_consensus    ATTTAATATTTTTTTAGAAAAGTATTTACTTTTAAATCAAAATTTTGCATTATAATAGTAATTGTTAATAACAAAATA [18954]

cp32-4_gi|11497149|B31_GB      AGGAGTAAAAAATGAAAGATTTTTTAATCACCACAAAAAATCCCACTTGCCACAACAAACACCAACACAAGTTAATAT [19032]
cp32-4_un10_B31_PacBio         AGGAGTAAAAAATGAAAGATTTTTTAATCACCACAAAAAATCCCACTTGCCACAACAAACACCAACACAAGTTAATAT [19032]
cp32-4_B31_TS_MP_FG_consensus  AGGAGTAAAAAATGAAAGATTTTTTAATCACCACAAAAAATCCCACTTGCCACAACAAACACCAACACAAGTTAATAT [19032]
cp32-4_B31_NX_Pl_consensus     AGGAGTAAAAAATGAAAGATTTTTTAATCACCACAAAAAATCCCACTTGCCACAACAAACACCAACACAAGTTAATAT [19032]
cp32-4_PAli_NX_MP_FG_consensus AGGAGTAAAAAATGAAAGATTTTTTAATCACCACAAAAAATCCCACTTGCCACAACAAACACCAACACAAGTTAATAT [19032]
cp32-4_PAli_NX_Pl_consensus    AGGAGTAAAAAATGAAAGATTTTTTAATCACCACAAAAAATCCCACTTGCCACAACAAACACCAACACAAGTTAATAT [19032]
cp32-4_PAbe_NX_MP_FG_consensus AGGAGTAAAAAATGAAAGATTTTTTAATCACCACAAAAAATCCCACTTGCCACAACAAACACCAACACAAGTTAATAT [19032]
cp32-4_PAbe_NX_Pl_consensus    AGGAGTAAAAAATGAAAGATTTTTTAATCACCACAAAAAATCCCACTTGCCACAACAAACACCAACACAAGTTAATAT [19032]

cp32-4_gi|11497149|B31_GB      ATCTTACATCAACAGTAGATTTTCTAAACAAAAAAGATAAGAAATATACACAACAAAACATACTCTATTACTATAATA [19110]
cp32-4_un10_B31_PacBio         ATCTTACATCAACAGTAGATTTTCTAAACAAAAAAGATAAGAAATATACACAACAAAACATACTCTATTACTATAATA [19110]
cp32-4_B31_TS_MP_FG_consensus  ATCTTACATCAACAGTAGATTTTCTAAACAAAAAAGATAAGAAATATACACAACAAAACATACTCTATTACTATAATA [19110]
cp32-4_B31_NX_Pl_consensus     ATCTTACATCAACAGTAGATTTTCTAAACAAAAAAGATAAGAAATATACACAACAAAACATACTCTATTACTATAATA [19110]
cp32-4_PAli_NX_MP_FG_consensus ATCTTACATCAACAGTAGATTTTCTAAACAAAAAAGATAAGAAATATACACAACAAAACATACTCTATTACTATAATA [19110]
cp32-4_PAli_NX_Pl_consensus    ATCTTACATCAACAGTAGATTTTCTAAACAAAAAAGATAAGAAATATACACAACAAAACATACTCTATTACTATAATA [19110]
cp32-4_PAbe_NX_MP_FG_consensus ATCTTACATCAACAGTAGATTTTCTAAACAAAAAAGATAAGAAATATACACAACAAAACATACTCTATTACTATAATA [19110]
cp32-4_PAbe_NX_Pl_consensus    ATCTTACATCAACAGTAGATTTTCTAAACAAAAAAGATAAGAAATATACACAACAAAACATACTCTATTACTATAATA [19110]

cp32-4_gi|11497149|B31_GB      AAAATCTAAAAAGAAATGGTTTGGCTCCCACTACACTGAGAACAATGCAAAATTATCTTTATAAATTAGAAAAAGTAT [19188]
cp32-4_un10_B31_PacBio         AAAATCTAAAAAGAAATGGTTTGGCTCCCACTACACTGAGAACAATGCAAAATTATCTTTATAAATTAGAAAAAGTAT [19188]
cp32-4_B31_TS_MP_FG_consensus  AAAATCTAAAAAGAAATGGTTTGGCTCCCACTACACTGAGAACAATGCAAAATTATCTTTATAAATTAGAAAAAGTAT [19188]
cp32-4_B31_NX_Pl_consensus     AAAATCTAAAAAGAAATGGTTTGGCTCCCACTACTTTAAGAACAATGCAAAACTATCTTTACAAATTAGAAAAAGTAT [19188]
cp32-4_PAli_NX_MP_FG_consensus AAAATCTAAAAAGAAATGGTTTGGCTCCCACTACACTGAGAACAATGCAAAATTATCTTTATAAATTAGAAAAAGTAT [19188]
cp32-4_PAli_NX_Pl_consensus    AAAATCTAAAAAGAAATGGTTTGGCTCCCACTACACTGAGAACAATGCAAAATTATCTTTATAAATTAGAAAAAGTAT [19188]
cp32-4_PAbe_NX_MP_FG_consensus AAAATCTAAAAAGAAATGGTTTGGCTCCCACTACACTGAGAACAATGCAAAATTATCTTTATAAATTAGAAAAAGTAT [19188]
cp32-4_PAbe_NX_Pl_consensus    AAAATCTAAAAAGAAATGGTTTGGCTCCCACTACACTGAGAACAATGCAAAATTATCTTTATAAATTAGAAAAAGTAT [19188]

cp32-4_gi|11497149|B31_GB      TAAAAGTCACAACGAATTACTACCAACACATGGGTGTAAATTGTGGAACTGAAATTTACTATAAGCTAAAGTATCCTA [19266]
cp32-4_un10_B31_PacBio         TAAAAGTCACAACGAATTACTACCAACACATGGGTGTAAATTGTGGAACTGAAATTTACTATAAGCTAAAGTATCCTA [19266]
cp32-4_B31_TS_MP_FG_consensus  TAAAAGTCACAACGAATTACTACCAACACATGGGTGTAAATTGTGGAACTGAAATTTACTATAAGCTAAAGTATCCTA [19266]
cp32-4_B31_NX_Pl_consensus     TAAAAGTTACAACTAATTACTACCAACACATGGGTGTAAATTGTGGAACTGAAATTTACTATAAGCTAAAGTATCCTA [19266]
cp32-4_PAli_NX_MP_FG_consensus TAAAAGTCACAACGAATTACTACCAACACATGGGTGTAAATTGTGGAACTGAAATTTACTATAAGCTAAAGTATCCTA [19266]
cp32-4_PAli_NX_Pl_consensus    TAAAAGTCACAACGAATTACTACCAACACATGGGTGTAAATTGTGGAACTGAAATTTACTATAAGCTAAAGTATCCTA [19266]
cp32-4_PAbe_NX_MP_FG_consensus TAAAAGTCACAACGAATTACTACCAACACATGGGTGTAAATTGTGGAACTGAAATTTACTATAAGCTAAAGTATCCTA [19266]
cp32-4_PAbe_NX_Pl_consensus    TAAAAGTCACAACGAATTACTACCAACACATGGGTGTAAATTGTGGAACTGAAATTTACTATAAGCTAAAGTATCCTA [19266]

cp32-4_gi|11497149|B31_GB      AAAAAGAATGTTACCAGAAAATCAACAAGTACTTTAAAGAACGAAAAAACTCTAGATTTAAATCTAGAGTTAATAACC [19344]
cp32-4_un10_B31_PacBio         AAAAAGAATGTTACCAGAAAATCAACAAGTACTTTAAAGAACGAAAAAACTCTAGATTTAAATCTAGAGTTAATAACC [19344]
cp32-4_B31_TS_MP_FG_consensus  AAAAAGAATGTTACCAGAAAATCAACAAGTACTTTAAAGAACGAAAAAACTCTAGATTTAAATCTAGAGTTAATAACC [19344]
cp32-4_B31_NX_Pl_consensus     AAAAAGAATGTTACCAGAAAATCAACAAGTACTTTAAAGAACGAAAAAACTCTAGATTTAAATCTAGAGTTAATAACC [19344]
cp32-4_PAli_NX_MP_FG_consensus AAAAAGAATGTTACCAGAAAATCAACAAGTACTTTAAAGAACGAAAAAACTCTAGATTTAAATCTAGAGTTAATAACC [19344]
cp32-4_PAli_NX_Pl_consensus    AAAAAGAATGTTACCAGAAAATCAACAAGTACTTTAAAGAACGAAAAAACTCTAGATTTAAATCTAGAGTTAATAACC [19344]
cp32-4_PAbe_NX_MP_FG_consensus AAAAAGAATGTTACCAGAAAATCAACAAGTACTTTAAAGAACGAAAAAACTCTAGATTTAAATCTAGAGTTAATAACC [19344]
cp32-4_PAbe_NX_Pl_consensus    AAAAAGAATGTTACCAGAAAATCAACAAGTACTTTAAAGAACGAAAAAACTCTAGATTTAAATCTAGAGTTAATAACC [19344]

cp32-4_gi|11497149|B31_GB      ATTTTAAAGACAATGTTTCTAAAAATAGTAGTGTAAATTCAGTGGAGTGTTTAAGTAATAAAAATAATATAAAAGAAG [19422]
cp32-4_un10_B31_PacBio         ATTTTAAAGACAATGTTTCTAAAAATAGTAGTGTAAATTCAGTGGAGTGTTTAAGTAATAAAAATAATATAAAAGAAG [19422]
cp32-4_B31_TS_MP_FG_consensus  ATTTTAAAGACAATGTTTCTAAAAATAGTAGTGTAAATTCAGTGGAGTGTTTAAGTAATAAAAATAATATAAAAGAAG [19422]
cp32-4_B31_NX_Pl_consensus     ATTTTAAAGACAATGTTTCTAAAAATAGTAGTGTAAATTCAGTGGAGTGTTTAAGTAATAAAAATAATATAAAAGAAG [19422]
cp32-4_PAli_NX_MP_FG_consensus ATTTTAAAGACAATGTTTCTAAAAATAGTAGTGTAAATTTAGTGGAGTGTTTAAGTAATAAAAATAATATAAAAGAAG [19422]
cp32-4_PAli_NX_Pl_consensus    ATTTTAAAGACAATGTTTCTAAAAATAGTAGTGTAAATTCAGTGGAGTGTTTAAGTAATAAAAATAATATAAAAGAAG [19422]
cp32-4_PAbe_NX_MP_FG_consensus ATTTTAAAGACAATGTTTCTAAAAATAGTAGTGTAAATTCAGTGGAGTGTTTAAGTAATAAAAATAATATAAAAGAAG [19422]
cp32-4_PAbe_NX_Pl_consensus    ATTTTAAAGACAATGTTTCTAAAAATAGTAGTGTAAATTCAGTGGAGTGTTTAAGTAATAAAAATAATATAAAAGAAG [19422]

cp32-4_gi|11497149|B31_GB      AAAGAAAGATTAAAGAAATAGAAAAATATCAACTAAGAAATTATTTCAATAATTGTAACTTTAAAACGGAAGAAGCTC [19500]
cp32-4_un10_B31_PacBio         AAAGAAAGATTAAAGAAATAGAAAAATATCAACTAAGAAATTATTTCAATAATTGTAACTTTAAAACGGAAGAAGCTC [19500]
cp32-4_B31_TS_MP_FG_consensus  AAAGAAAGATTAAAGAAATAGAAAAATATCAACTAAGAAATTATTTCAATAATTGTAACTTTAAAACGGAAGAAGCTC [19500]
cp32-4_B31_NX_Pl_consensus     AAAGAAAGATTAAAGAAATAGAAAAATATCAACTAAGAAATTATTTCAATAATTGTAACTTTAAAACGGAAGAAGCTC [19500]
cp32-4_PAli_NX_MP_FG_consensus AAAGAAAGATTAAAGAAATAGAAAAATATCAACTAAGAAATTATTTCAATAATTGTAACTTTAAAACGGAAGAAGCTC [19500]
cp32-4_PAli_NX_Pl_consensus    AAAGAAAGATTAAAGAAATAGAAAAATATCAACTAAGAAATTATTTCAATAATTGTAACTTTAAAACGGAAGAAGCTC [19500]
cp32-4_PAbe_NX_MP_FG_consensus AAAGAAAGATTAAAGAAATAGAAAAATATCAACTAAGAAATTATTTCAATAATTGTAACTTTAAAACGGAAGAAGCTC [19500]
cp32-4_PAbe_NX_Pl_consensus    AAAGAAAGATTAAAGAAATAGAAAAATATCAACTAAGAAATTATTTCAATAATTGTAACTTTAAAACGGAAGAAGCTC [19500]

cp32-4_gi|11497149|B31_GB      TTTCTATTTTGTATTTAAATACTGATAAAGATACTAAGATTGAGGCAATAAATATCTTAAAACAAAATGAAATTGCCC [19578]
cp32-4_un10_B31_PacBio         TTTCTATTTTGTATTTAAATACTGATAAAGATACTAAGATTGAGGCAATAAATATCTTAAAACAAAATGAAATTGCCC [19578]
cp32-4_B31_TS_MP_FG_consensus  TTTCTATTTTGTATTTAAATACTGATAAAGATACTAAGATTGAGGCAATAAATATCTTAAAACAAAATGAAATTGCCC [19578]
cp32-4_B31_NX_Pl_consensus     TTTCTATTTTGTATTTAAATACTGATAAAGATACTAAGATTGAGGCAATAAATATCTTAAAACAAAATGAAATTGCCC [19578]
cp32-4_PAli_NX_MP_FG_consensus TTTCTATTTTGTATTTAAATACTGATAAAGATACTAAGATTGAGGCAATAAATATCTTAAAACAAAATGAAATTGCCC [19578]
cp32-4_PAli_NX_Pl_consensus    TTTCTATTTTGTATTTAAATACTGATAAAGATACTAAGATTGAGGCAATAAATATCTTAAAACAAAATGAAATTGCCC [19578]
cp32-4_PAbe_NX_MP_FG_consensus TTTCTATTTTGTATTTAAATACTGATAAAGATACTAAGATTGAGGCAATAAATATCTTAAAACAAAATGAAATTGCCC [19578]
cp32-4_PAbe_NX_Pl_consensus    TTTCTATTTTGTATTTAAATACTGATAAAGATACTAAGATTGAGGCAATAAATATCTTAAAACAAAATGAAATTGCCC [19578]

cp32-4_gi|11497149|B31_GB      TAATAAAAAAATTCAATATAAAAAAATCTTGCATGAAAGAAAAACAAAAAAAATTAAAAGAAATTCTATACAACACTA [19656]
cp32-4_un10_B31_PacBio         TAATAAAAAAATTCAATATAAAAAAATCTTGCATGAAAGAAAAACAAAAAAAATTAAAAGAAATTCTATACAACACTA [19656]
cp32-4_B31_TS_MP_FG_consensus  TAATAAAAAAATTCAATATAAAAAAATCTTGCATGAAAGAAAAACAAAAAAAATTAAAAGAAATTCTATACAACACTA [19656]
cp32-4_B31_NX_Pl_consensus     TAATAAAAAAATTCAATATAAAAAAATCTTGCATGAAAGAAAAACAAAAAAAATTAAAAGAAATTCTATACAACACTA [19656]
cp32-4_PAli_NX_MP_FG_consensus TAATAAAAAAATTCAATATAAAAAAATCTTGCATGAAAGAAAAACAAAAAAAATTAAAAGAAATTCTATACAACACTA [19656]
cp32-4_PAli_NX_Pl_consensus    TAATAAAAAAATTCAATATAAAAAAATCTTGCATGAAAGAAAAACAAAAAAAATTAAAAGAAATTCTATACAACACTA [19656]
cp32-4_PAbe_NX_MP_FG_consensus TAATAAAAAAATTCAATATAAAAAAATCTTGCATGAAAGAAAAACAAAAAAAATTAAAAGAAATTCTATACAACACTA [19656]
cp32-4_PAbe_NX_Pl_consensus    TAATAAAAAAATTCAATATAAAAAAATCTTGCATGAAAGAAAAACAAAAAAAATTAAAAGAAATTCTATACAACACTA [19656]

cp32-4_gi|11497149|B31_GB      GGAAAAAATTAGAAGAAAACGGGTACAATCCCAAACAATTAGAAATAAATTTACAAAAAGTATACGAAAATTACAAAT [19734]
cp32-4_un10_B31_PacBio         GGAAAAAATTAGAAGAAAACGGGTACAATCCCAAACAATTAGAAATAAATTTACAAAAAGTATACGAAAATTACAAAT [19734]
cp32-4_B31_TS_MP_FG_consensus  GGAAAAAATTAGAAGAAAACGGGTACAATCCCAAACAATTAGAAATAAATTTACAAAAAGTATACGAAAATTACAAAT [19734]
cp32-4_B31_NX_Pl_consensus     GGAAAAAATTAGAAGAAAACGGGTACAATCCCAAACAATTAGAAATAAATTTACAAAAAGTATACGAAAATTACAAAT [19734]
cp32-4_PAli_NX_MP_FG_consensus GGAAAAAATTAGAAGAAAACGGGTATAATCCCAAACAATTAGAAATAAATTTACAAAAAGTATACGAAAATTACAAAT [19734]
cp32-4_PAli_NX_Pl_consensus    GGAAAAAATTAGAAGAAAACGGGTACAATCCCAAACAATTAGAAATAAATTTACAAAAAGTATACGAAAATTACAAAT [19734]
cp32-4_PAbe_NX_MP_FG_consensus GGAAAAAATTAGAAGAAAACGGGTACAATCCCAAACAATTAGAAATAAATTTACAAAAAGTATACGAAAATTACAAAT [19734]
cp32-4_PAbe_NX_Pl_consensus    GGAAAAAATTAGAAGAAAACGGGTACAATCCCAAACAATTAGAAATAAATTTACAAAAAGTATACGAAAATTACAAAT [19734]

cp32-4_gi|11497149|B31_GB      ACAAGCCCCATTTTATTATTGAAAATCATAAATATAATGATTTAGGTTACATAAAACGTAAATTAGAAAAGTCGATTG [19812]
cp32-4_un10_B31_PacBio         ACAAGCCCCATTTTATTATTGAAAATCATAAATATAATGATTTAGGTTACATAAAACGTAAATTAGAAAAGTCGATTG [19812]
cp32-4_B31_TS_MP_FG_consensus  ACAAGCCCCATTTTATTATTGAAAATCATAAATATAATGATTTAGGTTACATAAAACGTAAATTAGAAAAGTCGATTG [19812]
cp32-4_B31_NX_Pl_consensus     ACAAGCCCCATTTTATTATTGAAAATCATAAATATAATGATTTAGGTTACATAAAACGTAAATTAGAAAAGTCGATTG [19812]
```

```
cp32-4_PAli_NX_MP_FG_consensus ACAAGCCCCATTTTATTATTGAAAATCATAAATATAATGATTTAGGTTACATAAAACGTAAATTAGAAAAGTCGATTG [19812]
cp32-4_PAli_NX_Pl_consensus    ACAAGCCCCATTTTATTATTGAAAATCATAAATATAATGATTTAGGTTACATAAAACGTAAATTAGAAAAGTCGATTG [19812]
cp32-4_PAbe_NX_MP_FG_consensus ACAAGCCCCATTTTATTATTGAAAATCATAAATATAATGATTTAGGTTACATAAAACGTAAATTAGAAAAGTCGATTG [19812]
cp32-4_PAbe_NX_Pl_consensus    ACAAGCCCCATTTTATTATTGAAAATCATAAATATAATGATTTAGGTTACATAAAACGTAAATTAGAAAAGTCGATTG [19812]

cp32-4_gi|11497149|B31_GB      AAAGAAAAAAAGAAAATTCTCAACAAAATTATCAAAATTTAAAGGAAAATATTTTCAATATCCTTATTGAACAACTAA [19890]
cp32-4_un10_B31_PacBio         AAAGAAAAAAAGAAAATTCTCAACAAAATTATCAAAATTTAAAGGAAAATATTTTCAATATCCTTATTGAACAACTAA [19890]
cp32-4_B31_TS_MP_FG_consensus  AAAGAAAAAAAGAAAATTCTCAACAAAATTATCAAAATTTAAAGGAAAATATTTTCAATATCCTTATTGAACAACTAA [19890]
cp32-4_B31_NX_Pl_consensus     AAAGAAAAAAAGAAAATTCTCAACAAAATTATCAAAATTTAAAGGAAAATATTTTCAATATCCTTATTGAACAACTAA [19890]
cp32-4_PAli_NX_MP_FG_consensus AAAGAAAAAAAGAAAATTCTCAACAAAATTATCAAAATTTAAAGGAAAATATTTTCAATATCCTTATTGAACAACTAA [19890]
cp32-4_PAli_NX_Pl_consensus    AAAGAAAAAAAGAAAATTCTCAACAAAATTATCAAAATTTAAAGGAAAATATTTTCAATATCCTTATTGAACAACTAA [19890]
cp32-4_PAbe_NX_MP_FG_consensus AAAGAAAAAAAGAAAATTCTCAACAAAATTATCAAAATTTAAAGGAAAATATTTTCAATATCCTTATTGAACAACTAA [19890]
cp32-4_PAbe_NX_Pl_consensus    AAAGAAAAAAAGAAAATTCTCAACAAAATTATCAAAATTTAAAGGAAAATATTTTCAATATCCTTATTGAACAACTAA [19890]

cp32-4_gi|11497149|B31_GB      AAAAAGAAGTAAATATTGAAATTCTAAAGCCAATTATAAAAGAATATTTGAATAACCAAAAGAAAATAGAATACAATA [19968]
cp32-4_un10_B31_PacBio         AAAAAGAAGTAAATATTGAAATTCTAAAGCCAATTATAAAAGAATATTTGAATAACCAAAAGAAAATAGAATACAATA [19968]
cp32-4_B31_TS_MP_FG_consensus  AAAAAGAAGTAAATATTGAAATTCTAAAGCCAATTATAAAAGAATATTTGAATAACCAAAAGAAAATAGAATACAATA [19968]
cp32-4_B31_NX_Pl_consensus     AAAAAGAAGTAAATATTGAAATTCTAAAGCCAATTATAAAAGAATATTTGAATAACCAAAAGAAAATAGAATACAATA [19968]
cp32-4_PAli_NX_MP_FG_consensus AAAAAGAAGTAAATATTGAAATTCTAAAGCCAATTATAAAAGAATATTTGAATAACCAAAAGAAAATAGAATACAATA [19968]
cp32-4_PAli_NX_Pl_consensus    AAAAAGAAGTAAATATTGAAATTCTAAAGCCAATTATAAAAGAATATTTGAATAACCAAAAGAAAATAGAATACAATA [19968]
cp32-4_PAbe_NX_MP_FG_consensus AAAAAGAAGTAAATATTGAAATTCTAAAGCCAATTATAAAAGAATATTTGAATAACCAAAAGAAAATAGAATACAATA [19968]
cp32-4_PAbe_NX_Pl_consensus    AAAAAGAAGTAAATATTGAAATTCTAAAGCCAATTATAAAAGAATATTTGAATAACCAAAAGAAAATAGAATACAATA [19968]

cp32-4_gi|11497149|B31_GB      AAGTATTTGGTATATATCATCTTGAATTATTAGAAATAATAAAAAATGAAAAAAATTCTTTAACCACAGAAGAATTTA [20046]
cp32-4_un10_B31_PacBio         AAGTATTTGGTATATATCATCTTGAATTATTAGAAATAATAAAAAATGAAAAAAATTCTTTAACCACAGAAGAATTTA [20046]
cp32-4_B31_TS_MP_FG_consensus  AAGTATTTGGTATATATCATCTTGAATTATTAGAAATAATAAAAAATGAAAAAAATTCTTTAACCACAGAAGAATTTA [20046]
cp32-4_B31_NX_Pl_consensus     AAGTATTTGGTATATATCATCTTGAATTATTAGAAATAATAAAAAATGAAAAAAATTCTTTAACCACAGAAGAATTTA [20046]
cp32-4_PAli_NX_MP_FG_consensus AAGTATTTGGTATATATCATCTTGAATTATTAGAAATAATAAAAAATGAAAAAAATTCTTTAACCACAGAAGAATTTA [20046]
cp32-4_PAli_NX_Pl_consensus    AAGTATTTGGTATATATCATCTTGAATTATTAGAAATAATAAAAAATGAAAAAAATTCTTTAACCACAGAAGAATTTA [20046]
cp32-4_PAbe_NX_MP_FG_consensus AAGTATTTGGTATATATCATCTTGAATTATTAGAAATAATAAAAAATGAAAAAAATTCTTTAACCACAGAAGAATTTA [20046]
cp32-4_PAbe_NX_Pl_consensus    AAGTATTTGGTATATATCATCTTGAATTATTAGAAATAATAAAAAATGAAAAAAATTCTTTAACCACAGAAGAATTTA [20046]

cp32-4_gi|11497149|B31_GB      GCATAAAGGCCGTATGAGGATTTAACATGGAAAATGCACCAGAACCTATTGAAACTGTAAAAAAGGGTAAATGTAAGG [20124]
cp32-4_un10_B31_PacBio         GCATAAAGGCCGTATGAGGATTTAACATGGAAAATGCACCAGAACCTATTGAAACTGTAAAAAAGGGTAAATGTAAGG [20124]
cp32-4_B31_TS_MP_FG_consensus  GCATAAAGGCCGTATGAGGATTTAACATGGAAAATGCACCAGAACCTATTGAAACTGTAAAAAAGGGTAAATGTAAGG [20124]
cp32-4_B31_NX_Pl_consensus     GCATAAAGGCCGTATGAGGATTTAACATGGAAAATGCACCAGAACCTATTGAAACTGTAAAAAAGGGTAAATGTAAGG [20124]
cp32-4_PAli_NX_MP_FG_consensus GCATAAAGGCCGTATGAGGATTTAACATGGAAAATGCACCAGAACCTATTGAAACTGTAAAAAAGGGTAAATGTAAGG [20124]
cp32-4_PAli_NX_Pl_consensus    GCATAAAGGCCGTATGAGGATTTAACATGGAAAATGCACCAGAACCTATTGAAACTGTAAAAAAGGGTAAATGTAAGG [20124]
cp32-4_PAbe_NX_MP_FG_consensus GCATAAAGGCCGTATGAGGATTTAACATGGAAAATGCACCAGAACCTATTGAAACTGTAAAAAAGGGTAAATGTAAGG [20124]
cp32-4_PAbe_NX_Pl_consensus    GCATAAAGGCCGTATGAGGATTTAACATGGAAAATGCACCAGAACCTATTGAAACTGTAAAAAAGGGTAAATGTAAGG [20124]

cp32-4_gi|11497149|B31_GB      TTGAATGCCAAAATAAAGAACGCTTTATTTTAATTGAAAAAGAAAATGGTAAAGCAATGTATCATACAAAAATAATGA [20202]
cp32-4_un10_B31_PacBio         TTGAATGCCAAAATAAAGAACGCTTTATTTTAATTGAAAAAGAAAATGGTAAAGCAATGTATCATACAAAAATAATGA [20202]
cp32-4_B31_TS_MP_FG_consensus  TTGAATGCCAAAATAAAGAACGCTTTATTTTAATTGAAAAAGAAAATGGTAAAGCAATGTATCATACAAAAATAATGA [20202]
cp32-4_B31_NX_Pl_consensus     TTGAATGCCAAAATAAAGAACGCTTTATTTTAATTGAAAAAGAAAATGGTAAAGCAATGTATCATACAAAAATAATGA [20202]
cp32-4_PAli_NX_MP_FG_consensus TTGAATGCCAAAATAAAGAACGCTTTATTTTAATTGAAAAAGAAAATGGTAAAGCAATGTATCATACAAAAATAATGA [20202]
cp32-4_PAli_NX_Pl_consensus    TTGAATGCCAAAATAAAGAACGCTTTATTTTAATTGAAAAAGAAAATGGTAAAGCAATGTATCATACAAAAATAATGA [20202]
cp32-4_PAbe_NX_MP_FG_consensus TTGAATGCCAAAATAAAGAACGCTTTATTTTAATTGAAAAAGAAAATGGTAAAGCAATGTATCATACAAAAATAATGA [20202]
cp32-4_PAbe_NX_Pl_consensus    TTGAATGCCAAAATAAAGAACGCTTTATTTTAATTGAAAAAGAAAATGGTAAAGCAATGTATCATACAAAAATAATGA [20202]

cp32-4_gi|11497149|B31_GB      TGGATATTTATAAATTTGGAGTTTATGAGAAAAAACACGAATTTAGATTATCATTGAGGGCCTTATTTAATGGGGAAA [20280]
cp32-4_un10_B31_PacBio         TGGATATTTATAAATTTGGAGTTTATGAGAAAAAACACGAATTTAGATTATCATTGAGGGCCTTATTTAATGGGGAAA [20280]
cp32-4_B31_TS_MP_FG_consensus  TGGATATTTATAAATTTGGAGTTTATGAGAAAAAACACGAATTTAGATTATCATTGAGGGCCTTATTTAATGGGGAAA [20280]
cp32-4_B31_NX_Pl_consensus     TGGATATTTATAAATTTGGAGTTTATGAGAAAAAACACGAATTTAGATTATCATTGAGGGCCTTATTTAATGGGGAAA [20280]
cp32-4_PAli_NX_MP_FG_consensus TGGATATTTATAAATTTGGAGTTTATGAGAAAAAACACGAATTTAGATTATCATTGAGGGCCTTATTTAATGGGGAAA [20280]
cp32-4_PAli_NX_Pl_consensus    TGGATATTTATAAATTTGGAGTTTATGAGAAAAAACACGAATTTAGATTATCATTGAGGGCCTTATTTAATGGGGAAA [20280]
cp32-4_PAbe_NX_MP_FG_consensus TGGATATTTATAAATTTGGAGTTTATGAGAAAAAACACGAATTTAGATTATCATTGAGGGCCTTATTTAATGGGGAAA [20280]
cp32-4_PAbe_NX_Pl_consensus    TGGATATTTATAAATTTGGAGTTTATGAGAAAAAACACGAATTTAGATTATCATTGAGGGCCTTATTTAATGGGGAAA [20280]

cp32-4_gi|11497149|B31_GB      GGATTGTTGAAGAAACTCATTTGTACCCAATTAAAGAAGGAGATAAGTTTATTGGTATTTTTTATGGCTACAGAAAAC [20358]
cp32-4_un10_B31_PacBio         GGATTGTTGAAGAAACTCATTTGTACCCAATTAAAGAAGGAGATAAGTTTATTGGTATTTTTTATGGCTACAGAAAAC [20358]
cp32-4_B31_TS_MP_FG_consensus  GGATTGTTGAAGAAACTCATTTGTACCCAATTAAAGAAGGAGATAAGTTTATTGGTATTTTTTATGGCTACAGAAAAC [20358]
cp32-4_B31_NX_Pl_consensus     GGATTGTTGAAGAAACTCATTTGTACCCAATTAAAGAAGGAGATAAGTTTATTGGTATTTTTTATGGCTACAGAAAAC [20358]
cp32-4_PAli_NX_MP_FG_consensus GGATTGTTGAAGAAACTCATTTGTACCCAATTAAAGAAGGAGATAAGTTTATTGGTATTTTTTATGGCTACAGAAAAC [20358]
cp32-4_PAli_NX_Pl_consensus    GGATTGTTGAAGAAACTCATTTGTACCCAATTAAAGAAGGAGATAAGTTTATTGGTATTTTTTATGGCTACAGAAAAC [20358]
cp32-4_PAbe_NX_MP_FG_consensus GGATTGTTGAAGAAACTCATTTGTACCCAATTAAAGAAGGAGATAAGTTTATTGGTATTTTTTATGGCTACAGAAAAC [20358]
cp32-4_PAbe_NX_Pl_consensus    GGATTGTTGAAGAAACTCATTTGTACCCAATTAAAGAAGGAGATAAGTTTATTGGTATTTTTTATGGCTACAGAAAAC [20358]

cp32-4_gi|11497149|B31_GB      CAATAAAAAAGCCATTAATAAAGTATCAAATAAACGGAACTAGAAAAGCATATGCATTAGCAAGGGCATATTATATGG [20436]
cp32-4_un10_B31_PacBio         CAATAAAAAAGCCATTAATAAAGTATCAAATAAACGGAACTAGAAAAGCATATGCATTAGCAAGGGCATATTATATGG [20436]
cp32-4_B31_TS_MP_FG_consensus  CAATAAAAAAGCCATTAATAAAGTATCAAATAAACGGAACTAGAAAAGCATATGCATTAGCAAGGGCATATTATATGG [20436]
cp32-4_B31_NX_Pl_consensus     CAATAAAAAAGCCATTAATAAAGTATCAAATAAACGGAACTAGAAAAGCATATGCATTAGCAAGGGCATATTATATGG [20436]
cp32-4_PAli_NX_MP_FG_consensus CAATAAAAAAGCCATTAATAAAGTATCAAATAAACGGAACTAGAAAAGCATATGCATTAGCAAGGGCATATTATATGG [20436]
cp32-4_PAli_NX_Pl_consensus    CAATAAAAAAGCCATTAATAAAGTATCAAATAAACGGAACTAGAAAAGCATATGCATTAGCAAGGGCATATTATATGG [20436]
cp32-4_PAbe_NX_MP_FG_consensus CAATAAAAAAGCCATTAATAAAGTATCAAATAAACGGAACTAGAAAAGCATATGCATTAGCAAGGGCATATTATATGG [20436]
cp32-4_PAbe_NX_Pl_consensus    CAATAAAAAAGCCATTAATAAAGTATCAAATAAACGGAACTAGAAAAGCATATGCATTAGCAAGGGCATATTATATGG [20436]

cp32-4_gi|11497149|B31_GB      AATTTAGATTTAAAGCCGGAAGTGTATTTTGCTATTTCAAGGGATTATATCGATTATTAGATAAAAAAAGAACAAATA [20514]
cp32-4_un10_B31_PacBio         AATTTAGATTTAAAGCCGGAAGTGTATTTTGCTATTTCAAGGGATTATATCGATTATTAGATAAAAAAAGAACAAATA [20514]
cp32-4_B31_TS_MP_FG_consensus  AATTTAGATTTAAAGCCGGAAGTGTATTTTGCTATTTCAAGGGATTATATCGATTATTAGATAAAAAAAGAACAAATA [20514]
cp32-4_B31_NX_Pl_consensus     AATTTAGATTTAAAGCCGGAAGTGTATTTTGCTATTTCAAGGGATTATATCGATTATTAGATAAAAAAAGAACAAATA [20514]
cp32-4_PAli_NX_MP_FG_consensus AATTTAGATTTAAAGCCGGAAGTGTATTTTGCTATTTCAAGGGATTATATCGATTATTAGATAAAAAAAGAACAAATA [20514]
cp32-4_PAli_NX_Pl_consensus    AATTTAGATTTAAAGCCGGAAGTGTATTTTGCTATTTCAAGGGATTATATCGATTATTAGATAAAAAAAGAACAAATA [20514]
cp32-4_PAbe_NX_MP_FG_consensus AATTTAGATTTAAAGCCGGAAGTGTATTTTGCTATTTCAAGGGATTATATCGATTATTAGATAAAAAAAGAACAAATA [20514]
cp32-4_PAbe_NX_Pl_consensus    AATTTAGATTTAAAGCCGGAAGTGTATTTTGCTATTTCAAGGGATTATATCGATTATTAGATAAAAAAAGAACAAATA [20514]

cp32-4_gi|11497149|B31_GB      ATCATTACAACAAAGTTTTATTTAGTATGTTTACAGATTTAGAACAACAAGTATATAAATTTTATGGGAAAAAAATACC [20592]
cp32-4_un10_B31_PacBio         ATCATTACAACAAAGTTTTATTTAGTATGTTTACAGATTTAGAACAACAAGTATATAAATTTTATGGGAAAAAAATACC [20592]
cp32-4_B31_TS_MP_FG_consensus  ATCATTACAACAAAGTTTTATTTAGTATGTTTACAGATTTAGAACAACAAGTATATAAATTTTATGGGAAAAAAATACC [20592]
cp32-4_B31_NX_Pl_consensus     ATCATTACAACAAAGTTTTATTTAGTATGTTTACAGATTTAGAACAACAAGTATATAAATTTTATGGGAAAAAAATACC [20592]
cp32-4_PAli_NX_MP_FG_consensus ATCATTACAACAAAGTTTTATTTAGTATGTTTACAGATTTAGAACAACAAGTATATAAATTTTATGGGAAAAAAATACC [20592]
cp32-4_PAli_NX_Pl_consensus    ATCATTACAACAAAGTTTTATTTAGTATGTTTACAGATTTAGAACAACAAGTATATAAATTTTATGGGAAAAAAATACC [20592]
cp32-4_PAbe_NX_MP_FG_consensus ATCATTACAACAAAGTTTTATTTAGTATGTTTACAGATTTAGAACAACAAGTATATAAATTTTATGGGAAAAAAATACC [20592]
cp32-4_PAbe_NX_Pl_consensus    ATCATTACAACAAAGTTTTATTTAGTATGTTTACAGATTTAGAACAACAAGTATATAAATTTTATGGGAAAAAAATACC [20592]

cp32-4_gi|11497149|B31_GB      CAGAACAAGGACCGTTAATAAAATGGATAATAAAAAACCTAAAATAATAACAATAGCGTCAATTAAGGGCGGTGTTGG [20670]
cp32-4_un10_B31_PacBio         CAGAACAAGGACCGTTAATAAAATGGATAATAAAAAACCTAAAATAATAACAATAGCGTCAATTAAGGGCGGTGTTGG [20670]
cp32-4_B31_TS_MP_FG_consensus  CAGAACAAGGACCGTTAATAAAATGGATAATAAAAAACCTAAAATAATAACAATAGCGTCAATTAAGGGCGGTGTTGG [20670]
cp32-4_B31_NX_Pl_consensus     CAGAACAAGGACCGTTAATAAAATGGATAATAAAAAACCTAAAATAATAACAATAGCGTCAATTAAGGGCGGTGTTGG [20670]
cp32-4_PAli_NX_MP_FG_consensus CAGAACAAGGACCGTTAATAAAATGGATAATAAAAAACCTAAAATAATAACAATAGCGTCAATTAAGGGCGGTGTTGG [20670]
cp32-4_PAli_NX_Pl_consensus    CAGAACAAGGACCGTTAATAAAATGGATAATAAAAAACCTAAAATAATAACAATAGCGTCAATTAAGGGCGGTGTTGG [20670]
cp32-4_PAbe_NX_MP_FG_consensus CAGAACAAGGACCGTTAATAAAATGGATAATAAAAAACCTAAAATAATAACAATAGCGTCAATTAAGGGCGGTGTTGG [20670]
cp32-4_PAbe_NX_Pl_consensus    CAGAACAAGGACCGTTAATAAAATGGATAATAAAAAACCTAAAATAATAACAATAGCGTCAATTAAGGGCGGTGTTGG [20670]

cp32-4_gi|11497149|B31_GB      CAAAAGCATGTTGTCAATTATATTTTCATATATTTTAAGCGAAATGAACAATAAAGTGTTGATTGTAGATTTAGATCC [20748]
cp32-4_un10_B31_PacBio         CAAAAGCATGTTGTCAATTATATTTTCATATATTTTAAGCGAAATGAACAATAAAGTGTTGATTGTAGATTTAGATCC [20748]
cp32-4_B31_TS_MP_FG_consensus  CAAAAGCATGTTGTCAATTATATTTTCATATATTTTAAGCGAAATGAACAATAAAGTGTTGATTGTAGATTTAGATCC [20748]
cp32-4_B31_NX_Pl_consensus     CAAAAGCATGTTGTCAATTATATTTTCATATATTTTAAGCGAAATGAACAATAAAGTGTTGATTGTAGATTTAGATCC [20748]
cp32-4_PAli_NX_MP_FG_consensus CAAAAGCATGTTGTCAATTATATTTTCATATATTTTAAGCGAAATGAACAATAAAGTGTTGATTGTAGATTTAGATCC [20748]
```

```
cp32-4_PAli_NX_Pl_consensus    CAAAAGCATGTTGTCAATTATATTTTCATATATTTTAAGCGAAATGAACAATAAAGTGTTGATTGTAGATTTAGATCC [20748]
cp32-4_PAbe_NX_MP_FG_consensus CAAAAGCATGTTGTCAATTATATTTTCATATATTTTAAGCGAAATGAACAATAAAGTGTTGATTGTAGATTTAGATCC [20748]
cp32-4_PAbe_NX_Pl_consensus    CAAAAGCATGTTGTCAATTATATTTTCATATATTTTAAGCGAAATGAACAATAAAGTGTTGATTGTAGATTTAGATCC [20748]

cp32-4_gi|11497149|B31_GB      TCAAAATAGTTTAACCAGTTATTTTTTACAATATATCAGAAATATTGAATTGAATAATGTTTATTATCTTTTAAAAAG [20826]
cp32-4_un10_B31_PacBio         TCAAAATAGTTTAACCAGTTATTTTTTACAATATATCAGAAATATTGAATTGAATAATGTTTATTATCTTTTAAAAAG [20826]
cp32-4_B31_TS_MP_FG_consensus  TCAAAATAGTTTAACCAGTTATTTTTTACAATATATCAGAAATATTGAATTGAATAATGTTTATTATCTTTTAAAAAG [20826]
cp32-4_B31_NX_Pl_consensus     TCAAAATAGTTTAACCAGTTATTTTTTACAATATATCAGAAATATTGAATTGAATAATGTTTATTATCTTTTAAAAAG [20826]
cp32-4_PAli_NX_MP_FG_consensus TCAAAATAGTTTAACCAGTTATTTTTTACAATATATCAGAAATATTGAATTGAATAATGTTTATTATCTTTTAAAAAG [20826]
cp32-4_PAli_NX_Pl_consensus    TCAAAATAGTTTAACCAGTTATTTTTTACAATATATCAGAAATATTGAATTGAATAATGTTTATTATCTTTTAAAAAG [20826]
cp32-4_PAbe_NX_MP_FG_consensus TCAAAATAGTTTAACCAGTTATTTTTTACAATATATCAGAAATATTGAATTGAATAATGTTTATTATCTTTTAAAAAG [20826]
cp32-4_PAbe_NX_Pl_consensus    TCAAAATAGTTTAACCAGTTATTTTTTACAATATATCAGAAATATTGAATTGAATAATGTTTATTATCTTTTAAAAAG [20826]

cp32-4_gi|11497149|B31_GB      AGATCAAAACATTGCTTTTAATGAATATATAAATTCAATAAATAATAATATGTATATTATTCCGGCCCATCCAATTTT [20904]
cp32-4_un10_B31_PacBio         AGATCAAAACATTGCTTTTAATGAATATATAAATTCAATAAATAATAATATGTATATTATTCCGGCCCATCCAATTTT [20904]
cp32-4_B31_TS_MP_FG_consensus  AGATCAAAACATTGCTTTTAATGAATATATAAATTCAATAAATAATAATATGTATATTATTCCGGCCCATCCAATTTT [20904]
cp32-4_B31_NX_Pl_consensus     AGATCAAAACATTGCTTTTAATGAATATATAAATTCAATAAATAATAATATGTATATTATTCCGGCCCATCCAATTTT [20904]
cp32-4_PAli_NX_MP_FG_consensus AGATCAAAACATTGCTTTTAATGAATATATAAATTCAATAAATAATAATATGTATATTATTCCGGCCCATCCAATTTT [20904]
cp32-4_PAli_NX_Pl_consensus    AGATCAAAACATTGCTTTTAATGAATATATAAATTCAATAAATAATAATATGTATATTATTCCGGCCCATCCAATTTT [20904]
cp32-4_PAbe_NX_MP_FG_consensus AGATCAAAACATTGCTTTTAATGAATATATAAATTCAATAAATAATAATATGTATATTATTCCGGCCCATCCAATTTT [20904]
cp32-4_PAbe_NX_Pl_consensus    AGATCAAAACATTGCTTTTAATGAATATATAAATTCAATAAATAATAATATGTATATTATTCCGGCCCATCCAATTTT [20904]

cp32-4_gi|11497149|B31_GB      ATGTAAATTTGAAAAAGGAGATATTCCTTATAAAGAGCTTATGTTAGAACATATATTTGATAAAAATTTACATTATTA [20982]
cp32-4_un10_B31_PacBio         ATGTAAATTTGAAAAAGGAGATATTCCTTATAAAGAGCTTATGTTAGAACATATATTTGATAAAAATTTACATTATTA [20982]
cp32-4_B31_TS_MP_FG_consensus  ATGTAAATTTGAAAAAGGAGATATTCCTTATAAAGAGCTTATGTTAGAACATATATTTGATAAAAATTTACATTATTA [20982]
cp32-4_B31_NX_Pl_consensus     ATGTAAATTTGAAAAAGGAGATATTCCTTATAAAGAGCTTATGTTAGAACATATATTTGATAAAAATTTACATTATTA [20982]
cp32-4_PAli_NX_MP_FG_consensus ATGTAAATTTGAAAAAGGAGATATTCCTTATAAAGAGCTTATGTTAGAACATATATTTGATAAAAATTTACATTATTA [20982]
cp32-4_PAli_NX_Pl_consensus    ATGTAAATTTGAAAAAGGAGATATTCCTTATAAAGAGCTTATGTTAGAACATATATTTGATAAAAATTTACATTATTA [20982]
cp32-4_PAbe_NX_MP_FG_consensus ATGTAAATTTGAAAAAGGAGATATTCCTTATAAAGAGCTTATGTTAGAACATATATTTGATAAAAATTTACATTATTA [20982]
cp32-4_PAbe_NX_Pl_consensus    ATGTAAATTTGAAAAAGGAGATATTCCTTATAAAGAGCTTATGTTAGAACATATATTTGATAAAAATTTACATTATTA [20982]

cp32-4_gi|11497149|B31_GB      TAATTTTGATTATGTAGTAATTGATACTCCCCCTAGTTTAAGTTCTTTATTGTTTAATGCGTTAAACATTACACATAA [21060]
cp32-4_un10_B31_PacBio         TAATTTTGATTATGTAGTAATTGATACTCCCCCTAGTTTAAGTTCTTTATTGTTTAATGCGTTAAACATTACACATAA [21060]
cp32-4_B31_TS_MP_FG_consensus  TAATTTTGATTATGTAGTAATTGATACTCCCCCTAGTTTAAGTTCTTTATTGTTTAATGCGTTAAACATTACACATAA [21060]
cp32-4_B31_NX_Pl_consensus     TAATTTTGATTATGTAGTAATTGATACTCCCCCTAGTTTAAGTTCTTTATTGTTTAATGCGTTAAACATTACACATAA [21060]
cp32-4_PAli_NX_MP_FG_consensus TAATTTTGATTATGTAGTAATTGATACTCCCCCTAGTTTAAGTTCTTTATTGTTTAATGCGTTAAACATTACACATAA [21060]
cp32-4_PAli_NX_Pl_consensus    TAATTTTGATTATGTAGTAATTGATACTCCCCCTAGTTTAAGTTCTTTATTGTTTAATGCGTTAAACATTACACATAA [21060]
cp32-4_PAbe_NX_MP_FG_consensus TAATTTTGATTATGTAGTAATTGATACTCCCCCTAGTTTAAGTTCTTTATTGTTTAATGCGTTAAACATTACACATAA [21060]
cp32-4_PAbe_NX_Pl_consensus    TAATTTTGATTATGTAGTAATTGATACTCCCCCTAGTTTAAGTTCTTTATTGTTTAATGCGTTAAACATTACACATAA [21060]

cp32-4_gi|11497149|B31_GB      AGTTATAATACCTATTCAAGCAGAAAGGTGGTCTGTAGAGTCTTTGCCAATATTAATGAATGAAATAAAGGAAGTTGA [21138]
cp32-4_un10_B31_PacBio         AGTTATAATACCTATTCAAGCAGAAAGGTGGTCTGTAGAGTCTTTGCCAATATTAATGAATGAAATAAAGGAAGTTGA [21138]
cp32-4_B31_TS_MP_FG_consensus  AGTTATAATACCTATTCAAGCAGAAAGGTGGTCTGTAGAGTCTTTGCCAATATTAATGAATGAAATAAAGGAAGTTGA [21138]
cp32-4_B31_NX_Pl_consensus     AGTTATAATACCTATTCAAGCAGAAAGGTGGTCTGTAGAGTCTTTGCCAATATTAATGAATGAAATAAAGGAAGTTGA [21138]
cp32-4_PAli_NX_MP_FG_consensus AGTTATAATACCTATTCAAGCAGAAAGGTGGTCTGTAGAGTCTTTGCCAATATTAATGAATGAAATAAAGGAAGTTGA [21138]
cp32-4_PAli_NX_Pl_consensus    AGTTATAATACCTATTCAAGCAGAAAGGTGGTCTGTAGAGTCTTTGCCAATATTAATGAATGAAATAAAGGAAGTTGA [21138]
cp32-4_PAbe_NX_MP_FG_consensus AGTTATAATACCTATTCAAGCAGAAAGGTGGTCTGTAGAGTCTTTGCCAATATTAATGAATGAAATAAAGGAAGTTGA [21138]
cp32-4_PAbe_NX_Pl_consensus    AGTTATAATACCTATTCAAGCAGAAAGGTGGTCTGTAGAGTCTTTGCCAATATTAATGAATGAAATAAAGGAAGTTGA [21138]

cp32-4_gi|11497149|B31_GB      AATAATAAGAAAGAAAAATATTGATGTGTTAATAATAGAAAATCAATTTATAAAAAATAGAAATACGTATAAAGATAT [21216]
cp32-4_un10_B31_PacBio         AATAATAAGAAAGAAAAATATTGATGTGTTAATAATAGAAAATCAATTTATAAAAAATAGAAATACGTATAAAGATAT [21216]
cp32-4_B31_TS_MP_FG_consensus  AATAATAAGAAAGAAAAATATTGATGTGTTAATAATAGAAAATCAATTTATAAAAAATAGAAATACGTATAAAGATAT [21216]
cp32-4_B31_NX_Pl_consensus     AATAATAAGAAAGAAAAATATTGATGTGTTAATAATAGAAAATCAATTTATAAAAAATAGAAATACGTATAAAGATAT [21216]
cp32-4_PAli_NX_MP_FG_consensus AATAATAAGAAAGAAAAATATTGATGTGTTAATAATAGAAAATCAATTTATAAAAAATAGAAATACGTATAAAGATAT [21216]
cp32-4_PAli_NX_Pl_consensus    AATAATAAGAAAGAAAAATATTGATGTGTTAATAATAGAAAATCAATTTATAAAAAATAGAAATACGTATAAAGATAT [21216]
cp32-4_PAbe_NX_MP_FG_consensus AATAATAAGAAAGAAAAATATTGATGTGTTAATAATAGAAAATCAATTTATAAAAAATAGAAATACGTATAAAGATAT [21216]
cp32-4_PAbe_NX_Pl_consensus    AATAATAAGAAAGAAAAATATTGATGTGTTAATAATAGAAAATCAATTTATAAAAAATAGAAATACGTATAAAGATAT [21216]

cp32-4_gi|11497149|B31_GB      TGAAAGTATTTTGCAGTCAGAATACAAAGATCTTATTAAAGGAAGAGTTCATTTTTATAATTCAATTAAGGTTTTTAT [21294]
cp32-4_un10_B31_PacBio         TGAAAGTATTTTGCAGTCAGAATACAAAGATCTTATTAAAGGAAGAGTTCATTTTTATAATTCAATTAAGGTTTTTAT [21294]
cp32-4_B31_TS_MP_FG_consensus  TGAAAGTATTTTGCAGTCAGAATACAAAGATCTTATTAAAGGAAGAGTTCATTTTTATAATTCAATTAAGGTTTTTAT [21294]
cp32-4_B31_NX_Pl_consensus     TGAAAGTATTTTGCAGTCAGAATACAAAGATCTTATTAAAGGAAGAGTTCATTTTTATAATTCAATTAAGGTTTTTAT [21294]
cp32-4_PAli_NX_MP_FG_consensus TGAAAGTATTTTGCAGTCAGAATACAAAGATCTTATTAAAGGAAGAGTTCATTTTTATAATTCAATTAAGGTTTTTAT [21294]
cp32-4_PAli_NX_Pl_consensus    TGAAAGTATTTTGCAGTCAGAATACAAAGATCTTATTAAAGGAAGAGTTCATTTTTATAATTCAATTAAGGTTTTTAT [21294]
cp32-4_PAbe_NX_MP_FG_consensus TGAAAGTATTTTGCAGTCAGAATACAAAGATCTTATTAAAGGAAGAGTTCATTTTTATAATTCAATTAAGGTTTTTAT [21294]
cp32-4_PAbe_NX_Pl_consensus    TGAAAGTATTTTGCAGTCAGAATACAAAGATCTTATTAAAGGAAGAGTTCATTTTTATAATTCAATTAAGGTTTTTAT [21294]

cp32-4_gi|11497149|B31_GB      AAATGAATTGAAAGAGCCTAATAATAAAGAAATTTATTATCAAGAAATAAAAAAAATATTAAACAATATGTTTTAAAA [21372]
cp32-4_un10_B31_PacBio         AAATGAATTGAAAGAGCCTAATAATAAAGAAATTTATTATCAAGAAATAAAAAAAATATTAAACAATATGTTTTAAAA [21372]
cp32-4_B31_TS_MP_FG_consensus  AAATGAATTGAAAGAGCCTAATAATAAAGAAATTTATTATCAAGAAATAAAAAAAATATTAAACAATATGTTTTAAAA [21372]
cp32-4_B31_NX_Pl_consensus     AAATGAATTGAAAGAGCCTAATAATAAAGAAATTTATTATCAAGAAATAAAAAAAATATTAAACAATATGTTTTAAAA [21372]
cp32-4_PAli_NX_MP_FG_consensus AAATGAATTGAAAGAGCCTAATAATAAAGAAATTTATTATCAAGAAATAAAAAAAATATTAAACAATATGTTTTAAAA [21372]
cp32-4_PAli_NX_Pl_consensus    AAATGAATTGAAAGAGCCTAATAATAAAGAAATTTATTATCAAGAAATAAAAAAAATATTAAACAATATGTTTTAAAA [21372]
cp32-4_PAbe_NX_MP_FG_consensus AAATGAATTGAAAGAGCCTAATAATAAAGAAATTTATTATCAAGAAATAAAAAAAATATTAAACAATATGTTTTAAAA [21372]
cp32-4_PAbe_NX_Pl_consensus    AAATGAATTGAAAGAGCCTAATAATAAAGAAATTTATTATCAAGAAATAAAAAAAATATTAAACAATATGTTTTAAAA [21372]

cp32-4_gi|11497149|B31_GB      GTCTTCCGGAAGACTTTTAAAAATAAATCAGAAACTTAGGAGGATTAAGTGTCAAAGTTAAAAGTAAAAAAAATATTA [21450]
cp32-4_un10_B31_PacBio         GTCTTCCGGAAGACTTTTAAAAATAAATCAGAAACTTAGGAGGATTAAGTGTCAAAGTTAAAAGTAAAAAAAATATTA [21450]
cp32-4_B31_TS_MP_FG_consensus  GTCTTCCGGAAGACTTTTAAAAATAAATCAGAAACTTAGGAGGATTAAGTGTCAAAGTTAAAAGTAAAAAAAATATTA [21450]
cp32-4_B31_NX_Pl_consensus     GTCTTCCGGAAGACTTTTAAAAATAAATCAGAAACTTAGGAGGATTAAGTGTCAAAGTTAAAAGTAAAAAAAATATTA [21450]
cp32-4_PAli_NX_MP_FG_consensus GTCTTCCGGAAGACTTTTAAAAATAAATCAGAAACTTAGGAGGATTAAGTGTCAAAGTTAAAAGTAAAAAAAATATTA [21450]
cp32-4_PAli_NX_Pl_consensus    GTCTTCCGGAAGACTTTTAAAAATAAATCAGAAACTTAGGAGGATTAAGTGTCAAAGTTAAAAGTAAAAAAAATATTA [21450]
cp32-4_PAbe_NX_MP_FG_consensus GTCTTCCGGAAGACTTTTAAAAATAAATCAGAAACTTAGGAGGATTAAGTGTCAAAGTTAAAAGTAAAAAAAATATTA [21450]
cp32-4_PAbe_NX_Pl_consensus    GTCTTCCGGAAGACTTTTAAAAATAAATCAGAAACTTAGGAGGATTAAGTGTCAAAGTTAAAAGTAAAAAAAATATTA [21450]

cp32-4_gi|11497149|B31_GB      TTAAATAATAGAATTGAGGATGTTCAAGAATTTGAAAATGAGGAACTTGAATATAAAAGCTATAAGGATCAACTTCGT [21528]
cp32-4_un10_B31_PacBio         TTAAATAATAGAATTGAGGATGTTCAAGAATTTGAAAATGAGGAACTTGAATATAAAAGCTATAAGGATCAACTTCGT [21528]
cp32-4_B31_TS_MP_FG_consensus  TTAAATAATAGAATTGAGGATGTTCAAGAATTTGAAAATGAGGAACTTGAATATAAAAGCTATAAGGATCAACTTCGT [21528]
cp32-4_B31_NX_Pl_consensus     TTAAATAATAGAATTGAGGATGTTCAAGAATTTGAAAATGAGGAACTTGAATATAAAAGCTATAAGGATCAACTTCGT [21528]
cp32-4_PAli_NX_MP_FG_consensus TTAAATAATAGAATTGAGGATGTTCAAGAATTTGAAAATGAGGAACTTGAATATAAAAGCTATAAGGATCAACTTCGT [21528]
cp32-4_PAli_NX_Pl_consensus    TTAAATAATAGAATTGAGGATGTTCAAGAATTTGAAAATGAGGAACTTGAATATAAAAGCTATAAGGATCAACTTCGT [21528]
cp32-4_PAbe_NX_MP_FG_consensus TTAAATAATAGAATTGAGGATGTTCAAGAATTTGAAAATGAGGAACTTGAATATAAAAGCTATAAGGATCAACTTCGT [21528]
cp32-4_PAbe_NX_Pl_consensus    TTAAATAATAGAATTGAGGATGTTCAAGAATTTGAAAATGAGGAACTTGAATATAAAAGCTATAAGGATCAACTTCGT [21528]

cp32-4_gi|11497149|B31_GB      AGAATTACTGTTGATGATGTTGAGAATAAAATTAAAACAATGAAAATCCTATATAAAATAAGAGAAAAAAAACTTTAT [21606]
cp32-4_un10_B31_PacBio         AGAATTACTGTTGATGATGTTGAGAATAAAATTAAAACAATGAAAATCCTATATAAAATAAGAGAAAAAAAACTTTAT [21606]
cp32-4_B31_TS_MP_FG_consensus  AGAATTACTGTTGATGATGTTGAGAATAAAATTAAAACAATGAAAATCCTATATAAAATAAGAGAAAAAAAACTTTAT [21606]
cp32-4_B31_NX_Pl_consensus     AGAATTACTGTTGATGATGTTGAGAATAAAATTAAAACAATGAAAATCCTATATAAAATAAGAGAAAAAAAACTTTAT [21606]
cp32-4_PAli_NX_MP_FG_consensus AGAATTACTGTTGATGATGTTGAGAATAAAATTAAAACAATGAAAATCCTATATAAAATAAGAGAAAAAAAACTTTAT [21606]
cp32-4_PAli_NX_Pl_consensus    AGAATTACTGTTGATGATGTTGAGAATAAAATTAAAACAATGAAAATCCTATATAAAATAAGAGAAAAAAAACTTTAT [21606]
cp32-4_PAbe_NX_MP_FG_consensus AGAATTACTGTTGATGATGTTGAGAATAAAATTAAAACAATGAAAATCCTATATAAAATAAGAGAAAAAAAACTTTAT [21606]
cp32-4_PAbe_NX_Pl_consensus    AGAATTACTGTTGATGATGTTGAGAATAAAATTAAAACAATGAAAATCCTATATAAAATAAGAGAAAAAAAACTTTAT [21606]

cp32-4_gi|11497149|B31_GB      TTGATTGATGGTTATAAAAAATTTGAAGACTTTTTGTCAGAATTTATAATATCTAGAAGTCAGGCTTTTTTATACCTT [21684]
cp32-4_un10_B31_PacBio         TTGATTGATGGTTATAAAAAATTTGAAGACTTTTTGTCAGAATTTATAATATCTAGAAGTCAGGCTTTTTTATACCTT [21684]
cp32-4_B31_TS_MP_FG_consensus  TTGATTGATGGTTATAAAAAATTTGAAGACTTTTTGTCAGAATTTATAATATCTAGAAGTCAGGCTTTTTTATACCTT [21684]
cp32-4_B31_NX_Pl_consensus     TTGATTGATGGTTATAAAAAATTTGAAGACTTTTTGTCAGAATTTATAATATCTAGAAGTCAGGCTTTTTTATACCTT [21684]
cp32-4_PAli_NX_MP_FG_consensus TTGATTGATGGTTATAAAAAATTTGAAGACTTTTTGTCAGAATTTATAATATCTAGAAGTCAGGCTTTTTTATACCTT [21684]
cp32-4_PAli_NX_Pl_consensus    TTGATTGATGGTTATAAAAAATTTGAAGACTTTTTGTCAGAATTTATAATATCTAGAAGTCAGGCTTTTTTATACCTT [21684]
```

```
cp32-4_PAbe_NX_MP_FG_consensus TTGATTGATGGTTATAAAAAATTTGAAGACTTTTTGTCAGAATTTATAATATCTAGAAGTCAGGCTTTTTTATACCTT [21684]
cp32-4_PAbe_NX_Pl_consensus    TTGATTGATGGTTATAAAAAATTTGAAGACTTTTTGTCAGAATTTATAATATCTAGAAGTCAGGCTTTTTTATACCTT [21684]

cp32-4_gi|11497149|B31_GB      AAAATTTATAGAAAAGTATTAGAGGGGAGCATATCAATAAATGATATTAAAGAAAAAGGCTTAAAGGGTGTATATAGA [21762]
cp32-4_un10_B31_PacBio         AAAATTTATAGAAAAGTATTAGAGGGGAGCATATCAATAAATGATATTAAAGAAAAAGGCTTAAAGGGTGTATATAGA [21762]
cp32-4_B31_TS_MP_FG_consensus  AAAATTTATAGAAAAGTATTAGAGGGGAGCATATCAATAAATGATATTAAAGAAAAAGGCTTAAAGGGTGTATATAGA [21762]
cp32-4_B31_NX_Pl_consensus     AAAATTTATAGAAAAGTATTAGAGGGGAGCATATCAATAAATGATATTAAAGAAAAAGGCTTAAAGGGTGTATATAGA [21762]
cp32-4_PAli_NX_MP_FG_consensus AAAATTTATAGAAAAGTATTAGAGGGGAGCATATCAATAAATGATATTAAAGAAAAAGGCTTAAAGGGTGTATATAGA [21762]
cp32-4_PAli_NX_Pl_consensus    AAAATTTATAGAAAAGTATTAGAGGGGAGCATATCAATAAATGATATTAAAGAAAAAGGCTTAAAGGGTGTATATAGA [21762]
cp32-4_PAbe_NX_MP_FG_consensus AAAATTTATAGAAAAGTATTAGAGGGGAGCATATCAATAAATGATATTAAAGAAAAAGGCTTAAAGGGTGTATATAGA [21762]
cp32-4_PAbe_NX_Pl_consensus    AAAATTTATAGAAAAGTATTAGAGGGGAGCATATCAATAAATGATATTAAAGAAAAAGGCTTAAAGGGTGTATATAGA [21762]

cp32-4_gi|11497149|B31_GB      AATATATTAAACGTAGAAATTAAAGAGGATAAGTCAAAGCAAAATCCAATAAAACCCCTAAGATTTCAACTTAAAAAA [21840]
cp32-4_un10_B31_PacBio         AATATATTAAACGTAGAAATTAAAGAGGATAAGTCAAAGCAAAATCCAATAAAACCCCTAAGATTTCAACTTAAAAAA [21840]
cp32-4_B31_TS_MP_FG_consensus  AATATATTAAACGTAGAAATTAAAGAGGATAAGTCAAAGCAAAATCCAATAAAACCCCTAAGATTTCAGCTTAAGAAA [21840]
cp32-4_B31_NX_Pl_consensus     AATATATTAAACGTAGAAATTAAAGAGGATAAGTCAAAGCAAAATCCAATAAAACCCCTAAGATTTCAGCTTAAGAAA [21840]
cp32-4_PAli_NX_MP_FG_consensus AATATATTAAACGTAGAAATTAAAGAGGATAAGTCAAA-CAAAATTGGATAAAACCCCTAAGATTTCAGCTTAAGAAA [21840]
cp32-4_PAli_NX_Pl_consensus    AATATATTAAACGTAGAAATTAAAGAGGATAAGTCAAAGCAAAATCCAATAAAACCCCTAAGATTTCAACTTAAAAAA [21840]
cp32-4_PAbe_NX_MP_FG_consensus AATATATTAAACGTAGAAATTAAAGAGGATAAGTCAAA-CAAAATTGGATAAAACCCCTAAGATTTCAGCTTAAGAAA [21840]
cp32-4_PAbe_NX_Pl_consensus    AATATATTAAACGTAGAAATTAAAGAGGATAAGTCAAAGCAAAATCCAATAAAACCCCTAAGATTTCAACTTAAAAAA [21840]

cp32-4_gi|11497149|B31_GB      CAGGAAAGTTATAATTTTTATAAAAAAAATGCCAAATTTACTAGTTTTATGATGGACGAGATTTTTGAAAATCAAAAA [21918]
cp32-4_un10_B31_PacBio         CAGGAAAGTTATAATTTTTATAAAAAAAATGCCAAATTTACTAGTTTTATGATGGACGAGATTTTTGAAAATCAAAAA [21918]
cp32-4_B31_TS_MP_FG_consensus  CAAGAAAGCTATGATTTTTATAAAAAGCATGCTAAATTTACAAGTTTTATGATGGATGAGATTTTTGAAAATCAAAAA [21918]
cp32-4_B31_NX_Pl_consensus     CAAGAAAGCTATGATTTTTATAAAAAGCATGCTAAATTTACAAGTTTTATGATGGATGAGATTTTTGAAAATCAAAAA [21918]
cp32-4_PAli_NX_MP_FG_consensus CAAGAAAGCTATGATTTTTATAAAAAGCATGCTAAATTTACAAGTTTTATGATGGATGAGATTTTTGAAAATCAAAAA [21918]
cp32-4_PAli_NX_Pl_consensus    CAGGAAAGTTATAATTTTTATAAAAAAAATGCCAAATTTACAAGTTTTATGATGGATGAGATTTTTGAAAATCAAAAA [21918]
cp32-4_PAbe_NX_MP_FG_consensus CAAGAAAGCTATGATTTTTATAAAAAGCATGCTAAATTTACAAGTTTTATGATGGATGAGATTTTTGAAAATCAAAAA [21918]
cp32-4_PAbe_NX_Pl_consensus    CAGGAAAGTTATGATTTTTATAAAAAGCATGCTAAATTTACAAGTTTTATGATGGATGAGATTTTTGAAAATCAAAAA [21918]

cp32-4_gi|11497149|B31_GB      GATTTGATTAATAAACTTTTAAAAAAATATAAAGAATTAAAAGGATAATAAAGGGGATTTTATGAGTAATTTAGCCTA [21996]
cp32-4_un10_B31_PacBio         GATTTGATTAATAAACTTTTAAAAAAATATAAAGAATTAAAAGGATAATAAAGGGGATTTTATGAGTAATTTAGCCTA [21996]
cp32-4_B31_TS_MP_FG_consensus  GATTTTCTTAATAAACTTTTAAAAAAATATAAAGAATTAAAAGGATAATAAAGGGGATCTTATGAGTAATTTAGCCTA [21996]
cp32-4_B31_NX_Pl_consensus     GATTTTCTTAATAAACTTTTAAAAAAATATAAAGAATTAAAAGGATAATAAAGGGGATCTTATGAGTAATTTAGCCTA [21996]
cp32-4_PAli_NX_MP_FG_consensus GATTTTCTTAATAAACTTTTAAAAAAATATAAAGAATTAAAAGGATAATAAAGGGGATCTTATGAGTAATTTAGCCTA [21996]
cp32-4_PAli_NX_Pl_consensus    GATTTTCTTAATAAACTTTTAAAAAAATATAAAGAATTAAAAGGATAATAAAGGGGATCTTATGAGTAATTTAGCCTA [21996]
cp32-4_PAbe_NX_MP_FG_consensus GATTTTCTTAATAAACTTTTAAAAAAATATAAAGAATTAAAAGGATAATAAAGGGGATCTTATGAGTAATTTAGCCTA [21996]
cp32-4_PAbe_NX_Pl_consensus    GATTTTCTTAATAAACTTTTAAAAAAATATAAAGAATTAAAAGGATAATAAAGGGGATCTTATGAGTAATTTAGCCTA [21996]

cp32-4_gi|11497149|B31_GB      CAATGCTCTTAAAATAGAAAATATAAGGTTAGAATTTTTAAATAAGGGGTTTAGTGAAGAGGCAATAGATTTTGTTTT [22074]
cp32-4_un10_B31_PacBio         CAATGCTCTTAAAATAGAAAATATAAGGTTAGAATTTTTAAATAAGGGGTTTAGTGAAGAGGCAATAGATTTTGTTTT [22074]
cp32-4_B31_TS_MP_FG_consensus  CAATACTCTTAAAATAGAAAATATAAGGTTAGAATTTTTAAATAAAGGGTTTAGTGAAGAGGCAATAGATTTTGTTTT [22074]
cp32-4_B31_NX_Pl_consensus     CAATACTCTTAAAATAGAAAATATAAGGTTAGAATTTTTAAATAAAGGGTTTAGTGAAGAGGCAATAGATTTTGTTTT [22074]
cp32-4_PAli_NX_MP_FG_consensus CAATACTCTTAAAATAGAAAATATAAGGTTAGAATTTTTAAATAAAGGGTTTAGTGAAGAGGCAATAGATTTTGTTTT [22074]
cp32-4_PAli_NX_Pl_consensus    CAATGCTCTTAAAATAGAAAATATAAGGTTAGAATTTTTAAATAAAGGGTTTAGTGAAGAGGCAATAGATTTTGTTTT [22074]
cp32-4_PAbe_NX_MP_FG_consensus CAATACTCTTAAAATAGAAAATATAAGGTTAGAATTTTTAAATAAAGGGTTTAGTGAAGAGGCAATAGATTTTGTTTT [22074]
cp32-4_PAbe_NX_Pl_consensus    CAATACTCTTAAAATAGAAAATATAAGGTTAGAATTTTTAAATAAAGGGTTTAGTGAAGAGGCAATAGATTTTGTTTT [22074]

cp32-4_gi|11497149|B31_GB      ACTTCAGAACGACAATTACAACTTTGAAGTTTTAAAGGAGAAAATGAATTCTTTAGAACAACAAATAATTAATGTAGA [22152]
cp32-4_un10_B31_PacBio         ACTTCAGAACGACAATTACAACTTTGAAGTTTTAAAGGAGAAAATGAATTCTTTAGAACAACAAATAATTAATGTAGA [22152]
cp32-4_B31_TS_MP_FG_consensus  ACTTCAGAATGACAATTACAACTTTGAAGTTTTAAAGGAGAAAATGAATTCTTTAGAACAACAAATAATTAATGTAGA [22152]
cp32-4_B31_NX_Pl_consensus     ACTTCAGAATGACAATTACAACTTTGAAGTTTTAAAGGAGAAAATGAATTCTTTAGAACAACAAATAATTAATGTAGA [22152]
cp32-4_PAli_NX_MP_FG_consensus ACTTCAGAATGACAATTACAACTTTGAAGTTTTAAAGGAGAAAATGAATTCTTTAGAACAACAAATAATTAATGTAGA [22152]
cp32-4_PAli_NX_Pl_consensus    ACTTCAGAATGACAATTACAACTTTGAAGTTTTAAAGGAGAAAATGAATTCTTTAGAACAACAAATAATTAATGTAGA [22152]
cp32-4_PAbe_NX_MP_FG_consensus ACTTCAGAATGACAATTACAACTTTGAAGTTTTAAAGGAGAAAATGAATTCTTTAGAACAACAAATAATTAATGTAGA [22152]
cp32-4_PAbe_NX_Pl_consensus    ACTTCAGAATGACAATTACAACTTTGAAGTTTTAAAGGAGAAAATGAATTCTTTAGAACAACAAATAATTAATGTAGA [22152]

cp32-4_gi|11497149|B31_GB      AAAGAATTTTCAAAAAGATATAGATAGTGTAAATACTAAAATAGATAGTGTAGAAAAAACTTTGCAAAAAGATATATC [22230]
cp32-4_un10_B31_PacBio         AAAGAATTTTCAAAAAGATATAGATAGTGTAAATACTAAAATAGATAGTGTAGAAAAAACTTTGCAAAAAGATATATC [22230]
cp32-4_B31_TS_MP_FG_consensus  AAAGAATTTTCAAAAAGATGTAGACAGTATTTATATTAAAATAGATAGTGTAGAAAAAACTTTGCAAAAAGATATATC [22230]
cp32-4_B31_NX_Pl_consensus     AAAGAATTTTCAAAAAGATGTAGACAGTATTTATATTAAAATAGATAGTGTAGAAAAAACTTTGCAAAAAGATATATC [22230]
cp32-4_PAli_NX_MP_FG_consensus AAAGAATTTTCAAAAAGATGTAGACAGTATTTATATTAAAATAGATAGTGTAGAAAAAACTTTGCAAAAAGATATATC [22230]
cp32-4_PAli_NX_Pl_consensus    AAAGAATTTTCAAAAAGATATAGATAGTGTAAATACTAAAATAGATAGTGTAGAAAAAACTTTGCAAAAAGATATATC [22230]
cp32-4_PAbe_NX_MP_FG_consensus AAAGAATTTTCAAAAAGATGTAGACAGTATTTATATTAAAATAGATAGTGTAGAAAAAACTTTGCAAAAAGATATATC [22230]
cp32-4_PAbe_NX_Pl_consensus    AAAGAATTTTCAAAAAGATGTAGACAGTATTTATATTAAAATAGATAGTGTAGAAAAAACTTTGCAAAAAGATATATC [22230]

cp32-4_gi|11497149|B31_GB      TAGTCTAGACAATAAAATAGATATTTAAAAAAATGAACTTAATGCAAGCAATAGAACAATACAAGTAATTCTAATAAT [22308]
cp32-4_un10_B31_PacBio         TAGTCTAGACAATAAAATAGATATTTAAAAAAATGAACTTAATGCAAGCAATAGAACAATACAAGTAATTCTAATAAT [22308]
cp32-4_B31_TS_MP_FG_consensus  TAGTCTAGACAATAAAATAGATATTTAAAAAAATGAACTTAATGCAAGCAATAGAACAATACAAGTAATTCTAATAAT [22308]
cp32-4_B31_NX_Pl_consensus     TAGTCTAGACAATAAAATAGATATTTAAAAAAATGAACTTAATGCAAGCAATAGAACAATACAAGTAATGCTAATAGC [22308]
cp32-4_PAli_NX_MP_FG_consensus TAGTCTAGACAATAAAATAGATATTTAAAAAAATGAACTTAATGCAAGCAATAGAACAATACAAGTAATGCTAATAGC [22308]
cp32-4_PAli_NX_Pl_consensus    TAGTCTAGACAATAAAATAGATATTTAAAAAAATGAACTTAATGCAAGCAATAGAACAATACAAGTAATTCTAATAAT [22308]
cp32-4_PAbe_NX_MP_FG_consensus TAGTCTAGACAATAAAATAGATATTTAAAAAAATGAACTTAATGCAAGCAATAGAACAATACAAGTAATGCTAATAGC [22308]
cp32-4_PAbe_NX_Pl_consensus    TAGTCTAGACAATAAAATAGATATTTAAAAAAATGAACTTAATGCAAGCAATAGAACAATACAAGTAATTCTAATAAT [22308]

cp32-4_gi|11497149|B31_GB      GGGAATAACGTTTGCCCCAATTATCTATTCTATATTTAATAAGTGTTTTTTCAATTGAAAATGATTAAAATTTTATAA [22386]
cp32-4_un10_B31_PacBio         GGGAATAACGTTTGCCCCAATTATCTATTCTATATTTAATAAGTGTTTTTTCAATTGAAAATGATTAAAATTTTATAA [22386]
cp32-4_B31_TS_MP_FG_consensus  GGGAATAACGCTTGCCCCAATTATTTATTCTATATTCAATAAGTATTTTTTCAATTGAGAATGATTAAAATTTTATAA [22386]
cp32-4_B31_NX_Pl_consensus     AGGAATAACGCTTGCCCCAATTATTTATTCTATATTCAATAAGTATTTTTTCAATTGAGAATGATTAAAATTTTATAA [22386]
cp32-4_PAli_NX_MP_FG_consensus AGGAATAACGCTTGCCCCAATTATTTATTCTATATTCAATAAGTATTTTTTCAATTGAGAATGATTAAAATTTTATAA [22386]
cp32-4_PAli_NX_Pl_consensus    GGGAATAACGTTTGCCCCAATTATCTATTCTATATTTAATAAGTGTTTTTTCAATTGAAAATGATTAAAATTTTATAA [22386]
cp32-4_PAbe_NX_MP_FG_consensus AGGAATAACGCTTGCCCCAATTATTTATTCTATATTCAATAAGTATTTTTTCAATTGAGAATGATTAAAATTTTATAA [22386]
cp32-4_PAbe_NX_Pl_consensus    GGGAATAACGCTTGCCCCAATTATTTATTCTATATTCAATAAGTATTTTTTCAATTGAGAATGATTAAAATTTTATAA [22386]

cp32-4_gi|11497149|B31_GB      AGTAATAAGTTAGTATATAGCTCTAAAGTAGAACTTATTTGAATTTTTTAACAAGATAATTTAAATAGGTTCTTTTAT [22464]
cp32-4_un10_B31_PacBio         AGTAATAAGTTAGTATATAGCTCTAAAGTAGAACTTATTTGAATTTTTTAACAAGATAATTTAAATAGGTTCTTTTAT [22464]
cp32-4_B31_TS_MP_FG_consensus  AGTAATAAGTTAGTATATAGCTCTAAAGTAGAACTTATTTGAATTTTTTAACAAGAGAATTTAAATAGGTTCTTTTAT [22464]
cp32-4_B31_NX_Pl_consensus     AGTAATAAGTTAGTATATAGCTCTAAAGTAGAACTTATTTGAATTTTTTAACAAGAGAATTTAAATAGGTTCTTTTAT [22464]
cp32-4_PAli_NX_MP_FG_consensus AGTAATAAGTTAGTATATAGCTCTAAAGTAGAACTTATTTGAATTTTTTAACAAGAGAATTTAAATAGGTTCTTTTAT [22464]
cp32-4_PAli_NX_Pl_consensus    AGTAATAAGTTAGTATATAGCTCTAAAGTAGAACTTATTTGAATTTTTTAACAAGATAATTTAAATAGGTTCTTTTAT [22464]
cp32-4_PAbe_NX_MP_FG_consensus AGTAATAAGTTAGTATATAGCTCTAAAGTAGAACTTATTTGAATTTTTTAACAAGAGAATTTAAATAGGTTCTTTTAT [22464]
cp32-4_PAbe_NX_Pl_consensus    AGTAATAAGTTAGTATATAGCTCTAAAGTAGAACTTATTTGAATTTTTTAACAAGAGAATTTAAATAGGTTCTTTTAT [22464]

cp32-4_gi|11497149|B31_GB      TTTAATAAATACAAAATAATTTTAATTCTAAATTGAACTGAATTTAATTGTTTAGTGAGTTTATCTAAAATAAATTAA [22542]
cp32-4_un10_B31_PacBio         TTTAATAAATACAAAATAATTTTAATTCTAAATTGAACTGAATTTAATTGTTTAGTGAGTTTATCTAAAATAAATTAA [22542]
cp32-4_B31_TS_MP_FG_consensus  TTTAACAAATACAAATTGATTTTAATTCTAAATTGAACTGAATTTAATTGTTTAGTGAGTTTATCTAAAATAAATTAA [22542]
cp32-4_B31_NX_Pl_consensus     TTTAACAAATACAAATTGATTTTAATTCTAAATTGAACTGAATTTAATTGTTTAGTGAGTTTATCTAAAATAAATTAA [22542]
cp32-4_PAli_NX_MP_FG_consensus TTTAACAAATACAAATTGATTTTAATTCTAAATTGAACTGAATTTAATTGTTTAGTGAGTTTATCTAAAATAAATTAA [22542]
cp32-4_PAli_NX_Pl_consensus    TTTAATAAATACAAAATAATTTTAATTCTAAATTGAACTGAATTTAATTGTTTAGTGAGTTTATCTAAAATAAATTAA [22542]
cp32-4_PAbe_NX_MP_FG_consensus TTTAACAAATACAAATTGATTTTAATTCTAAATTGAACTGAATTTAATTGTTTAGTGAGTTTATCTAAAATAAATTAA [22542]
cp32-4_PAbe_NX_Pl_consensus    TTTAACAAATACAAATTGATTTTAATTCTAAATTGAACTGAATTTAATTGTTTAGTGAGTTTATCTAAAATAAATTAA [22542]

cp32-4_gi|11497149|B31_GB      GCTAAGCCCGCGGCTTTATTAAGCTCTTTAACATGAGAATTTAATAAAGCTTTTATTTATTATAATAATTTCTGTAAA [22620]
cp32-4_un10_B31_PacBio         GCTAAGCCCGCGGCTTTATTAAGCTCTTTAACATGAGAATTTAATAAAGCTTTTATTTATTATAATAATTTCTGTAAA [22620]
cp32-4_B31_TS_MP_FG_consensus  GCTAAGCCCGCGGCTTTATTAAGCTCTTTAACATGAGAATTTAATAAAGCTTTTATTTATTATAATAATTTCTGTAAA [22620]
cp32-4_B31_NX_Pl_consensus     GCTAAGCCCGCGGCTTTATTAAGCTCTTTAACATGAGAATTTAATAAAGCTTTTATTTATTATAATAATTTCTGTAAA [22620]
cp32-4_PAli_NX_MP_FG_consensus GCTAAGCCCGCGGCTTTATTAAGCTCTTTAACATGAGAATTTAATAAAGCTTTTATTTATTATAATAATTTCTGTAAA [22620]
cp32-4_PAli_NX_Pl_consensus    GCTAAGCCCGCGGCTTTATTAAGCTCTTTAACATGAGAATTTAATAAAGCTTTTATTTATTATAATAATTTCTGTAAA [22620]
cp32-4_PAbe_NX_MP_FG_consensus GCTAAGCCCGCGGCTTTATTAAGCTCTTTAACATGAGAATTTAATAAAGCTTTTATTTATTATAATAATTTCTGTAAA [22620]
```

```
cp32-4_PAbe_NX_Pl_consensus    GCTAAGCCCGCGGCTTTATTAAGCTCTTTAACATGAGAATTTAATAAAGCTTTTATTTATTATAATAATTTCTGTAAA [22620]

cp32-4_gi|11497149|B31_GB      AAGCCTGACAAAAATAGTTTTAGTTATACATATGTATATGTATAACTAAAAAAATATATTGCTATCAAAAAAATCCAA [22698]
cp32-4_un10_B31_PacBio         AAGCCTGACAAAAATAGTTTTAGTTATACATATGTATATGTATAACTAAAAAAATATATTGCTATCAAAAAAATCCAA [22698]
cp32-4_B31_TS_MP_FG_consensus  AAGCCTGACAAAAATAGTTTTTGTTATACATATGTATATGTATAGCTAAAAAAATATATTGCTATCAAAAAAATCCAA [22698]
cp32-4_B31_NX_Pl_consensus     AAGCCTGACAAAAATAGTTTTTGTTATACATATGTATATGTATAGCTAAAAAAATATATTGCTATCAAAAAAATCCAA [22698]
cp32-4_PAli_NX_MP_FG_consensus AAGCCTGACAAAAATAGTTTTTGTTATACATATGTATATGTATAGCTAAAAAAATATATTGCTATCAAAAAAATCCAA [22698]
cp32-4_PAli_NX_Pl_consensus    AAGCCTGACAAAAATAGTTTTAGTTATACATATGTATATGTATAACTAAAAAAATATATTGCTATCAAAAAAATCCAA [22698]
cp32-4_PAbe_NX_MP_FG_consensus AAGCCTGACAAAAATAGTTTTTGTTATACATATGTATATGTATAGCTAAAAAAATATATTGCTATCAAAAAAATCCAA [22698]
cp32-4_PAbe_NX_Pl_consensus    AAGCCTGACAAAAATAGTTTTAGTTATACATATGTATATGTATAGCTAAAAAAATATATTGCTATCAAAAAAATCCAA [22698]

cp32-4_gi|11497149|B31_GB      TTAAGTTGGGTTTAGCTAAGTTCTTTAACAAGAGAATTTAAATAAGCCCAACTAATTTTTTGTAAAATTTTTTGTAAA [22776]
cp32-4_un10_B31_PacBio         TTAAGTTGGGTTTAGCTAAGTTCTTTAACAAGAGAATTTAAATAAGCCCAACTAATTTTTTGTAAAATTTTTTGTAAA [22776]
cp32-4_B31_TS_MP_FG_consensus  TTAAGTTGGGTTTAGCTAAGTTCTTTAACAAGAGAATTTAAATAAGCCCAACTAATTTTTTGTAAAATTTTTTGTAAA [22776]
cp32-4_B31_NX_Pl_consensus     TTAAGTTGGGTTTAGCTAAGTTCTTTAACAAGAGAATTTAAATAAGCCCAACTAATTTTTTGTAAAATTTTTTGTAAA [22776]
cp32-4_PAli_NX_MP_FG_consensus TTAAGTTGGGTTTAGCTAAGTTCTTTAACAAGAGAATTTAAATAAGCCCAACTAATTTTTTGTAAAATTTTTTGTAAA [22776]
cp32-4_PAli_NX_Pl_consensus    TTAAGTTGGGTTTAGCTAAGTTCTTTAACAAGAGAATTTAAATAAGCCCAACTAATTTTTTGTAAAATTTTTTGTAAA [22776]
cp32-4_PAbe_NX_MP_FG_consensus TTAAGTTGGGTTTAGCTAAGTTCTTTAACAAGAGAATTTAAATAAGCCCAACTAATTTTTTGTAAAATTTTTTGTAAA [22776]
cp32-4_PAbe_NX_Pl_consensus    TTAAGTTGGGTTTAGCTAAGTTCTTTAACAAGAGAATTTAAATAAGCCCAACTAATTTTTTGTAAAATTTTTTGTAAA [22776]

cp32-4_gi|11497149|B31_GB      AAAGTTGGCAAAAATAGTTTTTGCTATATAATTATTTATTACAAAATAA-GGAGGAAAAAGATGGAAAATCTTTCAAA [22854]
cp32-4_un10_B31_PacBio         AAAGTTGGCAAAAATAGTTTTTGCTATATAATTATTTATTACAAAATAA-GGAGGAAAAAGATGGAAAATCTTTCAAA [22854]
cp32-4_B31_TS_MP_FG_consensus  AAAGTTGGCAAAAATAGTTTTTGCTATATAATTATTTATTACAAAATAA-GGAGGAAAAAGATGGAAAATCTTTCAAA [22854]
cp32-4_B31_NX_Pl_consensus     AAAGTTGGCAAAAATAGTTTTTGCTATATATTTATTTAATACCAATTAAAGGAGGAAAAAGATGGAAAATCTTTCAAA [22854]
cp32-4_PAli_NX_MP_FG_consensus AAAGTTGGCAAAAATAGTTTTTGCTATATAATTATTTATTACAAAATAA-GGAGGAAAAAGATGGAAAATCTTTCAAA [22854]
cp32-4_PAli_NX_Pl_consensus    AAAGTTGGCAAAAATAGTTTTTGCTATATAATTATTTATTACAAAATAA-GGAGGAAAAAGATGGAAAATCTTTCAAA [22854]
cp32-4_PAbe_NX_MP_FG_consensus AAAGTTGGCAAAAATAGTTTTTGCTATATAATTATTTATTACAAAATAA-GGAGGAAAAAGATGGAAAATCTTTCAAA [22854]
cp32-4_PAbe_NX_Pl_consensus    AAAGTTGGCAAAAATAGTTTTTGCTATATAATTATTTATTACAAAATAA-GGAGGAAAAAGATGGAAAATCTTTCAAA [22854]

cp32-4_gi|11497149|B31_GB      CAATAATAATCCACAAGAAAATATTCAAGGAGATCTCAAAATGATAAGTGTTAATCAACAAAGTTTTACTGGATGCGA [22932]
cp32-4_un10_B31_PacBio         CAATAATAATCCACAAGAAAATATTCAAGGAGATCTCAAAATGATAAGTGTTAATCAACAAAGTTTTACTGGATGCGA [22932]
cp32-4_B31_TS_MP_FG_consensus  CAATAATAATCCACAAGAAAATATTCAAGGAGATCTCAAAATGATAAGTGTTAATCAACAAAGTTTTACTGGATGCGA [22932]
cp32-4_B31_NX_Pl_consensus     CAATAATAATCCACAAGAAAATATTCAAGGAGATCTCAATATGACAAGAATTAATCAAAAAAGTTTTACTGGATGTGA [22932]
cp32-4_PAli_NX_MP_FG_consensus CAATAATAATCCACAAGAAAATATTCAAGGAGATCTCAAAATGATAAGTGTTAATCAACAAAGTTTTACTGGATGCGA [22932]
cp32-4_PAli_NX_Pl_consensus    CAATAATAATCCACAAGAAAATATTCAAGGAGATCTCAAAATGATAAGTGTTAATCAACAAAGTTTTACTGGATGCGA [22932]
cp32-4_PAbe_NX_MP_FG_consensus CAATAATAATCCACAAGAAAATATTCAAGGAGATCTCAAAATGATAAGTGTTAATCAACAAAGTTTTACTGGATGCGA [22932]
cp32-4_PAbe_NX_Pl_consensus    CAATAATAATCCACAAGAAAATATTCAAGGAGATCTCAAAATGATAAGTGTTAATCAACAAAGTTTTACTGGATGCGA [22932]

cp32-4_gi|11497149|B31_GB      AATATTTGAGGAAAAATCTTCTCCCATTAAAGAAAAAAGTAAATTAAGTAAGATAGGTAAGAAATTACCCGGAATAAG [23010]
cp32-4_un10_B31_PacBio         AATATTTGAGGAAAAATCTTCTCCCATTAAAGAAAAAAGTAAATTAAGTAAGATAGGTAAGAAATTACCCGGAATAAG [23010]
cp32-4_B31_TS_MP_FG_consensus  AATATTTGAGGAAAAATCTTCTCCCATTAAAGAAAAAAGTAAATTAAGTAAGATAGGTAAGAAATTACCCGGAATAAG [23010]
cp32-4_B31_NX_Pl_consensus     AATAATTGAGGAAAAATCTTCTCCCATTAAAGAAAAAAGTAAATTAAGTAAGATAGGTAAGAAATTACCCGGAATAAG [23010]
cp32-4_PAli_NX_MP_FG_consensus AATATTTGAGGAAAAATCTTCTCCCATTAAAGAAAAAAGTAAATTAAGTAAGATAGGTAAGAAATTACCCGGAATAAG [23010]
cp32-4_PAli_NX_Pl_consensus    AATATTTGAGGAAAAATCTTCTCCCATTAAAGAAAAAAGTAAATTAAGTAAGATAGGTAAGAAATTACCCGGAATAAG [23010]
cp32-4_PAbe_NX_MP_FG_consensus AATATTTGAGGAAAAATCTTCTCCCATTAAAGAAAAAAGTAAATTAAGTAAGATAGGTAAGAAATTACCCGGAATAAG [23010]
cp32-4_PAbe_NX_Pl_consensus    AATATTTGAGGAAAAATCTTCTCCCATTAAAGAAAAAAGTAAATTAAGTAAGATAGGTAAGAAATTACCCGGAATAAG [23010]

cp32-4_gi|11497149|B31_GB      CAGTCAAGAATGTTTTAGATTTAATCGAAATATTGATTTTAGTCTTCAAAGAAACAAGTTAGATAAATACGGTGCTAG [23088]
cp32-4_un10_B31_PacBio         CAGTCAAGAATGTTTTAGATTTAATCGAAATATTGATTTTAGTCTTCAAAGAAACAAGTTAGATAAATACGGTGCTAG [23088]
cp32-4_B31_TS_MP_FG_consensus  CAGTCAAGAATGTTTTAGATTTAATCGAAATATTGATTTTAGTCTTCAAAGAAACAAGTTAGATAAATACGGTGCTAG [23088]
cp32-4_B31_NX_Pl_consensus     CAGTCAAGAATGTTTTAGATTTAATCGAAATATTGATTTTAGTCTTCAAAGAAACAAGTTAGATAAATACGGTGCTAG [23088]
cp32-4_PAli_NX_MP_FG_consensus CAGTCAAGAATGTTTTAGATTTAATCGAAATATTGATTTTAGTCTTCAAAGAAACAAGTTAGATAAATACGGTGCTAG [23088]
cp32-4_PAli_NX_Pl_consensus    CAGTCAAGAATGTTTTAGATTTAATCGAAATATTGATTTTAGTCTTCAAAGAAACAAGTTAGATAAATACGGTGCTAG [23088]
cp32-4_PAbe_NX_MP_FG_consensus CAGTCAAGAATGTTTTAGATTTAATCGAAATATTGATTTTAGTCTTCAAAGAAACAAGTTAGATAAATACGGTGCTAG [23088]
cp32-4_PAbe_NX_Pl_consensus    CAGTCAAGAATGTTTTAGATTTAATCGAAATATTGATTTTAGTCTTCAAAGAAACAAGTTAGATAAATACGGTGCTAG [23088]

cp32-4_gi|11497149|B31_GB      TGAAGTAGGTAATATTCTTGTTGGAGGTGCTGGGCTGAAAGATTTAATGATAAACAGAGTGCTTAAATATTTTGATAT [23166]
cp32-4_un10_B31_PacBio         TGAAGTAGGTAATATTCTTGTTGGAGGTGCTGGGCTGAAAGATTTAATGATAAACAGAGTGCTTAAATATTTTGATAT [23166]
cp32-4_B31_TS_MP_FG_consensus  TGAAGTAGGTAATATTCTTGTTGGAGGTGCTGGGCTGAAAGATTTAATGATAAACAGAGTGCTTAAATATTTTGATAT [23166]
cp32-4_B31_NX_Pl_consensus     TGAAGTAGGTAATATTCTTGTTGGAGGTGCTGGGCTGAAAGATTTAATGATAAACAGAGTGCTTAAATATTTTGATAT [23166]
cp32-4_PAli_NX_MP_FG_consensus TGAAGTAGGTAATATTCTTGTTGGAGGTGCTGGGCTGAAAGATTTAATGATAAACAGAGTGCTTAAATATTTTGATAT [23166]
cp32-4_PAli_NX_Pl_consensus    TGAAGTAGGTAATATTCTTGTTGGAGGTGCTGGGCTGAAAGATTTAATGATAAACAGAGTGCTTAAATATTTTGATAT [23166]
cp32-4_PAbe_NX_MP_FG_consensus TGAAGTAGGTAATATTCTTGTTGGAGGTGCTGGGCTGAAAGATTTAATGATAAACAGAGTGCTTAAATATTTTGATAT [23166]
cp32-4_PAbe_NX_Pl_consensus    TGAAGTAGGTAATATTCTTGTTGGAGGTGCTGGGCTGAAAGATTTAATGATAAACAGAGTGCTTAAATATTTTGATAT [23166]

cp32-4_gi|11497149|B31_GB      GAGTCTACCTTTTGAAGAGAATTTATATATGCTCAAGGGCAAAGAGTTAGAGAATTTAGGATTTAGAGAATTTGTTAA [23244]
cp32-4_un10_B31_PacBio         GAGTCTACCTTTTGAAGAGAATTTATATATGCTCAAGGGCAAAGAGTTAGAGAATTTAGGATTTAGAGAATTTGTTAA [23244]
cp32-4_B31_TS_MP_FG_consensus  GAGTCTACCTTTTGAAGAGAATTTATATATGCTCAAGGGCAAAGAGTTAGAGAATTTAGGATTTAGAGAATTTGTTAA [23244]
cp32-4_B31_NX_Pl_consensus     GAGTCTACCTTTTGAAGAGAATTTATATATGCTCAAGGGCAAAGAGTTAGAGAATTTAGGATTTAGAGAATTTGTTAA [23244]
cp32-4_PAli_NX_MP_FG_consensus GAGTCTACCTTTTGAAGAGAATTTATATATGCTCAAGGGCAAAGAGTTAGAGAATTTAGGATTTAGAGAATTTGTTAA [23244]
cp32-4_PAli_NX_Pl_consensus    GAGTCTACCTTTTGAAGAGAATTTATATATGCTCAAGGGCAAAGAGTTAGAGAATTTAGGATTTAGAGAATTTGTTAA [23244]
cp32-4_PAbe_NX_MP_FG_consensus GAGTCTACCTTTTGAAGAGAATTTATATATGCTCAAGGGCAAAGAGTTAGAGAATTTAGGATTTAGAGAATTTGTTAA [23244]
cp32-4_PAbe_NX_Pl_consensus    GAGTCTACCTTTTGAAGAGAATTTATATATGCTCAAGGGCAAAGAGTTAGAGAATTTAGGATTTAGAGAATTTGTTAA [23244]

cp32-4_gi|11497149|B31_GB      AGCACATAGTGATAATATTAATGTTTTGTATAAAAACAAATATGCCAATGGAGTTGATAAGTATAACTATTTCAAAAA [23322]
cp32-4_un10_B31_PacBio         AGCACATAGTGATAATATTAATGTTTTGTATAAAAACAAATATGCCAATGGAGTTGATAAGTATAACTATTTCAAAAA [23322]
cp32-4_B31_TS_MP_FG_consensus  AGCACATAGTGATAATATTAATGTTTTGTATAAAAACAAATATGCCAATGGAGTTGATAAGTATAACTATTTCAAAAA [23322]
cp32-4_B31_NX_Pl_consensus     AGCACATAGTGATAATATTAATGTTTTGTATAAAAACAAATATGCCAATGGAGTTGATAAGTATAACTATTTCAAAAA [23322]
cp32-4_PAli_NX_MP_FG_consensus AGCACATAGTGATAATATTAATGTTTTGTATAAAAACAAATATGCCAATGGAGTTGATAAGTATAACTATTTCAAAAA [23322]
cp32-4_PAli_NX_Pl_consensus    AGCACATAGTGATAATATTAATGTTTTGTATAAAAACAAATATGCCAATGGAGTTGATAAGTATAACTATTTCAAAAA [23322]
cp32-4_PAbe_NX_MP_FG_consensus AGCACATAGTGATAATATTAATGTTTTGTATAAAAACAAATATGCCAATGGAGTTGATAAGTATAACTATTTCAAAAA [23322]
cp32-4_PAbe_NX_Pl_consensus    AGCACATAGTGATAATATTAATGTTTTGTATAAAAACAAATATGCCAATGGAGTTGATAAGTATAACTATTTCAAAAA [23322]

cp32-4_gi|11497149|B31_GB      AATGGGTAGTTCACAAACTTTAGTGGGCTCAACAATTGATGGCTGGTTTATTAATAATAATGGCGATTTAGAACTATT [23400]
cp32-4_un10_B31_PacBio         AATGGGTAGTTCACAAACTTTAGTGGGCTCAACAATTGATGGCTGGTTTATTAATAATAATGGCGATTTAGAACTATT [23400]
cp32-4_B31_TS_MP_FG_consensus  AATGGGTAGTTCACAAACTTTAGTGGGCTCAACAATTGATGGCTGGTTTATTAATAATAATGGCGATTTAGAACTATT [23400]
cp32-4_B31_NX_Pl_consensus     AATGGGTAGTTCACAAACTTTAGTGGGCTCAACAATTGATGGCTGGTTTATTAATAATAATGGCGATTTAGAACTATT [23400]
cp32-4_PAli_NX_MP_FG_consensus AATGGGTAGTTCACAAACTTTAGTGGGCTCAACAATTGATGGCTGGTTTATTAATAATAATGGCGATTTAGAACTATT [23400]
cp32-4_PAli_NX_Pl_consensus    AATGGGTAGTTCACAAACTTTAGTGGGCTCAACAATTGATGGCTGGTTTATTAATAATAATGGCGATTTAGAACTATT [23400]
cp32-4_PAbe_NX_MP_FG_consensus AATGGGTAGTTCACAAACTTTAGTGGGCTCAACAATTGATGGCTGGTTTATTAATAATAATGGCGATTTAGAACTATT [23400]
cp32-4_PAbe_NX_Pl_consensus    AATGGGTAGTTCACAAACTTTAGTGGGCTCAACAATTGATGGCTGGTTTATTAATAATAATGGCGATTTAGAACTATT [23400]

cp32-4_gi|11497149|B31_GB      AGAGATTAAAAGTAGCGACTCTCATTATATGAGTAGTGCTATTGCTGAGTACAATAAAAATGGCAATTTTTTAAGCAG [23478]
cp32-4_un10_B31_PacBio         AGAGATTAAAAGTAGCGACTCTCATTATATGAGTAGTGCTATTGCTGAGTACAATAAAAATGGCAATTTTTTAAGCAG [23478]
cp32-4_B31_TS_MP_FG_consensus  AGAGATTAAAAGTAGCGACTCTCATTATATGAGTAGTGCTATTGCTGAGTACAATAAAAATGGCAATTTTTTAAGCAG [23478]
cp32-4_B31_NX_Pl_consensus     AGAGATTAAAAGTAGCGACTCTCATTATATGAGTAGTGCTATTGCTGAGTACAATAAAAATGGCAATTTTTTAAGCAG [23478]
cp32-4_PAli_NX_MP_FG_consensus AGAGATTAAAAGTAGCGACTCTCATTATATGAGTAGTGCTATTGCTGAGTACAATAAAAATGGCAATTTTTTAAGCAG [23478]
cp32-4_PAli_NX_Pl_consensus    AGAGATTAAAAGTAGCGACTCTCATTATATGAGTAGTGCTATTGCTGAGTACAATAAAAATGGCAATTTTTTAAGCAG [23478]
cp32-4_PAbe_NX_MP_FG_consensus AGAGATTAAAAGTAGCGACTCTCATTATATGAGTAGTGCTATTGCTGAGTACAATAAAAATGGCAATTTTTTAAGCAG [23478]
cp32-4_PAbe_NX_Pl_consensus    AGAGATTAAAAGTAGCGACTCTCATTATATGAGTAGTGCTATTGCTGAGTACAATAAAAATGGCAATTTTTTAAGCAG [23478]

cp32-4_gi|11497149|B31_GB      TAAATATTTTTTCAAATATTATGTACAAGCACAAATGCAGCTAGCATGCACTGGGCTTGAGTATTGTAATTTGTTCTT [23556]
cp32-4_un10_B31_PacBio         TAAATATTTTTTCAAATATTATGTACAAGCACAAATGCAGCTAGCATGCACTGGGCTTGAGTATTGTAATTTGTTCTT [23556]
cp32-4_B31_TS_MP_FG_consensus  TAAATATTTTTTCAAATATTATGTACAAGCACAAATGCAGCTAGCATGCACTGGGCTTGAGTATTGTAATTTGTTCTT [23556]
cp32-4_B31_NX_Pl_consensus     TAAATATTTTTTCAAATATTATGTACAAGCACAAATGCAGCTAGCATGCACTGGGCTTGAGTATTGTAATTTGTTCTT [23556]
cp32-4_PAli_NX_MP_FG_consensus TAAATATTTTTTCAAATATTATGTACAAGCACAAATGCAGCTAGCATGCACTGGGCTTGAGTATTGTAATTTGTTCTT [23556]
cp32-4_PAli_NX_Pl_consensus    TAAATATTTTTTCAAATATTATGTACAAGCACAAATGCAGCTAGCATGCACTGGGCTTGAGTATTGTAATTTGTTCTT [23556]
cp32-4_PAbe_NX_MP_FG_consensus TAAATATTTTTTCAAATATTATGTACAAGCACAAATGCAGCTAGCATGCACTGGGCTTGAGTATTGTAATTTGTTCTT [23556]
cp32-4_PAbe_NX_Pl_consensus    TAAATATTTTTTCAAATATTATGTACAAGCACAAATGCAGCTAGCATGCACTGGGCTTGAGTATTGTAATTTGTTCTT [23556]
```

```
cp32-4_gi|11497149|B31_GB      TTTAATAGATGCTGCACCAATTAACTGTAAGATTAAAAGAGATGAGGCCTTAATATCAAAAGTGTTTGAATTTGTTAA [23634]
cp32-4_un10_B31_PacBio         TTTAATAGATGCTGCACCAATTAACTGTAAGATTAAAAGAGATGAGGCCTTAATATCAAAAGTGTTTGAATTTGTTAA [23634]
cp32-4_B31_TS_MP_FG_consensus  TTTAATAGATGCTGCACCAATTAACTGTAAGATTAAAAGAGATGAGGCCTTAATATCAAAAGTGTTTGAATTTGTTAA [23634]
cp32-4_B31_NX_Pl_consensus     TTTAATAGATGCTGCACCAATTAACTGTAAGATTAAAAGAGATGAGGCCTTAATATCAAAAGTGTTTGAATTTGTTAA [23634]
cp32-4_PAli_NX_MP_FG_consensus TTTAATAGATGCTGCACCAATTAACTGTAAGATTAAAAGAGATGAGGCCTTAATATCAAAAGTGTTTGAATTTGTTAA [23634]
cp32-4_PAli_NX_Pl_consensus    TTTAATAGATGCTGCACCAATTAACTGTAAGATTAAAAGAGATGAGGCCTTAATATCAAAAGTGTTTGAATTTGTTAA [23634]
cp32-4_PAbe_NX_MP_FG_consensus TTTAATAGATGCTGCACCAATTAACTGTAAGATTAAAAGAGATGAGGCCTTAATATCAAAAGTGTTTGAATTTGTTAA [23634]
cp32-4_PAbe_NX_Pl_consensus    TTTAATAGATGCTGCACCAATTAACTGTAAGATTAAAAGAGATGAGGCCTTAATATCAAAAGTGTTTGAATTTGTTAA [23634]

cp32-4_gi|11497149|B31_GB      TAAATGTGAATTAGAAATTATAAATTTAAAAAAAGATATTTATAGTAACTATAGAGACGATTACTTAATGGCACATAA [23712]
cp32-4_un10_B31_PacBio         TAAATGTGAATTAGAAATTATAAATTTAAAAAAAGATATTTATAGTAACTATAGAGACGATTACTTAATGGCACATAA [23712]
cp32-4_B31_TS_MP_FG_consensus  TAAATGTGAATTAGAAATTATAAATTTAAAAAAAGATATTTATAGTAACTATAGAGACGATTACTTAATGGCACATAA [23712]
cp32-4_B31_NX_Pl_consensus     TAAATGTGAATTAGAAATTATAAATTTAAAAAAAGATATTTATAGTAACTATAGAGACGATTACTTAATGGCACATAA [23712]
cp32-4_PAli_NX_MP_FG_consensus TAAATGTGAATTAGAAATTATAAATTTAAAAAAAGATATTTATAGTAACTATAGAGACGATTACTTAATGGCACATAA [23712]
cp32-4_PAli_NX_Pl_consensus    TAAATGTGAATTAGAAATTATAAATTTAAAAAAAGATATTTATAGTAACTATAGAGACGATTACTTAATGGCACATAA [23712]
cp32-4_PAbe_NX_MP_FG_consensus TAAATGTGAATTAGAAATTATAAATTTAAAAAAAGATATTTATAGTAACTATAGAGACGATTACTTAATGGCACATAA [23712]
cp32-4_PAbe_NX_Pl_consensus    TAAATGTGAATTAGAAATTATAAATTTAAAAAAAGATATTTATAGTAACTATAGAGACGATTACTTAATGGCACATAA [23712]

cp32-4_gi|11497149|B31_GB      TTTTAATGAGGATACGTTTATAAAACTTGTTGAAGATTTAGTAGAAAGGAGTGATTTTTATAGTTCTGGAGTTGAGTT [23790]
cp32-4_un10_B31_PacBio         TTTTAATGAGGATACGTTTATAAAACTTGTTGAAGATTTAGTAGAAAGGAGTGATTTTTATAGTTCTGGAGTTGAGTT [23790]
cp32-4_B31_TS_MP_FG_consensus  TTTTAATGAGGATACGTTTATAAAACTTGTTGAAGATTTAGTAGAAAGGAGTGATTTTTATAGTTCTGGAGTTGAGTT [23790]
cp32-4_B31_NX_Pl_consensus     TTTTAATGAGGATACGTTTATAAAACTTGTTGAAGATTTAGTAGAAAGGAGTGATTTTTATAGTTCTGGAGTTGAGTT [23790]
cp32-4_PAli_NX_MP_FG_consensus TTTTAATGAGGATACGTTTATAAAACTTGTTGAAGATTTAGTAGAAAGGAGTGATTTTTATAGTTCTGGAGTTGAGTT [23790]
cp32-4_PAli_NX_Pl_consensus    TTTTAATGAGGATACGTTTATAAAACTTGTTGAAGATTTAGTAGAAAGGAGTGATTTTTATAGTTCTGGAGTTGAGTT [23790]
cp32-4_PAbe_NX_MP_FG_consensus TTTTAATGAGGATACGTTTATAAAACTTGTTGAAGATTTAGTAGAAAGGAGTGATTTTTATAGTTCTGGAGTTGAGTT [23790]
cp32-4_PAbe_NX_Pl_consensus    TTTTAATGAGGATACGTTTATAAAACTTGTTGAAGATTTAGTAGAAAGGAGTGATTTTTATAGTTCTGGAGTTGAGTT [23790]

cp32-4_gi|11497149|B31_GB      TGATTGGGCAAGAGAATTTATAGAATATGTTGATTGTACAGACCTTGAAATTAAGGATAATCAATCTGCTGAAAATCT [23868]
cp32-4_un10_B31_PacBio         TGATTGGGCAAGAGAATTTATAGAATATGTTGATTGTACAGACCTTGAAATTAAGGATAATCAATCTGCTGAAAATCT [23868]
cp32-4_B31_TS_MP_FG_consensus  TGATTGGGCAAGAGAATTTATAGAATATGTTGATTGTACAGACCTTGAAATTAAGGATAATCAATCTGCTGAAAATCT [23868]
cp32-4_B31_NX_Pl_consensus     TGATTGGGCAAGAGAATTTATAGAATATGTTGATTGTACAGACCTTGAAATTAAGGATAATCAATCTGCTGAAAATCT [23868]
cp32-4_PAli_NX_MP_FG_consensus TGATTGGGCAAGAGAATTTATAGAATATGTTGATTGTACAGACCTTGAAATTAAGGATAATCAATCTGCTGAAAATCT [23868]
cp32-4_PAli_NX_Pl_consensus    TGATTGGGCAAGAGAATTTATAGAATATGTTGATTGTACAGACCTTGAAATTAAGGATAATCAATCTGCTGAAAATCT [23868]
cp32-4_PAbe_NX_MP_FG_consensus TGATTGGGCAAGAGAATTTATAGAATATGTTGATTGTACAGACCTTGAAATTAAGGATAATCAATCTGCTGAAAATCT [23868]
cp32-4_PAbe_NX_Pl_consensus    TGATTGGGCAAGAGAATTTATAGAATATGTTGATTGTACAGACCTTGAAATTAAGGATAATCAATCTGCTGAAAATCT [23868]

cp32-4_gi|11497149|B31_GB      TGCGTATGATTTAATGGAGATTGATAGTCTACAAAAAGAATTAAATAGAATCCAAAACGAAAATAAAAAAAGAGAAAA [23946]
cp32-4_un10_B31_PacBio         TGCGTATGATTTAATGGAGATTGATAGTCTACAAAAAGAATTAAATAGAATCCAAAACGAAAATAAAAAAAGAGAAAA [23946]
cp32-4_B31_TS_MP_FG_consensus  TGCGTATGATTTAATGGAGATTGATAGTCTACAAAAAGAATTAAATAGAATCCAAAACGAAAATAAAAAAAGAGAAAA [23946]
cp32-4_B31_NX_Pl_consensus     TGCGTATGATTTAATGGAGATTGATAGTCTACAAAAAGAATTAAATAGAATCCAAAACGAAAATAAAAAAAGAGAAAA [23946]
cp32-4_PAli_NX_MP_FG_consensus TGCGTATGATTTAATGGAGATTGATAGTCTACAAAAAGAATTAAATAGAATCCAAAACGAAAATAAAAAAAGAGAAAA [23946]
cp32-4_PAli_NX_Pl_consensus    TGCGTATGATTTAATGGAGATTGATAGTCTACAAAAAGAATTAAATAGAATCCAAAACGAAAATAAAAAAAGAGAAAA [23946]
cp32-4_PAbe_NX_MP_FG_consensus TGCGTATGATTTAATGGAGATTGATAGTCTACAAAAAGAATTAAATAGAATCCAAAACGAAAATAAAAAAAGAGAAAA [23946]
cp32-4_PAbe_NX_Pl_consensus    TGCGTATGATTTAATGGAGATTGATAGTCTACAAAAAGAATTAAATAGAATCCAAAACGAAAATAAAAAAAGAGAAAA [23946]

cp32-4_gi|11497149|B31_GB      GCCCATTAAAGATAGGTTGAAGATGCTAATTTACAATATTACGAATACATATCCACTAATTGAGCAGTTAAATTATAA [24024]
cp32-4_un10_B31_PacBio         GCCCATTAAAGATAGGTTGAAGATGCTAATTTACAATATTACGAATACATATCCACTAATTGAGCAGTTAAATTATAA [24024]
cp32-4_B31_TS_MP_FG_consensus  GCCCATTAAAGATAGGTTGAAGATGCTAATTTACAATATTACGAATACATATCCACTAATTGAGCAGTTAAATTATAA [24024]
cp32-4_B31_NX_Pl_consensus     GCCCATTAAAGATAGGTTGAAGATGCTAATTTACAATATTACGAATACATATCCACTAATTGAGCAGTTAAATTATAG [24024]
cp32-4_PAli_NX_MP_FG_consensus GCCCATTAAAGATAGGTTGAAGATGCTAATTTACAATATTACGAATACATATCCACTAATTGAGCAGTTAAATTATAA [24024]
cp32-4_PAli_NX_Pl_consensus    GCCCATTAAAGATAGGTTGAAGATGCTAATTTACAATATTACGAATACATATCCACTAATTGAGCAGTTAAATTATAA [24024]
cp32-4_PAbe_NX_MP_FG_consensus GCCCATTAAAGATAGGTTGAAGATGCTAATTTACAATATTACGAATACATATCCACTAATTGAGCAGTTAAATTATAA [24024]
cp32-4_PAbe_NX_Pl_consensus    GCCCATTAAAGATAGGTTGAAGATGCTAATTTACAATATTACGAATACATATCCACTAATTGAGCAGTTAAATTATAA [24024]

cp32-4_gi|11497149|B31_GB      ATTTGGAGAGTTTGTGTTTACTCTTGACCCTAAGAAAAGGGCAATATCAGATAGATTAAAGGGACTACTACCAACAAG [24102]
cp32-4_un10_B31_PacBio         ATTTGGAGAGTTTGTGTTTACTCTTGACCCTAAGAAAAGGGCAATATCAGATAGATTAAAGGGACTACTACCAACAAG [24102]
cp32-4_B31_TS_MP_FG_consensus  ATTTGGAGAGTTTGTGTTTACTCTTGACCCTAAGAAAAGGGCAATATCAGATAGATTAAAGGGACTACTACCAACAAG [24102]
cp32-4_B31_NX_Pl_consensus     GTTTAGAGAATTTGTGTTCACGTTTGACTCTAAGAAAAGGGCAATATCAGACAGATTAAGTGGTTTATTGCCAACAAG [24102]
cp32-4_PAli_NX_MP_FG_consensus ATTTGGAGAGTTTGTGTTTACTCTTGACCCTAAGAAAAGGGCAATATCAGATAGATTAAAGGGACTACTACCAACAAG [24102]
cp32-4_PAli_NX_Pl_consensus    ATTTGGAGAGTTTGTGTTTACTCTTGACCCTAAGAAAAGGGCAATATCAGATAGATTAAAGGGACTACTACCAACAAG [24102]
cp32-4_PAbe_NX_MP_FG_consensus ATTTGGAGAGTTTGTGTTTACTCTTGACCCTAAGAAAAGGGCAATATCAGATAGATTAAAGGGACTACTACCAACAAG [24102]
cp32-4_PAbe_NX_Pl_consensus    ATTTGGAGAGTTTGTGTTTACTCTTGACCCTAAGAAAAGGGCAATATCAGATAGATTAAAGGGACTACTACCAACAAG [24102]

cp32-4_gi|11497149|B31_GB      TGGTGCAGTATTTTTCCCTAGCAATATAGCATTTGCAAATAGTGTTAGTGTCCCCATGTGAATGGGTGCACTAAAAAA [24180]
cp32-4_un10_B31_PacBio         TGGTGCAGTATTTTTCCCTAGCAATATAGCATTTGCAAATAGTGTTAGTGTCCCCATGTGAATGGGTGCACTAAAAAA [24180]
cp32-4_B31_TS_MP_FG_consensus  TGGTGCAGTATTTTTCCCTAGCAATATAGCATTTGCAAATAGTGTTAGTGTCCCCATGTGAATGGGTGCACTAAAAAA [24180]
cp32-4_B31_NX_Pl_consensus     TGAAACATTGCTTTTCCCAAGCAATATAGCATTTGCAAATAGTGTTAGTGTCCCCATGTGAATGGGTGCACTAAAAAA [24180]
cp32-4_PAli_NX_MP_FG_consensus TGGTGCAGTATTTTTCCCTAGCAATATAGCATTTGCAAATAGTGTTAGTGTCCCCATGTGAATGGGTGCACTAAAAAA [24180]
cp32-4_PAli_NX_Pl_consensus    TGGTGCAGTATTTTTCCCTAGCAATATAGCATTTGCAAATAGTGTTAGTGTCCCCATGTGAATGGGTGCACTAAAAAA [24180]
cp32-4_PAbe_NX_MP_FG_consensus TGGTGCAGTATTTTTCCCTAGCAATATAGCATTTGCAAATAGTGTTAGTGTCCCCATGTGAATGGGTGCACTAAAAAA [24180]
cp32-4_PAbe_NX_Pl_consensus    TGGTGCAGTATTTTTCCCTAGCAATATAGCATTTGCAAATAGTGTTAGTGTCCCCATGTGAATGGGTGCACTAAAAAA [24180]

cp32-4_gi|11497149|B31_GB      TTAAAAAAAATAAATTTAATATAGGAGGATTAATTAATGCTTATTAATAAAATAAAACAAGACAATAGAACTTTACGC [24258]
cp32-4_un10_B31_PacBio         TTAAAAAAAATAAATTTAATATAGGAGGATTAATTAATGCTTATTAATAAAATAAAACAAGACAATAGAACTTTACGC [24258]
cp32-4_B31_TS_MP_FG_consensus  TTAAAAAAAATAAATTTAATATAGGAGGATTAATTAATGCTTATTAATAAAATAAAACAAGACAATAGAACTTTACGC [24258]
cp32-4_B31_NX_Pl_consensus     TTAAAAAAAATAAATTTAATATAGGAGGATTAATTAATGCTTATTAATAAAATAAAACAAGACAATAGAACTTTACGC [24258]
cp32-4_PAli_NX_MP_FG_consensus TTAAAAAAAATAAATTTAATATAGGAGGATTAATTAATGCTTATTAATAAAATAAAACAAGACAATAGAACTTTACGC [24258]
cp32-4_PAli_NX_Pl_consensus    TTAAAAAAAATAAATTTAATATAGGAGGATTAATTAATGCTTATTAATAAAATAAAACAAGACAATAGAACTTTACGC [24258]
cp32-4_PAbe_NX_MP_FG_consensus TTAAAAAAAATAAATTTAATATAGGAGGATTAATTAATGCTTATTAATAAAATAAAACAAGACAATAGAACTTTACGC [24258]
cp32-4_PAbe_NX_Pl_consensus    TTAAAAAAAATAAATTTAATATAGGAGGATTAATTAATGCTTATTAATAAAATAAAACAAGACAATAGAACTTTACGC [24258]

cp32-4_gi|11497149|B31_GB      CCAGAGATACAAAGGTGGGGCTGTTACTTTTTGTGTCTACATTATTATACAAGTCTATTTAAGAAACGTGAATTTAAT [24336]
cp32-4_un10_B31_PacBio         CCAGAGATACAAAGGTGGGGCTGTTACTTTTTGTGTCTACATTATTATACAAGTCTATTTAAGAAACGTGAATTTAAT [24336]
cp32-4_B31_TS_MP_FG_consensus  CCAGAGATACAAAGGTGGGGCTGTTACTTTTTGTGTCTACATTATTATACAAGTCTATTTAAGAAACGTGAATTTAAT [24336]
cp32-4_B31_NX_Pl_consensus     CCAGAGATACAAAGGTGGGGCTGTTACTTTTTGTGTCTACATTATTATACAAGTCTATTTAAGAAACGTGAATTTAAT [24336]
cp32-4_PAli_NX_MP_FG_consensus CCAGAGATACAAAGGTGGGGCTGTTACTTTTTGTGTCTACATTATTATACAAGTCTATTTAAGAAACGTGAATTTAAT [24336]
cp32-4_PAli_NX_Pl_consensus    CCAGAGATACAAAGGTGGGGCTGTTACTTTTTGTGTCTACATTATTATACAAGTCTATTTAAGAAACGTGAATTTAAT [24336]
cp32-4_PAbe_NX_MP_FG_consensus CCAGAGATACAAAGGTGGGGCTGTTACTTTTTGTGTCTACATTATTATACAAGTCTATTTAAGAAACGTGAATTTAAT [24336]
cp32-4_PAbe_NX_Pl_consensus    CCAGAGATACAAAGGTGGGGCTGTTACTTTTTGTGTCTACATTATTATACAAGTCTATTTAAGAAACGTGAATTTAAT [24336]

cp32-4_gi|11497149|B31_GB      GTTTATGAGATAAATGCAGCGTATTATAGATTTATAGGACTTGGATATATTAAGAGCAATTGTTTTATTATAAATCCA [24414]
cp32-4_un10_B31_PacBio         GTTTATGAGATAAATGCAGCGTATTATAGATTTATAGGACTTGGATATATTAAGAGCAATTGTTTTATTATAAATCCA [24414]
cp32-4_B31_TS_MP_FG_consensus  GTTTATGAGATAAATGCAGCGTATTATAGATTTATAGGACTTGGATATATTAAGAGCAATTGTTTTATTATAAATCCA [24414]
cp32-4_B31_NX_Pl_consensus     GTTTATGAGATAAATGCAGCGTATTATAGATTTATAGGACTTGGATATATTAAGAGCAATTGTTTTATTATAAATCCA [24414]
cp32-4_PAli_NX_MP_FG_consensus GTTTATGAGATAAATGCAGCGTATTATAGATTTATAGGACTTGGATATATTAAGAGCAATTGTTTTATTATAAATCCA [24414]
cp32-4_PAli_NX_Pl_consensus    GTTTATGAGATAAATGCAGCGTATTATAGATTTATAGGACTTGGATATATTAAGAGCAATTGTTTTATTATAAATCCA [24414]
cp32-4_PAbe_NX_MP_FG_consensus GTTTATGAGATAAATGCAGCGTATTATAGATTTATAGGACTTGGATATATTAAGAGCAATTGTTTTATTATAAATCCA [24414]
cp32-4_PAbe_NX_Pl_consensus    GTTTATGAGATAAATGCAGCGTATTATAGATTTATAGGACTTGGATATATTAAGAGCAATTGTTTTATTATAAATCCA [24414]

cp32-4_gi|11497149|B31_GB      TGTATGATACTTAATTACTACGGAATTAGAAGTAGTGTGAGATATGAGTCTTTAAATTATTTAGGTGCAGCCAATGAA [24492]
cp32-4_un10_B31_PacBio         TGTATGATACTTAATTACTACGGAATTAGAAGTAGTGTGAGATATGAGTCTTTAAATTATTTAGGTGCAGCCAATGAA [24492]
cp32-4_B31_TS_MP_FG_consensus  TGTATGATACTTAATTACTACGGAATTAGAAGTAGTGTGAGATATGAGTCTTTAAATTATTTAGGTGCAGCCAATGAA [24492]
cp32-4_B31_NX_Pl_consensus     TGTATGATACTTAATTACTACGGAATTAGGAGTAGCGTGAGATATGAGTCTTTAAATTATTTAGGTGCAGCAAATGAA [24492]
cp32-4_PAli_NX_MP_FG_consensus TGTATGATACTTAATTACTACGGAATTAGAAGTAGTGTGAGATATGAGTCTTTAAATTATTTAGGTGCAGCAAATGAA [24492]
cp32-4_PAli_NX_Pl_consensus    TGTATGATACTTAATTACTACGGAATTAGAAGTAGTGTGAGATATGAGTCTTTAAATTATTTAGGTGCAGCCAATGAA [24492]
cp32-4_PAbe_NX_MP_FG_consensus TGTATGATACTTAATTACTACGGAATTAGAAGTAGTGTGAGATATGAGTCTTTAAATTATTTAGGTGCAGCCAATGAA [24492]
cp32-4_PAbe_NX_Pl_consensus    TGTATGATACTTAATTACTACGGAATTAGAAGTAGCGTGAGATATGAGTCTTTAAATTATTTAGGTGCAGCAAATGAA [24492]
```

```
cp32-4_gi|11497149|B31_GB      TTTGAAATAAGCGAAGTTAAAATCGATAAGGTTAATGGATATCACTTTATAGCAACAAAAAATAAAGAAATATTATAT [24570]
cp32-4_un10_B31_PacBio         TTTGAAATAAGCGAAGTTAAAATCGATAAGGTTAATGGATATCACTTTATAGCAACAAAAAATAAAGAAATATTATAT [24570]
cp32-4_B31_TS_MP_FG_consensus  TTTGAAATAAGCGAAGTTAAAATCGATAAGGTTAATGGATATCACTTTATAGCAACAAAAAATAAAGAAATATTATAT [24570]
cp32-4_B31_NX_Pl_consensus     TTTGAAATAAGCGAAGTTAAAATCGATAAGGTTAATGGGTATCACTTTATAGCAACAAAAAATAAAGAAATATTATAT [24570]
cp32-4_PAli_NX_MP_FG_consensus TTTGAAATAAGCGAAGTTAAAATCGATAAGGTTAATGGGTATCACTTTATAGCAACAAAAAATAAAGAAATATTATAT [24570]
cp32-4_PAli_NX_Pl_consensus    TTTGAAATAAGCGAAGTTAAAATCGATAAGGTTAATGGATATCACTTTATAGCAACAAAAAATAAAGAAATATTATAT [24570]
cp32-4_PAbe_NX_MP_FG_consensus TTTGAAATAAGCGAAGTTAAAATCGATAAGGTTAATGGATATCACTTTATAGCAACAAAAAATAAAGAAATATTATAT [24570]
cp32-4_PAbe_NX_Pl_consensus    TTTGAAATAAGCGAAGTTAAAATCGATAAGGTTAATGGGTATCACTTTATAGCAACAAAAAATAAAGAAATATTATAT [24570]

cp32-4_gi|11497149|B31_GB      GATTCACTTGACTTGAAACCTCGTGGAAAAATATTTAAAGTAACTTCAAAGCGTATATTTAAACTGAAATAGTTTTAC [24648]
cp32-4_un10_B31_PacBio         GATTCACTTGACTTGAAACCTCGTGGAAAAATATTTAAAGTAACTTCAAAGCGTATATTTAAACTGAAATAGTTTTAC [24648]
cp32-4_B31_TS_MP_FG_consensus  GATTCACTTGACTTGAAACCTCGTGGAAAAATATTTAAAGTAACTTCAAAGCGTATATTTAAACTGAAATAGTTTTAC [24648]
cp32-4_B31_NX_Pl_consensus     GATTCACTTGACTTGAAACCTCGTGGAAAAATATTTAAAGTAACTTCAAAGCGTATATTTAAACTGAAATAGTTTTAC [24648]
cp32-4_PAli_NX_MP_FG_consensus GATTCACTTGATTTAAAACCTCGTGGAAAAATATTTAAAGTAACTTCAAAGCGTATATTTAAACTGAAATAGTTTTAC [24648]
cp32-4_PAli_NX_Pl_consensus    GATTCACTTGACTTGAAACCTCGTGGAAAAATATTTAAAGTAACTTCAAAGCGTATATTTAAACTGAAATAGTTTTAC [24648]
cp32-4_PAbe_NX_MP_FG_consensus GATTCACTTGACTTGAAACCTCGTGGAAAAATATTTAAAGTAACTTCAAAGCGTATATTTAAACTGAAATAGTTTTAC [24648]
cp32-4_PAbe_NX_Pl_consensus    GATTCACTTGACTTGAAACCTCGTGGAAAAATATTTAAAGTAACTTCAAAGCGTATATTTAAACTGAAATAGTTTTAC [24648]

cp32-4_gi|11497149|B31_GB      TAAAGTTTAAGGCACTTTTTAGCACATTCATAAGCTTGAATTTTATTAGCAGAAGAGAGTCCGTAGATATTATCAATT [24726]
cp32-4_un10_B31_PacBio         TAAAGTTTAAGGCACTTTTTAGCACATTCATAAGCTTGAATTTTATTAGCAGAAGAGAGTCCGTAGATATTATCAATT [24726]
cp32-4_B31_TS_MP_FG_consensus  TAAAGTTTAAGGCACTTTTTAGCACATTCATAAGCTTGAATTTTATTAGCAGAAGAGAGTCCGTAGATATTATCAATT [24726]
cp32-4_B31_NX_Pl_consensus     TAAAGTTTAAGGCACTTTTTAGCACATTCATAAGCTTGAATTTTATTAGCAGAAGAGAGTCCGTAGATATTATCAATT [24726]
cp32-4_PAli_NX_MP_FG_consensus TAAAGTTTAAGGCACTTTTTAGCACATTCATAAGCTTGAATTTTATTAGCAGAAGAGAGTCCGTAGATATTATCAATT [24726]
cp32-4_PAli_NX_Pl_consensus    TAAAGTTTAAGGCACTTTTTAGCACATTCATAAGCTTGAATTTTATTAGCAGAAGAGAGTCCGTAGATATTATCAATT [24726]
cp32-4_PAbe_NX_MP_FG_consensus TAAAGTTTAAGGCACTTTTTAGCACATTCATAAGCTTGAATTTTATTAGCAGAAGAGAGTCCGTAGATATTATCAATT [24726]
cp32-4_PAbe_NX_Pl_consensus    TAAAGTTTAAGGCACTTTTTAGCACATTCATAAGCTTGAATTTTATTAGCAGAAGAGAGTCCGTAGATATTATCAATT [24726]

cp32-4_gi|11497149|B31_GB      TCATTGGTTGAAGAATATTTCATAAGTTCTTTAATTTGAAAAGAATTGTAGCCGTTAGATTTCAAATTTGAAATAAAT [24804]
cp32-4_un10_B31_PacBio         TCATTGGTTGAAGAATATTTCATAAGTTCTTTAATTTGAAAAGAATTGTAGCCGTTAGATTTCAAATTTGAAATAAAT [24804]
cp32-4_B31_TS_MP_FG_consensus  TCATTGGTTGAAGAATATTTCATAAGTTCTTTAATTTGAAAAGAATTGTAGCCGTTAGATTTCAAATTTGAAATAAAT [24804]
cp32-4_B31_NX_Pl_consensus     TCATTGGTTGAAGAATATTTCATAAGTTCTTTAATTTGAAAAGAATTGTAGCCGTTAGATTTCAAATTTGAAATAAAT [24804]
cp32-4_PAli_NX_MP_FG_consensus TCATTGGTTGAAGAATATTTCATAAGTTCTTTAATTTGAAAAGAATTGTAGCCGTTAGATTTCAAATTTGAAATAAAT [24804]
cp32-4_PAli_NX_Pl_consensus    TCATTGGTTGAAGAATATTTCATAAGTTCTTTAATTTGAAAAGAATTGTAGCCGTTAGATTTCAAATTTGAAATAAAT [24804]
cp32-4_PAbe_NX_MP_FG_consensus TCATTGGTTGAAGAATATTTCATAAGTTCTTTAATTTGAAAAGAATTGTAGCCGTTAGATTTCAAATTTGAAATAAAT [24804]
cp32-4_PAbe_NX_Pl_consensus    TCATTGGTTGAAGAATATTTCATAAGTTCTTTAATTTGAAAAGAATTGTAGCCGTTAGATTTCAAATTTGAAATAAAT [24804]

cp32-4_gi|11497149|B31_GB      AAATTTCGACATAGATGGAGAGATTTATTTACACGAAATCCCGATTTTTTAAGAAGATTTTTGAATTTTTTAGAAATA [24882]
cp32-4_un10_B31_PacBio         AAATTTCGACATAGATGGAGAGATTTATTTACACGAAATCCCGATTTTTTAAGAAGATTTTTGAATTTTTTAGAAATA [24882]
cp32-4_B31_TS_MP_FG_consensus  AAATTTCGACATAGATGGAGAGATTTATTTACACGAAATCCCGATTTTTTAAGAAGATTTTTGAATTTTTTAGAAATA [24882]
cp32-4_B31_NX_Pl_consensus     AAATTTCGACATAGATGGAGAGATTTATTTACACGAAATCCCGATTTTTTAAGAAGATTTTTGAATTTTTTAGAAATA [24882]
cp32-4_PAli_NX_MP_FG_consensus AAATTTCGACATAGATGGAGAGATTTATTTACACGAAATCCCGATTTTTTAAGAAGATTTTTGAATTTTTTAGAAATA [24882]
cp32-4_PAli_NX_Pl_consensus    AAATTTCGACATAGATGGAGAGATTTATTTACACGAAATCCCGATTTTTTAAGAAGATTTTTGAATTTTTTAGAAATA [24882]
cp32-4_PAbe_NX_MP_FG_consensus AAATTTCGACATAGATGGAGAGATTTATTTACACGAAATCCCGATTTTTTAAGAAGATTTTTGAATTTTTTAGAAATA [24882]
cp32-4_PAbe_NX_Pl_consensus    AAATTTCGACATAGATGGAGAGATTTATTTACACGAAATCCCGATTTTTTAAGAAGATTTTTGAATTTTTTAGAAATA [24882]

cp32-4_gi|11497149|B31_GB      TGGACAATATCAATTTGGTTATCTTTAAACTTATGTTTGTTCTTTTGAAAAAGATAAGTACGTCGCGAGTCAAGAGTT [24960]
cp32-4_un10_B31_PacBio         TGGACAATATCAATTTGGTTATCTTTAAACTTATGTTTGTTCTTTTGAAAAAGATAAGTACGTCGCGAGTCAAGAGTT [24960]
cp32-4_B31_TS_MP_FG_consensus  TGGACAATATCAATTTGGTTATCTTTAAACTTATGTTTGTTCTTTTGAAAAAGATAAGTACGTCGCGAGTCAAGAGTT [24960]
cp32-4_B31_NX_Pl_consensus     TGGACAATATCAATTTGGTTATCTTTAAACTTATGTTTGTTCTTTTGAAAAAGATAAGTACGTCGCGAGTCAAGAGTT [24960]
cp32-4_PAli_NX_MP_FG_consensus TGGACAATATCAATTTGGTTATCTTTAAACTTATGTTTGTTCTTTTGAAAAAGATAAGTACGTCGCGAGTCAAGAGTT [24960]
cp32-4_PAli_NX_Pl_consensus    TGGACAATATCAATTTGGTTATCTTTAAACTTATGTTTGTTCTTTTGAAAAAGATAAGTACGTCGCGAGTCAAGAGTT [24960]
cp32-4_PAbe_NX_MP_FG_consensus TGGACAATATCAATTTGGTTATCTTTAAACTTATGTTTGTTCTTTTGAAAAAGATAAGTACGTCGCGAGTCAAGAGTT [24960]
cp32-4_PAbe_NX_Pl_consensus    TGGACAATATCAATTTGGTTATCTTTAAACTTATGTTTGTTCTTTTGAAAAAGATAAGTACGTCGCGAGTCAAGAGTT [24960]

cp32-4_gi|11497149|B31_GB      TTTTCTTGGAAATGATTTTTATGTGCTGTTTGGATAGCCTCGAACTCTTCTGAGTTGATGACAATTTCTCTAATACAA [25038]
cp32-4_un10_B31_PacBio         TTTTCTTGGAAATGATTTTTATGTGCTGTTTGGATAGCCTCGAACTCTTCTGAGTTGATGACAATTTCTCTAATACAA [25038]
cp32-4_B31_TS_MP_FG_consensus  TTTTCTTGGAAATGATTTTTATGTGCTGTTTGGATAGCCTCGAACTCTTCTGAGTTGATGACAATTTCTCTAATACAA [25038]
cp32-4_B31_NX_Pl_consensus     TTTTCTTGGAAATGATTTTTATGTGCTGTTTGGATAGCCTCGAACTCTTCTGAGTTGATGACAATTTCTCTAATACAA [25038]
cp32-4_PAli_NX_MP_FG_consensus TTTTCTTGGAAATGATTTTTATGTGCTGTTTGGATAGCCTCGAACTCTTCTGAGTTGATGACAATTTCTCTAATACAA [25038]
cp32-4_PAli_NX_Pl_consensus    TTTTCTTGGAAATGATTTTTATGTGCTGTTTGGATAGCCTCGAACTCTTCTGAGTTGATGACAATTTCTCTAATACAA [25038]
cp32-4_PAbe_NX_MP_FG_consensus TTTTCTTGGAAATGATTTTTATGTGCTGTTTGGATAGCCTCGAACTCTTCTGAGTTGATGACAATTTCTCTAATACAA [25038]
cp32-4_PAbe_NX_Pl_consensus    TTTTCTTGGAAATGATTTTTATGTGCTGTTTGGATAGCCTCGAACTCTTCTGAGTTGATGACAATTTCTCTAATACAA [25038]

cp32-4_gi|11497149|B31_GB      GAGGTATTTCTTTTTTTTGCCACATTTACTTTAATAGTATATAAAGTTTTTCCGGTTTTGCTTAAAAAAGTTGAAATA [25116]
cp32-4_un10_B31_PacBio         GAGGTATTTCTTTTTTTTGCCACATTTACTTTAATAGTATATAAAGTTTTTCCGGTTTTGCTTAAAAAAGTTGAAATA [25116]
cp32-4_B31_TS_MP_FG_consensus  GAGGTATTTCTTTTTTTTGCCACATTTACTTTAATAGTATATAAAGTTTTTCCGGTTTTGCTTAAAAAAGTTGAAATA [25116]
cp32-4_B31_NX_Pl_consensus     GAGGTATTTCTTTTTTTTGCCACATTTACTTTAATAGTATATAAAGTTTTTCCGGTTTTGCTTAAAAAAGTTGAAATA [25116]
cp32-4_PAli_NX_MP_FG_consensus GAGGTATTTCTTTTTTTTGCCACATTTACTTTAATAGTATATAAAGTTTTTCCGGTTTTGCTTAAAAAAGTTGAAATA [25116]
cp32-4_PAli_NX_Pl_consensus    GAGGTATTTCTTTTTTTTGCCACATTTACTTTAATAGTATATAAAGTTTTTCCGGTTTTGCTTAAAAAAGTTGAAATA [25116]
cp32-4_PAbe_NX_MP_FG_consensus GAGGTATTTCTTTTTTTTGCCACATTTACTTTAATAGTATATAAAGTTTTTCCGGTTTTGCTTAAAAAAGTTGAAATA [25116]
cp32-4_PAbe_NX_Pl_consensus    GAGGTATTTCTTTTTTTTGCCACATTTACTTTAATAGTATATAAAGTTTTTCCGGTTTTGCTTAAAAAAGTTGAAATA [25116]

cp32-4_gi|11497149|B31_GB      TCTTGCATTTTTACTTTTTGCATTTCAGTGCCTCTACATCCACTTATTAAGAGTAGATGCACAAACCAACCAGAAATT [25194]
cp32-4_un10_B31_PacBio         TCTTGCATTTTTACTTTTTGCATTTCAGTGCCTCTACATCCACTTATTAAGAGTAGATGCACAAACCAACCAGAAATT [25194]
cp32-4_B31_TS_MP_FG_consensus  TCTTGCATTTTTACTTTTTGCATTTCAGTGCCTCTACATCCACTTATTAAGAGTAGATGCACAAACCAACCAGAAATT [25194]
cp32-4_B31_NX_Pl_consensus     TCTTGCATTTTTACTTTTTGCATTTCAGTGCCTCTACATCCACTTATTAAGAGTAGATGCACAAACCAACCAGAAATT [25194]
cp32-4_PAli_NX_MP_FG_consensus TCTTGCATTTTTACTTTTTGCATTTCAGTGCCTCTACATCCACTTATTAAGAGTAGATGCACAAACCAACCAGAAATT [25194]
cp32-4_PAli_NX_Pl_consensus    TCTTGCATTTTTACTTTTTGCATTTCAGTGCCTCTACATCCACTTATTAAGAGTAGATGCACAAACCAACCAGAAATT [25194]
cp32-4_PAbe_NX_MP_FG_consensus TCTTGCATTTTTACTTTTTGCATTTCAGTGCCTCTACATCCACTTATTAAGAGTAGATGCACAAACCAACCAGAAATT [25194]
cp32-4_PAbe_NX_Pl_consensus    TCTTGCATTTTTACTTTTTGCATTTCAGTGCCTCTACATCCACTTATTAAGAGTAGATGCACAAACCAACCAGAAATT [25194]

cp32-4_gi|11497149|B31_GB      GGGTCAATTTGTTTTAGGGTTTTGGCACATTTTAGAATTAATTTAATACTTTTAGGGGTCAAATAAAACTTAGGAGTT [25272]
cp32-4_un10_B31_PacBio         GGGTCAATTTGTTTTAGGGTTTTGGCACATTTTAGAATTAATTTAATACTTTTAGGGGTCAAATAAAACTTAGGAGTT [25272]
cp32-4_B31_TS_MP_FG_consensus  GGGTCAATTTGTTTTAGGGTTTTGGCACATTTTAGAATTAATTTAATACTTTTAGGGGTCAAATAAAACTTAGGAGTT [25272]
cp32-4_B31_NX_Pl_consensus     GGGTCAATTTGTTTTAGGGTTTTGGCACATTTTAGAATTAATTTAATACTTTTAGGGGTCAAATAAAACTTAGGAGTT [25272]
cp32-4_PAli_NX_MP_FG_consensus GGGTCAATTTGTTTTAGGGTTTTGGCACATTTTAGAATTAATTTAATACTTTTAGGGGTCAAATAAAACTTAGGAGTT [25272]
cp32-4_PAli_NX_Pl_consensus    GGGTCAATTTGTTTTAGGGTTTTGGCACATTTTAGAATTAATTTAATACTTTTAGGGGTCAAATAAAACTTAGGAGTT [25272]
cp32-4_PAbe_NX_MP_FG_consensus GGGTCAATTTGTTTTAGGGTTTTGGCACATTTTAGAATTAATTTAATACTTTTAGGGGTCAAATAAAACTTAGGAGTT [25272]
cp32-4_PAbe_NX_Pl_consensus    GGGTCAATTTGTTTTAGGGTTTTGGCACATTTTAGAATTAATTTAATACTTTTAGGGGTCAAATAAAACTTAGGAGTT [25272]

cp32-4_gi|11497149|B31_GB      GGTTTTGAATTTTTTTTCTTACGTTTAGGTGAATTTTCTAGAGATTTAAGAATTTTATTTTCATTTTCTAGAATTTTG [25350]
cp32-4_un10_B31_PacBio         GGTTTTGAATTTTTTTTCTTACGTTTAGGTGAATTTTCTAGAGATTTAAGAATTTTATTTTCATTTTCTAGAATTTTG [25350]
cp32-4_B31_TS_MP_FG_consensus  GGTTTTGAATTTTTTTTCTTACGTTTAGGTGAATTTTCTAGAGATTTAAGAATTTTATTTTCATTTTCTAGAATTTTG [25350]
cp32-4_B31_NX_Pl_consensus     GGTTTTGAATTTTTTTTCTTACGTTTAGGTGAATTTTCTAGAGATTTAAGAATTTTATTTTCATTTTCTAGAATTTTG [25350]
cp32-4_PAli_NX_MP_FG_consensus GGTTTTGAATTTTTTTTCTTACGTTTAGGTGAATTTTCTAGAGATTTAAGAATTTTATTTTCATTTTCTAGAATTTTG [25350]
cp32-4_PAli_NX_Pl_consensus    GGTTTTGAATTTTTTTTCTTACGTTTAGGTGAATTTTCTAGAGATTTAAGAATTTTATTTTCATTTTCTAGAATTTTG [25350]
cp32-4_PAbe_NX_MP_FG_consensus GGTTTTGAATTTTTTTTCTTACGTTTAGGTGAATTTTCTAGAGATTTAAGAATTTTATTTTCATTTTCTAGAATTTTG [25350]
cp32-4_PAbe_NX_Pl_consensus    GGTTTTGAATTTTTTTTCTTACGTTTAGGTGAATTTTCTAGAGATTTAAGAATTTTATTTTCATTTTCTAGAATTTTG [25350]

cp32-4_gi|11497149|B31_GB      TTTTTATTTAATGCATTTTGGTATTCTTGAAATAGTTTGAGCGTAAAATCCATATTGAAATTATTTAAATTAAAAGAA [25428]
cp32-4_un10_B31_PacBio         TTTTTATTTAATGCATTTTGGTATTCTTGAAATAGTTTGAGCGTAAAATCCATATTGAAATTATTTAAATTAAAAGAA [25428]
cp32-4_B31_TS_MP_FG_consensus  TTTTTATTTAATGCATTTTGGTATTCTTGAAATAGTTTGAGCGTAAAATCCATATTGAAATTATTTAAATTAAAAGAA [25428]
cp32-4_B31_NX_Pl_consensus     TTTTTATTTAATGCATTTTGGTATTCTTGAAATAGTTTGAGCGTAAAATCCATATTGAAATTATTTAAATTAAAAGAA [25428]
cp32-4_PAli_NX_MP_FG_consensus TTTTTATTTAATGCATTTTGGTATTCTTGAAATAGTTTGAGCGTAAAATCCATATTGAAATTATTTAAATTAAAAGAA [25428]
cp32-4_PAli_NX_Pl_consensus    TTTTTATTTAATGCATTTTGGTATTCTTGAAATAGTTTGAGCGTAAAATCCATATTGAAATTATTTAAATTAAAAGAA [25428]
cp32-4_PAbe_NX_MP_FG_consensus TTTTTATTTAATGCATTTTGGTATTCTTGAAATAGTTTGAGCGTAAAATCCATATTGAAATTATTTAAATTAAAAGAA [25428]
cp32-4_PAbe_NX_Pl_consensus    TTTTTATTTAATGCATTTTGGTATTCTTGAAATAGTTTGAGCGTAAAATCCATATTGAAATTATTTAAATTAAAAGAA [25428]

cp32-4_gi|11497149|B31_GB      TTATTAGTATCCATAAGACAAGCTCCTTATAAGTGTTACTTTTAAATTAAGTAAAAGTAATAAAAATAGATGAAAAAT [25506]
```

```
cp32-4_un10_B31_PacBio         TTATTAGTATCCATAAGACAAGCTCCTTATAAGTGTTACTTTTAAATTAAGTAAAAGTAATAAAAATAGATGAAAAAT [25506]
cp32-4_B31_TS_MP_FG_consensus  TTATTAGTATCCATAAGACAAGCTCCTTATAAGTGTTACTTTTAAATTAAGTAAAAGTAATAAAAATAGATGAAAAAT [25506]
cp32-4_B31_NX_Pl_consensus     TTATTAGTATCCATAAGACAAGCTCCTTATAAGTGTTACTTTTAAATTAAGTAAAAGTAATAAAAATAGATGAAAAAT [25506]
cp32-4_PAli_NX_MP_FG_consensus TTATTAGTATCCATAAGACAAGCTCCTTATAAGTGTTACTTTTAAATTAAGTAAAAGTAATAAAAATAGATGAAAAAT [25506]
cp32-4_PAli_NX_Pl_consensus    TTATTAGTATCCATAAGACAAGCTCCTTATAAGTGTTACTTTTAAATTAAGTAAAAGTAATAAAAATAGATGAAAAAT [25506]
cp32-4_PAbe_NX_MP_FG_consensus TTATTAGTATCCATAAGACAAGCTCCTTATAAGTGTTACTTTTAAATTAAGTAAAAGTAATAAAAATAGATGAAAAAT [25506]
cp32-4_PAbe_NX_Pl_consensus    TTATTAGTATCCATAAGACAAGCTCCTTATAAGTGTTACTTTTAAATTAAGTAAAAGTAATAAAAATAGATGAAAAAT [25506]

cp32-4_gi|11497149|B31_GB      GTAATTTATATTGTACCAAAAACTAAAAATTTTAGTCAAATTTTGTGAGTTCTCATTGCATGCAAAATCTGGGCTGTA [25584]
cp32-4_un10_B31_PacBio         GTAATTTATATTGTACCAAAAACTAAAAATTTTAGTCAAATTTTGTGAGTTCTCATTGCATGCAAAATCTGGGCTGTA [25584]
cp32-4_B31_TS_MP_FG_consensus  GTAATTTATATTGTACCAAAAACTAAAAATTTTAGTCAAATTTTGTGAGTTCTCATTGCATGCAAAATCTGGGCTGTA [25584]
cp32-4_B31_NX_Pl_consensus     GTAATTTATATTGTACCAAAAACTAAAAATTTTAGTCAAATTTTGTGAGTTCTCATTGCATGCAAAATCTGGGCTGTA [25584]
cp32-4_PAli_NX_MP_FG_consensus GTAATTTATATTGTACCAAAAACTAAAAATTTTAGTCAAATTTTGTGAGTTCTCATTGCATGCAAAATCTGGGCTGTA [25584]
cp32-4_PAli_NX_Pl_consensus    GTAATTTATATTGTACCAAAAACTAAAAATTTTAGTCAAATTTTGTGAGTTCTCATTGCATGCAAAATCTGGGCTGTA [25584]
cp32-4_PAbe_NX_MP_FG_consensus GTAATTTATATTGTACCAAAAACTAAAAATTTTAGTCAAATTTTGTGAGTTCTCATTGCATGCAAAATCTGGGCTGTA [25584]
cp32-4_PAbe_NX_Pl_consensus    GTAATTTATATTGTACCAAAAACTAAAAATTTTAGTCAAATTTTGTGAGTTCTCATTGCATGCAAAATCTGGGCTGTA [25584]

cp32-4_gi|11497149|B31_GB      GAGCAGGTGGGATAAACAGAAGAGGCAATTTTTAAGGGTGGTACACAAGAAAGATACAATACTTTGTGTAATATATAG [25662]
cp32-4_un10_B31_PacBio         GAGCAGGTGGGATAAACAGAAGAGGCAATTTTTAAGGGTGGTACACAAGAAAGATACAATACTTTGTGTAATATATAG [25662]
cp32-4_B31_TS_MP_FG_consensus  GAGCAGGTGGGATAAACAGAAGAGGCAATTTTTAAGGGTGGTACACAAGAAAGATACAATACTTTGTGTAATATATAG [25662]
cp32-4_B31_NX_Pl_consensus     GAGCAGGTGGGATAAACAGAAGAGGCAATTTTTAAGGGTGGTACACAAGAAAGATACAATACTTTGTGTAATATATAG [25662]
cp32-4_PAli_NX_MP_FG_consensus GAGCAGGTGGGATAAACAGAAGAGGCAATTTTTAAGGGTGGTACACAAGAAAGATACAATACTTTGTGTAATATATAG [25662]
cp32-4_PAli_NX_Pl_consensus    GAGCAGGTGGGATAAACAGAAGAGGCAATTTTTAAGGGTGGTACACAAGAAAGATACAATACTTTGTGTAATATATAG [25662]
cp32-4_PAbe_NX_MP_FG_consensus GAGCAGGTGGGATAAACAGAAGAGGCAATTTTTAAGGGTGGTACACAAGAAAGATACAATACTTTGTGTAATATATAG [25662]
cp32-4_PAbe_NX_Pl_consensus    GAGCAGGTGGGATAAACAGAAGAGGCAATTTTTAAGGGTGGTACACAAGAAAGATACAATACTTTGTGTAATATATAG [25662]

cp32-4_gi|11497149|B31_GB      CAAAGACTTTGAAATTTAATTTGTATGTGTTTTGTAGTCTCTTATAATGAGTAGTGCATTTGCAATGGAGAGATTTTA [25740]
cp32-4_un10_B31_PacBio         CAAAGACTTTGAAATTTAATTTGTATGTGTTTTGTAGTCTCTTATAATGAGTAGTGCATTTGCAATGGAGAGATTTTA [25740]
cp32-4_B31_TS_MP_FG_consensus  CAAAGACTTTGAAATTTAATTTGTATGTGTTTTGTAGTCTCTTATAATGAGTAGTGCATTTGCAATGGAGAGATTTTA [25740]
cp32-4_B31_NX_Pl_consensus     CAAAGACTTTGAAATTTAATTTGTATGTGTTTTGTAGTCTCTTATAATGAGTAGTGCATTTGCAATGGAGAGATTTTA [25740]
cp32-4_PAli_NX_MP_FG_consensus CAAAGACTTTGAAATTTAATTTGTATGTGTTTTGTAGTCTCTTATAATGAGTAGTGCATTTGCAATGGAGAGATTTTA [25740]
cp32-4_PAli_NX_Pl_consensus    CAAAGACTTTGAAATTTAATTTGTATGTGTTTTGTAGTCTCTTATAATGAGTAGTGCATTTGCAATGGAGAGATTTTA [25740]
cp32-4_PAbe_NX_MP_FG_consensus CAAAGACTTTGAAATTTAATTTGTATGTGTTTTGTAGTCTCTTATAATGAGTAGTGCATTTGCAATGGAGAGATTTTA [25740]
cp32-4_PAbe_NX_Pl_consensus    CAAAGACTTTGAAATTTAATTTGTATGTGTTTTGTAGTCTCTTATAATGAGTAGTGCATTTGCAATGGAGAGATTTTA [25740]

cp32-4_gi|11497149|B31_GB      TGAGTTTGATTAAAATTACATTTTAGTTTTGTAACAATGTAATTCGAAATGTAACAAAATTATATATTTAAATCTTTG [25818]
cp32-4_un10_B31_PacBio         TGAGTTTGATTAAAATTACATTTTAGTTTTGTAACAATGTAATTCGAAATGTAACAAAATTATATATTTAAATCTTTG [25818]
cp32-4_B31_TS_MP_FG_consensus  TGAGTTTGATTAAAATTACATTTTAGTTTTGTAACAATGTAATTCGAAATGTAACAAAATTATATATTTAAATCTTTG [25818]
cp32-4_B31_NX_Pl_consensus     TGAGTTTGATTAAAATTACATTTTAGTTTTGTAACAATGTAATTCGAAATGTAACAAAATTATATATTTAAATCTTTG [25818]
cp32-4_PAli_NX_MP_FG_consensus TGAGTTTGATTAAAATTACATTTTAGTTTTGTAACAATGTAATTCGAAATGTAACAAAATTATATATTTAAATCTTTG [25818]
cp32-4_PAli_NX_Pl_consensus    TGAGTTTGATTAAAATTACATTTTAGTTTTGTAACAATGTAATTCGAAATGTAACAAAATTATATATTTAAATCTTTG [25818]
cp32-4_PAbe_NX_MP_FG_consensus TGAGTTTGATTAAAATTACATTTTAGTTTTGTAACAATGTAATTCGAAATGTAACAAAATTATATATTTAAATCTTTG [25818]
cp32-4_PAbe_NX_Pl_consensus    TGAGTTTGATTAAAATTACATTTTAGTTTTGTAACAATGTAATTCGAAATGTAACAAAATTATATATTTAAATCTTTG [25818]

cp32-4_gi|11497149|B31_GB      AAATATTGCAATTATTAGCTGTTGTGGTAATATTGGGACTTATGGAGTAACTTATGAATAAGAAAATGAAAAATTTAA [25896]
cp32-4_un10_B31_PacBio         AAATATTGCAATTATTAGCTGTTGTGGTAATATTGGGACTTATGGAGTAACTTATGAATAAGAAAATGAAAAATTTAA [25896]
cp32-4_B31_TS_MP_FG_consensus  AAATATTGCAATTATTAGCTGTTGTGGTAATATTGGGACTTATGGAGTAACTTATGAATAAGAAAATGAAAAATTTAA [25896]
cp32-4_B31_NX_Pl_consensus     AAATATTGCAATTATTAGCTGTTGTGGTAATATTGGGACTTATGGAGTAACTTATGAATAAGAAAATGAAAAATTTAA [25896]
cp32-4_PAli_NX_MP_FG_consensus AAATATTGCAATTATTAGCTGTTGTGGTAATATTGGGACTTATGGAGTAACTTATGAATAAGAAAATGAAAAATTTAA [25896]
cp32-4_PAli_NX_Pl_consensus    AAATATTGCAATTATTAGCTGTTGTGGTAATATTGGGACTTATGGAGTAACTTATGAATAAGAAAATGAAAAATTTAA [25896]
cp32-4_PAbe_NX_MP_FG_consensus AAATATTGCAATTATTAGCTGTTGTGGTAATATTGGGACTTATGGAGTAACTTATGAATAAGAAAATGAAAAATTTAA [25896]
cp32-4_PAbe_NX_Pl_consensus    AAATATTGCAATTATTAGCTGTTGTGGTAATATTGGGACTTATGGAGTAACTTATGAATAAGAAAATGAAAAATTTAA [25896]

cp32-4_gi|11497149|B31_GB      TTATTTGTGCAGTTTTTGTTTTGATAATTTCTTGTAAAAATAATACTTTATCTTTATATGATGAGCAAAGTATTGGCT [25974]
cp32-4_un10_B31_PacBio         TTATTTGTGCAGTTTTTGTTTTGATAATTTCTTGTAAAAATAATACTTTATCTTTATATGATGAGCAAAGTATTGGCT [25974]
cp32-4_B31_TS_MP_FG_consensus  TTATTTGTGCAGTTTTTGTTTTGATAATTTCTTGTAAAAATAATACTTTATCTTTATATGATGAGCAAAGTATTGGCT [25974]
cp32-4_B31_NX_Pl_consensus     TTATTTGTGCAGTTTTTGTTTTGATAATTTCTTGTAAAAATAATACTTTATCTTTATATGATGAGCAAAGTATTGGCT [25974]
cp32-4_PAli_NX_MP_FG_consensus TTATTTGTGCAGTTTTTGTTTTGATAATTTCTTGTAAAAATAATACTTTATCTTTATATGATGAGCAAAGTATTGGCT [25974]
cp32-4_PAli_NX_Pl_consensus    TTATTTGTGCAGTTTTTGTTTTGATAATTTCTTGTAAAAATAATACTTTATCTTTATATGATGAGCAAAGTATTGGCT [25974]
cp32-4_PAbe_NX_MP_FG_consensus TTATTTGTGCAGTTTTTGTTTTGATAATTTCTTGTAAAAATAATACTTTATCTTTATATGATGAGCAAAGTATTGGCT [25974]
cp32-4_PAbe_NX_Pl_consensus    TTATTTGTGCAGTTTTTGTTTTGATAATTTCTTGTAAAAATAATACTTTATCTTTATATGATGAGCAAAGTATTGGCT [25974]

cp32-4_gi|11497149|B31_GB      AGGTAAAATTTTGAAAAATAATTAGAAGGGGAACCCCATGAACAATGTTTCAGAAAAAAAATCAAGAAATGCAAAATAA [26052]
cp32-4_un10_B31_PacBio         AGGTAAAATTTTGAAAAATAATTAGAAGGGGAACCCCATGAACAATGTTTCAGAAAAAAAATCAAGAAATGCAAAATAA [26052]
cp32-4_B31_TS_MP_FG_consensus  AGGTAAAATTTTGAAAAATAATTAGAAGGGGAACCCCATGAACAATGTTTCAGAAAAAAAATCAAGAAATGCAAAATAA [26052]
cp32-4_B31_NX_Pl_consensus     AGGTAAAATTTTGAAAAATAATTAGAAGGGGAACCCCATGAACAATGTTTCAGAAAAAAAATCAAGAAATGCAAAATAA [26052]
cp32-4_PAli_NX_MP_FG_consensus AGGTAAAATTTTGAAAAATAATTAGAAGGGGAACCCCATGAACAATGTTTCAGAAAAAAAATCAAGAAATGCAAAATAA [26052]
cp32-4_PAli_NX_Pl_consensus    AGGTAAAATTTTGAAAAATAATTAGAAGGGGAACCCCATGAACAATGTTTCAGAAAAAAAATCAAGAAATGCAAAATAA [26052]
cp32-4_PAbe_NX_MP_FG_consensus AGGTAAAATTTTGAAAAATAATTAGAAGGGGAACCCCATGAACAATGTTTCAGAAAAAAAATCAAGAAATGCAAAATAA [26052]
cp32-4_PAbe_NX_Pl_consensus    AGGTAAAATTTTGAAAAATAATTAGAAGGGGAACCCCATGAACAATGTTTCAGAAAAAAAATCAAGAAATGCAAAATAA [26052]

cp32-4_gi|11497149|B31_GB      TATTCAAGCAAAAATAAGCTTCAGAAAAGATATGAAAACCCTAAGAATGAATTTATCAGGGATTAATAAGAATTCTAA [26130]
cp32-4_un10_B31_PacBio         TATTCAAGCAAAAATAAGCTTCAGAAAAGATATGAAAACCCTAAGAATGAATTTATCAGGGATTAATAAGAATTCTAA [26130]
cp32-4_B31_TS_MP_FG_consensus  TATTCAAGCAAAAATAAGCTTCAGAAAAGATATGAAAACCCTAAGAATGAATTTATCAGGGATTAATAAGAATTCTAA [26130]
cp32-4_B31_NX_Pl_consensus     TATTCAAGCAAAAATAAGCTTCAGAAAAGATATGAAAACCCTAAGAATGAATTTATCAGGGATTAATAAGAATTCTAA [26130]
cp32-4_PAli_NX_MP_FG_consensus TATTCAAGCAAAAATAAGCTTCAGAAAAGATATGAAAACCCTAAGAATGAATTTATCAGGGATTAATAAGAATTCTAA [26130]
cp32-4_PAli_NX_Pl_consensus    TATTCAAGCAAAAATAAGCTTCAGAAAAGATATGAAAACCCTAAGAATGAATTTATCAGGGATTAATAAGAATTCTAA [26130]
cp32-4_PAbe_NX_MP_FG_consensus TATTCAAGCAAAAATAAGCTTCAGAAAAGATATGAAAACCCTAAGAATGAATTTATCAGGGATTAATAAGAATTCTAA [26130]
cp32-4_PAbe_NX_Pl_consensus    TATTCAAGCAAAAATAAGCTTCAGAAAAGATATGAAAACCCTAAGAATGAATTTATCAGGGATTAATAAGAATTCTAA [26130]

cp32-4_gi|11497149|B31_GB      GGGGTACGAGTATAATTATCGAAGTTTAGATGACATAGTTAGAGAAATTAAAAACGTTATTGATAAGCATAATTTGGA [26208]
cp32-4_un10_B31_PacBio         GGGGTACGAGTATAATTATCGAAGTTTAGATGACATAGTTAGAGAAATTAAAAACGTTATTGATAAGCATAATTTGGA [26208]
cp32-4_B31_TS_MP_FG_consensus  GGGGTACGAGTATAATTATCGAAGTTTAGATGACATAGTTAGAGAAATTAAAAACGTTATTGATAAGCATAATTTGGA [26208]
cp32-4_B31_NX_Pl_consensus     GGGGTACGAGTATAATTATCGAAGTTTAGATGACATAGTTAGAGAAATTAAAAACGTTATTGATAAGCATAATTTGGA [26208]
cp32-4_PAli_NX_MP_FG_consensus GGGGTACGAGTATAATTATCGAAGTTTAGATGACATAGTTAGAGAAATTAAAAACGTTATTGATAAGCATAATTTGGA [26208]
cp32-4_PAli_NX_Pl_consensus    GGGGTACGAGTATAATTATCGAAGTTTAGATGACATAGTTAGAGAAATTAAAAACGTTATTGATAAGCATAATTTGGA [26208]
cp32-4_PAbe_NX_MP_FG_consensus GGGGTACGAGTATAATTATCGAAGTTTAGATGACATAGTTAGAGAAATTAAAAACGTTATTGATAAGCATAATTTGGA [26208]
cp32-4_PAbe_NX_Pl_consensus    GGGGTACGAGTATAATTATCGAAGTTTAGATGACATAGTTAGAGAAATTAAAAACGTTATTGATAAGCATAATTTGGA [26208]

cp32-4_gi|11497149|B31_GB      TCTTTTTTTTACGCAAGATCCAATTTCTGTAGAGGGGCAATATGGCATAGTTGATTATATTAGGACGACATTCTACAG [26286]
cp32-4_un10_B31_PacBio         TCTTTTTTTTACGCAAGATCCAATTTCTGTAGAGGGGCAATATGGCATAGTTGATTATATTAGGACGACATTCTACAG [26286]
cp32-4_B31_TS_MP_FG_consensus  TCTTTTTTTTACGCAAGATCCAATTTCTGTAGAGGGGCAATATGGCATAGTTGATTATATTAGGACGACATTCTACAG [26286]
cp32-4_B31_NX_Pl_consensus     TCTTTTTTTTACGCAAGATCCAATTTCTGTAGAGGGGCAATATGGCATAGTTGATTATATTAGGACGACATTCTACAG [26286]
cp32-4_PAli_NX_MP_FG_consensus TCTTTTTTTTACGCAAGATCCAATTTCTGTAGAGGGGCAATATGGCATAGTTGATTATATTAGGACGACATTCTACAG [26286]
cp32-4_PAli_NX_Pl_consensus    TCTTTTTTTTACGCAAGATCCAATTTCTGTAGAGGGGCAATATGGCATAGTTGATTATATTAGGACGACATTCTACAG [26286]
cp32-4_PAbe_NX_MP_FG_consensus TCTTTTTTTTACGCAAGATCCAATTTCTGTAGAGGGGCAATATGGCATAGTTGATTATATTAGGACGACATTCTACAG [26286]
cp32-4_PAbe_NX_Pl_consensus    TCTTTTTTTTACGCAAGATCCAATTTCTGTAGAGGGGCAATATGGCATAGTTGATTATATTAGGACGACATTCTACAG [26286]

cp32-4_gi|11497149|B31_GB      TACAAGCACTGTGTACAAATACTCATTTGATACGCCAACTTTTACAGAAAAATTACAATGGACCAATGAAAACAGCTC [26364]
cp32-4_un10_B31_PacBio         TACAAGCACTGTGTACAAATACTCATTTGATACGCCAACTTTTACAGAAAAATTACAATGGACCAATGAAAACAGCTC [26364]
cp32-4_B31_TS_MP_FG_consensus  TACAAGCACTGTGTACAAATACTCATTTGATACGCCAACTTTTACAGAAAAATTACAATGGACCAATGAAAACAGCTC [26364]
cp32-4_B31_NX_Pl_consensus     TACAAGCACTGTGTACAAATACTCATTTGATACGCCAACTTTTACAGAAAAATTACAATGGACCAATGAAAACAGCTC [26364]
cp32-4_PAli_NX_MP_FG_consensus TACAAGCACTGTGTACAAATACTCATTTGATACGCCAACTTTTACAGAAAAATTACAATGGACCAATGAAAACAGCTC [26364]
cp32-4_PAli_NX_Pl_consensus    TACAAGCACTGTGTACAAATACTCATTTGATACGCCAACTTTTACAGAAAAATTACAATGGACCAATGAAAACAGCTC [26364]
cp32-4_PAbe_NX_MP_FG_consensus TACAAGCACTGTGTACAAATACTCATTTGATACGCCAACTTTTACAGAAAAATTACAATGGACCAATGAAAACAGCTC [26364]
cp32-4_PAbe_NX_Pl_consensus    TACAAGCACTGTGTACAAATACTCATTTGATACGCCAACTTTTACAGAAAAATTACAATGGACCAATGAAAACAGCTC [26364]

cp32-4_gi|11497149|B31_GB      TAAAAATGGAAAGACCGTACCCCAAATGGTTGGAGCAGCTATTACTTATTTCAGAAGGTGCGCTTTAGTTGGATATCT [26442]
cp32-4_un10_B31_PacBio         TAAAAATGGAAAGACCGTACCCCAAATGGTTGGAGCAGCTATTACTTATTTCAGAAGGTGCGCTTTAGTTGGATATCT [26442]
```

```
cp32-4_B31_TS_MP_FG_consensus  TAAAAATGGAAAGACCGTACCCCAAATGGTTGGAGCAGCTATTACTTATTTCAGAAGGTGCGCTTTAGTTGGATATCT [26442]
cp32-4_B31_NX_Pl_consensus     TAAAAATGGAAAGACCGTACCCCAAATGGTTGGAGCAGCTATTACTTATTTCAGAAGGTGCGCTTTAGTTGGATATCT [26442]
cp32-4_PAli_NX_MP_FG_consensus TAAAAATGGAAAGACCGTACCCCAAATGGTTGGAGCAGCTATTACTTATTTCAGAAGGTGCGCTTTAGTTGGATATCT [26442]
cp32-4_PAli_NX_Pl_consensus    TAAAAATGGAAAGACCGTACCCCAAATGGTTGGAGCAGCTATTACTTATTTCAGAAGGTGCGCTTTAGTTGGATATCT [26442]
cp32-4_PAbe_NX_MP_FG_consensus TAAAAATGGAAAGACCGTACCCCAAATGGTTGGAGCAGCTATTACTTATTTCAGAAGGTGCGCTTTAGTTGGATATCT [26442]
cp32-4_PAbe_NX_Pl_consensus    TAAAAATGGAAAGACCGTACCCCAAATGGTTGGAGCAGCTATTACTTATTTCAGAAGGTGCGCTTTAGTTGGATATCT [26442]

cp32-4_gi|11497149|B31_GB      TCGCATAAAAACTGACGAATTTTTTGATTTTAATGTATATATTAAATATAAAAAAACAAATATCGGCAATTGGATAGA [26520]
cp32-4_un10_B31_PacBio         TCGCATAAAAACTGACGAATTTTTTGATTTTAATGTATATATTAAATATAAAAAAACAAATATCGGCAATTGGATAGA [26520]
cp32-4_B31_TS_MP_FG_consensus  TCGCATAAAAACTGACGAATTTTTTGATTTTAATGTATATATTAAATATAAAAAAACAAATATCGGCAATTGGATAGA [26520]
cp32-4_B31_NX_Pl_consensus     TCGCATAAAAACTGACGAATTTTTTGATTTTAATGTATATATTAAATATAAAAAAACAAATATCGGCAATTGGATAGA [26520]
cp32-4_PAli_NX_MP_FG_consensus TCGCATAAAAACTGACGAATTTTTTGATTTTAATGTATATATTAAATATAAAAAAACAAATATCGGCAATTGGATAGA [26520]
cp32-4_PAli_NX_Pl_consensus    TCGCATAAAAACTGACGAATTTTTTGATTTTAATGTATATATTAAATATAAAAAAACAAATATCGGCAATTGGATAGA [26520]
cp32-4_PAbe_NX_MP_FG_consensus TCGCATAAAAACTGACGAATTTTTTGATTTTAATGTATATATTAAATATAAAAAAACAAATATCGGCAATTGGATAGA [26520]
cp32-4_PAbe_NX_Pl_consensus    TCGCATAAAAACTGACGAATTTTTTGATTTTAATGTATATATTAAATATAAAAAAACAAATATCGGCAATTGGATAGA [26520]

cp32-4_gi|11497149|B31_GB      TTTAGGAACTTTAGATCTAATAAAAGAAGAATATAGTATTGTAACAGATTTAAATGTTGGGGGGCACACGGCTAGATT [26598]
cp32-4_un10_B31_PacBio         TTTAGGAACTTTAGATCTAATAAAAGAAGAATATAGTATTGTAACAGATTTAAATGTTGGGGGGCACACGGCTAGATT [26598]
cp32-4_B31_TS_MP_FG_consensus  TTTAGGAACTTTAGATCTAATAAAAGAAGAATATAGTATTGTAACAGATTTAAATGTTGGGGGGCACACGGCTAGATT [26598]
cp32-4_B31_NX_Pl_consensus     TTTAGGAACTTTAGATCTAATAAAAGAAGAATATAGTATTGTAACAGATTTAAATGTTGGGGGGCACACGGCTAGATT [26598]
cp32-4_PAli_NX_MP_FG_consensus TTTAGGAACTTTAGATCTAATAAAAGAAGAATATAGTATTGTAACAGATTTAAATGTTGGGGGGCACACGGCTAGATT [26598]
cp32-4_PAli_NX_Pl_consensus    TTTAGGAACTTTAGATCTAATAAAAGAAGAATATAGTATTGTAACAGATTTAAATGTTGGGGGGCACACGGCTAGATT [26598]
cp32-4_PAbe_NX_MP_FG_consensus TTTAGGAACTTTAGATCTAATAAAAGAAGAATATAGTATTGTAACAGATTTAAATGTTGGGGGGCACACGGCTAGATT [26598]
cp32-4_PAbe_NX_Pl_consensus    TTTAGGAACTTTAGATCTAATAAAAGAAGAATATAGTATTGTAACAGATTTAAATGTTGGGGGGCACACGGCTAGATT [26598]

cp32-4_gi|11497149|B31_GB      ATTTTCAGTAAAAGAGTCAGAAATTAACAACTTTGTAAATTCAATGATTAGAGGAGGAGCATTTAATGCGGATTATTA [26676]
cp32-4_un10_B31_PacBio         ATTTTCAGTAAAAGAGTCAGAAATTAACAACTTTGTAAATTCAATGATTAGAGGAGGAGCATTTAATGCGGATTATTA [26676]
cp32-4_B31_TS_MP_FG_consensus  ATTTTCAGTAAAAGAGTCAGAAATTAACAACTTTGTAAATTCAATGATTAGAGGAGGAGCATTTAATGCGGATTATTA [26676]
cp32-4_B31_NX_Pl_consensus     ATTTTCAGTAAAAGAGTCAGAAATTAACAACTTTGTAAATTCAATGATTAGAGGAGGAGCATTTAATGCGGATTATTA [26676]
cp32-4_PAli_NX_MP_FG_consensus ATTTTCAGTAAAAGAGTCAGAAATTAACAACTTTGTAAATTCAATGATTAGAGGAGGAGCATTTAATGCGGATTATTA [26676]
cp32-4_PAli_NX_Pl_consensus    ATTTTCAGTAAAAGAGTCAGAAATTAACAACTTTGTAAATTCAATGATTAGAGGAGGAGCATTTAATGCGGATTATTA [26676]
cp32-4_PAbe_NX_MP_FG_consensus ATTTTCAGTAAAAGAGTCAGAAATTAACAACTTTGTAAATTCAATGATTAGAGGAGGAGCATTTAATGCGGATTATTA [26676]
cp32-4_PAbe_NX_Pl_consensus    ATTTTCAGTAAAAGAGTCAGAAATTAACAACTTTGTAAATTCAATGATTAGAGGAGGAGCATTTAATGCGGATTATTA [26676]

cp32-4_gi|11497149|B31_GB      TGGTTATCAAGAAGGAGAAGATTATAAAATCAAAAACATAGAAACAAAAATAGAAACTATTAATGACTCTCAATTTAT [26754]
cp32-4_un10_B31_PacBio         TGGTTATCAAGAAGGAGAAGATTATAAAATCAAAAACATAGAAACAAAAATAGAAACTATTAATGACTCTCAATTTAT [26754]
cp32-4_B31_TS_MP_FG_consensus  TGGTTATCAAGAAGGAGAAGATTATAAAATCAAAAACATAGAAACAAAAATAGAAACTATTAATGACTCTCAATTTAT [26754]
cp32-4_B31_NX_Pl_consensus     TGGTTATCAAGAAGGAGAAGATTATAAAATCAAAAACATAGAAACAAAAATAGAAACTATTAATGACTCTCAATTTAT [26754]
cp32-4_PAli_NX_MP_FG_consensus TGGTTATCAAGAAGGAGAAGATTATAAAATCAAAAACATAGAAACAAAAATAGAAACTATTAATGACTCTCAATTTAT [26754]
cp32-4_PAli_NX_Pl_consensus    TGGTTATCAAGAAGGAGAAGATTATAAAATCAAAAACATAGAAACAAAAATAGAAACTATTAATGACTCTCAATTTAT [26754]
cp32-4_PAbe_NX_MP_FG_consensus TGGTTATCAAGAAGGAGAAGATTATAAAATCAAAAACATAGAAACAAAAATAGAAACTATTAATGACTCTCAATTTAT [26754]
cp32-4_PAbe_NX_Pl_consensus    TGGTTATCAAGAAGGAGAAGATTATAAAATCAAAAACATAGAAACAAAAATAGAAACTATTAATGACTCTCAATTTAT [26754]

cp32-4_gi|11497149|B31_GB      TACATTTTTAGGAAGAGATAATACATATGCAATTTTATTAGAGGAATTTAAGATGAATTTAAAAAGAATTTGAAATAA [26832]
cp32-4_un10_B31_PacBio         TACATTTTTAGGAAGAGATAATACATATGCAATTTTATTAGAGGAATTTAAGATGAATTTAAAAAGAATTTGAAATAA [26832]
cp32-4_B31_TS_MP_FG_consensus  TACATTTTTAGGAAGAGATAATACATATGCAATTTTATTAGAGGAATTTAAGATGAATTTAAAAAGAATTTGAAATAA [26832]
cp32-4_B31_NX_Pl_consensus     TACATTTTTAGGAAGAGATAATACATATGCAATTTTATTAGAGGAATTTAAGATGAATTTAAAAAGAATTTGAAATAA [26832]
cp32-4_PAli_NX_MP_FG_consensus TACATTTTTAGGAAGAGATAATACATATGCAATTTTATTAGAGGAATTTAAGATGAATTTAAAAAGAATTTGAAATAA [26832]
cp32-4_PAli_NX_Pl_consensus    TACATTTTTAGGAAGAGATAATACATATGCAATTTTATTAGAGGAATTTAAGATGAATTTAAAAAGAATTTGAAATAA [26832]
cp32-4_PAbe_NX_MP_FG_consensus TACATTTTTAGGAAGAGATAATACATATGCAATTTTATTAGAGGAATTTAAGATGAATTTAAAAAGAATTTGAAATAA [26832]
cp32-4_PAbe_NX_Pl_consensus    TACATTTTTAGGAAGAGATAATACATATGCAATTTTATTAGAGGAATTTAAGATGAATTTAAAAAGAATTTGAAATAA [26832]

cp32-4_gi|11497149|B31_GB      ACGCTTACAACATAGGAGACAATCTTTATGAATAAAAAAATAAAAATGTTTATTATTTGTGCTATTTTTATGCTGATA [26910]
cp32-4_un10_B31_PacBio         ACGCTTACAACATAGGAGACAATCTTTATGAATAAAAAAATAAAAATGTTTATTATTTGTGCTATTTTTATGCTGATA [26910]
cp32-4_B31_TS_MP_FG_consensus  ACGCTTACAACATAGGAGACAATCTTTATGAATAAAAAAATAAAAATGTTTATTATTTGTGCTATTTTTATGCTGATA [26910]
cp32-4_B31_NX_Pl_consensus     ACGCTTACAACATAGGAGACAATCTTTATGAATAAAAAAATAAAAATGTTTATTATTTGTGCTATTTTTATGCTGATA [26910]
cp32-4_PAli_NX_MP_FG_consensus ACGCTTACAACATAGGAGACAATCTTTATGAATAAAAAAATAAAAATGTTTATTATTTGTGCTATTTTTATGCTGATA [26910]
cp32-4_PAli_NX_Pl_consensus    ACGCTTACAACATAGGAGACAATCTTTATGAATAAAAAAATAAAAATGTTTATTATTTGTGCTATTTTTATGCTGATA [26910]
cp32-4_PAbe_NX_MP_FG_consensus ACGCTTACAACATAGGAGACAATCTTTATGAATAAAAAAATAAAAATGTTTATTATTTGTGCTATTTTTATGCTGATA [26910]
cp32-4_PAbe_NX_Pl_consensus    ACGCTTACAACATAGGAGACAATCTTTATGAATAAAAAAATAAAAATGTTTATTATTTGTGCTATTTTTATGCTGATA [26910]

cp32-4_gi|11497149|B31_GB      AGTTCTTGTAAGAATGATGTAACTAGTAAAGATTTAGAAGGGGCGGTGAAAGATTTAGAAAGTTCAGAACAAAATGTA [26988]
cp32-4_un10_B31_PacBio         AGTTCTTGTAAGAATGATGTAACTAGTAAAGATTTAGAAGGGGCGGTGAAAGATTTAGAAAGTTCAGAACAAAATGTA [26988]
cp32-4_B31_TS_MP_FG_consensus  AGTTCTTGTAAGAATGATGTAACTAGTAAAGATTTAGAAGGGGCGGTGAAAGATTTAGAAAGTTCAGAACAAAATGTA [26988]
cp32-4_B31_NX_Pl_consensus     AGTTCTTGTAAGAATGATGTAACTAGTAAAGATTTAGAAGGGGCGGTGAAAGATTTAGAAAGTTCAGAACAAAATGTA [26988]
cp32-4_PAli_NX_MP_FG_consensus AGTTCTTGTAAGAATGATGTAACTAGTAAAGATTTAGAAGGGGCGGTGAAAGATTTAGAAAGTTCAGAACAAAATGTA [26988]
cp32-4_PAli_NX_Pl_consensus    AGTTCTTGTAAGAATGATGTAACTAGTAAAGATTTAGAAGGGGCGGTGAAAGATTTAGAAAGTTCAGAACAAAATGTA [26988]
cp32-4_PAbe_NX_MP_FG_consensus AGTTCTTGTAAGAATGATGTAACTAGTAAAGATTTAGAAGGGGCGGTGAAAGATTTAGAAAGTTCAGAACAAAATGTA [26988]
cp32-4_PAbe_NX_Pl_consensus    AGTTCTTGTAAGAATGATGTAACTAGTAAAGATTTAGAAGGGGCGGTGAAAGATTTAGAAAGTTCAGAACAAAATGTA [26988]

cp32-4_gi|11497149|B31_GB      AAAAAAACAGAACAAGAGATAAAAAAACAAGTTGAAGGATTTTTAGAAATTTTAGAGACAAAAGATTTAAACACATTA [27066]
cp32-4_un10_B31_PacBio         AAAAAAACAGAACAAGAGATAAAAAAACAAGTTGAAGGATTTTTAGAAATTTTAGAGACAAAAGATTTAAACACATTA [27066]
cp32-4_B31_TS_MP_FG_consensus  AAAAAAACAGAACAAGAGATAAAAAAACAAGTTGAAGGATTTTTAGAAATTTTAGAGACAAAAGATTTAAACACATTA [27066]
cp32-4_B31_NX_Pl_consensus     AAAAAAACAGAACAAGAGATAAAAAAACAAGTTGAAGGATTTTTAGAAATTTTAGAGACAAAAGATTTAAACACATTA [27066]
cp32-4_PAli_NX_MP_FG_consensus AAAAAAACAGAACAAGAGATAAAAAAACAAGTTGAAGGATTTTTAGAAATTTTAGAGACAAAAGATTTAAACACATTA [27066]
cp32-4_PAli_NX_Pl_consensus    AAAAAAACAGAACAAGAGATAAAAAAACAAGTTGAAGGATTTTTAGAAATTTTAGAGACAAAAGATTTAAACACATTA [27066]
cp32-4_PAbe_NX_MP_FG_consensus AAAAAAACAGAACAAGAGATAAAAAAACAAGTTGAAGGATTTTTAGAAATTTTAGAGACAAAAGATTTAAACACATTA [27066]
cp32-4_PAbe_NX_Pl_consensus    AAAAAAACAGAACAAGAGATAAAAAAACAAGTTGAAGGATTTTTAGAAATTTTAGAGACAAAAGATTTAAACACATTA [27066]

cp32-4_gi|11497149|B31_GB      GATACAAAAGAAATTGAAAAACAAATTCAAGAATTAAAGAATAAGATAGAAAAATTAGACTCTAAAAAAACTTCTATT [27144]
cp32-4_un10_B31_PacBio         GATACAAAAGAAATTGAAAAACAAATTCAAGAATTAAAGAATAAGATAGAAAAATTAGACTCTAAAAAAACTTCTATT [27144]
cp32-4_B31_TS_MP_FG_consensus  GATACAAAAGAAATTGAAAAACAAATTCAAGAATTAAAGAATAAGATAGAAAAATTAGACTCTAAAAAAACTTCTATT [27144]
cp32-4_B31_NX_Pl_consensus     GATACAAAAGAAATTGAAAAACAAATTCAAGAATTAAAGAATAAGATAGAAAAATTAGACTCTAAAAAAACTTCTATT [27144]
cp32-4_PAli_NX_MP_FG_consensus GATACAAAAGAAATTGAAAAACAAATTCAAGAATTAAAGAATAAGATAGAAAAATTAGACTCTAAAAAAACTTCTATT [27144]
cp32-4_PAli_NX_Pl_consensus    GATACAAAAGAAATTGAAAAACAAATTCAAGAATTAAAGAATAAGATAGAAAAATTAGACTCTAAAAAAACTTCTATT [27144]
cp32-4_PAbe_NX_MP_FG_consensus GATACAAAAGAAATTGAAAAACAAATTCAAGAATTAAAGAATAAGATAGAAAAATTAGACTCTAAAAAAACTTCTATT [27144]
cp32-4_PAbe_NX_Pl_consensus    GATACAAAAGAAATTGAAAAACAAATTCAAGAATTAAAGAATAAGATAGAAAAATTAGACTCTAAAAAAACTTCTATT [27144]

cp32-4_gi|11497149|B31_GB      GAAACATATTCTGGGTATGAAGAAAAAATAAACAAAATAAAAGAAAAATTAAACGGAAAAGGACTTGAAGATAAATTA [27222]
cp32-4_un10_B31_PacBio         GAAACATATTCTGGGTATGAAGAAAAAATAAACAAAATAAAAGAAAAATTAAACGGAAAAGGACTTGAAGATAAATTA [27222]
cp32-4_B31_TS_MP_FG_consensus  GAAACATATTCTGGGTATGAAGAAAAAATAAACAAAATAAAAGAAAAATTAAACGGAAAAGGACTTGAAGATAAATTA [27222]
cp32-4_B31_NX_Pl_consensus     GAAACATATTCTGGGTATGAAGAAAAAATAAACAAAATAAAAGAAAAATTAAACGGAAAAGGACTTGAAGATAAATTA [27222]
cp32-4_PAli_NX_MP_FG_consensus GAAACATATTCTGGGTATGAAGAAAAAATAAACAAAATAAAAGAAAAATTAAACGGAAAAGGACTTGAAGATAAATTA [27222]
cp32-4_PAli_NX_Pl_consensus    GAAACATATTCTGGGTATGAAGAAAAAATAAACAAAATAAAAGAAAAATTAAACGGAAAAGGACTTGAAGATAAATTA [27222]
cp32-4_PAbe_NX_MP_FG_consensus GAAACATATTCTGGGTATGAAGAAAAAATAAACAAAATAAAAGAAAAATTAAACGGAAAAGGACTTGAAGATAAATTA [27222]
cp32-4_PAbe_NX_Pl_consensus    GAAACATATTCTGGGTATGAAGAAAAAATAAACAAAATAAAAGAAAAATTAAACGGAAAAGGACTTGAAGATAAATTA [27222]

cp32-4_gi|11497149|B31_GB      AATGAACTTTCAGAGAGCTTAAAAAAGAAAAAAGAGGAGAGAAAAAAAGCTTTACAAGAGGCTAAAAAGAAATTTGAA [27300]
cp32-4_un10_B31_PacBio         AATGAACTTTCAGAGAGCTTAAAAAAGAAAAAAGAGGAGAGAAAAAAAGCTTTACAAGAGGCTAAAAAGAAATTTGAA [27300]
cp32-4_B31_TS_MP_FG_consensus  AATGAACTTTCAGAGAGCTTAAAAAAGAAAAAAGAGGAGAGAAAAAAAGCTTTACAAGAGGCTAAAAAGAAATTTGAA [27300]
cp32-4_B31_NX_Pl_consensus     AATGAACTTTCAGAGAGCTTAAAAAAGAAAAAAGAGGAGAGAAAAAAAGCTTTACAAGAGGCTAAAAAGAAATTTGAA [27300]
cp32-4_PAli_NX_MP_FG_consensus AATGAACTTTCAGAGAGCTTAAAAAAGAAAAAAGAGGAGAGAAAAAAAGCTTTACAAGAGGCTAAAAAGAAATTTGAA [27300]
cp32-4_PAli_NX_Pl_consensus    AATGAACTTTCAGAGAGCTTAAAAAAGAAAAAAGAGGAGAGAAAAAAAGCTTTACAAGAGGCTAAAAAGAAATTTGAA [27300]
cp32-4_PAbe_NX_MP_FG_consensus AATGAACTTTCAGAGAGCTTAAAAAAGAAAAAAGAGGAGAGAAAAAAAGCTTTACAAGAGGCTAAAAAGAAATTTGAA [27300]
cp32-4_PAbe_NX_Pl_consensus    AATGAACTTTCAGAGAGCTTAAAAAAGAAAAAAGAGGAGAGAAAAAAAGCTTTACAAGAGGCTAAAAAGAAATTTGAA [27300]

cp32-4_gi|11497149|B31_GB      GAGTATAAAAACCAAGCTGAATCTGCAACTGGAGTAACGCATGGTTCTCAAGTCCAAAGACAAGGTGGTGTTGGATTA [27378]
cp32-4_un10_B31_PacBio         GAGTATAAAAACCAAGCTGAATCTGCAACTGGAGTAACGCATGGTTCTCAAGTCCAAAGACAAGGTGGTGTTGGATTA [27378]
cp32-4_B31_TS_MP_FG_consensus  GAGTATAAAAACCAAGCTGAATCTGCAACTGGAGTAACGCATGGTTCTCAAGTCCAAAGACAAGGTGGTGTTGGATTA [27378]
```

```
cp32-4_B31_NX_Pl_consensus     GAGTATAAAAACCAAGCTGAATCTGCAACTGGAGTAACGCATGGTTCTCAAGTCCAAAGACAAGGTGGTGTTGGATTA [27378]
cp32-4_PAli_NX_MP_FG_consensus GAGTATAAAAACCAAGCTGAATCTGCAACTGGAGTAACGCATGGTTCTCAAGTCCAAAGACAAGGTGGTGTTGGATTA [27378]
cp32-4_PAli_NX_Pl_consensus    GAGTATAAAAACCAAGCTGAATCTGCAACTGGAGTAACGCATGGTTCTCAAGTCCAAAGACAAGGTGGTGTTGGATTA [27378]
cp32-4_PAbe_NX_MP_FG_consensus GAGTATAAAAACCAAGCTGAATCTGCAACTGGAGTAACGCATGGTTCTCAAGTCCAAAGACAAGGTGGTGTTGGATTA [27378]
cp32-4_PAbe_NX_Pl_consensus    GAGTATAAAAACCAAGCTGAATCTGCAACTGGAGTAACGCATGGTTCTCAAGTCCAAAGACAAGGTGGTGTTGGATTA [27378]

cp32-4_gi|11497149|B31_GB      CAAGCTTGGCAGTGTGCTAATAGTTTGGGGTTTAAAAATATGACTAGTGGTAATAATACTAGCGATATGACCAATGAA [27456]
cp32-4_un10_B31_PacBio         CAAGCTTGGCAGTGTGCTAATAGTTTGGGGTTTAAAAATATGACTAGTGGTAATAATACTAGCGATATGACCAATGAA [27456]
cp32-4_B31_TS_MP_FG_consensus  CAAGCTTGGCAGTGTGCTAATAGTTTGGGGTTTAAAAATATGACTAGTGGTAATAATACTAGCGATATGACCAATGAA [27456]
cp32-4_B31_NX_Pl_consensus     CAAGCTTGGCAGTGTGCTAATAGTTTGGGGTTTAAAAATATGACTAGTGGTAATAATACTAGCGATATGACCAATGAA [27456]
cp32-4_PAli_NX_MP_FG_consensus CAAGCTTGGCAGTGTGCTAATAGTTTGGGGTTTAAAAATATGACTAGTGGTAATAATACTAGCGATATGACCAATGAA [27456]
cp32-4_PAli_NX_Pl_consensus    CAAGCTTGGCAGTGTGCTAATAGTTTGGGGTTTAAAAATATGACTAGTGGTAATAATACTAGCGATATGACCAATGAA [27456]
cp32-4_PAbe_NX_MP_FG_consensus CAAGCTTGGCAGTGTGCTAATAGTTTGGGGTTTAAAAATATGACTAGTGGTAATAATACTAGCGATATGACCAATGAA [27456]
cp32-4_PAbe_NX_Pl_consensus    CAAGCTTGGCAGTGTGCTAATAGTTTGGGGTTTAAAAATATGACTAGTGGTAATAATACTAGCGATATGACCAATGAA [27456]

cp32-4_gi|11497149|B31_GB      GTTATAACTAATTCGCTTAAAAAGATTGAAGAAGAACTTAAAAATATTGGAGAAACTGTAGAAGGTAAAAAAGAATAA [27534]
cp32-4_un10_B31_PacBio         GTTATAACTAATTCGCTTAAAAAGATTGAAGAAGAACTTAAAAATATTGGAGAAACTGTAGAAGGTAAAAAAGAATAA [27534]
cp32-4_B31_TS_MP_FG_consensus  GTTATAACTAATTCGCTTAAAAAGATTGAAGAAGAACTTAAAAATATTGGAGAAACTGTAGAAGGTAAAAAAGAATAA [27534]
cp32-4_B31_NX_Pl_consensus     GTTATAACTAATTCGCTTAAAAAGATTGAAGAAGAACTTAAAAATATTGGAGAAACTGTAGAAGGTAAAAAAGAATAA [27534]
cp32-4_PAli_NX_MP_FG_consensus GTTATAACTAATTCGCTTAAAAAGATTGAAGAAGAACTTAAAAATATTGGAGAAACTGTAGAAGGTAAAAAAGAATAA [27534]
cp32-4_PAli_NX_Pl_consensus    GTTATAACTAATTCGCTTAAAAAGATTGAAGAAGAACTTAAAAATATTGGAGAAACTGTAGAAGGTAAAAAAGAATAA [27534]
cp32-4_PAbe_NX_MP_FG_consensus GTTATAACTAATTCGCTTAAAAAGATTGAAGAAGAACTTAAAAATATTGGAGAAACTGTAGAAGGTAAAAAAGAATAA [27534]
cp32-4_PAbe_NX_Pl_consensus    GTTATAACTAATTCGCTTAAAAAGATTGAAGAAGAACTTAAAAATATTGGAGAAACTGTAGAAGGTAAAAAAGAATAA [27534]

cp32-4_gi|11497149|B31_GB      AGAAAAAGTTTTTATTGTTAATCGAATTTAGAAAAACAAGGCCAATTGATAGTCTTGTTTTTCTATTTAAGCTATATC [27612]
cp32-4_un10_B31_PacBio         AGAAAAAGTTTTTATTGTTAATCGAATTTAGAAAAACAAGGCCAATTGATAGTCTTGTTTTTCTATTTAAGCTATATC [27612]
cp32-4_B31_TS_MP_FG_consensus  AGAAAAAGTTTTTATTGTTAATCGAATTTAGAAAAACAAGGCCAATTGATAGTCTTGTTTTTCTATTTAAGCTATATC [27612]
cp32-4_B31_NX_Pl_consensus     AGAAAAAGTTTTTATTGTTAATCGAATTTAGAAAAACAAGGCCAATTGATAGTCTTGTTTTTCTATTTAAGCTATATC [27612]
cp32-4_PAli_NX_MP_FG_consensus AGAAAAAGTTTTTATTGTTAATCGAATTTAGAAAAACAAGGCCAATTGATAGTCTTGTTTTTCTATTTAAGCTATATC [27612]
cp32-4_PAli_NX_Pl_consensus    AGAAAAAGTTTTTATTGTTAATCGAATTTAGAAAAACAAGGCCAATTGATAGTCTTGTTTTTCTATTTAAGCTATATC [27612]
cp32-4_PAbe_NX_MP_FG_consensus AGAAAAAGTTTTTATTGTTAATCGAATTTAGAAAAACAAGGCCAATTGATAGTCTTGTTTTTCTATTTAAGCTATATC [27612]
cp32-4_PAbe_NX_Pl_consensus    AGAAAAAGTTTTTATTGTTAATCGAATTTAGAAAAACAAGGCCAATTGATAGTCTTGTTTTTCTATTTAAGCTATATC [27612]

cp32-4_gi|11497149|B31_GB      ACTTATGAAGTGAAAATGCCAAAAACTATTGTTAAAAATATTGTTTATTTATATACTCTCTAGAGTTTTGATGTATAT [27690]
cp32-4_un10_B31_PacBio         ACTTATGAAGTGAAAATGCCAAAAACTATTGTTAAAAATATTGTTTATTTATATACTCTCTAGAGTTTTGATGTATAT [27690]
cp32-4_B31_TS_MP_FG_consensus  ACTTATGAAGTGAAAATGCCAAAAACTATTGTTAAAAATATTGTTTATTTATATACTCTCTAGAGTTTTGATGTATAT [27690]
cp32-4_B31_NX_Pl_consensus     ACTTATGAAGTGAAAATGCCAAAAACTATTGTTAAAAATATTGTTTATTTATATACTCTCTAGAGTTTTGATGTATAT [27690]
cp32-4_PAli_NX_MP_FG_consensus ACTTATGAAGTGAAAATGCCAAAAACTATTGTTAAAAATATTGTTTATTTATATACTCTCTAGAGTTTTGATGTATAT [27690]
cp32-4_PAli_NX_Pl_consensus    ACTTATGAAGTGAAAATGCCAAAAACTATTGTTAAAAATATTGTTTATTTATATACTCTCTAGAGTTTTGATGTATAT [27690]
cp32-4_PAbe_NX_MP_FG_consensus ACTTATGAAGTGAAAATGCCAAAAACTATTGTTAAAAATATTGTTTATTTATATACTCTCTAGAGTTTTGATGTATAT [27690]
cp32-4_PAbe_NX_Pl_consensus    ACTTATGAAGTGAAAATGCCAAAAACTATTGTTAAAAATATTGTTTATTTATATACTCTCTAGAGTTTTGATGTATAT [27690]

cp32-4_gi|11497149|B31_GB      AAATGAAATTTCAGATTTTTATGATTGTTTGAGCCCAGGCACAAAAAAGGAAATAAGCAAACTTTATGGAGTTAAGCA [27768]
cp32-4_un10_B31_PacBio         AAATGAAATTTCAGATTTTTATGATTGTTTGAGCCCAGGCACAAAAAAGGAAATAAGCAAACTTTATGGAGTTAAGCA [27768]
cp32-4_B31_TS_MP_FG_consensus  AAATGAAATTTCAGATTTTTATGATTGTTTGAGCCCAGGCACAAAAAAGGAAATAAGCAAACTTTATGGAGTTAAGCA [27768]
cp32-4_B31_NX_Pl_consensus     AAATGAAATTTCAGATTTTTATGATTGTTTGAGCCCAGGCACAAAAAAGGAAATAAGCAAACTTTATGGAGTTAAGCA [27768]
cp32-4_PAli_NX_MP_FG_consensus AAATGAAATTTCAGATTTTTATGATTGTTTGAGCCCAGGCACAAAAAAGGAAATAAGCAAACTTTATGGAGTTAAGCA [27768]
cp32-4_PAli_NX_Pl_consensus    AAATGAAATTTCAGATTTTTATGATTGTTTGAGCCCAGGCACAAAAAAGGAAATAAGCAAACTTTATGGAGTTAAGCA [27768]
cp32-4_PAbe_NX_MP_FG_consensus AAATGAAATTTCAGATTTTTATGATTGTTTGAGCCCAGGCACAAAAAAGGAAATAAGCAAACTTTATGGAGTTAAGCA [27768]
cp32-4_PAbe_NX_Pl_consensus    AAATGAAATTTCAGATTTTTATGATTGTTTGAGCCCAGGCACAAAAAAGGAAATAAGCAAACTTTATGGAGTTAAGCA [27768]

cp32-4_gi|11497149|B31_GB      ATTAACTCTGGAACAAAAAAAGGATTTTTATAGAGGTTTTGTATCAATACAAGAATACAAAAGAAAAACCGGAAAAAG [27846]
cp32-4_un10_B31_PacBio         ATTAACTCTGGAACAAAAAAAGGATTTTTATAGAGGTTTTGTATCAATACAAGAATACAAAAGAAAAACCGGAAAAAG [27846]
cp32-4_B31_TS_MP_FG_consensus  ATTAACTCTGGAACAAAAAAAGGATTTTTATAGAGGTTTTGTATCAATACAAGAATACAAAAGAAAAACCGGAAAAAG [27846]
cp32-4_B31_NX_Pl_consensus     ATTAACTCTGGAACAAAAAAAGGATTTTTATAGAGGTTTTGTATCAATACAAGAATACAAAAGAAAAACCGGAAAAAG [27846]
cp32-4_PAli_NX_MP_FG_consensus ATTAACTCTGGAACAAAAAAAGGATTTTTATAGAGGTTTTGTATCAATACAAGAATACAAAAGAAAAACCGGAAAAAG [27846]
cp32-4_PAli_NX_Pl_consensus    ATTAACTCTGGAACAAAAAAAGGATTTTTATAGAGGTTTTGTATCAATACAAGAATACAAAAGAAAAACCGGAAAAAG [27846]
cp32-4_PAbe_NX_MP_FG_consensus ATTAACTCTGGAACAAAAAAAGGATTTTTATAGAGGTTTTGTATCAATACAAGAATACAAAAGAAAAACCGGAAAAAG [27846]
cp32-4_PAbe_NX_Pl_consensus    ATTAACTCTGGAACAAAAAAAGGATTTTTATAGAGGTTTTGTATCAATACAAGAATACAAAAGAAAAACCGGAAAAAG [27846]

cp32-4_gi|11497149|B31_GB      TATTGATGAAATTATAAATGGTATTATAAACCCTGCAAAAAATTTTATTAAAGATGTTCTGAAAGATAAGCATATAAT [27924]
cp32-4_un10_B31_PacBio         TATTGATGAAATTATAAATGGTATTATAAACCCTGCAAAAAATTTTATTAAAGATGTTCTGAAAGATAAGCATATAAT [27924]
cp32-4_B31_TS_MP_FG_consensus  TATTGATGAAATTATAAATGGTATTATAAACCCTGCAAAAAATTTTATTAAAGATGTTCTGAAAGATAAGCATATAAT [27924]
cp32-4_B31_NX_Pl_consensus     TATTGATGAAATTATAAATGGTATTATAAACCCTGCAAAAAATTTTATTAAAGATGTTCTGAAAGATAAGCATATAAT [27924]
cp32-4_PAli_NX_MP_FG_consensus TATTGATGAAATTATAAATGGTATTATAAACCCTGCAAAAAATTTTATTAAAGATGTTCTGAAAGATAAGCATATAAT [27924]
cp32-4_PAli_NX_Pl_consensus    TATTGATGAAATTATAAATGGTATTATAAACCCTGCAAAAAATTTTATTAAAGATGTTCTGAAAGATAAGCATATAAT [27924]
cp32-4_PAbe_NX_MP_FG_consensus TATTGATGAAATTATAAATGGTATTATAAACCCTGCAAAAAATTTTATTAAAGATGTTCTGAAAGATAAGCATATAAT [27924]
cp32-4_PAbe_NX_Pl_consensus    TATTGATGAAATTATAAATGGTATTATAAACCCTGCAAAAAATTTTATTAAAGATGTTCTGAAAGATAAGCATATAAT [27924]

cp32-4_gi|11497149|B31_GB      AGAAAAGTATAAAAAATTTCCAAAATATGAAGTTTGATTGTAGCTATAAGAAAGGAATGCTAGAAAAAATGTTTGAAAAA [28002]
cp32-4_un10_B31_PacBio         AGAAAAGTATAAAAAATTTCCAAAATATGAAGTTTGATTGTAGCTATAAGAAAGGAATGCTAGAAAAAATGTTTGAAAAA [28002]
cp32-4_B31_TS_MP_FG_consensus  AGAAAAGTATAAAAAATTTCCAAAATATGAAGTTTGATTGTAGCTATAAGAAAGGAATGCTAGAAAAAATGTTTGAAAAA [28002]
cp32-4_B31_NX_Pl_consensus     AGAAAAGTATAAAAAATTTCCAAAATATGAAGTTTGATTGTAGCTATAAGAAAGGAATGCTAGAAAAAATGTTTGAAAAA [28002]
cp32-4_PAli_NX_MP_FG_consensus AGAAAAGTATAAAAAATTTCCAAAATATGAAGTTTGATTGTAGCTATAAGAAAGGAATGCTAGAAAAAATGTTTGAAAAA [28002]
cp32-4_PAli_NX_Pl_consensus    AGAAAAGTATAAAAAATTTCCAAAATATGAAGTTTGATTGTAGCTATAAGAAAGGAATGCTAGAAAAAATGTTTGAAAAA [28002]
cp32-4_PAbe_NX_MP_FG_consensus AGAAAAGTATAAAAAATTTCCAAAATATGAAGTTTGATTGTAGCTATAAGAAAGGAATGCTAGAAAAAATGTTTGAAAAA [28002]
cp32-4_PAbe_NX_Pl_consensus    AGAAAAGTATAAAAAATTTCCAAAATATGAAGTTTGATTGTAGCTATAAGAAAGGAATGCTAGAAAAAATGTTTGAAAAA [28002]

cp32-4_gi|11497149|B31_GB      AATGGGAGAGAAATATTCCGACCGGTTTTTGAGTATTGTTTCTGATATTATGGATGAGATTGGTAGAAAAGATCCAAA [28080]
cp32-4_un10_B31_PacBio         AATGGGAGAGAAATATTCCGACCGGTTTTTGAGTATTGTTTCTGATATTATGGATGAGATTGGTAGAAAAGATCCAAA [28080]
cp32-4_B31_TS_MP_FG_consensus  AATGGGAGAGAAATATTCCGACCGGTTTTTGAGTATTGTTTCTGATATTATGGATGAGATTGGTAGAAAAGATCCAAA [28080]
cp32-4_B31_NX_Pl_consensus     AATGGGAGAGAAATATTCCGACCGGTTTTTGAGTATTGTTTCTGATATTATGGATGAGATTGGTAGAAAAGATCCAAA [28080]
cp32-4_PAli_NX_MP_FG_consensus AATGGGAGAGAAATATTCCGACCGGTTTTTGAGTATTGTTTCTGATATTATGGATGAGATTGGTAGAAAAGATCCAAA [28080]
cp32-4_PAli_NX_Pl_consensus    AATGGGAGAGAAATATTCCGACCGGTTTTTGAGTATTGTTTCTGATATTATGGATGAGATTGGTAGAAAAGATCCAAA [28080]
cp32-4_PAbe_NX_MP_FG_consensus AATGGGAGAGAAATATTCCGACCGGTTTTTGAGTATTGTTTCTGATATTATGGATGAGATTGGTAGAAAAGATCCAAA [28080]
cp32-4_PAbe_NX_Pl_consensus    AATGGGAGAGAAATATTCCGACCGGTTTTTGAGTATTGTTTCTGATATTATGGATGAGATTGGTAGAAAAGATCCAAA [28080]

cp32-4_gi|11497149|B31_GB      ACGAGAATCAATAGACGGGGTTCTTAACTTTGTAGATATATTATTTATAATCATGCACTACTATGATAAGGGAATTTG [28158]
cp32-4_un10_B31_PacBio         ACGAGAATCAATAGACGGGGTTCTTAACTTTGTAGATATATTATTTATAATCATGCACTACTATGATAAGGGAATTTG [28158]
cp32-4_B31_TS_MP_FG_consensus  ACGAGAATCAATAGACGGGGTTCTTAACTTTGTAGATATATTATTTATAATCATGCACTACTATGATAAGGGAATTTG [28158]
cp32-4_B31_NX_Pl_consensus     ACGAGAATCAATAGACGGGGTTCTTAACTTTGTAGATATATTATTTATAATCATGCACTACTATGATAAGGGAATTTG [28158]
cp32-4_PAli_NX_MP_FG_consensus ACGAGAATCAATAGACGGGGTTCTTAACTTTGTAGATATATTATTTATAATCATGCACTACTATGATAAGGGAATTTG [28158]
cp32-4_PAli_NX_Pl_consensus    ACGAGAATCAATAGACGGGGTTCTTAACTTTGTAGATATATTATTTATAATCATGCACTACTATGATAAGGGAATTTG [28158]
cp32-4_PAbe_NX_MP_FG_consensus ACGAGAATCAATAGACGGGGTTCTTAACTTTGTAGATATATTATTTATAATCATGCACTACTATGATAAGGGAATTTG [28158]
cp32-4_PAbe_NX_Pl_consensus    ACGAGAATCAATAGACGGGGTTCTTAACTTTGTAGATATATTATTTATAATCATGCACTACTATGATAAGGGAATTTG [28158]

cp32-4_gi|11497149|B31_GB      CACAAGGAAGTATCTTATAAAAACAATAAAAGATTTTTCCAAATTGGTATAGTCAAGTTGTAGCAAAATTTAATAAAT [28236]
cp32-4_un10_B31_PacBio         CACAAGGAAGTATCTTATAAAAACAATAAAAGATTTTTCCAAATTGGTATAGTCAAGTTGTAGCAAAATTTAATAAAT [28236]
cp32-4_B31_TS_MP_FG_consensus  CACAAGGAAGTATCTTATAAAAACAATAAAAGATTTTTCCAAATTGGTATAGTCAAGTTGTAGCAAAATTTAATAAAT [28236]
cp32-4_B31_NX_Pl_consensus     CACAAGGAAGTATCTTATAAAAACAATAAAAGATTTTTCCAAATTGGTATAGTCAAGTTGTAGCAAAATTTAATAAAT [28236]
cp32-4_PAli_NX_MP_FG_consensus CACAAGGAAGTATCTTATAAAAACAATAAAAGATTTTTCCAAATTGGTATAGTCAAGTTGTAGCAAAATTTAATAAAT [28236]
cp32-4_PAli_NX_Pl_consensus    CACAAGGAAGTATCTTATAAAAACAATAAAAGATTTTTCCAAATTGGTATAGTCAAGTTGTAGCAAAATTTAATAAAT [28236]
cp32-4_PAbe_NX_MP_FG_consensus CACAAGGAAGTATCTTATAAAAACAATAAAAGATTTTTCCAAATTGGTATAGTCAAGTTGTAGCAAAATTTAATAAAT [28236]
cp32-4_PAbe_NX_Pl_consensus    CACAAGGAAGTATCTTATAAAAACAATAAAAGATTTTTCCAAATTGGTATAGTCAAGTTGTAGCAAAATTTAATAAAT [28236]

cp32-4_gi|11497149|B31_GB      TAGTTAATCGCTATTTCTAAATCAATAAAAGTCAATAAATATCAAACATCTGTCAATAGATATCAATAAGATAATAGT [28314]
cp32-4_un10_B31_PacBio         TAGTTAATCGCTATTTCTAAATCAATAAAAGTCAATAAATATCAAACATCTGTCAATAGATATCAATAAGATAATAGT [28314]
cp32-4_B31_TS_MP_FG_consensus  TAGTTAATCGCTATTTCTAAATCAATAAAAGTCAATAAATATCAAACATCTGTCAATAGATATCAATAAGATAATAGT [28314]
cp32-4_B31_NX_Pl_consensus     TAGTTAATCGCTATTTCTAAATCAATAAAAGTCAATAAATATCAAACATCTGTCAATAGATATCAATAAGATAATAGT [28314]
```

```
cp32-4_PAli_NX_MP_FG_consensus TAGTTAATCGCTATTTCTAAATCAATAAAAGTCAATAAATATCAAACATCTGTCAATAGATATCAATAAGATAATAGT [28314]
cp32-4_PAli_NX_Pl_consensus    TAGTTAATCGCTATTTCTAAATCAATAAAAGTCAATAAATATCAAACATCTGTCAATAGATATCAATAAGATAATAGT [28314]
cp32-4_PAbe_NX_MP_FG_consensus TAGTTAATCGCTATTTCTAAATCAATAAAAGTCAATAAATATCAAACATCTGTCAATAGATATCAATAAGATAATAGT [28314]
cp32-4_PAbe_NX_Pl_consensus    TAGTTAATCGCTATTTCTAAATCAATAAAAGTCAATAAATATCAAACATCTGTCAATAGATATCAATAAGATAATAGT [28314]

cp32-4_gi|11497149|B31_GB      TAATGTTAATAATTAATAAATTGAATTACTATTTAGAGTAATAAGTTGTTAATTTTGTTATTTTAGGAGAATTTTCTT [28392]
cp32-4_un10_B31_PacBio         TAATGTTAATAATTAATAAATTGAATTACTATTTAGAGTAATAAGTTGTTAATTTTGTTATTTTAGGAGAATTTTCTT [28392]
cp32-4_B31_TS_MP_FG_consensus  TAATGTTAATAATTAATAAATTGAATTACTATTTAGAGTAATAAGTTGTTAATTTTGTTATTTTAGGAGAATTTTCTT [28392]
cp32-4_B31_NX_Pl_consensus     TAATGTTAATAATTAACAAATTGCTTTACTATTTAGAGTAATAAATTGTTAATTTTGTTATTTTAGGAGAATTTCTTT [28392]
cp32-4_PAli_NX_MP_FG_consensus TAATGTTAATAATTAATAAATTGAATTACTATTTAGAGTAATAAGTTGTTAATTTTGTTATTTTAGGAGAATTTTCTT [28392]
cp32-4_PAli_NX_Pl_consensus    TAATGTTAATAATTAATAAATTGAATTACTATTTAGAGTAATAAGTTGTTAATTTTGTTATTTTAGGAGAATTTTCTT [28392]
cp32-4_PAbe_NX_MP_FG_consensus TAATGTTAATAATTAATAAATTGAATTACTATTTAGAGTAATAAGTTGTTAATTTTGTTATTTTAGGAGAATTTTCTT [28392]
cp32-4_PAbe_NX_Pl_consensus    TAATGTTAATAATTAATAAATTGAATTACTATTTAGAGTAATAAGTTGTTAATTTTGTTATTTTAGGAGAATTTTCTT [28392]

cp32-4_gi|11497149|B31_GB      GAAAAGAGCCAAAAGGTCTTTTAATGATTATGTTGCATACTTTAGAGAAGGATCGTTAAGTGATAAAGAAATAGCAGC [28470]
cp32-4_un10_B31_PacBio         GAAAAGAGCCAAAAGGTCTTTTAATGATTATGTTGCATACTTTAGAGAAGGATCGTTAAGTGATAAAGAAATAGCAGC [28470]
cp32-4_B31_TS_MP_FG_consensus  GAAAAGAGCCAAAAGGTCTTTTAATGATTATGTTGCATACTTTAGAGAAGGATCGTTAAGTGATAAAGAAATAGCAGC [28470]
cp32-4_B31_NX_Pl_consensus     GAAAAGAGCCAAAAGGTCTTTTGATGATTATGTTGCGTATTTTAGCGAGGGTTCATTAAGTGATGGAGAAATAGCAGC [28470]
cp32-4_PAli_NX_MP_FG_consensus GAAAAGAGCCAAAAGGTCTTTTAATGATTATGTTGCATACTTTAGAGAAGGATCGTTAAGTGATAAAGAAATAGCAGC [28470]
cp32-4_PAli_NX_Pl_consensus    GAAAAGAGCCAAAAGGTCTTTTAATGATTATGTTGCATACTTTAGAGAAGGATCGTTAAGTGATAAAGAAATAGCAGC [28470]
cp32-4_PAbe_NX_MP_FG_consensus GAAAAGAGCCAAAAGGTCTTTTAATGATTATGTTGCATACTTTAGAGAAGGATCGTTAAGTGATAAAGAAATAGCAGC [28470]
cp32-4_PAbe_NX_Pl_consensus    GAAAAGAGCCAAAAGGTCTTTTAATGATTATGTTGCATACTTTAGAGAAGGATCGTTAAGTGATAAAGAAATAGCAGC [28470]

cp32-4_gi|11497149|B31_GB      TAAACTAAGGGTTTCTAAAGTAAATGTGTGGAGAATGAGACAAAAGTGGGAAAGTGGAGAAACTTCTGTTAGCGAGGA [28548]
cp32-4_un10_B31_PacBio         TAAACTAAGGGTTTCTAAAGTAAATGTGTGGAGAATGAGACAAAAGTGGGAAAGTGGAGAAACTTCTGTTAGCGAGGA [28548]
cp32-4_B31_TS_MP_FG_consensus  TAAACTAAGGGTTTCTAAAGTAAATGTGTGGAGAATGAGACAAAAGTGGGAAAGTGGAGAAACTTCTGTTAGCGAGGA [28548]
cp32-4_B31_NX_Pl_consensus     TAAACTAAGGGTTTCTAAAGTAAATGTGTGGAGAATGAGACAAAAGTGGGAAAGTGGAGAAACTTCTGTTAGCGAGGA [28548]
cp32-4_PAli_NX_MP_FG_consensus TAAACTAAGGGTTTCTAAAGTAAATGTGTGGAGAATGAGACAAAAGTGGGAAAGTGGAGAAACTTCTGTTAGCGAGGA [28548]
cp32-4_PAli_NX_Pl_consensus    TAAACTAAGGGTTTCTAAAGTAAATGTGTGGAGAATGAGACAAAAGTGGGAAAGTGGAGAAACTTCTGTTAGCGAGGA [28548]
cp32-4_PAbe_NX_MP_FG_consensus TAAACTAAGGGTTTCTAAAGTAAATGTGTGGAGAATGAGACAAAAGTGGGAAAGTGGAGAAACTTCTGTTAGCGAGGA [28548]
cp32-4_PAbe_NX_Pl_consensus    TAAACTAAGGGTTTCTAAAGTAAATGTGTGGAGAATGAGACAAAAGTGGGAAAGTGGAGAAACTTCTGTTAGCGAGGA [28548]

cp32-4_gi|11497149|B31_GB      CTCTAGACTAACAATTAGTGAAGATACTTTTGAACACCTTTTGTCGCAAACCTTTAGATCAGAAGTTAACGCTAGGAA [28626]
cp32-4_un10_B31_PacBio         CTCTAGACTAACAATTAGTGAAGATACTTTTGAACACCTTTTGTCGCAAACCTTTAGATCAGAAGTTAACGCTAGGAA [28626]
cp32-4_B31_TS_MP_FG_consensus  CTCTAGACTAACAATTAGTGAAGATACTTTTGAACACCTTTTGTCGCAAACCTTTAGATCAGAAGTTAACGCTAGGAA [28626]
cp32-4_B31_NX_Pl_consensus     CTCTAGACTAACAATTAGTGAAGATACTTTTGAACACCTTTTGTCGCAAACCTTTAGATCAGAAGTTAACGCTAGGAA [28626]
cp32-4_PAli_NX_MP_FG_consensus CTCTAGACTAACAATTAGTGAAGATACTTTTGAACACCTTTTGTCGCAAACCTTTAGATCAGAAGTTAACGCTAGGAA [28626]
cp32-4_PAli_NX_Pl_consensus    CTCTAGACTAACAATTAGTGAAGATACTTTTGAACACCTTTTGTCGCAAACCTTTAGATCAGAAGTTAACGCTAGGAA [28626]
cp32-4_PAbe_NX_MP_FG_consensus CTCTAGACTAACAATTAGTGAAGATACTTTTGAACACCTTTTGTCGCAAACCTTTAGATCAGAAGTTAACGCTAGGAA [28626]
cp32-4_PAbe_NX_Pl_consensus    CTCTAGACTAACAATTAGTGAAGATACTTTTGAACACCTTTTGTCGCAAACCTTTAGATCAGAAGTTAACGCTAGGAA [28626]

cp32-4_gi|11497149|B31_GB      AGTTAGAAGCGAATTGGATTTAGAGCGAGCCAATTTAGAATTAGGGTTTATACATGCATTTAAGCGATATTCTAGTGT [28704]
cp32-4_un10_B31_PacBio         AGTTAGAAGCGAATTGGATTTAGAGCGAGCCAATTTAGAATTAGGGTTTATACATGCATTTAAGCGATATTCTAGTGT [28704]
cp32-4_B31_TS_MP_FG_consensus  AGTTAGAAGCGAATTGGATTTAGAGCGAGCCAATTTAGAATTAGGGTTTATACATGCATTTAAGCGATATTCTAGTGT [28704]
cp32-4_B31_NX_Pl_consensus     AGTTAGAAGCGAATTGGATTTAGAGCGAGCCAATTTAGAATTAGGGTTTATACATGCATTTAAGCGATATTCTAGTGT [28704]
cp32-4_PAli_NX_MP_FG_consensus AGTTAGAAGCGAATTGGATTTAGAGCGAGCCAATTTAGAATTAGGGTTTATACATGCATTTAAGCGATATTCTAGTGT [28704]
cp32-4_PAli_NX_Pl_consensus    AGTTAGAAGCGAATTGGATTTAGAGCGAGCCAATTTAGAATTAGGGTTTATACATGCATTTAAGCGATATTCTAGTGT [28704]
cp32-4_PAbe_NX_MP_FG_consensus AGTTAGAAGCGAATTGGATTTAGAGCGAGCCAATTTAGAATTAGGGTTTATACATGCATTTAAGCGATATTCTAGTGT [28704]
cp32-4_PAbe_NX_Pl_consensus    AGTTAGAAGCGAATTGGATTTAGAGCGAGCCAATTTAGAATTAGGGTTTATACATGCATTTAAGCGATATTCTAGTGT [28704]

cp32-4_gi|11497149|B31_GB      TGAGCTTTTTAGTATGCATACTAAAATAGAAAATTTAAGAGCCGAAATTGATGCTTTAAATAAAGCAAGTAGTAAAAA [28782]
cp32-4_un10_B31_PacBio         TGAGCTTTTTAGTATGCATACTAAAATAGAAAATTTAAGAGCCGAAATTGATGCTTTAAATAAAGCAAGTAGTAAAAA [28782]
cp32-4_B31_TS_MP_FG_consensus  TGAGCTTTTTAGTATGCATACTAAAATAGAAAATTTAAGAGCCGAAATTGATGCTTTAAATAAAGCAAGTAGTAAAAA [28782]
cp32-4_B31_NX_Pl_consensus     TGAGCTTTTTAGTATGCATACTAAAATAGAAAATTTAAGAGCCGAAATTGATGCTTTAAATAAAGCAAGTAGTAAAAA [28782]
cp32-4_PAli_NX_MP_FG_consensus TGAGCTTTTTAGTATGCATACTAAAATAGAAAATTTAAGAGCCGAAATTGATGCTTTAAATAAAGCAAGTAGTAAAAA [28782]
cp32-4_PAli_NX_Pl_consensus    TGAGCTTTTTAGTATGCATACTAAAATAGAAAATTTAAGAGCCGAAATTGATGCTTTAAATAAAGCAAGTAGTAAAAA [28782]
cp32-4_PAbe_NX_MP_FG_consensus TGAGCTTTTTAGTATGCATACTAAAATAGAAAATTTAAGAGCCGAAATTGATGCTTTAAATAAAGCAAGTAGTAAAAA [28782]
cp32-4_PAbe_NX_Pl_consensus    TGAGCTTTTTAGTATGCATACTAAAATAGAAAATTTAAGAGCCGAAATTGATGCTTTAAATAAAGCAAGTAGTAAAAA [28782]

cp32-4_gi|11497149|B31_GB      AAACAAGCAAGTTGTTAATGGAGAAATTAATTCTTTAAAAAGTGAGCTTGATGAATATGTAAAGGAGTGCTCAATAAG [28860]
cp32-4_un10_B31_PacBio         AAACAAGCAAGTTGTTAATGGAGAAATTAATTCTTTAAAAAGTGAGCTTGATGAATATGTAAAGGAGTGCTCAATAAG [28860]
cp32-4_B31_TS_MP_FG_consensus  AAACAAGCAAGTTGTTAATGGAGAAATTAATTCTTTAAAAAGTGAGCTTGATGAATATGTAAAGGAGTGCTCAATAAG [28860]
cp32-4_B31_NX_Pl_consensus     AAACAAGCAAGTTGTTAATGGAGAAATTAATTCTTTAAAAAGTGAGCTTGATGAATATGTAAAGGAGTGCTCAATAAG [28860]
cp32-4_PAli_NX_MP_FG_consensus AAACAAGCAAGTTGTTAATGGAGAAATTAATTCTTTAAAAAGTGAGCTTGATGAATATGTAAAGGAGTGCTCAATAAG [28860]
cp32-4_PAli_NX_Pl_consensus    AAACAAGCAAGTTGTTAATGGAGAAATTAATTCTTTAAAAAGTGAGCTTGATGAATATGTAAAGGAGTGCTCAATAAG [28860]
cp32-4_PAbe_NX_MP_FG_consensus AAACAAGCAAGTTGTTAATGGAGAAATTAATTCTTTAAAAAGTGAGCTTGATGAATATGTAAAGGAGTGCTCAATAAG [28860]
cp32-4_PAbe_NX_Pl_consensus    AAACAAGCAAGTTGTTAATGGAGAAATTAATTCTTTAAAAAGTGAGCTTGATGAATATGTAAAGGAGTGCTCAATAAG [28860]

cp32-4_gi|11497149|B31_GB      AGAAATGGAGCTTTACTATGAATGTATGAAAAAACTTTCTTCTGCTCATGAAGTTGAGAATAAAGGCAATTACAAAAG [28938]
cp32-4_un10_B31_PacBio         AGAAATGGAGCTTTACTATGAATGTATGAAAAAACTTTCTTCTGCTCATGAAGTTGAGAATAAAGGCAATTACAAAAG [28938]
cp32-4_B31_TS_MP_FG_consensus  AGAAATGGAGCTTTACTATGAATGTATGAAAAAACTTTCTTCTGCTCATGAAGTTGAGAATAAAGGCAATTACAAAAG [28938]
cp32-4_B31_NX_Pl_consensus     AGAAATGGAGCTTTACTATGAATGTATGAAAAAACTTTCTTCTGCTCATGAAGTTGAGAATAAAGGCAATTACAAAAG [28938]
cp32-4_PAli_NX_MP_FG_consensus AGAAATGGAGCTTTACTATGAATGTATGAAAAAACTTTCTTCTGCTCATGAAGTTGAGAATAAAGGCAATTACAAAAG [28938]
cp32-4_PAli_NX_Pl_consensus    AGAAATGGAGCTTTACTATGAATGTATGAAAAAACTTTCTTCTGCTCATGAAGTTGAGAATAAAGGCAATTACAAAAG [28938]
cp32-4_PAbe_NX_MP_FG_consensus AGAAATGGAGCTTTACTATGAATGTATGAAAAAACTTTCTTCTGCTCATGAAGTTGAGAATAAAGGCAATTACAAAAG [28938]
cp32-4_PAbe_NX_Pl_consensus    AGAAATGGAGCTTTACTATGAATGTATGAAAAAACTTTCTTCTGCTCATGAAGTTGAGAATAAAGGCAATTACAAAAG [28938]

cp32-4_gi|11497149|B31_GB      TTATAAAGGGTACAAGTGAACTTATATCAAACAAAACTTTTTACAACACTACAAAAGGAATACAAAAATAAATATGGA [29016]
cp32-4_un10_B31_PacBio         TTATAAAGGGTACAAGTGAACTTATATCAAACAAAACTTTTTACAACACTACAAAAGGAATACAAAAATAAATATGGA [29016]
cp32-4_B31_TS_MP_FG_consensus  TTATAAAGGGTACAAGTGAACTTATATCAAACAAAACTTTTTACAACACTACAAAAGGAATACAAAAATAAATATGGA [29016]
cp32-4_B31_NX_Pl_consensus     TTATAAAGGGTACAAGTGAACTTATATCAAACAAAACTTTTTACAACACTACAAAAGGAATACAAAAATAAATATGGA [29016]
cp32-4_PAli_NX_MP_FG_consensus TTATAAAGGGTACAAGTGAACTTATATCAAACAAAACTTTTTACAACACTACAAAAGGAATACAAAAATAAATATGGA [29016]
cp32-4_PAli_NX_Pl_consensus    TTATAAAGGGTACAAGTGAACTTATATCAAACAAAACTTTTTACAACACTACAAAAGGAATACAAAAATAAATATGGA [29016]
cp32-4_PAbe_NX_MP_FG_consensus TTATAAAGGGTACAAGTGAACTTATATCAAACAAAACTTTTTACAACACTACAAAAGGAATACAAAAATAAATATGGA [29016]
cp32-4_PAbe_NX_Pl_consensus    TTATAAAGGGTACAAGTGAACTTATATCAAACAAAACTTTTTACAACACTACAAAAGGAATACAAAAATAAATATGGA [29016]

cp32-4_gi|11497149|B31_GB      GTTGACATATCACAATTTGTAAAGCTAACAAATTCTTCAATTAATTTTGATAAGTTTGAAAAAGAACAGTTAACCATA [29094]
cp32-4_un10_B31_PacBio         GTTGACATATCACAATTTGTAAAGCTAACAAATTCTTCAATTAATTTTGATAAGTTTGAAAAAGAACAGTTAACCATA [29094]
cp32-4_B31_TS_MP_FG_consensus  GTTGACATATCACAATTTGTAAAGCTAACAAATTCTTCAATTAATTTTGATAAGTTTGAAAAAGAACAGTTAACCATA [29094]
cp32-4_B31_NX_Pl_consensus     GTTGACATATCACAATTTGTAAAGCTAACAAATTCTTCAATTAATTTTGATAAGTTTGAAAAAGAACAGTTAACCATA [29094]
cp32-4_PAli_NX_MP_FG_consensus GTTGACATATCACAATTTGTAAAGCTAACAAATTCTTCAATTAATTTTGATAAGTTTGAAAAAGAACAGTTAACCATA [29094]
cp32-4_PAli_NX_Pl_consensus    GTTGACATATCACAATTTGTAAAGCTAACAAATTCTTCAATTAATTTTGATAAGTTTGAAAAAGAACAGTTAACCATA [29094]
cp32-4_PAbe_NX_MP_FG_consensus GTTGACATATCACAATTTGTAAAGCTAACAAATTCTTCAATTAATTTTGATAAGTTTGAAAAAGAACAGTTAACCATA [29094]
cp32-4_PAbe_NX_Pl_consensus    GTTGACATATCACAATTTGTAAAGCTAACAAATTCTTCAATTAATTTTGATAAGTTTGAAAAAGAACAGTTAACCATA [29094]

cp32-4_gi|11497149|B31_GB      AAACAAAAAAATGTTATAAAAAGTATTCAAAAAAATAATGAAAAGAAGATTATACTCAGCGGCGGCATAGCTAGCGGC [29172]
cp32-4_un10_B31_PacBio         AAACAAAAAAATGTTATAAAAAGTATTCAAAAAAATAATGAAAAGAAGATTATACTCAGCGGCGGCATAGCTAGCGGC [29172]
cp32-4_B31_TS_MP_FG_consensus  AAACAAAAAAATGTTATAAAAAGTATTCAAAAAAATAATGAAAAGAAGATTATACTCAGCGGCGGCATAGCTAGCGGC [29172]
cp32-4_B31_NX_Pl_consensus     AAACAAAAAAATGTTATAAAAAGTATTCAAAAAAATAATGAAAAGAAGATTATACTCAGCGGCGGCATAGCTAGCGGC [29172]
cp32-4_PAli_NX_MP_FG_consensus AAACAAAAAAATGTTATAAAAAGTATTCAAAAAAATAATGAAAAGAAGATTATACTCAGCGGCGGCATAGCTAGCGGC [29172]
cp32-4_PAli_NX_Pl_consensus    AAACAAAAAAATGTTATAAAAAGTATTCAAAAAAATAATGAAAAGAAGATTATACTCAGCGGCGGCATAGCTAGCGGC [29172]
cp32-4_PAbe_NX_MP_FG_consensus AAACAAAAAAATGTTATAAAAAGTATTCAAAAAAATAATGAAAAGAAGATTATACTCAGCGGCGGCATAGCTAGCGGC [29172]
cp32-4_PAbe_NX_Pl_consensus    AAACAAAAAAATGTTATAAAAAGTATTCAAAAAAATAATGAAAAGAAGATTATACTCAGCGGCGGCATAGCTAGCGGC [29172]

cp32-4_gi|11497149|B31_GB      AAAACGTATCTTGCATGTTATCTTTTTCTCAAAAGTTTAATTGAAAATAAAAAGTTATACTCTGGTGATATTAATAAT [29250]
cp32-4_un10_B31_PacBio         AAAACGTATCTTGCATGTTATCTTTTTCTCAAAAGTTTAATTGAAAATAAAAAGTTATACTCTGGTGATATTAATAAT [29250]
cp32-4_B31_TS_MP_FG_consensus  AAAACGTATCTTGCATGTTATCTTTTTCTCAAAAGTTTAATTGAAAATAAAAAGTTATACTCTGGTGATATTAATAAT [29250]
cp32-4_B31_NX_Pl_consensus     AAAACGTATCTTGCATGTTATCTTTTTCTCAAAAGTTTAATTGAAAATAAAAAGTTATACTCTGGTGATATTAATAAT [29250]
cp32-4_PAli_NX_MP_FG_consensus AAAACGTATCTTGCATGTTATCTTTTTCTCAAAAGTTTAATTGAAAATAAAAAGTTATACTCTGGTGATATTAATAAT [29250]
```

```
cp32-4_PAli_NX_Pl_consensus    AAAACGTATCTTGCATGTTATCTTTTTCTCAAAAGTTTAATTGAAAATAAAAAGTTATACTCTGGTGATATTAATAAT [29250]
cp32-4_PAbe_NX_MP_FG_consensus AAAACGTATCTTGCATGTTATCTTTTTCTCAAAAGTTTAATTGAAAATAAAAAGTTATACTCTGGTGATATTAATAAT [29250]
cp32-4_PAbe_NX_Pl_consensus    AAAACGTATCTTGCATGTTATCTTTTTCTCAAAAGTTTAATTGAAAATAAAAAGTTATACTCTGGTGATATTAATAAT [29250]

cp32-4_gi|11497149|B31_GB      TTTATTATTGGGAATTCACAACGCTCAGTTGAAGTTAATGTTTTGGGACAATTTGAAAAGCTATGTAAACTTCTTAAA [29328]
cp32-4_un10_B31_PacBio         TTTATTATTGGGAATTCACAACGCTCAGTTGAAGTTAATGTTTTGGGACAATTTGAAAAGCTATGTAAACTTCTTAAA [29328]
cp32-4_B31_TS_MP_FG_consensus  TTTATTATTGGGAATTCACAACGCTCAGTTGAAGTTAATGTTTTGGGACAATTTGAAAAGCTATGTAAACTTCTTAAA [29328]
cp32-4_B31_NX_Pl_consensus     TTTATTATTGGGAATTCACAACGCTCAGTTGAAGTTAATGTTTTGGGACAATTTGAAAAGCTATGTAAACTTCTTAAA [29328]
cp32-4_PAli_NX_MP_FG_consensus TTTATTATTGGGAATTCACAACGCTCAGTTGAAGTTAATGTTTTGGGACAATTTGAAAAGCTATGTAAACTTCTTAAA [29328]
cp32-4_PAli_NX_Pl_consensus    TTTATTATTGGGAATTCACAACGCTCAGTTGAAGTTAATGTTTTGGGACAATTTGAAAAGCTATGTAAACTTCTTAAA [29328]
cp32-4_PAbe_NX_MP_FG_consensus TTTATTATTGGGAATTCACAACGCTCAGTTGAAGTTAATGTTTTGGGACAATTTGAAAAGCTATGTAAACTTCTTAAA [29328]
cp32-4_PAbe_NX_Pl_consensus    TTTATTATTGGGAATTCACAACGCTCAGTTGAAGTTAATGTTTTGGGACAATTTGAAAAGCTATGTAAACTTCTTAAA [29328]

cp32-4_gi|11497149|B31_GB      ATTCCTTATATCCCAAGACATACAAATAATTCATATATATTAATTGATTCACTTCGTATTAATCTATATGGAGGAGAT [29406]
cp32-4_un10_B31_PacBio         ATTCCTTATATCCCAAGACATACAAATAATTCATATATATTAATTGATTCACTTCGTATTAATCTATATGGAGGAGAT [29406]
cp32-4_B31_TS_MP_FG_consensus  ATTCCTTATATCCCAAGACATACAAATAATTCATATATATTAATTGATTCACTTCGTATTAATCTATATGGAGGAGAT [29406]
cp32-4_B31_NX_Pl_consensus     ATTCCTTATATCCCAAGACATACAAATAATTCATATATATTAATTGATTCACTTCGTATTAATCTATATGGAGGAGAT [29406]
cp32-4_PAli_NX_MP_FG_consensus ATTCCTTATATCCCAAGACATACAAATAATTCATATATATTAATTGATTCACTTCGTATTAATCTATATGGAGGAGAT [29406]
cp32-4_PAli_NX_Pl_consensus    ATTCCTTATATCCCAAGACATACAAATAATTCATATATATTAATTGATTCACTTCGTATTAATCTATATGGAGGAGAT [29406]
cp32-4_PAbe_NX_MP_FG_consensus ATTCCTTATATCCCAAGACATACAAATAATTCATATATATTAATTGATTCACTTCGTATTAATCTATATGGAGGAGAT [29406]
cp32-4_PAbe_NX_Pl_consensus    ATTCCTTATATCCCAAGACATACAAATAATTCATATATATTAATTGATTCACTTCGTATTAATCTATATGGAGGAGAT [29406]

cp32-4_gi|11497149|B31_GB      AAGGCAAGTGATTTTGAAAGATTTAGAGGCAGTAATTCGGCACTTATTTTTGTTAATGAGGCTACTACTTTACACAAG [29484]
cp32-4_un10_B31_PacBio         AAGGCAAGTGATTTTGAAAGATTTAGAGGCAGTAATTCGGCACTTATTTTTGTTAATGAGGCTACTACTTTACACAAG [29484]
cp32-4_B31_TS_MP_FG_consensus  AAGGCAAGTGATTTTGAAAGATTTAGAGGCAGTAATTCGGCACTTATTTTTGTTAATGAGGCTACTACTTTACACAAG [29484]
cp32-4_B31_NX_Pl_consensus     AAGGCAAGTGATTTTGAAAGATTTAGAGGCAGTAATTCGGCACTTATTTTTGTTAATGAGGCTACTACTTTACACAAG [29484]
cp32-4_PAli_NX_MP_FG_consensus AAGGCAAGTGATTTTGAAAGATTTAGAGGCAGTAATTCGGCACTTATTTTTGTTAATGAGGCTACTACTTTACACAAG [29484]
cp32-4_PAli_NX_Pl_consensus    AAGGCAAGTGATTTTGAAAGATTTAGAGGCAGTAATTCGGCACTTATTTTTGTTAATGAGGCTACTACTTTACACAAG [29484]
cp32-4_PAbe_NX_MP_FG_consensus AAGGCAAGTGATTTTGAAAGATTTAGAGGCAGTAATTCGGCACTTATTTTTGTTAATGAGGCTACTACTTTACACAAG [29484]
cp32-4_PAbe_NX_Pl_consensus    AAGGCAAGTGATTTTGAAAGATTTAGAGGCAGTAATTCGGCACTTATTTTTGTTAATGAGGCTACTACTTTACACAAG [29484]

cp32-4_gi|11497149|B31_GB      CAAACTTTAGAGGAGGTCTTAAAAAGACTTAGGTGCGGACAAGAAACTATTATTTTTGATACTAATCCTGATCATCCA [29562]
cp32-4_un10_B31_PacBio         CAAACTTTAGAGGAGGTCTTAAAAAGACTTAGGTGCGGACAAGAAACTATTATTTTTGATACTAATCCTGATCATCCA [29562]
cp32-4_B31_TS_MP_FG_consensus  CAAACTTTAGAGGAGGTCTTAAAAAGACTTAGGTGCGGACAAGAAACTATTATTTTTGATACTAATCCTGATCATCCA [29562]
cp32-4_B31_NX_Pl_consensus     CAAACTTTAGAGGAGGTCTTAAAAAGACTTAGGTGCGGACAAGAAACTATTATTTTTGATACTAATCCTGATCATCCA [29562]
cp32-4_PAli_NX_MP_FG_consensus CAAACTTTAGAGGAGGTCTTAAAAAGACTTAGGTGCGGACAAGAAACTATTATTTTTGATACTAATCCTGATCATCCA [29562]
cp32-4_PAli_NX_Pl_consensus    CAAACTTTAGAGGAGGTCTTAAAAAGACTTAGGTGCGGACAAGAAACTATTATTTTTGATACTAATCCTGATCATCCA [29562]
cp32-4_PAbe_NX_MP_FG_consensus CAAACTTTAGAGGAGGTCTTAAAAAGACTTAGGTGCGGACAAGAAACTATTATTTTTGATACTAATCCTGATCATCCA [29562]
cp32-4_PAbe_NX_Pl_consensus    CAAACTTTAGAGGAGGTCTTAAAAAGACTTAGGTGCGGACAAGAAACTATTATTTTTGATACTAATCCTGATCATCCA [29562]

cp32-4_gi|11497149|B31_GB      GAACACTATTTTAAAACCGATTATATTGATAATATAGCGACATTTAAGACATATAATTTTACAACTTATGATAATGTG [29640]
cp32-4_un10_B31_PacBio         GAACACTATTTTAAAACCGATTATATTGATAATATAGCGACATTTAAGACATATAATTTTACAACTTATGATAATGTG [29640]
cp32-4_B31_TS_MP_FG_consensus  GAACACTATTTTAAAACCGATTATATTGATAATATAGCGACATTTAAGACATATAATTTTACAACTTATGATAATGTG [29640]
cp32-4_B31_NX_Pl_consensus     GAACACTATTTTAAAACCGATTATATTGATAATATAGCGACATTTAAGACATATAATTTTACAACTTATGATAATGTG [29640]
cp32-4_PAli_NX_MP_FG_consensus GAACACTATTTTAAAACCGATTATATTGATAATATAGCGACATTTAAGACATATAATTTTACAACTTATGATAATGTG [29640]
cp32-4_PAli_NX_Pl_consensus    GAACACTATTTTAAAACCGATTATATTGATAATATAGCGACATTTAAGACATATAATTTTACAACTTATGATAATGTG [29640]
cp32-4_PAbe_NX_MP_FG_consensus GAACACTATTTTAAAACCGATTATATTGATAATATAGCGACATTTAAGACATATAATTTTACAACTTATGATAATGTG [29640]
cp32-4_PAbe_NX_Pl_consensus    GAACACTATTTTAAAACCGATTATATTGATAATATAGCGACATTTAAGACATATAATTTTACAACTTATGATAATGTG [29640]

cp32-4_gi|11497149|B31_GB      CTACTTAGTAAAGGATTTATCGAAACACAAGAAAAACTCTATAAAGATATACCATCATATAAAGCAAGAGTTTTGCTA [29718]
cp32-4_un10_B31_PacBio         CTACTTAGTAAAGGATTTATCGAAACACAAGAAAAACTCTATAAAGATATACCATCATATAAAGCAAGAGTTTTGCTA [29718]
cp32-4_B31_TS_MP_FG_consensus  CTACTTAGTAAAGGATTTATCGAAACACAAGAAAAACTCTATAAAGATATACCATCATATAAAGCAAGAGTTTTGCTA [29718]
cp32-4_B31_NX_Pl_consensus     CTACTTAGTAAAGGATTTATCGAAACACAAGAAAAACTCTATAAAGATATACCATCATATAAAGCAAGAGTTTTGCTA [29718]
cp32-4_PAli_NX_MP_FG_consensus CTACTTAGTAAAGGATTTATCGAAACACAAGAAAAACTCTATAAAGATATACCATCATATAAAGCAAGAGTTTTGCTA [29718]
cp32-4_PAli_NX_Pl_consensus    CTACTTAGTAAAGGATTTATCGAAACACAAGAAAAACTCTATAAAGATATACCATCATATAAAGCAAGAGTTTTGCTA [29718]
cp32-4_PAbe_NX_MP_FG_consensus CTACTTAGTAAAGGATTTATCGAAACACAAGAAAAACTCTATAAAGATATACCATCATATAAAGCAAGAGTTTTGCTA [29718]
cp32-4_PAbe_NX_Pl_consensus    CTACTTAGTAAAGGATTTATCGAAACACAAGAAAAACTCTATAAAGATATACCATCATATAAAGCAAGAGTTTTGCTA [29718]

cp32-4_gi|11497149|B31_GB      GGTGAGTGGATAGCAAGCACCGATTCAATTTTTACACAAATAAATATTACTAATGATTATGTATTTACTAGCCCGATA [29796]
cp32-4_un10_B31_PacBio         GGTGAGTGGATAGCAAGCACCGATTCAATTTTTACACAAATAAATATTACTAATGATTATGTATTTACTAGCCCGATA [29796]
cp32-4_B31_TS_MP_FG_consensus  GGTGAGTGGATAGCAAGCACCGATTCAATTTTTACACAAATAAATATTACTAATGATTATGTATTTACTAGCCCGATA [29796]
cp32-4_B31_NX_Pl_consensus     GGTGAGTGGATAGCAAGCACCGATTCAATTTTTACACAAATAAATATTACTAATGATTATGTATTTACTAGCCCGATA [29796]
cp32-4_PAli_NX_MP_FG_consensus GGTGAGTGGATAGCAAGCACCGATTCAATTTTTACACAAATAAATATTACTAATGATTATGTATTTACTAGCCCGATA [29796]
cp32-4_PAli_NX_Pl_consensus    GGTGAGTGGATAGCAAGCACCGATTCAATTTTTACACAAATAAATATTACTAATGATTATGTATTTACTAGCCCGATA [29796]
cp32-4_PAbe_NX_MP_FG_consensus GGTGAGTGGATAGCAAGCACCGATTCAATTTTTACACAAATAAATATTACTAATGATTATGTATTTACTAGCCCGATA [29796]
cp32-4_PAbe_NX_Pl_consensus    GGTGAGTGGATAGCAAGCACCGATTCAATTTTTACACAAATAAATATTACTAATGATTATGTATTTACTAGCCCGATA [29796]

cp32-4_gi|11497149|B31_GB      GCATATTTAGACCCAGCATTTAGTGTTGGAGGGGATAACACTGCATTATGTGTTATGGAGCGAGTTGATGATAAGTAT [29874]
cp32-4_un10_B31_PacBio         GCATATTTAGACCCAGCATTTAGTGTTGGAGGGGATAACACTGCATTATGTGTTATGGAGCGAGTTGATGATAAGTAT [29874]
cp32-4_B31_TS_MP_FG_consensus  GCATATTTAGACCCAGCATTTAGTGTTGGAGGGGATAACACTGCATTATGTGTTATGGAGCGAGTTGATGATAAGTAT [29874]
cp32-4_B31_NX_Pl_consensus     GCATATTTAGACCCAGCATTTAGTGTTGGAGGGGATAACACTGCATTATGTGTTATGGAGCGAGTTGATGATAAGTAT [29874]
cp32-4_PAli_NX_MP_FG_consensus GCATATTTAGACCCAGCATTTAGTGTTGGAGGGGATAACACTGCATTATGTGTTATGGAGCGAGTTGATGATAAGTAT [29874]
cp32-4_PAli_NX_Pl_consensus    GCATATTTAGACCCAGCATTTAGTGTTGGAGGGGATAACACTGCATTATGTGTTATGGAGCGAGTTGATGATAAGTAT [29874]
cp32-4_PAbe_NX_MP_FG_consensus GCATATTTAGACCCAGCATTTAGTGTTGGAGGGGATAACACTGCATTATGTGTTATGGAGCGAGTTGATGATAAGTAT [29874]
cp32-4_PAbe_NX_Pl_consensus    GCATATTTAGACCCAGCATTTAGTGTTGGAGGGGATAACACTGCATTATGTGTTATGGAGCGAGTTGATGATAAGTAT [29874]

cp32-4_gi|11497149|B31_GB      TATGCTTTTGTATTTCAAGACCAACGACCAGCCAATGATCCTTATATTATGAATATGGTAAAGACCGTTATAGAAAAT [29952]
cp32-4_un10_B31_PacBio         TATGCTTTTGTATTTCAAGACCAACGACCAGCCAATGATCCTTATATTATGAATATGGTAAAGACCGTTATAGAAAAT [29952]
cp32-4_B31_TS_MP_FG_consensus  TATGCTTTTGTATTTCAAGACCAACGACCAGCCAATGATCCTTATATTATGAATATGGTAAAGACCGTTATAGAAAAT [29952]
cp32-4_B31_NX_Pl_consensus     TATGCTTTTGTATTTCAAGACCAACGACCAGCCAATGATCCTTATATTATGAATATGGTAAAGACCGTTATAGAAAAT [29952]
cp32-4_PAli_NX_MP_FG_consensus TATGCTTTTGTATTTCAAGACCAACGACCAGCCAATGATCCTTATATTATGAATATGGTAAAGACCGTTATAGAAAAT [29952]
cp32-4_PAli_NX_Pl_consensus    TATGCTTTTGTATTTCAAGACCAACGACCAGCCAATGATCCTTATATTATGAATATGGTAAAGACCGTTATAGAAAAT [29952]
cp32-4_PAbe_NX_MP_FG_consensus TATGCTTTTGTATTTCAAGACCAACGACCAGCCAATGATCCTTATATTATGAATATGGTAAAGACCGTTATAGAAAAT [29952]
cp32-4_PAbe_NX_Pl_consensus    TATGCTTTTGTATTTCAAGACCAACGACCAGCCAATGATCCTTATATTATGAATATGGTAAAGACCGTTATAGAAAAT [29952]

cp32-4_gi|11497149|B31_GB      TTCAATGTGCATACACTGTATTTAGAGGATAGAGATAATACAAAAGGTGCTGGTGGATTGACCCGCGAATACATCTTG [30030]
cp32-4_un10_B31_PacBio         TTCAATGTGCATACACTGTATTTAGAGGATAGAGATAATACAAAAGGTGCTGGTGGATTGACCCGCGAATACATCTTG [30030]
cp32-4_B31_TS_MP_FG_consensus  TTCAATGTGCATACACTGTATTTAGAGGATAGAGATAATACAAAAGGTGCTGGTGGATTGACCCGCGAATACATCTTG [30030]
cp32-4_B31_NX_Pl_consensus     TTCAATGTGCATACACTGTATTTAGAGGATAGAGATAATACAAAAGGTGCTGGTGGATTGACCCGCGAATACATCTTG [30030]
cp32-4_PAli_NX_MP_FG_consensus TTCAATGTGCATACACTGTATTTAGAGGATAGAGATAATACAAAAGGTGCTGGTGGATTGACCCGCGAATACATCTTG [30030]
cp32-4_PAli_NX_Pl_consensus    TTCAATGTGCATACACTGTATTTAGAGGATAGAGATAATACAAAAGGTGCTGGTGGATTGACCCGCGAATACATCTTG [30030]
cp32-4_PAbe_NX_MP_FG_consensus TTCAATGTGCATACACTGTATTTAGAGGATAGAGATAATACAAAAGGTGCTGGTGGATTGACCCGCGAATACATCTTG [30030]
cp32-4_PAbe_NX_Pl_consensus    TTCAATGTGCATACACTGTATTTAGAGGATAGAGATAATACAAAAGGTGCTGGTGGATTGACCCGCGAATACATCTTG [30030]

cp32-4_gi|11497149|B31_GB      CTAAGAAATAATATAAGCCAATATTTTAGAATTGTTCCAGTTAAGCCAAAGTCTAATAAATTTAGCAGAATAACAACG [30108]
cp32-4_un10_B31_PacBio         CTAAGAAATAATATAAGCCAATATTTTAGAATTGTTCCAGTTAAGCCAAAGTCTAATAAATTTAGCAGAATAACAACG [30108]
cp32-4_B31_TS_MP_FG_consensus  CTAAGAAATAATATAAGCCAATATTTTAGAATTGTTCCAGTTAAGCCAAAGTCTAATAAATTTAGCAGAATAACAACG [30108]
cp32-4_B31_NX_Pl_consensus     CTAAGAAATAATATAAGCCAATATTTTAGAATTGTTCCAGTTAAGCCAAAGTCTAATAAATTTAGCAGAATAACAACG [30108]
cp32-4_PAli_NX_MP_FG_consensus CTAAGAAATAATATAAGCCAATATTTTAGAATTGTTCCAGTTAAGCCAAAGTCTAATAAATTTAGCAGAATAACAACG [30108]
cp32-4_PAli_NX_Pl_consensus    CTAAGAAATAATATAAGCCAATATTTTAGAATTGTTCCAGTTAAGCCAAAGTCTAATAAATTTAGCAGAATAACAACG [30108]
cp32-4_PAbe_NX_MP_FG_consensus CTAAGAAATAATATAAGCCAATATTTTAGAATTGTTCCAGTTAAGCCAAAGTCTAATAAATTTAGCAGAATAACAACG [30108]
cp32-4_PAbe_NX_Pl_consensus    CTAAGAAATAATATAAGCCAATATTTTAGAATTGTTCCAGTTAAGCCAAAGTCTAATAAATTTAGCAGAATAACAACG [30108]

cp32-4_gi|11497149|B31_GB      GTAATTACGCCGTTTACTTATAAGAAACTTTACATTACAAAGTACAGTAGTTCTTCTGTATTTAATGATATTTATTCG [30186]
cp32-4_un10_B31_PacBio         GTAATTACGCCGTTTACTTATAAGAAACTTTACATTACAAAGTACAGTAGTTCTTCTGTATTTAATGATATTTATTCG [30186]
cp32-4_B31_TS_MP_FG_consensus  GTAATTACGCCGTTTACTTATAAGAAACTTTACATTACAAAGTACAGTAGTTCTTCTGTATTTAATGATATTTATTCG [30186]
cp32-4_B31_NX_Pl_consensus     GTAATTACGCCGTTTACTTATAAGAAACTTTACATTACAAAGTACAGTAGTTCTTCTGTATTTAATGATATTTATTCG [30186]
cp32-4_PAli_NX_MP_FG_consensus GTAATTACGCCGTTTACTTATAAGAAACTTTACATTACAAAGTACAGTAGTTCTTCTGTATTTAATGATATTTATTCG [30186]
cp32-4_PAli_NX_Pl_consensus    GTAATTACGCCGTTTACTTATAAGAAACTTTACATTACAAAGTACAGTAGTTCTTCTGTATTTAATGATATTTATTCG [30186]
```

```
cp32-4_PAbe_NX_MP_FG_consensus GTAATTACGCCGTTTACTTATAAGAAACTTTACATTACAAAGTACAGTAGTTCTTCTGTATTTAATGATATTTATTCG [30186]
cp32-4_PAbe_NX_Pl_consensus    GTAATTACGCCGTTTACTTATAAGAAACTTTACATTACAAAGTACAGTAGTTCTTCTGTATTTAATGATATTTATTCG [30186]

cp32-4_gi|11497149|B31_GB      TATAAGGGGGATAGCAAAACCCATGATGATGCTCTTGATGCAATGTCTGCAGCATATTTGATGTTGTCTTTAGGATAT [30264]
cp32-4_un10_B31_PacBio         TATAAGGGGGATAGCAAAACCCATGATGATGCTCTTGATGCAATGTCTGCAGCATATTTGATGTTGTCTTTAGGATAT [30264]
cp32-4_B31_TS_MP_FG_consensus  TATAAGGGGGATAGCAAAACCCATGATGATGCTCTTGATGCAATGTCTGCAGCATATTTGATGTTGTCTTTAGGATAT [30264]
cp32-4_B31_NX_Pl_consensus     TATAAGGGGGATAGCAAAACCCATGATGATGCTCTTGATGCAATGTCTGCAGCATATTTGATGTTGTCTTTAGGATAT [30264]
cp32-4_PAli_NX_MP_FG_consensus TATAAGGGGGATAGCAAAACCCATGATGATGCTCTTGATGCAATGTCTGCAGCATATTTGATGTTGTCTTTAGGATAT [30264]
cp32-4_PAli_NX_Pl_consensus    TATAAGGGGGATAGCAAAACCCATGATGATGCTCTTGATGCAATGTCTGCAGCATATTTGATGTTGTCTTTAGGATAT [30264]
cp32-4_PAbe_NX_MP_FG_consensus TATAAGGGGGATAGCAAAACCCATGATGATGCTCTTGATGCAATGTCTGCAGCATATTTGATGTTGTCTTTAGGATAT [30264]
cp32-4_PAbe_NX_Pl_consensus    TATAAGGGGGATAGCAAAACCCATGATGATGCTCTTGATGCAATGTCTGCAGCATATTTGATGTTGTCTTTAGGATAT [30264]

cp32-4_gi|11497149|B31_GB      AGAGAGCGAAGTGTTCACTTTGGCAATCAAAGATTTTTGTAA------------------------------------ [30342]
cp32-4_un10_B31_PacBio         AGAGAGCGAAGTGTTCACTTTGGCAATCAAAGATTTTTGTAA------------------------------------ [30342]
cp32-4_B31_TS_MP_FG_consensus  AGAGAGCGAAGTGTTCACTTTGGCAATCAAAGATTTTTGTAA------------------------------------ [30342]
cp32-4_B31_NX_Pl_consensus     AGAGAGCGAAGTGTTCACTTTGGCAATCAAAGATTTTTGTAA------------------------------------ [30342]
cp32-4_PAli_NX_MP_FG_consensus AGAGAGCGAAGTGTTCACTTTGGCAATCAAAGATTTTTGTAA------------------------------------ [30342]
cp32-4_PAli_NX_Pl_consensus    AGAGAGCGAAGTGTTCACTTTGGCAATCAAAGATTTTTGTAA------------------------------------ [30342]
cp32-4_PAbe_NX_MP_FG_consensus AGAGAGCGAAGTGTTCACTTTGGCAATCAAAGATTTTTGTAA------------------------------------ [30342]
cp32-4_PAbe_NX_Pl_consensus    AGAGAGCGAAGTGTTCACTTTGGCAATCAAAGATTTTTGTAA------------------------------------ [30342]
```

```
#NEXUS
[ Title ]
begin data;
   dimensions ntax=8 nchar=67548;
   format missing=? gap=- matchchar=. datatype=nucleotide interleave=yes;
   matrix

[!Domain=Data property=Coding CodonStart=1;]
cp32-9_gi|11497325|B31_GB      ATTTTATTGACAAAAATATTAGTTTTTGCTATCATACATCTAATTTAATAAAGAGAAGTAAAAAGGTGTGTGATTTAA [   78]
cp32-9_un7_B31_PacBio          ATTTTATTGACAAAAATATTAGTTTTTGCTATCATACATCTAATTTAATAAAGAGAAGTAAAA-GGTGTGTGATTTAA [   78]
cp32-9_B31_TS_MP_FG_consensus  ATTTTATTGACAAAAATATTAGTTTTTGCTATCATACATCTAATTTAATAAAGAGAAGTAAAAAGGTGTGTGATTTAA [   78]
cp32-9_B31_NX_Pl_consensus     ATTTTATTGACAAAAATATTAGTTTTTGCTATCATACATCTAATTTAATAAAGAGAAGTAAAAAGGTGTGTGATTTAA [   78]
cp32-9_PAli_NX_MP_FG_consensus ATTTTATTGACAAAAATATTAGTTTTTGCTATCATACATCTAATTTAATAAAGAGAAGTAAAAAGGTGTGTGATTTAA [   78]
cp32-9_PAli_NX_Pl_consensus    ATTTTATTGACAAAAATATTAGTTTTTGCTATCATACATCTAATTTAATAAAGAGAAGTAAAAAGGTGTGTGATTTAA [   78]
cp32-9_PAbe_NX_MP_FG_consensus ATTTTATTGACAAAAATATTAGTTTTTGCTATCATACATCTAATTTAATAAAGAGAAGTAAAAAGGTGTGTGATTTAA [   78]
cp32-9_PAbe_NX_Pl_consensus    ATTTTATTGACAAAAATATTAGTTTTTGCTATCATACATCTAATTTAATAAAGAGAAGTAAAAAGGTGTGTGATTTAA [   78]

cp32-9_gi|11497325|B31_GB      GAAAAACAAAACTAATAGATAAAATAAGTTCACTAGAACTATACAAATACTCAATATTTTTTAGAAATTACATTGAAA [  156]
cp32-9_un7_B31_PacBio          GAAAAACAAAACTAATAGATAAAATAAGTTCACTAGAACTATACAAATACTCAATATTTTTTAGAAATTACATTGAAA [  156]
cp32-9_B31_TS_MP_FG_consensus  GAAAAACAAAACTAATAGATAAAATAAGTTCACTAGAACTATACAAATACTCAATATTTTTTAGAAATTACATTGAAA [  156]
cp32-9_B31_NX_Pl_consensus     GAAAAACAAAACTAATAGATAAAATAAGTTCACTAGAACTATACAAATACTCAATATTTTTTAGAAATTACATTGAAA [  156]
cp32-9_PAli_NX_MP_FG_consensus GAAAAACAAAACTAATAGATAAAATAAGTTCACTAGAACTATACAAATACTCAATATTTTTTAGAAATTACATTGAAA [  156]
cp32-9_PAli_NX_Pl_consensus    GAAAAACAAAACTAATAGATAAAATAAGTTCACTAGAACTATACAAATACTCAATATTTTTTAGAAATTACATTGAAA [  156]
cp32-9_PAbe_NX_MP_FG_consensus GAAAAACAAAACTAATAGATAAAATAAGTTCACTAGAACTATACAAATACTCAATATTTTTTAGAAATTACATTGAAA [  156]
cp32-9_PAbe_NX_Pl_consensus    GAAAAACAAAACTAATAGATAAAATAAGTTCACTAGAACTATACAAATACTCAATATTTTTTAGAAATTACATTGAAA [  156]

cp32-9_gi|11497325|B31_GB      ATGTAGCAGAAGATTGCCTCAAGAATGGACTTGTTCTTGAGAGTGCTGACCACAATGTTAGTGAGGTTGAACTTGCTA [  234]
cp32-9_un7_B31_PacBio          ATGTAGCAGAAGATTGCCTCAAGAATGGACTTGTTCTTGAGAGTGCTGACCACAATGTTAGTGAGGTTGAACTTGCTA [  234]
cp32-9_B31_TS_MP_FG_consensus  ATGTAGCAGAAGATTGCCTCAAGAATGGACTTGTTCTTGAGAGTGCTGACCACAATGTTAGTGAGGTTGAACTTGCTA [  234]
cp32-9_B31_NX_Pl_consensus     ATGTAGCAGAAGATTGCCTCAAGAATGGACTTGTTCTTGAGAGTGCTGACCACAATGTTAGTGAGGTTGAACTTGCTA [  234]
cp32-9_PAli_NX_MP_FG_consensus ATGTAGCAGAAGATTGCCTCAAGAATGGACTTGTTCTTGAGAGTGCTGACCACAATGTTAGTGAGGTTGAACTTGCTA [  234]
cp32-9_PAli_NX_Pl_consensus    ATGTAGCAGAAGATTGCCTCAAGAATGGACTTGTTCTTGAGAGTGCTGACCACAATGTTAGTGAGGTTGAACTTGCTA [  234]
cp32-9_PAbe_NX_MP_FG_consensus ATGTAGCAGAAGATTGCCTCAAGAATGGACTTGTTCTTGAGAGTGCTGACCACAATGTTAGTGAGGTTGAACTTGCTA [  234]
cp32-9_PAbe_NX_Pl_consensus    ATGTAGCAGAAGATTGCCTCAAGAATGGACTTGTTCTTGAGAGTGCTGACCACAATGTTAGTGAGGTTGAACTTGCTA [  234]

cp32-9_gi|11497325|B31_GB      GGTTAAAGGTACAGCTTAAGAATGCTCTGCTTAATTGTATTATAAGCTACCGTTTTCATGGGATTGGCTATGTTTTAG [  312]
cp32-9_un7_B31_PacBio          GGTTAAAGGTACAGCTTAAGAATGCTCTGCTTAATTGTATTATAAGCTACCGTTTTCATGGGATTGGCTATGTTTTAG [  312]
cp32-9_B31_TS_MP_FG_consensus  GGTTAAAGGTACAGCTTAAGAATGCTCTGCTTAATTGTATTATAAGCTACCGTTTTCATGGGATTGGCTATGTTTTAG [  312]
cp32-9_B31_NX_Pl_consensus     GGTTAAAGGTACAGCTTAAGAATGCTCTGCTTAATTGTATTATAAGCTACCGTTTTCATGGGATTGGCTATGTTTTAG [  312]
cp32-9_PAli_NX_MP_FG_consensus GGTTAAAGGTACAGCTTAAGAATGCTCTGCTTAATTGTATTATAAGCTACCGTTTTCATGGGATTGGCTATGTTTTAG [  312]
cp32-9_PAli_NX_Pl_consensus    GGTTAAAGGTACAGCTTAAGAATGCTCTGCTTAATTGTATTATAAGCTACCGTTTTCATGGGATTGGCTATGTTTTAG [  312]
cp32-9_PAbe_NX_MP_FG_consensus GGTTAAAGGTACAGCTTAAGAATGCTCTGCTTAATTGTATTATAAGCTACCGTTTTCATGGGATTGGCTATGTTTTAG [  312]
cp32-9_PAbe_NX_Pl_consensus    GGTTAAAGGTACAGCTTAAGAATGCTCTGCTTAATTGTATTATAAGCTACCGTTTTCATGGGATTGGCTATGTTTTAG [  312]

cp32-9_gi|11497325|B31_GB      TAAAAACCAAAGATACTCTAATAGATCTCGAACAACCCGTTAATATAGAATTACCCATTGGTTTTGAATACCTTGATT [  390]
cp32-9_un7_B31_PacBio          TAAAAACCAAAGATACTCTAATAGATCTCGAACAACCCGTTAATATAGAATTACCCATTGGTTTTGAATACCTTGATT [  390]
cp32-9_B31_TS_MP_FG_consensus  TAAAAACCAAAGATACTCTAATAGATCTCGAACAACCCGTTAATATAGAATTACCCATTGGTTTTGAATACCTTGATT [  390]
cp32-9_B31_NX_Pl_consensus     TAAAAACCAAAGATACTCTAATAGATCTCGAACAACCCGTTAATATAGAATTACCCATTGGTTTTGAATACCTTGATT [  390]
cp32-9_PAli_NX_MP_FG_consensus TAAAAACCAAAGATACTCTAATAGATCTCGAACAACCCGTTAATATAGAATTACCCATTGGTTTTGAATACCTTGATT [  390]
cp32-9_PAli_NX_Pl_consensus    TAAAAACCAAAGATACTCTAATAGATCTCGAACAACCCGTTAATATAGAATTACCCATTGGTTTTGAATACCTTGATT [  390]
cp32-9_PAbe_NX_MP_FG_consensus TAAAAACCAAAGATACTCTAATAGATCTCGAACAACCCGTTAATATAGAATTACCCATTGGTTTTGAATACCTTGATT [  390]
cp32-9_PAbe_NX_Pl_consensus    TAAAAACCAAAGATACTCTAATAGATCTCGAACAACCCGTTAATATAGAATTACCCATTGGTTTTGAATACCTTGATT [  390]

cp32-9_gi|11497325|B31_GB      ATGAATATGTAAGAGATTTGGGAGTTGATTTTGATCATATAACCTATAAAGTAAAATCCAACAATAAGAACAATTCTT [  468]
cp32-9_un7_B31_PacBio          ATGAATATGTAAGAGATTTGGGAGTTGATTTTGATCATATAACCTATAAAGTAAAATCCAACAATAAGAACAATTCTT [  468]
cp32-9_B31_TS_MP_FG_consensus  ATGAATATGTAAGAGATTTGGGAGTTGATTTTGATCATATAACCTATAAAGTAAAATCCAACAATAAGAACAATTCTT [  468]
cp32-9_B31_NX_Pl_consensus     ATGAATATGTAAGAGATTTGGGAGTTGATTTTGATCATATAACCTATAAAGTAAAATCCAACAATAAGAACAATTCTT [  468]
cp32-9_PAli_NX_MP_FG_consensus ATGAATATGTAAGAGATTTGGGAGTTGATTTTGATCATATAACCTATAAAGTAAAATCCAACAATAAGAACAATTCTT [  468]
cp32-9_PAli_NX_Pl_consensus    ATGAATATGTAAGAGATTTGGGAGTTGATTTTGATCATATAACCTATAAAGTAAAATCCAACAATAAGAACAATTCTT [  468]
cp32-9_PAbe_NX_MP_FG_consensus ATGAATATGTAAGAGATTTGGGAGTTGATTTTGATCATATAACCTATAAAGTAAAATCCAACAATAAGAACAATTCTT [  468]
cp32-9_PAbe_NX_Pl_consensus    ATGAATATGTAAGAGATTTGGGAGTTGATTTTGATCATATAACCTATAAAGTAAAATCCAACAATAAGAACAATTCTT [  468]

cp32-9_gi|11497325|B31_GB      TAGACGCAGTTAAAATACATAAAAGTCGACTTATCATATATGAAAACTTTGATTATATCTTAAAAAGATATGTTCCGT [  546]
cp32-9_un7_B31_PacBio          TAGACGCAGTTAAAATACATAAAAGTCGACTTATCATATATGAAAACTTTGATTATATCTTAAAAAGATATGTTCCGT [  546]
cp32-9_B31_TS_MP_FG_consensus  TAGACGCAGTTAAAATACATAAAAGTCGACTTATCATATATGAAAACTTTGATTATATCTTAAAAAGATATGTTCCGT [  546]
cp32-9_B31_NX_Pl_consensus     TAGACGCAGTTAAAATACATAAAAGTCGACTTATCATATATGAAAACTTTGATTATATCTTAAAAAGATATGTTCCGT [  546]
cp32-9_PAli_NX_MP_FG_consensus TAGACGCAGTTAAAATACATAAAAGTCGACTTATCATATATGAAAACTTTGATTATATCTTAAAAAGATATGTTCCGT [  546]
cp32-9_PAli_NX_Pl_consensus    TAGACGCAGTTAAAATACATAAAAGTCGACTTATCATATATGAAAACTTTGATTATATCTTAAAAAGATATGTTCCGT [  546]
cp32-9_PAbe_NX_MP_FG_consensus TAGACGCAGTTAAAATACATAAAAGTCGACTTATCATATATGAAAACTTTGATTATATCTTAAAAAGATATGTTCCGT [  546]
cp32-9_PAbe_NX_Pl_consensus    TAGACGCAGTTAAAATACATAAAAGTCGACTTATCATATATGAAAACTTTGATTATATCTTAAAAAGATATGTTCCGT [  546]

cp32-9_gi|11497325|B31_GB      GTTATACCGAAAGCTTTTTACTAGATATTTATTTATTTGAAAAGATATACGTAGAAATAGAAAGACGTATTGAAAACC [  624]
cp32-9_un7_B31_PacBio          GTTATACCGAAAGCTTTTTACTAGATATTTATTTATTTGAAAAGATATACGTAGAAATAGAAAGACGTATTGAAAACC [  624]
cp32-9_B31_TS_MP_FG_consensus  GTTATACCGAAAGCTTTTTACTAGATATTTATTTATTTGAAAAGATATACGTAGAAATAGAAAGACGTATTGAAAACC [  624]
cp32-9_B31_NX_Pl_consensus     GTTATACCGAAAGCTTTTTACTAGATATTTATTTATTTGAAAAGATATACGTAGAAATAGAAAGACGTATTGAAAACC [  624]
cp32-9_PAli_NX_MP_FG_consensus GTTATACCGAAAGCTTTTTACTAGATATTTATTTATTTGAAAAGATATACGTAGAAATAGAAAGACGTATTGAAAACC [  624]
cp32-9_PAli_NX_Pl_consensus    GTTATACCGAAAGCTTTTTACTAGATATTTATTTATTTGAAAAGATATACGTAGAAATAGAAAGACGTATTGAAAACC [  624]
cp32-9_PAbe_NX_MP_FG_consensus GTTATACCGAAAGCTTTTTACTAGATATTTATTTATTTGAAAAGATATACGTAGAAATAGAAAGACGTATTGAAAACC [  624]
cp32-9_PAbe_NX_Pl_consensus    GTTATACCGAAAGCTTTTTACTAGATATTTATTTATTTGAAAAGATATACGTAGAAATAGAAAGACGTATTGAAAACC [  624]

cp32-9_gi|11497325|B31_GB      ACAATTTTTTGTTTTACAAAGATGAATCTTTAGTACAACTACAAGACGCGCTTTCTAGTGCAACAACTTCTTTAAGTG [  702]
cp32-9_un7_B31_PacBio          ACAATTTTTTGTTTTACAAAGATGAATCTTTAGTACAACTACAAGACGCGCTTTCTAGTGCAACAACTTCTTTAAGTG [  702]
cp32-9_B31_TS_MP_FG_consensus  ACAATTTTTTGTTTTACAAAGATGAATCTTTAGTACAACTACAAGACGCGCTTTCTAGTGCAACAACTTCTTTAAGTG [  702]
cp32-9_B31_NX_Pl_consensus     ACAATTTTTTGTTTTACAAAGATGAATCTTTAGTACAACTACAAGACGCGCTTTCTAGTGCAACAACTTCTTTAAGTG [  702]
cp32-9_PAli_NX_MP_FG_consensus ACAATTTTTTGTTTTACAAAGATGAATCTTTAGTACAACTACAAGACGCGCTTTCTAGTGCAACAACTTCTTTAAGTG [  702]
cp32-9_PAli_NX_Pl_consensus    ACAATTTTTTGTTTTACAAAGATGAATCTTTAGTACAACTACAAGACGCGCTTTCTAGTGCAACAACTTCTTTAAGTG [  702]
cp32-9_PAbe_NX_MP_FG_consensus ACAATTTTTTGTTTTACAAAGATGAATCTTTAGTACAACTACAAGACGCGCTTTCTAGTGCAACAACTTCTTTAAGTG [  702]
cp32-9_PAbe_NX_Pl_consensus    ACAATTTTTTGTTTTACAAAGATGAATCTTTAGTACAACTACAAGACGCGCTTTCTAGTGCAACAACTTCTTTAAGTG [  702]

cp32-9_gi|11497325|B31_GB      CACTTACTAAGAGCAGCAATAATGATGGGGGAAGTGGCATTTTATCTTCTTTTTTGAGAAAACAAAATTCAAACAATC [  780]
cp32-9_un7_B31_PacBio          CACTTACTAAGAGCAGCAATAATGATGGGGGAAGTGGCATTTTATCTTCTTTTTTGAGAAAACAAAATTCAAACAATC [  780]
cp32-9_B31_TS_MP_FG_consensus  CACTTACTAAGAGCAGCAATAATGATGGGGGAAGTGGCATTTTATCTTCTTTTTTGAGAAAACAAAATTCAAACAATC [  780]
cp32-9_B31_NX_Pl_consensus     CACTTACTAAGAGCAGCAATAATGATGGGGGAAGTGGCATTTTATCTTCTTTTTTGAGAAAACAAAATTCAAACAATC [  780]
cp32-9_PAli_NX_MP_FG_consensus CACTTACTAAGAGCAGCAATAATGATGGGGGAAGTGGCATTTTATCTTCTTTTTTGAGAAAACAAAATTCAAACAATC [  780]
cp32-9_PAli_NX_Pl_consensus    CACTTACTAAGAGCAGCAATAATGATGGGGGAAGTGGCATTTTATCTTCTTTTTTGAGAAAACAAAATTCAAACAATC [  780]
cp32-9_PAbe_NX_MP_FG_consensus CACTTACTAAGAGCAGCAATAATGATGGGGGAAGTGGCATTTTATCTTCTTTTTTGAGAAAACAAAATTCAAACAATC [  780]
cp32-9_PAbe_NX_Pl_consensus    CACTTACTAAGAGCAGCAATAATGATGGGGGAAGTGGCATTTTATCTTCTTTTTTGAGAAAACAAAATTCAAACAATC [  780]

cp32-9_gi|11497325|B31_GB      ATAGTAAAGATATTTCTAATTTAAGAAACCTTAATGACTCATTATCACAGGAGCTTGCTAGGCTAAAAAGCAATCTAA [  858]
cp32-9_un7_B31_PacBio          ATAGTAAAGATATTTCTAATTTAAGAAACCTTAATGACTCATTATCACAGGAGCTTGCTAGGCTAAAAAGCAATCTAA [  858]
cp32-9_B31_TS_MP_FG_consensus  ATAGTAAAGATATTTCTAATTTAAGAAACCTTAATGACTCATTATCACAGGAGCTTGCTAGGCTAAAAAGCAATCTAA [  858]
cp32-9_B31_NX_Pl_consensus     ATAGTAAAGATATTTCTAATTTAAGAAACCTTAATGACTCATTATCACAGGAGCTTGCTAGGCTAAAAAGCAATCTAA [  858]
cp32-9_PAli_NX_MP_FG_consensus ATAGTAAAGATATTTCTAATTTAAGAAACCTTAATGACTCATTATCACAGGAGCTTGCTAGGCTAAAAAGCAATCTAA [  858]
cp32-9_PAli_NX_Pl_consensus    ATAGTAAAGATATTTCTAATTTAAGAAACCTTAATGACTCATTATCACAGGAGCTTGCTAGGCTAAAAAGCAATCTAA [  858]
cp32-9_PAbe_NX_MP_FG_consensus ATAGTAAAGATATTTCTAATTTAAGAAACCTTAATGACTCATTATCACAGGAGCTTGCTAGGCTAAAAAGCAATCTAA [  858]
cp32-9_PAbe_NX_Pl_consensus    ATAGTAAAGATATTTCTAATTTAAGAAACCTTAATGACTCATTATCACAGGAGCTTGCTAGGCTAAAAAGCAATCTAA [  858]

cp32-9_gi|11497325|B31_GB      ATAATGAGGGAATGTTTTATACGGCCACCCTTAGTGCTAGTTTAGAGGTTATTAAATACGATCTTAGTTACTTAAGGG [  936]
cp32-9_un7_B31_PacBio          ATAATGAGGGAATGTTTTATACGGCCACCCTTAGTGCTAGTTTAGAGGTTATTAAATACGATCTTAGTTACTTAAGGG [  936]
```

```
cp32-9_B31_TS_MP_FG_consensus  ATAATGAGGGAATGTTTTATACGGCCACCCTTAGTGCTAGTTTAGAGGTTATTAAATACGATCTTAGTTACTTAAGGG [  936]
cp32-9_B31_NX_Pl_consensus     ATAATGAGGGAATGTTTTATACGGCCACCCTTAGTGCTAGTTTAGAGGTTATTAAATACGATCTTAGTTACTTAAGGG [  936]
cp32-9_PAli_NX_MP_FG_consensus ATAATGAGGGAATGTTTTATACGGCCACCCTTAGTGCTAGTTTAGAGGTTATTAAATACGATCTTAGTTACTTAAGGG [  936]
cp32-9_PAli_NX_Pl_consensus    ATAATGAGGGAATGTTTTATACGGCCACCCTTAGTGCTAGTTTAGAGGTTATTAAATACGATCTTAGTTACTTAAGGG [  936]
cp32-9_PAbe_NX_MP_FG_consensus ATAATGAGGGAATGTTTTATACGGCCACCCTTAGTGCTAGTTTAGAGGTTATTAAATACGATCTTAGTTACTTAAGGG [  936]
cp32-9_PAbe_NX_Pl_consensus    ATAATGAGGGAATGTTTTATACGGCCACCCTTAGTGCTAGTTTAGAGGTTATTAAATACGATCTTAGTTACTTAAGGG [  936]

cp32-9_gi|11497325|B31_GB      AGGCTTTAGCATTAATTAAGGCAAAAATTGGTGCAGATACTAAAGAGCCCTTAACCAGAAGCTTTAATGAACAGGCTA [ 1014]
cp32-9_un7_B31_PacBio          AGGCTTTAGCATTAATTAAGGCAAAAATTGGTGCAGATACTAAAGAGCCCTTAACCAGAAGCTTTAATGAACAGGCTA [ 1014]
cp32-9_B31_TS_MP_FG_consensus  AGGCTTTAGCATTAATTAAGGCAAAAATTGGTGCAGATACTAAAGAGCCCTTAACCAGAAGCTTTAATGAACAGGCTA [ 1014]
cp32-9_B31_NX_Pl_consensus     AGGCTTTAGCATTAATTAAGGCAAAAATTGGTGCAGATACTAAAGAGCCCTTAACCAGAAGCTTTAATGAACAGGCTA [ 1014]
cp32-9_PAli_NX_MP_FG_consensus AGGCTTTAGCATTAATTAAGGCAAAAATTGGTGCAGATACTAAAGAGCCCTTAACCAGAAGCTTTAATGAACAGGCTA [ 1014]
cp32-9_PAli_NX_Pl_consensus    AGGCTTTAGCATTAATTAAGGCAAAAATTGGTGCAGATACTAAAGAGCCCTTAACCAGAAGCTTTAATGAACAGGCTA [ 1014]
cp32-9_PAbe_NX_MP_FG_consensus AGGCTTTAGCATTAATTAAGGCAAAAATTGGTGCAGATACTAAAGAGCCCTTAACCAGAAGCTTTAATGAACAGGCTA [ 1014]
cp32-9_PAbe_NX_Pl_consensus    AGGCTTTAGCATTAATTAAGGCAAAAATTGGTGCAGATACTAAAGAGCCCTTAACCAGAAGCTTTAATGAACAGGCTA [ 1014]

cp32-9_gi|11497325|B31_GB      AAGGATTAGGAAATGGTGGTAAAGGTGATAGGAGTAATTATTACGATTTTCTCAAAGGTGTGCAAGAACAAATTGAGA [ 1092]
cp32-9_un7_B31_PacBio          AAGGATTAGGAAATGGTGGTAAAGGTGATAGGAGTAATTATTACGATTTTCTCAAAGGTGTGCAAGAACAAATTGAGA [ 1092]
cp32-9_B31_TS_MP_FG_consensus  AAGGATTAGGAAATGGTGGTAAAGGTGATAGGAGTAATTATTACGATTTTCTCAAAGGTGTGCAAGAACAAATTGAGA [ 1092]
cp32-9_B31_NX_Pl_consensus     AAGGATTAGGAAATGGTGGTAAAGGTGATAGGAGTAATTATTACGATTTTCTCAAAGGTGTGCAAGAACAAATTGAGA [ 1092]
cp32-9_PAli_NX_MP_FG_consensus AAGGATTAGGAAATGGTGGTAAAGGTGATAGGAGTAATTATTACGATTTTCTCAAAGGTGTGCAAGAACAAATTGAGA [ 1092]
cp32-9_PAli_NX_Pl_consensus    AAGGATTAGGAAATGGTGGTAAAGGTGATAGGAGTAATTATTACGATTTTCTCAAAGGTGTGCAAGAACAAATTGAGA [ 1092]
cp32-9_PAbe_NX_MP_FG_consensus AAGGATTAGGAAATGGTGGTAAAGGTGATAGGAGTAATTATTACGATTTTCTCAAAGGTGTGCAAGAACAAATTGAGA [ 1092]
cp32-9_PAbe_NX_Pl_consensus    AAGGATTAGGAAATGGTGGTAAAGGTGATAGGAGTAATTATTACGATTTTCTCAAAGGTGTGCAAGAACAAATTGAGA [ 1092]

cp32-9_gi|11497325|B31_GB      ACTCTTGTAATTTAAAACTTACAAAGTATTTTGGGCTTGATATGAAATTTAATTCGCTGATTATGTTAAGTGAAGAAC [ 1170]
cp32-9_un7_B31_PacBio          ACTCTTGTAATTTAAAACTTACAAAGTATTTTGGGCTTGATATGAAATTTAATTCGCTGATTATGTTAAGTGAAGAAC [ 1170]
cp32-9_B31_TS_MP_FG_consensus  ACTCTTGTAATTTAAAACTTACAAAGTATTTTGGGCTTGATATGAAATTTAATTCGCTGATTATGTTAAGTGAAGAAC [ 1170]
cp32-9_B31_NX_Pl_consensus     ACTCTTGTAATTTAAAACTTACAAAGTATTTTGGGCTTGATATGAAATTTAATTCGCTGATTATGTTAAGTGAAGAAC [ 1170]
cp32-9_PAli_NX_MP_FG_consensus ACTCTTGTAATTTAAAACTTACAAAGTATTTTGGGCTTGATATGAAATTTAATTCGCTGATTATGTTAAGTGAAGAAC [ 1170]
cp32-9_PAli_NX_Pl_consensus    ACTCTTGTAATTTAAAACTTACAAAGTATTTTGGGCTTGATATGAAATTTAATTCGCTGATTATGTTAAGTGAAGAAC [ 1170]
cp32-9_PAbe_NX_MP_FG_consensus ACTCTTGTAATTTAAAACTTACAAAGTATTTTGGGCTTGATATGAAATTTAATTCGCTGATTATGTTAAGTGAAGAAC [ 1170]
cp32-9_PAbe_NX_Pl_consensus    ACTCTTGTAATTTAAAACTTACAAAGTATTTTGGGCTTGATATGAAATTTAATTCGCTGATTATGTTAAGTGAAGAAC [ 1170]

cp32-9_gi|11497325|B31_GB      AAAAAGTGGAAAGAGATATAAAGCTAATTGAGCTTTACAGTAAATATAACCAGCTTATACAAAGTAGCTCCTTTGATA [ 1248]
cp32-9_un7_B31_PacBio          AAAAAGTGGAAAGAGATATAAAGCTAATTGAGCTTTACAGTAAATATAACCAGCTTATACAAAGTAGCTCCTTTGATA [ 1248]
cp32-9_B31_TS_MP_FG_consensus  AAAAAGTGGAAAGAGATATAAAGCTAATTGAGCTTTACAGTAAATATAACCAGCTTATACAAAGTAGCTCCTTTGATA [ 1248]
cp32-9_B31_NX_Pl_consensus     AAAAAGTGGAAAGAGATATAAAGCTAATTGAGCTTTACAGTAAATATAACCAGCTTATACAAAGTAGCTCCTTTGATA [ 1248]
cp32-9_PAli_NX_MP_FG_consensus AAAAAGTGGAAAGAGATATAAAGCTAATTGAGCTTTACAGTAAATATAACCAGCTTATACAAAGTAGCTCCTTTGATA [ 1248]
cp32-9_PAli_NX_Pl_consensus    AAAAAGTGGAAAGAGATATAAAGCTAATTGAGCTTTACAGTAAATATAACCAGCTTATACAAAGTAGCTCCTTTGATA [ 1248]
cp32-9_PAbe_NX_MP_FG_consensus AAAAAGTGGAAAGAGATATAAAGCTAATTGAGCTTTACAGTAAATATAACCAGCTTATACAAAGTAGCTCCTTTGATA [ 1248]
cp32-9_PAbe_NX_Pl_consensus    AAAAAGTGGAAAGAGATATAAAGCTAATTGAGCTTTACAGTAAATATAACCAGCTTATACAAAGTAGCTCCTTTGATA [ 1248]

cp32-9_gi|11497325|B31_GB      ATGAGGAGTTAGCGATTTTAAAAGAGAAATTATTCTCATTTTGAGAAAAGGAGTTAAAAGATGACTGAGAAAGAAGAA [ 1326]
cp32-9_un7_B31_PacBio          ATGAGGAGTTAGCGATTTTAAAAGAGAAATTATTCTCATTTTGAGAAAAGGAGTTAAAAGATGACTGAGAAAGAAGAA [ 1326]
cp32-9_B31_TS_MP_FG_consensus  ATGAGGAGTTAGCGATTTTAAAAGAGAAATTATTCTCATTTTGAGAAAAGGAGTTAAAAGATGACTGAGAAAGAAGAA [ 1326]
cp32-9_B31_NX_Pl_consensus     ATGAGGAGTTAGCGATTTTAAAAGAGAAATTATTCTCATTTTGAGAAAAGGAGTTAAAAGATGACTGAGAAAGAAGAA [ 1326]
cp32-9_PAli_NX_MP_FG_consensus ATGAGGAGTTAGCGATTTTAAAAGAGAAATTATTCTCATTTTGAGAAAAGGAGTTAAAAGATGACTGAGAAAGAAGAA [ 1326]
cp32-9_PAli_NX_Pl_consensus    ATGAGGAGTTAGCGATTTTAAAAGAGAAATTATTCTCATTTTGAGAAAAGGAGTTAAAAGATGACTGAGAAAGAAGAA [ 1326]
cp32-9_PAbe_NX_MP_FG_consensus ATGAGGAGTTAGCGATTTTAAAAGAGAAATTATTCTCATTTTGAGAAAAGGAGTTAAAAGATGACTGAGAAAGAAGAA [ 1326]
cp32-9_PAbe_NX_Pl_consensus    ATGAGGAGTTAGCGATTTTAAAAGAGAAATTATTCTCATTTTGAGAAAAGGAGTTAAAAGATGACTGAGAAAGAAGAA [ 1326]

cp32-9_gi|11497325|B31_GB      AAAGAAGACCTGCAAGCACAAGATAAAGAAGAGCAGCAAAATAAGGCTGATACTAAAGTTATAAGTGCGCAGGAATTT [ 1404]
cp32-9_un7_B31_PacBio          AAAGAAGACCTGCAAGCACAAGATAAAGAAGAGCAGCAAAATAAGGCTGATACTAAAGTTATAAGTGCGCAGGAATTT [ 1404]
cp32-9_B31_TS_MP_FG_consensus  AAAGAAGACCTGCAAGCACAAGATAAAGAAGAGCAGCAAAATAAGGCTGATACTAAAGTTATAAGTGCGCAGGAATTT [ 1404]
cp32-9_B31_NX_Pl_consensus     AAAGAAGACCTGCAAGCACAAGATAAAGAAGAGCAGCAAAATAAGGCTGATACTAAAGTTATAAGTGCGCAGGAATTT [ 1404]
cp32-9_PAli_NX_MP_FG_consensus AAAGAAGACCTGCAAGCACAAGATAAAGAAGAGCAGCAAAATAAGGCTGATACTAAAGTTATAAGTGCGCAGGAATTT [ 1404]
cp32-9_PAli_NX_Pl_consensus    AAAGAAGACCTGCAAGCACAAGATAAAGAAGAGCAGCAAAATAAGGCTGATACTAAAGTTATAAGTGCGCAGGAATTT [ 1404]
cp32-9_PAbe_NX_MP_FG_consensus AAAGAAGACCTGCAAGCACAAGATAAAGAAGAGCAGCAAAATAAGGCTGATACTAAAGTTATAAGTGCGCAGGAATTT [ 1404]
cp32-9_PAbe_NX_Pl_consensus    AAAGAAGACCTGCAAGCACAAGATAAAGAAGAGCAGCAAAATAAGGCTGATACTAAAGTTATAAGTGCGCAGGAATTT [ 1404]

cp32-9_gi|11497325|B31_GB      GAAGAGTACATGCGCTTTAAAGAGCAAGCAAATAGTAAATCCAAAGAGACAAGTCGAGATTTAAGTATAAATGAACGA [ 1482]
cp32-9_un7_B31_PacBio          GAAGAGTACATGCGCTTTAAAGAGCAAGCAAATAGTAAATCCAAAGAGACAAGTCGAGATTTAAGTATAAATGAACGA [ 1482]
cp32-9_B31_TS_MP_FG_consensus  GAAGAGTACATGCGCTTTAAAGAGCAAGCAAATAGTAAATCCAAAGAGACAAGTCGAGATTTAAGTATAAATGAACGA [ 1482]
cp32-9_B31_NX_Pl_consensus     GAAGAGTACATGCGCTTTAAAGAGCAAGCAAATAGTAAATCCAAAGAGACAAGTCGAGATTTAAGTATAAATGAACGA [ 1482]
cp32-9_PAli_NX_MP_FG_consensus GAAGAGTACATGCGCTTTAAAGAGCAAGCAAATAGTAAATCCAAAGAGACAAGTCGAGATTTAAGTATAAATGAACGA [ 1482]
cp32-9_PAli_NX_Pl_consensus    GAAGAGTACATGCGCTTTAAAGAGCAAGCAAATAGTAAATCCAAAGAGACAAGTCGAGATTTAAGTATAAATGAACGA [ 1482]
cp32-9_PAbe_NX_MP_FG_consensus GAAGAGTACATGCGCTTTAAAGAGCAAGCAAATAGTAAATCCAAAGAGACAAGTCGAGATTTAAGTATAAATGAACGA [ 1482]
cp32-9_PAbe_NX_Pl_consensus    GAAGAGTACATGCGCTTTAAAGAGCAAGCAAATAGTAAATCCAAAGAGACAAGTCGAGATTTAAGTATAAATGAACGA [ 1482]

cp32-9_gi|11497325|B31_GB      ATAACAAAAGAACTCGCAGAAGTTGAAGAGAGGGAGCGTATTAAAAAGCAATTATTGCTAGAGGCTGAGCGCATAAAT [ 1560]
cp32-9_un7_B31_PacBio          ATAACAAAAGAACTCGCAGAAGTTGAAGAGAGGGAGCGTATTAAAAAGCAATTATTGCTAGAGGCTGAGCGCATAAAT [ 1560]
cp32-9_B31_TS_MP_FG_consensus  ATAACAAAAGAACTCGCAGAAGTTGAAGAGAGGGAGCGTATTAAAAAGCAATTATTGCTAGAGGCTGAGCGCATAAAT [ 1560]
cp32-9_B31_NX_Pl_consensus     ATAACAAAAGAACTCGCAGAAGTTGAAGAGCGGGAGCGTATTGAAAAGCAATTATTGCTAGAGGCTGAGCGAATTAAT [ 1560]
cp32-9_PAli_NX_MP_FG_consensus ATAACAAAAGAACTCGCAGAAGTTGAAGAGAGGGAGCGTATTAAAAAGCAATTATTGCTAGAGGCTGAGCGCATAAAT [ 1560]
cp32-9_PAli_NX_Pl_consensus    ATAACAAAAGAACTCGCAGAAGTTGAAGAGAGGGAGCGTATTAAAAAGCAATTATTGCTAGAGGCTGAGCGCATAAAT [ 1560]
cp32-9_PAbe_NX_MP_FG_consensus ATAACAAAAGAACTCGCAGAAGTTGAAGAGCGGGAGCGTATTGAAAAGCAATTATTGCTAGAGGCTGAGCGAATTAAT [ 1560]
cp32-9_PAbe_NX_Pl_consensus    ATAACAAAAGAACTCGCAGAAGTTGAAGAGAGGGAGCGTATTGAAAAGCAATTATTGCTAGAGGCTGAGCGCATAAAT [ 1560]

cp32-9_gi|11497325|B31_GB      GAAATTGATACACTTGCAAAAGCACATCTTAGCAATCATTTCAATAAGGAGGTGTTGCTTGCAAAAGGATACACATTA [ 1638]
cp32-9_un7_B31_PacBio          GAAATTGATACACTTGCAAAAGCACATCTTAGCAATCATTTCAATAAGGAGGTGTTGCTTGCAAAAGGATACACATTA [ 1638]
cp32-9_B31_TS_MP_FG_consensus  GAAATTGATACACTTGCAAAAGCACATCTTAGCAATCATTTCAATAAGGAGGTGTTGCTTGCAAAAGGATACACATTA [ 1638]
cp32-9_B31_NX_Pl_consensus     GAAATTGATACACTTGCAAAAGCACATCTTAGCAATCATTTCAATAAGGAGGTGTTGCTTGCAAAAGGATACACATTA [ 1638]
cp32-9_PAli_NX_MP_FG_consensus GAAATTGATACACTTGCAAAAGCACATCTTAGCAATCATTTCAATAAGGAGGTGTTGCTTGCAAAAGGATACACATTA [ 1638]
cp32-9_PAli_NX_Pl_consensus    GAAATTGATACACTTGCAAAAGCACATCTTAGCAATCATTTCAATAAGGAGGTGTTGCTTGCAAAAGGATACACATTA [ 1638]
cp32-9_PAbe_NX_MP_FG_consensus GAAATTGATACACTTGCAAAAGCACATCTTAGCAATCATTTCAATAAGGAGGTGTTGCTTGCAAAAGGATACACATTA [ 1638]
cp32-9_PAbe_NX_Pl_consensus    GAAATTGATACACTTGCAAAAGCACATCTTAGCAATCATTTCAATAAGGAGGTGTTGCTTGCAAAAGGATACACATTA [ 1638]

cp32-9_gi|11497325|B31_GB      AAAGACATTATGCAAGCACAACGTAGAGAACTTGTGCGCAAGTTCGTTCCAATTGAGCAAATTAAAGCTATTGCCAAA [ 1716]
cp32-9_un7_B31_PacBio          AAAGACATTATGCAAGCACAACGTAGAGAACTTGTGCGCAAGTTCGTTCCAATTGAGCAAATTAAAGCTATTGCCAAA [ 1716]
cp32-9_B31_TS_MP_FG_consensus  AAAGACATTATGCAAGCACAACGTAGAGAACTTGTGCGCAAGTTCGTTCCAATTGAGCAAATTAAAGCTATTGCCAAA [ 1716]
cp32-9_B31_NX_Pl_consensus     AAAGACATTATGCAAGCACAACGTAGAGAACTTGTGCGCAAGTTCGTTCCAATTGAGCAAATTAAAGCTATTGCCAAA [ 1716]
cp32-9_PAli_NX_MP_FG_consensus AAAGACATTATGCAAGCACAACGTAGAGAACTTGTGCGCAAGTTCGTTCCAATTGAGCAAATTAAAGCTATTGCCAAA [ 1716]
cp32-9_PAli_NX_Pl_consensus    AAAGACATTATGCAAGCACAACGTAGAGAACTTGTGCGCAAGTTCGTTCCAATTGAGCAAATTAAAGCTATTGCCAAA [ 1716]
cp32-9_PAbe_NX_MP_FG_consensus AAAGACATTATGCAAGCACAACGTAGAGAACTTGTGCGCAAGTTCGTTCCAATTGAGCAAATTAAAGCTATTGCCAAA [ 1716]
cp32-9_PAbe_NX_Pl_consensus    AAAGACATTATGCAAGCACAACGTAGAGAACTTGTGCGCAAGTTCGTTCCAATTGAGCAAATTAAAGCTATTGCCAAA [ 1716]

cp32-9_gi|11497325|B31_GB      GTATCAGACATAAGTCATATCGATGGAGAGATGTTAGAGCAACTTGTTTCTTTAGCAAAAGTGAATATTAAATTAAGA [ 1794]
cp32-9_un7_B31_PacBio          GTATCAGACATAAGTCATATCGATGGAGAGATGTTAGAGCAACTTGTTTCTTTAGCAAAAGTGAATATTAAATTAAGA [ 1794]
cp32-9_B31_TS_MP_FG_consensus  GTATCAGACATAAGTCATATCGATGGAGAGATGTTAGAGCAACTTGTTTCTTTAGCAAAAGTGAATATTAAATTAAGA [ 1794]
cp32-9_B31_NX_Pl_consensus     GTATCAGACATAAGTCATATCGATGGAGAGATGTTAGAGCAACTTGTTTCTTTAGCAAAAGTGAATATTAAATTAAGA [ 1794]
cp32-9_PAli_NX_MP_FG_consensus GTATCAGACATAAGTCATATCGATGGAGAGATGTTAGAGCAACTTGTTTCTTTAGCAAAAGTGAATATTAAATTAAGA [ 1794]
cp32-9_PAli_NX_Pl_consensus    GTATCAGACATAAGTCATATCGATGGAGAGATGTTAGAGCAACTTGTTTCTTTAGCAAAAGTGAATATTAAATTAAGA [ 1794]
cp32-9_PAbe_NX_MP_FG_consensus GTATCAGACATAAGTCATATCGATGGAGAGATGTTAGAGCAACTTGTTTCTTTAGCAAAAGTGAATATTAAATTAAGA [ 1794]
cp32-9_PAbe_NX_Pl_consensus    GTATCAGACATAAGTCATATCGATGGAGAGATGTTAGAGCAACTTGTTTCTTTAGCAAAAGTGAATATTAAATTAAGA [ 1794]

cp32-9_gi|11497325|B31_GB      AAAACTGCGAGTAGCAATTTTTCTTCTGTTGACTCTGTCAGAGGCAATATTGTTGTTAAATCAGAAGAGAGAGCAAGT [ 1872]
cp32-9_un7_B31_PacBio          AAAACTGCGAGTAGCAATTTTTCTTCTGTTGACTCTGTCAGAGGCAATATTGTTGTTAAATCAGAAGAGAGAGCAAGT [ 1872]
cp32-9_B31_TS_MP_FG_consensus  AAAACTGCGAGTAGCAATTTTTCTTCTGTTGACTCTGTCAGAGGCAATATTGTTGTTAAATCAGAAGAGAGAGCAAGT [ 1872]
```

```
cp32-9_B31_NX_Pl_consensus     AAAACTGCGAGTAGCAATTTTTCTTCTGTTGACTCTGTCAGAGGCAATATTGTTGTTAAATCAGAAGAGAGAGCAAGT [ 1872]
cp32-9_PAli_NX_MP_FG_consensus AAAACTGCGAGTAGCAATTTTTCTTCTGTTGACTCTGTCAGAGGCAATATTGTTGTTAAATCAGAAGAGAGAGCAAGT [ 1872]
cp32-9_PAli_NX_Pl_consensus    AAAACTGCGAGTAGCAATTTTTCTTCTGTTGACTCTGTCAGAGGCAATATTGTTGTTAAATCAGAAGAGAGAGCAAGT [ 1872]
cp32-9_PAbe_NX_MP_FG_consensus AAAACTGCGAGTAGCAATTTTTCTTCTGTTGACTCTGTCAGAGGCAATATTGTTGTTAAATCAGAAGAGAGAGCAAGT [ 1872]
cp32-9_PAbe_NX_Pl_consensus    AAAACTGCGAGTAGCAATTTTTCTTCTGTTGACTCTGTCAGAGGCAATATTGTTGTTAAATCAGAAGAGAGAGCAAGT [ 1872]

cp32-9_gi|11497325|B31_GB      TTGCTTGATTCTAATTTTGTACCTATTAATTTCACAGAATTTGTACAAGCGATAAGTAATACTTACAAGCAAAGACGA [ 1950]
cp32-9_un7_B31_PacBio          TTGCTTGATTCTAATTTTGTACCTATTAATTTCACAGAATTTGTACAAGCGATAAGTAATACTTACAAGCAAAGACGA [ 1950]
cp32-9_B31_TS_MP_FG_consensus  TTGCTTGATTCTAATTTTGTACCTATTAATTTCACAGAATTTGTACAAGCGATAAGTAATACTTACAAGCAAAGACGA [ 1950]
cp32-9_B31_NX_Pl_consensus     TTGCTTGATTCTAATTTTGTACCTATTAATTTCACAGAATTTGTACAAGCGATAAGTAATACTTACAAGCAAAGACGA [ 1950]
cp32-9_PAli_NX_MP_FG_consensus TTGCTTGATTCTAATTTTGTACCTATTAATTTCACAGAATTTGTACAAGCGATAAGTAATACTTACAAGCAAAGACGA [ 1950]
cp32-9_PAli_NX_Pl_consensus    TTGCTTGATTCTAATTTTGTACCTATTAATTTCACAGAATTTGTACAAGCGATAAGTAATACTTACAAGCAAAGACGA [ 1950]
cp32-9_PAbe_NX_MP_FG_consensus TTGCTTGATTCTAATTTTGTACCTATTAATTTCACAGAATTTGTACAAGCGATAAGTAATACTTACAAGCAAAGACGA [ 1950]
cp32-9_PAbe_NX_Pl_consensus    TTGCTTGATTCTAATTTTGTACCTATTAATTTCACAGAATTTGTACAAGCGATAAGTAATACTTACAAGCAAAGACGA [ 1950]

cp32-9_gi|11497325|B31_GB      ATTCAATTTTATGAAAATCTAAAAAGACATAAAAGAACAAGTATTGCTTAAAGGAGTTTTTAATGAGCGATGGTATTA [ 2028]
cp32-9_un7_B31_PacBio          ATTCAATTTTATGAAAATCTAAAAAGACATAAAAGAACAAGTATTGCTTAAAGGAGTTTTTAATGAGCGATGGTATTA [ 2028]
cp32-9_B31_TS_MP_FG_consensus  ATTCAATTTTATGAAAATCTAAAAAGACATAAAAGAACAAGTATTGCTTAAAGGAGTTTTTAATGAGCGATGGTATTA [ 2028]
cp32-9_B31_NX_Pl_consensus     ATTCAATTTTATGAAAATCTAAAAAGACATAAAAGAACAAGTATTGCTTAAAGGAGTTTTTAATGAGCGATGGTATTA [ 2028]
cp32-9_PAli_NX_MP_FG_consensus ATTCAATTTTATGAAAATCTAAAAAGACATAAAAGAACAAGTATTGCTTAAAGGAGTTTTTAATGAGCGATGGTATTA [ 2028]
cp32-9_PAli_NX_Pl_consensus    ATTCAATTTTATGAAAATCTAAAAAGACATAAAAGAACAAGTATTGCTTAAAGGAGTTTTTAATGAGCGATGGTATTA [ 2028]
cp32-9_PAbe_NX_MP_FG_consensus ATTCAATTTTATGAAAATCTAAAAAGACATAAAAGAACAAGTATTGCTTAAAGGAGTTTTTAATGAGCGATGGTATTA [ 2028]
cp32-9_PAbe_NX_Pl_consensus    ATTCAATTTTATGAAAATCTAAAAAGACATAAAAGAACAAGTATTGCTTAAAGGAGTTTTTAATGAGCGATGGTATTA [ 2028]

cp32-9_gi|11497325|B31_GB      CAAAAATAAAAGAAGAGTTTGATAAAAAAGTTGCAGAAATTAAAGCATTAATGAAAAATCCTCAGCAAGATGCTGGTT [ 2106]
cp32-9_un7_B31_PacBio          CAAAAATAAAAGAAGAGTTTGATAAAAAAGTTGCAGAAATTAAAGCATTAATGAAAAATCCTCAGCAAGATGCTGGTT [ 2106]
cp32-9_B31_TS_MP_FG_consensus  CAAAAATAAAAGAAGAGTTTGATAAAAAAGTTGCAGAAATTAAAGCATTAATGAAAAATCCTCAGCAAGATGCTGGTT [ 2106]
cp32-9_B31_NX_Pl_consensus     CAAAAATAAAAGAAGAGTTTGATAAAAAAGTTGCAGAAATTAAAGCATTAATGAAAAATCCTCAGCAAGATGCTGGTT [ 2106]
cp32-9_PAli_NX_MP_FG_consensus CAAAAATAAAAGAAGAGTTTGATAAAAAAGTTGCAGAAATTAAAGCATTAATGAAAAATCCTCAGCAAGATGCTGGTT [ 2106]
cp32-9_PAli_NX_Pl_consensus    CAAAAATAAAAGAAGAGTTTGATAAAAAAGTTGCAGAAATTAAAGCATTAATGAAAAATCCTCAGCAAGATGCTGGTT [ 2106]
cp32-9_PAbe_NX_MP_FG_consensus CAAAAATAAAAGAAGAGTTTGATAAAAAAGTTGCAGAAATTAAAGCATTAATGAAAAATCCTCAGCAAGATGCTGGTT [ 2106]
cp32-9_PAbe_NX_Pl_consensus    CAAAAATAAAAGAAGAGTTTGATAAAAAAGTTGCAGAAATTAAAGCATTAATGAAAAATCCTCAGCAAGATGCTGGTT [ 2106]

cp32-9_gi|11497325|B31_GB      TGCTTAGGAATTCTGTAGACTTTAGAGATAAAAATCTAATTTACTCCAATTCGGATGGAGTTTTTACTAGTAGTAAAG [ 2184]
cp32-9_un7_B31_PacBio          TGCTTAGGAATTCTGTAGACTTTAGAGATAAAAATCTAATTTACTCCAATTCGGATGGAGTTTTTACTAGTAGTAAAG [ 2184]
cp32-9_B31_TS_MP_FG_consensus  TGCTTAGGAATTCTGTAGACTTTAGAGATAAAAATCTAATTTACTCCAATTCGGATGGAGTTTTTACTAGTAGTAAAG [ 2184]
cp32-9_B31_NX_Pl_consensus     TGCTTAGGAATTCTGTAGACTTTAGAGATAAAAATCTAATTTACTCCAATTCGGATGGAGTTTTTACTAGTAGTAAAG [ 2184]
cp32-9_PAli_NX_MP_FG_consensus TGCTTAGGAATTCTGTAGACTTTAGAGATAAAAATCTAATTTACTCCAATTCGGATGGAGTTTTTACTAGTAGTAAAG [ 2184]
cp32-9_PAli_NX_Pl_consensus    TGCTTAGGAATTCTGTAGACTTTAGAGATAAAAATCTAATTTACTCCAATTCGGATGGAGTTTTTACTAGTAGTAAAG [ 2184]
cp32-9_PAbe_NX_MP_FG_consensus TGCTTAGGAATTCTGTAGACTTTAGAGATAAAAATCTAATTTACTCCAATTCGGATGGAGTTTTTACTAGTAGTAAAG [ 2184]
cp32-9_PAbe_NX_Pl_consensus    TGCTTAGGAATTCTGTAGACTTTAGAGATAAAAATCTAATTTACTCCAATTCGGATGGAGTTTTTACTAGTAGTAAAG [ 2184]

cp32-9_gi|11497325|B31_GB      ACAAAATAGAAAATTATCCTGCTAAAGGGTATCCATACAAGCGTGGAGTCAAGCTTAGTTTTAGTGCAGATGGTACAA [ 2262]
cp32-9_un7_B31_PacBio          ACAAAATAGAAAATTATCCTGCTAAAGGGTATCCATACAAGCGTGGAGTCAAGCTTAGTTTTAGTGCAGATGGTACAA [ 2262]
cp32-9_B31_TS_MP_FG_consensus  ACAAAATAGAAAATTATCCTGCTAAAGGGTATCCATACAAGCGTGGAGTCAAGCTTAGTTTTAGTGCAGATGGTACAA [ 2262]
cp32-9_B31_NX_Pl_consensus     ACAAAATAGAAAATTATCCTGCTAAAGGGTATCCATACAAGCGTGGAGTCAAGCTTAGTTTTAGTGCAGATGGTACAA [ 2262]
cp32-9_PAli_NX_MP_FG_consensus ACAAAATAGAAAATTATCCTGCTAAAGGGTATCCATACAAGCGTGGAGTCAAGCTTAGTTTTAGTGCAGATGGTACAA [ 2262]
cp32-9_PAli_NX_Pl_consensus    ACAAAATAGAAAATTATCCTGCTAAAGGGTATCCATACAAGCGTGGAGTCAAGCTTAGTTTTAGTGCAGATGGTACAA [ 2262]
cp32-9_PAbe_NX_MP_FG_consensus ACAAAATAGAAAATTATCCTGCTAAAGGGTATCCATACAAGCGTGGAGTCAAGCTTAGTTTTAGTGCAGATGGTACAA [ 2262]
cp32-9_PAbe_NX_Pl_consensus    ACAAAATAGAAAATTATCCTGCTAAAGGGTATCCATACAAGCGTGGAGTCAAGCTTAGTTTTAGTGCAGATGGTACAA [ 2262]

cp32-9_gi|11497325|B31_GB      CAGAACTAGAAGTTGAGGCTGGTGGTGGGGATGACTTGTACGGAATATGCACTGATATAGATGAGTTTACTGGCATGG [ 2340]
cp32-9_un7_B31_PacBio          CAGAACTAGAAGTTGAGGCTGGTGGTGGGGATGACTTGTACGGAATATGCACTGATATAGATGAGTTTACTGGCATGG [ 2340]
cp32-9_B31_TS_MP_FG_consensus  CAGAACTAGAAGTTGAGGCTGGTGGTGGGGATGACTTGTACGGAATATGCACTGATATAGATGAGTTTACTGGCATGG [ 2340]
cp32-9_B31_NX_Pl_consensus     CAGAACTAGAAGTTGAGGCTGGTGGTGGGGATGACTTGTACGGAATATGCACTGATATAGATGAGTTTACTGGCATGG [ 2340]
cp32-9_PAli_NX_MP_FG_consensus CAGAACTAGAAGTTGAGGCTGGTGGTGGGGATGACTTGTACGGAATATGCACTGATATAGATGAGTTTACTGGCATGG [ 2340]
cp32-9_PAli_NX_Pl_consensus    CAGAACTAGAAGTTGAGGCTGGTGGTGGGGATGACTTGTACGGAATATGCACTGATATAGATGAGTTTACTGGCATGG [ 2340]
cp32-9_PAbe_NX_MP_FG_consensus CAGAACTAGAAGTTGAGGCTGGTGGTGGGGATGACTTGTACGGAATATGCACTGATATAGATGAGTTTACTGGCATGG [ 2340]
cp32-9_PAbe_NX_Pl_consensus    CAGAACTAGAAGTTGAGGCTGGTGGTGGGGATGACTTGTACGGAATATGCACTGATATAGATGAGTTTACTGGCATGG [ 2340]

cp32-9_gi|11497325|B31_GB      CAACTGTAGTTCCAATTATAAATAACTTCACGGGGTATTTAACATTTAAGAAAAATGGACAAAATGGTGTGAATCCGG [ 2418]
cp32-9_un7_B31_PacBio          CAACTGTAGTTCCAATTATAAATAACTTCACGGGGTATTTAACATTTAAGAAAAATGGACAAAATGGTGTGAATCCGG [ 2418]
cp32-9_B31_TS_MP_FG_consensus  CAACTGTAGTTCCAATTATAAATAACTTCACGGGGTATTTAACATTTAAGAAAAATGGACAAAATGGTGTGAATCCGG [ 2418]
cp32-9_B31_NX_Pl_consensus     CAACTGTAGTTCCAATTATAAATAACTTCACGGGGTATTTAACATTTAAGAAAAATGGACAAAATGGTGTGAATCCGG [ 2418]
cp32-9_PAli_NX_MP_FG_consensus CAACTGTAGTTCCAATTATAAATAACTTCACGGGGTATTTAACATTTAAGAAAAATGGACAAAATGGTGTGAATCCGG [ 2418]
cp32-9_PAli_NX_Pl_consensus    CAACTGTAGTTCCAATTATAAATAACTTCACGGGGTATTTAACATTTAAGAAAAATGGACAAAATGGTGTGAATCCGG [ 2418]
cp32-9_PAbe_NX_MP_FG_consensus CAACTGTAGTTCCAATTATAAATAACTTCACGGGGTATTTAACATTTAAGAAAAATGGACAAAATGGTGTGAATCCGG [ 2418]
cp32-9_PAbe_NX_Pl_consensus    CAACTGTAGTTCCAATTATAAATAACTTCACGGGGTATTTAACATTTAAGAAAAATGGACAAAATGGTGTGAATCCGG [ 2418]

cp32-9_gi|11497325|B31_GB      GTGATAAGCTGCATTTTAATGCACAAGGAGAGCTTGAAAAGAATGGGGGAAATGATAAATCTGTTAATGCTATAGCAC [ 2496]
cp32-9_un7_B31_PacBio          GTGATAAGCTGCATTTTAATGCACAAGGAGAGCTTGAAAAGAATGGGGGAAATGATAAATCTGTTAATGCTATAGCAC [ 2496]
cp32-9_B31_TS_MP_FG_consensus  GTGATAAGCTGCATTTTAATGCACAAGGAGAGCTTGAAAAGAATGGGGGAAATGATAAATCTGTTAATGCTATAGCAC [ 2496]
cp32-9_B31_NX_Pl_consensus     GTGATAAGCTGCATTTTAATGCACAAGGAGAGCTTGAAAAGAATGGGGGAAATGATAAATCTGTTAATGCTATAGCAC [ 2496]
cp32-9_PAli_NX_MP_FG_consensus GTGATAAGCTGCATTTTAATGCACAAGGAGAGCTTGAAAAGAATGGGGGAAATGATAAATCTGTTAATGCTATAGCAC [ 2496]
cp32-9_PAli_NX_Pl_consensus    GTGATAAGCTGCATTTTAATGCACAAGGAGAGCTTGAAAAGAATGGGGGAAATGATAAATCTGTTAATGCTATAGCAC [ 2496]
cp32-9_PAbe_NX_MP_FG_consensus GTGATAAGCTGCATTTTAATGCACAAGGAGAGCTTGAAAAGAATGGGGGAAATGATAAATCTGTTAATGCTATAGCAC [ 2496]
cp32-9_PAbe_NX_Pl_consensus    GTGATAAGCTGCATTTTAATGCACAAGGAGAGCTTGAAAAGAATGGGGGAAATGATAAATCTGTTAATGCTATAGCAC [ 2496]

cp32-9_gi|11497325|B31_GB      TTTCAAAAGTACATAAATTAACCGAAGAGTTATCCATAGTGCTTGCTAGTGTTTTTGGGAATAGAGCTTTAAAAGGTA [ 2574]
cp32-9_un7_B31_PacBio          TTTCAAAAGTACATAAATTAACCGAAGAGTTATCCATAGTGCTTGCTAGTGTTTTTGGGAATAGAGCTTTAAAAGGTA [ 2574]
cp32-9_B31_TS_MP_FG_consensus  TTTCAAAAGTACATAAATTAACCGAAGAGTTATCCATAGTGCTTGCTAGTGTTTTTGGGAATAGAGCTTTAAAAGGTA [ 2574]
cp32-9_B31_NX_Pl_consensus     TTTCAAAAGTACATAAATTAACCGAAGAGTTATCCATAGTGCTTGCTAGTGTTTTTGGGAATAGAGCTTTAAAAGGTA [ 2574]
cp32-9_PAli_NX_MP_FG_consensus TTTCAAAAGTACATAAATTAACCGAAGAGTTATCCATAGTGCTTGCTAGTGTTTTTGGGAATAGAGCTTTAAAAGGTA [ 2574]
cp32-9_PAli_NX_Pl_consensus    TTTCAAAAGTACATAAATTAACCGAAGAGTTATCCATAGTGCTTGCTAGTGTTTTTGGGAATAGAGCTTTAAAAGGTA [ 2574]
cp32-9_PAbe_NX_MP_FG_consensus TTTCAAAAGTACATAAATTAACCGAAGAGTTATCCATAGTGCTTGCTAGTGTTTTTGGGAATAGAGCTTTAAAAGGTA [ 2574]
cp32-9_PAbe_NX_Pl_consensus    TTTCAAAAGTACATAAATTAACCGAAGAGTTATCCATAGTGCTTGCTAGTGTTTTTGGGAATAGAGCTTTAAAAGGTA [ 2574]

cp32-9_gi|11497325|B31_GB      ATTAAATTATGGCTTTAAAAGGCAAAGGGCAAGCTAAATCTCCTAATGTTCATGATAATCCACAATTAGGGTTAGAAT [ 2652]
cp32-9_un7_B31_PacBio          ATTAAATTATGGCTTTAAAAGGCAAAGGGCAAGCTAAATCTCCTAATGTTCATGATAATCCACAATTAGGGTTAGAAT [ 2652]
cp32-9_B31_TS_MP_FG_consensus  ATTAAATTATGGCTTTAAAAGGCAAAGGGCAAGCTAAATCTCCTAATGTTCATGATAATCCACAATTAGGGTTAGAAT [ 2652]
cp32-9_B31_NX_Pl_consensus     ATTAAATTATGGCTTTAAAAGGCAAAGGGCAAGCTAAATCTCCTAATGTTCATGATAATCCACAATTAGGGTTAGAAT [ 2652]
cp32-9_PAli_NX_MP_FG_consensus ATTAAATTATGGCTTTAAAAGGCAAAGGGCAAGCTAAATCTCCTAATGTTCATGATAATCCACAATTAGGGTTAGAAT [ 2652]
cp32-9_PAli_NX_Pl_consensus    ATTAAATTATGGCTTTAAAAGGCAAAGGGCAAGCTAAATCTCCTAATGTTCATGATAATCCACAATTAGGGTTAGAAT [ 2652]
cp32-9_PAbe_NX_MP_FG_consensus ATTAAATTATGGCTTTAAAAGGCAAAGGGCAAGCTAAATCTCCTAATGTTCATGATAATCCACAATTAGGGTTAGAAT [ 2652]
cp32-9_PAbe_NX_Pl_consensus    ATTAAATTATGGCTTTAAAAGGCAAAGGGCAAGCTAAATCTCCTAATGTTCATGATAATCCACAATTAGGGTTAGAAT [ 2652]

cp32-9_gi|11497325|B31_GB      CAGAAATTCCAGTTGCTCCTAGATCTAAACGTCAAGCAAGACAGGCTGAAGAAGCACAAGCAAAAGATCCTTATTTAG [ 2730]
cp32-9_un7_B31_PacBio          CAGAAATTCCAGTTGCTCCTAGATCTAAACGTCAAGCAAGACAGGCTGAAGAAGCACAAGCAAAAGATCCTTATTTAG [ 2730]
cp32-9_B31_TS_MP_FG_consensus  CAGAAATTCCAGTTGCTCCTAGATCTAAACGTCAAGCAAGACAGGCTGAAGAAGCACAAGCAAAAGATCCTTATTTAG [ 2730]
cp32-9_B31_NX_Pl_consensus     CAGAAATTCCAGTTGCTCCTAGATCTAAACGTCAAGCAAGACAGGCTGAAGAAGCACAAGCAAAAGATCCTTATTTAG [ 2730]
cp32-9_PAli_NX_MP_FG_consensus CAGAAATTCCAGTTGCTCCTAGATCTAAACGTCAAGCAAGACAGGCTGAAGAAGCACAAGCAAAAGATCCTTATTTAG [ 2730]
cp32-9_PAli_NX_Pl_consensus    CAGAAATTCCAGTTGCTCCTAGATCTAAACGTCAAGCAAGACAGGCTGAAGAAGCACAAGCAAAAGATCCTTATTTAG [ 2730]
cp32-9_PAbe_NX_MP_FG_consensus CAGAAATTCCAGTTGCTCCTAGATCTAAACGTCAAGCAAGACAGGCTGAAGAAGCACAAGCAAAAGATCCTTATTTAG [ 2730]
cp32-9_PAbe_NX_Pl_consensus    CAGAAATTCCAGTTGCTCCTAGATCTAAACGTCAAGCAAGACAGGCTGAAGAAGCACAAGCAAAAGATCCTTATTTAG [ 2730]

cp32-9_gi|11497325|B31_GB      ATTCAGTTAAAGAACTTGACGATGTTCTTTTAAAATTTAAAAAATATTCAAAATCAATGAGTTCGATTGAAAATAAGG [ 2808]
cp32-9_un7_B31_PacBio          ATTCAGTTAAAGAACTTGACGATGTTCTTTTAAAATTTAAAAAATATTCAAAATCAATGAGTTCGATTGAAAATAAGG [ 2808]
cp32-9_B31_TS_MP_FG_consensus  ATTCAGTTAAAGAACTTGACGATGTTCTTTTAAAATTTAAAAAATATTCAAAATCAATGAGTTCGATTGAAAATAAGG [ 2808]
cp32-9_B31_NX_Pl_consensus     ATTCAGTTAAAGAACTTGACGATGTTCTTTTAAAATTTAAAAAATATTCAAAATCAATGAGTTCGATTGAAAATAAGG [ 2808]
```

```
cp32-9_PAli_NX_MP_FG_consensus ATTCAGTTAAAGAACTTGACGATGTTCTTTTAAAATTTAAAAAATATTCAAAATCAATGAGTTCGATTGAAAATAAGG [ 2808]
cp32-9_PAli_NX_Pl_consensus    ATTCAGTTAAAGAACTTGACGATGTTCTTTTAAAATTTAAAAAATATTCAAAATCAATGAGTTCGATTGAAAATAAGG [ 2808]
cp32-9_PAbe_NX_MP_FG_consensus ATTCAGTTAAAGAACTTGACGATGTTCTTTTAAAATTTAAAAAATATTCAAAATCAATGAGTTCGATTGAAAATAAGG [ 2808]
cp32-9_PAbe_NX_Pl_consensus    ATTCAGTTAAAGAACTTGACGATGTTCTTTTAAAATTTAAAAAATATTCAAAATCAATGAGTTCGATTGAAAATAAGG [ 2808]

cp32-9_gi|11497325|B31_GB      TTTTTAGTAGTTCGGGTGGTTGTTTTAAATCAAAGAATGAGCGAGTTAATGCTTATTCTTTTACATATTCAAGCTTTG [ 2886]
cp32-9_un7_B31_PacBio          TTTTTAGTAGTTCGGGTGGTTGTTTTAAATCAAAGAATGAGCGAGTTAATGCTTATTCTTTTACATATTCAAGCTTTG [ 2886]
cp32-9_B31_TS_MP_FG_consensus  TTTTTAGTAGTTCGGGTGGTTGTTTTAAATCAAAGAATGAGCGAGTTAATGCTTATTCTTTTACATATTCAAGCTTTG [ 2886]
cp32-9_B31_NX_Pl_consensus     TTTTTAGTAGTTCGGGTGGTTGTTTTAAATCAAAGAATGAGCGAGTTAATGCTTATTCTTTTACATATTCAAGCTTTG [ 2886]
cp32-9_PAli_NX_MP_FG_consensus TTTTTAGTAGTTCGGGTGGTTGTTTTAAATCAAAGAATGAGCGAGTTAATGCTTATTCTTTTACATATTCAAGCTTTG [ 2886]
cp32-9_PAli_NX_Pl_consensus    TTTTTAGTAGTTCGGGTGGTTGTTTTAAATCAAAGAATGAGCGAGTTAATGCTTATTCTTTTACATATTCAAGCTTTG [ 2886]
cp32-9_PAbe_NX_MP_FG_consensus TTTTTAGTAGTTCGGGTGGTTGTTTTAAATCAAAGAATGAGCGAGTTAATGCTTATTCTTTTACATATTCAAGCTTTG [ 2886]
cp32-9_PAbe_NX_Pl_consensus    TTTTTAGTAGTTCGGGTGGTTGTTTTAAATCAAAGAATGAGCGAGTTAATGCTTATTCTTTTACATATTCAAGCTTTG [ 2886]

cp32-9_gi|11497325|B31_GB      CAGACAAAATAGAAGAATACCTTTATGATCCAGCAAATAGTTTTCCATATAAGCGTGGGGTTAAACTTGTTCCAAAAG [ 2964]
cp32-9_un7_B31_PacBio          CAGACAAA-TAGAAGAATACCTTTATGATCCAGCAAATAGTTTTCCATATAAGCGTGGGGTTAAACTTGTTCCAAAAG [ 2964]
cp32-9_B31_TS_MP_FG_consensus  CAGACAAAATAGAAGAATACCTTTATGATCCAGCAAATAGTTTTCCATATAAGCGTGGGGTTAAACTTGTTCCAAAAG [ 2964]
cp32-9_B31_NX_Pl_consensus     CAGACAAAATAGAAGAATACCTTTATGATCCAGCAAATAGTTTTCCATATAAGCGTGGGGTTAAACTTGTTCCAAAAG [ 2964]
cp32-9_PAli_NX_MP_FG_consensus CAGACAAAATAGAAGAATACCTTTATGATCCAGCAAATAGTTTTCCATATAAGCGTGGGGTTAAACTTGTTCCAAAAG [ 2964]
cp32-9_PAli_NX_Pl_consensus    CAGACAAAATAGAAGAATACCTTTATGATCCAGCAAATAGTTTTCCATATAAGCGTGGGGTTAAACTTGTTCCAAAAG [ 2964]
cp32-9_PAbe_NX_MP_FG_consensus CAGACAAAATAGAAGAATACCTTTATGATCCAGCAAATAGTTTTCCATATAAGCGTGGGGTTAAACTTGTTCCAAAAG [ 2964]
cp32-9_PAbe_NX_Pl_consensus    CAGACAAAATAGAAGAATACCTTTATGATCCAGCAAATAGTTTTCCATATAAGCGTGGGGTTAAACTTGTTCCAAAAG [ 2964]

cp32-9_gi|11497325|B31_GB      AGAATTCTATATATGTTGAAGTTGGTGCTGATACTGATATGTATGGGATATGTGTAGATGTATGTGAGTTTAGTAGTA [ 3042]
cp32-9_un7_B31_PacBio          AGAATTCTATATATGTTGAAGTTGGTGCTGATACTGATATGTATGGGATATGTGTAGATGTATGTGAGTTTAGTAGTA [ 3042]
cp32-9_B31_TS_MP_FG_consensus  AGAATTCTATATATGTTGAAGTTGGTGCTGATACTGATATGTATGGGATATGTGTAGATGTATGTGAGTTTAGTAGTA [ 3042]
cp32-9_B31_NX_Pl_consensus     AGAATTCTATATATGTTGAAGTTGGTGCTGATACTGATATGTATGGGATATGTGTAGATGTATGTGAGTTTAGTAGTA [ 3042]
cp32-9_PAli_NX_MP_FG_consensus AGAATTCTATATATGTTGAAGTTGGTGCTGATACTGATATGTATGGGATATGTGTAGATGTATGTGAGTTTAGTAGTA [ 3042]
cp32-9_PAli_NX_Pl_consensus    AGAATTCTATATATGTTGAAGTTGGTGCTGATACTGATATGTATGGGATATGTGTAGATGTATGTGAGTTTAGTAGTA [ 3042]
cp32-9_PAbe_NX_MP_FG_consensus AGAATTCTATATATGTTGAAGTTGGTGCTGATACTGATATGTATGGGATATGTGTAGATGTATGTGAGTTTAGTAGTA [ 3042]
cp32-9_PAbe_NX_Pl_consensus    AGAATTCTATATATGTTGAAGTTGGTGCTGATACTGATATGTATGGGATATGTGTAGATGTATGTGAGTTTAGTAGTA [ 3042]

cp32-9_gi|11497325|B31_GB      CTGCGTATGTATTACCAATTACGAATAACTTTGAAGGGTATCTTGTTACAAGAAATCCGAGTATAAAAATGGGAGAAA [ 3120]
cp32-9_un7_B31_PacBio          CTGCGTATGTATTACCAATTACGAATAACTTTGAAGGGTATCTTGTTACAAGAAATCCGAGTATAAAAATGGGAGAAA [ 3120]
cp32-9_B31_TS_MP_FG_consensus  CTGCGTATGTATTACCAATTACGAATAACTTTGAAGGGTATCTTGTTACAAGAAATCCGAGTATAAAAATGGGAGAAA [ 3120]
cp32-9_B31_NX_Pl_consensus     CTGCGTATGTATTACCAATTACGAATAACTTTGAAGGGTATCTTGTTACAAGAAATCCGAGTATAAAAATGGGAGAAA [ 3120]
cp32-9_PAli_NX_MP_FG_consensus CTGCGTATGTATTACCAATTACGAATAACTTTGAAGGGTATCTTGTTACAAGAAATCCGAGTATAAAAATGGGAGAAA [ 3120]
cp32-9_PAli_NX_Pl_consensus    CTGCGTATGTATTACCAATTACGAATAACTTTGAAGGGTATCTTGTTACAAGAAATCCGAGTATAAAAATGGGAGAAA [ 3120]
cp32-9_PAbe_NX_MP_FG_consensus CTGCGTATGTATTACCAATTACGAATAACTTTGAAGGGTATCTTGTTACAAGAAATCCGAGTATAAAAATGGGAGAAA [ 3120]
cp32-9_PAbe_NX_Pl_consensus    CTGCGTATGTATTACCAATTACGAATAACTTTGAAGGGTATCTTGTTACAAGAAATCCGAGTATAAAAATGGGAGAAA [ 3120]

cp32-9_gi|11497325|B31_GB      TATTGGATATAAATAACAATGGGGTTATTATCAAGGCTGGTGGTGGGCCACCAACCGCAATTAATGCATATGCCCTCT [ 3198]
cp32-9_un7_B31_PacBio          TATTGGATATAAATAACAATGGGGTTATTATCAAGGCTGGTGGTGGGCCACCAACCGCAATTAATGCATATGCCCTCT [ 3198]
cp32-9_B31_TS_MP_FG_consensus  TATTGGATATAAATAACAATGGGGTTATTATCAAGGCTGGTGGTGGGCCACCAACCGCAATTAATGCATATGCCCTCT [ 3198]
cp32-9_B31_NX_Pl_consensus     TATTGGATATAAATAACAATGGGGTTATTATCAAGGCTGGTGGTGGGCCACCAACCGCAATTAATGCATATGCCCTCT [ 3198]
cp32-9_PAli_NX_MP_FG_consensus TATTGGATATAAATAACAATGGGGTTATTATCAAGGCTGGTGGTGGGCCACCAACCGCAATTAATGCATATGCCCTCT [ 3198]
cp32-9_PAli_NX_Pl_consensus    TATTGGATATAAATAACAATGGGGTTATTATCAAGGCTGGTGGTGGGCCACCAACCGCAATTAATGCATATGCCCTCT [ 3198]
cp32-9_PAbe_NX_MP_FG_consensus TATTGGATATAAATAACAATGGGGTTATTATCAAGGCTGGTGGTGGGCCACCAACCGCAATTAATGCATATGCCCTCT [ 3198]
cp32-9_PAbe_NX_Pl_consensus    TATTGGATATAAATAACAATGGGGTTATTATCAAGGCTGGTGGTGGGCCACCAACCGCAATTAATGCATATGCCCTCT [ 3198]

cp32-9_gi|11497325|B31_GB      CTGATTCATTTACAATCAATTTTGCACCCGAAGATGAACATCAAGATCAAGCTAGATATCCCAAACAAGAGTATTCTA [ 3276]
cp32-9_un7_B31_PacBio          CTGATTCATTTACAATCAATTTTGCACCCGAAGATGAACATCAAGATCAAGCTAGATATCCCAAACAAGAGTATTCTA [ 3276]
cp32-9_B31_TS_MP_FG_consensus  CTGATTCATTTACAATCAATTTTGCACCCGAAGATGAACATCAAGATCAAGCTAGATATCCCAAACAAGAGTATTCTA [ 3276]
cp32-9_B31_NX_Pl_consensus     CTGATTCATTTACAATCAATTTTGCACCCGAAGATGAACATCAAGATCAAGCTAGATATCCCAAACAAGAGTATTCTA [ 3276]
cp32-9_PAli_NX_MP_FG_consensus CTGATTCATTTACAATCAATTTTGCACCCGAAGATGAACATCAAGATCAAGCTAGATATCCCAAACAAGAGTATTCTA [ 3276]
cp32-9_PAli_NX_Pl_consensus    CTGATTCATTTACAATCAATTTTGCACCCGAAGATGAACATCAAGATCAAGCTAGATATCCCAAACAAGAGTATTCTA [ 3276]
cp32-9_PAbe_NX_MP_FG_consensus CTGATTCATTTACAATCAATTTTGCACCCGAAGATGAACATCAAGATCAAGCTAGATATCCCAAACAAGAGTATTCTA [ 3276]
cp32-9_PAbe_NX_Pl_consensus    CTGATTCATTTACAATCAATTTTGCACCCGAAGATGAACATCAAGATCAAGCTAGATATCCCAAACAAGAGTATTCTA [ 3276]

cp32-9_gi|11497325|B31_GB      TTAATTTGATAAAAGTTGCAATTTTTGGCAATAGAGGCCTTGAGAAAACAGTAACACCTGAAGCTGGTGGTTAAGCCT [ 3354]
cp32-9_un7_B31_PacBio          TTAATTTGATAAAAGTTGCAATTTTTGGCAATAGAGGCCTTGAGAAAACAGTAACACCTGAAGCTGGTGGTTAAGCCT [ 3354]
cp32-9_B31_TS_MP_FG_consensus  TTAATTTGATAAAAGTTGCAATTTTTGGCAATAGAGGCCTTGAGAAAACAGTAACACCTGAAGCTGGTGGTTAAGCCT [ 3354]
cp32-9_B31_NX_Pl_consensus     TTAATTTGATAAAAGTTGCAATTTTTGGCAATAGAGGCCTTGAGAAAACAGTAACACCTGAAGCTGGTGGTTAAGCCT [ 3354]
cp32-9_PAli_NX_MP_FG_consensus TTAATTTGATAAAAGTTGCAATTTTTGGCAATAGAGGCCTTGAGAAAACAGTAACACCTGAAGCTGGTGGTTAAGCCT [ 3354]
cp32-9_PAli_NX_Pl_consensus    TTAATTTGATAAAAGTTGCAATTTTTGGCAATAGAGGCCTTGAGAAAACAGTAACACCTGAAGCTGGTGGTTAAGCCT [ 3354]
cp32-9_PAbe_NX_MP_FG_consensus TTAATTTGATAAAAGTTGCAATTTTTGGCAATAGAGGCCTTGAGAAAACAGTAACACCTGAAGCTGGTGGTTAAGCCT [ 3354]
cp32-9_PAbe_NX_Pl_consensus    TTAATTTGATAAAAGTTGCAATTTTTGGCAATAGAGGCCTTGAGAAAACAGTAACACCTGAAGCTGGTGGTTAAGCCT [ 3354]

cp32-9_gi|11497325|B31_GB      TGGGCAATAAAAGGAGTTAAACAAATGGCAGACACAACGCAATTATTAAAAGATTATCAAGATAAACGAAGTAAACTT [ 3432]
cp32-9_un7_B31_PacBio          TGGGCAATAAAAGGAGTTAAACAAATGGCAGACACAACGCAATTATTAAAAGATTATCAAGATAAACGAAGTAAACTT [ 3432]
cp32-9_B31_TS_MP_FG_consensus  TGGGCAATAAAAGGAGTTAAACAAATGGCAGACACAACGCAATTATTAAAAGATTATCAAGATAAACGAAGTAAACTT [ 3432]
cp32-9_B31_NX_Pl_consensus     TGGGCAATAAAAGGAGTTAAACAAATGGCAGACACAACGCAATTATTAAAAGATTATCAAGATAAACGAAGTAAACTT [ 3432]
cp32-9_PAli_NX_MP_FG_consensus TGGGCAATAAAAGGAGTTAAACAAATGGCAGACACAACGCAATTATTAAAAGATTATCAAGATAAACGAAGTAAACTT [ 3432]
cp32-9_PAli_NX_Pl_consensus    TGGGCAATAAAAGGAGTTAAACAAATGGCAGACACAACGCAATTATTAAAAGATTATCAAGATAAACGAAGTAAACTT [ 3432]
cp32-9_PAbe_NX_MP_FG_consensus TGGGCAATAAAAGGAGTTAAACAAATGGCAGACACAACGCAATTATTAAAAGATTATCAAGATAAACGAAGTAAACTT [ 3432]
cp32-9_PAbe_NX_Pl_consensus    TGGGCAATAAAAGGAGTTAAACAAATGGCAGACACAACGCAATTATTAAAAGATTATCAAGATAAACGAAGTAAACTT [ 3432]

cp32-9_gi|11497325|B31_GB      GAAAAGTTTATGAAAAATCCCCAGTATGACGCTGGTTTGCTTAGCAATTCTGTAGAGTTTAGAGATAAAAACATACAA [ 3510]
cp32-9_un7_B31_PacBio          GAAAAGTTTATGAAAAATCCCCAGTATGACGCTGGTTTGCTTAGCAATTCTGTAGAGTTTAGAGATAAAAACATACAA [ 3510]
cp32-9_B31_TS_MP_FG_consensus  GAAAAGTTTATGAAAAATCCCCAGTATGACGCTGGTTTGCTTAGCAATTCTGTAGAGTTTAGAGATAAAAACATACAA [ 3510]
cp32-9_B31_NX_Pl_consensus     GAAAAGTTTATGAAAAATCCCCAGTATGACGCTGGTTTGCTTAGCAATTCTGTAGAGTTTAGAGATAAAAACATACAA [ 3510]
cp32-9_PAli_NX_MP_FG_consensus GAAAAGTTTATGAAAAATCCCCAGTATGACGCTGGTTTGCTTAGCAATTCTGTAGAGTTTAGAGATAAAAACATACAA [ 3510]
cp32-9_PAli_NX_Pl_consensus    GAAAAGTTTATGAAAAATCCCCAGTATGACGCTGGTTTGCTTAGCAATTCTGTAGAGTTTAGAGATAAAAACATACAA [ 3510]
cp32-9_PAbe_NX_MP_FG_consensus GAAAAGTTTATGAAAAATCCCCAGTATGACGCTGGTTTGCTTAGCAATTCTGTAGAGTTTAGAGATAAAAACATACAA [ 3510]
cp32-9_PAbe_NX_Pl_consensus    GAAAAGTTTATGAAAAATCCCCAGTATGACGCTGGTTTGCTTAGCAATTCTGTAGAGTTTAGAGATAAAAACATACAA [ 3510]

cp32-9_gi|11497325|B31_GB      TTTTTTGCCTCTGGAGGCACTAGAACCAGCAAATTTGACAAATTAGAAAATCATCCATTTTTAGGATATCCATATAAG [ 3588]
cp32-9_un7_B31_PacBio          TTTTTTGCCTCTGGAGGCACTAGAACCAGCAAATTTGACAAATTAGAAAATCATCCATTTTTAGGATATCCATATAAG [ 3588]
cp32-9_B31_TS_MP_FG_consensus  TTTTTTGCCTCTGGAGGCACTAGAACCAGCAAATTTGACAAATTAGAAAATCATCCATTTTTAGGATATCCATATAAG [ 3588]
cp32-9_B31_NX_Pl_consensus     TTTTTTGCCTCTGGAGGCACTAGAACCAGCAAATTTGACAAATTAGAAAATCATCCATTTTTAGGATATCCATATAAG [ 3588]
cp32-9_PAli_NX_MP_FG_consensus TTTTTTGCCTCTGGAGGCACTAGAACCAGCAAATTTGACAAATTAGAAAATCATCCATTTTTAGGATATCCATATAAG [ 3588]
cp32-9_PAli_NX_Pl_consensus    TTTTTTGCCTCTGGAGGCACTAGAACCAGCAAATTTGACAAATTAGAAAATCATCCATTTTTAGGATATCCATATAAG [ 3588]
cp32-9_PAbe_NX_MP_FG_consensus TTTTTTGCCTCTGGAGGCACTAGAACCAGCAAATTTGACAAATTAGAAAATCATCCATTTTTAGGATATCCATATAAG [ 3588]
cp32-9_PAbe_NX_Pl_consensus    TTTTTTGCCTCTGGAGGCACTAGAACCAGCAAATTTGACAAATTAGAAAATCATCCATTTTTAGGATATCCATATAAG [ 3588]

cp32-9_gi|11497325|B31_GB      CGTGGGGTAAAAAGAGTTATTCAAGAGGAACAAGAGAATCAAATTCACTATGAACCCCACGTTGAGGCTGGTGGTGGG [ 3666]
cp32-9_un7_B31_PacBio          CGTGGGGTAAAAAGAGTTATTCAAGAGGAACAAGAGAATCAAATTCACTATGAACCCCACGTTGAGGCTGGTGGTGGG [ 3666]
cp32-9_B31_TS_MP_FG_consensus  CGTGGGGTAAAAAGAGTTATTCAAGAGGAACAAGAGAATCAAATTCACTATGAACCCCACGTTGAGGCTGGTGGTGGG [ 3666]
cp32-9_B31_NX_Pl_consensus     CGTGGGGTAAAAAGAGTTATTCAAGAGGAACAAGAGAATCAAATTCACTATGAACCCCACGTTGAGGCTGGTGGTGGG [ 3666]
cp32-9_PAli_NX_MP_FG_consensus CGTGGGGTAAAAAGAGTTATTCAAGAGGAACAAGAGAATCAAATTCACTATGAACCCCACGTTGAGGCTGGTGGTGGG [ 3666]
cp32-9_PAli_NX_Pl_consensus    CGTGGGGTAAAAAGAGTTATTCAAGAGGAACAAGAGAATCAAATTCACTATGAACCCCACGTTGAGGCTGGTGGTGGG [ 3666]
cp32-9_PAbe_NX_MP_FG_consensus CGTGGGGTAAAAAGAGTTATTCAAGAGGAACAAGAGAATCAAATTCACTATGAACCCCACGTTGAGGCTGGTGGTGGG [ 3666]
cp32-9_PAbe_NX_Pl_consensus    CGTGGGGTAAAAAGAGTTATTCAAGAGGAACAAGAGAATCAAATTCACTATGAACCCCACGTTGAGGCTGGTGGTGGG [ 3666]

cp32-9_gi|11497325|B31_GB      GATGACTTGTACGGAATATGCACTGATATAGATGAGTTTAGCAAAACAGCCACTATTGTTCCAATTACAAATAATTTT [ 3744]
cp32-9_un7_B31_PacBio          GATGACTTGTACGGAATATGCACTGATATAGATGAGTTTAGCAAAACAGCCACTATTGTTCCAATTACAAATAATTTT [ 3744]
cp32-9_B31_TS_MP_FG_consensus  GATGACTTGTACGGAATATGCACTGATATAGATGAGTTTAGCAAAACAGCCACTATTGTTCCAATTACAAATAATTTT [ 3744]
cp32-9_B31_NX_Pl_consensus     GATGACTTGTACGGAATATGCACTGATATAGATGAGTTTAGCAAAACAGCCACTATTGTTCCAATTACAAATAATTTT [ 3744]
cp32-9_PAli_NX_MP_FG_consensus GATGACTTGTACGGAATATGCACTGATATAGATGAGTTTAGCAAAACAGCCACTATTGTTCCAATTACAAATAATTTT [ 3744]
```

```
cp32-9_PAli_NX_Pl_consensus    GATGACTTGTACGGAATATGCACTGATATAGATGAGTTTAGCAAAACAGCCACTATTGTTCCAATTACAAATAATTTT [ 3744]
cp32-9_PAbe_NX_MP_FG_consensus GATGACTTGTACGGAATATGCACTGATATAGATGAGTTTAGCAAAACAGCCACTATTGTTCCAATTACAAATAATTTT [ 3744]
cp32-9_PAbe_NX_Pl_consensus    GATGACTTGTACGGAATATGCACTGATATAGATGAGTTTAGCAAAACAGCCACTATTGTTCCAATTACAAATAATTTT [ 3744]

cp32-9_gi|11497325|B31_GB      GAGGGTTATTTAGTAGCAAAAGATTCTACGCTTAAAGTAAAAGACAAACTTGTTTTAATAAAGACGGTGCTCTTGAAA [ 3822]
cp32-9_un7_B31_PacBio          GAGGGTTATTTAGTAGCAAAAGATTCTACGCTTAAAGTAAAAGACAAACTTGTTTTAATAAAGACGGTGCTCTTGAAA [ 3822]
cp32-9_B31_TS_MP_FG_consensus  GAGGGTTATTTAGTAGCAAAAGATTCTACGCTTAAAGTAAAAGACAAACTTGTTTTAATAAAGACGGTGCTCTTGAAA [ 3822]
cp32-9_B31_NX_Pl_consensus     GAGGGTTATTTAGTAGCAAAAGATTCTACGCTTAAAGTAAAAGACAAACTTGTTTTAATAAAGACGGTGCTCTTGAAA [ 3822]
cp32-9_PAli_NX_MP_FG_consensus GAGGGTTATTTAGTAGCAAAAGATTCTACGCTTAAAGTAAAAGACAAACTTGTTTTAATAAAGACGGTGCTCTTGAAA [ 3822]
cp32-9_PAli_NX_Pl_consensus    GAGGGTTATTTAGTAGCAAAAGATTCTACGCTTAAAGTAAAAGACAAACTTGTTTTAATAAAGACGGTGCTCTTGAAA [ 3822]
cp32-9_PAbe_NX_MP_FG_consensus GAGGGTTATTTAGTAGCAAAAGATTCTACGCTTAAAGTAAAAGACAAACTTGTTTTAATAAAGACGGTGCTCTTGAAA [ 3822]
cp32-9_PAbe_NX_Pl_consensus    GAGGGTTATTTAGTAGCAAAAGATTCTACGCTTAAAGTAAAAGACAAACTTGTTTTAATAAAGACGGTGCTCTTGAAA [ 3822]

cp32-9_gi|11497325|B31_GB      AGGTGACTGGAGCACCACCAAATAAGGCAACTATTAATGCAATAGCGTTGTCTGATGCAAAACAAATTAGTAACGATG [ 3900]
cp32-9_un7_B31_PacBio          AGGTGACTGGAGCACCACCAAATAAGGCAACTATTAATGCAATAGCGTTGTCTGATGCAAAACAAATTAGTAACGATG [ 3900]
cp32-9_B31_TS_MP_FG_consensus  AGGTGACTGGAGCACCACCAAATAAGGCAACTATTAATGCAATAGCGTTGTCTGATGCAAAACAAATTAGTAACGATG [ 3900]
cp32-9_B31_NX_Pl_consensus     AGGTGACTGGAGCACCACCAAATAAGGCAACTATTAATGCAATAGCGTTGTCTGATGCAAAACAAATTAGTAACGATG [ 3900]
cp32-9_PAli_NX_MP_FG_consensus AGGTGACTGGAGCACCACCAAATAAGGCAACTATTAATGCAATAGCGTTGTCTGATGCAAAACAAATTAGTAACGATG [ 3900]
cp32-9_PAli_NX_Pl_consensus    AGGTGACTGGAGCACCACCAAATAAGGCAACTATTAATGCAATAGCGTTGTCTGATGCAAAACAAATTAGTAACGATG [ 3900]
cp32-9_PAbe_NX_MP_FG_consensus AGGTGACTGGAGCACCACCAAATAAGGCAACTATTAATGCAATAGCGTTGTCTGATGCAAAACAAATTAGTAACGATG [ 3900]
cp32-9_PAbe_NX_Pl_consensus    AGGTGACTGGAGCACCACCAAATAAGGCAACTATTAATGCAATAGCGTTGTCTGATGCAAAACAAATTAGTAACGATG [ 3900]

cp32-9_gi|11497325|B31_GB      TTTATTTAATAAAAGTAGCAGTATTTGGAAATAAAGCTGTAAGTAAAAATTAATGATATTTGGGAGGATTTAATATGG [ 3978]
cp32-9_un7_B31_PacBio          TTTATTTAATAAAAGTAGCAGTATTTGGAAATAAAGCTGTAAGTAAAAATTAATGATATTTGGGAGGATTTAATATGG [ 3978]
cp32-9_B31_TS_MP_FG_consensus  TTTATTTAATAAAAGTAGCAGTATTTGGAAATAAAGCTGTAAGTAAAAATTAATGATATTTGGGAGGATTTAATATGG [ 3978]
cp32-9_B31_NX_Pl_consensus     TTTATTTAATAAAAGTAGCAGTATTTGGAAATAAAGCTGTAAGTAAAAATTAATGATATTTGGGAGGATTTAATATGG [ 3978]
cp32-9_PAli_NX_MP_FG_consensus TTTATTTAATAAAAGTAGCAGTATTTGGAAATAAAGCTGTAAGTAAAAATTAATGATATTTGGGAGGATTTAATATGG [ 3978]
cp32-9_PAli_NX_Pl_consensus    TTTATTTAATAAAAGTAGCAGTATTTGGAAATAAAGCTGTAAGTAAAAATTAATGATATTTGGGAGGATTTAATATGG [ 3978]
cp32-9_PAbe_NX_MP_FG_consensus TTTATTTAATAAAAGTAGCAGTATTTGGAAATAAAGCTGTAAGTAAAAATTAATGATATTTGGGAGGATTTAATATGG [ 3978]
cp32-9_PAbe_NX_Pl_consensus    TTTATTTAATAAAAGTAGCAGTATTTGGAAATAAAGCTGTAAGTAAAAATTAATGATATTTGGGAGGATTTAATATGG [ 3978]

cp32-9_gi|11497325|B31_GB      AATTATTTGATGAAAATTATTATGCAAAAGCTGTGGCTAATATTATAGGGGAAGTTAAAGATCCTATAATGTATAAAT [ 4056]
cp32-9_un7_B31_PacBio          AATTATTTGATGAAAATTATTATGCAAAAGCTGTGGCTAATATTATAGGGGAAGTTAAAGATCCTATAATGTATAAAT [ 4056]
cp32-9_B31_TS_MP_FG_consensus  AATTATTTGATGAAAATTATTATGCAAAAGCTGTGGCTAATATTATAGGGGAAGTTAAAGATCCTATAATGTATAAAT [ 4056]
cp32-9_B31_NX_Pl_consensus     AATTATTTGATGAAAATTATTATGCAAAAGCTGTGGCTAATATTATAGGGGAAGTTAAAGATCCTATAATGTATAAAT [ 4056]
cp32-9_PAli_NX_MP_FG_consensus AATTATTTGATGAAAATTATTATGCAAAAGCTGTGGCTAATATTATAGGGGAAGTTAAAGATCCTATAATGTATAAAT [ 4056]
cp32-9_PAli_NX_Pl_consensus    AATTATTTGATGAAAATTATTATGCAAAAGCTGTGGCTAATATTATAGGGGAAGTTAAAGATCCTATAATGTATAAAT [ 4056]
cp32-9_PAbe_NX_MP_FG_consensus AATTATTTGATGAAAATTATTATGCAAAAGCTGTGGCTAATATTATAGGGGAAGTTAAAGATCCTATAATGTATAAAT [ 4056]
cp32-9_PAbe_NX_Pl_consensus    AATTATTTGATGAAAATTATTATGCAAAAGCTGTGGCTAATATTATAGGGGAAGTTAAAGATCCTATAATGTATAAAT [ 4056]

cp32-9_gi|11497325|B31_GB      GGTTTTCGCCTGATCAAATTGAAGATGTTGATCTTCAGATGGGCTACCAAAGAACTGTAAAGTGGGATGCGTTTTTAA [ 4134]
cp32-9_un7_B31_PacBio          GGTTTTCGCCTGATCAAATTGAAGATGTTGATCTTCAGATGGGCTACCAAAGAACTGTAAAGTGGGATGCGTTTTTAA [ 4134]
cp32-9_B31_TS_MP_FG_consensus  GGTTTTCGCCTGATCAAATTGAAGATGTTGATCTTCAGATGGGCTACCAAAGAACTGTAAAGTGGGATGCGTTTTTAA [ 4134]
cp32-9_B31_NX_Pl_consensus     GGTTTTCGCCTGATCAAATTGAAGATGTTGATCTTCAGATGGGCTACCAAAGAACTGTAAAGTGGGATGCGTTTTTAA [ 4134]
cp32-9_PAli_NX_MP_FG_consensus GGTTTTCGCCTGATCAAATTGAAGATGTTGATCTTCAGATGGGCTACCAAAGAACTGTAAAGTGGGATGCGTTTTTAA [ 4134]
cp32-9_PAli_NX_Pl_consensus    GGTTTTCGCCTGATCAAATTGAAGATGTTGATCTTCAGATGGGCTACCAAAGAACTGTAAAGTGGGATGCGTTTTTAA [ 4134]
cp32-9_PAbe_NX_MP_FG_consensus GGTTTTCGCCTGATCAAATTGAAGATGTTGATCTTCAGATGGGCTACCAAAGAACTGTAAAGTGGGATGCGTTTTTAA [ 4134]
cp32-9_PAbe_NX_Pl_consensus    GGTTTTCGCCTGATCAAATTGAAGATGTTGATCTTCAGATGGGCTACCAAAGAACTGTAAAGTGGGATGCGTTTTTAA [ 4134]

cp32-9_gi|11497325|B31_GB      ATGCTAATCCTACAACGATTGCTAATGAGGTTAATACTATTTCAACTATTGGATTTAGTTCTGAAGTGGTAAGACTTA [ 4212]
cp32-9_un7_B31_PacBio          ATGCTAATCCTACAACGATTGCTAATGAGGTTAATACTATTTCAACTATTGGATTTAGTTCTGAAGTGGTAAGACTTA [ 4212]
cp32-9_B31_TS_MP_FG_consensus  ATGCTAATCCTACAACGATTGCTAATGAGGTTAATACTATTTCAACTATTGGATTTAGTTCTGAAGTGGTAAGACTTA [ 4212]
cp32-9_B31_NX_Pl_consensus     ATGCTAATCCTACAACGATTGCTAATGAGGTTAATACTATTTCAACTATTGGATTTAGTTCTGAAGTGGTAAGACTTA [ 4212]
cp32-9_PAli_NX_MP_FG_consensus ATGCTAATCCTACAACGATTGCTAATGAGGTTAATACTATTTCAACTATTGGATTTAGTTCTGAAGTGGTAAGACTTA [ 4212]
cp32-9_PAli_NX_Pl_consensus    ATGCTAATCCTACAACGATTGCTAATGAGGTTAATACTATTTCAACTATTGGATTTAGTTCTGAAGTGGTAAGACTTA [ 4212]
cp32-9_PAbe_NX_MP_FG_consensus ATGCTAATCCTACAACGATTGCTAATGAGGTTAATACTATTTCAACTATTGGATTTAGTTCTGAAGTGGTAAGACTTA [ 4212]
cp32-9_PAbe_NX_Pl_consensus    ATGCTAATCCTACAACGATTGCTAATGAGGTTAATACTATTTCAACTATTGGATTTAGTTCTGAAGTGGTAAGACTTA [ 4212]

cp32-9_gi|11497325|B31_GB      ATTACTTAAAATTACAGTACAAATTTAGGCACTTAAAGCAGGCGTCTGAAAAATTTTACACTTCAGACTCATATCTTG [ 4290]
cp32-9_un7_B31_PacBio          ATTACTTAAAATTACAGTACAAATTTAGGCACTTAAAGCAGGCGTCTGAAAAATTTTACACTTCAGACTCATATCTTG [ 4290]
cp32-9_B31_TS_MP_FG_consensus  ATTACTTAAAATTACAGTACAAATTTAGGCACTTAAAGCAGGCGTCTGAAAAATTTTACACTTCAGACTCATATCTTG [ 4290]
cp32-9_B31_NX_Pl_consensus     ATTACTTAAAATTACAGTACAAATTTAGGCACTTAAAGCAGGCGTCTGAAAAATTTTACACTTCAGACTCATATCTTG [ 4290]
cp32-9_PAli_NX_MP_FG_consensus ATTACTTAAAATTACAGTACAAATTTAGGCACTTAAAGCAGGCGTCTGAAAAATTTTACACTTCAGACTCATATCTTG [ 4290]
cp32-9_PAli_NX_Pl_consensus    ATTACTTAAAATTACAGTACAAATTTAGGCACTTAAAGCAGGCGTCTGAAAAATTTTACACTTCAGACTCATATCTTG [ 4290]
cp32-9_PAbe_NX_MP_FG_consensus ATTACTTAAAATTACAGTACAAATTTAGGCACTTAAAGCAGGCGTCTGAAAAATTTTACACTTCAGACTCATATCTTG [ 4290]
cp32-9_PAbe_NX_Pl_consensus    ATTACTTAAAATTACAGTACAAATTTAGGCACTTAAAGCAGGCGTCTGAAAAATTTTACACTTCAGACTCATATCTTG [ 4290]

cp32-9_gi|11497325|B31_GB      GTGACATTAATAACAATTTACTTCCTTTCTCTCAAGCCTATAAGCTTGCAAGTAGCGAGATTATTAAACTTATTAATC [ 4368]
cp32-9_un7_B31_PacBio          GTGACATTAATAACAATTTACTTCCTTTCTCTCAAGCCTATAAGCTTGCAAGTAGCGAGATTATTAAACTTATTAATC [ 4368]
cp32-9_B31_TS_MP_FG_consensus  GTGACATTAATAACAATTTACTTCCTTTCTCTCAAGCCTATAAGCTTGCAAGTAGCGAGATTATTAAACTTATTAATC [ 4368]
cp32-9_B31_NX_Pl_consensus     GTGACATTAATAACAATTTACTTCCTTTCTCTCAAGCCTATAAGCTTGCAAGTAGCGAGATTATTAAACTTATTAATC [ 4368]
cp32-9_PAli_NX_MP_FG_consensus GTGACATTAATAACAATTTACTTCCTTTCTCTCAAGCCTATAAGCTTGCAAGTAGCGAGATTATTAAACTTATTAATC [ 4368]
cp32-9_PAli_NX_Pl_consensus    GTGACATTAATAACAATTTACTTCCTTTCTCTCAAGCCTATAAGCTTGCAAGTAGCGAGATTATTAAACTTATTAATC [ 4368]
cp32-9_PAbe_NX_MP_FG_consensus GTGACATTAATAACAATTTACTTCCTTTCTCTCAAGCCTATAAGCTTGCAAGTAGCGAGATTATTAAACTTATTAATC [ 4368]
cp32-9_PAbe_NX_Pl_consensus    GTGACATTAATAACAATTTACTTCCTTTCTCTCAAGCCTATAAGCTTGCAAGTAGCGAGATTATTAAACTTATTAATC [ 4368]

cp32-9_gi|11497325|B31_GB      ACTTTGTACTAACAGGCACTGTTTCAATTCAAAAAGATGGAAAAAATCAAAAACGTCTACTTCCCAATATGTATGGAC [ 4446]
cp32-9_un7_B31_PacBio          ACTTTGTACTAACAGGCACTGTTTCAATTCAAAAAGATGGAAAAAATCAAAAACGTCTACTTCCCAATATGTATGGAC [ 4446]
cp32-9_B31_TS_MP_FG_consensus  ACTTTGTACTAACAGGCACTGTTTCAATTCAAAAAGATGGAAAAAATCAAAAACGTCTACTTCCCAATATGTATGGAC [ 4446]
cp32-9_B31_NX_Pl_consensus     ACTTTGTACTAACAGGCACTGTTTCAATTCAAAAAGATGGAAAAAATCAAAAACGTCTACTTCCCAATATGTATGGAC [ 4446]
cp32-9_PAli_NX_MP_FG_consensus ACTTTGTACTAACAGGCACTGTTTCAATTCAAAAAGATGGAAAAAATCAAAAACGTCTACTTCCCAATATGTATGGAC [ 4446]
cp32-9_PAli_NX_Pl_consensus    ACTTTGTACTAACAGGCACTGTTTCAATTCAAAAAGATGGAAAAAATCAAAAACGTCTACTTCCCAATATGTATGGAC [ 4446]
cp32-9_PAbe_NX_MP_FG_consensus ACTTTGTACTAACAGGCACTGTTTCAATTCAAAAAGATGGAAAAAATCAAAAACGTCTACTTCCCAATATGTATGGAC [ 4446]
cp32-9_PAbe_NX_Pl_consensus    ACTTTGTACTAACAGGCACTGTTTCAATTCAAAAAGATGGAAAAAATCAAAAACGTCTACTTCCCAATATGTATGGAC [ 4446]

cp32-9_gi|11497325|B31_GB      TTCTTAATATGCCACATCAATCAAGTAAAAGAAGAGGTTGAAAGTGCTAATAAGGACAAAATGGATAAAATATTTGAA [ 4524]
cp32-9_un7_B31_PacBio          TTCTTAATATGCCACATCAATCAAGTAAAAGAAGAGGTTGAAAGTGCTAATAAGGACAAAATGGATAAAATATTTGAA [ 4524]
cp32-9_B31_TS_MP_FG_consensus  TTCTTAATATGCCACATCAATCAAGTAAAAGAAGAGGTTGAAAGTGCTAATAAGGACAAAATGGATAAAATATTTGAA [ 4524]
cp32-9_B31_NX_Pl_consensus     TTCTTAATATGCCACATCAATCAAGTAAAAGAAGAGGTTGAAAGTGCTAATAAGGACAAAATGGATAAAATATTTGAA [ 4524]
cp32-9_PAli_NX_MP_FG_consensus TTCTTAATATGCCACATCAATCAAGTAAAAGAAGAGGTTGAAAGTGCTAATAAGGACAAAATGGATAAAATATTTGAA [ 4524]
cp32-9_PAli_NX_Pl_consensus    TTCTTAATATGCCACATCAATCAAGTAAAAGAAGAGGTTGAAAGTGCTAATAAGGACAAAATGGATAAAATATTTGAA [ 4524]
cp32-9_PAbe_NX_MP_FG_consensus TTCTTAATATGCCACATCAATCAAGTAAAAGAAGAGGTTGAAAGTGCTAATAAGGACAAAATGGATAAAATATTTGAA [ 4524]
cp32-9_PAbe_NX_Pl_consensus    TTCTTAATATGCCACATCAATCAAGTAAAAGAAGAGGTTGAAAGTGCTAATAAGGACAAAATGGATAAAATATTTGAA [ 4524]

cp32-9_gi|11497325|B31_GB      AAAATTGAGGCGGGACTTTCAAAGTTAGAGCTAGGAGACGAATTTTCTACACCTATGATGGTAATAGTTGACCCTACA [ 4602]
cp32-9_un7_B31_PacBio          AAAATTGAGGCGGGACTTTCAAAGTTAGAGCTAGGAGACGAATTTTCTACACCTATGATGGTAATAGTTGACCCTACA [ 4602]
cp32-9_B31_TS_MP_FG_consensus  AAAATTGAGGCGGGACTTTCAAAGTTAGAGCTAGGAGACGAATTTTCTACACCTATGATGGTAATAGTTGACCCTACA [ 4602]
cp32-9_B31_NX_Pl_consensus     AAAATTGAGGCGGGACTTTCAAAGTTAGAGCTAGGAGACGAATTTTCTACACCTATGATGGTAATAGTTGACCCTACA [ 4602]
cp32-9_PAli_NX_MP_FG_consensus AAAATTGAGGCGGGACTTTCAAAGTTAGAGCTAGGAGACGAATTTTCTACACCTATGATGGTAATAGTTGACCCTACA [ 4602]
cp32-9_PAli_NX_Pl_consensus    AAAATTGAGGCGGGACTTTCAAAGTTAGAGCTAGGAGACGAATTTTCTACACCTATGATGGTAATAGTTGACCCTACA [ 4602]
cp32-9_PAbe_NX_MP_FG_consensus AAAATTGAGGCGGGACTTTCAAAGTTAGAGCTAGGAGACGAATTTTCTACACCTATGATGGTAATAGTTGACCCTACA [ 4602]
cp32-9_PAbe_NX_Pl_consensus    AAAATTGAGGCGGGACTTTCAAAGTTAGAGCTAGGAGACGAATTTTCTACACCTATGATGGTAATAGTTGACCCTACA [ 4602]

cp32-9_gi|11497325|B31_GB      ACTTCTCTCAAACTCGTAAAGCCATACGCAGCAGCACAGGGTGCAGCAAGTAGCTGCGAAAAATGGGAGGATGTTTTG [ 4680]
cp32-9_un7_B31_PacBio          ACTTCTCTCAAACTCGTAAAGCCATACGCAGCAGCACAGGGTGCAGCAAGTAGCTGCGAAAAATGGGAGGATGTTTTG [ 4680]
cp32-9_B31_TS_MP_FG_consensus  ACTTCTCTCAAACTCGTAAAGCCATACGCAGCAGCACAGGGTGCAGCAAGTAGCTGCGAAAAATGGGAGGATGTTTTG [ 4680]
cp32-9_B31_NX_Pl_consensus     ACTTCTCTCAAACTCGTAAAGCCATACGCAGCAGCACAGGGTGCAGCAAGTAGCTGCGAAAAATGGGAGGATGTTTTG [ 4680]
cp32-9_PAli_NX_MP_FG_consensus ACTTCTCTCAAACTCGTAAAGCCATACGCAGCAGCACAGGGTGCAGCAAGTAGCTGCGAAAAATGGGAGGATGTTTTG [ 4680]
cp32-9_PAli_NX_Pl_consensus    ACTTCTCTCAAACTCGTAAAGCCATACGCAGCAGCACAGGGTGCAGCAAGTAGCTGCGAAAAATGGGAGGATGTTTTG [ 4680]
```

```
cp32-9_PAbe_NX_MP_FG_consensus ACTTCTCTCAAACTCGTAAAGCCATACGCAGCAGCACAGGGTGCAGCAAGTAGCTGCGAAAAATGGGAGGATGTTTTG [ 4680]
cp32-9_PAbe_NX_Pl_consensus    ACTTCTCTCAAACTCGTAAAGCCATACGCAGCAGCACAGGGTGCAGCAAGTAGCTGCGAAAAATGGGAGGATGTTTTG [ 4680]

cp32-9_gi|11497325|B31_GB      ATTCAAACTATCAAGGCTATTAATAATAGAGAAGATGTCTACATTGAAACTTCAAACTTGCTAAAACATCAAATACTT [ 4758]
cp32-9_un7_B31_PacBio          ATTCAAACTATCAAGGCTATTAATAATAGAGAAGATGTCTACATTGAAACTTCAAACTTGCTAAAACATCAAATACTT [ 4758]
cp32-9_B31_TS_MP_FG_consensus  ATTCAAACTATCAAGGCTATTAATAATAGAGAAGATGTCTACATTGAAACTTCAAACTTGCTAAAACATCAAATACTT [ 4758]
cp32-9_B31_NX_Pl_consensus     ATTCAAACTATCAAGGCTATTAATAATAGAGAAGATGTCTACATTGAAACTTCAAACTTGCTAAAACATCAAATACTT [ 4758]
cp32-9_PAli_NX_MP_FG_consensus ATTCAAACTATCAAGGCTATTAATAATAGAGAAGATGTCTACATTGAAACTTCAAACTTGCTAAAACATCAAATACTT [ 4758]
cp32-9_PAli_NX_Pl_consensus    ATTCAAACTATCAAGGCTATTAATAATAGAGAAGATGTCTACATTGAAACTTCAAACTTGCTAAAACATCAAATACTT [ 4758]
cp32-9_PAbe_NX_MP_FG_consensus ATTCAAACTATCAAGGCTATTAATAATAGAGAAGATGTCTACATTGAAACTTCAAACTTGCTAAAACATCAAATACTT [ 4758]
cp32-9_PAbe_NX_Pl_consensus    ATTCAAACTATCAAGGCTATTAATAATAGAGAAGATGTCTACATTGAAACTTCAAACTTGCTAAAACATCAAATACTT [ 4758]

cp32-9_gi|11497325|B31_GB      ATTTACCCATTAAACCCAGAGCTTATTAAGTTTAAACCTAGCAAGTATATGTTACCTACACCAAATGAACAAGTGGAT [ 4836]
cp32-9_un7_B31_PacBio          ATTTACCCATTAAACCCAGAGCTTATTAAGTTTAAACCTAGCAAGTATATGTTACCTACACCAAATGAACAAGTGGAT [ 4836]
cp32-9_B31_TS_MP_FG_consensus  ATTTACCCATTAAACCCAGAGCTTATTAAGTTTAAACCTAGCAAGTATATGTTACCTACACCAAATGAACAAGTGGAT [ 4836]
cp32-9_B31_NX_Pl_consensus     ATTTACCCATTAAACCCAGAGCTTATTAAGTTTAAACCTAGCAAGTATATGTTACCTACACCAAATGAACAAGTGGAT [ 4836]
cp32-9_PAli_NX_MP_FG_consensus ATTTACCCATTAAACCCAGAGCTTATTAAGTTTAAACCTAGCAAGTATATGTTACCTACACCAAATGAACAAGTGGAT [ 4836]
cp32-9_PAli_NX_Pl_consensus    ATTTACCCATTAAACCCAGAGCTTATTAAGTTTAAACCTAGCAAGTATATGTTACCTACACCAAATGAACAAGTGGAT [ 4836]
cp32-9_PAbe_NX_MP_FG_consensus ATTTACCCATTAAACCCAGAGCTTATTAAGTTTAAACCTAGCAAGTATATGTTACCTACACCAAATGAACAAGTGGAT [ 4836]
cp32-9_PAbe_NX_Pl_consensus    ATTTACCCATTAAACCCAGAGCTTATTAAGTTTAAACCTAGCAAGTATATGTTACCTACACCAAATGAACAAGTGGAT [ 4836]

cp32-9_gi|11497325|B31_GB      AAAGATTCAACTGATATTGCTCATTCATACATTGATTTTGTTTTAGGAGGGCTACTTGCTACTAGAAAAACTATTTTG [ 4914]
cp32-9_un7_B31_PacBio          AAAGATTCAACTGATATTGCTCATTCATACATTGATTTTGTTT-AGGAGGGCTACTTGCTACTAGAAAAACTATTTTG [ 4914]
cp32-9_B31_TS_MP_FG_consensus  AAAGATTCAACTGATATTGCTCATTCATACATTGATTTTGTTTTAGGAGGGCTACTTGCTACTAGAAAAACTATTTTG [ 4914]
cp32-9_B31_NX_Pl_consensus     AAAGATTCAACTGATATTGCTCATTCATACATTGATTTTGTTTTAGGAGGGCTACTTGCTACTAGAAAAACTATTTTG [ 4914]
cp32-9_PAli_NX_MP_FG_consensus AAAGATTCAACTGATATTGCTCATTCATACATTGATTTTGTTTTAGGAGGGCTACTTGCTACTAGAAAAACTATTTTG [ 4914]
cp32-9_PAli_NX_Pl_consensus    AAAGATTCAACTGATATTGCTCATTCATACATTGATTTTGTTTTAGGAGGGCTACTTGCTACTAGAAAAACTATTTTG [ 4914]
cp32-9_PAbe_NX_MP_FG_consensus AAAGATTCAACTGATATTGCTCATTCATACATTGATTTTGTTTTAGGAGGGCTACTTGCTACTAGAAAAACTATTTTG [ 4914]
cp32-9_PAbe_NX_Pl_consensus    AAAGATTCAACTGATATTGCTCATTCATACATTGATTTTGTTTTAGGAGGGCTACTTGCTACTAGAAAAACTATTTTG [ 4914]

cp32-9_gi|11497325|B31_GB      CAAGTACATATTAAACAAAGTTAAAAACATAAGGTAAATCGAAGTGAGCGAACAAAAAAACTTACAAACACAAGTTGA [ 4992]
cp32-9_un7_B31_PacBio          CAAGTACATATTAAACAAAGTTAAAAACATAAGGTAAATCGAAGTGAGCGAACAAAAAAACTTACAAACACAAGTTGA [ 4992]
cp32-9_B31_TS_MP_FG_consensus  CAAGTACATATTAAACAAAGTTAAAAACATAAGGTAAATCGAAGTGAGCGAACAAAAAAACTTACAAACACAAGTTGA [ 4992]
cp32-9_B31_NX_Pl_consensus     CAAGTACATATTAAACAAAGTTAAAAACATAAGGTAAATCGAAGTGAGCGAACAAAAAAACTTACAAACACAAGTTGA [ 4992]
cp32-9_PAli_NX_MP_FG_consensus CAAGTACATATTAAACAAAGTTAAAAACATAAGGTAAATCGAAGTGAGCGAACAAAAAAACTTACAAACACAAGTTGA [ 4992]
cp32-9_PAli_NX_Pl_consensus    CAAGTACATATTAAACAAAGTTAAAAACATAAGGTAAATCGAAGTGAGCGAACAAAAAAACTTACAAACACAAGTTGA [ 4992]
cp32-9_PAbe_NX_MP_FG_consensus CAAGTACATATTAAACAAAGTTAAAAACATAAGGTAAATCGAAGTGAGCGAACAAAAAAACTTACAAACACAAGTTGA [ 4992]
cp32-9_PAbe_NX_Pl_consensus    CAAGTACATATTAAACAAAGTTAAAAACATAAGGTAAATCGAAGTGAGCGAACAAAAAAACTTACAAACACAAGTTGA [ 4992]

cp32-9_gi|11497325|B31_GB      GGCTGAAGAAGAACTTTTGGTAACAAAACTTCATTCCGAAGTGTTATTGTTACTAGGAATAGACAAACTTGCATTAAG [ 5070]
cp32-9_un7_B31_PacBio          GGCTGAAGAAGAACTTTTGGTAACAAAACTTCATTCCGAAGTGTTATTGTTACTAGGAATAGACAAACTTGCATTAAG [ 5070]
cp32-9_B31_TS_MP_FG_consensus  GGCTGAAGAAGAACTTTTGGTAACAAAACTTCATTCCGAAGTGTTATTGTTACTAGGAATAGACAAACTTGCATTAAG [ 5070]
cp32-9_B31_NX_Pl_consensus     GGCTGAAGAAGAACTTTTGGTAACAAAACTTCATTCCGAAGTGTTATTGTTACTAGGAATAGACAAACTTGCATTAAG [ 5070]
cp32-9_PAli_NX_MP_FG_consensus GGCTGAAGAAGAACTTTTGGTAACAAAACTTCATTCCGAAGTGTTATTGTTACTAGGAATAGACAAACTTGCATTAAG [ 5070]
cp32-9_PAli_NX_Pl_consensus    GGCTGAAGAAGAACTTTTGGTAACAAAACTTCATTCCGAAGTGTTATTGTTACTAGGAATAGACAAACTTGCATTAAG [ 5070]
cp32-9_PAbe_NX_MP_FG_consensus GGCTGAAGAAGAACTTTTGGTAACAAAACTTCATTCCGAAGTGTTATTGTTACTAGGAATAGACAAACTTGCATTAAG [ 5070]
cp32-9_PAbe_NX_Pl_consensus    GGCTGAAGAAGAACTTTTGGTAACAAAACTTCATTCCGAAGTGTTATTGTTACTAGGAATAGACAAACTTGCATTAAG [ 5070]

cp32-9_gi|11497325|B31_GB      CAGACAAAATTTTCTACTTCATTTATCTTTACTTCAAGCTATTCTAGTAACACGTGGTATTGATGCTAGTTCACTTAC [ 5148]
cp32-9_un7_B31_PacBio          CAGACAAAATTTTCTACTTCATTTATCTTTACTTCAAGCTATTCTAGTAACACGTGGTATTGATGCTAGTTCACTTAC [ 5148]
cp32-9_B31_TS_MP_FG_consensus  CAGACAAAATTTTCTACTTCATTTATCTTTACTTCAAGCTATTCTAGTAACACGTGGTATTGATGCTAGTTCACTTAC [ 5148]
cp32-9_B31_NX_Pl_consensus     CAGACAAAATTTTCTACTTCATTTATCTTTACTTCAAGCTATTCTAGTAACACGTGGTATTGATGCTAGTTCACTTAC [ 5148]
cp32-9_PAli_NX_MP_FG_consensus CAGACAAAATTTTCTACTTCATTTATCTTTACTTCAAGCTATTCTAGTAACACGTGGTATTGATGCTAGTTCACTTAC [ 5148]
cp32-9_PAli_NX_Pl_consensus    CAGACAAAATTTTCTACTTCATTTATCTTTACTTCAAGCTATTCTAGTAACACGTGGTATTGATGCTAGTTCACTTAC [ 5148]
cp32-9_PAbe_NX_MP_FG_consensus CAGACAAAATTTTCTACTTCATTTATCTTTACTTCAAGCTATTCTAGTAACACGTGGTATTGATGCTAGTTCACTTAC [ 5148]
cp32-9_PAbe_NX_Pl_consensus    CAGACAAAATTTTCTACTTCATTTATCTTTACTTCAAGCTATTCTAGTAACACGTGGTATTGATGCTAGTTCACTTAC [ 5148]

cp32-9_gi|11497325|B31_GB      ATATGAACAAATATTTTTACTTACCTTTTACCATATGGGTTGTCAATTAAGAAAACAGGGAGTTGTTCGAGAATTTGA [ 5226]
cp32-9_un7_B31_PacBio          ATATGAACAAATATTTTTACTTACCTTTTACCATATGGGTTGTCAATTAAGAAAACAGGGAGTTGTTCGAGAATTTGA [ 5226]
cp32-9_B31_TS_MP_FG_consensus  ATATGAACAAATATTTTTACTTACCTTTTACCATATGGGTTGTCAATTAAGAAAACAGGGAGTTGTTCGAGAATTTGA [ 5226]
cp32-9_B31_NX_Pl_consensus     ATATGAACAAATATTTTTACTTACCTTTTACCATATGGGTTGTCAATTAAGAAAACAGGGAGTTGTTCGAGAATTTGA [ 5226]
cp32-9_PAli_NX_MP_FG_consensus ATATGAACAAATATTTTTACTTACCTTTTACCATATGGGTTGTCAATTAAGAAAACAGGGAGTTGTTCGAGAATTTGA [ 5226]
cp32-9_PAli_NX_Pl_consensus    ATATGAACAAATATTTTTACTTACCTTTTACCATATGGGTTGTCAATTAAGAAAACAGGGAGTTGTTCGAGAATTTGA [ 5226]
cp32-9_PAbe_NX_MP_FG_consensus ATATGAACAAATATTTTTACTTACCTTTTACCATATGGGTTGTCAATTAAGAAAACAGGGAGTTGTTCGAGAATTTGA [ 5226]
cp32-9_PAbe_NX_Pl_consensus    ATATGAACAAATATTTTTACTTACCTTTTACCATATGGGTTGTCAATTAAGAAAACAGGGAGTTGTTCGAGAATTTGA [ 5226]

cp32-9_gi|11497325|B31_GB      ATTTGATAGGATCAAAAAAGAGAAATTCAATGAACTTGAACTTGATTATTATCCTAGTAGCAGTGGAGGCGAAGAAGG [ 5304]
cp32-9_un7_B31_PacBio          ATTTGATAGGATCAAAAAAGAGAAATTCAATGAACTTGAACTTGATTATTATCCTAGTAGCAGTGGAGGCGAAGAAGG [ 5304]
cp32-9_B31_TS_MP_FG_consensus  ATTTGATAGGATCAAAAAAGAGAAATTCAATGAACTTGAACTTGATTATTATCCTAGTAGCAGTGGAGGCGAAGAAGG [ 5304]
cp32-9_B31_NX_Pl_consensus     ATTTGATAGGATCAAAAAAGAGAAATTCAATGAACTTGAACTTGATTATTATCCTAGTAGCAGTGGAGGCGAAGAAGG [ 5304]
cp32-9_PAli_NX_MP_FG_consensus ATTTGATAGGATCAAAAAAGAGAAATTCAATGAACTTGAACTTGATTATTATCCTAGTAGCAGTGGAGGCGAAGAAGG [ 5304]
cp32-9_PAli_NX_Pl_consensus    ATTTGATAGGATCAAAAAAGAGAAATTCAATGAACTTGAACTTGATTATTATCCTAGTAGCAGTGGAGGCGAAGAAGG [ 5304]
cp32-9_PAbe_NX_MP_FG_consensus ATTTGATAGGATCAAAAAAGAGAAATTCAATGAACTTGAACTTGATTATTATCCTAGTAGCAGTGGAGGCGAAGAAGG [ 5304]
cp32-9_PAbe_NX_Pl_consensus    ATTTGATAGGATCAAAAAAGAGAAATTCAATGAACTTGAACTTGATTATTATCCTAGTAGCAGTGGAGGCGAAGAAGG [ 5304]

cp32-9_gi|11497325|B31_GB      TGGCGAGGGGGGTTGTGGCTCAAACAAGAGTTTTTGTTCACAACCTGATGCATTTTTAGAAAAACTAAAAAGAGAAAC [ 5382]
cp32-9_un7_B31_PacBio          TGGCGAGGGGGGTTGTGGCTCAAACAAGAGTTTTTGTTCACAACCTGATGCATTTTTAGAAAAACTAAAAAGAGAAAC [ 5382]
cp32-9_B31_TS_MP_FG_consensus  TGGCGAGGGGGGTTGTGGCTCAAACAAGAGTTTTTGTTCACAACCTGATGCATTTTTAGAAAAACTAAAAAGAGAAAC [ 5382]
cp32-9_B31_NX_Pl_consensus     TGGCGAGGGGGGTTGTGGCTCAAACAAGAGTTTTTGTTCACAACCTGATGCATTTTTAGAAAAACTAAAAAGAGAAAC [ 5382]
cp32-9_PAli_NX_MP_FG_consensus TGGCGAGGGGGGTTGTGGCTCAAACAAGAGTTTTTGTTCACAACCTGATGCATTTTTAGAAAAACTAAAAAGAGAAAC [ 5382]
cp32-9_PAli_NX_Pl_consensus    TGGCGAGGGGGGTTGTGGCTCAAACAAGAGTTTTTGTTCACAACCTGATGCATTTTTAGAAAAACTAAAAAGAGAAAC [ 5382]
cp32-9_PAbe_NX_MP_FG_consensus TGGCGAGGGGGGTTGTGGCTCAAACAAGAGTTTTTGTTCACAACCTGATGCATTTTTAGAAAAACTAAAAAGAGAAAC [ 5382]
cp32-9_PAbe_NX_Pl_consensus    TGGCGAGGGGGGTTGTGGCTCAAACAAGAGTTTTTGTTCACAACCTGATGCATTTTTAGAAAAACTAAAAAGAGAAAC [ 5382]

cp32-9_gi|11497325|B31_GB      TTCAACGCCATCTTGTGTGGGGGTTGTCTAATGAATGGTGTTAGGAAAAGACTTTCTGATATGTCATTTCGCATGATC [ 5460]
cp32-9_un7_B31_PacBio          TTCAACGCCATCTTGTGTGGGGGTTGTCTAATGAATGGTGTTAGGAAAAGACTTTCTGATATGTCATTTCGCATGATC [ 5460]
cp32-9_B31_TS_MP_FG_consensus  TTCAACGCCATCTTGTGTGGGGGTTGTCTAATGAATGGTGTTAGGAAAAGACTTTCTGATATGTCATTTCGCATGATC [ 5460]
cp32-9_B31_NX_Pl_consensus     TTCAACGCCATCTTGTGTGGGGGTTGTCTAATGAATGGTGTTAGGAAAAGACTTTCTGATATGTCATTTCGCATGATC [ 5460]
cp32-9_PAli_NX_MP_FG_consensus TTCAACGCCATCTTGTGTGGGGGTTGTCTAATGAATGGTGTTAGGAAAAGACTTTCTGATATGTCATTTCGCATGATC [ 5460]
cp32-9_PAli_NX_Pl_consensus    TTCAACGCCATCTTGTGTGGGGGTTGTCTAATGAATGGTGTTAGGAAAAGACTTTCTGATATGTCATTTCGCATGATC [ 5460]
cp32-9_PAbe_NX_MP_FG_consensus TTCAACGCCATCTTGTGTGGGGGTTGTCTAATGAATGGTGTTAGGAAAAGACTTTCTGATATGTCATTTCGCATGATC [ 5460]
cp32-9_PAbe_NX_Pl_consensus    TTCAACGCCATCTTGTGTGGGGGTTGTCTAATGAATGGTGTTAGGAAAAGACTTTCTGATATGTCATTTCGCATGATC [ 5460]

cp32-9_gi|11497325|B31_GB      AACGTATTTAAGGATCCTAAACCCTTAAAGTTTTATAAAGGTACTGTTGTAAAGCTTGAAAATGATTCTTCTTATCAG [ 5538]
cp32-9_un7_B31_PacBio          AACGTATTTAAGGATCCTAAACCCTTAAAGTTTTATAAAGGTACTGTTGTAAAGCTTGAAAATGATTCTTCTTATCAG [ 5538]
cp32-9_B31_TS_MP_FG_consensus  AACGTATTTAAGGATCCTAAACCCTTAAAGTTTTATAAAGGTACTGTTGTAAAGCTTGAAAATGATTCTTCTTATCAG [ 5538]
cp32-9_B31_NX_Pl_consensus     AACGTATTTAAGGATCCTAAACCCTTAAAGTTTTATAAAGGTACTGTTGTAAAGCTTGAAAATGATTCTTCTTATCAG [ 5538]
cp32-9_PAli_NX_MP_FG_consensus AACGTATTTAAGGATCCTAAACCCTTAAAGTTTTATAAAGGTACTGTTGTAAAGCTTGAAAATGATTCTTCTTATCAG [ 5538]
cp32-9_PAli_NX_Pl_consensus    AACGTATTTAAGGATCCTAAACCCTTAAAGTTTTATAAAGGTACTGTTGTAAAGCTTGAAAATGATTCTTCTTATCAG [ 5538]
cp32-9_PAbe_NX_MP_FG_consensus AACGTATTTAAGGATCCTAAACCCTTAAAGTTTTATAAAGGTACTGTTGTAAAGCTTGAAAATGATTCTTCTTATCAG [ 5538]
cp32-9_PAbe_NX_Pl_consensus    AACGTATTTAAGGATCCTAAACCCTTAAAGTTTTATAAAGGTACTGTTGTAAAGCTTGAAAATGATTCTTCTTATCAG [ 5538]

cp32-9_gi|11497325|B31_GB      AGAGTATTTGATAAAAATAAGTACACTGAATTCGCAGGAGTTATTATTGACATAAAGCCACAAGAACTTGCAATTCTT [ 5616]
cp32-9_un7_B31_PacBio          AGAGTATTTGATAAAAATAAGTACACTGAATTCGCAGGAGTTATTATTGACATAAAGCCACAAGAACTTGCAATTCTT [ 5616]
cp32-9_B31_TS_MP_FG_consensus  AGAGTATTTGATAAAAATAAGTACACTGAATTCGCAGGAGTTATTATTGACATAAAGCCACAAGAACTTGCAATTCTT [ 5616]
cp32-9_B31_NX_Pl_consensus     AGAGTATTTGATAAAAATAAGTACACTGAATTCGCAGGAGTTATTATTGACATAAAGCCACAAGAACTTGCAATTCTT [ 5616]
cp32-9_PAli_NX_MP_FG_consensus AGAGTATTTGATAAAAATAAGTACACTGAATTCGCAGGAGTTATTATTGACATAAAGCCACAAGAACTTGCAATTCTT [ 5616]
cp32-9_PAli_NX_Pl_consensus    AGAGTATTTGATAAAAATAAGTACACTGAATTCGCAGGAGTTATTATTGACATAAAGCCACAAGAACTTGCAATTCTT [ 5616]
cp32-9_PAbe_NX_MP_FG_consensus AGAGTATTTGATAAAAATAAGTACACTGAATTCGCAGGAGTTATTATTGACATAAAGCCACAAGAACTTGCAATTCTT [ 5616]
```

```
cp32-9_PAbe_NX_Pl_consensus    AGAGTATTTGATAAAAATAAGTACACTGAATTCGCAGGAGTTATTATTGACATAAAGCCACAAGAACTTGCAATTCTT [ 5616]

cp32-9_gi|11497325|B31_GB      TATGATTCTGATATGTCTGATATTCAAGGATATTCCAAACTTTACACATATCAAGACCTTAACTATGAACTAAAAGAC [ 5694]
cp32-9_un7_B31_PacBio          TATGATTCTGATATGTCTGATATTCAAGGATATTCCAAACTTTACACATATCAAGACCTTAACTATGAACTAAAAGAC [ 5694]
cp32-9_B31_TS_MP_FG_consensus  TATGATTCTGATATGTCTGATATTCAAGGATATTCCAAACTTTACACATATCAAGACCTTAACTATGAACTAAAAGAC [ 5694]
cp32-9_B31_NX_Pl_consensus     TATGATTCTGATATGTCTGATATTCAAGGATATTCCAAACTTTACACATATCAAGACCTTAACTATGAACTAAAAGAC [ 5694]
cp32-9_PAli_NX_MP_FG_consensus TATGATTCTGATATGTCTGATATTCAAGGATATTCCAAACTTTACACATATCAAGACCTTAACTATGAACTAAAAGAC [ 5694]
cp32-9_PAli_NX_Pl_consensus    TATGATTCTGATATGTCTGATATTCAAGGATATTCCAAACTTTACACATATCAAGACCTTAACTATGAACTAAAAGAC [ 5694]
cp32-9_PAbe_NX_MP_FG_consensus TATGATTCTGATATGTCTGATATTCAAGGATATTCCAAACTTTACACATATCAAGACCTTAACTATGAACTAAAAGAC [ 5694]
cp32-9_PAbe_NX_Pl_consensus    TATGATTCTGATATGTCTGATATTCAAGGATATTCCAAACTTTACACATATCAAGACCTTAACTATGAACTAAAAGAC [ 5694]

cp32-9_gi|11497325|B31_GB      CGAATATCAATTGCAGATTTAGTTTACTTTGAAATATTTAGTATTGACTCTTCAATCGGATATTTTACTTTGGTTTTA [ 5772]
cp32-9_un7_B31_PacBio          CGAATATCAATTGCAGATTTAGTTTACTTTGAAATATTTAGTATTGACTCTTCAATCGGATATTTTACTTTGGTTTTA [ 5772]
cp32-9_B31_TS_MP_FG_consensus  CGAATATCAATTGCAGATTTAGTTTACTTTGAAATATTTAGTATTGACTCTTCAATCGGATATTTTACTTTGGTTTTA [ 5772]
cp32-9_B31_NX_Pl_consensus     CGAATATCAATTGCAGATTTAGTTTACTTTGAAATATTTAGTATTGACTCTTCAATCGGATATTTTACTTTGGTTTTA [ 5772]
cp32-9_PAli_NX_MP_FG_consensus CGAATATCAATTGCAGATTTAGTTTACTTTGAAATATTTAGTATTGACTCTTCAATCGGATATTTTACTTTGGTTTTA [ 5772]
cp32-9_PAli_NX_Pl_consensus    CGAATATCAATTGCAGATTTAGTTTACTTTGAAATATTTAGTATTGACTCTTCAATCGGATATTTTACTTTGGTTTTA [ 5772]
cp32-9_PAbe_NX_MP_FG_consensus CGAATATCAATTGCAGATTTAGTTTACTTTGAAATATTTAGTATTGACTCTTCAATCGGATATTTTACTTTGGTTTTA [ 5772]
cp32-9_PAbe_NX_Pl_consensus    CGAATATCAATTGCAGATTTAGTTTACTTTGAAATATTTAGTATTGACTCTTCAATCGGATATTTTACTTTGGTTTTA [ 5772]

cp32-9_gi|11497325|B31_GB      AAGGAATTTATATGGACAAACTAGAATTTAAAATGGAATTGGAAATTGGGTGGTTTGGTGGTCGTGCAGGTATTGCTA [ 5850]
cp32-9_un7_B31_PacBio          AAGGAATTTATATGGACAAACTAGAATTTAAAATGGAATTGGAAATTGGGTGGTTTGGTGGTCGTGCAGGTATTGCTA [ 5850]
cp32-9_B31_TS_MP_FG_consensus  AAGGAATTTATATGGACAAACTAGAATTTAAAATGGAATTGGAAATTGGGTGGTTTGGTGGTCGTGCAGGTATTGCTA [ 5850]
cp32-9_B31_NX_Pl_consensus     AAGGAATTTATATGGACAAACTAGAATTTAAAATGGAATTGGAAATTGGGTGGTTTGGTGGTCGTGCAGGTATTGCTA [ 5850]
cp32-9_PAli_NX_MP_FG_consensus AAGGAATTTATATGGACAAACTAGAATTTAAAATGGAATTGGAAATTGGGTGGTTTGGTGGTCGTGCAGGTATTGCTA [ 5850]
cp32-9_PAli_NX_Pl_consensus    AAGGAATTTATATGGACAAACTAGAATTTAAAATGGAATTGGAAATTGGGTGGTTTGGTGGTCGTGCAGGTATTGCTA [ 5850]
cp32-9_PAbe_NX_MP_FG_consensus AAGGAATTTATATGGACAAACTAGAATTTAAAATGGAATTGGAAATTGGGTGGTTTGGTGGTCGTGCAGGTATTGCTA [ 5850]
cp32-9_PAbe_NX_Pl_consensus    AAGGAATTTATATGGACAAACTAGAATTTAAAATGGAATTGGAAATTGGGTGGTTTGGTGGTCGTGCAGGTATTGCTA [ 5850]

cp32-9_gi|11497325|B31_GB      AAATGCATGAAAAAGGGAGTAGCAATTTACCAGCAAGAAAACATTTAACCAAAATTGCTAGTAGTTCTGAGTTTAGAG [ 5928]
cp32-9_un7_B31_PacBio          AAATGCATGAAAAAGGGAGTAGCAATTTACCAGCAAGAAAACATTTAACCAAAATTGCTAGTAGTTCTGAGTTTAGAG [ 5928]
cp32-9_B31_TS_MP_FG_consensus  AAATGCATGAAAAAGGGAGTAGCAATTTACCAGCAAGAAAACATTTAACCAAAATTGCTAGTAGTTCTGAGTTTAGAG [ 5928]
cp32-9_B31_NX_Pl_consensus     AAATGCATGAAAAAGGGAGTAGCAATTTACCAGCAAGAAAACATTTAACCAAAATTGCTAGTAGTTCTGAGTTTAGAG [ 5928]
cp32-9_PAli_NX_MP_FG_consensus AAATGCATGAAAAAGGGAGTAGCAATTTACCAGCAAGAAAACATTTAACCAAAATTGCTAGTAGTTCTGAGTTTAGAG [ 5928]
cp32-9_PAli_NX_Pl_consensus    AAATGCATGAAAAAGGGAGTAGCAATTTACCAGCAAGAAAACATTTAACCAAAATTGCTAGTAGTTCTGAGTTTAGAG [ 5928]
cp32-9_PAbe_NX_MP_FG_consensus AAATGCATGAAAAAGGGAGTAGCAATTTACCAGCAAGAAAACATTTAACCAAAATTGCTAGTAGTTCTGAGTTTAGAG [ 5928]
cp32-9_PAbe_NX_Pl_consensus    AAATGCATGAAAAAGGGAGTAGCAATTTACCAGCAAGAAAACATTTAACCAAAATTGCTAGTAGTTCTGAGTTTAGAG [ 5928]

cp32-9_gi|11497325|B31_GB      AATATATCAATAATAGCTATATAAATTCTAAGTTTAATCTTGACCCTAAATCAGGAATGGAGGCTATTGGACAAGCTT [ 6006]
cp32-9_un7_B31_PacBio          AATATATCAATAATAGCTATATAAATTCTAAGTTTAATCTTGACCCTAAATCAGGAATGGAGGCTATTGGACAAGCTT [ 6006]
cp32-9_B31_TS_MP_FG_consensus  AATATATCAATAATAGCTATATAAATTCTAAGTTTAATCTTGACCCTAAATCAGGAATGGAGGCTATTGGACAAGCTT [ 6006]
cp32-9_B31_NX_Pl_consensus     AATATATCAATAATAGCTATATAAATTCTAAGTTTAATCTTGACCCTAAATCAGGAATGGAGGCTATTGGACAAGCTT [ 6006]
cp32-9_PAli_NX_MP_FG_consensus AATATATCAATAATAGCTATATAAATTCTAAGTTTAATCTTGACCCTAAATCAGGAATGGAGGCTATTGGACAAGCTT [ 6006]
cp32-9_PAli_NX_Pl_consensus    AATATATCAATAATAGCTATATAAATTCTAAGTTTAATCTTGACCCTAAATCAGGAATGGAGGCTATTGGACAAGCTT [ 6006]
cp32-9_PAbe_NX_MP_FG_consensus AATATATCAATAATAGCTATATAAATTCTAAGTTTAATCTTGACCCTAAATCAGGAATGGAGGCTATTGGACAAGCTT [ 6006]
cp32-9_PAbe_NX_Pl_consensus    AATATATCAATAATAGCTATATAAATTCTAAGTTTAATCTTGACCCTAAATCAGGAATGGAGGCTATTGGACAAGCTT [ 6006]

cp32-9_gi|11497325|B31_GB      TTATAAGGTACTATGAAAATTATCTACTATCAGCACAAGTCACTCCAGCCTTAAAGGCTAATACAATCAAAAGTAAGT [ 6084]
cp32-9_un7_B31_PacBio          TTATAAGGTACTATGAAAATTATCTACTATCAGCACAAGTCACTCCAGCCTTAAAGGCTAATACAATCAAAAGTAAGT [ 6084]
cp32-9_B31_TS_MP_FG_consensus  TTATAAGGTACTATGAAAATTATCTACTATCAGCACAAGTCACTCCAGCCTTAAAGGCTAATACAATCAAAAGTAAGT [ 6084]
cp32-9_B31_NX_Pl_consensus     TTATAAGGTACTATGAAAATTATCTACTATCAGCACAAGTCACTCCAGCCTTAAAGGCTAATACAATCAAAAGTAAGT [ 6084]
cp32-9_PAli_NX_MP_FG_consensus TTATAAGGTACTATGAAAATTATCTACTATCAGCACAAGTCACTCCAGCCTTAAAGGCTAATACAATCAAAAGTAAGT [ 6084]
cp32-9_PAli_NX_Pl_consensus    TTATAAGGTACTATGAAAATTATCTACTATCAGCACAAGTCACTCCAGCCTTAAAGGCTAATACAATCAAAAGTAAGT [ 6084]
cp32-9_PAbe_NX_MP_FG_consensus TTATAAGGTACTATGAAAATTATCTACTATCAGCACAAGTCACTCCAGCCTTAAAGGCTAATACAATCAAAAGTAAGT [ 6084]
cp32-9_PAbe_NX_Pl_consensus    TTATAAGGTACTATGAAAATTATCTACTATCAGCACAAGTCACTCCAGCCTTAAAGGCTAATACAATCAAAAGTAAGT [ 6084]

cp32-9_gi|11497325|B31_GB      TTAAAAGGGGCAGTAACACTGCAGCAATTCCACTTGTTGATACAGCCAAAATGTTATCAGAGATTACTTATAAGGTAA [ 6162]
cp32-9_un7_B31_PacBio          TTAAAAGGGGCAGTAACACTGCAGCAATTCCACTTGTTGATACAGCCAAAATGTTATCAGAGATTACTTATAAGGTAA [ 6162]
cp32-9_B31_TS_MP_FG_consensus  TTAAAAGGGGCAGTAACACTGCAGCAATTCCACTTGTTGATACAGCCAAAATGTTATCAGAGATTACTTATAAGGTAA [ 6162]
cp32-9_B31_NX_Pl_consensus     TTAAAAGGGGCAGTAACACTGCAGCAATTCCACTTGTTGATACAGCCAAAATGTTATCAGAGATTACTTATAAGGTAA [ 6162]
cp32-9_PAli_NX_MP_FG_consensus TTAAAAGGGGCAGTAACACTGCAGCAATTCCACTTGTTGATACAGCCAAAATGTTATCAGAGATTACTTATAAGGTAA [ 6162]
cp32-9_PAli_NX_Pl_consensus    TTAAAAGGGGCAGTAACACTGCAGCAATTCCACTTGTTGATACAGCCAAAATGTTATCAGAGATTACTTATAAGGTAA [ 6162]
cp32-9_PAbe_NX_MP_FG_consensus TTAAAAGGGGCAGTAACACTGCAGCAATTCCACTTGTTGATACAGCCAAAATGTTATCAGAGATTACTTATAAGGTAA [ 6162]
cp32-9_PAbe_NX_Pl_consensus    TTAAAAGGGGCAGTAACACTGCAGCAATTCCACTTGTTGATACAGCCAAAATGTTATCAGAGATTACTTATAAGGTAA [ 6162]

cp32-9_gi|11497325|B31_GB      CACTTGAATGATTTTCACTTTAGATATGGTATTAAATCATTTAACTCAAATATTTAAAGGGTTTAAGGCGTATGCAAC [ 6240]
cp32-9_un7_B31_PacBio          CACTTGAATGATTTTCACTTTAGATATGGTATTAAATCATTTAACTCAAATATTTAAAGGGTTTAAGGCGTATGCAAC [ 6240]
cp32-9_B31_TS_MP_FG_consensus  CACTTGAATGATTTTCACTTTAGATATGGTATTAAATCATTTAACTCAAATATTTAAAGGGTTTAAGGCGTATGCAAC [ 6240]
cp32-9_B31_NX_Pl_consensus     CACTTGAATGATTTTCACTTTAGATATGGTATTAAATCATTTAACTCAAATATTTAAAGGGTTTAAGGCGTATGCAAC [ 6240]
cp32-9_PAli_NX_MP_FG_consensus CACTTGAATGATTTTCACTTTAGATATGGTATTAAATCATTTAACTCAAATATTTAAAGGGTTTAAGGCGTATGCAAC [ 6240]
cp32-9_PAli_NX_Pl_consensus    CACTTGAATGATTTTCACTTTAGATATGGTATTAAATCATTTAACTCAAATATTTAAAGGGTTTAAGGCGTATGCAAC [ 6240]
cp32-9_PAbe_NX_MP_FG_consensus CACTTGAATGATTTTCACTTTAGATATGGTATTAAATCATTTAACTCAAATATTTAAAGGGTTTAAGGCGTATGCAAC [ 6240]
cp32-9_PAbe_NX_Pl_consensus    CACTTGAATGATTTTCACTTTAGATATGGTATTAAATCATTTAACTCAAATATTTAAAGGGTTTAAGGCGTATGCAAC [ 6240]

cp32-9_gi|11497325|B31_GB      TGAAAATAATTTTGAGTGCGATATCATAAATACCTACAATCATCCATATCTTTCAAAAATCACAGCTGCTAGCTCAAA [ 6318]
cp32-9_un7_B31_PacBio          TGAAAATAATTTTGAGTGCGATATCATAAATACCTACAATCATCCATATCTTTCAAAAATCACAGCTGCTAGCTCAAA [ 6318]
cp32-9_B31_TS_MP_FG_consensus  TGAAAATAATTTTGAGTGCGATATCATAAATACCTACAATCATCCATATCTTTCAAAAATCACAGCTGCTAGCTCAAA [ 6318]
cp32-9_B31_NX_Pl_consensus     TGAAAATAATTTTGAGTGCGATATCATAAATACCTACAATCATCCATATCTTTCAAAAATCACAGCTGCTAGCTCAAA [ 6318]
cp32-9_PAli_NX_MP_FG_consensus TGAAAATAATTTTGAGTGCGATATCATAAATACCTACAATCATCCATATCTTTCAAAAATCACAGCTGCTAGCTCAAA [ 6318]
cp32-9_PAli_NX_Pl_consensus    TGAAAATAATTTTGAGTGCGATATCATAAATACCTACAATCATCCATATCTTTCAAAAATCACAGCTGCTAGCTCAAA [ 6318]
cp32-9_PAbe_NX_MP_FG_consensus TGAAAATAATTTTGAGTGCGATATCATAAATACCTACAATCATCCATATCTTTCAAAAATCACAGCTGCTAGCTCAAA [ 6318]
cp32-9_PAbe_NX_Pl_consensus    TGAAAATAATTTTGAGTGCGATATCATAAATACCTACAATCATCCATATCTTTCAAAAATCACAGCTGCTAGCTCAAA [ 6318]

cp32-9_gi|11497325|B31_GB      TATAATAGCATTGAAATTTGATGGTACAGAAAATCTATTTGATCATAATTATAAAGCCGGTGTATTTTATGAAAATGC [ 6396]
cp32-9_un7_B31_PacBio          TATAATAGCATTGAAATTTGATGGTACAGAAAATCTATTTGATCATAATTATAAAGCCGGTGTATTTTATGAAAATGC [ 6396]
cp32-9_B31_TS_MP_FG_consensus  TATAATAGCATTGAAATTTGATGGTACAGAAAATCTATTTGATCATAATTATAAAGCCGGTGTATTTTATGAAAATGC [ 6396]
cp32-9_B31_NX_Pl_consensus     TATAATAGCATTGAAATTTGATGGTACAGAAAATCTATTTGATCATAATTATAAAGCCGGTGTATTTTATGAAAATGC [ 6396]
cp32-9_PAli_NX_MP_FG_consensus TATAATAGCATTGAAATTTGATGGTACAGAAAATCTATTTGATCATAATTATAAAGCCGGTGTATTTTATGAAAATGC [ 6396]
cp32-9_PAli_NX_Pl_consensus    TATAATAGCATTGAAATTTGATGGTACAGAAAATCTATTTGATCATAATTATAAAGCCGGTGTATTTTATGAAAATGC [ 6396]
cp32-9_PAbe_NX_MP_FG_consensus TATAATAGCATTGAAATTTGATGGTACAGAAAATCTATTTGATCATAATTATAAAGCCGGTGTATTTTATGAAAATGC [ 6396]
cp32-9_PAbe_NX_Pl_consensus    TATAATAGCATTGAAATTTGATGGTACAGAAAATCTATTTGATCATAATTATAAAGCCGGTGTATTTTATGAAAATGC [ 6396]

cp32-9_gi|11497325|B31_GB      TTTGGAATTTAGTATAAATTTTCAAATATATATTATTGCAATAGTGTTAAACGCCAAAGACTTTGACGCTAATTCACG [ 6474]
cp32-9_un7_B31_PacBio          TTTGGAATTTAGTATAAATTTTCAAATATATATTATTGCAATAGTGTTAAACGCCAAAGACTTTGACGCTAATTCACG [ 6474]
cp32-9_B31_TS_MP_FG_consensus  TTTGGAATTTAGTATAAATTTTCAAATATATATTATTGCAATAGTGTTAAACGCCAAAGACTTTGACGCTAATTCACG [ 6474]
cp32-9_B31_NX_Pl_consensus     TTTGGAATTTAGTATAAATTTTCAAATATATATTATTGCAATAGTGTTAAACGCCAAAGACTTTGACGCTAATTCACG [ 6474]
cp32-9_PAli_NX_MP_FG_consensus TTTGGAATTTAGTATAAATTTTCAAATATATATTATTGCAATAGTGTTAAACGCCAAAGACTTTGACGCTAATTCACG [ 6474]
cp32-9_PAli_NX_Pl_consensus    TTTGGAATTTAGTATAAATTTTCAAATATATATTATTGCAATAGTGTTAAACGCCAAAGACTTTGACGCTAATTCACG [ 6474]
cp32-9_PAbe_NX_MP_FG_consensus TTTGGAATTTAGTATAAATTTTCAAATATATATTATTGCAATAGTGTTAAACGCCAAAGACTTTGACGCTAATTCACG [ 6474]
cp32-9_PAbe_NX_Pl_consensus    TTTGGAATTTAGTATAAATTTTCAAATATATATTATTGCAATAGTGTTAAACGCCAAAGACTTTGACGCTAATTCACG [ 6474]

cp32-9_gi|11497325|B31_GB      CATGTTAATGCTTTATAGTATGCTTAGTGACTTTCTACACAATAAAGCTCATAAGTATACTTTGCCCAGTCTACAACC [ 6552]
cp32-9_un7_B31_PacBio          CATGTTAATGCTTTATAGTATGCTTAGTGACTTTCTACACAATAAAGCTCATAAGTATACTTTGCCCAGTCTACAACC [ 6552]
cp32-9_B31_TS_MP_FG_consensus  CATGTTAATGCTTTATAGTATGCTTAGTGACTTTCTACACAATAAAGCTCATAAGTATACTTTGCCCAGTCTACAACC [ 6552]
cp32-9_B31_NX_Pl_consensus     CATGTTAATGCTTTATAGTATGCTTAGTGACTTTCTACACAATAAAGCTCATAAGTATACTTTGCCCAGTCTACAACC [ 6552]
cp32-9_PAli_NX_MP_FG_consensus CATGTTAATGCTTTATAGTATGCTTAGTGACTTTCTACACAATAAAGCTCATAAGTATACTTTGCCCAGTCTACAACC [ 6552]
cp32-9_PAli_NX_Pl_consensus    CATGTTAATGCTTTATAGTATGCTTAGTGACTTTCTACACAATAAAGCTCATAAGTATACTTTGCCCAGTCTACAACC [ 6552]
cp32-9_PAbe_NX_MP_FG_consensus CATGTTAATGCTTTATAGTATGCTTAGTGACTTTCTACACAATAAAGCTCATAAGTATACTTTGCCCAGTCTACAACC [ 6552]
cp32-9_PAbe_NX_Pl_consensus    CATGTTAATGCTTTATAGTATGCTTAGTGACTTTCTACACAATAAAGCTCATAAGTATACTTTGCCCAGTCTACAACC [ 6552]
```

```
cp32-9_gi|11497325|B31_GB       CGAATATATTAATAAAATTAACTTCTACATTTACCCAACATCTAATATGCAAACAGTTGGACTGATTAATTTAGGCAC [ 6630]
cp32-9_un7_B31_PacBio           CGAATATATTAATAAAATTAACTTCTACATTTACCCAACATCTAATATGCAAACAGTTGGACTGATTAATTTAGGCAC [ 6630]
cp32-9_B31_TS_MP_FG_consensus   CGAATATATTAATAAAATTAACTTCTACATTTACCCAACATCTAATATGCAAACAGTTGGACTGATTAATTTAGGCAC [ 6630]
cp32-9_B31_NX_Pl_consensus      CGAATATATTAATAAAATTAACTTCTACATTTACCCAACATCTAATATGCAAACAGTTGGACTGATTAATTTAGGCAC [ 6630]
cp32-9_PAli_NX_MP_FG_consensus  CGAATATATTAATAAAATTAACTTCTACATTTACCCAACATCTAATATGCAAACAGTTGGACTGATTAATTTAGGCAC [ 6630]
cp32-9_PAli_NX_Pl_consensus     CGAATATATTAATAAAATTAACTTCTACATTTACCCAACATCTAATATGCAAACAGTTGGACTGATTAATTTAGGCAC [ 6630]
cp32-9_PAbe_NX_MP_FG_consensus  CGAATATATTAATAAAATTAACTTCTACATTTACCCAACATCTAATATGCAAACAGTTGGACTGATTAATTTAGGCAC [ 6630]
cp32-9_PAbe_NX_Pl_consensus     CGAATATATTAATAAAATTAACTTCTACATTTACCCAACATCTAATATGCAAACAGTTGGACTGATTAATTTAGGCAC [ 6630]

cp32-9_gi|11497325|B31_GB       AAAATATAGCAACCATGCATACAGTGCATCTATAGCATTCAATGCTAGTGTAAAAGCAATCGAAATTTTAAAGGAGGA [ 6708]
cp32-9_un7_B31_PacBio           AAAATATAGCAACCATGCATACAGTGCATCTATAGCATTCAATGCTAGTGTAAAAGCAATCGAAATTTTAAAGGAGGA [ 6708]
cp32-9_B31_TS_MP_FG_consensus   AAAATATAGCAACCATGCATACAGTGCATCTATAGCATTCAATGCTAGTGTAAAAGCAATCGAAATTTTAAAGGAGGA [ 6708]
cp32-9_B31_NX_Pl_consensus      AAAATATAGCAACCATGCATACAGTGCATCTATAGCATTCAATGCTAGTGTAAAAGCAATCGAAATTTTAAAGGAGGA [ 6708]
cp32-9_PAli_NX_MP_FG_consensus  AAAATATAGCAACCATGCATACAGTGCATCTATAGCATTCAATGCTAGTGTAAAAGCAATCGAAATTTTAAAGGAGGA [ 6708]
cp32-9_PAli_NX_Pl_consensus     AAAATATAGCAACCATGCATACAGTGCATCTATAGCATTCAATGCTAGTGTAAAAGCAATCGAAATTTTAAAGGAGGA [ 6708]
cp32-9_PAbe_NX_MP_FG_consensus  AAAATATAGCAACCATGCATACAGTGCATCTATAGCATTCAATGCTAGTGTAAAAGCAATCGAAATTTTAAAGGAGGA [ 6708]
cp32-9_PAbe_NX_Pl_consensus     AAAATATAGCAACCATGCATACAGTGCATCTATAGCATTCAATGCTAGTGTAAAAGCAATCGAAATTTTAAAGGAGGA [ 6708]

cp32-9_gi|11497325|B31_GB       ATACGAAATTGCCGCAAGATACAATTAGTGTAAGTTTTATTGACTCTAGGATTCAGACTAGTAGGCCCAATTATTATA [ 6786]
cp32-9_un7_B31_PacBio           ATACGAAATTGCCGCAAGATACAATTAGTGTAAGTTTTATTGACTCTAGGATTCAGACTAGTAGGCCCAATTATTATA [ 6786]
cp32-9_B31_TS_MP_FG_consensus   ATACGAAATTGCCGCAAGATACAATTAGTGTAAGTTTTATTGACTCTAGGATTCAGACTAGTAGGCCCAATTATTATA [ 6786]
cp32-9_B31_NX_Pl_consensus      ATACGAAATTGCCGCAAGATACAATTAGTGTAAGTTTTATTGACTCTAGGATTCAGACTAGTAGGCCCAATTATTATA [ 6786]
cp32-9_PAli_NX_MP_FG_consensus  ATACGAAATTGCCGCAAGATACAATTAGTGTAAGTTTTATTGACTCTAGGATTCAGACTAGTAGGCCCAATTATTATA [ 6786]
cp32-9_PAli_NX_Pl_consensus     ATACGAAATTGCCGCAAGATACAATTAGTGTAAGTTTTATTGACTCTAGGATTCAGACTAGTAGGCCCAATTATTATA [ 6786]
cp32-9_PAbe_NX_MP_FG_consensus  ATACGAAATTGCCGCAAGATACAATTAGTGTAAGTTTTATTGACTCTAGGATTCAGACTAGTAGGCCCAATTATTATA [ 6786]
cp32-9_PAbe_NX_Pl_consensus     ATACGAAATTGCCGCAAGATACAATTAGTGTAAGTTTTATTGACTCTAGGATTCAGACTAGTAGGCCCAATTATTATA [ 6786]

cp32-9_gi|11497325|B31_GB       ATCCACTTTTGGTTTACAAAACAGCTAAAATCAAAGTTAACAAAGATGCTGCTAGCTATAAAATATTGAATTTAACCG [ 6864]
cp32-9_un7_B31_PacBio           ATCCACTTTTGGTTTACAAAACAGCTAAAATCAAAGTTAACAAAGATGCTGCTAGCTATAAAATATTGAATTTAACCG [ 6864]
cp32-9_B31_TS_MP_FG_consensus   ATCCACTTTTGGTTTACAAAACAGCTAAAATCAAAGTTAACAAAGATGCTGCTAGCTATAAAATATTGAATTTAACCG [ 6864]
cp32-9_B31_NX_Pl_consensus      ATCCACTTTTGGTTTACAAAACAGCTAAAATCAAAGTTAACAAAGATGCTGCTAGCTATAAAATATTGAATTTAACCG [ 6864]
cp32-9_PAli_NX_MP_FG_consensus  ATCCACTTTTGGTTTACAAAACAGCTAAAATCAAAGTTAACAAAGATGCTGCTAGCTATAAAATATTGAATTTAACCG [ 6864]
cp32-9_PAli_NX_Pl_consensus     ATCCACTTTTGGTTTACAAAACAGCTAAAATCAAAGTTAACAAAGATGCTGCTAGCTATAAAATATTGAATTTAACCG [ 6864]
cp32-9_PAbe_NX_MP_FG_consensus  ATCCACTTTTGGTTTACAAAACAGCTAAAATCAAAGTTAACAAAGATGCTGCTAGCTATAAAATATTGAATTTAACCG [ 6864]
cp32-9_PAbe_NX_Pl_consensus     ATCCACTTTTGGTTTACAAAACAGCTAAAATCAAAGTTAACAAAGATGCTGCTAGCTATAAAATATTGAATTTAACCG [ 6864]

cp32-9_gi|11497325|B31_GB       TTAATAATTATGAAAAACAAATTGAAACTTTAGAAAAAGAGAATGGAAATGAAGAAGATCAGTTTGGAAAAGAAAAAA [ 6942]
cp32-9_un7_B31_PacBio           TTAATAATTATGAAAAACAAATTGAAACTTTAGAAAAAGAGAATGGAAATGAAGAAGATCAGTTTGGAAAAGAAAAAA [ 6942]
cp32-9_B31_TS_MP_FG_consensus   TTAATAATTATGAAAAACAAATTGAAACTTTAGAAAAAGAGAATGGAAATGAAGAAGATCAGTTTGGAAAAGAAAAAA [ 6942]
cp32-9_B31_NX_Pl_consensus      TTAATAATTATGAAAAACAAATTGAAACTTTAGAAAAAGAGAATGGAAATGAAGAAGATCAGTTTGGAAAAGAAAAAA [ 6942]
cp32-9_PAli_NX_MP_FG_consensus  TTAATAATTATGAAAAACAAATTGAAACTTTAGAAAAAGAGAATGGAAATGAAGAAGATCAGTTTGGAAAAGAAAAAA [ 6942]
cp32-9_PAli_NX_Pl_consensus     TTAATAATTATGAAAAACAAATTGAAACTTTAGAAAAAGAGAATGGAAATGAAGAAGATCAGTTTGGAAAAGAAAAAA [ 6942]
cp32-9_PAbe_NX_MP_FG_consensus  TTAATAATTATGAAAAACAAATTGAAACTTTAGAAAAAGAGAATGGAAATGAAGAAGATCAGTTTGGAAAAGAAAAAA [ 6942]
cp32-9_PAbe_NX_Pl_consensus     TTAATAATTATGAAAAACAAATTGAAACTTTAGAAAAAGAGAATGGAAATGAAGAAGATCAGTTTGGAAAAGAAAAAA [ 6942]

cp32-9_gi|11497325|B31_GB       CACTGCTTAAAACTGCAATGTCAAATTTTTTCAATTCAAGCGAAGAATCGTTAAAATCAGCCGATCTTTTCATTTATA [ 7020]
cp32-9_un7_B31_PacBio           CACTGCTTAAAACTGCAATGTCAAATTTTTTCAATTCAAGCGAAGAATCGTTAAAATCAGCCGATCTTTTCATTTATA [ 7020]
cp32-9_B31_TS_MP_FG_consensus   CACTGCTTAAAACTGCAATGTCAAATTTTTTCAATTCAAGCGAAGAATCGTTAAAATCAGCCGATCTTTTCATTTATA [ 7020]
cp32-9_B31_NX_Pl_consensus      CACTGCTTAAAACTGCAATGTCAAATTTTTTCAATTCAAGCGAAGAATCGTTAAAATCAGCCGATCTTTTCATTTATA [ 7020]
cp32-9_PAli_NX_MP_FG_consensus  CACTGCTTAAAACTGCAATGTCAAATTTTTTCAATTCAAGCGAAGAATCGTTAAAATCAGCCGATCTTTTCATTTATA [ 7020]
cp32-9_PAli_NX_Pl_consensus     CACTGCTTAAAACTGCAATGTCAAATTTTTTCAATTCAAGCGAAGAATCGTTAAAATCAGCCGATCTTTTCATTTATA [ 7020]
cp32-9_PAbe_NX_MP_FG_consensus  CACTGCTTAAAACTGCAATGTCAAATTTTTTCAATTCAAGCGAAGAATCGTTAAAATCAGCCGATCTTTTCATTTATA [ 7020]
cp32-9_PAbe_NX_Pl_consensus     CACTGCTTAAAACTGCAATGTCAAATTTTTTCAATTCAAGCGAAGAATCGTTAAAATCAGCCGATCTTTTCATTTATA [ 7020]

cp32-9_gi|11497325|B31_GB       AGGATAAGCCTGAAGAGCTAAAAAATTATCTTAAAGTACATAGACACACTTTTGTTGTACTTATTAACACTGAGGGGG [ 7098]
cp32-9_un7_B31_PacBio           AGGATAAGCCTGAAGAGCTAAAAAATTATCTTAAAGTACATAGACACACTTTTGTTGTACTTATTAACACTGAGGGGG [ 7098]
cp32-9_B31_TS_MP_FG_consensus   AGGATAAGCCTGAAGAGCTAAAAAATTATCTTAAAGTACATAGACACACTTTTGTTGTACTTATTAACACTGAGGGGG [ 7098]
cp32-9_B31_NX_Pl_consensus      AGGATAAGCCTGAAGAGCTAAAAAATTATCTTAAAGTACATAGACACACTTTTGTTGTACTTATTAACACTGAGGGGG [ 7098]
cp32-9_PAli_NX_MP_FG_consensus  AGGATAAGCCTGAAGAGCTAAAAAATTATCTTAAAGTACATAGACACACTTTTGTTGTACTTATTAACACTGAGGGGG [ 7098]
cp32-9_PAli_NX_Pl_consensus     AGGATAAGCCTGAAGAGCTAAAAAATTATCTTAAAGTACATAGACACACTTTTGTTGTACTTATTAACACTGAGGGGG [ 7098]
cp32-9_PAbe_NX_MP_FG_consensus  AGGATAAGCCTGAAGAGCTAAAAAATTATCTTAAAGTACATAGACACACTTTTGTTGTACTTATTAACACTGAGGGGG [ 7098]
cp32-9_PAbe_NX_Pl_consensus     AGGATAAGCCTGAAGAGCTAAAAAATTATCTTAAAGTACATAGACACACTTTTGTTGTACTTATTAACACTGAGGGGG [ 7098]

cp32-9_gi|11497325|B31_GB       ATGCGTCAGATGATGGACTTAAAATTTACAAAGATGACTATAATAAATTCAAAATGCCTTCAACTTTTTTTGTATTCT [ 7176]
cp32-9_un7_B31_PacBio           ATGCGTCAGATGATGGACTTAAAATTTACAAAGATGACTATAATAAATTCAAAATGCCTTCAACTTTTTTTGTATTCT [ 7176]
cp32-9_B31_TS_MP_FG_consensus   ATGCGTCAGATGATGGACTTAAAATTTACAAAGATGACTATAATAAATTCAAAATGCCTTCAACTTTTTTTGTATTCT [ 7176]
cp32-9_B31_NX_Pl_consensus      ATGCGTCAGATGATGGACTTAAAATTTACAAAGATGACTATAATAAATTCAAAATGCCTTCAACTTTTTTTGTATTCT [ 7176]
cp32-9_PAli_NX_MP_FG_consensus  ATGCGTCAGATGATGGACTTAAAATTTACAAAGATGACTATAATAAATTCAAAATGCCTTCAACTTTTTTTGTATTCT [ 7176]
cp32-9_PAli_NX_Pl_consensus     ATGCGTCAGATGATGGACTTAAAATTTACAAAGATGACTATAATAAATTCAAAATGCCTTCAACTTTTTTTGTATTCT [ 7176]
cp32-9_PAbe_NX_MP_FG_consensus  ATGCGTCAGATGATGGACTTAAAATTTACAAAGATGACTATAATAAATTCAAAATGCCTTCAACTTTTTTTGTATTCT [ 7176]
cp32-9_PAbe_NX_Pl_consensus     ATGCGTCAGATGATGGACTTAAAATTTACAAAGATGACTATAATAAATTCAAAATGCCTTCAACTTTTTTTGTATTCT [ 7176]

cp32-9_gi|11497325|B31_GB       CGACTAAAGAACAAGAAATAAAAGAACTATTTAAAGATAAAGGCAATACTGAAAAAGAAAGAAATATTGCTGTTTACA [ 7254]
cp32-9_un7_B31_PacBio           CGACTAAAGAACAAGAAATAAAAGAACTATTTAAAGATAAAGGCAATACTGAAAAAGAAAGAAATATTGCTGTTTACA [ 7254]
cp32-9_B31_TS_MP_FG_consensus   CGACTAAAGAACAAGAAATAAAAGAACTATTTAAAGATAAAGGCAATACTGAAAAAGAAAGAAATATTGCTGTTTACA [ 7254]
cp32-9_B31_NX_Pl_consensus      CGACTAAAGAACAAGAAATAAAAGAACTATTTAAAGATAAAGGCAATACTGAAAAAGAAAGAAATATTGCTGTTTACA [ 7254]
cp32-9_PAli_NX_MP_FG_consensus  CGACTAAAGAACAAGAAATAAAAGAACTATTTAAAGATAAAGGCAATACTGAAAAAGAAAGAAATATTGCTGTTTACA [ 7254]
cp32-9_PAli_NX_Pl_consensus     CGACTAAAGAACAAGAAATAAAAGAACTATTTAAAGATAAAGGCAATACTGAAAAAGAAAGAAATATTGCTGTTTACA [ 7254]
cp32-9_PAbe_NX_MP_FG_consensus  CGACTAAAGAACAAGAAATAAAAGAACTATTTAAAGATAAAGGCAATACTGAAAAAGAAAGAAATATTGCTGTTTACA [ 7254]
cp32-9_PAbe_NX_Pl_consensus     CGACTAAAGAACAAGAAATAAAAGAACTATTTAAAGATAAAGGCAATACTGAAAAAGAAAGAAATATTGCTGTTTACA [ 7254]

cp32-9_gi|11497325|B31_GB       GTAACAATAAAGACAATTTACACCTTAAATTTATAAGTCAATATCTCCATCAAGCTAGTATTTTTCATGCTGTAAATC [ 7332]
cp32-9_un7_B31_PacBio           GTAACAATAAAGACAATTTACACCTTAAATTTATAAGTCAATATCTCCATCAAGCTAGTATTTTTCATGCTGTAAATC [ 7332]
cp32-9_B31_TS_MP_FG_consensus   GTAACAATAAAGACAATTTACACCTTAAATTTATAAGTCAATATCTCCATCAAGCTAGTATTTTTCATGCTGTAAATC [ 7332]
cp32-9_B31_NX_Pl_consensus      GTAACAATAAAGACAATTTACACCTTAAATTTATAAGTCAATATCTCCATCAAGCTAGTATTTTTCATGCTGTAAATC [ 7332]
cp32-9_PAli_NX_MP_FG_consensus  GTAACAATAAAGACAATTTACACCTTAAATTTATAAGTCAATATCTCCATCAAGCTAGTATTTTTCATGCTGTAAATC [ 7332]
cp32-9_PAli_NX_Pl_consensus     GTAACAATAAAGACAATTTACACCTTAAATTTATAAGTCAATATCTCCATCAAGCTAGTATTTTTCATGCTGTAAATC [ 7332]
cp32-9_PAbe_NX_MP_FG_consensus  GTAACAATAAAGACAATTTACACCTTAAATTTATAAGTCAATATCTCCATCAAGCTAGTATTTTTCATGCTGTAAATC [ 7332]
cp32-9_PAbe_NX_Pl_consensus     GTAACAATAAAGACAATTTACACCTTAAATTTATAAGTCAATATCTCCATCAAGCTAGTATTTTTCATGCTGTAAATC [ 7332]

cp32-9_gi|11497325|B31_GB       CTTATAGCATGCCGCTGGCTGCTACACCACTTGTTGATGATACTGTAATTGGAAAGTTGCGAACTGCAAAAATCAACT [ 7410]
cp32-9_un7_B31_PacBio           CTTATAGCATGCCGCTGGCTGCTACACCACTTGTTGATGATACTGTAATTGGAAAGTTGCGAACTGCAAAAATCAACT [ 7410]
cp32-9_B31_TS_MP_FG_consensus   CTTATAGCATGCCGCTGGCTGCTACACCACTTGTTGATGATACTGTAATTGGAAAGTTGCGAACTGCAAAAATCAACT [ 7410]
cp32-9_B31_NX_Pl_consensus      CTTATAGCATGCCGCTGGCTGCTACACCACTTGTTGATGATACTGTAATTGGAAAGTTGCGAACTGCAAAAATCAACT [ 7410]
cp32-9_PAli_NX_MP_FG_consensus  CTTATAGCATGCCGCTGGCTGCTACACCACTTGTTGATGATACTGTAATTGGAAAGTTGCGAACTGCAAAAATCAACT [ 7410]
cp32-9_PAli_NX_Pl_consensus     CTTATAGCATGCCGCTGGCTGCTACACCACTTGTTGATGATACTGTAATTGGAAAGTTGCGAACTGCAAAAATCAACT [ 7410]
cp32-9_PAbe_NX_MP_FG_consensus  CTTATAGCATGCCGCTGGCTGCTACACCACTTGTTGATGATACTGTAATTGGAAAGTTGCGAACTGCAAAAATCAACT [ 7410]
cp32-9_PAbe_NX_Pl_consensus     CTTATAGCATGCCGCTGGCTGCTACACCACTTGTTGATGATACTGTAATTGGAAAGTTGCGAACTGCAAAAATCAACT [ 7410]

cp32-9_gi|11497325|B31_GB       TTTATTCACTTCTTAATGAAACTGGGCTTGATGGTTTACCTGCCTTTAAAGAAGGTGTTGACCTAGCTGGAAGTACAA [ 7488]
cp32-9_un7_B31_PacBio           TTTATTCACTTCTTAATGAAACTGGGCTTGATGGTTTACCTGCCTTTAAAGAAGGTGTTGACCTAGCTGGAAGTACAA [ 7488]
cp32-9_B31_TS_MP_FG_consensus   TTTATTCACTTCTTAATGAAACTGGGCTTGATGGTTTACCTGCCTTTAAAGAAGGTGTTGACCTAGCTGGAAGTACAA [ 7488]
cp32-9_B31_NX_Pl_consensus      TTTATTCACTTCTTAATGAAACTGGGCTTGATGGTTTACCTGCCTTTAAAGAAGGTGTTGACCTAGCTGGAAGTACAA [ 7488]
cp32-9_PAli_NX_MP_FG_consensus  TTTATTCACTTCTTAATGAAACTGGGCTTGATGGTTTACCTGCCTTTAAAGAAGGTGTTGACCTAGCTGGAAGTACAA [ 7488]
cp32-9_PAli_NX_Pl_consensus     TTTATTCACTTCTTAATGAAACTGGGCTTGATGGTTTACCTGCCTTTAAAGAAGGTGTTGACCTAGCTGGAAGTACAA [ 7488]
cp32-9_PAbe_NX_MP_FG_consensus  TTTATTCACTTCTTAATGAAACTGGGCTTGATGGTTTACCTGCCTTTAAAGAAGGTGTTGACCTAGCTGGAAGTACAA [ 7488]
cp32-9_PAbe_NX_Pl_consensus     TTTATTCACTTCTTAATGAAACTGGGCTTGATGGTTTACCTGCCTTTAAAGAAGGTGTTGACCTAGCTGGAAGTACAA [ 7488]
```

```
cp32-9_gi|11497325|B31_GB      TAGACGAACAATTTACATACCACTATATAAAAAACGAAGCGATTATTGAGCTTATTAAAATTTGGAACAAAAACAATA [ 7566]
cp32-9_un7_B31_PacBio          TAGACGAACAATTTACATACCACTATATAAAAAACGAAGCGATTATTGAGCTTATTAAAATTTGGAACAAAAACAATA [ 7566]
cp32-9_B31_TS_MP_FG_consensus  TAGACGAACAATTTACATACCACTATATAAAAAACGAAGCGATTATTGAGCTTATTAAAATTTGGAACAAAAACAATA [ 7566]
cp32-9_B31_NX_Pl_consensus     TAGACGAACAATTTACATACCACTATATAAAAAACGAAGCGATTATTGAGCTTATTAAAATTTGGAACAAAAACAATA [ 7566]
cp32-9_PAli_NX_MP_FG_consensus TAGACGAACAATTTACATACCACTATATAAAAAACGAAGCGATTATTGAGCTTATTAAAATTTGGAACAAAAACAATA [ 7566]
cp32-9_PAli_NX_Pl_consensus    TAGACGAACAATTTACATACCACTATATAAAAAACGAAGCGATTATTGAGCTTATTAAAATTTGGAACAAAAACAATA [ 7566]
cp32-9_PAbe_NX_MP_FG_consensus TAGACGAACAATTTACATACCACTATATAAAAAACGAAGCGATTATTGAGCTTATTAAAATTTGGAACAAAAACAATA [ 7566]
cp32-9_PAbe_NX_Pl_consensus    TAGACGAACAATTTACATACCACTATATAAAAAACGAAGCGATTATTGAGCTTATTAAAATTTGGAACAAAAACAATA [ 7566]

cp32-9_gi|11497325|B31_GB      GGCAAAATAGCAAATTATCTGCACTGCAGCTTAGTGGAGCTAGAGACAATGCATATACTTCAGCAATTGAATGTTTAC [ 7644]
cp32-9_un7_B31_PacBio          GGCAAAATAGCAAATTATCTGCACTGCAGCTTAGTGGAGCTAGAGACAATGCATATACTTCAGCAATTGAATGTT-AC [ 7644]
cp32-9_B31_TS_MP_FG_consensus  GGCAAAATAGCAAATTATCTGCACTGCAGCTTAGTGGAGCTAGAGACAATGCATATACTTCAGCAATTGAATGTTTAC [ 7644]
cp32-9_B31_NX_Pl_consensus     GGCAAAATAGCAAATTATCTGCACTGCAGCTTAGTGGAGCTAGAGACAATGCATATACTTCAGCAATTGAATGTTTAC [ 7644]
cp32-9_PAli_NX_MP_FG_consensus GGCAAAATAGCAAATTATCTGCACTGCAGCTTAGTGGAGCTAGAGACAATGCATATACTTCAGCAATTGAATGTTTAC [ 7644]
cp32-9_PAli_NX_Pl_consensus    GGCAAAATAGCAAATTATCTGCACTGCAGCTTAGTGGAGCTAGAGACAATGCATATACTTCAGCAATTGAATGTTTAC [ 7644]
cp32-9_PAbe_NX_MP_FG_consensus GGCAAAATAGCAAATTATCTGCACTGCAGCTTAGTGGAGCTAGAGACAATGCATATACTTCAGCAATTGAATGTTTAC [ 7644]
cp32-9_PAbe_NX_Pl_consensus    GGCAAAATAGCAAATTATCTGCACTGCAGCTTAGTGGAGCTAGAGACAATGCATATACTTCAGCAATTGAATGTTTAC [ 7644]

cp32-9_gi|11497325|B31_GB      TGAAAAGGTTTGTGGATAGAGGACTGATTATTGAGTACAAAAATTTAAATCTTACTCTTTCTCCTACCCCGCAACTTA [ 7722]
cp32-9_un7_B31_PacBio          TGAAAAGGTTTGTGGATAGAGGACTGATTATTGAGTACAAAAATTTAAATCTTACTCTTTCTCCTACCCCGCAACTTA [ 7722]
cp32-9_B31_TS_MP_FG_consensus  TGAAAAGGTTTGTGGATAGAGGACTGATTATTGAGTACAAAAATTTAAATCTTACTCTTTCTCCTACCCCGCAACTTA [ 7722]
cp32-9_B31_NX_Pl_consensus     TGAAAAGGTTTGTGGATAGAGGACTGATTATTGAGTACAAAAATTTAAATCTTACTCTTTCTCCTACCCCGCAACTTA [ 7722]
cp32-9_PAli_NX_MP_FG_consensus TGAAAAGGTTTGTGGATAGAGGACTGATTATTGAGTACAAAAATTTAAATCTTACTCTTTCTCCTACCCCGCAACTTA [ 7722]
cp32-9_PAli_NX_Pl_consensus    TGAAAAGGTTTGTGGATAGAGGACTGATTATTGAGTACAAAAATTTAAATCTTACTCTTTCTCCTACCCCGCAACTTA [ 7722]
cp32-9_PAbe_NX_MP_FG_consensus TGAAAAGGTTTGTGGATAGAGGACTGATTATTGAGTACAAAAATTTAAATCTTACTCTTTCTCCTACCCCGCAACTTA [ 7722]
cp32-9_PAbe_NX_Pl_consensus    TGAAAAGGTTTGTGGATAGAGGACTGATTATTGAGTACAAAAATTTAAATCTTACTCTTTCTCCTACCCCGCAACTTA [ 7722]

cp32-9_gi|11497325|B31_GB      AATTAGAACTTGGCGTGAATATTACTTATAACTTTAGCATTAATGCTGTTGCTTTAGTAATTACTACTCAAGATATAG [ 7800]
cp32-9_un7_B31_PacBio          AATTAGAACTTGGCGTGAATATTACTTATAACTTTAGCATTAATGCTGTTGCTTTAGTAATTACTACTCAAGATATAG [ 7800]
cp32-9_B31_TS_MP_FG_consensus  AATTAGAACTTGGCGTGAATATTACTTATAACTTTAGCATTAATGCTGTTGCTTTAGTAATTACTACTCAAGATATAG [ 7800]
cp32-9_B31_NX_Pl_consensus     AATTAGAACTTGGCGTGAATATTACTTATAACTTTAGCATTAATGCTGTTGCTTTAGTAATTACTACTCAAGATATAG [ 7800]
cp32-9_PAli_NX_MP_FG_consensus AATTAGAACTTGGCGTGAATATTACTTATAACTTTAGCATTAATGCTGTTGCTTTAGTAATTACTACTCAAGATATAG [ 7800]
cp32-9_PAli_NX_Pl_consensus    AATTAGAACTTGGCGTGAATATTACTTATAACTTTAGCATTAATGCTGTTGCTTTAGTAATTACTACTCAAGATATAG [ 7800]
cp32-9_PAbe_NX_MP_FG_consensus AATTAGAACTTGGCGTGAATATTACTTATAACTTTAGCATTAATGCTGTTGCTTTAGTAATTACTACTCAAGATATAG [ 7800]
cp32-9_PAbe_NX_Pl_consensus    AATTAGAACTTGGCGTGAATATTACTTATAACTTTAGCATTAATGCTGTTGCTTTAGTAATTACTACTCAAGATATAG [ 7800]

cp32-9_gi|11497325|B31_GB      TTTGATTATCAAAACAGCTTAAGTGCTTAAAAGGGGGTCTAAAAATGCAATTTTATGATTTAAGAGAAGTTTATTTTT [ 7878]
cp32-9_un7_B31_PacBio          TTTGATTATCAAAACAGCTTAAGTGCTTAAAAGGGGGTCTAAAAATGCAATTTTATGATTTAAGAGAAGTTTATTTTT [ 7878]
cp32-9_B31_TS_MP_FG_consensus  TTTGATTATCAAAACAGCTTAAGTGCTTAAAAGGGGGTCTAAAAATGCAATTTTATGATTTAAGAGAAGTTTATTTTT [ 7878]
cp32-9_B31_NX_Pl_consensus     TTTGATTATCAAAACAGCTTAAGTGCTTAAAAGGGGGTCTAAAAATGCAATTTTATGATTTAAGAGAAGTTTATTTTT [ 7878]
cp32-9_PAli_NX_MP_FG_consensus TTTGATTATCAAAACAGCTTAAGTGCTTAAAAGGGGGTCTAAAAATGCAATTTTATGATTTAAGAGAAGTTTATTTTT [ 7878]
cp32-9_PAli_NX_Pl_consensus    TTTGATTATCAAAACAGCTTAAGTGCTTAAAAGGGGGTCTAAAAATGCAATTTTATGATTTAAGAGAAGTTTATTTTT [ 7878]
cp32-9_PAbe_NX_MP_FG_consensus TTTGATTATCAAAACAGCTTAAGTGCTTAAAAGGGGGTCTAAAAATGCAATTTTATGATTTAAGAGAAGTTTATTTTT [ 7878]
cp32-9_PAbe_NX_Pl_consensus    TTTGATTATCAAAACAGCTTAAGTGCTTAAAAGGGGGTCTAAAAATGCAATTTTATGATTTAAGAGAAGTTTATTTTT [ 7878]

cp32-9_gi|11497325|B31_GB      CAATTGGTGGTACGCAGTTACATAGTGGCAAGCTAGAGCTTACAAGCGAACCTACAACAAGAGCAGTGATTAGTAGTG [ 7956]
cp32-9_un7_B31_PacBio          CAATTGGTGGTACGCAGTTACATAGTGGCAAGCTAGAGCTTACAAGCGAACCTACAACAAGAGCAGTGATTAGTAGTG [ 7956]
cp32-9_B31_TS_MP_FG_consensus  CAATTGGTGGTACGCAGTTACATAGTGGCAAGCTAGAGCTTACAAGCGAACCTACAACAAGAGCAGTGATTAGTAGTG [ 7956]
cp32-9_B31_NX_Pl_consensus     CAATTGGTGGTACGCAGTTACATAGTGGCAAGCTAGAGCTTACAAGCGAACCTACAACAAGAGCAGTGATTAGTAGTG [ 7956]
cp32-9_PAli_NX_MP_FG_consensus CAATTGGTGGTACGCAGTTACATAGTGGCAAGCTAGAGCTTACAAGCGAACCTACAACAAGAGCAGTGATTAGTAGTG [ 7956]
cp32-9_PAli_NX_Pl_consensus    CAATTGGTGGTACGCAGTTACATAGTGGCAAGCTAGAGCTTACAAGCGAACCTACAACAAGAGCAGTGATTAGTAGTG [ 7956]
cp32-9_PAbe_NX_MP_FG_consensus CAATTGGTGGTACGCAGTTACATAGTGGCAAGCTAGAGCTTACAAGCGAACCTACAACAAGAGCAGTGATTAGTAGTG [ 7956]
cp32-9_PAbe_NX_Pl_consensus    CAATTGGTGGTACGCAGTTACATAGTGGCAAGCTAGAGCTTACAAGCGAACCTACAACAAGAGCAGTGATTAGTAGTG [ 7956]

cp32-9_gi|11497325|B31_GB      AAGATAAAGGTATGCCTGTAATAAGCTTAAGAGATCCCAAAACAATAACTTATGTTTTCAACATTGAAGTGACACTAG [ 8034]
cp32-9_un7_B31_PacBio          AAGATAAAGGTATGCCTGTAATAAGCTTAAGAGATCCCAAAACAATAACTTATGTTTTCAACATTGAAGTGACACTAG [ 8034]
cp32-9_B31_TS_MP_FG_consensus  AAGATAAAGGTATGCCTGTAATAAGCTTAAGAGATCCCAAAACAATAACTTATGTTTTCAACATTGAAGTGACACTAG [ 8034]
cp32-9_B31_NX_Pl_consensus     AAGATAAAGGTATGCCTGTAATAAGCTTAAGAGATCCCAAAACAATAACTTATGTTTTCAACATTGAAGTGACACTAG [ 8034]
cp32-9_PAli_NX_MP_FG_consensus AAGATAAAGGTATGCCTGTAATAAGCTTAAGAGATCCCAAAACAATAACTTATGTTTTCAACATTGAAGTGACACTAG [ 8034]
cp32-9_PAli_NX_Pl_consensus    AAGATAAAGGTATGCCTGTAATAAGCTTAAGAGATCCCAAAACAATAACTTATGTTTTCAACATTGAAGTGACACTAG [ 8034]
cp32-9_PAbe_NX_MP_FG_consensus AAGATAAAGGTATGCCTGTAATAAGCTTAAGAGATCCCAAAACAATAACTTATGTTTTCAACATTGAAGTGACACTAG [ 8034]
cp32-9_PAbe_NX_Pl_consensus    AAGATAAAGGTATGCCTGTAATAAGCTTAAGAGATCCCAAAACAATAACTTATGTTTTCAACATTGAAGTGACACTAG [ 8034]

cp32-9_gi|11497325|B31_GB      GTAGTCATGACTACATTTTGTTAACTGAACTTTCTGATGAACAGTTTTACAACATGGATGTGAGAAAAGAGGATAAAA [ 8112]
cp32-9_un7_B31_PacBio          GTAGTCATGACTACATTTTGTTAACTGAACTTTCTGATGAACAGTTTTACAACATGGATGTGAGAAAAGAGGATAAAA [ 8112]
cp32-9_B31_TS_MP_FG_consensus  GTAGTCATGACTACATTTTGTTAACTGAACTTTCTGATGAACAGTTTTACAACATGGATGTGAGAAAAGAGGATAAAA [ 8112]
cp32-9_B31_NX_Pl_consensus     GTAGTCATGACTACATTTTGTTAACTGAACTTTCTGATGAACAGTTTTACAACATGGATGTGAGAAAAGAGGATAAAA [ 8112]
cp32-9_PAli_NX_MP_FG_consensus GTAGTCATGACTACATTTTGTTAACTGAACTTTCTGATGAACAGTTTTACAACATGGATGTGAGAAAAGAGGATAAAA [ 8112]
cp32-9_PAli_NX_Pl_consensus    GTAGTCATGACTACATTTTGTTAACTGAACTTTCTGATGAACAGTTTTACAACATGGATGTGAGAAAAGAGGATAAAA [ 8112]
cp32-9_PAbe_NX_MP_FG_consensus GTAGTCATGACTACATTTTGTTAACTGAACTTTCTGATGAACAGTTTTACAACATGGATGTGAGAAAAGAGGATAAAA [ 8112]
cp32-9_PAbe_NX_Pl_consensus    GTAGTCATGACTACATTTTGTTAACTGAACTTTCTGATGAACAGTTTTACAACATGGATGTGAGAAAAGAGGATAAAA [ 8112]

cp32-9_gi|11497325|B31_GB      TGCTTGATTTAGTATTCAATGATAGAATTGCTACCAAAATTATTTCTAACTATGCAATTTTTACTGAAGAGCCTTCAA [ 8190]
cp32-9_un7_B31_PacBio          TGCTTGATTTAGTATTCAATGATAGAATTGCTACCAAAATTATTTCTAACTATGCAATTTTTACTGAAGAGCCTTCAA [ 8190]
cp32-9_B31_TS_MP_FG_consensus  TGCTTGATTTAGTATTCAATGATAGAATTGCTACCAAAATTATTTCTAACTATGCAATTTTTACTGAAGAGCCTTCAA [ 8190]
cp32-9_B31_NX_Pl_consensus     TGCTTGATTTAGTATTCAATGATAGAATTGCTACCAAAATTATTTCTAACTATGCAATTTTTACTGAAGAGCCTTCAA [ 8190]
cp32-9_PAli_NX_MP_FG_consensus TGCTTGATTTAGTATTCAATGATAGAATTGCTACCAAAATTATTTCTAACTATGCAATTTTTACTGAAGAGCCTTCAA [ 8190]
cp32-9_PAli_NX_Pl_consensus    TGCTTGATTTAGTATTCAATGATAGAATTGCTACCAAAATTATTTCTAACTATGCAATTTTTACTGAAGAGCCTTCAA [ 8190]
cp32-9_PAbe_NX_MP_FG_consensus TGCTTGATTTAGTATTCAATGATAGAATTGCTACCAAAATTATTTCTAACTATGCAATTTTTACTGAAGAGCCTTCAA [ 8190]
cp32-9_PAbe_NX_Pl_consensus    TGCTTGATTTAGTATTCAATGATAGAATTGCTACCAAAATTATTTCTAACTATGCAATTTTTACTGAAGAGCCTTCAA [ 8190]

cp32-9_gi|11497325|B31_GB      GAAGTTATTCTGCTGAGGCCGAAAAAGTATCTTTTGAAATTAGGGCTATTAATTGCCAAAAAACTAAACCAAACAACA [ 8268]
cp32-9_un7_B31_PacBio          GAAGTTATTCTGCTGAGGCCGAAAAAGTATCTTTTGAAATTAGGGCTATTAATTGCCAAAAAACTAAACCAAACAACA [ 8268]
cp32-9_B31_TS_MP_FG_consensus  GAAGTTATTCTGCTGAGGCCGAAAAAGTATCTTTTGAAATTAGGGCTATTAATTGCCAAAAAACTAAACCAAACAACA [ 8268]
cp32-9_B31_NX_Pl_consensus     GAAGTTATTCTGCTGAGGCCGAAAAAGTATCTTTTGAAATTAGGGCTATTAATTGCCAAAAAACTAAACCAAACAACA [ 8268]
cp32-9_PAli_NX_MP_FG_consensus GAAGTTATTCTGCTGAGGCCGAAAAAGTATCTTTTGAAATTAGGGCTATTAATTGCCAAAAAACTAAACCAAACAACA [ 8268]
cp32-9_PAli_NX_Pl_consensus    GAAGTTATTCTGCTGAGGCCGAAAAAGTATCTTTTGAAATTAGGGCTATTAATTGCCAAAAAACTAAACCAAACAACA [ 8268]
cp32-9_PAbe_NX_MP_FG_consensus GAAGTTATTCTGCTGAGGCCGAAAAAGTATCTTTTGAAATTAGGGCTATTAATTGCCAAAAAACTAAACCAAACAACA [ 8268]
cp32-9_PAbe_NX_Pl_consensus    GAAGTTATTCTGCTGAGGCCGAAAAAGTATCTTTTGAAATTAGGGCTATTAATTGCCAAAAAACTAAACCAAACAACA [ 8268]

cp32-9_gi|11497325|B31_GB      CTTAAAAGGAGAGTCTTATTATGATAATGAGATATAAAATGAAAATTTTAACTAAAAATAAAACTTATGAATATCCAC [ 8346]
cp32-9_un7_B31_PacBio          CTTAAAAGGAGAGTCTTATTATGATAATGAGATATAAAATGAAAATTTTAACTAAAAATAAAACTTATGAATATCCAC [ 8346]
cp32-9_B31_TS_MP_FG_consensus  CTTAAAAGGAGAGTCTTATTATGATAATGAGATATAAAATGAAAATTTTAACTAAAAATAAAACTTATGAATATCCAC [ 8346]
cp32-9_B31_NX_Pl_consensus     CTTAAAAGGAGAGTCTTATTATGATAATGAGATATAAAATGAAAATTTTAACTAAAAATAAAACTTATGAATATCCAC [ 8346]
cp32-9_PAli_NX_MP_FG_consensus CTTAAAAGGAGAGTCTTATTATGATAATGAGATATAAAATGAAAATTTTAACTAAAAATAAAACTTATGAATATCCAC [ 8346]
cp32-9_PAli_NX_Pl_consensus    CTTAAAAGGAGAGTCTTATTATGATAATGAGATATAAAATGAAAATTTTAACTAAAAATAAAACTTATGAATATCCAC [ 8346]
cp32-9_PAbe_NX_MP_FG_consensus CTTAAAAGGAGAGTCTTATTATGATAATGAGATATAAAATGAAAATTTTAACTAAAAATAAAACTTATGAATATCCAC [ 8346]
cp32-9_PAbe_NX_Pl_consensus    CTTAAAAGGAGAGTCTTATTATGATAATGAGATATAAAATGAAAATTTTAACTAAAAATAAAACTTATGAATATCCAC [ 8346]

cp32-9_gi|11497325|B31_GB      TAAAAGTATTACCAGTCTATGAATGGGATAAAGGTGCTAGGATTTAATCAAAGTGACGCTGTTTTAAAGCTTAATGAG [ 8424]
cp32-9_un7_B31_PacBio          TAAAAGTATTACCAGTCTATGAATGGGATAAAGGTGCTAGGATTTAATCAAAGTGACGCTGTTTTAAAGCTTAATGAG [ 8424]
cp32-9_B31_TS_MP_FG_consensus  TAAAAGTATTACCAGTCTATGAATGGGATAAAGGTGCTAGGATTTAATCAAAGTGACGCTGTTTTAAAGCTTAATGAG [ 8424]
cp32-9_B31_NX_Pl_consensus     TAAAAGTATTACCAGTCTATGAATGGGATAAAGGTGCTAGGATTTAATCAAAGTGACGCTGTTTTAAAGCTTAATGAG [ 8424]
cp32-9_PAli_NX_MP_FG_consensus TAAAAGTATTACCAGTCTATGAATGGGATAAAGGTGCTAGGATTTAATCAAAGTGACGCTGTTTTAAAGCTTAATGAG [ 8424]
cp32-9_PAli_NX_Pl_consensus    TAAAAGTATTACCAGTCTATGAATGGGATAAAGGTGCTAGGATTTAATCAAAGTGACGCTGTTTTAAAGCTTAATGAG [ 8424]
cp32-9_PAbe_NX_MP_FG_consensus TAAAAGTATTACCAGTCTATGAATGGGATAAAGGTGCTAGGATTTAATCAAAGTGACGCTGTTTTAAAGCTTAATGAG [ 8424]
cp32-9_PAbe_NX_Pl_consensus    TAAAAGTATTACCAGTCTATGAATGGGATAAAGGTGCTAGGATTTAATCAAAGTGACGCTGTTTTAAAGCTTAATGAG [ 8424]

cp32-9_gi|11497325|B31_GB      GTTAAATACTTAAGAGAAATCACAAGCTTAATGATAAGTCCAAAATTTTTAGACGAATTATATGTGATTTTGGATCAA [ 8502]
```

```
cp32-9_un7_B31_PacBio          GTTAAATACTTAAGAGAAATCACAAGCTTAATGATAAGTCCAAAATTTTTAGACGAATTATATGTGATTTTGGATCAA [ 8502]
cp32-9_B31_TS_MP_FG_consensus  GTTAAATACTTAAGAGAAATCACAAGCTTAATGATAAGTCCAAAATTTTTAGACGAATTATATGTGATTTTGGATCAA [ 8502]
cp32-9_B31_NX_Pl_consensus     GTTAAATACTTAAGAGAAATCACAAGCTTAATGATAAGTCCAAAATTTTTAGACGAATTATATGTGATTTTGGATCAA [ 8502]
cp32-9_PAli_NX_MP_FG_consensus GTTAAATACTTAAGAGAAATCACAAGCTTAATGATAAGTCCAAAATTTTTAGACGAATTATATGTGATTTTGGATCAA [ 8502]
cp32-9_PAli_NX_Pl_consensus    GTTAAATACTTAAGAGAAATCACAAGCTTAATGATAAGTCCAAAATTTTTAGACGAATTATATGTGATTTTGGATCAA [ 8502]
cp32-9_PAbe_NX_MP_FG_consensus GTTAAATACTTAAGAGAAATCACAAGCTTAATGATAAGTCCAAAATTTTTAGACGAATTATATGTGATTTTGGATCAA [ 8502]
cp32-9_PAbe_NX_Pl_consensus    GTTAAATACTTAAGAGAAATCACAAGCTTAATGATAAGTCCAAAATTTTTAGACGAATTATATGTGATTTTGGATCAA [ 8502]

cp32-9_gi|11497325|B31_GB      AATAGAGAATTTATTTCTTATTATAAGGACTATCTTGTTGCAATAATTTACACTGCACAATTTAATACTTTTCATTTA [ 8580]
cp32-9_un7_B31_PacBio          AATAGAGAATTTATTTCTTATTATAAGGACTATCTTGTTGCAATAATTTACACTGCACAATTTAATACTTTTCATTTA [ 8580]
cp32-9_B31_TS_MP_FG_consensus  AATAGAGAATTTATTTCTTATTATAAGGACTATCTTGTTGCAATAATTTACACTGCACAATTTAATACTTTTCATTTA [ 8580]
cp32-9_B31_NX_Pl_consensus     AATAGAGAATTTATTTCTTATTATAAGGACTATCTTGTTGCAATAATTTACACTGCACAATTTAATACTTTTCATTTA [ 8580]
cp32-9_PAli_NX_MP_FG_consensus AATAGAGAATTTATTTCTTATTATAAGGACTATCTTGTTGCAATAATTTACACTGCACAATTTAATACTTTTCATTTA [ 8580]
cp32-9_PAli_NX_Pl_consensus    AATAGAGAATTTATTTCTTATTATAAGGACTATCTTGTTGCAATAATTTACACTGCACAATTTAATACTTTTCATTTA [ 8580]
cp32-9_PAbe_NX_MP_FG_consensus AATAGAGAATTTATTTCTTATTATAAGGACTATCTTGTTGCAATAATTTACACTGCACAATTTAATACTTTTCATTTA [ 8580]
cp32-9_PAbe_NX_Pl_consensus    AATAGAGAATTTATTTCTTATTATAAGGACTATCTTGTTGCAATAATTTACACTGCACAATTTAATACTTTTCATTTA [ 8580]

cp32-9_gi|11497325|B31_GB      GACAATGATCTAAAAAAGCCCGCTTTAGTATATTTGAGTGAGTATGAAAATAATGTTGGTGATTTTGTTGCTTTTGAC [ 8658]
cp32-9_un7_B31_PacBio          GACAATGATCTAAAAAAGCCCGCTTTAGTATATTTGAGTGAGTATGAAAATAATGTTGGTGATTTTGTTGCTTT-GAC [ 8658]
cp32-9_B31_TS_MP_FG_consensus  GACAATGATCTAAAAAAGCCCGCTTTAGTATATTTGAGTGAGTATGAAAATAATGTTGGTGATTTTGTTGCTTTTGAC [ 8658]
cp32-9_B31_NX_Pl_consensus     GACAATGATCTAAAAAAGCCCGCTTTAGTATATTTGAGTGAGTATGAAAATAATGTTGGTGATTTTGTTGCTTTTGAC [ 8658]
cp32-9_PAli_NX_MP_FG_consensus GACAATGATCTAAAAAAGCCCGCTTTAGTATATTTGAGTGAGTATGAAAATAATGTTGGTGATTTTGTTGCTTTTGAC [ 8658]
cp32-9_PAli_NX_Pl_consensus    GACAATGATCTAAAAAAGCCCGCTTTAGTATATTTGAGTGAGTATGAAAATAATGTTGGTGATTTTGTTGCTTTTGAC [ 8658]
cp32-9_PAbe_NX_MP_FG_consensus GACAATGATCTAAAAAAGCCCGCTTTAGTATATTTGAGTGAGTATGAAAATAATGTTGGTGATTTTGTTGCTTTTGAC [ 8658]
cp32-9_PAbe_NX_Pl_consensus    GACAATGATCTAAAAAAGCCCGCTTTAGTATATTTGAGTGAGTATGAAAATAATGTTGGTGATTTTGTTGCTTTTGAC [ 8658]

cp32-9_gi|11497325|B31_GB      TATATTAATGAAAATTTTGATTATGAAAAAGTAGCCACTTCGCTTTCATCAATTACATCAAATTCCAATGCGCTGGTT [ 8736]
cp32-9_un7_B31_PacBio          TATATTAATGAAAATTTTGATTATGAAAAAGTAGCCACTTCGCTTTCATCAATTACATCAAATTCCAATGCGCTGGTT [ 8736]
cp32-9_B31_TS_MP_FG_consensus  TATATTAATGAAAATTTTGATTATGAAAAAGTAGCCACTTCGCTTTCATCAATTACATCAAATTCCAATGCGCTGGTT [ 8736]
cp32-9_B31_NX_Pl_consensus     TATATTAATGAAAATTTTGATTATGAAAAAGTAGCCACTTCGCTTTCATCAATTACATCAAATTCCAATGCGCTGGTT [ 8736]
cp32-9_PAli_NX_MP_FG_consensus TATATTAATGAAAATTTTGATTATGAAAAAGTAGCCACTTCGCTTTCATCAATTACATCAAATTCCAATGCGCTGGTT [ 8736]
cp32-9_PAli_NX_Pl_consensus    TATATTAATGAAAATTTTGATTATGAAAAAGTAGCCACTTCGCTTTCATCAATTACATCAAATTCCAATGCGCTGGTT [ 8736]
cp32-9_PAbe_NX_MP_FG_consensus TATATTAATGAAAATTTTGATTATGAAAAAGTAGCCACTTCGCTTTCATCAATTACATCAAATTCCAATGCGCTGGTT [ 8736]
cp32-9_PAbe_NX_Pl_consensus    TATATTAATGAAAATTTTGATTATGAAAAAGTAGCCACTTCGCTTTCATCAATTACATCAAATTCCAATGCGCTGGTT [ 8736]

cp32-9_gi|11497325|B31_GB      GCTAAATGAGCAAAAGAAATAGAGATATTGATAAAGCTATTGCAAGTCTTGATGAGACTAGAAAAAAAATATTTTAACT [ 8814]
cp32-9_un7_B31_PacBio          GCTAAATGAGCAAAAGAAATAGAGATATTGATAAAGCTATTGCAAGTCTTGATGAGACTAGAAAAAAAATATTTTAACT [ 8814]
cp32-9_B31_TS_MP_FG_consensus  GCTAAATGAGCAAAAGAAATAGAGATATTGATAAAGCTATTGCAAGTCTTGATGAGACTAGAAAAAAAATATTTTAACT [ 8814]
cp32-9_B31_NX_Pl_consensus     GCTAAATGAGCAAAAGAAATAGAGATATTGATAAAGCTATTGCAAGTCTTGATGAGACTAGAAAAAAAATATTTTAACT [ 8814]
cp32-9_PAli_NX_MP_FG_consensus GCTAAATGAGCAAAAGAAATAGAGATATTGATAAAGCTATTGCAAGTCTTGATGAGACTAGAAAAAAAATATTTTAACT [ 8814]
cp32-9_PAli_NX_Pl_consensus    GCTAAATGAGCAAAAGAAATAGAGATATTGATAAAGCTATTGCAAGTCTTGATGAGACTAGAAAAAAAATATTTTAACT [ 8814]
cp32-9_PAbe_NX_MP_FG_consensus GCTAAATGAGCAAAAGAAATAGAGATATTGATAAAGCTATTGCAAGTCTTGATGAGACTAGAAAAAAAATATTTTAACT [ 8814]
cp32-9_PAbe_NX_Pl_consensus    GCTAAATGAGCAAAAGAAATAGAGATATTGATAAAGCTATTGCAAGTCTTGATGAGACTAGAAAAAAAATATTTTAACT [ 8814]

cp32-9_gi|11497325|B31_GB      TGCTTGACGAGATTAAGAACGATAAATACTTTTTCCCAGTAATTATGAATATTTGCTCATACTACTCGGTTAAAAAAT [ 8892]
cp32-9_un7_B31_PacBio          TGCTTGACGAGATTAAGAACGATAAATACTTTTTCCCAGTAATTATGAATATTTGCTCATACTACTCGGTTAAAAAAT [ 8892]
cp32-9_B31_TS_MP_FG_consensus  TGCTTGACGAGATTAAGAACGATAAATACTTTTTCCCAGTAATTATGAATATTTGCTCATACTACTCGGTTAAAAAAT [ 8892]
cp32-9_B31_NX_Pl_consensus     TGCTTGACGAGATTAAGAACGATAAATACTTTTTCCCAGTAATTATGAATATTTGCTCATACTACTCGGTTAAAAAAT [ 8892]
cp32-9_PAli_NX_MP_FG_consensus TGCTTGACGAGATTAAGAACGATAAATACTTTTTCCCAGTAATTATGAATATTTGCTCATACTACTCGGTTAAAAAAT [ 8892]
cp32-9_PAli_NX_Pl_consensus    TGCTTGACGAGATTAAGAACGATAAATACTTTTTCCCAGTAATTATGAATATTTGCTCATACTACTCGGTTAAAAAAT [ 8892]
cp32-9_PAbe_NX_MP_FG_consensus TGCTTGACGAGATTAAGAACGATAAATACTTTTTCCCAGTAATTATGAATATTTGCTCATACTACTCGGTTAAAAAAT [ 8892]
cp32-9_PAbe_NX_Pl_consensus    TGCTTGACGAGATTAAGAACGATAAATACTTTTTCCCAGTAATTATGAATATTTGCTCATACTACTCGGTTAAAAAAT [ 8892]

cp32-9_gi|11497325|B31_GB      TGCCTTATGACGAGCTTTTAGAAGTCAATAGACTTGCTGAGATTAAATTAGAAAAAGAATTGTATGAATTAATTTTAA [ 8970]
cp32-9_un7_B31_PacBio          TGCCTTATGACGAGCTTTTAGAAGTCAATAGACTTGCTGAGATTAAATTAGAAAAAGAATTGTATGAATTAATTTTAA [ 8970]
cp32-9_B31_TS_MP_FG_consensus  TGCCTTATGACGAGCTTTTAGAAGTCAATAGACTTGCTGAGATTAAATTAGAAAAAGAATTGTATGAATTAATTTTAA [ 8970]
cp32-9_B31_NX_Pl_consensus     TGCCTTATGACGAGCTTTTAGAAGTCAATAGACTTGCTGAGATTAAATTAGAAAAAGAATTGTATGAATTAATTTTAA [ 8970]
cp32-9_PAli_NX_MP_FG_consensus TGCCTTATGACGAGCTTTTAGAAGTCAATAGACTTGCTGAGATTAAATTAGAAAAAGAATTGTATGAATTAATTTTAA [ 8970]
cp32-9_PAli_NX_Pl_consensus    TGCCTTATGACGAGCTTTTAGAAGTCAATAGACTTGCTGAGATTAAATTAGAAAAAGAATTGTATGAATTAATTTTAA [ 8970]
cp32-9_PAbe_NX_MP_FG_consensus TGCCTTATGACGAGCTTTTAGAAGTCAATAGACTTGCTGAGATTAAATTAGAAAAAGAATTGTATGAATTAATTTTAA [ 8970]
cp32-9_PAbe_NX_Pl_consensus    TGCCTTATGACGAGCTTTTAGAAGTCAATAGACTTGCTGAGATTAAATTAGAAAAAGAATTGTATGAATTAATTTTAA [ 8970]

cp32-9_gi|11497325|B31_GB      GCAAGTGAGGACTTAGTGGGCGACAAATTCACCATTAAATTTAAAGGTATTCTTGATCATGCTGCAACAAAAAAGGCC [ 9048]
cp32-9_un7_B31_PacBio          GCAAGTGAGGACTTAGTGGGCGACAAATTCACCATTAAATTTAAAGGTATTCTTGATCATGCTGCAACAAAAAAGGCC [ 9048]
cp32-9_B31_TS_MP_FG_consensus  GCAAGTGAGGACTTAGTGGGCGACAAATTCACCATTAAATTTAAAGGTATTCTTGATCATGCTGCAACAAAAAAGGCC [ 9048]
cp32-9_B31_NX_Pl_consensus     GCAAGTGAGGACTTAGTGGGCGACAAATTCACCATTAAATTTAAAGGTATTCTTGATCATGCTGCAACAAAAAAGGCC [ 9048]
cp32-9_PAli_NX_MP_FG_consensus GCAAGTGAGGACTTAGTGGGCGACAAATTCACCATTAAATTTAAAGGTATTCTTGATCATGCTGCAACAAAAAAGGCC [ 9048]
cp32-9_PAli_NX_Pl_consensus    GCAAGTGAGGACTTAGTGGGCGACAAATTCACCATTAAATTTAAAGGTATTCTTGATCATGCTGCAACAAAAAAGGCC [ 9048]
cp32-9_PAbe_NX_MP_FG_consensus GCAAGTGAGGACTTAGTGGGCGACAAATTCACCATTAAATTTAAAGGTATTCTTGATCATGCTGCAACAAAAAAGGCC [ 9048]
cp32-9_PAbe_NX_Pl_consensus    GCAAGTGAGGACTTAGTGGGCGACAAATTCACCATTAAATTTAAAGGTATTCTTGATCATGCTGCAACAAAAAAGGCC [ 9048]

cp32-9_gi|11497325|B31_GB      ATTGAACAAGATATTTCTAAAATGGGAAAATATCTTAAACCTAAAAAATCTAGTTTGGGTAGCACTAAAGATATTGTA [ 9126]
cp32-9_un7_B31_PacBio          ATTGAACAAGATATTTCTAAAATGGGAAA-TATCTTAAACCTAAAAAATCTAGTTTGGGTAGCACTAAAGATATTGTA [ 9126]
cp32-9_B31_TS_MP_FG_consensus  ATTGAACAAGATATTTCTAAAATGGGAAAATATCTTAAACCTAAAAAATCTAGTTTGGGTAGCACTAAAGATATTGTA [ 9126]
cp32-9_B31_NX_Pl_consensus     ATTGAACAAGATATTTCTAAAATGGGAAAATATCTTAAACCTAAAAAATCTAGTTTGGGTAGCACTAAAGATATTGTA [ 9126]
cp32-9_PAli_NX_MP_FG_consensus ATTGAACAAGATATTTCTAAAATGGGAAAATATCTTAAACCTAAAAAATCTAGTTTGGGTAGCACTAAAGATATTGTA [ 9126]
cp32-9_PAli_NX_Pl_consensus    ATTGAACAAGATATTTCTAAAATGGGAAAATATCTTAAACCTAAAAAATCTAGTTTGGGTAGCACTAAAGATATTGTA [ 9126]
cp32-9_PAbe_NX_MP_FG_consensus ATTGAACAAGATATTTCTAAAATGGGAAAATATCTTAAACCTAAAAAATCTAGTTTGGGTAGCACTAAAGATATTGTA [ 9126]
cp32-9_PAbe_NX_Pl_consensus    ATTGAACAAGATATTTCTAAAATGGGAAAATATCTTAAACCTAAAAAATCTAGTTTGGGTAGCACTAAAGATATTGTA [ 9126]

cp32-9_gi|11497325|B31_GB      AAAAATAATTTGTCGGACAAGAAAAAAGAACTTAGTAGACAATCTAAATTTGAAAGCTTAAGAGAGCGTGTTGAGAAA [ 9204]
cp32-9_un7_B31_PacBio          AAAAATAATTTGTCGGACAAGAAAAAAGAACTTAGTAGACAATCTAAATTTGAAAGCTTAAGAGAGCGTGTTGAGAAA [ 9204]
cp32-9_B31_TS_MP_FG_consensus  AAAAATAATTTGTCGGACAAGAAAAAAGAACTTAGTAGACAATCTAAATTTGAAAGCTTAAGAGAGCGTGTTGAGAAA [ 9204]
cp32-9_B31_NX_Pl_consensus     AAAAATAATTTGTCGGACAAGAAAAAAGAACTTAGTAGACAATCTAAATTTGAAAGCTTAAGAGAGCGTGTTGAGAAA [ 9204]
cp32-9_PAli_NX_MP_FG_consensus AAAAATAATTTGTCGGACAAGAAAAAAGAACTTAGTAGACAATCTAAATTTGAAAGCTTAAGAGAGCGTGTTGAGAAA [ 9204]
cp32-9_PAli_NX_Pl_consensus    AAAAATAATTTGTCGGACAAGAAAAAAGAACTTAGTAGACAATCTAAATTTGAAAGCTTAAGAGAGCGTGTTGAGAAA [ 9204]
cp32-9_PAbe_NX_MP_FG_consensus AAAAATAATTTGTCGGACAAGAAAAAAGAACTTAGTAGACAATCTAAATTTGAAAGCTTAAGAGAGCGTGTTGAGAAA [ 9204]
cp32-9_PAbe_NX_Pl_consensus    AAAAATAATTTGTCGGACAAGAAAAAAGAACTTAGTAGACAATCTAAATTTGAAAGCTTAAGAGAGCGTGTTGAGAAA [ 9204]

cp32-9_gi|11497325|B31_GB      TATAGACTTACACAAACTAAAAAACTTATAAAACAGGGCATGGGGTTTGAGAAAGCTAGAAAAGAGGCTTTCAGAAGA [ 9282]
cp32-9_un7_B31_PacBio          TATAGACTTACACAAACTAAAAAACTTATAAAACAGGGCATGGGGTTTGAGAAAGCTAGAAAAGAGGCTTTCAGAAGA [ 9282]
cp32-9_B31_TS_MP_FG_consensus  TATAGACTTACACAAACTAAAAAACTTATAAAACAGGGCATGGGGTTTGAGAAAGCTAGAAAAGAGGCTTTCAGAAGA [ 9282]
cp32-9_B31_NX_Pl_consensus     TATAGACTTACACAAACTAAAAAACTTATAAAACAGGGCATGGGGTTTGAGAAAGCTAGAAAAGAGGCTTTCAGAAGA [ 9282]
cp32-9_PAli_NX_MP_FG_consensus TATAGACTTACACAAACTAAAAAACTTATAAAACAGGGCATGGGGTTTGAGAAAGCTAGAAAAGAGGCTTTCAGAAGA [ 9282]
cp32-9_PAli_NX_Pl_consensus    TATAGACTTACACAAACTAAAAAACTTATAAAACAGGGCATGGGGTTTGAGAAAGCTAGAAAAGAGGCTTTCAGAAGA [ 9282]
cp32-9_PAbe_NX_MP_FG_consensus TATAGACTTACACAAACTAAAAAACTTATAAAACAGGGCATGGGGTTTGAGAAAGCTAGAAAAGAGGCTTTCAGAAGA [ 9282]
cp32-9_PAbe_NX_Pl_consensus    TATAGACTTACACAAACTAAAAAACTTATAAAACAGGGCATGGGGTTTGAGAAAGCTAGAAAAGAGGCTTTCAGAAGA [ 9282]

cp32-9_gi|11497325|B31_GB      TCTTTAATGTCTGATAGAGACAAAAGGCGTCTTGAGTATAAAGAACTTGCAAAAGAATCAAAAGCAAAAAGTAAAATG [ 9360]
cp32-9_un7_B31_PacBio          TCTTTAATGTCTGATAGAGACAAAAGGCGTCTTGAGTATAAAGAACTTGCAAAAGAATCAAAAGCAAAAAGTAAAATG [ 9360]
cp32-9_B31_TS_MP_FG_consensus  TCTTTAATGTCTGATAGAGACAAAAGGCGTCTTGAGTATAAAGAACTTGCAAAAGAATCAAAAGCAAAAAGTAAAATG [ 9360]
cp32-9_B31_NX_Pl_consensus     TCTTTAATGTCTGATAGAGACAAAAGGCGTCTTGAGTATAAAGAACTTGCAAAAGAATCAAAAGCAAAAAGTAAAATG [ 9360]
cp32-9_PAli_NX_MP_FG_consensus TCTTTAATGTCTGATAGAGACAAAAGGCGTCTTGAGTATAAAGAACTTGCAAAAGAATCAAAAGCAAAAAGTAAAATG [ 9360]
cp32-9_PAli_NX_Pl_consensus    TCTTTAATGTCTGATAGAGACAAAAGGCGTCTTGAGTATAAAGAACTTGCAAAAGAATCAAAAGCAAAAAGTAAAATG [ 9360]
cp32-9_PAbe_NX_MP_FG_consensus TCTTTAATGTCTGATAGAGACAAAAGGCGTCTTGAGTATAAAGAACTTGCAAAAGAATCAAAAGCAAAAAGTAAAATG [ 9360]
cp32-9_PAbe_NX_Pl_consensus    TCTTTAATGTCTGATAGAGACAAAAGGCGTCTTGAGTATAAAGAACTTGCAAAAGAATCAAAAGCAAAAAGTAAAATG [ 9360]

cp32-9_gi|11497325|B31_GB      TTAGCGGCCTCTCAAGGAAAAGGACTTGTTGCCAAAATTGCTATAGGTAGTGCCCTAGGGAATATCATTAGCAACGCT [ 9438]
cp32-9_un7_B31_PacBio          TTAGCGGCCTCTCAAGGAAAAGGACTTGTTGCCAAAATTGCTATAGGTAGTGCCCTAGGGAATATCATTAGCAACGCT [ 9438]
```

```
cp32-9_B31_TS_MP_FG_consensus  TTAGCGGCCTCTCAAGGAAAAGGACTTGTTGCCAAAATTGCTATAGGTAGTGCCCTAGGGAATATCATTAGCAACGCT [ 9438]
cp32-9_B31_NX_Pl_consensus     TTAGCGGCCTCTCAAGGAAAAGGACTTGTTGCCAAAATTGCTATAGGTAGTGCCCTAGGGAATATCATTAGCAACGCT [ 9438]
cp32-9_PAli_NX_MP_FG_consensus TTAGCGGCCTCTCAAGGAAAAGGACTTGTTGCCAAAATTGCTATAGGTAGTGCCCTAGGGAATATCATTAGCAACGCT [ 9438]
cp32-9_PAli_NX_Pl_consensus    TTAGCGGCCTCTCAAGGAAAAGGACTTGTTGCCAAAATTGCTATAGGTAGTGCCCTAGGGAATATCATTAGCAACGCT [ 9438]
cp32-9_PAbe_NX_MP_FG_consensus TTAGCGGCCTCTCAAGGAAAAGGACTTGTTGCCAAAATTGCTATAGGTAGTGCCCTAGGGAATATCATTAGCAACGCT [ 9438]
cp32-9_PAbe_NX_Pl_consensus    TTAGCGGCCTCTCAAGGAAAAGGACTTGTTGCCAAAATTGCTATAGGTAGTGCCCTAGGGAATATCATTAGCAACGCT [ 9438]

cp32-9_gi|11497325|B31_GB      ATGAGTAAAGTTGGAGGAGGCCTTTTAGGTTTTGCTAAAAAATCGGTTGAAGAAGACACCAAAACAAAAAGAACACAA [ 9516]
cp32-9_un7_B31_PacBio          ATGAGTAAAGTTGGAGGAGGCCTTTTAGGTTTTGCTAAAAAATCGGTTGAAGAAGACACCAAAACAAAAAGAACACAA [ 9516]
cp32-9_B31_TS_MP_FG_consensus  ATGAGTAAAGTTGGAGGAGGCCTTTTAGGTTTTGCTAAAAAATCGGTTGAAGAAGACACCAAAACAAAAAGAACACAA [ 9516]
cp32-9_B31_NX_Pl_consensus     ATGAGTAAAGTTGGAGGAGGCCTTTTAGGTTTTGCTAAAAAATCGGTTGAAGAAGACACCAAAACAAAAAGAACACAA [ 9516]
cp32-9_PAli_NX_MP_FG_consensus ATGAGTAAAGTTGGAGGAGGCCTTTTAGGTTTTGCTAAAAAATCGGTTGAAGAAGACACCAAAACAAAAAGAACACAA [ 9516]
cp32-9_PAli_NX_Pl_consensus    ATGAGTAAAGTTGGAGGAGGCCTTTTAGGTTTTGCTAAAAAATCGGTTGAAGAAGACACCAAAACAAAAAGAACACAA [ 9516]
cp32-9_PAbe_NX_MP_FG_consensus ATGAGTAAAGTTGGAGGAGGCCTTTTAGGTTTTGCTAAAAAATCGGTTGAAGAAGACACCAAAACAAAAAGAACACAA [ 9516]
cp32-9_PAbe_NX_Pl_consensus    ATGAGTAAAGTTGGAGGAGGCCTTTTAGGTTTTGCTAAAAAATCGGTTGAAGAAGACACCAAAACAAAAAGAACACAA [ 9516]

cp32-9_gi|11497325|B31_GB      CTTCTCAATAAAGCGTTTTATGGTGATCCAAAAGAGAAAGAGAGTCTTTTAAAGATTATTGGCGGAATGAAGGGATTT [ 9594]
cp32-9_un7_B31_PacBio          CTTCTCAATAAAGCGTTTTATGGTGATCCAAAAGAGAAAGAGAGTCTTTTAA-GATTATTGGCGGAATGAAGGGATTT [ 9594]
cp32-9_B31_TS_MP_FG_consensus  CTTCTCAATAAAGCGTTTTATGGTGATCCAAAAGAGAAAGAGAGTCTTTTAAAGATTATTGGCGGAATGAAGGGATTT [ 9594]
cp32-9_B31_NX_Pl_consensus     CTTCTCAATAAAGCGTTTTATGGTGATCCAAAAGAGAAAGAGAGTCTTTTAAAGATTATTGGCGGAATGAAGGGATTT [ 9594]
cp32-9_PAli_NX_MP_FG_consensus CTTCTCAATAAAGCGTTTTATGGTGATCCAAAAGAGAAAGAGAGTCTTTTAAAGATTATTGGCGGAATGAAGGGATTT [ 9594]
cp32-9_PAli_NX_Pl_consensus    CTTCTCAATAAAGCGTTTTATGGTGATCCAAAAGAGAAAGAGAGTCTTTTAAAGATTATTGGCGGAATGAAGGGATTT [ 9594]
cp32-9_PAbe_NX_MP_FG_consensus CTTCTCAATAAAGCGTTTTATGGTGATCCAAAAGAGAAAGAGAGTCTTTTAAAGATTATTGGCGGAATGAAGGGATTT [ 9594]
cp32-9_PAbe_NX_Pl_consensus    CTTCTCAATAAAGCGTTTTATGGTGATCCAAAAGAGAAAGAGAGTCTTTTAAAGATTATTGGCGGAATGAAGGGATTT [ 9594]

cp32-9_gi|11497325|B31_GB      GAGCGCGACCTAGAAAAAGAAGAATTCTTAAATCAAGCAAGTGTCTTTAAGGGTACTTTAAGGGACTTAGATATGTTA [ 9672]
cp32-9_un7_B31_PacBio          GAGCGCGACCTAGAAAAAGAAGAATTCTTAAATCAAGCAAGTGTCTTTAAGGGTACTTTAAGG-ACTTAGATATGTTA [ 9672]
cp32-9_B31_TS_MP_FG_consensus  GAGCGCGACCTAGAAAAAGAAGAATTCTTAAATCAAGCAAGTGTCTTTAAGGGTACTTTAAGGGACTTAGATATGTTA [ 9672]
cp32-9_B31_NX_Pl_consensus     GAGCGCGACCTAGAAAAAGAAGAATTCTTAAATCAAGCAAGTGTCTTTAAGGGTACTTTAAGGGACTTAGATATGTTA [ 9672]
cp32-9_PAli_NX_MP_FG_consensus GAGCGCGACCTAGAAAAAGAAGAATTCTTAAATCAAGCAAGTGTCTTTAAGGGTACTTTAAGGGACTTAGATATGTTA [ 9672]
cp32-9_PAli_NX_Pl_consensus    GAGCGCGACCTAGAAAAAGAAGAATTCTTAAATCAAGCAAGTGTCTTTAAGGGTACTTTAAGGGACTTAGATATGTTA [ 9672]
cp32-9_PAbe_NX_MP_FG_consensus GAGCGCGACCTAGAAAAAGAAGAATTCTTAAATCAAGCAAGTGTCTTTAAGGGTACTTTAAGGGACTTAGATATGTTA [ 9672]
cp32-9_PAbe_NX_Pl_consensus    GAGCGCGACCTAGAAAAAGAAGAATTCTTAAATCAAGCAAGTGTCTTTAAGGGTACTTTAAGGGACTTAGATATGTTA [ 9672]

cp32-9_gi|11497325|B31_GB      AATGAAACTAATTTGAAAAACGCAGTAGAATTTGCAGCTATGCTTAAATCCAGTGGTGCTATGAGCAGCGAAGATGCA [ 9750]
cp32-9_un7_B31_PacBio          AATGAAACTAATTTGAAAAACGCAGTAGAATTTGCAGCTATGCTTAA-TCCAGTGGTGCTATGAGCAGCGAAGATGCA [ 9750]
cp32-9_B31_TS_MP_FG_consensus  AATGAAACTAATTTGAAAAACGCAGTAGAATTTGCAGCTATGCTTAAATCCAGTGGTGCTATGAGCAGCGAAGATGCA [ 9750]
cp32-9_B31_NX_Pl_consensus     AATGAAACTAATTTGAAAAACGCAGTAGAATTTGCAGCTATGCTTAAATCCAGTGGTGCTATGAGCAGCGAAGATGCA [ 9750]
cp32-9_PAli_NX_MP_FG_consensus AATGAAACTAATTTGAAAAACGCAGTAGAATTTGCAGCTATGCTTAAATCCAGTGGTGCTATGAGCAGCGAAGATGCA [ 9750]
cp32-9_PAli_NX_Pl_consensus    AATGAAACTAATTTGAAAAACGCAGTAGAATTTGCAGCTATGCTTAAATCCAGTGGTGCTATGAGCAGCGAAGATGCA [ 9750]
cp32-9_PAbe_NX_MP_FG_consensus AATGAAACTAATTTGAAAAACGCAGTAGAATTTGCAGCTATGCTTAAATCCAGTGGTGCTATGAGCAGCGAAGATGCA [ 9750]
cp32-9_PAbe_NX_Pl_consensus    AATGAAACTAATTTGAAAAACGCAGTAGAATTTGCAGCTATGCTTAAATCCAGTGGTGCTATGAGCAGCGAAGATGCA [ 9750]

cp32-9_gi|11497325|B31_GB      GTAAAGGCTGTTAATAGTGTTCTTGGGGGTGATGGAAGTGAGCTTTTTGATCTACTAAAAAAGTCGGGCGTTGGCGAC [ 9828]
cp32-9_un7_B31_PacBio          GTAAAGGCTGTTAATAGTGT-CTTGGGGGTGATGGAAGTGAGCTTTTTGATCTACTAAAAAAGTCGGGCGTTGGCGAC [ 9828]
cp32-9_B31_TS_MP_FG_consensus  GTAAAGGCTGTTAATAGTGTTCTTGGGGGTGATGGAAGTGAGCTTTTTGATCTACTAAAAAAGTCGGGCGTTGGCGAC [ 9828]
cp32-9_B31_NX_Pl_consensus     GTAAAGGCTGTTAATAGTGTTCTTGGGGGTGATGGAAGTGAGCTTTTTGATCTACTAAAAAAGTCGGGCGTTGGCGAC [ 9828]
cp32-9_PAli_NX_MP_FG_consensus GTAAAGGCTGTTAATAGTGTTCTTGGGGGTGATGGAAGTGAGCTTTTTGATCTACTAAAAAAGTCGGGCGTTGGCGAC [ 9828]
cp32-9_PAli_NX_Pl_consensus    GTAAAGGCTGTTAATAGTGTTCTTGGGGGTGATGGAAGTGAGCTTTTTGATCTACTAAAAAAGTCGGGCGTTGGCGAC [ 9828]
cp32-9_PAbe_NX_MP_FG_consensus GTAAAGGCTGTTAATAGTGTTCTTGGGGGTGATGGAAGTGAGCTTTTTGATCTACTAAAAAAGTCGGGCGTTGGCGAC [ 9828]
cp32-9_PAbe_NX_Pl_consensus    GTAAAGGCTGTTAATAGTGTTCTTGGGGGTGATGGAAGTGAGCTTTTTGATCTACTAAAAAAGTCGGGCGTTGGCGAC [ 9828]

cp32-9_gi|11497325|B31_GB      AAATACATAGAAGATGCCAAAAGGGCTTGGCAAGGCGGGGCTGAGGTAGATTTAGAGTCCAGAATTACCAAGATGATG [ 9906]
cp32-9_un7_B31_PacBio          AAATACATAGAAGATGCCAAAAGGGCTTGGCAAGGCGGGGCTGAGGTAGATTTAGAGTCCAGAATTACCAAGATGATG [ 9906]
cp32-9_B31_TS_MP_FG_consensus  AAATACATAGAAGATGCCAAAAGGGCTTGGCAAGGCGGGGCTGAGGTAGATTTAGAGTCCAGAATTACCAAGATGATG [ 9906]
cp32-9_B31_NX_Pl_consensus     AAATACATAGAAGATGCCAAAAGGGCTTGGCAAGGCGGGGCTGAGGTAGATTTAGAGTCCAGAATTACCAAGATGATG [ 9906]
cp32-9_PAli_NX_MP_FG_consensus AAATACATAGAAGATGCCAAAAGGGCTTGGCAAGGCGGGGCTGAGGTAGATTTAGAGTCCAGAATTACCAAGATGATG [ 9906]
cp32-9_PAli_NX_Pl_consensus    AAATACATAGAAGATGCCAAAAGGGCTTGGCAAGGCGGGGCTGAGGTAGATTTAGAGTCCAGAATTACCAAGATGATG [ 9906]
cp32-9_PAbe_NX_MP_FG_consensus AAATACATAGAAGATGCCAAAAGGGCTTGGCAAGGCGGGGCTGAGGTAGATTTAGAGTCCAGAATTACCAAGATGATG [ 9906]
cp32-9_PAbe_NX_Pl_consensus    AAATACATAGAAGATGCCAAAAGGGCTTGGCAAGGCGGGGCTGAGGTAGATTTAGAGTCCAGAATTACCAAGATGATG [ 9906]

cp32-9_gi|11497325|B31_GB      GAAATGTTCGAGGATTTTAAATCTTTCGGCCTTACAAAAAAAGCCAATACTGCTGAGATTATTCAAAGTAATTTGGCC [ 9984]
cp32-9_un7_B31_PacBio          GAAATGTTCGAGGATTTTAAATCTTTCGGCCTTACAAAAAA-GCCAATACTGCTGAGATTATTCAAAGTAATTTGGCC [ 9984]
cp32-9_B31_TS_MP_FG_consensus  GAAATGTTCGAGGATTTTAAATCTTTCGGCCTTACAAAAAAAGCCAATACTGCTGAGATTATTCAAAGTAATTTGGCC [ 9984]
cp32-9_B31_NX_Pl_consensus     GAAATGTTCGAGGATTTTAAATCTTTCGGCCTTACAAAAAAAGCCAATACTGCTGAGATTATTCAAAGTAATTTGGCC [ 9984]
cp32-9_PAli_NX_MP_FG_consensus GAAATGTTCGAGGATTTTAAATCTTTCGGCCTTACAAAAAAAGCCAATACTGCTGAGATTATTCAAAGTAATTTGGCC [ 9984]
cp32-9_PAli_NX_Pl_consensus    GAAATGTTCGAGGATTTTAAATCTTTCGGCCTTACAAAAAAAGCCAATACTGCTGAGATTATTCAAAGTAATTTGGCC [ 9984]
cp32-9_PAbe_NX_MP_FG_consensus GAAATGTTCGAGGATTTTAAATCTTTCGGCCTTACAAAAAAAGCCAATACTGCTGAGATTATTCAAAGTAATTTGGCC [ 9984]
cp32-9_PAbe_NX_Pl_consensus    GAAATGTTCGAGGATTTTAAATCTTTCGGCCTTACAAAAAAAGCCAATACTGCTGAGATTATTCAAAGTAATTTGGCC [ 9984]

cp32-9_gi|11497325|B31_GB      TCAGCTGAGCAAACTCTTCAAAACTTAACCACTACTGTCTTGGACCCATTACTTGACCTCATTAATAAGATAACTAAT [10062]
cp32-9_un7_B31_PacBio          TCAGCTGAGCAAACTCTTCAAAACTTAACCACTACTGTCTTGGACCCATTACTTGACCTCATTAATAAGATAACTAAT [10062]
cp32-9_B31_TS_MP_FG_consensus  TCAGCTGAGCAAACTCTTCAAAACTTAACCACTACTGTCTTGGACCCATTACTTGACCTCATTAATAAGATAACTAAT [10062]
cp32-9_B31_NX_Pl_consensus     TCAGCTGAGCAAACTCTTCAAAACTTAACCACTACTGTCTTGGACCCATTACTTGACCTCATTAATAAGATAACTAAT [10062]
cp32-9_PAli_NX_MP_FG_consensus TCAGCTGAGCAAACTCTTCAAAACTTAACCACTACTGTCTTGGACCCATTACTTGACCTCATTAATAAGATAACTAAT [10062]
cp32-9_PAli_NX_Pl_consensus    TCAGCTGAGCAAACTCTTCAAAACTTAACCACTACTGTCTTGGACCCATTACTTGACCTCATTAATAAGATAACTAAT [10062]
cp32-9_PAbe_NX_MP_FG_consensus TCAGCTGAGCAAACTCTTCAAAACTTAACCACTACTGTCTTGGACCCATTACTTGACCTCATTAATAAGATAACTAAT [10062]
cp32-9_PAbe_NX_Pl_consensus    TCAGCTGAGCAAACTCTTCAAAACTTAACCACTACTGTCTTGGACCCATTACTTGACCTCATTAATAAGATAACTAAT [10062]

cp32-9_gi|11497325|B31_GB      TACTTTAAAGACTTTACGTTTGAAACACATATTATTGAACCCATAATTAATGGCATTAAAAGTATTTTTAATCTTAAT [10140]
cp32-9_un7_B31_PacBio          TACTTTAAAGACTTTACGTTTGAAACACATATTATTGAACCCATAATTAATGGCATTAAAAGTATTTTTAATCTTAAT [10140]
cp32-9_B31_TS_MP_FG_consensus  TACTTTAAAGACTTTACGTTTGAAACACATATTATTGAACCCATAATTAATGGCATTAAAAGTATTTTTAATCTTAAT [10140]
cp32-9_B31_NX_Pl_consensus     TACTTTAAAGACTTTACGTTTGAAACACATATTATTGAACCCATAATTAATGGCATTAAAAGTATTTTTAATCTTAAT [10140]
cp32-9_PAli_NX_MP_FG_consensus TACTTTAAAGACTTTACGTTTGAAACACATATTATTGAACCCATAATTAATGGCATTAAAAGTATTTTTAATCTTAAT [10140]
cp32-9_PAli_NX_Pl_consensus    TACTTTAAAGACTTTACGTTTGAAACACATATTATTGAACCCATAATTAATGGCATTAAAAGTATTTTTAATCTTAAT [10140]
cp32-9_PAbe_NX_MP_FG_consensus TACTTTAAAGACTTTACGTTTGAAACACATATTATTGAACCCATAATTAATGGCATTAAAAGTATTTTTAATCTTAAT [10140]
cp32-9_PAbe_NX_Pl_consensus    TACTTTAAAGACTTTACGTTTGAAACACATATTATTGAACCCATAATTAATGGCATTAAAAGTATTTTTAATCTTAAT [10140]

cp32-9_gi|11497325|B31_GB      TATTTCTTTGCAAAATTAAAATCGATGCTACCTGGATGGATGGGCGGAGATGAGGGTGCGGCTCTAAAAAAACTACAA [10218]
cp32-9_un7_B31_PacBio          TATTTCTTTGCAAAATTAAAATCGATGCTACCTGGATGGATGGGCGGAGATGAGGGTGCGGCTCTAAAAAAACTACAA [10218]
cp32-9_B31_TS_MP_FG_consensus  TATTTCTTTGCAAAATTAAAATCGATGCTACCTGGATGGATGGGCGGAGATGAGGGTGCGGCTCTAAAAAAACTACAA [10218]
cp32-9_B31_NX_Pl_consensus     TATTTCTTTGCAAAATTAAAATCGATGCTACCTGGATGGATGGGCGGAGATGAGGGTGCGGCTCTAAAAAAACTACAA [10218]
cp32-9_PAli_NX_MP_FG_consensus TATTTCTTTGCAAAATTAAAATCGATGCTACCTGGATGGATGGGCGGAGATGAGGGTGCGGCTCTAAAAAAACTACAA [10218]
cp32-9_PAli_NX_Pl_consensus    TATTTCTTTGCAAAATTAAAATCGATGCTACCTGGATGGATGGGCGGAGATGAGGGTGCGGCTCTAAAAAAACTACAA [10218]
cp32-9_PAbe_NX_MP_FG_consensus TATTTCTTTGCAAAATTAAAATCGATGCTACCTGGATGGATGGGCGGAGATGAGGGTGCGGCTCTAAAAAAACTACAA [10218]
cp32-9_PAbe_NX_Pl_consensus    TATTTCTTTGCAAAATTAAAATCGATGCTACCTGGATGGATGGGCGGAGATGAGGGTGCGGCTCTAAAAAAACTACAA [10218]

cp32-9_gi|11497325|B31_GB      GAAGAAATTCAAAATCAAGACAATGCTAACAGCACACCATAATTTTTACAAAAGGTAATTACTTATGACAAGTAACAA [10296]
cp32-9_un7_B31_PacBio          GAAGAAATTCAAAATCAAGACAATGCTAACAGCACACCATAATTTTTACAAAAGGTAATTACTTATGACAAGTAACAA [10296]
cp32-9_B31_TS_MP_FG_consensus  GAAGAAATTCAAAATCAAGACAATGCTAACAGCACACCATAATTTTTACAAAAGGTAATTACTTATGACAAGTAACAA [10296]
cp32-9_B31_NX_Pl_consensus     GAAGAAATTCAAAATCAAGACAATGCTAACAGCACACCATAATTTTTACAAAAGGTAATTACTTATGACAAGTAACAA [10296]
cp32-9_PAli_NX_MP_FG_consensus GAAGAAATTCAAAATCAAGACAATGCTAACAGCACACCATAATTTTTACAAAAGGTAATTACTTATGACAAGTAACAA [10296]
cp32-9_PAli_NX_Pl_consensus    GAAGAAATTCAAAATCAAGACAATGCTAACAGCACACCATAATTTTTACAAAAGGTAATTACTTATGACAAGTAACAA [10296]
cp32-9_PAbe_NX_MP_FG_consensus GAAGAAATTCAAAATCAAGACAATGCTAACAGCACACCATAATTTTTACAAAAGGTAATTACTTATGACAAGTAACAA [10296]
cp32-9_PAbe_NX_Pl_consensus    GAAGAAATTCAAAATCAAGACAATGCTAACAGCACACCATAATTTTTACAAAAGGTAATTACTTATGACAAGTAACAA [10296]

cp32-9_gi|11497325|B31_GB      AAAAATTGCTAACAATGCAGCTAACAAAATAGATATTAATAATAAAATTACTAACAATCATGATATTGAAAA-GAAAA [10374]
cp32-9_un7_B31_PacBio          AAAAATTGCTAACAATGCAGCTAACAAAATAGATATTAATAATAAAATTACTAACAATCATGATATTGAAAAAGAAAA [10374]
cp32-9_B31_TS_MP_FG_consensus  AAAAATTGCTAACAATGCAGCTAACAAAATAGATATTAATAATAAAATTACTAACAATCATGATATTGAAAA-GAAAA [10374]
```

```
cp32-9_B31_NX_Pl_consensus     AAAAATTGCTAACAATGCAGCTAACAAAATAGATATTAATAATAAAATTACTAACAATCATGATATTGAAAA-GAAAA [10374]
cp32-9_PAli_NX_MP_FG_consensus AAAAATTGCTAACAATGCAGCTAACAAAATAGATATTAATAATAAAATTACTAACAATCATGATATTGAAAA-GAAAA [10374]
cp32-9_PAli_NX_Pl_consensus    AAAAATTGCTAACAATGCAGCTAACAAAATAGATATTAATAATAAAATTACTAACAATCATGATATTGAAAA-GAAAA [10374]
cp32-9_PAbe_NX_MP_FG_consensus AAAAATTGCTAACAATGCAGCTAACAAAATAGATATTAATAATAAAATTACTAACAATCATGATATTGAAAA-GAAAA [10374]
cp32-9_PAbe_NX_Pl_consensus    AAAAATTGCTAACAATGCAGCTAACAAAATAGATATTAATAATAAAATTACTAACAATCATGATATTGAAAA-GAAAA [10374]

cp32-9_gi|11497325|B31_GB      AAATCGAGGAAAAAATTAATGATATTGAAAAGAAAAAAATCGAGGAAAAAATTAATGATATTGAAAAGAAAAAA-TCG [10452]
cp32-9_un7_B31_PacBio          AAATCGAGGAAAAAATTAATGATATTGAAAAGAAAAAAATCGAGGAAAAAATTAATGATATTGAAAAGAAAAAA-TCG [10452]
cp32-9_B31_TS_MP_FG_consensus  AAATCGAGGAAAAAATTAATGATATTGAAAAGAAAAAAATCGAGGAAAAAATTAATGATATTGAAAAGAAAAAA-TCG [10452]
cp32-9_B31_NX_Pl_consensus     AAATCGAGGAAAAAATTAATGATATTGAAAAGAAAAAAATCGAGGAAAAAATTAATGATATTGAAAAGAAAAAA-TCG [10452]
cp32-9_PAli_NX_MP_FG_consensus AAATCGAGGAAAAAATTAATGATATTGAAAAGAAAGAAATCGGGGAAA??????ATGATATTGAAAAGAAAAAAATCG [10452]
cp32-9_PAli_NX_Pl_consensus    AAATCGAGGAAAAAATTAATGATATTGAAAAGAAAGAAATCGGGGAAAA------TGATATTGAAAAGAAAAAAATCG [10452]
cp32-9_PAbe_NX_MP_FG_consensus AAATCGAGGAAAAAATTAATGATATTGAAAAGAAAAAAATCGAGGAAAAAATTAATGATATTGAAAAGAAAAAA-TCG [10452]
cp32-9_PAbe_NX_Pl_consensus    AAATCGAGGAAAAAATTAATGATATTGAAAAGAAAAAAATCGAGGAAAAAATTAATGATATTGAAAAGAAAAAA-TCG [10452]

cp32-9_gi|11497325|B31_GB      AGGAAAAAATTAATGATATTGAAAAGAAAGAAATCGGGGAAATTACTCGAATAATAAGAGATGTAATAACCCAAATAT [10530]
cp32-9_un7_B31_PacBio          AGGAAAAAATTAATGATATTGAAAAGAAAGAAATCGGGGAAATTACTCGAATAATAAGAGATGTAATAACCCAAATAT [10530]
cp32-9_B31_TS_MP_FG_consensus  AGGAAAAAATTAATGATATTGAAAAGAAAGAAATCGGGGAAATTACTCGAATAATAAGAGATGTAATAACCCAAATAT [10530]
cp32-9_B31_NX_Pl_consensus     AGGAAAAAATTAATGATATTGAAAAGAAAGAAATCGGGGAAATTACTCGAATAATAAGAGATGTAATAACCCAAATAT [10530]
cp32-9_PAli_NX_MP_FG_consensus AGGAAAAAATTAATGATATTGAAAAGAAAGAAATCGGGGAAATTACTCGAATAATAAGAGATGTAATAACCCAAATAT [10530]
cp32-9_PAli_NX_Pl_consensus    AGGAAAAAATTAATGATATTGAAAAGAAAGAAATCGGGGAAATTACTCGAATAATAAGAGATGTAATAACCCAAATAT [10530]
cp32-9_PAbe_NX_MP_FG_consensus AGGAAAAAATTAATGATATTGAAAAGAAAGAAATCGGGGAAATTACTCGAATAATAAGAGATGTAATAACCCAAATAT [10530]
cp32-9_PAbe_NX_Pl_consensus    AGGAAAAAATTAATGATATTGAAAAGAAAGAAATCGGGGAAATTACTCGAATAATAAGAGATGTAATAACCCAAATAT [10530]

cp32-9_gi|11497325|B31_GB      TTGCCCTTTTCGGAGCAGATAATTTTTTAGTGTTATTTCCTAGAATGGATCTAAAAGGTTTTGGATATGTTCCTCAAT [10608]
cp32-9_un7_B31_PacBio          TTGCCCTTTTCGGAGCAGATAATTTTTTAGTGTTATTTCCTAGAATGGATCTAAAAGGTTTTGGATATGTTCCTCAAT [10608]
cp32-9_B31_TS_MP_FG_consensus  TTGCCCTTTTCGGAGCAGATAATTTTTTAGTGTTATTTCCTAGAATGGATCTAAAAGGTTTTGGATATGTTCCTCAAT [10608]
cp32-9_B31_NX_Pl_consensus     TTGCCCTTTTCGGAGCAGATAATTTTTTAGTGTTATTTCCTAGAATGGATCTAAAAGGTTTTGGATATGTTCCTCAAT [10608]
cp32-9_PAli_NX_MP_FG_consensus TTGCCCTTTTCGGAGCAGATAATTTTTTAGTGTTATTTCCTAGAATGGATCTAAAAGGTTTTGGATATGTTCCTCAAT [10608]
cp32-9_PAli_NX_Pl_consensus    TTGCCCTTTTCGGAGCAGATAATTTTTTAGTGTTATTTCCTAGAATGGATCTAAAAGGTTTTGGATATGTTCCTCAAT [10608]
cp32-9_PAbe_NX_MP_FG_consensus TTGCCCTTTTCGGAGCAGATAATTTTTTAGTGTTATTTCCTAGAATGGATCTAAAAGGTTTTGGATATGTTCCTCAAT [10608]
cp32-9_PAbe_NX_Pl_consensus    TTGCCCTTTTCGGAGCAGATAATTTTTTAGTGTTATTTCCTAGAATGGATCTAAAAGGTTTTGGATATGTTCCTCAAT [10608]

cp32-9_gi|11497325|B31_GB      TGTTTTTTATAAAACCAAAAAATGAACTCATAACACGCACTTACAATACTAATTGTTCTAAAAGACCAGTTATCAACT [10686]
cp32-9_un7_B31_PacBio          -GTTTTTTATAAAACCAAAAAATGAACTCATAACACGCACTTACAATACTAATTGTTCTAAAAGACCAGTTATCAACT [10686]
cp32-9_B31_TS_MP_FG_consensus  TGTTTTTTATAAAACCAAAAAATGAACTCATAACACGCACTTACAATACTAATTGTTCTAAAAGACCAGTTATCAACT [10686]
cp32-9_B31_NX_Pl_consensus     TGTTTTTTATAAAACCAAAAAATGAACTCATAACACGCACTTACAATACTAATTGTTCTAAAAGACCAGTTATCAACT [10686]
cp32-9_PAli_NX_MP_FG_consensus TGTTTTTTATAAAACCAAAAAATGAACTCATAACACGCACTTACAATACTAATTGTTCTAAAAGACCAGTTATCAACT [10686]
cp32-9_PAli_NX_Pl_consensus    TGTTTTTTATAAAACCAAAAAATGAACTCATAACACGCACTTACAATACTAATTGTTCTAAAAGACCAGTTATCAACT [10686]
cp32-9_PAbe_NX_MP_FG_consensus TGTTTTTTATAAAACCAAAAAATGAACTCATAACACGCACTTACAATACTAATTGTTCTAAAAGACCAGTTATCAACT [10686]
cp32-9_PAbe_NX_Pl_consensus    TGTTTTTTATAAAACCAAAAAATGAACTCATAACACGCACTTACAATACTAATTGTTCTAAAAGACCAGTTATCAACT [10686]

cp32-9_gi|11497325|B31_GB      ATTATGATAGAAAAGCGGAATATGTAAGCTACAATCCGGTAATGACTGGTGAAAACATATCATTAAACGGTGGAATAC [10764]
cp32-9_un7_B31_PacBio          ATTATGATAGAAAAGCGGAATATGTAAGCTACAATCCGGTAATGACTGGTGAAAACATATCATTAAACGGTGGAATAC [10764]
cp32-9_B31_TS_MP_FG_consensus  ATTATGATAGAAAAGCGGAATATGTAAGCTACAATCCGGTAATGACTGGTGAAAACATATCATTAAACGGTGGAATAC [10764]
cp32-9_B31_NX_Pl_consensus     ATTATGATAGAAAAGCGGAATATGTAAGCTACAATCCGGTAATGACTGGTGAAAACATATCATTAAACGGTGGAATAC [10764]
cp32-9_PAli_NX_MP_FG_consensus ATTATGATAGAAAAGCGGAATATGTAAGCTACAATCCGGTAATGACTGGTGAAAACATATCATTAAACGGTGGAATAC [10764]
cp32-9_PAli_NX_Pl_consensus    ATTATGATAGAAAAGCGGAATATGTAAGCTACAATCCGGTAATGACTGGTGAAAACATATCATTAAACGGTGGAATAC [10764]
cp32-9_PAbe_NX_MP_FG_consensus ATTATGATAGAAAAGCGGAATATGTAAGCTACAATCCGGTAATGACTGGTGAAAACATATCATTAAACGGTGGAATAC [10764]
cp32-9_PAbe_NX_Pl_consensus    ATTATGATAGAAAAGCGGAATATGTAAGCTACAATCCGGTAATGACTGGTGAAAACATATCATTAAACGGTGGAATAC [10764]

cp32-9_gi|11497325|B31_GB      TAACATCCTTATATAAGGATATGCTTTCTTTACTTAAAATGACTGTTTTTGGCAATACTATGCTACGTTTTGACGCGC [10842]
cp32-9_un7_B31_PacBio          TAACATCCTTATATAAGGATATGCTTTCTT-ACTTAAAATGACTGTTTTTGGCAATACTATGCTACGTTTTGACGCGC [10842]
cp32-9_B31_TS_MP_FG_consensus  TAACATCCTTATATAAGGATATGCTTTCTTTACTTAAAATGACTGTTTTTGGCAATACTATGCTACGTTTTGACGCGC [10842]
cp32-9_B31_NX_Pl_consensus     TAACATCCTTATATAAGGATATGCTTTCTTTACTTAAAATGACTGTTTTTGGCAATACTATGCTACGTTTTGACGCGC [10842]
cp32-9_PAli_NX_MP_FG_consensus TAACATCCTTATATAAGGATATGCTTTCTTTACTTAAAATGACTGTTTTTGGCAATACTATGCTACGTTTTGACGCGC [10842]
cp32-9_PAli_NX_Pl_consensus    TAACATCCTTATATAAGGATATGCTTTCTTTACTTAAAATGACTGTTTTTGGCAATACTATGCTACGTTTTGACGCGC [10842]
cp32-9_PAbe_NX_MP_FG_consensus TAACATCCTTATATAAGGATATGCTTTCTTTACTTAAAATGACTGTTTTTGGCAATACTATGCTACGTTTTGACGCGC [10842]
cp32-9_PAbe_NX_Pl_consensus    TAACATCCTTATATAAGGATATGCTTTCTTTACTTAAAATGACTGTTTTTGGCAATACTATGCTACGTTTTGACGCGC [10842]

cp32-9_gi|11497325|B31_GB      ATCTCGCAAAAGAACAACTAGCAAACAGACTTCAAGCTCAAGTTCCTTTTAGTATCTACAGTCCAACTTTTGGCCTAA [10920]
cp32-9_un7_B31_PacBio          ATCTCGCAAAAGAACAACTAGCAAACAGACTTCAAGCTCAAGTTCCTTTTAGTATCTACAGTCCAACTTTTGGCCTAA [10920]
cp32-9_B31_TS_MP_FG_consensus  ATCTCGCAAAAGAACAACTAGCAAACAGACTTCAAGCTCAAGTTCCTTTTAGTATCTACAGTCCAACTTTTGGCCTAA [10920]
cp32-9_B31_NX_Pl_consensus     ATCTCGCAAAAGAACAACTAGCAAACAGACTTCAAGCTCAAGTTCCTTTTAGTATCTACAGTCCAACTTTTGGCCTAA [10920]
cp32-9_PAli_NX_MP_FG_consensus ATCTCGCAAAAGAACAACTAGCAAACAGACTTCAAGCTCAAGTTCCTTTTAGTATCTACAGTCCAACTTTTGGCCTAA [10920]
cp32-9_PAli_NX_Pl_consensus    ATCTCGCAAAAGAACAACTAGCAAACAGACTTCAAGCTCAAGTTCCTTTTAGTATCTACAGTCCAACTTTTGGCCTAA [10920]
cp32-9_PAbe_NX_MP_FG_consensus ATCTCGCAAAAGAACAACTAGCAAACAGACTTCAAGCTCAAGTTCCTTTTAGTATCTACAGTCCAACTTTTGGCCTAA [10920]
cp32-9_PAbe_NX_Pl_consensus    ATCTCGCAAAAGAACAACTAGCAAACAGACTTCAAGCTCAAGTTCCTTTTAGTATCTACAGTCCAACTTTTGGCCTAA [10920]

cp32-9_gi|11497325|B31_GB      AAGAATTGGCTGTAATTACAAGTCTTTCGTTTAAGGACACTCCTTTCATTGACGAAGTTGAGGTTAGTCTATCAATAG [10998]
cp32-9_un7_B31_PacBio          AAGAATTGGCTGTAATTACAAGTCTTTCGTTTAAGGACACTCCTTTCATTGACGAAGTTGAGGTTAGTCTATCAATAG [10998]
cp32-9_B31_TS_MP_FG_consensus  AAGAATTGGCTGTAATTACAAGTCTTTCGTTTAAGGACACTCCTTTCATTGACGAAGTTGAGGTTAGTCTATCAATAG [10998]
cp32-9_B31_NX_Pl_consensus     AAGAATTGGCTGTAATTACAAGTCTTTCGTTTAAGGACACTCCTTTCATTGACGAAGTTGAGGTTAGTCTATCAATAG [10998]
cp32-9_PAli_NX_MP_FG_consensus AAGAATTGGCTGTAATTACAAGTCTTTCGTTTAAGGACACTCCTTTCATTGACGAAGTTGAGGTTAGTCTATCAATAG [10998]
cp32-9_PAli_NX_Pl_consensus    AAGAATTGGCTGTAATTACAAGTCTTTCGTTTAAGGACACTCCTTTCATTGACGAAGTTGAGGTTAGTCTATCAATAG [10998]
cp32-9_PAbe_NX_MP_FG_consensus AAGAATTGGCTGTAATTACAAGTCTTTCGTTTAAGGACACTCCTTTCATTGACGAAGTTGAGGTTAGTCTATCAATAG [10998]
cp32-9_PAbe_NX_Pl_consensus    AAGAATTGGCTGTAATTACAAGTCTTTCGTTTAAGGACACTCCTTTCATTGACGAAGTTGAGGTTAGTCTATCAATAG [10998]

cp32-9_gi|11497325|B31_GB      AAATAGTAAAAACATTTGCATTGGAAAAATATAAAGGATAAAAAATGCTGTTACTACAATATGATTTTAAAATTGAGT [11076]
cp32-9_un7_B31_PacBio          AAATAGTAAAAACATTTGCATTGGAAAAATATAAAGGATAAAAAATGCTGTTACTACAATATGATTTTAAAATTGAGT [11076]
cp32-9_B31_TS_MP_FG_consensus  AAATAGTAAAAACATTTGCATTGGAAAAATATAAAGGATAAAAAATGCTGTTACTACAATATGATTTTAAAATTGAGT [11076]
cp32-9_B31_NX_Pl_consensus     AAATAGTAAAAACATTTGCATTGGAAAAATATAAAGGATAAAAAATGCTGTTACTACAATATGATTTTAAAATTGAGT [11076]
cp32-9_PAli_NX_MP_FG_consensus AAATAGTAAAAACATTTGCATTGGAAAAATATAAAGGATAAAAAATGCTGTTACTACAATATGATTTTAAAATTGAGT [11076]
cp32-9_PAli_NX_Pl_consensus    AAATAGTAAAAACATTTGCATTGGAAAAATATAAAGGATAAAAAATGCTGTTACTACAATATGATTTTAAAATTGAGT [11076]
cp32-9_PAbe_NX_MP_FG_consensus AAATAGTAAAAACATTTGCATTGGAAAAATATAAAGGATAAAAAATGCTGTTACTACAATATGATTTTAAAATTGAGT [11076]
cp32-9_PAbe_NX_Pl_consensus    AAATAGTAAAAACATTTGCATTGGAAAAATATAAAGGATAAAAAATGCTGTTACTACAATATGATTTTAAAATTGAGT [11076]

cp32-9_gi|11497325|B31_GB      TCTACAATGTAGATACATCAAAAAAATCCCCTGATGGAATTCCTTTCGCCGAAGAAATTCCTAAAATTATCATCAATA [11154]
cp32-9_un7_B31_PacBio          TCTACAATGTAGATACATCAAAAAAATCCCCTGATGGAATTCCTTTCGCCGAAGAAATTCCTAAAATTATCATCAATA [11154]
cp32-9_B31_TS_MP_FG_consensus  TCTACAATGTAGATACATCAAAAAAATCCCCTGATGGAATTCCTTTCGCCGAAGAAATTCCTAAAATTATCATCAATA [11154]
cp32-9_B31_NX_Pl_consensus     TCTACAATGTAGATACATCAAAAAAATCCCCTGATGGAATTCCTTTCGCCGAAGAAATTCCTAAAATTATCATCAATA [11154]
cp32-9_PAli_NX_MP_FG_consensus TCTACAATGTAGATACATCAAAAAAATCCCCTGATGGAATTCCTTTCGCCGAAGAAATTCCTAAAATTATCATCAATA [11154]
cp32-9_PAli_NX_Pl_consensus    TCTACAATGTAGATACATCAAAAAAATCCCCTGATGGAATTCCTTTCGCCGAAGAAATTCCTAAAATTATCATCAATA [11154]
cp32-9_PAbe_NX_MP_FG_consensus TCTACAATGTAGATACATCAAAAAAATCCCCTGATGGAATTCCTTTCGCCGAAGAAATTCCTAAAATTATCATCAATA [11154]
cp32-9_PAbe_NX_Pl_consensus    TCTACAATGTAGATACATCAAAAAAATCCCCTGATGGAATTCCTTTCGCCGAAGAAATTCCTAAAATTATCATCAATA [11154]

cp32-9_gi|11497325|B31_GB      CACAAGATGGAATTCATATTGATATTTCCATATCCAACGTGTATTCAAATATTCATACTATAAGTTCCAAACAAGCAA [11232]
cp32-9_un7_B31_PacBio          CACAAGATGGAATTCATATTGATATTTCCATATCCAACGTGTATTCAAATATTCATACTATAAGTTCCAAACAAGCAA [11232]
cp32-9_B31_TS_MP_FG_consensus  CACAAGATGGAATTCATATTGATATTTCCATATCCAACGTGTATTCAAATATTCATACTATAAGTTCCAAACAAGCAA [11232]
cp32-9_B31_NX_Pl_consensus     CACAAGATGGAATTCATATTGATATTTCCATATCCAACGTGTATTCAAATATTCATACTATAAGTTCCAAACAAGCAA [11232]
cp32-9_PAli_NX_MP_FG_consensus CACAAGATGGAATTCATATTGATATTTCCATATCCAACGTGTATTCAAATATTCATACTATAAGTTCCAAACAAGCAA [11232]
cp32-9_PAli_NX_Pl_consensus    CACAAGATGGAATTCATATTGATATTTCCATATCCAACGTGTATTCAAATATTCATACTATAAGTTCCAAACAAGCAA [11232]
cp32-9_PAbe_NX_MP_FG_consensus CACAAGATGGAATTCATATTGATATTTCCATATCCAACGTGTATTCAAATATTCATACTATAAGTTCCAAACAAGCAA [11232]
cp32-9_PAbe_NX_Pl_consensus    CACAAGATGGAATTCATATTGATATTTCCATATCCAACGTGTATTCAAATATTCATACTATAAGTTCCAAACAAGCAA [11232]

cp32-9_gi|11497325|B31_GB      AAGTCGTACTTTGGAATCTTCCCTTAGACCTCACCGACGACATTAAATTTGGAGATATAGTAAAAATATATTATAAGA [11310]
cp32-9_un7_B31_PacBio          AAGTCGTACTTTGGAATCTTCCCTTAGACCTCACCGACGACATTAAATTTGGAGATATAGTAAAAATATATTATAAGA [11310]
cp32-9_B31_TS_MP_FG_consensus  AAGTCGTACTTTGGAATCTTCCCTTAGACCTCACCGACGACATTAAATTTGGAGATATAGTAAAAATATATTATAAGA [11310]
cp32-9_B31_NX_Pl_consensus     AAGTCGTACTTTGGAATCTTCCCTTAGACCTCACCGACGACATTAAATTTGGAGATATAGTAAAAATATATTATAAGA [11310]
```

```
cp32-9_PAli_NX_MP_FG_consensus AAGTCGTACTTTGGAATCTTCCCTTAGACCTCACCGACGACATTAAATTTGGAGATATAGTAAAAATATATTATAAGA [11310]
cp32-9_PAli_NX_Pl_consensus    AAGTCGTACTTTGGAATCTTCCCTTAGACCTCACCGACGACATTAAATTTGGAGATATAGTAAAAATATATTATAAGA [11310]
cp32-9_PAbe_NX_MP_FG_consensus AAGTCGTACTTTGGAATCTTCCCTTAGACCTCACCGACGACATTAAATTTGGAGATATAGTAAAAATATATTATAAGA [11310]
cp32-9_PAbe_NX_Pl_consensus    AAGTCGTACTTTGGAATCTTCCCTTAGACCTCACCGACGACATTAAATTTGGAGATATAGTAAAAATATATTATAAGA [11310]

cp32-9_gi|11497325|B31_GB      AATTTGCTCATGAAAAAAATTTTGATTTCATAATGGCAGGAACTTTAGGACCTCCTATGAGCACTGATTATCCGGGTG [11388]
cp32-9_un7_B31_PacBio          AATTTGCTCATGAAAAAAATTTTGATTTCATAATGGCAGGAACTTTAGGACCTCCTATGAGCACTGATTATCCGGGTG [11388]
cp32-9_B31_TS_MP_FG_consensus  AATTTGCTCATGAAAAAAATTTTGATTTCATAATGGCAGGAACTTTAGGACCTCCTATGAGCACTGATTATCCGGGTG [11388]
cp32-9_B31_NX_Pl_consensus     AATTTGCTCATGAAAAAAATTTTGATTTCATAATGGCAGGAACTTTAGGACCTCCTATGAGCACTGATTATCCGGGTG [11388]
cp32-9_PAli_NX_MP_FG_consensus AATTTGCTCATGAAAAAAATTTTGATTTCATAATGGCAGGAACTTTAGGACCTCCTATGAGCACTGATTATCCGGGTG [11388]
cp32-9_PAli_NX_Pl_consensus    AATTTGCTCATGAAAAAAATTTTGATTTCATAATGGCAGGAACTTTAGGACCTCCTATGAGCACTGATTATCCGGGTG [11388]
cp32-9_PAbe_NX_MP_FG_consensus AATTTGCTCATGAAAAAAATTTTGATTTCATAATGGCAGGAACTTTAGGACCTCCTATGAGCACTGATTATCCGGGTG [11388]
cp32-9_PAbe_NX_Pl_consensus    AATTTGCTCATGAAAAAAATTTTGATTTCATAATGGCAGGAACTTTAGGACCTCCTATGAGCACTGATTATCCGGGTG [11388]

cp32-9_gi|11497325|B31_GB      GGGATTTTAGTGTAGATCTTGATGTTCGTTTATTAACTAAAAGCAATTTCTTCAATCGCGAATTGGCAGGCAAAGAAG [11466]
cp32-9_un7_B31_PacBio          GGGATTTTAGTGTAGATCTTGATGTTCGTTTATTAACTAAAAGCAATTTCTTCAATCGCGAATTGGCAGGCAAAGAAG [11466]
cp32-9_B31_TS_MP_FG_consensus  GGGATTTTAGTGTAGATCTTGATGTTCGTTTATTAACTAAAAGCAATTTCTTCAATCGCGAATTGGCAGGCAAAGAAG [11466]
cp32-9_B31_NX_Pl_consensus     GGGATTTTAGTGTAGATCTTGATGTTCGTTTATTAACTAAAAGCAATTTCTTCAATCGCGAATTGGCAGGCAAAGAAG [11466]
cp32-9_PAli_NX_MP_FG_consensus GGGATTTTAGTGTAGATCTTGATGTTCGTTTATTAACTAAAAGCAATTTCTTCAATCGCGAATTGGCAGGCAAAGAAG [11466]
cp32-9_PAli_NX_Pl_consensus    GGGATTTTAGTGTAGATCTTGATGTTCGTTTATTAACTAAAAGCAATTTCTTCAATCGCGAATTGGCAGGCAAAGAAG [11466]
cp32-9_PAbe_NX_MP_FG_consensus GGGATTTTAGTGTAGATCTTGATGTTCGTTTATTAACTAAAAGCAATTTCTTCAATCGCGAATTGGCAGGCAAAGAAG [11466]
cp32-9_PAbe_NX_Pl_consensus    GGGATTTTAGTGTAGATCTTGATGTTCGTTTATTAACTAAAAGCAATTTCTTCAATCGCGAATTGGCAGGCAAAGAAG [11466]

cp32-9_gi|11497325|B31_GB      GCAAAAACTTTAAAGGCAAAACGGTGCAGGAGGCAATAGAATCTGTATTTCCCAATCGCAATATCCTTAATATGGATG [11544]
cp32-9_un7_B31_PacBio          GCAAAAACTTTAAAGGCAAAACGGTGCAGGAGGCAATAGAATCTGTATTTCCCAATCGCAATATCCTTAATATGGATG [11544]
cp32-9_B31_TS_MP_FG_consensus  GCAAAAACTTTAAAGGCAAAACGGTGCAGGAGGCAATAGAATCTGTATTTCCCAATCGCAATATCCTTAATATGGATG [11544]
cp32-9_B31_NX_Pl_consensus     GCAAAAACTTTAAAGGCAAAACGGTGCAGGAGGCAATAGAATCTGTATTTCCCAATCGCAATATCCTTAATATGGATG [11544]
cp32-9_PAli_NX_MP_FG_consensus GCAAAAACTTTAAAGGCAAAACGGTGCAGGAGGCAATAGAATCTGTATTTCCCAATCGCAATATCCTTAATATGGATG [11544]
cp32-9_PAli_NX_Pl_consensus    GCAAAAACTTTAAAGGCAAAACGGTGCAGGAGGCAATAGAATCTGTATTTCCCAATCGCAATATCCTTAATATGGATG [11544]
cp32-9_PAbe_NX_MP_FG_consensus GCAAAAACTTTAAAGGCAAAACGGTGCAGGAGGCAATAGAATCTGTATTTCCCAATCGCAATATCCTTAATATGGATG [11544]
cp32-9_PAbe_NX_Pl_consensus    GCAAAAACTTTAAAGGCAAAACGGTGCAGGAGGCAATAGAATCTGTATTTCCCAATCGCAATATCCTTAATATGGATG [11544]

cp32-9_gi|11497325|B31_GB      AAAAAGATTGTCTTAAAATTATTGACAAAGATATTTATGCCACAACACCAAAAGAGTTTATTGACAAAATAAAAGGAA [11622]
cp32-9_un7_B31_PacBio          AAAAAGATTGTCTTAAAATTATTGACAAAGATATTTATGCCACAACACCAAAAGAGTTTATTGACAAAATAAAAGGAA [11622]
cp32-9_B31_TS_MP_FG_consensus  AAAAAGATTGTCTTAAAATTATTGACAAAGATATTTATGCCACAACACCAAAAGAGTTTATTGACAAAATAAAAGGAA [11622]
cp32-9_B31_NX_Pl_consensus     AAAAAGATTGTCTTAAAATTATTGACAAAGATATTTATGCCACAACACCAAAAGAGTTTATTGACAAAATAAAAGGAA [11622]
cp32-9_PAli_NX_MP_FG_consensus AAAAAGATTGTCTTAAAATTATTGACAAAGATATTTATGCCACAACACCAAAAGAGTTTATTGACAAAATAAAAGGAA [11622]
cp32-9_PAli_NX_Pl_consensus    AAAAAGATTGTCTTAAAATTATTGACAAAGATATTTATGCCACAACACCAAAAGAGTTTATTGACAAAATAAAAGGAA [11622]
cp32-9_PAbe_NX_MP_FG_consensus AAAAAGATTGTCTTAAAATTATTGACAAAGATATTTATGCCACAACACCAAAAGAGTTTATTGACAAAATAAAAGGAA [11622]
cp32-9_PAbe_NX_Pl_consensus    AAAAAGATTGTCTTAAAATTATTGACAAAGATATTTATGCCACAACACCAAAAGAGTTTATTGACAAAATAAAAGGAA [11622]

cp32-9_gi|11497325|B31_GB      CATACGCTCATAACGTAATAGCCGATATTGGTACTGGTTTACGGGGATATGAATGCTATCTGATATTTACTAATTATA [11700]
cp32-9_un7_B31_PacBio          CATACGCTCATAACGTAATAGCCGATATTGGTACTGGTTTACGGGGATATGAATGCTATCTGATATTTACTAATTATA [11700]
cp32-9_B31_TS_MP_FG_consensus  CATACGCTCATAACGTAATAGCCGATATTGGTACTGGTTTACGGGGATATGAATGCTATCTGATATTTACTAATTATA [11700]
cp32-9_B31_NX_Pl_consensus     CATACGCTCATAACGTAATAGCCGATATTGGTACTGGTTTACGGGGATATGAATGCTATCTGATATTTACTAATTATA [11700]
cp32-9_PAli_NX_MP_FG_consensus CATACGCTCATAACGTAATAGCCGATATTGGTACTGGTTTACGGGGATATGAATGCTATCTGATATTTACTAATTATA [11700]
cp32-9_PAli_NX_Pl_consensus    CATACGCTCATAACGTAATAGCCGATATTGGTACTGGTTTACGGGGATATGAATGCTATCTGATATTTACTAATTATA [11700]
cp32-9_PAbe_NX_MP_FG_consensus CATACGCTCATAACGTAATAGCCGATATTGGTACTGGTTTACGGGGATATGAATGCTATCTGATATTTACTAATTATA [11700]
cp32-9_PAbe_NX_Pl_consensus    CATACGCTCATAACGTAATAGCCGATATTGGTACTGGTTTACGGGGATATGAATGCTATCTGATATTTACTAATTATA [11700]

cp32-9_gi|11497325|B31_GB      TAAAAAAGGGGGAAAATGTCCACTACGAGGCATTAGAAGACTATGGGCTTGAATTTATACCACAACAAGAAATTACTT [11778]
cp32-9_un7_B31_PacBio          TAAAAAAGGGGGAAAATGTCCACTACGAGGCATTAGAAGACTATGGGCTTGAATTTATACCACAACAAGAAATTACTT [11778]
cp32-9_B31_TS_MP_FG_consensus  TAAAAAAGGGGGAAAATGTCCACTACGAGGCATTAGAAGACTATGGGCTTGAATTTATACCACAACAAGAAATTACTT [11778]
cp32-9_B31_NX_Pl_consensus     TAAAAAAGGGGGAAAATGTCCACTACGAGGCATTAGAAGACTATGGGCTTGAATTTATACCACAACAAGAAATTACTT [11778]
cp32-9_PAli_NX_MP_FG_consensus TAAAAAAGGGGGAAAATGTCCACTACGAGGCATTAGAAGACTATGGGCTTGAATTTATACCACAACAAGAAATTACTT [11778]
cp32-9_PAli_NX_Pl_consensus    TAAAAAAGGGGGAAAATGTCCACTACGAGGCATTAGAAGACTATGGGCTTGAATTTATACCACAACAAGAAATTACTT [11778]
cp32-9_PAbe_NX_MP_FG_consensus TAAAAAAGGGGGAAAATGTCCACTACGAGGCATTAGAAGACTATGGGCTTGAATTTATACCACAACAAGAAATTACTT [11778]
cp32-9_PAbe_NX_Pl_consensus    TAAAAAAGGGGGAAAATGTCCACTACGAGGCATTAGAAGACTATGGGCTTGAATTTATACCACAACAAGAAATTACTT [11778]

cp32-9_gi|11497325|B31_GB      TGGGCACAACACTCAAAAAAAAATCTTATATTTTGGAACGCAAAAACATTTTTCACACATAAGTTAAATGTTGGAGATA [11856]
cp32-9_un7_B31_PacBio          TGGGCACAACACTCAAAAAAAAATCTTATATTTTGGAACGCAAAAACATTTTTCACACATAAGTTAAATGTTGGAGATA [11856]
cp32-9_B31_TS_MP_FG_consensus  TGGGCACAACACTCAAAAAAAAATCTTATATTTTGGAACGCAAAAACATTTTTCACACATAAGTTAAATGTTGGAGATA [11856]
cp32-9_B31_NX_Pl_consensus     TGGGCACAACACTCAAAAAAAAATCTTATATTTTGGAACGCAAAAACATTTTTCACACATAAGTTAAATGTTGGAGATA [11856]
cp32-9_PAli_NX_MP_FG_consensus TGGGCACAACACTCAAAAAAAAATCTTATATTTTGGAACGCAAAAACATTTTTCACACATAAGTTAAATGTTGGAGATA [11856]
cp32-9_PAli_NX_Pl_consensus    TGGGCACAACACTCAAAAAAAAATCTTATATTTTGGAACGCAAAAACATTTTTCACACATAAGTTAAATGTTGGAGATA [11856]
cp32-9_PAbe_NX_MP_FG_consensus TGGGCACAACACTCAAAAAAAAATCTTATATTTTGGAACGCAAAAACATTTTTCACACATAAGTTAAATGTTGGAGATA [11856]
cp32-9_PAbe_NX_Pl_consensus    TGGGCACAACACTCAAAAAAAAATCTTATATTTTGGAACGCAAAAACATTTTTCACACATAAGTTAAATGTTGGAGATA [11856]

cp32-9_gi|11497325|B31_GB      AAGTCTCATTTATTGATGGACTAGGGAAAATGATAAAAACCACTATAAAAGAAACAAGTGCAAGGCTTAGCAATACAG [11934]
cp32-9_un7_B31_PacBio          AAGTCTCATTTATTGATGGACTAGGGAAAATGATAAAAACCACTATAAAAGAAACAAGTGCAAGGCTTAGCAATACAG [11934]
cp32-9_B31_TS_MP_FG_consensus  AAGTCTCATTTATTGATGGACTAGGGAAAATGATAAAAACCACTATAAAAGAAACAAGTGCAAGGCTTAGCAATACAG [11934]
cp32-9_B31_NX_Pl_consensus     AAGTCTCATTTATTGATGGACTAGGGAAAATGATAAAAACCACTATAAAAGAAACAAGTGCAAGGCTTAGCAATACAG [11934]
cp32-9_PAli_NX_MP_FG_consensus AAGTCTCATTTATTGATGGACTAGGGAAAATGATAAAAACCACTATAAAAGAAACAAGTGCAAGGCTTAGCAATACAG [11934]
cp32-9_PAli_NX_Pl_consensus    AAGTCTCATTTATTGATGGACTAGGGAAAATGATAAAAACCACTATAAAAGAAACAAGTGCAAGGCTTAGCAATACAG [11934]
cp32-9_PAbe_NX_MP_FG_consensus AAGTCTCATTTATTGATGGACTAGGGAAAATGATAAAAACCACTATAAAAGAAACAAGTGCAAGGCTTAGCAATACAG [11934]
cp32-9_PAbe_NX_Pl_consensus    AAGTCTCATTTATTGATGGACTAGGGAAAATGATAAAAACCACTATAAAAGAAACAAGTGCAAGGCTTAGCAATACAG [11934]

cp32-9_gi|11497325|B31_GB      GAGAGTGTTCATTAATATTAAAGTTAGAAGATGATTCTAATACAAAACGTAAAAAATAAAGGGGACTAGAATTAGAAT [12012]
cp32-9_un7_B31_PacBio          GAGAGTGTTCATTAATATTAAAGTTAGAAGATGATTCTAATACAAAACGTAAAAAATAAAGGGGACTAGAATTAGAAT [12012]
cp32-9_B31_TS_MP_FG_consensus  GAGAGTGTTCATTAATATTAAAGTTAGAAGATGATTCTAATACAAAACGTAAAAAATAAAGGGGACTAGAATTAGAAT [12012]
cp32-9_B31_NX_Pl_consensus     GAGAGTGTTCATTAATATTAAAGTTAGAAGATGATTCTAATACAAAACGTAAAAAATAAAGGGGACTAGAATTAGAAT [12012]
cp32-9_PAli_NX_MP_FG_consensus GAGAGTGTTCATTAATATTAAAGTTAGAAGATGATTCTAATACAAAACGTAAAAAATAAAGGGGACTAGAATTAGAAT [12012]
cp32-9_PAli_NX_Pl_consensus    GAGAGTGTTCATTAATATTAAAGTTAGAAGATGATTCTAATACAAAACGTAAAAAATAAAGGGGACTAGAATTAGAAT [12012]
cp32-9_PAbe_NX_MP_FG_consensus GAGAGTGTTCATTAATATTAAAGTTAGAAGATGATTCTAATACAAAACGTAAAAAATAAAGGGGACTAGAATTAGAAT [12012]
cp32-9_PAbe_NX_Pl_consensus    GAGAGTGTTCATTAATATTAAAGTTAGAAGATGATTCTAATACAAAACGTAAAAAATAAAGGGGACTAGAATTAGAAT [12012]

cp32-9_gi|11497325|B31_GB      GAATGAAGACTATGAAATTTACAGAATGAATCAACGCCTTTATGGCCAGGCATTGGCTCAAGAAGACCTTAAAAATTG [12090]
cp32-9_un7_B31_PacBio          GAATGAAGACTATGAAATTTACAGAATGAATCAACGCCTTTATGGCCAGGCATTGGCTCAAGAAGACCTTAAAAATTG [12090]
cp32-9_B31_TS_MP_FG_consensus  GAATGAAGACTATGAAATTTACAGAATGAATCAACGCCTTTATGGCCAGGCATTGGCTCAAGAAGACCTTAAAAATTG [12090]
cp32-9_B31_NX_Pl_consensus     GAATGAAGACTATGAAATTTACAGAATGAATCAACGCCTTTATGGCCAGGCATTGGCTCAAGAAGACCTTAAAAATTG [12090]
cp32-9_PAli_NX_MP_FG_consensus GAATGAAGACTATGAAATTTACAGAATGAATCAACGCCTTTATGGCCAGGCATTGGCTCAAGAAGACCTTAAAAATTG [12090]
cp32-9_PAli_NX_Pl_consensus    GAATGAAGACTATGAAATTTACAGAATGAATCAACGCCTTTATGGCCAGGCATTGGCTCAAGAAGACCTTAAAAATTG [12090]
cp32-9_PAbe_NX_MP_FG_consensus GAATGAAGACTATGAAATTTACAGAATGAATCAACGCCTTTATGGCCAGGCATTGGCTCAAGAAGACCTTAAAAATTG [12090]
cp32-9_PAbe_NX_Pl_consensus    GAATGAAGACTATGAAATTTACAGAATGAATCAACGCCTTTATGGCCAGGCATTGGCTCAAGAAGACCTTAAAAATTG [12090]

cp32-9_gi|11497325|B31_GB      GATTTATTCAAACATTTTTATAATTAAAATTGGCACTGTAAAGGGGTTTAAACATCAAACTTAAGAAGCTATTGTTAC [12168]
cp32-9_un7_B31_PacBio          GATTTATTCAAACATTTTTATAATTAAAATTGGCACTGTAAAGGGGTTTAAACATCAAACTTAAGAAGCTATTGTTAC [12168]
cp32-9_B31_TS_MP_FG_consensus  GATTTATTCAAACATTTTTATAATTAAAATTGGCACTGTAAAGGGGTTTAAACATCAAACTTAAGAAGCTATTGTTAC [12168]
cp32-9_B31_NX_Pl_consensus     GATTTATTCAAACATTTTTATAATTAAAATTGGCACTGTAAAGGGGTTTAAACATCAAACTTAAGAAGCTATTGTTAC [12168]
cp32-9_PAli_NX_MP_FG_consensus GATTTATTCAAACATTTTTATAATTAAAATTGGCACTGTAAAGGGGTTTAAACATCAAACTTAAGAAGCTATTGTTAC [12168]
cp32-9_PAli_NX_Pl_consensus    GATTTATTCAAACATTTTTATAATTAAAATTGGCACTGTAAAGGGGTTTAAACATCAAACTTAAGAAGCTATTGTTAC [12168]
cp32-9_PAbe_NX_MP_FG_consensus GATTTATTCAAACATTTTTATAATTAAAATTGGCACTGTAAAGGGGTTTAAACATCAAACTTAAGAAGCTATTGTTAC [12168]
cp32-9_PAbe_NX_Pl_consensus    GATTTATTCAAACATTTTTATAATTAAAATTGGCACTGTAAAGGGGTTTAAACATCAAACTTAAGAAGCTATTGTTAC [12168]

cp32-9_gi|11497325|B31_GB      AATACCCGAATTTGAAGATTTAGAAATTCACACAAAAAATATTTCTAATATCCGTTTAGAACTATCAAAAGGTGATAA [12246]
cp32-9_un7_B31_PacBio          AATACCCGAATTTGAAGATTTAGAAATTCACACAAAAAATATTTCTAATATCCGTTTAGAACTATCAAAAGGTGATAA [12246]
cp32-9_B31_TS_MP_FG_consensus  AATACCCGAATTTGAAGATTTAGAAATTCACACAAAAAATATTTCTAATATCCGTTTAGAACTATCAAAAGGTGATAA [12246]
cp32-9_B31_NX_Pl_consensus     AATACCCGAATTTGAAGATTTAGAAATTCACACAAAAAATATTTCTAATATCCGTTTAGAACTATCAAAAGGTGATAA [12246]
cp32-9_PAli_NX_MP_FG_consensus AATACCCGAATTTGAAGATTTAGAAATTCACACAAAAAATATTTCTAATATCCGTTTAGAACTATCAAAAGGTGATAA [12246]
```

```
cp32-9_PAli_NX_Pl_consensus    AATACCCGAATTTGAAGATTTAGAAATTCACACAAAAAATATTTCTAATATCCGTTTAGAACTATCAAAAGGTGATAA [12246]
cp32-9_PAbe_NX_MP_FG_consensus AATACCCGAATTTGAAGATTTAGAAATTCACACAAAAAATATTTCTAATATCCGTTTAGAACTATCAAAAGGTGATAA [12246]
cp32-9_PAbe_NX_Pl_consensus    AATACCCGAATTTGAAGATTTAGAAATTCACACAAAAAATATTTCTAATATCCGTTTAGAACTATCAAAAGGTGATAA [12246]

cp32-9_gi|11497325|B31_GB      CGTTTTACTGCTTCAATCAAGCGTTAATATTTTTGATAAAAATAACGATATTCACTTTGACAAACATCACTTTTATAT [12324]
cp32-9_un7_B31_PacBio          CGTTTTACTGCTTCAATCAAGCGTTAATATTTTTGATAAAAATAACGATATTCACTTTGACAAACATCACTTTTATAT [12324]
cp32-9_B31_TS_MP_FG_consensus  CGTTTTACTGCTTCAATCAAGCGTTAATATTTTTGATAAAAATAACGATATTCACTTTGACAAACATCACTTTTATAT [12324]
cp32-9_B31_NX_Pl_consensus     CGTTTTACTGCTTCAATCAAGCGTTAATATTTTTGATAAAAATAACGATATTCACTTTGACAAACATCACTTTTATAT [12324]
cp32-9_PAli_NX_MP_FG_consensus CGTTTTACTGCTTCAATCAAGCGTTAATATTTTTGATAAAAATAACGATATTCACTTTGACAAACATCACTTTTATAT [12324]
cp32-9_PAli_NX_Pl_consensus    CGTTTTACTGCTTCAATCAAGCGTTAATATTTTTGATAAAAATAACGATATTCACTTTGACAAACATCACTTTTATAT [12324]
cp32-9_PAbe_NX_MP_FG_consensus CGTTTTACTGCTTCAATCAAGCGTTAATATTTTTGATAAAAATAACGATATTCACTTTGACAAACATCACTTTTATAT [12324]
cp32-9_PAbe_NX_Pl_consensus    CGTTTTACTGCTTCAATCAAGCGTTAATATTTTTGATAAAAATAACGATATTCACTTTGACAAACATCACTTTTATAT [12324]

cp32-9_gi|11497325|B31_GB      ACTTAGTGCAATTAGCCCAAAGACTTTAAATCTAATCTCTGATACTGTTAAAATTAAAGCAAACAATAATATTGAAAT [12402]
cp32-9_un7_B31_PacBio          ACTTAGTGCAATTAGCCCAAAGACTTTAAATCTAATCTCTGATACTGTTAAAATTAAAGCAAACAATAATATTGAAAT [12402]
cp32-9_B31_TS_MP_FG_consensus  ACTTAGTGCAATTAGCCCAAAGACTTTAAATCTAATCTCTGATACTGTTAAAATTAAAGCAAACAATAATATTGAAAT [12402]
cp32-9_B31_NX_Pl_consensus     ACTTAGTGCAATTAGCCCAAAGACTTTAAATCTAATCTCTGATACTGTTAAAATTAAAGCAAACAATAATATTGAAAT [12402]
cp32-9_PAli_NX_MP_FG_consensus ACTTAGTGCAATTAGCCCAAAGACTTTAAATCTAATCTCTGATACTGTTAAAATTAAAGCAAACAATAATATTGAAAT [12402]
cp32-9_PAli_NX_Pl_consensus    ACTTAGTGCAATTAGCCCAAAGACTTTAAATCTAATCTCTGATACTGTTAAAATTAAAGCAAACAATAATATTGAAAT [12402]
cp32-9_PAbe_NX_MP_FG_consensus ACTTAGTGCAATTAGCCCAAAGACTTTAAATCTAATCTCTGATACTGTTAAAATTAAAGCAAACAATAATATTGAAAT [12402]
cp32-9_PAbe_NX_Pl_consensus    ACTTAGTGCAATTAGCCCAAAGACTTTAAATCTAATCTCTGATACTGTTAAAATTAAAGCAAACAATAATATTGAAAT [12402]

cp32-9_gi|11497325|B31_GB      AGCTAACCAAACAACTAGCTTAAAAACAATTCTCAAAAATATTGTAAGTGCTATTGAGGGTATAAAAATAGCAACCAC [12480]
cp32-9_un7_B31_PacBio          AGCTAACCAAACAACTAGCTTAAAAACAATTCTCAAAAATATTGTAAGTGCTATTGAGGGTATAAAAATAGCAACCAC [12480]
cp32-9_B31_TS_MP_FG_consensus  AGCTAACCAAACAACTAGCTTAAAAACAATTCTCAAAAATATTGTAAGTGCTATTGAGGGTATAAAAATAGCAACCAC [12480]
cp32-9_B31_NX_Pl_consensus     AGCTAACCAAACAACTAGCTTAAAAACAATTCTCAAAAATATTGTAAGTGCTATTGAGGGTATAAAAATAGCAACCAC [12480]
cp32-9_PAli_NX_MP_FG_consensus AGCTAACCAAACAACTAGCTTAAAAACAATTCTCAAAAATATTGTAAGTGCTATTGAGGGTATAAAAATAGCAACCAC [12480]
cp32-9_PAli_NX_Pl_consensus    AGCTAACCAAACAACTAGCTTAAAAACAATTCTCAAAAATATTGTAAGTGCTATTGAGGGTATAAAAATAGCAACCAC [12480]
cp32-9_PAbe_NX_MP_FG_consensus AGCTAACCAAACAACTAGCTTAAAAACAATTCTCAAAAATATTGTAAGTGCTATTGAGGGTATAAAAATAGCAACCAC [12480]
cp32-9_PAbe_NX_Pl_consensus    AGCTAACCAAACAACTAGCTTAAAAACAATTCTCAAAAATATTGTAAGTGCTATTGAGGGTATAAAAATAGCAACCAC [12480]

cp32-9_gi|11497325|B31_GB      ACAAGGAGGCCCAGTAATTGAACCAGTCAGCCTAAAAATAGCAACCACTAAAATTAATTATGATATTAACAGTTTGTT [12558]
cp32-9_un7_B31_PacBio          ACAAGGAGGCCCAGTAATTGAACCAGTCAGCCTAAAAATAGCAACCACTAAAATTAATTATGATATTAACAGTTTGTT [12558]
cp32-9_B31_TS_MP_FG_consensus  ACAAGGAGGCCCAGTAATTGAACCAGTCAGCCTAAAAATAGCAACCACTAAAATTAATTATGATATTAACAGTTTGTT [12558]
cp32-9_B31_NX_Pl_consensus     ACAAGGAGGCCCAGTAATTGAACCAGTCAGCCTAAAAATAGCAACCACTAAAATTAATTATGATATTAACAGTTTGTT [12558]
cp32-9_PAli_NX_MP_FG_consensus ACAAGGAGGCCCAGTAATTGAACCAGTCAGCCTAAAAATAGCAACCACTAAAATTAATTATGATATTAACAGTTTGTT [12558]
cp32-9_PAli_NX_Pl_consensus    ACAAGGAGGCCCAGTAATTGAACCAGTCAGCCTAAAAATAGCAACCACTAAAATTAATTATGATATTAACAGTTTGTT [12558]
cp32-9_PAbe_NX_MP_FG_consensus ACAAGGAGGCCCAGTAATTGAACCAGTCAGCCTAAAAATAGCAACCACTAAAATTAATTATGATATTAACAGTTTGTT [12558]
cp32-9_PAbe_NX_Pl_consensus    ACAAGGAGGCCCAGTAATTGAACCAGTCAGCCTAAAAATAGCAACCACTAAAATTAATTATGATATTAACAGTTTGTT [12558]

cp32-9_gi|11497325|B31_GB      CAAGTAATTTTTGCTAATTATGGTATAATTAATAGTATGGATTTAAGATTAGGCAATAATTTTGAATTGGTATTTAAT [12636]
cp32-9_un7_B31_PacBio          CAAGTAATTTTTGCTAATTATGGTATAATTAATAGTATGGATTTAAGATTAGGCAATAATTTTGAATTGGTATTTAAT [12636]
cp32-9_B31_TS_MP_FG_consensus  CAAGTAATTTTTGCTAATTATGGTATAATTAATAGTATGGATTTAAGATTAGGCAATAATTTTGAATTGGTATTTAAT [12636]
cp32-9_B31_NX_Pl_consensus     CAAGTAATTTTTGCTAATTATGGTATAATTAATAGTATGGATTTAAGATTAGGCAATAATTTTGAATTGGTATTTAAT [12636]
cp32-9_PAli_NX_MP_FG_consensus CAAGTAATTTTTGCTAATTATGGTATAATTAATAGTATGGATTTAAGATTAGGCAATAATTTTGAATTGGTATTTAAT [12636]
cp32-9_PAli_NX_Pl_consensus    CAAGTAATTTTTGCTAATTATGGTATAATTAATAGTATGGATTTAAGATTAGGCAATAATTTTGAATTGGTATTTAAT [12636]
cp32-9_PAbe_NX_MP_FG_consensus CAAGTAATTTTTGCTAATTATGGTATAATTAATAGTATGGATTTAAGATTAGGCAATAATTTTGAATTGGTATTTAAT [12636]
cp32-9_PAbe_NX_Pl_consensus    CAAGTAATTTTTGCTAATTATGGTATAATTAATAGTATGGATTTAAGATTAGGCAATAATTTTGAATTGGTATTTAAT [12636]

cp32-9_gi|11497325|B31_GB      AACGATTTATCACTTGTTGATGGAATTGAAGAACAAAAACAAAGATTTTTGATATTTTTAAAAACCTTAAGGGGTAGT [12714]
cp32-9_un7_B31_PacBio          AACGATTTATCACTTGTTGATGGAATTGAAGAACAAAAACAAAGATTTTTGATATTTTTAAAAACCTTAAGGGGTAGT [12714]
cp32-9_B31_TS_MP_FG_consensus  AACGATTTATCACTTGTTGATGGAATTGAAGAACAAAAACAAAGATTTTTGATATTTTTAAAAACCTTAAGGGGTAGT [12714]
cp32-9_B31_NX_Pl_consensus     AACGATTTATCACTTGTTGATGGAATTGAAGAACAAAAACAAAGATTTTTGATATTTTTAAAAACCTTAAGGGGTAGT [12714]
cp32-9_PAli_NX_MP_FG_consensus AACGATTTATCACTTGTTGATGGAATTGAAGAACAAAAACAAAGATTTTTGATATTTTTAAAAACCTTAAGGGGTAGT [12714]
cp32-9_PAli_NX_Pl_consensus    AACGATTTATCACTTGTTGATGGAATTGAAGAACAAAAACAAAGATTTTTGATATTTTTAAAAACCTTAAGGGGTAGT [12714]
cp32-9_PAbe_NX_MP_FG_consensus AACGATTTATCACTTGTTGATGGAATTGAAGAACAAAAACAAAGATTTTTGATATTTTTAAAAACCTTAAGGGGTAGT [12714]
cp32-9_PAbe_NX_Pl_consensus    AACGATTTATCACTTGTTGATGGAATTGAAGAACAAAAACAAAGATTTTTGATATTTTTAAAAACCTTAAGGGGTAGT [12714]

cp32-9_gi|11497325|B31_GB      TTAAGCTATGCTCCTCATTGGGGATTGGACTATTTCTTGCTTTTAAAACTGTTAAAAATTAACAATCTTCACGCTGTA [12792]
cp32-9_un7_B31_PacBio          TTAAGCTATGCTCCTCATTGGGGATTGGACTATTTCTTGCTTTTAAAACTGTTAAAAATTAACAATCTTCACGCTGTA [12792]
cp32-9_B31_TS_MP_FG_consensus  TTAAGCTATGCTCCTCATTGGGGATTGGACTATTTCTTGCTTTTAAAACTGTTAAAAATTAACAATCTTCACGCTGTA [12792]
cp32-9_B31_NX_Pl_consensus     TTAAGCTATGCTCCTCATTGGGGATTGGACTATTTCTTGCTTTTAAAACTGTTAAAAATTAACAATCTTCACGCTGTA [12792]
cp32-9_PAli_NX_MP_FG_consensus TTAAGCTATGCTCCTCATTGGGGATTGGACTATTTCTTGCTTTTAAAACTGTTAAAAATTAACAATCTTCACGCTGTA [12792]
cp32-9_PAli_NX_Pl_consensus    TTAAGCTATGCTCCTCATTGGGGATTGGACTATTTCTTGCTTTTAAAACTGTTAAAAATTAACAATCTTCACGCTGTA [12792]
cp32-9_PAbe_NX_MP_FG_consensus TTAAGCTATGCTCCTCATTGGGGATTGGACTATTTCTTGCTTTTAAAACTGTTAAAAATTAACAATCTTCACGCTGTA [12792]
cp32-9_PAbe_NX_Pl_consensus    TTAAGCTATGCTCCTCATTGGGGATTGGACTATTTCTTGCTTTTAAAACTGTTAAAAATTAACAATCTTCACGCTGTA [12792]

cp32-9_gi|11497325|B31_GB      AAAAATTATTTTCATGAAATATCTAAAGAGCTTAACTTAGATTTAATAAATATTTCAACTACTATACAAGACAATAAA [12870]
cp32-9_un7_B31_PacBio          AAAAATTATTTTCATGAAATATCTAAAGAGCTTAACTTAGATTTAATAAATATTTCAACTACTATACAAGACAATAAA [12870]
cp32-9_B31_TS_MP_FG_consensus  AAAAATTATTTTCATGAAATATCTAAAGAGCTTAACTTAGATTTAATAAATATTTCAACTACTATACAAGACAATAAA [12870]
cp32-9_B31_NX_Pl_consensus     AAAAATTATTTTCATGAAATATCTAAAGAGCTTAACTTAGATTTAATAAATATTTCAACTACTATACAAGACAATAAA [12870]
cp32-9_PAli_NX_MP_FG_consensus AAAAATTATTTTCATGAAATATCTAAAGAGCTTAACTTAGATTTAATAAATATTTCAACTACTATACAAGACAATAAA [12870]
cp32-9_PAli_NX_Pl_consensus    AAAAATTATTTTCATGAAATATCTAAAGAGCTTAACTTAGATTTAATAAATATTTCAACTACTATACAAGACAATAAA [12870]
cp32-9_PAbe_NX_MP_FG_consensus AAAAATTATTTTCATGAAATATCTAAAGAGCTTAACTTAGATTTAATAAATATTTCAACTACTATACAAGACAATAAA [12870]
cp32-9_PAbe_NX_Pl_consensus    AAAAATTATTTTCATGAAATATCTAAAGAGCTTAACTTAGATTTAATAAATATTTCAACTACTATACAAGACAATAAA [12870]

cp32-9_gi|11497325|B31_GB      GCGCATATATCCTTTTTTTTCTCAGGCGATGTTTTGAATATGGAGTTTAATTTATGAGCATCGTTTTTGATTCTGATT [12948]
cp32-9_un7_B31_PacBio          GCGCATATATCCTTTTTTTTCTCAGGCGATGTTTTGAATATGGAGTTTAATTTATGAGCATCGTTTTTGATTCTGATT [12948]
cp32-9_B31_TS_MP_FG_consensus  GCGCATATATCCTTTTTTTTCTCAGGCGATGTTTTGAATATGGAGTTTAATTTATGAGCATCGTTTTTGATTCTGATT [12948]
cp32-9_B31_NX_Pl_consensus     GCGCATATATCCTTTTTTTTCTCAGGCGATGTTTTGAATATGGAGTTTAATTTATGAGCATCGTTTTTGATTCTGATT [12948]
cp32-9_PAli_NX_MP_FG_consensus GCGCATATATCCTTTTTTTTCTCAGGCGATGTTTTGAATATGGAGTTTAATTTATGAGCATCGTTTTTGATTCTGATT [12948]
cp32-9_PAli_NX_Pl_consensus    GCGCATATATCCTTTTTTTTCTCAGGCGATGTTTTGAATATGGAGTTTAATTTATGAGCATCGTTTTTGATTCTGATT [12948]
cp32-9_PAbe_NX_MP_FG_consensus GCGCATATATCCTTTTTTTTCTCAGGCGATGTTTTGAATATGGAGTTTAATTTATGAGCATCGTTTTTGATTCTGATT [12948]
cp32-9_PAbe_NX_Pl_consensus    GCGCATATATCCTTTTTTTTCTCAGGCGATGTTTTGAATATGGAGTTTAATTTATGAGCATCGTTTTTGATTCTGATT [12948]

cp32-9_gi|11497325|B31_GB      TTGGCATTTTAAAACGTACAATTAAGGATATTGTAAGATCGAAAAGAGAATATTTGCGTGTAAATTATGGGATTAATA [13026]
cp32-9_un7_B31_PacBio          TTGGCATTTTAAAACGTACAATTAAGGATATTGTAAGATCGAAAAGAGAATATTTGCGTGTAAATTATGGGATTAATA [13026]
cp32-9_B31_TS_MP_FG_consensus  TTGGCATTTTAAAACGTACAATTAAGGATATTGTAAGATCGAAAAGAGAATATTTGCGTGTAAATTATGGGATTAATA [13026]
cp32-9_B31_NX_Pl_consensus     TTGGCATTTTAAAACGTACAATTAAGGATATTGTAAGATCGAAAAGAGAATATTTGCGTGTAAATTATGGGATTAATA [13026]
cp32-9_PAli_NX_MP_FG_consensus TTGGCATTTTAAAACGTACAATTAAGGATATTGTAAGATCGAAAAGAGAATATTTGCGTGTAAATTATGGGATTAATA [13026]
cp32-9_PAli_NX_Pl_consensus    TTGGCATTTTAAAACGTACAATTAAGGATATTGTAAGATCGAAAAGAGAATATTTGCGTGTAAATTATGGGATTAATA [13026]
cp32-9_PAbe_NX_MP_FG_consensus TTGGCATTTTAAAACGTACAATTAAGGATATTGTAAGATCGAAAAGAGAATATTTGCGTGTAAATTATGGGATTAATA [13026]
cp32-9_PAbe_NX_Pl_consensus    TTGGCATTTTAAAACGTACAATTAAGGATATTGTAAGATCGAAAAGAGAATATTTGCGTGTAAATTATGGGATTAATA [13026]

cp32-9_gi|11497325|B31_GB      TTGATGATAATCAAAGCTCAATTTATAACATTATTGCGTCTTCTTTAGCATTAATTGAAGAAGAAATAATTAATGAGC [13104]
cp32-9_un7_B31_PacBio          TTGATGATAATCAAAGCTCAATTTATAACATTATTGCGTCTTCTTTAGCATTAATTGAAGAAGAAATAATTAATGAGC [13104]
cp32-9_B31_TS_MP_FG_consensus  TTGATGATAATCAAAGCTCAATTTATAACATTATTGCGTCTTCTTTAGCATTAATTGAAGAAGAAATAATTAATGAGC [13104]
cp32-9_B31_NX_Pl_consensus     TTGATGATAATCAAAGCTCAATTTATAACATTATTGCGTCTTCTTTAGCATTAATTGAAGAAGAAATAATTAATGAGC [13104]
cp32-9_PAli_NX_MP_FG_consensus TTGATGATAATCAAAGCTCAATTTATAACATTATTGCGTCTTCTTTAGCATTAATTGAAGAAGAAATAATTAATGAGC [13104]
cp32-9_PAli_NX_Pl_consensus    TTGATGATAATCAAAGCTCAATTTATAACATTATTGCGTCTTCTTTAGCATTAATTGAAGAAGAAATAATTAATGAGC [13104]
cp32-9_PAbe_NX_MP_FG_consensus TTGATGATAATCAAAGCTCAATTTATAACATTATTGCGTCTTCTTTAGCATTAATTGAAGAAGAAATAATTAATGAGC [13104]
cp32-9_PAbe_NX_Pl_consensus    TTGATGATAATCAAAGCTCAATTTATAACATTATTGCGTCTTCTTTAGCATTAATTGAAGAAGAAATAATTAATGAGC [13104]

cp32-9_gi|11497325|B31_GB      TTAATCTCTTTTTTTCTAAAATGAAACCGGGTGGCACTTATTGGGCTGCTATTGAAGAACACATTTCTTCCAAAAGCA [13182]
cp32-9_un7_B31_PacBio          TTAATCTCTTTTTTTCTAAAATGAAACCGGGTGGCACTTATTGGGCTGCTATTGAAGAACACATTTCTTCCAAAAGCA [13182]
cp32-9_B31_TS_MP_FG_consensus  TTAATCTCTTTTTTTCTAAAATGAAACCGGGTGGCACTTATTGGGCTGCTATTGAAGAACACATTTCTTCCAAAAGCA [13182]
cp32-9_B31_NX_Pl_consensus     TTAATCTCTTTTTTTCTAAAATGAAACCGGGTGGCACTTATTGGGCTGCTATTGAAGAACACATTTCTTCCAAAAGCA [13182]
cp32-9_PAli_NX_MP_FG_consensus TTAATCTCTTTTTTTCTAAAATGAAACCGGGTGGCACTTATTGGGCTGCTATTGAAGAACACATTTCTTCCAAAAGCA [13182]
cp32-9_PAli_NX_Pl_consensus    TTAATCTCTTTTTTTCTAAAATGAAACCGGGTGGCACTTATTGGGCTGCTATTGAAGAACACATTTCTTCCAAAAGCA [13182]
```

```
cp32-9_PAbe_NX_MP_FG_consensus TTAATCTCTTTTTTTCTAAAATGAAACCGGGTGGCACTTATTGGGCTGCTATTGAAGAACACATTTCTTCCAAAAGCA [13182]
cp32-9_PAbe_NX_Pl_consensus    TTAATCTCTTTTTTTCTAAAATGAAACCGGGTGGCACTTATTGGGCTGCTATTGAAGAACACATTTCTTCCAAAAGCA [13182]

cp32-9_gi|11497325|B31_GB      CAACTTACAGCGCGGTTCGCACGGCTTTACTTAATCTTGATGAGGTTGAGTACACTAATATTAAAAGTGCAGCTGGTA [13260]
cp32-9_un7_B31_PacBio          CAACTTACAGCGCGGTTCGCACGGCTTTACTTAATCTTGATGAGGTTGAGTACACTAATATTAAAAGTGCAGCTGGTA [13260]
cp32-9_B31_TS_MP_FG_consensus  CAACTTACAGCGCGGTTCGCACGGCTTTACTTAATCTTGATGAGGTTGAGTACACTAATATTAAAAGTGCAGCTGGTA [13260]
cp32-9_B31_NX_Pl_consensus     CAACTTACAGCGCGGTTCGCACGGCTTTACTTAATCTTGATGAGGTTGAGTACACTAATATTAAAAGTGCAGCTGGTA [13260]
cp32-9_PAli_NX_MP_FG_consensus CAACTTACAGCGCGGTTCGCACGGCTTTACTTAATCTTGATGAGGTTGAGTACACTAATATTAAAAGTGCAGCTGGTA [13260]
cp32-9_PAli_NX_Pl_consensus    CAACTTACAGCGCGGTTCGCACGGCTTTACTTAATCTTGATGAGGTTGAGTACACTAATATTAAAAGTGCAGCTGGTA [13260]
cp32-9_PAbe_NX_MP_FG_consensus CAACTTACAGCGCGGTTCGCACGGCTTTACTTAATCTTGATGAGGTTGAGTACACTAATATTAAAAGTGCAGCTGGTA [13260]
cp32-9_PAbe_NX_Pl_consensus    CAACTTACAGCGCGGTTCGCACGGCTTTACTTAATCTTGATGAGGTTGAGTACACTAATATTAAAAGTGCAGCTGGTA [13260]

cp32-9_gi|11497325|B31_GB      AAGCCAACATATATCTAATTCTAAAGGAAACTTTACTAGACACTAGTAAATCTAACATTAATAGTTCTGAATTTAAAG [13338]
cp32-9_un7_B31_PacBio          AAGCCAACATATATCTAATTCTAAAGGAAACTTTACTAGACACTAGTAAATCTAACATTAATAGTTCTGAATTTAAAG [13338]
cp32-9_B31_TS_MP_FG_consensus  AAGCCAACATATATCTAATTCTAAAGGAAACTTTACTAGACACTAGTAAATCTAACATTAATAGTTCTGAATTTAAAG [13338]
cp32-9_B31_NX_Pl_consensus     AAGCCAACATATATCTAATTCTAAAGGAAACTTTACTAGACACTAGTAAATCTAACATTAATAGTTCTGAATTTAAAG [13338]
cp32-9_PAli_NX_MP_FG_consensus AAGCCAACATATATCTAATTCTAAAGGAAACTTTACTAGACACTAGTAAATCTAACATTAATAGTTCTGAATTTAAAG [13338]
cp32-9_PAli_NX_Pl_consensus    AAGCCAACATATATCTAATTCTAAAGGAAACTTTACTAGACACTAGTAAATCTAACATTAATAGTTCTGAATTTAAAG [13338]
cp32-9_PAbe_NX_MP_FG_consensus AAGCCAACATATATCTAATTCTAAAGGAAACTTTACTAGACACTAGTAAATCTAACATTAATAGTTCTGAATTTAAAG [13338]
cp32-9_PAbe_NX_Pl_consensus    AAGCCAACATATATCTAATTCTAAAGGAAACTTTACTAGACACTAGTAAATCTAACATTAATAGTTCTGAATTTAAAG [13338]

cp32-9_gi|11497325|B31_GB      CAAAACTTTGGGAAACATTATATCTAACAACTCCTAGTGGTACTTTACTTGAGGGAGACATAGAAATTGATGGTCTCA [13416]
cp32-9_un7_B31_PacBio          CAAAACTTTGGGAAACATTATATCTAACAACTCCTAGTGGTACTTTACTTGAGGGAGACATAGAAATTGATGGTCTCA [13416]
cp32-9_B31_TS_MP_FG_consensus  CAAAACTTTGGGAAACATTATATCTAACAACTCCTAGTGGTACTTTACTTGAGGGAGACATAGAAATTGATGGTCTCA [13416]
cp32-9_B31_NX_Pl_consensus     CAAAACTTTGGGAAACATTATATCTAACAACTCCTAGTGGTACTTTACTTGAGGGAGACATAGAAATTGATGGTCTCA [13416]
cp32-9_PAli_NX_MP_FG_consensus CAAAACTTTGGGAAACATTATATCTAACAACTCCTAGTGGTACTTTACTTGAGGGAGACATAGAAATTGATGGTCTCA [13416]
cp32-9_PAli_NX_Pl_consensus    CAAAACTTTGGGAAACATTATATCTAACAACTCCTAGTGGTACTTTACTTGAGGGAGACATAGAAATTGATGGTCTCA [13416]
cp32-9_PAbe_NX_MP_FG_consensus CAAAACTTTGGGAAACATTATATCTAACAACTCCTAGTGGTACTTTACTTGAGGGAGACATAGAAATTGATGGTCTCA [13416]
cp32-9_PAbe_NX_Pl_consensus    CAAAACTTTGGGAAACATTATATCTAACAACTCCTAGTGGTACTTTACTTGAGGGAGACATAGAAATTGATGGTCTCA [13416]

cp32-9_gi|11497325|B31_GB      ATTCAACTGGACAACGTAAATCCTATAAAATATCACTAGGGAAAAGAAAATATGTTTATATGAAAGTAAAGTATAAAC [13494]
cp32-9_un7_B31_PacBio          ATTCAACTGGACAACGTAAATCCTATAAAATATCACTAGGGAAAAGAAAATATGTTTATATGAAAGTAAAGTATAAAC [13494]
cp32-9_B31_TS_MP_FG_consensus  ATTCAACTGGACAACGTAAATCCTATAAAATATCACTAGGGAAAAGAAAATATGTTTATATGAAAGTAAAGTATAAAC [13494]
cp32-9_B31_NX_Pl_consensus     ATTCAACTGGACAACGTAAATCCTATAAAATATCACTAGGGAAAAGAAAATATGTTTATATGAAAGTAAAGTATAAAC [13494]
cp32-9_PAli_NX_MP_FG_consensus ATTCAACTGGACAACGTAAATCCTATAAAATATCACTAGGGAAAAGAAAATATGTTTATATGAAAGTAAAGTATAAAC [13494]
cp32-9_PAli_NX_Pl_consensus    ATTCAACTGGACAACGTAAATCCTATAAAATATCACTAGGGAAAAGAAAATATGTTTATATGAAAGTAAAGTATAAAC [13494]
cp32-9_PAbe_NX_MP_FG_consensus ATTCAACTGGACAACGTAAATCCTATAAAATATCACTAGGGAAAAGAAAATATGTTTATATGAAAGTAAAGTATAAAC [13494]
cp32-9_PAbe_NX_Pl_consensus    ATTCAACTGGACAACGTAAATCCTATAAAATATCACTAGGGAAAAGAAAATATGTTTATATGAAAGTAAAGTATAAAC [13494]

cp32-9_gi|11497325|B31_GB      TTGACCTTAAAAACTATCTCTACTTAAACATAGACTCTCAAATTAGGGACATTTATTCTAGGATTATTTTAAATAACT [13572]
cp32-9_un7_B31_PacBio          TTGACCTTAAAAACTATCTCTACTTAAACATAGACTCTCAAATTAGGGACATTTATTCTAGGATTATTTTAAATAACT [13572]
cp32-9_B31_TS_MP_FG_consensus  TTGACCTTAAAAACTATCTCTACTTAAACATAGACTCTCAAATTAGGGACATTTATTCTAGGATTATTTTAAATAACT [13572]
cp32-9_B31_NX_Pl_consensus     TTGACCTTAAAAACTATCTCTACTTAAACATAGACTCTCAAATTAGGGACATTTATTCTAGGATTATTTTAAATAACT [13572]
cp32-9_PAli_NX_MP_FG_consensus TTGACCTTAAAAACTATCTCTACTTAAACATAGACTCTCAAATTAGGGACATTTATTCTAGGATTATTTTAAATAACT [13572]
cp32-9_PAli_NX_Pl_consensus    TTGACCTTAAAAACTATCTCTACTTAAACATAGACTCTCAAATTAGGGACATTTATTCTAGGATTATTTTAAATAACT [13572]
cp32-9_PAbe_NX_MP_FG_consensus TTGACCTTAAAAACTATCTCTACTTAAACATAGACTCTCAAATTAGGGACATTTATTCTAGGATTATTTTAAATAACT [13572]
cp32-9_PAbe_NX_Pl_consensus    TTGACCTTAAAAACTATCTCTACTTAAACATAGACTCTCAAATTAGGGACATTTATTCTAGGATTATTTTAAATAACT [13572]

cp32-9_gi|11497325|B31_GB      ATTCTGATATGGGAATTAGCTTTGAATATCAAGACTTTTTTGCTCCAGTTAATGAAGTTAAAGGAATTAAGTTTATGG [13650]
cp32-9_un7_B31_PacBio          ATTCTGATATGGGAATTAGCTTTGAATATCAAGACTTTTTTGCTCCAGTTAATGAAGTTAAAGGAATTAAGTTTATGG [13650]
cp32-9_B31_TS_MP_FG_consensus  ATTCTGATATGGGAATTAGCTTTGAATATCAAGACTTTTTTGCTCCAGTTAATGAAGTTAAAGGAATTAAGTTTATGG [13650]
cp32-9_B31_NX_Pl_consensus     ATTCTGATATGGGAATTAGCTTTGAATATCAAGACTTTTTTGCTCCAGTTAATGAAGTTAAAGGAATTAAGTTTATGG [13650]
cp32-9_PAli_NX_MP_FG_consensus ATTCTGATATGGGAATTAGCTTTGAATATCAAGACTTTTTTGCTCCAGTTAATGAAGTTAAAGGAATTAAGTTTATGG [13650]
cp32-9_PAli_NX_Pl_consensus    ATTCTGATATGGGAATTAGCTTTGAATATCAAGACTTTTTTGCTCCAGTTAATGAAGTTAAAGGAATTAAGTTTATGG [13650]
cp32-9_PAbe_NX_MP_FG_consensus ATTCTGATATGGGAATTAGCTTTGAATATCAAGACTTTTTTGCTCCAGTTAATGAAGTTAAAGGAATTAAGTTTATGG [13650]
cp32-9_PAbe_NX_Pl_consensus    ATTCTGATATGGGAATTAGCTTTGAATATCAAGACTTTTTTGCTCCAGTTAATGAAGTTAAAGGAATTAAGTTTATGG [13650]

cp32-9_gi|11497325|B31_GB      AAATAAGTGCTTGTATTAAAGATACAGACACTGAGAGTATTACAAAAATTGGTGATAGCGATTTTAAAAAAAAATCAAG [13728]
cp32-9_un7_B31_PacBio          AAATAAGTGCTTGTATTAAAGATACAGACACTGAGAGTATTACAAAAATTGGTGATAGCGATTTTAAAAAAAAATCAAG [13728]
cp32-9_B31_TS_MP_FG_consensus  AAATAAGTGCTTGTATTAAAGATACAGACACTGAGAGTATTACAAAAATTGGTGATAGCGATTTTAAAAAAAAATCAAG [13728]
cp32-9_B31_NX_Pl_consensus     AAATAAGTGCTTGTATTAAAGATACAGACACTGAGAGTATTACAAAAATTGGTGATAGCGATTTTAAAAAAAAATCAAG [13728]
cp32-9_PAli_NX_MP_FG_consensus AAATAAGTGCTTGTATTAAAGATACAGACACTGAGAGTATTACAAAAATTGGTGATAGCGATTTTAAAAAAAAATCAAG [13728]
cp32-9_PAli_NX_Pl_consensus    AAATAAGTGCTTGTATTAAAGATACAGACACTGAGAGTATTACAAAAATTGGTGATAGCGATTTTAAAAAAAAATCAAG [13728]
cp32-9_PAbe_NX_MP_FG_consensus AAATAAGTGCTTGTATTAAAGATACAGACACTGAGAGTATTACAAAAATTGGTGATAGCGATTTTAAAAAAAAATCAAG [13728]
cp32-9_PAbe_NX_Pl_consensus    AAATAAGTGCTTGTATTAAAGATACAGACACTGAGAGTATTACAAAAATTGGTGATAGCGATTTTAAAAAAAAATCAAG [13728]

cp32-9_gi|11497325|B31_GB      ATATTGCCATTAATGATGACACAATGCTACTTTTCAATACGACAGATAGATTGCTTATTGATATTGGATAGTTAACAA [13806]
cp32-9_un7_B31_PacBio          ATATTGCCATTAATGATGACACAATGCTACTTTTCAATACGACAGATAGATTGCTTATTGATATTGGATAGTTAACAA [13806]
cp32-9_B31_TS_MP_FG_consensus  ATATTGCCATTAATGATGACACAATGCTACTTTTCAATACGACAGATAGATTGCTTATTGATATTGGATAGTTAACAA [13806]
cp32-9_B31_NX_Pl_consensus     ATATTGCCATTAATGATGACACAATGCTACTTTTCAATACGACAGATAGATTGCTTATTGATATTGGATAGTTAACAA [13806]
cp32-9_PAli_NX_MP_FG_consensus ATATTGCCATTAATGATGACACAATGCTACTTTTCAATACGACAGATAGATTGCTTATTGATATTGGATAGTTAACAA [13806]
cp32-9_PAli_NX_Pl_consensus    ATATTGCCATTAATGATGACACAATGCTACTTTTCAATACGACAGATAGATTGCTTATTGATATTGGATAGTTAACAA [13806]
cp32-9_PAbe_NX_MP_FG_consensus ATATTGCCATTAATGATGACACAATGCTACTTTTCAATACGACAGATAGATTGCTTATTGATATTGGATAGTTAACAA [13806]
cp32-9_PAbe_NX_Pl_consensus    ATATTGCCATTAATGATGACACAATGCTACTTTTCAATACGACAGATAGATTGCTTATTGATATTGGATAGTTAACAA [13806]

cp32-9_gi|11497325|B31_GB      ATATGAAAATACCCAATCTTTTCAAAAACACCGAAATTCATAAATTTATACGTACAGAAACAGAATATGCACAAGCAT [13884]
cp32-9_un7_B31_PacBio          ATATGAAAATACCCAATCTTTTCAAAAACACCGAAATTCATAAATTTATACGTACAGAAACAGAATATGCACAAGCAT [13884]
cp32-9_B31_TS_MP_FG_consensus  ATATGAAAATACCCAATCTTTTCAAAAACACCGAAATTCATAAATTTATACGTACAGAAACAGAATATGCACAAGCAT [13884]
cp32-9_B31_NX_Pl_consensus     ATATGAAAATACCCAATCTTTTCAAAAACACCGAAATTCATAAATTTATACGTACAGAAACAGAATATGCACAAGCAT [13884]
cp32-9_PAli_NX_MP_FG_consensus ATATGAAAATACCCAATCTTTTCAAAAACACCGAAATTCATAAATTTATACGTACAGAAACAGAATATGCACAAGCAT [13884]
cp32-9_PAli_NX_Pl_consensus    ATATGAAAATACCCAATCTTTTCAAAAACACCGAAATTCATAAATTTATACGTACAGAAACAGAATATGCACAAGCAT [13884]
cp32-9_PAbe_NX_MP_FG_consensus ATATGAAAATACCCAATCTTTTCAAAAACACCGAAATTCATAAATTTATACGTACAGAAACAGAATATGCACAAGCAT [13884]
cp32-9_PAbe_NX_Pl_consensus    ATATGAAAATACCCAATCTTTTCAAAAACACCGAAATTCATAAATTTATACGTACAGAAACAGAATATGCACAAGCAT [13884]

cp32-9_gi|11497325|B31_GB      TGCTTAATGAACTTAAGTCCCTTAATTCCAACTTCATGTCCATTAATGTAATAGAAAATATAAAATCAAGATATATTG [13962]
cp32-9_un7_B31_PacBio          TGCTTAATGAACTTAAGTCCCTTAATTCCAACTTCATGTCCATTAATGTAATAGAAAATATAAAATCAAGATATATTG [13962]
cp32-9_B31_TS_MP_FG_consensus  TGCTTAATGAACTTAAGTCCCTTAATTCCAACTTCATGTCCATTAATGTAATAGAAAATATAAAATCAAGATATATTG [13962]
cp32-9_B31_NX_Pl_consensus     TGCTTAATGAACTTAAGTCCCTTAATTCCAACTTCATGTCCATTAATGTAATAGAAAATATAAAATCAAGATATATTG [13962]
cp32-9_PAli_NX_MP_FG_consensus TGCTTAATGAACTTAAGTCCCTTAATTCCAACTTCATGTCCATTAATGTAATAGAAAATATAAAATCAAGATATATTG [13962]
cp32-9_PAli_NX_Pl_consensus    TGCTTAATGAACTTAAGTCCCTTAATTCCAACTTCATGTCCATTAATGTAATAGAAAATATAAAATCAAGATATATTG [13962]
cp32-9_PAbe_NX_MP_FG_consensus TGCTTAATGAACTTAAGTCCCTTAATTCCAACTTCATGTCCATTAATGTAATAGAAAATATAAAATCAAGATATATTG [13962]
cp32-9_PAbe_NX_Pl_consensus    TGCTTAATGAACTTAAGTCCCTTAATTCCAACTTCATGTCCATTAATGTAATAGAAAATATAAAATCAAGATATATTG [13962]

cp32-9_gi|11497325|B31_GB      CAATATGGATATCTCAAGTTTTATCTATCTTTTATGCAAAAACTCAAACTTTACAAAGTATTACAAGCAATATTAATA [14040]
cp32-9_un7_B31_PacBio          CAATATGGATATCTCAAGTTTTATCTATCTTTTATGCAAAAACTCAAACTTTACAAAGTATTACAAGCAATATTAATA [14040]
cp32-9_B31_TS_MP_FG_consensus  CAATATGGATATCTCAAGTTTTATCTATCTTTTATGCAAAAACTCAAACTTTACAAAGTATTACAAGCAATATTAATA [14040]
cp32-9_B31_NX_Pl_consensus     CAATATGGATATCTCAAGTTTTATCTATCTTTTATGCAAAAACTCAAACTTTACAAAGTATTACAAGCAATATTAATA [14040]
cp32-9_PAli_NX_MP_FG_consensus CAATATGGATATCTCAAGTTTTATCTATCTTTTATGCAAAAACTCAAACTTTACAAAGTATTACAAGCAATATTAATA [14040]
cp32-9_PAli_NX_Pl_consensus    CAATATGGATATCTCAAGTTTTATCTATCTTTTATGCAAAAACTCAAACTTTACAAAGTATTACAAGCAATATTAATA [14040]
cp32-9_PAbe_NX_MP_FG_consensus CAATATGGATATCTCAAGTTTTATCTATCTTTTATGCAAAAACTCAAACTTTACAAAGTATTACAAGCAATATTAATA [14040]
cp32-9_PAbe_NX_Pl_consensus    CAATATGGATATCTCAAGTTTTATCTATCTTTTATGCAAAAACTCAAACTTTACAAAGTATTACAAGCAATATTAATA [14040]

cp32-9_gi|11497325|B31_GB      GTGTTATTTTTGCTTTACGTCATATTGGCACTGATGAGTCATTTAGACTGATTTTCAAGACCTTTTTAAATGTGGATA [14118]
cp32-9_un7_B31_PacBio          GTGTTATTTTTGCTTTACGTCATATTGGCACTGATGAGTCATTTAGACTGATTTTCAAGACCTTTTTAAATGTGGATA [14118]
cp32-9_B31_TS_MP_FG_consensus  GTGTTATTTTTGCTTTACGTCATATTGGCACTGATGAGTCATTTAGACTGATTTTCAAGACCTTTTTAAATGTGGATA [14118]
cp32-9_B31_NX_Pl_consensus     GTGTTATTTTTGCTTTACGTCATATTGGCACTGATGAGTCATTTAGACTGATTTTCAAGACCTTTTTAAATGTGGATA [14118]
cp32-9_PAli_NX_MP_FG_consensus GTGTTATTTTTGCTTTACGTCATATTGGCACTGATGAGTCATTTAGACTGATTTTCAAGACCTTTTTAAATGTGGATA [14118]
cp32-9_PAli_NX_Pl_consensus    GTGTTATTTTTGCTTTACGTCATATTGGCACTGATGAGTCATTTAGACTGATTTTCAAGACCTTTTTAAATGTGGATA [14118]
cp32-9_PAbe_NX_MP_FG_consensus GTGTTATTTTTGCTTTACGTCATATTGGCACTGATGAGTCATTTAGACTGATTTTCAAGACCTTTTTAAATGTGGATA [14118]
```

```
cp32-9_PAbe_NX_Pl_consensus    GTGTTATTTTTGCTTTACGTCATATTGGCACTGATGAGTCATTTAGACTGATTTTCAAGACCTTTTTAAATGTGGATA [14118]

cp32-9_gi|11497325|B31_GB      TTGAAGTTACTACTCCTGAAGCTGGGGTTATTGATATCTCTTTAAAAGGGGTAATAAAAACAAACTTTACTACATTTA [14196]
cp32-9_un7_B31_PacBio          TTGAAGTTACTACTCCTGAAGCTGGGGTTATTGATATCTCTTTAAAAGGGGTAATAAAAACAAACTTTACTACATTTA [14196]
cp32-9_B31_TS_MP_FG_consensus  TTGAAGTTACTACTCCTGAAGCTGGGGTTATTGATATCTCTTTAAAAGGGGTAATAAAAACAAACTTTACTACATTTA [14196]
cp32-9_B31_NX_Pl_consensus     TTGAAGTTACTACTCCTGAAGCTGGGGTTATTGATATCTCTTTAAAAGGGGTAATAAAAACAAACTTTACTACATTTA [14196]
cp32-9_PAli_NX_MP_FG_consensus TTGAAGTTACTACTCCTGAAGCTGGGGTTATTGATATCTCTTTAAAAGGGGTAATAAAAACAAACTTTACTACATTTA [14196]
cp32-9_PAli_NX_Pl_consensus    TTGAAGTTACTACTCCTGAAGCTGGGGTTATTGATATCTCTTTAAAAGGGGTAATAAAAACAAACTTTACTACATTTA [14196]
cp32-9_PAbe_NX_MP_FG_consensus TTGAAGTTACTACTCCTGAAGCTGGGGTTATTGATATCTCTTTAAAAGGGGTAATAAAAACAAACTTTACTACATTTA [14196]
cp32-9_PAbe_NX_Pl_consensus    TTGAAGTTACTACTCCTGAAGCTGGGGTTATTGATATCTCTTTAAAAGGGGTAATAAAAACAAACTTTACTACATTTA [14196]

cp32-9_gi|11497325|B31_GB      TTTCGCCTAGCACTAAGAAAGGAAAACGACTAAAAAAAGATAATTCTTAGAGAAAAGAAGCCGGGATACGCTGCATCT [14274]
cp32-9_un7_B31_PacBio          TTTCGCCTAGCACTAAGAAAGGAAAACGACTAAAAAAAGATAATTCTTAGAGAAAAGAAGCCGGGATACGCTGCATCT [14274]
cp32-9_B31_TS_MP_FG_consensus  TTTCGCCTAGCACTAAGAAAGGAAAACGACTAAAAAAAGATAATTCTTAGAGAAAAGAAGCCGGGATACGCTGCATCT [14274]
cp32-9_B31_NX_Pl_consensus     TTTCGCCTAGCACTAAGAAAGGAAAACGACTAAAAAAAGATAATTCTTAGAGAAAAGAAGCCGGGATACGCTGCATCT [14274]
cp32-9_PAli_NX_MP_FG_consensus TTTCGCCTAGCACTAAGAAAGGAAAACGACTAAAAAAAGATAATTCTTAGAGAAAAGAAGCCGGGATACGCTGCATCT [14274]
cp32-9_PAli_NX_Pl_consensus    TTTCGCCTAGCACTAAGAAAGGAAAACGACTAAAAAAAGATAATTCTTAGAGAAAAGAAGCCGGGATACGCTGCATCT [14274]
cp32-9_PAbe_NX_MP_FG_consensus TTTCGCCTAGCACTAAGAAAGGAAAACGACTAAAAAAAGATAATTCTTAGAGAAAAGAAGCCGGGATACGCTGCATCT [14274]
cp32-9_PAbe_NX_Pl_consensus    TTTCGCCTAGCACTAAGAAAGGAAAACGACTAAAAAAAGATAATTCTTAGAGAAAAGAAGCCGGGATACGCTGCATCT [14274]

cp32-9_gi|11497325|B31_GB      AAAAAAGCTTTAGTATTTAACTCACTTCCTAAAGGCTATGATCATTCAATTTATGCTTTTATAAAGAGAATTATTCCT [14352]
cp32-9_un7_B31_PacBio          AAAAAAGCTTTAGTATTTAACTCACTTCCTAAAGGCTATGATCATTCAATTTATGCTTTTATAAAGAGAATTATTCCT [14352]
cp32-9_B31_TS_MP_FG_consensus  AAAAAAGCTTTAGTATTTAACTCACTTCCTAAAGGCTATGATCATTCAATTTATGCTTTTATAAAGAGAATTATTCCT [14352]
cp32-9_B31_NX_Pl_consensus     AAAAAAGCTTTAGTATTTAACTCACTTCCTAAAGGCTATGATCATTCAATTTATGCTTTTATAAAGAGAATTATTCCT [14352]
cp32-9_PAli_NX_MP_FG_consensus AAAAAAGCTTTAGTATTTAACTCACTTCCTAAAGGCTATGATCATTCAATTTATGCTTTTATAAAGAGAATTATTCCT [14352]
cp32-9_PAli_NX_Pl_consensus    AAAAAAGCTTTAGTATTTAACTCACTTCCTAAAGGCTATGATCATTCAATTTATGCTTTTATAAAGAGAATTATTCCT [14352]
cp32-9_PAbe_NX_MP_FG_consensus AAAAAAGCTTTAGTATTTAACTCACTTCCTAAAGGCTATGATCATTCAATTTATGCTTTTATAAAGAGAATTATTCCT [14352]
cp32-9_PAbe_NX_Pl_consensus    AAAAAAGCTTTAGTATTTAACTCACTTCCTAAAGGCTATGATCATTCAATTTATGCTTTTATAAAGAGAATTATTCCT [14352]

cp32-9_gi|11497325|B31_GB      ATTGGTAGAGTTCTCAAAATTAATAATACAGATGGTAACAATATTATAACTTTTAACAACTAAGGAGGTTTTATGGCT [14430]
cp32-9_un7_B31_PacBio          ATTGGTAGAGTTCTCAAAATTAATAATACAGATGGTAACAATATTATAACTTTTAACAACTAAGGAGGTTTTATGGCT [14430]
cp32-9_B31_TS_MP_FG_consensus  ATTGGTAGAGTTCTCAAAATTAATAATACAGATGGTAACAATATTATAACTTTTAACAACTAAGGAGGTTTTATGGCT [14430]
cp32-9_B31_NX_Pl_consensus     ATTGGTAGAGTTCTCAAAATTAATAATACAGATGGTAACAATATTATAACTTTTAACAACTAAGGAGGTTTTATGGCT [14430]
cp32-9_PAli_NX_MP_FG_consensus ATTGGTAGAGTTCTCAAAATTAATAATACAGATGGTAACAATATTATAACTTTTAACAACTAAGGAGGTTTTATGGCT [14430]
cp32-9_PAli_NX_Pl_consensus    ATTGGTAGAGTTCTCAAAATTAATAATACAGATGGTAACAATATTATAACTTTTAACAACTAAGGAGGTTTTATGGCT [14430]
cp32-9_PAbe_NX_MP_FG_consensus ATTGGTAGAGTTCTCAAAATTAATAATACAGATGGTAACAATATTATAACTTTTAACAACTAAGGAGGTTTTATGGCT [14430]
cp32-9_PAbe_NX_Pl_consensus    ATTGGTAGAGTTCTCAAAATTAATAATACAGATGGTAACAATATTATAACTTTTAACAACTAAGGAGGTTTTATGGCT [14430]

cp32-9_gi|11497325|B31_GB      GATGATCAAGAAAAAATTACTAATTGATGAAGAAGAAACGGTTCAAATAAAAGATTTAAATAAGGTTACGACCGTTAAC [14508]
cp32-9_un7_B31_PacBio          GATGATCAAGAAAAAATTACTAATTGATGAAGAAGAAACGGTTCAAATAAAAGATTTAAATAAGGTTACGACCGTTAAC [14508]
cp32-9_B31_TS_MP_FG_consensus  GATGATCAAGAAAAAATTACTAATTGATGAAGAAGAAACGGTTCAAATAAAAGATTTAAATAAGGTTACGACCGTTAAC [14508]
cp32-9_B31_NX_Pl_consensus     GATGATCAAGAAAAAATTACTAATTGATGAAGAAGAAACGGTTCAAATAAAAGATTTAAATAAGGTTACGACCGTTAAC [14508]
cp32-9_PAli_NX_MP_FG_consensus GATGATCAAGAAAAAATTACTAATTGATGAAGAAGAAACGGTTCAAATAAAAGATTTAAATAAGGTTACGACCGTTAAC [14508]
cp32-9_PAli_NX_Pl_consensus    GATGATCAAGAAAAAATTACTAATTGATGAAGAAGAAACGGTTCAAATAAAAGATTTAAATAAGGTTACGACCGTTAAC [14508]
cp32-9_PAbe_NX_MP_FG_consensus GATGATCAAGAAAAAATTACTAATTGATGAAGAAGAAACGGTTCAAATAAAAGATTTAAATAAGGTTACGACCGTTAAC [14508]
cp32-9_PAbe_NX_Pl_consensus    GATGATCAAGAAAAAATTACTAATTGATGAAGAAGAAACGGTTCAAATAAAAGATTTAAATAAGGTTACGACCGTTAAC [14508]

cp32-9_gi|11497325|B31_GB      AATACTGATCTTTTACTGCTTGATGATGGAGCTGCAAGCAGTAATGCTATCACCTTTAAAAACTTTTTAGATGCTTCT [14586]
cp32-9_un7_B31_PacBio          AATACTGATCTTTTACTGCTTGATGATGGAGCTGCAAGCAGTAATGCTATCACCTTTAAAAACTTTTTAGATGCTTCT [14586]
cp32-9_B31_TS_MP_FG_consensus  AATACTGATCTTTTACTGCTTGATGATGGAGCTGCAAGCAGTAATGCTATCACCTTTAAAAACTTTTTAGATGCTTCT [14586]
cp32-9_B31_NX_Pl_consensus     AATACTGATCTTTTACTGCTTGATGATGGAGCTGCAAGCAGTAATGCTATCACCTTTAAAAACTTTTTAGATGCTTCT [14586]
cp32-9_PAli_NX_MP_FG_consensus AATACTGATCTTTTACTGCTTGATGATGGAGCTGCAAGCAGTAATGCTATCACCTTTAAAAACTTTTTAGATGCTTCT [14586]
cp32-9_PAli_NX_Pl_consensus    AATACTGATCTTTTACTGCTTGATGATGGAGCTGCAAGCAGTAATGCTATCACCTTTAAAAACTTTTTAGATGCTTCT [14586]
cp32-9_PAbe_NX_MP_FG_consensus AATACTGATCTTTTACTGCTTGATGATGGAGCTGCAAGCAGTAATGCTATCACCTTTAAAAACTTTTTAGATGCTTCT [14586]
cp32-9_PAbe_NX_Pl_consensus    AATACTGATCTTTTACTGCTTGATGATGGAGCTGCAAGCAGTAATGCTATCACCTTTAAAAACTTTTTAGATGCTTCT [14586]

cp32-9_gi|11497325|B31_GB      AAAGACAAAATATTTAAAGGAGAAGGATTAGACTATTTTAAGCAGATAATTAAGTCTACAATTGCCGAAGAACTTGCA [14664]
cp32-9_un7_B31_PacBio          AAAGACAAAATATTTAAAGGAGAAGGATTAGACTATTTTAAGCAGATAATTAAGTCTACAATTGCCGAAGAACTTGCA [14664]
cp32-9_B31_TS_MP_FG_consensus  AAAGACAAAATATTTAAAGGAGAAGGATTAGACTATTTTAAGCAGATAATTAAGTCTACAATTGCCGAAGAACTTGCA [14664]
cp32-9_B31_NX_Pl_consensus     AAAGACAAAATATTTAAAGGAGAAGGATTAGACTATTTTAAGCAGATAATTAAGTCTACAATTGCCGAAGAACTTGCA [14664]
cp32-9_PAli_NX_MP_FG_consensus AAAGACAAAATATTTAAAGGAGAAGGATTAGACTATTTTAAGCAGATAATTAAGTCTACAATTGCCGAAGAACTTGCA [14664]
cp32-9_PAli_NX_Pl_consensus    AAAGACAAAATATTTAAAGGAGAAGGATTAGACTATTTTAAGCAGATAATTAAGTCTACAATTGCCGAAGAACTTGCA [14664]
cp32-9_PAbe_NX_MP_FG_consensus AAAGACAAAATATTTAAAGGAGAAGGATTAGACTATTTTAAGCAGATAATTAAGTCTACAATTGCCGAAGAACTTGCA [14664]
cp32-9_PAbe_NX_Pl_consensus    AAAGACAAAATATTTAAAGGAGAAGGATTAGACTATTTTAAGCAGATAATTAAGTCTACAATTGCCGAAGAACTTGCA [14664]

cp32-9_gi|11497325|B31_GB      GCCAATACAGATTTTGTAGAAACAATTTATGCTAAAATAACGGACAAATTAATTAACAACGATTCTACTAATATTTCT [14742]
cp32-9_un7_B31_PacBio          GCCAATACAGATTTTGTAGAAACAATTTATGCTAAAATAACGGACAAATTAATTAACAACGATTCTACTAATATTTCT [14742]
cp32-9_B31_TS_MP_FG_consensus  GCCAATACAGATTTTGTAGAAACAATTTATGCTAAAATAACGGACAAATTAATTAACAACGATTCTACTAATATTTCT [14742]
cp32-9_B31_NX_Pl_consensus     GCCAATACAGATTTTGTAGAAACAATTTATGCTAAAATAACGGACAAATTAATTAACAACGATTCTACTAATATTTCT [14742]
cp32-9_PAli_NX_MP_FG_consensus GCCAATACAGATTTTGTAGAAACAATTTATGCTAAAATAACGGACAAATTAATTAACAACGATTCTACTAATATTTCT [14742]
cp32-9_PAli_NX_Pl_consensus    GCCAATACAGATTTTGTAGAAACAATTTATGCTAAAATAACGGACAAATTAATTAACAACGATTCTACTAATATTTCT [14742]
cp32-9_PAbe_NX_MP_FG_consensus GCCAATACAGATTTTGTAGAAACAATTTATGCTAAAATAACGGACAAATTAATTAACAACGATTCTACTAATATTTCT [14742]
cp32-9_PAbe_NX_Pl_consensus    GCCAATACAGATTTTGTAGAAACAATTTATGCTAAAATAACGGACAAATTAATTAACAACGATTCTACTAATATTTCT [14742]

cp32-9_gi|11497325|B31_GB      AACCTTTTTAGTAAAATTAAATCACGCCTTACAAATAGCATATCATCAGCCACTCTATCTAGAAATGATGATCTTTTG [14820]
cp32-9_un7_B31_PacBio          AACCTTTTTAGTAAAATTAAATCACGCCTTACAAATAGCATATCATCAGCCACTCTATCTAGAAATGATGATCTTTTG [14820]
cp32-9_B31_TS_MP_FG_consensus  AACCTTTTTAGTAAAATTAAATCACGCCTTACAAATAGCATATCATCAGCCACTCTATCTAGAAATGATGATCTTTTG [14820]
cp32-9_B31_NX_Pl_consensus     AACCTTTTTAGTAAAATTAAATCACGCCTTACAAATAGCATATCATCAGCCACTCTATCTAGAAATGATGATCTTTTG [14820]
cp32-9_PAli_NX_MP_FG_consensus AACCTTTTTAGTAAAATTAAATCACGCCTTACAAATAGCATATCATCAGCCACTCTATCTAGAAATGATGATCTTTTG [14820]
cp32-9_PAli_NX_Pl_consensus    AACCTTTTTAGTAAAATTAAATCACGCCTTACAAATAGCATATCATCAGCCACTCTATCTAGAAATGATGATCTTTTG [14820]
cp32-9_PAbe_NX_MP_FG_consensus AACCTTTTTAGTAAAATTAAATCACGCCTTACAAATAGCATATCATCAGCCACTCTATCTAGAAATGATGATCTTTTG [14820]
cp32-9_PAbe_NX_Pl_consensus    AACCTTTTTAGTAAAATTAAATCACGCCTTACAAATAGCATATCATCAGCCACTCTATCTAGAAATGATGATCTTTTG [14820]

cp32-9_gi|11497325|B31_GB      ATAATGTCTTCTTCAAGTATTCAAAAAACACCCGTTCCAAAACAACTGCTTGGAGCACCATCAGATTTTTCACATGGA [14898]
cp32-9_un7_B31_PacBio          ATAATGTCTTCTTCAAGTATTCAAAAAACACCCGTTCCAAAACAACTGCTTGGAGCACCATCAGATTTTTCACATGGA [14898]
cp32-9_B31_TS_MP_FG_consensus  ATAATGTCTTCTTCAAGTATTCAAAAAACACCCGTTCCAAAACAACTGCTTGGAGCACCATCAGATTTTTCACATGGA [14898]
cp32-9_B31_NX_Pl_consensus     ATAATGTCTTCTTCAAGTATTCAAAAAACACCCGTTCCAAAACAACTGCTTGGAGCACCATCAGATTTTTCACATGGA [14898]
cp32-9_PAli_NX_MP_FG_consensus ATAATGTCTTCTTCAAGTATTCAAAAAACACCCGTTCCAAAACAACTGCTTGGAGCACCATCAGATTTTTCACATGGA [14898]
cp32-9_PAli_NX_Pl_consensus    ATAATGTCTTCTTCAAGTATTCAAAAAACACCCGTTCCAAAACAACTGCTTGGAGCACCATCAGATTTTTCACATGGA [14898]
cp32-9_PAbe_NX_MP_FG_consensus ATAATGTCTTCTTCAAGTATTCAAAAAACACCCGTTCCAAAACAACTGCTTGGAGCACCATCAGATTTTTCACATGGA [14898]
cp32-9_PAbe_NX_Pl_consensus    ATAATGTCTTCTTCAAGTATTCAAAAAACACCCGTTCCAAAACAACTGCTTGGAGCACCATCAGATTTTTCACATGGA [14898]

cp32-9_gi|11497325|B31_GB      AGAAATTTTGCATACGGACCAACGCTTTATTCGTCTGACTACAAGAACAAATCAATAACTATTAAGTTGGAGCACACT [14976]
cp32-9_un7_B31_PacBio          AGAAATTTTGCATACGGACCAACGCTTTATTCGTCTGACTACAAGAACAAATCAATAACTATTAAGTTGGAGCACACT [14976]
cp32-9_B31_TS_MP_FG_consensus  AGAAATTTTGCATACGGACCAACGCTTTATTCGTCTGACTACAAGAACAAATCAATAACTATTAAGTTGGAGCACACT [14976]
cp32-9_B31_NX_Pl_consensus     AGAAATTTTGCATACGGACCAACGCTTTATTCGTCTGACTACAAGAACAAATCAATAACTATTAAGTTGGAGCACACT [14976]
cp32-9_PAli_NX_MP_FG_consensus AGAAATTTTGCATACGGACCAACGCTTTATTCGTCTGACTACAAGAACAAATCAATAACTATTAAGTTGGAGCACACT [14976]
cp32-9_PAli_NX_Pl_consensus    AGAAATTTTGCATACGGACCAACGCTTTATTCGTCTGACTACAAGAACAAATCAATAACTATTAAGTTGGAGCACACT [14976]
cp32-9_PAbe_NX_MP_FG_consensus AGAAATTTTGCATACGGACCAACGCTTTATTCGTCTGACTACAAGAACAAATCAATAACTATTAAGTTGGAGCACACT [14976]
cp32-9_PAbe_NX_Pl_consensus    AGAAATTTTGCATACGGACCAACGCTTTATTCGTCTGACTACAAGAACAAATCAATAACTATTAAGTTGGAGCACACT [14976]

cp32-9_gi|11497325|B31_GB      AATAATGCAACTCTTATTTTTTATAAATATAAGGATAATGACCCCATTTACCTTGATATTGAGCTTGACGCAGAACAC [15054]
cp32-9_un7_B31_PacBio          AATAATGCAACTCTTATTTTTTATAAATATAAGGATAATGACCCCATTTACCTTGATATTGAGCTTGACGCAGAACAC [15054]
cp32-9_B31_TS_MP_FG_consensus  AATAATGCAACTCTTATTTTTTATAAATATAAGGATAATGACCCCATTTACCTTGATATTGAGCTTGACGCAGAACAC [15054]
cp32-9_B31_NX_Pl_consensus     AATAATGCAACTCTTATTTTTTATAAATATAAGGATAATGACCCCATTTACCTTGATATTGAGCTTGACGCAGAACAC [15054]
cp32-9_PAli_NX_MP_FG_consensus AATAATGCAACTCTTATTTTTTATAAATATAAGGATAATGACCCCATTTACCTTGATATTGAGCTTGACGCAGAACAC [15054]
cp32-9_PAli_NX_Pl_consensus    AATAATGCAACTCTTATTTTTTATAAATATAAGGATAATGACCCCATTTACCTTGATATTGAGCTTGACGCAGAACAC [15054]
cp32-9_PAbe_NX_MP_FG_consensus AATAATGCAACTCTTATTTTTTATAAATATAAGGATAATGACCCCATTTACCTTGATATTGAGCTTGACGCAGAACAC [15054]
cp32-9_PAbe_NX_Pl_consensus    AATAATGCAACTCTTATTTTTTATAAATATAAGGATAATGACCCCATTTACCTTGATATTGAGCTTGACGCAGAACAC [15054]
```

```
cp32-9_gi|11497325|B31_GB      TACAGTAATAGCGAGAGTAAAGTACTTTATCTAAGATATTCCGATGAAAGTGAAACAAGTATGGCTTATGAGCAATCA [15132]
cp32-9_un7_B31_PacBio          TACAGTAATAGCGAGAGTAAAGTACTTTATCTAAGATATTCCGATGAAAGTGAAACAAGTATGGCTTATGAGCAATCA [15132]
cp32-9_B31_TS_MP_FG_consensus  TACAGTAATAGCGAGAGTAAAGTACTTTATCTAAGATATTCCGATGAAAGTGAAACAAGTATGGCTTATGAGCAATCA [15132]
cp32-9_B31_NX_Pl_consensus     TACAGTAATAGCGAGAGTAAAGTACTTTATCTAAGATATTCCGATGAAAGTGAAACAAGTATGGCTTATGAGCAATCA [15132]
cp32-9_PAli_NX_MP_FG_consensus TACAGTAATAGCGAGAGTAAAGTACTTTATCTAAGATATTCCGATGAAAGTGAAACAAGTATGGCTTATGAGCAATCA [15132]
cp32-9_PAli_NX_Pl_consensus    TACAGTAATAGCGAGAGTAAAGTACTTTATCTAAGATATTCCGATGAAAGTGAAACAAGTATGGCTTATGAGCAATCA [15132]
cp32-9_PAbe_NX_MP_FG_consensus TACAGTAATAGCGAGAGTAAAGTACTTTATCTAAGATATTCCGATGAAAGTGAAACAAGTATGGCTTATGAGCAATCA [15132]
cp32-9_PAbe_NX_Pl_consensus    TACAGTAATAGCGAGAGTAAAGTACTTTATCTAAGATATTCCGATGAAAGTGAAACAAGTATGGCTTATGAGCAATCA [15132]

cp32-9_gi|11497325|B31_GB      GACGCTCCCCCAAGGACTATCTAGTAGATTTCCCATGTATAAAGGATGGTATATTCAAAAAAGAGCCTACCTATCAGG [15210]
cp32-9_un7_B31_PacBio          GACGCTCCCCCAAGGACTATCTAGTAGATTTCCCATGTATAAAGGATGGTATATTCAAAAAAGAGCCTACCTATCAGG [15210]
cp32-9_B31_TS_MP_FG_consensus  GACGCTCCCCCAAGGACTATCTAGTAGATTTCCCATGTATAAAGGATGGTATATTCAAAAAAGAGCCTACCTATCAGG [15210]
cp32-9_B31_NX_Pl_consensus     GACGCTCCCCCAAGGACTATCTAGTAGATTTCCCATGTATAAAGGATGGTATATTCAAAAAAGAGCCTACCTATCAGG [15210]
cp32-9_PAli_NX_MP_FG_consensus GACGCTCCCCCAAGGACTATCTAGTAGATTTCCCATGTATAAAGGATGGTATATTCAAAAAAGAGCCTACCTATCAGG [15210]
cp32-9_PAli_NX_Pl_consensus    GACGCTCCCCCAAGGACTATCTAGTAGATTTCCCATGTATAAAGGATGGTATATTCAAAAAAGAGCCTACCTATCAGG [15210]
cp32-9_PAbe_NX_MP_FG_consensus GACGCTCCCCCAAGGACTATCTAGTAGATTTCCCATGTATAAAGGATGGTATATTCAAAAAAGAGCCTACCTATCAGG [15210]
cp32-9_PAbe_NX_Pl_consensus    GACGCTCCCCCAAGGACTATCTAGTAGATTTCCCATGTATAAAGGATGGTATATTCAAAAAAGAGCCTACCTATCAGG [15210]

cp32-9_gi|11497325|B31_GB      GGGCCCCATCCCGCAGCTTTAAAAGCTGCAAACTTTTTTAGCAAAAATTACGATTTTGATATAATGTATATACAAATA [15288]
cp32-9_un7_B31_PacBio          GGGCCCCATCCCGCAGCTTTAAAAGCTGCAAACTTTTTTAGCAAAAATTACGATTTTGATATAATGTATATACAAATA [15288]
cp32-9_B31_TS_MP_FG_consensus  GGGCCCCATCCCGCAGCTTTAAAAGCTGCAAACTTTTTTAGCAAAAATTACGATTTTGATATAATGTATATACAAATA [15288]
cp32-9_B31_NX_Pl_consensus     GGGCCCCATCCCGCAGCTTTAAAAGCTGCAAACTTTTTTAGCAAAAATTACGATTTTGATATAATGTATATACAAATA [15288]
cp32-9_PAli_NX_MP_FG_consensus GGGCCCCATCCCGCAGCTTTAAAAGCTGCAAACTTTTTTAGCAAAAATTACGATTTTGATATAATGTATATACAAATA [15288]
cp32-9_PAli_NX_Pl_consensus    GGGCCCCATCCCGCAGCTTTAAAAGCTGCAAACTTTTTTAGCAAAAATTACGATTTTGATATAATGTATATACAAATA [15288]
cp32-9_PAbe_NX_MP_FG_consensus GGGCCCCATCCCGCAGCTTTAAAAGCTGCAAACTTTTTTAGCAAAAATTACGATTTTGATATAATGTATATACAAATA [15288]
cp32-9_PAbe_NX_Pl_consensus    GGGCCCCATCCCGCAGCTTTAAAAGCTGCAAACTTTTTTAGCAAAAATTACGATTTTGATATAATGTATATACAAATA [15288]

cp32-9_gi|11497325|B31_GB      AAAAATTAAAATAAAGGATTAAAAAATGGATACTATTAAATTAACAGAACTTCTTATCAATTTAAATGAAATTAAACT [15366]
cp32-9_un7_B31_PacBio          AAAAATTAAAATAAAGGATTAAAAAATGGATACTATTAAATTAACAGAACTTCTTATCAATTTAAATGAAATTAAACT [15366]
cp32-9_B31_TS_MP_FG_consensus  AAAAATTAAAATAAAGGATTAAAAAATGGATACTATTAAATTAACAGAACTTCTTATCAATTTAAATGAAATTAAACT [15366]
cp32-9_B31_NX_Pl_consensus     AAAAATTAAAATAAAGGATTAAAAAATGGATACTATTAAATTAACAGAACTTCTTATCAATTTAAATGAAATTAAACT [15366]
cp32-9_PAli_NX_MP_FG_consensus AAAAATTAAAATAAAGGATTAAAAAATGGATACTATTAAATTAACAGAACTTCTTATCAATTTAAATGAAATTAAACT [15366]
cp32-9_PAli_NX_Pl_consensus    AAAAATTAAAATAAAGGATTAAAAAATGGATACTATTAAATTAACAGAACTTCTTATCAATTTAAATGAAATTAAACT [15366]
cp32-9_PAbe_NX_MP_FG_consensus AAAAATTAAAATAAAGGATTAAAAAATGGATACTATTAAATTAACAGAACTTCTTATCAATTTAAATGAAATTAAACT [15366]
cp32-9_PAbe_NX_Pl_consensus    AAAAATTAAAATAAAGGATTAAAAAATGGATACTATTAAATTAACAGAACTTCTTATCAATTTAAATGAAATTAAACT [15366]

cp32-9_gi|11497325|B31_GB      TATAGCCGTAATGATTTTTGTAACAGTATTGGTTTTAGGAGTGTTAATTCTTCTCAAGCCTTTATTAAAAGACATATT [15444]
cp32-9_un7_B31_PacBio          TATAGCCGTAATGATTTTTGTAACAGTATTGGTTTTAGGAGTGTTAATTCTTCTCAAGCCTTTATTAAAAGACATATT [15444]
cp32-9_B31_TS_MP_FG_consensus  TATAGCCGTAATGATTTTTGTAACAGTATTGGTTTTAGGAGTGTTAATTCTTCTCAAGCCTTTATTAAAAGACATATT [15444]
cp32-9_B31_NX_Pl_consensus     TATAGCCGTAATGATTTTTGTAACAGTATTGGTTTTAGGAGTGTTAATTCTTCTCAAGCCTTTATTAAAAGACATATT [15444]
cp32-9_PAli_NX_MP_FG_consensus TATAGCCGTAATGATTTTTGTAACAGTATTGGTTTTAGGAGTGTTAATTCTTCTCAAGCCTTTATTAAAAGACATATT [15444]
cp32-9_PAli_NX_Pl_consensus    TATAGCCGTAATGATTTTTGTAACAGTATTGGTTTTAGGAGTGTTAATTCTTCTCAAGCCTTTATTAAAAGACATATT [15444]
cp32-9_PAbe_NX_MP_FG_consensus TATAGCCGTAATGATTTTTGTAACAGTACTGGTTTTAGGAGTGTTAATTCTTCTCAAGCCTTTATTAAAAGACATATT [15444]
cp32-9_PAbe_NX_Pl_consensus    TATAGCCGTAATGATTTTTGTAACAGTATTGGTTTTAGGAGTGTTAATTCTTCTCAAGCCTTTATTAAAAGACATATT [15444]

cp32-9_gi|11497325|B31_GB      GACTATTGTAATAGGCAAGATTTTTAAGAATGGCAATGGTAATGGCAAAAATCACATTAAAAAAAGAGATTAACTTAT [15522]
cp32-9_un7_B31_PacBio          GACTATTGTAATAGGCAAGATTTTTAAGAATGGCAATGGTAATGGCAAAAATCACATTAAAAAAAGAGATTAACTTAT [15522]
cp32-9_B31_TS_MP_FG_consensus  GACTATTGTAATAGGCAAGATTTTTAAGAATGGCAATGGTAATGGCAAAAATCACATTAAAAAAAGAGATTAACTTAT [15522]
cp32-9_B31_NX_Pl_consensus     GACTATTGTAATAGGCAAGATTTTTAAGAATGGCAATGGTAATGGCAAAAATCACATTAAAAAAAGAGATTAACTTAT [15522]
cp32-9_PAli_NX_MP_FG_consensus GACTATTGTAATAGGCAAGATTTTTAAGAATGGCAATGGTAATGGCAAAAATCACATTAAAAAAAGAGATTAACTTAT [15522]
cp32-9_PAli_NX_Pl_consensus    GACTATTGTAATAGGCAAGATTTTTAAGAATGGCAATGGTAATGGCAAAAATCACATTAAAAAAAGAGATTAACTTAT [15522]
cp32-9_PAbe_NX_MP_FG_consensus GACTATTGTAATAGGCAAGATTTTTAAGAATGGCAATGGTAATGGCAAAAATCACATTAAAAAAAGAGATTAATTTAT [15522]
cp32-9_PAbe_NX_Pl_consensus    GACTATTGTAATAGGCAAGATTTTTAAGAATGGCAATGGTAATGGCAAAAATCACATTAAAAAAAGAGATTAACTTAT [15522]

cp32-9_gi|11497325|B31_GB      GAAATTATCCAAAGATAATGTTGAGCTTGGACTTACGTCTTTATCAACCCTTATTGATATATTTTCTAAATTTGAAGA [15600]
cp32-9_un7_B31_PacBio          GAAATTATCCAAAGATAATGTTGAGCTTGGACTTACGTCTTTATCAACCCTTATTGATATATTTTCTAAATTTGAAGA [15600]
cp32-9_B31_TS_MP_FG_consensus  GAAATTATCCAAAGATAATGTTGAGCTTGGACTTACGTCTTTATCAACCCTTATTGATATATTTTCTAAATTTGAAGA [15600]
cp32-9_B31_NX_Pl_consensus     GAAATTATCCAAAGATAATGTTGAGCTTGGACTTACGTCTTTATCAACCCTTATTGATATATTTTCTAAATTTGAAGA [15600]
cp32-9_PAli_NX_MP_FG_consensus GAAATTATCCAAAGATAATGTTGAGCTTGGACTTACGTCTTTATCAACCCTTATTGATATATTTTCTAAATTTGAAGA [15600]
cp32-9_PAli_NX_Pl_consensus    GAAATTATCCAAAGATAATGTTGAGCTTGGACTTACGTCTTTATCAACCCTTATTGATATATTTTCTAAATTTGAAGA [15600]
cp32-9_PAbe_NX_MP_FG_consensus GAAATTATCCAAAGATAATGTTGAGCTTGGACTTACGTCTTTATCAACCCTTATTGATATATTTTCTAAATTTGAAGA [15600]
cp32-9_PAbe_NX_Pl_consensus    GAAATTATCCAAAGATAATGTTGAGCTTGGACTTACGTCTTTATCAACCCTTATTGATATATTTTCTAAATTTGAAGA [15600]

cp32-9_gi|11497325|B31_GB      TGAATTTGATGAAATTGCACATAAAGGATTCTTTTTGGTTTATGAGCTGTATTCTCATTATAAATTAATCTATACAGC [15678]
cp32-9_un7_B31_PacBio          TGAATTTGATGAAATTGCACATAAAGGATTCTTTTTGGTTTATGAGCTGTATTCTCATTATAAATTAATCTATACAGC [15678]
cp32-9_B31_TS_MP_FG_consensus  TGAATTTGATGAAATTGCACATAAAGGATTCTTTTTGGTTTATGAGCTGTATTCTCATTATAAATTAATCTATACAGC [15678]
cp32-9_B31_NX_Pl_consensus     TGAATTTGATGAAATTGCACATAAAGGATTCTTTTTGGTTTATGAGCTGTATTCTCATTATAAATTAATCTATACAGC [15678]
cp32-9_PAli_NX_MP_FG_consensus TGAATTTGATGAAATTGCACATAAAGGATTCTTTTTGGTTTATGAGCTGTATTCTCATTATAAATTAATCTATACAGC [15678]
cp32-9_PAli_NX_Pl_consensus    TGAATTTGATGAAATTGCACATAAAGGATTCTTTTTGGTTTATGAGCTGTATTCTCATTATAAATTAATCTATACAGC [15678]
cp32-9_PAbe_NX_MP_FG_consensus TGAATTTGATAAAATTGCACATAAAGGATTCTTTTTGGTTTATGAGCTGTATTCTCATTATAAATTAATCTATACAGC [15678]
cp32-9_PAbe_NX_Pl_consensus    TGAATTTGATGAAATTGCACATAAAGGATTCTTTTTGGTTTATGAGCTGTATTCTCATTATAAATTAATCTATACAGC [15678]

cp32-9_gi|11497325|B31_GB      AAATATGGAAAGACTTGAGAGTGCATTAACCCCAGCAATAAATGCGGCACTCGCTCCATTAAATGAAAAAATCAATCA [15756]
cp32-9_un7_B31_PacBio          AAATATGGAAAGACTTGAGAGTGCATTAACCCCAGCAATAAATGCGGCACTCGCTCCATTAAATGAAAAAATCAATCA [15756]
cp32-9_B31_TS_MP_FG_consensus  AAATATGGAAAGACTTGAGAGTGCATTAACCCCAGCAATAAATGCGGCACTCGCTCCATTAAATGAAAAAATCAATCA [15756]
cp32-9_B31_NX_Pl_consensus     AAATATGGAAAGACTTGAGAGTGCATTAACCCCAGCAATAAATGCGGCACTCGCTCCATTAAATGAAAAAATCAATCA [15756]
cp32-9_PAli_NX_MP_FG_consensus AAATATGGAAAGACTTGAGAGTGCATTAACCCCAGCAATAAATGCGGCACTCGCTCCATTAAATGAAAAAATCAATCA [15756]
cp32-9_PAli_NX_Pl_consensus    AAATATGGAAAGACTTGAGAGTGCATTAACCCCAGCAATAAATGCGGCACTCGCTCCATTAAATGAAAAAATCAATCA [15756]
cp32-9_PAbe_NX_MP_FG_consensus AAATATGGAAAGACTTGAGAGTGCATTAACCCCAGCAATAAATGCGGCACTCGCTCCATTAAATGAAAAAATCAATCA [15756]
cp32-9_PAbe_NX_Pl_consensus    AAATATGGAAAGACTTGAGAGTGCATTAACCCCAGCAATAAATGCGGCACTCGCTCCATTAAATGAAAAAATCAATCA [15756]

cp32-9_gi|11497325|B31_GB      ATGCATTGACTTAGTTAATTCTGATGAAAAAAATCTCAAAATATCTAATGATCTGAAATTCAATCAGGAAGGAAAACC [15834]
cp32-9_un7_B31_PacBio          ATGCATTGACTTAGTTAATTCTGATGAAAAAAATCTCAAAATATCTAATGATCTGAAATTCAATCAGGAAGGAAAACC [15834]
cp32-9_B31_TS_MP_FG_consensus  ATGCATTGACTTAGTTAATTCTGATGAAAAAAATCTCAAAATATCTAATGATCTGAAATTCAATCAGGAAGGAAAACC [15834]
cp32-9_B31_NX_Pl_consensus     ATGCATTGACTTAGTTAATTCTGATGAAAAAAATCTCAAAATATCTAATGATCTGAAATTCAATCAGGAAGGAAAACC [15834]
cp32-9_PAli_NX_MP_FG_consensus ATGCATTGACTTAGTTAATTCTGATGAAAAAAATCTCAAAATATCTAATGATCTGAAATTCAATCAGGAAGGAAAACC [15834]
cp32-9_PAli_NX_Pl_consensus    ATGCATTGACTTAGTTAATTCTGATGAAAAAAATCTCAAAATATCTAATGATCTGAAATTCAATCAGGAAGGAAAACC [15834]
cp32-9_PAbe_NX_MP_FG_consensus ATGCATTGACTTAATTAATTCTGATGAAAAAAATCTCAAAATATCTAATGATCTGAAATTCAATCAGGAAGGAAAACC [15834]
cp32-9_PAbe_NX_Pl_consensus    ATGCATTGACTTAGTTAATTCTGATGAAAAAAATCTCAAAATATCTAATGATCTGAAATTCAATCAGGAAGGAAAACC [15834]

cp32-9_gi|11497325|B31_GB      TATCTATAAGGAAAGAACAAATAATGCAAAATAACACTATTGGTTTAGGACTTAATTTACTATCCAGCTTAACTAACA [15912]
cp32-9_un7_B31_PacBio          TATCTATAAGGAAAGAACAAATAATGCAAAATAACACTATTGGTTTAGGACTTAATTTACTATCCAGCTTAACTAACA [15912]
cp32-9_B31_TS_MP_FG_consensus  TATCTATAAGGAAAGAACAAATAATGCAAAATAACACTATTGGTTTAGGACTTAATTTACTATCCAGCTTAACTAACA [15912]
cp32-9_B31_NX_Pl_consensus     TATCTATAAGGAAAGAACAAATAATGCAAAATAACACTATTGGTTTAGGACTTAATTTACTATCCAGCTTAACTAACA [15912]
cp32-9_PAli_NX_MP_FG_consensus TATCTATAAGGAAAGAACAAATAATGCAAAATAACACTATTGGTTTAGGACTTAATTTACTATCCAGCTTAACTAACA [15912]
cp32-9_PAli_NX_Pl_consensus    TATCTATAAGGAAAGAACAAATAATGCAAAATAACACTATTGGTTTAGGACTTAATTTACTATCCAGCTTAACTAACA [15912]
cp32-9_PAbe_NX_MP_FG_consensus TATCTATAAGGAAAGAACAAATAATGCAAAATAACACTATTGGTTTAGGACTTAATTTACTATCCAGCTTAACTAACA [15912]
cp32-9_PAbe_NX_Pl_consensus    TATCTATAAGGAAAGAACAAATAATGCAAAATAACACTATTGGTTTAGGACTTAATTTACTATCCAGCTTAACTAACA [15912]

cp32-9_gi|11497325|B31_GB      TAGCTAAAACTGATACAAACATAGATCATAATTACATTAATACTTTTAGTAAAGTAATAGATTTTTTCTACAAAACAT [15990]
cp32-9_un7_B31_PacBio          TAGCTAAAACTGATACAAACATAGATCATAATTACATTAATACTTTTAGTAAAGTAATAGATTTTTTCTACAAAACAT [15990]
cp32-9_B31_TS_MP_FG_consensus  TAGCTAAAACTGATACAAACATAGATCATAATTACATTAATACTTTTAGTAAAGTAATAGATTTTTTCTACAAAACAT [15990]
cp32-9_B31_NX_Pl_consensus     TAGCTAAAACTGATACAAACATAGATCATAATTACATTAATACTTTTAGTAAAGTAATAGATTTTTTCTACAAAACAT [15990]
cp32-9_PAli_NX_MP_FG_consensus TAGCTAAAACTGATACAAACATAGATCATAATTACATTAATACTTTTAGTAAAGTAATAGATTTTTTCTACAAAACAT [15990]
cp32-9_PAli_NX_Pl_consensus    TAGCTAAAACTGATACAAACATAGATCATAATTACATTAATACTTTTAGTAAAGTAATAGATTTTTTCTACAAAACAT [15990]
cp32-9_PAbe_NX_MP_FG_consensus TAGCTAAAACTGATACAAACATAGATCATAATTACATTAATACTTTTAGTAAAGTAATAGATTTTTTCTACAAAACAT [15990]
cp32-9_PAbe_NX_Pl_consensus    TAGCTAAAACTGATACAAACATAGATCATAATTACATTAATACTTTTAGTAAAGTAATAGATTTTTTCTACAAAACAT [15990]
```

```
cp32-9_gi|11497325|B31_GB      ATATAAGCACACTAAAATCTATGGAAACAGCTGAGTCAACTAAAATATTTGAAGAAATACAAGACATTTTAAAATATA [16068]
cp32-9_un7_B31_PacBio          ATATAAGCACACTAAAATCTATGGAAACAGCTGAGTCAACTAAAATATTTGAAGAAATACAAGACATTTTAAAATATA [16068]
cp32-9_B31_TS_MP_FG_consensus  ATATAAGCACACTAAAATCTATGGAAACAGCTGAGTCAACTAAAATATTTGAAGAAATACAAGACATTTTAAAATATA [16068]
cp32-9_B31_NX_Pl_consensus     ATATAAGCACACTAAAATCTATGGAAACAGCTGAGTCAACTAAAATATTTGAAGAAATACAAGACATTTTAAAATATA [16068]
cp32-9_PAli_NX_MP_FG_consensus ATATAAGCACACTAAAATCTATGGAAACAGCTGAGTCAACTAAAATATTTGAAGAAATACAAGACATTTTAAAATATA [16068]
cp32-9_PAli_NX_Pl_consensus    ATATAAGCACACTAAAATCTATGGAAACAGCTGAGTCAACTAAAATATTTGAAGAAATACAAGACATTTTAAAATATA [16068]
cp32-9_PAbe_NX_MP_FG_consensus ATATAAGCACACTAAAATCTATGGAAACAGCTGAGTCAACTAAAATATTTGAAGAAATACAAGACATTTTAAAATATA [16068]
cp32-9_PAbe_NX_Pl_consensus    ATATAAGCACACTAAAATCTATGGAAACAGCTGAGTCAACTAAAATATTTGAAGAAATACAAGACATTTTAAAATATA [16068]

cp32-9_gi|11497325|B31_GB      ACATTGAGATAATAGAGGCTATCTCTACTGATAAAAGCAAAAGAATTATCACTTCACTTAAAGCAACACGTAACAAAA [16146]
cp32-9_un7_B31_PacBio          ACATTGAGATAATAGAGGCTATCTCTACTGATAAAAGCAAAAGAATTATCACTTCACTTAAAGCAACACGTAACAAAA [16146]
cp32-9_B31_TS_MP_FG_consensus  ACATTGAGATAATAGAGGCTATCTCTACTGATAAAAGCAAAAGAATTATCACTTCACTTAAAGCAACACGTAACAAAA [16146]
cp32-9_B31_NX_Pl_consensus     ACATTGAGATAATAGAGGCTATCTCTACTGATAAAAGCAAAAGAATTATCACTTCACTTAAAGCAACACGTAACAAAA [16146]
cp32-9_PAli_NX_MP_FG_consensus ACATTGAGATAATAGAGGCTATCTCTACTGATAAAAGCAAAAGAATTATCACTTCACTTAAAGCAACACGTAACAAAA [16146]
cp32-9_PAli_NX_Pl_consensus    ACATTGAGATAATAGAGGCTATCTCTACTGATAAAAGCAAAAGAATTATCACTTCACTTAAAGCAACACGTAACAAAA [16146]
cp32-9_PAbe_NX_MP_FG_consensus ACATTGAGATAATAGAGGCTATCTCTACTGATAAAAGCAAAAGAATTATCACTTCACTTAAAGCAACACGTAACAAAA [16146]
cp32-9_PAbe_NX_Pl_consensus    ACATTGAGATAATAGAGGCTATCTCTACTGATAAAAGCAAAAGAATTATCACTTCACTTAAAGCAACACGTAACAAAA [16146]

cp32-9_gi|11497325|B31_GB      TCATGAAAGAATATATCAAAATACTTAAAAGAGGTGAAAATGCTTAAAAGATTGCATTGTCTACTAATTGCTTTGCTA [16224]
cp32-9_un7_B31_PacBio          TCATGAAAGAATATATCAAAATACTTAAAAGAGGTGAAAATGCTTAAAAGATTGCATTGTCTACTAATTGCTTTGCTA [16224]
cp32-9_B31_TS_MP_FG_consensus  TCATGAAAGAATATATCAAAATACTTAAAAGAGGTGAAAATGCTTAAAAGATTGCATTGTCTACTAATTGCTTTGCTA [16224]
cp32-9_B31_NX_Pl_consensus     TCATGAAAGAATATATCAAAATACTTAAAAGAGGTGAAAATGCTTAAAAGATTGCATTGTCTACTAATTGCTTTGCTA [16224]
cp32-9_PAli_NX_MP_FG_consensus TCATGAAAGAATATATCAAAATACTTAAAAGAGGTGAAAATGCTTAAAAGATTGCATTGTCTACTAATTGCTTTGCTA [16224]
cp32-9_PAli_NX_Pl_consensus    TCATGAAAGAATATATCAAAATACTTAAAAGAGGTGAAAATGCTTAAAAGATTGCATTGTCTACTAATTGCTTTGCTA [16224]
cp32-9_PAbe_NX_MP_FG_consensus TCATGAAAGAATATATCAAAATACTTAAAAGAGGTGAAAATGCTTAAAAGATTGCATTGTCTACTAATTGCTTTGCTA [16224]
cp32-9_PAbe_NX_Pl_consensus    TCATGAAAGAATATATCAAAATACTTAAAAGAGGTGAAAATGCTTAAAAGATTGCATTGTCTACTAATTGCTTTGCTA [16224]

cp32-9_gi|11497325|B31_GB      CTATGTTGCACCACTATTGCCAACCTACCAGAAGAGCCAAACCCGCCAATTATTCCAACACTAAAATCTTTAGCTAAA [16302]
cp32-9_un7_B31_PacBio          CTATGTTGCACCACTATTGCCAACCTACCAGAAGAGCCAAACCCGCCAATTATTCCAACACTAAAATCTTTAGCTAAA [16302]
cp32-9_B31_TS_MP_FG_consensus  CTATGTTGCACCACTATTGCCAACCTACCAGAAGAGCCAAACCCGCCAATTATTCCAACACTAAAATCTTTAGCTAAA [16302]
cp32-9_B31_NX_Pl_consensus     CTATGTTGCACCACTATTGCCAACCTACCAGAAGAGCCAAACCCGCCAATTATTCCAACACTAAAATCTTTAGCTAAA [16302]
cp32-9_PAli_NX_MP_FG_consensus CTATGTTGCACCACTATTGCCAACCTACCAGAAGAGCCAAACCCGCCAATTATTCCAACACTAAAATCTTTAGCTAAA [16302]
cp32-9_PAli_NX_Pl_consensus    CTATGTTGCACCACTATTGCCAACCTACCAGAAGAGCCAAACCCGCCAATTATTCCAACACTAAAATCTTTAGCTAAA [16302]
cp32-9_PAbe_NX_MP_FG_consensus CTATGTTGCACCACTATTGCCAACCTACCAGAAGAGCCAAACCCGCCAATTATTCCAACACTAAAATCTTTAGCTAAA [16302]
cp32-9_PAbe_NX_Pl_consensus    CTATGTTGCACCACTATTGCCAACCTACCAGAAGAGCCAAACCCGCCAATTATTCCAACACTAAAATCTTTAGCTAAA [16302]

cp32-9_gi|11497325|B31_GB      TATGAAACACAACTTTCAGAGTATGTTATTTACCTAGTAACATTTTTAGCTAAAACAAAAGTTAAAGTTAATGATCCA [16380]
cp32-9_un7_B31_PacBio          TATGAAACACAACTTTCAGAGTATGTTATTTACCTAGTAACATTTTTAGCTAAAACAAAAGTTAAAGTTAATGATCCA [16380]
cp32-9_B31_TS_MP_FG_consensus  TATGAAACACAACTTTCAGAGTATGTTATTTACCTAGTAACATTTTTAGCTAAAACAAAAGTTAAAGTTAATGATCCA [16380]
cp32-9_B31_NX_Pl_consensus     TATGAAACACAACTTTCAGAGTATGTTATTTACCTAGTAACATTTTTAGCTAAAACAAAAGTTAAAGTTAATGATCCA [16380]
cp32-9_PAli_NX_MP_FG_consensus TATGAAACACAACTTTCAGAGTATGTTATTTACCTAGTAACATTTTTAGCTAAAACAAAAGTTAAAGTTAATGATCCA [16380]
cp32-9_PAli_NX_Pl_consensus    TATGAAACACAACTTTCAGAGTATGTTATTTACCTAGTAACATTTTTAGCTAAAACAAAAGTTAAAGTTAATGATCCA [16380]
cp32-9_PAbe_NX_MP_FG_consensus TATGAAACACAACTTTCAGAGTATGTTATTTACCTAGTAACATTTTTAGCTAAAACAAAAGTTAAAGTTAATGATCCA [16380]
cp32-9_PAbe_NX_Pl_consensus    TATGAAACACAACTTTCAGAGTATGTTATTTACCTAGTAACATTTTTAGCTAAAACAAAAGTTAAAGTTAATGATCCA [16380]

cp32-9_gi|11497325|B31_GB      AATTATCCAGAATATCCTTATCCAGACTTATCAGCACTAAAAGACGAACACTCCATAACTGCGGTAAGACATAATATC [16458]
cp32-9_un7_B31_PacBio          AATTATCCAGAATATCCTTATCCAGACTTATCAGCACTAAAAGACGAACACTCCATAACTGCGGTAAGACATAATATC [16458]
cp32-9_B31_TS_MP_FG_consensus  AATTATCCAGAATATCCTTATCCAGACTTATCAGCACTAAAAGACGAACACTCCATAACTGCGGTAAGACATAATATC [16458]
cp32-9_B31_NX_Pl_consensus     AATTATCCAGAATATCCTTATCCAGACTTATCAGCACTAAAAGACGAACACTCCATAACTGCGGTAAGACATAATATC [16458]
cp32-9_PAli_NX_MP_FG_consensus AATTATCCAGAATATCCTTATCCAGACTTATCAGCACTAAAAGACGAACACTCCATAACTGCGGTAAGACATAATATC [16458]
cp32-9_PAli_NX_Pl_consensus    AATTATCCAGAATATCCTTATCCAGACTTATCAGCACTAAAAGACGAACACTCCATAACTGCGGTAAGACATAATATC [16458]
cp32-9_PAbe_NX_MP_FG_consensus AATTATCCAGAATATCCTTATCCAGACTTATCAGCACTAAAAGACGAACACTCCATAACTGCGGTAAGACATAATATC [16458]
cp32-9_PAbe_NX_Pl_consensus    AATTATCCAGAATATCCTTATCCAGACTTATCAGCACTAAAAGACGAACACTCCATAACTGCGGTAAGACATAATATC [16458]

cp32-9_gi|11497325|B31_GB      AACATATATTTAGAGTACATTAAAAAAACAAAACCAATAGCGGAAAAAGTCTATAATAAATATTCCCAGTTAAAAATG [16536]
cp32-9_un7_B31_PacBio          AACATATATTTAGAGTACATTAAAAAAACAAAACCAATAGCGGAAAAAGTCTATAATAAATATTCCCAGTTAAAAATG [16536]
cp32-9_B31_TS_MP_FG_consensus  AACATATATTTAGAGTACATTAAAAAAACAAAACCAATAGCGGAAAAAGTCTATAATAAATATTCCCAGTTAAAAATG [16536]
cp32-9_B31_NX_Pl_consensus     AACATATATTTAGAGTACATTAAAAAAACAAAACCAATAGCGGAAAAAGTCTATAATAAATATTCCCAGTTAAAAATG [16536]
cp32-9_PAli_NX_MP_FG_consensus AACATATATTTAGAGTACATTAAAAAAACAAAACCAATAGCGGAAAAAGTCTATAATAAATATTCCCAGTTAAAAATG [16536]
cp32-9_PAli_NX_Pl_consensus    AACATATATTTAGAGTACATTAAAAAAACAAAACCAATAGCGGAAAAAGTCTATAATAAATATTCCCAGTTAAAAATG [16536]
cp32-9_PAbe_NX_MP_FG_consensus AACATATATTTAGAGTACATTAAAAAAACAAAACCAATAGCGGAAAAAGTCTATAATAAATATTCCCAGTTAAAAATG [16536]
cp32-9_PAbe_NX_Pl_consensus    AACATATATTTAGAGTACATTAAAAAAACAAAACCAATAGCGGAAAAAGTCTATAATAAATATTCCCAGTTAAAAATG [16536]

cp32-9_gi|11497325|B31_GB      TAAATTACAAAAAGGTTTTTCTTGCAAGAAATTCTATTTTACATATAAAAATCTCTAAAGCCAATTAATCTAAAATAG [16614]
cp32-9_un7_B31_PacBio          TAAATTACAAAAAGGTTTTTCTTGCAAGAAATTCTATTTTACATATAAAAATCTCTAAAGCCAATTAATCTAAAATAG [16614]
cp32-9_B31_TS_MP_FG_consensus  TAAATTACAAAAAGGTTTTTCTTGCAAGAAATTCTATTTTACATATAAAAATCTCTAAAGCCAATTAATCTAAAATAG [16614]
cp32-9_B31_NX_Pl_consensus     TAAATTACAAAAAGGTTTTTCTTGCAAGAAATTCTATTTTACATATAAAAATCTCTAAAGCCAATTAATCTAAAATAG [16614]
cp32-9_PAli_NX_MP_FG_consensus TAAATTACAAAAAGGTTTTTCTTGCAAGAAATTCTATTTTACATATAAAAATCTCTAAAGCCAATTAATCTAAAATAG [16614]
cp32-9_PAli_NX_Pl_consensus    TAAATTACAAAAAGGTTTTTCTTGCAAGAAATTCTATTTTACATATAAAAATCTCTAAAGCCAATTAATCTAAAATAG [16614]
cp32-9_PAbe_NX_MP_FG_consensus TAAATTACAAAAAGGTTTTTCTTGCAAGAAATTCTATTTTACATATAAAAATCTCTAAAGCCAATTAATCTAAAATAG [16614]
cp32-9_PAbe_NX_Pl_consensus    TAAATTACAAAAAGGTTTTTCTTGCAAGAAATTCTATTTTACATATAAAAATCTCTAAAGCCAATTAATCTAAAATAG [16614]

cp32-9_gi|11497325|B31_GB      TGTATAATATGACCATAAGGGAAAATTTTATGGAAACAGTGTCAACAAATATTGCAAGTGTAACTCAAGAACAAATAT [16692]
cp32-9_un7_B31_PacBio          TGTATAATATGACCATAAGGGAAAATTTTATGGAAACAGTGTCAACAAATATTGCAAGTGTAACTCAAGAACAAATAT [16692]
cp32-9_B31_TS_MP_FG_consensus  TGTATAATATGACCATAAGGGAAAATTTTATGGAAACAGTGTCAACAAATATTGCAAGTGTAACTCAAGAACAAATAT [16692]
cp32-9_B31_NX_Pl_consensus     TGTATAATATGACCATAAGGGAAAATTTTATGGAAACAGTGTCAACAAATATTGCAAGTGTAACTCAAGAACAAATAT [16692]
cp32-9_PAli_NX_MP_FG_consensus TGTATAATATGACCATAAGGGAAAATTTTATGGAAACAGTGTCAACAAATATTGCAAGTGTAACTCAAGAACAAATAT [16692]
cp32-9_PAli_NX_Pl_consensus    TGTATAATATGACCATAAGGGAAAATTTTATGGAAACAGTGTCAACAAATATTGCAAGTGTAACTCAAGAACAAATAT [16692]
cp32-9_PAbe_NX_MP_FG_consensus TGTATAATATGACCATAAGGGAAAATTTTATGGAAACAGTGTCAACAAATATTGCAAGTGTAACTCAAGAACAAATAT [16692]
cp32-9_PAbe_NX_Pl_consensus    TGTATAATATGACCATAAGGGAAAATTTTATGGAAACAGTGTCAACAAATATTGCAAGTGTAACTCAAGAACAAATAT [16692]

cp32-9_gi|11497325|B31_GB      ATAAAGAATTTATTAGACTGGGCATGGAACAATTAATAGCACAAGATTTATCTAAAAGATATTATCACAATGAGCTAA [16770]
cp32-9_un7_B31_PacBio          ATAAAGAATTTATTAGACTGGGCATGGAACAATTAATAGCACAAGATTTATCTAAAAGATATTATCACAATGAGCTAA [16770]
cp32-9_B31_TS_MP_FG_consensus  ATAAAGAATTTATTAGACTGGGCATGGAACAATTAATAGCACAAGATTTATCTAAAAGATATTATCACAATGAGCTAA [16770]
cp32-9_B31_NX_Pl_consensus     ATAAAGAATTTATTAGACTGGGCATGGAACAATTAATAGCACAAGATTTATCTAAAAGATATTATCACAATGAGCTAA [16770]
cp32-9_PAli_NX_MP_FG_consensus ATAAAGAATTTATTAGACTGGGCATGGAACAATTAATAGCACAAGATTTATCTAAAAGATATTATCACAATGAGCTAA [16770]
cp32-9_PAli_NX_Pl_consensus    ATAAAGAATTTATTAGACTGGGCATGGAACAATTAATAGCACAAGATTTATCTAAAAGATATTATCACAATGAGCTAA [16770]
cp32-9_PAbe_NX_MP_FG_consensus ATAAAGAATTTATTAGACTGGGCATGGAACAATTAATAGCACAAGATTTATCTAAAAGATATTATCACAATGAGCTAA [16770]
cp32-9_PAbe_NX_Pl_consensus    ATAAAGAATTTATTAGACTGGGCATGGAACAATTAATAGCACAAGATTTATCTAAAAGATATTATCACAATGAGCTAA [16770]

cp32-9_gi|11497325|B31_GB      CATATAGAGATTTAGAAAATTTAGAAAAACAATTTGATATAAAATTTGATAATCTTATTTCTAAAATAGATAGTGTCA [16848]
cp32-9_un7_B31_PacBio          CATATAGAGATTTAGAAAATTTAGAAAAACAATTTGATATAAAATTTGATAATCTTATTTCTAAAATAGATAGTGTCA [16848]
cp32-9_B31_TS_MP_FG_consensus  CATATAGAGATTTAGAAAATTTAGAAAAACAATTTGATATAAAATTTGATAATCTTATTTCTAAAATAGATAGTGTCA [16848]
cp32-9_B31_NX_Pl_consensus     CATATAGAGATTTAGAAAATTTAGAAAAACAATTTGATATAAAATTTGATAATCTTATTTCTAAAATAGATAGTGTCA [16848]
cp32-9_PAli_NX_MP_FG_consensus CATATAGAGATTTAGAAAATTTAGAAAAACAATTTGATATAAAATTTGATAATCTTATTTCTAAAATAGATAGTGTCA [16848]
cp32-9_PAli_NX_Pl_consensus    CATATAGAGATTTAGAAAATTTAGAAAAACAATTTGATATAAAATTTGATAATCTTATTTCTAAAATAGATAGTGTCA [16848]
cp32-9_PAbe_NX_MP_FG_consensus CATATAGAGATTTAGAAAATTTAGAAAAACAATTTGATATAAAATTTGATAATCTTATTTCTAAAATAGATAGTGTCA [16848]
cp32-9_PAbe_NX_Pl_consensus    CATATAGAGATTTAGAAAATTTAGAAAAACAATTTGATATAAAATTTGATAATCTTATTTCTAAAATAGATAGTGTCA [16848]

cp32-9_gi|11497325|B31_GB      AAAGCGAACTTAATACTAAAATAGACAATGTAGAAAAGAATTTAAATCTAAAAATAGATGGTTTAGATACTAAGATAG [16926]
cp32-9_un7_B31_PacBio          AAAGCGAACTTAATACTAAAATAGACAATGTAGAAAAGAATTTAAATCTAAAAATAGATGGTTTAGATACTAAGATAG [16926]
cp32-9_B31_TS_MP_FG_consensus  AAAGCGAACTTAATACTAAAATAGACAATGTAGAAAAGAATTTAAATCTAAAAATAGATGGTTTAGATACTAAGATAG [16926]
cp32-9_B31_NX_Pl_consensus     AAAGCGAACTTAATACTAAAATAGACAATGTAGAAAAGAATTTAAATCTAAAAATAGATGGTTTAGATACTAAGATAG [16926]
cp32-9_PAli_NX_MP_FG_consensus AAAGCGAACTTAATACTAAAATAGACAATGTAGAAAAGAATTTAAATCTAAAAATAGATGGTTTAGATACTAAGATAG [16926]
cp32-9_PAli_NX_Pl_consensus    AAAGCGAACTTAATACTAAAATAGACAATGTAGAAAAGAATTTAAATCTAAAAATAGATGGTTTAGATACTAAGATAG [16926]
cp32-9_PAbe_NX_MP_FG_consensus AAAGCGAACTTAATACTAAAATAGACAATGTAGAAAAGAATTTAAATCTAAAAATAGATGGTTTAGATACTAAGATAG [16926]
cp32-9_PAbe_NX_Pl_consensus    AAAGCGAACTTAATACTAAAATAGACAATGTAGAAAAGAATTTAAATCTAAAAATAGATGGTTTAGATACTAAGATAG [16926]

cp32-9_gi|11497325|B31_GB      ATACTGTAGAAAAGAATTTAAATCTAAAAATAGATGGTTTAGATACTAAGATAGATACTGTAGAAAAGAATTTAAATG [17004]
```

```
cp32-9_un7_B31_PacBio          ATACTGTAGAAAAGAATTTAAATCTAAAAATAGATGGTTTAGATACTAAGATAGATACTGTAGAAAAGAATTTAAATG [17004]
cp32-9_B31_TS_MP_FG_consensus  ATACTGTAGAAAAGAATTTAAATCTAAAAATAGATGGTTTAGATACTAAGATAGATACTGTAGAAAAGAATTTAAATG [17004]
cp32-9_B31_NX_Pl_consensus     ATACTGTAGAAAAGAATTTAAATCTAAAAATAGATGGTTTAGATACTAAGATAGATACTGTAGAAAAGAATTTAAATG [17004]
cp32-9_PAli_NX_MP_FG_consensus ATACTGTAGAAAAGAATTTAAATCTAAAAATAGATGGTTTAGATACTAAGATAGATACTGTAGAAAAGAATTTAAATG [17004]
cp32-9_PAli_NX_Pl_consensus    ATACTGTAGAAAAGAATTTAAATCTAAAAATAGATGGTTTAGATACTAAGATAGATACTGTAGAAAAGAATTTAAATG [17004]
cp32-9_PAbe_NX_MP_FG_consensus ATACTGTAGAAAAGAATTTAAATCTAAAAATAGATGGTTTAGATACTAAGATAGATACTGTAGAAAAGAATTTAAATG [17004]
cp32-9_PAbe_NX_Pl_consensus    ATACTGTAGAAAAGAATTTAAATCTAAAAATAGATGGTTTAGATACTAAGATAGATACTGTAGAAAAGAATTTAAATG [17004]

cp32-9_gi|11497325|B31_GB      TCAAAATAGATAGTGTTAAAAGCGAACTTAATGCTAAGATCGATAGTGTTAAAAATGAACTTACTGCTAAAATAGACA [17082]
cp32-9_un7_B31_PacBio          TCAAAATAGATAGTGTTAAAAGCGAACTTAATGCTAAGATCGATAGTGTTAAAAATGAACT-ACTGCTAAAATAGACA [17082]
cp32-9_B31_TS_MP_FG_consensus  TCAAAATAGATAGTGTTAAAAGCGAACTTAATGCTAAGATCGATAGTGTTAAAAATGAACTTACTGCTAAAATAGACA [17082]
cp32-9_B31_NX_Pl_consensus     TCAAAATAGATAGTGTTAAAAGCGAACTTAATGCTAAGATCGATAGTGTTAAAAATGAACTTACTGCTAAAATAGACA [17082]
cp32-9_PAli_NX_MP_FG_consensus TCAAAATAGATAGTGTTAAAAGCGAACTTAATGCTAAGATCGATAGTGTTAAAAATGAACTTACTGCTAAAATAGACA [17082]
cp32-9_PAli_NX_Pl_consensus    TCAAAATAGATAGTGTTAAAAGCGAACTTAATGCTAAGATCGATAGTGTTAAAAATGAACTTACTGCTAAAATAGACA [17082]
cp32-9_PAbe_NX_MP_FG_consensus TCAAAATAGATAGTGTTAAAAGCGAACTTAATGCTAAGATCGATAGTGTTAAAAATGAACTTACTGCTAAAATAGACA [17082]
cp32-9_PAbe_NX_Pl_consensus    TCAAAATAGATAGTGTTAAAAGCGAACTTAATGCTAAGATCGATAGTGTTAAAAATGAACTTACTGCTAAAATAGACA [17082]

cp32-9_gi|11497325|B31_GB      ATGTAGAAAAGAATTTAATGTCTCTTTCAGAAATGCTTAAATGGGTATTGGGAATTATGGGAGCAATGTCTATCACAA [17160]
cp32-9_un7_B31_PacBio          ATGTAGAAAAGAATTTAATGTCTCTTTCAGAAATGCTTAAATGGGTATTGGGAATTATGGGAGCAATGTCTATCACAA [17160]
cp32-9_B31_TS_MP_FG_consensus  ATGTAGAAAAGAATTTAATGTCTCTTTCAGAAATGCTTAAATGGGTATTGGGAATTATGGGAGCAATGTCTATCACAA [17160]
cp32-9_B31_NX_Pl_consensus     ATGTAGAAAAGAATTTAATGTCTCTTTCAGAAATGCTTAAATGGGTATTGGGAATTATGGGAGCAATGTCTATCACAA [17160]
cp32-9_PAli_NX_MP_FG_consensus ATGTAGAAAAGAATTTAATGTCTCTTTCAGAAATGCTTAAATGGGTATTGGGAATTATGGGAGCAATGTCTATCACAA [17160]
cp32-9_PAli_NX_Pl_consensus    ATGTAGAAAAGAATTTAATGTCTCTTTCAGAAATGCTTAAATGGGTATTGGGAATTATGGGAGCAATGTCTATCACAA [17160]
cp32-9_PAbe_NX_MP_FG_consensus ATGTAGAAAAGAATTTAATGTCTCTTTCAGAAATGCTTAAATGGGTATTGGGAATTATGGGAGCAATGTCTATCACAA [17160]
cp32-9_PAbe_NX_Pl_consensus    ATGTAGAAAAGAATTTAATGTCTCTTTCAGAAATGCTTAAATGGGTATTGGGAATTATGGGAGCAATGTCTATCACAA [17160]

cp32-9_gi|11497325|B31_GB      TGATAGCAGGGCTAATATTTGCTTTCATTTCTAAATAGCTACTACCTCACTTAAAACATATCAATTCAAATAATAATT [17238]
cp32-9_un7_B31_PacBio          TGATAGCAGGGCTAATATTTGCTTTCATTTCTAAATAGCTACTACCTCACTTAAAACATATCAATTCAAATAATAATT [17238]
cp32-9_B31_TS_MP_FG_consensus  TGATAGCAGGGCTAATATTTGCTTTCATTTCTAAATAGCTACTACCTCACTTAAAACATATCAATTCAAATAATAATT [17238]
cp32-9_B31_NX_Pl_consensus     TGATAGCAGGGCTAATATTTGCTTTCATTTCTAAATAGCTACTACCTCACTTAAAACATATCAATTCAAATAATAATT [17238]
cp32-9_PAli_NX_MP_FG_consensus TGATAGCAGGGCTAATATTTGCTTTCATTTCTAAATAGCTACTACCTCACTTAAAACATATCAATTCAAATAATAATT [17238]
cp32-9_PAli_NX_Pl_consensus    TGATAGCAGGGCTAATATTTGCTTTCATTTCTAAATAGCTACTACCTCACTTAAAACATATCAATTCAAATAATAATT [17238]
cp32-9_PAbe_NX_MP_FG_consensus TGATAGCAGGGCTAATATTTGCTTTCATTTCTAAATAGCTACTACCTCACTTAAAACATATCAATTCAAATAATAATT [17238]
cp32-9_PAbe_NX_Pl_consensus    TGATAGCAGGGCTAATATTTGCTTTCATTTCTAAATAGCTACTACCTCACTTAAAACATATCAATTCAAATAATAATT [17238]

cp32-9_gi|11497325|B31_GB      GTTTATCCAATATTTAGTATTAATAATTGATTAAGTGAATATTAATAATAAAAAAAGGAATAACAATGAAAATTATCA [17316]
cp32-9_un7_B31_PacBio          GTTTATCCAATATTTAGTATTAATAATTGATTAAGTGAATATTAATAATAAAAAAAGGAATAACAATGAAAATTATCA [17316]
cp32-9_B31_TS_MP_FG_consensus  GTTTATCCAATATTTAGTATTAATAATTGATTAAGTGAATATTAATAATAAAAAAAGGAATAACAATGAAAATTATCA [17316]
cp32-9_B31_NX_Pl_consensus     GTTTATCCAATATTTAGTATTAATAATTGATTAAGTGAATATTAATAATAAAAAAAGGAATAACAATGAAAATTATCA [17316]
cp32-9_PAli_NX_MP_FG_consensus GTTTATCCAATATTTAGTATTAATAATTGATTAAGTGAATATTAATAATAAAAAAAGGAATAACAATGAAAATTATCA [17316]
cp32-9_PAli_NX_Pl_consensus    GTTTATCCAATATTTAGTATTAATAATTGATTAAGTGAATATTAATAATAAAAAAAGGAATAACAATGAAAATTATCA [17316]
cp32-9_PAbe_NX_MP_FG_consensus GTTTATCCAATATTTAGTATTAATAATTGATTAAGTGAATATTAATAATAAAAAAAGGAATAACAATGAAAATTATCA [17316]
cp32-9_PAbe_NX_Pl_consensus    GTTTATCCAATATTTAGTATTAATAATTGATTAAGTGAATATTAATAATAAAAAAAGGAATAACAATGAAAATTATCA [17316]

cp32-9_gi|11497325|B31_GB      ACATATTATTTTGTTTATTTTTACTAATGCTAAACAGCTGTAATTCTAATGATACTAATACTAGCCAAACAAAAAGTA [17394]
cp32-9_un7_B31_PacBio          ACATATTATTTTGTTTATTTTTACTAATGCTAAACAGCTGTAATTCTAATGATACTAATACTAGCCAAACAAAAAGTA [17394]
cp32-9_B31_TS_MP_FG_consensus  ACATATTATTTTGTTTATTTTTACTAATGCTAAACAGCTGTAATTCTAATGATACTAATACTAGCCAAACAAAAAGTA [17394]
cp32-9_B31_NX_Pl_consensus     ACATATTATTTTGTTTATTTTTACTAATGCTAAACAGCTGTAATTCTAATGATACTAATACTAGCCAAACAAAAAGTA [17394]
cp32-9_PAli_NX_MP_FG_consensus ACATATTATTTTGTTTATTTTTACTAATGCTAAACAGCTGTAATTCTAATGATACTAATACTAGCCAAACAAAAAGTA [17394]
cp32-9_PAli_NX_Pl_consensus    ACATATTATTTTGTTTATTTTTACTAATGCTAAACAGCTGTAATTCTAATGATACTAATACTAGCCAAACAAAAAGTA [17394]
cp32-9_PAbe_NX_MP_FG_consensus ACATATTATTTTGTTTATTTTTACTAATGCTAAACAGCTGTAATTCTAATGATACTAATACTAGCCAAACAAAAAGTA [17394]
cp32-9_PAbe_NX_Pl_consensus    ACATATTATTTTGTTTATTTTTACTAATGCTAAACAGCTGTAATTCTAATGATACTAATACTAGCCAAACAAAAAGTA [17394]

cp32-9_gi|11497325|B31_GB      GACAAAAACGTGATTTAACCCAAAAAGAAGCAACACAAGAAAAACCAAAATCTAAAGAAGACCTGCTTAGAGAAAAGC [17472]
cp32-9_un7_B31_PacBio          GACAAAAACGTGATTTAACCCAAAAAGAAGCAACACAAGAAAAACCAAAATCTAAAGAAGACCTGCTTAGAGAAAAGC [17472]
cp32-9_B31_TS_MP_FG_consensus  GACAAAAACGTGATTTAACCCAAAAAGAAGCAACACAAGAAAAACCAAAATCTAAAGAAGACCTGCTTAGAGAAAAGC [17472]
cp32-9_B31_NX_Pl_consensus     GACAAAAACGTGATTTAACCCAAAAAGAAGCAACACAAGAAAAACCAAAATCTAAAGAAGACCTGCTTAGAGAAAAGC [17472]
cp32-9_PAli_NX_MP_FG_consensus GACAAAAACGTGATTTAACCCAAAAAGAAGCAACACAAGAAAAACCAAAATCTAAAGAAGACCTGCTTAGAGAAAAGC [17472]
cp32-9_PAli_NX_Pl_consensus    GACAAAAACGTGATTTAACCCAAAAAGAAGCAACACAAGAAAAACCAAAATCTAAAGAAGACCTGCTTAGAGAAAAGC [17472]
cp32-9_PAbe_NX_MP_FG_consensus GACAAAAACGTGATTTAACCCAAAAAGAAGCAACACAAGAAAAACCAAAATCTAAAGAAGACCTGCTTAGAGAAAAGC [17472]
cp32-9_PAbe_NX_Pl_consensus    GACAAAAACGTGATTTAACCCAAAAAGAAGCAACACAAGAAAAACCAAAATCTAAAGAAGACCTGCTTAGAGAAAAGC [17472]

cp32-9_gi|11497325|B31_GB      TATCTGAAGACCAAAAAACACATCTTGACTGGTTAAAAACCGCTTTAACTGGTGCTGGAGAATTTGATAAATTTTTAG [17550]
cp32-9_un7_B31_PacBio          TATCTGAAGACCAAAAAACACATCTTGACTGGTTAAAAACCGCTTTAACTGGTGCTGGAGAATTTGATAAATTTTTAG [17550]
cp32-9_B31_TS_MP_FG_consensus  TATCTGAAGACCAAAAAACACATCTTGACTGGTTAAAAACCGCTTTAACTGGTGCTGGAGAATTTGATAAATTTTTAG [17550]
cp32-9_B31_NX_Pl_consensus     TATCTGAAGACCAAAAAACACATCTTGACTGGTTAAAAACCGCTTTAACTGGTGCTGGAGAATTTGATAAATTTTTAG [17550]
cp32-9_PAli_NX_MP_FG_consensus TATCTGAAGACCAAAAAACACATCTTGACTGGTTAAAAACCGCTTTAACTGGTGCTGGAGAATTTGATAAATTTTTAG [17550]
cp32-9_PAli_NX_Pl_consensus    TATCTGAAGACCAAAAAACACATCTTGACTGGTTAAAAACCGCTTTAACTGGTGCTGGAGAATTTGATAAATTTTTAG [17550]
cp32-9_PAbe_NX_MP_FG_consensus TATCTGAAGACCAAAAAACACATCTTGACTGGTTAAAAACCGCTTTAACTGGTGCTGGAGAATTTGATAAATTTTTAG [17550]
cp32-9_PAbe_NX_Pl_consensus    TATCTGAAGACCAAAAAACACATCTTGACTGGTTAAAAACCGCTTTAACTGGTGCTGGAGAATTTGATAAATTTTTAG [17550]

cp32-9_gi|11497325|B31_GB      GATATGACGAAGACAAAATAAAAGGTGCACTTAATCATATAAAGAGTGAACTTGATAAGTGTACTGGGGATAATTCTG [17628]
cp32-9_un7_B31_PacBio          GATATGACGAAGACAAAATAAAAGGTGCACTTAATCATATAAAGAGTGAACTTGATAAGTGTACTGGGGATAATTCTG [17628]
cp32-9_B31_TS_MP_FG_consensus  GATATGACGAAGACAAAATAAAAGGTGCACTTAATCATATAAAGAGTGAACTTGATAAGTGTACTGGGGATAATTCTG [17628]
cp32-9_B31_NX_Pl_consensus     GATATGACGAAGACAAAATAAAAGGTGCACTTAATCATATAAAGAGTGAACTTGATAAGTGTACTGGGGATAATTCTG [17628]
cp32-9_PAli_NX_MP_FG_consensus GATATGACGAAGACAAAATAAAAGGTGCACTTAATCATATAAAGAGTGAACTTGATAAGTGTACTGGGGATAATTCTG [17628]
cp32-9_PAli_NX_Pl_consensus    GATATGACGAAGACAAAATAAAAGGTGCACTTAATCATATAAAGAGTGAACTTGATAAGTGTACTGGGGATAATTCTG [17628]
cp32-9_PAbe_NX_MP_FG_consensus GATATGACGAAGACAAAATAAAAGGTGCACTTAATCATATAAAGAGTGAACTTGATAAGTGTACTGGGGATAATTCTG [17628]
cp32-9_PAbe_NX_Pl_consensus    GATATGACGAAGACAAAATAAAAGGTGCACTTAATCATATAAAGAGTGAACTTGATAAGTGTACTGGGGATAATTCTG [17628]

cp32-9_gi|11497325|B31_GB      AACAACAAAAAAGCACCTTCAAAGAGGTGGTTAAGGGGGCTCTTGGTGGCGGTATAGATAGTTTTGCAACTAGTGCAA [17706]
cp32-9_un7_B31_PacBio          AACAACAAAAAAGCACCTTCAAAGAGGTGGTTAAGGGGGCTCTTGGTGGCGGTATAGATAGTTTTGCAACTAGTGCAA [17706]
cp32-9_B31_TS_MP_FG_consensus  AACAACAAAAAAGCACCTTCAAAGAGGTGGTTAAGGGGGCTCTTGGTGGCGGTATAGATAGTTTTGCAACTAGTGCAA [17706]
cp32-9_B31_NX_Pl_consensus     AACAACAAAAAAGCACCTTCAAAGAGGTGGTTAAGGGGGCTCTTGGTGGCGGTATAGATAGTTTTGCAACTAGTGCAA [17706]
cp32-9_PAli_NX_MP_FG_consensus AACAACAAAAAAGCACCTTCAAAGAGGTGGTTAAGGGGGCTCTTGGTGGCGGTATAGATAGTTTTGCAACTAGTGCAA [17706]
cp32-9_PAli_NX_Pl_consensus    AACAACAAAAAAGCACCTTCAAAGAGGTGGTTAAGGGGGCTCTTGGTGGCGGTATAGATAGTTTTGCAACTAGTGCAA [17706]
cp32-9_PAbe_NX_MP_FG_consensus AACAACAAAAAAGCACCTTCAAAGAGGTGGTTAAGGGGGCTCTTGGTGGCGGTATAGATAGTTTTGCAACTAGTGCAA [17706]
cp32-9_PAbe_NX_Pl_consensus    AACAACAAAAAAGCACCTTCAAAGAGGTGGTTAAGGGGGCTCTTGGTGGCGGTATAGATAGTTTTGCAACTAGTGCAA [17706]

cp32-9_gi|11497325|B31_GB      GTAGTACCTGCCAAGCTCAGCAATAATATCGCAGCCCCCATGTGGGGGCTTCACCATATAAGAATTCGCTATGCTGCA [17784]
cp32-9_un7_B31_PacBio          GTAGTACCTGCCAAGCTCAGCAATAATATCGCAGCCCCCATGTGGGGGCTTCACCATATAAGAATTCGCTATGCTGCA [17784]
cp32-9_B31_TS_MP_FG_consensus  GTAGTACCTGCCAAGCTCAGCAATAATATCGCAGCCCCCATGTGGGGGCTTCACCATATAAGAATTCGCTATGCTGCA [17784]
cp32-9_B31_NX_Pl_consensus     GTAGTACCTGCCAAGCTCAGCAATAATATCGCAGCCCCCATGTGGGGGCTTCACCATATAAGAATTCGCTATGCTGCA [17784]
cp32-9_PAli_NX_MP_FG_consensus GTAGTACCTGCCAAGCTCAGCAATAATATCGCAGCCCCCATGTGGGGGCTTCACCATATAAGAATTCGCTATGCTGCA [17784]
cp32-9_PAli_NX_Pl_consensus    GTAGTACCTGCCAAGCTCAGCAATAATATCGCAGCCCCCATGTGGGGGCTTCACCATATAAGAATTCGCTATGCTGCA [17784]
cp32-9_PAbe_NX_MP_FG_consensus GTAGTACCTGCCAAGCTCAGCAATAATATCGCAGCCCCCATGTGGGGGCTTCACCATATAAGAATTCGCTATGCTGCA [17784]
cp32-9_PAbe_NX_Pl_consensus    GTAGTACCTGCCAAGCTCAGCAATAATATCGCAGCCCCCATGTGGGGGCTTCACCATATAAGAATTCGCTATGCTGCA [17784]

cp32-9_gi|11497325|B31_GB      AATATGCAATCAAACCGTTCATATTCTTTTAAGACACTAAAGAATATTAATGGACTAGGGCCATAAGCAGTCCTCTTA [17862]
cp32-9_un7_B31_PacBio          AATATGCAATCAAACCGTTCATATTCTTTTAAGACACTAAAGAATATTAATGGACTAGGGCCATAAGCAGTCCTCTTA [17862]
cp32-9_B31_TS_MP_FG_consensus  AATATGCAATCAAACCGTTCATATTCTTTTAAGACACTAAAGAATATTAATGGACTAGGGCCATAAGCAGTCCTCTTA [17862]
cp32-9_B31_NX_Pl_consensus     AATATGCAATCAAACCGTTCATATTCTTTTAAGACACTAAAGAATATTAATGGACTAGGGCCATAAGCAGTCCTCTTA [17862]
cp32-9_PAli_NX_MP_FG_consensus AATATGCAATCAAACCGTTCATATTCTTTTAAGACACTAAAGAATATTAATGGACTAGGGCCATAAGCAGTCCTCTTA [17862]
cp32-9_PAli_NX_Pl_consensus    AATATGCAATCAAACCGTTCATATTCTTTTAAGACACTAAAGAATATTAATGGACTAGGGCCATAAGCAGTCCTCTTA [17862]
cp32-9_PAbe_NX_MP_FG_consensus AATATGCAATCAAACCGTTCATATTCTTTTAAGACACTAAAGAATATTAATGGACTAGGGCCATAAGCAGTCCTCTTA [17862]
cp32-9_PAbe_NX_Pl_consensus    AATATGCAATCAAACCGTTCATATTCTTTTAAGACACTAAAGAATATTAATGGACTAGGGCCATAAGCAGTCCTCTTA [17862]

cp32-9_gi|11497325|B31_GB      AACGACACAAACTTTTCAAAATCTTGTAAATCCTTTAATCTATTATTTTTCTTGAAATAGTTTTTTATAATCTCCGCC [17940]
cp32-9_un7_B31_PacBio          AACGACACAAACTTTTCAAAATCTTGTAAATCCTTTAATCTATTATTTTTCTTGAAATAGTTTTTTATAATCTCCGCC [17940]
```

```
cp32-9_B31_TS_MP_FG_consensus  AACGACACAAACTTTTCAAAATCTTGTAAATCCTTTAATCTATTATTTTTCTTGAAATAGTTTTTTATAATCTCCGCC [17940]
cp32-9_B31_NX_Pl_consensus     AACGACACAAACTTTTCAAAATCTTGTAAATCCTTTAATCTATTATTTTTCTTGAAATAGTTTTTTATAATCTCCGCC [17940]
cp32-9_PAli_NX_MP_FG_consensus AACGACACAAACTTTTCAAAATCTTGTAAATCCTTTAATCTATTATTTTTCTTGAAATAGTTTTTTATAATCTCCGCC [17940]
cp32-9_PAli_NX_Pl_consensus    AACGACACAAACTTTTCAAAATCTTGTAAATCCTTTAATCTATTATTTTTCTTGAAATAGTTTTTTATAATCTCCGCC [17940]
cp32-9_PAbe_NX_MP_FG_consensus AACGACACAAACTTTTCAAAATCTTGTAAATCCTTTAATCTATTATTTTTCTTGAAATAGTTTTTTATAATCTCCGCC [17940]
cp32-9_PAbe_NX_Pl_consensus    AACGACACAAACTTTTCAAAATCTTGTAAATCCTTTAATCTATTATTTTTCTTGAAATAGTTTTTTATAATCTCCGCC [17940]

cp32-9_gi|11497325|B31_GB      CAATAGTTTATACTTTTAAAATCACTATTTTTCTCAAAATACGAGATTAAATCAGATTCAATTTTCTTGATATCATCA [18018]
cp32-9_un7_B31_PacBio          CAATAGTTTATACTTTTAAAATCACTATTTTTCTCAAAATACGAGATTAAATCAGATTCAATTTTCTTGATATCATCA [18018]
cp32-9_B31_TS_MP_FG_consensus  CAATAGTTTATACTTTTAAAATCACTATTTTTCTCAAAATACGAGATTAAATCAGATTCAATTTTCTTGATATCATCA [18018]
cp32-9_B31_NX_Pl_consensus     CAATAGTTTATACTTTTAAAATCACTATTTTTCTCAAAATACGAGATTAAATCAGATTCAATTTTCTTGATATCATCA [18018]
cp32-9_PAli_NX_MP_FG_consensus CAATAGTTTATACTTTTAAAATCACTATTTTTCTCAAAATACGAGATTAAATCAGATTCAATTTTCTTGATATCATCA [18018]
cp32-9_PAli_NX_Pl_consensus    CAATAGTTTATACTTTTAAAATCACTATTTTTCTCAAAATACGAGATTAAATCAGATTCAATTTTCTTGATATCATCA [18018]
cp32-9_PAbe_NX_MP_FG_consensus CAATAGTTTATACTTTTAAAATCACTATTTTTCTCAAAATACGAGATTAAATCAGATTCAATTTTCTTGATATCATCA [18018]
cp32-9_PAbe_NX_Pl_consensus    CAATAGTTTATACTTTTAAAATCACTATTTTTCTCAAAATACGAGATTAAATCAGATTCAATTTTCTTGATATCATCA [18018]

cp32-9_gi|11497325|B31_GB      ACATTATTGGGGTCTATATTCTGAATAAAGCTTATTTTTTGAATAATTGAGTTTATATTATCTTTTGTTGTAGGGCTA [18096]
cp32-9_un7_B31_PacBio          ACATTATTGGGGTCTATATTCTGAATAAAGCTTATTTTTTGAATAATTGAGTTTATATTATCTTTTGTTGTAGGGCTA [18096]
cp32-9_B31_TS_MP_FG_consensus  ACATTATTGGGGTCTATATTCTGAATAAAGCTTATTTTTTGAATAATTGAGTTTATATTATCTTTTGTTGTAGGGCTA [18096]
cp32-9_B31_NX_Pl_consensus     ACATTATTGGGGTCTATATTCTGAATAAAGCTTATTTTTTGAATAATTGAGTTTATATTATCTTTTGTTGTAGGGCTA [18096]
cp32-9_PAli_NX_MP_FG_consensus ACATTATTGGGGTCTATATTCTGAATAAAGCTTATTTTTTGAATAATTGAGTTTATATTATCTTTTGTTGTAGGGCTA [18096]
cp32-9_PAli_NX_Pl_consensus    ACATTATTGGGGTCTATATTCTGAATAAAGCTTATTTTTTGAATAATTGAGTTTATATTATCTTTTGTTGTAGGGCTA [18096]
cp32-9_PAbe_NX_MP_FG_consensus ACATTATTGGGGTCTATATTCTGAATAAAGCTTATTTTTTGAATAATTGAGTTTATATTATCTTTTGTTGTAGGGCTA [18096]
cp32-9_PAbe_NX_Pl_consensus    ACATTATTGGGGTCTATATTCTGAATAAAGCTTATTTTTTGAATAATTGAGTTTATATTATCTTTTGTTGTAGGGCTA [18096]

cp32-9_gi|11497325|B31_GB      TTTACCCAATCTTTTATATTAGACAACGCTTCTTTAAAAACACTGTAATAATAAAACCTATCTTTTTTTTATTCTTGC [18174]
cp32-9_un7_B31_PacBio          TTTACCCAATCTTTTATATTAGACAACGCTTCTTTAAAAACACTGTAATAATAAAACCTATCTTTTTTTTATTCTTGC [18174]
cp32-9_B31_TS_MP_FG_consensus  TTTACCCAATCTTTTATATTAGACAACGCTTCTTTAAAAACACTGTAATAATAAAACCTATCTTTTTTTTATTCTTGC [18174]
cp32-9_B31_NX_Pl_consensus     TTTACCCAATCTTTTATATTAGACAACGCTTCTTTAAAAACACTGTAATAATAAAACCTATCTTTTTTTTATTCTTGC [18174]
cp32-9_PAli_NX_MP_FG_consensus TTTACCCAATCTTTTATATTAGACAACGCTTCTTTAAAAACACTGTAATAATAAAACCTATCTTTTTTTTATTCTTGC [18174]
cp32-9_PAli_NX_Pl_consensus    TTTACCCAATCTTTTATATTAGACAACGCTTCTTTAAAAACACTGTAATAATAAAACCTATCTTTTTTTTATTCTTGC [18174]
cp32-9_PAbe_NX_MP_FG_consensus TTTACCCAATCTTTTATATTAGACAACGCTTCTTTAAAAACACTGTAATAATAAAACCTATCTTTTTTTTATTCTTGC [18174]
cp32-9_PAbe_NX_Pl_consensus    TTTACCCAATCTTTTATATTAGACAACGCTTCTTTAAAAACACTGTAATAATAAAACCTATCTTTTTTTTATTCTTGC [18174]

cp32-9_gi|11497325|B31_GB      TTAATATCTCTTTTCTGGTTTTGAATAATATTATTTTTCTGAATTTGATTAATCTCTTGTTTTTGTTCTCTTTTTTGT [18252]
cp32-9_un7_B31_PacBio          TTAATATCTCTTTTCTGGTTTTGAATAATATTATTTTTCTGAATTTGATTAATCTCTTGTTTTTGTTCTCTTTTTTGT [18252]
cp32-9_B31_TS_MP_FG_consensus  TTAATATCTCTTTTCTGGTTTTGAATAATATTATTTTTCTGAATTTGATTAATCTCTTGTTTTTGTTCTCTTTTTTGT [18252]
cp32-9_B31_NX_Pl_consensus     TTAATATCTCTTTTCTGGTTTTGAATAATATTATTTTTCTGAATTTGATTAATCTCTTGTTTTTGTTCTCTTTTTTGT [18252]
cp32-9_PAli_NX_MP_FG_consensus TTAATATCTCTTTTCTGGTTTTGAATAATATTATTTTTCTGAATTTGATTAATCTCTTGTTTTTGTTCTCTTTTTTGT [18252]
cp32-9_PAli_NX_Pl_consensus    TTAATATCTCTTTTCTGGTTTTGAATAATATTATTTTTCTGAATTTGATTAATCTCTTGTTTTTGTTCTCTTTTTTGT [18252]
cp32-9_PAbe_NX_MP_FG_consensus TTAATATCTCTTTTCTGGTTTTGAATAATATTATTTTTCTGAATTTGATTAATCTCTTGTTTTTGTTCTCTTTTTTGT [18252]
cp32-9_PAbe_NX_Pl_consensus    TTAATATCTCTTTTCTGGTTTTGAATAATATTATTTTTCTGAATTTGATTAATCTCTTGTTTTTGTTCTCTTTTTTGT [18252]

cp32-9_gi|11497325|B31_GB      TCTTACTTTTGATTAACACTAACTTGTTTGTTAGGCATAGAATTTCTGTTTTCATAATTATTGTAAATAGGTGCTGCA [18330]
cp32-9_un7_B31_PacBio          TCTTACTTTTGATTAACACTAACTTGTTTGTTAGGCATAGAATTTCTGTTTTCATAATTATTGTAAATAGGTGCTGCA [18330]
cp32-9_B31_TS_MP_FG_consensus  TCTTACTTTTGATTAACACTAACTTGTTTGTTAGGCATAGAATTTCTGTTTTCATAATTATTGTAAATAGGTGCTGCA [18330]
cp32-9_B31_NX_Pl_consensus     TCTTACTTTTGATTAACACTAACTTGTTTGTTAGGCATAGAATTTCTGTTTTCATAATTATTGTAAATAGGTGCTGCA [18330]
cp32-9_PAli_NX_MP_FG_consensus TCTTACTTTTGATTAACACTAACTTGTTTGTTAGGCATAGAATTTCTGTTTTCATAATTATTGTAAATAGGTGCTGCA [18330]
cp32-9_PAli_NX_Pl_consensus    TCTTACTTTTGATTAACACTAACTTGTTTGTTAGGCATAGAATTTCTGTTTTCATAATTATTGTAAATAGGTGCTGCA [18330]
cp32-9_PAbe_NX_MP_FG_consensus TCTTACTTTTGATTAACACTAACTTGTTTGTTAGGCATAGAATTTCTGTTTTCATAATTATTGTAAATAGGTGCTGCA [18330]
cp32-9_PAbe_NX_Pl_consensus    TCTTACTTTTGATTAACACTAACTTGTTTGTTAGGCATAGAATTTCTGTTTTCATAATTATTGTAAATAGGTGCTGCA [18330]

cp32-9_gi|11497325|B31_GB      TCAGTATCCATTTCGCTTTTTATATTAAGACATGCAACTAAAGCGTATCTTTTGAAATAAGTAATAGCTGAACCAACA [18408]
cp32-9_un7_B31_PacBio          TCAGTATCCATTTCGCTTTTTATATTAAGACATGCAACTAAAGCGTATCTTTTGAAATAAGTAATAGCTGAACCAACA [18408]
cp32-9_B31_TS_MP_FG_consensus  TCAGTATCCATTTCGCTTTTTATATTAAGACATGCAACTAAAGCGTATCTTTTGAAATAAGTAATAGCTGAACCAACA [18408]
cp32-9_B31_NX_Pl_consensus     TCAGTATCCATTTCGCTTTTTATATTAAGACATGCAACTAAAGCGTATCTTTTGAAATAAGTAATAGCTGAACCAACA [18408]
cp32-9_PAli_NX_MP_FG_consensus TCAGTATCCATTTCGCTTTTTATATTAAGACATGCAACTAAAGCGTATCTTTTGAAATAAGTAATAGCTGAACCAACA [18408]
cp32-9_PAli_NX_Pl_consensus    TCAGTATCCATTTCGCTTTTTATATTAAGACATGCAACTAAAGCGTATCTTTTGAAATAAGTAATAGCTGAACCAACA [18408]
cp32-9_PAbe_NX_MP_FG_consensus TCAGTATCCATTTCGCTTTTTATATTAAGACATGCAACTAAAGCGTATCTTTTGAAATAAGTAATAGCTGAACCAACA [18408]
cp32-9_PAbe_NX_Pl_consensus    TCAGTATCCATTTCGCTTTTTATATTAAGACATGCAACTAAAGCGTATCTTTTGAAATAAGTAATAGCTGAACCAACA [18408]

cp32-9_gi|11497325|B31_GB      AATTGTGGCAATGTATTTACATTTTTAGAACCATTTTCACTGTTCCATTGCAAATTTTCTGTAAGAATTGGGGTATCA [18486]
cp32-9_un7_B31_PacBio          AATTGTGGCAATGTATTTACATTTTTAGAACCATTTTCACTGTTCCATTGCAAATTTTCTGTAAGAATTGGGGTATCA [18486]
cp32-9_B31_TS_MP_FG_consensus  AATTGTGGCAATGTATTTACATTTTTAGAACCATTTTCACTGTTCCATTGCAAATTTTCTGTAAGAATTGGGGTATCA [18486]
cp32-9_B31_NX_Pl_consensus     AATTGTGGCAATGTATTTACATTTTTAGAACCATTTTCACTGTTCCATTGCAAATTTTCTGTAAGAATTGGGGTATCA [18486]
cp32-9_PAli_NX_MP_FG_consensus AATTGTGGCAATGTATTTACATTTTTAGAACCATTTTCACTGTTCCATTGCAAATTTTCTGTAAGAATTGGGGTATCA [18486]
cp32-9_PAli_NX_Pl_consensus    AATTGTGGCAATGTATTTACATTTTTAGAACCATTTTCACTGTTCCATTGCAAATTTTCTGTAAGAATTGGGGTATCA [18486]
cp32-9_PAbe_NX_MP_FG_consensus AATTGTGGCAATGTATTTACATTTTTAGAACCATTTTCACTGTTCCATTGCAAATTTTCTGTAAGAATTGGGGTATCA [18486]
cp32-9_PAbe_NX_Pl_consensus    AATTGTGGCAATGTATTTACATTTTTAGAACCATTTTCACTGTTCCATTGCAAATTTTCTGTAAGAATTGGGGTATCA [18486]

cp32-9_gi|11497325|B31_GB      AATGACTCTCTATACCCACTGCTTGTGCTGTAGAATGTGGTCCTAATAACATGAACTATACCATATGGATCATGTGTA [18564]
cp32-9_un7_B31_PacBio          AATGACTCTCTATACCCACTGCTTGTGCTGTAGAATGTGGTCCTAATAACATGAACTATACCATATGGATCATGTGTA [18564]
cp32-9_B31_TS_MP_FG_consensus  AATGACTCTCTATACCCACTGCTTGTGCTGTAGAATGTGGTCCTAATAACATGAACTATACCATATGGATCATGTGTA [18564]
cp32-9_B31_NX_Pl_consensus     AATGACTCTCTATACCCACTGCTTGTGCTGTAGAATGTGGTCCTAATAACATGAACTATACCATATGGATCATGTGTA [18564]
cp32-9_PAli_NX_MP_FG_consensus AATGACTCTCTATACCCACTGCTTGTGCTGTAGAATGTGGTCCTAATAACATGAACTATACCATATGGATCATGTGTA [18564]
cp32-9_PAli_NX_Pl_consensus    AATGACTCTCTATACCCACTGCTTGTGCTGTAGAATGTGGTCCTAATAACATGAACTATACCATATGGATCATGTGTA [18564]
cp32-9_PAbe_NX_MP_FG_consensus AATGACTCTCTATACCCACTGCTTGTGCTGTAGAATGTGGTCCTAATAACATGAACTATACCATATGGATCATGTGTA [18564]
cp32-9_PAbe_NX_Pl_consensus    AATGACTCTCTATACCCACTGCTTGTGCTGTAGAATGTGGTCCTAATAACATGAACTATACCATATGGATCATGTGTA [18564]

cp32-9_gi|11497325|B31_GB      AAAGTTGGAAATTGCCTAAACATAAGATCCAAATTATGCTTTTCAATAACGTTTTCAATTTCTTCTAATATGTCATTG [18642]
cp32-9_un7_B31_PacBio          AAAGTTGGAAATTGCCTAAACATAAGATCCAAATTATGCTTTTCAATAACGTTTTCAATTTCTTCTAATATGTCATTG [18642]
cp32-9_B31_TS_MP_FG_consensus  AAAGTTGGAAATTGCCTAAACATAAGATCCAAATTATGCTTTTCAATAACGTTTTCAATTTCTTCTAATATGTCATTG [18642]
cp32-9_B31_NX_Pl_consensus     AAAGTTGGAAATTGCCTAAACATAAGATCCAAATTATGCTTTTCAATAACGTTTTCAATTTCTTCTAATATGTCATTG [18642]
cp32-9_PAli_NX_MP_FG_consensus AAAGTTGGAAATTGCCTAAACATAAGATCCAAATTATGCTTTTCAATAACGTTTTCAATTTCTTCTAATATGTCATTG [18642]
cp32-9_PAli_NX_Pl_consensus    AAAGTTGGAAATTGCCTAAACATAAGATCCAAATTATGCTTTTCAATAACGTTTTCAATTTCTTCTAATATGTCATTG [18642]
cp32-9_PAbe_NX_MP_FG_consensus AAAGTTGGAAATTGCCTAAACATAAGATCCAAATTATGCTTTTCAATAACGTTTTCAATTTCTTCTAATATGTCATTG [18642]
cp32-9_PAbe_NX_Pl_consensus    AAAGTTGGAAATTGCCTAAACATAAGATCCAAATTATGCTTTTCAATAACGTTTTCAATTTCTTCTAATATGTCATTG [18642]

cp32-9_gi|11497325|B31_GB      AAATCCTGATACTTATATCCGTACCCTTTAAGACTTTTGTCAATACGTGGCAAGTTCTTCATTAGGGTTTTCATATCA [18720]
cp32-9_un7_B31_PacBio          AAATCCTGATACTTATATCCGTACCCTTTAAGACTTTTGTCAATACGTGGCAAGTTCTTCATTAGGGTTTTCATATCA [18720]
cp32-9_B31_TS_MP_FG_consensus  AAATCCTGATACTTATATCCGTACCCTTTAAGACTTTTGTCAATACGTGGCAAGTTCTTCATTAGGGTTTTCATATCA [18720]
cp32-9_B31_NX_Pl_consensus     AAATCCTGATACTTATATCCGTACCCTTTAAGACTTTTGTCAATACGTGGCAAGTTCTTCATTAGGGTTTTCATATCA [18720]
cp32-9_PAli_NX_MP_FG_consensus AAATCCTGATACTTATATCCGTACCCTTTAAGACTTTTGTCAATACGTGGCAAGTTCTTCATTAGGGTTTTCATATCA [18720]
cp32-9_PAli_NX_Pl_consensus    AAATCCTGATACTTATATCCGTACCCTTTAAGACTTTTGTCAATACGTGGCAAGTTCTTCATTAGGGTTTTCATATCA [18720]
cp32-9_PAbe_NX_MP_FG_consensus AAATCCTGATACTTATATCCGTACCCTTTAAGACTTTTGTCAATACGTGGCAAGTTCTTCATTAGGGTTTTCATATCA [18720]
cp32-9_PAbe_NX_Pl_consensus    AAATCCTGATACTTATATCCGTACCCTTTAAGACTTTTGTCAATACGTGGCAAGTTCTTCATTAGGGTTTTCATATCA [18720]

cp32-9_gi|11497325|B31_GB      TTTAAGAATTTAATTTCTGCTTGAATATTTTCTTGTGGATTATTATTGTTTTAAAGATTTTCCATCTTTTTCCTCCGT [18798]
cp32-9_un7_B31_PacBio          TTTAAGAATTTAATTTCTGCTTGAATATTTTCTTGTGGATTATTATTGTTTTAAAGATTTTCCATCTTTTTCCTCCGT [18798]
cp32-9_B31_TS_MP_FG_consensus  TTTAAGAATTTAATTTCTGCTTGAATATTTTCTTGTGGATTATTATTGTTTTAAAGATTTTCCATCTTTTTCCTCCGT [18798]
cp32-9_B31_NX_Pl_consensus     TTTAAGAATTTAATTTCTGCTTGAATATTTTCTTGTGGATTATTATTGTTTTAAAGATTTTCCATCTTTTTCCTCCGT [18798]
cp32-9_PAli_NX_MP_FG_consensus TTTAAGAATTTAATTTCTGCTTGAATATTTTCTTGTGGATTATTATTGTTTTAAAGATTTTCCATCTTTTTCCTCCGT [18798]
cp32-9_PAli_NX_Pl_consensus    TTTAAGAATTTAATTTCTGCTTGAATATTTTCTTGTGGATTATTATTGTTTTAAAGATTTTCCATCTTTTTCCTCCGT [18798]
cp32-9_PAbe_NX_MP_FG_consensus TTTAAGAATTTAATTTCTGCTTGAATATTTTCTTGTGGATTATTATTGTTTTAAAGATTTTCCATCTTTTTCCTCCGT [18798]
cp32-9_PAbe_NX_Pl_consensus    TTTAAGAATTTAATTTCTGCTTGAATATTTTCTTGTGGATTATTATTGTTTTAAAGATTTTCCATCTTTTTCCTCCGT [18798]

cp32-9_gi|11497325|B31_GB      TTATATGTATTAATAAATATAAGTATATAGCAAAAACTATTTTTGCCAACTTCTTTACAAAAAATTTTTACAAAAAGT [18876]
cp32-9_un7_B31_PacBio          TTATATGTATTAATAAATATAAGTATATAGCAAAAACTATTTTTGCCAACTTCTTTACAAAAAATTTTTACAAAAAGT [18876]
cp32-9_B31_TS_MP_FG_consensus  TTATATGTATTAATAAATATAAGTATATAGCAAAAACTATTTTTGCCAACTTCTTTACAAAAAATTTTTACAAAAAGT [18876]
```

```
cp32-9_B31_NX_Pl_consensus     TTATATGTATTAATAAATATAAGTATATAGCAAAAACTATTTTTGCCAACTTCTTTACAAAAAATTTTTACAAAAAGT [18876]
cp32-9_PAli_NX_MP_FG_consensus TTATATGTATTAATAAATATAAGTATATAGCAAAAACTATTTTTGCCAACTTCTTTACAAAAAATTTTTACAAAAAGT [18876]
cp32-9_PAli_NX_Pl_consensus    TTATATGTATTAATAAATATAAGTATATAGCAAAAACTATTTTTGCCAACTTCTTTACAAAAAATTTTTACAAAAAGT [18876]
cp32-9_PAbe_NX_MP_FG_consensus TTATATGTATTAATAAATATAAGTATATAGCAAAAACTATTTTTGCCAACTTCTTTACAAAAAATTTTTACAAAAAGT [18876]
cp32-9_PAbe_NX_Pl_consensus    TTATATGTATTAATAAATATAAGTATATAGCAAAAACTATTTTTGCCAACTTCTTTACAAAAAATTTTTACAAAAAGT [18876]

cp32-9_gi|11497325|B31_GB      GCGGGCTTAGCTAAATTCTCTTGTTAAAGAACTTAGTTAAGCCCATATCTTTATTTCTTGTAAATTCATATAAACGGT [18954]
cp32-9_un7_B31_PacBio          GCGGGCTTAGCTAAATTCTCTTGTTAAAGAACTTAGTTAAGCCCATATCTTTATTTCTTGTAAATTCATATAAACGGT [18954]
cp32-9_B31_TS_MP_FG_consensus  GCGGGCTTAGCTAAATTCTCTTGTTAAAGAACTTAGTTAAGCCCATATCTTTATTTCTTGTAAATTCATATAAACGGT [18954]
cp32-9_B31_NX_Pl_consensus     GCGGGCTTAGCTAAATTCTCTTGTTAAAGAACTTAGTTAAGCCCATATCTTTATTTCTTGTAAATTCATATAAACGGT [18954]
cp32-9_PAli_NX_MP_FG_consensus GCGGGCTTAGCTAAATTCTCTTGTTAAAGAACTTAGTTAAGCCCATATCTTTATTTCTTGTAAATTCATATAAACGGT [18954]
cp32-9_PAli_NX_Pl_consensus    GCGGGCTTAGCTAAATTCTCTTGTTAAAGAACTTAGTTAAGCCCATATCTTTATTTCTTGTAAATTCATATAAACGGT [18954]
cp32-9_PAbe_NX_MP_FG_consensus GCGGGCTTAGCTAAATTCTCTTGTTAAAGAACTTAGTTAAGCCCATATCTTTATTTCTTGTAAATTCATATAAACGGT [18954]
cp32-9_PAbe_NX_Pl_consensus    GCGGGCTTAGCTAAATTCTCTTGTTAAAGAACTTAGTTAAGCCCATATCTTTATTTCTTGTAAATTCATATAAACGGT [18954]

cp32-9_gi|11497325|B31_GB      AGAAAAAATGCGAACATTTTACTACGCTATTTGTAGTATAAGTCAACTTAGAATTAAAATCAATTTATATTTTTACTA [19032]
cp32-9_un7_B31_PacBio          AGAAAAAATGCGAACATTTTACTACGCTATTTGTAGTATAAGTCAACTTAGAATTAAAATCAATTTATATTTTTACTA [19032]
cp32-9_B31_TS_MP_FG_consensus  AGAAAAAATGCGAACATTTTACTACGCTATTTGTAGTATAAGTCAACTTAGAATTAAAATCAATTTATATTTTTACTA [19032]
cp32-9_B31_NX_Pl_consensus     AGAAAAAATGCGAACATTTTACTACGCTATTTGTAGTATAAGTCAACTTAGAATTAAAATCAATTTATATTTTTACTA [19032]
cp32-9_PAli_NX_MP_FG_consensus AGAAAAAATGCGAACATTTTACTACGCTATTTGTAGTATAAGTCAACTTAGAATTAAAATCAATTTATATTTTTACTA [19032]
cp32-9_PAli_NX_Pl_consensus    AGAAAAAATGCGAACATTTTACTACGCTATTTGTAGTATAAGTCAACTTAGAATTAAAATCAATTTATATTTTTACTA [19032]
cp32-9_PAbe_NX_MP_FG_consensus AGAAAAAATGCGAACATTTTACTACGCTATTTGTAGTATAAGTCAACTTAGAATTAAAATCAATTTATATTTTTACTA [19032]
cp32-9_PAbe_NX_Pl_consensus    AGAAAAAATGCGAACATTTTACTACGCTATTTGTAGTATAAGTCAACTTAGAATTAAAATCAATTTATATTTTTACTA [19032]

cp32-9_gi|11497325|B31_GB      AATTACAAATTTTTATATTAATTTAACAAAATTAATAATTAAAATTTAATATTTTTTTAGAAAAGTATTTACTTTTAA [19110]
cp32-9_un7_B31_PacBio          AATTACAAATTTTTATATTAATTTAACAAAATTAATAATTAAAATTTAATATTTTTTTAGAAAAGTATTTACTTTTAA [19110]
cp32-9_B31_TS_MP_FG_consensus  AATTACAAATTTTTATATTAATTTAACAAAATTAATAATTAAAATTTAATATTTTTTTAGAAAAGTATTTACTTTTAA [19110]
cp32-9_B31_NX_Pl_consensus     AATTACAAATTTTTATATTAATTTAACAAAATTAATAATTAAAATTTAATATTTTTTTAGAAAAGTATTTACTTTTAA [19110]
cp32-9_PAli_NX_MP_FG_consensus AATTACAAATTTTTATATTAATTTAACAAAATTAATAATTAAAATTTAATATTTTTTTAGAAAAGTATTTACTTTTAA [19110]
cp32-9_PAli_NX_Pl_consensus    AATTACAAATTTTTATATTAATTTAACAAAATTAATAATTAAAATTTAATATTTTTTTAGAAAAGTATTTACTTTTAA [19110]
cp32-9_PAbe_NX_MP_FG_consensus AATTACAAATTTTTATATTAATTTAACAAAATTAATAATTAAAATTTAATATTTTTTTAGAAAAGTATTTACTTTTAA [19110]
cp32-9_PAbe_NX_Pl_consensus    AATTACAAATTTTTATATTAATTTAACAAAATTAATAATTAAAATTTAATATTTTTTTAGAAAAGTATTTACTTTTAA [19110]

cp32-9_gi|11497325|B31_GB      ATCAAAATTTTGCACTATATTAGTAATTACTAATACATGTAAACGGAGAAAAAAAAATGCAAAATTTCCCAAATAAAAC [19188]
cp32-9_un7_B31_PacBio          ATCAAAATTTTGCACTATATTAGTAATTACTAATACATGTAAACGGAGAAAAAAAA-TGCAAAATTTCCCAAATAAAAC [19188]
cp32-9_B31_TS_MP_FG_consensus  ATCAAAATTTTGCACTATATTAGTAATTACTAATACATGTAAACGGAGAAAAAAAAATGCAAAATTTCCCAAATAAAAC [19188]
cp32-9_B31_NX_Pl_consensus     ATCAAAATTTTGCACTATATTAGTAATTACTAATACATGTAAACGGAGAAAAAAAAATGCAAAATTTCCCAAATAAAAC [19188]
cp32-9_PAli_NX_MP_FG_consensus ATCAAAATTTTGCACTATATTAGTAATTACTAATACATGTAAACGGAGAAAAAAAAATGCAAAATTTCCCAAATAAAAC [19188]
cp32-9_PAli_NX_Pl_consensus    ATCAAAATTTTGCACTATATTAGTAATTACTAATACATGTAAACGGAGAAAAAAAAATGCAAAATTTCCCAAATAAAAC [19188]
cp32-9_PAbe_NX_MP_FG_consensus ATCAAAATTTTGCACTATATTAGTAATTACTAATACATGTAAACGGAGAAAAAAAAATGCAAAATTTCCCAAATAAAAC [19188]
cp32-9_PAbe_NX_Pl_consensus    ATCAAAATTTTGCACTATATTAGTAATTACTAATACATGTAAACGGAGAAAAAAAAATGCAAAATTTCCCAAATAAAAC [19188]

cp32-9_gi|11497325|B31_GB      AAAAATTCCAACTTGCCACAACAAACACCAACACAAATTAATATCTCTTACTTCAACGCTAGATTTTCTAAACAAAAA [19266]
cp32-9_un7_B31_PacBio          AAAAATTCCAACTTGCCACAACAAACACCAACACAAATTAATATCTCTTACTTCAACGCTAGATTTTCTAAACAAAAA [19266]
cp32-9_B31_TS_MP_FG_consensus  AAAAATTCCAACTTGCCACAACAAACACCAACACAAATTAATATCTCTTACTTCAACGCTAGATTTTCTAAACAAAAA [19266]
cp32-9_B31_NX_Pl_consensus     AAAAATTCCAACTTGCCACAACAAACACCAACACAAATTAATATCTCTTACTTCAACGCTAGATTTTCTAAACAAAAA [19266]
cp32-9_PAli_NX_MP_FG_consensus AAAAATTCCAACTTGCCACAACAAACACCAACACAAATTAATATCTCTTACTTCAACGCTAGATTTTCTAAACAAAAA [19266]
cp32-9_PAli_NX_Pl_consensus    AAAAATTCCAACTTGCCACAACAAACACCAACACAAATTAATATCTCTTACTTCAACGCTAGATTTTCTAAACAAAAA [19266]
cp32-9_PAbe_NX_MP_FG_consensus AAAAATTCCAACTTGCCACAACAAACACCAACACAAATTAATATCTCTTACTTCAACGCTAGATTTTCTAAACAAAAA [19266]
cp32-9_PAbe_NX_Pl_consensus    AAAAATTCCAACTTGCCACAACAAACACCAACACAAATTAATATCTCTTACTTCAACGCTAGATTTTCTAAACAAAAA [19266]

cp32-9_gi|11497325|B31_GB      AGATAAAAAATACACACAACAAAACATACTCTACTGCTTTAACGAAAATCTAAAAAGAAATGGGCTAGCTCCCACTAC [19344]
cp32-9_un7_B31_PacBio          AGATAAAAAATACACACAACAAAACATACTCTACTGCTTTAACGAAAATCTAAAAAGAAATGGGCTAGCTCCCACTAC [19344]
cp32-9_B31_TS_MP_FG_consensus  AGATAAAAAATACACACAACAAAACATACTCTACTGCTTTAACGAAAATCTAAAAAGAAATGGGCTAGCTCCCACTAC [19344]
cp32-9_B31_NX_Pl_consensus     AGATAAAAAATACACACAACAAAACATACTCTACTGCTTTAACGAAAATCTAAAAAGAAATGGGCTAGCTCCCACTAC [19344]
cp32-9_PAli_NX_MP_FG_consensus AGATAAAAAATACACACAACAAAACATACTCTACTGCTTTAACGAAAATCTAAAAAGAAATGGGCTAGCTCCCACTAC [19344]
cp32-9_PAli_NX_Pl_consensus    AGATAAAAAATACACACAACAAAACATACTCTACTGCTTTAACGAAAATCTAAAAAGAAATGGGCTAGCTCCCACTAC [19344]
cp32-9_PAbe_NX_MP_FG_consensus AGATAAAAAATACACACAACAAAACATACTCTACTGCTTTAACGAAAATCTAAAAAGAAATGGGCTAGCTCCCACTAC [19344]
cp32-9_PAbe_NX_Pl_consensus    AGATAAAAAATACACACAACAAAACATACTCTACTGCTTTAACGAAAATCTAAAAAGAAATGGGCTAGCTCCCACTAC [19344]

cp32-9_gi|11497325|B31_GB      ACTAAGAACAATGCAAAATTATCTTTACAAATTAGAAAAAGTACTAAAAGTTACAACTAATTACTACCAACACATGGG [19422]
cp32-9_un7_B31_PacBio          ACTAAGAACAATGCAAAATTATCTTTACAAATTAGAAAAAGTACTAAAAGTTACAACTAATTACTACCAACACATGGG [19422]
cp32-9_B31_TS_MP_FG_consensus  ACTAAGAACAATGCAAAATTATCTTTACAAATTAGAAAAAGTACTAAAAGTTACAACTAATTACTACCAACACATGGG [19422]
cp32-9_B31_NX_Pl_consensus     ACTAAGAACAATGCAAAATTATCTTTACAAATTAGAAAAAGTACTAAAAGTTACAACTAATTACTACCAACACATGGG [19422]
cp32-9_PAli_NX_MP_FG_consensus ACTAAGAACAATGCAAAATTATCTTTACAAATTAGAAAAAGTACTAAAAGTTACAACTAATTACTACCAACACATGGG [19422]
cp32-9_PAli_NX_Pl_consensus    ACTAAGAACAATGCAAAATTATCTTTACAAATTAGAAAAAGTACTAAAAGTTACAACTAATTACTACCAACACATGGG [19422]
cp32-9_PAbe_NX_MP_FG_consensus ACTAAGAACAATGCAAAATTATCTTTACAAATTAGAAAAAGTACTAAAAGTTACAACTAATTACTACCAACACATGGG [19422]
cp32-9_PAbe_NX_Pl_consensus    ACTAAGAACAATGCAAAATTATCTTTACAAATTAGAAAAAGTACTAAAAGTTACAACTAATTACTACCAACACATGGG [19422]

cp32-9_gi|11497325|B31_GB      TGTAAATTGTGGAACTGAAATTTACTATAAACTAAAGTATCCCAAAAAAGAATGTTACCAGAAAATCAACAAGTACTT [19500]
cp32-9_un7_B31_PacBio          TGTAAATTGTGGAACTGAAATTTACTATAAACTAAAGTATCCCAAAAAAGAATGTTACCAGAAAATCAACAAGTACTT [19500]
cp32-9_B31_TS_MP_FG_consensus  TGTAAATTGTGGAACTGAAATTTACTATAAACTAAAGTATCCCAAAAAAGAATGTTACCAGAAAATCAACAAGTACTT [19500]
cp32-9_B31_NX_Pl_consensus     TGTAAATTGTGGAACTGAAATTTACTATAAACTAAAGTATCCCAAAAAAGAATGTTACCAGAAAATCAACAAGTACTT [19500]
cp32-9_PAli_NX_MP_FG_consensus TGTAAATTGTGGAACTGAAATTTACTATAAACTAAAGTATCCCAAAAAAGAATGTTACCAGAAAATCAACAAGTACTT [19500]
cp32-9_PAli_NX_Pl_consensus    TGTAAATTGTGGAACTGAAATTTACTATAAACTAAAGTATCCCAAAAAAGAATGTTACCAGAAAATCAACAAGTACTT [19500]
cp32-9_PAbe_NX_MP_FG_consensus TGTAAATTGTGGAACTGAAATTTACTATAAACTAAAGTATCCCAAAAAAGAATGTTACCAGAAAATCAACAAGTACTT [19500]
cp32-9_PAbe_NX_Pl_consensus    TGTAAATTGTGGAACTGAAATTTACTATAAACTAAAGTATCCCAAAAAAGAATGTTACCAGAAAATCAACAAGTACTT [19500]

cp32-9_gi|11497325|B31_GB      TAAAGAACGAAAAAACTCTAGATTTAAATCTAGAGTTAATAACCATTTTAAAGACAATGTTTCTAAAAATAGTAGTGT [19578]
cp32-9_un7_B31_PacBio          TAAAGAACGAAAAAACTCTAGATTTAAATCTAGAGTTAATAACCATTTTAAAGACAATGTTTCTAAAAATAGTAGTGT [19578]
cp32-9_B31_TS_MP_FG_consensus  TAAAGAACGAAAAAACTCTAGATTTAAATCTAGAGTTAATAACCATTTTAAAGACAATGTTTCTAAAAATAGTAGTGT [19578]
cp32-9_B31_NX_Pl_consensus     TAAAGAACGAAAAAACTCTAGATTTAAATCTAGAGTTAATAACCATTTTAAAGACAATGTTTCTAAAAATAGTAGTGT [19578]
cp32-9_PAli_NX_MP_FG_consensus TAAAGAACGAAAAAACTCTAGATTTAAATCTAGAGTTAATAACCATTTTAAAGACAATGTTTCTAAAAATAGTAGTGT [19578]
cp32-9_PAli_NX_Pl_consensus    TAAAGAACGAAAAAACTCTAGATTTAAATCTAGAGTTAATAACCATTTTAAAGACAATGTTTCTAAAAATAGTAGTGT [19578]
cp32-9_PAbe_NX_MP_FG_consensus TAAAGAACGAAAAAACTCTAGATTTAAATCTAGAGTTAATAACCATTTTAAAGACAATGTTTCTAAAAATAGTAGTGT [19578]
cp32-9_PAbe_NX_Pl_consensus    TAAAGAACGAAAAAACTCTAGATTTAAATCTAGAGTTAATAACCATTTTAAAGACAATGTTTCTAAAAATAGTAGTGT [19578]

cp32-9_gi|11497325|B31_GB      AAATTCAGTGGAGTGTTTAAATAATAAAAATAATAATACAAAAGAAGAAAGAAAGATTAAAGAAATAGAAAAATATCA [19656]
cp32-9_un7_B31_PacBio          AAATTCAGTGGAGTGTTTAAATAATAAAAATAATAATACAAAAGAAGAAAGAAAGATTAAAGAAATAGAAAAATATCA [19656]
cp32-9_B31_TS_MP_FG_consensus  AAATTCAGTGGAGTGTTTAAATAATAAAAATAATAATACAAAAGAAGAAAGAAAGATTAAAGAAATAGAAAAATATCA [19656]
cp32-9_B31_NX_Pl_consensus     AAATTCAGTGGAGTGTTTAAATAATAAAAATAATAATACAAAAGAAGAAAGAAAGATTAAAGAAATAGAAAAATATCA [19656]
cp32-9_PAli_NX_MP_FG_consensus AAATTCAGTGGAGTGTTTAAATAATAAAAATAATAATACAAAAGAAGAAAGAAAGATTAAAGAAATAGAAAAATATCA [19656]
cp32-9_PAli_NX_Pl_consensus    AAATTCAGTGGAGTGTTTAAATAATAAAAATAATAATACAAAAGAAGAAAGAAAGATTAAAGAAATAGAAAAATATCA [19656]
cp32-9_PAbe_NX_MP_FG_consensus AAATTCAGTGGAGTGTTTAAATAATAAAAATAATAATACAAAAGAAGAAAGAAAGATTAAAGAAATAGAAAAATATCA [19656]
cp32-9_PAbe_NX_Pl_consensus    AAATTCAGTGGAGTGTTTAAATAATAAAAATAATAATACAAAAGAAGAAAGAAAGATTAAAGAAATAGAAAAATATCA [19656]

cp32-9_gi|11497325|B31_GB      ACTAAGAAATTATTTCAATAATTGTAACTTTAAAACGGAAGAAGCTCTTTCTATTTTGTATTTAAATATTGATAAAGA [19734]
cp32-9_un7_B31_PacBio          ACTAAGAAATTATTTCAATAATTGTAACTTTAAAACGGAAGAAGCTCTTTCTATTTTGTATTTAAATATTGATAAAGA [19734]
cp32-9_B31_TS_MP_FG_consensus  ACTAAGAAATTATTTCAATAATTGTAACTTTAAAACGGAAGAAGCTCTTTCTATTTTGTATTTAAATATTGATAAAGA [19734]
cp32-9_B31_NX_Pl_consensus     ACTAAGAAATTATTTCAATAATTGTAACTTTAAAACGGAAGAAGCTCTTTCTATTTTGTATTTAAATATTGATAAAGA [19734]
cp32-9_PAli_NX_MP_FG_consensus ACTAAGAAATTATTTCAATAATTGTAACTTTAAAACGGAAGAAGCTCTTTCTATTTTGTATTTAAATATTGATAAAGA [19734]
cp32-9_PAli_NX_Pl_consensus    ACTAAGAAATTATTTCAATAATTGTAACTTTAAAACGGAAGAAGCTCTTTCTATTTTGTATTTAAATATTGATAAAGA [19734]
cp32-9_PAbe_NX_MP_FG_consensus ACTAAGAAATTATTTCAATAATTGTAACTTTAAAACGGAAGAAGCTCTTTCTATTTTGTATTTAAATATTGATAAAGA [19734]
cp32-9_PAbe_NX_Pl_consensus    ACTAAGAAATTATTTCAATAATTGTAACTTTAAAACGGAAGAAGCTCTTTCTATTTTGTATTTAAATATTGATAAAGA [19734]

cp32-9_gi|11497325|B31_GB      TACTAAGATTGAGGCAATAAATATCTTAAAACAAAATGAAATTGCCCTAATAAAAAAATTCAATATAAAAAAATCTTG [19812]
cp32-9_un7_B31_PacBio          TACTAAGATTGAGGCAATAAATATCTTAAAACAAAATGAAATTGCCCTAATAAAAAAATTCAATATAAAAAAATCTTG [19812]
cp32-9_B31_TS_MP_FG_consensus  TACTAAGATTGAGGCAATAAATATCTTAAAACAAAATGAAATTGCCCTAATAAAAAAATTCAATATAAAAAAATCTTG [19812]
cp32-9_B31_NX_Pl_consensus     TACTAAGATTGAGGCAATAAATATCTTAAAACAAAATGAAATTGCCCTAATAAAAAAATTCAATATAAAAAAATCTTG [19812]
```

```
cp32-9_PAli_NX_MP_FG_consensus TACTAAGATTGAGGCAATAAATATCTTAAAACAAAATGAAATTGCCCTAATAAAAAAATTCAATATAAAAAAATCTTG [19812]
cp32-9_PAli_NX_Pl_consensus    TACTAAGATTGAGGCAATAAATATCTTAAAACAAAATGAAATTGCCCTAATAAAAAAATTCAATATAAAAAAATCTTG [19812]
cp32-9_PAbe_NX_MP_FG_consensus TACTAAGATTGAGGCAATAAATATCTTAAAACAAAATGAAATTGCCCTAATAAAAAAATTCAATATAAAAAAATCTTG [19812]
cp32-9_PAbe_NX_Pl_consensus    TACTAAGATTGAGGCAATAAATATCTTAAAACAAAATGAAATTGCCCTAATAAAAAAATTCAATATAAAAAAATCTTG [19812]

cp32-9_gi|11497325|B31_GB      CATGAAAGAAAAACAAAAAAAATTAAAAGAAATTCTACACAACACTAGGAAAAAATTAGAAGAAAACGGGTACAATCC [19890]
cp32-9_un7_B31_PacBio          CATGAAAGAAAAACAAAAAAAATTAAAAGAAATTCTACACAACACTAGGAAAAAATTAGAAGAAAACGGGTACAATCC [19890]
cp32-9_B31_TS_MP_FG_consensus  CATGAAAGAAAAACAAAAAAAATTAAAAGAAATTCTACACAACACTAGGAAAAAATTAGAAGAAAACGGGTACAATCC [19890]
cp32-9_B31_NX_Pl_consensus     CATGAAAGAAAAACAAAAAAAATTAAAAGAAATTCTACACAACACTAGGAAAAAATTAGAAGAAAACGGGTACAATCC [19890]
cp32-9_PAli_NX_MP_FG_consensus CATGAAAGAAAAACAAAAAAAATTAAAAGAAATTCTACACAACACTAGGAAAAAATTAGAAGAAAACGGGTACAATCC [19890]
cp32-9_PAli_NX_Pl_consensus    CATGAAAGAAAAACAAAAAAAATTAAAAGAAATTCTACACAACACTAGGAAAAAATTAGAAGAAAACGGGTACAATCC [19890]
cp32-9_PAbe_NX_MP_FG_consensus CATGAAAGAAAAACAAAAAAAATTAAAAGAAATTCTACACAACACTAGGAAAAAATTAGAAGAAAACGGGTACAATCC [19890]
cp32-9_PAbe_NX_Pl_consensus    CATGAAAGAAAAACAAAAAAAATTAAAAGAAATTCTACACAACACTAGGAAAAAATTAGAAGAAAACGGGTACAATCC [19890]

cp32-9_gi|11497325|B31_GB      CAAACAATTAGAAATAAATTTACAAAAAGTATACGAAAATTACAAATATAAGCCCCACTTTATTATTGAAAATCATAA [19968]
cp32-9_un7_B31_PacBio          CAAACAATTAGAAATAAATTTACAAAAAGTATACGAAAATTACAAATATAAGCCCCACTTTATTATTGAAAATCATAA [19968]
cp32-9_B31_TS_MP_FG_consensus  CAAACAATTAGAAATAAATTTACAAAAAGTATACGAAAATTACAAATATAAGCCCCACTTTATTATTGAAAATCATAA [19968]
cp32-9_B31_NX_Pl_consensus     CAAACAATTAGAAATAAATTTACAAAAAGTATACGAAAATTACAAATATAAGCCCCACTTTATTATTGAAAATCATAA [19968]
cp32-9_PAli_NX_MP_FG_consensus CAAACAATTAGAAATAAATTTACAAAAAGTATACGAAAATTACAAATATAAGCCCCACTTTATTATTGAAAATCATAA [19968]
cp32-9_PAli_NX_Pl_consensus    CAAACAATTAGAAATAAATTTACAAAAAGTATACGAAAATTACAAATATAAGCCCCACTTTATTATTGAAAATCATAA [19968]
cp32-9_PAbe_NX_MP_FG_consensus CAAACAATTAGAAATAAATTTACAAAAAGTATACGAAAATTACAAATATAAGCCCCACTTTATTATTGAAAATCATAA [19968]
cp32-9_PAbe_NX_Pl_consensus    CAAACAATTAGAAATAAATTTACAAAAAGTATACGAAAATTACAAATATAAGCCCCACTTTATTATTGAAAATCATAA [19968]

cp32-9_gi|11497325|B31_GB      ATATAGCGATTTAAACAATATAAAGCGTAAATTAGAAAAGTCGATTGAAAGAAAAAAAGAAAATTCTCAACAAAATTA [20046]
cp32-9_un7_B31_PacBio          ATATAGCGATTTAAACAATATAAAGCGTAAATTAGAAAAGTCGATTGAAAGAAAAAAAGAAAATTCTCAACAAAATTA [20046]
cp32-9_B31_TS_MP_FG_consensus  ATATAGCGATTTAAACAATATAAAGCGTAAATTAGAAAAGTCGATTGAAAGAAAAAAAGAAAATTCTCAACAAAATTA [20046]
cp32-9_B31_NX_Pl_consensus     ATATAGCGATTTAAACAATATAAAGCGTAAATTAGAAAAGTCGATTGAAAGAAAAAAAGAAAATTCTCAACAAAATTA [20046]
cp32-9_PAli_NX_MP_FG_consensus ATATAGCGATTTAAACAATATAAAGCGTAAATTAGAAAAGTCGATTGAAAGAAAAAAAGAAAATTCTCAACAAAATTA [20046]
cp32-9_PAli_NX_Pl_consensus    ATATAGCGATTTAAACAATATAAAGCGTAAATTAGAAAAGTCGATTGAAAGAAAAAAAGAAAATTCTCAACAAAATTA [20046]
cp32-9_PAbe_NX_MP_FG_consensus ATATAGCGATTTAAACAATATAAAGCGTAAATTAGAAAAGTCGATTGAAAGAAAAAAAGAAAATTCTCAACAAAATTA [20046]
cp32-9_PAbe_NX_Pl_consensus    ATATAGCGATTTAAACAATATAAAGCGTAAATTAGAAAAGTCGATTGAAAGAAAAAAAGAAAATTCTCAACAAAATTA [20046]

cp32-9_gi|11497325|B31_GB      TCAAAATTTAAAGGAAAATATTTTCAATATCCTTATTGAACAACTAAAAAAAGAAACAAATATTGAAATTCTAAAGCC [20124]
cp32-9_un7_B31_PacBio          TCAAAATTTAAAGGAAAATATTTTCAATATCCTTATTGAACAACTAAAAAAAGAAACAAATATTGAAATTCTAAAGCC [20124]
cp32-9_B31_TS_MP_FG_consensus  TCAAAATTTAAAGGAAAATATTTTCAATATCCTTATTGAACAACTAAAAAAAGAAACAAATATTGAAATTCTAAAGCC [20124]
cp32-9_B31_NX_Pl_consensus     TCAAAATTTAAAGGAAAATATTTTCAATATCCTTATTGAACAACTAAAAAAAGAAACAAATATTGAAATTCTAAAGCC [20124]
cp32-9_PAli_NX_MP_FG_consensus TCAAAATTTAAAGGAAAATATTTTCAATATCCTTATTGAACAACTAAAAAAAGAAACAAATATTGAAATTCTAAAGCC [20124]
cp32-9_PAli_NX_Pl_consensus    TCAAAATTTAAAGGAAAATATTTTCAATATCCTTATTGAACAACTAAAAAAAGAAACAAATATTGAAATTCTAAAGCC [20124]
cp32-9_PAbe_NX_MP_FG_consensus TCAAAATTTAAAGGAAAATATTTTCAATATCCTTATTGAACAACTAAAAAAAGAAACAAATATTGAAATTCTAAAGCC [20124]
cp32-9_PAbe_NX_Pl_consensus    TCAAAATTTAAAGGAAAATATTTTCAATATCCTTATTGAACAACTAAAAAAAGAAACAAATATTGAAATTCTAAAGCC [20124]

cp32-9_gi|11497325|B31_GB      AATTACCAAAAAATATTTGAATAACCAAAAGAAAATAGAATACAATAAAGTATTTGGTACATATTATCTTGAATTATT [20202]
cp32-9_un7_B31_PacBio          AATTACCAAAAAATATTTGAATAACCAAAAGAAAATAGAATACAATAAAGTATTTGGTACATATTATCTTGAATTATT [20202]
cp32-9_B31_TS_MP_FG_consensus  AATTACCAAAAAATATTTGAATAACCAAAAGAAAATAGAATACAATAAAGTATTTGGTACATATTATCTTGAATTATT [20202]
cp32-9_B31_NX_Pl_consensus     AATTACCAAAAAATATTTGAATAACCAAAAGAAAATAGAATACAATAAAGTATTTGGTACATATTATCTTGAATTATT [20202]
cp32-9_PAli_NX_MP_FG_consensus AATTACCAAAAAATATTTGAATAACCAAAAGAAAATAGAATACAATAAAGTATTTGGTACATATTATCTTGAATTATT [20202]
cp32-9_PAli_NX_Pl_consensus    AATTACCAAAAAATATTTGAATAACCAAAAGAAAATAGAATACAATAAAGTATTTGGTACATATTATCTTGAATTATT [20202]
cp32-9_PAbe_NX_MP_FG_consensus AATTACCAAAAAATATTTGAATAACCAAAAGAAAATAGAATACAATAAAGTATTTGGTACATATTATCTTGAATTATT [20202]
cp32-9_PAbe_NX_Pl_consensus    AATTACCAAAAAATATTTGAATAACCAAAAGAAAATAGAATACAATAAAGTATTTGGTACATATTATCTTGAATTATT [20202]

cp32-9_gi|11497325|B31_GB      GGAAATAATAAGAAAACAAAAAAATTCTTTAACCACAGAAGAATTTAACATAAAGGCTATATGAGGATTTAAATATGG [20280]
cp32-9_un7_B31_PacBio          GGAAATAATAAGAAAACAAAAAAATTCTTTAACCACAGAAGAATTTAACATAAAGGCTATATGAGGATTTAAATATGG [20280]
cp32-9_B31_TS_MP_FG_consensus  GGAAATAATAAGAAAACAAAAAAATTCTTTAACCACAGAAGAATTTAACATAAAGGCTATATGAGGATTTAAATATGG [20280]
cp32-9_B31_NX_Pl_consensus     GGAAATAATAAGAAAACAAAAAAATTCTTTAACCACAGAAGAATTTAACATAAAGGCTATATGAGGATTTAAATATGG [20280]
cp32-9_PAli_NX_MP_FG_consensus GGAAATAATAAGAAAACAAAAAAATTCTTTAACCACAGAAGAATTTAACATAAAGGCTATATGAGGATTTAAATATGG [20280]
cp32-9_PAli_NX_Pl_consensus    GGAAATAATAAGAAAACAAAAAAATTCTTTAACCACAGAAGAATTTAACATAAAGGCTATATGAGGATTTAAATATGG [20280]
cp32-9_PAbe_NX_MP_FG_consensus GGAAATAATAAGAAAACAAAAAAATTCTTTAACCACAGAAGAATTTAACATAAAGGCTATATGAGGATTTAAATATGG [20280]
cp32-9_PAbe_NX_Pl_consensus    GGAAATAATAAGAAAACAAAAAAATTCTTTAACCACAGAAGAATTTAACATAAAGGCTATATGAGGATTTAAATATGG [20280]

cp32-9_gi|11497325|B31_GB      AAAGTGCAATGGAGCCTATTGAAACTATAAAAAAGGGCAAATGTAAAGTTGAATGTCAAAATAAAGAACGCTTTATTT [20358]
cp32-9_un7_B31_PacBio          AAAGTGCAATGGAGCCTATTGAAACTATAAAAAAGGGCAAATGTAAAGTTGAATGTCAAAATAAAGAACGCTTTATTT [20358]
cp32-9_B31_TS_MP_FG_consensus  AAAGTGCAATGGAGCCTATTGAAACTATAAAAAAGGGCAAATGTAAAGTTGAATGTCAAAATAAAGAACGCTTTATTT [20358]
cp32-9_B31_NX_Pl_consensus     AAAGTGCAATGGAGCCTATTGAAACTATAAAAAAGGGCAAATGTAAAGTTGAATGTCAAAATAAAGAACGCTTTATTT [20358]
cp32-9_PAli_NX_MP_FG_consensus AAAGTGCAATGGAGCCTATTGAAACTATAAAAAAGGGCAAATGTAAAGTTGAATGTCAAAATAAAGAACGCTTTATTT [20358]
cp32-9_PAli_NX_Pl_consensus    AAAGTGCAATGGAGCCTATTGAAACTATAAAAAAGGGCAAATGTAAAGTTGAATGTCAAAATAAAGAACGCTTTATTT [20358]
cp32-9_PAbe_NX_MP_FG_consensus AAAGTGCAATGGAGCCTATTGAAACTATAAAAAAGGGCAAATGTAAAGTTGAATGTCAAAATAAAGAACGCTTTATTT [20358]
cp32-9_PAbe_NX_Pl_consensus    AAAGTGCAATGGAGCCTATTGAAACTATAAAAAAGGGCAAATGTAAAGTTGAATGTCAAAATAAAGAACGCTTTATTT [20358]

cp32-9_gi|11497325|B31_GB      TAATTGAAAAAGAAAATGGTAAAGCAATGTACCATACAAAAATAATGATGGACATTTACAAATTTGGAGTATATGAAA [20436]
cp32-9_un7_B31_PacBio          TAATTGAAAAAGAAAATGGTAAAGCAATGTACCATACAAAAATAATGATGGACATTTACAAATTTGGAGTATATGAAA [20436]
cp32-9_B31_TS_MP_FG_consensus  TAATTGAAAAAGAAAATGGTAAAGCAATGTACCATACAAAAATAATGATGGACATTTACAAATTTGGAGTATATGAAA [20436]
cp32-9_B31_NX_Pl_consensus     TAATTGAAAAAGAAAATGGTAAAGCAATGTACCATACAAAAATAATGATGGACATTTACAAATTTGGAGTATATGAAA [20436]
cp32-9_PAli_NX_MP_FG_consensus TAATTGAAAAAGAAAATGGTAAAGCAATGTACCATACAAAAATAATGATGGACATTTACAAATTTGGAGTATATGAAA [20436]
cp32-9_PAli_NX_Pl_consensus    TAATTGAAAAAGAAAATGGTAAAGCAATGTACCATACAAAAATAATGATGGACATTTACAAATTTGGAGTATATGAAA [20436]
cp32-9_PAbe_NX_MP_FG_consensus TAATTGAAAAAGAAAATGGTAAAGCAATGTACCATACAAAAATAATGATGGACATTTACAAATTTGGAGTATATGAAA [20436]
cp32-9_PAbe_NX_Pl_consensus    TAATTGAAAAAGAAAATGGTAAAGCAATGTACCATACAAAAATAATGATGGACATTTACAAATTTGGAGTATATGAAA [20436]

cp32-9_gi|11497325|B31_GB      AAAAACATGAATTTAGACTATCATTAAGGGCCCTATTTAATGGGGAAAGAATTGTTGAAGAAACTCACTTATACCCAA [20514]
cp32-9_un7_B31_PacBio          AAAAACATGAATTTAGACTATCATTAAGGGCCCTATTTAATGGGGAAAGAATTGTTGAAGAAACTCACTTATACCCAA [20514]
cp32-9_B31_TS_MP_FG_consensus  AAAAACATGAATTTAGACTATCATTAAGGGCCCTATTTAATGGGGAAAGAATTGTTGAAGAAACTCACTTATACCCAA [20514]
cp32-9_B31_NX_Pl_consensus     AAAAACATGAATTTAGACTATCATTAAGGGCCCTATTTAATGGGGAAAGAATTGTTGAAGAAACTCACTTATACCCAA [20514]
cp32-9_PAli_NX_MP_FG_consensus AAAAACATGAATTTAGACTATCATTAAGGGCCCTATTTAATGGGGAAAGAATTGTTGAAGAAACTCACTTATACCCAA [20514]
cp32-9_PAli_NX_Pl_consensus    AAAAACATGAATTTAGACTATCATTAAGGGCCCTATTTAATGGGGAAAGAATTGTTGAAGAAACTCACTTATACCCAA [20514]
cp32-9_PAbe_NX_MP_FG_consensus AAAAACATGAATTTAGACTATCATTAAGGGCCCTATTTAATGGGGAAAGAATTGTTGAAGAAACTCACTTATACCCAA [20514]
cp32-9_PAbe_NX_Pl_consensus    AAAAACATGAATTTAGACTATCATTAAGGGCCCTATTTAATGGGGAAAGAATTGTTGAAGAAACTCACTTATACCCAA [20514]

cp32-9_gi|11497325|B31_GB      TTAAAGAAGGAGATAAGTTTATTGGTATTTTTTATGGCTATAGAAAACCAATCAAAAAACCTTTAATAAAGTATAAAA [20592]
cp32-9_un7_B31_PacBio          TTAAAGAAGGAGATAAGTTTATTGGTATTTTTTATGGCTATAGAAAACCAATCAAAAAACCTTTAATAAAGTATAAAA [20592]
cp32-9_B31_TS_MP_FG_consensus  TTAAAGAAGGAGATAAGTTTATTGGTATTTTTTATGGCTATAGAAAACCAATCAAAAAACCTTTAATAAAGTATAAAA [20592]
cp32-9_B31_NX_Pl_consensus     TTAAAGAAGGAGATAAGTTTATTGGTATTTTTTATGGCTATAGAAAACCAATCAAAAAACCTTTAATAAAGTATAAAA [20592]
cp32-9_PAli_NX_MP_FG_consensus TTAAAGAAGGAGATAAGTTTATTGGTATTTTTTATGGCTATAGAAAACCAATCAAAAAACCTTTAATAAAGTATAAAA [20592]
cp32-9_PAli_NX_Pl_consensus    TTAAAGAAGGAGATAAGTTTATTGGTATTTTTTATGGCTATAGAAAACCAATCAAAAAACCTTTAATAAAGTATAAAA [20592]
cp32-9_PAbe_NX_MP_FG_consensus TTAAAGAAGGAGATAAGTTTATTGGTATTTTTTATGGCTATAGAAAACCAATCAAAAAACCTTTAATAAAGTATAAAA [20592]
cp32-9_PAbe_NX_Pl_consensus    TTAAAGAAGGAGATAAGTTTATTGGTATTTTTTATGGCTATAGAAAACCAATCAAAAAACCTTTAATAAAGTATAAAA [20592]

cp32-9_gi|11497325|B31_GB      TAAATGGAACTAGAAAAGCATATGCATTAGCAAGAGCATATTATATGGAATTTAGATTTAAAGCCGGAAGTGTTTTTT [20670]
cp32-9_un7_B31_PacBio          TAAATGGAACTAGAAAAGCATATGCATTAGCAAGAGCATATTATATGGAATTTAGATTTAAAGCCGGAAGTGTTTTTT [20670]
cp32-9_B31_TS_MP_FG_consensus  TAAATGGAACTAGAAAAGCATATGCATTAGCAAGAGCATATTATATGGAATTTAGATTTAAAGCCGGAAGTGTTTTTT [20670]
cp32-9_B31_NX_Pl_consensus     TAAATGGAACTAGAAAAGCATATGCATTAGCAAGAGCATATTATATGGAATTTAGATTTAAAGCCGGAAGTGTTTTTT [20670]
cp32-9_PAli_NX_MP_FG_consensus TAAATGGAACTAGAAAAGCATATGCATTAGCAAGAGCATATTATATGGAATTTAGATTTAAAGCCGGAAGTGTTTTTT [20670]
cp32-9_PAli_NX_Pl_consensus    TAAATGGAACTAGAAAAGCATATGCATTAGCAAGAGCATATTATATGGAATTTAGATTTAAAGCCGGAAGTGTTTTTT [20670]
cp32-9_PAbe_NX_MP_FG_consensus TAAATGGAACTAGAAAAGCATATGCATTAGCAAGAGCATATTATATGGAATTTAGATTTAAAGCCGGAAGTGTTTTTT [20670]
cp32-9_PAbe_NX_Pl_consensus    TAAATGGAACTAGAAAAGCATATGCATTAGCAAGAGCATATTATATGGAATTTAGATTTAAAGCCGGAAGTGTTTTTT [20670]

cp32-9_gi|11497325|B31_GB      GCTATTTTAAGGGGCTATATCGTTTATTGGATAAAAAAAGAACAAATAATCATTACAACAAAGTTTTATTTAGTATGT [20748]
cp32-9_un7_B31_PacBio          GCTATTTTAAGGGGCTATATCGTTTATTGGATAAAAAAAGAACAAATAATCATTACAATAAAGTTTTATTTAGTATGT [20748]
cp32-9_B31_TS_MP_FG_consensus  GCTATTTTAAGGGGCTATATCGTTTATTGGATAAAAAAAGAACAAATAATCATTACAATAAAGTTTTATTTAGTATGT [20748]
cp32-9_B31_NX_Pl_consensus     GCTATTTTAAGGGGCTATATCGTTTATTGGATAAAAAAAGAACAAATAATCATTACAATAAAGTTTTATTTAGTATGT [20748]
cp32-9_PAli_NX_MP_FG_consensus GCTATTTTAAGGGGCTATATCGTTTATTGGATAAAAAAAGAACAAATAATCATTACAATAAAGTTTTATTTAGTATGT [20748]
```

```
cp32-9_PAli_NX_Pl_consensus    GCTATTTTAAGGGGCTATATCGTTTATTGGATAAAAAAAGAACAAATAATCATTACAATAAAGTTTTATTTAGTATGT [20748]
cp32-9_PAbe_NX_MP_FG_consensus GCTATTTTAAGGGGCTATATCGTTTATTGGATAAAAAAAGAACAAATAATCATTACAATAAAGTTTTATTTAGTATGT [20748]
cp32-9_PAbe_NX_Pl_consensus    GCTATTTTAAGGGGCTATATCGTTTATTGGATAAAAAAAGAACAAATAATCATTACAATAAAGTTTTATTTAGTATGT [20748]

cp32-9_gi|11497325|B31_GB      TTGCAGATTTAGAACAACAAGTATATAAATTTTATGGGAAAAAAATACCCGGAGCAAGGACCGTTAATAAAATGGATAA [20826]
cp32-9_un7_B31_PacBio          TTGCAGATTTAGAACAACAAGTATATAAATTTTATGGGAAAAAAATACCCGGAGCAAGGACCGTTAATAAAATGGATAA [20826]
cp32-9_B31_TS_MP_FG_consensus  TTGCAGATTTAGAACAACAAGTATATAAATTTTATGGGAAAAAAATACCCGGAGCAAGGACCGTTAATAAAATGGATAA [20826]
cp32-9_B31_NX_Pl_consensus     TTGCAGATTTAGAACAACAAGTATATAAATTTTATGGGAAAAAAATACCCGGAGCAAGGACCGTTAATAAAATGGATAA [20826]
cp32-9_PAli_NX_MP_FG_consensus TTGCAGATTTAGAACAACAAGTATATAAATTTTATGGGAAAAAAATACCCGGAGCAAGGACCGTTAATAAAATGGATAA [20826]
cp32-9_PAli_NX_Pl_consensus    TTGCAGATTTAGAACAACAAGTATATAAATTTTATGGGAAAAAAATACCCGGAGCAAGGACCGTTAATAAAATGGATAA [20826]
cp32-9_PAbe_NX_MP_FG_consensus TTGCAGATTTAGAACAACAAGTATATAAATTTTATGGGAAAAAAATACCCGGAGCAAGGACCGTTAATAAAATGGATAA [20826]
cp32-9_PAbe_NX_Pl_consensus    TTGCAGATTTAGAACAACAAGTATATAAATTTTATGGGAAAAAAATACCCGGAGCAAGGACCGTTAATAAAATGGATAA [20826]

cp32-9_gi|11497325|B31_GB      TAAAAAACCTAAAATAATAACAATAGCGTCAATCAAGGGCGGTGTTGGTAAAAGCACAAGTGCCTTATTTTATGGCAA [20904]
cp32-9_un7_B31_PacBio          TAAAAAACCTAAAATAATAACAATAGCGTCAATCAAGGGCGGTGTTGGTAAAAGCACAAGTGCCTTATTTTATGGCAA [20904]
cp32-9_B31_TS_MP_FG_consensus  TAAAAAACCTAAAATAATAACAATAGCGTCAATCAAGGGCGGTGTTGGTAAAAGCACAAGTGCCTTATTTTATGGCAA [20904]
cp32-9_B31_NX_Pl_consensus     TAAAAAACCTAAAATAATAACAATAGCGTCAATCAAGGGCGGTGTTGGTAAAAGCACAAGTGCCTTATTTTATGGCAA [20904]
cp32-9_PAli_NX_MP_FG_consensus TAAAAAACCTAAAATAATAACAATAGCGTCAATCAAGGGCGGTGTTGGTAAAAGCACAAGTGCCTTATTTTATGGCAA [20904]
cp32-9_PAli_NX_Pl_consensus    TAAAAAACCTAAAATAATAACAATAGCGTCAATCAAGGGCGGTGTTGGTAAAAGCACAAGTGCCTTATTTTATGGCAA [20904]
cp32-9_PAbe_NX_MP_FG_consensus TAAAAAACCTAAAATAATAACAATAGCGTCAATCAAGGGCGGTGTTGGTAAAAGCACAAGTGCCTTATTTTATGGCAA [20904]
cp32-9_PAbe_NX_Pl_consensus    TAAAAAACCTAAAATAATAACAATAGCGTCAATCAAGGGCGGTGTTGGTAAAAGCACAAGTGCCTTATTTTATGGCAA [20904]

cp32-9_gi|11497325|B31_GB      TATTTTAGCTAAAGAAAGACATAAAGTATTGATAATTGATAGTGATCCACAGGCCAGTATTACTAGTTACTTTTTGTT [20982]
cp32-9_un7_B31_PacBio          TATTTTAGCTAAAGAAAGACATAAAGTATTGATAATTGATAGTGATCCACAGGCCAGTATTACTAGTTACTTTTTGTT [20982]
cp32-9_B31_TS_MP_FG_consensus  TATTTTAGCTAAAGAAAGACATAAAGTATTGATAATTGATAGTGATCCACAGGCCAGTATTACTAGTTACTTTTTGTT [20982]
cp32-9_B31_NX_Pl_consensus     TATTTTAGCTAAAGAAAGACATAAAGTATTGATAATTGATAGTGATCCACAGGCCAGTATTACTAGTTACTTTTTGTT [20982]
cp32-9_PAli_NX_MP_FG_consensus TATTTTAGCTAAAGAAAGACATAAAGTATTGATAATTGATAGTGATCCACAGGCCAGTATTACTAGTTACTTTTTGTT [20982]
cp32-9_PAli_NX_Pl_consensus    TATTTTAGCTAAAGAAAGACATAAAGTATTGATAATTGATAGTGATCCACAGGCCAGTATTACTAGTTACTTTTTGTT [20982]
cp32-9_PAbe_NX_MP_FG_consensus TATTTTAGCTAAAGAAAGACATAAAGTATTGATAATTGATAGTGATCCACAGGCCAGTATTACTAGTTACTTTTTGTT [20982]
cp32-9_PAbe_NX_Pl_consensus    TATTTTAGCTAAAGAAAGACATAAAGTATTGATAATTGATAGTGATCCACAGGCCAGTATTACTAGTTACTTTTTGTT [20982]

cp32-9_gi|11497325|B31_GB      TAAATTAAAAGAACAAAATGTGAATGTCGAAAATTACAATCTTTATGAAGTTTTTAAACAAAGAAAATATATAGAAAA [21060]
cp32-9_un7_B31_PacBio          TAAATTAAAAGAACAAAATGTGAATGTCGAAAATTACAATCTTTATGAAGTTTTTAAACAAAGAAAATATATAGAAAA [21060]
cp32-9_B31_TS_MP_FG_consensus  TAAATTAAAAGAACAAAATGTGAATGTCGAAAATTACAATCTTTATGAAGTTTTTAAACAAAGAAAATATATAGAAAA [21060]
cp32-9_B31_NX_Pl_consensus     TAAATTAAAAGAACAAAATGTGAATGTCGAAAATTACAATCTTTATGAAGTTTTTAAACAAAGAAAATATATAGAAAA [21060]
cp32-9_PAli_NX_MP_FG_consensus TAAATTAAAAGAACAAAATGTGAATGTCGAAAATTACAATCTTTATGAAGTTTTTAAACAAAGAAAATATATAGAAAA [21060]
cp32-9_PAli_NX_Pl_consensus    TAAATTAAAAGAACAAAATGTGAATGTCGAAAATTACAATCTTTATGAAGTTTTTAAACAAAGAAAATATATAGAAAA [21060]
cp32-9_PAbe_NX_MP_FG_consensus TAAATTAAAAGAACAAAATGTGAATGTCGAAAATTACAATCTTTATGAAGTTTTTAAACAAAGAAAATATATAGAAAA [21060]
cp32-9_PAbe_NX_Pl_consensus    TAAATTAAAAGAACAAAATGTGAATGTCGAAAATTACAATCTTTATGAAGTTTTTAAACAAAGAAAATATATAGAAAA [21060]

cp32-9_gi|11497325|B31_GB      TTGCATTTTTACAGTATCTAATTGTTTAGATATAATTCCCAGTTCCTTAGAATTATCTGTTTTTAATTCAGAAAGCAT [21138]
cp32-9_un7_B31_PacBio          TTGCATTTTTACAGTATCTAATTGTTTAGATATAATTCCCAGTTCCTTAGAATTATCTGTTTTTAATTCAGAAAGCAT [21138]
cp32-9_B31_TS_MP_FG_consensus  TTGCATTTTTACAGTATCTAATTGTTTAGATATAATTCCCAGTTCCTTAGAATTATCTGTTTTTAATTCAGAAAGCAT [21138]
cp32-9_B31_NX_Pl_consensus     TTGCATTTTTACAGTATCTAATTGTTTAGATATAATTCCCAGTTCCTTAGAATTATCTGTTTTTAATTCAGAAAGCAT [21138]
cp32-9_PAli_NX_MP_FG_consensus TTGCATTTTTACAGTATCTAATTGTTTAGATATAATTCCCAGTTCCTTAGAATTATCTGTTTTTAATTCAGAAAGCAT [21138]
cp32-9_PAli_NX_Pl_consensus    TTGCATTTTTACAGTATCTAATTGTTTAGATATAATTCCCAGTTCCTTAGAATTATCTGTTTTTAATTCAGAAAGCAT [21138]
cp32-9_PAbe_NX_MP_FG_consensus TTGCATTTTTACAGTATCTAATTGTTTAGATATAATTCCCAGTTCCTTAGAATTATCTGTTTTTAATTCAGAAAGCAT [21138]
cp32-9_PAbe_NX_Pl_consensus    TTGCATTTTTACAGTATCTAATTGTTTAGATATAATTCCCAGTTCCTTAGAATTATCTGTTTTTAATTCAGAAAGCAT [21138]

cp32-9_gi|11497325|B31_GB      ACCATTACAAGACAACCTTTTAGAAAAAAGACTTTTGACTATTAAATCTAAATATGATTATGTGATAATCGATACAAA [21216]
cp32-9_un7_B31_PacBio          ACCATTACAAGACAACCTTTTAGAAAAAAGACTTTTGACTATTAAATCTAAATATGATTATGTGATAATCGATACAAA [21216]
cp32-9_B31_TS_MP_FG_consensus  ACCATTACAAGACAACCTTTTAGAAAAAAGACTTTTGACTATTAAATCTAAATATGATTATGTGATAATCGATACAAA [21216]
cp32-9_B31_NX_Pl_consensus     ACCATTACAAGACAACCTTTTAGAAAAAAGACTTTTGACTATTAAATCTAAATATGATTATGTGATAATCGATACAAA [21216]
cp32-9_PAli_NX_MP_FG_consensus ACCATTACAAGACAACCTTTTAGAAAAAAGACTTTTGACTATTAAATCTAAATATGATTATGTGATAATCGATACAAA [21216]
cp32-9_PAli_NX_Pl_consensus    ACCATTACAAGACAACCTTTTAGAAAAAAGACTTTTGACTATTAAATCTAAATATGATTATGTGATAATCGATACAAA [21216]
cp32-9_PAbe_NX_MP_FG_consensus ACCATTACAAGACAACCTTTTAGAAAAAAGACTTTTGACTATTAAATCTAAATATGATTATGTGATAATCGATACAAA [21216]
cp32-9_PAbe_NX_Pl_consensus    ACCATTACAAGACAACCTTTTAGAAAAAAGACTTTTGACTATTAAATCTAAATATGATTATGTGATAATCGATACAAA [21216]

cp32-9_gi|11497325|B31_GB      TCCCAGCTTAGGACATCTTTTAAACAATGCTTTAGTAATTACCAATTATTTAATAATACCAATTAATTCCGATTTATG [21294]
cp32-9_un7_B31_PacBio          TCCCAGCTTAGGACATCTTTTAAACAATGCTTTAGTAATTACCAATTATTTAATAATACCAATTAATTCCGATTTATG [21294]
cp32-9_B31_TS_MP_FG_consensus  TCCCAGCTTAGGACATCTTTTAAACAATGCTTTAGTAATTACCAATTATTTAATAATACCAATTAATTCCGATTTATG [21294]
cp32-9_B31_NX_Pl_consensus     TCCCAGCTTAGGACATCTTTTAAACAATGCTTTAGTAATTACCAATTATTTAATAATACCAATTAATTCCGATTTATG [21294]
cp32-9_PAli_NX_MP_FG_consensus TCCCAGCTTAGGACATCTTTTAAACAATGCTTTAGTAATTACCAATTATTTAATAATACCAATTAATTCCGATTTATG [21294]
cp32-9_PAli_NX_Pl_consensus    TCCCAGCTTAGGACATCTTTTAAACAATGCTTTAGTAATTACCAATTATTTAATAATACCAATTAATTCCGATTTATG [21294]
cp32-9_PAbe_NX_MP_FG_consensus TCCCAGCTTAGGACATCTTTTAAACAATGCTTTAGTAATTACCAATTATTTAATAATACCAATTAATTCCGATTTATG [21294]
cp32-9_PAbe_NX_Pl_consensus    TCCCAGCTTAGGACATCTTTTAAACAATGCTTTAGTAATTACCAATTATTTAATAATACCAATTAATTCCGATTTATG [21294]

cp32-9_gi|11497325|B31_GB      GGCAGTTGAAAGTATAGATCTAATATTAGATGCAATAAATAAAGTTTATAGAAATGATATTACACCTTATTTTTTAGT [21372]
cp32-9_un7_B31_PacBio          GGCAGTTGAAAGTATAGATCTAATATTAGATGCAATAAATAAAGTTTATAGAAATGATATTACACCTTATTTTTTAGT [21372]
cp32-9_B31_TS_MP_FG_consensus  GGCAGTTGAAAGTATAGATCTAATATTAGATGCAATAAATAAAGTTTATAGAAATGATATTACACCTTATTTTTTAGT [21372]
cp32-9_B31_NX_Pl_consensus     GGCAGTTGAAAGTATAGATCTAATATTAGATGCAATAAATAAAGTTTATAGAAATGATATTACACCTTATTTTTTAGT [21372]
cp32-9_PAli_NX_MP_FG_consensus GGCAGTTGAAAGTATAGATCTAATATTAGATGCAATAAATAAAGTTTATAGAAATGATATTACACCTTATTTTTTAGT [21372]
cp32-9_PAli_NX_Pl_consensus    GGCAGTTGAAAGTATAGATCTAATATTAGATGCAATAAATAAAGTTTATAGAAATGATATTACACCTTATTTTTTAGT [21372]
cp32-9_PAbe_NX_MP_FG_consensus GGCAGTTGAAAGTATAGATCTAATATTAGATGCAATAAATAAAGTTTATAGAAATGATATTACACCTTATTTTTTAGT [21372]
cp32-9_PAbe_NX_Pl_consensus    GGCAGTTGAAAGTATAGATCTAATATTAGATGCAATAAATAAAGTTTATAGAAATGATATTACACCTTATTTTTTAGT [21372]

cp32-9_gi|11497325|B31_GB      GACGGGGGCACTAGAGAGACAAAACATAGATAAGGAAATAATATTTAATTTGGAGAATAGATATAAAGAAAATCTAAT [21450]
cp32-9_un7_B31_PacBio          GACGGGGGCACTAGAGAGACAAAACATAGATAAGGAAATAATATTTAATTTGGAGAATAGATATAAAGAAAATCTAAT [21450]
cp32-9_B31_TS_MP_FG_consensus  GACGGGGGCACTAGAGAGACAAAACATAGATAAGGAAATAATATTTAATTTGGAGAATAGATATAAAGAAAATCTAAT [21450]
cp32-9_B31_NX_Pl_consensus     GACGGGGGCACTAGAGAGACAAAACATAGATAAGGAAATAATATTTAATTTGGAGAATAGATATAAAGAAAATCTAAT [21450]
cp32-9_PAli_NX_MP_FG_consensus GACGGGGGCACTAGAGAGACAAAACATAGATAAGGAAATAATATTTAATTTGGAGAATAGATATAAAGAAAATCTAAT [21450]
cp32-9_PAli_NX_Pl_consensus    GACGGGGGCACTAGAGAGACAAAACATAGATAAGGAAATAATATTTAATTTGGAGAATAGATATAAAGAAAATCTAAT [21450]
cp32-9_PAbe_NX_MP_FG_consensus GACGGGGGCACTAGAGAGACAAAACATAGATAAGGAAATAATATTTAATTTGGAGAATAGATATAAAGAAAATCTAAT [21450]
cp32-9_PAbe_NX_Pl_consensus    GACGGGGGCACTAGAGAGACAAAACATAGATAAGGAAATAATATTTAATTTGGAGAATAGATATAAAGAAAATCTAAT [21450]

cp32-9_gi|11497325|B31_GB      AGGAGTTATTCCTAAAAGAGATGATATCAAAAAAGTGCTGTTTTATAGAAAAGAATTTTCTTCAAAAACAGACTATTA [21528]
cp32-9_un7_B31_PacBio          AGGAGTTATTCCTAAAAGAGATGATATCAAAAAAGTGCTGTTTTATAGAAAAGAATTTTCTTCAAAAACAGACTATTA [21528]
cp32-9_B31_TS_MP_FG_consensus  AGGAGTTATTCCTAAAAGAGATGATATCAAAAAAGTGCTGTTTTATAGAAAAGAATTTTCTTCAAAAACAGACTATTA [21528]
cp32-9_B31_NX_Pl_consensus     AGGAGTTATTCCTAAAAGAGATGATATCAAAAAAGTGCTGTTTTATAGAAAAGAATTTTCTTCAAAAACAGACTATTA [21528]
cp32-9_PAli_NX_MP_FG_consensus AGGAGTTATTCCTAAAAGAGATGATATCAAAAAAGTGCTGTTTTATAGAAAAGAATTTTCTTCAAAAACAGACTATTA [21528]
cp32-9_PAli_NX_Pl_consensus    AGGAGTTATTCCTAAAAGAGATGATATCAAAAAAGTGCTGTTTTATAGAAAAGAATTTTCTTCAAAAACAGACTATTA [21528]
cp32-9_PAbe_NX_MP_FG_consensus AGGAGTTATTCCTAAAAGAGATGATATCAAAAAAGTGCTGTTTTATAGAAAAGAATTTTCTTCAAAAACAGACTATTA [21528]
cp32-9_PAbe_NX_Pl_consensus    AGGAGTTATTCCTAAAAGAGATGATATCAAAAAAGTGCTGTTTTATAGAAAAGAATTTTCTTCAAAAACAGACTATTA [21528]

cp32-9_gi|11497325|B31_GB      TCAAGAATATAAAAAATCTTTAGATAAAATGTTAAAAATAAAATAACAAATAAAATATATCCAGTAATGGACAAATAA [21606]
cp32-9_un7_B31_PacBio          TCAAGAATATAAAAAATCTTTAGATAAAATGTTAAAAATAAAATAACAAATAAAATATATCCAGTAATGGACAAATAA [21606]
cp32-9_B31_TS_MP_FG_consensus  TCAAGAATATAAAAAATCTTTAGATAAAATGTTAAAAATAAAATAACAAATAAAATATATCCAGTAATGGACAAATAA [21606]
cp32-9_B31_NX_Pl_consensus     TCAAGAATATAAAAAATCTTTAGATAAAATGTTAAAAATAAAATAACAAATAAAATATATCCAGTAATGGACAAATAA [21606]
cp32-9_PAli_NX_MP_FG_consensus TCAAGAATATAAAAAATCTTTAGATAAAATGTTAAAAATAAAATAACAAATAAAATATATCCAGTAATGGACAAATAA [21606]
cp32-9_PAli_NX_Pl_consensus    TCAAGAATATAAAAAATCTTTAGATAAAATGTTAAAAATAAAATAACAAATAAAATATATCCAGTAATGGACAAATAA [21606]
cp32-9_PAbe_NX_MP_FG_consensus TCAAGAATATAAAAAATCTTTAGATAAAATGTTAAAAATAAAATAACAAATAAAATATATCCAGTAATGGACAAATAA [21606]
cp32-9_PAbe_NX_Pl_consensus    TCAAGAATATAAAAAATCTTTAGATAAAATGTTAAAAATAAAATAACAAATAAAATATATCCAGTAATGGACAAATAA [21606]

cp32-9_gi|11497325|B31_GB      GGAGTTTGCATGAGCATTAAAAATAAAATGATAATAACCAAAAGAATAGATATAAAGGAAAATATGTCTAAAATGGAG [21684]
cp32-9_un7_B31_PacBio          GGAGTTTGCATGAGCATTAAAAATAAAATGATAATAACCAAAAGAATAGATATAAAGGAAAATATGTCTAAAATGGAG [21684]
cp32-9_B31_TS_MP_FG_consensus  GGAGTTTGCATGAGCATTAAAAATAAAATGATAATAACCAAAAGAATAGATATAAAGGAAAATATGTCTAAAATGGAG [21684]
cp32-9_B31_NX_Pl_consensus     GGAGTTTGCATGAGCATTAAAAATAAAATGATAATAACCAAAAGAATAGATATAAAGGAAAATATGTCTAAAATGGAG [21684]
cp32-9_PAli_NX_MP_FG_consensus GGAGTTTGCATGAGCATTAAAAATAAAATGATAATAACCAAAAGAATAGATATAAAGGAAAATATGTCTAAAATGGAG [21684]
cp32-9_PAli_NX_Pl_consensus    GGAGTTTGCATGAGCATTAAAAATAAAATGATAATAACCAAAAGAATAGATATAAAGGAAAATATGTCTAAAATGGAG [21684]
```

```
cp32-9_PAbe_NX_MP_FG_consensus GGAGTTTGCATGAGCATTAAAAATAAAATGATAATAACCAAAAGAATAGATATAAAGGAAAATATGTCTAAAATGGAG [21684]
cp32-9_PAbe_NX_Pl_consensus    GGAGTTTGCATGAGCATTAAAAATAAAATGATAATAACCAAAAGAATAGATATAAAGGAAAATATGTCTAAAATGGAG [21684]

cp32-9_gi|11497325|B31_GB      TCATTAGAAGAAATTCATAAAGAAGAATATTTGAGATTAAAAGACAAATTAAAAACTCTAACAACGGATGATATTTAT [21762]
cp32-9_un7_B31_PacBio          TCATTAGAAGAAATTCATAAAGAAGAATATTTGAGATTAAAAGACAAATTAAAAACTCTAACAACGGATGATATTTAT [21762]
cp32-9_B31_TS_MP_FG_consensus  TCATTAGAAGAAATTCATAAAGAAGAATATTTGAGATTAAAAGACAAATTAAAAACTCTAACAACGGATGATATTTAT [21762]
cp32-9_B31_NX_Pl_consensus     TCATTAGAAGAAATTCATAAAGAAGAATATTTGAGATTAAAAGACAAATTAAAAACTCTAACAACGGATGATATTTAT [21762]
cp32-9_PAli_NX_MP_FG_consensus TCATTAGAAGAAATTCATAAAGAAGAATATTTGAGATTAAAAGACAAATTAAAAACTCTAACAACGGATGATATTTAT [21762]
cp32-9_PAli_NX_Pl_consensus    TCATTAGAAGAAATTCATAAAGAAGAATATTTGAGATTAAAAGACAAATTAAAAACTCTAACAACGGATGATATTTAT [21762]
cp32-9_PAbe_NX_MP_FG_consensus TCATTAGAAGAAATTCATAAAGAAGAATATTTGAGATTAAAAGACAAATTAAAAACTCTAACAACGGATGATATTTAT [21762]
cp32-9_PAbe_NX_Pl_consensus    TCATTAGAAGAAATTCATAAAGAAGAATATTTGAGATTAAAAGACAAATTAAAAACTCTAACAACGGATGATATTTAT [21762]

cp32-9_gi|11497325|B31_GB      AATAAAATAGAAACAGCAAAAATATTAAATGCGATTAATCAAAAAAAACTGTAT-TTTTAGACGGATATAAAAATTTT [21840]
cp32-9_un7_B31_PacBio          AATAAAATAGAAACAGCAAAAATATTAAATGCGATTAATCAAAAAAAACTGTATATTTTAGACGGATATAAAAATTTT [21840]
cp32-9_B31_TS_MP_FG_consensus  AATAAAATAGAAACAGCAAAAATATTAAATGCGATTAATCAAAAAAAACTGTATATTTTAGACGGATATAAAAATTTT [21840]
cp32-9_B31_NX_Pl_consensus     AATAAAATAGAAACAGCAAAAATATTAAATGCGATTAATCAAAAAAAACTGTATATTTTAGACGGATATAAAAATTTT [21840]
cp32-9_PAli_NX_MP_FG_consensus AATAAAATAGAAACAGCAAAAATATTAAATGCGATTAATCAAAAAAAACTGTATATTTTAGACGGATATAAAAATTTT [21840]
cp32-9_PAli_NX_Pl_consensus    AATAAAATAGAAACAGCAAAAATATTAAATGCGATTAATCAAAAAAAACTGTATATTTTAGACGGATATAAAAATTTT [21840]
cp32-9_PAbe_NX_MP_FG_consensus AATAAAATAGAAACAGCAAAAATATTAAATGCGATTAATCAAAAAAAACTGTATATTTTAGACGGATATAAAAATTTT [21840]
cp32-9_PAbe_NX_Pl_consensus    AATAAAATAGAAACAGCAAAAATATTAAATGCGATTAATCAAAAAAAACTGTATATTTTAGACGGATATAAAAATTTT [21840]

cp32-9_gi|11497325|B31_GB      TATAGCTTTTTAGCTGATTTTAAAATCGCTAAATCTCAAGCATATAAATATATAAAAATAGTATCGGGCGTAGAAAAA [21918]
cp32-9_un7_B31_PacBio          TATAGCTTTTTAGCTGATTTTAAAATCGCTAAATCTCAAGCATATAAATATATAAAAATAGTATCGGGCGTAGAAAAA [21918]
cp32-9_B31_TS_MP_FG_consensus  TATAGCTTTTTAGCTGATTTTAAAATCGCTAAATCTCAAGCATATAAATATATAAAAATAGTATCGGGCGTAGAAAAA [21918]
cp32-9_B31_NX_Pl_consensus     TATAGCTTTTTAGCTGATTTTAAAATCGCTAAATCTCAAGCATATAAATATATAAAAATAGTATCGGGCGTAGAAAAA [21918]
cp32-9_PAli_NX_MP_FG_consensus TATAGCTTTTTAGCTGATTTTAAAATCGCTAAATCTCAAGCATATAAATATATAAAAATAGTATCGGGCGTAGAAAAA [21918]
cp32-9_PAli_NX_Pl_consensus    TATAGCTTTTTAGCTGATTTTAAAATCGCTAAATCTCAAGCATATAAATATATAAAAATAGTATCGGGCGTAGAAAAA [21918]
cp32-9_PAbe_NX_MP_FG_consensus TATAGCTTTTTAGCTGATTTTAAAATCGCTAAATCTCAAGCATATAAATATATAAAAATAGTATCGGGCGTAGAAAAA [21918]
cp32-9_PAbe_NX_Pl_consensus    TATAGCTTTTTAGCTGATTTTAAAATCGCTAAATCTCAAGCATATAAATATATAAAAATAGTATCGGGCGTAGAAAAA [21918]

cp32-9_gi|11497325|B31_GB      GGTATTATTGACTATAATTTTATTGCTAATAATGGCATTGAAAAAACAATTAAACAATTGGAAAGTAACAATGTTATT [21996]
cp32-9_un7_B31_PacBio          GGTATTATTGACTATAATTTTATTGCTAATAATGGCATTGAAAAAACAATTAAACAATTGGAAAGTAACAATGTTATT [21996]
cp32-9_B31_TS_MP_FG_consensus  GGTATTATTGACTATAATTTTATTGCTAATAATGGCATTGAAAAAACAATTAAACAATTGGAAAGTAACAATGTTATT [21996]
cp32-9_B31_NX_Pl_consensus     GGTATTATTGACTATAATTTTATTGCTAATAATGGCATTGAAAAAACAATTAAACAATTGGAAAGTAACAATGTTATT [21996]
cp32-9_PAli_NX_MP_FG_consensus GGTATTATTGACTATAATTTTATTGCTAATAATGGCATTGAAAAAACAATTAAACAATTGGAAAGTAACAATGTTATT [21996]
cp32-9_PAli_NX_Pl_consensus    GGTATTATTGACTATAATTTTATTGCTAATAATGGCATTGAAAAAACAATTAAACAATTGGAAAGTAACAATGTTATT [21996]
cp32-9_PAbe_NX_MP_FG_consensus GGTATTATTGACTATAATTTTATTGCTAATAATGGCATTGAAAAAACAATTAAACAATTGGAAAGTAACAATGTTATT [21996]
cp32-9_PAbe_NX_Pl_consensus    GGTATTATTGACTATAATTTTATTGCTAATAATGGCATTGAAAAAACAATTAAACAATTGGAAAGTAACAATGTTATT [21996]

cp32-9_gi|11497325|B31_GB      AAAAAATCTAGGCAAAATCCAATAAAGCCTTTAAGGTTTCAACTTAAAAAGCAAGAAAGTTATGATTTTTATAAAAAA [22074]
cp32-9_un7_B31_PacBio          AAAAAATCTAGGCAAAATCCAATAAAGCCTTTAAGGTTTCAACTTAAAAAGCAAGAAAGTTATGATTTTTATAAAAAA [22074]
cp32-9_B31_TS_MP_FG_consensus  AAAAAATCTAGGCAAAATCCAATAAAGCCTTTAAGGTTTCAACTTAAAAAGCAAGAAAGTTATGATTTTTATAAAAAA [22074]
cp32-9_B31_NX_Pl_consensus     AAAAAATCTAGGCAAAATCCAATAAAGCCTTTAAGGTTTCAACTTAAAAAGCAAGAAAGTTATGATTTTTATAAAAAA [22074]
cp32-9_PAli_NX_MP_FG_consensus AAAAAATCTAGGCAAAATCCAATAAAGCCTTTAAGGTTTCAACTTAAAAAGCAAGAAAGTTATGATTTTTATAAAAAA [22074]
cp32-9_PAli_NX_Pl_consensus    AAAAAATCTAGGCAAAATCCAATAAAGCCTTTAAGGTTTCAACTTAAAAAGCAAGAAAGTTATGATTTTTATAAAAAA [22074]
cp32-9_PAbe_NX_MP_FG_consensus AAAAAATCTAGGCAAAATCCAATAAAGCCTTTAAGGTTTCAACTTAAAAAGCAAGAAAGTTATGATTTTTATAAAAAA [22074]
cp32-9_PAbe_NX_Pl_consensus    AAAAAATCTAGGCAAAATCCAATAAAGCCTTTAAGGTTTCAACTTAAAAAGCAAGAAAGTTATGATTTTTATAAAAAA [22074]

cp32-9_gi|11497325|B31_GB      AATGGGAAGTTTACTGGGTTTTTATTGGAAGAACTTCTTGAAAGTCAAACAGATTTGATTAATAAGCTTTTAAAAAAA [22152]
cp32-9_un7_B31_PacBio          AATGGGAAGTTTACTGGGTTTTTATTGGAAGAACTTCTTGAAAGTCAAACAGATTTGATTAATAAGCTTTTAAAAAAA [22152]
cp32-9_B31_TS_MP_FG_consensus  AATGGGAAGTTTACTGGGTTTTTATTGGAAGAACTTCTTGAAAGTCAAACAGATTTGATTAATAAGCTTTTAAAAAAA [22152]
cp32-9_B31_NX_Pl_consensus     AATGGGAAGTTTACTGGGTTTTTATTGGAAGAACTTCTTGAAAGTCAAACAGATTTGATTAATAAGCTTTTAAAAAAA [22152]
cp32-9_PAli_NX_MP_FG_consensus AATGGGAAGTTTACTGGGTTTTTATTGGAAGAACTTCTTGAAAGTCAAACAGATTTGATTAATAAGCTTTTAAAAAAA [22152]
cp32-9_PAli_NX_Pl_consensus    AATGGGAAGTTTACTGGGTTTTTATTGGAAGAACTTCTTGAAAGTCAAACAGATTTGATTAATAAGCTTTTAAAAAAA [22152]
cp32-9_PAbe_NX_MP_FG_consensus AATGGGAAGTTTACTGGGTTTTTATTGGAAGAACTTCTTGAAAGTCAAACAGATTTGATTAATAAGCTTTTAAAAAAA [22152]
cp32-9_PAbe_NX_Pl_consensus    AATGGGAAGTTTACTGGGTTTTTATTGGAAGAACTTCTTGAAAGTCAAACAGATTTGATTAATAAGCTTTTAAAAAAA [22152]

cp32-9_gi|11497325|B31_GB      TATAAACAATTAAAAGGATATTAAGAGGATTTTATGAGAAATTTGGTGCACAGAACATATGATATAGAAAGCATAAAA [22230]
cp32-9_un7_B31_PacBio          TATAAACAATTAAAAGGATATTAAGAGGATTTTATGAGAAATTTGGTGCACAGAACATATGATATAGAAAGCATAAAA [22230]
cp32-9_B31_TS_MP_FG_consensus  TATAAACAATTAAAAGGATATTAAGAGGATTTTATGAGAAATTTGGTGCACAGAACATATGATATAGAAAGCATAAAA [22230]
cp32-9_B31_NX_Pl_consensus     TATAAACAATTAAAAGGATATTAAGAGGATTTTATGAGAAATTTGGTGCACAGAACATATGATATAGAAAGCATAAAA [22230]
cp32-9_PAli_NX_MP_FG_consensus TATAAACAATTAAAAGGATATTAAGAGGATTTTATGAGAAATTTGGTGCACAGAACATATGATATAGAAAGCATAAAA [22230]
cp32-9_PAli_NX_Pl_consensus    TATAAACAATTAAAAGGATATTAAGAGGATTTTATGAGAAATTTGGTGCACAGAACATATGATATAGAAAGCATAAAA [22230]
cp32-9_PAbe_NX_MP_FG_consensus TATAAACAATTAAAAGGATATTAAGAGGATTTTATGAGAAATTTGGTGCACAGAACATATGATATAGAAAGCATAAAA [22230]
cp32-9_PAbe_NX_Pl_consensus    TATAAACAATTAAAAGGATATTAAGAGGATTTTATGAGAAATTTGGTGCACAGAACATATGATATAGAAAGCATAAAA [22230]

cp32-9_gi|11497325|B31_GB      AATGAATTTTTAAACATAGGATTTAGTGAAGAGGCAATAGATTTTGTTTTTCTTCATAATGATAATTACAACTATGAG [22308]
cp32-9_un7_B31_PacBio          AATGAATTTTTAAACATAGGATTTAGTGAAGAGGCAATAGATTTTGTTTTTCTTCATAATGATAATTACAACTATGAG [22308]
cp32-9_B31_TS_MP_FG_consensus  AATGAATTTTTAAACATAGGATTTAGTGAAGAGGCAATAGATTTTGTTTTTCTTCATAATGATAATTACAACTATGAG [22308]
cp32-9_B31_NX_Pl_consensus     AATGAATTTTTAAACATAGGATTTAGTGAAGAGGCAATAGATTTTGTTTTTCTTCATAATGATAATTACAACTATGAG [22308]
cp32-9_PAli_NX_MP_FG_consensus AATGAATTTTTAAACATAGGATTTAGTGAAGAGGCAATAGATTTTGTTTTTCTTCATAATGATAATTACAACTATGAG [22308]
cp32-9_PAli_NX_Pl_consensus    AATGAATTTTTAAACATAGGATTTAGTGAAGAGGCAATAGATTTTGTTTTTCTTCATAATGATAATTACAACTATGAG [22308]
cp32-9_PAbe_NX_MP_FG_consensus AATGAATTTTTAAACATAGGATTTAGTGAAGAGGCAATAGATTTTGTTTTTCTTCATAATGATAATTACAACTATGAG [22308]
cp32-9_PAbe_NX_Pl_consensus    AATGAATTTTTAAACATAGGATTTAGTGAAGAGGCAATAGATTTTGTTTTTCTTCATAATGATAATTACAACTATGAG [22308]

cp32-9_gi|11497325|B31_GB      GTTTTAAAAGAGAAAATAATTGATGTAGAAAAGAATTTGCAAAAAGACATATCTAGTTTAGATACTAAGATAGATAAT [22386]
cp32-9_un7_B31_PacBio          GTTTTAAAAGAGAAAATAATTGATGTAGAAAAGAATTTGCAAAAAGACATATCTAGTTTAGATACTAAGATAGATAAT [22386]
cp32-9_B31_TS_MP_FG_consensus  GTTTTAAAAGAGAAAATAATTGATGTAGAAAAGAATTTGCAAAAAGACATATCTAGTTTAGATACTAAGATAGATAAT [22386]
cp32-9_B31_NX_Pl_consensus     GTTTTAAAAGAGAAAATAATTGATGTAGAAAAGAATTTGCAAAAAGACATATCTAGTTTAGATACTAAGATAGATAAT [22386]
cp32-9_PAli_NX_MP_FG_consensus GTTTTAAAAGAGAAAATAATTGATGTAGAAAAGAATTTGCAAAAAGACATATCTAGTTTAGATACTAAGATAGATAAT [22386]
cp32-9_PAli_NX_Pl_consensus    GTTTTAAAAGAGAAAATAATTGATGTAGAAAAGAATTTGCAAAAAGACATATCTAGTTTAGATACTAAGATAGATAAT [22386]
cp32-9_PAbe_NX_MP_FG_consensus GTTTTAAAAGAGAAAATAATTGATGTAGAAAAGAATTTGCAAAAAGACATATCTAGTTTAGATACTAAGATAGATAAT [22386]
cp32-9_PAbe_NX_Pl_consensus    GTTTTAAAAGAGAAAATAATTGATGTAGAAAAGAATTTGCAAAAAGACATATCTAGTTTAGATACTAAGATAGATAAT [22386]

cp32-9_gi|11497325|B31_GB      GTAGAAAAGAATTTAAATGTTAAGATAGATAATGTAGAGAAGAATTTAAATATTAAAATAGATAGTGTTAAAAATGAA [22464]
cp32-9_un7_B31_PacBio          GTAGAAAAGAATTTAAATGTTAAGATAGATAATGTAGAGAAGAATTTAAATATTAAAATAGATAGTGTTAAAAATGAA [22464]
cp32-9_B31_TS_MP_FG_consensus  GTAGAAAAGAATTTAAATGTTAAGATAGATAATGTAGAGAAGAATTTAAATATTAAAATAGATAGTGTTAAAAATGAA [22464]
cp32-9_B31_NX_Pl_consensus     GTAGAAAAGAATTTAAATGTTAAGATAGATAATGTAGAGAAGAATTTAAATATTAAAATAGATAGTGTTAAAAATGAA [22464]
cp32-9_PAli_NX_MP_FG_consensus GTAGAAAAGAATTTAAATGTTAAGATAGATAATGTAGAGAAGAATTTAAATATTAAAATAGATAGTGTTAAAAATGAA [22464]
cp32-9_PAli_NX_Pl_consensus    GTAGAAAAGAATTTAAATGTTAAGATAGATAATGTAGAGAAGAATTTAAATATTAAAATAGATAGTGTTAAAAATGAA [22464]
cp32-9_PAbe_NX_MP_FG_consensus GTAGAAAAGAATTTAAATGTTAAGATAGATAATGTAGAGAAGAATTTAAATATTAAAATAGATAGTGTTAAAAATGAA [22464]
cp32-9_PAbe_NX_Pl_consensus    GTAGAAAAGAATTTAAATGTTAAGATAGATAATGTAGAGAAGAATTTAAATATTAAAATAGATAGTGTTAAAAATGAA [22464]

cp32-9_gi|11497325|B31_GB      CTTAATTCTAAAATAGATAGTTTAGATACTAAGATAGATAATGTAGAAAAAACTTTGCAAAAAGATATATCTAGCTTG [22542]
cp32-9_un7_B31_PacBio          CTTAATTCTAAAATAGATAGTTTAGATACTAAGATAGATAATGTAGAAAAAACTTTGCAAAAAGATATATCTAGCTTG [22542]
cp32-9_B31_TS_MP_FG_consensus  CTTAATTCTAAAATAGATAGTTTAGATACTAAGATAGATAATGTAGAAAAAACTTTGCAAAAAGATATATCTAGCTTG [22542]
cp32-9_B31_NX_Pl_consensus     CTTAATTCTAAAATAGATAGTTTAGATACTAAGATAGATAATGTAGAAAAAACTTTGCAAAAAGATATATCTAGCTTG [22542]
cp32-9_PAli_NX_MP_FG_consensus CTTAATTCTAAAATAGATAGTTTAGATACTAAGATAGATAATGTAGAAAAAACTTTGCAAAAAGATATATCTAGCTTG [22542]
cp32-9_PAli_NX_Pl_consensus    CTTAATTCTAAAATAGATAGTTTAGATACTAAGATAGATAATGTAGAAAAAACTTTGCAAAAAGATATATCTAGCTTG [22542]
cp32-9_PAbe_NX_MP_FG_consensus CTTAATTCTAAAATAGATAGTTTAGATACTAAGATAGATAATGTAGAAAAAACTTTGCAAAAAGATATATCTAGCTTG [22542]
cp32-9_PAbe_NX_Pl_consensus    CTTAATTCTAAAATAGATAGTTTAGATACTAAGATAGATAATGTAGAAAAAACTTTGCAAAAAGATATATCTAGCTTG [22542]

cp32-9_gi|11497325|B31_GB      AATACTAAAATAGATAGTGTAGAAAAAACCTTACAAAAGGATATATTTAGCCTAGATAATAAAATAAATGTTTTAAAA [22620]
cp32-9_un7_B31_PacBio          AATACTAAAATAGATAGTGTAGAAAAAACCTTACAAAAGGATATATTTAGCCTAGATAATAAAATAAATGTTTTAAAA [22620]
cp32-9_B31_TS_MP_FG_consensus  AATACTAAAATAGATAGTGTAGAAAAAACCTTACAAAAGGATATATTTAGCCTAGATAATAAAATAAATGTTTTAAAA [22620]
cp32-9_B31_NX_Pl_consensus     AATACTAAAATAGATAGTGTAGAAAAAACCTTACAAAAGGATATATTTAGCCTAGATAATAAAATAAATGTTTTAAAA [22620]
cp32-9_PAli_NX_MP_FG_consensus AATACTAAAATAGATAGTGTAGAAAAAACCTTACAAAAGGATATATTTAGCCTAGATAATAAAATAAATGTTTTAAAA [22620]
cp32-9_PAli_NX_Pl_consensus    AATACTAAAATAGATAGTGTAGAAAAAACCTTACAAAAGGATATATTTAGCCTAGATAATAAAATAAATGTTTTAAAA [22620]
cp32-9_PAbe_NX_MP_FG_consensus AATACTAAAATAGATAGTGTAGAAAAAACCTTACAAAAGGATATATTTAGCCTAGATAATAAAATAAATGTTTTAAAA [22620]
```

```
cp32-9_PAbe_NX_Pl_consensus    AATACTAAAATAGATAGTGTAGAAAAAACCTTACAAAAGGATATATTTAGCCTAGATAATAAAATAAATGTTTTAAAA [22620]

cp32-9_gi|11497325|B31_GB      AACGAACTTACTGCAAGTAATAGAACAATACAAGTAATTTTAATAATGGGAATAACGCTTGCTCCAATTATTTATTCT [22698]
cp32-9_un7_B31_PacBio          AACGAACTTACTGCAAGTAATAGAACAATACAAGTAATTTTAATAATGGGAATAACGCTTGCTCCAATTATTTATTCT [22698]
cp32-9_B31_TS_MP_FG_consensus  AACGAACTTACTGCAAGTAATAGAACAATACAAGTAATTTTAATAATGGGAATAACGCTTGCTCCAATTATTTATTCT [22698]
cp32-9_B31_NX_Pl_consensus     AACGAACTTACTGCAAGTAATAGAACAATACAAGTAATTTTAATAATGGGAATAACGCTTGCCCCAATTATTTATTCT [22698]
cp32-9_PAli_NX_MP_FG_consensus AACGAACTTACTGCAAGTAATAGAACAATACAAGTAATTTTAATAATGGGAATAACGCTTGCTCCAATTATTTATTCT [22698]
cp32-9_PAli_NX_Pl_consensus    AACGAACTTACTGCAAGTAATAGAACAATACAAGTAATTTTAATAATGGGAATAACGCTTGCTCCAATTATTTATTCT [22698]
cp32-9_PAbe_NX_MP_FG_consensus AACGAACTTACTGCAAGTAATAGAACAATACAAGTAATGCTAATAGCAGGAATAACGCTTGCCCCAATTATTTATTCT [22698]
cp32-9_PAbe_NX_Pl_consensus    AACGAACTTACTGCAAGTAATAGAACAATACAAGTAATTTTAATAATGGGAATAACGCTTGCTCCAATTATTTATTCT [22698]

cp32-9_gi|11497325|B31_GB      ATATTCAATAAGCATTTTTTAAATTAAGAATGATTAAAATTTTATAAAGTAATAAGTTAGTATATAGCTTTAAAGTAG [22776]
cp32-9_un7_B31_PacBio          ATATTCAATAAGCATTTTTTAAATTAAGAATGATTAAAATTTTATAAAGTAATAAGTTAGTATATAGCTTTAAAGTAG [22776]
cp32-9_B31_TS_MP_FG_consensus  ATATTCAATAAGCATTTTTTAAATTAAGAATGATTAAAATTTTATAAAGTAATAAGTTAGTATATAGCTTTAAAGTAG [22776]
cp32-9_B31_NX_Pl_consensus     ATATTCAATAAGTATTTTTTCAATTGAGAATGATTAAAATTTTATAAAGTAATAAGTTAGTATATAGCTCTAAAGTAG [22776]
cp32-9_PAli_NX_MP_FG_consensus ATATTCAATAAGTATTTTTTCAATTGAGAATGATTAAAATTTTATAAAGTAATAAGTTAGTATATAGCTCTAAAGTAG [22776]
cp32-9_PAli_NX_Pl_consensus    ATATTCAATAAGCATTTTTTAAATTAAGAATGATTAAAATTTTATAAAGTAATAAGTTAGTATATAGCTTTAAAGTAG [22776]
cp32-9_PAbe_NX_MP_FG_consensus ATATTCAATAAGTATTTTTTCAATTGAGAATGATTAAAATTTTATAAAGTAATAAGTTAGTATATAGCTCTAAAGTAG [22776]
cp32-9_PAbe_NX_Pl_consensus    ATATTCAATAAGTATTTTTTCAATTGAGAATGATTAAAATTTTATAAAGTAATAAGTTAGTATATAGCTCTAAAGTAG [22776]

cp32-9_gi|11497325|B31_GB      AACTTATTTGAATTTTTTAACAAGAGAATTTAAATAGGTTCTTTTATTTTAACAAATACAAAATAATTTTAATTCTAA [22854]
cp32-9_un7_B31_PacBio          AACTTATTTGAATTTTTTAACAAGAGAATTTAAATAGGTTCTTTTATTTTAACAAATACAAAATAATTTTAATTCTAA [22854]
cp32-9_B31_TS_MP_FG_consensus  AACTTATTTGAATTTTTTAACAAGAGAATTTAAATAGGTTCTTTTATTTTAACAAATACAAAATAATTTTAATTCTAA [22854]
cp32-9_B31_NX_Pl_consensus     AACTTATTTGAATTTTTTAACAAGAGAATTTAAATAGGTTCTTTTATTTTAACAAATACAAATTGATTTTAATTCTAA [22854]
cp32-9_PAli_NX_MP_FG_consensus AACTTATTTGAATTTTTTAACAAGAGAATTTAAATAGGTTCTTTTATTTTAACAAATACAAATTGATTTTAATTCTAA [22854]
cp32-9_PAli_NX_Pl_consensus    AACTTATTTGAATTTTTTAACAAGAGAATTTAAATAGGTTCTTTTATTTTAACAAATACAAAATAATTTTAATTCTAA [22854]
cp32-9_PAbe_NX_MP_FG_consensus AACTTATTTGAATTTTTTAACAAGAGAATTTAAATAGGTTCTTTTATTTTAACAAATACAAAATGATTTTAATTCTAA [22854]
cp32-9_PAbe_NX_Pl_consensus    AACTTATTTGAATTTTTTAACAAGAGAATTTAAATAGGTTCTTTTATTTTAACAAATACAAAATAATTTTAATTCTAA [22854]

cp32-9_gi|11497325|B31_GB      ATTGAACTGAATTTAATTGTTTAGTGAGTTTATCTAAAATAAATTGAGCTAAGCCAGCGGCTTTCTTAAGCTCTTTAA [22932]
cp32-9_un7_B31_PacBio          ATTGAACTGAATTTAATTGTTTAGTGAGTTTATCTAAAATAAATTGAGCTAAGCCAGCGGCTTTCTTAAGCTCTTTAA [22932]
cp32-9_B31_TS_MP_FG_consensus  ATTGAACTGAATTTAATTGTTTAGTGAGTTTATCTAAAATAAATTGAGCTAAGCCAGCGGCTTTCTTAAGCTCTTTAA [22932]
cp32-9_B31_NX_Pl_consensus     ATTGAACTGAATTTAATTGTTTAGTGAGTTTATCTAAAATAAATTGAGCTAAGCCAGCGGCTTTCTTAAGCTCTTTAA [22932]
cp32-9_PAli_NX_MP_FG_consensus ATTGAACTGAATTTAATTGTTTAGTGAGTTTATCTAAAATAAATTGAGCTAAGCCAGCGGCTTTCTTAAGCTCTTTAA [22932]
cp32-9_PAli_NX_Pl_consensus    ATTGAACTGAATTTAATTGTTTAGTGAGTTTATCTAAAATAAATTGAGCTAAGCCAGCGGCTTTCTTAAGCTCTTTAA [22932]
cp32-9_PAbe_NX_MP_FG_consensus ATTGAACTGAATTTAATTGTTTAGTGAGTTTATCTAAAATAAATTGAGCTAAGCCAGCGGCTTTCTTAAGCTCTTTAA [22932]
cp32-9_PAbe_NX_Pl_consensus    ATTGAACTGAATTTAATTGTTTAGTGAGTTTATCTAAAATAAATTGAGCTAAGCCAGCGGCTTTCTTAAGCTCTTTAA [22932]

cp32-9_gi|11497325|B31_GB      CATGAGAATTTAATAAAGCTTTTATTTATTATAATAATTTCTGTAAAAAGCCTGACAAAAATAATTTTTGTTATACAT [23010]
cp32-9_un7_B31_PacBio          CATGAGAATTTAATAAAGCTTTTATTTATTATAATAATTTCTGTAAAAAGCCTGACAAAAATAATTTTTGTTATACAT [23010]
cp32-9_B31_TS_MP_FG_consensus  CATGAGAATTTAATAAAGCTTTTATTTATTATAATAATTTCTGTAAAAAGCCTGACAAAAATAATTTTTGTTATACAT [23010]
cp32-9_B31_NX_Pl_consensus     CATGAGAATTTAATAAAGCTTTTATTTATTATAATAATTTCTGTAAAAAGCCTGACAAAAATAATTTTTGTTATACAT [23010]
cp32-9_PAli_NX_MP_FG_consensus CATGAGAATTTAATAAAGCTTTTATTTATTATAATAATTTCTGTAAAAAGCCTGACAAAAATAATTTTTGTTATACAT [23010]
cp32-9_PAli_NX_Pl_consensus    CATGAGAATTTAATAAAGCTTTTATTTATTATAATAATTTCTGTAAAAAGCCTGACAAAAATAATTTTTGTTATACAT [23010]
cp32-9_PAbe_NX_MP_FG_consensus CATGAGAATTTAATAAAGCTTTTATTTATTATAATAATTTCTGTAAAAAGCCTGACAAAAATAATTTTTGTTATACAT [23010]
cp32-9_PAbe_NX_Pl_consensus    CATGAGAATTTAATAAAGCTTTTATTTATTATAATAATTTCTGTAAAAAGCCTGACAAAAATAATTTTTGTTATACAT [23010]

cp32-9_gi|11497325|B31_GB      ATGTATATGTATAGCTAAAAAAATATATTGCTATCAAAAAAATCCAATTAAGTTGGGTTTAGCTAAGTTCTTTAACAA [23088]
cp32-9_un7_B31_PacBio          ATGTATATGTATAGCTAAAAAAATATATTGCTATCAAAAAAATCCAATTAAGTTGGGTTTAGCTAAGTTCTTTAACAA [23088]
cp32-9_B31_TS_MP_FG_consensus  ATGTATATGTATAGCTAAAAAAATATATTGCTATCAAAAAAATCCAATTAAGTTGGGTTTAGCTAAGTTCTTTAACAA [23088]
cp32-9_B31_NX_Pl_consensus     ATGTATATGTATAGCTAAAAAAATATATTGCTATCAAAAAAATCCAATTAAGTTGGGTTTAGCTAAGTTCTTTAACAA [23088]
cp32-9_PAli_NX_MP_FG_consensus ATGTATATGTATAGCTAAAAAAATATATTGCTATCAAAAAAATCCAATTAAGTTGGGTTTAGCTAAGTTCTTTAACAA [23088]
cp32-9_PAli_NX_Pl_consensus    ATGTATATGTATAGCTAAAAAAATATATTGCTATCAAAAAAATCCAATTAAGTTGGGTTTAGCTAAGTTCTTTAACAA [23088]
cp32-9_PAbe_NX_MP_FG_consensus ATGTATATGTATAGCTAAAAAAATATATTGCTATCAAAAAAATCCAATTAAGTTGGGTTTAGCTAAGTTCTTTAACAA [23088]
cp32-9_PAbe_NX_Pl_consensus    ATGTATATGTATAGCTAAAAAAATATATTGCTATCAAAAAAATCCAATTAAGTTGGGTTTAGCTAAGTTCTTTAACAA [23088]

cp32-9_gi|11497325|B31_GB      GAGAATTTAAATAAGTCCAACTATTTTTTTGTAAAAATTTTTGTAAAAAAGTTGTCAAAAATAGTTTTTGCTATGTAA [23166]
cp32-9_un7_B31_PacBio          GAGAATTTAAATAAGTCCAACTATTTTTTTGTAAAAATTTTTGTAAAAAAGTTGTCAAAAATAGTTTTTGCTATGTAA [23166]
cp32-9_B31_TS_MP_FG_consensus  GAGAATTTAAATAAGTCCAACTATTTTTTTGTAAAAATTTTTGTAAAAAAGTTGTCAAAAATAGTTTTTGCTATGTAA [23166]
cp32-9_B31_NX_Pl_consensus     GAGAATTTAAATAAGTCCAACTATTTTTTTGTAAAAATTTTTGTAAAAAAGTTGTCAAAAATAGTTTTTGCTATGTAA [23166]
cp32-9_PAli_NX_MP_FG_consensus GAGAATTTAAATAAGTCCAACTATTTTTTTGTAAAAATTTTTGTAAAAAAGTTGTCAAAAATAGTTTTTGCTATGTAA [23166]
cp32-9_PAli_NX_Pl_consensus    GAGAATTTAAATAAGTCCAACTATTTTTTTGTAAAAATTTTTGTAAAAAAGTTGTCAAAAATAGTTTTTGCTATGTAA [23166]
cp32-9_PAbe_NX_MP_FG_consensus GAGAATTTAAATAAGTCCAACTATTTTTTTGTAAAAATTTTTGTAAAAAAGTTGTCAAAAATAGTTTTTGCTATGTAA [23166]
cp32-9_PAbe_NX_Pl_consensus    GAGAATTTAAATAAGTCCAACTATTTTTTTGTAAAAATTTTTGTAAAAAAGTTGTCAAAAATAGTTTTTGCTATGTAA [23166]

cp32-9_gi|11497325|B31_GB      TTATTTATTACAAAATAAGGAGGAAAAAGATGGAAAATCTTTCAAACAATAATAATCCACAAGAAAATATTCAAGGAG [23244]
cp32-9_un7_B31_PacBio          TTATTTATTACAAAATAAGGAGGAAAAAGATGGAAAATCTTTCAAACAATAATAATCCACAAGAAAATATTCAAGGAG [23244]
cp32-9_B31_TS_MP_FG_consensus  TTATTTATTACAAAATAAGGAGGAAAAAGATGGAAAATCTTTCAAACAATAATAATCCACAAGAAAATATTCAAGGAG [23244]
cp32-9_B31_NX_Pl_consensus     TTATTTATTACAAAATAAGGAGGAAAAAGATGGAAAATCTTTCAAACAATAATAATCCACAAGAAAATATTCAAGGAG [23244]
cp32-9_PAli_NX_MP_FG_consensus TTATTTATTACAAAATAAGGAGGAAAAAGATGGAAAATCTTTCAAACAATAATAATCCACAAGAAAATATTCAAGGAG [23244]
cp32-9_PAli_NX_Pl_consensus    TTATTTATTACAAAATAAGGAGGAAAAAGATGGAAAATCTTTCAAACAATAATAATCCACAAGAAAATATTCAAGGAG [23244]
cp32-9_PAbe_NX_MP_FG_consensus TTATTTATTACAAAATAAGGAGGAAAAAGATGGAAAATCTTTCAAACAATAATAATCCACAAGAAAATATTCAAGGAG [23244]
cp32-9_PAbe_NX_Pl_consensus    TTATTTATTACAAAATAAGGAGGAAAAAGATGGAAAATCTTTCAAACAATAATAATCCACAAGAAAATATTCAAGGAG [23244]

cp32-9_gi|11497325|B31_GB      AGCTCAAAATGATAAGTATTAATCAACAAAGTTTTACTGGTTGTGAAATATTTGAGGAAAAATCTTCTCCCATTAAAG [23322]
cp32-9_un7_B31_PacBio          AGCTCAAAATGATAAGTATTAATCAACAAAGTTTTACTGGTTGTGAAATATTTGAGGAAAAATCTTCTCCCATTAAAG [23322]
cp32-9_B31_TS_MP_FG_consensus  AGCTCAAAATGATAAGTATTAATCAACAAAGTTTTACTGGTTGTGAAATATTTGAGGAAAAATCTTCTCCCATTAAAG [23322]
cp32-9_B31_NX_Pl_consensus     AGCTCAAAATGATAAGTATTAATCAACAAAGTTTTACTGGTTGTGAAATATTTGAGGAAAAATCTTCTCCCATTAAAG [23322]
cp32-9_PAli_NX_MP_FG_consensus AGCTCAAAATGATAAGTATTAATCAACAAAGTTTTACTGGTTGTGAAATATTTGAGGAAAAATCTTCTCCCATTAAAG [23322]
cp32-9_PAli_NX_Pl_consensus    AGCTCAAAATGATAAGTATTAATCAACAAAGTTTTACTGGTTGTGAAATATTTGAGGAAAAATCTTCTCCCATTAAAG [23322]
cp32-9_PAbe_NX_MP_FG_consensus AGCTCAAAATGATAAGTATTAATCAACAAAGTTTTACTGGTTGTGAAATATTTGAGGAAAAATCTTCTCCCATTAAAG [23322]
cp32-9_PAbe_NX_Pl_consensus    AGCTCAAAATGATAAGTATTAATCAACAAAGTTTTACTGGTTGTGAAATATTTGAGGAAAAATCTTCTCCCATTAAAG [23322]

cp32-9_gi|11497325|B31_GB      AAAAAAGTAAATTAAGTAAGATAGGTAAGAAATTACCAGGAATAAGTAGTCAAGAATGTTTTAGATTTAATCGCAATA [23400]
cp32-9_un7_B31_PacBio          AAAAAAGTAAATTAAGTAAGATAGGTAAGAAATTACCAGGAATAAGTAGTCAAGAATGTTTTAGATTTAATCGCAATA [23400]
cp32-9_B31_TS_MP_FG_consensus  AAAAAAGTAAATTAAGTAAGATAGGTAAGAAATTACCAGGAATAAGTAGTCAAGAATGTTTTAGATTTAATCGCAATA [23400]
cp32-9_B31_NX_Pl_consensus     AAAAAAGTAAATTAAGTAAGATAGGTAAGAAATTACCAGGAATAAGTAGTCAAGAATGTTTTAGATTTAATCGCAATA [23400]
cp32-9_PAli_NX_MP_FG_consensus AAAAAAGTAAATTAAGTAAGATAGGTAAGAAATTACCAGGAATAAGTAGTCAAGAATGTTTTAGATTTAATCGCAATA [23400]
cp32-9_PAli_NX_Pl_consensus    AAAAAAGTAAATTAAGTAAGATAGGTAAGAAATTACCAGGAATAAGTAGTCAAGAATGTTTTAGATTTAATCGCAATA [23400]
cp32-9_PAbe_NX_MP_FG_consensus AAAAAAGTAAATTAAGTAAGATAGGTAAGAAATTACCAGGAATAAGTAGTCAAGAATGTTTTAGATTTAATCGCAATA [23400]
cp32-9_PAbe_NX_Pl_consensus    AAAAAAGTAAATTAAGTAAGATAGGTAAGAAATTACCAGGAATAAGTAGTCAAGAATGTTTTAGATTTAATCGCAATA [23400]

cp32-9_gi|11497325|B31_GB      TTGATTTTAGTGTGCAAAGAAACAAGTTAGATAAATACGGTGCTAGTGAAGTAGGTAATATTCTTGTTGGAGGTGCTG [23478]
cp32-9_un7_B31_PacBio          TTGATTTTAGTGTGCAAAGAAACAAGTTAGATAAATACGGTGCTAGTGAAGTAGGTAATATTCTTGTTGGAGGTGCTG [23478]
cp32-9_B31_TS_MP_FG_consensus  TTGATTTTAGTGTGCAAAGAAACAAGTTAGATAAATACGGTGCTAGTGAAGTAGGTAATATTCTTGTTGGAGGTGCTG [23478]
cp32-9_B31_NX_Pl_consensus     TTGATTTTAGTGTGCAAAGAAACAAGTTAGATAAATACGGTGCTAGTGAAGTAGGTAATATTCTTGTTGGAGGTGCTG [23478]
cp32-9_PAli_NX_MP_FG_consensus TTGATTTTAGTGTGCAAAGAAACAAGTTAGATAAATACGGTGCTAGTGAAGTAGGTAATATTCTTGTTGGAGGTGCTG [23478]
cp32-9_PAli_NX_Pl_consensus    TTGATTTTAGTGTGCAAAGAAACAAGTTAGATAAATACGGTGCTAGTGAAGTAGGTAATATTCTTGTTGGAGGTGCTG [23478]
cp32-9_PAbe_NX_MP_FG_consensus TTGATTTTAGTGTGCAAAGAAACAAGTTAGATAAATACGGTGCTAGTGAAGTAGGTAATATTCTTGTTGGAGGTGCTG [23478]
cp32-9_PAbe_NX_Pl_consensus    TTGATTTTAGTGTGCAAAGAAACAAGTTAGATAAATACGGTGCTAGTGAAGTAGGTAATATTCTTGTTGGAGGTGCTG [23478]

cp32-9_gi|11497325|B31_GB      GGCTGAAAGATTTAATGATAAACAGAGTGCTTAAATATTTTGATATGAGCCTATCTTTTGAAGAGAATTTATATATGC [23556]
cp32-9_un7_B31_PacBio          GGCTGAAAGATTTAATGATAAACAGAGTGCTTAAATATTTTGATATGAGCCTATCTTTTGAAGAGAATTTATATATGC [23556]
cp32-9_B31_TS_MP_FG_consensus  GGCTGAAAGATTTAATGATAAACAGAGTGCTTAAATATTTTGATATGAGCCTATCTTTTGAAGAGAATTTATATATGC [23556]
cp32-9_B31_NX_Pl_consensus     GGCTGAAAGATTTAATGATAAACAGAGTGCTTAAATATTTTGATATGAGCCTATCTTTTGAAGAGAATTTATATATGC [23556]
cp32-9_PAli_NX_MP_FG_consensus GGCTGAAAGATTTAATGATAAACAGAGTGCTTAAATATTTTGATATGAGCCTATCTTTTGAAGAGAATTTATATATGC [23556]
cp32-9_PAli_NX_Pl_consensus    GGCTGAAAGATTTAATGATAAACAGAGTGCTTAAATATTTTGATATGAGCCTATCTTTTGAAGAGAATTTATATATGC [23556]
cp32-9_PAbe_NX_MP_FG_consensus GGCTGAAAGATTTAATGATAAACAGAGTGCTTAAATATTTTGATATGAGCCTATCTTTTGAAGAGAATTTATATATGC [23556]
cp32-9_PAbe_NX_Pl_consensus    GGCTGAAAGATTTAATGATAAACAGAGTGCTTAAATATTTTGATATGAGCCTATCTTTTGAAGAGAATTTATATATGC [23556]
```

```
cp32-9_gi|11497325|B31_GB      TCAAGGGCAAAGAGTTAGAGAATTTAGGATTTAGAGAGTTTGTTAAAGCATACGGTGATAATATTGATATTTTATATA [23634]
cp32-9_un7_B31_PacBio          TCAAGGGCAAAGAGTTAGAGAATTTAGGATTTAGAGAGTTTGTTAAAGCATACGGTGATAATATTGATATTTTATATA [23634]
cp32-9_B31_TS_MP_FG_consensus  TCAAGGGCAAAGAGTTAGAGAATTTAGGATTTAGAGAGTTTGTTAAAGCATACGGTGATAATATTGATATTTTATATA [23634]
cp32-9_B31_NX_Pl_consensus     TCAAGGGCAAAGAGTTAGAGAATTTAGGATTTAGAGAGTTTGTTAAAGCATACGGTGATAATATTGATATTTTATATA [23634]
cp32-9_PAli_NX_MP_FG_consensus TCAAGGGCAAAGAGTTAGAGAATTTAGGATTTAGAGAGTTTGTTAAAGCATACGGTGATAATATTGATATTTTATATA [23634]
cp32-9_PAli_NX_Pl_consensus    TCAAGGGCAAAGAGTTAGAGAATTTAGGATTTAGAGAGTTTGTTAAAGCATACGGTGATAATATTGATATTTTATATA [23634]
cp32-9_PAbe_NX_MP_FG_consensus TCAAGGGCAAAGAGTTAGAGAATTTAGGATTTAGAGAGTTTGTTAAAGCATACGGTGATAATATTGATATTTTATATA [23634]
cp32-9_PAbe_NX_Pl_consensus    TCAAGGGCAAAGAGTTAGAGAATTTAGGATTTAGAGAGTTTGTTAAAGCATACGGTGATAATATTGATATTTTATATA [23634]

cp32-9_gi|11497325|B31_GB      AAAATAAATATGCCAACGGTGTTGATAAGTATAATTATTTCAAAAAAATGGGCAGTTCAGAAACTTTAGTGGGCTCAA [23712]
cp32-9_un7_B31_PacBio          AAAATAAATATGCCAACGGTGTTGATAAGTATAATTATTTCAAAAAAATGGGCAGTTCAGAAACTTTAGTGGGCTCAA [23712]
cp32-9_B31_TS_MP_FG_consensus  AAAATAAATATGCCAACGGTGTTGATAAGTATAATTATTTCAAAAAAATGGGCAGTTCAGAAACTTTAGTGGGCTCAA [23712]
cp32-9_B31_NX_Pl_consensus     AAAATAAATATGCCAACGGTGTTGATAAGTATAATTATTTCAAAAAAATGGGCAGTTCAGAAACTTTAGTGGGCTCAA [23712]
cp32-9_PAli_NX_MP_FG_consensus AAAATAAATATGCCAACGGTGTTGATAAGTATAATTATTTCAAAAAAATGGGCAGTTCAGAAACTTTAGTGGGCTCAA [23712]
cp32-9_PAli_NX_Pl_consensus    AAAATAAATATGCCAACGGTGTTGATAAGTATAATTATTTCAAAAAAATGGGCAGTTCAGAAACTTTAGTGGGCTCAA [23712]
cp32-9_PAbe_NX_MP_FG_consensus AAAATAAATATGCCAACGGTGTTGATAAGTATAATTATTTCAAAAAAATGGGCAGTTCAGAAACTTTAGTGGGCTCAA [23712]
cp32-9_PAbe_NX_Pl_consensus    AAAATAAATATGCCAACGGTGTTGATAAGTATAATTATTTCAAAAAAATGGGCAGTTCAGAAACTTTAGTGGGCTCAA [23712]

cp32-9_gi|11497325|B31_GB      CAATTGATGGCTGGTTTATTAATAATAATGGCGATTTAGAACTATTAGAGATTAAAAGTAGCGACTCTAATTATATGA [23790]
cp32-9_un7_B31_PacBio          CAATTGATGGCTGGTTTATTAATAATAATGGCGATTTAGAACTATTAGAGATTAAAAGTAGCGACTCTAATTATATGA [23790]
cp32-9_B31_TS_MP_FG_consensus  CAATTGATGGCTGGTTTATTAATAATAATGGCGATTTAGAACTATTAGAGATTAAAAGTAGCGACTCTAATTATATGA [23790]
cp32-9_B31_NX_Pl_consensus     CAATTGATGGCTGGTTTATTAATAATAATGGCGATTTAGAACTATTAGAGATTAAAAGTAGCGACTCTAATTATATGA [23790]
cp32-9_PAli_NX_MP_FG_consensus CAATTGATGGCTGGTTTATTAATAATAATGGCGATTTAGAACTATTAGAGATTAAAAGTAGCGACTCTAATTATATGA [23790]
cp32-9_PAli_NX_Pl_consensus    CAATTGATGGCTGGTTTATTAATAATAATGGCGATTTAGAACTATTAGAGATTAAAAGTAGCGACTCTAATTATATGA [23790]
cp32-9_PAbe_NX_MP_FG_consensus CAATTGATGGCTGGTTTATTAATAATAATGGCGATTTAGAACTATTAGAGATTAAAAGTAGCGACTCTAATTATATGA [23790]
cp32-9_PAbe_NX_Pl_consensus    CAATTGATGGCTGGTTTATTAATAATAATGGCGATTTAGAACTATTAGAGATTAAAAGTAGCGACTCTAATTATATGA [23790]

cp32-9_gi|11497325|B31_GB      GTAGTGCTATTGCTGAGGACAATAAAAATGGCAATTTTTTAAGCAGTAAATATTTTTTCAAATATTATGTACAAGCAC [23868]
cp32-9_un7_B31_PacBio          GTAGTGCTATTGCTGAGGACAATAAAAATGGCAATTTTTTAAGCAGTAAATATTTTTTCAAATATTATGTACAAGCAC [23868]
cp32-9_B31_TS_MP_FG_consensus  GTAGTGCTATTGCTGAGGACAATAAAAATGGCAATTTTTTAAGCAGTAAATATTTTTTCAAATATTATGTACAAGCAC [23868]
cp32-9_B31_NX_Pl_consensus     GTAGTGCTATTGCTGAGGACAATAAAAATGGCAATTTTTTAAGCAGTAAATATTTTTTCAAATATTATGTACAAGCAC [23868]
cp32-9_PAli_NX_MP_FG_consensus GTAGTGCTATTGCTGAGGACAATAAAAATGGCAATTTTTTAAGCAGTAAATATTTTTTCAAATATTATGTACAAGCAC [23868]
cp32-9_PAli_NX_Pl_consensus    GTAGTGCTATTGCTGAGGACAATAAAAATGGCAATTTTTTAAGCAGTAAATATTTTTTCAAATATTATGTACAAGCAC [23868]
cp32-9_PAbe_NX_MP_FG_consensus GTAGTGCTATTGCTGAGGACAATAAAAATGGCAATTTTTTAAGCAGTAAATATTTTTTCAAATATTATGTACAAGCAC [23868]
cp32-9_PAbe_NX_Pl_consensus    GTAGTGCTATTGCTGAGGACAATAAAAATGGCAATTTTTTAAGCAGTAAATATTTTTTCAAATATTATGTACAAGCAC [23868]

cp32-9_gi|11497325|B31_GB      AAATACAGCTAGCATGCACTGGGCTTAAGTATTGTAATTTGTTCTTTTTAATAGATGCTGCACCAATTAACTGTAAGA [23946]
cp32-9_un7_B31_PacBio          AAATACAGCTAGCATGCACTGGGCTTAAGTATTGTAATTTGTTCTTTTTAATAGATGCTGCACCAATTAACTGTAAGA [23946]
cp32-9_B31_TS_MP_FG_consensus  AAATACAGCTAGCATGCACTGGGCTTAAGTATTGTAATTTGTTCTTTTTAATAGATGCTGCACCAATTAACTGTAAGA [23946]
cp32-9_B31_NX_Pl_consensus     AAATACAGCTAGCATGCACTGGGCTTAAGTATTGTAATTTGTTCTTTTTAATAGATGCTGCACCAATTAACTGTAAGA [23946]
cp32-9_PAli_NX_MP_FG_consensus AAATACAGCTAGCATGCACTGGGCTTAAGTATTGTAATTTGTTCTTTTTAATAGATGCTGCACCAATTAACTGTAAGA [23946]
cp32-9_PAli_NX_Pl_consensus    AAATACAGCTAGCATGCACTGGGCTTAAGTATTGTAATTTGTTCTTTTTAATAGATGCTGCACCAATTAACTGTAAGA [23946]
cp32-9_PAbe_NX_MP_FG_consensus AAATACAGCTAGCATGCACTGGGCTTAAGTATTGTAATTTGTTCTTTTTAATAGATGCTGCACCAATTAACTGTAAGA [23946]
cp32-9_PAbe_NX_Pl_consensus    AAATACAGCTAGCATGCACTGGGCTTAAGTATTGTAATTTGTTCTTTTTAATAGATGCTGCACCAATTAACTGTAAGA [23946]

cp32-9_gi|11497325|B31_GB      TTAAAAGAGATGAGGCCTTAATATCAAAAGTGTTTGAATTTGTTAATAAATGTGAATTAGAAATTATAAATTTGAAAA [24024]
cp32-9_un7_B31_PacBio          TTAAAAGAGATGAGGCCTTAATATCAAAAGTGTTTGAATTTGTTAATAAATGTGAATTAGAAATTATAAATTTGAAAA [24024]
cp32-9_B31_TS_MP_FG_consensus  TTAAAAGAGATGAGGCCTTAATATCAAAAGTGTTTGAATTTGTTAATAAATGTGAATTAGAAATTATAAATTTGAAAA [24024]
cp32-9_B31_NX_Pl_consensus     TTAAAAGAGATGAGGCCTTAATATCAAAAGTGTTTGAATTTGTTAATAAATGTGAATTAGAAATTATAAATTTGAAAA [24024]
cp32-9_PAli_NX_MP_FG_consensus TTAAAAGAGATGAGGCCTTAATATCAAAAGTGTTTGAATTTGTTAATAAATGTGAATTAGAAATTATAAATTTGAAAA [24024]
cp32-9_PAli_NX_Pl_consensus    TTAAAAGAGATGAGGCCTTAATATCAAAAGTGTTTGAATTTGTTAATAAATGTGAATTAGAAATTATAAATTTGAAAA [24024]
cp32-9_PAbe_NX_MP_FG_consensus TTAAAAGAGATGAGGCCTTAATATCAAAAGTGTTTGAATTTGTTAATAAATGTGAATTAGAAATTATAAATTTGAAAA [24024]
cp32-9_PAbe_NX_Pl_consensus    TTAAAAGAGATGAGGCCTTAATATCAAAAGTGTTTGAATTTGTTAATAAATGTGAATTAGAAATTATAAATTTGAAAA [24024]

cp32-9_gi|11497325|B31_GB      AAGATATTTATAGTAACTATAGAGAGGAATACTTAATGGCACATAATTTTAACGAGGATACGTTTATAAAACTTGTTG [24102]
cp32-9_un7_B31_PacBio          AAGATATTTATAGTAACTATAGAGAGGAATACTTAATGGCACATAATTTTAACGAGGATACGTTTATAAAACTTGTTG [24102]
cp32-9_B31_TS_MP_FG_consensus  AAGATATTTATAGTAACTATAGAGAGGAATACTTAATGGCACATAATTTTAACGAGGATACGTTTATAAAACTTGTTG [24102]
cp32-9_B31_NX_Pl_consensus     AAGATATTTATAGTAACTATAGAGAGGAATACTTAATGGCACATAATTTTAACGAGGATACGTTTATAAAACTTGTTG [24102]
cp32-9_PAli_NX_MP_FG_consensus AAGATATTTATAGTAACTATAGAGAGGAATACTTAATGGCACATAATTTTAACGAGGATACGTTTATAAAACTTGTTG [24102]
cp32-9_PAli_NX_Pl_consensus    AAGATATTTATAGTAACTATAGAGAGGAATACTTAATGGCACATAATTTTAACGAGGATACGTTTATAAAACTTGTTG [24102]
cp32-9_PAbe_NX_MP_FG_consensus AAGATATTTATAGTAACTATAGAGAGGAATACTTAATGGCACATAATTTTAACGAGGATACGTTTATAAAACTTGTTG [24102]
cp32-9_PAbe_NX_Pl_consensus    AAGATATTTATAGTAACTATAGAGAGGAATACTTAATGGCACATAATTTTAACGAGGATACGTTTATAAAACTTGTTG [24102]

cp32-9_gi|11497325|B31_GB      AAGATTTAGTAGAAAGGAGTGATTTTTATAGTTCTGAAGTTGAGTTTGATTGGGTAAGAGAATTTATAGAATATGTTG [24180]
cp32-9_un7_B31_PacBio          AAGATTTAGTAGAAAGGAGTGATTTTTATAGTTCTGAAGTTGAGTTTGATTGGGTAAGAGAATTTATAGAATATGTTG [24180]
cp32-9_B31_TS_MP_FG_consensus  AAGATTTAGTAGAAAGGAGTGATTTTTATAGTTCTGAAGTTGAGTTTGATTGGGTAAGAGAATTTATAGAATATGTTG [24180]
cp32-9_B31_NX_Pl_consensus     AAGATTTAGTAGAAAGGAGTGATTTTTATAGTTCTGAAGTTGAGTTTGATTGGGTAAGAGAATTTATAGAATATGTTG [24180]
cp32-9_PAli_NX_MP_FG_consensus AAGATTTAGTAGAAAGGAGTGATTTTTATAGTTCTGAAGTTGAGTTTGATTGGGTAAGAGAATTTATAGAATATGTTG [24180]
cp32-9_PAli_NX_Pl_consensus    AAGATTTAGTAGAAAGGAGTGATTTTTATAGTTCTGAAGTTGAGTTTGATTGGGTAAGAGAATTTATAGAATATGTTG [24180]
cp32-9_PAbe_NX_MP_FG_consensus AAGATTTAGTAGAAAGGAGTGATTTTTATAGTTCTGAAGTTGAGTTTGATTGGGTAAGAGAATTTATAGAATATGTTG [24180]
cp32-9_PAbe_NX_Pl_consensus    AAGATTTAGTAGAAAGGAGTGATTTTTATAGTTCTGAAGTTGAGTTTGATTGGGTAAGAGAATTTATAGAATATGTTG [24180]

cp32-9_gi|11497325|B31_GB      ATTGTGCAGACCTTGAAATTAAGGATAATCAATTTGCTGAAAATCTTGCGTATGATTTAATGGAGATTGATAGTCTAC [24258]
cp32-9_un7_B31_PacBio          ATTGTGCAGACCTTGAAATTAAGGATAATCAATTTGCTGAAAATCTTGCGTATGATTTAATGGAGATTGATAGTCTAC [24258]
cp32-9_B31_TS_MP_FG_consensus  ATTGTGCAGACCTTGAAATTAAGGATAATCAATTTGCTGAAAATCTTGCGTATGATTTAATGGAGATTGATAGTCTAC [24258]
cp32-9_B31_NX_Pl_consensus     ATTGTGTAGAGCTTGAAATTACAGATAGGCAAGTTGCTGAAAATGTTGCATGTGATCTAATGGAAGTTGATAGCGCAA [24258]
cp32-9_PAli_NX_MP_FG_consensus ATTGTGCAGACCTTGAAATTAAGGATAATCAATTTGCTGAAAATCTTGCGTATGATTTAATGGAGATTGATAGTCTAC [24258]
cp32-9_PAli_NX_Pl_consensus    ATTGTGCAGACCTTGAAATTAAGGATAATCAATTTGCTGAAAATCTTGCGTATGATTTAATGGAGATTGATAGTCTAC [24258]
cp32-9_PAbe_NX_MP_FG_consensus ATTGTGCAGACCTTGAAATTAAGGATAATCAATTTGCTGAAAATCTTGCGTATGATTTAATGGAGATTGATAGTCTAC [24258]
cp32-9_PAbe_NX_Pl_consensus    ATTGTGCAGACCTTGAAATTAAGGATAATCAATTTGCTGAAAATCTTGCATGTGATCTAATGGAAGTTGATAGCGCAA [24258]

cp32-9_gi|11497325|B31_GB      AAAAAGAATTAAATAGAATCCAAAACGAAAATAAAAAAAGAGAAAAGCCCATTAAAGATAGGTTGAAGATGCTAATTT [24336]
cp32-9_un7_B31_PacBio          AAAAAGAATTAAATAGAATCCAAAACGAAAATAAAAAAAGAGAAAAGCCCATTAAAGATAGGTTGAAGATGCTAATTT [24336]
cp32-9_B31_TS_MP_FG_consensus  AAAAAGAATTAAATAGAATCCAAAACGAAAATAAAAAAAGAGAAAAGCCCATTAAAGATAGGTTGAAGATGCTAATTT [24336]
cp32-9_B31_NX_Pl_consensus     AAAAAGAATTAAACAAAATGCAAAACGAACACAAAAAAAGAGAAAAGTATCTTAAAGATAGGTTGAAGATGAAAATCT [24336]
cp32-9_PAli_NX_MP_FG_consensus AAAAAGAATTAAATAGAATCCAAAACGAAAATAAAAAAAGAGAAAAGCCCATTAAAGATAGGTTGAAGATGCTAATTT [24336]
cp32-9_PAli_NX_Pl_consensus    AAAAAGAATTAAATAGAATCCAAAACGAAAATAAAAAAAGAGAAAAGCCCATTAAAGATAGGTTGAAGATGCTAATTT [24336]
cp32-9_PAbe_NX_MP_FG_consensus AAAAAGAATTAAATAGAATCCAAAACGAAAATAAAAAAAGAGAAAAGCCCATTAAAGATAGGTTGAAGATGCTAATTT [24336]
cp32-9_PAbe_NX_Pl_consensus    AAAAAGAATTAAACAAAATGCAAAACGAACACAAAAAAAGAGAAAAGTATCTTAAAGATAGGTTGAAGATGCTAATTT [24336]

cp32-9_gi|11497325|B31_GB      ACAATATTACGAATACATATCCACTAATTGAGCAGTTAAATTATAAATTTGGAGAGTTTGTGTTTACTCTTGACCCTA [24414]
cp32-9_un7_B31_PacBio          ACAATATTACGAATACATATCCACTAATTGAGCAGTTAAATTATAAATTTGGAGAGTTTGTGTTTACTCTTGACCCTA [24414]
cp32-9_B31_TS_MP_FG_consensus  ACAATATTACGAATATATGTCCACTAATTGAGCATGTAAATTATAGGTTTAGAGAATTTGTGTTCACGTTTGACTCTA [24414]
cp32-9_B31_NX_Pl_consensus     ACAATATTACGAATACATATCCACTAATTGAGCAGTTAAATTATAAATTTGGAGAGTTTGTGTTTACTCTTGACCCTA [24414]
cp32-9_PAli_NX_MP_FG_consensus ACAATATTACGAATATATGTCCACTAATTGAGCATGTAAATTATAGGTTTAGAGAATTTGTGTTCACGTTTGACTCTA [24414]
cp32-9_PAli_NX_Pl_consensus    ACAATATTACGAATACATATCCACTAATTGAGCAGTTAAATTATAAATTTGGAGAGTTTGTGTTTACTCTTGACCCTA [24414]
cp32-9_PAbe_NX_MP_FG_consensus ACAATATTACGAATACATATCCACTAATTGAGCATGTAAATTATAGGTTTAGAGAATTTGTGTTCACGTTTGACTCTA [24414]
cp32-9_PAbe_NX_Pl_consensus    ACAATATTACGAATACATATCCACTAATTGAGCAGTTAAATTATAAATTTGGAGAGTTTGTGTTTACTCTTGACCCTA [24414]

cp32-9_gi|11497325|B31_GB      AGAAAAGGGCAATATCAGATAGATTAAAGGGACTACTACCAACAAGTGGTACAGTATTTTTCCCTAGCAATATAGCAT [24492]
cp32-9_un7_B31_PacBio          AGAAAAGGGCAATATCAGATAGATTAAAGGGACTACTACCAACAAGTGGTACAGTATTTTTCCCTAGCAATATAGCAT [24492]
cp32-9_B31_TS_MP_FG_consensus  AGAAAAGGGCAATATCAGACAGATTAAGTGGTTTATTGCCAACAAGTGAAACATTGCTTTTCCCAAGCAATATAGCAT [24492]
cp32-9_B31_NX_Pl_consensus     AGAAAAGGGCAATATCAGACAGATTAAGTGGTTTATTGCCAACAAGTGAAACATTGCTTTTCCCAAGCAATATAGCAT [24492]
cp32-9_PAli_NX_MP_FG_consensus AGAAAAGGGCAATATCAGACAGATTAAGTGGTTTATTGCCAACAAGTGAAACATTGCTTTTCCCAAGCAATATAGCAT [24492]
cp32-9_PAli_NX_Pl_consensus    AGAAAAGGGCAATATCAGATAGATTAAAGGGACTACTACCAACAAGTGGTACAGTATTTTTCCCTAGCAATATAGCAT [24492]
cp32-9_PAbe_NX_MP_FG_consensus AGAAAAGGGCAATATCAGACAGATTAAGTGGTTTATTGCCAACAAGTGAAACATTGCTTTTCCCAAGCAATATAGCAT [24492]
cp32-9_PAbe_NX_Pl_consensus    AGAAAAGGGCAATATCAGATAGATTAAAGGGACTACTACCAACAAGTGAAACATTGCTTTTCCCAAGCAATATAGCAT [24492]
```

```
cp32-9_gi|11497325|B31_GB      TTGCAAATAGTGTCAGTGTCCCCATGTGAATGGGAGCACTAAAAAATTAAAAAAATAAATTTAATATAGGAGGATTAA [24570]
cp32-9_un7_B31_PacBio          TTGCAAATAGTGTCAGTGTCCCCATGTGAATGGGAGCACTAAAAAATTAAAAAAATAAATTTAATATAGGAGGATTAA [24570]
cp32-9_B31_TS_MP_FG_consensus  TTGCAAATAGTGTTAGTGTCCCCATGTGAATGGGAGCACTAAAAAATTAAAGAAATAA-TTTAATATAGGAGGATTAA [24570]
cp32-9_B31_NX_Pl_consensus     TTGCAAATAGTGTTAGTGTCCCCATGTGAATGGGAGCACTAAAAAATTAAAGAAATAA-TTTAATATAGGAGGATTAA [24570]
cp32-9_PAli_NX_MP_FG_consensus TTGCAAATAGTGTTAGTGTCCCCATGTGAATGGGAGCACTAAAAAATTAAAGAAATAA-TTTAATATAGGAGGATTAA [24570]
cp32-9_PAli_NX_Pl_consensus    TTGCAAATAGTGTCAGTGTCCCCATGTGAATGGGAGCACTAAAAAATTAAAAAAATAAATTTAATATAGGAGGATTAA [24570]
cp32-9_PAbe_NX_MP_FG_consensus TTGCAAATAGTGTTAGTGTCCCCATGTGAATGGGAGCACTAAAAAATTAAAGAAATAA-TTTAATATAGGAGGATTAA [24570]
cp32-9_PAbe_NX_Pl_consensus    TTGCAAATAGTGTTAGTGTCCCCATGTGAATGGGAGCACTAAAAAATTAAAGAAATAA-TTTAATATAGGAGGATTAA [24570]

cp32-9_gi|11497325|B31_GB      TTAATGCTTATTAATAAAATAAAACAAGATAATAGAACTTTACGCCCAGAGATACAAAGGTGGGGTTGTTACTTTTTG [24648]
cp32-9_un7_B31_PacBio          TTAATGCTTATTAATAAAATAAAACAAGATAATAGAACTTTACGCCCAGAGATACAAAGGTGGGGTTGTTACTTTTTG [24648]
cp32-9_B31_TS_MP_FG_consensus  TTAATGCTTATTAATAAAATAAAACAAGATAATAGAACTTTACGCCCAGAGATACAAAGGTGGGGTTGTTACTTTTTG [24648]
cp32-9_B31_NX_Pl_consensus     TTAATGCTTATTAATAAAATAAAACAAGATAATAGAACTTTACGCCCAGAGATACAAAGGTGGGGTTGTTACTTTTTG [24648]
cp32-9_PAli_NX_MP_FG_consensus TTAATGCTTATTAATAAAATAAAACAAGATAATAGAACTTTACGCCCAGAGATACAAAGGTGGGGTTGTTACTTTTTG [24648]
cp32-9_PAli_NX_Pl_consensus    TTAATGCTTATTAATAAAATAAAACAAGATAATAGAACTTTACGCCCAGAGATACAAAGGTGGGGTTGTTACTTTTTG [24648]
cp32-9_PAbe_NX_MP_FG_consensus TTAATGCTTATTAATAAAATAAAACAAGATAATAGAACTTTACGCCCAGAGATACAAAGGTGGGGTTGTTACTTTTTG [24648]
cp32-9_PAbe_NX_Pl_consensus    TTAATGCTTATTAATAAAATAAAACAAGATAATAGAACTTTACGCCCAGAGATACAAAGGTGGGGTTGTTACTTTTTG [24648]

cp32-9_gi|11497325|B31_GB      TGTCTACATTATTATACAAGCCTATTTAAGAAATGTGAATTTAATGCTTATGAGATAAATGCAGCGTATTATAGATTT [24726]
cp32-9_un7_B31_PacBio          TGTCTACATTATTATACAAGCCTATTTAAGAAATGTGAATTTAATGCTTATGAGATAAATGCAGCGTATTATAGATTT [24726]
cp32-9_B31_TS_MP_FG_consensus  TGTCTACATTATTATACAAGCCTATTTAAGAAATGTGAATTTAATGCTTATGAGATAAATGCAGCGTATTATAGATTT [24726]
cp32-9_B31_NX_Pl_consensus     TGTCTACATTATTATACAAGCCTATTTAAGAAATGTGAATTTAATGCTTATGAGATAAATGCAGCGTATTATAGATTT [24726]
cp32-9_PAli_NX_MP_FG_consensus TGTCTACATTATTATACAAGCCTATTTAAGAAATGTGAATTTAATGCTTATGAGATAAATGCAGCGTATTATAGATTT [24726]
cp32-9_PAli_NX_Pl_consensus    TGTCTACATTATTATACAAGCCTATTTAAGAAATGTGAATTTAATGCTTATGAGATAAATGCAGCGTATTATAGATTT [24726]
cp32-9_PAbe_NX_MP_FG_consensus TGTCTACATTATTATACAAGCCTATTTAAGAAATGTGAATTTAATGCTTATGAGATAAATGCAGCGTATTATAGATTT [24726]
cp32-9_PAbe_NX_Pl_consensus    TGTCTACATTATTATACAAGCCTATTTAAGAAATGTGAATTTAATGCTTATGAGATAAATGCAGCGTATTATAGATTT [24726]

cp32-9_gi|11497325|B31_GB      ATAGGACTTGGGTACATTAAGAGTAATTGTTTTATTATAAATCCATGTATGATACTTAATTATTACGGAATTAGAAGT [24804]
cp32-9_un7_B31_PacBio          ATAGGACTTGGGTACATTAAGAGTAATTGTTTTATTATAAATCCATGTATGATACTTAATTATTACGGAATTAGAAGT [24804]
cp32-9_B31_TS_MP_FG_consensus  ATAGGACTTGGGTACATTAAGAGTAATTGTTTTATTATAAATCCATGTATGATACTTAATTATTACGGAATTAGAAGT [24804]
cp32-9_B31_NX_Pl_consensus     ATAGGACTTGGGTACATTAAGAGTAATTGTTTTATTATAAATCCATGTATGATACTTAATTATTACGGAATTAGAAGT [24804]
cp32-9_PAli_NX_MP_FG_consensus ATAGGACTTGGGTACATTAAGAGTAATTGTTTTATTATAAATCCATGTATGATACTTAATTATTACGGAATTAGAAGT [24804]
cp32-9_PAli_NX_Pl_consensus    ATAGGACTTGGGTACATTAAGAGTAATTGTTTTATTATAAATCCATGTATGATACTTAATTATTACGGAATTAGAAGT [24804]
cp32-9_PAbe_NX_MP_FG_consensus ATAGGACTTGGGTACATTAAGAGTAATTGTTTTATTATAAATCCATGTATGATACTTAATTATTACGGAATTAGAAGT [24804]
cp32-9_PAbe_NX_Pl_consensus    ATAGGACTTGGGTACATTAAGAGTAATTGTTTTATTATAAATCCATGTATGATACTTAATTATTACGGAATTAGAAGT [24804]

cp32-9_gi|11497325|B31_GB      AGTGTGAGATATGAGTCTTTAAATTATTTAGGTGCAGCCAATGAATTTGAAATAAGCGAAGTTAAAATCGATAAGGTT [24882]
cp32-9_un7_B31_PacBio          AGTGTGAGATATGAGTCTTTAAATTATTTAGGTGCAGCCAATGAATTTGAAATAAGCGAAGTTAAAATCGATAAGGTT [24882]
cp32-9_B31_TS_MP_FG_consensus  AGTGTGAGATATGAGTCTTTAAATTATTTAGGTGCAGCCAATGAATTTGAAATAAGCGAAGTTAAAATCGATAAGGTT [24882]
cp32-9_B31_NX_Pl_consensus     AGTGTGAGATATGAGTCTTTAAATTATTTAGGTGCAGCCAATGAATTTGAAATAAGCGAAGTTAAAATCGATAAGGTT [24882]
cp32-9_PAli_NX_MP_FG_consensus AGTGTGAGATATGAGTCTTTAAATTATTTAGGTGCAGCCAATGAATTTGAAATAAGCGAAGTTAAAATCGATAAGGTT [24882]
cp32-9_PAli_NX_Pl_consensus    AGTGTGAGATATGAGTCTTTAAATTATTTAGGTGCAGCCAATGAATTTGAAATAAGCGAAGTTAAAATCGATAAGGTT [24882]
cp32-9_PAbe_NX_MP_FG_consensus AGTGTGAGATATGAGTCTTTAAATTATTTAGGTGCAGCCAATGAATTTGAAATAAGCGAAGTTAAAATCGATAAGGTT [24882]
cp32-9_PAbe_NX_Pl_consensus    AGTGTGAGATATGAGTCTTTAAATTATTTAGGTGCAGCCAATGAATTTGAAATAAGCGAAGTTAAAATCGATAAGGTT [24882]

cp32-9_gi|11497325|B31_GB      AATGGATATCACTTTATAGCAACAAAAAATAAAGAAATATTATATGATTCACTTGACTTGAAACCTCGTGGGAAAATA [24960]
cp32-9_un7_B31_PacBio          AATGGATATCACTTTATAGCAACAAAAA-TAAAGAAATATTATATGATTCACTTGACTTGAAACCTCGTGGGAAAATA [24960]
cp32-9_B31_TS_MP_FG_consensus  AATGGATATCACTTTATAGCAACAAAAAATAAAGAAATATTATATGATTCACTTGACTTGAAACCTCGTGGGAAAATA [24960]
cp32-9_B31_NX_Pl_consensus     AATGGATATCACTTTATAGCAACAAAAAATAAAGAAATATTATATGATTCACTTGACTTGAAACCTCGTGGGAAAATA [24960]
cp32-9_PAli_NX_MP_FG_consensus AATGGATATCACTTTATAGCAACAAAAAATAAAGAAATATTATATGATTCACTTGACTTGAAACCTCGTGGGAAAATA [24960]
cp32-9_PAli_NX_Pl_consensus    AATGGATATCACTTTATAGCAACAAAAAATAAAGAAATATTATATGATTCACTTGACTTGAAACCTCGTGGGAAAATA [24960]
cp32-9_PAbe_NX_MP_FG_consensus AATGGATATCACTTTATAGCAACAAAAAATAAAGAAATATTATATGATTCACTTGACTTGAAACCTCGTGGGAAAATA [24960]
cp32-9_PAbe_NX_Pl_consensus    AATGGATATCACTTTATAGCAACAAAAAATAAAGAAATATTATATGATTCACTTGACTTGAAACCTCGTGGGAAAATA [24960]

cp32-9_gi|11497325|B31_GB      TTTAAAGTAACTTCAAAGCGTATATTTAGACTAAAGTAGTTTTTTTAAAGTTTAAGGTTATTTTGCATGCATTTATAA [25038]
cp32-9_un7_B31_PacBio          TTTAAAGTAACTTCAAAGCGTATATTTAGACTAAAGTAGTTTTTTTAAAGTTTAAGGTTATTTTGCATGCATTTATAA [25038]
cp32-9_B31_TS_MP_FG_consensus  TTTAAAGTAACTTCAAAGCGTATATTTAGACTAAAGTAGTTTTTTTAAAGTTTAAGGTTATTTTTCATGCATTTATAA [25038]
cp32-9_B31_NX_Pl_consensus     TTTAAAGTAACTTCAAAGCGTATATTTAGACTAAAGTAGTTTTTTTAAAGTTTAAGGTTATTTTGCATGCATTTATAA [25038]
cp32-9_PAli_NX_MP_FG_consensus TTTAAAGTAACTTCAAAGCGTATATTTAGACTAAAGTAGTTTTTTTAAAGTTTAAGGTTATTTTGCATGCATTTATAA [25038]
cp32-9_PAli_NX_Pl_consensus    TTTAAAGTAACTTCAAAGCGTATATTTAGACTAAAGTAGTTTTTTTAAAGTTTAAGGTTATTTTGCATGCATTTATAA [25038]
cp32-9_PAbe_NX_MP_FG_consensus TTTAAAGTAACTTCAAAGCGTATATTTAGACTAAAGTAGTTTTTTTAAAGTTTAAGGTTATTTTGCATGCATTTATAA [25038]
cp32-9_PAbe_NX_Pl_consensus    TTTAAAGTAACTTCAAAGCGTATATTTAGACTAAAGTAGTTTTTTTAAAGTTTAAGGTTATTTTGCATGCATTTATAA [25038]

cp32-9_gi|11497325|B31_GB      GCGTGAATTTTATTAGCAGCAGAGAGTCCATAAATATTATCAATTTCGGAAGTTGAATGATATTTCATAAGTTCTTTA [25116]
cp32-9_un7_B31_PacBio          GCGTGAATTTTATTAGCAGCAGAGAGTCCATAAATATTATCAATTTCGGAAGTTGAATGATATTTCATAAGTTCTTTA [25116]
cp32-9_B31_TS_MP_FG_consensus  GCGTGAATTTTATTAGCAGCAGAGAGGCCGTAGATATTATCAATTTCGGAAGTTGAATGATATTTCATAAGTTCTTTA [25116]
cp32-9_B31_NX_Pl_consensus     GCGTGAATTTTATTAGCAGCAGAGAGTCCATAAATATTATCAATTTCGGAAGTTGAATGATATTTCATAAGTTCTTTA [25116]
cp32-9_PAli_NX_MP_FG_consensus GCGTGAATTTTATTAGCAGCAGAGAGTCCGTAGATATTATCAATTTCGGAAGTTGAATGATATTTCATAAGTTCTTTA [25116]
cp32-9_PAli_NX_Pl_consensus    GCGTGAATTTTATTAGCAGCAGAGAGTCCATAAATATTATCAATTTCGGAAGTTGAATGATATTTCATAAGTTCTTTA [25116]
cp32-9_PAbe_NX_MP_FG_consensus GCGTGAATTTTATTAGCAGCAGAGAGTCCATAAATATTATCAATTTCGGAAGTTGAATGATATTTCATAAGTTCTTTA [25116]
cp32-9_PAbe_NX_Pl_consensus    GCGTGAATTTTATTAGCAGCAGAGAGTCCATAAATATTATCAATTTCGGAAGTTGAATGATATTTCATAAGTTCTTTA [25116]

cp32-9_gi|11497325|B31_GB      ATTTGAAAAGAATTATAACCATTAGATTTTAAATATGAAATAAACAAATTTCTAAATAAATGAAGTGATTTATTAGCA [25194]
cp32-9_un7_B31_PacBio          ATTTGAAAAGAATTATAACCATTAGATTTTAAATATGAAATAAACAAATTTCTAAATAAATGAAGTGATTTATTAGCA [25194]
cp32-9_B31_TS_MP_FG_consensus  ATTTGGAAAGAATTATAACCATTAGATTTTAAATATGAAATAAACAAATTTCTAAATAAATGAAGTGATTTATTAGCA [25194]
cp32-9_B31_NX_Pl_consensus     ATTTGGAAAGAATTATAACCATTAGATTTTAAATATGAAATAAACAAATTTCTAAATAAATGAAGTGATTTATTAGCA [25194]
cp32-9_PAli_NX_MP_FG_consensus ATTTGGAAAGAATTATAACCATTAGATTTTAAATATGAAATAAACAAATTTCTAAATAAATGAAGTGATTTATTAGCA [25194]
cp32-9_PAli_NX_Pl_consensus    ATTTGAAAAGAATTATAACCATTAGATTTTAAATATGAAATAAACAAATTTCTAAATAAATGAAGTGATTTATTAGCA [25194]
cp32-9_PAbe_NX_MP_FG_consensus ATTTGGAAAGAATTATAACCATTAGATTTTAAATATGAAATAAACAAATTTCTAAATAAATGAAGTGATTTATTAGCA [25194]
cp32-9_PAbe_NX_Pl_consensus    ATTTGAAAAGAATTATAACCATTAGATTTTAAATATGAAATAAACAAATTTCTAAATAAATGAAGTGATTTATTAGCA [25194]

cp32-9_gi|11497325|B31_GB      CGAAATCCTGACTTTTTGAGAAGATTTTTGAATTTATTAGAAATATGGATAATGCTAATTTGATTATCTTTAAATTTA [25272]
cp32-9_un7_B31_PacBio          CGAAATCCTGACTTTTTGAGAAGATTTTTGAATTTATTAGAAATATGGATAATGCTAATTTGATTATCTTTAAATTTA [25272]
cp32-9_B31_TS_MP_FG_consensus  CGAAATCCTGACTTTTTGAGAAGATTTTTGAATTTATTAGAAATATGGATAATGCTAATTTGATTATCTTTAAATTTA [25272]
cp32-9_B31_NX_Pl_consensus     CGAAATCCTGACTTTTTGAGAAGATTTTTGAATTTATTAGAAATATGGATAATGCTAATTTGATTATCTTTAAATTTA [25272]
cp32-9_PAli_NX_MP_FG_consensus CGAAATCCTGACTTTTTGAGAAGATTTTTGAATTTATTAGAAATATGGATAATGCTAATTTGATTATCTTTAAATTTA [25272]
cp32-9_PAli_NX_Pl_consensus    CGAAATCCTGACTTTTTGAGAAGATTTTTGAATTTATTAGAAATATGGATAATGCTAATTTGATTATCTTTAAATTTA [25272]
cp32-9_PAbe_NX_MP_FG_consensus CGAAATCCTGACTTTTTGAGAAGATTTTTGAATTTATTAGAAATATGGATAATGCTAATTTGATTATCTTTAAATTTA [25272]
cp32-9_PAbe_NX_Pl_consensus    CGAAATCCTGACTTTTTGAGAAGATTTTTGAATTTATTAGAAATATGGATAATGCTAATTTGATTATCTTTAAATTTA [25272]

cp32-9_gi|11497325|B31_GB      TGTTTAGTTTTTTGGAAAAGATAAGTACGCCGAGAGTCAAGATTTTTTTCATTAAAGTAATTTTCGTGAGCTTTTTGA [25350]
cp32-9_un7_B31_PacBio          TGTTTAGTTTTTTGGAAAAGATAAGTACGCCGAGAGTCAAGATTTTTTTCATTAAAGTAATTTTCGTGAGCTTTTTGA [25350]
cp32-9_B31_TS_MP_FG_consensus  TGTTTAGTTTTTTGGAAAAGATAAGTACGCCGAGGGTCAAGATTTTTTTCATTAAAGTAATTTTCGTGAGCTTTTTGA [25350]
cp32-9_B31_NX_Pl_consensus     TGTTTAGTTTTTTGGAAAAGATAAGTACGCCGAGGGTCAAGATTTTTTTCATTAAAGTAATTTTCGTGAGCTTTTTGA [25350]
cp32-9_PAli_NX_MP_FG_consensus TGTTTAGTTTTTTGGAAAAGATAAGTACGCCGAGGGTCAAGATTTTTTTCATTAAAGTAATTTTCGTGAGCTTTTTGA [25350]
cp32-9_PAli_NX_Pl_consensus    TGTTTAGTTTTTTGGAAAAGATAAGTACGCCGAGGGTCAAGATTTTTTTCATTAAAGTAATTTTCGTGAGCTTTTTGA [25350]
cp32-9_PAbe_NX_MP_FG_consensus TGTTTAGTTTTTTGGAAAAGATAAGTACGCCGAGGGTCAAGATTTTTTTCATTAAAGTAATTTTCGTGAGCTTTTTGA [25350]
cp32-9_PAbe_NX_Pl_consensus    TGTTTAGTTTTTTGGAAAAGATAAGTACGCCGAGGGTCAAGATTTTTTTCATTAAAGTAATTTTCGTGAGCTTTTTGA [25350]

cp32-9_gi|11497325|B31_GB      ATAGCATCAAATTCTACAGATTTTATGACAATTTCTCTAATGCAAGTGACATTTCTTTTTTTAGCTACATTTACTTTT [25428]
cp32-9_un7_B31_PacBio          ATAGCATCAAATTCTACAGATTTTATGACAATTTCTCTAATGCAAGTGACATTTCTTTTTTTAGCTACATTTACTTTT [25428]
cp32-9_B31_TS_MP_FG_consensus  ATAGCATCAAACTCTACAGATTTTATGACAATTTCTCTAATACAAGTGACATTTCTTTTTTTAGCTACATTTACTTTT [25428]
cp32-9_B31_NX_Pl_consensus     ATAGCATCAAACTCTACAGATTTTATGACAATTTCTCTAATACAAGTGACATTTCTTTTTTTAGCTACATTTACTTTT [25428]
cp32-9_PAli_NX_MP_FG_consensus ATAGCATCAAACTCTACAGATTTTATGACAATTTCTCTAATACAAGTGACATTTCTTTTTTTAGCTACATTTACTTTT [25428]
cp32-9_PAli_NX_Pl_consensus    ATAGCATCAAATTCTACAGATTTTATGACAATTTCTCTAATACAAGTGACATTTCTTTTTTTAGCTACATTTACTTTT [25428]
cp32-9_PAbe_NX_MP_FG_consensus ATAGCATCAAACTCTACAGATTTTATGACAATTTCTCTAATACAAGTGACATTTCTTTTTTTAGCTACATTTACTTTT [25428]
cp32-9_PAbe_NX_Pl_consensus    ATAGCATCAAACTCTACAGATTTTATGACAATTTCTCTAATACAAGTGACATTTCTTTTTTTAGCTACATTTACTTTT [25428]

cp32-9_gi|11497325|B31_GB      ATATTGTAAAAAGTTTCTCCAGTTTTGCTTAAAAAGGGAGTAATATCTTGCATTTTTACTTTTTGCAGCTCGGCACCT [25506]
```

```
cp32-9_un7_B31_PacBio          ATATTGTAAAAAGTTTCTCCAGTTTTGCTTAAAAAGGGAGTAATATCTTGCATTTTTACTTTTTGCAGCTCGGCACCT [25506]
cp32-9_B31_TS_MP_FG_consensus  ATATTGTAAAAAGTTTCTCCAGTTTTGCTTAAAAAGGGAGTAATATCTTGTATTTTTACTTTTTGTAGTTCAGCACCT [25506]
cp32-9_B31_NX_Pl_consensus     ATATTGTAAAAAGTTTCTCCAGTTTTGCTTAAAAAGGGAGTAATATCTTGTATTTTTACTTTTTGTAGTTCAGCACCT [25506]
cp32-9_PAli_NX_MP_FG_consensus ATATTGTAAAAAGTTTCTCCAGTTTTGCTTAAAAAGGGAGTAATATCTTGTATTTTTACTTTTTGTAGTTCAGCACCT [25506]
cp32-9_PAli_NX_Pl_consensus    ATATTGTAAAAAGTTTCTCCAGTTTTGCTTAAAAAGGGAGTAATATCTTGCATTTTTACTTTTTGTAGTTCAGCACCT [25506]
cp32-9_PAbe_NX_MP_FG_consensus ATATTGTAAAAAGTTTCTCCAGTTTTGCTTAAAAAGGGAGTAATATCTTGTATTTTTACTTTTTGTAGTTCAGCACCT [25506]
cp32-9_PAbe_NX_Pl_consensus    ATATTGTAAAAAGTTTCTCCAGTTTTGCTTAAAAAGGGAGTAATATCTTGTATTTTTACTTTTTGTAGTTCAGCACCT [25506]

cp32-9_gi|11497325|B31_GB      CTGCAGCCACTTATTGCCAGTAGATTTAAAAACCAACCAGAAATTGGGTCAATTTATTTTAATGTTTTAACACATTTT [25584]
cp32-9_un7_B31_PacBio          CTGCAGCCACTTATTGCCAGTAGATTTAAAAACCAACCAGAAATTGGGTCAATTTATTTTAATGTTTTAACACATTTT [25584]
cp32-9_B31_TS_MP_FG_consensus  CTGCAGCCACTTATTGCGAGTAGATGTAAAAACCAACCAGAAATCGGGTCAATTTGTTTTAATGTTTTAACGCATTTT [25584]
cp32-9_B31_NX_Pl_consensus     CTGCAGCCACTTATTGCGAGTAGATGTAAAAACCAACCAGAAATCGGGTCAATTTGTTTTAATGTTTTAACGCATTTT [25584]
cp32-9_PAli_NX_MP_FG_consensus CTGCAGCCACTTATTGCGAGTAGATGTAAAAACCAACCAGAAATCGGGTCAATTTGTTTTAATGTTTTAACGCATTTT [25584]
cp32-9_PAli_NX_Pl_consensus    CTGCAGCCACTTATTGCGAGTAGATGTAAAAACCAACCAGAAATCGGGTCAATTTGTTTTAATGTTTTAACGCATTTT [25584]
cp32-9_PAbe_NX_MP_FG_consensus CTGCAGCCACTTATTGCGAGTAGATGTAAAAACCAACCAGAAATCGGGTCAATTTGTTTTAATGTTTTAACGCATTTT [25584]
cp32-9_PAbe_NX_Pl_consensus    CTGCAGCCACTTATTGCGAGTAGATGTAAAAACCAACCAGAAATCGGGTCAATTTGTTTTAATGTTTTAACGCATTTT [25584]

cp32-9_gi|11497325|B31_GB      TCAATTATTTTGATAATTTTTTGATTCAAATAAAACTTTGGAGTTGGTTTTGAAGCTTTTTTAGTAGGCTTAGAAGAA [25662]
cp32-9_un7_B31_PacBio          TCAATTATTTTGATAATTTTTTGATTCAAATAAAACTTTGGAGTTGGTTTTGAAGCTTTTTTAGTAGGCTTAGAAGAA [25662]
cp32-9_B31_TS_MP_FG_consensus  TCAATTATTTTGATAATTTTTTGATTCAAATAAAACTTTGGAGTTGGTTTTGAAGCTTTTTTAGTAGGCTTAGAAGAA [25662]
cp32-9_B31_NX_Pl_consensus     TCAATTATTTTGATAATTTTTTGATTCAAATAAAACTTTGGAGTTGGTTTTGAAGCTTTTTTAGTAGGCTTAGAAGAA [25662]
cp32-9_PAli_NX_MP_FG_consensus TCAATTATTTTGATAATTTTTTGATTCAAATAAAACTTTGGAGTTGGTTTTGAAGCTTTTTTAGTAGGCTTAGAAGAA [25662]
cp32-9_PAli_NX_Pl_consensus    TCAATTATTTTGATAATTTTTTGATTCAAATAAAACTTTGGAGTTGGTTTTGAAGCTTTTTTAGTAGGCTTAGAAGAA [25662]
cp32-9_PAbe_NX_MP_FG_consensus TCAATTATTTTGATAATTTTTTGATTCAAATAAAACTTTGGAGTTGGTTTTGAAGCTTTTTTAGTAGGCTTAGAAGAA [25662]
cp32-9_PAbe_NX_Pl_consensus    TCAATTATTTTGATAATTTTTTGATTCAAATAAAACTTTGGAGTTGGTTTTGAAGCTTTTTTAGTAGGCTTAGAAGAA [25662]

cp32-9_gi|11497325|B31_GB      ATTTTTAGTGAATTTTTAAGAATTTTGTTTTCATTTAATACATTTTGATAGTCTTTTAAGAATTTGTGCATCAAATCT [25740]
cp32-9_un7_B31_PacBio          ATTTTTAGTGAATTTTTAAGAATTTTGTTTTCATTTAATACATTTTGATAGTCTTTTAAGAATTTGTGCATCAAATCT [25740]
cp32-9_B31_TS_MP_FG_consensus  ATTTTTAGTGAATTTTTAAGAATTTTGTTTTCATTTAATACATTTTGATAGTCTTTTAAGAATTTGTGCATCAAATCT [25740]
cp32-9_B31_NX_Pl_consensus     ATTTTTAGTGAATTTTTAAGAATTTTGTTTTCATTTAATACATTTTGATAGTCTTTTAAGAATTTGTGCATCAAATCT [25740]
cp32-9_PAli_NX_MP_FG_consensus ATTTTTAGTGAATTTTTAAGAATTTTGTTTTCATTTAATACATTTTGATAGTCTTTTAAGAATTTGTGCATCAAATCT [25740]
cp32-9_PAli_NX_Pl_consensus    ATTTTTAGTGAATTTTTAAGAATTTTGTTTTCATTTAATACATTTTGATAGTCTTTTAAGAATTTGTGCATCAAATCT [25740]
cp32-9_PAbe_NX_MP_FG_consensus ATTTTTAGTGAATTTTTAAGAATTTTATTTTCATTTAGCACATTTTGATAGTCTTTTAAGAATTTGTGCATCAAATCT [25740]
cp32-9_PAbe_NX_Pl_consensus    ATTTTTAGTGAATTTTTAAGAATTTTGTTTTCATTTAATACATTTTGATAGTCTTTTAAGAATTTGTGCATCAAATCT [25740]

cp32-9_gi|11497325|B31_GB      GTATCGAAATTATTTAAATTAAAATAATTATTAGTATCCATAAAATCCTCTCCTTTGAAGTGTTACTTTTAAATTAAG [25818]
cp32-9_un7_B31_PacBio          GTATCGAAATTATTTAAATTAAAATAATTATTAGTATCCATAAAATCCTCTCCTTTGAAGTGTTACTTTTAAATTAAG [25818]
cp32-9_B31_TS_MP_FG_consensus  GTATCGAAATTATTTAAATTAAAATAATTATTAGTGTCCATAAAATCCTCTCCTTATAAGTGTTACTTTTAAATTAAG [25818]
cp32-9_B31_NX_Pl_consensus     GTATCGAAATTATTTAAATTAAAATAATTATTAGTATCCATAAAATCCTCTCCTTTGAAGTGTTACTTTTAAATTAAG [25818]
cp32-9_PAli_NX_MP_FG_consensus GTATCGAAATTATTTAAATTAAAATAATTATTAGTATCCATAAAATCCTCTCCTTATAAGTGTTACTTTTAAATTAAG [25818]
cp32-9_PAli_NX_Pl_consensus    GTATCGAAATTATTTAAATTAAAATAATTATTAGTATCCATAAAATCCTCTCCTTTGAAGTGTTACTTTTAAATTAAG [25818]
cp32-9_PAbe_NX_MP_FG_consensus GTATCGAAATTATTTAAATTAAAATAATTATTAGTGTCCATAAAATCCTCTCCTTATAAGTGTTACTTTTAAATTAAG [25818]
cp32-9_PAbe_NX_Pl_consensus    GTATCGAAATTATTTAAATTAAAATAATTATTAGTATCCATAAAATCCTCTCCTTTGAAGTGTTACTTTTAAATTAAG [25818]

cp32-9_gi|11497325|B31_GB      TAAAAGTAATAAAAAATTTAATAAAAAATGTAATTTATATTTTATCAAAACCCTAAAATTTTAGTCAAATTTATGGTGTT [25896]
cp32-9_un7_B31_PacBio          TAAAAGTAATAAAAAATTTAATAAAAAATGTAATTTATATTTTATCAAAACCCTAAAATTTTAGTCAAATTTATGGTGTT [25896]
cp32-9_B31_TS_MP_FG_consensus  TAAAAGTAATAAAAAATTTAATAAAAAATGTAATTTATATTTTATCAAAAACATAAAATTTTAGTCAAATTTATGGTGTT [25896]
cp32-9_B31_NX_Pl_consensus     TAAAAGTAATAAAAAATTTAATAAAAAATGTAATTTATATTTTATCAAAAACATAAAATTTTAGTCAAATTTATGGTGTT [25896]
cp32-9_PAli_NX_MP_FG_consensus TAAAAGTAATAAAAAATTTAATAAAAAATGTAATTTATATTTTATCAAAAACATAAAATTTTAGTCAAATTTATGGTGTT [25896]
cp32-9_PAli_NX_Pl_consensus    TAAAAGTAATAAAAAATTTAATAAAAAATGTAATTTATATTTTATCAAAACCCTAAAATTTTAGTCAAATTTATGGTGTT [25896]
cp32-9_PAbe_NX_MP_FG_consensus TAAAAGTAATAAAAAATTTAATAAAAAATGTAATTTATATTTTATCAAAAACATAAAATTTTAGTCAAATTTATGGTGTT [25896]
cp32-9_PAbe_NX_Pl_consensus    TAAAAGTAATAAAAAATTTAATAAAAAATGTAATTTATATTTTATCAAAACCCTAAAATTTTAGTCAAATTTATGGTGTT [25896]

cp32-9_gi|11497325|B31_GB      CTCATTGCATGCGAAATTTGGGTTGTGGAGTGGCTGTGATAAACAGAAGAGGCAATTTTTAAGGGGTGGACTTAAGAA [25974]
cp32-9_un7_B31_PacBio          CTCATTGCATGCGAAATTTGGGTTGTGGAGTGGCTGTGATAAACAGAAGAGGCAATTTTTAAGGGGTGGACTTAAGAA [25974]
cp32-9_B31_TS_MP_FG_consensus  CTCATTGCATGAGAAATTTGTGTTGTGGAGTGGCTGTGATAAACAGAAGAGGCAATTTTTAAGGGGTGGACTTAAGAA [25974]
cp32-9_B31_NX_Pl_consensus     CTCATTGCATGAGAAATTTGTGTTGTGGAGTGGCTGTGATAAACAGAAGAGGCAATTTTTAAGGGGTGGACTTAAGAA [25974]
cp32-9_PAli_NX_MP_FG_consensus CTCATTGCATGAGAAATTTGTGTTGTGGAGTGGCTGTGATAAACAGAAGAGGCAATTTTTAAGGGGTGGACTTAAGAA [25974]
cp32-9_PAli_NX_Pl_consensus    CTCATTGCATGCGAAATTTGGGTTGTGGAGTGGCTGTGATAAACAGAAGAGGCAATTTTTAAGGGGTGGACTTAAGAA [25974]
cp32-9_PAbe_NX_MP_FG_consensus CTCATTGCATGAGAAATTTGTGTTGTGGAGTGGCTGTGATAAACAGAAGAGGCAATTTTTAAGGGGTGGACTTAAGAA [25974]
cp32-9_PAbe_NX_Pl_consensus    CTCATTGCATGAGAAATTTGTGTTGTGGAGTGGCTGTGATAAACAGAAGAGGCAATTTTTAAGGGGTGGACTTAAGAA [25974]

cp32-9_gi|11497325|B31_GB      AGACATGATACTTTAAGTGATATATAGCAAAGACTTTGAAATTTAATTTGTATGTGTTTTGTAGTCTTTTGTAATGAG [26052]
cp32-9_un7_B31_PacBio          AGACATGATACTTTAAGTGATATATAGCAAAGACTTTGAAATTTAATTTGTATGTGTTTTGTAGTCTTTTGTAATGAG [26052]
cp32-9_B31_TS_MP_FG_consensus  AGACTAGATACTTTAAGTGATATATAGCAAAGACTTTGAAATTTAAGTTGTATGTGTTTTATAGTCTCTTGTAATGAG [26052]
cp32-9_B31_NX_Pl_consensus     AGACTAGATACTTTAAGTGATATATAGCAAAGACTTTGAAATTTAAGTTGTATGTGTTTTATAGTCTCTTGTAATGAG [26052]
cp32-9_PAli_NX_MP_FG_consensus AGACTAGATACTTTAAGTGATATATAGCAAAGACTTTGAAATTTAAGTTGTATGTGTTTTATAGTCTCTTGTAATGAG [26052]
cp32-9_PAli_NX_Pl_consensus    AGACATGATACTTTAAGTGATATATAGCAAAGACTTTGAAATTTAATTTGTATGTGTTTTGTAGTCTTTTGTAATGAG [26052]
cp32-9_PAbe_NX_MP_FG_consensus AGACTAGATACTTTAAGTGATATATAGCAAAGACTTTGAAATTTAAGTTGTATGTGTTTTATAGTCTTTTGTAATGAG [26052]
cp32-9_PAbe_NX_Pl_consensus    AGACTAGATACTTTAAGTGATATATAGCAAAGACTTTGAAATTTAAGTTGTATGTGTTTTATAGTCTTTTGTAATGAG [26052]

cp32-9_gi|11497325|B31_GB      CCGGGCATTTGCAATGGAGAGATTTTTGGGAGTTGGTTAAAATTACATTTGAGTTTTGTTAATATGTAATAGCTGAAT [26130]
cp32-9_un7_B31_PacBio          CCGGGCATTTGCAATGGAGAGATTTTTGGGAGTTGGTTAAAATTACATTTGAGTTTTGTTAATATGTAATAGCTGAAT [26130]
cp32-9_B31_TS_MP_FG_consensus  TAGTGCATTTGCAATGGAGAGATTTTTGGGAGTTGGTTAAAATTACATTTGAGTTTTGTTAAAATGTAACAGCTGAAT [26130]
cp32-9_B31_NX_Pl_consensus     TAGTGCATTTGCAATGGAGAGATTTTTGGGAGTTGGTTAAAATTACATTTGAGTTTTGTTAAAATGTAACAGCTGAAT [26130]
cp32-9_PAli_NX_MP_FG_consensus TAGTGCATTTGCAATGGAGAGATTTTTGGGAGTTGGTTAAAATTACATTTGAGTTTTGTTAAAATGTAATAGCTGAAT [26130]
cp32-9_PAli_NX_Pl_consensus    CCGGGCATTTGCAATGGAGAGATTTTTGGGAGTTGGTTAAAATTACATTTGAGTTTTGTTAATATGTAATAGCTGAAT [26130]
cp32-9_PAbe_NX_MP_FG_consensus CAGGGCATTTGCAATGGAGAGATTTTTGGGAGTTGGTTAAAATTACATTTGAGTTTTGTTAAAATGTAACAGCTGAAT [26130]
cp32-9_PAbe_NX_Pl_consensus    CAGGGCATTTGCAATGGAGAGATTTTTGGGAGTTGGTTAAAATTACATTTGAGTTTTGTTAAAATGTAACAGCTGAAT [26130]

cp32-9_gi|11497325|B31_GB      GTAACAAAATTATATATTTAAATCTTTGAAATATTGCAATTATTAGCTGTTGTGGTATGATTAGGACTTATGGAGAAA [26208]
cp32-9_un7_B31_PacBio          GTAACAAAATTATATATTTAAATCTTTGAAATATTGCAATTATTAGCTGTTGTGGTATGATTAGGACTTATGGAGAAA [26208]
cp32-9_B31_TS_MP_FG_consensus  GTAACAAAATTATATATTTAAATCTTTGAAATATTGCAATTATTAGCTGTTGTGGTATGATTAGGACTTATGGAGAAA [26208]
cp32-9_B31_NX_Pl_consensus     GTAACAAAATTATATATTTAAATCTTTGAAATATTGCAATTATTAGCTGTTGTGGTATGATTAGGACTTATGGAGAAA [26208]
cp32-9_PAli_NX_MP_FG_consensus GTAACAAAATTATATATTTAAATCTTTGAAATATTGCAATTATTAGCTGTTGTGGTATGATTAGGACTTATGGAGAAA [26208]
cp32-9_PAli_NX_Pl_consensus    GTAACAAAATTATATATTTAAATCTTTGAAATATTGCAATTATTAGCTGTTGTGGTATGATTAGGACTTATGGAGAAA [26208]
cp32-9_PAbe_NX_MP_FG_consensus GTAACAAAATTATATATTTAAATCTTTGAAATATTGCAATTATTAGCTGTTGTGGTATGATTAGGACTTATGGAGAAA [26208]
cp32-9_PAbe_NX_Pl_consensus    GTAACAAAATTATATATTTAAATCTTTGAAATATTGCAATTATTAGCTGTTGTGGTATGATTAGGACTTATGGAGAAA [26208]

cp32-9_gi|11497325|B31_GB      TTTATGAATAAGAAAATGAAAATGTTTATTGTTTGTGCTGTTTTTATACTTATAGGTGCTTGCAAAATTCATACTTCA [26286]
cp32-9_un7_B31_PacBio          TTTATGAATAAGAAAATGAAAATGTTTATTGTTTGTGCTGTTTTTATACTTATAGGTGCTTGCAAAATTCATACTTCA [26286]
cp32-9_B31_TS_MP_FG_consensus  TTTATGAATAAGAAAATGAAAATGTTTATTGTTTGTGCTGTTTTTATACTTATAGGTGCTTGCAAAATTCATACTTCA [26286]
cp32-9_B31_NX_Pl_consensus     TTTATGAATAAGAAAATGAAAATGTTTATTGTTTGTGCTGTTTTTATACTTATAGGTGCTTGCAAAATTCATACTTCA [26286]
cp32-9_PAli_NX_MP_FG_consensus TTTATGAATAAGAAAATGAAAATGTTTATTGTTTGTGCTGTTTTTATACTTATAGGTGCTTGCAAAATTCATACTTCA [26286]
cp32-9_PAli_NX_Pl_consensus    TTTATGAATAAGAAAATGAAAATGTTTATTGTTTGTGCTGTTTTTATACTTATAGGTGCTTGCAAAATTCATACTTCA [26286]
cp32-9_PAbe_NX_MP_FG_consensus TTTATGAATAAGAAAATGAAAATGTTTATTGTTTGTGCTGTTTTTATACTTATAGGTGCTTGCAAAATTCATACTTCA [26286]
cp32-9_PAbe_NX_Pl_consensus    TTTATGAATAAGAAAATGAAAATGTTTATTGTTTGTGCTGTTTTTATACTTATAGGTGCTTGCAAAATTCATACTTCA [26286]

cp32-9_gi|11497325|B31_GB      TATGATGAGCAAAGTAGTGGTGAGATAAACCATACTTTATATGATGAGCAAAGTAATGGTGAGTTAAAACTTAAAAAA [26364]
cp32-9_un7_B31_PacBio          TATGATGAGCAAAGTAGTGGTGAGATAAACCATACTTTATATGATGAGCAAAGTAATGGTGAGTTAAAACTTAAAAAA [26364]
cp32-9_B31_TS_MP_FG_consensus  TATGATGAGCAAAGTAGTGGTGAGATAAACCATACTTTATATGATGAGCAAAGTAATGGTGAGTTAAAACTTAAAAAA [26364]
cp32-9_B31_NX_Pl_consensus     TATGATGAGCAAAGTAGTGGTGAGATAAACCATACTTTATATGATGAGCAAAGTAATGGTGAGTTAAAACTTAAAAAA [26364]
cp32-9_PAli_NX_MP_FG_consensus TATGATGAGCAAAGTAGTGGTGAGATAAACCATACTTTATATGATGAGCAAAGTAATGGTGAGTTAAAACTTAAAAAA [26364]
cp32-9_PAli_NX_Pl_consensus    TATGATGAGCAAAGTAGTGGTGAGATAAACCATACTTTATATGATGAGCAAAGTAATGGTGAGTTAAAACTTAAAAAA [26364]
cp32-9_PAbe_NX_MP_FG_consensus TATGATGAGCAAAGTAGTGGTGAGATAAACCATACTTTATATGATGAGCAAAGTAATGGTGAGTTAAAACTTAAAAAA [26364]
cp32-9_PAbe_NX_Pl_consensus    TATGATGAGCAAAGTAGTGGTGAGATAAACCATACTTTATATGATGAGCAAAGTAATGGTGAGTTAAAACTTAAAAAA [26364]

cp32-9_gi|11497325|B31_GB      ATAGAATTCTCTAAATTTACTGTAAAAATTAAAAATAAAGATAATAATAGTAACTGGACAGACCTAGGAGATTTAGTT [26442]
cp32-9_un7_B31_PacBio          ATAGAATTCTCTAAATTTACTGTAAAAATTAAAAATAAAGATAATAATAGTAACTGGACAGACCTAGGAGATTTAGTT [26442]
```

```
cp32-9_B31_TS_MP_FG_consensus  ATAGAATTCTCTAAATTTACTGTAAAAATTAAAAATAAAGATAATAATAGTAACTGGACAGACCTAGGAGATTTAGTT [26442]
cp32-9_B31_NX_Pl_consensus     ATAGAATTCTCTAAATTTACTGTAAAAATTAAAAATAAAGATAATAATAGTAACTGGACAGACCTAGGAGATTTAGTT [26442]
cp32-9_PAli_NX_MP_FG_consensus ATAGAATTCTCTAAATTTACTGTAAAAATTAAAAATAAAGATAATAATAGTAACTGGACAGACCTAGGAGATTTAGTT [26442]
cp32-9_PAli_NX_Pl_consensus    ATAGAATTCTCTAAATTTACTGTAAAAATTAAAAATAAAGATAATAATAGTAACTGGACAGACCTAGGAGATTTAGTT [26442]
cp32-9_PAbe_NX_MP_FG_consensus ATAGAATTCTCTAAATTTACTGTAAAAATTAAAAATAAAGATAATAATAGTAACTGGACAGACCTAGGAGATTTAGTT [26442]
cp32-9_PAbe_NX_Pl_consensus    ATAGAATTCTCTAAATTTACTGTAAAAATTAAAAATAAAGATAATAATAGTAACTGGACAGACCTAGGAGATTTAGTT [26442]

cp32-9_gi|11497325|B31_GB      GTAAGAAAAGAAGAAAATGGTATTGATACGGGTTTAAACGCTGGGGGACATTCGGCTACATTCTTTTCATTAAAAGAA [26520]
cp32-9_un7_B31_PacBio          GTAAGAAAAGAAGAAAATGGTATTGATACGGGTTTAAACGCTGGGGGACATTCGGCTACATTCTTTTCATTAAAAGAA [26520]
cp32-9_B31_TS_MP_FG_consensus  GTAAGAAAAGAAGAAAATGGTATTGATACGGGTTTAAACGCTGGGGGACATTCGGCTACATTCTTTTCATTAAAAGAA [26520]
cp32-9_B31_NX_Pl_consensus     GTAAGAAAAGAAGAAAATGGTATTGATACGGGTTTAAACGCTGGGGGACATTCGGCTACATTCTTTTCATTAAAAGAA [26520]
cp32-9_PAli_NX_MP_FG_consensus GTAAGAAAAGAAGAAAATGGTATTGATACGGGTTTAAACGCTGGGGGACATTCGGCTACATTCTTTTCATTAAAAGAA [26520]
cp32-9_PAli_NX_Pl_consensus    GTAAGAAAAGAAGAAAATGGTATTGATACGGGTTTAAACGCTGGGGGACATTCGGCTACATTCTTTTCATTAAAAGAA [26520]
cp32-9_PAbe_NX_MP_FG_consensus GTAAGAAAAGAAGAAAATGGTATTGATACGGGTTTAAACGCTGGGGGACATTCGGCTACATTCTTTTCATTAAAAGAA [26520]
cp32-9_PAbe_NX_Pl_consensus    GTAAGAAAAGAAGAAAATGGTATTGATACGGGTTTAAACGCTGGGGGACATTCGGCTACATTCTTTTCATTAAAAGAA [26520]

cp32-9_gi|11497325|B31_GB      TCAGAAGTTAATAACTTTATAAAAGCAATGACTAAAGGCGGATCATTTAAAACTAGTTTGTATTATGGATATAAGTAC [26598]
cp32-9_un7_B31_PacBio          TCAGAAGTTAATAACTTTATAAAAGCAATGACTAAAGGCGGATCATTTAAAACTAGTTTGTATTATGGATATAAGTAC [26598]
cp32-9_B31_TS_MP_FG_consensus  TCAGAAGTTAATAACTTTATAAAAGCAATGACTAAAGGCGGATCATTTAAAACTAGTTTGTATTATGGATATAAGTAC [26598]
cp32-9_B31_NX_Pl_consensus     TCAGAAGTTAATAACTTTATAAAAGCAATGACTAAAGGCGGATCATTTAAAACTAGTTTGTATTATGGATATAAGTAC [26598]
cp32-9_PAli_NX_MP_FG_consensus TCAGAAGTTAATAACTTTATAAAAGCAATGACTAAAGGCGGATCATTTAAAACTAGTTTGTATTATGGATATAAGTAC [26598]
cp32-9_PAli_NX_Pl_consensus    TCAGAAGTTAATAACTTTATAAAAGCAATGACTAAAGGCGGATCATTTAAAACTAGTTTGTATTATGGATATAAGTAC [26598]
cp32-9_PAbe_NX_MP_FG_consensus TCAGAAGTTAATAACTTTATAAAAGCAATGACTAAAGGCGGATCATTTAAAACTAGTTTGTATTATGGATATAAGTAC [26598]
cp32-9_PAbe_NX_Pl_consensus    TCAGAAGTTAATAACTTTATAAAAGCAATGACTAAAGGCGGATCATTTAAAACTAGTTTGTATTATGGATATAAGTAC [26598]

cp32-9_gi|11497325|B31_GB      GAACAAAGTAGTGCAAATGGTATCCAAAACAAAGAGATCATAACAAAAATAGAAAGTATTAATGGTGCTGAACATATT [26676]
cp32-9_un7_B31_PacBio          GAACAAAGTAGTGCAAATGGTATCCAAAACAAAGAGATCATAACAAAAATAGAAAGTATTAATGGTGCTGAACATATT [26676]
cp32-9_B31_TS_MP_FG_consensus  GAACAAAGTAGTGCAAATGGTATCCAAAACAAAGAGATCATAACAAAAATAGAAAGTATTAATGGTGCTGAACATATT [26676]
cp32-9_B31_NX_Pl_consensus     GAACAAAGTAGTGCAAATGGTATCCAAAACAAAGAGATCATAACAAAAATAGAAAGTATTAATGGTGCTGAACATATT [26676]
cp32-9_PAli_NX_MP_FG_consensus GAACAAAGTAGTGCAAATGGTATCCAAAACAAAGAGATCATAACAAAAATAGAAAGTATTAATGGTGCTGAACATATT [26676]
cp32-9_PAli_NX_Pl_consensus    GAACAAAGTAGTGCAAATGGTATCCAAAACAAAGAGATCATAACAAAAATAGAAAGTATTAATGGTGCTGAACATATT [26676]
cp32-9_PAbe_NX_MP_FG_consensus GAACAAAGTAGTGCAAATGGTATCCAAAACAAAGAGATCATAACAAAAATAGAAAGTATTAATGGTGCTGAACATATT [26676]
cp32-9_PAbe_NX_Pl_consensus    GAACAAAGTAGTGCAAATGGTATCCAAAACAAAGAGATCATAACAAAAATAGAAAGTATTAATGGTGCTGAACATATT [26676]

cp32-9_gi|11497325|B31_GB      GCGTTTTTAGGAGATAAAATTAATAACGGTGTGGGGGGAGATAAAACAGCTGAATATGCAATACCACTAGAAGTGCTT [26754]
cp32-9_un7_B31_PacBio          GCGTTTTTAGGAGATAAA-TTAATAACGGTGTGGGGGGAGATAAAACAGCTGAATATGCAATACCACTAGAAGTGCTT [26754]
cp32-9_B31_TS_MP_FG_consensus  GCGTTTTTAGGAGATAAAATTAATAACGGTGTGGGGGGAGATAAAACAGCTGAATATGCAATACCACTAGAAGTGCTT [26754]
cp32-9_B31_NX_Pl_consensus     GCGTTTTTAGGAGATAAAATTAATAACGGTGTGGGGGGAGATAAAACAGCTGAATATGCAATACCACTAGAAGTGCTT [26754]
cp32-9_PAli_NX_MP_FG_consensus GCGTTTTTAGGAGATAAAATTAATAACGGTGTGGGGGGAGATAAAACAGCTGAATATGCAATACCACTAGAAGTGCTT [26754]
cp32-9_PAli_NX_Pl_consensus    GCGTTTTTAGGAGATAAAATTAATAACGGTGTGGGGGGAGATAAAACAGCTGAATATGCAATACCACTAGAAGTGCTT [26754]
cp32-9_PAbe_NX_MP_FG_consensus GCGTTTTTAGGAGATAAAATTAATAACGGTGTGGGGGGAGATAAAACAGCTGAATATGCAATACTACTAGAAGATCTT [26754]
cp32-9_PAbe_NX_Pl_consensus    GCGTTTTTAGGAGATAAAATTAATAACGGTGTGGGGGGAGATAAAACAGCTGAATATGCAATACCACTAGAAGTGCTT [26754]

cp32-9_gi|11497325|B31_GB      AAAAAAAATTTAAAATAGAATTTAGAAATATAGGAGAGAATAATTATGAATAAAAAAACATTGATTATTTGTGCTGTT [26832]
cp32-9_un7_B31_PacBio          AAAAAAA-TTTAAAATAGAATTTAGAAATATAGGAGAGAATAATTATGAATAAAAAAACATTGATTATTTGTGCTGTT [26832]
cp32-9_B31_TS_MP_FG_consensus  AAAAAAAATTTAAAATAGAATTTAGAAATATAGGAGAGAATAATTATGAATAAAAAAACATTGATTATTTGTGCTGTT [26832]
cp32-9_B31_NX_Pl_consensus     AAAAAAAATTTAAAATAGAAGTTAGAAGTATAGGGGAGAACAATTATGAATCAAAAAGCATTTATTATTTGCGCTGTT [26832]
cp32-9_PAli_NX_MP_FG_consensus AAAAAAAATTTAAAATAGAATTTAGAAATATAGGAGAGAATAATTATGAATAAAAAAACATTGATTATTTGTGCTGTT [26832]
cp32-9_PAli_NX_Pl_consensus    AAAAAAAATTTAAAATAGAATTTAGAAATATAGGAGAGAATAATTATGAATAAAAAAACATTGATTATTTGTGCTGTT [26832]
cp32-9_PAbe_NX_MP_FG_consensus AAAAAAAATTTAAAATAGAAGTTAGAAGTATAGGGGAGAACAATTATGAATCAAAAAGCATTTATTATTTGCGCTGTT [26832]
cp32-9_PAbe_NX_Pl_consensus    AAAAAAAATTTAAAATAGAATTTAGAAATATAGGAGAGAACAATTATGAATCAAAAAGCATTTATTATTTGCGCTGTT [26832]

cp32-9_gi|11497325|B31_GB      TTTGCGCTGATAATTTCTTGCAAGAATTTTGCAACTGGTAAAGATATAAAACAAAATTCAGAAGGGAAAATTAAAGGA [26910]
cp32-9_un7_B31_PacBio          TTTGCGCTGATAATTTCTTGCAAGAATTTTGCAACTGGTAAAGATATAAAACAAAATTCAGAAGGGAAAATTAAAGGA [26910]
cp32-9_B31_TS_MP_FG_consensus  TTTGCGCTGATAATTTCTTGCAAGAATTTTGCAACTGGTAAAGATATAAAACAAAATTCAGAAGGGAAAATTAAAGGA [26910]
cp32-9_B31_NX_Pl_consensus     TTTGCGCTGATAATTTCTTGCAAGAATTATGCAACTAGTAAAGATTTAAAACAAAATCCAGAAGGGAAAATTAAAGGA [26910]
cp32-9_PAli_NX_MP_FG_consensus TTTGCGCTGATAATTTCTTGCAAGAATTTTGCAACTGGTAAAGATATAAAACAAAATTCAGAAGGGAAAATTAAAGGA [26910]
cp32-9_PAli_NX_Pl_consensus    TTTGCGCTGATAATTTCTTGCAAGAATTTTGCAACTGGTAAAGATATAAAACAAAATTCAGAAGGGAAAATTAAAGGA [26910]
cp32-9_PAbe_NX_MP_FG_consensus TTTGCGCTGATAATTTCTTGCAAGAATTATGCAACTAGTAAAGATTTAAAACAAAATCCAGAAGGGAAAATTAAAGGA [26910]
cp32-9_PAbe_NX_Pl_consensus    TTTGCGCTGATAATTTCTTGCAAGAATTATGCAACTAGTAAAGATTTAAAACAAAATCCAGAAGGGAAAATTAAAGGA [26910]

cp32-9_gi|11497325|B31_GB      TTTGTAAATAAGATTTTAGATCCAGTAAAGGATAAAATTGCTTCAAGTGGTACAAAAGTAGATGAAGTAGCAAAAAAA [26988]
cp32-9_un7_B31_PacBio          TTTGTAAATAAGATTTTAGATCCAGTAAAGGATAAAATTGCTTCAAGTGGTACAAAAGTAGATGAAGTAGCAAAAAAA [26988]
cp32-9_B31_TS_MP_FG_consensus  TTTGTAAATAAGATTTTAGATCCAGTAAAGGATAAAATTGCTTCAAGTGGTACAAAAGTAGATGAAGTAGCAAAAAAA [26988]
cp32-9_B31_NX_Pl_consensus     TTTTTAGATAAGATTTTAGATCCAACAAAGGATAAAATTACTTCAATTGGGCCAAAAGTAGATGAAGTAGCAAAAAAA [26988]
cp32-9_PAli_NX_MP_FG_consensus TTTGTAAATAAGATTTTAGATCCAGTAAAGGATAAAATTGCTTCAAGTGGTACAAAAGTAGATGAAGTAGCAAAAAAA [26988]
cp32-9_PAli_NX_Pl_consensus    TTTGTAAATAAGATTTTAGATCCAGTAAAGGATAAAATTGCTTCAAGTGGTACAAAAGTAGATGAAGTAGCAAAAAAA [26988]
cp32-9_PAbe_NX_MP_FG_consensus TTTGTT-------TTTTAGATCCAACAAAGGATAAAATTACTTCAATTGGGCCAAAAGTAGATGAAGTAGCAAAAAAA [26988]
cp32-9_PAbe_NX_Pl_consensus    TTTTTAGATAAGATTTTAGATCCAGTAAAGGATAAAATTACTTCAATTGGGCCAAAAGTAGATGAAGTAGCAAAAAAA [26988]

cp32-9_gi|11497325|B31_GB      TTACAAGAAGAAGAAAAAGAAGAATTAATGCAGGGCGATGATCCTAATGGCAGTGGAATAAATCCGCCACCAGTATTG [27066]
cp32-9_un7_B31_PacBio          TTACAAGAAGAAGAAAAAGAAGAATTAATGCAGGGCGATGATCCTAATGGCAGTGGAATAAATCCGCCACCAGTATTG [27066]
cp32-9_B31_TS_MP_FG_consensus  TTACAAGAAGAAGAAAAAGAAGAATTAATGCAGGGCGATGATCCTAATGGCAGTGGAATAAATCCGCCACCAGTATTG [27066]
cp32-9_B31_NX_Pl_consensus     TTGAAACAAGAAGAAAGAGA---ATTAATGCAGGGCGATGATCCTAATGGCAGCGGAATAAATCCGCCACCAACATTA [27066]
cp32-9_PAli_NX_MP_FG_consensus TTACAAGAAGAAGAAAAAGAAGAATTAATGCAGGGCGATGATCCTAATGGCAGTGGAATAAATCCGCCACCAGTATTG [27066]
cp32-9_PAli_NX_Pl_consensus    TTACAAGAAGAAGAAAAAGAAGAATTAATGCAGGGCGATGATCCTAATGGCAGTGGAATAAATCCGCCACCAGTATTG [27066]
cp32-9_PAbe_NX_MP_FG_consensus TTGAAACAAGAAGAAAGAGA---ATTAATGCAGGGCGATGATCCTAATGGCAGCGGAATAAATCCGCCACCAGCATTG [27066]
cp32-9_PAbe_NX_Pl_consensus    TTGAAACAAGAAGAAAGAGA---ATTAATGCAGGGCGATGATCCTAATGGCAGCGGAATAAATCCGCCACCAGTATTG [27066]

cp32-9_gi|11497325|B31_GB      CCGGAAAATATTCACAATAATGCATTAGTATTAAAAGCAATAGAACAAAGTGATGGTCAACAAGAAAAAAAAGTAGAA [27144]
cp32-9_un7_B31_PacBio          CCGGAAAATATTCACAATAATGCATTAGTATTAAAAGCAATAGAACAAAGTGATGGTCAACAAGAAAAAAAAGTAGAA [27144]
cp32-9_B31_TS_MP_FG_consensus  CCGGAAAATATTCACAATAATGCATTAGTATTAAAAGCAATAGAACAAAGTGATGGTCAACAAGAAAAAAAAGTAGAA [27144]
cp32-9_B31_NX_Pl_consensus     CTAGAAAATATTCACAATAATGCATTAGTATTAAAAGCAATAGAACAAAGTGATGGTCAACAAGAAAAAAAAGTAGAA [27144]
cp32-9_PAli_NX_MP_FG_consensus CCGGAAAATATTCACAATAATGCATTAGTATTAAAAGCAATAGAACAAAGTGATGGTCAACAAGAAAAAAAAGTAGAA [27144]
cp32-9_PAli_NX_Pl_consensus    CCGGAAAATATTCACAATAATGCATTAGTATTAAAAGCAATAGAACAAAGTGATGGTCAACAAGAAAAAAAAGTAGAA [27144]
cp32-9_PAbe_NX_MP_FG_consensus CCAGAAAATATTCACAATAATGCATTAGTATTAAAAGCAATAGAACAAAGTGATGGTCAACAAGAAAAAAAAGTAGAA [27144]
cp32-9_PAbe_NX_Pl_consensus    CTAGAAAATATTCACAATAATGCATTAGTATTAAAAGCAATAGAACAAAGTGATGGTCAACAAGAAAAAAAAGTAGAA [27144]

cp32-9_gi|11497325|B31_GB      GAAGCTGAAGCTAAAGTTGAAGAAAATAAAGAAAAACAAGAGAATACAGAAGAAAACATTAAAGAAAAAGAAATAATA [27222]
cp32-9_un7_B31_PacBio          GAAGCTGAAGCTAAAGTTGAAGAAAATAAAGAAAAACAAGAGAATACAGAAGAAA-CATTAAAGAAAAAGAAATAATA [27222]
cp32-9_B31_TS_MP_FG_consensus  GAAGCTGAAGCTAAAGTTGAAGAAAATAAAGAAAAACAAGAGAATACAGAAGAAAACATTAAAGAAAAAGAAATAATA [27222]
cp32-9_B31_NX_Pl_consensus     GAAGCTGAAGCTAAAGTTGAAGAAAATAAAGAAAAACAAGAGAATACAGAAGAAAACATTAAAGAAAAAGAAATAATA [27222]
cp32-9_PAli_NX_MP_FG_consensus GAAGCTGAAGCTAAAGTTGAAGAAAATAAAGAAAAACAAGAGAATACAGAAGAAAACATTAAAGAAAAAGAAATAATA [27222]
cp32-9_PAli_NX_Pl_consensus    GAAGCTGAAGCTAAAGTTGAAGAAAATAAAGAAAAACAAGAGAATACAGAAGAAAACATTAAAGAAAAAGAAATAATA [27222]
cp32-9_PAbe_NX_MP_FG_consensus GAAGCTGAAGCTAAAGTTGAAGAAAATAAAGAAAAACAAGAGAATACAGAAGAAAACATTAAAGAAAAAGAAATAATA [27222]
cp32-9_PAbe_NX_Pl_consensus    GAAGCTGAAGCTAAAGTTGAAGAAAATAAAGAAAAACAAGAGAATACAGAAGAAAACATTAAAGAAAAAGAAATAATA [27222]

cp32-9_gi|11497325|B31_GB      GACGAACAAAACAAACAAGAATTAGCTAAAGCTAAAGAAGAAGAACAACAAAAAGAACAAAAAAGACATCAAGAAGAG [27300]
cp32-9_un7_B31_PacBio          GACGAACAAAACAAACAAGAATTAGCTAAAGCTAAAGAAGAAGAACAACAAAAAGAACAAAAAAGACATCAAGAAGAG [27300]
cp32-9_B31_TS_MP_FG_consensus  GACGAACAAAACAAACAAGAATTAGCTAAAGCTAAAGAAGAAGAACAACAAAAAGAACAAAAAAGACATCAAGAAGAG [27300]
cp32-9_B31_NX_Pl_consensus     GACGAACAAAACAAACAAGAATTAGCTAAAGCTAAAGAAGAAGAACAACAAAAAGAACAAAAAAGACATCAAGAAGAG [27300]
cp32-9_PAli_NX_MP_FG_consensus GACGAACAAAACAAACAAGAATTAGCTAAAGCTAAAGAAGAAGAACAACAAAAAGAACAAAAAAGACATCAAGAAGAG [27300]
cp32-9_PAli_NX_Pl_consensus    GACGAACAAAACAAACAAGAATTAGCTAAAGCTAAAGAAGAAGAACAACAAAAAGAACAAAAAAGACATCAAGAAGAG [27300]
cp32-9_PAbe_NX_MP_FG_consensus GACGAACAAAACAAACAAGAATTAGCTAAAGCTAAAGAAGAAGAACAACAAAAAGAACAAAAAAGACATCAAGAAGAG [27300]
cp32-9_PAbe_NX_Pl_consensus    GACGAACAAAACAAACAAGAATTAGCTAAAGCTAAAGAAGAAGAACAACAAAAAGAACAAAAAAGACATCAAGAAGAG [27300]

cp32-9_gi|11497325|B31_GB      CAACAAAGAAAAGCTAAAGCAGAAAAAGAAAAAAGAGAAAGAGAAGAGGCAGAACAACAAAAACGACAACAAGAAGAG [27378]
cp32-9_un7_B31_PacBio          CAACAAAGAAAAGCTAAAGCAGAAAAAGAAAAAAGAGAAAGAGAAGAGGCAGAACAACAAAAACGACAACAAGAAGAG [27378]
cp32-9_B31_TS_MP_FG_consensus  CAACAAAGAAAAGCTAAAGCAGAAAAAGAAAAAAGAGAAAGAGAAGAGGCAGAACAACAAAAACGACAACAAGAAGAG [27378]
```

```
cp32-9_B31_NX_Pl_consensus     CAACAAAGAAAAGCTAAAGCAGAAAAAGAAAAAAGAGAAAGAGAAGAGGCAGAACAACAAAAACGACAACAAGAAGAG [27378]
cp32-9_PAli_NX_MP_FG_consensus CAACAAAGAAAAGCTAAAGCAGAAAAAGAAAAAAGAGAAAGAGAAGAGGCAGAACAACAAAAACGACAACAAGAAGAG [27378]
cp32-9_PAli_NX_Pl_consensus    CAACAAAGAAAAGCTAAAGCAGAAAAAGAAAAAAGAGAAAGAGAAGAGGCAGAACAACAAAAACGACAACAAGAAGAG [27378]
cp32-9_PAbe_NX_MP_FG_consensus CAACAAAGAAAAGCTAAAGCAGAAAAAGAAAAAAGAGAAAGAGAAGAGGCAGAACAACAAAAACGACAACAAGAAGAG [27378]
cp32-9_PAbe_NX_Pl_consensus    CAACAAAGAAAAGCTAAAGCAGAAAAAGAAAAAAGAGAAAGAGAAGAGGCAGAACAACAAAAACGACAACAAGAAGAG [27378]

cp32-9_gi|11497325|B31_GB      GAAGAAAAAAAGGCAAGTTGATAACCAAATTAAAACACTTATAGCTAAAATAGATGAGATCAATGAAAATATTGATGTT [27456]
cp32-9_un7_B31_PacBio          GAAGAAAAAAAGGCAAGTTGATAACCAAATTAAAACACTTATAGCTAAAATAGATGAGATCAATGAAAATATTGATGTT [27456]
cp32-9_B31_TS_MP_FG_consensus  GAAGAAAAAAAGGCAAGTTGATAACCAAATTAAAACACTTATAGCTAAAATAGATGAGATCAATGAAAATATTGATGTT [27456]
cp32-9_B31_NX_Pl_consensus     GAAGAAAAAAAGGCAAGTTGATAACCAAATTAAAACACTTATAGCTAAAATAGATGAGATCAATGAAAATATTGATGTT [27456]
cp32-9_PAli_NX_MP_FG_consensus GAAGAAAAAAAGGCAAGTTGATAACCAAATTAAAACACTTATAGCTAAAATAGATGAGATCAATGAAAATATTGATGTT [27456]
cp32-9_PAli_NX_Pl_consensus    GAAGAAAAAAAGGCAAGTTGATAACCAAATTAAAACACTTATAGCTAAAATAGATGAGATCAATGAAAATATTGATGTT [27456]
cp32-9_PAbe_NX_MP_FG_consensus GAAGAAAAAAAGGCAAGTTGATAACCAAATTAAAACACTTATAGCTAAAATAGATGAGATCAATGAAAATATTGATGTT [27456]
cp32-9_PAbe_NX_Pl_consensus    GAAGAAAAAAAGGCAAGTTGATAACCAAATTAAAACACTTATAGCTAAAATAGATGAGATCAATGAAAATATTGATGTT [27456]

cp32-9_gi|11497325|B31_GB      ATAAAATGGCAAACGACTGTAGGCCCACAAGGCGTTATAGATAGAATTACTGGGCCTGTGTATGATGATTTTACCAAT [27534]
cp32-9_un7_B31_PacBio          ATAAAATGGCAAACGACTGTAGGCCCACAAGGCGTTATAGATAGAATTACTGGGCCTGTGTATGATGATTTTACCAAT [27534]
cp32-9_B31_TS_MP_FG_consensus  ATAAAATGGCAAACGACTGTAGGCCCACAAGGCGTTATAGATAGAATTACTGGGCCTGTGTATGATGATTTTACCAAT [27534]
cp32-9_B31_NX_Pl_consensus     ATAAAATGGCAAACGACTGTAGGCCCACAAGGCGTTATAGATAGAATTACTGGGCCTGTGTATGATGATTTTACCAAT [27534]
cp32-9_PAli_NX_MP_FG_consensus ATAAAATGGCAAACGACTGTAGGCCCACAAGGCGTTATAGATAGAATTACTGGGCCTGTGTATGATGATTTTACCAAT [27534]
cp32-9_PAli_NX_Pl_consensus    ATAAAATGGCAAACGACTGTAGGCCCACAAGGCGTTATAGATAGAATTACTGGGCCTGTGTATGATGATTTTACCAAT [27534]
cp32-9_PAbe_NX_MP_FG_consensus ATAAAATGGCAAACGACTGTAGGCCCACAAGGCGTTATAGATAGAATTACTGGGCCTGTGTATGATGATTTTACCAAT [27534]
cp32-9_PAbe_NX_Pl_consensus    ATAAAATGGCAAACGACTGTAGGCCCACAAGGCGTTATAGATAGAATTACTGGGCCTGTGTATGATGATTTTACCAAT [27534]

cp32-9_gi|11497325|B31_GB      GGCAATAATTCTATACGCGAAACTTGGGAGGGGTTAGAAGAGGAATCAGAAGACGAAGGATTAGGAAAATTATTGAAA [27612]
cp32-9_un7_B31_PacBio          GGCAATAATTCTATACGCGAAACTTGGGAGGGGTTAGAAGAGGAATCAGAAGACGAAGGATTAGGAAAATTATTGAAA [27612]
cp32-9_B31_TS_MP_FG_consensus  GGCAATAATTCTATACGCGAAACTTGGGAGGGGTTAGAAGAGGAATCAGAAGACGAAGGATTAGGAAAATTATTGAAA [27612]
cp32-9_B31_NX_Pl_consensus     GGCAATAATTCTATACGCGAAACTTGGGAGGGGTTAGAAGAGGAATCAGAAGACGAAGGATTAGGAAAATTATTGAAA [27612]
cp32-9_PAli_NX_MP_FG_consensus GGCAATAATTCTATACGCGAAACTTGGGAGGGGTTAGAAGAGGAATCAGAAGACGAAGGATTAGGAAAATTATTGAAA [27612]
cp32-9_PAli_NX_Pl_consensus    GGCAATAATTCTATACGCGAAACTTGGGAGGGGTTAGAAGAGGAATCAGAAGACGAAGGATTAGGAAAATTATTGAAA [27612]
cp32-9_PAbe_NX_MP_FG_consensus GGCAATAATTCTATACGCGAAACTTGGGAGGGGTTAGAAGAGGAATCAGAAGACGAAGGATTAGGAAAATTATTGAAA [27612]
cp32-9_PAbe_NX_Pl_consensus    GGCAATAATTCTATACGCGAAACTTGGGAGGGGTTAGAAGAGGAATCAGAAGACGAAGGATTAGGAAAATTATTGAAA [27612]

cp32-9_gi|11497325|B31_GB      GAATTGAGTGATGCTAGGGACGCGCTAAGAACTAAATTAAATGAAGGCAATAAACCATATACTGGTTACGAAGAGCCT [27690]
cp32-9_un7_B31_PacBio          GAATTGAGTGATGCTAGGGACGCGCTAAGAACTAAATTAAATGAAGGCAATAAACCATATACTGGTTACGAAGAGCCT [27690]
cp32-9_B31_TS_MP_FG_consensus  GAATTGAGTGATGCTAGGGACGCGCTAAGAACTAAATTAAATGAAGGCAATAAACCATATACTGGTTACGAAGAGCCT [27690]
cp32-9_B31_NX_Pl_consensus     GAATTGAGTGATGCTAGGGACGCGCTAAGAACTAAATTAAATGAAGGCAATAAACCATATACTGGTTACGAAGAGCCT [27690]
cp32-9_PAli_NX_MP_FG_consensus GAATTGAGTGATGCTAGGGACGCGCTAAGAACTAAATTAAATGAAGGCAATAAACCATATACTGGTTACGAAGAGCCT [27690]
cp32-9_PAli_NX_Pl_consensus    GAATTGAGTGATGCTAGGGACGCGCTAAGAACTAAATTAAATGAAGGCAATAAACCATATACTGGTTACGAAGAGCCT [27690]
cp32-9_PAbe_NX_MP_FG_consensus GAATTGAGTGATGCTAGGGACGCGCTAAGAACTAAATTAAATGAAGGCAATAAACCATATACTGGTTACGAAGAGCCT [27690]
cp32-9_PAbe_NX_Pl_consensus    GAATTGAGTGATGCTAGGGACGCGCTAAGAACTAAATTAAATGAAGGCAATAAACCATATACTGGTTACGAAGAGCCT [27690]

cp32-9_gi|11497325|B31_GB      AAGTTAAAAGAAAGTGTAAATGTTAGCGAAATTAAAGAAGATTTAGAAAAATTAAAATCAAAATTAGAAGAAGTTAAA [27768]
cp32-9_un7_B31_PacBio          AAGTTAAAAGAAAGTGTAAATGTTAGCGAAATTAAAGAAGATTTAGAAAAATTAAAATCAAAATTAGAAGAAGTTAAA [27768]
cp32-9_B31_TS_MP_FG_consensus  AAGTTAAAAGAAAGTGTAAATGTTAGCGAAATTAAAGAAGATTTAGAAAAATTAAAATCAAAATTAGAAGAAGTTAAA [27768]
cp32-9_B31_NX_Pl_consensus     AAGTTAAAAGAAAGTGTAAATGTTAGCGAAATTAAAGAAGATTTAGAAAAATTAAAATCAAAATTAGAAGAAGTTAAA [27768]
cp32-9_PAli_NX_MP_FG_consensus AAGTTAAAAGAAAGTGTAAATGTTAGCGAAATTAAAGAAGATTTAGAAAAATTAAAATCAAAATTAGAAGAAGTTAAA [27768]
cp32-9_PAli_NX_Pl_consensus    AAGTTAAAAGAAAGTGTAAATGTTAGCGAAATTAAAGAAGATTTAGAAAAATTAAAATCAAAATTAGAAGAAGTTAAA [27768]
cp32-9_PAbe_NX_MP_FG_consensus AAGTTAAAAGAAAGTGTAAATGTTAGCGAAATTAAAGAAGATTTAGAAAAATTAAAATCAAAATTAGAAGAAGTTAAA [27768]
cp32-9_PAbe_NX_Pl_consensus    AAGTTAAAAGAAAGTGTAAATGTTAGCGAAATTAAAGAAGATTTAGAAAAATTAAAATCAAAATTAGAAGAAGTTAAA [27768]

cp32-9_gi|11497325|B31_GB      AAATATCTTAAAGATAGTTCTAAATTTGAAGAAATTAAAGGATACATCAGTGACAGTCAGTAATTATATTGGATGCTT [27846]
cp32-9_un7_B31_PacBio          AAATATCTTAAAGATAGTTCTAAATTTGAAGAAATTAAAGGATACATCAGTGACAGTCAGTAATTATATTGGATGCTT [27846]
cp32-9_B31_TS_MP_FG_consensus  AAATATCTTAAAGATAGTTCTAAATTTGAAGAAATTAAAGGATACATCAGTGACAGTCAGTAATTATATTGGATGCTT [27846]
cp32-9_B31_NX_Pl_consensus     AAATATCTTAAAGATAGTTCTAAATTTGAAGAAATTAAAGGATACATCAGTGACAGTCAGTAATTATATTGGATGCTT [27846]
cp32-9_PAli_NX_MP_FG_consensus AAATATCTTAAAGATAGTTCTAAATTTGAAGAAATTAAAGGATACATCAGTGACAGTCAGTAATTATATTGGATGCTT [27846]
cp32-9_PAli_NX_Pl_consensus    AAATATCTTAAAGATAGTTCTAAATTTGAAGAAATTAAAGGATACATCAGTGACAGTCAGTAATTATATTGGATGCTT [27846]
cp32-9_PAbe_NX_MP_FG_consensus AAATATCTTAAAGATAGTTCTAAATTTGAAGAAATTAAAGGATACATCAGTGACAGTCAGTAATTATATTGGATGCTT [27846]
cp32-9_PAbe_NX_Pl_consensus    AAATATCTTAAAGATAGTTCTAAATTTGAAGAAATTAAAGGATACATCAGTGACAGTCAGTAATTATATTGGATGCTT [27846]

cp32-9_gi|11497325|B31_GB      TTAGGTGTAACTAAATTTGCGTATACAAAGTAACAGCTAGTAGAAAAGTTCACTGGCTGTTATTTTTTTGTAGATTTC [27924]
cp32-9_un7_B31_PacBio          TTAGGTGTAACTAAATTTGCGTATACAAAGTAACAGCTAGTAGAAAAGTTCACTGGCTGTTATTTTTTTGTAGATTTC [27924]
cp32-9_B31_TS_MP_FG_consensus  TTAGGTGTAACTAAATTTGCGTATACAAAGTAACAGCTAGTAGAAAAGTTCACTGGCTGTTATTTTTTTGTAGATTTC [27924]
cp32-9_B31_NX_Pl_consensus     TTAGGTGTAACTAAATTTGCGTATACAAAGTAACAGCTAGTAGAAAAGTTCACTGGCTGTTATTTTTTTGTAGATTTC [27924]
cp32-9_PAli_NX_MP_FG_consensus TTAGGTGTAACTAAATTTGCGTATACAAAGTAACAGCTAGTAGAAAAGTTCACTGGCTGTTATTTTTTTGTAGATTTC [27924]
cp32-9_PAli_NX_Pl_consensus    TTAGGTGTAACTAAATTTGCGTATACAAAGTAACAGCTAGTAGAAAAGTTCACTGGCTGTTATTTTTTTGTAGATTTC [27924]
cp32-9_PAbe_NX_MP_FG_consensus TTAGGTGTAACTAAATTTGCGTATACAAAGTAACAGCTAGTAGAAAAGTTCACTGGCTGTTATTTTTTTGTAGATTTC [27924]
cp32-9_PAbe_NX_Pl_consensus    TTAGGTGTAACTAAATTTGCGTATACAAAGTAACAGCTAGTAGAAAAGTTCACTGGCTGTTATTTTTTTGTAGATTTC [27924]

cp32-9_gi|11497325|B31_GB      ATTGTTATGAATATAGAAATGTTTTCTATCAAAACTTTCATTTAAAAAATGCCAAAAACTATTGCTCAAAATATTGTT [28002]
cp32-9_un7_B31_PacBio          ATTGTTATGAATATAGAAATGTTTTCTATCAAAACTTTCATTTAAAAAATGCCAAAAACTATTGCTCAAAATATTGTT [28002]
cp32-9_B31_TS_MP_FG_consensus  ATTGTTATGAATATAGAAATGTTTTCTATCAAAACTTTCATTTAAAAAATGCCAAAAACTATTGCTCAAAATATTGTT [28002]
cp32-9_B31_NX_Pl_consensus     ATTGTTATGAATATAGAAATGTTTTCTATCAAAACTTTCATTTAAAAAATGCCAAAAACTATTGCTCAAAATATTGTT [28002]
cp32-9_PAli_NX_MP_FG_consensus ATTGTTATGAATATAGAAATGTTTTCTATCAAAACTTTCATTTAAAAAATGCCAAAAACTATTGCTCAAAATATTGTT [28002]
cp32-9_PAli_NX_Pl_consensus    ATTGTTATGAATATAGAAATGTTTTCTATCAAAACTTTCATTTAAAAAATGCCAAAAACTATTGCTCAAAATATTGTT [28002]
cp32-9_PAbe_NX_MP_FG_consensus ATTGTTATGAATATAGAAATGTTTTCTATCAAAACTTTCATTTAAAAAATGCCAAAAACTATTGCTCAAAATATTGTT [28002]
cp32-9_PAbe_NX_Pl_consensus    ATTGTTATGAATATAGAAATGTTTTCTATCAAAACTTTCATTTAAAAAATGCCAAAAACTATTGCTCAAAATATTGTT [28002]

cp32-9_gi|11497325|B31_GB      TATTTATATACTATCTATAATTAATATGCATACTAATGAAATTTCAAATTTTTATGATAATCTTAGTGAAGATTTAAA [28080]
cp32-9_un7_B31_PacBio          TATTTATATACTATCTATAATTAATATGCATACTAATGAAATTTCAAATTTTTATGATAATCTTAGTGAAGATTTAAA [28080]
cp32-9_B31_TS_MP_FG_consensus  TATTTATATACTATCTATAATTAATATGCATACTAATGAAATTTCAAATTTTTATGATAATCTTAGTGAAGATTTAAA [28080]
cp32-9_B31_NX_Pl_consensus     TATTTATATACTATCTATAATTAATATGCATACTAATGAAATTTCAAATTTTTATGATAATCTTAGTGAAGATTTAAA [28080]
cp32-9_PAli_NX_MP_FG_consensus TATTTATATACTATCTATAATTAATATGCATACTAATGAAATTTCAAATTTTTATGATAATCTTAGTGAAGATTTAAA [28080]
cp32-9_PAli_NX_Pl_consensus    TATTTATATACTATCTATAATTAATATGCATACTAATGAAATTTCAAATTTTTATGATAATCTTAGTGAAGATTTAAA [28080]
cp32-9_PAbe_NX_MP_FG_consensus TATTTATATACTATCTATAATTAATATGCATACTAATGAAATTTCAAATTTTTATGATAATCTTAGTGAAGATTTAAA [28080]
cp32-9_PAbe_NX_Pl_consensus    TATTTATATACTATCTATAATTAATATGCATACTAATGAAATTTCAAATTTTTATGATAATCTTAGTGAAGATTTAAA [28080]

cp32-9_gi|11497325|B31_GB      AAAGTCAATAAATAAACTTTATGAAACCGAACAAGCAACGCAAGAACAAAAAAGTCAAATGTATAGCTCTTATAAAGC [28158]
cp32-9_un7_B31_PacBio          AAAGTCAATAAATAAACTTTATGAAACCGAACAAGCAACGCAAGAACAAAAAAGTCAAATGTATAGCTCTTATAAAGC [28158]
cp32-9_B31_TS_MP_FG_consensus  AAAGTCAATAAATAAACTTTATGAAACCGAACAAGCAACGCAAGAACAAAAAAGTCAAATGTATAGCTCTTATAAAGC [28158]
cp32-9_B31_NX_Pl_consensus     AAAGTCAATAAATAAACTTTATGAAACCGAACAAGCAACGCAAGAACAAAAAAGTCAAATGTATAGCTCTTATAAAGC [28158]
cp32-9_PAli_NX_MP_FG_consensus AAAGTCAATAAATAAACTTTATGAAACCGAACAAGCAACGCAAGAACAAAAAAGTCAAATGTATAGCTCTTATAAAGC [28158]
cp32-9_PAli_NX_Pl_consensus    AAAGTCAATAAATAAACTTTATGAAACCGAACAAGCAACGCAAGAACAAAAAAGTCAAATGTATAGCTCTTATAAAGC [28158]
cp32-9_PAbe_NX_MP_FG_consensus AAAGTCAATAAATAAACTTTATGAAACCGAACAAGCAACGCAAGAACAAAAAAGTCAAATGTATAGCTCTTATAAAGC [28158]
cp32-9_PAbe_NX_Pl_consensus    AAAGTCAATAAATAAACTTTATGAAACCGAACAAGCAACGCAAGAACAAAAAAGTCAAATGTATAGCTCTTATAAAGC [28158]

cp32-9_gi|11497325|B31_GB      AGCGCAAGAATACGGAATAAAAACTGGAAAAAGCACTGAGGAAATAATAAATGACATTATAGACCCTACAAAAAAAAT [28236]
cp32-9_un7_B31_PacBio          AGCGCAAGAATACGGAATAAAAACTGGAAAAAGCACTGAGGAAATAATAAATGACATTATAGACCCTACAAAAAAA-T [28236]
cp32-9_B31_TS_MP_FG_consensus  AGCGCAAGAATACGGAATAAAAACTGGAAAAAGCACTGAGGAAATAATAAATGACATTATAGACCCTACAAAAAAAAT [28236]
cp32-9_B31_NX_Pl_consensus     AGCGCAAGAATACGGAATAAAAACTGGAAAAAGCACTGAGGAAATAATAAATGACATTATAGACCCTACAAAAAAAAT [28236]
cp32-9_PAli_NX_MP_FG_consensus AGCGCAAGAATACGGAATAAAAACTGGAAAAAGCACTGAGGAAATAATAAATGACATTATAGACCCTACAAAAAAAAT [28236]
cp32-9_PAli_NX_Pl_consensus    AGCGCAAGAATACGGAATAAAAACTGGAAAAAGCACTGAGGAAATAATAAATGACATTATAGACCCTACAAAAAAAAT [28236]
cp32-9_PAbe_NX_MP_FG_consensus AGCGCAAGAATACGGAATAAAAACTGGAAAAAGCACTGAGGAAATAATAAATGACATTATAGACCCTACAAAAAAAAT [28236]
cp32-9_PAbe_NX_Pl_consensus    AGCGCAAGAATACGGAATAAAAACTGGAAAAAGCACTGAGGAAATAATAAATGACATTATAGACCCTACAAAAAAAAT [28236]

cp32-9_gi|11497325|B31_GB      TATTAAAGACGTTCTGAAAAATAAATATTTAATACAAAAATATAAAAATTCCAAAAATATGCAAGTTGATTATAGCGA [28314]
cp32-9_un7_B31_PacBio          TATTAAAGACGTTCTGAAAAATAAATATTTAATAAAAAAATATAAAAATTCCAAAAATATGCAAGTTGATTATAGCGA [28314]
cp32-9_B31_TS_MP_FG_consensus  TATTAAAGACGTTCTGAAAAATAAATATTTAATAAAAAAATATAAAAATTCCAAAAATATGCAAGTTGATTATAGCGA [28314]
cp32-9_B31_NX_Pl_consensus     TATTAAAGACGTTCTGAAAAATAAATATTTAATAAAAAAATATAAAAATTCCAAAAATATGCAAGTTGATTATAGCGA [28314]
```

```
cp32-9_PAli_NX_MP_FG_consensus TATTAAAGACGTTCTGAAAAATAAATATTTAATAAAAAAATATAAAAATTCCAAAAATATGCAAGTTGATTATAGCGA [28314]
cp32-9_PAli_NX_Pl_consensus    TATTAAAGACGTTCTGAAAAATAAATATTTAATAAAAAAATATAAAAATTCCAAAAATATGCAAGTTGATTATAGCGA [28314]
cp32-9_PAbe_NX_MP_FG_consensus TATTAAAGACGTTCTGAAAAATAAATATTTAATAAAAAAATATAAAAATTCCAAAAATATGCAAGTTGATTATAGCGA [28314]
cp32-9_PAbe_NX_Pl_consensus    TATTAAAGACGTTCTGAAAAATAAATATTTAATAAAAAAATATAAAAATTCCAAAAATATGCAAGTTGATTATAGCGA [28314]

cp32-9_gi|11497325|B31_GB      CAAGAAAGGGATGCTAAAGAAATGTTTGAAAAAATTAGGAGAGTATGATTCCATGATATTTTTGGGGGCTGTTTATGG [28392]
cp32-9_un7_B31_PacBio          CAAGAAAGGGATGCTAAAGAAATGTTTGAAAAAATTAGGAGAGTATGATTCCATGATATTTTTGGGGGCTGTTTATGG [28392]
cp32-9_B31_TS_MP_FG_consensus  CAAGAAAGGGATGCTAAAGAAATGTTTGAAAAAATTAGGAGAGTATGATTCCATGATATTTTTGGGGGCTGTTTATGG [28392]
cp32-9_B31_NX_Pl_consensus     CAAGAAAGGGATGCTAAAGAAATGTTTGAAAAAATTAGGAGAGTATGATTCCATGATATTTTTGGGGGCTGTTTATGG [28392]
cp32-9_PAli_NX_MP_FG_consensus CAAGAAAGGGATGCTAAAGAAATGTTTGAAAAAATTAGGAGAGTATGATTCCATGATATTTTTGGGGGCTGTTTATGG [28392]
cp32-9_PAli_NX_Pl_consensus    CAAGAAAGGGATGCTAAAGAAATGTTTGAAAAAATTAGGAGAGTATGATTCCATGATATTTTTGGGGGCTGTTTATGG [28392]
cp32-9_PAbe_NX_MP_FG_consensus CAAGAAAGGGATGCTAAAGAAATGTTTGAAAAAATTAGGAGAGTATGATTCCATGATATTTTTGGGGGCTGTTTATGG [28392]
cp32-9_PAbe_NX_Pl_consensus    CAAGAAAGGGATGCTAAAGAAATGTTTGAAAAAATTAGGAGAGTATGATTCCATGATATTTTTGGGGGCTGTTTATGG [28392]

cp32-9_gi|11497325|B31_GB      TATTCTTAACCATATTTATCAAAAAGTTTCAAAACAATATCAATTAATTATGATCAACGAATTTAAAGATATACTATT [28470]
cp32-9_un7_B31_PacBio          TATTCTTAACCATATTTATCAAAAAGTTTCAAAACAATATCAATTAATTATGATCAACGAATTTAAAGATATACTATT [28470]
cp32-9_B31_TS_MP_FG_consensus  TATTCTTAACCATATTTATCAAAAAGTTTCAAAACAATATCAATTAATTATGATCAACGAATTTAAAGATATACTATT [28470]
cp32-9_B31_NX_Pl_consensus     TATTCTTAACCATATTTATCAAAAAGTTTCAAAACAATATCAATTAATTATGATCAACGAATTTAAAGATATACTATT [28470]
cp32-9_PAli_NX_MP_FG_consensus TATTCTTAACCATATTTATCAAAAAGTTTCAAAACAATATCAATTAATTATGATCAACGAATTTAAAGATATACTATT [28470]
cp32-9_PAli_NX_Pl_consensus    TATTCTTAACCATATTTATCAAAAAGTTTCAAAACAATATCAATTAATTATGATCAACGAATTTAAAGATATACTATT [28470]
cp32-9_PAbe_NX_MP_FG_consensus TATTCTTAACCATATTTATCAAAAAGTTTCAAAACAATATCAATTAATTATGATCAACGAATTTAAAGATATACTATT [28470]
cp32-9_PAbe_NX_Pl_consensus    TATTCTTAACCATATTTATCAAAAAGTTTCAAAACAATATCAATTAATTATGATCAACGAATTTAAAGATATACTATT [28470]

cp32-9_gi|11497325|B31_GB      TATAAGGATACACAACTATGATAAGAGAATTTTTACAAGCGATGATCTAATTAAAACAATAAAAGATTTTCCCTAATT [28548]
cp32-9_un7_B31_PacBio          TATAAGGATACACAACTATGATAAGAGAATTTTTACAAGCGATGATCTAATTAAAACAATAAAAGATTTTCCCTAATT [28548]
cp32-9_B31_TS_MP_FG_consensus  TATAAGGATACACAACTATGATAAGAGAATTTTTACAAGCGATGATCTAATTAAAACAATAAAAGATTTTCCCTAATT [28548]
cp32-9_B31_NX_Pl_consensus     TATAAGGATACACAACTATGATAAGAGAATTTTTACAAGCGATGATCTAATTAAAACAATAAAAGATTTTCCCTAATT [28548]
cp32-9_PAli_NX_MP_FG_consensus TATAAGGATACACAACTATGATAAGAGAATTTTTACAAGCGATGATCTAATTAAAACAATAAAAGATTTTCCCTAATT [28548]
cp32-9_PAli_NX_Pl_consensus    TATAAGGATACACAACTATGATAAGAGAATTTTTACAAGCGATGATCTAATTAAAACAATAAAAGATTTTCCCTAATT [28548]
cp32-9_PAbe_NX_MP_FG_consensus TATAAGGATACACAACTATGATAAGAGAATTTTTACAAGCGATGATCTAATTAAAACAATAAAAGATTTTCCCTAATT [28548]
cp32-9_PAbe_NX_Pl_consensus    TATAAGGATACACAACTATGATAAGAGAATTTTTACAAGCGATGATCTAATTAAAACAATAAAAGATTTTCCCTAATT [28548]

cp32-9_gi|11497325|B31_GB      TATATAGTCAAGTTGTTGCAAAATTCAATAAATTAGTTAATTGCTATTTCTAAATCAATAAAAGTCAAAAAAATACCA [28626]
cp32-9_un7_B31_PacBio          TATATAGTCAAGTTGTTGCAAAATTCAATAAATTAGTTAATTGCTATTTCTAAATCAATAAAAGTCAAAAAAATACCA [28626]
cp32-9_B31_TS_MP_FG_consensus  TATATAGTCAAGTTGTTGCAAAATTCAATAAATTAGTTAATTGCTATTTCTAAATCAATAAAAGTCAAAAAAATACCA [28626]
cp32-9_B31_NX_Pl_consensus     TATATAGTCAAGTTGTTGCAAAATTCAATAAATTAGTTAATTGCTATTTCTAAATCAATAAAAGTCAAAAAAATACCA [28626]
cp32-9_PAli_NX_MP_FG_consensus TATATAGTCAAGTTGTTGCAAAATTCAATAAATTAGTTAATTGCTATTTCTAAATCAATAAAAGTCAAAAAAATACCA [28626]
cp32-9_PAli_NX_Pl_consensus    TATATAGTCAAGTTGTTGCAAAATTCAATAAATTAGTTAATTGCTATTTCTAAATCAATAAAAGTCAAAAAAATACCA [28626]
cp32-9_PAbe_NX_MP_FG_consensus TATATAGTCAAGTTGTTGCAAAATTCAATAAATTAGTTAATTGCTATTTCTAAATCAATAAAAGTCAAAAAAATACCA [28626]
cp32-9_PAbe_NX_Pl_consensus    TATATAGTCAAGTTGTTGCAAAATTCAATAAATTAGTTAATTGCTATTTCTAAATCAATAAAAGTCAAAAAAATACCA [28626]

cp32-9_gi|11497325|B31_GB      AACACCTGTCAATAGATATCAATAAGATAATAGTTAATGTTAACAATTAACAAATTGATTTACTATTTAGAGTAACAA [28704]
cp32-9_un7_B31_PacBio          AACACCTGTCAATAGATATCAATAAGATAATAGTTAATGTTAACAATTAACAAATTGATTTACTATTTAGAGTAACAA [28704]
cp32-9_B31_TS_MP_FG_consensus  AACACCTGTCAATAGATATCAATAAGATAATAGTTAATGTTAACAATTAACAAATTGATTTACTATTTAGAGTAACAA [28704]
cp32-9_B31_NX_Pl_consensus     AACACCTGTCAATAGATATCAATAAGATAATAGTTAATGTTAACAATTAACAAATTGATTTACTATTTAGAGTAACAA [28704]
cp32-9_PAli_NX_MP_FG_consensus AACACCTGTCAATAGATATCAATAAGATAATAGTTAATGTTAACAATTAACAAATTGATTTACTATTTAGAGTAACAA [28704]
cp32-9_PAli_NX_Pl_consensus    AACACCTGTCAATAGATATCAATAAGATAATAGTTAATGTTAACAATTAACAAATTGATTTACTATTTAGAGTAACAA [28704]
cp32-9_PAbe_NX_MP_FG_consensus AACACCTGTCAATAGATATCAATAAGATAATAGTTAATGTTAACAATTAACAAATTGATTTACTATTTAGAGTAACAA [28704]
cp32-9_PAbe_NX_Pl_consensus    AACACCTGTCAATAGATATCAATAAGATAATAGTTAATGTTAACAATTAACAAATTGATTTACTATTTAGAGTAACAA [28704]

cp32-9_gi|11497325|B31_GB      TTTGTTAATTTAGTTATTTTAGGAGAAATTGTTTTGAAAAGAGCTAAAAGGTCTTTTAATGATTATGTTGCATACTTT [28782]
cp32-9_un7_B31_PacBio          TTTGTTAATTTAGTTATTTTAGGAGAAATTGTTTTGAAAAGAGCTAAAAGGTCTTTTAATGATTATGTTGCATACTTT [28782]
cp32-9_B31_TS_MP_FG_consensus  TTTGTTAATTTAGTTATTTTAGGAGAAATTGTTTTGAAAAGAGCTAAAAGGTCTTTTAATGATTATGTTGCATACTTT [28782]
cp32-9_B31_NX_Pl_consensus     TTTGTTAATTTAGTTATTTTAGGAGAAATTGTTTTGAAAAGAGCTAAAAGGTCTTTTAATGATTATGTTGCATACTTT [28782]
cp32-9_PAli_NX_MP_FG_consensus TTTGTTAATTTAGTTATTTTAGGAGAAATTGTTTTGAAAAGAGCTAAAAGGTCTTTTAATGATTATGTTGCATACTTT [28782]
cp32-9_PAli_NX_Pl_consensus    TTTGTTAATTTAGTTATTTTAGGAGAAATTGTTTTGAAAAGAGCTAAAAGGTCTTTTAATGATTATGTTGCATACTTT [28782]
cp32-9_PAbe_NX_MP_FG_consensus TTTGTTAATTTAGTTATTTTAGGAGAAATTGTTTTGAAAAGAGCTAAAAGGTCTTTTAATGATTATGTTGCATACTTT [28782]
cp32-9_PAbe_NX_Pl_consensus    TTTGTTAATTTAGTTATTTTAGGAGAAATTGTTTTGAAAAGAGCTAAAAGGTCTTTTAATGATTATGTTGCATACTTT [28782]

cp32-9_gi|11497325|B31_GB      AGAGAAGGATCGTTAGATGATAGAGAAATATCGGTTAAATTGGGAGTTTCTAGGGTAAATGTGTGGAGAATGAGACAA [28860]
cp32-9_un7_B31_PacBio          AGAGAAGGATCGTTAGATGATAGAGAAATATCGGTTAAATTGGGAGTTTCTAGGGTAAATGTGTGGAGAATGAGACAA [28860]
cp32-9_B31_TS_MP_FG_consensus  AGAGAAGGATCGTTAGATGATAGAGAAATATCGGTTAAATTGGGAGTTTCTAGGGTAAATGTGTGGAGAATGAGACAA [28860]
cp32-9_B31_NX_Pl_consensus     AGAGAAGGATCGTTAGATGATAGAGAAATATCGGTTAAATTGGGAGTTTCTAGGGTAAATGTGTGGAGAATGAGACAA [28860]
cp32-9_PAli_NX_MP_FG_consensus AGAGAAGGATCGTTAGATGATAGAGAAATATCGGTTAAATTGGGAGTTTCTAGGGTAAATGTGTGGAGAATGAGACAA [28860]
cp32-9_PAli_NX_Pl_consensus    AGAGAAGGATCGTTAGATGATAGAGAAATATCGGTTAAATTGGGAGTTTCTAGGGTAAATGTGTGGAGAATGAGACAA [28860]
cp32-9_PAbe_NX_MP_FG_consensus AGAGAAGGATCGTTAGATGATAGAGAAATATCGGTTAAATTGGGAGTTTCTAGGGTAAATGTGTGGAGAATGAGACAA [28860]
cp32-9_PAbe_NX_Pl_consensus    AGAGAAGGATCGTTAGATGATAGAGAAATATCGGTTAAATTGGGAGTTTCTAGGGTAAATGTGTGGAGAATGAGACAA [28860]

cp32-9_gi|11497325|B31_GB      AAATGGGAAAGTGGAGAAACTTCTGTTAGCGAGGATTCTAGAGTAACAATTAGTGAAGATACTTTTGAACACCTTTTG [28938]
cp32-9_un7_B31_PacBio          AAATGGGAAAGTGGAGAAACTTCTGTTAGCGAGGATTCTAGAGTAACAATTAGTGAAGATACTTTTGAACACCTTTTG [28938]
cp32-9_B31_TS_MP_FG_consensus  AAATGGGAAAGTGGAGAAACTTCTGTTAGCGAGGATTCTAGAGTAACAATTAGTGAAGATACTTTTGAACACCTTTTG [28938]
cp32-9_B31_NX_Pl_consensus     AAATGGGAAAGTGGAGAAACTTCTGTTAGCGAGGATTCTAGAGTAACAATTAGTGAAGATACTTTTGAACACCTTTTG [28938]
cp32-9_PAli_NX_MP_FG_consensus AAATGGGAAAGTGGAGAAACTTCTGTTAGCGAGGATTCTAGAGTAACAATTAGTGAAGATACTTTTGAACACCTTTTG [28938]
cp32-9_PAli_NX_Pl_consensus    AAATGGGAAAGTGGAGAAACTTCTGTTAGCGAGGATTCTAGAGTAACAATTAGTGAAGATACTTTTGAACACCTTTTG [28938]
cp32-9_PAbe_NX_MP_FG_consensus AAATGGGAAAGTGGAGAAACTTCTGTTAGCGAGGACTCTAGAGTAACAATTAGTGAAGATACTTTTGAACACCTTTTG [28938]
cp32-9_PAbe_NX_Pl_consensus    AAATGGGAAAGTGGAGAAACTTCTGTTAGCGAGGATTCTAGAGTAACAATTAGTGAAGATACTTTTGAACACCTTTTG [28938]

cp32-9_gi|11497325|B31_GB      TCGCAAACCTTTAGATCAGAAGTTAACGCTAGGAAAGTTAGAAGCGAATTGGATTTAGAGCGGGCTAATTTAGAATTA [29016]
cp32-9_un7_B31_PacBio          TCGCAAACCTTTAGATCAGAAGTTAACGCTAGGAAAGTTAGAAGCGAATTGGATTTAGAGCGGGCTAATTTAGAATTA [29016]
cp32-9_B31_TS_MP_FG_consensus  TCGCAAACCTTTAGATCAGAAGTTAACGCTAGGAAAGTTAGAAGCGAATTGGATTTAGAGCGGGCTAATTTAGAATTA [29016]
cp32-9_B31_NX_Pl_consensus     TCGCAAACCTTTAGATCAGAAGTTAACGCTAGGAAAGTTAGAAGCGAATTGGATTTAGAGCGGGCTAATTTAGAATTA [29016]
cp32-9_PAli_NX_MP_FG_consensus TCGCAAACCTTTAGATCAGAAGTTAACGCTAGGAAAGTTAGAAGCGAATTGGATTTAGAGCGGGCTAATTTAGAATTA [29016]
cp32-9_PAli_NX_Pl_consensus    TCGCAAACCTTTAGATCAGAAGTTAACGCTAGGAAAGTTAGAAGCGAATTGGATTTAGAGCGGGCTAATTTAGAATTA [29016]
cp32-9_PAbe_NX_MP_FG_consensus TCGCAAACCTTTAGATCAGAAGTTAACGCTAGGAAAGTTAGAAGCGAATTGGATTTAGAGCGGGCTAATTTAGAATTA [29016]
cp32-9_PAbe_NX_Pl_consensus    TCGCAAACCTTTAGATCAGAAGTTAACGCTAGGAAAGTTAGAAGCGAATTGGATTTAGAGCGGGCTAATTTAGAATTA [29016]

cp32-9_gi|11497325|B31_GB      GGATTTATAAATGCATTTAAGCAATATTCTAGTGTTGAGCTTGTTAGTATGCATACTAAAATAGAAAATTTAAGAGCC [29094]
cp32-9_un7_B31_PacBio          GGATTTATAAATGCATTTAAGCAATATTCTAGTGTTGAGCTTGTTAGTATGCATACTAAAATAGAAAATTTAAGAGCC [29094]
cp32-9_B31_TS_MP_FG_consensus  GGATTTATAAATGCATTTAAGCAATATTCTAGTGTTGAGCTTGTTAGTATGCATACTAAAATAGAAAATTTAAGAGCC [29094]
cp32-9_B31_NX_Pl_consensus     GGATTTATAAATGCATTTAAGCAATATTCTAGTGTTGAGCTTGTTAGTATGCATACTAAAATAGAAAATTTAAGAGCC [29094]
cp32-9_PAli_NX_MP_FG_consensus GGATTTATAAATGCATTTAAGCAATATTCTAGTGTTGAGCTTGTTAGTATGCATACTAAAATAGAAAATTTAAGAGCC [29094]
cp32-9_PAli_NX_Pl_consensus    GGATTTATAAATGCATTTAAGCAATATTCTAGTGTTGAGCTTGTTAGTATGCATACTAAAATAGAAAATTTAAGAGCC [29094]
cp32-9_PAbe_NX_MP_FG_consensus GGATTTATAAATGCATTTAAGCAATATTCTAGTGTTGAGCTTGTTAGTATGCATACTAAAATAGAAAATTTAAGAGCC [29094]
cp32-9_PAbe_NX_Pl_consensus    GGATTTATAAATGCATTTAAGCAATATTCTAGTGTTGAGCTTGTTAGTATGCATACTAAAATAGAAAATTTAAGAGCC [29094]

cp32-9_gi|11497325|B31_GB      GAAATTGACGCTTTAAATAAAGCAAGTAGTAAAAAAAACAAGCAAGTTGTTAATGGAGAAATTAATTCTTTAAAAAGC [29172]
cp32-9_un7_B31_PacBio          GAAATTGACGCTTTAAATAAAGCAAGTAGTAAAAAAAACAAGCAAGTTGTTAATGGAGAAATTAATTCTTTAAAAAGC [29172]
cp32-9_B31_TS_MP_FG_consensus  GAAATTGACGCTTTAAATAAAGCAAGTAGTAAAAAAAACAAGCAAGTTGTTAATGGAGAAATTAATTCTTTAAAAAGC [29172]
cp32-9_B31_NX_Pl_consensus     GAAATTGACGCTTTAAATAAAGCAAGTAGTAAAAAAAACAAGCAAGTTGTTAATGGAGAAATTAATTCTTTAAAAAGC [29172]
cp32-9_PAli_NX_MP_FG_consensus GAAATTGACGCTTTAAATAAAGCAAGTAGTAAAAAAAACAAGCAAGTTGTTAATGGAGAAATTAATTCTTTAAAAAGC [29172]
cp32-9_PAli_NX_Pl_consensus    GAAATTGACGCTTTAAATAAAGCAAGTAGTAAAAAAAACAAGCAAGTTGTTAATGGAGAAATTAATTCTTTAAAAAGC [29172]
cp32-9_PAbe_NX_MP_FG_consensus GAAATTGACGCTTTAAATAAAGCAAGTAGTAAAAAAAACAATCAAGTTGTTAATGGAGAAATTAATTCTTTAAAAAGC [29172]
cp32-9_PAbe_NX_Pl_consensus    GAAATTGACGCTTTAAATAAAGCAAGTAGTAAAAAAAACAAGCAAGTTGTTAATGGAGAAATTAATTCTTTAAAAAGC [29172]

cp32-9_gi|11497325|B31_GB      GAGCTTGATGAATATGTAAAGGAGTGCTCAATAAGAGAAATGGAGCTTTACTATGAATGTATGAAAAAACTTACAACT [29250]
cp32-9_un7_B31_PacBio          GAGCTTGATGAATATGTAAAGGAGTGCTCAATAAGAGAAATGGAGCTTTACTATGAATGTATGAAAAAACTTACAACT [29250]
cp32-9_B31_TS_MP_FG_consensus  GAGCTTGATGAATATGTAAAGGAGTGCTCAATAAGAGAAATGGAGCTTTACTATGAATGTATGAAAAAACTTACAACT [29250]
cp32-9_B31_NX_Pl_consensus     GAGCTTGATGAATATGTAAAGGAGTGCTCAATAAGAGAAATGGAGCTTTACTATGAATGTATGAAAAAACTTACAACT [29250]
cp32-9_PAli_NX_MP_FG_consensus GAGCTTGATGAATATGTAAAGGAGTGCTCAATAAGAGAAATGGAGCTTTACTATGAATGTATGAAAAAACTTACAACT [29250]
```

```
cp32-9_PAli_NX_Pl_consensus    GAGCTTGATGAATATGTAAAGGAGTGCTCAATAAGAGAAATGGAGCTTTACTATGAATGTATGAAAAAACTTACAACT [29250]
cp32-9_PAbe_NX_MP_FG_consensus GAGCTTGATGAATATATAAAGGAGTGCTCAATAAGAGAAATGGAGCTTTACTATGAATGTATGAAAAAACTTACAACT [29250]
cp32-9_PAbe_NX_Pl_consensus    GAGCTTGATGAATATGTAAAGGAGTGCTCAATAAGAGAAATGGAGCTTTACTATGAATGTATGAAAAAACTTACAACT [29250]

cp32-9_gi|11497325|B31_GB      GCTCATGAAGCTGAAAGTAAAAGCAACTACAAAAATAGTAAAGGGAACAAGTGAACTTATATCAAACAAAACTTTTTA [29328]
cp32-9_un7_B31_PacBio          GCTCATGAAGCTGAAAGTAAAAGCAACTACAAAAATAGTAAAGGGAACAAGTGAACTTATATCAAACAAAACTTTTTA [29328]
cp32-9_B31_TS_MP_FG_consensus  GCTCATGAAGCTGAAAGTAAAAGCAACTACAAAAATAGTAAAGGGAACAAGTGAACTTATATCAAACAAAACTTTTTA [29328]
cp32-9_B31_NX_Pl_consensus     GCTCATGAAGCTGAAAGTAAAAGCAACTACAAAAATAGTAAAGGGAACAAGTGAACTTATATCAAACAAAACTTTTTA [29328]
cp32-9_PAli_NX_MP_FG_consensus GCTCATGAAGCTGAAAGTAAAAGCAACTACAAAAATAGTAAAGGGAACAAGTGAACTTATATCAAACAAAACTTTTTA [29328]
cp32-9_PAli_NX_Pl_consensus    GCTCATGAAGCTGAAAGTAAAAGCAACTACAAAAATAGTAAAGGGAACAAGTGAACTTATATCAAACAAAACTTTTTA [29328]
cp32-9_PAbe_NX_MP_FG_consensus GCTCATGAAGCTGAAAGTAAAAGCAACTACAAAAATAGTAAAGGGAACAAGTGAACTTATATCAAACAAAACTTTTTA [29328]
cp32-9_PAbe_NX_Pl_consensus    GCTCATGAAGCTGAAAGTAAAAGCAACTACAAAAATAGTAAAGGGAACAAGTGAACTTATATCAAACAAAACTTTTTA [29328]

cp32-9_gi|11497325|B31_GB      CAACACTACAAAAGGAATACAAAAATAAATATGGAGTTGACATATCACAATTTGTAAAGCTAGCAAATTCTTCAATTA [29406]
cp32-9_un7_B31_PacBio          CAACACTACAAAAGGAATACAAAA-TAAATATGGAGTTGACATATCACAATTTGTAAAGCTAGCAAATTCTTCAATTA [29406]
cp32-9_B31_TS_MP_FG_consensus  CAACACTACAAAAGGAATACAAAAATAAATATGGAGTTGACATATCACAATTTGTAAAGCTAGCAAATTCTTCAATTA [29406]
cp32-9_B31_NX_Pl_consensus     CAACACTACAAAAGGAATACAAAAATAAATATGGAGTTGACATATCACAATTTGTAAAGCTAGCAAATTCTTCAATTA [29406]
cp32-9_PAli_NX_MP_FG_consensus CAACACTACAAAAGGAATACAAAAATAAATATGGAGTTGACATATCACAATTTGTAAAGCTAGCAAATTCTTCAATTA [29406]
cp32-9_PAli_NX_Pl_consensus    CAACACTACAAAAGGAATACAAAAATAAATATGGAGTTGACATATCACAATTTGTAAAGCTAGCAAATTCTTCAATTA [29406]
cp32-9_PAbe_NX_MP_FG_consensus CAACACTACAAAAGGAATACAAAAATAAATATGGAGTTGACATATCACAATTTGTAAAGCTAGCAAATTCTTCAATTA [29406]
cp32-9_PAbe_NX_Pl_consensus    CAACACTACAAAAGGAATACAAAAATAAATATGGAGTTGACATATCACAATTTGTAAAGCTAGCAAATTCTTCAATTA [29406]

cp32-9_gi|11497325|B31_GB      ATTTTGATAAGTTTGAAGAAAAACAGTTAACTTTAAAACAAAAAAATGTGATAAAAAGTATTAAAAAGAATAATGAAA [29484]
cp32-9_un7_B31_PacBio          ATTTTGATAAGTTTGAAGAAAAACAGTTAACTTTAAAACAAAAAAATGTGATAAAAAGTATTAAAAAGAATAATGAAA [29484]
cp32-9_B31_TS_MP_FG_consensus  ATTTTGATAAGTTTGAAGAAAAACAGTTAACTTTAAAACAAAAAAATGTGATAAAAAGTATTAAAAAGAATAATGAAA [29484]
cp32-9_B31_NX_Pl_consensus     ATTTTGATAAGTTTGAAGAAAAACAGTTAACTTTAAAACAAAAAAATGTGATAAAAAGTATTAAAAAGAATAATGAAA [29484]
cp32-9_PAli_NX_MP_FG_consensus ATTTTGATAAGTTTGAAGAAAAACAGTTAACTTTAAAACAAAAAAATGTGATAAAAAGTATTAAAAAGAATAATGAAA [29484]
cp32-9_PAli_NX_Pl_consensus    ATTTTGATAAGTTTGAAGAAAAACAGTTAACTTTAAAACAAAAAAATGTGATAAAAAGTATTAAAAAGAATAATGAAA [29484]
cp32-9_PAbe_NX_MP_FG_consensus ATTTTGATAAGTTTGAAGAAAAACAGTTAACTTTAAAACAAAAAAATGTGATAAAAAGTATTAAAAAGAATAATGAAA [29484]
cp32-9_PAbe_NX_Pl_consensus    ATTTTGATAAGTTTGAAGAAAAACAGTTAACTTTAAAACAAAAAAATGTGATAAAAAGTATTAAAAAGAATAATGAAA [29484]

cp32-9_gi|11497325|B31_GB      AAAAGATTATACTCAGCGGAGGCATAGCTAGTGGCAAAACGTATCTTGCATGTTATCTTTTTCTAAAAAGTTTAATTG [29562]
cp32-9_un7_B31_PacBio          AAA-GATTATACTCAGCGGAGGCATAGCTAGTGGCAAAACGTATCTTGCATGTTATCTTTTTCTAAAAAGTTTAATTG [29562]
cp32-9_B31_TS_MP_FG_consensus  AAAAGATTATACTCAGCGGAGGCATAGCTAGTGGCAAAACGTATCTTGCATGTTATCTTTTTCTAAAAAGTTTAATTG [29562]
cp32-9_B31_NX_Pl_consensus     AAAAGATTATACTCAGCGGAGGCATAGCTAGTGGCAAAACGTATCTTGCATGTTATCTTTTTCTAAAAAGTTTAATTG [29562]
cp32-9_PAli_NX_MP_FG_consensus AAAAGATTATACTCAGCGGAGGCATAGCTAGTGGCAAAACGTATCTTGCATGTTATCTTTTTCTAAAAAGTTTAATTG [29562]
cp32-9_PAli_NX_Pl_consensus    AAAAGATTATACTCAGCGGAGGCATAGCTAGTGGCAAAACGTATCTTGCATGTTATCTTTTTCTAAAAAGTTTAATTG [29562]
cp32-9_PAbe_NX_MP_FG_consensus AAAAGATTATACTCAGCGGAGGCATAGCTAGTGGCAAAACGTATCTTGCATGTTATCTTTTTCTAAAAAGTTTAATTG [29562]
cp32-9_PAbe_NX_Pl_consensus    AAAAGATTATACTCAGCGGAGGCATAGCTAGTGGCAAAACGTATCTTGCATGTTATCTTTTTCTAAAAAGTTTAATTG [29562]

cp32-9_gi|11497325|B31_GB      AAAATAAAAAGTTATACTCTAGTGATACTAATAATTTCATTATTGGGAATTCACAACGCTCAGTTGAAGTTAATGTTT [29640]
cp32-9_un7_B31_PacBio          AAAATAAAAAGTTATACTCTAGTGATACTAATAATTTCATTATTGGGAATTCACAACGCTCAGTTGAAGTTAATGTTT [29640]
cp32-9_B31_TS_MP_FG_consensus  AAAATAAAAAGTTATACTCTAGTGATACTAATAATTTCATTATTGGGAATTCACAACGCTCAGTTGAAGTTAATGTTT [29640]
cp32-9_B31_NX_Pl_consensus     AAAATAAAAAGTTATACTCTAGTGATACTAATAATTTCATTATTGGGAATTCACAACGCTCAGTTGAAGTTAATGTTT [29640]
cp32-9_PAli_NX_MP_FG_consensus AAAATAAAAAGTTATACTCTAGTGATACTAATAATTTCATTATTGGGAATTCACAACGCTCAGTTGAAGTTAATGTTT [29640]
cp32-9_PAli_NX_Pl_consensus    AAAATAAAAAGTTATACTCTAGTGATACTAATAATTTCATTATTGGGAATTCACAACGCTCAGTTGAAGTTAATGTTT [29640]
cp32-9_PAbe_NX_MP_FG_consensus AAAATAAAAAGTTATACTCTAGTGATACTAATAATTTCATTATTGGGAATTCACAACGCTCAGTTGAAGTTAATGTTT [29640]
cp32-9_PAbe_NX_Pl_consensus    AAAATAAAAAGTTATACTCTAGTGATACTAATAATTTCATTATTGGGAATTCACAACGCTCAGTTGAAGTTAATGTTT [29640]

cp32-9_gi|11497325|B31_GB      TAGGACAATTTGAAAAGCTATGTAAACTTCTTAAAATTCCTTATATCCCAAGACATACAAATAATTCATATATTCTGA [29718]
cp32-9_un7_B31_PacBio          TAGGACAATTTGAAAAGCTATGTAAACTTCTTAAAATTCCTTATATCCCAAGACATACAAATAATTCATATATTCTGA [29718]
cp32-9_B31_TS_MP_FG_consensus  TAGGACAATTTGAAAAGCTATGTAAACTTCTTAAAATTCCTTATATCCCAAGACATACAAATAATTCATATATTCTGA [29718]
cp32-9_B31_NX_Pl_consensus     TAGGACAATTTGAAAAGCTATGTAAACTTCTTAAAATTCCTTATATCCCAAGACATACAAATAATTCATATATTCTGA [29718]
cp32-9_PAli_NX_MP_FG_consensus TAGGACAATTTGAAAAGCTATGTAAACTTCTTAAAATTCCTTATATCCCAAGACATACAAATAATTCATATATTCTGA [29718]
cp32-9_PAli_NX_Pl_consensus    TAGGACAATTTGAAAAGCTATGTAAACTTCTTAAAATTCCTTATATCCCAAGACATACAAATAATTCATATATTCTGA [29718]
cp32-9_PAbe_NX_MP_FG_consensus TAGGACAATTTGAAAAGCTATGTAAACTTCTTAAAATTCCTTATATCCCAAGACATACAAATAATTCATATATTCTGA [29718]
cp32-9_PAbe_NX_Pl_consensus    TAGGACAATTTGAAAAGCTATGTAAACTTCTTAAAATTCCTTATATCCCAAGACATACAAATAATTCATATATTCTGA [29718]

cp32-9_gi|11497325|B31_GB      TTGATTCACTACGTATTAATCTATATGGTGGAGATAAGGCAAGTGATTTTGAAAGATTTAGGGGAAGTAATTCAGCAC [29796]
cp32-9_un7_B31_PacBio          TTGATTCACTACGTATTAATCTATATGGTGGAGATAAGGCAAGTGATTTTGAAAGATTTAGGGGAAGTAATTCAGCAC [29796]
cp32-9_B31_TS_MP_FG_consensus  TTGATTCACTACGTATTAATCTATATGGTGGAGATAAGGCAAGTGATTTTGAAAGATTTAGGGGAAGTAATTCAGCAC [29796]
cp32-9_B31_NX_Pl_consensus     TTGATTCACTACGTATTAATCTATATGGTGGAGATAAGGCAAGTGATTTTGAAAGATTTAGGGGAAGTAATTCAGCAC [29796]
cp32-9_PAli_NX_MP_FG_consensus TTGATTCACTACGTATTAATCTATATGGTGGAGATAAGGCAAGTGATTTTGAAAGATTTAGGGGAAGTAATTCAGCAC [29796]
cp32-9_PAli_NX_Pl_consensus    TTGATTCACTACGTATTAATCTATATGGTGGAGATAAGGCAAGTGATTTTGAAAGATTTAGGGGAAGTAATTCAGCAC [29796]
cp32-9_PAbe_NX_MP_FG_consensus TTGATTCACTACGTATTAATCTATATGGTGGAGATAAGGCAAGTGTTTTTGAAAGATTTAGGGGAAGTAATTCAGCAC [29796]
cp32-9_PAbe_NX_Pl_consensus    TTGATTCACTACGTATTAATCTATATGGTGGAGATAAGGCAAGTGATTTTGAAAGATTTAGGGGAAGTAATTCAGCAC [29796]

cp32-9_gi|11497325|B31_GB      TTATTTTTGTTAATGAGGCTACAACTTTACACAAGCAAACTTTAGAGGAGGTCTTAAAAAGACTAAGATGCGGGCAAG [29874]
cp32-9_un7_B31_PacBio          TTATTTTTGTTAATGAGGCTACAACTTTACACAAGCAAACTTTAGAGGAGGTCTTAAAAAGACTAAGATGCGGGCAAG [29874]
cp32-9_B31_TS_MP_FG_consensus  TTATTTTTGTTAATGAGGCTACAACTTTACACAAGCAAACTTTAGAGGAGGTCTTAAAAAGACTAAGATGCGGGCAAG [29874]
cp32-9_B31_NX_Pl_consensus     TTATTTTTGTGAATGAGGCTACAACTTTACACAAGCAAACTTTAGAGGAGGTCTTAAAAAGACTAAGATGCGGGCAAG [29874]
cp32-9_PAli_NX_MP_FG_consensus TTATTTTTGTTAATGAGGCTACAACTTTACACAAGCAAACTTTAGAGGAGGTCTTAAAAAGACTAAGATGCGGGCAAG [29874]
cp32-9_PAli_NX_Pl_consensus    TTATTTTTGTTAATGAGGCTACAACTTTACACAAGCAAACTTTAGAGGAGGTCTTAAAAAGACTAAGATGCGGGCAAG [29874]
cp32-9_PAbe_NX_MP_FG_consensus TTATTTTTGTGAATGAGGCTACAACTTTACACAAGCAAACTTTAGAGGAGGTCTTAAAAAGACTAAGATGCGGGCAAG [29874]
cp32-9_PAbe_NX_Pl_consensus    TTATTTTTGTGAATGAGGCTACAACTTTACACAAGCAAACTTTAGAGGAGGTCTTAAAAAGACTAAGATGCGGGCAAG [29874]

cp32-9_gi|11497325|B31_GB      AAACTATTATTTTTGATACTAATCCTGATCATCCAGAACACTATTTTAAAACCGATTATATTGATAATATAGCGACAT [29952]
cp32-9_un7_B31_PacBio          AAACTATTATTTTTGATACTAATCCTGATCATCCAGAACACTATTTTAAAACCGATTATATTGATAATATAGCGACAT [29952]
cp32-9_B31_TS_MP_FG_consensus  AAACTATTATTTTTGATACTAATCCTGATCATCCAGAACACTATTTTAAAACCGATTATATTGATAATATAGCGACAT [29952]
cp32-9_B31_NX_Pl_consensus     AAACTATTATTTTTGATACTAATCCTGATCATCCAGAACACTATTTTAAAACCGATTATATTGATAATATAGCGACAT [29952]
cp32-9_PAli_NX_MP_FG_consensus AAACTATTATTTTTGATACTAATCCTGATCATCCAGAACACTATTTTAAAACCGATTATATTGATAATATAGCGACAT [29952]
cp32-9_PAli_NX_Pl_consensus    AAACTATTATTTTTGATACTAATCCTGATCATCCAGAACACTATTTTAAAACCGATTATATTGATAATATAGCGACAT [29952]
cp32-9_PAbe_NX_MP_FG_consensus AAACTATTATTTTTGATACTAATCCTGATCATCCAGAACACTATTTTAAAACCGATTATATTGATAATATAGCGACAT [29952]
cp32-9_PAbe_NX_Pl_consensus    AAACTATTATTTTTGATACTAATCCTGATCATCCAGAACACTATTTTAAAACCGATTATATTGATAATATAGCGACAT [29952]

cp32-9_gi|11497325|B31_GB      TTAAGATATATAATTTTACAACTTATGATAATGTGCTACTTAGTAAAGGATTTATCGAAACACAAGAAAAACTCTATA [30030]
cp32-9_un7_B31_PacBio          TTAAGATATATAATTTTACAACTTATGATAATGTGCTACTTAGTAAAGGATTTATCGAAACACAAGAAAAACTCTATA [30030]
cp32-9_B31_TS_MP_FG_consensus  TTAAGATATATAATTTTACAACTTATGATAATGTGCTACTTAGTAAAGGATTTATCGAAACACAAGAAAAACTCTATA [30030]
cp32-9_B31_NX_Pl_consensus     TTAAGATATATAATTTTACAACTTATGATAATGTGCTACTTAGTAAAGGATTTATCGAAACACAAGAAAAACTCTATA [30030]
cp32-9_PAli_NX_MP_FG_consensus TTAAGATATATAATTTTACAACTTATGATAATGTGCTACTTAGTAAAGGATTTATCGAAACACAAGAAAAACTCTATA [30030]
cp32-9_PAli_NX_Pl_consensus    TTAAGATATATAATTTTACAACTTATGATAATGTGCTACTTAGTAAAGGATTTATCGAAACACAAGAAAAACTCTATA [30030]
cp32-9_PAbe_NX_MP_FG_consensus TTAAGATATATAATTTTACAACTTATGATAATGTGCTACTTAGTAAAGGATTTATCGAAACACAAGAAAAACTCTATA [30030]
cp32-9_PAbe_NX_Pl_consensus    TTAAGATATATAATTTTACAACTTATGATAATGTGCTACTTAGTAAAGGATTTATCGAAACACAAGAAAAACTCTATA [30030]

cp32-9_gi|11497325|B31_GB      AAGATATACCATCATATAAAGCAAGAGTTTTGCTAGGTGAGTGGATAGCAAGCACTGATTCAATTTTTACACAAATAA [30108]
cp32-9_un7_B31_PacBio          AAGATATACCATCATATAAAGCAAGAGTTTTGCTAGGTGAGTGGATAGCAAGCACTGATTCAATTTTTACACAAATAA [30108]
cp32-9_B31_TS_MP_FG_consensus  AAGATATACCATCATATAAAGCAAGAGTTTTGCTAGGTGAGTGGATAGCAAGCACTGATTCAATTTTTACACAAATAA [30108]
cp32-9_B31_NX_Pl_consensus     AAGATATACCATCATATAAAGCAAGAGTTTTGCTAGGTGAGTGGATAGCAAGCACTGATTCAATTTTTACACAAATAA [30108]
cp32-9_PAli_NX_MP_FG_consensus AAGATATACCATCATATAAAGCAAGAGTTTTGCTAGGTGAGTGGATAGCAAGCACTGATTCAATTTTTACACAAATAA [30108]
cp32-9_PAli_NX_Pl_consensus    AAGATATACCATCATATAAAGCAAGAGTTTTGCTAGGTGAGTGGATAGCAAGCACTGATTCAATTTTTACACAAATAA [30108]
cp32-9_PAbe_NX_MP_FG_consensus AAGATATACCATCATATAAAGCAAGAGTTTTGCTAGGTGAGTGGATAGCAAGCACTGATTCAATTTTTACACAAATAA [30108]
cp32-9_PAbe_NX_Pl_consensus    AAGATATACCATCATATAAAGCAAGAGTTTTGCTAGGTGAGTGGATAGCAAGCACTGATTCAATTTTTACACAAATAA [30108]

cp32-9_gi|11497325|B31_GB      ATATTACTGATGATTATATATTTACTAGCCCGATAGCATATTTAGACCCAGCATTTAGTGTTGGAGGGGATAACACTG [30186]
cp32-9_un7_B31_PacBio          ATATTACTGATGATTATATATTTACTAGCCCGATAGCATATTTAGACCCAGCATTTAGTGTTGGAGGGGATAACACTG [30186]
cp32-9_B31_TS_MP_FG_consensus  ATATTACTGATGATTATATATTTACTAGCCCGATAGCATATTTAGACCCAGCATTTAGTGTTGGAGGGGATAACACTG [30186]
cp32-9_B31_NX_Pl_consensus     ATATTACTGATGATTATATATTTACTAGCCCGATAGCATATTTAGACCCAGCATTTAGTGTTGGAGGGGATAACACTG [30186]
cp32-9_PAli_NX_MP_FG_consensus ATATTACTGATGATTATATATTTACTAGCCCGATAGCATATTTAGACCCAGCATTTAGTGTTGGAGGGGATAACACTG [30186]
cp32-9_PAli_NX_Pl_consensus    ATATTACTGATGATTATATATTTACTAGCCCGATAGCATATTTAGACCCAGCATTTAGTGTTGGAGGGGATAACACTG [30186]
```

```
cp32-9_PAbe_NX_MP_FG_consensus ATATTACTGATGATTATATATTTACTAGCCCGATAGCATATTTAGACCCAGCATTTAGTGTTGGAGGGGATAACACTG [30186]
cp32-9_PAbe_NX_Pl_consensus    ATATTACTGATGATTATATATTTACTAGCCCGATAGCATATTTAGACCCAGCATTTAGTGTTGGAGGGGATAACACTG [30186]

cp32-9_gi|11497325|B31_GB      CATTATGTGTTATGGAGCGAGTTGATGATAAGTATTATGCTTTTGTATTTCAAGACCAACGACCAGCCAATGATCCTT [30264]
cp32-9_un7_B31_PacBio          CATTATGTGTTATGGAGCGAGTTGATGATAAGTATTATGCTTTTGTATTTCAAGACCAACGACCAGCCAATGATCCTT [30264]
cp32-9_B31_TS_MP_FG_consensus  CATTATGTGTTATGGAGCGAGTTGATGATAAGTATTATGCTTTTGTATTTCAAGACCAACGACCAGCCAATGATCCTT [30264]
cp32-9_B31_NX_Pl_consensus     CATTATGTGTTATGGAGCGAGTTGATGATAAGTATTATGCTTTTGTATTTCAAGACCAACGACCAGCCAATGATCCTT [30264]
cp32-9_PAli_NX_MP_FG_consensus CATTATGTGTTATGGAGCGAGTTGATGATAAGTATTATGCTTTTGTATTTCAAGACCAACGACCAGCCAATGATCCTT [30264]
cp32-9_PAli_NX_Pl_consensus    CATTATGTGTTATGGAGCGAGTTGATGATAAGTATTATGCTTTTGTATTTCAAGACCAACGACCAGCCAATGATCCTT [30264]
cp32-9_PAbe_NX_MP_FG_consensus CATTATGTGTTATGGAGCGAGTTGATGATAAGTATTATGCTTTTGTATTTCAAGACCAACGACCAGCCAATGATCCTT [30264]
cp32-9_PAbe_NX_Pl_consensus    CATTATGTGTTATGGAGCGAGTTGATGATAAGTATTATGCTTTTGTATTTCAAGACCAACGACCAGCCAATGATCCTT [30264]

cp32-9_gi|11497325|B31_GB      ATATTATGAATATGGTAAAGACCGTTATAGAAAATTTCAATGTGCATACACTGTATTTAGAGGATAGAGATAATACAA [30342]
cp32-9_un7_B31_PacBio          ATATTATGAATATGGTAAAGACCGTTATAGAAAATTTCAATGTGCATACACTGTATTTAGAGGATAGAGATAATACAA [30342]
cp32-9_B31_TS_MP_FG_consensus  ATATTATGAATATGGTAAAGACCGTTATAGAAAATTTCAATGTGCATACACTGTATTTAGAGGATAGAGATAATACAA [30342]
cp32-9_B31_NX_Pl_consensus     ATATTATGAATATGGTAAAGACCGTTATAGAAAATTTCAATGTGCATACACTGTATTTAGAGGATAGAGATAATACAA [30342]
cp32-9_PAli_NX_MP_FG_consensus ATATTATGAATATGGTAAAGACCGTTATAGAAAATTTCAATGTGCATACACTGTATTTAGAGGATAGAGATAATACAA [30342]
cp32-9_PAli_NX_Pl_consensus    ATATTATGAATATGGTAAAGACCGTTATAGAAAATTTCAATGTGCATACACTGTATTTAGAGGATAGAGATAATACAA [30342]
cp32-9_PAbe_NX_MP_FG_consensus ATATTATGAATATGGTAAAGACCGTTATAGAAAATTTCAATGTGCATACACTGTATTTAGAGGATAGAGATAATACAA [30342]
cp32-9_PAbe_NX_Pl_consensus    ATATTATGAATATGGTAAAGACCGTTATAGAAAATTTCAATGTGCATACACTGTATTTAGAGGATAGAGATAATACAA [30342]

cp32-9_gi|11497325|B31_GB      AAGGTGCTGGTGGATTGACCCGCGAATACATCTTGCTAAGAAATAATATAAGCCAATATTTTAGAATTGTTCCAGTTA [30420]
cp32-9_un7_B31_PacBio          AAGGTGCTGGTGGATTGACCCGCGAATACATCTTGCTAAGAAATAATATAAGCCAATATTTTAGAATTGTTCCAGTTA [30420]
cp32-9_B31_TS_MP_FG_consensus  AAGGTGCTGGTGGATTGACCCGCGAATACATCTTGCTAAGAAATAATATAAGCCAATATTTTAGAATTGTTCCAGTTA [30420]
cp32-9_B31_NX_Pl_consensus     AAGGTGCTGGTGGATTGACCCGCGAATACATCTTGCTAAGAAATAATATAAGCCAATATTTTAGAATTGTTCCAGTTA [30420]
cp32-9_PAli_NX_MP_FG_consensus AAGGTGCTGGTGGATTGACCCGCGAATACATCTTGCTAAGAAATAATATAAGCCAATATTTTAGAATTGTTCCAGTTA [30420]
cp32-9_PAli_NX_Pl_consensus    AAGGTGCTGGTGGATTGACCCGCGAATACATCTTGCTAAGAAATAATATAAGCCAATATTTTAGAATTGTTCCAGTTA [30420]
cp32-9_PAbe_NX_MP_FG_consensus AAGGTGCTGGTGGATTGACCCGCGAATACATCTTGCTAAGAAATAATATAAGCCAATATTTTAGAATTGTTCCAGTTA [30420]
cp32-9_PAbe_NX_Pl_consensus    AAGGTGCTGGTGGATTGACCCGCGAATACATCTTGCTAAGAAATAATATAAGCCAATATTTTAGAATTGTTCCAGTTA [30420]

cp32-9_gi|11497325|B31_GB      AGCCAAAGTCTAATAAATTTAGCAGAATAACAATGTTAATTACGCCGTTTACTTACAAAAAACTTTATATTACAAAGT [30498]
cp32-9_un7_B31_PacBio          AGCCAAAGTCTAATAAATTTAGCAGAATAACAATGTTAATTACGCCGTTTACTTACAAAAAACTTTATATTACAAAGT [30498]
cp32-9_B31_TS_MP_FG_consensus  AGCCAAAGTCTAATAAATTTAGCAGAATAACAATGTTAATTACGCCGTTTACTTACAAAAAACTTTATATTACAAAGT [30498]
cp32-9_B31_NX_Pl_consensus     AGCCAAAGTCTAATAAATTTAGCAGAATAACAATGTTAATTACGCCGTTTACTTACAAAAAACTTTATATTACAAAGT [30498]
cp32-9_PAli_NX_MP_FG_consensus AGCCAAAGTCTAATAAATTTAGCAGAATAACAATGTTAATTACGCCGTTTACTTACAAAAAACTTTATATTACAAAGT [30498]
cp32-9_PAli_NX_Pl_consensus    AGCCAAAGTCTAATAAATTTAGCAGAATAACAATGTTAATTACGCCGTTTACTTACAAAAAACTTTATATTACAAAGT [30498]
cp32-9_PAbe_NX_MP_FG_consensus AGCCAAAGTCTAATAAATTTAGCAGAATAACAATGTTAATTACGCCGTTTACTTACAAAAAACTTTATATTACAAAGT [30498]
cp32-9_PAbe_NX_Pl_consensus    AGCCAAAGTCTAATAAATTTAGCAGAATAACAATGTTAATTACGCCGTTTACTTACAAAAAACTTTATATTACAAAGT [30498]

cp32-9_gi|11497325|B31_GB      ACAGTAGTTCTTCTGTATTTAATGATATTTATTCGTATAAGGGGGATAGCAAAACCCATGATGATGCTCTTGATGCAA [30576]
cp32-9_un7_B31_PacBio          ACAGTAGTTCTTCTGTATTTAATGATATTTATTCGTATAAGGGGGATAGCAAAACCCATGATGATGCTCTTGATGCAA [30576]
cp32-9_B31_TS_MP_FG_consensus  ACAGTAGTTCTTCTGTATTTAATGATATTTATTCGTATAAGGGGGATAGCAAAACCCATGATGATGCTCTTGATGCAA [30576]
cp32-9_B31_NX_Pl_consensus     ACAGTAGTTCTTCTGTATTTAATGATATTTATTCGTATAAGGGGGATAGCAAAACCCATGATGATGCTCTTGATGCAA [30576]
cp32-9_PAli_NX_MP_FG_consensus ACAGTAGTTCTTCTGTATTTAATGATATTTATTCGTATAAGGGGGATAGCAAAACCCATGATGATGCTCTTGATGCAA [30576]
cp32-9_PAli_NX_Pl_consensus    ACAGTAGTTCTTCTGTATTTAATGATATTTATTCGTATAAGGGGGATAGCAAAACCCATGATGATGCTCTTGATGCAA [30576]
cp32-9_PAbe_NX_MP_FG_consensus ACAGTAGTTCTTCTGTATTTAATGATATTTATTCGTATAAGGGGGATAGCAAAACCCATGATGATGCTCTTGATGCAA [30576]
cp32-9_PAbe_NX_Pl_consensus    ACAGTAGTTCTTCTGTATTTAATGATATTTATTCGTATAAGGGGGATAGCAAAACCCATGATGATGCTCTTGATGCAA [30576]

cp32-9_gi|11497325|B31_GB      TATCTGCAGCATATTTGATGTTGTCTTTAGGATATAGAGAGCGAAGTGTTCACTTTGGCAATCAAAGATTTTTGTAA- [30654]
cp32-9_un7_B31_PacBio          TATCTGCAGCATATTTGATGTTGTCTTTAGGATATAGAGAGCGAAGTGTTCACTTTGGCAATCAAAGATTTTTGTAA- [30654]
cp32-9_B31_TS_MP_FG_consensus  TATCTGCAGCATATTTGATGTTGTCTTTAGGATATAGAGAGCGAAGTGTTCACTTTGGCAATCAAAGATTTTTGTAA- [30654]
cp32-9_B31_NX_Pl_consensus     TATCTGCAGCATATTTGATGTTGTCTTTAGGATATAGAGAGCGAAGTGTTCACTTTGGCAATCAAAGATTTTTGTAA- [30654]
cp32-9_PAli_NX_MP_FG_consensus TATCTGCAGCATATTTGATGTTGTCTTTAGGATATAGAGAGCGAAGTGTTCACTTTGGCAATCAAAGATTTTTGTAA- [30654]
cp32-9_PAli_NX_Pl_consensus    TATCTGCAGCATATTTGATGTTGTCTTTAGGATATAGAGAGCGAAGTGTTCACTTTGGCAATCAAAGATTTTTGTAA- [30654]
cp32-9_PAbe_NX_MP_FG_consensus TATCTGCAGCATATTTGATGTTGTCTTTAGGATATAGAGAGCGAAGTGTTCACTTTGGCAATCAAAGATTTTTGTAA- [30654]
cp32-9_PAbe_NX_Pl_consensus    TATCTGCAGCATATTTGATGTTGTCTTTAGGATATAGAGAGCGAAGTGTTCACTTTGGCAATCAAAGATTTTTGTAA- [30654]
```

```
#NEXUS
[ Title ]
begin data;
   dimensions ntax=8 nchar=34664;
   format missing=? gap=- matchchar=. datatype=nucleotide interleave=yes;
   matrix

[!Domain=Data property=Coding CodonStart=1;]
lp17_gi|365823332|B31_GB     ------------------------------------------------------------------------------ [   78]
lp17_un5_B31_PacBio          TTTGATTTGTCAAAGGAAAAGTAGGAGTTATTATTTTTCTAATTTAAAAATTGAACTTTGTACTAAATAAATATTATA [   78]
lp17_B31_TS_MP_FG_consensus  ------------------------------------------------------------------------------ [   78]
lp17_B31_NX_Pl_consensus     ------------------------------------------------------------------------------ [   78]
lp17_PAli_NX_MP_FG_consensus ------------------------------------------------------------------------------ [   78]
lp17_PAli_NX_Pl_consensus    ------------------------------------------------------------------------------ [   78]
lp17_PAbe_NX_MP_FG_consensus ------------------------------------------------------------------------------ [   78]
lp17_PAbe_NX_Pl_consensus    ------------------------------------------------------------------------------ [   78]

lp17_gi|365823332|B31_GB     ------------------------------------------------------------------------------ [  156]
lp17_un5_B31_PacBio          TATATAATATTTATTTAGTACAAAGTTCAATTTTTAAATTAGAAAAATAATAACTCCTACTTTTCCTTTGACAAATCA [  156]
lp17_B31_TS_MP_FG_consensus  ------------------------------------------------------------------------------ [  156]
lp17_B31_NX_Pl_consensus     ------------------------------------------------------------------------------ [  156]
lp17_PAli_NX_MP_FG_consensus ------------------------------------------------------------------------------ [  156]
lp17_PAli_NX_Pl_consensus    ------------------------------------------------------------------------------ [  156]
lp17_PAbe_NX_MP_FG_consensus ------------------------------------------------------------------------------ [  156]
lp17_PAbe_NX_Pl_consensus    ------------------------------------------------------------------------------ [  156]

lp17_gi|365823332|B31_GB     --TTACTTATAATAGCTATATACTACGAAATATACAAAAGAAAAATATATAAATGCCGTTTATATAAAATTACTTGGA [  234]
lp17_un5_B31_PacBio          AATTACTTATAATAGCTATATACTACGAAATATACAAAAGAAAAATATATAAATGCCGTTTATATAAAATTACTTGGA [  234]
lp17_B31_TS_MP_FG_consensus  --TTACTTATAATAGCTATATACTACGAAATATACAAAAGAAAAATATATAAATGCCGTTTATATAAAATTACTTGGA [  234]
lp17_B31_NX_Pl_consensus     --TTACTTATAATAGCTATATACTACGAAATATACAAAAGAAAAATATATAAATGCCGTTTATATAAAATTACTTGGA [  234]
lp17_PAli_NX_MP_FG_consensus --TTACTTATAATAGCTATATACTACGAAATATACAAAAGAAAAATATATAAATGCCGTTTATATAAAATTACTTGGA [  234]
lp17_PAli_NX_Pl_consensus    --TTACTTATAATAGCTATATACTACGAAATATACAAAAGAAAAATATATAAATGCCGTTTATATAAAATTACTTGGA [  234]
lp17_PAbe_NX_MP_FG_consensus --TTACTTATAATAGCTATATACTACGAAATATACAAAAGAAAAATATATAAATGCCGTTTATATAAAATTACTTGGA [  234]
lp17_PAbe_NX_Pl_consensus    --TTACTTATAATAGCTATATACTACGAAATATACAAAAGAAAAATATATAAATGCCGTTTATATAAAATTACTTGGA [  234]

lp17_gi|365823332|B31_GB     TTAGAAAAACAGGGAAAATTATTTCCTATAAACCATCAAAAAATATAAAAACTTTCAAAAAGTATGAGGGAAAAAGTA [  312]
lp17_un5_B31_PacBio          TTAGAAAAACAGGGAAAATTATTTCCTATAAACCATCAAAAAATATAAAAACTTTCAAAAAGTATGAGGGAAAAAGTA [  312]
lp17_B31_TS_MP_FG_consensus  TTAGAAAAACAGGGAAAATTATTTCCTATAAACCATCAAAAAATATAAAAACTTTCAAAAAGTATGAGGGAAAAAGTA [  312]
lp17_B31_NX_Pl_consensus     TTAGAAAAACAGGGAAAATTATTTCCTATAAACCATCAAAAAATATAAAAACTTTCAAAAAGTATGAGGGAAAAAGTA [  312]
lp17_PAli_NX_MP_FG_consensus TTAGAAAAACAGGGAAAATTATTTCCTATAAACCATCAAAAAATATAAAAACTTTCAAAAAGTATGAGGGAAAAAGTA [  312]
lp17_PAli_NX_Pl_consensus    TTAGAAAAACAGGGAAAATTATTTCCTATAAACCATCAAAAAATATAAAAACTTTCAAAAAGTATGAGGGAAAAAGTA [  312]
lp17_PAbe_NX_MP_FG_consensus TTAGAAAAACAGGGAAAATTATTTCCTATAAACCATCAAAAAATATAAAAACTTTCAAAAAGTATGAGGGAAAAAGTA [  312]
lp17_PAbe_NX_Pl_consensus    TTAGAAAAACAGGGAAAATTATTTCCTATAAACCATCAAAAAATATAAAAACTTTCAAAAAGTATGAGGGAAAAAGTA [  312]

lp17_gi|365823332|B31_GB     TTAATTTACATCCCCCCAAAAGAAAAAAATTTGAAAATATTTTATAACACCAAACTTTTAAAAAGTAACAACCCTAACA [  390]
lp17_un5_B31_PacBio          TTAATTTACATCCCCCCAAAAGAAAAAAATTTGAAAATATTTTATAACACCAAACTTTTAAAAAGTAACAACCCTAACA [  390]
lp17_B31_TS_MP_FG_consensus  TTAATTTACATCCCCCCAAAAGAAAAAAATTTGAAAATATTTTATAACACCAAACTTTTAAAAAGTAACAACCCTAACA [  390]
lp17_B31_NX_Pl_consensus     TTAATTTACATCCCCCCAAAAGAAAAAAATTTGAAAATATTTTATAACACCAAACTTTTAAAAAGTAACAACCCTAACA [  390]
lp17_PAli_NX_MP_FG_consensus TTAATTTACATCCCCCCAAAAGAAAAAAATTTGAAAATATTTTATAACACCAAACTTTTAAAAAGTAACAACCCTAACA [  390]
lp17_PAli_NX_Pl_consensus    TTAATTTACATCCCCCCAAAAGAAAAAAATTTGAAAATATTTTATAACACCAAACTTTTAAAAAGTAACAACCCTAACA [  390]
lp17_PAbe_NX_MP_FG_consensus TTAATTTACATCCCCCCAAAAGAAAAAAATTTGAAAATATTTTATAACACCAAACTTTTAAAAAGTAACAACCCTAACA [  390]
lp17_PAbe_NX_Pl_consensus    TTAATTTACATCCCCCCAAAAGAAAAAAATTTGAAAATATTTTATAACACCAAACTTTTAAAAAGTAACAACCCTAACA [  390]

lp17_gi|365823332|B31_GB     AATATTTTCTCAAGTGCAATCTATTAAAATTGGTCAAAATGCCATAACGTTTGAAGAGCTTGCAATCAAATATATAAA [  468]
lp17_un5_B31_PacBio          AATATTTTCTCAAGTGCAATCTATTAAAATTGGTCAAAATGCCATAACGTTTGAAGAGCTTGCAATCAAATATATAAA [  468]
lp17_B31_TS_MP_FG_consensus  AATATTTTCTCAAGTGCAATCTATTAAAATTGGTCAAAATGCCATAACGTTTGAAGAGCTTGCAATCAAATATATAAA [  468]
lp17_B31_NX_Pl_consensus     AATATTTTCTCAAGTGCAATCTATTAAAATTGGTCAAAATGCCATAACGTTTGAAGAGCTTGCAATCAAATATATAAA [  468]
lp17_PAli_NX_MP_FG_consensus AATATTTTCTCAAGTGCAATCTATTAAAATTGGTCAAAATGCCATAACGTTTGAAGAGCTTGCAATCAAATATATAAA [  468]
lp17_PAli_NX_Pl_consensus    AATATTTTCTCAAGTGCAATCTATTAAAATTGGTCAAAATGCCATAACGTTTGAAGAGCTTGCAATCAAATATATAAA [  468]
lp17_PAbe_NX_MP_FG_consensus AATATTTTCTCAAGTGCAATCTATTAAAATTGGTCAAAATGCCATAACGTTTGAAGAGCTTGCAATCAAATATATAAA [  468]
lp17_PAbe_NX_Pl_consensus    AATATTTTCTCAAGTGCAATCTATTAAAATTGGTCAAAATGCCATAACGTTTGAAGAGCTTGCAATCAAATATATAAA [  468]

lp17_gi|365823332|B31_GB     AATCCAATAATATTGCCTAGCAAATCTTATTATTTTAAAATTTTAAAAAAACAAATCCTAACTATGTCTTAAATCTGG [  546]
lp17_un5_B31_PacBio          AATCCAATAATATTGCCTAGCAAATCTTATTATTTTAAAATTTTAAAAAAACAAATCCTAACTATGTCTTAAATCTGG [  546]
lp17_B31_TS_MP_FG_consensus  AATCCAATAATATTGCCTAGCAAATCTTATTATTTTAAAATTTTAAAAAAACAAATCCTAACTATGTCTTAAATCTGG [  546]
lp17_B31_NX_Pl_consensus     AATCCAATAATATTGCCTAGCAAATCTTATTATTTTAAAATTTTAAAAAAACAAATCCTAACTATGTCTTAAATCTGG [  546]
lp17_PAli_NX_MP_FG_consensus AATCCAATAATATTGCCTAGCAAATCTTATTATTTTAAAATTTTAAAAAAACAAATCCTAACTATGTCTTAAATCTGG [  546]
lp17_PAli_NX_Pl_consensus    AATCCAATAATATTGCCTAGCAAATCTTATTATTTTAAAATTTTAAAAAAACAAATCCTAACTATGTCTTAAATCTGG [  546]
lp17_PAbe_NX_MP_FG_consensus AATCCAATAATATTGCCTAGCAAATCTTATTATTTTAAAATTTTAAAAAAACAAATCCTAACTATGTCTTAAATCTGG [  546]
lp17_PAbe_NX_Pl_consensus    AATCCAATAATATTGCCTAGCAAATCTTATTATTTTAAAATTTTAAAAAAACAAATCCTAACTATGTCTTAAATCTGG [  546]

lp17_gi|365823332|B31_GB     ATCAATATAATTTATTTAAAACATTAAATAAAGATTATATAGATAATATACTTATTATTTTTAATTCAAAGCCTAGAA [  624]
lp17_un5_B31_PacBio          ATCAATATAATTTATTTAAAACATTAAATAAAGATTATATAGATAATATACTTATTATTTTTAATTCAAAGCCTAGAA [  624]
lp17_B31_TS_MP_FG_consensus  ATCAATATAATTTATTTAAAACATTAAATAAAGATTATATAGATAATATACTTATTATTTTTAATTCAAAGCCTAGAA [  624]
lp17_B31_NX_Pl_consensus     ATCAATATAATTTATTTAAAACATTAAATAAAGATTATATAGATAATATACTTATTATTTTTAATTCAAAGCCTAGAA [  624]
lp17_PAli_NX_MP_FG_consensus ATCAATATAATTTATTTAAAACATTAAATAAAGATTATATAGATAATATACTTATTATTTTTAATTCAAAGCCTAGAA [  624]
lp17_PAli_NX_Pl_consensus    ATCAATATAATTTATTTAAAACATTAAATAAAGATTATATAGATAATATACTTATTATTTTTAATTCAAAGCCTAGAA [  624]
lp17_PAbe_NX_MP_FG_consensus ATCAATATAATTTATTTAAAACATTAAATAAAGATTATATAGATAATATACTTATTATTTTTAATTCAAAGCCTAGAA [  624]
lp17_PAbe_NX_Pl_consensus    ATCAATATAATTTATTTAAAACATTAAATAAAGATTATATAGATAATATACTTATTATTTTTAATTCAAAGCCTAGAA [  624]

lp17_gi|365823332|B31_GB     GAGAAAAAAAGAATGCCCATTAAATGTAACAAGATAAGTAAAAAAGAGGAAACAAAAAAAATTTTTTGTGTGGAAATTT [  702]
lp17_un5_B31_PacBio          GAGAAAAAAAGAATGCCCATTAAATGTAACAAGATAAGTAAAAAAGAGGAAACAAAAAAAATTTTTTGTGTGGAAATTT [  702]
lp17_B31_TS_MP_FG_consensus  GAGAAAAAAAGAATGCCCATTAAATGTAACAAGATAAGTAAAAAAGAGGAAACAAAAAAAATTTTTTGTGTGGAAATTT [  702]
lp17_B31_NX_Pl_consensus     GAGAAAAAAAGAATGCCCATTAAATGTAACAAGATAAGTAAAAAAGAGGAAACAAAAAAAATTTTTTGTGTGGAAATTT [  702]
lp17_PAli_NX_MP_FG_consensus GAGAAAAAAAGAATGCCCATTAAATGTAACAAGATAAGTAAAAAAGAGGAAACAAAAAAAATTTTTTGTGTGGAAATTT [  702]
lp17_PAli_NX_Pl_consensus    GAGAAAAAAAGAATGCCCATTAAATGTAACAAGATAAGTAAAAAAGAGGAAACAAAAAAAATTTTTTGTGTGGAAATTT [  702]
lp17_PAbe_NX_MP_FG_consensus GAGAAAAAAAGAATGCCCATTAAATGTAACAAGATAAGTAAAAAAGAGGAAACAAAAAAAATTTTTTGTGTGGAAATTT [  702]
lp17_PAbe_NX_Pl_consensus    GAGAAAAAAAGAATGCCCATTAAATGTAACAAGATAAGTAAAAAAGAGGAAACAAAAAAAATTTTTTGTGTGGAAATTT [  702]

lp17_gi|365823332|B31_GB     TTCAAAAAAATAACATAGTTAAAAAGGCATTTAAAAACACTCTCAAATATTAAAATTATAACATGCATTTATATAAAT [  780]
lp17_un5_B31_PacBio          TTCAAAAAAATAACATAGTTAAAAAGGCATTTAAAAACACTCTCAAATATTAAAATTATAACATGCATTTATATAAAT [  780]
lp17_B31_TS_MP_FG_consensus  TTCAAAAAAATAACATAGTTAAAAAGGCATTTAAAAACACTCTCAAATATTAAAATTATAACATGCATTTATATAAAT [  780]
lp17_B31_NX_Pl_consensus     TTCAAAAAAATAACATAGTTAAAAAGGCATTTAAAAACACTCTCAAATATTAAAATTATAACATGCATTTATATAAAT [  780]
lp17_PAli_NX_MP_FG_consensus TTCAAAAAAATAACATAGTTAAAAAGGCATTTAAAAACACTCTCAAATATTAAAATTATAACATGCATTTATATAAAT [  780]
lp17_PAli_NX_Pl_consensus    TTCAAAAAAATAACATAGTTAAAAAGGCATTTAAAAACACTCTCAAATATTAAAATTATAACATGCATTTATATAAAT [  780]
lp17_PAbe_NX_MP_FG_consensus TTCAAAAAAATAACATAGTTAAAAAGGCATTTAAAAACACTCTCAAATATTAAAATTATAACATGCATTTATATAAAT [  780]
lp17_PAbe_NX_Pl_consensus    TTCAAAAAAATAACATAGTTAAAAAGGCATTTAAAAACACTCTCAAATATTAAAATTATAACATGCATTTATATAAAT [  780]

lp17_gi|365823332|B31_GB     GCTACAGAACTATCGATAAACAAAAATGGAATTTTTTAAAAATTGAGATAGACTAAAATTGAAGGAGAATTAAAATTT [  858]
lp17_un5_B31_PacBio          GCTACAGAACTATCGATAAACAAAAATGGAATTTTTTAAAAATTGAGATAGACTAAAATTGAAGGAGAATTAAAATTT [  858]
lp17_B31_TS_MP_FG_consensus  GCTACAGAACTATCGATAAACAAAAATGGAATTTTTTAAAAATTGAGATAGACTAAAATTGAAGGAGAATTAAAATTT [  858]
lp17_B31_NX_Pl_consensus     GCTACAGAACTATCGATAAACAAAAATGGAATTTTTTAAAAATTGAGATAGACTAAAATTGAAGGAGAATTAAAATTT [  858]
lp17_PAli_NX_MP_FG_consensus GCTACAGAACTATCGATAAACAAAAATGGAATTTTTTAAAAATTGAGATAGACTAAAATTGAAGGAGAATTAAAATTT [  858]
lp17_PAli_NX_Pl_consensus    GCTACAGAACTATCGATAAACAAAAATGGAATTTTTTAAAAATTGAGATAGACTAAAATTGAAGGAGAATTAAAATTT [  858]
lp17_PAbe_NX_MP_FG_consensus GCTACAGAACTATCGATAAACAAAAATGGAATTTTTTAAAAATTGAGATAGACTAAAATTGAAGGAGAATTAAAATTT [  858]
lp17_PAbe_NX_Pl_consensus    GCTACAGAACTATCGATAAACAAAAATGGAATTTTTTAAAAATTGAGATAGACTAAAATTGAAGGAGAATTAAAATTT [  858]

lp17_gi|365823332|B31_GB     TTATGACAGCAATAATCGTGTATTCATGCTTGACTATGTGTGTAATATATTTTCACTTGCAACTAAAAACATTTTTCA [  936]
lp17_un5_B31_PacBio          TTATGACAGCAATAATCGTGTATTCATGCTTGACTATGTGTGTAATATATTTTCACTTGCAACTAAAAACATTTTTCA [  936]
```

```
lp17_B31_TS_MP_FG_consensus  TTATGACAGCAATAATCGTGTATTCATGCTTGACTATGTGTGTAATATATTTTCACTTGCAACTAAAAACATTTTTCA [  936]
lp17_B31_NX_Pl_consensus     TTATGACAGCAATAATCGTGTATTCATGCTTGACTATGTGTGTAATATATTTTCACTTGCAACTAAAAACATTTTTCA [  936]
lp17_PAli_NX_MP_FG_consensus TTATGACAGCAATAATCGTGTATTCATGCTTGACTATGTGTGTAATATATTTTCACTTGCAACTAAAAACATTTTTCA [  936]
lp17_PAli_NX_Pl_consensus    TTATGACAGCAATAATCGTGTATTCATGCTTGACTATGTGTGTAATATATTTTCACTTGCAACTAAAAACATTTTTCA [  936]
lp17_PAbe_NX_MP_FG_consensus TTATGACAGCAATAATCGTGTATTCATGCTTGACTATGTGTGTAATATATTTTCACTTGCAACTAAAAACATTTTTCA [  936]
lp17_PAbe_NX_Pl_consensus    TTATGACAGCAATAATCGTGTATTCATGCTTGACTATGTGTGTAATATATTTTCACTTGCAACTAAAAACATTTTTCA [  936]

lp17_gi|365823332|B31_GB     CAAAATTAATCAGATTTTGTAAAAAATGTTTTGATATTTTTTTATTATTAATAGAAATGTTAAAATTAATTTTTTATC [ 1014]
lp17_un5_B31_PacBio          CAAAATTAATCAGATTTTGTAAAAAATGTTTTGATATTTTTTTATTATTAATAGAAATGTTAAAATTAATTTTTTATC [ 1014]
lp17_B31_TS_MP_FG_consensus  CAAAATTAATCAGATTTTGTAAAAAATGTTTTGATATTTTTTTATTATTAATAGAAATGTTAAAATTAATTTTTTATC [ 1014]
lp17_B31_NX_Pl_consensus     CAAAATTAATCAGATTTTGTAAAAAATGTTTTGATATTTTTTTATTATTAATAGAAATGTTAAAATTAATTTTTTATC [ 1014]
lp17_PAli_NX_MP_FG_consensus CAAAATTAATCAGATTTTGTAAAAAATGTTTTGATATTTTTTTATTATTAATAGAAATGTTAAAATTAATTTTTTATC [ 1014]
lp17_PAli_NX_Pl_consensus    CAAAATTAATCAGATTTTGTAAAAAATGTTTTGATATTTTTTTATTATTAATAGAAATGTTAAAATTAATTTTTTATC [ 1014]
lp17_PAbe_NX_MP_FG_consensus CAAAATTAATCAGATTTTGTAAAAAATGTTTTGATATTTTTTTATTATTAATAGAAATGTTAAAATTAATTTTTTATC [ 1014]
lp17_PAbe_NX_Pl_consensus    CAAAATTAATCAGATTTTGTAAAAAATGTTTTGATATTTTTTTATTATTAATAGAAATGTTAAAATTAATTTTTTATC [ 1014]

lp17_gi|365823332|B31_GB     TATTAATTATTAATAATAAGTTTTATATATTTATTATAATATCAATAGCTTTAATTACAATCAATACAATGATTTAAC [ 1092]
lp17_un5_B31_PacBio          TATTAATTATTAATAATAAGTTTTATATATTTATTATAATATCAATAGCTTTAATTACAATCAATACAATGATTTAAC [ 1092]
lp17_B31_TS_MP_FG_consensus  TATTAATTATTAATAATAAGTTTTATATATTTATTATAATATCAATAGCTTTAATTACAATCAATACAATGATTTAAC [ 1092]
lp17_B31_NX_Pl_consensus     TATTAATTATTAATAATAAGTTTTATATATTTATTATAATATCAATAGCTTTAATTACAATCAATACAATGATTTAAC [ 1092]
lp17_PAli_NX_MP_FG_consensus TATTAATTATTAATAATAAGTTTTATATATTTATTATAATATCAATAGCTTTAATTACAATCAATACAATGATTTAAC [ 1092]
lp17_PAli_NX_Pl_consensus    TATTAATTATTAATAATAAGTTTTATATATTTATTATAATATCAATAGCTTTAATTACAATCAATACAATGATTTAAC [ 1092]
lp17_PAbe_NX_MP_FG_consensus TATTAATTATTAATAATAAGTTTTATATATTTATTATAATATCAATAGCTTTAATTACAATCAATACAATGATTTAAC [ 1092]
lp17_PAbe_NX_Pl_consensus    TATTAATTATTAATAATAAGTTTTATATATTTATTATAATATCAATAGCTTTAATTACAATCAATACAATGATTTAAC [ 1092]

lp17_gi|365823332|B31_GB     TTATTGTATTGATTGTAATTAAGCCAAATGTATCCAACAAGCTAACTACTATTTCTAAATCAAAGATCATTAAAATAA [ 1170]
lp17_un5_B31_PacBio          TTATTGTATTGATTGTAATTAAGCCAAATGTATCCAACAAGCTAACTACTATTTCTAAATCAAAGATCATTAAAATAA [ 1170]
lp17_B31_TS_MP_FG_consensus  TTATTGTATTGATTGTAATTAAGCCAAATGTATCCAACAAGCTAACTACTATTTCTAAATCAAAGATCATTAAAATAA [ 1170]
lp17_B31_NX_Pl_consensus     TTATTGTATTGATTGTAATTAAGCCAAATGTATCCAACAAGCTAACTACTATTTCTAAATCAAAGATCATTAAAATAA [ 1170]
lp17_PAli_NX_MP_FG_consensus TTATTGTATTGATTGTAATTAAGCCAAATGTATCCAACAAGCTAACTACTATTTCTAAATCAAAGATCATTAAAATAA [ 1170]
lp17_PAli_NX_Pl_consensus    TTATTGTATTGATTGTAATTAAGCCAAATGTATCCAACAAGCTAACTACTATTTCTAAATCAAAGATCATTAAAATAA [ 1170]
lp17_PAbe_NX_MP_FG_consensus TTATTGTATTGATTGTAATTAAGCCAAATGTATCCAACAAGCTAACTACTATTTCTAAATCAAAGATCATTAAAATAA [ 1170]
lp17_PAbe_NX_Pl_consensus    TTATTGTATTGATTGTAATTAAGCCAAATGTATCCAACAAGCTAACTACTATTTCTAAATCAAAGATCATTAAAATAA [ 1170]

lp17_gi|365823332|B31_GB     CTTATTGCTTCATCTCATATGATAACTTTTTAAATTATTTTTTATTAAGACCGGCCATCCGCCAAAACATAATCCCCC [ 1248]
lp17_un5_B31_PacBio          CTTATTGCTTCATCTCATATGATAACTTTTTAAATTATTTTTTATTAAGACCGGCCATCCGCCAAAACATAATCCCCC [ 1248]
lp17_B31_TS_MP_FG_consensus  CTTATTGCTTCATCTCATATGATAACTTTTTAAATTATTTTTTATTAAGACCGGCCATCCGCCAAAACATAATCCCCC [ 1248]
lp17_B31_NX_Pl_consensus     CTTATTGCTTCATCTCATATGATAACTTTTTAAATTATTTTTTATTAAGACCGGCCATCCGCCAAAACATAATCCCCC [ 1248]
lp17_PAli_NX_MP_FG_consensus CTTATTGCTTCATCTCATATGATAACTTTTTAAATTATTTTTTATTAAGACCGGCCATCCGCCAAAACATAATCCCCC [ 1248]
lp17_PAli_NX_Pl_consensus    CTTATTGCTTCATCTCATATGATAACTTTTTAAATTATTTTTTATTAAGACCGGCCATCCGCCAAAACATAATCCCCC [ 1248]
lp17_PAbe_NX_MP_FG_consensus CTTATTGCTTCATCTCATATGATAACTTTTTAAATTATTTTTTATTAAGACCGGCCATCCGCCAAAACATAATCCCCC [ 1248]
lp17_PAbe_NX_Pl_consensus    CTTATTGCTTCATCTCATATGATAACTTTTTAAATTATTTTTTATTAAGACCGGCCATCCGCCAAAACATAATCCCCC [ 1248]

lp17_gi|365823332|B31_GB     AAACCTTACTTTAGGAAGTTCTTACCTTGGTTTTTTATTTCTTTATAAAAAGAAAGTCTTAAGATTTAAAATTGCATT [ 1326]
lp17_un5_B31_PacBio          AAACCTTACTTTAGGAAGTTCTTACCTTGGTTTTTTATTTCTTTATAAAAAGAAAGTCTTAAGATTTAAAATTGCATT [ 1326]
lp17_B31_TS_MP_FG_consensus  AAACCTTACTTTAGGAAGTTCTTACCTTGGTTTTTTATTTCTTTATAAAAAGAAAGTCTTAAGATTTAAAATTGCATT [ 1326]
lp17_B31_NX_Pl_consensus     AAACCTTACTTTAGGAAGTTCTTACCTTGGTTTTTTATTTCTTTATAAAAAGAAAGTCTTAAGATTTAAAATTGCATT [ 1326]
lp17_PAli_NX_MP_FG_consensus AAACCTTACTTTAGGAAGTTCTTACCTTGGTTTTTTATTTCTTTATAAAAAGAAAGTCTTAAGATTTAAAATTGCATT [ 1326]
lp17_PAli_NX_Pl_consensus    AAACCTTACTTTAGGAAGTTCTTACCTTGGTTTTTTATTTCTTTATAAAAAGAAAGTCTTAAGATTTAAAATTGCATT [ 1326]
lp17_PAbe_NX_MP_FG_consensus AAACCTTACTTTAGGAAGTTCTTACCTTGGTTTTTTATTTCTTTATAAAAAGAAAGTCTTAAGATTTAAAATTGCATT [ 1326]
lp17_PAbe_NX_Pl_consensus    AAACCTTACTTTAGGAAGTTCTTACCTTGGTTTTTTATTTCTTTATAAAAAGAAAGTCTTAAGATTTAAAATTGCATT [ 1326]

lp17_gi|365823332|B31_GB     TAAATCTCTATCATACATATTGAAACTTTACTAAAACATTCCTTTTTGTATAAAATTAATTGATTAAATTCCCTATCA [ 1404]
lp17_un5_B31_PacBio          TAAATCTCTATCATACATATTGAAACTTTACTAAAACATTCCTTTTTGTATAAAATTAATTGATTAAATTCCCTATCA [ 1404]
lp17_B31_TS_MP_FG_consensus  TAAATCTCTATCATACATATTGAAACTTTACTAAAACATTCCTTTTTGTATAAAATTAATTGATTAAATTCCCTATCA [ 1404]
lp17_B31_NX_Pl_consensus     TAAATCTCTATCATACATATTGAAACTTTACTAAAACATTCCTTTTTGTATAAAATTAATTGATTAAATTCCCTATCA [ 1404]
lp17_PAli_NX_MP_FG_consensus TAAATCTCTATCATACATATTGAAACTTTACTAAAACATTCCTTTTTGTATAAAATTAATTGATTAAATTCCCTATCA [ 1404]
lp17_PAli_NX_Pl_consensus    TAAATCTCTATCATACATATTGAAACTTTACTAAAACATTCCTTTTTGTATAAAATTAATTGATTAAATTCCCTATCA [ 1404]
lp17_PAbe_NX_MP_FG_consensus TAAATCTCTATCATACATATTGAAACTTTACTAAAACATTCCTTTTTGTATAAAATTAATTGATTAAATTCCCTATCA [ 1404]
lp17_PAbe_NX_Pl_consensus    TAAATCTCTATCATACATATTGAAACTTTACTAAAACATTCCTTTTTGTATAAAATTAATTGATTAAATTCCCTATCA [ 1404]

lp17_gi|365823332|B31_GB     TTATGTTTTTATAATTGACTAAAAAGTAATAAGACAATTTACATAAAAAGTCTTTTCTTTGAATTTAATTTAAACTTT [ 1482]
lp17_un5_B31_PacBio          TTATGTTTTTATAATTGACTAAAAAGTAATAAGACAATTTACATAAAAAGTCTTTTCTTTGAATTTAATTTAAACTTT [ 1482]
lp17_B31_TS_MP_FG_consensus  TTATGTTTTTATAATTGACTAAAAAGTAATAAGACAATTTACATAAAAAGTCTTTTCTTTGAATTTAATTTAAACTTT [ 1482]
lp17_B31_NX_Pl_consensus     TTATGTTTTTATAATTGACTAAAAAGTAATAAGACAATTTACATAAAAAGTCTTTTCTTTGAATTTAATTTAAACTTT [ 1482]
lp17_PAli_NX_MP_FG_consensus TTATGTTTTTATAATTGACTAAAAAGTAATAAGACAATTTACATAAAAAGTCTTTTCTTTGAATTTAATTTAAACTTT [ 1482]
lp17_PAli_NX_Pl_consensus    TTATGTTTTTATAATTGACTAAAAAGTAATAAGACAATTTACATAAAAAGTCTTTTCTTTGAATTTAATTTAAACTTT [ 1482]
lp17_PAbe_NX_MP_FG_consensus TTATGTTTTTATAATTGACTAAAAAGTAATAAGACAATTTACATAAAAAGTCTTTTCTTTGAATTTAATTTAAACTTT [ 1482]
lp17_PAbe_NX_Pl_consensus    TTATGTTTTTATAATTGACTAAAAAGTAATAAGACAATTTACATAAAAAGTCTTTTCTTTGAATTTAATTTAAACTTT [ 1482]

lp17_gi|365823332|B31_GB     TCTTATACAAATTAGCAACCCTTAATCTAGATTTAGCTCTATTAATAGCGCCCTTTGTCTTTTTAATAGTTTTCTTCC [ 1560]
lp17_un5_B31_PacBio          TCTTATACAAATTAGCAACCCTTAATCTAGATTTAGCTCTATTAATAGCGCCCTTTGTCTTTTTAATAGTTTTCTTCC [ 1560]
lp17_B31_TS_MP_FG_consensus  TCTTATACAAATTAGCAACCCTTAATCTAGATTTAGCTCTATTAATAGCGCCCTTTGTCTTTTTAATAGTTTTCTTCC [ 1560]
lp17_B31_NX_Pl_consensus     TCTTATACAAATTAGCAACCCTTAATCTAGATTTAGCTCTATTAATAGCGCCCTTTGTCTTTTTAATAGTTTTCTTCC [ 1560]
lp17_PAli_NX_MP_FG_consensus TCTTATACAAATTAGCAACCCTTAATCTAGATTTAGCTCTATTAATAGCGCCCTTTGTCTTTTTAATAGTTTTCTTCC [ 1560]
lp17_PAli_NX_Pl_consensus    TCTTATACAAATTAGCAACCCTTAATCTAGATTTAGCTCTATTAATAGCGCCCTTTGTCTTTTTAATAGTTTTCTTCC [ 1560]
lp17_PAbe_NX_MP_FG_consensus TCTTATACAAATTAGCAACCCTTAATCTAGATTTAACTCTATTAATAGCGCCCTTTGTCTTTTTAATAGTTTTCTTCC [ 1560]
lp17_PAbe_NX_Pl_consensus    TCTTATACAAATTAGCAACCCTTAATCTAGATTTAGCTCTATTAATAGCGCCCTTTGTCTTTTTAATAGTTTTCTTCC [ 1560]

lp17_gi|365823332|B31_GB     AGTATTTTTAAGTTTACTTTTATTTTTTATAAATATTTAGAATGATTGTTTTTTTACTTTCACGACTCTTTTTTATTA [ 1638]
lp17_un5_B31_PacBio          AGTATTTTTAAGTTTACTTTTATTTTTTATAAATATTTAGAATGATTGTTTTTTTACTTTCACGACTCTTTTTTATTA [ 1638]
lp17_B31_TS_MP_FG_consensus  AGTATTTTTAAGTTTACTTTTATTTTTTATAAATATTTAGAATGATTGTTTTTTTACTTTCACGACTCTTTTTTATTA [ 1638]
lp17_B31_NX_Pl_consensus     AGTATTTTTAAGTTTACTTTTATTTTTTATAAATATTTAGAATGATTGTTTTTTTACTTTCACGACTCTTTTTTATTA [ 1638]
lp17_PAli_NX_MP_FG_consensus AGTATTTTTAAGTTTACTTTTATTTTTTATAAATATTTAGAATGATTGTTTTTTTACTTTCACGACTCTTTTTTATTA [ 1638]
lp17_PAli_NX_Pl_consensus    AGTATTTTTAAGTTTACTTTTATTTTTTATAAATATTTAGAATGATTGTTTTTTTACTTTCACGACTCTTTTTTATTA [ 1638]
lp17_PAbe_NX_MP_FG_consensus AGTATTTTTAAGTTTACTTTTATTTTTTATAAATATTTAGAATGATTGTTTTTTTACTTTCACGACTCTTTTTTATTA [ 1638]
lp17_PAbe_NX_Pl_consensus    AGTATTTTTAAGTTTACTTTTATTTTTTATAAATATTTAGAATGATTGTTTTTTTACTTTCACGACTCTTTTTTATTA [ 1638]

lp17_gi|365823332|B31_GB     TTTTTTTTATTGTTATTTTTAATATTTAAGCTCTCAACTACTACTGAAATATAATATTTATTATCGGTATTTTTTCTA [ 1716]
lp17_un5_B31_PacBio          TTTTTTTTATTGTTATTTTTAATATTTAAGCTCTCAACTACTACTGAAATATAATATTTATTATCGGTATTTTTTCTA [ 1716]
lp17_B31_TS_MP_FG_consensus  TTTTTTTTATTGTTATTTTTAATATTTAAGCTCTCAACTACTACTGAAATATAATATTTATTATCGGTATTTTTTCTA [ 1716]
lp17_B31_NX_Pl_consensus     TTTTTTTTATTGTTATTTTTAATATTTAAGCTCTCAACTACTACTGAAATATAATATTTATTATCGGTATTTTTTCTA [ 1716]
lp17_PAli_NX_MP_FG_consensus TTTTTTTTATTGTTATTTTTAATATTTAAGCTCTCAACTACTACTGAAATATAATATTTATTATCGGTATTTTTTCTA [ 1716]
lp17_PAli_NX_Pl_consensus    TTTTTTTTATTGTTATTTTTAATATTTAAGCTCTCAACTACTACTGAAATATAATATTTATTATCGGTATTTTTTCTA [ 1716]
lp17_PAbe_NX_MP_FG_consensus TTTTTTTTATTGTTATTTTTAATATTTAAGCTCTCAACTACTACTGAAATATAATATTTATTATCGGTATTTTTTCTA [ 1716]
lp17_PAbe_NX_Pl_consensus    TTTTTTTTATTGTTATTTTTAATATTTAAGCTCTCAACTACTACTGAAATATAATATTTATTATCGGTATTTTTTCTA [ 1716]

lp17_gi|365823332|B31_GB     CTACTAAATTTTTAATAACTTTATTATTATAATGATCCTATGTAGACATAACTTTACAAATCCTATTTTAGATAAGCT [ 1794]
lp17_un5_B31_PacBio          CTACTAAATTTTTAATAACTTTATTATTATAATGATCCTATGTAGACATAACTTTACAAATCCTATTTTAGATAAGCT [ 1794]
lp17_B31_TS_MP_FG_consensus  CTACTAAATTTTTAATAACTTTATTATTATAATGATCCTATGTAGACATAACTTTACAAATCCTATTTTAGATAAGCT [ 1794]
lp17_B31_NX_Pl_consensus     CTACTAAATTTTTAATAACTTTATTATTATAATGATCCTATGTAGACATAACTTTACAAATCCTATTTTAGATAAGCT [ 1794]
lp17_PAli_NX_MP_FG_consensus CTACTAAATTTTTAATAACTTTATTATTATAATGATCCTATGTAGACATAACTTTACAAATCCTATTTTAGATAAGCT [ 1794]
lp17_PAli_NX_Pl_consensus    CTACTAAATTTTTAATAACTTTATTATTATAATGATCCTATGTAGACATAACTTTACAAATCCTATTTTAGATAAGCT [ 1794]
lp17_PAbe_NX_MP_FG_consensus CTACTAAATTTTTAATAACTTTATTATTATAATGATCCTATGTAGACATAACTTTACAAATCCTATTTTAGATAAGCT [ 1794]
lp17_PAbe_NX_Pl_consensus    CTACTAAATTTTTAATAACTTTATTATTATAATGATCCTATGTAGACATAACTTTACAAATCCTATTTTAGATAAGCT [ 1794]

lp17_gi|365823332|B31_GB     TTATATTCATTTTCTATTCTTATTGGGTTTTTCATTTTATATTTAGGAAATCCTTACGTTTTGCTTCCCTTTTTAATT [ 1872]
lp17_un5_B31_PacBio          TTATATTCATTTTCTATTCTTATTGGGTTTTTCATTTTATATTTAGGAAATCCTTACGTTTTGCTTCCCTTTTTAATT [ 1872]
lp17_B31_TS_MP_FG_consensus  TTATATTCATTTTCTATTCTTATTGGGTTTTTCATTTTATATTTAGGAAATCCTTACGTTTTGCTTCCCTTTTTAATT [ 1872]
```

```
lp17_B31_NX_Pl_consensus     TTATATTCATTTTCTATTCTTATTGGGTTTTTCATTTTATATTTAGGAAATCCTTACGTTTTGCTTCCCTTTTTAATT [ 1872]
lp17_PAli_NX_MP_FG_consensus TTATATTCATTTTCTATTCTTATTGGGTTTTTCATTTTATATTTAGGAAATCCTTACGTTTTGCTTCCCTTTTTAATT [ 1872]
lp17_PAli_NX_Pl_consensus    TTATATTCATTTTCTATTCTTATTGGGTTTTTCATTTTATATTTAGGAAATCCTTACGTTTTGCTTCCCTTTTTAATT [ 1872]
lp17_PAbe_NX_MP_FG_consensus TTATATTCATTTTCTATTCTTATTGGGTTTTTCATTTTATATTTAGGAAATCCTTACGTTTTGCTTCCCTTTTTAATT [ 1872]
lp17_PAbe_NX_Pl_consensus    TTATATTCATTTTCTATTCTTATTGGGTTTTTCATTTTATATTTAGGAAATCCTTACGTTTTGCTTCCCTTTTTAATT [ 1872]

lp17_gi|365823332|B31_GB     TATCTAAAAAAATTATTATCTGCGAGGCTTTAATCAATCCATGCACAAAAAAGAGTTAAAGTATTAATCTCCTTTAAGA [ 1950]
lp17_un5_B31_PacBio          TATCTAAAAAAATTATTATCTGCGAGGCTTTAATCAATCCATGCACAAAAAAGAGTTAAAGTATTAATCTCCTTTAAGA [ 1950]
lp17_B31_TS_MP_FG_consensus  TATCTAAAAAAATTATTATCTGCGAGGCTTTAATCAATCCATGCACAAAAAAGAGTTAAAGTATTAATCTCCTTTAAGA [ 1950]
lp17_B31_NX_Pl_consensus     TATCTAAAAAAATTATTATCTGCGAGGCTTTAATCAATCCATGCACAAAAAAGAGTTAAAGTATTAATCTCCTTTAAGA [ 1950]
lp17_PAli_NX_MP_FG_consensus TATCTAAAAAAATTATTATCTGCGAGGCTTTAATCAATCCATGCACAAAAAAGAGTTAAAGTATTAATCTCCTTTAAGA [ 1950]
lp17_PAli_NX_Pl_consensus    TATCTAAAAAAATTATTATCTGCGAGGCTTTAATCAATCCATGCACAAAAAAGAGTTAAAGTATTAATCTCCTTTAAGA [ 1950]
lp17_PAbe_NX_MP_FG_consensus TATCTAAAAAAATTATTATCTGCGAGGCTTTAATCAATCCATGCACAAAAAAGAGTTAAAGTATTAATCTCCTTTAAGA [ 1950]
lp17_PAbe_NX_Pl_consensus    TATCTAAAAAAATTATTATCTGCGAGGCTTTAATCAATCCATGCACAAAAAAGAGTTAAAGTATTAATCTCCTTTAAGA [ 1950]

lp17_gi|365823332|B31_GB     GTGAAAATTTATTTTTATATTTGCCTAGATTAATGCTAAAACTTTTCTTGTTTTTTTATAAGAATCTTTCCTATCACT [ 2028]
lp17_un5_B31_PacBio          GTGAAAATTTATTTTTATATTTGCCTAGATTAATGCTAAAACTTTTCTTGTTTTTTTATAAGAATCTTTCCTATCACT [ 2028]
lp17_B31_TS_MP_FG_consensus  GTGAAAATTTATTTTTATATTTGCCTAGATTAATGCTAAAACTTTTCTTGTTTTTTTATAAGAATCTTTCCTATCACT [ 2028]
lp17_B31_NX_Pl_consensus     GTGAAAATTTATTTTTATATTTGCCTAGATTAATGCTAAAACTTTTCTTGTTTTTTTATAAGAATCTTTCCTATCACT [ 2028]
lp17_PAli_NX_MP_FG_consensus GTGAAAATTTATTTTTATATTTGCCTAGATTAATGCTAAAACTTTTCTTGTTTTTTTATAAGAATCTTTCCTATCACT [ 2028]
lp17_PAli_NX_Pl_consensus    GTGAAAATTTATTTTTATATTTGCCTAGATTAATGCTAAAACTTTTCTTGTTTTTTTATAAGAATCTTTCCTATCACT [ 2028]
lp17_PAbe_NX_MP_FG_consensus GTGAAAATTTATTTTTATATTTGCCTAGATTAATGCTAAAACTTTTCTTGTTTTTTTATAAGAATCTTTCCTATCACT [ 2028]
lp17_PAbe_NX_Pl_consensus    GTGAAAATTTATTTTTATATTTGCCTAGATTAATGCTAAAACTTTTCTTGTTTTTTTATAAGAATCTTTCCTATCACT [ 2028]

lp17_gi|365823332|B31_GB     TAACATTTTGTCATACAAAAATCTTACAAATCTAAATACTTTTAAAAATTTTTTTGATTAGTGTTAGAATATATTCTG [ 2106]
lp17_un5_B31_PacBio          TAACATTTTGTCATACAAAAATCTTACAAATCTAAATACTTTTAAAAATTTTTTTGATTAGTGTTAGAATATATTCTG [ 2106]
lp17_B31_TS_MP_FG_consensus  TAACATTTTGTCATACAAAAATCTTACAAATCTAAATACTTTTAAAAATTTTTTTGATTAGTGTTAGAATATATTCTG [ 2106]
lp17_B31_NX_Pl_consensus     TAACATTTTGTCATACAAAAATCTTACAAATCTAAATACTTTTAAAAATTTTTTTGATTAGTGTTAGAATATATTCTG [ 2106]
lp17_PAli_NX_MP_FG_consensus TAACATTTTGTCATACAAAAATCTTACAAATCTAAATACTTTTAAAAATTTTTTTGATTAGTGTTAGAATATATTCTG [ 2106]
lp17_PAli_NX_Pl_consensus    TAACATTTTGTCATACAAAAATCTTACAAATCTAAATACTTTTAAAAATTTTTTTGATTAGTGTTAGAATATATTCTG [ 2106]
lp17_PAbe_NX_MP_FG_consensus TAACATTTTGTCATACAAAAATCTTACAAATCTAAATACTTTTAAAAATTTTTTTGATTAGTGTTAGAATATATTCTG [ 2106]
lp17_PAbe_NX_Pl_consensus    TAACATTTTGTCATACAAAAATCTTACAAATCTAAATACTTTTAAAAATTTTTTTGATTAGTGTTAGAATATATTCTG [ 2106]

lp17_gi|365823332|B31_GB     TTTTTACAAACTTTATCGGCACTTATATACTCTATTATTTTATAATAATTACTTAGTGTATATAGAAATGAGATAGCT [ 2184]
lp17_un5_B31_PacBio          TTTTTACAAACTTTATCGGCACTTATATACTCTATTATTTTATAATAATTACTTAGTGTATATAGAAATGAGATAGCT [ 2184]
lp17_B31_TS_MP_FG_consensus  TTTTTACAAACTTTATCGGCACTTATATACTCTATTATTTTATAATAATTACTTAGTGTATATAGAAATGAGATAGCT [ 2184]
lp17_B31_NX_Pl_consensus     TTTTTACAAACTTTATCGGCACTTATATACTCTATTATTTTATAATAATTACTTAGTGTATATAGAAATGAGATAGCT [ 2184]
lp17_PAli_NX_MP_FG_consensus TTTTTACAAACTTTATCGGCACTTATATACTCTATTATTTTATAATAATTACTTAGTGTATATAGAAATGAGATAGCT [ 2184]
lp17_PAli_NX_Pl_consensus    TTTTTACAAACTTTATCGGCACTTATATACTCTATTATTTTATAATAATTACTTAGTGTATATAGAAATGAGATAGCT [ 2184]
lp17_PAbe_NX_MP_FG_consensus TTTTTACAAACTTTATCGGCACTTATATACTCTATTATTTTATAATAATTACTTAGTGTATATAGAAATGAGATAGCT [ 2184]
lp17_PAbe_NX_Pl_consensus    TTTTTACAAACTTTATCGGCACTTATATACTCTATTATTTTATAATAATTACTTAGTGTATATAGAAATGAGATAGCT [ 2184]

lp17_gi|365823332|B31_GB     TTTAATAATCACTGCATATATTTAGTTAATTATCCCCCCCCCCCGAGTACTAGTTACTAACCATAGACACAAATGATG [ 2262]
lp17_un5_B31_PacBio          TTTAATAATCACTGCATATATTTAGTTAATTATCCCCCCCCCC-GAGTACTAGTTACTAACCATAGACACAAATGATG [ 2262]
lp17_B31_TS_MP_FG_consensus  TTTAATAATCACTGCATATATTTAGTTAATTATCCCCCCCCCCCGAGTACTAGTTACTAACCATAGACACAAATGATG [ 2262]
lp17_B31_NX_Pl_consensus     TTTAATAATCACTGCATATATTTAGTTAATTATCCCCCCCCCCCGAGTACTAGTTACTAACCATAGACACAAATGATG [ 2262]
lp17_PAli_NX_MP_FG_consensus TTTAATAATCACTGCATATATTTAGTTAATTATCCCCCCCCCCCGAGTACTAGTTACTAACCATAGACACAAATGATG [ 2262]
lp17_PAli_NX_Pl_consensus    TTTAATAATCACTGCATATATTTAGTTAATTATCCCCCCCCCCCGAGTACTAGTTACTAACCATAGACACAAATGATG [ 2262]
lp17_PAbe_NX_MP_FG_consensus TTTAATAATCACTGCATATATTTAGTTAATTATCCCCCCCCCCCGAGTACTAGTTACTAACCATAGACACAAATGATG [ 2262]
lp17_PAbe_NX_Pl_consensus    TTTAATAATCACTGCATATATTTAGTTAATTATCCCCCCCCCC-GAGTACTAGTTACTAACCATAGACACAAATGATG [ 2262]

lp17_gi|365823332|B31_GB     AGCTTATGCCCTGTCTCACAAAATAATACTTTTATATTCCTTATGGAAAATAACCATTAATGAATTAAATTGCAATAA [ 2340]
lp17_un5_B31_PacBio          AGCTTATGCCCTGTCTCACAAAATAATACTTTTATATTCCTTATGGAAAATAACCATTAATGAATTAAATTGCAATAA [ 2340]
lp17_B31_TS_MP_FG_consensus  AGCTTATGCCCTGTCTCACAAAATAATACTTTTATATTCCTTATGGAAAATAACCATTAATGAATTAAATTGCAATAA [ 2340]
lp17_B31_NX_Pl_consensus     AGCTTATGCCCTGTCTCACAAAATAATACTTTTATATTCCTTATGGAAAATAACCATTAATGAATTAAATTGCAATAA [ 2340]
lp17_PAli_NX_MP_FG_consensus AGCTTATGCCCTGTCTCACAAAATAATACTTTTATATTCCTTATGGAAAATAACCATTAATGAATTAAATTGCAATAA [ 2340]
lp17_PAli_NX_Pl_consensus    AGCTTATGCCCTGTCTCACAAAATAATACTTTTATATTCCTTATGGAAAATAACCATTAATGAATTAAATTGCAATAA [ 2340]
lp17_PAbe_NX_MP_FG_consensus AGCTTATGCCCTGTCTCACAAAATAATACTTTTATATTCCTTATGGAAAATAACCATTAATGAATTAAATTGCAATAA [ 2340]
lp17_PAbe_NX_Pl_consensus    AGCTTATGCCCTGTCTCACAAAATAATATTTTTATATTCCTTATGGAAAATAACCATTAATGAATTAAATTGCAATAA [ 2340]

lp17_gi|365823332|B31_GB     AGATCACATTGCCAATATTCAATCTTCTAAATTCATCAATAATTTAATACAATATCTTCATCAAAGCTTATAAGAAAA [ 2418]
lp17_un5_B31_PacBio          AGATCACATTGCCAATATTCAATCTTCTAAATTCATCAATAATTTAATACAATATCTTCATCAAAGCTTATAAGAAAA [ 2418]
lp17_B31_TS_MP_FG_consensus  AGATCACATTGCCAATATTCAATCTTCTAAATTCATCAATAATTTAATACAATATCTTCATCAAAGCTTATAAGAAAA [ 2418]
lp17_B31_NX_Pl_consensus     AGATCACATTGCCAATATTCAATCTTCTAAATTCATCAATAATTTAATACAATATCTTCATCAAAGCTTATAAGAAAA [ 2418]
lp17_PAli_NX_MP_FG_consensus AGATCACATTGCCAATATTCAATCTTCTAAATTCATCAATAATTTAATACAATATCTTCATCAAAGCTTATAAGAAAA [ 2418]
lp17_PAli_NX_Pl_consensus    AGATCACATTGCCAATATTCAATCTTCTAAATTCATCAATAATTTAATACAATATCTTCATCAAAGCTTATAAGAAAA [ 2418]
lp17_PAbe_NX_MP_FG_consensus AGATCACATTGCCAATATTCAATCTTCTAAATTCATCAATAATTTAATACAATATCTTCATCAAAGCTTATAAGAAAA [ 2418]
lp17_PAbe_NX_Pl_consensus    AGATCACATTGCCAATATTCAATCTTCTAAATTCATCAATAATTTAATACAATATCTTCATCAAAGCTTATAAGAAAA [ 2418]

lp17_gi|365823332|B31_GB     AATATTCTAATTATTTATGCATTGCTGAAAATCTTATTTTTAATCTAAAAACTACTGACTTATTTATACTAGAGAGAA [ 2496]
lp17_un5_B31_PacBio          AATATTCTAATTATTTATGCATTGCTGAAAATCTTATTTTTAATCTAAAAACTACTGACTTATTTATACTAGAGAGAA [ 2496]
lp17_B31_TS_MP_FG_consensus  AATATTCTAATTATTTATGCATTGCTGAAAATCTTATTTTTAATCTAAAAACTACTGACTTATTTATACTAGAGAGAA [ 2496]
lp17_B31_NX_Pl_consensus     AATATTCTAATTATTTATGCATTGCTGAAAATCTTATTTTTAATCTAAAAACTACTGACTTATTTATACTAGAGAGAA [ 2496]
lp17_PAli_NX_MP_FG_consensus AATATTCTAATTATTTATGCATTGCTGAAAATCTTATTTTTAATCTAAAAACTACTGACTTATTTATACTAGAGAGAA [ 2496]
lp17_PAli_NX_Pl_consensus    AATATTCTAATTATTTATGCATTGCTGAAAATCTTATTTTTAATCTAAAAACTACTGACTTATTTATACTAGAGAGAA [ 2496]
lp17_PAbe_NX_MP_FG_consensus AATATTCTAATTATTTATGCATTGCTGAAAATCTTATTTTTAATCTAAAAACTACTGACTTATTTATACTAGAGAGAA [ 2496]
lp17_PAbe_NX_Pl_consensus    AATATTCTAATTATTTATGCATTGCTGAAAATCTTATTTTTAATCTAAAAACTACTGACTTATTTATACTAGAGAGAA [ 2496]

lp17_gi|365823332|B31_GB     AGTTTTTCCGATATGATCAAAAAGCATATTCGGAATCAAAATAAATCCATTTACTAACAAATTCATATCTACCTAAAT [ 2574]
lp17_un5_B31_PacBio          AGTTTTTCCGATATGATCAAAAAGCATATTCGGAATCAAAATAAATCCATTTACTAACAAATTCATATCTACCTAAAT [ 2574]
lp17_B31_TS_MP_FG_consensus  AGTTTTTCCGATATGATCAAAAAGCATATTCGGAATCAAAATAAATCCATTTACTAACAAATTCATATCTACCTAAAT [ 2574]
lp17_B31_NX_Pl_consensus     AGTTTTTCCGATATGATCAAAAAGCATATTCGGAATCAAAATAAATCCATTTACTAACAAATTCATATCTACCTAAAT [ 2574]
lp17_PAli_NX_MP_FG_consensus AGTTTTTCCGATATGATCAAAAAGCATATTCGGAATCAAAATAAATCCATTTACTAACAAATTCATATCTACCTAAAT [ 2574]
lp17_PAli_NX_Pl_consensus    AGTTTTTCCGATATGATCAAAAAGCATATTCGGAATCAAAATAAATCCATTTACTAACAAATTCATATCTACCTAAAT [ 2574]
lp17_PAbe_NX_MP_FG_consensus AGTTTTTCCGATATGATCAAAAAGCATATTCGGAATCAAAATAAATCCATTTACTAACAAATTCATATCTACCTAAAT [ 2574]
lp17_PAbe_NX_Pl_consensus    AGTTTTTCCGATATGATCAAAAAGCATATTCGGAATCAAAATAAATCCATTTACTAACAAATTCATATCTACCTAAAT [ 2574]

lp17_gi|365823332|B31_GB     TTCACAGAAATCATAGGTAGATAATTCTTTATTATTTTTTTGTTAAAAATTTAAATAACAAAATTATTATTTTTTAGA [ 2652]
lp17_un5_B31_PacBio          TTCACAGAAATCATAGGTAGATAATTCTTTATTATTTTTTTGTTAAAAATTTAAATAACAAAATTATTATTTTTTAGA [ 2652]
lp17_B31_TS_MP_FG_consensus  TTCACAGAAATCATAGGTAGATAATTCTTTATTATTTTTTTGTTAAAAATTTAAATAACAAAATTATTATTTTTTAGA [ 2652]
lp17_B31_NX_Pl_consensus     TTCACAGAAATCATAGGTAGATAATTCTTTATTATTTTTTTGTTAAAAATTTAAATAACAAAATTATTATTTTTTAGA [ 2652]
lp17_PAli_NX_MP_FG_consensus TTCACAGAAATCATAGGTAGATAATTCTTTATTATTTTTTTGTTAAAAATTTAAATAACAAAATTATTATTTTTTAGA [ 2652]
lp17_PAli_NX_Pl_consensus    TTCACAGAAATCATAGGTAGATAATTCTTTATTATTTTTTTGTTAAAAATTTAAATAACAAAATTATTATTTTTTAGA [ 2652]
lp17_PAbe_NX_MP_FG_consensus TTCACAGAAATCATAGGTAGATAATTCTTTATTATTTTTTTGTTAAAAATTTAAATAACAAAATTATTATTTTTTAGA [ 2652]
lp17_PAbe_NX_Pl_consensus    TTCACAGAAATCATAGGTAGATAATTCTTTATTATTTTTTTGTTAAAAATTTAAATAACAAAATTATTATTTTTTAGA [ 2652]

lp17_gi|365823332|B31_GB     AGCTTCTATATATTCCATAATTAAAGTTCTTATAAAACCAGAAGCTGTTAATCCCTTTTTGTCTAAAATAGAATAAAA [ 2730]
lp17_un5_B31_PacBio          AGCTTCTATATATTCCATAATTAAAGTTCTTATAAAACCAGAAGCTGTTAATCCCTTTTTGTCTAAAATAGAATAAAA [ 2730]
lp17_B31_TS_MP_FG_consensus  AGCTTCTATATATTCCATAATTAAAGTTCTTATAAAACCAGAAGCTGTTAATCCCTTTTTGTCTAAAATAGAATAAAA [ 2730]
lp17_B31_NX_Pl_consensus     AGCTTCTATATATTCCATAATTAAAGTTCTTATAAAACCAGAAGCTGTTAATCCCTTTTTGTCTAAAATAGAATAAAA [ 2730]
lp17_PAli_NX_MP_FG_consensus AGCTTCTATATATTCCATAATTAAAGTTCTTATAAAACCAGAAGCTGTTAATCCCTTTTTGTCTAAAATAGAATAAAA [ 2730]
lp17_PAli_NX_Pl_consensus    AGCTTCTATATATTCCATAATTAAAGTTCTTATAAAACCAGAAGCTGTTAATCCCTTTTTGTCTAAAATAGAATAAAA [ 2730]
lp17_PAbe_NX_MP_FG_consensus AGCTTCTATATATTCCATAATTAAAGTTCTTATAAAACCAGAAGCTGTTAATCCCTTTTTGTCTAAAATAGAATAAAA [ 2730]
lp17_PAbe_NX_Pl_consensus    AGCTTCTATATATTCCATAATTAAAGTTCTTATAAAACCAGAAGCTGTTAATCCCTTTTTGTCTAAAATAGAATAAAA [ 2730]

lp17_gi|365823332|B31_GB     TTCAATCCAATATTCATATTTCAGTCCTACACTTATTTGCTTGATCGTCATTTTTGATCTAATAGGCTTAACATTTGG [ 2808]
lp17_un5_B31_PacBio          TTCAATCCAATATTCATATTTCAGTCCTACACTTATTTGCTTGATCGTCATTTTTGATCTAATAGGCTTAACATTTGG [ 2808]
lp17_B31_TS_MP_FG_consensus  TTCAATCCAATATTCATATTTCAGTCCTACACTTATTTGCTTGATCGTCATTTTTGATCTAATAGGCTTAACATTTGG [ 2808]
lp17_B31_NX_Pl_consensus     TTCAATCCAATATTCATATTTCAGTCCTACACTTATTTGCTTGATCGTCATTTTTGATCTAATAGGCTTAACATTTGG [ 2808]
```

```
lp17_PAli_NX_MP_FG_consensus TTCAATCCAATATTCATATTTCAGTCCTACACTTATTTGCTTGATCGTCATTTTTGATCTAATAGGCTTAACATTTGG [ 2808]
lp17_PAli_NX_Pl_consensus    TTCAATCCAATATTCATATTTCAGTCCTACACTTATTTGCTTGATCGTCATTTTTGATCTAATAGGCTTAACATTTGG [ 2808]
lp17_PAbe_NX_MP_FG_consensus TTCAATCCAATATTCATATTTCAGTCCTACACTTATTTGCTTGATCGTCATTTTTGATCTAATAGGCTTAACATTTGG [ 2808]
lp17_PAbe_NX_Pl_consensus    TTCAATCCAATATTCATATTTCAGTCCTACACTTATTTGCTTGATCGTCATTTTTGATCTAATAGGCTTAACATTTGG [ 2808]

lp17_gi|365823332|B31_GB      AATGTTATTGCTAATAACATCATTATCACGAAAAGCAAATTTAGATTTGCTAACATTTTGTTTAATAATTTCTTCTAA [ 2886]
lp17_un5_B31_PacBio           AATGTTATTGCTAATAACATCATTATCACGAAAAGCAAATTTAGATTTGCTAACATTTTGTTTAATAATTTCTTCTAA [ 2886]
lp17_B31_TS_MP_FG_consensus   AATGTTATTGCTAATAACATCATTATCACGAAAAGCAAATTTAGATTTGCTAACATTTTGTTTAATAATTTCTTCTAA [ 2886]
lp17_B31_NX_Pl_consensus      AATGTTATTGCTAATAACATCATTATCACGAAAAGCAAATTTAGATTTGCTAACATTTTGTTTAATAATTTCTTCTAA [ 2886]
lp17_PAli_NX_MP_FG_consensus AATGTTATTGCTAATAACATCATTATCACGAAAAGCAAATTTAGATTTGCTAACATTTTGTTTAATAATTTCTTCTAA [ 2886]
lp17_PAli_NX_Pl_consensus     AATGTTATTGCTAATAACATCATTATCACGAAAAGCAAATTTAGATTTGCTAACATTTTGTTTAATAATTTCTTCTAA [ 2886]
lp17_PAbe_NX_MP_FG_consensus AATGTTATTGCTAATAACATCATTATCACGAAAAGCAAATTTAGATTTGCTAACATTTTGTTTAATAATTTCTTCTAA [ 2886]
lp17_PAbe_NX_Pl_consensus     AATGTTATTGCTAATAACATCATTATCACGAAAAGCAAATTTAGATTTGCTAACATTTTGTTTAATAATTTCTTCTAA [ 2886]

lp17_gi|365823332|B31_GB      ATTAACCTCTTTGCTTATAGGCATAAAGCATCTCCCAATCTTGAAAGTTCTAAGACAGATCTACTCTCCGGATAATAA [ 2964]
lp17_un5_B31_PacBio           ATTAACCTCTTTGCTTATAGGCATAAAGCATCTCCCAATCTTGAAAGTTCTAAGACAGATCTACTCTCCGGATAATAA [ 2964]
lp17_B31_TS_MP_FG_consensus   ATTAACCTCTTTGCTTATAGGCATAAAGCATCTCCCAATCTTGAAAGTTCTAAGACAGATCTACTCTCCGGATAATAA [ 2964]
lp17_B31_NX_Pl_consensus      ATTAACCTCTTTGCTTATAGGCATAAAGCATCTCCCAATCTTGAAAGTTCTAAGACAGATCTACTCTCCGGATAATAA [ 2964]
lp17_PAli_NX_MP_FG_consensus ATTAACCTCTTTGCTTATAGGCATAAAGCATCTCCCAATCTTGAAAGTTCTAAGACAGATCTACTCTCCGGATAATAA [ 2964]
lp17_PAli_NX_Pl_consensus     ATTAACCTCTTTGCTTATAGGCATAAAGCATCTCCCAATCTTGAAAGTTCTAAGACAGATCTACTCTCCGGATAATAA [ 2964]
lp17_PAbe_NX_MP_FG_consensus ATTAACCTCTTTGCTTATAGGCATAAAGCATCTCCCAATCTTGAAAGTTCTAAGACAGATCTACTCTCCGGATAATAA [ 2964]
lp17_PAbe_NX_Pl_consensus     ATTAACCTCTTTGCTTATAGGCATAAAGCATCTCCCAATCTTGAAAGTTCTAAGACAGATCTACTCTCCGGATAATAA [ 2964]

lp17_gi|365823332|B31_GB      TCAAAAATCGACTTCTTATATAGCTGAGATTCTGCTATTTTAGCATCTTGTCCAACCTCATAAAGATCATATCCAAAA [ 3042]
lp17_un5_B31_PacBio           TCAAAAATCGACTTCTTATATAGCTGAGATTCTGCTATTTTAGCATCTTGTCCAACCTCATAAAGATCATATCCAAAA [ 3042]
lp17_B31_TS_MP_FG_consensus   TCAAAAATCGACTTCTTATATAGCTGAGATTCTGCTATTTTAGCATCTTGTCCAACCTCATAAAGATCATATCCAAAA [ 3042]
lp17_B31_NX_Pl_consensus      TCAAAAATCGACTTCTTATATAGCTGAGATTCTGCTATTTTAGCATCTTGTCCAACCTCATAAAGATCATATCCAAAA [ 3042]
lp17_PAli_NX_MP_FG_consensus TCAAAAATCGACTTCTTATATAGCTGAGATTCTGCTATTTTAGCATCTTGTCCAACCTCATAAAGATCATATCCAAAA [ 3042]
lp17_PAli_NX_Pl_consensus     TCAAAAATCGACTTCTTATATAGCTGAGATTCTGCTATTTTAGCATCTTGTCCAACCTCATAAAGATCATATCCAAAA [ 3042]
lp17_PAbe_NX_MP_FG_consensus TCAAAAATCGACTTCTTATATAGCTGAGATTCTGCTATTTTAGCATCTTGTCCAACCTCATAAAGATCATATCCAAAA [ 3042]
lp17_PAbe_NX_Pl_consensus     TCAAAAATCGACTTCTTATATAGCTGAGATTCTGCTATTTTAGCATCTTGTCCAACCTCATAAAGATCATATCCAAAA [ 3042]

lp17_gi|365823332|B31_GB      GTTTTAAATTGCCTTAGATGCAAATTATGTCTTTTAAAACTTTTATTAAGCATATTGCAAATAATCTTCTCATGTTTA [ 3120]
lp17_un5_B31_PacBio           GTTTTAAATTGCCTTAGATGCAAATTATGTCTTTTAAAACTTTTATTAAGCATATTGCAAATAATCTTCTCATGTTTA [ 3120]
lp17_B31_TS_MP_FG_consensus   GTTTTAAATTGCCTTAGATGCAAATTATGTCTTTTAAAACTTTTATTAAGCATATTGCAAATAATCTTCTCATGTTTA [ 3120]
lp17_B31_NX_Pl_consensus      GTTTTAAATTGCCTTAGATGCAAATTATGTCTTTTAAAACTTTTATTAAGCATATTGCAAATAATCTTCTCATGTTTA [ 3120]
lp17_PAli_NX_MP_FG_consensus GTTTTAAATTGCCTTAGATGCAAATTATGTCTTTTAAAACTTTTATTAAGCATATTGCAAATAATCTTCTCATGTTTA [ 3120]
lp17_PAli_NX_Pl_consensus     GTTTTAAATTGCCTTAGATGCAAATTATGTCTTTTAAAACTTTTATTAAGCATATTGCAAATAATCTTCTCATGTTTA [ 3120]
lp17_PAbe_NX_MP_FG_consensus GTTTTAAATTGCCTTAGATGCAAATTATGTCTTTTAAAACTTTTATTAAGCATATTGCAAATAATCTTCTCATGTTTA [ 3120]
lp17_PAbe_NX_Pl_consensus     GTTTTAAATTGCCTTAGATGCAAATTATGTCTTTTAAAACTTTTATTAAGCATATTGCAAATAATCTTCTCATGTTTA [ 3120]

lp17_gi|365823332|B31_GB      ACCTTTTTTCTATAAGATTTTAACAAAGACTCAAACTCTTCTTTAAAAATATTAATTCCTTCAAGGCTTAAAAATTCT [ 3198]
lp17_un5_B31_PacBio           ACCTTTTTTCTATAAGATTTTAACAAAGACTCAAACTCTTCTTTAAAAATATTAATTCCTTCAAGGCTTAAAAATTCT [ 3198]
lp17_B31_TS_MP_FG_consensus   ACCTTTTTTCTATAAGATTTTAACAAAGACTCAAACTCTTCTTTAAAAATATTAATTCCTTCAAGGCTTAAAAATTCT [ 3198]
lp17_B31_NX_Pl_consensus      ACCTTTTTTCTATAAGATTTTAACAAAGACTCAAACTCTTCTTTAAAAATATTAATTCCTTCAAGGCTTAAAAATTCT [ 3198]
lp17_PAli_NX_MP_FG_consensus ACCTTTTTTCTATAAGATTTTAACAAAGACTCAAACTCTTCTTTAAAAATATTAATTCCTTCAAGGCTTAAAAATTCT [ 3198]
lp17_PAli_NX_Pl_consensus     ACCTTTTTTCTATAAGATTTTAACAAAGACTCAAACTCTTCTTTAAAAATATTAATTCCTTCAAGGCTTAAAAATTCT [ 3198]
lp17_PAbe_NX_MP_FG_consensus ACCTTTTTTCTATAAGATTTTAACAAAGACTCAAACTCTTCTTTAAAAATATTAATTCCTTCAAGGCTTAAAAATTCT [ 3198]
lp17_PAbe_NX_Pl_consensus     ACCTTTTTTCTATAAGATTTTAACAAAGACTCAAACTCTTCTTTAAAAATATTAATTCCTTCAAGGCTTAAAAATTCT [ 3198]

lp17_gi|365823332|B31_GB      GGGGTCAGTGGAGTAATAACTTCACACATTGCAAGAATAATTCTTCGCTCCCAAAGCTCAAAACTGGGAGATAAATCA [ 3276]
lp17_un5_B31_PacBio           GGGGTCAGTGGAGTAATAACTTCACACATTGCAAGAATAATTCTTCGCTCCCAAAGCTCAAAACTGGGAGATAAATCA [ 3276]
lp17_B31_TS_MP_FG_consensus   GGGGTCAGTGGAGTAATAACTTCACACATTGCAAGAATAATTCTTCGCTCCCAAAGCTCAAAACTGGGAGATAAATCA [ 3276]
lp17_B31_NX_Pl_consensus      GGGGTCAGTGGAGTAATAACTTCACACATTGCAAGAATAATTCTTCGCTCCCAAAGCTCAAAACTGGGAGATAAATCA [ 3276]
lp17_PAli_NX_MP_FG_consensus GGGGTCAGTGGAGTAATAACTTCACACATTGCAAGAATAATTCTTCGCTCCCAAAGCTCAAAACTGGGAGATAAATCA [ 3276]
lp17_PAli_NX_Pl_consensus     GGGGTCAGTGGAGTAATAACTTCACACATTGCAAGAATAATTCTTCGCTCCCAAAGCTCAAAACTGGGAGATAAATCA [ 3276]
lp17_PAbe_NX_MP_FG_consensus GGGGTCAGTGGAGTAATAACTTCACACATTGCAAGAATAATTCTTCGCTCCCAAAGCTCAAAACTGGGAGATAAATCA [ 3276]
lp17_PAbe_NX_Pl_consensus     GGGGTCAGTGGAGTAATAACTTCACACATTGCAAGAATAATTCTTCGCTCCCAAAGCTCAAAACTGGGAGATAAATCA [ 3276]

lp17_gi|365823332|B31_GB      AAAATTGCAAATTCAAATCCCAATTTCTCCAATTCCAAACAAAAATCATCTATCAAATATGGAAAATCTTGCAATTCA [ 3354]
lp17_un5_B31_PacBio           AAAATTGCAAATTCAAATCCCAATTTCTCCAATTCCAAACAAAAATCATCTATCAAATATGGAAAATCTTGCAATTCA [ 3354]
lp17_B31_TS_MP_FG_consensus   AAAATTGCAAATTCAAATCCCAATTTCTCCAATTCCAAACAAAAATCATCTATCAAATATGGAAAATCTTGCAATTCA [ 3354]
lp17_B31_NX_Pl_consensus      AAAATTGCAAATTCAAATCCCAATTTCTCCAATTCCAAACAAAAATCATCTATCAAATATGGAAAATCTTGCAATTCA [ 3354]
lp17_PAli_NX_MP_FG_consensus AAAATTGCAAATTCAAATCCCAATTTCTCCAATTCCAAACAAAAATCATCTATCAAATATGGAAAATCTTGCAATTCA [ 3354]
lp17_PAli_NX_Pl_consensus     AAAATTGCAAATTCAAATCCCAATTTCTCCAATTCCAAACAAAAATCATCTATCAAATATGGAAAATCTTGCAATTCA [ 3354]
lp17_PAbe_NX_MP_FG_consensus AAAATTGCAAATTCAAATCCCAATTTCTCCAATTCCAAACAAAAATCATCTATCAAATATGGAAAATCTTGCAATTCA [ 3354]
lp17_PAbe_NX_Pl_consensus     AAAATTGCAAATTCAAATCCCAATTTCTCCAATTCCAAACAAAAATCATCTATCAAATATGGAAAATCTTGCAATTCA [ 3354]

lp17_gi|365823332|B31_GB      TGTTGCACATCTCTTCTAAAAGTTCCACTCGGCACACATGGCAAAATATAAAAATTTTTTTGTATTTGTCTTACTACT [ 3432]
lp17_un5_B31_PacBio           TGTTGCACATCTCTTCTAAAAGTTCCACTCGGCACACATGGCAAAATATAAAAATTTTTTTGTATTTGTCTTACTACT [ 3432]
lp17_B31_TS_MP_FG_consensus   TGTTGCACATCTCTTCTAAAAGTTCCACTCGGCACACATGGCAAAATATAAAAATTTTTTTGTATTTGTCTTACTACT [ 3432]
lp17_B31_NX_Pl_consensus      TGTTGCACATCTCTTCTAAAAGTTCCACTCGGCACACATGGCAAAATATAAAAATTTTTTTGTATTTGTCTTACTACT [ 3432]
lp17_PAli_NX_MP_FG_consensus TGTTGCACATCTCTTCTAAAAGTTCCACTCGGCACACATGGCAAAATATAAAAATTTTTTTGTATTTGTCTTACTACT [ 3432]
lp17_PAli_NX_Pl_consensus     TGTTGCACATCTCTTCTAAAAGTTCCACTCGGCACACATGGCAAAATATAAAAATTTTTTTGTATTTGTCTTACTACT [ 3432]
lp17_PAbe_NX_MP_FG_consensus TGTTGCACATCTCTTCTAAAAGTTCCACTCGGCACACATGGCAAAATATAAAAATTTTTTTGTATTTGTCTTACTACT [ 3432]
lp17_PAbe_NX_Pl_consensus     TGTTGCACATCTCTTCTAAAAGTTCCACTCGGCACACATGGCAAAATATAAAAATTTTTTTGTATTTGTCTTACTACT [ 3432]

lp17_gi|365823332|B31_GB      TGATCTACATCCATCTTCTTCAAAAGAAAATCTTTAATATCCAGCTTAAGAATTTCATGATTAAGAAACCATGTAGAA [ 3510]
lp17_un5_B31_PacBio           TGATCTACATCCATCTTCTTCAAAAGAAAATCTTTAATATCCAGCTTAAGAATTTCATGATTAAGAAACCATGTAGAA [ 3510]
lp17_B31_TS_MP_FG_consensus   TGATCTACATCCATCTTCTTCAAAAGAAAATCTTTAATATCCAGCTTAAGAATTTCATGATTAAGAAACCATGTAGAA [ 3510]
lp17_B31_NX_Pl_consensus      TGATCTACATCCATCTTCTTCAAAAGAAAATCTTTAATATCCAGCTTAAGAATTTCATGATTAAGAAACCATGTAGAA [ 3510]
lp17_PAli_NX_MP_FG_consensus TGATCTACATCCATCTTCTTCAAAAGAAAATCTTTAATATCCAGCTTAAGAATTTCATGATTAAGAAACCATGTAGAA [ 3510]
lp17_PAli_NX_Pl_consensus     TGATCTACATCCATCTTCTTCAAAAGAAAATCTTTAATATCCAGCTTAAGAATTTCATGATTAAGAAACCATGTAGAA [ 3510]
lp17_PAbe_NX_MP_FG_consensus TGATCTACATCCATCTTCTTCAAAAGAAAATCTTTAATATCCAGCTTAAGAATTTCATGATTAAGAAACCATGTAGAA [ 3510]
lp17_PAbe_NX_Pl_consensus     TGATCTACATCCATCTTCTTCAAAAGAAAATCTTTAATATCCAGCTTAAGAATTTCATGATTAAGAAACCATGTAGAA [ 3510]

lp17_gi|365823332|B31_GB      GAACTTGCTTGCTGTATATCACAATCAACTAATATAACTTTTTTTGTTTTAGATAAATAACTTGCAATATTTCCGCTT [ 3588]
lp17_un5_B31_PacBio           GAACTTGCTTGCTGTATATCACAATCAACTAATATAACTTTTTTTGTTTTAGATAAATAACTTGCAATATTTCCGCTT [ 3588]
lp17_B31_TS_MP_FG_consensus   GAACTTGCTTGCTGTATATCACAATCAACTAATATAACTTTTTTTGTTTTAGATAAATAACTTGCAATATTTCCGCTT [ 3588]
lp17_B31_NX_Pl_consensus      GAACTTGCTTGCTGTATATCACAATCAACTAATATAACTTTTTTTGTTTTAGATAAATAACTTGCAATATTTCCGCTT [ 3588]
lp17_PAli_NX_MP_FG_consensus GAACTTGCTTGCTGTATATCACAATCAACTAATATAACTTTTTTTGTTTTAGATAAATAACTTGCAATATTTCCGCTT [ 3588]
lp17_PAli_NX_Pl_consensus     GAACTTGCTTGCTGTATATCACAATCAACTAATATAACTTTTTTTGTTTTAGATAAATAACTTGCAATATTTCCGCTT [ 3588]
lp17_PAbe_NX_MP_FG_consensus GAACTTGCTTGCTGTATATCACAATCAACTAATATAACTTTTTTTGTTTTAGATAAATAACTTGCAATATTTCCGCTT [ 3588]
lp17_PAbe_NX_Pl_consensus     GAACTTGCTTGCTGTATATCACAATCAACTAATATAACTTTTTTTGTTTTAGATAAATAACTTGCAATATTTCCGCTT [ 3588]

lp17_gi|365823332|B31_GB      AAGGTAGTTTTCCCAACACCACCTTTTTGAATATGAAATGCTATTTTTTTCATACAAACTTCCTTTTAATTATTTTAT [ 3666]
lp17_un5_B31_PacBio           AAGGTAGTTTTCCCAACACCACCTTTTTGAATATGAAATGCTATTTTTTTCATACAAACTTCCTTTTAATTATTTTAT [ 3666]
lp17_B31_TS_MP_FG_consensus   AAGGTAGTTTTCCCAACACCACCTTTTTGAATATGAAATGCTATTTTTTTCATACAAACTTCCTTTTAATTATTTTAT [ 3666]
lp17_B31_NX_Pl_consensus      AAGGTAGTTTTCCCAACACCACCTTTTTGAATATGAAATGCTATTTTTTTCATACAAACTTCCTTTTAATTATTTTAT [ 3666]
lp17_PAli_NX_MP_FG_consensus AAGGTAGTTTTCCCAACACCACCTTTTTGAATATGAAATGCTATTTTTTTCATACAAACTTCCTTTTAATTATTTTAT [ 3666]
lp17_PAli_NX_Pl_consensus     AAGGTAGTTTTCCCAACACCACCTTTTTGAATATGAAATGCTATTTTTTTCATACAAACTTCCTTTTAATTATTTTAT [ 3666]
lp17_PAbe_NX_MP_FG_consensus AAGGTAGTTTTCCCAACACCACCTTTTTGAATATGAAATGCTATTTTTTTCATACAAACTTCCTTTTAATTATTTTAT [ 3666]
lp17_PAbe_NX_Pl_consensus     AAGGTAGTTTTCCCAACACCACCTTTTTGAATATGAAATGCTATTTTTTTCATACAAACTTCCTTTTAATTATTTTAT [ 3666]

lp17_gi|365823332|B31_GB      ATCACATATTAATTATTTTATATCACATATTAATTATTTTATATCACATATTAATTATTTTATATCACATATTAATTA [ 3744]
lp17_un5_B31_PacBio           ATCACATATTAATTATTTTATATCACATATTAATTATTTTATATCACATATTAATTATTTTATATCACATATTAATTA [ 3744]
lp17_B31_TS_MP_FG_consensus   ATCACATATTAATTATTTTATATCACATATTAATTATTTTATATCACATATTAATTATTTTATATCACATATTAATTA [ 3744]
lp17_B31_NX_Pl_consensus      ATCACATATTAATTATTTTATATCACATATTAATTATTTTATATCACATATTAATTATTTTATATCACATATTAATTA [ 3744]
lp17_PAli_NX_MP_FG_consensus ATCACATATTAATTATTTTATATCACATATTAATTATTTTATATCACATATTAATTATTTTATATCACATATTAATTA [ 3744]
```

```
lp17_PAli_NX_Pl_consensus    ATCACATATTAATTATTTTATATCACATATTAATTATTTTATATCACATATTAATTATTTTATATCACATATTAATTA [ 3744]
lp17_PAbe_NX_MP_FG_consensus ATCACATATTAATTATTTTATATCACATATTAATTATTTTATATCACATATTAATTATTTTATATCACATATTAATTA [ 3744]
lp17_PAbe_NX_Pl_consensus    ATCACATATTAATTATTTTATATCACATATTAATTATTTTATATCACATATTAATTATTTTATATCACATATTAATTA [ 3744]

lp17_gi|365823332|B31_GB     TTTTATATCACATATTAATTATTTTATATCACATATTAATTATTTTATATCACATATTAATTATTTTATATCACATAT [ 3822]
lp17_un5_B31_PacBio          TTTTATATCACATATTAATTATTTTATATCACATATTAATTATTTTATATCACATATTAATTATTTTATATCACATAT [ 3822]
lp17_B31_TS_MP_FG_consensus  TTTTATATCACATATTAATTATTTTATATCACATATTAATTATTTTATATCACATATTAATTATTTTATATCACATAT [ 3822]
lp17_B31_NX_Pl_consensus     TTTTATATCACATATTAATTATTTTATATCACATATTAATTATTTTATATCACATATTAATTATTTTATATCACATAT [ 3822]
lp17_PAli_NX_MP_FG_consensus TTTTATATCACATATTAATTATTTTATATCACATATTAATTATTTTATATCACATATTAATTATTTTATATCACATAT [ 3822]
lp17_PAli_NX_Pl_consensus    TTTTATATCACATATTAATTATTTTATATCACATATTAATTATTTTATATCACATATTAATTATTTTATATCACATAT [ 3822]
lp17_PAbe_NX_MP_FG_consensus TTTTATATCACATATTAATTATTTTATATCACATATTAATTATTTTATATCACATATTAATTATTTTATATCACATAT [ 3822]
lp17_PAbe_NX_Pl_consensus    TTTTATATCACATATTAATTATTTTATATCACATATTAATTATTTTATATCACATATTAATTATTTTATATCACATAT [ 3822]

lp17_gi|365823332|B31_GB     TAATTACTTTAATCTCTACCTATGAAGCCCCTTTCACAAAATTTTTTAATGAATTAGATCTGATGTGGCCTTAAAAGC [ 3900]
lp17_un5_B31_PacBio          TAATTACTTTAATCTCTACCTATGAAGCCCCTTTCACAAAATTTTTTAATGAATTAGATCTGATGTGGCCTTAAAAGC [ 3900]
lp17_B31_TS_MP_FG_consensus  TAATTACTTTAATCTCTACCTATGAAGCCCCTTTCACAAAATTTTTTAATGAATTAGATCTGATGTGGCCTTAAAAGC [ 3900]
lp17_B31_NX_Pl_consensus     TAATTACTTTAATCTCTACCTATGAAGCCCCTTTCACAAAATTTTTTAATGAATTAGATCTGATGTGGCCTTAAAAGC [ 3900]
lp17_PAli_NX_MP_FG_consensus TAATTACTTTAATCTCTACCTATGAAGCCCCTTTCACAAAATTTTTTAATGAATTAGATCTGATGTGGCCTTAAAAGC [ 3900]
lp17_PAli_NX_Pl_consensus    TAATTACTTTAATCTCTACCTATGAAGCCCCTTTCACAAAATTTTTTAATGAATTAGATCTGATGTGGCCTTAAAAGC [ 3900]
lp17_PAbe_NX_MP_FG_consensus TAATTACTTTAATCTCTACCTATGAAGCCCCTTTCACAAAATTTTTTAATGAATTAGATCTGATGTGGCCTTAAAAGC [ 3900]
lp17_PAbe_NX_Pl_consensus    TAATTACTTTAATCTCTACCTATGAAGCCCCTTTCACAAAATTTTTTAATGAATTAGATCTGATGTGGCCTTAAAAGC [ 3900]

lp17_gi|365823332|B31_GB     TAGCAAACTACTATCTCTATATTCACGGCTTTTGTCTCATTCTACGATAACTTTTTAGTTCTTTTTATATCTTATTTT [ 3978]
lp17_un5_B31_PacBio          TAGCAAACTACTATCTCTATATTCACGGCTTTTGTCTCATTCTACGATAACTTTTTAGTTCTTTTTATATCTTATTTT [ 3978]
lp17_B31_TS_MP_FG_consensus  TAGCAAACTACTATCTCTATATTCACGGCTTTTGTCTCATTCTACGATAACTTTTTAGTTCTTTTTATATCTTATTTT [ 3978]
lp17_B31_NX_Pl_consensus     TAGCAAACTACTATCTCTATATTCACGGCTTTTGTCTCATTCTACGATAACTTTTTAGTTCTTTTTATATCTTATTTT [ 3978]
lp17_PAli_NX_MP_FG_consensus TAGCAAACTACTATCTCTATATTCACGGCTTTTGTCTCATTCTACGATAACTTTTTAGTTCTTTTTATATCTTATTTT [ 3978]
lp17_PAli_NX_Pl_consensus    TAGCAAACTACTATCTCTATATTCACGGCTTTTGTCTCATTCTACGATAACTTTTTAGTTCTTTTTATATCTTATTTT [ 3978]
lp17_PAbe_NX_MP_FG_consensus TAGCAAACTACTATCTCTATATTCACGGCTTTTGTCTCATTCTACGATAACTTTTTAGTTCTTTTTATATCTTATTTT [ 3978]
lp17_PAbe_NX_Pl_consensus    TAGCAAACTACTATCTCTATATTCACGGCTTTTGTCTCATTCTACGATAACTTTTTAGTTCTTTTTATATCTTATTTT [ 3978]

lp17_gi|365823332|B31_GB     CACACCAATAGCATGATTAACAAGCCTAAATTCGGAAAATTCCTGTCTTAGCTTTTATTTCTTTATAGCAATAAAACT [ 4056]
lp17_un5_B31_PacBio          CACACCAATAGCATGATTAACAAGCCTAAATTCGGAAAATTCCTGTCTTAGCTTTTATTTCTTTATAGCAATAAAACT [ 4056]
lp17_B31_TS_MP_FG_consensus  CACACCAATAGCATGATTAACAAGCCTAAATTCGGAAAATTCCTGTCTTAGCTTTTATTTCTTTATAGCAATAAAACT [ 4056]
lp17_B31_NX_Pl_consensus     CACACCAATAGCATGATTAACAAGCCTAAATTCGGAAAATTCCTGTCTTAGCTTTTATTTCTTTATAGCAATAAAACT [ 4056]
lp17_PAli_NX_MP_FG_consensus CACACCAATAGCATGATTAACAAGCCTAAATTCGGAAAATTCCTGTCTTAGCTTTTATTTCTTTATAGCAATAAAACT [ 4056]
lp17_PAli_NX_Pl_consensus    CACACCAATAGCATGATTAACAAGCCTAAATTCGGAAAATTCCTGTCTTAGCTTTTATTTCTTTATAGCAATAAAACT [ 4056]
lp17_PAbe_NX_MP_FG_consensus CACACCAATAGCATGATTAACAAGCCTAAATTCGGAAAATTCCTGTCTTAGCTTTTATTTCTTTATAGCAATAAAACT [ 4056]
lp17_PAbe_NX_Pl_consensus    CACACCAATAGCATGATTAACAAGCCTAAATTCGGAAAATTCCTGTCTTAGCTTTTATTTCTTTATAGCAATAAAACT [ 4056]

lp17_gi|365823332|B31_GB     TAAGATTTAGAATTGCATTTATATCTTTATTATACAAAACGCTGTAAGTTTACCTAGCACCACTTAATTTTAGAATTG [ 4134]
lp17_un5_B31_PacBio          TAAGATTTAGAATTGCATTTATATCTTTATTATACAAAACGCTGTAAGTTTACCTAGCACCACTTAATTTTAGAATTG [ 4134]
lp17_B31_TS_MP_FG_consensus  TAAGATTTAGAATTGCATTTATATCTTTATTATACAAAACGCTGTAAGTTTACCTAGCACCACTTAATTTTAGAATTG [ 4134]
lp17_B31_NX_Pl_consensus     TAAGATTTAGAATTGCATTTATATCTTTATTATACAAAACGCTGTAAGTTTACCTAGCACCACTTAATTTTAGAATTG [ 4134]
lp17_PAli_NX_MP_FG_consensus TAAGATTTAGAATTGCATTTATATCTTTATTATACAAAACGCTGTAAGTTTACCTAGCACCACTTAATTTTAGAATTG [ 4134]
lp17_PAli_NX_Pl_consensus    TAAGATTTAGAATTGCATTTATATCTTTATTATACAAAACGCTGTAAGTTTACCTAGCACCACTTAATTTTAGAATTG [ 4134]
lp17_PAbe_NX_MP_FG_consensus TAAGATTTAGAATTGCATTTATATCTTTATTATACAAAACGCTGTAAGTTTACCTAGCACCACTTAATTTTAGAATTG [ 4134]
lp17_PAbe_NX_Pl_consensus    TAAGATTTAGAATTGCATTTATATCTTTATTATACAAAACGCTGTAAGTTTACCTAGCACCACTTAATTTTAGAATTG [ 4134]

lp17_gi|365823332|B31_GB     TATTTTTAACACCACAATTACAAATAGCTTGCTTGATGAAAAATATATACCTACTTTATACAAAAAGGATCCATACCA [ 4212]
lp17_un5_B31_PacBio          TATTTTTAACACCACAATTACAAATAGCTTGCTTGATGAAAAATATATACCTACTTTATACAAAAAGGATCCATACCA [ 4212]
lp17_B31_TS_MP_FG_consensus  TATTTTTAACACCACAATTACAAATAGCTTGCTTGATGAAAAATATATACCTACTTTATACAAAAAGGATCCATACCA [ 4212]
lp17_B31_NX_Pl_consensus     TATTTTTAACACCACAATTACAAATAGCTTGCTTGATGAAAAATATATACCTACTTTATACAAAAAGGATCCATACCA [ 4212]
lp17_PAli_NX_MP_FG_consensus TATTTTTAACACCACAATTACAAATAGCTTGCTTGATGAAAAATATATACCTACTTTATACAAAAAGGATCCATACCA [ 4212]
lp17_PAli_NX_Pl_consensus    TATTTTTAACACCACAATTACAAATAGCTTGCTTGATGAAAAATATATACCTACTTTATACAAAAAGGATCCATACCA [ 4212]
lp17_PAbe_NX_MP_FG_consensus TATTTTTAACACCACAATTACAAATAGCTTGCTTGATGAAAAATATATACCTACTTTATACAAAAAGGATCCATACCA [ 4212]
lp17_PAbe_NX_Pl_consensus    TATTTTTAACACCACAATTACAAATAGCTTGCTTGATGAAAAATATATACCTACTTTATACAAAAAGGATCCATACCA [ 4212]

lp17_gi|365823332|B31_GB     TTCTGATTTATACTATAATCGTCTTACAAGTTTATAACATCCTAAATTATTAATACTTTACCCAAACATTCACATATC [ 4290]
lp17_un5_B31_PacBio          TTCTGATTTATACTATAATCGTCTTACAAGTTTATAACATCCTAAATTATTAATACTTTACCCAAACATTCACATATC [ 4290]
lp17_B31_TS_MP_FG_consensus  TTCTGATTTATACTATAATCGTCTTACAAGTTTATAACATCCTAAATTATTAATACTTTACCCAAACATTCACATATC [ 4290]
lp17_B31_NX_Pl_consensus     TTCTGATTTATACTATAATCGTCTTACAAGTTTATAACATCCTAAATTATTAATACTTTACCCAAACATTCACATATC [ 4290]
lp17_PAli_NX_MP_FG_consensus TTCTGATTTATACTATAATCGTCTTACAAGTTTATAACATCCTAAATTATTAATACTTTACCCAAACATTCACATATC [ 4290]
lp17_PAli_NX_Pl_consensus    TTCTGATTTATACTATAATCGTCTTACAAGTTTATAACATCCTAAATTATTAATACTTTACCCAAACATTCACATATC [ 4290]
lp17_PAbe_NX_MP_FG_consensus TTCTGATTTATACTATAATCGTCTTACAAGTTTATAACATCCTAAATTATTAATACTTTACCCAAACATTCACATATC [ 4290]
lp17_PAbe_NX_Pl_consensus    TTCTGATTTATACTATAATCGTCTTACAAGTTTATAACATCCTAAATTATTAATACTTTACCCAAACATTCACATATC [ 4290]

lp17_gi|365823332|B31_GB     TTTAATTAATAAGTTCTCTATTGCTATATTTTTATAAATAACTATAAAGCAAAAAATACCTTGTGTAAAAAGGTTTTT [ 4368]
lp17_un5_B31_PacBio          TTTAATTAATAAGTTCTCTATTGCTATATTTTTATAAATAACTATAAAGCAAAAAATACCTTGTGTAAAAAGGTTTTT [ 4368]
lp17_B31_TS_MP_FG_consensus  TTTAATTAATAAGTTCTCTATTGCTATATTTTTATAAATAACTATAAAGCAAAAAATACCTTGTGTAAAAAGGTTTTT [ 4368]
lp17_B31_NX_Pl_consensus     TTTAATTAATAAGTTCTCTATTGCTATATTTTTATAAATAACTATAAAGCAAAAAATACCTTGTGTAAAAAGGTTTTT [ 4368]
lp17_PAli_NX_MP_FG_consensus TTTAATTAATAAGTTCTCTATTGCTATATTTTTATAAATAACTATAAAGCAAAAAATACCTTGTGTAAAAAGGTTTTT [ 4368]
lp17_PAli_NX_Pl_consensus    TTTAATTAATAAGTTCTCTATTGCTATATTTTTATAAATAACTATAAAGCAAAAAATACCTTGTGTAAAAAGGTTTTT [ 4368]
lp17_PAbe_NX_MP_FG_consensus TTTAATTAATAAGTTCTCTATTGCTATATTTTTATAAATAACTATAAAGCAAAAAATACCTTGTGTAAAAAGGTTTTT [ 4368]
lp17_PAbe_NX_Pl_consensus    TTTAATTAATAAGTTCTCTATTGCTATATTTTTATAAATAACTATAAAGCAAAAAATACCTTGTGTAAAAAGGTTTTT [ 4368]

lp17_gi|365823332|B31_GB     TGATTTACAATTTTCTCATGTAACTTAGTAATTCTTAATATAGATTTAGCTCCATTAATAGAAGTTTTTTGTTTTTTT [ 4446]
lp17_un5_B31_PacBio          TGATTTACAATTTTCTCATGTAACTTAGTAATTCTTAATATAGATTTAGCTCCATTAATAGAAGTTTTTTGTTTTTTT [ 4446]
lp17_B31_TS_MP_FG_consensus  TGATTTACAATTTTCTCATGTAACTTAGTAATTCTTAATATAGATTTAGCTCCATTAATAGAAGTTTTTTGTTTTTTT [ 4446]
lp17_B31_NX_Pl_consensus     TGATTTACAATTTTCTCATGTAACTTAGTAATTCTTAATATAGATTTAGCTCCATTAATAGAAGTTTTTTGTTTTTTT [ 4446]
lp17_PAli_NX_MP_FG_consensus TGATTTACAATTTTCTCATGTAACTTAGTAATTCTTAATATAGATTTAGCTCCATTAATAGAAGTTTTTTGTTTTTTT [ 4446]
lp17_PAli_NX_Pl_consensus    TGATTTACAATTTTCTCATGTAACTTAGTAATTCTTAATATAGATTTAGCTCCATTAATAGAAGTTTTTTGTTTTTTT [ 4446]
lp17_PAbe_NX_MP_FG_consensus TGATTTACAATTTTCTCATGTAACTTAGTAATTCTTAATATAGATTTAGCTCCATTAATAGAAGTTTTTTGTTTTTTT [ 4446]
lp17_PAbe_NX_Pl_consensus    TGATTTACAATTTTCTCATGTAACTTAGTAATTCTTAATATAGATTTAGCTCCATTAATAGAAGTTTTTTGTTTTTTT [ 4446]

lp17_gi|365823332|B31_GB     GATAATTTTTTTAGTATTCTTTAAGTTTATTTTCGTTGTTTTTTTATTATTAGCTTTAAAAGCTTGCTTATTTTTCTT [ 4524]
lp17_un5_B31_PacBio          GATAATTTTTTTAGTATTCTTTAAGTTTATTTTCGTTGTTTTTTTATTATTAGCTTTAAAAGCTTGCTTATTTTTCTT [ 4524]
lp17_B31_TS_MP_FG_consensus  GATAATTTTTTTAGTATTCTTTAAGTTTATTTTCGTTGTTTTTTTATTATTAGCTTTAAAAGCTTGCTTATTTTTCTT [ 4524]
lp17_B31_NX_Pl_consensus     GATAATTTTTTTAGTATTCTTTAAGTTTATTTTCGTTGTTTTTTTATTATTAGCTTTAAAAGCTTGCTTATTTTTCTT [ 4524]
lp17_PAli_NX_MP_FG_consensus GATAATTTTTTTAGTATTCTTTAAGTTTATTTTCGTTGTTTTTTTATTATTAGCTTTAAAAGCTTGCTTATTTTTCTT [ 4524]
lp17_PAli_NX_Pl_consensus    GATAATTTTTTTAGTATTCTTTAAGTTTATTTTCGTTGTTTTTTTATTATTAGCTTTAAAAGCTTGCTTATTTTTCTT [ 4524]
lp17_PAbe_NX_MP_FG_consensus GATAATTTTTTTAGTATTCTTTAAGTTTATTTTCGTTGTTTTTTTATTATTAGCTTTAAAAGCTTGCTTATTTTTCTT [ 4524]
lp17_PAbe_NX_Pl_consensus    GATAATTTTTTTAGTATTCTTTAAGTTTATTTTCGTTGTTTTTTTATTATTAGCTTTAAAAGCTTGCTTATTTTTCTT [ 4524]

lp17_gi|365823332|B31_GB     GCTCTTATGTTTAAGAAATCCTTGTGTTCTATCCCTCTTTAAATTTCTCTAAAAGAATTTATATATAGAGCTTAAGTC [ 4602]
lp17_un5_B31_PacBio          GCTCTTATGTTTAAGAAATCCTTGTGTTCTATCCCTCTTTAAATTTCTCTAAAAGAATTTATATATAGAGCTTAAGTC [ 4602]
lp17_B31_TS_MP_FG_consensus  GCTCTTATGTTTAAGAAATCCTTGTGTTCTATCCCTCTTTAAATTTCTCTAAAAGAATTTATATATAGAGCTTAAGTC [ 4602]
lp17_B31_NX_Pl_consensus     GCTCTTATGTTTAAGAAATCCTTGTGTTCTATCCCTCTTTAAATTTCTCTAAAAGAATTTATATATAGAGCTTAAGTC [ 4602]
lp17_PAli_NX_MP_FG_consensus GCTCTTATGTTTAAGAAATCCTTGTGTTCTATCCCTCTTTAAATTTCTCTAAAAGAATTTATATATAGAGCTTAAGTC [ 4602]
lp17_PAli_NX_Pl_consensus    GCTCTTATGTTTAAGAAATCCTTGTGTTCTATCCCTCTTTAAATTTCTCTAAAAGAATTTATATATAGAGCTTAAGTC [ 4602]
lp17_PAbe_NX_MP_FG_consensus GCTCTTATGTTTAAGAAATCCTTGTGTTCTATCCCTCTTTAAATTTCTCTAAAAGAATTTATATATAGAGCTTAAGTC [ 4602]
lp17_PAbe_NX_Pl_consensus    GCTCTTATGTTTAAGAAATCCTTGTGTTCTATCCCTCTTTAAATTTCTCTAAAAGAATTTATATATAGAGCTTAAGTC [ 4602]

lp17_gi|365823332|B31_GB     AATCCATGCATACAAAAGGCCAAACTATAAACTTTTTTTAGCAATAGAAGCTCTTATTTATATTTATTTGAACAAGTA [ 4680]
lp17_un5_B31_PacBio          AATCCATGCATACAAAAGGCCAAACTATAAACTTTTTTTAGCAATAGAAGCTCTTATTTATATTTATTTGAACAAGTA [ 4680]
lp17_B31_TS_MP_FG_consensus  AATCCATGCATACAAAAGGCCAAACTATAAACTTTTTTTAGCAATAGAAGCTCTTATTTATATTTATTTGAACAAGTA [ 4680]
lp17_B31_NX_Pl_consensus     AATCCATGCATACAAAAGGCCAAACTATAAACTTTTTTTAGCAATAGAAGCTCTTATTTATATTTATTTGAACAAGTA [ 4680]
lp17_PAli_NX_MP_FG_consensus AATCCATGCATACAAAAGGCCAAACTATAAACTTTTTTTAGCAATAGAAGCTCTTATTTATATTTATTTGAACAAGTA [ 4680]
lp17_PAli_NX_Pl_consensus    AATCCATGCATACAAAAGGCCAAACTATAAACTTTTTTTAGCAATAGAAGCTCTTATTTATATTTATTTGAACAAGTA [ 4680]
```

```
lp17_PAbe_NX_MP_FG_consensus AATCCATGCATACAAAAGGCCAAACTATAAACTTTTTTTAGCAATAGAAGCTCTTATTTATATTTATTTGAACAAGTA [ 4680]
lp17_PAbe_NX_Pl_consensus    AATCCATGCATACAAAAGGCCAAACTATAAACTTTTTTTAGCAATAGAAGCTCTTATTTATATTTATTTGAACAAGTA [ 4680]

lp17_gi|365823332|B31_GB     ATAAGATTTTGCTTATTTTTTTTAATAAAAATCTATCTTGCCGCTTTAACACTGTATTATATAAAAATCCTACACATC [ 4758]
lp17_un5_B31_PacBio          ATAAGATTTTGCTTATTTTTTTTAATAAAAATCTATCTTGCCGCTTTAACACTGTATTATATAAAAATCCTACACATC [ 4758]
lp17_B31_TS_MP_FG_consensus  ATAAGATTTTGCTTATTTTTTTTAATAAAAATCTATCTTGCCGCTTTAACACTGTATTATATAAAAATCCTACACATC [ 4758]
lp17_B31_NX_Pl_consensus     ATAAGATTTTGCTTATTTTTTTTAATAAAAATCTATCTTGCCGCTTTAACACTGTATTATATAAAAATCCTACACATC [ 4758]
lp17_PAli_NX_MP_FG_consensus ATAAGATTTTGCTTATTTTTTTTAATAAAAATCTATCTTGCCGCTTTAACACTGTATTATATAAAAATCCTACACATC [ 4758]
lp17_PAli_NX_Pl_consensus    ATAAGATTTTGCTTATTTTTTTTAATAAAAATCTATCTTGCCGCTTTAACACTGTATTATATAAAAATCCTACACATC [ 4758]
lp17_PAbe_NX_MP_FG_consensus ATAAGATTTTGCTTATTTTTTTTAATAAAAATCTATCTTGCCGCTTTAACACTGTATTATATAAAAATCCTACACATC [ 4758]
lp17_PAbe_NX_Pl_consensus    ATAAGATTTTGCTTATTTTTTTTAATAAAAATCTATCTTGCCGCTTTAACACTGTATTATATAAAAATCCTACACATC [ 4758]

lp17_gi|365823332|B31_GB     TAAATACTTTTAAAAAGTATTTCTTTTGGTTGGTGTTAAAATATATTCTGTACTTATAAATGCCAACCTTCTCTTTGC [ 4836]
lp17_un5_B31_PacBio          TAAATACTTTTAAAAAGTATTTCTTTTGGTTGGTGTTAAAATATATTCTGTACTTATAAATGCCAACCTTCTCTTTGC [ 4836]
lp17_B31_TS_MP_FG_consensus  TAAATACTTTTAAAAAGTATTTCTTTTGGTTGGTGTTAAAATATATTCTGTACTTATAAATGCCAACCTTCTCTTTGC [ 4836]
lp17_B31_NX_Pl_consensus     TAAATACTTTTAAAAAGTATTTCTTTTGGTTGGTGTTAAAATATATTCTGTACTTATAAATGCCAACCTTCTCTTTGC [ 4836]
lp17_PAli_NX_MP_FG_consensus TAAATACTTTTAAAAAGTATTTCTTTTGGTTGGTGTTAAAATATATTCTGTACTTATAAATGCCAACCTTCTCTTTGC [ 4836]
lp17_PAli_NX_Pl_consensus    TAAATACTTTTAAAAAGTATTTCTTTTGGTTGGTGTTAAAATATATTCTGTACTTATAAATGCCAACCTTCTCTTTGC [ 4836]
lp17_PAbe_NX_MP_FG_consensus TAAATACTTTTAAAAAGTATTTCTTTTGGTTGGTGTTAAAATATATTCTGTACTTATAAATGCCAACCTTCTCTTTGC [ 4836]
lp17_PAbe_NX_Pl_consensus    TAAATACTTTTAAAAAGTATTTCTTTTGGTTGGTGTTAAAATATATTCTGTACTTATAAATGCCAACCTTCTCTTTGC [ 4836]

lp17_gi|365823332|B31_GB     CAAATAATACTTATTTTATGTTCTTTATAGAAAAAAGAACTTTTATTAAAAAAATTTAAATTATGATAAAAATAAAAT [ 4914]
lp17_un5_B31_PacBio          CAAATAATACTTATTTTATGTTCTTTATAGAAAAAAGAACTTTTATTAAAAAAATTTAAATTATGATAAAAATAAAAT [ 4914]
lp17_B31_TS_MP_FG_consensus  CAAATAATACTTATTTTATGTTCTTTATAGAAAAAAGAACTTTTATTAAAAAAATTTAAATTATGATAAAAATAAAAT [ 4914]
lp17_B31_NX_Pl_consensus     CAAATAATACTTATTTTATGTTCTTTATAGAAAAAAGAACTTTTATTAAAAAAATTTAAATTATGATAAAAATAAAAT [ 4914]
lp17_PAli_NX_MP_FG_consensus CAAATAATACTTATTTTATGTTCTTTATAGAAAAAAGAACTTTTATTAAAAAAATTTAAATTATGATAAAAATAAAAT [ 4914]
lp17_PAli_NX_Pl_consensus    CAAATAATACTTATTTTATGTTCTTTATAGAAAAAAGAACTTTTATTAAAAAAATTTAAATTATGATAAAAATAAAAT [ 4914]
lp17_PAbe_NX_MP_FG_consensus CAAATAATACTTATTTTATGTTCTTTATAGAAAAAAGAACTTTTATTAAAAAAATTTAAATTATGATAAAAATAAAAT [ 4914]
lp17_PAbe_NX_Pl_consensus    CAAATAATACTTATTTTATGTTCTTTATAGAAAAAAGAACTTTTATTAAAAAAATTTAAATTATGATAAAAATAAAAT [ 4914]

lp17_gi|365823332|B31_GB     TCATTTATCACCAGAATTTACTTACAATATTTAACCTTCTAAATTAATTAATAATCTAAAAACGGCACCTAAGCTATT [ 4992]
lp17_un5_B31_PacBio          TCATTTATCACCAGAATTTACTTACAATATTTAACCTTCTAAATTAATTAATAATCTAAAAACGGCACCTAAGCTATT [ 4992]
lp17_B31_TS_MP_FG_consensus  TCATTTATCACCAGAATTTACTTACAATATTTAACCTTCTAAATTAATTAATAATCTAAAAACGGCACCTAAGCTATT [ 4992]
lp17_B31_NX_Pl_consensus     TCATTTATCACCAGAATTTACTTACAATATTTAACCTTCTAAATTAATTAATAATCTAAAAACGGCACCTAAGCTATT [ 4992]
lp17_PAli_NX_MP_FG_consensus TCATTTATCACCAGAATTTACTTACAATATTTAACCTTCTAAATTAATTAATAATCTAAAAACGGCACCTAAGCTATT [ 4992]
lp17_PAli_NX_Pl_consensus    TCATTTATCACCAGAATTTACTTACAATATTTAACCTTCTAAATTAATTAATAATCTAAAAACGGCACCTAAGCTATT [ 4992]
lp17_PAbe_NX_MP_FG_consensus TCATTTATCACCAGAATTTACTTACAATATTTAACCTTCTAAATTAATTAATAATCTAAAAACGGCACCTAAGCTATT [ 4992]
lp17_PAbe_NX_Pl_consensus    TCATTTATCACCAGAATTTACTTACAATATTTAACCTTCTAAATTAATTAATAATCTAAAAACGGCACCTAAGCTATT [ 4992]

lp17_gi|365823332|B31_GB     GTCTTATCTATATTAGAAGCCTCTACCGATATCATAAAAAGTATATTCAGAGCTAAAAATAAATCTATTTTACAAACC [ 5070]
lp17_un5_B31_PacBio          GTCTTATCTATATTAGAAGCCTCTACCGATATCATAAAAAGTATATTCAGAGCTAAAAATAAATCTATTTTACAAACC [ 5070]
lp17_B31_TS_MP_FG_consensus  GTCTTATCTATATTAGAAGCCTCTACCGATATCATAAAAAGTATATTCAGAGCTAAAAATAAATCTATTTTACAAACC [ 5070]
lp17_B31_NX_Pl_consensus     GTCTTATCTATATTAGAAGCCTCTACCGATATCATAAAAAGTATATTCAGAGCTAAAAATAAATCTATTTTACAAACC [ 5070]
lp17_PAli_NX_MP_FG_consensus GTCTTATCTATATTAGAAGCCTCTACCGATATCATAAAAAGTATATTCAGAGCTAAAAATAAATCTATTTTACAAACC [ 5070]
lp17_PAli_NX_Pl_consensus    GTCTTATCTATATTAGAAGCCTCTACCGATATCATAAAAAGTATATTCAGAGCTAAAAATAAATCTATTTTACAAACC [ 5070]
lp17_PAbe_NX_MP_FG_consensus GTCTTATCTATATTAGAAGCCTCTACCGATATCATAAAAAGTATATTCAGAGCTAAAAATAAATCTATTTTACAAACC [ 5070]
lp17_PAbe_NX_Pl_consensus    GTCTTATCTATATTAGAAGCCTCTACCGATATCATAAAAAGTATATTCAGAGCTAAAAATAAATCTATTTTACAAACC [ 5070]

lp17_gi|365823332|B31_GB     TACCCCCCGCCCAAATTTATTTTGAAATTTAGGCGGGCAATTCTTCATTATTCTTTATTAAATTTATAAAAAGTCCTA [ 5148]
lp17_un5_B31_PacBio          TACCCCCCGCCCAAATTTATTTTGAAATTTAGGCGGGCAATTCTTCATTATTCTTTATTAAATTTATAAAAAGTCCTA [ 5148]
lp17_B31_TS_MP_FG_consensus  TACCCCCCGCCCAAATTTATTTTGAAATTTAGGCGGGCAATTCTTCATTATTCTTTATTAAATTTATAAAAAGTCCTA [ 5148]
lp17_B31_NX_Pl_consensus     TACCCCCCGCCCAAATTTATTTTGAAATTTAGGCGGGCAATTCTTCATTATTCTTTATTAAATTTATAAAAAGTCCTA [ 5148]
lp17_PAli_NX_MP_FG_consensus TACCCCCCGCCCAAATTTATTTTGAAATTTAGGCGGGCAATTCTTCATTATTCTTTATTAAATTTATAAAAAGTCCTA [ 5148]
lp17_PAli_NX_Pl_consensus    TACCCCCCGCCCAAATTTATTTTGAAATTTAGGCGGGCAATTCTTCATTATTCTTTATTAAATTTATAAAAAGTCCTA [ 5148]
lp17_PAbe_NX_MP_FG_consensus TACCCCCCGCCCAAATTTATTTTGAAATTTAGGCGGGCAATTCTTCATTATTCTTTATTAAATTTATAAAAAGTCCTA [ 5148]
lp17_PAbe_NX_Pl_consensus    TACCCCCCGCCCAAATTTATTTTGAAATTTAGGCGGGCAATTCTTCATTATTCTTTATTAAATTTATAAAAAGTCCTA [ 5148]

lp17_gi|365823332|B31_GB     AGAAATTTCTCTCTTAAGGACTTTTTATAAATGGTTAAAGGTTAAAGATAAGTTAAAATCTACAACAGTTTAGAAAAA [ 5226]
lp17_un5_B31_PacBio          AGAAATTTCTCTCTTAAGGACTTTTTATAAATGGTTAAAGGTTAAAGATAAGTTAAAATCTACAACAGTTTAGAAAAA [ 5226]
lp17_B31_TS_MP_FG_consensus  AGAAATTTCTCTCTTAAGGACTTTTTATAAATGGTTAAAGGTTAAAGATAAGTTAAAATCTACAACAGTTTAGAAAAA [ 5226]
lp17_B31_NX_Pl_consensus     AGAAATTTCTCTCTTAAGGACTTTTTATAAATGGTTAAAGGTTAAAGATAAGTTAAAATCTACAACAGTTTAGAAAAA [ 5226]
lp17_PAli_NX_MP_FG_consensus AGAAATTTCTCTCTTAAGGACTTTTTATAAATGGTTAAAGGTTAAAGATAAGTTAAAATCTACAACAGTTTAGAAAAA [ 5226]
lp17_PAli_NX_Pl_consensus    AGAAATTTCTCTCTTAAGGACTTTTTATAAATGGTTAAAGGTTAAAGATAAGTTAAAATCTACAACAGTTTAGAAAAA [ 5226]
lp17_PAbe_NX_MP_FG_consensus AGAAATTTCTCTCTTAAGGACTTTTTATAAATGGTTAAAGGTTAAAGATAAGTTAAAATCTACAACAGTTTAGAAAAA [ 5226]
lp17_PAbe_NX_Pl_consensus    AGAAATTTCTCTCTTAAGGACTTTTTATAAATGGTTAAAGGTTAAAGATAAGTTAAAATCTACAACAGTTTAGAAAAA [ 5226]

lp17_gi|365823332|B31_GB     AAACAAAATTAAAAATATTTTTTTTATTTATACTATGTATTAATTGATATATAATATCAATTAATACATAGTATTATA [ 5304]
lp17_un5_B31_PacBio          AAACAAAATTAAAAATATTTTTTTTATTTATACTATGTATTAATTGATATATAATATCAATTAATACATAGTATTATA [ 5304]
lp17_B31_TS_MP_FG_consensus  AAACAAAATTAAAAATATTTTTTTTATTTATACTATGTATTAATTGATATATAATATCAATTAATACATAGTATTATA [ 5304]
lp17_B31_NX_Pl_consensus     AAACAAAATTAAAAATATTTTTTTTATTTATACTATGTATTAATTGATATATAATATCAATTAATACATAGTATTATA [ 5304]
lp17_PAli_NX_MP_FG_consensus AAACAAAATTAAAAATATTTTTTTTATTTATACTATGTATTAATTGATATATAATATCAATTAATACATAGTATTATA [ 5304]
lp17_PAli_NX_Pl_consensus    AAACAAAATTAAAAATATTTTTTTTATTTATACTATGTATTAATTGATATATAATATCAATTAATACATAGTATTATA [ 5304]
lp17_PAbe_NX_MP_FG_consensus AAACAAAATTAAAAATATTTTTTTTATTTATACTATGTATTAATTGATATATAATATCAATTAATACATAGTATTATA [ 5304]
lp17_PAbe_NX_Pl_consensus    AAACAAAATTAAAAATATTTTTTTTATTTATACTATGTATTAATTGATATATAATATCAATTAATACATAGTATTATA [ 5304]

lp17_gi|365823332|B31_GB     TTATATTAATATATTTTTAAAGGATAAAAATGCAAAAAGAAATAACAATAAACTATAATGAATATACAATTGGGGGCA [ 5382]
lp17_un5_B31_PacBio          TTATATTAATATATTTTTAAAGGATAAAAATGCAAAAAGAAATAACAATAAACTATAATGAATATACAATTGGGGGCA [ 5382]
lp17_B31_TS_MP_FG_consensus  TTATATTAATATATTTTTAAAGGATAAAAATGCAAAAAGAAATAACAATAAACTATAATGAATATACAATTGGGGGCA [ 5382]
lp17_B31_NX_Pl_consensus     TTATATTAATATATTTTTAAAGGATAAAAATGCAAAAAGAAATAACAATAAACTATAATGAATATACAATTGGGGGCA [ 5382]
lp17_PAli_NX_MP_FG_consensus TTATATTAATATATTTTTAAAGGATAAAAATGCAAAAAGAAATAACAATAAACTATAATGAATATACAATTGGGGGCA [ 5382]
lp17_PAli_NX_Pl_consensus    TTATATTAATATATTTTTAAAGGATAAAAATGCAAAAAGAAATAACAATAAACTATAATGAATATACAATTGGGGGCA [ 5382]
lp17_PAbe_NX_MP_FG_consensus TTATATTAATATATTTTTAAAGGATAAAAATGCAAAAAGAAATAACAATAAACTATAATGAATATACAATTGGGGGCA [ 5382]
lp17_PAbe_NX_Pl_consensus    TTATATTAATATATTTTTAAAGGATAAAAATGCAAAAAGAAATAACAATAAACTATAATGAATATACAATTGGGGGCA [ 5382]

lp17_gi|365823332|B31_GB     TAAAAGGAACTTTCTTTGGAAATTCTCAATATGAAGCTAATTATCGAGCAAAAATAAATGGCTTTATAATTGACTTTT [ 5460]
lp17_un5_B31_PacBio          TAAAAGGAACTTTCTTTGGAAATTCTCAATATGAAGCTAATTATCGAGCAAAAATAAATGGCTTTATAATTGACTTTT [ 5460]
lp17_B31_TS_MP_FG_consensus  TAAAAGGAACTTTCTTTGGAAATTCTCAATATGAAGCTAATTATCGAGCAAAAATAAATGGCTTTATAATTGACTTTT [ 5460]
lp17_B31_NX_Pl_consensus     TAAAAGGAACTTTCTTTGGAAATTCTCAATATGAAGCTAATTATCGAGCAAAAATAAATGGCTTTATAATTGACTTTT [ 5460]
lp17_PAli_NX_MP_FG_consensus TAAAAGGAACTTTCTTTGGAAATTCTCAATATGAAGCTAATTATCGAGCAAAAATAAATGGCTTTATAATTGACTTTT [ 5460]
lp17_PAli_NX_Pl_consensus    TAAAAGGAACTTTCTTTGGAAATTCTCAATATGAAGCTAATTATCGAGCAAAAATAAATGGCTTTATAATTGACTTTT [ 5460]
lp17_PAbe_NX_MP_FG_consensus TAAAAGGAACTTTCTTTGGAAATTCTCAATATGAAGCTAATTATCGAGCAAAAATAAATGGCTTTATAATTGACTTTT [ 5460]
lp17_PAbe_NX_Pl_consensus    TAAAAGGAACTTTCTTTGGAAATTCTCAATATGAAGCTAATTATCGAGCAAAAATAAATGGCTTTATAATTGACTTTT [ 5460]

lp17_gi|365823332|B31_GB     TTAAAATTCCCATAAGTTTAAAGAAAAAAATATGAACTTAATATTAAAGCCCTTAGTGATCCTAATTTTTCATCCACAA [ 5538]
lp17_un5_B31_PacBio          TTAAAATTCCCATAAGTTTAAAGAAAAAAATATGAACTTAATATTAAAGCCCTTAGTGATCCTAATTTTTCATCCACAA [ 5538]
lp17_B31_TS_MP_FG_consensus  TTAAAATTCCCATAAGTTTAAAGAAAAAAATATGAACTTAATATTAAAGCCCTTAGTGATCCTAATTTTTCATCCACAA [ 5538]
lp17_B31_NX_Pl_consensus     TTAAAATTCCCATAAGTTTAAAGAAAAAAATATGAACTTAATATTAAAGCCCTTAGTGATCCTAATTTTTCATCCACAA [ 5538]
lp17_PAli_NX_MP_FG_consensus TTAAAATTCCCATAAGTTTAAAGAAAAAAATATGAACTTAATATTAAAGCCCTTAGTGATCCTAATTTTTCATCCACAA [ 5538]
lp17_PAli_NX_Pl_consensus    TTAAAATTCCCATAAGTTTAAAGAAAAAAATATGAACTTAATATTAAAGCCCTTAGTGATCCTAATTTTTCATCCACAA [ 5538]
lp17_PAbe_NX_MP_FG_consensus TTAAAATTCCCATAAGTTTAAAGAAAAAAATATGAACTTAATATTAAAGCCCTTAGTGATCCTAATTTTTCATCCACAA [ 5538]
lp17_PAbe_NX_Pl_consensus    TTAAAATTCCCATAAGTTTAAAGAAAAAAATATGAACTTAATATTAAAGCCCTTAGTGATCCTAATTTTTCATCCACAA [ 5538]

lp17_gi|365823332|B31_GB     ATATTGCAATGAATTGCATAAATACTTTCAAGCTTATAGTTGATATTGTTAATATGCAAACCGGTGAAAATTACGATT [ 5616]
lp17_un5_B31_PacBio          ATATTGCAATGAATTGCATAAATACTTTCAAGCTTATAGTTGATATTGTTAATATGCAAACCGGTGAAAATTACGATT [ 5616]
lp17_B31_TS_MP_FG_consensus  ATATTGCAATGAATTGCATAAATACTTTCAAGCTTATAGTTGATATTGTTAATATGCAAACCGGTGAAAATTACGATT [ 5616]
lp17_B31_NX_Pl_consensus     ATATTGCAATGAATTGCATAAATACTTTCAAGCTTATAGTTGATATTGTTAATATGCAAACCGGTGAAAATTACGATT [ 5616]
lp17_PAli_NX_MP_FG_consensus ATATTGCAATGAATTGCATAAATACTTTCAAGCTTATAGTTGATATTGTTAATATGCAAACCGGTGAAAATTACGATT [ 5616]
lp17_PAli_NX_Pl_consensus    ATATTGCAATGAATTGCATAAATACTTTCAAGCTTATAGTTGATATTGTTAATATGCAAACCGGTGAAAATTACGATT [ 5616]
lp17_PAbe_NX_MP_FG_consensus ATATTGCAATGAATTGCATAAATACTTTCAAGCTTATAGTTGATATTGTTAATATGCAAACCGGTGAAAATTACGATT [ 5616]
```

```
lp17_PAbe_NX_Pl_consensus    ATATTGCAATGAATTGCATAAATACTTTCAAGCTTATAGTTGATATTGTTAATATGCAAACCGGTGAAAATTACGATT [ 5616]

lp17_gi|365823332|B31_GB     ATGACACATTTATTACCAAAACAGACACAGAAAAAATGCTTAAATATGGAACAAAAATAATTGCCGCACTAGCAAGGC [ 5694]
lp17_un5_B31_PacBio          ATGACACATTTATTACCAAAACAGACACAGAAAAAATGCTTAAATATGGAACAAAAATAATTGCCGCACTAGCAAGGC [ 5694]
lp17_B31_TS_MP_FG_consensus  ATGACACATTTATTACCAAAACAGACACAGAAAAAATGCTTAAATATGGAACAAAAATAATTGCCGCACTAGCAAGGC [ 5694]
lp17_B31_NX_Pl_consensus     ATGACACATTTATTACCAAAACAGACACAGAAAAAATGCTTAAATATGGAACAAAAATAATTGCCGCACTAGCAAGGC [ 5694]
lp17_PAli_NX_MP_FG_consensus ATGACACATTTATTACCAAAACAGACACAGAAAAAATGCTTAAATATGGAACAAAAATAATTGCCGCACTAGCAAGGC [ 5694]
lp17_PAli_NX_Pl_consensus    ATGACACATTTATTACCAAAACAGACACAGAAAAAATGCTTAAATATGGAACAAAAATAATTGCCGCACTAGCAAGGC [ 5694]
lp17_PAbe_NX_MP_FG_consensus ATGACACATTTATTACCAAAACAGACACAGAAAAAATGCTTAAATATGGAACAAAAATAATTGCCGCACTAGCAAGGC [ 5694]
lp17_PAbe_NX_Pl_consensus    ATGACACATTTATTACCAAAACAGACACAGAAAAAATGCTTAAATATGGAACAAAAATAATTGCCGCACTAGCAAGGC [ 5694]

lp17_gi|365823332|B31_GB     ACTTCGACGAACAAAACAAAACCAACTTTAATGAAAGCTATTATGAATGGGAAAAAGGCTGGATAGATAAAAAATGGA [ 5772]
lp17_un5_B31_PacBio          ACTTCGACGAACAAAACAAAACCAACTTTAATGAAAGCTATTATGAATGGGAAAAAGGCTGGATAGATAAAAAATGGA [ 5772]
lp17_B31_TS_MP_FG_consensus  ACTTCGACGAACAAAACAAAACCAACTTTAATGAAAGCTATTATGAATGGGAAAAAGGCTGGATAGATAAAAAATGGA [ 5772]
lp17_B31_NX_Pl_consensus     ACTTCGACGAACAAAACAAAACCAACTTTAATGAAAGCTATTATGAATGGGAAAAAGGCTGGATAGATAAAAAATGGA [ 5772]
lp17_PAli_NX_MP_FG_consensus ACTTCGACGAACAAAACAAAACCAACTTTAATGAAAGCTATTATGAATGGGAAAAAGGCTGGATAGATAAAAAATGGA [ 5772]
lp17_PAli_NX_Pl_consensus    ACTTCGACGAACAAAACAAAACCAACTTTAATGAAAGCTATTATGAATGGGAAAAAGGCTGGATAGATAAAAAATGGA [ 5772]
lp17_PAbe_NX_MP_FG_consensus ACTTCGACGAACAAAACAAAACCAACTTTAATGAAAGCTATTATGAATGGGAAAAAGGCTGGATAGATAAAAAATGGA [ 5772]
lp17_PAbe_NX_Pl_consensus    ACTTCGACGAACAAAACAAAACCAACTTTAATGAAAGCTATTATGAATGGGAAAAAGGCTGGATAGATAAAAAATGGA [ 5772]

lp17_gi|365823332|B31_GB     TAAATTATGAACCAACTGCAGAAGAAATTAAAGAAATTCAAATAATGAATCAAAAATTAAATCCTTTAAAACTCAAAC [ 5850]
lp17_un5_B31_PacBio          TAAATTATGAACCAACTGCAGAAGAAATTAAAGAAATTCAAATAATGAATCAAAAATTAAATCCTTTAAAACTCAAAC [ 5850]
lp17_B31_TS_MP_FG_consensus  TAAATTATGAACCAACTGCAGAAGAAATTAAAGAAATTCAAATAATGAATCAAAAATTAAATCCTTTAAAACTCAAAC [ 5850]
lp17_B31_NX_Pl_consensus     TAAATTATGAACCAACTGCAGAAGAAATTAAAGAAATTCAAATAATGAATCAAAAATTAAATCCTTTAAAACTCAAAC [ 5850]
lp17_PAli_NX_MP_FG_consensus TAAATTATGAACCAACTGCAGAAGAAATTAAAGAAATTCAAATAATGAATCAAAAATTAAATCCTTTAAAACTCAAAC [ 5850]
lp17_PAli_NX_Pl_consensus    TAAATTATGAACCAACTGCAGAAGAAATTAAAGAAATTCAAATAATGAATCAAAAATTAAATCCTTTAAAACTCAAAC [ 5850]
lp17_PAbe_NX_MP_FG_consensus TAAATTATGAACCAACTGCAGAAGAAATTAAAGAAATTCAAATAATGAATCAAAAATTAAATCCTTTAAAACTCAAAC [ 5850]
lp17_PAbe_NX_Pl_consensus    TAAATTATGAACCAACTGCAGAAGAAATTAAAGAAATTCAAATAATGAATCAAAAATTAAATCCTTTAAAACTCAAAC [ 5850]

lp17_gi|365823332|B31_GB     ATAGAAAAAAAAAACCTTAATAACGGCCAAATAAGGCTTTTACAAGCCATCAATAAAATTGAAGATCAATCACAACAAC [ 5928]
lp17_un5_B31_PacBio          ATAGAAAAAAAAAACCTTAATAACGGCCAAATAAGGCTTTTACAAGCCATCAATAAAATTGAAGATCAATCACAACAAC [ 5928]
lp17_B31_TS_MP_FG_consensus  ATAGAAAAAAAAAACCTTAATAACGGCCAAATAAGGCTTTTACAAGCCATCAATAAAATTGAAGATCAATCACAACAAC [ 5928]
lp17_B31_NX_Pl_consensus     ATAGAAAAAAAAAACCTTAATAACGGCCAAATAAGGCTTTTACAAGCCATCAATAAAATTGAAGATCAATCACAACAAC [ 5928]
lp17_PAli_NX_MP_FG_consensus ATAGAAAAAAAAAACCTTAATAACGGCCAAATAAGGCTTTTACAAGCCATCAATAAAATTGAAGATCAATCACAACAAC [ 5928]
lp17_PAli_NX_Pl_consensus    ATAGAAAAAAAAAACCTTAATAACGGCCAAATAAGGCTTTTACAAGCCATCAATAAAATTGAAGATCAATCACAACAAC [ 5928]
lp17_PAbe_NX_MP_FG_consensus ATAGAAAAAAAAAACCTTAATAACGGCCAAATAAGGCTTTTACAAGCCATCAATAAAATTGAAGATCAATCACAACAAC [ 5928]
lp17_PAbe_NX_Pl_consensus    ATAGAAAAAAAAAACCTTAATAACGGCCAAATAAGGCTTTTACAAGCCATCAATAAAATTGAAGATCAATCACAACAAC [ 5928]

lp17_gi|365823332|B31_GB     AGGCTACTAAATCAAATAGCAAAACTAAAAAATTAAAAGGAAATCATGGGGAAAAAACCAAAATTTAACATTTTACTT [ 6006]
lp17_un5_B31_PacBio          AGGCTACTAAATCAAATAGCAAAACTAAAAAATTAAAAGGAAATCATGGGGAAAAAACCAAAATTTAACATTTTACTT [ 6006]
lp17_B31_TS_MP_FG_consensus  AGGCTACTAAATCAAATAGCAAAACTAAAAAATTAAAAGGAAATCATGGGGAAAAAACCAAAATTTAACATTTTACTT [ 6006]
lp17_B31_NX_Pl_consensus     AGGCTACTAAATCAAATAGCAAAACTAAAAAATTAAAAGGAAATCATGGGGAAAAAACCAAAATTTAACATTTTACTT [ 6006]
lp17_PAli_NX_MP_FG_consensus AGGCTACTAAATCAAATAGCAAAACTAAAAAATTAAAAGGAAATCATGGGGAAAAAACCAAAATTTAACATTTTACTT [ 6006]
lp17_PAli_NX_Pl_consensus    AGGCTACTAAATCAAATAGCAAAACTAAAAAATTAAAAGGAAATCATGGGGAAAAAACCAAAATTTAACATTTTACTT [ 6006]
lp17_PAbe_NX_MP_FG_consensus AGGCTACTAAATCAAATAGCAAAACTAAAAAATTAAAAGGAAATCATGGGGAAAAAACCAAAATTTAACATTTTACTT [ 6006]
lp17_PAbe_NX_Pl_consensus    AGGCTACTAAATCAAATAGCAAAACTAAAAAATTAAAAGGAAATCATGGGGAAAAAACCAAAATTTAACATTTTACTT [ 6006]

lp17_gi|365823332|B31_GB     AAAAGCGTTTAATTTTATTTATTATTATTAAATAATATTTACAAAATTAAAAAAATAAAATATTATATATGTAAGGTA [ 6084]
lp17_un5_B31_PacBio          AAAAGCGTTTAATTTTATTTATTATTATTAAATAATATTTACAAAATTAAAAAAATAAAATATTATATATGTAAGGTA [ 6084]
lp17_B31_TS_MP_FG_consensus  AAAAGCGTTTAATTTTATTTATTATTATTAAATAATATTTACAAAATTAAAAAAATAAAATATTATATATGTAAGGTA [ 6084]
lp17_B31_NX_Pl_consensus     AAAAGCGTTTAATTTTATTTATTATTATTAAATAATATTTACAAAATTAAAAAAATAAAATATTATATATGTAAGGTA [ 6084]
lp17_PAli_NX_MP_FG_consensus AAAAGCGTTTAATTTTATTTATTATTATTAAATAATATTTACAAAATTAAAAAAATAAAATATTATATATGTAAGGTA [ 6084]
lp17_PAli_NX_Pl_consensus    AAAAGCGTTTAATTTTATTTATTATTATTAAATAATATTTACAAAATTAAAAAAATAAAATATTATATATGTAAGGTA [ 6084]
lp17_PAbe_NX_MP_FG_consensus AAAAGCGTTTAATTTTATTTATTATTATTAAATAATATTTACAAAATTAAAAAAATAAAATATTATATATGTAAGGTA [ 6084]
lp17_PAbe_NX_Pl_consensus    AAAAGCGTTTAATTTTATTTATTATTATTAAATAATATTTACAAAATTAAAAAAATAAAATATTATATATGTAAGGTA [ 6084]

lp17_gi|365823332|B31_GB     GAAAACGATCTACCAATAGTCCCCAACACCCCTATTAATCCTCTCCAAATAAGATCGGGGGTAGGGACTTCTTTTTTA [ 6162]
lp17_un5_B31_PacBio          GAAAACGATCTACCAATAGTCCCCAACACCCCTATTAATCCTCTCCAAATAAGATCGGGGGTAGGGACTTCTTTTTTA [ 6162]
lp17_B31_TS_MP_FG_consensus  GAAAACGATCTACCAATAGTCCCCAACACCCCTATTAATCCTCTCCAAATAAGATCGGGGGTAGGGACTTCTTTTTTA [ 6162]
lp17_B31_NX_Pl_consensus     GAAAACGATCTACCAATAGTCCCCAACACCCCTATTAATCCTCTCCAAATAAGATCGGGGGTAGGGACTTCTTTTTTA [ 6162]
lp17_PAli_NX_MP_FG_consensus GAAAACGATCTACCAATAGTCCCCAACACCCCTATTAATCCTCTCCAAATAAGATCGGGGGTAGGGACTTCTTTTTTA [ 6162]
lp17_PAli_NX_Pl_consensus    GAAAACGATCTACCAATAGTCCCCAACACCCCTATTAATCCTCTCCAAATAAGATCGGGGGTAGGGACTTCTTTTTTA [ 6162]
lp17_PAbe_NX_MP_FG_consensus GAAAACGATCTACCAATAGTCCCCAACACCCCTATTAATCCTCTCCAAATAAGATCGGGGGTAGGGACTTCTTTTTTA [ 6162]
lp17_PAbe_NX_Pl_consensus    GAAAACGATCTACCAATAGTCCCCAACACCCCTATTAATCCTCTCCAAATAAGATCGGGGGTAGGGACTTCTTTTTTA [ 6162]

lp17_gi|365823332|B31_GB     AAAAAATAAAAACTAAAATATTTGGTGCCTAAATTAATAAGTTTATAGCAAATAAGAAAAATGAAAATCTAAAAAACA [ 6240]
lp17_un5_B31_PacBio          AAAAAATAAAAACTAAAATATTTGGTGCCTAAATTAATAAGTTTATAGCAAATAAGAAAAATGAAAATCTAAAAAACA [ 6240]
lp17_B31_TS_MP_FG_consensus  AAAAAATAAAAACTAAAATATTTGGTGCCTAAATTAATAAGTTTATAGCAAATAAGAAAAATGAAAATCTAAAAAACA [ 6240]
lp17_B31_NX_Pl_consensus     AAAAAATAAAAACTAAAATATTTGGTGCCTAAATTAATAAGTTTATAGCAAATAAGAAAAATGAAAATCTAAAAAACA [ 6240]
lp17_PAli_NX_MP_FG_consensus AAAAAATAAAAACTAAAATATTTGGTGCCTAAATTAATAAGTTTATAGCAAATAAGAAAAATGAAAATCTAAAAAACA [ 6240]
lp17_PAli_NX_Pl_consensus    AAAAAATAAAAACTAAAATATTTGGTGCCTAAATTAATAAGTTTATAGCAAATAAGAAAAATGAAAATCTAAAAAACA [ 6240]
lp17_PAbe_NX_MP_FG_consensus AAAAAATAAAAACTAAAATATTTGGTGCCTAAATTAATAAGTTTATAGCAAATAAGAAAAATGAAAATCTAAAAAACA [ 6240]
lp17_PAbe_NX_Pl_consensus    AAAAAATAAAAACTAAAATATTTGGTGCCTAAATTAATAAGTTTATAGCAAATAAGAAAAATGAAAATCTAAAAAACA [ 6240]

lp17_gi|365823332|B31_GB     TAAATTAACTAAAAATTTGCATTGTGAATATAAAATTTTAACAATGCTTTAAGATTTAAATTACTATTTCTAAATCAA [ 6318]
lp17_un5_B31_PacBio          TAAATTAACTAAAAATTTGCATTGTGAATATAAAATTTTAACAATGCTTTAAGATTTAAATTACTATTTCTAAATCAA [ 6318]
lp17_B31_TS_MP_FG_consensus  TAAATTAACTAAAAATTTGCATTGTGAATATAAAATTTTAACAATGCTTTAAGATTTAAATTACTATTTCTAAATCAA [ 6318]
lp17_B31_NX_Pl_consensus     TAAATTAACTAAAAATTTGCATTGTGAATATAAAATTTTAACAATGCTTTAAGATTTAAATTACTATTTCTAAATCAA [ 6318]
lp17_PAli_NX_MP_FG_consensus TAAATTAACTAAAAATTTGCATTGTGAATATAAAATTTTAACAATGCTTTAAGATTTAAATTACTATTTCTAAATCAA [ 6318]
lp17_PAli_NX_Pl_consensus    TAAATTAACTAAAAATTTGCATTGTGAATATAAAATTTTAACAATGCTTTAAGATTTAAATTACTATTTCTAAATCAA [ 6318]
lp17_PAbe_NX_MP_FG_consensus TAAATTAACTAAAAATTTGCATTGTGAATATAAAATTTTAACAATGCTTTAAGATTTAAATTACTATTTCTAAATCAA [ 6318]
lp17_PAbe_NX_Pl_consensus    TAAATTAACTAAAAATTTGCATTGTGAATATAAAATTTTAACAATGCTTTAAGATTTAAATTACTATTTCTAAATCAA [ 6318]

lp17_gi|365823332|B31_GB     AGATTATAGAAACAAATCCTCGATTTATTTCATGGCAGCTTTCTAAACTATTTAAAAGAACAGTATTTACCAATACAT [ 6396]
lp17_un5_B31_PacBio          AGATTATAGAAACAAATCCTCGATTTATTTCATGGCAGCTTTCTAAACTATTTAAAAGAACAGTATTTACCAATACAT [ 6396]
lp17_B31_TS_MP_FG_consensus  AGATTATAGAAACAAATCCTCGATTTATTTCATGGCAGCTTTCTAAACTATTTAAAAGAACAGTATTTACCAATACAT [ 6396]
lp17_B31_NX_Pl_consensus     AGATTATAGAAACAAATCCTCGATTTATTTCATGGCAGCTTTCTAAACTATTTAAAAGAACAGTATTTACCAATACAT [ 6396]
lp17_PAli_NX_MP_FG_consensus AGATTATAGAAACAAATCCTCGATTTATTTCATGGCAGCTTTCTAAACTATTTAAAAGAACAGTATTTACCAATACAT [ 6396]
lp17_PAli_NX_Pl_consensus    AGATTATAGAAACAAATCCTCGATTTATTTCATGGCAGCTTTCTAAACTATTTAAAAGAACAGTATTTACCAATACAT [ 6396]
lp17_PAbe_NX_MP_FG_consensus AGATTATAGAAACAAATCCTCGATTTATTTCATGGCAGCTTTCTAAACTATTTAAAAGAACAGTATTTACCAATACAT [ 6396]
lp17_PAbe_NX_Pl_consensus    AGATTATAGAAACAAATCCTCGATTTATTTCATGGCAGCTTTCTAAACTATTTAAAAGAACAGTATTTACCAATACAT [ 6396]

lp17_gi|365823332|B31_GB     GATCTAGCCTTACTTAAGGCAGCTCCTGTCTTAGCTTTTATTTCTTTATAAATAAGACTTAAGATTTGGATTTGAATT [ 6474]
lp17_un5_B31_PacBio          GATCTAGCCTTACTTAAGGCAGCTCCTGTCTTAGCTTTTATTTCTTTATAAATAAGACTTAAGATTTGGATTTGAATT [ 6474]
lp17_B31_TS_MP_FG_consensus  GATCTAGCCTTACTTAAGGCAGCTCCTGTCTTAGCTTTTATTTCTTTATAAATAAGACTTAAGATTTGGATTTGAATT [ 6474]
lp17_B31_NX_Pl_consensus     GATCTAGCCTTACTTAAGGCAGCTCCTGTCTTAGCTTTTATTTCTTTATAAATAAGACTTAAGATTTGGATTTGAATT [ 6474]
lp17_PAli_NX_MP_FG_consensus GATCTAGCCTTACTTAAGGCAGCTCCTGTCTTAGCTTTTATTTCTTTATAAATAAGACTTAAGATTTGGATTTGAATT [ 6474]
lp17_PAli_NX_Pl_consensus    GATCTAGCCTTACTTAAGGCAGCTCCTGTCTTAGCTTTTATTTCTTTATAAATAAGACTTAAGATTTGGATTTGAATT [ 6474]
lp17_PAbe_NX_MP_FG_consensus GATCTAGCCTTACTTAAGGCAGCTCCTGTCTTAGCTTTTATTTCTTTATAAATAAGACTTAAGATTTGGATTTGAATT [ 6474]
lp17_PAbe_NX_Pl_consensus    GATCTAGCCTTACTTAAGGCAGCTCCTGTCTTAGCTTTTATTTCTTTATAAATAAGACTTAAGATTTGGATTTGAATT [ 6474]

lp17_gi|365823332|B31_GB     TAGATTTCTATCACTTAGAACTACAACTGCCAAAAGGCCAACTGGTTTCTCTTAATTTTAGAGTTGTATTTTTAATAC [ 6552]
lp17_un5_B31_PacBio          TAGATTTCTATCACTTAGAACTACAACTGCCAAAAGGCCAACTGGTTTCTCTTAATTTTAGAGTTGTATTTTTAATAC [ 6552]
lp17_B31_TS_MP_FG_consensus  TAGATTTCTATCACTTAGAACTACAACTGCCAAAAGGCCAACTGGTTTCTCTTAATTTTAGAGTTGTATTTTTAATAC [ 6552]
lp17_B31_NX_Pl_consensus     TAGATTTCTATCACTTAGAACTACAACTGCCAAAAGGCCAACTGGTTTCTCTTAATTTTAGAGTTGTATTTTTAATAC [ 6552]
lp17_PAli_NX_MP_FG_consensus TAGATTTCTATCACTTAGAACTACAACTGCCAAAAGGCCAACTGGTTTCTCTTAATTTTAGAGTTGTATTTTTAATAC [ 6552]
lp17_PAli_NX_Pl_consensus    TAGATTTCTATCACTTAGAACTACAACTGCCAAAAGGCCAACTGGTTTCTCTTAATTTTAGAGTTGTATTTTTAATAC [ 6552]
lp17_PAbe_NX_MP_FG_consensus TAGATTTCTATCACTTAGAACTACAACTGCCAAAAGGCCAACTGGTTTCTCTTAATTTTAGAGTTGTATTTTTAATAC [ 6552]
lp17_PAbe_NX_Pl_consensus    TAGATTTCTATCACTTAGAACTACAACTGCCAAAAGGCCAACTGGTTTCTCTTAATTTTAGAGTTGTATTTTTAATAC [ 6552]
```

```
lp17_gi|365823332|B31_GB     CATAATTACTACATAGTTTACTTAATAGGAAACATCTATCTGCTTTATGTAAATAAAATCCATAACAACTTAATTTAT [ 6630]
lp17_un5_B31_PacBio          CATAATTACTACATAGTTTACTTAATAGGAAACATCTATCTGCTTTATGTAAATAAAATCCATAACAACTTAATTTAT [ 6630]
lp17_B31_TS_MP_FG_consensus  CATAATTACTACATAGTTTACTTAATAGGAAACATCTATCTGCTTTATGTAAATAAAATCCATAACAACTTAATTTAT [ 6630]
lp17_B31_NX_Pl_consensus     CATAATTACTACATAGTTTACTTAATAGGAAACATCTATCTGCTTTATGTAAATAAAATCCATAACAACTTAATTTAT [ 6630]
lp17_PAli_NX_MP_FG_consensus CATAATTACTACATAGTTTACTTAATAGGAAACATCTATCTGCTTTATGTAAATAAAATCCATAACAACTTAATTTAT [ 6630]
lp17_PAli_NX_Pl_consensus    CATAATTACTACATAGTTTACTTAATAGGAAACATCTATCTGCTTTATGTAAATAAAATCCATAACAACTTAATTTAT [ 6630]
lp17_PAbe_NX_MP_FG_consensus CATAATTACTACATAGTTTACTTAATAGGAAACATCTATCTGCTTTATGTAAATAAAATCCATAACAACTTAATTTAT [ 6630]
lp17_PAbe_NX_Pl_consensus    CATAATTACTACATAGTTTACTTAATAGGAAACATCTATCTGCTTTATGTAAATAAAATCCATAACAACTTAATTTAT [ 6630]

lp17_gi|365823332|B31_GB     ATAATAATTATCTTGCGACTCTCTATACCACCATAAACCACCAATACTTTTCTTTTGCTATACAAAAATCTTACAGAT [ 6708]
lp17_un5_B31_PacBio          ATAATAATTATCTTGCGACTCTCTATACCACCATAAACCACCAATACTTTTCTTTTGCTATACAAAAATCTTACAGAT [ 6708]
lp17_B31_TS_MP_FG_consensus  ATAATAATTATCTTGCGACTCTCTATACCACCATAAACCACCAATACTTTTCTTTTGCTATACAAAAATCTTACAGAT [ 6708]
lp17_B31_NX_Pl_consensus     ATAATAATTATCTTGCGACTCTCTATACCACCATAAACCACCAATACTTTTCTTTTGCTATACAAAAATCTTACAGAT [ 6708]
lp17_PAli_NX_MP_FG_consensus ATAATAATTATCTTGCGACTCTCTATACCACCATAAACCACCAATACTTTTCTTTTGCTATACAAAAATCTTACAGAT [ 6708]
lp17_PAli_NX_Pl_consensus    ATAATAATTATCTTGCGACTCTCTATACCACCATAAACCACCAATACTTTTCTTTTGCTATACAAAAATCTTACAGAT [ 6708]
lp17_PAbe_NX_MP_FG_consensus ATAATAATTATCTTGCGACTCTCTATACCACCATAAACCACCAATACTTTTCTTTTGCTATACAAAAATCTTACAGAT [ 6708]
lp17_PAbe_NX_Pl_consensus    ATAATAATTATCTTGCGACTCTCTATACCACCATAAACCACCAATACTTTTCTTTTGCTATACAAAAATCTTACAGAT [ 6708]

lp17_gi|365823332|B31_GB     CTAAATACTTTTGAAAATTTCTTTTGGGTTTAGAATATCAATAATTAATTTTCAATTTTTATTTAAAAGAATGATAAT [ 6786]
lp17_un5_B31_PacBio          CTAAATACTTTTGAAAATTTCTTTTGGGTTTAGAATATCAATAATTAATTTTCAATTTTTATTTAAAAGAATGATAAT [ 6786]
lp17_B31_TS_MP_FG_consensus  CTAAATACTTTTGAAAATTTCTTTTGGGTTTAGAATATCAATAATTAATTTTCAATTTTTATTTAAAAGAATGATAAT [ 6786]
lp17_B31_NX_Pl_consensus     CTAAATACTTTTGAAAATTTCTTTTGGGTTTAGAATATCAATAATTAATTTTCAATTTTTATTTAAAAGAATGATAAT [ 6786]
lp17_PAli_NX_MP_FG_consensus CTAAATACTTTTGAAAATTTCTTTTGGGTTTAGAATATCAATAATTAATTTTCAATTTTTATTTAAAAGAATGATAAT [ 6786]
lp17_PAli_NX_Pl_consensus    CTAAATACTTTTGAAAATTTCTTTTGGGTTTAGAATATCAATAATTAATTTTCAATTTTTATTTAAAAGAATGATAAT [ 6786]
lp17_PAbe_NX_MP_FG_consensus CTAAATACTTTTGAAAATTTCTTTTGGGTTTAGAATATCAATAATTAATTTTCAATTTTTATTTAAAAGAATGATAAT [ 6786]
lp17_PAbe_NX_Pl_consensus    CTAAATACTTTTGAAAATTTCTTTTGGGTTTAGAATATCAATAATTAATTTTCAATTTTTATTTAAAAGAATGATAAT [ 6786]

lp17_gi|365823332|B31_GB     AATTTAATGCATTATTTAGGGTTTATGAAATAATATTTTTGCATTTCTTATTCGTTTATATTTCTTTATAAGAATATA [ 6864]
lp17_un5_B31_PacBio          AATTTAATGCATTATTTAGGGTTTATGAAATAATATTTTTGCATTTCTTATTCGTTTATATTTCTTTATAAGAATATA [ 6864]
lp17_B31_TS_MP_FG_consensus  AATTTAATGCATTATTTAGGGTTTATGAAATAATATTTTTGCATTTCTTATTCGTTTATATTTCTTTATAAGAATATA [ 6864]
lp17_B31_NX_Pl_consensus     AATTTAATGCATTATTTAGGGTTTATGAAATAATATTTTTGCATTTCTTATTCGTTTATATTTCTTTATAAGAATATA [ 6864]
lp17_PAli_NX_MP_FG_consensus AATTTAATGCATTATTTAGGGTTTATGAAATAATATTTTTGCATTTCTTATTCGTTTATATTTCTTTATAAGAATATA [ 6864]
lp17_PAli_NX_Pl_consensus    AATTTAATGCATTATTTAGGGTTTATGAAATAATATTTTTGCATTTCTTATTCGTTTATATTTCTTTATAAGAATATA [ 6864]
lp17_PAbe_NX_MP_FG_consensus AATTTAATGCATTATTTAGGGTTTATGAAATAATATTTTTGCATTTCTTATTCGTTTATATTTCTTTATAAGAATATA [ 6864]
lp17_PAbe_NX_Pl_consensus    AATTTAATGCATTATTTAGGGTTTATGAAATAATATTTTTGCATTTCTTATTCGTTTATATTTCTTTATAAGAATATA [ 6864]

lp17_gi|365823332|B31_GB     TAATTTTGTTCGAATTCTGCTTTAAAAAATGGTTAATTTAGAATATAATTCTAATGGCTAAAATGCTAAAAATGGATA [ 6942]
lp17_un5_B31_PacBio          TAATTTTGTTCGAATTCTGCTTTAAAAAATGGTTAATTTAGAATATAATTCTAATGGCTAAAATGCTAAAAATGGATA [ 6942]
lp17_B31_TS_MP_FG_consensus  TAATTTTGTTCGAATTCTGCTTTAAAAAATGGTTAATTTAGAATATAATTCTAATGGCTAAAATGCTAAAAATGGATA [ 6942]
lp17_B31_NX_Pl_consensus     TAATTTTGTTCGAATTCTGCTTTAAAAAATGGTTAATTTAGAATATAATTCTAATGGCTAAAATGCTAAAAATGGATA [ 6942]
lp17_PAli_NX_MP_FG_consensus TAATTTTGTTCGAATTCTGCTTTAAAAAATGGTTAATTTAGAATATAATTCTAATGGCTAAAATGCTAAAAATGGATA [ 6942]
lp17_PAli_NX_Pl_consensus    TAATTTTGTTCGAATTCTGCTTTAAAAAATGGTTAATTTAGAATATAATTCTAATGGCTAAAATGCTAAAAATGGATA [ 6942]
lp17_PAbe_NX_MP_FG_consensus TAATTTTGTTCGAATTCTGCTTTAAAAAATGGTTAATTTAGAATATAATTCTAATGGCTAAAATGCTAAAAATGGATA [ 6942]
lp17_PAbe_NX_Pl_consensus    TAATTTTGTTCGAATTCTGCTTTAAAAAATGGTTAATTTAGAATATAATTCTAATGGCTAAAATGCTAAAAATGGATA [ 6942]

lp17_gi|365823332|B31_GB     TAAATTTGGACTAATTTTATTATATTGAAAAACAAATTATCTGTATATACTACAATAATGCTTAACTTTAAATTTTTA [ 7020]
lp17_un5_B31_PacBio          TAAATTTGGACTAATTTTATTATATTGAAAAACAAATTATCTGTATATACTACAATAATGCTTAACTTTAAATTTTTA [ 7020]
lp17_B31_TS_MP_FG_consensus  TAAATTTGGACTAATTTTATTATATTGAAAAACAAATTATCTGTATATACTACAATAATGCTTAACTTTAAATTTTTA [ 7020]
lp17_B31_NX_Pl_consensus     TAAATTTGGACTAATTTTATTATATTGAAAAACAAATTATCTGTATATACTACAATAATGCTTAACTTTAAATTTTTA [ 7020]
lp17_PAli_NX_MP_FG_consensus TAAATTTGGACTAATTTTATTATATTGAAAAACAAATTATCTGTATATACTACAATAATGCTTAACTTTAAATTTTTA [ 7020]
lp17_PAli_NX_Pl_consensus    TAAATTTGGACTAATTTTATTATATTGAAAAACAAATTATCTGTATATACTACAATAATGCTTAACTTTAAATTTTTA [ 7020]
lp17_PAbe_NX_MP_FG_consensus TAAATTTGGACTAATTTTATTATATTGAAAAACAAATTATCTGTATATACTACAATAATGCTTAACTTTAAATTTTTA [ 7020]
lp17_PAbe_NX_Pl_consensus    TAAATTTGGACTAATTTTATTATATTGAAAAACAAATTATCTGTATATACTACAATAATGCTTAACTTTAAATTTTTA [ 7020]

lp17_gi|365823332|B31_GB     AAATGTGTTTACTTGTGCTTTATGGTTTTTGTAAGGTTAATTTTAATAATCAAATTTAGGGGAAAGAAATTTATGAAT [ 7098]
lp17_un5_B31_PacBio          AAATGTGTTTACTTGTGCTTTATGGTTTTTGTAAGGTTAATTTTAATAATCAAATTTAGGGGAAAGAAATTTATGAAT [ 7098]
lp17_B31_TS_MP_FG_consensus  AAATGTGTTTACTTGTGCTTTATGGTTTTTGTAAGGTTAATTTTAATAATCAAATTTAGGGGAAAGAAATTTATGAAT [ 7098]
lp17_B31_NX_Pl_consensus     AAATGTGTTTACTTGTGCTTTATGGTTTTTGTAAGGTTAATTTTAATAATCAAATTTAGGGGAAAGAAATTTATGAAT [ 7098]
lp17_PAli_NX_MP_FG_consensus AAATGTGTTTACTTGTGCTTTATGGTTTTTGTAAGGTTAATTTTAATAATCAAATTTAGGGGAAAGAAATTTATGAAT [ 7098]
lp17_PAli_NX_Pl_consensus    AAATGTGTTTACTTGTGCTTTATGGTTTTTGTAAGGTTAATTTTAATAATCAAATTTAGGGGAAAGAAATTTATGAAT [ 7098]
lp17_PAbe_NX_MP_FG_consensus AAATGTGTTTACTTGTGCTTTATGGTTTTTGTAAGGTTAATTTTAATAATCAAATTTAGGGGAAAGAAATTTATGAAT [ 7098]
lp17_PAbe_NX_Pl_consensus    AAATGTGTTTACTTGTGCTTTATGGTTTTTGTAAGGTTAATTTTAATAATCAAATTTAGGGGAAAGAAATTTATGAAT [ 7098]

lp17_gi|365823332|B31_GB     AGAAAATTTGTTATTTCATTATTATTTATAATATTAACTTTTTTATTAATATTGGGTTGTGATTTATCAATAAATAAC [ 7176]
lp17_un5_B31_PacBio          AGAAAATTTGTTATTTCATTATTATTTATAATATTAACTTTTTTATTAATATTGGGTTGTGATTTATCAATAAATAAC [ 7176]
lp17_B31_TS_MP_FG_consensus  AGAAAATTTGTTATTTCATTATTATTTATAATATTAACTTTTTTATTAATATTGGGTTGTGATTTATCAATAAATAAC [ 7176]
lp17_B31_NX_Pl_consensus     AGAAAATTTGTTATTTCATTATTATTTATAATATTAACTTTTTTATTAATATTGGGTTGTGATTTATCAATAAATAAC [ 7176]
lp17_PAli_NX_MP_FG_consensus AGAAAATTTGTTATTTCATTATTATTTATAATATTAACTTTTTTATTAATATTGGGTTGTGATTTATCAATAAATAAC [ 7176]
lp17_PAli_NX_Pl_consensus    AGAAAATTTGTTATTTCATTATTATTTATAATATTAACTTTTTTATTAATATTGGGTTGTGATTTATCAATAAATAAC [ 7176]
lp17_PAbe_NX_MP_FG_consensus AGAAAATTTGTTATTTCATTATTATTTATAATATTAACTTTTTTATTAATATTGGGTTGTGATTTATCAATAAATAAC [ 7176]
lp17_PAbe_NX_Pl_consensus    AGAAAATTTGTTATTTCATTATTATTTATAATATTAACTTTTTTATTAATATTGGGTTGTGATTTATCAATAAATAAC [ 7176]

lp17_gi|365823332|B31_GB     GATCGAAACAAGATAGATGGGGCTTCTCATTTTAAAAAGAAATATATGGATAATTTGAATTATCAATGTTTAAGCAAA [ 7254]
lp17_un5_B31_PacBio          GATCGAAACAAGATAGATGGGGCTTCTCATTTTAAAAAGAAATATATGGATAATTTGAATTATCAATGTTTAAGCAAA [ 7254]
lp17_B31_TS_MP_FG_consensus  GATCGAAACAAGATAGATGGGGCTTCTCATTTTAAAAAGAAATATATGGATAATTTGAATTATCAATGTTTAAGCAAA [ 7254]
lp17_B31_NX_Pl_consensus     GATCGAAACAAGATAGATGGGGCTTCTCATTTTAAAAAGAAATATATGGATAATTTGAATTATCAATGTTTAAGCAAA [ 7254]
lp17_PAli_NX_MP_FG_consensus GATCGAAACAAGATAGATGGGGCTTCTCATTTTAAAAAGAAATATATGGATAATTTGAATTATCAATGTTTAAGCAAA [ 7254]
lp17_PAli_NX_Pl_consensus    GATCGAAACAAGATAGATGGGGCTTCTCATTTTAAAAAGAAATATATGGATAATTTGAATTATCAATGTTTAAGCAAA [ 7254]
lp17_PAbe_NX_MP_FG_consensus GATCGAAACAAGATAGATGGGGCTTCTCATTTTAAAAAGAAATATATGGATAATTTGAATTATCAATGTTTAAGCAAA [ 7254]
lp17_PAbe_NX_Pl_consensus    GATCGAAACAAGATAGATGGGGCTTCTCATTTTAAAAAGAAATATATGGATAATTTGAATTATCAATGTTTAAGCAAA [ 7254]

lp17_gi|365823332|B31_GB     AAAGAGTCTGAGGCTAAAAATTCTCAAATTAAACTGGATGAGAATAATAATAAAAATCATTTTTATTCTTCCAGAGTA [ 7332]
lp17_un5_B31_PacBio          AAAGAGTCTGAGGCTAAAAATTCTCAAATTAAACTGGATGAGAATAATAATAAAAATCATTTTTATTCTTCCAGAGTA [ 7332]
lp17_B31_TS_MP_FG_consensus  AAAGAGTCTGAGGCTAAAAATTCTCAAATTAAACTGGATGAGAATAATAATAAAAATCATTTTTATTCTTCCAGAGTA [ 7332]
lp17_B31_NX_Pl_consensus     AAAGAGTCTGAGGCTAAAAATTCTCAAATTAAACTGGATGAGAATAATAATAAAAATCATTTTTATTCTTCCAGAGTA [ 7332]
lp17_PAli_NX_MP_FG_consensus AAAGAGTCTGAGGCTAAAAATTCTCAAATTAAACTGGATGAGAATAATAATAAAAATCATTTTTATTCTTCCAGAGTA [ 7332]
lp17_PAli_NX_Pl_consensus    AAAGAGTCTGAGGCTAAAAATTCTCAAATTAAACTGGATGAGAATAATAATAAAAATCATTTTTATTCTTCCAGAGTA [ 7332]
lp17_PAbe_NX_MP_FG_consensus AAAGAGTCTGAGGCTAAAAATTCTCAAATTAAACTGGATGAGAATAATAATAAAAATCATTTTTATTCTTCCAGAGTA [ 7332]
lp17_PAbe_NX_Pl_consensus    AAAGAGTCTGAGGCTAAAAATTCTCAAATTAAACTGGATGAGAATAATAATAAAAATCATTTTTATTCTTCCAGAGTA [ 7332]

lp17_gi|365823332|B31_GB     TCTAATGTTTCAAATTACTATGATAGAACTCATATATCTTGCAAAAAAAATGATTGATTTTTTTTGAAAAATAAAAGC [ 7410]
lp17_un5_B31_PacBio          TCTAATGTTTCAAATTACTATGATAGAACTCATATATCTTGCAAAAAAAATGATTGATTTTTTTTGAAAAATAAAAGC [ 7410]
lp17_B31_TS_MP_FG_consensus  TCTAATGTTTCAAATTACTATGATAGAACTCATATATCTTGCAAAAAAAATGATTGATTTTTTTTGAAAAATAAAAGC [ 7410]
lp17_B31_NX_Pl_consensus     TCTAATGTTTCAAATTACTATGATAGAACTCATATATCTTGCAAAAAAAATGATTGATTTTTTTTGAAAAATAAAAGC [ 7410]
lp17_PAli_NX_MP_FG_consensus TCTAATGTTTCAAATTACTATGATAGAACTCATATATCTTGCAAAAAAAATGATTGATTTTTTTTGAAAAATAAAAGC [ 7410]
lp17_PAli_NX_Pl_consensus    TCTAATGTTTCAAATTACTATGATAGAACTCATATATCTTGCAAAAAAAATGATTGATTTTTTTTGAAAAATAAAAGC [ 7410]
lp17_PAbe_NX_MP_FG_consensus TCTAATGTTTCAAATTACTATGATAGAACTCATATATCTTGCAAAAAAAATGATTGATTTTTTTTGAAAAATAAAAGC [ 7410]
lp17_PAbe_NX_Pl_consensus    TCTAATGTTTCAAATTACTATGATAGAACTCATATATCTTGCAAAAAAAATGATTGATTTTTTTTGAAAAATAAAAGC [ 7410]

lp17_gi|365823332|B31_GB     ATGCTTTGTTTACAATCTGATTTTAAAAAATTTTAGTTTAGTTGGTAAATATGAGTTTTTCAGTGTCTTTTTAATAGA [ 7488]
lp17_un5_B31_PacBio          ATGCTTTGTTTACAATCTGATTTTAAAAAATTTTAGTTTAGTTGGTAAATATGAGTTTTTCAGTGTCTTTTTAATAGA [ 7488]
lp17_B31_TS_MP_FG_consensus  ATGCTTTGTTTACAATCTGATTTTAAAAAATTTTAGTTTAGTTGGTAAATATGAGTTTTTCAGTGTCTTTTTAATAGA [ 7488]
lp17_B31_NX_Pl_consensus     ATGCTTTGTTTACAATCTGATTTTAAAAAATTTTAGTTTAGTTGGTAAATATGAGTTTTTCAGTGTCTTTTTAATAGA [ 7488]
lp17_PAli_NX_MP_FG_consensus ATGCTTTGTTTACAATCTGATTTTAAAAAATTTTAGTTTAGTTGGTAAATATGAGTTTTTCAGTGTCTTTTTAATAGA [ 7488]
lp17_PAli_NX_Pl_consensus    ATGCTTTGTTTACAATCTGATTTTAAAAAATTTTAGTTTAGTTGGTAAATATGAGTTTTTCAGTGTCTTTTTAATAGA [ 7488]
lp17_PAbe_NX_MP_FG_consensus ATGCTTTGTTTACAATCTGATTTTAAAAAATTTTAGTTTAGTTGGTAAATATGAGTTTTTCAGTGTCTTTTTAATAGA [ 7488]
lp17_PAbe_NX_Pl_consensus    ATGCTTTGTTTACAATCTGATTTTAAAAAATTTTAGTTTAGTTGGTAAATATGAGTTTTTCAGTGTCTTTTTAATAGA [ 7488]
```

```
lp17_gi|365823332|B31_GB     AATGCATATATATATATGAACTGTAAGAAAATATATTAAATGTTCCTAAAGTAAAAAAATTCTTAGAATAGGGCCTAT [ 7566]
lp17_un5_B31_PacBio          AATGCATATATATATATGAACTGTAAGAAAATATATTAAATGTTCCTAAAGTAAAAAAATTCTTAGAATAGGGCCTAT [ 7566]
lp17_B31_TS_MP_FG_consensus  AATGCATATATATATATGAACTGTAAGAAAATATATTAAATGTTCCTAAAGTAAAAAAATTCTTAGAATAGGGCCTAT [ 7566]
lp17_B31_NX_Pl_consensus     AATGCATATATATATATGAACTGTAAGAAAATATATTAAATGTTCCTAAAGTAAAAAAATTCTTAGAATAGGGCCTAT [ 7566]
lp17_PAli_NX_MP_FG_consensus AATGCATATATATATATGAACTGTAAGAAAATATATTAAATGTTCCTAAAGTAAAAAAATTCTTAGAATAGGGCCTAT [ 7566]
lp17_PAli_NX_Pl_consensus    AATGCATATATATATATGAACTGTAAGAAAATATATTAAATGTTCCTAAAGTAAAAAAATTCTTAGAATAGGGCCTAT [ 7566]
lp17_PAbe_NX_MP_FG_consensus AATGCATATATATATATGAACTGTAAGAAAATATATTAAATGTTCCTAAAGTAAAAAAATTCTTAGAATAGGGCCTAT [ 7566]
lp17_PAbe_NX_Pl_consensus    AATGCATATATATATATGAACTGTAAGAAAATATATTAAATGTTCCTAAAGTAAAAAAATTCTTAGAATAGGGCCTAT [ 7566]

lp17_gi|365823332|B31_GB     ATATAATAATACTTTTTATATATGTAAATAGCAATTAATTTTGATTAATTTTAATTTTGCTGATTCTTTTAAAACCTT [ 7644]
lp17_un5_B31_PacBio          ATATAATAATACTTTTTATATATGTAAATAGCAATTAATTTTGATTAATTTTAATTTTGCTGATTCTTTTAAAACCTT [ 7644]
lp17_B31_TS_MP_FG_consensus  ATATAATAATACTTTTTATATATGTAAATAGCAATTAATTTTGATTAATTTTAATTTTGCTGATTCTTTTAAAACCTT [ 7644]
lp17_B31_NX_Pl_consensus     ATATAATAATACTTTTTATATATGTAAATAGCAATTAATTTTGATTAATTTTAATTTTGCTGATTCTTTTAAAACCTT [ 7644]
lp17_PAli_NX_MP_FG_consensus ATATAATAATACTTTTTATATATGTAAATAGCAATTAATTTTGATTAATTTTAATTTTGCTGATTCTTTTAAAACCTT [ 7644]
lp17_PAli_NX_Pl_consensus    ATATAATAATACTTTTTATATATGTAAATAGCAATTAATTTTGATTAATTTTAATTTTGCTGATTCTTTTAAAACCTT [ 7644]
lp17_PAbe_NX_MP_FG_consensus ATATAATAATACTTTTTATATATGTAAATAGCAATTAATTTTGATTAATTTTAATTTTGCTGATTCTTTTAAAACCTT [ 7644]
lp17_PAbe_NX_Pl_consensus    ATATAATAATACTTTTTATATATGTAAATAGCAATTAATTTTGATTAATTTTAATTTTGCTGATTCTTTTAAAACCTT [ 7644]

lp17_gi|365823332|B31_GB     TACTGTTAGAAAAAAATAATACTTACTCTTTTTTCGTTATTAATAATCTTTTTATTTTCTTTTTCATAATTACTTCTAT [ 7722]
lp17_un5_B31_PacBio          TACTGTTAGAAAAAAATAATACTTACTCTTTTTTCGTTATTAATAATCTTTTTATTTTCTTTTTCATAATTACTTCTAT [ 7722]
lp17_B31_TS_MP_FG_consensus  TACTGTTAGAAAAAAATAATACTTACTCTTTTTTCGTTATTAATAATCTTTTTATTTTCTTTTTCATAATTACTTCTAT [ 7722]
lp17_B31_NX_Pl_consensus     TACTGTTAGAAAAAAATAATACTTACTCTTTTTTCGTTATTAATAATCTTTTTATTTTCTTTTTCATAATTACTTCTAT [ 7722]
lp17_PAli_NX_MP_FG_consensus TACTGTTAGAAAAAAATAATACTTACTCTTTTTTCGTTATTAATAATCTTTTTATTTTCTTTTTCATAATTACTTCTAT [ 7722]
lp17_PAli_NX_Pl_consensus    TACTGTTAGAAAAAAATAATACTTACTCTTTTTTCGTTATTAATAATCTTTTTATTTTCTTTTTCATAATTACTTCTAT [ 7722]
lp17_PAbe_NX_MP_FG_consensus TACTGTTAGAAAAAAATAATACTTACTCTTTTTTCGTTATTAATAATCTTTTTATTTTCTTTTTCATAATTACTTCTAT [ 7722]
lp17_PAbe_NX_Pl_consensus    TACTGTTAGAAAAAAATAATACTTACTCTTTTTTCGTTATTAATAATCTTTTTATTTTCTTTTTCATAATTACTTCTAT [ 7722]

lp17_gi|365823332|B31_GB     ATTGTTTTTTTAAATTTTTATTTAAAAACGTTTTTTTAAATCTTTTTTCCCAAATTTCATAAAAGTCATTTGTTCTGT [ 7800]
lp17_un5_B31_PacBio          ATTGTTTTTTTAAATTTTTATTTAAAAACGTTTTTTTAAATCTTTTTTCCCAAATTTCATAAAAGTCATTTGTTCTGT [ 7800]
lp17_B31_TS_MP_FG_consensus  ATTGTTTTTTTAAATTTTTATTTAAAAACGTTTTTTTAAATCTTTTTTCCCAAATTTCATAAAAGTCATTTGTTCTGT [ 7800]
lp17_B31_NX_Pl_consensus     ATTGTTTTTTTAAATTTTTATTTAAAAACGTTTTTTTAAATCTTTTTTCCCAAATTTCATAAAAGTCATTTGTTCTGT [ 7800]
lp17_PAli_NX_MP_FG_consensus ATTGTTTTTTTAAATTTTTATTTAAAAACGTTTTTTTAAATCTTTTTTCCCAAATTTCATAAAAGTCATTTGTTCTGT [ 7800]
lp17_PAli_NX_Pl_consensus    ATTGTTTTTTTAAATTTTTATTTAAAAACGTTTTTTTAAATCTTTTTTCCCAAATTTCATAAAAGTCATTTGTTCTGT [ 7800]
lp17_PAbe_NX_MP_FG_consensus ATTGTTTTTTTAAATTTTTATTTAAAAACGTTTTTTTAAATCTTTTTTCCCAAATTTCATAAAAGTCATTTGTTCTGT [ 7800]
lp17_PAbe_NX_Pl_consensus    ATTGTTTTTTTAAATTTTTATTTAAAAACGTTTTTTTAAATCTTTTTTCCCAAATTTCATAAAAGTCATTTGTTCTGT [ 7800]

lp17_gi|365823332|B31_GB     CAGTTTTTCCATTCATCATCCATATTTTTTTACTATACTTATTTTTAAACTCTTTTATAAAATGATTTGAAATGTCTT [ 7878]
lp17_un5_B31_PacBio          CAGTTTTTCCATTCATCATCCATATTTTTTTACTATACTTATTTTTAAACTCTTTTATAAAATGATTTGAAATGTCTT [ 7878]
lp17_B31_TS_MP_FG_consensus  CAGTTTTTCCATTCATCATCCATATTTTTTTACTATACTTATTTTTAAACTCTTTTATAAAATGATTTGAAATGTCTT [ 7878]
lp17_B31_NX_Pl_consensus     CAGTTTTTCCATTCATCATCCATATTTTTTTACTATACTTATTTTTAAACTCTTTTATAAAATGATTTGAAATGTCTT [ 7878]
lp17_PAli_NX_MP_FG_consensus CAGTTTTTCCATTCATCATCCATATTTTTTTACTATACTTATTTTTAAACTCTTTTATAAAATGATTTGAAATGTCTT [ 7878]
lp17_PAli_NX_Pl_consensus    CAGTTTTTCCATTCATCATCCATATTTTTTTACTATACTTATTTTTAAACTCTTTTATAAAATGATTTGAAATGTCTT [ 7878]
lp17_PAbe_NX_MP_FG_consensus CAGTTTTTCCATTCATCATCCATATTTTTTTACTATACTTATTTTTAAACTCTTTTATAAAATGATTTGAAATGTCTT [ 7878]
lp17_PAbe_NX_Pl_consensus    CAGTTTTTCCATTCATCATCCATATTTTTTTACTATACTTATTTTTAAACTCTTTTATAAAATGATTTGAAATGTCTT [ 7878]

lp17_gi|365823332|B31_GB     CAATTTTATATTCTTCCTTATATTTTTCTATTGCCAACTTTAAGTTTCTTAATGCATTTATATAGGTTTTTTCATTGT [ 7956]
lp17_un5_B31_PacBio          CAATTTTATATTCTTCCTTATATTTTTCTATTGCCAACTTTAAGTTTCTTAATGCATTTATATAGGTTTTTTCATTGT [ 7956]
lp17_B31_TS_MP_FG_consensus  CAATTTTATATTCTTCCTTATATTTTTCTATTGCCAACTTTAAGTTTCTTAATGCATTTATATAGGTTTTTTCATTGT [ 7956]
lp17_B31_NX_Pl_consensus     CAATTTTATATTCTTCCTTATATTTTTCTATTGCCAACTTTAAGTTTCTTAATGCATTTATATAGGTTTTTTCATTGT [ 7956]
lp17_PAli_NX_MP_FG_consensus CAATTTTATATTCTTCCTTATATTTTTCTATTGCCAACTTTAAGTTTCTTAATGCATTTATATAGGTTTTTTCATTGT [ 7956]
lp17_PAli_NX_Pl_consensus    CAATTTTATATTCTTCCTTATATTTTTCTATTGCCAACTTTAAGTTTCTTAATGCATTTATATAGGTTTTTTCATTGT [ 7956]
lp17_PAbe_NX_MP_FG_consensus CAATTTTATATTCTTCCTTATATTTTTCTATTGCCAACTTTAAGTTTCTTAATGCATTTATATAGGTTTTTTCATTGT [ 7956]
lp17_PAbe_NX_Pl_consensus    CAATTTTATATTCTTCCTTATATTTTTCTATTGCCAACTTTAAGTTTCTTAATGCATTTATATAGGTTTTTTCATTGT [ 7956]

lp17_gi|365823332|B31_GB     TGCTTATTTTAAGTATTTTTGTTATTTGCATTTTTTCTATTTTGTGAACTTCTACTAACTTGTTTGCAATATATTCTT [ 8034]
lp17_un5_B31_PacBio          TGCTTATTTTAAGTATTTTTGTTATTTGCATTTTTTCTATTTTGTGAACTTCTACTAACTTGTTTGCAATATATTCTT [ 8034]
lp17_B31_TS_MP_FG_consensus  TGCTTATTTTAAGTATTTTTGTTATTTGCATTTTTTCTATTTTGTGAACTTCTACTAACTTGTTTGCAATATATTCTT [ 8034]
lp17_B31_NX_Pl_consensus     TGCTTATTTTAAGTATTTTTGTTATTTGCATTTTTTCTATTTTGTGAACTTCTACTAACTTGTTTGCAATATATTCTT [ 8034]
lp17_PAli_NX_MP_FG_consensus TGCTTATTTTAAGTATTTTTGTTATTTGCATTTTTTCTATTTTGTGAACTTCTACTAACTTGTTTGCAATATATTCTT [ 8034]
lp17_PAli_NX_Pl_consensus    TGCTTATTTTAAGTATTTTTGTTATTTGCATTTTTTCTATTTTGTGAACTTCTACTAACTTGTTTGCAATATATTCTT [ 8034]
lp17_PAbe_NX_MP_FG_consensus TGCTTATTTTAAGTATTTTTGTTATTTGCATTTTTTCTATTTTGTGAACTTCTACTAACTTGTTTGCAATATATTCTT [ 8034]
lp17_PAbe_NX_Pl_consensus    TGCTTATTTTAAGTATTTTTGTTATTTGCATTTTTTCTATTTTGTGAACTTCTACTAACTTGTTTGCAATATATTCTT [ 8034]

lp17_gi|365823332|B31_GB     TGTAAGAAATTTTATTTATTTTTCCATTATTTTTTTCTATAGAATTCTCTATCTTATTTATACCTTTTATACCTTTAG [ 8112]
lp17_un5_B31_PacBio          TGTAAGAAATTTTATTTATTTTTCCATTATTTTTTTCTATAGAATTCTCTATCTTATTTATACCTTTTATACCTTTAG [ 8112]
lp17_B31_TS_MP_FG_consensus  TGTAAGAAATTTTATTTATTTTTCCATTATTTTTTTCTATAGAATTCTCTATCTTATTTATACCTTTTATACCTTTAG [ 8112]
lp17_B31_NX_Pl_consensus     TGTAAGAAATTTTATTTATTTTTCCATTATTTTTTTCTATAGAATTCTCTATCTTATTTATACCTTTTATACCTTTAG [ 8112]
lp17_PAli_NX_MP_FG_consensus TGTAAGAAATTTTATTTATTTTTCCATTATTTTTTTCTATAGAATTCTCTATCTTATTTATACCTTTTATACCTTTAG [ 8112]
lp17_PAli_NX_Pl_consensus    TGTAAGAAATTTTATTTATTTTTCCATTATTTTTTTCTATAGAATTCTCTATCTTATTTATACCTTTTATACCTTTAG [ 8112]
lp17_PAbe_NX_MP_FG_consensus TGTAAGAAATTTTATTTATTTTTCCATTATTTTTTTCTATAGAATTCTCTATCTTATTTATACCTTTTATACCTTTAG [ 8112]
lp17_PAbe_NX_Pl_consensus    TGTAAGAAATTTTATTTATTTTTCCATTATTTTTTTCTATAGAATTCTCTATCTTATTTATACCTTTTATACCTTTAG [ 8112]

lp17_gi|365823332|B31_GB     GTATAACATCCGCAATTGATTCATCTGCAATTGAGCTTTTAGGTTTTATATTTCTTATATTATTCTTGTTTATAGTAT [ 8190]
lp17_un5_B31_PacBio          GTATAACATCCGCAATTGATTCATCTGCAATTGAGCTTTTAGGTTTTATATTTCTTATATTATTCTTGTTTATAGTAT [ 8190]
lp17_B31_TS_MP_FG_consensus  GTATAACATCCGCAATTGATTCATCTGCAATTGAGCTTTTAGGTTTTATATTTCTTATATTATTCTTGTTTATAGTAT [ 8190]
lp17_B31_NX_Pl_consensus     GTATAACATCCGCAATTGATTCATCTGCAATTGAGCTTTTAGGTTTTATATTTCTTATATTATTCTTGTTTATAGTAT [ 8190]
lp17_PAli_NX_MP_FG_consensus GTATAACATCCGCAATTGATTCATCTGCAATTGAGCTTTTAGGTTTTATATTTCTTATATTATTCTTGTTTATAGTAT [ 8190]
lp17_PAli_NX_Pl_consensus    GTATAACATCCGCAATTGATTCATCTGCAATTGAGCTTTTAGGTTTTATATTTCTTATATTATTCTTGTTTATAGTAT [ 8190]
lp17_PAbe_NX_MP_FG_consensus GTATAACATCCGCAATTGATTCATCTGCAATTGAGCTTTTAGGTTTTATATTTCTTATATTATTCTTGTTTATAGTAT [ 8190]
lp17_PAbe_NX_Pl_consensus    GTATAACATCCGCAATTGATTCATCTGCAATTGAGCTTTTAGGTTTTATATTTCTTATATTATTCTTGTTTATAGTAT [ 8190]

lp17_gi|365823332|B31_GB     TGTTGATTTCCTTGAAAAAATCAGATACTATTTTTTTATCTTTTAAATATTCTTTTATTTCTTTATTAATTGCTTCCT [ 8268]
lp17_un5_B31_PacBio          TGTTGATTTCCTTGAAAAAATCAGATACTATTTTTTTATCTTTTAAATATTCTTTTATTTCTTTATTAATTGCTTCCT [ 8268]
lp17_B31_TS_MP_FG_consensus  TGTTGATTTCCTTGAAAAAATCAGATACTATTTTTTTATCTTTTAAATATTCTTTTATTTCTTTATTAATTGCTTCCT [ 8268]
lp17_B31_NX_Pl_consensus     TGTTGATTTCCTTGAAAAAATCAGATACTATTTTTTTATCTTTTAAATATTCTTTTATTTCTTTATTAATTGCTTCCT [ 8268]
lp17_PAli_NX_MP_FG_consensus TGTTGATTTCCTTGAAAAAATCAGATACTATTTTTTTATCTTTTAAATATTCTTTTATTTCTTTATTAATTGCTTCCT [ 8268]
lp17_PAli_NX_Pl_consensus    TGTTGATTTCCTTGAAAAAATCAGATACTATTTTTTTATCTTTTAAATATTCTTTTATTTCTTTATTAATTGCTTCCT [ 8268]
lp17_PAbe_NX_MP_FG_consensus TGTTGATTTCCTTGAAAAAATCAGATACTATTTTTTTATCTTTTAAATATTCTTTTATTTCTTTATTAATTGCTTCCT [ 8268]
lp17_PAbe_NX_Pl_consensus    TGTTGATTTCCTTGAAAAAATCAGATACTATTTTTTTATCTTTTAAATATTCTTTTATTTCTTTATTAATTGCTTCCT [ 8268]

lp17_gi|365823332|B31_GB     GAATAATTATAAAGCGGTGTTTCCAAAGATTTTTGTTTTGTATATAAAATGCAAAGCTTCCGTTATCTTTGCCTAAAT [ 8346]
lp17_un5_B31_PacBio          GAATAATTATAAAGCGGTGTTTCCAAAGATTTTTGTTTTGTATATAAAATGCAAAGCTTCCGTTATCTTTGCCTAAAT [ 8346]
lp17_B31_TS_MP_FG_consensus  GAATAATTATAAAGCGGTGTTTCCAAAGATTTTTGTTTTGTATATAAAATGCAAAGCTTCCGTTATCTTTGCCTAAAT [ 8346]
lp17_B31_NX_Pl_consensus     GAATAATTATAAAGCGGTGTTTCCAAAGATTTTTGTTTTGTATATAAAATGCAAAGCTTCCGTTATCTTTGCCTAAAT [ 8346]
lp17_PAli_NX_MP_FG_consensus GAATAATTATAAAGCGGTGTTTCCAAAGATTTTTGTTTTGTATATAAAATGCAAAGCTTCCGTTATCTTTGCCTAAAT [ 8346]
lp17_PAli_NX_Pl_consensus    GAATAATTATAAAGCGGTGTTTCCAAAGATTTTTGTTTTGTATATAAAATGCAAAGCTTCCGTTATCTTTGCCTAAAT [ 8346]
lp17_PAbe_NX_MP_FG_consensus GAATAATTATAAAGCGGTGTTTCCAAAGATTTTTGTTTTGTATATAAAATGCAAAGCTTCCGTTATCTTTGCCTAAAT [ 8346]
lp17_PAbe_NX_Pl_consensus    GAATAATTATAAAGCGGTGTTTCCAAAGATTTTTGTTTTGTATATAAAATGCAAAGCTTCCGTTATCTTTGCCTAAAT [ 8346]

lp17_gi|365823332|B31_GB     GTTTTAGTTTGGTTTTTATTAAGAGTATTTGATTTAAAAATGATATATCATATTCTAAGGTCCTGATTGATATTTTTT [ 8424]
lp17_un5_B31_PacBio          GTTTTAGTTTGGTTTTTATTAAGAGTATTTGATTTAAAAATGATATATCATATTCTAAGGTCCTGATTGATATTTTTT [ 8424]
lp17_B31_TS_MP_FG_consensus  GTTTTAGTTTGGTTTTTATTAAGAGTATTTGATTTAAAAATGATATATCATATTCTAAGGTCCTGATTGATATTTTTT [ 8424]
lp17_B31_NX_Pl_consensus     GTTTTAGTTTGGTTTTTATTAAGAGTATTTGATTTAAAAATGATATATCATATTCTAAGGTCCTGATTGATATTTTTT [ 8424]
lp17_PAli_NX_MP_FG_consensus GTTTTAGTTTGGTTTTTATTAAGAGTATTTGATTTAAAAATGATATATCATATTCTAAGGTCCTGATTGATATTTTTT [ 8424]
lp17_PAli_NX_Pl_consensus    GTTTTAGTTTGGTTTTTATTAAGAGTATTTGATTTAAAAATGATATATCATATTCTAAGGTCCTGATTGATATTTTTT [ 8424]
lp17_PAbe_NX_MP_FG_consensus GTTTTAGTTTGGTTTTTATTAAGAGTATTTGATTTAAAAATGATATATCATATTCTAAGGTCCTGATTGATATTTTTT [ 8424]
lp17_PAbe_NX_Pl_consensus    GTTTTAGTTTGGTTTTTATTAAGAGTATTTGATTTAAAAATGATATATCATATTCTAAGGTCCTGATTGATATTTTTT [ 8424]

lp17_gi|365823332|B31_GB     TATTATCTTTTGCAAGGCATTTATTAACTATATTATGTATTTCTATTGTGGAATATGTTTTAAGTTTGTTAGATTTAT [ 8502]
```

```
lp17_un5_B31_PacBio          TATTATCTTTTGCAAGGCATTTATTAACTATATTATGTATTTCTATTGTGGAATATGTTTTAAGTTTGTTAGATTTAT [ 8502]
lp17_B31_TS_MP_FG_consensus  TATTATCTTTTGCAAGGCATTTATTAACTATATTATGTATTTCTATTGTGGAATATGTTTTAAGTTTGTTAGATTTAT [ 8502]
lp17_B31_NX_Pl_consensus     TATTATCTTTTGCAAGGCATTTATTAACTATATTATGTATTTCTATTGTGGAATATGTTTTAAGTTTGTTAGATTTAT [ 8502]
lp17_PAli_NX_MP_FG_consensus TATTATCTTTTGCAAGGCATTTATTAACTATATTATGTATTTCTATTGTGGAATATGTTTTAAGTTTGTTAGATTTAT [ 8502]
lp17_PAli_NX_Pl_consensus    TATTATCTTTTGCAAGGCATTTATTAACTATATTATGTATTTCTATTGTGGAATATGTTTTAAGTTTGTTAGATTTAT [ 8502]
lp17_PAbe_NX_MP_FG_consensus TATTATCTTTTGCAAGGCATTTATTAACTATATTATGTATTTCTATTGTGGAATATGTTTTAAGTTTGTTAGATTTAT [ 8502]
lp17_PAbe_NX_Pl_consensus    TATTATCTTTTGCAAGGCATTTATTAACTATATTATGTATTTCTATTGTGGAATATGTTTTAAGTTTGTTAGATTTAT [ 8502]

lp17_gi|365823332|B31_GB     AGTATTGTTTGTTTTTAAGGTCAATTGCCCAGCATACTTTGAGTATTCGTTGGTATATGGCTCTTTTTTTAGTTAAAT [ 8580]
lp17_un5_B31_PacBio          AGTATTGTTTGTTTTTAAGGTCAATTGCCCAGCATACTTTGAGTATTCGTTGGTATATGGCTCTTTTTTTAGTTAAAT [ 8580]
lp17_B31_TS_MP_FG_consensus  AGTATTGTTTGTTTTTAAGGTCAATTGCCCAGCATACTTTGAGTATTCGTTGGTATATGGCTCTTTTTTTAGTTAAAT [ 8580]
lp17_B31_NX_Pl_consensus     AGTATTGTTTGTTTTTAAGGTCAATTGCCCAGCATACTTTGAGTATTCGTTGGTATATGGCTCTTTTTTTAGTTAAAT [ 8580]
lp17_PAli_NX_MP_FG_consensus AGTATTGTTTGTTTTTAAGGTCAATTGCCCAGCATACTTTGAGTATTCGTTGGTATATGGCTCTTTTTTTAGTTAAAT [ 8580]
lp17_PAli_NX_Pl_consensus    AGTATTGTTTGTTTTTAAGGTCAATTGCCCAGCATACTTTGAGTATTCGTTGGTATATGGCTCTTTTTTTAGTTAAAT [ 8580]
lp17_PAbe_NX_MP_FG_consensus AGTATTGTTTGTTTTTAAGGTCAATTGCCCAGCATACTTTGAGTATTCGTTGGTATATGGCTCTTTTTTTAGTTAAAT [ 8580]
lp17_PAbe_NX_Pl_consensus    AGTATTGTTTGTTTTTAAGGTCAATTGCCCAGCATACTTTGAGTATTCGTTGGTATATGGCTCTTTTTTTAGTTAAAT [ 8580]

lp17_gi|365823332|B31_GB     ATGATTTTAGCCTTGAGCGTTTTAATTCTATTAAATTAGAGCTTGCGTTTTTTTGATTGTACTTTATAATTTCTTCTA [ 8658]
lp17_un5_B31_PacBio          ATGATTTTAGCCTTGAGCGTTTTAATTCTATTAAATTAGAGCTTGCGTTTTTTTGATTGTACTTTATAATTTCTTCTA [ 8658]
lp17_B31_TS_MP_FG_consensus  ATGATTTTAGCCTTGAGCGTTTTAATTCTATTAAATTAGAGCTTGCGTTTTTTTGATTGTACTTTATAATTTCTTCTA [ 8658]
lp17_B31_NX_Pl_consensus     ATGATTTTAGCCTTGAGCGTTTTAATTCTATTAAATTAGAGCTTGCGTTTTTTTGATTGTACTTTATAATTTCTTCTA [ 8658]
lp17_PAli_NX_MP_FG_consensus ATGATTTTAGCCTTGAGCGTTTTAATTCTATTAAATTAGAGCTTGCGTTTTTTTGATTGTACTTTATAATTTCTTCTA [ 8658]
lp17_PAli_NX_Pl_consensus    ATGATTTTAGCCTTGAGCGTTTTAATTCTATTAAATTAGAGCTTGCGTTTTTTTGATTGTACTTTATAATTTCTTCTA [ 8658]
lp17_PAbe_NX_MP_FG_consensus ATGATTTTAGCCTTGAGCGTTTTAATTCTATTAAATTAGAGCTTGCGTTTTTTTGATTGTACTTTATAATTTCTTCTA [ 8658]
lp17_PAbe_NX_Pl_consensus    ATGATTTTAGCCTTGAGCGTTTTAATTCTATTAAATTAGAGCTTGCGTTTTTTTGATTGTACTTTATAATTTCTTCTA [ 8658]

lp17_gi|365823332|B31_GB     ATGTTATGAGATTATTAAAATTTGTATTGACATTATTTTTTATTTTTATTATCATTATCTTATCCTGGCTTCTGAAGT [ 8736]
lp17_un5_B31_PacBio          ATGTTATGAGATTATTAAAATTTGTATTGACATTATTTTTTATTTTTATTATCATTATCTTATCCTGGCTTCTGAAGT [ 8736]
lp17_B31_TS_MP_FG_consensus  ATGTTATGAGATTATTAAAATTTGTATTGACATTATTTTTTATTTTTATTATCATTATCTTATCCTGGCTTCTGAAGT [ 8736]
lp17_B31_NX_Pl_consensus     ATGTTATGAGATTATTAAAATTTGTATTGACATTATTTTTTATTTTTATTATCATTATCTTATCCTGGCTTCTGAAGT [ 8736]
lp17_PAli_NX_MP_FG_consensus ATGTTATGAGATTATTAAAATTTGTATTGACATTATTTTTTATTTTTATTATCATTATCTTATCCTGGCTTCTGAAGT [ 8736]
lp17_PAli_NX_Pl_consensus    ATGTTATGAGATTATTAAAATTTGTATTGACATTATTTTTTATTTTTATTATCATTATCTTATCCTGGCTTCTGAAGT [ 8736]
lp17_PAbe_NX_MP_FG_consensus ATGTTATGAGATTATTAAAATTTGTATTGACATTATTTTTTATTTTTATTATCATTATCTTATCCTGGCTTCTGAAGT [ 8736]
lp17_PAbe_NX_Pl_consensus    ATGTTATGAGATTATTAAAATTTGTATTGACATTATTTTTTATTTTTATTATCATTATCTTATCCTGGCTTCTGAAGT [ 8736]

lp17_gi|365823332|B31_GB     TAAATTTGTTGTGGTTTCAGAAGCTTTTTCAATTATTTGGCTTGCATTCTAAATTCTTACTGCATCACAAACGAAATA [ 8814]
lp17_un5_B31_PacBio          TAAATTTGTTGTGGTTTCAGAAGCTTTTTCAATTATTTGGCTTGCATTCTAAATTCTTACTGCATCACAAACGAAATA [ 8814]
lp17_B31_TS_MP_FG_consensus  TAAATTTGTTGTGGTTTCAGAAGCTTTTTCAATTATTTGGCTTGCATTCTAAATTCTTACTGCATCACAAACGAAATA [ 8814]
lp17_B31_NX_Pl_consensus     TAAATTTGTTGTGGTTTCAGAAGCTTTTTCAATTATTTGGCTTGCATTCTAAATTCTTACTGCATCACAAACGAAATA [ 8814]
lp17_PAli_NX_MP_FG_consensus TAAATTTGTTGTGGTTTCAGAAGCTTTTTCAATTATTTGGCTTGCATTCTAAATTCTTACTGCATCACAAACGAAATA [ 8814]
lp17_PAli_NX_Pl_consensus    TAAATTTGTTGTGGTTTCAGAAGCTTTTTCAATTATTTGGCTTGCATTCTAAATTCTTACTGCATCACAAACGAAATA [ 8814]
lp17_PAbe_NX_MP_FG_consensus TAAATTTGTTGTGGTTTCAGAAGCTTTTTCAATTATTTGGCTTGCATTCTAAATTCTTACTGCATCACAAACGAAATA [ 8814]
lp17_PAbe_NX_Pl_consensus    TAAATTTGTTGTGGTTTCAGAAGCTTTTTCAATTATTTGGCTTGCATTCTAAATTCTTACTGCATCACAAACGAAATA [ 8814]

lp17_gi|365823332|B31_GB     ATTAGCTTTTTACTATATTCTATAGCAATGTTTTTAAAATGTCAATATGATAGATTATAAAATATGACCCATCTTTTT [ 8892]
lp17_un5_B31_PacBio          ATTAGCTTTTTACTATATTCTATAGCAATGTTTTTAAAATGTCAATATGATAGATTATAAAATATGACCCATCTTTTT [ 8892]
lp17_B31_TS_MP_FG_consensus  ATTAGCTTTTTACTATATTCTATAGCAATGTTTTTAAAATGTCAATATGATAGATTATAAAATATGACCCATCTTTTT [ 8892]
lp17_B31_NX_Pl_consensus     ATTAGCTTTTTACTATATTCTATAGCAATGTTTTTAAAATGTCAATATGATAGATTATAAAATATGACCCATCTTTTT [ 8892]
lp17_PAli_NX_MP_FG_consensus ATTAGCTTTTTACTATATTCTATAGCAATGTTTTTAAAATGTCAATATGATAGATTATAAAATATGACCCATCTTTTT [ 8892]
lp17_PAli_NX_Pl_consensus    ATTAGCTTTTTACTATATTCTATAGCAATGTTTTTAAAATGTCAATATGATAGATTATAAAATATGACCCATCTTTTT [ 8892]
lp17_PAbe_NX_MP_FG_consensus ATTAGCTTTTTACTATATTCTATAGCAATGTTTTTAAAATGTCAATATGATAGATTATAAAATATGACCCATCTTTTT [ 8892]
lp17_PAbe_NX_Pl_consensus    ATTAGCTTTTTACTATATTCTATAGCAATGTTTTTAAAATGTCAATATGATAGATTATAAAATATGACCCATCTTTTT [ 8892]

lp17_gi|365823332|B31_GB     TGAAGCAATTTGTATTTCGTGTTTTGTAGATTTTCGGCTCATTATTTTTTTTGCTTGTTCAATTCTTAATTGAATTGT [ 8970]
lp17_un5_B31_PacBio          TGAAGCAATTTGTATTTCGTGTTTTGTAGATTTTCGGCTCATTATTTTTTTTGCTTGTTCAATTCTTAATTGAATTGT [ 8970]
lp17_B31_TS_MP_FG_consensus  TGAAGCAATTTGTATTTCGTGTTTTGTAGATTTTCGGCTCATTATTTTTTTTGCTTGTTCAATTCTTAATTGAATTGT [ 8970]
lp17_B31_NX_Pl_consensus     TGAAGCAATTTGTATTTCGTGTTTTGTAGATTTTCGGCTCATTATTTTTTTTGCTTGTTCAATTCTTAATTGAATTGT [ 8970]
lp17_PAli_NX_MP_FG_consensus TGAAGCAATTTGTATTTCGTGTTTTGTAGATTTTCGGCTCATTATTTTTTTTGCTTGTTCAATTCTTAATTGAATTGT [ 8970]
lp17_PAli_NX_Pl_consensus    TGAAGCAATTTGTATTTCGTGTTTTGTAGATTTTCGGCTCATTATTTTTTTTGCTTGTTCAATTCTTAATTGAATTGT [ 8970]
lp17_PAbe_NX_MP_FG_consensus TGAAGCAATTTGTATTTCGTGTTTTGTAGATTTTCGGCTCATTATTTTTTTTGCTTGTTCAATTCTTAATTGAATTGT [ 8970]
lp17_PAbe_NX_Pl_consensus    TGAAGCAATTTGTATTTCGTGTTTTGTAGATTTTCGGCTCATTATTTTTTTTGCTTGTTCAATTCTTAATTGAATTGT [ 8970]

lp17_gi|365823332|B31_GB     TTCTTTCATGCTATTTGTAATAGGTTTGGGTCTTCTAAAGTCCATTAGAAAATATGTGCAAAAATCTTCTAAAAGGTA [ 9048]
lp17_un5_B31_PacBio          TTCTTTCATGCTATTTGTAATAGGTTTGGGTCTTCTAAAGTCCATTAGAAAATATGTGCAAAAATCTTCTAAAAGGTA [ 9048]
lp17_B31_TS_MP_FG_consensus  TTCTTTCATGCTATTTGTAATAGGTTTGGGTCTTCTAAAGTCCATTAGAAAATATGTGCAAAAATCTTCTAAAAGGTA [ 9048]
lp17_B31_NX_Pl_consensus     TTCTTTCATGCTATTTGTAATAGGTTTGGGTCTTCTAAAGTCCATTAGAAAATATGTGCAAAAATCTTCTAAAAGGTA [ 9048]
lp17_PAli_NX_MP_FG_consensus TTCTTTCATGCTATTTGTAATAGGTTTGGGTCTTCTAAAGTCCATTAGAAAATATGTGCAAAAATCTTCTAAAAGGTA [ 9048]
lp17_PAli_NX_Pl_consensus    TTCTTTCATGCTATTTGTAATAGGTTTGGGTCTTCTAAAGTCCATTAGAAAATATGTGCAAAAATCTTCTAAAAGGTA [ 9048]
lp17_PAbe_NX_MP_FG_consensus TTCTTTCATGCTATTTGTAATAGGTTTGGGTCTTCTAAAGTCCATTAGAAAATATGTGCAAAAATCTTCTAAAAGGTA [ 9048]
lp17_PAbe_NX_Pl_consensus    TTCTTTCATGCTATTTGTAATAGGTTTGGGTCTTCTAAAGTCCATTAGAAAATATGTGCAAAAATCTTCTAAAAGGTA [ 9048]

lp17_gi|365823332|B31_GB     AGGACGGTTGTCTACATGGATTGCGCATTTTAAAATTTTTGATTTTTTAGATATAGTATTTTTGCTAATTGAAAGATT [ 9126]
lp17_un5_B31_PacBio          AGGACGGTTGTCTACATGGATTGCGCATTTTAAAATTTTTGATTTTTTAGATATAGTATTTTTGCTAATTGAAAGATT [ 9126]
lp17_B31_TS_MP_FG_consensus  AGGACGGTTGTCTACATGGATTGCGCATTTTAAAATTTTTGATTTTTTAGATATAGTATTTTTGCTAATTGAAAGATT [ 9126]
lp17_B31_NX_Pl_consensus     AGGACGGTTGTCTACATGGATTGCGCATTTTAAAATTTTTGATTTTTTAGATATAGTATTTTTGCTAATTGAAAGATT [ 9126]
lp17_PAli_NX_MP_FG_consensus AGGACGGTTGTCTACATGGATTGCGCATTTTAAAATTTTTGATTTTTTAGATATAGTATTTTTGCTAATTGAAAGATT [ 9126]
lp17_PAli_NX_Pl_consensus    AGGACGGTTGTCTACATGGATTGCGCATTTTAAAATTTTTGATTTTTTAGATATAGTATTTTTGCTAATTGAAAGATT [ 9126]
lp17_PAbe_NX_MP_FG_consensus AGGACGGTTGTCTACATGGATTGCGCATTTTAAAATTTTTGATTTTTTAGATATAGTATTTTTGCTAATTGAAAGATT [ 9126]
lp17_PAbe_NX_Pl_consensus    AGGACGGTTGTCTACATGGATTGCGCATTTTAAAATTTTTGATTTTTTAGATATAGTATTTTTGCTAATTGAAAGATT [ 9126]

lp17_gi|365823332|B31_GB     ATAAGAATCTTCTAATATATTTTGGAATTCTTTTAAAGAATAAAGTCTTACACCGTTAATACTTAACATAAATACCTC [ 9204]
lp17_un5_B31_PacBio          ATAAGAATCTTCTAATATATTTTGGAATTCTTTTAAAGAATAAAGTCTTACACCGTTAATACTTAACATAAATACCTC [ 9204]
lp17_B31_TS_MP_FG_consensus  ATAAGAATCTTCTAATATATTTTGGAATTCTTTTAAAGAATAAAGTCTTACACCGTTAATACTTAACATAAATACCTC [ 9204]
lp17_B31_NX_Pl_consensus     ATAAGAATCTTCTAATATATTTTGGAATTCTTTTAAAGAATAAAGTCTTACACCGTTAATACTTAACATAAATACCTC [ 9204]
lp17_PAli_NX_MP_FG_consensus ATAAGAATCTTCTAATATATTTTGGAATTCTTTTAAAGAATAAAGTCTTACACCGTTAATACTTAACATAAATACCTC [ 9204]
lp17_PAli_NX_Pl_consensus    ATAAGAATCTTCTAATATATTTTGGAATTCTTTTAAAGAATAAAGTCTTACACCGTTAATACTTAACATAAATACCTC [ 9204]
lp17_PAbe_NX_MP_FG_consensus ATAAGAATCTTCTAATATATTTTGGAATTCTTTTAAAGAATAAAGTCTTACACCGTTAATACTTAACATAAATACCTC [ 9204]
lp17_PAbe_NX_Pl_consensus    ATAAGAATCTTCTAATATATTTTGGAATTCTTTTAAAGAATAAAGTCTTACACCGTTAATACTTAACATAAATACCTC [ 9204]

lp17_gi|365823332|B31_GB     ATAATTTCATTTTATTATATATTGCATGTTAGTTTCAAGGATGTTAGGTTATATGTTGAATACTTTTGTTAGGGTGTT [ 9282]
lp17_un5_B31_PacBio          ATAATTTCATTTTATTATATATTGCATGTTAGTTTCAAGGATGTTAGGTTATATGTTGAATACTTTTGTTAGGGTGTT [ 9282]
lp17_B31_TS_MP_FG_consensus  ATAATTTCATTTTATTATATATTGCATGTTAGTTTCAAGGATGTTAGGTTATATGTTGAATACTTTTGTTAGGGTGTT [ 9282]
lp17_B31_NX_Pl_consensus     ATAATTTCATTTTATTATATATTGCATGTTAGTTTCAAGGATGTTAGGTTATATGTTGAATACTTTTGTTAGGGTGTT [ 9282]
lp17_PAli_NX_MP_FG_consensus ATAATTTCATTTTATTATATATTGCATGTTAGTTTCAAGGATGTTAGGTTATATGTTGAATACTTTTGTTAGGGTGTT [ 9282]
lp17_PAli_NX_Pl_consensus    ATAATTTCATTTTATTATATATTGCATGTTAGTTTCAAGGATGTTAGGTTATATGTTGAATACTTTTGTTAGGGTGTT [ 9282]
lp17_PAbe_NX_MP_FG_consensus ATAATTTCATTTTATTATATATTGCATGTTAGTTTCAAGGATGTTAGGTTATATGTTGAATACTTTTGTTAGGGTGTT [ 9282]
lp17_PAbe_NX_Pl_consensus    ATAATTTCATTTTATTATATATTGCATGTTAGTTTCAAGGATGTTAGGTTATATGTTGAATACTTTTGTTAGGGTGTT [ 9282]

lp17_gi|365823332|B31_GB     TGTCAAGAGCTTATTAGATTATGTAGCTTGGGCAGATTGCAGTAATTTGTCTTTCCAAAAAAAAATTACAATAATGTG [ 9360]
lp17_un5_B31_PacBio          TGTCAAGAGCTTATTAGATTATGTAGCTTGGGCAGATTGCAGTAATTTGTCTTTCCAAAAAAAAATTACAATAATGTG [ 9360]
lp17_B31_TS_MP_FG_consensus  TGTCAAGAGCTTATTAGATTATGTAGCTTGGGCAGATTGCAGTAATTTGTCTTTCCAAAAAAAAATTACAATAATGTG [ 9360]
lp17_B31_NX_Pl_consensus     TGTCAAGAGCTTATTAGATTATGTAGCTTGGGCAGATTGCAGTAATTTGTCTTTCCAAAAAAAAATTACAATAATGTG [ 9360]
lp17_PAli_NX_MP_FG_consensus TGTCAAGAGCTTATTAGATTATGTAGCTTGGGCAGATTGCAGTAATTTGTCTTTCCAAAAAAAAATTACAATAATGTG [ 9360]
lp17_PAli_NX_Pl_consensus    TGTCAAGAGCTTATTAGATTATGTAGCTTGGGCAGATTGCAGTAATTTGTCTTTCCAAAAAAAAATTACAATAATGTG [ 9360]
lp17_PAbe_NX_MP_FG_consensus TGTCAAGAGCTTATTAGATTATGTAGCTTGGGCAGATTGCAGTAATTTGTCTTTCCAAAAAAAAATTACAATAATGTG [ 9360]
lp17_PAbe_NX_Pl_consensus    TGTCAAGAGCTTATTAGATTATGTAGCTTGGGCAGATTGCAGTAATTTGTCTTTCCAAAAAAAAATTACAATAATGTG [ 9360]

lp17_gi|365823332|B31_GB     ATATTTTTAAGAAATGGGGTTACATTTGAAGCTTGGTTTAAGTATTTTTTTTCAATTTTATCTTCTAATAAGGTGATA [ 9438]
lp17_un5_B31_PacBio          ATATTTTTAAGAAATGGGGTTACATTTGAAGCTTGGTTTAAGTATTTTTTTTCAATTTTATCTTCTAATAAGGTGATA [ 9438]
```

```
lp17_B31_TS_MP_FG_consensus  ATATTTTTAAGAAATGGGGTTACATTTGAAGCTTGGTTTAAGTATTTTTTTTCAATTTTATCTTCTAATAAGGTGATA [ 9438]
lp17_B31_NX_Pl_consensus     ATATTTTTAAGAAATGGGGTTACATTTGAAGCTTGGTTTAAGTATTTTTTTTCAATTTTATCTTCTAATAAGGTGATA [ 9438]
lp17_PAli_NX_MP_FG_consensus ATATTTTTAAGAAATGGGGTTACATTTGAAGCTTGGTTTAAGTATTTTTTTTCAATTTTATCTTCTAATAAGGTGATA [ 9438]
lp17_PAli_NX_Pl_consensus    ATATTTTTAAGAAATGGGGTTACATTTGAAGCTTGGTTTAAGTATTTTTTTTCAATTTTATCTTCTAATAAGGTGATA [ 9438]
lp17_PAbe_NX_MP_FG_consensus ATATTTTTAAGAAATGGGGTTACATTTGAAGCTTGGTTTAAGTATTTTTTTTCAATTTTATCTTCTAATAAGGTGATA [ 9438]
lp17_PAbe_NX_Pl_consensus    ATATTTTTAAGAAATGGGGTTACATTTGAAGCTTGGTTTAAGTATTTTTTTTCAATTTTATCTTCTAATAAGGTGATA [ 9438]

lp17_gi|365823332|B31_GB     TCGATTCTGATACAGCTATTTTCTTGTATAATATTGATGTCATTTAATATAAAGTCTAAGTCATTTAAGTTTAAATTT [ 9516]
lp17_un5_B31_PacBio          TCGATTCTGATACAGCTATTTTCTTGTATAATATTGATGTCATTTAATATAAAGTCTAAGTCATTTAAGTTTAAATTT [ 9516]
lp17_B31_TS_MP_FG_consensus  TCGATTCTGATACAGCTATTTTCTTGTATAATATTGATGTCATTTAATATAAAGTCTAAGTCATTTAAGTTTAAATTT [ 9516]
lp17_B31_NX_Pl_consensus     TCGATTCTGATACAGCTATTTTCTTGTATAATATTGATGTCATTTAATATAAAGTCTAAGTCATTTAAGTTTAAATTT [ 9516]
lp17_PAli_NX_MP_FG_consensus TCGATTCTGATACAGCTATTTTCTTGTATAATATTGATGTCATTTAATATAAAGTCTAAGTCATTTAAGTTTAAATTT [ 9516]
lp17_PAli_NX_Pl_consensus    TCGATTCTGATACAGCTATTTTCTTGTATAATATTGATGTCATTTAATATAAAGTCTAAGTCATTTAAGTTTAAATTT [ 9516]
lp17_PAbe_NX_MP_FG_consensus TCGATTCTGATACAGCTATTTTCTTGTATAATATTGATGTCATTTAATATAAAGTCTAAGTCATTTAAGTTTAAATTT [ 9516]
lp17_PAbe_NX_Pl_consensus    TCGATTCTGATACAGCTATTTTCTTGTATAATATTGATGTCATTTAATATAAAGTCTAAGTCATTTAAGTTTAAATTT [ 9516]

lp17_gi|365823332|B31_GB     TTAATCAAAAAATTTGTTCTTTCTAAGAAGATTAACTTTATTTTACCTCCCAACCCATTCTCATTCTCAATAAAAGAT [ 9594]
lp17_un5_B31_PacBio          TTAATCAAAAAATTTGTTCTTTCTAAGAAGATTAACTTTATTTTACCTCCCAACCCATTCTCATTCTCAATAAAAGAT [ 9594]
lp17_B31_TS_MP_FG_consensus  TTAATCAAAAAATTTGTTCTTTCTAAGAAGATTAACTTTATTTTACCTCCCAACCCATTCTCATTCTCAATAAAAGAT [ 9594]
lp17_B31_NX_Pl_consensus     TTAATCAAAAAATTTGTTCTTTCTAAGAAGATTAACTTTATTTTACCTCCCAACCCATTCTCATTCTCAATAAAAGAT [ 9594]
lp17_PAli_NX_MP_FG_consensus TTAATCAAAAAATTTGTTCTTTCTAAGAAGATTAACTTTATTTTACCTCCCAACCCATTCTCATTCTCAATAAAAGAT [ 9594]
lp17_PAli_NX_Pl_consensus    TTAATCAAAAAATTTGTTCTTTCTAAGAAGATTAACTTTATTTTACCTCCCAACCCATTCTCATTCTCAATAAAAGAT [ 9594]
lp17_PAbe_NX_MP_FG_consensus TTAATCAAAAAATTTGTTCTTTCTAAGAAGATTAACTTTATTTTACCTCCCAACCCATTCTTATTCTCAATAAAAGAT [ 9594]
lp17_PAbe_NX_Pl_consensus    TTAATCAAAAAATTTGTTCTTTCTAAGAAGATTAACTTTATTTTACCTCCCAACCCATTCTCATTCTCAATAAAAGAT [ 9594]

lp17_gi|365823332|B31_GB     CTTAATGTTTTTAGAATTTTTTGTTTATTGTTGATAGTTTTTATTTGATTTGAATTTGTTAAAACTAGTTCTTCGTAG [ 9672]
lp17_un5_B31_PacBio          CTTAATGTTTTTAGAATTTTTTGTTTATTGTTGATAGTTTTTATTTGATTTGAATTTGTTAAAACTAGTTCTTCGTAG [ 9672]
lp17_B31_TS_MP_FG_consensus  CTTAATGTTTTTAGAATTTTTTGTTTATTGTTGATAGTTTTTATTTGATTTGAATTTGTTAAAACTAGTTCTTCGTAG [ 9672]
lp17_B31_NX_Pl_consensus     CTTAATGTTTTTAGAATTTTTTGTTTATTGTTGATAGTTTTTATTTGATTTGAATTTGTTAAAACTAGTTCTTCGTAG [ 9672]
lp17_PAli_NX_MP_FG_consensus CTTAATGTTTTTAGAATTTTTTGTTTATTGTTGATAGTTTTTATTTGATTTGAATTTGTTAAAACTAGTTCTTCGTAG [ 9672]
lp17_PAli_NX_Pl_consensus    CTTAATGTTTTTAGAATTTTTTGTTTATTGTTGATAGTTTTTATTTGATTTGAATTTGTTAAAACTAGTTCTTCGTAG [ 9672]
lp17_PAbe_NX_MP_FG_consensus CTTAATGTTTTTAGAATTTTTTGTTTATTGTTGATAGTTTTTATTTGATTTGAATTTGTTAAAACTAGTTCTTCGTAG [ 9672]
lp17_PAbe_NX_Pl_consensus    CTTAATGTTTTTAGAATTTTTTGTTTATTGTTGATAGTTTTTATTTGATTTGAATTTGTTAAAACTAGTTCTTCGTAG [ 9672]

lp17_gi|365823332|B31_GB     ATTTTTGTGCTTAGATTTAGCAACATTCTATTCGTTGTTTTAGTGTTCGTATTTAAATTTTCATTAGTTTTTATATCT [ 9750]
lp17_un5_B31_PacBio          ATTTTTGTGCTTAGATTTAGCAACATTCTATTCGTTGTTTTAGTGTTCGTATTTAAATTTTCATTAGTTTTTATATCT [ 9750]
lp17_B31_TS_MP_FG_consensus  ATTTTTGTGCTTAGATTTAGCAACATTCTATTCGTTGTTTTAGTGTTCGTATTTAAATTTTCATTAGTTTTTATATCT [ 9750]
lp17_B31_NX_Pl_consensus     ATTTTTGTGCTTAGATTTAGCAACATTCTATTCGTTGTTTTAGTGTTCGTATTTAAATTTTCATTAGTTTTTATATCT [ 9750]
lp17_PAli_NX_MP_FG_consensus ATTTTTGTGCTTAGATTTAGCAACATTCTATTCGTTGTTTTAGTGTTCGTATTTAAATTTTCATTAGTTTTTATATCT [ 9750]
lp17_PAli_NX_Pl_consensus    ATTTTTGTGCTTAGATTTAGCAACATTCTATTCGTTGTTTTAGTGTTCGTATTTAAATTTTCATTAGTTTTTATATCT [ 9750]
lp17_PAbe_NX_MP_FG_consensus ATTTTTGTGCTTAGATTTAGCAACATTCTATTCGTTGTTTTAGTGTTCGTATTTAAATTTTCATTAGTTTTTATATCT [ 9750]
lp17_PAbe_NX_Pl_consensus    ATTTTTGTGCTTAGATTTAGCAACATTCTATTCGTTGTTTTAGTGTTCGTATTTAAATTTTCATTAGTTTTTATATCT [ 9750]

lp17_gi|365823332|B31_GB     GCGGTATTAAGAGTTTTAATATTTGAATAGCTGATTTTTGTTAAAAACGGAAGTTCAATAGTCTTATCGATGCCTTTT [ 9828]
lp17_un5_B31_PacBio          GCGGTATTAAGAGTTTTAATATTTGAATAGCTGATTTTTGTTAAAAACGGAAGTTCAATAGTCTTATCGATGCCTTTT [ 9828]
lp17_B31_TS_MP_FG_consensus  GCGGTATTAAGAGTTTTAATATTTGAATAGCTGATTTTTGTTAAAAACGGAAGTTCAATAGTCTTATCGATGCCTTTT [ 9828]
lp17_B31_NX_Pl_consensus     GCGGTATTAAGAGTTTTAATATTTGAATAGCTGATTTTTGTTAAAAACGGAAGTTCAATAGTCTTATCGATGCCTTTT [ 9828]
lp17_PAli_NX_MP_FG_consensus GCGGTATTAAGAGTTTTAATATTTGAATAGCTGATTTTTGTTAAAAACGGAAGTTCAATAGTCTTATCGATGCCTTTT [ 9828]
lp17_PAli_NX_Pl_consensus    GCGGTATTAAGAGTTTTAATATTTGAATAGCTGATTTTTGTTAAAAACGGAAGTTCAATAGTCTTATCGATGCCTTTT [ 9828]
lp17_PAbe_NX_MP_FG_consensus GCGGTATTAAGAGTTTTAATATTTGAATAGCTGATTTTTGTTAAAAACGGAAGTTTAATAGTCTTATCGATGCCTTTT [ 9828]
lp17_PAbe_NX_Pl_consensus    GCGGTATTAAGAGTTTTAATATTTGAATAGCTGATTTTTGTTAAAAACGGAAGTTCAATAGTCTTATCGATGCCTTTT [ 9828]

lp17_gi|365823332|B31_GB     AATAGTAAATTAACTTTTTGGTTGTTTGCTTTGTTTTCAAAAAGGTAAGTATTGTTATTTTTTATAATAGGAATAAAC [ 9906]
lp17_un5_B31_PacBio          AATAGTAAATTAACTTTTTGGTTGTTTGCTTTGTTTTCAAAAAGGTAAGTATTGTTATTTTTTATAATAGGAATAAAC [ 9906]
lp17_B31_TS_MP_FG_consensus  AATAGTAAATTAACTTTTTGGTTGTTTGCTTTGTTTTCAAAAAGGTAAGTATTGTTATTTTTTATAATAGGAATAAAC [ 9906]
lp17_B31_NX_Pl_consensus     AATAGTAAATTAACTTTTTGGTTGTTTGCTTTGTTTTCAAAAAGGTAAGTATTGTTATTTTTTATAATAGGAATAAAC [ 9906]
lp17_PAli_NX_MP_FG_consensus AATAGTAAATTAACTTTTTGGTTGTTTGCTTTGTTTTCAAAAAGGTAAGTATTGTTATTTTTTATAATAGGAATAAAC [ 9906]
lp17_PAli_NX_Pl_consensus    AATAGTAAATTAACTTTTTGGTTGTTTGCTTTGTTTTCAAAAAGGTAAGTATTGTTATTTTTTATAATAGGAATAAAC [ 9906]
lp17_PAbe_NX_MP_FG_consensus AATAGTAAATTAACTTTTTGGTTGTTTGCTTTGTTTTCAAAAAGGTAAGTATTGTTATTTTTTATAATAGGAATAAAC [ 9906]
lp17_PAbe_NX_Pl_consensus    AATAGTAAATTAACTTTTTGGTTGTTTGCTTTGTTTTCAAAAAGGTAAGTATTGTTATTTTTTATAATAGGAATAAAC [ 9906]

lp17_gi|365823332|B31_GB     GCATTTTCTATATTTTCTTTTCTATAAATAGATCCAAGTATTTTAAGTTCGATTAAGTAAATTTCATCTTTATTGATA [ 9984]
lp17_un5_B31_PacBio          GCATTTTCTATATTTTCTTTTCTATAAATAGATCCAAGTATTTTAAGTTCGATTAAGTAAATTTCATCTTTATTGATA [ 9984]
lp17_B31_TS_MP_FG_consensus  GCATTTTCTATATTTTCTTTTCTATAAATAGATCCAAGTATTTTAAGTTCGATTAAGTAAATTTCATCTTTATTGATA [ 9984]
lp17_B31_NX_Pl_consensus     GCATTTTCTATATTTTCTTTTCTATAAATAGATCCAAGTATTTTAAGTTCGATTAAGTAAATTTCATCTTTATTGATA [ 9984]
lp17_PAli_NX_MP_FG_consensus GCATTTTCTATATTTTCTTTTCTATAAATAGATCCAAGTATTTTAAGTTCGATTAAGTAAATTTCATCTTTATTGATA [ 9984]
lp17_PAli_NX_Pl_consensus    GCATTTTCTATATTTTCTTTTCTATAAATAGATCCAAGTATTTTAAGTTCGATTAAGTAAATTTCATCTTTATTGATA [ 9984]
lp17_PAbe_NX_MP_FG_consensus GCATTTTCTATATTTTCTTTTCTATAAATAGATCCAAGTATTTTAAGTTCGATTAAGTAAATTTCATCTTTATTGATA [ 9984]
lp17_PAbe_NX_Pl_consensus    GCATTTTCTATATTTTCTTTTCTATAAATAGATCCAAGTATTTTAAGTTCGATTAAGTAAATTTCATCTTTATTGATA [ 9984]

lp17_gi|365823332|B31_GB     GCTTTTAAGTCAATTGTGTAAACTTTGACTACCATCATGTCATAAACTATTTTTAGTTTTTCTTCACTTACATAGTTA [10062]
lp17_un5_B31_PacBio          GCTTTTAAGTCAATTGTGTAAACTTTGACTACCATCATGTCATAAACTATTTTTAGTTTTTCTTCACTTACATAGTTA [10062]
lp17_B31_TS_MP_FG_consensus  GCTTTTAAGTCAATTGTGTAAACTTTGACTACCATCATGTCATAAACTATTTTTAGTTTTTCTTCACTTACATAGTTA [10062]
lp17_B31_NX_Pl_consensus     GCTTTTAAGTCAATTGTGTAAACTTTGACTACCATCATGTCATAAACTATTTTTAGTTTTTCTTCACTTACATAGTTA [10062]
lp17_PAli_NX_MP_FG_consensus GCTTTTAAGTCAATTGTGTAAACTTTGACTACCATCATGTCATAAACTATTTTTAGTTTTTCTTCACTTACATAGTTA [10062]
lp17_PAli_NX_Pl_consensus    GCTTTTAAGTCAATTGTGTAAACTTTGACTACCATCATGTCATAAACTATTTTTAGTTTTTCTTCACTTACATAGTTA [10062]
lp17_PAbe_NX_MP_FG_consensus GCTTTTAAGTCAATTGTGTAAACTTTGACTACCATCATGTCATAAACTATTTTTAGTTTTTCTTCACTTACATAGTTA [10062]
lp17_PAbe_NX_Pl_consensus    GCTTTTAAGTCAATTGTGTAAACTTTGACTACCATCATGTCATAAACTATTTTTAGTTTTTCTTCACTTACATAGTTA [10062]

lp17_gi|365823332|B31_GB     ATATAAAATTCAAGTTTTCCCTTTAGAATGTCTTGTGGTAATTTAGAAAGTTCATTTGACCTTAATTGTAGACTAATA [10140]
lp17_un5_B31_PacBio          ATATAAAATTCAAGTTTTCCCTTTAGAATGTCTTGTGGTAATTTAGAAAGTTCATTTGACCTTAATTGTAGACTAATA [10140]
lp17_B31_TS_MP_FG_consensus  ATATAAAATTCAAGTTTTCCCTTTAGAATGTCTTGTGGTAATTTAGAAAGTTCATTTGACCTTAATTGTAGACTAATA [10140]
lp17_B31_NX_Pl_consensus     ATATAAAATTCAAGTTTTCCCTTTAGAATGTCTTGTGGTAATTTAGAAAGTTCATTTGACCTTAATTGTAGACTAATA [10140]
lp17_PAli_NX_MP_FG_consensus ATATAAAATTCAAGTTTTCCCTTTAGAATGTCTTGTGGTAATTTAGAAAGTTCATTTGACCTTAATTGTAGACTAATA [10140]
lp17_PAli_NX_Pl_consensus    ATATAAAATTCAAGTTTTCCCTTTAGAATGTCTTGTGGTAATTTAGAAAGTTCATTTGACCTTAATTGTAGACTAATA [10140]
lp17_PAbe_NX_MP_FG_consensus ATATAAAATTCAAGTTTTCCCTTTAGAATGTCTTGTGGTAATTTAGAAAGTTCATTTGACCTTAATTGTAGACTAATA [10140]
lp17_PAbe_NX_Pl_consensus    ATATAAAATTCAAGTTTTCCCTTTAGAATGTCTTGTGGTAATTTAGAAAGTTCATTTGACCTTAATTGTAGACTAATA [10140]

lp17_gi|365823332|B31_GB     ATTCCGCCTTTAAATGTTTTAAGTGCTATATTCTCTTTTGAATTTGTTTGAATAATTTTTTCAAAATTAAGTTCAAAT [10218]
lp17_un5_B31_PacBio          ATTCCGCCTTTAAATGTTTTAAGTGCTATATTCTCTTTTGAATTTGTTTGAATAATTTTTTCAAAATTAAGTTCAAAT [10218]
lp17_B31_TS_MP_FG_consensus  ATTCCGCCTTTAAATGTTTTAAGTGCTATATTCTCTTTTGAATTTGTTTGAATAATTTTTTCAAAATTAAGTTCAAAT [10218]
lp17_B31_NX_Pl_consensus     ATTCCGCCTTTAAATGTTTTAAGTGCTATATTCTCTTTTGAATTTGTTTGAATAATTTTTTCAAAATTAAGTTCAAAT [10218]
lp17_PAli_NX_MP_FG_consensus ATTCCGCCTTTAAATGTTTTAAGTGCTATATTCTCTTTTGAATTTGTTTGAATAATTTTTTCAAAATTAAGTTCAAAT [10218]
lp17_PAli_NX_Pl_consensus    ATTCCGCCTTTAAATGTTTTAAGTGCTATATTCTCTTTTGAATTTGTTTGAATAATTTTTTCAAAATTAAGTTCAAAT [10218]
lp17_PAbe_NX_MP_FG_consensus ATTCCGCCTTTAAATGTTTTAAGTGCTATATTCTCTTTTGAATTTGTTTGAATAATTTTTTCAAAATTAAGTTCAAAT [10218]
lp17_PAbe_NX_Pl_consensus    ATTCCGCCTTTAAATGTTTTAAGTGCTATATTCTCTTTTGAATTTGTTTGAATAATTTTTTCAAAATTAAGTTCAAAT [10218]

lp17_gi|365823332|B31_GB     TTAAAAGCTATTGGGTTGATAATGAAGATTTTTTTGTTTTTTACAAAGTAAGTATTAATAATTGACCTTGGGTCAGTA [10296]
lp17_un5_B31_PacBio          TTAAAAGCTATTGGGTTGATAATGAAGATTTTTTTGTTTTTTACAAAGTAAGTATTAATAATTGACCTTGGGTCAGTA [10296]
lp17_B31_TS_MP_FG_consensus  TTAAAAGCTATTGGGTTGATAATGAAGATTTTTTTGTTTTTTACAAAGTAAGTATTAATAATTGACCTTGGGTCAGTA [10296]
lp17_B31_NX_Pl_consensus     TTAAAAGCTATTGGGTTGATAATGAAGATTTTTTTGTTTTTTACAAAGTAAGTATTAATAATTGACCTTGGGTCAGTA [10296]
lp17_PAli_NX_MP_FG_consensus TTAAAAGCTATTGGGTTGATAATGAAGATTTTTTTGTTTTTTACAAAGTAAGTATTAATAATTGACCTTGGGTCAGTA [10296]
lp17_PAli_NX_Pl_consensus    TTAAAAGCTATTGGGTTGATAATGAAGATTTTTTTGTTTTTTACAAAGTAAGTATTAATAATTGACCTTGGGTCAGTA [10296]
lp17_PAbe_NX_MP_FG_consensus TTAAAAGCTATTGGGTTGATAATGAAGATTTTTTTGTTTTTTACAAAGTAAGTATTAATAATTGACCTTGGGTCAGTA [10296]
lp17_PAbe_NX_Pl_consensus    TTAAAAGCTATTGGGTTGATAATGAAGATTTTTTTGTTTTTTACAAAGTAAGTATTAATAATTGACCTTGGGTCAGTA [10296]

lp17_gi|365823332|B31_GB     TACACGATTTCTCCTATAGATGCACTTTGAAATTTATATAAATTGGGATTAGATATAATCTAATCTTTAAATTGGTTT [10374]
lp17_un5_B31_PacBio          TACACGATTTCTCCTATAGATGCACTTTGAAATTTATATAAATTGGGATTAGATATAATCTAATCTTTAAATTGGTTT [10374]
lp17_B31_TS_MP_FG_consensus  TACACGATTTCTCCTATAGATGCACTTTGAAATTTATATAAATTGGGATTAGATATAATCTAATCTTTAAATTGGTTT [10374]
```

```
lp17_B31_NX_Pl_consensus      TACACGATTTCTCCTATAGATGCACTTTGAAATTTATATAAATTGGGATTAGATATAATCTAATCTTTAAATTGGTTT [10374]
lp17_PAli_NX_MP_FG_consensus  TACACGATTTCTCCTATAGATGCACTTTGAAATTTATATAAATTGGGATTAGATATAATCTAATCTTTAAATTGGTTT [10374]
lp17_PAli_NX_Pl_consensus     TACACGATTTCTCCTATAGATGCACTTTGAAATTTATATAAATTGGGATTAGATATAATCTAATCTTTAAATTGGTTT [10374]
lp17_PAbe_NX_MP_FG_consensus  TACACGATTTCTCCTATAGATGCACTTTGAAATTTATATAAATTGGGATTAGATATAATCTAATCTTTAAATTGGTTT [10374]
lp17_PAbe_NX_Pl_consensus     TACACGATTTCTCCTATAGATGCACTTTGAAATTTATATAAATTGGGATTAGATATAATCTAATCTTTAAATTGGTTT [10374]

lp17_gi|365823332|B31_GB      TTGTTAGAACAACACCTAATAATAATAGTTTTTTAATTTTAATTCAAGCTCTAAAATTAGAATGTTTTTATTTGAATT [10452]
lp17_un5_B31_PacBio           TTGTTAGAACAACACCTAATAATAATAGTTTTTTAATTTTAATTCAAGCTCTAAAATTAGAATGTTTTTATTTGAATT [10452]
lp17_B31_TS_MP_FG_consensus   TTGTTAGAACAACACCTAATAATAATAGTTTTTTAATTTTAATTCAAGCTCTAAAATTAGAATGTTTTTATTTGAATT [10452]
lp17_B31_NX_Pl_consensus      TTGTTAGAACAACACCTAATAATAATAGTTTTTTAATTTTAATTCAAGCTCTAAAATTAGAATGTTTTTATTTGAATT [10452]
lp17_PAli_NX_MP_FG_consensus  TTGTTAGAACAACACCTAATAATAATAGTTTTTTAATTTTAATTCAAGCTCTAAAATTAGAATGTTTTTATTTGAATT [10452]
lp17_PAli_NX_Pl_consensus     TTGTTAGAACAACACCTAATAATAATAGTTTTTTAATTTTAATTCAAGCTCTAAAATTAGAATGTTTTTATTTGAATT [10452]
lp17_PAbe_NX_MP_FG_consensus  TTGTTAGAACAACACCTAATAATAATAGTTTTTTAATTTTAATTCAAGCTCTAAAATTAGAATGTTTTTATTTGAATT [10452]
lp17_PAbe_NX_Pl_consensus     TTGTTAGAACAACACCTAATAATAATAGTTTTTTAATTTTAATTCAAGCTCTAAAATTAGAATGTTTTTATTTGAATT [10452]

lp17_gi|365823332|B31_GB      AATTATATTATTTAATTTTCTAATTTTTGAGAAATTTTTTTAAAATAAATATAAGATAAACTAAGGGGGGTTTTAATG [10530]
lp17_un5_B31_PacBio           AATTATATTATTTAATTTTCTAATTTTTGAGAAATTTTTTTAAAATAAATATAAGATAAACTAAGGGGGGTTTTAATG [10530]
lp17_B31_TS_MP_FG_consensus   AATTATATTATTTAATTTTCTAATTTTTGAGAAATTTTTTTAAAATAAATATAAGATAAACTAAGGGGGGTTTTAATG [10530]
lp17_B31_NX_Pl_consensus      AATTATATTATTTAATTTTCTAATTTTTGAGAAATTTTTTTAAAATAAATATAAGATAAACTAAGGGGGGTTTTAATG [10530]
lp17_PAli_NX_MP_FG_consensus  AATTATATTATTTAATTTTCTAATTTTTGAGAAATTTTTTTAAAATAAATATAAGATAAACTAAGGGGGGTTTTAATG [10530]
lp17_PAli_NX_Pl_consensus     AATTATATTATTTAATTTTCTAATTTTTGAGAAATTTTTTTAAAATAAATATAAGATAAACTAAGGGGGGTTTTAATG [10530]
lp17_PAbe_NX_MP_FG_consensus  AATTATATTATTTAATTTTCTAATTTTTGAGAAATTTTTTTAAAATAAATATAAGATAAACTAAGGGGGGTTTTAATG [10530]
lp17_PAbe_NX_Pl_consensus     AATTATATTATTTAATTTTCTAATTTTTGAGAAATTTTTTTAAAATAAATATAAGATAAACTAAGGGGGGTTTTAATG [10530]

lp17_gi|365823332|B31_GB      AATTCAAAATTTATTTTAAAGTATTTTATTTTGGCGTTTTTTTTAGTTTCTTGTCAAACTTATCAAATAGCTTATGAT [10608]
lp17_un5_B31_PacBio           AATTCAAAATTTATTTTAAAGTATTTTATTTTGGCGTTTTTTTTAGTTTCTTGTCAAACTTATCAAATAGCTTATGAT [10608]
lp17_B31_TS_MP_FG_consensus   AATTCAAAATTTATTTTAAAGTATTTTATTTTGGCGTTTTTTTTAGTTTCTTGTCAAACTTATCAAATAGCTTATGAT [10608]
lp17_B31_NX_Pl_consensus      AATTCAAAATTTATTTTAAAGTATTTTATTTTGGCGTTTTTTTTAGTTTCTTGTCAAACTTATCAAATAGCTTATGAT [10608]
lp17_PAli_NX_MP_FG_consensus  AATTCAAAATTTATTTTAAAGTATTTTATTTTGGCGTTTTTTTTAGTTTCTTGTCAAACTTATCAAATAGCTTATGAT [10608]
lp17_PAli_NX_Pl_consensus     AATTCAAAATTTATTTTAAAGTATTTTATTTTGGCGTTTTTTTTAGTTTCTTGTCAAACTTATCAAATAGCTTATGAT [10608]
lp17_PAbe_NX_MP_FG_consensus  AATTCAAAATTTATTTTAAAGTATTTTATTTTGGCGTTTTTTTTAGTTTCTTGTCAAACTTATCAAATAGCTTATGAT [10608]
lp17_PAbe_NX_Pl_consensus     AATTCAAAATTTATTTTAAAGTATTTTATTTTGGCGTTTTTTTTAGTTTCTTGTCAAACTTATCAAATAGCTTATGAT [10608]

lp17_gi|365823332|B31_GB      AGGTTTTCTCAAGTATTAGATTCACAATATGATATTGGGGTAAATTATTCTAGAGATGGAATATTTAAGTCTGTAATT [10686]
lp17_un5_B31_PacBio           AGGTTTTCTCAAGTATTAGATTCACAATATGATATTGGGGTAAATTATTCTAGAGATGGAATATTTAAGTCTGTAATT [10686]
lp17_B31_TS_MP_FG_consensus   AGGTTTTCTCAAGTATTAGATTCACAATATGATATTGGGGTAAATTATTCTAGAGATGGAATATTTAAGTCTGTAATT [10686]
lp17_B31_NX_Pl_consensus      AGGTTTTCTCAAGTATTAGATTCACAATATGATATTGGGGTAAATTATTCTAGAGATGGAATATTTAAGTCTGTAATT [10686]
lp17_PAli_NX_MP_FG_consensus  AGGTTTTCTCAAGTATTAGATTCACAATATGATATTGGGGTAAATTATTCTAGAGATGGAATATTTAAGTCTGTAATT [10686]
lp17_PAli_NX_Pl_consensus     AGGTTTTCTCAAGTATTAGATTCACAATATGATATTGGGGTAAATTATTCTAGAGATGGAATATTTAAGTCTGTAATT [10686]
lp17_PAbe_NX_MP_FG_consensus  AGGTTTTCTCAAGTATTAGATTCACAATATGATATTGGGGTAAATTATTCTAGAGATGGAATATTTAAGTCTGTAATT [10686]
lp17_PAbe_NX_Pl_consensus     AGGTTTTCTCAAGTATTAGATTCACAATATGATATTGGGGTAAATTATTCTAGAGATGGAATATTTAAGTCTGTAATT [10686]

lp17_gi|365823332|B31_GB      TCTATTAAATATGATAAATTGAAAAATAAAAGGGAATATTTTATTTTAGTTAGAGTTGAATCTAGAAATTCTAGTCAA [10764]
lp17_un5_B31_PacBio           TCTATTAAATATGATAAATTGAAAAATAAAAGGGAATATTTTATTTTAGTTAGAGTTGAATCTAGAAATTCTAGTCAA [10764]
lp17_B31_TS_MP_FG_consensus   TCTATTAAATATGATAAATTGAAAAATAAAAGGGAATATTTTATTTTAGTTAGAGTTGAATCTAGAAATTCTAGTCAA [10764]
lp17_B31_NX_Pl_consensus      TCTATTAAATATGATAAATTGAAAAATAAAAGGGAATATTTTATTTTAGTTAGAGTTGAATCTAGAAATTCTAGTCAA [10764]
lp17_PAli_NX_MP_FG_consensus  TCTATTAAATATGATAAATTGAAAAATAAAAGGGAATATTTTATTTTAGTTAGAGTTGAATCTAGAAATTCTAGTCAA [10764]
lp17_PAli_NX_Pl_consensus     TCTATTAAATATGATAAATTGAAAAATAAAAGGGAATATTTTATTTTAGTTAGAGTTGAATCTAGAAATTCTAGTCAA [10764]
lp17_PAbe_NX_MP_FG_consensus  TCTATTAAATATGATAAATTGAAAAATAAAAGGGAATATTTTATTTTAGTTAGAGTTGAATCTAGAAATTCTAGTCAA [10764]
lp17_PAbe_NX_Pl_consensus     TCTATTAAATATGATAAATTGAAAAATAAAAGGGAATATTTTATTTTAGTTAGAGTTGAATCTAGAAATTCTAGTCAA [10764]

lp17_gi|365823332|B31_GB      ATAAAGCCCGAAAAGATAATAACAGATACTAGATTTGAGGCTAAAGGAGAGTTGGTTTCAGAGGATAGCAAACGTGTT [10842]
lp17_un5_B31_PacBio           ATAAAGCCCGAAAAGATAATAACAGATACTAGATTTGAGGCTAAAGGAGAGTTGGTTTCAGAGGATAGCAAACGTGTT [10842]
lp17_B31_TS_MP_FG_consensus   ATAAAGCCCGAAAAGATAATAACAGATACTAGATTTGAGGCTAAAGGAGAGTTGGTTTCAGAGGATAGCAAACGTGTT [10842]
lp17_B31_NX_Pl_consensus      ATAAAGCCCGAAAAGATAATAACAGATACTAGATTTGAGGCTAAAGGAGAGTTGGTTTCAGAGGATAGCAAACGTGTT [10842]
lp17_PAli_NX_MP_FG_consensus  ATAAAGCCCGAAAAGATAATAACAGATACTAGATTTGAGGCTAAAGGAGAGTTGGTTTCAGAGGATAGCAAACGTGTT [10842]
lp17_PAli_NX_Pl_consensus     ATAAAGCCCGAAAAGATAATAACAGATACTAGATTTGAGGCTAAAGGAGAGTTGGTTTCAGAGGATAGCAAACGTGTT [10842]
lp17_PAbe_NX_MP_FG_consensus  ATAAAGCCCGAAAAGATAATAACAGATACTAGATTTGAGGCTAAAGGAGAGTTGGTTTCAGAGGATAGCAAACGTGTT [10842]
lp17_PAbe_NX_Pl_consensus     ATAAAGCCCGAAAAGATAATAACAGATACTAGATTTGAGGCTAAAGGAGAGTTGGTTTCAGAGGATAGCAAACGTGTT [10842]

lp17_gi|365823332|B31_GB      GTTTATTATAACGATTTTTATGATTCATATTTTCCTTATGATTACACAACAGTGATTACTGAAAAAAATATTAAGGTA [10920]
lp17_un5_B31_PacBio           GTTTATTATAACGATTTTTATGATTCATATTTTCCTTATGATTACACAACAGTGATTACTGAAAAAAATATTAAGGTA [10920]
lp17_B31_TS_MP_FG_consensus   GTTTATTATAACGATTTTTATGATTCATATTTTCCTTATGATTACACAACAGTGATTACTGAAAAAAATATTAAGGTA [10920]
lp17_B31_NX_Pl_consensus      GTTTATTATAACGATTTTTATGATTCATATTTTCCTTATGATTACACAACAGTGATTACTGAAAAAAATATTAAGGTA [10920]
lp17_PAli_NX_MP_FG_consensus  GTTTATTATAACGATTTTTATGATTCATATTTTCCTTATGATTACACAACAGTGATTACTGAAAAAAATATTAAGGTA [10920]
lp17_PAli_NX_Pl_consensus     GTTTATTATAACGATTTTTATGATTCATATTTTCCTTATGATTACACAACAGTGATTACTGAAAAAAATATTAAGGTA [10920]
lp17_PAbe_NX_MP_FG_consensus  GTTTATTATAACGATTTTTATGATTCATATTTTCCTTATGATTACACAACAGTGATTACTGAAAAAAATATTAAGGTA [10920]
lp17_PAbe_NX_Pl_consensus     GTTTATTATAACGATTTTTATGATTCATATTTTCCTTATGATTACACAACAGTGATTACTGAAAAAAATATTAAGGTA [10920]

lp17_gi|365823332|B31_GB      GAAATTTATAAATTTATTATTTCAGAAAGCGAATTTATAAGATTTGTTGCCTTGGGAAATGATAATATAGCAGCATTT [10998]
lp17_un5_B31_PacBio           GAAATTTATAAATTTATTATTTCAGAAAGCGAATTTATAAGATTTGTTGCCTTGGGAAATGATAATATAGCAGCATTT [10998]
lp17_B31_TS_MP_FG_consensus   GAAATTTATAAATTTATTATTTCAGAAAGCGAATTTATAAGATTTGTTGCCTTGGGAAATGATAATATAGCAGCATTT [10998]
lp17_B31_NX_Pl_consensus      GAAATTTATAAATTTATTATTTCAGAAAGCGAATTTATAAGATTTGTTGCCTTGGGAAATGATAATATAGCAGCATTT [10998]
lp17_PAli_NX_MP_FG_consensus  GAAATTTATAAATTTATTATTTCAGAAAGCGAATTTATAAGATTTGTTGCCTTGGGAAATGATAATATAGCAGCATTT [10998]
lp17_PAli_NX_Pl_consensus     GAAATTTATAAATTTATTATTTCAGAAAGCGAATTTATAAGATTTGTTGCCTTGGGAAATGATAATATAGCAGCATTT [10998]
lp17_PAbe_NX_MP_FG_consensus  GAAATTTATAAATTTATTATTTCAGAAAGCGAATTTATAAGATTTGTTGCCTTGGGAAATGATAATATAGCAGCATTT [10998]
lp17_PAbe_NX_Pl_consensus     GAAATTTATAAATTTATTATTTCAGAAAGCGAATTTATAAGATTTGTTGCCTTGGGAAATGATAATATAGCAGCATTT [10998]

lp17_gi|365823332|B31_GB      AGAATCTATGCGTTCAGAAATGATGTTATTGTAAGTTTTAATAAGATTCCTTTTAAAAAGTTTTTGGATGATTTTAAT [11076]
lp17_un5_B31_PacBio           AGAATCTATGCGTTCAGAAATGATGTTATTGTAAGTTTTAATAAGATTCCTTTTAAAAAGTTTTTGGATGATTTTAAT [11076]
lp17_B31_TS_MP_FG_consensus   AGAATCTATGCGTTCAGAAATGATGTTATTGTAAGTTTTAATAAGATTCCTTTTAAAAAGTTTTTGGATGATTTTAAT [11076]
lp17_B31_NX_Pl_consensus      AGAATCTATGCGTTCAGAAATGATGTTATTGTAAGTTTTAATAAGATTCCTTTTAAAAAGTTTTTGGATGATTTTAAT [11076]
lp17_PAli_NX_MP_FG_consensus  AGAATCTATGCGTTCAGAAATGATGTTATTGTAAGTTTTAATAAGATTCCTTTTAAAAAGTTTTTGGATGATTTTAAT [11076]
lp17_PAli_NX_Pl_consensus     AGAATCTATGCGTTCAGAAATGATGTTATTGTAAGTTTTAATAAGATTCCTTTTAAAAAGTTTTTGGATGATTTTAAT [11076]
lp17_PAbe_NX_MP_FG_consensus  AGAATCTATGCGTTCAGAAATGATGTTATTGTAAGTTTTAATAAGATTCCTTTTAAAAAGTTTTTGGATGATTTTAAT [11076]
lp17_PAbe_NX_Pl_consensus     AGAATCTATGCGTTCAGAAATGATGTTATTGTAAGTTTTAATAAGATTCCTTTTAAAAAGTTTTTGGATGATTTTAAT [11076]

lp17_gi|365823332|B31_GB      TCAAAAGTTAAATTACTTGGTGGCAGTTGACTTAAATTTAAATAATTTTTAAGCCCAAGTTAAGTAATTATTTGTTAA [11154]
lp17_un5_B31_PacBio           TCAAAAGTTAAATTACTTGGTGGCAGTTGACTTAAATTTAAATAATTTTTAAGCCCAAGTTAAGTAATTATTTGTTAA [11154]
lp17_B31_TS_MP_FG_consensus   TCAAAAGTTAAATTACTTGGTGGCAGTTGACTTAAATTTAAATAATTTTTAAGCCCAAGTTAAGTAATTATTTGTTAA [11154]
lp17_B31_NX_Pl_consensus      TCAAAAGTTAAATTACTTGGTGGCAGTTGACTTAAATTTAAATAATTTTTAAGCCCAAGTTAAGTAATTATTTGTTAA [11154]
lp17_PAli_NX_MP_FG_consensus  TCAAAAGTTAAATTACTTGGTGGCAGTTGACTTAAATTTAAATAATTTTTAAGCCCAAGTTAAGTAATTATTTGTTAA [11154]
lp17_PAli_NX_Pl_consensus     TCAAAAGTTAAATTACTTGGTGGCAGTTGACTTAAATTTAAATAATTTTTAAGCCCAAGTTAAGTAATTATTTGTTAA [11154]
lp17_PAbe_NX_MP_FG_consensus  TCAAAAGTTAAATTACTTGGTGGCAGTTGACTTAAATTTAAATAATTTTTAAGCCCAAGTTAAGTAATTATTTGTTAA [11154]
lp17_PAbe_NX_Pl_consensus     TCAAAAGTTAAATTACTTGGTGGCAGTTGACTTAAATTTAAATAATTTTTAAGCCCAAGTTAAGTAATTATTTGTTAA [11154]

lp17_gi|365823332|B31_GB      AAAGGATATAGATTAAGGGTGTTTAGTATAAACAAGGTTTTTGATATTAGTTTATTATAATATCGACTTTAAAATGTT [11232]
lp17_un5_B31_PacBio           AAAGGATATAGATTAAGGGTGTTTAGTATAAACAAGGTTTTTGATATTAGTTTATTATAATATCGACTTTAAAATGTT [11232]
lp17_B31_TS_MP_FG_consensus   AAAGGATATAGATTAAGGGTGTTTAGTATAAACAAGGTTTTTGATATTAGTTTATTATAATATCGACTTTAAAATGTT [11232]
lp17_B31_NX_Pl_consensus      AAAGGATATAGATTAAGGGTGTTTAGTATAAACAAGGTTTTTGATATTAGTTTATTATAATATCGACTTTAAAATGTT [11232]
lp17_PAli_NX_MP_FG_consensus  AAAGGATATAGATTAAGGGTGTTTAGTATAAACAAGGTTTTTGATATTAGTTTATTATAATATCGACTTTAAAATGTT [11232]
lp17_PAli_NX_Pl_consensus     AAAGGATATAGATTAAGGGTGTTTAGTATAAACAAGGTTTTTGATATTAGTTTATTATAATATCGACTTTAAAATGTT [11232]
lp17_PAbe_NX_MP_FG_consensus  AAAGGATATAGATTAAGGGTGTTTAGTATAAACAAGGTTTTTGATATTAGTTTATTATAATATCGACTTTAAAATGTT [11232]
lp17_PAbe_NX_Pl_consensus     AAAGGATATAGATTAAGGGTGTTTAGTATAAACAAGGTTTTTGATATTAGTTTATTATAATATCGACTTTAAAATGTT [11232]

lp17_gi|365823332|B31_GB      TTTAATATCTAATCTTTTGAAAAATTAATAAATTCTAGTATCGCTTTTTCATTTCTCAGCAAGAATAGCCCTTCCCTT [11310]
lp17_un5_B31_PacBio           TTTAATATCTAATCTTTTGAAAAATTAATAAATTCTAGTATCGCTTTTTCATTTCTCAGCAAGAATAGCCCTTCCCTT [11310]
lp17_B31_TS_MP_FG_consensus   TTTAATATCTAATCTTTTGAAAAATTAATAAATTCTAGTATCGCTTTTTCATTTCTCAGCAAGAATAGCCCTTCCCTT [11310]
lp17_B31_NX_Pl_consensus      TTTAATATCTAATCTTTTGAAAAATTAATAAATTCTAGTATCGCTTTTTCATTTCTCAGCAAGAATAGCCCTTCCCTT [11310]
```

```
lp17_PAli_NX_MP_FG_consensus TTTAATATCTAATCTTTTGAAAAATTAATAAATTCTAGTATCGCTTTTTCATTTCTCAGCAAGAATAGCCCTTCCCTT [11310]
lp17_PAli_NX_Pl_consensus    TTTAATATCTAATCTTTTGAAAAATTAATAAATTCTAGTATCGCTTTTTCATTTCTCAGCAAGAATAGCCCTTCCCTT [11310]
lp17_PAbe_NX_MP_FG_consensus TTTAATATCTAATCTTTTGAAAAATTAATAAATTCTAGTATCGCTTTTTCATTTCTCAGCAAGAATAGCCCTTCCCTT [11310]
lp17_PAbe_NX_Pl_consensus    TTTAATATCTAATCTTTTGAAAAATTAATAAATTCTAGTATCGCTTTTTCATTTCTCAGCAAGAATAGCCCTTCCCTT [11310]

lp17_gi|365823332|B31_GB     ATTCCATTATGGAATTCTTCAAAGGAAATTAAGATTGTTTTTAAATTTTTATCTTTATCATCTATATTGTCGTAATAA [11388]
lp17_un5_B31_PacBio          ATTCCATTATGGAATTCTTCAAAGGAAATTAAGATTGTTTTTAAATTTTTATCTTTATCATCTATATTGTCGTAATAA [11388]
lp17_B31_TS_MP_FG_consensus  ATTCCATTATGGAATTCTTCAAAGGAAATTAAGATTGTTTTTAAATTTTTATCTTTATCATCTATATTGTCGTAATAA [11388]
lp17_B31_NX_Pl_consensus     ATTCCATTATGGAATTCTTCAAAGGAAATTAAGATTGTTTTTAAATTTTTATCTTTATCATCTATATTGTCGTAATAA [11388]
lp17_PAli_NX_MP_FG_consensus ATTCCATTATGGAATTCTTCAAAGGAAATTAAGATTGTTTTTAAATTTTTATCTTTATCATCTATATTGTCGTAATAA [11388]
lp17_PAli_NX_Pl_consensus    ATTCCATTATGGAATTCTTCAAAGGAAATTAAGATTGTTTTTAAATTTTTATCTTTATCATCTATATTGTCGTAATAA [11388]
lp17_PAbe_NX_MP_FG_consensus ATTCCATTATGGAATTCTTCAAAGGAAATTAAGATTGTTTTTAAATTTTTATCTTTATCATCTATATTGTCGTAATAA [11388]
lp17_PAbe_NX_Pl_consensus    ATTCCATTATGGAATTCTTCAAAGGAAATTAAGATTGTTTTTAAATTTTTATCTTTATCATCTATATTGTCGTAATAA [11388]

lp17_gi|365823332|B31_GB     AATATAAAGTAAGGTCTTCCAGTATTATCTTTAAAGCCAAGAAAATTTACAAATATTGCTGTTTCTTGAAAATCTGTT [11466]
lp17_un5_B31_PacBio          AATATAAAGTAAGGTCTTCCAGTATTATCTTTAAAGCCAAGAAAATTTACAAATATTGCTGTTTCTTGAAAATCTGTT [11466]
lp17_B31_TS_MP_FG_consensus  AATATAAAGTAAGGTCTTCCAGTATTATCTTTAAAGCCAAGAAAATTTACAAATATTGCTGTTTCTTGAAAATCTGTT [11466]
lp17_B31_NX_Pl_consensus     AATATAAAGTAAGGTCTTCCAGTATTATCTTTAAAGCCAAGAAAATTTACAAATATTGCTGTTTCTTGAAAATCTGTT [11466]
lp17_PAli_NX_MP_FG_consensus AATATAAAGTAAGGTCTTCCAGTATTATCTTTAAAGCCAAGAAAATTTACAAATATTGCTGTTTCTTGAAAATCTGTT [11466]
lp17_PAli_NX_Pl_consensus    AATATAAAGTAAGGTCTTCCAGTATTATCTTTAAAGCCAAGAAAATTTACAAATATTGCTGTTTCTTGAAAATCTGTT [11466]
lp17_PAbe_NX_MP_FG_consensus AATATAAAGTAAGGTCTTCCAGTATTATCTTTAAAGCCAAGAAAATTTACAAATATTGCTGTTTCTTGAAAATCTGTT [11466]
lp17_PAbe_NX_Pl_consensus    AATATAAAGTAAGGTCTTCCAGTATTATCTTTAAAGCCAAGAAAATTTACAAATATTGCTGTTTCTTGAAAATCTGTT [11466]

lp17_gi|365823332|B31_GB     TTTAAATTGTATGAGTGACCATTTTTGTATAAAAAAAATTCCATTTCCTTCAAGATTTTTAATTTCTAAATTATTGATT [11544]
lp17_un5_B31_PacBio          TTTAAATTGTATGAGTGACCATTTTTGTATAAAAAAAATTCCATTTCCTTCAAGATTTTTAATTTCTAAATTATTGATT [11544]
lp17_B31_TS_MP_FG_consensus  TTTAAATTGTATGAGTGACCATTTTTGTATAAAAAAAATTCCATTTCCTTCAAGATTTTTAATTTCTAAATTATTGATT [11544]
lp17_B31_NX_Pl_consensus     TTTAAATTGTATGAGTGACCATTTTTGTATAAAAAAAATTCCATTTCCTTCAAGATTTTTAATTTCTAAATTATTGATT [11544]
lp17_PAli_NX_MP_FG_consensus TTTAAATTGTATGAGTGACCATTTTTGTATAAAAAAAATTCCATTTCCTTCAAGATTTTTAATTTCTAAATTATTGATT [11544]
lp17_PAli_NX_Pl_consensus    TTTAAATTGTATGAGTGACCATTTTTGTATAAAAAAAATTCCATTTCCTTCAAGATTTTTAATTTCTAAATTATTGATT [11544]
lp17_PAbe_NX_MP_FG_consensus TTTAAATTGTATGAGTGACCATTTTTGTATAAAAAAAATTCCATTTCCTTCAAGATTTTTAATTTCTAAATTATTGATT [11544]
lp17_PAbe_NX_Pl_consensus    TTTAAATTGTATGAGTGACCATTTTTGTATAAAAAAAATTCCATTTCCTTCAAGATTTTTAATTTCTAAATTATTGATT [11544]

lp17_gi|365823332|B31_GB     GTATTTTTAATTTTAGCAAAACTCTCAATTGCTTGTAAAATATATTTTATTGATGAAAATTTGGTATAGTATATGCGG [11622]
lp17_un5_B31_PacBio          GTATTTTTAATTTTAGCAAAACTCTCAATTGCTTGTAAAATATATTTTATTGATGAAAATTTGGTATAGTATATGCGG [11622]
lp17_B31_TS_MP_FG_consensus  GTATTTTTAATTTTAGCAAAACTCTCAATTGCTTGTAAAATATATTTTATTGATGAAAATTTGGTATAGTATATGCGG [11622]
lp17_B31_NX_Pl_consensus     GTATTTTTAATTTTAGCAAAACTCTCAATTGCTTGTAAAATATATTTTATTGATGAAAATTTGGTATAGTATATGCGG [11622]
lp17_PAli_NX_MP_FG_consensus GTATTTTTAATTTTAGCAAAACTCTCAATTGCTTGTAAAATATATTTTATTGATGAAAATTTGGTATAGTATATGCGG [11622]
lp17_PAli_NX_Pl_consensus    GTATTTTTAATTTTAGCAAAACTCTCAATTGCTTGTAAAATATATTTTATTGATGAAAATTTGGTATAGTATATGCGG [11622]
lp17_PAbe_NX_MP_FG_consensus GTATTTTTAATTTTAGCAAAACTCTCAATTGCTTGTAAAATATATTTTATTGATGAAAATTTGGTATAGTATATGCGG [11622]
lp17_PAbe_NX_Pl_consensus    GTATTTTTAATTTTAGCAAAACTCTCAATTGCTTGTAAAATATATTTTATTGATGAAAATTTGGTATAGTATATGCGG [11622]

lp17_gi|365823332|B31_GB     TTATTGATTTGAAATTCTAAAAAGATTCCGCATTCATTACCGGCTCTAATTACATCTTGCTCATTTTTAATAGAAGTT [11700]
lp17_un5_B31_PacBio          TTATTGATTTGAAATTCTAAAAAGATTCCGCATTCATTACCGGCTCTAATTACATCTTGCTCATTTTTAATAGAAGTT [11700]
lp17_B31_TS_MP_FG_consensus  TTATTGATTTGAAATTCTAAAAAGATTCCGCATTCATTACCGGCTCTAATTACATCTTGCTCATTTTTAATAGAAGTT [11700]
lp17_B31_NX_Pl_consensus     TTATTGATTTGAAATTCTAAAAAGATTCCGCATTCATTACCGGCTCTAATTACATCTTGCTCATTTTTAATAGAAGTT [11700]
lp17_PAli_NX_MP_FG_consensus TTATTGATTTGAAATTCTAAAAAGATTCCGCATTCATTACCGGCTCTAATTACATCTTGCTCATTTTTAATAGAAGTT [11700]
lp17_PAli_NX_Pl_consensus    TTATTGATTTGAAATTCTAAAAAGATTCCGCATTCATTACCGGCTCTAATTACATCTTGCTCATTTTTAATAGAAGTT [11700]
lp17_PAbe_NX_MP_FG_consensus TTATTGATTTGAAATTCTAAAAAGATTCCGCATTCATTACCGGCTCTAATTACATCTTGCTCATTTTTAATAGAAGTT [11700]
lp17_PAbe_NX_Pl_consensus    TTATTGATTTGAAATTCTAAAAAGATTCCGCATTCATTACCGGCTCTAATTACATCTTGCTCATTTTTAATAGAAGTT [11700]

lp17_gi|365823332|B31_GB     GCAATTTTTTTGTCAATAACAAGATTGATTTTGAATGTAGAAATATGGTTTAAGGTTTTTACATTGTTAATTGCTAAA [11778]
lp17_un5_B31_PacBio          GCAATTTTTTTGTCAATAACAAGATTGATTTTGAATGTAGAAATATGGTTTAAGGTTTTTACATTGTTAATTGCTAAA [11778]
lp17_B31_TS_MP_FG_consensus  GCAATTTTTTTGTCAATAACAAGATTGATTTTGAATGTAGAAATATGGTTTAAGGTTTTTACATTGTTAATTGCTAAA [11778]
lp17_B31_NX_Pl_consensus     GCAATTTTTTTGTCAATAACAAGATTGATTTTGAATGTAGAAATATGGTTTAAGGTTTTTACATTGTTAATTGCTAAA [11778]
lp17_PAli_NX_MP_FG_consensus GCAATTTTTTTGTCAATAACAAGATTGATTTTGAATGTAGAAATATGGTTTAAGGTTTTTACATTGTTAATTGCTAAA [11778]
lp17_PAli_NX_Pl_consensus    GCAATTTTTTTGTCAATAACAAGATTGATTTTGAATGTAGAAATATGGTTTAAGGTTTTTACATTGTTAATTGCTAAA [11778]
lp17_PAbe_NX_MP_FG_consensus GCAATTTTTTTGTCAATAACAAGATTGATTTTGAATGTAGAAATATGGTTTAAGGTTTTTACATTGTTAATTGCTAAA [11778]
lp17_PAbe_NX_Pl_consensus    GCAATTTTTTTGTCAATAACAAGATTGATTTTGAATGTAGAAATATGGTTTAAGGTTTTTACATTGTTAATTGCTAAA [11778]

lp17_gi|365823332|B31_GB     TTTTCTGCTAGATAGACTTTATAATTAATAAATATCCCTTTTAGATTTTTAATTTTAGCTTGTTTAAATTCATAGTTT [11856]
lp17_un5_B31_PacBio          TTTTCTGCTAGATAGACTTTATAATTAATAAATATCCCTTTTAGATTTTTAATTTTAGCTTGTTTAAATTCATAGTTT [11856]
lp17_B31_TS_MP_FG_consensus  TTTTCTGCTAGATAGACTTTATAATTAATAAATATCCCTTTTAGATTTTTAATTTTAGCTTGTTTAAATTCATAGTTT [11856]
lp17_B31_NX_Pl_consensus     TTTTCTGCTAGATAGACTTTATAATTAATAAATATCCCTTTTAGATTTTTAATTTTAGCTTGTTTAAATTCATAGTTT [11856]
lp17_PAli_NX_MP_FG_consensus TTTTCTGCTAGATAGACTTTATAATTAATAAATATCCCTTTTAGATTTTTAATTTTAGCTTGTTTAAATTCATAGTTT [11856]
lp17_PAli_NX_Pl_consensus    TTTTCTGCTAGATAGACTTTATAATTAATAAATATCCCTTTTAGATTTTTAATTTTAGCTTGTTTAAATTCATAGTTT [11856]
lp17_PAbe_NX_MP_FG_consensus TTTTCTGCTAGATAGACTTTATAATTAATAAATATCCCTTTTAGATTTTTAATTTTAGCTTGTTTAAATTCATAGTTT [11856]
lp17_PAbe_NX_Pl_consensus    TTTTCTGCTAGATAGACTTTATAATTAATAAATATCCCTTTTAGATTTTTAATTTTAGCTTGTTTAAATTCATAGTTT [11856]

lp17_gi|365823332|B31_GB     ATATTTTGAGCATATATTGCCAAATTTTTAATCAAAAATATTAAGAATATAAAAGTTTTATTTTTCATTATACAACTT [11934]
lp17_un5_B31_PacBio          ATATTTTGAGCATATATTGCCAAATTTTTAATCAAAAATATTAAGAATATAAAAGTTTTATTTTTCATTATACAACTT [11934]
lp17_B31_TS_MP_FG_consensus  ATATTTTGAGCATATATTGCCAAATTTTTAATCAAAAATATTAAGAATATAAAAGTTTTATTTTTCATTATACAACTT [11934]
lp17_B31_NX_Pl_consensus     ATATTTTGAGCATATATTGCCAAATTTTTAATCAAAAATATTAAGAATATAAAAGTTTTATTTTTCATTATACAACTT [11934]
lp17_PAli_NX_MP_FG_consensus ATATTTTGAGCATATATTGCCAAATTTTTAATCAAAAATATTAAGAATATAAAAGTTTTATTTTTCATTATACAACTT [11934]
lp17_PAli_NX_Pl_consensus    ATATTTTGAGCATATATTGCCAAATTTTTAATCAAAAATATTAAGAATATAAAAGTTTTATTTTTCATTATACAACTT [11934]
lp17_PAbe_NX_MP_FG_consensus ATATTTTGAGCATATATTGCCAAATTTTTAATCAAAAATATTAAGAATATAAAAGTTTTATTTTTCATTATACAACTT [11934]
lp17_PAbe_NX_Pl_consensus    ATATTTTGAGCATATATTGCCAAATTTTTAATCAAAAATATTAAGAATATAAAAGTTTTATTTTTCATTATACAACTT [11934]

lp17_gi|365823332|B31_GB     ATTATATGATAGCAGTCTTGAATAATTTTAGTAAACCTTTTTTATAGATTAAGTCTGAAATTTATAAAAAATTTTAGTT [12012]
lp17_un5_B31_PacBio          ATTATATGATAGCAGTCTTGAATAATTTTAGTAAACCTTTTTTATAGATTAAGTCTGAAATTTATAAAAAATTTTAGTT [12012]
lp17_B31_TS_MP_FG_consensus  ATTATATGATAGCAGTCTTGAATAATTTTAGTAAACCTTTTTTATAGATTAAGTCTGAAATTTATAAAAAATTTTAGTT [12012]
lp17_B31_NX_Pl_consensus     ATTATATGATAGCAGTCTTGAATAATTTTAGTAAACCTTTTTTATAGATTAAGTCTGAAATTTATAAAAAATTTTAGTT [12012]
lp17_PAli_NX_MP_FG_consensus ATTATATGATAGCAGTCTTGAATAATTTTAGTAAACCTTTTTTATAGATTAAGTCTGAAATTTATAAAAAATTTTAGTT [12012]
lp17_PAli_NX_Pl_consensus    ATTATATGATAGCAGTCTTGAATAATTTTAGTAAACCTTTTTTATAGATTAAGTCTGAAATTTATAAAAAATTTTAGTT [12012]
lp17_PAbe_NX_MP_FG_consensus ATTATATGATAGCAGTCTTGAATAATTTTAGTAAACCTTTTTTATAGATTAAGTCTGAAATTTATAAAAAATTTTAGTT [12012]
lp17_PAbe_NX_Pl_consensus    ATTATATGATAGCAGTCTTGAATAATTTTAGTAAACCTTTTTTATAGATTAAGTCTGAAATTTATAAAAAATTTTAGTT [12012]

lp17_gi|365823332|B31_GB     ATAAAAGTAGTTTTTGTTGATTTTTTAGAGAATATGTCTTTCAATATTTTGCAGAGATTTGTGTTAGATGATAATCTA [12090]
lp17_un5_B31_PacBio          ATAAAAGTAGTTTTTGTTGATTTTTTAGAGAATATGTCTTTCAATATTTTGCAGAGATTTGTGTTAGATGATAATCTA [12090]
lp17_B31_TS_MP_FG_consensus  ATAAAAGTAGTTTTTGTTGATTTTTTAGAGAATATGTCTTTCAATATTTTGCAGAGATTTGTGTTAGATGATAATCTA [12090]
lp17_B31_NX_Pl_consensus     ATAAAAGTAGTTTTTGTTGATTTTTTAGAGAATATGTCTTTCAATATTTTGCAGAGATTTGTGTTAGATGATAATCTA [12090]
lp17_PAli_NX_MP_FG_consensus ATAAAAGTAGTTTTTGTTGATTTTTTAGAGAATATGTCTTTCAATATTTTGCAGAGATTTGTGTTAGATGATAATCTA [12090]
lp17_PAli_NX_Pl_consensus    ATAAAAGTAGTTTTTGTTGATTTTTTAGAGAATATGTCTTTCAATATTTTGCAGAGATTTGTGTTAGATGATAATCTA [12090]
lp17_PAbe_NX_MP_FG_consensus ATAAAAGTAGTTTTTGTTGATTTTTTAGAGAATATGTCTTTCAATATTTTGCAGAGATTTGTGTTAGATGATAATCTA [12090]
lp17_PAbe_NX_Pl_consensus    ATAAAAGTAGTTTTTGTTGATTTTTTAGAGAATATGTCTTTCAATATTTTGCAGAGATTTGTGTTAGATGATAATCTA [12090]

lp17_gi|365823332|B31_GB     CTATTTATAAATCAAGGATTATAAAAACAGCTTTTTCCTTTATTCTGGATAGCTATACAATAATTGTCTTACAATTTC [12168]
lp17_un5_B31_PacBio          CTATTTATAAATCAAGGATTATAAAAACAGCTTTTTCCTTTATTCTGGATAGCTATACAATAATTGTCTTACAATTTC [12168]
lp17_B31_TS_MP_FG_consensus  CTATTTATAAATCAAGGATTATAAAAACAGCTTTTTCCTTTATTCTGGATAGCTATACAATAATTGTCTTACAATTTC [12168]
lp17_B31_NX_Pl_consensus     CTATTTATAAATCAAGGATTATAAAAACAGCTTTTTCCTTTATTCTGGATAGCTATACAATAATTGTCTTACAATTTC [12168]
lp17_PAli_NX_MP_FG_consensus CTATTTATAAATCAAGGATTATAAAAACAGCTTTTTCCTTTATTCTGGATAGCTATACAATAATTGTCTTACAATTTC [12168]
lp17_PAli_NX_Pl_consensus    CTATTTATAAATCAAGGATTATAAAAACAGCTTTTTCCTTTATTCTGGATAGCTATACAATAATTGTCTTACAATTTC [12168]
lp17_PAbe_NX_MP_FG_consensus CTATTTATAAATCAAGGATTATAAAAACAGCTTTTTCCTTTATTCTGGATAGCTATACAATAATTGTCTTACAATTTC [12168]
lp17_PAbe_NX_Pl_consensus    CTATTTATAAATCAAGGATTATAAAAACAGCTTTTTCCTTTATTCTGGATAGCTATACAATAATTGTCTTACAATTTC [12168]

lp17_gi|365823332|B31_GB     ATGCAATTCTAAATCATTAATACTCTTTCTAAACATTCCTTTTTGTATAGCTTTAATTTATAGTTTTCTATTGCTATG [12246]
lp17_un5_B31_PacBio          ATGCAATTCTAAATCATTAATACTCTTTCTAAACATTCCTTTTTGTATAGCTTTAATTTATAGTTTTCTATTGCTATG [12246]
lp17_B31_TS_MP_FG_consensus  ATGCAATTCTAAATCATTAATACTCTTTCTAAACATTCCTTTTTGTATAGCTTTAATTTATAGTTTTCTATTGCTATG [12246]
lp17_B31_NX_Pl_consensus     ATGCAATTCTAAATCATTAATACTCTTTCTAAACATTCCTTTTTGTATAGCTTTAATTTATAGTTTTCTATTGCTATG [12246]
lp17_PAli_NX_MP_FG_consensus ATGCAATTCTAAATCATTAATACTCTTTCTAAACATTCCTTTTTGTATAGCTTTAATTTATAGTTTTCTATTGCTATG [12246]
```

```
lp17_PAli_NX_Pl_consensus    ATGCAATTCTAAATCATTAATACTCTTTCTAAACATTCCTTTTTGTATAGCTTTAATTTATAGTTTTCTATTGCTATG [12246]
lp17_PAbe_NX_MP_FG_consensus ATGCAATTCTAAATCATTAATACTCTTTCTAAACATTCCTTTTTGTATAGCTTTAATTTATAGTTTTCTATTGCTATG [12246]
lp17_PAbe_NX_Pl_consensus    ATGCAATTCTAAATCATTAATACTCTTTCTAAACATTCCTTTTTGTATAGCTTTAATTTATAGTTTTCTATTGCTATG [12246]

lp17_gi|365823332|B31_GB     TTTTTATAATTAGCCATAAATATAGGCATAAATGTGTAAATTCTAAGCTTTCATCTTTTTTTATCCAAATAATACTTT [12324]
lp17_un5_B31_PacBio          TTTTTATAATTAGCCATAAATATAGGCATAAATGTGTAAATTCTAAGCTTTCATCTTTTTTTATCCAAATAATACTTT [12324]
lp17_B31_TS_MP_FG_consensus  TTTTTATAATTAGCCATAAATATAGGCATAAATGTGTAAATTCTAAGCTTTCATCTTTTTTTATCCAAATAATACTTT [12324]
lp17_B31_NX_Pl_consensus     TTTTTATAATTAGCCATAAATATAGGCATAAATGTGTAAATTCTAAGCTTTCATCTTTTTTTATCCAAATAATACTTT [12324]
lp17_PAli_NX_MP_FG_consensus TTTTTATAATTAGCCATAAATATAGGCATAAATGTGTAAATTCTAAGCTTTCATCTTTTTTTATCCAAATAATACTTT [12324]
lp17_PAli_NX_Pl_consensus    TTTTTATAATTAGCCATAAATATAGGCATAAATGTGTAAATTCTAAGCTTTCATCTTTTTTTATCCAAATAATACTTT [12324]
lp17_PAbe_NX_MP_FG_consensus TTTTTATAATTAGCCATAAATATAGGCATAAATGTGTAAATTCTAAGCTTTCATCTTTTTTTATCCAAATAATACTTT [12324]
lp17_PAbe_NX_Pl_consensus    TTTTTATAATTAGCCATAAATATAGGCATAAATGTGTAAATTCTAAGCTTTCATCTTTTTTTATCCAAATAATACTTT [12324]

lp17_gi|365823332|B31_GB     ATTTTCTGTTCTGTGTAGAAAAGAACCCTTAATAAATCCAATTATGACAATGATTACATTCATTTATTATTAGAATTT [12402]
lp17_un5_B31_PacBio          ATTTTCTGTTCTGTGTAGAAAAGAACCCTTAATAAATCCAATTATGACAATGATTACATTCATTTATTATTAGAATTT [12402]
lp17_B31_TS_MP_FG_consensus  ATTTTCTGTTCTGTGTAGAAAAGAACCCTTAATAAATCCAATTATGACAATGATTACATTCATTTATTATTAGAATTT [12402]
lp17_B31_NX_Pl_consensus     ATTTTCTGTTCTGTGTAGAAAAGAACCCTTAATAAATCCAATTATGACAATGATTACATTCATTTATTATTAGAATTT [12402]
lp17_PAli_NX_MP_FG_consensus ATTTTCTGTTCTGTGTAGAAAAGAACCCTTAATAAATCCAATTATGACAATGATTACATTCATTTATTATTAGAATTT [12402]
lp17_PAli_NX_Pl_consensus    ATTTTCTGTTCTGTGTAGAAAAGAACCCTTAATAAATCCAATTATGACAATGATTACATTCATTTATTATTAGAATTT [12402]
lp17_PAbe_NX_MP_FG_consensus ATTTTCTGTTCTGTGTAGAAAAGAACCCTTAATAAATCCAATTATGACAATGATTACATTCATTTATTATTAGAATTT [12402]
lp17_PAbe_NX_Pl_consensus    ATTTTCTGTTCTGTGTAGAAAAGAACCCTTAATAAATCCAATTATGACAATGATTACATTCATTTATTATTAGAATTT [12402]

lp17_gi|365823332|B31_GB     GCCTCCAATATTTAACTTTCTAAATTCATCAATAACCCAAAGATAGTAATCTTAAAGATTTATAAGAAAAAATATTTT [12480]
lp17_un5_B31_PacBio          GCCTCCAATATTTAACTTTCTAAATTCATCAATAACCCAAAGATAGTAATCTTAAAGATTTATAAGAAAAAATATTTT [12480]
lp17_B31_TS_MP_FG_consensus  GCCTCCAATATTTAACTTTCTAAATTCATCAATAACCCAAAGATAGTAATCTTAAAGATTTATAAGAAAAAATATTTT [12480]
lp17_B31_NX_Pl_consensus     GCCTCCAATATTTAACTTTCTAAATTCATCAATAACCCAAAGATAGTAATCTTAAAGATTTATAAGAAAAAATATTTT [12480]
lp17_PAli_NX_MP_FG_consensus GCCTCCAATATTTAACTTTCTAAATTCATCAATAACCCAAAGATAGTAATCTTAAAGATTTATAAGAAAAAATATTTT [12480]
lp17_PAli_NX_Pl_consensus    GCCTCCAATATTTAACTTTCTAAATTCATCAATAACCCAAAGATAGTAATCTTAAAGATTTATAAGAAAAAATATTTT [12480]
lp17_PAbe_NX_MP_FG_consensus GCCTCCAATATTTAACTTTCTAAATTCATCAATAACCCAAAGATAGTAATCTTAAAGATTTATAAGAAAAAATATTTT [12480]
lp17_PAbe_NX_Pl_consensus    GCCTCCAATATTTAACTTTCTAAATTCATCAATAACCCAAAGATAGTAATCTTAAAGATTTATAAGAAAAAATATTTT [12480]

lp17_gi|365823332|B31_GB     ATTTATTTATATGAGCATTACTTAAGCCATTATTCTAGCTTTGAATAATATTTGATCTTCTCTGCAAAAATCTAATTT [12558]
lp17_un5_B31_PacBio          ATTTATTTATATGAGCATTACTTAAGCCATTATTCTAGCTTTGAATAATATTTGATCTTCTCTGCAAAAATCTAATTT [12558]
lp17_B31_TS_MP_FG_consensus  ATTTATTTATATGAGCATTACTTAAGCCATTATTCTAGCTTTGAATAATATTTGATCTTCTCTGCAAAAATCTAATTT [12558]
lp17_B31_NX_Pl_consensus     ATTTATTTATATGAGCATTACTTAAGCCATTATTCTAGCTTTGAATAATATTTGATCTTCTCTGCAAAAATCTAATTT [12558]
lp17_PAli_NX_MP_FG_consensus ATTTATTTATATGAGCATTACTTAAGCCATTATTCTAGCTTTGAATAATATTTGATCTTCTCTGCAAAAATCTAATTT [12558]
lp17_PAli_NX_Pl_consensus    ATTTATTTATATGAGCATTACTTAAGCCATTATTCTAGCTTTGAATAATATTTGATCTTCTCTGCAAAAATCTAATTT [12558]
lp17_PAbe_NX_MP_FG_consensus ATTTATTTATATGAGCATTACTTAAGCCATTATTCTAGCTTTGAATAATATTTGATCTTCTCTGCAAAAATCTAATTT [12558]
lp17_PAbe_NX_Pl_consensus    ATTTATTTATATGAGCATTACTTAAGCCATTATTCTAGCTTTGAATAATATTTGATCTTCTCTGCAAAAATCTAATTT [12558]

lp17_gi|365823332|B31_GB     AAAAAAAAATTTTAAAAATACATGGTAATACTCAATTATTGAACTAATAATTGAGTATTAAATATTCTCCTTTTTTAA [12636]
lp17_un5_B31_PacBio          AAAAAAAAATTTTAAAAATACATGGTAATACTCAATTATTGAACTAATAATTGAGTATTAAATATTCTCCTTTTTTAA [12636]
lp17_B31_TS_MP_FG_consensus  AAAAAAAAATTTTAAAAATACATGGTAATACTCAATTATTGAACTAATAATTGAGTATTAAATATTCTCCTTTTTTAA [12636]
lp17_B31_NX_Pl_consensus     AAAAAAAAATTTTAAAAATACATGGTAATACTCAATTATTGAACTAATAATTGAGTATTAAATATTCTCCTTTTTTAA [12636]
lp17_PAli_NX_MP_FG_consensus AAAAAAAAATTTTAAAAATACATGGTAATACTCAATTATTGAACTAATAATTGAGTATTAAATATTCTCCTTTTTTAA [12636]
lp17_PAli_NX_Pl_consensus    AAAAAAAAATTTTAAAAATACATGGTAATACTCAATTATTGAACTAATAATTGAGTATTAAATATTCTCCTTTTTTAA [12636]
lp17_PAbe_NX_MP_FG_consensus AAAAAAAAATTTTAAAAATACATGGTAATACTCAATTATTGAACTAATAATTGAGTATTAAATATTCTCCTTTTTTAA [12636]
lp17_PAbe_NX_Pl_consensus    AAAAAAAAATTTTAAAAATACATGGTAATACTCAATTATTGAACTAATAATTGAGTATTAAATATTCTCCTTTTTTAA [12636]

lp17_gi|365823332|B31_GB     AATTAAAAGAATTTATTATCAATATTTACTTCATACCATACATTCTTTTAAATAAACCTCTTATCTTTAAGGGGTTTT [12714]
lp17_un5_B31_PacBio          AATTAAAAGAATTTATTATCAATATTTACTTCATACCATACATTCTTTTAAATAAACCTCTTATCTTTAAGGGGTTTT [12714]
lp17_B31_TS_MP_FG_consensus  AATTAAAAGAATTTATTATCAATATTTACTTCATACCATACATTCTTTTAAATAAACCTCTTATCTTTAAGGGGTTTT [12714]
lp17_B31_NX_Pl_consensus     AATTAAAAGAATTTATTATCAATATTTACTTCATACCATACATTCTTTTAAATAAACCTCTTATCTTTAAGGGGTTTT [12714]
lp17_PAli_NX_MP_FG_consensus AATTAAAAGAATTTATTATCAATATTTACTTCATACCATACATTCTTTTAAATAAACCTCTTATCTTTAAGGGGTTTT [12714]
lp17_PAli_NX_Pl_consensus    AATTAAAAGAATTTATTATCAATATTTACTTCATACCATACATTCTTTTAAATAAACCTCTTATCTTTAAGGGGTTTT [12714]
lp17_PAbe_NX_MP_FG_consensus AATTAAAAGAATTTATTATCAATATTTACTTCATACCATACATTCTTTTAAATAAACCTCTTATCTTTAAGGGGTTTT [12714]
lp17_PAbe_NX_Pl_consensus    AATTAAAAGAATTTATTATCAATATTTACTTCATACCATACATTCTTTTAAATAAACCTCTTATCTTTAAGGGGTTTT [12714]

lp17_gi|365823332|B31_GB     CTTTTTATTAAATCTTTAAGATTACTATCTTTGGGTTATTGATGAATTTAGAAAGTTAAATATTGGAGGCAAATTCTA [12792]
lp17_un5_B31_PacBio          CTTTTTATTAAATCTTTAAGATTACTATCTTTGGGTTATTGATGAATTTAGAAAGTTAAATATTGGAGGCAAATTCTA [12792]
lp17_B31_TS_MP_FG_consensus  CTTTTTATTAAATCTTTAAGATTACTATCTTTGGGTTATTGATGAATTTAGAAAGTTAAATATTGGAGGCAAATTCTA [12792]
lp17_B31_NX_Pl_consensus     CTTTTTATTAAATCTTTAAGATTACTATCTTTGGGTTATTGATGAATTTAGAAAGTTAAATATTGGAGGCAAATTCTA [12792]
lp17_PAli_NX_MP_FG_consensus CTTTTTATTAAATCTTTAAGATTACTATCTTTGGGTTATTGATGAATTTAGAAAGTTAAATATTGGAGGCAAATTCTA [12792]
lp17_PAli_NX_Pl_consensus    CTTTTTATTAAATCTTTAAGATTACTATCTTTGGGTTATTGATGAATTTAGAAAGTTAAATATTGGAGGCAAATTCTA [12792]
lp17_PAbe_NX_MP_FG_consensus CTTTTTATTAAATCTTTAAGATTACTATCTTTGGGTTATTGATGAATTTAGAAAGTTAAATATTGGAGGCAAATTCTA [12792]
lp17_PAbe_NX_Pl_consensus    CTTTTTATTAAATCTTTAAGATTACTATCTTTGGGTTATTGATGAATTTAGAAAGTTAAATATTGGAGGCAAATTCTA [12792]

lp17_gi|365823332|B31_GB     ATAATAAATGAATGTAATCATTGTCATAATTGGATTTATTAAGGGTTCTTTTACATAAAGAATTTGCTATGCAATTTT [12870]
lp17_un5_B31_PacBio          ATAATAAATGAATGTAATCATTGTCATAATTGGATTTATTAAGGGTTCTTTTACATAAAGAATTTGCTATGCAATTTT [12870]
lp17_B31_TS_MP_FG_consensus  ATAATAAATGAATGTAATCATTGTCATAATTGGATTTATTAAGGGTTCTTTTACATAAAGAATTTGCTATGCAATTTT [12870]
lp17_B31_NX_Pl_consensus     ATAATAAATGAATGTAATCATTGTCATAATTGGATTTATTAAGGGTTCTTTTACATAAAGAATTTGCTATGCAATTTT [12870]
lp17_PAli_NX_MP_FG_consensus ATAATAAATGAATGTAATCATTGTCATAATTGGATTTATTAAGGGTTCTTTTACATAAAGAATTTGCTATGCAATTTT [12870]
lp17_PAli_NX_Pl_consensus    ATAATAAATGAATGTAATCATTGTCATAATTGGATTTATTAAGGGTTCTTTTACATAAAGAATTTGCTATGCAATTTT [12870]
lp17_PAbe_NX_MP_FG_consensus ATAATAAATGAATGTAATCATTGTCATAATTGGATTTATTAAGGGTTCTTTTACATAAAGAATTTGCTATGCAATTTT [12870]
lp17_PAbe_NX_Pl_consensus    ATAATAAATGAATGTAATCATTGTCATAATTGGATTTATTAAGGGTTCTTTTACATAAAGAATTTGCTATGCAATTTT [12870]

lp17_gi|365823332|B31_GB     AATTTTTGTTTGTAATCGCCAATTATTAAAAATAAAATTATTAAAGGGAATTAATGTAAAATTAATTACCTTTAAATA [12948]
lp17_un5_B31_PacBio          AATTTTTGTTTGTAATCGCCAATTATTAAAAATAAAATTATTAAAGGGAATTAATGTAAAATTAATTACCTTTAAATA [12948]
lp17_B31_TS_MP_FG_consensus  AATTTTTGTTTGTAATCGCCAATTATTAAAAATAAAATTATTAAAGGGAATTAATGTAAAATTAATTACCTTTAAATA [12948]
lp17_B31_NX_Pl_consensus     AATTTTTGTTTGTAATCGCCAATTATTAAAAATAAAATTATTAAAGGGAATTAATGTAAAATTAATTACCTTTAAATA [12948]
lp17_PAli_NX_MP_FG_consensus AATTTTTGTTTGTAATCGCCAATTATTAAAAATAAAATTATTAAAGGGAATTAATGTAAAATTAATTACCTTTAAATA [12948]
lp17_PAli_NX_Pl_consensus    AATTTTTGTTTGTAATCGCCAATTATTAAAAATAAAATTATTAAAGGGAATTAATGTAAAATTAATTACCTTTAAATA [12948]
lp17_PAbe_NX_MP_FG_consensus AATTTTTGTTTGTAATCGCCAATTATTAAAAATAAAATTATTAAAGGGAATTAATGTAAAATTAATTACCTTTAAATA [12948]
lp17_PAbe_NX_Pl_consensus    AATTTTTGTTTGTAATCGCCAATTATTAAAAATAAAATTATTAAAGGGAATTAATGTAAAATTAATTACCTTTAAATA [12948]

lp17_gi|365823332|B31_GB     TACTTCTTTTAGAGAATATATCATTACTAATATTAATCTATTACTAATGATATTATTAAATTAAAGATCTATAGAAAC [13026]
lp17_un5_B31_PacBio          TACTTCTTTTAGAGAATATATCATTACTAATATTAATCTATTACTAATGATATTATTAAATTAAAGATCTATAGAAAC [13026]
lp17_B31_TS_MP_FG_consensus  TACTTCTTTTAGAGAATATATCATTACTAATATTAATCTATTACTAATGATATTATTAAATTAAAGATCTATAGAAAC [13026]
lp17_B31_NX_Pl_consensus     TACTTCTTTTAGAGAATATATCATTACTAATATTAATCTATTACTAATGATATTATTAAATTAAAGATCTATAGAAAC [13026]
lp17_PAli_NX_MP_FG_consensus TACTTCTTTTAGAGAATATATCATTACTAATATTAATCTATTACTAATGATATTATTAAATTAAAGATCTATAGAAAC [13026]
lp17_PAli_NX_Pl_consensus    TACTTCTTTTAGAGAATATATCATTACTAATATTAATCTATTACTAATGATATTATTAAATTAAAGATCTATAGAAAC [13026]
lp17_PAbe_NX_MP_FG_consensus TACTTCTTTTAGAGAATATATCATTACTAATATTAATCTATTACTAATGATATTATTAAATTAAAGATCTATAGAAAC [13026]
lp17_PAbe_NX_Pl_consensus    TACTTCTTTTAGAGAATATATCATTACTAATATTAATCTATTACTAATGATATTATTAAATTAAAGATCTATAGAAAC [13026]

lp17_gi|365823332|B31_GB     ATCAGTTTACTTATTATTAGAATTAAAGTTTATGTAAATTATAGGTTAAAAATTTTTTGTAATCTTTAGTAAGAATGT [13104]
lp17_un5_B31_PacBio          ATCAGTTTACTTATTATTAGAATTAAAGTTTATGTAAATTATAGGTTAAAAATTTTTTGTAATCTTTAGTAAGAATGT [13104]
lp17_B31_TS_MP_FG_consensus  ATCAGTTTACTTATTATTAGAATTAAAGTTTATGTAAATTATAGGTTAAAAATTTTTTGTAATCTTTAGTAAGAATGT [13104]
lp17_B31_NX_Pl_consensus     ATCAGTTTACTTATTATTAGAATTAAAGTTTATGTAAATTATAGGTTAAAAATTTTTTGTAATCTTTAGTAAGAATGT [13104]
lp17_PAli_NX_MP_FG_consensus ATCAGTTTACTTATTATTAGAATTAAAGTTTATGTAAATTATAGGTTAAAAATTTTTTGTAATCTTTAGTAAGAATGT [13104]
lp17_PAli_NX_Pl_consensus    ATCAGTTTACTTATTATTAGAATTAAAGTTTATGTAAATTATAGGTTAAAAATTTTTTGTAATCTTTAGTAAGAATGT [13104]
lp17_PAbe_NX_MP_FG_consensus ATCAGTTTACTTATTATTAGAATTAAAGTTTATGTAAATTATAGGTTAAAAATTTTTTGTAATCTTTAGTAAGAATGT [13104]
lp17_PAbe_NX_Pl_consensus    ATCAGTTTACTTATTATTAGAATTAAAGTTTATGTAAATTATAGGTTAAAAATTTTTTGTAATCTTTAGTAAGAATGT [13104]

lp17_gi|365823332|B31_GB     TAAACCTATCTTTTATTAAAGACAGGCTTTGTATTATAACAATTTCAATATGCAATTTAAAGGCTACTATATATTGAA [13182]
lp17_un5_B31_PacBio          TAAACCTATCTTTTATTAAAGACAGGCTTTGTATTATAACAATTTCAATATGCAATTTAAAGGCTACTATATATTGAA [13182]
lp17_B31_TS_MP_FG_consensus  TAAACCTATCTTTTATTAAAGACAGGCTTTGTATTATAACAATTTCAATATGCAATTTAAAGGCTACTATATATTGAA [13182]
lp17_B31_NX_Pl_consensus     TAAACCTATCTTTTATTAAAGACAGGCTTTGTATTATAACAATTTCAATATGCAATTTAAAGGCTACTATATATTGAA [13182]
lp17_PAli_NX_MP_FG_consensus TAAACCTATCTTTTATTAAAGACAGGCTTTGTATTATAACAATTTCAATATGCAATTTAAAGGCTACTATATATTGAA [13182]
lp17_PAli_NX_Pl_consensus    TAAACCTATCTTTTATTAAAGACAGGCTTTGTATTATAACAATTTCAATATGCAATTTAAAGGCTACTATATATTGAA [13182]
```

```
lp17_PAbe_NX_MP_FG_consensus TAAACCTATCTTTTATTAAAGACAGGCTTTGTATTATAACAATTTCAATATGCAATTTAAAGGCTACTATATATTGAA [13182]
lp17_PAbe_NX_Pl_consensus    TAAACCTATCTTTTATTAAAGACAGGCTTTGTATTATAACAATTTCAATATGCAATTTAAAGGCTACTATATATTGAA [13182]

lp17_gi|365823332|B31_GB     TATATGTCATTTTTTAGTTTTTCTGCTTCTTTTCTTTTTTCTGAGACTTGATTGCAAATATCTTCTGTATGAGATTTA [13260]
lp17_un5_B31_PacBio          TATATGTCATTTTTTAGTTTTTCTGCTTCTTTTCTTTTTTCTGAGACTTGATTGCAAATATCTTCTGTATGAGATTTA [13260]
lp17_B31_TS_MP_FG_consensus  TATATGTCATTTTTTAGTTTTTCTGCTTCTTTTCTTTTTTCTGAGACTTGATTGCAAATATCTTCTGTATGAGATTTA [13260]
lp17_B31_NX_Pl_consensus     TATATGTCATTTTTTAGTTTTTCTGCTTCTTTTCTTTTTTCTGAGACTTGATTGCAAATATCTTCTGTATGAGATTTA [13260]
lp17_PAli_NX_MP_FG_consensus TATATGTCATTTTTTAGTTTTTCTGCTTCTTTTCTTTTTTCTGAGACTTGATTGCAAATATCTTCTGTATGAGATTTA [13260]
lp17_PAli_NX_Pl_consensus    TATATGTCATTTTTTAGTTTTTCTGCTTCTTTTCTTTTTTCTGAGACTTGATTGCAAATATCTTCTGTATGAGATTTA [13260]
lp17_PAbe_NX_MP_FG_consensus TATATGTCATTTTTTAGTTTTTCTGCTTCTTTTCTTTTTTCTGAGACTTGATTGCAAATATCTTCTGTATGAGATTTA [13260]
lp17_PAbe_NX_Pl_consensus    TATATGTCATTTTTTAGTTTTTCTGCTTCTTTTCTTTTTTCTGAGACTTGATTGCAAATATCTTCTGTATGAGATTTA [13260]

lp17_gi|365823332|B31_GB     AGTGCATTTTCATCTGTTTGTATGTTATTTATATTATTTTGATAGTCTAATAAGAGTTGCTGCATCATTTCTGAGACT [13338]
lp17_un5_B31_PacBio          AGTGCATTTTCATCTGTTTGTATGTTATTTATATTATTTTGATAGTCTAATAAGAGTTGCTGCATCATTTCTGAGACT [13338]
lp17_B31_TS_MP_FG_consensus  AGTGCATTTTCATCTGTTTGTATGTTATTTATATTATTTTGATAGTCTAATAAGAGTTGCTGCATCATTTCTGAGACT [13338]
lp17_B31_NX_Pl_consensus     AGTGCATTTTCATCTGTTTGTATGTTATTTATATTATTTTGATAGTCTAATAAGAGTTGCTGCATCATTTCTGAGACT [13338]
lp17_PAli_NX_MP_FG_consensus AGTGCATTTTCATCTGTTTGTATGTTATTTATATTATTTTGATAGTCTAATAAGAGTTGCTGCATCATTTCTGAGACT [13338]
lp17_PAli_NX_Pl_consensus    AGTGCATTTTCATCTGTTTGTATGTTATTTATATTATTTTGATAGTCTAATAAGAGTTGCTGCATCATTTCTGAGACT [13338]
lp17_PAbe_NX_MP_FG_consensus AGTGCATTTTCATCTGTTTGTATGTTATTTATATTATTTTGATAGTCTAATAAGAGTTGCTGCATCATTTCTGAGACT [13338]
lp17_PAbe_NX_Pl_consensus    AGTGCATTTTCATCTGTTTGTATGTTATTTATATTATTTTGATAGTCTAATAAGAGTTGCTGCATCATTTCTGAGACT [13338]

lp17_gi|365823332|B31_GB     GTTGTTTTTGTAGATAAAAATTTTCCCAAATAATATTTAAGCAATACAAGATTTAGTAAAGCGTATATTTTTATTTTT [13416]
lp17_un5_B31_PacBio          GTTGTTTTTGTAGATAAAAATTTTCCCAAATAATATTTAAGCAATACAAGATTTAGTAAAGCGTATATTTTTATTTTT [13416]
lp17_B31_TS_MP_FG_consensus  GTTGTTTTTGTAGATAAAAATTTTCCCAAATAATATTTAAGCAATACAAGATTTAGTAAAGCGTATATTTTTATTTTT [13416]
lp17_B31_NX_Pl_consensus     GTTGTTTTTGTAGATAAAAATTTTCCCAAATAATATTTAAGCAATACAAGATTTAGTAAAGCGTATATTTTTATTTTT [13416]
lp17_PAli_NX_MP_FG_consensus GTTGTTTTTGTAGATAAAAATTTTCCCAAATAATATTTAAGCAATACAAGATTTAGTAAAGCGTATATTTTTATTTTT [13416]
lp17_PAli_NX_Pl_consensus    GTTGTTTTTGTAGATAAAAATTTTCCCAAATAATATTTAAGCAATACAAGATTTAGTAAAGCGTATATTTTTATTTTT [13416]
lp17_PAbe_NX_MP_FG_consensus GTTGTTTTTGTAGATAAAAATTTTCCCAAATAATATTTAAGCAATACAAGATTTAGTAAAGCGTATATTTTTATTTTT [13416]
lp17_PAbe_NX_Pl_consensus    GTTGTTTTTGTAGATAAAAATTTTCCCAAATAATATTTAAGCAATACAAGATTTAGTAAAGCGTATATTTTTATTTTT [13416]

lp17_gi|365823332|B31_GB     TGATTTAATTTCAGTTATTTTTAGGATTTTTATTATTTTAATCATATTTTCTTTATCAATATTTAATGTTAATAAAAT [13494]
lp17_un5_B31_PacBio          TGATTTAATTTCAGTTATTTTTAGGATTTTTATTATTTTAATCATATTTTCTTTATCAATATTTAATGTTAATAAAAT [13494]
lp17_B31_TS_MP_FG_consensus  TGATTTAATTTCAGTTATTTTTAGGATTTTTATTATTTTAATCATATTTTCTTTATCAATATTTAATGTTAATAAAAT [13494]
lp17_B31_NX_Pl_consensus     TGATTTAATTTCAGTTATTTTTAGGATTTTTATTATTTTAATCATATTTTCTTTATCAATATTTAATGTTAATAAAAT [13494]
lp17_PAli_NX_MP_FG_consensus TGATTTAATTTCAGTTATTTTTAGGATTTTTATTATTTTAATCATATTTTCTTTATCAATATTTAATGTTAATAAAAT [13494]
lp17_PAli_NX_Pl_consensus    TGATTTAATTTCAGTTATTTTTAGGATTTTTATTATTTTAATCATATTTTCTTTATCAATATTTAATGTTAATAAAAT [13494]
lp17_PAbe_NX_MP_FG_consensus TGATTTAATTTCAGTTATTTTTAGGATTTTTATTATTTTAATCATATTTTCTTTATCAATATTTAATGTTAATAAAAT [13494]
lp17_PAbe_NX_Pl_consensus    TGATTTAATTTCAGTTATTTTTAGGATTTTTATTATTTTAATCATATTTTCTTTATCAATATTTAATGTTAATAAAAT [13494]

lp17_gi|365823332|B31_GB     TGAAATAATTTCTTTACATAAAAAGTCACATTTATTGAAATGCTTTATTACTTGATACTTTTCTATTTCGTTAATTTT [13572]
lp17_un5_B31_PacBio          TGAAATAATTTCTTTACATAAAAAGTCACATTTATTGAAATGCTTTATTACTTGATACTTTTCTATTTCGTTAATTTT [13572]
lp17_B31_TS_MP_FG_consensus  TGAAATAATTTCTTTACATAAAAAGTCACATTTATTGAAATGCTTTATTACTTGATACTTTTCTATTTCGTTAATTTT [13572]
lp17_B31_NX_Pl_consensus     TGAAATAATTTCTTTACATAAAAAGTCACATTTATTGAAATGCTTTATTACTTGATACTTTTCTATTTCGTTAATTTT [13572]
lp17_PAli_NX_MP_FG_consensus TGAAATAATTTCTTTACATAAAAAGTCACATTTATTGAAATGCTTTATTACTTGATACTTTTCTATTTCGTTAATTTT [13572]
lp17_PAli_NX_Pl_consensus    TGAAATAATTTCTTTACATAAAAAGTCACATTTATTGAAATGCTTTATTACTTGATACTTTTCTATTTCGTTAATTTT [13572]
lp17_PAbe_NX_MP_FG_consensus TGAAATAATTTCTTTACATAAAAAGTCACATTTATTGAAATGCTTTATTACTTGATACTTTTCTATTTCGTTAATTTT [13572]
lp17_PAbe_NX_Pl_consensus    TGAAATAATTTCTTTACATAAAAAGTCACATTTATTGAAATGCTTTATTACTTGATACTTTTCTATTTCGTTAATTTT [13572]

lp17_gi|365823332|B31_GB     TCTTTCTTCTTTTATATTATTATTACAATTCTCCAATTGTACACTACCCATTTTTGTATCAGAATTTTTATTAAAATA [13650]
lp17_un5_B31_PacBio          TCTTTCTTCTTTTATATTATTATTACAATTCTCCAATTGTACACTACCCATTTTTGTATCAGAATTTTTATTAAAATA [13650]
lp17_B31_TS_MP_FG_consensus  TCTTTCTTCTTTTATATTATTATTACAATTCTCCAATTGTACACTACCCATTTTTGTATCAGAATTTTTATTAAAATA [13650]
lp17_B31_NX_Pl_consensus     TCTTTCTTCTTTTATATTATTATTACAATTCTCCAATTGTACACTACCCATTTTTGTATCAGAATTTTTATTAAAATA [13650]
lp17_PAli_NX_MP_FG_consensus TCTTTCTTCTTTTATATTATTATTACAATTCTCCAATTGTACACTACCCATTTTTGTATCAGAATTTTTATTAAAATA [13650]
lp17_PAli_NX_Pl_consensus    TCTTTCTTCTTTTATATTATTATTACAATTCTCCAATTGTACACTACCCATTTTTGTATCAGAATTTTTATTAAAATA [13650]
lp17_PAbe_NX_MP_FG_consensus TCTTTCTTCTTTTATATTATTATTACAATTCTCCAATTGTACACTACCCATTTTTGTATCAGAATTTTTATTAAAATA [13650]
lp17_PAbe_NX_Pl_consensus    TCTTTCTTCTTTTATATTATTATTACAATTCTCCAATTGTACACTACCCATTTTTGTATCAGAATTTTTATTAAAATA [13650]

lp17_gi|365823332|B31_GB     GTTGGCAACTCTATTTTGAAATCTTTTTTCTTTTTTTTCTTTAAAGTGTTGGTTTATCTTATGGTAACAATCTTTTTT [13728]
lp17_un5_B31_PacBio          GTTGGCAACTCTATTTTGAAATCTTTTTTCTTTTTTTT-CTTTAAAGTGTTGGTTTATCTTATGGTAACAATCTTTTTT [13728]
lp17_B31_TS_MP_FG_consensus  GTTGGCAACTCTATTTTGAAATCTTTTTTCTTTTTTTTCTTTAAAGTGTTGGTTTATCTTATGGTAACAATCTTTTTT [13728]
lp17_B31_NX_Pl_consensus     GTTGGCAACTCTATTTTGAAATCTTTTTTCTTTTTTTTCTTTAAAGTGTTGGTTTATCTTATGGTAACAATCTTTTTT [13728]
lp17_PAli_NX_MP_FG_consensus GTTGGCAACTCTATTTTGAAATCTTTTTTCTTTTTTTTCTTTAAAGTGTTGGTTTATCTTATGGTAACAATCTTTTTT [13728]
lp17_PAli_NX_Pl_consensus    GTTGGCAACTCTATTTTGAAATCTTTTTTCTTTTTTTTCTTTAAAGTGTTGGTTTATCTTATGGTAACAATCTTTTTT [13728]
lp17_PAbe_NX_MP_FG_consensus GTTGGCAACTCTATTTTGAAATCTTTTTTCTTTTTTTTCTTTAAAGTGTTGGTTTATCTTATGGTAACAATCTTTTTT [13728]
lp17_PAbe_NX_Pl_consensus    GTTGGCAACTCTATTTTGAAATCTTTTTTCTTTTTTTTCTTTAAAGTGTTGGTTTATCTTATGGTAACAATCTTTTTT [13728]

lp17_gi|365823332|B31_GB     AGGATAATTAAGCTTATAATAAATTTCTGTACCCGAATTTACCCCCATATGTTGATAGTAATTTGTTGTGACTTTTAT [13806]
lp17_un5_B31_PacBio          AGGATAATTAAGCTTATAATAAATTTCTGTACCCGAATTTACCCCCATATGTTGATAGTAATTTGTTGTGACTTTTAT [13806]
lp17_B31_TS_MP_FG_consensus  AGGATAATTAAGCTTATAATAAATTTCTGTACCCGAATTTACCCCCATATGTTGATAGTAATTTGTTGTGACTTTTAT [13806]
lp17_B31_NX_Pl_consensus     AGGATAATTAAGCTTATAATAAATTTCTGTACCCGAATTTACCCCCATATGTTGATAGTAATTTGTTGTGACTTTTAT [13806]
lp17_PAli_NX_MP_FG_consensus AGGATAATTAAGCTTATAATAAATTTCTGTACCCGAATTTACCCCCATATGTTGATAGTAATTTGTTGTGACTTTTAT [13806]
lp17_PAli_NX_Pl_consensus    AGGATAATTAAGCTTATAATAAATTTCTGTACCCGAATTTACCCCCATATGTTGATAGTAATTTGTTGTGACTTTTAT [13806]
lp17_PAbe_NX_MP_FG_consensus AGGATAATTAAGCTTATAATAAATTTCTGTACCCGAATTTACCCCCATATGTTGATAGTAATTTGTTGTGACTTTTAT [13806]
lp17_PAbe_NX_Pl_consensus    AGGATAATTAAGCTTATAATAAATTTCTGTACCCGAATTTACCCCCATATGTTGATAGTAATTTGTTGTGACTTTTAT [13806]

lp17_gi|365823332|B31_GB     TTCTTTTTGTAGTCTATAAATATACTTTTGCATAGTTCTTAGTGTAGAAATAGTTTGCCCGTTTTTTATAGATTTTCA [13884]
lp17_un5_B31_PacBio          TTCTTTTTGTAGTCTATAAATATACTTTTGCATAGTTCTTAGTGTAGAAATAGTTTGCCCGTTTTTTATAGATTTTCA [13884]
lp17_B31_TS_MP_FG_consensus  TTCTTTTTGTAGTCTATAAATATACTTTTGCATAGTTCTTAGTGTAGAAATAGTTTGCCCGTTTTTTATAGATTTTCA [13884]
lp17_B31_NX_Pl_consensus     TTCTTTTTGTAGTCTATAAATATACTTTTGCATAGTTCTTAGTGTAGAAATAGTTTGCCCGTTTTTTATAGATTTTCA [13884]
lp17_PAli_NX_MP_FG_consensus TTCTTTTTGTAGTCTATAAATATACTTTTGCATAGTTCTTAGTGTAGAAATAGTTTGCCCGTTTTTTATAGATTTTCA [13884]
lp17_PAli_NX_Pl_consensus    TTCTTTTTGTAGTCTATAAATATACTTTTGCATAGTTCTTAGTGTAGAAATAGTTTGCCCGTTTTTTATAGATTTTCA [13884]
lp17_PAbe_NX_MP_FG_consensus TTCTTTTTGTAGTCTATAAATATACTTTTGCATAGTTCTTAGTGTAGAAATAGTTTGCCCGTTTTTTATAGATTTTCA [13884]
lp17_PAbe_NX_Pl_consensus    TTCTTTTTGTAGTCTATAAATATACTTTTGCATAGTTCTTAGTGTAGAAATAGTTTGCCCGTTTTTTATAGATTTTCA [13884]

lp17_gi|365823332|B31_GB     TTGAAATAATATAATATGGTTTTTGGGGTATATTTTAGATTTTTGGTATTTAAATAGCTTGTAGAAATAAGAACTATC [13962]
lp17_un5_B31_PacBio          TTGAAATAATATAATATGGTTTTTGGGGTATATTTTAGATTTTTGGTATTTAAATAGCTTGTAGAAATAAGAACTATC [13962]
lp17_B31_TS_MP_FG_consensus  TTGAAATAATATAATATGGTTTTTGGGGTATATTTTAGATTTTTGGTATTTAAATAGCTTGTAGAAATAAGAACTATC [13962]
lp17_B31_NX_Pl_consensus     TTGAAATAATATAATATGGTTTTTGGGGTATATTTTAGATTTTTGGTATTTAAATAGCTTGTAGAAATAAGAACTATC [13962]
lp17_PAli_NX_MP_FG_consensus TTGAAATAATATAATATGGTTTTTGGGGTATATTTTAGATTTTTGGTATTTAAATAGCTTGTAGAAATAAGAACTATC [13962]
lp17_PAli_NX_Pl_consensus    TTGAAATAATATAATATGGTTTTTGGGGTATATTTTAGATTTTTGGTATTTAAATAGCTTGTAGAAATAAGAACTATC [13962]
lp17_PAbe_NX_MP_FG_consensus TTGAAATAATATAATATGGTTTTTGGGGTATATTTTAGATTTTTGGTATTTAAATAGCTTGTAGAAATAAGAACTATC [13962]
lp17_PAbe_NX_Pl_consensus    TTGAAATAATATAATATGGTTTTTGGGGTATATTTTAGATTTTTGGTATTTAAATAGCTTGTAGAAATAAGAACTATC [13962]

lp17_gi|365823332|B31_GB     AATTTATATTTATATTGGTTATAATTTAGAATATTACTAGATTAATATATCTAGACTTTATTTTCTATTTAATATACA [14040]
lp17_un5_B31_PacBio          AATTTATATTTATATTGGTTATAATTTAGAATATTACTAGATTAATATATCTAGACTTTATTTTCTATTTAATATACA [14040]
lp17_B31_TS_MP_FG_consensus  AATTTATATTTATATTGGTTATAATTTAGAATATTACTAGATTAATATATCTAGACTTTATTTTCTATTTAATATACA [14040]
lp17_B31_NX_Pl_consensus     AATTTATATTTATATTGGTTATAATTTAGAATATTACTAGATTAATATATCTAGACTTTATTTTCTATTTAATATACA [14040]
lp17_PAli_NX_MP_FG_consensus AATTTATATTTATATTGGTTATAATTTAGAATATTACTAGATTAATATATCTAGACTTTATTTTCTATTTAATATACA [14040]
lp17_PAli_NX_Pl_consensus    AATTTATATTTATATTGGTTATAATTTAGAATATTACTAGATTAATATATCTAGACTTTATTTTCTATTTAATATACA [14040]
lp17_PAbe_NX_MP_FG_consensus AATTTATATTTATATTGGTTATAATTTAGAATATTACTAGATTAATATATCTAGACTTTATTTTCTATTTAATATACA [14040]
lp17_PAbe_NX_Pl_consensus    AATTTATATTTATATTGGTTATAATTTAGAATATTACTAGATTAATATATCTAGACTTTATTTTCTATTTAATATACA [14040]

lp17_gi|365823332|B31_GB     ATTAATTAGGAAGCATTATGTGCTCCAAATGGATGATAACCAGATAAAGGGCTTTAAGTGGCTTAAGGAAGATAAGTT [14118]
lp17_un5_B31_PacBio          ATTAATTAGGAAGCATTATGTGCTCCAAATGGATGATAACCAGATAAAGGGCTTTAAGTGGCTTAAGGAAGATAAGTT [14118]
lp17_B31_TS_MP_FG_consensus  ATTAATTAGGAAGCATTATGTGCTCCAAATGGATGATAACCAGATAAAGGGCTTTAAGTGGCTTAAGGAAGATAAGTT [14118]
lp17_B31_NX_Pl_consensus     ATTAATTAGGAAGCATTATGTGCTCCAAATGGATGATAACCAGATAAAGGGCTTTAAGTGGCTTAAGGAAGATAAGTT [14118]
lp17_PAli_NX_MP_FG_consensus ATTAATTAGGAAGCATTATGTGCTCCAAATGGATGATAACCAGATAAAGGGCTTTAAGTGGCTTAAGGAAGATAAGTT [14118]
lp17_PAli_NX_Pl_consensus    ATTAATTAGGAAGCATTATGTGCTCCAAATGGATGATAACCAGATAAAGGGCTTTAAGTGGCTTAAGGAAGATAAGTT [14118]
lp17_PAbe_NX_MP_FG_consensus ATTAATTAGGAAGCATTATGTGCTCCAAATGGATGATAACCAGATAAAGGGCTTTAAGTGGCTTAAGGAAGATAAGTT [14118]
```

```
lp17_PAbe_NX_Pl_consensus    ATTAATTAGGAAGCATTATGTGCTCCAAATGGATGATAACCAGATAAAGGGCTTTAAGTGGCTTAAGGAAGATAAGTT [14118]

lp17_gi|365823332|B31_GB     ACTTAAAGCCCTTATCGCATTTATACTAATTTCCCTAATTTACGTTTTATTTTGTTTAGAGCGTTATATTATTAATTT [14196]
lp17_un5_B31_PacBio          ACTTAAAGCCCTTATCGCATTTATACTAATTTCCCTAATTTACGTTTTATTTTGTTTAGAGCGTTATATTATTAATTT [14196]
lp17_B31_TS_MP_FG_consensus  ACTTAAAGCCCTTATCGCATTTATACTAATTTCCCTAATTTACGTTTTATTTTGTTTAGAGCGTTATATTATTAATTT [14196]
lp17_B31_NX_Pl_consensus     ACTTAAAGCCCTTATCGCATTTATACTAATTTCCCTAATTTACGTTTTATTTTGTTTAGAGCGTTATATTATTAATTT [14196]
lp17_PAli_NX_MP_FG_consensus ACTTAAAGCCCTTATCGCATTTATACTAATTTCCCTAATTTACGTTTTATTTTGTTTAGAGCGTTATATTATTAATTT [14196]
lp17_PAli_NX_Pl_consensus    ACTTAAAGCCCTTATCGCATTTATACTAATTTCCCTAATTTACGTTTTATTTTGTTTAGAGCGTTATATTATTAATTT [14196]
lp17_PAbe_NX_MP_FG_consensus ACTTAAAGCCCTTATCGCATTTATACTAATTTCCCTAATTTACGTTTTATTTTGTTTAGAGCGTTATATTATTAATTT [14196]
lp17_PAbe_NX_Pl_consensus    ACTTAAAGCCCTTATCGCATTTATACTAATTTCCCTAATTTACGTTTTATTTTGTTTAGAGCGTTATATTATTAATTT [14196]

lp17_gi|365823332|B31_GB     TTAATCATAAAATGGAGGATTAGTTTTGTAATTGTATATATTTTTAGCTTTAATTGTTCTTTATTAGGCCTTTAATAC [14274]
lp17_un5_B31_PacBio          TTAATCATAAAATGGAGGATTAGTTTTGTAATTGTATATATTTTTAGCTTTAATTGTTCTTTATTAGGCCTTTAATAC [14274]
lp17_B31_TS_MP_FG_consensus  TTAATCATAAAATGGAGGATTAGTTTTGTAATTGTATATATTTTTAGCTTTAATTGTTCTTTATTAGGCCTTTAATAC [14274]
lp17_B31_NX_Pl_consensus     TTAATCATAAAATGGAGGATTAGTTTTGTAATTGTATATATTTTTAGCTTTAATTGTTCTTTATTAGGCCTTTAATAC [14274]
lp17_PAli_NX_MP_FG_consensus TTAATCATAAAATGGAGGATTAGTTTTGTAATTGTATATATTTTTAGCTTTAATTGTTCTTTATTAGGCCTTTAATAC [14274]
lp17_PAli_NX_Pl_consensus    TTAATCATAAAATGGAGGATTAGTTTTGTAATTGTATATATTTTTAGCTTTAATTGTTCTTTATTAGGCCTTTAATAC [14274]
lp17_PAbe_NX_MP_FG_consensus TTAATCATAAAATGGAGGATTAGTTTTGTAATTGTATATATTTTTAGCTTTAATTGTTCTTTATTAGGCCTTTAATAC [14274]
lp17_PAbe_NX_Pl_consensus    TTAATCATAAAATGGAGGATTAGTTTTGTAATTGTATATATTTTTAGCTTTAATTGTTCTTTATTAGGCCTTTAATAC [14274]

lp17_gi|365823332|B31_GB     TCTAAAGTATTATGCTATTTCGCAAAAATAATTTTCTGGATTATATTAGCTTACATATTAGGATGAGAATTATAAATT [14352]
lp17_un5_B31_PacBio          TCTAAAGTATTATGCTATTTCGCAAAAATAATTTTCTGGATTATATTAGCTTACATATTAGGATGAGAATTATAAATT [14352]
lp17_B31_TS_MP_FG_consensus  TCTAAAGTATTATGCTATTTCGCAAAAATAATTTTCTGGATTATATTAGCTTACATATTAGGATGAGAATTATAAATT [14352]
lp17_B31_NX_Pl_consensus     TCTAAAGTATTATGCTATTTCGCAAAAATAATTTTCTGGATTATATTAGCTTACATATTAGGATGAGAATTATAAATT [14352]
lp17_PAli_NX_MP_FG_consensus TCTAAAGTATTATGCTATTTCGCAAAAATAATTTTCTGGATTATATTAGCTTACATATTAGGATGAGAATTATAAATT [14352]
lp17_PAli_NX_Pl_consensus    TCTAAAGTATTATGCTATTTCGCAAAAATAATTTTCTGGATTATATTAGCTTACATATTAGGATGAGAATTATAAATT [14352]
lp17_PAbe_NX_MP_FG_consensus TCTAAAGTATTATGCTATTTCGCAAAAATAATTTTCTGGATTATATTAGCTTACATATTAGGATGAGAATTATAAATT [14352]
lp17_PAbe_NX_Pl_consensus    TCTAAAGTATTATGCTATTTCGCAAAAATAATTTTCTGGATTATATTAGCTTACATATTAGGATGAGAATTATAAATT [14352]

lp17_gi|365823332|B31_GB     TTAGTGCAACAACCTTCTACAGGGTAAAGGAGTGTATAAAGGCCTCTAAAGCTTTCATTTTCTTTATTTGTGGAACAT [14430]
lp17_un5_B31_PacBio          TTAGTGCAACAACCTTCTACAGGGTAAAGGAGTGTATAAAGGCCTCTAAAGCTTTCATTTTCTTTATTTGTGGAACAT [14430]
lp17_B31_TS_MP_FG_consensus  TTAGTGCAACAACCTTCTACAGGGTAAAGGAGTGTATAAAGGCCTCTAAAGCTTTCATTTTCTTTATTTGTGGAACAT [14430]
lp17_B31_NX_Pl_consensus     TTAGTGCAACAACCTTCTACAGGGTAAAGGAGTGTATAAAGGCCTCTAAAGCTTTCATTTTCTTTATTTGTGGAACAT [14430]
lp17_PAli_NX_MP_FG_consensus TTAGTGCAACAACCTTCTACAGGGTAAAGGAGTGTATAAAGGCCTCTAAAGCTTTCATTTTCTTTATTTGTGGAACAT [14430]
lp17_PAli_NX_Pl_consensus    TTAGTGCAACAACCTTCTACAGGGTAAAGGAGTGTATAAAGGCCTCTAAAGCTTTCATTTTCTTTATTTGTGGAACAT [14430]
lp17_PAbe_NX_MP_FG_consensus TTAGTGCAACAACCTTCTACAGGGTAAAGGAGTGTATAAAGGCCTCTAAAGCTTTCATTTTCTTTATTTGTGGAACAT [14430]
lp17_PAbe_NX_Pl_consensus    TTAGTGCAACAACCTTCTACAGGGTAAAGGAGTGTATAAAGGCCTCTAAAGCTTTCATTTTCTTTATTTGTGGAACAT [14430]

lp17_gi|365823332|B31_GB     TTGAAGATTACTTTTGGATTTTTTCTAATATTTCAAGATATTCTAACAGGGTTTTAGAATCAACTTCATGTTGATTAG [14508]
lp17_un5_B31_PacBio          TTGAAGATTACTTTTGGATTTTTTCTAATATTTCAAGATATTCTAACAGGGTTTTAGAATCAACTTCATGTTGATTAG [14508]
lp17_B31_TS_MP_FG_consensus  TTGAAGATTACTTTTGGATTTTTTCTAATATTTCAAGATATTCTAACAGGGTTTTAGAATCAACTTCATGTTGATTAG [14508]
lp17_B31_NX_Pl_consensus     TTGAAGATTACTTTTGGATTTTTTCTAATATTTCAAGATATTCTAACAGGGTTTTAGAATCAACTTCATGTTGATTAG [14508]
lp17_PAli_NX_MP_FG_consensus TTGAAGATTACTTTTGGATTTTTTCTAATATTTCAAGATATTCTAACAGGGTTTTAGAATCAACTTCATGTTGATTAG [14508]
lp17_PAli_NX_Pl_consensus    TTGAAGATTACTTTTGGATTTTTTCTAATATTTCAAGATATTCTAACAGGGTTTTAGAATCAACTTCATGTTGATTAG [14508]
lp17_PAbe_NX_MP_FG_consensus TTGAAGATTACTTTTGGATTTTTTCTAATATTTCAAGATATTCTAACAGGGTTTTAGAATCAACTTCATGTTGATTAG [14508]
lp17_PAbe_NX_Pl_consensus    TTGAAGATTACTTTTGGATTTTTTCTAATATTTCAAGATATTCTAACAGGGTTTTAGAATCAACTTCATGTTGATTAG [14508]

lp17_gi|365823332|B31_GB     TTTTAGCTAGAGCCTTATTAAACTTGCTTCTTATTAAGGAATAAGCATAATTTTTATTTGCACTATGGAATAATTTTT [14586]
lp17_un5_B31_PacBio          TTTTAGCTAGAGCCTTATTAAACTTGCTTCTTATTAAGGAATAAGCATAATTTTTATTTGCACTATGGAATAATTTTT [14586]
lp17_B31_TS_MP_FG_consensus  TTTTAGCTAGAGCCTTATTAAACTTGCTTCTTATTAAGGAATAAGCATAATTTTTATTTGCACTATGGAATAATTTTT [14586]
lp17_B31_NX_Pl_consensus     TTTTAGCTAGAGCCTTATTAAACTTGCTTCTTATTAAGGAATAAGCATAATTTTTATTTGCACTATGGAATAATTTTT [14586]
lp17_PAli_NX_MP_FG_consensus TTTTAGCTAGAGCCTTATTAAACTTGCTTCTTATTAAGGAATAAGCATAATTTTTATTTGCACTATGGAATAATTTTT [14586]
lp17_PAli_NX_Pl_consensus    TTTTAGCTAGAGCCTTATTAAACTTGCTTCTTATTAAGGAATAAGCATAATTTTTATTTGCACTATGGAATAATTTTT [14586]
lp17_PAbe_NX_MP_FG_consensus TTTTAGCTAGAGCCTTATTAAACTTGCTTCTTATTAAGGAATAAGCATAATTTTTATTTGCACTATGGAATAATTTTT [14586]
lp17_PAbe_NX_Pl_consensus    TTTTAGCTAGAGCCTTATTAAACTTGCTTCTTATTAAGGAATAAGCATAATTTTTATTTGCACTATGGAATAATTTTT [14586]

lp17_gi|365823332|B31_GB     TATTCATATGATCTAGTGTTTTGACAAGAATGATTAACTTATTCTTAGCAAATTAAATTTGCTTTGTTATTCTTGCCA [14664]
lp17_un5_B31_PacBio          TATTCATATGATCTAGTGTTTTGACAAGAATGATTAACTTATTCTTAGCAAATTAAATTTGCTTTGTTATTCTTGCCA [14664]
lp17_B31_TS_MP_FG_consensus  TATTCATATGATCTAGTGTTTTGACAAGAATGATTAACTTATTCTTAGCAAATTAAATTTGCTTTGTTATTCTTGCCA [14664]
lp17_B31_NX_Pl_consensus     TATTCATATGATCTAGTGTTTTGACAAGAATGATTAACTTATTCTTAGCAAATTAAATTTGCTTTGTTATTCTTGCCA [14664]
lp17_PAli_NX_MP_FG_consensus TATTCATATGATCTAGTGTTTTGACAAGAATGATTAACTTATTCTTAGCAAATTAAATTTGCTTTGTTATTCTTGCCA [14664]
lp17_PAli_NX_Pl_consensus    TATTCATATGATCTAGTGTTTTGACAAGAATGATTAACTTATTCTTAGCAAATTAAATTTGCTTTGTTATTCTTGCCA [14664]
lp17_PAbe_NX_MP_FG_consensus TATTCATATGATCTAGTGTTTTGACAAGAATGATTAACTTATTCTTAGCAAATTAAATTTGCTTTGTTATTCTTGCCA [14664]
lp17_PAbe_NX_Pl_consensus    TATTCATATGATCTAGTGTTTTGACAAGAATGATTAACTTATTCTTAGCAAATTAAATTTGCTTTGTTATTCTTGCCA [14664]

lp17_gi|365823332|B31_GB     ATGTATTGTTAATTACTCCATCCATAATGAATTAGCCCCCTATTATATTAAATTTATATTATAAATATAGCACAGTAT [14742]
lp17_un5_B31_PacBio          ATGTATTGTTAATTACTCCATCCATAATGAATTAGCCCCCTATTATATTAAATTTATATTATAAATATAGCACAGTAT [14742]
lp17_B31_TS_MP_FG_consensus  ATGTATTGTTAATTACTCCATCCATAATGAATTAGCCCCCTATTATATTAAATTTATATTATAAATATAGCACAGTAT [14742]
lp17_B31_NX_Pl_consensus     ATGTATTGTTAATTACTCCATCCATAATGAATTAGCCCCCTATTATATTAAATTTATATTATAAATATAGCACAGTAT [14742]
lp17_PAli_NX_MP_FG_consensus ATGTATTGTTAATTACTCCATCCATAATGAATTAGCCCCCTATTATATTAAATTTATATTATAAATATAGCACAGTAT [14742]
lp17_PAli_NX_Pl_consensus    ATGTATTGTTAATTACTCCATCCATAATGAATTAGCCCCCTATTATATTAAATTTATATTATAAATATAGCACAGTAT [14742]
lp17_PAbe_NX_MP_FG_consensus ATGTATTGTTAATTACTCCATCCATAATGAATTAGCCCCCTATTATATTAAATTTATATTATAAATATAGCACAGTAT [14742]
lp17_PAbe_NX_Pl_consensus    ATGTATTGTTAATTACTCCATCCATAATGAATTAGCCCCCTATTATATTAAATTTATATTATAAATATAGCACAGTAT [14742]

lp17_gi|365823332|B31_GB     TTTTTTAAATTTTTTTAGCGTAAAACAATATATTTCTAAAGTTTTGCATAAGCTTATTTTATAATGTATTTATAATTA [14820]
lp17_un5_B31_PacBio          TTTTTTAAATTTTTTTAGCGTAAAACAATATATTTCTAAAGTTTTGCATAAGCTTATTTTATAATGTATTTATAATTA [14820]
lp17_B31_TS_MP_FG_consensus  TTTTTTAAATTTTTTTAGCGTAAAACAATATATTTCTAAAGTTTTGCATAAGCTTATTTTATAATGTATTTATAATTA [14820]
lp17_B31_NX_Pl_consensus     TTTTTTAAATTTTTTTAGCGTAAAACAATATATTTCTAAAGTTTTGCATAAGCTTATTTTATAATGTATTTATAATTA [14820]
lp17_PAli_NX_MP_FG_consensus TTTTTTAAATTTTTTTAGCGTAAAACAATATATTTCTAAAGTTTTGCATAAGCTTATTTTATAATGTATTTATAATTA [14820]
lp17_PAli_NX_Pl_consensus    TTTTTTAAATTTTTTTAGCGTAAAACAATATATTTCTAAAGTTTTGCATAAGCTTATTTTATAATGTATTTATAATTA [14820]
lp17_PAbe_NX_MP_FG_consensus TTTTTTAAATTTTTTTAGCGTAAAACAATATATTTCTAAAGTTTTGCATAAGCTTATTTTATAATGTATTTATAATTA [14820]
lp17_PAbe_NX_Pl_consensus    TTTTTTAAATTTTTTTAGCGTAAAACAATATATTTCTAAAGTTTTGCATAAGCTTATTTTATAATGTATTTATAATTA [14820]

lp17_gi|365823332|B31_GB     AGTAGTAGTGATTTTTTGTAAAAATTGTTTTAATTCTTCATAGAATAATACGTAGTAGTCTTTTATTTTTAATAAAAC [14898]
lp17_un5_B31_PacBio          AGTAGTAGTGATTTTTTGTAAAAATTGTTTTAATTCTTCATAGAATAATACGTAGTAGTCTTTTATTTTTAATAAAAC [14898]
lp17_B31_TS_MP_FG_consensus  AGTAGTAGTGATTTTTTGTAAAAATTGTTTTAATTCTTCATAGAATAATACGTAGTAGTCTTTTATTTTTAATAAAAC [14898]
lp17_B31_NX_Pl_consensus     AGTAGTAGTGATTTTTTGTAAAAATTGTTTTAATTCTTCATAGAATAATACGTAGTAGTCTTTTATTTTTAATAAAAC [14898]
lp17_PAli_NX_MP_FG_consensus AGTAGTAGTGATTTTTTGTAAAAATTGTTTTAATTCTTCATAGAATAATACGTAGTAGTCTTTTATTTTTAATAAAAC [14898]
lp17_PAli_NX_Pl_consensus    AGTAGTAGTGATTTTTTGTAAAAATTGTTTTAATTCTTCATAGAATAATACGTAGTAGTCTTTTATTTTTAATAAAAC [14898]
lp17_PAbe_NX_MP_FG_consensus AGTAGTAGTGATTTTTTGTAAAAATTGTTTTAATTCTTCATAGAATAATACGTAGTAGTCTTTTATTTTTAATAAAAC [14898]
lp17_PAbe_NX_Pl_consensus    AGTAGTAGTGATTTTTTGTAAAAATTGTTTTAATTCTTCATAGAATAATACGTAGTAGTCTTTTATTTTTAATAAAAC [14898]

lp17_gi|365823332|B31_GB     TCTTTCTAAGGAGCAATTAACTTTATAATAAATTATAGATTTAGGATTTTTCTTAAAATAAAGTTTGACTATGACGGT [14976]
lp17_un5_B31_PacBio          TCTTTCTAAGGAGCAATTAACTTTATAATAAATTATAGATTTAGGATTTTTCTTAAAATAAAGTTTGACTATGACGGT [14976]
lp17_B31_TS_MP_FG_consensus  TCTTTCTAAGGAGCAATTAACTTTATAATAAATTATAGATTTAGGATTTTTCTTAAAATAAAGTTTGACTATGACGGT [14976]
lp17_B31_NX_Pl_consensus     TCTTTCTAAGGAGCAATTAACTTTATAATAAATTATAGATTTAGGATTTTTCTTAAAATAAAGTTTGACTATGACGGT [14976]
lp17_PAli_NX_MP_FG_consensus TCTTTCTAAGGAGCAATTAACTTTATAATAAATTATAGATTTAGGATTTTTCTTAAAATAAAGTTTGACTATGACGGT [14976]
lp17_PAli_NX_Pl_consensus    TCTTTCTAAGGAGCAATTAACTTTATAATAAATTATAGATTTAGGATTTTTCTTAAAATAAAGTTTGACTATGACGGT [14976]
lp17_PAbe_NX_MP_FG_consensus TCTTTCTAAGGAGCAATTAACTTTATAATAAATTATAGATTTAGGATTTTTCTTAAAATAAAGTTTGACTATGACGGT [14976]
lp17_PAbe_NX_Pl_consensus    TCTTTCTAAGGAGCAATTAACTTTATAATAAATTATAGATTTAGGATTTTTCTTAAAATAAAGTTTGACTATGACGGT [14976]

lp17_gi|365823332|B31_GB     TATTTCTTTTTCTAATTTGCGTTTTATTTATATATTTTTTAGTTTTAATTGTTCTTTATTAGGCCTATGAGACACTAT [15054]
lp17_un5_B31_PacBio          TATTTCTTTTTCTAATTTGCGTTTTATTTATATATTTTTTAGTTTTAATTGTTCTTTATTAGGCCTATGAGACACTAT [15054]
lp17_B31_TS_MP_FG_consensus  TATTTCTTTTTCTAATTTGCGTTTTATTTATATATTTTTTAGTTTTAATTGTTCTTTATTAGGCCTATGAGACACTAT [15054]
lp17_B31_NX_Pl_consensus     TATTTCTTTTTCTAATTTGCGTTTTATTTATATATTTTTTAGTTTTAATTGTTCTTTATTAGGCCTATGAGACACTAT [15054]
lp17_PAli_NX_MP_FG_consensus TATTTCTTTTTCTAATTTGCGTTTTATTTATATATTTTTTAGTTTTAATTGTTCTTTATTAGGCCTATGAGACACTAT [15054]
lp17_PAli_NX_Pl_consensus    TATTTCTTTTTCTAATTTGCGTTTTATTTATATATTTTTTAGTTTTAATTGTTCTTTATTAGGCCTATGAGACACTAT [15054]
lp17_PAbe_NX_MP_FG_consensus TATTTCTTTTTCTAATTTGCGTTTTATTTATATATTTTTTAGTTTTAATTGTTCTTTATTAGGCCTATGAGACACTAT [15054]
lp17_PAbe_NX_Pl_consensus    TATTTCTTTTTCTAATTTGCGTTTTATTTATATATTTTTTAGTTTTAATTGTTCTTTATTAGGCCTATGAGACACTAT [15054]
```

```
lp17_gi|365823332|B31_GB     AATACATTTGCAATACCTGATTTTTTAATTAATTTGGGATATATAATTTAAAATATGGTATCACCCCCATTATACACC [15132]
lp17_un5_B31_PacBio          AATACATTTGCAATACCTGATTTTTTAATTAATTTGGGATATATAATTTAAAATATGGTATCACCCCCATTATACACC [15132]
lp17_B31_TS_MP_FG_consensus  AATACATTTGCAATACCTGATTTTTTAATTAATTTGGGATATATAATTTAAAATATGGTATCACCCCCATTATACACC [15132]
lp17_B31_NX_Pl_consensus     AATACATTTGCAATACCTGATTTTTTAATTAATTTGGGATATATAATTTAAAATATGGTATCACCCCCATTATACACC [15132]
lp17_PAli_NX_MP_FG_consensus AATACATTTGCAATACCTGATTTTTTAATTAATTTGGGATATATAATTTAAAATATGGTATCACCCCCATTATACACC [15132]
lp17_PAli_NX_Pl_consensus    AATACATTTGCAATACCTGATTTTTTAATTAATTTGGGATATATAATTTAAAATATGGTATCACCCCCATTATACACC [15132]
lp17_PAbe_NX_MP_FG_consensus AATACATTTGCAATACCTGATTTTTTAATTAATTTGGGATATATAATTTAAAATATGGTATCACCCCCATTATACACC [15132]
lp17_PAbe_NX_Pl_consensus    AATACATTTGCAATACCTGATTTTTTAATTAATTTGGGATATATAATTTAAAATATGGTATCACCCCCATTATACACC [15132]

lp17_gi|365823332|B31_GB     TTAAAACAACTTTAGATTAAGGGGAGAAACACCTTTAGTAATATACTAAAGATGTTTCTCTTTTTAATATCATTATAT [15210]
lp17_un5_B31_PacBio          TTAAAACAACTTTAGATTAAGGGGAGAAACACCTTTAGTAATATACTAAAGATGTTTCTCTTTTTAATATCATTATAT [15210]
lp17_B31_TS_MP_FG_consensus  TTAAAACAACTTTAGATTAAGGGGAGAAACACCTTTAGTAATATACTAAAGATGTTTCTCTTTTTAATATCATTATAT [15210]
lp17_B31_NX_Pl_consensus     TTAAAACAACTTTAGATTAAGGGGAGAAACACCTTTAGTAATATACTAAAGATGTTTCTCTTTTTAATATCATTATAT [15210]
lp17_PAli_NX_MP_FG_consensus TTAAAACAACTTTAGATTAAGGGGAGAAACACCTTTAGTAATATACTAAAGATGTTTCTCTTTTTAATATCATTATAT [15210]
lp17_PAli_NX_Pl_consensus    TTAAAACAACTTTAGATTAAGGGGAGAAACACCTTTAGTAATATACTAAAGATGTTTCTCTTTTTAATATCATTATAT [15210]
lp17_PAbe_NX_MP_FG_consensus TTAAAACAACTTTAGATTAAGGGGAGAAACACCTTTAGTAATATACTAAAGATGTTTCTCTTTTTAATATCATTATAT [15210]
lp17_PAbe_NX_Pl_consensus    TTAAAACAACTTTAGATTAAGGGGAGAAACACCTTTAGTAATATACTAAAGATGTTTCTCTTTTTAATATCATTATAT [15210]

lp17_gi|365823332|B31_GB     AGTCTATTTATAGTAGTTCATAATTTTTGTGTAACATATTTTTAAAGGATAATTGATTATGTAGTAAAGTTTGAATAT [15288]
lp17_un5_B31_PacBio          AGTCTATTTATAGTAGTTCATAATTTTTGTGTAACATATTTTTAAAGGATAATTGATTATGTAGTAAAGTTTGAATAT [15288]
lp17_B31_TS_MP_FG_consensus  AGTCTATTTATAGTAGTTCATAATTTTTGTGTAACATATTTTTAAAGGATAATTGATTATGTAGTAAAGTTTGAATAT [15288]
lp17_B31_NX_Pl_consensus     AGTCTATTTATAGTAGTTCATAATTTTTGTGTAACATATTTTTAAAGGATAATTGATTATGTAGTAAAGTTTGAATAT [15288]
lp17_PAli_NX_MP_FG_consensus AGTCTATTTATAGTAGTTCATAATTTTTGTGTAACATATTTTTAAAGGATAATTGATTATGTAGTAAAGTTTGAATAT [15288]
lp17_PAli_NX_Pl_consensus    AGTCTATTTATAGTAGTTCATAATTTTTGTGTAACATATTTTTAAAGGATAATTGATTATGTAGTAAAGTTTGAATAT [15288]
lp17_PAbe_NX_MP_FG_consensus AGTCTATTTATAGTAGTTCATAATTTTTGTGTAACATATTTTTAAAGGATAATTGATTATGTAGTAAAGTTTGAATAT [15288]
lp17_PAbe_NX_Pl_consensus    AGTCTATTTATAGTAGTTCATAATTTTTGTGTAACATATTTTTAAAGGATAATTGATTATGTAGTAAAGTTTGAATAT [15288]

lp17_gi|365823332|B31_GB     ATTTTTTTCTACTTTATATTTATTAGTTATAATTTTTAGGTTGTTTTCTAAAAAATCGAGATACTTTATTAATAAGTC [15366]
lp17_un5_B31_PacBio          ATTTTTTTCTACTTTATATTTATTAGTTATAATTTTTAGGTTGTTTTCTAAAAAATCGAGATACTTTATTAATAAGTC [15366]
lp17_B31_TS_MP_FG_consensus  ATTTTTTTCTACTTTATATTTATTAGTTATAATTTTTAGGTTGTTTTCTAAAAAATCGAGATACTTTATTAATAAGTC [15366]
lp17_B31_NX_Pl_consensus     ATTTTTTTCTACTTTATATTTATTAGTTATAATTTTTAGGTTGTTTTCTAAAAAATCGAGATACTTTATTAATAAGTC [15366]
lp17_PAli_NX_MP_FG_consensus ATTTTTTTCTACTTTATATTTATTAGTTATAATTTTTAGGTTGTTTTCTAAAAAATCGAGATACTTTATTAATAAGTC [15366]
lp17_PAli_NX_Pl_consensus    ATTTTTTTCTACTTTATATTTATTAGTTATAATTTTTAGGTTGTTTTCTAAAAAATCGAGATACTTTATTAATAAGTC [15366]
lp17_PAbe_NX_MP_FG_consensus ATTTTTTTCTACTTTATATTTATTAGTTATAATTTTTAGGTTGTTTTCTAAAAAATCGAGATACTTTATTAATAAGTC [15366]
lp17_PAbe_NX_Pl_consensus    ATTTTTTTCTACTTTATATTTATTAGTTATAATTTTTAGGTTGTTTTCTAAAAAATCGAGATACTTTATTAATAAGTC [15366]

lp17_gi|365823332|B31_GB     TATGTCTATTTCAATATCTTTACTAGTATTGCAATATCTATCTATGTTAAAAGTAAACGCACGGCGTATAAAGCCCCT [15444]
lp17_un5_B31_PacBio          TATGTCTATTTCAATATCTTTACTAGTATTGCAATATCTATCTATGTTAAAAGTAAACGCACGGCGTATAAAGCCCCT [15444]
lp17_B31_TS_MP_FG_consensus  TATGTCTATTTCAATATCTTTACTAGTATTGCAATATCTATCTATGTTAAAAGTAAACGCACGGCGTATAAAGCCCCT [15444]
lp17_B31_NX_Pl_consensus     TATGTCTATTTCAATATCTTTACTAGTATTGCAATATCTATCTATGTTAAAAGTAAACGCACGGCGTATAAAGCCCCT [15444]
lp17_PAli_NX_MP_FG_consensus TATGTCTATTTCAATATCTTTACTAGTATTGCAATATCTATCTATGTTAAAAGTAAACGCACGGCGTATAAAGCCCCT [15444]
lp17_PAli_NX_Pl_consensus    TATGTCTATTTCAATATCTTTACTAGTATTGCAATATCTATCTATGTTAAAAGTAAACGCACGGCGTATAAAGCCCCT [15444]
lp17_PAbe_NX_MP_FG_consensus TATGTCTATTTCAATATCTTTACTAGTATTGCAATATCTATCTATGTTAAAAGTAAACGCACGGCGTATAAAGCCCCT [15444]
lp17_PAbe_NX_Pl_consensus    TATGTCTATTTCAATATCTTTACTAGTATTGCAATATCTATCTATGTTAAAAGTAAACGCACGGCGTATAAAGCCCCT [15444]

lp17_gi|365823332|B31_GB     ACTATAGTATCCAATATTTTTTGAATTTAGGTCAATGTTGTTTAGTGTGTAAATAAGAATTATTAAGTGTGAAGATAG [15522]
lp17_un5_B31_PacBio          ACTATAGTATCCAATATTTTTTGAATTTAGGTCAATGTTGTTTAGTGTGTAAATAAGAATTATTAAGTGTGAAGATAG [15522]
lp17_B31_TS_MP_FG_consensus  ACTATAGTATCCAATATTTTTTGAATTTAGGTCAATGTTGTTTAGTGTGTAAATAAGAATTATTAAGTGTGAAGATAG [15522]
lp17_B31_NX_Pl_consensus     ACTATAGTATCCAATATTTTTTGAATTTAGGTCAATGTTGTTTAGTGTGTAAATAAGAATTATTAAGTGTGAAGATAG [15522]
lp17_PAli_NX_MP_FG_consensus ACTATAGTATCCAATATTTTTTGAATTTAGGTCAATGTTGTTTAGTGTGTAAATAAGAATTATTAAGTGTGAAGATAG [15522]
lp17_PAli_NX_Pl_consensus    ACTATAGTATCCAATATTTTTTGAATTTAGGTCAATGTTGTTTAGTGTGTAAATAAGAATTATTAAGTGTGAAGATAG [15522]
lp17_PAbe_NX_MP_FG_consensus ACTATAGTATCCAATATTTTTTGAATTTAGGTCAATGTTGTTTAGTGTGTAAATAAGAATTATTAAGTGTGAAGATAG [15522]
lp17_PAbe_NX_Pl_consensus    ACTATAGTATCCAATATTTTTTGAATTTAGGTCAATGTTGTTTAGTGTGTAAATAAGAATTATTAAGTGTGAAGATAG [15522]

lp17_gi|365823332|B31_GB     CCTATTTTTGCTATTCATACTTAATATTTCTTTTAAAAATTTTTCCAAATAGTGCCCCAGTAATTTTTATTATTTATC [15600]
lp17_un5_B31_PacBio          CCTATTTTTGCTATTCATACTTAATATTTCTTTTAAAAATTTTTCCAAATAGTGCCCCAGTAATTTTTATTATTTATC [15600]
lp17_B31_TS_MP_FG_consensus  CCTATTTTTGCTATTCATACTTAATATTTCTTTTAAAAATTTTTCCAAATAGTGCCCCAGTAATTTTTATTATTTATC [15600]
lp17_B31_NX_Pl_consensus     CCTATTTTTGCTATTCATACTTAATATTTCTTTTAAAAATTTTTCCAAATAGTGCCCCAGTAATTTTTATTATTTATC [15600]
lp17_PAli_NX_MP_FG_consensus CCTATTTTTGCTATTCATACTTAATATTTCTTTTAAAAATTTTTCCAAATAGTGCCCCAGTAATTTTTATTATTTATC [15600]
lp17_PAli_NX_Pl_consensus    CCTATTTTTGCTATTCATACTTAATATTTCTTTTAAAAATTTTTCCAAATAGTGCCCCAGTAATTTTTATTATTTATC [15600]
lp17_PAbe_NX_MP_FG_consensus CCTATTTTTGCTATTCATACTTAATATTTCTTTTAAAAATTTTTCCAAATAGTGCCCCAGTAATTTTTATTATTTATC [15600]
lp17_PAbe_NX_Pl_consensus    CCTATTTTTGCTATTCATACTTAATATTTCTTTTAAAAATTTTTCCAAATAGTGCCCCAGTAATTTTTATTATTTATC [15600]

lp17_gi|365823332|B31_GB     AAATAAAAATATATGTCTTTACATTTATATTTCTAAATTGCTTAATTTGCAAAGAAATATTTTTTACGATTAAATAGT [15678]
lp17_un5_B31_PacBio          AAATAAAAATATATGTCTTTACATTTATATTTCTAAATTGCTTAATTTGCAAAGAAATATTTTTTACGATTAAATAGT [15678]
lp17_B31_TS_MP_FG_consensus  AAATAAAAATATATGTCTTTACATTTATATTTCTAAATTGCTTAATTTGCAAAGAAATATTTTTTACGATTAAATAGT [15678]
lp17_B31_NX_Pl_consensus     AAATAAAAATATATGTCTTTACATTTATATTTCTAAATTGCTTAATTTGCAAAGAAATATTTTTTACGATTAAATAGT [15678]
lp17_PAli_NX_MP_FG_consensus AAATAAAAATATATGTCTTTACATTTATATTTCTAAATTGCTTAATTTGCAAAGAAATATTTTTTACGATTAAATAGT [15678]
lp17_PAli_NX_Pl_consensus    AAATAAAAATATATGTCTTTACATTTATATTTCTAAATTGCTTAATTTGCAAAGAAATATTTTTTACGATTAAATAGT [15678]
lp17_PAbe_NX_MP_FG_consensus AAATAAAAATATATGTCTTTACATTTATATTTCTAAATTGCTTAATTTGCAAAGAAATATTTTTTACGATTAAATAGT [15678]
lp17_PAbe_NX_Pl_consensus    AAATAAAAATATATGTCTTTACATTTATATTTCTAAATTGCTTAATTTGCAAAGAAATATTTTTTACGATTAAATAGT [15678]

lp17_gi|365823332|B31_GB     AGTAGGATAGTTTAGTTCTAACCGTAGTTTTAGTTTATCTGCTATTGTTTGATAGTAGTCTTGTATTGTTGAACGAAA [15756]
lp17_un5_B31_PacBio          AGTAGGATAGTTTAGTTCTAACCGTAGTTTTAGTTTATCTGCTATTGTTTGATAGTAGTCTTGTATTGTTGAACGAAA [15756]
lp17_B31_TS_MP_FG_consensus  AGTAGGATAGTTTAGTTCTAACCGTAGTTTTAGTTTATCTGCTATTGTTTGATAGTAGTCTTGTATTGTTGAACGAAA [15756]
lp17_B31_NX_Pl_consensus     AGTAGGATAGTTTAGTTCTAACCGTAGTTTTAGTTTATCTGCTATTGTTTGATAGTAGTCTTGTATTGTTGAACGAAA [15756]
lp17_PAli_NX_MP_FG_consensus AGTAGGATAGTTTAGTTCTAACCGTAGTTTTAGTTTATCTGCTATTGTTTGATAGTAGTCTTGTATTGTTGAACGAAA [15756]
lp17_PAli_NX_Pl_consensus    AGTAGGATAGTTTAGTTCTAACCGTAGTTTTAGTTTATCTGCTATTGTTTGATAGTAGTCTTGTATTGTTGAACGAAA [15756]
lp17_PAbe_NX_MP_FG_consensus AGTAGGATAGTTTAGTTCTAACCGTAGTTTTAGTTTATCTGCTATTGTTTGATAGTAGTCTTGTATTGTTGAACGAAA [15756]
lp17_PAbe_NX_Pl_consensus    AGTAGGATAGTTTAGTTCTAACCGTAGTTTTAGTTTATCTGCTATTGTTTGATAGTAGTCTTGTATTGTTGAACGAAA [15756]

lp17_gi|365823332|B31_GB     CACTTTAAGGGGGATTAATTTTATAATAAATCATATATTTGGACTGGTTTTTAAAAGATAGTTTTACTATGGTTTTTG [15834]
lp17_un5_B31_PacBio          CACTTTAAGGGGGATTAATTTTATAATAAATCATATATTTGGACTGGTTTTTAAAAGATAGTTTTACTATGGTTTTTG [15834]
lp17_B31_TS_MP_FG_consensus  CACTTTAAGGGGGATTAATTTTATAATAAATCATATATTTGGACTGGTTTTTAAAAGATAGTTTTACTATGGTTTTTG [15834]
lp17_B31_NX_Pl_consensus     CACTTTAAGGGGGATTAATTTTATAATAAATCATATATTTGGACTGGTTTTTAAAAGATAGTTTTACTATGGTTTTTG [15834]
lp17_PAli_NX_MP_FG_consensus CACTTTAAGGGGGATTAATTTTATAATAAATCATATATTTGGACTGGTTTTTAAAAGATAGTTTTACTATGGTTTTTG [15834]
lp17_PAli_NX_Pl_consensus    CACTTTAAGGGGGATTAATTTTATAATAAATCATATATTTGGACTGGTTTTTAAAAGATAGTTTTACTATGGTTTTTG [15834]
lp17_PAbe_NX_MP_FG_consensus CACTTTAAGGGGGATTAATTTTATAATAAATCATATATTTGGACTGGTTTTTAAAAGATAGTTTTACTATGGTTTTTG [15834]
lp17_PAbe_NX_Pl_consensus    CACTTTAAGGGGGATTAATTTTATAATAAATCATATATTTGGACTGGTTTTTAAAAGATAGTTTTACTATGGTTTTTG [15834]

lp17_gi|365823332|B31_GB     TTTTTTCTAATATATCAAGATATTTATTCATTGTTGGTAGATCGCAAGAATTTTTATTATCTTTTTTAAGAATTTCGT [15912]
lp17_un5_B31_PacBio          TTTTTTCTAATATATCAAGATATTTATTCATTGTTGGTAGATCGCAAGAATTTTTATTATCTTTTTTAAGAATTTCGT [15912]
lp17_B31_TS_MP_FG_consensus  TTTTTTCTAATATATCAAGATATTTATTCATTGTTGGTAGATCGCAAGAATTTTTATTATCTTTTTTAAGAATTTCGT [15912]
lp17_B31_NX_Pl_consensus     TTTTTTCTAATATATCAAGATATTTATTCATTGTTGGTAGATCGCAAGAATTTTTATTATCTTTTTTAAGAATTTCGT [15912]
lp17_PAli_NX_MP_FG_consensus TTTTTTCTAATATATCAAGATATTTATTCATTGTTGGTAGATCGCAAGAATTTTTATTATCTTTTTTAAGAATTTCGT [15912]
lp17_PAli_NX_Pl_consensus    TTTTTTCTAATATATCAAGATATTTATTCATTGTTGGTAGATCGCAAGAATTTTTATTATCTTTTTTAAGAATTTCGT [15912]
lp17_PAbe_NX_MP_FG_consensus TTTTTTCTAATATATCAAGATATTTATTCATTGTTGGTAGATCGCAAGAATTTTTATTATCTTTTTTAAGAATTTCGT [15912]
lp17_PAbe_NX_Pl_consensus    TTTTTTCTAATATATCAAGATATTTATTCATTGTTGGTAGATCGCAAGAATTTTTATTATCTTTTTTAAGAATTTCGT [15912]

lp17_gi|365823332|B31_GB     TGTAAAATTTTAATAGTTTTTTTGAGAATAATTATTCAATTCTAGATTTAATAATGTATTGTCACATAAAGCAGGCAT [15990]
lp17_un5_B31_PacBio          TGTAAAATTTTAATAGTTTTTTTGAGAATAATTATTCAATTCTAGATTTAATAATGTATTGTCACATAAAGCAGGCAT [15990]
lp17_B31_TS_MP_FG_consensus  TGTAAAATTTTAATAGTTTTTTTGAGAATAATTATTCAATTCTAGATTTAATAATGTATTGTCACATAAAGCAGGCAT [15990]
lp17_B31_NX_Pl_consensus     TGTAAAATTTTAATAGTTTTTTTGAGAATAATTATTCAATTCTAGATTTAATAATGTATTGTCACATAAAGCAGGCAT [15990]
lp17_PAli_NX_MP_FG_consensus TGTAAAATTTTAATAGTTTTTTTGAGAATAATTATTCAATTCTAGATTTAATAATGTATTGTCACATAAAGCAGGCAT [15990]
lp17_PAli_NX_Pl_consensus    TGTAAAATTTTAATAGTTTTTTTGAGAATAATTATTCAATTCTAGATTTAATAATGTATTGTCACATAAAGCAGGCAT [15990]
lp17_PAbe_NX_MP_FG_consensus TGTAAAATTTTAATAGTTTTTTTGAGAATAATTATTCAATTCTAGATTTAATAATGTATTGTCACATAAAGCAGGCAT [15990]
lp17_PAbe_NX_Pl_consensus    TGTAAAATTTTAATAGTTTTTTTGAGAATAATTATTCAATTCTAGATTTAATAATGTATTGTCACATAAAGCAGGCAT [15990]
```

```
lp17_gi|365823332|B31_GB     ACCCCAGTAAACTCAATTTCTAATTGATGGAGTTGTTTTTCTATTTCTGATTGGTTATTTATTATTTCTAATTGTGTT [16068]
lp17_un5_B31_PacBio          ACCCCAGTAAACTCAATTTCTAATTGATGGAGTTGTTTTTCTATTTCTGATTGGTTATTTATTATTTCTAATTGTGTT [16068]
lp17_B31_TS_MP_FG_consensus  ACCCCAGTAAACTCAATTTCTAATTGATGGAGTTGTTTTTCTATTTCTGATTGGTTATTTATTATTTCTAATTGTGTT [16068]
lp17_B31_NX_Pl_consensus     ACCCCAGTAAACTCAATTTCTAATTGATGGAGTTGTTTTTCTATTTCTGATTGGTTATTTATTATTTCTAATTGTGTT [16068]
lp17_PAli_NX_MP_FG_consensus ACCCCAGTAAACTCAATTTCTAATTGATGGAGTTGTTTTTCTATTTCTGATTGGTTATTTATTATTTCTAATTGTGTT [16068]
lp17_PAli_NX_Pl_consensus    ACCCCAGTAAACTCAATTTCTAATTGATGGAGTTGTTTTTCTATTTCTGATTGGTTATTTATTATTTCTAATTGTGTT [16068]
lp17_PAbe_NX_MP_FG_consensus ACCCCAGTAAACTCAATTTCTAATTGATGGAGTTGTTTTTCTATTTCTGATTGGTTATTTATTATTTCTAATTGTGTT [16068]
lp17_PAbe_NX_Pl_consensus    ACCCCAGTAAACTCAATTTCTAATTGATGGAGTTGTTTTTCTATTTCTGATTGGTTATTTATTATTTCTAATTGTGTT [16068]

lp17_gi|365823332|B31_GB     TTTTCTATTTCTGAGCACTTGTTTTTTTGATTTTTTTTCATAATATTATAGTATATAAAAAAAATAGTATTAAAATTT [16146]
lp17_un5_B31_PacBio          TTTTCTATTTCTGAGCACTTGTTTTTTTGATTTTTTTTCATAATATTATAGTATATAAAAAAAATAGTATTAAAATTT [16146]
lp17_B31_TS_MP_FG_consensus  TTTTCTATTTCTGAGCACTTGTTTTTTTGATTTTTTTTCATAATATTATAGTATATAAAAAAAATAGTATTAAAATTT [16146]
lp17_B31_NX_Pl_consensus     TTTTCTATTTCTGAGCACTTGTTTTTTTGATTTTTTTTCATAATATTATAGTATATAAAAAAAATAGTATTAAAATTT [16146]
lp17_PAli_NX_MP_FG_consensus TTTTCTATTTCTGAGCACTTGTTTTTTTGATTTTTTTTCATAATATTATAGTATATAAAAAAAATAGTATTAAAATTT [16146]
lp17_PAli_NX_Pl_consensus    TTTTCTATTTCTGAGCACTTGTTTTTTTGATTTTTTTTCATAATATTATAGTATATAAAAAAAATAGTATTAAAATTT [16146]
lp17_PAbe_NX_MP_FG_consensus TTTTCTATTTCTGAGCACTTGTTTTTTTGATTTTTTTTCATAATATTATAGTATATAAAAAAAATAGTATTAAAATTT [16146]
lp17_PAbe_NX_Pl_consensus    TTTTCTATTTCTGAGCACTTGTTTTTTTGATTTTTTTTCATAATATTATAGTATATAAAAAAAATAGTATTAAAATTT [16146]

lp17_gi|365823332|B31_GB     TCAAGAATATTGGTAATTTATAAAGATTTTTTATAAGTTGCTAATAATTCTTAACAAATTGTCTCCATTTATTATGTA [16224]
lp17_un5_B31_PacBio          TCAAGAATATTGGTAATTTATAAAGATTTTTTATAAGTTGCTAATAATTCTTAACAAATTGTCTCCATTTATTATGTA [16224]
lp17_B31_TS_MP_FG_consensus  TCAAGAATATTGGTAATTTATAAAGATTTTTTATAAGTTGCTAATAATTCTTAACAAATTGTCTCCATTTATTATGTA [16224]
lp17_B31_NX_Pl_consensus     TCAAGAATATTGGTAATTTATAAAGATTTTTTATAAGTTGCTAATAATTCTTAACAAATTGTCTCCATTTATTATGTA [16224]
lp17_PAli_NX_MP_FG_consensus TCAAGAATATTGGTAATTTATAAAGATTTTTTATAAGTTGCTAATAATTCTTAACAAATTGTCTCCATTTATTATGTA [16224]
lp17_PAli_NX_Pl_consensus    TCAAGAATATTGGTAATTTATAAAGATTTTTTATAAGTTGCTAATAATTCTTAACAAATTGTCTCCATTTATTATGTA [16224]
lp17_PAbe_NX_MP_FG_consensus TCAAGAATATTGGTAATTTATAAAGATTTTTTATAAGTTGCTAATAATTCTTAACAAATTGTCTCCATTTATTATGTA [16224]
lp17_PAbe_NX_Pl_consensus    TCAAGAATATTGGTAATTTATAAAGATTTTTTATAAGTTGCTAATAATTCTTAACAAATTGTCTCCATTTATTATGTA [16224]

lp17_gi|365823332|B31_GB     GTTGGTATAGTAATAACTACTACTACTATCAGCATTATTGTTAATGCTAGGCATGATTAAATGTATGTATTCTTTAAA [16302]
lp17_un5_B31_PacBio          GTTGGTATAGTAATAACTACTACTACTATCAGCATTATTGTTAATGCTAGGCATGATTAAATGTATGTATTCTTTAAA [16302]
lp17_B31_TS_MP_FG_consensus  GTTGGTATAGTAATAACTACTACTACTATCAGCATTATTGTTAATGCTAGGCATGATTAAATGTATGTATTCTTTAAA [16302]
lp17_B31_NX_Pl_consensus     GTTGGTATAGTAATAACTACTACTACTATCAGCATTATTGTTAATGCTAGGCATGATTAAATGTATGTATTCTTTAAA [16302]
lp17_PAli_NX_MP_FG_consensus GTTGGTATAGTAATAACTACTACTACTATCAGCATTATTGTTAATGCTAGGCATGATTAAATGTATGTATTCTTTAAA [16302]
lp17_PAli_NX_Pl_consensus    GTTGGTATAGTAATAACTACTACTACTATCAGCATTATTGTTAATGCTAGGCATGATTAAATGTATGTATTCTTTAAA [16302]
lp17_PAbe_NX_MP_FG_consensus GTTGGTATAGTAATAACTACTACTACTATCAGCATTATTGTTAATGCTAGGCATGATTAAATGTATGTATTCTTTAAA [16302]
lp17_PAbe_NX_Pl_consensus    GTTGGTATAGTAATAACTACTACTACTATCAGCATTATTGTTAATGCTAGGCATGATTAAATGTATGTATTCTTTAAA [16302]

lp17_gi|365823332|B31_GB     ATTGTCGACATGAATATGCGAGTCTTTGTTGTTTTCTTTTTGGGTTTGATTTTCTCTAACATCTTTAGCCCTAAAACT [16380]
lp17_un5_B31_PacBio          ATTGTCGACATGAATATGCGAGTCTTTGTTGTTTTCTTTTTGGGTTTGATTTTCTCTAACATCTTTAGCCCTAAAACT [16380]
lp17_B31_TS_MP_FG_consensus  ATTGTCGACATGAATATGCGAGTCTTTGTTGTTTTCTTTTTGGGTTTGATTTTCTCTAACATCTTTAGCCCTAAAACT [16380]
lp17_B31_NX_Pl_consensus     ATTGTCGACATGAATATGCGAGTCTTTGTTGTTTTCTTTTTGGGTTTGATTTTCTCTAACATCTTTAGCCCTAAAACT [16380]
lp17_PAli_NX_MP_FG_consensus ATTGTCGACATGAATATGCGAGTCTTTGTTGTTTTCTTTTTGGGTTTGATTTTCTCTAACATCTTTAGCCCTAAAACT [16380]
lp17_PAli_NX_Pl_consensus    ATTGTCGACATGAATATGCGAGTCTTTGTTGTTTTCTTTTTGGGTTTGATTTTCTCTAACATCTTTAGCCCTAAAACT [16380]
lp17_PAbe_NX_MP_FG_consensus ATTGTCGACATGAATATGCGAGTCTTTGTTGTTTTCTTTTTGGGTTTGATTTTCTCTAACATCTTTAGCCCTAAAACT [16380]
lp17_PAbe_NX_Pl_consensus    ATTGTCGACATGAATATGCGAGTCTTTGTTGTTTTCTTTTTGGGTTTGATTTTCTCTAACATCTTTAGCCCTAAAACT [16380]

lp17_gi|365823332|B31_GB     TCCTCCATATTGAATTGCATCTTTAAGTCGAGAAATATTCCCATTTGGAATTTCTAAAAGCTTTTTAGTTTCTGTGCC [16458]
lp17_un5_B31_PacBio          TCCTCCATATTGAATTGCATCTTTAAGTCGAGAAATATTCCCATTTGGAATTTCTAAAAGCTTTTTAGTTTCTGTGCC [16458]
lp17_B31_TS_MP_FG_consensus  TCCTCCATATTGAATTGCATCTTTAAGTCGAGAAATATTCCCATTTGGAATTTCTAAAAGCTTTTTAGTTTCTGTGCC [16458]
lp17_B31_NX_Pl_consensus     TCCTCCATATTGAATTGCATCTTTAAGTCGAGAAATATTCCCATTTGGAATTTCTAAAAGCTTTTTAGTTTCTGTGCC [16458]
lp17_PAli_NX_MP_FG_consensus TCCTCCATATTGAATTGCATCTTTAAGTCGAGAAATATTCCCATTTGGAATTTCTAAAAGCTTTTTAGTTTCTGTGCC [16458]
lp17_PAli_NX_Pl_consensus    TCCTCCATATTGAATTGCATCTTTAAGTCGAGAAATATTCCCATTTGGAATTTCTAAAAGCTTTTTAGTTTCTGTGCC [16458]
lp17_PAbe_NX_MP_FG_consensus TCCTCCATATTGAATTGCATCTTTAAGTCGAGAAATATTCCCATTTGGAATTTCTAAAAGCTTTTTAGTTTCTGTGCC [16458]
lp17_PAbe_NX_Pl_consensus    TCCTCCATATTGAATTGCATCTTTAAGTCGAGAAATATTCCCATTTGGAATTTCTAAAAGCTTTTTAGTTTCTGTGCC [16458]

lp17_gi|365823332|B31_GB     ACTTTCTTTAGTCATCCCATTAATAATAATATTTCCGTCAACTTGTTTTTCTATTTTTAAGATAGTTTGAATATAAGG [16536]
lp17_un5_B31_PacBio          ACTTTCTTTAGTCATCCCATTAATAATAATATTTCCGTCAACTTGTTTTTCTATTTTTAAGATAGTTTGAATATAAGG [16536]
lp17_B31_TS_MP_FG_consensus  ACTTTCTTTAGTCATCCCATTAATAATAATATTTCCGTCAACTTGTTTTTCTATTTTTAAGATAGTTTGAATATAAGG [16536]
lp17_B31_NX_Pl_consensus     ACTTTCTTTAGTCATCCCATTAATAATAATATTTCCGTCAACTTGTTTTTCTATTTTTAAGATAGTTTGAATATAAGG [16536]
lp17_PAli_NX_MP_FG_consensus ACTTTCTTTAGTCATCCCATTAATAATAATATTTCCGTCAACTTGTTTTTCTATTTTTAAGATAGTTTGAATATAAGG [16536]
lp17_PAli_NX_Pl_consensus    ACTTTCTTTAGTCATCCCATTAATAATAATATTTCCGTCAACTTGTTTTTCTATTTTTAAGATAGTTTGAATATAAGG [16536]
lp17_PAbe_NX_MP_FG_consensus ACTTTCTTTAGTCATCCCATTAATAATAATATTTCCGTCAACTTGTTTTTCTATTTTTAAGATAGTTTGAATATAAGG [16536]
lp17_PAbe_NX_Pl_consensus    ACTTTCTTTAGTCATCCCATTAATAATAATATTTCCGTCAACTTGTTTTTCTATTTTTAAGATAGTTTGAATATAAGG [16536]

lp17_gi|365823332|B31_GB     TGGGTAGTTAATAGTAGAATTAATATTATCATTTTGGTTAGTAATAATGGTTGAACGTGTATCTTTTAACGCTTGAAT [16614]
lp17_un5_B31_PacBio          TGGGTAGTTAATAGTAGAATTAATATTATCATTTTGGTTAGTAATAATGGTTGAACGTGTATCTTTTAACGCTTGAAT [16614]
lp17_B31_TS_MP_FG_consensus  TGGGTAGTTAATAGTAGAATTAATATTATCATTTTGGTTAGTAATAATGGTTGAACGTGTATCTTTTAACGCTTGAAT [16614]
lp17_B31_NX_Pl_consensus     TGGGTAGTTAATAGTAGAATTAATATTATCATTTTGGTTAGTAATAATGGTTGAACGTGTATCTTTTAACGCTTGAAT [16614]
lp17_PAli_NX_MP_FG_consensus TGGGTAGTTAATAGTAGAATTAATATTATCATTTTGGTTAGTAATAATGGTTGAACGTGTATCTTTTAACGCTTGAAT [16614]
lp17_PAli_NX_Pl_consensus    TGGGTAGTTAATAGTAGAATTAATATTATCATTTTGGTTAGTAATAATGGTTGAACGTGTATCTTTTAACGCTTGAAT [16614]
lp17_PAbe_NX_MP_FG_consensus TGGGTAGTTAATAGTAGAATTAATATTATCATTTTGGTTAGTAATAATGGTTGAACGTGTATCTTTTAACGCTTGAAT [16614]
lp17_PAbe_NX_Pl_consensus    TGGGTAGTTAATAGTAGAATTAATATTATCATTTTGGTTAGTAATAATGGTTGAACGTGTATCTTTTAACGCTTGAAT [16614]

lp17_gi|365823332|B31_GB     TTCGTTTGAAAATTGAATATTTGTTTGACGACTCATTTATTTGATATTCGCTTATTTTTTCTTTATTTGACATGTTTA [16692]
lp17_un5_B31_PacBio          TTCGTTTGAAAATTGAATATTTGTTTGACGACTCATTTATTTGATATTCGCTTATTTTTTCTTTATTTGACATGTTTA [16692]
lp17_B31_TS_MP_FG_consensus  TTCGTTTGAAAATTGAATATTTGTTTGACGACTCATTTATTTGATATTCGCTTATTTTTTCTTTATTTGACATGTTTA [16692]
lp17_B31_NX_Pl_consensus     TTCGTTTGAAAATTGAATATTTGTTTGACGACTCATTTATTTGATATTCGCTTATTTTTTCTTTATTTGACATGTTTA [16692]
lp17_PAli_NX_MP_FG_consensus TTCGTTTGAAAATTGAATATTTGTTTGACGACTCATTTATTTGATATTCGCTTATTTTTTCTTTATTTGACATGTTTA [16692]
lp17_PAli_NX_Pl_consensus    TTCGTTTGAAAATTGAATATTTGTTTGACGACTCATTTATTTGATATTCGCTTATTTTTTCTTTATTTGACATGTTTA [16692]
lp17_PAbe_NX_MP_FG_consensus TTCGTTTGAAAATTGAATATTTGTTTGACGACTCATTTATTTGATATTCGCTTATTTTTTCTTTATTTGACATGTTTA [16692]
lp17_PAbe_NX_Pl_consensus    TTCGTTTGAAAATTGAATATTTGTTTGACGACTCATTTATTTGATATTCGCTTATTTTTTCTTTATTTGACATGTTTA [16692]

lp17_gi|365823332|B31_GB     GTGGATTATTTTGATCAGAATTACTTAAATTACATGAAAAGACTAATAAAATAATGAATATCAATAATTTGTTCATGG [16770]
lp17_un5_B31_PacBio          GTGGATTATTTTGATCAGAATTACTTAAATTACATGAAAAGACTAATAAAATAATGAATATCAATAATTTGTTCATGG [16770]
lp17_B31_TS_MP_FG_consensus  GTGGATTATTTTGATCAGAATTACTTAAATTACATGAAAAGACTAATAAAATAATGAATATCAATAATTTGTTCATGG [16770]
lp17_B31_NX_Pl_consensus     GTGGATTATTTTGATCAGAATTACTTAAATTACATGAAAAGACTAATAAAATAATGAATATCAATAATTTGTTCATGG [16770]
lp17_PAli_NX_MP_FG_consensus GTGGATTATTTTGATCAGAATTACTTAAATTACATGAAAAGACTAATAAAATAATGAATATCAATAATTTGTTCATGG [16770]
lp17_PAli_NX_Pl_consensus    GTGGATTATTTTGATCAGAATTACTTAAATTACATGAAAAGACTAATAAAATAATGAATATCAATAATTTGTTCATGG [16770]
lp17_PAbe_NX_MP_FG_consensus GTGGATTATTTTGATCAGAATTACTTAAATTACATGAAAAGACTAATAAAATAATGAATATCAATAATTTGTTCATGG [16770]
lp17_PAbe_NX_Pl_consensus    GTGGATTATTTTGATCAGAATTACTTAAATTACATGAAAAGACTAATAAAATAATGAATATCAATAATTTGTTCATGG [16770]

lp17_gi|365823332|B31_GB     ATATTATAGCTCCTATTAAACTTGCAAAAACCGCTTATAATATTATAGATCAGGTTTATTGATAATCAATAAACCTGA [16848]
lp17_un5_B31_PacBio          ATATTATAGCTCCTATTAAACTTGCAAAAACCGCTTATAATATTATAGATCAGGTTTATTGATAATCAATAAACCTGA [16848]
lp17_B31_TS_MP_FG_consensus  ATATTATAGCTCCTATTAAACTTGCAAAAACCGCTTATAATATTATAGATCAGGTTTATTGATAATCAATAAACCTGA [16848]
lp17_B31_NX_Pl_consensus     ATATTATAGCTCCTATTAAACTTGCAAAAACCGCTTATAATATTATAGATCAGGTTTATTGATAATCAATAAACCTGA [16848]
lp17_PAli_NX_MP_FG_consensus ATATTATAGCTCCTATTAAACTTGCAAAAACCGCTTATAATATTATAGATCAGGTTTATTGATAATCAATAAACCTGA [16848]
lp17_PAli_NX_Pl_consensus    ATATTATAGCTCCTATTAAACTTGCAAAAACCGCTTATAATATTATAGATCAGGTTTATTGATAATCAATAAACCTGA [16848]
lp17_PAbe_NX_MP_FG_consensus ATATTATAGCTCCTATTAAACTTGCAAAAACCGCTTATAATATTATAGATCAGGTTTATTGATAATCAATAAACCTGA [16848]
lp17_PAbe_NX_Pl_consensus    ATATTATAGCTCCTATTAAACTTGCAAAAACCGCTTATAATATTATAGATCAGGTTTATTGATAATCAATAAACCTGA [16848]

lp17_gi|365823332|B31_GB     TCAACGATTATTCTAATCGTTTTAAAAAAAACAAGAGAATAAAATTGCAAAAATTAAAGTTTTATCATTTCGGGTTAA [16926]
lp17_un5_B31_PacBio          TCAACGATTATTCTAATCGTTTTAAAAAAA-CAAGAGAATAAAATTGCAAAAATTAAAGTTTTATCATTTCGGGTTAA [16926]
lp17_B31_TS_MP_FG_consensus  TCAACGATTATTCTAATCGTTTTAAAAAAAACAAGAGAATAAAATTGCAAAAATTAAAGTTTTATCATTTCGGGTTAA [16926]
lp17_B31_NX_Pl_consensus     TCAACGATTATTCTAATCGTTTTAAAAAAAACAAGAGAATAAAATTGCAAAAATTAAAGTTTTATCATTTCGGGTTAA [16926]
lp17_PAli_NX_MP_FG_consensus TCAACGATTATTCTAATCGTTTTAAAAAAAACAAGAGAATAAAATTGCAAAAATTAAAGTTTTATCATTTCGGGTTAA [16926]
lp17_PAli_NX_Pl_consensus    TCAACGATTATTCTAATCGTTTTAAAAAAAACAAGAGAATAAAATTGCAAAAATTAAAGTTTTATCATTTCGGGTTAA [16926]
lp17_PAbe_NX_MP_FG_consensus TCAACGATTATTCTAATCGTTTTAAAAAAAACAAGAGAATAAAATTGCAAAAATTAAAGTTTTATCATTTCGGGTTAA [16926]
lp17_PAbe_NX_Pl_consensus    TCAACGATTATTCTAATCGTTTTAAAAAAAACAAGAGAATAAAATTGCAAAAATTAAAGTTTTATCATTTCGGGTTAA [16926]

lp17_gi|365823332|B31_GB     AGTTTAGTATTTTCAAGTTAAAGTTAGCAATTTAAAGGGTAAAGTTTTGAGTC------------------------- [17004]
```

```
lp17_un5_B31_PacBio          AGTTTAGTATTTTCAAGTTAAAGTTAGCAATTTAAAGGGTAAAGTTTTGAGTCAAAATACTCTATACTAATAAAAAAT [17004]
lp17_B31_TS_MP_FG_consensus  AGTTTAGTATTTTCAAGTTAAAGTTAGCAATTTAAAGGGTAAAGTTTTGAGTC------------------------- [17004]
lp17_B31_NX_Pl_consensus     AGTTTAGTATTTTCAAGTTAAAGTTAGCAATTTAAAGGGTAAAGTTTTGAGTC------------------------- [17004]
lp17_PAli_NX_MP_FG_consensus AGTTTAGTATTTTCAAGTTAAAGTTAGCAATTTAAAGGGTAAAGTTTTGAGTC------------------------- [17004]
lp17_PAli_NX_Pl_consensus    AGTTTAGTATTTTCAAGTTAAAGTTAGCAATTTAAAGGGTAAAGTTTTGAGTC------------------------- [17004]
lp17_PAbe_NX_MP_FG_consensus AGTTTAGTATTTTCAAGTTAAAGTTAGCAATTTAAAGGGTAAAGTTTTGAGTC------------------------- [17004]
lp17_PAbe_NX_Pl_consensus    AGTTTAGTATTTTCAAGTTAAAGTTAGCAATTTAAAGGGTAAAGTTTTGAGTC------------------------- [17004]

lp17_gi|365823332|B31_GB     ------------------------------------------------------------------------------ [17082]
lp17_un5_B31_PacBio          TATATATATAATTTTTTATTAGTATAGAGTATTTT------------------------------------------- [17082]
lp17_B31_TS_MP_FG_consensus  ------------------------------------------------------------------------------ [17082]
lp17_B31_NX_Pl_consensus     ------------------------------------------------------------------------------ [17082]
lp17_PAli_NX_MP_FG_consensus ------------------------------------------------------------------------------ [17082]
lp17_PAli_NX_Pl_consensus    ------------------------------------------------------------------------------ [17082]
lp17_PAbe_NX_MP_FG_consensus ------------------------------------------------------------------------------ [17082]
lp17_PAbe_NX_Pl_consensus    ------------------------------------------------------------------------------ [17082]

#NEXUS
[ Title ]
begin data;
   dimensions ntax=8 nchar=37857;
   format missing=? gap=- matchchar=. datatype=nucleotide interleave=yes;
   matrix

[!Domain=Data property=Coding CodonStart=1;]
lp36_gi|11496779|B31_GB      AACTACTAATTTATATAATCTTTTCACTTAACATTTTGTTATACAAAAATCTTAAATATCTAAATACTTTTGAAAAAT [   78]
lp36_un11_B31_PacBio         ------------------------------------------------------------------CTTTTGAAAAAT [   78]
lp36_B31_TS_MP_FG_consensus  AACTACTAATTTATATAATCTTTTCACTTAACATTTTGTTATACAAAAATCTTAAATATCTAAATACTTTTGAAAAAT [   78]
lp36_B31_NX_Pl_consensus     AACTACTAATTTATATAATCTTTTCACTTAACATTTTGTTATACAAAAATCTTAAATATCTAAATACTTTTGAAAAAT [   78]
lp36_PAli_NX_MP_FG_consensus AACTACTAATTTATATAATCTTTTCACTTAACATTTTGTTATACAAAAATCTTAAATATCTAAATACTTTTGAAAAAT [   78]
lp36_PAli_NX_Pl_consensus    AACTACTAATTTATATAATCTTTTCACTTAACATTTTGTTATACAAAAATCTTAAATATCTAAATACTTTTGAAAAAT [   78]
lp36_PAbe_NX_MP_FG_consensus AACTACTAATTTATATAATCTTTTCACTTAACATTTTGTTATACAAAAATCTTAAATATCTAAATACTTTTGAAAAAT [   78]
lp36_PAbe_NX_Pl_consensus    AACTACTAATTTATATAATCTTTTCACTTAACATTTTGTTATACAAAAATCTTAAATATCTAAATACTTTTGAAAAAT [   78]

lp36_gi|11496779|B31_GB      ATTTTTATTTACAATTTATTGTAATTAGAACAAATTGATATATAATATCAATTAAGAATAATTATTATTTATAATAAT [  156]
lp36_un11_B31_PacBio         ATTTTTATTTACAATTTATTGTAATTAGAACAAATTGATATATAATATCAATTAAGAATAATTATTATTTATAATAAT [  156]
lp36_B31_TS_MP_FG_consensus  ATTTTTATTTACAATTTATTGTAATTAGAACAAATTGATATATAATATCAATTAAGAATAATTATTATTTATAATAAT [  156]
lp36_B31_NX_Pl_consensus     ATTTTTATTTACAATTTATTGTAATTAGAACAAATTGATATATAATATCAATTAAGAATAATTATTATTTATAATAAT [  156]
lp36_PAli_NX_MP_FG_consensus ATTTTTATTTACAATTTATTGTAATTAGAACAAATTGATATATAATATCAATTAAGAATAATTATTATTTATAATAAT [  156]
lp36_PAli_NX_Pl_consensus    ATTTTTATTTACAATTTATTGTAATTAGAACAAATTGATATATAATATCAATTAAGAATAATTATTATTTATAATAAT [  156]
lp36_PAbe_NX_MP_FG_consensus ATTTTTATTTACAATTTATTGTAATTAGAACAAATTGATATATAATATCAATTAAGAATAATTATTATTTATAATAAT [  156]
lp36_PAbe_NX_Pl_consensus    ATTTTTATTTACAATTTATTGTAATTAGAACAAATTGATATATAATATCAATTAAGAATAATTATTATTTATAATAAT [  156]

lp36_gi|11496779|B31_GB      TATTCTTAATTGATAAAAGGAGAATATTTTTATGAGAAAAAGTTTGTTTTTATATGCATTATTAATGGGAGGATTGAT [  234]
lp36_un11_B31_PacBio         TATTCTTAATTGATAAAAGGAGAATATTTTTATGAGAAAAAGTTTGTTTTTATATGCATTATTAATGGGAGGATTGAT [  234]
lp36_B31_TS_MP_FG_consensus  TATTCTTAATTGATAAAAGGAGAATATTTTTATGAGAAAAAGTTTGTTTTTATATGCATTATTAATGGGAGGATTGAT [  234]
lp36_B31_NX_Pl_consensus     TATTCTTAATTGATAAAAGGAGAATATTTTTATGAGAAAAAGTTTGTTTTTATATGCATTATTAATGGGAGGATTGAT [  234]
lp36_PAli_NX_MP_FG_consensus TATTCTTAATTGATAAAAGGAGAATATTTTTATGAGAAAAAGTTTGTTTTTATATGCATTATTAATGGGAGGATTGAT [  234]
lp36_PAli_NX_Pl_consensus    TATTCTTAATTGATAAAAGGAGAATATTTTTATGAGAAAAAGTTTGTTTTTATATGCATTATTAATGGGAGGATTGAT [  234]
lp36_PAbe_NX_MP_FG_consensus TATTCTTAATTGATAAAAGGAGAATATTTTTATGAGAAAAAGTTTGTTTTTATATGCATTATTAATGGGAGGATTGAT [  234]
lp36_PAbe_NX_Pl_consensus    TATTCTTAATTGATAAAAGGAGAATATTTTTATGAGAAAAAGTTTGTTTTTATATGCATTATTAATGGGAGGATTGAT [  234]

lp36_gi|11496779|B31_GB      GTCTTGTAATCTAGATTCCAAATTATCTAGTAACAAAGAACAAAAAAATAACAATAATGTAAAAGAAGTTTCGGATAG [  312]
lp36_un11_B31_PacBio         GTCTTGTAATCTAGATTCCAAATTATCTAGTAACAAAGAACAAAAAAATAACAATAATGTAAAAGAAGTTTCGGATAG [  312]
lp36_B31_TS_MP_FG_consensus  GTCTTGTAATCTAGATTCCAAATTATCTAGTAACAAAGAACAAAAAAATAACAATAATGTAAAAGAAGTTTCGGATAG [  312]
lp36_B31_NX_Pl_consensus     GTCTTGTAATCTAGATTCCAAATTATCTAGTAACAAAGAACAAAAAAATAACAATAATGTAAAAGAAGTTTCGGATAG [  312]
lp36_PAli_NX_MP_FG_consensus GTCTTGTAATCTAGATTCCAAATTATCTAGTAACAAAGAACAAAAAAATAACAATAATGTAAAAGAAGTTTCGGATAG [  312]
lp36_PAli_NX_Pl_consensus    GTCTTGTAATCTAGATTCCAAATTATCTAGTAACAAAGAACAAAAAAATAACAATAATGTAAAAGAAGTTTCGGATAG [  312]
lp36_PAbe_NX_MP_FG_consensus GTCTTGTAATCTAGATTCCAAATTATCTAGTAACAAAGAACAAAAAAATAACAATAATGTAAAAGAAGTTTCGGATAG [  312]
lp36_PAbe_NX_Pl_consensus    GTCTTGTAATCTAGATTCCAAATTATCTAGTAACAAAGAACAAAAAAATAACAATAATGTAAAAGAAGTTTCGGATAG [  312]

lp36_gi|11496779|B31_GB      TGTTCAAGAAGATGGTCTTAATGATTTATATAATAATCAAGAAAAGCAAAAAAGCTTTACTAAAAATTTTGGAGAACG [  390]
lp36_un11_B31_PacBio         TGTTCAAGAAGATGGTCTTAATGATTTATATAATAATCAAGAAAAGCAAAAAAGCTTTACTAAAAATTTTGGAGAACG [  390]
lp36_B31_TS_MP_FG_consensus  TGTTCAAGAAGATGGTCTTAATGATTTATATAATAATCAAGAAAAGCAAAAAAGCTTTACTAAAAATTTTGGAGAACG [  390]
lp36_B31_NX_Pl_consensus     TGTTCAAGAAGATGGTCTTAATGATTTATATAATAATCAAGAAAAGCAAAAAAGCTTTACTAAAAATTTTGGAGAACG [  390]
lp36_PAli_NX_MP_FG_consensus TGTTCAAGAAGATGGTCTTAATGATTTATATAATAATCAAGAAAAGCAAAAAAGCTTTACTAAAAATTTTGGAGAACG [  390]
lp36_PAli_NX_Pl_consensus    TGTTCAAGAAGATGGTCTTAATGATTTATATAATAATCAAGAAAAGCAAAAAAGCTTTACTAAAAATTTTGGAGAACG [  390]
lp36_PAbe_NX_MP_FG_consensus TGTTCAAGAAGATGGTCTTAATGATTTATATAATAATCAAGAAAAGCAAAAAAGCTTTACTAAAAATTTTGGAGAACG [  390]
lp36_PAbe_NX_Pl_consensus    TGTTCAAGAAGATGGTCTTAATGATTTATATAATAATCAAGAAAAGCAAAAAAGCTTTACTAAAAATTTTGGAGAACG [  390]

lp36_gi|11496779|B31_GB      GAAATATGAGGATTTAATTAATCCTATAGAGCCTATAATACCTTCAGAATCACCAAAGAATAAGGCTAATATACCAAA [  468]
lp36_un11_B31_PacBio         GAAATATGAGGATTTAATTAATCCTATAGAGCCTATAATACCTTCAGAATCACCAAAGAATAAGGCTAATATACCAAA [  468]
lp36_B31_TS_MP_FG_consensus  GAAATATGAGGATTTAATTAATCCTATAGAGCCTATAATACCTTCAGAATCACCAAAGAATAAGGCTAATATACCAAA [  468]
lp36_B31_NX_Pl_consensus     GAAATATGAGGATTTAATTAATCCTATAGAGCCTATAATACCTTCAGAATCACCAAAGAATAAGGCTAATATACCAAA [  468]
lp36_PAli_NX_MP_FG_consensus GAAATATGAGGATTTAATTAATCCTATAGAGCCTATAATACCTTCAGAATCACCAAAGAATAAGGCTAATATACCAAA [  468]
lp36_PAli_NX_Pl_consensus    GAAATATGAGGATTTAATTAATCCTATAGAGCCTATAATACCTTCAGAATCACCAAAGAATAAGGCTAATATACCAAA [  468]
lp36_PAbe_NX_MP_FG_consensus GAAATATGAGGATTTAATTAATCCTATAGAGCCTATAATACCTTCAGAATCACCAAAGAATAAGGCTAATATACCAAA [  468]
lp36_PAbe_NX_Pl_consensus    GAAATATGAGGATTTAATTAATCCTATAGAGCCTATAATACCTTCAGAATCACCAAAGAATAAGGCTAATATACCAAA [  468]

lp36_gi|11496779|B31_GB      TATTTCAATTGCGCATACTGAAAAAAAAGAGACAAAAAAGGAGAATTTAATCCCTTCTACTAATGAAGAAAAGGAAGC [  546]
lp36_un11_B31_PacBio         TATTTCAATTGCGCATACTGAAAAAAAAGAGACAAAAAAGGAGAATTTAATCCCTTCTACTAATGAAGAAAAGGAAGC [  546]
lp36_B31_TS_MP_FG_consensus  TATTTCAATTGCGCATACTGAAAAAAAAGAGACAAAAAAGGAGAATTTAATCCCTTCTACTAATGAAGAAAAGGAAGC [  546]
lp36_B31_NX_Pl_consensus     TATTTCAATTGCGCATACTGAAAAAAAAGAGACAAAAAAGGAGAATTTAATCCCTTCTACTAATGAAGAAAAGGAAGC [  546]
lp36_PAli_NX_MP_FG_consensus TATTTCAATTGCGCATACTGAAAAAAAAGAGACAAAAAAGGAGAATTTAATCCCTTCTACTAATGAAGAAAAGGAAGC [  546]
lp36_PAli_NX_Pl_consensus    TATTTCAATTGCGCATACTGAAAAAAAAGAGACAAAAAAGGAGAATTTAATCCCTTCTACTAATGAAGAAAAGGAAGC [  546]
lp36_PAbe_NX_MP_FG_consensus TATTTCAATTGCGCATACTGAAAAAAAAGAGACAAAAAAGGAGAATTTAATCCCTTCTACTAATGAAGAAAAGGAAGC [  546]
lp36_PAbe_NX_Pl_consensus    TATTTCAATTGCGCATACTGAAAAAAAAGAGACAAAAAAGGAGAATTTAATCCCTTCTACTAATGAAGAAAAGGAAGC [  546]

lp36_gi|11496779|B31_GB      TGATGCAGCAATTAAATATTTAGAAGAAAATATTCTTAAAAACTCTAAATTTTCTGAATTAATTAGAGAAGTACGTGT [  624]
lp36_un11_B31_PacBio         TGATGCAGCAATTAAATATTTAGAAGAAAATATTCTTAAAAACTCTAAATTTTCTGAATTAATTAGAGAAGTACGTGT [  624]
lp36_B31_TS_MP_FG_consensus  TGATGCAGCAATTAAATATTTAGAAGAAAATATTCTTAAAAACTCTAAATTTTCTGAATTAATTAGAGAAGTACGTGT [  624]
lp36_B31_NX_Pl_consensus     TGATGCAGCAATTAAATATTTAGAAGAAAATATTCTTAAAAACTCTAAATTTTCTGAATTAATTAGAGAAGTACGTGT [  624]
lp36_PAli_NX_MP_FG_consensus TGATGCAGCAATTAAATATTTAGAAGAAAATATTCTTAAAAACTCTAAATTTTCTGAATTAATTAGAGAAGTACGTGT [  624]
lp36_PAli_NX_Pl_consensus    TGATGCAGCAATTAAATATTTAGAAGAAAATATTCTTAAAAACTCTAAATTTTCTGAATTAATTAGAGAAGTACGTGT [  624]
lp36_PAbe_NX_MP_FG_consensus TGATGCAGCAATTAAATATTTAGAAGAAAATATTCTTAAAAACTCTAAATTTTCTGAATTAATTAGAGAAGTACGTGT [  624]
lp36_PAbe_NX_Pl_consensus    TGATGCAGCAATTAAATATTTAGAAGAAAATATTCTTAAAAACTCTAAATTTTCTGAATTAATTAGAGAAGTACGTGT [  624]

lp36_gi|11496779|B31_GB      AATTAAAGATGAATATGCTTTAATAAAAGCTGATTTGTATGATGTAATTGGAAAGATTAACAATAAAAAAACATCATT [  702]
lp36_un11_B31_PacBio         AATTAAAGATGAATATGCTTTAATAAAAGCTGATTTGTATGATGTAATTGGAAAGATTAACAATAAAAAAACATCATT [  702]
lp36_B31_TS_MP_FG_consensus  AATTAAAGATGAATATGCTTTAATAAAAGCTGATTTGTATGATGTAATTGGAAAGATTAACAATAAAAAAACATCATT [  702]
lp36_B31_NX_Pl_consensus     AATTAAAGATGAATATGCTTTAATAAAAGCTGATTTGTATGATGTAATTGGAAAGATTAACAATAAAAAAACATCATT [  702]
lp36_PAli_NX_MP_FG_consensus AATTAAAGATGAATATGCTTTAATAAAAGCTGATTTGTATGATGTAATTGGAAAGATTAACAATAAAAAAACATCATT [  702]
lp36_PAli_NX_Pl_consensus    AATTAAAGATGAATATGCTTTAATAAAAGCTGATTTGTATGATGTAATTGGAAAGATTAACAATAAAAAAACATCATT [  702]
lp36_PAbe_NX_MP_FG_consensus AATTAAAGATGAATATGCTTTAATAAAAGCTGATTTGTATGATGTAATTGGAAAGATTAACAATAAAAAAACATCATT [  702]
lp36_PAbe_NX_Pl_consensus    AATTAAAGATGAATATGCTTTAATAAAAGCTGATTTGTATGATGTAATTGGAAAGATTAACAATAAAAAAACATCATT [  702]

lp36_gi|11496779|B31_GB      AATGGAGAATCCTAAGAACAATAGAGATAAGATAAATAAATTAACACAATTGTTGCAAAATAATTTAAAGATAGATAG [  780]
lp36_un11_B31_PacBio         AATGGAGAATCCTAAGAACAATAGAGATAAGATAAATAAATTAACACAATTGTTGCAAAATAATTTAAAGATAGATAG [  780]
lp36_B31_TS_MP_FG_consensus  AATGGAGAATCCTAAGAACAATAGAGATAAGATAAATAAATTAACACAATTGTTGCAAAATAATTTAAAGATAGATAG [  780]
```

```
lp36_B31_NX_Pl_consensus     AATGGAGAATCCTAAGAACAATAGAGATAAGATAAATAAATTAACACAATTGTTGCAAAATAATTTAAAGATAGATAG [  780]
lp36_PAli_NX_MP_FG_consensus AATGGAGAATCCTAAGAACAATAGAGATAAGATAAATAAATTAACACAATTGTTGCAAAATAATTTAAAGATAGATAG [  780]
lp36_PAli_NX_Pl_consensus    AATGGAGAATCCTAAGAACAATAGAGATAAGATAAATAAATTAACACAATTGTTGCAAAATAATTTAAAGATAGATAG [  780]
lp36_PAbe_NX_MP_FG_consensus AATGGAGAATCCTAAGAACAATAGAGATAAGATAAATAAATTAACACAATTGTTGCAAAATAATTTAAAGATAGATAG [  780]
lp36_PAbe_NX_Pl_consensus    AATGGAGAATCCTAAGAACAATAGAGATAAGATAAATAAATTAACACAATTGTTGCAAAATAATTTAAAGATAGATAG [  780]

lp36_gi|11496779|B31_GB      TGAACTTGAGCAGCTTATAAATATGATTGATATGGCAGAAAATGAAATAAGCTCTGCGGCTTTCTTTTTTGACAACGC [  858]
lp36_un11_B31_PacBio         TGAACTTGAGCAGCTTATAAATATGATTGATATGGCAGAAAATGAAATAAGCTCTGCGGCTTTCTTTTT-GACAACGC [  858]
lp36_B31_TS_MP_FG_consensus  TGAACTTGAGCAGCTTATAAATATGATTGATATGGCAGAAAATGAAATAAGCTCTGCGGCTTTCTTTTTTGACAACGC [  858]
lp36_B31_NX_Pl_consensus     TGAACTTGAGCAGCTTATAAATATGATTGATATGGCAGAAAATGAAATAAGCTCTGCGGCTTTCTTTTTTGACAACGC [  858]
lp36_PAli_NX_MP_FG_consensus TGAACTTGAGCAGCTTATAAATATGATTGATATGGCAGAAAATGAAATAAGCTCTGCGGCTTTCTTTTTTGACAACGC [  858]
lp36_PAli_NX_Pl_consensus    TGAACTTGAGCAGCTTATAAATATGATTGATATGGCAGAAAATGAAATAAGCTCTGCGGCTTTCTTTTTTGACAACGC [  858]
lp36_PAbe_NX_MP_FG_consensus TGAACTTGAGCAGCTTATAAATATGATTGATATGGCAGAAAATGAAATAAGCTCTGCGGCTTTCTTTTTTGACAACGC [  858]
lp36_PAbe_NX_Pl_consensus    TGAACTTGAGCAGCTTATAAATATGATTGATATGGCAGAAAATGAAATAAGCTCTGCGGCTTTCTTTTTTGACAACGC [  858]

lp36_gi|11496779|B31_GB      TCAGAAAAGGTTAAAAGAAAGCATTATTAAAAGATTAGAGAGTAAAAATAATAGATCTTATGCATTAAAATTGTCTAG [  936]
lp36_un11_B31_PacBio         TCAGAAAAGGTTAAAAGAAAGCATTATTAAAAGATTAGAGAGTAAAAATAATAGATCTTATGCATTAAAATTGTCTAG [  936]
lp36_B31_TS_MP_FG_consensus  TCAGAAAAGGTTAAAAGAAAGCATTATTAAAAGATTAGAGAGTAAAAATAATAGATCTTATGCATTAAAATTGTCTAG [  936]
lp36_B31_NX_Pl_consensus     TCAGAAAAGGTTAAAAGAAAGCATTATTAAAAGATTAGAGAGTAAAAATAATAGATCTTATGCATTAAAATTGTCTAG [  936]
lp36_PAli_NX_MP_FG_consensus TCAGAAAAGGTTAAAAGAAAGCATTATTAAAAGATTAGAGAGTAAAAATAATAGATCTTATGCATTAAAATTGTCTAG [  936]
lp36_PAli_NX_Pl_consensus    TCAGAAAAGGTTAAAAGAAAGCATTATTAAAAGATTAGAGAGTAAAAATAATAGATCTTATGCATTAAAATTGTCTAG [  936]
lp36_PAbe_NX_MP_FG_consensus TCAGAAAAGGTTAAAAGAAAGCATTATTAAAAGATTAGAGAGTAAAAATAATAGATCTTATGCATTAAAATTGTCTAG [  936]
lp36_PAbe_NX_Pl_consensus    TCAGAAAAGGTTAAAAGAAAGCATTATTAAAAGATTAGAGAGTAAAAATAATAGATCTTATGCATTAAAATTGTCTAG [  936]

lp36_gi|11496779|B31_GB      ACAGGCTTTAAGTGACGCAAGAAGTGCTTTAAGTAATTTAGAATCTTTTGCCTCTAAAAGAATTGAACCAATGGTGAG [ 1014]
lp36_un11_B31_PacBio         ACAGGCTTTAAGTGACGCAAGAAGTGCTTTAAGTAATTTAGAATCTTTTGCCTCTAAAAGAATTGAACCAATGGTGAG [ 1014]
lp36_B31_TS_MP_FG_consensus  ACAGGCTTTAAGTGACGCAAGAAGTGCTTTAAGTAATTTAGAATCTTTTGCCTCTAAAAGAATTGAACCAATGGTGAG [ 1014]
lp36_B31_NX_Pl_consensus     ACAGGCTTTAAGTGACGCAAGAAGTGCTTTAAGTAATTTAGAATCTTTTGCCTCTAAAAGAATTGAACCAATGGTGAG [ 1014]
lp36_PAli_NX_MP_FG_consensus ACAGGCTTTAAGTGACGCAAGAAGTGCTTTAAGTAATTTAGAATCTTTTGCCTCTAAAAGAATTGAACCAATGGTGAG [ 1014]
lp36_PAli_NX_Pl_consensus    ACAGGCTTTAAGTGACGCAAGAAGTGCTTTAAGTAATTTAGAATCTTTTGCCTCTAAAAGAATTGAACCAATGGTGAG [ 1014]
lp36_PAbe_NX_MP_FG_consensus ACAGGCTTTAAGTGACGCAAGAAGTGCTTTAAGTAATTTAGAATCTTTTGCCTCTAAAAGAATTGAACCAATGGTGAG [ 1014]
lp36_PAbe_NX_Pl_consensus    ACAGGCTTTAAGTGACGCAAGAAGTGCTTTAAGTAATTTAGAATCTTTTGCCTCTAAAAGAATTGAACCAATGGTGAG [ 1014]

lp36_gi|11496779|B31_GB      AAAGGAAGAAATAAAAGAGCTTATTAAACATGCAAAAACTGTTTTAGAAAGTCTCAATAAAAAATAACAAAGAATATT [ 1092]
lp36_un11_B31_PacBio         AAAGGAAGAAATAAAAGAGCTTATTAAACATGCAAAAACTGTTTTAGAAAGTCTCAATAAAAAATAACAAAGAATATT [ 1092]
lp36_B31_TS_MP_FG_consensus  AAAGGAAGAAATAAAAGAGCTTATTAAACATGCAAAAACTGTTTTAGAAAGTCTCAATAAAAAATAACAAAGAATATT [ 1092]
lp36_B31_NX_Pl_consensus     AAAGGAAGAAATAAAAGAGCTTATTAAACATGCAAAAACTGTTTTAGAAAGTCTCAATAAAAAATAACAAAGAATATT [ 1092]
lp36_PAli_NX_MP_FG_consensus AAAGGAAGAAATAAAAGAGCTTATTAAACATGCAAAAACTGTTTTAGAAAGTCTCAATAAAAAATAACAAAGAATATT [ 1092]
lp36_PAli_NX_Pl_consensus    AAAGGAAGAAATAAAAGAGCTTATTAAACATGCAAAAACTGTTTTAGAAAGTCTCAATAAAAAATAACAAAGAATATT [ 1092]
lp36_PAbe_NX_MP_FG_consensus AAAGGAAGAAATAAAAGAGCTTATTAAACATGCAAAAACTGTTTTAGAAAGTCTCAATAAAAAATAACAAAGAATATT [ 1092]
lp36_PAbe_NX_Pl_consensus    AAAGGAAGAAATAAAAGAGCTTATTAAACATGCAAAAACTGTTTTAGAAAGTCTCAATAAAAAATAACAAAGAATATT [ 1092]

lp36_gi|11496779|B31_GB      CCACTTATAATTTCTATGAAATTTAGGTGGAAATAAATTTACAAAGTAAATTAAATAATTTCAGTGTCTGTGTGTACC [ 1170]
lp36_un11_B31_PacBio         CCACTTATAATTTCTATGAAATTTAGGTGGAAATAAATTTACAAAGTAAATTAAATAATTTCAGTGTCTGTGTGTACC [ 1170]
lp36_B31_TS_MP_FG_consensus  CCACTTATAATTTCTATGAAATTTAGGTGGAAATAAATTTACAAAGTAAATTAAATAATTTCAGTGTCTGTGTGTACC [ 1170]
lp36_B31_NX_Pl_consensus     CCACTTATAATTTCTATGAAATTTAGGTGGAAATAAATTTACAAAGTAAATTAAATAATTTCAGTGTCTGTGTGTACC [ 1170]
lp36_PAli_NX_MP_FG_consensus CCACTTATAATTTCTATGAAATTTAGGTGGAAATAAATTTACAAAGTAAATTAAATAATTTCAGTGTCTGTGTGTACC [ 1170]
lp36_PAli_NX_Pl_consensus    CCACTTATAATTTCTATGAAATTTAGGTGGAAATAAATTTACAAAGTAAATTAAATAATTTCAGTGTCTGTGTGTACC [ 1170]
lp36_PAbe_NX_MP_FG_consensus CCACTTATAATTTCTATGAAATTTAGGTGGAAATAAATTTACAAAGTAAATTAAATAATTTCAGTGTCTGTGTGTACC [ 1170]
lp36_PAbe_NX_Pl_consensus    CCACTTATAATTTCTATGAAATTTAGGTGGAAATAAATTTACAAAGTAAATTAAATAATTTCAGTGTCTGTGTGTACC [ 1170]

lp36_gi|11496779|B31_GB      AAACTCAGACTTTCACAAACAGAGGCTCATCAAATCTATTTACGCTGCATTATGCTTATCCATTCTTCTTAAATAATT [ 1248]
lp36_un11_B31_PacBio         AAACTCAGACTTTCACAAACAGAGGCTCATCAAATCTATTTACGCTGCATTATGCTTATCCATTCTTCTTAAATAATT [ 1248]
lp36_B31_TS_MP_FG_consensus  AAACTCAGACTTTCACAAACAGAGGCTCATCAAATCTATTTACGCTGCATTATGCTTATCCATTCTTCTTAAATAATT [ 1248]
lp36_B31_NX_Pl_consensus     AAACTCAGACTTTCACAAACAGAGGCTCATCAAATCTATTTACGCTGCATTATGCTTATCCATTCTTCTTAAATAATT [ 1248]
lp36_PAli_NX_MP_FG_consensus AAACTCAGACTTTCACAAACAGAGGCTCATCAAATCTATTTACGCTGCATTATGCTTATCCATTCTTCTTAAATAATT [ 1248]
lp36_PAli_NX_Pl_consensus    AAACTCAGACTTTCACAAACAGAGGCTCATCAAATCTATTTACGCTGCATTATGCTTATCCATTCTTCTTAAATAATT [ 1248]
lp36_PAbe_NX_MP_FG_consensus AAACTCAGACTTTCACAAACAGAGGCTCATCAAATCTATTTACGCTGCATTATGCTTATCCATTCTTCTTAAATAATT [ 1248]
lp36_PAbe_NX_Pl_consensus    AAACTCAGACTTTCACAAACAGAGGCTCATCAAATCTATTTACGCTGCATTATGCTTATCCATTCTTCTTAAATAATT [ 1248]

lp36_gi|11496779|B31_GB      CTCATATCCTTTTATTAAAGATGAAATATAATCTTCTCCTTTTTTGTTTTTGCAAACCTTAAAATCATTAAGCAAAGT [ 1326]
lp36_un11_B31_PacBio         CTCATATCCTTTTATTAAAGATGAAATATAATCTTCTCCTTTTTTGTTTTTGCAAACCTTAAAATCATTAAGCAAAGT [ 1326]
lp36_B31_TS_MP_FG_consensus  CTCATATCCTTTTATTAAAGATGAAATATAATCTTCTCCTTTTTTGTTTTTGCAAACCTTAAAATCATTAAGCAAAGT [ 1326]
lp36_B31_NX_Pl_consensus     CTCATATCCTTTTATTAAAGATGAAATATAATCTTCTCCTTTTTTGTTTTTGCAAACCTTAAAATCATTAAGCAAAGT [ 1326]
lp36_PAli_NX_MP_FG_consensus CTCATATCCTTTTATTAAAGATGAAATATAATCTTCTCCTTTTTTGTTTTTGCAAACCTTAAAATCATTAAGCAAAGT [ 1326]
lp36_PAli_NX_Pl_consensus    CTCATATCCTTTTATTAAAGATGAAATATAATCTTCTCCTTTTTTGTTTTTGCAAACCTTAAAATCATTAAGCAAAGT [ 1326]
lp36_PAbe_NX_MP_FG_consensus CTCATATCCTTTTATTAAAGATGAAATATAATCTTCTCCTTTTTTGTTTTTGCAAACCTTAAAATCATTAAGCAAAGT [ 1326]
lp36_PAbe_NX_Pl_consensus    CTCATATCCTTTTATTAAAGATGAAATATAATCTTCTCCTTTTTTGTTTTTGCAAACCTTAAAATCATTAAGCAAAGT [ 1326]

lp36_gi|11496779|B31_GB      TACAAAATCTTCTTTGGTTAACGAGTAAAGACTGGCCACAATGAAATTATTTTCATTTTCTTTTTCTTTAAAGAATTC [ 1404]
lp36_un11_B31_PacBio         TACAAAATCTTCTTTGGTTAACGAGTAAAGACTGGCCACAATGAAATTATTTTCATTTTCTTTTTCTTTAAAGAATTC [ 1404]
lp36_B31_TS_MP_FG_consensus  TACAAAATCTTCTTTGGTTAACGAGTAAAGACTGGCCACAATGAAATTATTTTCATTTTCTTTTTCTTTAAAGAATTC [ 1404]
lp36_B31_NX_Pl_consensus     TACAAAATCTTCTTTGGTTAACGAGTAAAGACTGGCCACAATGAAATTATTTTCATTTTCTTTTTCTTTAAAGAATTC [ 1404]
lp36_PAli_NX_MP_FG_consensus TACAAAATCTTCTTTGGTTAACGAGTAAAGACTGGCCACAATGAAATTATTTTCATTTTCTTTTTCTTTAAAGAATTC [ 1404]
lp36_PAli_NX_Pl_consensus    TACAAAATCTTCTTTGGTTAACGAGTAAAGACTGGCCACAATGAAATTATTTTCATTTTCTTTTTCTTTAAAGAATTC [ 1404]
lp36_PAbe_NX_MP_FG_consensus TACAAAATCTTCTTTGGTTAACGAGTAAAGACTGGCCACAATGAAATTATTTTCATTTTCTTTTTCTTTAAAGAATTC [ 1404]
lp36_PAbe_NX_Pl_consensus    TACAAAATCTTCTTTGGTTAACGAGTAAAGACTGGCCACAATGAAATTATTTTCATTTTCTTTTTCTTTAAAGAATTC [ 1404]

lp36_gi|11496779|B31_GB      ATCTTTGGGGTCTAGATTTAACATCTTGTCAACTTCTTCTTTGCCAAACTCAAAATATTCTAAGTAAAGCAAATATTT [ 1482]
lp36_un11_B31_PacBio         ATCTTTGGGGTCTAGATTTAACATCTTGTCAACTTCTTCTTTGCCAAACTCAAAATATTCTAAGTAAAGCAAATATTT [ 1482]
lp36_B31_TS_MP_FG_consensus  ATCTTTGGGGTCTAGATTTAACATCTTGTCAACTTCTTCTTTGCCAAACTCAAAATATTCTAAGTAAAGCAAATATTT [ 1482]
lp36_B31_NX_Pl_consensus     ATCTTTGGGGTCTAGATTTAACATCTTGTCAACTTCTTCTTTGCCAAACTCAAAATATTCTAAGTAAAGCAAATATTT [ 1482]
lp36_PAli_NX_MP_FG_consensus ATCTTTGGGGTCTAGATTTAACATCTTGTCAACTTCTTCTTTGCCAAACTCAAAATATTCTAAGTAAAGCAAATATTT [ 1482]
lp36_PAli_NX_Pl_consensus    ATCTTTGGGGTCTAGATTTAACATCTTGTCAACTTCTTCTTTGCCAAACTCAAAATATTCTAAGTAAAGCAAATATTT [ 1482]
lp36_PAbe_NX_MP_FG_consensus ATCTTTGGGGTCTAGATTTAACATCTTGTCAACTTCTTCTTTGCCAAACTCAAAATATTCTAAGTAAAGCAAATATTT [ 1482]
lp36_PAbe_NX_Pl_consensus    ATCTTTGGGGTCTAGATTTAACATCTTGTCAACTTCTTCTTTGCCAAACTCAAAATATTCTAAGTAAAGCAAATATTT [ 1482]

lp36_gi|11496779|B31_GB      AAAGTTATCGGGATCATTTTTAACTATTAGCAAGGAAGTGTTTTTAGCTAAAGTTAAGTATAAAGAGTTATTTAAAAT [ 1560]
lp36_un11_B31_PacBio         AAAGTTATCGGGATCATTTTTAACTATTAGCAAGGAAGTGTTTTTAGCTAAAGTTAAGTATAAAGAGTTATTTAAAAT [ 1560]
lp36_B31_TS_MP_FG_consensus  AAAGTTATCGGGATCATTTTTAACTATTAGCAAGGAAGTGTTTTTAGCTAAAGTTAAGTATAAAGAGTTATTTAAAAT [ 1560]
lp36_B31_NX_Pl_consensus     AAAGTTATCGGGATCATTTTTAACTATTAGCAAGGAAGTGTTTTTAGCTAAAGTTAAGTATAAAGAGTTATTTAAAAT [ 1560]
lp36_PAli_NX_MP_FG_consensus AAAGTTATCGGGATCATTTTTAACTATTAGCAAGGAAGTGTTTTTAGCTAAAGTTAAGTATAAAGAGTTATTTAAAAT [ 1560]
lp36_PAli_NX_Pl_consensus    AAAGTTATCGGGATCATTTTTAACTATTAGCAAGGAAGTGTTTTTAGCTAAAGTTAAGTATAAAGAGTTATTTAAAAT [ 1560]
lp36_PAbe_NX_MP_FG_consensus AAAGTTATCGGGATCATTTTTAACTATTAGCAAGGAAGTGTTTTTAGCTAAAGTTAAGTATAAAGAGTTATTTAAAAT [ 1560]
lp36_PAbe_NX_Pl_consensus    AAAGTTATCGGGATCATTTTTAACTATTAGCAAGGAAGTGTTTTTAGCTAAAGTTAAGTATAAAGAGTTATTTAAAAT [ 1560]

lp36_gi|11496779|B31_GB      CTCATCCTCTTCGGGTTGAGGCATTGGGCATTGATATAAACATGATTTTAGCACATTTGAGTTAACAAATCTTCTAAT [ 1638]
lp36_un11_B31_PacBio         CTCATCCTCTTCGGGTTGAGGCATTGGGCATTGATATAAACATGATTTTAGCACATTTGAGTTAACAAATCTTCTAAT [ 1638]
lp36_B31_TS_MP_FG_consensus  CTCATCCTCTTCGGGTTGAGGCATTGGGCATTGATATAAACATGATTTTAGCACATTTGAGTTAACAAATCTTCTAAT [ 1638]
lp36_B31_NX_Pl_consensus     CTCATCCTCTTCGGGTTGAGGCATTGGGCATTGATATAAACATGATTTTAGCACATTTGAGTTAACAAATCTTCTAAT [ 1638]
lp36_PAli_NX_MP_FG_consensus CTCATCCTCTTCGGGTTGAGGCATTGGGCATTGATATAAACATGATTTTAGCACATTTGAGTTAACAAATCTTCTAAT [ 1638]
lp36_PAli_NX_Pl_consensus    CTCATCCTCTTCGGGTTGAGGCATTGGGCATTGATATAAACATGATTTTAGCACATTTGAGTTAACAAATCTTCTAAT [ 1638]
lp36_PAbe_NX_MP_FG_consensus CTCATCCTCTTCGGGTTGAGGCATTGGGCATTGATATAAACATGATTTTAGCACATTTGAGTTAACAAATCTTCTAAT [ 1638]
lp36_PAbe_NX_Pl_consensus    CTCATCCTCTTCGGGTTGAGGCATTGGGCATTGATATAAACATGATTTTAGCACATTTGAGTTAACAAATCTTCTAAT [ 1638]

lp36_gi|11496779|B31_GB      TATAAAGTCAAACACAAATGAGTTAAAAATTGATATAATAAATAATTTTTTATAAAGTGATATTGGTATTTCCTCATA [ 1716]
lp36_un11_B31_PacBio         TATAAAGTCAAACACAAATGAGTTAAAAATTGATATAATAAATAATTTTTTATAAAGTGATATTGGTATTTCCTCATA [ 1716]
lp36_B31_TS_MP_FG_consensus  TATAAAGTCAAACACAAATGAGTTAAAAATTGATATAATAAATAATTTTTTATAAAGTGATATTGGTATTTCCTCATA [ 1716]
lp36_B31_NX_Pl_consensus     TATAAAGTCAAACACAAATGAGTTAAAAATTGATATAATAAATAATTTTTTATAAAGTGATATTGGTATTTCCTCATA [ 1716]
```

```
lp36_PAli_NX_MP_FG_consensus TATAAAGTCAAACACAAATGAGTTAAAAATTGATATAATAAATAATTTTTTATAAAGTGATATTGGTATTTCCTCATA [ 1716]
lp36_PAli_NX_Pl_consensus    TATAAAGTCAAACACAAATGAGTTAAAAATTGATATAATAAATAATTTTTTATAAAGTGATATTGGTATTTCCTCATA [ 1716]
lp36_PAbe_NX_MP_FG_consensus TATAAAGTCAAACACAAATGAGTTAAAAATTGATATAATAAATAATTTTTTATAAAGTGATATTGGTATTTCCTCATA [ 1716]
lp36_PAbe_NX_Pl_consensus    TATAAAGTCAAACACAAATGAGTTAAAAATTGATATAATAAATAATTTTTTATAAAGTGATATTGGTATTTCCTCATA [ 1716]

lp36_gi|11496779|B31_GB      ATTTATATATATTGAATTCACGCAATAACAATTTTTAGGAGAAAGTGTGCTAATCATCGTTCTTTCATTTGTGTTACT [ 1794]
lp36_un11_B31_PacBio         ATTTATATATATTGAATTCACGCAATAACAATTTTTAGGAGAAAGTGTGCTAATCATCGTTCTTTCATTTGTGTTACT [ 1794]
lp36_B31_TS_MP_FG_consensus  ATTTATATATATTGAATTCACGCAATAACAATTTTTAGGAGAAAGTGTGCTAATCATCGTTCTTTCATTTGTGTTACT [ 1794]
lp36_B31_NX_Pl_consensus     ATTTATATATATTGAATTCACGCAATAACAATTTTTAGGAGAAAGTGTGCTAATCATCGTTCTTTCATTTGTGTTACT [ 1794]
lp36_PAli_NX_MP_FG_consensus ATTTATATATATTGAATTCACGCAATAACAATTTTTAGGAGAAAGTGTGCTAATCATCGTTCTTTCATTTGTGTTACT [ 1794]
lp36_PAli_NX_Pl_consensus    ATTTATATATATTGAATTCACGCAATAACAATTTTTAGGAGAAAGTGTGCTAATCATCGTTCTTTCATTTGTGTTACT [ 1794]
lp36_PAbe_NX_MP_FG_consensus ATTTATATATATTGAATTCACGCAATAACAATTTTTAGGAGAAAGTGTGCTAATCATCGTTCTTTCATTTGTGTTACT [ 1794]
lp36_PAbe_NX_Pl_consensus    ATTTATATATATTGAATTCACGCAATAACAATTTTTAGGAGAAAGTGTGCTAATCATCGTTCTTTCATTTGTGTTACT [ 1794]

lp36_gi|11496779|B31_GB      TGCAATTACCCTATAGAATACTCTTTCGGCTTGATATTGGCTGTCTTTCATTAATACTTTTTCTAAGTCTTTTTTATC [ 1872]
lp36_un11_B31_PacBio         TGCAATTACCCTATAGAATACTCTTTCGGCTTGATATTGGCTGTCTTTCATTAATACTTTTTCTAAGTCTTTTTTATC [ 1872]
lp36_B31_TS_MP_FG_consensus  TGCAATTACCCTATAGAATACTCTTTCGGCTTGATATTGGCTGTCTTTCATTAATACTTTTTCTAAGTCTTTTTTATC [ 1872]
lp36_B31_NX_Pl_consensus     TGCAATTACCCTATAGAATACTCTTTCGGCTTGATATTGGCTGTCTTTCATTAATACTTTTTCTAAGTCTTTTTTATC [ 1872]
lp36_PAli_NX_MP_FG_consensus TGCAATTACCCTATAGAATACTCTTTCGGCTTGATATTGGCTGTCTTTCATTAATACTTTTTCTAAGTCTTTTTTATC [ 1872]
lp36_PAli_NX_Pl_consensus    TGCAATTACCCTATAGAATACTCTTTCGGCTTGATATTGGCTGTCTTTCATTAATACTTTTTCTAAGTCTTTTTTATC [ 1872]
lp36_PAbe_NX_MP_FG_consensus TGCAATTACCCTATAGAATACTCTTTCGGCTTGATATTGGCTGTCTTTCATTAATACTTTTTCTAAGTCTTTTTTATC [ 1872]
lp36_PAbe_NX_Pl_consensus    TGCAATTACCCTATAGAATACTCTTTCGGCTTGATATTGGCTGTCTTTCATTAATACTTTTTCTAAGTCTTTTTTATC [ 1872]

lp36_gi|11496779|B31_GB      TATCCATAGTAATTTAGAACTTTCTTTTGCAGCTCTATCTTCAAAAAATCTTGAATTAAATTGGTGAATATTGGCTCC [ 1950]
lp36_un11_B31_PacBio         TATCCATAGTAATTTAGAACTTTCTTTTGCAGCTCTATCTTCAAAAAATCTTGAATTAAATTGGTGAATATTGGCTCC [ 1950]
lp36_B31_TS_MP_FG_consensus  TATCCATAGTAATTTAGAACTTTCTTTTGCAGCTCTATCTTCAAAAAATCTTGAATTAAATTGGTGAATATTGGCTCC [ 1950]
lp36_B31_NX_Pl_consensus     TATCCATAGTAATTTAGAACTTTCTTTTGCAGCTCTATCTTCAAAAAATCTTGAATTAAATTGGTGAATATTGGCTCC [ 1950]
lp36_PAli_NX_MP_FG_consensus TATCCATAGTAATTTAGAACTTTCTTTTGCAGCTCTATCTTCAAAAAATCTTGAATTAAATTGGTGAATATTGGCTCC [ 1950]
lp36_PAli_NX_Pl_consensus    TATCCATAGTAATTTAGAACTTTCTTTTGCAGCTCTATCTTCAAAAAATCTTGAATTAAATTGGTGAATATTGGCTCC [ 1950]
lp36_PAbe_NX_MP_FG_consensus TATCCATAGTAATTTAGAACTTTCTTTTGCAGCTCTATCTTCAAAAAATCTTGAATTAAATTGGTGAATATTGGCTCC [ 1950]
lp36_PAbe_NX_Pl_consensus    TATCCATAGTAATTTAGAACTTTCTTTTGCAGCTCTATCTTCAAAAAATCTTGAATTAAATTGGTGAATATTGGCTCC [ 1950]

lp36_gi|11496779|B31_GB      AGAATACAGAAATATAAATTTTTCATTATTATATTCTTTATACAGTGCCTTATACTTTGTTAAATTTAGTCCCTCTCT [ 2028]
lp36_un11_B31_PacBio         AGAATACAGAAATATAAATTTTTCATTATTATATTCTTTATACAGTGCCTTATACTTTGTTAAATTTAGTCCCTCTCT [ 2028]
lp36_B31_TS_MP_FG_consensus  AGAATACAGAAATATAAATTTTTCATTATTATATTCTTTATACAGTGCCTTATACTTTGTTAAATTTAGTCCCTCTCT [ 2028]
lp36_B31_NX_Pl_consensus     AGAATACAGAAATATAAATTTTTCATTATTATATTCTTTATACAGTGCCTTATACTTTGTTAAATTTAGTCCCTCTCT [ 2028]
lp36_PAli_NX_MP_FG_consensus AGAATACAGAAATATAAATTTTTCATTATTATATTCTTTATACAGTGCCTTATACTTTGTTAAATTTAGTCCCTCTCT [ 2028]
lp36_PAli_NX_Pl_consensus    AGAATACAGAAATATAAATTTTTCATTATTATATTCTTTATACAGTGCCTTATACTTTGTTAAATTTAGTCCCTCTCT [ 2028]
lp36_PAbe_NX_MP_FG_consensus AGAATACAGAAATATAAATTTTTCATTATTATATTCTTTATACAGTGCCTTATACTTTGTTAAATTTAGTCCCTCTCT [ 2028]
lp36_PAbe_NX_Pl_consensus    AGAATACAGAAATATAAATTTTTCATTATTATATTCTTTATACAGTGCCTTATACTTTGTTAAATTTAGTCCCTCTCT [ 2028]

lp36_gi|11496779|B31_GB      AAAATCAATATACTCTTGAATAAGAGTATCAAATCGACTAAACATTTTATTAACAAGCGTGAACTCAGCGCTATCTCT [ 2106]
lp36_un11_B31_PacBio         AAAATCAATATACTCTTGAATAAGAGTATCAAATCGACTAAACATTTTATTAACAAGCGTGAACTCAGCGCTATCTCT [ 2106]
lp36_B31_TS_MP_FG_consensus  AAAATCAATATACTCTTGAATAAGAGTATCAAATCGACTAAACATTTTATTAACAAGCGTGAACTCAGCGCTATCTCT [ 2106]
lp36_B31_NX_Pl_consensus     AAAATCAATATACTCTTGAATAAGAGTATCAAATCGACTAAACATTTTATTAACAAGCGTGAACTCAGCGCTATCTCT [ 2106]
lp36_PAli_NX_MP_FG_consensus AAAATCAATATACTCTTGAATAAGAGTATCAAATCGACTAAACATTTTATTAACAAGCGTGAACTCAGCGCTATCTCT [ 2106]
lp36_PAli_NX_Pl_consensus    AAAATCAATATACTCTTGAATAAGAGTATCAAATCGACTAAACATTTTATTAACAAGCGTGAACTCAGCGCTATCTCT [ 2106]
lp36_PAbe_NX_MP_FG_consensus AAAATCAATATACTCTTGAATAAGAGTATCAAATCGACTAAACATTTTATTAACAAGCGTGAACTCAGCGCTATCTCT [ 2106]
lp36_PAbe_NX_Pl_consensus    AAAATCAATATACTCTTGAATAAGAGTATCAAATCGACTAAACATTTTATTAACAAGCGTGAACTCAGCGCTATCTCT [ 2106]

lp36_gi|11496779|B31_GB      AAATTCTATTATGGACTCTTGAATAGGAGATAGCCTTTTGATTTGAGCTATATCCAATTCGATTCCCTTATAAGCATT [ 2184]
lp36_un11_B31_PacBio         AAATTCTATTATGGACTCTTGAATAGGAGATAGCCTTTTGATTTGAGCTATATCCAATTCGATTCCCTTATAAGCATT [ 2184]
lp36_B31_TS_MP_FG_consensus  AAATTCTATTATGGACTCTTGAATAGGAGATAGCCTTTTGATTTGAGCTATATCCAATTCGATTCCCTTATAAGCATT [ 2184]
lp36_B31_NX_Pl_consensus     AAATTCTATTATGGACTCTTGAATAGGAGATAGCCTTTTGATTTGAGCTATATCCAATTCGATTCCCTTATAAGCATT [ 2184]
lp36_PAli_NX_MP_FG_consensus AAATTCTATTATGGACTCTTGAATAGGAGATAGCCTTTTGATTTGAGCTATATCCAATTCGATTCCCTTATAAGCATT [ 2184]
lp36_PAli_NX_Pl_consensus    AAATTCTATTATGGACTCTTGAATAGGAGATAGCCTTTTGATTTGAGCTATATCCAATTCGATTCCCTTATAAGCATT [ 2184]
lp36_PAbe_NX_MP_FG_consensus AAATTCTATTATGGACTCTTGAATAGGAGATAGCCTTTTGATTTGAGCTATATCCAATTCGATTCCCTTATAAGCATT [ 2184]
lp36_PAbe_NX_Pl_consensus    AAATTCTATTATGGACTCTTGAATAGGAGATAGCCTTTTGATTTGAGCTATATCCAATTCGATTCCCTTATAAGCATT [ 2184]

lp36_gi|11496779|B31_GB      TTCATTACCCTCTTTCAGATCACTGGTTATTTCTTTCATGATATTATCGCTACTCTGGATAACAAATTTTACTCTAAA [ 2262]
lp36_un11_B31_PacBio         TTCATTACCCTCTTTCAGATCACTGGTTATTTCTTTCATGATATTATCGCTACTCTGGATAACAAATTTTACTCTAAA [ 2262]
lp36_B31_TS_MP_FG_consensus  TTCATTACCCTCTTTCAGATCACTGGTTATTTCTTTCATGATATTATCGCTACTCTGGATAACAAATTTTACTCTAAA [ 2262]
lp36_B31_NX_Pl_consensus     TTCATTACCCTCTTTCAGATCACTGGTTATTTCTTTCATGATATTATCGCTACTCTGGATAACAAATTTTACTCTAAA [ 2262]
lp36_PAli_NX_MP_FG_consensus TTCATTACCCTCTTTCAGATCACTGGTTATTTCTTTCATGATATTATCGCTACTCTGGATAACAAATTTTACTCTAAA [ 2262]
lp36_PAli_NX_Pl_consensus    TTCATTACCCTCTTTCAGATCACTGGTTATTTCTTTCATGATATTATCGCTACTCTGGATAACAAATTTTACTCTAAA [ 2262]
lp36_PAbe_NX_MP_FG_consensus TTCATTACCCTCTTTCAGATCACTGGTTATTTCTTTCATGATATTATCGCTACTCTGGATAACAAATTTTACTCTAAA [ 2262]
lp36_PAbe_NX_Pl_consensus    TTCATTACCCTCTTTCAGATCACTGGTTATTTCTTTCATGATATTATCGCTACTCTGGATAACAAATTTTACTCTAAA [ 2262]

lp36_gi|11496779|B31_GB      TTTCGTTGTGGGTTCTTTACTATTACTGATCTGAAATATTGCAAATTTGAAGCCAGGGGTCATGGCCCTAAACCCTTT [ 2340]
lp36_un11_B31_PacBio         TTTCGTTGTGGGTTCTTTACTATTACTGATCTGAAATATTGCAAATTTGAAGCCAGGGGTCATGGCCCTAAACCCTTT [ 2340]
lp36_B31_TS_MP_FG_consensus  TTTCGTTGTGGGTTCTTTACTATTACTGATCTGAAATATTGCAAATTTGAAGCCAGGGGTCATGGCCCTAAACCCTTT [ 2340]
lp36_B31_NX_Pl_consensus     TTTCGTTGTGGGTTCTTTACTATTACTGATCTGAAATATTGCAAATTTGAAGCCAGGGGTCATGGCCCTAAACCCTTT [ 2340]
lp36_PAli_NX_MP_FG_consensus TTTCGTTGTGGGTTCTTTACTATTACTGATCTGAAATATTGCAAATTTGAAGCCAGGGGTCATGGCCCTAAACCCTTT [ 2340]
lp36_PAli_NX_Pl_consensus    TTTCGTTGTGGGTTCTTTACTATTACTGATCTGAAATATTGCAAATTTGAAGCCAGGGGTCATGGCCCTAAACCCTTT [ 2340]
lp36_PAbe_NX_MP_FG_consensus TTTCGTTGTGGGTTCTTTACTATTACTGATCTGAAATATTGCAAATTTGAAGCCAGGGGTCATGGCCCTAAACCCTTT [ 2340]
lp36_PAbe_NX_Pl_consensus    TTTCGTTGTGGGTTCTTTACTATTACTGATCTGAAATATTGCAAATTTGAAGCCAGGGGTCATGGCCCTAAACCCTTT [ 2340]

lp36_gi|11496779|B31_GB      CTGATTTTCAAACTGGTATATATAATTAAGCTTGTAATTTGAAAATATATGCTGCCTCAGTGCTTTAGAGCCGGATTC [ 2418]
lp36_un11_B31_PacBio         CTGATTTTCAAACTGGTATATATAATTAAGCTTGTAATTTGAAAATATATGCTGCCTCAGTGCTTTAGAGCCGGATTC [ 2418]
lp36_B31_TS_MP_FG_consensus  CTGATTTTCAAACTGGTATATATAATTAAGCTTGTAATTTGAAAATATATGCTGCCTCAGTGCTTTAGAGCCGGATTC [ 2418]
lp36_B31_NX_Pl_consensus     CTGATTTTCAAACTGGTATATATAATTAAGCTTGTAATTTGAAAATATATGCTGCCTCAGTGCTTTAGAGCCGGATTC [ 2418]
lp36_PAli_NX_MP_FG_consensus CTGATTTTCAAACTGGTATATATAATTAAGCTTGTAATTTGAAAATATATGCTGCCTCAGTGCTTTAGAGCCGGATTC [ 2418]
lp36_PAli_NX_Pl_consensus    CTGATTTTCAAACTGGTATATATAATTAAGCTTGTAATTTGAAAATATATGCTGCCTCAGTGCTTTAGAGCCGGATTC [ 2418]
lp36_PAbe_NX_MP_FG_consensus CTGATTTTCAAACTGGTATATATAATTAAGCTTGTAATTTGAAAATATATGCTGCCTCAGTGCTTTAGAGCCGGATTC [ 2418]
lp36_PAbe_NX_Pl_consensus    CTGATTTTCAAACTGGTATATATAATTAAGCTTGTAATTTGAAAATATATGCTGCCTCAGTGCTTTAGAGCCGGATTC [ 2418]

lp36_gi|11496779|B31_GB      GCTCCACAAACTGGAAGGAGTCAAATAAGTTAAGTTGCCGCCGGGCTTCACTAGCTTCAAATTGAAAGCCGTAAAATA [ 2496]
lp36_un11_B31_PacBio         GCTCCACAAACTGGAAGGAGTCAAATAAGTTAAGTTGCCGCCGGGCTTCACTAGCTTCAAATTGAAAGCCGTAAAATA [ 2496]
lp36_B31_TS_MP_FG_consensus  GCTCCACAAACTGGAAGGAGTCAAATAAGTTAAGTTGCCGCCGGGCTTCACTAGCTTCAAATTGAAAGCCGTAAAATA [ 2496]
lp36_B31_NX_Pl_consensus     GCTCCACAAACTGGAAGGAGTCAAATAAGTTAAGTTGCCGCCGGGCTTCACTAGCTTCAAATTGAAAGCCGTAAAATA [ 2496]
lp36_PAli_NX_MP_FG_consensus GCTCCACAAACTGGAAGGAGTCAAATAAGTTAAGTTGCCGCCGGGCTTCACTAGCTTCAAATTGAAAGCCGTAAAATA [ 2496]
lp36_PAli_NX_Pl_consensus    GCTCCACAAACTGGAAGGAGTCAAATAAGTTAAGTTGCCGCCGGGCTTCACTAGCTTCAAATTGAAAGCCGTAAAATA [ 2496]
lp36_PAbe_NX_MP_FG_consensus GCTCCACAAACTGGAAGGAGTCAAATAAGTTAAGTTGCCGCCGGGCTTCACTAGCTTCAAATTGAAAGCCGTAAAATA [ 2496]
lp36_PAbe_NX_Pl_consensus    GCTCCACAAACTGGAAGGAGTCAAATAAGTTAAGTTGCCGCCGGGCTTCACTAGCTTCAAATTGAAAGCCGTAAAATA [ 2496]

lp36_gi|11496779|B31_GB      TCTAAAAAGATTCGGACCCCCCCACTAGAAAATTCTCTGAACACCAACCTATACATATTATTCAAAGTTTTCAAACTG [ 2574]
lp36_un11_B31_PacBio         TCTAAAAAGATTCGGACCCCCCCACTAGAAAATTCTCTGAACACCAACCTATACATATTATTCAAAGTTTTCAAACTG [ 2574]
lp36_B31_TS_MP_FG_consensus  TCTAAAAAGATTCGGACCCCCCCACTAGAAAATTCTCTGAACACCAACCTATACATATTATTCAAAGTTTTCAAACTG [ 2574]
lp36_B31_NX_Pl_consensus     TCTAAAAAGATTCGGACCCCCCCACTAGAAAATTCTCTGAACACCAACCTATACATATTATTCAAAGTTTTCAAACTG [ 2574]
lp36_PAli_NX_MP_FG_consensus TCTAAAAAGATTCGGACCCCCCCACTAGAAAATTCTCTGAACACCAACCTATACATATTATTCAAAGTTTTCAAACTG [ 2574]
lp36_PAli_NX_Pl_consensus    TCTAAAAAGATTCGGACCCCCCCACTAGAAAATTCTCTGAACACCAACCTATACATATTATTCAAAGTTTTCAAACTG [ 2574]
lp36_PAbe_NX_MP_FG_consensus TCTAAAAAGATTCGGACCCCCCCACTAGAAAATTCTCTGAACACCAACCTATACATATTATTCAAAGTTTTCAAACTG [ 2574]
lp36_PAbe_NX_Pl_consensus    TCTAAAAAGATTCGGACCCCCCCACTAGAAAATTCTCTGAACACCAACCTATACATATTATTCAAAGTTTTCAAACTG [ 2574]

lp36_gi|11496779|B31_GB      TTCTTTTCCTCATAGCGCTCGATACTCAAAGGATGATTCTCTTTACTAAGTAGTTCTTGTTTTATCTTATTTTGTTCT [ 2652]
lp36_un11_B31_PacBio         TTCTTTTCCTCATAGCGCTCGATACTCAAAGGATGATTCTCTTTACTAAGTAGTTCTTGTTTTATCTTATTTTGTTCT [ 2652]
lp36_B31_TS_MP_FG_consensus  TTCTTTTCCTCATAGCGCTCGATACTCAAAGGATGATTCTCTTTACTAAGTAGTTCTTGTTTTATCTTATTTTGTTCT [ 2652]
lp36_B31_NX_Pl_consensus     TTCTTTTCCTCATAGCGCTCGATACTCAAAGGATGATTCTCTTTACTAAGTAGTTCTTGTTTTATCTTATTTTGTTCT [ 2652]
lp36_PAli_NX_MP_FG_consensus TTCTTTTCCTCATAGCGCTCGATACTCAAAGGATGATTCTCTTTACTAAGTAGTTCTTGTTTTATCTTATTTTGTTCT [ 2652]
```

```
lp36_PAli_NX_Pl_consensus    TTCTTTTCCTCATAGCGCTCGATACTCAAAGGATGATTCTCTTTACTAAGTAGTTCTTGTTTTATCTTATTTTGTTCT [ 2652]
lp36_PAbe_NX_MP_FG_consensus TTCTTTTCCTCATAGCGCTCGATACTCAAAGGATGATTCTCTTTACTAAGTAGTTCTTGTTTTATCTTATTTTGTTCT [ 2652]
lp36_PAbe_NX_Pl_consensus    TTCTTTTCCTCATAGCGCTCGATACTCAAAGGATGATTCTCTTTACTAAGTAGTTCTTGTTTTATCTTATTTTGTTCT [ 2652]

lp36_gi|11496779|B31_GB      TTTATGCTTAGTTTTCTGTAGTTAAGAACATGTTTTGATAAAAATTCAGATTCATCAAACTTAGTTTTCTCCCATGGA [ 2730]
lp36_un11_B31_PacBio         TTTATGCTTAGTTTTCTGTAGTTAAGAACATGTTTTGATAAAAATTCAGATTCATCAAACTTAGTTTTCTCCCATGGA [ 2730]
lp36_B31_TS_MP_FG_consensus  TTTATGCTTAGTTTTCTGTAGTTAAGAACATGTTTTGATAAAAATTCAGATTCATCAAACTTAGTTTTCTCCCATGGA [ 2730]
lp36_B31_NX_Pl_consensus     TTTATGCTTAGTTTTCTGTAGTTAAGAACATGTTTTGATAAAAATTCAGATTCATCAAACTTAGTTTTCTCCCATGGA [ 2730]
lp36_PAli_NX_MP_FG_consensus TTTATGCTTAGTTTTCTGTAGTTAAGAACATGTTTTGATAAAAATTCAGATTCATCAAACTTAGTTTTCTCCCATGGA [ 2730]
lp36_PAli_NX_Pl_consensus    TTTATGCTTAGTTTTCTGTAGTTAAGAACATGTTTTGATAAAAATTCAGATTCATCAAACTTAGTTTTCTCCCATGGA [ 2730]
lp36_PAbe_NX_MP_FG_consensus TTTATGCTTAGTTTTCTGTAGTTAAGAACATGTTTTGATAAAAATTCAGATTCATCAAACTTAGTTTTCTCCCATGGA [ 2730]
lp36_PAbe_NX_Pl_consensus    TTTATGCTTAGTTTTCTGTAGTTAAGAACATGTTTTGATAAAAATTCAGATTCATCAAACTTAGTTTTCTCCCATGGA [ 2730]

lp36_gi|11496779|B31_GB      GGATTTCCAATTACAATATCAAATCCTTCTTGAATATCTGGAAACTCAATTCCATAGTGAAAAAATTTATAATAACTA [ 2808]
lp36_un11_B31_PacBio         GGATTTCCAATTACAATATCAAATCCTTCTTGAATATCTGGAAACTCAATTCCATAGTGAAAAAATTTATAATAACTA [ 2808]
lp36_B31_TS_MP_FG_consensus  GGATTTCCAATTACAATATCAAATCCTTCTTGAATATCTGGAAACTCAATTCCATAGTGAAAAAATTTATAATAACTA [ 2808]
lp36_B31_NX_Pl_consensus     GGATTTCCAATTACAATATCAAATCCTTCTTGAATATCTGGAAACTCAATTCCATAGTGAAAAAATTTATAATAACTA [ 2808]
lp36_PAli_NX_MP_FG_consensus GGATTTCCAATTACAATATCAAATCCTTCTTGAATATCTGGAAACTCAATTCCATAGTGAAAAAATTTATAATAACTA [ 2808]
lp36_PAli_NX_Pl_consensus    GGATTTCCAATTACAATATCAAATCCTTCTTGAATATCTGGAAACTCAATTCCATAGTGAAAAAATTTATAATAACTA [ 2808]
lp36_PAbe_NX_MP_FG_consensus GGATTTCCAATTACAATATCAAATCCTTCTTGAATATCTGGAAACTCAATTCCATAGTGAAAAAATTTATAATAACTA [ 2808]
lp36_PAbe_NX_Pl_consensus    GGATTTCCAATTACAATATCAAATCCTTCTTGAATATCTGGAAACTCAATTCCATAGTGAAAAAATTTATAATAACTA [ 2808]

lp36_gi|11496779|B31_GB      CTTAATTTTCTAATTTTTTTCTATTTTTTTCTTTATCTTCACTAGAAATTTTACTGTCTAAAATATTTCCAATCAAATT [ 2886]
lp36_un11_B31_PacBio         CTTAATTTTCTAATTTTTTTCTATTTTTTTCTTTATCTTCACTAGAAATTTTACTGTCTAAAATATTTCCAATCAAATT [ 2886]
lp36_B31_TS_MP_FG_consensus  CTTAATTTTCTAATTTTTTTCTATTTTTTTCTTTATCTTCACTAGAAATTTTACTGTCTAAAATATTTCCAATCAAATT [ 2886]
lp36_B31_NX_Pl_consensus     CTTAATTTTCTAATTTTTTTCTATTTTTTTCTTTATCTTCACTAGAAATTTTACTGTCTAAAATATTTCCAATCAAATT [ 2886]
lp36_PAli_NX_MP_FG_consensus CTTAATTTTCTAATTTTTTTCTATTTTTTTCTTTATCTTCACTAGAAATTTTACTGTCTAAAATATTTCCAATCAAATT [ 2886]
lp36_PAli_NX_Pl_consensus    CTTAATTTTCTAATTTTTTTCTATTTTTTTCTTTATCTTCACTAGAAATTTTACTGTCTAAAATATTTCCAATCAAATT [ 2886]
lp36_PAbe_NX_MP_FG_consensus CTTAATTTTCTAATTTTTTTCTATTTTTTTCTTTATCTTCACTAGAAATTTTACTGTCTAAAATATTTCCAATCAAATT [ 2886]
lp36_PAbe_NX_Pl_consensus    CTTAATTTTCTAATTTTTTTCTATTTTTTTCTTTATCTTCACTAGAAATTTTACTGTCTAAAATATTTCCAATCAAATT [ 2886]

lp36_gi|11496779|B31_GB      AATTACGGTTGTAATATCACTAAATTTTATATTCAAAGATTTATCAAAAGACAACGAATAAAGTTTAATTAAAGAAAA [ 2964]
lp36_un11_B31_PacBio         AATTACGGTTGTAATATCACTAAATTTTATATTCAAAGATTTATCAAAAGACAACGAATAAAGTTTAATTAAAGAAAA [ 2964]
lp36_B31_TS_MP_FG_consensus  AATTACGGTTGTAATATCACTAAATTTTATATTCAAAGATTTATCAAAAGACAACGAATAAAGTTTAATTAAAGAAAA [ 2964]
lp36_B31_NX_Pl_consensus     AATTACGGTTGTAATATCACTAAATTTTATATTCAAAGATTTATCAAAAGACAACGAATAAAGTTTAATTAAAGAAAA [ 2964]
lp36_PAli_NX_MP_FG_consensus AATTACGGTTGTAATATCACTAAATTTTATATTCAAAGATTTATCAAAAGACAACGAATAAAGTTTAATTAAAGAAAA [ 2964]
lp36_PAli_NX_Pl_consensus    AATTACGGTTGTAATATCACTAAATTTTATATTCAAAGATTTATCAAAAGACAACGAATAAAGTTTAATTAAAGAAAA [ 2964]
lp36_PAbe_NX_MP_FG_consensus AATTACGGTTGTAATATCACTAAATTTTATATTCAAAGATTTATCAAAAGACAACGAATAAAGTTTAATTAAAGAAAA [ 2964]
lp36_PAbe_NX_Pl_consensus    AATTACGGTTGTAATATCACTAAATTTTATATTCAAAGATTTATCAAAAGACAACGAATAAAGTTTAATTAAAGAAAA [ 2964]

lp36_gi|11496779|B31_GB      TATTATTCTTAAATTATTTATATTTTCATTTTCCTGATATTCTTTGTATATCTTTTTAGATTTTTCTAAATCTTCTTT [ 3042]
lp36_un11_B31_PacBio         TATTATTCTTAAATTATTTATATTTTCATTTTCCTGATATTCTTTGTATATCTTTTTAGATTTTTCTAAATCTTCTTT [ 3042]
lp36_B31_TS_MP_FG_consensus  TATTATTCTTAAATTATTTATATTTTCATTTTCCTGATATTCTTTGTATATCTTTTTAGATTTTTCTAAATCTTCTTT [ 3042]
lp36_B31_NX_Pl_consensus     TATTATTCTTAAATTATTTATATTTTCATTTTCCTGATATTCTTTGTATATCTTTTTAGATTTTTCTAAATCTTCTTT [ 3042]
lp36_PAli_NX_MP_FG_consensus TATTATTCTTAAATTATTTATATTTTCATTTTCCTGATATTCTTTGTATATCTTTTTAGATTTTTCTAAATCTTCTTT [ 3042]
lp36_PAli_NX_Pl_consensus    TATTATTCTTAAATTATTTATATTTTCATTTTCCTGATATTCTTTGTATATCTTTTTAGATTTTTCTAAATCTTCTTT [ 3042]
lp36_PAbe_NX_MP_FG_consensus TATTATTCTTAAATTATTTATATTTTCATTTTCCTGATATTCTTTGTATATCTTTTTAGATTTTTCTAAATCTTCTTT [ 3042]
lp36_PAbe_NX_Pl_consensus    TATTATTCTTAAATTATTTATATTTTCATTTTCCTGATATTCTTTGTATATCTTTTTAGATTTTTCTAAATCTTCTTT [ 3042]

lp36_gi|11496779|B31_GB      AATGGTATAATTAATACCTTTGATTTTTTGATGGATATCTTCTAAAATAGTCATAATTTCGTTAATCTTTTTTACAAC [ 3120]
lp36_un11_B31_PacBio         AATGGTATAATTAATACCTTTGATTTTTTGATGGATATCTTCTAAAATAGTCATAATTTCGTTAATCTTTTTTACAAC [ 3120]
lp36_B31_TS_MP_FG_consensus  AATGGTATAATTAATACCTTTGATTTTTTGATGGATATCTTCTAAAATAGTCATAATTTCGTTAATCTTTTTTACAAC [ 3120]
lp36_B31_NX_Pl_consensus     AATGGTATAATTAATACCTTTGATTTTTTGATGGATATCTTCTAAAATAGTCATAATTTCGTTAATCTTTTTTACAAC [ 3120]
lp36_PAli_NX_MP_FG_consensus AATGGTATAATTAATACCTTTGATTTTTTGATGGATATCTTCTAAAATAGTCATAATTTCGTTAATCTTTTTTACAAC [ 3120]
lp36_PAli_NX_Pl_consensus    AATGGTATAATTAATACCTTTGATTTTTTGATGGATATCTTCTAAAATAGTCATAATTTCGTTAATCTTTTTTACAAC [ 3120]
lp36_PAbe_NX_MP_FG_consensus AATGGTATAATTAATACCTTTGATTTTTTGATGGATATCTTCTAAAATAGTCATAATTTCGTTAATCTTTTTTACAAC [ 3120]
lp36_PAbe_NX_Pl_consensus    AATGGTATAATTAATACCTTTGATTTTTTGATGGATATCTTCTAAAATAGTCATAATTTCGTTAATCTTTTTTACAAC [ 3120]

lp36_gi|11496779|B31_GB      TGACAAGCAGTTACCTCTAAATTCGTTATCCAAAATATTCAAAAATTCATCCTTGGTATACCCTAAAAGGGCATTTCC [ 3198]
lp36_un11_B31_PacBio         TGACAAGCAGTTACCTCTAAATTCGTTATCCAAAATATTCAAAAATTCATCCTTGGTATACCCTAAAAGGGCATTTCC [ 3198]
lp36_B31_TS_MP_FG_consensus  TGACAAGCAGTTACCTCTAAATTCGTTATCCAAAATATTCAAAAATTCATCCTTGGTATACCCTAAAAGGGCATTTCC [ 3198]
lp36_B31_NX_Pl_consensus     TGACAAGCAGTTACCTCTAAATTCGTTATCCAAAATATTCAAAAATTCATCCTTGGTATACCCTAAAAGGGCATTTCC [ 3198]
lp36_PAli_NX_MP_FG_consensus TGACAAGCAGTTACCTCTAAATTCGTTATCCAAAATATTCAAAAATTCATCCTTGGTATACCCTAAAAGGGCATTTCC [ 3198]
lp36_PAli_NX_Pl_consensus    TGACAAGCAGTTACCTCTAAATTCGTTATCCAAAATATTCAAAAATTCATCCTTGGTATACCCTAAAAGGGCATTTCC [ 3198]
lp36_PAbe_NX_MP_FG_consensus TGACAAGCAGTTACCTCTAAATTCGTTATCCAAAATATTCAAAAATTCATCCTTGGTATACCCTAAAAGGGCATTTCC [ 3198]
lp36_PAbe_NX_Pl_consensus    TGACAAGCAGTTACCTCTAAATTCGTTATCCAAAATATTCAAAAATTCATCCTTGGTATACCCTAAAAGGGCATTTCC [ 3198]

lp36_gi|11496779|B31_GB      TACTTTTATGTGATGTTTAATCAAGCTTAGTGGCGTTTCAAAAAAATAAAGGTATTAATCCATAAACCTAACATTGCG [ 3276]
lp36_un11_B31_PacBio         TACTTTTATGTGATGTTTAATCAAGCTTAGTGGCGTTTCAAAAAAATAAAGGTATTAATCCATAAACCTAACATTGCG [ 3276]
lp36_B31_TS_MP_FG_consensus  TACTTTTATGTGATGTTTAATCAAGCTTAGTGGCGTTTCAAAAAAATAAAGGTATTAATCCATAAACCTAACATTGCG [ 3276]
lp36_B31_NX_Pl_consensus     TACTTTTATGTGATGTTTAATCAAGCTTAGTGGCGTTTCAAAAAAATAAAGGTATTAATCCATAAACCTAACATTGCG [ 3276]
lp36_PAli_NX_MP_FG_consensus TACTTTTATGTGATGTTTAATCAAGCTTAGTGGCGTTTCAAAAAAATAAAGGTATTAATCCATAAACCTAACATTGCG [ 3276]
lp36_PAli_NX_Pl_consensus    TACTTTTATGTGATGTTTAATCAAGCTTAGTGGCGTTTCAAAAAAATAAAGGTATTAATCCATAAACCTAACATTGCG [ 3276]
lp36_PAbe_NX_MP_FG_consensus TACTTTTATGTGATGTTTAATCAAGCTTAGTGGCGTTTCAAAAAAATAAAGGTATTAATCCATAAACCTAACATTGCG [ 3276]
lp36_PAbe_NX_Pl_consensus    TACTTTTATGTGATGTTTAATCAAGCTTAGTGGCGTTTCAAAAAAATAAAGGTATTAATCCATAAACCTAACATTGCG [ 3276]

lp36_gi|11496779|B31_GB      ATTTGTACAGATATATGATCAATATCAACTCCGTAAATACATTTTCCAAGCAACATTCTTTTAAGAACGGTCTTCCGG [ 3354]
lp36_un11_B31_PacBio         ATTTGTACAGATATATGATCAATATCAACTCCGTAAATACATTTTCCAAGCAACATTCTTTTAAGAACGGTCTTCCGG [ 3354]
lp36_B31_TS_MP_FG_consensus  ATTTGTACAGATATATGATCAATATCAACTCCGTAAATACATTTTCCAAGCAACATTCTTTTAAGAACGGTCTTCCGG [ 3354]
lp36_B31_NX_Pl_consensus     ATTTGTACAGATATATGATCAATATCAACTCCGTAAATACATTTTCCAAGCAACATTCTTTTAAGAACGGTCTTCCGG [ 3354]
lp36_PAli_NX_MP_FG_consensus ATTTGTACAGATATATGATCAATATCAACTCCGTAAATACATTTTCCAAGCAACATTCTTTTAAGAACGGTCTTCCGG [ 3354]
lp36_PAli_NX_Pl_consensus    ATTTGTACAGATATATGATCAATATCAACTCCGTAAATACATTTTCCAAGCAACATTCTTTTAAGAACGGTCTTCCGG [ 3354]
lp36_PAbe_NX_MP_FG_consensus ATTTGTACAGATATATGATCAATATCAACTCCGTAAATACATTTTCCAAGCAACATTCTTTTAAGAACGGTCTTCCGG [ 3354]
lp36_PAbe_NX_Pl_consensus    ATTTGTACAGATATATGATCAATATCAACTCCGTAAATACATTTTCCAAGCAACATTCTTTTAAGAACGGTCTTCCGG [ 3354]

lp36_gi|11496779|B31_GB      CTTATACTGTCTTGAATATTATATTTTTTAGCTTTATTAATAACATTTCTATACTCCATATCTAATTTTTTTTTACAT [ 3432]
lp36_un11_B31_PacBio         CTTATACTGTCTTGAATATTATATTTTTTAGCTTTATTAATAACATTTCTATACTCCATATCTAATTTTTTTTTACAT [ 3432]
lp36_B31_TS_MP_FG_consensus  CTTATACTGTCTTGAATATTATATTTTTTAGCTTTATTAATAACATTTCTATACTCCATATCTAATTTTTTTTTACAT [ 3432]
lp36_B31_NX_Pl_consensus     CTTATACTGTCTTGAATATTATATTTTTTAGCTTTATTAATAACATTTCTATACTCCATATCTAATTTTTTTTTACAT [ 3432]
lp36_PAli_NX_MP_FG_consensus CTTATACTGTCTTGAATATTATATTTTTTAGCTTTATTAATAACATTTCTATACTCCATATCTAATTTTTTTTTACAT [ 3432]
lp36_PAli_NX_Pl_consensus    CTTATACTGTCTTGAATATTATATTTTTTAGCTTTATTAATAACATTTCTATACTCCATATCTAATTTTTTTTTACAT [ 3432]
lp36_PAbe_NX_MP_FG_consensus CTTATACTGTCTTGAATATTATATTTTTTAGCTTTATTAATAACATTTCTATACTCCATATCTAATTTTTTTTTACAT [ 3432]
lp36_PAbe_NX_Pl_consensus    CTTATACTGTCTTGAATATTATATTTTTTAGCTTTATTAATAACATTTCTATACTCCATATCTAATTTTTTTTTACAT [ 3432]

lp36_gi|11496779|B31_GB      CTTCAAATTTATCTAGCTCGTACCACACTTTTTCTGTTAAGTAATCTAGACAAGAAATTAAAAAATTTCCCGATCCAC [ 3510]
lp36_un11_B31_PacBio         CTTCAAATTTATCTAGCTCGTACCACACTTTTTCTGTTAAGTAATCTAGACAAGAAATTAAAAAATTTCCCGATCCAC [ 3510]
lp36_B31_TS_MP_FG_consensus  CTTCAAATTTATCTAGCTCGTACCACACTTTTTCTGTTAAGTAATCTAGACAAGAAATTAAAAAATTTCCCGATCCAC [ 3510]
lp36_B31_NX_Pl_consensus     CTTCAAATTTATCTAGCTCGTACCACACTTTTTCTGTTAAGTAATCTAGACAAGAAATTAAAAAATTTCCCGATCCAC [ 3510]
lp36_PAli_NX_MP_FG_consensus CTTCAAATTTATCTAGCTCGTACCACACTTTTTCTGTTAAGTAATCTAGACAAGAAATTAAAAAATTTCCCGATCCAC [ 3510]
lp36_PAli_NX_Pl_consensus    CTTCAAATTTATCTAGCTCGTACCACACTTTTTCTGTTAAGTAATCTAGACAAGAAATTAAAAAATTTCCCGATCCAC [ 3510]
lp36_PAbe_NX_MP_FG_consensus CTTCAAATTTATCTAGCTCGTACCACACTTTTTCTGTTAAGTAATCTAGACAAGAAATTAAAAAATTTCCCGATCCAC [ 3510]
lp36_PAbe_NX_Pl_consensus    CTTCAAATTTATCTAGCTCGTACCACACTTTTTCTGTTAAGTAATCTAGACAAGAAATTAAAAAATTTCCCGATCCAC [ 3510]

lp36_gi|11496779|B31_GB      AAGAATTATCAATGATTTTTATATCTAAAGGGGATTTAGTTTTAAGCCGTTCTTCAATTGAAGACACAACCATAAATT [ 3588]
lp36_un11_B31_PacBio         AAGAATTATCAATGATTTTTATATCTAAAGGGGATTTAGTTTTAAGCCGTTCTTCAATTGAAGACACAACCATAAATT [ 3588]
lp36_B31_TS_MP_FG_consensus  AAGAATTATCAATGATTTTTATATCTAAAGGGGATTTAGTTTTAAGCCGTTCTTCAATTGAAGACACAACCATAAATT [ 3588]
lp36_B31_NX_Pl_consensus     AAGAATTATCAATGATTTTTATATCTAAAGGGGATTTAGTTTTAAGCCGTTCTTCAATTGAAGACACAACCATAAATT [ 3588]
lp36_PAli_NX_MP_FG_consensus AAGAATTATCAATGATTTTTATATCTAAAGGGGATTTAGTTTTAAGCCGTTCTTCAATTGAAGACACAACCATAAATT [ 3588]
lp36_PAli_NX_Pl_consensus    AAGAATTATCAATGATTTTTATATCTAAAGGGGATTTAGTTTTAAGCCGTTCTTCAATTGAAGACACAACCATAAATT [ 3588]
```

```
lp36_PAbe_NX_MP_FG_consensus AAGAATTATCAATGATTTTTATATCTAAAGGGGATTTAGTTTTAAGCCGTTCTTCAATTGAAGACACAACCATAAATT [ 3588]
lp36_PAbe_NX_Pl_consensus    AAGAATTATCAATGATTTTTATATCTAAAGGGGATTTAGTTTTAAGCCGTTCTTCAATTGAAGACACAACCATAAATT [ 3588]

lp36_gi|11496779|B31_GB      CCGTCAAATCTTCTGGGGTGTAATATGCTCCATTTTTCTTTCTGTCAAGAGATCTAGATGCAAGATAAATATTGCCTT [ 3666]
lp36_un11_B31_PacBio         CCGTCAAATCTTCTGGGGTGTAATATGCTCCATTTTTCTTTCTGTCAAGAGATCTAGATGCAAGATAAATATTGCCTT [ 3666]
lp36_B31_TS_MP_FG_consensus  CCGTCAAATCTTCTGGGGTGTAATATGCTCCATTTTTCTTTCTGTCAAGAGATCTAGATGCAAGATAAATATTGCCTT [ 3666]
lp36_B31_NX_Pl_consensus     CCGTCAAATCTTCTGGGGTGTAATATGCTCCATTTTTCTTTCTGTCAAGAGATCTAGATGCAAGATAAATATTGCCTT [ 3666]
lp36_PAli_NX_MP_FG_consensus CCGTCAAATCTTCTGGGGTGTAATATGCTCCATTTTTCTTTCTGTCAAGAGATCTAGATGCAAGATAAATATTGCCTT [ 3666]
lp36_PAli_NX_Pl_consensus    CCGTCAAATCTTCTGGGGTGTAATATGCTCCATTTTTCTTTCTGTCAAGAGATCTAGATGCAAGATAAATATTGCCTT [ 3666]
lp36_PAbe_NX_MP_FG_consensus CCGTCAAATCTTCTGGGGTGTAATATGCTCCATTTTTCTTTCTGTCAAGAGATCTAGATGCAAGATAAATATTGCCTT [ 3666]
lp36_PAbe_NX_Pl_consensus    CCGTCAAATCTTCTGGGGTGTAATATGCTCCATTTTTCTTTCTGTCAAGAGATCTAGATGCAAGATAAATATTGCCTT [ 3666]

lp36_gi|11496779|B31_GB      TATAATAGGTAGCAACTTTGTTTACTTTCTTGTTTTTAAGCTCTTCTTCAGAATAAATAAAATAAGTTCCATTTTTAA [ 3744]
lp36_un11_B31_PacBio         TATAATAGGTAGCAACTTTGTTTACTTTCTTGTTTTTAAGCTCTTCTTCAGAATAAATAAAATAAGTTCCATTTTTAA [ 3744]
lp36_B31_TS_MP_FG_consensus  TATAATAGGTAGCAACTTTGTTTACTTTCTTGTTTTTAAGCTCTTCTTCAGAATAAATAAAATAAGTTCCATTTTTAA [ 3744]
lp36_B31_NX_Pl_consensus     TATAATAGGTAGCAACTTTGTTTACTTTCTTGTTTTTAAGCTCTTCTTCAGAATAAATAAAATAAGTTCCATTTTTAA [ 3744]
lp36_PAli_NX_MP_FG_consensus TATAATAGGTAGCAACTTTGTTTACTTTCTTGTTTTTAAGCTCTTCTTCAGAATAAATAAAATAAGTTCCATTTTTAA [ 3744]
lp36_PAli_NX_Pl_consensus    TATAATAGGTAGCAACTTTGTTTACTTTCTTGTTTTTAAGCTCTTCTTCAGAATAAATAAAATAAGTTCCATTTTTAA [ 3744]
lp36_PAbe_NX_MP_FG_consensus TATAATAGGTAGCAACTTTGTTTACTTTCTTGTTTTTAAGCTCTTCTTCAGAATAAATAAAATAAGTTCCATTTTTAA [ 3744]
lp36_PAbe_NX_Pl_consensus    TATAATAGGTAGCAACTTTGTTTACTTTCTTGTTTTTAAGCTCTTCTTCAGAATAAATAAAATAAGTTCCATTTTTAA [ 3744]

lp36_gi|11496779|B31_GB      GAATGCGGTGAATAGTAGTAAACCAATATCTATCCTAGTGTATTATGGCCTATAATAGGCCCAATAAAGAATATTTTG [ 3822]
lp36_un11_B31_PacBio         GAATGCGGTGAATAGTAGTAAACCAATATCTATCCTAGTGTATTATGGCCTATAATAGGCCCAATAAAGAATATTTTG [ 3822]
lp36_B31_TS_MP_FG_consensus  GAATGCGGTGAATAGTAGTAAACCAATATCTATCCTAGTGTATTATGGCCTATAATAGGCCCAATAAAGAATATTTTG [ 3822]
lp36_B31_NX_Pl_consensus     GAATGCGGTGAATAGTAGTAAACCAATATCTATCCTAGTGTATTATGGCCTATAATAGGCCCAATAAAGAATATTTTG [ 3822]
lp36_PAli_NX_MP_FG_consensus GAATGCGGTGAATAGTAGTAAACCAATATCTATCCTAGTGTATTATGGCCTATAATAGGCCCAATAAAGAATATTTTG [ 3822]
lp36_PAli_NX_Pl_consensus    GAATGCGGTGAATAGTAGTAAACCAATATCTATCCTAGTGTATTATGGCCTATAATAGGCCCAATAAAGAATATTTTG [ 3822]
lp36_PAbe_NX_MP_FG_consensus GAATGCGGTGAATAGTAGTAAACCAATATCTATCCTAGTGTATTATGGCCTATAATAGGCCCAATAAAGAATATTTTG [ 3822]
lp36_PAbe_NX_Pl_consensus    GAATGCGGTGAATAGTAGTAAACCAATATCTATCCTAGTGTATTATGGCCTATAATAGGCCCAATAAAGAATATTTTG [ 3822]

lp36_gi|11496779|B31_GB      ATCTATAAAGGACTATATGTCTTTTATGGATTTAGAACATGAATAATTATTTAATTACCAATATTAAGCTAAATACAA [ 3900]
lp36_un11_B31_PacBio         ATCTATAAAGGACTATATGTCTTTTATGGATTTAGAACATGAATAATTATTTAATTACCAATATTAAGCTAAATACAA [ 3900]
lp36_B31_TS_MP_FG_consensus  ATCTATAAAGGACTATATGTCTTTTATGGATTTAGAACATGAATAATTATTTAATTACCAATATTAAGCTAAATACAA [ 3900]
lp36_B31_NX_Pl_consensus     ATCTATAAAGGACTATATGTCTTTTATGGATTTAGAACATGAATAATTATTTAATTACCAATATTAAGCTAAATACAA [ 3900]
lp36_PAli_NX_MP_FG_consensus ATCTATAAAGGACTATATGTCTTTTATGGATTTAGAACATGAATAATTATTTAATTACCAATATTAAGCTAAATACAA [ 3900]
lp36_PAli_NX_Pl_consensus    ATCTATAAAGGACTATATGTCTTTTATGGATTTAGAACATGAATAATTATTTAATTACCAATATTAAGCTAAATACAA [ 3900]
lp36_PAbe_NX_MP_FG_consensus ATCTATAAAGGACTATATGTCTTTTATGGATTTAGAACATGAATAATTATTTAATTACCAATATTAAGCTAAATACAA [ 3900]
lp36_PAbe_NX_Pl_consensus    ATCTATAAAGGACTATATGTCTTTTATGGATTTAGAACATGAATAATTATTTAATTACCAATATTAAGCTAAATACAA [ 3900]

lp36_gi|11496779|B31_GB      AATAAATCCAAATATATCTTTAAATTCTAGAAAATTTTTAATCTAATAAAAATTATTATTATAGTGCCCCAAACAAAA [ 3978]
lp36_un11_B31_PacBio         AATAAATCCAAATATATCTTTAAATTCTAGAAAATTTTTAATCTAATAAAAATTATTATTATAGTGCCCCAAACAAAA [ 3978]
lp36_B31_TS_MP_FG_consensus  AATAAATCCAAATATATCTTTAAATTCTAGAAAATTTTTAATCTAATAAAAATTATTATTATAGTGCCCCAAACAAAA [ 3978]
lp36_B31_NX_Pl_consensus     AATAAATCCAAATATATCTTTAAATTCTAGAAAATTTTTAATCTAATAAAAATTATTATTATAGTGCCCCAAACAAAA [ 3978]
lp36_PAli_NX_MP_FG_consensus AATAAATCCAAATATATCTTTAAATTCTAGAAAATTTTTAATCTAATAAAAATTATTATTATAGTGCCCCAAACAAAA [ 3978]
lp36_PAli_NX_Pl_consensus    AATAAATCCAAATATATCTTTAAATTCTAGAAAATTTTTAATCTAATAAAAATTATTATTATAGTGCCCCAAACAAAA [ 3978]
lp36_PAbe_NX_MP_FG_consensus AATAAATCCAAATATATCTTTAAATTCTAGAAAATTTTTAATCTAATAAAAATTATTATTATAGTGCCCCAAACAAAA [ 3978]
lp36_PAbe_NX_Pl_consensus    AATAAATCCAAATATATCTTTAAATTCTAGAAAATTTTTAATCTAATAAAAATTATTATTATAGTGCCCCAAACAAAA [ 3978]

lp36_gi|11496779|B31_GB      TAAAACTTAAATTGGGGAAATTAGTGTTACATAAAATGAATTTCCATAGAAGATTTTTTTAAATTGGAATGCTTTAAT [ 4056]
lp36_un11_B31_PacBio         TAAAACTTAAATTGGGGAAATTAGTGTTACATAAAATGAATTTCCATAGAAGATTTTTTTAAATTGGAATGCTTTAAT [ 4056]
lp36_B31_TS_MP_FG_consensus  TAAAACTTAAATTGGGGAAATTAGTGTTACATAAAATGAATTTCCATAGAAGATTTTTTTAAATTGGAATGCTTTAAT [ 4056]
lp36_B31_NX_Pl_consensus     TAAAACTTAAATTGGGGAAATTAGTGTTACATAAAATGAATTTCCATAGAAGATTTTTTTAAATTGGAATGCTTTAAT [ 4056]
lp36_PAli_NX_MP_FG_consensus TAAAACTTAAATTGGGGAAATTAGTGTTACATAAAATGAATTTCCATAGAAGATTTTTTTAAATTGGAATGCTTTAAT [ 4056]
lp36_PAli_NX_Pl_consensus    TAAAACTTAAATTGGGGAAATTAGTGTTACATAAAATGAATTTCCATAGAAGATTTTTTTAAATTGGAATGCTTTAAT [ 4056]
lp36_PAbe_NX_MP_FG_consensus TAAAACTTAAATTGGGGAAATTAGTGTTACATAAAATGAATTTCCATAGAAGATTTTTTTAAATTGGAATGCTTTAAT [ 4056]
lp36_PAbe_NX_Pl_consensus    TAAAACTTAAATTGGGGAAATTAGTGTTACATAAAATGAATTTCCATAGAAGATTTTTTTAAATTGGAATGCTTTAAT [ 4056]

lp36_gi|11496779|B31_GB      AACGTTATTTGACATGCAATATTAATGAAACGATTGCCAATCTTCTGAAAAGAAGATGAAAAAAGGTTTTTATATAGC [ 4134]
lp36_un11_B31_PacBio         AACGTTATTTGACATGCAATATTAATGAAACGATTGCCAATCTTCTGAAAAGAAGATGAAAAAAGGTTTTTATATAGC [ 4134]
lp36_B31_TS_MP_FG_consensus  AACGTTATTTGACATGCAATATTAATGAAACGATTGCCAATCTTCTGAAAAGAAGATGAAAAAAGGTTTTTATATAGC [ 4134]
lp36_B31_NX_Pl_consensus     AACGTTATTTGACATGCAATATTAATGAAACGATTGCCAATCTTCTGAAAAGAAGATGAAAAAAGGTTTTTATATAGC [ 4134]
lp36_PAli_NX_MP_FG_consensus AACGTTATTTGACATGCAATATTAATGAAACGATTGCCAATCTTCTGAAAAGAAGATGAAAAAAGGTTTTTATATAGC [ 4134]
lp36_PAli_NX_Pl_consensus    AACGTTATTTGACATGCAATATTAATGAAACGATTGCCAATCTTCTGAAAAGAAGATGAAAAAAGGTTTTTATATAGC [ 4134]
lp36_PAbe_NX_MP_FG_consensus AACGTTATTTGACATGCAATATTAATGAAACGATTGCCAATCTTCTGAAAAGAAGATGAAAAAAGGTTTTTATATAGC [ 4134]
lp36_PAbe_NX_Pl_consensus    AACGTTATTTGACATGCAATATTAATGAAACGATTGCCAATCTTCTGAAAAGAAGATGAAAAAAGGTTTTTATATAGC [ 4134]

lp36_gi|11496779|B31_GB      ATTTATAACTTATAAAATGGTGTAAAATACTCAATGGGATTGAATTGATTTAAACTAAACTAAGGAAATTTAGCAAAG [ 4212]
lp36_un11_B31_PacBio         ATTTATAACTTATAAAATGGTGTAAAATACTCAATGGGATTGAATTGATTTAAACTAAACTAAGGAAATTTAGCAAAG [ 4212]
lp36_B31_TS_MP_FG_consensus  ATTTATAACTTATAAAATGGTGTAAAATACTCAATGGGATTGAATTGATTTAAACTAAACTAAGGAAATTTAGCAAAG [ 4212]
lp36_B31_NX_Pl_consensus     ATTTATAACTTATAAAATGGTGTAAAATACTCAATGGGATTGAATTGATTTAAACTAAACTAAGGAAATTTAGCAAAG [ 4212]
lp36_PAli_NX_MP_FG_consensus ATTTATAACTTATAAAATGGTGTAAAATACTCAATGGGATTGAATTGATTTAAACTAAACTAAGGAAATTTAGCAAAG [ 4212]
lp36_PAli_NX_Pl_consensus    ATTTATAACTTATAAAATGGTGTAAAATACTCAATGGGATTGAATTGATTTAAACTAAACTAAGGAAATTTAGCAAAG [ 4212]
lp36_PAbe_NX_MP_FG_consensus ATTTATAACTTATAAAATGGTGTAAAATACTCAATGGGATTGAATTGATTTAAACTAAACTAAGGAAATTTAGCAAAG [ 4212]
lp36_PAbe_NX_Pl_consensus    ATTTATAACTTATAAAATGGTGTAAAATACTCAATGGGATTGAATTGATTTAAACTAAACTAAGGAAATTTAGCAAAG [ 4212]

lp36_gi|11496779|B31_GB      ATAAATTAAGTAATAATAAATATAAAAATTCTTATATTTATTCAAAAATTTGAAAAAATGATCTAGGTTTTAAAAAAA [ 4290]
lp36_un11_B31_PacBio         ATAAATTAAGTAATAATAAATATAAAAATTCTTATATTTATTCAAAAATTTGAAAAAATGATCTAGGTTTTAAAAAAA [ 4290]
lp36_B31_TS_MP_FG_consensus  ATAAATTAAGTAATAATAAATATAAAAATTCTTATATTTATTCAAAAATTTGAAAAAATGATCTAGGTTTTAAAAAAA [ 4290]
lp36_B31_NX_Pl_consensus     ATAAATTAAGTAATAATAAATATAAAAATTCTTATATTTATTCAAAAATTTGAAAAAATGATCTAGGTTTTAAAAAAA [ 4290]
lp36_PAli_NX_MP_FG_consensus ATAAATTAAGTAATAATAAATATAAAAATTCTTATATTTATTCAAAAATTTGAAAAAATGATCTAGGTTTTAAAAAAA [ 4290]
lp36_PAli_NX_Pl_consensus    ATAAATTAAGTAATAATAAATATAAAAATTCTTATATTTATTCAAAAATTTGAAAAAATGATCTAGGTTTTAAAAAAA [ 4290]
lp36_PAbe_NX_MP_FG_consensus ATAAATTAAGTAATAATAAATATAAAAATTCTTATATTTATTCAAAAATTTGAAAAAATGATCTAGGTTTTAAAAAAA [ 4290]
lp36_PAbe_NX_Pl_consensus    ATAAATTAAGTAATAATAAATATAAAAATTCTTATATTTATTCAAAAATTTGAAAAAATGATCTAGGTTTTAAAAAAA [ 4290]

lp36_gi|11496779|B31_GB      AGACATTAAAACTAGATTGACTGATAGACATACTCAAAAGATTTTTTGTATCTAATCAAATAATTTAAAATAATTAAG [ 4368]
lp36_un11_B31_PacBio         AGACATTAAAACTAGATTGACTGATAGACATACTCAAAAGATTTTTTGTATCTAATCAAATAATTTAAAATAATTAAG [ 4368]
lp36_B31_TS_MP_FG_consensus  AGACATTAAAACTAGATTGACTGATAGACATACTCAAAAGATTTTTTGTATCTAATCAAATAATTTAAAATAATTAAG [ 4368]
lp36_B31_NX_Pl_consensus     AGACATTAAAACTAGATTGACTGATAGACATACTCAAAAGATTTTTTGTATCTAATCAAATAATTTAAAATAATTAAG [ 4368]
lp36_PAli_NX_MP_FG_consensus AGACATTAAAACTAGATTGACTGATAGACATACTCAAAAGATTTTTTGTATCTAATCAAATAATTTAAAATAATTAAG [ 4368]
lp36_PAli_NX_Pl_consensus    AGACATTAAAACTAGATTGACTGATAGACATACTCAAAAGATTTTTTGTATCTAATCAAATAATTTAAAATAATTAAG [ 4368]
lp36_PAbe_NX_MP_FG_consensus AGACATTAAAACTAGATTGACTGATAGACATACTCAAAAGATTTTTTGTATCTAATCAAATAATTTAAAATAATTAAG [ 4368]
lp36_PAbe_NX_Pl_consensus    AGACATTAAAACTAGATTGACTGATAGACATACTCAAAAGATTTTTTGTATCTAATCAAATAATTTAAAATAATTAAG [ 4368]

lp36_gi|11496779|B31_GB      TTTATGATAAATTTTAGTTCCAAAATTTAATCCCAATATTCAAATCCGCAGACATTTTTTAGTATGTGTTCTATGGTA [ 4446]
lp36_un11_B31_PacBio         TTTATGATAAATTTTAGTTCCAAAATTTAATCCCAATATTCAAATCCGCAGACATTTTTTAGTATGTGTTCTATGGTA [ 4446]
lp36_B31_TS_MP_FG_consensus  TTTATGATAAATTTTAGTTCCAAAATTTAATCCCAATATTCAAATCCGCAGACATTTTTTAGTATGTGTTCTATGGTA [ 4446]
lp36_B31_NX_Pl_consensus     TTTATGATAAATTTTAGTTCCAAAATTTAATCCCAATATTCAAATCCGCAGACATTTTTTAGTATGTGTTCTATGGTA [ 4446]
lp36_PAli_NX_MP_FG_consensus TTTATGATAAATTTTAGTTCCAAAATTTAATCCCAATATTCAAATCCGCAGACATTTTTTAGTATGTGTTCTATGGTA [ 4446]
lp36_PAli_NX_Pl_consensus    TTTATGATAAATTTTAGTTCCAAAATTTAATCCCAATATTCAAATCCGCAGACATTTTTTAGTATGTGTTCTATGGTA [ 4446]
lp36_PAbe_NX_MP_FG_consensus TTTATGATAAATTTTAGTTCCAAAATTTAATCCCAATATTCAAATCCGCAGACATTTTTTAGTATGTGTTCTATGGTA [ 4446]
lp36_PAbe_NX_Pl_consensus    TTTATGATAAATTTTAGTTCCAAAATTTAATCCCAATATTCAAATCCGCAGACATTTTTTAGTATGTGTTCTATGGTA [ 4446]

lp36_gi|11496779|B31_GB      ACTTTTTGAGATTTTAGAGAATGATTTGAAGTGATTTTGACAGTTTTTAATAGAAAGGTTTCGGTTCTAATTATAAAA [ 4524]
lp36_un11_B31_PacBio         ACTTTTTGAGATTTTAGAGAATGATTTGAAGTGATTTTGACAGTTTTTAATAGAAAGGTTTCGGTTCTAATTATAAAA [ 4524]
lp36_B31_TS_MP_FG_consensus  ACTTTTTGAGATTTTAGAGAATGATTTGAAGTGATTTTGACAGTTTTTAATAGAAAGGTTTCGGTTCTAATTATAAAA [ 4524]
lp36_B31_NX_Pl_consensus     ACTTTTTGAGATTTTAGAGAATGATTTGAAGTGATTTTGACAGTTTTTAATAGAAAGGTTTCGGTTCTAATTATAAAA [ 4524]
lp36_PAli_NX_MP_FG_consensus ACTTTTTGAGATTTTAGAGAATGATTTGAAGTGATTTTGACAGTTTTTAATAGAAAGGTTTCGGTTCTAATTATAAAA [ 4524]
lp36_PAli_NX_Pl_consensus    ACTTTTTGAGATTTTAGAGAATGATTTGAAGTGATTTTGACAGTTTTTAATAGAAAGGTTTCGGTTCTAATTATAAAA [ 4524]
lp36_PAbe_NX_MP_FG_consensus ACTTTTTGAGATTTTAGAGAATGATTTGAAGTGATTTTGACAGTTTTTAATAGAAAGGTTTCGGTTCTAATTATAAAA [ 4524]
```

```
lp36_PAbe_NX_Pl_consensus    ACTTTTTGAGATTTTAGAGAATGATTTGAAGTGATTTTGACAGTTTTTAATAGAAAGGTTTCGGTTCTAATTATAAAA [ 4524]

lp36_gi|11496779|B31_GB      TTTAAAACGGCAACTTTGTCAATAGTCGAGGATCCCTTTTAAAGATTGATTTTTTTGAATATTTCGTTAAGATTTTTG [ 4602]
lp36_un11_B31_PacBio         TTTAAAACGGCAACTTTGTCAATAGTCGAGGATCCCTTTTAAAGATTGATTTTTTTGAATATTTCGTTAAGATTTTTG [ 4602]
lp36_B31_TS_MP_FG_consensus  TTTAAAACGGCAACTTTGTCAATAGTCGAGGATCCCTTTTAAAGATTGATTTTTTTGAATATTTCGTTAAGATTTTTG [ 4602]
lp36_B31_NX_Pl_consensus     TTTAAAACGGCAACTTTGTCAATAGTCGAGGATCCCTTTTAAAGATTGATTTTTTTGAATATTTCGTTAAGATTTTTG [ 4602]
lp36_PAli_NX_MP_FG_consensus TTTAAAACGGCAACTTTGTCAATAGTCGAGGATCCCTTTTAAAGATTGATTTTTTTGAATATTTCGTTAAGATTTTTG [ 4602]
lp36_PAli_NX_Pl_consensus    TTTAAAACGGCAACTTTGTCAATAGTCGAGGATCCCTTTTAAAGATTGATTTTTTTGAATATTTCGTTAAGATTTTTG [ 4602]
lp36_PAbe_NX_MP_FG_consensus TTTAAAACGGCAACTTTGTCAATAGTCGAGGATCCCTTTTAAAGATTGATTTTTTTGAATATTTCGTTAAGATTTTTG [ 4602]
lp36_PAbe_NX_Pl_consensus    TTTAAAACGGCAACTTTGTCAATAGTCGAGGATCCCTTTTAAAGATTGATTTTTTTGAATATTTCGTTAAGATTTTTG [ 4602]

lp36_gi|11496779|B31_GB      ACAATGAAATTGATAATCGGTCTGGAGCATGGCAAATACCTTTGGTCTTTCTAAAAAAATAATCATATTGAATTAGAA [ 4680]
lp36_un11_B31_PacBio         ACAATGAAATTGATAATCGGTCTGGAGCATGGCAAATACCTTTGGTCTTTCTAAAAAAATAATCATATTGAATTAGAA [ 4680]
lp36_B31_TS_MP_FG_consensus  ACAATGAAATTGATAATCGGTCTGGAGCATGGCAAATACCTTTGGTCTTTCTAAAAAAATAATCATATTGAATTAGAA [ 4680]
lp36_B31_NX_Pl_consensus     ACAATGAAATTGATAATCGGTCTGGAGCATGGCAAATACCTTTGGTCTTTCTAAAAAAATAATCATATTGAATTAGAA [ 4680]
lp36_PAli_NX_MP_FG_consensus ACAATGAAATTGATAATCGGTCTGGAGCATGGCAAATACCTTTGGTCTTTCTAAAAAAATAATCATATTGAATTAGAA [ 4680]
lp36_PAli_NX_Pl_consensus    ACAATGAAATTGATAATCGGTCTGGAGCATGGCAAATACCTTTGGTCTTTCTAAAAAAATAATCATATTGAATTAGAA [ 4680]
lp36_PAbe_NX_MP_FG_consensus ACAATGAAATTGATAATCGGTCTGGAGCATGGCAAATACCTTTGGTCTTTCTAAAAAAATAATCATATTGAATTAGAA [ 4680]
lp36_PAbe_NX_Pl_consensus    ACAATGAAATTGATAATCGGTCTGGAGCATGGCAAATACCTTTGGTCTTTCTAAAAAAATAATCATATTGAATTAGAA [ 4680]

lp36_gi|11496779|B31_GB      AATTTTAATAAAAGTTGAGACACGAATCTTTGAGTATGAACCCGAACTTGTGACATAAAAATTTTTGAAAGTCAATTT [ 4758]
lp36_un11_B31_PacBio         AATTTTAATAAAAGTTGAGACACGAATCTTTGAGTATGAACCCGAACTTGTGACATAAAAATTTTTGAAAGTCAATTT [ 4758]
lp36_B31_TS_MP_FG_consensus  AATTTTAATAAAAGTTGAGACACGAATCTTTGAGTATGAACCCGAACTTGTGACATAAAAATTTTTGAAAGTCAATTT [ 4758]
lp36_B31_NX_Pl_consensus     AATTTTAATAAAAGTTGAGACACGAATCTTTGAGTATGAACCCGAACTTGTGACATAAAAATTTTTGAAAGTCAATTT [ 4758]
lp36_PAli_NX_MP_FG_consensus AATTTTAATAAAAGTTGAGACACGAATCTTTGAGTATGAACCCGAACTTGTGACATAAAAATTTTTGAAAGTCAATTT [ 4758]
lp36_PAli_NX_Pl_consensus    AATTTTAATAAAAGTTGAGACACGAATCTTTGAGTATGAACCCGAACTTGTGACATAAAAATTTTTGAAAGTCAATTT [ 4758]
lp36_PAbe_NX_MP_FG_consensus AATTTTAATAAAAGTTGAGACACGAATCTTTGAGTATGAACCCGAACTTGTGACATAAAAATTTTTGAAAGTCAATTT [ 4758]
lp36_PAbe_NX_Pl_consensus    AATTTTAATAAAAGTTGAGACACGAATCTTTGAGTATGAACCCGAACTTGTGACATAAAAATTTTTGAAAGTCAATTT [ 4758]

lp36_gi|11496779|B31_GB      CAGATCAATTCCATTCACGGTATCGATAATATCTAATAATATTGTTTTTATATTTTAAAATTCATTCTTTTTTGCAAA [ 4836]
lp36_un11_B31_PacBio         CAGATCAATTCCATTCACGGTATCGATAATATCTAATAATATTGTTTTTATATTTTAAAATTCATTCTTTTTTGCAAA [ 4836]
lp36_B31_TS_MP_FG_consensus  CAGATCAATTCCATTCACGGTATCGATAATATCTAATAATATTGTTTTTATATTTTAAAATTCATTCTTTTTTGCAAA [ 4836]
lp36_B31_NX_Pl_consensus     CAGATCAATTCCATTCACGGTATCGATAATATCTAATAATATTGTTTTTATATTTTAAAATTCATTCTTTTTTGCAAA [ 4836]
lp36_PAli_NX_MP_FG_consensus CAGATCAATTCCATTCACGGTATCGATAATATCTAATAATATTGTTTTTATATTTTAAAATTCATTCTTTTTTGCAAA [ 4836]
lp36_PAli_NX_Pl_consensus    CAGATCAATTCCATTCACGGTATCGATAATATCTAATAATATTGTTTTTATATTTTAAAATTCATTCTTTTTTGCAAA [ 4836]
lp36_PAbe_NX_MP_FG_consensus CAGATCAATTCCATTCACGGTATCGATAATATCTAATAATATTGTTTTTATATTTTAAAATTCATTCTTTTTTGCAAA [ 4836]
lp36_PAbe_NX_Pl_consensus    CAGATCAATTCCATTCACGGTATCGATAATATCTAATAATATTGTTTTTATATTTTAAAATTCATTCTTTTTTGCAAA [ 4836]

lp36_gi|11496779|B31_GB      AGTTTCGACAATATTTAAAATGCTATTTATTGAAAGCTCATCAAGAATGAAATTCACTATGTTATCTATATTTTGATT [ 4914]
lp36_un11_B31_PacBio         AGTTTCGACAATATTTAAAATGCTATTTATTGAAAGCTCATCAAGAATGAAATTCACTATGTTATCTATATTTTGATT [ 4914]
lp36_B31_TS_MP_FG_consensus  AGTTTCGACAATATTTAAAATGCTATTTATTGAAAGCTCATCAAGAATGAAATTCACTATGTTATCTATATTTTGATT [ 4914]
lp36_B31_NX_Pl_consensus     AGTTTCGACAATATTTAAAATGCTATTTATTGAAAGCTCATCAAGAATGAAATTCACTATGTTATCTATATTTTGATT [ 4914]
lp36_PAli_NX_MP_FG_consensus AGTTTCGACAATATTTAAAATGCTATTTATTGAAAGCTCATCAAGAATGAAATTCACTATGTTATCTATATTTTGATT [ 4914]
lp36_PAli_NX_Pl_consensus    AGTTTCGACAATATTTAAAATGCTATTTATTGAAAGCTCATCAAGAATGAAATTCACTATGTTATCTATATTTTGATT [ 4914]
lp36_PAbe_NX_MP_FG_consensus AGTTTCGACAATATTTAAAATGCTATTTATTGAAAGCTCATCAAGAATGAAATTCACTATGTTATCTATATTTTGATT [ 4914]
lp36_PAbe_NX_Pl_consensus    AGTTTCGACAATATTTAAAATGCTATTTATTGAAAGCTCATCAAGAATGAAATTCACTATGTTATCTATATTTTGATT [ 4914]

lp36_gi|11496779|B31_GB      TTGCTTTCACTGTTTAGATGCATAAGGAAAATAGTATATATGATGGTTTGGGCTCTTCTAAAGTTAGTGTTTGTTCTA [ 4992]
lp36_un11_B31_PacBio         TTGCTTTCACTGTTTAGATGCATAAGGAAAATAGTATATATGATGGTTTGGGCTCTTCTAAAGTTAGTGTTTGTTCTA [ 4992]
lp36_B31_TS_MP_FG_consensus  TTGCTTTCACTGTTTAGATGCATAAGGAAAATAGTATATATGATGGTTTGGGCTCTTCTAAAGTTAGTGTTTGTTCTA [ 4992]
lp36_B31_NX_Pl_consensus     TTGCTTTCACTGTTTAGATGCATAAGGAAAATAGTATATATGATGGTTTGGGCTCTTCTAAAGTTAGTGTTTGTTCTA [ 4992]
lp36_PAli_NX_MP_FG_consensus TTGCTTTCACTGTTTAGATGCATAAGGAAAATAGTATATATGATGGTTTGGGCTCTTCTAAAGTTAGTGTTTGTTCTA [ 4992]
lp36_PAli_NX_Pl_consensus    TTGCTTTCACTGTTTAGATGCATAAGGAAAATAGTATATATGATGGTTTGGGCTCTTCTAAAGTTAGTGTTTGTTCTA [ 4992]
lp36_PAbe_NX_MP_FG_consensus TTGCTTTCACTGTTTAGATGCATAAGGAAAATAGTATATATGATGGTTTGGGCTCTTCTAAAGTTAGTGTTTGTTCTA [ 4992]
lp36_PAbe_NX_Pl_consensus    TTGCTTTCACTGTTTAGATGCATAAGGAAAATAGTATATATGATGGTTTGGGCTCTTCTAAAGTTAGTGTTTGTTCTA [ 4992]

lp36_gi|11496779|B31_GB      TTTGTCATTAAAAAAAAAAAGACTCTGGTTTTGTTTGCAAACAAACTTGCTAGGTTTTTGATATTGTTTACTATAGGA [ 5070]
lp36_un11_B31_PacBio         TTTGTCATTAAAAAAAAAAAGACTCTGGTTTTGTTTGCAAACAAACTTGCTAGGTTTTTGATATTGTTTACTATAGGA [ 5070]
lp36_B31_TS_MP_FG_consensus  TTTGTCATTAAAAAAAAAAAGACTCTGGTTTTGTTTGCAAACAAACTTGCTAGGTTTTTGATATTGTTTACTATAGGA [ 5070]
lp36_B31_NX_Pl_consensus     TTTGTCATTAAAAAAAAAAAGACTCTGGTTTTGTTTGCAAACAAACTTGCTAGGTTTTTGATATTGTTTACTATAGGA [ 5070]
lp36_PAli_NX_MP_FG_consensus TTTGTCATTAAAAAAAAAAAGACTCTGGTTTTGTTTGCAAACAAACTTGCTAGGTTTTTGATATTGTTTACTATAGGA [ 5070]
lp36_PAli_NX_Pl_consensus    TTTGTCATTAAAAAAAAAAAGACTCTGGTTTTGTTTGCAAACAAACTTGCTAGGTTTTTGATATTGTTTACTATAGGA [ 5070]
lp36_PAbe_NX_MP_FG_consensus TTTGTCATTAAAAAAAAAAAGACTCTGGTTTTGTTTGCAAACAAACTTGCTAGGTTTTTGATATTGTTTACTATAGGA [ 5070]
lp36_PAbe_NX_Pl_consensus    TTTGTCATTAAAAAAAAAAAGACTCTGGTTTTGTTTGCAAACAAACTTGCTAGGTTTTTGATATTGTTTACTATAGGA [ 5070]

lp36_gi|11496779|B31_GB      GGTTTTTGAAATTTTTGATTATTCATTATATTTTAACCTCATATTATGGAATAATTTGATTATATTCAATGGTTTTTA [ 5148]
lp36_un11_B31_PacBio         GGTTTTTGAAATTTTTGATTATTCATTATATTTTAACCTCATATTATGGAATAATTTGATTATATTCAATGGTTTTTA [ 5148]
lp36_B31_TS_MP_FG_consensus  GGTTTTTGAAATTTTTGATTATTCATTATATTTTAACCTCATATTATGGAATAATTTGATTATATTCAATGGTTTTTA [ 5148]
lp36_B31_NX_Pl_consensus     GGTTTTTGAAATTTTTGATTATTCATTATATTTTAACCTCATATTATGGAATAATTTGATTATATTCAATGGTTTTTA [ 5148]
lp36_PAli_NX_MP_FG_consensus GGTTTTTGAAATTTTTGATTATTCATTATATTTTAACCTCATATTATGGAATAATTTGATTATATTCAATGGTTTTTA [ 5148]
lp36_PAli_NX_Pl_consensus    GGTTTTTGAAATTTTTGATTATTCATTATATTTTAACCTCATATTATGGAATAATTTGATTATATTCAATGGTTTTTA [ 5148]
lp36_PAbe_NX_MP_FG_consensus GGTTTTTGAAATTTTTGATTATTCATTATATTTTAACCTCATATTATGGAATAATTTGATTATATTCAATGGTTTTTA [ 5148]
lp36_PAbe_NX_Pl_consensus    GGTTTTTGAAATTTTTGATTATTCATTATATTTTAACCTCATATTATGGAATAATTTGATTATATTCAATGGTTTTTA [ 5148]

lp36_gi|11496779|B31_GB      TAAAAACCCCAAAATTCCTTTGAAAAAAATTTCAAGAGGATTGCGGGTGTTTTTTTTGAATAGAAAATTACTTTGGGA [ 5226]
lp36_un11_B31_PacBio         TAAAAACCCCAAAATTCCTTTGAAAAAAATTTCAAGAGGATTGCGGGTGTTTTTTTTGAATAGAAAATTACTTTGGGA [ 5226]
lp36_B31_TS_MP_FG_consensus  TAAAAACCCCAAAATTCCTTTGAAAAAAATTTCAAGAGGATTGCGGGTGTTTTTTTTGAATAGAAAATTACTTTGGGA [ 5226]
lp36_B31_NX_Pl_consensus     TAAAAACCCCAAAATTCCTTTGAAAAAAATTTCAAGAGGATTGCGGGTGTTTTTTTTGAATAGAAAATTACTTTGGGA [ 5226]
lp36_PAli_NX_MP_FG_consensus TAAAAACCCCAAAATTCCTTTGAAAAAAATTTCAAGAGGATTGCGGGTGTTTTTTTTGAATAGAAAATTACTTTGGGA [ 5226]
lp36_PAli_NX_Pl_consensus    TAAAAACCCCAAAATTCCTTTGAAAAAAATTTCAAGAGGATTGCGGGTGTTTTTTTTGAATAGAAAATTACTTTGGGA [ 5226]
lp36_PAbe_NX_MP_FG_consensus TAAAAACCCCAAAATTCCTTTGAAAAAAATTTCAAGAGGATTGCGGGTGTTTTTTTTGAATAGAAAATTACTTTGGGA [ 5226]
lp36_PAbe_NX_Pl_consensus    TAAAAACCCCAAAATTCCTTTGAAAAAAATTTCAAGAGGATTGCGGGTGTTTTTTTTGAATAGAAAATTACTTTGGGA [ 5226]

lp36_gi|11496779|B31_GB      TTTTAGTTGAAATTTTTAGAATTTTTCTTTTTATTAGAATTGTAAACCAAAATGCCCAGTTTTAATTATTAAAGCACA [ 5304]
lp36_un11_B31_PacBio         TTTTAGTTGAAATTTTTAGAATTTTTCTTTTTATTAGAATTGTAAACCAAAATGCCCAGTTTTAATTATTAAAGCACA [ 5304]
lp36_B31_TS_MP_FG_consensus  TTTTAGTTGAAATTTTTAGAATTTTTCTTTTTATTAGAATTGTAAACCAAAATGCCCAGTTTTAATTATTAAAGCACA [ 5304]
lp36_B31_NX_Pl_consensus     TTTTAGTTGAAATTTTTAGAATTTTTCTTTTTATTAGAATTGTAAACCAAAATGCCCAGTTTTAATTATTAAAGCACA [ 5304]
lp36_PAli_NX_MP_FG_consensus TTTTAGTTGAAATTTTTAGAATTTTTCTTTTTATTAGAATTGTAAACCAAAATGCCCAGTTTTAATTATTAAAGCACA [ 5304]
lp36_PAli_NX_Pl_consensus    TTTTAGTTGAAATTTTTAGAATTTTTCTTTTTATTAGAATTGTAAACCAAAATGCCCAGTTTTAATTATTAAAGCACA [ 5304]
lp36_PAbe_NX_MP_FG_consensus TTTTAGTTGAAATTTTTAGAATTTTTCTTTTTATTAGAATTGTAAACCAAAATGCCCAGTTTTAATTATTAAAGCACA [ 5304]
lp36_PAbe_NX_Pl_consensus    TTTTAGTTGAAATTTTTAGAATTTTTCTTTTTATTAGAATTGTAAACCAAAATGCCCAGTTTTAATTATTAAAGCACA [ 5304]

lp36_gi|11496779|B31_GB      AATGTATGGCCATTGCTGCATTCTCTATTTTATTGGCTGCCTCACTCAAAGCTGCCAATATTTTAGTATATTTGTCGT [ 5382]
lp36_un11_B31_PacBio         AATGTATGGCCATTGCTGCATTCTCTATTTTATTGGCTGCCTCACTCAAAGCTGCCAATATTTTAGTATATTTGTCGT [ 5382]
lp36_B31_TS_MP_FG_consensus  AATGTATGGCCATTGCTGCATTCTCTATTTTATTGGCTGCCTCACTCAAAGCTGCCAATATTTTAGTATATTTGTCGT [ 5382]
lp36_B31_NX_Pl_consensus     AATGTATGGCCATTGCTGCATTCTCTATTTTATTGGCTGCCTCACTCAAAGCTGCCAATATTTTAGTATATTTGTCGT [ 5382]
lp36_PAli_NX_MP_FG_consensus AATGTATGGCCATTGCTGCATTCTCTATTTTATTGGCTGCCTCACTCAAAGCTGCCAATATTTTAGTATATTTGTCGT [ 5382]
lp36_PAli_NX_Pl_consensus    AATGTATGGCCATTGCTGCATTCTCTATTTTATTGGCTGCCTCACTCAAAGCTGCCAATATTTTAGTATATTTGTCGT [ 5382]
lp36_PAbe_NX_MP_FG_consensus AATGTATGGCCATTGCTGCATTCTCTATTTTATTGGCTGCCTCACTCAAAGCTGCCAATATTTTAGTATATTTGTCGT [ 5382]
lp36_PAbe_NX_Pl_consensus    AATGTATGGCCATTGCTGCATTCTCTATTTTATTGGCTGCCTCACTCAAAGCTGCCAATATTTTAGTATATTTGTCGT [ 5382]

lp36_gi|11496779|B31_GB      CTTTTTTATCTTTGAGTTCGGTGCTCACGCCCTTGAAATAGGTTTCCACATTGGTCATAAGAGTTTTTATGCACTTTT [ 5460]
lp36_un11_B31_PacBio         CTTTTTTATCTTTGAGTTCGGTGCTCACGCCCTTGAAATAGGTTTCCACATTGGTCATAAGAGTTTTTATGCACTTTT [ 5460]
lp36_B31_TS_MP_FG_consensus  CTTTTTTATCTTTGAGTTCGGTGCTCACGCCCTTGAAATAGGTTTCCACATTGGTCATAAGAGTTTTTATGCACTTTT [ 5460]
lp36_B31_NX_Pl_consensus     CTTTTTTATCTTTGAGTTCGGTGCTCACGCCCTTGAAATAGGTTTCCACATTGGTCATAAGAGTTTTTATGCACTTTT [ 5460]
lp36_PAli_NX_MP_FG_consensus CTTTTTTATCTTTGAGTTCGGTGCTCACGCCCTTGAAATAGGTTTCCACATTGGTCATAAGAGTTTTTATGCACTTTT [ 5460]
lp36_PAli_NX_Pl_consensus    CTTTTTTATCTTTGAGTTCGGTGCTCACGCCCTTGAAATAGGTTTCCACATTGGTCATAAGAGTTTTTATGCACTTTT [ 5460]
lp36_PAbe_NX_MP_FG_consensus CTTTTTTATCTTTGAGTTCGGTGCTCACGCCCTTGAAATAGGTTTCCACATTGGTCATAAGAGTTTTTATGCACTTTT [ 5460]
lp36_PAbe_NX_Pl_consensus    CTTTTTTATCTTTGAGTTCGGTGCTCACGCCCTTGAAATAGGTTTCCACATTGGTCATAAGAGTTTTTATGCACTTTT [ 5460]
```

```
lp36_gi|11496779|B31_GB       TGGCTTCAATTTTACTACCATTTTCTAATGGTACCTTTATCGCTGAATTTTCAGCATTAAACCTATTTACAACGGTTT [ 5538]
lp36_un11_B31_PacBio          TGGCTTCAATTTTACTACCATTTTCTAATGGTACCTTTATCGCTGAATTTTCAGCATTAAACCTATTTACAACGGTTT [ 5538]
lp36_B31_TS_MP_FG_consensus   TGGCTTCAATTTTACTACCATTTTCTAATGGTACCTTTATCGCTGAATTTTCAGCATTAAACCTATTTACAACGGTTT [ 5538]
lp36_B31_NX_Pl_consensus      TGGCTTCAATTTTACTACCATTTTCTAATGGTACCTTTATCGCTGAATTTTCAGCATTAAACCTATTTACAACGGTTT [ 5538]
lp36_PAli_NX_MP_FG_consensus TGGCTTCAATTTTACTACCATTTTCTAATGGTACCTTTATCGCTGAATTTTCAGCATTAAACCTATTTACAACGGTTT [ 5538]
lp36_PAli_NX_Pl_consensus     TGGCTTCAATTTTACTACCATTTTCTAATGGTACCTTTATCGCTGAATTTTCAGCATTAAACCTATTTACAACGGTTT [ 5538]
lp36_PAbe_NX_MP_FG_consensus TGGCTTCAATTTTACTACCATTTTCTAATGGTACCTTTATCGCTGAATTTTCAGCATTAAACCTATTTACAACGGTTT [ 5538]
lp36_PAbe_NX_Pl_consensus     TGGCTTCAATTTTACTACCATTTTCTAATGGTACCTTTATCGCTGAATTTTCAGCATTAAACCTATTTACAACGGTTT [ 5538]

lp36_gi|11496779|B31_GB       TAAGTTCATTGTATTTTTGGGTACTTTTATCACCATTGCTACTTACCGATATTTTTAGCAACTCATCCAGTAATTTCA [ 5616]
lp36_un11_B31_PacBio          TAAGTTCATTGTATTTTTGGGTACTTTTATCACCATTGCTACTTACCGATATTTTTAGCAACTCATCCAGTAATTTCA [ 5616]
lp36_B31_TS_MP_FG_consensus   TAAGTTCATTGTATTTTTGGGTACTTTTATCACCATTGCTACTTACCGATATTTTTAGCAACTCATCCAGTAATTTCA [ 5616]
lp36_B31_NX_Pl_consensus      TAAGTTCATTGTATTTTTGGGTACTTTTATCACCATTGCTACTTACCGATATTTTTAGCAACTCATCCAGTAATTTCA [ 5616]
lp36_PAli_NX_MP_FG_consensus TAAGTTCATTGTATTTTTGGGTACTTTTATCACCATTGCTACTTACCGATATTTTTAGCAACTCATCCAGTAATTTCA [ 5616]
lp36_PAli_NX_Pl_consensus     TAAGTTCATTGTATTTTTGGGTACTTTTATCACCATTGCTACTTACCGATATTTTTAGCAACTCATCCAGTAATTTCA [ 5616]
lp36_PAbe_NX_MP_FG_consensus TAAGTTCATTGTATTTTTGGGTACTTTTATCACCATTGCTACTTACCGATATTTTTAGCAACTCATCCAGTAATTTCA [ 5616]
lp36_PAbe_NX_Pl_consensus     TAAGTTCATTGTATTTTTGGGTACTTTTATCACCATTGCTACTTACCGATATTTTTAGCAACTCATCCAGTAATTTCA [ 5616]

lp36_gi|11496779|B31_GB       CCATCTTTAGTCCATTCTTCAAATTACTTTTGGCTGCATAGGTAGCATTATAACCTATATTAATTAGTGTTTGAAAAA [ 5694]
lp36_un11_B31_PacBio          CCATCTTTAGTCCATTCTTCAAATTACTTTTGGCTGCATAGGTAGCATTATAACCTATATTAATTAGTGTTTGAAAAA [ 5694]
lp36_B31_TS_MP_FG_consensus   CCATCTTTAGTCCATTCTTCAAATTACTTTTGGCTGCATAGGTAGCATTATAACCTATATTAATTAGTGTTTGAAAAA [ 5694]
lp36_B31_NX_Pl_consensus      CCATCTTTAGTCCATTCTTCAAATTACTTTTGGCTGCATAGGTAGCATTATAACCTATATTAATTAGTGTTTGAAAAA [ 5694]
lp36_PAli_NX_MP_FG_consensus CCATCTTTAGTCCATTCTTCAAATTACTTTTGGCTGCATAGGTAGCATTATAACCTATATTAATTAGTGTTTGAAAAA [ 5694]
lp36_PAli_NX_Pl_consensus     CCATCTTTAGTCCATTCTTCAAATTACTTTTGGCTGCATAGGTAGCATTATAACCTATATTAATTAGTGTTTGAAAAA [ 5694]
lp36_PAbe_NX_MP_FG_consensus CCATCTTTAGTCCATTCTTCAAATTACTTTTGGCTGCATAGGTAGCATTATAACCTATATTAATTAGTGTTTGAAAAA [ 5694]
lp36_PAbe_NX_Pl_consensus     CCATCTTTAGTCCATTCTTCAAATTACTTTTGGCTGCATAGGTAGCATTATAACCTATATTAATTAGTGTTTGAAAAA [ 5694]

lp36_gi|11496779|B31_GB       CCGAATCAATTTTTTCTGATGAATTCTTAATCAATTTGATTAAGCGAGGAGTATCCTTTACTTCTATGTTTTTACTTT [ 5772]
lp36_un11_B31_PacBio          CCGAATCAATTTTTTCTGATGAATTCTTAATCAATTTGATTAAGCGAGGAGTATCCTTTACTTCTATGTTTTTACTTT [ 5772]
lp36_B31_TS_MP_FG_consensus   CCGAATCAATTTTTTCTGATGAATTCTTAATCAATTTGATTAAGCGAGGAGTATCCTTTACTTCTATGTTTTTACTTT [ 5772]
lp36_B31_NX_Pl_consensus      CCGAATCAATTTTTTCTGATGAATTCTTAATCAATTTGATTAAGCGAGGAGTATCCTTTACTTCTATGTTTTTACTTT [ 5772]
lp36_PAli_NX_MP_FG_consensus CCGAATCAATTTTTTCTGATGAATTCTTAATCAATTTGATTAAGCGAGGAGTATCCTTTACTTCTATGTTTTTACTTT [ 5772]
lp36_PAli_NX_Pl_consensus     CCGAATCAATTTTTTCTGATGAATTCTTAATCAATTTGATTAAGCGAGGAGTATCCTTTACTTCTATGTTTTTACTTT [ 5772]
lp36_PAbe_NX_MP_FG_consensus CCGAATCAATTTTTTCTGATGAATTCTTAATCAATTTGATTAAGCGAGGAGTATCCTTTACTTCTATGTTTTTACTTT [ 5772]
lp36_PAbe_NX_Pl_consensus     CCGAATCAATTTTTTCTGATGAATTCTTAATCAATTTGATTAAGCGAGGAGTATCCTTTACTTCTATGTTTTTACTTT [ 5772]

lp36_gi|11496779|B31_GB       CCTTGGTATTTTTTTGATCTTCAATTTCTTTTTTGGATTTTTCAGAATCATCTTGAGCTTCCTTTGCTAAATTTTCTA [ 5850]
lp36_un11_B31_PacBio          CCTTGGTATTTTTTTGATCTTCAATTTCTTTTTTGGATTTTTCAGAATCATCTTGAGCTTCCTTTGCTAAATTTTCTA [ 5850]
lp36_B31_TS_MP_FG_consensus   CCTTGGTATTTTTTTGATCTTCAATTTCTTTTTTGGATTTTTCAGAATCATCTTGAGCTTCCTTTGCTAAATTTTCTA [ 5850]
lp36_B31_NX_Pl_consensus      CCTTGGTATTTTTTTGATCTTCAATTTCTTTTTTGGATTTTTCAGAATCATCTTGAGCTTCCTTTGCTAAATTTTCTA [ 5850]
lp36_PAli_NX_MP_FG_consensus CCTTGGTATTTTTTTGATCTTCAATTTCTTTTTTGGATTTTTCAGAATCATCTTGAGCTTCCTTTGCTAAATTTTCTA [ 5850]
lp36_PAli_NX_Pl_consensus     CCTTGGTATTTTTTTGATCTTCAATTTCTTTTTTGGATTTTTCAGAATCATCTTGAGCTTCCTTTGCTAAATTTTCTA [ 5850]
lp36_PAbe_NX_MP_FG_consensus CCTTGGTATTTTTTTGATCTTCAATTTCTTTTTTGGATTTTTCAGAATCATCTTGAGCTTCCTTTGCTAAATTTTCTA [ 5850]
lp36_PAbe_NX_Pl_consensus     CCTTGGTATTTTTTTGATCTTCAATTTCTTTTTTGGATTTTTCAGAATCATCTTGAGCTTCCTTTGCTAAATTTTCTA [ 5850]

lp36_gi|11496779|B31_GB       GCTCTTCCGGAGTTAATTTTGTGATTTTTTCCGAACTTAGACCTGTAGCTTCACTTATCTCATCTAATACTTGATCAA [ 5928]
lp36_un11_B31_PacBio          GCTCTTCCGGAGTTAATTTTGTGATTTTTTCCGAACTTAGACCTGTAGCTTCACTTATCTCATCTAATACTTGATCAA [ 5928]
lp36_B31_TS_MP_FG_consensus   GCTCTTCCGGAGTTAATTTTGTGATTTTTTCCGAACTTAGACCTGTAGCTTCACTTATCTCATCTAATACTTGATCAA [ 5928]
lp36_B31_NX_Pl_consensus      GCTCTTCCGGAGTTAATTTTGTGATTTTTTCCGAACTTAGACCTGTAGCTTCACTTATCTCATCTAATACTTGATCAA [ 5928]
lp36_PAli_NX_MP_FG_consensus GCTCTTCCGGAGTTAATTTTGTGATTTTTTCCGAACTTAGACCTGTAGCTTCACTTATCTCATCTAATACTTGATCAA [ 5928]
lp36_PAli_NX_Pl_consensus     GCTCTTCCGGAGTTAATTTTGTGATTTTTTCCGAACTTAGACCTGTAGCTTCACTTATCTCATCTAATACTTGATCAA [ 5928]
lp36_PAbe_NX_MP_FG_consensus GCTCTTCCGGAGTTAATTTTGTGATTTTTTCCGAACTTAGACCTGTAGCTTCACTTATCTCATCTAATACTTGATCAA [ 5928]
lp36_PAbe_NX_Pl_consensus     GCTCTTCCGGAGTTAATTTTGTGATTTTTTCCGAACTTAGACCTGTAGCTTCACTTATCTCATCTAATACTTGATCAA [ 5928]

lp36_gi|11496779|B31_GB       CAGATGTTAGTGCTGATTTACTTTCTGCAGTTGCTTCATCAATGGGATTGTCTACATGCCATTTACATGAAATTATTA [ 6006]
lp36_un11_B31_PacBio          CAGATGTTAGTGCTGATTTACTTTCTGCAGTTGCTTCATCAATGGGATTGTCTACATGCCATTTACATGAAATTATTA [ 6006]
lp36_B31_TS_MP_FG_consensus   CAGATGTTAGTGCTGATTTACTTTCTGCAGTTGCTTCATCAATGGGATTGTCTACATGCCATTTACATGAAATTATTA [ 6006]
lp36_B31_NX_Pl_consensus      CAGATGTTAGTGCTGATTTACTTTCTGCAGTTGCTTCATCAATGGGATTGTCTACATGCCATTTACATGAAATTATTA [ 6006]
lp36_PAli_NX_MP_FG_consensus CAGATGTTAGTGCTGATTTACTTTCTGCAGTTGCTTCATCAATGGGATTGTCTACATGCCATTTACATGAAATTATTA [ 6006]
lp36_PAli_NX_Pl_consensus     CAGATGTTAGTGCTGATTTACTTTCTGCAGTTGCTTCATCAATGGGATTGTCTACATGCCATTTACATGAAATTATTA [ 6006]
lp36_PAbe_NX_MP_FG_consensus CAGATGTTAGTGCTGATTTACTTTCTGCAGTTGCTTCATCAATGGGATTGTCTACATGCCATTTACATGAAATTATTA [ 6006]
lp36_PAbe_NX_Pl_consensus     CAGATGTTAGTGCTGATTTACTTTCTGCAGTTGCTTCATCAATGGGATTGTCTACATGCCATTTACATGAAATTATTA [ 6006]

lp36_gi|11496779|B31_GB       GCAGTATAGATATTGCCAATATTAGTTTACTCATTTGTAAACACCTCCTTGTAGTGTAATATTTAATATATTTAGTGA [ 6084]
lp36_un11_B31_PacBio          GCAGTATAGATATTGCCAATATTAGTTTACTCATTTGTAAACACCTCCTTGTAGTGTAATATTTAATATATTTAGTGA [ 6084]
lp36_B31_TS_MP_FG_consensus   GCAGTATAGATATTGCCAATATTAGTTTACTCATTTGTAAACACCTCCTTGTAGTGTAATATTTAATATATTTAGTGA [ 6084]
lp36_B31_NX_Pl_consensus      GCAGTATAGATATTGCCAATATTAGTTTACTCATTTGTAAACACCTCCTTGTAGTGTAATATTTAATATATTTAGTGA [ 6084]
lp36_PAli_NX_MP_FG_consensus GCAGTATAGATATTGCCAATATTAGTTTACTCATTTGTAAACACCTCCTTGTAGTGTAATATTTAATATATTTAGTGA [ 6084]
lp36_PAli_NX_Pl_consensus     GCAGTATAGATATTGCCAATATTAGTTTACTCATTTGTAAACACCTCCTTGTAGTGTAATATTTAATATATTTAGTGA [ 6084]
lp36_PAbe_NX_MP_FG_consensus GCAGTATAGATATTGCCAATATTAGTTTACTCATTTGTAAACACCTCCTTGTAGTGTAATATTTAATATATTTAGTGA [ 6084]
lp36_PAbe_NX_Pl_consensus     GCAGTATAGATATTGCCAATATTAGTTTACTCATTTGTAAACACCTCCTTGTAGTGTAATATTTAATATATTTAGTGA [ 6084]

lp36_gi|11496779|B31_GB       ATTATAGCTCAAAATTCATTTCTACCTTAACTGCAAAAATCTTTACAATTAACTTGTGGGTAAAGATTTTTTTAATTC [ 6162]
lp36_un11_B31_PacBio          ATTATAGCTCAAAATTCATTTCTACCTTAACTGCAAAAATCTTTACAATTAACTTGTGGGTAAAGATTTTTTTAATTC [ 6162]
lp36_B31_TS_MP_FG_consensus   ATTATAGCTCAAAATTCATTTCTACCTTAACTGCAAAAATCTTTACAATTAACTTGTGGGTAAAGATTTTTTTAATTC [ 6162]
lp36_B31_NX_Pl_consensus      ATTATAGCTCAAAATTCATTTCTACCTTAACTGCAAAAATCTTTACAATTAACTTGTGGGTAAAGATTTTTTTAATTC [ 6162]
lp36_PAli_NX_MP_FG_consensus ATTATAGCTCAAAATTCATTTCTACCTTAACTGCAAAAATCTTTACAATTAACTTGTGGGTAAAGATTTTTTTAATTC [ 6162]
lp36_PAli_NX_Pl_consensus     ATTATAGCTCAAAATTCATTTCTACCTTAACTGCAAAAATCTTTACAATTAACTTGTGGGTAAAGATTTTTTTAATTC [ 6162]
lp36_PAbe_NX_MP_FG_consensus ATTATAGCTCAAAATTCATTTCTACCTTAACTGCAAAAATCTTTACAATTAACTTGTGGGTAAAGATTTTTTTAATTC [ 6162]
lp36_PAbe_NX_Pl_consensus     ATTATAGCTCAAAATTCATTTCTACCTTAACTGCAAAAATCTTTACAATTAACTTGTGGGTAAAGATTTTTTTAATTC [ 6162]

lp36_gi|11496779|B31_GB       TGGTTTGGGAAATTCTAATTTTTTCTTAGAGGCATTACGCCTTCTTTGATTTTTTTATAATTTTTTGGATTCACAAGA [ 6240]
lp36_un11_B31_PacBio          TGGTTTGGGAAATTCTAATTTTTTCTTAGAGGCATTACGCCTTCTTTGATTTTTTTATAATTTTTTGGATTCACAAGA [ 6240]
lp36_B31_TS_MP_FG_consensus   TGGTTTGGGAAATTCTAATTTTTTCTTAGAGGCATTACGCCTTCTTTGATTTTTTTATAATTTTTTGGATTCACAAGA [ 6240]
lp36_B31_NX_Pl_consensus      TGGTTTGGGAAATTCTAATTTTTTCTTAGAGGCATTACGCCTTCTTTGATTTTTTTATAATTTTTTGGATTCACAAGA [ 6240]
lp36_PAli_NX_MP_FG_consensus TGGTTTGGGAAATTCTAATTTTTTCTTAGAGGCATTACGCCTTCTTTGATTTTTTTATAATTTTTTGGATTCACAAGA [ 6240]
lp36_PAli_NX_Pl_consensus     TGGTTTGGGAAATTCTAATTTTTTCTTAGAGGCATTACGCCTTCTTTGATTTTTTTATAATTTTTTGGATTCACAAGA [ 6240]
lp36_PAbe_NX_MP_FG_consensus TGGTTTGGGAAATTCTAATTTTTTCTTAGAGGCATTACGCCTTCTTTGATTTTTTTATAATTTTTTGGATTCACAAGA [ 6240]
lp36_PAbe_NX_Pl_consensus     TGGTTTGGGAAATTCTAATTTTTTCTTAGAGGCATTACGCCTTCTTTGATTTTTTTATAATTTTTTGGATTCACAAGA [ 6240]

lp36_gi|11496779|B31_GB       TATTTTTTGAGCTTTTCTAAGAATTTTTTTATTTTTTTCAAATCTTATGTAATTTTTCTTATTTTGACTGTGTTTCGG [ 6318]
lp36_un11_B31_PacBio          TATTTTTTGAGCTTTTCTAAGAATTTTTTTATTTTTTTCAAATCTTATGTAATTTTTCTTATTTTGACTGTGTTTCGG [ 6318]
lp36_B31_TS_MP_FG_consensus   TATTTTTTGAGCTTTTCTAAGAATTTTTTTATTTTTTTCAAATCTTATGTAATTTTTCTTATTTTGACTGTGTTTCGG [ 6318]
lp36_B31_NX_Pl_consensus      TATTTTTTGAGCTTTTCTAAGAATTTTTTTATTTTTTTCAAATCTTATGTAATTTTTCTTATTTTGACTGTGTTTCGG [ 6318]
lp36_PAli_NX_MP_FG_consensus TATTTTTTGAGCTTTTCTAAGAATTTTTTTATTTTTTTCAAATCTTATGTAATTTTTCTTATTTTGACTGTGTTTCGG [ 6318]
lp36_PAli_NX_Pl_consensus     TATTTTTTGAGCTTTTCTAAGAATTTTTTTATTTTTTTCAAATCTTATGTAATTTTTCTTATTTTGACTGTGTTTCGG [ 6318]
lp36_PAbe_NX_MP_FG_consensus TATTTTTTGAGCTTTTCTAAGAATTTTTTTATTTTTTTCAAATCTTATGTAATTTTTCTTATTTTGACTGTGTTTCGG [ 6318]
lp36_PAbe_NX_Pl_consensus     TATTTTTTGAGCTTTTCTAAGAATTTTTTTATTTTTTTCAAATCTTATGTAATTTTTCTTATTTTGACTGTGTTTCGG [ 6318]

lp36_gi|11496779|B31_GB       TCTTGATCAGCACTCAACTATTTTGATTTTTAACTTTTAAATATCTTATGAAATTTATGAATAACAAAATTGGAAATT [ 6396]
lp36_un11_B31_PacBio          TCTTGATCAGCACTCAACTATTTTGATTTTTAACTTTTAAATATCTTATGAAATTTATGAATAACAAAATTGGAAATT [ 6396]
lp36_B31_TS_MP_FG_consensus   TCTTGATCAGCACTCAACTATTTTGATTTTTAACTTTTAAATATCTTATGAAATTTATGAATAACAAAATTGGAAATT [ 6396]
lp36_B31_NX_Pl_consensus      TCTTGATCAGCACTCAACTATTTTGATTTTTAACTTTTAAATATCTTATGAAATTTATGAATAACAAAATTGGAAATT [ 6396]
lp36_PAli_NX_MP_FG_consensus TCTTGATCAGCACTCAACTATTTTGATTTTTAACTTTTAAATATCTTATGAAATTTATGAATAACAAAATTGGAAATT [ 6396]
lp36_PAli_NX_Pl_consensus     TCTTGATCAGCACTCAACTATTTTGATTTTTAACTTTTAAATATCTTATGAAATTTATGAATAACAAAATTGGAAATT [ 6396]
lp36_PAbe_NX_MP_FG_consensus TCTTGATCAGCACTCAACTATTTTGATTTTTAACTTTTAAATATCTTATGAAATTTATGAATAACAAAATTGGAAATT [ 6396]
lp36_PAbe_NX_Pl_consensus     TCTTGATCAGCACTCAACTATTTTGATTTTTAACTTTTAAATATCTTATGAAATTTATGAATAACAAAATTGGAAATT [ 6396]
```

```
lp36_gi|11496779|B31_GB      ATCAAGTTCTTAAAAGTTATTTGAAGTATACAATTCAAAAACAAATGGAAATTGACGAAATAATTTCAAATTTAAGAG [ 6474]
lp36_un11_B31_PacBio         ATCAAGTTCTTAAAAGTTATTTGAAGTATACAATTCAAAAACAAATGGAAATTGACGAAATAATTTCAAATTTAAGAG [ 6474]
lp36_B31_TS_MP_FG_consensus  ATCAAGTTCTTAAAAGTTATTTGAAGTATACAATTCAAAAACAAATGGAAATTGACGAAATAATTTCAAATTTAAGAG [ 6474]
lp36_B31_NX_Pl_consensus     ATCAAGTTCTTAAAAGTTATTTGAAGTATACAATTCAAAAACAAATGGAAATTGACGAAATAATTTCAAATTTAAGAG [ 6474]
lp36_PAli_NX_MP_FG_consensus ATCAAGTTCTTAAAAGTTATTTGAAGTATACAATTCAAAAACAAATGGAAATTGACGAAATAATTTCAAATTTAAGAG [ 6474]
lp36_PAli_NX_Pl_consensus    ATCAAGTTCTTAAAAGTTATTTGAAGTATACAATTCAAAAACAAATGGAAATTGACGAAATAATTTCAAATTTAAGAG [ 6474]
lp36_PAbe_NX_MP_FG_consensus ATCAAGTTCTTAAAAGTTATTTGAAGTATACAATTCAAAAACAAATGGAAATTGACGAAATAATTTCAAATTTAAGAG [ 6474]
lp36_PAbe_NX_Pl_consensus    ATCAAGTTCTTAAAAGTTATTTGAAGTATACAATTCAAAAACAAATGGAAATTGACGAAATAATTTCAAATTTAAGAG [ 6474]

lp36_gi|11496779|B31_GB      AATTTGGCAGTCCATATATCTATGAAAGCAATGTTGGAAAAAATATTTTTTTTGTTATAAAGCAGTTCATGGGTTTAT [ 6552]
lp36_un11_B31_PacBio         AATTTGGCAGTCCATATATCTATGAAAGCAATGTTGGAAAAAATATTTTTTTTGTTATAAAGCAGTTCATGGGTTTAT [ 6552]
lp36_B31_TS_MP_FG_consensus  AATTTGGCAGTCCATATATCTATGAAAGCAATGTTGGAAAAAATATTTTTTTTGTTATAAAGCAGTTCATGGGTTTAT [ 6552]
lp36_B31_NX_Pl_consensus     AATTTGGCAGTCCATATATCTATGAAAGCAATGTTGGAAAAAATATTTTTTTTGTTATAAAGCAGTTCATGGGTTTAT [ 6552]
lp36_PAli_NX_MP_FG_consensus AATTTGGCAGTCCATATATCTATGAAAGCAATGTTGGAAAAAATATTTTTTTTGTTATAAAGCAGTTCATGGGTTTAT [ 6552]
lp36_PAli_NX_Pl_consensus    AATTTGGCAGTCCATATATCTATGAAAGCAATGTTGGAAAAAATATTTTTTTTGTTATAAAGCAGTTCATGGGTTTAT [ 6552]
lp36_PAbe_NX_MP_FG_consensus AATTTGGCAGTCCATATATCTATGAAAGCAATGTTGGAAAAAATATTTTTTTTGTTATAAAGCAGTTCATGGGTTTAT [ 6552]
lp36_PAbe_NX_Pl_consensus    AATTTGGCAGTCCATATATCTATGAAAGCAATGTTGGAAAAAATATTTTTTTTGTTATAAAGCAGTTCATGGGTTTAT [ 6552]

lp36_gi|11496779|B31_GB      TTTTAGTAATCGATTTTGGCACTATGATTGGGATATTGGGAATATACTTTTCAATATTTGCCCCATTTTACCTCAATT [ 6630]
lp36_un11_B31_PacBio         TTTTAGTAATCGATTTTGGCACTATGATTGGGATATTGGGAATATACTTTTCAATATTTGCCCCATTTTACCTCAATT [ 6630]
lp36_B31_TS_MP_FG_consensus  TTTTAGTAATCGATTTTGGCACTATGATTGGGATATTGGGAATATACTTTTCAATATTTGCCCCATTTTACCTCAATT [ 6630]
lp36_B31_NX_Pl_consensus     TTTTAGTAATCGATTTTGGCACTATGATTGGGATATTGGGAATATACTTTTCAATATTTGCCCCATTTTACCTCAATT [ 6630]
lp36_PAli_NX_MP_FG_consensus TTTTAGTAATCGATTTTGGCACTATGATTGGGATATTGGGAATATACTTTTCAATATTTGCCCCATTTTACCTCAATT [ 6630]
lp36_PAli_NX_Pl_consensus    TTTTAGTAATCGATTTTGGCACTATGATTGGGATATTGGGAATATACTTTTCAATATTTGCCCCATTTTACCTCAATT [ 6630]
lp36_PAbe_NX_MP_FG_consensus TTTTAGTAATCGATTTTGGCACTATGATTGGGATATTGGGAATATACTTTTCAATATTTGCCCCATTTTACCTCAATT [ 6630]
lp36_PAbe_NX_Pl_consensus    TTTTAGTAATCGATTTTGGCACTATGATTGGGATATTGGGAATATACTTTTCAATATTTGCCCCATTTTACCTCAATT [ 6630]

lp36_gi|11496779|B31_GB      TTTTGGATATTTTTAATTAATAGGCATTTATGAGATTTTTTTAAATTCACAAATAGGGCGCACATTTAGTAAATAGTT [ 6708]
lp36_un11_B31_PacBio         TTTTGGATATTTTTAATTAATAGGCATTTATGAGATTTTTTTAAATTCACAAATAGGGCGCACATTTAGTAAATAGTT [ 6708]
lp36_B31_TS_MP_FG_consensus  TTTTGGATATTTTTAATTAATAGGCATTTATGAGATTTTTTTAAATTCACAAATAGGGCGCACATTTAGTAAATAGTT [ 6708]
lp36_B31_NX_Pl_consensus     TTTTGGATATTTTTAATTAATAGGCATTTATGAGATTTTTTTAAATTCACAAATAGGGCGCACATTTAGTAAATAGTT [ 6708]
lp36_PAli_NX_MP_FG_consensus TTTTGGATATTTTTAATTAATAGGCATTTATGAGATTTTTTTAAATTCACAAATAGGGCGCACATTTAGTAAATAGTT [ 6708]
lp36_PAli_NX_Pl_consensus    TTTTGGATATTTTTAATTAATAGGCATTTATGAGATTTTTTTAAATTCACAAATAGGGCGCACATTTAGTAAATAGTT [ 6708]
lp36_PAbe_NX_MP_FG_consensus TTTTGGATATTTTTAATTAATAGGCATTTATGAGATTTTTTTAAATTCACAAATAGGGCGCACATTTAGTAAATAGTT [ 6708]
lp36_PAbe_NX_Pl_consensus    TTTTGGATATTTTTAATTAATAGGCATTTATGAGATTTTTTTAAATTCACAAATAGGGCGCACATTTAGTAAATAGTT [ 6708]

lp36_gi|11496779|B31_GB      GCAAGAACCCTTTGCCAAAGAAAGAAAATATTTTTCTCCTTTTTTATTTTTTAATGTGTTAAAATCATTAAGTAAGAT [ 6786]
lp36_un11_B31_PacBio         GCAAGAACCCTTTGCCAAAGAAAGAAAATATTTTTCTCCTTTTTTATTTTTTAATGTGTTAAAATCATTAAGTAAGAT [ 6786]
lp36_B31_TS_MP_FG_consensus  GCAAGAACCCTTTGCCAAAGAAAGAAAATATTTTTCTCCTTTTTTATTTTTTAATGTGTTAAAATCATTAAGTAAGAT [ 6786]
lp36_B31_NX_Pl_consensus     GCAAGAACCCTTTGCCAAAGAAAGAAAATATTTTTCTCCTTTTTTATTTTTTAATGTGTTAAAATCATTAAGTAAGAT [ 6786]
lp36_PAli_NX_MP_FG_consensus GCAAGAACCCTTTGCCAAAGAAAGAAAATATTTTTCTCCTTTTTTATTTTTTAATGTGTTAAAATCATTAAGTAAGAT [ 6786]
lp36_PAli_NX_Pl_consensus    GCAAGAACCCTTTGCCAAAGAAAGAAAATATTTTTCTCCTTTTTTATTTTTTAATGTGTTAAAATCATTAAGTAAGAT [ 6786]
lp36_PAbe_NX_MP_FG_consensus GCAAGAACCCTTTGCCAAAGAAAGAAAATATTTTTCTCCTTTTTTATTTTTTAATGTGTTAAAATCATTAAGTAAGAT [ 6786]
lp36_PAbe_NX_Pl_consensus    GCAAGAACCCTTTGCCAAAGAAAGAAAATATTTTTCTCCTTTTTTATTTTTTAATGTGTTAAAATCATTAAGTAAGAT [ 6786]

lp36_gi|11496779|B31_GB      TTTAAATTCTTTTATAGTTAAAAAATAAGACCGGCTACAATAAAATTATTTTCTTTTTCTTTAAAGAATTCGTCTTTG [ 6864]
lp36_un11_B31_PacBio         TTTAAATTCTTTTATAGTTAAAAAATAAGACCGGCTACAATAAAATTATTTTCTTTTTCTTTAAAGAATTCGTCTTTG [ 6864]
lp36_B31_TS_MP_FG_consensus  TTTAAATTCTTTTATAGTTAAAAAATAAGACCGGCTACAATAAAATTATTTTCTTTTTCTTTAAAGAATTCGTCTTTG [ 6864]
lp36_B31_NX_Pl_consensus     TTTAAATTCTTTTATAGTTAAAAAATAAGACCGGCTACAATAAAATTATTTTCTTTTTCTTTAAAGAATTCGTCTTTG [ 6864]
lp36_PAli_NX_MP_FG_consensus TTTAAATTCTTTTATAGTTAAAAAATAAGACCGGCTACAATAAAATTATTTTCTTTTTCTTTAAAGAATTCGTCTTTG [ 6864]
lp36_PAli_NX_Pl_consensus    TTTAAATTCTTTTATAGTTAAAAAATAAGACCGGCTACAATAAAATTATTTTCTTTTTCTTTAAAGAATTCGTCTTTG [ 6864]
lp36_PAbe_NX_MP_FG_consensus TTTAAATTCTTTTATAGTTAAAAAATAAGACCGGCTACAATAAAATTATTTTCTTTTTCTTTAAAGAATTCGTCTTTG [ 6864]
lp36_PAbe_NX_Pl_consensus    TTTAAATTCTTTTATAGTTAAAAAATAAGACCGGCTACAATAAAATTATTTTCTTTTTCTTTAAAGAATTCGTCTTTG [ 6864]

lp36_gi|11496779|B31_GB      GAGTTCAAATTTAGCATTTTATTTATTTTTGCTTTACTAAGCTTAAAATGTTCTAAATAAAGTAAAATTAGAAAAATT [ 6942]
lp36_un11_B31_PacBio         GAGTTCAAATTTAGCATTTTATTTATTTTTGCTTTACTAAGCTTAAAATGTTCTAAATAAAGTAAAATTAGAAAAATT [ 6942]
lp36_B31_TS_MP_FG_consensus  GAGTTCAAATTTAGCATTTTATTTATTTTTGCTTTACTAAGCTTAAAATGTTCTAAATAAAGTAAAATTAGAAAAATT [ 6942]
lp36_B31_NX_Pl_consensus     GAGTTCAAATTTAGCATTTTATTTATTTTTGCTTTACTAAGCTTAAAATGTTCTAAATAAAGTAAAATTAGAAAAATT [ 6942]
lp36_PAli_NX_MP_FG_consensus GAGTTCAAATTTAGCATTTTATTTATTTTTGCTTTACTAAGCTTAAAATGTTCTAAATAAAGTAAAATTAGAAAAATT [ 6942]
lp36_PAli_NX_Pl_consensus    GAGTTCAAATTTAGCATTTTATTTATTTTTGCTTTACTAAGCTTAAAATGTTCTAAATAAAGTAAAATTAGAAAAATT [ 6942]
lp36_PAbe_NX_MP_FG_consensus GAGTTCAAATTTAGCATTTTATTTATTTTTGCTTTACTAAGCTTAAAATGTTCTAAATAAAGTAAAATTAGAAAAATT [ 6942]
lp36_PAbe_NX_Pl_consensus    GAGTTCAAATTTAGCATTTTATTTATTTTTGCTTTACTAAGCTTAAAATGTTCTAAATAAAGTAAAATTAGAAAAATT [ 6942]

lp36_gi|11496779|B31_GB      TTCAAGATCATTTTTGGATTATACATTTGTACCAATTCCACAAACTGTTTCTGCTGGGAATAACAAGCTCTCCCATTT [ 7020]
lp36_un11_B31_PacBio         TTCAAGATCATTTTTGGATTATACATTTGTACCAATTCCACAAACTGTTTCTGCTGGGAATAACAAGCTCTCCCATTT [ 7020]
lp36_B31_TS_MP_FG_consensus  TTCAAGATCATTTTTGGATTATACATTTGTACCAATTCCACAAACTGTTTCTGCTGGGAATAACAAGCTCTCCCATTT [ 7020]
lp36_B31_NX_Pl_consensus     TTCAAGATCATTTTTGGATTATACATTTGTACCAATTCCACAAACTGTTTCTGCTGGGAATAACAAGCTCTCCCATTT [ 7020]
lp36_PAli_NX_MP_FG_consensus TTCAAGATCATTTTTGGATTATACATTTGTACCAATTCCACAAACTGTTTCTGCTGGGAATAACAAGCTCTCCCATTT [ 7020]
lp36_PAli_NX_Pl_consensus    TTCAAGATCATTTTTGGATTATACATTTGTACCAATTCCACAAACTGTTTCTGCTGGGAATAACAAGCTCTCCCATTT [ 7020]
lp36_PAbe_NX_MP_FG_consensus TTCAAGATCATTTTTGGATTATACATTTGTACCAATTCCACAAACTGTTTCTGCTGGGAATAACAAGCTCTCCCATTT [ 7020]
lp36_PAbe_NX_Pl_consensus    TTCAAGATCATTTTTGGATTATACATTTGTACCAATTCCACAAACTGTTTCTGCTGGGAATAACAAGCTCTCCCATTT [ 7020]

lp36_gi|11496779|B31_GB      TTATAAGTTTTTAAGCCTTTATATTTGGATTGCTACGAATTAATGTTGTCAACATTATCTTTTAGTAGCATTAAAATT [ 7098]
lp36_un11_B31_PacBio         TTATAAGTTTTTAAGCCTTTATATTTGGATTGCTACGAATTAATGTTGTCAACATTATCTTTTAGTAGCATTAAAATT [ 7098]
lp36_B31_TS_MP_FG_consensus  TTATAAGTTTTTAAGCCTTTATATTTGGATTGCTACGAATTAATGTTGTCAACATTATCTTTTAGTAGCATTAAAATT [ 7098]
lp36_B31_NX_Pl_consensus     TTATAAGTTTTTAAGCCTTTATATTTGGATTGCTACGAATTAATGTTGTCAACATTATCTTTTAGTAGCATTAAAATT [ 7098]
lp36_PAli_NX_MP_FG_consensus TTATAAGTTTTTAAGCCTTTATATTTGGATTGCTACGAATTAATGTTGTCAACATTATCTTTTAGTAGCATTAAAATT [ 7098]
lp36_PAli_NX_Pl_consensus    TTATAAGTTTTTAAGCCTTTATATTTGGATTGCTACGAATTAATGTTGTCAACATTATCTTTTAGTAGCATTAAAATT [ 7098]
lp36_PAbe_NX_MP_FG_consensus TTATAAGTTTTTAAGCCTTTATATTTGGATTGCTACGAATTAATGTTGTCAACATTATCTTTTAGTAGCATTAAAATT [ 7098]
lp36_PAbe_NX_Pl_consensus    TTATAAGTTTTTAAGCCTTTATATTTGGATTGCTACGAATTAATGTTGTCAACATTATCTTTTAGTAGCATTAAAATT [ 7098]

lp36_gi|11496779|B31_GB      TCATTAATTTATAATTTTGTATACTTTTTAGGATTACTATTATCCTTTTAGGATCAATGATTTTAAATTTACTGCTAG [ 7176]
lp36_un11_B31_PacBio         TCATTAATTTATAATTTTGTATACTTTTTAGGATTACTATTATCCTTTTAGGATCAATGATTTTAAATTTACTGCTAG [ 7176]
lp36_B31_TS_MP_FG_consensus  TCATTAATTTATAATTTTGTATACTTTTTAGGATTACTATTATCCTTTTAGGATCAATGATTTTAAATTTACTGCTAG [ 7176]
lp36_B31_NX_Pl_consensus     TCATTAATTTATAATTTTGTATACTTTTTAGGATTACTATTATCCTTTTAGGATCAATGATTTTAAATTTACTGCTAG [ 7176]
lp36_PAli_NX_MP_FG_consensus TCATTAATTTATAATTTTGTATACTTTTTAGGATTACTATTATCCTTTTAGGATCAATGATTTTAAATTTACTGCTAG [ 7176]
lp36_PAli_NX_Pl_consensus    TCATTAATTTATAATTTTGTATACTTTTTAGGATTACTATTATCCTTTTAGGATCAATGATTTTAAATTTACTGCTAG [ 7176]
lp36_PAbe_NX_MP_FG_consensus TCATTAATTTATAATTTTGTATACTTTTTAGGATTACTATTATCCTTTTAGGATCAATGATTTTAAATTTACTGCTAG [ 7176]
lp36_PAbe_NX_Pl_consensus    TCATTAATTTATAATTTTGTATACTTTTTAGGATTACTATTATCCTTTTAGGATCAATGATTTTAAATTTACTGCTAG [ 7176]

lp36_gi|11496779|B31_GB      GTTTACTCTTCAATAAGGGAATCTTCAATTCTTTATCGCCGAAAGGAGATTGTTAACTAAATCAAAAATCTTCTTATC [ 7254]
lp36_un11_B31_PacBio         GTTTACTCTTCAATAAGGGAATCTTCAATTCTTTATCGCCGAAAGGAGATTGTTAACTAAATCAAAAATCTTCTTATC [ 7254]
lp36_B31_TS_MP_FG_consensus  GTTTACTCTTCAATAAGGGAATCTTCAATTCTTTATCGCCGAAAGGAGATTGTTAACTAAATCAAAAATCTTCTTATC [ 7254]
lp36_B31_NX_Pl_consensus     GTTTACTCTTCAATAAGGGAATCTTCAATTCTTTATCGCCGAAAGGAGATTGTTAACTAAATCAAAAATCTTCTTATC [ 7254]
lp36_PAli_NX_MP_FG_consensus GTTTACTCTTCAATAAGGGAATCTTCAATTCTTTATCGCCGAAAGGAGATTGTTAACTAAATCAAAAATCTTCTTATC [ 7254]
lp36_PAli_NX_Pl_consensus    GTTTACTCTTCAATAAGGGAATCTTCAATTCTTTATCGCCGAAAGGAGATTGTTAACTAAATCAAAAATCTTCTTATC [ 7254]
lp36_PAbe_NX_MP_FG_consensus GTTTACTCTTCAATAAGGGAATCTTCAATTCTTTATCGCCGAAAGGAGATTGTTAACTAAATCAAAAATCTTCTTATC [ 7254]
lp36_PAbe_NX_Pl_consensus    GTTTACTCTTCAATAAGGGAATCTTCAATTCTTTATCGCCGAAAGGAGATTGTTAACTAAATCAAAAATCTTCTTATC [ 7254]

lp36_gi|11496779|B31_GB      CCTGACTTTCACTTTTGAGGATTTTTATAGTCTAAGCTAAATATTTGCACTATAACTACACCGCCTAAAGGAGTTTAC [ 7332]
lp36_un11_B31_PacBio         CCTGACTTTCACTTTTGAGGATTTTTATAGTCTAAGCTAAATATTTGCACTATAACTACACCGCCTAAAGGAGTTTAC [ 7332]
lp36_B31_TS_MP_FG_consensus  CCTGACTTTCACTTTTGAGGATTTTTATAGTCTAAGCTAAATATTTGCACTATAACTACACCGCCTAAAGGAGTTTAC [ 7332]
lp36_B31_NX_Pl_consensus     CCTGACTTTCACTTTTGAGGATTTTTATAGTCTAAGCTAAATATTTGCACTATAACTACACCGCCTAAAGGAGTTTAC [ 7332]
lp36_PAli_NX_MP_FG_consensus CCTGACTTTCACTTTTGAGGATTTTTATAGTCTAAGCTAAATATTTGCACTATAACTACACCGCCTAAAGGAGTTTAC [ 7332]
lp36_PAli_NX_Pl_consensus    CCTGACTTTCACTTTTGAGGATTTTTATAGTCTAAGCTAAATATTTGCACTATAACTACACCGCCTAAAGGAGTTTAC [ 7332]
lp36_PAbe_NX_MP_FG_consensus CCTGACTTTCACTTTTGAGGATTTTTATAGTCTAAGCTAAATATTTGCACTATAACTACACCGCCTAAAGGAGTTTAC [ 7332]
lp36_PAbe_NX_Pl_consensus    CCTGACTTTCACTTTTGAGGATTTTTATAGTCTAAGCTAAATATTTGCACTATAACTACACCGCCTAAAGGAGTTTAC [ 7332]

lp36_gi|11496779|B31_GB      AAATGAGTAAACTAATATTGGCAATATCTATACTGCTAATAATTTCATGTAAATGGTATGTAGACAATACCATTGATG [ 7410]
```

```
lp36_un11_B31_PacBio         AAATGAGTAAACTAATATTGGCAATATCTATACTGCTAATAATTTCATGTAAATGGTATGTAGACAATACCATTGATG [ 7410]
lp36_B31_TS_MP_FG_consensus  AAATGAGTAAACTAATATTGGCAATATCTATACTGCTAATAATTTCATGTAAATGGTATGTAGACAATACCATTGATG [ 7410]
lp36_B31_NX_Pl_consensus     AAATGAGTAAACTAATATTGGCAATATCTATACTGCTAATAATTTCATGTAAATGGTATGTAGACAATACCATTGATG [ 7410]
lp36_PAli_NX_MP_FG_consensus AAATGAGTAAACTAATATTGGCAATATCTATACTGCTAATAATTTCATGTAAATGGTATGTAGACAATACCATTGATG [ 7410]
lp36_PAli_NX_Pl_consensus    AAATGAGTAAACTAATATTGGCAATATCTATACTGCTAATAATTTCATGTAAATGGTATGTAGACAATACCATTGATG [ 7410]
lp36_PAbe_NX_MP_FG_consensus AAATGAGTAAACTAATATTGGCAATATCTATACTGCTAATAATTTCATGTAAATGGTATGTAGACAATACCATTGATG [ 7410]
lp36_PAbe_NX_Pl_consensus    AAATGAGTAAACTAATATTGGCAATATCTATACTGCTAATAATTTCATGTAAATGGTATGTAGACAATACCATTGATG [ 7410]

lp36_gi|11496779|B31_GB      AAGCAACTGTAGAAAGTAAATCAGCACTAACATCTATTGATCAAGTATTAGATGAGATAAGTGAAGCCACAGGCCTAA [ 7488]
lp36_un11_B31_PacBio         AAGCAACTGTAGAAAGTAAATCAGCACTAACATCTATTGATCAAGTATTAGATGAGATAAGTGAAGCCACAGGCCTAA [ 7488]
lp36_B31_TS_MP_FG_consensus  AAGCAACTGTAGAAAGTAAATCAGCACTAACATCTATTGATCAAGTATTAGATGAGATAAGTGAAGCCACAGGCCTAA [ 7488]
lp36_B31_NX_Pl_consensus     AAGCAACTGTAGAAAGTAAATCAGCACTAACATCTATTGATCAAGTATTAGATGAGATAAGTGAAGCCACAGGCCTAA [ 7488]
lp36_PAli_NX_MP_FG_consensus AAGCAACTGTAGAAAGTAAATCAGCACTAACATCTATTGATCAAGTATTAGATGAGATAAGTGAAGCCACAGGCCTAA [ 7488]
lp36_PAli_NX_Pl_consensus    AAGCAACTGTAGAAAGTAAATCAGCACTAACATCTATTGATCAAGTATTAGATGAGATAAGTGAAGCCACAGGCCTAA [ 7488]
lp36_PAbe_NX_MP_FG_consensus AAGCAACTGTAGAAAGTAAATCAGCACTAACATCTATTGATCAAGTATTAGATGAGATAAGTGAAGCCACAGGCCTAA [ 7488]
lp36_PAbe_NX_Pl_consensus    AAGCAACTGTAGAAAGTAAATCAGCACTAACATCTATTGATCAAGTATTAGATGAGATAAGTGAAGCCACAGGCCTAA [ 7488]

lp36_gi|11496779|B31_GB      GTTCGGAAAAAATCACAAAATTAACTCCGGAAGAGCTAGAAAATTTAGCAAAGGAAGCTCAAGATGACTCTGAAAAAT [ 7566]
lp36_un11_B31_PacBio         GTTCGGAAAAAATCACAAAATTAACTCCGGAAGAGCTAGAAAATTTAGCAAAGGAAGCTCAAGATGACTCTGAAAAAT [ 7566]
lp36_B31_TS_MP_FG_consensus  GTTCGGAAAAAATCACAAAATTAACTCCGGAAGAGCTAGAAAATTTAGCAAAGGAAGCTCAAGATGACTCTGAAAAAT [ 7566]
lp36_B31_NX_Pl_consensus     GTTCGGAAAAAATCACAAAATTAACTCCGGAAGAGCTAGAAAATTTAGCAAAGGAAGCTCAAGATGACTCTGAAAAAT [ 7566]
lp36_PAli_NX_MP_FG_consensus GTTCGGAAAAAATCACAAAATTAACTCCGGAAGAGCTAGAAAATTTAGCAAAGGAAGCTCAAGATGACTCTGAAAAAT [ 7566]
lp36_PAli_NX_Pl_consensus    GTTCGGAAAAAATCACAAAATTAACTCCGGAAGAGCTAGAAAATTTAGCAAAGGAAGCTCAAGATGACTCTGAAAAAT [ 7566]
lp36_PAbe_NX_MP_FG_consensus GTTCGGAAAAAATCACAAAATTAACTCCGGAAGAGCTAGAAAATTTAGCAAAGGAAGCTCAAGATGACTCTGAAAAAT [ 7566]
lp36_PAbe_NX_Pl_consensus    GTTCGGAAAAAATCACAAAATTAACTCCGGAAGAGCTAGAAAATTTAGCAAAGGAAGCTCAAGATGACTCTGAAAAAT [ 7566]

lp36_gi|11496779|B31_GB      CCAAAAAAGAAATTGAAGATCAAAAAAATACCAAGGAAAGTAAAAACATAGAAGTAAAGGATACTCCTCGCTTAATCA [ 7644]
lp36_un11_B31_PacBio         CCAAAAAAGAAATTGAAGATCAAAAAAATACCAAGGAAAGTAAAAACATAGAAGTAAAGGATACTCCTCGCTTAATCA [ 7644]
lp36_B31_TS_MP_FG_consensus  CCAAAAAAGAAATTGAAGATCAAAAAAATACCAAGGAAAGTAAAAACATAGAAGTAAAGGATACTCCTCGCTTAATCA [ 7644]
lp36_B31_NX_Pl_consensus     CCAAAAAAGAAATTGAAGATCAAAAAAATACCAAGGAAAGTAAAAACATAGAAGTAAAGGATACTCCTCGCTTAATCA [ 7644]
lp36_PAli_NX_MP_FG_consensus CCAAAAAAGAAATTGAAGATCAAAAAAATACCAAGGAAAGTAAAAACATAGAAGTAAAGGATACTCCTCGCTTAATCA [ 7644]
lp36_PAli_NX_Pl_consensus    CCAAAAAAGAAATTGAAGATCAAAAAAATACCAAGGAAAGTAAAAACATAGAAGTAAAGGATACTCCTCGCTTAATCA [ 7644]
lp36_PAbe_NX_MP_FG_consensus CCAAAAAAGAAATTGAAGATCAAAAAAATACCAAGGAAAGTAAAAACATAGAAGTAAAGGATACTCCTCGCTTAATCA [ 7644]
lp36_PAbe_NX_Pl_consensus    CCAAAAAAGAAATTGAAGATCAAAAAAATACCAAGGAAAGTAAAAACATAGAAGTAAAGGATACTCCTCGCTTAATCA [ 7644]

lp36_gi|11496779|B31_GB      AATTGATAAAGAATTCATCAGAAAAAAATTGATTCGGTTTTTCAAACACTAATTAATATAGGTTATAATGCTACCTATG [ 7722]
lp36_un11_B31_PacBio         AATTGATAAAGAATTCATCAGAAAAAAATTGATTCGGTTTTTCAAACACTAATTAATATAGGTTATAATGCTACCTATG [ 7722]
lp36_B31_TS_MP_FG_consensus  AATTGATAAAGAATTCATCAGAAAAAAATTGATTCGGTTTTTCAAACACTAATTAATATAGGTTATAATGCTACCTATG [ 7722]
lp36_B31_NX_Pl_consensus     AATTGATAAAGAATTCATCAGAAAAAAATTGATTCGGTTTTTCAAACACTAATTAATATAGGTTATAATGCTACCTATG [ 7722]
lp36_PAli_NX_MP_FG_consensus AATTGATAAAGAATTCATCAGAAAAAAATTGATTCGGTTTTTCAAACACTAATTAATATAGGTTATAATGCTACCTATG [ 7722]
lp36_PAli_NX_Pl_consensus    AATTGATAAAGAATTCATCAGAAAAAAATTGATTCGGTTTTTCAAACACTAATTAATATAGGTTATAATGCTACCTATG [ 7722]
lp36_PAbe_NX_MP_FG_consensus AATTGATAAAGAATTCATCAGAAAAAAATTGATTCGGTTTTTCAAACACTAATTAATATAGGTTATAATGCTACCTATG [ 7722]
lp36_PAbe_NX_Pl_consensus    AATTGATAAAGAATTCATCAGAAAAAAATTGATTCGGTTTTTCAAACACTAATTAATATAGGTTATAATGCTACCTATG [ 7722]

lp36_gi|11496779|B31_GB      CAGCCAAAAGTAATTTGAAGAATGGACTAAAGATGGTGAAATTACTGGATGAGTTGCTAAAAATATCGGTAAGTAGCA [ 7800]
lp36_un11_B31_PacBio         CAGCCAAAAGTAATTTGAAGAATGGACTAAAGATGGTGAAATTACTGGATGAGTTGCTAAAAATATCGGTAAGTAGCA [ 7800]
lp36_B31_TS_MP_FG_consensus  CAGCCAAAAGTAATTTGAAGAATGGACTAAAGATGGTGAAATTACTGGATGAGTTGCTAAAAATATCGGTAAGTAGCA [ 7800]
lp36_B31_NX_Pl_consensus     CAGCCAAAAGTAATTTGAAGAATGGACTAAAGATGGTGAAATTACTGGATGAGTTGCTAAAAATATCGGTAAGTAGCA [ 7800]
lp36_PAli_NX_MP_FG_consensus CAGCCAAAAGTAATTTGAAGAATGGACTAAAGATGGTGAAATTACTGGATGAGTTGCTAAAAATATCGGTAAGTAGCA [ 7800]
lp36_PAli_NX_Pl_consensus    CAGCCAAAAGTAATTTGAAGAATGGACTAAAGATGGTGAAATTACTGGATGAGTTGCTAAAAATATCGGTAAGTAGCA [ 7800]
lp36_PAbe_NX_MP_FG_consensus CAGCCAAAAGTAATTTGAAGAATGGACTAAAGATGGTGAAATTACTGGATGAGTTGCTAAAAATATCGGTAAGTAGCA [ 7800]
lp36_PAbe_NX_Pl_consensus    CAGCCAAAAGTAATTTGAAGAATGGACTAAAGATGGTGAAATTACTGGATGAGTTGCTAAAAATATCGGTAAGTAGCA [ 7800]

lp36_gi|11496779|B31_GB      ATGGTGATAAAAGTACCCAAAAATACAATGAACTTAAAACCGTTGTAAATAAGTTTAATGCTGAAAATTCGGTAAGCG [ 7878]
lp36_un11_B31_PacBio         ATGGTGATAAAAGTACCCAAAAATACAATGAACTTAAAACCGTTGTAAATAAGTTTAATGCTGAAAATTCGGTAAGCG [ 7878]
lp36_B31_TS_MP_FG_consensus  ATGGTGATAAAAGTACCCAAAAATACAATGAACTTAAAACCGTTGTAAATAAGTTTAATGCTGAAAATTCGGTAAGCG [ 7878]
lp36_B31_NX_Pl_consensus     ATGGTGATAAAAGTACCCAAAAATACAATGAACTTAAAACCGTTGTAAATAAGTTTAATGCTGAAAATTCGGTAAGCG [ 7878]
lp36_PAli_NX_MP_FG_consensus ATGGTGATAAAAGTACCCAAAAATACAATGAACTTAAAACCGTTGTAAATAAGTTTAATGCTGAAAATTCGGTAAGCG [ 7878]
lp36_PAli_NX_Pl_consensus    ATGGTGATAAAAGTACCCAAAAATACAATGAACTTAAAACCGTTGTAAATAAGTTTAATGCTGAAAATTCGGTAAGCG [ 7878]
lp36_PAbe_NX_MP_FG_consensus ATGGTGATAAAAGTACCCAAAAATACAATGAACTTAAAACCGTTGTAAATAAGTTTAATGCTGAAAATTCGGTAAGCG [ 7878]
lp36_PAbe_NX_Pl_consensus    ATGGTGATAAAAGTACCCAAAAATACAATGAACTTAAAACCGTTGTAAATAAGTTTAATGCTGAAAATTCGGTAAGCG [ 7878]

lp36_gi|11496779|B31_GB      TTTCTTTTAAAGAACATTCAAACAGTAAAATTGAAACTAAAAAATGTATTCAAACTCTTATGAAAAATGTAGAAACAT [ 7956]
lp36_un11_B31_PacBio         TTTCTTTTAAAGAACATTCAAACAGTAAAATTGAAACTAAAAAATGTATTCAAACTCTTATGAAAAATGTAGAAACAT [ 7956]
lp36_B31_TS_MP_FG_consensus  TTTCTTTTAAAGAACATTCAAACAGTAAAATTGAAACTAAAAAATGTATTCAAACTCTTATGAAAAATGTAGAAACAT [ 7956]
lp36_B31_NX_Pl_consensus     TTTCTTTTAAAGAACATTCAAACAGTAAAATTGAAACTAAAAAATGTATTCAAACTCTTATGAAAAATGTAGAAACAT [ 7956]
lp36_PAli_NX_MP_FG_consensus TTTCTTTTAAAGAACATTCAAACAGTAAAATTGAAACTAAAAAATGTATTCAAACTCTTATGAAAAATGTAGAAACAT [ 7956]
lp36_PAli_NX_Pl_consensus    TTTCTTTTAAAGAACATTCAAACAGTAAAATTGAAACTAAAAAATGTATTCAAACTCTTATGAAAAATGTAGAAACAT [ 7956]
lp36_PAbe_NX_MP_FG_consensus TTTCTTTTAAAGAACATTCAAACAGTAAAATTGAAACTAAAAAATGTATTCAAACTCTTATGAAAAATGTAGAAACAT [ 7956]
lp36_PAbe_NX_Pl_consensus    TTTCTTTTAAAGAACATTCAAACAGTAAAATTGAAACTAAAAAATGTATTCAAACTCTTATGAAAAATGTAGAAACAT [ 7956]

lp36_gi|11496779|B31_GB      ACTTTGAAGGTGTATGCAGCGAACTTAAAAACAAAAATGATGGTGAGTACGAAAAAACATTGACAACTTTAAGCTAAG [ 8034]
lp36_un11_B31_PacBio         ACTTTGAAGGTGTATGCAGCGAACTTAAAAACAAAAATGATGGTGAGTACGAAAAAACATTGACAACTTTAAGCTAAG [ 8034]
lp36_B31_TS_MP_FG_consensus  ACTTTGAAGGTGTATGCAGCGAACTTAAAAACAAAAATGATGGTGAGTACGAAAAAACATTGACAACTTTAAGCTAAG [ 8034]
lp36_B31_NX_Pl_consensus     ACTTTGAAGGTGTATGCAGCGAACTTAAAAACAAAAATGATGGTGAGTACGAAAAAACATTGACAACTTTAAGCTAAG [ 8034]
lp36_PAli_NX_MP_FG_consensus ACTTTGAAGGTGTATGCAGCGAACTTAAAAACAAAAATGATGGTGAGTACGAAAAAACATTGACAACTTTAAGCTAAG [ 8034]
lp36_PAli_NX_Pl_consensus    ACTTTGAAGGTGTATGCAGCGAACTTAAAAACAAAAATGATGGTGAGTACGAAAAAACATTGACAACTTTAAGCTAAG [ 8034]
lp36_PAbe_NX_MP_FG_consensus ACTTTGAAGGTGTATGCAGCGAACTTAAAAACAAAAATGATGGTGAGTACGAAAAAACATTGACAACTTTAAGCTAAG [ 8034]
lp36_PAbe_NX_Pl_consensus    ACTTTGAAGGTGTATGCAGCGAACTTAAAAACAAAAATGATGGTGAGTACGAAAAAACATTGACAACTTTAAGCTAAG [ 8034]

lp36_gi|11496779|B31_GB      CAGCCAACAAGATAAAGAGTACAGCAATAAATATATATAGCTATGCTTCAATTAGGTGTGTAGATCGCGTCATGGTAT [ 8112]
lp36_un11_B31_PacBio         CAGCCAACAAGATAAAGAGTACAGCAATAAATATATATAGCTATGCTTCAATTAGGTGTGTAGATCGCGTCATGGTAT [ 8112]
lp36_B31_TS_MP_FG_consensus  CAGCCAACAAGATAAAGAGTACAGCAATAAATATATATAGCTATGCTTCAATTAGGTGTGTAGATCGCGTCATGGTAT [ 8112]
lp36_B31_NX_Pl_consensus     CAGCCAACAAGATAAAGAGTACAGCAATAAATATATATAGCTATGCTTCAATTAGGTGTGTAGATCGCGTCATGGTAT [ 8112]
lp36_PAli_NX_MP_FG_consensus CAGCCAACAAGATAAAGAGTACAGCAATAAATATATATAGCTATGCTTCAATTAGGTGTGTAGATCGCGTCATGGTAT [ 8112]
lp36_PAli_NX_Pl_consensus    CAGCCAACAAGATAAAGAGTACAGCAATAAATATATATAGCTATGCTTCAATTAGGTGTGTAGATCGCGTCATGGTAT [ 8112]
lp36_PAbe_NX_MP_FG_consensus CAGCCAACAAGATAAAGAGTACAGCAATAAATATATATAGCTATGCTTCAATTAGGTGTGTAGATCGCGTCATGGTAT [ 8112]
lp36_PAbe_NX_Pl_consensus    CAGCCAACAAGATAAAGAGTACAGCAATAAATATATATAGCTATGCTTCAATTAGGTGTGTAGATCGCGTCATGGTAT [ 8112]

lp36_gi|11496779|B31_GB      GAAACCGCAAGCTATTTATATGGGTTTTTAGAAAAAATAGTTCGCCAAATATCAATCCAAATAATAAGAAACGGTTGT [ 8190]
lp36_un11_B31_PacBio         GAAACCGCAAGCTATTTATATGGGTTTTTAGAAAAAATAGTTCGCCAAATATCAATCCAAATAATAAGAAACGGTTGT [ 8190]
lp36_B31_TS_MP_FG_consensus  GAAACCGCAAGCTATTTATATGGGTTTTTAGAAAAAATAGTTCGCCAAATATCAATCCAAATAATAAGAAACGGTTGT [ 8190]
lp36_B31_NX_Pl_consensus     GAAACCGCAAGCTATTTATATGGGTTTTTAGAAAAAATAGTTCGCCAAATATCAATCCAAATAATAAGAAACGGTTGT [ 8190]
lp36_PAli_NX_MP_FG_consensus GAAACCGCAAGCTATTTATATGGGTTTTTAGAAAAAATAGTTCGCCAAATATCAATCCAAATAATAAGAAACGGTTGT [ 8190]
lp36_PAli_NX_Pl_consensus    GAAACCGCAAGCTATTTATATGGGTTTTTAGAAAAAATAGTTCGCCAAATATCAATCCAAATAATAAGAAACGGTTGT [ 8190]
lp36_PAbe_NX_MP_FG_consensus GAAACCGCAAGCTATTTATATGGGTTTTTAGAAAAAATAGTTCGCCAAATATCAATCCAAATAATAAGAAACGGTTGT [ 8190]
lp36_PAbe_NX_Pl_consensus    GAAACCGCAAGCTATTTATATGGGTTTTTAGAAAAAATAGTTCGCCAAATATCAATCCAAATAATAAGAAACGGTTGT [ 8190]

lp36_gi|11496779|B31_GB      AACGATTCTTAATATTTTTTTTGGATAACGTTCTTGATCGCCCAAGCTTCTATCAATTGGAAGAAATTCAAAATATCC [ 8268]
lp36_un11_B31_PacBio         AACGATTCTTAATATTTTTTTTGGATAACGTTCTTGATCGCCCAAGCTTCTATCAATTGGAAGAAATTCAAAATATCC [ 8268]
lp36_B31_TS_MP_FG_consensus  AACGATTCTTAATATTTTTTTTGGATAACGTTCTTGATCGCCCAAGCTTCTATCAATTGGAAGAAATTCAAAATATCC [ 8268]
lp36_B31_NX_Pl_consensus     AACGATTCTTAATATTTTTTTTGGATAACGTTCTTGATCGCCCAAGCTTCTATCAATTGGAAGAAATTCAAAATATCC [ 8268]
lp36_PAli_NX_MP_FG_consensus AACGATTCTTAATATTTTTTTTGGATAACGTTCTTGATCGCCCAAGCTTCTATCAATTGGAAGAAATTCAAAATATCC [ 8268]
lp36_PAli_NX_Pl_consensus    AACGATTCTTAATATTTTTTTTGGATAACGTTCTTGATCGCCCAAGCTTCTATCAATTGGAAGAAATTCAAAATATCC [ 8268]
lp36_PAbe_NX_MP_FG_consensus AACGATTCTTAATATTTTTTTTGGATAACGTTCTTGATCGCCCAAGCTTCTATCAATTGGAAGAAATTCAAAATATCC [ 8268]
lp36_PAbe_NX_Pl_consensus    AACGATTCTTAATATTTTTTTTGGATAACGTTCTTGATCGCCCAAGCTTCTATCAATTGGAAGAAATTCAAAATATCC [ 8268]

lp36_gi|11496779|B31_GB      TTGATTTGCGTTTTTAATTTTCCCCAATTTTGAACTTGAATGTTTTGCAAATTCCAAAGCCGCTAATTTCGCATTTCT [ 8346]
lp36_un11_B31_PacBio         TTGATTTGCGTTTTTAATTTTCCCCAATTTTGAACTTGAATGTTTTGCAAATTCCAAAGCCGCTAATTTCGCATTTCT [ 8346]
```

```
lp36_B31_TS_MP_FG_consensus  TTGATTTGCGTTTTTAATTTTCCCCAATTTTGAACTTGAATGTTTTGCAAATTCCAAAGCCGCTAATTTCGCATTTCT [ 8346]
lp36_B31_NX_Pl_consensus     TTGATTTGCGTTTTTAATTTTCCCCAATTTTGAACTTGAATGTTTTGCAAATTCCAAAGCCGCTAATTTCGCATTTCT [ 8346]
lp36_PAli_NX_MP_FG_consensus TTGATTTGCGTTTTTAATTTTCCCCAATTTTGAACTTGAATGTTTTGCAAATTCCAAAGCCGCTAATTTCGCATTTCT [ 8346]
lp36_PAli_NX_Pl_consensus    TTGATTTGCGTTTTTAATTTTCCCCAATTTTGAACTTGAATGTTTTGCAAATTCCAAAGCCGCTAATTTCGCATTTCT [ 8346]
lp36_PAbe_NX_MP_FG_consensus TTGATTTGCGTTTTTAATTTTCCCCAATTTTGAACTTGAATGTTTTGCAAATTCCAAAGCCGCTAATTTCGCATTTCT [ 8346]
lp36_PAbe_NX_Pl_consensus    TTGATTTGCGTTTTTAATTTTCCCCAATTTTGAACTTGAATGTTTTGCAAATTCCAAAGCCGCTAATTTCGCATTTCT [ 8346]

lp36_gi|11496779|B31_GB      AATTGAATCTGCTAACATTTCGGGCTTTATATCATTAATATTGTCAAAGTAATATCTTGGCCCCCCACTATTACTAAT [ 8424]
lp36_un11_B31_PacBio         AATTGAATCTGCTAACATTTCGGGCTTTATATCATTAATATTGTCAAAGTAATATCTTGGCCCCCCACTATTACTAAT [ 8424]
lp36_B31_TS_MP_FG_consensus  AATTGAATCTGCTAACATTTCGGGCTTTATATCATTAATATTGTCAAAGTAATATCTTGGCCCCCCACTATTACTAAT [ 8424]
lp36_B31_NX_Pl_consensus     AATTGAATCTGCTAACATTTCGGGCTTTATATCATTAATATTGTCAAAGTAATATCTTGGCCCCCCACTATTACTAAT [ 8424]
lp36_PAli_NX_MP_FG_consensus AATTGAATCTGCTAACATTTCGGGCTTTATATCATTAATATTGTCAAAGTAATATCTTGGCCCCCCACTATTACTAAT [ 8424]
lp36_PAli_NX_Pl_consensus    AATTGAATCTGCTAACATTTCGGGCTTTATATCATTAATATTGTCAAAGTAATATCTTGGCCCCCCACTATTACTAAT [ 8424]
lp36_PAbe_NX_MP_FG_consensus AATTGAATCTGCTAACATTTCGGGCTTTATATCATTAATATTGTCAAAGTAATATCTTGGCCCCCCACTATTACTAAT [ 8424]
lp36_PAbe_NX_Pl_consensus    AATTGAATCTGCTAACATTTCGGGCTTTATATCATTAATATTGTCAAAGTAATATCTTGGCCCCCCACTATTACTAAT [ 8424]

lp36_gi|11496779|B31_GB      TAATATACCTTGATTATAAAGCTCAGCAATATTTTTTTCTGCTGCTTCCATTTTCTCAATATTTTTTGTATGAACAGT [ 8502]
lp36_un11_B31_PacBio         TAATATACCTTGATTATAAAGCTCAGCAATATTTTTTTCTGCTGCTTCCATTTTCTCAATATTTTTTGTATGAACAGT [ 8502]
lp36_B31_TS_MP_FG_consensus  TAATATACCTTGATTATAAAGCTCAGCAATATTTTTTTCTGCTGCTTCCATTTTCTCAATATTTTTTGTATGAACAGT [ 8502]
lp36_B31_NX_Pl_consensus     TAATATACCTTGATTATAAAGCTCAGCAATATTTTTTTCTGCTGCTTCCATTTTCTCAATATTTTTTGTATGAACAGT [ 8502]
lp36_PAli_NX_MP_FG_consensus TAATATACCTTGATTATAAAGCTCAGCAATATTTTTTTCTGCTGCTTCCATTTTCTCAATATTTTTTGTATGAACAGT [ 8502]
lp36_PAli_NX_Pl_consensus    TAATATACCTTGATTATAAAGCTCAGCAATATTTTTTTCTGCTGCTTCCATTTTCTCAATATTTTTTGTATGAACAGT [ 8502]
lp36_PAbe_NX_MP_FG_consensus TAATATACCTTGATTATAAAGCTCAGCAATATTTTTTTCTGCTGCTTCCATTTTCTCAATATTTTTTGTATGAACAGT [ 8502]
lp36_PAbe_NX_Pl_consensus    TAATATACCTTGATTATAAAGCTCAGCAATATTTTTTTCTGCTGCTTCCATTTTCTCAATATTTTTTGTATGAACAGT [ 8502]

lp36_gi|11496779|B31_GB      TAAAGATATATATGCTCTATACTTATAAAGAGATTCTTTGTAAGTCTCTTCATTAAATTCCATAAATCCCATTTTTAT [ 8580]
lp36_un11_B31_PacBio         TAAAGATATATATGCTCTATACTTATAAAGAGATTCTTTGTAAGTCTCTTCATTAAATTCCATAAATCCCATTTTTAT [ 8580]
lp36_B31_TS_MP_FG_consensus  TAAAGATATATATGCTCTATACTTATAAAGAGATTCTTTGTAAGTCTCTTCATTAAATTCCATAAATCCCATTTTTAT [ 8580]
lp36_B31_NX_Pl_consensus     TAAAGATATATATGCTCTATACTTATAAAGAGATTCTTTGTAAGTCTCTTCATTAAATTCCATAAATCCCATTTTTAT [ 8580]
lp36_PAli_NX_MP_FG_consensus TAAAGATATATATGCTCTATACTTATAAAGAGATTCTTTGTAAGTCTCTTCATTAAATTCCATAAATCCCATTTTTAT [ 8580]
lp36_PAli_NX_Pl_consensus    TAAAGATATATATGCTCTATACTTATAAAGAGATTCTTTGTAAGTCTCTTCATTAAATTCCATAAATCCCATTTTTAT [ 8580]
lp36_PAbe_NX_MP_FG_consensus TAAAGATATATATGCTCTATACTTATAAAGAGATTCTTTGTAAGTCTCTTCATTAAATTCCATAAATCCCATTTTTAT [ 8580]
lp36_PAbe_NX_Pl_consensus    TAAAGATATATATGCTCTATACTTATAAAGAGATTCTTTGTAAGTCTCTTCATTAAATTCCATAAATCCCATTTTTAT [ 8580]

lp36_gi|11496779|B31_GB      ATGGTCTTCGCTAAATCCATGTTTTAAAAAAAAACTTTTAATCTTCGATAAGCTTAAATTATTTGCTTTATTAATATC [ 8658]
lp36_un11_B31_PacBio         ATGGTCTTCGCTAAATCCATGTTTTAAAAAAAAACTTTTAATCTTCGATAAGCTTAAATTATTTGCTTTATTAATATC [ 8658]
lp36_B31_TS_MP_FG_consensus  ATGGTCTTCGCTAAATCCATGTTTTAAAAAAAAACTTTTAATCTTCGATAAGCTTAAATTATTTGCTTTATTAATATC [ 8658]
lp36_B31_NX_Pl_consensus     ATGGTCTTCGCTAAATCCATGTTTTAAAAAAAAACTTTTAATCTTCGATAAGCTTAAATTATTTGCTTTATTAATATC [ 8658]
lp36_PAli_NX_MP_FG_consensus ATGGTCTTCGCTAAATCCATGTTTTAAAAAAAAACTTTTAATCTTCGATAAGCTTAAATTATTTGCTTTATTAATATC [ 8658]
lp36_PAli_NX_Pl_consensus    ATGGTCTTCGCTAAATCCATGTTTTAAAAAAAAACTTTTAATCTTCGATAAGCTTAAATTATTTGCTTTATTAATATC [ 8658]
lp36_PAbe_NX_MP_FG_consensus ATGGTCTTCGCTAAATCCATGTTTTAAAAAAAAACTTTTAATCTTCGATAAGCTTAAATTATTTGCTTTATTAATATC [ 8658]
lp36_PAbe_NX_Pl_consensus    ATGGTCTTCGCTAAATCCATGTTTTAAAAAAAAACTTTTAATCTTCGATAAGCTTAAATTATTTGCTTTATTAATATC [ 8658]

lp36_gi|11496779|B31_GB      ATTTATAGTGTTACCGGTTAAGCTGTATCTAAGCCCCCAACTAGAAGACGTTGATAAAATTTCTCTTTCGCTAAGACC [ 8736]
lp36_un11_B31_PacBio         ATTTATAGTGTTACCGGTTAAGCTGTATCTAAGCCCCCAACTAGAAGACGTTGATAAAATTTCTCTTTCGCTAAGACC [ 8736]
lp36_B31_TS_MP_FG_consensus  ATTTATAGTGTTACCGGTTAAGCTGTATCTAAGCCCCCAACTAGAAGACGTTGATAAAATTTCTCTTTCGCTAAGACC [ 8736]
lp36_B31_NX_Pl_consensus     ATTTATAGTGTTACCGGTTAAGCTGTATCTAAGCCCCCAACTAGAAGACGTTGATAAAATTTCTCTTTCGCTAAGACC [ 8736]
lp36_PAli_NX_MP_FG_consensus ATTTATAGTGTTACCGGTTAAGCTGTATCTAAGCCCCCAACTAGAAGACGTTGATAAAATTTCTCTTTCGCTAAGACC [ 8736]
lp36_PAli_NX_Pl_consensus    ATTTATAGTGTTACCGGTTAAGCTGTATCTAAGCCCCCAACTAGAAGACGTTGATAAAATTTCTCTTTCGCTAAGACC [ 8736]
lp36_PAbe_NX_MP_FG_consensus ATTTATAGTGTTACCGGTTAAGCTGTATCTAAGCCCCCAACTAGAAGACGTTGATAAAATTTCTCTTTCGCTAAGACC [ 8736]
lp36_PAbe_NX_Pl_consensus    ATTTATAGTGTTACCGGTTAAGCTGTATCTAAGCCCCCAACTAGAAGACGTTGATAAAATTTCTCTTTCGCTAAGACC [ 8736]

lp36_gi|11496779|B31_GB      CTTTACCGTAATATAATTTTCATTTTTGGTGCCAATGTTTTTTATTCCGTGAGAAATTATAATTGATGATATAAACAA [ 8814]
lp36_un11_B31_PacBio         CTTTACCGTAATATAATTTTCATTTTTGGTGCCAATGTTTTTTATTCCGTGAGAAATTATAATTGATGATATAAACAA [ 8814]
lp36_B31_TS_MP_FG_consensus  CTTTACCGTAATATAATTTTCATTTTTGGTGCCAATGTTTTTTATTCCGTGAGAAATTATAATTGATGATATAAACAA [ 8814]
lp36_B31_NX_Pl_consensus     CTTTACCGTAATATAATTTTCATTTTTGGTGCCAATGTTTTTTATTCCGTGAGAAATTATAATTGATGATATAAACAA [ 8814]
lp36_PAli_NX_MP_FG_consensus CTTTACCGTAATATAATTTTCATTTTTGGTGCCAATGTTTTTTATTCCGTGAGAAATTATAATTGATGATATAAACAA [ 8814]
lp36_PAli_NX_Pl_consensus    CTTTACCGTAATATAATTTTCATTTTTGGTGCCAATGTTTTTTATTCCGTGAGAAATTATAATTGATGATATAAACAA [ 8814]
lp36_PAbe_NX_MP_FG_consensus CTTTACCGTAATATAATTTTCATTTTTGGTGCCAATGTTTTTTATTCCGTGAGAAATTATAATTGATGATATAAACAA [ 8814]
lp36_PAbe_NX_Pl_consensus    CTTTACCGTAATATAATTTTCATTTTTGGTGCCAATGTTTTTTATTCCGTGAGAAATTATAATTGATGATATAAACAA [ 8814]

lp36_gi|11496779|B31_GB      AAGTAACGAAAATAACAAGAAATATATTTCTTTTCCTCTAAACATAGATTGACCTCCTAAAAGCAATAATATATTAAA [ 8892]
lp36_un11_B31_PacBio         AAGTAACGAAAATAACAAGAAATATATTTCTTTTCCTCTAAACATAGATTGACCTCCTAAAAGCAATAATATATTAAA [ 8892]
lp36_B31_TS_MP_FG_consensus  AAGTAACGAAAATAACAAGAAATATATTTCTTTTCCTCTAAACATAGATTGACCTCCTAAAAGCAATAATATATTAAA [ 8892]
lp36_B31_NX_Pl_consensus     AAGTAACGAAAATAACAAGAAATATATTTCTTTTCCTCTAAACATAGATTGACCTCCTAAAAGCAATAATATATTAAA [ 8892]
lp36_PAli_NX_MP_FG_consensus AAGTAACGAAAATAACAAGAAATATATTTCTTTTCCTCTAAACATAGATTGACCTCCTAAAAGCAATAATATATTAAA [ 8892]
lp36_PAli_NX_Pl_consensus    AAGTAACGAAAATAACAAGAAATATATTTCTTTTCCTCTAAACATAGATTGACCTCCTAAAAGCAATAATATATTAAA [ 8892]
lp36_PAbe_NX_MP_FG_consensus AAGTAACGAAAATAACAAGAAATATATTTCTTTTCCTCTAAACATAGATTGACCTCCTAAAAGCAATAATATATTAAA [ 8892]
lp36_PAbe_NX_Pl_consensus    AAGTAACGAAAATAACAAGAAATATATTTCTTTTCCTCTAAACATAGATTGACCTCCTAAAAGCAATAATATATTAAA [ 8892]

lp36_gi|11496779|B31_GB      TTACTGTATTTAAATTTATTTTAACATATTGCTTAATATTAATAAATCCGAATACATTTTAGTAGATCATAATTTTAT [ 8970]
lp36_un11_B31_PacBio         TTACTGTATTTAAATTTATTTTAACATATTGCTTAATATTAATAAATCCGAATACATTTTAGTAGATCATAATTTTAT [ 8970]
lp36_B31_TS_MP_FG_consensus  TTACTGTATTTAAATTTATTTTAACATATTGCTTAATATTAATAAATCCGAATACATTTTAGTAGATCATAATTTTAT [ 8970]
lp36_B31_NX_Pl_consensus     TTACTGTATTTAAATTTATTTTAACATATTGCTTAATATTAATAAATCCGAATACATTTTAGTAGATCATAATTTTAT [ 8970]
lp36_PAli_NX_MP_FG_consensus TTACTGTATTTAAATTTATTTTAACATATTGCTTAATATTAATAAATCCGAATACATTTTAGTAGATCATAATTTTAT [ 8970]
lp36_PAli_NX_Pl_consensus    TTACTGTATTTAAATTTATTTTAACATATTGCTTAATATTAATAAATCCGAATACATTTTAGTAGATCATAATTTTAT [ 8970]
lp36_PAbe_NX_MP_FG_consensus TTACTGTATTTAAATTTATTTTAACATATTGCTTAATATTAATAAATCCGAATACATTTTAGTAGATCATAATTTTAT [ 8970]
lp36_PAbe_NX_Pl_consensus    TTACTGTATTTAAATTTATTTTAACATATTGCTTAATATTAATAAATCCGAATACATTTTAGTAGATCATAATTTTAT [ 8970]

lp36_gi|11496779|B31_GB      GACCTTGCTAAAGACAATGTTTTATAAAAAAGAAAAGCCAAGATAGCTGCATAATTTAAGTTAATTGAAGCAATTAAA [ 9048]
lp36_un11_B31_PacBio         GACCTTGCTAAAGACAATGTTTTATAAAAAAGAAAAGCCAAGATAGCTGCATAATTTAAGTTAATTGAAGCAATTAAA [ 9048]
lp36_B31_TS_MP_FG_consensus  GACCTTGCTAAAGACAATGTTTTATAAAAAAGAAAAGCCAAGATAGCTGCATAATTTAAGTTAATTGAAGCAATTAAA [ 9048]
lp36_B31_NX_Pl_consensus     GACCTTGCTAAAGACAATGTTTTATAAAAAAGAAAAGCCAAGATAGCTGCATAATTTAAGTTAATTGAAGCAATTAAA [ 9048]
lp36_PAli_NX_MP_FG_consensus GACCTTGCTAAAGACAATGTTTTATAAAAAAGAAAAGCCAAGATAGCTGCATAATTTAAGTTAATTGAAGCAATTAAA [ 9048]
lp36_PAli_NX_Pl_consensus    GACCTTGCTAAAGACAATGTTTTATAAAAAAGAAAAGCCAAGATAGCTGCATAATTTAAGTTAATTGAAGCAATTAAA [ 9048]
lp36_PAbe_NX_MP_FG_consensus GACCTTGCTAAAGACAATGTTTTATAAAAAAGAAAAGCCAAGATAGCTGCATAATTTAAGTTAATTGAAGCAATTAAA [ 9048]
lp36_PAbe_NX_Pl_consensus    GACCTTGCTAAAGACAATGTTTTATAAAAAAGAAAAGCCAAGATAGCTGCATAATTTAAGTTAATTGAAGCAATTAAA [ 9048]

lp36_gi|11496779|B31_GB      AGCCATCATGATATTAAAGCAAGAAGTTATTTTATAATCTTGTGTTTAGAAATAGCACATAATTATTTAACATTTCAA [ 9126]
lp36_un11_B31_PacBio         AGCCATCATGATATTAAAGCAAGAAGTTATTTTATAATCTTGTGTTTAGAAATAGCACATAATTATTTAACATTTCAA [ 9126]
lp36_B31_TS_MP_FG_consensus  AGCCATCATGATATTAAAGCAAGAAGTTATTTTATAATCTTGTGTTTAGAAATAGCACATAATTATTTAACATTTCAA [ 9126]
lp36_B31_NX_Pl_consensus     AGCCATCATGATATTAAAGCAAGAAGTTATTTTATAATCTTGTGTTTAGAAATAGCACATAATTATTTAACATTTCAA [ 9126]
lp36_PAli_NX_MP_FG_consensus AGCCATCATGATATTAAAGCAAGAAGTTATTTTATAATCTTGTGTTTAGAAATAGCACATAATTATTTAACATTTCAA [ 9126]
lp36_PAli_NX_Pl_consensus    AGCCATCATGATATTAAAGCAAGAAGTTATTTTATAATCTTGTGTTTAGAAATAGCACATAATTATTTAACATTTCAA [ 9126]
lp36_PAbe_NX_MP_FG_consensus AGCCATCATGATATTAAAGCAAGAAGTTATTTTATAATCTTGTGTTTAGAAATAGCACATAATTATTTAACATTTCAA [ 9126]
lp36_PAbe_NX_Pl_consensus    AGCCATCATGATATTAAAGCAAGAAGTTATTTTATAATCTTGTGTTTAGAAATAGCACATAATTATTTAACATTTCAA [ 9126]

lp36_gi|11496779|B31_GB      AATATTGCAAAAAAATCATTTTTAAATTATATTTAATTTTATAATGATTACTATTTAATATTTATTATAAGTTTGTAT [ 9204]
lp36_un11_B31_PacBio         AATATTGCAAAAAAATCATTTTTAAATTATATTTAATTTTATAATGATTACTATTTAATATTTATTATAAGTTTGTAT [ 9204]
lp36_B31_TS_MP_FG_consensus  AATATTGCAAAAAAATCATTTTTAAATTATATTTAATTTTATAATGATTACTATTTAATATTTATTATAAGTTTGTAT [ 9204]
lp36_B31_NX_Pl_consensus     AATATTGCAAAAAAATCATTTTTAAATTATATTTAATTTTATAATGATTACTATTTAATATTTATTATAAGTTTGTAT [ 9204]
lp36_PAli_NX_MP_FG_consensus AATATTGCAAAAAAATCATTTTTAAATTATATTTAATTTTATAATGATTACTATTTAATATTTATTATAAGTTTGTAT [ 9204]
lp36_PAli_NX_Pl_consensus    AATATTGCAAAAAAATCATTTTTAAATTATATTTAATTTTATAATGATTACTATTTAATATTTATTATAAGTTTGTAT [ 9204]
lp36_PAbe_NX_MP_FG_consensus AATATTGCAAAAAAATCATTTTTAAATTATATTTAATTTTATAATGATTACTATTTAATATTTATTATAAGTTTGTAT [ 9204]
lp36_PAbe_NX_Pl_consensus    AATATTGCAAAAAAATCATTTTTAAATTATATTTAATTTTATAATGATTACTATTTAATATTTATTATAAGTTTGTAT [ 9204]

lp36_gi|11496779|B31_GB      TATGGCTTTGCAGATATTCTGTAAAACCATTTTAAGGGCTTTATTTATGAAAAAAAATTAATATTATTAATCAATATT [ 9282]
lp36_un11_B31_PacBio         TATGGCTTTGCAGATATTCTGTAAAACCATTTTAAGGGCTTTATTTATGAAAAAAAATTAATATTATTAATCAATATT [ 9282]
lp36_B31_TS_MP_FG_consensus  TATGGCTTTGCAGATATTCTGTAAAACCATTTTAAGGGCTTTATTTATGAAAAAAAATTAATATTATTAATCAATATT [ 9282]
```

```
lp36_B31_NX_Pl_consensus      TATGGCTTTGCAGATATTCTGTAAAACCATTTTAAGGGCTTTATTTATGAAAAAAAATTAATATTATTAATCAATATT [ 9282]
lp36_PAli_NX_MP_FG_consensus  TATGGCTTTGCAGATATTCTGTAAAACCATTTTAAGGGCTTTATTTATGAAAAAAAATTAATATTATTAATCAATATT [ 9282]
lp36_PAli_NX_Pl_consensus     TATGGCTTTGCAGATATTCTGTAAAACCATTTTAAGGGCTTTATTTATGAAAAAAAATTAATATTATTAATCAATATT [ 9282]
lp36_PAbe_NX_MP_FG_consensus  TATGGCTTTGCAGATATTCTGTAAAACCATTTTAAGGGCTTTATTTATGAAAAAAAATTAATATTATTAATCAATATT [ 9282]
lp36_PAbe_NX_Pl_consensus     TATGGCTTTGCAGATATTCTGTAAAACCATTTTAAGGGCTTTATTTATGAAAAAAAATTAATATTATTAATCAATATT [ 9282]

lp36_gi|11496779|B31_GB       TTTTTATTTATTTCATATCAAAACGATGGTTTAAATAAAAATAAGGCTAGCAAAAATCATAATTATAAAAAACAACTT [ 9360]
lp36_un11_B31_PacBio          TTTTTATTTATTTCATATCAAAACGATGGTTTAAATAAAAATAAGGCTAGCAAAAATCATAATTATAAAAAACAACTT [ 9360]
lp36_B31_TS_MP_FG_consensus   TTTTTATTTATTTCATATCAAAACGATGGTTTAAATAAAAATAAGGCTAGCAAAAATCATAATTATAAAAAACAACTT [ 9360]
lp36_B31_NX_Pl_consensus      TTTTTATTTATTTCATATCAAAACGATGGTTTAAATAAAAATAAGGCTAGCAAAAATCATAATTATAAAAAACAACTT [ 9360]
lp36_PAli_NX_MP_FG_consensus  TTTTTATTTATTTCATATCAAAACGATGGTTTAAATAAAAATAAGGCTAGCAAAAATCATAATTATAAAAAACAACTT [ 9360]
lp36_PAli_NX_Pl_consensus     TTTTTATTTATTTCATATCAAAACGATGGTTTAAATAAAAATAAGGCTAGCAAAAATCATAATTATAAAAAACAACTT [ 9360]
lp36_PAbe_NX_MP_FG_consensus  TTTTTATTTATTTCATATCAAAACGATGGTTTAAATAAAAATAAGGCTAGCAAAAATCATAATTATAAAAAACAACTT [ 9360]
lp36_PAbe_NX_Pl_consensus     TTTTTATTTATTTCATATCAAAACGATGGTTTAAATAAAAATAAGGCTAGCAAAAATCATAATTATAAAAAACAACTT [ 9360]

lp36_gi|11496779|B31_GB       GGGAAAAAAATATGTGCGGGGAGAAAAGAATGAACTTTTATCTTGGATAAATAATTTTGTAGAAGTTAATAATAAAGCC [ 9438]
lp36_un11_B31_PacBio          GGGAAAAAAATATGTGCGGGGAGAAAAGAATGAACTTTTATCTTGGATAAATAATTTTGTAGAAGTTAATAATAAAGCC [ 9438]
lp36_B31_TS_MP_FG_consensus   GGGAAAAAAATATGTGCGGGGAGAAAAGAATGAACTTTTATCTTGGATAAATAATTTTGTAGAAGTTAATAATAAAGCC [ 9438]
lp36_B31_NX_Pl_consensus      GGGAAAAAAATATGTGCGGGGAGAAAAGAATGAACTTTTATCTTGGATAAATAATTTTGTAGAAGTTAATAATAAAGCC [ 9438]
lp36_PAli_NX_MP_FG_consensus  GGGAAAAAAATATGTGCGGGGAGAAAAGAATGAACTTTTATCTTGGATAAATAATTTTGTAGAAGTTAATAATAAAGCC [ 9438]
lp36_PAli_NX_Pl_consensus     GGGAAAAAAATATGTGCGGGGAGAAAAGAATGAACTTTTATCTTGGATAAATAATTTTGTAGAAGTTAATAATAAAGCC [ 9438]
lp36_PAbe_NX_MP_FG_consensus  GGGAAAAAAATATGTGCGGGGAGAAAAGAATGAACTTTTATCTTGGATAAATAATTTTGTAGAAGTTAATAATAAAGCC [ 9438]
lp36_PAbe_NX_Pl_consensus     GGGAAAAAAATATGTGCGGGGAGAAAAGAATGAACTTTTATCTTGGATAAATAATTTTGTAGAAGTTAATAATAAAGCC [ 9438]

lp36_gi|11496779|B31_GB       ATAGATAATTCTTTTGAAAAATTCAAAAGTGAGCCATCTAATCAATATAATCTTCCATTTAAAAATGTTAGTTGGTAT [ 9516]
lp36_un11_B31_PacBio          ATAGATAATTCTTTTGAAAAATTCAAAAGTGAGCCATCTAATCAATATAATCTTCCATTTAAAAATGTTAGTTGGTAT [ 9516]
lp36_B31_TS_MP_FG_consensus   ATAGATAATTCTTTTGAAAAATTCAAAAGTGAGCCATCTAATCAATATAATCTTCCATTTAAAAATGTTAGTTGGTAT [ 9516]
lp36_B31_NX_Pl_consensus      ATAGATAATTCTTTTGAAAAATTCAAAAGTGAGCCATCTAATCAATATAATCTTCCATTTAAAAATGTTAGTTGGTAT [ 9516]
lp36_PAli_NX_MP_FG_consensus  ATAGATAATTCTTTTGAAAAATTCAAAAGTGAGCCATCTAATCAATATAATCTTCCATTTAAAAATGTTAGTTGGTAT [ 9516]
lp36_PAli_NX_Pl_consensus     ATAGATAATTCTTTTGAAAAATTCAAAAGTGAGCCATCTAATCAATATAATCTTCCATTTAAAAATGTTAGTTGGTAT [ 9516]
lp36_PAbe_NX_MP_FG_consensus  ATAGATAATTCTTTTGAAAAATTCAAAAGTGAGCCATCTAATCAATATAATCTTCCATTTAAAAATGTTAGTTGGTAT [ 9516]
lp36_PAbe_NX_Pl_consensus     ATAGATAATTCTTTTGAAAAATTCAAAAGTGAGCCATCTAATCAATATAATCTTCCATTTAAAAATGTTAGTTGGTAT [ 9516]

lp36_gi|11496779|B31_GB       ACACTAAGGAAAACACTTGCTGATAATGTAGAAGAATCTAAAAAGGCTAGAGGGTGTTTGTATGCGGTTTTAGATTAC [ 9594]
lp36_un11_B31_PacBio          ACACTAAGGAAAACACTTGCTGATAATGTAGAAGAATCTAAAAAGGCTAGAGGGTGTTTGTATGCGGTTTTAGATTAC [ 9594]
lp36_B31_TS_MP_FG_consensus   ACACTAAGGAAAACACTTGCTGATAATGTAGAAGAATCTAAAAAGGCTAGAGGGTGTTTGTATGCGGTTTTAGATTAC [ 9594]
lp36_B31_NX_Pl_consensus      ACACTAAGGAAAACACTTGCTGATAATGTAGAAGAATCTAAAAAGGCTAGAGGGTGTTTGTATGCGGTTTTAGATTAC [ 9594]
lp36_PAli_NX_MP_FG_consensus  ACACTAAGGAAAACACTTGCTGATAATGTAGAAGAATCTAAAAAGGCTAGAGGGTGTTTGTATGCGGTTTTAGATTAC [ 9594]
lp36_PAli_NX_Pl_consensus     ACACTAAGGAAAACACTTGCTGATAATGTAGAAGAATCTAAAAAGGCTAGAGGGTGTTTGTATGCGGTTTTAGATTAC [ 9594]
lp36_PAbe_NX_MP_FG_consensus  ACACTAAGGAAAACACTTGCTGATAATGTAGAAGAATCTAAAAAGGCTAGAGGGTGTTTGTATGCGGTTTTAGATTAC [ 9594]
lp36_PAbe_NX_Pl_consensus     ACACTAAGGAAAACACTTGCTGATAATGTAGAAGAATCTAAAAAGGCTAGAGGGTGTTTGTATGCGGTTTTAGATTAC [ 9594]

lp36_gi|11496779|B31_GB       AATGAAAGCGAGTTGAAAGAATTAGGATTAATTTTAAATTCAAATTTACATAGCATTAAGAAGATTGCTAATAAAATT [ 9672]
lp36_un11_B31_PacBio          AATGAAAGCGAGTTGAAAGAATTAGGATTAATTTTAAATTCAAATTTACATAGCATTAAGAAGATTGCTAATAAAATT [ 9672]
lp36_B31_TS_MP_FG_consensus   AATGAAAGCGAGTTGAAAGAATTAGGATTAATTTTAAATTCAAATTTACATAGCATTAAGAAGATTGCTAATAAAATT [ 9672]
lp36_B31_NX_Pl_consensus      AATGAAAGCGAGTTGAAAGAATTAGGATTAATTTTAAATTCAAATTTACATAGCATTAAGAAGATTGCTAATAAAATT [ 9672]
lp36_PAli_NX_MP_FG_consensus  AATGAAAGCGAGTTGAAAGAATTAGGATTAATTTTAAATTCAAATTTACATAGCATTAAGAAGATTGCTAATAAAATT [ 9672]
lp36_PAli_NX_Pl_consensus     AATGAAAGCGAGTTGAAAGAATTAGGATTAATTTTAAATTCAAATTTACATAGCATTAAGAAGATTGCTAATAAAATT [ 9672]
lp36_PAbe_NX_MP_FG_consensus  AATGAAAGCGAGTTGAAAGAATTAGGATTAATTTTAAATTCAAATTTACATAGCATTAAGAAGATTGCTAATAAAATT [ 9672]
lp36_PAbe_NX_Pl_consensus     AATGAAAGCGAGTTGAAAGAATTAGGATTAATTTTAAATTCAAATTTACATAGCATTAAGAAGATTGCTAATAAAATT [ 9672]

lp36_gi|11496779|B31_GB       ATGCAAAATGCGGGCATAGAATTCCAATTTAGATTTGAGGCAATAATGAGGCTGTTACTTGATAAAAAAAAACAACCTA [ 9750]
lp36_un11_B31_PacBio          ATGCAAAATGCGGGCATAGAATTCCAATTTAGATTTGAGGCAATAATGAGGCTGTTACTTGATAAAAAAAAACAACCTA [ 9750]
lp36_B31_TS_MP_FG_consensus   ATGCAAAATGCGGGCATAGAATTCCAATTTAGATTTGAGGCAATAATGAGGCTGTTACTTGATAAAAAAAAACAACCTA [ 9750]
lp36_B31_NX_Pl_consensus      ATGCAAAATGCGGGCATAGAATTCCAATTTAGATTTGAGGCAATAATGAGGCTGTTACTTGATAAAAAAAAACAACCTA [ 9750]
lp36_PAli_NX_MP_FG_consensus  ATGCAAAATGCGGGCATAGAATTCCAATTTAGATTTGAGGCAATAATGAGGCTGTTACTTGATAAAAAAAAACAACCTA [ 9750]
lp36_PAli_NX_Pl_consensus     ATGCAAAATGCGGGCATAGAATTCCAATTTAGATTTGAGGCAATAATGAGGCTGTTACTTGATAAAAAAAAACAACCTA [ 9750]
lp36_PAbe_NX_MP_FG_consensus  ATGCAAAATGCGGGCATAGAATTCCAATTTAGATTTGAGGCAATAATGAGGCTGTTACTTGATAAAAAAAAACAACCTA [ 9750]
lp36_PAbe_NX_Pl_consensus     ATGCAAAATGCGGGCATAGAATTCCAATTTAGATTTGAGGCAATAATGAGGCTGTTACTTGATAAAAAAAAACAACCTA [ 9750]

lp36_gi|11496779|B31_GB       ATGTTATTAAATATTCCTGATCTTAAAAGGATTAAAATTTGTCTTGAAAATTTTTTATCTTTAAGAACAACCTTTAGA [ 9828]
lp36_un11_B31_PacBio          ATGTTATTAAATATTCCTGATCTTAAAAGGATTAAAATTTGTCTTGAAAATTTTTTATCTTTAAGAACAACCTTTAGA [ 9828]
lp36_B31_TS_MP_FG_consensus   ATGTTATTAAATATTCCTGATCTTAAAAGGATTAAAATTTGTCTTGAAAATTTTTTATCTTTAAGAACAACCTTTAGA [ 9828]
lp36_B31_NX_Pl_consensus      ATGTTATTAAATATTCCTGATCTTAAAAGGATTAAAATTTGTCTTGAAAATTTTTTATCTTTAAGAACAACCTTTAGA [ 9828]
lp36_PAli_NX_MP_FG_consensus  ATGTTATTAAATATTCCTGATCTTAAAAGGATTAAAATTTGTCTTGAAAATTTTTTATCTTTAAGAACAACCTTTAGA [ 9828]
lp36_PAli_NX_Pl_consensus     ATGTTATTAAATATTCCTGATCTTAAAAGGATTAAAATTTGTCTTGAAAATTTTTTATCTTTAAGAACAACCTTTAGA [ 9828]
lp36_PAbe_NX_MP_FG_consensus  ATGTTATTAAATATTCCTGATCTTAAAAGGATTAAAATTTGTCTTGAAAATTTTTTATCTTTAAGAACAACCTTTAGA [ 9828]
lp36_PAbe_NX_Pl_consensus     ATGTTATTAAATATTCCTGATCTTAAAAGGATTAAAATTTGTCTTGAAAATTTTTTATCTTTAAGAACAACCTTTAGA [ 9828]

lp36_gi|11496779|B31_GB       GAGCTTGTTAAACAATTATTAAATGATTATAACAGCAATAAAGAGTCCATCAAGTCTGAAAATATCAAATTGGGAGAT [ 9906]
lp36_un11_B31_PacBio          GAGCTTGTTAAACAATTATTAAATGATTATAACAGCAATAAAGAGTCCATCAAGTCTGAAAATATCAAATTGGGAGAT [ 9906]
lp36_B31_TS_MP_FG_consensus   GAGCTTGTTAAACAATTATTAAATGATTATAACAGCAATAAAGAGTCCATCAAGTCTGAAAATATCAAATTGGGAGAT [ 9906]
lp36_B31_NX_Pl_consensus      GAGCTTGTTAAACAATTATTAAATGATTATAACAGCAATAAAGAGTCCATCAAGTCTGAAAATATCAAATTGGGAGAT [ 9906]
lp36_PAli_NX_MP_FG_consensus  GAGCTTGTTAAACAATTATTAAATGATTATAACAGCAATAAAGAGTCCATCAAGTCTGAAAATATCAAATTGGGAGAT [ 9906]
lp36_PAli_NX_Pl_consensus     GAGCTTGTTAAACAATTATTAAATGATTATAACAGCAATAAAGAGTCCATCAAGTCTGAAAATATCAAATTGGGAGAT [ 9906]
lp36_PAbe_NX_MP_FG_consensus  GAGCTTGTTAAACAATTATTAAATGATTATAACAGCAATAAAGAGTCCATCAAGTCTGAAAATATCAAATTGGGAGAT [ 9906]
lp36_PAbe_NX_Pl_consensus     GAGCTTGTTAAACAATTATTAAATGATTATAACAGCAATAAAGAGTCCATCAAGTCTGAAAATATCAAATTGGGAGAT [ 9906]

lp36_gi|11496779|B31_GB       TATTTAAATAAAGCTTTTTCAGATATTGTTTTAAGCATGGATAAAAATCCTATAAATCTAGAAAAAGAGATTCGAAAT [ 9984]
lp36_un11_B31_PacBio          TATTTAAATAAAGCTTTTTCAGATATTGTTTTAAGCATGGATAAAAATCCTATAAATCTAGAAAAAGAGATTCGAAAT [ 9984]
lp36_B31_TS_MP_FG_consensus   TATTTAAATAAAGCTTTTTCAGATATTGTTTTAAGCATGGATAAAAATCCTATAAATCTAGAAAAAGAGATTCGAAAT [ 9984]
lp36_B31_NX_Pl_consensus      TATTTAAATAAAGCTTTTTCAGATATTGTTTTAAGCATGGATAAAAATCCTATAAATCTAGAAAAAGAGATTCGAAAT [ 9984]
lp36_PAli_NX_MP_FG_consensus  TATTTAAATAAAGCTTTTTCAGATATTGTTTTAAGCATGGATAAAAATCCTATAAATCTAGAAAAAGAGATTCGAAAT [ 9984]
lp36_PAli_NX_Pl_consensus     TATTTAAATAAAGCTTTTTCAGATATTGTTTTAAGCATGGATAAAAATCCTATAAATCTAGAAAAAGAGATTCGAAAT [ 9984]
lp36_PAbe_NX_MP_FG_consensus  TATTTAAATAAAGCTTTTTCAGATATTGTTTTAAGCATGGATAAAAATCCTATAAATCTAGAAAAAGAGATTCGAAAT [ 9984]
lp36_PAbe_NX_Pl_consensus     TATTTAAATAAAGCTTTTTCAGATATTGTTTTAAGCATGGATAAAAATCCTATAAATCTAGAAAAAGAGATTCGAAAT [ 9984]

lp36_gi|11496779|B31_GB       ATTATTGTTATTTAATTTTAAATTTAATGTAAAACCTTTTTATAAAAATCAATTAAAAATTGTTTGATACTTTACCAC [10062]
lp36_un11_B31_PacBio          ATTATTGTTATTTAATTTTAAATTTAATGTAAAACCTTTTTATAAAAATCAATTAAAAATTGTTTGATACTTTACCAC [10062]
lp36_B31_TS_MP_FG_consensus   ATTATTGTTATTTAATTTTAAATTTAATGTAAAACCTTTTTATAAAAATCAATTAAAAATTGTTTGATACTTTACCAC [10062]
lp36_B31_NX_Pl_consensus      ATTATTGTTATTTAATTTTAAATTTAATGTAAAACCTTTTTATAAAAATCAATTAAAAATTGTTTGATACTTTACCAC [10062]
lp36_PAli_NX_MP_FG_consensus  ATTATTGTTATTTAATTTTAAATTTAATGTAAAACCTTTTTATAAAAATCAATTAAAAATTGTTTGATACTTTACCAC [10062]
lp36_PAli_NX_Pl_consensus     ATTATTGTTATTTAATTTTAAATTTAATGTAAAACCTTTTTATAAAAATCAATTAAAAATTGTTTGATACTTTACCAC [10062]
lp36_PAbe_NX_MP_FG_consensus  ATTATTGTTATTTAATTTTAAATTTAATGTAAAACCTTTTTATAAAAATCAATTAAAAATTGTTTGATACTTTACCAC [10062]
lp36_PAbe_NX_Pl_consensus     ATTATTGTTATTTAATTTTAAATTTAATGTAAAACCTTTTTATAAAAATCAATTAAAAATTGTTTGATACTTTACCAC [10062]

lp36_gi|11496779|B31_GB       ATAATCTTTGCTGGTATCAAAATTAGAATTGCTAGCTATTTTTTATTTAAATTTTTTCTATTTATTTTACCCAAAAAA [10140]
lp36_un11_B31_PacBio          ATAATCTTTGCTGGTATCAAAATTAGAATTGCTAGCTATTTTTTATTTAAATTTTTTCTATTTATTTTACCCAAAAAA [10140]
lp36_B31_TS_MP_FG_consensus   ATAATCTTTGCTGGTATCAAAATTAGAATTGCTAGCTATTTTTTATTTAAATTTTTTCTATTTATTTTACCCAAAAAA [10140]
lp36_B31_NX_Pl_consensus      ATAATCTTTGCTGGTATCAAAATTAGAATTGCTAGCTATTTTTTATTTAAATTTTTTCTATTTATTTTACCCAAAAAA [10140]
lp36_PAli_NX_MP_FG_consensus  ATAATCTTTGCTGGTATCAAAATTAGAATTGCTAGCTATTTTTTATTTAAATTTTTTCTATTTATTTTACCCAAAAAA [10140]
lp36_PAli_NX_Pl_consensus     ATAATCTTTGCTGGTATCAAAATTAGAATTGCTAGCTATTTTTTATTTAAATTTTTTCTATTTATTTTACCCAAAAAA [10140]
lp36_PAbe_NX_MP_FG_consensus  ATAATCTTTGCTGGTATCAAAATTAGAATTGCTAGCTATTTTTTATTTAAATTTTTTCTATTTATTTTACCCAAAAAA [10140]
lp36_PAbe_NX_Pl_consensus     ATAATCTTTGCTGGTATCAAAATTAGAATTGCTAGCTATTTTTTATTTAAATTTTTTCTATTTATTTTACCCAAAAAA [10140]

lp36_gi|11496779|B31_GB       TTATCTTCGATTTTTATCGTATATAACAAATATCGACGATTTTTATTTTTTCTAAATACAGTTGCAATTAAATATACT [10218]
lp36_un11_B31_PacBio          TTATCTTCGATTTTTATCGTATATAACAAATATCGACGATTTTTATTTTTTCTAAATACAGTTGCAATTAAATATACT [10218]
lp36_B31_TS_MP_FG_consensus   TTATCTTCGATTTTTATCGTATATAACAAATATCGACGATTTTTATTTTTTCTAAATACAGTTGCAATTAAATATACT [10218]
lp36_B31_NX_Pl_consensus      TTATCTTCGATTTTTATCGTATATAACAAATATCGACGATTTTTATTTTTTCTAAATACAGTTGCAATTAAATATACT [10218]
```

```
lp36_PAli_NX_MP_FG_consensus TTATCTTCGATTTTTATCGTATATAACAAATATCGACGATTTTTATTTTTTCTAAATACAGTTGCAATTAAATATACT [10218]
lp36_PAli_NX_Pl_consensus    TTATCTTCGATTTTTATCGTATATAACAAATATCGACGATTTTTATTTTTTCTAAATACAGTTGCAATTAAATATACT [10218]
lp36_PAbe_NX_MP_FG_consensus TTATCTTCGATTTTTATCGTATATAACAAATATCGACGATTTTTATTTTTTCTAAATACAGTTGCAATTAAATATACT [10218]
lp36_PAbe_NX_Pl_consensus    TTATCTTCGATTTTTATCGTATATAACAAATATCGACGATTTTTATTTTTTCTAAATACAGTTGCAATTAAATATACT [10218]

lp36_gi|11496779|B31_GB      GGCACTTTGCGCTATAATTTAAGTTTAAAGATTTTAAGGTAGAGTATATTATGAACAAAAAGTTTTTAGGAGAGGATA [10296]
lp36_un11_B31_PacBio         GGCACTTTGCGCTATAATTTAAGTTTAAAGATTTTAAGGTAGAGTATATTATGAACAAAAAGTTTTTAGGAGAGGATA [10296]
lp36_B31_TS_MP_FG_consensus  GGCACTTTGCGCTATAATTTAAGTTTAAAGATTTTAAGGTAGAGTATATTATGAACAAAAAGTTTTTAGGAGAGGATA [10296]
lp36_B31_NX_Pl_consensus     GGCACTTTGCGCTATAATTTAAGTTTAAAGATTTTAAGGTAGAGTATATTATGAACAAAAAGTTTTTAGGAGAGGATA [10296]
lp36_PAli_NX_MP_FG_consensus GGCACTTTGCGCTATAATTTAAGTTTAAAGATTTTAAGGTAGAGTATATTATGAACAAAAAGTTTTTAGGAGAGGATA [10296]
lp36_PAli_NX_Pl_consensus    GGCACTTTGCGCTATAATTTAAGTTTAAAGATTTTAAGGTAGAGTATATTATGAACAAAAAGTTTTTAGGAGAGGATA [10296]
lp36_PAbe_NX_MP_FG_consensus GGCACTTTGCGCTATAATTTAAGTTTAAAGATTTTAAGGTAGAGTATATTATGAACAAAAAGTTTTTAGGAGAGGATA [10296]
lp36_PAbe_NX_Pl_consensus    GGCACTTTGCGCTATAATTTAAGTTTAAAGATTTTAAGGTAGAGTATATTATGAACAAAAAGTTTTTAGGAGAGGATA [10296]

lp36_gi|11496779|B31_GB      AATAATGGATTTATTTAAAATTGAAGCTAATTATATTGACATCTTTAATAAAGAAATTTATCCAGCTAGTATAGCGAT [10374]
lp36_un11_B31_PacBio         AATAATGGATTTATTTAAAATTGAAGCTAATTATATTGACATCTTTAATAAAGAAATTTATCCAGCTAGTATAGCGAT [10374]
lp36_B31_TS_MP_FG_consensus  AATAATGGATTTATTTAAAATTGAAGCTAATTATATTGACATCTTTAATAAAGAAATTTATCCAGCTAGTATAGCGAT [10374]
lp36_B31_NX_Pl_consensus     AATAATGGATTTATTTAAAATTGAAGCTAATTATATTGACATCTTTAATAAAGAAATTTATCCAGCTAGTATAGCGAT [10374]
lp36_PAli_NX_MP_FG_consensus AATAATGGATTTATTTAAAATTGAAGCTAATTATATTGACATCTTTAATAAAGAAATTTATCCAGCTAGTATAGCGAT [10374]
lp36_PAli_NX_Pl_consensus    AATAATGGATTTATTTAAAATTGAAGCTAATTATATTGACATCTTTAATAAAGAAATTTATCCAGCTAGTATAGCGAT [10374]
lp36_PAbe_NX_MP_FG_consensus AATAATGGATTTATTTAAAATTGAAGCTAATTATATTGACATCTTTAATAAAGAAATTTATCCAGCTAGTATAGCGAT [10374]
lp36_PAbe_NX_Pl_consensus    AATAATGGATTTATTTAAAATTGAAGCTAATTATATTGACATCTTTAATAAAGAAATTTATCCAGCTAGTATAGCGAT [10374]

lp36_gi|11496779|B31_GB      TGCAAACGGTCATATTGCGAGCATAGAAAAAATTAATGCGACATTGGATGAATATGTGCTGCCAGGATTTATTGATGC [10452]
lp36_un11_B31_PacBio         TGCAAACGGTCATATTGCGAGCATAGAAAAAATTAATGCGACATTGGATGAATATGTGCTGCCAGGATTTATTGATGC [10452]
lp36_B31_TS_MP_FG_consensus  TGCAAACGGTCATATTGCGAGCATAGAAAAAATTAATGCGACATTGGATGAATATGTGCTGCCAGGATTTATTGATGC [10452]
lp36_B31_NX_Pl_consensus     TGCAAACGGTCATATTGCGAGCATAGAAAAAATTAATGCGACATTGGATGAATATGTGCTGCCAGGATTTATTGATGC [10452]
lp36_PAli_NX_MP_FG_consensus TGCAAACGGTCATATTGCGAGCATAGAAAAAATTAATGCGACATTGGATGAATATGTGCTGCCAGGATTTATTGATGC [10452]
lp36_PAli_NX_Pl_consensus    TGCAAACGGTCATATTGCGAGCATAGAAAAAATTAATGCGACATTGGATGAATATGTGCTGCCAGGATTTATTGATGC [10452]
lp36_PAbe_NX_MP_FG_consensus TGCAAACGGTCATATTGCGAGCATAGAAAAAATTAATGCGACATTGGATGAATATGTGCTGCCAGGATTTATTGATGC [10452]
lp36_PAbe_NX_Pl_consensus    TGCAAACGGTCATATTGCGAGCATAGAAAAAATTAATGCGACATTGGATGAATATGTGCTGCCAGGATTTATTGATGC [10452]

lp36_gi|11496779|B31_GB      GCATATACATATAGAGAGTTCTTTTCTTGTTCCATCAAACTTTGCTCATTTAGTAGTTGCACACGGCACTGTGGCTAC [10530]
lp36_un11_B31_PacBio         GCATATACATATAGAGAGTTCTTTTCTTGTTCCATCAAACTTTGCTCATTTAGTAGTTGCACACGGCACTGTGGCTAC [10530]
lp36_B31_TS_MP_FG_consensus  GCATATACATATAGAGAGTTCTTTTCTTGTTCCATCAAACTTTGCTCATTTAGTAGTTGCACACGGCACTGTGGCTAC [10530]
lp36_B31_NX_Pl_consensus     GCATATACATATAGAGAGTTCTTTTCTTGTTCCATCAAACTTTGCTCATTTAGTAGTTGCACACGGCACTGTGGCTAC [10530]
lp36_PAli_NX_MP_FG_consensus GCATATACATATAGAGAGTTCTTTTCTTGTTCCATCAAACTTTGCTCATTTAGTAGTTGCACACGGCACTGTGGCTAC [10530]
lp36_PAli_NX_Pl_consensus    GCATATACATATAGAGAGTTCTTTTCTTGTTCCATCAAACTTTGCTCATTTAGTAGTTGCACACGGCACTGTGGCTAC [10530]
lp36_PAbe_NX_MP_FG_consensus GCATATACATATAGAGAGTTCTTTTCTTGTTCCATCAAACTTTGCTCATTTAGTAGTTGCACACGGCACTGTGGCTAC [10530]
lp36_PAbe_NX_Pl_consensus    GCATATACATATAGAGAGTTCTTTTCTTGTTCCATCAAACTTTGCTCATTTAGTAGTTGCACACGGCACTGTGGCTAC [10530]

lp36_gi|11496779|B31_GB      AATAAGCGATCCTCATGAAATAGCCAATGTTAATGGTATTGATGGCATCAATTTTATGATAAATAATTCTAAAAAAAC [10608]
lp36_un11_B31_PacBio         AATAAGCGATCCTCATGAAATAGCCAATGTTAATGGTATTGATGGCATCAATTTTATGATAAATAATTCTAAAAAAAC [10608]
lp36_B31_TS_MP_FG_consensus  AATAAGCGATCCTCATGAAATAGCCAATGTTAATGGTATTGATGGCATCAATTTTATGATAAATAATTCTAAAAAAAC [10608]
lp36_B31_NX_Pl_consensus     AATAAGCGATCCTCATGAAATAGCCAATGTTAATGGTATTGATGGCATCAATTTTATGATAAATAATTCTAAAAAAAC [10608]
lp36_PAli_NX_MP_FG_consensus AATAAGCGATCCTCATGAAATAGCCAATGTTAATGGTATTGATGGCATCAATTTTATGATAAATAATTCTAAAAAAAC [10608]
lp36_PAli_NX_Pl_consensus    AATAAGCGATCCTCATGAAATAGCCAATGTTAATGGTATTGATGGCATCAATTTTATGATAAATAATTCTAAAAAAAC [10608]
lp36_PAbe_NX_MP_FG_consensus AATAAGCGATCCTCATGAAATAGCCAATGTTAATGGTATTGATGGCATCAATTTTATGATAAATAATTCTAAAAAAAC [10608]
lp36_PAbe_NX_Pl_consensus    AATAAGCGATCCTCATGAAATAGCCAATGTTAATGGTATTGATGGCATCAATTTTATGATAAATAATTCTAAAAAAAC [10608]

lp36_gi|11496779|B31_GB      CGAGTTTAAATTTTTTTTTGGAGCTCCTTCTTGTGTGCCAGCGTTGTCACAAGAATTTGAAACTTCAGGATATGTATT [10686]
lp36_un11_B31_PacBio         CGAGTTTAAATTTTTTTTTGGAGCTCCTTCTTGTGTGCCAGCGTTGTCACAAGAATTTGAAACTTCAGGATATGTATT [10686]
lp36_B31_TS_MP_FG_consensus  CGAGTTTAAATTTTTTTTTGGAGCTCCTTCTTGTGTGCCAGCGTTGTCACAAGAATTTGAAACTTCAGGATATGTATT [10686]
lp36_B31_NX_Pl_consensus     CGAGTTTAAATTTTTTTTTGGAGCTCCTTCTTGTGTGCCAGCGTTGTCACAAGAATTTGAAACTTCAGGATATGTATT [10686]
lp36_PAli_NX_MP_FG_consensus CGAGTTTAAATTTTTTTTTGGAGCTCCTTCTTGTGTGCCAGCGTTGTCACAAGAATTTGAAACTTCAGGATATGTATT [10686]
lp36_PAli_NX_Pl_consensus    CGAGTTTAAATTTTTTTTTGGAGCTCCTTCTTGTGTGCCAGCGTTGTCACAAGAATTTGAAACTTCAGGATATGTATT [10686]
lp36_PAbe_NX_MP_FG_consensus CGAGTTTAAATTTTTTTTTGGAGCTCCTTCTTGTGTGCCAGCGTTGTCACAAGAATTTGAAACTTCAGGATATGTATT [10686]
lp36_PAbe_NX_Pl_consensus    CGAGTTTAAATTTTTTTTTGGAGCTCCTTCTTGTGTGCCAGCGTTGTCACAAGAATTTGAAACTTCAGGATATGTATT [10686]

lp36_gi|11496779|B31_GB      AAATGATAAAGATATAGATGAATTGATGAAATTAGATGATATTTACTATTTGGCTGAAGTAATGGATTTTAAAGGCGT [10764]
lp36_un11_B31_PacBio         AAATGATAAAGATATAGATGAATTGATGAAATTAGATGATATTTACTATTTGGCTGAAGTAATGGATTTTAAAGGCGT [10764]
lp36_B31_TS_MP_FG_consensus  AAATGATAAAGATATAGATGAATTGATGAAATTAGATGATATTTACTATTTGGCTGAAGTAATGGATTTTAAAGGCGT [10764]
lp36_B31_NX_Pl_consensus     AAATGATAAAGATATAGATGAATTGATGAAATTAGATGATATTTACTATTTGGCTGAAGTAATGGATTTTAAAGGCGT [10764]
lp36_PAli_NX_MP_FG_consensus AAATGATAAAGATATAGATGAATTGATGAAATTAGATGATATTTACTATTTGGCTGAAGTAATGGATTTTAAAGGCGT [10764]
lp36_PAli_NX_Pl_consensus    AAATGATAAAGATATAGATGAATTGATGAAATTAGATGATATTTACTATTTGGCTGAAGTAATGGATTTTAAAGGCGT [10764]
lp36_PAbe_NX_MP_FG_consensus AAATGATAAAGATATAGATGAATTGATGAAATTAGATGATATTTACTATTTGGCTGAAGTAATGGATTTTAAAGGCGT [10764]
lp36_PAbe_NX_Pl_consensus    AAATGATAAAGATATAGATGAATTGATGAAATTAGATGATATTTACTATTTGGCTGAAGTAATGGATTTTAAAGGCGT [10764]

lp36_gi|11496779|B31_GB      GATTAACAAAGATATTGAAATTATAAATAAAATAAATTCTGCATTAAAGCGTAATAAGGTTGTTGATGGGCATGCTCC [10842]
lp36_un11_B31_PacBio         GATTAACAAAGATATTGAAATTATAAATAAAATAAATTCTGCATTAAAGCGTAATAAGGTTGTTGATGGGCATGCTCC [10842]
lp36_B31_TS_MP_FG_consensus  GATTAACAAAGATATTGAAATTATAAATAAAATAAATTCTGCATTAAAGCGTAATAAGGTTGTTGATGGGCATGCTCC [10842]
lp36_B31_NX_Pl_consensus     GATTAACAAAGATATTGAAATTATAAATAAAATAAATTCTGCATTAAAGCGTAATAAGGTTGTTGATGGGCATGCTCC [10842]
lp36_PAli_NX_MP_FG_consensus GATTAACAAAGATATTGAAATTATAAATAAAATAAATTCTGCATTAAAGCGTAATAAGGTTGTTGATGGGCATGCTCC [10842]
lp36_PAli_NX_Pl_consensus    GATTAACAAAGATATTGAAATTATAAATAAAATAAATTCTGCATTAAAGCGTAATAAGGTTGTTGATGGGCATGCTCC [10842]
lp36_PAbe_NX_MP_FG_consensus GATTAACAAAGATATTGAAATTATAAATAAAATAAATTCTGCATTAAAGCGTAATAAGGTTGTTGATGGGCATGCTCC [10842]
lp36_PAbe_NX_Pl_consensus    GATTAACAAAGATATTGAAATTATAAATAAAATAAATTCTGCATTAAAGCGTAATAAGGTTGTTGATGGGCATGCTCC [10842]

lp36_gi|11496779|B31_GB      TGGTTTGTCCCCCAATTTAACTTTAAAGTATGCATCTTCAGGCATTAGCACTGATCATGAATGTTTAACAATAGAAGA [10920]
lp36_un11_B31_PacBio         TGGTTTGTCCCCCAATTTAACTTTAAAGTATGCATCTTCAGGCATTAGCACTGATCATGAATGTTTAACAATAGAAGA [10920]
lp36_B31_TS_MP_FG_consensus  TGGTTTGTCCCCCAATTTAACTTTAAAGTATGCATCTTCAGGCATTAGCACTGATCATGAATGTTTAACAATAGAAGA [10920]
lp36_B31_NX_Pl_consensus     TGGTTTGTCCCCCAATTTAACTTTAAAGTATGCATCTTCAGGCATTAGCACTGATCATGAATGTTTAACAATAGAAGA [10920]
lp36_PAli_NX_MP_FG_consensus TGGTTTGTCCCCCAATTTAACTTTAAAGTATGCATCTTCAGGCATTAGCACTGATCATGAATGTTTAACAATAGAAGA [10920]
lp36_PAli_NX_Pl_consensus    TGGTTTGTCCCCCAATTTAACTTTAAAGTATGCATCTTCAGGCATTAGCACTGATCATGAATGTTTAACAATAGAAGA [10920]
lp36_PAbe_NX_MP_FG_consensus TGGTTTGTCCCCCAATTTAACTTTAAAGTATGCATCTTCAGGCATTAGCACTGATCATGAATGTTTAACAATAGAAGA [10920]
lp36_PAbe_NX_Pl_consensus    TGGTTTGTCCCCCAATTTAACTTTAAAGTATGCATCTTCAGGCATTAGCACTGATCATGAATGTTTAACAATAGAAGA [10920]

lp36_gi|11496779|B31_GB      TGCAAGATATAAATTATCTTTGGGCATGAAAATACTAATTAGAGAAGGAAGTGCCGCTAAAAATTTTGAATCTTTGCA [10998]
lp36_un11_B31_PacBio         TGCAAGATATAAATTATCTTTGGGCATGAAAATACTAATTAGAGAAGGAAGTGCCGCTAAAAATTTTGAATCTTTGCA [10998]
lp36_B31_TS_MP_FG_consensus  TGCAAGATATAAATTATCTTTGGGCATGAAAATACTAATTAGAGAAGGAAGTGCCGCTAAAAATTTTGAATCTTTGCA [10998]
lp36_B31_NX_Pl_consensus     TGCAAGATATAAATTATCTTTGGGCATGAAAATACTAATTAGAGAAGGAAGTGCCGCTAAAAATTTTGAATCTTTGCA [10998]
lp36_PAli_NX_MP_FG_consensus TGCAAGATATAAATTATCTTTGGGCATGAAAATACTAATTAGAGAAGGAAGTGCCGCTAAAAATTTTGAATCTTTGCA [10998]
lp36_PAli_NX_Pl_consensus    TGCAAGATATAAATTATCTTTGGGCATGAAAATACTAATTAGAGAAGGAAGTGCCGCTAAAAATTTTGAATCTTTGCA [10998]
lp36_PAbe_NX_MP_FG_consensus TGCAAGATATAAATTATCTTTGGGCATGAAAATACTAATTAGAGAAGGAAGTGCCGCTAAAAATTTTGAATCTTTGCA [10998]
lp36_PAbe_NX_Pl_consensus    TGCAAGATATAAATTATCTTTGGGCATGAAAATACTAATTAGAGAAGGAAGTGCCGCTAAAAATTTTGAATCTTTGCA [10998]

lp36_gi|11496779|B31_GB      TCCTTTGATTAGTGAATGTTCTAAAAAATATTGTGATTCCTTAATGTTTTGTTTTGATGATGCACACCCAAATGACAT [11076]
lp36_un11_B31_PacBio         TCCTTTGATTAGTGAATGTTCTAAAAAATATTGTGATTCCTTAATGTTTTGTTTTGATGATGCACACCCAAATGACAT [11076]
lp36_B31_TS_MP_FG_consensus  TCCTTTGATTAGTGAATGTTCTAAAAAATATTGTGATTCCTTAATGTTTTGTTTTGATGATGCACACCCAAATGACAT [11076]
lp36_B31_NX_Pl_consensus     TCCTTTGATTAGTGAATGTTCTAAAAAATATTGTGATTCCTTAATGTTTTGTTTTGATGATGCACACCCAAATGACAT [11076]
lp36_PAli_NX_MP_FG_consensus TCCTTTGATTAGTGAATGTTCTAAAAAATATTGTGATTCCTTAATGTTTTGTTTTGATGATGCACACCCAAATGACAT [11076]
lp36_PAli_NX_Pl_consensus    TCCTTTGATTAGTGAATGTTCTAAAAAATATTGTGATTCCTTAATGTTTTGTTTTGATGATGCACACCCAAATGACAT [11076]
lp36_PAbe_NX_MP_FG_consensus TCCTTTGATTAGTGAATGTTCTAAAAAATATTGTGATTCCTTAATGTTTTGTTTTGATGATGCACACCCAAATGACAT [11076]
lp36_PAbe_NX_Pl_consensus    TCCTTTGATTAGTGAATGTTCTAAAAAATATTGTGATTCCTTAATGTTTTGTTTTGATGATGCACACCCAAATGACAT [11076]

lp36_gi|11496779|B31_GB      TCTAAATGGACATATTAATTTAATAGTAGCTCGTGCAATAAAGCATGGGCACGACTTTTTTGATGTTTTGAAAATAGC [11154]
lp36_un11_B31_PacBio         TCTAAATGGACATATTAATTTAATAGTAGCTCGTGCAATAAAGCATGGGCACGACTTTTTTGATGTTTTGAAAATAGC [11154]
lp36_B31_TS_MP_FG_consensus  TCTAAATGGACATATTAATTTAATAGTAGCTCGTGCAATAAAGCATGGGCACGACTTTTTTGATGTTTTGAAAATAGC [11154]
lp36_B31_NX_Pl_consensus     TCTAAATGGACATATTAATTTAATAGTAGCTCGTGCAATAAAGCATGGGCACGACTTTTTTGATGTTTTGAAAATAGC [11154]
lp36_PAli_NX_MP_FG_consensus TCTAAATGGACATATTAATTTAATAGTAGCTCGTGCAATAAAGCATGGGCACGACTTTTTTGATGTTTTGAAAATAGC [11154]
```

```
lp36_PAli_NX_Pl_consensus    TCTAAATGGACATATTAATTTAATAGTAGCTCGTGCAATAAAGCATGGGCACGACTTTTTTGATGTTTTGAAAATAGC [11154]
lp36_PAbe_NX_MP_FG_consensus TCTAAATGGACATATTAATTTAATAGTAGCTCGTGCAATAAAGCATGGGCACGACTTTTTTGATGTTTTGAAAATAGC [11154]
lp36_PAbe_NX_Pl_consensus    TCTAAATGGACATATTAATTTAATAGTAGCTCGTGCAATAAAGCATGGGCACGACTTTTTTGATGTTTTGAAAATAGC [11154]

lp36_gi|11496779|B31_GB      ATGCATTAATCCGGTTTTACACTATAAAATCCCAGTGGGGTTGTTAAGAATAGGAGATCCTGCTGATTTTATAATTAC [11232]
lp36_un11_B31_PacBio         ATGCATTAATCCGGTTTTACACTATAAAATCCCAGTGGGGTTGTTAAGAATAGGAGATCCTGCTGATTTTATAATTAC [11232]
lp36_B31_TS_MP_FG_consensus  ATGCATTAATCCGGTTTTACACTATAAAATCCCAGTGGGGTTGTTAAGAATAGGAGATCCTGCTGATTTTATAATTAC [11232]
lp36_B31_NX_Pl_consensus     ATGCATTAATCCGGTTTTACACTATAAAATCCCAGTGGGGTTGTTAAGAATAGGAGATCCTGCTGATTTTATAATTAC [11232]
lp36_PAli_NX_MP_FG_consensus ATGCATTAATCCGGTTTTACACTATAAAATCCCAGTGGGGTTGTTAAGAATAGGAGATCCTGCTGATTTTATAATTAC [11232]
lp36_PAli_NX_Pl_consensus    ATGCATTAATCCGGTTTTACACTATAAAATCCCAGTGGGGTTGTTAAGAATAGGAGATCCTGCTGATTTTATAATTAC [11232]
lp36_PAbe_NX_MP_FG_consensus ATGCATTAATCCGGTTTTACACTATAAAATCCCAGTGGGGTTGTTAAGAATAGGAGATCCTGCTGATTTTATAATTAC [11232]
lp36_PAbe_NX_Pl_consensus    ATGCATTAATCCGGTTTTACACTATAAAATCCCAGTGGGGTTGTTAAGAATAGGAGATCCTGCTGATTTTATAATTAC [11232]

lp36_gi|11496779|B31_GB      AAAAGATATCAAAACATTCAAAATAAATAAAACCTACATTAATGGTAAATTGGTTTTTAATGATGGAATTTCACTTAT [11310]
lp36_un11_B31_PacBio         AAAAGATATCAAAACATTCAAAATAAATAAAACCTACATTAATGGTAAATTGGTTTTTAATGATGGAATTTCACTTAT [11310]
lp36_B31_TS_MP_FG_consensus  AAAAGATATCAAAACATTCAAAATAAATAAAACCTACATTAATGGTAAATTGGTTTTTAATGATGGAATTTCACTTAT [11310]
lp36_B31_NX_Pl_consensus     AAAAGATATCAAAACATTCAAAATAAATAAAACCTACATTAATGGTAAATTGGTTTTTAATGATGGAATTTCACTTAT [11310]
lp36_PAli_NX_MP_FG_consensus AAAAGATATCAAAACATTCAAAATAAATAAAACCTACATTAATGGTAAATTGGTTTTTAATGATGGAATTTCACTTAT [11310]
lp36_PAli_NX_Pl_consensus    AAAAGATATCAAAACATTCAAAATAAATAAAACCTACATTAATGGTAAATTGGTTTTTAATGATGGAATTTCACTTAT [11310]
lp36_PAbe_NX_MP_FG_consensus AAAAGATATCAAAACATTCAAAATAAATAAAACCTACATTAATGGTAAATTGGTTTTTAATGATGGAATTTCACTTAT [11310]
lp36_PAbe_NX_Pl_consensus    AAAAGATATCAAAACATTCAAAATAAATAAAACCTACATTAATGGTAAATTGGTTTTTAATGATGGAATTTCACTTAT [11310]

lp36_gi|11496779|B31_GB      CCCATTAATAAATGAAATTCCTATAAACAATTTTAATTGTAGCAAAAAATCTATTTCGGATTTTAAATTTTCCACTAA [11388]
lp36_un11_B31_PacBio         CCCATTAATAAATGAAATTCCTATAAACAATTTTAATTGTAGCAAAAAATCTATTTCGGATTTTAAATTTTCCACTAA [11388]
lp36_B31_TS_MP_FG_consensus  CCCATTAATAAATGAAATTCCTATAAACAATTTTAATTGTAGCAAAAAATCTATTTCGGATTTTAAATTTTCCACTAA [11388]
lp36_B31_NX_Pl_consensus     CCCATTAATAAATGAAATTCCTATAAACAATTTTAATTGTAGCAAAAAATCTATTTCGGATTTTAAATTTTCCACTAA [11388]
lp36_PAli_NX_MP_FG_consensus CCCATTAATAAATGAAATTCCTATAAACAATTTTAATTGTAGCAAAAAATCTATTTCGGATTTTAAATTTTCCACTAA [11388]
lp36_PAli_NX_Pl_consensus    CCCATTAATAAATGAAATTCCTATAAACAATTTTAATTGTAGCAAAAAATCTATTTCGGATTTTAAATTTTCCACTAA [11388]
lp36_PAbe_NX_MP_FG_consensus CCCATTAATAAATGAAATTCCTATAAACAATTTTAATTGTAGCAAAAAATCTATTTCGGATTTTAAATTTTCCACTAA [11388]
lp36_PAbe_NX_Pl_consensus    CCCATTAATAAATGAAATTCCTATAAACAATTTTAATTGTAGCAAAAAATCTATTTCGGATTTTAAATTTTCCACTAA [11388]

lp36_gi|11496779|B31_GB      AAATAAGATGATCCCAGTAATCAAATGCATCAGCAACCAAATTATTACTCATAAAACTATGATTGATAGCAATTTATT [11466]
lp36_un11_B31_PacBio         AAATAAGATGATCCCAGTAATCAAATGCATCAGCAACCAAATTATTACTCATAAAACTATGATTGATAGCAATTTATT [11466]
lp36_B31_TS_MP_FG_consensus  AAATAAGATGATCCCAGTAATCAAATGCATCAGCAACCAAATTATTACTCATAAAACTATGATTGATAGCAATTTATT [11466]
lp36_B31_NX_Pl_consensus     AAATAAGATGATCCCAGTAATCAAATGCATCAGCAACCAAATTATTACTCATAAAACTATGATTGATAGCAATTTATT [11466]
lp36_PAli_NX_MP_FG_consensus AAATAAGATGATCCCAGTAATCAAATGCATCAGCAACCAAATTATTACTCATAAAACTATGATTGATAGCAATTTATT [11466]
lp36_PAli_NX_Pl_consensus    AAATAAGATGATCCCAGTAATCAAATGCATCAGCAACCAAATTATTACTCATAAAACTATGATTGATAGCAATTTATT [11466]
lp36_PAbe_NX_MP_FG_consensus AAATAAGATGATCCCAGTAATCAAATGCATCAGCAACCAAATTATTACTCATAAAACTATGATTGATAGCAATTTATT [11466]
lp36_PAbe_NX_Pl_consensus    AAATAAGATGATCCCAGTAATCAAATGCATCAGCAACCAAATTATTACTCATAAAACTATGATTGATAGCAATTTATT [11466]

lp36_gi|11496779|B31_GB      GGCCCCAGATTTTCAATCTAATATTGCCGAGGATATTTTAAAAATAGCTATAATAAATCGATATAAAGACAATAGTAA [11544]
lp36_un11_B31_PacBio         GGCCCCAGATTTTCAATCTAATATTGCCGAGGATATTTTAAAAATAGCTATAATAAATCGATATAAAGACAATAGTAA [11544]
lp36_B31_TS_MP_FG_consensus  GGCCCCAGATTTTCAATCTAATATTGCCGAGGATATTTTAAAAATAGCTATAATAAATCGATATAAAGACAATAGTAA [11544]
lp36_B31_NX_Pl_consensus     GGCCCCAGATTTTCAATCTAATATTGCCGAGGATATTTTAAAAATAGCTATAATAAATCGATATAAAGACAATAGTAA [11544]
lp36_PAli_NX_MP_FG_consensus GGCCCCAGATTTTCAATCTAATATTGCCGAGGATATTTTAAAAATAGCTATAATAAATCGATATAAAGACAATAGTAA [11544]
lp36_PAli_NX_Pl_consensus    GGCCCCAGATTTTCAATCTAATATTGCCGAGGATATTTTAAAAATAGCTATAATAAATCGATATAAAGACAATAGTAA [11544]
lp36_PAbe_NX_MP_FG_consensus GGCCCCAGATTTTCAATCTAATATTGCCGAGGATATTTTAAAAATAGCTATAATAAATCGATATAAAGACAATAGTAA [11544]
lp36_PAbe_NX_Pl_consensus    GGCCCCAGATTTTCAATCTAATATTGCCGAGGATATTTTAAAAATAGCTATAATAAATCGATATAAAGACAATAGTAA [11544]

lp36_gi|11496779|B31_GB      AATTTCTATAGGATTTATAAAAAATTTTGGAATAAGAAACGGCGCCATAGGAAGTACAGTTGCTCATGATTCTCACAA [11622]
lp36_un11_B31_PacBio         AATTTCTATAGGATTTATAAAAAATTTTGGAATAAGAAACGGCGCCATAGGAAGTACAGTTGCTCATGATTCTCACAA [11622]
lp36_B31_TS_MP_FG_consensus  AATTTCTATAGGATTTATAAAAAATTTTGGAATAAGAAACGGCGCCATAGGAAGTACAGTTGCTCATGATTCTCACAA [11622]
lp36_B31_NX_Pl_consensus     AATTTCTATAGGATTTATAAAAAATTTTGGAATAAGAAACGGCGCCATAGGAAGTACAGTTGCTCATGATTCTCACAA [11622]
lp36_PAli_NX_MP_FG_consensus AATTTCTATAGGATTTATAAAAAATTTTGGAATAAGAAACGGCGCCATAGGAAGTACAGTTGCTCATGATTCTCACAA [11622]
lp36_PAli_NX_Pl_consensus    AATTTCTATAGGATTTATAAAAAATTTTGGAATAAGAAACGGCGCCATAGGAAGTACAGTTGCTCATGATTCTCACAA [11622]
lp36_PAbe_NX_MP_FG_consensus AATTTCTATAGGATTTATAAAAAATTTTGGAATAAGAAACGGCGCCATAGGAAGTACAGTTGCTCATGATTCTCACAA [11622]
lp36_PAbe_NX_Pl_consensus    AATTTCTATAGGATTTATAAAAAATTTTGGAATAAGAAACGGCGCCATAGGAAGTACAGTTGCTCATGATTCTCACAA [11622]

lp36_gi|11496779|B31_GB      CATCATACTTGTTGGAAGCAATGATGAATATTTGTGTAAAGCGGCAAATACAATCATCCAGAATAAGGGGGGTTTATG [11700]
lp36_un11_B31_PacBio         CATCATACTTGTTGGAAGCAATGATGAATATTTGTGTAAAGCGGCAAATACAATCATCCAGAATAAGGGGGGTTTATG [11700]
lp36_B31_TS_MP_FG_consensus  CATCATACTTGTTGGAAGCAATGATGAATATTTGTGTAAAGCGGCAAATACAATCATCCAGAATAAGGGGGGTTTATG [11700]
lp36_B31_NX_Pl_consensus     CATCATACTTGTTGGAAGCAATGATGAATATTTGTGTAAAGCGGCAAATACAATCATCCAGAATAAGGGGGGTTTATG [11700]
lp36_PAli_NX_MP_FG_consensus CATCATACTTGTTGGAAGCAATGATGAATATTTGTGTAAAGCGGCAAATACAATCATCCAGAATAAGGGGGGTTTATG [11700]
lp36_PAli_NX_Pl_consensus    CATCATACTTGTTGGAAGCAATGATGAATATTTGTGTAAAGCGGCAAATACAATCATCCAGAATAAGGGGGGTTTATG [11700]
lp36_PAbe_NX_MP_FG_consensus CATCATACTTGTTGGAAGCAATGATGAATATTTGTGTAAAGCGGCAAATACAATCATCCAGAATAAGGGGGGTTTATG [11700]
lp36_PAbe_NX_Pl_consensus    CATCATACTTGTTGGAAGCAATGATGAATATTTGTGTAAAGCGGCAAATACAATCATCCAGAATAAGGGGGGTTTATG [11700]

lp36_gi|11496779|B31_GB      TGCACTAAACAATGAAAAAACTATAATAATGGAACTTCCTATTTCTGGATTAATGAGTACTCTTTCAGCCGAAAGAGT [11778]
lp36_un11_B31_PacBio         TGCACTAAACAATGAAAAAACTATAATAATGGAACTTCCTATTTCTGGATTAATGAGTACTCTTTCAGCCGAAAGAGT [11778]
lp36_B31_TS_MP_FG_consensus  TGCACTAAACAATGAAAAAACTATAATAATGGAACTTCCTATTTCTGGATTAATGAGTACTCTTTCAGCCGAAAGAGT [11778]
lp36_B31_NX_Pl_consensus     TGCACTAAACAATGAAAAAACTATAATAATGGAACTTCCTATTTCTGGATTAATGAGTACTCTTTCAGCCGAAAGAGT [11778]
lp36_PAli_NX_MP_FG_consensus TGCACTAAACAATGAAAAAACTATAATAATGGAACTTCCTATTTCTGGATTAATGAGTACTCTTTCAGCCGAAAGAGT [11778]
lp36_PAli_NX_Pl_consensus    TGCACTAAACAATGAAAAAACTATAATAATGGAACTTCCTATTTCTGGATTAATGAGTACTCTTTCAGCCGAAAGAGT [11778]
lp36_PAbe_NX_MP_FG_consensus TGCACTAAACAATGAAAAAACTATAATAATGGAACTTCCTATTTCTGGATTAATGAGTACTCTTTCAGCCGAAAGAGT [11778]
lp36_PAbe_NX_Pl_consensus    TGCACTAAACAATGAAAAAACTATAATAATGGAACTTCCTATTTCTGGATTAATGAGTACTCTTTCAGCCGAAAGAGT [11778]

lp36_gi|11496779|B31_GB      AGCTTCACAATATATAAAATTAAATGATTTTTGTAAAAATGTTTTAGGCTCTCGGCTTGATGATCCTCTAATGACTTT [11856]
lp36_un11_B31_PacBio         AGCTTCACAATATATAAAATTAAATGATTTTTGTAAAAATGTTTTAGGCTCTCGGCTTGATGATCCTCTAATGACTTT [11856]
lp36_B31_TS_MP_FG_consensus  AGCTTCACAATATATAAAATTAAATGATTTTTGTAAAAATGTTTTAGGCTCTCGGCTTGATGATCCTCTAATGACTTT [11856]
lp36_B31_NX_Pl_consensus     AGCTTCACAATATATAAAATTAAATGATTTTTGTAAAAATGTTTTAGGCTCTCGGCTTGATGATCCTCTAATGACTTT [11856]
lp36_PAli_NX_MP_FG_consensus AGCTTCACAATATATAAAATTAAATGATTTTTGTAAAAATGTTTTAGGCTCTCGGCTTGATGATCCTCTAATGACTTT [11856]
lp36_PAli_NX_Pl_consensus    AGCTTCACAATATATAAAATTAAATGATTTTTGTAAAAATGTTTTAGGCTCTCGGCTTGATGATCCTCTAATGACTTT [11856]
lp36_PAbe_NX_MP_FG_consensus AGCTTCACAATATATAAAATTAAATGATTTTTGTAAAAATGTTTTAGGCTCTCGGCTTGATGATCCTCTAATGACTTT [11856]
lp36_PAbe_NX_Pl_consensus    AGCTTCACAATATATAAAATTAAATGATTTTTGTAAAAATGTTTTAGGCTCTCGGCTTGATGATCCTCTAATGACTTT [11856]

lp36_gi|11496779|B31_GB      GTCTTTTATGTCTTTAACGGTGGTTCCCCATTTAAAGATAAATGATAAAGGACTGTTTGATGTTGATTCTTTTTGTTT [11934]
lp36_un11_B31_PacBio         GTCTTTTATGTCTTTAACGGTGGTTCCCCATTTAAAGATAAATGATAAAGGACTGTTTGATGTTGATTCTTTTTGTTT [11934]
lp36_B31_TS_MP_FG_consensus  GTCTTTTATGTCTTTAACGGTGGTTCCCCATTTAAAGATAAATGATAAAGGACTGTTTGATGTTGATTCTTTTTGTTT [11934]
lp36_B31_NX_Pl_consensus     GTCTTTTATGTCTTTAACGGTGGTTCCCCATTTAAAGATAAATGATAAAGGACTGTTTGATGTTGATTCTTTTTGTTT [11934]
lp36_PAli_NX_MP_FG_consensus GTCTTTTATGTCTTTAACGGTGGTTCCCCATTTAAAGATAAATGATAAAGGACTGTTTGATGTTGATTCTTTTTGTTT [11934]
lp36_PAli_NX_Pl_consensus    GTCTTTTATGTCTTTAACGGTGGTTCCCCATTTAAAGATAAATGATAAAGGACTGTTTGATGTTGATTCTTTTTGTTT [11934]
lp36_PAbe_NX_MP_FG_consensus GTCTTTTATGTCTTTAACGGTGGTTCCCCATTTAAAGATAAATGATAAAGGACTGTTTGATGTTGATTCTTTTTGTTT [11934]
lp36_PAbe_NX_Pl_consensus    GTCTTTTATGTCTTTAACGGTGGTTCCCCATTTAAAGATAAATGATAAAGGACTGTTTGATGTTGATTCTTTTTGTTT [11934]

lp36_gi|11496779|B31_GB      TGTAGATTATTAATTTTAAATTATTATAAAAAGGCAAAAAAAGATTAAAAAATTAAGCGATAAAATTGTAGAAGGAAC [12012]
lp36_un11_B31_PacBio         TGTAGATTATTAATTTTAAATTATTATAAAAAGGCAAAAAAAGATTAAAAAATTAAGCGATAAAATTGTAGAAGGAAC [12012]
lp36_B31_TS_MP_FG_consensus  TGTAGATTATTAATTTTAAATTATTATAAAAAGGCAAAAAAAGATTAAAAAATTAAGCGATAAAATTGTAGAAGGAAC [12012]
lp36_B31_NX_Pl_consensus     TGTAGATTATTAATTTTAAATTATTATAAAAAGGCAAAAAAAGATTAAAAAATTAAGCGATAAAATTGTAGAAGGAAC [12012]
lp36_PAli_NX_MP_FG_consensus TGTAGATTATTAATTTTAAATTATTATAAAAAGGCAAAAAAAGATTAAAAAATTAAGCGATAAAATTGTAGAAGGAAC [12012]
lp36_PAli_NX_Pl_consensus    TGTAGATTATTAATTTTAAATTATTATAAAAAGGCAAAAAAAGATTAAAAAATTAAGCGATAAAATTGTAGAAGGAAC [12012]
lp36_PAbe_NX_MP_FG_consensus TGTAGATTATTAATTTTAAATTATTATAAAAAGGCAAAAAAAGATTAAAAAATTAAGCGATAAAATTGTAGAAGGAAC [12012]
lp36_PAbe_NX_Pl_consensus    TGTAGATTATTAATTTTAAATTATTATAAAAAGGCAAAAAAAGATTAAAAAATTAAGCGATAAAATTGTAGAAGGAAC [12012]

lp36_gi|11496779|B31_GB      TCAAAAGTTTTTTGTCTGTTATTATCTATTAAGGATTATTAATTGTGTGTCTGGCAGTTTTTTATTTTAAAAATTTTA [12090]
lp36_un11_B31_PacBio         TCAAAAGTTTTTTGTCTGTTATTATCTATTAAGGATTATTAATTGTGTGTCTGGCAGTTTTTTATTTTAAAAATTTTA [12090]
lp36_B31_TS_MP_FG_consensus  TCAAAAGTTTTTTGTCTGTTATTATCTATTAAGGATTATTAATTGTGTGTCTGGCAGTTTTTTATTTTAAAAATTTTA [12090]
lp36_B31_NX_Pl_consensus     TCAAAAGTTTTTTGTCTGTTATTATCTATTAAGGATTATTAATTGTGTGTCTGGCAGTTTTTTATTTTAAAAATTTTA [12090]
lp36_PAli_NX_MP_FG_consensus TCAAAAGTTTTTTGTCTGTTATTATCTATTAAGGATTATTAATTGTGTGTCTGGCAGTTTTTTATTTTAAAAATTTTA [12090]
lp36_PAli_NX_Pl_consensus    TCAAAAGTTTTTTGTCTGTTATTATCTATTAAGGATTATTAATTGTGTGTCTGGCAGTTTTTTATTTTAAAAATTTTA [12090]
```

```
lp36_PAbe_NX_MP_FG_consensus TCAAAAGTTTTTTGTCTGTTATTATCTATTAAGGATTATTAATTGTGTGTCTGGCAGTTTTTTATTTTAAAAATTTTA [12090]
lp36_PAbe_NX_Pl_consensus    TCAAAAGTTTTTTGTCTGTTATTATCTATTAAGGATTATTAATTGTGTGTCTGGCAGTTTTTTATTTTAAAAATTTTA [12090]

lp36_gi|11496779|B31_GB      TGCATTAAATCCTTAAGCAAAAAGTAGTTTTTGATTGCTGTTTGGAGGCACAAGCACCATAAATAATATCCTGTAATA [12168]
lp36_un11_B31_PacBio         TGCATTAAATCCTTAAGCAAAAAGTAGTTTTTGATTGCTGTTTGGAGGCACAAGCACCATAAATAATATCCTGTAATA [12168]
lp36_B31_TS_MP_FG_consensus  TGCATTAAATCCTTAAGCAAAAAGTAGTTTTTGATTGCTGTTTGGAGGCACAAGCACCATAAATAATATCCTGTAATA [12168]
lp36_B31_NX_Pl_consensus     TGCATTAAATCCTTAAGCAAAAAGTAGTTTTTGATTGCTGTTTGGAGGCACAAGCACCATAAATAATATCCTGTAATA [12168]
lp36_PAli_NX_MP_FG_consensus TGCATTAAATCCTTAAGCAAAAAGTAGTTTTTGATTGCTGTTTGGAGGCACAAGCACCATAAATAATATCCTGTAATA [12168]
lp36_PAli_NX_Pl_consensus    TGCATTAAATCCTTAAGCAAAAAGTAGTTTTTGATTGCTGTTTGGAGGCACAAGCACCATAAATAATATCCTGTAATA [12168]
lp36_PAbe_NX_MP_FG_consensus TGCATTAAATCCTTAAGCAAAAAGTAGTTTTTGATTGCTGTTTGGAGGCACAAGCACCATAAATAATATCCTGTAATA [12168]
lp36_PAbe_NX_Pl_consensus    TGCATTAAATCCTTAAGCAAAAAGTAGTTTTTGATTGCTGTTTGGAGGCACAAGCACCATAAATAATATCCTGTAATA [12168]

lp36_gi|11496779|B31_GB      ACGCAAAACGTTACTATTGATTTAATTTATGGAAACCATATGTGTAAATATTGATGCATCTTCTCAATAAATAACATT [12246]
lp36_un11_B31_PacBio         ACGCAAAACGTTACTATTGATTTAATTTATGGAAACCATATGTGTAAATATTGATGCATCTTCTCAATAAATAACATT [12246]
lp36_B31_TS_MP_FG_consensus  ACGCAAAACGTTACTATTGATTTAATTTATGGAAACCATATGTGTAAATATTGATGCATCTTCTCAATAAATAACATT [12246]
lp36_B31_NX_Pl_consensus     ACGCAAAACGTTACTATTGATTTAATTTATGGAAACCATATGTGTAAATATTGATGCATCTTCTCAATAAATAACATT [12246]
lp36_PAli_NX_MP_FG_consensus ACGCAAAACGTTACTATTGATTTAATTTATGGAAACCATATGTGTAAATATTGATGCATCTTCTCAATAAATAACATT [12246]
lp36_PAli_NX_Pl_consensus    ACGCAAAACGTTACTATTGATTTAATTTATGGAAACCATATGTGTAAATATTGATGCATCTTCTCAATAAATAACATT [12246]
lp36_PAbe_NX_MP_FG_consensus ACGCAAAACGTTACTATTGATTTAATTTATGGAAACCATATGTGTAAATATTGATGCATCTTCTCAATAAATAACATT [12246]
lp36_PAbe_NX_Pl_consensus    ACGCAAAACGTTACTATTGATTTAATTTATGGAAACCATATGTGTAAATATTGATGCATCTTCTCAATAAATAACATT [12246]

lp36_gi|11496779|B31_GB      TATACCCCCCGGTTAAAATAAACTATTGTTTGTCCAAGTCTTAATCTTAAGAAACAAATTAATTAATTCTTTAGCTCC [12324]
lp36_un11_B31_PacBio         TATACCCCCCGGTTAAAATAAACTATTGTTTGTCCAAGTCTTAATCTTAAGAAACAAATTAATTAATTCTTTAGCTCC [12324]
lp36_B31_TS_MP_FG_consensus  TATACCCCCCGGTTAAAATAAACTATTGTTTGTCCAAGTCTTAATCTTAAGAAACAAATTAATTAATTCTTTAGCTCC [12324]
lp36_B31_NX_Pl_consensus     TATACCCCCCGGTTAAAATAAACTATTGTTTGTCCAAGTCTTAATCTTAAGAAACAAATTAATTAATTCTTTAGCTCC [12324]
lp36_PAli_NX_MP_FG_consensus TATACCCCCCGGTTAAAATAAACTATTGTTTGTCCAAGTCTTAATCTTAAGAAACAAATTAATTAATTCTTTAGCTCC [12324]
lp36_PAli_NX_Pl_consensus    TATACCCCCCGGTTAAAATAAACTATTGTTTGTCCAAGTCTTAATCTTAAGAAACAAATTAATTAATTCTTTAGCTCC [12324]
lp36_PAbe_NX_MP_FG_consensus TATACCCCCCGGTTAAAATAAACTATTGTTTGTCCAAGTCTTAATCTTAAGAAACAAATTAATTAATTCTTTAGCTCC [12324]
lp36_PAbe_NX_Pl_consensus    TATACCCCCCGGTTAAAATAAACTATTGTTTGTCCAAGTCTTAATCTTAAGAAACAAATTAATTAATTCTTTAGCTCC [12324]

lp36_gi|11496779|B31_GB      CTTAGAACCCGAGCTTATATAAAAACTAGTGAAATTTTTTTTATCTCATTACATCTAAAAATAAATCAATAAAAAATC [12402]
lp36_un11_B31_PacBio         CTTAGAACCCGAGCTTATATAAAAACTAGTGAAATTTTTTTTATCTCATTACATCTAAAAATAAATCAATAAAAAATC [12402]
lp36_B31_TS_MP_FG_consensus  CTTAGAACCCGAGCTTATATAAAAACTAGTGAAATTTTTTTTATCTCATTACATCTAAAAATAAATCAATAAAAAATC [12402]
lp36_B31_NX_Pl_consensus     CTTAGAACCCGAGCTTATATAAAAACTAGTGAAATTTTTTTTATCTCATTACATCTAAAAATAAATCAATAAAAAATC [12402]
lp36_PAli_NX_MP_FG_consensus CTTAGAACCCGAGCTTATATAAAAACTAGTGAAATTTTTTTTATCTCATTACATCTAAAAATAAATCAATAAAAAATC [12402]
lp36_PAli_NX_Pl_consensus    CTTAGAACCCGAGCTTATATAAAAACTAGTGAAATTTTTTTTATCTCATTACATCTAAAAATAAATCAATAAAAAATC [12402]
lp36_PAbe_NX_MP_FG_consensus CTTAGAACCCGAGCTTATATAAAAACTAGTGAAATTTTTTTTATCTCATTACATCTAAAAATAAATCAATAAAAAATC [12402]
lp36_PAbe_NX_Pl_consensus    CTTAGAACCCGAGCTTATATAAAAACTAGTGAAATTTTTTTTATCTCATTACATCTAAAAATAAATCAATAAAAAATC [12402]

lp36_gi|11496779|B31_GB      ATTTTTGTTTCGGCTCCGCTTTTAATAGCATCCTTATGAGATTATATTTTTGCACTTATATTTTTTTATTACCTCTATA [12480]
lp36_un11_B31_PacBio         ATTTTTGTTTCGGCTCCGCTTTTAATAGCATCCTTATGAGATTATATTTTTGCACTTATATTTTTTTATTACCTCTATA [12480]
lp36_B31_TS_MP_FG_consensus  ATTTTTGTTTCGGCTCCGCTTTTAATAGCATCCTTATGAGATTATATTTTTGCACTTATATTTTTTTATTACCTCTATA [12480]
lp36_B31_NX_Pl_consensus     ATTTTTGTTTCGGCTCCGCTTTTAATAGCATCCTTATGAGATTATATTTTTGCACTTATATTTTTTTATTACCTCTATA [12480]
lp36_PAli_NX_MP_FG_consensus ATTTTTGTTTCGGCTCCGCTTTTAATAGCATCCTTATGAGATTATATTTTTGCACTTATATTTTTTTATTACCTCTATA [12480]
lp36_PAli_NX_Pl_consensus    ATTTTTGTTTCGGCTCCGCTTTTAATAGCATCCTTATGAGATTATATTTTTGCACTTATATTTTTTTATTACCTCTATA [12480]
lp36_PAbe_NX_MP_FG_consensus ATTTTTGTTTCGGCTCCGCTTTTAATAGCATCCTTATGAGATTATATTTTTGCACTTATATTTTTTTATTACCTCTATA [12480]
lp36_PAbe_NX_Pl_consensus    ATTTTTGTTTCGGCTCCGCTTTTAATAGCATCCTTATGAGATTATATTTTTGCACTTATATTTTTTTATTACCTCTATA [12480]

lp36_gi|11496779|B31_GB      AAAATTTTATTTTAAAAAAAGATATTTATTAATTATAATATCAATATTATTGGGAAATAGTTAATAAAAAAATTAAACA [12558]
lp36_un11_B31_PacBio         AAAATTTTATTTTAAAAAAAGATATTTATTAATTATAATATCAATATTATTGGGAAATAGTTAATAAAAAAATTAAACA [12558]
lp36_B31_TS_MP_FG_consensus  AAAATTTTATTTTAAAAAAAGATATTTATTAATTATAATATCAATATTATTGGGAAATAGTTAATAAAAAAATTAAACA [12558]
lp36_B31_NX_Pl_consensus     AAAATTTTATTTTAAAAAAAGATATTTATTAATTATAATATCAATATTATTGGGAAATAGTTAATAAAAAAATTAAACA [12558]
lp36_PAli_NX_MP_FG_consensus AAAATTTTATTTTAAAAAAAGATATTTATTAATTATAATATCAATATTATTGGGAAATAGTTAATAAAAAAATTAAACA [12558]
lp36_PAli_NX_Pl_consensus    AAAATTTTATTTTAAAAAAAGATATTTATTAATTATAATATCAATATTATTGGGAAATAGTTAATAAAAAAATTAAACA [12558]
lp36_PAbe_NX_MP_FG_consensus AAAATTTTATTTTAAAAAAAGATATTTATTAATTATAATATCAATATTATTGGGAAATAGTTAATAAAAAAATTAAACA [12558]
lp36_PAbe_NX_Pl_consensus    AAAATTTTATTTTAAAAAAAGATATTTATTAATTATAATATCAATATTATTGGGAAATAGTTAATAAAAAAATTAAACA [12558]

lp36_gi|11496779|B31_GB      TATACTTTTAAATAAAGTAAATATTAAATTAAGGAGTTATAAATTGAAAAAATATATTATCAATTTAAGTTTATGTTT [12636]
lp36_un11_B31_PacBio         TATACTTTTAAATAAAGTAAATATTAAATTAAGGAGTTATAAATTGAAAAAATATATTATCAATTTAAGTTTATGTTT [12636]
lp36_B31_TS_MP_FG_consensus  TATACTTTTAAATAAAGTAAATATTAAATTAAGGAGTTATAAATTGAAAAAATATATTATCAATTTAAGTTTATGTTT [12636]
lp36_B31_NX_Pl_consensus     TATACTTTTAAATAAAGTAAATATTAAATTAAGGAGTTATAAATTGAAAAAATATATTATCAATTTAAGTTTATGTTT [12636]
lp36_PAli_NX_MP_FG_consensus TATACTTTTAAATAAAGTAAATATTAAATTAAGGAGTTATAAATTGAAAAAATATATTATCAATTTAAGTTTATGTTT [12636]
lp36_PAli_NX_Pl_consensus    TATACTTTTAAATAAAGTAAATATTAAATTAAGGAGTTATAAATTGAAAAAATATATTATCAATTTAAGTTTATGTTT [12636]
lp36_PAbe_NX_MP_FG_consensus TATACTTTTAAATAAAGTAAATATTAAATTAAGGAGTTATAAATTGAAAAAATATATTATCAATTTAAGTTTATGTTT [12636]
lp36_PAbe_NX_Pl_consensus    TATACTTTTAAATAAAGTAAATATTAAATTAAGGAGTTATAAATTGAAAAAATATATTATCAATTTAAGTTTATGTTT [12636]

lp36_gi|11496779|B31_GB      ATTATTGCTTTCATGCAATCTTTTTTCAAAAGATTCTCGATCACGCCAAAAATACAATTTTAAAGTACCCGCTAAATC [12714]
lp36_un11_B31_PacBio         ATTATTGCTTTCATGCAATCTTTTTTCAAAAGATTCTCGATCACGCCAAAAATACAATTTTAAAGTACCCGCTAAATC [12714]
lp36_B31_TS_MP_FG_consensus  ATTATTGCTTTCATGCAATCTTTTTTCAAAAGATTCTCGATCACGCCAAAAATACAATTTTAAAGTACCCGCTAAATC [12714]
lp36_B31_NX_Pl_consensus     ATTATTGCTTTCATGCAATCTTTTTTCAAAAGATTCTCGATCACGCCAAAAATACAATTTTAAAGTACCCGCTAAATC [12714]
lp36_PAli_NX_MP_FG_consensus ATTATTGCTTTCATGCAATCTTTTTTCAAAAGATTCTCGATCACGCCAAAAATACAATTTTAAAGTACCCGCTAAATC [12714]
lp36_PAli_NX_Pl_consensus    ATTATTGCTTTCATGCAATCTTTTTTCAAAAGATTCTCGATCACGCCAAAAATACAATTTTAAAGTACCCGCTAAATC [12714]
lp36_PAbe_NX_MP_FG_consensus ATTATTGCTTTCATGCAATCTTTTTTCAAAAGATTCTCGATCACGCCAAAAATACAATTTTAAAGTACCCGCTAAATC [12714]
lp36_PAbe_NX_Pl_consensus    ATTATTGCTTTCATGCAATCTTTTTTCAAAAGATTCTCGATCACGCCAAAAATACAATTTTAAAGTACCCGCTAAATC [12714]

lp36_gi|11496779|B31_GB      GGTTTCAAATCCTATCAATAAAGAAAATATAGACACTGAAAAAGGTACTAACACTACACTTTGCATAAAAGAAAAAGA [12792]
lp36_un11_B31_PacBio         GGTTTCAAATCCTATCAATAAAGAAAATATAGACACTGAAAAAGGTACTAACACTACACTTTGCATAAAAGAAAAAGA [12792]
lp36_B31_TS_MP_FG_consensus  GGTTTCAAATCCTATCAATAAAGAAAATATAGACACTGAAAAAGGTACTAACACTACACTTTGCATAAAAGAAAAAGA [12792]
lp36_B31_NX_Pl_consensus     GGTTTCAAATCCTATCAATAAAGAAAATATAGACACTGAAAAAGGTACTAACACTACACTTTGCATAAAAGAAAAAGA [12792]
lp36_PAli_NX_MP_FG_consensus GGTTTCAAATCCTATCAATAAAGAAAATATAGACACTGAAAAAGGTACTAACACTACACTTTGCATAAAAGAAAAAGA [12792]
lp36_PAli_NX_Pl_consensus    GGTTTCAAATCCTATCAATAAAGAAAATATAGACACTGAAAAAGGTACTAACACTACACTTTGCATAAAAGAAAAAGA [12792]
lp36_PAbe_NX_MP_FG_consensus GGTTTCAAATCCTATCAATAAAGAAAATATAGACACTGAAAAAGGTACTAACACTACACTTTGCATAAAAGAAAAAGA [12792]
lp36_PAbe_NX_Pl_consensus    GGTTTCAAATCCTATCAATAAAGAAAATATAGACACTGAAAAAGGTACTAACACTACACTTTGCATAAAAGAAAAAGA [12792]

lp36_gi|11496779|B31_GB      TAGCAGAATTATAATTAAAGATTGTATTAATAATCAAGAGCTTTTTAAAGTAAAATCTAAAAGAAGATATGATTTTAA [12870]
lp36_un11_B31_PacBio         TAGCAGAATTATAATTAAAGATTGTATTAATAATCAAGAGCTTTTTAAAGTAAAATCTAAAAGAAGATATGATTTTAA [12870]
lp36_B31_TS_MP_FG_consensus  TAGCAGAATTATAATTAAAGATTGTATTAATAATCAAGAGCTTTTTAAAGTAAAATCTAAAAGAAGATATGATTTTAA [12870]
lp36_B31_NX_Pl_consensus     TAGCAGAATTATAATTAAAGATTGTATTAATAATCAAGAGCTTTTTAAAGTAAAATCTAAAAGAAGATATGATTTTAA [12870]
lp36_PAli_NX_MP_FG_consensus TAGCAGAATTATAATTAAAGATTGTATTAATAATCAAGAGCTTTTTAAAGTAAAATCTAAAAGAAGATATGATTTTAA [12870]
lp36_PAli_NX_Pl_consensus    TAGCAGAATTATAATTAAAGATTGTATTAATAATCAAGAGCTTTTTAAAGTAAAATCTAAAAGAAGATATGATTTTAA [12870]
lp36_PAbe_NX_MP_FG_consensus TAGCAGAATTATAATTAAAGATTGTATTAATAATCAAGAGCTTTTTAAAGTAAAATCTAAAAGAAGATATGATTTTAA [12870]
lp36_PAbe_NX_Pl_consensus    TAGCAGAATTATAATTAAAGATTGTATTAATAATCAAGAGCTTTTTAAAGTAAAATCTAAAAGAAGATATGATTTTAA [12870]

lp36_gi|11496779|B31_GB      AAAAGCCATGCTTCTTGGAATTCAAACAGCTTTAAAAGTTATAAATATTGGCAATAATAATAAAAAAATTAACTTCCAT [12948]
lp36_un11_B31_PacBio         AAAAGCCATGCTTCTTGGAATTCAAACAGCTTTAAAAGTTATAAATATTGGCAATAATAATAAAAAAATTAACTTCCAT [12948]
lp36_B31_TS_MP_FG_consensus  AAAAGCCATGCTTCTTGGAATTCAAACAGCTTTAAAAGTTATAAATATTGGCAATAATAATAAAAAAATTAACTTCCAT [12948]
lp36_B31_NX_Pl_consensus     AAAAGCCATGCTTCTTGGAATTCAAACAGCTTTAAAAGTTATAAATATTGGCAATAATAATAAAAAAATTAACTTCCAT [12948]
lp36_PAli_NX_MP_FG_consensus AAAAGCCATGCTTCTTGGAATTCAAACAGCTTTAAAAGTTATAAATATTGGCAATAATAATAAAAAAATTAACTTCCAT [12948]
lp36_PAli_NX_Pl_consensus    AAAAGCCATGCTTCTTGGAATTCAAACAGCTTTAAAAGTTATAAATATTGGCAATAATAATAAAAAAATTAACTTCCAT [12948]
lp36_PAbe_NX_MP_FG_consensus AAAAGCCATGCTTCTTGGAATTCAAACAGCTTTAAAAGTTATAAATATTGGCAATAATAATAAAAAAATTAACTTCCAT [12948]
lp36_PAbe_NX_Pl_consensus    AAAAGCCATGCTTCTTGGAATTCAAACAGCTTTAAAAGTTATAAATATTGGCAATAATAATAAAAAAATTAACTTCCAT [12948]

lp36_gi|11496779|B31_GB      AAAAAAACATAATGATCATATTTTATTAGAATTTAAAGATAATAAGATATATATAATTCGATTATCTGAACTTAAAAA [13026]
lp36_un11_B31_PacBio         AAAAAAACATAATGATCATATTTTATTAGAATTTAAAGATAATAAGATATATATAATTCGATTATCTGAACTTAAAAA [13026]
lp36_B31_TS_MP_FG_consensus  AAAAAAACATAATGATCATATTTTATTAGAATTTAAAGATAATAAGATATATATAATTCGATTATCTGAACTTAAAAA [13026]
lp36_B31_NX_Pl_consensus     AAAAAAACATAATGATCATATTTTATTAGAATTTAAAGATAATAAGATATATATAATTCGATTATCTGAACTTAAAAA [13026]
lp36_PAli_NX_MP_FG_consensus AAAAAAACATAATGATCATATTTTATTAGAATTTAAAGATAATAAGATATATATAATTCGATTATCTGAACTTAAAAA [13026]
lp36_PAli_NX_Pl_consensus    AAAAAAACATAATGATCATATTTTATTAGAATTTAAAGATAATAAGATATATATAATTCGATTATCTGAACTTAAAAA [13026]
lp36_PAbe_NX_MP_FG_consensus AAAAAAACATAATGATCATATTTTATTAGAATTTAAAGATAATAAGATATATATAATTCGATTATCTGAACTTAAAAA [13026]
```

```
lp36_PAbe_NX_Pl_consensus    AAAAAAACATAATGATCATATTTTATTAGAATTTAAAGATAATAAGATATATATAATTCGATTATCTGAACTTAAAAA [13026]

lp36_gi|11496779|B31_GB      ACATTTACTAAAAAGCAAGAAAAAACCACTATTAGGAAGTCCGATACCGGGCGGCGGAGATGCAGAATTTGTAGATGA [13104]
lp36_un11_B31_PacBio         ACATTTACTAAAAAGCAAGAAAAAACCACTATTAGGAAGTCCGATACCGGGCGGCGGAGATGCAGAATTTGTAGATGA [13104]
lp36_B31_TS_MP_FG_consensus  ACATTTACTAAAAAGCAAGAAAAAACCACTATTAGGAAGTCCGATACCGGGCGGCGGAGATGCAGAATTTGTAGATGA [13104]
lp36_B31_NX_Pl_consensus     ACATTTACTAAAAAGCAAGAAAAAACCACTATTAGGAAGTCCGATACCGGGCGGCGGAGATGCAGAATTTGTAGATGA [13104]
lp36_PAli_NX_MP_FG_consensus ACATTTACTAAAAAGCAAGAAAAAACCACTATTAGGAAGTCCGATACCGGGCGGCGGAGATGCAGAATTTGTAGATGA [13104]
lp36_PAli_NX_Pl_consensus    ACATTTACTAAAAAGCAAGAAAAAACCACTATTAGGAAGTCCGATACCGGGCGGCGGAGATGCAGAATTTGTAGATGA [13104]
lp36_PAbe_NX_MP_FG_consensus ACATTTACTAAAAAGCAAGAAAAAACCACTATTAGGAAGTCCGATACCGGGCGGCGGAGATGCAGAATTTGTAGATGA [13104]
lp36_PAbe_NX_Pl_consensus    ACATTTACTAAAAAGCAAGAAAAAACCACTATTAGGAAGTCCGATACCGGGCGGCGGAGATGCAGAATTTGTAGATGA [13104]

lp36_gi|11496779|B31_GB      TCCTGATGGCAGAATAGAAGCAGAATTAGAGGCAGAACAAGAGCAAGAGATGTTAGATAGAGAAGATTTTGGAGACGA [13182]
lp36_un11_B31_PacBio         TCCTGATGGCAGAATAGAAGCAGAATTAGAGGCAGAACAAGAGCAAGAGATGTTAGATAGAGAAGATTTTGGAGACGA [13182]
lp36_B31_TS_MP_FG_consensus  TCCTGATGGCAGAATAGAAGCAGAATTAGAGGCAGAACAAGAGCAAGAGATGTTAGATAGAGAAGATTTTGGAGACGA [13182]
lp36_B31_NX_Pl_consensus     TCCTGATGGCAGAATAGAAGCAGAATTAGAGGCAGAACAAGAGCAAGAGATGTTAGATAGAGAAGATTTTGGAGACGA [13182]
lp36_PAli_NX_MP_FG_consensus TCCTGATGGCAGAATAGAAGCAGAATTAGAGGCAGAACAAGAGCAAGAGATGTTAGATAGAGAAGATTTTGGAGACGA [13182]
lp36_PAli_NX_Pl_consensus    TCCTGATGGCAGAATAGAAGCAGAATTAGAGGCAGAACAAGAGCAAGAGATGTTAGATAGAGAAGATTTTGGAGACGA [13182]
lp36_PAbe_NX_MP_FG_consensus TCCTGATGGCAGAATAGAAGCAGAATTAGAGGCAGAACAAGAGCAAGAGATGTTAGATAGAGAAGATTTTGGAGACGA [13182]
lp36_PAbe_NX_Pl_consensus    TCCTGATGGCAGAATAGAAGCAGAATTAGAGGCAGAACAAGAGCAAGAGATGTTAGATAGAGAAGATTTTGGAGACGA [13182]

lp36_gi|11496779|B31_GB      AGAAGACGAAGAATTAGAAGAAGAAATATTTGGAAAAGAAAAACCTAACAATTGAAACTAAAATACTACATTTCAATA [13260]
lp36_un11_B31_PacBio         AGAAGACGAAGAATTAGAAGAAGAAATATTTGGAAAAGAAAAACCTAACAATTGAAACTAAAATACTACATTTCAATA [13260]
lp36_B31_TS_MP_FG_consensus  AGAAGACGAAGAATTAGAAGAAGAAATATTTGGAAAAGAAAAACCTAACAATTGAAACTAAAATACTACATTTCAATA [13260]
lp36_B31_NX_Pl_consensus     AGAAGACGAAGAATTAGAAGAAGAAATATTTGGAAAAGAAAAACCTAACAATTGAAACTAAAATACTACATTTCAATA [13260]
lp36_PAli_NX_MP_FG_consensus AGAAGACGAAGAATTAGAAGAAGAAATATTTGGAAAAGAAAAACCTAACAATTGAAACTAAAATACTACATTTCAATA [13260]
lp36_PAli_NX_Pl_consensus    AGAAGACGAAGAATTAGAAGAAGAAATATTTGGAAAAGAAAAACCTAACAATTGAAACTAAAATACTACATTTCAATA [13260]
lp36_PAbe_NX_MP_FG_consensus AGAAGACGAAGAATTAGAAGAAGAAATATTTGGAAAAGAAAAACCTAACAATTGAAACTAAAATACTACATTTCAATA [13260]
lp36_PAbe_NX_Pl_consensus    AGAAGACGAAGAATTAGAAGAAGAAATATTTGGAAAAGAAAAACCTAACAATTGAAACTAAAATACTACATTTCAATA [13260]

lp36_gi|11496779|B31_GB      AAATAAAGAAGCCCCTAAAAATAGGGGCTTCTTTATTTTATTGAAATGTAACTTGCTAAAGCCACTGTAAATCTAAAG [13338]
lp36_un11_B31_PacBio         AAATAAAGAAGCCCCTAAAAATAGGGGCTTCTTTATTTTATTGAAATGTAACTTGCTAAAGCCACTGTAAATCTAAAG [13338]
lp36_B31_TS_MP_FG_consensus  AAATAAAGAAGCCCCTAAAAATAGGGGCTTCTTTATTTTATTGAAATGTAACTTGCTAAAGCCACTGTAAATCTAAAG [13338]
lp36_B31_NX_Pl_consensus     AAATAAAGAAGCCCCTAAAAATAGGGGCTTCTTTATTTTATTGAAATGTAACTTGCTAAAGCCACTGTAAATCTAAAG [13338]
lp36_PAli_NX_MP_FG_consensus AAATAAAGAAGCCCCTAAAAATAGGGGCTTCTTTATTTTATTGAAATGTAACTTGCTAAAGCCACTGTAAATCTAAAG [13338]
lp36_PAli_NX_Pl_consensus    AAATAAAGAAGCCCCTAAAAATAGGGGCTTCTTTATTTTATTGAAATGTAACTTGCTAAAGCCACTGTAAATCTAAAG [13338]
lp36_PAbe_NX_MP_FG_consensus AAATAAAGAAGCCCCTAAAAATAGGGGCTTCTTTATTTTATTGAAATGTAACTTGCTAAAGCCACTGTAAATCTAAAG [13338]
lp36_PAbe_NX_Pl_consensus    AAATAAAGAAGCCCCTAAAAATAGGGGCTTCTTTATTTTATTGAAATGTAACTTGCTAAAGCCACTGTAAATCTAAAG [13338]

lp36_gi|11496779|B31_GB      AATATCACTTAATTTTAGATTTGTATTTTTAATACTACAATTGATAAGCTTCTAATTCAAATATTTAGAATTTTTTAT [13416]
lp36_un11_B31_PacBio         AATATCACTTAATTTTAGATTTGTATTTTTAATACTACAATTGATAAGCTTCTAATTCAAATATTTAGAATTTTTTAT [13416]
lp36_B31_TS_MP_FG_consensus  AATATCACTTAATTTTAGATTTGTATTTTTAATACTACAATTGATAAGCTTCTAATTCAAATATTTAGAATTTTTTAT [13416]
lp36_B31_NX_Pl_consensus     AATATCACTTAATTTTAGATTTGTATTTTTAATACTACAATTGATAAGCTTCTAATTCAAATATTTAGAATTTTTTAT [13416]
lp36_PAli_NX_MP_FG_consensus AATATCACTTAATTTTAGATTTGTATTTTTAATACTACAATTGATAAGCTTCTAATTCAAATATTTAGAATTTTTTAT [13416]
lp36_PAli_NX_Pl_consensus    AATATCACTTAATTTTAGATTTGTATTTTTAATACTACAATTGATAAGCTTCTAATTCAAATATTTAGAATTTTTTAT [13416]
lp36_PAbe_NX_MP_FG_consensus AATATCACTTAATTTTAGATTTGTATTTTTAATACTACAATTGATAAGCTTCTAATTCAAATATTTAGAATTTTTTAT [13416]
lp36_PAbe_NX_Pl_consensus    AATATCACTTAATTTTAGATTTGTATTTTTAATACTACAATTGATAAGCTTCTAATTCAAATATTTAGAATTTTTTAT [13416]

lp36_gi|11496779|B31_GB      AAGTTTAAATCAATTAGTTTTTTGATGCTAAAATTCAACTTCGTACAATAAAAATAATTAATTTATGGTTAATTTTAT [13494]
lp36_un11_B31_PacBio         AAGTTTAAATCAATTAGTTTTTTGATGCTAAAATTCAACTTCGTACAATAAAAATAATTAATTTATGGTTAATTTTAT [13494]
lp36_B31_TS_MP_FG_consensus  AAGTTTAAATCAATTAGTTTTTTGATGCTAAAATTCAACTTCGTACAATAAAAATAATTAATTTATGGTTAATTTTAT [13494]
lp36_B31_NX_Pl_consensus     AAGTTTAAATCAATTAGTTTTTTGATGCTAAAATTCAACTTCGTACAATAAAAATAATTAATTTATGGTTAATTTTAT [13494]
lp36_PAli_NX_MP_FG_consensus AAGTTTAAATCAATTAGTTTTTTGATGCTAAAATTCAACTTCGTACAATAAAAATAATTAATTTATGGTTAATTTTAT [13494]
lp36_PAli_NX_Pl_consensus    AAGTTTAAATCAATTAGTTTTTTGATGCTAAAATTCAACTTCGTACAATAAAAATAATTAATTTATGGTTAATTTTAT [13494]
lp36_PAbe_NX_MP_FG_consensus AAGTTTAAATCAATTAGTTTTTTGATGCTAAAATTCAACTTCGTACAATAAAAATAATTAATTTATGGTTAATTTTAT [13494]
lp36_PAbe_NX_Pl_consensus    AAGTTTAAATCAATTAGTTTTTTGATGCTAAAATTCAACTTCGTACAATAAAAATAATTAATTTATGGTTAATTTTAT [13494]

lp36_gi|11496779|B31_GB      ATTTTTAAGATCATCAAAACCAATTAGGTAAAATTCTTTATAATTTTTATTTCTAAAATCCAGCAATTAGCAGAATAG [13572]
lp36_un11_B31_PacBio         ATTTTTAAGATCATCAAAACCAATTAGGTAAAATTCTTTATAATTTTTATTTCTAAAATCCAGCAATTAGCAGAATAG [13572]
lp36_B31_TS_MP_FG_consensus  ATTTTTAAGATCATCAAAACCAATTAGGTAAAATTCTTTATAATTTTTATTTCTAAAATCCAGCAATTAGCAGAATAG [13572]
lp36_B31_NX_Pl_consensus     ATTTTTAAGATCATCAAAACCAATTAGGTAAAATTCTTTATAATTTTTATTTCTAAAATCCAGCAATTAGCAGAATAG [13572]
lp36_PAli_NX_MP_FG_consensus ATTTTTAAGATCATCAAAACCAATTAGGTAAAATTCTTTATAATTTTTATTTCTAAAATCCAGCAATTAGCAGAATAG [13572]
lp36_PAli_NX_Pl_consensus    ATTTTTAAGATCATCAAAACCAATTAGGTAAAATTCTTTATAATTTTTATTTCTAAAATCCAGCAATTAGCAGAATAG [13572]
lp36_PAbe_NX_MP_FG_consensus ATTTTTAAGATCATCAAAACCAATTAGGTAAAATTCTTTATAATTTTTATTTCTAAAATCCAGCAATTAGCAGAATAG [13572]
lp36_PAbe_NX_Pl_consensus    ATTTTTAAGATCATCAAAACCAATTAGGTAAAATTCTTTATAATTTTTATTTCTAAAATCCAGCAATTAGCAGAATAG [13572]

lp36_gi|11496779|B31_GB      CGATTTACAGTATATTTTTTAAAGCGCTTTCAGCTTCCCTATAATAAATTTCTTTCAAAGAAGGCTCTAAAAGGTCAT [13650]
lp36_un11_B31_PacBio         CGATTTACAGTATATTTTTTAAAGCGCTTTCAGCTTCCCTATAATAAATTTCTTTCAAAGAAGGCTCTAAAAGGTCAT [13650]
lp36_B31_TS_MP_FG_consensus  CGATTTACAGTATATTTTTTAAAGCGCTTTCAGCTTCCCTATAATAAATTTCTTTCAAAGAAGGCTCTAAAAGGTCAT [13650]
lp36_B31_NX_Pl_consensus     CGATTTACAGTATATTTTTTAAAGCGCTTTCAGCTTCCCTATAATAAATTTCTTTCAAAGAAGGCTCTAAAAGGTCAT [13650]
lp36_PAli_NX_MP_FG_consensus CGATTTACAGTATATTTTTTAAAGCGCTTTCAGCTTCCCTATAATAAATTTCTTTCAAAGAAGGCTCTAAAAGGTCAT [13650]
lp36_PAli_NX_Pl_consensus    CGATTTACAGTATATTTTTTAAAGCGCTTTCAGCTTCCCTATAATAAATTTCTTTCAAAGAAGGCTCTAAAAGGTCAT [13650]
lp36_PAbe_NX_MP_FG_consensus CGATTTACAGTATATTTTTTAAAGCGCTTTCAGCTTCCCTATAATAAATTTCTTTCAAAGAAGGCTCTAAAAGGTCAT [13650]
lp36_PAbe_NX_Pl_consensus    CGATTTACAGTATATTTTTTAAAGCGCTTTCAGCTTCCCTATAATAAATTTCTTTCAAAGAAGGCTCTAAAAGGTCAT [13650]

lp36_gi|11496779|B31_GB      TTATAAAAACTTTTATACTATTTGAAAAGTGAATTTTACCTTTTATATATTTGCCATACTTTTCATAAAGCACTTCTT [13728]
lp36_un11_B31_PacBio         TTATAAAAACTTTTATACTATTTGAAAAGTGAATTTTACCTTTTATATATTTGCCATACTTTTCATAAAGCACTTCTT [13728]
lp36_B31_TS_MP_FG_consensus  TTATAAAAACTTTTATACTATTTGAAAAGTGAATTTTACCTTTTATATATTTGCCATACTTTTCATAAAGCACTTCTT [13728]
lp36_B31_NX_Pl_consensus     TTATAAAAACTTTTATACTATTTGAAAAGTGAATTTTACCTTTTATATATTTGCCATACTTTTCATAAAGCACTTCTT [13728]
lp36_PAli_NX_MP_FG_consensus TTATAAAAACTTTTATACTATTTGAAAAGTGAATTTTACCTTTTATATATTTGCCATACTTTTCATAAAGCACTTCTT [13728]
lp36_PAli_NX_Pl_consensus    TTATAAAAACTTTTATACTATTTGAAAAGTGAATTTTACCTTTTATATATTTGCCATACTTTTCATAAAGCACTTCTT [13728]
lp36_PAbe_NX_MP_FG_consensus TTATAAAAACTTTTATACTATTTGAAAAGTGAATTTTACCTTTTATATATTTGCCATACTTTTCATAAAGCACTTCTT [13728]
lp36_PAbe_NX_Pl_consensus    TTATAAAAACTTTTATACTATTTGAAAAGTGAATTTTACCTTTTATATATTTGCCATACTTTTCATAAAGCACTTCTT [13728]

lp36_gi|11496779|B31_GB      CTACCTCTTTTAAGGTATTCCTATTTTTAATAAATTGGTTTTCTATAATAGAAATATTATATCTTTTGTTTTTAATGA [13806]
lp36_un11_B31_PacBio         CTACCTCTTTTAAGGTATTCCTATTTTTAATAAATTGGTTTTCTATAATAGAAATATTATATCTTTTGTTTTTAATGA [13806]
lp36_B31_TS_MP_FG_consensus  CTACCTCTTTTAAGGTATTCCTATTTTTAATAAATTGGTTTTCTATAATAGAAATATTATATCTTTTGTTTTTAATGA [13806]
lp36_B31_NX_Pl_consensus     CTACCTCTTTTAAGGTATTCCTATTTTTAATAAATTGGTTTTCTATAATAGAAATATTATATCTTTTGTTTTTAATGA [13806]
lp36_PAli_NX_MP_FG_consensus CTACCTCTTTTAAGGTATTCCTATTTTTAATAAATTGGTTTTCTATAATAGAAATATTATATCTTTTGTTTTTAATGA [13806]
lp36_PAli_NX_Pl_consensus    CTACCTCTTTTAAGGTATTCCTATTTTTAATAAATTGGTTTTCTATAATAGAAATATTATATCTTTTGTTTTTAATGA [13806]
lp36_PAbe_NX_MP_FG_consensus CTACCTCTTTTAAGGTATTCCTATTTTTAATAAATTGGTTTTCTATAATAGAAATATTATATCTTTTGTTTTTAATGA [13806]
lp36_PAbe_NX_Pl_consensus    CTACCTCTTTTAAGGTATTCCTATTTTTAATAAATTGGTTTTCTATAATAGAAATATTATATCTTTTGTTTTTAATGA [13806]

lp36_gi|11496779|B31_GB      TCTGAATATTATTTATTGTTTCTGTTAAAATAGAAAAGCTTTCTATTGACCATCTTTCTACTTGAACTGGGATTATAA [13884]
lp36_un11_B31_PacBio         TCTGAATATTATTTATTGTTTCTGTTAAAATAGAAAAGCTTTCTATTGACCATCTTTCTACTTGAACTGGGATTATAA [13884]
lp36_B31_TS_MP_FG_consensus  TCTGAATATTATTTATTGTTTCTGTTAAAATAGAAAAGCTTTCTATTGACCATCTTTCTACTTGAACTGGGATTATAA [13884]
lp36_B31_NX_Pl_consensus     TCTGAATATTATTTATTGTTTCTGTTAAAATAGAAAAGCTTTCTATTGACCATCTTTCTACTTGAACTGGGATTATAA [13884]
lp36_PAli_NX_MP_FG_consensus TCTGAATATTATTTATTGTTTCTGTTAAAATAGAAAAGCTTTCTATTGACCATCTTTCTACTTGAACTGGGATTATAA [13884]
lp36_PAli_NX_Pl_consensus    TCTGAATATTATTTATTGTTTCTGTTAAAATAGAAAAGCTTTCTATTGACCATCTTTCTACTTGAACTGGGATTATAA [13884]
lp36_PAbe_NX_MP_FG_consensus TCTGAATATTATTTATTGTTTCTGTTAAAATAGAAAAGCTTTCTATTGACCATCTTTCTACTTGAACTGGGATTATAA [13884]
lp36_PAbe_NX_Pl_consensus    TCTGAATATTATTTATTGTTTCTGTTAAAATAGAAAAGCTTTCTATTGACCATCTTTCTACTTGAACTGGGATTATAA [13884]

lp36_gi|11496779|B31_GB      TATGGTCTGTAACATTTAGAGCATTCTTTAAAAGGAAATTCCTACTAGGAGAAGTATCTATTATAATATAATCAAAAT [13962]
lp36_un11_B31_PacBio         TATGGTCTGTAACATTTAGAGCATTCTTTAAAAGGAAATTCCTACTAGGAGAAGTATCTATTATAATATAATCAAAAT [13962]
lp36_B31_TS_MP_FG_consensus  TATGGTCTGTAACATTTAGAGCATTCTTTAAAAGGAAATTCCTACTAGGAGAAGTATCTATTATAATATAATCAAAAT [13962]
lp36_B31_NX_Pl_consensus     TATGGTCTGTAACATTTAGAGCATTCTTTAAAAGGAAATTCCTACTAGGAGAAGTATCTATTATAATATAATCAAAAT [13962]
lp36_PAli_NX_MP_FG_consensus TATGGTCTGTAACATTTAGAGCATTCTTTAAAAGGAAATTCCTACTAGGAGAAGTATCTATTATAATATAATCAAAAT [13962]
lp36_PAli_NX_Pl_consensus    TATGGTCTGTAACATTTAGAGCATTCTTTAAAAGGAAATTCCTACTAGGAGAAGTATCTATTATAATATAATCAAAAT [13962]
lp36_PAbe_NX_MP_FG_consensus TATGGTCTGTAACATTTAGAGCATTCTTTAAAAGGAAATTCCTACTAGGAGAAGTATCTATTATAATATAATCAAAAT [13962]
lp36_PAbe_NX_Pl_consensus    TATGGTCTGTAACATTTAGAGCATTCTTTAAAAGGAAATTCCTACTAGGAGAAGTATCTATTATAATATAATCAAAAT [13962]
```

```
lp36_gi|11496779|B31_GB      CAAAACTTTTGGTGCTCTTATTTAATCTAAATTCTAAAATAATTTCTTTATAATCTATATCATCCGTGTTAAATTTTT [14040]
lp36_un11_B31_PacBio         CAAAACTTTTGGTGCTCTTATTTAATCTAAATTCTAAAATAATTTCTTTATAATCTATATCATCCGTGTTAAATTTTT [14040]
lp36_B31_TS_MP_FG_consensus  CAAAACTTTTGGTGCTCTTATTTAATCTAAATTCTAAAATAATTTCTTTATAATCTATATCATCCGTGTTAAATTTTT [14040]
lp36_B31_NX_Pl_consensus     CAAAACTTTTGGTGCTCTTATTTAATCTAAATTCTAAAATAATTTCTTTATAATCTATATCATCCGTGTTAAATTTTT [14040]
lp36_PAli_NX_MP_FG_consensus CAAAACTTTTGGTGCTCTTATTTAATCTAAATTCTAAAATAATTTCTTTATAATCTATATCATCCGTGTTAAATTTTT [14040]
lp36_PAli_NX_Pl_consensus    CAAAACTTTTGGTGCTCTTATTTAATCTAAATTCTAAAATAATTTCTTTATAATCTATATCATCCGTGTTAAATTTTT [14040]
lp36_PAbe_NX_MP_FG_consensus CAAAACTTTTGGTGCTCTTATTTAATCTAAATTCTAAAATAATTTCTTTATAATCTATATCATCCGTGTTAAATTTTT [14040]
lp36_PAbe_NX_Pl_consensus    CAAAACTTTTGGTGCTCTTATTTAATCTAAATTCTAAAATAATTTCTTTATAATCTATATCATCCGTGTTAAATTTTT [14040]

lp36_gi|11496779|B31_GB      CTAAAATAGGATGAGAGGGAATTATAGAAATAAATTCATTAATTTTATTTTCACATTTATCAAAATACGTATTTCCTT [14118]
lp36_un11_B31_PacBio         CTAAAATAGGATGAGAGGGAATTATAGAAATAAATTCATTAATTTTATTTTCACATTTATCAAAATACGTATTTCCTT [14118]
lp36_B31_TS_MP_FG_consensus  CTAAAATAGGATGAGAGGGAATTATAGAAATAAATTCATTAATTTTATTTTCACATTTATCAAAATACGTATTTCCTT [14118]
lp36_B31_NX_Pl_consensus     CTAAAATAGGATGAGAGGGAATTATAGAAATAAATTCATTAATTTTATTTTCACATTTATCAAAATACGTATTTCCTT [14118]
lp36_PAli_NX_MP_FG_consensus CTAAAATAGGATGAGAGGGAATTATAGAAATAAATTCATTAATTTTATTTTCACATTTATCAAAATACGTATTTCCTT [14118]
lp36_PAli_NX_Pl_consensus    CTAAAATAGGATGAGAGGGAATTATAGAAATAAATTCATTAATTTTATTTTCACATTTATCAAAATACGTATTTCCTT [14118]
lp36_PAbe_NX_MP_FG_consensus CTAAAATAGGATGAGAGGGAATTATAGAAATAAATTCATTAATTTTATTTTCACATTTATCAAAATACGTATTTCCTT [14118]
lp36_PAbe_NX_Pl_consensus    CTAAAATAGGATGAGAGGGAATTATAGAAATAAATTCATTAATTTTATTTTCACATTTATCAAAATACGTATTTCCTT [14118]

lp36_gi|11496779|B31_GB      TTAAAAATTCATAAACGTTATACTTTTTAATACTTGAAATATACTTATTAAAATAAGAAGTTAAAGAATTTTGTGGAT [14196]
lp36_un11_B31_PacBio         TTAAAAATTCATAAACGTTATACTTTTTAATACTTGAAATATACTTATTAAAATAAGAAGTTAAAGAATTTTGTGGAT [14196]
lp36_B31_TS_MP_FG_consensus  TTAAAAATTCATAAACGTTATACTTTTTAATACTTGAAATATACTTATTAAAATAAGAAGTTAAAGAATTTTGTGGAT [14196]
lp36_B31_NX_Pl_consensus     TTAAAAATTCATAAACGTTATACTTTTTAATACTTGAAATATACTTATTAAAATAAGAAGTTAAAGAATTTTGTGGAT [14196]
lp36_PAli_NX_MP_FG_consensus TTAAAAATTCATAAACGTTATACTTTTTAATACTTGAAATATACTTATTAAAATAAGAAGTTAAAGAATTTTGTGGAT [14196]
lp36_PAli_NX_Pl_consensus    TTAAAAATTCATAAACGTTATACTTTTTAATACTTGAAATATACTTATTAAAATAAGAAGTTAAAGAATTTTGTGGAT [14196]
lp36_PAbe_NX_MP_FG_consensus TTAAAAATTCATAAACGTTATACTTTTTAATACTTGAAATATACTTATTAAAATAAGAAGTTAAAGAATTTTGTGGAT [14196]
lp36_PAbe_NX_Pl_consensus    TTAAAAATTCATAAACGTTATACTTTTTAATACTTGAAATATACTTATTAAAATAAGAAGTTAAAGAATTTTGTGGAT [14196]

lp36_gi|11496779|B31_GB      CTAAATCAATCAGTAGCACTTTTTTCCCCAATTCTTTTAAAACATAAGAAAAAAGTATAGAAAGTGAACTTTTGCCGA [14274]
lp36_un11_B31_PacBio         CTAAATCAATCAGTAGCACTTTTTTCCCCAATTCTTTTAAAACATAAGAAAAAAGTATAGAAAGTGAACTTTTGCCGA [14274]
lp36_B31_TS_MP_FG_consensus  CTAAATCAATCAGTAGCACTTTTTTCCCCAATTCTTTTAAAACATAAGAAAAAAGTATAGAAAGTGAACTTTTGCCGA [14274]
lp36_B31_NX_Pl_consensus     CTAAATCAATCAGTAGCACTTTTTTCCCCAATTCTTTTAAAACATAAGAAAAAAGTATAGAAAGTGAACTTTTGCCGA [14274]
lp36_PAli_NX_MP_FG_consensus CTAAATCAATCAGTAGCACTTTTTTCCCCAATTCTTTTAAAACATAAGAAAAAAGTATAGAAAGTGAACTTTTGCCGA [14274]
lp36_PAli_NX_Pl_consensus    CTAAATCAATCAGTAGCACTTTTTTCCCCAATTCTTTTAAAACATAAGAAAAAAGTATAGAAAGTGAACTTTTGCCGA [14274]
lp36_PAbe_NX_MP_FG_consensus CTAAATCAATCAGTAGCACTTTTTTCCCCAATTCTTTTAAAACATAAGAAAAAAGTATAGAAAGTGAACTTTTGCCGA [14274]
lp36_PAbe_NX_Pl_consensus    CTAAATCAATCAGTAGCACTTTTTTCCCCAATTCTTTTAAAACATAAGAAAAAAGTATAGAAAGTGAACTTTTGCCGA [14274]

lp36_gi|11496779|B31_GB      CTCCTCCTTTTAAACTTGCAAGTGTAATTACAATTGGTTTTTTTTGATCCATTTATTTATAACCCCCCCATCTTTTAG [14352]
lp36_un11_B31_PacBio         CTCCTCCTTTTAAACTTGCAAGTGTAATTACAATTGGTTTTTTTTGATCCATTTATTTATAACCCCCCCATCTTTTAG [14352]
lp36_B31_TS_MP_FG_consensus  CTCCTCCTTTTAAACTTGCAAGTGTAATTACAATTGGTTTTTTTTGATCCATTTATTTATAACCCCCCCATCTTTTAG [14352]
lp36_B31_NX_Pl_consensus     CTCCTCCTTTTAAACTTGCAAGTGTAATTACAATTGGTTTTTTTTGATCCATTTATTTATAACCCCCCCATCTTTTAG [14352]
lp36_PAli_NX_MP_FG_consensus CTCCTCCTTTTAAACTTGCAAGTGTAATTACAATTGGTTTTTTTTGATCCATTTATTTATAACCCCCCCATCTTTTAG [14352]
lp36_PAli_NX_Pl_consensus    CTCCTCCTTTTAAACTTGCAAGTGTAATTACAATTGGTTTTTTTTGATCCATTTATTTATAACCCCCCCATCTTTTAG [14352]
lp36_PAbe_NX_MP_FG_consensus CTCCTCCTTTTAAACTTGCAAGTGTAATTACAATTGGTTTTTTTTGATCCATTTATTTATAACCCCCCCATCTTTTAG [14352]
lp36_PAbe_NX_Pl_consensus    CTCCTCCTTTTAAACTTGCAAGTGTAATTACAATTGGTTTTTTTTGATCCATTTATTTATAACCCCCCCATCTTTTAG [14352]

lp36_gi|11496779|B31_GB      TTTTTTATTATAAAATTCATATACTTTTTTTTCCATTTTTTTCAAATGTGATAAATTGAATTGATAATACTCGGTAGT [14430]
lp36_un11_B31_PacBio         TTTTTTATTATAAAATTCATATACTTTTTTTTCCATTTTTTTCAAATGTGATAAATTGAATTGATAATACTCGGTAGT [14430]
lp36_B31_TS_MP_FG_consensus  TTTTTTATTATAAAATTCATATACTTTTTTTTCCATTTTTTTCAAATGTGATAAATTGAATTGATAATACTCGGTAGT [14430]
lp36_B31_NX_Pl_consensus     TTTTTTATTATAAAATTCATATACTTTTTTTTCCATTTTTTTCAAATGTGATAAATTGAATTGATAATACTCGGTAGT [14430]
lp36_PAli_NX_MP_FG_consensus TTTTTTATTATAAAATTCATATACTTTTTTTTCCATTTTTTTCAAATGTGATAAATTGAATTGATAATACTCGGTAGT [14430]
lp36_PAli_NX_Pl_consensus    TTTTTTATTATAAAATTCATATACTTTTTTTTCCATTTTTTTCAAATGTGATAAATTGAATTGATAATACTCGGTAGT [14430]
lp36_PAbe_NX_MP_FG_consensus TTTTTTATTATAAAATTCATATACTTTTTTTTCCATTTTTTTCAAATGTGATAAATTGAATTGATAATACTCGGTAGT [14430]
lp36_PAbe_NX_Pl_consensus    TTTTTTATTATAAAATTCATATACTTTTTTTTCCATTTTTTTCAAATGTGATAAATTGAATTGATAATACTCGGTAGT [14430]

lp36_gi|11496779|B31_GB      TTCTTTATTTTTTCTTAAAAGTGTTCTTAAAGATTGAACATAGGCTTTAATAGAGCCATTTTTAAAGACAAACTCTAT [14508]
lp36_un11_B31_PacBio         TTCTTTATTTTTTCTTAAAAGTGTTCTTAAAGATTGAACATAGGCTTTAATAGAGCCATTTTTAAAGACAAACTCTAT [14508]
lp36_B31_TS_MP_FG_consensus  TTCTTTATTTTTTCTTAAAAGTGTTCTTAAAGATTGAACATAGGCTTTAATAGAGCCATTTTTAAAGACAAACTCTAT [14508]
lp36_B31_NX_Pl_consensus     TTCTTTATTTTTTCTTAAAAGTGTTCTTAAAGATTGAACATAGGCTTTAATAGAGCCATTTTTAAAGACAAACTCTAT [14508]
lp36_PAli_NX_MP_FG_consensus TTCTTTATTTTTTCTTAAAAGTGTTCTTAAAGATTGAACATAGGCTTTAATAGAGCCATTTTTAAAGACAAACTCTAT [14508]
lp36_PAli_NX_Pl_consensus    TTCTTTATTTTTTCTTAAAAGTGTTCTTAAAGATTGAACATAGGCTTTAATAGAGCCATTTTTAAAGACAAACTCTAT [14508]
lp36_PAbe_NX_MP_FG_consensus TTCTTTATTTTTTCTTAAAAGTGTTCTTAAAGATTGAACATAGGCTTTAATAGAGCCATTTTTAAAGACAAACTCTAT [14508]
lp36_PAbe_NX_Pl_consensus    TTCTTTATTTTTTCTTAAAAGTGTTCTTAAAGATTGAACATAGGCTTTAATAGAGCCATTTTTAAAGACAAACTCTAT [14508]

lp36_gi|11496779|B31_GB      ATAATAGAGCTTTTTTATTGCATAAGTTTTATTATTTTCTTTTTTAAGAAAAAAGGGTCTATCAAGTTTATCATGCTT [14586]
lp36_un11_B31_PacBio         ATAATAGAGCTTTTTTATTGCATAAGTTTTATTATTTTCTTTTTTAAGAAAAAAGGGTCTATCAAGTTTATCATGCTT [14586]
lp36_B31_TS_MP_FG_consensus  ATAATAGAGCTTTTTTATTGCATAAGTTTTATTATTTTCTTTTTTAAGAAAAAAGGGTCTATCAAGTTTATCATGCTT [14586]
lp36_B31_NX_Pl_consensus     ATAATAGAGCTTTTTTATTGCATAAGTTTTATTATTTTCTTTTTTAAGAAAAAAGGGTCTATCAAGTTTATCATGCTT [14586]
lp36_PAli_NX_MP_FG_consensus ATAATAGAGCTTTTTTATTGCATAAGTTTTATTATTTTCTTTTTTAAGAAAAAAGGGTCTATCAAGTTTATCATGCTT [14586]
lp36_PAli_NX_Pl_consensus    ATAATAGAGCTTTTTTATTGCATAAGTTTTATTATTTTCTTTTTTAAGAAAAAAGGGTCTATCAAGTTTATCATGCTT [14586]
lp36_PAbe_NX_MP_FG_consensus ATAATAGAGCTTTTTTATTGCATAAGTTTTATTATTTTCTTTTTTAAGAAAAAAGGGTCTATCAAGTTTATCATGCTT [14586]
lp36_PAbe_NX_Pl_consensus    ATAATAGAGCTTTTTTATTGCATAAGTTTTATTATTTTCTTTTTTAAGAAAAAAGGGTCTATCAAGTTTATCATGCTT [14586]

lp36_gi|11496779|B31_GB      ATATTTTATTTCTAGGAACTTATCATTTTCTCTTAAAGGAAATAAATTGAAGAAAAGAAAATCTTCATTGTTATTAAA [14664]
lp36_un11_B31_PacBio         ATATTTTATTTCTAGGAACTTATCATTTTCTCTTAAAGGAAATAAATTGAAGAAAAGAAAATCTTCATTGTTATTAAA [14664]
lp36_B31_TS_MP_FG_consensus  ATATTTTATTTCTAGGAACTTATCATTTTCTCTTAAAGGAAATAAATTGAAGAAAAGAAAATCTTCATTGTTATTAAA [14664]
lp36_B31_NX_Pl_consensus     ATATTTTATTTCTAGGAACTTATCATTTTCTCTTAAAGGAAATAAATTGAAGAAAAGAAAATCTTCATTGTTATTAAA [14664]
lp36_PAli_NX_MP_FG_consensus ATATTTTATTTCTAGGAACTTATCATTTTCTCTTAAAGGAAATAAATTGAAGAAAAGAAAATCTTCATTGTTATTAAA [14664]
lp36_PAli_NX_Pl_consensus    ATATTTTATTTCTAGGAACTTATCATTTTCTCTTAAAGGAAATAAATTGAAGAAAAGAAAATCTTCATTGTTATTAAA [14664]
lp36_PAbe_NX_MP_FG_consensus ATATTTTATTTCTAGGAACTTATCATTTTCTCTTAAAGGAAATAAATTGAAGAAAAGAAAATCTTCATTGTTATTAAA [14664]
lp36_PAbe_NX_Pl_consensus    ATATTTTATTTCTAGGAACTTATCATTTTCTCTTAAAGGAAATAAATTGAAGAAAAGAAAATCTTCATTGTTATTAAA [14664]

lp36_gi|11496779|B31_GB      TTCTTGAAAAGTAAGTCTTAGCCTTTTTCCTTTGTTTGTAATTTTAAAACTAACTAAATGTTTAAATATTTTTGTACA [14742]
lp36_un11_B31_PacBio         TTCTTGAAAAGTAAGTCTTAGCCTTTTTCCTTTGTTTGTAATTTTAAAACTAACTAAATGTTTAAATATTTTTGTACA [14742]
lp36_B31_TS_MP_FG_consensus  TTCTTGAAAAGTAAGTCTTAGCCTTTTTCCTTTGTTTGTAATTTTAAAACTAACTAAATGTTTAAATATTTTTGTACA [14742]
lp36_B31_NX_Pl_consensus     TTCTTGAAAAGTAAGTCTTAGCCTTTTTCCTTTGTTTGTAATTTTAAAACTAACTAAATGTTTAAATATTTTTGTACA [14742]
lp36_PAli_NX_MP_FG_consensus TTCTTGAAAAGTAAGTCTTAGCCTTTTTCCTTTGTTTGTAATTTTAAAACTAACTAAATGTTTAAATATTTTTGTACA [14742]
lp36_PAli_NX_Pl_consensus    TTCTTGAAAAGTAAGTCTTAGCCTTTTTCCTTTGTTTGTAATTTTAAAACTAACTAAATGTTTAAATATTTTTGTACA [14742]
lp36_PAbe_NX_MP_FG_consensus TTCTTGAAAAGTAAGTCTTAGCCTTTTTCCTTTGTTTGTAATTTTAAAACTAACTAAATGTTTAAATATTTTTGTACA [14742]
lp36_PAbe_NX_Pl_consensus    TTCTTGAAAAGTAAGTCTTAGCCTTTTTCCTTTGTTTGTAATTTTAAAACTAACTAAATGTTTAAATATTTTTGTACA [14742]

lp36_gi|11496779|B31_GB      GTATATTTTTCTACTATCTATTTCTTCAATTCTATTGAATATAATTCTTTTTTCTATATCACTTTTTTTGTTTCTGAG [14820]
lp36_un11_B31_PacBio         GTATATTTTTCTACTATCTATTTCTTCAATTCTATTGAATATAATTCTTTTTTCTATATCACTTTTTTTGTTTCTGAG [14820]
lp36_B31_TS_MP_FG_consensus  GTATATTTTTCTACTATCTATTTCTTCAATTCTATTGAATATAATTCTTTTTTCTATATCACTTTTTTTGTTTCTGAG [14820]
lp36_B31_NX_Pl_consensus     GTATATTTTTCTACTATCTATTTCTTCAATTCTATTGAATATAATTCTTTTTTCTATATCACTTTTTTTGTTTCTGAG [14820]
lp36_PAli_NX_MP_FG_consensus GTATATTTTTCTACTATCTATTTCTTCAATTCTATTGAATATAATTCTTTTTTCTATATCACTTTTTTTGTTTCTGAG [14820]
lp36_PAli_NX_Pl_consensus    GTATATTTTTCTACTATCTATTTCTTCAATTCTATTGAATATAATTCTTTTTTCTATATCACTTTTTTTGTTTCTGAG [14820]
lp36_PAbe_NX_MP_FG_consensus GTATATTTTTCTACTATCTATTTCTTCAATTCTATTGAATATAATTCTTTTTTCTATATCACTTTTTTTGTTTCTGAG [14820]
lp36_PAbe_NX_Pl_consensus    GTATATTTTTCTACTATCTATTTCTTCAATTCTATTGAATATAATTCTTTTTTCTATATCACTTTTTTTGTTTCTGAG [14820]

lp36_gi|11496779|B31_GB      TTTTTCAAGCAAATTACTCAAATTTCAATCCCTTTGACAAAATTTTAGATAAAATATTATTGGCTATTGTACCAGCAT [14898]
lp36_un11_B31_PacBio         TTTTTCAAGCAAATTACTCAAATTTCAATCCCTTTGACAAAATTTTAGATAAAATATTATTGGCTATTGTACCAGCAT [14898]
lp36_B31_TS_MP_FG_consensus  TTTTTCAAGCAAATTACTCAAATTTCAATCCCTTTGACAAAATTTTAGATAAAATATTATTGGCTATTGTACCAGCAT [14898]
lp36_B31_NX_Pl_consensus     TTTTTCAAGCAAATTACTCAAATTTCAATCCCTTTGACAAAATTTTAGATAAAATATTATTGGCTATTGTACCAGCAT [14898]
lp36_PAli_NX_MP_FG_consensus TTTTTCAAGCAAATTACTCAAATTTCAATCCCTTTGACAAAATTTTAGATAAAATATTATTGGCTATTGTACCAGCAT [14898]
lp36_PAli_NX_Pl_consensus    TTTTTCAAGCAAATTACTCAAATTTCAATCCCTTTGACAAAATTTTAGATAAAATATTATTGGCTATTGTACCAGCAT [14898]
lp36_PAbe_NX_MP_FG_consensus TTTTTCAAGCAAATTACTCAAATTTCAATCCCTTTGACAAAATTTTAGATAAAATATTATTGGCTATTGTACCAGCAT [14898]
lp36_PAbe_NX_Pl_consensus    TTTTTCAAGCAAATTACTCAAATTTCAATCCCTTTGACAAAATTTTAGATAAAATATTATTGGCTATTGTACCAGCAT [14898]
```

```
lp36_gi|11496779|B31_GB      GTCCTGAAAAGCTAGTTATTTTATCTGCTTTCCATTTAGTAGTATAAATATCTTTAAATTCTCCTGCAAGAGTATAAA [14976]
lp36_un11_B31_PacBio         GTCCTGAAAAGCTAGTTATTTTATCTGCTTTCCATTTAGTAGTATAAATATCTTTAAATTCTCCTGCAAGAGTATAAA [14976]
lp36_B31_TS_MP_FG_consensus  GTCCTGAAAAGCTAGTTATTTTATCTGCTTTCCATTTAGTAGTATAAATATCTTTAAATTCTCCTGCAAGAGTATAAA [14976]
lp36_B31_NX_Pl_consensus     GTCCTGAAAAGCTAGTTATTTTATCTGCTTTCCATTTAGTAGTATAAATATCTTTAAATTCTCCTGCAAGAGTATAAA [14976]
lp36_PAli_NX_MP_FG_consensus GTCCTGAAAAGCTAGTTATTTTATCTGCTTTCCATTTAGTAGTATAAATATCTTTAAATTCTCCTGCAAGAGTATAAA [14976]
lp36_PAli_NX_Pl_consensus    GTCCTGAAAAGCTAGTTATTTTATCTGCTTTCCATTTAGTAGTATAAATATCTTTAAATTCTCCTGCAAGAGTATAAA [14976]
lp36_PAbe_NX_MP_FG_consensus GTCCTGAAAAGCTAGTTATTTTATCTGCTTTCCATTTAGTAGTATAAATATCTTTAAATTCTCCTGCAAGAGTATAAA [14976]
lp36_PAbe_NX_Pl_consensus    GTCCTGAAAAGCTAGTTATTTTATCTGCTTTCCATTTAGTAGTATAAATATCTTTAAATTCTCCTGCAAGAGTATAAA [14976]

lp36_gi|11496779|B31_GB      AATCGGTTTTATAGGGTGCAATTTTTCTCATAAAGTGGATTTTATGCTTATAAATGTTTAATTTGGACAAGAAAAACT [15054]
lp36_un11_B31_PacBio         AATCGGTTTTATAGGGTGCAATTTTTCTCATAAAGTGGATTTTATGCTTATAAATGTTTAATTTGGACAAGAAAAACT [15054]
lp36_B31_TS_MP_FG_consensus  AATCGGTTTTATAGGGTGCAATTTTTCTCATAAAGTGGATTTTATGCTTATAAATGTTTAATTTGGACAAGAAAAACT [15054]
lp36_B31_NX_Pl_consensus     AATCGGTTTTATAGGGTGCAATTTTTCTCATAAAGTGGATTTTATGCTTATAAATGTTTAATTTGGACAAGAAAAACT [15054]
lp36_PAli_NX_MP_FG_consensus AATCGGTTTTATAGGGTGCAATTTTTCTCATAAAGTGGATTTTATGCTTATAAATGTTTAATTTGGACAAGAAAAACT [15054]
lp36_PAli_NX_Pl_consensus    AATCGGTTTTATAGGGTGCAATTTTTCTCATAAAGTGGATTTTATGCTTATAAATGTTTAATTTGGACAAGAAAAACT [15054]
lp36_PAbe_NX_MP_FG_consensus AATCGGTTTTATAGGGTGCAATTTTTCTCATAAAGTGGATTTTATGCTTATAAATGTTTAATTTGGACAAGAAAAACT [15054]
lp36_PAbe_NX_Pl_consensus    AATCGGTTTTATAGGGTGCAATTTTTCTCATAAAGTGGATTTTATGCTTATAAATGTTTAATTTGGACAAGAAAAACT [15054]

lp36_gi|11496779|B31_GB      CTTTAATATCTTTTACCTCATAATCATATTTTAAATAATTTTTAAAATCTTTATAGTTTAATAAAGTGTTTTTTAGTG [15132]
lp36_un11_B31_PacBio         CTTTAATATCTTTTACCTCATAATCATATTTTAAATAATTTTTAAAATCTTTATAGTTTAATAAAGTGTTTTTTAGTG [15132]
lp36_B31_TS_MP_FG_consensus  CTTTAATATCTTTTACCTCATAATCATATTTTAAATAATTTTTAAAATCTTTATAGTTTAATAAAGTGTTTTTTAGTG [15132]
lp36_B31_NX_Pl_consensus     CTTTAATATCTTTTACCTCATAATCATATTTTAAATAATTTTTAAAATCTTTATAGTTTAATAAAGTGTTTTTTAGTG [15132]
lp36_PAli_NX_MP_FG_consensus CTTTAATATCTTTTACCTCATAATCATATTTTAAATAATTTTTAAAATCTTTATAGTTTAATAAAGTGTTTTTTAGTG [15132]
lp36_PAli_NX_Pl_consensus    CTTTAATATCTTTTACCTCATAATCATATTTTAAATAATTTTTAAAATCTTTATAGTTTAATAAAGTGTTTTTTAGTG [15132]
lp36_PAbe_NX_MP_FG_consensus CTTTAATATCTTTTACCTCATAATCATATTTTAAATAATTTTTAAAATCTTTATAGTTTAATAAAGTGTTTTTTAGTG [15132]
lp36_PAbe_NX_Pl_consensus    CTTTAATATCTTTTACCTCATAATCATATTTTAAATAATTTTTAAAATCTTTATAGTTTAATAAAGTGTTTTTTAGTG [15132]

lp36_gi|11496779|B31_GB      TTTTAATTGGGTTTTTTGTATTAGAAACTATGGATTTAATTTTTTTTAAAAAAGAGTTTTCTATAGAATTCTTATTAT [15210]
lp36_un11_B31_PacBio         TTTTAATTGGGTTTTTTGTATTAGAAACTATGGATTTAATTTTTTTTAAAAAAGAGTTTTCTATAGAATTCTTATTAT [15210]
lp36_B31_TS_MP_FG_consensus  TTTTAATTGGGTTTTTTGTATTAGAAACTATGGATTTAATTTTTTTTAAAAAAGAGTTTTCTATAGAATTCTTATTAT [15210]
lp36_B31_NX_Pl_consensus     TTTTAATTGGGTTTTTTGTATTAGAAACTATGGATTTAATTTTTTTTAAAAAAGAGTTTTCTATAGAATTCTTATTAT [15210]
lp36_PAli_NX_MP_FG_consensus TTTTAATTGGGTTTTTTGTATTAGAAACTATGGATTTAATTTTTTTTAAAAAAGAGTTTTCTATAGAATTCTTATTAT [15210]
lp36_PAli_NX_Pl_consensus    TTTTAATTGGGTTTTTTGTATTAGAAACTATGGATTTAATTTTTTTTAAAAAAGAGTTTTCTATAGAATTCTTATTAT [15210]
lp36_PAbe_NX_MP_FG_consensus TTTTAATTGGGTTTTTTGTATTAGAAACTATGGATTTAATTTTTTTTAAAAAAGAGTTTTCTATAGAATTCTTATTAT [15210]
lp36_PAbe_NX_Pl_consensus    TTTTAATTGGGTTTTTTGTATTAGAAACTATGGATTTAATTTTTTTTAAAAAAGAGTTTTCTATAGAATTCTTATTAT [15210]

lp36_gi|11496779|B31_GB      ATATATTTATATATTGTTTATTATATACAGTGATATTTTCAGTGATATTTTGAGGTTTACAATGCTCTTTCTTTTCCC [15288]
lp36_un11_B31_PacBio         ATATATTTATATATTGTTTATTATATACAGTGATATTTTCAGTGATATTTTGAGGTTTACAATGCTCTTTCTTTTCCC [15288]
lp36_B31_TS_MP_FG_consensus  ATATATTTATATATTGTTTATTATATACAGTGATATTTTCAGTGATATTTTGAGGTTTACAATGCTCTTTCTTTTCCC [15288]
lp36_B31_NX_Pl_consensus     ATATATTTATATATTGTTTATTATATACAGTGATATTTTCAGTGATATTTTGAGGTTTACAATGCTCTTTCTTTTCCC [15288]
lp36_PAli_NX_MP_FG_consensus ATATATTTATATATTGTTTATTATATACAGTGATATTTTCAGTGATATTTTGAGGTTTACAATGCTCTTTCTTTTCCC [15288]
lp36_PAli_NX_Pl_consensus    ATATATTTATATATTGTTTATTATATACAGTGATATTTTCAGTGATATTTTGAGGTTTACAATGCTCTTTCTTTTCCC [15288]
lp36_PAbe_NX_MP_FG_consensus ATATATTTATATATTGTTTATTATATACAGTGATATTTTCAGTGATATTTTGAGGTTTACAATGCTCTTTCTTTTCCC [15288]
lp36_PAbe_NX_Pl_consensus    ATATATTTATATATTGTTTATTATATACAGTGATATTTTCAGTGATATTTTGAGGTTTACAATGCTCTTTCTTTTCCC [15288]

lp36_gi|11496779|B31_GB      TTAAAACATCTTTTTCTTTCTTTACTTTTTTAATTAAGTCTGATTTGGTGTTGCTAAAATATGTGTCGATCAATTTAT [15366]
lp36_un11_B31_PacBio         TTAAAACATCTTTTTCTTTCTTTACTTTTTTAATTAAGTCTGATTTGGTGTTGCTAAAATATGTGTCGATCAATTTAT [15366]
lp36_B31_TS_MP_FG_consensus  TTAAAACATCTTTTTCTTTCTTTACTTTTTTAATTAAGTCTGATTTGGTGTTGCTAAAATATGTGTCGATCAATTTAT [15366]
lp36_B31_NX_Pl_consensus     TTAAAACATCTTTTTCTTTCTTTACTTTTTTAATTAAGTCTGATTTGGTGTTGCTAAAATATGTGTCGATCAATTTAT [15366]
lp36_PAli_NX_MP_FG_consensus TTAAAACATCTTTTTCTTTCTTTACTTTTTTAATTAAGTCTGATTTGGTGTTGCTAAAATATGTGTCGATCAATTTAT [15366]
lp36_PAli_NX_Pl_consensus    TTAAAACATCTTTTTCTTTCTTTACTTTTTTAATTAAGTCTGATTTGGTGTTGCTAAAATATGTGTCGATCAATTTAT [15366]
lp36_PAbe_NX_MP_FG_consensus TTAAAACATCTTTTTCTTTCTTTACTTTTTTAATTAAGTCTGATTTGGTGTTGCTAAAATATGTGTCGATCAATTTAT [15366]
lp36_PAbe_NX_Pl_consensus    TTAAAACATCTTTTTCTTTCTTTACTTTTTTAATTAAGTCTGATTTGGTGTTGCTAAAATATGTGTCGATCAATTTAT [15366]

lp36_gi|11496779|B31_GB      ATGCAATAGGGGATGCCTTATATATACAGAGCTTTCCTCTAAATTCTCCGAAGTTATTTGAAAATGTTAGCATTCTTT [15444]
lp36_un11_B31_PacBio         ATGCAATAGGGGATGCCTTATATATACAGAGCTTTCCTCTAAATTCTCCGAAGTTATTTGAAAATGTTAGCATTCTTT [15444]
lp36_B31_TS_MP_FG_consensus  ATGCAATAGGGGATGCCTTATATATACAGAGCTTTCCTCTAAATTCTCCGAAGTTATTTGAAAATGTTAGCATTCTTT [15444]
lp36_B31_NX_Pl_consensus     ATGCAATAGGGGATGCCTTATATATACAGAGCTTTCCTCTAAATTCTCCGAAGTTATTTGAAAATGTTAGCATTCTTT [15444]
lp36_PAli_NX_MP_FG_consensus ATGCAATAGGGGATGCCTTATATATACAGAGCTTTCCTCTAAATTCTCCGAAGTTATTTGAAAATGTTAGCATTCTTT [15444]
lp36_PAli_NX_Pl_consensus    ATGCAATAGGGGATGCCTTATATATACAGAGCTTTCCTCTAAATTCTCCGAAGTTATTTGAAAATGTTAGCATTCTTT [15444]
lp36_PAbe_NX_MP_FG_consensus ATGCAATAGGGGATGCCTTATATATACAGAGCTTTCCTCTAAATTCTCCGAAGTTATTTGAAAATGTTAGCATTCTTT [15444]
lp36_PAbe_NX_Pl_consensus    ATGCAATAGGGGATGCCTTATATATACAGAGCTTTCCTCTAAATTCTCCGAAGTTATTTGAAAATGTTAGCATTCTTT [15444]

lp36_gi|11496779|B31_GB      TTTCCAATGCTTTTAGCTTAACCAAAAATCTTAAATCTATTCTTAGAGTATTAATTGTAGTTTCTTTTTTATTCTCTC [15522]
lp36_un11_B31_PacBio         TTTCCAATGCTTTTAGCTTAACCAAAAATCTTAAATCTATTCTTAGAGTATTAATTGTAGTTTCTTTTTTATTCTCTC [15522]
lp36_B31_TS_MP_FG_consensus  TTTCCAATGCTTTTAGCTTAACCAAAAATCTTAAATCTATTCTTAGAGTATTAATTGTAGTTTCTTTTTTATTCTCTC [15522]
lp36_B31_NX_Pl_consensus     TTTCCAATGCTTTTAGCTTAACCAAAAATCTTAAATCTATTCTTAGAGTATTAATTGTAGTTTCTTTTTTATTCTCTC [15522]
lp36_PAli_NX_MP_FG_consensus TTTCCAATGCTTTTAGCTTAACCAAAAATCTTAAATCTATTCTTAGAGTATTAATTGTAGTTTCTTTTTTATTCTCTC [15522]
lp36_PAli_NX_Pl_consensus    TTTCCAATGCTTTTAGCTTAACCAAAAATCTTAAATCTATTCTTAGAGTATTAATTGTAGTTTCTTTTTTATTCTCTC [15522]
lp36_PAbe_NX_MP_FG_consensus TTTCCAATGCTTTTAGCTTAACCAAAAATCTTAAATCTATTCTTAGAGTATTAATTGTAGTTTCTTTTTTATTCTCTC [15522]
lp36_PAbe_NX_Pl_consensus    TTTCCAATGCTTTTAGCTTAACCAAAAATCTTAAATCTATTCTTAGAGTATTAATTGTAGTTTCTTTTTTATTCTCTC [15522]

lp36_gi|11496779|B31_GB      TATGTATATTTGAGTTTAGCACTCGAAGTATATCTCCTTGATGATGAACCCGAATTTCTTTTAGTTTTTCAGGGTCAA [15600]
lp36_un11_B31_PacBio         TATGTATATTTGAGTTTAGCACTCGAAGTATATCTCCTTGATGATGAACCCGAATTTCTTTTAGTTTTTCAGGGTCAA [15600]
lp36_B31_TS_MP_FG_consensus  TATGTATATTTGAGTTTAGCACTCGAAGTATATCTCCTTGATGATGAACCCGAATTTCTTTTAGTTTTTCAGGGTCAA [15600]
lp36_B31_NX_Pl_consensus     TATGTATATTTGAGTTTAGCACTCGAAGTATATCTCCTTGATGATGAACCCGAATTTCTTTTAGTTTTTCAGGGTCAA [15600]
lp36_PAli_NX_MP_FG_consensus TATGTATATTTGAGTTTAGCACTCGAAGTATATCTCCTTGATGATGAACCCGAATTTCTTTTAGTTTTTCAGGGTCAA [15600]
lp36_PAli_NX_Pl_consensus    TATGTATATTTGAGTTTAGCACTCGAAGTATATCTCCTTGATGATGAACCCGAATTTCTTTTAGTTTTTCAGGGTCAA [15600]
lp36_PAbe_NX_MP_FG_consensus TATGTATATTTGAGTTTAGCACTCGAAGTATATCTCCTTGATGATGAACCCGAATTTCTTTTAGTTTTTCAGGGTCAA [15600]
lp36_PAbe_NX_Pl_consensus    TATGTATATTTGAGTTTAGCACTCGAAGTATATCTCCTTGATGATGAACCCGAATTTCTTTTAGTTTTTCAGGGTCAA [15600]

lp36_gi|11496779|B31_GB      AATGAATTCTGTGTATTGATTCATTATATTTTTTTTCAAACTCTTTATTTAAGTAAATAATAACCGAAATTATTTTTT [15678]
lp36_un11_B31_PacBio         AATGAATTCTGTGTATTGATTCATTATATTTTTTTTCAAACTCTTTATTTAAGTAAATAATAACCGAAATTATTTTTT [15678]
lp36_B31_TS_MP_FG_consensus  AATGAATTCTGTGTATTGATTCATTATATTTTTTTTCAAACTCTTTATTTAAGTAAATAATAACCGAAATTATTTTTT [15678]
lp36_B31_NX_Pl_consensus     AATGAATTCTGTGTATTGATTCATTATATTTTTTTTCAAACTCTTTATTTAAGTAAATAATAACCGAAATTATTTTTT [15678]
lp36_PAli_NX_MP_FG_consensus AATGAATTCTGTGTATTGATTCATTATATTTTTTTTCAAACTCTTTATTTAAGTAAATAATAACCGAAATTATTTTTT [15678]
lp36_PAli_NX_Pl_consensus    AATGAATTCTGTGTATTGATTCATTATATTTTTTTTCAAACTCTTTATTTAAGTAAATAATAACCGAAATTATTTTTT [15678]
lp36_PAbe_NX_MP_FG_consensus AATGAATTCTGTGTATTGATTCATTATATTTTTTTTCAAACTCTTTATTTAAGTAAATAATAACCGAAATTATTTTTT [15678]
lp36_PAbe_NX_Pl_consensus    AATGAATTCTGTGTATTGATTCATTATATTTTTTTTCAAACTCTTTATTTAAGTAAATAATAACCGAAATTATTTTTT [15678]

lp36_gi|11496779|B31_GB      TAAGTTTACAGTCTTTTGAAAGTAATTTATTAGATTTATTGGTATTAATTTGATGGCTATAACTAGGTATAACGGCTT [15756]
lp36_un11_B31_PacBio         TAAGTTTACAGTCTTTTGAAAGTAATTTATTAGATTTATTGGTATTAATTTGATGGCTATAACTAGGTATAACGGCTT [15756]
lp36_B31_TS_MP_FG_consensus  TAAGTTTACAGTCTTTTGAAAGTAATTTATTAGATTTATTGGTATTAATTTGATGGCTATAACTAGGTATAACGGCTT [15756]
lp36_B31_NX_Pl_consensus     TAAGTTTACAGTCTTTTGAAAGTAATTTATTAGATTTATTGGTATTAATTTGATGGCTATAACTAGGTATAACGGCTT [15756]
lp36_PAli_NX_MP_FG_consensus TAAGTTTACAGTCTTTTGAAAGTAATTTATTAGATTTATTGGTATTAATTTGATGGCTATAACTAGGTATAACGGCTT [15756]
lp36_PAli_NX_Pl_consensus    TAAGTTTACAGTCTTTTGAAAGTAATTTATTAGATTTATTGGTATTAATTTGATGGCTATAACTAGGTATAACGGCTT [15756]
lp36_PAbe_NX_MP_FG_consensus TAAGTTTACAGTCTTTTGAAAGTAATTTATTAGATTTATTGGTATTAATTTGATGGCTATAACTAGGTATAACGGCTT [15756]
lp36_PAbe_NX_Pl_consensus    TAAGTTTACAGTCTTTTGAAAGTAATTTATTAGATTTATTGGTATTAATTTGATGGCTATAACTAGGTATAACGGCTT [15756]

lp36_gi|11496779|B31_GB      TCATTTTTTGTCCCTCAGCTTAATGTGTGTTAATTTACAGTTAAAAATTATAGTATATAAAATAATAAATTTCAATCT [15834]
lp36_un11_B31_PacBio         TCATTTTTTGTCCCTCAGCTTAATGTGTGTTAATTTACAGTTAAAAATTATAGTATATAAAATAATAAATTTCAATCT [15834]
lp36_B31_TS_MP_FG_consensus  TCATTTTTTGTCCCTCAGCTTAATGTGTGTTAATTTACAGTTAAAAATTATAGTATATAAAATAATAAATTTCAATCT [15834]
lp36_B31_NX_Pl_consensus     TCATTTTTTGTCCCTCAGCTTAATGTGTGTTAATTTACAGTTAAAAATTATAGTATATAAAATAATAAATTTCAATCT [15834]
lp36_PAli_NX_MP_FG_consensus TCATTTTTTGTCCCTCAGCTTAATGTGTGTTAATTTACAGTTAAAAATTATAGTATATAAAATAATAAATTTCAATCT [15834]
lp36_PAli_NX_Pl_consensus    TCATTTTTTGTCCCTCAGCTTAATGTGTGTTAATTTACAGTTAAAAATTATAGTATATAAAATAATAAATTTCAATCT [15834]
lp36_PAbe_NX_MP_FG_consensus TCATTTTTTGTCCCTCAGCTTAATGTGTGTTAATTTACAGTTAAAAATTATAGTATATAAAATAATAAATTTCAATCT [15834]
lp36_PAbe_NX_Pl_consensus    TCATTTTTTGTCCCTCAGCTTAATGTGTGTTAATTTACAGTTAAAAATTATAGTATATAAAATAATAAATTTCAATCT [15834]

lp36_gi|11496779|B31_GB      TCTGTTAAAATTAAAGCCAAAAACCATTTTAAGAATATTTAAGCTAATTGGGAGAGGGGGTGATTTTTTGACAAAAAA [15912]
```

```
lp36_un11_B31_PacBio         TCTGTTAAAATTAAAGCCAAAAACCATTTTAAGAATATTTAAGCTAATTGGGAGAGGGGGTGATTTTTTGACAAAAAA [15912]
lp36_B31_TS_MP_FG_consensus  TCTGTTAAAATTAAAGCCAAAAACCATTTTAAGAATATTTAAGCTAATTGGGAGAGGGGGTGATTTTTTGACAAAAAA [15912]
lp36_B31_NX_Pl_consensus     TCTGTTAAAATTAAAGCCAAAAACCATTTTAAGAATATTTAAGCTAATTGGGAGAGGGGGTGATTTTTTGACAAAAAA [15912]
lp36_PAli_NX_MP_FG_consensus TCTGTTAAAATTAAAGCCAAAAACCATTTTAAGAATATTTAAGCTAATTGGGAGAGGGGGTGATTTTTTGACAAAAAA [15912]
lp36_PAli_NX_Pl_consensus    TCTGTTAAAATTAAAGCCAAAAACCATTTTAAGAATATTTAAGCTAATTGGGAGAGGGGGTGATTTTTTGACAAAAAA [15912]
lp36_PAbe_NX_MP_FG_consensus TCTGTTAAAATTAAAGCCAAAAACCATTTTAAGAATATTTAAGCTAATTGGGAGAGGGGGTGATTTTTTGACAAAAAA [15912]
lp36_PAbe_NX_Pl_consensus    TCTGTTAAAATTAAAGCCAAAAACCATTTTAAGAATATTTAAGCTAATTGGGAGAGGGGGTGATTTTTTGACAAAAAA [15912]

lp36_gi|11496779|B31_GB      GCATTTACTATTATGCAGTAGCTAGAATGAAGCTTTTTAATAGGCTCAATTAAGAATAGAATAATAAATAACAACCAG [15990]
lp36_un11_B31_PacBio         GCATTTACTATTATGCAGTAGCTAGAATGAAGCTTTTTAATAGGCTCAATTAAGAATAGAATAATAAATAACAACCAG [15990]
lp36_B31_TS_MP_FG_consensus  GCATTTACTATTATGCAGTAGCTAGAATGAAGCTTTTTAATAGGCTCAATTAAGAATAGAATAATAAATAACAACCAG [15990]
lp36_B31_NX_Pl_consensus     GCATTTACTATTATGCAGTAGCTAGAATGAAGCTTTTTAATAGGCTCAATTAAGAATAGAATAATAAATAACAACCAG [15990]
lp36_PAli_NX_MP_FG_consensus GCATTTACTATTATGCAGTAGCTAGAATGAAGCTTTTTAATAGGCTCAATTAAGAATAGAATAATAAATAACAACCAG [15990]
lp36_PAli_NX_Pl_consensus    GCATTTACTATTATGCAGTAGCTAGAATGAAGCTTTTTAATAGGCTCAATTAAGAATAGAATAATAAATAACAACCAG [15990]
lp36_PAbe_NX_MP_FG_consensus GCATTTACTATTATGCAGTAGCTAGAATGAAGCTTTTTAATAGGCTCAATTAAGAATAGAATAATAAATAACAACCAG [15990]
lp36_PAbe_NX_Pl_consensus    GCATTTACTATTATGCAGTAGCTAGAATGAAGCTTTTTAATAGGCTCAATTAAGAATAGAATAATAAATAACAACCAG [15990]

lp36_gi|11496779|B31_GB      GGTGTAAATAATCAAAACAATCTAAAAAAATAGATTTTCTTTAACCACTATTTATATAATCTTTGATTTAGAAATATC [16068]
lp36_un11_B31_PacBio         GGTGTAAATAATCAAAACAATCTAAAAAAATAGATTTTCTTTAACCACTATTTATATAATCTTTGATTTAGAAATATC [16068]
lp36_B31_TS_MP_FG_consensus  GGTGTAAATAATCAAAACAATCTAAAAAAATAGATTTTCTTTAACCACTATTTATATAATCTTTGATTTAGAAATATC [16068]
lp36_B31_NX_Pl_consensus     GGTGTAAATAATCAAAACAATCTAAAAAAATAGATTTTCTTTAACCACTATTTATATAATCTTTGATTTAGAAATATC [16068]
lp36_PAli_NX_MP_FG_consensus GGTGTAAATAATCAAAACAATCTAAAAAAATAGATTTTCTTTAACCACTATTTATATAATCTTTGATTTAGAAATATC [16068]
lp36_PAli_NX_Pl_consensus    GGTGTAAATAATCAAAACAATCTAAAAAAATAGATTTTCTTTAACCACTATTTATATAATCTTTGATTTAGAAATATC [16068]
lp36_PAbe_NX_MP_FG_consensus GGTGTAAATAATCAAAACAATCTAAAAAAATAGATTTTCTTTAACCACTATTTATATAATCTTTGATTTAGAAATATC [16068]
lp36_PAbe_NX_Pl_consensus    GGTGTAAATAATCAAAACAATCTAAAAAAATAGATTTTCTTTAACCACTATTTATATAATCTTTGATTTAGAAATATC [16068]

lp36_gi|11496779|B31_GB      CTCTTGTATTGTTCAGAACCGAACGATTTTATTTGTGAATAATTATTGACACAAATTTTAACTTGTGTCAATAATTAT [16146]
lp36_un11_B31_PacBio         CTCTTGTATTGTTCAGAACCGAACGATTTTATTTGTGAATAATTATTGACACAAATTTTAACTTGTGTCAATAATTAT [16146]
lp36_B31_TS_MP_FG_consensus  CTCTTGTATTGTTCAGAACCGAACGATTTTATTTGTGAATAATTATTGACACAAATTTTAACTTGTGTCAATAATTAT [16146]
lp36_B31_NX_Pl_consensus     CTCTTGTATTGTTCAGAACCGAACGATTTTATTTGTGAATAATTATTGACACAAATTTTAACTTGTGTCAATAATTAT [16146]
lp36_PAli_NX_MP_FG_consensus CTCTTGTATTGTTCAGAACCGAACGATTTTATTTGTGAATAATTATTGACACAAATTTTAACTTGTGTCAATAATTAT [16146]
lp36_PAli_NX_Pl_consensus    CTCTTGTATTGTTCAGAACCGAACGATTTTATTTGTGAATAATTATTGACACAAATTTTAACTTGTGTCAATAATTAT [16146]
lp36_PAbe_NX_MP_FG_consensus CTCTTGTATTGTTCAGAACCGAACGATTTTATTTGTGAATAATTATTGACACAAATTTTAACTTGTGTCAATAATTAT [16146]
lp36_PAbe_NX_Pl_consensus    CTCTTGTATTGTTCAGAACCGAACGATTTTATTTGTGAATAATTATTGACACAAATTTTAACTTGTGTCAATAATTAT [16146]

lp36_gi|11496779|B31_GB      TCATATATTAGTGATATTAGATTTATTATGTGTTTTTGTCTCATGTTATTAGTCTAAACTTATTTTTTAAATGCAAGT [16224]
lp36_un11_B31_PacBio         TCATATATTAGTGATATTAGATTTATTATGTGTTTTTGTCTCATGTTATTAGTCTAAACTTATTTTTTAAATGCAAGT [16224]
lp36_B31_TS_MP_FG_consensus  TCATATATTAGTGATATTAGATTTATTATGTGTTTTTGTCTCATGTTATTAGTCTAAACTTATTTTTTAAATGCAAGT [16224]
lp36_B31_NX_Pl_consensus     TCATATATTAGTGATATTAGATTTATTATGTGTTTTTGTCTCATGTTATTAGTCTAAACTTATTTTTTAAATGCAAGT [16224]
lp36_PAli_NX_MP_FG_consensus TCATATATTAGTGATATTAGATTTATTATGTGTTTTTGTCTCATGTTATTAGTCTAAACTTATTTTTTAAATGCAAGT [16224]
lp36_PAli_NX_Pl_consensus    TCATATATTAGTGATATTAGATTTATTATGTGTTTTTGTCTCATGTTATTAGTCTAAACTTATTTTTTAAATGCAAGT [16224]
lp36_PAbe_NX_MP_FG_consensus TCATATATTAGTGATATTAGATTTATTATGTGTTTTTGTCTCATGTTATTAGTCTAAACTTATTTTTTAAATGCAAGT [16224]
lp36_PAbe_NX_Pl_consensus    TCATATATTAGTGATATTAGATTTATTATGTGTTTTTGTCTCATGTTATTAGTCTAAACTTATTTTTTAAATGCAAGT [16224]

lp36_gi|11496779|B31_GB      TAATGTTAAATACATATAAATATCTGCTTTTAAAGCAATAGGAGTGCAAAATGATAGGGAAAGAAAGTAAAAAGAAAA [16302]
lp36_un11_B31_PacBio         TAATGTTAAATACATATAAATATCTGCTTTTAAAGCAATAGGAGTGCAAAATGATAGGGAAAGAAAGTAAAAAGAAAA [16302]
lp36_B31_TS_MP_FG_consensus  TAATGTTAAATACATATAAATATCTGCTTTTAAAGCAATAGGAGTGCAAAATGATAGGGAAAGAAAGTAAAAAGAAAA [16302]
lp36_B31_NX_Pl_consensus     TAATGTTAAATACATATAAATATCTGCTTTTAAAGCAATAGGAGTGCAAAATGATAGGGAAAGAAAGTAAAAAGAAAA [16302]
lp36_PAli_NX_MP_FG_consensus TAATGTTAAATACATATAAATATCTGCTTTTAAAGCAATAGGAGTGCAAAATGATAGGGAAAGAAAGTAAAAAGAAAA [16302]
lp36_PAli_NX_Pl_consensus    TAATGTTAAATACATATAAATATCTGCTTTTAAAGCAATAGGAGTGCAAAATGATAGGGAAAGAAAGTAAAAAGAAAA [16302]
lp36_PAbe_NX_MP_FG_consensus TAATGTTAAATACATATAAATATCTGCTTTTAAAGCAATAGGAGTGCAAAATGATAGGGAAAGAAAGTAAAAAGAAAA [16302]
lp36_PAbe_NX_Pl_consensus    TAATGTTAAATACATATAAATATCTGCTTTTAAAGCAATAGGAGTGCAAAATGATAGGGAAAGAAAGTAAAAAGAAAA [16302]

lp36_gi|11496779|B31_GB      ATAAAAAAGAGATCATTTTAAATGATAGATTTGAAGATTTTATTGATAAAAGTCAAAATTTAAATAATAATCAAGATG [16380]
lp36_un11_B31_PacBio         ATAAAAAAGAGATCATTTTAAATGATAGATTTGAAGATTTTATTGATAAAAGTCAAAATTTAAATAATAATCAAGATG [16380]
lp36_B31_TS_MP_FG_consensus  ATAAAAAAGAGATCATTTTAAATGATAGATTTGAAGATTTTATTGATAAAAGTCAAAATTTAAATAATAATCAAGATG [16380]
lp36_B31_NX_Pl_consensus     ATAAAAAAGAGATCATTTTAAATGATAGATTTGAAGATTTTATTGATAAAAGTCAAAATTTAAATAATAATCAAGATG [16380]
lp36_PAli_NX_MP_FG_consensus ATAAAAAAGAGATCATTTTAAATGATAGATTTGAAGATTTTATTGATAAAAGTCAAAATTTAAATAATAATCAAGATG [16380]
lp36_PAli_NX_Pl_consensus    ATAAAAAAGAGATCATTTTAAATGATAGATTTGAAGATTTTATTGATAAAAGTCAAAATTTAAATAATAATCAAGATG [16380]
lp36_PAbe_NX_MP_FG_consensus ATAAAAAAGAGATCATTTTAAATGATAGATTTGAAGATTTTATTGATAAAAGTCAAAATTTAAATAATAATCAAGATG [16380]
lp36_PAbe_NX_Pl_consensus    ATAAAAAAGAGATCATTTTAAATGATAGATTTGAAGATTTTATTGATAAAAGTCAAAATTTAAATAATAATCAAGATG [16380]

lp36_gi|11496779|B31_GB      AAAATTTAATAATTTATAATGATCTAAAAGACCAATTAAAGCTTAATTTAAAAGATGATATTGACAATAAAATTCAAA [16458]
lp36_un11_B31_PacBio         AAAATTTAATAATTTATAATGATCTAAAAGACCAATTAAAGCTTAATTTAAAAGATGATATTGACAATAAAATTCAAA [16458]
lp36_B31_TS_MP_FG_consensus  AAAATTTAATAATTTATAATGATCTAAAAGACCAATTAAAGCTTAATTTAAAAGATGATATTGACAATAAAATTCAAA [16458]
lp36_B31_NX_Pl_consensus     AAAATTTAATAATTTATAATGATCTAAAAGACCAATTAAAGCTTAATTTAAAAGATGATATTGACAATAAAATTCAAA [16458]
lp36_PAli_NX_MP_FG_consensus AAAATTTAATAATTTATAATGATCTAAAAGACCAATTAAAGCTTAATTTAAAAGATGATATTGACAATAAAATTCAAA [16458]
lp36_PAli_NX_Pl_consensus    AAAATTTAATAATTTATAATGATCTAAAAGACCAATTAAAGCTTAATTTAAAAGATGATATTGACAATAAAATTCAAA [16458]
lp36_PAbe_NX_MP_FG_consensus AAAATTTAATAATTTATAATGATCTAAAAGACCAATTAAAGCTTAATTTAAAAGATGATATTGACAATAAAATTCAAA [16458]
lp36_PAbe_NX_Pl_consensus    AAAATTTAATAATTTATAATGATCTAAAAGACCAATTAAAGCTTAATTTAAAAGATGATATTGACAATAAAATTCAAA [16458]

lp36_gi|11496779|B31_GB      GAATGAAAATTCTATATGAAATTAAACAAAAAAAGCTTTACAAATATGATGGTTTTAAAAGCTTTGAACAATTTATAA [16536]
lp36_un11_B31_PacBio         GAATGAAAATTCTATATGAAATTAAACAAAAAAAGCTTTACAAATATGATGGTTTTAAAAGCTTTGAACAATTTATAA [16536]
lp36_B31_TS_MP_FG_consensus  GAATGAAAATTCTATATGAAATTAAACAAAAAAAGCTTTACAAATATGATGGTTTTAAAAGCTTTGAACAATTTATAA [16536]
lp36_B31_NX_Pl_consensus     GAATGAAAATTCTATATGAAATTAAACAAAAAAAGCTTTACAAATATGATGGTTTTAAAAGCTTTGAACAATTTATAA [16536]
lp36_PAli_NX_MP_FG_consensus GAATGAAAATTCTATATGAAATTAAACAAAAAAAGCTTTACAAATATGATGGTTTTAAAAGCTTTGAACAATTTATAA [16536]
lp36_PAli_NX_Pl_consensus    GAATGAAAATTCTATATGAAATTAAACAAAAAAAGCTTTACAAATATGATGGTTTTAAAAGCTTTGAACAATTTATAA [16536]
lp36_PAbe_NX_MP_FG_consensus GAATGAAAATTCTATATGAAATTAAACAAAAAAAGCTTTACAAATATGATGGTTTTAAAAGCTTTGAACAATTTATAA [16536]
lp36_PAbe_NX_Pl_consensus    GAATGAAAATTCTATATGAAATTAAACAAAAAAAGCTTTACAAATATGATGGTTTTAAAAGCTTTGAACAATTTATAA [16536]

lp36_gi|11496779|B31_GB      AATCTTTTATAATTGCAAAAACCCAGGCTTATACATATTTAAAAGTTTATGAAAAGGTTTTAGAAGGTGTTATTTCTA [16614]
lp36_un11_B31_PacBio         AATCTTTTATAATTGCAAAAACCCAGGCTTATACATATTTAAAAGTTTATGAAAAGGTTTTAGAAGGTGTTATTTCTA [16614]
lp36_B31_TS_MP_FG_consensus  AATCTTTTATAATTGCAAAAACCCAGGCTTATACATATTTAAAAGTTTATGAAAAGGTTTTAGAAGGTGTTATTTCTA [16614]
lp36_B31_NX_Pl_consensus     AATCTTTTATAATTGCAAAAACCCAGGCTTATACATATTTAAAAGTTTATGAAAAGGTTTTAGAAGGTGTTATTTCTA [16614]
lp36_PAli_NX_MP_FG_consensus AATCTTTTATAATTGCAAAAACCCAGGCTTATACATATTTAAAAGTTTATGAAAAGGTTTTAGAAGGTGTTATTTCTA [16614]
lp36_PAli_NX_Pl_consensus    AATCTTTTATAATTGCAAAAACCCAGGCTTATACATATTTAAAAGTTTATGAAAAGGTTTTAGAAGGTGTTATTTCTA [16614]
lp36_PAbe_NX_MP_FG_consensus AATCTTTTATAATTGCAAAAACCCAGGCTTATACATATTTAAAAGTTTATGAAAAGGTTTTAGAAGGTGTTATTTCTA [16614]
lp36_PAbe_NX_Pl_consensus    AATCTTTTATAATTGCAAAAACCCAGGCTTATACATATTTAAAAGTTTATGAAAAGGTTTTAGAAGGTGTTATTTCTA [16614]

lp36_gi|11496779|B31_GB      TTGATAAAATTAAGGAAGCGGGGTTTAATAGCATATATCACGCCATACAAAAACAAGGGTTATCAGAAATAAATCAAG [16692]
lp36_un11_B31_PacBio         TTGATAAAATTAAGGAAGCGGGGTTTAATAGCATATATCACGCCATACAAAAACAAGGGTTATCAGAAATAAATCAAG [16692]
lp36_B31_TS_MP_FG_consensus  TTGATAAAATTAAGGAAGCGGGGTTTAATAGCATATATCACGCCATACAAAAACAAGGGTTATCAGAAATAAATCAAG [16692]
lp36_B31_NX_Pl_consensus     TTGATAAAATTAAGGAAGCGGGGTTTAATAGCATATATCACGCCATACAAAAACAAGGGTTATCAGAAATAAATCAAG [16692]
lp36_PAli_NX_MP_FG_consensus TTGATAAAATTAAGGAAGCGGGGTTTAATAGCATATATCACGCCATACAAAAACAAGGGTTATCAGAAATAAATCAAG [16692]
lp36_PAli_NX_Pl_consensus    TTGATAAAATTAAGGAAGCGGGGTTTAATAGCATATATCACGCCATACAAAAACAAGGGTTATCAGAAATAAATCAAG [16692]
lp36_PAbe_NX_MP_FG_consensus TTGATAAAATTAAGGAAGCGGGGTTTAATAGCATATATCACGCCATACAAAAACAAGGGTTATCAGAAATAAATCAAG [16692]
lp36_PAbe_NX_Pl_consensus    TTGATAAAATTAAGGAAGCGGGGTTTAATAGCATATATCACGCCATACAAAAACAAGGGTTATCAGAAATAAATCAAG [16692]

lp36_gi|11496779|B31_GB      AAGATATTAATACAAACAACAAAGAAAATATCCCAATTAGAATTTTAATAAAAGATAATGAATTATATGATTTTTGTA [16770]
lp36_un11_B31_PacBio         AAGATATTAATACAAACAACAAAGAAAATATCCCAATTAGAATTTTAATAAAAGATAATGAATTATATGATTTTTGTA [16770]
lp36_B31_TS_MP_FG_consensus  AAGATATTAATACAAACAACAAAGAAAATATCCCAATTAGAATTTTAATAAAAGATAATGAATTATATGATTTTTGTA [16770]
lp36_B31_NX_Pl_consensus     AAGATATTAATACAAACAACAAAGAAAATATCCCAATTAGAATTTTAATAAAAGATAATGAATTATATGATTTTTGTA [16770]
lp36_PAli_NX_MP_FG_consensus AAGATATTAATACAAACAACAAAGAAAATATCCCAATTAGAATTTTAATAAAAGATAATGAATTATATGATTTTTGTA [16770]
lp36_PAli_NX_Pl_consensus    AAGATATTAATACAAACAACAAAGAAAATATCCCAATTAGAATTTTAATAAAAGATAATGAATTATATGATTTTTGTA [16770]
lp36_PAbe_NX_MP_FG_consensus AAGATATTAATACAAACAACAAAGAAAATATCCCAATTAGAATTTTAATAAAAGATAATGAATTATATGATTTTTGTA [16770]
lp36_PAbe_NX_Pl_consensus    AAGATATTAATACAAACAACAAAGAAAATATCCCAATTAGAATTTTAATAAAAGATAATGAATTATATGATTTTTGTA [16770]

lp36_gi|11496779|B31_GB      GAAAAGATACCAAAAGAGTATATTTTGTTTTAAAGGAGATTTTTAAAAATAAAAAAGATATTTTATCGGAGATTATAA [16848]
lp36_un11_B31_PacBio         GAAAAGATACCAAAAGAGTATATTTTGTTTTAAAGGAGATTTTTAAAAATAAAAAAGATATTTTATCGGAGATTATAA [16848]
```

```
lp36_B31_TS_MP_FG_consensus  GAAAAGATACCAAAAGAGTATATTTTGTTTTAAAGGAGATTTTTAAAAATAAAAAAGATATTTTATCGGAGATTATAA [16848]
lp36_B31_NX_Pl_consensus     GAAAAGATACCAAAAGAGTATATTTTGTTTTAAAGGAGATTTTTAAAAATAAAAAAGATATTTTATCGGAGATTATAA [16848]
lp36_PAli_NX_MP_FG_consensus GAAAAGATACCAAAAGAGTATATTTTGTTTTAAAGGAGATTTTTAAAAATAAAAAAGATATTTTATCGGAGATTATAA [16848]
lp36_PAli_NX_Pl_consensus    GAAAAGATACCAAAAGAGTATATTTTGTTTTAAAGGAGATTTTTAAAAATAAAAAAGATATTTTATCGGAGATTATAA [16848]
lp36_PAbe_NX_MP_FG_consensus GAAAAGATACCAAAAGAGTATATTTTGTTTTAAAGGAGATTTTTAAAAATAAAAAAGATATTTTATCGGAGATTATAA [16848]
lp36_PAbe_NX_Pl_consensus    GAAAAGATACCAAAAGAGTATATTTTGTTTTAAAGGAGATTTTTAAAAATAAAAAAGATATTTTATCGGAGATTATAA [16848]

lp36_gi|11496779|B31_GB      TTAAATATGATAATTCTAAAAAGAAAAGGAATAAATAAATTTTCAAGGGTTATTGACTTTAATGCTTAATTAATGATA [16926]
lp36_un11_B31_PacBio         TTAAATATGATAATTCTAAAAAGAAAAGGAATAAATAAATTTTCAAGGGTTATTGACTTTAATGCTTAATTAATGATA [16926]
lp36_B31_TS_MP_FG_consensus  TTAAATATGATAATTCTAAAAAGAAAAGGAATAAATAAATTTTCAAGGGTTATTGACTTTAATGCTTAATTAATGATA [16926]
lp36_B31_NX_Pl_consensus     TTAAATATGATAATTCTAAAAAGAAAAGGAATAAATAAATTTTCAAGGGTTATTGACTTTAATGCTTAATTAATGATA [16926]
lp36_PAli_NX_MP_FG_consensus TTAAATATGATAATTCTAAAAAGAAAAGGAATAAATAAATTTTCAAGGGTTATTGACTTTAATGCTTAATTAATGATA [16926]
lp36_PAli_NX_Pl_consensus    TTAAATATGATAATTCTAAAAAGAAAAGGAATAAATAAATTTTCAAGGGTTATTGACTTTAATGCTTAATTAATGATA [16926]
lp36_PAbe_NX_MP_FG_consensus TTAAATATGATAATTCTAAAAAGAAAAGGAATAAATAAATTTTCAAGGGTTATTGACTTTAATGCTTAATTAATGATA [16926]
lp36_PAbe_NX_Pl_consensus    TTAAATATGATAATTCTAAAAAGAAAAGGAATAAATAAATTTTCAAGGGTTATTGACTTTAATGCTTAATTAATGATA [16926]

lp36_gi|11496779|B31_GB      CTCTATTACTTCTAATAGGCTTAAGTTGTTTTGCCTAGTTTAATGTCGAAAAGATGCCCCGAAAAGGGTTTTTTTCAT [17004]
lp36_un11_B31_PacBio         CTCTATTACTTCTAATAGGCTTAAGTTGTTTTGCCTAGTTTAATGTCGAAAAGATGCCCCGAAAAGGGTTTTTTTCAT [17004]
lp36_B31_TS_MP_FG_consensus  CTCTATTACTTCTAATAGGCTTAAGTTGTTTTGCCTAGTTTAATGTCGAAAAGATGCCCCGAAAAGGGTTTTTTTCAT [17004]
lp36_B31_NX_Pl_consensus     CTCTATTACTTCTAATAGGCTTAAGTTGTTTTGCCTAGTTTAATGTCGAAAAGATGCCCCGAAAAGGGTTTTTTTCAT [17004]
lp36_PAli_NX_MP_FG_consensus CTCTATTACTTCTAATAGGCTTAAGTTGTTTTGCCTAGTTTAATGTCGAAAAGATGCCCCGAAAAGGGTTTTTTTCAT [17004]
lp36_PAli_NX_Pl_consensus    CTCTATTACTTCTAATAGGCTTAAGTTGTTTTGCCTAGTTTAATGTCGAAAAGATGCCCCGAAAAGGGTTTTTTTCAT [17004]
lp36_PAbe_NX_MP_FG_consensus CTCTATTACTTCTAATAGGCTTAAGTTGTTTTGCCTAGTTTAATGTCGAAAAGATGCCCCGAAAAGGGTTTTTTTCAT [17004]
lp36_PAbe_NX_Pl_consensus    CTCTATTACTTCTAATAGGCTTAAGTTGTTTTGCCTAGTTTAATGTCGAAAAGATGCCCCGAAAAGGGTTTTTTTCAT [17004]

lp36_gi|11496779|B31_GB      TTTTAAATCTAAATTTTGGAATTTGACATTGAATGCTAGGTTTTTTAACTTTAAATCCCTGTTGCATGTCCGAGTAAT [17082]
lp36_un11_B31_PacBio         TTTTAAATCTAAATTTTGGAATTTGACATTGAATGCTAGGTTTTTTAACTTTAAATCCCTGTTGCATGTCCGAGTAAT [17082]
lp36_B31_TS_MP_FG_consensus  TTTTAAATCTAAATTTTGGAATTTGACATTGAATGCTAGGTTTTTTAACTTTAAATCCCTGTTGCATGTCCGAGTAAT [17082]
lp36_B31_NX_Pl_consensus     TTTTAAATCTAAATTTTGGAATTTGACATTGAATGCTAGGTTTTTTAACTTTAAATCCCTGTTGCATGTCCGAGTAAT [17082]
lp36_PAli_NX_MP_FG_consensus TTTTAAATCTAAATTTTGGAATTTGACATTGAATGCTAGGTTTTTTAACTTTAAATCCCTGTTGCATGTCCGAGTAAT [17082]
lp36_PAli_NX_Pl_consensus    TTTTAAATCTAAATTTTGGAATTTGACATTGAATGCTAGGTTTTTTAACTTTAAATCCCTGTTGCATGTCCGAGTAAT [17082]
lp36_PAbe_NX_MP_FG_consensus TTTTAAATCTAAATTTTGGAATTTGACATTGAATGCTAGGTTTTTTAACTTTAAATCCCTGTTGCATGTCCGAGTAAT [17082]
lp36_PAbe_NX_Pl_consensus    TTTTAAATCTAAATTTTGGAATTTGACATTGAATGCTAGGTTTTTTAACTTTAAATCCCTGTTGCATGTCCGAGTAAT [17082]

lp36_gi|11496779|B31_GB      TAATAAGCTCTCTATCACTATGTTTTTATAATTATCTACAAAGTAAAGAAATAATAATTTATGTAAAAAATCTTTTCT [17160]
lp36_un11_B31_PacBio         TAATAAGCTCTCTATCACTATGTTTTTATAATTATCTACAAAGTAAAGAAATAATAATTTATGTAAAAAATCTTTTCT [17160]
lp36_B31_TS_MP_FG_consensus  TAATAAGCTCTCTATCACTATGTTTTTATAATTATCTACAAAGTAAAGAAATAATAATTTATGTAAAAAATCTTTTCT [17160]
lp36_B31_NX_Pl_consensus     TAATAAGCTCTCTATCACTATGTTTTTATAATTATCTACAAAGTAAAGAAATAATAATTTATGTAAAAAATCTTTTCT [17160]
lp36_PAli_NX_MP_FG_consensus TAATAAGCTCTCTATCACTATGTTTTTATAATTATCTACAAAGTAAAGAAATAATAATTTATGTAAAAAATCTTTTCT [17160]
lp36_PAli_NX_Pl_consensus    TAATAAGCTCTCTATCACTATGTTTTTATAATTATCTACAAAGTAAAGAAATAATAATTTATGTAAAAAATCTTTTCT [17160]
lp36_PAbe_NX_MP_FG_consensus TAATAAGCTCTCTATCACTATGTTTTTATAATTATCTACAAAGTAAAGAAATAATAATTTATGTAAAAAATCTTTTCT [17160]
lp36_PAbe_NX_Pl_consensus    TAATAAGCTCTCTATCACTATGTTTTTATAATTATCTACAAAGTAAAGAAATAATAATTTATGTAAAAAATCTTTTCT [17160]

lp36_gi|11496779|B31_GB      TTGATTTGAAATTTTCTTATATAGCCTAGCCTCTTAATTTAGATTTGTTTATATTAATAGAACCTTTTTGTTTTTTGA [17238]
lp36_un11_B31_PacBio         TTGATTTGAAATTTTCTTATATAGCCTAGCCTCTTAATTTAGATTTGTTTATATTAATAGAACCTTTTTGTTTTTTGA [17238]
lp36_B31_TS_MP_FG_consensus  TTGATTTGAAATTTTCTTATATAGCCTAGCCTCTTAATTTAGATTTGTTTATATTAATAGAACCTTTTTGTTTTTTGA [17238]
lp36_B31_NX_Pl_consensus     TTGATTTGAAATTTTCTTATATAGCCTAGCCTCTTAATTTAGATTTGTTTATATTAATAGAACCTTTTTGTTTTTTGA [17238]
lp36_PAli_NX_MP_FG_consensus TTGATTTGAAATTTTCTTATATAGCCTAGCCTCTTAATTTAGATTTGTTTATATTAATAGAACCTTTTTGTTTTTTGA [17238]
lp36_PAli_NX_Pl_consensus    TTGATTTGAAATTTTCTTATATAGCCTAGCCTCTTAATTTAGATTTGTTTATATTAATAGAACCTTTTTGTTTTTTGA [17238]
lp36_PAbe_NX_MP_FG_consensus TTGATTTGAAATTTTCTTATATAGCCTAGCCTCTTAATTTAGATTTGTTTATATTAATAGAACCTTTTTGTTTTTTGA [17238]
lp36_PAbe_NX_Pl_consensus    TTGATTTGAAATTTTCTTATATAGCCTAGCCTCTTAATTTAGATTTGTTTATATTAATAGAACCTTTTTGTTTTTTGA [17238]

lp36_gi|11496779|B31_GB      TAATTTTCTTTGGTATTTATTAAGCCTATTTTCATTTTTTGTTACATATTTAGGATGATTTGTTTTTTCACTTTCACT [17316]
lp36_un11_B31_PacBio         TAATTTTCTTTGGTATTTATTAAGCCTATTTTCATTTTTTGTTACATATTTAGGATGATTTGTTTTTTCACTTTCACT [17316]
lp36_B31_TS_MP_FG_consensus  TAATTTTCTTTGGTATTTATTAAGCCTATTTTCATTTTTTGTTACATATTTAGGATGATTTGTTTTTTCACTTTCACT [17316]
lp36_B31_NX_Pl_consensus     TAATTTTCTTTGGTATTTATTAAGCCTATTTTCATTTTTTGTTACATATTTAGGATGATTTGTTTTTTCACTTTCACT [17316]
lp36_PAli_NX_MP_FG_consensus TAATTTTCTTTGGTATTTATTAAGCCTATTTTCATTTTTTGTTACATATTTAGGATGATTTGTTTTTTCACTTTCACT [17316]
lp36_PAli_NX_Pl_consensus    TAATTTTCTTTGGTATTTATTAAGCCTATTTTCATTTTTTGTTACATATTTAGGATGATTTGTTTTTTCACTTTCACT [17316]
lp36_PAbe_NX_MP_FG_consensus TAATTTTCTTTGGTATTTATTAAGCCTATTTTCATTTTTTGTTACATATTTAGGATGATTTGTTTTTTCACTTTCACT [17316]
lp36_PAbe_NX_Pl_consensus    TAATTTTCTTTGGTATTTATTAAGCCTATTTTCATTTTTTGTTACATATTTAGGATGATTTGTTTTTTCACTTTCACT [17316]

lp36_gi|11496779|B31_GB      ACTTGCTAAAAAATTGTAAATCAAAGTCTTTGATTTATTTTTTAATTTTGGGAGGGGATTATTATGAATAAAAACCGG [17394]
lp36_un11_B31_PacBio         ACTTGCTAAAAAATTGTAAATCAAAGTCTTTGATTTATTTTTTAATTTTGGGAGGGGATTATTATGAATAAAAACCGG [17394]
lp36_B31_TS_MP_FG_consensus  ACTTGCTAAAAAATTGTAAATCAAAGTCTTTGATTTATTTTTTAATTTTGGGAGGGGATTATTATGAATAAAAACCGG [17394]
lp36_B31_NX_Pl_consensus     ACTTGCTAAAAAATTGTAAATCAAAGTCTTTGATTTATTTTTTAATTTTGGGAGGGGATTATTATGAATAAAAACCGG [17394]
lp36_PAli_NX_MP_FG_consensus ACTTGCTAAAAAATTGTAAATCAAAGTCTTTGATTTATTTTTTAATTTTGGGAGGGGATTATTATGAATAAAAACCGG [17394]
lp36_PAli_NX_Pl_consensus    ACTTGCTAAAAAATTGTAAATCAAAGTCTTTGATTTATTTTTTAATTTTGGGAGGGGATTATTATGAATAAAAACCGG [17394]
lp36_PAbe_NX_MP_FG_consensus ACTTGCTAAAAAATTGTAAATCAAAGTCTTTGATTTATTTTTTAATTTTGGGAGGGGATTATTATGAATAAAAACCGG [17394]
lp36_PAbe_NX_Pl_consensus    ACTTGCTAAAAAATTGTAAATCAAAGTCTTTGATTTATTTTTTAATTTTGGGAGGGGATTATTATGAATAAAAACCGG [17394]

lp36_gi|11496779|B31_GB      GTTATTACTTGCGTTGTTTTTGCACTGATAAGTTCTTTTAAAAATTTTTCACAGCTTTGTTGCTTATATAGTTCTGAG [17472]
lp36_un11_B31_PacBio         GTTATTACTTGCGTTGTTTTTGCACTGATAAGTTCTTTTAAAAATTTTTCACAGCTTTGTTGCTTATATAGTTCTGAG [17472]
lp36_B31_TS_MP_FG_consensus  GTTATTACTTGCGTTGTTTTTGCACTGATAAGTTCTTTTAAAAATTTTTCACAGCTTTGTTGCTTATATAGTTCTGAG [17472]
lp36_B31_NX_Pl_consensus     GTTATTACTTGCGTTGTTTTTGCACTGATAAGTTCTTTTAAAAATTTTTCACAGCTTTGTTGCTTATATAGTTCTGAG [17472]
lp36_PAli_NX_MP_FG_consensus GTTATTACTTGCGTTGTTTTTGCACTGATAAGTTCTTTTAAAAATTTTTCACAGCTTTGTTGCTTATATAGTTCTGAG [17472]
lp36_PAli_NX_Pl_consensus    GTTATTACTTGCGTTGTTTTTGCACTGATAAGTTCTTTTAAAAATTTTTCACAGCTTTGTTGCTTATATAGTTCTGAG [17472]
lp36_PAbe_NX_MP_FG_consensus GTTATTACTTGCGTTGTTTTTGCACTGATAAGTTCTTTTAAAAATTTTTCACAGCTTTGTTGCTTATATAGTTCTGAG [17472]
lp36_PAbe_NX_Pl_consensus    GTTATTACTTGCGTTGTTTTTGCACTGATAAGTTCTTTTAAAAATTTTTCACAGCTTTGTTGCTTATATAGTTCTGAG [17472]

lp36_gi|11496779|B31_GB      CAAGATGCTTTTAAAGAAGAAATTAACAAAAGACTCTATAATTGTAATTTTAAAAATGAAAAGGATAAAAACATTTTT [17550]
lp36_un11_B31_PacBio         CAAGATGCTTTTAAAGAAGAAATTAACAAAAGACTCTATAATTGTAATTTTAAAAATGAAAAGGATAAAAACATTTTT [17550]
lp36_B31_TS_MP_FG_consensus  CAAGATGCTTTTAAAGAAGAAATTAACAAAAGACTCTATAATTGTAATTTTAAAAATGAAAAGGATAAAAACATTTTT [17550]
lp36_B31_NX_Pl_consensus     CAAGATGCTTTTAAAGAAGAAATTAACAAAAGACTCTATAATTGTAATTTTAAAAATGAAAAGGATAAAAACATTTTT [17550]
lp36_PAli_NX_MP_FG_consensus CAAGATGCTTTTAAAGAAGAAATTAACAAAAGACTCTATAATTGTAATTTTAAAAATGAAAAGGATAAAAACATTTTT [17550]
lp36_PAli_NX_Pl_consensus    CAAGATGCTTTTAAAGAAGAAATTAACAAAAGACTCTATAATTGTAATTTTAAAAATGAAAAGGATAAAAACATTTTT [17550]
lp36_PAbe_NX_MP_FG_consensus CAAGATGCTTTTAAAGAAGAAATTAACAAAAGACTCTATAATTGTAATTTTAAAAATGAAAAGGATAAAAACATTTTT [17550]
lp36_PAbe_NX_Pl_consensus    CAAGATGCTTTTAAAGAAGAAATTAACAAAAGACTCTATAATTGTAATTTTAAAAATGAAAAGGATAAAAACATTTTT [17550]

lp36_gi|11496779|B31_GB      TTGTTTATGCTTCACAAATTGGAGAGCGTTTAAGAAAAGTTCGGATTAAAGAAAAAAGTGTTTTTTAGATACTCTTTA [17628]
lp36_un11_B31_PacBio         TTGTTTATGCTTCACAAATTGGAGAGCGTTTAAGAAAAGTTCGGATTAAAGAAAAAAGTGTTTTTTAGATACTCTTTA [17628]
lp36_B31_TS_MP_FG_consensus  TTGTTTATGCTTCACAAATTGGAGAGCGTTTAAGAAAAGTTCGGATTAAAGAAAAAAGTGTTTTTTAGATACTCTTTA [17628]
lp36_B31_NX_Pl_consensus     TTGTTTATGCTTCACAAATTGGAGAGCGTTTAAGAAAAGTTCGGATTAAAGAAAAAAGTGTTTTTTAGATACTCTTTA [17628]
lp36_PAli_NX_MP_FG_consensus TTGTTTATGCTTCACAAATTGGAGAGCGTTTAAGAAAAGTTCGGATTAAAGAAAAAAGTGTTTTTTAGATACTCTTTA [17628]
lp36_PAli_NX_Pl_consensus    TTGTTTATGCTTCACAAATTGGAGAGCGTTTAAGAAAAGTTCGGATTAAAGAAAAAAGTGTTTTTTAGATACTCTTTA [17628]
lp36_PAbe_NX_MP_FG_consensus TTGTTTATGCTTCACAAATTGGAGAGCGTTTAAGAAAAGTTCGGATTAAAGAAAAAAGTGTTTTTTAGATACTCTTTA [17628]
lp36_PAbe_NX_Pl_consensus    TTGTTTATGCTTCACAAATTGGAGAGCGTTTAAGAAAAGTTCGGATTAAAGAAAAAAGTGTTTTTTAGATACTCTTTA [17628]

lp36_gi|11496779|B31_GB      ATCAAGAGGTGTCTTATAAAAAAATAATAAATCTAATTAATTGAGAATTGATAAAAAGTGAGAAATAAGTTTAAAAGC [17706]
lp36_un11_B31_PacBio         ATCAAGAGGTGTCTTATAAAAAAATAATAAATCTAATTAATTGAGAATTGATAAAAAGTGAGAAATAAGTTTAAAAGC [17706]
lp36_B31_TS_MP_FG_consensus  ATCAAGAGGTGTCTTATAAAAAAATAATAAATCTAATTAATTGAGAATTGATAAAAAGTGAGAAATAAGTTTAAAAGC [17706]
lp36_B31_NX_Pl_consensus     ATCAAGAGGTGTCTTATAAAAAAATAATAAATCTAATTAATTGAGAATTGATAAAAAGTGAGAAATAAGTTTAAAAGC [17706]
lp36_PAli_NX_MP_FG_consensus ATCAAGAGGTGTCTTATAAAAAAATAATAAATCTAATTAATTGAGAATTGATAAAAAGTGAGAAATAAGTTTAAAAGC [17706]
lp36_PAli_NX_Pl_consensus    ATCAAGAGGTGTCTTATAAAAAAATAATAAATCTAATTAATTGAGAATTGATAAAAAGTGAGAAATAAGTTTAAAAGC [17706]
lp36_PAbe_NX_MP_FG_consensus ATCAAGAGGTGTCTTATAAAAAAATAATAAATCTAATTAATTGAGAATTGATAAAAAGTGAGAAATAAGTTTAAAAGC [17706]
lp36_PAbe_NX_Pl_consensus    ATCAAGAGGTGTCTTATAAAAAAATAATAAATCTAATTAATTGAGAATTGATAAAAAGTGAGAAATAAGTTTAAAAGC [17706]

lp36_gi|11496779|B31_GB      TTATTCTAAATATAAATTATTTATAGTCAAGTAAATATTAAAATAAGTTTTGGTCGACTTGCTCTAAGATTAGTTTTT [17784]
lp36_un11_B31_PacBio         TTATTCTAAATATAAATTATTTATAGTCAAGTAAATATTAAAATAAGTTTTGGTCGACTTGCTCTAAGATTAGTTTTT [17784]
lp36_B31_TS_MP_FG_consensus  TTATTCTAAATATAAATTATTTATAGTCAAGTAAATATTAAAATAAGTTTTGGTCGACTTGCTCTAAGATTAGTTTTT [17784]
```

```
lp36_B31_NX_Pl_consensus      TTATTCTAAATATAAATTATTTATAGTCAAGTAAATATTAAAATAAGTTTTGGTCGACTTGCTCTAAGATTAGTTTTT [17784]
lp36_PAli_NX_MP_FG_consensus TTATTCTAAATATAAATTATTTATAGTCAAGTAAATATTAAAATAAGTTTTGGTCGACTTGCTCTAAGATTAGTTTTT [17784]
lp36_PAli_NX_Pl_consensus     TTATTCTAAATATAAATTATTTATAGTCAAGTAAATATTAAAATAAGTTTTGGTCGACTTGCTCTAAGATTAGTTTTT [17784]
lp36_PAbe_NX_MP_FG_consensus TTATTCTAAATATAAATTATTTATAGTCAAGTAAATATTAAAATAAGTTTTGGTCGACTTGCTCTAAGATTAGTTTTT [17784]
lp36_PAbe_NX_Pl_consensus     TTATTCTAAATATAAATTATTTATAGTCAAGTAAATATTAAAATAAGTTTTGGTCGACTTGCTCTAAGATTAGTTTTT [17784]

lp36_gi|11496779|B31_GB       TTAATTAAAAAAAAACTAATCTTAGAGAAGAGTATTGTGAGTATCAAGAAGGGCTAAAAAGGATTTGTAAATAACATGA [17862]
lp36_un11_B31_PacBio          TTAATTAAAAAAAAACTAATCTTAGAGAAGAGTATTGTGAGTATCAAGAAGGGCTAAAAAGGATTTGTAAATAACATGA [17862]
lp36_B31_TS_MP_FG_consensus   TTAATTAAAAAAAAACTAATCTTAGAGAAGAGTATTGTGAGTATCAAGAAGGGCTAAAAAGGATTTGTAAATAACATGA [17862]
lp36_B31_NX_Pl_consensus      TTAATTAAAAAAAAACTAATCTTAGAGAAGAGTATTGTGAGTATCAAGAAGGGCTAAAAAGGATTTGTAAATAACATGA [17862]
lp36_PAli_NX_MP_FG_consensus TTAATTAAAAAAAAACTAATCTTAGAGAAGAGTATTGTGAGTATCAAGAAGGGCTAAAAAGGATTTGTAAATAACATGA [17862]
lp36_PAli_NX_Pl_consensus     TTAATTAAAAAAAAACTAATCTTAGAGAAGAGTATTGTGAGTATCAAGAAGGGCTAAAAAGGATTTGTAAATAACATGA [17862]
lp36_PAbe_NX_MP_FG_consensus TTAATTAAAAAAAAACTAATCTTAGAGAAGAGTATTGTGAGTATCAAGAAGGGCTAAAAAGGATTTGTAAATAACATGA [17862]
lp36_PAbe_NX_Pl_consensus     TTAATTAAAAAAAAACTAATCTTAGAGAAGAGTATTGTGAGTATCAAGAAGGGCTAAAAAGGATTTGTAAATAACATGA [17862]

lp36_gi|11496779|B31_GB       TGGTTTAAACATAAACAATGAAAGCCGATTACATTATAAAAACAAATGATCCAAATGTATCTCTTTATAAAGAACTGT [17940]
lp36_un11_B31_PacBio          TGGTTTAAACATAAACAATGAAAGCCGATTACATTATAAAAACAAATGATCCAAATGTATCTCTTTATAAAGAACTGT [17940]
lp36_B31_TS_MP_FG_consensus   TGGTTTAAACATAAACAATGAAAGCCGATTACATTATAAAAACAAATGATCCAAATGTATCTCTTTATAAAGAACTGT [17940]
lp36_B31_NX_Pl_consensus      TGGTTTAAACATAAACAATGAAAGCCGATTACATTATAAAAACAAATGATCCAAATGTATCTCTTTATAAAGAACTGT [17940]
lp36_PAli_NX_MP_FG_consensus TGGTTTAAACATAAACAATGAAAGCCGATTACATTATAAAAACAAATGATCCAAATGTATCTCTTTATAAAGAACTGT [17940]
lp36_PAli_NX_Pl_consensus     TGGTTTAAACATAAACAATGAAAGCCGATTACATTATAAAAACAAATGATCCAAATGTATCTCTTTATAAAGAACTGT [17940]
lp36_PAbe_NX_MP_FG_consensus TGGTTTAAACATAAACAATGAAAGCCGATTACATTATAAAAACAAATGATCCAAATGTATCTCTTTATAAAGAACTGT [17940]
lp36_PAbe_NX_Pl_consensus     TGGTTTAAACATAAACAATGAAAGCCGATTACATTATAAAAACAAATGATCCAAATGTATCTCTTTATAAAGAACTGT [17940]

lp36_gi|11496779|B31_GB       AAAGAGACTTTATAAGAAAAGAAAGTATTAATAGTTTAAAAGACTTTTTTATTTTTATAAAAAAATAAACTTTCTTCAA [18018]
lp36_un11_B31_PacBio          AAAGAGACTTTATAAGAAAAGAAAGTATTAATAGTTTAAAAGACTTTTTTATTTTTATAAAAAAATAAACTTTCTTCAA [18018]
lp36_B31_TS_MP_FG_consensus   AAAGAGACTTTATAAGAAAAGAAAGTATTAATAGTTTAAAAGACTTTTTTATTTTTATAAAAAAATAAACTTTCTTCAA [18018]
lp36_B31_NX_Pl_consensus      AAAGAGACTTTATAAGAAAAGAAAGTATTAATAGTTTAAAAGACTTTTTTATTTTTATAAAAAAATAAACTTTCTTCAA [18018]
lp36_PAli_NX_MP_FG_consensus AAAGAGACTTTATAAGAAAAGAAAGTATTAATAGTTTAAAAGACTTTTTTATTTTTATAAAAAAATAAACTTTCTTCAA [18018]
lp36_PAli_NX_Pl_consensus     AAAGAGACTTTATAAGAAAAGAAAGTATTAATAGTTTAAAAGACTTTTTTATTTTTATAAAAAAATAAACTTTCTTCAA [18018]
lp36_PAbe_NX_MP_FG_consensus AAAGAGACTTTATAAGAAAAGAAAGTATTAATAGTTTAAAAGACTTTTTTATTTTTATAAAAAAATAAACTTTCTTCAA [18018]
lp36_PAbe_NX_Pl_consensus     AAAGAGACTTTATAAGAAAAGAAAGTATTAATAGTTTAAAAGACTTTTTTATTTTTATAAAAAAATAAACTTTCTTCAA [18018]

lp36_gi|11496779|B31_GB       TAGATGATAATTCAACAGAAGCAAATATAGAGTCTTTACTAAAATCTATTTTCGAAGAACTAAATTATTCAGTAGAAC [18096]
lp36_un11_B31_PacBio          TAGATGATAATTCAACAGAAGCAAATATAGAGTCTTTACTAAAATCTATTTTCGAAGAACTAAATTATTCAGTAGAAC [18096]
lp36_B31_TS_MP_FG_consensus   TAGATGATAATTCAACAGAAGCAAATATAGAGTCTTTACTAAAATCTATTTTCGAAGAACTAAATTATTCAGTAGAAC [18096]
lp36_B31_NX_Pl_consensus      TAGATGATAATTCAACAGAAGCAAATATAGAGTCTTTACTAAAATCTATTTTCGAAGAACTAAATTATTCAGTAGAAC [18096]
lp36_PAli_NX_MP_FG_consensus TAGATGATAATTCAACAGAAGCAAATATAGAGTCTTTACTAAAATCTATTTTCGAAGAACTAAATTATTCAGTAGAAC [18096]
lp36_PAli_NX_Pl_consensus     TAGATGATAATTCAACAGAAGCAAATATAGAGTCTTTACTAAAATCTATTTTCGAAGAACTAAATTATTCAGTAGAAC [18096]
lp36_PAbe_NX_MP_FG_consensus TAGATGATAATTCAACAGAAGCAAATATAGAGTCTTTACTAAAATCTATTTTCGAAGAACTAAATTATTCAGTAGAAC [18096]
lp36_PAbe_NX_Pl_consensus     TAGATGATAATTCAACAGAAGCAAATATAGAGTCTTTACTAAAATCTATTTTCGAAGAACTAAATTATTCAGTAGAAC [18096]

lp36_gi|11496779|B31_GB       AACAAAAAGGTGGGCAAATAGAAGGAGTAAAATCCAAAGTAGATATACTACTTTTTGAAAATGATAAAGACAAAGTAG [18174]
lp36_un11_B31_PacBio          AACAAAAAGGTGGGCAAATAGAAGGAGTAAAATCCAAAGTAGATATACTACTTTTTGAAAATGATAAAGACAAAGTAG [18174]
lp36_B31_TS_MP_FG_consensus   AACAAAAAGGTGGGCAAATAGAAGGAGTAAAATCCAAAGTAGATATACTACTTTTTGAAAATGATAAAGACAAAGTAG [18174]
lp36_B31_NX_Pl_consensus      AACAAAAAGGTGGGCAAATAGAAGGAGTAAAATCCAAAGTAGATATACTACTTTTTGAAAATGATAAAGACAAAGTAG [18174]
lp36_PAli_NX_MP_FG_consensus AACAAAAAGGTGGGCAAATAGAAGGAGTAAAATCCAAAGTAGATATACTACTTTTTGAAAATGATAAAGACAAAGTAG [18174]
lp36_PAli_NX_Pl_consensus     AACAAAAAGGTGGGCAAATAGAAGGAGTAAAATCCAAAGTAGATATACTACTTTTTGAAAATGATAAAGACAAAGTAG [18174]
lp36_PAbe_NX_MP_FG_consensus AACAAAAAGGTGGGCAAATAGAAGGAGTAAAATCCAAAGTAGATATACTACTTTTTGAAAATGATAAAGACAAAGTAG [18174]
lp36_PAbe_NX_Pl_consensus     AACAAAAAGGTGGGCAAATAGAAGGAGTAAAATCCAAAGTAGATATACTACTTTTTGAAAATGATAAAGACAAAGTAG [18174]

lp36_gi|11496779|B31_GB       ATTTTAATAAAAAATTAGAAGAAGCTAAAAAAATAATTCCCATATTTTCAACTGAAGATATCTTACTTATAGCGGAAG [18252]
lp36_un11_B31_PacBio          ATTTTAATAAAAAATTAGAAGAAGCTAAAAAAATAATTCCCATATTTTCAACTGAAGATATCTTACTTATAGCGGAAG [18252]
lp36_B31_TS_MP_FG_consensus   ATTTTAATAAAAAATTAGAAGAAGCTAAAAAAATAATTCCCATATTTTCAACTGAAGATATCTTACTTATAGCGGAAG [18252]
lp36_B31_NX_Pl_consensus      ATTTTAATAAAAAATTAGAAGAAGCTAAAAAAATAATTCCCATATTTTCAACTGAAGATATCTTACTTATAGCGGAAG [18252]
lp36_PAli_NX_MP_FG_consensus ATTTTAATAAAAAATTAGAAGAAGCTAAAAAAATAATTCCCATATTTTCAACTGAAGATATCTTACTTATAGCGGAAG [18252]
lp36_PAli_NX_Pl_consensus     ATTTTAATAAAAAATTAGAAGAAGCTAAAAAAATAATTCCCATATTTTCAACTGAAGATATCTTACTTATAGCGGAAG [18252]
lp36_PAbe_NX_MP_FG_consensus ATTTTAATAAAAAATTAGAAGAAGCTAAAAAAATAATTCCCATATTTTCAACTGAAGATATCTTACTTATAGCGGAAG [18252]
lp36_PAbe_NX_Pl_consensus     ATTTTAATAAAAAATTAGAAGAAGCTAAAAAAATAATTCCCATATTTTCAACTGAAGATATCTTACTTATAGCGGAAG [18252]

lp36_gi|11496779|B31_GB       TTAAGCATCCAGTATTTAGTTTTGATGCTAAAGATAAGGTAAAAGAAGCAGAAGATCAGCTATATAGATGTCTAAATC [18330]
lp36_un11_B31_PacBio          TTAAGCATCCAGTATTTAGTTTTGATGCTAAAGATAAGGTAAAAGAAGCAGAAGATCAGCTATATAGATGTCTAAATC [18330]
lp36_B31_TS_MP_FG_consensus   TTAAGCATCCAGTATTTAGTTTTGATGCTAAAGATAAGGTAAAAGAAGCAGAAGATCAGCTATATAGATGTCTAAATC [18330]
lp36_B31_NX_Pl_consensus      TTAAGCATCCAGTATTTAGTTTTGATGCTAAAGATAAGGTAAAAGAAGCAGAAGATCAGCTATATAGATGTCTAAATC [18330]
lp36_PAli_NX_MP_FG_consensus TTAAGCATCCAGTATTTAGTTTTGATGCTAAAGATAAGGTAAAAGAAGCAGAAGATCAGCTATATAGATGTCTAAATC [18330]
lp36_PAli_NX_Pl_consensus     TTAAGCATCCAGTATTTAGTTTTGATGCTAAAGATAAGGTAAAAGAAGCAGAAGATCAGCTATATAGATGTCTAAATC [18330]
lp36_PAbe_NX_MP_FG_consensus TTAAGCATCCAGTATTTAGTTTTGATGCTAAAGATAAGGTAAAAGAAGCAGAAGATCAGCTATATAGATGTCTAAATC [18330]
lp36_PAbe_NX_Pl_consensus     TTAAGCATCCAGTATTTAGTTTTGATGCTAAAGATAAGGTAAAAGAAGCAGAAGATCAGCTATATAGATGTCTAAATC [18330]

lp36_gi|11496779|B31_GB       AATATCAAAAACATTATGGAATACTTTCAAATGGAAAGGTATGGAGCTTATATGATAAATCTAAGACTTTATGGAGAA [18408]
lp36_un11_B31_PacBio          AATATCAAAAACATTATGGAATACTTTCAAATGGAAAGGTATGGAGCTTATATGATAAATCTAAGACTTTATGGAGAA [18408]
lp36_B31_TS_MP_FG_consensus   AATATCAAAAACATTATGGAATACTTTCAAATGGAAAGGTATGGAGCTTATATGATAAATCTAAGACTTTATGGAGAA [18408]
lp36_B31_NX_Pl_consensus      AATATCAAAAACATTATGGAATACTTTCAAATGGAAAGGTATGGAGCTTATATGATAAATCTAAGACTTTATGGAGAA [18408]
lp36_PAli_NX_MP_FG_consensus AATATCAAAAACATTATGGAATACTTTCAAATGGAAAGGTATGGAGCTTATATGATAAATCTAAGACTTTATGGAGAA [18408]
lp36_PAli_NX_Pl_consensus     AATATCAAAAACATTATGGAATACTTTCAAATGGAAAGGTATGGAGCTTATATGATAAATCTAAGACTTTATGGAGAA [18408]
lp36_PAbe_NX_MP_FG_consensus AATATCAAAAACATTATGGAATACTTTCAAATGGAAAGGTATGGAGCTTATATGATAAATCTAAGACTTTATGGAGAA [18408]
lp36_PAbe_NX_Pl_consensus     AATATCAAAAACATTATGGAATACTTTCAAATGGAAAGGTATGGAGCTTATATGATAAATCTAAGACTTTATGGAGAA [18408]

lp36_gi|11496779|B31_GB       AAAAGATATATTGAATTTGATTTTTCTAAAATTGAAGACAAAAAAGAATACAAATAACAAGAATGGTTTATTTTTATTT [18486]
lp36_un11_B31_PacBio          AAAAGATATATTGAATTTGATTTTTCTAAAATTGAAGACAAAAAAGAATACAAATAACAAGAATGGTTTATTTTTATTT [18486]
lp36_B31_TS_MP_FG_consensus   AAAAGATATATTGAATTTGATTTTTCTAAAATTGAAGACAAAAAAGAATACAAATAACAAGAATGGTTTATTTTTATTT [18486]
lp36_B31_NX_Pl_consensus      AAAAGATATATTGAATTTGATTTTTCTAAAATTGAAGACAAAAAAGAATACAAATAACAAGAATGGTTTATTTTTATTT [18486]
lp36_PAli_NX_MP_FG_consensus AAAAGATATATTGAATTTGATTTTTCTAAAATTGAAGACAAAAAAGAATACAAATAACAAGAATGGTTTATTTTTATTT [18486]
lp36_PAli_NX_Pl_consensus     AAAAGATATATTGAATTTGATTTTTCTAAAATTGAAGACAAAAAAGAATACAAATAACAAGAATGGTTTATTTTTATTT [18486]
lp36_PAbe_NX_MP_FG_consensus AAAAGATATATTGAATTTGATTTTTCTAAAATTGAAGACAAAAAAGAATACAAATAACAAGAATGGTTTATTTTTATTT [18486]
lp36_PAbe_NX_Pl_consensus     AAAAGATATATTGAATTTGATTTTTCTAAAATTGAAGACAAAAAAGAATACAAATAACAAGAATGGTTTATTTTTATTT [18486]

lp36_gi|11496779|B31_GB       AGCTATATTATAAGAAAAGAAAGCTACCTAAAAACAAGTAATGTAATAGCAGTTGAAAAAGAGCAAATAGTTAAAGAA [18564]
lp36_un11_B31_PacBio          AGCTATATTATAAGAAAAGAAAGCTACCTAAAAACAAGTAATGTAATAGCAGTTGAAAAAGAGCAAATAGTTAAAGAA [18564]
lp36_B31_TS_MP_FG_consensus   AGCTATATTATAAGAAAAGAAAGCTACCTAAAAACAAGTAATGTAATAGCAGTTGAAAAAGAGCAAATAGTTAAAGAA [18564]
lp36_B31_NX_Pl_consensus      AGCTATATTATAAGAAAAGAAAGCTACCTAAAAACAAGTAATGTAATAGCAGTTGAAAAAGAGCAAATAGTTAAAGAA [18564]
lp36_PAli_NX_MP_FG_consensus AGCTATATTATAAGAAAAGAAAGCTACCTAAAAACAAGTAATGTAATAGCAGTTGAAAAAGAGCAAATAGTTAAAGAA [18564]
lp36_PAli_NX_Pl_consensus     AGCTATATTATAAGAAAAGAAAGCTACCTAAAAACAAGTAATGTAATAGCAGTTGAAAAAGAGCAAATAGTTAAAGAA [18564]
lp36_PAbe_NX_MP_FG_consensus AGCTATATTATAAGAAAAGAAAGCTACCTAAAAACAAGTAATGTAATAGCAGTTGAAAAAGAGCAAATAGTTAAAGAA [18564]
lp36_PAbe_NX_Pl_consensus     AGCTATATTATAAGAAAAGAAAGCTACCTAAAAACAAGTAATGTAATAGCAGTTGAAAAAGAGCAAATAGTTAAAGAA [18564]

lp36_gi|11496779|B31_GB       AAAGATATAATTCAAAAAACTCTTAGAGAGATGCTTTATGAGAAGCCTGATGACTCTATAGTATTTAAAATTGTAAAA [18642]
lp36_un11_B31_PacBio          AAAGATATAATTCAAAAAACTCTTAGAGAGATGCTTTATGAGAAGCCTGATGACTCTATAGTATTTAAAATTGTAAAA [18642]
lp36_B31_TS_MP_FG_consensus   AAAGATATAATTCAAAAAACTCTTAGAGAGATGCTTTATGAGAAGCCTGATGACTCTATAGTATTTAAAATTGTAAAA [18642]
lp36_B31_NX_Pl_consensus      AAAGATATAATTCAAAAAACTCTTAGAGAGATGCTTTATGAGAAGCCTGATGACTCTATAGTATTTAAAATTGTAAAA [18642]
lp36_PAli_NX_MP_FG_consensus AAAGATATAATTCAAAAAACTCTTAGAGAGATGCTTTATGAGAAGCCTGATGACTCTATAGTATTTAAAATTGTAAAA [18642]
lp36_PAli_NX_Pl_consensus     AAAGATATAATTCAAAAAACTCTTAGAGAGATGCTTTATGAGAAGCCTGATGACTCTATAGTATTTAAAATTGTAAAA [18642]
lp36_PAbe_NX_MP_FG_consensus AAAGATATAATTCAAAAAACTCTTAGAGAGATGCTTTATGAGAAGCCTGATGACTCTATAGTATTTAAAATTGTAAAA [18642]
lp36_PAbe_NX_Pl_consensus     AAAGATATAATTCAAAAAACTCTTAGAGAGATGCTTTATGAGAAGCCTGATGACTCTATAGTATTTAAAATTGTAAAA [18642]

lp36_gi|11496779|B31_GB       AAAATATATATGACAAAGAATTTAAAGTATCAGACAAAGAATTTACTCGTTATATTTTGGCTAGCATACTTAAAGAGG [18720]
lp36_un11_B31_PacBio          AAAATATATATGACAAAGAATTTAAAGTATCAGACAAAGAATTTACTCGTTATATTTTGGCTAGCATACTTAAAGAGG [18720]
lp36_B31_TS_MP_FG_consensus   AAAATATATATGACAAAGAATTTAAAGTATCAGACAAAGAATTTACTCGTTATATTTTGGCTAGCATACTTAAAGAGG [18720]
lp36_B31_NX_Pl_consensus      AAAATATATATGACAAAGAATTTAAAGTATCAGACAAAGAATTTACTCGTTATATTTTGGCTAGCATACTTAAAGAGG [18720]
```

```
lp36_PAli_NX_MP_FG_consensus AAAATATATATGACAAAGAATTTAAAGTATCAGACAAAGAATTTACTCGTTATATTTTGGCTAGCATACTTAAAGAGG [18720]
lp36_PAli_NX_Pl_consensus    AAAATATATATGACAAAGAATTTAAAGTATCAGACAAAGAATTTACTCGTTATATTTTGGCTAGCATACTTAAAGAGG [18720]
lp36_PAbe_NX_MP_FG_consensus AAAATATATATGACAAAGAATTTAAAGTATCAGACAAAGAATTTACTCGTTATATTTTGGCTAGCATACTTAAAGAGG [18720]
lp36_PAbe_NX_Pl_consensus    AAAATATATATGACAAAGAATTTAAAGTATCAGACAAAGAATTTACTCGTTATATTTTGGCTAGCATACTTAAAGAGG [18720]

lp36_gi|11496779|B31_GB      CAATTATTTTTATTTTAAGAATATTTTTTATTGCATATATTTAAGACAACGACAGTTTTAAGAAAATTTTAGAAGAAA [18798]
lp36_un11_B31_PacBio         CAATTATTTTTATTTTAAGAATATTTTTTATTGCATATATTTAAGACAACGACAGTTTTAAGAAAATTTTAGAAGAAA [18798]
lp36_B31_TS_MP_FG_consensus  CAATTATTTTTATTTTAAGAATATTTTTTATTGCATATATTTAAGACAACGACAGTTTTAAGAAAATTTTAGAAGAAA [18798]
lp36_B31_NX_Pl_consensus     CAATTATTTTTATTTTAAGAATATTTTTTATTGCATATATTTAAGACAACGACAGTTTTAAGAAAATTTTAGAAGAAA [18798]
lp36_PAli_NX_MP_FG_consensus CAATTATTTTTATTTTAAGAATATTTTTTATTGCATATATTTAAGACAACGACAGTTTTAAGAAAATTTTAGAAGAAA [18798]
lp36_PAli_NX_Pl_consensus    CAATTATTTTTATTTTAAGAATATTTTTTATTGCATATATTTAAGACAACGACAGTTTTAAGAAAATTTTAGAAGAAA [18798]
lp36_PAbe_NX_MP_FG_consensus CAATTATTTTTATTTTAAGAATATTTTTTATTGCATATATTTAAGACAACGACAGTTTTAAGAAAATTTTAGAAGAAA [18798]
lp36_PAbe_NX_Pl_consensus    CAATTATTTTTATTTTAAGAATATTTTTTATTGCATATATTTAAGACAACGACAGTTTTAAGAAAATTTTAGAAGAAA [18798]

lp36_gi|11496779|B31_GB      ACAAGCTATACAGATCTTCTATATCTTTTAGATATTTTTTTATGATTAAAATACAAAAAAGAAATTAGGATATAAAAA [18876]
lp36_un11_B31_PacBio         ACAAGCTATACAGATCTTCTATATCTTTTAGATATTTTTTTATGATTAAAATACAAAAAAGAAATTAGGATATAAAAA [18876]
lp36_B31_TS_MP_FG_consensus  ACAAGCTATACAGATCTTCTATATCTTTTAGATATTTTTTTATGATTAAAATACAAAAAAGAAATTAGGATATAAAAA [18876]
lp36_B31_NX_Pl_consensus     ACAAGCTATACAGATCTTCTATATCTTTTAGATATTTTTTTATGATTAAAATACAAAAAAGAAATTAGGATATAAAAA [18876]
lp36_PAli_NX_MP_FG_consensus ACAAGCTATACAGATCTTCTATATCTTTTAGATATTTTTTTATGATTAAAATACAAAAAAGAAATTAGGATATAAAAA [18876]
lp36_PAli_NX_Pl_consensus    ACAAGCTATACAGATCTTCTATATCTTTTAGATATTTTTTTATGATTAAAATACAAAAAAGAAATTAGGATATAAAAA [18876]
lp36_PAbe_NX_MP_FG_consensus ACAAGCTATACAGATCTTCTATATCTTTTAGATATTTTTTTATGATTAAAATACAAAAAAGAAATTAGGATATAAAAA [18876]
lp36_PAbe_NX_Pl_consensus    ACAAGCTATACAGATCTTCTATATCTTTTAGATATTTTTTTATGATTAAAATACAAAAAAGAAATTAGGATATAAAAA [18876]

lp36_gi|11496779|B31_GB      ATAATAACAATTTTTAATTTACTTGATAAGGGAAGTGATTCAATAAAGTTCCCCGTATTTAATGGAGGATTATTTGTA [18954]
lp36_un11_B31_PacBio         ATAATAACAATTTTTAATTTACTTGATAAGGGAAGTGATTCAATAAAGTTCCCCGTATTTAATGGAGGATTATTTGTA [18954]
lp36_B31_TS_MP_FG_consensus  ATAATAACAATTTTTAATTTACTTGATAAGGGAAGTGATTCAATAAAGTTCCCCGTATTTAATGGAGGATTATTTGTA [18954]
lp36_B31_NX_Pl_consensus     ATAATAACAATTTTTAATTTACTTGATAAGGGAAGTGATTCAATAAAGTTCCCCGTATTTAATGGAGGATTATTTGTA [18954]
lp36_PAli_NX_MP_FG_consensus ATAATAACAATTTTTAATTTACTTGATAAGGGAAGTGATTCAATAAAGTTCCCCGTATTTAATGGAGGATTATTTGTA [18954]
lp36_PAli_NX_Pl_consensus    ATAATAACAATTTTTAATTTACTTGATAAGGGAAGTGATTCAATAAAGTTCCCCGTATTTAATGGAGGATTATTTGTA [18954]
lp36_PAbe_NX_MP_FG_consensus ATAATAACAATTTTTAATTTACTTGATAAGGGAAGTGATTCAATAAAGTTCCCCGTATTTAATGGAGGATTATTTGTA [18954]
lp36_PAbe_NX_Pl_consensus    ATAATAACAATTTTTAATTTACTTGATAAGGGAAGTGATTCAATAAAGTTCCCCGTATTTAATGGAGGATTATTTGTA [18954]

lp36_gi|11496779|B31_GB      GAAGATAAGGTTAAATATTTGAATAATGAAAGTTTGCTAAGTATTAGTGAACTTGAAGAAATACTTGTTAAAATACTC [19032]
lp36_un11_B31_PacBio         GAAGATAAGGTTAAATATTTGAATAATGAAAGTTTGCTAAGTATTAGTGAACTTGAAGAAATACTTGTTAAAATACTC [19032]
lp36_B31_TS_MP_FG_consensus  GAAGATAAGGTTAAATATTTGAATAATGAAAGTTTGCTAAGTATTAGTGAACTTGAAGAAATACTTGTTAAAATACTC [19032]
lp36_B31_NX_Pl_consensus     GAAGATAAGGTTAAATATTTGAATAATGAAAGTTTGCTAAGTATTAGTGAACTTGAAGAAATACTTGTTAAAATACTC [19032]
lp36_PAli_NX_MP_FG_consensus GAAGATAAGGTTAAATATTTGAATAATGAAAGTTTGCTAAGTATTAGTGAACTTGAAGAAATACTTGTTAAAATACTC [19032]
lp36_PAli_NX_Pl_consensus    GAAGATAAGGTTAAATATTTGAATAATGAAAGTTTGCTAAGTATTAGTGAACTTGAAGAAATACTTGTTAAAATACTC [19032]
lp36_PAbe_NX_MP_FG_consensus GAAGATAAGGTTAAATATTTGAATAATGAAAGTTTGCTAAGTATTAGTGAACTTGAAGAAATACTTGTTAAAATACTC [19032]
lp36_PAbe_NX_Pl_consensus    GAAGATAAGGTTAAATATTTGAATAATGAAAGTTTGCTAAGTATTAGTGAACTTGAAGAAATACTTGTTAAAATACTC [19032]

lp36_gi|11496779|B31_GB      TTCTTTGAAGAAAAAAAATATTAAAGATGAAAAATTTGTAGAGTATTCAAGGTTAGATCCCAAAAGTTTTGGAGAATTA [19110]
lp36_un11_B31_PacBio         TTCTTTGAAGAAAAAAAATATTAAAGATGAAAAATTTGTAGAGTATTCAAGGTTAGATCCCAAAAGTTTTGGAGAATTA [19110]
lp36_B31_TS_MP_FG_consensus  TTCTTTGAAGAAAAAAAATATTAAAGATGAAAAATTTGTAGAGTATTCAAGGTTAGATCCCAAAAGTTTTGGAGAATTA [19110]
lp36_B31_NX_Pl_consensus     TTCTTTGAAGAAAAAAAATATTAAAGATGAAAAATTTGTAGAGTATTCAAGGTTAGATCCCAAAAGTTTTGGAGAATTA [19110]
lp36_PAli_NX_MP_FG_consensus TTCTTTGAAGAAAAAAAATATTAAAGATGAAAAATTTGTAGAGTATTCAAGGTTAGATCCCAAAAGTTTTGGAGAATTA [19110]
lp36_PAli_NX_Pl_consensus    TTCTTTGAAGAAAAAAAATATTAAAGATGAAAAATTTGTAGAGTATTCAAGGTTAGATCCCAAAAGTTTTGGAGAATTA [19110]
lp36_PAbe_NX_MP_FG_consensus TTCTTTGAAGAAAAAAAATATTAAAGATGAAAAATTTGTAGAGTATTCAAGGTTAGATCCCAAAAGTTTTGGAGAATTA [19110]
lp36_PAbe_NX_Pl_consensus    TTCTTTGAAGAAAAAAAATATTAAAGATGAAAAATTTGTAGAGTATTCAAGGTTAGATCCCAAAAGTTTTGGAGAATTA [19110]

lp36_gi|11496779|B31_GB      TACGAAACGCTACTTGAATATGGCCTAAGAATTGCAGATACTACTGTTCATCGTATTATTTAAGACGGGATTTATCTC [19188]
lp36_un11_B31_PacBio         TACGAAACGCTACTTGAATATGGCCTAAGAATTGCAGATACTACTGTTCATCGTATTATTTAAGACGGGATTTATCTC [19188]
lp36_B31_TS_MP_FG_consensus  TACGAAACGCTACTTGAATATGGCCTAAGAATTGCAGATACTACTGTTCATCGTATTATTTAAGACGGGATTTATCTC [19188]
lp36_B31_NX_Pl_consensus     TACGAAACGCTACTTGAATATGGCCTAAGAATTGCAGATACTACTGTTCATCGTATTATTTAAGACGGGATTTATCTC [19188]
lp36_PAli_NX_MP_FG_consensus TACGAAACGCTACTTGAATATGGCCTAAGAATTGCAGATACTACTGTTCATCGTATTATTTAAGACGGGATTTATCTC [19188]
lp36_PAli_NX_Pl_consensus    TACGAAACGCTACTTGAATATGGCCTAAGAATTGCAGATACTACTGTTCATCGTATTATTTAAGACGGGATTTATCTC [19188]
lp36_PAbe_NX_MP_FG_consensus TACGAAACGCTACTTGAATATGGCCTAAGAATTGCAGATACTACTGTTCATCGTATTATTTAAGACGGGATTTATCTC [19188]
lp36_PAbe_NX_Pl_consensus    TACGAAACGCTACTTGAATATGGCCTAAGAATTGCAGATACTACTGTTCATCGTATTATTTAAGACGGGATTTATCTC [19188]

lp36_gi|11496779|B31_GB      ATTCGTACCGAAGAAGAGCTTGCAAACAAGAAAGTAAACAAAGTTGCTACTTACATACAAAGAATATGAGGAAAGTGA [19266]
lp36_un11_B31_PacBio         ATTCGTACCGAAGAAGAGCTTGCAAACAAGAAAGTAAACAAAGTTGCTACTTACATACAAAGAATATGAGGAAAGTGA [19266]
lp36_B31_TS_MP_FG_consensus  ATTCGTACCGAAGAAGAGCTTGCAAACAAGAAAGTAAACAAAGTTGCTACTTACATACAAAGAATATGAGGAAAGTGA [19266]
lp36_B31_NX_Pl_consensus     ATTCGTACCGAAGAAGAGCTTGCAAACAAGAAAGTAAACAAAGTTGCTACTTACATACAAAGAATATGAGGAAAGTGA [19266]
lp36_PAli_NX_MP_FG_consensus ATTCGTACCGAAGAAGAGCTTGCAAACAAGAAAGTAAACAAAGTTGCTACTTACATACAAAGAATATGAGGAAAGTGA [19266]
lp36_PAli_NX_Pl_consensus    ATTCGTACCGAAGAAGAGCTTGCAAACAAGAAAGTAAACAAAGTTGCTACTTACATACAAAGAATATGAGGAAAGTGA [19266]
lp36_PAbe_NX_MP_FG_consensus ATTCGTACCGAAGAAGAGCTTGCAAACAAGAAAGTAAACAAAGTTGCTACTTACATACAAAGAATATGAGGAAAGTGA [19266]
lp36_PAbe_NX_Pl_consensus    ATTCGTACCGAAGAAGAGCTTGCAAACAAGAAAGTAAACAAAGTTGCTACTTACATACAAAGAATATGAGGAAAGTGA [19266]

lp36_gi|11496779|B31_GB      ATATATAGATAATTTAAGAATAATATTTTCTTTAATTAAACTTTATTTATTATCTTTTGACAAATCTTTAAACATAGA [19344]
lp36_un11_B31_PacBio         ATATATAGATAATTTAAGAATAATATTTTCTTTAATTAAACTTTATTTATTATCTTTTGACAAATCTTTAAACATAGA [19344]
lp36_B31_TS_MP_FG_consensus  ATATATAGATAATTTAAGAATAATATTTTCTTTAATTAAACTTTATTTATTATCTTTTGACAAATCTTTAAACATAGA [19344]
lp36_B31_NX_Pl_consensus     ATATATAGATAATTTAAGAATAATATTTTCTTTAATTAAACTTTATTTATTATCTTTTGACAAATCTTTAAACATAGA [19344]
lp36_PAli_NX_MP_FG_consensus ATATATAGATAATTTAAGAATAATATTTTCTTTAATTAAACTTTATTTATTATCTTTTGACAAATCTTTAAACATAGA [19344]
lp36_PAli_NX_Pl_consensus    ATATATAGATAATTTAAGAATAATATTTTCTTTAATTAAACTTTATTTATTATCTTTTGACAAATCTTTAAACATAGA [19344]
lp36_PAbe_NX_MP_FG_consensus ATATATAGATAATTTAAGAATAATATTTTCTTTAATTAAACTTTATTTATTATCTTTTGACAAATCTTTAAACATAGA [19344]
lp36_PAbe_NX_Pl_consensus    ATATATAGATAATTTAAGAATAATATTTTCTTTAATTAAACTTTATTTATTATCTTTTGACAAATCTTTAAACATAGA [19344]

lp36_gi|11496779|B31_GB      GAACTAGACTCACTCCAAAGAGCCTAAGGAACCAAATAAGTTAAATTACCCCCTGGTTTTATTAGTTTCAAATTAAAT [19422]
lp36_un11_B31_PacBio         GAACTAGACTCACTCCAAAGAGCCTAAGGAACCAAATAAGTTAAATTACCCCCTGGTTTTATTAGTTTCAAATTAAAT [19422]
lp36_B31_TS_MP_FG_consensus  GAACTAGACTCACTCCAAAGAGCCTAAGGAACCAAATAAGTTAAATTACCCCCTGGTTTTATTAGTTTCAAATTAAAT [19422]
lp36_B31_NX_Pl_consensus     GAACTAGACTCACTCCAAAGAGCCTAAGGAACCAAATAAGTTAAATTACCCCCTGGTTTTATTAGTTTCAAATTAAAT [19422]
lp36_PAli_NX_MP_FG_consensus GAACTAGACTCACTCCAAAGAGCCTAAGGAACCAAATAAGTTAAATTACCCCCTGGTTTTATTAGTTTCAAATTAAAT [19422]
lp36_PAli_NX_Pl_consensus    GAACTAGACTCACTCCAAAGAGCCTAAGGAACCAAATAAGTTAAATTACCCCCTGGTTTTATTAGTTTCAAATTAAAT [19422]
lp36_PAbe_NX_MP_FG_consensus GAACTAGACTCACTCCAAAGAGCCTAAGGAACCAAATAAGTTAAATTACCCCCTGGTTTTATTAGTTTCAAATTAAAT [19422]
lp36_PAbe_NX_Pl_consensus    GAACTAGACTCACTCCAAAGAGCCTAAGGAACCAAATAAGTTAAATTACCCCCTGGTTTTATTAGTTTCAAATTAAAT [19422]

lp36_gi|11496779|B31_GB      GCAACAAAGTATCTAAAAAGATTTGGATCCCCACCACTAGCAAAGTCTTTAAAATCACTTTTATAAATATTTATTAAT [19500]
lp36_un11_B31_PacBio         GCAACAAAGTATCTAAAAAGATTTGGATCCCCACCACTAGCAAAGTCTTTAAAATCACTTTTATAAATATTTATTAAT [19500]
lp36_B31_TS_MP_FG_consensus  GCAACAAAGTATCTAAAAAGATTTGGATCCCCACCACTAGCAAAGTCTTTAAAATCACTTTTATAAATATTTATTAAT [19500]
lp36_B31_NX_Pl_consensus     GCAACAAAGTATCTAAAAAGATTTGGATCCCCACCACTAGCAAAGTCTTTAAAATCACTTTTATAAATATTTATTAAT [19500]
lp36_PAli_NX_MP_FG_consensus GCAACAAAGTATCTAAAAAGATTTGGATCCCCACCACTAGCAAAGTCTTTAAAATCACTTTTATAAATATTTATTAAT [19500]
lp36_PAli_NX_Pl_consensus    GCAACAAAGTATCTAAAAAGATTTGGATCCCCACCACTAGCAAAGTCTTTAAAATCACTTTTATAAATATTTATTAAT [19500]
lp36_PAbe_NX_MP_FG_consensus GCAACAAAGTATCTAAAAAGATTTGGATCCCCACCACTAGCAAAGTCTTTAAAATCACTTTTATAAATATTTATTAAT [19500]
lp36_PAbe_NX_Pl_consensus    GCAACAAAGTATCTAAAAAGATTTGGATCCCCACCACTAGCAAAGTCTTTAAAATCACTTTTATAAATATTTATTAAT [19500]

lp36_gi|11496779|B31_GB      AGCGCTTATACTGCTTTTTTCTTCATTGTATTCAGTATTCAAATAATAATTGTCTTTACTAAGTATTTCTTGCTTTAT [19578]
lp36_un11_B31_PacBio         AGCGCTTATACTGCTTTTTTCTTCATTGTATTCAGTATTCAAATAATAATTGTCTTTACTAAGTATTTCTTGCTTTAT [19578]
lp36_B31_TS_MP_FG_consensus  AGCGCTTATACTGCTTTTTTCTTCATTGTATTCAGTATTCAAATAATAATTGTCTTTACTAAGTATTTCTTGCTTTAT [19578]
lp36_B31_NX_Pl_consensus     AGCGCTTATACTGCTTTTTTCTTCATTGTATTCAGTATTCAAATAATAATTGTCTTTACTAAGTATTTCTTGCTTTAT [19578]
lp36_PAli_NX_MP_FG_consensus AGCGCTTATACTGCTTTTTTCTTCATTGTATTCAGTATTCAAATAATAATTGTCTTTACTAAGTATTTCTTGCTTTAT [19578]
lp36_PAli_NX_Pl_consensus    AGCGCTTATACTGCTTTTTTCTTCATTGTATTCAGTATTCAAATAATAATTGTCTTTACTAAGTATTTCTTGCTTTAT [19578]
lp36_PAbe_NX_MP_FG_consensus AGCGCTTATACTGCTTTTTTCTTCATTGTATTCAGTATTCAAATAATAATTGTCTTTACTAAGTATTTCTTGCTTTAT [19578]
lp36_PAbe_NX_Pl_consensus    AGCGCTTATACTGCTTTTTTCTTCATTGTATTCAGTATTCAAATAATAATTGTCTTTACTAAGTATTTCTTGCTTTAT [19578]

lp36_gi|11496779|B31_GB      TTTATTTTGTTCTTTTATGCTTAGTCTTCTGTAGCTAGGAATATGTTTTGAGAAGAATTCTGATTCATTAAACTTAGT [19656]
lp36_un11_B31_PacBio         TTTATTTTGTTCTTTTATGCTTAGTCTTCTGTAGCTAGGAATATGTTTTGAGAAGAATTCTGATTCATTAAACTTAGT [19656]
lp36_B31_TS_MP_FG_consensus  TTTATTTTGTTCTTTTATGCTTAGTCTTCTGTAGCTAGGAATATGTTTTGAGAAGAATTCTGATTCATTAAACTTAGT [19656]
lp36_B31_NX_Pl_consensus     TTTATTTTGTTCTTTTATGCTTAGTCTTCTGTAGCTAGGAATATGTTTTGAGAAGAATTCTGATTCATTAAACTTAGT [19656]
lp36_PAli_NX_MP_FG_consensus TTTATTTTGTTCTTTTATGCTTAGTCTTCTGTAGCTAGGAATATGTTTTGAGAAGAATTCTGATTCATTAAACTTAGT [19656]
```

```
lp36_PAli_NX_Pl_consensus    TTTATTTTGTTCTTTTATGCTTAGTCTTCTGTAGCTAGGAATATGTTTTGAGAAGAATTCTGATTCATTAAACTTAGT [19656]
lp36_PAbe_NX_MP_FG_consensus TTTATTTTGTTCTTTTATGCTTAGTCTTCTGTAGCTAGGAATATGTTTTGAGAAGAATTCTGATTCATTAAACTTAGT [19656]
lp36_PAbe_NX_Pl_consensus    TTTATTTTGTTCTTTTATGCTTAGTCTTCTGTAGCTAGGAATATGTTTTGAGAAGAATTCTGATTCATTAAACTTAGT [19656]

lp36_gi|11496779|B31_GB      TTTCTCCCATGGTGGATTTCTAATTACAATATCAAATCCTTCCTGAACATCTGGAAACTCAATTCCATAGTGGAAAAA [19734]
lp36_un11_B31_PacBio         TTTCTCCCATGGTGGATTTCTAATTACAATATCAAATCCTTCCTGAACATCTGGAAACTCAATTCCATAGTGGAAAAA [19734]
lp36_B31_TS_MP_FG_consensus  TTTCTCCCATGGTGGATTTCTAATTACAATATCAAATCCTTCCTGAACATCTGGAAACTCAATTCCATAGTGGAAAAA [19734]
lp36_B31_NX_Pl_consensus     TTTCTCCCATGGTGGATTTCTAATTACAATATCAAATCCTTCCTGAACATCTGGAAACTCAATTCCATAGTGGAAAAA [19734]
lp36_PAli_NX_MP_FG_consensus TTTCTCCCATGGTGGATTTCTAATTACAATATCAAATCCTTCCTGAACATCTGGAAACTCAATTCCATAGTGGAAAAA [19734]
lp36_PAli_NX_Pl_consensus    TTTCTCCCATGGTGGATTTCTAATTACAATATCAAATCCTTCCTGAACATCTGGAAACTCAATTCCATAGTGGAAAAA [19734]
lp36_PAbe_NX_MP_FG_consensus TTTCTCCCATGGTGGATTTCTAATTACAATATCAAATCCTTCCTGAACATCTGGAAACTCAATTCCATAGTGGAAAAA [19734]
lp36_PAbe_NX_Pl_consensus    TTTCTCCCATGGTGGATTTCTAATTACAATATCAAATCCTTCCTGAACATCTGGAAACTCAATTCCATAGTGGAAAAA [19734]

lp36_gi|11496779|B31_GB      TTTTATAGTAGCTACTTAATTTTCTAATTTTCTTCATTTTTTCTTTATCCCCACTAGAAGGCTTATTCCCCAAAATAT [19812]
lp36_un11_B31_PacBio         TTTTATAGTAGCTACTTAATTTTCTAATTTTCTTCATTTTTTCTTTATCCCCACTAGAAGGCTTATTCCCCAAAATAT [19812]
lp36_B31_TS_MP_FG_consensus  TTTTATAGTAGCTACTTAATTTTCTAATTTTCTTCATTTTTTCTTTATCCCCACTAGAAGGCTTATTCCCCAAAATAT [19812]
lp36_B31_NX_Pl_consensus     TTTTATAGTAGCTACTTAATTTTCTAATTTTCTTCATTTTTTCTTTATCCCCACTAGAAGGCTTATTCCCCAAAATAT [19812]
lp36_PAli_NX_MP_FG_consensus TTTTATAGTAGCTACTTAATTTTCTAATTTTCTTCATTTTTTCTTTATCCCCACTAGAAGGCTTATTCCCCAAAATAT [19812]
lp36_PAli_NX_Pl_consensus    TTTTATAGTAGCTACTTAATTTTCTAATTTTCTTCATTTTTTCTTTATCCCCACTAGAAGGCTTATTCCCCAAAATAT [19812]
lp36_PAbe_NX_MP_FG_consensus TTTTATAGTAGCTACTTAATTTTCTAATTTTCTTCATTTTTTCTTTATCCCCACTAGAAGGCTTATTCCCCAAAATAT [19812]
lp36_PAbe_NX_Pl_consensus    TTTTATAGTAGCTACTTAATTTTCTAATTTTCTTCATTTTTTCTTTATCCCCACTAGAAGGCTTATTCCCCAAAATAT [19812]

lp36_gi|11496779|B31_GB      TTTCAATTAAACTGATTATATCTGCAATATCACTAAATTCTAGTTCATTAAGAAAGCATATATTTGCTAATTATAAAC [19890]
lp36_un11_B31_PacBio         TTTCAATTAAACTGATTATATCTGCAATATCACTAAATTCTAGTTCATTAAGAAAGCATATATTTGCTAATTATAAAC [19890]
lp36_B31_TS_MP_FG_consensus  TTTCAATTAAACTGATTATATCTGCAATATCACTAAATTCTAGTTCATTAAGAAAGCATATATTTGCTAATTATAAAC [19890]
lp36_B31_NX_Pl_consensus     TTTCAATTAAACTGATTATATCTGCAATATCACTAAATTCTAGTTCATTAAGAAAGCATATATTTGCTAATTATAAAC [19890]
lp36_PAli_NX_MP_FG_consensus TTTCAATTAAACTGATTATATCTGCAATATCACTAAATTCTAGTTCATTAAGAAAGCATATATTTGCTAATTATAAAC [19890]
lp36_PAli_NX_Pl_consensus    TTTCAATTAAACTGATTATATCTGCAATATCACTAAATTCTAGTTCATTAAGAAAGCATATATTTGCTAATTATAAAC [19890]
lp36_PAbe_NX_MP_FG_consensus TTTCAATTAAACTGATTATATCTGCAATATCACTAAATTCTAGTTCATTAAGAAAGCATATATTTGCTAATTATAAAC [19890]
lp36_PAbe_NX_Pl_consensus    TTTCAATTAAACTGATTATATCTGCAATATCACTAAATTCTAGTTCATTAAGAAAGCATATATTTGCTAATTATAAAC [19890]

lp36_gi|11496779|B31_GB      TTAACTATATTTATCAATTTCAAAATCAAAAAAGATTTAAAGATGTGGTAGCACTTTTTAAATTTGCAATATTTCAAC [19968]
lp36_un11_B31_PacBio         TTAACTATATTTATCAATTTCAAAATCAAAAAAGATTTAAAGATGTGGTAGCACTTTTTAAATTTGCAATATTTCAAC [19968]
lp36_B31_TS_MP_FG_consensus  TTAACTATATTTATCAATTTCAAAATCAAAAAAGATTTAAAGATGTGGTAGCACTTTTTAAATTTGCAATATTTCAAC [19968]
lp36_B31_NX_Pl_consensus     TTAACTATATTTATCAATTTCAAAATCAAAAAAGATTTAAAGATGTGGTAGCACTTTTTAAATTTGCAATATTTCAAC [19968]
lp36_PAli_NX_MP_FG_consensus TTAACTATATTTATCAATTTCAAAATCAAAAAAGATTTAAAGATGTGGTAGCACTTTTTAAATTTGCAATATTTCAAC [19968]
lp36_PAli_NX_Pl_consensus    TTAACTATATTTATCAATTTCAAAATCAAAAAAGATTTAAAGATGTGGTAGCACTTTTTAAATTTGCAATATTTCAAC [19968]
lp36_PAbe_NX_MP_FG_consensus TTAACTATATTTATCAATTTCAAAATCAAAAAAGATTTAAAGATGTGGTAGCACTTTTTAAATTTGCAATATTTCAAC [19968]
lp36_PAbe_NX_Pl_consensus    TTAACTATATTTATCAATTTCAAAATCAAAAAAGATTTAAAGATGTGGTAGCACTTTTTAAATTTGCAATATTTCAAC [19968]

lp36_gi|11496779|B31_GB      TTAGTAATACTAAAACCTGCACATCAAGTTTTAAAGCAAAATTTATGATTCAGGGCAATGATAATTGATAAAGAATAT [20046]
lp36_un11_B31_PacBio         TTAGTAATACTAAAACCTGCACATCAAGTTTTAAAGCAAAATTTATGATTCAGGGCAATGATAATTGATAAAGAATAT [20046]
lp36_B31_TS_MP_FG_consensus  TTAGTAATACTAAAACCTGCACATCAAGTTTTAAAGCAAAATTTATGATTCAGGGCAATGATAATTGATAAAGAATAT [20046]
lp36_B31_NX_Pl_consensus     TTAGTAATACTAAAACCTGCACATCAAGTTTTAAAGCAAAATTTATGATTCAGGGCAATGATAATTGATAAAGAATAT [20046]
lp36_PAli_NX_MP_FG_consensus TTAGTAATACTAAAACCTGCACATCAAGTTTTAAAGCAAAATTTATGATTCAGGGCAATGATAATTGATAAAGAATAT [20046]
lp36_PAli_NX_Pl_consensus    TTAGTAATACTAAAACCTGCACATCAAGTTTTAAAGCAAAATTTATGATTCAGGGCAATGATAATTGATAAAGAATAT [20046]
lp36_PAbe_NX_MP_FG_consensus TTAGTAATACTAAAACCTGCACATCAAGTTTTAAAGCAAAATTTATGATTCAGGGCAATGATAATTGATAAAGAATAT [20046]
lp36_PAbe_NX_Pl_consensus    TTAGTAATACTAAAACCTGCACATCAAGTTTTAAAGCAAAATTTATGATTCAGGGCAATGATAATTGATAAAGAATAT [20046]

lp36_gi|11496779|B31_GB      AAAGGTCTGATTACAGTTCGAATTCTAAGAATGAAATCAGCATATGATAAGATGGTAGCTACATTTAAACTTTTTTCA [20124]
lp36_un11_B31_PacBio         AAAGGTCTGATTACAGTTCGAATTCTAAGAATGAAATCAGCATATGATAAGATGGTAGCTACATTTAAACTTTTTTCA [20124]
lp36_B31_TS_MP_FG_consensus  AAAGGTCTGATTACAGTTCGAATTCTAAGAATGAAATCAGCATATGATAAGATGGTAGCTACATTTAAACTTTTTTCA [20124]
lp36_B31_NX_Pl_consensus     AAAGGTCTGATTACAGTTCGAATTCTAAGAATGAAATCAGCATATGATAAGATGGTAGCTACATTTAAACTTTTTTCA [20124]
lp36_PAli_NX_MP_FG_consensus AAAGGTCTGATTACAGTTCGAATTCTAAGAATGAAATCAGCATATGATAAGATGGTAGCTACATTTAAACTTTTTTCA [20124]
lp36_PAli_NX_Pl_consensus    AAAGGTCTGATTACAGTTCGAATTCTAAGAATGAAATCAGCATATGATAAGATGGTAGCTACATTTAAACTTTTTTCA [20124]
lp36_PAbe_NX_MP_FG_consensus AAAGGTCTGATTACAGTTCGAATTCTAAGAATGAAATCAGCATATGATAAGATGGTAGCTACATTTAAACTTTTTTCA [20124]
lp36_PAbe_NX_Pl_consensus    AAAGGTCTGATTACAGTTCGAATTCTAAGAATGAAATCAGCATATGATAAGATGGTAGCTACATTTAAACTTTTTTCA [20124]

lp36_gi|11496779|B31_GB      ATTTTATTTAGAAGTTATTTTTTAAGATCTGTTTATAAAATTTAATAAAAAGTACTTTGTTCACCCTCTTGATAGCAC [20202]
lp36_un11_B31_PacBio         ATTTTATTTAGAAGTTATTTTTTAAGATCTGTTTATAAAATTTAATAAAAAGTACTTTGTTCACCCTCTTGATAGCAC [20202]
lp36_B31_TS_MP_FG_consensus  ATTTTATTTAGAAGTTATTTTTTAAGATCTGTTTATAAAATTTAATAAAAAGTACTTTGTTCACCCTCTTGATAGCAC [20202]
lp36_B31_NX_Pl_consensus     ATTTTATTTAGAAGTTATTTTTTAAGATCTGTTTATAAAATTTAATAAAAAGTACTTTGTTCACCCTCTTGATAGCAC [20202]
lp36_PAli_NX_MP_FG_consensus ATTTTATTTAGAAGTTATTTTTTAAGATCTGTTTATAAAATTTAATAAAAAGTACTTTGTTCACCCTCTTGATAGCAC [20202]
lp36_PAli_NX_Pl_consensus    ATTTTATTTAGAAGTTATTTTTTAAGATCTGTTTATAAAATTTAATAAAAAGTACTTTGTTCACCCTCTTGATAGCAC [20202]
lp36_PAbe_NX_MP_FG_consensus ATTTTATTTAGAAGTTATTTTTTAAGATCTGTTTATAAAATTTAATAAAAAGTACTTTGTTCACCCTCTTGATAGCAC [20202]
lp36_PAbe_NX_Pl_consensus    ATTTTATTTAGAAGTTATTTTTTAAGATCTGTTTATAAAATTTAATAAAAAGTACTTTGTTCACCCTCTTGATAGCAC [20202]

lp36_gi|11496779|B31_GB      TTATTTTAAATTTTTAAAAAGAAAGAGAAATTCAATGAATTGATAAATAATAGTAAATATTAGTTTAGTATTATTACA [20280]
lp36_un11_B31_PacBio         TTATTTTAAATTTTTAAAAAGAAAGAGAAATTCAATGAATTGATAAATAATAGTAAATATTAGTTTAGTATTATTACA [20280]
lp36_B31_TS_MP_FG_consensus  TTATTTTAAATTTTTAAAAAGAAAGAGAAATTCAATGAATTGATAAATAATAGTAAATATTAGTTTAGTATTATTACA [20280]
lp36_B31_NX_Pl_consensus     TTATTTTAAATTTTTAAAAAGAAAGAGAAATTCAATGAATTGATAAATAATAGTAAATATTAGTTTAGTATTATTACA [20280]
lp36_PAli_NX_MP_FG_consensus TTATTTTAAATTTTTAAAAAGAAAGAGAAATTCAATGAATTGATAAATAATAGTAAATATTAGTTTAGTATTATTACA [20280]
lp36_PAli_NX_Pl_consensus    TTATTTTAAATTTTTAAAAAGAAAGAGAAATTCAATGAATTGATAAATAATAGTAAATATTAGTTTAGTATTATTACA [20280]
lp36_PAbe_NX_MP_FG_consensus TTATTTTAAATTTTTAAAAAGAAAGAGAAATTCAATGAATTGATAAATAATAGTAAATATTAGTTTAGTATTATTACA [20280]
lp36_PAbe_NX_Pl_consensus    TTATTTTAAATTTTTAAAAAGAAAGAGAAATTCAATGAATTGATAAATAATAGTAAATATTAGTTTAGTATTATTACA [20280]

lp36_gi|11496779|B31_GB      ATTAAAATTGTAATAATTTTGCCTCTTTTTGATAAGAAAAGTTTATTAATTTTTGGAATCCGACTTAAAATGATTTAA [20358]
lp36_un11_B31_PacBio         ATTAAAATTGTAATAATTTTGCCTCTTTTTGATAAGAAAAGTTTATTAATTTTTGGAATCCGACTTAAAATGATTTAA [20358]
lp36_B31_TS_MP_FG_consensus  ATTAAAATTGTAATAATTTTGCCTCTTTTTGATAAGAAAAGTTTATTAATTTTTGGAATCCGACTTAAAATGATTTAA [20358]
lp36_B31_NX_Pl_consensus     ATTAAAATTGTAATAATTTTGCCTCTTTTTGATAAGAAAAGTTTATTAATTTTTGGAATCCGACTTAAAATGATTTAA [20358]
lp36_PAli_NX_MP_FG_consensus ATTAAAATTGTAATAATTTTGCCTCTTTTTGATAAGAAAAGTTTATTAATTTTTGGAATCCGACTTAAAATGATTTAA [20358]
lp36_PAli_NX_Pl_consensus    ATTAAAATTGTAATAATTTTGCCTCTTTTTGATAAGAAAAGTTTATTAATTTTTGGAATCCGACTTAAAATGATTTAA [20358]
lp36_PAbe_NX_MP_FG_consensus ATTAAAATTGTAATAATTTTGCCTCTTTTTGATAAGAAAAGTTTATTAATTTTTGGAATCCGACTTAAAATGATTTAA [20358]
lp36_PAbe_NX_Pl_consensus    ATTAAAATTGTAATAATTTTGCCTCTTTTTGATAAGAAAAGTTTATTAATTTTTGGAATCCGACTTAAAATGATTTAA [20358]

lp36_gi|11496779|B31_GB      AGTTAGTATTAACTTTAAAGGAGAGAAAGCATGAAAAAAGTTAAAAGTAAATATTTGGCTTTGGGATTACTTTTTGGT [20436]
lp36_un11_B31_PacBio         AGTTAGTATTAACTTTAAAGGAGAGAAAGCATGAAAAAAGTTAAAAGTAAATATTTGGCTTTGGGATTACTTTTTGGT [20436]
lp36_B31_TS_MP_FG_consensus  AGTTAGTATTAACTTTAAAGGAGAGAAAGCATGAAAAAAGTTAAAAGTAAATATTTGGCTTTGGGATTACTTTTTGGT [20436]
lp36_B31_NX_Pl_consensus     AGTTAGTATTAACTTTAAAGGAGAGAAAGCATGAAAAAAGTTAAAAGTAAATATTTGGCTTTGGGATTACTTTTTGGT [20436]
lp36_PAli_NX_MP_FG_consensus AGTTAGTATTAACTTTAAAGGAGAGAAAGCATGAAAAAAGTTAAAAGTAAATATTTGGCTTTGGGATTACTTTTTGGT [20436]
lp36_PAli_NX_Pl_consensus    AGTTAGTATTAACTTTAAAGGAGAGAAAGCATGAAAAAAGTTAAAAGTAAATATTTGGCTTTGGGATTACTTTTTGGT [20436]
lp36_PAbe_NX_MP_FG_consensus AGTTAGTATTAACTTTAAAGGAGAGAAAGCATGAAAAAAGTTAAAAGTAAATATTTGGCTTTGGGATTACTTTTTGGT [20436]
lp36_PAbe_NX_Pl_consensus    AGTTAGTATTAACTTTAAAGGAGAGAAAGCATGAAAAAAGTTAAAAGTAAATATTTGGCTTTGGGATTACTTTTTGGT [20436]

lp36_gi|11496779|B31_GB      TTTATAAGTTGTGATTTATTCATAAGATATGAAATGAAAGAGGAATCCCCTGGCTTATTTGATAAGGGAAACTCTATT [20514]
lp36_un11_B31_PacBio         TTTATAAGTTGTGATTTATTCATAAGATATGAAATGAAAGAGGAATCCCCTGGCTTATTTGATAAGGGAAACTCTATT [20514]
lp36_B31_TS_MP_FG_consensus  TTTATAAGTTGTGATTTATTCATAAGATATGAAATGAAAGAGGAATCCCCTGGCTTATTTGATAAGGGAAACTCTATT [20514]
lp36_B31_NX_Pl_consensus     TTTATAAGTTGTGATTTATTCATAAGATATGAAATGAAAGAGGAATCCCCTGGCTTATTTGATAAGGGAAACTCTATT [20514]
lp36_PAli_NX_MP_FG_consensus TTTATAAGTTGTGATTTATTCATAAGATATGAAATGAAAGAGGAATCCCCTGGCTTATTTGATAAGGGAAACTCTATT [20514]
lp36_PAli_NX_Pl_consensus    TTTATAAGTTGTGATTTATTCATAAGATATGAAATGAAAGAGGAATCCCCTGGCTTATTTGATAAGGGAAACTCTATT [20514]
lp36_PAbe_NX_MP_FG_consensus TTTATAAGTTGTGATTTATTCATAAGATATGAAATGAAAGAGGAATCCCCTGGCTTATTTGATAAGGGAAACTCTATT [20514]
lp36_PAbe_NX_Pl_consensus    TTTATAAGTTGTGATTTATTCATAAGATATGAAATGAAAGAGGAATCCCCTGGCTTATTTGATAAGGGAAACTCTATT [20514]

lp36_gi|11496779|B31_GB      TTAGAGACTAGCGAGGAATCTATTAAAAAGCCTATGAATAAGAAAGGTAAAGGTAAGATTGCTAGAAAGAAAGGCAAA [20592]
lp36_un11_B31_PacBio         TTAGAGACTAGCGAGGAATCTATTAAAAAGCCTATGAATAAGAAAGGTAAAGGTAAGATTGCTAGAAAGAAAGGCAAA [20592]
lp36_B31_TS_MP_FG_consensus  TTAGAGACTAGCGAGGAATCTATTAAAAAGCCTATGAATAAGAAAGGTAAAGGTAAGATTGCTAGAAAGAAAGGCAAA [20592]
lp36_B31_NX_Pl_consensus     TTAGAGACTAGCGAGGAATCTATTAAAAAGCCTATGAATAAGAAAGGTAAAGGTAAGATTGCTAGAAAGAAAGGCAAA [20592]
lp36_PAli_NX_MP_FG_consensus TTAGAGACTAGCGAGGAATCTATTAAAAAGCCTATGAATAAGAAAGGTAAAGGTAAGATTGCTAGAAAGAAAGGCAAA [20592]
lp36_PAli_NX_Pl_consensus    TTAGAGACTAGCGAGGAATCTATTAAAAAGCCTATGAATAAGAAAGGTAAAGGTAAGATTGCTAGAAAGAAAGGCAAA [20592]
```

```
lp36_PAbe_NX_MP_FG_consensus TTAGAGACTAGCGAGGAATCTATTAAAAAGCCTATGAATAAGAAAGGTAAAGGTAAGATTGCTAGAAAGAAAGGCAAA [20592]
lp36_PAbe_NX_Pl_consensus    TTAGAGACTAGCGAGGAATCTATTAAAAAGCCTATGAATAAGAAAGGTAAAGGTAAGATTGCTAGAAAGAAAGGCAAA [20592]

lp36_gi|11496779|B31_GB      AGCAAGGTTTCTAGAAAAGAACCGTATATTCATAGTTTAAAAAGGGACTCTGCTAATAAAAGCAATTTTTTACAAAAA [20670]
lp36_un11_B31_PacBio         AGCAAGGTTTCTAGAAAAGAACCGTATATTCATAGTTTAAAAAGGGACTCTGCTAATAAAAGCAATTTTTTACAAAAA [20670]
lp36_B31_TS_MP_FG_consensus  AGCAAGGTTTCTAGAAAAGAACCGTATATTCATAGTTTAAAAAGGGACTCTGCTAATAAAAGCAATTTTTTACAAAAA [20670]
lp36_B31_NX_Pl_consensus     AGCAAGGTTTCTAGAAAAGAACCGTATATTCATAGTTTAAAAAGGGACTCTGCTAATAAAAGCAATTTTTTACAAAAA [20670]
lp36_PAli_NX_MP_FG_consensus AGCAAGGTTTCTAGAAAAGAACCGTATATTCATAGTTTAAAAAGGGACTCTGCTAATAAAAGCAATTTTTTACAAAAA [20670]
lp36_PAli_NX_Pl_consensus    AGCAAGGTTTCTAGAAAAGAACCGTATATTCATAGTTTAAAAAGGGACTCTGCTAATAAAAGCAATTTTTTACAAAAA [20670]
lp36_PAbe_NX_MP_FG_consensus AGCAAGGTTTCTAGAAAAGAACCGTATATTCATAGTTTAAAAAGGGACTCTGCTAATAAAAGCAATTTTTTACAAAAA [20670]
lp36_PAbe_NX_Pl_consensus    AGCAAGGTTTCTAGAAAAGAACCGTATATTCATAGTTTAAAAAGGGACTCTGCTAATAAAAGCAATTTTTTACAAAAA [20670]

lp36_gi|11496779|B31_GB      AATGTAATTTTAGAGGAAGAAAGTTTAAAAACTGAATTATTAAAAGAGCAATCTGAGACTAGAAAAGAAAAAAATACAA [20748]
lp36_un11_B31_PacBio         AATGTAATTTTAGAGGAAGAAAGTTTAAAAACTGAATTATTAAAAGAGCAATCTGAGACTAGAAAAGAAAAAAATACAA [20748]
lp36_B31_TS_MP_FG_consensus  AATGTAATTTTAGAGGAAGAAAGTTTAAAAACTGAATTATTAAAAGAGCAATCTGAGACTAGAAAAGAAAAAAATACAA [20748]
lp36_B31_NX_Pl_consensus     AATGTAATTTTAGAGGAAGAAAGTTTAAAAACTGAATTATTAAAAGAGCAATCTGAGACTAGAAAAGAAAAAAATACAA [20748]
lp36_PAli_NX_MP_FG_consensus AATGTAATTTTAGAGGAAGAAAGTTTAAAAACTGAATTATTAAAAGAGCAATCTGAGACTAGAAAAGAAAAAAATACAA [20748]
lp36_PAli_NX_Pl_consensus    AATGTAATTTTAGAGGAAGAAAGTTTAAAAACTGAATTATTAAAAGAGCAATCTGAGACTAGAAAAGAAAAAAATACAA [20748]
lp36_PAbe_NX_MP_FG_consensus AATGTAATTTTAGAGGAAGAAAGTTTAAAAACTGAATTATTAAAAGAGCAATCTGAGACTAGAAAAGAAAAAAATACAA [20748]
lp36_PAbe_NX_Pl_consensus    AATGTAATTTTAGAGGAAGAAAGTTTAAAAACTGAATTATTAAAAGAGCAATCTGAGACTAGAAAAGAAAAAAATACAA [20748]

lp36_gi|11496779|B31_GB      AAACAACAAGATGAATATAAAGGGATGACTCAAGGAAGTTTAAATTCCCTTAGCGGTGAAAGTGGTGAATTGGAGGAG [20826]
lp36_un11_B31_PacBio         AAACAACAAGATGAATATAAAGGGATGACTCAAGGAAGTTTAAATTCCCTTAGCGGTGAAAGTGGTGAATTGGAGGAG [20826]
lp36_B31_TS_MP_FG_consensus  AAACAACAAGATGAATATAAAGGGATGACTCAAGGAAGTTTAAATTCCCTTAGCGGTGAAAGTGGTGAATTGGAGGAG [20826]
lp36_B31_NX_Pl_consensus     AAACAACAAGATGAATATAAAGGGATGACTCAAGGAAGTTTAAATTCCCTTAGCGGTGAAAGTGGTGAATTGGAGGAG [20826]
lp36_PAli_NX_MP_FG_consensus AAACAACAAGATGAATATAAAGGGATGACTCAAGGAAGTTTAAATTCCCTTAGCGGTGAAAGTGGTGAATTGGAGGAG [20826]
lp36_PAli_NX_Pl_consensus    AAACAACAAGATGAATATAAAGGGATGACTCAAGGAAGTTTAAATTCCCTTAGCGGTGAAAGTGGTGAATTGGAGGAG [20826]
lp36_PAbe_NX_MP_FG_consensus AAACAACAAGATGAATATAAAGGGATGACTCAAGGAAGTTTAAATTCCCTTAGCGGTGAAAGTGGTGAATTGGAGGAG [20826]
lp36_PAbe_NX_Pl_consensus    AAACAACAAGATGAATATAAAGGGATGACTCAAGGAAGTTTAAATTCCCTTAGCGGTGAAAGTGGTGAATTGGAGGAG [20826]

lp36_gi|11496779|B31_GB      CCTATTGAAAGTAATGAAATTGATCTTACTATAGATTCTGATTTAAGGCCAAAGAGTTCCTTACAAGGCATTGCAGGA [20904]
lp36_un11_B31_PacBio         CCTATTGAAAGTAATGAAATTGATCTTACTATAGATTCTGATTTAAGGCCAAAGAGTTCCTTACAAGGCATTGCAGGA [20904]
lp36_B31_TS_MP_FG_consensus  CCTATTGAAAGTAATGAAATTGATCTTACTATAGATTCTGATTTAAGGCCAAAGAGTTCCTTACAAGGCATTGCAGGA [20904]
lp36_B31_NX_Pl_consensus     CCTATTGAAAGTAATGAAATTGATCTTACTATAGATTCTGATTTAAGGCCAAAGAGTTCCTTACAAGGCATTGCAGGA [20904]
lp36_PAli_NX_MP_FG_consensus CCTATTGAAAGTAATGAAATTGATCTTACTATAGATTCTGATTTAAGGCCAAAGAGTTCCTTACAAGGCATTGCAGGA [20904]
lp36_PAli_NX_Pl_consensus    CCTATTGAAAGTAATGAAATTGATCTTACTATAGATTCTGATTTAAGGCCAAAGAGTTCCTTACAAGGCATTGCAGGA [20904]
lp36_PAbe_NX_MP_FG_consensus CCTATTGAAAGTAATGAAATTGATCTTACTATAGATTCTGATTTAAGGCCAAAGAGTTCCTTACAAGGCATTGCAGGA [20904]
lp36_PAbe_NX_Pl_consensus    CCTATTGAAAGTAATGAAATTGATCTTACTATAGATTCTGATTTAAGGCCAAAGAGTTCCTTACAAGGCATTGCAGGA [20904]

lp36_gi|11496779|B31_GB      TCAAACTCTATTTCATACACTGATGAAATAGAGGAAGAGGATTATGATCAGTATTATTTAGATGAATATGATGAAGAG [20982]
lp36_un11_B31_PacBio         TCAAACTCTATTTCATACACTGATGAAATAGAGGAAGAGGATTATGATCAGTATTATTTAGATGAATATGATGAAGAG [20982]
lp36_B31_TS_MP_FG_consensus  TCAAACTCTATTTCATACACTGATGAAATAGAGGAAGAGGATTATGATCAGTATTATTTAGATGAATATGATGAAGAG [20982]
lp36_B31_NX_Pl_consensus     TCAAACTCTATTTCATACACTGATGAAATAGAGGAAGAGGATTATGATCAGTATTATTTAGATGAATATGATGAAGAG [20982]
lp36_PAli_NX_MP_FG_consensus TCAAACTCTATTTCATACACTGATGAAATAGAGGAAGAGGATTATGATCAGTATTATTTAGATGAATATGATGAAGAG [20982]
lp36_PAli_NX_Pl_consensus    TCAAACTCTATTTCATACACTGATGAAATAGAGGAAGAGGATTATGATCAGTATTATTTAGATGAATATGATGAAGAG [20982]
lp36_PAbe_NX_MP_FG_consensus TCAAACTCTATTTCATACACTGATGAAATAGAGGAAGAGGATTATGATCAGTATTATTTAGATGAATATGATGAAGAG [20982]
lp36_PAbe_NX_Pl_consensus    TCAAACTCTATTTCATACACTGATGAAATAGAGGAAGAGGATTATGATCAGTATTATTTAGATGAATATGATGAAGAG [20982]

lp36_gi|11496779|B31_GB      GATGAAGAGGAAATAAGATTAAGCAATCGATATCAATCTTATCTAGAAGGTGTTAAATATAATGTAGATTCAGCAATT [21060]
lp36_un11_B31_PacBio         GATGAAGAGGAAATAAGATTAAGCAATCGATATCAATCTTATCTAGAAGGTGTTAAATATAATGTAGATTCAGCAATT [21060]
lp36_B31_TS_MP_FG_consensus  GATGAAGAGGAAATAAGATTAAGCAATCGATATCAATCTTATCTAGAAGGTGTTAAATATAATGTAGATTCAGCAATT [21060]
lp36_B31_NX_Pl_consensus     GATGAAGAGGAAATAAGATTAAGCAATCGATATCAATCTTATCTAGAAGGTGTTAAATATAATGTAGATTCAGCAATT [21060]
lp36_PAli_NX_MP_FG_consensus GATGAAGAGGAAATAAGATTAAGCAATCGATATCAATCTTATCTAGAAGGTGTTAAATATAATGTAGATTCAGCAATT [21060]
lp36_PAli_NX_Pl_consensus    GATGAAGAGGAAATAAGATTAAGCAATCGATATCAATCTTATCTAGAAGGTGTTAAATATAATGTAGATTCAGCAATT [21060]
lp36_PAbe_NX_MP_FG_consensus GATGAAGAGGAAATAAGATTAAGCAATCGATATCAATCTTATCTAGAAGGTGTTAAATATAATGTAGATTCAGCAATT [21060]
lp36_PAbe_NX_Pl_consensus    GATGAAGAGGAAATAAGATTAAGCAATCGATATCAATCTTATCTAGAAGGTGTTAAATATAATGTAGATTCAGCAATT [21060]

lp36_gi|11496779|B31_GB      CAAACAATTACTAAGATATATAATACTTATACATTATTTTCAACAAAGCTAACCCAAATGTATTCTACACGCCTTGAC [21138]
lp36_un11_B31_PacBio         CAAACAATTACTAAGATATATAATACTTATACATTATTTTCAACAAAGCTAACCCAAATGTATTCTACACGCCTTGAC [21138]
lp36_B31_TS_MP_FG_consensus  CAAACAATTACTAAGATATATAATACTTATACATTATTTTCAACAAAGCTAACCCAAATGTATTCTACACGCCTTGAC [21138]
lp36_B31_NX_Pl_consensus     CAAACAATTACTAAGATATATAATACTTATACATTATTTTCAACAAAGCTAACCCAAATGTATTCTACACGCCTTGAC [21138]
lp36_PAli_NX_MP_FG_consensus CAAACAATTACTAAGATATATAATACTTATACATTATTTTCAACAAAGCTAACCCAAATGTATTCTACACGCCTTGAC [21138]
lp36_PAli_NX_Pl_consensus    CAAACAATTACTAAGATATATAATACTTATACATTATTTTCAACAAAGCTAACCCAAATGTATTCTACACGCCTTGAC [21138]
lp36_PAbe_NX_MP_FG_consensus CAAACAATTACTAAGATATATAATACTTATACATTATTTTCAACAAAGCTAACCCAAATGTATTCTACACGCCTTGAC [21138]
lp36_PAbe_NX_Pl_consensus    CAAACAATTACTAAGATATATAATACTTATACATTATTTTCAACAAAGCTAACCCAAATGTATTCTACACGCCTTGAC [21138]

lp36_gi|11496779|B31_GB      AACTTTGCTAAAGCCAAAGCTAAAGAAGAAGCTGCAAAGTTTACAAAAGAAGACCTTGAAAAAAATTTCAAGACCTTA [21216]
lp36_un11_B31_PacBio         AACTTTGCTAAAGCCAAAGCTAAAGAAGAAGCTGCAAAGTTTACAAAAGAAGACCTTGAAAAAAATTTCAAGACCTTA [21216]
lp36_B31_TS_MP_FG_consensus  AACTTTGCTAAAGCCAAAGCTAAAGAAGAAGCTGCAAAGTTTACAAAAGAAGACCTTGAAAAAAATTTCAAGACCTTA [21216]
lp36_B31_NX_Pl_consensus     AACTTTGCTAAAGCCAAAGCTAAAGAAGAAGCTGCAAAGTTTACAAAAGAAGACCTTGAAAAAAATTTCAAGACCTTA [21216]
lp36_PAli_NX_MP_FG_consensus AACTTTGCTAAAGCCAAAGCTAAAGAAGAAGCTGCAAAGTTTACAAAAGAAGACCTTGAAAAAAATTTCAAGACCTTA [21216]
lp36_PAli_NX_Pl_consensus    AACTTTGCTAAAGCCAAAGCTAAAGAAGAAGCTGCAAAGTTTACAAAAGAAGACCTTGAAAAAAATTTCAAGACCTTA [21216]
lp36_PAbe_NX_MP_FG_consensus AACTTTGCTAAAGCCAAAGCTAAAGAAGAAGCTGCAAAGTTTACAAAAGAAGACCTTGAAAAAAATTTCAAGACCTTA [21216]
lp36_PAbe_NX_Pl_consensus    AACTTTGCTAAAGCCAAAGCTAAAGAAGAAGCTGCAAAGTTTACAAAAGAAGACCTTGAAAAAAATTTCAAGACCTTA [21216]

lp36_gi|11496779|B31_GB      TTAAATTATATTCAAGTAAGTGTAAAGACTGCAGCAAATTTTGTATACATAAATGACACGCATGCAAAAAGGAAATTA [21294]
lp36_un11_B31_PacBio         TTAAATTATATTCAAGTAAGTGTAAAGACTGCAGCAAATTTTGTATACATAAATGACACGCATGCAAAAAGGAAATTA [21294]
lp36_B31_TS_MP_FG_consensus  TTAAATTATATTCAAGTAAGTGTAAAGACTGCAGCAAATTTTGTATACATAAATGACACGCATGCAAAAAGGAAATTA [21294]
lp36_B31_NX_Pl_consensus     TTAAATTATATTCAAGTAAGTGTAAAGACTGCAGCAAATTTTGTATACATAAATGACACGCATGCAAAAAGGAAATTA [21294]
lp36_PAli_NX_MP_FG_consensus TTAAATTATATTCAAGTAAGTGTAAAGACTGCAGCAAATTTTGTATACATAAATGACACGCATGCAAAAAGGAAATTA [21294]
lp36_PAli_NX_Pl_consensus    TTAAATTATATTCAAGTAAGTGTAAAGACTGCAGCAAATTTTGTATACATAAATGACACGCATGCAAAAAGGAAATTA [21294]
lp36_PAbe_NX_MP_FG_consensus TTAAATTATATTCAAGTAAGTGTAAAGACTGCAGCAAATTTTGTATACATAAATGACACGCATGCAAAAAGGAAATTA [21294]
lp36_PAbe_NX_Pl_consensus    TTAAATTATATTCAAGTAAGTGTAAAGACTGCAGCAAATTTTGTATACATAAATGACACGCATGCAAAAAGGAAATTA [21294]

lp36_gi|11496779|B31_GB      GAGAACATTGAAGCAGAAATAAAAACTTTAATTGCAAAGATCAAAGAACAATCTAATTTATACGAAGCATATAAAGCA [21372]
lp36_un11_B31_PacBio         GAGAACATTGAAGCAGAAATAAAAACTTTAATTGCAAAGATCAAAGAACAATCTAATTTATACGAAGCATATAAAGCA [21372]
lp36_B31_TS_MP_FG_consensus  GAGAACATTGAAGCAGAAATAAAAACTTTAATTGCAAAGATCAAAGAACAATCTAATTTATACGAAGCATATAAAGCA [21372]
lp36_B31_NX_Pl_consensus     GAGAACATTGAAGCAGAAATAAAAACTTTAATTGCAAAGATCAAAGAACAATCTAATTTATACGAAGCATATAAAGCA [21372]
lp36_PAli_NX_MP_FG_consensus GAGAACATTGAAGCAGAAATAAAAACTTTAATTGCAAAGATCAAAGAACAATCTAATTTATACGAAGCATATAAAGCA [21372]
lp36_PAli_NX_Pl_consensus    GAGAACATTGAAGCAGAAATAAAAACTTTAATTGCAAAGATCAAAGAACAATCTAATTTATACGAAGCATATAAAGCA [21372]
lp36_PAbe_NX_MP_FG_consensus GAGAACATTGAAGCAGAAATAAAAACTTTAATTGCAAAGATCAAAGAACAATCTAATTTATACGAAGCATATAAAGCA [21372]
lp36_PAbe_NX_Pl_consensus    GAGAACATTGAAGCAGAAATAAAAACTTTAATTGCAAAGATCAAAGAACAATCTAATTTATACGAAGCATATAAAGCA [21372]

lp36_gi|11496779|B31_GB      ATAGTAACGTCAATCTTATTAATGAGGGATTCTCTTAAAGAAGTGCAAGGTATCATTGACAAGAATGGCGTTTGGTAC [21450]
lp36_un11_B31_PacBio         ATAGTAACGTCAATCTTATTAATGAGGGATTCTCTTAAAGAAGTGCAAGGTATCATTGACAAGAATGGCGTTTGGTAC [21450]
lp36_B31_TS_MP_FG_consensus  ATAGTAACGTCAATCTTATTAATGAGGGATTCTCTTAAAGAAGTGCAAGGTATCATTGACAAGAATGGCGTTTGGTAC [21450]
lp36_B31_NX_Pl_consensus     ATAGTAACGTCAATCTTATTAATGAGGGATTCTCTTAAAGAAGTGCAAGGTATCATTGACAAGAATGGCGTTTGGTAC [21450]
lp36_PAli_NX_MP_FG_consensus ATAGTAACGTCAATCTTATTAATGAGGGATTCTCTTAAAGAAGTGCAAGGTATCATTGACAAGAATGGCGTTTGGTAC [21450]
lp36_PAli_NX_Pl_consensus    ATAGTAACGTCAATCTTATTAATGAGGGATTCTCTTAAAGAAGTGCAAGGTATCATTGACAAGAATGGCGTTTGGTAC [21450]
lp36_PAbe_NX_MP_FG_consensus ATAGTAACGTCAATCTTATTAATGAGGGATTCTCTTAAAGAAGTGCAAGGTATCATTGACAAGAATGGCGTTTGGTAC [21450]
lp36_PAbe_NX_Pl_consensus    ATAGTAACGTCAATCTTATTAATGAGGGATTCTCTTAAAGAAGTGCAAGGTATCATTGACAAGAATGGCGTTTGGTAC [21450]

lp36_gi|11496779|B31_GB      TAATATTAATTTTTTTAATTTAAAACAGGCTACATAATATGTAAATATGTAGCTTGTTTAAAGTAAAATAATTAAAGT [21528]
lp36_un11_B31_PacBio         TAATATTAATTTTTTTAATTTAAAACAGGCTACATAATATGTAAATATGTAGCTTGTTTAAAGTAAAATAATTAAAGT [21528]
lp36_B31_TS_MP_FG_consensus  TAATATTAATTTTTTTAATTTAAAACAGGCTACATAATATGTAAATATGTAGCTTGTTTAAAGTAAAATAATTAAAGT [21528]
lp36_B31_NX_Pl_consensus     TAATATTAATTTTTTTAATTTAAAACAGGCTACATAATATGTAAATATGTAGCTTGTTTAAAGTAAAATAATTAAAGT [21528]
lp36_PAli_NX_MP_FG_consensus TAATATTAATTTTTTTAATTTAAAACAGGCTACATAATATGTAAATATGTAGCTTGTTTAAAGTAAAATAATTAAAGT [21528]
lp36_PAli_NX_Pl_consensus    TAATATTAATTTTTTTAATTTAAAACAGGCTACATAATATGTAAATATGTAGCTTGTTTAAAGTAAAATAATTAAAGT [21528]
lp36_PAbe_NX_MP_FG_consensus TAATATTAATTTTTTTAATTTAAAACAGGCTACATAATATGTAAATATGTAGCTTGTTTAAAGTAAAATAATTAAAGT [21528]
```

```
lp36_PAbe_NX_Pl_consensus    TAATATTAATTTTTTTAATTTAAAACAGGCTACATAATATGTAAATATGTAGCTTGTTTAAAGTAAAATAATTAAAGT [21528]

lp36_gi|11496779|B31_GB      TCTAGTTGTAAAAAAGTATTGTGGATAAGAAAATGGATTTCGTCAATTTACAAAAGGTATATTAACTGATTTAGATAA [21606]
lp36_un11_B31_PacBio         TCTAGTTGTAAAAAAGTATTGTGGATAAGAAAATGGATTTCGTCAATTTACAAAAGGTATATTAACTGATTTAGATAA [21606]
lp36_B31_TS_MP_FG_consensus  TCTAGTTGTAAAAAAGTATTGTGGATAAGAAAATGGATTTCGTCAATTTACAAAAGGTATATTAACTGATTTAGATAA [21606]
lp36_B31_NX_Pl_consensus     TCTAGTTGTAAAAAAGTATTGTGGATAAGAAAATGGATTTCGTCAATTTACAAAAGGTATATTAACTGATTTAGATAA [21606]
lp36_PAli_NX_MP_FG_consensus TCTAGTTGTAAAAAAGTATTGTGGATAAGAAAATGGATTTCGTCAATTTACAAAAGGTATATTAACTGATTTAGATAA [21606]
lp36_PAli_NX_Pl_consensus    TCTAGTTGTAAAAAAGTATTGTGGATAAGAAAATGGATTTCGTCAATTTACAAAAGGTATATTAACTGATTTAGATAA [21606]
lp36_PAbe_NX_MP_FG_consensus TCTAGTTGTAAAAAAGTATTGTGGATAAGAAAATGGATTTCGTCAATTTACAAAAGGTATATTAACTGATTTAGATAA [21606]
lp36_PAbe_NX_Pl_consensus    TCTAGTTGTAAAAAAGTATTGTGGATAAGAAAATGGATTTCGTCAATTTACAAAAGGTATATTAACTGATTTAGATAA [21606]

lp36_gi|11496779|B31_GB      AAGTCAAAAATATTGTTTTGATTTATATCAGAATTGGTAGATTGCCATGTTTTAAAGTAAGTATTTAGAATAGCTTTT [21684]
lp36_un11_B31_PacBio         AAGTCAAAAATATTGTTTTGATTTATATCAGAATTGGTAGATTGCCATGTTTTAAAGTAAGTATTTAGAATAGCTTTT [21684]
lp36_B31_TS_MP_FG_consensus  AAGTCAAAAATATTGTTTTGATTTATATCAGAATTGGTAGATTGCCATGTTTTAAAGTAAGTATTTAGAATAGCTTTT [21684]
lp36_B31_NX_Pl_consensus     AAGTCAAAAATATTGTTTTGATTTATATCAGAATTGGTAGATTGCCATGTTTTAAAGTAAGTATTTAGAATAGCTTTT [21684]
lp36_PAli_NX_MP_FG_consensus AAGTCAAAAATATTGTTTTGATTTATATCAGAATTGGTAGATTGCCATGTTTTAAAGTAAGTATTTAGAATAGCTTTT [21684]
lp36_PAli_NX_Pl_consensus    AAGTCAAAAATATTGTTTTGATTTATATCAGAATTGGTAGATTGCCATGTTTTAAAGTAAGTATTTAGAATAGCTTTT [21684]
lp36_PAbe_NX_MP_FG_consensus AAGTCAAAAATATTGTTTTGATTTATATCAGAATTGGTAGATTGCCATGTTTTAAAGTAAGTATTTAGAATAGCTTTT [21684]
lp36_PAbe_NX_Pl_consensus    AAGTCAAAAATATTGTTTTGATTTATATCAGAATTGGTAGATTGCCATGTTTTAAAGTAAGTATTTAGAATAGCTTTT [21684]

lp36_gi|11496779|B31_GB      ACTATTAAGCTTGCATACAAAATGGTATTGTCAGCTTGATTCCAATCTTAATTTCATTTTATCAACTCAAATTAAGAT [21762]
lp36_un11_B31_PacBio         ACTATTAAGCTTGCATACAAAATGGTATTGTCAGCTTGATTCCAATCTTAATTTCATTTTATCAACTCAAATTAAGAT [21762]
lp36_B31_TS_MP_FG_consensus  ACTATTAAGCTTGCATACAAAATGGTATTGTCAGCTTGATTCCAATCTTAATTTCATTTTATCAACTCAAATTAAGAT [21762]
lp36_B31_NX_Pl_consensus     ACTATTAAGCTTGCATACAAAATGGTATTGTCAGCTTGATTCCAATCTTAATTTCATTTTATCAACTCAAATTAAGAT [21762]
lp36_PAli_NX_MP_FG_consensus ACTATTAAGCTTGCATACAAAATGGTATTGTCAGCTTGATTCCAATCTTAATTTCATTTTATCAACTCAAATTAAGAT [21762]
lp36_PAli_NX_Pl_consensus    ACTATTAAGCTTGCATACAAAATGGTATTGTCAGCTTGATTCCAATCTTAATTTCATTTTATCAACTCAAATTAAGAT [21762]
lp36_PAbe_NX_MP_FG_consensus ACTATTAAGCTTGCATACAAAATGGTATTGTCAGCTTGATTCCAATCTTAATTTCATTTTATCAACTCAAATTAAGAT [21762]
lp36_PAbe_NX_Pl_consensus    ACTATTAAGCTTGCATACAAAATGGTATTGTCAGCTTGATTCCAATCTTAATTTCATTTTATCAACTCAAATTAAGAT [21762]

lp36_gi|11496779|B31_GB      TGGAATCAAATGAAACAAAAAAAGTGAATAAGAATTCATCAAAAGAAATTCGCAAATATGTTAAAGGATTACTTAATA [21840]
lp36_un11_B31_PacBio         TGGAATCAAATGAAACAAAAAAAGTGAATAAGAATTCATCAAAAGAAATTCGCAAATATGTTAAAGGATTACTTAATA [21840]
lp36_B31_TS_MP_FG_consensus  TGGAATCAAATGAAACAAAAAAAGTGAATAAGAATTCATCAAAAGAAATTCGCAAATATGTTAAAGGATTACTTAATA [21840]
lp36_B31_NX_Pl_consensus     TGGAATCAAATGAAACAAAAAAAGTGAATAAGAATTCATCAAAAGAAATTCGCAAATATGTTAAAGGATTACTTAATA [21840]
lp36_PAli_NX_MP_FG_consensus TGGAATCAAATGAAACAAAAAAAGTGAATAAGAATTCATCAAAAGAAATTCGCAAATATGTTAAAGGATTACTTAATA [21840]
lp36_PAli_NX_Pl_consensus    TGGAATCAAATGAAACAAAAAAAGTGAATAAGAATTCATCAAAAGAAATTCGCAAATATGTTAAAGGATTACTTAATA [21840]
lp36_PAbe_NX_MP_FG_consensus TGGAATCAAATGAAACAAAAAAAGTGAATAAGAATTCATCAAAAGAAATTCGCAAATATGTTAAAGGATTACTTAATA [21840]
lp36_PAbe_NX_Pl_consensus    TGGAATCAAATGAAACAAAAAAAGTGAATAAGAATTCATCAAAAGAAATTCGCAAATATGTTAAAGGATTACTTAATA [21840]

lp36_gi|11496779|B31_GB      TTGTTAAAAATATTGTGCCTGTTACAACTAAAAGGTTAAAGAACTATATTTAAAATTGTGCAGGGTATCTAATGAAGA [21918]
lp36_un11_B31_PacBio         TTGTTAAAAATATTGTGCCTGTTACAACTAAAAGGTTAAAGAACTATATTTAAAATTGTGCAGGGTATCTAATGAAGA [21918]
lp36_B31_TS_MP_FG_consensus  TTGTTAAAAATATTGTGCCTGTTACAACTAAAAGGTTAAAGAACTATATTTAAAATTGTGCAGGGTATCTAATGAAGA [21918]
lp36_B31_NX_Pl_consensus     TTGTTAAAAATATTGTGCCTGTTACAACTAAAAGGTTAAAGAACTATATTTAAAATTGTGCAGGGTATCTAATGAAGA [21918]
lp36_PAli_NX_MP_FG_consensus TTGTTAAAAATATTGTGCCTGTTACAACTAAAAGGTTAAAGAACTATATTTAAAATTGTGCAGGGTATCTAATGAAGA [21918]
lp36_PAli_NX_Pl_consensus    TTGTTAAAAATATTGTGCCTGTTACAACTAAAAGGTTAAAGAACTATATTTAAAATTGTGCAGGGTATCTAATGAAGA [21918]
lp36_PAbe_NX_MP_FG_consensus TTGTTAAAAATATTGTGCCTGTTACAACTAAAAGGTTAAAGAACTATATTTAAAATTGTGCAGGGTATCTAATGAAGA [21918]
lp36_PAbe_NX_Pl_consensus    TTGTTAAAAATATTGTGCCTGTTACAACTAAAAGGTTAAAGAACTATATTTAAAATTGTGCAGGGTATCTAATGAAGA [21918]

lp36_gi|11496779|B31_GB      ATAATGGCCCCTTGATACTTTGGATATCATTACTATACTATTTAATAAGAGAAAAATTTATTTATCACTCAATATTAA [21996]
lp36_un11_B31_PacBio         ATAATGGCCCCTTGATACTTTGGATATCATTACTATACTATTTAATAAGAGAAAAATTTATTTATCACTCAATATTAA [21996]
lp36_B31_TS_MP_FG_consensus  ATAATGGCCCCTTGATACTTTGGATATCATTACTATACTATTTAATAAGAGAAAAATTTATTTATCACTCAATATTAA [21996]
lp36_B31_NX_Pl_consensus     ATAATGGCCCCTTGATACTTTGGATATCATTACTATACTATTTAATAAGAGAAAAATTTATTTATCACTCAATATTAA [21996]
lp36_PAli_NX_MP_FG_consensus ATAATGGCCCCTTGATACTTTGGATATCATTACTATACTATTTAATAAGAGAAAAATTTATTTATCACTCAATATTAA [21996]
lp36_PAli_NX_Pl_consensus    ATAATGGCCCCTTGATACTTTGGATATCATTACTATACTATTTAATAAGAGAAAAATTTATTTATCACTCAATATTAA [21996]
lp36_PAbe_NX_MP_FG_consensus ATAATGGCCCCTTGATACTTTGGATATCATTACTATACTATTTAATAAGAGAAAAATTTATTTATCACTCAATATTAA [21996]
lp36_PAbe_NX_Pl_consensus    ATAATGGCCCCTTGATACTTTGGATATCATTACTATACTATTTAATAAGAGAAAAATTTATTTATCACTCAATATTAA [21996]

lp36_gi|11496779|B31_GB      GCTTGTTTTTTCCCCTATTTAATATTTTACTATCCAATTTAAATTTACTTGATATTTACTTATATAAAACAATTGAAT [22074]
lp36_un11_B31_PacBio         GCTTGTTTTTTCCCCTATTTAATATTTTACTATCCAATTTAAATTTACTTGATATTTACTTATATAAAACAATTGAAT [22074]
lp36_B31_TS_MP_FG_consensus  GCTTGTTTTTTCCCCTATTTAATATTTTACTATCCAATTTAAATTTACTTGATATTTACTTATATAAAACAATTGAAT [22074]
lp36_B31_NX_Pl_consensus     GCTTGTTTTTTCCCCTATTTAATATTTTACTATCCAATTTAAATTTACTTGATATTTACTTATATAAAACAATTGAAT [22074]
lp36_PAli_NX_MP_FG_consensus GCTTGTTTTTTCCCCTATTTAATATTTTACTATCCAATTTAAATTTACTTGATATTTACTTATATAAAACAATTGAAT [22074]
lp36_PAli_NX_Pl_consensus    GCTTGTTTTTTCCCCTATTTAATATTTTACTATCCAATTTAAATTTACTTGATATTTACTTATATAAAACAATTGAAT [22074]
lp36_PAbe_NX_MP_FG_consensus GCTTGTTTTTTCCCCTATTTAATATTTTACTATCCAATTTAAATTTACTTGATATTTACTTATATAAAACAATTGAAT [22074]
lp36_PAbe_NX_Pl_consensus    GCTTGTTTTTTCCCCTATTTAATATTTTACTATCCAATTTAAATTTACTTGATATTTACTTATATAAAACAATTGAAT [22074]

lp36_gi|11496779|B31_GB      TTTGGATTTTATATGCGGAGCGACTAAAGCTTGTAAAGCTTATGAAAGTATTAATTTAAAACTAGGTTAGAAAAGCAA [22152]
lp36_un11_B31_PacBio         TTTGGATTTTATATGCGGAGCGACTAAAGCTTGTAAAGCTTATGAAAGTATTAATTTAAAACTAGGTTAGAAAAGCAA [22152]
lp36_B31_TS_MP_FG_consensus  TTTGGATTTTATATGCGGAGCGACTAAAGCTTGTAAAGCTTATGAAAGTATTAATTTAAAACTAGGTTAGAAAAGCAA [22152]
lp36_B31_NX_Pl_consensus     TTTGGATTTTATATGCGGAGCGACTAAAGCTTGTAAAGCTTATGAAAGTATTAATTTAAAACTAGGTTAGAAAAGCAA [22152]
lp36_PAli_NX_MP_FG_consensus TTTGGATTTTATATGCGGAGCGACTAAAGCTTGTAAAGCTTATGAAAGTATTAATTTAAAACTAGGTTAGAAAAGCAA [22152]
lp36_PAli_NX_Pl_consensus    TTTGGATTTTATATGCGGAGCGACTAAAGCTTGTAAAGCTTATGAAAGTATTAATTTAAAACTAGGTTAGAAAAGCAA [22152]
lp36_PAbe_NX_MP_FG_consensus TTTGGATTTTATATGCGGAGCGACTAAAGCTTGTAAAGCTTATGAAAGTATTAATTTAAAACTAGGTTAGAAAAGCAA [22152]
lp36_PAbe_NX_Pl_consensus    TTTGGATTTTATATGCGGAGCGACTAAAGCTTGTAAAGCTTATGAAAGTATTAATTTAAAACTAGGTTAGAAAAGCAA [22152]

lp36_gi|11496779|B31_GB      TCTTAGATTTAGTAATGAAAAACTCGATACATTAACTTTTTTCGTAAATGTATAAAATCAGCGCATTTTAATTTTTAT [22230]
lp36_un11_B31_PacBio         TCTTAGATTTAGTAATGAAAAACTCGATACATTAACTTTTTTCGTAAATGTATAAAATCAGCGCATTTTAATTTTTAT [22230]
lp36_B31_TS_MP_FG_consensus  TCTTAGATTTAGTAATGAAAAACTCGATACATTAACTTTTTTCGTAAATGTATAAAATCAGCGCATTTTAATTTTTAT [22230]
lp36_B31_NX_Pl_consensus     TCTTAGATTTAGTAATGAAAAACTCGATACATTAACTTTTTTCGTAAATGTATAAAATCAGCGCATTTTAATTTTTAT [22230]
lp36_PAli_NX_MP_FG_consensus TCTTAGATTTAGTAATGAAAAACTCGATACATTAACTTTTTTCGTAAATGTATAAAATCAGCGCATTTTAATTTTTAT [22230]
lp36_PAli_NX_Pl_consensus    TCTTAGATTTAGTAATGAAAAACTCGATACATTAACTTTTTTCGTAAATGTATAAAATCAGCGCATTTTAATTTTTAT [22230]
lp36_PAbe_NX_MP_FG_consensus TCTTAGATTTAGTAATGAAAAACTCGATACATTAACTTTTTTCGTAAATGTATAAAATCAGCGCATTTTAATTTTTAT [22230]
lp36_PAbe_NX_Pl_consensus    TCTTAGATTTAGTAATGAAAAACTCGATACATTAACTTTTTTCGTAAATGTATAAAATCAGCGCATTTTAATTTTTAT [22230]

lp36_gi|11496779|B31_GB      TGAAAAGGAGATGTATCCCAAATAGAAGAGTATGTTAAAAGGTTTTTATTCAAACTGAAAAATATGGGTAAAGTTAAA [22308]
lp36_un11_B31_PacBio         TGAAAAGGAGATGTATCCCAAATAGAAGAGTATGTTAAAAGGTTTTTATTCAAACTGAAAAATATGGGTAAAGTTAAA [22308]
lp36_B31_TS_MP_FG_consensus  TGAAAAGGAGATGTATCCCAAATAGAAGAGTATGTTAAAAGGTTTTTATTCAAACTGAAAAATATGGGTAAAGTTAAA [22308]
lp36_B31_NX_Pl_consensus     TGAAAAGGAGATGTATCCCAAATAGAAGAGTATGTTAAAAGGTTTTTATTCAAACTGAAAAATATGGGTAAAGTTAAA [22308]
lp36_PAli_NX_MP_FG_consensus TGAAAAGGAGATGTATCCCAAATAGAAGAGTATGTTAAAAGGTTTTTATTCAAACTGAAAAATATGGGTAAAGTTAAA [22308]
lp36_PAli_NX_Pl_consensus    TGAAAAGGAGATGTATCCCAAATAGAAGAGTATGTTAAAAGGTTTTTATTCAAACTGAAAAATATGGGTAAAGTTAAA [22308]
lp36_PAbe_NX_MP_FG_consensus TGAAAAGGAGATGTATCCCAAATAGAAGAGTATGTTAAAAGGTTTTTATTCAAACTGAAAAATATGGGTAAAGTTAAA [22308]
lp36_PAbe_NX_Pl_consensus    TGAAAAGGAGATGTATCCCAAATAGAAGAGTATGTTAAAAGGTTTTTATTCAAACTGAAAAATATGGGTAAAGTTAAA [22308]

lp36_gi|11496779|B31_GB      AAGCCGCTTAGCTATATTAAATATGGTAGAGATAATCAGCTTAATGTAGAAGGTAAAGATAGAAATCATATAGATGTG [22386]
lp36_un11_B31_PacBio         AAGCCGCTTAGCTATATTAAATATGGTAGAGATAATCAGCTTAATGTAGAAGGTAAAGATAGAAATCATATAGATGTG [22386]
lp36_B31_TS_MP_FG_consensus  AAGCCGCTTAGCTATATTAAATATGGTAGAGATAATCAGCTTAATGTAGAAGGTAAAGATAGAAATCATATAGATGTG [22386]
lp36_B31_NX_Pl_consensus     AAGCCGCTTAGCTATATTAAATATGGTAGAGATAATCAGCTTAATGTAGAAGGTAAAGATAGAAATCATATAGATGTG [22386]
lp36_PAli_NX_MP_FG_consensus AAGCCGCTTAGCTATATTAAATATGGTAGAGATAATCAGCTTAATGTAGAAGGTAAAGATAGAAATCATATAGATGTG [22386]
lp36_PAli_NX_Pl_consensus    AAGCCGCTTAGCTATATTAAATATGGTAGAGATAATCAGCTTAATGTAGAAGGTAAAGATAGAAATCATATAGATGTG [22386]
lp36_PAbe_NX_MP_FG_consensus AAGCCGCTTAGCTATATTAAATATGGTAGAGATAATCAGCTTAATGTAGAAGGTAAAGATAGAAATCATATAGATGTG [22386]
lp36_PAbe_NX_Pl_consensus    AAGCCGCTTAGCTATATTAAATATGGTAGAGATAATCAGCTTAATGTAGAAGGTAAAGATAGAAATCATATAGATGTG [22386]

lp36_gi|11496779|B31_GB      AAGGAGCATCATAGAGATAAAGGGGGATTTGCTAATGTTTTTGATAATTATTTTATGTTTTCAAATCCCCCATCTCTT [22464]
lp36_un11_B31_PacBio         AAGGAGCATCATAGAGATAAAGGGGGATTTGCTAATGTTTTTGATAATTATTTTATGTTTTCAAATCCCCCATCTCTT [22464]
lp36_B31_TS_MP_FG_consensus  AAGGAGCATCATAGAGATAAAGGGGGATTTGCTAATGTTTTTGATAATTATTTTATGTTTTCAAATCCCCCATCTCTT [22464]
lp36_B31_NX_Pl_consensus     AAGGAGCATCATAGAGATAAAGGGGGATTTGCTAATGTTTTTGATAATTATTTTATGTTTTCAAATCCCCCATCTCTT [22464]
lp36_PAli_NX_MP_FG_consensus AAGGAGCATCATAGAGATAAAGGGGGATTTGCTAATGTTTTTGATAATTATTTTATGTTTTCAAATCCCCCATCTCTT [22464]
lp36_PAli_NX_Pl_consensus    AAGGAGCATCATAGAGATAAAGGGGGATTTGCTAATGTTTTTGATAATTATTTTATGTTTTCAAATCCCCCATCTCTT [22464]
lp36_PAbe_NX_MP_FG_consensus AAGGAGCATCATAGAGATAAAGGGGGATTTGCTAATGTTTTTGATAATTATTTTATGTTTTCAAATCCCCCATCTCTT [22464]
lp36_PAbe_NX_Pl_consensus    AAGGAGCATCATAGAGATAAAGGGGGATTTGCTAATGTTTTTGATAATTATTTTATGTTTTCAAATCCCCCATCTCTT [22464]
```

```
lp36_gi|11496779|B31_GB      TAAGTGGATAGCAATAATGGAGAATTGAGTGCATTAGACTTTCTCCTATTAATAGCAATCCTGTGAAGATAAGACGTT [22542]
lp36_un11_B31_PacBio         TAAGTGGATAGCAATAATGGAGAATTGAGTGCATTAGACTTTCTCCTATTAATAGCAATCCTGTGAAGATAAGACGTT [22542]
lp36_B31_TS_MP_FG_consensus  TAAGTGGATAGCAATAATGGAGAATTGAGTGCATTAGACTTTCTCCTATTAATAGCAATCCTGTGAAGATAAGACGTT [22542]
lp36_B31_NX_Pl_consensus     TAAGTGGATAGCAATAATGGAGAATTGAGTGCATTAGACTTTCTCCTATTAATAGCAATCCTGTGAAGATAAGACGTT [22542]
lp36_PAli_NX_MP_FG_consensus TAAGTGGATAGCAATAATGGAGAATTGAGTGCATTAGACTTTCTCCTATTAATAGCAATCCTGTGAAGATAAGACGTT [22542]
lp36_PAli_NX_Pl_consensus    TAAGTGGATAGCAATAATGGAGAATTGAGTGCATTAGACTTTCTCCTATTAATAGCAATCCTGTGAAGATAAGACGTT [22542]
lp36_PAbe_NX_MP_FG_consensus TAAGTGGATAGCAATAATGGAGAATTGAGTGCATTAGACTTTCTCCTATTAATAGCAATCCTGTGAAGATAAGACGTT [22542]
lp36_PAbe_NX_Pl_consensus    TAAGTGGATAGCAATAATGGAGAATTGAGTGCATTAGACTTTCTCCTATTAATAGCAATCCTGTGAAGATAAGACGTT [22542]

lp36_gi|11496779|B31_GB      GAATGACATTTAAAATGTATGCCAGAGATTTAGAGATTCACATTAATGAATATTACTACTTTTTAGTGTTGCTATTAT [22620]
lp36_un11_B31_PacBio         GAATGACATTTAAAATGTATGCCAGAGATTTAGAGATTCACATTAATGAATATTACTACTTTTTAGTGTTGCTATTAT [22620]
lp36_B31_TS_MP_FG_consensus  GAATGACATTTAAAATGTATGCCAGAGATTTAGAGATTCACATTAATGAATATTACTACTTTTTAGTGTTGCTATTAT [22620]
lp36_B31_NX_Pl_consensus     GAATGACATTTAAAATGTATGCCAGAGATTTAGAGATTCACATTAATGAATATTACTACTTTTTAGTGTTGCTATTAT [22620]
lp36_PAli_NX_MP_FG_consensus GAATGACATTTAAAATGTATGCCAGAGATTTAGAGATTCACATTAATGAATATTACTACTTTTTAGTGTTGCTATTAT [22620]
lp36_PAli_NX_Pl_consensus    GAATGACATTTAAAATGTATGCCAGAGATTTAGAGATTCACATTAATGAATATTACTACTTTTTAGTGTTGCTATTAT [22620]
lp36_PAbe_NX_MP_FG_consensus GAATGACATTTAAAATGTATGCCAGAGATTTAGAGATTCACATTAATGAATATTACTACTTTTTAGTGTTGCTATTAT [22620]
lp36_PAbe_NX_Pl_consensus    GAATGACATTTAAAATGTATGCCAGAGATTTAGAGATTCACATTAATGAATATTACTACTTTTTAGTGTTGCTATTAT [22620]

lp36_gi|11496779|B31_GB      TTGACTATACTTTAGAGAAAAGTATTTGATTATTATTAAATACAAGAGCTTAATTACACTATTTGAAAATTGCGAGCA [22698]
lp36_un11_B31_PacBio         TTGACTATACTTTAGAGAAAAGTATTTGATTATTATTAAATACAAGAGCTTAATTACACTATTTGAAAATTGCGAGCA [22698]
lp36_B31_TS_MP_FG_consensus  TTGACTATACTTTAGAGAAAAGTATTTGATTATTATTAAATACAAGAGCTTAATTACACTATTTGAAAATTGCGAGCA [22698]
lp36_B31_NX_Pl_consensus     TTGACTATACTTTAGAGAAAAGTATTTGATTATTATTAAATACAAGAGCTTAATTACACTATTTGAAAATTGCGAGCA [22698]
lp36_PAli_NX_MP_FG_consensus TTGACTATACTTTAGAGAAAAGTATTTGATTATTATTAAATACAAGAGCTTAATTACACTATTTGAAAATTGCGAGCA [22698]
lp36_PAli_NX_Pl_consensus    TTGACTATACTTTAGAGAAAAGTATTTGATTATTATTAAATACAAGAGCTTAATTACACTATTTGAAAATTGCGAGCA [22698]
lp36_PAbe_NX_MP_FG_consensus TTGACTATACTTTAGAGAAAAGTATTTGATTATTATTAAATACAAGAGCTTAATTACACTATTTGAAAATTGCGAGCA [22698]
lp36_PAbe_NX_Pl_consensus    TTGACTATACTTTAGAGAAAAGTATTTGATTATTATTAAATACAAGAGCTTAATTACACTATTTGAAAATTGCGAGCA [22698]

lp36_gi|11496779|B31_GB      TAATATGTGTTTTTTTATTTTAGCACAAGTTTTTTAGACTTTCTAAAAAAGTTAAAAAAAAAGAAGATGCTGAGTAAT [22776]
lp36_un11_B31_PacBio         TAATATGTGTTTTTTTATTTTAGCACAAGTTTTTTAGACTTTCTAAAAAAGTTAAAAAAAAAGAAGATGCTGAGTAAT [22776]
lp36_B31_TS_MP_FG_consensus  TAATATGTGTTTTTTTATTTTAGCACAAGTTTTTTAGACTTTCTAAAAAAGTTAAAAAAAAAGAAGATGCTGAGTAAT [22776]
lp36_B31_NX_Pl_consensus     TAATATGTGTTTTTTTATTTTAGCACAAGTTTTTTAGACTTTCTAAAAAAGTTAAAAAAAAAGAAGATGCTGAGTAAT [22776]
lp36_PAli_NX_MP_FG_consensus TAATATGTGTTTTTTTATTTTAGCACAAGTTTTTTAGACTTTCTAAAAAAGTTAAAAAAAAAGAAGATGCTGAGTAAT [22776]
lp36_PAli_NX_Pl_consensus    TAATATGTGTTTTTTTATTTTAGCACAAGTTTTTTAGACTTTCTAAAAAAGTTAAAAAAAAAGAAGATGCTGAGTAAT [22776]
lp36_PAbe_NX_MP_FG_consensus TAATATGTGTTTTTTTATTTTAGCACAAGTTTTTTAGACTTTCTAAAAAAGTTAAAAAAAAAGAAGATGCTGAGTAAT [22776]
lp36_PAbe_NX_Pl_consensus    TAATATGTGTTTTTTTATTTTAGCACAAGTTTTTTAGACTTTCTAAAAAAGTTAAAAAAAAAGAAGATGCTGAGTAAT [22776]

lp36_gi|11496779|B31_GB      TTGTATAGTTCTTTTAAGATTTTCATTTAAGTAATAAATTATATTATCTTGGTATACTTCTAATATTTACCCATTAAT [22854]
lp36_un11_B31_PacBio         TTGTATAGTTCTTTTAAGATTTTCATTTAAGTAATAAATTATATTATCTTGGTATACTTCTAATATTTACCCATTAAT [22854]
lp36_B31_TS_MP_FG_consensus  TTGTATAGTTCTTTTAAGATTTTCATTTAAGTAATAAATTATATTATCTTGGTATACTTCTAATATTTACCCATTAAT [22854]
lp36_B31_NX_Pl_consensus     TTGTATAGTTCTTTTAAGATTTTCATTTAAGTAATAAATTATATTATCTTGGTATACTTCTAATATTTACCCATTAAT [22854]
lp36_PAli_NX_MP_FG_consensus TTGTATAGTTCTTTTAAGATTTTCATTTAAGTAATAAATTATATTATCTTGGTATACTTCTAATATTTACCCATTAAT [22854]
lp36_PAli_NX_Pl_consensus    TTGTATAGTTCTTTTAAGATTTTCATTTAAGTAATAAATTATATTATCTTGGTATACTTCTAATATTTACCCATTAAT [22854]
lp36_PAbe_NX_MP_FG_consensus TTGTATAGTTCTTTTAAGATTTTCATTTAAGTAATAAATTATATTATCTTGGTATACTTCTAATATTTACCCATTAAT [22854]
lp36_PAbe_NX_Pl_consensus    TTGTATAGTTCTTTTAAGATTTTCATTTAAGTAATAAATTATATTATCTTGGTATACTTCTAATATTTACCCATTAAT [22854]

lp36_gi|11496779|B31_GB      AAAGCTAGTTTGGCTTAACAAATAAAAACCAATAAATATTTAAAAAATGATTAATTTAGAATATAATTTCTATAGCTA [22932]
lp36_un11_B31_PacBio         AAAGCTAGTTTGGCTTAACAAATAAAAACCAATAAATATTTAAAAAATGATTAATTTAGAATATAATTTCTATAGCTA [22932]
lp36_B31_TS_MP_FG_consensus  AAAGCTAGTTTGGCTTAACAAATAAAAACCAATAAATATTTAAAAAATGATTAATTTAGAATATAATTTCTATAGCTA [22932]
lp36_B31_NX_Pl_consensus     AAAGCTAGTTTGGCTTAACAAATAAAAACCAATAAATATTTAAAAAATGATTAATTTAGAATATAATTTCTATAGCTA [22932]
lp36_PAli_NX_MP_FG_consensus AAAGCTAGTTTGGCTTAACAAATAAAAACCAATAAATATTTAAAAAATGATTAATTTAGAATATAATTTCTATAGCTA [22932]
lp36_PAli_NX_Pl_consensus    AAAGCTAGTTTGGCTTAACAAATAAAAACCAATAAATATTTAAAAAATGATTAATTTAGAATATAATTTCTATAGCTA [22932]
lp36_PAbe_NX_MP_FG_consensus AAAGCTAGTTTGGCTTAACAAATAAAAACCAATAAATATTTAAAAAATGATTAATTTAGAATATAATTTCTATAGCTA [22932]
lp36_PAbe_NX_Pl_consensus    AAAGCTAGTTTGGCTTAACAAATAAAAACCAATAAATATTTAAAAAATGATTAATTTAGAATATAATTTCTATAGCTA [22932]

lp36_gi|11496779|B31_GB      AAGCAAAAAATAAATATAAATTTGGACTAGTTTTATTACATAAAAAGATAAAATTAGTCATGCTTGTTGCAATAGAGC [23010]
lp36_un11_B31_PacBio         AAGCAAAAAATAAATATAAATTTGGACTAGTTTTATTACATAAAAAGATAAAATTAGTCATGCTTGTTGCAATAGAGC [23010]
lp36_B31_TS_MP_FG_consensus  AAGCAAAAAATAAATATAAATTTGGACTAGTTTTATTACATAAAAAGATAAAATTAGTCATGCTTGTTGCAATAGAGC [23010]
lp36_B31_NX_Pl_consensus     AAGCAAAAAATAAATATAAATTTGGACTAGTTTTATTACATAAAAAGATAAAATTAGTCATGCTTGTTGCAATAGAGC [23010]
lp36_PAli_NX_MP_FG_consensus AAGCAAAAAATAAATATAAATTTGGACTAGTTTTATTACATAAAAAGATAAAATTAGTCATGCTTGTTGCAATAGAGC [23010]
lp36_PAli_NX_Pl_consensus    AAGCAAAAAATAAATATAAATTTGGACTAGTTTTATTACATAAAAAGATAAAATTAGTCATGCTTGTTGCAATAGAGC [23010]
lp36_PAbe_NX_MP_FG_consensus AAGCAAAAAATAAATATAAATTTGGACTAGTTTTATTACATAAAAAGATAAAATTAGTCATGCTTGTTGCAATAGAGC [23010]
lp36_PAbe_NX_Pl_consensus    AAGCAAAAAATAAATATAAATTTGGACTAGTTTTATTACATAAAAAGATAAAATTAGTCATGCTTGTTGCAATAGAGC [23010]

lp36_gi|11496779|B31_GB      CTACTATCTATCCCGCGAGGTATATTTATTTATATTGCTTTTTAGTTTTTGTAAAGTGACTTTTAATTATTAAAATCT [23088]
lp36_un11_B31_PacBio         CTACTATCTATCCCGCGAGGTATATTTATTTATATTGCTTTTTAGTTTTTGTAAAGTGACTTTTAATTATTAAAATCT [23088]
lp36_B31_TS_MP_FG_consensus  CTACTATCTATCCCGCGAGGTATATTTATTTATATTGCTTTTTAGTTTTTGTAAAGTGACTTTTAATTATTAAAATCT [23088]
lp36_B31_NX_Pl_consensus     CTACTATCTATCCCGCGAGGTATATTTATTTATATTGCTTTTTAGTTTTTGTAAAGTGACTTTTAATTATTAAAATCT [23088]
lp36_PAli_NX_MP_FG_consensus CTACTATCTATCCCGCGAGGTATATTTATTTATATTGCTTTTTAGTTTTTGTAAAGTGACTTTTAATTATTAAAATCT [23088]
lp36_PAli_NX_Pl_consensus    CTACTATCTATCCCGCGAGGTATATTTATTTATATTGCTTTTTAGTTTTTGTAAAGTGACTTTTAATTATTAAAATCT [23088]
lp36_PAbe_NX_MP_FG_consensus CTACTATCTATCCCGCGAGGTATATTTATTTATATTGCTTTTTAGTTTTTGTAAAGTGACTTTTAATTATTAAAATCT [23088]
lp36_PAbe_NX_Pl_consensus    CTACTATCTATCCCGCGAGGTATATTTATTTATATTGCTTTTTAGTTTTTGTAAAGTGACTTTTAATTATTAAAATCT [23088]

lp36_gi|11496779|B31_GB      AAGGAGAAGAGATTTATAAACAAAAAATTTTCTATTTCATTATTATCTACAATATTAGCCTTCTTGTTAGTATTAGGT [23166]
lp36_un11_B31_PacBio         AAGGAGAAGAGATTTATAAACAAAAAATTTTCTATTTCATTATTATCTACAATATTAGCCTTCTTGTTAGTATTAGGT [23166]
lp36_B31_TS_MP_FG_consensus  AAGGAGAAGAGATTTATAAACAAAAAATTTTCTATTTCATTATTATCTACAATATTAGCCTTCTTGTTAGTATTAGGT [23166]
lp36_B31_NX_Pl_consensus     AAGGAGAAGAGATTTATAAACAAAAAATTTTCTATTTCATTATTATCTACAATATTAGCCTTCTTGTTAGTATTAGGT [23166]
lp36_PAli_NX_MP_FG_consensus AAGGAGAAGAGATTTATAAACAAAAAATTTTCTATTTCATTATTATCTACAATATTAGCCTTCTTGTTAGTATTAGGT [23166]
lp36_PAli_NX_Pl_consensus    AAGGAGAAGAGATTTATAAACAAAAAATTTTCTATTTCATTATTATCTACAATATTAGCCTTCTTGTTAGTATTAGGT [23166]
lp36_PAbe_NX_MP_FG_consensus AAGGAGAAGAGATTTATAAACAAAAAATTTTCTATTTCATTATTATCTACAATATTAGCCTTCTTGTTAGTATTAGGT [23166]
lp36_PAbe_NX_Pl_consensus    AAGGAGAAGAGATTTATAAACAAAAAATTTTCTATTTCATTATTATCTACAATATTAGCCTTCTTGTTAGTATTAGGT [23166]

lp36_gi|11496779|B31_GB      TGTGATTTGTCAAGCAATAATGCTGAAAACATGTTGTATCTAATATCCTTGCTTTCTTTTAAAGCTTCTGCTAAAGCA [23244]
lp36_un11_B31_PacBio         TGTGATTTGTCAAGCAATAATGCTGAAAACATGTTGTATCTAATATCCTTGCTTTCTTTTAAAGCTTCTGCTAAAGCA [23244]
lp36_B31_TS_MP_FG_consensus  TGTGATTTGTCAAGCAATAATGCTGAAAACATGTTGTATCTAATATCCTTGCTTTCTTTTAAAGCTTCTGCTAAAGCA [23244]
lp36_B31_NX_Pl_consensus     TGTGATTTGTCAAGCAATAATGCTGAAAACATGTTGTATCTAATATCCTTGCTTTCTTTTAAAGCTTCTGCTAAAGCA [23244]
lp36_PAli_NX_MP_FG_consensus TGTGATTTGTCAAGCAATAATGCTGAAAACATGTTGTATCTAATATCCTTGCTTTCTTTTAAAGCTTCTGCTAAAGCA [23244]
lp36_PAli_NX_Pl_consensus    TGTGATTTGTCAAGCAATAATGCTGAAAACATGTTGTATCTAATATCCTTGCTTTCTTTTAAAGCTTCTGCTAAAGCA [23244]
lp36_PAbe_NX_MP_FG_consensus TGTGATTTGTCAAGCAATAATGCTGAAAACATGTTGTATCTAATATCCTTGCTTTCTTTTAAAGCTTCTGCTAAAGCA [23244]
lp36_PAbe_NX_Pl_consensus    TGTGATTTGTCAAGCAATAATGCTGAAAACATGTTGTATCTAATATCCTTGCTTTCTTTTAAAGCTTCTGCTAAAGCA [23244]

lp36_gi|11496779|B31_GB      TCATCAGCCTTGTTATTTGTATCTCTAAAAATAACACAAGCTTTTTCTAAAGAATAAGTTGCATCAACATAGCAAACA [23322]
lp36_un11_B31_PacBio         TCATCAGCCTTGTTATTTGTATCTCTAAAAATAACACAAGCTTTTTCTAAAGAATAAGTTGCATCAACATAGCAAACA [23322]
lp36_B31_TS_MP_FG_consensus  TCATCAGCCTTGTTATTTGTATCTCTAAAAATAACACAAGCTTTTTCTAAAGAATAAGTTGCATCAACATAGCAAACA [23322]
lp36_B31_NX_Pl_consensus     TCATCAGCCTTGTTATTTGTATCTCTAAAAATAACACAAGCTTTTTCTAAAGAATAAGTTGCATCAACATAGCAAACA [23322]
lp36_PAli_NX_MP_FG_consensus TCATCAGCCTTGTTATTTGTATCTCTAAAAATAACACAAGCTTTTTCTAAAGAATAAGTTGCATCAACATAGCAAACA [23322]
lp36_PAli_NX_Pl_consensus    TCATCAGCCTTGTTATTTGTATCTCTAAAAATAACACAAGCTTTTTCTAAAGAATAAGTTGCATCAACATAGCAAACA [23322]
lp36_PAbe_NX_MP_FG_consensus TCATCAGCCTTGTTATTTGTATCTCTAAAAATAACACAAGCTTTTTCTAAAGAATAAGTTGCATCAACATAGCAAACA [23322]
lp36_PAbe_NX_Pl_consensus    TCATCAGCCTTGTTATTTGTATCTCTAAAAATAACACAAGCTTTTTCTAAAGAATAAGTTGCATCAACATAGCAAACA [23322]

lp36_gi|11496779|B31_GB      TTAAGAGAAGCATAACTACTCTTAACCTTGCTAATGGCTTTATGCAAGTTATACAATAAGGAATACTTAACTTTATTA [23400]
lp36_un11_B31_PacBio         TTAAGAGAAGCATAACTACTCTTAACCTTGCTAATGGCTTTATGCAAGTTATACAATAAGGAATACTTAACTTTATTA [23400]
lp36_B31_TS_MP_FG_consensus  TTAAGAGAAGCATAACTACTCTTAACCTTGCTAATGGCTTTATGCAAGTTATACAATAAGGAATACTTAACTTTATTA [23400]
lp36_B31_NX_Pl_consensus     TTAAGAGAAGCATAACTACTCTTAACCTTGCTAATGGCTTTATGCAAGTTATACAATAAGGAATACTTAACTTTATTA [23400]
lp36_PAli_NX_MP_FG_consensus TTAAGAGAAGCATAACTACTCTTAACCTTGCTAATGGCTTTATGCAAGTTATACAATAAGGAATACTTAACTTTATTA [23400]
lp36_PAli_NX_Pl_consensus    TTAAGAGAAGCATAACTACTCTTAACCTTGCTAATGGCTTTATGCAAGTTATACAATAAGGAATACTTAACTTTATTA [23400]
lp36_PAbe_NX_MP_FG_consensus TTAAGAGAAGCATAACTACTCTTAACCTTGCTAATGGCTTTATGCAAGTTATACAATAAGGAATACTTAACTTTATTA [23400]
lp36_PAbe_NX_Pl_consensus    TTAAGAGAAGCATAACTACTCTTAACCTTGCTAATGGCTTTATGCAAGTTATACAATAAGGAATACTTAACTTTATTA [23400]
```

```
lp36_gi|11496779|B31_GB      GATCTAGGAGTAGTTCTAGCTTCTTTTAAATAAGAGGCAGCTGTGCTAAGCAAGCATTCCATATTACTAAGGCTTACT [23478]
lp36_un11_B31_PacBio         GATCTAGGAGTAGTTCTAGCTTCTTTTAAATAAGAGGCAGCTGTGCTAAGCAAGCATTCCATATTACTAAGGCTTACT [23478]
lp36_B31_TS_MP_FG_consensus  GATCTAGGAGTAGTTCTAGCTTCTTTTAAATAAGAGGCAGCTGTGCTAAGCAAGCATTCCATATTACTAAGGCTTACT [23478]
lp36_B31_NX_Pl_consensus     GATCTAGGAGTAGTTCTAGCTTCTTTTAAATAAGAGGCAGCTGTGCTAAGCAAGCATTCCATATTACTAAGGCTTACT [23478]
lp36_PAli_NX_MP_FG_consensus GATCTAGGAGTAGTTCTAGCTTCTTTTAAATAAGAGGCAGCTGTGCTAAGCAAGCATTCCATATTACTAAGGCTTACT [23478]
lp36_PAli_NX_Pl_consensus    GATCTAGGAGTAGTTCTAGCTTCTTTTAAATAAGAGGCAGCTGTGCTAAGCAAGCATTCCATATTACTAAGGCTTACT [23478]
lp36_PAbe_NX_MP_FG_consensus GATCTAGGAGTAGTTCTAGCTTCTTTTAAATAAGAGGCAGCTGTGCTAAGCAAGCATTCCATATTACTAAGGCTTACT [23478]
lp36_PAbe_NX_Pl_consensus    GATCTAGGAGTAGTTCTAGCTTCTTTTAAATAAGAGGCAGCTGTGCTAAGCAAGCATTCCATATTACTAAGGCTTACT [23478]

lp36_gi|11496779|B31_GB      TTTATTTCATCTAGTTCTGTATCTAGTCTAATAAACTCTTCTACATAAGAGTCGACTTCCCTCTTAATATCTCTAGCT [23556]
lp36_un11_B31_PacBio         TTTATTTCATCTAGTTCTGTATCTAGTCTAATAAACTCTTCTACATAAGAGTCGACTTCCCTCTTAATATCTCTAGCT [23556]
lp36_B31_TS_MP_FG_consensus  TTTATTTCATCTAGTTCTGTATCTAGTCTAATAAACTCTTCTACATAAGAGTCGACTTCCCTCTTAATATCTCTAGCT [23556]
lp36_B31_NX_Pl_consensus     TTTATTTCATCTAGTTCTGTATCTAGTCTAATAAACTCTTCTACATAAGAGTCGACTTCCCTCTTAATATCTCTAGCT [23556]
lp36_PAli_NX_MP_FG_consensus TTTATTTCATCTAGTTCTGTATCTAGTCTAATAAACTCTTCTACATAAGAGTCGACTTCCCTCTTAATATCTCTAGCT [23556]
lp36_PAli_NX_Pl_consensus    TTTATTTCATCTAGTTCTGTATCTAGTCTAATAAACTCTTCTACATAAGAGTCGACTTCCCTCTTAATATCTCTAGCT [23556]
lp36_PAbe_NX_MP_FG_consensus TTTATTTCATCTAGTTCTGTATCTAGTCTAATAAACTCTTCTACATAAGAGTCGACTTCCCTCTTAATATCTCTAGCT [23556]
lp36_PAbe_NX_Pl_consensus    TTTATTTCATCTAGTTCTGTATCTAGTCTAATAAACTCTTCTACATAAGAGTCGACTTCCCTCTTAATATCTCTAGCT [23556]

lp36_gi|11496779|B31_GB      TCGCTCCCTTGGTTATTAACTTGCTGAGGAGTAGGGCTACTATTATTATTTTGTATGTTAGTTTGCATGTCTTTAATC [23634]
lp36_un11_B31_PacBio         TCGCTCCCTTGGTTATTAACTTGCTGAGGAGTAGGGCTACTATTATTATTTTGTATGTTAGTTTGCATGTCTTTAATC [23634]
lp36_B31_TS_MP_FG_consensus  TCGCTCCCTTGGTTATTAACTTGCTGAGGAGTAGGGCTACTATTATTATTTTGTATGTTAGTTTGCATGTCTTTAATC [23634]
lp36_B31_NX_Pl_consensus     TCGCTCCCTTGGTTATTAACTTGCTGAGGAGTAGGGCTACTATTATTATTTTGTATGTTAGTTTGCATGTCTTTAATC [23634]
lp36_PAli_NX_MP_FG_consensus TCGCTCCCTTGGTTATTAACTTGCTGAGGAGTAGGGCTACTATTATTATTTTGTATGTTAGTTTGCATGTCTTTAATC [23634]
lp36_PAli_NX_Pl_consensus    TCGCTCCCTTGGTTATTAACTTGCTGAGGAGTAGGGCTACTATTATTATTTTGTATGTTAGTTTGCATGTCTTTAATC [23634]
lp36_PAbe_NX_MP_FG_consensus TCGCTCCCTTGGTTATTAACTTGCTGAGGAGTAGGGCTACTATTATTATTTTGTATGTTAGTTTGCATGTCTTTAATC [23634]
lp36_PAbe_NX_Pl_consensus    TCGCTCCCTTGGTTATTAACTTGCTGAGGAGTAGGGCTACTATTATTATTTTGTATGTTAGTTTGCATGTCTTTAATC [23634]

lp36_gi|11496779|B31_GB      ATATCTTTGCATACTGCCTGCATCTTGCTCTATCTGTATTCTTGCCCCTTAACACCTTATCATTAAAGAATATATCAC [23712]
lp36_un11_B31_PacBio         ATATCTTTGCATACTGCCTGCATCTTGCTCTATCTGTATTCTTGCCCCTTAACACCTTATCATTAAAGAATATATCAC [23712]
lp36_B31_TS_MP_FG_consensus  ATATCTTTGCATACTGCCTGCATCTTGCTCTATCTGTATTCTTGCCCCTTAACACCTTATCATTAAAGAATATATCAC [23712]
lp36_B31_NX_Pl_consensus     ATATCTTTGCATACTGCCTGCATCTTGCTCTATCTGTATTCTTGCCCCTTAACACCTTATCATTAAAGAATATATCAC [23712]
lp36_PAli_NX_MP_FG_consensus ATATCTTTGCATACTGCCTGCATCTTGCTCTATCTGTATTCTTGCCCCTTAACACCTTATCATTAAAGAATATATCAC [23712]
lp36_PAli_NX_Pl_consensus    ATATCTTTGCATACTGCCTGCATCTTGCTCTATCTGTATTCTTGCCCCTTAACACCTTATCATTAAAGAATATATCAC [23712]
lp36_PAbe_NX_MP_FG_consensus ATATCTTTGCATACTGCCTGCATCTTGCTCTATCTGTATTCTTGCCCCTTAACACCTTATCATTAAAGAATATATCAC [23712]
lp36_PAbe_NX_Pl_consensus    ATATCTTTGCATACTGCCTGCATCTTGCTCTATCTGTATTCTTGCCCCTTAACACCTTATCATTAAAGAATATATCAC [23712]

lp36_gi|11496779|B31_GB      ATTTGTAAAAATAGCTTGTATTATTTGATACAAGCTATAAGGCTAAGAGAAAAGTAAATAAGTAAGTGGGCTTGGTTA [23790]
lp36_un11_B31_PacBio         ATTTGTAAAAATAGCTTGTATTATTTGATACAAGCTATAAGGCTAAGAGAAAAGTAAATAAGTAAGTGGGCTTGGTTA [23790]
lp36_B31_TS_MP_FG_consensus  ATTTGTAAAAATAGCTTGTATTATTTGATACAAGCTATAAGGCTAAGAGAAAAGTAAATAAGTAAGTGGGCTTGGTTA [23790]
lp36_B31_NX_Pl_consensus     ATTTGTAAAAATAGCTTGTATTATTTGATACAAGCTATAAGGCTAAGAGAAAAGTAAATAAGTAAGTGGGCTTGGTTA [23790]
lp36_PAli_NX_MP_FG_consensus ATTTGTAAAAATAGCTTGTATTATTTGATACAAGCTATAAGGCTAAGAGAAAAGTAAATAAGTAAGTGGGCTTGGTTA [23790]
lp36_PAli_NX_Pl_consensus    ATTTGTAAAAATAGCTTGTATTATTTGATACAAGCTATAAGGCTAAGAGAAAAGTAAATAAGTAAGTGGGCTTGGTTA [23790]
lp36_PAbe_NX_MP_FG_consensus ATTTGTAAAAATAGCTTGTATTATTTGATACAAGCTATAAGGCTAAGAGAAAAGTAAATAAGTAAGTGGGCTTGGTTA [23790]
lp36_PAbe_NX_Pl_consensus    ATTTGTAAAAATAGCTTGTATTATTTGATACAAGCTATAAGGCTAAGAGAAAAGTAAATAAGTAAGTGGGCTTGGTTA [23790]

lp36_gi|11496779|B31_GB      AAAAAGAGAATAAGGACTAGACTTACTTAAGTAACCAAGTAAGTTTTTAATTTTAAGGAGAGGAGTTTTAATATGAGG [23868]
lp36_un11_B31_PacBio         AAAAAGAGAATAAGGACTAGACTTACTTAAGTAACCAAGTAAGTTTTTAATTTTAAGGAGAGGAGTTTTAATATGAGG [23868]
lp36_B31_TS_MP_FG_consensus  AAAAAGAGAATAAGGACTAGACTTACTTAAGTAACCAAGTAAGTTTTTAATTTTAAGGAGAGGAGTTTTAATATGAGG [23868]
lp36_B31_NX_Pl_consensus     AAAAAGAGAATAAGGACTAGACTTACTTAAGTAACCAAGTAAGTTTTTAATTTTAAGGAGAGGAGTTTTAATATGAGG [23868]
lp36_PAli_NX_MP_FG_consensus AAAAAGAGAATAAGGACTAGACTTACTTAAGTAACCAAGTAAGTTTTTAATTTTAAGGAGAGGAGTTTTAATATGAGG [23868]
lp36_PAli_NX_Pl_consensus    AAAAAGAGAATAAGGACTAGACTTACTTAAGTAACCAAGTAAGTTTTTAATTTTAAGGAGAGGAGTTTTAATATGAGG [23868]
lp36_PAbe_NX_MP_FG_consensus AAAAAGAGAATAAGGACTAGACTTACTTAAGTAACCAAGTAAGTTTTTAATTTTAAGGAGAGGAGTTTTAATATGAGG [23868]
lp36_PAbe_NX_Pl_consensus    AAAAAGAGAATAAGGACTAGACTTACTTAAGTAACCAAGTAAGTTTTTAATTTTAAGGAGAGGAGTTTTAATATGAGG [23868]

lp36_gi|11496779|B31_GB      ATATATTTGTGTTTATGTAGTGTTCTTTTAGCGACCTCTTGTAAGTTATTTTATTCAAATGTAGGAACTGGGCCAGCA [23946]
lp36_un11_B31_PacBio         ATATATTTGTGTTTATGTAGTGTTCTTTTAGCGACCTCTTGTAAGTTATTTTATTCAAATGTAGGAACTGGGCCAGCA [23946]
lp36_B31_TS_MP_FG_consensus  ATATATTTGTGTTTATGTAGTGTTCTTTTAGCGACCTCTTGTAAGTTATTTTATTCAAATGTAGGAACTGGGCCAGCA [23946]
lp36_B31_NX_Pl_consensus     ATATATTTGTGTTTATGTAGTGTTCTTTTAGCGACCTCTTGTAAGTTATTTTATTCAAATGTAGGAACTGGGCCAGCA [23946]
lp36_PAli_NX_MP_FG_consensus ATATATTTGTGTTTATGTAGTGTTCTTTTAGCGACCTCTTGTAAGTTATTTTATTCAAATGTAGGAACTGGGCCAGCA [23946]
lp36_PAli_NX_Pl_consensus    ATATATTTGTGTTTATGTAGTGTTCTTTTAGCGACCTCTTGTAAGTTATTTTATTCAAATGTAGGAACTGGGCCAGCA [23946]
lp36_PAbe_NX_MP_FG_consensus ATATATTTGTGTTTATGTAGTGTTCTTTTAGCGACCTCTTGTAAGTTATTTTATTCAAATGTAGGAACTGGGCCAGCA [23946]
lp36_PAbe_NX_Pl_consensus    ATATATTTGTGTTTATGTAGTGTTCTTTTAGCGACCTCTTGTAAGTTATTTTATTCAAATGTAGGAACTGGGCCAGCA [23946]

lp36_gi|11496779|B31_GB      GAAGCATAAAAGACTAGTAAGGCCGAACCTGCTTTTGATCTTGCTGTTGTTAAAGAAAAGGGTGTAGGTAAGTAGTCT [24024]
lp36_un11_B31_PacBio         GAAGCATAAAAGACTAGTAAGGCCGAACCTGCTTTTGATCTTGCTGTTGTTAAAGAAAAGGGTGTAGGTAAGTAGTCT [24024]
lp36_B31_TS_MP_FG_consensus  GAAGCATAAAAGACTAGTAAGGCCGAACCTGCTTTTGATCTTGCTGTTGTTAAAGAAAAGGGTGTAGGTAAGTAGTCT [24024]
lp36_B31_NX_Pl_consensus     GAAGCATAAAAGACTAGTAAGGCCGAACCTGCTTTTGATCTTGCTGTTGTTAAAGAAAAGGGTGTAGGTAAGTAGTCT [24024]
lp36_PAli_NX_MP_FG_consensus GAAGCATAAAAGACTAGTAAGGCCGAACCTGCTTTTGATCTTGCTGTTGTTAAAGAAAAGGGTGTAGGTAAGTAGTCT [24024]
lp36_PAli_NX_Pl_consensus    GAAGCATAAAAGACTAGTAAGGCCGAACCTGCTTTTGATCTTGCTGTTGTTAAAGAAAAGGGTGTAGGTAAGTAGTCT [24024]
lp36_PAbe_NX_MP_FG_consensus GAAGCATAAAAGACTAGTAAGGCCGAACCTGCTTTTGATCTTGCTGTTGTTAAAGAAAAGGGTGTAGGTAAGTAGTCT [24024]
lp36_PAbe_NX_Pl_consensus    GAAGCATAAAAGACTAGTAAGGCCGAACCTGCTTTTGATCTTGCTGTTGTTAAAGAAAAGGGTGTAGGTAAGTAGTCT [24024]

lp36_gi|11496779|B31_GB      AAATAAGAGAGTAAGGCATTAGGCAAAGATGGGCTTGAAAAGATGGCACAAGAGGTAAAGGATGTAGCTAGTGATACT [24102]
lp36_un11_B31_PacBio         AAATAAGAGAGTAAGGCATTAGGCAAAGATGGGCTTGAAAAGATGGCACAAGAGGTAAAGGATGTAGCTAGTGATACT [24102]
lp36_B31_TS_MP_FG_consensus  AAATAAGAGAGTAAGGCATTAGGCAAAGATGGGCTTGAAAAGATGGCACAAGAGGTAAAGGATGTAGCTAGTGATACT [24102]
lp36_B31_NX_Pl_consensus     AAATAAGAGAGTAAGGCATTAGGCAAAGATGGGCTTGAAAAGATGGCACAAGAGGTAAAGGATGTAGCTAGTGATACT [24102]
lp36_PAli_NX_MP_FG_consensus AAATAAGAGAGTAAGGCATTAGGCAAAGATGGGCTTGAAAAGATGGCACAAGAGGTAAAGGATGTAGCTAGTGATACT [24102]
lp36_PAli_NX_Pl_consensus    AAATAAGAGAGTAAGGCATTAGGCAAAGATGGGCTTGAAAAGATGGCACAAGAGGTAAAGGATGTAGCTAGTGATACT [24102]
lp36_PAbe_NX_MP_FG_consensus AAATAAGAGAGTAAGGCATTAGGCAAAGATGGGCTTGAAAAGATGGCACAAGAGGTAAAGGATGTAGCTAGTGATACT [24102]
lp36_PAbe_NX_Pl_consensus    AAATAAGAGAGTAAGGCATTAGGCAAAGATGGGCTTGAAAAGATGGCACAAGAGGTAAAGGATGTAGCTAGTGATACT [24102]

lp36_gi|11496779|B31_GB      AAGGTTAAGGTACTAGATAGCATGACAAAAGGCGGTAGTAGCAAAATACCAATAAGTGAAGTACCTTCTCTTGTAAGC [24180]
lp36_un11_B31_PacBio         AAGGTTAAGGTACTAGATAGCATGACAAAAGGCGGTAGTAGCAAAATACCAATAAGTGAAGTACCTTCTCTTGTAAGC [24180]
lp36_B31_TS_MP_FG_consensus  AAGGTTAAGGTACTAGATAGCATGACAAAAGGCGGTAGTAGCAAAATACCAATAAGTGAAGTACCTTCTCTTGTAAGC [24180]
lp36_B31_NX_Pl_consensus     AAGGTTAAGGTACTAGATAGCATGACAAAAGGCGGTAGTAGCAAAATACCAATAAGTGAAGTACCTTCTCTTGTAAGC [24180]
lp36_PAli_NX_MP_FG_consensus AAGGTTAAGGTACTAGATAGCATGACAAAAGGCGGTAGTAGCAAAATACCAATAAGTGAAGTACCTTCTCTTGTAAGC [24180]
lp36_PAli_NX_Pl_consensus    AAGGTTAAGGTACTAGATAGCATGACAAAAGGCGGTAGTAGCAAAATACCAATAAGTGAAGTACCTTCTCTTGTAAGC [24180]
lp36_PAbe_NX_MP_FG_consensus AAGGTTAAGGTACTAGATAGCATGACAAAAGGCGGTAGTAGCAAAATACCAATAAGTGAAGTACCTTCTCTTGTAAGC [24180]
lp36_PAbe_NX_Pl_consensus    AAGGTTAAGGTACTAGATAGCATGACAAAAGGCGGTAGTAGCAAAATACCAATAAGTGAAGTACCTTCTCTTGTAAGC [24180]

lp36_gi|11496779|B31_GB      CAAATTAAAGAAACAGCTAGAAGGATAGAGGCAGGTGTATCTGCATTAATATCTTCAGGATAAATGTAGCAGGAGCAC [24258]
lp36_un11_B31_PacBio         CAAATTAAAGAAACAGCTAGAAGGATAGAGGCAGGTGTATCTGCATTAATATCTTCAGGATAAATGTAGCAGGAGCAC [24258]
lp36_B31_TS_MP_FG_consensus  CAAATTAAAGAAACAGCTAGAAGGATAGAGGCAGGTGTATCTGCATTAATATCTTCAGGATAAATGTAGCAGGAGCAC [24258]
lp36_B31_NX_Pl_consensus     CAAATTAAAGAAACAGCTAGAAGGATAGAGGCAGGTGTATCTGCATTAATATCTTCAGGATAAATGTAGCAGGAGCAC [24258]
lp36_PAli_NX_MP_FG_consensus CAAATTAAAGAAACAGCTAGAAGGATAGAGGCAGGTGTATCTGCATTAATATCTTCAGGATAAATGTAGCAGGAGCAC [24258]
lp36_PAli_NX_Pl_consensus    CAAATTAAAGAAACAGCTAGAAGGATAGAGGCAGGTGTATCTGCATTAATATCTTCAGGATAAATGTAGCAGGAGCAC [24258]
lp36_PAbe_NX_MP_FG_consensus CAAATTAAAGAAACAGCTAGAAGGATAGAGGCAGGTGTATCTGCATTAATATCTTCAGGATAAATGTAGCAGGAGCAC [24258]
lp36_PAbe_NX_Pl_consensus    CAAATTAAAGAAACAGCTAGAAGGATAGAGGCAGGTGTATCTGCATTAATATCTTCAGGATAAATGTAGCAGGAGCAC [24258]

lp36_gi|11496779|B31_GB      TTAAAGATAATCTTAAGACAGGGATAAGTAGCATATGCTTACTATAAAAATATTAGAAATGGCAAAAGAAAGTGGCCT [24336]
lp36_un11_B31_PacBio         TTAAAGATAATCTTAAGACAGGGATAAGTAGCATATGCTTACTATAAAAATATTAGAAATGGCAAAAGAAAGTGGCCT [24336]
lp36_B31_TS_MP_FG_consensus  TTAAAGATAATCTTAAGACAGGGATAAGTAGCATATGCTTACTATAAAAATATTAGAAATGGCAAAAGAAAGTGGCCT [24336]
lp36_B31_NX_Pl_consensus     TTAAAGATAATCTTAAGACAGGGATAAGTAGCATATGCTTACTATAAAAATATTAGAAATGGCAAAAGAAAGTGGCCT [24336]
lp36_PAli_NX_MP_FG_consensus TTAAAGATAATCTTAAGACAGGGATAAGTAGCATATGCTTACTATAAAAATATTAGAAATGGCAAAAGAAAGTGGCCT [24336]
lp36_PAli_NX_Pl_consensus    TTAAAGATAATCTTAAGACAGGGATAAGTAGCATATGCTTACTATAAAAATATTAGAAATGGCAAAAGAAAGTGGCCT [24336]
lp36_PAbe_NX_MP_FG_consensus TTAAAGATAATCTTAAGACAGGGATAAGTAGCATATGCTTACTATAAAAATATTAGAAATGGCAAAAGAAAGTGGCCT [24336]
lp36_PAbe_NX_Pl_consensus    TTAAAGATAATCTTAAGACAGGGATAAGTAGCATATGCTTACTATAAAAATATTAGAAATGGCAAAAGAAAGTGGCCT [24336]

lp36_gi|11496779|B31_GB      TAAGCATGAAGATGATGATTATGGTTTGCGTGATAAGGTAAATAAGGTAATAGAAGAATTTAGAAGAGGAAAAGAAGG [24414]
```

```
lp36_un11_B31_PacBio         TAAGCATGAAGATGATGATTATGGTTTGCGTGATAAGGTAAATAAGGTAATAGAAGAATTTAGAAGAGGAAAAGAAGG [24414]
lp36_B31_TS_MP_FG_consensus  TAAGCATGAAGATGATGATTATGGTTTGCGTGATAAGGTAAATAAGGTAATAGAAGAATTTAGAAGAGGAAAAGAAGG [24414]
lp36_B31_NX_Pl_consensus     TAAGCATGAAGATGATGATTATGGTTTGCGTGATAAGGTAAATAAGGTAATAGAAGAATTTAGAAGAGGAAAAGAAGG [24414]
lp36_PAli_NX_MP_FG_consensus TAAGCATGAAGATGATGATTATGGTTTGCGTGATAAGGTAAATAAGGTAATAGAAGAATTTAGAAGAGGAAAAGAAGG [24414]
lp36_PAli_NX_Pl_consensus    TAAGCATGAAGATGATGATTATGGTTTGCGTGATAAGGTAAATAAGGTAATAGAAGAATTTAGAAGAGGAAAAGAAGG [24414]
lp36_PAbe_NX_MP_FG_consensus TAAGCATGAAGATGATGATTATGGTTTGCGTGATAAGGTAAATAAGGTAATAGAAGAATTTAGAAGAGGAAAAGAAGG [24414]
lp36_PAbe_NX_Pl_consensus    TAAGCATGAAGATGATGATTATGGTTTGCGTGATAAGGTAAATAAGGTAATAGAAGAATTTAGAAGAGGAAAAGAAGG [24414]

lp36_gi|11496779|B31_GB      TGGTCTTGTAGCATTTTCTCTTAAAGTAGAAGGCGCTAAGAAAAACGGTGAAGTTGTTAAGGAGTGTGTAACTGGGTT [24492]
lp36_un11_B31_PacBio         TGGTCTTGTAGCATTTTCTCTTAAAGTAGAAGGCGCTAAGAAAAACGGTGAAGTTGTTAAGGAGTGTGTAACTGGGTT [24492]
lp36_B31_TS_MP_FG_consensus  TGGTCTTGTAGCATTTTCTCTTAAAGTAGAAGGCGCTAAGAAAAACGGTGAAGTTGTTAAGGAGTGTGTAACTGGGTT [24492]
lp36_B31_NX_Pl_consensus     TGGTCTTGTAGCATTTTCTCTTAAAGTAGAAGGCGCTAAGAAAAACGGTGAAGTTGTTAAGGAGTGTGTAACTGGGTT [24492]
lp36_PAli_NX_MP_FG_consensus TGGTCTTGTAGCATTTTCTCTTAAAGTAGAAGGCGCTAAGAAAAACGGTGAAGTTGTTAAGGAGTGTGTAACTGGGTT [24492]
lp36_PAli_NX_Pl_consensus    TGGTCTTGTAGCATTTTCTCTTAAAGTAGAAGGCGCTAAGAAAAACGGTGAAGTTGTTAAGGAGTGTGTAACTGGGTT [24492]
lp36_PAbe_NX_MP_FG_consensus TGGTCTTGTAGCATTTTCTCTTAAAGTAGAAGGCGCTAAGAAAAACGGTGAAGTTGTTAAGGAGTGTGTAACTGGGTT [24492]
lp36_PAbe_NX_Pl_consensus    TGGTCTTGTAGCATTTTCTCTTAAAGTAGAAGGCGCTAAGAAAAACGGTGAAGTTGTTAAGGAGTGTGTAACTGGGTT [24492]

lp36_gi|11496779|B31_GB      AATGGGAAAAGTAGAAAGCCTTTTTGGCGATATTAAAGGAAATGGCGATGGTGTTGCTCAAGAGTTGATGCAAGAAGG [24570]
lp36_un11_B31_PacBio         AATGGGAAAAGTAGAAAGCCTTTTTGGCGATATTAAAGGAAATGGCGATGGTGTTGCTCAAGAGTTGATGCAAGAAGG [24570]
lp36_B31_TS_MP_FG_consensus  AATGGGAAAAGTAGAAAGCCTTTTTGGCGATATTAAAGGAAATGGCGATGGTGTTGCTCAAGAGTTGATGCAAGAAGG [24570]
lp36_B31_NX_Pl_consensus     AATGGGAAAAGTAGAAAGCCTTTTTGGCGATATTAAAGGAAATGGCGATGGTGTTGCTCAAGAGTTGATGCAAGAAGG [24570]
lp36_PAli_NX_MP_FG_consensus AATGGGAAAAGTAGAAAGCCTTTTTGGCGATATTAAAGGAAATGGCGATGGTGTTGCTCAAGAGTTGATGCAAGAAGG [24570]
lp36_PAli_NX_Pl_consensus    AATGGGAAAAGTAGAAAGCCTTTTTGGCGATATTAAAGGAAATGGCGATGGTGTTGCTCAAGAGTTGATGCAAGAAGG [24570]
lp36_PAbe_NX_MP_FG_consensus AATGGGAAAAGTAGAAAGCCTTTTTGGCGATATTAAAGGAAATGGCGATGGTGTTGCTCAAGAGTTGATGCAAGAAGG [24570]
lp36_PAbe_NX_Pl_consensus    AATGGGAAAAGTAGAAAGCCTTTTTGGCGATATTAAAGGAAATGGCGATGGTGTTGCTCAAGAGTTGATGCAAGAAGG [24570]

lp36_gi|11496779|B31_GB      TAAGAGTGAAGAAAAACGGTTTAGTAAGCCATTAAGCTTAATAAGAGAAGTTGTGTGTGATATAACAGAAGCATGCAA [24648]
lp36_un11_B31_PacBio         TAAGAGTGAAGAAAAACGGTTTAGTAAGCCATTAAGCTTAATAAGAGAAGTTGTGTGTGATATAACAGAAGCATGCAA [24648]
lp36_B31_TS_MP_FG_consensus  TAAGAGTGAAGAAAAACGGTTTAGTAAGCCATTAAGCTTAATAAGAGAAGTTGTGTGTGATATAACAGAAGCATGCAA [24648]
lp36_B31_NX_Pl_consensus     TAAGAGTGAAGAAAAACGGTTTAGTAAGCCATTAAGCTTAATAAGAGAAGTTGTGTGTGATATAACAGAAGCATGCAA [24648]
lp36_PAli_NX_MP_FG_consensus TAAGAGTGAAGAAAAACGGTTTAGTAAGCCATTAAGCTTAATAAGAGAAGTTGTGTGTGATATAACAGAAGCATGCAA [24648]
lp36_PAli_NX_Pl_consensus    TAAGAGTGAAGAAAAACGGTTTAGTAAGCCATTAAGCTTAATAAGAGAAGTTGTGTGTGATATAACAGAAGCATGCAA [24648]
lp36_PAbe_NX_MP_FG_consensus TAAGAGTGAAGAAAAACGGTTTAGTAAGCCATTAAGCTTAATAAGAGAAGTTGTGTGTGATATAACAGAAGCATGCAA [24648]
lp36_PAbe_NX_Pl_consensus    TAAGAGTGAAGAAAAACGGTTTAGTAAGCCATTAAGCTTAATAAGAGAAGTTGTGTGTGATATAACAGAAGCATGCAA [24648]

lp36_gi|11496779|B31_GB      GCGACTTATTTATGACCTGTAGAATGAATAGTAGCAGTTCTTAACTGCTATTCTTTATTGTTATTTTTTAAAGCCAAG [24726]
lp36_un11_B31_PacBio         GCGACTTATTTATGACCTGTAGAATGAATAGTAGCAGTTCTTAACTGCTATTCTTTATTGTTATTTTTTAAAGCCAAG [24726]
lp36_B31_TS_MP_FG_consensus  GCGACTTATTTATGACCTGTAGAATGAATAGTAGCAGTTCTTAACTGCTATTCTTTATTGTTATTTTTTAAAGCCAAG [24726]
lp36_B31_NX_Pl_consensus     GCGACTTATTTATGACCTGTAGAATGAATAGTAGCAGTTCTTAACTGCTATTCTTTATTGTTATTTTTTAAAGCCAAG [24726]
lp36_PAli_NX_MP_FG_consensus GCGACTTATTTATGACCTGTAGAATGAATAGTAGCAGTTCTTAACTGCTATTCTTTATTGTTATTTTTTAAAGCCAAG [24726]
lp36_PAli_NX_Pl_consensus    GCGACTTATTTATGACCTGTAGAATGAATAGTAGCAGTTCTTAACTGCTATTCTTTATTGTTATTTTTTAAAGCCAAG [24726]
lp36_PAbe_NX_MP_FG_consensus GCGACTTATTTATGACCTGTAGAATGAATAGTAGCAGTTCTTAACTGCTATTCTTTATTGTTATTTTTTAAAGCCAAG [24726]
lp36_PAbe_NX_Pl_consensus    GCGACTTATTTATGACCTGTAGAATGAATAGTAGCAGTTCTTAACTGCTATTCTTTATTGTTATTTTTTAAAGCCAAG [24726]

lp36_gi|11496779|B31_GB      CTTCTCTATTCTATAAAAATAGAAGCCAGAAGCTTAGTCTAGATTATCCTATAATCTAGTTTTAGGGGTTCTTATGTC [24804]
lp36_un11_B31_PacBio         CTTCTCTATTCTATAAAAATAGAAGCCAGAAGCTTAGTCTAGATTATCCTATAATCTAGTTTTAGGGGTTCTTATGTC [24804]
lp36_B31_TS_MP_FG_consensus  CTTCTCTATTCTATAAAAATAGAAGCCAGAAGCTTAGTCTAGATTATCCTATAATCTAGTTTTAGGGGTTCTTATGTC [24804]
lp36_B31_NX_Pl_consensus     CTTCTCTATTCTATAAAAATAGAAGCCAGAAGCTTAGTCTAGATTATCCTATAATCTAGTTTTAGGGGTTCTTATGTC [24804]
lp36_PAli_NX_MP_FG_consensus CTTCTCTATTCTATAAAAATAGAAGCCAGAAGCTTAGTCTAGATTATCCTATAATCTAGTTTTAGGGGTTCTTATGTC [24804]
lp36_PAli_NX_Pl_consensus    CTTCTCTATTCTATAAAAATAGAAGCCAGAAGCTTAGTCTAGATTATCCTATAATCTAGTTTTAGGGGTTCTTATGTC [24804]
lp36_PAbe_NX_MP_FG_consensus CTTCTCTATTCTATAAAAATAGAAGCCAGAAGCTTAGTCTAGATTATCCTATAATCTAGTTTTAGGGGTTCTTATGTC [24804]
lp36_PAbe_NX_Pl_consensus    CTTCTCTATTCTATAAAAATAGAAGCCAGAAGCTTAGTCTAGATTATCCTATAATCTAGTTTTAGGGGTTCTTATGTC [24804]

lp36_gi|11496779|B31_GB      TAGTTTGCTATGAGCTATCAGAGAACTAGTAAAGTATAATTTTGTAATTAGGAAATATAGAAATTTATCAATAAATAT [24882]
lp36_un11_B31_PacBio         TAGTTTGCTATGAGCTATCAGAGAACTAGTAAAGTATAATTTTGTAATTAGGAAATATAGAAATTTATCAATAAATAT [24882]
lp36_B31_TS_MP_FG_consensus  TAGTTTGCTATGAGCTATCAGAGAACTAGTAAAGTATAATTTTGTAATTAGGAAATATAGAAATTTATCAATAAATAT [24882]
lp36_B31_NX_Pl_consensus     TAGTTTGCTATGAGCTATCAGAGAACTAGTAAAGTATAATTTTGTAATTAGGAAATATAGAAATTTATCAATAAATAT [24882]
lp36_PAli_NX_MP_FG_consensus TAGTTTGCTATGAGCTATCAGAGAACTAGTAAAGTATAATTTTGTAATTAGGAAATATAGAAATTTATCAATAAATAT [24882]
lp36_PAli_NX_Pl_consensus    TAGTTTGCTATGAGCTATCAGAGAACTAGTAAAGTATAATTTTGTAATTAGGAAATATAGAAATTTATCAATAAATAT [24882]
lp36_PAbe_NX_MP_FG_consensus TAGTTTGCTATGAGCTATCAGAGAACTAGTAAAGTATAATTTTGTAATTAGGAAATATAGAAATTTATCAATAAATAT [24882]
lp36_PAbe_NX_Pl_consensus    TAGTTTGCTATGAGCTATCAGAGAACTAGTAAAGTATAATTTTGTAATTAGGAAATATAGAAATTTATCAATAAATAT [24882]

lp36_gi|11496779|B31_GB      TGATCTTGTAGTTAGAAGCAGACAAGTGAAGTAATGATAATGAATCTTGTTAAGACAAGCAATACTAATACTAAAAGA [24960]
lp36_un11_B31_PacBio         TGATCTTGTAGTTAGAAGCAGACAAGTGAAGTAATGATAATGAATCTTGTTAAGACAAGCAATACTAATACTAAAAGA [24960]
lp36_B31_TS_MP_FG_consensus  TGATCTTGTAGTTAGAAGCAGACAAGTGAAGTAATGATAATGAATCTTGTTAAGACAAGCAATACTAATACTAAAAGA [24960]
lp36_B31_NX_Pl_consensus     TGATCTTGTAGTTAGAAGCAGACAAGTGAAGTAATGATAATGAATCTTGTTAAGACAAGCAATACTAATACTAAAAGA [24960]
lp36_PAli_NX_MP_FG_consensus TGATCTTGTAGTTAGAAGCAGACAAGTGAAGTAATGATAATGAATCTTGTTAAGACAAGCAATACTAATACTAAAAGA [24960]
lp36_PAli_NX_Pl_consensus    TGATCTTGTAGTTAGAAGCAGACAAGTGAAGTAATGATAATGAATCTTGTTAAGACAAGCAATACTAATACTAAAAGA [24960]
lp36_PAbe_NX_MP_FG_consensus TGATCTTGTAGTTAGAAGCAGACAAGTGAAGTAATGATAATGAATCTTGTTAAGACAAGCAATACTAATACTAAAAGA [24960]
lp36_PAbe_NX_Pl_consensus    TGATCTTGTAGTTAGAAGCAGACAAGTGAAGTAATGATAATGAATCTTGTTAAGACAAGCAATACTAATACTAAAAGA [24960]

lp36_gi|11496779|B31_GB      TGACTTAGAAAGAACTTTGATTTTGTCACTAGCTTTGACAACCTATCTTATTAGCTAAAATGTGTGTTACTATTAATA [25038]
lp36_un11_B31_PacBio         TGACTTAGAAAGAACTTTGATTTTGTCACTAGCTTTGACAACCTATCTTATTAGCTAAAATGTGTGTTACTATTAATA [25038]
lp36_B31_TS_MP_FG_consensus  TGACTTAGAAAGAACTTTGATTTTGTCACTAGCTTTGACAACCTATCTTATTAGCTAAAATGTGTGTTACTATTAATA [25038]
lp36_B31_NX_Pl_consensus     TGACTTAGAAAGAACTTTGATTTTGTCACTAGCTTTGACAACCTATCTTATTAGCTAAAATGTGTGTTACTATTAATA [25038]
lp36_PAli_NX_MP_FG_consensus TGACTTAGAAAGAACTTTGATTTTGTCACTAGCTTTGACAACCTATCTTATTAGCTAAAATGTGTGTTACTATTAATA [25038]
lp36_PAli_NX_Pl_consensus    TGACTTAGAAAGAACTTTGATTTTGTCACTAGCTTTGACAACCTATCTTATTAGCTAAAATGTGTGTTACTATTAATA [25038]
lp36_PAbe_NX_MP_FG_consensus TGACTTAGAAAGAACTTTGATTTTGTCACTAGCTTTGACAACCTATCTTATTAGCTAAAATGTGTGTTACTATTAATA [25038]
lp36_PAbe_NX_Pl_consensus    TGACTTAGAAAGAACTTTGATTTTGTCACTAGCTTTGACAACCTATCTTATTAGCTAAAATGTGTGTTACTATTAATA [25038]

lp36_gi|11496779|B31_GB      GTAGATATTAAGGATGTTAAATATAGATAACAAAAGTAGTTAAATTGCAGTAAAGGAGGGTTATATGAGTAATTTTGC [25116]
lp36_un11_B31_PacBio         GTAGATATTAAGGATGTTAAATATAGATAACAAAAGTAGTTAAATTGCAGTAAAGGAGGGTTATATGAGTAATTTTGC [25116]
lp36_B31_TS_MP_FG_consensus  GTAGATATTAAGGATGTTAAATATAGATAACAAAAGTAGTTAAATTGCAGTAAAGGAGGGTTATATGAGTAATTTTGC [25116]
lp36_B31_NX_Pl_consensus     GTAGATATTAAGGATGTTAAATATAGATAACAAAAGTAGTTAAATTGCAGTAAAGGAGGGTTATATGAGTAATTTTGC [25116]
lp36_PAli_NX_MP_FG_consensus GTAGATATTAAGGATGTTAAATATAGATAACAAAAGTAGTTAAATTGCAGTAAAGGAGGGTTATATGAGTAATTTTGC [25116]
lp36_PAli_NX_Pl_consensus    GTAGATATTAAGGATGTTAAATATAGATAACAAAAGTAGTTAAATTGCAGTAAAGGAGGGTTATATGAGTAATTTTGC [25116]
lp36_PAbe_NX_MP_FG_consensus GTAGATATTAAGGATGTTAAATATAGATAACAAAAGTAGTTAAATTGCAGTAAAGGAGGGTTATATGAGTAATTTTGC [25116]
lp36_PAbe_NX_Pl_consensus    GTAGATATTAAGGATGTTAAATATAGATAACAAAAGTAGTTAAATTGCAGTAAAGGAGGGTTATATGAGTAATTTTGC [25116]

lp36_gi|11496779|B31_GB      ATGTGAAGCTAAACCCAAATATGAGTATGTAAAGCAGGTATTTCTTGATAAGGATTTTCCAGAAGATGTAGTTGATTA [25194]
lp36_un11_B31_PacBio         ATGTGAAGCTAAACCCAAATATGAGTATGTAAAGCAGGTATTTCTTGATAAGGATTTTCCAGAAGATGTAGTTGATTA [25194]
lp36_B31_TS_MP_FG_consensus  ATGTGAAGCTAAACCCAAATATGAGTATGTAAAGCAGGTATTTCTTGATAAGGATTTTCCAGAAGATGTAGTTGATTA [25194]
lp36_B31_NX_Pl_consensus     ATGTGAAGCTAAACCCAAATATGAGTATGTAAAGCAGGTATTTCTTGATAAGGATTTTCCAGAAGATGTAGTTGATTA [25194]
lp36_PAli_NX_MP_FG_consensus ATGTGAAGCTAAACCCAAATATGAGTATGTAAAGCAGGTATTTCTTGATAAGGATTTTCCAGAAGATGTAGTTGATTA [25194]
lp36_PAli_NX_Pl_consensus    ATGTGAAGCTAAACCCAAATATGAGTATGTAAAGCAGGTATTTCTTGATAAGGATTTTCCAGAAGATGTAGTTGATTA [25194]
lp36_PAbe_NX_MP_FG_consensus ATGTGAAGCTAAACCCAAATATGAGTATGTAAAGCAGGTATTTCTTGATAAGGATTTTCCAGAAGATGTAGTTGATTA [25194]
lp36_PAbe_NX_Pl_consensus    ATGTGAAGCTAAACCCAAATATGAGTATGTAAAGCAGGTATTTCTTGATAAGGATTTTCCAGAAGATGTAGTTGATTA [25194]

lp36_gi|11496779|B31_GB      TGTGCTACTTCGTAGTTCAAATTATGTATATGAAAACCTAGAAAGTAGCATGAGTATGCTAGAAAAGGAAATGAATAA [25272]
lp36_un11_B31_PacBio         TGTGCTACTTCGTAGTTCAAATTATGTATATGAAAACCTAGAAAGTAGCATGAGTATGCTAGAAAAGGAAATGAATAA [25272]
lp36_B31_TS_MP_FG_consensus  TGTGCTACTTCGTAGTTCAAATTATGTATATGAAAACCTAGAAAGTAGCATGAGTATGCTAGAAAAGGAAATGAATAA [25272]
lp36_B31_NX_Pl_consensus     TGTGCTACTTCGTAGTTCAAATTATGTATATGAAAACCTAGAAAGTAGCATGAGTATGCTAGAAAAGGAAATGAATAA [25272]
lp36_PAli_NX_MP_FG_consensus TGTGCTACTTCGTAGTTCAAATTATGTATATGAAAACCTAGAAAGTAGCATGAGTATGCTAGAAAAGGAAATGAATAA [25272]
lp36_PAli_NX_Pl_consensus    TGTGCTACTTCGTAGTTCAAATTATGTATATGAAAACCTAGAAAGTAGCATGAGTATGCTAGAAAAGGAAATGAATAA [25272]
lp36_PAbe_NX_MP_FG_consensus TGTGCTACTTCGTAGTTCAAATTATGTATATGAAAACCTAGAAAGTAGCATGAGTATGCTAGAAAAGGAAATGAATAA [25272]
lp36_PAbe_NX_Pl_consensus    TGTGCTACTTCGTAGTTCAAATTATGTATATGAAAACCTAGAAAGTAGCATGAGTATGCTAGAAAAGGAAATGAATAA [25272]

lp36_gi|11496779|B31_GB      AGCTAGAGATGAGTTTAGAAGTGGAATAGGGAAGTTAGATGAAAGAATAGGCAAGTTAGATGAAAAGGTAGAAAAAGT [25350]
lp36_un11_B31_PacBio         AGCTAGAGATGAGTTTAGAAGTGGAATAGGGAAGTTAGATGAAAGAATAGGCAAGTTAGATGAAAAGGTAGAAAAAGT [25350]
```

```
lp36_B31_TS_MP_FG_consensus  AGCTAGAGATGAGTTTAGAAGTGGAATAGGGAAGTTAGATGAAAGAATAGGCAAGTTAGATGAAAAGGTAGAAAAAGT [25350]
lp36_B31_NX_Pl_consensus     AGCTAGAGATGAGTTTAGAAGTGGAATAGGGAAGTTAGATGAAAGAATAGGCAAGTTAGATGAAAAGGTAGAAAAAGT [25350]
lp36_PAli_NX_MP_FG_consensus AGCTAGAGATGAGTTTAGAAGTGGAATAGGGAAGTTAGATGAAAGAATAGGCAAGTTAGATGAAAAGGTAGAAAAAGT [25350]
lp36_PAli_NX_Pl_consensus    AGCTAGAGATGAGTTTAGAAGTGGAATAGGGAAGTTAGATGAAAGAATAGGCAAGTTAGATGAAAAGGTAGAAAAAGT [25350]
lp36_PAbe_NX_MP_FG_consensus AGCTAGAGATGAGTTTAGAAGTGGAATAGGGAAGTTAGATGAAAGAATAGGCAAGTTAGATGAAAAGGTAGAAAAAGT [25350]
lp36_PAbe_NX_Pl_consensus    AGCTAGAGATGAGTTTAGAAGTGGAATAGGGAAGTTAGATGAAAGAATAGGCAAGTTAGATGAAAAGGTAGAAAAAGT [25350]

lp36_gi|11496779|B31_GB      AAGAAGTGAGCTTTCTGCAGAGATAAAGACAGTAAGAAGTGAGCTTAAGGGTGAGATAGTAAAGTTAGATGAAAGAAT [25428]
lp36_un11_B31_PacBio         AAGAAGTGAGCTTTCTGCAGAGATAAAGACAGTAAGAAGTGAGCTTAAGGGTGAGATAGTAAAGTTAGATGAAAGAAT [25428]
lp36_B31_TS_MP_FG_consensus  AAGAAGTGAGCTTTCTGCAGAGATAAAGACAGTAAGAAGTGAGCTTAAGGGTGAGATAGTAAAGTTAGATGAAAGAAT [25428]
lp36_B31_NX_Pl_consensus     AAGAAGTGAGCTTTCTGCAGAGATAAAGACAGTAAGAAGTGAGCTTAAGGGTGAGATAGTAAAGTTAGATGAAAGAAT [25428]
lp36_PAli_NX_MP_FG_consensus AAGAAGTGAGCTTTCTGCAGAGATAAAGACAGTAAGAAGTGAGCTTAAGGGTGAGATAGTAAAGTTAGATGAAAGAAT [25428]
lp36_PAli_NX_Pl_consensus    AAGAAGTGAGCTTTCTGCAGAGATAAAGACAGTAAGAAGTGAGCTTAAGGGTGAGATAGTAAAGTTAGATGAAAGAAT [25428]
lp36_PAbe_NX_MP_FG_consensus AAGAAGTGAGCTTTCTGCAGAGATAAAGACAGTAAGAAGTGAGCTTAAGGGTGAGATAGTAAAGTTAGATGAAAGAAT [25428]
lp36_PAbe_NX_Pl_consensus    AAGAAGTGAGCTTTCTGCAGAGATAAAGACAGTAAGAAGTGAGCTTAAGGGTGAGATAGTAAAGTTAGATGAAAGAAT [25428]

lp36_gi|11496779|B31_GB      AGAAAAAGTAAGAAGTGAGCTTAAGGGTGAGATAGTAAAGTTAGATGAAAGAATAGGCAAGTTAGATGAAAAAAATAAA [25506]
lp36_un11_B31_PacBio         AGAAAAAGTAAGAAGTGAGCTTAAGGGTGAGATAGTAAAGTTAGATGAAAGAATAGGCAAGTTAGATGAAAAAAATAAA [25506]
lp36_B31_TS_MP_FG_consensus  AGAAAAAGTAAGAAGTGAGCTTAAGGGTGAGATAGTAAAGTTAGATGAAAGAATAGGCAAGTTAGATGAAAAAAATAAA [25506]
lp36_B31_NX_Pl_consensus     AGAAAAAGTAAGAAGTGAGCTTAAGGGTGAGATAGTAAAGTTAGATGAAAGAATAGGCAAGTTAGATGAAAAAAATAAA [25506]
lp36_PAli_NX_MP_FG_consensus AGAAAAAGTAAGAAGTGAGCTTAAGGGTGAGATAGTAAAGTTAGATGAAAGAATAGGCAAGTTAGATGAAAAAAATAAA [25506]
lp36_PAli_NX_Pl_consensus    AGAAAAAGTAAGAAGTGAGCTTAAGGGTGAGATAGTAAAGTTAGATGAAAGAATAGGCAAGTTAGATGAAAAAAATAAA [25506]
lp36_PAbe_NX_MP_FG_consensus AGAAAAAGTAAGAAGTGAGCTTAAGGGTGAGATAGTAAAGTTAGATGAAAGAATAGGCAAGTTAGATGAAAAAAATAAA [25506]
lp36_PAbe_NX_Pl_consensus    AGAAAAAGTAAGAAGTGAGCTTAAGGGTGAGATAGTAAAGTTAGATGAAAGAATAGGCAAGTTAGATGAAAAAAATAAA [25506]

lp36_gi|11496779|B31_GB      TACAAATCATAAAGAGCTTATAGGGTTGTTTAAGGAAATAAGAAGTGAGAACAACTCGCATATAAAAAGCTTGATATA [25584]
lp36_un11_B31_PacBio         TACAAATCATAAAGAGCTTATAGGGTTGTTTAAGGAAATAAGAAGTGAGAACAACTCGCATATAAAAAGCTTGATATA [25584]
lp36_B31_TS_MP_FG_consensus  TACAAATCATAAAGAGCTTATAGGGTTGTTTAAGGAAATAAGAAGTGAGAACAACTCGCATATAAAAAGCTTGATATA [25584]
lp36_B31_NX_Pl_consensus     TACAAATCATAAAGAGCTTATAGGGTTGTTTAAGGAAATAAGAAGTGAGAACAACTCGCATATAAAAAGCTTGATATA [25584]
lp36_PAli_NX_MP_FG_consensus TACAAATCATAAAGAGCTTATAGGGTTGTTTAAGGAAATAAGAAGTGAGAACAACTCGCATATAAAAAGCTTGATATA [25584]
lp36_PAli_NX_Pl_consensus    TACAAATCATAAAGAGCTTATAGGGTTGTTTAAGGAAATAAGAAGTGAGAACAACTCGCATATAAAAAGCTTGATATA [25584]
lp36_PAbe_NX_MP_FG_consensus TACAAATCATAAAGAGCTTATAGGGTTGTTTAAGGAAATAAGAAGTGAGAACAACTCGCATATAAAAAGCTTGATATA [25584]
lp36_PAbe_NX_Pl_consensus    TACAAATCATAAAGAGCTTATAGGGTTGTTTAAGGAAATAAGAAGTGAGAACAACTCGCATATAAAAAGCTTGATATA [25584]

lp36_gi|11496779|B31_GB      TCCATTTTATTGGATATTAGGAATATTTATTCCATCAGTAGTGGGAATGTTTTTATATTTACTGCAAAAATAGTAATT [25662]
lp36_un11_B31_PacBio         TCCATTTTATTGGATATTAGGAATATTTATTCCATCAGTAGTGGGAATGTTTTTATATTTACTGCAAAAATAGTAATT [25662]
lp36_B31_TS_MP_FG_consensus  TCCATTTTATTGGATATTAGGAATATTTATTCCATCAGTAGTGGGAATGTTTTTATATTTACTGCAAAAATAGTAATT [25662]
lp36_B31_NX_Pl_consensus     TCCATTTTATTGGATATTAGGAATATTTATTCCATCAGTAGTGGGAATGTTTTTATATTTACTGCAAAAATAGTAATT [25662]
lp36_PAli_NX_MP_FG_consensus TCCATTTTATTGGATATTAGGAATATTTATTCCATCAGTAGTGGGAATGTTTTTATATTTACTGCAAAAATAGTAATT [25662]
lp36_PAli_NX_Pl_consensus    TCCATTTTATTGGATATTAGGAATATTTATTCCATCAGTAGTGGGAATGTTTTTATATTTACTGCAAAAATAGTAATT [25662]
lp36_PAbe_NX_MP_FG_consensus TCCATTTTATTGGATATTAGGAATATTTATTCCATCAGTAGTGGGAATGTTTTTATATTTACTGCAAAAATAGTAATT [25662]
lp36_PAbe_NX_Pl_consensus    TCCATTTTATTGGATATTAGGAATATTTATTCCATCAGTAGTGGGAATGTTTTTATATTTACTGCAAAAATAGTAATT [25662]

lp36_gi|11496779|B31_GB      AGTGTAAGTGTACTTATCCATTTTAGCATTAATTTATTCTTAACAGTATTATTATGATCAAGAATGGTCTAAGAGGCA [25740]
lp36_un11_B31_PacBio         AGTGTAAGTGTACTTATCCATTTTAGCATTAATTTATTCTTAACAGTATTATTATGATCAAGAATGGTCTAAGAGGCA [25740]
lp36_B31_TS_MP_FG_consensus  AGTGTAAGTGTACTTATCCATTTTAGCATTAATTTATTCTTAACAGTATTATTATGATCAAGAATGGTCTAAGAGGCA [25740]
lp36_B31_NX_Pl_consensus     AGTGTAAGTGTACTTATCCATTTTAGCATTAATTTATTCTTAACAGTATTATTATGATCAAGAATGGTCTAAGAGGCA [25740]
lp36_PAli_NX_MP_FG_consensus AGTGTAAGTGTACTTATCCATTTTAGCATTAATTTATTCTTAACAGTATTATTATGATCAAGAATGGTCTAAGAGGCA [25740]
lp36_PAli_NX_Pl_consensus    AGTGTAAGTGTACTTATCCATTTTAGCATTAATTTATTCTTAACAGTATTATTATGATCAAGAATGGTCTAAGAGGCA [25740]
lp36_PAbe_NX_MP_FG_consensus AGTGTAAGTGTACTTATCCATTTTAGCATTAATTTATTCTTAACAGTATTATTATGATCAAGAATGGTCTAAGAGGCA [25740]
lp36_PAbe_NX_Pl_consensus    AGTGTAAGTGTACTTATCCATTTTAGCATTAATTTATTCTTAACAGTATTATTATGATCAAGAATGGTCTAAGAGGCA [25740]

lp36_gi|11496779|B31_GB      AGATAATTTTTAAAAAAGCCTAAGATACAAGTACTTAGGCTTTTTTAAAATAAGAAGATATTGTTGTGCTAGTTATAT [25818]
lp36_un11_B31_PacBio         AGATAATTTTTAAAAAAGCCTAAGATACAAGTACTTAGGCTTTTTTAAAATAAGAAGATATTGTTGTGCTAGTTATAT [25818]
lp36_B31_TS_MP_FG_consensus  AGATAATTTTTAAAAAAGCCTAAGATACAAGTACTTAGGCTTTTTTAAAATAAGAAGATATTGTTGTGCTAGTTATAT [25818]
lp36_B31_NX_Pl_consensus     AGATAATTTTTAAAAAAGCCTAAGATACAAGTACTTAGGCTTTTTTAAAATAAGAAGATATTGTTGTGCTAGTTATAT [25818]
lp36_PAli_NX_MP_FG_consensus AGATAATTTTTAAAAAAGCCTAAGATACAAGTACTTAGGCTTTTTTAAAATAAGAAGATATTGTTGTGCTAGTTATAT [25818]
lp36_PAli_NX_Pl_consensus    AGATAATTTTTAAAAAAGCCTAAGATACAAGTACTTAGGCTTTTTTAAAATAAGAAGATATTGTTGTGCTAGTTATAT [25818]
lp36_PAbe_NX_MP_FG_consensus AGATAATTTTTAAAAAAGCCTAAGATACAAGTACTTAGGCTTTTTTAAAATAAGAAGATATTGTTGTGCTAGTTATAT [25818]
lp36_PAbe_NX_Pl_consensus    AGATAATTTTTAAAAAAGCCTAAGATACAAGTACTTAGGCTTTTTTAAAATAAGAAGATATTGTTGTGCTAGTTATAT [25818]

lp36_gi|11496779|B31_GB      TGATTTGGCATCCTTTTCTAAGTCATTAAGTATAGTAGCAAGGCTACCTTTACTATCATCATTCATATAAATTAATGT [25896]
lp36_un11_B31_PacBio         TGATTTGGCATCCTTTTCTAAGTCATTAAGTATAGTAGCAAGGCTACCTTTACTATCATCATTCATATAAATTAATGT [25896]
lp36_B31_TS_MP_FG_consensus  TGATTTGGCATCCTTTTCTAAGTCATTAAGTATAGTAGCAAGGCTACCTTTACTATCATCATTCATATAAATTAATGT [25896]
lp36_B31_NX_Pl_consensus     TGATTTGGCATCCTTTTCTAAGTCATTAAGTATAGTAGCAAGGCTACCTTTACTATCATCATTCATATAAATTAATGT [25896]
lp36_PAli_NX_MP_FG_consensus TGATTTGGCATCCTTTTCTAAGTCATTAAGTATAGTAGCAAGGCTACCTTTACTATCATCATTCATATAAATTAATGT [25896]
lp36_PAli_NX_Pl_consensus    TGATTTGGCATCCTTTTCTAAGTCATTAAGTATAGTAGCAAGGCTACCTTTACTATCATCATTCATATAAATTAATGT [25896]
lp36_PAbe_NX_MP_FG_consensus TGATTTGGCATCCTTTTCTAAGTCATTAAGTATAGTAGCAAGGCTACCTTTACTATCATCATTCATATAAATTAATGT [25896]
lp36_PAbe_NX_Pl_consensus    TGATTTGGCATCCTTTTCTAAGTCATTAAGTATAGTAGCAAGGCTACCTTTACTATCATCATTCATATAAATTAATGT [25896]

lp36_gi|11496779|B31_GB      TTTGTGTATTAGTTTAATGATAAGATAAGCTTGAGCTACCTTCTCTTTAGTAATCCCATCAATAGTAACGCTAGAAAG [25974]
lp36_un11_B31_PacBio         TTTGTGTATTAGTTTAATGATAAGATAAGCTTGAGCTACCTTCTCTTTAGTAATCCCATCAATAGTAACGCTAGAAAG [25974]
lp36_B31_TS_MP_FG_consensus  TTTGTGTATTAGTTTAATGATAAGATAAGCTTGAGCTACCTTCTCTTTAGTAATCCCATCAATAGTAACGCTAGAAAG [25974]
lp36_B31_NX_Pl_consensus     TTTGTGTATTAGTTTAATGATAAGATAAGCTTGAGCTACCTTCTCTTTAGTAATCCCATCAATAGTAACGCTAGAAAG [25974]
lp36_PAli_NX_MP_FG_consensus TTTGTGTATTAGTTTAATGATAAGATAAGCTTGAGCTACCTTCTCTTTAGTAATCCCATCAATAGTAACGCTAGAAAG [25974]
lp36_PAli_NX_Pl_consensus    TTTGTGTATTAGTTTAATGATAAGATAAGCTTGAGCTACCTTCTCTTTAGTAATCCCATCAATAGTAACGCTAGAAAG [25974]
lp36_PAbe_NX_MP_FG_consensus TTTGTGTATTAGTTTAATGATAAGATAAGCTTGAGCTACCTTCTCTTTAGTAATCCCATCAATAGTAACGCTAGAAAG [25974]
lp36_PAbe_NX_Pl_consensus    TTTGTGTATTAGTTTAATGATAAGATAAGCTTGAGCTACCTTCTCTTTAGTAATCCCATCAATAGTAACGCTAGAAAG [25974]

lp36_gi|11496779|B31_GB      ATTGCTACCAAGATGGCCTTTAGCCTTACCACATAAAGTCTTTAGCTTATCTTTGATGTTCTTTAAGTTTATAAATTT [26052]
lp36_un11_B31_PacBio         ATTGCTACCAAGATGGCCTTTAGCCTTACCACATAAAGTCTTTAGCTTATCTTTGATGTTCTTTAAGTTTATAAATTT [26052]
lp36_B31_TS_MP_FG_consensus  ATTGCTACCAAGATGGCCTTTAGCCTTACCACATAAAGTCTTTAGCTTATCTTTGATGTTCTTTAAGTTTATAAATTT [26052]
lp36_B31_NX_Pl_consensus     ATTGCTACCAAGATGGCCTTTAGCCTTACCACATAAAGTCTTTAGCTTATCTTTGATGTTCTTTAAGTTTATAAATTT [26052]
lp36_PAli_NX_MP_FG_consensus ATTGCTACCAAGATGGCCTTTAGCCTTACCACATAAAGTCTTTAGCTTATCTTTGATGTTCTTTAAGTTTATAAATTT [26052]
lp36_PAli_NX_Pl_consensus    ATTGCTACCAAGATGGCCTTTAGCCTTACCACATAAAGTCTTTAGCTTATCTTTGATGTTCTTTAAGTTTATAAATTT [26052]
lp36_PAbe_NX_MP_FG_consensus ATTGCTACCAAGATGGCCTTTAGCCTTACCACATAAAGTCTTTAGCTTATCTTTGATGTTCTTTAAGTTTATAAATTT [26052]
lp36_PAbe_NX_Pl_consensus    ATTGCTACCAAGATGGCCTTTAGCCTTACCACATAAAGTCTTTAGCTTATCTTTGATGTTCTTTAAGTTTATAAATTT [26052]

lp36_gi|11496779|B31_GB      AGCATCAAACTCTTGTTCTGTCTGATAACCCAATTTCTTACGCTGATCAATCTTATCTTTTGCTTCTATAAGACTACT [26130]
lp36_un11_B31_PacBio         AGCATCAAACTCTTGTTCTGTCTGATAACCCAATTTCTTACGCTGATCAATCTTATCTTTTGCTTCTATAAGACTACT [26130]
lp36_B31_TS_MP_FG_consensus  AGCATCAAACTCTTGTTCTGTCTGATAACCCAATTTCTTACGCTGATCAATCTTATCTTTTGCTTCTATAAGACTACT [26130]
lp36_B31_NX_Pl_consensus     AGCATCAAACTCTTGTTCTGTCTGATAACCCAATTTCTTACGCTGATCAATCTTATCTTTTGCTTCTATAAGACTACT [26130]
lp36_PAli_NX_MP_FG_consensus AGCATCAAACTCTTGTTCTGTCTGATAACCCAATTTCTTACGCTGATCAATCTTATCTTTTGCTTCTATAAGACTACT [26130]
lp36_PAli_NX_Pl_consensus    AGCATCAAACTCTTGTTCTGTCTGATAACCCAATTTCTTACGCTGATCAATCTTATCTTTTGCTTCTATAAGACTACT [26130]
lp36_PAbe_NX_MP_FG_consensus AGCATCAAACTCTTGTTCTGTCTGATAACCCAATTTCTTACGCTGATCAATCTTATCTTTTGCTTCTATAAGACTACT [26130]
lp36_PAbe_NX_Pl_consensus    AGCATCAAACTCTTGTTCTGTCTGATAACCCAATTTCTTACGCTGATCAATCTTATCTTTTGCTTCTATAAGACTACT [26130]

lp36_gi|11496779|B31_GB      AAGCTTTGTAATCTCATAAATAACATCTTTAAGAGATTGGTTTAGTGTGTCTTTAGTAGAATTATTGTTGTCTTCATA [26208]
lp36_un11_B31_PacBio         AAGCTTTGTAATCTCATAAATAACATCTTTAAGAGATTGGTTTAGTGTGTCTTTAGTAGAATTATTGTTGTCTTCATA [26208]
lp36_B31_TS_MP_FG_consensus  AAGCTTTGTAATCTCATAAATAACATCTTTAAGAGATTGGTTTAGTGTGTCTTTAGTAGAATTATTGTTGTCTTCATA [26208]
lp36_B31_NX_Pl_consensus     AAGCTTTGTAATCTCATAAATAACATCTTTAAGAGATTGGTTTAGTGTGTCTTTAGTAGAATTATTGTTGTCTTCATA [26208]
lp36_PAli_NX_MP_FG_consensus AAGCTTTGTAATCTCATAAATAACATCTTTAAGAGATTGGTTTAGTGTGTCTTTAGTAGAATTATTGTTGTCTTCATA [26208]
lp36_PAli_NX_Pl_consensus    AAGCTTTGTAATCTCATAAATAACATCTTTAAGAGATTGGTTTAGTGTGTCTTTAGTAGAATTATTGTTGTCTTCATA [26208]
lp36_PAbe_NX_MP_FG_consensus AAGCTTTGTAATCTCATAAATAACATCTTTAAGAGATTGGTTTAGTGTGTCTTTAGTAGAATTATTGTTGTCTTCATA [26208]
lp36_PAbe_NX_Pl_consensus    AAGCTTTGTAATCTCATAAATAACATCTTTAAGAGATTGGTTTAGTGTGTCTTTAGTAGAATTATTGTTGTCTTCATA [26208]

lp36_gi|11496779|B31_GB      ATTTTCAATACCTTTTTCAAGATCTTTTAATTTATGGGAAATGCTATAAGCCTCAAGCCCAGCCATCTTAACAAGATT [26286]
lp36_un11_B31_PacBio         ATTTTCAATACCTTTTTCAAGATCTTTTAATTTATGGGAAATGCTATAAGCCTCAAGCCCAGCCATCTTAACAAGATT [26286]
lp36_B31_TS_MP_FG_consensus  ATTTTCAATACCTTTTTCAAGATCTTTTAATTTATGGGAAATGCTATAAGCCTCAAGCCCAGCCATCTTAACAAGATT [26286]
```

```
lp36_B31_NX_Pl_consensus      ATTTTCAATACCTTTTTCAAGATCTTTTAATTTATGGGAAATGCTATAAGCCTCAAGCCCAGCCATCTTAACAAGATT [26286]
lp36_PAli_NX_MP_FG_consensus ATTTTCAATACCTTTTTCAAGATCTTTTAATTTATGGGAAATGCTATAAGCCTCAAGCCCAGCCATCTTAACAAGATT [26286]
lp36_PAli_NX_Pl_consensus     ATTTTCAATACCTTTTTCAAGATCTTTTAATTTATGGGAAATGCTATAAGCCTCAAGCCCAGCCATCTTAACAAGATT [26286]
lp36_PAbe_NX_MP_FG_consensus ATTTTCAATACCTTTTTCAAGATCTTTTAATTTATGGGAAATGCTATAAGCCTCAAGCCCAGCCATCTTAACAAGATT [26286]
lp36_PAbe_NX_Pl_consensus     ATTTTCAATACCTTTTTCAAGATCTTTTAATTTATGGGAAATGCTATAAGCCTCAAGCCCAGCCATCTTAACAAGATT [26286]

lp36_gi|11496779|B31_GB       AGTTAAGTCAGAAGTAGTTTGCATGAATTTTTGAAGTCTTGCTTTCTTTTCTATTTCTCCTCTCTTCCTCATATTGTG [26364]
lp36_un11_B31_PacBio          AGTTAAGTCAGAAGTAGTTTGCATGAATTTTTGAAGTCTTGCTTTCTTTTCTATTTCTCCTCTCTTCCTCATATTGTG [26364]
lp36_B31_TS_MP_FG_consensus  AGTTAAGTCAGAAGTAGTTTGCATGAATTTTTGAAGTCTTGCTTTCTTTTCTATTTCTCCTCTCTTCCTCATATTGTG [26364]
lp36_B31_NX_Pl_consensus      AGTTAAGTCAGAAGTAGTTTGCATGAATTTTTGAAGTCTTGCTTTCTTTTCTATTTCTCCTCTCTTCCTCATATTGTG [26364]
lp36_PAli_NX_MP_FG_consensus AGTTAAGTCAGAAGTAGTTTGCATGAATTTTTGAAGTCTTGCTTTCTTTTCTATTTCTCCTCTCTTCCTCATATTGTG [26364]
lp36_PAli_NX_Pl_consensus     AGTTAAGTCAGAAGTAGTTTGCATGAATTTTTGAAGTCTTGCTTTCTTTTCTATTTCTCCTCTCTTCCTCATATTGTG [26364]
lp36_PAbe_NX_MP_FG_consensus AGTTAAGTCAGAAGTAGTTTGCATGAATTTTTGAAGTCTTGCTTTCTTTTCTATTTCTCCTCTCTTCCTCATATTGTG [26364]
lp36_PAbe_NX_Pl_consensus     AGTTAAGTCAGAAGTAGTTTGCATGAATTTTTGAAGTCTTGCTTTCTTTTCTATTTCTCCTCTCTTCCTCATATTGTG [26364]

lp36_gi|11496779|B31_GB       CTTTAATCTTATCAAGCTCTTGTTGTTTCTGTCTTTGCTGCTCTTCTAGAATAGCTTTTCTTTTCTTCTTTAATTTTT [26442]
lp36_un11_B31_PacBio          CTTTAATCTTATCAAGCTCTTGTTGTTTCTGTCTTTGCTGCTCTTCTAGAATAGCTTTTCTTTTCTTCTTTAATTTTT [26442]
lp36_B31_TS_MP_FG_consensus  CTTTAATCTTATCAAGCTCTTGTTGTTTCTGTCTTTGCTGCTCTTCTAGAATAGCTTTTCTTTTCTTCTTTAATTTTT [26442]
lp36_B31_NX_Pl_consensus      CTTTAATCTTATCAAGCTCTTGTTGTTTCTGTCTTTGCTGCTCTTCTAGAATAGCTTTTCTTTTCTTCTTTAATTTTT [26442]
lp36_PAli_NX_MP_FG_consensus CTTTAATCTTATCAAGCTCTTGTTGTTTCTGTCTTTGCTGCTCTTCTAGAATAGCTTTTCTTTTCTTCTTTAATTTTT [26442]
lp36_PAli_NX_Pl_consensus     CTTTAATCTTATCAAGCTCTTGTTGTTTCTGTCTTTGCTGCTCTTCTAGAATAGCTTTTCTTTTCTTCTTTAATTTTT [26442]
lp36_PAbe_NX_MP_FG_consensus CTTTAATCTTATCAAGCTCTTGTTGTTTCTGTCTTTGCTGCTCTTCTAGAATAGCTTTTCTTTTCTTCTTTAATTTTT [26442]
lp36_PAbe_NX_Pl_consensus     CTTTAATCTTATCAAGCTCTTGTTGTTTCTGTCTTTGCTGCTCTTCTAGAATAGCTTTTCTTTTCTTCTTTAATTTTT [26442]

lp36_gi|11496779|B31_GB       TCCTTTATAGCTTCAAGAGTTTCTTTAATAAGAGCATCTTTTTTTGAGTCTAAGTTTGTAGCACTTTTTGTATGTTCT [26520]
lp36_un11_B31_PacBio          TCCTTTATAGCTTCAAGAGTTTCTTTAATAAGAGCATCTTTTTTTGAGTCTAAGTTTGTAGCACTTTTTGTATGTTCT [26520]
lp36_B31_TS_MP_FG_consensus  TCCTTTATAGCTTCAAGAGTTTCTTTAATAAGAGCATCTTTTTTTGAGTCTAAGTTTGTAGCACTTTTTGTATGTTCT [26520]
lp36_B31_NX_Pl_consensus      TCCTTTATAGCTTCAAGAGTTTCTTTAATAAGAGCATCTTTTTTTGAGTCTAAGTTTGTAGCACTTTTTGTATGTTCT [26520]
lp36_PAli_NX_MP_FG_consensus TCCTTTATAGCTTCAAGAGTTTCTTTAATAAGAGCATCTTTTTTTGAGTCTAAGTTTGTAGCACTTTTTGTATGTTCT [26520]
lp36_PAli_NX_Pl_consensus     TCCTTTATAGCTTCAAGAGTTTCTTTAATAAGAGCATCTTTTTTTGAGTCTAAGTTTGTAGCACTTTTTGTATGTTCT [26520]
lp36_PAbe_NX_MP_FG_consensus TCCTTTATAGCTTCAAGAGTTTCTTTAATAAGAGCATCTTTTTTTGAGTCTAAGTTTGTAGCACTTTTTGTATGTTCT [26520]
lp36_PAbe_NX_Pl_consensus     TCCTTTATAGCTTCAAGAGTTTCTTTAATAAGAGCATCTTTTTTTGAGTCTAAGTTTGTAGCACTTTTTGTATGTTCT [26520]

lp36_gi|11496779|B31_GB       TGTGTTGAATAAGCAGGGTTTGTGAGTTCTTTTTCTTGTGAAGAATTGTCTAGGCTTCTTTTTTCTATGTTTTTTTAT [26598]
lp36_un11_B31_PacBio          TGTGTTGAATAAGCAGGGTTTGTGAGTTCTTTTTCTTGTGAAGAATTGTCTAGGCTTCTTTTTTCTATGTTTTTTTAT [26598]
lp36_B31_TS_MP_FG_consensus  TGTGTTGAATAAGCAGGGTTTGTGAGTTCTTTTTCTTGTGAAGAATTGTCTAGGCTTCTTTTTTCTATGTTTTTTTAT [26598]
lp36_B31_NX_Pl_consensus      TGTGTTGAATAAGCAGGGTTTGTGAGTTCTTTTTCTTGTGAAGAATTGTCTAGGCTTCTTTTTTCTATGTTTTTTTAT [26598]
lp36_PAli_NX_MP_FG_consensus TGTGTTGAATAAGCAGGGTTTGTGAGTTCTTTTTCTTGTGAAGAATTGTCTAGGCTTCTTTTTTCTATGTTTTTTTAT [26598]
lp36_PAli_NX_Pl_consensus     TGTGTTGAATAAGCAGGGTTTGTGAGTTCTTTTTCTTGTGAAGAATTGTCTAGGCTTCTTTTTTCTATGTTTTTTTAT [26598]
lp36_PAbe_NX_MP_FG_consensus TGTGTTGAATAAGCAGGGTTTGTGAGTTCTTTTTCTTGTGAAGAATTGTCTAGGCTTCTTTTTTCTATGTTTTTTTAT [26598]
lp36_PAbe_NX_Pl_consensus     TGTGTTGAATAAGCAGGGTTTGTGAGTTCTTTTTCTTGTGAAGAATTGTCTAGGCTTCTTTTTTCTATGTTTTTTTAT [26598]

lp36_gi|11496779|B31_GB       CTATTTCTTGTTTTTGTGAAACACCTGGGTTCAATTGTTGTAATTGAGCTTGCTTATTAAAGCTATCGTCTTGTTTTA [26676]
lp36_un11_B31_PacBio          CTATTTCTTGTTTTTGTGAAACACCTGGGTTCAATTGTTGTAATTGAGCTTGCTTATTAAAGCTATCGTCTTGTTTTA [26676]
lp36_B31_TS_MP_FG_consensus  CTATTTCTTGTTTTTGTGAAACACCTGGGTTCAATTGTTGTAATTGAGCTTGCTTATTAAAGCTATCGTCTTGTTTTA [26676]
lp36_B31_NX_Pl_consensus      CTATTTCTTGTTTTTGTGAAACACCTGGGTTCAATTGTTGTAATTGAGCTTGCTTATTAAAGCTATCGTCTTGTTTTA [26676]
lp36_PAli_NX_MP_FG_consensus CTATTTCTTGTTTTTGTGAAACACCTGGGTTCAATTGTTGTAATTGAGCTTGCTTATTAAAGCTATCGTCTTGTTTTA [26676]
lp36_PAli_NX_Pl_consensus     CTATTTCTTGTTTTTGTGAAACACCTGGGTTCAATTGTTGTAATTGAGCTTGCTTATTAAAGCTATCGTCTTGTTTTA [26676]
lp36_PAbe_NX_MP_FG_consensus CTATTTCTTGTTTTTGTGAAACACCTGGGTTCAATTGTTGTAATTGAGCTTGCTTATTAAAGCTATCGTCTTGTTTTA [26676]
lp36_PAbe_NX_Pl_consensus     CTATTTCTTGTTTTTGTGAAACACCTGGGTTCAATTGTTGTAATTGAGCTTGCTTATTAAAGCTATCGTCTTGTTTTA [26676]

lp36_gi|11496779|B31_GB       TGTGTGTTTGATTAGATAATTTGATTTCTAATTTGTCTTCTAATTGCTTTTTTAAAGCTTCCAAAGAAGTAATTTTAT [26754]
lp36_un11_B31_PacBio          TGTGTGTTTGATTAGATAATTTGATTTCTAATTTGTCTTCTAATTGCTTTTTTAAAGCTTCCAAAGAAGTAATTTTAT [26754]
lp36_B31_TS_MP_FG_consensus  TGTGTGTTTGATTAGATAATTTGATTTCTAATTTGTCTTCTAATTGCTTTTTTAAAGCTTCCAAAGAAGTAATTTTAT [26754]
lp36_B31_NX_Pl_consensus      TGTGTGTTTGATTAGATAATTTGATTTCTAATTTGTCTTCTAATTGCTTTTTTAAAGCTTCCAAAGAAGTAATTTTAT [26754]
lp36_PAli_NX_MP_FG_consensus TGTGTGTTTGATTAGATAATTTGATTTCTAATTTGTCTTCTAATTGCTTTTTTAAAGCTTCCAAAGAAGTAATTTTAT [26754]
lp36_PAli_NX_Pl_consensus     TGTGTGTTTGATTAGATAATTTGATTTCTAATTTGTCTTCTAATTGCTTTTTTAAAGCTTCCAAAGAAGTAATTTTAT [26754]
lp36_PAbe_NX_MP_FG_consensus TGTGTGTTTGATTAGATAATTTGATTTCTAATTTGTCTTCTAATTGCTTTTTTAAAGCTTCCAAAGAAGTAATTTTAT [26754]
lp36_PAbe_NX_Pl_consensus     TGTGTGTTTGATTAGATAATTTGATTTCTAATTTGTCTTCTAATTGCTTTTTTAAAGCTTCCAAAGAAGTAATTTTAT [26754]

lp36_gi|11496779|B31_GB       CATCAATATTTGGAATATCTTCTGAAGAAATCTGATTAATTTCATACAATTTTTCTTTTAAAAGATTTTCTATTGCAA [26832]
lp36_un11_B31_PacBio          CATCAATATTTGGAATATCTTCTGAAGAAATCTGATTAATTTCATACAATTTTTCTTTTAAAAGATTTTCTATTGCAA [26832]
lp36_B31_TS_MP_FG_consensus  CATCAATATTTGGAATATCTTCTGAAGAAATCTGATTAATTTCATACAATTTTTCTTTTAAAAGATTTTCTATTGCAA [26832]
lp36_B31_NX_Pl_consensus      CATCAATATTTGGAATATCTTCTGAAGAAATCTGATTAATTTCATACAATTTTTCTTTTAAAAGATTTTCTATTGCAA [26832]
lp36_PAli_NX_MP_FG_consensus CATCAATATTTGGAATATCTTCTGAAGAAATCTGATTAATTTCATACAATTTTTCTTTTAAAAGATTTTCTATTGCAA [26832]
lp36_PAli_NX_Pl_consensus     CATCAATATTTGGAATATCTTCTGAAGAAATCTGATTAATTTCATACAATTTTTCTTTTAAAAGATTTTCTATTGCAA [26832]
lp36_PAbe_NX_MP_FG_consensus CATCAATATTTGGAATATCTTCTGAAGAAATCTGATTAATTTCATACAATTTTTCTTTTAAAAGATTTTCTATTGCAA [26832]
lp36_PAbe_NX_Pl_consensus     CATCAATATTTGGAATATCTTCTGAAGAAATCTGATTAATTTCATACAATTTTTCTTTTAAAAGATTTTCTATTGCAA [26832]

lp36_gi|11496779|B31_GB       TCTCTTGTGGTAGTGCAGTAGCAACATCTTTGCTACTAAAGGCAGTATTGCCAGTAGTTGAATTTTCTAATAAAGGAG [26910]
lp36_un11_B31_PacBio          TCTCTTGTGGTAGTGCAGTAGCAACATCTTTGCTACTAAAGGCAGTATTGCCAGTAGTTGAATTTTCTAATAAAGGAG [26910]
lp36_B31_TS_MP_FG_consensus  TCTCTTGTGGTAGTGCAGTAGCAACATCTTTGCTACTAAAGGCAGTATTGCCAGTAGTTGAATTTTCTAATAAAGGAG [26910]
lp36_B31_NX_Pl_consensus      TCTCTTGTGGTAGTGCAGTAGCAACATCTTTGCTACTAAAGGCAGTATTGCCAGTAGTTGAATTTTCTAATAAAGGAG [26910]
lp36_PAli_NX_MP_FG_consensus TCTCTTGTGGTAGTGCAGTAGCAACATCTTTGCTACTAAAGGCAGTATTGCCAGTAGTTGAATTTTCTAATAAAGGAG [26910]
lp36_PAli_NX_Pl_consensus     TCTCTTGTGGTAGTGCAGTAGCAACATCTTTGCTACTAAAGGCAGTATTGCCAGTAGTTGAATTTTCTAATAAAGGAG [26910]
lp36_PAbe_NX_MP_FG_consensus TCTCTTGTGGTAGTGCAGTAGCAACATCTTTGCTACTAAAGGCAGTATTGCCAGTAGTTGAATTTTCTAATAAAGGAG [26910]
lp36_PAbe_NX_Pl_consensus     TCTCTTGTGGTAGTGCAGTAGCAACATCTTTGCTACTAAAGGCAGTATTGCCAGTAGTTGAATTTTCTAATAAAGGAG [26910]

lp36_gi|11496779|B31_GB       CATAATTGCTAGAATCATAAACTAAAGTGTCTGAATTAAAAAAATCATTAGATTTTAGTGAAGAGAGCTTATCACTTA [26988]
lp36_un11_B31_PacBio          CATAATTGCTAGAATCATAAACTAAAGTGTCTGAATTAAAAAAATCATTAGATTTTAGTGAAGAGAGCTTATCACTTA [26988]
lp36_B31_TS_MP_FG_consensus  CATAATTGCTAGAATCATAAACTAAAGTGTCTGAATTAAAAAAATCATTAGATTTTAGTGAAGAGAGCTTATCACTTA [26988]
lp36_B31_NX_Pl_consensus      CATAATTGCTAGAATCATAAACTAAAGTGTCTGAATTAAAAAAATCATTAGATTTTAGTGAAGAGAGCTTATCACTTA [26988]
lp36_PAli_NX_MP_FG_consensus CATAATTGCTAGAATCATAAACTAAAGTGTCTGAATTAAAAAAATCATTAGATTTTAGTGAAGAGAGCTTATCACTTA [26988]
lp36_PAli_NX_Pl_consensus     CATAATTGCTAGAATCATAAACTAAAGTGTCTGAATTAAAAAAATCATTAGATTTTAGTGAAGAGAGCTTATCACTTA [26988]
lp36_PAbe_NX_MP_FG_consensus CATAATTGCTAGAATCATAAACTAAAGTGTCTGAATTAAAAAAATCATTAGATTTTAGTGAAGAGAGCTTATCACTTA [26988]
lp36_PAbe_NX_Pl_consensus     CATAATTGCTAGAATCATAAACTAAAGTGTCTGAATTAAAAAAATCATTAGATTTTAGTGAAGAGAGCTTATCACTTA [26988]

lp36_gi|11496779|B31_GB       AATCTTTAGATATTGAGCAACTAAGTATAAGAGATATTAGATATATATATTGGTAAAAATAAGTTTGTTAAGCTTTAT [27066]
lp36_un11_B31_PacBio          AATCTTTAGATATTGAGCAACTAAGTATAAGAGATATTAGATATATATATTGGTAAAAATAAGTTTGTTAAGCTTTAT [27066]
lp36_B31_TS_MP_FG_consensus  AATCTTTAGATATTGAGCAACTAAGTATAAGAGATATTAGATATATATATTGGTAAAAATAAGTTTGTTAAGCTTTAT [27066]
lp36_B31_NX_Pl_consensus      AATCTTTAGATATTGAGCAACTAAGTATAAGAGATATTAGATATATATATTGGTAAAAATAAGTTTGTTAAGCTTTAT [27066]
lp36_PAli_NX_MP_FG_consensus AATCTTTAGATATTGAGCAACTAAGTATAAGAGATATTAGATATATATATTGGTAAAAATAAGTTTGTTAAGCTTTAT [27066]
lp36_PAli_NX_Pl_consensus     AATCTTTAGATATTGAGCAACTAAGTATAAGAGATATTAGATATATATATTGGTAAAAATAAGTTTGTTAAGCTTTAT [27066]
lp36_PAbe_NX_MP_FG_consensus AATCTTTAGATATTGAGCAACTAAGTATAAGAGATATTAGATATATATATTGGTAAAAATAAGTTTGTTAAGCTTTAT [27066]
lp36_PAbe_NX_Pl_consensus     AATCTTTAGATATTGAGCAACTAAGTATAAGAGATATTAGATATATATATTGGTAAAAATAAGTTTGTTAAGCTTTAT [27066]

lp36_gi|11496779|B31_GB       AAGCATCTCCTATAAGTATACTTTTTTAAAGTAATATGTTAATTTTTAAGATTCAATTTTTGTTATAGTAACAATATA [27144]
lp36_un11_B31_PacBio          AAGCATCTCCTATAAGTATACTTTTTTAAAGTAATATGTTAATTTTTAAGATTCAATTTTTGTTATAGTAACAATATA [27144]
lp36_B31_TS_MP_FG_consensus  AAGCATCTCCTATAAGTATACTTTTTTAAAGTAATATGTTAATTTTTAAGATTCAATTTTTGTTATAGTAACAATATA [27144]
lp36_B31_NX_Pl_consensus      AAGCATCTCCTATAAGTATACTTTTTTAAAGTAATATGTTAATTTTTAAGATTCAATTTTTGTTATAGTAACAATATA [27144]
lp36_PAli_NX_MP_FG_consensus AAGCATCTCCTATAAGTATACTTTTTTAAAGTAATATGTTAATTTTTAAGATTCAATTTTTGTTATAGTAACAATATA [27144]
lp36_PAli_NX_Pl_consensus     AAGCATCTCCTATAAGTATACTTTTTTAAAGTAATATGTTAATTTTTAAGATTCAATTTTTGTTATAGTAACAATATA [27144]
lp36_PAbe_NX_MP_FG_consensus AAGCATCTCCTATAAGTATACTTTTTTAAAGTAATATGTTAATTTTTAAGATTCAATTTTTGTTATAGTAACAATATA [27144]
lp36_PAbe_NX_Pl_consensus     AAGCATCTCCTATAAGTATACTTTTTTAAAGTAATATGTTAATTTTTAAGATTCAATTTTTGTTATAGTAACAATATA [27144]

lp36_gi|11496779|B31_GB       GTGCGTAACTAGAAAGAAACATTTAATTTTAAAAATTAATGATTAAGCAGGACGGGACGTATAGAAGTGGAAGAGTAA [27222]
lp36_un11_B31_PacBio          GTGCGTAACTAGAAAGAAACATTTAATTTTAAAAATTAATGATTAAGCAGGACGGGACGTATAGAAGTGGAAGAGTAA [27222]
lp36_B31_TS_MP_FG_consensus  GTGCGTAACTAGAAAGAAACATTTAATTTTAAAAATTAATGATTAAGCAGGACGGGACGTATAGAAGTGGAAGAGTAA [27222]
lp36_B31_NX_Pl_consensus      GTGCGTAACTAGAAAGAAACATTTAATTTTAAAAATTAATGATTAAGCAGGACGGGACGTATAGAAGTGGAAGAGTAA [27222]
```

```
lp36_PAli_NX_MP_FG_consensus GTGCGTAACTAGAAAGAAACATTTAATTTTAAAAATTAATGATTAAGCAGGACGGGACGTATAGAAGTGGAAGAGTAA [27222]
lp36_PAli_NX_Pl_consensus    GTGCGTAACTAGAAAGAAACATTTAATTTTAAAAATTAATGATTAAGCAGGACGGGACGTATAGAAGTGGAAGAGTAA [27222]
lp36_PAbe_NX_MP_FG_consensus GTGCGTAACTAGAAAGAAACATTTAATTTTAAAAATTAATGATTAAGCAGGACGGGACGTATAGAAGTGGAAGAGTAA [27222]
lp36_PAbe_NX_Pl_consensus    GTGCGTAACTAGAAAGAAACATTTAATTTTAAAAATTAATGATTAAGCAGGACGGGACGTATAGAAGTGGAAGAGTAA [27222]

lp36_gi|11496779|B31_GB      ATCTAGGATAAATGAATTACTTTACCAGGGAGCTTAAAATATTTATCTTAGAAATAATGGAGTTGTTTTATATGTGCA [27300]
lp36_un11_B31_PacBio         ATCTAGGATAAATGAATTACTTTACCAGGGAGCTTAAAATATTTATCTTAGAAATAATGGAGTTGTTTTATATGTGCA [27300]
lp36_B31_TS_MP_FG_consensus  ATCTAGGATAAATGAATTACTTTACCAGGGAGCTTAAAATATTTATCTTAGAAATAATGGAGTTGTTTTATATGTGCA [27300]
lp36_B31_NX_Pl_consensus     ATCTAGGATAAATGAATTACTTTACCAGGGAGCTTAAAATATTTATCTTAGAAATAATGGAGTTGTTTTATATGTGCA [27300]
lp36_PAli_NX_MP_FG_consensus ATCTAGGATAAATGAATTACTTTACCAGGGAGCTTAAAATATTTATCTTAGAAATAATGGAGTTGTTTTATATGTGCA [27300]
lp36_PAli_NX_Pl_consensus    ATCTAGGATAAATGAATTACTTTACCAGGGAGCTTAAAATATTTATCTTAGAAATAATGGAGTTGTTTTATATGTGCA [27300]
lp36_PAbe_NX_MP_FG_consensus ATCTAGGATAAATGAATTACTTTACCAGGGAGCTTAAAATATTTATCTTAGAAATAATGGAGTTGTTTTATATGTGCA [27300]
lp36_PAbe_NX_Pl_consensus    ATCTAGGATAAATGAATTACTTTACCAGGGAGCTTAAAATATTTATCTTAGAAATAATGGAGTTGTTTTATATGTGCA [27300]

lp36_gi|11496779|B31_GB      AAAAAGCCAGACTATTTTTGTTAAAATCCAAAAGCTTCCTTATTTATAAGTAAGTGTCATGTCTTCTTTGAAGCTACA [27378]
lp36_un11_B31_PacBio         AAAAAGCCAGACTATTTTTGTTAAAATCCAAAAGCTTCCTTATTTATAAGTAAGTGTCATGTCTTCTTTGAAGCTACA [27378]
lp36_B31_TS_MP_FG_consensus  AAAAAGCCAGACTATTTTTGTTAAAATCCAAAAGCTTCCTTATTTATAAGTAAGTGTCATGTCTTCTTTGAAGCTACA [27378]
lp36_B31_NX_Pl_consensus     AAAAAGCCAGACTATTTTTGTTAAAATCCAAAAGCTTCCTTATTTATAAGTAAGTGTCATGTCTTCTTTGAAGCTACA [27378]
lp36_PAli_NX_MP_FG_consensus AAAAAGCCAGACTATTTTTGTTAAAATCCAAAAGCTTCCTTATTTATAAGTAAGTGTCATGTCTTCTTTGAAGCTACA [27378]
lp36_PAli_NX_Pl_consensus    AAAAAGCCAGACTATTTTTGTTAAAATCCAAAAGCTTCCTTATTTATAAGTAAGTGTCATGTCTTCTTTGAAGCTACA [27378]
lp36_PAbe_NX_MP_FG_consensus AAAAAGCCAGACTATTTTTGTTAAAATCCAAAAGCTTCCTTATTTATAAGTAAGTGTCATGTCTTCTTTGAAGCTACA [27378]
lp36_PAbe_NX_Pl_consensus    AAAAAGCCAGACTATTTTTGTTAAAATCCAAAAGCTTCCTTATTTATAAGTAAGTGTCATGTCTTCTTTGAAGCTACA [27378]

lp36_gi|11496779|B31_GB      CCAGCTAAGATTCTCTACTACTTAAAGCAGCTTCATGTGCTTTATTTAACTCTTCAAACTCTTTATTTGCTTGATCCA [27456]
lp36_un11_B31_PacBio         CCAGCTAAGATTCTCTACTACTTAAAGCAGCTTCATGTGCTTTATTTAACTCTTCAAACTCTTTATTTGCTTGATCCA [27456]
lp36_B31_TS_MP_FG_consensus  CCAGCTAAGATTCTCTACTACTTAAAGCAGCTTCATGTGCTTTATTTAACTCTTCAAACTCTTTATTTGCTTGATCCA [27456]
lp36_B31_NX_Pl_consensus     CCAGCTAAGATTCTCTACTACTTAAAGCAGCTTCATGTGCTTTATTTAACTCTTCAAACTCTTTATTTGCTTGATCCA [27456]
lp36_PAli_NX_MP_FG_consensus CCAGCTAAGATTCTCTACTACTTAAAGCAGCTTCATGTGCTTTATTTAACTCTTCAAACTCTTTATTTGCTTGATCCA [27456]
lp36_PAli_NX_Pl_consensus    CCAGCTAAGATTCTCTACTACTTAAAGCAGCTTCATGTGCTTTATTTAACTCTTCAAACTCTTTATTTGCTTGATCCA [27456]
lp36_PAbe_NX_MP_FG_consensus CCAGCTAAGATTCTCTACTACTTAAAGCAGCTTCATGTGCTTTATTTAACTCTTCAAACTCTTTATTTGCTTGATCCA [27456]
lp36_PAbe_NX_Pl_consensus    CCAGCTAAGATTCTCTACTACTTAAAGCAGCTTCATGTGCTTTATTTAACTCTTCAAACTCTTTATTTGCTTGATCCA [27456]

lp36_gi|11496779|B31_GB      TCTTGTCTTTAAGTTTTTCTTGTTTCTGCTTAGCAGTCTTAAGGCTAACTTTAGCCCTGCCCATTGCTTCTTTAGCAT [27534]
lp36_un11_B31_PacBio         TCTTGTCTTTAAGTTTTTCTTGTTTCTGCTTAGCAGTCTTAAGGCTAACTTTAGCCCTGCCCATTGCTTCTTTAGCAT [27534]
lp36_B31_TS_MP_FG_consensus  TCTTGTCTTTAAGTTTTTCTTGTTTCTGCTTAGCAGTCTTAAGGCTAACTTTAGCCCTGCCCATTGCTTCTTTAGCAT [27534]
lp36_B31_NX_Pl_consensus     TCTTGTCTTTAAGTTTTTCTTGTTTCTGCTTAGCAGTCTTAAGGCTAACTTTAGCCCTGCCCATTGCTTCTTTAGCAT [27534]
lp36_PAli_NX_MP_FG_consensus TCTTGTCTTTAAGTTTTTCTTGTTTCTGCTTAGCAGTCTTAAGGCTAACTTTAGCCCTGCCCATTGCTTCTTTAGCAT [27534]
lp36_PAli_NX_Pl_consensus    TCTTGTCTTTAAGTTTTTCTTGTTTCTGCTTAGCAGTCTTAAGGCTAACTTTAGCCCTGCCCATTGCTTCTTTAGCAT [27534]
lp36_PAbe_NX_MP_FG_consensus TCTTGTCTTTAAGTTTTTCTTGTTTCTGCTTAGCAGTCTTAAGGCTAACTTTAGCCCTGCCCATTGCTTCTTTAGCAT [27534]
lp36_PAbe_NX_Pl_consensus    TCTTGTCTTTAAGTTTTTCTTGTTTCTGCTTAGCAGTCTTAAGGCTAACTTTAGCCCTGCCCATTGCTTCTTTAGCAT [27534]

lp36_gi|11496779|B31_GB      CATTCATATAATAGTGGTATCGAGCATATTGGTAACCTTGAGTATTGCTATTATTTAACGCTTCTTGTAAAGCATCTT [27612]
lp36_un11_B31_PacBio         CATTCATATAATAGTGGTATCGAGCATATTGGTAACCTTGAGTATTGCTATTATTTAACGCTTCTTGTAAAGCATCTT [27612]
lp36_B31_TS_MP_FG_consensus  CATTCATATAATAGTGGTATCGAGCATATTGGTAACCTTGAGTATTGCTATTATTTAACGCTTCTTGTAAAGCATCTT [27612]
lp36_B31_NX_Pl_consensus     CATTCATATAATAGTGGTATCGAGCATATTGGTAACCTTGAGTATTGCTATTATTTAACGCTTCTTGTAAAGCATCTT [27612]
lp36_PAli_NX_MP_FG_consensus CATTCATATAATAGTGGTATCGAGCATATTGGTAACCTTGAGTATTGCTATTATTTAACGCTTCTTGTAAAGCATCTT [27612]
lp36_PAli_NX_Pl_consensus    CATTCATATAATAGTGGTATCGAGCATATTGGTAACCTTGAGTATTGCTATTATTTAACGCTTCTTGTAAAGCATCTT [27612]
lp36_PAbe_NX_MP_FG_consensus CATTCATATAATAGTGGTATCGAGCATATTGGTAACCTTGAGTATTGCTATTATTTAACGCTTCTTGTAAAGCATCTT [27612]
lp36_PAbe_NX_Pl_consensus    CATTCATATAATAGTGGTATCGAGCATATTGGTAACCTTGAGTATTGCTATTATTTAACGCTTCTTGTAAAGCATCTT [27612]

lp36_gi|11496779|B31_GB      CTGCTTTTCTATTTGCATACTCAAAATCGTGCTTAGAACTTTTTAAGGCACTTACTGCATCTGAATAATTAGAGTTAG [27690]
lp36_un11_B31_PacBio         CTGCTTTTCTATTTGCATACTCAAAATCGTGCTTAGAACTTTTTAAGGCACTTACTGCATCTGAATAATTAGAGTTAG [27690]
lp36_B31_TS_MP_FG_consensus  CTGCTTTTCTATTTGCATACTCAAAATCGTGCTTAGAACTTTTTAAGGCACTTACTGCATCTGAATAATTAGAGTTAG [27690]
lp36_B31_NX_Pl_consensus     CTGCTTTTCTATTTGCATACTCAAAATCGTGCTTAGAACTTTTTAAGGCACTTACTGCATCTGAATAATTAGAGTTAG [27690]
lp36_PAli_NX_MP_FG_consensus CTGCTTTTCTATTTGCATACTCAAAATCGTGCTTAGAACTTTTTAAGGCACTTACTGCATCTGAATAATTAGAGTTAG [27690]
lp36_PAli_NX_Pl_consensus    CTGCTTTTCTATTTGCATACTCAAAATCGTGCTTAGAACTTTTTAAGGCACTTACTGCATCTGAATAATTAGAGTTAG [27690]
lp36_PAbe_NX_MP_FG_consensus CTGCTTTTCTATTTGCATACTCAAAATCGTGCTTAGAACTTTTTAAGGCACTTACTGCATCTGAATAATTAGAGTTAG [27690]
lp36_PAbe_NX_Pl_consensus    CTGCTTTTCTATTTGCATACTCAAAATCGTGCTTAGAACTTTTTAAGGCACTTACTGCATCTGAATAATTAGAGTTAG [27690]

lp36_gi|11496779|B31_GB      CATAAGCATGGCTGCTACTAACTTTGCGAATAGCTTGATCAAGTTTAGGCAATAAGGTTGGGTTAGCTTTATTAGATT [27768]
lp36_un11_B31_PacBio         CATAAGCATGGCTGCTACTAACTTTGCGAATAGCTTGATCAAGTTTAGGCAATAAGGTTGGGTTAGCTTTATTAGATT [27768]
lp36_B31_TS_MP_FG_consensus  CATAAGCATGGCTGCTACTAACTTTGCGAATAGCTTGATCAAGTTTAGGCAATAAGGTTGGGTTAGCTTTATTAGATT [27768]
lp36_B31_NX_Pl_consensus     CATAAGCATGGCTGCTACTAACTTTGCGAATAGCTTGATCAAGTTTAGGCAATAAGGTTGGGTTAGCTTTATTAGATT [27768]
lp36_PAli_NX_MP_FG_consensus CATAAGCATGGCTGCTACTAACTTTGCGAATAGCTTGATCAAGTTTAGGCAATAAGGTTGGGTTAGCTTTATTAGATT [27768]
lp36_PAli_NX_Pl_consensus    CATAAGCATGGCTGCTACTAACTTTGCGAATAGCTTGATCAAGTTTAGGCAATAAGGTTGGGTTAGCTTTATTAGATT [27768]
lp36_PAbe_NX_MP_FG_consensus CATAAGCATGGCTGCTACTAACTTTGCGAATAGCTTGATCAAGTTTAGGCAATAAGGTTGGGTTAGCTTTATTAGATT [27768]
lp36_PAbe_NX_Pl_consensus    CATAAGCATGGCTGCTACTAACTTTGCGAATAGCTTGATCAAGTTTAGGCAATAAGGTTGGGTTAGCTTTATTAGATT [27768]

lp36_gi|11496779|B31_GB      TTCTAGCATTATTTAAAAAAGAACTAGCATTGTCAACTTTACTTTTTATATTATTAAGTCTTGACTTTATTGTTTCTA [27846]
lp36_un11_B31_PacBio         TTCTAGCATTATTTAAAAAAGAACTAGCATTGTCAACTTTACTTTTTATATTATTAAGTCTTGACTTTATTGTTTCTA [27846]
lp36_B31_TS_MP_FG_consensus  TTCTAGCATTATTTAAAAAAGAACTAGCATTGTCAACTTTACTTTTTATATTATTAAGTCTTGACTTTATTGTTTCTA [27846]
lp36_B31_NX_Pl_consensus     TTCTAGCATTATTTAAAAAAGAACTAGCATTGTCAACTTTACTTTTTATATTATTAAGTCTTGACTTTATTGTTTCTA [27846]
lp36_PAli_NX_MP_FG_consensus TTCTAGCATTATTTAAAAAAGAACTAGCATTGTCAACTTTACTTTTTATATTATTAAGTCTTGACTTTATTGTTTCTA [27846]
lp36_PAli_NX_Pl_consensus    TTCTAGCATTATTTAAAAAAGAACTAGCATTGTCAACTTTACTTTTTATATTATTAAGTCTTGACTTTATTGTTTCTA [27846]
lp36_PAbe_NX_MP_FG_consensus TTCTAGCATTATTTAAAAAAGAACTAGCATTGTCAACTTTACTTTTTATATTATTAAGTCTTGACTTTATTGTTTCTA [27846]
lp36_PAbe_NX_Pl_consensus    TTCTAGCATTATTTAAAAAAGAACTAGCATTGTCAACTTTACTTTTTATATTATTAAGTCTTGACTTTATTGTTTCTA [27846]

lp36_gi|11496779|B31_GB      AATCTGTAGTTATTTTATTAACTTCTTCTAAAGAGGTATAAATTTCTTGTATAATGTTTCTAGCTTCACTAGTTTCCC [27924]
lp36_un11_B31_PacBio         AATCTGTAGTTATTTTATTAACTTCTTCTAAAGAGGTATAAATTTCTTGTATAATGTTTCTAGCTTCACTAGTTTCCC [27924]
lp36_B31_TS_MP_FG_consensus  AATCTGTAGTTATTTTATTAACTTCTTCTAAAGAGGTATAAATTTCTTGTATAATGTTTCTAGCTTCACTAGTTTCCC [27924]
lp36_B31_NX_Pl_consensus     AATCTGTAGTTATTTTATTAACTTCTTCTAAAGAGGTATAAATTTCTTGTATAATGTTTCTAGCTTCACTAGTTTCCC [27924]
lp36_PAli_NX_MP_FG_consensus AATCTGTAGTTATTTTATTAACTTCTTCTAAAGAGGTATAAATTTCTTGTATAATGTTTCTAGCTTCACTAGTTTCCC [27924]
lp36_PAli_NX_Pl_consensus    AATCTGTAGTTATTTTATTAACTTCTTCTAAAGAGGTATAAATTTCTTGTATAATGTTTCTAGCTTCACTAGTTTCCC [27924]
lp36_PAbe_NX_MP_FG_consensus AATCTGTAGTTATTTTATTAACTTCTTCTAAAGAGGTATAAATTTCTTGTATAATGTTTCTAGCTTCACTAGTTTCCC [27924]
lp36_PAbe_NX_Pl_consensus    AATCTGTAGTTATTTTATTAACTTCTTCTAAAGAGGTATAAATTTCTTGTATAATGTTTCTAGCTTCACTAGTTTCCC [27924]

lp36_gi|11496779|B31_GB      TAGAGTTATTATTAACTTGTTGAGGTTGTCTATTAGTATGTTTTTCACTAGCATCGCTAGCAACGGAAGGGTTTGTGT [28002]
lp36_un11_B31_PacBio         TAGAGTTATTATTAACTTGTTGAGGTTGTCTATTAGTATGTTTTTCACTAGCATCGCTAGCAACGGAAGGGTTTGTGT [28002]
lp36_B31_TS_MP_FG_consensus  TAGAGTTATTATTAACTTGTTGAGGTTGTCTATTAGTATGTTTTTCACTAGCATCGCTAGCAACGGAAGGGTTTGTGT [28002]
lp36_B31_NX_Pl_consensus     TAGAGTTATTATTAACTTGTTGAGGTTGTCTATTAGTATGTTTTTCACTAGCATCGCTAGCAACGGAAGGGTTTGTGT [28002]
lp36_PAli_NX_MP_FG_consensus TAGAGTTATTATTAACTTGTTGAGGTTGTCTATTAGTATGTTTTTCACTAGCATCGCTAGCAACGGAAGGGTTTGTGT [28002]
lp36_PAli_NX_Pl_consensus    TAGAGTTATTATTAACTTGTTGAGGTTGTCTATTAGTATGTTTTTCACTAGCATCGCTAGCAACGGAAGGGTTTGTGT [28002]
lp36_PAbe_NX_MP_FG_consensus TAGAGTTATTATTAACTTGTTGAGGTTGTCTATTAGTATGTTTTTCACTAGCATCGCTAGCAACGGAAGGGTTTGTGT [28002]
lp36_PAbe_NX_Pl_consensus    TAGAGTTATTATTAACTTGTTGAGGTTGTCTATTAGTATGTTTTTCACTAGCATCGCTAGCAACGGAAGGGTTTGTGT [28002]

lp36_gi|11496779|B31_GB      TTACAATACCATCTTTTTGTATACCTAAATTATTGTTTTTTTTACTTTCTTTTGCTTCTTTTAGGTTCTTTTCATTAT [28080]
lp36_un11_B31_PacBio         TTACAATACCATCTTTTTGTATACCTAAATTATTGTTTTTTTTACTTTCTTTTGCTTCTTTTAGGTTCTTTTCATTAT [28080]
lp36_B31_TS_MP_FG_consensus  TTACAATACCATCTTTTTGTATACCTAAATTATTGTTTTTTTTACTTTCTTTTGCTTCTTTTAGGTTCTTTTCATTAT [28080]
lp36_B31_NX_Pl_consensus     TTACAATACCATCTTTTTGTATACCTAAATTATTGTTTTTTTTACTTTCTTTTGCTTCTTTTAGGTTCTTTTCATTAT [28080]
lp36_PAli_NX_MP_FG_consensus TTACAATACCATCTTTTTGTATACCTAAATTATTGTTTTTTTTACTTTCTTTTGCTTCTTTTAGGTTCTTTTCATTAT [28080]
lp36_PAli_NX_Pl_consensus    TTACAATACCATCTTTTTGTATACCTAAATTATTGTTTTTTTTACTTTCTTTTGCTTCTTTTAGGTTCTTTTCATTAT [28080]
lp36_PAbe_NX_MP_FG_consensus TTACAATACCATCTTTTTGTATACCTAAATTATTGTTTTTTTTACTTTCTTTTGCTTCTTTTAGGTTCTTTTCATTAT [28080]
lp36_PAbe_NX_Pl_consensus    TTACAATACCATCTTTTTGTATACCTAAATTATTGTTTTTTTTACTTTCTTTTGCTTCTTTTAGGTTCTTTTCATTAT [28080]

lp36_gi|11496779|B31_GB      TTTGTTCTTGATTATTTACTTCCTTATAACTAATACTTCTAGCTTTACGACCACTTCTTTTGTTGTCTTTTATAAGAG [28158]
lp36_un11_B31_PacBio         TTTGTTCTTGATTATTTACTTCCTTATAACTAATACTTCTAGCTTTACGACCACTTCTTTTGTTGTCTTTTATAAGAG [28158]
lp36_B31_TS_MP_FG_consensus  TTTGTTCTTGATTATTTACTTCCTTATAACTAATACTTCTAGCTTTACGACCACTTCTTTTGTTGTCTTTTATAAGAG [28158]
lp36_B31_NX_Pl_consensus     TTTGTTCTTGATTATTTACTTCCTTATAACTAATACTTCTAGCTTTACGACCACTTCTTTTGTTGTCTTTTATAAGAG [28158]
lp36_PAli_NX_MP_FG_consensus TTTGTTCTTGATTATTTACTTCCTTATAACTAATACTTCTAGCTTTACGACCACTTCTTTTGTTGTCTTTTATAAGAG [28158]
```

```
lp36_PAli_NX_Pl_consensus    TTTGTTCTTGATTATTTACTTCCTTATAACTAATACTTCTAGCTTTACGACCACTTCTTTTGTTGTCTTTTATAAGAG [28158]
lp36_PAbe_NX_MP_FG_consensus TTTGTTCTTGATTATTTACTTCCTTATAACTAATACTTCTAGCTTTACGACCACTTCTTTTGTTGTCTTTTATAAGAG [28158]
lp36_PAbe_NX_Pl_consensus    TTTGTTCTTGATTATTTACTTCCTTATAACTAATACTTCTAGCTTTACGACCACTTCTTTTGTTGTCTTTTATAAGAG [28158]

lp36_gi|11496779|B31_GB      ATTTATCTACAGCTTCATATAGCTTGCAAGAGATAAAGCTTAAAAATAAACTGAATATTAATACTTTAATCATTAAAT [28236]
lp36_un11_B31_PacBio         ATTTATCTACAGCTTCATATAGCTTGCAAGAGATAAAGCTTAAAAATAAACTGAATATTAATACTTTAATCATTAAAT [28236]
lp36_B31_TS_MP_FG_consensus  ATTTATCTACAGCTTCATATAGCTTGCAAGAGATAAAGCTTAAAAATAAACTGAATATTAATACTTTAATCATTAAAT [28236]
lp36_B31_NX_Pl_consensus     ATTTATCTACAGCTTCATATAGCTTGCAAGAGATAAAGCTTAAAAATAAACTGAATATTAATACTTTAATCATTAAAT [28236]
lp36_PAli_NX_MP_FG_consensus ATTTATCTACAGCTTCATATAGCTTGCAAGAGATAAAGCTTAAAAATAAACTGAATATTAATACTTTAATCATTAAAT [28236]
lp36_PAli_NX_Pl_consensus    ATTTATCTACAGCTTCATATAGCTTGCAAGAGATAAAGCTTAAAAATAAACTGAATATTAATACTTTAATCATTAAAT [28236]
lp36_PAbe_NX_MP_FG_consensus ATTTATCTACAGCTTCATATAGCTTGCAAGAGATAAAGCTTAAAAATAAACTGAATATTAATACTTTAATCATTAAAT [28236]
lp36_PAbe_NX_Pl_consensus    ATTTATCTACAGCTTCATATAGCTTGCAAGAGATAAAGCTTAAAAATAAACTGAATATTAATACTTTAATCATTAAAT [28236]

lp36_gi|11496779|B31_GB      TCATTGTGCTAACTTTATGCAAAAATAGAGTATTATTGTAATACAATATCATTATATTACATTTATTATTAGTAATCA [28314]
lp36_un11_B31_PacBio         TCATTGTGCTAACTTTATGCAAAAATAGAGTATTATTGTAATACAATATCATTATATTACATTTATTATTAGTAATCA [28314]
lp36_B31_TS_MP_FG_consensus  TCATTGTGCTAACTTTATGCAAAAATAGAGTATTATTGTAATACAATATCATTATATTACATTTATTATTAGTAATCA [28314]
lp36_B31_NX_Pl_consensus     TCATTGTGCTAACTTTATGCAAAAATAGAGTATTATTGTAATACAATATCATTATATTACATTTATTATTAGTAATCA [28314]
lp36_PAli_NX_MP_FG_consensus TCATTGTGCTAACTTTATGCAAAAATAGAGTATTATTGTAATACAATATCATTATATTACATTTATTATTAGTAATCA [28314]
lp36_PAli_NX_Pl_consensus    TCATTGTGCTAACTTTATGCAAAAATAGAGTATTATTGTAATACAATATCATTATATTACATTTATTATTAGTAATCA [28314]
lp36_PAbe_NX_MP_FG_consensus TCATTGTGCTAACTTTATGCAAAAATAGAGTATTATTGTAATACAATATCATTATATTACATTTATTATTAGTAATCA [28314]
lp36_PAbe_NX_Pl_consensus    TCATTGTGCTAACTTTATGCAAAAATAGAGTATTATTGTAATACAATATCATTATATTACATTTATTATTAGTAATCA [28314]

lp36_gi|11496779|B31_GB      AGAATATGAGTTATATGTTAAGCTTATAGCATAGAGCTTTTTTCTTTAAAGCTGAGCTATCTAAGATTAGTATTATTT [28392]
lp36_un11_B31_PacBio         AGAATATGAGTTATATGTTAAGCTTATAGCATAGAGCTTTTTTCTTTAAAGCTGAGCTATCTAAGATTAGTATTATTT [28392]
lp36_B31_TS_MP_FG_consensus  AGAATATGAGTTATATGTTAAGCTTATAGCATAGAGCTTTTTTCTTTAAAGCTGAGCTATCTAAGATTAGTATTATTT [28392]
lp36_B31_NX_Pl_consensus     AGAATATGAGTTATATGTTAAGCTTATAGCATAGAGCTTTTTTCTTTAAAGCTGAGCTATCTAAGATTAGTATTATTT [28392]
lp36_PAli_NX_MP_FG_consensus AGAATATGAGTTATATGTTAAGCTTATAGCATAGAGCTTTTTTCTTTAAAGCTGAGCTATCTAAGATTAGTATTATTT [28392]
lp36_PAli_NX_Pl_consensus    AGAATATGAGTTATATGTTAAGCTTATAGCATAGAGCTTTTTTCTTTAAAGCTGAGCTATCTAAGATTAGTATTATTT [28392]
lp36_PAbe_NX_MP_FG_consensus AGAATATGAGTTATATGTTAAGCTTATAGCATAGAGCTTTTTTCTTTAAAGCTGAGCTATCTAAGATTAGTATTATTT [28392]
lp36_PAbe_NX_Pl_consensus    AGAATATGAGTTATATGTTAAGCTTATAGCATAGAGCTTTTTTCTTTAAAGCTGAGCTATCTAAGATTAGTATTATTT [28392]

lp36_gi|11496779|B31_GB      AATAAGCAGCTTCATATGCTTTATTTAGCTCTTCAAAGTCTGCATTTGCCTGATACATATTAGAATTAAGATATTCTT [28470]
lp36_un11_B31_PacBio         AATAAGCAGCTTCATATGCTTTATTTAGCTCTTCAAAGTCTGCATTTGCCTGATACATATTAGAATTAAGATATTCTT [28470]
lp36_B31_TS_MP_FG_consensus  AATAAGCAGCTTCATATGCTTTATTTAGCTCTTCAAAGTCTGCATTTGCCTGATACATATTAGAATTAAGATATTCTT [28470]
lp36_B31_NX_Pl_consensus     AATAAGCAGCTTCATATGCTTTATTTAGCTCTTCAAAGTCTGCATTTGCCTGATACATATTAGAATTAAGATATTCTT [28470]
lp36_PAli_NX_MP_FG_consensus AATAAGCAGCTTCATATGCTTTATTTAGCTCTTCAAAGTCTGCATTTGCCTGATACATATTAGAATTAAGATATTCTT [28470]
lp36_PAli_NX_Pl_consensus    AATAAGCAGCTTCATATGCTTTATTTAGCTCTTCAAAGTCTGCATTTGCCTGATACATATTAGAATTAAGATATTCTT [28470]
lp36_PAbe_NX_MP_FG_consensus AATAAGCAGCTTCATATGCTTTATTTAGCTCTTCAAAGTCTGCATTTGCCTGATACATATTAGAATTAAGATATTCTT [28470]
lp36_PAbe_NX_Pl_consensus    AATAAGCAGCTTCATATGCTTTATTTAGCTCTTCAAAGTCTGCATTTGCCTGATACATATTAGAATTAAGATATTCTT [28470]

lp36_gi|11496779|B31_GB      GTTTATGCTTAGCGTTATCTAGCATCCTTTTAGCCCTCTCCATTGCATCTTTAGCGTCATTTATAAAAGAATGATAAT [28548]
lp36_un11_B31_PacBio         GTTTATGCTTAGCGTTATCTAGCATCCTTTTAGCCCTCTCCATTGCATCTTTAGCGTCATTTATAAAAGAATGATAAT [28548]
lp36_B31_TS_MP_FG_consensus  GTTTATGCTTAGCGTTATCTAGCATCCTTTTAGCCCTCTCCATTGCATCTTTAGCGTCATTTATAAAAGAATGATAAT [28548]
lp36_B31_NX_Pl_consensus     GTTTATGCTTAGCGTTATCTAGCATCCTTTTAGCCCTCTCCATTGCATCTTTAGCGTCATTTATAAAAGAATGATAAT [28548]
lp36_PAli_NX_MP_FG_consensus GTTTATGCTTAGCGTTATCTAGCATCCTTTTAGCCCTCTCCATTGCATCTTTAGCGTCATTTATAAAAGAATGATAAT [28548]
lp36_PAli_NX_Pl_consensus    GTTTATGCTTAGCGTTATCTAGCATCCTTTTAGCCCTCTCCATTGCATCTTTAGCGTCATTTATAAAAGAATGATAAT [28548]
lp36_PAbe_NX_MP_FG_consensus GTTTATGCTTAGCGTTATCTAGCATCCTTTTAGCCCTCTCCATTGCATCTTTAGCGTCATTTATAAAAGAATGATAAT [28548]
lp36_PAbe_NX_Pl_consensus    GTTTATGCTTAGCGTTATCTAGCATCCTTTTAGCCCTCTCCATTGCATCTTTAGCGTCATTTATAAAAGAATGATAAT [28548]

lp36_gi|11496779|B31_GB      GACCGTTATACCATTCATAATTTTCCTTTGAAGCATTTTATGCCTCAACTGCCTTTCTCTTTGCATCTTTAAAGGCAG [28626]
lp36_un11_B31_PacBio         GACCGTTATACCATTCATAATTTTCCTTTGAAGCATTTTATGCCTCAACTGCCTTTCTCTTTGCATCTTTAAAGGCAG [28626]
lp36_B31_TS_MP_FG_consensus  GACCGTTATACCATTCATAATTTTCCTTTGAAGCATTTTATGCCTCAACTGCCTTTCTCTTTGCATCTTTAAAGGCAG [28626]
lp36_B31_NX_Pl_consensus     GACCGTTATACCATTCATAATTTTCCTTTGAAGCATTTTATGCCTCAACTGCCTTTCTCTTTGCATCTTTAAAGGCAG [28626]
lp36_PAli_NX_MP_FG_consensus GACCGTTATACCATTCATAATTTTCCTTTGAAGCATTTTATGCCTCAACTGCCTTTCTCTTTGCATCTTTAAAGGCAG [28626]
lp36_PAli_NX_Pl_consensus    GACCGTTATACCATTCATAATTTTCCTTTGAAGCATTTTATGCCTCAACTGCCTTTCTCTTTGCATCTTTAAAGGCAG [28626]
lp36_PAbe_NX_MP_FG_consensus GACCGTTATACCATTCATAATTTTCCTTTGAAGCATTTTATGCCTCAACTGCCTTTCTCTTTGCATCTTTAAAGGCAG [28626]
lp36_PAbe_NX_Pl_consensus    GACCGTTATACCATTCATAATTTTCCTTTGAAGCATTTTATGCCTCAACTGCCTTTCTCTTTGCATCTTTAAAGGCAG [28626]

lp36_gi|11496779|B31_GB      TATCAGCTATTCCCAGGGAATTAAATGCATCATTATAACAAATGATAAAAGAAGTATGACTACTCTTAACCTTAGCAA [28704]
lp36_un11_B31_PacBio         TATCAGCTATTCCCAGGGAATTAAATGCATCATTATAACAAATGATAAAAGAAGTATGACTACTCTTAACCTTAGCAA [28704]
lp36_B31_TS_MP_FG_consensus  TATCAGCTATTCCCAGGGAATTAAATGCATCATTATAACAAATGATAAAAGAAGTATGACTACTCTTAACCTTAGCAA [28704]
lp36_B31_NX_Pl_consensus     TATCAGCTATTCCCAGGGAATTAAATGCATCATTATAACAAATGATAAAAGAAGTATGACTACTCTTAACCTTAGCAA [28704]
lp36_PAli_NX_MP_FG_consensus TATCAGCTATTCCCAGGGAATTAAATGCATCATTATAACAAATGATAAAAGAAGTATGACTACTCTTAACCTTAGCAA [28704]
lp36_PAli_NX_Pl_consensus    TATCAGCTATTCCCAGGGAATTAAATGCATCATTATAACAAATGATAAAAGAAGTATGACTACTCTTAACCTTAGCAA [28704]
lp36_PAbe_NX_MP_FG_consensus TATCAGCTATTCCCAGGGAATTAAATGCATCATTATAACAAATGATAAAAGAAGTATGACTACTCTTAACCTTAGCAA [28704]
lp36_PAbe_NX_Pl_consensus    TATCAGCTATTCCCAGGGAATTAAATGCATCATTATAACAAATGATAAAAGAAGTATGACTACTCTTAACCTTAGCAA [28704]

lp36_gi|11496779|B31_GB      TAGCTTGGTGCAGGCTAAGCAATAAGGTTTGATTATCTTGATTAGATTTAGGTGCTTTTCTAGCGTTCTCTAAATAAG [28782]
lp36_un11_B31_PacBio         TAGCTTGGTGCAGGCTAAGCAATAAGGTTTGATTATCTTGATTAGATTTAGGTGCTTTTCTAGCGTTCTCTAAATAAG [28782]
lp36_B31_TS_MP_FG_consensus  TAGCTTGGTGCAGGCTAAGCAATAAGGTTTGATTATCTTGATTAGATTTAGGTGCTTTTCTAGCGTTCTCTAAATAAG [28782]
lp36_B31_NX_Pl_consensus     TAGCTTGGTGCAGGCTAAGCAATAAGGTTTGATTATCTTGATTAGATTTAGGTGCTTTTCTAGCGTTCTCTAAATAAG [28782]
lp36_PAli_NX_MP_FG_consensus TAGCTTGGTGCAGGCTAAGCAATAAGGTTTGATTATCTTGATTAGATTTAGGTGCTTTTCTAGCGTTCTCTAAATAAG [28782]
lp36_PAli_NX_Pl_consensus    TAGCTTGGTGCAGGCTAAGCAATAAGGTTTGATTATCTTGATTAGATTTAGGTGCTTTTCTAGCGTTCTCTAAATAAG [28782]
lp36_PAbe_NX_MP_FG_consensus TAGCTTGGTGCAGGCTAAGCAATAAGGTTTGATTATCTTGATTAGATTTAGGTGCTTTTCTAGCGTTCTCTAAATAAG [28782]
lp36_PAbe_NX_Pl_consensus    TAGCTTGGTGCAGGCTAAGCAATAAGGTTTGATTATCTTGATTAGATTTAGGTGCTTTTCTAGCGTTCTCTAAATAAG [28782]

lp36_gi|11496779|B31_GB      AATTAGCTGTATTAAGCAAGCTTTCTATATTATCAAGTATTTGTTTTATAGTTTCTAAATCTTTTCTTAGTCTAGCGC [28860]
lp36_un11_B31_PacBio         AATTAGCTGTATTAAGCAAGCTTTCTATATTATCAAGTATTTGTTTTATAGTTTCTAAATCTTTTCTTAGTCTAGCGC [28860]
lp36_B31_TS_MP_FG_consensus  AATTAGCTGTATTAAGCAAGCTTTCTATATTATCAAGTATTTGTTTTATAGTTTCTAAATCTTTTCTTAGTCTAGCGC [28860]
lp36_B31_NX_Pl_consensus     AATTAGCTGTATTAAGCAAGCTTTCTATATTATCAAGTATTTGTTTTATAGTTTCTAAATCTTTTCTTAGTCTAGCGC [28860]
lp36_PAli_NX_MP_FG_consensus AATTAGCTGTATTAAGCAAGCTTTCTATATTATCAAGTATTTGTTTTATAGTTTCTAAATCTTTTCTTAGTCTAGCGC [28860]
lp36_PAli_NX_Pl_consensus    AATTAGCTGTATTAAGCAAGCTTTCTATATTATCAAGTATTTGTTTTATAGTTTCTAAATCTTTTCTTAGTCTAGCGC [28860]
lp36_PAbe_NX_MP_FG_consensus AATTAGCTGTATTAAGCAAGCTTTCTATATTATCAAGTATTTGTTTTATAGTTTCTAAATCTTTTCTTAGTCTAGCGC [28860]
lp36_PAbe_NX_Pl_consensus    AATTAGCTGTATTAAGCAAGCTTTCTATATTATCAAGTATTTGTTTTATAGTTTCTAAATCTTTTCTTAGTCTAGCGC [28860]

lp36_gi|11496779|B31_GB      ACTCTTCTGTAGAAGATTCAATTTCCTTCATAATATTACTATCTTCATTAGCTTTACTGGATTCACTATTAACTTGTT [28938]
lp36_un11_B31_PacBio         ACTCTTCTGTAGAAGATTCAATTTCCTTCATAATATTACTATCTTCATTAGCTTTACTGGATTCACTATTAACTTGTT [28938]
lp36_B31_TS_MP_FG_consensus  ACTCTTCTGTAGAAGATTCAATTTCCTTCATAATATTACTATCTTCATTAGCTTTACTGGATTCACTATTAACTTGTT [28938]
lp36_B31_NX_Pl_consensus     ACTCTTCTGTAGAAGATTCAATTTCCTTCATAATATTACTATCTTCATTAGCTTTACTGGATTCACTATTAACTTGTT [28938]
lp36_PAli_NX_MP_FG_consensus ACTCTTCTGTAGAAGATTCAATTTCCTTCATAATATTACTATCTTCATTAGCTTTACTGGATTCACTATTAACTTGTT [28938]
lp36_PAli_NX_Pl_consensus    ACTCTTCTGTAGAAGATTCAATTTCCTTCATAATATTACTATCTTCATTAGCTTTACTGGATTCACTATTAACTTGTT [28938]
lp36_PAbe_NX_MP_FG_consensus ACTCTTCTGTAGAAGATTCAATTTCCTTCATAATATTACTATCTTCATTAGCTTTACTGGATTCACTATTAACTTGTT [28938]
lp36_PAbe_NX_Pl_consensus    ACTCTTCTGTAGAAGATTCAATTTCCTTCATAATATTACTATCTTCATTAGCTTTACTGGATTCACTATTAACTTGTT [28938]

lp36_gi|11496779|B31_GB      GGGGAAGACTGCTGCTACTATTATCATTTTGCATATCTGCAACAACTAAATGATTTTTGTCTATGTTTTGTTTCATAT [29016]
lp36_un11_B31_PacBio         GGGGAAGACTGCTGCTACTATTATCATTTTGCATATCTGCAACAACTAAATGATTTTTGTCTATGTTTTGTTTCATAT [29016]
lp36_B31_TS_MP_FG_consensus  GGGGAAGACTGCTGCTACTATTATCATTTTGCATATCTGCAACAACTAAATGATTTTTGTCTATGTTTTGTTTCATAT [29016]
lp36_B31_NX_Pl_consensus     GGGGAAGACTGCTGCTACTATTATCATTTTGCATATCTGCAACAACTAAATGATTTTTGTCTATGTTTTGTTTCATAT [29016]
lp36_PAli_NX_MP_FG_consensus GGGGAAGACTGCTGCTACTATTATCATTTTGCATATCTGCAACAACTAAATGATTTTTGTCTATGTTTTGTTTCATAT [29016]
lp36_PAli_NX_Pl_consensus    GGGGAAGACTGCTGCTACTATTATCATTTTGCATATCTGCAACAACTAAATGATTTTTGTCTATGTTTTGTTTCATAT [29016]
lp36_PAbe_NX_MP_FG_consensus GGGGAAGACTGCTGCTACTATTATCATTTTGCATATCTGCAACAACTAAATGATTTTTGTCTATGTTTTGTTTCATAT [29016]
lp36_PAbe_NX_Pl_consensus    GGGGAAGACTGCTGCTACTATTATCATTTTGCATATCTGCAACAACTAAATGATTTTTGTCTATGTTTTGTTTCATAT [29016]

lp36_gi|11496779|B31_GB      AAGCATTATTTGTACTTCTAGGCCTGCGACTTCTACTCTCATTGCTGCTTCCTAAAGTATTAAAAGACTTGTTGTTGT [29094]
lp36_un11_B31_PacBio         AAGCATTATTTGTACTTCTAGGCCTGCGACTTCTACTCTCATTGCTGCTTCCTAAAGTATTAAAAGACTTGTTGTTGT [29094]
lp36_B31_TS_MP_FG_consensus  AAGCATTATTTGTACTTCTAGGCCTGCGACTTCTACTCTCATTGCTGCTTCCTAAAGTATTAAAAGACTTGTTGTTGT [29094]
lp36_B31_NX_Pl_consensus     AAGCATTATTTGTACTTCTAGGCCTGCGACTTCTACTCTCATTGCTGCTTCCTAAAGTATTAAAAGACTTGTTGTTGT [29094]
lp36_PAli_NX_MP_FG_consensus AAGCATTATTTGTACTTCTAGGCCTGCGACTTCTACTCTCATTGCTGCTTCCTAAAGTATTAAAAGACTTGTTGTTGT [29094]
lp36_PAli_NX_Pl_consensus    AAGCATTATTTGTACTTCTAGGCCTGCGACTTCTACTCTCATTGCTGCTTCCTAAAGTATTAAAAGACTTGTTGTTGT [29094]
```

```
lp36_PAbe_NX_MP_FG_consensus AAGCATTATTTGTACTTCTAGGCCTGCGACTTCTACTCTCATTGCTGCTTCCTAAAGTATTAAAAGACTTGTTGTTGT [29094]
lp36_PAbe_NX_Pl_consensus    AAGCATTATTTGTACTTCTAGGCCTGCGACTTCTACTCTCATTGCTGCTTCCTAAAGTATTAAAAGACTTGTTGTTGT [29094]

lp36_gi|11496779|B31_GB      CTAGTATGTCTGTAACCTGCTCAGCGTTGTCTGCAAGATTATCATATAGGTTACAAGAGAGGATATTTGGAATTGAAA [29172]
lp36_un11_B31_PacBio         CTAGTATGTCTGTAACCTGCTCAGCGTTGTCTGCAAGATTATCATATAGGTTACAAGAGAGGATATTTGGAATTGAAA [29172]
lp36_B31_TS_MP_FG_consensus  CTAGTATGTCTGTAACCTGCTCAGCGTTGTCTGCAAGATTATCATATAGGTTACAAGAGAGGATATTTGGAATTGAAA [29172]
lp36_B31_NX_Pl_consensus     CTAGTATGTCTGTAACCTGCTCAGCGTTGTCTGCAAGATTATCATATAGGTTACAAGAGAGGATATTTGGAATTGAAA [29172]
lp36_PAli_NX_MP_FG_consensus CTAGTATGTCTGTAACCTGCTCAGCGTTGTCTGCAAGATTATCATATAGGTTACAAGAGAGGATATTTGGAATTGAAA [29172]
lp36_PAli_NX_Pl_consensus    CTAGTATGTCTGTAACCTGCTCAGCGTTGTCTGCAAGATTATCATATAGGTTACAAGAGAGGATATTTGGAATTGAAA [29172]
lp36_PAbe_NX_MP_FG_consensus CTAGTATGTCTGTAACCTGCTCAGCGTTGTCTGCAAGATTATCATATAGGTTACAAGAGAGGATATTTGGAATTGAAA [29172]
lp36_PAbe_NX_Pl_consensus    CTAGTATGTCTGTAACCTGCTCAGCGTTGTCTGCAAGATTATCATATAGGTTACAAGAGAGGATATTTGGAATTGAAA [29172]

lp36_gi|11496779|B31_GB      CTAAAGTGGATAAAATAAATAATTTAGCAATTAAATTCATCTATCTTAGTACCTCTTCTTAGAATCTGATGATATTTA [29250]
lp36_un11_B31_PacBio         CTAAAGTGGATAAAATAAATAATTTAGCAATTAAATTCATCTATCTTAGTACCTCTTCTTAGAATCTGATGATATTTA [29250]
lp36_B31_TS_MP_FG_consensus  CTAAAGTGGATAAAATAAATAATTTAGCAATTAAATTCATCTATCTTAGTACCTCTTCTTAGAATCTGATGATATTTA [29250]
lp36_B31_NX_Pl_consensus     CTAAAGTGGATAAAATAAATAATTTAGCAATTAAATTCATCTATCTTAGTACCTCTTCTTAGAATCTGATGATATTTA [29250]
lp36_PAli_NX_MP_FG_consensus CTAAAGTGGATAAAATAAATAATTTAGCAATTAAATTCATCTATCTTAGTACCTCTTCTTAGAATCTGATGATATTTA [29250]
lp36_PAli_NX_Pl_consensus    CTAAAGTGGATAAAATAAATAATTTAGCAATTAAATTCATCTATCTTAGTACCTCTTCTTAGAATCTGATGATATTTA [29250]
lp36_PAbe_NX_MP_FG_consensus CTAAAGTGGATAAAATAAATAATTTAGCAATTAAATTCATCTATCTTAGTACCTCTTCTTAGAATCTGATGATATTTA [29250]
lp36_PAbe_NX_Pl_consensus    CTAAAGTGGATAAAATAAATAATTTAGCAATTAAATTCATCTATCTTAGTACCTCTTCTTAGAATCTGATGATATTTA [29250]

lp36_gi|11496779|B31_GB      TGCTTTATTCTTAAAAAATAAATATATCACATTTATAAAAATAGAAAAATAGTGAATGCTAAATGCAAACCAAACTAT [29328]
lp36_un11_B31_PacBio         TGCTTTATTCTTAAAAAATAAATATATCACATTTATAAAAATAGAAAAATAGTGAATGCTAAATGCAAACCAAACTAT [29328]
lp36_B31_TS_MP_FG_consensus  TGCTTTATTCTTAAAAAATAAATATATCACATTTATAAAAATAGAAAAATAGTGAATGCTAAATGCAAACCAAACTAT [29328]
lp36_B31_NX_Pl_consensus     TGCTTTATTCTTAAAAAATAAATATATCACATTTATAAAAATAGAAAAATAGTGAATGCTAAATGCAAACCAAACTAT [29328]
lp36_PAli_NX_MP_FG_consensus TGCTTTATTCTTAAAAAATAAATATATCACATTTATAAAAATAGAAAAATAGTGAATGCTAAATGCAAACCAAACTAT [29328]
lp36_PAli_NX_Pl_consensus    TGCTTTATTCTTAAAAAATAAATATATCACATTTATAAAAATAGAAAAATAGTGAATGCTAAATGCAAACCAAACTAT [29328]
lp36_PAbe_NX_MP_FG_consensus TGCTTTATTCTTAAAAAATAAATATATCACATTTATAAAAATAGAAAAATAGTGAATGCTAAATGCAAACCAAACTAT [29328]
lp36_PAbe_NX_Pl_consensus    TGCTTTATTCTTAAAAAATAAATATATCACATTTATAAAAATAGAAAAATAGTGAATGCTAAATGCAAACCAAACTAT [29328]

lp36_gi|11496779|B31_GB      AAATTTGAAAGAAGTAGAGTAGTGCTTGTAAAGGCAAGAGGGAAGTGAAATAGTTAATTTAGGTAAGCCGATATATAA [29406]
lp36_un11_B31_PacBio         AAATTTGAAAGAAGTAGAGTAGTGCTTGTAAAGGCAAGAGGGAAGTGAAATAGTTAATTTAGGTAAGCCGATATATAA [29406]
lp36_B31_TS_MP_FG_consensus  AAATTTGAAAGAAGTAGAGTAGTGCTTGTAAAGGCAAGAGGGAAGTGAAATAGTTAATTTAGGTAAGCCGATATATAA [29406]
lp36_B31_NX_Pl_consensus     AAATTTGAAAGAAGTAGAGTAGTGCTTGTAAAGGCAAGAGGGAAGTGAAATAGTTAATTTAGGTAAGCCGATATATAA [29406]
lp36_PAli_NX_MP_FG_consensus AAATTTGAAAGAAGTAGAGTAGTGCTTGTAAAGGCAAGAGGGAAGTGAAATAGTTAATTTAGGTAAGCCGATATATAA [29406]
lp36_PAli_NX_Pl_consensus    AAATTTGAAAGAAGTAGAGTAGTGCTTGTAAAGGCAAGAGGGAAGTGAAATAGTTAATTTAGGTAAGCCGATATATAA [29406]
lp36_PAbe_NX_MP_FG_consensus AAATTTGAAAGAAGTAGAGTAGTGCTTGTAAAGGCAAGAGGGAAGTGAAATAGTTAATTTAGGTAAGCCGATATATAA [29406]
lp36_PAbe_NX_Pl_consensus    AAATTTGAAAGAAGTAGAGTAGTGCTTGTAAAGGCAAGAGGGAAGTGAAATAGTTAATTTAGGTAAGCCGATATATAA [29406]

lp36_gi|11496779|B31_GB      GCTTTTCTAGTAAAGATGGGATTATATATGAGTAGAGATATTAAAGAAACTAAAGCCTACACTGGAAGTTTTAAAGCT [29484]
lp36_un11_B31_PacBio         GCTTTTCTAGTAAAGATGGGATTATATATGAGTAGAGATATTAAAGAAACTAAAGCCTACACTGGAAGTTTTAAAGCT [29484]
lp36_B31_TS_MP_FG_consensus  GCTTTTCTAGTAAAGATGGGATTATATATGAGTAGAGATATTAAAGAAACTAAAGCCTACACTGGAAGTTTTAAAGCT [29484]
lp36_B31_NX_Pl_consensus     GCTTTTCTAGTAAAGATGGGATTATATATGAGTAGAGATATTAAAGAAACTAAAGCCTACACTGGAAGTTTTAAAGCT [29484]
lp36_PAli_NX_MP_FG_consensus GCTTTTCTAGTAAAGATGGGATTATATATGAGTAGAGATATTAAAGAAACTAAAGCCTACACTGGAAGTTTTAAAGCT [29484]
lp36_PAli_NX_Pl_consensus    GCTTTTCTAGTAAAGATGGGATTATATATGAGTAGAGATATTAAAGAAACTAAAGCCTACACTGGAAGTTTTAAAGCT [29484]
lp36_PAbe_NX_MP_FG_consensus GCTTTTCTAGTAAAGATGGGATTATATATGAGTAGAGATATTAAAGAAACTAAAGCCTACACTGGAAGTTTTAAAGCT [29484]
lp36_PAbe_NX_Pl_consensus    GCTTTTCTAGTAAAGATGGGATTATATATGAGTAGAGATATTAAAGAAACTAAAGCCTACACTGGAAGTTTTAAAGCT [29484]

lp36_gi|11496779|B31_GB      TGAATATATTTTCTATTTCTTTTTTGCAAGAATTTCCCTGTTGAAGATAATCAATAGCTTTTTGTGTATATTCAATGC [29562]
lp36_un11_B31_PacBio         TGAATATATTTTCTATTTCTTTTTTGCAAGAATTTCCCTGTTGAAGATAATCAATAGCTTTTTGTGTATATTCAATGC [29562]
lp36_B31_TS_MP_FG_consensus  TGAATATATTTTCTATTTCTTTTTTGCAAGAATTTCCCTGTTGAAGATAATCAATAGCTTTTTGTGTATATTCAATGC [29562]
lp36_B31_NX_Pl_consensus     TGAATATATTTTCTATTTCTTTTTTGCAAGAATTTCCCTGTTGAAGATAATCAATAGCTTTTTGTGTATATTCAATGC [29562]
lp36_PAli_NX_MP_FG_consensus TGAATATATTTTCTATTTCTTTTTTGCAAGAATTTCCCTGTTGAAGATAATCAATAGCTTTTTGTGTATATTCAATGC [29562]
lp36_PAli_NX_Pl_consensus    TGAATATATTTTCTATTTCTTTTTTGCAAGAATTTCCCTGTTGAAGATAATCAATAGCTTTTTGTGTATATTCAATGC [29562]
lp36_PAbe_NX_MP_FG_consensus TGAATATATTTTCTATTTCTTTTTTGCAAGAATTTCCCTGTTGAAGATAATCAATAGCTTTTTGTGTATATTCAATGC [29562]
lp36_PAbe_NX_Pl_consensus    TGAATATATTTTCTATTTCTTTTTTGCAAGAATTTCCCTGTTGAAGATAATCAATAGCTTTTTGTGTATATTCAATGC [29562]

lp36_gi|11496779|B31_GB      ATGCAGAAGCAAAGTCTATAGCTCTGTATGCTAAATTTTTTGCATGTTGCATATCACGTCCGTTAAAACTGCCACCGA [29640]
lp36_un11_B31_PacBio         ATGCAGAAGCAAAGTCTATAGCTCTGTATGCTAAATTTTTTGCATGTTGCATATCACGTCCGTTAAAACTGCCACCGA [29640]
lp36_B31_TS_MP_FG_consensus  ATGCAGAAGCAAAGTCTATAGCTCTGTATGCTAAATTTTTTGCATGTTGCATATCACGTCCGTTAAAACTGCCACCGA [29640]
lp36_B31_NX_Pl_consensus     ATGCAGAAGCAAAGTCTATAGCTCTGTATGCTAAATTTTTTGCATGTTGCATATCACGTCCGTTAAAACTGCCACCGA [29640]
lp36_PAli_NX_MP_FG_consensus ATGCAGAAGCAAAGTCTATAGCTCTGTATGCTAAATTTTTTGCATGTTGCATATCACGTCCGTTAAAACTGCCACCGA [29640]
lp36_PAli_NX_Pl_consensus    ATGCAGAAGCAAAGTCTATAGCTCTGTATGCTAAATTTTTTGCATGTTGCATATCACGTCCGTTAAAACTGCCACCGA [29640]
lp36_PAbe_NX_MP_FG_consensus ATGCAGAAGCAAAGTCTATAGCTCTGTATGCTAAATTTTTTGCATGTTGCATATCACGTCCGTTAAAACTGCCACCGA [29640]
lp36_PAbe_NX_Pl_consensus    ATGCAGAAGCAAAGTCTATAGCTCTGTATGCTAAATTTTTTGCATGTTGCATATCACGTCCGTTAAAACTGCCACCGA [29640]

lp36_gi|11496779|B31_GB      ACCGGTATGAATTGCCATTGTATAGTCTTTTATAAATAGATTCTGCTAGTTTATCCTTAGCCTCATCTAGCATGCCTT [29718]
lp36_un11_B31_PacBio         ACCGGTATGAATTGCCATTGTATAGTCTTTTATAAATAGATTCTGCTAGTTTATCCTTAGCCTCATCTAGCATGCCTT [29718]
lp36_B31_TS_MP_FG_consensus  ACCGGTATGAATTGCCATTGTATAGTCTTTTATAAATAGATTCTGCTAGTTTATCCTTAGCCTCATCTAGCATGCCTT [29718]
lp36_B31_NX_Pl_consensus     ACCGGTATGAATTGCCATTGTATAGTCTTTTATAAATAGATTCTGCTAGTTTATCCTTAGCCTCATCTAGCATGCCTT [29718]
lp36_PAli_NX_MP_FG_consensus ACCGGTATGAATTGCCATTGTATAGTCTTTTATAAATAGATTCTGCTAGTTTATCCTTAGCCTCATCTAGCATGCCTT [29718]
lp36_PAli_NX_Pl_consensus    ACCGGTATGAATTGCCATTGTATAGTCTTTTATAAATAGATTCTGCTAGTTTATCCTTAGCCTCATCTAGCATGCCTT [29718]
lp36_PAbe_NX_MP_FG_consensus ACCGGTATGAATTGCCATTGTATAGTCTTTTATAAATAGATTCTGCTAGTTTATCCTTAGCCTCATCTAGCATGCCTT [29718]
lp36_PAbe_NX_Pl_consensus    ACCGGTATGAATTGCCATTGTATAGTCTTTTATAAATAGATTCTGCTAGTTTATCCTTAGCCTCATCTAGCATGCCTT [29718]

lp36_gi|11496779|B31_GB      TAGCAGATACCCAAGAATTAGTTGCTTGCTCCATAAAAGAACGCATTTCTTGTAATTTAGGCATAATAGTCTTTATTT [29796]
lp36_un11_B31_PacBio         TAGCAGATACCCAAGAATTAGTTGCTTGCTCCATAAAAGAACGCATTTCTTGTAATTTAGGCATAATAGTCTTTATTT [29796]
lp36_B31_TS_MP_FG_consensus  TAGCAGATACCCAAGAATTAGTTGCTTGCTCCATAAAAGAACGCATTTCTTGTAATTTAGGCATAATAGTCTTTATTT [29796]
lp36_B31_NX_Pl_consensus     TAGCAGATACCCAAGAATTAGTTGCTTGCTCCATAAAAGAACGCATTTCTTGTAATTTAGGCATAATAGTCTTTATTT [29796]
lp36_PAli_NX_MP_FG_consensus TAGCAGATACCCAAGAATTAGTTGCTTGCTCCATAAAAGAACGCATTTCTTGTAATTTAGGCATAATAGTCTTTATTT [29796]
lp36_PAli_NX_Pl_consensus    TAGCAGATACCCAAGAATTAGTTGCTTGCTCCATAAAAGAACGCATTTCTTGTAATTTAGGCATAATAGTCTTTATTT [29796]
lp36_PAbe_NX_MP_FG_consensus TAGCAGATACCCAAGAATTAGTTGCTTGCTCCATAAAAGAACGCATTTCTTGTAATTTAGGCATAATAGTCTTTATTT [29796]
lp36_PAbe_NX_Pl_consensus    TAGCAGATACCCAAGAATTAGTTGCTTGCTCCATAAAAGAACGCATTTCTTGTAATTTAGGCATAATAGTCTTTATTT [29796]

lp36_gi|11496779|B31_GB      TTCTATTCAAAGGAGATGCGTCTAAATTAGAGAATAACAAGTTTGATAAAGTTTCATAATCTTTTTCAAACTCAAGGA [29874]
lp36_un11_B31_PacBio         TTCTATTCAAAGGAGATGCGTCTAAATTAGAGAATAACAAGTTTGATAAAGTTTCATAATCTTTTTCAAACTCAAGGA [29874]
lp36_B31_TS_MP_FG_consensus  TTCTATTCAAAGGAGATGCGTCTAAATTAGAGAATAACAAGTTTGATAAAGTTTCATAATCTTTTTCAAACTCAAGGA [29874]
lp36_B31_NX_Pl_consensus     TTCTATTCAAAGGAGATGCGTCTAAATTAGAGAATAACAAGTTTGATAAAGTTTCATAATCTTTTTCAAACTCAAGGA [29874]
lp36_PAli_NX_MP_FG_consensus TTCTATTCAAAGGAGATGCGTCTAAATTAGAGAATAACAAGTTTGATAAAGTTTCATAATCTTTTTCAAACTCAAGGA [29874]
lp36_PAli_NX_Pl_consensus    TTCTATTCAAAGGAGATGCGTCTAAATTAGAGAATAACAAGTTTGATAAAGTTTCATAATCTTTTTCAAACTCAAGGA [29874]
lp36_PAbe_NX_MP_FG_consensus TTCTATTCAAAGGAGATGCGTCTAAATTAGAGAATAACAAGTTTGATAAAGTTTCATAATCTTTTTCAAACTCAAGGA [29874]
lp36_PAbe_NX_Pl_consensus    TTCTATTCAAAGGAGATGCGTCTAAATTAGAGAATAACAAGTTTGATAAAGTTTCATAATCTTTTTCAAACTCAAGGA [29874]

lp36_gi|11496779|B31_GB      GTGTATTGTTTAATTTGTTTTCAGGATTAGCATGATTAACAATTTGCGTGGCATTTTTAATTTCTGAAAGAGATGTTT [29952]
lp36_un11_B31_PacBio         GTGTATTGTTTAATTTGTTTTCAGGATTAGCATGATTAACAATTTGCGTGGCATTTTTAATTTCTGAAAGAGATGTTT [29952]
lp36_B31_TS_MP_FG_consensus  GTGTATTGTTTAATTTGTTTTCAGGATTAGCATGATTAACAATTTGCGTGGCATTTTTAATTTCTGAAAGAGATGTTT [29952]
lp36_B31_NX_Pl_consensus     GTGTATTGTTTAATTTGTTTTCAGGATTAGCATGATTAACAATTTGCGTGGCATTTTTAATTTCTGAAAGAGATGTTT [29952]
lp36_PAli_NX_MP_FG_consensus GTGTATTGTTTAATTTGTTTTCAGGATTAGCATGATTAACAATTTGCGTGGCATTTTTAATTTCTGAAAGAGATGTTT [29952]
lp36_PAli_NX_Pl_consensus    GTGTATTGTTTAATTTGTTTTCAGGATTAGCATGATTAACAATTTGCGTGGCATTTTTAATTTCTGAAAGAGATGTTT [29952]
lp36_PAbe_NX_MP_FG_consensus GTGTATTGTTTAATTTGTTTTCAGGATTAGCATGATTAACAATTTGCGTGGCATTTTTAATTTCTGAAAGAGATGTTT [29952]
lp36_PAbe_NX_Pl_consensus    GTGTATTGTTTAATTTGTTTTCAGGATTAGCATGATTAACAATTTGCGTGGCATTTTTAATTTCTGAAAGAGATGTTT [29952]

lp36_gi|11496779|B31_GB      GCTCATACTCTTCTTGTGCAAATTCATCTAAGTTAGCTTTAATTTCTTCTTCTGAAGGAATAGCAGGAGTTTTACTTG [30030]
lp36_un11_B31_PacBio         GCTCATACTCTTCTTGTGCAAATTCATCTAAGTTAGCTTTAATTTCTTCTTCTGAAGGAATAGCAGGAGTTTTACTTG [30030]
lp36_B31_TS_MP_FG_consensus  GCTCATACTCTTCTTGTGCAAATTCATCTAAGTTAGCTTTAATTTCTTCTTCTGAAGGAATAGCAGGAGTTTTACTTG [30030]
lp36_B31_NX_Pl_consensus     GCTCATACTCTTCTTGTGCAAATTCATCTAAGTTAGCTTTAATTTCTTCTTCTGAAGGAATAGCAGGAGTTTTACTTG [30030]
lp36_PAli_NX_MP_FG_consensus GCTCATACTCTTCTTGTGCAAATTCATCTAAGTTAGCTTTAATTTCTTCTTCTGAAGGAATAGCAGGAGTTTTACTTG [30030]
lp36_PAli_NX_Pl_consensus    GCTCATACTCTTCTTGTGCAAATTCATCTAAGTTAGCTTTAATTTCTTCTTCTGAAGGAATAGCAGGAGTTTTACTTG [30030]
lp36_PAbe_NX_MP_FG_consensus GCTCATACTCTTCTTGTGCAAATTCATCTAAGTTAGCTTTAATTTCTTCTTCTGAAGGAATAGCAGGAGTTTTACTTG [30030]
```

```
lp36_PAbe_NX_Pl_consensus    GCTCATACTCTTCTTGTGCAAATTCATCTAAGTTAGCTTTAATTTCTTCTTCTGAAGGAATAGCAGGAGTTTTACTTG [30030]

lp36_gi|11496779|B31_GB      TTGTAGTAAGGCTATTTTTAAAATTACTTTGTTCAATTGGATCATGTTGTGGAGTAGCTGATTTATTTTCTTCCATCT [30108]
lp36_un11_B31_PacBio         TTGTAGTAAGGCTATTTTTAAAATTACTTTGTTCAATTGGATCATGTTGTGGAGTAGCTGATTTATTTTCTTCCATCT [30108]
lp36_B31_TS_MP_FG_consensus  TTGTAGTAAGGCTATTTTTAAAATTACTTTGTTCAATTGGATCATGTTGTGGAGTAGCTGATTTATTTTCTTCCATCT [30108]
lp36_B31_NX_Pl_consensus     TTGTAGTAAGGCTATTTTTAAAATTACTTTGTTCAATTGGATCATGTTGTGGAGTAGCTGATTTATTTTCTTCCATCT [30108]
lp36_PAli_NX_MP_FG_consensus TTGTAGTAAGGCTATTTTTAAAATTACTTTGTTCAATTGGATCATGTTGTGGAGTAGCTGATTTATTTTCTTCCATCT [30108]
lp36_PAli_NX_Pl_consensus    TTGTAGTAAGGCTATTTTTAAAATTACTTTGTTCAATTGGATCATGTTGTGGAGTAGCTGATTTATTTTCTTCCATCT [30108]
lp36_PAbe_NX_MP_FG_consensus TTGTAGTAAGGCTATTTTTAAAATTACTTTGTTCAATTGGATCATGTTGTGGAGTAGCTGATTTATTTTCTTCCATCT [30108]
lp36_PAbe_NX_Pl_consensus    TTGTAGTAAGGCTATTTTTAAAATTACTTTGTTCAATTGGATCATGTTGTGGAGTAGCTGATTTATTTTCTTCCATCT [30108]

lp36_gi|11496779|B31_GB      TTACATTATTTTGAGCATGCGAGTCATTTTGTATTATTGCTGGACTTGAATTTGGTAGGGATTCTTTATTATTAGCAC [30186]
lp36_un11_B31_PacBio         TTACATTATTTTGAGCATGCGAGTCATTTTGTATTATTGCTGGACTTGAATTTGGTAGGGATTCTTTATTATTAGCAC [30186]
lp36_B31_TS_MP_FG_consensus  TTACATTATTTTGAGCATGCGAGTCATTTTGTATTATTGCTGGACTTGAATTTGGTAGGGATTCTTTATTATTAGCAC [30186]
lp36_B31_NX_Pl_consensus     TTACATTATTTTGAGCATGCGAGTCATTTTGTATTATTGCTGGACTTGAATTTGGTAGGGATTCTTTATTATTAGCAC [30186]
lp36_PAli_NX_MP_FG_consensus TTACATTATTTTGAGCATGCGAGTCATTTTGTATTATTGCTGGACTTGAATTTGGTAGGGATTCTTTATTATTAGCAC [30186]
lp36_PAli_NX_Pl_consensus    TTACATTATTTTGAGCATGCGAGTCATTTTGTATTATTGCTGGACTTGAATTTGGTAGGGATTCTTTATTATTAGCAC [30186]
lp36_PAbe_NX_MP_FG_consensus TTACATTATTTTGAGCATGCGAGTCATTTTGTATTATTGCTGGACTTGAATTTGGTAGGGATTCTTTATTATTAGCAC [30186]
lp36_PAbe_NX_Pl_consensus    TTACATTATTTTGAGCATGCGAGTCATTTTGTATTATTGCTGGACTTGAATTTGGTAGGGATTCTTTATTATTAGCAC [30186]

lp36_gi|11496779|B31_GB      CAGGATCATCTGACATATGTTGATTAGAACCTACTTGTAAGATTGGATCATAAGTTTTTTTATTTGATGATGTAACGT [30264]
lp36_un11_B31_PacBio         CAGGATCATCTGACATATGTTGATTAGAACCTACTTGTAAGATTGGATCATAAGTTTTTTTATTTGATGATGTAACGT [30264]
lp36_B31_TS_MP_FG_consensus  CAGGATCATCTGACATATGTTGATTAGAACCTACTTGTAAGATTGGATCATAAGTTTTTTTATTTGATGATGTAACGT [30264]
lp36_B31_NX_Pl_consensus     CAGGATCATCTGACATATGTTGATTAGAACCTACTTGTAAGATTGGATCATAAGTTTTTTTATTTGATGATGTAACGT [30264]
lp36_PAli_NX_MP_FG_consensus CAGGATCATCTGACATATGTTGATTAGAACCTACTTGTAAGATTGGATCATAAGTTTTTTTATTTGATGATGTAACGT [30264]
lp36_PAli_NX_Pl_consensus    CAGGATCATCTGACATATGTTGATTAGAACCTACTTGTAAGATTGGATCATAAGTTTTTTTATTTGATGATGTAACGT [30264]
lp36_PAbe_NX_MP_FG_consensus CAGGATCATCTGACATATGTTGATTAGAACCTACTTGTAAGATTGGATCATAAGTTTTTTTATTTGATGATGTAACGT [30264]
lp36_PAbe_NX_Pl_consensus    CAGGATCATCTGACATATGTTGATTAGAACCTACTTGTAAGATTGGATCATAAGTTTTTTTATTTGATGATGTAACGT [30264]

lp36_gi|11496779|B31_GB      CTTTAGGAGATTCAATTTCCTTACTATTTTTATCTACTGAAGTATTAGTAGTATTTTTATTTTCTTGATGATCTTTTA [30342]
lp36_un11_B31_PacBio         CTTTAGGAGATTCAATTTCCTTACTATTTTTATCTACTGAAGTATTAGTAGTATTTTTATTTTCTTGATGATCTTTTA [30342]
lp36_B31_TS_MP_FG_consensus  CTTTAGGAGATTCAATTTCCTTACTATTTTTATCTACTGAAGTATTAGTAGTATTTTTATTTTCTTGATGATCTTTTA [30342]
lp36_B31_NX_Pl_consensus     CTTTAGGAGATTCAATTTCCTTACTATTTTTATCTACTGAAGTATTAGTAGTATTTTTATTTTCTTGATGATCTTTTA [30342]
lp36_PAli_NX_MP_FG_consensus CTTTAGGAGATTCAATTTCCTTACTATTTTTATCTACTGAAGTATTAGTAGTATTTTTATTTTCTTGATGATCTTTTA [30342]
lp36_PAli_NX_Pl_consensus    CTTTAGGAGATTCAATTTCCTTACTATTTTTATCTACTGAAGTATTAGTAGTATTTTTATTTTCTTGATGATCTTTTA [30342]
lp36_PAbe_NX_MP_FG_consensus CTTTAGGAGATTCAATTTCCTTACTATTTTTATCTACTGAAGTATTAGTAGTATTTTTATTTTCTTGATGATCTTTTA [30342]
lp36_PAbe_NX_Pl_consensus    CTTTAGGAGATTCAATTTCCTTACTATTTTTATCTACTGAAGTATTAGTAGTATTTTTATTTTCTTGATGATCTTTTA [30342]

lp36_gi|11496779|B31_GB      ATGCTAATGCAAACTGTTCTATAGATTTTTTAGATAGAAAACCAGAACAACTTTCAAATAAAAAAAGAGAACTAAATA [30420]
lp36_un11_B31_PacBio         ATGCTAATGCAAACTGTTCTATAGATTTTTTAGATAGAAAACCAGAACAACTTTCAAATAAAAAAAGAGAACTAAATA [30420]
lp36_B31_TS_MP_FG_consensus  ATGCTAATGCAAACTGTTCTATAGATTTTTTAGATAGAAAACCAGAACAACTTTCAAATAAAAAAAGAGAACTAAATA [30420]
lp36_B31_NX_Pl_consensus     ATGCTAATGCAAACTGTTCTATAGATTTTTTAGATAGAAAACCAGAACAACTTTCAAATAAAAAAAGAGAACTAAATA [30420]
lp36_PAli_NX_MP_FG_consensus ATGCTAATGCAAACTGTTCTATAGATTTTTTAGATAGAAAACCAGAACAACTTTCAAATAAAAAAAGAGAACTAAATA [30420]
lp36_PAli_NX_Pl_consensus    ATGCTAATGCAAACTGTTCTATAGATTTTTTAGATAGAAAACCAGAACAACTTTCAAATAAAAAAAGAGAACTAAATA [30420]
lp36_PAbe_NX_MP_FG_consensus ATGCTAATGCAAACTGTTCTATAGATTTTTTAGATAGAAAACCAGAACAACTTTCAAATAAAAAAAGAGAACTAAATA [30420]
lp36_PAbe_NX_Pl_consensus    ATGCTAATGCAAACTGTTCTATAGATTTTTTAGATAGAAAACCAGAACAACTTTCAAATAAAAAAAGAGAACTAAATA [30420]

lp36_gi|11496779|B31_GB      CTAAATTAGGTATAATAAAAATTTTTATTAAACATAATCGCATAATATTTATATTTCCTTTTTAATAAAACTTTGTTA [30498]
lp36_un11_B31_PacBio         CTAAATTAGGTATAATAAAAATTTTTATTAAACATAATCGCATAATATTTATATTTCCTTTTTAATAAAACTTTGTTA [30498]
lp36_B31_TS_MP_FG_consensus  CTAAATTAGGTATAATAAAAATTTTTATTAAACATAATCGCATAATATTTATATTTCCTTTTTAATAAAACTTTGTTA [30498]
lp36_B31_NX_Pl_consensus     CTAAATTAGGTATAATAAAAATTTTTATTAAACATAATCGCATAATATTTATATTTCCTTTTTAATAAAACTTTGTTA [30498]
lp36_PAli_NX_MP_FG_consensus CTAAATTAGGTATAATAAAAATTTTTATTAAACATAATCGCATAATATTTATATTTCCTTTTTAATAAAACTTTGTTA [30498]
lp36_PAli_NX_Pl_consensus    CTAAATTAGGTATAATAAAAATTTTTATTAAACATAATCGCATAATATTTATATTTCCTTTTTAATAAAACTTTGTTA [30498]
lp36_PAbe_NX_MP_FG_consensus CTAAATTAGGTATAATAAAAATTTTTATTAAACATAATCGCATAATATTTATATTTCCTTTTTAATAAAACTTTGTTA [30498]
lp36_PAbe_NX_Pl_consensus    CTAAATTAGGTATAATAAAAATTTTTATTAAACATAATCGCATAATATTTATATTTCCTTTTTAATAAAACTTTGTTA [30498]

lp36_gi|11496779|B31_GB      TGTACTTATATAGCAAATAATATCTATATTAATAATAATTATAATATAAAAATAAAGATATTAAAGGTAATTCATGTA [30576]
lp36_un11_B31_PacBio         TGTACTTATATAGCAAATAATATCTATATTAATAATAATTATAATATAAAAATAAAGATATTAAAGGTAATTCATGTA [30576]
lp36_B31_TS_MP_FG_consensus  TGTACTTATATAGCAAATAATATCTATATTAATAATAATTATAATATAAAAATAAAGATATTAAAGGTAATTCATGTA [30576]
lp36_B31_NX_Pl_consensus     TGTACTTATATAGCAAATAATATCTATATTAATAATAATTATAATATAAAAATAAAGATATTAAAGGTAATTCATGTA [30576]
lp36_PAli_NX_MP_FG_consensus TGTACTTATATAGCAAATAATATCTATATTAATAATAATTATAATATAAAAATAAAGATATTAAAGGTAATTCATGTA [30576]
lp36_PAli_NX_Pl_consensus    TGTACTTATATAGCAAATAATATCTATATTAATAATAATTATAATATAAAAATAAAGATATTAAAGGTAATTCATGTA [30576]
lp36_PAbe_NX_MP_FG_consensus TGTACTTATATAGCAAATAATATCTATATTAATAATAATTATAATATAAAAATAAAGATATTAAAGGTAATTCATGTA [30576]
lp36_PAbe_NX_Pl_consensus    TGTACTTATATAGCAAATAATATCTATATTAATAATAATTATAATATAAAAATAAAGATATTAAAGGTAATTCATGTA [30576]

lp36_gi|11496779|B31_GB      ATTATTTAAAAAATAATGACTTTTTAAGTCAAACTATAAAGTTAAGAGAAAATAAAGTGTTGTTTAGAAAAAGAGTAT [30654]
lp36_un11_B31_PacBio         ATTATTTAAAAAATAATGACTTTTTAAGTCAAACTATAAAGTTAAGAGAAAATAAAGTGTTGTTTAGAAAAAGAGTAT [30654]
lp36_B31_TS_MP_FG_consensus  ATTATTTAAAAAATAATGACTTTTTAAGTCAAACTATAAAGTTAAGAGAAAATAAAGTGTTGTTTAGAAAAAGAGTAT [30654]
lp36_B31_NX_Pl_consensus     ATTATTTAAAAAATAATGACTTTTTAAGTCAAACTATAAAGTTAAGAGAAAATAAAGTGTTGTTTAGAAAAAGAGTAT [30654]
lp36_PAli_NX_MP_FG_consensus ATTATTTAAAAAATAATGACTTTTTAAGTCAAACTATAAAGTTAAGAGAAAATAAAGTGTTGTTTAGAAAAAGAGTAT [30654]
lp36_PAli_NX_Pl_consensus    ATTATTTAAAAAATAATGACTTTTTAAGTCAAACTATAAAGTTAAGAGAAAATAAAGTGTTGTTTAGAAAAAGAGTAT [30654]
lp36_PAbe_NX_MP_FG_consensus ATTATTTAAAAAATAATGACTTTTTAAGTCAAACTATAAAGTTAAGAGAAAATAAAGTGTTGTTTAGAAAAAGAGTAT [30654]
lp36_PAbe_NX_Pl_consensus    ATTATTTAAAAAATAATGACTTTTTAAGTCAAACTATAAAGTTAAGAGAAAATAAAGTGTTGTTTAGAAAAAGAGTAT [30654]

lp36_gi|11496779|B31_GB      TATAAACAAAGAAGCCAAACCTCATAAAGTATTGTCTGGCTTGTCTTTAATATTTTACTTTTACTTATCTAGAGTCCA [30732]
lp36_un11_B31_PacBio         TATAAACAAAGAAGCCAAACCTCATAAAGTATTGTCTGGCTTGTCTTTAATATTTTACTTTTACTTATCTAGAGTCCA [30732]
lp36_B31_TS_MP_FG_consensus  TATAAACAAAGAAGCCAAACCTCATAAAGTATTGTCTGGCTTGTCTTTAATATTTTACTTTTACTTATCTAGAGTCCA [30732]
lp36_B31_NX_Pl_consensus     TATAAACAAAGAAGCCAAACCTCATAAAGTATTGTCTGGCTTGTCTTTAATATTTTACTTTTACTTATCTAGAGTCCA [30732]
lp36_PAli_NX_MP_FG_consensus TATAAACAAAGAAGCCAAACCTCATAAAGTATTGTCTGGCTTGTCTTTAATATTTTACTTTTACTTATCTAGAGTCCA [30732]
lp36_PAli_NX_Pl_consensus    TATAAACAAAGAAGCCAAACCTCATAAAGTATTGTCTGGCTTGTCTTTAATATTTTACTTTTACTTATCTAGAGTCCA [30732]
lp36_PAbe_NX_MP_FG_consensus TATAAACAAAGAAGCCAAACCTCATAAAGTATTGTCTGGCTTGTCTTTAATATTTTACTTTTACTTATCTAGAGTCCA [30732]
lp36_PAbe_NX_Pl_consensus    TATAAACAAAGAAGCCAAACCTCATAAAGTATTGTCTGGCTTGTCTTTAATATTTTACTTTTACTTATCTAGAGTCCA [30732]

lp36_gi|11496779|B31_GB      TATCTTGCAATTTTTTATATATATCATTTAACTCTTGAAAGTCTTTATTTGTCTTAGTCATATTTTCATTAAGTTCTT [30810]
lp36_un11_B31_PacBio         TATCTTGCAATTTTTTATATATATCATTTAACTCTTGAAAGTCTTTATTTGTCTTAGTCATATTTTCATTAAGTTCTT [30810]
lp36_B31_TS_MP_FG_consensus  TATCTTGCAATTTTTTATATATATCATTTAACTCTTGAAAGTCTTTATTTGTCTTAGTCATATTTTCATTAAGTTCTT [30810]
lp36_B31_NX_Pl_consensus     TATCTTGCAATTTTTTATATATATCATTTAACTCTTGAAAGTCTTTATTTGTCTTAGTCATATTTTCATTAAGTTCTT [30810]
lp36_PAli_NX_MP_FG_consensus TATCTTGCAATTTTTTATATATATCATTTAACTCTTGAAAGTCTTTATTTGTCTTAGTCATATTTTCATTAAGTTCTT [30810]
lp36_PAli_NX_Pl_consensus    TATCTTGCAATTTTTTATATATATCATTTAACTCTTGAAAGTCTTTATTTGTCTTAGTCATATTTTCATTAAGTTCTT [30810]
lp36_PAbe_NX_MP_FG_consensus TATCTTGCAATTTTTTATATATATCATTTAACTCTTGAAAGTCTTTATTTGTCTTAGTCATATTTTCATTAAGTTCTT [30810]
lp36_PAbe_NX_Pl_consensus    TATCTTGCAATTTTTTATATATATCATTTAACTCTTGAAAGTCTTTATTTGTCTTAGTCATATTTTCATTAAGTTCTT [30810]

lp36_gi|11496779|B31_GB      TTTGCTTATTCTTAGCAACCTCTAGCAAACTTTTAGTACTACTCATTGAAGCTTTAGCATCAGCCATAAATTGGTGGT [30888]
lp36_un11_B31_PacBio         TTTGCTTATTCTTAGCAACCTCTAGCAAACTTTTAGTACTACTCATTGAAGCTTTAGCATCAGCCATAAATTGGTGGT [30888]
lp36_B31_TS_MP_FG_consensus  TTTGCTTATTCTTAGCAACCTCTAGCAAACTTTTAGTACTACTCATTGAAGCTTTAGCATCAGCCATAAATTGGTGGT [30888]
lp36_B31_NX_Pl_consensus     TTTGCTTATTCTTAGCAACCTCTAGCAAACTTTTAGTACTACTCATTGAAGCTTTAGCATCAGCCATAAATTGGTGGT [30888]
lp36_PAli_NX_MP_FG_consensus TTTGCTTATTCTTAGCAACCTCTAGCAAACTTTTAGTACTACTCATTGAAGCTTTAGCATCAGCCATAAATTGGTGGT [30888]
lp36_PAli_NX_Pl_consensus    TTTGCTTATTCTTAGCAACCTCTAGCAAACTTTTAGTACTACTCATTGAAGCTTTAGCATCAGCCATAAATTGGTGGT [30888]
lp36_PAbe_NX_MP_FG_consensus TTTGCTTATTCTTAGCAACCTCTAGCAAACTTTTAGTACTACTCATTGAAGCTTTAGCATCAGCCATAAATTGGTGGT [30888]
lp36_PAbe_NX_Pl_consensus    TTTGCTTATTCTTAGCAACCTCTAGCAAACTTTTAGTACTACTCATTGAAGCTTTAGCATCAGCCATAAATTGGTGGT [30888]

lp36_gi|11496779|B31_GB      AGCCAGCATAGTAGTAGCTCTCATGCCTTGAAGCATTGCTATTACTTAATGCCTCTTCTAAAGCCCGATCTGCTTTTC [30966]
lp36_un11_B31_PacBio         AGCCAGCATAGTAGTAGCTCTCATGCCTTGAAGCATTGCTATTACTTAATGCCTCTTCTAAAGCCCGATCTGCTTTTC [30966]
lp36_B31_TS_MP_FG_consensus  AGCCAGCATAGTAGTAGCTCTCATGCCTTGAAGCATTGCTATTACTTAATGCCTCTTCTAAAGCCCGATCTGCTTTTC [30966]
lp36_B31_NX_Pl_consensus     AGCCAGCATAGTAGTAGCTCTCATGCCTTGAAGCATTGCTATTACTTAATGCCTCTTCTAAAGCCCGATCTGCTTTTC [30966]
lp36_PAli_NX_MP_FG_consensus AGCCAGCATAGTAGTAGCTCTCATGCCTTGAAGCATTGCTATTACTTAATGCCTCTTCTAAAGCCCGATCTGCTTTTC [30966]
lp36_PAli_NX_Pl_consensus    AGCCAGCATAGTAGTAGCTCTCATGCCTTGAAGCATTGCTATTACTTAATGCCTCTTCTAAAGCCCGATCTGCTTTTC [30966]
lp36_PAbe_NX_MP_FG_consensus AGCCAGCATAGTAGTAGCTCTCATGCCTTGAAGCATTGCTATTACTTAATGCCTCTTCTAAAGCCCGATCTGCTTTTC [30966]
lp36_PAbe_NX_Pl_consensus    AGCCAGCATAGTAGTAGCTCTCATGCCTTGAAGCATTGCTATTACTTAATGCCTCTTCTAAAGCCCGATCTGCTTTTC [30966]
```

```
lp36_gi|11496779|B31_GB      TTTGTGCATACTCAAAATCGTTCTTAGCTCTTTTTAAAGCAGCAATAGCATCATTGCAATGAGTATCAGCAGAAGCAT [31044]
lp36_un11_B31_PacBio         TTTGTGCATACTCAAAATCGTTCTTAGCTCTTTTTAAAGCAGCAATAGCATCATTGCAATGAGTATCAGCAGAAGCAT [31044]
lp36_B31_TS_MP_FG_consensus  TTTGTGCATACTCAAAATCGTTCTTAGCTCTTTTTAAAGCAGCAATAGCATCATTGCAATGAGTATCAGCAGAAGCAT [31044]
lp36_B31_NX_Pl_consensus     TTTGTGCATACTCAAAATCGTTCTTAGCTCTTTTTAAAGCAGCAATAGCATCATTGCAATGAGTATCAGCAGAAGCAT [31044]
lp36_PAli_NX_MP_FG_consensus TTTGTGCATACTCAAAATCGTTCTTAGCTCTTTTTAAAGCAGCAATAGCATCATTGCAATGAGTATCAGCAGAAGCAT [31044]
lp36_PAli_NX_Pl_consensus    TTTGTGCATACTCAAAATCGTTCTTAGCTCTTTTTAAAGCAGCAATAGCATCATTGCAATGAGTATCAGCAGAAGCAT [31044]
lp36_PAbe_NX_MP_FG_consensus TTTGTGCATACTCAAAATCGTTCTTAGCTCTTTTTAAAGCAGCAATAGCATCATTGCAATGAGTATCAGCAGAAGCAT [31044]
lp36_PAbe_NX_Pl_consensus    TTTGTGCATACTCAAAATCGTTCTTAGCTCTTTTTAAAGCAGCAATAGCATCATTGCAATGAGTATCAGCAGAAGCAT [31044]

lp36_gi|11496779|B31_GB      GATTACTCTTAACCTTAGCAATAGCTTCTTCTAGGCTAGGCAATAAGGCTAAGTTAGCTTTACTAGATCCCACACCTC [31122]
lp36_un11_B31_PacBio         GATTACTCTTAACCTTAGCAATAGCTTCTTCTAGGCTAGGCAATAAGGCTAAGTTAGCTTTACTAGATCCCACACCTC [31122]
lp36_B31_TS_MP_FG_consensus  GATTACTCTTAACCTTAGCAATAGCTTCTTCTAGGCTAGGCAATAAGGCTAAGTTAGCTTTACTAGATCCCACACCTC [31122]
lp36_B31_NX_Pl_consensus     GATTACTCTTAACCTTAGCAATAGCTTCTTCTAGGCTAGGCAATAAGGCTAAGTTAGCTTTACTAGATCCCACACCTC [31122]
lp36_PAli_NX_MP_FG_consensus GATTACTCTTAACCTTAGCAATAGCTTCTTCTAGGCTAGGCAATAAGGCTAAGTTAGCTTTACTAGATCCCACACCTC [31122]
lp36_PAli_NX_Pl_consensus    GATTACTCTTAACCTTAGCAATAGCTTCTTCTAGGCTAGGCAATAAGGCTAAGTTAGCTTTACTAGATCCCACACCTC [31122]
lp36_PAbe_NX_MP_FG_consensus GATTACTCTTAACCTTAGCAATAGCTTCTTCTAGGCTAGGCAATAAGGCTAAGTTAGCTTTACTAGATCCCACACCTC [31122]
lp36_PAbe_NX_Pl_consensus    GATTACTCTTAACCTTAGCAATAGCTTCTTCTAGGCTAGGCAATAAGGCTAAGTTAGCTTTACTAGATCCCACACCTC [31122]
```
[truncated: 1,343,252 more chars]
